# Origin and evolution of fungal HECT ubiquitin ligases

**DOI:** 10.1038/s41598-018-24914-x

**Published:** 2018-04-23

**Authors:** Ignacio Marín

**Affiliations:** Instituto de Biomedicina de Valencia, Consejo Superior de Investigaciones Científicas, (IBV-CSIC), Valencia, Spain

## Abstract

Ubiquitin ligases (E3s) are basic components of the eukaryotic ubiquitination system. In this work, the emergence and diversification of fungal HECT ubiquitin ligases is described. Phylogenetic and structural data indicate that six HECT subfamilies (RSP5, TOM1, UFD4, HUL4, HUL4A and HUL5) existed in the common ancestor of all fungi. These six subfamilies have evolved very conservatively, with only occasional losses and duplications in particular fungal lineages. However, an early, drastic reduction in the number of HECT genes occurred in microsporidians, in parallel to the reduction of their genomes. A significant correlation between the total number of genes and the number of HECT-encoding genes present in fungi has been observed. However, transitions from unicellularity to multicellularity or vice versa apparently had no effect on the evolution of this family. Likely orthologs or co-orthologs of all fungal HECT genes have been detected in animals. Four genes are deduced to be present in the common ancestor of fungi, animals and plants. Protein-protein interactions detected in both the yeast *Saccharomyces cerevisiae* and humans suggest that some ancient functions of HECT proteins have been conserved since the animals/fungi split.

## Introduction

Protein ubiquitination is involved in the control of multiple essential functions in all eukaryotic species^1–3^. Given its importance, there is a significant interest in understanding the evolution of the ubiquitination system, from its early origin^4–6^ to its complex patterns of diversification in eukaryotic phyla, in which the ubiquitination machinery typically involves hundreds of proteins^7^. Among them, the most diverse are ubiquitin ligases (E3s), the enzymes that transfer ubiquitin to target proteins. Ubiquitin ligases have been classified into different classes according to structural and functional features: Ring Finger-containing E3s (including U-box ubiquitin ligases), Cullin-containing E3 complexes, RBR ubiquitin ligases and HECT E3s^8–10^. In addition of many works analyzing the ubiquitin ligases of particular species, detailed studies of the evolution of several E3 gene families in the animal and plant kingdoms have been performed^11–24^. However, similar studies are lacking in other groups, most particularly in fungi. Only a few studies focused on the evolution of E3 families have included fungal species, most often the model yeasts *Saccharomyces cerevisiae* and *Schizosaccharomyces pombe*^7,11–13,18,24,25^. This is quite surprising, because fungi have several interesting features that may provide a decisive contribution to the understanding of the evolution of ubiquitination. First, general studies indicated that fungi have a particularly simple ubiquitination machinery^7^, a fact that remains unexplained. Second, very complete evolutionary studies can be performed, given the large number and diversity of fungal genomes that have been sequenced, which include unicellular and multicellular species^26,27^, related taxa in which significant gene amplifications and reductions have been detected^28,29^ and lineages in which whole genome duplications occurred^30,31^. Also, the fact that fungi are quite close relatives of animals may improve our understanding of the emergence and diversification of the ubiquitination system in this last group.

Here, a comprehensive analysis of the diversity of the HECT ubiquitin ligase protein family in fungi is performed in order to answer the following questions: (1) When the current diversity of fungal HECT genes emerged. In this study, microsporidia and cryptomycota will be considered as fungi, although whether they are better classified as the closest relatives of fungi *sensu stricto* remains an unsolved taxonomic problem, which depends on how the kingdom is defined^32^; (2) Whether amplifications or reductions of the HECT family have occurred, associated to general changes in genome complexity; (3) Whether the transitions from unicellularity and multicellularity (or vice versa) have had any effect on the sets of HECT genes; (4) How fungal HECT ubiquitin ligases relate to those present in animals and whether conservation of the functions of orthologous genes in both phyla exist; and, (5) Using comparative data from animals, plants and fungi, to establish the HECT genes already present in the ancestor of the three kingdoms. A general view of the evolution of these ubiquitin ligases in fungi, as well as insights on their early evolution in all eukaryotes, are thus obtained.

## Results

### Global diversity of fungal HECT ubiquitin ligases is low

**Figure 1:**
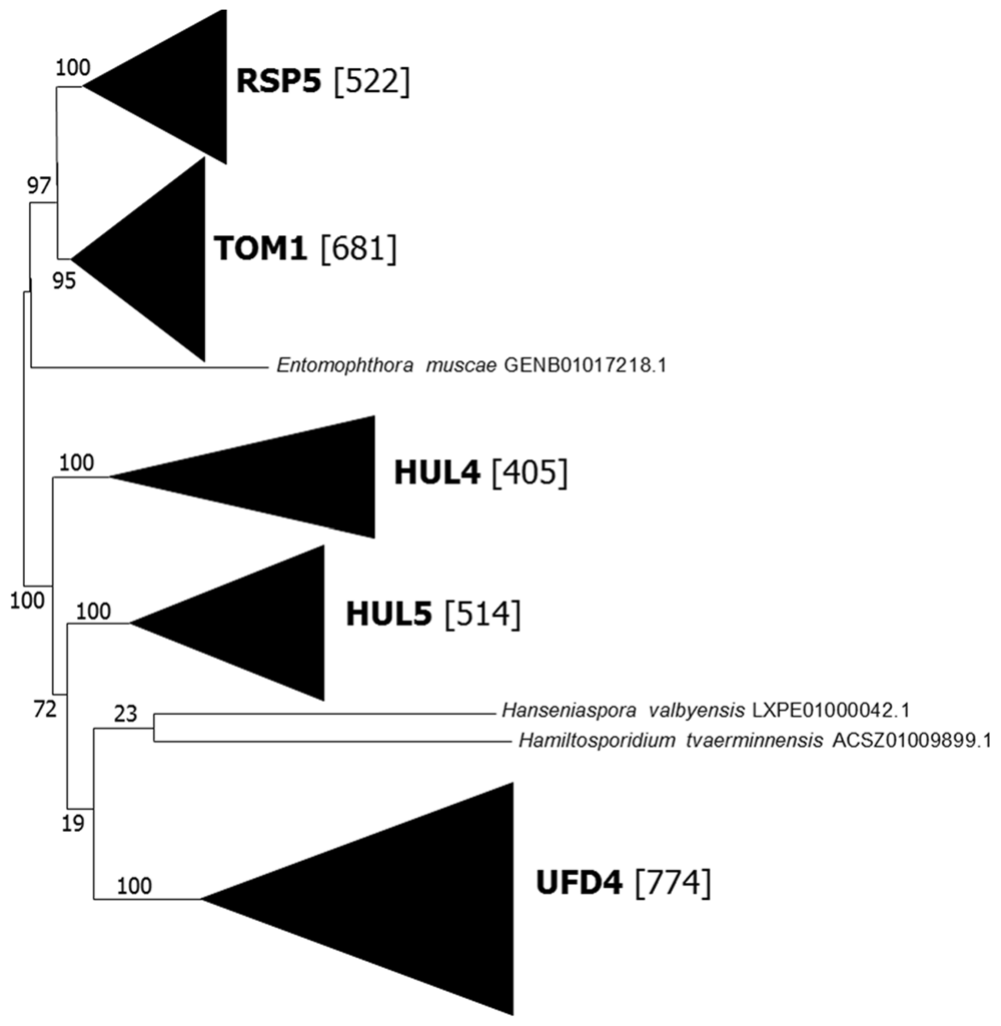
ML phylogenetic analysis of a dataset of 2899 HECT domain sequences. The best tree, shown here, was obtained with the LG + R10 model of sequence evolution^58^ and perturbation strength = 0.5 (see Methods). In brackets, the number of sequences included in each of the five highly-supported groups, named according to the *Saccharomyces cerevisiae* genes included in each of them.

As indicated in the Methods section, 5455 hits were detected in sequence databases when they were queried with the *Saccharomyces cerevisiae* HECT family proteins and a final dataset of 2899 HECT domains was obtained after eliminating duplicated and partial sequences. These 2899 sequences were aligned (Supplementary File 1) and a prospective maximum-likelihood phylogenetic analysis performed. It determined the presence of five well-supported groups, each of them including one of the five genes present in *S*. *cerevisiae* (Fig. 1). Only three sequences were excluded from these groups, being one of them, supposedly from the fly parasite *Entomophthora muscae* (GENB01017218.1), a contaminant derived from the host fly. The other two were true divergent sequences. These results suggest that the diversity of HECT proteins in fungi is low, similar to the diversity detected in plants^23^ but much lower than that observed in animals^20^.

### A detailed analysis of 39 model species refines the classification of fungal HECTs

The results just described must be considered incomplete for two reasons. First, these searches may have unearthed only a fraction of the fungal HECT proteins. If some of them are extremely divergent, they may be so different from the *S*. *cerevisiae* sequences used to query the databases as to remain undetectable. Second, some significant sequences could have been detected but only partially retrieved and therefore subsequently eliminated. In order to avoid these problems, specific searches were performed for 38 species that comprehend the main fungal taxa (Table 1). As expected, several sequences that had been eliminated as partial were detected, correctly reconstructed and added to the dataset. In addition, very interestingly, a few additional, highly divergent sequences were also found.

**Table 1:** HECT genes and total number of genes present in 38 model fungal species and the slime mold *Fonticula alba* (red).

Species	Taxonomic group	*RSP5*	*TOM1*	*HUL5*	*UFD4*	*HUL4*	*HUL4A*	*HUL4*-*like*	Other	No. HECT genes	Total No. genes
*Aspergillus niger*	Ascomycota, pezizomycotina, eurotiomycetes	1	1	1	1	1	1	0	0	**6**	**10785**
*Penicillium chrysogenum*	Ascomycota, pezizomycotina, eurotiomycetes	1	1	1	1	1	1	0	0	**6**	**11460**
*Coccidiodes immitis*	Ascomycota, pezizomycotina, eurotiomycetes	1	1	1	1	1	1	0	0	**6**	**9905**
*Pyrenophora tritici*-*repentis*	Ascomycota, pezizomycotina, dothiomycetes	1	1	1	1	0	1	0	0	**5**	**12300**
*Mycosphaerella graminicola*	Ascomycota, pezizomycotina, dothiomycetes	1	1	1	1	1	1	0	0	**6**	**10964**
*Chaetomium globosum*	Ascomycota, pezizomycotina, sordariomycetes	1	1	1	1	0	1	0	0	**5**	**11232**
*Fusarium verticilloides*	Ascomycota, pezizomycotina, sordariomycetes	1	1	1	1	1	1	0	0	**6**	**16012**
*Sclerotinia sclerotiorum*	Ascomycota, pezizomycotina, leotiomycetes	1	1	1	1	1	1	0	0	**6**	**14637**
*Tuber melanoporum*	Ascomycota, pezizomycotina, pezyzomycetes	1	1	1	1	1	1	0	0	**6**	**7496**
*Saccharomyces cerevisiae*	Ascomycota, saccharomycotina	1	1	1	1	1	0	0	0	**5**	**5949**
*Kluyveromyces lactis*	Ascomycota, saccharomycotina	1	1	1	1	1	0	0	0	**5**	**5379**
*Candida albicans*	Ascomycota, saccharomycotina	1	1	1	1	1	0	0	0	**5**	**6263**
*Yarrowia lipolytica*	Ascomycota, saccharomycotina	1	1	1	1	1	0	0	0	**5**	**7086**
*Schizosaccharomyces pombe*	Ascomycota, taphrinomycotina	3	1	1	1	1	0	0	0	**7**	**6953**
*Pneumocystis carinii*	Ascomycota, taphrinomycotina	1	1	1	1	0	0	0	0	**4**	**3695**
*Laccaria bicolor*	Basidiomycota, agaricomycotina	2	1	1	0	0	0	0	0	**4**	**18264**
*Cryptococcus neoformans var*. *grubii*	Basidiomycota, agaricomycotina	1	1	1	1	0	0	0	0	**4**	**6617**
*Puccinia graminis*	Basidiomycota, pucciniomycotina	1	1	1	1	1	0	0	0	**5**	**16309**
*Ustilago maydis*	Basidiomycota, ustilaginomycotina	1	1	1	1	0	0	0	1	**5**	**6910**
*Mortierella alpina*	Mucoromycotina	1	1	2	1	1	2	0	0	**8**	**9909**
*Rhizopus oryzae*	Mucoromycotina	2	2	2	1	1	2	0	0	**10**	**14134**
*Phycomyces blakesleeanus*	Mucoromycotina	2	1	2	1	1	1	0	0	**8**	**16850**
*Entomophthora muscae*	Zoopagomycota	1	1	1	1	0	1	0	0	**5**	**?**
*Piromyces finnis*	Neocallimastigomycota	1	1	0	1	0	1	0	0	**4**	**10978**
*Pecoramyces ruminatium*	Neocallimastigomycota	7	1	0	1	0	1	0	0	**10**	**16347**
*Anaeromyces robustus*	Neocallimastigomycota	2	1	1	2	0	1	0	0	**7**	**13083**
*Spizellomyces punctatus*	Euchytrids, spizellomycetales	1	1	2	1	1	1	0	0	**7**	**9169**
*Allomyces macrogynus*	Blastocladiomycota	3	1	1	2	1	2	0	0	**10**	**19282**
*Encephalitozoon cuniculi*	Microsporidia	0	1	0	0	0	0	1	0	**2**	**2029**
*Ordospora colligata*	Microsporidia	0	1	0	0	0	0	1	0	**2**	**1879**
*Nosema ceranae*	Microsporidia	0	1	0	0	0	0	1	2	**4**	**2678**
*Hamiltosporidium tvaerminnensis*	Microsporidia	0	0	0	0	0	0	1	4	**5**	**2174**
*Nematocida parisii*	Microsporidia	0	1	0	0	0	0	1	1	**3**	**2724**
*Mitosporidium daphniae*	Microsporidia	1	1	0	0	1	0	0	3	**6**	**3330**
*Rozella allomycis*	Cryptomycota	1	1	1	1	0	1	0	1	**6**	**6350**
*Fonticula alba*	Cristidiscoidea, Fonticulida	1	1	0	1	0	0	0	2	**5**	**6457**
	**Total number of genes:**	**45**	**36**	**31**	**31**	**19**	**22**	**5**	**14**	**203**	

**Figure 2:**
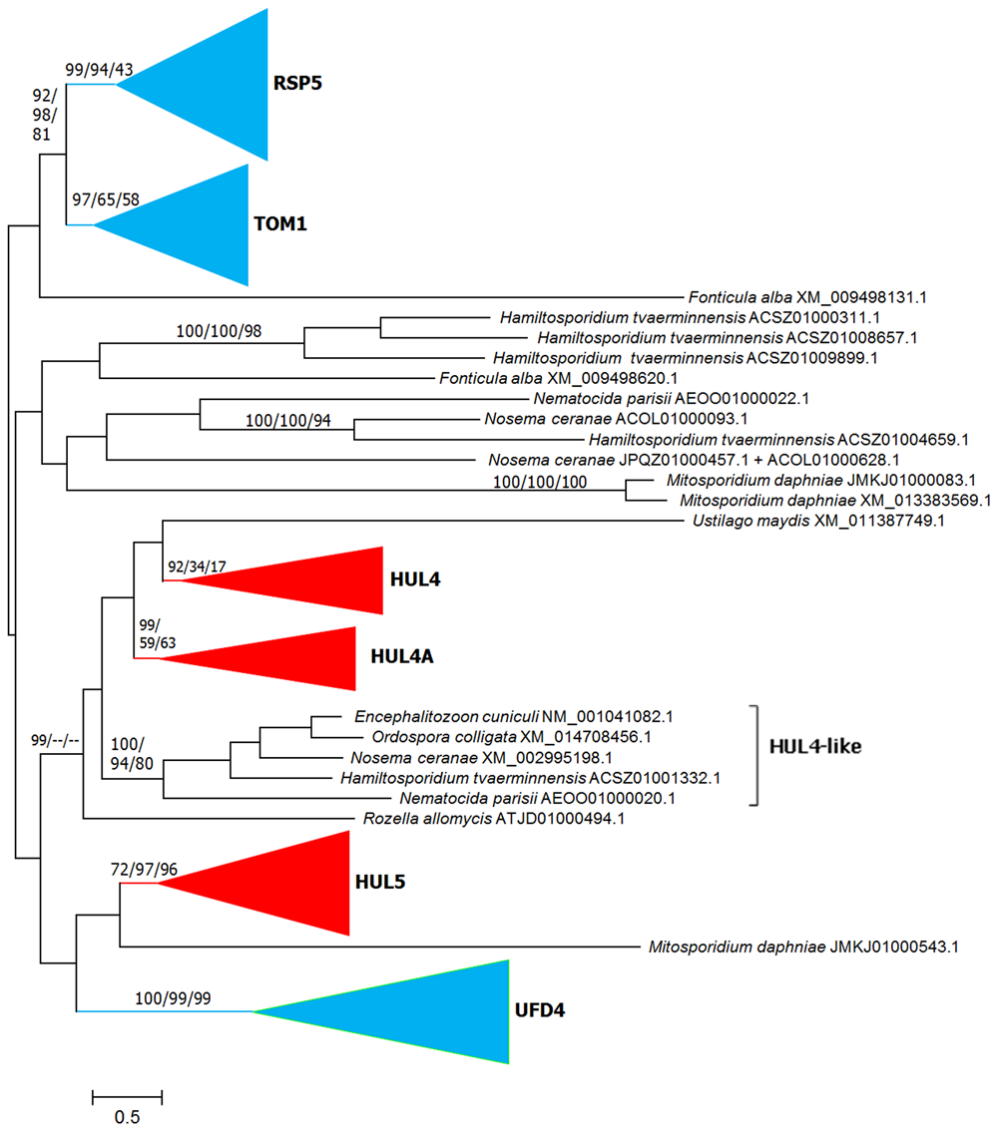
ML tree based on the analysis of 203 sequences from fungi and *Fonticula alba*. The LG + R9 model with perturbation strength = 0.8 provided the top ML value. NJ and MP results were so similar that they are also shown here. Numbers above the branches refer to ML/NJ/MP bootstrap support. Groups that include *Fonticula alba* sequences are indicated in blue and those that only include fungal sequences, in red. The branch that comprises all the HUL4-related sequences was supported by ML analysis but not by NJ or MP analyses (indicated as 99/−/−).

**Figure 3:**
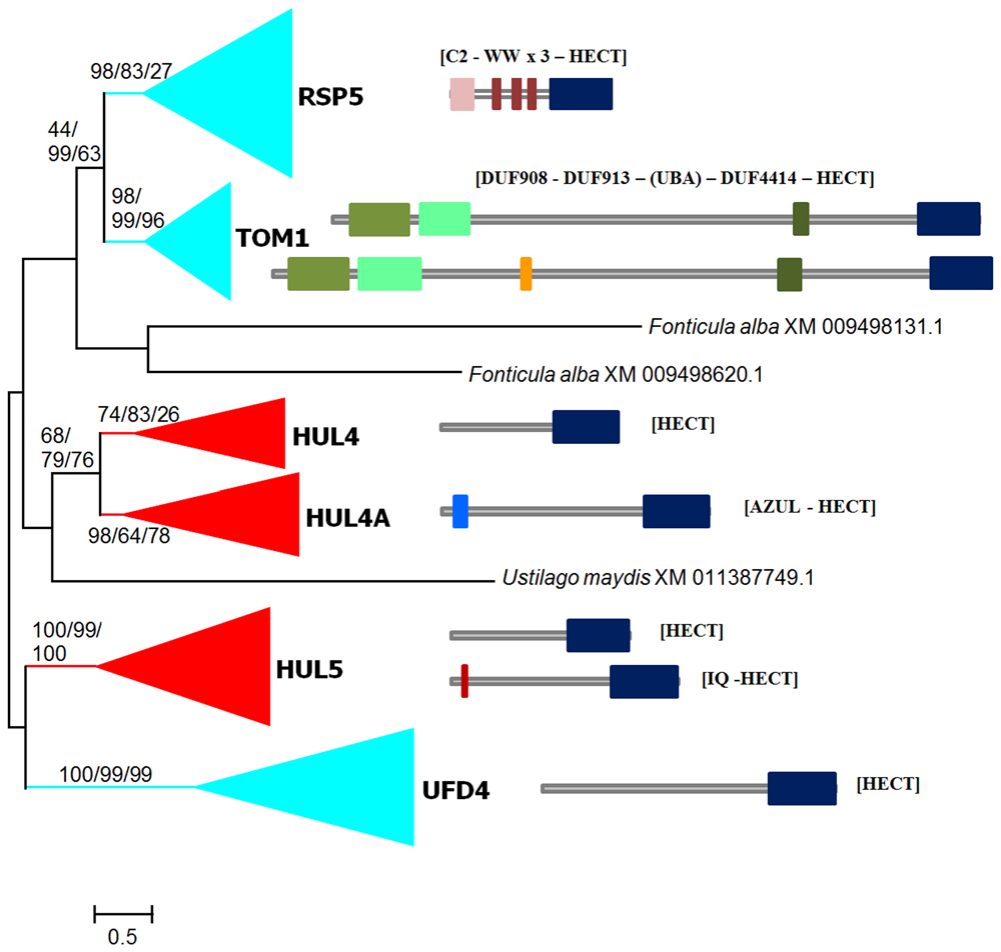
ML tree of fungi *sensu stricto* and *Fonticula* sequences. In this case, the LG + R8 model with perturbation strength = 0.5 provided the best ML value. Numbers refer to bootstrap support (as in Fig. 2: ML/NJ/MP). At the right, eight examples of the most typical structures found in fungal HECT proteins. Domains are indicated as colored boxes. From top to bottom: *Saccharomyces cerevisiae* RSP5, *S*. *cerevisiae* TOM1, *Laccaria bicolor* TOM1 (Accession number XM_001877693.1), *S*. *cerevisiae* HUL4, *Coccidioides immitis* HUL4A (XM_001247392.2), *S*. *cerevisiae* HUL5, *Fusarium verticilloides* HUL5 (XM_018889203.1) and *S*. *cerevisiae* UFD4. Figures are drawn to scale; the HECT domains shown correspond to 300–310 amino acids.

The final dataset for the 38 species contained 198 sequences. Five HECTs detected in the slime mold *Fonticula alba* were added to this dataset. *Fonticula* belongs to the Cristidiscoidea^33^, considered the sister group of the fungi *sensu stricto*/microsporidia/cryptomycota evolutionary lineage^32,34^ and it is thus a useful outgroup. Figure 2 shows a summary of the maximum likelihood (ML), neighbor-joining (NJ) and maximum parsimony (MP) trees obtained from the alignment of the 203 sequences (Supplementary File 2). It is obvious that Figs 1 and 2 are almost identical, except that Fig. 2 includes a few additional, highly divergent sequences, all but two coming either from *Fonticula* or from early-diverging fungal species, such as microsporidia or *Rozella*, and that the HUL4 group detected in our original analyses (Fig. 1) appears now subdivided into three subgroups. The name HUL4 was kept for the one that contains the *S*. *cerevisiae* HUL4 gene, while the other two were named HUL4A and HUL4-like. The latter includes just five microsporidian sequences. The differences between Figs 1 and 2 are caused by the presence of two highly divergent sequences (those from *Ustilago* and *Rozella* in Fig. 2), which were absent in the original dataset. The branch corresponding to the original HUL4 group plus these two additional sequences still had a very significant support in ML analyses (bootstrap = 99%) but it was not recovered either in NJ or MP analyses. Additional trees (Fig. 3) were generated for the *sensu stricto* fungal lineages, i. e. eliminating the sequences from microsporidia and *Rozella*, As expected, all the remnant sequences but the atypical one from *Ustilago* just mentioned, were included in the groups already observed in Fig. 2.

### Patterns of HECT genes diversification in fungi

**Figure 4:**
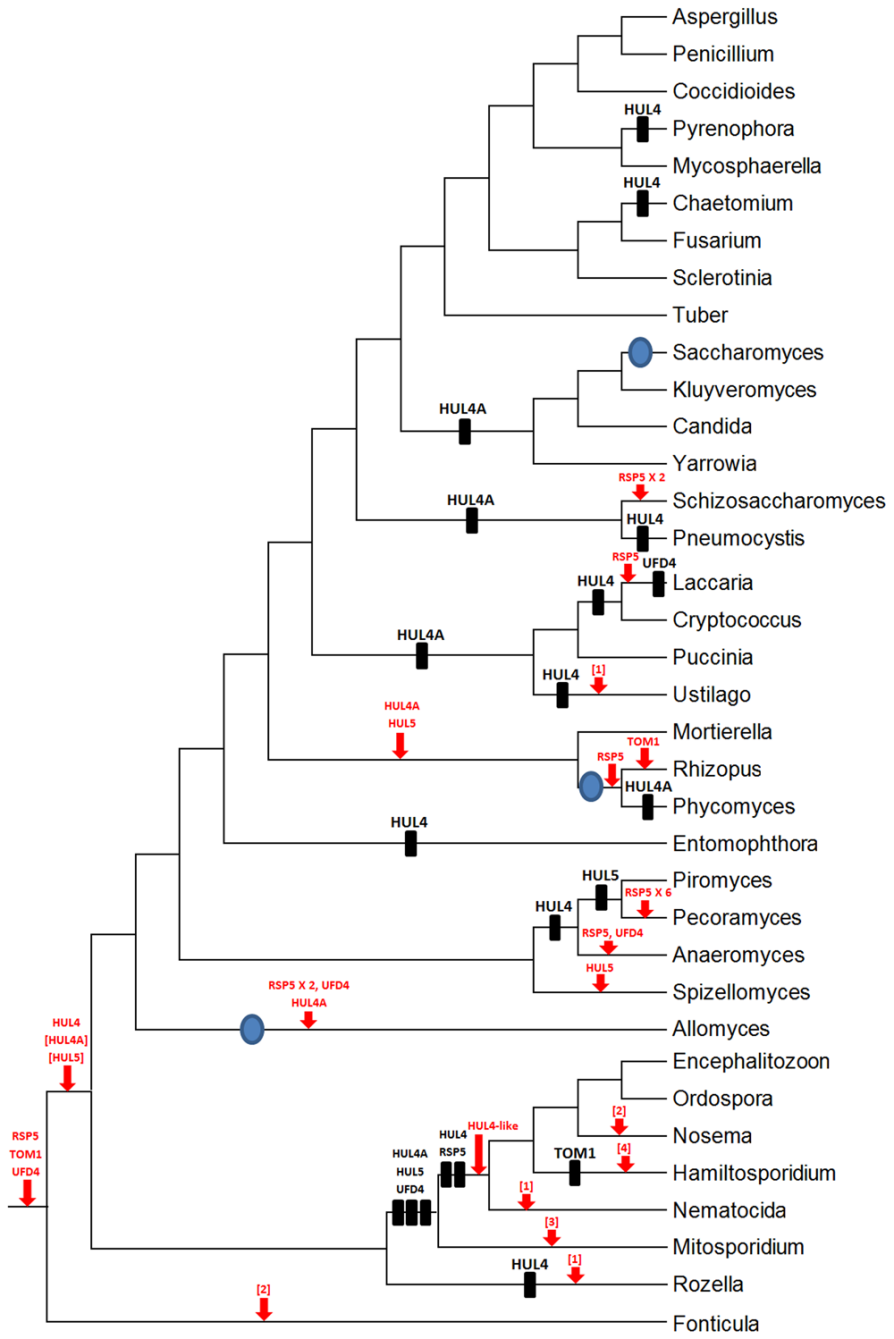
The most parsimonious hypothesis that explains the distribution of genes into classes summarized in Table 1. Whole genome duplications^30,64^ are indicated as blue ellipses, black rectangles indicate gene losses and red arrows are gene emergences or duplications. The emergences of the HUL4A and HUL5 genes, after the *Fonticula*/fungi split, are indicated in brackets because animal data indicate an earlier origin for these genes that it is shown here (see main text). The topology of the tree was obtained from refs^29,35,65–68^.

For the 39 model species analyzed, the distribution of genes among the groups indicated in Figs 2 and 3 are detailed in Table 1. The number of HECT genes in these species ranges from 2 to 10 (average: 5.2). Most fungi contain one or a few genes of the RSP5, TOM1, HUL4, HUL4A, HUL5 and UFD4 groups. The most prominent exception regards microsporidians, which lack several or even all of those genes. On the other hand, only a large amplification has been observed: seven RSP5 sequences were detected in *Pecoramyces ruminatium* (Table 1). Data in that Table can be summarized in an evolutionary context. Figure 4 shows the most parsimonious explanation for the pattern of gene presence/absence detailed in Table 1. Information in that figure can be used to obtain a classification of HECT sequences into subfamilies, as follows: (1) At least three groups, RSP5, TOM1 and UFD4, emerged before the fungi/*Fonticula* split and other three (HUL4, HUL4A and HUL5) were also present in the last common ancestor of all fungi. These results indicate that HUL4 and HUL4A sequences are better classified into two different subfamilies, as was already suggested by the trees summarized in Figs 2 and 3; (2) Microsporidian HUL4-like genes may be highly modified HUL4 genes, given that the supposed appearance of these HUL4-like genes coincides in time (i. e. occurs in the same branch of the tree) with the apparent loss of HUL4; and, (3) Identical logic can be applied to the two peculiar HUL4-related sequences detected in *Ustilago* and *Rozella* (Fig. 2), which can also be interpreted as extremely divergent HUL4 genes. Thus, only six HECT subfamilies must be defined: RSP5, TOM1, UFD4, HUL4, HUL4A and HUL5, with just a few sequences of *Fonticula* and microsporidia (isolated from these subfamilies; see Fig. 2) not being assignable to any of them.

Figure 4 indicates that some losses and duplications have occurred in particular lineages. The genes most frequently lost are HUL4 and HUL4A and the most commonly duplicated gene is RSP5, which is moreover the only one for which 3 or more paralogs are detected in some species (Table 1). As already indicated, an obvious early reduction of the number of canonical HECT genes is detected in microsporidians, which may be related to the know genome simplifications occurred in this group of parasites^29,35^. It is also clear that whole genome duplications (blue ellipses in Fig. 3) may have contributed to the emergence of new HECT genes in some species (as in *Allomyces macrogynus*), but not in others (e. g. *Saccharomyces cerevisiae*). A significant correlation between the number of HECT genes and the total number of genes in a given species (Table 1) was observed. The Spearmann rank order correlation coefficient is 0.576 (*p* = 0.0003) when all species are considered (except *Entomophthora muscae*, for which, to our knowledge, no estimation of the number of genes is available). This correlation is significant even when microsporidians are excluded (Spearmann coefficient = 0.428, *p* = 0.02), indicating that the general trends of genomic reductions/amplifications have had an impact on the conservation or loss of HECT genes. On the contrary, the transitions to multicellularity or back from multicellularity to unicellularity giving rise to yeasts such as *S*. *cerevisiae* or *S*. *pombe*^27^ did not have a significant impact on the evolution of the HECT family. It is particularly significant that the emergence of all fungal HECT subfamilies occurred before multicellular fungi arose^27^.

### Comparative analysis of animal and fungal HECT genes

**Figure 5:**
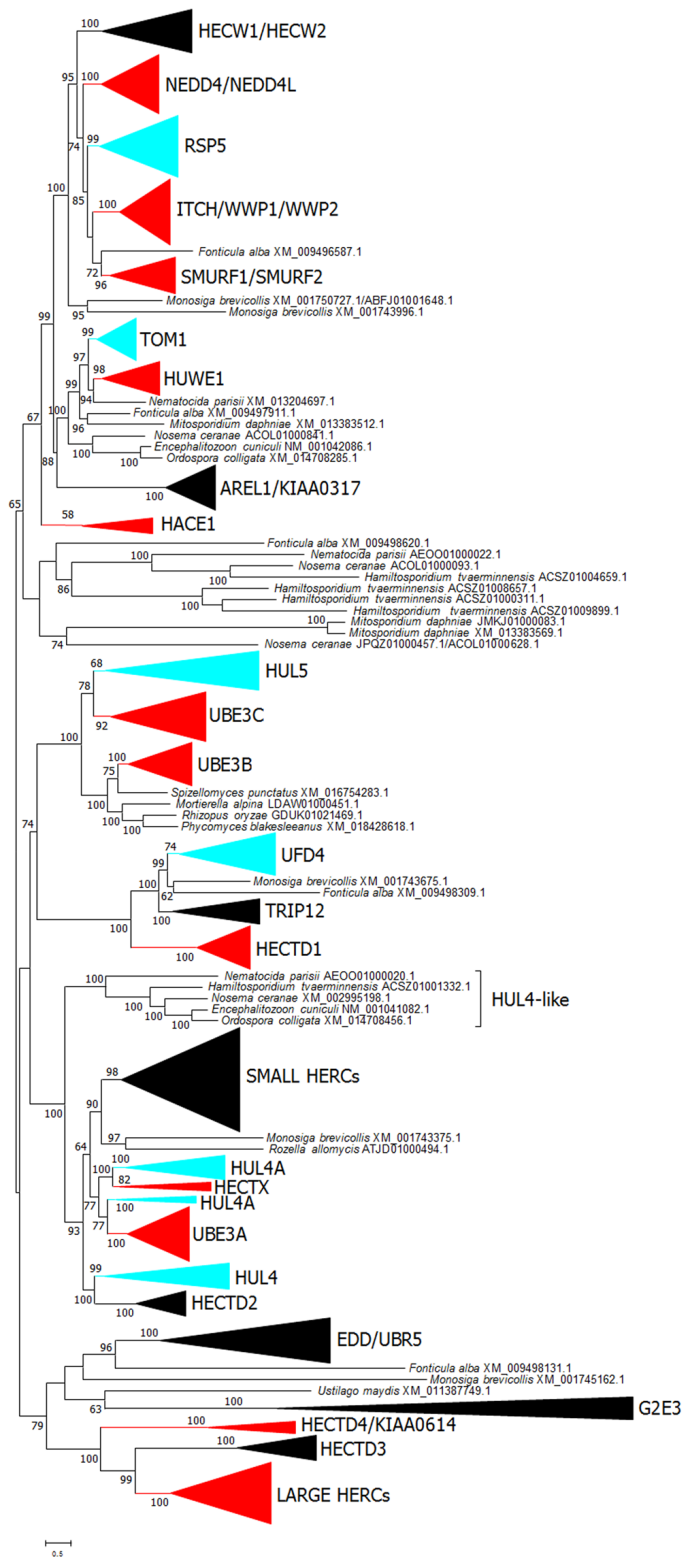
Maximum Likelihood phylogenetic tree of fungal and animal HECT sequences. The best ML value was obtained with the LG + R10 model of sequence evolution and perturbation strength = 0.5. Numbers indicate ultrafast bootstrap support. Blue: fungal groups. In all cases but HUL4A, they correspond to the fungal HECT subfamilies shown in Fig. 2. HUL4A subfamily sequences are here divided into two groups. Red: animal subfamilies; these groups include a sequence of the choanoflagellate *Monosiga brevicollis*. Black: animal subfamilies for which no orthologous choanoflagellate sequences were found.

**Figure 6:**
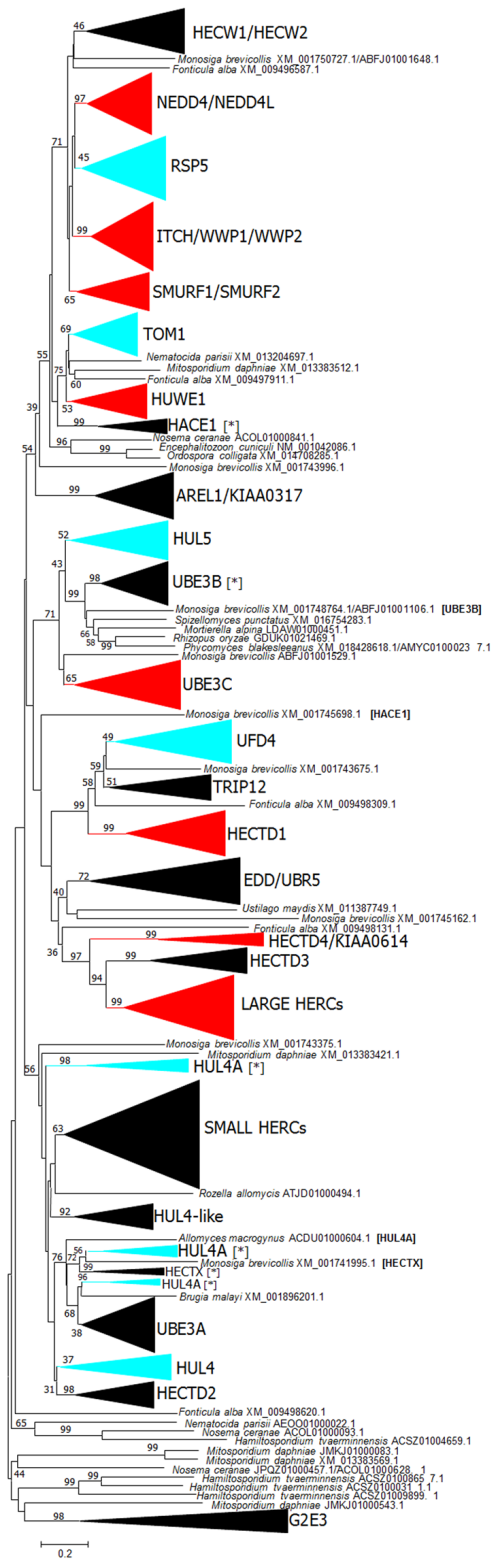
Neighbor-Joining tree of fungal and animal HECTs. Numbers refer to bootstrap support. Color conventions as in Fig. 5. There are six differences with that Figure, which are indicated with asterisks. For example, HACE1 [*] indicates that at least one sequence included in the HACE1 subfamily in Fig. 5 is missing in the corresponding group in Fig. 6. In this case, the difference consists in just one sequence, *Monosiga brevicollis* XM_001745698.1, which can be found below in the tree. Four sequences that in this tree appear isolated but were included in one of the subfamilies in Fig. 5 have the name of the corresponding subfamily indicated within brackets. Notice that HUL4A sequences are divided here into three groups instead of two, as in the previous figure.

A comparison between the sets of HECT genes in metazoa and fungi may provide additional insights on their early evolution. Figures 5 and 6 respectively show the maximum likelihood (ML) and neighbor-joining (NJ) trees corresponding to an alignment (Supplementary File 3) of the 203 sequences described above, plus a dataset already used in previous studies^20,23,36^, which includes HECT sequences of animals and the choanoflagellate *Monosiga brevicollis* as an outgroup. Both trees are largely congruent and show that all fungal genes have close animal relatives. Thus, fungal RSP5 genes appear in a well-supported branch together with the nine animal genes of the NEDD4 subfamily (see ref.^20^ for details). Similarly, TOM1 appears together with animal HUWE1, in both trees with significant statistical support (see Figs 5 and 6). Also, HUL5 is related to the UBE3B/3C subfamily genes. However, in this case which of the animal genes is the most similar to HUL5 is unclear, because both trees provide different answers. Finally, the fungi HUL4 and HUL4A genes appear close in the trees to the animal UBE3A, HECTD2, HECTX and Small HERC subfamilies (Figs 5 and 6). Again, the exact relationships of the two fungal genes with the animal genes are unclear, all branches having low bootstrap support in one or both trees. Moreover, the HUL4A sequences appear divided into two or three groups, depending on the particular analysis performed.

Animal HECT proteins often have additional, subfamily-specific protein domains^20^. Because the acquisition of domains is a rare event, the presence of the same domain in proteins of different lineages, such as animals and fungi, may be used as an additional kind of evidence to establish orthology relationships. A total of 1096 predicted full-length proteins out of the 2899 sequences in our original dataset (characterized by having accession numbers starting with “XP_” or “NP_” in Supplementary File 1) were retrieved and searched for domains present in the Pfam database (see Methods). Out of 226 RSP5 sequences analyzed, 207 had the structure [C2 domain − 3 × WW domain − HECT domain] (see Fig. 3 for a scheme; from now on, protein structures are indicated in brackets, “3×” indicates three identical domains in tandem). This structure is very similar to the one most typical of animal NEDD4 subfamily proteins, [C2 − 4 × WW − HECT], thus confirming the close relationship among RSP5 and NEDD4 subfamily genes detected in the phylogenetic trees (Figs 5 and 6). Most of the 19 remnant RSP5 sequences had related but simpler structures ([3 × WW − H], [2 × WW − H], etc. where H refers to the HECT domain). These exceptions may be in some cases real, but most likely correspond to incorrectly annotated proteins (which typically are either artificially truncated proteins or very long proteins produced by the spurious fusion of two adjacent genes). The [C2 − N × WW − HECT] structure is also present in the choanoflagellate *Monosiga brevicollis*^20^ but not in plant HECTs^23^.

Similarly, 204 full-length TOM1 sequences were obtained. For 134 of them, the structure [DUF908 – DUF913 – DUF4414 – HECT] (DUF: “Domain of Unknown Function”) was obtained, 37 had the structure [DUF908 – DUF913 – UBA – DUF4414 – HECT] (examples of these two main structures are shown in Fig. 3) and the other 33 corresponded either to potentially truncated proteins lacking some of those domains or to proteins with two DUF908 or DUF913 domains instead of a single one. These results support that fungal TOM1 genes and animal or choanoflagellate HUWE1 genes^20^ are orthologs, as suggested by the phylogenetic trees (Figs 5 and 6), because HUWE1 proteins have a very similar domain composition (e. g. [DUF908 – DUF913 – UBA – WWE - DUF4414 – HECT] in human HUWE1). In addition, plant Subfamily V proteins, which also appear together with animal HUWE1 genes in phylogenetic trees^23^ also have a [DUF908 – DUF913 – UBA – DUF4414 – HECT] domain composition. Thus, it can be concluded that fungal TOM1, animal/choanoflagellate HUWE1 and plant Subfamily V genes all derive from an ancient gene, present in the common ancestor of the three kingdoms.

A total of 200 putative full-length HUL5 sequences were analyzed. In Pfam searches, 189 contained just the HECT domain, 5 had a [IQ – HECT] structure and 6 had other structures. The finding of some sequences with IQ domains was very interesting because that domain is present in both animal UBE3B and UBE3C proteins^20^, the closest relatives of HUL5 (Figs 5 and 6), and also in the very similar Subfamily II and Subfamily III proteins in plants^23^. However, the fact that apparently only a few sequences contained that domain was puzzling. It was observed that InterProScan analyses (see Methods), which use domain definitions of different structural databases, detected additional IQ domains in HUL5 proteins. This occurred when the Prosite definition of that domain, instead of the Pfam definition, was used. When all the HUL5 proteins were scanned for IQ domains in Prosite, it turned out that those domains were detected in 116 of them (see example in Fig. 3). In conclusion, these results together with the findings described before^23^ indicate the presence of an ancient gene, progenitor of fungal HUL5, animal UBE3B and UBE3C and plant Subfamily II and III genes, in the common ancestor of the three kingdoms.

A set of 258 HUL4, HUL4A and HUL4-like full-length proteins was obtained. 170 were found to contain only a HECT domain, 84 had an [AZUL – HECT] structure (Fig. 3) and only 4 had other structures. The AZUL domain is typical of UBE3A proteins and was here found only in HUL4A sequences. These [AZUL – H] structures have never been detected in plants^23^, but, very significantly, Pfam searches showed that they can be found in some proteins in alveolates (*Tetrahymena thermophila*, *Oxytricha trifallax*) or parabasalians (*Trichomonas vaginalis*), which suggests a very ancient origin. Neither HUL4 proteins nor animal HECTD2 proteins, which appear as very close in Figs 5 and 6, have any detectable domains besides, of course, the HECT domain. The same is true for animal HECTX proteins, which are also similar to HUL4 and HUL4A proteins (Figs 5 and 6). Therefore, structures do not provide in these cases additional information to assign orthology relationships. Interestingly, plants also have genes, those of Subfamily IV, which encode proteins very similar to HECTD2, UBE3A and HECTX^23^. These proteins also lack additional domains. Thus, although the exact orthology relationships cannot be determined in this case, it is reasonable to hypothesize that a gene encoding proteins with [H] or [AZUL – H] structures was present in the ancestor of plants, animals and fungi from which all these modern genes descend, as already suggested before^23^.

Finally, 208 full-length UFD4 sequences were obtained. All but four had the simplest possible structure, containing only the HECT domain. The four exceptions corresponded to annotation errors that put together two adjacent genes, In both ML and NJ analyses, UFD4 appear close to animal TRIP12 and HECTD1, in a strongly supported branch (Figs 5 and 6). Because the animal proteins contain additional domains, structural data cannot be used in this case to confirm orthology^20^. However, significantly, plants also have genes (Subfamily I) that encode proteins very similar to TRIP12 and HECTD1^23^. Thus, it is again a reasonable hypothesis that these genes in fungi, animals and plants are co-orthologs, all deriving from a single common ancestral gene, as also proposed before^23^.

Assuming that all these results are correct, the conclusions derived from the patterns of presence/absence of genes in fungal species that were summarized in Fig. 4 should be slightly modified. Adding the new information, it is most likely that HUL4A and HUL5 are older than it was deduced using only fungal data. The comparisons with animal HECTs strongly suggest that these genes were present in the common ancestor of fungi and *Fonticula alba* and subsequently lost in the lineage that gave rise to the latter species.

### Conservation of HECT protein-protein interactions in fungi and animals

Indirect evidence for functional conservation may be inferred from comparative protein-protein interaction data. All well-supported interacting partners characterized so far for *Saccharomyces cerevisiae* HECT proteins (found in at least two independent experiments) were downloaded from BioGRID (see Methods). A total number of 101 partners, namely 85 interactors with RSP5, 12 with TOM1 and 4 with UFD4, were obtained, while no interactions were recovered for either HUL4 or HUL5. In 21 cases, the human orthologs of those interactors were also found to interact with the corresponding human HECT proteins (Table 2). These are therefore ancient protein-protein interactions, conserved in both *S*. *cerevisiae* and *H*. *sapiens*. It is significant that these potentially conserved interactions involved RSP5 in yeasts and the orthologous proteins of the human NEDD4 subfamily in seventeen of these 21 cases.

**Table 2:** Conservation of HECT-interacting proteins.

*S*. *cerevisiae* HECTs	*S*. *cerevisiae* interacting proteins	*Homo sapiens* HECTs	*H*. *sapiens* interacting proteins	Function of HECT-interacting proteins in yeast	References
RSP5	UBC1	NEDD4L, ITCH, SMURF1, WWP2	UBE2K	Ubiquitin-conjugating enzyme	^69^
RSP5	UBC4, UBC5	NEDD4, NEDD4L, HECW1, HECW2, ITCH, SMURF1, SMURF2, WWP1, WWP2	UBE2D1, UBE2D2, UBE2D3, UBE2E1, UBE2E3	Ubiquitin-conjugating enzymes	^69^
RSP5	UBC6	NEDD4L, HECW1, ITCH, WWP2	UBE2J2	Ubiquitin-conjugating enzyme	^69^
RSP5	UBI4	HECW1	UBC	Polyubiquitin	^70^
RSP5	RPN10	NEDD4, NEDD4L, HUWE1, SMURF1	PSMD4	26S proteasome regulatory subunit	^71^
RSP5	RPO21	NEDD4, WWP1, WWP2, ITCH, SMURF1	POLR2A	DNA-directed RNA polymerase II subunit	^72^
RSP5	RPB2	NEDD4, HECW2, ITCH, WWP1	POLR2B/RPB2	DNA-directed RNA polymerase II subunit	^72^
RSP5	RPB3	NEDD4, ITCH, WWP1, WWP2	POLR2C	DNA-directed RNA polymerase II subunit	^72^
RSP5	RPB5	NEDD4, ITCH, WWP2	POLR2E	DNA-directed RNA polymerases I, II, and III subunit	^72^
RSP5	RPB7	WWP1	POLR2G	DNA-directed RNA polymerase II subunit	^72^
RSP5	SEC7	ITCH	ARFGEF2	Vesicular transport protein, guanine exchange factor	^73^
RSP5	HSE1	ITCH	STAM1, STAM2	Vacuolar protein-sorting machinery	^74^
RSP5	HSP82	WWP1	HSP90AA1	ATP-dependent molecular chaperone	^75^
RSP5	LHP1	SMURF1	SSB	tRNA maturation control	^76^
RSP5	NFI1, SIZ1	SMURF2	PIAS3	Sumo conjugation	^77^
TOM1	MLC1	HUWE1	MYL6B	Myosin light chain	^78^
TOM1	HSP82, HSC82	HUWE1	HSP90AA1, HSP90AB1, HSP90B1	ATP-dependent molecular chaperones	^75^
UFD4	UBC4	TRIP12	UBE2D1, UBE2D4	Ubiquitin-conjugating enzyme	^69^

## Discussion

The results described here for the fungal HECT genes, together with those obtained before for animal and plant HECTs^20,23^ provide a general view of the patterns of evolution of this family. It has been concluded that the common ancestor of all fungi had six different HECT genes: RSP5, TOM1, UFD4, HUL4, HUL4A and HUL5 (Fig. 4). These six genes were already present before the split between the microsporidian and *Rozella* lineages and the lineage that gave rise to all fungi *sensu stricto* (Fig. 4), so that conclusion holds no matter how the kingdom fungi is defined^32^. Unusual genes that may not be orthologous to any of those six have been found only either in very early diverging species (microsporidians, *Rozella*) or, in a single case, in the basidiomycete *Ustilago maydis* (Table 1). However, as already indicated, some of these uncommon genes probably are highly divergent members of the HUL4 subfamily.

The six ancestral genes have been conserved in most fungal species; only occasional, independent losses or duplications are observed in particular lineages (Fig. 4). The exception to this rule are microsporidians, in which severe genome reductions occurred associated to their intracellular lifestyle^29,35^, which are reflected in multiple losses of HECT genes (Table 1). It is interesting that, after a general reduction early in microsporidian evolution, lineage-specific expansions of multiple gene families, similar to those deduced to have occurred for microsporidian HECTs (see arrows in Fig. 3), have been described^37^. As indicated above, the number of HECT genes correlates with the total number of genes in the fungal genomes analyzed, so whole-genome duplications, genome amplifications and genome reductions may all have impinged on HECT gene numbers, as occurs in other protein families (e. g. ref.^38^). On the contrary, the transitions from unicellularity to multicellularity or vice versa does not seem to have had a significant impact on the number of HECT genes present. Not only multicellular species, such as *Laccaria bicolor*, *Tuber melanosporum*, etc., do not have more HECT genes than the unicellular ancestor of all fungi, but also yeasts as *S*. *pombe* have actually increased that number respect to its multicellular ancestors (Fig. 3)^39,40^. This situation is very different from what it was found in animals, in which multiple new subfamilies emerged in multicellular organisms after the split of animals from unicellular choanoflagellates^20^, but agrees well with the pattern observed in plants^23^. It can be concluded that changes in the number or diversity of HECT genes is not necessarily required for the transition from unicellular to multicellular organisms (or vice versa) to occur.

In fungi, RSP5 is the most conserved gene (Table 1, Fig. 4). Only some microsporidians lack an RSP5 ortholog, and a single event must be postulated to explain that fact (Fig. 4). In *S*. *cerevisiae*, RSP5 is the only HECT gene whose deletions cause lethality and RSP5 protein perform fundamental roles in multiple cellular process^41,42^. This central metabolic position of RSP5, if general in all fungi, may explain why losses of this gene are so uncommon (Fig. 4). On the contrary, the genes that are most commonly lost are HUL4A and HUL4. The roles of the first are totally unknown. For HUL4, the functional information is very limited. In *S*. *cerevisiae*, it may have a specific role in sporulation^43,44^.

Protein-protein interaction data provide clues about long-range functional conservation. Seven of the 21 cases described in Table 2 involve proteins that belong to the ubiquitination or proteasome machinery, being ubiquitin-conjugating (E2) enzymes, the most frequently characterized conserved partners of HECTs. All these interactions are easily explained by conservation of the fundamental ubiquitination machinery since the common ancestor of animals and fungi. In addition, some interactions already studied in detail from a functional point of view and that correspond to known functions of these proteins (e. g. RSP5 and NEDD4 - RNA polymerase II^45,46^; RSP5/ITCH - SEC. 7/ARFGEF2^47^; RSP5/SMURF2 - SIZ1/PIAS3)^48,49^ were also found. For some of the other (e. g. RSP5-HSE1)^50^, the functional connection was known in yeasts but not, to our knowledge, in humans or other mammals. These results all support similar roles for fungal RSP5 and animal NEDD4 proteins, emerged before the two lineages split. Data in Table 2 also suggest a potential functional redundancy or collaboration of NEDD4 subfamily members regarding ubiquitination of RNA polymerase subunits. The only work that tackled this issue detected that only NEDD4, but not WWP1, WWP2 or SMURF1, was able to ubiquitinate RNA Pol II *in vivo* and NEDD4 and SMURF1, but not WWP1 or WWP2, were able to perform that reaction in an *in vitro* assay^46^. Additional research seems advisable, given that Table 2 results suggest that as many as six NEDD4 subfamily proteins may interact and potentially ubiquitinate RNA Pol II units.

As described in detail in the Results section, it has been deduced that the common ancestor of plants, animals and fungi already had four genes from which all observed today in those three kingdoms are derived. For TOM1 and HUL5, both high sequence similarity and similar protein domains have been detected in plant and animal orthologs (Figs 5 and 6; ref.^23^). For three other fungal genes, UFD4, HUL4 and HUL4A, it can also be proposed that orthologs/co-orthologs in animals and plants exist, all of them originating from two ancestral genes (see above). A caveat of this hypothesis is that it is mostly based on sequence similarity. Only the presence of an AZUL domain in some fungal HUL4A and in animal UBE3A proteins offers additional support for the corresponding genes being orthologous. Finally, RSP5 is clearly the fungal ortholog of the genes of the animal NEDD4 subfamily, a fact supported by sequence similarity, structural data and also functional data (Table 2). In plants, no RSP5/NEDD4 genes are present^23^.

It is interesting to compare these conclusions with those obtained in a previous work in which a “genomic survey”, i. e. a limited sampling of HECT sequences of all eukaryotes, was performed^25^. Those authors suggested that at least six genes were present in the last eukaryotic common ancestor (which they assumed was the same as the common ancestor of plants, animals and fungi, although this is not totally clear)^51,52^ and no less than nine in the ancestor of all fungi. It is simple to point out some basic mistakes in that study: (1) Extremely shallow and erroneous species sampling. We already showed in a previous work^23^ that the conclusions of those authors regarding plant species were incorrect, because they totally missed one of the subfamilies of plant HECTs, as well as an additional angiosperm-specific HECT lineage, due to poor species sampling. Regarding fungi, they considered only eight species, and, moreover, they failed to include any early-diverging species (microsporidia, cryptomycota) or any fungi-related protozoan lineage. Thus, they totally missed the HUL4-like genes and the highly divergent *Fonticula*-specific and microsporidian-specific genes characterized in this study; (2) Improper use of statistical support in phylogenetic trees. It can be observed by simply inspecting their figures that many of the critical branches from which they derived their main conclusions lack significant support. For example, two groups of sequences that they called “classes” and that they interpreted as indicating the existence of two corresponding genes present in the ancestor of all eukaryotes, had bootstrap support in ML analyses as low as 6% and 8%, respectively. The same occurs for about half of the groups that they call “subfamilies”, supposedly groups of orthologs in different eukaryotic taxa. On top of this, support was deemed to be sufficient or not haphazardly. Thus, a “class” was defined with as low as 60% Bayesian support and 8% ML support but the orthologous group HUL5/UBE3C/Plant subfamilies II + III, which they detected with a higher support (respectively, 70% and 11%) was not deemed significant, which led them to erroneously conclude that genes that not only have very similar sequences, but also all encode complex [DUF908 – DUF913 – (UBA) – DUF4414 – HECT] proteins, emerged three times independently; and, (3) No attempt to establish the true phylogenetic range of the genes analyzed. For example, they concluded that there are seven subfamilies of HECT genes in fungi instead of six, because they confused a *HUL5* duplicate which is mucoromycota-specific and absent in all other fungi (see Fig. 3) with an ancient *UBE3B* ortholog.

It is easy to appreciate that combining a very shallow species sampling with lack of rigour when assessing the statistical support of phylogenetic trees leads to a systematic bias in the interpretation of the data. This occurs because any sequence found in just one model species, or a few closely related ones, will be assumed to already exist when the taxon that includes that/those species emerged (as an example, the mucoromycota-specific HUL5 genes just mentioned were implicitly assumed to be already present in the ancestor of all fungi). Of course, the alternative explanation, i. e. that the duplication is very recent, simply cannot be tested if species sampling is too superficial. Now, if a mistaken interpretation of recent duplicates as ancient genes is mixed together with a permissive acceptance of minimal statistic support for tree branches, then: (1) paralogous genes emerged in different groups will be often confused as orthologs; (2) all the genes merged together in a false orthology group will be assumed to be much older than actually are; and, (3) if the error involves distantly-related species, that “gene” will show an evolutionarily patchy distribution, with many independent losses having to be hypothesized to explain its presence in such distant relatives. Such a systematic bias explains why it was concluded by those authors that so many HECT genes were present early in eukaryotic evolution, leading them to necessarily compensate, in order to fit the data, by hypothesizing abundant losses of HECT genes, with a considerable number of them being lost many times independently. This extreme dispensability of multiple HECT family genes is not only biologically implausible, but also not supported at all by our three specific, complete studies in animals, plants and fungi (refs^20,23^ and this work). The (obvious) corollary is that analyzing the available information in full and in depth is advisable if the goal is to truly understand the evolution of a complex gene family.

## Methods

Because the HECT domain is long (about 350 amino acids) and evolutionarily well conserved, it is easy to generate comprehensive databases of HECT ubiquitin ligases in any given lineage. Fungal HECT sequences were obtained using TblastN searches against the nr, wgs, est, tsa, gss, and htgs databases of the National Center for Biotechnology Information (NCBI; http://www.ncbi.nlm.nih.gov/), which were queried with the sequences of the HECT domains of *Saccharomyces cerevisiae* proteins. From 5455 positive hits, a final dataset of 2899 HECT domain sequences was obtained once duplicated and truncated sequences were eliminated. This dataset was aligned using ClustalX 2.1^53^ and manually corrected using GeneDoc 2.7^54^. The final alignment can be found in Supplementary File 1. Additional species-specific searches for 38 fungi and the protozoan *Fonticula alba* (see Table 1) were performed screening the same databases, but this time using as query sequences members of the five main groups detected when preliminary phylogenetic analyses were performed (Fig. 1) that belonged to species that were distantly related to *S*. *cerevisiae* according to fungi phylogenies (microsporidians, *Allomyces*, *Piromyces*, etc; Fig. 4). These searches quickly become saturated, i. e. the same sequences were repeatedly detected, indicating that no further HECT sequences were present in the databases. All these potential HECT domain sequences were examined in detail, combining protein alignments and searches in the Pfam database of protein domains (http://pfam.xfam.org/) to determine whether they indeed corresponded to bona fide HECT domain sequences. To obtain the full-length domains, it was sometimes necessary (especially when small exons, difficult to detect in TblastN searches, existed), to retrieve, examine and conceptually translate the corresponding nucleotide sequences. The final dataset for the 39 selected species (203 sequences) was aligned following the same methods described above. That alignment can be found in Supplementary File 2.

Phylogenetic trees were obtained with the programs IQ-TREE 1.5.5 (for Maximum Likelihood, ML, analyses)^55^, Mega 7.0.26 (Neighbor-Joining, NJ)^56^ and PAUP* 4.0 beta 10 (Maximum Parsimony, MP)^57^. The best model for ML analyses was determined using ModelFinder^58^. In our hands, single IQ-TREE analyses with default parameters (i. e. 1000 ultrafast bootstrap replicates^59^, 100 iterations of the nearest-neighbor interchange algorithm, perturbation strength = 0.5) generated clearly erroneous trees, corresponding to local optima. Therefore, and following the recommendations described in ref.^55^, searches were replicated 10 times with each of two different perturbation strengths (0.5 and 0.8) and the number of unsuccessful iterations to stop the tree search was increased to 500 in all cases. For each ML analysis, 1000 ultrafast bootstrap replicates were obtained. For NJ analyses, 1000 bootstrap replicates were also obtained. For MP analyses, which requires longer computation times, the tree bisection-recognition (TBR) search was used and 100 bootstrap replicates were generated. For the combined analysis of animal and fungal HECT, in addition of the ML and NJ trees shown in Figs 4 and 5, a maximum parsimony analysis was performed. However, due to limitations of the MP heuristic searches when so many sequences are analyzed, it did not provide significant support for the internal branches of the tree (not shown).

Most protein domain searches were implemented using the Pfam batch search (http://pfam.xfam.org/search#tabview=tab1). Only in particular cases, indicated in the text, the InterProScan searches (https://www.ebi.ac.uk/interpro/search/sequence-search) or the ScanProsite batch search at Prosite (https://prosite.expasy.org/scanprosite/) were used. The Pfam and InterPro (https://www.ebi.ac.uk/interpro/) databases were searched for particular combinations of domains. The total number of genes for each species (right column in Table 1) were obtained from the corresponding genome pages at the NCBI (https://www.ncbi.nlm.nih.gov/genome/) except for *Mortierella alpina*^60^, *Rhizopus oryzae*^61^, *Pecoramyces ruminatium*^62^ and *Hamiltosporidium tvaerminnensis*^63^. Correlation analyses were performed using SigmaPlot 13 (http://www.sigmaplot.co.uk). Finally, protein-protein interaction data for both *Saccharomyces cerevisiae* and *Homo sapiens* (Table 2) were downloaded from BioGRID (https://thebiogrid.org/) in October 2017. The *S*. *cerevisiae* proteins that were found to interact with HECT ubiquitin ligases in at least two experiments were compared using TBlastP with all *H*. *sapiens* proteins to detect potential orthologs. When significant sequence similarity was detected (minimal Expect value < e^−10^), the literature was scanned for studies indicating that those were true orthologous genes (“References” in Table 2).

## Electronic supplementary material

Sequence files in FASTA format
