# Supplementary material for: Origin and evolution of fungal HECT ubiquitin ligases
Source: Sci Rep. 2018 Apr 23;8:6419. doi: 10.1038/s41598-018-24914-x (PMC5913265; doi:10.1038/s41598-018-24914-x)
Supplement: Supplementary file 1 — Sequence files in FASTA format [file 41598_2018_24914_MOESM1_ESM.pdf]

Supplementary information
Article: Origin and evolution of fungal HECT ubiquitin ligases
Author: Ignacio Marín

Supplementary File 1

```
>Lachancea_meyersii_FJUM01000010.1 .
IKVRRKNI-FEDAYQEI--------------MRQTPEDLKK-RLMIKFDG-------EEG
LDYGGVSREF---FFLL----SHEMFNPFY--------CLFEYSAHDNY-----------
----------------TIQINPKS------------------------------------
--GINPEHLNYFKFIGRVVGLGVFHRRFLDAFFVG--ALYKMMLH---------------
--------KKVVLQDMEG----------------------VDAEVHNS--------LKWI
LDN------------------------------------------------SID-GIL--
-DLTFSADDET---------------------------FGE-LMTVD-LKAD--------
GKNVE-------------------------------------------------------
------------------------------VTDENKR-EYVEL-FTQWKIYNR-VQEQFK
-AFMDG---FNELIPE--DLVN-VFDER-ELELLIGG-----------------------
----IAEID-----------VEDW----KKNTDYRG-YQES-------------------
------------------------DETI-KWFWKAIS--------------------EWD
NEQKARLLQF-------TTGTSRIP------------VNG-----FK-DLQGSD--GPRR
FTIEKAGEV-------------------------QQLPKSHTCF-NR-VDLPPY-TDYES
LKQKLT-LAVEE-TIG-
>Lachancea_lanzarotensis_CDLU01000012.1 .
IKVRRKNI-FEDAYQEI--------------MRQTPEDLKK-RLMIKFDG-------EEG
LDYGGVSREF---FFLL----SHEMFNPFY--------CLFEYSAHDNY-----------
----------------TIQINPKS------------------------------------
--GINPEHLNYFKFIGRVVGLGVFHRRFLDAFFVG--ALYKMMLH---------------
--------KKVVLQDMEG----------------------VDAEVHNS--------LKWI
LDN------------------------------------------------SID-GVL--
-DLTFSADDET---------------------------FGE-LITVD-LKAD--------
GKNLD-------------------------------------------------------
------------------------------VTDENKK-EYVEL-FTQWKIYNR-VQEQFK
-AFMDG---FNELIPE--DLVN-VFDER-ELELLIGG-----------------------
----IAEID-----------VEDW----KKNTDYRG-YQES-------------------
------------------------DETI-KWFWKAIS--------------------EWD
NEQKARLLQF-------TTGTSRIP------------VNG-----FK-DLQGSD--GPRR
FTIEKAGEV-------------------------QQLPKSHTCF-NR-VDLPPY-TDYES
LKQKLT-LAVEE-TIG-
>3_Lachancea_dasiensis_FJUP01000011.1 .
IKVRRKNI-FEDAYQEI--------------MRQTPEDLKK-RLMIKFDG-------EEG
LDYGGVSREF---FFLL----SHEMFNPFY--------CLFEYSAHDNY-----------
----------------TIQINPKS------------------------------------
--GINPEHLNYFKFIGRVVGLGVFHRRFLDAFFVG--ALYKMMLH---------------
--------KKVVLQDMEG----------------------VDAEVHNS--------LKWI
LEN------------------------------------------------SID-GIL--
-DLTFSADDES---------------------------FGE-LTTVD-LKAD--------
GRNIE-------------------------------------------------------
------------------------------VTDENKK-EYVEL-FTQWKIYNR-VQEQFK
-AFMDG---FNELIPE--DLVN-VFDER-ELELLIGG-----------------------
----IAEID-----------VEDW----KKHTDYRG-YQES-------------------
------------------------DETI-KWFWKAIS--------------------EWD
NEQKARLLQF-------TTGTSRIP------------VNG-----FK-DLQGSD--GPRR
FTIEKAGEV-------------------------QQLPKSHTCF-NR-VDLPPY-NDYES
LKQKLT-LAVEE-TIG-
>Lachancea_nothofagi_FJUQ01000026.1 .
IKVRRKNI-FEDAYQEI--------------MRQTPEDLKK-RLMIKFDG-------EEG
LDYGGVSREF---FFLL----SHEMFNPFY--------CLFEYSAHDNY-----------
----------------TIQINPKS------------------------------------
```

--GINPEHLNYFKFIGRVVGLGVFHRRFLDAFFVG--ALYKMMLH---------------
--------KKVVLQDMEG----------------------VDAEVHNS--------LKWI
LEN--NID-GIL--
-DLTFSADDET---------------------------FGE-LMTVD-LKPE--------
GRDIE---
------------------------------VTDENKK-EYVEL-FTQWKIYNR-VQEQFK
-AFMDG---FNELIPE--DLVN-VFDER-ELELLIGG-----------------------
----IAEID-----------VEDW----KKHTDYRG-YQES-------------------
------------------------DETI-KWFWKAIS--------------------EWD
NEQKARLLQF-------TTGTSRIP------------VNG-----FK-DLQGSD--GPRR
FTIEKAGEI-------------------------QQLPKSHTCF-NR-VDLPPY-DDYES
LKQKLT-LAVEE-TIG-
>Lachancea_waltii_AADM01000132.1 .
IKVRRKNI-FEDAYQEI--------------MRQTPEDLKK-RLMIKFDG-------EEG
LDYGGVSREF---FFLL----SHEMFNPFY--------CLFEYSAHDNY-----------
----------------TIQINPKS------------------------------------
--GINPEHLNYFKFIGRVVGLGVFHRRFLDAFFVG--ALYKMMLH---------------
--------KKVVLQDMEG----------------------VDAEVYNS--------LKWI
LEN--SIE-GVL--
-DLTFSADDET---------------------------FGE-VMTVD-LKPD--------
GRNIE---
------------------------------VTDENKK-EYVEL-FTQWKTCSR-VQEQFK
-AFMDG---FNELIPE--DLVN-VFDER-ELELLIGG-----------------------
----IAEID-----------VEDW----KKHTDYRG-YQES-------------------
------------------------DETI-KWFWKAIS--------------------EWD
NEQKARLLQF-------TTGTSRIP------------VNG-----FK-DLQGSD--GPRR
FTIEKAGEI-------------------------QQLPKSHTCF-NR-VDLPPY-DDYES
LKQKLT-LAVEE-TIG-
>Lachancea_thermotolerans_XM_002553734.1 .
IKVRRKNI-FEDAYQEI--------------MRQTPEDLKK-RLMIKFDG-------EEG
LDYGGVSREF---FFLL----SHEMFNPFY--------CLFEYSAHDNY-----------
----------------TIQINPKS------------------------------------
--GINPEHLNYFKFIGRVVGLGVFHRRFLDAFFVG--ALYKMMLH---------------
--------KKVVLQDMEG----------------------VDAEVYNS--------LKWI
LEN--SID-GIL--
-DLTFSADDET---------------------------FGE-VVTVD-LKPD--------
GRNIE---
------------------------------VTDENKK-EYVEL-FTQWKTCSR-VQEQFK
-AFMDG---FNELIPE--DLVN-VFDER-ELELLIGG-----------------------
----IAEID-----------VEDW----KKHTDYRG-YQES-------------------
------------------------DETV-KWFWKAIS--------------------EWD
NEQRARLLQF-------TTGTSRIP------------VNG-----FK-DLQGSD--GPRR
FTIEKAGEV-------------------------QQLPKSHTCF-NR-VDLPPY-GDYET
LKQKLT-LAVEE-TIGF
>Lachancea_quebecensis_CZLH01000087.1 .
IKVRRKNI-FEDAYQEI--------------MRQTPEDLKK-RLMIKFDG-------EEG
LDYGGVSREF---FFLL----SHEMFNPFY--------CLFEYSAHDNY-----------
----------------TIQINPKS------------------------------------
--GINPEHLNYFKFIGRVVGLGVFHRRFLDAFFVG--ALYKMMLH---------------
--------KKVVLQDMEG----------------------VDAEVYNS--------LKWI
LEN--SID-GIL--
-DLTFSADDET---------------------------FGE-VVTVD-LKPD--------
GRNIE---
------------------------------VTDENKK-EYVEL-FTQWKTCSR-VQEQFK
-AFMDG---FNELIPE--DLVN-VFDER-ELELLIGG-----------------------
----IAEID-----------VEDW----KKHTDYRG-YQES-------------------
------------------------DETV-KWFWKAIS--------------------EWD
NEQRARLLQF-------TTGTSRIP------------VNG-----FK-DLQGSD--GPRR

FTIEKAGEV-------------------------QQLPKSHTCF-NR-VDLPPY-GDYET
LKQKLT-LAVEE-TIG-
>Lachancea_mirantina_FJUN01000004.1 .
IKVRRKNI-FEDAYQEI--------------MRQTPEDLKK-RLMIKFDG-------EEG
LDYGGVSREF---FFLL----SHEMFNPFY--------CLFEYSAHDNY-----------
----------------TIQINPNS------------------------------------
--GINPEHLNYFKFIGRVVGLGVFHRRFLDAFFVG--ALYKMMLH---------------
--------KKVVLQDMEG----------------------VDAEVHNS--------LKWI
LEN--SID-GVL--
-DLTFSADDER---------------------------FGE-VVTVD-LKPN--------
GRDLE---
------------------------------VTDENKK-EYVDL-FTQWKIANR-VEEQFK
-AFMDG---FNELIPE--DLVN-VFDER-ELELLIGG-----------------------
----IAEID-----------VEDW----KKHTDYRG-YQES-------------------
------------------------DETV-KWFWKIIS--------------------EWD
NEQKARLLQF-------TTGTSRIP------------VNG-----FK-DLQGSD--GPRR
FTIEKAGEV-------------------------QQLPKSHTCF-NR-VDLPPY-ADYES
LKQKLT-LAVEE-TIG-
>Lachancea_kluyveri_AACE03000008.2 .
IKVRRKNI-FEDAYQEI--------------MRQTPEDLKK-RLMIKFDG-------EEG
LDYGGVSREF---FFLL----SHEMFNPFY--------CLFEYSAHDNY-----------
----------------TIQINPNS------------------------------------
--GINPEHLNYFKFIGRVVGLGVFHRRFLDAFFVG--ALYKMMLH---------------
--------KKVVLQDMEG----------------------VDAEVYNS--------LKWI
LEN--SID-GIL--
-DLTFSADDER---------------------------FGQ-VVTVD-LKSD--------
GRDIE---
------------------------------VLDDNKK-EYIEL-FTQWKICDR-VEEQFK
-AFMDG---FNELIPE--DLVN-VFDER-ELELLIGG-----------------------
----IAEID-----------VEDW----KKHTDYRG-YQES-------------------
------------------------DETI-KWFWQVIS--------------------EWD
NEQKARLLQF-------TTGTSRIP------------VNG-----FK-DLQGSD--GPRR
FTIEKAGEV-------------------------QQLPKSHTCF-NR-VDLPPY-PDYET
LKQKLT-LAVEE-TIG-
>Lachancea_fermentati_FJUO01000036.1 .
IKVRRKNI-FEDAYQEI--------------MRQTPEDLKK-RLMIKFDG-------EEG
LDYGGVSREF---FFLL----SHEMFNPFY--------CLFEYSAHDNY-----------
----------------TIQINPNS------------------------------------
--GINPEHLNYFKFIGRVVGLGVFHRRFLDAFFVG--ALYKMMLH---------------
--------KKVVLQDMEG----------------------VDAEVYNS--------LKWI
LEN--SID-GVL--
-DLTFSADDER---------------------------FGE-VVTVD-LKPD--------
GRNIE---
------------------------------VTDENKK-EYIEL-FTQWKIYNR-VEEQFK
-AFMDG---FNELIPE--DLVN-VFDER-ELELLIGG-----------------------
----IAEID-----------VEDW----KKHTDYRG-YQES-------------------
------------------------DVTV-KWFWKVIS--------------------EWD
NEQKARLLQF-------TTGTSRIP------------VNG-----FK-DLQGSD--GPRR
FTIEKAGEI-------------------------QQLPKSHTCF-NR-VDLPPY-PDYET
LKQKLT-LAVEE-TIG-
>Lachancea_cidri_FJUT01000024.1 .
IKVRRKNI-FEDAYQEI--------------MRQTPEDLKK-RLMIKFDG-------EEG
LDYGGVSREF---FFLL----SHEMFNPFY--------CLFEYSAHDNY-----------
----------------TIQVNPNS------------------------------------
--AINPEHLNYFKFIGRVVGLGVFHRRFLDAFFVG--ALYKMMLH---------------
--------KKVVLQDMEG----------------------VDAEVYNS--------LKWI
LEN--SID-GIL--
-DLTFSADDER---------------------------FGE-VVTVD-LKPD--------

GRNIE---
------------------------------VTDENKK-EYIEL-FTQWKIYNR-VEEQFK
-AFMDG---FNELIPE--DLVN-VFDER-ELELLIGG-----------------------
----IAEID-----------VEDW----KKHTDYRG-YQES-------------------
------------------------DVTV-KWFWKVIS--------------------EWD
NEQKARLLQF-------TTGTSRIP------------VNG-----FK-DLQGSD--GPRR
FTIEKAGEI-------------------------QQLPKSHTCF-NR-VDLPPY-PDYDT
LKQKLT-LAVEE-TIG-
>Eremothecium_sinecaudum_XM_018132685.1 .
IRVRRKHI-FEDAYQEI--------------MRQTPEDLKK-RLMIKFDS-------EEG
LDYGGVSREF---FFLL----SHEMFNPFY--------CLFEYSSHDNY-----------
----------------TIQINPNS------------------------------------
--GINPEHLNYFKFIGRVVGLGIFHRRFLDAFFVG--ALYKMMLR---------------
--------KKVVLQDMEG----------------------VDAEVYNS--------LKWI
LEN--SID-GIL--
-DLTFSADDER---------------------------FGE-VVTVD-LKPG--------
GRSIE---
------------------------------VTDENKK-EYVEL-FTQWKICTR-VEAQLK
-AFMDG---FNELIPE--DLVN-VFDGR-ELELLIGG-----------------------
----IAEID-----------VEDW----KKHTDYRG-YQES-------------------
------------------------DEVI-RWFWRCIS--------------------EWD
NEQKARLLQF-------TTGTSRIP------------VNG-----FK-DLQGSD--GPRR
FTIEKAGEV-------------------------QQLPKSHTCF-NR-VDLPAY-PDYET
LKQKLT-LAVEE-TIGF
>Eremothecium_cymbalariae_XM_003648433.1 .
IRVRRKHI-FEDAYQEI--------------MRQTPEDLKK-RLMIKFDG-------EEG
LDYGGVSREF---FFLL----SHEMFNPFY--------CLFEYSSHDNY-----------
----------------TIQINPNS------------------------------------
--GINPEHLNYFKFIGRVVGLGVFHRRFLDAFFIG--ALYKMMLR---------------
--------KKVVLQDMEG----------------------VDSDVYNS--------LKWI
LEN--SID-GIL--
-DLTFSADDER---------------------------FGE-LVTVD-LKPK--------
DRNID---
------------------------------VTDENKK-EYVEL-FTQWKICSR-VEEQFK
-AFIDG---FNELIPE--DLVN-VFDER-ELELLIGG-----------------------
----IAEID-----------VEDW----KKHTDYRG-YQES-------------------
------------------------DEVI-KWFWKCIS--------------------EWD
NEQKARLLQF-------TTGTSRIP------------VNG-----FK-DLQGSD--GPRR
FTIEKAGEV-------------------------QQLPKSHTCF-NR-VDLPSY-PDYET
LKQKLT-LAVEE-TIGF
>Ashbya_gossypii_CP002708.1 .
IRVRRKNI-FEDAYQEI--------------MRQSPDDLKK-RLMIKFDG-------EEG
LDYGGVSREF---FFLL----SHEMFNPFY--------CLFEYSSHDNY-----------
----------------TIQINPNS------------------------------------
--GINPEHLNYFKFIGRVVGLGVFHRRFLDAFFVG--ALYKMMLR---------------
--------KKVVLQDMEG----------------------VDSEVYNS--------LKWI
LEN--SIA-GIL--
-DLTFSADDER---------------------------FGE-VVTVD-LKPN--------
GRNID---
------------------------------VTDDNKK-EYVEL-FTQWKICSR-VEDQFK
-AFIDG---FNELIPE--DLVN-VFDER-ELELLIGG-----------------------
----IAEID-----------VEDW----KKHTDYRG-YQES-------------------
------------------------DEVI-KWFWKCIS--------------------EWD
NEQKARLLQF-------TTGTSRIP------------VNG-----FK-DLQGSD--GPRR
FTIEKAGEV-------------------------QQLPKSHTCF-NR-VDLPAY-HDYDT
LKQKLT-LAVEE-TIG-
>Eremothecium_coryli_AZAH01000002.1 .
IRVRRKNI-FEDAYQEI--------------MRQTPEDLKK-RLMIKFDG-------EEG

LDYGGVSREF---FFLL----SHEMFNPFY--------CLFEYSSHDNY-----------
----------------TIQINPNS------------------------------------
--GINPEHLNYFKFIGRVVGLGVFHRRFLDAFFVG--ALYKMMLR---------------
--------KKVVLQDMEG----------------------VDVEVYNS--------LKWI
LEN--SID-GIL--
-DLTFSADDER---------------------------FGE-VVTVD-LKPD--------
GRNID---
------------------------------VTDENKK-EYVEL-FTQWKICTR-VQDQFK
-AFIDG---FNELIPE--DLVN-VFDGR-ELELLIGG-----------------------
----IAEID-----------VDDW----KKHTDYRG-YQES-------------------
------------------------DEVI-KWFWKCIS--------------------EWD
NEQKARLLQF-------TTGTSRIP------------VNG-----FK-DLQGSD--GPRR
FTIEKTGEV-------------------------QQLPKSHTCF-NR-VDLPAY-PDYAT
LKQKLT-LAVEE-TIG-
>Kluyveromyces_aestuarii_AEAS01000334.1 .
IRVRRKNI-FEDSYQEI--------------MRQTPEDLKK-RLMIKFDG-------EEG
LDYGGVSREF---FFLL----SHEMFNPFY--------CLFEYSAHDNY-----------
----------------TIQINPNS------------------------------------
--GINPEHLNYFKFIGRVVGLGVFHRRFLDAFFVG--ALYKMMLR---------------
--------KKVVLQDMEG----------------------VDSEVYNS--------LKWI
LDN--SID-GIL--
-DLTFSVDDER---------------------------FGE-LVIVD-LKPN--------
GKEIE---
------------------------------VTDENKK-EYVEL-YTQWRIVDR-VQEQFR
-AFMDG---FNELVPE--DLVN-VFDER-ELELLIGG-----------------------
----IAEID-----------VEDW----KKHTDYRG-YQES-------------------
------------------------DEVI-QWFWKCVT--------------------EWD
NEQKARLLQF-------TTGTSRIP------------VNG-----FK-DLQGSD--GPRR
FTIEKAGEA-------------------------QQLPKSHTCF-NR-VDLPPY-NDYES
FKQKLT-LAVEE-TIG-
>Kluyveromyces_lactis_XM_454562.1 .
IRVRRKNI-FEDSYQEI--------------MRQTPEDLKK-RLMIKFDG-------EEG
LDYGGVSREF---FFLL----SHEMFNPFY--------CLFEYSAHDNY-----------
----------------TIQINPNS------------------------------------
--GINPEHLNYFKFIGRVVGLGVFHRRFLDAFFVG--ALYKMMLR---------------
--------KKVVLQDMEG----------------------VDSEVYNS--------LKWI
LEN--SID-GIL--
-DLTFNVDDER---------------------------FGE-LVVVD-LKPN--------
GREIE---
------------------------------VTDENKK-EYVEL-YTQWRIADR-VQEQFK
-AFMDG---FNELVPE--DLVN-VFDER-ELELLIGG-----------------------
----IAEID-----------VEDW----KKHTDYRG-YQES-------------------
------------------------DEVI-QWFWKCIT--------------------EWD
NEQRARLLQF-------TTGTSRIP------------VNG-----FK-DLQGSD--GPRR
FTIEKAGEV-------------------------QQLPKSHTCF-NR-VDLPPY-TDYES
FKQKLT-LAVEE-TIG-
>Kluyveromyces_marxianus_CP015061.1 .
IRVRRKNI-FEDAYQEI--------------MRQTPEDLKK-RLMIKFDG-------EEG
LDYGGVSREF---FFLL----SHEMFNPFY--------CLFEYSAHDNY-----------
----------------TIQINPNS------------------------------------
--GINPEHLNYFKFIGRVVGLGVFHRRFLDAFFVG--ALYKMMLR---------------
--------KKVVLQDMEG----------------------VDSEVYNS--------LKWI
LEN--SID-GIL--
-DLTFSVDDER---------------------------FGE-LVVID-LKPN--------
GREIE---
------------------------------VTDENKK-EYVEL-YTQWRIADR-VQEQFK
-AFMDG---FNELVPE--DLVN-VFDER-ELELLIGG-----------------------
----IAEID-----------VEDW----KKHTDYRG-YQES-------------------

------------------------DEVI-QWFWKCIS--------------------EWD
NEQRARLLQF-------TTGTSRIP------------VNG-----FK-DLQGSD--GPRR
FTIEKAGEI-------------------------QQLPKSHTCF-NR-VDLPPY-TDYES
FKQKLT-LAVEE-TIGF
>Kluyveromyces_dobzhanskii_CCBQ010000016.1 .
IRVRRKNI-FEDAYQEI--------------MRQTPEDLKK-RLMIKFDG-------EEG
LDYGGVSREF---FFLL----SHEMFNPFY--------CLFEYSAHDNY-----------
----------------TIQINPNS------------------------------------
--GINPEHLNYFKFIGRVVGLGVFHRRFLDAFFVG--ALYKMMLR---------------
--------KKVVLQDMEG----------------------VDSEVYNS--------LKWI
LEN--SID-GIL--
-DLTFNVDDER---------------------------FGE-LVVID-LKPN--------
GREIE---
------------------------------VTDENKK-EYVEL-YTQWRIADR-VQEQFK
-AFMDG---FNELVPE--DLVN-VFDER-ELELLIGG-----------------------
----IAEID-----------VEDW----KKHTDYRG-YQES-------------------
------------------------DEVI-QWFWKCIT--------------------EWD
NEQRARLLQF-------TTGTSRIP------------VNG-----FK-DLQGSD--GPRR
FTIEKAGEI-------------------------QQLPKSHTCF-NR-VDLPPY-TDYDS
FKQKLT-LAVEE-TIG-
>Naumovozyma_castellii_XM_003674862.1 .
IKVRRKNI-FEDAYQEI--------------MRQSPEDLKK-RLMIKFDG-------EEG
LDYGGVSREF---FFLL----SHEMFNPFY--------CLFEYSAHDNY-----------
----------------TIQINPNS------------------------------------
--GINPEHLNYFKFIGRVVGLGVFHRRFLDAFFVG--ALYKMMLR---------------
--------KKVILQDMEG----------------------VDADVYNS--------LNWM
LEN--SID-GVL--
-DLTFSADDER---------------------------FGE-VVTVD-LKEN--------
GRDIE---
------------------------------VTDENKK-EYVEL-FAQWKIVDR-VQEQFR
-AFMDG---FNELIPE--DLVT-VFDER-ELELLIGG-----------------------
----IAEID-----------VEDW----KKHTDYRG-YQES-------------------
------------------------DEVV-KWFWKCIS--------------------EWD
NEQRARLLQF-------TTGTSRIP------------VNG-----FK-DLQGSD--GPRR
FTIEKAGEA-------------------------QQLPKSHTCF-NR-VDLPVY-EDYDN
LKQKLT-LAVEE-TIGF
>Naumovozyma_dairenensis_XM_003668476.1 .
IKVRRKNI-FEDAYQEI--------------MRQTPEDLKK-RLMIKFDG-------EEG
LDYGGVSREF---FFLL----SHEMFNPFY--------CLFEYSAHDNY-----------
----------------TIQINPNS------------------------------------
--GINPEHLNYFKFIGRVVGLGVFHRRFLDAFFVG--ALYKMMLR---------------
--------KKVILQDMEG----------------------VDADVYNS--------LNWM
LEN--SID-GVL--
-DLTFSADDER---------------------------FGE-VVTVD-LKEN--------
GRNIE---
------------------------------VTDENKK-EYVEL-FAQWKIVDR-VQEQFR
-AFMDG---FNELIPE--DLVT-VFDER-ELELLIGG-----------------------
----IAEID-----------VEDW----KKHTDYRG-YQES-------------------
------------------------DDVI-KWFWKCIS--------------------SWD
NEQRARLLQF-------TTGTSRIP------------VNG-----FK-DLQGSD--GPRR
FTIEKAGEV-------------------------QQLPKSHTCF-NR-VDLPPY-DDFDN
MKQKLT-LAVEE-TIGF
>Torulaspora_delbrueckii_XM_003682073.1 .
IKVRRKNI-FEDAYQEI--------------MRQTPEDLKK-RLMIKFDG-------EEG
LDYGGVSREF---FFLL----SHEMFNPFY--------CLFEYSAHDNY-----------
----------------TIQINPNS------------------------------------
--GINPEHLNYFKFIGRVVGLGVFHRRFLDAFFVG--ALYKMMLR---------------
--------KKVVLQDMEG----------------------VDAEVHNS--------LKWM

```
LEN------------------------------------------------SID-GIL--
-DLTFSADDER---------------------------FGE-LLTVD-LKPK--------
GRTIE-------------------------------------------------------
------------------------------VTDENKK-EYVEL-FAQWKIVDR-VQEQFR
-AFMDG---FNELIPE--DLVT-VFDER-ELELLIGG-----------------------
----IAEID-----------VEDW----KKHTDYRG-YQES-------------------
------------------------DEVV-KWFWKCIT--------------------EWD
NEQRARLLQF-------TTGTSRIP------------VNG-----FK-DLQGSD--GPRR
FTIEKAGEQ-------------------------GQLPKSHTCF-NR-VDLPPY-DDYDS
LKQKLT-LAVEE-TIGF
>Zygosaccharomyces_rouxii_XM_002498758.1 .
IKVRRKNI-FEDAYQEI--------------MRQTPEDLKK-RLMIKFDG-------EEG
LDYGGVSREF---FFLL----SHEMFNPFY--------CLFEYSAHDNY-----------
----------------TIQINPNS------------------------------------
--GVNPEHLNYFKFIGRVVGLGVFHRRFLDAFFVG--ALYKMMLR---------------
--------KKVVLQDMEG----------------------VDAEVHNS--------LKWI
LEN------------------------------------------------SID-GIL--
-DLTFSADDES---------------------------FGE-IHTID-LKPN--------
GRNIE-------------------------------------------------------
------------------------------VIDENKK-EYVEL-FSQWKIVDR-VKEQFR
-AFMDG---FNELIPE--DLVT-VFDER-ELELLIGG-----------------------
----IAEID-----------VEDW----KKHTDYRG-YQES-------------------
------------------------DEVI-KWFWRCIG--------------------SWD
NEQRARLLQF-------TTGTSRIP------------VNG-----FK-DLQGSD--GPRR
FTIEKAGEI-------------------------KHLPKSHTCF-NR-VDLPPY-DDFES
LDKKLT-LAVEE-TIGF
>Zygosaccharomyces_bailii_HG316459.1_a.
IKVRRKNI-FEDAYQEI--------------MRQTPEDLKK-RLMIKFDG-------EEG
LDYGGVSREF---FFLL----SHEMFNPFY--------CLFEYSAHDNY-----------
----------------TIQINPNS------------------------------------
--GVNPEHLNYFKFIGRVVGLGVFHRRFLDAFFVG--ALYKMMLR---------------
--------KKVVLQDMEG----------------------VDAEVHNS--------LKWI
LEN------------------------------------------------SID-GIL--
-DLTFSADDES---------------------------FGE-IQTVD-LKPN--------
GRNIE-------------------------------------------------------
------------------------------VNDENKK-EYVEL-FSQWKIVDR-VKEQFR
-AFMDG---FNELIPE--DLVT-VFDER-ELELLIGG-----------------------
----IAEID-----------VEDW----KKHTDYRG-YQES-------------------
------------------------DEVI-KWFWTCVS--------------------EWD
NEQRARLLQF-------TTGTSRIP------------VNG-----FK-DLQGSD--GPRR
FTIEKAGEI-------------------------RHLPKSHTCF-NR-VDLPPY-EDYDN
LKQKLT-IAVEE-TVG-
>Zygosaccharomyces_parabailii_CP019504.1 .
IKVRRKNI-FEDAYQEI--------------MRQTPEDLKK-RLMIKFDG-------EEG
LDYGGVSREF---FFLL----SHEMFNPFY--------CLFEYSAHDNY-----------
----------------TIQINPNS------------------------------------
--GVNPEHLNYFKFIGRVVGLGVFHRRFLDAFFVG--ALYKMMLR---------------
--------KKVVLQDMEG----------------------VDAEVHNS--------LKWI
LEN------------------------------------------------SID-GIL--
-DLTFSADDES---------------------------FGE-IQTVD-LKPN--------
GRNIE-------------------------------------------------------
------------------------------VNDENKK-EYVEL-FSQWKIVDR-VKEQFR
-AFMDG---FNELIPE--DLVT-VFDER-ELELLIGG-----------------------
----IAEID-----------VEDW----KKHTDYRG-YQES-------------------
------------------------DEVI-KWFWTCVS--------------------EWD
NEQRARLLQF-------TTGTSRIP------------VNG-----FK-DLQGSD--GPRR
FTIEKAGEI-------------------------RHLPKSHTCF-NR-VDLPPY-EDYDN
LKQKLT-IAVEE-TVGF
```

>Tetrapisispora_phaffii_XM_003685130.1 .
IKVRRKNI-FEDAYQEI--------------MRQTPEDLKK-RLMIKFDG-------EEG
LDYGGVSREF---FFLL----SHEMFNPFY--------CLFEYSAHDNY-----------
----------------TIQINPNS------------------------------------
--AINPEHLNYFKFIGRVVGLGVFHRRFLDAFFVG--ALYKMILR---------------
--------KKVILQDMEG----------------------VDAEVHNS--------LKWM
LEN--DIE-GIL--
-DLTFSADDER---------------------------FGE-LVTID-LKPD--------
GRNIE---
------------------------------VTNENKK-EYIEL-YTQWKIYDR-VQEQFK
-AFMDG---FNELIPE--DLVT-VFDER-ELELLIGG-----------------------
----IAEID-----------VEDW----KKHTDYRG-YQES-------------------
------------------------DEVI-QWFWKCIT--------------------EWD
NEQRARLLQF-------TTGTSRIP------------VNG-----FK-DLQGSD--GPRR
FTIEKAGES-------------------------NQLPKSHTCF-NR-VDLPPY-DVYDS
LKQKLT-LAVEE-TIGF
>Vanderwaltozyma_polyspora_XM_001645943.1 .
IKVRRKNI-FEDSYQEI--------------MRQTPEDLKK-RLMIKFDG-------EEG
LDYGGVSREF---FFLL----SHEMFNPFY--------CLFEYSAHDNY-----------
----------------TIQINPNS------------------------------------
--GINPEHLNYFKFIGRVVGLGIFHRRFLDAFFVG--ALYKMILR---------------
--------KKVILQDMEG----------------------VDAEVNNS--------LKWM
LEN--SIE-GIL--
-DLTFSADDER---------------------------FGE-LVTVD-LKPD--------
GRNIE---
------------------------------VTDENKK-EYVEL-YTQWRIVDR-VQEQFK
-AFMDG---FNELIPE--DLIT-VFDER-ELELLIGG-----------------------
----IAEID-----------IEDW----KKHTDYRG-YQES-------------------
------------------------DEVV-QWFWKCVG--------------------EWD
NEQRARLLQF-------TTGTSRIP------------VNG-----FK-DLQGSD--GPRR
FTIEKAGES-------------------------MQLPKSHTCF-NR-VDLPPY-DDFES
MRQKMT-LAVEE-TIGF
>2_Kazachstania_saulgeensis_FXLY01000003.1 .
IKVRRKNI-FEDAYQEI--------------MRQTPEDLKK-RLMIKFDG-------EEG
LDYGGVSREF---FFLL----SHEMFNPFY--------CLFEYSAHDNY-----------
----------------TIQINPNS------------------------------------
--GINPEHLNYFKFIGRVVGLGVFHRRFLDAFFVG--ALYKMMLR---------------
--------KKVMLQDMEG----------------------VDAEVYNS--------LNWM
LEN--SID-GVL--
-DLTFSADDER---------------------------FGE-VVTVD-LKPN--------
GRNIE---
------------------------------VTDENKK-EYVEL-FSQWKIVDR-VQEQLK
-AFMDG---FNELIPE--DLVT-VFDER-ELELLIGG-----------------------
----IAEID-----------IEDW----KKHTDYRG-YQES-------------------
------------------------DDAI-QWFWKTVS--------------------EWD
NEQRARLLQF-------TTGTSRIP------------VNG-----FK-DLQGSD--GPRR
FTIEKAGEI-------------------------QQLPKSHTCF-NR-VDLPPY-TDAEA
LKQKLT-LAVEE-TIG-
>Tetrapisispora_blattae_XM_004181688.1 .
IKVRRKNI-FEDAYQEI--------------MRQTPEDLKK-RLMIKFDG-------EEG
LDYGGVSREF---FFLL----SHEMFNPFY--------CLFEYSAHDNY-----------
----------------TIQINPNS------------------------------------
--AINPEHLNYFKFIGRVVGLGVFHRRFLDAFFVG--ALYKMMLR---------------
--------KKVVLQDMEG----------------------VDNEVYNS--------LKWI
LDN--SIE-GIL--
-DLTFSADDER---------------------------FGE-VITVD-LKEN--------
GRNIE---
------------------------------VTDENKK-EYIEL-FTQWKIVDR-VQEQLK

-AFMDG---FNELIPE--DLVT-VFDER-ELELLIGG-----------------------
----IAEID-----------IEDW----KKHTDYRG-YQES-------------------
------------------------DEVI-QWFWKCVG--------------------EWD
NEQRARLLQF-------TTGTSRIP------------VNG-----FK-DLQGSD--GPRR
FTIEKAGES-------------------------NQLPKSHTCF-NR-VDLPPY-TDYDS
MKQKLT-LAVEE-TIGF
>Kazachstania_africana_XM_003955594.1 .
IKVRRKNI-FEDAYQEI--------------MRQTPEDLKK-RLMIKFDG-------EEG
LDYGGVSREF---FFLL----SHEMFNPFY--------CLFEYSAHDNY-----------
----------------TIQINPNS------------------------------------
--AINPEHLNYFKFIGRVVGLGVFHRRFLDAFFVG--ALYKMMLR---------------
--------KKVVLQDMEG----------------------VDADVHNS--------LNWT
LEN--SID-GVL--
-DLTFSADDER---------------------------FGE-VVTVD-LKEN--------
GRDIE---
------------------------------VTDENKK-EYIEL-YTQWKIVDR-VQEQFK
-AFMDG---FNELIPE--DLVT-VFDER-ELELLIGG-----------------------
----IAEID-----------IEDW----KKHTDYRG-YQES-------------------
------------------------DEVI-QWFWKCVT--------------------EWD
NEQKARLLQF-------TTGTSRIP------------VNG-----FK-DLQGSD--GPRR
FTIEKAGEV-------------------------QQLPKSHTCF-NR-VDLPPY-ADYES
LRQKLT-LAVEE-TIGF
>Kazachstania_naganishii_HE978317.1 .
IKVRRNNI-FEDAYQEI--------------MRQTPEDLKK-RLMIKFDG-------EEG
LDYGGVSREF---FFLL----SHEMFNPFY--------CLFEYSAHDNY-----------
----------------TIQINPNS------------------------------------
--GINPEHLNYFKFIGRVVGLGVFHRRFLDAFFVG--ALYKMMLR---------------
--------KKVVIQDMEG----------------------VDADVYNS--------LNWT
LEN--SID-GVL--
-DLTFSADDER---------------------------FGE-VVTVD-LKPE--------
GRDVE---
------------------------------VTDENKK-EYVDL-YTQWRIVDR-VQEQFK
-AFMDG---FNELIPE--DLVT-VFDER-ELELLIGG-----------------------
----IAEID-----------IEDW----KKHTDYRG-YQES-------------------
------------------------DEVV-QWFWKCIS--------------------EWD
NEQRARLLQF-------TTGTSRIP------------VNG-----FK-DLQGSD--GPRR
FTIEKAGEE-------------------------KQLPKSHTCF-NR-VDLPPY-TDFEA
MKQKMS-LAVEE-TIGF
>Nakaseomyces_bacillisporus_CAPX01000181.1 .
IKVRRKNI-FEDAYQEI--------------MRQTPEDLKK-RLMIKFDG-------EEG
LDYGGVSREF---FFLL----SHEMFNPFY--------CLFEYSAHDNY-----------
----------------TIQINSNS------------------------------------
--GINPEHLNYFKFIGRVVGLGVFHRRFLDAFFVA--ALYKMMLR---------------
--------KKVVLQDMEG----------------------VDADVYNS--------LNWM
LEN--EIE-GVL--
-DLTFSADDER---------------------------FGE-IVTVD-LKPN--------
GRNIE---
------------------------------VTDENKK-EYVEL-YTQWRISDR-VKEQFR
-AFMDG---FNELIPE--DLVT-VFDER-ELEFLIGG-----------------------
----IAEID-----------IEDW----KKHTDYRG-YQES-------------------
------------------------DEVI-QWFWKCVT--------------------EWD
NEQRARLLQF-------TTGTSRIP------------VNG-----FK-DLQGSD--GPRR
FTIEKAGES-------------------------QQLPKSHTCF-NR-VDLPPY-VDYES
MQKKLT-LAVEE-TIG-
>Candida_castellii_CAPW01000080.1 .
IKVRRNNI-FEDAYQEI--------------MRQTPEDLKK-RLMIKFDG-------EEG
LDYGGVSREF---FFLL----SHEMFNPFY--------CLFEYSAHDNY-----------
----------------TIQINSNS------------------------------------

```
--GINPEHLNYFKFIGRVVGLGVFHRRFLDAFFVA--ALYKMMLS---------------
--------KKVVLQDMEG----------------------VDADVYNS--------LQWM
IDN-------------------------------------------------SIE-GVL--
-DLTFSADDER---------------------------FGE-VVTVD-LKPD--------
GRNIE-------------------------------------------------------
------------------------------VTDENKK-EYVEL-YTQWRISNR-VQEQFK
-AFMDG---FNELIPE--DLVT-VFDER-ELELLIGG-----------------------
----IAEID-----------IEDW----KKHTDYRG-YQES-------------------
------------------------DEVV-QWFWKCVS--------------------EWD
NEQRARLLQF-------TTGTSRIP------------VNG-----FK-DLQGSD--GPRR
FTIEKAGET-------------------------QQLPKSHTCF-NR-VDLPPY-DDFES
MQKKLT-LAVEE-TIG-
>Candida_nivariensis_CAPV01000038.1 .
IKVRRKNI-FEDAYQEI--------------MRQTPEDLKK-RLMIKFDG-------EEG
LDYGGVSREF---FFLL----SHEMFNPFY--------CLFEYSAHDNY-----------
----------------TIQINSNS------------------------------------
--GINPEHLNYFKFIGRVVGLGVFHRRFLDAFFVG--ALYKMMLR---------------
--------KKVVLQDMEG----------------------VDADVYNS--------LNWM
LEN-------------------------------------------------SID-GVL--
-DLTFSADDER---------------------------FGE-VVTVD-LKPD--------
GRNIE-------------------------------------------------------
------------------------------VTDENKK-EYVEL-YTQWRIVDR-VQEQFK
-AFMDG---FNELIPE--DLVT-VFDER-ELELLIGG-----------------------
----IAEID-----------IEDW----KKHTDYRG-YQES-------------------
------------------------DEVI-QWFWKCVS--------------------EWD
NEQRARLLQF-------TTGTSRIP------------VNG-----FK-DLQGSD--GPRR
FTIEKAGEV-------------------------EQLPKSHTCF-NR-VDLPPY-ADFDS
MAKKLT-LAVEE-TIG-
>Candida_bracarensis_CAPU01000047.1 .
IKVRRKNI-FEDAYQEI--------------MRQTPEDLKK-RLMIKFDG-------EEG
LDYGGVSREF---FFLL----SHEMFNPFY--------CLFEYSAHDNY-----------
----------------TIQINSNS------------------------------------
--GINPEHLNYFKFIGRVVGLGVFHRRFLDAFFVG--ALYKMMLR---------------
--------KKVVLQDMEG----------------------VDADVYNS--------LNWM
LEN-------------------------------------------------SID-GVL--
-DLTFSADDER---------------------------FGE-VVTVD-LKPD--------
GRNIE-------------------------------------------------------
------------------------------VTDENKK-EYVEL-YTQWRIVDR-VEEQFK
-AFMDG---FNELIPE--DLVT-VFDER-ELELLIGG-----------------------
----IAEID-----------IEDW----KKHTDYRG-YQES-------------------
------------------------DEVI-QWFWKCVS--------------------DWD
NEQRARLLQF-------TTGTSRIP------------VNG-----FK-DLQGSD--GPRR
FTIEKAGEV-------------------------EQLPKSHTCF-NR-VDLPPY-ADYDS
MAKKLT-LAVEE-TIG-
>Nakaseomyces_delphensis_CAPT01000076.1 .
IKVRRKNI-FEDAYQEI--------------MRQTPEDLKK-RLMIKFDG-------EEG
LDYGGVSREF---FFLL----SHEMFNPFY--------CLFEYSAHDNY-----------
----------------TIQINSNS------------------------------------
--GINPEHLNYFKFIGRVVGLGVFHRRFLDAFFVG--ALYKMMLR---------------
--------KKVVLQDMEG----------------------VDADVYNS--------LNWM
LGN-------------------------------------------------SID-GIL--
-ELTFSADDER---------------------------FGE-VVTVD-LKPD--------
GRNIE-------------------------------------------------------
------------------------------VTDENKK-EYVEL-YTQWRVLDR-VQDQFK
-AFMDG---FNELIPE--DLVT-VFDER-ELELLIGG-----------------------
----IAEID-----------IEDW----KKHTDYRG-YQES-------------------
------------------------DEVI-QWFWKCVS--------------------EWD
NEQRARLLQF-------TTGTSRIP------------VNG-----FK-DLQGSD--GPRR
```

FTIEKAGEV-------------------------EQLPKSHTCF-NR-VDLPPY-ADYDS
MAKKLT-LAVEE-TIG-
>Candida_glabrata_CR380957.2 .
IKVRRNNI-FEDAYQEI--------------MRQTPEDLKK-RLMIKFDG-------EEG
LDYGGVSREF---FFLL----SHEMFNPFY--------CLFEYSAHDNY-----------
----------------TIQINSNS------------------------------------
--GINPEHLNYFKFIGRVVGLGVFHRRFLDAFFVG--ALYKMMLR---------------
--------KKVALQDMEG----------------------VDADVYNS--------LKWM
LEN--SID-GVL--
-DLTFSADDER---------------------------FGE-VVTVD-LKPD--------
GRNIE---
------------------------------VTDEVKK-EYVEL-YSQWRIVDR-VSEQFK
-AFMDG---FNELIPE--DLVT-VFDER-ELELLIGG-----------------------
----IAEID-----------IEDW----KKHTDYRG-YQES-------------------
------------------------DEVI-QWFWKCVS--------------------EWD
NEQRARLLQF-------TTGTSRIP------------VNG-----FK-DLQGSD--GPRR
FTIEKAGEV-------------------------EQLPKSHTCF-NR-VDLPPY-ADYDS
MAKKLT-LAVEE-TIGF
>Saccharomyces_mikatae_AABZ01000008.1 .
IKVRRKNI-FEDAYQEI--------------MRQTPEDLKK-RLMIKFDG-------EEG
LDYGGVSREF---FFLL----SHEMFNPFY--------CLFEYSAYDNY-----------
----------------TIQINPNS------------------------------------
--GINPEHLNYFKFIGRVVGLGVFHRRFLDAFFVG--ALYKMMLR---------------
--------KKVVLQDMEG----------------------VDAEVYNS--------LNWM
LEN--SID-GVL--
-DLTFSADDER---------------------------FGE-VVTVD-LKPD--------
GRNIE---
------------------------------VTDGNKK-EYVEL-YTQWRIVDR-VQEQFK
-AFMDG---FNELIPE--DLVT-VFDER-ELELLIGG-----------------------
----IAEID-----------IEDW----KKHTDYRG-YQES-------------------
------------------------DEVI-QWFWKCVS--------------------EWD
NEQRARLLQF-------TTGTSRIP------------VNG-----FK-DLQGSD--GPRR
FTIEKAGEI-------------------------QQLPKSHTCF-NR-VDLPQY-VDYDS
MKQKLT-LAVEE-TIGF
>Saccharomyces_cerevisiae_NM_001179019.2 .
IKVRRKNI-FEDAYQEI--------------MRQTPEDLKK-RLMIKFDG-------EEG
LDYGGVSREF---FFLL----SHEMFNPFY--------CLFEYSAYDNY-----------
----------------TIQINPNS------------------------------------
--GINPEHLNYFKFIGRVVGLGVFHRRFLDAFFVG--ALYKMMLR---------------
--------KKVVLQDMEG----------------------VDAEVYNS--------LNWM
LEN--SID-GVL--
-DLTFSADDER---------------------------FGE-VVTVD-LKPD--------
GRNIE---
------------------------------VTDGNKK-EYVEL-YTQWRIVDR-VQEQFK
-AFMDG---FNELIPE--DLVT-VFDER-ELELLIGG-----------------------
----IAEID-----------IEDW----KKHTDYRG-YQES-------------------
------------------------DEVI-QWFWKCVS--------------------EWD
NEQRARLLQF-------TTGTSRIP------------VNG-----FK-DLQGSD--GPRR
FTIEKAGEV-------------------------QQLPKSHTCF-NR-VDLPQY-VDYDS
MKQKLT-LAVEE-TIGF
>Saccharomyces_kudriavzevii_AACI02000191.1 .
IKVRRKNI-FEDAYQEI--------------MRQTPEDLKK-RLMIKFDG-------EEG
LDYGGVSREF---FFLL----SHEMFNPFY--------CLFEYSAYDNY-----------
----------------TIQINPNS------------------------------------
--GINPEHLNYFKFIGRVVGLGVFHRRFLDAFFVG--ALYKMMLR---------------
--------KKVVLQDMEG----------------------VDAEVYNS--------LNWM
LEN--SID-GVL--
-DLTFSADDER---------------------------FGE-VVTVD-LKPD--------

GRNIE---
------------------------------VTDGNKK-EYVEL-YTQWRIVDR-VQEQFK
-AFMDG---FNELIPE--DLVT-VFDER-ELELLIGG-----------------------
----IAEID-----------IEDW----KKHTDYRG-YQES-------------------
------------------------DEVI-QWFWKCVS--------------------EWD
NEQRARLLQF-------TTGTSRIP------------VNG-----FK-DLQGSD--GPRR
FTIEKAGEV-------------------------QQLPKSHTCF-NR-VDLPQY-VDYDS
MKQKLT-LAVEE-TIGF
>Saccharomyces_paradoxus_CP020314.1 .
IKVRRKNI-FEDAYQEI--------------MRQTPEDLKK-RLMIKFDG-------EEG
LDYGGVSREF---FFLL----SHEMFNPFY--------CLFEYSAYDNY-----------
----------------TIQINPNS------------------------------------
--GINPEHLNYFKFIGRVVGLGVFHRRFLDAFFVG--ALYKMMLR---------------
--------KKVVLQDMEG----------------------VDAEVYNS--------LNWM
LEN--SID-GVL--
-DLTFSADDER---------------------------FGE-VVTVD-LKPD--------
GRNIE---
------------------------------VTDGNKK-EYVEL-YTQWRIVDR-VQEQFK
-AFMDG---FNELIPE--DLVT-VFDER-ELELLIGG-----------------------
----IAEID-----------IEDW----KKHTDYRG-YQES-------------------
------------------------DEVI-QWFWKCVS--------------------EWD
NEQRARLLQF-------TTGTSRIP------------VNG-----FK-DLQGSD--GPRR
FTIEKAGEV-------------------------QQLPKSHTCF-NR-VDLPQY-VDYDS
MKQKLT-LAVEE-TIGF
>Hanseniaspora_osmophila_LPNM01000008.1 .
IKVRRKNI-FEDAYQEV--------------MRQSPEDLKK-RLMIEFEG-------EEG
VDYGGVSREF---FFLL----SHEMFNPIY--------CLFEYSSHDNY-----------
----------------TIQINPNS------------------------------------
--KINPEHLNYFKFIGRVVGLGVFHRRFLDAFFIG--ALYKMLLK---------------
--------KKVVLQDMEG----------------------VDHEVYNS--------LKWI
MEN--SIE-GIL--
-DLTFSIDDDN---------------------------FGE-IVTID-LKPD--------
GRNIE---
------------------------------VTDENKK-EYVEL-FTQWKICDR-VHDQLQ
-AFMDG---FNELIPE--ELVT-VFDER-ELELLIGG-----------------------
----IAEID-----------VEDW----KKHTDYRG-YQES-------------------
------------------------DQTV-QWFWKIIT--------------------EWD
NEQKARLLQF-------TTGTSRIP------------VNG-----FK-DLQGSD--GPRR
FTIEKAGEP-------------------------SQLPKSHTCF-NR-VDLPPY-DDYES
MKQKLT-WAVEE-TIG-
>Hanseniaspora_vineae_JFAV02000158.1 .
IKVRRKNI-FEDAYQEI--------------MRQSPEDLKK-RLMIEFEG-------EEG
VDYGGVSREF---FFLL----SHEMFNPIY--------CLFEYSSHDNY-----------
----------------TIQINPNS------------------------------------
--KINPEHLNYFKFIGRVVGLGVFHRRFLDAFFIG--ALYKMLLK---------------
--------KKVVLQDMEG----------------------VDHEVYNS--------LKWI
LEN--SIE-GIL--
-DLTFSIDDDN---------------------------FGE-IVTID-LKPD--------
GRNIE---
------------------------------VTDENKK-EYVEL-FTQWKICDR-VHDQLQ
-AFMDG---FNELIPE--ELVT-VFDER-ELELLIGG-----------------------
----IAEID-----------VEDW----KKHTDYRG-YQES-------------------
------------------------DQTV-QWFWKIIT--------------------EWD
NEQKARLLQF-------TTGTSRIP------------VNG-----FK-DLQGSD--GPRR
FTIEKAGEP-------------------------SQLPKSHTCF-NR-VDLPPY-DDYDS
MKQKLT-WAVEE-TIG-
>Hanseniaspora_valbyensis_LXPE01000029.1 .
IKVQRSNI-FEDAYQEI--------------MRQSPEDLKK-RLMIEFDG-------EEG

```
VDYGGVSREF---FFLL----SHEMFNPVY--------CLFEYSSHDNY-----------
----------------TIQINPNS------------------------------------
--GINPEHLNYFKFIGRVVGLGVFHRRFLDAFFIG--ALYKMLLK---------------
--------KKVGLQDMEG----------------------VDHEVYNS--------LKWM
MEN------------------------------------------------SID-GIL--
-DLTFAVDDEV---------------------------FGE-IVQVE-LKPG--------
GKDIE-------------------------------------------------------
------------------------------VTDENKK-EYIEL-FTQWKISDR-VSAQLK
-AFMDG---FNELIPE--DLVT-VFDER-ELELLIGG-----------------------
----ISEID-----------VEDW----KKHTDYRG-YQEG-------------------
------------------------DQTV-QWFWKIVS--------------------EWD
NESKARLLQF-------TTGTSRIP------------VNG-----FK-DLQGSD--GPRR
FTIEKAGEI-------------------------SQLPKSHTCF-NR-VDLPEY-DDYES
MKRKLT-WAVEE-TIG-
>Hanseniaspora_uvarum_LPNN01000005.1 .
VKVKRSNI-FEDAYQEI--------------MRQSPEDLKK-RLMIEFDG-------EEG
VDYGGVSREF---FFLL----SHEMFNPVY--------CLFEYSSHDNY-----------
----------------TIQINPNS------------------------------------
--GVNPEHLNYFKFIGRVVGLGVYHRRFLDAFFIG--AFYKLLLR---------------
--------KKVGLQDMES----------------------VDNEVYNS--------LKWM
MEN------------------------------------------------EIN-GIL--
-DLTFAVDDEV---------------------------FGE-IVQVE-LKPG--------
GKDIE-------------------------------------------------------
------------------------------VTDDNKK-EYVEL-FTQWKIHDR-VSAQLK
-AFMDG---FNELIPE--DLIT-VFDER-ELELLIGG-----------------------
----ISEID-----------VEDW----KKHTDYRG-YQEG-------------------
------------------------DQTV-QWFWKIVS--------------------EWD
NESKARLLQF-------TTGTSRIP------------VNG-----FK-DLQGSD--GPRR
FTIEKAGEA-------------------------DQLPKSHTCF-NR-VDLPNY-DDYES
MKRKLT-WAVEE-TIG-
>Hanseniaspora_guilliermondiiFQNF01000033. 1..
IKVKRSNI-FEDAYQEI--------------MRQSPEDLKK-RLMIEFDG-------EEG
VDYGGVSREF---FFLL----SHEMFNPIY--------CLFEYSSHDNY-----------
----------------TIQINPNS------------------------------------
--GVNPEHLNYFKFIGRVVGLGVYHRRFLDAFFIG--AFYKLLLR---------------
--------KKVGLQDMES----------------------VDNEVYNS--------LKWM
MEN------------------------------------------------EID-GIL--
-DLTFAVDDEV---------------------------FGE-IVQVE-LKPG--------
GKDIE-------------------------------------------------------
------------------------------VTDENKK-EYVEL-FTQWKIHDR-VSAQLK
-AFMDG---FNELIPE--DLIT-VFDER-ELELLIGG-----------------------
----ISEID-----------VEDW----KKHTDYRG-YQES-------------------
------------------------DQTV-QWFWKIVS--------------------EWD
NESKARLLQF-------TTGTSRIP------------VNG-----FK-DLQGSD--GPRK
FTIEKAGET-------------------------DQLPKSHTCF-NR-VDLPNY-DDYES
MKRKLT-WAVEE-TIG-
>Hanseniaspora_opuntiae_LPNL01000003.1 .
IKVKRSNI-FEDAYQEI--------------MRQSPEDLKK-RLMIEFDG-------EEG
VDYGGVSREF---FFLL----SHEMFNPVY--------CLFEYSSHDNY-----------
----------------TIQINPNS------------------------------------
--GVNPEHLNYFKFIGRVVGLGVYHRRFLDAFFIG--AFYKLLLR---------------
--------KKVGLQDMES----------------------VDNEVYNS--------LKWM
MEN------------------------------------------------EID-GIL--
-DLTFAVDDEV---------------------------FGE-IVQVE-LKPG--------
GKDIE-------------------------------------------------------
------------------------------VTDENKK-EYVDL-FTQWKIHDR-VSAQLK
-AFMDG---FNELIPE--DLIT-VFDER-ELELLIGG-----------------------
----ISEID-----------VEDW----KKHTDYRG-YQES-------------------
```

------------------------DQTV-QWFWKIVS--------------------EWD
NESKARLLQF-------TTGTSRIP------------VNG-----FK-DLQGSD--GPRK
FTIEKAGET-------------------------DQLPKSHTCF-NR-VDLPCY-DDYES
MKRKLT-WAVEE-TIG-
>Cyberlindnera_jadinii_XM_020217040.1 .
IKVRRDHI-FEDSYQEI--------------MRQTPEDLKK-RLMIKFEG-------EEG
LDYGGVSREF---FFLL----SHEMFNPFY--------CLFEYSAHDNY-----------
----------------TLQINPNS------------------------------------
--GINPEHLNYFKFIGRVVGLGVFHRRFLDAFFVG--ALYKMMLH---------------
--------KKVVLQDMEG----------------------VDAEFYRS--------LKWM
LEN--DIQ-GIL--
-DLTFSAEEER---------------------------FGE-MHEVD-LKPN--------
GREIE---
------------------------------VTNDNKK-EYVEL-ITEWKIYKC-VEEQFK
-AFMDG---FNELIPE--ELVN-VFDER-ELELLIGG-----------------------
----IAEID-----------VDDW----KKHTDYRG-YQES-------------------
------------------------DEVI-QWFWKCIS--------------------EWD
NEQRARLLQF-------TTGTSRIP------------VNG-----FK-DLQGSD--GPRR
FTIEKAGEP-------------------------NQLPKSHTCF-NR-VDLPPY-VDYDS
MKQKMS-LAVEE-TVGF
>Cyberlindnera_fabianii_LK052886.1 .
IKVRRDHI-FEDSYQEI--------------MRQTPEDLKK-RLMIKFDG-------EEG
LDYGGVSREF---FFLL----SHEMFNPFY--------CLFEYSAHDNY-----------
----------------TLQINPNS------------------------------------
--GINPEHLNYFKFIGRVVGLGVFHRRFLDAFFVG--ALYKMMLH---------------
--------KKVVLQDMEG----------------------VDAEFYRS--------LKWM
LEN--DID-GVL--
-DLTFSAEEER---------------------------FGE-MREVD-LKPN--------
GAKIE---
------------------------------VTNDNKK-EYVEL-ITEWKIYKC-VEEQFK
-SFMDG---FNELIPE--DLVN-VFDER-ELELLIGG-----------------------
----IAEID-----------VEDW----KKHTDYRG-YQES-------------------
------------------------DEVI-QWFWKCIG--------------------EWD
NEQRARLLQF-------TTGTSRIP------------VNG-----FK-DLQGSD--GPRR
FTIEKAGEA-------------------------NQLPKSHTCF-NR-VDLPPY-VDYES
MKQKMS-LAVEE-TVGF
>Wickerhamomyces_anomalus_XM_019186123.1 .
IKVRRDHI-FEDSYQEI--------------MRQTPEDLKK-RLMIKFDG-------EEG
LDYGGVSREF---FFLL----SHEMFNPFY--------CLFEYSAHDNY-----------
----------------TLQINPNS------------------------------------
--NINPEHLNYFKFIGRVVGLGVFHRRFLDAFFVG--ALYKMMLH---------------
--------KKVILQDMEG----------------------VDAEFYRS--------LKWT
LDN--DID-GVL--
-DLTFSAEDER---------------------------FGE-LITVD-LKPK--------
GRDIE---
------------------------------VTNENKK-EYVEL-ITEWRIYKR-VEEQFK
-SFMDG---FNELIPE--DLVN-VFDER-ELELLIGG-----------------------
----IAEID-----------VEDW----KKHTDYRG-YQET-------------------
------------------------DEVI-QWFWKCIT--------------------EWD
NEQKARLLQF-------TTGTSRIP------------VNG-----FK-DLQGSD--GPRR
FTIEKAGES-------------------------NQLPKSHTCF-NR-VDLPPY-TDYEA
MKQKLS-LAVEE-TVGF
>Wickerhamomyces_ciferrii_XM_011276811.1 .
IKVRRDHI-FEDSYQEI--------------MRQTPEDLKK-RLMIKFDG-------EEG
LDYGGVSREF---FFLL----SHEMFNPFY--------CLFEYSAHDNY-----------
----------------TLQINPNS------------------------------------
--NINPEHLNYFKFIGRVVGLGVFHRRFLDAFFVA--ALYKMMLH---------------
--------KKVILQDMEG----------------------VDAEFYRS--------LKWT

LEN--DID-GVL--
-DLTFSAEDER---------------------------FGE-IVTID-LKEG--------
GADIE---
------------------------------VTNENKK-EYVEL-ITEWRIYKR-VEEQFK
-AFMDG---FNELIPE--DLVN-VFDER-ELELLIGG-----------------------
----IAEID-----------VEDW----KKHTDYRG-YQET-------------------
------------------------DEVI-QWFWKCIS--------------------EWD
NEQKARLLQF-------TTGTSRIP------------VNG-----FK-DLQGSD--GPRR
FTIEKAGES-------------------------NQLPKSHTCF-NR-VDLPPY-TDYEA
MKHKMS-LAVEE-TVGF
>Saccharomycopsis_malanga_BCGJ01000003.1 .
IKVRRDHI-FDDAYQEI--------------MRQTPEELKK-RLMIRFEG-------EEG
LDYGGVSREF---FFLL----SHEMFNPQY--------CLFEYAAYDNY-----------
----------------TLQINPNS------------------------------------
--GINSEHLNYFKFIGRVVGLCIFHRRFLDTFFVG--AFYKMMLH---------------
--------KKIVLQDMEG----------------------IDAEYYNS--------LVWM
LEN--DIT-DVL--
-YLNFSAEEQR---------------------------FGE-TVIYD-LKEN--------
GRNIE---
------------------------------VTEENKK-EYVEL-ITEWKIYKK-VEEQFK
-AFLDG---FNELIPE--DLVN-VFDER-ELELLIGG-----------------------
----ISDID-----------VEDW----KKHTDYRS-YQES-------------------
------------------------DQVI-QWFWEIVS--------------------SWD
NEKRARLLQF-------TTGTSRIP------------VNG-----FK-DLQGSD--GPRR
FTIEKAGEA-------------------------NQLPKSHTCF-NR-VDLPPY-SDKES
LSQKLT-LAVEE-TMG-
>Saccharomycopsis_fibuligera_CP012820.1 .
IKVRRDHI-FDDAYQEI--------------MRQTPEELKK-RLMIRFEA-------EEG
LDYGGVSREF---FFLL----SHEMFNPQY--------CLFEYAAYDNY-----------
----------------TLQINPNS------------------------------------
--GINNEHLNYFKFIGRVVGLCIFHRRFLDTFFVG--AFYKMMLH---------------
--------KKIVLQDMEG----------------------IDAEYYNS--------LVWM
LEN--DIT-DVL--
-YLTFSAEEQR---------------------------FGE-TVIYD-LKPD--------
GRNIE---
------------------------------VTEENKK-EYVEL-ITEWKIYKK-VEEQFK
-AFLTG---FNELIPE--DLVN-VFDER-ELELLIGG-----------------------
----ISDID-----------VDDW----KKHTDYRG-YQES-------------------
------------------------DQVV-RWFWEIVT--------------------KWD
NEKRARLLQF-------TTGTSRIP------------VNG-----FK-DLQGSD--GPRR
FTIEKAGEA-------------------------KQLPKSHTCF-NR-VDLPPY-TDINA
LEQKLT-LAVEE-TMGF
>Kuraishia_capsulata_CBUD020000028.1 .
IKVRRSNL-FEDAFQEI--------------MRQTPEDLKK-RLMIKFDG-------EEG
LDYGGVSREF---FQLV----SHEMFNPFY--------CLFQYATIDNY-----------
----------------TLQINPNS------------------------------------
--GINPEHLNYFKFIGRAVGLGVFHRRFLDAFFVG--AMYKMMLG---------------
--------KKIVLQDLEG----------------------IDNEMYKS--------LTWM
LEN--DID-NVL--
-FETFSVEDDR---------------------------FGE-RVTID-LKPN--------
GREIE---
------------------------------VTNDNKR-EYVEL-KTEWMISKP-IEQQFK
-AFMDG---FNELIPQ--DLVS-VFDER-ELELLIGG-----------------------
----LAEID-----------VEDW----KKHTDYRG-YQES-------------------
------------------------DEVI-QWFWKCIT--------------------EWE
SEQRARLLQF-------TTGTSRIP------------VNG-----FK-DLQGSD--GPRR
FTIEKAGES-------------------------NQLPKSHTCF-NR-VDLPPY-KDYES
LRQKLT-IAVEE-TVG-

>Candida_ethanolica_ANNA01000420.1 .
IKVRRDCL-LEDSFREV--------------MRQTPEDLKK-RLMIKFEG-------EEG
LDYGGVSREF---FQQL----SHEVFNPAY--------GLFKYASSDNY-----------
----------------TLQINPNS------------------------------------
--NINPEHLNYFKFVGRVVGLAVFHRRFLDAFFVG--AMYKMMLR---------------
--------KKIVLQDLES----------------------VDSEMYKS--------LCWM
LEN--DIT-DIL--
-YETFSVEEDR---------------------------FGE-KVTID-LKPN--------
GRNIE---
------------------------------VTNENKR-EFVEL-KTEWIISKP-VEGQFR
-SFMEG---FNELIPQ--ELVQ-VFDER-ELELLIGG-----------------------
----LAEID-----------IEDW----KKHTDYRG-YQES-------------------
------------------------DEVI-QWFWKAVS--------------------EWE
SEQRARLLQF-------TTGTSRIP------------VNG-----FK-DLQGSD--GPRR
FTIEKAGEI-------------------------DQLPKSHTCF-NR-VDLPPY-KDYDT
LKKKLT-IAVEN-TIG-
>Pichia_membranifaciens_XM_019163985.1 .
IKVRRDCL-LEDSFREV--------------MRQTPEDLKK-RLMIKFEG-------EEG
LDYGGVSREF---FQQL----SHEVFNPAY--------GLFKYASSDNY-----------
----------------TLQINPNS------------------------------------
--NINPEHLNYFKFVGRVVGLAVFHRRFLDAFFVG--AMYKMMLR---------------
--------KKIVLQDLES----------------------VDSEMYKS--------LCWM
LEN--DIT-DIL--
-YESFSVEEDR---------------------------FGE-KVTID-LKPN--------
GRDVE---
------------------------------VTNENKR-EFVEL-KTEWIISKP-VEGQFR
-SFMEG---FNELIPQ--ELVQ-VFDER-ELELLIGG-----------------------
----LAEID-----------IDDW----KKHTDYRG-YQES-------------------
------------------------DEVI-QWFWKAVS--------------------EWE
SEQRARLLQF-------TTGTSRIP------------VNG-----FK-DLQGSD--GPRR
FTIEKAGEI-------------------------DQLPKSHTCF-NR-VDLPPY-KDYDA
LKKKLT-IAVEN-TIGF
>Pichia_kudriavzevii_XM_020687502.1 .
IKIRRERL-LEDSFREV--------------MRQTPEDLKK-RLMIKFEG-------EEG
LDYGGVSREF---FQQL----SHEVFNPAY--------GLFKYASSDNY-----------
----------------TLQINPNS------------------------------------
--NINPEHLNYFKFVGRVVGLAVFHRRFLDAFFVG--AMYKMMLR---------------
--------KKIVLQDLES----------------------VDSEMYKS--------LCWM
LEN--DIT-DIL--
-YETFSVEEDR---------------------------FGE-TVTID-LKPN--------
GREVE---
------------------------------VTNENKN-EFVEL-KTEWIISKP-VENQFR
-SFMEG---FNELIPQ--ELVQ-VFDER-ELELLIGG-----------------------
----LAEID-----------VEDW----KKHTDYRG-YQES-------------------
------------------------DEVI-QWFWKAVT--------------------EWE
SEQRARLLQF-------TTGTSRIP------------VNG-----FK-DLQGSD--GPRR
FTIEKAGEI-------------------------DQLPKSHTCF-NR-VDLPPY-KDYES
LKKKLT-IAVEN-TVGF
>Brettanomyces_bruxellensis_MDGX01000319.1 .
IKVRRSNL-MEDSYREV--------------MRQTPEDLKK-RLMIKFDG-------EEG
LDYGGVSREF---FQQL----SHEIFNPIY--------CLFKYASSDNY-----------
----------------TLQINPDS------------------------------------
--GINPEHLSYFKFIGRTVGLGVFHRRFLDAFFVG--AMYKMMLH---------------
--------KKILLQDLES----------------------VDSEMYRS--------LCWM
LAN--DIT-DVI--
-FETFSVEVDH---------------------------FGK-KEVID-LKPD--------
GRHID---
------------------------------VTNDNKK-EFVEL-KTEWIISKP-IEQQFK

```
-AFMDG---FNELIPE--DLVQ-VFDER-ELELLIGG-----------------------
----LSDID-----------IDDW----KRHTDYRG-YQES-------------------
------------------------DQVI-EWFWKCIA--------------------EWE
SDQRARLLQF-------ATGTSRIP------------VNG-----FK-DLQGSD--GPRR
FTVEKAGDP-------------------------DQLPKSHTCF-NR-IDLPPY-KDYES
LRKKLT-MAVEE-TVG-
>Brettanomyces_anomalus_MDSB01000003.1 .
IKVRRNNL-MEDSYREV--------------MRQTPEDLKK-RLMIKFDG-------EEG
LDYGGVSREF---FQQL----SHEIFNPIY--------CLFKYASSDNY-----------
----------------TLQINPDS------------------------------------
--GINPEHLSYFKFIGRTVGLGVFHRRFLDAFFVG--AMYKMMLH---------------
--------KKIVLQDLES----------------------VDSEMYRS--------LCWM
LKN-----------------------------------------------DIT-DVI--
-FETFSVEVDH---------------------------FGK-KEVID-LKPD--------
GQNIE-------------------------------------------------------
------------------------------VTNENKK-EFVEL-KTEWIISKP-IEQQFK
-AFMDG---FNELIPE--DLVQ-VFDER-ELELLIGG-----------------------
----LSDID-----------IDDW----KRHTDYRG-YQES-------------------
------------------------DQVI-EWLWKCIA--------------------EWE
SDQRARLLQF-------ATGTSRIP------------VNG-----FK-DLQGSD--GPRR
FTVEKAGDP-------------------------DQLPKSHTCF-NR-IDLPPY-KDYES
LRKKLT-MAVEE-TVG-
>Brettanomyces_custersianus_MDVK01000008.1 .
IKVQRNSL-MEDAYRET--------------MRQTPEDLKK-RLMIKFEG-------EEG
LDYGGVSREF---FQQL----SHEIFNPVY--------CLFKYASSDNY-----------
----------------TLQINPES------------------------------------
--GINPEHLSYFKFIGRVVGLGVFHRRFLDAFFVG--AMYKMMLH---------------
--------KKIVLEDLES----------------------VDAEMYRS--------LCWM
LKN-----------------------------------------------DID-GII--
-FETFSVEVDH---------------------------FGK-KEVID-LKPN--------
GRNID-------------------------------------------------------
------------------------------VTNENKK-DFVQL-KTEWLISKP-VEEQFK
-AFMDG---FNELIPE--DLVQ-VFDER-ELELLIGG-----------------------
----LTDID-----------IEDW----RKHTDYRG-YQES-------------------
------------------------DQVI-QWFWKCIA--------------------EWE
GDQRARLLQF-------TTGTSRIP------------VNG-----FK-DLQGSD--GPRR
FTIEKAGDP-------------------------DQLPKSHTCF-NR-IDLPPY-KDYES
LKKKLT-MAVEE-TVG-
>Brettanomyces_naardenensis_MDSA01000004.1 .
IKVRRENL-MEDSYREV--------------MRQTPEDLKK-RLMIKFDG-------EEG
LDYGGVSREF---FQQL----SHEMFNPVY--------CLFKYASSDNY-----------
----------------TLQINPDS------------------------------------
--GVNPEHLSYFKFIGRTVGLGVFHRRFLDAFFVG--AMYKMMLH---------------
--------KKIVLQDLES----------------------VDSEMYKS--------LCWM
FEN-----------------------------------------------DIT-GII--
-FETFSVEVDH---------------------------FGE-KKTID-LKPN--------
GREIE-------------------------------------------------------
------------------------------VTNENKK-EFVEL-KTEWIISRP-IEQQFK
-SFMEG---FNELIPE--ELVQ-VFDEK-ELELLIGG-----------------------
----LSDID-----------VEDW----KKHTDYRG-YQES-------------------
------------------------DEVI-QWFWKCVA--------------------EWE
SDQRARLLQF-------TTGTSRIP------------VNG-----FK-DLQGSD--GPRR
FTIEKAGDP-------------------------DQLPKSHTCF-NR-VDLPPY-TDYES
LKKKLT-MAVEE-TVG-
>Candida_succiphila_BCGL01000003.1 .
IKVRRNNL-MEDSFRDI--------------MRQTPEDLKK-RLMIKFEG-------EEG
LDYGGVSREF---FQQL----SHEMFNPFY--------CLFQYATSDNY-----------
----------------TLQINPNS------------------------------------
```

--GVNPEHLNYFKFIGRTVGLGVFHRRFLDAFFVG--AMYKMMLH---------------
--------KKIVLQDLES----------------------VDAEMYRS--------LCWM
LEN--DID-GII--
-FETFSVEYER---------------------------FGE-TVIFD-LKEN--------
GRNIE---
------------------------------VTNDNKK-EFAEL-KTEWMISKP-VEQQFK
-AFMDG---FNELIPQ--ELVS-VFDER-ELELLIGG-----------------------
----LADID-----------IEDW----KKHTDYRG-YQES-------------------
------------------------DEVI-QWFWKCVS--------------------EWE
SEQRARLLQF-------TTGTSRIP------------VNG-----FK-DLQGSD--GPRR
FTIEKAGES-------------------------DQLPKSHTCF-NR-VDLPPY-KDYES
LRKKLT-IAVEE-TIG-
>Candida_arabinofermentans_LWUO01000002.1 .
IKVRRDHL-MEDAFRDV--------------MRQTPEDLKK-RLMIKFEG-------EEG
LDYGGVSREF---FQQL----SHEMFNPIY--------CLFQYATSDNY-----------
----------------TLQINPSS------------------------------------
--GVNSEHLNYFKFIGRTVGLGVFHRRFLDAFFVG--AMYKMMLH---------------
--------KKIVLQDLES----------------------VDVELYRS--------LCWM
LDN--DIT-DII--
-YETFSIEEDR---------------------------FGE-KVVID-LKPD--------
GRNID---
------------------------------VTNENKR-EYVEL-KTEWIISKP-VEAQFK
-AFMDG---FNEMIPQ--ELVS-VFDER-ELELLIGG-----------------------
----LAAID-----------IEDW----KKHTDYRG-YQES-------------------
------------------------DEVI-QWFWKCIS--------------------EWE
GEQRARLLQF-------TTGTSRIP------------VNG-----FK-DLQGSD--GPRR
FTIEKAGES-------------------------DQLPKSHTCF-NR-VDLPPY-KDYES
LKKKLT-IAVEE-TIG-
>Ogataea_methanolica_IAAJ01004670.1 .
IKVRRDHL-MEDAFRDV--------------MRQTPEDLKK-RLMIKFEG-------EEG
LDYGGVSREF---FQQL----SHEMFNPIY--------CLFQYATSDNY-----------
----------------TLQINPSS------------------------------------
--GVNSEHLNYFKFIGRTVGLGVFHRRFLDAFFVG--AMYKMMLH---------------
--------KKIVLQDLES----------------------VDVELYRS--------LCWM
LDN--DIT-DII--
-YESFCIEEDR---------------------------FGE-KVVID-LIPG--------
GRDIE---
------------------------------VTNENKR-EYVEL-KTEWIISKP-VEAQFK
-AFMDG---FNEMIPQ--ELVS-VFDER-ELELLIGG-----------------------
----LADID-----------IDDW----KKHTDYRG-YQES-------------------
------------------------DEVI-QWFWRCVA--------------------EWE
GEQKARLLQF-------TTGTSRIP------------VNG-----FK-DLQGSD--GPRR
FTIEKAGES-------------------------DQLPKSHTCF-NR-VDLPPY-KDYES
LKKKLT-IAVEE-TIGF
>Candida_boidinii_MSSB01000260.1 .
IKVRRDRL-MEDAFRDV--------------MRQTPEDLKK-RLMIKFEG-------EEG
LDYGGVSREF---FQQL----SHEMFNPFY--------CLFQYASSDNY-----------
----------------TLQINPNS------------------------------------
--NVNPEHLNYFKFIGRTVGLGVFHRRFLDAFFVG--AMYKMMLH---------------
--------KKIVLQDLES----------------------VDSEMYKS--------LCWM
LEN--DID-GII--
-FETFSIEDDK---------------------------FGE-KVVID-LKPN--------
GRDIE---
------------------------------VTNENKR-EYVEL-KTEWMISKP-VEQQFK
-AFMDG---FNELIPQ--ELVS-VFDER-ELELLIGG-----------------------
----LADID-----------IEDW----KKHTDYRG-YQES-------------------
------------------------DEVI-QWFWKCVS--------------------EWE
SEQRARLLQF-------TTGTSRIP------------VNG-----FK-DLQGSD--GPRR

FTIEKAGES-------------------------DQLPKSHTCF-NR-VDLPPY-KDYES
LKKKLT-IAVEE-TIG-
>Ogataea_parapolymorpha_XM_014077788.1 .
IKVRRDHL-MEDAFRDI--------------MRQTPEDLKK-RLMIKFEG-------EEG
LDYGGVSREF---FQQL----SHEMFNPFY--------CLFQYASSDNY-----------
----------------TLQINPNS------------------------------------
--GVNPENLTYFKFIGRTVGLGVFHRRFLDAFFVG--AMYKMMLH---------------
--------KKIVLQDLES----------------------VDAEMYKS--------LCWM
LEN---DIT-DVI--
-YETFSIEEDR---------------------------FGE-KVIID-LKPN--------
GRNIE---
------------------------------VTNENKR-EYVEL-KTEWIISKP-VEAQFK
-AFMDG---FNELIPQ--ELVS-VFDER-ELELLIGG-----------------------
----LADID-----------IEDW----KKHTDYRG-YQES-------------------
------------------------DEVI-QWFWKCVS--------------------EWE
GEQRARLLQF-------TTGTSRIP------------VNG-----FK-DLQGSD--GPRR
FTIEKAGEP-------------------------DQLPKSHTCF-NR-VDLPPY-KDYES
LKKKLT-IAVEE-TIGF
>Ogataea_polymorpha_XM_018353826.1 .
IKVRRDHL-MEDAFRDI--------------MRQTPEDLKK-RLMIKFEG-------EEG
LDYGGVSREF---FQQL----SHEMFNPFY--------CLFQYASSDNY-----------
----------------TLQINPNS------------------------------------
--GVNPEHLTYFKFIGRTVGLGVFHRRFLDAFFVG--AMYKMMLH---------------
--------KKIVLQDLES----------------------VDAEMYKS--------LCWM
LEN---DIT-DVI--
-YETFSIEEDR---------------------------FGE-KVIID-LKPN--------
GRNIE---
------------------------------VTNENKR-EYVEL-KTEWIISKP-VEAQFK
-AFMDG---FNELIPQ--ELVS-VFDER-ELELLIGG-----------------------
----LADID-----------IEDW----KKHTDYRG-YQES-------------------
------------------------DEVI-QWFWKCVS--------------------EWE
GEQRARLLQF-------TTGTSRIP------------VNG-----FK-DLQGSD--GPRR
FTIEKAGEP-------------------------DQLPKSHTCF-NR-VDLPPY-KDYES
LKKKLT-IAVEE-TIGF
>Ambrosiozyma_monospora_BCIP01000001.1 .
IKVRRDHL-MEDAFRDI--------------MRQTPEDLKK-RLMIKFEG-------EEG
LDYGGVSREF---FQQL----SHEMFNPFY--------CLFQYASSDNY-----------
----------------TLQINPNS------------------------------------
--DVNPEHLNYFKFIGRTVGLGVFHRRFLDAFFVG--AMYKMMLH---------------
--------KKIVLQDLES----------------------VDAELYKS--------LCWM
LDN---DIT-DII--
-YESFSVEEDR---------------------------FGE-KVTID-LKPN--------
GRDIE---
------------------------------VTNENKR-EYVEL-KTEWIISKP-VEQQFK
-AFMDG---FNELIPQ--ELVS-VFDER-ELELLIGG-----------------------
----LADID-----------IEDW----KKHTDYRG-YQES-------------------
------------------------DEVI-QWFWKCVS--------------------EWE
GEQRARLLQF-------TTGTSRIP------------VNG-----FK-DLQGSD--GPRR
FTIEKAGEP-------------------------DQLPKSHTCF-NR-VDLPPY-RDYES
LKKKLT-IAVEE-TVG-
>Ambrosiozyma_kashinagacola_IAAG01012186.1 .
IKVRRDHL-MEDAFRDI--------------MRQTPEDLKK-RLMIKFEG-------EEG
LDYGGVSREF---FQQL----SHEMFNPFY--------CLFQYASSDNY-----------
----------------TLQINPNS------------------------------------
--GVNPEHLNYFKFIGRTVGLGVFHRRFLDAFFVG--AMYKMMLH---------------
--------KKIVLQDLES----------------------VDAELYKS--------MCWM
LDN---DIT-DII--
-YESFSVEEDR---------------------------FGE-KVVID-LKPN--------

GRDIE---
------------------------------VTNENKR-EYVEL-KTEWIISKP-VEQQFK
-AFMDG---FNELIPQ--ELVS-VFDER-ELELLIGG-----------------------
----LADID-----------IDDW----KKHTDYRG-YQES-------------------
------------------------DEVI-QWFWRCVS--------------------EWE
GEQRARLLQF-------TTGTSRIP------------VNG-----FK-DLQGSD--GPRR
FTIEKAGEA-------------------------DQLPKSHTCF-NR-VDLPPY-KDYES
LKKKLT-IAVEE-TIGF
>Ascoidea_rubescens_XM_020193493.1 .
IKVRRNNI-FEDAYQEI--------------MRQTSDDLKK-RLMIKFDG-------EEG
LDYGGVSREF---FFLL----SHEMFNPSY--------GLFEYSAHDNY-----------
----------------TLQINPHS------------------------------------
--NINPEHLNYFKFIGRVVGLCVFHRRFLDTFFVG--ALYKMMLR---------------
--------RKVVLQDMEG----------------------VDAEYYNS--------LKWI
LDN--DVT-DLG--
--FTFSIEDER---------------------------FGE-VKTIE-LKPD--------
GDNVD---
------------------------------VGESNKK-EYVEL-LTEFKIYKR-VEDQFK
-AFVDG---FNELIPP--DLVN-VFDER-ELELLIGG-----------------------
----ISEID-----------VEDW----KKHTDYRG-YQES-------------------
------------------------DQVI-QWFWQTIK--------------------EWD
NEQRARLLQF-------TTGTSRIP------------VNG-----FK-DLQGSD--GPRR
FTIEKAGEP-------------------------NQLPKSHTCF-NR-VDLPPY-ADIDA
LKQKLS-LAVEE-TVGF
>Ascoidea_asiatica_BCKQ01000023.1 .
IKVKRSNI-FEDSYQEI--------------MRQTSEDLKK-RLMIKFDG-------EEG
LDYGGVSREF---FFLL----SHEMFNPSY--------GLFEYSAHDNY-----------
----------------TLQINPHS------------------------------------
--SINPEHLNYFKFIGRVVGLCVFHRRFLDAFFIG--ALYKMMLR---------------
--------RKVVLQDMEG----------------------VDAEYYNS--------LKWI
VEN--DVA-DLG--
--FTFSIEDER---------------------------FGQ-VTTIE-LKPD--------
GDNID---
------------------------------VTEDNKK-EYVEL-LTEFKIYKR-VEEQFK
-AFVDG---FNELIPA--DLVN-VFDER-ELELLIGG-----------------------
----ISEID-----------VEDW----KKHTDYRG-YQES-------------------
------------------------DEVI-QWFWKTIK--------------------EWD
NEQRARLLQF-------TTGTSRIP------------VNG-----FK-DLQGSD--GPRR
FTIEKAGES-------------------------NQLPKSHTCF-NR-VDLPPY-ADAES
LAKKLS-LAVEE-TVG-
>Candida_maltosa_AOGT01002063.1 .
IKIRRDHI-FEDSYQEI--------------MRQTPEDLKK-RLMIKFDG-------EEG
LDYGGVSREF---FFLL----SHDMFNPFY--------CLFEYSSHDNY-----------
----------------TLQINPNS------------------------------------
--GINPEHLNYFKFIGRVVGLGVFHRRFLDAFFVG--ALYKMMLH---------------
--------KKVVLQDMEG----------------------VDSEFYRS--------LKWI
LDN--DIT-GIL--
-DLTFSAEEER---------------------------FGE-VVEVD-LKPN--------
GREIE---
------------------------------VTEDNKA-EYVQM-VTEWRISKR-VEDQFQ
-AFISG---FNELIPH--DLIN-VFDER-ELELLIGG-----------------------
----LASID-----------VLDW----KANTDYRG-YSES-------------------
------------------------DVTV-QLFWKVVE--------------------EWD
LEQKARLLQF-------TTGTSRIP------------VNG-----FK-DLQGSD--GPRR
FTIEKAGEP-------------------------NQLPKSHTCF-NR-VDLPPY-TDYES
LKQKLT-LACEE-TVG-
>Komagataella_pastoris_FBUB01000013.1 .
IKVRRSHI-FEDSYQEI--------------MRQSPEDLKK-RLMIKFDG-------EEG

```
LDYGGVSREF---FFLL----SHDMFNPFY--------CLFEYSTHDNY-----------
----------------TLQINPNS------------------------------------
--GINPEHLNYFKFIGRVVGLGIFHRRFLDAFFVG--ALYKMILH---------------
--------KKVILQDMEG----------------------VDAEFYRS--------LKWI
LEN---------------------------------------------DIT-DVL--
-DLTFSAEDER---------------------------FGE-IVTVD-LKEG--------
GRDIE-------------------------------------------------------
------------------------------VTEENKM-EYVEL-ITEWKIHRR-VEQQFK
-AFMDG---FNELIPQ--ELIN-VFDER-ELELLIGG-----------------------
----IADVD-----------VEDW----KKHTDYRG-YQES-------------------
------------------------DEVI-QWFWKCVT--------------------EWD
KEQKARLLQF-------TTGTSRIP------------VNG-----FK-DLQGSD--GPRR
FTIEKAGES-------------------------NQLPKSHTCF-NR-VDLPPY-KDYGS
LKQKLT-LAVEE-TVG-
>Komagataella_phaffii_CP014716.1_a.
IKVRRSHI-FEDSYQEI--------------MRQSPEDLKK-RLMIKFDG-------EEG
LDYGGVSREF---FFLL----SHDMFNPFY--------CLFEYSTHDNY-----------
----------------TLQINPNS------------------------------------
--GINPEHLNYFKFIGRVVGLGIFHRRFLDAFFVG--ALYKMILH---------------
--------KKVILQDMEG----------------------VDAEFYRS--------LKWI
LEN---------------------------------------------DIT-DVL--
-DLTFSAEDER---------------------------FGE-IVTVD-LKEG--------
GRDIE-------------------------------------------------------
------------------------------VTEENKM-EYVEL-ITEWKIHRR-VEQQFK
-AFMDG---FNELIPQ--ELIN-VFDER-ELELLIGG-----------------------
----IADVD-----------VEDW----KKHTDYRG-YQES-------------------
------------------------DEVI-QWFWKCVT--------------------EWD
KEQKARLLQF-------TTGTSRIP------------VNG-----FK-DLQGSD--GPRR
FTIEKAGES-------------------------NQLPKSHTCF-NR-VDLPPY-KDYGS
LKQKLT-LAVEE-TVGF
>Nakazawaea_peltata_BCGQ01000001.1 .
IKVRRSHI-FEDSYQEI--------------MRQTPEDLKK-RLMIKFDG-------EEG
LDYGGVSREF---FFLL----SHDMFNPFY--------CLFEYSSHDNY-----------
----------------TLQINPNS------------------------------------
--GVNPEHLNYFKFIGRVVGLGVFHRRFLDLFFVG--ALYKMMLR---------------
--------KKVVLQDMEG----------------------VDAEFYRS--------LQWI
LDN---------------------------------------------DIT-DIL--
-ELTFSAEDER---------------------------FGE-IVTID-LVEN--------
GRNIE-------------------------------------------------------
------------------------------VTEENKR-EYVEK-ITEWKIYER-VQEQFK
-LFMDG---FNELIPQ--DLVN-VFDER-ELELLIGG-----------------------
----LLEID-----------VEDW----KKHTDYRG-YQEN-------------------
------------------------DEVI-QWFWRCIK--------------------EWD
SEQKARLLQF-------TTGTSRIP------------VNG-----FK-DLQGSD--GPRR
FTIEKAGES-------------------------NQLPKSHTCF-NR-VDLPPY-KDYES
LKQKLT-LAVEE-TVG-
>Pachysolen_tannophilus_CAHV01000033.1 .
IKVRRTHI-FEDSYQEI--------------MRQTPEDLKK-RLMIKFDG-------EEG
LDYGGVSREF---FFLL----SHDMFNPFY--------CLFEYSSHDNY-----------
----------------TLQINPNS------------------------------------
--GINPEHLNYFKFIGRVVGLGVFHRRFLDAFFVG--ALYKMMLR---------------
--------KKVVLQDMEG----------------------VDAEFYRS--------LKWI
IEN---------------------------------------------DIT-DVL--
-DLTFSAEDER---------------------------FGE-IVTVD-LKEN--------
GRNIE-------------------------------------------------------
------------------------------VTEENKR-AYVEL-ITEWKIFKR-VEQQFK
-AFIDG---FNELIPQ--DLVN-VFDER-ELELLIGG-----------------------
----LAEID-----------VEDW----KKHTDYRG-YQES-------------------
```

```
------------------------DEVI-QWFWRCIK--------------------EWD
SEQKARLLQF-------TTGTSRIP------------VNG-----FK-DLQGSD--GPRR
FTIEKAGEP-------------------------NQLPKSHTCF-NR-VDLPPY-KDYES
LKQKLT-LAVEE-TVG-
>Priceomyces_haplophilus_BCIF01000002.1 .
IKVRRDFI-FEDSYQEI--------------MRQTPEDLKK-RLMIKFDA-------EEG
LDYGGVSREF---FFLL----SHDMFNPFY--------CLFEYSSHDNY-----------
----------------TLQINANS------------------------------------
--GINPEHLNYFKFIGRVVGLGVFHRRFLDAFFVG--ALYKMMLN---------------
--------KKVVLQDMEG----------------------VDAEFYRS--------LKWI
LDN------------------------------------------------DIT-DVL--
-ELTFSTEDER---------------------------FGE-IVEIE-LKPG--------
GKDIE-------------------------------------------------------
------------------------------VTEENKH-EYVEL-ISEWKISKR-VEEQFQ
-AFAAG---FNELIPQ--DLVN-VFDER-ELELLIGG-----------------------
----LAEID-----------VDDW----AKHTDYRG-YQES-------------------
------------------------DQVV-EWFWQCIK--------------------EWD
SEQKARLLQF-------TTGTSRIP------------VNG-----FK-DLQGSD--GPRR
FTIEKAGGP-------------------------DQLPKSHTCF-NR-VDLPPY-TSYES
LKQKLT-LAVEE-TVG-
>Babjeviella_inositovora_XM_019127592.1 .
IKVRRNHI-FEDSYQEI--------------MRQTPEDLKK-RLMIKFDG-------EEG
LDYGGVSREY---FFLL----SHDMFNPFY--------CLFEYSSHDNY-----------
----------------TLQINTNS------------------------------------
--GINPEHLNYFKFIGRVVGLGVFHRRFLDAFFVG--ALYKMMLH---------------
--------KKVVLQDMEG----------------------VDSEFYRS--------LKWI
LDN------------------------------------------------DIT-DVL--
-DLTFSADDER---------------------------FGE-VLTVD-LKAG--------
GRDIE-------------------------------------------------------
------------------------------VTEENKR-EYVEL-ITEWKISRR-VDEQFR
-AFMDG---FNELIPE--ELVN-VFDER-ELELLIGG-----------------------
----LAEID-----------VEDW----KKHTDYRG-YQET-------------------
------------------------DQVI-QWFWQCIK--------------------DWD
SEQKARLLQF-------TTGTSRIP------------VNG-----FK-DLQGSD--GPRR
FTIEKAGEP-------------------------GQLPKSHTCF-NR-VDLPPY-TDYET
MKQKLT-LAVEE-TVGF
>Metschnikowia_bicuspidata_XM_018857478.1 .
IKVRRDHI-FEDSYQEI--------------MRQTPDDLKK-RLMIKFDG-------EEG
LDYGGVSREF---FFLL----SHDMFNPFY--------CLFEYSSHDNY-----------
----------------TLQINPNS------------------------------------
--GINPEHLNYFKFIGRVVGLGVFHRRFLDAFFVG--ALYKMMLH---------------
--------KKVVLQDMEG----------------------VDAEFYRS--------LQWI
IDN------------------------------------------------DIT-DVL--
-DLTFSAEDDQ---------------------------FGQ-VVEVD-LKAG--------
GRDIE-------------------------------------------------------
------------------------------VTEDNKH-EYVEL-ISEWKISRR-IQEQFK
-AFIDG---FNELIPQ--ELVN-VFDER-ELELLIGG-----------------------
----LAEID-----------VDDW----KKHTDYRG-YQES-------------------
------------------------DQVV-QWFWRCIK--------------------EWD
SEQKARLLQF-------TTGTSRIP------------VNG-----FK-DLQGSD--GPRR
FTIEKAGEP-------------------------QQLPKSHTCF-NR-VDLPPY-TNYEA
LKQKLT-LAVEE-TVGF
>Metschnikowia_australis_MVNQ01000026.1 .
IKVRRDHI-FEDSYQEI--------------MRQTPDDLKK-RLMIKFDG-------EEG
LDYGGVSREF---FFLL----SHDMFNPFY--------CLFEYSSHDNY-----------
----------------TLQINPNS------------------------------------
--AINPEHLNYFKFIGRVVGLGVFHRRFLDAFFVG--ALYKMMLH---------------
--------KKVVLQDMEG----------------------VDAEFYRS--------LQWI
```

IDN--DIT-DVL--
-DLTFSAEDDQ---------------------------FGQ-VVEVD-LKDG--------
GRDIE---
------------------------------VTEDNKR-EYVEL-VCEWKISRR-IQEQFK
-AFIDG---FNELIPQ--ELVN-VFDER-ELELLIGG-----------------------
----LAEID-----------VDDW----KKHTDYRG-YQES-------------------
------------------------DQVV-QWFWRCIK--------------------EWD
SEQKARLLQF-------TTGTSRIP------------VNG-----FK-DLQGSD--GPRR
FTIEKAGEP-------------------------QQLPKSHTCF-NR-VDLPPY-TNYEA
LKQKLT-LAVEE-TVG-
>Metschnikowia_fructicola_ANFW02000045.1 .
IKVRRDHI-FEDSYQEI--------------MRQTPDDLKK-RLMIKFDG-------EEG
LDYGGVSREF---FFLL----SHDMFNPFY--------CLFEYSSHDNY-----------
----------------TLQINPNS------------------------------------
--GINPEHLNYFKFIGRVVGLGVFHRRFLDAFFVG--ALYKMMLH---------------
--------KKVVLQDMEG----------------------VDAEFYRS--------LQWI
IDN--DIT-DVL--
-ELTFSAEDDK---------------------------FGQ-VVEVD-LKPG--------
GRDIE---
------------------------------VTQENKH-EYVEL-ICEWKIHRR-IEEQFK
-AFIDG---FNELIPQ--ELVN-VFDER-ELELLIGG-----------------------
----LADID-----------VDDW----KKHTDYRG-YQES-------------------
------------------------DQVV-QWFWRCIK--------------------EWD
SEQKARLLQF-------TTGTSRIP------------VNG-----FK-DLQGSD--GPRR
FTIEKAGEP-------------------------QQLPKSHTCF-NR-VDLPPY-TNYEA
LKQKLT-LAVEE-TVG-
>Candida_auris_XM_018310771.1 .
IKVRRDHI-FEDSYQEI--------------MRQTPEDLKK-RLMIKFDG-------EEG
LDYGGVSREF---FFLL----SHDMFNPFY--------CLFEYSSHDNY-----------
----------------TLQINPNS------------------------------------
--GINPEHLNYFKFIGRVVGLGVFHRRFLDAFFVG--ALYKMMLR---------------
--------KKVCLQDMEG----------------------VDAEFFRS--------LQWI
CDN--DIT-NVL--
-DLTFSAEDDK---------------------------FGE-IVEVD-LKPN--------
GRNIE---
------------------------------VTEENKH-EYVEL-ISEWKIHKR-IELQFQ
-AFIDG---FNELIPH--ELVN-VFDER-ELELLIGG-----------------------
----LAEID-----------VEDW----KKHTDYRG-YQET-------------------
------------------------DQVI-QWFWQCIK--------------------EWD
SEQKARLLQF-------TTGTSRIP------------VNG-----FK-DLQGSD--GPRR
FTIEKAGEP-------------------------NQLPKSHTCF-NR-VDLPPY-TSYES
MKQKLT-LAVEE-TVGF
>Clavispora_lusitaniae_XM_002616928.1 .
IKVRRDHI-FEDSYQEI--------------MRQTPDDLKK-RLMIKFDG-------EEG
LDYGGVSREF---FFLL----SHDMFNPFY--------CLFEYSSHDNY-----------
----------------TLQINPNS------------------------------------
--GINPEHLNYFKFIGRVVGLGVFHRRFLDAFFVG--ALYKMMLR---------------
--------KKVVLQDMEG----------------------VDAEFYRS--------LQWI
IDN--DIT-GVL--
-DLTFSAEDDK---------------------------FGQ-IVEVD-LKPG--------
GRDIE---
------------------------------VTEENKH-EYVEL-ICEWKIYKR-IEEQFK
-AFIDG---FNELIPQ--ELVN-VFDER-ELELLIGG-----------------------
----LAEID-----------VEDW----KKHTDYRG-YQEN-------------------
------------------------DQVI-QWFWKCIK--------------------EWD
SEQKARLLQF-------TTGTSRIP------------VNG-----FK-DLQGSD--GPRR
FTIEKAGEP-------------------------NQLPKSHTCF-NR-VDLPPY-TSYES
LKQKLT-LAVEE-TVGF

>Candida_intermedia_LT635767.1 .
IKVRRDHI-FEDSYQEI--------------MRQTPDDLKK-RLMIKFDG-------EEG
LDYGGVSREF---FFLL----SHDMFNPFY--------CLFEYSSHDNY-----------
----------------TLQINPNS------------------------------------
--GINPEHLNYFKFIGRVVGLGVFHRRFLDAFFVG--ALYKMMLR---------------
--------KKVVLQDMEG----------------------VDAEFYRS--------LQWM
VDN---DIT-DVL--
-DLTFSAEDDK---------------------------FGE-IVEFD-LKPN--------
GRNIE---
------------------------------VTEENKH-EYVEL-ICEWKIYKR-IEEQFK
-AFIDG---FNELIPQ--ELVN-VFDER-ELELLIGG-----------------------
----LAEID-----------VEDW----KKHTDYRG-YQEN-------------------
------------------------DQVI-QWFWKCIK--------------------EWD
SEQKARLLQF-------TTGTSRIP------------VNG-----FK-DLQGSD--GPRR
FTIEKAGES-------------------------NQLPKSHTCF-NR-VDLPPY-TSYES
LKQKLT-LAVEE-TVGF
>Pichia_sorbitophila_FO082051.1 .
IKVRRDHI-FEDSYQEI--------------MRQTPEDLKK-RLMIKFDG-------EEG
LDYGGVSREF---FFLL----SHDMFNPFY--------CLFEYSSHDNY-----------
----------------TLQINPHS------------------------------------
--GINPEHLNYFKFIGRVVGLGVFHRRFLDAFFIG--ALYKMMLR---------------
--------KKVVLQDMEG----------------------VDAEFYRS--------LKWI
CDN---DIT-DVL--
-DLTFTAEDDR---------------------------FGE-IVEVD-LKPG--------
GRDIE---
------------------------------VTEENKH-EYVEL-ISDWKIVKR-VEEQFK
-AFMDG---FNELIPQ--ELVN-VFDER-ELELLIGG-----------------------
----LAEID-----------VEDW----KKHTDYRG-YQES-------------------
------------------------DQVI-QWFWQCIK--------------------EWD
SEQKARLLQF-------TTGTSRIP------------VNG-----FK-DLQGSD--GPRR
FTIEKAGEP-------------------------NQLPKSHTCF-NR-VDLPPY-SNYES
LKQKLT-LAVEE-TVGF
>Candida_orthopsilosis_XM_021355946.1 .
IKVRRDHI-FEDSYQEI--------------MRQTPEDLKK-RLMIKFDG-------EEG
LDYGGVSREF---FFLL----SHDMFNPFY--------CLFEYSSHDNY-----------
----------------TLQINPNS------------------------------------
--SINPEHLNYFKFIGRVVGLGVFHRRFLDAFFVG--ALYKMMLH---------------
--------KKVVLQDMEG----------------------VDAEFYRS--------LKWI
LDN---DIT-DIL--
-DLTFSTEEEI---------------------------FGE-RVEVD-LKPD--------
GKNIE---
------------------------------VTEENKH-EYVEL-ITEWRISKR-VEEQFK
-AFIDG---FNELIPQ--ELVN-VFDER-ELELLIGG-----------------------
----LAEID-----------TVDW----KKHTDYRG-YQEL-------------------
------------------------DQVI-QWFWKCIN--------------------EWD
SEQKARLLQF-------TTGTSRIP------------VNG-----FK-DLQGSD--GPRR
FTIEKAGEP-------------------------NQLPKSHTCF-NR-VDLPPY-ADYES
LKQKLS-LAVEE-TVG-
>Candida_orthopsilosis_XM_003868858.1 .
IKVRRDHI-FEDSYQEI--------------MRQTPEDLKK-RLMIKFDG-------EEG
LDYGGVSREF---FFLL----SHDMFNPFY--------CLFEYSSHDNY-----------
----------------TLQINPNS------------------------------------
--SINPEHLNYFKFIGRVVGLGVFHRRFLDAFFVG--ALYKMMLH---------------
--------KKVVLQDMEG----------------------VDAEFYRS--------LKWI
LDN---DIT-DIL--
-DLTFSTEEEI---------------------------FGE-RVEVD-LKPD--------
GKNIE---
------------------------------VTEENKH-EYVEL-ITEWRISKR-VEEQFK

-AFIDG---FNELIPQ--ELVN-VFDER-ELELLIGG-----------------------
----LAEID-----------TVDW----KKHTDYRG-YQEL-------------------
------------------------DQVI-QWFWKCIN--------------------EWD
SEQKARLLQF-------TTGTSRIP------------VNG-----FK-DLQGSD--GPRR
FTIEKAGEP-------------------------NQLPKSHTCF-NR-VDLPPY-ADYES
LKQKLS-LAVEE-TVGF
>Candida_parapsilosis_HE605208.1 .
IKVRRDHI-FEDSYQEI--------------MRQTPEDLKK-RLMIKFDG-------EEG
LDYGGVSREF---FFLL----SHDMFNPFY--------CLFEYSSHDNY-----------
----------------TLQINPNS------------------------------------
--SINPEHLNYFKFIGRVVGLGVFHRRFLDAFFVG--ALYKMMLH---------------
--------KKVVLQDMEG----------------------VDAEFYRS--------LKWI
LDN--DIT-DIL--
-DLTFSTEEEI---------------------------FGE-RVEVD-LKPD--------
GKNIE---
------------------------------VTEENKH-EYVEL-ITEWRISKR-VEEQFK
-AFIDG---FNELIPQ--ELVN-VFDER-ELELLIGG-----------------------
----LAEID-----------TADW----KKHTDYRG-YQEL-------------------
------------------------DQVI-QWFWKCIN--------------------EWD
SEQKARLLQF-------TTGTSRIP------------VNG-----FK-DLQGSD--GPRR
FTIEKAGEP-------------------------NQLPKSHTCF-NR-VDLPPY-ADYES
LKQKLS-LAVEE-TVGF
>Lodderomyces_elongisporus_XM_001525802.1 .
IKVRRDHI-FEDSYQEI--------------MRQTPEDLKK-RLMIKFDG-------EEG
LDYGGVSREF---FFLL----SHDMFNPFY--------CLFEYSSHDNY-----------
----------------TLQINPNS------------------------------------
--GINPEHLNYFKFIGRVVGLGVFHRRFLDAFFVG--ALYKMMLH---------------
--------KKPMLQDMEG----------------------VDAEFYRS--------LKWI
LDN--DIT-DIL--
-DLTFSTEEEK---------------------------FGE-RVEVD-LKPN--------
GKDIE---
------------------------------VTEDNKQ-EYVEL-ITEWRISKR-VEEQFR
-AFIDG---FNELIPQ--DLVN-VFDER-ELELLIGG-----------------------
----LAEID-----------VEDW----KKHTDYRG-YQES-------------------
------------------------DQVI-QWFWKCIN--------------------EWD
SEQKARLLQF-------TTGTSRIP------------VNG-----FK-DLQGSD--GPRR
FTIEKAGEP-------------------------NQLPKSHTCF-NR-VDLPPY-ADYAS
LKQKLS-LAVEE-TVGF
>Candida_sojae_LMTL01000109.1 .
IKVRRDHI-FEDSYQEI--------------MRQTPEDLKK-RLMIKFDG-------EEG
LDYGGVSREF---FFLL----SHDMFNPFY--------CLFEYSSHDNY-----------
----------------TLQINPNS------------------------------------
--AINPEHLNYFKFIGRVVGLGVFHRRFLDAFFVG--ALYKMMLH---------------
--------KKVVLQDMEG----------------------VDAEFYRS--------LKWI
LDN--DIT-DIL--
-ELTFSAEEES---------------------------FGE-IVEVD-LKPG--------
GRDIE---
------------------------------VTEENKH-EYVEL-ITEWRISKR-VEEQFK
-AFIDG---FNELIPQ--ELVN-VFDER-ELELLIGG-----------------------
----LADID-----------CDDW----KKHTDYRG-YQES-------------------
------------------------DQVI-QWFWKCIS--------------------EWD
SEQKARLLQF-------TTGTSRIP------------VNG-----FK-DLQGSD--GPRR
FTIEKAGEP-------------------------NQLPKSHTCF-NR-VDLPPY-TDYET
LKLKLT-FAIEE-TLG-
>Candida_tropicalis_XM_002547491.1 .
IKVRRDHI-FEDSYQEI--------------MRQTPEDLKK-RLMIKFDG-------EEG
LDYGGVSREF---FFLL----SHDMFNPFY--------CLFEYSSHDNY-----------
----------------TLQINPNS------------------------------------

--GINPEHLNYFKFIGRVVGLGVFHRRFLDAFFVG--ALYKMMLH---------------
--------KKVVLQDMEG----------------------VDAEFYRS--------LKWI
LDN--DIT-DIL--
-ELTFSAEEES---------------------------FGE-IVEVD-LKPG--------
GRDIE---
------------------------------VTEENKH-EYVEL-ITEWRISKR-VEEQFK
-AFIDG---FNELIPQ--ELVN-VFDER-ELELLIGG-----------------------
----LADID-----------CEDW----KKHTDYRG-YQES-------------------
------------------------DQVI-QWFWKCIG--------------------EWD
SEQKARLLQF-------TTGTSRIP------------VNG-----FK-DLQGSD--GPRR
FTIEKAGEP-------------------------NQLPKSHTCF-NR-VDLPPY-TDYET
LKLKLT-FAIEE-TLGF
>Candida_dubliniensis_XM_002418692.1 .
IKVRRDHI-FEDSYQEI--------------MRQTPEDLKK-RLMIKFDG-------EEG
LDYGGVSREF---FFLL----SHDMFNPFY--------CLFEYSSHDNY-----------
----------------TLQINPNS------------------------------------
--GINPEHLNYFKFIGRVVGLGVFHRRFLDAFFVG--ALYKMMLH---------------
--------KKVVLQDMEG----------------------VDAEFYRS--------LKWI
LDN--DIT-DIL--
-DLTFSAEEES---------------------------FGE-IVEVD-LKPG--------
GRDIE---
------------------------------VTEENKH-EYVEL-ITEWRISKR-VEEQFK
-AFIDG---FNELIPQ--ELVN-VFDER-ELELLIGG-----------------------
----LAEID-----------CEDW----KKHTDYRG-YQEN-------------------
------------------------DQVI-QWFWKCVN--------------------EWD
SEQKARLLQF-------TTGTSRIP------------VNG-----FK-DLQGSD--GPRR
FTIEKAGEA-------------------------NQLPKSHTCF-NR-VDLPPY-TDYES
LKQKLT-LAVEE-TVGF
>Candida_albicans_XM_709377.2 .
IKVRRDHI-FEDSYQEI--------------MRQTPEDLKK-RLMIKFDG-------EEG
LDYGGVSREF---FFLL----SHDMFNPFY--------CLFEYSSHDNY-----------
----------------TLQINPNS------------------------------------
--GINPEHLNYFKFIGRVVGLGVFHRRFLDAFFVG--ALYKMMLH---------------
--------KKVVLQDMEG----------------------VDAEFYRS--------LKWI
LDN--DIT-GIL--
-DLTFSAEEES---------------------------FGE-IVEVD-LKPG--------
GRDIE---
------------------------------VTEENKH-EYVEL-ITEWRISKR-VEEQFK
-AFIDG---FNELIPQ--ELVN-VFDER-ELELLIGG-----------------------
----LAEID-----------CEDW----KKHTDYRG-YQEN-------------------
------------------------DQVI-QWFWKCIN--------------------EWD
SEQKARLLQF-------TTGTSRIP------------VNG-----FK-DLQGSD--GPRR
FTIEKAGEA-------------------------NQLPKSHTCF-NR-VDLPPY-TDYES
LKQKLT-LAVEE-TVGF
>Candida_aaseri_LKAN01000053.1 .
IKVRRDHI-FEDSYQEI--------------MRQTPEDLKK-RLMIKFDG-------EEG
LDYGGVSREF---FFLL----SHDMFNPFY--------CLFEYSSHDNY-----------
----------------TLQINPNS------------------------------------
--GINPEHLNYFKFIGRVVGLGVFHRRFLDAFFVG--ALYKMMLH---------------
--------KKVVLQDMEG----------------------VDAEFYRS--------LKWI
LDN--DIT-DVL--
-DLTFSAEDER---------------------------FGE-IVEID-LKPD--------
GRNIE---
------------------------------VTEENKH-EYVEL-ITEWRISKR-VEEQFK
-AFITG---FNELIPQ--ELVN-VFDER-ELELLIGG-----------------------
----LADID-----------VEDW----KKHTDYRG-YQES-------------------
------------------------DQVI-QWFWQCIK--------------------EWD
SEQKARLLQF-------TTGTSRIP------------VNG-----FK-DLQGSD--GPRR

FTIEKAGEA-------------------------NQLPKSHTCF-NR-VDLPPY-TDYES
LKQKLT-LAVEE-TVG-
>Candida_tenuis_XM_006685682.1 .
ITVRRDHI-FEDSYQEI--------------MRQTPEDLKK-RLMIKFDG-------EEG
LDYGGVSREF---FFLL----SHDMFNPFY--------CLFEYSSHDNY-----------
----------------TLQINPNS------------------------------------
--GINPEHLNYFKFIGRVVGLGVFHRRFLDAFFVG--ALYKMMLR---------------
--------KKVVLQDMEG----------------------VDAEFYRS--------LKWI
LDN--DIT-DVL--
-DLTFSAEDER---------------------------FGE-IVEVD-LKED--------
GRNIE---
------------------------------VTEENKH-EYVEF-ITEWKIYKR-VEEQFK
-AFISG---FNELIPQ--ELVN-VFDER-ELELLIGG-----------------------
----LAEID-----------VEDW----KKHTDYRG-YQES-------------------
------------------------DQVI-QWFWQCIK--------------------EWD
SEQKARLLQF-------TTGTSRIP------------VNG-----FK-DLQGSD--GPRR
FTIEKAGES-------------------------NQLPKSHTCF-NR-VDLPPY-TDYES
LKQKLT-LAVEE-TVGF
>Hyphopichia_burtonii_XM_020222920.1 .
IKVRRDHI-FEDSYQEI--------------MRQTPEDLKK-RLMIKFDG-------EEG
LDYGGVSREF---FFLL----SHDMFNPFY--------CLFEYSSHDNY-----------
----------------TLQINPNS------------------------------------
--GINPEHLNYFKFIGRVVGLGVFHRRFLDAFFVG--ALYKMMLH---------------
--------KKVVLQDMEG----------------------VDAEFYRS--------LKWI
CDN--DIT-DIL--
-DLTFSAEDDK---------------------------FGE-IVEVE-LKPG--------
GKDIE---
------------------------------VTEDNKH-EYVEL-ISEWKISKR-VEEQFK
-AFING---FNELIPQ--ELVN-VFDER-ELELLIGG-----------------------
----LAEID-----------IEDW----KKHTDYRG-YQEN-------------------
------------------------DQVI-QWFWKCIK--------------------EWD
SEQKARLLQF-------TTGTSRIP------------VNG-----FK-DLQGSD--GPRR
FTIEKAGEP-------------------------NQLPKSHTCF-NR-VDLPSY-TNYES
LKQKLT-LAVEE-TVGF
>Candida_homilentoma_BCGB01000008.1 .
IKVRRDHI-FEDSYQEI--------------MRQTPEDLKK-RLMIKFDG-------EEG
LDYGGVSREF---FFLL----SHDMFNPFY--------CLFEYSSHDNY-----------
----------------TLQINPNS------------------------------------
--GINPEHLNYFKFIGRVVGLGVFHRRFLDAFFVG--ALYKMMLH---------------
--------KKVVLQDMEG----------------------VDAEFYRS--------LRWI
LDN--DIT-DVL--
-DLTFSAEDDK---------------------------FGE-IVEVD-LKPG--------
GRDIE---
------------------------------VTEENKH-EYVEL-ISEWKISKR-VEEQFK
-AFMTG---FNELIPQ--ELVN-VFDER-ELELLIGG-----------------------
----LAEID-----------IEDW----KKHTDYRG-YQEN-------------------
------------------------DQVI-QWFWRCIK--------------------EWD
SEQKARLLQF-------TTGTSRIP------------VNG-----FK-DLQGSD--GPRR
FTIEKAGES-------------------------NQLPKSHTCF-NR-VDLPPY-TNYES
LKQKLT-LAVEE-TVG-
>Debaryomyces_fabryi_XM_015610677.1 .
IKVRRDHI-FEDSYQEI--------------MRQTPEDLKK-RLMIKFDG-------EEG
LDYGGVSREF---FFLL----SHDMFNPFY--------CLFEYSSHDNY-----------
----------------TLQINPNS------------------------------------
--GINPEHLNYFKFIGRVVGLGVFHRRFLDAFFVG--ALYKMMLR---------------
--------KKVVLQDMEG----------------------VDAEFYRS--------LKWI
CDN--DIT-DIL--
-DLTFSAEDER---------------------------FGE-IVEVD-LKPG--------

GAQIE---
------------------------------VTEENKH-EYVEL-ISEWKISKR-VEEQFK
-AFIDG---FNELIPQ--ELVN-VFDER-ELELLIGG-----------------------
----LAEID-----------IEDW----KKHTDYRG-YQES-------------------
------------------------DQVI-QWFWRCIK--------------------EWD
SEQKARLLQF-------TTGTSRIP------------VNG-----FK-DLQGSD--GPRR
FTIEKAGES-------------------------NQLPKSHTCF-NR-VDLPPY-TNYES
LKQKLS-LAVEE-TVGF
>Debaryomyces_hansenii_XM_458629.1 .
IKVRRDHI-FEDSYQEI--------------MRQTPEDLKK-RLMIKFDG-------EEG
LDYGGVSREF---FFLL----SHDMFNPFY--------CLFEYSSHDNY-----------
----------------TLQINPNS------------------------------------
--GINPEHLNYFKFIGRVVGLGVFHRRFLDAFFVG--ALYKMMLR---------------
--------KKVVLQDMEG----------------------VDAEFYRS--------LKWI
CDN--DIT-DIL--
-DLTFSAEDER---------------------------FGE-IVEVD-LKPG--------
GTQIE---
------------------------------VTQENKH-EYVEL-ISEWKISKR-VEEQFK
-AFIDG---FNELIPQ--ELVN-VFDER-ELELLIGG-----------------------
----LAEID-----------IEDW----KKHTDYRG-YQEN-------------------
------------------------DQVI-QWFWRCIK--------------------EWD
SEQKARLLQF-------TTGTSRIP------------VNG-----FK-DLQGSD--GPRR
FTIEKAGES-------------------------NQLPKSHTCF-NR-VDLPPY-TNYES
LKQKLS-LAVEE-TVG-
>Sugiyamaella_xylanicola_MQSX01000033.1 .
IKVRRDHI-FEDSYQEI--------------MRQTPEDLKK-RLMIKFDG-------EEG
LDYGGVSREF---FFLL----SHDMFNPFY--------CLFEYSSHDNY-----------
----------------TLQINPNS------------------------------------
--GINPEHLNYFKFIGRVVGLGVFHRRFLDAFFVG--ALYKMMLH---------------
--------KKVILQDMEG----------------------VDAEFYRS--------LKWI
CDN--DIT-DIL--
-DLTFSAEDER---------------------------FGE-IVEVD-LKPG--------
GANIE---
------------------------------VTEENKH-EYVEL-ISEWKIYKR-VEEQFK
-AFIDG---FNELIPQ--ELVN-VFDER-ELELLIGG-----------------------
----LAEID-----------EKDW----KKHTDYRG-YQES-------------------
------------------------DQVI-QWFWKCIS--------------------EWD
SEQKARLLQF-------TTGTSRIP------------VNG-----FK-DLQGSD--GPRR
FTIEKAGEP-------------------------NQLPKSHTCF-NR-VDLPPY-TDYES
LKQKLT-LAVEE-TVG-
>Millerozyma_acaciae_BCKO01000006.1 .
IKVRRDHI-FEDSYQEI--------------MRQPPEDLKK-RLMIKFDG-------EEG
LDYGGVSREF---FFLL----SHDMFNPFY--------CLFEYSSHDNY-----------
----------------TLQINPNS------------------------------------
--GINPEHLNYFKFIGRVVGLGVFHRRFLDAFFVG--ALYKMMLR---------------
--------KKVVLQDMEG----------------------VDAEFYRS--------LKWI
CDN--DIT-GIL--
-DLTFSAEDER---------------------------FGE-IVEVD-LKPN--------
GRNIE---
------------------------------VTEENKH-EYVEL-ISDWKIVKR-VEEQFK
-AFIDG---FNELIPQ--ELVN-VFDER-ELELLIGG-----------------------
----LAEID-----------IDDW----KKHTDYKG-YQES-------------------
------------------------DQVI-QWFWRCIK--------------------EWD
SEQKARLLQF-------TTGTSRIP------------VNG-----FK-DLQGSD--GPRR
FTIEKAGEP-------------------------NQLPKSHTCF-NR-VDLPPY-TNYES
LKQKLS-LAVEE-TVG-
>Spathaspora_hagerdaliae_LQHL01001349.1 .
IKVRRDHI-FEDSYQEI--------------MRQTPEDLKK-RLMIKFDG-------EEG

```
LDYGGVSREF---FFLL----SHDMFNPFY--------CLFEYSSHDNY-----------
----------------TLQINPNS------------------------------------
--AINPEHLNYFKFIGRVVGLGVFHRRFLDAFFVG--ALYKMMLH---------------
--------KKVVLQDMEG----------------------VDAEFYRS--------LKWI
LDN------------------------------------------------DIT-GIL--
-DLTFSAEDER---------------------------FGE-IVEVD-LKPD--------
GRNIE-------------------------------------------------------
------------------------------VTEENKH-EYVEY-ITEWRISRR-VEEQFK
-AFIDG---FNELIPQ--ELVN-VFDER-ELELLIGG-----------------------
----LAEID-----------IEDW----KKHTDYRG-YQES-------------------
------------------------DQVI-QWFWRCIK--------------------EWD
SEQKARLLQF-------TTGTSRIP------------VNG-----FK-DLQGSD--GPRR
FTIEKAGES-------------------------NQLPKSHTCF-NR-VDLPPY-ADYES
LKQKLT-LA--------
>Spathaspora_gorwiae_LQMZ01002119.1 .
IKVRRDHI-FEDSYQEI--------------MRQTPEDLKK-RLMIKFDG-------EEG
LDYGGVSREF---FFLL----SHDMFNPFY--------CLFEYSSHDNY-----------
----------------TLQINPNS------------------------------------
--AINPEHLNYFKFIGRVVGLGVFHRRFLDAFFVG--ALYKMMLH---------------
--------KKVVLQDMEG----------------------VDAEFYRS--------LKWI
LDN------------------------------------------------DIT-GIL--
-DLTFSAEDER---------------------------FGE-IVEVD-LKPD--------
GRNIE-------------------------------------------------------
------------------------------VTEENKH-EYVEY-ITEWRISKR-VEEQFK
-AFIDG---FNELIPQ--ELVN-VFDER-ELELLIGG-----------------------
----LAEID-----------IEDW----KKHTDYRG-YQES-------------------
------------------------DQVI-QWFWRCIK--------------------EWD
SEQKARLLQF-------TTGTSRIP------------VNG-----FK-DLQGSD--GPRR
FTIEKAGEA-------------------------NQLPKSHTCF-NR-VDLPPY-ADYES
LKQKLT-LAVEE-TVG-
>Spathaspora_xylofermentans_NDXA01000022.1 .
IKVRRDHI-FEDSYQEI--------------MRQTPEDLKK-RLMIKFDG-------EEG
LDYGGVSREF---FFLL----SHDMFNPFY--------CLFEYSSHDNY-----------
----------------TLQINPNS------------------------------------
--GINPEHLNYFKFIGRVVGLGVFHRRFLDAFFVG--ALYKMMLH---------------
--------KKVVLQDMEG----------------------VDAEFYRS--------LKWI
LDN------------------------------------------------DIT-DIL--
-DLTFSAEDER---------------------------FGE-IVEVD-LKPD--------
GRNIE-------------------------------------------------------
------------------------------VTEENKH-EYVEY-ITEWKISKR-VEEQFK
-AFIDG---FNELIPQ--DLVN-VFDER-ELELLIGG-----------------------
----LAEID-----------VDDW----KKHTDYRG-YQES-------------------
------------------------DQVI-QWFWKCIK--------------------EWD
SEQKARLLQF-------TTGTSRIP------------VNG-----FK-DLQGSD--GPRR
FTIEKAGEP-------------------------NQLPKSHTCF-NR-VDLPPY-ADYES
LKQKLT-LAVEE-TVG-
>Candida_carpophila_BCGK01000001.1 .
IKVRRDHI-FEDSYQEI--------------MRQTPEDLKK-RLMIKFEG-------EEG
LDYGGVSREF---FFLL----SHDMFNPFY--------CLFEYSSHDNY-----------
----------------TLQFNPNS------------------------------------
--GINPEHLNYFKFIGRVVGLGVFHRRFLDAFFVG--ALYKMMLR---------------
--------KKVVLQDMEG----------------------VDAEFYRS--------LKWI
LDN------------------------------------------------DIT-GIL--
-DLTFSAEDER---------------------------FGE-IVEVD-LKEN--------
GRNIE-------------------------------------------------------
------------------------------VTEENKH-EYVEL-ISEWKIYKR-VEEQFK
-AFIDG---FNELIPQ--ELVN-VFDER-ELELLIGG-----------------------
----LAEID-----------VEDW----KKHTDYRG-YQES-------------------
```

------------------------DQVI-QWFWKCIN--------------------EWD
SEQKARLLQF-------TTGTSRIP------------VNG-----FK-DLQGSD--GPRR
FTIEKAGEA-------------------------NQLPKSHTCF-NR-VDLPPY-SDYAS
LKQKLT-LAVEE-TVG-
>Meyerozyma_guilliermondii_XM_001485835.1 .
IKVRRDHI-FEDSYQEI--------------MRQTPEDLKK-RLMIKFDG-------EEG
LDYGGVSREF---FFLL----SHDMFNPFY--------CLFEYSSHDNY-----------
----------------TLQFNPNS------------------------------------
--GINPEHLNYFKFIGRVVGLGVFHRRFLDAFFVG--ALYKMMLH---------------
--------KKVVLQDMEG----------------------VDAEFFRS--------LKWI
LDN--DIT-GIL--
-DLTFSAEDER---------------------------FGE-IVEVD-LKEN--------
GRNIE---
------------------------------VTEENKH-EYVEL-ISEWKIYKR-VEEQFK
-AFIDG---FNELIPQ--ELVN-VFDER-ELELLIGG-----------------------
----LAEID-----------VEDW----KKHTDYRG-YQES-------------------
------------------------DQVI-QWFWKCIK--------------------EWD
SEQKARLLQF-------TTGTSRIP------------VNG-----FK-DLQGSD--GPRR
FTIEKAGEP-------------------------NQLPKSHTCF-NR-VDLPPY-ADYAS
LKQKLT-LAVEE-TVGF
>Wickerhamia_fluorescens_BCGE01000002.1 .
IKVRRDHI-FEDSYQEI--------------MRQTPEDLKK-RLMIKFDG-------EEG
LDYGGVSREF---FFLL----SHDMFNPFY--------CLFEYSSHDNY-----------
----------------TLQINPNS------------------------------------
--GINPEHLSYFKFIGRVVGLGVFHRRFLDAFFVG--ALYKMMLH---------------
--------KKVVLQDMEG----------------------VDAEFYRS--------LKWI
CDN--DIT-GIL--
-DLTFSAEDER---------------------------FGE-IVEVD-LKPG--------
GRDIE---
------------------------------VTEENKL-EYVEL-ISEWKISKR-VEEQFK
-AFIDG---FNELIPQ--ELVN-VFDER-ELELLIGG-----------------------
----LADID-----------VEDW----KKHTDYRG-FQEN-------------------
------------------------DQVI-QWFWQCIK--------------------EWD
SEQKARLLQF-------TTGTSRIP------------VNG-----FK-DLQGSD--GPRR
FTIEKAGEA-------------------------NQLPKSHTCF-NR-VDLPPY-KDYES
LKQKLT-LAVEE-TVG-
>Candida_tanzawaensis_XM_020207405.1 .
IKVRRDHI-FEDSYQEI--------------MRQTPEDLKK-RLMIKFDG-------EEG
LDYGGVSREF---FFLL----SHDMFNPFY--------CLFEYSSHDNY-----------
----------------TLQINPNS------------------------------------
--GINPEHLSYFKFIGRVVGLGVFHRRFLDAFFVG--ALYKMMLH---------------
--------KKVVLQDMEG----------------------VDAEFYRS--------LKWI
CDN--DIT-DIL--
-DLTFSAEDER---------------------------FGE-IVEVD-LKPG--------
GRDIE---
------------------------------VTEENKH-EYVEL-ISEWKISKR-VEEQFK
-AFIDG---FNELIPQ--ELVN-VFDER-ELELLIGG-----------------------
----LAEID-----------VEDW----KKHTDYRG-YQES-------------------
------------------------DQVI-QWFWKIIK--------------------EWD
SEQKARLLQF-------TTGTSRIP------------VNG-----FK-DLQGSD--GPRR
FTIEKAGEA-------------------------NQLPKSHTCF-NR-VDLPPY-IDYDS
LKQKLT-LAVEE-TVGF
>Scheffersomyces_lignosus_BCGS01000004.1 .
IKVRRDHI-FEDSYQEI--------------MRQTPEDLKK-RLMIKFDG-------EEG
LDYGGVSREF---FFLL----SHDMFNPFY--------CLFEYSSHDNY-----------
----------------TLQINPNS------------------------------------
--GINPEHLNYFKFIGRVVGLGVFHRRFLDAFFVG--ALYKMMLH---------------
--------KKVVLQDMEG----------------------VDAEFYRS--------LKWI

LDN--DIT-DVL--
-DLTFSAEDER---------------------------FGE-IVEVD-LKPG--------
GRDIE---
------------------------------VTEENKH-EYVEY-ISEWKISKR-VEEQFR
-AFIDG---FNELIPQ--ELVN-VFDER-ELELLIGG-----------------------
----LAEID-----------VEDW----KKHTDYRG-YQES-------------------
------------------------DQVI-QWFWKCIK--------------------EWD
SEQKARLLQF-------TTGTSRIP------------VNG-----FK-DLQGSD--GPRR
FTIEKAGES-------------------------NQLPKSHTCF-NR-VDLPPY-LDYES
LRQKLT-LAVEE-TVG-
>Scheffersomyces_stipitis_XM_001384106.1 .
IKVRRDHI-FEDSYQEI--------------MRQTPEDLKK-RLMIKFDG-------EEG
LDYGGVSREF---FFLL----SHDMFNPFY--------CLFEYSSHDNY-----------
----------------TLQINPNS------------------------------------
--AINPEHLNYFKFIGRVVGLGVFHRRFLDAFFVG--ALYKMMLH---------------
--------KKVILQDMEG----------------------VDAEFYRS--------LKWI
LDN--DIT-DVL--
-DLTFSAEDER---------------------------FGE-IVEVD-LKPG--------
GRDIE---
------------------------------VTEENKH-EYVEF-ISEWKISKR-VEEQFK
-AFIDG---FNELIPQ--ELVN-VFDER-ELELLIGG-----------------------
----LAEID-----------VEDW----KKHTDYRG-YQES-------------------
------------------------DQVI-QWFWKCIK--------------------EWD
SEQKARLLQF-------TTGTSRIP------------VNG-----FK-DLQGSD--GPRR
FTIEKAGEP-------------------------NQLPKSHTCF-NR-VDLPPY-NDYES
LKQKLT-LAVEE-TVGF
>Spathaspora_passalidarum_XM_007374372.1 .
IKVRRDHI-FEDSYQEI--------------MRQTPEDLKK-RLMIKFDG-------EEG
LDYGGVSREF---FFLL----SHDMFNPFY--------CLFEYSSHDNY-----------
----------------TLQINPNS------------------------------------
--GINPEHLNYFKFIGRVVGLGVFHRRFLDAFFVG--ALYKMMLH---------------
--------KKVVLQDMEG----------------------VDAEFYRS--------LKWI
LDN--DIT-DIL--
-DLTFSAEDER---------------------------FGE-IVEVD-LKPG--------
GRDIE---
------------------------------VTEENKH-EYVEL-ISEWKISKR-VEEQFK
-AFIDG---FNELIPQ--ELVN-VFDER-ELELLIGG-----------------------
----LAEID-----------VEDW----KKHTDYRG-YQES-------------------
------------------------DQVI-QWFWKCIG--------------------EWD
SEQKARLLQF-------TTGTSRIP------------VNG-----FK-DLQGSD--GPRR
FTIEKAGES-------------------------NQLPKSHTCF-NR-VDLPPY-ADYES
LKQKLT-LAVEE-TVGF
>Spathaspora_arborariae_AYLH01000327.1 .
IKVRREHI-FEDSYQEI--------------MRQTPEDLKK-RLMIKFDG-------EEG
LDYGGVSREF---FFLL----SHDMFNPFY--------CLFEYSSHDNY-----------
----------------TLQINPNS------------------------------------
--GINPEHLNYFKFIGRVVGLGVFHRRFLDAFFVG--ALYKMMLH---------------
--------KKVVLQDMEG----------------------VDAEFYRS--------LKWI
LDN--DIT-DIL--
-DLTFSAEDER---------------------------FGE-IVEVD-LKPG--------
GRDIE---
------------------------------VTESNKH-EYVEL-ISEWKISKR-VEEQFK
-AFIDG---FNELIPQ--ELVN-VFDER-ELELLIGG-----------------------
----LAEID-----------IEDW----KKHTDYRG-YQEL-------------------
------------------------DQVI-QWFWKCVT--------------------EWD
SEQKARLLQF-------TTGTSRIP------------VNG-----FK-DLQGSD--GPRR
FTIEKAGES-------------------------NQLPKSHTCF-NR-VDLPPY-SDYES
LKQKLT-LAVEE-TVG-

```
>Spathaspora_girioi_LQMS01002266.1 .
IKVRRDHI-FEDSYQEI--------------MRQTPEDLKK-RLMIKFDG-------EEG
LDYGGVSREF---FFLL----SHDMFNPFY--------CLFEYSSHDNY-----------
----------------TLQINPNS------------------------------------
--GINPEHLNYFKFIGRVVGLGVFHRRFLDAFFVG--ALYKMMLH---------------
--------KKVVLQDMEG----------------------VDAEFYRS--------LKWI
LDN-------------------------------------------------DIT-DIL--
-DLTFSAEDER--------------------------FGE-IVEVD-LKEG--------
GRDIE-------------------------------------------------------
------------------------------VTEENKH-EYVEL-ISEWKISRR-VEEQFK
-AFIDG---FNELIPQ--ELVN-VFDER-ELELLIGG----------------------
----LAEID-----------IEDW----KKHTDYRG-YQES-------------------
------------------------DQII-QWFWKCIT--------------------EWD
SEQKARLLQF-------TTGTSRIP------------VNG-----FK-DLQGSD--GPRR
FTIEKAGEA-------------------------NQLPKSHTCF-NR-VDLPPY-VDYES
LRQKLT-LAVEE-TVG-
>Nadsonia_fulvescens_LXPB01000102.1 .
VKVRRDKI-FEDSYQEV--------------MRQTPEDLKK-RLMIKFDG-------EEG
LDYGGVSREY---FFLL----SHEMFNPFY--------GLFEYSAHDNY-----------
----------------TLQINPRS------------------------------------
--GINPEHLNYFKFIGRIVGLAVFHRRFLDAFFIG--AFYKMMLN---------------
--------KRVFLDDMEG----------------------VDHDYHRN--------LNWM
LDN-------------------------------------------------DIT-DIL--
-ELTFSVEEDQ--------------------------FGE-LVTLD-LKPN--------
GRNID-------------------------------------------------------
------------------------------VTNENKQ-EYVEL-VTEWKISRR-IKEQFE
-AFITG---FHELIPA--DLVN-VFDER-ELELLIGG----------------------
----LSEID-----------IQDW----KKHTDYRG-YSEK-------------------
------------------------DQVI-EWFWQCIE--------------------SMD
NEQKSRLLQF-------TTGTSRIP------------VNG-----FK-DLQGSD--GPRR
FTIEKAGES-------------------------TQLPKSHTCF-NR-VDLPKY-EDYET
LKTKLI-MAVEE-TMG-
>Tortispora_caseinolytica_LSKT01000055.1 .
LKVRRDHI-FEDSYQEV--------------MRQSPQDLKK-RLMIKFEG-------EEG
LDYGGVSREF---FFLL----SHEMFNPFY--------CLFEYSAHDNY-----------
----------------TLQINPHS------------------------------------
--GINPEHLNYFKFIGRIVGLAIFHRRFLDAFFVG--AFYKMLLR---------------
--------KKVGLIDMEG----------------------IDADVYRN--------LQWT
LEN-------------------------------------------------DIT-DIL--
-DLTFSAEDDK--------------------------FGE-LVTID-LKPN--------
GRNIP-------------------------------------------------------
------------------------------VTNENKK-EYIDL-ISEWRIHKR-VEEQFE
-AFITG---FNELIPQ--ELIN-VFDDK-ELELLIGG----------------------
----IADID-----------VDDW----KKHTDYRG-YTET-------------------
------------------------DDVI-QWFWKCIR--------------------SWD
SEKKSRLLQF-------TTGTSRIP------------VNG-----FK-DLQGSD--GPRR
FTIEKAGEI-------------------------NQLPKSHTCF-NR-VDLPPY-KDYET
LEQKLS-LAVEE-TMG-
>Lipomyces_starkeyi_LSGR01000180.1 .
IKVRRNHI-FEDSYAEI--------------MRQSPQDLKK-RLMIKFEG-------EEG
LDYGGVSREF---FFLL----SHEMFNPFY--------CLFEYSAHDNY-----------
----------------TLQINPHS------------------------------------
--GINPEHLNYFKFIGRVVGLGIFHRRFLDAFFIG--AFYKMVLK---------------
--------KRVVLTDMEG----------------------VDADFYRN--------LSWT
LDN-------------------------------------------------DIT-DVL--
-ELTFSTEDDR--------------------------FGE-LVTVD-LKPN--------
GRNIP-------------------------------------------------------
------------------------------VSNENKR-EYVEL-ITEWRISKR-VEEQFK
```

-AFMSG---FNELIPQ--ELVN-VFDER-ELELLIGG----------------------
----LADID-----------VDDW----KKHTDYRG-YSES-------------------
------------------------DEVI-QWFWKVXR--------------------TWD
SEQKSRLLQF-------TTGTSRVP------------VNG-----FK-DLQGSD--GPRR
FTIEKAGEI-------------------------QHLPKSHTCF-NR-VDLPAY-KDYQS
LVQKLT-LAVEE-TVG-
>Galactomyces_candidum_CCBN010000009.1 .
VKVRRDHI-FEDSYQEI--------------MRQTPADLKK-RLMIKFDG-------EEG
LDYGGVSREF---FFLL----SHEMFNPFY--------CLFEYSAHDNY-----------
----------------TLQINPHS------------------------------------
--GINPEHLNYFKFIGRVVGLAIFHRRFLDAFFIG--AFYKMILR---------------
--------KKVVLNDMEG----------------------VDAEYFRN--------LEWT
LNN--DIE-DIL--
-ELTFSVEDDQ--------------------------FGE-IVTVD-LKPN--------
GRDIP---
------------------------------VTNENKR-EYVEL-VTEWKISRR-VEEQFK
-SFMSG---FNELIPQ--ELIN-VFDER-ELELLIGG----------------------
----ISEID-----------VDDW----KKHTDYRG-YSEN-------------------
------------------------DEVI-QWFWKCVR--------------------SWD
SEQKSRLLQF-------TTGTSRIP------------VNG-----FK-DLQGSD--GPRR
FTIEKAGET-------------------------ESLPKSHTCF-NR-VDLPLY-NSYES
LVHKLT-LAVEE-TVG-
>Saprochaete_clavata_CBXB010000094.1 .
IKVRRNHI-FEDSYQEI--------------MRQSPTDLKK-RLMIKFDG-------EEG
LDYGGVSREF---FFLL----SHEMFNPFY--------CLFEYSAHDNY-----------
----------------TLQINPHS------------------------------------
--GINPEHLNYFRFIGRVVGLAIFHKRFLDGFFIS--AFYKMILN---------------
--------KKVVLSDMEG----------------------VDADFYRN--------LEWT
LNN--DID-GIL--
-DLTFSVEDDQ--------------------------FGK-IVTVD-LKPG--------
GRDIP---
------------------------------VTNENKR-EYVEL-VTEWKIQRR-VEEQFK
-NFMTG---FNELIPQ--ELIN-VFDER-ELELLIGG----------------------
----IAEID-----------VDDW----KKHTDYRG-YTEN-------------------
------------------------DEVI-QWFWKCVR--------------------SWD
SEQKSRLLQF-------TTGTSRIP------------VNG-----FK-DLQGSD--GPRR
FTIEKAGEP-------------------------NQLPKSHTCF-NR-VDLPPY-KDYES
LQQKLL-MAVEE-TVG-
>Yarrowia_lipolytica_HG934063.1 .
IKVRRDHI-FEDSYQEI--------------MRQTPQDLQK-RLMIKFDG-------EEG
LDYGGVSREF---FFLL----SHEMFNPFY--------CLFEYSAHDNY-----------
----------------TLQINPHS------------------------------------
--GINPEHLNYFKFIGRCVGLAIFHRRFLDAFFIG--AFYKMILK---------------
--------KKVMLEDMEG----------------------VDADYHRN--------LEWA
LDN--DIT-DVL--
-DLTFSVEDDQ--------------------------FGE-IVTID-LKPD--------
GRNIE---
------------------------------VTNDNKI-EYVEL-VTEWRISKR-VEEQFQ
-AFVSG---FYELIPQ--ELVN-VFDER-ELELLIGG----------------------
----IADID-----------VDDW----KKHTDYRG-YSES-------------------
------------------------DEVI-KWFWQCIR--------------------SWD
SEQKSRLLQF-------TTGTSRIP------------VNG-----FK-DLQGSD--GPRR
FTIEKAGEA-------------------------QHLPKSHTCF-NR-VDLPPY-KNYED
LVKKLS-MAVEE-TVG-
>Blastobotrys_adeninivorans_CBZY010000004. 1..
IKVRRDHI-FEDSYQEI--------------MRQSPQDLKK-RLMIKFDG-------EEG
LDYGGVSREF---FFLL----SHEMFNPFY--------CLFEYSAHDNY-----------
----------------TLQINPHS------------------------------------

```
--GVNPEHLNYFRFIGRCVGLAIFHRRFLDAFFIG--AFYKMILR---------------
--------KKVVLADMDG----------------------VDAEFYRN--------LEWT
LDN------------------------------------------------DIT-DIL--
-ELTFSIEDDQ---------------------------FGE-IKTID-LKPG--------
GRDIP-------------------------------------------------------
------------------------------VTNDNKN-EYVEL-VTEWKISKR-VEEQFN
-AFVTG---FNELIPQ--ELIN-VFDER-ELELLIGG-----------------------
----IADID-----------VDDW----KKHTDYRG-YSES-------------------
------------------------DEVV-QWFWKCIR--------------------NMD
AEQKSRLLQF-------TTGTSRIP------------VNG-----FK-DLQGSD--GPRR
FTIEKTGEP-------------------------QHLPKSHTCF-NR-VDLPPY-TDYDT
LVQKLN-LAVEE-TVG-
>Sugiyamaella_lignohabitans_XM_018882785.1 .
VKVRRDHI-FEDSYQEI--------------MRQSPQDLKK-RLMIKFDG-------EEG
LDYGGVSREF---FFLL----SHEMFNPFY--------CLFEYSAHDNY-----------
----------------TLQINPHS------------------------------------
--GINPEHLNYFKFIGRVVGLAVFHRRFLDAFFIG--AFYKMILR---------------
--------KRVVLEDMEG----------------------VDAEFYRN--------LEWM
LDH------------------------------------------------DIT-DVL--
-DLTFSIEDDQ---------------------------FGE-IVTVD-LKPN--------
GRNLE-------------------------------------------------------
------------------------------VTNDNKL-EYVEA-VTEWKISKR-VEEQFQ
-AFVTG---FNELIPQ--DLVN-VFDER-ELELLIGG-----------------------
----IAEID-----------VDDW----KKHTDYRG-YSES-------------------
------------------------DEVV-QWFWKCVR--------------------SWD
SEQKSRLLQF-------TTGTSRIP------------VNG-----FK-DLQGSD--GPRR
FTIEKAGEA-------------------------GHLPKSHTCF-NR-VDLPPY-KDYES
LVQKLG-LAVEE-TVGF
>Candida_infanticola_LWLF01000021.1 .
IKVRRDHI-FEDSYQEI--------------MRQSPQDLKK-RLMIKFDG-------EEG
LDYGGVSREY---FFLL----SHEMFNPFY--------CLFEYSAHDNY-----------
----------------TLQINPHS------------------------------------
--EINPEHLNYFKFIGRVVGLAVFHRRFLDAFFIG--AFYKMMLQ---------------
--------KKVVVEDMEG----------------------VDAEFYRN--------LEWT
LDN------------------------------------------------DIT-DIL--
-DLTFSVDDDH---------------------------FGE-VVTVD-LKEG--------
GRDIP-------------------------------------------------------
------------------------------VTNDNKL-EYVEL-VTQWKIERR-VQKQFQ
-AFITG---FNELIPE--DLVN-VFDER-ELELLIGG-----------------------
----ISEID-----------VDDW----KKHTDYRG-YSEN-------------------
------------------------DEVI-QWFWKCIR--------------------NWD
SEQKSRLLQF-------TTGTSRIP------------VNG-----FK-DLQGSD--GPRR
FTIEKANDS-------------------------QYLPKSHTCF-NR-VDLPPY-KDYET
LVQKLS-IAVEE-TVG-
>2_Candida_versatilis_BCJV01000001.1 .
IKVRRDHI-FEDSYQEI--------------MRQTPQDLKK-RLMIKFDG-------EEG
LDYGGVSREY---FFLL----SHEMFNPFY--------CLFEYSAHDNY-----------
----------------TLQINPHS------------------------------------
--GVNPEHLNYFKFIGRVVGLAVFHRRFLDAFFIS--AFYKMILG---------------
--------KRIVVEDMEG----------------------VDADFHRN--------LEWM
LDN------------------------------------------------DIT-DVL--
-DLNFTVEDDN---------------------------FGE-VETID-LKPD--------
GTNIP-------------------------------------------------------
------------------------------VTNDNKL-EYVEL-ITQWRIETR-VQEQFR
-AFITG---FNELIPE--DLVN-VFDER-ELELLIGG-----------------------
----ISDID-----------IDDW----KKHTDYRG-YSES-------------------
------------------------DEVI-QWFWKCVQ--------------------EWD
AEQKSRLLQF-------TTGTSRIP------------VNG-----FK-DLQGSD--GPRR
```

FTLEKAGDA-------------------------RYLPKSHTCF-NR-VDLPPY-KDYDT
LKKKLG-IAVEE-TVG-
>2_Wickerhamiella_domercqiae_BCGM01000002.1 .
IKVRRDHI-FEDSYQEI--------------MRQTPQDLKQ-RLMIKFDG-------EEG
LDYGGVSREY---FFLL----SHEMFNPFY--------GLFEYSAHDNY-----------
----------------TLQINPHS------------------------------------
--GINPEHLNYFKFIGRVVGLAVFHRRFLDAFFIG--AFYKMMLA---------------
--------KKVVVEDMEG----------------------VDAEFHRN--------LEWM
LDN--DIT-DVL--
-DLTFAIEDDN---------------------------FGH-VASIE-LKPG--------
GKDIP---
------------------------------VTNDNKL-EYVEL-VTQWKIEKR-VAEQFK
-AFQHG---FNELIPE--DLVN-VFDER-ELELLIGG-----------------------
----ISEID-----------VDDW----KKHTDYRG-YSES-------------------
------------------------DEVI-QWFWECIR--------------------SWD
SEQKSRLLQF-------TTGTSRIP------------VNG-----FK-DLQGSD--GPRR
FTIEKAGDS-------------------------RHLPKSHTCF-NR-VDLPPY-KEYDT
LVKKLS-IAVEE-TVG-
>Starmerella_bacillaris_MWPI01000009.1 .
IKVRRDHI-FEDSYQEI--------------MRQTPQDLKQ-RLMIKFDG-------EEG
LDYGGVSREY---FFLL----SHEMFNPFY--------GLFEYSAHDNY-----------
----------------TLQINPHS------------------------------------
--GINPEHLNYFKFIGRVVGLAVFHRRFLDAFFIG--AFYKMILA---------------
--------KKVVFEDMEG----------------------VDAEFHRN--------LEWM
LDN--DIT-DVL--
-DLTFSVEDDN---------------------------FGQ-KQELD-LKPD--------
GRNIA---
------------------------------VTNENKL-EYVEL-VTQWKIETR-VSEQFK
-AFRSG---FNELIPE--DLVN-VFDER-ELELLIGG-----------------------
----ISEID-----------VDDW----KKHTDYRG-YSEN-------------------
------------------------DEVI-QWFWKCIR--------------------SWD
SEQKSRLLQF-------TTGTSRIP------------VNG-----FK-DLQGSD--GPRR
FTIEKAGDS-------------------------RHLPKSHTCF-NR-VDLPQY-KDYDT
LVKKLS-IAVEE-TVG-
>Candida_apicola_LBNK01000023.1 .
VKVRRSHI-FEDSYQEI--------------MRQTPQDLKQ-RLMIKFDG-------EEG
LDYGGVSREY---FFLL----SHEMFNPFY--------GLFEYSAHDNY-----------
----------------TLQINPHS------------------------------------
--SINPEHLNYFKFIGRVVGLAVFHRRFLDAFFIG--AFYKMMLD---------------
--------KKVVVEDMEG----------------------VDADFYRN--------LEWM
LDN--DIT-DIL--
-DLTFSVEDDN---------------------------FGQ-KQELE-LKPG--------
GAEIP---
------------------------------VTNENKL-EYVEL-VTRWKIETR-VQDQFK
-AFKTG---FNELIPE--DLVN-VFDER-ELELLIGG-----------------------
----ISEID-----------VDDW----KTHTDYRG-YSEN-------------------
------------------------DEVI-QWFWKCIA--------------------LWD
SEQKSRLLQF-------TTGTSRIP------------VNG-----FK-DLQGSD--GPRR
FTIEKAGDS-------------------------RYLPKSHTCF-NR-VDLPPY-KDFDT
LVKKLG-IAVEE-TVG-
>Starmerella_bombicola_BBSW01000026.1 .
IKVRRNRI-FEDSYQEI--------------MRQTPQDLKQ-RLMIKFEG-------EEG
LDYGGVSREY---FFLL----SHEMFNPFY--------GLFEYSAHDNY-----------
----------------TLQINPHS------------------------------------
--SINPEHLNYFKFIGRVLGLAVFHRRFLDAFFIG--AFYKMILG---------------
--------KKVVVEDMEG----------------------VDATFHRN--------LEWM
LDN--DIT-DVL--
-DLTFSIEDDN---------------------------FGE-KQQLE-LKPG--------

GKGIE---
------------------------------VTNENKL-EYVEL-VTQWKIERR-ISEQLT
-AFKTG---FNELIPE--DLVN-VFDER-ELELLIGG-----------------------
----ISDID-----------VEDW----KKHTDYRG-YSEN-------------------
------------------------DQVI-QWFWKCIK--------------------EWD
PEQKSRLLQF-------TTGTSRIP------------VNG-----FK-DLQGSD--GPRR
FTIEKAGDE-------------------------RHLPKSHTCF-NR-VDLPEY-HDFDT
LVKKLG-IAVEE-TVG-
>Sporopachydermia_quercuum_BCGN01000002.1 .
VKVRRDHI-FEDSYQEI--------------MRQTPTDLKK-RLMIKFDG-------EEG
LDYGGVSREF---FFLL----SHEMFNPFY--------CLFEYSAHDNY-----------
----------------TLQINPHS------------------------------------
--AINPEHLNYFKFIGRVVGLAIFHRRFLDAFFIG--AFYKMILR---------------
--------KKVTLADMEG----------------------VDSDIFRN--------LTWT
LEN--DVE-GLD--
--FTFSVEDDK---------------------------FGE-IVTVD-LKPG--------
GRDII---
------------------------------VTNDNKK-EYVDL-MTEWRITKR-VEEQFN
-AFISG---FNELIPP--DLVN-VFDER-ELELLIGG-----------------------
----LSEID-----------VEDW----KKHTDYRG-YTES-------------------
------------------------DQVI-QWFWELMK--------------------SWD
AEQKSRVLQF-------CTGTSRIP------------VNG-----FK-DLQGSD--GPRR
FTIEKAGEI-------------------------TQLPKSHTCF-NR-IDLPPY-DNIED
LRKKLT-LAVEE-TIG-
>Alloascoidea_hylecoeti_BCKZ01000002.1 .
VKVRREHI-FEDSYQEI--------------MRQSPADLKK-RLMIKFDG-------EEG
LDYGGVSREY---FFLL----SHEMFNPFY--------CLFEYSAHDNY-----------
----------------TLQINPHS------------------------------------
--SINPEHLNYFKFIGRVVGLAVFHRRFLDAFFIG--AFYKMILR---------------
--------KPVSLADMEG----------------------VDAEIFKN--------LTWV
LEN--DIT-DIL--
-EMTFSVEDDQ---------------------------FGV-IVTID-LKED--------
GRNIE---
------------------------------VTNENKH-EYVAL-MTKWRIEKR-VDEQFQ
-SFIAG---FNELIPQ--DLVN-VFDER-ELELLIGG-----------------------
----LAEID-----------IEDW----KKHTDYRG-YTES-------------------
------------------------DQVV-QWFWKLME--------------------SWD
SEQKSRMLQF-------TTGTSRIP------------VNG-----FK-DLQGSD--GPRR
FTIEKSGEI-------------------------TQLPKSHTCF-NR-IDLPQY-DDFES
MKQKMT-LAVEE-TIG-
>Dactylellina_haptotyla_XM_011116532.1 .
VKVRRNHI-FEDSYAEI--------------MRQHPNDLKK-RLMVKFDG-------EDG
LDYGGLSREF---FFLL----SHEMFNPFY--------CLFEYSAHDNY-----------
----------------TLQINPHS------------------------------------
--GINPEHLNYFKFIGRVVGLAIFHRRFLDAFFIG--AFYKMILR---------------
--------KKVALADMEG----------------------VDADFHRN--------LTWT
LEN--DIT-DIL--
-DLTFSTEDNR---------------------------FGE-TVTID-LKPN--------
GRDIE---
------------------------------VTNENKK-EYIDL-ITEWRISKR-VEEQFN
-AFITG---FNELIPP--DLIN-VFDER-ELELLIGG-----------------------
----IADID-----------VDDW----KKHTDYRG-YTET-------------------
------------------------DEVI-QNFWKCVR--------------------SWD
AEQKSRLLQF-------TTGTSRIP------------VNG-----FK-DLQGSD--GPRR
FTIEKAGEQ-------------------------LHLPKSHTCF-NR-LDLPPY-KTYDA
LAQKLA-LAVEE-TMGF
>Arthrobotrys_oligospora_XM_011128307.1 .
VKVRRNHI-FEDSYAEI--------------MRQHPNDLKK-RLMVKFDG-------EDG

LDYGGLSREF---FFLL----SHEMFNPFY--------CLFEYSAHDNY-----------
----------------TLQINPHS------------------------------------
--GINPEHLNYFKFIGRVVGLAIFHRRFLDAFFIG--AFYKMILR---------------
--------KKVSLADMEG----------------------VDADFHRN--------LTWT
LEN--DIT-DIL--
-DLTFSTEDNR---------------------------FGE-TVTID-LKPN--------
GRDIE---
------------------------------VTNENKK-EYIDL-ITEWRISKR-VEEQFN
-AFITG---FNELIPP--DLVN-VFDER-ELELLIGG-----------------------
----IADID-----------VDDW----KKHTDYRG-YTET-------------------
------------------------DEVI-QNFWKCVR--------------------SWD
AEQKSRLLQF-------TTGTSRIP------------VNG-----FK-DLQGSD--GPRR
FTIEKAGEQ-------------------------LHLPKSHTCF-NR-LDLPPY-KSYDA
LAQKLA-LAVEE-TMGF
>Tuber_melanosporum_XM_002842107.1 .
VKVRRNHI-FEDSYAEI--------------MRQTPNDLKK-RLMVKFDG-------EDG
LDYGGLSREF---FFLL----SHEMFNPFY--------CLFEYSAHDNY-----------
----------------TLQINPHS------------------------------------
--GINPEHLNYFKFIGRVVGLAIFHRRFLDAFFIG--AFYKMILK---------------
--------KKVVLADMEG----------------------VDADFHRN--------LTWM
LEN--DIT-DIL--
-DLTFSTEDSR---------------------------FGE-TVTID-LKPN--------
GQNIE---
------------------------------VTNDNKR-EYVDL-VTGWRIEKR-VQEQFK
-AFVDG---FHDLIPA--DLIN-VFDER-ELELLIGG-----------------------
----IADID-----------VEDW----KKHTDYRG-YTES-------------------
------------------------DDCV-R---------------------------SWD
AEQKSRLLQF-------TTGTSRIP------------VNG-----FK-DLQGSD--GPRR
FTIEKAGDI-------------------------GQLPKSHTCF-NR-LDLPPY-KSFDV
LNQKLS-LAVEE-TMGF
>Pyrenophora_tritici_repentis_AAXI01000200 1..
VKVRRTHI-FEDSYHEI--------------MRQSAADLKK-RLMIKFDG-------EDG
LDYGGLSREF---FFLL----SHEMFNPFY--------CLFEYSAHDNY-----------
----------------TLQINPHS------------------------------------
--GINPEHLNYFKFIGRVVGLAIFHRRFLDAFFIG--AFYKMILR---------------
--------KKVALQDMEG----------------------VDADFHRN--------LEWM
LXN--DIT-DAL--
-ELTFATDDER---------------------------FGE-TVSIE-LKPG--------
GDEIE---
------------------------------VTNENKH-EYVEX-ITEWRIQKR-VEEQFQ
-AFITG---FHELIPA--DLVN-VFDER-ELELLIGG-----------------------
----IADID-----------VEDW----KKHTDYRG-YTEN-------------------
------------------------DEVI-QNFWKCIR--------------------SWD
AEQKSRLLQF-------ATGTSRIP------------VNG-----FK-DLQGSD--GPRR
FTIEKAGEP-------------------------NQLPKSHTXF-NR-LDLPPY-KTFEA
LNQKLT-IAVEE-TVG-
>Pyrenophora_teres_XM_003299960.1 .
VKVRRTHI-FEDSYHEI--------------MRQSAADLKK-RLMIKFDG-------EDG
LDYGGLSREF---FFLL----SHEMFNPFY--------CLFEYSAHDNY-----------
----------------TLQINPHS------------------------------------
--GINPEHLNYFKFIGRVVGLAIFHRRFLDAFFIG--AFYKMILR---------------
--------KKVALQDMEG----------------------VDADFHRN--------LEWM
LNN--DIT-DAL--
-ELTFATDDER---------------------------FGE-TVSIE-LKPG--------
GDEIE---
------------------------------VTNENKH-EYVEL-ITEWRIQKR-VEEQFQ
-AFITG---FHELIPA--DLVN-VFDER-ELELLIGG-----------------------
----IADID-----------VEDW----KKHTDYRG-YTEN-------------------

------------------------DEVI-QNFWKCIR--------------------SWD
AEQKSRLLQF-------ATGTSRIP------------VNG-----FK-DLQGSD--GPRR
FTIEKAGEP-------------------------NQLPKSHTCF-NR-LDLPPY-KTFEA
LNQKLT-IAVEE-TVGF
>Leptosphaeria_maculans_NW_003533848.1 .
VKVRRTHI-FEDSYHEI--------------MRQSAADLKK-RLMIKFDG-------EDG
LDYGGLSREF---FFLL----SHEMFNPFY--------CLFEYSAHDNY-----------
----------------TLQINPHS------------------------------------
--GINPEHLNYFKFIGRVVGLAIFHRRFLDAFFIG--AFYKMILR---------------
--------KKVALQDMEG----------------------VDADFHRN--------LEWM
LXN--DIT-DAL--
-ELTFATDDER---------------------------FGE-TVSIE-LKPG--------
GDEIE---
------------------------------VTNENKH-EYVEX-ITEWRIQKR-VEEQFQ
-AFITG---FHELIPA--DLVN-VFDER-ELELLIGG-----------------------
----IADID-----------VEDW----KKHTDYRG-YTEN-------------------
------------------------DEVI-QNFWKCIR--------------------SWD
AEQKSRLLQF-------ATGTSRIP------------VNG-----FK-DLQGSD--GPRR
FTIEKAGEP-------------------------NQLPKSHTWF-NR-LDLPPY-KTYEA
LNNKLT-IAVEE-TVGF
>Leptosphaeria_biglobosa_FO905859.1 .
VKVRRTHI-FEDSYHEI--------------MRQSAADLKK-RLMIKFDG-------EDG
LDYGGLSREF---FFLL----SHEMFNPFY--------CLFEYSAHDNY-----------
----------------TLQINPHS------------------------------------
--GINPEHLNYFKFIGRVVGLAIFHRRFLDAFFIG--AFYKMILR---------------
--------KKVALQDMEG----------------------VDADFHRN--------LEWM
LXN--DIT-DAL--
-ELTFATDDER---------------------------FGE-TVSIE-LKPG--------
GDDIE---
------------------------------VTNENKH-EYVEL-ITEWRIQKR-VEEQFQ
-AFITG---FHELIPA--DLVN-VFDER-ELELLIGG-----------------------
----IADID-----------VEDW----KKHTDYRG-YTEN-------------------
------------------------DEVI-QNFWKCIR--------------------SWD
AEQKSRLLQF-------ATGTSRIP------------VNG-----FK-DLQGSD--GPRR
FTIEKAGEP-------------------------NQLPKSHTWX-NR-LDLPAY-KTYEA
LNGKLT-IAVEE-TVGF
>Alternaria_alternata_XM_018530720.1 .
VKVRRTHI-FEDSYHEI--------------MRQSAADLKK-RLMIKFDG-------EDG
LDYGGLSREF---FFLL----SHEMFNPFY--------CLFEYSAHDNY-----------
----------------TLQINPHS------------------------------------
--GINPEHLNYFKFIGRVVGLAIFHRRFLDAFFIG--AFYKMILR---------------
--------KKVALQDMEG----------------------VDADFHRN--------LEWM
LNN--DIT-DAL--
-ELTFATDDER---------------------------FGE-TVSIE-LKPG--------
GDNIE---
------------------------------VTNENKH-EYVEL-ITEWRIQKR-VEEQFQ
-AFITG---FHELIPA--DLVN-VFDER-ELELLIGG-----------------------
----IADID-----------VDDW----KKHTDYRG-YTEN-------------------
------------------------DEVI-QNFWKCIR--------------------SWD
AEQKSRLLQF-------ATGTSRIP------------VNG-----FK-DLQGSD--GPRR
FTIEKAGEP-------------------------NQLPKSHTCF-NR-LDLPPY-KTFEA
LNQKLT-IAVEE-TVGF
>Alternaria_brassicicola_ACIW01001067.1_R .
VKVRRTHI-FEDSYHEI--------------MRQSAADLKK-RLMIKFDG-------EDG
LDYGGLSREF---FFLL----SHEMFNPFY--------CLFEYSAHDNY-----------
----------------TLQINPHS------------------------------------
--GINPEHLNYFKFIGRVVGLAIFHRRFLDAFFIG--AFYKMILR---------------
--------KKVALQDMEG----------------------VDADFHRN--------LEWM

```
LXN------------------------------------------------DIT-DAL--
-ELTFATDDER---------------------------FGE-TVSIE-LKPG--------
GDNIE-------------------------------------------------------
------------------------------VTNENKH-EYVEY-VTEWRIQKR-VEEQFQ
-AFITG---FHELIPA--DLVN-VFDER-ELELLIGG-----------------------
----IADID-----------VDDW----KKHTDYRG-YTEN-------------------
------------------------DEVI-QNFWKCIR--------------------SWD
AEQKSRLLQF-------ATGTSRIP------------VNG-----FK-DLQGSD--GPRR
FTIEKAGEP-------------------------NQLPKSHTWYFNR-LDLPPY-KTFEA
LNQKLT-IAVEE-----
>Ascochyta_rabiei_JYNV01000142.1 .
VKVRRTHI-FEDSYHEI--------------MRQSAADLKK-RLMIKFDG-------EDG
LDYGGLSREF---FFLL----SHEMFNPFY--------CLFEYSAHDNY-----------
----------------TLQINPHS------------------------------------
--GINPEHLNYFKFIGRVVGLSIFHRRFLDAFFIG--AFYKMILR---------------
--------KKVALQDMEG----------------------VDADFHRN--------LEWM
LNN------------------------------------------------DIT-DAL--
-ELTFATDDER---------------------------FGE-TVSIE-LKPG--------
GDDIE-------------------------------------------------------
------------------------------VTNENKH-EYVEX-ITEWRIQKR-VEEQFN
-AFLSG---FNELVPP--ELVN-VFDER-ELELLIGG-----------------------
----IADID-----------VEDW----KKHTDYRG-YTEN-------------------
------------------------DEVI-QNFWKCIR--------------------SWD
AEQKSRLLQF-------ATGTSRIP------------VNG-----FK-DLQGSD--GPRR
FTIEKAGEP-------------------------NQLP----------------------
-----------------
>Phaeosphaeria_nodorum_XM_001795456.1 .
VKVRRTHI-FEDSYHEV--------------MRQSAADLKK-RLMIKFDG-------EDG
LDYGGLSREF---FFLL----SHEMFNPFY--------CLFEYSAHDNY-----------
----------------TLQINPHS------------------------------------
--GINPEHLNYFKFIGRVVGLAIFHRRFLDAFFIG--AFYKMILR---------------
--------KKVSLQDMEG----------------------VDADFHRN--------LEWM
LNN------------------------------------------------DIT-DAL--
-ELTFATDDER---------------------------FGE-TVSIE-LKPG--------
GEEIE-------------------------------------------------------
------------------------------VTNENKG-EYVEL-ITEWRIQKR-VEEQFQ
-AFIAG---FHELIPA--DLVN-VFDER-ELELLIGG-----------------------
----IADID-----------VEDW----KKHTDYRG-YTEN-------------------
------------------------DEVI-QNFWKCIR--------------------SWD
AEQKSRLLQF-------ATGTSRIP------------VNG-----FK-DLQGSD--GPRR
FTIEKAGEP-------------------------NQLPKSHTCF-NR-LDLPPY-KTFEA
LNQKLT-IAVEE-TVGF
>Pyrenochaeta_lycopersici_GAJI01012090.1 .
VKVRRTHI-FEDSYHEI--------------MRQSAADLKK-RLMIKFDG-------EDG
LDYGGLSREF---FFLL----SHEMFNPFY--------CLFEYSAHDNY-----------
----------------TLQINPHS------------------------------------
--GINPEHLNYFKFIGRVVGLAIFHRRFLDAFFIG--AFYKMILR---------------
--------KKVSLQDMEG----------------------VDADFHRN--------LEWM
LNN------------------------------------------------DIT-DAL--
-ELTFATDDER---------------------------FGE-TVSIE-LKPG--------
GENIE-------------------------------------------------------
------------------------------VTNENKG-EYVEL-ITEWRIQKR-VEDQFQ
-AFVTG---FHELIPA--DLVN-VFDER-ELELLIGG-----------------------
----IAEID-----------VDDW----KKHTDYRG-YTEN-------------------
------------------------DEVI-QNFWKCIR--------------------SWD
AEQKSRLLQF-------ATGTSRIP------------VNG-----FK-DLQGSD--GPRR
FTIEKAGEP-------------------------NQLPKSHTCF-NR-LDLPAY-KTFEA
LNGKLT-IAVEE-TVGF
```

>Cochliobolus_sativus_XM_007705905.1 .
VKVRRSHI-FEDSYHEI--------------MRQSAADLKK-RLMIKFDG-------EDG
LDYGGLSREF---FFLL----SHEMFNPFY--------CLFEYSAHDNY-----------
----------------TLQINPHS------------------------------------
--GINPEHLNYFKFIGRVVGLAIFHRRFLDAFFIG--AFYKMILK---------------
--------KKVSLQDMEG----------------------VDADFHRN--------LEWM
LNN---DIT-DAL--
-ELTFSTDDER---------------------------FGE-TVSIE-LKPG--------
GENIE---
-------------------------------VTNENKH-EYVEL-ITEWRIQKR-VEEQFQ
-AFITG---FHELIPA--DLVN-VFDER-ELELLIGG-----------------------
----IADID-----------VDDW----KKHTDYRG-YTEN-------------------
------------------------DEVI-QNFWKCIR--------------------SWD
AEQKSRLLQF-------ATGTSRIP------------VNG-----FK-DLQGSD--GPRR
FTIEKAGEP-------------------------NQLPKSHTCF-NR-LDLPPY-KTYEV
LNQKLT-IAVEE-TVGF
>Setosphaeria_turcica_XM_008022069.1 .
VKVRRSHI-FEDSYHEI--------------MRQSAADLKK-RLMIKFDG-------EDG
LDYGGLSREF---FFLL----SHEMFNPFY--------CLFEYSAHDNY-----------
----------------TLQINPHS------------------------------------
--GINPEHLNYFKFIGRVVGLAIFHRRFLDAFFIG--AFYKMILK---------------
--------KKVSLQDMEG----------------------VDADFHRN--------LEWM
LNN---DIT-DAL--
-ELTFSTDDER---------------------------FGE-TVSIE-LKPG--------
GENIE---
-------------------------------VTNENKH-EYVEL-ITEWRIQKR-VEEQFQ
-AFITG---FHELIPA--DLVN-VFDER-ELELLIGG-----------------------
----IADID-----------VDDW----KKHTDYRG-YTEN-------------------
------------------------DEVI-QNFWKCIR--------------------SWD
AEQKSRLLQF-------ATGTSRIP------------VNG-----FK-DLQGSD--GPRR
FTIEKAGEP-------------------------NQLPKSHTCF-NR-LDLPPY-KTFEA
LNQKLT-IAVEE-TVGF
>Bipolaris_victoriae_XM_014696558.1 .
VKVRRSHI-FEDSYHEI--------------MRQSAADLKK-RLMIKFDG-------EDG
LDYGGLSREF---FFLL----SHEMFNPFY--------CLFEYSAHDNY-----------
----------------TLQINPHS------------------------------------
--GINPEHLNYFKFIGRVVGLAIFHRRFLDAFFIG--AFYKMILK---------------
--------KKVSLQDMEG----------------------VDADFHRN--------LEWM
LNN---DIT-DAL--
-ELTFSTDDER---------------------------FGE-TVSIE-LKPG--------
GENIE---
-------------------------------VTNENKH-EYVEL-ITEWRIQKR-VEEQFQ
-AFITG---FHELIPA--DLVN-VFDER-ELELLIGG-----------------------
----IADID-----------VDDW----KKHTDYRG-YTEN-------------------
------------------------DEVI-QNFWKCIR--------------------SWD
AEQKSRLLQF-------ATGTSRIP------------VNG-----FK-DLQGSD--GPRR
FTIEKAGEP-------------------------NQLPKSHTCF-NR-LDLPPY-KTYEA
LNQKLT-IAVEE-TVGF
>Paraphaeosphaeria_sporulosa_XM_018178255.1 .
VKVRRTHI-FEDSYHEI--------------MRQSAADLKK-RLMIKFDG-------EDG
LDYGGLSREF---FFLL----SHEMFNPFY--------CLFEYSAHDNY-----------
----------------TLQINPHS------------------------------------
--GINPEHLNYFKFIGRVVGLAIFHRRFLDAFFIG--AFYKMILR---------------
--------KKVALGDMEG----------------------VDADFHRN--------LEWM
LNN---DIT-DAL--
-ELTFSTDDER---------------------------FGE-TVSIE-LKPG--------
GEDIE---
-------------------------------VTNENKK-EYVDL-ITEWRIQKR-VEEQFN

-AFISG---FHELIPA--DLVN-VFDER-ELELLIGG-----------------------
----IADID-----------VEDW----KKHTDYRG-YTEN-------------------
------------------------DEVV-QHFWKCIR--------------------SWD
AEQKSRLLQF-------ATGTSRIP------------VNG-----FK-DLQGSD--GPRR
FTIEKAGEP-------------------------TQLPKSHTCF-NR-LDLPPY-KSFEQ
LNGKLT-IAVEE-TVGF
>Neofusicoccum_parvum_XM_007584966.1 .
VKVRRSHI-FEDSYHEV--------------MRQSASDLKK-RLMIKFDG-------EDG
LDYGGLSREF---FFLL----SHEMFNPFY--------CLFEYSAHDNY-----------
----------------TLQINPHS------------------------------------
--GINPEHLNYFKFIGRVVGLAIFHRRFLDAFFIS--AFYKMILK---------------
--------KKITLQDMEG----------------------VDADFHRN--------LQWT
MDN--DID-GVL--
-DLTFSTDDER---------------------------FGE-TVTID-LKPG--------
GSEIE---
------------------------------VTNDNKR-EYVEL-ITEWRIQKR-VDEQFN
-AFITG---FHELIPA--DLIT-VFDER-ELELLIGG-----------------------
----IADID-----------VDDW----KKHTDYRG-YTES-------------------
------------------------DEVI-QNFWKKSR-----------------------
------LLQF-------ATGTSRIP------------VNG-----FK-DLQGSD--GPRR
FTIEKAGEV-------------------------TQLPKSHTCF-NR-LDLPPY-KTYEA
LNQKLT-IAVEE-TVGF
>Diplodia_corticola_XM_020278467.1 .
VKVRRSHI-FEDSYHEV--------------MRQSAGDLKK-RLMIKFDG-------EDG
LDYGGLSREF---FFLL----SHEMFNPFY--------CLFEYSAHDNY-----------
----------------TLQINPHS------------------------------------
--GINPEHLNYFKFIGRVVGLAIFHRRFLDAFFIS--AFYKMILK---------------
--------KKITLQDMEG----------------------VDADFHRN--------LQWT
MDN--DID-DVL--
-DLTFSTDDER---------------------------FGE-TVTID-LKPG--------
GRDIE---
------------------------------VTNDNKR-EYVEL-ITEWRIQKR-VEEQFN
-AFITG---FHELIPA--DLIT-VFDER-ELELLIGG-----------------------
----IADID-----------VEDW----KKHTDYRG-YTES-------------------
------------------------DEVI-QNFWKCIR--------------------SWD
AEQKSRLLQF-------ATGTSRIP------------VNG-----FK-DLQGSD--GPRR
FTIEKAGEI-------------------------TQLPKSHTCF-NR-LDLPPY-KTYEA
LNQKLT-IAVEE-TVGF
>Verruconis_gallopava_XM_016357139.1 .
IKVRRTHI-FEDSYHEI--------------MRQSATDLKK-RLMIKFDG-------EDG
LDYGGLSREF---FFLL----SHEMFNPFY--------CLFEYSAHDNY-----------
----------------TLQINPHS------------------------------------
--GINPEHLNYFKFIGRVVGLAIFHRRFLDAFFIG--AFYKMILR---------------
--------KKVTLQDMEG----------------------VDADFHRN--------LTWT
LDN--DID-GVL--
-DLTFSTDDER---------------------------FGE-TVTID-LKEN--------
GRNIE---
------------------------------VTNENKK-EYIDL-ITEWRIQKR-VSEQFN
-AFITG---FNELIPP--DLVN-VFDER-ELELLIGG-----------------------
----IADID-----------VDDW----KKHTDYRG-YTES-------------------
------------------------DQVV-QDFWKCIR--------------------SWD
AEQKSRLLQF-------ATGTSRIP------------VNG-----FK-DLQGSD--GPRR
FTIEKAGEP-------------------------GQLPKSHTCF-NR-IDLPPY-KSYEQ
LVQKLT-IAVEE-TVGF
>Coniosporium_apollinis_XM_007779947.1 .
VKVRRSHI-FEDSYHEI--------------MRQSAGDLKK-RLMIKFDG-------EDG
LDYGGLSREF---FFLL----SHEMFNPFY--------CLFEYSAHDNY-----------
----------------TLQINPHS------------------------------------

--GINPEHLNYFKFIGRVVGLAIFHRRFLDAFFIG--AFYKMILR---------------
--------KKVSLQDMEG----------------------VDADFHRN--------LTWT
LDN--DID-GIL--
-DLTFSTDDER---------------------------FGE-TVTID-LKPN--------
GRNIE---
------------------------------VTNENKK-EYIDL-ITEWRIQKR-VEEQFN
-AFITG---FNELIPP--DLVN-VFDER-ELELLIGG-----------------------
----IADID-----------VEDW----KKHTDYRG-YTES-------------------
------------------------DEVV-QNFWKCIR--------------------SWD
AEQKSRLLQF-------ATGTSRIP------------VNG-----FK-DLQGSD--GPRR
FTIEKAGEV-------------------------TQLPKSHTCF-NR-LDLPPY-KTYEA
LNGKLT-IAVEE-TVGF
>Xylona_heveae_XM_018334809.1 .
VKVRRSHI-FDDAYGEI--------------MRQSATDLKK-RLMIKFDG-------EDG
LDYGGLSREF---FFLL----SHEMFNPFY--------CLFEYSAHDNY-----------
----------------TLQINPHS------------------------------------
--GINPEHLSYFKFIGRVVGLAIFHRRFLDAFFIG--AFYKMILR---------------
--------KKVTLADMEG----------------------VDADFHRN--------LTWT
LEN--DID-GIL--
-DLTFSTDDER---------------------------FGE-TVTID-LKPN--------
GRNID---
------------------------------VTNENKK-EYIDL-ITEWRIQKR-VEEQFN
-AFVTG---FNELIPP--DLIN-VFDER-ELELLIGG-----------------------
----IADID-----------VEDW----KKHTDYRG-YTES-------------------
------------------------DEVI-QNFWKCIR--------------------SWD
AEQKSRLLQF-------ATGTSRIP------------VNG-----FK-DLQGSD--GPRR
FTIEKSGEI-------------------------TQLPKSHTCF-NR-LDLPPY-KTFEV
LNQKLG-IAVEE-TVGF
>Zymoseptoria_pseudotritici_GCJY01001058.1 .
VKVRRTHI-FEDSYAEI--------------MRQSPNDLKK-RLMIKFDG-------EDG
LDYGGLSREF---FFLL----SHEMFNPFY--------CLFEYSAHDNY-----------
----------------TLQINPHS------------------------------------
--GINPEHLGYFKFIGRVVGLAIFHRRFLDAFFIG--AFYKMILR---------------
--------KKVNLLDMEG----------------------VDAEFHRT--------LSWA
MEN--DIT-DVI--
-YSTFSVEDER---------------------------FGE-KVTVD-LKPG--------
GRDIE---
------------------------------VDNDNKK-EYVEL-ITEWRIQKR-VEEQFN
-AFVAG---FHELIPA--DLVN-VFDER-ELELLIGG-----------------------
----IADID-----------VDDW----KKHTDYRG-YTES-------------------
------------------------DVVV-QNFWKCIR--------------------GWD
AEQKSRLLQF-------ATGTSRIP------------VNG-----FK-DLQGSD--GPRR
FTIEKSGEE-------------------------TQLPKSHTCF-NR-LDLPPY-KSYEA
LNTKLT-WAVEE-TVGF
>Zymoseptoria_brevis_GCVP01014422.1 .
VKVRRTHI-FEDSYAEI--------------MRQSPNDLKK-RLMIKFDG-------EDG
LDYGGLSREF---FFLL----SHEMFNPFY--------CLFEYSAHDNY-----------
----------------TLQINPHS------------------------------------
--GINPEHLGYFKFIGRVVGLAIFHRRFLDAFFIG--AFYKMILR---------------
--------KKVNLLDMEG----------------------VDAEFHRT--------LSWA
MEN--DIT-DVI--
-YSTFSVEDER---------------------------FGE-KVTVD-LKPG--------
GRDIE---
------------------------------VDNDNKK-EYVEL-ITEWRIQKR-VEEQFN
-AFVAG---FHELIPA--DLVN-VFDER-ELELLIGG-----------------------
----IADID-----------VDDW----KKHTDYRG-YTES-------------------
------------------------DVVV-QNFWKCIR--------------------GWD
AEQKSRLLQF-------ATGTSRIP------------VNG-----FK-DLQGSD--GPRR

FTIEKSGEE-------------------------TQLPKSHTCF-NR-LDLPPY-KSYEV
LNTKLT-WAVEE-TVGF
>Mycosphaerella_graminicola_XM_003848197.1 .
VKVRRTHI-FEDSYAEI--------------MRQSPNDLKK-RLMIKFDG-------EDG
LDYGGLSREF---FFLL----SHEMFNPFY--------CLFEYSAHDNY-----------
----------------TLQINPHS------------------------------------
--GINPEHLGYFKFIGRVVGLAIFHRRFLDAFFIG--AFYKMILR---------------
--------KKVNLLDMEG----------------------VDAEFHRT--------LSWA
MEN--DIT-DVI--
-YSTFSVEDER---------------------------FGE-KVTVD-LKPG--------
GRDIE---
------------------------------VDNDNKK-EYVEL-ITEWRIQKR-VEEQFN
-AFVAG---FHELIPA--DLVN-VFDER-ELELLIGG-----------------------
----IADID-----------VDDW----KKHTDYRG-YTES-------------------
------------------------DLVV-QNFWKCIR--------------------GWD
AEQKSRLLQF-------ATGTSRIP------------VNG-----FK-DLQGSD--GPRR
FTIEKSGEE-------------------------TQLPKSHTCF-NR-LDLPPY-KSYEA
LNTKLT-WAVEE-TVGF
>Zymoseptoria_tritici_GCJU01009155.1 .
VKVRRTHI-FEDSYAEI--------------MRQSPNDLKK-RLMIKFDG-------EDG
LDYGGLSREF---FFLL----SHEMFNPFY--------CLFEYSAHDNY-----------
----------------TLQINPHS------------------------------------
--GINPEHLGYFKFIGRVVGLAIFHRRFLDAFFIG--AFYKMILR---------------
--------KKVNLLDMEG----------------------VDAEFHRT--------LSWA
MEN--DIT-DVI--
-YSTFSVEDER---------------------------FGE-KVTVD-LKPG--------
GRDIE---
------------------------------VDNDNKK-EYVEL-ITEWRIQKR-VEEQFN
-AFVAG---FHELIPA--DLVN-VFDER-ELELLIGG-----------------------
----IADID-----------VDDW----KKHTDYRG-YTES-------------------
------------------------DLVV-QNFWKCIR--------------------GWD
AEQKSRLLQF-------ATGTSRIP------------VNG-----FK-DLQGSD--GPRR
FTIEKSGEE-------------------------TQLPKSHTCF-NR-LDLPPY-KSYEA
LNTKLT-WAVEE-TVGF
>Zymoseptoria_passerinii_AFIY01000187.1_R .
VKVRRTHI-FEDSYAEI--------------MRQSPNDLKK-RLMIKFDG-------EDG
LDYGGLSXEF---FFLL----SHEMFNPFY--------CLFEYSAHDNY-----------
----------------TLQINPHS------------------------------------
--GINPEHLGYFKFIGRVVGLAIFHRRFLDAFFIG--AFYKMILR---------------
--------KKVNLIDMEG----------------------VDAEFHRT--------LTWT
MEN--DIT-DVI--
-YSTFSVEDER---------------------------FGE-KVTVD-LKPG--------
GRDIE---
------------------------------VDNDNKK-EYVEL-ITEWRIQKR-VEEQFN
-AFVAG---FHELIPA--DLVN-VFDER-ELELLIGG-----------------------
----IADID-----------VDDW----KKHTDYRG-YTES-------------------
------------------------DVVV-QNFWQCIR--------------------GWD
AEQKSRLLQF-------ATGTSRIP------------VNG-----FK-DLQGSD--GPRR
FTIEKSGEE-------------------------TQLPKSHTCF-NR-LDLPPY-KSYEA
LNTKLT-WAVEE-----
>Sphaerulina_musiva_XM_016907261.1 .
VKVRRTHI-FEDSYAEI--------------MRQSPNDLKK-RLMIKFDG-------EDG
LDYGGLSREF---FFLL----SHEMFNPFY--------CLFEYSAHDNY-----------
----------------TLQINPHS------------------------------------
--GINPEHLGYFKFIGRVVGLAIFHRRFLDAFFIG--AFYKMILK---------------
--------KKVNLQDMEG----------------------VDAEFHRT--------LTWT
MEN--DIT-DVI--
-YSTFSVEDER---------------------------FGE-KVTVD-LKPG--------

GRDIE---
------------------------------VTNENKK-EYVEL-ITEWRIQKR-VDEQFN
-AFVTG---FHELIPA--DLVN-VFDER-ELELLIGG-----------------------
----IADID-----------VDDW----KKHTDYRG-YTEN-------------------
------------------------DAVV-QNFWKVVR--------------------AWD
AEQKSRLLQF-------ATGTSRIP------------VNG-----FK-DLQGSD--GPRR
FTIEKSGEE-------------------------NQLPKSHTCF-NR-LDLPPY-KSFDA
LSQKLV-WAVEE-TVGF
>Pseudocerc_fijiensis_XM_007926643.1 .
VKVRRTHI-FEDSYAEI--------------MRQSPNDLKK-RLMIKFDG-------EDG
LDYGGLSREF---FFLL----SHEMFNPFY--------CLFEYSAHDNY-----------
----------------TLQINPHS------------------------------------
--GINPEHLGYFKFIGRVVGLAIFHRRFLDAFFIG--AFYKMILR---------------
--------KKVNLQDMEG----------------------VDAEFHRT--------LTWA
MDN--DIT-DVI--
-YSTFSVEDER---------------------------FGE-KVTVD-LKPG--------
GRDIE---
------------------------------VTNENKK-EYVEL-ITEWRIQKR-VEEQFN
-AFVTG---FHELIPA--DLVN-VFDER-ELELLIGG-----------------------
----IADID-----------VDDW----KKHTDYRG-YTEN-------------------
------------------------DVVI-QNFWKVVR--------------------SWD
AEQKSRLLQF-------ATGTSRIP------------VNG-----FK-DLQGSD--GPRR
FTIEKSGEE-------------------------TQLPKSHTCF-NR-LDLPPY-KSYDA
LTQKLV-WAVEE-TVGF
>Mycosphaerella_eumusae_GDIK01002888.1 .
VKVRRTHI-FEDSYAEI--------------MRQSPNDLKK-RLMIKFDG-------EDG
LDYGGLSREF---FFLL----SHEMFNPFY--------CLFEYSAHDNY-----------
----------------TLQINPHS------------------------------------
--GINPEHLGYFKFIGRVVGLAIFHRRFLDAFFIG--AFYKMILR---------------
--------KKVNLQDMEG----------------------VDAEFHRT--------LTWA
MDN--DIT-DVI--
-YSTFSVEDER---------------------------FGE-KVTVD-LKPG--------
GRDIE---
------------------------------VTNENKK-EYVEL-ITEWRIQKR-VEEQFN
-AFVTG---FHELIPA--DLVN-VFDER-ELELLIGG-----------------------
----IADID-----------VDDW----KKHTDYRG-YTEN-------------------
------------------------DVVI-QNFWKVVR--------------------SWD
AEQKSRLLQF-------ATGTSRIP------------VNG-----FK-DLQGSD--GPRR
FTIEKSGEE-------------------------TQLPKSHTCF-NR-LDLPPY-KSYDA
LTQKLV-WAVEE-TVGF
>Cladosporium_sphaerospermum_AIIA01011974.1_R .
VKVRRSHI-FEDSYAEI--------------MRQSPNDLKK-RLMIKFDG-------EDG
LDYGGLSXEF---FFLL----SHEMFNPFY--------CLFEYSAHDNY-----------
----------------TLQINPHS------------------------------------
--GINPEHLGYFKFIGRVVGLAIFHRRFLDAFFIG--AFYKMILK---------------
--------KKVALVDMEG----------------------VDAEFHRT--------LTWT
MEN--DIT-DVI--
-YSTFSVDDER---------------------------FGE-KVIVD-LKEG--------
GRDIE---
------------------------------VTNENKK-EYVEX-ITEWRIQKR-VEEQFN
-AFVQG---FHELIPA--DLVN-VFDER-ELELLIGG-----------------------
----IADID-----------VDDW----KKHTDYRG-YTES-------------------
------------------------DLVV-QNFWXVIR--------------------SWD
AEQKSRLLQF-------ATGTSRIP------------VNG-----FK-DLQGSD--GPRR
FTIEKSGEE-------------------------SQLPKSHTCF-NR-LDLPPY-KSYDA
LQQKLT-WAVEE-----
>Baudoinia_panamericana_XM_007677203.1 .
VKVRRSHI-FEDSYAEI--------------MRQSPNDLKK-RLMIKFDG-------EDG

LDYGGLSREF---FFLL----SHEMFNPFY--------CLFEYSAHDNY-----------
----------------TLQINPHS------------------------------------
--GINPEHLGYFKFIGRVVGLAIFHRRFLDAFFIG--AFYKMILR---------------
--------KKVALQDMEG----------------------VDAEFHRT--------LSWA
MDN--DIT-DVI--
-YSTFSVEDER---------------------------FGE-KVTVE-LKPG--------
GKDIE---
------------------------------VTNENKK-EYVEL-ITEWRIQKR-VEEQFN
-AFIAG---FHELIPA--DLVN-VFDER-ELELLIGG-----------------------
----IADID-----------VDDW----KKHTDYRG-YTES-------------------
------------------------DAVI-TNFWKCIR--------------------SWD
AEQKSRLLQF-------ATGTSRIP------------VNG-----FK-DLQGSD--GPRR
FTIEKSGEE-------------------------GQLPKSHTCF-NR-LDLPPY-KTFDA
LQQKLL-WAVEE-TVGF
>Acidomyces_richmondensis_JOOL01001181.1 .
VKVRRTHI-FEDSYAEI--------------MRQSPSDLKK-RLMIKFDG-------EDG
LDYGGLSREF---FFLL----SHEMFNPFY--------CLFEYSAHDNY-----------
----------------TLQINPHS------------------------------------
--GINPEHLGYFKFIGRVVGLAIFHRRFLDAFFIG--AFYKMILK---------------
--------KKVTLQDMEG----------------------VDAEFHRT--------LSWA
MEN--DIT-DVI--
-YSTFSVEDER---------------------------FGE-KVTVE-LKPG--------
GRDIE---
------------------------------VTNENKK-EYVEX-ITEWRIQKR-VEEQFN
-AFITG---FHELIPA--DLIN-VFDER-ELELLIGG-----------------------
----IADID-----------VDDW----KKHTDYRG-YTES-------------------
------------------------DPVI-QNFWKCIR--------------------SLD
AEQKSRLLQF-------ATGTSRIP------------VNG-----FK-DLQGSD--GPRR
FTIEKSGEE-------------------------GQLPKSHTCF-NR-LDLPPY-RTFEA
LRDKLM-WAVEE-TVG-
>Aureobasidium_subglaciale_XM_013484019.1 .
VKVRRTHI-FEDSYAEI--------------MRQSPSDLKK-RLMIKFDG-------EDG
LDYGGLSREF---FFLL----SHEMFNPFY--------CLFEYSAIDNY-----------
----------------TLQINPHS------------------------------------
--GINPEHLGYFKFIGRVVGLAIFHRRFLDAFFIG--AFYKMILR---------------
--------KKVSLADMEG----------------------VDADFYRT--------LSWT
LDN--SVE-NVI--
-FEHFCVEDEV---------------------------FGE-KVTID-LKPG--------
GRDIE---
------------------------------VTDDNKR-EYVEL-VTEWRIQKR-VDEQFN
-AFIQG---FHELIPS--DLVN-VFDER-ELELLIGG-----------------------
----ISEID-----------VDDW----AKNTDYRG-YDEK-------------------
------------------------DPVI-QNFWKCIR--------------------TWD
SEQKSRLLQF-------ATGTSRIP------------VNG-----FK-DLQGSD--GPRR
FTIEKSGEE-------------------------GQLPKSHTCF-NR-LDLPPY-KTYEA
LNAKLI-WAVEE-TVGF
>Aureobasidium_namibiae_XM_013576587.1 .
VKIRRSHI-FEDSYAEI--------------MRQSPSDLKK-RLMIKFDG-------EDG
LDYGGLSREF---FFLL----SHEMFNPFY--------CLFEYSAIDNY-----------
----------------TLQINPHS------------------------------------
--GINPEHLGYFKFIGRVVGLAIFHRRFLDAFFIG--AFYKMILR---------------
--------KKVSLADMEG----------------------VDADFYRT--------LSWT
LDN--SVE-NVI--
-FEHFCVEDEV---------------------------FGE-KVTID-LKPG--------
GRDIE---
------------------------------VTDDNKR-EYVEL-VTEWRIQKR-VEEQFN
-AFIQG---FHELIPS--DLVN-VFDER-ELELLIGG-----------------------
----ISEID-----------VDDW----AKNTDYRG-YDEK-------------------

------------------------DPVI-QNFWKCIR--------------------TWD
SEQKSRLLQF-------ATGTSRIP------------VNG-----FK-DLQGSD--GPRR
FTIEKSGEE-------------------------GQLPKSHTCF-NR-LDLPPY-KTYEA
LNAKLL-WAVEE-TVGF
>Aureobasidium_melanogenum_GEEO01008061.1 .
VKVRRSHI-FEDSYAEI--------------MRQSPSDLKK-RLMIKFDG-------EDG
LDYGGLSREF---FFLL----SHEMFNPFY--------CLFEYSAIDNY-----------
----------------TLQINPHS------------------------------------
--GINPEHLGYFKFIGRVVGLAIFHRRFLDAFFIG--AFYKMILR---------------
--------KKVSLADMEG----------------------VDADFYRT--------LSWT
LDN--SVE-NVI--
-FEHFCVEDEV---------------------------FGE-KVTID-LKPG--------
GRDIE---
------------------------------VTDENKR-EYVEL-VTEWRIQKR-VEEQFN
-AFIQG---FHELIPS--DLVN-VFDER-ELELLIGG-----------------------
----ISEID-----------VDDW----AKNTDYRG-YDEK-------------------
------------------------DPVI-QNFWKCIR--------------------TWD
SEQKSRLLQF-------ATGTSRIP------------VNG-----FK-DLQGSD--GPRR
FTIEKSGEE-------------------------GQLPKSHTCF-NR-LDLPPY-KTYEA
LNAKLL-WAVEE-TVGF
>Cladophialophora_immunda_XM_016390254.1 .
IKVRRGAI-FEDSYAEI--------------MRQSATDLKK-RLMIKFDG-------EDG
LDYGGLSREF---FFLL----SHEMFNPFY--------CLFEYSAHDNY-----------
----------------TLQINPHS------------------------------------
--GINPEHLNYFKFIGRVVGLAIFHRRFLDSFFIG--AFYKMMLR---------------
--------KKVTINDMEG----------------------VDEEYHKN--------LTWM
LEN--DIT-DVL--
-DQTFSIEDEQ---------------------------FGE-TKTID-LKPG--------
GRDIA---
------------------------------VTNENKR-EYVEL-LTEWKIQKR-VEEQFN
-AFITG---FNELIPA--DLVN-VFDER-ELELLIGG-----------------------
----IADID-----------VDDW----KKHTDYRG-YQEQ-------------------
------------------------DEVI-QNFWKVIR--------------------SWD
AEQKSRLLQF-------ATGTSRIP------------VNG-----FK-DLQGSD--GPRR
FTIEKAGEV-------------------------NALPKSHTCF-NR-LDLPPY-KSYDA
LQSKLS-TAVEE-TLGF
>Cladophialophora_psammophila_XM_007749474.1 .
IKVRRGAI-FEDSYAEI--------------MRQSATDLKK-RLMIKFDG-------EDG
LDYGGLSREF---FFLL----SHEMFNPFY--------CLFEYSAHDNY-----------
----------------TLQINPHS------------------------------------
--GINPEHLNYFKFIGRVVGLAIFHRRFLDSFFIG--AFYKMMLR---------------
--------KKVAINDMEG----------------------VDEEYHKN--------LTWM
LEN--DIT-DVL--
-DQTFSIEDEQ---------------------------FGE-TKTID-LKPG--------
GRDIA---
------------------------------VTNENKR-EYVEL-LTEWKIQKR-VEEQFN
-AFITG---FNELIPA--DLVN-VFDER-ELELLIGG-----------------------
----IADID-----------VDDW----KKHTDYRG-YQEQ-------------------
------------------------DEVI-QNFWKVIR--------------------SWD
AEQKSRLLQF-------ATGTSRIP------------VNG-----FK-DLQGSD--GPRR
FTIEKAGEV-------------------------NALPKSHTCF-NR-LDLPPY-KTYDA
LQSKLS-TAVEE-TLGF
>Cladophialophora_bantiana_XM_016761507.1 .
IKVRRGAI-FEDSYAEI--------------MRQSATDLKK-RLMIKFDG-------EDG
LDYGGLSREF---FFLL----SHEMFNPFY--------CLFEYSAHDNY-----------
----------------TLQINPHS------------------------------------
--GINPEHLNYFKFIGRVVGLAIFHRRFLDSFFIG--AFYKMMLR---------------
--------KKVTINDMEG----------------------VDEEYHKN--------LTWM

LEN--DIT-DVL--
-DQTFSIEDEQ---------------------------FGE-TKTID-LKPG--------
GRDIA---
------------------------------VTNENKR-EYVEL-LTEWKIQKR-VEEQFN
-AFITG---FNELIPA--DLVN-VFDER-ELELLIGG-----------------------
----IADID-----------VDDW----KKHTDYRG-YQEQ-------------------
------------------------DEVI-QNFWKVIR--------------------SWD
AEQKSRLLQF-------ATGTSRIP------------VNG-----FK-DLQGSD--GPRR
FTIEKAGEI-------------------------NALPKSHTCF-NR-LDLPPY-KTYDA
LQSKLS-TAVEE-TLGF
>Fonsecaea_pedrosoi_XM_013434127.1 .
IKVRRGAI-FEDSYAEI--------------MRQSATDLKK-RLMIKFDG-------EDG
LDYGGLSREF---FFLL----SHEMFNPFY--------CLFEYSAHDNY-----------
----------------TLQINPHS------------------------------------
--GINPEHLNYFKFIGRVVGLAIFHRRFLDSFFIG--AFYKMMLR---------------
--------KKVTINDMEG----------------------VDEEYHKN--------LTWM
LEN--DIT-DVL--
-DQTFSIEDEQ---------------------------FGE-TKTID-LKPG--------
GRDIA---
------------------------------VTNENKR-EYVEL-LTEWKIQKR-VEEQFN
-AFITG---FNELIPA--DLVN-VFDER-ELELLIGG-----------------------
----IADID-----------VDDW----KKHTDYRG-YQEQ-------------------
------------------------DEVI-QNFWKVIR--------------------SWD
AEQKSRLLQF-------ATGTSRIP------------VNG-----FK-DLQGSD--GPRR
FTIEKAGEI-------------------------NALPKSHTCF-NR-LDLPPY-KTYDA
LQSKLS-TAVEE-TLG-
>Fonsecaea_erecta_XM_018837303.1 .
IKVRRGAI-FEDSFAEI--------------MRQSATDLKK-RLMIKFDG-------EDG
LDYGGLSREF---FFLL----SHEMFNPFY--------CLFEYSAHDNY-----------
----------------TLQINPHS------------------------------------
--GINPEHLNYFKFIGRVVGLAIFHRRFLDSFFIG--AFYKMMLR---------------
--------KKVTINDMEG----------------------VDEEYHKN--------LTWM
LEN--DIT-DVL--
-DQTFSIEDEQ---------------------------FGE-TKTID-LKPG--------
GRDIP---
------------------------------VTNENKR-EYVEL-LTEWKIQKR-VEEQFN
-AFITG---FNELIPA--DLVN-VFDER-ELELLIGG-----------------------
----IADID-----------VDDW----KKHTDYRG-YQEQ-------------------
------------------------DEVI-QNFWKVIR--------------------SWD
AEQKSRLLQF-------ATGTSRIP------------VNG-----FK-DLQGSD--GPRR
FTIEKAGEV-------------------------NALPKSHTCF-NR-LDLPPY-KTYDA
LQSKLS-TAVEE-TLGF
>Fonsecaea_multimorphosa_XM_016780286.1 .
IKVRRGAI-FEDSYAEI--------------MRQSATDLKK-RLMIKFDG-------EDG
LDYGGLSREF---FFLL----SHEMFNPFY--------CLFEYSAHDNY-----------
----------------TLQINPHS------------------------------------
--GINPEHLNYFKFIGRVVGLAIFHRRFLDSFFIG--AFYKMMLR---------------
--------KKVTINDMEG----------------------VDEEYHKN--------LTWM
LEN--DIT-DVL--
-DQTFSIEDEQ---------------------------FGE-TKTID-LKPG--------
GRDLP---
------------------------------VTNENKR-EYVEL-LTEWKIQKR-VEEQFN
-AFITG---FNELIPA--DLVN-VFDER-ELELLIGG-----------------------
----IADID-----------VDDW----KKHTDYRG-YQEQ-------------------
------------------------DEVI-QNFWKVIR--------------------SWD
AEQKSRLLQF-------ATGTSRIP------------VNG-----FK-DLQGSD--GPRR
FTIEKAGEI-------------------------NALPKSHTCF-NR-LDLPPY-KTYEA
LQGKLS-TAVEE-TLGF

>Cladophialophora_yegresii_XM_007760027.1 .
IKVRRGAI-FEDSYAEI--------------MRQSATDLKK-RLMIKFDG-------EDG
LDYGGLSREF---FFLL----SHEMFNPFY--------CLFEYSAHDNY-----------
----------------TLQINPHS------------------------------------
--GINPEHLNYFKFIGRVVGLAIFHRRFLDSFFIG--AFYKMMLR---------------
--------KKVTINDMEG----------------------VDEEYHKN--------LTWM
LDN--DIT-DVL--
-DQTFSIEDEQ---------------------------FGE-TKTID-LKPG--------
GRDIP---
------------------------------VTNENKR-EYVEL-LTEWKIQKR-VEEQFN
-AFITG---FNELIPA--DLVN-VFDER-ELELLIGG-----------------------
----IADID-----------VDDW----KKHTDYRG-YQEQ-------------------
------------------------DEVI-QNFWKVIR--------------------SWD
AEQKSRLLQF-------ATGTSRIP------------VNG-----FK-DLQGSD--GPRR
FTIEKAGEI-------------------------NALPKSHTCF-NR-LDLPPY-KTYEA
LNGKLS-TAVEE-TLGF
>Rhinocladiella_mackenziei_XM_013416193.1 .
IKVRRGAI-FEDSYAEI--------------MRQSATDLKK-RLMIKFDG-------EDG
LDYGGLSREF---FFLL----SHEMFNPFY--------CLFEYSAHDNY-----------
----------------TLQINPHS------------------------------------
--GINPEHLNYFKFIGRVVGLAIFHRRFLDSFFIG--AFYKMMLR---------------
--------KKVTINDMEG----------------------VDEEYHKN--------LTWT
LEN--DIT-DIL--
-DQTFSIEDEQ---------------------------FGE-TKTID-LKPG--------
GRDIQ---
------------------------------VTNENKR-EYVEL-VTEWKIQKR-VEEQFN
-AFITG---FNELIPA--DLVN-VFDER-ELELLIGG-----------------------
----IADID-----------VDDW----KKHTDYRG-YQEQ-------------------
------------------------DEVI-QNFWKVIR--------------------SWD
AEQKSRLLQF-------ATGTSRIP------------VNG-----FK-DLQGSD--GPRR
FTIEKAGEV-------------------------NALPKSHTCF-NR-LDLPPY-RSYDT
LNLKLS-TAVEE-TLGF
>Phialophora_attae_XM_018146824.1 .
VKVRRGNI-FEDSYAEI--------------MRQSATDLKK-RLMIKFDG-------EDG
LDYGGLSREF---FFLL----SHEMFNPFY--------CLFEYSAHDNY-----------
----------------TLQINPHS------------------------------------
--GINPEHLNYFKFIGRVS----------------------MMLR---------------
--------KKVAINDMEG----------------------VDEEYHKN--------LTWT
LEN--DIT-DIL--
-DQTFSVEDEQ---------------------------FGE-TKTID-LKPG--------
GRDIP---
------------------------------VTNENKR-EYVEL-VTQWKIQTR-VEEQFN
-AFITG---FNELIPA--DLVN-VFDER-ELELLIGG-----------------------
----IADID-----------VDDW----KKHTDYRG-YQEG-------------------
------------------------DEVI-QNFWKIIR--------------------TWD
AEQKSRLLQF-------ATGTSRIP------------VNG-----FK-DLQGSD--GPRR
FTIEKAGEI-------------------------NALPKSHTCF-NR-LDLPPY-KSHDV
LQSKLS-TAVEE-TLGF
>Cyphellophora_europaea_XM_008713043.1 .
VKVRRGNI-FEDSYAEI--------------MRQSATDLKK-RLMIKFDG-------EDG
LDYGGLSREF---FFLL----SHEMFNPFY--------CLFEYSAHDNY-----------
----------------TLQINPHS------------------------------------
--GINPEHLNYFKFIGRVVGLAIFHRRFLDSFFIG--AFYKMMLR---------------
--------KKVQINDMEG----------------------VDEEYHKN--------LTWT
LDN--DIT-DIL--
-DQTFSVEDEQ---------------------------FGE-TKTID-LKPG--------
GRDIP---
------------------------------VTNENKR-EYVEL-VTEWKIVKR-VEEQFN

-AFITG---FNELIPA--DLVN-VFDER-ELELLIGG-----------------------
----IADID-----------VDDW----KKHTDYRG-YQES-------------------
------------------------DEVV-QNFWKVIR--------------------TWD
AEQKSRLLQF-------ATGTSRIP------------VNG-----FK-DLQGSD--GPRR
FTIEKAGEI-------------------------NALPKSHTCF-NR-LDLPPY-KSYEV
LNSKLS-TAVEE-TLGF
>Exophiala_aquamarina_XM_013403525.1 .
IKVRRGAI-FEDSYAEI--------------MRQSATDLKK-RLMIKFDG-------EDG
LDYGGLSREF---FFLL----SHEMFNPFY--------CLFEYSAHDNY-----------
----------------TLQINPHS------------------------------------
--GINPEHLNYFKFIGRVVGLAIFHRRFLDSFFIG--AFYKMMLR---------------
--------KKVAINDMEG----------------------VDEEYHKN--------LTWT
LDN---DIT-DIL--
-DQTFAIEDEQ---------------------------FGE-TKTID-LKPG--------
GRDIP---
------------------------------VTNENKR-EYVEL-VTEWKIQKR-VEEQFN
-AFITG---FNELIPA--DLVN-VFDER-ELELLIGG-----------------------
----IADID-----------VEDW----KKHTDYRG-YQEQ-------------------
------------------------DQVI-QDFWKVIR--------------------TWD
AEQKSRLLQF-------ATGTSRIP------------VNG-----FK-DLQGSD--GPRR
FTIEKAGEI-------------------------NALPKSHTCF-NR-LDLPPY-KTYEV
LNSKLS-TAVEE-TLGF
>Exophiala_mesophila_XM_016373368.1 .
IKVRRGAI-FEDSYAEI--------------MRQSATDLKK-RLMIKFDG-------EDG
LDYGGLSREF---FFLL----SHEMFNPFY--------CLFEYSAHDNY-----------
----------------TLQINPHS------------------------------------
--GINPEHLNYFKFIGRVVGLAIFHRRFLDSFFIG--AFYKMMLR---------------
--------KKVAINDMEG----------------------VDEEYHKN--------LTWT
LDN---DIT-DIL--
-DQTFSIEDEQ---------------------------FGE-TKTID-LKPG--------
GRDIP---
------------------------------VTNENKR-EYVEL-VTEWKIQKR-VEEQFN
-AFITG---FNELIPA--DLVN-VFDER-ELELLIGG-----------------------
----IADID-----------VEDW----KKHTDYRG-YQEQ-------------------
------------------------DEVI-QHFWKVIR--------------------SWD
AEQKSRLLQF-------ATGTSRIP------------VNG-----FK-DLQGSD--GPRR
FTIEKAGEI-------------------------NALPKSHTCF-NR-LDLPPY-KSYDV
LNSKLS-TAVEE-TLGF
>Exophiala_xenobiotica_XM_013457981.1 .
IKVRRGNI-FEDSYAEI--------------MRQSATDLKK-RLMIKFDG-------EDG
LDYGGLSREF---FFLL----SHEMFNPFY--------CLFEYSAHDNY-----------
----------------TLQINPHS------------------------------------
--GINPEHLNYFKFIGRVVGLAIFHRRFLDSFFIG--AFYKMMLR---------------
--------KKVQIQDMEG----------------------VDEEYHKN--------LTWT
LEN---DIT-DIL--
-DQTFSIEDEQ---------------------------FGE-TKTID-LKPN--------
GQNIP---
------------------------------VTNENKR-EYVEL-VTEWKIVKR-VEEQFN
-AFITG---FNELIPA--DLVN-VFDER-ELELLIGG-----------------------
----IADID-----------VEDW----KKHTDYRG-YQEQ-------------------
------------------------DEVI-QNFWKVVR--------------------TWD
AEQKSRLLQF-------ATGTSRIP------------VNG-----FK-DLQGSD--GPRR
FTIEKAGEI-------------------------NALPKSHTCF-NR-LDLPPY-KTYDV
LNSKLS-TAVEE-TLGF
>Exophiala_oligosperma_XM_016403160.1 .
IKVRRGNI-FEDSYAEI--------------MRQSATDLKK-RLMIKFDG-------EDG
LDYGGLSREF---FFLL----SHEMFNPFY--------CLFEYSAHDNY-----------
----------------TLQINPHS------------------------------------

```
--GINPEHLNYFKFIGRVVGLAIFHRRFLDSFFIG--AFYKMMLR---------------
--------KKVQIQDMEG----------------------VDEEYHKN--------LTWT
LEN------------------------------------------------DIT-DIL--
-DQTFSIEDEQ---------------------------FGE-TKTID-LKPG--------
GRDIP-------------------------------------------------------
------------------------------VTNENKR-EYVEL-VTEWKIVKR-VEEQFN
-AFITG---FNELIPA--DLVN-VFDER-ELELLIGG-----------------------
----IADID-----------VDDW----KKHTDYRG-YQEQ-------------------
------------------------DEVI-QNFWKVVR--------------------TWD
AEQKSRLLQF-------ATGTSRIP------------VNG-----FK-DLQGSD--GPRR
FTIEKAGEI-------------------------NALPKSHTCF-NR-LDLPSY-KTYDV
LNSKLS-TAVEE-TLGF
>Exophiala_spinifera_XM_016376988.1 .
IKVRRGNI-FEDSYAEI--------------MRQSATDLKK-RLMIKFDG-------EDG
LDYGGLSREF---FFLL----SHEMFNPFY--------CLFEYSAHDNY-----------
----------------TLQINPHS------------------------------------
--GINPEHLNYFKFIGRVVGLAIFHRRFLDSFFIG--AFYKMMLR---------------
--------KKVQIQDMEG----------------------VDEEYHKN--------LTWT
LEN------------------------------------------------DIT-DIL--
-DQTFSIEDEQ---------------------------FGE-TKTID-LKPG--------
GRDIP-------------------------------------------------------
------------------------------VTNENKR-EYVEL-VTEWKIVKR-VEEQFN
-AFITG---FNELIPA--DLVN-VFDER-ELELLIGG-----------------------
----IADID-----------VDDW----KKHTDYRG-YQEQ-------------------
------------------------DEVI-QNFWKVVR--------------------TWD
AEQKSRLLQF-------ATGTSRIP------------VNG-----FK-DLQGSD--GPRR
FTIEKAGEI-------------------------NALPKSHTCF-NR-LDLPPY-KTYDV
LNSKLS-TAVEE-TLGF
>Exophiala_dermatitidis_XM_009157907.1 .
IKVRRGAI-FEDSYAEI--------------MRQSATDLKK-RLMIKFDG-------EDG
LDYGGLSREF---FFLL----SHEMFNPFY--------CLFEYSAHDNY-----------
----------------TLQINPHS------------------------------------
--GINPEHLNYFKFIGRVVGLAIFHRRFLDSFFIG--AFYKMMLR---------------
--------KKVTINDMEG----------------------VDEEYHKN--------LTWC
LEN------------------------------------------------DIT-DVL--
-DQTFSIEDEQ---------------------------FGE-TKTID-LKPN--------
GRNIP-------------------------------------------------------
------------------------------VTNENKR-EYVEL-VTEWKIVKR-VEEQFN
-AFITG---FNELIPQ--DLVN-VFDER-ELELLIGG-----------------------
----IADID-----------VDDW----KKHTDYRG-YQES-------------------
------------------------DEVI-QNFWKIVR--------------------SWD
AEQKSRLLQF-------ATGTSRIP------------VNG-----FK-DLQGSD--GPRR
FTIEKAGEI-------------------------NALPKSHTCF-NR-LDLPPY-KSYEV
LQQKLS-IAVEE-TLGF
>Capronia_coronata_XM_007727262.1 .
IKVRRGAI-FEDSYAEI--------------MRQSATDLKK-RLMIKFDG-------EDG
LDYGGLSREF---FFLL----SHEMFNPFY--------CLFEYSAHDNY-----------
----------------TLQINPHS------------------------------------
--GINPEHLNYFKFIGRVVGLAIFHRRFLDSFFIG--AFYKMMLR---------------
--------KKVTINDMEG----------------------VDEEYHKN--------LTWC
LEN------------------------------------------------DIT-DVL--
-DQTFSIEDEQ---------------------------FGE-TKTID-LKPD--------
GRNIP-------------------------------------------------------
------------------------------VTNENKR-EYVEL-VTEWKIVKR-VEEQFN
-AFITG---FNELIPQ--DLVN-VFDER-ELELLIGG-----------------------
----IADID-----------VDDW----KKHTDYRG-YQES-------------------
------------------------DEVI-QNFWKIVR--------------------TWD
AEQKSRLLQF-------ATGTSRIP------------VNG-----FK-DLQGSD--GPRR
```

FTIEKAGEI-------------------------NALPKSHTCF-NR-LDLPPY-KTYEA
LQQKLT-IAVEE-TLGF
>Capronia_epimyces_XM_007740094.1 .
IKVRRGAI-FEDSYAEI--------------MRQSATDLKK-RLMIKFDG-------EDG
LDYGGLSREF---FFLL----SHEMFNPFY--------CLFEYSAHDNY-----------
----------------TLQINPHS------------------------------------
--GINPEHLNYFKFIGRVVGLAIFHRRFLDSFFIG--AFYKMMLR---------------
--------KKVTINDMEG----------------------VDEEYHKN--------LTWC
LEN--DIT-DVL--
-DQTFSIEDEQ---------------------------FGE-TKTID-LKPD--------
GRNIP---
------------------------------VTNENKR-EYVEL-VTEWKIVKR-VEEQFN
-AFITG---FNELIPQ--DLVN-VFDER-ELELLIGG-----------------------
----IADID-----------VDDW----KKHTDYRG-YQES-------------------
------------------------DEVI-QNFWKIVR--------------------TWD
AEQKSRLLQF-------ATGTSRIP------------VNG-----FK-DLQGSD--GPRR
FTIEKAGEI-------------------------NALPKSHTCF-NR-LDLPPY-KSHEV
LQQKLT-IAVEE-TLGF
>Endocarpon_pusillum_XM_007788882.1 .
VKVRRGNI-FEDSYAEI--------------MRQSATDLKK-RLMIKFDG-------EDG
LDYGGLSREF---FFLL----SHEMFNPFY--------CLFEYSAHDNY-----------
----------------TLQINPHS------------------------------------
--GINPEHLNYFKFIGRVVGLAIFHRRFLDSFFIG--AFYKMMLR---------------
--------KKVQIQDMEG----------------------VDEDFHRN--------LTWM
LEN--DIE-NVV--
-DLTFSVDDDQ---------------------------FGE-TKTVD-LKPG--------
GSEIP---
------------------------------VTNQSKK-EYIEL-VTEWKIQKR-VSEQFN
-AFITG---FNELIPA--DLVN-VFDER-ELELLIGG-----------------------
----IADID-----------VDDW----KKHTDYRG-YQEQ-------------------
------------------------DEVI-QNFWKVIR--------------------TWD
AEQKSRLLQF-------ATGTSRIP------------VNG-----FK-DLQGSD--GPRR
FTIEKAGEV-------------------------NALPKSHTCF-NR-LDLPPY-KSYDT
LNSKLS-TAVEE-TLGF
>Byssoonygena_ceratinophila_GDRB01008470.1 .
VKVRRTAI-FEDSYAEI--------------MRQTPADLKK-RLMIKFDG-------EDG
LDYGGLSREF---FFLL----SHEMFNPFY--------CLFEYSAHDNY-----------
----------------TLQINPHS------------------------------------
--GVNPEHLNYFRFIGRVVGLAIFHRRFLDSFFIG--AFYKMMLR---------------
--------KKVTLQDMEG----------------------VDEDFHRN--------LTWT
LEN--DID-GVF--
-ELTFAVDDEQ---------------------------FGE-HKTID-LMPG--------
GRDIA---
------------------------------VTNENKR-QYVEL-VTEWKIQKR-VEEQFN
-AFIAG---FNELIPA--DLVN-VFDER-ELELLIGG-----------------------
----IADID-----------VEDW----KKHTDYRG-YQEQ-------------------
------------------------DEVI-QNFWKIIR--------------------TWD
AEQKSRLLQF-------ATGTSRIP------------VNG-----FK-DLQGSD--GPRR
FTIEKSGDV-------------------------NALPKSHTCF-NR-LDLPPY-KSHEA
LQHKLS-IAVEE-TVGF
>Uncinocarpus_reesii_XM_002585045.1 .
VKVRRGAI-FEDSYAEI--------------MRQSPADLKK-RLMIKFDG-------EDG
LDYGGLSREF---FFLL----SHEMFNPFY--------CLFEYSAHDNY-----------
----------------TLQINPHS------------------------------------
--GVNPEHLNYFRFIGRVVGLAIFHRRFLDSFFIG--AFYKMMLR---------------
--------KKVTLQDMEG----------------------VDEDFHRN--------LTWT
LEN--DIE-GVF--
-ELTFAVDDEQ---------------------------FGE-HKTID-LIPG--------

GRDIA---
------------------------------VTNENKR-QYVEL-VTEWKIQKR-VEEQFN
-AFIAG---FNELIPS--DLVN-VFDER-ELELLIGG-----------------------
----IADID-----------VEDW----KKHTDYRG-YQEQ-------------------
------------------------DEVI-QNFWKIIR--------------------SWD
AEQKSRLLQF-------ATGTSRIP------------VNG-----FK-DLQGSD--GPRR
FTIEKSGDI-------------------------NALPKSHTCF-NR-LDLPPY-KTHEA
LQNKLS-IAVEE-TVGF
>Amauroascus_niger_GDRA01004031.1 .
VKIRRGAI-FEDSYSEI--------------MRQSPADLKK-RLMIKFDG-------EDG
LDYGGLSREF---FFLL----SHEMFNPFY--------CLFEYSAHDNY-----------
----------------TLQINPHS------------------------------------
--GVNPEHLNYFRFIGRVVGLAIFHRRFLDSFFIG--AFYKMMLR---------------
--------KKVTLQDMEG----------------------VDEDFHRN--------LTWT
LEN--DID-GVF--
-ELTFAVDDEQ---------------------------FGE-HKTID-LIPG--------
GRDIP---
------------------------------VTNENKR-QYVEL-VTEWKIQKR-VEEQFN
-AFIAG---FNELIPA--DLVN-VFDER-ELELLIGG-----------------------
----IADID-----------VDDW----KKHTDYRG-YQEQ-------------------
------------------------DEVI-QNFWKIIR--------------------TWD
AEQKSRLLQF-------ATGTSRIP------------VNG-----FK-DLQGSD--GPRR
FTIEKSGDI-------------------------NALPKSHTCF-NR-LDLPPY-KTFDA
LLNKLS-IAVEE-TVGF
>Chrysosporium_queenslandicum_GDRC01004475.1 .
VKVRRGAI-FEDSYAEI--------------MRQSPSDLKK-RLMIKFDG-------EDG
LDYGGLSREF---FFLL----SHEMFNPFY--------CLFEYSAHDNY-----------
----------------TLQINPHS------------------------------------
--GVNPEHLNYFRFIGRVVGLAIFHRRFLDSFFIG--AFYKMMLR---------------
--------KKVTLQDMEG----------------------VDEDFHRN--------LTWT
LEN--DID-GVF--
-ELTFSVDDEQ---------------------------FGE-HRTID-LIPG--------
GRDIA---
------------------------------VTNENKR-QYVEL-VTEWKIQKR-VEEQFN
-AFIAG---FNELIPA--DLVN-VFDER-ELELLIGG-----------------------
----IADID-----------VEDW----KKHTDYRG-YQEQ-------------------
------------------------DEVI-QNFWKIIR--------------------TWD
AEQKSRLLQF-------ATGTSRIP------------VNG-----FK-DLQGSD--GPRR
FTIEKSGDI-------------------------NALPKSHTCF-NR-LDLPPY-KTYEA
LQNKLS-IAVEE-TVGF
>Amauroascus_mutatus_GDQZ01001260.1 .
VKVRRGAI-FEDSYAEI--------------MRQSPSDLKK-RLMIKFDG-------EDG
LDYGGLSREF---FFLL----SHEMFNPFY--------CLFEYSAHDNY-----------
----------------TLQINPHS------------------------------------
--GVNPEHLNYFRFIGRVVGLAIFHRRFLDSFFIG--AFYKMMLR---------------
--------KKVTLQDMEG----------------------VDEDYHRN--------LTWT
LEN--DIE-GVF--
-ELTFSVDDEQ---------------------------FGE-HKTID-LIPG--------
GRDIA---
------------------------------VTNENKR-QYVEL-VTEWKIQKR-VEEQFN
-SFITG---FNELIPA--DLVN-VFDER-ELELLIGG-----------------------
----IADID-----------VDDW----KKHTDYRG-YQEQ-------------------
------------------------DEVI-QNFWKIIR--------------------TWD
AEQKSRLLQF-------ATGTSRIP------------VNG-----FK-DLQGSD--GPRR
FTIEKSGDI-------------------------NALPKSHTCF-NR-LDLPPY-KTHEA
LQNKLS-IAVEE-TVGF
>Coccidioides_immitis_XM_001243223.2 .
VKIRRSAI-FEDSYAEI--------------MRQSPSDLKK-RLMIKFDG-------EDG

```
LDYGGLSREF---FFLL----SHEMFNPFY--------CLFEYSAHDNY-----------
----------------TLQINPHS------------------------------------
--GVNPEHLNYFRFIGRVVGLAIFHRRFLDSFFIG--AFYKMMLR---------------
--------KKVTLQDMEG----------------------VDEDFHRN--------LTWT
LEN------------------------------------------------DIE-GVF--
-ELTFAVDDEQ---------------------------FGE-HKTID-LIPN--------
GRDIA-------------------------------------------------------
------------------------------VTNENKR-QYVEL-VTEWKIQKR-VEEQFN
-AFITG---FNELIPA--DLVN-VFDER-ELELLIGG-----------------------
----IADID-----------VDDW----KKHTDYRG-YQEQ-------------------
------------------------DEVI-QNFWKIIR--------------------TWD
AEQKSRLLQF-------ATGTSRIP------------VNG-----FK-DLQGSD--GPRR
FTIEKSGDI-------------------------NALPKSHTCF-NR-LDLPPY-KTYEA
LQNKLS-IAVEE-TVGF
>Coccidioides_posadasii_XM_003070218.1 .
VKIRRSAI-FEDSYAEI--------------MRQSPSDLKK-RLMIKFDG-------EDG
LDYGGLSREF---FFLL----SHEMFNPFY--------CLFEYSAHDNY-----------
----------------TLQINPHS------------------------------------
--GVNPEHLNYFRFIGRVVGLAIFHRRFLDSFFIG--AFYKMMLR---------------
--------KKVTLQDMEG----------------------VDEDFHRN--------LTWT
LEN------------------------------------------------DIE-GVF--
-ELTFAVDDEQ---------------------------FGE-HKTID-LIPN--------
GRDIA-------------------------------------------------------
------------------------------VTNENKR-QYVEL-VTEWKIQKR-VEEQFN
-AFITG---FNELIPA--DLVN-VFDER-ELELLIGG-----------------------
----IADID-----------VDDW----KKHTDYRG-YQEQ-------------------
------------------------DEVI-QNFWKIIR--------------------TWD
AEQKSRLLQF-------ATGTSRIP------------VNG-----FK-DLQGSD--GPRR
FTIEKSGDI-------------------------NALPKSHTCF-NR-LDLPPY-KTYEA
LQNKLS-IAVEE-TVG-
>Arthroderma_otae_XM_002842617.1 .
VKVRRGAI-FEDSFAEI--------------MRQSPADLKK-RLMIKFDG-------EDG
LDYGGLSREF---FFLL----SHEMFNPFY--------CLFEYSAHDNY-----------
----------------TLQINPHS------------------------------------
--GINPEHLNYFKFIGRVVGLAIFHRRFLDSFFIG--AFYKMMLR---------------
--------KKVTLQDMEG----------------------VDEDFHRN--------LTWT
LKN------------------------------------------------DIE-GII--
-ELTFSIDDEQ---------------------------FGE-RRTID-LIPN--------
GRNIP-------------------------------------------------------
------------------------------VTNENKK-QYVEL-VTEWKIQKR-VEEQFN
-AFLAG---FHELIPA--DLVN-VFDER-ELELLIGG-----------------------
----IADIH-----------IDDW----KKHTDYRG-YQED-------------------
------------------------DEVI-QNFWKVIR--------------------SWD
AEQKSRLLQF-------ATGTSRIP------------VNG-----FK-DLQGSD--GPRR
FTIEKSGDI-------------------------NALPKSHTCF-NR-LDLPPY-KTYEA
LQNKLT-IAVEE-TLGF
>Trichophyton_tonsurans_ACPI01000168.1_R .
VKVRRGAI-FEDSFAEI--------------MRQSPADLKK-RLMIKFDG-------EDG
LDYGGLSREF---FFLL----SHEMFNPFY--------CLFEYSAHDNY-----------
----------------TLQINPHS------------------------------------
--GINPEHLNYFKFIGRVVGLAIFHRRFLDSFFIG--AFYKMMLR---------------
--------KKVTLQDMEG----------------------VDEDFHRN--------LTWT
LXN------------------------------------------------DID-GII--
-ELTFSIDDEQ---------------------------FGE-RRTID-LIPD--------
GRNIP-------------------------------------------------------
------------------------------VTNENKK-QYVEY-VTEWKIQKR-VEEQFN
-AFITG---FNELIPA--ELVN-VFDER-ELELLIGG-----------------------
----IADIH-----------IDDW----KKHTDYRG-YQED-------------------
```

```
------------------------DEVI-QNFWKVIR--------------------SWD
AEQKSRLLQF-------ATGTSRIP------------VNG-----FK-DLQGSD--GPRR
FTIEKSGDI-------------------------NALPKSHTXF-NR-LDLPPY-KTYEA
LQNKLT-IAVEE-----
>Nannizzia_gypsea_XM_003169049.1 .
VKVRRGAI-FEDSFAEI--------------MRQSPADLKK-RLMIKFDG-------EDG
LDYGGLSREF---FFLL----SHEMFNPFY--------CLFEYSAHDNY-----------
----------------TLQINPHS------------------------------------
--GINPEHLNYFKFIGRVVGLAIFHRRFLDSFFIG--AFYKMMLR---------------
--------KKVTLQDMEG----------------------VDEDFHRN--------LTWT
LNN------------------------------------------------DID-GII--
-ELTFSIDDEQ---------------------------FGE-RRTID-LIPD--------
GRNIP-------------------------------------------------------
------------------------------VTNENKK-QYVEL-VTEWKIQKR-VEEQFN
-AFITG---FNELIPA--ELVN-VFDER-ELELLIGG-----------------------
----IADIH-----------IDDW----KKHTDYRG-YQED-------------------
------------------------DEVI-QNFWKVIR--------------------GWD
AEQKSRLLQF-------ATGTSRIP------------VNG-----FK-DLQGSD--GPRR
FTIEKSGDI-------------------------NALPKSHTCF-NR-LDLPPY-KTYEA
LQNKLT-IAVEE-TLGF
>Trichophyton_rubrum_XM_003235785.1 .
VKVRRGAI-FEDSFAEI--------------MRQSPADLKK-RLMIKFDG-------EDG
LDYGGLSREF---FFLL----SHEMFNPFY--------CLFEYSAHDNY-----------
----------------TLQINPHS------------------------------------
--GINPEHLNYFKFIGRVVGLAIFHRRFLDSFFIG--AFYKMMLR---------------
--------KKVTLQDMEG----------------------VDEDFHRN--------LTWT
LNN------------------------------------------------DID-GII--
-ELTFSIDDEQ---------------------------FGE-RRTID-LIPD--------
GRNIP-------------------------------------------------------
------------------------------VTNENKK-QYVEL-VTEWKIQKR-VEEQFN
-AFITG---FNELIPA--ELVN-VFDER-ELELLIGG-----------------------
----IADIH-----------IDDW----KKHTDYRG-YQED-------------------
------------------------DEVI-QNFWKVIR--------------------SWD
AEQKSRLLQF-------ATGTSRIP------------VNG-----FK-DLQGSD--GPRR
FTIEKSGDI-------------------------NALPKSHTCF-NR-LDLPPY-KTYEA
LQNKLT-IAVEE-TLGF
>Arthroderma_benhamiae_XM_003013868.1 .
VKVRRGAI-FEDSFAEI--------------MRQSPADLKK-RLMIKFDG-------EDG
LDYGGLSREF---FFLL----SHEMFNPFY--------CLFEYSAHDNY-----------
----------------TLQINPHS------------------------------------
--GINPEHLNYFKFIGRVVGLAIFHRRFLDSFFIG--AFYKMMLR---------------
--------KKVTLQDMEG----------------------VDEDFHRN--------LTWT
LNN------------------------------------------------DID-GII--
-ELTFSIDDEQ---------------------------FGE-RRTID-LIPD--------
GRNIP-------------------------------------------------------
------------------------------VTNENKK-QYVEL-VTEWKIQKR-VEEQFN
-AFITG---FNELIPA--ELVN-VFDER-ELELLIGG-----------------------
----IADIH-----------IDDW----KKHTDYRG-YQED-------------------
------------------------DEVI-QNFWKVIR--------------------SWD
AEQKSRLLQF-------ATGTSRIP------------VNG-----FK-DLQGSD--GPRR
FTIEKSGDI-------------------------NALPKSHTXF-NR-LDLPPY-KTYEA
LQNKLT-IAVEE-TLGF
>Trichophyton_verrucosum_XM_003021448.1 .
VKVRRGAI-FEDSFAEI--------------MRQSPADLKK-RLMIKFDG-------EDG
LDYGGLSREF---FFLL----SHEMFNPFY--------CLFEYSAHDNY-----------
----------------TLQINPHS------------------------------------
--GINPEHLNYFKFIGRVVGLAIFHRRFLDSFFIG--AFYKMMLR---------------
--------KKVTLQDMEG----------------------VDEDFHRN--------LTWT
```

LNN--DID-GII--
-ELTFSIDDEQ---------------------------FGE-RRTID-LIPD--------
GRNIP---
------------------------------VTNENKK-QYVEL-VTEWKIQKR-VEEQFN
-AFITG---FNELIPA--ELVN-VFDER-ELELLIGG-----------------------
----IADIH-----------IDDW----KKHTDYRG-YQED-------------------
------------------------DEVI-QNFWKVIR--------------------SWD
AEQKSRLLQF-------ATGTSRIP------------VNG-----FK-DLQGSD--GPRR
FTIEKSGDI-------------------------NALPKSHTXF-NR-LDLPPY-KTYEA
LQNKLT-IAVEE-TLGF
>Ajellomyces_capsulatus_XM_001538504.1 .
VKVRRGAI-FEDSYAEI--------------MRQSASDLKK-RLMIKFDG-------EDG
LDYGGLSREF---FFLL----SHEMFNPFY--------CLFEYSAHDNY-----------
----------------TLQINPHS------------------------------------
--GINPEHLNYFKFIGRVVGLAIFHRRFLDSFFIG--AFYKMMLR---------------
--------KKVTLQDMEG----------------------VDEDFHRN--------LTWT
LEH--DIT-GVFD-
-ELTFSIDDDQ---------------------------FGE-RKTVD-LIPN--------
GSNIP---
------------------------------VTNENKK-QYVEL-ITEWKIQKR-VEEQFN
-AFITG---FNELIPA--DLVN-VFDER-ELELLIGG-----------------------
----IADID-----------VDDW----KKHTDYRG-YQEQ-------------------
------------------------DEVI-QNFWKVIR--------------------SWD
AEQKSRLLQF-------ATGTSRIP------------VNG-----FK-DLQGSD--GPRR
FTIEKSGEI-------------------------TALPKSHTCF-NR-LDLPPY-KTYEQ
LQHKLS-IAVEE-TLGF
>Ajellomyces_dermatitidis_XM_002627658.1 .
VKVRRGAI-FEDSYAEI--------------MRQSASDLKK-RLMIKFDG-------EDG
LDYGGLSREF---FFLL----SHEMFNPFY--------CLFEYSAHDNY-----------
----------------TLQINPHS------------------------------------
--GINPEHLNYFKFIGRVVGLAIFHRRFLDSFFIG--AFYKMMLR---------------
--------KKVTLQDMEG----------------------VDEDFHRN--------LTWT
LEH--DIT-GVFD-
-ELTFSIDDDQ---------------------------FGE-RKTVD-LIPN--------
GSNIP---
------------------------------VTNENKK-QYVEL-ITEWKIQKR-VEEQFN
-AFITG---FNELIPA--DLVN-VFDER-ELELLIGG-----------------------
----IADID-----------VDDW----KKHTDYRG-YQEQ-------------------
------------------------DEVI-QNFWKVIR--------------------SWD
SEQKSRLLQF-------ATGTSRIP------------VNG-----FK-DLQGSD--GPRR
FTIEKSGEI-------------------------TALPKSHTCF-NR-LDLPPY-KTYEQ
LQHKLS-IAVEE-TLGF
>Paracoccidioides_lutzii_XM_002794544.1 .
VKVRRGAI-FEDSYAEI--------------MRQSASDLKK-RLMIKFDG-------EDG
LDYGGLSREF---FFLL----SHEMFNPFY--------CLFEYSAHDNY-----------
----------------TLQINPHS------------------------------------
--GINPEHLNYFKFIGRVVGLAIFHRRFLDSFFIG--AFYKMMLR---------------
--------KKVTLQDMEG----------------------VDEDFHRN--------LTWT
LEN--DIT-GVFD-
-ELTFSIDDDQ---------------------------FGE-RKTVD-LIPN--------
GSNIP---
------------------------------VTNENKK-QYVEL-ITEWKIQKR-VEEQFN
-AFITG---FNELIPA--DLVN-VFDER-ELELLIGG-----------------------
----IADID-----------VDDW----KKHTDYRG-YQEQ-------------------
------------------------DEVI-QNFWKVIR--------------------TWD
AEQKSRLLQF-------ATGTSRIP------------VNG-----FK-DLQGSD--GPRR
FTIEKSGEI-------------------------SALPKSHTCF-NR-LDLPPY-KTYEQ
LQSKLT-IAVEE-TLGF

>Paracoccidioides_brasiliensis_XM_010760690.1 .
VKVRRGAI-FEDSYAEI--------------MRQSASDLKK-RLMIKFDG-------EDG
LDYGGLSREF---FFLL----SHEMFNPFY--------CLFEYSAHDNY-----------
----------------TLQINPHS------------------------------------
--GINPEHLNYFKFIGRVVGLAIFHRRFLDSFFIG--AFYKMMLR---------------
--------KKVTLQDMEG----------------------VDEDFHRN--------LTWT
LEN--DIT-GVFD-
-ELTFSIDDDQ---------------------------FGE-RKTVD-LIPN--------
GSNIP---
------------------------------VTNENKK-QYVEL-ITEWKIQKR-VEEQFN
-AFITG---FNELIPA--DLVN-VFDER-ELELLIGG-----------------------
----IADID-----------VDDW----KKHTDYRG-YQEQ-------------------
------------------------DEVI-QNFWKVIR--------------------TWD
AEQKSRLLQF-------ATGTSRIP------------VNG-----FK-DLQGSD--GPRR
FTIEKSGEI-------------------------TALPKSHTCF-NR-LDLPPY-KTYEQ
LQSKLT-IAVEE-TLGF
>Penicillium_marneffei_XM_002144166.1 .
IKVRRSNI-FEDSYAEI--------------MRQSASDLKK-RLMIKFDG-------EDG
LDYGGLSREY---FFLL----SHEMFNPFY--------CLFEYSAHDNY-----------
----------------TLQINPHS------------------------------------
--GINPEHLNYFKFIGRVVGLAIFHRRFLDSFFIG--AFYKMMLR---------------
--------KKVTLQDMEG----------------------VDEDFHRN--------LTWT
LEN--DIE-GIV--
-ELTFSIDDEK---------------------------FGE-RDTID-LIPD--------
GRNIP---
------------------------------VTNENKH-KYVEL-VTEWKIQKR-VEQQFN
-AFLSG---FNELIPQ--DLVN-VFDER-ELELLIGG-----------------------
----IADID-----------VDDW----KKHTDYRG-YQES-------------------
------------------------DEVI-QNFWKIIR--------------------SWD
AEQKSRLLQF-------ATGTSRIP------------VNG-----FK-DLQGSD--GPRR
FTIEKSGDP-------------------------NALPKSHTCF-NR-LDLPPY-KSHEA
LQQKLS-IAVEE-TLGF
>Talaromyces_atroroseus_XM_020268306.1 .
IKVRRSNI-FEDSYAEI--------------MRQSASDLKK-RLMIKFDG-------EDG
LDYGGLSREY---FFLL----SHEMFNPFY--------CLFEYSAHDNY-----------
----------------TLQINPHS------------------------------------
--GINPEHLNYFKFIGRVVGLAIFHRRFLDSFFIG--AFYKMMLR---------------
--------KKVTLQDMEG----------------------VDEDFHRN--------LSWT
LEN--DIE-GIV--
-ELTFSIDDEK---------------------------FGE-RDTID-LIPD--------
GRNIP---
------------------------------VTNENKH-KYVEL-VTEWKIQKR-VEEQFN
-AFISG---FNELIPQ--DLVN-VFDER-ELELLIGG-----------------------
----IADID-----------VDDW----KKHTDYRG-YQES-------------------
------------------------DEVI-QNFWKIIR--------------------SWD
AEQKSRLLQF-------ATGTSRIP------------VNG-----FK-DLQGSD--GPRR
FTIEKSGDP-------------------------GALPKSHTCF-NR-LDLPPY-KTNEA
LQQKLS-IAVEE-TLGF
>Talaromyces_stipitatus_XM_002341310.1 .
IKVRRSNI-FEDSYAEI--------------MRQSASDLKK-RLMIKFDG-------EDG
LDYGGLSREY---FFLL----SHEMFNPFY--------CLFEYSAHDNY-----------
----------------TLQINPHS------------------------------------
--GINPEHLNYFKFIGRVVGLAIFHRRFLDSFFIG--AFYKMMLR---------------
--------KKVTLQDMEG----------------------VDEDFHRN--------LTWT
LEN--DIE-GIV--
-ELTFSVDDEK---------------------------FGE-RETID-LIPD--------
GRNIP---
------------------------------VTNENKH-KYVEL-VTEWKIQKR-VEEQFN

-AFISG---FNELIPQ--DLVN-VFDER-ELELLIGG----------------------
----IADID-----------VDDW----KKHTDYRG-YQES-------------------
------------------------DEVI-QNFWKIIR--------------------SWD
AEQKSRLLQF-------ATGTSRIP------------VNG-----FK-DLQGSD--GPRR
FTIEKSGDP-------------------------NALPKSHTCF-NR-LDLPPY-KSNEV
LQQKLS-IAVEE-TLGF
>Rasamsonia_emersonii_XM_013469810.1 .
IKVRRNNI-FEDSYAEI--------------MRQSASDLKK-RLMIKFDG-------EDG
LDYGGLSREF---FFLL----SHEMFNPFY--------CLFEYSAHDNY-----------
----------------TLQINPHS------------------------------------
--GINPEHLNYFRFIGRVVGLAIFHRRFLDSFFIG--AFYKMMLR---------------
--------KKVTLQDMEG----------------------VDEDFHRN--------LTWT
LEN--DID-GVV--
-ELTFSVDDEK---------------------------FGE-RRTID-LIPD--------
GRNIP---
------------------------------VTNENKH-KYVEL-VTEWKIQKR-VEEQFN
-AFISG---FNELIPQ--DLVN-VFDER-ELELLIGG----------------------
----IADID-----------VDDW----KKHTDYRG-YTES-------------------
------------------------DEVI-QNFWKIIR--------------------SWD
AEQKSRLLQF-------ATGTSRIP------------VNG-----FK-DLQGSD--GPRR
FTIEKAGDI-------------------------NALPKSHTCF-NR-LDLPPY-KTYET
LQQKLT-IAVEE-TLGF
>Penicillium_chrysogenum_XM_002560600.1 .
VKVRRNNI-FEDSYAEI--------------MRQSASDLKK-RLMIKFDG-------EDG
LDYGGLSREF---FFLL----SHEMFNPFY--------CLFEYSAHDNY-----------
----------------TLQINPHS------------------------------------
--GVNPEHLNYFKFIGRVVGLAIFHRRFLDSFFIG--AFYKMMLR---------------
--------KKVSLQDMEG----------------------VDEDLHRN--------LAWT
LDN--DID-GIV--
-ELTFSVDDEK---------------------------FGE-RRTID-LIPG--------
GRDIP---
------------------------------VTNENKP-QYIEL-VTEWKIMKR-VEEQFD
-AFMSG---FNELIPP--DLVN-VFDER-ELELLIGG----------------------
----IADID-----------VEDW----KKHTDYRG-YQEQ-------------------
------------------------DEVI-QNFWKIVR--------------------TWD
AEQKSRLLQF-------TTGTSRIP------------VNG-----FK-DLQGSD--GPRR
FTIEKSGDP-------------------------AALPKSHTCF-NR-LDLPPY-KTHDA
LEHKMS-IAVEE-TLGF
>Penicillium_expansum_XM_016744811.1 .
VKVRRNNI-FEDSYAEI--------------MRQSASDLKK-RLMIKFDG-------EDG
LDYGGLSREF---FFLL----SHEMFNPFY--------CLFEYSAHDNY-----------
----------------TLQINPHS------------------------------------
--GVNPEHLNYFKFIGRVVGLAIFHRRFLDSFFIG--AFYKMMLR---------------
--------KKVSLQDMEG----------------------VDEDLHRN--------LAWT
LDN--DIN-GIV--
-ELTFSVDDEK---------------------------FGE-RRTID-LIPG--------
GRDIP---
------------------------------VTNENKP-QYIEL-VTEWKIMKR-VEEQFD
-AFMSG---FNELIPP--DLVN-VFDER-ELELLIGG----------------------
----IADID-----------VEDW----KKHTDYRG-YQEQ-------------------
------------------------DEVI-QNFWKIVR--------------------TWD
AEQKSRLLQF-------TTGTSRIP------------VNG-----FK-DLQGSD--GPRR
FTIEKSGDP-------------------------AALPKSHTCF-NR-LDLPPY-KTHDA
LEHKMS-IAVEE-TLGF
>Penicillium_digitatum_XM_014679607.1 .
VKVRRNNI-FEDSYAEI--------------MRQSASDLKK-RLMIKFDG-------EDG
LDYGGLSREF---FFLL----SHEMFNPFY--------CLFEYSAHDNY-----------
----------------TLQINPHS------------------------------------

```
--GVNPEHLNYFKFIGRVVGLAIFHRRFLDSFFIG--AFYKMMLR---------------
--------KKVSLQDMEG----------------------VDEDLHRN--------LAWT
LDN------------------------------------------------DID-GIV--
-ELTFSVDDEK---------------------------FGE-RRTID-LIPG--------
GRDIP-------------------------------------------------------
------------------------------VTNENKP-QYIEL-VTEWKIMKR-VEEQFD
-AFMSG---FNELIPP--DLVN-VFDER-ELELLIGG-----------------------
----IADID-----------VEDW----KKHTDYRG-YQEQ-------------------
------------------------DEVI-QNFWKIVR--------------------TWD
AEQKSRLLQF-------TTGTSRIP------------VNG-----FK-DLQGSD--GPRR
FTIEKSGDP-------------------------SALPKSHTCF-NR-LDLPPY-KTHEA
LEHKMS-IAVEE-TLGF
>Penicillium_raistrickii_GFHR01010945.1 .
VKVRRNNI-FEDSYAEI--------------MRQSASDLKK-RLMIKFDG-------EDG
LDYGGLSREF---FFLL----SHEMFNPFY--------CLFEYSAHDNY-----------
----------------TLQINPHS------------------------------------
--GVNPEHLNYFKFIGRVVGLAIFHRRFLDSFFIG--AFYKMMLR---------------
--------KKVTLQDMEG----------------------VDEDLHRN--------LAWT
LDN------------------------------------------------DIE-GIV--
-ELTFSVDDEK---------------------------FGE-RSTMD-LIPG--------
GRDIA-------------------------------------------------------
------------------------------VTNENKP-QYIEL-VTEWKIMKR-VEEQFN
-AFMSG---FNELIPP--DLVN-VFDER-ELELLIGG-----------------------
----IADID-----------VDDW----KKHTDYRG-YQEQ-------------------
------------------------DEVI-QNFWKIVR--------------------TWD
AEQKSRLLQF-------TTGTSRIP------------VNG-----FK-DLQGSD--GPRR
FTIEKSGDP-------------------------QALPKSHTCF-NR-LDLPPY-KTHDA
LEHKMS-IAVEE-TLGF
>Penicillium_janthinellum_GBSP01006180.1 .
VKVRRNNI-FEDSYAEI--------------MRQSASDLKK-RLMIKFDG-------EDG
LDYGGLSREF---FFLL----SHEMFNPFY--------CLFEYSAHDNY-----------
----------------TLQINPHS------------------------------------
--GVNPEHLNYFKFIGRVVGLAIFHRRFLDSFFIG--AFYKMMLR---------------
--------KKVTLQDMEG----------------------VDEDLHRN--------LAWT
LDN------------------------------------------------DIE-GIV--
-ELTFSVDDEK---------------------------FGE-RRTID-LIPG--------
GRDIP-------------------------------------------------------
------------------------------VTNENKP-QYIEL-VTEWKIMKR-VEEQFN
-AFMSG---FNELIPA--DLVN-VFDER-ELELLIGG-----------------------
----IADID-----------VDDW----KKHTDYRG-YQES-------------------
------------------------DEVI-QNFWKIVR--------------------SWD
AEQKSRLLQF-------TTGTSRIP------------VNG-----FK-DLQGSD--GPRR
FTIEKSGDP-------------------------SALPKSHTCF-NR-LDLPPY-KTHET
LEHKMS-IAVEE-TLGF
>Neosartorya_fischeri_XM_001264814.1 .
VKVRRNNI-FEDSYAEI--------------MRQSASDLKK-RLMIKFDG-------EDG
LDYGGLSREF---FFLL----SHEMFNPFY--------CLFEYSAHDNY-----------
----------------TLQINPHS------------------------------------
--GVNPEHLNYFKFIGRVVGLAIFHRRFLDSFFIG--AFYKMMLR---------------
--------KKVSLQDMEG----------------------VDEDLHRN--------LTWT
LDN------------------------------------------------DIE-GVL--
-ELTFSVDDEK---------------------------FGE-RRTID-LKPG--------
GRDIP-------------------------------------------------------
------------------------------VTNENKA-EYVEL-VTEWKIVKR-VEEQFN
-AFMSG---FNELIPA--DLVN-VFDER-ELELLIGG-----------------------
----IADID-----------VDDW----KKHTDYRG-YQES-------------------
------------------------DEVI-QNFWKVVR--------------------SWD
AEQKSRLLQF-------TTGTSRIP------------VNG-----FK-DLQGSD--GPRR
```

FTIEKSGDP-------------------------AALPKSHTCF-NR-LDLPPY-KSYET
LEHKMS-IAVEE-TLGF
>Aspergillus_fumigatus_XM_747224.1 .
VKVRRNNI-FEDSYAEI--------------MRQSASDLKK-RLMIKFDG-------EDG
LDYGGLSREF---FFLL----SHEMFNPFY--------CLFEYSAHDNY-----------
----------------TLQINPHS------------------------------------
--GVNPEHLNYFKFIGRVVGLAIFHRRFLDSFFIG--AFYKMMLR---------------
--------KKVSLQDMEG----------------------VDEDLHRN--------LTWT
LDN--DIE-GVL--
-ELTFSVDDEK---------------------------FGE-RRTID-LKPG--------
GRDIP---
------------------------------VTNENKA-EYVEL-VTEWKIVKR-VEEQFN
-AFMSG---FNELIPA--DLVN-VFDER-ELELLIGG-----------------------
----IADID-----------VDDW----KKHTDYRG-YQES-------------------
------------------------DEVI-QNFWKIVR--------------------SWD
AEQKSRLLQF-------TTGTSRIP------------VNG-----FK-DLQGSD--GPRR
FTIEKSGDP-------------------------AALPKSHTCF-NR-LDLPPY-KSYET
LEHKMS-IAVEE-TLGF
>Aspergillus_clavatus_XM_001269308.1 .
VKVRRNNI-FEDSYAEI--------------MRQSASDLKK-RLMIKFDG-------EDG
LDYGGLSREF---FFLL----SHEMFNPFY--------CLFEYSAHDNY-----------
----------------TLQINPHS------------------------------------
--GVNPEHLNYFKFIGRVVGLAIFHRRFLDSFFIG--AFYKMMLR---------------
--------KKVSLQDMEG----------------------VDEDLHRN--------LTWT
LDN--DIE-GVL--
-ELTFAVDDEK---------------------------FGE-RRTID-LKPG--------
GRDIP---
------------------------------VTNENKG-EYVEL-VTEWKIVKR-VEEQFN
-AFMSG---FNELIPA--DLVN-VFDER-ELELLIGG-----------------------
----IADID-----------VDDW----KKHTDYRG-YQES-------------------
------------------------DDVI-QNFWKVVR--------------------TWD
AEQKSRLLQF-------TTGTSRIP------------VNG-----FK-DLQGSD--GPRR
FTIEKSGDP-------------------------VALPKSHTCF-NR-LDLPPY-KTYET
LEHKMS-IAVEE-TLGF
>Aspergillus_aculeatus_XM_020202347.1 .
VKVRRNNI-FEDSYAEI--------------MRQSASDLKK-RLMIKFDG-------EDG
LDYGGLSREF---FFLL----SHEMFNPFY--------CLFEYSAHDNY-----------
----------------TLQINPHS------------------------------------
--GVNPEHLNYFKFIGRVVGLAIFHRRFLDSFFIG--AFYKMMLR---------------
--------KKVSLQDMEG----------------------VDEDLHRN--------LTWT
LEN--DID-GVL--
-ELTFAVDDEK---------------------------FGE-RTTVD-LKPG--------
GRDIP---
------------------------------VTNENKH-EYVEL-VTEWKIVKR-VEEQFN
-AFMSG---FNELIPA--DLVN-VFDER-ELELLIGG-----------------------
----IADID-----------VDDW----KKHTDYRG-YQEQ-------------------
------------------------DEVI-QNFWKIVR--------------------TWD
AEQKSRLLQF-------TTGTSRIP------------VNG-----FK-DLQGSD--GPRR
FTIEKSGDP-------------------------NALPKSHTCF-NR-LDLPPY-KTHEA
LEHKMS-IAVEE-TLGF
>Aspergillus_oryzae_XM_001727811.2 .
VKVRRNNI-FEDSYAEI--------------MRQSASDLKK-RLMIKFDG-------EDG
LDYGGLSREF---FFLL----SHEMFNPFY--------CLFEYSAHDNY-----------
----------------TLQINPHS------------------------------------
--GVNPEHLNYFKFIGRVVGLAIFHRRFLDSFFIG--AFYKMMLR---------------
--------KKVSLQDMEG----------------------VDEDLHRN--------LTWT
LDN--DIE-GII--
-ELTFAVDDEK---------------------------FGE-RRTID-LKPG--------

```
GRDIP------------------------------------------------------------
------------------------------VTNENKG-EYVEL-VTEWKIVKR-VEEQFN
-AFMSG---FNELIPA--DLVN-VFDER-ELELLIGG-----------------------
----IADID-----------VDDW----KKHTDYRG-YQES-------------------
------------------------DEVI-QNFWKIVR--------------------TWD
AEQKSRLLQF-------TTGTSRIP------------VNG-----FK-DLQGSD--GPRR
FTIEKSGDP-------------------------GALPKSHTCF-NR-LDLPPY-KTNDV
LEHKLS-IAVEE-TLGF
>Aspergillus_niger_XM_001392187.2 .
VKVRRNNI-FEDSYAEI--------------MRQSASDLKK-RLMIKFDG-------EDG
LDYGGLSREF---FFLL----SHEMFNPFY--------CLFEYSAHDNY-----------
----------------TLQINPHS------------------------------------
--GVNPEHLNYFKFIGRVVGLAIFHRRFLDSFFIG--AFYKMMLR---------------
--------KKVSLQDMEG---------------------VDEDLHRN--------LAWT
LEN------------------------------------------------DIE-GII--
-ELTFSVDDEK---------------------------FGE-RTTID-LKPG--------
GRDIP------------------------------------------------------------
------------------------------VTNENKG-EYVEL-VTEWKIVKR-VEEQFN
-AFMSG---FNELIPA--DLVN-VFDER-ELELLIGG-----------------------
----IADID-----------VDDW----KKHTDYRG-YQEQ-------------------
------------------------DEVI-QNFWKIVR--------------------TWD
AEQKSRLLQF-------TTGTSRIP------------VNG-----FK-DLQGSD--GPRR
FTIEKSGDP-------------------------IALPKSHTCF-NR-LDLPPY-KTHDV
LEHKLS-IAVEE-TLGF
>Aspergillus_kawachii_BACL01000172.1_R .
VKVRRNNI-FEDSYAEI--------------MRQSASDLKK-RLMIKFDG-------EDG
LDYGGLSREF---FFLL----SHEMFNPFY--------CLFEYSAHDNY-----------
----------------TLQINPHS------------------------------------
--GVNPEHLNYFKFIGRVVGLAIFHRRFLDSFFIG--AFYKMMLR---------------
--------KKVSLQDMEG---------------------VDEDLHRN--------LAWT
LXN------------------------------------------------DIE-GII--
-ELTFCVDDEK---------------------------FGE-RTTID-LKPG--------
GRDIP------------------------------------------------------------
------------------------------VTNENKH-EYVEX-VTEWKIVKR-VEEQFN
-AFMSG---FNELIPA--DLVN-VFDER-ELELLIGG-----------------------
----IADID-----------VDDW----KKHTDYRG-YQEQ-------------------
------------------------DEVI-QNFWKIVR--------------------TWD
AEQKSRLLQF-------TTGTSRIP------------VNG-----FK-DLQGSD--GPRR
FTIEKSGDP-------------------------IALPKSHT------------------
----------------
>Aspergillus_nidulans_XM_653851.1 .
VKVRRNNI-FEDSYAEI--------------MRQSASDLKK-RLMIKFDG-------EDG
LDYGGLSREF---FFLL----SHEMFNPFY--------CLFEYSAHDNY-----------
----------------TLQINPHS------------------------------------
--GVNPEHLNYFKFIGRVVGLAIFHRRFLDSFFIG--AFYKMMLR---------------
--------KKVSLQDMEG---------------------VDEDLHRN--------LTWT
LEN------------------------------------------------DIE-GII--
-DLTFTVDDEK---------------------------FGE-RRTIE-LKPG--------
GEDIP------------------------------------------------------------
------------------------------VTNENKH-EYVEL-VTEWKIVKR-VEEQFN
-AFMSG---FNELIPA--DLVN-VFDER-ELELLIGG-----------------------
----IADID-----------VDDW----KKHTDYRG-YQEQ-------------------
------------------------DEVI-QNFWKIVR--------------------TWD
AEQKSRLLQF-------TTGTSRIP------------VNG-----FK-DLQGSD--GPRR
FTIEKSGDP-------------------------IALPKSHTCF-NR-LDLPPY-KSHEV
LEHKLS-IAVEE-TLGF
>Aspergillus_terreus_XM_001217158.1 .
VKVRRNNI-FEDSYAEI--------------MRQSASDLKK-RLMIKFDG-------EDG
```

LDYGGLSREF---FFLL----SHEMFNPFY--------CLFEYSAHDNY-----------
----------------TLQINPHS------------------------------------
--GVNPEHLNYFKFIGRVVGLAIFHRRFLDSFFIG--AFYKMMLR---------------
--------KKVSLQDMEG----------------------VDEDLHRN--------LTWT
LDN--DIE-GII--
-ELTFAVDDEK---------------------------FGE-RRTID-LKPG--------
GRDIP---
------------------------------VTNENKH-EYVEL-VTEWKIVKR-VEEQFN
-AFMSG---FNELIPA--DLVN-VFDER-ELELLIGG-----------------------
----IADID-----------VDDW----KKHTDYRG-YQEQ-------------------
------------------------DEVI-QNFWKIVR--------------------TWD
AEQKSRLLQF-------TTGTSRIP------------VNG-----FK-DLQGSD--GPRR
FTIEKSGDP-------------------------AALPKSHTCF-NR-LDLPPY-KTHET
LEHKLS-IAVEE-TLGF
>Rutstroemia_sydowiana_JWJB01009999.1 .
VKIRRSHI-FEDSYAEI--------------MRQSATDLKK-RLMIKFDG-------EDG
LDYGGLSREF---FFLL----SHEMFNPFY--------CLFEYSAHDNY-----------
----------------TLQINPHS------------------------------------
--GINPEHLNYFKFIGRVVGLAIFHRRFLDAFFIG--ALYKMMLN---------------
--------KAVALSDMEG----------------------VDADFHRS--------LQWM
LDN--PIE-GVL--
-EQTFSTEDER---------------------------FGV-TNVED-LKPG--------
GRDID---
------------------------------VTDSNKK-EYVDL-MVKWRIQKR-IDEQFQ
-AFITG---FHELIPA--ELVN-VFDER-ELELLIGG-----------------------
----IAEID-----------VEDW----KKHTDYRG-YTES-------------------
------------------------DEVI-KFFWQTIR--------------------SWD
GEQKSRLLQF-------ATGTSRIP------------VNG-----FK-DLQGSD--GPRR
FTIEKQGEV-------------------------NNLPKSHTXF-NR-LDLPQY-KSLEQ
LQQKLT-MAVEE-TMG-
>Rutstroemia_echinophila_JWJA01005488.1 .
VKVRRSHI-FEDSYAEI--------------MRQSATDLKK-RLMIKFDG-------EDG
LDYGGLSREF---FFLL----SHEMFNPFY--------CLFEYSAHDNY-----------
----------------TLQINPHS------------------------------------
--GINPEHLNYFKFIGRVVGLAIFHRRFLDAFFIG--ALYKMMLN---------------
--------KAVALSDMEG----------------------VDADFHRS--------LQWM
LDN--PIE-GVL--
-EQTFSTEDER---------------------------FGV-TNVED-LKPG--------
GRDID---
------------------------------VTDSNKK-EYVDL-MVKWRIQKR-IDEQFQ
-AFITG---FHELIPA--ELVN-VFDER-ELELLIGG-----------------------
----IAEID-----------VEDW----KKHTDYRG-YTES-------------------
------------------------DEVI-KFFWQTIR--------------------SWD
GEQKSRLLQF-------ATGTSRIP------------VNG-----FK-DLQGSD--GPRR
FTIEKQGEV-------------------------NNLPKSHTXF-NR-LDLPQY-KTLEQ
LQQKLT-MAVEE-TMG-
>Calycina_herbarum_LLEY01000056.1 .
VKVRRSHI-FEDSYAEI--------------MRQSATDLKK-RLMIKFDG-------EDG
LDYGGLSREF---FFLL----SHEMFNPFY--------CLFEYSAHDNY-----------
----------------TLQINPHS------------------------------------
--GINPEHLNYFKFIGRVVGLAIFHRRFLDAFFVG--ALYKMMLH---------------
--------KAVALSDMEG----------------------VDADFHRS--------LQWM
LDN--PIE-GVL--
-EQTFSTEDER---------------------------FGV-TNVED-LKPG--------
GRDID---
------------------------------VTDVNKK-EYVDL-MVKWRIQKR-IDEQFQ
-AFING---FHELIPA--ELVN-VFDER-ELELLIGG-----------------------
----IAEID-----------VEDW----KKHTDYRG-YTES-------------------

------------------------DEVI-KFFWQTIR--------------------SWD
GEQKSRLLQF-------ATGTSRIP------------VNG-----FK-DLQGSD--GPRR
FTIEKQGEV-------------------------NNLPKSHTXF-NR-LDLPAY-KSLEQ
LQQKLT-MAVEE-TMG-
>Hymenoscyphus_fructigenus_LKUV01000055.1 .
VKVRRSHI-FEDSYAEI--------------MRQSATDLKK-RLMIKFDG-------EDG
LDYGGLSREF---FFLL----SHEMFNPFY--------CLFEYSAHDNY-----------
----------------TLQINPHS------------------------------------
--GINPEHLNYFKFIGRVVGLAIFHRRFLDAFFVG--ALYKMMLH---------------
--------KAVALSDMEG----------------------VDADFHRS--------LQWM
LDN--PIE-GVL--
-EQTFSTEDER---------------------------FGV-TNVED-LKPG--------
GRDID---
------------------------------VTDVNKK-EYVDL-MVKWRIQKR-IDEQFQ
-AFING---FHELIPA--ELVN-VFDER-ELELLIGG-----------------------
----IAEID-----------VEDW----KKHTDYRG-YTES-------------------
------------------------DEVI-KFFWQTIR--------------------SWD
GEQKSRLLQF-------ATGTSRIP------------VNG-----FK-DLQGSD--GPRR
FTIEKQGEV-------------------------NNLPKSHTXF-NR-LDLPAY-KSLEQ
LQQKLT-MAVEE-TMG-
>Glarea_lozoyensis_XM_008080276.1 .
VKVRRSHI-FEDSYAEI--------------MRQSATDLKK-RLMIKFDG-------EDG
LDYGGLSREF---FFLL----SHEMFNPFY--------CLFEYSAHDNY-----------
----------------TLQINPHS------------------------------------
--GINPEHLNYFKFIGRVVGLAIFHRRFLDAFFIG--ALYKMMLN---------------
--------KAVSLSDMEG----------------------VDADFHRS--------LQWM
LDN--PIE-GVL--
-EQTFSTEDER---------------------------FGV-TNVED-LKPG--------
GRDID---
------------------------------VTDANKK-EYVDL-MVKWRIQKR-IDEQFQ
-AFITG---FHELIPA--ELVN-VFDER-ELELLIGG-----------------------
----IAEID-----------VEDW----KKHTDYRG-YTES-------------------
------------------------DEVI-KFFWQTIR--------------------SWD
GEQKSRLLQF-------ATGTSRIP------------VNG-----FK-DLQGSD--GPRR
FTIEKQGEV-------------------------GNLPKSHTCF-NR-LDLPAY-KSLEQ
LQTKLT-MAVEE-TMGF
>Sclerotinia_homoeocarpa_JW824251.1 .
IKVRRSHI-FEDSYAEI--------------MRQSATDLKK-RLMIKFDG-------EDG
LDYGGLSREF---FFLL----SHEMFNPFY--------CLFEYSAHDNY-----------
----------------TLQINPHS------------------------------------
--GINPEHLNYFKFIGRVVGLAIFHRRFLDAFFIG--ALYKMMLN---------------
--------KAVSLSDMEG----------------------VDADFHRS--------LQWM
LDN--PIE-GVL--
-EQTFSTEDER---------------------------FGV-TQVED-LKPG--------
GRDID---
------------------------------VTDANKK-EYVDL-MVKWRIQKR-IDEQFQ
-AFITG---FHELIPA--ELVN-VFDER-ELELLIGG-----------------------
----IAEID-----------VEDW----KKHTDYRG-YTES-------------------
------------------------DEVI-KFFWQTIR--------------------SWD
GEQKSRLLQF-------ATGTSRIP------------VNG-----FK-DLQGSD--GPRR
FTIEKQGEV-------------------------NNLPKSHTCF-NR-LDLPPY-KSLEQ
LQTKLT-MAVEE-TMGF
>Cadophora_malorum_FKJQ01000133.1 .
VKVRRSHI-FEDSYAEI--------------MRQSATDLKK-RLMIKFDG-------EDG
LDYGGLSREF---FFLL----SHEMFNPFY--------CLFEYSAHDNY-----------
----------------TLQINPHS------------------------------------
--GINPEHLNYFKFIGRVVGLAIFHRRFLDAFFIG--ALYKMMLN---------------
--------KAVALSDMEG----------------------VDADFHRS--------LQWM

```
LDN-----------------------------------------------SIE-GVL--
-EQTFSTEDER---------------------------FGV-TNVED-LKPG--------
GRDIE-------------------------------------------------------
------------------------------VTDENKK-EYVDL-MVKWRIQKR-IDEQFQ
-AFITG---FHELIPA--ELVN-VFDER-ELELLIGG-----------------------
----IAEID-----------VEDW----KKHTDYRG-YTES-------------------
------------------------DEVI-KFFWQTIR--------------------SWD
GEQKSRLLQF-------ATGTSRIP------------VNG-----FK-DLQGSD--GPRR
FTIEKAGEF-------------------------NNLPKSHTXF-NR-LDLPPY-KSLEA
LQTKLT-MAVEE-TMG-
>Cairneyella_variabilis_AYLM01000130.1 .
VKVRRSHI-FEDSYAEI--------------MRQSATDLKK-RLMIKFDG-------EDG
LDYGGLSREF---FFLL----SHEMFNPFY--------CLFEYSAHDNY-----------
----------------TLQINPHS------------------------------------
--GINPEHLNYFKFIGRVVGLAIFHRRFLDAFFIG--ALYKMMLN---------------
--------KPVALSDMEG----------------------VDADFHRS--------LQWM
LDN-----------------------------------------------PIE-GVL--
-DQTFSTEDER---------------------------FGV-TNIED-LKPG--------
GRDID-------------------------------------------------------
------------------------------VTDENKK-EYVDL-MVKWRIQKR-IDDQFQ
-AFITG---FHELIPA--ELVN-VFDER-ELELLIGG-----------------------
----IAEID-----------VEDW----KKHTDYRG-YTES-------------------
------------------------DEVI-KFFWQTIR--------------------SWD
GEQKSRLLQF-------ATGTSRIP------------VNG-----FK-DLQGSD--GPRR
FTIEKAGEV-------------------------NNLPKSHTXF-NR-LDLPPY-KSLEA
LQTKLT-MAVEE-TMG-
>Phialocephala_subalpina_FJOG01000016.1 .
VKVRRSHI-FEDSYAEI--------------MRQSATDLKK-RLMIKFDG-------EDG
LDYGGLSREF---FFLL----SHEMFNPFY--------CLFEYSAHDNY-----------
----------------TLQINPHS------------------------------------
--GINPEHLNYFKFIGRVVGLAIFHRRFLDAFFIG--ALYKMMLN---------------
--------KAVALSDMEG----------------------VDADFHRS--------LQWM
LDN-----------------------------------------------PIE-GVL--
-DQTFSTEDER---------------------------FGQ-TNVED-LKPG--------
GRDIE-------------------------------------------------------
------------------------------VTDANKK-EYVDL-MVKWRIQKR-IDEQFQ
-AFITG---FHELIPA--ELVN-VFDER-ELELLIGG-----------------------
----IAEID-----------VEDW----KKHTDYRG-YTES-------------------
------------------------DEVI-KFFWQTIR--------------------SWD
GEQKSRLLQF-------ATGTSRIP------------VNG-----FK-DLQGSD--GPRR
FTIEKAGEV-------------------------NNLPKSHTXF-NR-LDLPPY-KSLEA
LQTKLT-MAVEE-TMG-
>Phialocephala_scopiformis_XM_018216928.1 .
VKVRRSHI-FEDSYAEI--------------MRQSATDLKK-RLMIKFDG-------EDG
LDYGGLSREF---FFLL----SHEMFNPFY--------CLFEYSAHDNY-----------
----------------TLQINPHS------------------------------------
--GINPEHLNYFKFIGRVVGLAIFHRRFLDAFFIG--ALYKMMLN---------------
--------KAVALSDMEG----------------------VDADFHRS--------LQWM
LDN-----------------------------------------------PIE-GVL--
-DQTFSTEDER---------------------------FGQ-TNVED-LKPG--------
GRDIE-------------------------------------------------------
------------------------------VTDANKK-EYVDL-MVKWRIQKR-IDEQFQ
-AFING---FHELIPA--ELVN-VFDER-ELELLIGG-----------------------
----IAEID-----------VEDW----KKHTDYRG-YTES-------------------
------------------------DEVI-KFFWQTIR--------------------SWD
GEQKSRLLQF-------ATGTSRIP------------VNG-----FK-DLQGSD--GPRR
FTIEKAGEV-------------------------NNLPKSHTCF-NR-LDLPPY-KSLEA
LQTKLT-MAVEE-TMGF
```

>Amorphotheca_resinae_JZSE01000128.1 .
VKVRRSHI-FEDSYAEI--------------MRQSATDLKK-RLMIKFDG-------EDG
LDYGGLSREF---FFLL----SHEMFNPFY--------CLFEYSAHDNY-----------
----------------TLQINPHS------------------------------------
--GINPEHLNYFKFIGRVVGLAIFHRRFLDAFFIG--ALYKMMLN---------------
--------KPVTLSDMEG----------------------VDADFHRS--------LQWM
LDN--PIE-GVL--
-EQTFSTEDER---------------------------FGV-TTVED-LKPG--------
GRDIE---
------------------------------VTDENKK-EYVDL-MVKWRIQKR-IDEQFQ
-AFITG---FHELIPA--ELVN-VFDER-ELELLIGG-----------------------
----IAEID-----------VDDW----KKHTDYRG-YTES-------------------
------------------------DDVI-KFFWQTIR--------------------SWD
GEQKSRLLQF-------ATGTSRIP------------VNG-----FK-DLQGSD--GPRR
FTIEKQGEI-------------------------NNLPKSHTXF-NR-LDLPAY-KTLEQ
LQTKLT-MAVEE-TMG-
>Ciborinia_camelliae_LGKQ01001380.1 .
VKIRRSHI-FEDSYAEI--------------MRQSATDLKK-RLMIKFDG-------EDG
LDYGGLSREF---FFLL----SHEMFNPFY--------CLFEYSAHDNY-----------
----------------TLQINPHS------------------------------------
--GINPEHLNYFKFIGRVVGLAIFHRRFLDAFFIG--ALYKMMLN---------------
--------KAVALQDMEG----------------------VDADFHRS--------LQWM
LDN--PIE-GVL--
-DQTFSTEDER---------------------------FGV-TNVED-LKPG--------
GRDIE---
------------------------------VTDENKK-EYVDL-MVKWRIQKR-IDEQFQ
-AFING---FHELIPA--ELVN-VFDER-ELELLIGG-----------------------
----IAEID-----------VDDW----KKHTDYRG-YTES-------------------
------------------------DEVI-KFFWQTIR--------------------SWD
GEQKSRLLQF-------ATGTSRIP------------VNG-----FK-DLQGSD--GPRR
FTIEKQGEA-------------------------NNLPKSHTXF-NR-LDLPPY-KTLEQ
LQTKLT-MAVEE-TMG-
>Sclerotinia_borealis_AYSA01000086.1 .
VKIRRSHI-FEDSYAEI--------------MRQSATDLKK-RLMIKFDG-------EDG
LDYGGLSREF---FFLL----SHEMFNPFY--------CLFEYSAHDNY-----------
----------------TLQINPHS------------------------------------
--GINPEHLNYFKFIGRVVGLAIFHRRFLDAFFIG--ALYKMMLN---------------
--------KAVALQDMEG----------------------VDADFHRS--------LQWM
LDN--PIE-GVL--
-DQTFSTEDER---------------------------FGV-TNVED-LKPD--------
GRDIE---
------------------------------VTDENKK-EYVDL-MVKWRIQKR-IDEQFQ
-AFING---FHELIPA--ELVN-VFDER-ELELLIGG-----------------------
----IAEID-----------VDDW----KKHTDYRG-YTES-------------------
------------------------DEVI-KFFWQTIR--------------------SWD
GEQKSRLLQF-------ATGTSRIP------------VNG-----FK-DLQGSD--GPRR
FTIEKQGEA-------------------------NNLPKSHTXF-NR-LDLPQY-KTLEQ
LQTKMT-MAVEE-TMG-
>Sclerotinia_sclerotiorum_XM_001595569.1 .
VKIRRSHI-FEDSYAEI--------------MRQSATDLKK-RLMIKFDG-------EDG
LDYGGLSREF---FFLL----SHEMFNPFY--------CLFEYSAHDNY-----------
----------------TLQINPHS------------------------------------
--GINPEHLNYFKFIGRVVGLAIFHRRFLDAFFIG--ALYKMMLS---------------
--------KAVSLQDMEG----------------------VDADFHRS--------LQWM
LDN--PIE-GVL--
-DQTFSTEDER---------------------------FGV-TNVED-LKPG--------
GRDIE---
------------------------------VTDENKK-EYVDL-MVKWRIQKR-IDEQFQ

-AFING---FHELIPA--ELVN-VFDER-ELELLIGG-----------------------
----IAEID-----------VDDW----KKHTDYRG-YTES-------------------
------------------------DEVI-KFFWQTIR--------------------SWD
GEQKSRLLQF-------ATGTSRIP------------VNG-----FK-DLQGSD--GPRR
FTIEKQGEP-------------------------NNLPKSHTCF-NR-LDLPPY-KTLEQ
LQTKLT-MAVEE-TMGF
>Botryotinia_fuckeliana_XM_001554417.1 .
VKIRRSHI-FEDSYAEI--------------MRQSATDLKK-RLMIKFDG-------EDG
LDYGGLSREF---FFLL----SHEMFNPFY--------CLFEYSAHDNY-----------
----------------TLQINPHS------------------------------------
--GINPEHLNYFKFIGRVVGLAIFHRRFLDAFFIG--ALYKMMLN---------------
--------KAVSLQDMEG----------------------VDADFHRS--------LQWM
LDN--PIE-GVL--
-DQTFSTEDER---------------------------FGV-TNVED-LKPG--------
GRDIE---
------------------------------VTDENKK-EYVDL-MVKWRIQKR-IDEQFQ
-AFITG---FHELIPA--ELVN-VFDER-ELELLIGG-----------------------
----IAEID-----------VDDW----KKHTDYRG-YTES-------------------
------------------------DEVI-KFFWQTIR--------------------SWD
GEQKSRLLQF-------ATGTSRIP------------VNG-----FK-DLQGSD--GPRR
FTIEKQGEP-------------------------NNLPKSHTCF-NR-LDLPPY-KNLEQ
LQTKLT-MAVEE-TMGF
>Botrytis_cinerea_CP009808.1_a.
VKIRRSHI-FEDSYAEI--------------MRQSATDLKK-RLMIKFDG-------EDG
LDYGGLSREF---FFLL----SHEMFNPFY--------CLFEYSAHDNY-----------
----------------TLQINPHS------------------------------------
--GINPEHLNYFKFIGRVVGLAIFHRRFLDAFFIG--ALYKMMLN---------------
--------KAVSLQDMEG----------------------VDADFHRS--------LQWM
LDN--PIE-GVL--
-DQTFSTEDER---------------------------FGV-TNVED-LKPG--------
GRDIE---
------------------------------VTDENKK-EYVDL-MVKWRIQKR-IDEQFQ
-AFITG---FHELIPA--ELVN-VFDER-ELELLIGG-----------------------
----IAEID-----------VDDW----KKHTDYRG-YTES-------------------
------------------------DEVI-KFFWQTIR--------------------SWD
GEQKSRLLQF-------ATGTSRIP------------VNG-----FK-DLQGSD--GPRR
FTIEKQGEP-------------------------NNLPKSHTXF-NR-LDLPPY-KNLEQ
LQTKLT-MAVEE-TMGF
>Podosphaera_xanthii_GEUO01000241.1 .
VKIRRSHI-FEDSYAEI--------------MRQSATDLKK-RLMIKFEG-------EDG
LDYGGLSREF---FFLL----SHEMFNPFY--------CLFEYSAHDNY-----------
----------------TLQINPHS------------------------------------
--GINPEHLNYFKFIGRVVGLAIFHRRFLDAFFIG--ALYKMMLS---------------
--------KPVSLSDMEG----------------------VDADFHRS--------LQWM
LDN--PIE-GIL--
-EQTFSTEDER---------------------------FGQ-MTVED-LKPG--------
GRDIE---
------------------------------VTDANKK-EYVDM-MVKWRIQKR-IDEQFQ
-AFIAG---FHELIPA--ELVN-VFDER-ELELLIGG-----------------------
----IAEID-----------VEDW----KKHTDYRG-YTES-------------------
------------------------DEVI-KFFWQTIR--------------------SWD
GEQKSRLLQF-------ATGTSRIP------------VNG-----FK-DLQGSD--GPRR
FTIEKAGEI-------------------------NNLPKSHTCF-NR-LDLPPY-KSLET
LQSKLT-MAVEE-TMGF
>Erysiphe_pisiCACM01012729.1 .
VKIRRSHI-FEDSYAEI--------------MRQSASDLKK-RLMIKFDG-------EDG
LDYGGLSREF---FFLL----SHEMFNPFY--------CLFEYSAHDNY-----------
----------------TLQINPHS------------------------------------

```
--GINPEHLNYFKFIGRVVGLAIFHRRFLDAFFIG--ALYKMMLN---------------
--------KAVSLSDMEG----------------------VDADFHRS--------LQWM
LDN-------------------------------------------------PIE-GVL--
-EQTFSTEDER----------------------------FGQ-TTIED-LKPG--------
GRDIE-------------------------------------------------------
------------------------------VTDSNKK-EYVEL-MVKWRIQKR-VDEQFQ
-AFITG---FHELIPS--ELVN-VFDER-ELELLIGG-----------------------
----IAEID-----------VEDW----KKHTDYRG-YTES-------------------
------------------------DEVI-KFFWQTIR--------------------SWD
AEQKSRLLQF-------ATGTSRIP------------VNG-----FK-DLQGSD--GPRR
FTIERAGEI-------------------------NNLPKSHTXF-NR-LDLPPY-KNLEM
LQSKLT-IAVEE-TMG-
>Marssonina_brunnea_XM_007295780.1 .
VKIRRSHI-FEDSYAEI--------------MRQSATDLKK-RLMIKFDG-------EDG
LDYGGLSREF---FFLL----SHEMFNPFY--------CLFEYSAHDNY-----------
----------------TLQINPHS------------------------------------
--GINPEHLNYFKFIGRVVGLAIFHRRFLDAFFIG--ALYKMMLN---------------
--------KAVSLPDMEG----------------------VDADFHRS--------LQWM
LDN-------------------------------------------------PIE-GVL--
-EQTFSTEDER----------------------------FGQ-TQVED-LKPD--------
GRDIE-------------------------------------------------------
------------------------------VTDENKK-EYVDL-MVNWRIKKR-IDEQFQ
-AFITG---FHELIPA--ELVN-VFDER-ELELLIGG-----------------------
----IAEID-----------VEDW----KKHTDYRG-YTES-------------------
------------------------DEVI-KFFWQTIR--------------------SWD
GEQKSRLLQF-------ATGTSRIP------------VNG-----FK-DLQGSD--GPRR
FTIEKAGEF-------------------------NNLPKSHTCF-NR-LDLPPY-KSLET
LQSKLT-MAVEE-TMGF
>Pseudogymnoascus_verrucosus_XM_018273646.1 .
IKARRSHI-FEDSYAEI--------------MRQSATDLKK-RLMIKFEG-------EDG
LDYGGVSREF---FFLL----SHEMFNPFY--------CLFEYSAHDNY-----------
----------------TLQINPHS------------------------------------
--GINPEHLNYFKFIGRVVGLAIFHRRFLDAFFIG--ALYKMMLH---------------
--------KPVSLQDMEG----------------------VDADFHRS--------LVWT
LEN-------------------------------------------------DIE-GVL--
-DQTFSTEDER----------------------------FGV-TSVED-LKPG--------
GRDIA-------------------------------------------------------
------------------------------VTNENKK-EYVDL-MIKWRIQKR-VDEQFQ
-AFVTG---FHELIPA--DLVN-VFDER-ELELLIGG-----------------------
----IAEID-----------VDDW----KKHTDYRG-YQES-------------------
------------------------DEVI-KFFWQTIK--------------------SWD
GEQKSRLLQF-------ATGTSRIP------------VNG-----FK-DLQGSD--GPRR
FTIEKAGDV-------------------------GNLPKSHTCF-NR-LDLPPY-KSLDA
LQGKLT-TAVEE-TMGF
>Pseudogymnoascus_destructans_XM_012890916.1 .
IKARRSHI-FEDSYAEI--------------MRQSATDLKK-RLMIKFEG-------EDG
LDYGGVSREF---FFLL----SHEMFNPFY--------CLFEYSAHDNY-----------
----------------TLQINPHS------------------------------------
--GINPEHLNYFKFIGRVVGLAIFHRRFLDAFFIG--ALYKMMLH---------------
--------KPVSLQDMEG----------------------VDADFHRS--------LVWT
LEN-------------------------------------------------DIE-GVL--
-DQTFSTEDER----------------------------FGV-TSVED-LKPG--------
GRDIA-------------------------------------------------------
------------------------------VTNENKK-EYVDL-MIKWRIQKR-VDEQFQ
-AFVSG---FHELIPA--DLVN-VFDER-ELELLIGG-----------------------
----IAEID-----------VDDW----KKHTDYRG-YQES-------------------
------------------------DEVI-KFFWQTIK--------------------SWD
GEQKSRLLQF-------ATGTSRIP------------VNG-----FK-DLQGSD--GPRR
```

FTIEKAGDV-------------------------GNLPKSHTCF-NR-LDLPPY-KSLDA
LQGKLT-TAVEE-TMGF
>Thielaviopsis_musarum_LKBB01000363.1 .
IKIRRSHI-FEDSFAEI--------------QRQTPTDLKK-RLMIKFDG-------EDG
LDYGGLSREF---FFLL----SHEMFNPFY--------CLFEYSAHDNY-----------
----------------TLQINPHS------------------------------------
--GINPEHLNYFRFIGRVVGLAIFHRRFLDAFFIG--ALYKMILN---------------
--------KAVALADMEG----------------------VDADFHRS--------LQWI
LDN--DISDGIL--
-EQTFSTEDER---------------------------FGT-IVVED-LITD--------
GRNID---
------------------------------VTNENKK-EYVDL-MVKWRIQKR-IEEQFK
-AFKEG---FSELIPQ--DLIN-VFDER-ELELLIGG-----------------------
----IAEID-----------VDDW----KKHTDYRG-YTEN-------------------
------------------------DEVI-QFFWQTIK--------------------GWD
GEQKSRLLQF-------ATGTSRIP------------VNG-----FK-DLQGSD--GPRR
FTIEKAGDP-------------------------NNLPKAH---------------TWVF
INQPY------------
>Thielaviopsis_paradoxa_BCHJ01000002.1 .
IKIRRSHI-FEDSFAEI--------------QRQTPTDLKK-RLMIKFDG-------EDG
LDYGGLSREF---FFLL----SHEMFNPFY--------CLFEYSAHDNY-----------
----------------TLQINPHS------------------------------------
--GINPEHLNYFRFIGRVVGLAIFHRRFLDAFFIG--ALYKMILN---------------
--------KAVSLADMEG----------------------VDADFHRS--------LQWI
LDN--DISDGIL--
-EQTFSTEDER---------------------------FGT-IVVED-LVPD--------
GRNID---
------------------------------VTNENKK-EYVDL-MVKWRIQKR-IEEQFK
-AFKEG---FSELIPQ--DLIN-VFDER-ELELLIGG-----------------------
----IAEID-----------VDDW----KKHTDYRG-YTEN-------------------
------------------------DEVI-QFFWQTIK--------------------GWD
GEQKSRLLQF-------ATGTSRIP------------VNG-----FK-DLQGSD--GPRR
FTIEKAGDP-------------------------NNLP----------------------

>Thielaviopsis_punctulata_LAEV01001069.1 .
IKIRRSHI-FEDSFAEI--------------QRQTPTDLKK-RLMIKFDG-------EDG
LDYGGLSREF---FFLL----SHEMFNPFY--------CLFEYSAHDNY-----------
----------------TLQINPHS------------------------------------
--GINPEHLNYFRFIGRVVGLAIFHRRFLDAFFIG--ALYKMILN---------------
--------KAVTLADMEG----------------------VDADFHRS--------LQWI
LDN--DISDGIL--
-EQTFSTEDER---------------------------FGT-IVVED-LIPN--------
GRNID---
------------------------------VTNENKK-EYVDL-MVKWRIQKR-IEEQFK
-AFKEG---FSELIPQ--DLIN-VFDER-ELELLIGG-----------------------
----IAEID-----------VDDW----KKHTDYRG-YTEN-------------------
------------------------DEVI-QFFWQTIK--------------------SWD
GEQKSRLLQF-------ATGTSRIP------------VNG-----FK-DLQGSD--GPRR
FTIEKAGDI-------------------------NNLPKAHTXF-NR-LDLPPY-KTLET
LQQKLT-IAVEE-TMG-
>Endoconidiophora_polonica_LXKZ01000665.1 .
IKVRRSHI-FEDSFAEI--------------QRQTPTELKK-RLMIKFDG-------EDG
LDYGGLSREF---FFLL----SHEMFNPFY--------CLFEYSAHDNY-----------
----------------TLQINPHS------------------------------------
--GINPEHLNYFRFIGRVVGLAIFHRRFLDAFFIG--ALYKMILG---------------
--------KAVSLADMEG----------------------VDADFHRS--------LQWI
LDN--DISDGIL--
-EQTFSTEDER---------------------------FGT-IVVED-LKEG--------

GRDIE---
------------------------------VTNENKK-EYVDL-MVKWRIQKR-IEEQFK
-AFKEG---FSELIPQ--ELIN-VFDER-ELELLIGG-----------------------
----IAEID-----------VDDW----KKHTDYRG-YTES-------------------
------------------------DEVI-QFFWQTIK--------------------GWD
GEQKSRLLQF-------ATGTSRIP------------VNG-----FK-DLQGSD--GPRR
FTIEKAGDI-------------------------NNLPKAHTXF-NR-LDLPPY-KSLEM
LQQKLT-IAVEE-TMG-
>Sporothrix_schenckii_XM_016731650.1 .
IKVRRSHI-FEDSFAEI--------------TRQTPHDLKK-RLMIKFDG-------EDG
LDYGGLSREF---FFLL----SHEMFNPFY--------CLFEYSAHDNY-----------
----------------TLQINPHS------------------------------------
--GINPEHLNYFKFIGRVVGLAIFHRRFLDAFFIG--ALYKMVLG---------------
--------KMVVLADMEG----------------------VDADFHRS--------LQWI
LDN--DISGGIL--
-EQTFSTEDER---------------------------FGV-LTVED-LIPG--------
GRDIE---
------------------------------VTNENKK-EYVDL-MVKWRIEKR-ISEQFQ
-SFREG---FNELIPQ--DLIN-VFDER-ELELLIGG-----------------------
----IAEID-----------VDDW----KKHTDYRG-YTES-------------------
------------------------DEVI-QFFWQTVR--------------------SWD
GEQKSRLLQF-------TTGTSRIP------------VNG-----FK-DLQGSD--GPRR
FTIEKAGDV-------------------------INLPKAHTCF-NR-LDLPPY-KNLET
LQGKMT-IAVEE-TMGF
>Grosmannia_clavigera_XM_014313120.1 .
IKVRRSHI-FEDSFAEI--------------TRQSPTDLKK-RLMIKFDG-------EDG
LDYGGLSREF---FFLL----SHEMFNPFY--------CLFEYSAHDNY-----------
----------------TLQINPHS------------------------------------
--GINPEHLNYFRFIGRVVGLAIFHRRFLDAFFIG--ALYKMILG---------------
--------KMVVLADMEG----------------------VDADFHRS--------LQWI
LDN--DISGGIL--
-EQTFSTEDER---------------------------FGV-ITVED-LIED--------
GRNID---
------------------------------VTNENKK-EYVDL-MVKWRIQKR-IAEQFQ
-AFKEG---FNELIPQ--DLIN-VFDER-ELELLMGG-----------------------
----IAEID-----------VDDW----KKHTDYRG-YTES-------------------
------------------------DDVI-QFFWQTVR--------------------SWD
GEQKSRLLQF-------TTGTSRIP------------VNG-----FK-DLQGSD--GPRR
FTIEKAGDI-------------------------QTLPKAHTCF-NR-LDLPPY-KNLEQ
LQGKMT-IAVEE-TMGF
>Colletotrichum_fioriniae_XM_007595341.1 .
IKVRRSHI-FEDSFAEI--------------TRQSATDLKK-RLMIKFDG-------EDG
LDYGGLSREF---FFLL----SHEMFNPFY--------CLFEYSAHDNY-----------
----------------TLQINPHS------------------------------------
--GINPEHLNYFKFIGRVVGLAIFHRRFLDAFFIG--ALYKMILG---------------
--------KSVVLADMEG----------------------VDADFHRS--------LQWM
LDN--DISGGIL--
-EQTFSTEDER---------------------------FGV-MTVED-LIPG--------
GRDID---
------------------------------VTNENKK-EYVDL-MVKWRIEKR-IAEQFQ
-AFKDG---FHELIPQ--DLVN-VFDER-ELELLIGG-----------------------
----IAEID-----------VDDW----KKHTDYRG-YTES-------------------
------------------------DEVI-QFFWQTIR--------------------SWD
GEQKSRLLQF-------ATGTSRIP------------VNG-----FK-DLQGSD--GPRR
FTIEKAGEI-------------------------NNLPKAHTCF-NR-LDLPPY-KSLEM
LQQKLT-IAVEE-TMG-
>Colletotrichum_higginsianum_XM_018304058.1 .
IKVRRSHI-FEDSFAEI--------------TRQSATDLKK-RLMIKFDG-------EDG

LDYGGLSREF---FFLL----SHEMFNPFY--------CLFEYSAHDNY-----------
----------------TLQINPHS------------------------------------
--GINPEHLNYFKFIGRVVGLAIFHRRFLDAFFIG--ALYKMILG---------------
--------KSVVLADMEG----------------------VDADFHRS--------LQWM
LDN--DISGGIL--
-EQTFSTEDER---------------------------FGV-MTVED-LIPG--------
GRDID---
------------------------------VTNENKK-EYVDL-MVKWRIEKR-IAEQFQ
-AFKDG---FHELIPQ--DLVN-VFDER-ELELLIGG-----------------------
----IAEID-----------VDDW----KKHTDYRG-YTES-------------------
------------------------DEVI-QFFWQTIR--------------------SWD
GEQKSRLLQF-------ATGTSRIP------------VNG-----FK-DLQGSD--GPRR
FTIEKAGEI-------------------------NNLPKAHTCF-NR-LDLPPY-KSLEM
LQQKLT-IAVEE-TMGF
>Colletotrichum_acutatum_LVCK01000927.1 .
IKVRRSHI-FEDSFAEI--------------TRQSATDLKK-RLMIKFDG-------EDG
LDYGGLSREF---FFLL----SHEMFNPFY--------CLFEYSAHDNY-----------
----------------TLQINPHS------------------------------------
--GINPEHLNYFKFIGRVVGLAIFHRRFLDAFFIG--ALYKMILG---------------
--------KSVVLADMEG----------------------VDADFHRS--------LQWM
LDN--DISGGIL--
-EQTFSTEDER---------------------------FGV-MTVED-LIPG--------
GRDID---
------------------------------VTNENKK-EYVDL-MVKWRIEKR-IAEQFQ
-AFKDG---FHELIPQ--DLVN-VFDER-ELELLIGG-----------------------
----IAEID-----------VDDW----KKHTDYRG-YTES-------------------
------------------------DEVI-QFFWQTIR--------------------SWD
GEQKSRLLQF-------ATGTSRIP------------VNG-----FK-DLQGSD--GPRR
FTIEKAGEI-------------------------NNLPKAHTXF-NR-LDLPPY-KSLEM
LQQKLT-IAVEE-TMG-
>Colletotrichum_simmondsii_JFBX01000642.1 .
IKVRRSHI-FEDSFAEI--------------TRQSATDLKK-RLMIKFDG-------EDG
LDYGGLSREF---FFLL----SHEMFNPFY--------CLFEYSAHDNY-----------
----------------TLQINPHS------------------------------------
--GINPEHLNYFKFIGRVVGLAIFHRRFLDAFFIG--ALYKMILG---------------
--------KSVVLADMEG----------------------VDADFHRS--------LQWM
LDN--DISGGIL--
-EQTFSTEDER---------------------------FGV-MTVED-LIPG--------
GRDID---
------------------------------VTNENKK-EYVDL-MVKWRIEKR-IAEQFQ
-AFKDG---FHELIPQ--DLVN-VFDER-ELELLIGG-----------------------
----IAEID-----------VDDW----KKHTDYRG-YTES-------------------
------------------------DEVI-QFFWQTIR--------------------SWD
GEQKSRLLQF-------ATGTSRIP------------VNG-----FK-DLQGSD--GPRR
FTIEKAGEI-------------------------NNLPKAHTXF-NR-LDLPPY-KSLEM
LQQKLT-IAVEE-TMG-
>Colletotrichum_nymphaeae_JEMN01001025.1 .
IKVRRSHI-FEDSFAEI--------------TRQSATDLKK-RLMIKFDG-------EDG
LDYGGLSREF---FFLL----SHEMFNPFY--------CLFEYSAHDNY-----------
----------------TLQINPHS------------------------------------
--GINPEHLNYFKFIGRVVGLAIFHRRFLDAFFIG--ALYKMILG---------------
--------KSVVLADMEG----------------------VDADFHRS--------LQWM
LDN--DISGGIL--
-EQTFSTEDER---------------------------FGV-MTVED-LIPG--------
GRDID---
------------------------------VTNENKK-EYVDL-MVKWRIEKR-IAEQFQ
-AFKDG---FHELIPQ--DLVN-VFDER-ELELLIGG-----------------------
----IAEID-----------VDDW----KKHTDYRG-YTES-------------------

------------------------DEVI-QFFWQTIR--------------------SWD
GEQKSRLLQF-------ATGTSRIP------------VNG-----FK-DLQGSD--GPRR
FTIEKAGEI-------------------------NNLPKAHTXF-NR-LDLPPY-KSLEM
LQQKLT-IAVEE-TMG-
>Colletotrichum_graminicola_XM_008100848.1 .
IKVRRSHI-FEDSFAEI--------------TRQSATDLKK-RLMIKFDG-------EDG
LDYGGLSREF---FFLL----SHEMFNPFY--------CLFEYSAHDNY-----------
----------------TLQINPHS------------------------------------
--GINPEHLNYFKFIGRVVGLAIFHRRFLDAFFIG--ALYKMILG---------------
--------KPVALADMEG----------------------VDADFHRS--------LQWM
LDN--DISGGIL--
-EQTFSTEDER---------------------------FGV-MTVED-LIPG--------
GRDID---
------------------------------VTNENKK-EYVDL-MVKWRIEKR-IAEQFQ
-AFKDG---FHELIPQ--DLVN-VFDER-ELELLIGG-----------------------
----IAEID-----------VDDW----KKHTDYRG-YTES-------------------
------------------------DEVI-QFFWQTIR--------------------SWD
GEQKSRLLQF-------ATGTSRIP------------VNG-----FK-DLQGSD--GPRR
FTIEKAGEI-------------------------NNLPKAHTCF-NR-LDLPPY-KSLEM
LQQKLT-IAVEE-TMGF
>Colletotrichum_gloeosporioides_XM_007275426.1 .
IKVRRSHI-FEDSFAEI--------------TRQSATDLKK-RLMIKFDG-------EDG
LDYGGLSREF---FFLL----SHEMFNPFY--------CLFEYSAHDNY-----------
----------------TLQINPHS------------------------------------
--GINPEHLNYFKFIGRVVGLAIFHRRFLDAFFIG--ALYKMILG---------------
--------KSVVLADMEG----------------------VDADFHRS--------LQWM
LDN--DISGGIL--
-EQTFSTEDER---------------------------FGV-MTVED-LIPN--------
GRNID---
------------------------------VTNENKK-EYVDL-MVKWRIEKR-IAEQFQ
-AFKDG---FHELIPQ--DLIN-VFDER-ELELLIGG-----------------------
----IAEID-----------VDDW----KKHTDYRG-YTES-------------------
------------------------DEVI-QFFWQTIR--------------------SWD
GEQKSRLLQF-------ATGTSRIP------------VNG-----FK-DLQGSD--GPRR
FTIEKAGEI-------------------------NNLPKAHTCF-NR-LDLPPY-KSLEM
LQQKLT-IAVEE-TMGF
>Colletotrichum_chlorophyti_MPGH01000162.1 .
IKVRRSHI-FEDSFAEI--------------TRQSATDLKK-RLMIKFDG-------EDG
LDYGGLSREF---FFLL----SHEMFNPFY--------CLFEYSAHDNY-----------
----------------TLQINPHS------------------------------------
--GINPEHLNYFKFIGRVVGLAIFHRRFLDAFFIG--ALYKMILG---------------
--------KSVVLADMEG----------------------VDADFHRS--------LQWM
LDN--DISGGIL--
-EQTFSTEDER---------------------------FGV-MTVED-LIPN--------
GRNID---
------------------------------VTNENKK-EYVDL-MVKWRIEKR-IAEQFQ
-AFKDG---FHELIPQ--DLIN-VFDER-ELELLIGG-----------------------
----IAEID-----------VDDW----KKHTDYRG-YTES-------------------
------------------------DEVI-QFFWQTIR--------------------SWD
GEQKSRLLQF-------ATGTSRIP------------VNG-----FK-DLQGSD--GPRR
FTIEKAGEI-------------------------NNLPKAHTXF-NR-LDLPPY-KSLEM
LQQKLT-IAVEE-TMG-
>Pestalotiopsis_fici_XM_007841709.1 .
IKVRRSHI-FEDSFAEI--------------SRQSATDLKK-RLMIKFDG-------EDG
LDYGGLSREF---FFLL----SHEMFNPFY--------CLFEYSAHDNY-----------
----------------TLQINPHS------------------------------------
--GINPEHLNYFKFIGRVVGLAIFHRRFLDAFFIG--ALYKMVLG---------------
--------KPVSLQDMEG----------------------VDADFHRS--------LQWM

LDN--DISGGIL--
-EQTFSTEDER---------------------------FGV-LTVED-LKPD--------
GRNIE---
------------------------------VTNENKK-EYVEL-MVKWRIEKR-IAEQFQ
-AFKDG---FHELIPQ--DLVN-VFDER-ELELLIGG-----------------------
----IAEID-----------VDDW----KKHTDYRG-YTES-------------------
------------------------DEVI-QFFWQTIR--------------------SWD
GEQKSRLLQF-------ATGTSRIP------------VNG-----FK-DLQGSD--GPRR
FTIEKAGEV-------------------------HNLPKAHTCF-NR-LDLPPY-KSLEA
LQQKLT-IAVEE-TMGF
>Verticillium_dahliae_XM_009651450.1 .
IKVRRSHI-FEDSFAEI--------------SRQSATDLKK-RLMIKFDG-------EDG
LDYGGLSREF---FFLL----SHEMFNPFY--------CLFEYSAHDNY-----------
----------------TLQINPHS------------------------------------
--GINPEHLNYFKFIGRVVGLAIFHRRFLDAFFIG--ALYKMILG---------------
--------KSVVLADMEG----------------------VDADFHRS--------LQWM
LDN--DISGGIL--
-EQTFSTEDER---------------------------FGV-ICVED-LKPD--------
GRNID---
------------------------------VDNDNKK-EYVDL-MVKWRIEKR-IAEQFQ
-AFQTG---FHELIPQ--DLIN-VFDER-ELELLIGG-----------------------
----IAEID-----------VDDW----KKHTDYRG-YTES-------------------
------------------------DEVV-QFFWQTIR--------------------SWD
GEQKSRLLQF-------ATGTSRIP------------VNG-----FK-DLQGSD--GPRR
FTIEKAGEV-------------------------NNLPKAHTCF-NR-LDLPPY-KNLEA
LQQKLT-IAVEE-TMGF
>Verticillium_alboatrum_XM_003008737.1 .
IKVRRSHI-FEDSFAEI--------------SRQSATDLKK-RLMIKFDG-------EDG
LDYGGLSREF---FFLL----SHEMFNPFY--------CLFEYSAHDNY-----------
----------------TLQINPHS------------------------------------
--GINPEHLNYFKFIGRVVGLAIFHRRFLDAFFIG--ALYKMILG---------------
--------KSVVLADMEG----------------------VDADFHRS--------LQWM
LDN--DISGGIL--
-EQTFSTEDER---------------------------FGV-ICVED-LKPD--------
GRNID---
------------------------------VDNDNKK-EYVDL-MVKWRIEKR-IAEQFQ
-AFQTG---FHELIPQ--DLIN-VFDER-ELELLIGG-----------------------
----IAEID-----------VDDW----KKHTDYRG-YTES-------------------
------------------------DEVV-QFFWQTIR--------------------SWD
GEQKSRLLQF-------ATGTSRIP------------VNG-----FK-DLQGSD--GPRR
FTIEKAGEV-------------------------NNLPKAHTCF-NR-LDLPPY-KNLEA
LQQKLT-IAVEE-TMGF
>Scedosporium_apiospermum_XM_016785637.1 .
IKVRRSHI-FEDSFAEI--------------QRQSPTDLKK-RLMIKFDG-------EDG
LDYGGLSREF---FFLL----SHEMFNPFY--------CLFEYSAHDNY-----------
----------------TLQINPHS------------------------------------
--GINPEHLNYFKFIGRVVGLAIYHRRFLDAFFIG--ALYKMILG---------------
--------KPVVLADMEG----------------------VDADFHRS--------LQWM
LDN--DISGGVL--
-EQTFSTEDER---------------------------FGV-VTEED-LKPN--------
GRNIE---
------------------------------VTNENKK-EYVDL-MVKWRIEKR-IAEQFQ
-AFKEG---FHELIPQ--DLIN-VFDER-ELELLIGG-----------------------
----IAEID-----------VDDW----KKHTDYRG-YTET-------------------
------------------------DEVI-QFFWQTIR--------------------SWD
GEQKSRLLQF-------ATGTSRIP------------VNG-----FK-DLQGSD--GPRR
FTIEKAGDI-------------------------NNLPKAHTCF-NR-LDLPQY-KSLEM
LQQKLT-IAVEE-TMGF

>Corollospora_maritima_GDFX01001143.1 .
IKVRRSHI-FEDSFAEI--------------QRQSPTDLKK-RLMIKFDG-------EDG
LDYGGLSREF---FFLL----SHEMFNPFY--------CLFEYSAHDNY-----------
----------------TLQINPHS------------------------------------
--GINPEHLNYFKFIGRVVGLAIFHRRFLDAFFIG--ALYKMILG---------------
--------KAVSLADMEG----------------------VDADFHRS--------LQWM
LDN--DISGGIL--
-EQTFSTEDER---------------------------FGV-VTVED-LIPN--------
GRNIE---
------------------------------VTNENKK-EYVDL-MVKWRIERR-IAEQFQ
-AFKDG---FHELIPQ--DLIN-VFDER-ELELLIGG-----------------------
----IAEID-----------VDDW----KKHTDYRG-YTEK-------------------
------------------------DEVI-QFFWQTIR--------------------SWD
GEQKSRLLQF-------ATGTSRIP------------VNG-----FK-DLQGSD--GPRR
FTIEKAGEI-------------------------TNLPKAHTCF-NR-LDLPPY-KSLEM
LQQKLT-IAVEE-TMGF
>Knoxdaviesia_capensis_LNGK01000002.1 .
IKVRRSHI-FADSFAEI--------------QRQSPTDLKK-RLMIKFDG-------EDG
LDYGGLSREF---FFLL----SHEMFNPFY--------CLFEYSAHDNY-----------
----------------TLQINPHS------------------------------------
--GINPEHLAYFKFIGRVVGLAIFHRRFLDAFFIG--ALYKMVLG---------------
--------KPVALPDMEG----------------------VDADFHRS--------LQWM
LDN--DISGGIL--
-EQTFSTEDER---------------------------FGV-ITVED-LIPG--------
GRDIE---
------------------------------VTNENKK-EYVDL-MVKWRIEKR-IAEQFT
-AFKEG---FQELIPQ--ELIN-VFDER-ELELLIGG-----------------------
----IAEID-----------VDDW----KKHTDYRG-YTES-------------------
------------------------DEVI-QYFWQTIR--------------------SWD
GEQKSRLLQF-------ATGTSRIP------------VNG-----FK-DLQGSD--GPRR
FTIEKAGEV-------------------------GNLPKAHTXF-NR-LDLPPY-KNLDM
LQQKLT-IAVEE-TMG-
>Togninia_minima_XM_007916715.1 .
IKVRRSHI-FEDSFAEI--------------SRQSATDLKK-RLMIKFDG-------EDG
LDYGGLSREF---FFLL----SHEMFNPFY--------CLFEYSAHDNY-----------
----------------TLQINPHS------------------------------------
--GINPEHLNYFKFIGRVVGLAIFHRRFLDAFFIG--ALYKMILG---------------
--------KSVQLADMEG----------------------VDADFHRS--------LQWM
LDN--DISGGIL--
-EQTFSTEDER---------------------------FGV-ITVED-LIPN--------
GRNID---
------------------------------VTNENKK-EYVDL-MVKWRIEKR-IAEQFQ
-AFKEG---FHELIPQ--DLIN-VFDER-ELELLIGG-----------------------
----IAEID-----------VDDW----KKHTDYRG-YTES-------------------
------------------------DEVI-QYFWQTIR--------------------SWD
GEQKSRLLQF-------ATGTSRIP------------VNG-----FK-DLQGSD--GPRR
FTIEKAGEI-------------------------TNLPKAHTCF-NR-LDLPPY-KNLDM
LQGKLT-IAVEE-TMGF
>Chaetomium_thermophilum_XM_006694928.1 .
IKVRRSHI-FEDAFAEI--------------SRQSATDLKK-RLMIKFDG-------EDG
LDYGGLSREF---FFLL----SHEMFNPFY--------CLFEYSAHDNY-----------
----------------TLQINPHS------------------------------------
--GINPEHLNYFKFIGRVVGLAIFHRRFLDAFFIT--AFYKMILG---------------
--------KPVTLADMEG----------------------VDADFHRS--------LQWM
LDN--DISGGII--
-EATFSTEDER---------------------------FGV-ITVED-LKPN--------
GRNIE---
------------------------------VTNENKR-EYVEL-MVKWRIQKR-VEEQFK

```
-AFKEG---FNELIPQ--DLIN-VFDER-ELELLIGG-----------------------
----IAEID-----------VDDW----KKHTDYRG-YTES-------------------
------------------------DEVI-QFFWQTVR--------------------SWD
SEQKSRLLQF-------TTGTSRIP------------VNG-----FK-DLQGSD--GPRR
FTIERAGDI-------------------------NNLPKAHTCF-NR-LDLPPY-KTLEQ
LQQKLT-MAVEE-TMGF
>Myceliophthora_thermophila_XM_003664458.1 .
IKVRRSHI-FEDSFAEI--------------SRQSPTDLKK-RLMIKFDG-------EDG
LDYGGLSREF---FFLL----SHEMFNPFY--------CLFEYSAHDNY-----------
----------------TLQINPHS------------------------------------
--GINPEHLNYFKFIGRVVGLAIFHRRFLDAFFIG--ALYKMILG---------------
--------KNVVLADMEG----------------------VDADFHRS--------LQWM
LDN------------------------------------------------DISGGIL--
-EQTFSTEDER---------------------------FGV-VTVED-LIPN--------
GRNIE-------------------------------------------------------
------------------------------VTNENKK-EYVDL-MVKWRIQKR-VSEQFE
-AFMEG---FHDLIPQ--DLIG-VFDER-ELELLIGG-----------------------
----IAEID-----------VDDW----KKHTDYRG-YTES-------------------
------------------------DKVI-QFFWQTVR--------------------SWD
GEQKSRLLQF-------TTGTSRIP------------VNG-----FK-DLQGSD--GPRR
FTIEKAGDI-------------------------TNLPKAHTCF-NR-LDLPPY-ETLEM
LQQKLT-MAVEE-TMGF
>Chaetomium_globosum_XM_001226583.1 .
IKVRRSHI-FEDSFAEI--------------SRQSATDLKK-RLMIKFDG-------EDG
LDYGGLSREF---FFLL----SHEMFNPFY--------CLFEYSAHDNY-----------
----------------TLQINPHS------------------------------------
--GINPEHLNYFKFIGRVVGLAIFHRRFLDAFFIG--ALYKMWM----------------
------------------------------------------------------------
LDN------------------------------------------------DISGGIL--
-EQTFSTEDER---------------------------FGV-ITVED-LIPN--------
GRNID-------------------------------------------------------
------------------------------VTNDNKK-EYVDL-MVKWRIQKR-IAEQFE
-AFKEG---FQDLIPQ--DLIN-VFDER-ELELLIGG-----------------------
----IAEID-----------VDDW----KKHTDYRG-YTES-------------------
------------------------DEVI-QFFWQTVR--------------------SWD
GEQKSRLLQF-------TTGTSRIP------------VNG-----FK-DLQGSD--GPRR
FTIEKAGEL-------------------------GNLPKAHTCF-NR-LDLPPY-KTLEM
LQGKLT-MAVEE-TMGF
>Daldinia_eschscholzii_AIID01007771.1 .
MKVRRSHI-FEDSFAEI--------------SRQSATDLKK-RLMIKFDG-------EDG
LDYGGLSREF---FFLL----SHEMFNPFY--------CLFEYSAHDNY-----------
----------------TLQINPHS------------------------------------
--GINPEHLNYFKFIGRVVGLAIFHRRFLDAFFIG--ALYKMILG---------------
--------KPVVLADMEG----------------------VDADFHRS--------LQWM
LDN------------------------------------------------DISGGIL--
-EQTFSTEDER---------------------------FGV-VTVED-LIPN--------
GRNID-------------------------------------------------------
------------------------------VTNENKK-EYVDL-MVKWRIEKR-ISEQFQ
-AFKEG---FHELIPQ--DLIN-VFDER-ELELLIGG-----------------------
----IADID-----------VDDW----KKHTDYRG-YTES-------------------
------------------------DEVI-QFFWQTVR--------------------SWD
GEQKSRLLQF-------TTGTSRIP------------VNG-----FK-DLQGSD--GPRR
FTIEKAGEV-------------------------GNLPKAHTXF-NR-LDLPPY-KSLEQ
LQSKLT-TAVEE-TMGF
>Hypoxylon_sp._MDCK01000009.1 .
MKVRRSHI-FEDSFAEI--------------SRQSATDLKK-RLMIKFDG-------EDG
LDYGGLSREF---FFLL----SHEMFNPFY--------CLFEYSAHDNY-----------
----------------TLQINPHS------------------------------------
```

--GINPEHLNYFKFIGRVVGLAIFHRRFLDAFFIG--ALYKMILG---------------
--------KPVVLADMEG----------------------VDADFHRS--------LQWM
LDN--DISGGIL--
-EQTFSTEDER---------------------------FGV-ITVED-LIPN--------
GRNIE---
------------------------------VTNENKK-EYVDL-MVKWRIEKR-ISEQFQ
-AFKEG---FHELIPQ--DLIG-VFDER-ELELLIGG-----------------------
----IADID-----------VDDW----KKHTDYRG-YTES-------------------
------------------------DETI-QFFWQTVR--------------------SWD
GEQKSRLLQF-------TTGTSRIP------------VNG-----FK-DLQGSD--GPRR
FTIEKAGEI-------------------------GNLPKAHTXF-NR-LDLPPY-KTLEQ
LQSKLT-TAVEE-TMG-
>Eutypa_lata_XM_007800325.1 .
IKVRRSHI-FEDSFAEI--------------SRQSATDLKK-RLMIKFDG-------EDG
LDYGGLSREF---FFLL----SHEMFNPFY--------CLFEYSAHDNY-----------
----------------TLQINPHS------------------------------------
--GINPEHLNYFKFIGRVVGLAIFHRRFLDAFFIG--ALYKMILG---------------
--------KGVTLSDMEG----------------------VDADFHRS--------LQWM
LDN--DISGGIL--
-EQTFSTEDER---------------------------FGV-MTEED-LIPN--------
GRNIE---
------------------------------VTNENKK-EYVDL-MVTWRIEKR-IAEQFK
-AFKEG---FHELIPQ--DLIN-VFDER-ELELLIGG-----------------------
----IAEID-----------VDDW----KKHTDYRG-YTES-------------------
------------------------DEVI-QFFWQTVR--------------------SWD
GEQKSRLLQF-------TTGTSRIP------------VNG-----FK-DLQGSD--GPRR
FTIEKAGDI-------------------------GNLPKAHTCF-NR-LDLPPY-KNLEQ
LQQKLT-IAVEE-TMGF
>Thielavia_terrestris_XM_003650838.1 .
IKVRRSHI-FEDSFAEI--------------SRLSATDLKK-RLMIKFDG-------EDG
LDYGGLSREF---FFLL----SHEMFNPFY--------CLFEYSAHDNY-----------
----------------TLQINPHS------------------------------------
--GINPEHLNYFKFIGRVVGLAIFHRRFLDAFFIG--ALYKMMLG---------------
--------KPVVLADMEG----------------------VDADFHRS--------LKWM
LEN--DISGGIL--
-EQTFSTEDER---------------------------FGV-VTVED-LIPN--------
GRNIE---
------------------------------VTNENKK-EYVDL-MVKWRIEKR-VAEQFA
-AFKSG---FEELIPQ--DLIN-VFDER-ELELLIGG-----------------------
----IAEID-----------VDDW----KKHTDYRG-YTES-------------------
------------------------DEVI-QFFWQTVR--------------------SWD
GEQKSRLLQF-------TTGTSRIP------------VNG-----FK-DLQGSD--GPRR
FTIEKAGEI-------------------------TNLPKAHTCF-NR-LDLPPY-KTLEM
LQQKLT-IAVEE-TMGF
>Magnaporthe_oryzae_XM_003715426.1 .
IKVRRSHI-FEDSFAEI--------------SRQSATDLKK-RLMIKFDG-------EDG
LDYGGLSREF---FFLL----SHEMFNPFY--------CLFEYSAHDNY-----------
----------------TLQINPHS------------------------------------
--GINPEHLNYFKFIGRVVGLAIFHRRFLDAFFIG--ALYKMVLG---------------
--------KSVALPDMEG----------------------VDADFHRS--------LQWM
LDN--DISGGIL--
-EQTFSTEDER---------------------------FGV-ITVED-LIPN--------
GRNID---
------------------------------VTNENKK-EYVDL-MVKWRIEKR-IAEQFE
-AFKTG---FHELIPQ--DLIN-VFDER-ELELLIGG-----------------------
----IAEID-----------VDDW----KKHTDYRG-YTEQ-------------------
------------------------DEVI-QFFWQTVR--------------------SWD
GEQKSRLLQF-------TTGTSRIP------------VNG-----FK-DLQGSD--GPRR

FTIEKAGEI-------------------------NNLPKAHTCF-NR-LDLPPY-KSLEM
LQQKMT-IAVEE-TMGF
>Magnaporthe_grisea_XM_367330.2 .
IKVRRSHI-FEDSFAEI--------------SRQSATDLKK-RLMIKFDG-------EDG
LDYGGLSREF---FFLL----SHEMFNPFY--------CLFEYSAHDNY-----------
----------------TLQINPHS------------------------------------
--GINPEHLNYFKFIGRVVGLAIFHRRFLDAFFIG--ALYKMVLG---------------
--------KSVALPDMEG----------------------VDADFHRS--------LQWM
LDN--DISGGIL--
-EQTFSTEDER---------------------------FGV-ITVED-LIPN--------
GRNID---
------------------------------VTNENKK-EYVDL-MVKWRIEKR-IAEQFE
-AFKTG---FHELIPQ--DLIN-VFDER-ELELLIGG-----------------------
----IAEID-----------VDDW----KKHTDYRG-YTEQ-------------------
------------------------DEVI-QFFWQTVR--------------------SWD
GEQKSRLLQF-------TTGTSRIP------------VNG-----FK-DLQGSD--GPRR
FTIEKAGEI-------------------------NNLPKAHTCF-NR-LDLPPY-KSLEM
LQQKMT-IAVEE-TMGF
>2_Falciphora_oryzae_JNVV01000001.1 .
IKVRRSHI-FEDSFAEI--------------SRQSATDLKK-RLMIKFDG-------EDG
LDYGGLSREF---FFLL----SHEMFNPFY--------CLFEYSAHDNY-----------
----------------TLQINPHS------------------------------------
--GINPEHLNYFKFIGRVVGLAIFHRRFLDAFFIG--ALYKMVLG---------------
--------KPVALADMEG----------------------VDADFHRS--------LQWM
IDN--DISGGIL--
-EQTFSTEDER---------------------------FGV-ITVED-LIPN--------
GRNID---
------------------------------VTNENKR-EYVDL-MVKWRIEKR-IAEQFE
-AFKVG---FHELIPI--DLIN-VFDER-ELELLIGG-----------------------
----IAEID-----------VDDW----KKHTDYRG-YTES-------------------
------------------------DEVI-QFFWQTVR--------------------SWD
GEQKSRLLQF-------TTGTSRIP------------VNG-----FK-DLQGSD--GPRR
FTIEKAGEI-------------------------NNLP----------------------

>Gaeumannomyces_graminis_XM_009218029.1 .
IKVRRSHI-FEDSFAEI--------------SRQSATDLKK-RLMIKFDG-------EDG
LDYGGLSREF---FFLL----SHEMFNPFY--------CLFEYSAHDNY-----------
----------------TLQINPHS------------------------------------
--GINPEHLNYFKFIGRVVGLAIFHRRFLDAFFIG--ALYKMVLG---------------
--------KAVALPDMEG----------------------VDADFHRS--------LQWM
IDN--DISGGIL--
-EQTFSTEDER---------------------------FGV-ITVED-LIPN--------
GRDID---
------------------------------VTNENKK-EYVDL-MVKWRIEKR-IAEQFE
-AFKVG---FHELIPI--DLIN-VFDER-ELELLIGG-----------------------
----IAEID-----------VDDW----KKHTDYRG-YTES-------------------
------------------------DEVI-QFFWQTVR--------------------SWD
GEQKSRLLQF-------TTGTSRIP------------VNG-----FK-DLQGSD--GPRR
FTIEKAGEI-------------------------NNLPKAHTCF-NR-LDLPPY-KSLEV
LQQKMT-IAVEE-TMGF
>Magnaporthiopsis_poae_ADBL01001730.1 .
IKVRRSHI-FEDSFAEI--------------SRQSATDLKK-RLMIKFDG-------EDG
LDYGGLSREF---FFLL----SHEMFNPFY--------CLFEYSAHDNY-----------
----------------TLQINPHS------------------------------------
--GINPEHLNYFKFIGRVVGLAIFHRRFLDAFFIG--ALYKMMLG---------------
--------KAVALPDMEG----------------------VDADFHRS--------LQWM
IDN--DISGGIL--
-EQTFSTEDER---------------------------FGV-ITVED-LIPN--------

GRNID---
------------------------------VTNENKK-EYVDL-MVKWRIEKR-IAEQFE
-AFKLG---FHELIPI--DLIN-VFDER-ELELLIGG-----------------------
----IAEID-----------VDDW----KKHTDYRG-YTES-------------------
------------------------DEVI-QFFWQTVR--------------------SWD
GEQKSRLLQF-------TTGTSRIP------------VNG-----FK-DLQGSD--GPRR
FTIEKAGEI-------------------------NNLPKAHTXF-NR-LDLPPY-KSLEV
LQQKMT-IAVEE-TMG-
>Podospora_anserina_XM_001903877.1 .
IKVRRSHI-FEDSFAEI--------------SRQSATDLKK-RLMIKFDG-------EDG
LDYGGLSREF---FFLL----SHEMFNPFY--------CLFEYSAHDNY-----------
----------------TLQINPHS------------------------------------
--GINPEHLNYFKFIGRVVGLAIFHRRFLDAFFIG--ALYKMVLG---------------
--------KAVVLADMEG----------------------VDADFHRS--------LQWI
LDN--DITDAGL--
-EMTFSTEDER---------------------------FGV-IAVED-LKPN--------
GRNID---
------------------------------VTEENKK-EYVDL-MVKWRIEKR-IAEQFQ
-AFKEG---FQELIPH--DLIN-VFDER-ELELLIGG-----------------------
----IAEID-----------VDDW----KKHTDYRG-YTES-------------------
------------------------DEVI-QFFWQTVR--------------------SWD
GEQKSRLLQF-------TTGTSRIP------------VNG-----FK-DLQGSD--GPRR
FTIEKAGEI-------------------------TNLPKAHTCF-NR-LDLPPY-KDLAM
LQNKLT-IAVEE-TMGF
>Neurospora_africana_CAPO020005133.1 .
IKVRRSHI-FEDSFAEI--------------TRQSATDLKK-RLMIKFDG-------EDG
LDYGGLSREF---FFLL----SHEMFNPFY--------CLFEYSAHDNY-----------
----------------TLQINPHS------------------------------------
--GINPEHLNYFKFIGRVVGLAIFHRRFLDAFFIG--ALYKMVLG---------------
--------KAVSLADMEG----------------------VDADFHRS--------LQWM
LDN--DIT-DVL--
-DATFSTEDER---------------------------FGV-ITEED-LKPN--------
GRNIA---
------------------------------VTNENKK-EYVDL-MVKWRIEKR-IEQQFQ
-AFKEG---FHELIPQ--DLIN-VFDER-ELELLIGG-----------------------
----IAEID-----------VDDW----KKHTDYRG-YTES-------------------
------------------------DEVI-QFFWQTVR--------------------SWD
GEQKSRLLQF-------TTGTSRIP------------VNG-----FK-DLQGSD--GPRR
FTIEKAGEI-------------------------TNLPKAHTXF-NR-LDLPPY-KSLEM
LQQKLT-IAVEE-TMG-
>Neurospora_sublineolata_CAPP020018472.1 .
IKVRRSHI-FEDSFAEI--------------SRQSATDLKK-RLMIKFDG-------EDG
LDYGGLSREF---FFLL----SHEMFNPFY--------CLFEYSAHDNY-----------
----------------TLQINPHS------------------------------------
--GINPEHLNYFKFIGRVVGLAIFHRRFLDAFFIG--ALYKMVLG---------------
--------KAVSLADMEG----------------------VDADFHRS--------LQWM
LDN--DIT-DVL--
-DATFSTEDER---------------------------FGV-ITEED-LIPD--------
GRNIA---
------------------------------VTNENKK-EYVDL-MVKWRIEKR-IEQQFR
-AFKEG---FHELIPQ--DLIN-VFDER-ELELLIGG-----------------------
----IAEID-----------VDDW----KKHTDYRG-YTES-------------------
------------------------DEVI-QFFWQTVR--------------------SWD
GEQKSRLLQF-------TTGTSRIP------------VNG-----FK-DLQGSD--GPRR
FTIEKAGEI-------------------------TNLPKAHTWY-VY-MK----------

>Neurospora_terricola_CAPR020001809.1 .
IKVRRSHI-FEDSFAEI--------------SRQSATDLKK-RLMIKFDG-------EDG

```
LDYGGLSREF---FFLL----SHEMFNPFY--------CLFEYSAHDNY-----------
----------------TLQINPHS------------------------------------
--GINPEHLNYFKFIGRVVGLAIFHRRFLDAFFIG--ALYKMVLG---------------
--------KAVSLADMEG----------------------VDADFHRS--------LQWM
LDN------------------------------------------------DIT-DVL--
-DATFSTEDER---------------------------FGV-ITEED-LIPN--------
GRNIA-------------------------------------------------------
------------------------------VTNENKK-EYVDL-MVKWRIEKR-IEQQFQ
-AFKEG---FHELIPQ--DLIN-VFDER-ELELLIGG-----------------------
----IAEID-----------VDDW----KKHTDYRG-YTES-------------------
------------------------DEVI-QFFWQTVR--------------------SWD
GEQKSRLLQF-------TTGTSRIP------------VNG-----FK-DLQGSD--GPRR
FTIEKAGE----------------------------------------------------
-----------------
>Sordaria_macrospora_XM_003344786.1 .
IKVRRSHI-FEDSFAEI--------------SRQSATDLKK-RLMIKFDG-------EDG
LDYGGLSREF---FFLL----SHEMFNPFY--------CLFEYSAHDNY-----------
----------------TLQINPHS------------------------------------
--GINPEHLNYFKFIGRVVGLAIFHRRFLDAFFIG--ALYKMVLG---------------
--------KAVSLADMEG----------------------VDADFHRS--------LQWM
LDN------------------------------------------------DIT-DVL--
-DATFSTEDER---------------------------FGV-ITEED-LIPN--------
GRNIA-------------------------------------------------------
------------------------------VTNENKK-EYVDL-MVKWRIEKR-IEQQFQ
-AFKEG---FHELIPQ--DLIN-VFDER-ELELLIGG-----------------------
----IAEID-----------VDDW----KKHTDYRG-YTES-------------------
------------------------DEVI-QFFWQTVR--------------------SWD
GEQKSRLLQF-------TTGTSRIP------------VNG-----FK-DLQGSD--GPRR
FTIEKAGEI-------------------------NNLPKAHTCF-NR-LDLPPY-KSLEM
LQQKLT-IAVEE-TMGF
>Neurospora_tetrasperma_XM_009856194.1 .
IKVRRSHI-FEDSFAEI--------------SRQSATDLKK-RLMIKFDG-------EDG
LDYGGLSREF---FFLL----SHEMFNPFY--------CLFEYSAHDNY-----------
----------------TLQINPHS------------------------------------
--GINPEHLNYFKFIGRVVGLAIFHRRFLDAFFIG--ALYKMVLG---------------
--------KAVSLADMEG----------------------VDADFHRS--------LQWM
LDN------------------------------------------------DIT-DVL--
-DATFSTEDER---------------------------FGV-ITEED-LIPN--------
GRNIA-------------------------------------------------------
------------------------------VTNENKK-EYVEL-MVKWRIEKR-IEQQFR
-AFKDG---FHELIPQ--DLIN-VFDER-ELELLIGG-----------------------
----IAEID-----------VDDW----KKHTDYRG-YTES-------------------
------------------------DEVI-QFFWQTVR--------------------SWD
GEQKSRLLQF-------TTGTSRIP------------VNG-----FK-DLQGSD--GPRR
FTIEKAGEI-------------------------TNLPKAHTCF-NR-LDLPPY-KSLEM
LQQKLT-IAVEE-TMG-
>Neurospora_crassa_XM_952493.3 .
IKVRRSHI-FEDSFAEI--------------SRQSATDLKK-RLMIKFDG-------EDG
LDYGGLSREF---FFLL----SHEMFNPFY--------CLFEYSAHDNY-----------
----------------TLQINPHS------------------------------------
--GINPEHLNYFKFIGRVVGLAIFHRRFLDAFFIG--ALYKMVLG---------------
--------KAVSLADMEG----------------------VDADFHRS--------LQWM
LDN------------------------------------------------DIT-DVL--
-DATFSTEDER---------------------------FGV-ITEED-LIPN--------
GRNIA-------------------------------------------------------
------------------------------VTNENKK-EYVEL-MVKWRIEKR-IEQQFR
-AFKDG---FHELIPQ--DLIN-VFDER-ELELLIGG-----------------------
----IAEID-----------VDDW----KKHTDYRG-YTES-------------------
```

------------------------DEVI-QFFWQTVR--------------------SWD
GEQKSRLLQF-------TTGTSRIP------------VNG-----FK-DLQGSD--GPRR
FTIEKAGEI-------------------------TNLPKAHTCF-NR-LDLPPY-KSLEM
LQQKLT-IAVEE-TMGF
>Diaporthe_ampelina_LWAD01000364.1 .
IKIRRSHI-FEDSFAEI--------------SRQSATDLKK-RLMIKFDG-------EDG
LDYGGLSREF---FFLL----SHEMFNPFY--------CLFEYSAHDNY-----------
----------------TLQINPHS------------------------------------
--GINPEHLNYFKFIGRVVGLAIFHRRFLDAFFIG--ALYKMILG---------------
--------KSVVLADMEG----------------------VDADFHRS--------LQWM
LDN--DISGGIL--
-EQTFSTEDER---------------------------FGV-ITVED-LIPG--------
GRDID---
------------------------------VTNDNKK-EYVDL-MVKWRIEKR-IAEQFQ
-AFREG---FHELIPQ--DLIN-VFDER-ELELLIGG-----------------------
----IAEID-----------VDDW----KKHTDYRG-YTES-------------------
------------------------DEVI-QFFWQTVR--------------------SWD
GEQKSRLLQF-------TTGTSRIP------------VNG-----FK-DLQGSD--GPRR
FTIEKAGEV-------------------------NNLPKAHTXF-NR-LDLPPY-KSLEQ
LQQKLT-IAVEE-TMG-
>Diaporthe_longicolla_AYRD02011485.1 .
IKIRRSHI-FEDSFAEI--------------SRQSATDLKK-RLMIKFDG-------EDG
LDYGGLSREF---FFLL----SHEMFNPFY--------CLFEYSAHDNY-----------
----------------TLQINPHS------------------------------------
--GINPEHLNYFKFIGRVVGLAIFHRRFLDAFFIG--ALYKMILG---------------
--------KSVVLADMEG----------------------VDADFHRS--------LQWM
LDN--DISGGIL--
-EQTFSTEDER---------------------------FGV-ITVED-LIPG--------
GRDID---
------------------------------VTNDNKK-EYVDL-MVKWRIEKR-IAEQFQ
-AFREG---FHELIPQ--DLIN-VFDER-ELELLIGG-----------------------
----IAEID-----------VDDW----KKHTDYRG-YTES-------------------
------------------------DEVI-QFFWQTVR--------------------SWD
GEQKSRLLQF-------TTGTSRIP------------VNG-----FK-DLQGSD--GPRR
FTIEKAGEV-------------------------NNLPKAHTXF-NR-LDLPPY-KSLEQ
LQQKLT-IAVEE-TMG-
>Diaporthe_aspalathi_LJJS01001699.1 .
IKIRRSHI-FEDSFAEI--------------SRQSATDLKK-RLMIKFDG-------EDG
LDYGGLSREF---FFLL----SHEMFNPFY--------CLFEYSAHDNY-----------
----------------TLQINPHS------------------------------------
--GINPEHLNYFKFIGRVVGLAIFHRRFLDAFFIG--ALYKMILG---------------
--------KSVVLADMEG----------------------VDADFHRS--------LQWM
LDN--DISGGIL--
-EQTFSTEDER---------------------------FGV-ITVED-LIPG--------
GRDID---
------------------------------VTNDNKK-EYVDL-MVKWRIEKR-IAEQFQ
-AFREG---FHELIPQ--DLIN-VFDER-ELELLIGG-----------------------
----IAEID-----------VDDW----KKHTDYRG-YTES-------------------
------------------------DEVI-QFFWQTVR--------------------SWD
GEQKSRLLQF-------TTGTSRIP------------VNG-----FK-DLQGSD--GPRR
FTIEKAGEV-------------------------NNLPKAHTXF-NR-LDLPPY-KSLEQ
LQQKLT-IAVEE-TMG-
>Diaporthe_helianthi_MAVT01006267.1 .
IKIRRSHI-FEDSFAEI--------------SRQSATDLKK-RLMIKFDG-------EDG
LDYGGLSREF---FFLL----SHEMFNPFY--------CLFEYSAHDNY-----------
----------------TLQINPHS------------------------------------
--GINPEHLNYFKFIGRVVGLAIFHRRFLDAFFIG--ALYKMILG---------------
--------KSVVLADMEG----------------------VDADFHRS--------LQWM

LDN--DISGGIL--
-EQTFSTEDER---------------------------FGV-ITVED-LIPG--------
GRDID---
------------------------------VTNDNKK-EYVDL-MVKWRIEKR-IAEQFQ
-AFREG---FHELIPQ--DLIN-VFDER-ELELLIGG-----------------------
----IAEID-----------VDDW----KKHTDYRG-YTES-------------------
------------------------DEVI-QFFWQTVR--------------------SWD
GEQKSRLLQF-------TTGTSRIP------------VNG-----FK-DLQGSD--GPRR
FTIEKAGEV-------------------------NNLPKAHTXF-NR-LDLPPY-KSLEQ
LQQKLT-IAVEE-TMG-
>Valsa_mali_JUIY01000894.1 .
IKVRRSHI-FEDSFAEI--------------SRQSATDLKK-RLMIKFDG-------EDG
LDYGGLSREF---FFLL----SHEMFNPFY--------CLFEYSAHDNY-----------
----------------TLQINPHS------------------------------------
--GINPEHLNYFKFIGRVVGLAIFHRRFLDAFFIG--ALYKMILG---------------
--------KSVVLADMEG----------------------VDADFHRS--------LQWM
LDN--DISGGIL--
-EQTFSTEDER---------------------------FGV-ITVED-LIPG--------
GRDID---
------------------------------VTNDNKK-EYVDL-MVKWRIEKR-IAEQFQ
-AFREG---FHELIPH--DLIN-VFDER-ELELLIGG-----------------------
----IAEID-----------VDDW----KKHTDYRG-YTES-------------------
------------------------DEVI-QNFWQTVR--------------------GWD
GEQKSRLLQF-------TTGTSRIP------------VNG-----FK-DLQGSD--GPRR
FTIEKAGEI-------------------------TNLPKAHTXF-NR-LDLPPY-KSLEQ
LQHKLT-IAVEE-TMG-
>Termitomyces_clypeatus_GAGE01002736.1 .
IKVRRSHI-FEDSFAEI--------------SRQSATDLKK-RLMIKFDG-------EDG
LDYGGLSREF---FFLL----SHEMFNPFY--------CLFEYSAHDNY-----------
----------------TLQINPHS------------------------------------
--GINPEHLNYFKFIGRVVGLAIFHRRFLDAFFIG--ALYKMMLG---------------
--------KAVQLADMEG----------------------VDADFHRS--------LQWM
LDN--DISGGIL--
-EQTFSTEDER---------------------------FGV-IHVED-LIPG--------
GRDID---
------------------------------VTNDNKK-EYVDL-MVKWRIEKR-IEEQFQ
-AFKEG---FHELIPQ--DLIN-VFDER-ELELLIGG-----------------------
----IAEID-----------VDDW----KKHTDYRG-YTES-------------------
------------------------DEVI-QFFWQTVR--------------------SWD
GEQKSRLLQF-------TTGTSRIP------------VNG-----FK-DLQGSD--GPRR
FTIEKAGEL-------------------------GNLPKAHTCF-NR-LDLPPY-KSLEA
L----------------
>Arthrinium_malaysianum_GEGW01000484.1 .
IKVRRSHI-FEDSFAEI--------------SRQSATDLKK-RLMIKFDG-------EDG
LDYGGLSREF---FFLL----SHEMFNPFY--------CLFEYSAHDNY-----------
----------------TLQINPHS------------------------------------
--GINPEHLNYFKFIGRVVGLAIFHRRFLDAFFIG--ALYKMMLG---------------
--------KAVQLADMEG----------------------VDADFHRS--------LQWM
LDN--DISGGIL--
-EQTFSTEDER---------------------------FGV-IHVED-LIPG--------
GRDID---
------------------------------VTNDNKK-EYVDL-MVKWRIEKR-IEEQFQ
-AFKEG---FHELIPQ--DLIN-VFDER-ELELLIGG-----------------------
----IAEID-----------VDDW----KKHTDYRG-YTES-------------------
------------------------DEVI-QFFWQTVR--------------------SWD
GEQKSRLLQF-------TTGTSRIP------------VNG-----FK-DLQGSD--GPRR
FTIEKAGEL-------------------------GNLPKAHTCF-NR-LDLPPY-KSLEA
LQQKLT-IAVEE-TMGF

```
>Stachybotrys_chlorohalonata_APWP01004264. 1..
IKVRRSHI-FEDSFAEI--------------TRQPATDLKK-RLMIKFDG-------EDG
LDYGGLSREF---FFLL----SHEMFNPFY--------CLFEYSAHDNY-----------
----------------TLQINPHS------------------------------------
--GINPEHLNYFKFIGRVVGLAIFHRRFLDAFFIG--ALYKMILG---------------
--------KAVALADMEG----------------------VDADFHRS--------LQWM
LDN-----------------------------------------------DISGGIL--
-EQTFSTEDER---------------------------FGV-LTVED-LIPD--------
GRNIE-------------------------------------------------------
------------------------------VTNENKK-EYVDL-MVKWRIEKR-IAEQFQ
-AFKDG---FHELIPH--DLIN-VFDER-ELELLIGG-----------------------
----IAEID-----------VDDW----KKHTDYRG-YTES-------------------
------------------------DEVV-QNFWQTVR--------------------SWD
GEQKSRLLQF-------TTGTSRIP------------VNG-----FK-DLQGSD--GPRR
FTIEKAGEI-------------------------NNLPKAHTXF-NR-LDLPPY-KSLEM
LQQKLT-IAVEE-TMG-
>Stachybotrys_chartarum_ASEQ01002806.1 .
IKVRRSHI-FEDSFAEI--------------TRQPATDLKK-RLMIKFDG-------EDG
LDYGGLSREF---FFLL----SHEMFNPFY--------CLFEYSAHDNY-----------
----------------TLQINPHS------------------------------------
--GINPEHLNYFKFIGRVVGLAIFHRRFLDAFFIG--ALYKMILG---------------
--------KAVALADMEG----------------------VDADFHRS--------LQWM
LDN-----------------------------------------------DISGGIL--
-EQTFSTEDER---------------------------FGV-LTVED-LIPD--------
GRNIE-------------------------------------------------------
------------------------------VTNENKK-EYVDL-MVKWRIEKR-IAEQFQ
-AFKDG---FHELIPH--DLIN-VFDER-ELELLIGG-----------------------
----IAEID-----------VDDW----KKHTDYRG-YTES-------------------
------------------------DEVV-QNFWQTVR--------------------SWD
GEQKSRLLQF-------TTGTSRIP------------VNG-----FK-DLQGSD--GPRR
FTIEKAGEI-------------------------NNLPKAHTXF-NR-LDLPPY-KSLEM
LQQKLT-IAVEE-TMG-
>Fusarium_graminearum_XM_011320923.1 .
IKVRRSHI-FEDSFAEI--------------TRQSATDLKK-RLMIKFDG-------EDG
LDYGGLSREF---FFLL----SHEMFNPFY--------CLFEYSAHDNY-----------
----------------TLQINPHS------------------------------------
--GINPEHLNYFKFIGRVVGLAIFHRRFLDAFFIG--ALYKMMLG---------------
--------KAVALADMEG----------------------VDADFHRS--------LQWM
LDN-----------------------------------------------DISGGIL--
-EQTFSTEDER---------------------------FGV-LTTED-LIPG--------
GRDIE-------------------------------------------------------
------------------------------VTNENKK-EYVDL-MVKWRIEKR-IAEQFQ
-AFKEG---FQELIPQ--DLIN-VFDER-ELELLIGG-----------------------
----IAEID-----------VDDW----KKHTDYRG-YTES-------------------
------------------------DEVV-QNFWATVR--------------------SWD
GEQKSRLLQF-------TTGTSRIP------------VNG-----FK-DLQGSD--GPRR
FTIEKAGEI-------------------------TNLPKAHTCF-NR-LDLPPY-KSLEM
LQQKLT-IAVEE-TMGF
>Fusarium_pseudograminearum_XM_009256718.1 .
IKVRRSHI-FEDSFAEI--------------TRQSATDLKK-RLMIKFDG-------EDG
LDYGGLSREF---FFLL----SHEMFNPFY--------CLFEYSAHDNY-----------
----------------TLQINPHS------------------------------------
--GINPEHLNYFKFIGRVVGLAIFHRRFLDAFFIG--ALYKMMLG---------------
--------KAVALADMEG----------------------VDADFHRS--------LQWM
LDN-----------------------------------------------DISGGIL--
-EQTFSTEDER---------------------------FGV-LTTED-LIPG--------
GRDIE-------------------------------------------------------
------------------------------VTNENKK-EYVDL-MVKWRIEKR-IAEQFQ
```

-AFKEG---FQELIPQ--DLIN-VFDER-ELELLIGG----------------------
----IAEID-----------VDDW----KKHTDYRG-YTES-------------------
------------------------DEVV-QNFWATVR--------------------SWD
GEQKSRLLQF-------TTGTSRIP------------VNG-----FK-DLQGSD--GPRR
FTIEKAGEI-------------------------TNLPKAHTCF-NR-LDLPPY-KSLEM
LQQKLT-IAVEE-TMG-
>2_Fusarium_poae_LYXU01000001.1 .
IKVRRSHI-FEDSFAEI--------------TRQSATDLKK-RLMIKFDG-------EDG
LDYGGLSREF---FFLL----SHEMFNPFY--------CLFEYSAHDNY-----------
----------------TLQINPHS------------------------------------
--GINPEHLNYFKFIGRVVGLAIFHRRFLDAFFIG--ALYKMMLG---------------
--------KAVALADMEG----------------------VDADFHRS--------LQWM
LDN---DISGGIL--
-EQTFSTEDER---------------------------FGV-LTTED-LIPG--------
GRDIE---
------------------------------VTNENKK-EYVDL-MVKWRIEKR-IAEQFQ
-AFKEG---FQELIPQ--DLIN-VFDER-ELELLIGG----------------------
----IAEID-----------VDDW----KKHTDYRG-YTES-------------------
------------------------DEVV-QNFWATVR--------------------SWD
GEQKSRLLQF-------TTGTSRIP------------VNG-----FK-DLQGSD--GPRR
FTIEKAGEI-------------------------TNLPKAHTXF-NR-LDLPPY-KSLEM
LQQKLT-IAVEE-TMG-
>Fusarium_sambucinum_LSRD01000016.1 .
IKVRRSHI-FEDSFAEI--------------TRQSATDLKK-RLMIKFDG-------EDG
LDYGGLSREF---FFLL----SHEMFNPFY--------CLFEYSAHDNY-----------
----------------TLQINPHS------------------------------------
--GINPEHLNYFKFIGRVVGLAIFHRRFLDAFFIG--ALYKMMLG---------------
--------KAVALADMEG----------------------VDADFHRS--------LQWM
LDN---DISGGIL--
-EQTFSTEDER---------------------------FGV-LTTED-LIPG--------
GRDIE---
------------------------------VTNENKK-EYVDL-MVKWRIEKR-IAEQFQ
-AFKEG---FQELIPQ--DLIN-VFDER-ELELLIGG----------------------
----IAEID-----------VDDW----KKHTDYRG-YTES-------------------
------------------------DEVV-QNFWATVR--------------------SWD
GEQKSRLLQF-------TTGTSRIP------------VNG-----FK-DLQGSD--GPRR
FTIEKAGEI-------------------------TNLPKAHTXF-NR-LDLPPY-KSLEM
LQQKLT-IAVEE-TMG-
>Fusarium_praegraminearum_LXHY01000057.1 .
IKVRRSHI-FEDSFAEI--------------TRQSATDLKK-RLMIKFDG-------EDG
LDYGGLSREF---FFLL----SHEMFNPFY--------CLFEYSAHDNY-----------
----------------TLQINPHS------------------------------------
--GINPEHLNYFKFIGRVVGLAIFHRRFLDAFFIG--ALYKMMLG---------------
--------KAVALADMEG----------------------VDADFHRS--------LQWM
LDN---DISGGIL--
-EQTFSTEDER---------------------------FGV-LTTED-LIPG--------
GRDIE---
------------------------------VTNENKK-EYVDL-MVKWRIEKR-IAEQFQ
-AFKEG---FQELIPQ--DLIN-VFDER-ELELLIGG----------------------
----IAEID-----------VDDW----KKHTDYRG-YTES-------------------
------------------------DEVV-QNFWATVR--------------------SWD
GEQKSRLLQF-------TTGTSRIP------------VNG-----FK-DLQGSD--GPRR
FTIEKAGEI-------------------------TNLPKAHTXF-NR-LDLPPY-KSLEM
LQQKLT-IAVEE-TMG-
>Fusarium_culmorum_LT598659.1 .
IKVRRSHI-FEDSFAEI--------------TRQSATDLKK-RLMIKFDG-------EDG
LDYGGLSREF---FFLL----SHEMFNPFY--------CLFEYSAHDNY-----------
----------------TLQINPHS------------------------------------

--GINPEHLNYFKFIGRVVGLAIFHRRFLDAFFIG--ALYKMMLG---------------
--------KAVALADMEG----------------------VDADFHRS--------LQWM
LDN--DISGGIL--
-EQTFSTEDER---------------------------FGV-LTTED-LIPG--------
GRDIE---
------------------------------VTNENKK-EYVDL-MVKWRIEKR-IAEQFQ
-AFKEG---FQELIPQ--DLIN-VFDER-ELELLIGG-----------------------
----IAEID-----------VDDW----KKHTDYRG-YTES-------------------
------------------------DEVV-QNFWATVR--------------------SWD
GEQKSRLLQF-------TTGTSRIP------------VNG-----FK-DLQGSD--GPRR
FTIEKAGEI-------------------------TNLPKAHTXF-NR-LDLPPY-KSLEM
LQQKLT-IAVEE-TMGF
>Fusarium_equiseti_CBMI010000342.1 .
IKVRRSHI-FEDSFAEI--------------TRQSATDLKK-RLMIKFDG-------EDG
LDYGGLSREF---FFLL----SHEMFNPFY--------CLFEYSAHDNY-----------
----------------TLQINPHS------------------------------------
--GINPEHLNYFKFIGRVVGLAIFHRRFLDAFFIG--ALYKMMLG---------------
--------KAVSLADMEG----------------------VDADFHRS--------LQWM
LDN--DISGGIL--
-EQTFSTEDER---------------------------FGV-LTTED-LIPG--------
GRDIE---
------------------------------VTNENKK-EYVDL-MVKWRIEKR-IAEQFQ
-AFKEG---FQELIPQ--DLIN-VFDER-ELELLIGG-----------------------
----IAEID-----------VDDW----KKHTDYRG-YTES-------------------
------------------------DEVV-QNFWATVR--------------------SWD
GEQKSRLLQF-------TTGTSRIP------------VNG-----FK-DLQGSD--GPRR
FTIEKAGEI-------------------------TNLPKAHTXF-NR-LDLPPY-KSLEM
LQQKLT-IAVEE-TMG-
>Fusarium_oxysporum_BCHB01000005.1 .
IKVRRSHI-FEDSFAEI--------------TRQSATDLKK-RLMIKFDG-------EDG
LDYGGLSREF---FFLL----SHEMFNPFY--------CLFEYSAHDNY-----------
----------------TLQINPHS------------------------------------
--GINPEHLNYFKFIGRVVGLAIFHRRFLDAFFIG--ALYKMMLG---------------
--------KAVALADMEG----------------------VDADFHRS--------LQWM
LDN--DISGGIL--
-EQTFSTEDER---------------------------FGV-MTTED-LIPD--------
GRNID---
------------------------------VTNENKK-EYVDL-MVKWRIEKR-IAEQFQ
-AFKEG---FQELIPQ--DLIN-VFDER-ELELLIGG-----------------------
----IAEID-----------VDDW----KKHTDYRG-YTES-------------------
------------------------DEVV-QNFWATVR--------------------SWD
GEQKSRLLQF-------TTGTSRIP------------VNG-----FK-DLQGSD--GPRR
FTIEKAGEI-------------------------TNLPKAHTXF-NR-LDLPPY-KSLEM
LQQKLT-IAVEE-TMG-
>Fusarium_verticillioides_XM_018890483.1 .
IKVRRSHI-FEDSFAEI--------------TRQSATDLKK-RLMIKFDG-------EDG
LDYGGLSREF---FFLL----SHEMFNPFY--------CLFEYSAHDNY-----------
----------------TLQINPHS------------------------------------
--GINPEHLNYFKFIGRVVGLAIFHRRFLDAFFIG--ALYKMMLG---------------
--------KAVALADMEG----------------------VDADFHRS--------LQWM
LDN--DISGGIL--
-EQTFSTEDER---------------------------FGV-MTTED-LIPD--------
GRNID---
------------------------------VTNENKK-EYVDL-MVKWRIEKR-IAEQFQ
-AFKEG---FQELIPQ--DLIN-VFDER-ELELLIGG-----------------------
----IAEID-----------VDDW----KKHTDYRG-YTES-------------------
------------------------DEVV-QNFWATVR--------------------SWD
GEQKSRLLQF-------TTGTSRIP------------VNG-----FK-DLQGSD--GPRR

FTIEKAGEI-------------------------TNLPKAHTCF-NR-LDLPPY-KSLEM
LQQKLT-IAVEE-TMGF
>Fusarium_fujikuroi_HF679027.1 .
IKVRRSHI-FEDSFAEI--------------TRQSATDLKK-RLMIKFDG-------EDG
LDYGGLSREF---FFLL----SHEMFNPFY--------CLFEYSAHDNY-----------
----------------TLQINPHS------------------------------------
--GINPEHLNYFKFIGRVVGLAIFHRRFLDAFFIG--ALYKMMLG---------------
--------KAVALADMEG----------------------VDADFHRS--------LQWM
LDN--DISGGIL--
-EQTFSTEDER---------------------------FGV-MTTED-LIPD--------
GRNID---
------------------------------VTNENKK-EYVDL-MVKWRIEKR-IAEQFQ
-AFKEG---FQELIPQ--DLIN-VFDER-ELELLIGG-----------------------
----IAEID-----------VDDW----KKHTDYRG-YTES-------------------
------------------------DEVV-QNFWATVR--------------------SWD
GEQKSRLLQF-------TTGTSRIP------------VNG-----FK-DLQGSD--GPRR
FTIEKAGEI-------------------------TNLPKAHTXF-NR-LDLPPY-KSLEM
LQQKLT-IAVEE-TMGF
>Nectria_haematococca_XM_003051059.1 .
IKVRRSHI-FEDSFAEI--------------TRQSATDLKK-RLMIKFDG-------EDG
LDYGGLSREF---FFLL----SHEMFNPFY--------CLFEYSAHDNY-----------
----------------TLQINPHS------------------------------------
--GINPEHLNYFKFIGRVVGLAIFHRRFLDAFFIG--ALYKMMLG---------------
--------KAVALADMEG----------------------VDADFHRS--------LQWM
LDN--DISGGIL--
-EQTFSTEDER---------------------------FGV-MTTED-LIPN--------
GRNID---
------------------------------VTNENKK-EYVDL-MVKWRIEKR-IAEQFQ
-AFKEG---FQELIPQ--DLIN-VFDER-ELELLIGG-----------------------
----IAEID-----------VDDW----KKHTDYRG-YTES-------------------
------------------------DEVV-QNFWATVR--------------------SWD
GEQKSRLLQF-------TTGTSRIP------------VNG-----FK-DLQGSD--GPRR
FTIEKAGEI-------------------------TNLPKAHTCF-NR-LDLPAY-KSLEM
LQQKLT-IAVEE-TMGF
>Fusarium_virguliforme_GBJV01007843.1 .
IKVRRSHI-FEDSFAEI--------------TRQSATDLKK-RLMIKFDG-------EDG
LDYGGLSREF---FFLL----SHEMFNPFY--------CLFEYSAHDNY-----------
----------------TLQINPHS------------------------------------
--GINPEHLNYFKFIGRVVGLAIFHRRFLDAFFIG--ALYKMMLG---------------
--------KAVALADMEG----------------------VDADFHRS--------LQWM
LDN--DISGGIL--
-EQTFSTEDER---------------------------FGV-MTTED-LIPN--------
GRNIE---
------------------------------VTNENKK-EYVDL-MVKWRIEKR-IAEQFQ
-AFKEG---FQELIPQ--DLIN-VFDER-ELELLIGG-----------------------
----IAEID-----------VDDW----KKHTDYRG-YTES-------------------
------------------------DEVV-QNFWATVR--------------------SWD
GEQKSRLLQF-------TTGTSRIP------------VNG-----FK-DLQGSD--GPRR
FTIEKAGEI-------------------------TNLPKAHTCF-NR-LDLPAY-KSLEM
LQQKLT-IAVEE-TMGF
>Fusarium_tucumaniae_MAEF01007474.1 .
IKVRRSHI-FEDSFAEI--------------TRQSATDLKK-RLMIKFDG-------EDG
LDYGGLSREF---FFLL----SHEMFNPFY--------CLFEYSAHDNY-----------
----------------TLQINPHS------------------------------------
--GINPEHLNYFKFIGRVVGLAIFHRRFLDAFFIG--ALYKMMLG---------------
--------KAVALADMEG----------------------VDADFHRS--------LQWM
LDN--DISGGIL--
-EQTFSTEDER---------------------------FGV-MTTED-LIPN--------

GRNIE---
------------------------------VTNENKK-EYVDL-MVKWRIEKR-IAEQFQ
-AFKEG---FQELIPQ--DLIN-VFDER-ELELLIGG-----------------------
----IAEID-----------VDDW----KKHTDYRG-YTES-------------------
------------------------DEVV-QNFWATVR--------------------SWD
GEQKSRLLQF-------TTGTSRIP------------VNG-----FK-DLQGSD--GPRR
FTIEKAGEI-------------------------TNLPKAHTXF-NR-LDLPAY-KSLEM
LQQKLT-IAVEE-TMG-
>Calonectria_pseudoreteaudii_MOCD01000024. 1..
IKVRRSHI-FEDSFAEI--------------TRQSATDLKK-RLMIKFDG-------EDG
LDYGGLSREF---FFLL----SHEMFNPFY--------CLFEYSAHDNY-----------
----------------TLQINPHS------------------------------------
--GINPEHLNYFKFIGRVVGLAIFHRRFLDAFFIG--ALYKMILG---------------
--------KAVALADMEG----------------------VDADFHRS--------LQWM
LDN--DISGGIL--
-EQTFSTEDER---------------------------FGV-MTTED-LIPD--------
GRNIE---
------------------------------VTNENKK-EYVDL-MVKWRIEKR-IAEQFQ
-AFKEG---FQELIPQ--DLIN-VFDER-ELELLIGG-----------------------
----IAEID-----------VDDW----KKHTDYRG-YTES-------------------
------------------------DEVV-QNFWATVR--------------------SWD
GEQKSRLLQF-------TTGTSRIP------------VNG-----FK-DLQGSD--GPRR
FTIEKAGEI-------------------------GNLPKAHTXF-NR-LDLPPY-KSLEQ
LQNKLT-IAVEE-TMG-
>Dactylonectria_macrodidyma_JYGD01000340.1 .
IKVRRSHI-FEDSFAEI--------------TRQSATDLKK-RLMIKFDG-------EDG
LDYGGLSREF---FFLL----SHEMFNPFY--------CLFEYSAHDNY-----------
----------------TLQINPHS------------------------------------
--GINPEHLNYFKFIGRVVGLAIFHRRFLDAFFIG--ALYKMMLG---------------
--------KAVALADMEG----------------------VDSDFHRS--------LQWM
LDN--DISGGIL--
-EQTFSTEDER---------------------------FGV-LTTED-LIPN--------
GRNID---
------------------------------VTNENKK-EYVDL-MVKWRIEKR-IAEQFQ
-AFKEG---FQELIPH--ELIN-VFDER-ELELLIGG-----------------------
----IAEID-----------VDDW----KKHTDYRG-YTES-------------------
------------------------DEVV-QNFWTTVR--------------------SWD
GEQKSRLLQF-------TTGTSRIP------------VNG-----FK-DLQGSD--GPRR
FTIEKAGEI-------------------------NNLP----------------------

>Neonectria_ditissima_LDPK01000189.1 .
IKVRRSHI-FEDSFAEI--------------TRQSATDLKK-RLMIKFDG-------EDG
LDYGGLSREF---FFLL----SHEMFNPFY--------CLFEYSAHDNY-----------
----------------TLQINPHS------------------------------------
--GINPEHLNYFKFIGRVVGLAIFHRRFLDAFFIG--ALYKMMLG---------------
--------KAVALADMEG----------------------VDADFHRS--------LQWM
LDN--DISGGIL--
-EQTFSTEDER---------------------------FGV-LTTED-LIPD--------
GRNID---
------------------------------VTNENKK-EYVDL-MVKWRIEKR-IAEQFQ
-AFKEG---FQELIPH--ELIN-VFDER-ELELLIGG-----------------------
----IAEID-----------VDDW----KKHTDYRG-YTES-------------------
------------------------DEVV-QNFWTTVR--------------------SWD
GEQKSRLLQF-------TTGTSRIP------------VNG-----FK-DLQGSD--GPRR
FTIEKAGEQ-------------------------NNLP----------------------

>Ilyonectria_destructans_MPHF01000006.1 .
IKVRRSHI-FEDSFAEI--------------TRQSATDLKK-RLMIKFDG-------EDG

LDYGGLSREF---FFLL----SHEMFNPFY--------CLFEYSAHDNY-----------
----------------TLQINPHS------------------------------------
--GINPEHLNYFKFIGRVVGLAIFHRRFLDAFFIG--ALYKMMLG---------------
--------KAVALADMEG----------------------VDADFHRS--------LQWM
LDN---DISGGIL--
-EQTFSTEDER---------------------------FGV-LTTED-LIPD--------
GRNID---
------------------------------VTNENKK-EYVDL-MVKWRIEKR-IAEQFQ
-AFKEG---FQELIPH--ELIN-VFDER-ELELLIGG-----------------------
----IAEID-----------VDDW----KKHTDYRG-YTES-------------------
------------------------DEVV-QNFWSTVR--------------------SWD
GEQKSRLLQF-------TTGTSRIP------------VNG-----FK-DLQGSD--GPRR
FTIEKAGEI-------------------------NNLPKAHTW-----LALP--------

>Trichoderma_virens_XM_014095335.1 .
IKVRRSHI-FEDSFAEI--------------TRQSATDLKK-RLMIKFDG-------EDG
LDYGGLSREF---FFLL----SHEMFNPFY--------CLFEYSAHDNY-----------
----------------TLQINPHS------------------------------------
--GINPEHLNYFKFIGRVVGMAIFHRRFLDAFFIG--ALYKMILG---------------
--------KGVTLADMEG----------------------VDADFHRS--------LQWM
LDN---DISGGIL--
-EQTFSTEDER---------------------------FGV-LTTED-LIPG--------
GRDIE---
------------------------------VTNENKK-EYVEL-MVKWRIEKR-IAEQFQ
-AFKEG---FQELIPQ--DLIN-VFDER-ELELLIGG-----------------------
----IAEID-----------VDDW----KKHTDYRG-YTES-------------------
------------------------DEVI-QNFWATVR--------------------SWD
GEQKSRLLQF-------TTGTSRIP------------VNG-----FK-DLQGSD--GPRR
FTIEKAGDL-------------------------SNLPKAHTCF-NR-IDLPAY-KTLET
LQQKLT-IAVEE-TMGF
>Trichoderma_reesei_XM_006963157.1 .
IKVRRSHI-FEDSFAEI--------------TRQSATDLKK-RLMIKFDG-------EDG
LDYGGLSREF---FFLL----SHEMFNPFY--------CLFEYSAHDNY-----------
----------------TLQINPHS------------------------------------
--GINPEHLNYFKFIGRVVGMAIFHRRFLDAFFIG--ALYKMILG---------------
--------KPVTLADMEG----------------------VDADFHRS--------LQWM
LDN---DISGGIL--
-EQTFSTEDER---------------------------FGV-MTTED-LIPG--------
GRDIE---
------------------------------VTNENKK-EYVEL-MVKWRIEKR-IAEQFQ
-AFKEG---FQELIPQ--DLIN-VFDER-ELELLIGG-----------------------
----IAEID-----------VDDW----KKHTDYRG-YTES-------------------
------------------------DEVI-QNFWATVR--------------------SWD
GEQKSRLLQF-------TTGTSRIP------------VNG-----FK-DLQGSD--GPRR
FTIEKAGDL-------------------------SNLPKAHTCF-NR-IDLPAY-KTLET
LQQKLT-IAVEE-TMGF
>Trichoderma_atroviride_XM_014084162.1 .
IKVRRSHI-FEDSFAEI--------------TRQSATDLKK-RLMIKFDG-------EDG
LDYGGLSREF---FFLL----SHEMFNPFY--------CLFEYSAHDNY-----------
----------------TLQINPHS------------------------------------
--GINPEHLNYFKFIGRVVGMAIFHRRFLDAFFIG--ALYKMILG---------------
--------KGVTLADMEG----------------------VDADFHRS--------LQWM
LDN---DISGGIL--
-EQTFSTEDER---------------------------FGV-LTTED-LIPG--------
GRDIE---
------------------------------VTNENKK-EYVDL-MVKWRIEKR-IAEQFQ
-AFKEG---FQELIPH--DLIN-VFDER-ELELLIGG-----------------------
----IAEID-----------VDDW----KKHTDYRG-YTES-------------------

------------------------DEVI-QNFWATVR--------------------SWD
GEQKSRLLQF-------TTGTSRIP------------VNG-----FK-DLQGSD--GPRR
FTIEKAGDL------------------------ANLPKAHTCF-NR-IDLPAY-KTLET
LQQKLT-IAVEE-TMGF
>Trichoderma_gamsii_XM_018806736.1 .
IKVRRSHI-FEDSFAEI--------------TRQSATDLKK-RLMIKFDG-------EDG
LDYGGLSREF---FFLL----SHEMFNPFY--------CLFEYSAHDNY-----------
----------------TLQINPHS------------------------------------
--GINPEHLNYFKFIGRVVGMAIFHRRFLDAFFIG--ALYKMVLG---------------
--------KGVTLADMEG----------------------VDADFHRS--------LQWM
LDN---DISGGIL--
-EQTFSTEDER---------------------------FGV-LTTED-LIPG--------
GRDIE---
------------------------------VTNENKK-EYVDL-MVKWRIEKR-IAEQFQ
-AFKEG---FQELIPH--DLIN-VFDER-ELELLIGG-----------------------
----IAEID-----------VDDW----KKHTDYRG-YTES-------------------
------------------------DEVI-QNFWATVR--------------------SWD
GEQKSRLLQF-------TTGTSRIP------------VNG-----FK-DLQGSD--GPRR
FTIEKAGDL------------------------NNLPKAHTCF-NR-IDLPPY-KTLET
LQQKLT-IAVEE-TMGF
>Isaria_fumosorosea_XM_018845584.1 .
VKVRRSHI-FEDSFAEI--------------TRQSATDLKK-RLMIKFDG-------EEG
LDYGGVSREF---FFLL----SHEMFNPFY--------CLFEYSAHDNY-----------
----------------TLQINPHS------------------------------------
--GINPEHLNYFKFIGRVVGLAIFHRRFLDAFFIG--ALYKMVLG---------------
--------KAVALADMEG----------------------VDADFHRS--------LQWM
LDN---DISGGIL--
-EQTFSTEDER---------------------------FGV-LTTED-LIPG--------
GRDIE---
------------------------------VTNENKK-QYVDL-MVKWRIEKR-IAEQFQ
-AFKEG---FHELIPQ--DLIN-VFDER-ELELLIGG-----------------------
----IAEID-----------VDDW----KKHTDYRG-YTES-------------------
------------------------DEVI-QNFWKTVR--------------------SWD
GEQKSRLLQF-------TTGTSRIP------------VNG-----FK-DLQGSD--GPRR
FTIEKAGEL------------------------ANLPKAHTCF-NR-IDLPPY-KDMET
LQQKLT-IAVEE-TMGF
>Cordyceps_militaris_XM_006666250.1 .
VKVRRSHI-FEDSFAEI--------------TRQSATDLKK-RLMIKFDG-------EEG
LDYGGVSREF---FFLL----SHEMFNPFY--------CLFEYSAHDNY-----------
----------------TLQINPHS------------------------------------
--GINPEHLNYFKFIGRVVGLAIFHRRFLDAFFIG--ALYKMVLG---------------
--------KAVALADMEG----------------------VDADFHRS--------LQWM
LDN---DISGGIL--
-EQTFSTEDER---------------------------FGV-LTTED-LIPG--------
GRDID---
------------------------------VTNDNKK-QYVDL-MVKWRIEKR-IAEQFQ
-AFKEG---FHELIPQ--DLIN-VFDDR-ELELLIGG-----------------------
----IAEID-----------VDDW----KKHTDYRG-YTES-------------------
------------------------DEVI-QNFWKTVR--------------------SWD
GEQKSRLLQF-------TTGTSRIP------------VNG-----FK-DLQGSD--GPRR
FTIEKAGEL------------------------VNLPKAHTCF-NR-IDLPPY-KDMET
LQQKLT-IAVEE-TMGF
>Beauveria_bassiana_XM_008596919.1 .
VKVRRSHI-FEDSFAEI--------------TRQSATDLKK-RLMIKFDG-------EEG
LDYGGVSREF---FFLL----SHEMFNPFY--------CLFEYSAHDNY-----------
----------------TLQINPHS------------------------------------
--GINPEHLNYFKFIGRVVGLAIFHRRFLDAFFIG--ALYKMVLG---------------
--------KAVSLADMEG----------------------VDADFHRS--------LQWM

LDN--DISGGIL--
-EQTFSTEDER---------------------------FGV-LTTED-LIPD--------
GRNIE---
------------------------------VTNENKK-QYVDL-MVKWRIEKR-IAEQFQ
-AFKEG---FHELIPI--DLIN-VFDER-ELELLIGG-----------------------
----IAEID-----------VDDW----KKHTDYRG-YTES-------------------
------------------------DEVI-QNFWKTVR--------------------SWD
GEQKSRLLQF-------TTGTSRIP------------VNG-----FK-DLQGSD--GPRR
FTIEKAGEL-------------------------VNLPKAHTCF-NR-IDLPPY-KDMET
LTQKLT-IAVEE-TMGF
>2_Cordyceps_confragosa_AZHF01000002.1 .
VKVRRSHI-FEDSFAEI--------------TRQSATDLKK-RLMIKFDG-------EEG
LDYGGVSREF---FFLL----SHEMFNPFY--------CLFEYSAHDNY-----------
----------------TLQINPHS------------------------------------
--GINPEHLNYFKFIGRVVGLAIFHRRFLDAFFIG--ALYKMVLG---------------
--------KAVALADMEG----------------------VDADFHRS--------LQWM
LDN--DISGGIL--
-EQTFSTEDER---------------------------FGV-MTTED-LIPD--------
GRNID---
------------------------------VTNENKK-QYVDL-MVKWRIEKR-IAEQFQ
-AFKEG---FHELIPH--DLIN-VFDER-ELELLIGG-----------------------
----IAEID-----------VDDW----KKHTDYRG-YTES-------------------
------------------------DEVI-QNFWKTVR--------------------SWD
GEQKSRLLQF-------TTGTSRIP------------VNG-----FK-DLQGSD--GPRR
FTIEKAGEL-------------------------VNLPKAHTWY----------------

>Torrubiella_hemipterigena_CDHN01000002.1 .
IKVRRSHL-FEDSFAEI--------------TRQSPMDLKK-RLMIKFDG-------EDG
LDYGGVSREF---FFLL----SHEMFNPFY--------CLFEYSAHDNY-----------
----------------TLQINPHS------------------------------------
--GINPEHLNYFKFIGRVVGLAIFHRRFLDAFFIG--ALYKMVLG---------------
--------KAVALADMEG----------------------VDADFHRS--------LQWM
LDN--DISGGIL--
-EQTFSTEDER---------------------------FGV-MTTED-LIPG--------
GRDIE---
------------------------------VTNENKK-EYVDL-MVKWRIEKR-IAEQFQ
-AFKEG---FQELIPQ--DLIN-VFDER-ELELLIGG-----------------------
----IAEID-----------VDDW----KKHTDYRG-YTES-------------------
------------------------DEVV-QNFWKTVR--------------------SWD
GEQKSRLLQF-------TTGTSRIP------------VNG-----FK-DLQGSD--GPRR
FTIEKAGEL-------------------------MNLPKAHTXF-NR-IDLPPY-KDLET
LQQKLT-IAVEE-TMG-
>Drechmeria_coniospora_JYHR01000002.1 .
IKVRRSHI-FEDSFAEI--------------TRQSPADLKK-RLMIKFDG-------EDG
LDYGGLSREF---FFLL----SHEMFNPFY--------CLFEYSAHDNY-----------
----------------TLQINPHS------------------------------------
--GINPEHLNYFKFIGRVVGLSIFHRRFLDAFFIG--ALYKMVLG---------------
--------KAVALADMEG----------------------VDADFHRS--------LQWM
LDN--DISGGIL--
-EQTFSTEDER---------------------------FGV-LTTED-LIPN--------
GRNID---
------------------------------VTNENKK-EYVDL-MVKWRIEKR-IAEQFH
-AFKEG---FQELIPQ--DLIN-VFDER-ELELLIGG-----------------------
----IAEID-----------VDDW----KKHTDYRG-YTES-------------------
------------------------DEVI-QNFWTTVR--------------------SWD
GEQKSRLLQF-------TTGTSRIP------------VNG-----FK-DLQGSD--GPRR
FTIEKAGEI-------------------------TNLPKAHTXF-NR-IDLPAY-KNMET
LQQKLT-IAVEE-TMG-

```
>Ophiocord_sinensis_GAGW01006528.1 .
IKVRRSHI-FEDSFAEI--------------TRQTPMDLKK-RLMIKFDG-------EDG
LDYGGLSREF---FFLL----SHEMFNPFY--------CLFEYSAHDNY-----------
----------------TLQINPHS------------------------------------
--GINPEHLNYFKFIGRVVGLAIFHRRFLDAFFIG--ALYKMVLG---------------
--------KSVALADMEG----------------------VDADFHRS--------LQWM
LDN------------------------------------------------DISGGIL--
-EQTFSTEDER---------------------------FGV-MTTED-LILN--------
GRNID-------------------------------------------------------
------------------------------VTNENKK-EYVDL-MVKWRIEKR-IAEQFQ
-AFKEG---FHELVPQ--DLIN-VFDER-ELELLIGG-----------------------
----IAEID-----------VDDW----KKHTDYRG-YTES-------------------
------------------------DEVI-QNFWQTVR--------------------SWD
GEQKSRLLQF-------TTGTSRIP------------VNG-----FK-DLQGSD--GPRR
FTIEKAGEI-------------------------VNLPKAHTCF-NR-IDLPAY-KNMET
LQQKLT-IAVEE-TMGF
>Purpureocillium_lilacinum_XM_018326570.1 .
IKVRRSHI-FEDSFAEI--------------TRQSPTDLKK-RLMIKFDG-------EDG
LDYGGLSREF---FFLL----SHEMFNPFY--------CLFEYSAHDNY-----------
----------------TLQINPHS------------------------------------
--GINPEHLNYFKFIGRVVGLAIFHRRFLDAFFIG--ALYKMVLG---------------
--------KAVALADMEG----------------------VDADFHRS--------LQWM
LDN------------------------------------------------DISGGIL--
-EQTFSTEDER---------------------------FGV-LTTED-LIPN--------
GRNIE-------------------------------------------------------
------------------------------VTNENKK-EYVDL-MVKWRIEKR-IAEQFQ
-AFKEG---FQELIPQ--DLIN-VFDER-ELELLIGG-----------------------
----IAEID-----------VDDW----KKHTDYRG-YTES-------------------
------------------------DEVI-QNFWTTVR--------------------SWD
GEQKSRLLQF-------TTGTSRIP------------VNG-----FK-DLQGSD--GPRR
FTIEKAGEI-------------------------VNLPKAHTCF-NR-IDLPPY-KNMET
LQQKLT-IAVEE-TMGF
>Pochonia_chlamydosporia_XM_018290478.1 .
IKVRREHI-FEDSFAEI--------------TRQSATDLKK-RLMIKFDG-------EDG
LDYGGLSREF---FFLL----SHEMFNPFY--------CLFEYSAHDNY-----------
----------------TLQINPHS------------------------------------
--GINPEHLNYFKFIGRVVGLAIFHRRFLDAFFIG--ALYKMVLG---------------
--------KAVALADMEG----------------------VDADFHRS--------LQWM
LDN------------------------------------------------DISGGIL--
-EQTFSTEDER---------------------------FGV-MTTED-LIPN--------
GRNIE-------------------------------------------------------
------------------------------VTNENKK-EYVDL-MVKWRIEKR-IAEQFQ
-AFKEG---FHELIPQ--DLIN-VFDER-ELELLIGG-----------------------
----IAEID-----------VDDW----KKHTDYRG-YTES-------------------
------------------------DEVI-QNFWQTVR--------------------SWD
GEQKSRLLQF-------TTGTSRIP------------VNG-----FK-DLQGSD--GPRR
FTIEKTGEL-------------------------TNLPKAHTCF-NR-IDLPPY-KTLET
LQQKLT-IAVEE-TMGF
>Metarhizium_anisopliae_JNNZ01000175.1 .
IKVRREHI-FEDSFAEI--------------TRQSATDLKK-RLMIKFDG-------EDG
LDYGGLSREF---FFLL----SHEMFNPFY--------CLFEYSAHDNY-----------
----------------TLQINPHS------------------------------------
--GINPEHLNYFKFIGRVVGLAIFHRRFLDAFFIG--ALYKMVLG---------------
--------KAVSLADMEG----------------------VDADFHRS--------LQWM
LDN------------------------------------------------DISGGIL--
-EQTFSTEDER---------------------------FGV-MTTED-LIPN--------
GRNIE-------------------------------------------------------
------------------------------VTNENKK-EYVDL-MVKWRIEKR-IAEQFQ
```

-AFKEG---FHELIPQ--DLIN-VFDER-ELELLIGG-----------------------
----IAEID-----------VDDW----KKHTDYRG-YTES-------------------
------------------------DEVI-QNFWQVVR--------------------SWD
GEQKSRLLQF-------TTGTSRIP------------VNG-----FK-DLQGSD--GPRR
FTIEKTGEL-------------------------TNLPKAHTSF-NR-IDLPPY-KSMDT
LQNKLT-IAVEE-TMG-
>Metarhizium_robertsii_JELW01000023.1 .
IKVRREHI-FEDSFAEI--------------TRQSATDLKK-RLMIKFDG-------EDG
LDYGGLSREF---FFLL----SHEMFNPFY--------CLFEYSAHDNY-----------
----------------TLQINPHS------------------------------------
--GINPEHLNYFKFIGRVVGLAIFHRRFLDAFFIG--ALYKMVLG---------------
--------KAVSLADMEG----------------------VDADFHRS--------LQWM
LDN--DISGGIL--
-EQTFSTEDER---------------------------FGV-MTTED-LIPN--------
GRNIE---
------------------------------VTNENKK-EYVDL-MVKWRIEKR-IAEQFQ
-AFKEG---FHELIPQ--DLIN-VFDER-ELELLIGG-----------------------
----IAEID-----------VDDW----KKHTDYRG-YTES-------------------
------------------------DEVI-QNFWQVVR--------------------SWD
GEQKSRLLQF-------TTGTSRIP------------VNG-----FK-DLQGSD--GPRR
FTIEKTGEL-------------------------TNLPKAHTSF-NR-IDLPPY-KSMDT
LQNKLT-IAVEE-TMGF
>Metarhizium_majus_XM_014720546.1 .
IKVRREHI-FEDSFAEI--------------TRQSATDLKK-RLMIKFDG-------EDG
LDYGGLSREF---FFLL----SHEMFNPFY--------CLFEYSAHDNY-----------
----------------TLQINPHS------------------------------------
--GINPEHLNYFKFIGRVVGLAIFHRRFLDAFFIG--ALYKMVLG---------------
--------KAVSLADMEG----------------------VDADFHRS--------LQWM
LDN--DISGGIL--
-EQTFSTEDER---------------------------FGV-MTTED-LIPN--------
GRNIE---
------------------------------VTNENKK-EYVDL-MVKWRIEKR-IAEQFQ
-AFKEG---FHELIPQ--DLIN-VFDER-ELELLIGG-----------------------
----IAEID-----------VDDW----KKHTDYRG-YTES-------------------
------------------------DEVI-QNFWQVVR--------------------SWD
GEQKSRLLQF-------TTGTSRIP------------VNG-----FK-DLQGSD--GPRR
FTIEKTGEL-------------------------TNLPKAHTCF-NR-IDLPPY-KSMDT
LQNKLT-IAVEE-TMG-
>Metarhizium_album_AZHE01000015.1 .
IKVRREHI-FEDSFAEI--------------TRQSATDLKK-RLMIKFDG-------EDG
LDYGGLSREF---FFLL----SHEMFNPFY--------CLFEYSAHDNY-----------
----------------TLQINPHS------------------------------------
--GINPEHLNYFKFIGRVVGLAIFHRRFLDAFFIG--ALYKMVLG---------------
--------KAVSLADMEG----------------------VDADFHRS--------LQWM
LDN--DISGGIL--
-EQTFSTEDER---------------------------FGV-MTTED-LIPN--------
GRNIE---
------------------------------VTNENKK-EYVDL-MVKWRIEKR-IAEQFQ
-AFKEG---FHELIPQ--DLIN-VFDER-ELELLIGG-----------------------
----IAEID-----------VDDW----KKHTDYRG-YTES-------------------
------------------------DEVI-QNFWQVVR--------------------SWD
GEQKSRLLQF-------TTGTSRIP------------VNG-----FK-DLQGSD--GPRR
FTIEKTGEL-------------------------TNLPKAHTSF-NR-IDLPPY-KNLDT
LQNKLT-IAVEE-TMG-
>Metarhizium_acridum_XM_007815881.1 .
IKVRREHI-FEDSFAEI--------------TRQSATDLKK-RLMIKFDG-------EDG
LDYGGLSREF---FFLL----SHEMFNPFY--------CLFEYSAHDNY-----------
----------------TLQINPHS------------------------------------

--GINPEHLNYFKFIGRVVGLAIFHRRFLDAFFIG--ALYKMVLG---------------
--------KPVSLADMEG----------------------VDADFHRS--------LQWM
LDN--DISGGIL--
-EQTFSTEDER---------------------------FGV-MTTED-LIPN--------
GRNIE---
------------------------------VTNENKK-EYVDL-MVKWRIEKR-IAEQFQ
-AFKEG---FHELIPQ--DLIN-VFDER-ELELLIGG-----------------------
----IAEID-----------VDDW----KKHTDYRG-YTES-------------------
------------------------DEVI-QNFWQVVR--------------------SWD
GEQKSRLLQF-------TTGTSRIP------------VNG-----FK-DLQGSD--GPRR
FTIEKTGEL-------------------------TNLPKAHTCF-NR-IDLPPY-KSLDS
LQNKLT-IAVEE-TMGF
>Periglandula_ipomoeae_AFRD01000212.1 .
IKVRREHI-FEDSFAEI--------------TRQSATDLKK-RLMIKFDG-------EDG
LDYGGLSREF---FFLL----SHEMFNPFY--------CLFEYSAHDNY-----------
----------------TLQINPHS------------------------------------
--GINPEHLNYFKFIGRVVGLAIFHRRFLDAFFIG--ALYKMALG---------------
--------KAVALADMEG----------------------VDADFHRS--------LQWM
LDN--DISGGIL--
-EQTFSTEDER---------------------------FGV-LMTED-LIPN--------
GRNIE---
------------------------------VTNENKK-EYVDL-MVKWRIEKR-IAEQFQ
-AFKEG---FHELIPQ--DLIN-VFDER-ELELLIGG-----------------------
----IAEID-----------VDDW----KKHTDYRG-YTES-------------------
------------------------DEVI-QNFWQVVR--------------------SWD
GEQKSRLLQF-------TTGTSRIP------------VNG-----FK-DLQGSD--GPRR
FTIEKTGEL-------------------------TNLPKAHTWY----------------

>Claviceps_paspali_AFRC01000126.1 .
IKVRREHI-FEDSFAEI--------------TRQSATDLKK-RLMIKFDG-------EDG
LDYGGLSREF---FFLL----SHEMFNPFY--------CLFEYSAHDNY-----------
----------------TLQINPHS------------------------------------
--GINPEHLNYFKFIGRVVGLAIFHRRFLDAFFIG--ALYKMVLG---------------
--------KAVALADMEG----------------------VDADFHRS--------LQWM
LDN--DISGGIL--
-EQTFSTEDER---------------------------FGV-LTTED-LIPN--------
GRNID---
------------------------------VTNENKK-EYVDL-MVKWRIEKR-IAEQFQ
-AFKEG---FHELIPQ--DLIN-VFDER-ELELLIGG-----------------------
----IAEID-----------VDDW----KKHTDYRG-YTES-------------------
------------------------DEVI-QNFWQVVR--------------------SWD
GEQKSRLLQF-------TTGTSRIP------------VNG-----FK-DLQGSD--GPRR
FTIEKTGEL-------------------------TNLPKAHTWY----------------

>Neotyphodium_gansuense_AFRE01000221.1_R .
IKVRREHI-FEDSFAEI--------------TRQSATDLKK-RLMIKFDG-------EDG
LDYGGLSREF---FFLL----SHEMFNPFY--------CLFEYSAHDNY-----------
----------------TLQINPHS------------------------------------
--GINPEHLNYFKFIGRVVGLAIFHRRFLDAFFIG--ALYKMVLG---------------
--------KAVALADMEG----------------------VDADFHRS--------LQWM
LDN--DISGGIL--
-EQTFSTEDER---------------------------FGV-LTTED-LIPN--------
GRNID---
------------------------------VTNENKK-EYVDL-MVKWRIEKR-IAEQFQ
-AFKEG---FHELIPQ--DLIN-VFDER-ELELLIGG-----------------------
----IAEID-----------VDDW----KKHTDYRG-YTES-------------------
------------------------DEVI-QNFWQVVR--------------------SWD
GEQKSRLLQF-------TTGTSRIP------------VNG-----FK-DLQGSD--GPRR

FTIEKTGEL-------------------------TNLPKAHTWY-NR-IDLPPY-KTLET
LQQKLT-IAVEE-----
>Claviceps_fusiformis_AFRA01001141.1 .
IKVRREHI-FEDSFAEI--------------TRQSATDLKK-RLMIKFDG-------EDG
LDYGGLSREF---FFLL----SHEMFNPFY--------CLFEYSAHDNY-----------
----------------TLQINPHS------------------------------------
--GINPEHLNYFKFIGRVVGLAIFHRRFLDAFFIG--ALYKMVLG---------------
--------KAVALADMEG----------------------VDADFHRS--------LQWM
LDN--DISGGIL--
-EQTFSTEDER---------------------------FGV-LTTED-LIPN--------
GRNID---
------------------------------VTNENKK-EYVDL-MVKWRIEKR-IAEQFQ
-AFKEG---FHELIPQ--DLIN-VFDER-ELELLIGG-----------------------
----IAEID-----------VDDW----KKHTDYRG-YTES-------------------
------------------------DEVI-QNFWHVVR--------------------SWD
GEQKSRLLQF-------TTGTSRIP------------VNG-----FK-DLQGSD--GPRR
FTIEKTGEL-------------------------TNLPKAHTWY----------------

>Aciculosporium_take_AFQZ01000456.1 .
IKVRREHI-FEDSFAEI--------------TRQSATDLKK-RLMIKFDG-------EDG
LDYGGLSREF---FFLL----SHEMFNPFY--------CLFEYSAHDNY-----------
----------------TLQINPHS------------------------------------
--GINPEHLNYFKFIGRVVGLAIFHRRFLDAFFIG--ALYKMVLG---------------
--------KAVSLADMEG----------------------VDADFHRS--------LQWM
LDN--DISGGIL--
-EQTFSTEDER---------------------------FGV-LTTED-LIPN--------
GRNID---
------------------------------VTNENKK-EYVDL-MVKWRIEKR-IAEQFQ
-AFKEG---FHELIPQ--DLIN-VFDER-ELELLIGG-----------------------
----IAEID-----------VDDW----KKHTDYRG-YTES-------------------
------------------------DEVI-QNFWQVVR--------------------SWD
GEQKSRLLQF-------TTGTSRIP------------VNG-----FK-DLQGSD--GPRR
FTIEKTGEL-------------------------TNLPKAHTWY----------------

>Balansia_obtecta_JFZS01000064.1 .
IKVRREHI-FEDSFAEI--------------TRQSATDLKK-RLMIKFDG-------EDG
LDYGGLSREF---FFLL----SHEMFNPFY--------CLFEYSAHDNY-----------
----------------TLQINPHS------------------------------------
--GINPEHLNYFKFIGRVVGLAIFHRRFLDAFFIG--ALYKMVLG---------------
--------KAVSLADMEG----------------------VDADFHRS--------LQWM
LDN--DISGGIL--
-EQTFSTEDER---------------------------FGV-LTTED-LVPN--------
GRNIE---
------------------------------VTNENKK-EYVDL-MVKWRIEKR-IAEQFQ
-AFKEG---FHELIPQ--DLIN-VFDER-ELELLIGG-----------------------
----IAEID-----------VDDW----KKHTDYRG-YTES-------------------
------------------------DEVI-QNFWQVVR--------------------SWD
GEQKSRLLQF-------TTGTSRIP------------VNG-----FK-DLQGSD--GPRR
FTIEKTGEL-------------------------TNLPKAHTXF-NR-IDLPPY-KTLET
LQQKLT-IAVEE-TMG-
>Atkinsonella_texensis_LBNC01000437.1 .
IKVRREHI-FEDSFAEI--------------TRQSATDLKK-RLMIKFDG-------EDG
LDYGGLSREF---FFLL----SHEMFNPFY--------CLFEYSAHDNY-----------
----------------TLQINPHS------------------------------------
--GINPEHLNYFKFIGRVVGLAIFHRRFLDAFFIG--ALYKMVLG---------------
--------KAVALADMEG----------------------VDADFHRS--------LQWM
LDN--DISGGIL--
-EQTFSTEDER---------------------------FGV-LTTED-LVPN--------

GRNIE---
------------------------------VTNENKK-EYVDL-MVKWRIEKR-IAEQFQ
-AFKEG---FHELIPQ--DLIN-VFDER-ELELLIGG-----------------------
----IAEID-----------VDDW----KKHTDYRG-YTES-------------------
------------------------DEVI-QNFWQVVR--------------------SWD
GEQKSRLLQF-------TTGTSRIP------------VNG-----FK-DLQGSD--GPRR
FTIEKTGEL-------------------------TNLPKAHTXF-NR-IDLPPY-KTLET
LQQKLT-IAVEE-TMG-
>Epichloe_amarillans_JFGZ01000122.1 .
IKVRREHI-FEDSFAEI--------------TRQSATDLKK-RLMIKFDG-------EDG
LDYGGLSREF---FFLL----SHEMFNPFY--------CLFEYSAHDNY-----------
----------------TLQINPHS------------------------------------
--GINPEHLNYFKFIGRVVGLAIFHRRFLDAFFIG--ALYKMVLG---------------
--------KAVALADMEG----------------------VDADFHRS--------LQWM
LDN--DISGGIL--
-EQTFSTEDER---------------------------FGV-LTTED-LIPN--------
GRNID---
------------------------------VTNENKK-EYVDL-MVKWRIEKR-IAEQFQ
-AFKEG---FHELIPQ--DLIN-VFDER-ELELLIGG-----------------------
----IAEID-----------VDDW----KKHTDYRG-YTES-------------------
------------------------DEVV-QNFWQVVR--------------------SWD
GEQKSRLLQF-------TTGTSRIP------------VNG-----FK-DLQGSD--GPRR
FTIEKTGEL-------------------------TNLPKAHTXF-NR-IDLPPY-KTLET
LQQKLT-IAVEE-TMGF
>Epichloe_festucae_ADFL02000387.1 .
IKVRREHI-FEDSFAEI--------------TRQSATDLKK-RLMIKFDG-------EDG
LDYGGLSREF---FFLL----SHEMFNPFY--------CLFEYSAHDNY-----------
----------------TLQINPHS------------------------------------
--GINPEHLNYFKFIGRVVGLAIFHRRFLDAFFIG--ALYKMVLG---------------
--------KAVALADMEG----------------------VDADFHRS--------LQWM
LDN--DISGGIL--
-EQTFSTEDER---------------------------FGV-LTTED-LIPN--------
GRNID---
------------------------------VTNENKK-EYVDL-MVKWRIEKR-IAEQFQ
-AFKEG---FHELIPQ--DLIN-VFDER-ELELLIGG-----------------------
----IAEID-----------VDDW----KKHTDYRG-YTES-------------------
------------------------DEVI-QNFWQVVR--------------------SWD
GEQKSRLLQF-------TTGTSRIP------------VNG-----FK-DLQGSD--GPRR
FTIEKTGEL-------------------------TNLPKAHTXF-NR-IDLPPY-KTLET
LQQKLT-IAVEE-TMGF
>Epichloe_typhina_AMDI01000444.1 .
IKVRREHI-FEDSFAEI--------------TRQSATDLKK-RLMIKFDG-------EDG
LDYGGLSREF---FFLL----SHEMFNPFY--------CLFEYSAHDNY-----------
----------------TLQINPHS------------------------------------
--GINPEHLNYFKFIGRVVGLAIFHRRFLDAFFIG--ALYKMVLG---------------
--------KAVALADMEG----------------------VDADFHRS--------LQWM
LDN--DISGGIL--
-EQTFSTEDER---------------------------FGV-LTTED-LIPN--------
GRNID---
------------------------------VTNENKK-EYVDL-MVKWRIEKR-IAEQFQ
-AFKEG---FHELIPQ--DLIN-VFDER-ELELLIGG-----------------------
----IAEID-----------VDDW----KKHTDYRG-YTES-------------------
------------------------DEVI-QNFWQVVR--------------------SWD
GEQKSRLLQF-------TTGTSRIP------------VNG-----FK-DLQGSD--GPRR
FTIEKTGEL-------------------------TNLPKAHTXF-NR-IDLPPY-KTLET
LQQKLT-IAVEE-TMG-
>Epichloe_bromicola_LBNH01001549.1 .
IKVRREHI-FEDSFAEI--------------TRQSATDLKK-RLMIKFDG-------EDG

```
LDYGGLSREF---FFLL----SHEMFNPFY--------CLFEYSAHDNY-----------
----------------TLQINPHS------------------------------------
--GINPEHLNYFKFIGRVVGLAIFHRRFLDAFFIG--ALYKMVLG---------------
--------KAVALADMEG----------------------VDADFHRS--------LQWM
LDN------------------------------------------------DISGGIL--
-EQTFSTEDER---------------------------FGV-LTTED-LIPN--------
GRNID-------------------------------------------------------
------------------------------VTNENKK-DYVDL-MVKWRIEKR-IAEQFQ
-AFKEG---FHELIPQ--DLIN-VFDER-ELELLIGG-----------------------
----IAEID-----------VDDW----KKHTDYRG-YTES-------------------
------------------------DEVI-QNFWQVVR--------------------SWD
GEQKSRLLQF-------TTGTSRIP------------VNG-----FK-DLQGSD--GPRR
FTIEKTGEL-------------------------TNLPKAHTXF-NR-IDLPPY-KTLET
LQQKLT-IAVEE-TMG-
>Epichloe_uncinata_LELE01001605.1 .
IKVRREHI-FEDSFAEI--------------TRQSATDLKK-RLMIKFDG-------EDG
LDYGGLSREF---FFLL----SHEMFNPFY--------CLFEYSAHDNY-----------
----------------TLQINPHS------------------------------------
--GINPEHLNYFKFIGRVVGLAIFHRRFLDAFFIG--ALYKMVLG---------------
--------KAVALADMEG----------------------VDADFHRS--------LQWM
LDN------------------------------------------------DISGGIL--
-EQTFSTEDER---------------------------FGV-LTTED-LIPN--------
GRNID-------------------------------------------------------
------------------------------VTNENKK-DYVDL-MVKWRIEKR-IAEQFQ
-AFKEG---FHELIPQ--DLIN-VFDER-ELELLIGG-----------------------
----IAEID-----------VDDW----KKHTDYRG-YTES-------------------
------------------------DEVI-QNFWQVVR--------------------SWD
GEQKSRLLQF-------TTGTSRIP------------VNG-----FK-DLQGSD--GPRR
FTIEKTGEL-------------------------TNLPKAHTXF-NR-IDLPPY-KTLET
LQQKLT-IAVEE-TMG-
>Saitoella_complicata_XM_019170113.1 .
IKVRRSHI-FEDSYAEI--------------MRQTATDLKK-RLMIKFDG-------EDG
LDYGGLSREF---FFLL----SHEMFNPFY--------CLFEYSAHDNY-----------
----------------TLQINPHS------------------------------------
--AINPEHLNYFKFIGRVLGLSIFHRRFLDAFFIV--SMYKMILR---------------
--------KKVTLADMES----------------------VDADFHRS--------LQWI
LDN------------------------------------------------PIQ-DVL--
-ELTFSTEDDR---------------------------FGE-VVTVD-LKPN--------
GRDIE-------------------------------------------------------
------------------------------VTDENKK-EYVEL-VTEWRISKR-VEEQFN
-HFMTG---FNELVPQ--ELIN-VFDER-ELELLIGG-----------------------
----IAEID-----------VDDW----KKHTDYRG-YTES-------------------
------------------------DEVV-QWFFKVVQ--------------------SWD
SEKRSRLLQF-------ATGTSRIP------------VGG-----FK-DLQGSD--GPRR
FTIEKAGNP-------------------------SQLPKAHTCF-NR-IDLPPY-NSYED
LLTKLT-YAVEE-TMGF
>Spizellomyces_punctatus_XM_016750836.1 .
VTVRRSNI-FEDAYSEI--------------MRYPAPELQK-RLMIKFHG-------EDG
LDYGGLSREF---FFLL----SHEMFNPFY--------CLFEYSAHDNY-----------
----------------TLQINPNS------------------------------------
--GVNPEHLNYFKFIGRVVGLAIFHQRFLDAFFIT--AFYKMILK---------------
--------KKIVMKDMES----------------------VDAEHWRS--------LQWM
LDN------------------------------------------------DIT-GVL--
-DLTFSAEEEV---------------------------FGV-VRTVD-LKPD--------
GQNIP-------------------------------------------------------
------------------------------VTEENKQ-EYVEL-IVEWRVCKR-VEEQFK
-AFQQG---FHELVPP--DLIT-VFDER-ELELLIGG-----------------------
----IADID-----------VDDW----KKHTDYRG-YQET-------------------
```

```
------------------------DEVV-QWFWKCVR--------------------SWD
SEKKARLLQF-------VTGTSRIP------------VNG-----FK-DLQGSD--GPRR
FTIEKAGEG-------------------------EQLPKSHTCF-NR-LDLPPY-RGMDI
LEKKLT-MAIEE-TIGF
>Homoloaphlyctis_polyrhiza_AFSM01007931.1_R .
VVVRRNTI-FEDSFSEI--------------MRIPANDLKK-RLMIKFQG-------EDG
LDYGGLSXEF---FFLL----SHEMFNPFY--------GLFEYSAHDNY-----------
----------------TLQINPHS------------------------------------
--AINPEHLNYFKFIGRVVGLAIFHQRFLDAFFIT--SFYKMILH---------------
--------KKVTLKDMES----------------------VDADFHRS--------LQWT
LXN------------------------------------------------SIE-GII--
-DSTFTVEDER---------------------------FGE-TITVE-LKPG--------
GKDIQ-------------------------------------------------------
------------------------------VTDENKA-EYVQL-MVEWRISRR-VEEQCR
-AFSEG---FHELIPS--NLIN-VFDER-EL-LLIGG-----------------------
----IAEID-----------VDDW----KKNTDYRG-YKED-------------------
------------------------DEVI-QWFWQSVR--------------------SWE
SEKKARLLQF-------VTGTSRIP------------VNG-----FK-DLQGSD--GPRR
FTIEKTGEI-------------------------EALPKSHTWF-NR-LDLPPY-KNEGV
FNNKLT-LAIEE-----
>Batrachochytrium_dendrobatidis_XM_006682088.1 .
VAVRRSNI-FEDSFTEI--------------MRVPAVDLKK-RLMIKFQG-------EDG
LDYGGLSREF---FFLL----SHEMFNPFY--------GLFEYSAHDNY-----------
----------------TLQINPHS------------------------------------
--GINPEHLNYFKFIGRVVGLAIFHQRFLDAFFIT--SFYKLILH---------------
--------KKISLKDMES----------------------VDADLYRS--------LNWT
LEN------------------------------------------------SIE-GVL--
-DLTFTAEDER---------------------------FGE-IVTVD-LKTD--------
GKDIV-------------------------------------------------------
------------------------------VTDENKA-EYIQL-ITEWRIGKR-VEEQTK
-AFSDG---FHELIPR--DLVN-VFDER-ELELLIGG-----------------------
----IADID-----------VDDW----KKHTDYRG-YTED-------------------
------------------------DEVI-QWFWKSVQ--------------------SWD
SEKKARLLQF-------VTGTSRIP------------VNG-----FK-DLQGSD--GPRR
FTIEKTGEI-------------------------ESLPKSHTCF-NR-LDLPPY-RSQSA
FNRKIT-LAIEE-TIGF
>Pseudozyma_hubeiensis_XM_012337611.1 .
IKIRRTHI-FEDSYAEI--------------MRQQPNDLKK-RLMIKFDG-------EDG
LDYGGLSREF---FFLL----SHEMFNPFY--------CLFEYSAHDNY-----------
----------------TLQINPHS------------------------------------
--GINPEHLNYFKFIGRVLGLAIFHRRFLDAYFIV--SFYKMILK---------------
--------KKITLSDLES----------------------VDADYHRS--------LQWM
LDN------------------------------------------------SIE-GIV--
-EETFTAVEDK---------------------------FGE-MVTVE-LKKG--------
GEEVD-------------------------------------------------------
------------------------------VTDENKK-EYVDL-MTEWRISKR-VEEQFK
-AFISG---FTELIPQ--DLIN-VFDER-ELELLIGG-----------------------
----MSEID-----------VDDW----KKFTDYRG-FTEQ-------------------
------------------------DQVV-QWFWQCVR--------------------AWP
TEKKSRLLQF-------ATGTSRIP------------VNG-----FK-DLQGSD--GPRR
FTIEKSGDV-------------------------NQLPKSHTCF-NR-IDLPPY-PSFET
LESKLA-LAIEE-GMGF
>Ustilago_vetiveriae_MAIM01000001.1 .
IKVRRTHI-FEDSYAEI--------------MRQQPNDLKK-RLMIKFDG-------EDG
LDYGGLSREF---FYLL----SHEMFNPFY--------CLFEYSAHDNY-----------
----------------TLQINPHS------------------------------------
--GINPEHLNYFKFIGRVLGLAIFHRRFLDAYFIV--SFYKMILK---------------
--------KKITLSDLES----------------------VDADYHRS--------LQWM
```

LDN--SIE-GIV--
-EETFTAVEDK---------------------------FGE-MVTVE-LKKG--------
GEEVD---
------------------------------VTDENKK-EYVDL-MTEWRISKR-VEEQFK
-AFISG---FTELIPQ--DLIN-VFDER-ELELLIGG-----------------------
----MSEID-----------VDDW----KKFTDYRG-FTEQ-------------------
------------------------DQVV-QWFWQCVR--------------------AWP
TEKKSRLLQF-------ATGTSRIP------------VNG-----FK-DLQGSD--GPRR
FTIEKSGDV-------------------------NQLPKSHTCF-NR-IDLPPY-PSFET
LESKLA-LAIEE-GMG-
>Ustilago_maydis_XM_011388165.1 .
IKVRRTHI-FEDSYAEI--------------MRQQPNDLKK-RLMIKFDG-------EDG
LDYGGLSREF---FFLL----SHEMFNPFY--------CLFEYSAHDNY-----------
----------------TLQINPHS------------------------------------
--GINPEHLNYFKFIGRVLGLAIFHRRFLDAYFIV--SFYKMILK---------------
--------KKITLSDLES----------------------VDADYHRS--------LQWM
LDN--SIE-GIV--
-EETFTAVEDK---------------------------FGE-MVTVE-LKKG--------
GEEVE---
------------------------------VTDENKK-EYVDL-MTEWRISKR-VEEQFK
-AFISG---FTELIPQ--DLIN-VFDER-ELELLIGG-----------------------
----MSEID-----------VDDW----KKFTDYRG-FTEQ-------------------
------------------------DQVV-QWFWQCVR--------------------AWP
TEKKSRLLQF-------ATGTSRIP------------VNG-----FK-DLQGSD--GPRR
FTIEKSGDV-------------------------NQLPKSHTCF-NR-IDLPPY-PSFET
LESKLA-LAIEE-GMGF
>Moesziomyces_aphidis_AWNI01000008.1 .
IKVRRTHI-FEDSYAEI--------------MRQQPNDLKK-RLMIKFDG-------EDG
LDYGGLSREF---FFLL----SHEMFNPFY--------CLFEYSAHDNY-----------
----------------TLQINPHS------------------------------------
--GINPEHLNYFKFIGRVLGLAIFHRRFLDAYFIV--SFYKMILK---------------
--------KKITLSDLES----------------------VDADYHRS--------LQWM
LDN--SIE-GIV--
-EETFTAVEDK---------------------------FGE-MVTIE-LKNG--------
GEEVE---
------------------------------VTDENKK-EYVEL-MTEWRISKR-VEEQFK
-AFISG---FTELIPQ--DLIN-VFDER-ELELLIGG-----------------------
----MSEID-----------VDDW----KKFTDYRG-FTEQ-------------------
------------------------DQVV-QWFWQCVR--------------------AWP
TEKKSRLLQF-------ATGTSRIP------------VNG-----FK-DLQGSD--GPRR
FTIEKSGDV-------------------------NQLPKSHTCF-NR-IDLPPY-PSFET
LESKLA-LAIEE-GMG-
>Pseudozyma_antarctica_XM_014803797.1 .
IKVRRTHI-FEDSYAEI--------------MRQQPNDLKK-RLMIKFDG-------EDG
LDYGGLSREF---FFLL----SHEMFNPFY--------CLFEYSAHDNY-----------
----------------TLQINPHS------------------------------------
--GINPEHLNYFKFIGRVLGLAIFHRRFLDAYFIV--SFYKMILK---------------
--------KKITLSDLES----------------------VDADYHRS--------LQWM
LDN--SIE-GIV--
-EETFTAVEDK---------------------------FGE-MVTIE-LKKG--------
GEEVE---
------------------------------VTDENKK-EYVEL-MTEWRISKR-VEEQFK
-AFISG---FTELIPQ--DLIN-VFDER-ELELLIGG-----------------------
----MSEID-----------VDDW----KKFTDYRG-FTEQ-------------------
------------------------DQVV-QWFWQCVR--------------------AWP
TEKKSRLLQF-------ATGTSRIP------------VNG-----FK-DLQGSD--GPRR
FTIEKSGDV-------------------------NQLPKSHTCF-NR-IDLPPY-PSFET
LESKLA-LAIEE-GMGF

>Pseudozyma_tsukubaensis_MAIP01000044.1 .
IKVRRTHI-FEDSYAEI--------------MRQQPNDLKK-RLMIKFDG-------EDG
LDYGGLSREF---FFLL----SHEMFNPFY--------CLFEYSAHDNY-----------
----------------TLQINPHS------------------------------------
--GINPEHLNYFKFIGRVLGLAIFHRRFLDAYFIV--SFYKMILK---------------
--------KKITLSDLES----------------------VDADYHRS--------LQWM
LNN--SIE-GIV--
-EETFTAVEDK---------------------------FGE-MVTVE-LKPG--------
GEEVE---
------------------------------VTDENKK-EYVEL-MTEWRISTR-VQEQFK
-AFISG---FTELIPQ--DLIN-VFDER-ELELLIGG-----------------------
----MSEID-----------CDDW----KKFTDYRG-FTEQ-------------------
------------------------DQVV-QWFWQCVR--------------------AWP
TEKKSRLLQF-------ATGTSRIP------------VNG-----FK-DLQGSD--GPRR
FTIEKSGDV-------------------------NQLPKSHTCF-NR-IDLPPY-PSFET
LESKLA-LAIEE-GMG-
>Sporisor_scitamineum_LK056662.1 .
IKVRRTHI-FEDSYAEI--------------MRQQPNDLKK-RLMIKFDG-------EDG
LDYGGLSREF---FFLL----SHEMFNPFY--------CLFEYSAHDNY-----------
----------------TLQINPHS------------------------------------
--GINPEHLNYFKFIGRVLGLAIFHRRFLDAYFIV--SFYKMILK---------------
--------KKITLNDLES----------------------VDADYHRS--------LQWM
LDN--SIE-GIV--
-EETFTAVEDK---------------------------FGE-MVTVE-LKPG--------
GEEVE---
------------------------------VTDENKK-EYVDL-MTEWRISKR-VEEQFK
-AFISG---FTELIPQ--DLIN-VFDER-ELELLIGG-----------------------
----MSEID-----------VDDW----KKFTDYRG-FTEQ-------------------
------------------------DQVV-QWFWQCVR--------------------AWP
TEKKSRLLQF-------ATGTSRIP------------VNG-----FK-DLQGSD--GPRR
FTIEKSGDV-------------------------NQLPKSHTCF-NR-IDLPPY-PSFET
LESKLA-LAIEE-GMGF
>Ustilago_trichophora_LVYE01000003.1 .
IKVRRTHI-FEDSYAEI--------------MRQQPNDLKK-RLMIKFDG-------EDG
LDYGGLSREF---FFLL----SHEMFNPFY--------CLFEYSAHDNY-----------
----------------TLQINPHS------------------------------------
--GINPEHLNYFKFIGRVLGLAIFHRRFLDAYFIV--SFYKMILK---------------
--------KKITLSDLES----------------------VDADYHRS--------LQWM
LDN--SIE-GIV--
-EETFTAVEDK---------------------------FGE-MNTVE-LKSG--------
GEEIE---
------------------------------VTDENKK-EYVEL-MTEWRISKR-VEEQFK
-AFISG---FTELIPQ--DLIN-VFDER-ELELLIGG-----------------------
----MSEID-----------VDDW----KKFTDYRG-FTEQ-------------------
------------------------DQVV-QWFWQCVR--------------------AWP
TEKKSRLLQF-------ATGTSRIP------------VNG-----FK-DLQGSD--GPRR
FTIEKSGDV-------------------------NQLPKSHTCF-NR-IDLPPY-PSFET
LESKLA-LAIEE-GMG-
>Ustilago_hordei_CAGI01000161.1 .
IKVRRTHI-FEDSYAEI--------------MRQQPNDLKK-RLMIKFDG-------EDG
LDYGGLSREF---FFLL----SHEMFNPFY--------CLFEYSAHDNY-----------
----------------TLQINPHS------------------------------------
--GINPEHLNYFKFIGRVLGLAIFHRRFLDAYFIV--SFYKMILK---------------
--------KKITLSDLES----------------------VDADYHRS--------LQWM
LDN--SIE-GIV--
-EETFTAVEDK---------------------------FGE-MVTVE-LKPG--------
GEEIE---
------------------------------VTDENKK-EYIEL-MTEWRISKR-VEEQFK

```
-AFISG---FTELIPQ--DLIN-VFDER-ELELLIGG-----------------------
----MSEID-----------VDDW----KKFTDYRG-FTEQ-------------------
------------------------DQVV-QWFWQCVR--------------------AWP
TEKKSRLLQF-------ATGTSRIP------------VNG-----FK-DLQGSD--GPRR
FTIEKSGDV-------------------------NQLPKSHTCF-NR-IDIPPY-PSFET
LESKLA-LAIEE-GMG-
>Ustilago_esculenta_JTLW01000016.1 .
IKVRRTHI-FEDSYAEI--------------MRQQPNDLKK-RLMIKFDG-------EDG
LDYGGLSREF---FFLL----SHEMFNPFY--------CLFEYSAHDNY-----------
----------------TLQINPHS------------------------------------
--GINPEHLNYFKFIGRVLGLAIFHRRFLDAYFIV--SFYKMILK---------------
--------KKITLSDLES----------------------VDADYHRS--------LQWM
LDN------------------------------------------------SIE-GIV--
-EETFTAVEDK---------------------------FGE-MVTVE-LKPG--------
GEEIE-------------------------------------------------------
------------------------------VTDENKK-DYVEL-MTEWRISKR-VEEQFK
-AFISG---FTELIPQ--DLIN-VFDER-ELELLIGG-----------------------
----MSEID-----------VDDW----KKFTDYRG-FTEQ-------------------
------------------------DQVV-QWFWQCVR--------------------AWP
TEKKSRLLQF-------ATGTSRIP------------VNG-----FK-DLQGSD--GPRR
FTIEKSGDV-------------------------NQLPKSHTCF-NR-IDLPPY-PSFET
LESKLA-LAIEE-GMG-
>Sporisor_reilianum_FQ311430.1 .
IKVRRTHI-FEDSYAEI--------------MRQQPNDLKK-RLMIKFDG-------EDG
LDYGGLSREF---FFLL----SHEMFNPFY--------CLFEYSAHDNY-----------
----------------TLQINPHS------------------------------------
--GINPEHLNYFKFIGRVLGLAIFHRRFLDAYFIV--SFYKMILK---------------
--------KKITLSDLES----------------------VDADYHRS--------LQWM
LDN------------------------------------------------SIE-GIV--
-EETFTAVEDK---------------------------FGE-MVTVE-LKPG--------
GEEVE-------------------------------------------------------
------------------------------VTDENKK-DYVDL-MTEWRISKR-VEEQFK
-AFISG---FTELIPQ--DLIN-VFDER-ELELLIGG-----------------------
----MSEID-----------VDDW----KKFTDYRG-FTEQ-------------------
------------------------DQVV-QWFWQCVR--------------------AWP
TEKKSRLLQF-------ATGTSRIP------------VNG-----FK-DLQGSD--GPRR
FTIEKSGDV-------------------------NQLPKSHTCF-NR-IDLPPY-PSFET
LESKLA-LAIEE-GMGF
>Ustilago_bromivora_LT558117.1 .
IKVRRTHI-FEDSYAEI--------------MRQQPNDLKK-RLMIKFDG-------EDG
LDYGGLSREF---FFLL----SHEMFNPFY--------CLFEYSAHDNY-----------
----------------TLQINPHS------------------------------------
--GINPEHLNYFKFIGRVLGLAIFHRRFLDAYFIV--SFYKMILK---------------
--------KKITLSDLES----------------------VDADYHRS--------LQWM
LDN------------------------------------------------SIE-GIV--
-EETFTAVEDK---------------------------FGE-MVTVE-LKPG--------
GEQVE-------------------------------------------------------
------------------------------VTDENKK-DYVEL-MTEWRISKR-VEEQFK
-AFISG---FTELIPQ--DLIN-VFDER-ELELLIGG-----------------------
----MSEID-----------VDDW----KKFTDYRG-FTEQ-------------------
------------------------DQVV-QWFWQCVR--------------------AWP
TEKKSRLLQF-------ATGTSRIP------------VNG-----FK-DLQGSD--GPRR
FTIEKSGDV-------------------------NQLPKSHTCF-NR-IDIPPY-PSFET
LESKLA-LAIEE-GMGF
>Kalmanozyma_brasiliensis_XM_016438044.1 .
IKVRRTHI-FEDSYAEI--------------MRQQPNDLKK-RLMIKFDG-------EDG
LDYGGLSREF---FFLL----SHEMFNPFY--------CLFEYSAHDNY-----------
----------------TLQINPHS------------------------------------
```

--GINPEHLNYFKFIGRVLGLAIFHRRFLDAYFIV--SFYKMILK---------------
--------KKITLSDLES----------------------VDADYHRS--------LQWM
LDN--SIE-GIV--
-EETFTAVEDK---------------------------FGE-MVTVE-LKPG--------
GEEVD---
------------------------------VTDENKK-DYVEL-MTEWRISKR-VEEQFK
-AFISG---FTELIPQ--DLIN-VFDER-ELELLIGG-----------------------
----MSEID-----------CDDW----KKFTDYRG-FTEQ-------------------
------------------------DQVV-QWFWQCVR--------------------AWP
TEKKSRLLQF-------ATGTSRIP------------VNG-----FK-DLQGSD--GPRR
FTIEKSGDV-------------------------NQLPKSHTCF-NR-IDLPPY-PSFET
LESKLA-LAIEE-GMGF
>Ustilago_cynodontis_LZZZ01000030.1 .
IKVRRTHI-FEDSYAEI--------------MRQQPNDLKK-RLMIKFDG-------EDG
LDYGGLSREF---FFLL----SHEMFNPFY--------CLFEYSAHDNY-----------
----------------TLQINPHS------------------------------------
--GINPEHLNYFKFIGRVLGLAIFHRRFLDAYFIV--SFYKMILK---------------
--------KKITLSDLES----------------------VDADYHRS--------LQWM
LDN--SIE-GIV--
-EETFTAVEDK---------------------------FGE-MVTVE-LKPG--------
GEEIE---
------------------------------VTDENKK-EYVEL-MTEWRISKR-VEEQFR
-AFISG---FTELIPQ--DLIN-VFDER-ELELLIGG-----------------------
----MSEID-----------CDDW----KKFTDYRG-FTEQ-------------------
------------------------DQVV-QWFWQCVR--------------------AWP
TEKKSRLLQF-------ATGTSRIP------------VNG-----FK-DLQGSD--GPRR
FTIEKSGDI-------------------------NQLPKSHTCF-NR-IDLPPY-PSFET
LESKLA-LAIEE-GMG-
>Ustilago_xerochloae_MAIN01000220.1 .
IKVRRTHI-FEDSYAEI--------------MRQQPNDLKK-RLMIKFDG-------EDG
LDYGGLSREF---FFLL----SHEMFNPFY--------CLFEYSAHDNY-----------
----------------TLQINPHS------------------------------------
--GINPEHLNYFKFIGRVLGLAIFHRRFLDAYFIV--SFYKMILK---------------
--------KKITLSDLES----------------------VDADYHRS--------LQWM
LDN--SIE-GIV--
-EETFTAVEDK---------------------------FGE-MVTVE-LKPG--------
GEEIE---
------------------------------VTDENKK-EYVEL-MTEWRISKR-VEEQFK
-AFISG---FTELIPQ--DLIN-VFDER-ELELLIGG-----------------------
----MSEID-----------CDDW----KKFTDYRG-FTEQ-------------------
------------------------DQVV-QWFWQCVR--------------------AWP
TEKKSRLLQF-------ATGTSRIP------------VNG-----FK-DLQGSD--GPRR
FTIEKSGDI-------------------------NQLPKSHTCF-NR-IDLPPY-PSFET
LESKLA-LAIEE-GMG-
>Sporisor_iseilematis_ciliati_MJEU01000012 1..
IKVRRTHI-FEDSYAEI--------------MRQQPNDLKK-RLMIKFDG-------EDG
LDYGGLSREF---FFLL----SHEMFNPFY--------CLFEYSAHDNY-----------
----------------TLQINPHS------------------------------------
--GINPEHLNYFKFIGRVLGLAIFHRRFLDAYFIV--SFYKMILK---------------
--------KKITLGDLES----------------------VDADYHRS--------LQWM
LDN--SIE-GIV--
-EETFTAVESK---------------------------FGE-MITVE-LKPG--------
GEIE--
------------------------------VTDENKK-EYIDL-MTEWRISKR-VEEQFK
-AFISG---FTELIPQ--DLIN-VFDER-ELELLIGG-----------------------
----MSEID-----------VDDW----KKFTDYRG-FTEQ-------------------
------------------------DQVV-QWFWQCVR--------------------AWP
TEKKSRLLQF-------ATGTSRIP------------VNG-----FK-DLQGSD--GPRR

```
FTIEKSGDV-------------------------NQLPKSHTCF-NR-IDLPPY-PSFET
LESKLA-LAIEE-GMG-
>Pseudozyma_flocculosa_XM_007877555.1 .
IKVRRTHI-FEDSYAEI--------------MRQQPNDLKK-RLMIKFDG-------EDG
LDYGGLSREF---FFLL----SHEMFNPFY--------CLFEYSAHDNY-----------
----------------TLQINPHS------------------------------------
--GINPEHLNYFKFIGRVLGLAIFHRRFLDAYFIV--SFYKMILK---------------
--------KKITLSDLES----------------------VDADYHRS--------LQWM
LDN--------------------------------------------------SIE-GVV--
-EETFTAVEDK---------------------------FGE-MVTVE-LRPG--------
GEEIE-------------------------------------------------------
------------------------------VTDENKK-EYVEL-MTEWRISRR-VEEQFK
-AFISG---FTELIPQ--DLIN-VFDER-ELELLIGG-----------------------
----MSEID-----------VDDW----KRFTDYRG-FTEQ-------------------
------------------------DQVV-QWFWQCVK--------------------AWP
AERKSRLLQF-------ATGTSRIP------------VNG-----FK-DLQGSD--GPRR
FTIEKSGEV-------------------------NQLPKSHTCF-NR-IDLPPY-PSFET
LESKLA-LAIEE-GMGF
>Anthracocystis_flocculosa_AOUS01000008.1 .
IKVRRTHI-FEDSYAEI--------------MRQQPNDLKK-RLMIKFDG-------EDG
LDYGGLSREF---FFLL----SHEMFNPFY--------CLFEYSAHDNY-----------
----------------TLQINPHS------------------------------------
--GINPEHLNYFKFIGRVLGLAIFHRRFLDAYFIV--SFYKMILK---------------
--------KKITLSDLES----------------------VDADYHRS--------LQWM
LDN--------------------------------------------------SIE-GVV--
-EETFTAVEDK---------------------------FGE-MVTVE-LRPG--------
GEEIE-------------------------------------------------------
------------------------------VTDENKK-EYVEL-MTEWRISRR-VEEQFK
-AFISG---FTELIPQ--DLIN-VFDER-ELELLIGG-----------------------
----MSEID-----------VDDW----KRFTDYRG-FTEQ-------------------
------------------------DQVV-QWFWQCVK--------------------AWP
AERKSRLLQF-------ATGTSRIP------------VNG-----FK-DLQGSD--GPRR
FTIEKSGEV-------------------------NQLPKSHTCF-NR-IDLPPY-PSFET
LESKLA-LAIEE-GMG-
>Melanopsichium_pennsylvanicum_HG529511.1 .
IKVRRSHI-FEDSYAEI--------------MRQQPNDLKK-RLMIKFDG-------EDG
LDYGGLSREF---FFLL----SHEMFNPFY--------CLFEYSAHDNY-----------
----------------TLQINPHS------------------------------------
--GINPEHLNYFKFIGRVLGLAIFHRRFLDAYFIV--SFYKMILN---------------
--------KKITLSDLES----------------------VDADYHRS--------LHWM
LDN--------------------------------------------------SIE-GIV--
-EETFTAVEDK---------------------------FGE-MNSVE-LKPG--------
GEEVE-------------------------------------------------------
------------------------------VTDENKK-EYVEL-MTEWRISKR-VEEQFK
-AFISG---FTELIPQ--DLIN-VFDER-ELELLIGG-----------------------
----MSEID-----------VDDW----KKFTDYRG-FTEQ-------------------
------------------------DQVV-QWFWQCVR--------------------AWP
TEKKSRLLQF-------ATGTSRIP------------VNG-----FK-DLQGSD--GPRR
FTIEKSGEV-------------------------NQLPKSHTCF-NR-IDLPPY-PSFET
LENKLA-LAIEE-GMGF
>Tilletiopsis_pallescens_BCHO01000023.1 .
VKVRRTHI-FEDSYAEI--------------MRQQPNDLKK-RLMIKFDG-------EDG
LDYGGLSREF---FFLL----SHEMFNPFY--------CLFEYSAHDNY-----------
----------------TLQINPHS------------------------------------
--GINPEHLNYFKFIGRVLGLAIFHRRFLDAYFIV--SFYKMILK---------------
--------KKITLGDLES----------------------VDADYHRS--------LQWM
LDN--------------------------------------------------PIE-GVV--
-DETFTAVEDK---------------------------FGE-MVTVE-LKPG--------
```

GEQVE---
------------------------------VTDENKK-EYIEL-MTEWRISRR-VEEQFK
-AFTSG---FTELIPQ--DLIN-VFDER-ELELLIGG-----------------------
----MSEID-----------VEDW----KRYTDYRG-FTEQ-------------------
------------------------DQVV-QWFWNCVK--------------------AWP
PERKSRLLQF-------ATGTSRIP------------VNG-----FK-DLQGSD--GPRR
FTLEKTSGDP------------------------NSLPKSHTCF-NR-IDLPSY-VSAEQ
LEQKLS-E---------
>Ceraceosorus_bombacis_CCYA01000258.1 .
IKVRRTHI-FEDSYAEI--------------MRQQPNDLKK-RLMIKFDG-------EEG
LDYGGLSREF---FFLL----SHEMFNPFY--------CLFEYSAHDNY-----------
----------------TLQINPHS------------------------------------
--GINPEHLNYFKFIGRVLGLAIFHRRFLDAYFIV--SFYKMILN---------------
--------KKITLADLES----------------------VDADYHRS--------LQWM
LNN--PIA-DVV--
-EETFTAVEDK---------------------------FGE-MVTVD-LKPG--------
GSEMD---
------------------------------VTDENKA-EYVEL-MTEWRIQKR-VEEQFK
-AFSSG---FTELIPQ--DLIN-VFDER-ELELLIGG-----------------------
----MSEID-----------IDDW----KRYTDYRG-FTEQ-------------------
------------------------DEVV-QWFWKCVK--------------------AWP
AERKSRLLQF-------ATGTSRIP------------VNG-----FK-DLQGSD--GPRR
FTIEKSGEI-------------------------TQLPKSHTCF-NR-IDLPPY-QSEKQ
LEDKLV-LAVEE-GLG-
>NWGS3B_2_Meira_nashicola_BCJU01000003.1 .
IKVRRTHI-FEDSYAEI--------------MRQQPNDLKK-RLMIKFDG-------EDG
LDYGGLSREF---FFLL----SHEMFNPFY--------CLFEYSAHDNY-----------
----------------TLQINPHS------------------------------------
--GVNPEHLNYFKFIGRVLGLAIFHRRFLDAYFIV--SFYKMILK---------------
--------KKIQLGDLEG----------------------VDADYYRS--------LQWM
LDN--SIV-DVV--
-DETFTAVEDK---------------------------FGE-IVTVP-LKPG--------
GEDIE---
------------------------------VTDENKK-EYIEL-MTEWRIVRR-VEEQFK
-AFISG---FTELIPQ--DLIN-VFDER-ELELLIGG-----------------------
----MSEID-----------VDDW----KRFTDYRG-FTEQ-------------------
------------------------DDVV-QWFWQCVR--------------------SWP
PERKSRLLQF-------ATGTSRIP------------VNG-----FK-DLQGSD--GPRR
FTIEKSGEV-------------------------TQLPKSHTCF-NR-IDLPPY-SSKEI
LEEKLV-WAVEE-TVG-
>Tilletiaria_anomala_XM_013387205.1 .
IKVRRTHI-FEDSYAEI--------------MRQQPNDLKK-RLMIKFEG-------EDG
LDYGGLSREF---FFLL----SHEMFNPFY--------CLFEYSAHDNY-----------
----------------TLQINPHS------------------------------------
--GINPEHLNYFKFIGRVLGLAIFHRRFLDAYFIV--SFYKMILK---------------
--------KKITLADLES----------------------VDADYHRS--------LQWM
LEN--PIE-GAI--
-DETFTAVEDK---------------------------FGE-MVTVE-LKPG--------
GADIP---
------------------------------VTDENKK-EYIDL-MTEYRISKR-VEEQFK
-AFTSG---FTELIPQ--DLIN-VFDER-ELELLIGG-----------------------
----MSEID-----------VDDW----KKWTDYRG-FTES-------------------
------------------------DEVV-QWFWQCVR--------------------SWP
AERKSRLLQF-------ATGTSRIP------------VNG-----FK-DLQGSD--GPRR
FTIEKSGEI-------------------------SALPKSHTCF-NR-MDLPPY-PNKEV
LEAKLT-LAIEE-GLHF
>Malassezia_furfur_LFGJ01001041.1 .
VKVRRTHI-FEDSYAEI--------------MRQQPNDLKK-RLMIKFEG-------EDA

LDYGGVSREF---FFLL----SHEMFNPFY--------CLFEYSAHDNY-----------
----------------TLQINPHS------------------------------------
--GINPEHLNYFKFIGRVLGLAIFHRRFLDAHFIV--SFYKMILK---------------
--------KKILLSDMES----------------------VDADYHRS--------LQWM
LDN--PIA-GIM--
-DETFSAIDDK---------------------------FGE-MITVE-LKPG--------
GEHIE---
------------------------------VTDENKR-EYVER-MVEWRIVKR-VEEQFR
-AFISG---FSELIPL--DLIN-VFDER-ELELLMGG-----------------------
----MSEID-----------VDDW----KRFTDYRG-FTEQ-------------------
------------------------DDVV-QWFWQCVQ--------------------KWP
AEQRSRLLQF-------ATGTSRIP------------VNG-----FK-DLQGSD--GPRR
FTIEKSGEV-------------------------NQLPKSHTCF-NR-IDLPPY-PSREV
LENKLV-LAIEE-G---
>Malassezia_obtusa_LFGC01001112.1 .
VKVRRTHI-FEDSYAEI--------------MRQQPNDLKK-RLMIKFEG-------EDA
LDYGGVSREF---FFLL----SHEMFNPFY--------CLFEYSAHDNY-----------
----------------TLQINPHS------------------------------------
--GINPEHLNYFKFIGRVLGLAIFHRRFLDAHFIV--SFYKMILK---------------
--------KKILLSDMES----------------------VDADYHRS--------LQWM
LDN--PIA-GIM--
-EETFSAIDDK---------------------------FGE-MITVE-LKPG--------
GEQIE---
------------------------------VTDENKR-EYVER-MVEWRIVKR-VEEQFR
-AFISG---FSELIPL--DLIN-VFDER-ELELLMGG-----------------------
----MSEID-----------VDDW----KRFTDYRG-FTEQ-------------------
------------------------DDVV-QWFWQCVQ--------------------KWP
AEQRSRLLQF-------ATGTSRIP------------VNG-----FK-DLQGSD--GPRR
FTIEKSGEI-------------------------NQLPKSHTCF-NR-IDLPPY-PSMEV
LENKLV-LAIEE-G---
>Malassezia_yamatoensis_LFCX01000016.1 .
VKVRRTHI-FEDSYAEI--------------MRQQPNDLKK-RLMIKFEG-------EDA
LDYGGVSREF---FFLL----SHEMFNPFY--------CLFEYSAHDNY-----------
----------------TLQINPHS------------------------------------
--GINPEHLNYFKFIGRVLGLAIFHRRFLDAHFIV--SFYKMILK---------------
--------KKILLSDMES----------------------VDADYHRS--------LQWM
LDN--PIE-GIM--
-DETFSAIDDK---------------------------FGE-MITVE-LKPG--------
GEQIE---
------------------------------VTDENKR-EYVER-MVEWRIMRR-VEEQFR
-AFISG---FSELIPL--DLIN-VFDER-ELELLMGG-----------------------
----MSEID-----------VDDW----KRFTDYRG-FTEQ-------------------
------------------------DDVV-QWFWQCVQ--------------------KWP
AEQRSRLLQF-------ATGTSRIP------------VNG-----FK-DLQGSD--GPRR
FTIEKSGAV-------------------------NQLPKSHTCF-NR-IDLPPY-PSKEV
LENKLV-LAIEE-G---
>Malassezia_slooffiae_LFGK01000848.1 .
VKVRRTHI-FEDSYAEI--------------MRQQPNDLKK-RLMIKFEG-------EDA
LDYGGVSREF---FFLL----SHEMFNPFY--------CLFEYSAHDNY-----------
----------------TLQINPHS------------------------------------
--GINPEHLNYFKFIGRVLGLAIFHRRFLDAHFIV--SFYKMILK---------------
--------KKITLADLES----------------------VDADYYRS--------LQWM
LDN--SIE-GIM--
-EETFSAVEDK---------------------------FGE-MITVE-LKPG--------
GEHVE---
------------------------------VTDENKR-EYVER-MIEWRIVKR-VEEQFR
-AFISG---FSELIPL--DLIN-VFDER-ELELLMGG-----------------------
----MSEID-----------VDDW----KRFTDYRG-FSEQ-------------------

------------------------DDVV-QWFWQCVQ--------------------KWP
AEQRSRLLQF-------ATGTSRIP------------VNG-----FK-DLQGSD--GPRR
FTIEKSGEI-------------------------NQLPKSHTCF-NR-IDLPPY-PSMEV
LENKLV-LAIEE-G---
>Malassezia_japonica_LFDB01000173.1 .
VKVRRTHI-FEDSYAEI--------------MRQQPNDLKK-RLMIKFEG-------EDA
LDYGGVSREF---FFLL----SHEMFNPFY--------CLFEYSAHDNY-----------
----------------TLQINPHS------------------------------------
--GINPEHLNYFKFIGRVLGLAIFHRRFLDAHFIV--SFYKMILK---------------
--------KKILLSDMES----------------------VDADYHRS--------LQWM
LDN--PIE-GIM--
-EESFSAIEDK---------------------------FGE-MITVE-LKPN--------
GENIE---
------------------------------VTDENKR-EYVER-MIEWRIVTR-VEEQFR
-AFISG---FSELIPL--DLIN-VFDER-ELELLMGG-----------------------
----MSEID-----------VDDW----KRFTDYRG-FSEQ-------------------
------------------------DDVV-QWFWQCVQ--------------------KWP
AEQRSRLLQF-------ATGTSRIP------------VNG-----FK-DLQGSD--GPRR
FTIEKSGEV-------------------------NQLPKSHTCF-NR-IDLPPY-PSMEV
LENKLV-LAIEE-G---
>Malassezia_cuniculi_LFFW01000021.1 .
VKVRRTHI-FEDSYAEI--------------MRQQPNDLKK-RLMIKFEG-------EDA
LDYGGVSREF---FFLL----SHEMFNPFY--------CLFEYSAHDNY-----------
----------------TLQINPHS------------------------------------
--GINPEHLNYFKFIGRVLGLAIFHRRFLDAHFIV--SFYKMILK---------------
--------KKITLSDLES----------------------VDADYHRS--------LQWM
LDN--PIE-GIV--
-EETFSAIEDK---------------------------FGE-LITIE-LKPG--------
GEDIP---
------------------------------VTDENKR-EYVEK-MIEWRIVRR-VEEQFR
-AFIAG---FSELIPL--DLIN-VFDER-ELELLMGG-----------------------
----MSEID-----------VDDW----KRFTDYRG-FSEQ-------------------
------------------------DDVV-KWFWECVQ--------------------KWP
AEQRSRLLQF-------ATGTSRIP------------VNG-----FK-DLQGSD--GPRR
FTIEKSGEI-------------------------NHLPKSHTCF-NR-IDLPPY-PSLEV
LENKLV-LAIEE-G---
>Malassezia_restricta_LFCZ01000064.1 .
VKVRRTHI-FEDSYAEI--------------MRQQPNDLKK-RLMIKFEG-------EDA
LDYGGVSREF---FFLL----SHEMFNPFY--------CLFEYSAHDNY-----------
----------------TLQINPHS------------------------------------
--GINPEHLNYFKFIGRVLGLAIFHRRFLDAHFIV--SFYKMILK---------------
--------KKITLADMES----------------------VDADYHRS--------LQWM
LDN--SIE-GVM--
-EETFSTLEDK---------------------------FGE-MVTVE-LKPG--------
GEHID---
------------------------------VTNENKH-EYVDC-MVEWRIIKR-VEEQFR
-AFISG---FSELIPL--DLIN-VFDER-ELELLMGG-----------------------
----MSEID-----------VDDW----KRFTDYRG-FTEQ-------------------
------------------------DDVV-QWFWQCVQ--------------------QWP
AEQRSRLLQF-------ATGTSRIP------------VNG-----FK-DLQGSD--GPRR
FTIEKSGEV-------------------------NQLPKSHTCF-NR-IDLPPY-PSKEV
LENKLV-LAIEE-G---
>Malassezia_globosa_XM_001732780.1 .
VKVRRTHI-FEDSYAEI--------------MRQQPNDLKK-RLMIKFEG-------EDA
LDYGGVSREF---FFLL----SHEMFNPFY--------CLFEYSAHDNY-----------
----------------TLQINPHS------------------------------------
--GINPEHLNYFKFIGRVLGLAIFHRRFLDAHFIV--SFYKMILK---------------
--------KKITLADMES----------------------VDADYHRS--------LQWM

LDN--SIE-GIM--
-EETFSTMEDK---------------------------FGE-MVTIE-LKPG--------
GEHID---
------------------------------VTNENKR-EYVER-MVEWRIVKR-VEEQFR
-AFISG---FSELIPL--DLIN-VFDER-ELELLMGG-----------------------
----MSEID-----------VDDW----KRFTDYRG-FTEQ-------------------
------------------------DDVV-QWFWQCVQ--------------------QWP
AEQRSRLLQF-------ATGTSRIP------------VNG-----FK-DLQGSD--GPRR
FTIEKSGEI-------------------------NQLPKSHTCF-NR-IDLPPY-PSKEV
LENKLV-LAIEE-GMRF
>Malassezia_pachydermatis_XM_018137262.1 .
VKVRRTHI-FEDSYAEI--------------MRQQPNDLKK-RLMIKFEG-------EDA
LDYGGVSREF---FFLL----SHEMFNPFY--------CLFEYSAHDNY-----------
----------------TLQINPHS------------------------------------
--GINPEHLNYFKFIGRVLGLAIFHRRFLDAHFIV--SFYKMILK---------------
--------KKITLSDMES----------------------VDADYYRS--------LQWM
LDN--PIE-GIM--
-EETFSTMEDK---------------------------FGE-MVTIE-LKPG--------
GEHID---
------------------------------VTDENKR-EYVER-MIEWRIVKR-VEEQFR
-AFISG---FSELIPL--DLIN-VFDER-ELELLMGG-----------------------
----MSEID-----------VDDW----KRFTDYRG-FTEQ-------------------
------------------------DNVV-QWFWECVT--------------------NWP
AEQRSRLLQF-------ATGTSRIP------------VNG-----FK-DLQGSD--GPRR
FTIEKSGEI-------------------------NQLPKSHTCF-NR-IDLPPY-PSKEV
LENKLV-LAIEE-GMRF
>Malassezia_caprae_LFFV01000131.1 .
VKVRRTHI-FEDSYAEI--------------MRQQPNDLKK-RLMIKFEG-------EDA
LDYGGVSREF---FFLL----SHEMFNPFY--------CLFEYSAHDNY-----------
----------------TLQINPHS------------------------------------
--GINPEHLNYFKFIGRVLGLAIFHRRFLDAHFIV--SFYKMILK---------------
--------KRITLSDMES----------------------VDADYYRS--------LQWM
LDN--PIA-GVM--
-EETFSTMEDK---------------------------FGE-MVTIE-LTPG--------
GENIE---
------------------------------VTDENKR-DYVER-MVEWRIVKR-VEEQFR
-AFISG---FSELIPL--DLIN-VFDER-ELELLMGG-----------------------
----MSEID-----------VDDW----KRFTDYRG-FTEQ-------------------
------------------------DDVV-QWFWQCIT--------------------QWP
AEQRSRLLQF-------ATGTSRIP------------VNG-----FK-DLQGSD--GPRR
FTIEKSGEI-------------------------NQLPKSHTCF-NR-IDLPPY-PTKEI
LENKLV-LAIEE-G---
>Malassezia_dermatis_LFFX01000099.1 .
VKVRRTHI-FEDSYAEI--------------MRQQPNDLKK-RLMIKFEG-------EDA
LDYGGVSREF---FFLL----SHEMFNPFY--------CLFEYSAHDNY-----------
----------------TLQINPHS------------------------------------
--GINPEHLNYFKFIGRVLGLAIFHRRFLDAHFIV--SFYKMILK---------------
--------KRITLSDMES----------------------VDADYYRS--------LQWM
LDN--PIA-GVM--
-EETFSTMEDK---------------------------FGE-MVTIE-LTPG--------
GENIE---
------------------------------VTDENKR-EYVER-MVEWRIVKR-VEEQFR
-AFISG---FSELIPL--DLIN-VFDER-ELELLMGG-----------------------
----MSEID-----------VDDW----KRFTDYRG-FTEQ-------------------
------------------------DDVV-QWFWQCIT--------------------QWP
AEQRSRLLQF-------ATGTSRIP------------VNG-----FK-DLQGSD--GPRR
FTIEKSGEI-------------------------NQLPKSHTCF-NR-IDLPPY-PSKEI
LENKLV-LAIEE-G---

```
>Malassezia_sympodialis_XM_018884654.1 .
VKVRRTHI-FEDSYAEI--------------MRQQPNDLKK-RLMIKFEG-------EDA
LDYGGVSREF---FFLL----SHEMFNPFY--------CLFEYSAHDNY-----------
----------------TLQINPHS------------------------------------
--GINPEHLNYFKFIGRVLGLAIFHRRFLDAHFIV--SFYKMILK---------------
--------KRITLSDMES----------------------VDADYYRS--------LQWM
LDN--------------------------------------------------PIA-GVM--
-EETFSTMEDK---------------------------FGE-MVTIE-LTPG--------
GENIE-------------------------------------------------------
------------------------------VTDENKR-EYVER-MVEWRIVKR-IEEQFR
-AFISG---FSELIPL--DLIN-VFDER-ELELLMGG-----------------------
----MSEID-----------VDDW----KRFTDYRG-FTEQ-------------------
------------------------DDVV-QWFWQCIT--------------------QWP
AEQRSRLLQF-------ATGTSRIP------------VNG-----FK-DLQGSD--GPRR
FTIEKSGEI-------------------------NQLPKSHTCF-NR-IDLPPY-PSKEI
LENKLV-LAIEE-G---
>Malassezia_nana_LFGD01000073.1 .
VKVRRTHI-FEDSYAEI--------------MRQQPNDLKK-RLMIKFEG-------EDA
LDYGGVSREF---FFLL----SHEMFNPFY--------CLFEYSAHDNY-----------
----------------TLQINPHS------------------------------------
--GINPEHLNYFKFIGRVLGLAIFHRRFLDAHFIV--SFYKMILK---------------
--------KRITLSDMES----------------------VDADYYRS--------LQWM
LDN--------------------------------------------------PIA-GVM--
-EETFSTMEDK---------------------------FGE-MVTIE-LTPG--------
GEHIE-------------------------------------------------------
------------------------------VTDENKR-EYVER-MVEWRIVKR-IEEQFR
-AFISG---FSELIPL--DLIN-VFDER-ELELLMGG-----------------------
----MSEID-----------VDDW----KRFTDYRG-FTEQ-------------------
------------------------DDVV-QWFWQCIT--------------------QWP
AEQRSRLLQF-------ATGTSRIP------------VNG-----FK-DLQGSD--GPRR
FTIEKSGEI-------------------------NQLPKSHTCF-NR-IDLPPY-PTKEI
LENKLV-LAIEE-G---
>Malassezia_equina_LFFY01000052.1 .
VKVRRTHI-FEDSYAEI--------------MRQQPNDLKK-RLMIKFEG-------EDA
LDYGGVSREF---FFLL----SHEMFNPFY--------CLFEYSAHDNY-----------
----------------TLQINPHS------------------------------------
--GINPEHLNYFKFIGRVLGLAIFHRRFLDAHFIV--SFYKMILK---------------
--------KRITLSDMES----------------------VDADYYRS--------LQWM
LDN--------------------------------------------------PIA-GVM--
-EETFSTMEDK---------------------------FGE-MVTIE-LTPG--------
GEHID-------------------------------------------------------
------------------------------VTDENKR-EYVER-MVEWRIVKR-IEEQFR
-AFISG---FSELIPL--DLIN-VFDER-ELELLMGG-----------------------
----MSEID-----------VDDW----KRFTDYRG-FTEQ-------------------
------------------------DDVV-QWFWQCIT--------------------QWP
AEQRSRLLQF-------ATGTSRIP------------VNG-----FK-DLQGSD--GPRR
FTIEKSGEI-------------------------NQLPKSHTCF-NR-IDLPPY-PTKEI
LENKLV-LAIEE-G---
>Tilletia_horrida_LAXH01000002.1 .
MKVRRSHI-FEDSYTQI--------------MMQAPNDLKK-RLMIKFEG-------EDG
LDYGGLSREF---FYLL----SHEMFNPFY--------CLFEYSAQDNY-----------
----------------TLQINPHS------------------------------------
--GINPEHLNYFKFIGRVLGLAIFHRRFLDAYFIV--SFYKMILK---------------
--------KKITLADLEA----------------------VDAEYHRS--------LQWM
LNN--------------------------------------------------DVT-DTV--
-YETFSTVEDT---------------------------FGE-HITVE-LKPG--------
GANIE-------------------------------------------------------
------------------------------VTNENKK-EYVEL-MTEWRISKR-VEEQFK
```

-AFMGG---FTELIPQ--DLIN-VFDER-ELELLIGG-----------------------
----MSEID-----------VDDW----KRFTDYRG-FTEQ-------------------
------------------------DEVV-QWFWQTVR--------------------AWP
PERKSRLLQF-------ATGTSRIP------------VNG-----FK-DLQGSD--GPRR
FTIEKSGEV-------------------------TALPKSHTCF-NR-IDLPPY-SSKED
LERKL------------
>Salmacisia_buchloeana_MOEQ01000150.1 .
MKVRRSHI-FEDSYTQI--------------MMQAPNDLKK-RLMIKFEG-------EDG
LDYGGLSREF---FYLL----SHEMFNPFY--------CLFEYSAQDNY-----------
----------------TLQINPHS------------------------------------
--GINPEHLNYFKFIGRVLGLAIFHRRFLDAYFIV--SFYKMILK---------------
--------KKITLADLEA----------------------VDAEYHRS--------LQWM
LNN--DIT-DAV--
-YETFTTSEDT---------------------------FGE-LVTVE-LKPG--------
GANID---
------------------------------VTNENKK-EYVEL-MTEWRISKR-VEEQFN
-AFMGG---FTELIPQ--DLIN-VFDER-ELELLIGG-----------------------
----MSEID-----------VDDW----KRFTDYRG-FTEQ-------------------
------------------------DDVV-QWFWQTVR--------------------AWP
PERKSRLLQF-------ATGTSRIP------------VNG-----FK-DLQGSD--GPRR
FTIEKAGEV-------------------------TSLPKSHTCF-NR-IDLPPY-STKED
LERKLI-IAVEE-TMG-
>Tilletia_walkeri_LWDG01000334.1 .
MKVRRSHI-FEDSYTQI--------------MMHAPNDLKK-RLMIKFEG-------EDG
LDYGGLSREF---FYLL----SHEMFNPFY--------CLFEYSAQDNY-----------
----------------TLQINPHS------------------------------------
--GINPEHLNYFKFIGRVLGLAIFHRRFLDAYFIV--SFYKMILK---------------
--------KKITLADLEA----------------------VDAEYHRS--------LQWM
LAN--DIT-DAV--
-YETFTTSEDT---------------------------FGE-LVTVD-LKPG--------
GADID---
------------------------------VTNDNKK-EYVEL-MTEWRISKR-VEEQFK
-AFMGG---FTELIPQ--DLIN-VFDER-ELELLIGG-----------------------
----MSEID-----------VDDW----KRFTDYRG-FTEQ-------------------
------------------------DEVV-QWFWAAVR--------------------AWP
PERKSRLLQF-------ATGTSRIP------------VNG-----FK-DLQGSD--GPRR
FTIEKAGEI-------------------------TSLPKSHTCF-NR-IDLPPY-SNKED
LERKLI-IAVEE-TMG-
>Tilletia_controversa_LWDE01000790.1 .
MKVRRSHI-FEDSYTQI--------------MMHAPNDLKK-RLMIKFEG-------EDG
LDYGGLSREF---FYLL----SHEMFNPFY--------CLFEYSAQDNY-----------
----------------TLQINPHS------------------------------------
--GINPEHLNYFKFIGRVLGLAIFHRRFLDAYFIV--SFYKMILK---------------
--------KKITLADLEA----------------------VDAEYHRS--------LQWM
LNN--DIT-DAV--
-FETFTTSEDT---------------------------FGE-LVTVE-LKPG--------
GAEID---
------------------------------VTNENKK-EYVEL-MTEWRISKR-VEEQFK
-AFMGG---FTELIPQ--DLIN-VFDER-ELELLIGG-----------------------
----MSEID-----------VDDW----KRFTDYRG-FTEQ-------------------
------------------------DDVV-QWFWAAVR--------------------SWP
PERKSRLLQF-------ATGTSRIP------------VNG-----FK-DLQGSD--GPRR
FTIEKSGEI-------------------------TSLPKSHTCF-NR-IDLPPY-SNKED
MERKLI-IAVEE-TMG-
>Rhodotorula_toruloides_XM_016416618.1 .
VKVRRTHI-FEDSYAEI--------------MRQTPNDLKK-RLMIKFDG-------EDG
LDYGGLSREF---FFLL----SHEMFNPFY--------CLFEYSAHDNY-----------
----------------TLQINPNS------------------------------------

--SVNPEHLNYFKFIGRVLALAVFHRRFLDAYFIT--ALYKMILK---------------
--------KKITLADMES----------------------VDAEMFRS--------LAWI
LEN--DIT-DVI--
-ENTFSAEDDR---------------------------FGE-TVTID-LKPG--------
GRDIE---
------------------------------VTNENKK-EYVQL-IVEWRIQGR-VKDQFS
-AFLSG---FNELIPQ--ELIN-VFDER-ELELLIGG-----------------------
----MSEID-----------VDDW----VKHTDYRG-FTQS-------------------
------------------------DEVV-QWFWKASAVK------------------SWP
TEKKSRLLQF-------ATGTSRIP------------VNG-----FK-DLQGSD--GPRR
FTLEKSGEI-------------------------TQLPKSHTCF-NR-LDLPPY-PDYET
LEKKLT-IAIEE-TMGF
>Rhodosporidium_toruloides_LK052942.1 .
VKVRRTHI-FEDSYAEI--------------MRQTPNDLKK-RLMIKFDG-------EDG
LDYGGLSREF---FFLL----SHEMFNPFY--------CLFEYSAHDNY-----------
----------------TLQINPNS------------------------------------
--SVNPEHLNYFKFIGRVLALAVFHRRFLDAYFIT--ALYKMILK---------------
--------KKITLADMES----------------------VDAEMFRS--------LAWI
LEN--DIT-DVI--
-ENTFSAEDDR---------------------------FGE-TVTID-LKPG--------
GRDIE---
------------------------------VTNENKK-EYVQL-IVEWRIQGR-VKDQFS
-AFLSG---FNELIPQ--ELIN-VFDER-ELQLLIGG-----------------------
----MSEID-----------VDDW----VKHTDYRG-FTQS-------------------
------------------------DEVV-QWFWQAVK--------------------SWP
TEKKSRLLQF-------ATGTSRIP------------VNG-----FK-DLQGSD--GPRR
FTLEKSGEI-------------------------TQLPKSHTCF-NR-LDLPPY-PDYET
CVVLLT-LCARQ-----
>Rhodotorula_graminis_XM_018418591.1 .
VKVRRTHI-FEDSYAEI--------------MRQTPNDLKK-RLMIKFDG-------EDG
LDYGGLSREF---FFLL----SHEMFNPFY--------CLFEYSAHDNY-----------
----------------TLQINPNS------------------------------------
--SINPEHLNYFKFIGRVLSLAVFHRRFLDAYFIT--ALYKMILK---------------
--------KKITLADMES----------------------VDAEMYRS--------LAWM
LEN--DIT-DVL--
-ENTFSAEDDR---------------------------FGE-TVTID-LKPD--------
GQHIE---
------------------------------VTNDNKK-EYVQL-IVEWRIQRR-VEDQFK
-AFLSG---FNELIPQ--ELIN-VFDER-ELELLIGG-----------------------
----MSEID-----------VDDW----QKHTDYRG-YQQT-------------------
------------------------DEVV-QWFWKAVK--------------------SWP
TEKKSRLLQF-------TTGTSRIP------------VNG-----FK-DLQGSD--GPRR
FTLEKSGEV-------------------------TQLPKSHTCF-NR-LDLPPY-PDYDT
LESKLA-FAIEN-TMGF
>Microbot_lychnidis_dioicae_CZCQ01000165. 1..
VKVRRSHI-FEDSYAEI--------------MRQTPNDLKK-RLMIKFDG-------EDG
LDYGGLSREF---FFLL----SHEMFNPFY--------CLFEYSAHDNY-----------
----------------TLQINPNS------------------------------------
--SINPEHLTYFKFIGRVLSLAVFHRRFLDAYFIT--ALYKMVLK---------------
--------KKITLQDMES----------------------VDAEMFRS--------LTWM
LEN--DIT-DVL--
-DNTFSAEDDR---------------------------FGE-TVTID-LKPG--------
GLDVA---
------------------------------VTNDNKK-EYVQL-VTEWRIQKR-VEDQFK
-AFLSG---FNELIPQ--ELIN-VFDER-ELELLIGG-----------------------
----MSEID-----------VDDW----MKHTDYRG-YTQA-------------------
------------------------DEVV-QWFWKAVK--------------------EWP
AEKKSRLLQF-------TTGTSRIP------------VNG-----FK-DLQGSD--GPRR

FTLEKSGEV-------------------------TQLPKSHTCF-NR-LDLPAY-PSFEV
LEQKLA-FAV-------
>Microbot_violaceum_FBRE01000014.1 .
VKVRRSHI-FEDSYAEI--------------MRQTPNDLKK-RLMIKFDG-------EDG
LDYGGLSREF---FFLL----SHEMFNPFY--------CLFEYSAHDNY-----------
----------------TLQINPNS------------------------------------
--SINPEHLNYFKFIGRVLSLAVFHRRFLDAYFIT--ALYKMVLK---------------
--------KKITLQDMES----------------------VDAEMFRS--------LTWM
LEN--DIT-DVL--
-DNTFSAEDDR---------------------------FGE-TVTIN-LKQG--------
GSDVA---
------------------------------VTNENKK-EYVQL-VTEWRIQKR-VEDQFK
-AFLSG---FNELIPQ--ELIN-VFDER-ELELLIGG-----------------------
----MSEID-----------VDDW----MKHTDYRG-YTQA-------------------
------------------------DEVV-QWFWKAVK--------------------EWP
AEKKSRLLQF-------TTGTSRIP------------VNG-----FK-DLQGSD--GPRR
FTLEKSGEV-------------------------TQLPKSHTCF-NR-LDLPAY-PSFEV
LEQKLA-FAVEN-TLG-
>Mixia_osmundae_XM_014710807.1 .
VKVRRNHI-FEDSYAEI--------------MRQTPNDLKK-RLMIRFDG-------EDG
LDYGGVSREF---FFLL----SHESFNPAY--------GLFEYAAIDSY-----------
----------------TLQINPHS------------------------------------
--GINPEHLNYFKFIGRCIGLAIFHRRFLDAYFIT--SFYKMILN---------------
--------KKIALTDMES----------------------VDAEIFRS--------MQWM
LDN--DVT-DVI--
-ENTFSVEEDK---------------------------FGE-VVITP-LKPG--------
GENIQ---
------------------------------VTEENKR-EYVDL-ITEWRIQRR-VEDQFK
-AFMSG---FNELIPQ--ELIN-VFDER-ELELLIGG-----------------------
----MSEID-----------VDDW----MRHTDYRG-YTQS-------------------
------------------------DPTV-QLFWQAIR--------------------SWP
AEKKSRLLQF-------STGTSRIP------------VNG-----FR-DLQGSD--GPRR
FTIEKSGDAL------------------------TQLPKSHTCF-NR-LDLPPY-TTLQQ
LDDRLT-YSLEN-TIGF
>Puccinia_graminis_XM_003889329.1 .
VKIRRSHI-FEDSYAEI--------------MRQQPNDLKK-RLMIKFDG-------EDG
LDYGGVSREF---FFLL----SHEMFNPFY--------CLFEYSAVDNY-----------
----------------TLQINPHS------------------------------------
--GVNPEHLNYFKFIGRVLALAIFHRRFLDAYFIT--SFYKMILK---------------
--------KKIALADMES----------------------VDAEIFRS--------LTWM
LEN--DIT-DVI--
-ENSFSVEDEK---------------------------FGE-VVTID-LREN--------
GRNIP---
------------------------------VTEANKK-DYIEL-ITQWRIEKR-VADQFK
-AFLSG---FHELIPQ--ELIN-VFDER-ELELLIGG-----------------------
----MSDID-----------VDDW----IKHTDYRG-YQPD-------------------
------------------------DQVI-KWFWQAVR--------------------AWP
AEKKSRLLQF-------TTGTSRIP------------VNG-----FK-DLQGSD--GPRR
FTIEKAGEI-------------------------TQLPKSHTCF-NR-LDLPPY-PNFDQ
LEQKIS-FAIEE-TLGF
>Puccinia_triticina_ADAS01001658.1 .
VKIRRSHI-FEDSYAEI--------------MRQQPNDLKK-RLMIKFDG-------EDG
LDYGGVXREF---FFLL----SHEMFNPFY--------CLFEYSAVDNY-----------
----------------TQQINPHS------------------------------------
--GVNPEHLNYFKFIGRVLALAIXXX-FLDAYFIT--SFYKMILK---------------
--------KKIALADMES----------------------VDAEIFRS--------LTWM
LXN--DIT-DVI--
-ENSFSVEDEK---------------------------FGE-VVTID-LKEN--------

GRNMP---
------------------------------VTEENKK-EYIEL-ITQWRIEKR-VVEQFR
-AFLSG---FHELIPQ--ELIN-VFDER-ELELLIGG-----------------------
----MSDID-----------VDDW----IKHTDYRG-YQPD-------------------
------------------------DQVI-KWFWQAVR--------------------AWP
AEKKSRLLQF-------TTGXSRIP------------VNG-----FK-DLQGSD--GPRR
FTIEKAGEI-------------------------TQLPKSHTCF-NR-LDLPPY-PNFD-

>Cronartium_ribicola_GBSG01001506.1 .
IKIRRSHI-FEDSYAEI--------------MRQQPNDLKK-RLMIKFDG-------EDG
LDYGGVSREF---FFLL----SHEMFNPFY--------CLFEYSAVDNY-----------
----------------TLQINPHS------------------------------------
--GVNPEHLNYFKFIGRVLALAIFHRRFLDAYFIT--SFYKMILK---------------
--------KRIALADMES---------------------VDAEIFRS---------LSWM
LDN--DIT-DVI--
-ENTFSVEDEK---------------------------FGE-VVTID-LKEE--------
GRDIP---
------------------------------VTEENKK-EYIDL-ITQWRIERR-VADQFK
-AFLTG---FNELIPQ--ELIN-VFDER-ELELLIGG-----------------------
----MSDID-----------VDDW----IKHTDYRG-YQQD-------------------
------------------------DQVI-KWFWQAVR--------------------AWP
AEKKSRLLQF-------TTGTSRIP------------VNG-----FK-DLQGSD--GPRR
FTIEKAGEI-------------------------SQLPKSHTCF-NR-LDLPPY-PNFDQ
LEQKIT-FAIEE-TLGF
>Melampsora_larici_populina_XM_007406701.1 .
VKIRRSHI-FEDSYAEI--------------MRQQPNDLKK-RLMIKFDG-------EDG
LDYGGVSREF---FFLL----SHEMFNPFY--------CLFEYSAVDNY-----------
----------------TLQINPHS------------------------------------
--GVNPEHLNYFKFIGRVLALAIFHRRFLDAYFIT--SFYKMILK---------------
--------KRIQLADMES---------------------VDAEIFRS---------LSWM
LDN--DIT-DVI--
-ENSFSVEDEK---------------------------FGE-VVTID-LKED--------
GRNIP---
------------------------------VTEENKK-EYIDL-ITQWRIEKR-VVDQFK
-AFLTG---FNELIPQ--DLIN-VFDER-ELELLIGG-----------------------
----MSDID-----------VDDW----IKHTDYRG-YQQD-------------------
------------------------DQVI-KWFWQAVR--------------------AWP
AEKKSRLLQF-------TTGTSRIP------------VNG-----FK-DLQGSD--GPRR
FTIEKAGEI-------------------------TQLPKSHTCF-NR-LDLPPY-PNFDQ
LEQKIT-FAIEE-TLGF
>Wallemia_sebi_XM_006955935.1 .
IKVRRNFI-FEDSYAEI--------------MRQTPNDLKK-RLMIKFEG-------EDG
LDYGGVSREF---FFLL----SHELFSPLY--------CLFEYSAHDNY-----------
----------------TLQINPNS------------------------------------
--SINPEHLNYFKFIGRCVGLAIFHRRFLDAYFIT--SFYKMILN---------------
--------KRIGLQDLES---------------------VDAELHRS---------MSWI
LDN--DIT-DIL--
-DNNFVADVET---------------------------FGE-IQSVP-LKEG--------
GEDIE---
------------------------------LNESNKK-EYVDL-MVQFRIVTR-VKDQFE
-AFMSG---FKELIPQ--DLIN-VFDER-ELELLIGG-----------------------
----MAEID-----------IDDW----KKHTDYRG-YTES-------------------
------------------------DEVV-QWFWNAIK--------------------SWP
TEKKSRLLQF-------TTGTSRIP------------VNG-----FK-DLQGSD--GPRR
FTIEKAGEI-------------------------NHLPKSHTCF-NR-IDLPPY-RNFEA
LEQKLS-TAVEE-TLGF
>NWGS3B_2_Wallemia_mellicola_AFQX01000003.1 .
IKVRRNFI-FEDSYAEI--------------MRQTPNDLKK-RLMIKFEG-------EDG

```
LDYGGVSREF---FFLL----SHELFSPLY--------CLFEYSAHDNY-----------
----------------TLQINPNS------------------------------------
--SINPEHLNYFKFIGRCVGLAIFHRRFLDAYFIT--SFYKMILN---------------
--------KRIGLQDLES----------------------VDAELHRS--------MSWI
LDN--------------------------------------------DIT-DIL--
-DNNFVADVET---------------------------FGE-IQSVP-LKEG--------
GEDIE-------------------------------------------------------
------------------------------LNESNKK-EYVDL-MVQFRIVTR-VKDQFE
-AFMSG---FKELIPQ--DLIN-VFDER-ELELLIGG-----------------------
----MAEID-----------IDDW----KKHTDYRG-YTES-------------------
------------------------DEVV-QWFWNAIK--------------------SWP
TEKKSRLLQF-------TTGTSRIP------------VNG-----FK-DLQGSD--GPRR
FTIEKAGEI-------------------------NHLPKSHTCF-NR-VSF---------
-----------------
>Wallemia_ichthyophaga_XM_009268008.1 .
IKVRRNHI-FEDSYAEI--------------MRQTPNDLKK-RLMIKFEG-------EDG
LDYGGVSREF---FFLL----SHELFSPLY--------CLFEYSAHDNY-----------
----------------TLQINPNS------------------------------------
--SINPEHLNYFKFIGRCVGLAIFHRRFLDAYFIT--SLYKMILN---------------
--------KRIGLQDLES----------------------VDAELHRS--------MSWI
LDN--------------------------------------------DIT-DIL--
-DNNFVADVET---------------------------FGE-IQSVP-LKEG--------
GEDVE-------------------------------------------------------
------------------------------LNESNKK-EYVDL-MVQFRIVTR-VKDQFE
-AFMSG---FKELIPQ--DLIN-VFDER-ELELLIGG-----------------------
----MAEID-----------IEDW----KKHTDYRG-YTES-------------------
------------------------DQVV-QWFWNVVK--------------------NWP
TEKKSRLLQF-------TTGTSRIP------------VNG-----FK-DLQGSD--GPRR
FTIEKAGEI-------------------------NHLPKSHTCF-NR-IDLPPY-RNFEA
LDQKLS-TAVEE-TLGF
>NWGS3B_2_Trichosporon_gracile_BCJO01000003.1 .
IKVSRDNI-FEGSYTEI--------------MRQSPSDLKK-RLMVKFEG-------EEG
LDYGGLSREF---FFLL----SHEMFNPFY--------CLFEYSAHDNY-----------
----------------TLQINPNS------------------------------------
--GVNPEHLNYFKFIGRVVGLGIFHRRFLDAYFIS--SFYKMILK---------------
--------KKITLTDLES----------------------VDAGLHRG--------LTWM
LEN--------------------------------------------DIT-DII--
-EDTFSITEEH---------------------------FGE-VVTVD-LKEG--------
GQNIE-------------------------------------------------------
------------------------------VTEDNKK-DYVDL-VTEYRISRR-VSEQFE
-AFMSG---FNELIPQ--ELIS-VFDER-ELELLIGG-----------------------
----MSEID-----------VDDW----QKHTDYRG-YNPS-------------------
------------------------DEVV-EWFWKIVR--------------------SWP
AERKARLLQF-------TTGTSRIP------------VNG-----FK-DLQGSD--GPRR
FTIEKAGEI-------------------------TQLPKSHTCF-NR-IDLPAY-KSYDT
LEQKLT-LA--------
>Trichosporon_laibachii_BCKV01000004.1 .
IKVSRDNI-FEGSYTEI--------------MRQSPNDLKK-RLMVKFEG-------EEG
LDYGGLSREF---FFLL----SHEMFNPFY--------CLFEYSAHDNY-----------
----------------TLQINPNS------------------------------------
--GVNPEHLNYFKFIGRVVGLGIFHRRFLDAYFIS--SFYKMILK---------------
--------KKITLTDLES----------------------VDAGLHRG--------LTWM
LDN--------------------------------------------DIT-DII--
-EDTFSITEEH---------------------------FGE-VVTVD-LKEN--------
GQNIE-------------------------------------------------------
------------------------------VTEDNKK-DYVDL-VTEYRISRR-VSEQFE
-AFMSG---FNELIPQ--ELIS-VFDER-ELELLIGG-----------------------
----MSEID-----------VDDW----QKHTDYRG-YNPS-------------------
```

------------------------DEVV-EWFWKIVR--------------------SWP
AERKARLLQF-------TTGTSRIP------------VNG-----FK-DLQGSD--GPRR
FTIEKAGEL-------------------------TQLPKSHTCF-NR-IDLPAY-KSYDT
LEQKLT-LA--------
>Trichosporon_brassicae_BCJI01000008.1 .
IKVSRDNI-FEGSYTEI--------------MRQSPNDLKK-RLMVKFEG-------EEG
LDYGGLSREF---FFLL----SHEMFNPFY--------CLFEYSAHDNY-----------
----------------TLQINPNS------------------------------------
--GVNPEHLNYFKFIGRVVGLGIFHRRFLDAYFIS--SFYKMILK---------------
--------KKITLTDLES----------------------VDAGLHRG--------LTWM
LEN--DIT-DII--
-EDTFSITEEH---------------------------FGE-VVTVD-LMEN--------
GQNIE---
------------------------------VNETNKD-KYVDL-VTEYRISRR-VSEQFE
-AFMSG---FNELIPQ--ELIS-VFDER-ELELLIGG-----------------------
----MSEID-----------VDDW----QKHTDYRG-YNPS-------------------
------------------------DEVV-EWFWQIVR--------------------SWP
AERKARLLQF-------TTGTSRIP------------VNG-----FK-DLQGSD--GPRR
FTIEKAGEI-------------------------TQLPKSHTCF-NR-IDLPAY-KSYDT
LEQKLT-LA--------
>Trichosporon_domesticum_BCFW01000009.1 .
IKVSRDNI-FEGSYTEI--------------MRQSPNDLKK-RLMVKFEG-------EEG
LDYGGLSREF---FFLL----SHEMFNPFY--------CLFEYSAHDNY-----------
----------------TLQINPNS------------------------------------
--GVNPEHLNYFKFIGRVVGLGIFHRRFLDAYFIS--SFYKMILK---------------
--------KKITLTDLES----------------------VDSALHRG--------LTWM
LEN--DIT-DII--
-EDTFSITEEH---------------------------FGE-VVTVD-LKEN--------
GQNID---
------------------------------VTEENKK-DYVDL-VTEYRISRR-VSEQFE
-AFMSG---FNELIPQ--ELIS-VFDER-ELELLIGG-----------------------
----MSEID-----------VDDW----QKHTDYRG-YNPS-------------------
------------------------DEVV-EWFWKIVR--------------------SWP
AERKARLLQF-------TTGTSRIP------------VNG-----FK-DLQGSD--GPRR
FTIEKAGEI-------------------------TQLPKSHTCF-NR-IDLPAY-KSYDT
LEQKLT-LA--------
>Cutaneotrichosp_cutaneum_LTAL01000814.1 .
IKVSRDNI-FEGSFTEI--------------MRQSPTDLKK-RLMIKFEG-------EEG
LDYGGLSREF---FFLL----SHEMFNPFY--------CLFEYSAHDNY-----------
----------------TLQINPNS------------------------------------
--GVNPEHLNYFKFIGRVVGLGIFHRRFLDAYFIS--SFYKMILK---------------
--------KKITLTDLES----------------------VDAGLHRG--------LTWM
LEN--DIT-DII--
-EDTFSITEEH---------------------------FGE-VITVD-LKEN--------
GHNID---
------------------------------VTEENKK-EYVDL-VTEYRISRR-VSEQFE
-AFMSG---FNELIPQ--ELIS-VFDER-ELELLIGG-----------------------
----MSEID-----------VDDW----QKHTDYRG-YNPS-------------------
------------------------DEVV-EWFWKIVR--------------------SWP
AERKARLLQF-------TTGTSRIP------------VNG-----FK-DLQGSD--GPRR
FTIEKAGEI-------------------------TQLPKSHTCF-NR-IDLPAY-KSYDT
LEQKLT-LA--------
>Trichosporon_veenhuisii_BCKJ01000006.1 .
IKVSRDNI-FEGSFTEI--------------MRQSPTDLKK-RLMVKFEG-------EEG
LDYGGLSREF---FFLL----SHEMFNPFY--------CLFEYSAHDNY-----------
----------------TLQINPNS------------------------------------
--GVNPEHLNYFKFIGRVVGLGIFHRRFLDAYFIS--SFYKMILK---------------
--------KKITLTDLES----------------------VDAGLHRG--------LTWM

LEN--DIT-DII--
-EDTFSITEEH---------------------------FGE-VVTVD-LKEN--------
GHNIE---
------------------------------VTEENKK-EYVDL-VTEYRISRR-VSEQFE
-AFMSG---FNELIPQ--ELIS-VFDER-ELELLIGG-----------------------
----MSEID-----------VDDW----QKHTDYRG-YNPS-------------------
------------------------DEVV-EWFWKIVR--------------------SWP
AERKARLLQF-------TTGTSRIP------------VNG-----FK-DLQGSD--GPRR
FTIEKAGEI-------------------------TQLPKSHTCF-NR-IDLPAY-KSYDT
LEQKLT-LA--------
>Trichosporon_porosum_BCJG01000001.1 .
IKVSRDNI-FEGSYTEI--------------MRQSPTDLKK-RLMVKFEG-------EDG
LDYGGLSREF---FFLL----SHEMFNPFY--------CLFEYSAHDNY-----------
----------------TLQINPNS------------------------------------
--GVNPEHLNYFKFIGRVVGLGIFHRRFLDAYFIS--SFYKMILK---------------
--------KKISLPDLES----------------------VDAGLHRG--------LTWM
LEN--DIT-DII--
-EDTFSITEEH---------------------------FGE-VVTVD-LKDG--------
GRDVE---
------------------------------VTELNKK-DYVDL-VTEYRIERR-VSEQFE
-AFMSG---FNELIPQ--ELIS-VFDER-ELELLIGG-----------------------
----MSEID-----------VDDW----QKHTDYRG-YNPS-------------------
------------------------DEVV-EWFWKIVR--------------------SWP
AERKARLLQF-------TTGTSRIP------------VNG-----FK-DLQGSD--GPRR
FTIEKAGEI-------------------------TQLPKSHTCF-NR-IDLPAY-KSYDT
LEQKLT-LA--------
>Trichosporon_gamsii_BCJN01000011.1 .
IKITRDNI-FEGAYTEI--------------MRQSPVELKK-RIMIKFEG-------EDG
LDYGGLSREF---FFLL----SHEMFNPFY--------CLFEYSAHDNY-----------
----------------TLQINPNS------------------------------------
--GVNPEHLNYFKFIGRVVGLGIFHRRYLDAYFIS--SFYKMILK---------------
--------KKISLPDLES----------------------VDAGLHRG--------LTWM
LEN--DIT-DII--
-EDTFSITEEH---------------------------FGE-VVTVD-LKDG--------
GRDVE---
------------------------------VTEDNKK-DYVDL-VTEYRIERR-VHEQFE
-AFMSG---FNELIPQ--ELIS-VFDER-ELELLIGG-----------------------
----MSEID-----------VADW----QKHTDYRG-YNPS-------------------
------------------------DEVV-EWFWKIVR--------------------SWP
AERKARLLQF-------TTGTSRIP------------VNG-----FK-DLQGSD--GPRR
FTIEKAGEI-------------------------TQLPKSHTCF-NR-IDLPAY-KSYDA
LEQKLT-LAVDE-----
>Tremella_mesenterica_XM_007007453.1 .
IKVSRENI-FEGSYTEI--------------MRQSPNDLKK-RLMIKFEG-------EDG
LDYGGLSREF---FFLL----SHEMFNPFY--------CLFEYSAHDNY-----------
----------------TLQINPNS------------------------------------
--GVNPEHLNYFKFIGRVVGLGIFHRRFLDAYFIV--SFYKMILK---------------
--------KKISLPDLES----------------------VDAGLFRG--------LMWM
LEN--DIT-DVI--
-EDTFSISEEH---------------------------FGE-LVTVD-LKEN--------
GRDIE---
------------------------------VTEENKK-EYVDL-VTEYRISRR-VAEQFE
-AFMSG---FNELIPQ--ELIN-VFDER-ELELLIGG-----------------------
----MSEID-----------VDDW----QKHTDYRG-YNPS-------------------
------------------------DEVV-EWFWKIVK--------------------AWP
AERKSRLLQF-------TTGTSRIP------------VNG-----FK-DLQGSD--GPRR
FTIEKAGEI-------------------------TQLPKSHTCF-NR-IDLPAY-KSYDV
LEQKLT-IAVEE-TLGF

>Vanrija_humicola_BCJF01000001.1 .
IKVSRENI-FEGSYTEI--------------MRQSPNDLKK-RLMIKFEG-------EDG
LDYGGLSREF---FFLL----SHEMFNPFY--------CLFEYSAHDNY-----------
----------------TLQINPNS------------------------------------
--GVNPEHLNYFKFIGRVVGLGIFHRRFLDAYFIV--SFYKMILK---------------
--------KKITLADLES----------------------VDAGLHRG--------LTWM
LEN--DIT-DVI--
-EDTFSISEEH---------------------------FGE-LVTID-LKPG--------
GRDIE---
------------------------------VTEENKK-DYVNY-VTEYRISRR-VSEQFE
-AFMSG---FNELIPQ--ELIN-VFDER-ELELLIGG-----------------------
----MSEID-----------VDDW----QKHTDYRG-YNPS-------------------
------------------------DEVV-EWFWKIVR--------------------SWP
AERKSRLLQF-------TTGTSRIP------------VNG-----FK-DLQGSD--GPRR
FTIEKAGEI-------------------------TQLPKSHTCF-NR-IDLPAY-KSYDT
LEQKLT-LAVEE-TLG-
>Trichosporon_cutaneum_BCKU01000005.1 .
IKVSRENI-FEGSYTEI--------------MRQSPNDLKK-RLMIKFEG-------EDG
LDYGGLSREF---FFLL----SHEMFNPFY--------CLFEYSAHDNY-----------
----------------TLQINPNS------------------------------------
--GVNPEHLNYFKFIGRVVGLGIFHRRFLDAYFIV--SFYKMILK---------------
--------KKITLADLES----------------------VDAELFRG--------LTWM
LEN--SID-GVI--
-EDTFSITEEH---------------------------FGE-LVTID-LKED--------
GHKID---
------------------------------VTEENKK-DYVDL-VTEYRISRR-VKEQFE
-AFMSG---FNELIPQ--ELIN-VFDER-ELELLIGG-----------------------
----MSEID-----------VDDW----QKHTDYRG-YNPS-------------------
------------------------DEVV-EWFWKIVR--------------------SWP
AERKSRLLQF-------TTGTSRIP------------VNG-----FK-DLQGSD--GPRR
FTIEKAGEI-------------------------TQLPKSHTCF-NR-IDLPAY-KSYDT
LEQKLT-LA--------
>Cutaneotrichosp_cutaneum_LRUG01000048.1 .
IKVSRENI-FEGSYTEI--------------MRQSPNDLKK-RLMIKFEG-------EDG
LDYGGLSREF---FFLL----SHEMFNPFY--------CLFEYSAHDNY-----------
----------------TLQINPNS------------------------------------
--GVNPEHLNYFKFIGRVVGLGIFHRRFLDAYFIV--SFYKMILK---------------
--------KKITLADLES----------------------VDAGLFRG--------LTWM
LEN--DIT-DVI--
-EDTFSITEEH---------------------------FGE-VVTID-LKDG--------
GHEID---
------------------------------VTEDNKK-EYVDL-VTEYRISRR-VKEQFE
-AFMSG---FNELIPQ--ELIN-VFDER-ELELLIGG-----------------------
----MSEID-----------VDDW----QKHTDYRG-YNPS-------------------
------------------------DEVV-EWFWKIVR--------------------SWP
AERKSRLLQF-------TTGTSRIP------------VNG-----FK-DLQGSD--GPRR
FTIEKAGEI-------------------------TQLPKSHTCF-NR-IDLPAY-KSYDT
LEQKLT-LA--------
>Cutaneotrichosp_oleaginosus_JZUH01000132. 1..
IKVSRENI-FEGSYTEI--------------MRQSPNDLKK-RLMIKFEG-------EDG
LDYGGLSREF---FFLL----SHEMFNPFY--------CLFEYSAHDNY-----------
----------------TLQINPNS------------------------------------
--GVNPEHLNYFKFIGRVVGLGIFHRRFLDAYFIV--SFYKMILK---------------
--------KKITLADLES----------------------VDAGLFRG--------LTWM
LEN--DIT-DVI--
-EDTFSITEEH---------------------------FGE-VVTID-LKEG--------
GREID---
------------------------------VTEENKK-DYVDL-VTEYRISRR-VKEQFE

-AFMSG---FNELIPQ--ELIN-VFDER-ELELLIGG----------------------
----MSEID-----------VDDW----QKHTDYRG-YNPS-------------------
------------------------DEVV-EWFWKIVR--------------------SWP
AERKSRLLQF-------TTGTSRIP------------VNG-----FK-DLQGSD--GPRR
FTIEKAGEV-------------------------TQLPKSHTCF-NR-IDLPAY-KSYDA
LEQKLT-LA--------
>Trichosporon_oleaginosus_XM_018424394.1 .
IKVSRENI-FEGSYTEI--------------MRQSPNDLKK-RLMIKFEG-------EDG
LDYGGLSREF---FFLL----SHEMFNPFY--------CLFEYSAHDNY-----------
----------------TLQINPNS------------------------------------
--GVNPEHLNYFKFIGRVVGLGIFHRRFLDAYFIV--SFYKMILK---------------
--------KKITLADLES----------------------VDAGLFRG--------LTWM
LEN---DIT-DVI--
-EDTFSITEEH---------------------------FGE-VVTID-LKEG--------
GREID---
------------------------------VTEENKK-DYVDL-VTEYRISRR-VKEQFE
-AFMSG---FNELIPQ--ELIN-VFDER-ELELLIGG----------------------
----MSEID-----------VDDW----QKHTDYRG-YNPS-------------------
------------------------DEVV-EWFWKIVR--------------------SWP
AERKSRLLQF-------TTGTSRIP------------VNG-----FK-DLQGSD--GPRR
FTIEKAGEV-------------------------TQLPKSHTCF-NR-IDLPAY-KSYDA
LEQKLT-LAVEE-TVGF
>Kwoniella_bestiolae_XM_019189408.1 .
IKVSRDNI-FEGSYTEI--------------MRQTPNDLKK-RLMIKFEG-------EDG
LDYGGLSREF---FFLL----SHEMFNPFY--------CLFEYSAHDNY-----------
----------------TLQINPNS------------------------------------
--GVNPEHLNYFKFIGRVVGLGIFHRRFLDAYFIV--SFYKMILK---------------
--------KKIALPDLES----------------------VDSGLHRG--------LTWM
LEN---DIT-DVI--
-EDTFSITEEH---------------------------FGE-VVTVD-LKED--------
GRNIE---
------------------------------VTEDNKK-DYVDL-VTEYRISRR-VSEQFD
-AFMSG---FNELIPQ--ELIN-VFDER-ELELLIGG----------------------
----MSEID-----------VDDW----MKHTDYRG-YNPS-------------------
------------------------DEVV-EWFWKIVR--------------------GWP
AERKSRLLQF-------TTGTSRIP------------VNG-----FK-DLQGSD--GPRR
FTIEKAGEP-------------------------TQLPKSHTCF-NR-IDLPAY-KTYEA
LEQKLT-IAVEE-TLGF
>Kwoniella_dejecticola_XM_018404285.1 .
IKVSRDNI-FEGSYTEI--------------MRQTPNDLKK-RLMIKFEG-------EDG
LDYGGLSREF---FFLL----SHEMFNPFY--------CLFEYSAHDNY-----------
----------------TLQINPNS------------------------------------
--GVNPEHLNYFKFIGRVVGLGIFHRRFLDAYFIV--SFYKMILK---------------
--------KKITLQDLES----------------------VDSGLHRG--------LTWM
LEN---DIT-DVI--
-EDTFSITEEH---------------------------FGE-VVTVD-LKDD--------
GRNIE---
------------------------------VTEDNKK-DYVDL-VTEYRISRR-VSEQFD
-AFMSG---FNELIPQ--ELIN-VFDER-ELELLIGG----------------------
----MSEID-----------VDDW----MKHTDYRG-YNPS-------------------
------------------------DEVV-EWFWKIVR--------------------GWP
AERKSRLLQF-------TTGTSRIP------------VNG-----FK-DLQGSD--GPRR
FTIEKAGEP-------------------------TQLPKSHTCF-NR-IDLPAY-KTYEA
LEQKLT-IAVEE-TLGF
>Kwoniella_mangroviensis_XM_019148046.1 .
IKVSRDNI-FEGSYTEI--------------MRQTPNDLKK-RLMIKFEG-------EDG
LDYGGLSREF---FFLL----SHEMFNPFY--------CLFEYSAHDNY-----------
----------------TLQINPNS------------------------------------

```
--GVNPEHLNYFKFIGRVVGLGIFHRRFLDAYFIV--SFYKMILK---------------
--------KKITLQDLES---------------------VDSGLHRG--------LTWM
LEN-------------------------------------------------DIT-DVI--
-EDTFSITEEH---------------------------FGE-VVTVD-LKED--------
GRNIE-------------------------------------------------------
------------------------------VTEENKK-DYVDL-VTEYRISRR-VSEQFD
-AFMSG---FNELIPQ--ELIN-VFDER-ELELLIGG-----------------------
----MSEID-----------VDDW----MKHTDYRG-YNPS-------------------
------------------------DEVV-EWFWKIVR--------------------GWP
AERKSRLLQF-------TTGTSRIP------------VNG-----FK-DLQGSD--GPRR
FTIEKAGEP-------------------------TQLPKSHTCF-NR-IDLPAY-KTYEA
LEQKLT-IAVEE-TLGF
>Kwoniella_pini_XM_019152448.1 .
IKVSRDNI-FEGSYTEI--------------MRQTPNDLKK-RLMIKFEG-------EDG
LDYGGLSREF---FFLL----SHEMFNPFY--------CLFEYSAHDNY-----------
----------------TLQINPNS------------------------------------
--GVNPEHLNYFKFIGRVVGLGIFHRRFLDAYFIV--SFYKMILK---------------
--------KKITLQDLES---------------------VDSGLHRG--------LTWM
LEN-------------------------------------------------DIT-DVI--
-EDTFSITEEH---------------------------FGE-VVTVD-LKEG--------
GRDIE-------------------------------------------------------
------------------------------VTEDNKK-DYVDL-VTEYRISRR-VSEQFE
-AFMSG---FNELIPQ--ELIN-VFDER-ELELLIGG-----------------------
----MSEID-----------VDDW----MKHTDYRG-YNPS-------------------
------------------------DEVV-EWFWKIVR--------------------GWP
AERKSRLLQF-------TTGTSRIP------------VNG-----FK-DLQGSD--GPRR
FTIEKAGEP-------------------------TQLPKSHTCF-NR-IDLPAY-KTYEA
LEQKLT-IAVEE-TLGF
>Tsuchiyaea_wingfieldii_XM_019178683.1 .
MKVSRENI-FEGSYTEI--------------MRQTPSDLKK-RLMIKFEG-------EDG
LDYGGLSREF---FFLL----SHEMFNPFY--------CLFEYSAHDNY-----------
----------------TLQINPNS------------------------------------
--GVNPEHLNYFKFIGRVVGLAIFHRRFLDAYFIV--SFYKMILG---------------
--------KKIALPDLES---------------------VDAGLFRG--------LTWM
LEN-------------------------------------------------DIT-GVI--
-EDTFSITEEH---------------------------FGE-VVTVD-LKEG--------
GRDVE-------------------------------------------------------
------------------------------VTQDNKK-DYVDL-VTEYRISRR-VSEQFQ
-AFMSG---FNELIPQ--ELIN-VFDER-ELELLIGG-----------------------
----MSEID-----------VDDW----QKHTDYRG-YNPS-------------------
------------------------DEVV-EWFWKCVR--------------------SWP
PERKSRLLQF-------TTGTSRIP------------VNG-----FK-DLQGSD--GPRR
FTIEKAGEV-------------------------TQLPKSHTCF-NR-IDLPAY-KSYDA
LEQKLT-IAVEE-TLGF
>Cryptococcus_amylolentus_XM_019140074.1 .
MKVSRENI-FEGSYTEI--------------MRQTPSDLKK-RLMIKFEG-------EDG
LDYGGLSREF---FFLL----SHEMFNPFY--------CLFEYSAHDNY-----------
----------------TLQINPNS------------------------------------
--GVNPEHLNYFKFIGRVVGLAIFHRRFLDAYFIV--SFYKMILG---------------
--------KKIALPDLES---------------------VDAGLFRG--------LTWM
LEN-------------------------------------------------DIT-GVI--
-EDTFSITEEH---------------------------FGE-VVTVD-LKDG--------
GRDIE-------------------------------------------------------
------------------------------VTQDNKK-DYVDL-VTEYRISRR-VSEQFQ
-AFMSG---FNELIPQ--ELIN-VFDER-ELELLIGG-----------------------
----MSEID-----------VDDW----QKHTDYRG-YNPS-------------------
------------------------DEVV-EWFWKCVR--------------------SWP
PERKSRLLQF-------TTGTSRIP------------VNG-----FK-DLQGSD--GPRR
```

FTIEKAGEV-------------------------TQLPKSHTCF-NR-IDLPAY-KSYDA
LEQKLT-IAVEE-TLGF
>Cryptococcus_gattii_XM_003195518.1 .
MKVSRDNI-FEGSFTEI--------------MRQTPNDLKK-RLMIKFEG-------EDG
LDYGGLSREF---FFLL----SHEMFNPFY--------CLFEYSAHDNY-----------
----------------TLQINPNS------------------------------------
--GVNPEHLNYFKFIGRVVGLGIFHRRFLDAYFIV--SFYKMILG---------------
--------KKIALQDLES----------------------VDAGLFRG--------LTWM
LEN--DIT-GVI--
-EDTFSITEEH---------------------------FGE-VVTVD-LKPG--------
GRDVE---
------------------------------VTEDNKK-DYVDL-VTEYRISKR-VSEQFQ
-AFMSG---FNELIPQ--ELIN-VFDER-ELELLIGG-----------------------
----MSEID-----------VDDW----QKHTDYRG-YNPS-------------------
------------------------DEVV-EWFWKIVK--------------------NWP
AEKKSRLLQF-------TTGTSRIP------------VNG-----FK-DLQGSD--GPRR
FTIEKAGEV-------------------------TQLPKSHTCF-NR-IDLPAY-KSYEA
LEQKLT-IAVEE-TVGF
>Cryptococcus_neoformans_XM_767448.1 .
MKVSRDNI-FEGSYTEI--------------MRQTPNDLKK-RLMIKFEG-------EDG
LDYGGLSREF---FFLL----SHEMFNPFY--------CLFEYSAHDNY-----------
----------------TLQINPNS------------------------------------
--GVNPEHLNYFKFIGRVVGLGIFHRRFLDAYFIV--SFYKMILG---------------
--------KKIALQDLES----------------------VDAGLFRG--------LTWM
LEN--DIT-GVI--
-EDTFSITEEH---------------------------FGE-VVTVD-LKPG--------
GRDVE---
------------------------------VTEDNKK-DYVDL-VTEYRISKR-VSEQFQ
-AFMSG---FNELIPQ--ELIN-VFDER-ELELLIGG-----------------------
----MSEID-----------VDDW----QKHTDYRG-YNPS-------------------
------------------------DEVV-EWFWKIVK--------------------NWP
AEKKSRLLQF-------TTGTSRIP------------VNG-----FK-DLQGSD--GPRR
FTIEKAGEV-------------------------TQLPKSHTCF-NR-IDLPAY-KSYEA
LEQKLT-IAVEE-TVGF
>Cryptococcus_neoformans_XM_012198185.1 .
MKVSRDNI-FEGSYTEI--------------MRQTPNDLKK-RLMIKFEG-------EDG
LDYGGLSREF---FFLL----SHEMFNPFY--------CLFEYSAHDNY-----------
----------------TLQINPNS------------------------------------
--GVNPEHLNYFKFIGRVVGLGIFHRRFLDAYFIV--SFYKMILG---------------
--------KKIALQDLES----------------------VDAGLFRG--------LTWM
LEN--DIT-GVI--
-EDTFSITEEH---------------------------FGE-VVTVD-LMPG--------
GRDVE---
------------------------------VTEDNKK-DYVDL-VTEYRISKR-VSEQFQ
-AFMSG---FNELIPQ--ELIN-VFDER-ELELLIGG-----------------------
----MSEID-----------VDDW----QKHTDYRG-YNPS-------------------
------------------------DEVV-EWFWKIVK--------------------NWP
AEKKSRLLQF-------TTGTSRIP------------VNG-----FK-DLQGSD--GPRR
FTIEKAGEV-------------------------TQLPKSHTCF-NR-IDLPAY-KSYEA
LEQKLT-IAVEE-TVGF
>Trichosporon_guehoae_BCJX01000016.1 .
IKVSRENI-FEGSYTEI--------------MRQSPNDLKK-RLMIKFEG-------EDG
LDYGGLSREF---FFLL----SHEMFNPFY--------CLFEYSAHDNY-----------
----------------TLQINPNS------------------------------------
--GVNPEHLNYFKFIGRVVGLGIFHRRFLDAYFIV--SFYKMILK---------------
--------KRITLADLES----------------------VDAGLYRG--------LTWM
LEN--DIT-DVI--
-EDTFSITEEH---------------------------FGE-VVVLD-LKPG--------

GQEVE---
------------------------------VTEENKK-EYVEL-VTEYRISRR-VSEQFE
-AFMSG---FNELIPQ--DLIN-VFDER-ELELLIGG-----------------------
----MSEID-----------VDDW----KKHTDYRG-YNPS-------------------
------------------------DEVV-EWFWKIVR--------------------GWP
AERKSRLLQF-------TTGTSRIP------------VNG-----FK-DLQGSD--GPRR
FTIEKAGEL-------------------------TQLPKSHTCF-NR-IDLPPY-KSYDT
LEQKLT-LA--------
>Cryptococcus_curvatus_BCJH01000011.1 .
IKVSRESL-FEGAYSEV--------------MRQTPTDLKK-RLMVKFEG-------EDG
LDYGGPSREF---FFLL----SHEMFNPFY--------CLFEYSAHDNY-----------
----------------TLQINPNS------------------------------------
--GVNPEHLKYFKFIGRCVGLGIFHRRFLDAYFIV--SFYKMILN---------------
--------KKITIADLES----------------------VDAGLYRG--------LMWM
LDN---DIT-DVI--
-EDTFSITEEH---------------------------FGE-VVTVD-LKEG--------
GQHID---
------------------------------VTEENKK-EYVEL-VTEYRISRR-VKEQFE
-AFMSG---FNELIPQ--ALIN-VFDER-ELELLIGG-----------------------
----MSEID-----------VDDW----QKHTDYRG-YSPA-------------------
------------------------DEVV-EWFWKIVR--------------------SWP
AERKSRLLQF-------TTGTSRIP------------VNG-----FK-DLQGSD--GPRR
FTIEKAGEV-------------------------NQLPKSHTCF-NR-IDLPAY-KSYEA
LEQKLT-LA--------
>NWGS3B_2_Trichosporon_ovoides_JXYN01000012.1 .
VKVSRSNI-FEGSYTEI--------------MRQTPNDLKK-RLMIKFEG-------EDG
LDYGGLSREF---FFLL----SHEMFNPFY--------CLFEYSAHDNY-----------
----------------TLQINPNS------------------------------------
--GVNPEHLNYFKFIGRVVGLGIFHRRFLDAYFIV--AFYKMILK---------------
--------KRISLADLES----------------------VDASLHRS--------LTWM
LEN---DIT-DII--
-EETFSITEEH---------------------------FGE-MVTVD-LKEN--------
GQHIE---
------------------------------VNEENKK-EYVDL-VTEYRISRR-VSQQFD
-SFMSG---FNEIIPQ--ELIN-VFDER-ELELLIGG-----------------------
----MSEID-----------VDDW----QKHTDYRG-YNPS-------------------
------------------------DEVI-EWFWKIVR--------------------SWP
AERKSRLLQF-------TTGTSRIP------------VNG-----FK-DLQGSD--GPRR
FTIEKAGEI-------------------------TQLPKSHTCF-NR-IDLPAY-KTYES
LEQKL------------
>Trichosporon_inkin_JXYM01000005.1 .
VKVSRNNI-FEGSYTEI--------------MRQTPNDLKK-RLMIKFEG-------EDG
LDYGGLSREF---FFLL----SHEMFNPFY--------CLFEYSAHDNY-----------
----------------TLQINPNS------------------------------------
--GVNPEHLNYFKFIGRVVGLGIFHRRFLDAYFIV--AFYKMILK---------------
--------KRISLADLES----------------------VDASLHRS--------LTWM
LEN---DIT-DII--
-EETFSITEEH---------------------------FGE-MVTVD-LKEN--------
GQHIE---
------------------------------VNEENKK-EYVDL-VTEYRISRR-VSQQFE
-SFMSG---FNEIIPQ--ELIN-VFDER-ELELLIGG-----------------------
----MSEID-----------VDDW----QKHTDYRG-YNPS-------------------
------------------------DEVI-EWFWKIVR--------------------SWP
AERKSRLLQF-------TTGTSRIP------------VNG-----FK-DLQGSD--GPRR
FTIEKAGEI-------------------------TQLPKSHTCF-NR-IDLPAY-KTYES
LEQKL------------
>Trichosporon_asahii_XM_014327021.1 .
VKVSRNNI-FEGSYTEI--------------MRQTPNDLKK-RLMIKFEG-------EDG

LDYGGLSREF---FFLL----SHEMFNPFY--------CLFEYSAHDNY-----------
----------------TLQINPNS------------------------------------
--GVNPEHLNYFKFIGRVVGLGIFHRRFLDAYFIV--AFYKMILK---------------
--------KRISLADLES----------------------VDASLHRS--------LTWM
LEN--DIT-DII--
-EETFSITEEH---------------------------FGE-MVTVD-LKEN--------
GQNIE---
------------------------------VNEDNKK-EYVDL-VTEYRISRR-VSQQFE
-SFMSG---FNEIIPQ--ELIN-VFDER-ELELLIGG-----------------------
----MSEID-----------VDDW----QKHTDYRG-YNPS-------------------
------------------------DEVI-EWFWKIVR--------------------SWP
AERKSRLLQF-------TTGTSRIP------------VNG-----FK-DLQGSD--GPRR
FTIEKAGEI-------------------------TQLPKSHTCF-NR-IDLPAY-KTYES
LEQKLT-IAVDE-TEGF
>Trichosporon_coremiiforme_JXYL01000002.1 .
VKVSRNNI-FEGSYTEI--------------MRQTPNDLKK-RLMIKFEG-------EDG
LDYGGLSREF---FFLL----SHEMFNPFY--------CLFEYSAHDNY-----------
----------------TLQINPNS------------------------------------
--GVNPEHLNYFKFIGRVVGLGIFHRRFLDAYFIV--AFYKMILK---------------
--------KRISLADLES----------------------VDASLHRS--------LTWM
LEN--DIT-DII--
-EETFSITEEH---------------------------FGE-MVTVD-LKEN--------
GQNIE---
------------------------------VNEDNKK-EYVDL-VTEYRISRR-VSQQFE
-SFMSG---FNEIIPQ--ELIN-VFDER-ELELLIGG-----------------------
----MSEID-----------VDDW----QKHTDYRG-YNPS-------------------
------------------------DEVI-EWFWKIVR--------------------SWP
AERKSRLLQF-------TTGTSRIP------------VNG-----FK-DLQGSD--GPRR
FTIEKAGEI-------------------------TQLPKSHTCF-NR-IDLPAY-KTYES
LEQKLT-IAVDE-TEG-
>Agrocybe_aegerita_JW842321.1 .
IKVRRTRV-LEDSYGAV--------------MALAGEDLKR-RLMVNFEG-------EDG
LDYGGVSREW---FFLL----SHEIFNPSY--------GLFEYSTHDNY-----------
----------------TLQINPAS------------------------------------
--GINPDHLSYFKFIGRCLGLAIFHRRFLDAYFVP--SFYKMILG---------------
--------KHMSLVDLEA----------------------VDADLHRS--------LVWM
LEN--DIT-DVL--
-DETFTTTEER---------------------------FGE-LVTIE-LRPG--------
GEDIP---
------------------------------LTEQNKK-EYVDA-VVAYRISKR-VKEQFD
-AFMEG---LLELIPR--ELIN-VFDER-ELELLIGG-----------------------
----MSEID-----------MDDW----TKFTDYRG-YEKT-------------------
------------------------DQVI-EWFWQCIR--------------------SWP
AEKKSRLLQF-------TTGTSRVP------------VNG-----FK-DLQGSD--GPRR
FTIEKSGDP-------------------------AGLPRSHTCF-NR-LDLPPY-QDYES
LESKLS-FAIEE-TEGF
>Hypsizygus_marmoreus_GBCL01008324.1 .
IKIRRTRV-LEDSYGAI--------------MAKTGEDLRR-RLMVSFEG-------EDG
LDYGGVSREW---FFLL----SHEIFNPSY--------GLFEYSTHDNY-----------
----------------TLQINPAS------------------------------------
--GINPDHLSYFKFIGRCLGLAIFHRRFLDAYFVP--SFYKMILG---------------
--------KHMSLADLES----------------------VDSDLHRS--------LVWM
LEN--DIT-DVL--
-DETFTVTEER---------------------------FGE-HVTIE-LKSG--------
GEDIA---
------------------------------VTEENKK-DYVDT-VVAYRISKR-VKEQFD
-AFMSG---LLELIPS--DLIG-VFDER-ELELLIGG-----------------------
----MSEID-----------MDDW----TKFTDYRG-YEKT-------------------

```
------------------------DQVI-EWFWQCIR--------------------SWP
AERKSRLLQF-------TTGTSRVP------------VNG-----FK-DLQGSD--GPRR
FTIEKSGDP-------------------------HGLPRSHTCF-NR-LDLPPY-QDYES
LETKLI-YAIEE-TEGF
>Laccaria_bicolor_XM_001876255.1 .
VKVRRSRV-LEDSYASV--------------MGFTGEDLKR-RLMVNFDG-------EDG
LDYGGVSREW---FFLL----SHEIFNPSY--------GLFEYSTHDNY-----------
----------------TLQINPAS------------------------------------
--GINPDHLSYFKFIGRCLGLAIFHRRFLDAYFVP--SFYKMILG---------------
--------KHMALADLES----------------------VDSDLHRS--------LVWM
LEN------------------------------------------------DIT-DVL--
-DETFTTAEER---------------------------FGE-LVTIE-LKPG--------
GEEVP-------------------------------------------------------
------------------------------VTEENKK-EYVDS-VVAYRISKR-VKEQFD
-AFMEG---LLELVPR--DLIN-VFDER-ELELLIGG-----------------------
----MSEID-----------MDDW----TKFTDYRG-YEKT-------------------
------------------------DQVI-EWFWQCIR--------------------SWP
AERKSRLLQF-------TTGTSRVP------------VNG-----FK-DLQGSD--GPRR
FTIEKSGDP-------------------------MGLPRSHTCF-NR-LDLPPY-QDYES
LETKLL-FAIEE-TEGF
>Moniliophthora_roreri_XM_007852050.1 .
VKVRRSRV-LEDSYTAV--------------MGHSGEDLKR-RLMVSFEG-------EDG
LDYGGVSREW---FFLL----SHEIFNPSY--------GLFEYSTHDNY-----------
----------------TLQINPAS------------------------------------
--GINPDHLSYFKFIGRCLGLAIFHRRFLDAYFVP--SFYKMMLG---------------
--------KPVSLGDLES----------------------VDADLHRS--------LLWM
LEN------------------------------------------------DIT-DVL--
-DETFTTTEDR---------------------------FGE-LVTID-LKPG--------
GADVP-------------------------------------------------------
------------------------------VTEENKK-EYVNA-IVDYRISKR-VKDQFD
-SFMEG---LLELIPV--DLLG-VFDER-ELELLIGG-----------------------
----MSEID-----------MDDW----SKFTDYRG-YEKT-------------------
------------------------DQVI-EWFWQCIR--------------------SWP
AERKSRLLQF-------TTGTSRVP------------VNG-----FK-DLQGSD--GPRR
FTIEKSGDP-------------------------QGLPRSHTCF-NR-LDLPPY-TDYES
LETKLL-YAIEE-TEGF
>Armillaria_ostoyae_GAHM01001742.1 .
IKVRRARI-LEDSYTAV--------------MAQNPADLKR-RLLVNFEG-------EDG
LDYGGVGREW---FFLL----SHEIFNPCY--------GLFEYSTHDNY-----------
----------------TLQINPAS------------------------------------
--GINPDHLSYFKFIGRCLGLAIFHRRFLDVYFVP--SFYKMMLR---------------
--------KHMALVDLES----------------------VDADLHRS--------LVWM
LEN------------------------------------------------DIT-DVL--
-DETFTTTENR---------------------------FGE-LVTVD-LKPG--------
GESIP-------------------------------------------------------
------------------------------VTEANKA-EYVDA-VVAYRISTR-VKEQFQ
-SFMDG---LLELIPE--DLVS-VFDER-ELELLIGG-----------------------
----MAEID-----------VDDW----TKFTDYRG-YEKT-------------------
------------------------DQVI-EWFWQCIR--------------------SWP
AERKSRLLQF-------TTGTSRVP------------VNG-----FK-DLQGSD--GPRR
FTIEKSGDP-------------------------QGLPRSHTCF-NR-LDLPPY-QDYES
LETKLI-YAIEE-TEGF
>Schizophyllum_commune_XM_003034528.1 .
IRVRRSHI-LEDSYAAV--------------MQHSGENLKR-RLMINFEG-------EDG
LDYGGVSREW---FFLI----SHEIFNPSY--------GLFEYSAHDNY-----------
----------------TLQINPAS------------------------------------
--GINPDHLSYFKFIGRVLGLTVFHRRFLDAYFVP--SIYKMILG---------------
--------KHMTLADLES----------------------IDADLHRS--------LNWM
```

```
LTN------------------------------------------------DIT-DVL--
-EETFSITEDR---------------------------FGE-LVTIE-LKPG--------
GENLE-------------------------------------------------------
------------------------------VTEANKK-EYVDC-VVDYRISRR-VKDQFD
-AFMEG---LLELIPM--DLLH-VFDER-ELELLIGG-----------------------
----MSEID-----------MDDW----TKFTDYRG-YEKT-------------------
------------------------DQVI-EWFWQCIR--------------------SWP
AERKSRLLQF-------TTGTSRVP------------VNG-----FK-DLQGSD--GPRR
FTIEKSGDP-------------------------AGLPRSHTCF-NR-LDLPPY-TDYES
LETKLI-YAIEE-TEGF
>Coprinopsis_cinerea_XM_001835680.2 .
IKVRRNRV-MEDSYSAI--------------MAQTGEDLKR-RLMVSFDG-------EDG
LDYGGVSREW---FFLL----SHEIFNPSY--------GLFEYSTHDNY-----------
----------------TLQINPAS------------------------------------
--GINPDHLSYFKFIGRVVGLAIFHRRFLDAYFVP--SFYKMILG---------------
--------KPMTLNDLEA----------------------VNAELHRS--------LKWM
LEN------------------------------------------------DIT-DVL--
-YETFSLTEER---------------------------FGE-MVTIE-LKPG--------
GEDIP-------------------------------------------------------
------------------------------VTEENKK-EYVDL-VVEYRISRR-IKEQFD
-AFMDG---LLELIPR--DLIN-VFDER-ELELLIGG-----------------------
----MSEID-----------MDDW----SKFTDYRG-YEKT-------------------
------------------------DQVI-EWFWQCIR--------------------SWP
AEKKARLLQF-------TTGTSRVP------------VNG-----FK-DLQGSD--GPRR
FTIEKSGDP-------------------------SGLPRSHTCF-NR-LDLPPY-EDYES
LEQKLS-FAIEE-TEGF
>Agaricus_bisporus_XM_007330072.1 .
LKVRRNNI-LEDSYGAI--------------MSHSGEDLKK-RLMVSFDN-------EDG
LDYGGVSREW---FFLL----SHEIFNPSY--------GLFEYSTYDNY-----------
----------------TLQINHAS------------------------------------
--GINPDHLSYFKFIGRTVGLAIFHRRFLDAYFVR--SLYKMILS---------------
--------KPVSITDLEA----------------------IDADLHRS--------LMWM
LEN------------------------------------------------DIT-DVL--
-DETFSQTEER---------------------------FGE-LITID-LKPG--------
GEHIE-------------------------------------------------------
------------------------------VTEENKK-EYVDL-VVQYRIARR-IKEQFG
-AFMEG---LLELIPK--DLIT-VFDER-ELELLIGG-----------------------
----MSEID-----------MDDW----TKFTDYRG-YEKT-------------------
------------------------DQVI-EWFWQCIR--------------------SWP
AERKARLLQF-------TTGTSRVP------------VNG-----FK-DLQGSD--GPRR
FTIEKSGDP-------------------------SGLPRSHTCF-NR-LDLPPY-PDFES
LESKLL-FAIEE-TEGF
>Leucocoprinus_gongylophorus_HAAN01002201.1 .
LKVRRNNV-LEDSYAAV--------------MAHSGEDLKK-RLMVSFDN-------EDG
LDYGGVSREW---FFLL----SHEIFNPSY--------GLFEYSTHDNY-----------
----------------TLQINHAS------------------------------------
--AINPDHLSYFKFIGRAVGLAIFHRRFLDAYFVP--SLYKMILG---------------
--------KHVSMADLEA----------------------VDADLHRS--------LTWM
LEN------------------------------------------------NIT-GIL--
-EETFSQTEER---------------------------FGE-FVTVE-LKPG--------
GEEID-------------------------------------------------------
------------------------------VTEENKK-EYVDL-VVEYRISKR-VTEQFG
-AFMDG---LLELTPK--ELIT-VFDER-ELELLIGG-----------------------
----MSEID-----------MDDW----TKFTDYRG-YEKT-------------------
------------------------DQVI-EWFWQCIR--------------------SWP
AERKARLLQF-------TTGTSRVP------------VNG-----FK-DLQGSD--GPRH
FTIEKSGDP-------------------------SGLPRSHTCF-NR-LDLPPY-PDYES
LESKLL-FAIEE-TEGF
```

>Gloeophyllum_trabeum_XM_007867425.1 .
ITLRRNMV-FEDSYSAV--------------MKFRGSDLKK-RLYIKFEG-------EEG
LDFGGVSREW---FFLL----SHEMFNPSY--------GLFQYSTHDNY-----------
----------------TLQINKAS------------------------------------
--GINPEHLDYFTFIGRCIGLAVFHRRFLDVYFVP--SFYKMMLG---------------
--------KRLTLADLEG----------------------VDADLHRS--------LVWM
LEN--DIT-DVL--
-DETFSTTEDR---------------------------FGE-MVTVP-LIPG--------
GEDIP---
------------------------------VTEENKR-EYVEA-LVDYRISKG-VKEQFD
-AFMSG---FVEIIPK--DMIS-VFNEH-ELEWLIGG-----------------------
----ISDVD-----------MDDW----AKYTDYRN-YEKT-------------------
------------------------DQVI-QWFWECVR--------------------KWP
TERKSRLLQF-------VTGTSRVP------------MNG-----FK-DLQGAD--GPRR
FTIEKWGDP-------------------------SQLPRSHTCF-NR-LDLPPY-QDYAS
LETKLL-FAIEE-TEGF
>Serpula_lacrymans_XM_007319506.1 .
MKLRRGRI-LEDSFSAV--------------MKMSGNDLKK-RLVIKFEG-------EDG
LDYGGVSREW---FFLL----SHEIFNPSY--------GLFEYSAHDNY-----------
----------------TLQINWSS------------------------------------
--GINPEHLTYFKFIGRCLGMAIFHRRFLDAYFVS--SFYKMILA---------------
--------KKATLADLEG----------------------VDADLHRG--------LVWM
LDN--DIT-DVL--
-EETFSTTEDR---------------------------FGE-IVTVE-LKPG--------
GADIP---
------------------------------VTEDNKK-EYVEA-VVLYRTMTR-VKEQFD
-AFSEG---FKELIPQ--ELID-VFDER-ELELLIGG-----------------------
----MSEID-----------MDDW----TKFTDYRG-YEKS-------------------
------------------------DQVI-EWFWQCIR--------------------SWP
AERKSRLLQF-------ATGTSRVP------------VNG-----FK-DLQGSD--GPRR
FTIEKFGDA-------------------------SQLPRSHTCF-NR-IDLPPY-EDYES
LEQKLT-FAIEE-TEGF
>Coniophora_puteana_XM_007770302.1 .
MRLRRNHI-LEDSFAAT--------------MRMSGNDLKK-RLVIRFEG-------EDG
LDYGGVSREW---FFLI----SHEVFDPAY--------GLFEYSAHDNY-----------
----------------TLQINWAS------------------------------------
--SINPEHITYFKFIGRCLGLAIFHKRFLDAYFVP--SFYKSILG---------------
--------KRTTLADLEG----------------------VDAELHRG--------MTWM
LEN--DIT-DVL--
-DETFTVTESR---------------------------FGE-MVEVE-LMPG--------
GADVP---
------------------------------VTENNKA-EYVEA-VIEYRTKTR-IQEQFT
-AFMEG---FREIIPG--ELLN-VFDER-ELELLIGG-----------------------
----MSDID-----------VDDW----NRYTDYRG-YQKD-------------------
------------------------DQVI-EWFWQCIR--------------------SWP
SERKSRLLQF-------ATGTSRVP------------VNG-----FK-DLQGSD--GPRR
FTIDKSGDP-------------------------SQLPRSHTCF-NR-IELPPY-EDYES
LERKLM-FAIDE-TEGF
>Boletopsis_grisea_GEZR01001371.1 .
LKVRRGSV-FEDSFGAV--------------MRLSKEEMRR-RLMVRFEG-------EDG
LDYGGVSREW---FFLL----SHEMFDPLY--------GLFTYSTMDSY-----------
----------------TLQINPAS------------------------------------
--YVNPEHLTYFKFVGRVIGMAIFHRRFLDAFFVP--CLYKMMLG---------------
--------KKVTLSDLEG----------------------VDADLHRG--------MTWM
LEN--DIT-NVL--
-EETFSVTEDR---------------------------FGE-LVTVE-LKPG--------
GSSIA---
------------------------------VTEENKK-EYVDD-VVHYRIIGR-VKEQFA

```
-SFLDG---FLEVIPK--SLVS-VFDER-ELELLIGG-----------------------
----MADID-----------MDDW----SKFTDYRG-YDKT-------------------
------------------------DQVI-SWFWQILR--------------------SWP
PEQKARLLQF-------TTGTSRVP------------VNG-----FK-DLQGSD--GPRR
FTIEKYGDS------------------------TKLPRSHTCF-NR-LDLPPY-DDPKT
LEDKLK-FAIEE-TEGF
>Heterobasidion_irregulare_XM_009553415.1 .
VRVRRGWV-FEDSFAAI--------------MRLKADDLRK-RLMVKFEG-------EDG
LDYGGVSREW---FFLL----SHEMFNPSY--------GLFEYSAHDNY-----------
----------------TLQINSAS------------------------------------
--GINPEHLEYFKFIGRCLGLSIFHHRFLDAYFVP--SLYKMILG---------------
--------KKVTLKDLEA----------------------VDAELHRG--------LTWM
LEN-----------------------------------------------NIT-DIL--
-DETFTTTEDR---------------------------FGE-LVTID-LKPD--------
GSNIA-------------------------------------------------------
------------------------------VTEANKA-EYVDL-VVEYRIHKR-VGDQFR
-AFMGG---FSEVIPQ--ELIT-VFDET-ELELLIGG-----------------------
----MSEID-----------MDDW----TKFTDYRG-YEKT-------------------
------------------------DQVI-EWFWQCLR--------------------SWP
TERKARLLQF-------TTGTSRVP------------VNG-----FK-DLQGSD--GPRR
FTIEKSGDP------------------------NGLPRSHTCF-NR-LDLPPY-QDYES
LERKLR-YAIDE-TEGF
>Stereum_hirsutum_XM_007305836.1 .
IRLRRGMV-FEDSFRAV--------------MRLKKEDLRK-RLVVRFEG-------EDG
LDYGGVSREW---FFLL----SHEMFNPSY--------GLFEYSAHDNY-----------
----------------TLQINPFS------------------------------------
--GINPEHLDYFKFIGRCLGLAVFHHRFLDAYFVP--SFYKMILG---------------
--------KKVTMKDLEA----------------------VDYELWRG--------LSWM
LDN-----------------------------------------------DIT-DVL--
-DETFSTTEDN---------------------------FGQ-LLTIP-LRPN--------
GEDIP-------------------------------------------------------
------------------------------VTEVNKA-EYVEL-LVDYRIRRR-VEEQFA
-AFMEG---FGEVVPL--ELIK-VFDEN-EVELLIGG-----------------------
----MSEID-----------MDDW----TKFTDYRG-YAKT-------------------
------------------------DQVI-SWFWEILR--------------------SWP
TERKARLLQF-------TTGTSRVP------------VNG-----FK-DLQGSD--GPRR
FTIEKSGDV------------------------NGLPRSHTCF-NR-LDLPEY-KEKEK
LEERLR-FAIEE-TEGF
>Piriformospora_indica_CAFZ01000086.1_R .
IKVRRNYI-FEDSYAEI--------------MRQSPNDLKK-RLMITFEG-------EPG
LDYGGVSREF---FFLL----SHEMFNPFY--------CLFEYSAHDNY-----------
----------------TLQISPAS------------------------------------
--GVNPEHLNYFKFIGRVVGLAIFHRRFLDAYFIT--SMYKMILH---------------
--------KKIALSDLES----------------------VDAELHRS--------MSWT
LXN-----------------------------------------------DIT-DVI--
-EETFSVQEER---------------------------FGE-LVTIE-LKPG--------
GADIP-------------------------------------------------------
------------------------------VTEENKK-EFVEY-MVEYRITKR-VQEQYT
-AFMEG---FNELIPQ--ELIN-VFDER-ELELLIGG-----------------------
----ISXVD-----------VDDW----AKFTDYRG-YSTD-------------------
------------------------DQVI-KWFWTCVR--------------------SWP
PERKSRLLQF-------VTGTSRIP------------VNG-----FK-DLQGSD--GPRR
FTIEKSGDP------------------------SQLPKSHTCF-NR-VSV----------
-----------------
>Fomitiporia_mediterranea_XM_007264026.1 .
IRIRRSHL-FEDSYAEI--------------MRQSPSELKK-RLMIKFDG-------EDG
LDYGGVSREF---FFLL----SHEMFNPFY--------CLFEYSAHDNY-----------
----------------TLQINPAS------------------------------------
```

--GVNPEHLNYFRFIGRCMGLAIFHRRFLDAYFIV--SFYKMILK---------------
--------KKVTLSDLES----------------------VDAELYRG--------LKWM
LEN--DIT-DVL--
-DETFSTTEER---------------------------FGE-IFTID-LCPN--------
GSTIP---
------------------------------VTEVNKG-EYVDA-VVNYRIAKR-VKEQFD
-AFMAG---INELVPQ--DLIM-VFDER-ELELLIGG-----------------------
----MSEID-----------VDDW----NKFTDYRG-YEVN-------------------
------------------------DTVI-QWFWKIVR--------------------AWP
PEKKSRLLQF-------ATGTSRIP------------VNG-----FK-DLQGSD--GPRR
FTIEKSGDP-------------------------SQLPKSHTCF-NR-IDLPPY-TNYEV
MEQKLT-LAVEE-TMGF
>Rigidoporus_microporus_GDMN01024079.1 .
IRIRRNHI-FEDSFTEI--------------MRQTPTDLKK-RLMIKFDG-------EDG
LDYGGLSREY---FFLL----SHEMFNPFY--------CLFEYSALDNY-----------
----------------TLQINPAS------------------------------------
--GINPEHLSYFKFIGRVMGLAVYHRRFLDAYFVV--GFYKMLLK---------------
--------KKVTISDLES----------------------VDAELYRG--------LKWM
LEN--DIT-DII--
-DETFTTTEER---------------------------FGE-LVTIE-LRPD--------
GEGIP---
------------------------------VTEENKA-EYVDA-VVHYRISKR-VQDQFE
-SFMSG---FNELVPQG--LIE-VFDER-ELELLIGG-----------------------
----MSEID-----------VDDW----ARFTDYRG-YDAN-------------------
------------------------DEVV-QWFWKIIR--------------------SWP
PEKKSHLLQF-------ATGTSRIP------------VNG-----FK-DLQGSD--GPRR
FTIEKSGDP-------------------------NGLPKSHTCF-NR-IDLPPY-TDYEV
LERKLT-LAVEE-TCGF
>Fomitiporia_mediterranea_XM_007262754.1 .
IKIRRNHI-FEDSYAEI--------------MRQTPADLKK-RLMIKFDG-------EDG
LDYGGVSREF---FFLL----SHEMFNPFY--------CLFEYSAHDNY-----------
----------------TLQINPAS------------------------------------
--GVNPEHLNYFKFIGRCLGLAIFHRRFLDAYFIV--SFYKMILK---------------
--------KKVTLADLES----------------------VDAELFRG--------LTWM
LEN--EIE-GVI--
-DETFTTAEER---------------------------FGE-MVTIE-LKPG--------
GADVA---
------------------------------VTDENKK-EYVDL-VVEYRIFKR-VQEQFE
-AFISG---FNELIPQ--ELIN-VFDER-ELELLIGG-----------------------
----MSEID-----------VDDW----AKYTDYRG-YEMN-------------------
------------------------DEVI-QWFWKCIR--------------------SWP
AERKSRLLQF-------TTGTSRIP------------VNG-----FK-DLQGSD--GPRR
FTIEKSGDP-------------------------SQLPKSHTCF-NR-LDLPPY-KDYES
LEQKLT-LAVEE-TVGF
>Auricularia_subglabra_XM_007345608.1 .
IKVRRNHI-FEDSYAEI--------------MRQTPNDLKK-RLMIKFDG-------EDG
LDYGGLAREF---FFLL----SHEMFNPFY--------CLFEYSAHDNY-----------
----------------TLQINPAS------------------------------------
--GVNPEHLNYFKFIGRCVGLGIFHRRFLDAYFIV--SFYKMILR---------------
--------KKITLSDLES----------------------VDAELFRG--------LTWM
LEN--DIT-DII--
-EETFTTTEDR---------------------------FGE-MVTID-LKPG--------
GADIP---
------------------------------VTEENKK-EYVEY-IIDYRIQKR-VREQFD
-AFMAG---FNELIPQ--ELIN-VFDER-ELELLIGG-----------------------
----ISEID-----------VDDW----CKFTDYRG-YEVN-------------------
------------------------DEVV-QWFWKCVR--------------------SWP
SERKSRLLQF-------ATGTSRIP------------VNG-----FK-DLQGSD--GPRR

FTIEKSGDP-------------------------SQLPKSHTCF-NR-IDLPPY-KDYAS
LEHKLT-LAVEE-TVGF
>Rhizoctonia_solani_JP279083.1 .
IKVRRNHI-FEDSYAEI--------------MRQTPSDLKK-RLMIKFDG-------EDG
LDYGGLSREF---FFLL----SHEMFNPFY--------CLFEYSAHDNY-----------
----------------TLQINPAS------------------------------------
--GVNPEHLNYFKFIGRCVGLGIFHRRFLDAYFIV--SFYKMILK---------------
--------KKITLADLES----------------------VDAELHRG--------MTWM
LEN--DIT-DVI--
-DETFTTVEER---------------------------FGE-LVTIE-LRPG--------
GADVE---
------------------------------VTEENKK-EYVEA-VIEYRIQKR-VKEQFD
-AFMAG---FSELIPQ--ELIN-VFDER-ELELLIGG-----------------------
----MSEID-----------VDDW----TKYTDYRG-YELN-------------------
------------------------DEVI-QWFWQCIR--------------------SWP
PERKSRLLQF-------ATGTSRIP------------VNG-----FK-DLQGSD--GPRR
FTIEKAGDP-------------------------SQLPKSHTCF-NR-IDLPPY-KDYKT
LEQKLT-MAVEE-TVGF
>Boletopsis_grisea_GEZR01000762.1 .
IKVRRNHI-FEDSYAEI--------------MRQTPNDLKK-RLMIKFDG-------EDG
LDYGGLSREF---FFLL----SHEMFNPFY--------CLFEYSAHDNY-----------
----------------TLQINPAS------------------------------------
--GVNPEHLNYFKFIGRCLGLGIFHRRFLDAYFIV--AFYKMVLK---------------
--------KKVVLADLES----------------------VDAELHRG--------LTWM
LNN--DIT-DLI--
-DDTFTTTEER---------------------------FGE-MVTIE-LKPG--------
GADVP---
------------------------------VTEDNKK-EYVEH-IVEYRISKR-VQEQFE
-AFMSG---FSELIPQ--ELVN-VFDER-ELELLIGG-----------------------
----MSEID-----------VDDW----SKFTDYRG-YEVS-------------------
------------------------DEVI-QWFWKCVR--------------------AWP
AERKSRLLQF-------ATGTSRIP------------VNG-----FK-DLQGSD--GPRR
FTIEKSGDP-------------------------SQLPKSHTCF-NR-IDLPPY-KDYAT
LEQKLT-LAVEE-TVGF
>Coriolopsis_gallica_GBYM01001394.1 .
IKVRRNHI-FEDSYAEI--------------MRQTPNDLKK-RLMIKFEG-------EDG
LDYGGLSREF---FFLL----SHEMFNPFY--------CLFEYSAHDNY-----------
----------------TLQINPAS------------------------------------
--GVNPEHLNYFKFIGRCLGLGIFHRRFLDAYFVT--AFYKMILR---------------
--------KKVTLADLES----------------------VDAELHRG--------LSWM
LEN--DIT-DVI--
-DETFTTTEER---------------------------FGE-MVTVE-LKPG--------
GADIP---
------------------------------VTEENKK-DYVNA-IVEYRISKR-VKEQFD
-AFMSG---FSELIPQ--DLIN-VFDER-ELELLIGG-----------------------
----MSEID-----------VDDW----IKFTDYRG-YEVN-------------------
------------------------DEVV-QWFWKCVR--------------------SWP
PERKSRLLQF-------ATGTSRIP------------VNG-----FK-DLQGSD--GPRR
FTIEKSGDP-------------------------NQLPKSHTCF-NR-IDLPPY-KDYET
LEQKLT-WAVEE-TVGF
>Dichomitus_squalens_XM_007364577.1 .
IKVRRNHI-FEDSYAEI--------------MRQTPNDLKK-RLMIKFEG-------EDG
LDYGGLSREF---FFLL----SHEMFNPFY--------CLFEYSAHDNY-----------
----------------TLQINPAS------------------------------------
--GVNPEHLNYFKFIGRCLGLGIFHRRFLDAYFVT--AFYKMILR---------------
--------KKVTLADLES----------------------VDAELHRG--------LTWM
LEN--DIT-DVI--
-DETFTTTEER---------------------------FGE-MVTVE-LKPG--------

GADIP---
------------------------------VTEENKK-DYVNA-IVEYRISKR-VKEQFD
-AFMSG---FSELIPQ--ELIN-VFDER-ELELLIGG-----------------------
----MSEID-----------VDDW----IKFTDYRG-YEVN-------------------
------------------------DEVV-QWFWKCVR--------------------SWP
PERKSRLLQF-------ATGTSRIP------------VNG-----FK-DLQGSD--GPRR
FTIEKSGDP-------------------------NQLPKSHTCF-NR-IDLPPY-KDYET
LEQKLT-WAVEE-TVGF
>Ganoderma_lucidum_AGAX01000004.1 .
IKVRRNHI-FEDSYAEI--------------MRQTPNDLKK-RLMIKFEG-------EDG
LDYGGLXREF---FFLL----SHEMFNPFY--------CLFEYSAHDNY-----------
----------------TLQINPAS------------------------------------
--GVNPEHLNYFKFIGRCLGLGIFHRRFLDAYFVT--AFYKMILR---------------
--------KKVIIADLES----------------------VDAELHRG--------LTWM
LXN--DIT-DVI--
-DETFTTTEER---------------------------FGE-MVTVE-LKPG--------
GADIP---
------------------------------VTEENKK-DYVSA-VVEYRISKR-VKEQFD
-AFMSG---FSELIPQ--ELIN-VFDDR-ELELLIGG-----------------------
----MSEID-----------VXDW----IKFTDYRG-YEVN-------------------
------------------------DEVV-QWFWKCVR--------------------SWP
AERKSRLLQF-------ATGTSRIP------------VNG-----FK-DLQGSD--GPRR
FTIEKSGDP-------------------------NQLPKSHTCF-NR-IDLPPY-KDYET
LEQKLT-WAV-------
>Trametes_versicolor_XM_008038762.1 .
IKVRRNHI-FEDSYAEI--------------MRQTPNDLKK-RLMIKFEG-------EDG
LDYGGLSREF---FFLL----SHEMFNPFY--------CLFEYSAHDNY-----------
----------------TLQINPAS------------------------------------
--GVNPEHLNYFKFIGRCLGLGIFHRRFLDAYFVT--AFYKMILR---------------
--------KKVTLADLES----------------------VDAELHRG--------LTWM
LEN--DIT-DVI--
-DETFTTVEER---------------------------FGE-MVTVE-LKPG--------
GGDVP---
------------------------------VTEDNKK-DYVLA-VVEYRISKR-VKEQFD
-AFMSG---FSELIPQ--DLIN-VFDER-ELELLIGG-----------------------
----MSEID-----------VDDW----MKYTDYRG-YEVN-------------------
------------------------DEVV-QWFWKCVR--------------------SWP
PERKSRLLQF-------ATGTSRIP------------VNG-----FK-DLQGSD--GPRR
FTIEKSGDP-------------------------NQLPKSHTCF-NR-IDLPPY-KDYET
LEQKLT-WAVEE-TVGF
>Trametes_hirsuta_CP019376.1 .
IKVRRNHI-FEDSYAEI--------------MRQTPNDLKK-RLMIKFEG-------EDG
LDYGGLSREF---FFLL----SHEMFNPFY--------CLFEYSAHDNY-----------
----------------TLQINPAS------------------------------------
--GVNPEHLNYFKFIGRCLGLGIFHRRFLDAYFVT--AFYKMILR---------------
--------KKVTLADLES----------------------VDAELHRG--------LTWM
LEN--DIT-DVI--
-DETFTTTEER---------------------------FGE-MVTVE-LKPG--------
GEEIP---
------------------------------VTEENKK-DYVNA-IVEYRISKR-VKEQFD
-AFMSG---FSELIPQ--DLIN-VFDER-ELELLIGG-----------------------
----MSEID-----------XDDW----MKYTDYRG-YEVN-------------------
------------------------DEVV-QWFWKCVR--------------------SWP
PERKSRLLQF-------ATGTSRIP------------VNG-----FK-DLQGSD--GPRR
FTIEKSGDP-------------------------NQLPKSHTCF-NR-IDLPPY-KDYET
LEQKLT-WAVE------
>Pycnoporus_coccineus_NCSW01000248.1 .
IKVRRNHI-FEDSYAEI--------------MRQTPNDLKK-RLMIKFEG-------EDG

LDYGGLSREF---FFLL----SHEMFNPFY--------CLFEYSAHDNY-----------
----------------TLQINPAS------------------------------------
--GVNPEHLNYFKFIGRVLGLGIFHRRFLDAYFVT--AFYKMILR---------------
--------KKVTLADLES----------------------VDAELHRG--------LTWM
LXN--DIT-DVI--
-DETFTTTEER---------------------------FGE-MVTVE-LKPG--------
GADIP---
------------------------------VTEENKK-EYVNL-IVEYRISKR-VKEQFD
-AFMSG---FSELIPQ--DLIN-VFDER-ELELLIGG-----------------------
----MSXTI-----------SDDW----MKYTDYRG-YEVN-------------------
------------------------DEVV-QWFWKCVR--------------------SWP
PERKSRLLQF-------ATGTSRIP------------VNG-----FK-DLQGSD--GPRR
FTIEKSGDP-------------------------NQLPKSHTCF-NR-IDLPPY-KDYET
LEQKLT-WAV-------
>Trametes_sanguinea_GAKI01000556.1 .
IKVRRNHI-FEDSYAEI--------------MRQTPNDLKK-RLMIKFEG-------EDG
LDYGGLSREF---FFLL----SHEMFNPFY--------CLFEYSAHDNY-----------
----------------TLQINPAS------------------------------------
--GVNPEHLNYFKFIGRVLGLGIFHRRFLDAYFVT--AFYKMILR---------------
--------KKVTLADLES----------------------VDAELHRG--------LTWM
LEN--DIT-DVI--
-DETFTTTEER---------------------------FGE-MVTVE-LKPG--------
GADIP---
------------------------------VTEENKK-EYVNL-IVEYRISKR-VKEQFD
-AFMSG---FSELIPQ--DLIN-VFDER-ELELLIGG-----------------------
----MSEID-----------VDDW----MKYTDYRG-YEVN-------------------
------------------------DEVV-QWFWKCVR--------------------SWP
PERKSRLLQF-------ATGTSRIP------------VNG-----FK-DLQGSD--GPRR
FTIEKSGDP-------------------------NQLPKSHTCF-NR-IDLPPY-KDYET
LEQKLT-WAVEE-TVGF
>Sparassis_latifolia_GELB01009027.1 .
IKVRRNHI-FEDSYAEI--------------MRQTPSDLKK-RLMIKFDG-------EDG
LDYGGLSREF---FFLL----SHEMFNPFY--------CLFEYSAHDNY-----------
----------------TLQINPAS------------------------------------
--GVNPEHLNYFKFIGRCLGLGIFHRRFLDAYFIT--AFYKMILK---------------
--------KKVTLADLES----------------------VDAELHRG--------LTWM
LQN--DIT-DVI--
-DETFTTTEER---------------------------FGE-MVTVE-LRPG--------
GADVP---
------------------------------VTEENKK-EYVNC-VVEYRISKR-VKEQFD
-AFMSG---FSELIPQ--DLIN-VFDER-ELELLIGG-----------------------
----MSEID-----------VDDW----SKFTDYRG-YEVS-------------------
------------------------DEVV-QWFWQCVR--------------------SWP
PERKSRLLQF-------ATGTSRIP------------VNG-----FK-DLQGSD--GPRR
FTIEKSGDP-------------------------SQLPKSHTCF-NR-IDLPPY-KTYNT
LEQKLT-LAVEE-TVGF
>Irpex_lacteus_GBRE01002240.1 .
IKVRRNHI-FEDSYAEI--------------MRQTPSDLKK-RLMIKFDG-------EDG
LDYGGLSREF---FFLL----SHEMFNPFY--------CLFEYSAHDNY-----------
----------------TLQINPAS------------------------------------
--GVNPEHLNYFKFIGRCLGLGIFHRRFLDAYFIT--AFYKMILK---------------
--------KKVTLADLES----------------------VDAELHRG--------LTWM
LEN--DIT-DII--
-DETFTTTEER---------------------------FGE-MVTVE-LKPD--------
GENIP---
------------------------------VTEENKK-DYVNC-VVEYRISRR-VKEQFD
-AFMSG---FSELIPQ--DLIN-VFDER-ELELLIGG-----------------------
----MSEID-----------VDDW----AKFTDYRG-YEVS-------------------

------------------------DEVV-QWFWKCVR--------------------SWP
PERKSRLLQF-------ATGTSRIP------------VNG-----FK-DLQGSD--GPRR
FTIEKSGDP-------------------------SQLPKSHTCF-NR-IDLPPY-RDYAT
LEQKLT-LAVEE-TVGF
>Fibroporia_radiculosa_XM_012327803.1 .
IKVRRNHI-FEDSYAEI--------------MRQTPSDLKK-RLMIKFDG-------EDG
LDYGGLSREF---FFLL----SHEMFNPFY--------CLFEYSAHDNY-----------
----------------TLQINPAS------------------------------------
--GVNPEHLNYFKFIGRCLGLGIFHRRFLDAYFIT--AFYKMILK---------------
--------KKVTLADLES----------------------VDAELHRG--------LTWM
LDN--DIT-DVI--
-DETFTTTEER---------------------------FGE-MVTVE-LKPG--------
GADVP---
------------------------------VTEDNKK-EYVDC-VVEYRISRR-VKEQFE
-AFMSG---FSELIPQ--DLIN-VFDER-ELELLIGG-----------------------
----MSEID-----------VDDW----SKYTDYRG-YEVN-------------------
------------------------DEVV-QWFWKCIR--------------------SWP
PERKSRLLQF-------ATGTSRIP------------VNG-----FK-DLQGSD--GPRR
FTIEKSGDP-------------------------SQLPKSHTCF-NR-IDLPPY-RDYET
LEQKLT-LAVEE-TVGF
>Phanerochaete_carnosa_XM_007396111.1 .
IKVRRNHI-FEDSYAEI--------------MRQTPNDLKK-RLMIKFDG-------EDG
LDYGGLSREF---FFLL----SHEMFNPFY--------CLFEYSAHDNY-----------
----------------TLQINPAS------------------------------------
--GVNPEHLNYFKFIGRCLGLGIFHRRFLDAYFIT--AFYKMILR---------------
--------KKVTLTDLES----------------------VDAELHRG--------LTWM
FEN--DIT-DVI--
-DETFTTTEER---------------------------FGE-MVTVE-LKPG--------
GADIA---
------------------------------VTEENKK-EYVDC-VVEYRISRR-VKDQFE
-AFMSG---FSELIPQ--DLVN-VFDER-ELELLIGG-----------------------
----MSEID-----------VDDW----AKFTDYRG-YEVN-------------------
------------------------DEVV-QWFWKCVR--------------------SWP
PERKSRLLQF-------ATGTSRIP------------VNG-----FK-DLQGSD--GPRR
FTIEKSGDP-------------------------SQLPKSHTCF-NR-IDLPPY-KDYAT
LEQKLT-LAVEE-TVGF
>Postia_placenta_ABWF01007561.1_R .
IKVRRNHI-FEDSYAEI--------------MRQTPNDLKK-RLMIKFDG-------EDG
LDYGGLSREF---FFLL----SHEMFNPFY--------CLFEYSAHDNY-----------
----------------TLQINPAS------------------------------------
--GVNPEHLNYFKFIGRCLGLGIFHRRFLDAYFIT--AFYKMILK---------------
--------KKVTLADLES----------------------VDAELHRG--------LTWM
LEN--DIT-DVI--
-DETFTTTEER---------------------------FGE-MVTVE-LKPG--------
GEDVP---
------------------------------VTEDNKK-EYVNC-VVEYRISRR-VKDQFE
-AFMSG---FSELIPQ--DLIN-VFDER-ELELLIGG-----------------------
----MSEID-----------VDDW----SKFTDYRG-YEVN-------------------
------------------------DEVI-QWFWKCVR--------------------SWP
PERKSRLLQF-------ATGTSRIP------------VNG-----FK-DLQGSD--GPRR
FTIEKSGDP-------------------------SQLPKSHTCF-NR-IDLPPY-KDYAT
LEQKLT-LAV-------
>Ceriporiopsis_subvermispora_AEOV01000246.1_R .
IKVRRNHI-FEDSYAEI--------------MRQSPNDLKK-RLMIKFDG-------EDG
LDYGGLSREF---FFLL----SHEMFNPFY--------CLFEYSAHDNY-----------
----------------TLQINPAS------------------------------------
--GVNPEHLNYFKFIGRCLGLGIFHRRFLDAYFIT--AFYKMILK---------------
--------KKVTLADLES----------------------VDAELHRG--------LTWM

LXN--DIT-DVI--
-DETFTTTEER---------------------------FGE-MVTVE-LKPG--------
GGDVP---
------------------------------VTEDNKK-DYVNH-VVEYRIARR-VKDQFE
-AFMSG---FSELIPQ--DLIN-VFDER-ELELLIGG-----------------------
----MSEID-----------VXDW----AKFTDYRG-YEVN-------------------
------------------------DEVV-QWFWKCVR--------------------SWP
PERKSRLLQF-------ATGTSRIP------------VNG-----FK-DLQGSD--GPRR
FTIEKSGDP-------------------------SQLPKSHTCF-NR-IDLPPY-KDYAT
LEQKLT-LAV-------
>Gloeophyllum_trabeum_XM_007864830.1 .
IKVRRNHI-FEDSYAEI--------------MRQTPNDLKK-RLMIKFDG-------EDG
LDYGGLSREF---FFLL----SHEMFNPFY--------CLFEYSAHDNY-----------
----------------TLQINPAS------------------------------------
--GVNPEHLNYFKFIGRCLGLSIFHRRFLDAYFIV--SFYKMILR---------------
--------KKVTLADLES----------------------VDADLHRG--------LTWM
LEN--DIT-DVI--
-DETFTTVEDR---------------------------FGE-MITVE-LKPG--------
GADIP---
------------------------------VTEENKK-EYVDL-IVEYRICKR-VKDQFE
-AFMSG---FSELIPM--DLIT-VFDER-ELELLIGG-----------------------
----MSEID-----------VDDW----TKYTDYRG-YEMN-------------------
------------------------DEVI-QWFWKCVR--------------------SWP
PERKSRLLQF-------ATGTSRIP------------VNG-----FK-DLQGSD--GPRR
FTIEKAGDP-------------------------SQLPKSHTCF-NR-IDLPPY-KDYNT
LEQKLT-LAVEE-TVGF
>Punctularia_strigosozonata_XM_007385107.1 .
IKVRRNHI-FEDSYAEI--------------MRQTPNDLKK-RLMIKFDG-------EDG
LDYGGLSREF---FFLL----SHEMFNPFY--------CLFEYSAHDNY-----------
----------------TLQINPAS------------------------------------
--GVNPEHLNYFKFIGRCLGLGIFHRRFLDAYFIV--SFYKMILK---------------
--------KKVTLADLES----------------------VDAELHRG--------LTWM
LEN--DIT-DVI--
-DETFTTVEDR---------------------------FGE-MVTVE-LKPG--------
GADVP---
------------------------------VTEENKR-EYVDC-VVEYRISRR-VKEQFD
-AFMSG---FSELIPL--DLIT-VFDER-ELELLIGG-----------------------
----MSEID-----------VDDW----TKYTDYRG-YEMN-------------------
------------------------DEVI-QWFWKCVK--------------------SWP
PERKSRLLQF-------ATGTSRIP------------VNG-----FK-DLQGSD--GPRR
FTIEKAGDP-------------------------SQLPKSHTCF-NR-IDLPPY-KDYDT
LEQKLT-LAVEE-TVGF
>Agaricus_bisporus_XM_007326212.1 .
IKVRRNHI-FEDSYAEI--------------MRQTPNDLKK-RLMIKFDG-------EDG
LDYGGLSREF---FFLL----SHEMFNPFY--------CLFEYSAHDNY-----------
----------------TLQINPAS------------------------------------
--GVNPEHLNYFKFIGRCLGLGIFHRRFLDAYFIV--SFYKMILK---------------
--------KKVTLADLES----------------------VDTELHRG--------LTWM
LDN--DIT-DVI--
-DETFTTTEDR---------------------------FGE-MVTVE-LKPG--------
GEEVP---
------------------------------VTEENKK-EYVEL-VVEYRIVRR-VKEQFD
-AFMSG---FSELIPL--ELVT-VFDER-ELELLIGG-----------------------
----MSEID-----------VDDW----TKYTDYRG-YEQS-------------------
------------------------DEVI-QWFWKCVR--------------------SWP
PERKSRLLQF-------ATGTSRIP------------VNG-----FK-DLQGSD--GPRR
FTIEKSGDP-------------------------SQLPKSHTCF-NR-IDLPPY-KDYAS
LEYKLT-LAVEE-TVGF

```
>Leucocoprinus_gongylophorus_HAAN01010744.1 .
IKVRRNHI-FEDSYAEI--------------MRQAPYDLKK-RLMIKFDG-------EDG
LDYGGLSREF---FFLL----SHEMFNPFY--------CLFEYSAHDNY-----------
----------------TLQINPAS------------------------------------
--GVNPEHLNYFKFIGRCLGLGIFHRRFLDAYFIV--SFYKMILK---------------
--------KKVTLSDLES----------------------VDAELHHG--------LTWM
LXN------------------------------------------------DIT-DII--
-DETFTTSEDR---------------------------FGE-MVTVE-LKPG--------
GADIQ-------------------------------------------------------
------------------------------VTEENKK-DYVDC-VVEYRISKR-VKEQFD
-AFMSG---FSELIPL--DLVT-VFDER-ELELLIGG-----------------------
----MSEID-----------VDDW----TKYTDYRG-YEQT-------------------
------------------------DDVI-QWFWKCVR--------------------SWP
PERKSRLLQF-------ATGTSRIP------------VNG-----FK-DLQGSD--GPRR
FTIEKSGDP-------------------------SQLPKSHTCF-NR-IDLPPY-KDYAS
LEQKLT-LAVEE-TVGF
>Schizophyllum_commune_XM_003035072.1 .
IKVRRNHI-FEDSYAEI--------------MRQTPNDLKK-RLMIKFDG-------EDG
LDYGGLSREF---FFLL----SHEMFNPFY--------CLFEYSAHDNY-----------
----------------TLQINPAS------------------------------------
--GVNPEHLNYFKFIGRCLGLGIFHRRFLDAYFIT--SFYKMILR---------------
--------KKVTLADLES----------------------VDAELYRG--------MVWM
LEN------------------------------------------------DIT-DII--
-DETFTTMEER---------------------------FGE-MVTIE-LKPG--------
GADVQ-------------------------------------------------------
------------------------------VTEENKK-EYVDL-IVEYRISKR-VKEQFE
-AFMSG---FSELIPL--DLIT-VFDER-ELELLIGG-----------------------
----MSEID-----------VDDW----TKFTDYRG-YEMN-------------------
------------------------DEVI-QWFWKCVR--------------------SWP
PERKSRLLQF-------ATGTSRIP------------VNG-----FK-DLQGSD--GPRR
FTIEKSGDP-------------------------SQLPKSHTCF-NR-IDLPPY-KDYAS
LEQKLT-LAVEE-TVGF
>Coprinopsis_cinerea_XM_001833568.2 .
IKIRRNHI-FEDSYAEI--------------MRQTPNDLKK-RLMIKFDG-------EDG
LDYGGLSREF---FFLL----SHEMFNPFY--------CLFEYSAHDNY-----------
----------------TLQINPAS------------------------------------
--GVNPEHLNYFKFIGRCLGLGIFHRRFLDAYFIV--SFYKMILR---------------
--------KKVTLSDLES----------------------VDAELHRG--------LTWM
LEN------------------------------------------------DIT-DII--
-DETFTTVEER---------------------------FGE-MVTID-LKPG--------
GSEVQ-------------------------------------------------------
------------------------------VTEENKK-EYVDC-VVEYRISKR-VKEQFE
-AFMSG---FSELIPQ--DLIT-VFDER-ELELLIGG-----------------------
----MSEID-----------VDDW----TKFTDYRG-YEVT-------------------
------------------------DEVI-QWFWKCVR--------------------SWP
PERKSRLLQF-------ATGTSRIP------------VNG-----FK-DLQGSD--GPRR
FTIEKSGDP-------------------------TMLPKSHTCF-NR-IDLPPY-KDYAT
LEQKLT-LAVEE-TVGF
>Stereum_hirsutum_XM_007301261.1 .
IKVRRNHI-FEDSYAEI--------------MRQTPNDLKK-RLMIKFDG-------EDG
LDYGGLSREF---FFLL----SHEMFNPFY--------CLFEYSAHDNY-----------
----------------TLQINPAS------------------------------------
--GVNPEHLNYFKFIGRCLGLGIFHRRFLDAYFIV--SFYKMILK---------------
--------KKVTLADLES----------------------VDAELHRG--------LTWM
LEN------------------------------------------------DIT-DII--
-DETFTTTEER---------------------------FGE-LVTID-LKPG--------
GADEP-------------------------------------------------------
------------------------------VTQDNKK-EYVDH-VVEYRISKR-VKEQFD
```

-AFMSG---FSELIPQ--DLIN-VFDER-ELELLIGG----------------------
----MSEID-----------VDDW----TKFTDYRG-YEVN-------------------
------------------------DEVI-QWFWKCVR--------------------SWP
PERKSRLLQF-------ATGTSRIP------------VNG-----FK-DLQGSD--GPRR
FTIEKAGDP-------------------------SQLPKSHTCF-NR-IDLPPY-KDYAS
LEQKLT-LAVD------
>Heterobasidion_irregulare_XM_009544025.1 .
IKVRRNHI-FEDSYAEI--------------MRQTPNDLKK-RLMIKFDG-------EDG
LDYGGLSREF---FFLL----SHEMFNPFY--------CLFEYSAHDNY-----------
----------------TLQINPAS------------------------------------
--GVNPEHLNYFKFIGRCLGLGIFHRRFLDAYFIV--SFYKMILK---------------
--------KKVTLADLES----------------------VDAELHRG--------LTWM
LDN---DIT-DII--
-DETFTTTEER---------------------------FGE-MVTIE-LKPG--------
GGDVP---
------------------------------VTQDNKK-EYVEH-VVEYRISKR-VKEQFD
-AFMSG---FSELIPQ--DLVN-VFDER-ELELLIGG----------------------
----MSEID-----------VDDW----TKFTDYRG-YEVN-------------------
------------------------DEVI-QWFWKCVR--------------------SWP
PERKSRLLQF-------ATGTSRIP------------VNG-----FK-DLQGSD--GPRR
FTIEKAGDP-------------------------SQLPKSHTCF-NR-IDLPPY-KDYAS
LEHKLT-LAVEE-TVGF
>Coniophora_puteana_XM_007766105.1 .
IKVRRNHI-FEDSYAEI--------------MRQTSNDLKK-RLMIKFDG-------EDG
LDYGGLSREF---FFLL----SHEMFNPFY--------CLFEYSAHDNY-----------
----------------TLQINPAS------------------------------------
--GVNPEHLNYFKFIGRCLGLGIFHRRFLDAYFIV--SFYKMILK---------------
--------KKVTLSDLES----------------------VDAELHRG--------LTWM
LEN---DIT-DVI--
-DETFTTTEER---------------------------FGE-MVTID-LKPG--------
GADMP---
------------------------------VTQDNKK-DYVDA-VVEYRISKR-VKEQFD
-AFMSG---FSELIPQ--DLIT-VFDER-ELELLIGG----------------------
----MSEID-----------VDDW----SKFTDYRG-YSLE-------------------
------------------------DEVI-QWFWKCVR--------------------SWP
PERKSRLLQF-------ATGTSRIP------------VNG-----FK-DLQGSD--GPRR
FTIEKSGDP-------------------------SQLPKSHTCF-NR-IDLPPY-KDYAS
LENKLT-LAVEE-TVGF
>Serpula_lacrymans_XM_007321237.1 .
IKVRRNHI-FEDSYAEI--------------MRQTPNDLKK-RLMIKFDG-------EDG
LDYGGLSREF---FFLL----SHEMFNPFY--------CLFEYSAHDNY-----------
----------------TLQINPAS------------------------------------
--GVNPEHLNYFKFIGRCLGLGIFHRRFLDAYFIV--SFYKMILK---------------
--------KKVTLSDLES----------------------VDAELHRG--------LTWM
LEN---DIT-DVI--
-DETFTTTEER---------------------------FGE-MVNVE-LKPG--------
GGDVT---
------------------------------VTEDNKK-DYVDA-VVEYRISKR-VKEQFE
-SFMSG---FSELIPQ--DLIT-VFDER-ELELLIGG----------------------
----MSEID-----------VDDW----TKFTDYRG-YEMN-------------------
------------------------DEVI-QWFWKCVR--------------------GWP
PERKSRLLQF-------ATGTSRIP------------VNG-----FK-DLQGSD--GPRR
FTIEKSGEP-------------------------SQLPKSHTCF-NR-IDLPPY-KDYAS
LEQKLT-LAVEE-TVGF
>Moniliophthora_roreri_XM_007849446.1 .
IKIRRNHI-FEDSYAEI--------------MRQTPNDLKK-RLMIKFDG-------EDG
LDYGGLSREF---FFLL----SHEMFNPFY--------CLFEYSAHDNY-----------
----------------TLQINPAS------------------------------------

--GVNPEHLNYFKFIGRCLGLGIFHRRFLDAYFIV--SFYKMILK---------------
--------KKVTLSDLES----------------------VDAELHRG--------MTWM
LDN--DIT-DII--
-DETFTTTEER---------------------------FGE-MVTIE-LKPG--------
GADIP---
------------------------------VTEENKK-EYVDA-IVEYRISKR-VKEQFD
-AFMSG---FSELIPQ--DLIT-VFDER-ELELLIGG-----------------------
----MSEID-----------VDDW----TKFTDYRG-YEMD-------------------
------------------------DEVI-QWFWKCVR--------------------SWP
PERKSRLLQF-------ATGTSRIP------------VNG-----FK-DLQGSD--GPRR
FTIEKSGDP-------------------------SQLPKSHTCF-NR-IDLPPY-KDYAS
LEQKLT-LAVEE-TVGF
>Agrocybe_aegerita_JW843518.1 .
IKIRRNHI-FEDSYAEI--------------MRQTPNDLKK-RLMIKFDG-------EDG
LDYGGLSREF---FFLL----SHEMFNPFY--------CLFEYSAHDNY-----------
----------------TLQINPAS------------------------------------
--GVNPEHLNYFKFIGRCLGLGIFHRRFLDAYFIV--SFYKMILK---------------
--------KKVTLSDLES----------------------VDAELHRG--------LTWM
LEN--DIT-DII--
-DETFTTTEER---------------------------FGE-MVTIE-LKPG--------
GADVP---
------------------------------VTEENKK-DYVDH-VVEYRISKR-VKDQFE
-AFMSG---FSELIPQ--DLIT-VFDER-ELELLIGG-----------------------
----MSEID-----------VDDW----TKFTDYRG-YEVN-------------------
------------------------DEVI-QWFWKCVR--------------------SWP
PERKSRLLQF-------ATGTSRIP------------VNG-----FK-DLQGSD--GPRR
FTIEKSGDP-------------------------SQLPKSHTCF-NR-IDLPPY-KDYAS
LEQKLT-LAVEE-TVGF
>Laccaria_bicolor_XM_001878532.1 .
IKIRRNHI-FEDSYAEI--------------MRQTPNDLKK-RLMIKFDG-------EDG
LDYGGLSREF---FFLL----SHEMFNPFY--------CLFEYSAHDNY-----------
----------------TLQINPAS------------------------------------
--GVNPEHLNYFKFIGRCLGLGIFHRRFLDAYFIV--SFYKMILK---------------
--------KKVTLSDLES----------------------VDAELHRG--------MTWM
LEN--DIT-DII--
-DETFTTTEER---------------------------FGE-MVTID-LKPG--------
GADVP---
------------------------------VTEENKK-EYVDH-VVDYRISKR-VNEQFE
-AFMSG---FSELIPQ--ELIT-VFDER-ELELLIGG-----------------------
----MSEID-----------VDDW----TKFTDYRG-YEMN-------------------
------------------------DEVI-QWFWKCVR--------------------SWP
PERKSRLLQF-------ATGTSRIP------------VNG-----FK-DLQGSD--GPRR
FTIEKSGDP-------------------------SQLPKSHTCF-NR-IDLPPY-KDYAS
LEHKLT-LAVEE-TVGF
>Hypsizygus_marmoreus_GBCL01000174.1 .
IKIRRNHI-FEDSYAEI--------------MRQTPNDLKK-RLMIKFDG-------EDG
LDYGGLSREF---FFLL----SHEMFNPFY--------CLFEYSAHDNY-----------
----------------TLQINPAS------------------------------------
--GVNPEHLNYFKFIGRCLGLGIFHRRFLDAYFIV--SFYKMILK---------------
--------KKVTLSDLES----------------------VDAELHRG--------MTWM
LEN--DIT-DII--
-DETFTTVEER---------------------------FGE-MVTIE-LKPG--------
GADIP---
------------------------------VTEENKK-EYVEH-VVDYRISKR-VKEQFE
-AFMSG---FSELIPQ--DLIT-VFDER-ELELLIGG-----------------------
----MSEID-----------VDDW----TKFTDYRG-YEMN-------------------
------------------------DDVI-QWFWKCVR--------------------SWP
PERKSRLLQF-------ATGTSRIP------------VNG-----FK-DLQGSD--GPRR

FTIEKSGDP-------------------------SQLPKSHTCF-NR-IDLPPY-KDYAS
LEQKLT-LAVEE-TVGF
>Armillaria_ostoyae_GAHM01015188.1 .
IKIRRNHI-FEDSYAEI--------------MRQTPNDLKK-RLMIKFDG-------EDG
LDYGGLSREF---FFLL----SHEMFNPFY--------CLFEYSAHDNY-----------
----------------TLQINPAS------------------------------------
--GVNPEHLNYFKFIGRCLGLGIFHRRFLDAYFIV--SFYKMILK---------------
--------KKVTLSDLES---------------------VDAELHRG--------MTWM
LEN--DIT-DVI--
-DETFTTTEER---------------------------FGE-MVTIE-LKPG--------
GADVP---
------------------------------VTQDNKK-EYVEH-VVEYRISKR-VKEQFE
-AFMSG---FSELIPQ--DLIT-VFDER-ELELLIGG-----------------------
----MSEID-----------VDDW----TKFTDYRG-YEMN-------------------
------------------------DEVI-QWFWKCVR--------------------SWP
PERKSRLLQF-------ATGTSRIP------------VNG-----FK-DLQGSD--GPRR
FTIEKSGDP-------------------------SQLPKSHTCF-NR-IDLPPY-KDYAS
LEQKLT-LAVEE-TVGF
>Mortierella_alpina_ADAG01000979.1 .
MKVRRSHI-FEDAYHEI--------------MRQSPTDLKK-RLMIKFEG-------EDG
LDYGGLSXEF---FFLL----SHEMFNPFY--------CLFEYSAHDNY-----------
----------------TLQINPHS------------------------------------
--SINSEHLNYFKFIGRVVGLAIFHRRLLDAFFIV--SFYKMILK---------------
--------KKVTLADLES---------------------VDADVYRN--------LNWL
LDD--DTAAETL--
-DTTFSTNDER---------------------------FGE-IVTID-LKEN--------
GRDIE---
------------------------------VTEENKK-EYVEL-MTEWRITRR-VEEQFK
-AFAEG---FHQLIPQ--ELVT-VFDER-ELELLMGG-----------------------
----ISEID-----------CDDW----KKHTDYRG-YTEQ-------------------
------------------------DEVV-QWFWKCIR--------------------SWD
SEKKARLLQF-------TTGTSRIP------------VNG-----FK-DLQGSD--GPRR
FTIEKAGEI-------------------------GQLPKSHTXF-NR-IDLPPY-KSYDV
LVNKLT-MAVEE-----
>Gigaspora_margarita_GBYF01010219.1 .
IKVRREFI-FEDAYSEI--------------MRQQPQDLKK-RLMIKFDG-------EDG
LDYGGLSREF---FFLL----SHKMFDPFY--------CLFEYSAHDNY-----------
----------------TLQINPHS------------------------------------
--GIDPQHLNHFKFIGRVVGLAIFHRRFLDAFFIT--SFYKMILK---------------
--------KKVGLADMES---------------------VDADFYRS--------LKWA
LES--NIT-DVL--
-DLTFSTEDER---------------------------FGE-VETVD-LKPN--------
GRNIQ---
------------------------------VTEENKK-EYVEL-ITEWRISKR-VEDQFK
-AFMEG---FNQLIPQ--ELIT-VFDER-ELELLIGG-----------------------
----IAEID-----------VDDW----KKHTDYRG-YTES-------------------
------------------------DDVI-QWFWKCVR--------------------SWD
GEKKSRLLQF-------TTGTSRIP------------VNG-----FK-DLQGSD--GPRR
FTIEKALDT-------------------------SQLPKSHTCF-NR-IDLPPY-KNYEA
LVSKLT-LAVEE-TVGF
>Absidia_caerulea_GFAW01015837.1 .
IKVRREHV-FEDSYNEI--------------MRQAPADLKK-RLMIKFDG-------EDG
LDYGGLSREF---FFLL----SHEMFNPFY--------CLFEYSAHDNY-----------
----------------TLQINPHS------------------------------------
--GINPEHLNYFKFIGRVVGLAIFHRRFLDAFFIV--SFYKMILN---------------
--------KRVTVADMES---------------------VDSEFYRS--------LMWI
LDN--DIT-DIL--
-ELTFSTDDDR---------------------------FGE-LVTVD-LKPG--------

```
GRDIE-------------------------------------------------------
------------------------------VTEENKK-EYVNL-ITEWRISKR-VDEQFQ
-AFKEG---FNQLIPQ--DLIN-VFDER-ELELLVGG-----------------------
----IAEID-----------VDDW----KKHTDYRG-YTEQ-------------------
------------------------DDVI-QWFWKCVR--------------------SWD
SEKKSRLLQF-------TTGTSRIP------------VNG-----FK-DLQGSD--GPRR
FTIEKSGEV-------------------------TQLPKAHTCF-NR-IDMPPY-KSYES
LVTKLT-LAVEE-TMGF
>Rhizopus_microsporus_JNER01004428.1 .
IKVRRDHI-FEDAYAEI--------------MRQVPADLKK-RLMIKFDG-------EDG
LDYGGLSREF---FFLL----SHEMFNPFY--------CLFEYSAHDNY-----------
----------------TLQINPHS------------------------------------
--GINPEHLNYFRFIGRVVGLAIFHRRFLDAFFIV--SFYKMILN---------------
--------KKVMVEDMES----------------------VDADFYRS--------LKWI
LXN------------------------------------------------DIT-DIL--
-DLTFSVDDDR---------------------------FGE-VVTVD-LKEN--------
GRDIE-------------------------------------------------------
------------------------------VTEENKK-EYVEX-VTEWRISKR-VEEQFK
-AFKEG---FNQLIPQ--DLIN-VFDER-ELELLIGG-----------------------
----IAEID-----------VDDW----KKHTDYRG-YTEQ-------------------
------------------------DDVI-QWFWKCIR--------------------SWD
SEKKSRLLQF-------TTGTSRIP------------VNG-----FK-DLQ---------
------------------------------------------------------------
-----------------
>Lichtheimia_ramosa_LK023357.1 .
IKVRREHI-FEDAYAEI--------------MRQAPADLRK-RLMIKFDG-------EDG
LDYGGLSREF---FFLL----SHEMFNPFY--------XLFEYSAHDNY-----------
----------------TLQINPHS------------------------------------
--GINPEHLNYFRFIGRVVGLAIFHRRFLDAFFIV--SFYKNILN---------------
--------KKVTVGDMES----------------------VDADFHRS--------LQWI
LXN------------------------------------------------DIT-DVL--
-DLTFSTDDDR---------------------------FGE-VVTVD-LKPG--------
GRDIE-------------------------------------------------------
------------------------------VTEENKK-EYIKL-ITEWRISKR-VEEQFK
-AFKEG---FNQLIPQ--DLVN-VFDER-ELELLIGG-----------------------
----IAEID-----------VDDW----KKHTDYRG-YTEQ-------------------
------------------------DDVI-QWFWKVVR--------------------SWD
SEKKSRLLQF-------TTGTSRIP------------VNG-----FK-DLQGSD--GPRR
FTIEKAGEV-------------------------TQLPKAHTXX-NR-IDMPPY-KSYDA
LVAKLT-LAVEE-TLGF
>Phycomyces_blakesleeanus_XM_018439955.1 .
IKVRRDHI-FEDAYAEV--------------MRQSPADLKK-RLMIKFEG-------EDG
LDYGGLSREF---FFLL----SHEMFNPFY--------CLFEYSAHDNY-----------
----------------TLQINPHS------------------------------------
--GINPEHLNYFRFIGRVVGLSIFHRRFLDAFFIV--SFYKMILN---------------
--------KRVAVVDMES----------------------VDAEFHRS--------LKWI
LDN------------------------------------------------DIT-DVL--
-DLTFSTDDDR---------------------------FGE-LVTVD-LKPD--------
GQNIE-------------------------------------------------------
------------------------------VTEENKK-EYVDL-ITEWRISKR-VEEQFK
-AFKDG---FNQLIPQ--DLIN-VFDER-ELELLIGG-----------------------
----IAEID-----------VDDW----KKHTDYRG-YTEQ-------------------
------------------------DDVI-QWFWKCVS--------------------SWD
SEKKSRLLQF-------TTGTSRIP------------VNG-----FK-DLQGSD--GPRR
FTIEKAGEV-------------------------TMLPKAHTCF-NR-IDMPPY-KTYES
LVAKLT-LAVEE-TMGF
>Phycomyces_blakesleeanus_XM_018443014.1 .
IKIRRETI-FEDAYAEV--------------MRQSPSDLKK-RLMIKFDS-------EDG
```

LDYGGLSREF---FFLL----SHEMFNPFY--------CLFEYSAHDNY-----------
----------------TLQINPHS------------------------------------
--GINPEHLNYFRFIGRVVGLSIFHRRFLDSFFIV--SFYKMILS---------------
--------KRVAVADMES----------------------VDAEYHRS--------LMWM
LNN--DIT-DVL--
-DLTFSTEDDR---------------------------FGE-TVTVD-LKPD--------
GQNIP---
------------------------------VTEENKK-EYVNL-VTEWRISRR-VEEQFK
-AFKEG---FNQLIPH--DLVN-VFDER-ELELLIGG-----------------------
----ISEID-----------VEDW----KKHTDYRG-YTEQ-------------------
------------------------DDVI-QWFWKCIK--------------------TWD
SEKKSRLLQF-------TTGTSRIP------------VNG-----FK-DLQGSD--GPRR
FTIEKSGEV-------------------------TQLPKAHTCF-NR-IDMPPY-KSYEA
LVAKLT-MAVEE-TVGF
>Mucor_irregularis_GFBC01014345.1 .
IKVRRDHI-FEDAYAEI--------------MRQAPADLKK-RLMIKFDG-------EDG
LDYGGLSREF---FFLL----SHEMFNPFY--------CLFEYSAHDNY-----------
----------------TLQINPHS------------------------------------
--GINPEHLNYFRFIGRVVGLSIFHRRFLDAFFIV--SFYKMILN---------------
--------KKILVADMES----------------------VDADFYRS--------LMWI
LNN--DIT-DVL--
-ELTFSTDDDR---------------------------FGE-VVTVD-LIPN--------
GENIE---
------------------------------VTEENKK-EYVNL-ITEWRIHKR-VEEQFK
-AFKEG---FNQLIPQ--DLVN-VFDER-ELELLIGG-----------------------
----IAEID-----------VDDW----KKHTDYRG-YTEQ-------------------
------------------------DDVI-QWFWKCIR--------------------TWD
SEKKSRLLQF-------TTGTSRIP------------VNG-----FK-DLQGSD--GPRR
FTIEKSGEV-------------------------TQLPKAHTCF-NR-IDMPPY-KSYET
LVAKLT-MAVEE-TVGF
>Choanephora_cucurbitarum_LUGH01000194.1 .
IKVRRDHI-FEDAYAEI--------------MRQAPADLKK-RLMIKFDG-------EDG
LDYGGLSREF---FFLL----SHEMFNPFY--------CLFEYSAHDNY-----------
----------------TLQINPHS------------------------------------
--GINPEHLNYFRFIGRVVGLSIFHRRFLDAFFIV--SFYKMILN---------------
--------KKILVADMES----------------------VDADFYRS--------LTWI
LNN--DIT-DIL--
-ELTFSTDDDR---------------------------FGE-MVTVD-LIPN--------
GQQIE---
------------------------------VTEENKK-EYVNL-ITEWRIHKR-VEEQFK
-AFKEG---FNQLIPQ--DLIN-VFDER-ELELLIGG-----------------------
----IAEID-----------VDDW----KKHTDYRG-YTEQ-------------------
------------------------DDVI-QWFWQCIR--------------------SWD
SEKKSRLLQF-------TTGTSRIP------------VNG-----FK-DLQGSD--GPRR
FTIEKSGEV-------------------------TQLPKAHTXF-NR-IDMPPY-KSYET
LVAKLT-MAVEE-TMG-
>Rhizopus_microsporus_CDGI01000652.1 .
IKIRRDHI-FEDAYAEI--------------MRQAPADLKK-RLMIKFDG-------EDG
LDYGGLSREF---FFLL----SHEMFNPFY--------CLFEYSAHDNY-----------
----------------TLQINPHS------------------------------------
--GINPEHLNYFRFIGRVVGLAIFHRRFLDAFFIV--SFYKMILN---------------
--------KKILVADMES----------------------VDAEFFRS--------LTWI
LEN--DIT-DVL--
-DLTFSTDDDR---------------------------FGE-VVTVD-LIPN--------
GQNIE---
------------------------------VTEENKK-EYVNL-ITEWRIHKR-VEEQFK
-AFKEG---FNQLIPQ--ELIN-VFDER-ELELLIGG-----------------------
----IAEID-----------VDDW----KKHTDYRG-YTEQ-------------------

------------------------DDVI-QWFWKCIR--------------------SWD
SEKKARLLQF-------TTGTSRIP------------VNG-----FK-DLQGSD--GPRR
FTIEKSGEI-------------------------TQLPKAHTXF-NR-IDMPPY-KSYET
LVAKLT-MAVEE-TVG-
>Rhizopus_oryzae_GDUK01018646.1 .
IKVRRDHI-FEDAYAEI--------------MRQVPADLKK-RLMIKFDG-------EDG
LDYGGLSREF---FFLL----SHEMFNPFY--------CLFEYSAHDNY-----------
----------------TLQINPHS------------------------------------
--GINPEHLNYFRFIGRVVGLSIFHRRFLDAFFIV--SFYKMVLN---------------
--------KKILVADMES----------------------VDAEFHRS--------LMWI
LDN--DIT-DIL--
-DLTFSTDDDR---------------------------FGE-VVTVD-LIPN--------
GQNIE---
------------------------------VTEENKK-EYVNL-ITEWRIHRR-VEEQFK
-AFKEG---FNQLIPQ--ELIN-VFDER-ELELLIGG-----------------------
----IAEID-----------VDDW----KKHTDYRG-YTEQ-------------------
------------------------DDVI-QWFWKCIR--------------------SWD
SEKKARLLQF-------TTGTSRIP------------VNG-----FK-DLQGSD--GPRR
FTIEKSGEI-------------------------TQLPKAHTCF-NR-IDMPPY-KSYET
LVAKLT-MAVEE-TVGF
>Mucor_irregularis_GFBC01025915.1 .
IKVRRGHI-FEDAYAEV--------------MRQVPADLKK-RLMIKFDG-------EDG
LDYGGVSREF---FFVL----SHEMFNPFY--------CLFEYSAHDNY-----------
----------------TLQINPHS------------------------------------
--GINPEHLNYFRFIGRVVGLSIFHRRFLDAFFIV--SFYKMVLN---------------
--------KKILVADMES----------------------VDAEYHRS--------LMWI
LDN--DIT-DIL--
-DLTFSTDDDR---------------------------FGE-VVTVD-LIPN--------
GQNIE---
------------------------------VTEENKK-EYVNL-ITEWRIHRR-VEEQFK
-AFKEG---FNQLIPQ--ELVN-VFDER-ELELLIGG-----------------------
----IAEID-----------VDDW----KKHTDYRG-YTEQ-------------------
------------------------DDVI-QWFWKCIR--------------------SWD
SEKKARLLQF-------TTGTSRIP------------VNG-----FK-DLQGSD--GPRR
FTIEKSGEI-------------------------TQLPKAHTCF-NR-IDMPPY-KSYET
LVAKLT-MA--------
>Rhizopus_delemar_JNEA01006929.1 .
IKVRRGHI-FEDAYAEV--------------MRQAPADLKK-RLMIKFDG-------EDG
LDYGGLSREF---FFLL----SHEMFNPFY--------CLFEYSAHDNY-----------
----------------TLQINPHS------------------------------------
--GINPEHLNYFRFIGRVVGLSIFHRRFLDAFFIV--SFYKMVLN---------------
--------KKILVADMES----------------------VDAEFHRS--------LMWI
LXN--DIT-DIL--
-DLTFSTDDDR---------------------------FGE-VVTVD-LIPN--------
GQNIE---
------------------------------VTEENKK-EYVNL-ITEWRIHRR-VEEQFK
-AFKEG---FNQLIPQ--ELIN-VFDER-ELELLIGG-----------------------
----IAEID-----------VDDW----KKHTDYRG-YTEQ-------------------
------------------------DDVI-QWFWKCIR--------------------SWD
SEKKARLLQF-------TTGTSRIP------------VNG-----FK-DLQGSD--GPRR
FTIEKSGEI-------------------------TQLPKAHTXF-NR-IDMPPY-KSYET
LVAKLT-MAVEE-TVG-
>Rhizopus_oryzae_GDUK01018644.1 .
IKVRRGHI-FEDAYAEV--------------MRQAPADLKK-RLMIKFDG-------EDG
LDYGGLSREF---FFLL----SHEMFNPFY--------CLFEYSAHDNY-----------
----------------TLQINPHS------------------------------------
--GINPEHLNYFRFIGRVVGLSIFHRRFLDAFFIV--SFYKMVLN---------------
--------KKILVADMES----------------------VDAEFHRS--------LMWI

```
LDN------------------------------------------------DIT-DIL--
-DLTFSTDDDR---------------------------FGE-VVTVD-LIPN--------
GQNIE-------------------------------------------------------
------------------------------VTEENKK-EYVNL-ITEWRIHRR-VEEQFK
-AFKEG---FNQLIPQ--ELIN-VFDER-ELELLIGG-----------------------
----IAEID-----------VDDW----KKHTDYRG-YTEQ-------------------
------------------------DDVI-QWFWKCIR--------------------SWD
SEKKARLLQF-------TTGTSRIP------------VNG-----FK-DLQGSD--GPRR
FTIEKSGEI-------------------------TQLPKAHTCF-NR-IDMPPY-KSYET
LVAKLT-MAVEE-TVG-
>Pneumocystis_carinii_XM_018371258.1 .
IKIRRNHI-FEDSYAEI--------------MRQSPNDLKK-RFMVKFDG-------EDG
LDYGGLSREF---FFLL----SHEMFNPFY--------CLFEYSSVDNY-----------
----------------TLQINPHS------------------------------------
--GINPEHLNYFKFIGRVLGLAIFHRRFLDAFFIV--SFYKMILK---------------
--------KKVTLADMES----------------------VDAEFFRS--------LTWI
LEN------------------------------------------------DIT-NVL--
-ELTFSTEDDR---------------------------FGE-VMTID-LKPN--------
GRNIE-------------------------------------------------------
------------------------------VTNDNKK-EYVEL-VAYWRVFKR-VEEQFN
-AFQSG---FNELIPH--ELIS-VFDER-ELELLIGG-----------------------
----ITEMD-----------MDDW----KKHTDYRG-YTES-------------------
------------------------DEII-QWFWKCVR--------------------SWD
SERKSRLLQF-------ITGTSRVP------------VNG-----FK-DLQGSD--GPRR
FTIERAGEI-------------------------TQLPKSHTCF-NR-VDLPQY-PTYEM
LVQKLT-LAVEE-TVGF
>Pneumocystis_murina_XM_007876305.1 .
IKIRRNHI-FEDSYAEI--------------MRQTPSDLKK-RFMVKFDG-------EDG
LDYGGLSREF---FFLL----SHEMFNPFY--------CLFEYSSVDNY-----------
----------------TLQINPHS------------------------------------
--GINPEHLNYFKFIGRVLGLAIFHRRFLDAFFIV--SFYKMILK---------------
--------KKVTLADMES----------------------VDAEFFRS--------LTWI
LEN------------------------------------------------DIA-NVL--
-ELTFSTEDDR---------------------------FGE-VMTID-LKPN--------
GRNIE-------------------------------------------------------
------------------------------VTNDNKK-EYVEL-VAYWRVFKR-VEEQFN
-AFQDG---FNELIPQ--ELIS-VFDER-ELELLIGG-----------------------
----ITEMD-----------MDDW----KKHTDYRG-YTES-------------------
------------------------DEII-QWFWKCVR--------------------SWD
SERKSRLLQF-------ITGTSRVP------------VNG-----FK-DLQGSD--GPRR
FTIEKAGEI-------------------------TQLPKSHTCF-NR-VDLPQY-PTYEM
LVQKLT-LAVEE-TVGF
>Pneumocystis_jirovecii_XM_018372941.1 .
IKVRRNRI-FEDSYAEI--------------MRHAPNDLKK-RFMVKFDG-------EDG
LDYGGLSREF---FFLL----SHEMFNPFY--------CLFEYSSVDNY-----------
----------------TLQINPHS------------------------------------
--GINPEHLNYFKFIGRILGLAIFHRRFLDAFFIV--SFYKMILR---------------
--------KKVTLADMES----------------------VDAEFFRS--------LTWI
LEN------------------------------------------------DIT-DIL--
-ELTFSTEDDR---------------------------FGE-VTTID-LKPN--------
GRNIQ-------------------------------------------------------
------------------------------VTNDNKK-EYVEL-VAYWRVFKR-VEEQFN
-AFQDG---FNELIPQ--ELIS-VFDER-ELELLIGG-----------------------
----ITEMD-----------MDDW----KKYTDYRG-YTES-------------------
------------------------DEVI-QWFWKCVR--------------------SWD
SEKKSRLLQF-------ITGTSRVP------------VNG-----FK-DLQGSD--GPRR
FTIEKAGEI-------------------------TQLPKSHTCF-NR-VDLPEY-PTYEM
LVQKLT-LAVEE-TIGF
```

```
>Taphrina_deformans_CAHR02000317.1 .
IKIRRDHI-FEDSYAEI--------------MRQSSGDLKK-RLMIKFEG-------EDG
LDYGGLSREF---FFVL----SHEMFNPFY--------CLFEYSAVDNY-----------
----------------TLQINPHS------------------------------------
--GINPEHLNYFKFIGRVLGLSIFHRRFVDAFFVV--SFYKMILS---------------
--------KKCSLADMES----------------------IDAEFFRS--------LTWI
LEN------------------------------------------------SID-GIL--
-DLTFSTEDDR---------------------------FGE-TVTLD-LKPN--------
GRDLE-------------------------------------------------------
------------------------------VTDENKS-EYVEL-VTEWRIQKR-IEEQFV
-AFKAG---FNELVPQ--ELIN-VFDER-ELELLIGG-----------------------
----IADMD-----------VDDW----KKHTDYRG-YTET-------------------
------------------------DEII-QMFFKLVT--------------------SWD
NEKRARLLQF-------ATGTSRIP------------IGG-----FK-DLQGSD--GPRR
FTIEKAGEV-------------------------TQLPKAHTCF-NR-IDLPPY-STYEE
LEKKLS-YSVDE-TMG-
>Taphrina_wiesneri_BAVU01000036.1 .
IKIRRDHI-FEDSYAEI--------------MRQSSGDLKK-RLMIKFEG-------EDG
LDYGGLSREF---FFVL----SHEMFNPFY--------CLFEYSAVDNY-----------
----------------TLQINPHS------------------------------------
--GINPEHLNYFKFIGRVLGLSIFHRRFVDAFFVV--SFYKMILS---------------
--------KKCTLADMES----------------------IDAEFFRS--------LTWI
LEN------------------------------------------------SID-GIL--
-DLTFSTEDDR---------------------------FGE-TVTLD-LKPN--------
GRDLE-------------------------------------------------------
------------------------------VTDENKS-EYVEL-VTEWRIQKR-IEEQFV
-AFKAG---FNELVPQ--ELIN-VFDER-ELELLIGG-----------------------
----IADMD-----------VDDW----KKHTDYRG-YTET-------------------
------------------------DEII-QMFFKLIT--------------------SWD
NEKRARLLQF-------ATGTSRIP------------IGG-----FK-DLQGSD--GPRR
FTIEKAGEV-------------------------TQLPKAHTCF-NR-IDLPPY-STYEE
LEKKLS-YSVDE-TMG-
>Taphrina_flavorubra_BAVW01000113.1 .
IKIRRDHI-FEDSYAEI--------------MRQSSGDLKK-RLMIKFEG-------EDG
LDYGGLSREF---FFVL----SHEMFNPFY--------CLFEYSAVDNY-----------
----------------TLQINPHS------------------------------------
--GINPEHLNYFKFIGRVLGLSIFHRRFVDAFFVV--SFYKMILS---------------
--------KKCTLADMES----------------------IDAEFFRS--------LTWI
LEN------------------------------------------------SID-GIL--
-DLTFSTEDDR---------------------------FGE-TVTLD-LKPN--------
GRDLE-------------------------------------------------------
------------------------------VTDENKT-EYVEL-VTEWRIQKR-IEEQFI
-AFKAG---FNELVPQ--ELIN-VFDER-ELELLIGG-----------------------
----IADMD-----------VDDW----KKHTDYRG-YTET-------------------
------------------------DEII-QMFFKLIT--------------------SWD
NEKRARLLQF-------ATGTSRIP------------IGG-----FK-DLQGSD--GPRR
FTIEKAGEV-------------------------TQLPKAHTCF-NR-IDLPPY-STYEE
LEKKLS-YSVDE-TMG-
>Taphrina_populina_BAVX01000022.1 .
IKIRRDHI-FEDSYAEI--------------MRQSSSDLKK-RLMIKFEG-------EDG
LDYGGLSREF---FFLL----SHEMFNPFY--------CLFEYSAVDNY-----------
----------------TLQINPHS------------------------------------
--GINPEHLNYFKFIGRVLGLSIFHRRFVDAFFVV--SFYKMILS---------------
--------KKCTLADMES----------------------IDAEFFRS--------LTWI
LEN------------------------------------------------SID-GVL--
-DLTFSTEDDR---------------------------FGE-TVTLD-LKQN--------
GRDIE-------------------------------------------------------
------------------------------VNDGNKA-EYVEL-VTEWRIQRR-IDEQFI
```

-AFKAG---FNELVPQ--ELIN-VFDER-ELELLIGG-----------------------
----IADMD-----------VDDW----KKHTDYRG-YTET-------------------
------------------------DEVI-QMFFKLIT--------------------SWD
NERRARLLQF-------ATGTSRIP------------IGG-----FK-DLQGSD--GPRR
FTIEKAGEV-------------------------TQLPKAHTCF-NR-IDLPPY-STYEE
LEKKLS-YSVDE-TMG-
>Protomyces_lactucaedebilis_MCFI01000034.1 .
IKVRRDHI-FEDSYAEI--------------MRQSSGDLKK-RLMIKFEG-------EDG
LDYGGLSREF---FFLL----SHEMFNPFY--------CLFEYSAVDNY-----------
----------------TLQMNPHS------------------------------------
--GINPEHLNYFKFIGRVLGLSIFHRRFVDAFFVV--SFYKMILN---------------
--------KKCTLADMES----------------------IDAEFFRS--------LTWM
LEN--SID-GIL--
-DLTFSTEDDR---------------------------FGE-VVTLD-LKPD--------
GQSIE---
------------------------------VTDENKS-EYVEL-VTEWRIQKR-IEEQFN
-AFKAG---FHELVPQ--ELIN-VFDER-ELELLIGG-----------------------
----IADMD-----------VDDW----KKHTDYRG-YTET-------------------
------------------------DDVV-QWFFKLIN--------------------TWD
NEKRARLLQF-------ATGTSRIP------------IGG-----FK-DLQGSD--GPRR
FTIEKSGET-------------------------TQLPKAHTCF-NR-IDLPPY-SSYEE
LEKKLS-FSVDE-SIG-
>Schizosacch_cryophilus_XM_013166371.1 .
IKVRRDHI-FEDSYAEI--------------MRNSAHDLKK-RLMIRFEG-------EDG
LDYGGLSREF---FFLL----SHKMFDPIY--------CLFEYSAVDNY-----------
----------------TLQINPHS------------------------------------
--GINPEHLNYFRFIGRVIGLAIFHRRFLDAFFVI--SLYKKLLQ---------------
--------KKVTLADMES----------------------IDADFYRS--------LKWV
LEN--DIT-GIL--
-DLTFSAEEDH---------------------------FGE-VRTIE-LKPN--------
GENIE---
------------------------------VTEENKK-EYVDL-VTEWRISKR-VEDQFN
-AFYAG---FVELVPP--DLVS-VFDER-ELELLIGG-----------------------
----ISDVD-----------INDW----KNHTEYRT-YSAS-------------------
------------------------DQVV-KWFWEIIS--------------------NWK
NEERSMLLQF-------ATGTSRIP------------VNG-----FR-DLQGSD--GPRK
FTIEKAGSP-------------------------DQLPVAHTCF-NR-LDLPEY-PTKAK
LEGKLS-LAIEN-TVGF
>Schizosacch_octosporus_XM_013162116.1 .
IKVRRDHI-FEDSYAEI--------------MRNSAQDLKK-RLMIRFEG-------EDG
LDYGGLSREF---FFLL----SHKMFDPIY--------CLFEYSAVDNY-----------
----------------TLQINPHS------------------------------------
--GINPEHLNYFRFIGRVIGLAIFHRRFLDAFFVV--SLYKKLLQ---------------
--------KKVTLADMES----------------------IDADFYRS--------LKWV
LEN--DIT-GIL--
-DLTFSAEEDH---------------------------FGE-VRTIE-LKPN--------
GENIE---
------------------------------VTEENKK-EYVDL-VTEWRISRR-VENQFN
-AFYAG---FVELVPP--DLVS-VFDER-ELELLIGG-----------------------
----ISDVD-----------IGDW----KSHTEYRT-YSSS-------------------
------------------------DQVV-KWFWEIIS--------------------NWK
NEERSMLLQF-------VTGTSRIP------------VNG-----FR-DLQGSD--GPRK
FTIEKAGSP-------------------------DQLPVAHTCF-NR-LDLPEY-PTKAK
LEGKLS-LAIEN-TVGF
>Schizosacch_pombe_NM_001021694.2 .
VKVRRDHI-FEDSYAEI--------------MRYSAHDLKK-RLMIRFDG-------EDG
LDYGGLSREF---FFLL----SHKMFDPIY--------CLFEYSAVDNY-----------
----------------TLQINPHS------------------------------------

--SINPEHLNYFRFIGRVIGLAIFHRRFLDAFFVV--SLYKKLLR---------------
--------KKVSLADMES----------------------IDAEFYRS--------LKWV
LEN--DIT-GIL--
-DLTFSVEEDH---------------------------FGE-VRTVE-LITN--------
GENIE---
------------------------------VTEENKK-KYVDL-VTEWRVSKR-VEQQFN
-AFYSG---FVELVSP--DLVN-VFDER-ELELLIGG-----------------------
----ISDVD-----------VEDW----KSHTEYRT-YIAT-------------------
------------------------DPVI-KWFWEIIA--------------------GWK
NEDRSKLLQF-------ATGTSRIP------------VNG-----FR-DLQGSD--GPRK
FTIEKAGTP-------------------------DQLPVAHTCF-NR-LDLPDY-PSKDT
LHEKLS-LAVEN-TVGF
>Schizosacch_japonicus_XM_002175654.2 .
IKVRREHI-FEDSYAEI--------------MRQSPIELKK-RLMIRFEG-------EDG
LDYGGLSREF---FFLL----SHKMFDPIY--------CLFEYSAVDNY-----------
----------------TLQINPHS------------------------------------
--SINPEHLNYFKFIGRVIGLAIFHRRFLDAFFVV--SLYKMLLR---------------
--------KKVTLADMES----------------------IDAEFYRS--------LKWI
LDN--DIT-GIL--
-DLTFIAEEDH---------------------------FGE-VRTVE-LKPN--------
GDQIE---
------------------------------VTEDNKK-EYVEL-VTQWRVTKR-VEEQFN
-AFYDG---FIDIIPP--ELIN-IFDER-ELELLIGG-----------------------
----ISDVD-----------VEDW----KTNTEYRT-YTST-------------------
------------------------DQVV-VWFWDIIS--------------------SWE
NEKRSRLLQF-------ATGTSRIP------------VNG-----FR-DLQGSD--GPRK
FTIEKAGSA-------------------------EQLPVAHTCF-NR-LDLPPY-ESKER
LDDRLT-MAIEN-TIGF
>Schizosacch_japonicus_XM_002175058.2 .
IKVRRDHI-FEDSYAEI--------------MRQSASDLKK-RLMIKFDG-------EDG
LDYGGLSREY---FYLL----SHEMFNPFY--------CLFEYSSVDNY-----------
----------------TLQINPHS------------------------------------
--GINPEHLNYFKFIGRVIGLAIFHRRFVDAFFVV--SFYKMILK---------------
--------KKVALSDMES----------------------MDAEYYRS--------LMWI
LNN--DIT-DIL--
-DLTFSVEDNC---------------------------FGE-VVTVD-LIPN--------
GRNIE---
------------------------------VTEENKQ-QYVEA-VTEWRIQTR-IQDQFR
-AFYEG---FSELIPH--ELVT-VFDER-ELELLIGG-----------------------
----ISEID-----------LEDW----KKYTEYRS-YTAN-------------------
------------------------DQVI-KWFWELIE--------------------EWD
NEKRSRLLQF-------TTGTSRIP------------VNG-----FK-DLQGSD--GPRK
FTIEKSGEP-------------------------TQLPKAHTCF-NR-LDLPDY-PTKQV
LDAKLS-LAIEE-TIGF
>Schizosacch_pombe_NM_001019819.2 .
IKVRRNHI-FEDSYAEI--------------MRQSATDLKK-RLMIKFDG-------EDG
LDYGGLSREY---FFLL----SHEMFNPFY--------CLFEYSSVDNY-----------
----------------TLQINPHS------------------------------------
--GINPEHLNYFKFIGRVIGLAIFHRRFVDAFFVV--SFYKMILQ---------------
--------KKVTLQDMES----------------------MDAEYYRS--------LVWI
LDN--DIT-GVL--
-DLTFSVEDNC---------------------------FGE-VVTID-LKPN--------
GRNIE---
------------------------------VTEENKR-EYVDL-VTVWRIQKR-IEEQFN
-AFHEG---FSELIPQ--ELIN-VFDER-ELELLIGG-----------------------
----ISEID-----------MEDW----KKHTDYRS-YSEN-------------------
------------------------DQII-KWFWELMD--------------------EWS
NEKKSRLLQF-------TTGTSRIP------------VNG-----FK-DLQGSD--GPRK

```
FTIEKAGEP-------------------------NKLPKAHTCF-NR-LDLPPY-TSKKD
LDHKLS-IAVEE-TIGF
>Schizosacch_cryophilus_XM_013168604.1 .
IKVRRNSI-FEDSYAEI--------------MRQSASDLKK-RLMIKFEG-------EDG
LDYGGLSREY---FYLL----SHEMFNPFY--------CLFEYSSMDNY-----------
----------------TLQINPHS------------------------------------
--GINPEHLNYFKFIGRVIGLAVFHRRFVDAFFVV--SFYKMILQ---------------
--------KKITLQDMES----------------------MDAEYYRS--------LVWI
LKN------------------------------------------------DIT-GIL--
-DLTFSVEDNC---------------------------FGE-VVTID-LKPD--------
GRNIE-------------------------------------------------------
------------------------------VTEENKK-EYVDL-VTVWRVQKR-IEDQFN
-AFHDG---FSELIPQ--DLIN-VFDER-ELELLIGG-----------------------
----ISEVD-----------MDDW----KKHTDYRS-YSDN-------------------
------------------------DQII-KWFWELMN--------------------EWS
NEKKSRLLQF-------TTGTSRIP------------VNG-----FK-DLQGSD--GPRR
FTIEKSGEP-------------------------SKLPKAHTCF-NR-LDLPPY-ISKHD
LDHKLS-IAVEE-TIGF
>Schizosacch_octosporus_XM_013161548.1 .
IKVRRTSI-FEDSYAEI--------------MRQSASDLKK-RLMIKFEG-------EDG
LDYGGLSREY---FYLL----SHEMFNPFY--------CLFEYSSMDNY-----------
----------------TLQINPHS------------------------------------
--GINPEHLNYFKFIGRVIGLAVFHRRFVDAFFVV--SFYKMILQ---------------
--------KKTTLQDMES----------------------MDAEYYRS--------LVWI
LKN------------------------------------------------DIT-GIL--
-DLTFSVEDNC---------------------------FGE-VVTID-LKPD--------
GRNIE-------------------------------------------------------
------------------------------VTEENKK-EYVDL-VTVWRVEKR-IEDQFN
-AFHEG---FSELIPQ--DLIN-VFDER-ELELLIGG-----------------------
----ISEVD-----------MDDW----LKHTDYRS-YCEN-------------------
------------------------DAVI-KWFWELMN--------------------EWS
NEKKSRLLQF-------TTGTSRIP------------VNG-----FK-DLQGSD--GPRR
FTIEKSGEA-------------------------SKLPKAHTCF-NR-LDLPPY-VSKQD
LDHKLS-IAVEE-TIGF
>Entomophthora_muscae_GEND01005691.1 .
IKVRRDHI-FEDAFVEV--------------MKHSPSDLKK-RLMIKFEG-------EDG
LDYGGLSREF---FYLL----SHEMFNPVY--------CLFQYSSHNNY-----------
----------------TLQINPYS------------------------------------
--AINPEHLNYFKFIGRVLGLAVFHRRFLDAFFVV--SIYKMILK---------------
--------KKTTLADMES----------------------IDVEYWRS--------LEWM
LNN------------------------------------------------DIE-DIL--
-ELDFSVEEDR---------------------------FGE-KVQID-LITD--------
GRNIP-------------------------------------------------------
------------------------------VTNENKR-EYVEL-VTQWKINNR-IREQFT
-AFFEG---FHQLIPD--DLIV-VFDER-ELELLIGG-----------------------
----IAEID-----------VEDW----KKHTDYRG-YTEQ-------------------
------------------------DEVI-QWFWKCVR--------------------TFD
NEKKSRLLQF-------TTGTSRIP------------VNG-----FK-DLQGSD--GPRR
FTIEKAGEI-------------------------AQLPKSHTCF-NR-IDLPPY-KSYDV
LVQKLT-FAVEE-TLGF
>Allomyces_macrogynus_ACDU01000955.1 .
IKVSRNAI-FEDSYNEI--------------MRLPVAELKK-KLVIIFDK-------EDG
LDYGGLSREF---FFLL----SHEMFNPFY--------CLFEYSAHDNY-----------
----------------TLQINPNS------------------------------------
--SINAEHLNYFRFIXRIVGLAIFHRRFLDAFFVV--SFYKTILK---------------
--------KKITLADMES----------------------VDADFHRS--------LEWM
LNN------------------------------------------------DIT-DIL--
-DLTMSTEDNR---------------------------FGE-VVTID-LVPG--------
```

GRDIP---
------------------------------VTEENKR-EYVEK-ITEWRIVKR-VEEQSN
-AFRQG---LFDFVPE--DLIT-VFDER-ELELLIGG-----------------------
----LAEID-----------VDDW----VKHTDYRG-YTES-------------------
------------------------DEVV-LWFWKCIR--------------------SWE
NEKRARFLQF-------ATGTSRIP------------VNG-----FK-DLQGSD--GPRR
FTIEKAAGDD------------------------QALPKAHTCF-NR-IDLPPY-KSYDV
LVQKLS-LAVEE-TMGF
>Linderina_pennispora_MCFD01000004.1 .
IKVSRDSV-FENSYNEI--------------MRVPVSELKK-RLMIKFDG-------EDG
LDYGGVSREY---FFLL----SHEMFNPQY--------CLFEYSAHDSY-----------
----------------TLQINPHS------------------------------------
--NINPEHLNYFRFIGRTMGLAIFHRRFLDAFFTS--SFYKMLLK---------------
--------KPVDLEDMQS----------------------VDVEIYNS--------LKWV
LEN--DVS-DMG--
--FTFSLDDDK---------------------------FGE-RVEVE-LKPG--------
GKDIE---
------------------------------VTEENKK-EYVQLN-VQYRVCDR-IKEQFE
-AFQAG---FHELIPE--DLIQ-VFDER-ELELLIGG-----------------------
----IAEID-----------IDDW----KKHTDYRG-YTES-------------------
------------------------DQVV-QWFWKAVQ--------------------EMD
SEHQARMLQF-------TTGTSRIP------------VNG-----FK-DLQGSD--GPRR
FTIEKAGDI-------------------------VALPKSHTCF-NR-IDLPPY-TEYET
LKSKII-LAIEN-TVG-
>Zancudomyces_culisetae_LSSK01001377.1 .
IKVVRGSV-LEDSFKEI--------------AACPVNELKK-RLMIKFDG-------EDG
LDYGGVSREF---FFLL----SHEMFNPLY--------CLFEYSAHDTY-----------
----------------SLQISPKS------------------------------------
--AINPDHLNYFKFIGRTVGLAIFHRRFLDAFFTS--SFYKMILK---------------
--------KPITLEDMQS----------------------VDDEIYRS--------LVWT
LEN--DIT-DMG--
--FTFSIDDCV---------------------------FGE-MVEVE-LKEG--------
GKDIA---
------------------------------VTEENKR-EWVDLN-VQYRICKR-IEPQFE
-KFMEG---FSELVPP--ELIQ-VFDER-ELELLIGG-----------------------
----LAETD-----------VDDW----KKHTDYRG-YTEA-------------------
------------------------DQVI-QWFWKCVR--------------------EMD
DEKRVRLLQFS-------TGTSRIP------------VNG-----FK-DLQGSD--GPRR
FTIEKAGTI-------------------------DSLPKSHTCF-NR-IDLPPY-PDYET
LIQKLT-MAVEN-TVG-
>Capniomyces_stellatus_LUVW01000022.1 .
IKVRRDTI-FEDSFNEI--------------LRPPVFELKK-RLMIKFDG-------EDG
LDYGGVSREF---FFLL----SHEMFNPQY--------CLFEYSAHDTY-----------
----------------SLQINPNS------------------------------------
--GINPEHLNYFKFIGRTVGLAIFHRRFLDAFFTS--SFYKMILK---------------
--------KPITLDDMQS----------------------VDDEIYRS--------LVWT
LQN--DVS-DMG--
--FTFSIDDNK---------------------------FGE-LTEVE-LKPG--------
GKDIA---
------------------------------VTQENKK-EWVDLN-IQYRICTR-IQPQFD
-SFLAG---FYELIPH--DMIQ-VFDER-ELELLIGG-----------------------
----LAETD-----------VDDW----KKHTDYRG-YTEA-------------------
------------------------DQVI-QWFWLCVK--------------------AMD
AEHQTRLLQF-------TTGTSRIP------------VNG-----FK-DLQGSD--GPRR
FTIEKTGSI-------------------------DSLPKSHTCF-NR-IDLPPY-PDYDT
LVHKLT-LAIEN-TVG-
>Smittium_mucronatum_LSSL01006007.1 .
IRVNRETI-FEDSYKEI--------------KVLPAIDLKK-RLMLKFEG-------EDG

LDYGGVSREF---FFLL----SHEMFNPQY--------CLFEYSAHDTY-----------
----------------TLQISPNS------------------------------------
--SINPDHLEYFRFIGRIVGLAIFHRRFLDAFFTT--SFYKMILN---------------
--------KPITLDDMQS----------------------VDDEIYRS--------LKWA
LEN--DIS-EMG--
--FTFSIEEAK---------------------------FGE-IVEVE-LKPG--------
GAEID---
------------------------------VTEENKK-EWVQLN-VQYRICDR-IKPQFD
-AFLTG---FNELIPQ--DMIQ-VFDER-ELELLIGG-----------------------
----LAETD-----------LDDW----KKHTDYRG-YTES-------------------
------------------------DQVI-QWFWLCIK--------------------NMD
DEHRTRLLQF-------TTGTSRIP------------VNG-----FK-DLQGSD--GPRR
FTIEKSGDI-------------------------GALPKSHTCF-NR-IDLPPY-PDYET
LAYKLN-LAIEN-TIG-
>Smittium_culicis_LSSN01001734.1 .
IKISRDTI-FEDSYNAI--------------KCLTPIDLKK-RLMLKFEG-------EDG
LDYGGVSREF---FFLL----SHEMFNPLY--------CLFEYSAHDTY-----------
----------------TLQISSNS------------------------------------
--SINPDHLEYFKFIGRIVGLAIFHRRFLDSFFTT--SFYKMILG---------------
--------KPITLDDMQS----------------------VDNEIYNS--------LKWA
LDN--DVT-GMD--
--FTFSVEESK---------------------------FGE-IVEVE-LKPG--------
GAEIE---
------------------------------VTEENKK-EWVQLN-VQYRICDR-IKPQFD
-AFLSG---FNELIPQ--DMIQ-VFDER-ELELLIGG-----------------------
----LAETD-----------IDDW----KKHTDYRG-YTES-------------------
------------------------DQVI-QWFWLCIK--------------------NMD
DEHRTRLLQF-------TTGTSRIP------------VNG-----FK-DLQGSD--GPRR
FTIEKSGDI-------------------------NSLPKSHTCF-NR-IDLPPY-PDYET
LSHKLN-LAIEN-TIG-
>Rhodotorula_graminis_XM_018413272.1 .
IIVRREHL-FEDAFAEM--------------MKYSGEDLRK-RLMVSFQG-------EEG
VDFGGVSREF---FFML----SHEIFNPSY--------CLFEPTEKTSY-----------
----------------TLQVNPNS------------------------------------
--SINEDHLSYFQFVGRCVGLAIFHRRFLDVHFST--AIYKTCLG---------------
--------RTIGLEDMAV----------------------IDQQMHQS--------LTWM
SEN--DIT-DVI--
-ELDFTAQHES---------------------------FGT-LETTE-LVPG--------
GADIA---
------------------------------ITEDNKH-EYIQL-LCQHRLKGR-VEQQLE
-AFKLG---LGEIVPL--KELE-VFDEK-EFELIVSG-----------------------
----VSELD-----------LTDW----EKHTDYRG-FHKD-------------------
------------------------DQLV-RWFWQVVG--------------------TWD
LEKKARLLQF-------TTGTSRVP------------VNG-----FK-DLQGSD--GPRR
FTLEKSGDI-------------------------SQLPKSHTCF-NR-LELAPY-PTYDM
LEQKLT-FAVEN-TLGF
>Rhodotorula_toruloides_XM_016416399.1 .
LIVRRANL-FEDAFGEV--------------MKYSPEDLKK-RLMVTFKG-------EEG
VDFGGVSREF---FFLL----SHAVFDPSY--------CLFEPTEKTSY-----------
----------------TLQIHPNS------------------------------------
--GINPEHLDYFTFVGRAVGMAIFHRRFLDAHFAT--SIYKACLD---------------
--------RPIGLEDMAT----------------------IDAQLWQS--------LTWM
AEN--DIT-DVL--
-DLDFTSQYES---------------------------FGT-LETCE-LMPD--------
GANVP---
------------------------------VTEENKH-EYIRL-LCEHRLKGR-VEAQLE
-ALKRG---LGEIVPL--KELR-VFDEK-ELELLIGG-----------------------
----VETID-----------VQDW----EAHTDYRG-YTAT-------------------

------------------------DQVV-RWFWQAVK--------------------SWP
AEKRSRLLQF-------STGTSRTP------------PNG-----FR-DLQGSD--GPRR
FTIEKAVGGK------------------------GALPKSHTCF-NR-IDLPPY-ESLEI
LESKLL-FALEEGASGF
>Capniomyces_stellatus_LUVW01000001.1 .
VDVKRDNL-FESSYQNI--------------MSIKVENLKK-ALKIKFDG-------EDG
LDYGGVSREY---FYLL----SHEMFNPEY--------CLFQYSDIDNY-----------
----------------TLQINPNS------------------------------------
--GINPEHLNYFNFIGRVIGLAIFHQKYLDAFFTS--QIYKSILR---------------
--------KPIEIEDMQS----------------------IDSEIYNS--------LVWV
LDN--DVT-ELG--
--LTFSIDDNQ---------------------------FGK-PVEIE-LKPN--------
GRNIA---
------------------------------VTNANKY-EYVSLS-INFRITDR-VKPQIN
-SLLEG---FHLFVPL--ELIQ-VFDEK-ELELLIGG-----------------------
----IAKID-----------MDDW----VKNTNYKN-YSET-------------------
------------------------DQVI-VWFWKCVK--------------------EMD
SELQIRLLQF-------TTGTSRIP------------VNG-----FK-DLYGSD--GPRL
FTIENTGSI-------------------------DSLPKSHTCF-NR-IDLPPY-KDYDT
LASKLT-LAIEN-TIG-
>Zancudomyces_culisetae_LSSK01000076.1 .
-KIDRNDI-VESAYKQI--------------MSMDVDDLKK-VLRIKIGD-------EEG
IDYGGVSREF---FYLL----SRSIFNPAY--------CLFQYAAIDNY-----------
----------------TLQINPQS------------------------------------
--GINPAHLDYFYFVGKIMGLAVFHQRYLDAFFTA--QIYKSILR---------------
--------KPIRVDDMQS----------------------IDPEIYNS--------LLWM
LDN--DVT-DLD--
--MTFIVEIDR---------------------------FGV-KEVIE-LVPD--------
GKNIV---
------------------------------VTNENKY-KFVDLN-VNFRIIDN-VKPQLD
-RLLAG---FHEFVPL--ELIQ-VFDER-EMEFVLGG-----------------------
----LATID-----------IDDW----KANTVYKN-YNEM-------------------
------------------------DMPV-VWFWKCVS--------------------EMN
SENRALLLQF-------VTGTSRVP------------VNG-----FK-DLYGSD--GPRK
FTIEKLGTS-------------------------SSLPKSHTCF-NR-IDLPPY-ESFDI
LVSKLT-LAIEN-TVG-
>Schizosacch_japonicus_XM_002172574.2 .
IKVERKNI-LHNAFDII--------------LRLPPEDLKM-KLLIRFKG-------EDG
LDYGGVSREF---FYLL----SHQMFDPST--------CLFEYSNAGDY-----------
----------------SLRISIHS------------------------------------
--CINEEHLSWFRFIGRVIGLAIFHRRYLDVCFVR--TLYKTLLG---------------
--------QSPEFEDLQY----------------------VDKDLYHS--------LSWI
RDN--HVD-ETL--
-CLTFSTTVDH---------------------------FGE-AVIYD-FKPN--------
GRNIS---
------------------------------VTEENKH-EFLKL-ASEWHMYKN-TEEQFQ
-SLKRG---LNEIISD--KDLR-IFDVD-ELGVLIGG-----------------------
----TSTID-----------VEDW----KRYTDYRS-YSES-------------------
------------------------DLTI-KLFWRLVS--------------------EWP
EEKRARLLQF-------TTGTSRIP------------LNG-----FK-DIHGSD--GPRK
FVIEKVGTV-------------------------DQLPHAHTCF-NR-LDLPPY-QTKQQ
LDRKLT-LAIEE-TAGF
>Schizosacch_pombe_NM_001019355.2 .
LKVSRATT-FEDAYDII--------------SKLSVSDMKK-KLLIRFRN-------EDG
LDYGGVSREF---FYIL----SHAIFNPGY--------SLFEYATDDNY-----------
----------------GLQISPLS------------------------------------
--SVNPDFRSYFRFVGRVMGLAIYHRRYLDVQFVL--PFYKRILQ---------------
--------KPLCLEDVKD----------------------VDEVYYES--------LKWI

```
KNN------------------------------------------------DVD-ESL--
-CLNFSVEENR---------------------------FGE-SVTVD-LIPN--------
GRNIA-------------------------------------------------------
------------------------------VNNQNKM-NYLKA-LTEHKLVTS-TEEQFN
-ALKGG---LNELIPD--SVLQ-IFNEN-ELDTLLNG-----------------------
----KRDID-----------VQDW----KRFTDYRS-YTET-------------------
------------------------DDIV-IWFWELLS--------------------EWS
PEKKAKLLQF-------ATGTSRLP------------LSG-----FK-DMHGSD--GPRK
FTIEKVGHI-------------------------SQLPKAHTCF-NR-LDIPPY-NSKEE
LEQKLT-IAIQE-TAGF
>Schizosacch_octosporus_XM_013162238.1 .
LRVSRATA-LEDAYDII--------------MKLSVQDMRK-RLLVRFRN-------EDG
LDYGGVSREF---FYIL----SHAVFDPSY--------SLFEYAAQDNY-----------
----------------GLQISPSS------------------------------------
--YFNPEHLTYFRFVGRIMGLAIFHRRYLDVQFVL--PFYKRLLQ---------------
--------KPLTMEDFKD----------------------IDEVYYES--------LKWI
REN------------------------------------------------KVD-DSL--
-CLNFSCEENR---------------------------LGD-SVTVD-LKTN--------
GRNLP-------------------------------------------------------
------------------------------VNDENKE-EYIQL-LTQYKLVRS-TQLQFD
-ALKGG---LNELIPD--SILF-LFNER-ELDLVLNG-----------------------
----KRDID-----------VEDW----KRYTDYRA-YTET-------------------
------------------------DRIA-IWFWEIVS--------------------QWP
TNKKEKLLQF-------STGTSRLP------------LGG-----FK-DLHGSD--GPRK
FTLENVGSV-------------------------SQLPKAHTCF-NR-LDIPPY-ESKEE
LERKLT-IAIEE-TAGF
>Schizosacch_cryophilus_XM_013166028.1 .
LRVNRATA-LEDAYDII--------------MKLSVEDMRK-RLLVRFRN-------EDG
LDYGGVSREF---FYIL----SHALFNPSY--------SLFEYATQDNY-----------
----------------GLRISPSS------------------------------------
--YVNPEHLTYFRFVGRIMGLAIFHRRYLDVQFVL--PFYKRLLQ---------------
--------KPMVMEDFRD----------------------IDEIYYES--------LNWI
RNN------------------------------------------------KVD-EFL--
-CLNFSCEENR---------------------------LGD-SVTID-LIPN--------
GRNIP-------------------------------------------------------
------------------------------VTDENKL-EYLRL-LTDYKLVRS-THMQFD
-ALKEG---LNELIPD--SVLL-LFDEN-ELDVLLNG-----------------------
----KRDID-----------VEDW----KRYTDYRA-YTET-------------------
------------------------DRIV-TWFWEIIS--------------------HWP
IEKKQKLLQF-------STGTSRLP------------LGG-----FK-DLHGSD--GPRK
FTLESIGSV-------------------------SQLPKAHTCF-NR-LDIPPY-ESKKE
LERKLT-IAIEE-TSGF
>Entomophthora_muscae_GEND01016179.1 .
LQIRRTHL-LEDAFRRI--------------MSANKKDLQRGRLAVLFDT-------EEG
LDYGGPSREF---FFLL----SRELFNPYY--------GLFEYSANDTY-----------
----------------TVQVSPLS------------------------------------
--AFVDNCHDWFRFSGRVLGLALVHQYLLDAFFTR--PFYKALLR---------------
--------LPVALSDLES----------------------LDNEFHQS--------LQWI
RDN------------------------------------------------DIGXIDL--
-GLTFCVTEEL---------------------------LGR-IVERE-LKPG--------
GKNXI-------------------------------------------------------
------------------------------VNEKNKK-EYLER-MIKWRLERG-VQEQTE
-SLVRG---FYEVVDS--RLVS-VFDAR-ELELVIAG-----------------------
----TAEID-----------INDW----RLNTEYRSGYHDN-------------------
------------------------HIVI-IWFWQVIE--------------------KFT
NEQRLRLLQF-------VTGTSSIP------------YEG-----FS-ALRGST--GPRR
FCIEKWGKP-------------------------NALPRAHTCF-NR-LDLPPY-PTPEL
LYEKLL-LAVEE-TNTF
```

>Mitosporidium_daphniae_XM_013382979.1 .
VPIRRDNL-LEDSFNVI--------------SNLTLSSLKK-KPQIIFEG-------EEG
LDYGGISREF---FFLL----AKEIFNPYY--------SLFEYSSQDNY-----------
----------------TLQISPGS------------------------------------
--YINPEHMAYFYFVGRILGLAAFHGFLIDAYFVP--AFYKRLLD---------------
------RAACTMLSDLEA----------------------IDSELYSS--------LIWM
LNN--PIH-DQI--
-FETMSVEEVRFG-------------------------GGD-PIIVD-LIPN--------
GSSIS---
------------------------------VTDDNKR-LYIEK-LVEWKTYKR-ISEQMH
-QINRG---FYEIVPR--ELIS-IFDAR-ELEVVH-------------------------

---SYP
AEKKIRLLQF-------VTGSSKLP------------LNG-----FR-DLQGSD--GPRK
FTIEKTLSPP------------------------SHLPVSHTCF-NR-LDLPSY-NSMEM
LKKRLD-KSLEN-ILGF
>Galactomyces_candidum_CCBN010000015.1 .
LKISRDRL-IETSFDAI--------------MSQSPKKLKK-RLCIKFCG-------EDG
VDQGGILKEF---FYHL----SRSLLDPGY--------GMFEHGSDSKD-----------
----------------SLQINPYY------------------------------------
-----TSSLDHYKFAGRLVGLAVFHGHLIDGYFGL--WFYRHILG---------------
--------KTVEIADLEL----------------------MDPEYYRS--------LKWM
RDN--DIE-GII--
-DNTFSIEEDV---------------------------QGK-LNTID-LKVD--------
GHNIS---
------------------------------VTNKNKK-EYLQL-VGEWRLYKR-SEVQFQ
-QFLEG---FHEFVPA--TITK-LLNEK-ELGLLVAG-----------------------
----HPRID-----------LDYW----RRHTKYGEGYTND-------------------
------------------------CHVI-NWFWACLE--------------------TWD
PEQHSRLLRF-------VTGSGRIP------------ANG-----FS-PMGYHS--EQLR
FTILRQPNP-------------------------KSLPTASTCT-SS-LYLPRY-HSYHD
LNKKLM-FAIEE-TIG-
>Alloascoidea_hylecoeti_BCKZ01000023.1 .
IDVSRDQI-FETSYASL--------------MSLPPKKLRKRKLQLTFAG-------ENG
VDIGGVSKDF---FHSI----SQVIFNPDF--------GLFEYASSENY-----------
----------------RLQINPDP-----------------------------------G
NKFTEIDRLSHFRFVGRIIGLCMFHHNYLNVSFVE--SLYKQLAG---------------
--------APVSISDLKI----------------------NDIQFYNS--------LMYI
LNN--DDV-EDL--
-ELNFSVDKMK---------------------------SGK-SKIID-LIDN--------
GRNVL---
------------------------------VTNKNKD-LYVEL-MTEWKLTKI-CHNFTK
-QLLKG---FHEVIPE--SALN-SVNGP-QLEYIICG-----------------------
----TAEID-----------IIDW----KENTILYK-YKET-------------------
------------------------DDVY-RWFWMCME--------------------SWN
MEQRAKVLQF-------TTGSTRLP------------VTG-----FK-DLPAGK--GHHK
FTIQKTSKV-------------------------RELPRSHVCF-NT-LVLPSY-SSYKE
LCDKLE-LAVQE-TEG-
>Pecoramyces_ruminatium_ASRE01009082.1 .
ITINRNEL-FSDAYKAI--------------MTISPDELKN-LLKIKYVG-------EEG
IDAGGLLRDF---FYNL----AKEIGNPDY--------LLFQYSSDNSY-----------
----------------DLNINKHS------------------------------------
-SRIHSNYLGYYKFVGRMLGLTILHKQTLPISFSI--LFYKKLLN---------------
--------KPVTFSDLKY----------------------IDSELYKN--------LNWL
KEN--DGS-ENL--
-FLTFELEETDC--------------------------FGQ-HNVVE-LKPN--------
GRNIN---
------------------------------VNDSNKN-EYIDL-IVQKKLKSN-DEEQMI

```
-AIREG---FYELIPN--DVSE-ILNEI-DLNYLISG-----------------------
----TNEID-----------INDW----KNNTIYIG-YNEN-------------------
------------------------DTTV-VNFWKCIE--------------------DFS
DEKRKRLLLF-------VTGNTKLP------------VTG-----FK-DLQGRNG-KIGH
FTIRKLGKI------------------------NDLPKSHTCC-NY-LSIPPY-TSYTQ
LKQKLL-FSITE-G---
>Nematocida_parisii_XM_013204697.1 .
LMVQRGAV-FEDTFHQL--------------MRLNGEQVRNAKFNIKFAG-------EEG
VDAGGLTREW---YSEL----SKEMFNANY--------ALFTPIGS--------------
----------------SYQPNHIS------------------------------------
--HINPEHLVYFKFIGRIIGKAVYDEMTVDCHFTR--AFYKRVLS---------------
--------IPVDLTDVEA----------------------LDPEFHRS--------LVWI
LEN------------------------------------------------DIE-NVL--
-EMTFSLEQDR---------------------------FGI-TEVID-LKEN--------
GRNIA-------------------------------------------------------
------------------------------VTNENKR-EYVEL-VCRFKLVRV-IERQLS
-AFAEG---FFEILDV--DMLR-MFNEK-ELELLISG-----------------------
----LPEID-----------VDDW----RNNTIYFG-YTSD-------------------
------------------------SQVI-RWYWRAVR--------------------NFS
MEERAKLLQF-------ATGTSKLP------------LEG-----FA-GLRCQN--GNQK
FQIHKASGGS-----------------------SRLPTAHTCF-NQ-LDLPEY-DSYEQ
LVKALL-FSLEECTSGF
>Nematocida_displodere_LTDL01000042.1 .
--VNRETI-FEDTFHQL--------------MQYSGKEVREGKFNIKFAE-------EEG
IDGGGLTREW---YSEL----SKEMFNPNY--------ALFTPIGL--------------
----------------AYQPNPNS------------------------------------
--YINPEHLLYFKFIGRIIGKAVHDGMNLDCYFTR--VFYKRILG---------------
--------INVDLTDMEA----------------------MDPEFHRS--------LKWI
EEN------------------------------------------------DIDSVGL--
-DLTFSIENDR---------------------------FGV-TEIVE-LKNN--------
GKNLL-------------------------------------------------------
------------------------------VTNANKK-EYVDL-ICTFKLIRM-VERQLA
-AFIEG---FVEVLDS--SLVK-MFDEK-ELELLISG-----------------------
----LPEID-----------VDDW----RNNTVYHG-YTAN-------------------
------------------------SQVI-RWFWRAVR--------------------NFS
VEERTKLLQF-------ATGTSKLP------------LEG-----FA-ALKGQN--GPQK
FQIHKASGSS-----------------------NRLPSAHTCF-NQ-LDLPEY-EEYEV
L----------------
>Edhazardia_aedis_AFBI03000025.1 .
IKVDRSKI-FEDSYSAI--------------MNQSPANLERSKLSIKFKE-------EEG
VDGGGLTREF---YQCL----SKEIVNPDY--------CLFVLNSDNI------------
----------------TYSINKSS------------------------------------
--YVNPEHLLYFRFVGRIIAKGIIDENYFDIHFTK--SFYKQILG---------------
--------KEPDFNDLES----------------------FDENFYRS--------MKWL
KNN------------------------------------------------SIQ-NIV--
-ELYFCEEYEE---------------------------FGE-RKTFN-LIEN--------
GDKIM-------------------------------------------------------
------------------------------VTDHNKD-LYISL-ISKFKMISG-CEKQTE
-AFVQG---FREILDQ--KLVN-LFDEK-ELELLISG-----------------------
----CPDLD-----------VDDW----RNNTAYHG-YKSN-------------------
------------------------DKEI-QWFWRAVR--------------------SFT
NENKAKLLQF-------VTGTSKLP------------IDG-----FS-ALRGSN--DLEK
FQIHLAHTG------------------------KLPSAHTCS-NQ-LDLPKY-DSYEE
LRKYLL-YAITECS---
>Nosema_ceranae_ACOL01000841.1 .
ITVRRSSI-LNDSFFQV--------------MRKSSKELKAKRIQIKFSG-------EQG
IDFGGLTKEW---LELV----MQEALKPDQ--------GLFVYSSDKRN-----------
----------------SLHPFKNS------------------------------------
```

--KIDPDHLSFFRFIGRMLAKIIIEGFNISIHFDK--SVYKYLLG---------------
--------LKCTLQDLEE----------------------IDPQFYNS--------LMWI
KNN--KIE-NIL--
-NLTFSVENNN---------------------------FGF-NEVID-LIPE--------
GRQVL---
------------------------------VTDDNKN-DYIDL-VVENRLIKS-VEKQLN
-AMREG---LFEMIDE--DNIC-IFNEK-ELELLICG-----------------------
----IPDIN-----------IDDW----KNNTEYIG-YTSH-------------------
------------------------SRNI-SWFWKAVE--------------------GFT
PEEKAKLLQF-------CTGSSRVP------------FEG-----FK-NLQSTN--GYQK
FSINKIST--------------------------NRLPSAHTCF-NQ-LDLPEY-TSYEE
LRKNIL-YAINECQGG-
>Ordospora_colligata_XM_014708285.1 .
VYVDRDDV-LRSSYFQV--------------MAKSPEDFRTKRFEIKLAG-------EEG
LDYGGITREW---LLLL----AKDLLDPNF--------ALFEFSTEDKT-----------
----------------VAVPCKNS------------------------------------
--HVNPEHLSYFKFVGRIMAKAIMEGYFLNLQLPK--FVYKHILG---------------
--------KTCGLDDLKS----------------------VDNEFYKS--------LIWI
RDH--SVD-ESL--
-GLMFSFTEVS---------------------------FGV-NITTD-LIDN--------
GRDVF---
------------------------------VSESNKK-EYVRV-AAWHRLFNG-IEAQLS
-ALKAG---IFEILGE--DALD-MFDEN-ELELLVCG-----------------------
----IPEIN-----------VDDW----KSNTLYYG-YTES-------------------
------------------------SKTI-IWFWKAVK--------------------CLD
SVQKAKLLQF-------ATGTSTLP------------FEG-----FS-HLQGNN--TIQK
FSIHKMPDRM------------------------DSLPTAHTCF-NQ-LVLPSY-SSYET
MLKCLT-TAINECSTGF
>Encephalitozoon_cuniculi_NM_001042086.1 .
VYVDRSDV-LRSSYFQV--------------MAKSPEEFRTRRLEIKLTG-------EEG
LDYGGLTREW---LVLL----AKDLLDPNF--------ALFEFATEDKT-----------
----------------TVVPCKNS------------------------------------
--YVNPEHLSYFKFVGRIIAKAIMDGNFINLHLSK--FIYQYILG---------------
--------KSCDLQDLES----------------------ADPEFHKS--------LVWI
RDN--PVD-KSL--
-GITFSFDDVS---------------------------FGV-HRTVE-LVEG--------
GAHVF---
------------------------------VDDSNKA-EYVKL-ATQYRLFNG-IELQLS
-ALKSG---LFEILGS--KALE-MFDES-ELELLICG-----------------------
----IPDID-----------VDDW----KNNTLYYG-YAEN-------------------
------------------------SKTV-IWFWRAVK--------------------SLD
SVSRAKLLQF-------VTGTSTLP------------FEG-----FS-HLQGNN--EVQK
FSIHKVSDRI------------------------DSLPTAHTCF-NQ-LVLPEY-SSYEN
LLKYLT-LAINECSTGF
>Encephalitozoon_intestinalis_XM_003073772.1 .
VYVDRNDV-LRSSYFQV--------------MAKSPEEFRTRRLEIKLTG-------EEG
LDYGGLTREW---LVLL----SKDFLDPNF--------ALFEFSTEDKT-----------
----------------VAVPCKNS------------------------------------
--YVNPEHLSYFKFVGRIMAKVIMDGNFINLYLPK--FIYKHILG---------------
--------KSCDLQDLES----------------------ADPEFHKS--------LVWI
RDN--SVG-KSL--
-GLTFSFDDVS---------------------------FGV-HRTVE-LVKG--------
GANIF---
------------------------------VDDSNKE-EYIKL-ATQYRLFDG-VELQLS
-ALKSG---LFEILGN--RALE-MFDEN-ELELLICG-----------------------
----IPDID-----------IDDW----KSNTLYYG-YTES-------------------
------------------------SKTV-IWFWKAVK--------------------SFD
SVNRARLLQF-------VTGTSTLP------------FEG-----FS-HLQGNN--EIQK

FSIHKISDRT------------------------DSLPTAHTCF-NQ-LVLPEY-SSYES
LLKYLM-LAINECSTGF
>Encephalitozoon_hellem_XM_003888164.1 .
VYVDRSDV-LRSSYFQV--------------MAKSPEEFRTRRLEIKLTG-------EEG
LDYGGLTREW---LVLL----AKDLLDPNF--------ALFEFSTEDKT-----------
----------------VAVPCKNS------------------------------------
--YVNPEHLSYFKFVGRIVAKAIMDGNFINLHLSK--FIYKHILG---------------
--------KSCDLQDLES---------------------ADPEFYKS--------LAWI
RDN--HVD-ESL--
-GLTFSFDDVS---------------------------FGI-HRTAE-LIKD--------
GANVF---
------------------------------VNDTNKA-EYISL-ATQYRLFNG-IELQLS
-ALKSG---LFEILGN--KALE-MFDEN-ELELLICG----------------------
----VPDID-----------VDDW----KSNTLYYG-YTES------------------
------------------------SKTI-IWFWKAVK--------------------SFD
SVNRAKLLQF-------VTGTSTLP------------FEG-----FS-HLQGNN--EVQK
FSIHRVSDRT------------------------DSLPTAHTCF-NQ-LVLPEY-TSYEN
LLRYLT-LAINECSTGF
>Encephalitozoon_romaleae_XM_009267164.1 .
VYVDRNDV-LRSSYFQV--------------MAKSPEEFRTRRLEIKLTG-------EEG
LDYGGLTREW---LVLL----AKDLLDPNF--------ALFEFSTGDKT-----------
----------------TTVPCKNS------------------------------------
--YVNPEHLSYFKFVGRIVAKAIMDGNFINLHLSK--FIYKHILG---------------
--------KNCDLQDLES---------------------TDPEFYKS--------LAWI
RDN--HVD-ESL--
-GLTFSFDDVN---------------------------FGI-HRTAE-LIEG--------
GANVF---
------------------------------VNDTNKT-EYINL-ATQYRLFNG-IELQLS
-ALKSG---LFEILGN--EALE-MFDEN-ELELLICG----------------------
----IPDID-----------IDDW----KSNTLYYG-YTES------------------
------------------------SKTV-IWFWKAVK--------------------SFN
SVNRAKLLQF-------VTGTSTLP------------FEG-----FS-HLQGNN--EVQK
FSIHRVSDRM------------------------DSLPTAHTCF-NQ-LVLPEY-SSYES
LLKYLT-LAINECSTGF
>Apophysomyces_trapeziformis_JNDP01001095. 1..
LNVRRQYV-FEDSYHQL--------------QGRTGNEIKYGKLNVRFYN-------EEG
MDAGGVSREW---FSVL----ARQMFDPNY--------ALFITSAADKL-----------
----------------TYQPNHAS------------------------------------
--CVNPDHLSFFKFVGRVIGKAIYDGRLLDAYFTR--SFYKHILG---------------
--------RPVDYRDVEA---------------------IDPEYYKS--------LVWM
LEN--DIT-DVI--
-DLTFSIETDD---------------------------FGT-TKTID-LKPG--------
GRDIP---
------------------------------VTEANKH-EYVTL-VTEQKLTNA-IKDQIN
-AFLQG---FHDIIPA--SLIK-IFNEQ-ELELLISG----------------------
----LPDID-----------IDDW----KNNTVYEGGYSAS------------------
------------------------SPQI-QWFWRAVR--------------------SFD
QEERAKLLQF-------ATGTSKVP------------LEG-----FA-HLQGSS--GVQK
FQIHKDFGGD------------------------NRLPSAHTXF-NQ-VDLPVY-NSYES
LRANLF-KAMSECSTGF
>Apophysomyces_elegans_JNDQ01001485.1 .
LNVRRQYV-FEDSYHQL--------------QGRTGNEIKYGKLNVRFYN-------EEG
MDAGGVSREW---FSVL----ARQMFDPNY--------ALFITSAADKL-----------
----------------TYQPNHAS------------------------------------
--CVNPDHLSFFKFVGRVIGKAIYDGRLLDAYFTR--SFYKHILG---------------
--------RPVDYRDVEA---------------------IDPEYYKS--------LVWM
LEN--DIT-DVI--
-DLTFSIETDD---------------------------FGT-TKTID-LKPG--------

```
GRDIP-------------------------------------------------------
------------------------------VTEANKH-EYVTL-VTEQKLTNA-IKDQIN
-AFLQG---FHDIIPA--SLIK-IFNEQ-ELELLISG-----------------------
----LPDID-----------IDDW----KNNTVYEGGYSAS-------------------
------------------------SPQI-QWFWRAVR--------------------SFD
QEERAKLLQF-------ATGTSKVP------------LEG-----FA-HLQGSS--GVQK
FQIHKDFGGD------------------------NRLPSAHTXF-NQ-VDLPVY-NSYES
LRANLF-KAMSECSTGF
>Saksenae_oblongispora_JNEV01000921.1 .
LNVRRQYV-FEDSYHQL--------------QGRTGNEIKYGKLNVRFYN-------EEG
MDAGGVSREW---FSVL----ARQMFDPNY--------ALFITSAADKL-----------
----------------TYQPNHAS------------------------------------
--IVNPDHLSFFKFVGRVIGKAIYDGRLLDAYFTR--SFYKHILS---------------
--------RSVDYRDVEA----------------------IDPEYYKS--------LVWM
LEN------------------------------------------------DIT-DVI--
-DLTFSIETDD---------------------------FGD-TKTID-LKPG--------
GRDIP-------------------------------------------------------
------------------------------VTEANKH-EYVTL-VTEQKLTNA-IKDQIN
-AFLQG---FHDIIPA--SLIQ-IFNEQ-ELELLISG-----------------------
----LPDID-----------IDDW----KNNTVYEGGYSAS-------------------
------------------------SPQI-QWFWRAVR--------------------SFD
QEERAKLLQF-------ATGTSKVP------------LEG-----FA-HLQGSS--GVQR
FQIHKDFGGD------------------------NRLPSAHTXF-NQ-VDLPVY-NSYES
LRANLF-KAMSECSTGF
>Saksenaea_vasiformis_JNDT01001937.1 .
LNVRRQYV-FEDSYHQL--------------QGRTGNEIKYGKLNVRFYN-------EEG
MDAGGVSREW---FSVL----ARQMFDPNY--------ALFITSAADKL-----------
----------------TYQPNHAS------------------------------------
--CVNPDHLSFFKFVGRVIGKAIYDGRLLDAYFTR--SFYKHILS---------------
--------RSVDYRDVEA----------------------IDPEYYKS--------LVWM
LEN------------------------------------------------DIT-DVI--
-DLTFSIETDD---------------------------FGD-TKTID-LKPG--------
GRDIP-------------------------------------------------------
------------------------------VTEANKH-EYVTL-VTEQKLTNA-IKDQIN
-AFLQG---FHDIIPA--SLIQ-IFNEQ-ELELLISG-----------------------
----LPDID-----------IDDW----KNNTVYEGGYSAS-------------------
------------------------SPQI-QWFWRAVR--------------------SFD
QEERAKLLQF-------ATGTSKVP------------LEG-----FA-HLQGSS--GVQR
FQIHKDFGGD------------------------NRLPSAHTXF-NQ-VDLPVY-NSYES
LRANLF-KAMSECSTGF
>Phycomyces_blakesleeanus_XM_018428041.1 .
LNVRRQYV-FEDSYHQL--------------QGRTGEEIKHGKLNVRFYD-------EEG
VDAGGVTREW---FSVL----ARQMFDPNY--------ALFITSAADKL-----------
----------------TYQPNRAS------------------------------------
--WVNPDHLSFFKFVGRLIGKAIYDGRLLDAYFTR--SFYKHILG---------------
--------RQVDYRDVEA----------------------IDPSYYKS--------LVWM
LEN------------------------------------------------NIT-DVV--
-DLTFSIDTDD---------------------------FGT-AKTID-LKPN--------
GRDIP-------------------------------------------------------
------------------------------VTEQNKH-EYVYL-VTEQKLTTA-IKDQIN
-AFLQG---FHDIIPA--SLIQ-IFNEQ-ELELLISG-----------------------
----LPDID-----------IDDW----RNNTDYET-YNVS-------------------
------------------------SIQI-QWFWRAVR--------------------SFD
QEERAKLLQF-------ATGTSKVP------------LKG-----FA-HLQGSS--GLQK
FQIHKDFGGE------------------------NRLPSAHTCF-NQ-IDLPMY-TSYES
LRANLF-KAINECSTGF
>Cunninghamella_elegans_JNDR01001010.1 .
LNVRRQFV-FEDSYHQL--------------QGRTGEEIKYGKLMVRFYD-------EEG
```

VDAGGVSREW---FSVL----ARQMFDPNY--------ALFIASAADKL-----------
----------------TYQPNRAS------------------------------------
--WVNPDHLSFFKFVGRVIGKAIYDGRLLDAYFTR--SFYKHILN---------------
--------RSVDYRDVEA----------------------IDPEYYKS--------LVWM
LDN--DIT-DVI--
-DLTFSMEMDD---------------------------FGT-TKVID-LKPG--------
GRDIP---
------------------------------VTEQNKR-EYVNL-VTEQKLSIA-IKDQIN
-AFLQG---FHDVIPA--SLIQ-IFNEQ-ELELLISG-----------------------
----LPDID-----------LDDW----KNNTVYET-YHAN-------------------
------------------------SPQV-QWFWRAVR--------------------SFD
QEERAKLLQF-------ATGTSKVP------------LEG-----FA-HLQGSG--GVQK
FQIHKDFGGD------------------------SRLPSAHTXF-NQ-IDLPMY-DSYSN
LRHNLF-KAINECATGF
>Absidia_repens_MCGE01000004.1 .
LNVRRQYV-FEDSYHQL--------------QGRTGEEIKFGKLMVRFYD-------EEG
VDAGGVSREW---FSVL----ARQMFDPNY--------ALFIASAADKL-----------
----------------TYQPNRAS------------------------------------
--WVNSDHLSFFKFVGRVIGKAIYDGRLLDAYFTR--SFYKHILN---------------
--------RSVDYRDVEA----------------------VDPEYYKS--------LVWM
LDN--DIT-DII--
-DLTFSMEMDD---------------------------FGT-TKVID-LKPG--------
GRDLP---
------------------------------VTEQNKH-EYVNL-VTAQKLTIA-IKDQIN
-AFLQG---FHDIIPA--SLIQ-IFNEQ-ELELLISG-----------------------
----LPDID-----------LDDW----KNNTEYET-YHST-------------------
------------------------SPQI-QWFWRAVR--------------------SFD
QEERAKLLQF-------ATGTSKVP------------LEG-----FA-HLQGSG--GVQK
FQIHKDFGGD------------------------SRLPSAHTXF-NQ-VDLPEY-DSYEN
LRSSLF-KAINECSTGF
>Gongronella_sp._LVWK01000733.1 .
LNVRRQYV-FEDSYQQL--------------QGRTGGEIKHGKLMVRFYD-------EEG
VDAGGVSREW---FSVL----ARQMFDPNY--------ALFIASAADKL-----------
----------------TYQPNRAS------------------------------------
--WVNADHLSYFKFVGRVIGKAIYDGRLLDAYFTR--SFYKHILN---------------
--------RSVDYRDVEA----------------------VDPEYYKS--------LVWM
LDN--DIT-DVI--
-DLTFSMEVDD---------------------------FGT-NKTVD-LKPG--------
GRDLP---
------------------------------VTEQNKH-EYVNL-VTAQKLTIA-IKDQIN
-AFLQG---FHDIISP--ELIQ-IFNEQ-ELELLISG-----------------------
----LPDID-----------LDDW----KNNTVYES-YHAN-------------------
------------------------SPQI-QWFWRAVR--------------------SFD
QEERAKLLQF-------ATGTSKVP------------LAG-----FA-HLQGSG--GVQK
FQIHKDFSGQ------------------------NRLPSAHTXF-NQ-VDLPEY-DSYES
LRANLF-KAINECSTG-
>Hesseltinella_vesiculosa_MCGT01000022.1 .
LNVRRQYV-FEDSYQQL--------------LGRPGRDIKHGKLMVRFYD-------EEG
VDAGGVSREW---FSVL----ARQMFDPNY--------ALFIASAADKL-----------
----------------TYQPNRAS------------------------------------
--YVNNDHLSYFKFVGRVIGKAIYDGRLLDAYFTR--SFYKHILN---------------
--------RSVDYRDVEA----------------------IDPEYYKS--------LVWM
LDN--DIT-DVI--
-DLTFSMEIDD---------------------------FGT-TKTVD-LKPG--------
GRDLA---
------------------------------VTEANKH-EYVNL-VTEQKLTIA-IRDQIN
-GFLQG---FHDIIPP--ELIQ-IFNEQ-ELELLISG-----------------------
----LPDID-----------LDDW----KNNTTYET-YHAN-------------------

------------------------SPQV-QWFWRAVR--------------------SFD
QEERAKLLQF-------STGTSKVP------------LNG-----FS-HLQGSG--GIQK
FQIHKDFGGE------------------------NRLPSAHTX--NQ-VDIPEY-DSYES
LRANLV-KAINECATGF
>Lichtheimia_ramosa_LK023327.1 .
LNVRRQYV-FEDSYHQL--------------LGRTGDEIKYGKLSVRFYD-------EEG
VDAGGVAREW---FSVL----SRQMFDPNY--------ALFITSAADKL-----------
----------------TYQPNRAS------------------------------------
--AINPDHLSYLKFVGRVIGKAIYDGRLMDAYFTR--SFYKHILN---------------
--------RSVDYRDVEA----------------------IDPEYYKS--------LVWM
LEN--DIT-DVI--
-DLTFSIETDD---------------------------FGT-TKEID-LKPG--------
GRDIP---
------------------------------VTEENKH-EYVAL-VTEQKLTLA-IKDQIN
-AFLQG---FHDIIPP--SLIQ-IFNEQ-ELELLISG-----------------------
----LPDID-----------IDDW----KANTEYQGGYSQG-------------------
------------------------SPQI-QWFWRAVR--------------------SFD
QEERAKLLQF-------ATGTSKVP------------LEG-----FS-QLQGSG--GIQR
FQIHKDYGGD------------------------SRLPSAHT------------------

>Lichtheimia_corymbifera_CBTN010000094.1 .
LNVRRQYV-FEDSYHQL--------------LGRTGDEIKYGKLSVRFYD-------EEG
VDAGGVAREW---FSVL----SRQMFDPNY--------ALFITSAADKL-----------
----------------TYQPNRAS------------------------------------
--AINPDHLSYLKFVGRVIGKAIYDGRLMDAYFTR--SFYKHILN---------------
--------RSVDYRDVEA----------------------IDPEYYKS--------LVWM
LEN--DIT-DVI--
-DLTFSIETDD---------------------------FGT-TKEID-LKPG--------
GRDIP---
------------------------------VTEENKH-EYVAL-VTEQKLTLA-IKDQIN
-AFLQG---FHDIIPP--SLIQ-IFNEQ-ELELLISG-----------------------
----LPDID-----------IDDW----KANTEYQGGYSQG-------------------
------------------------SPQI-QWFWRAVR--------------------SFD
QEERAKLLQF-------ATGTSKVP------------LEG-----FS-QLQGSG--GIQR
FQIHKDFGGE------------------------SRLPSAHTXF-NQ-IDLPEY-DSYDT
LRSSLF-KAINECSTGF
>Thermomucor_indicae_seudaticae_JSYX010003 .
LNVRRQYV-FEDSYHQL--------------LGRTGDEIKYGKLSVRFYD-------EEG
VDAGGVTREW---FSVL----ARQMFDPNY--------ALFITSAADKL-----------
----------------TYQPNRAS------------------------------------
--SVNPDHLSYLKFVGRVIGKAIYDGRLLDAYFTR--SFYKHILG---------------
--------RPVDYRDVEA----------------------VDPEYYKS--------LVWM
LEN--DIT-DVI--
-DLTFSIETDD---------------------------FGT-TKVVD-LKPG--------
GRDIP---
------------------------------VTEENKH-EYVAL-VTEQKLTTA-IKDQIN
-AFLQG---FHDIIPA--QLIQ-IFNEQ-ELELLISG-----------------------
----LPDID-----------IDDW----KNNTEYQG-YSPS-------------------
------------------------SPQI-QWFWRAVR--------------------SFD
QEERAKLLQF-------ATGTSKVP------------LEG-----FA-HLQGSG--GVQR
FQIHKDFGGE------------------------NRLPSAHTXF-NQ-IDLPEY-DSYET
LRANLF-KAINECSTGF
>Rhizomucor_miehei_AGBC01003546.1 .
LNVRRQYV-FEDSYHQL--------------LGRTGDEIKYGKLSVRFYD-------EEG
VDAGGVAREW---FSVL----ARQMFDPNY--------ALFITSAADKL-----------
----------------TYQPNRAS------------------------------------
--AINPDHLSYLKFVGRVIGKAIYDGRLLDAYFTR--SFYKHILG---------------
--------RPVDYRDVEA----------------------IDPEYYKS--------LVWM

LEN--DIT-DII--
-DLTFCIETDD---------------------------FGT-TKVID-LKPN--------
GRNIP---
------------------------------VTEENKH-EYVAL-VTEQKLTLA-IKDQIN
-AFLQG---FHDIIPA--HLIQ-IFNEQ-ELELLISG-----------------------
----LPDID-----------IDDW----KNNTEYQGGYNAS-------------------
------------------------SPQI-QWFWRAVR--------------------SFD
QEERAKLLQF-------ATGTSKVP------------LEG-----FA-HLQGSG--GVQR
FQIHKDFGGE------------------------NRLPSAHTXF-NQ-VDLPEY-DSYES
LRSNLF-KAINECSTGF
>Syncephalastrum_monosporum_JNEN01001246.1 .
LNVRRQYV-FEDSYHQL--------------LGRTGDEIKFGKLSVRFYD-------EEG
VDAGGVAREW---FSVL----ARQMFDPNY--------ALFITSAADKL-----------
----------------TYQPNRAS------------------------------------
--YVNPDHLSYFKFVGRVIGKAIYDGRLLDAYFTR--SFYKHILG---------------
--------RQVDYRDVEA----------------------IDPEYYKS--------LVWM
LEN--DIT-DVI--
-DLTFSLETDD---------------------------FGT-TEVID-LKPN--------
GRDIP---
------------------------------VTEENKH-EYVAL-VTEQKLTTA-IKDQIN
-AFLQG---FHDVIQA--PLIQ-IFNEQ-ELELLISG-----------------------
----MPDID-----------IDDW----KNNTEYQG-YTPA-------------------
------------------------SPQI-QWFWRAVR--------------------SFD
QEERAKLLQF-------ATGTSKVP------------LEG-----FT-QLQGSG--GVQK
FQIHKDFGGE------------------------NRLPSAHTWX-NQ-VDLPMY-DTYES
LRSNLF-KAINECSTGF
>Syncephalastrum_racemosum_JNDN01000703.1 .
LNVRRQYV-FEDSYHQL--------------LGRTGDEIKFGKLSVRFYD-------EEG
VDAGGVAREW---FSVL----ARQMFDPNY--------ALFITSAADKL-----------
----------------TYQPNRAS------------------------------------
--YVNPDHLSYFKFVGRVIGKAIYDGRLLDAYFTR--SFYKHILG---------------
--------RQVDYRDVEA----------------------IDPEYYKS--------LVWM
LEN--DIT-DII--
-DLTFSLETDD---------------------------FGT-TEVID-LKPN--------
GRDIP---
------------------------------VTEENKH-EYVAL-VTEQKLTTA-IKDQIN
-AFLQG---FHDVIQA--PLIQ-IFNEQ-ELELLISG-----------------------
----MPDID-----------IDDW----KNNTEYQG-YTPA-------------------
------------------------SPQI-QWFWRAVR--------------------SFD
QEERAKLLQF-------ATGTSKVP------------LEG-----FT-QLQGSG--GVQK
FQIHKDFGGE------------------------NRLPSAHTXF-NQ-VDLPMY-DTYES
LRSNLF-KAINECSTGF
>Cokeromyces_recurvatus_JNEH01001913.1 .
LNVRRHNV-FEDSYHQL--------------QGRTGDEFKHGKLAVRFYN-------EEG
VDAGGVSREW---FSVL----ARQMFDPNY--------ALFVKSAADKL-----------
----------------TYQPNRDS------------------------------------
--AVNPDHLSFFKFVGRIIGKAIYDGRLLDAYFTR--SFYKHILG---------------
--------RSVDYRDVEA----------------------LDPEYYKS--------LVWM
LEN--DIT-DII--
-DLTFSIETDY---------------------------FGT-KETVD-LKPN--------
GRNIP---
------------------------------VTEENKH-EYVAL-VTEQKLTTA-IKDQIN
-AFVQG---FHDIIPA--HLIQ-IFNEQ-ELELLISG-----------------------
----LPDID-----------IDDW----KNNTEYQG-YTSS-------------------
------------------------SPPV-QWFWRAVR--------------------SFD
QEERAKLLQF-------ATGTSKVP------------LEG-----FS-QLQGSN--GVQK
FQIHKDFGGE------------------------NRLPSAHTXF-NQ-IDLPQY-DSYES
LRTNLF-KAISECSTGF

>Rhizopus_oryzae_GDUK01028786.1 .
LNVRRQYV-FEDSYHQL--------------QGRTGDEIKYGKLSVLFYD-------EEG
LDAGGVTREW---FSVL----ARQMFDPNY--------ALFITSAADKL-----------
----------------TYQPNRAS------------------------------------
--AVNPDHLSFFKFVGRVIGKAIYDGRLLDAYFTR--SFYKHILG---------------
--------RPVDYRDVEA----------------------IDPEYYKS--------LVWM
LEN--DIT-DII--
-DLTFSIETDY---------------------------FGT-KETVD-LKPD--------
GRNIP---
------------------------------VTEANKH-EYVTL-VTEQKLTTA-IKDQIN
-AFVQG---FHDIIPA--HLIQ-IFNEQ-ELELLISG-----------------------
----LPDID-----------IDDW----KNNTEYEG-YSAS-------------------
------------------------SPPI-QWFWRAVR--------------------SFD
QEERAKLLQF-------ATGTSKVP------------LEG-----FA-HLQGSS--GIQK
FQIHKDFGGE------------------------KRLPSAHTCF-NQ-IDLPQY-DSYES
LRANLF-KAINECSTGF
>Mucor_irregularis_GFBC01014296.1 .
LNVRRQYV-FEDSYHQL--------------QGRTGDEIKYGKLSVLFYD-------EEG
LDAGGVTREW---FSVL----ARQMFDPNY--------ALFITSAADKL-----------
----------------TYQPNRAS------------------------------------
--AVNPDHLSFFKFVGRVIGKAIYDGRLLDAYFTR--SFYKHILG---------------
--------RPVDYRDVEA----------------------IDPEYYKS--------LVWM
LEN--DIT-DII--
-DLTFSIETDY---------------------------FGT-KETVD-LKPD--------
GRNIP---
------------------------------VTEANKH-EYVTL-VTEQKLTTA-IKDQIN
-AFVQG---FHDIIPA--HLIQ-IFNEQ-ELELLISG-----------------------
----LPDID-----------IDDW----KNNTEYEG-YSAS-------------------
------------------------SPPI-QWFWRAVR--------------------SFD
QEERAKLLQF-------ATGTSKVP------------LEG-----FA-HLQGSS--GIQK
FQIHKDFGGE------------------------KRLPSAHTCF-NQ-IDLPQY-DSYES
LRANLF-KAINEC----
>Rhizopus_stolonifer_JNDS01003012.1 .
LNVRRQYV-FEDSYHQL--------------QGRTGDEIKYGKLSVRFYD-------EEG
LDAGGVTREW---FSVL----ARQMFDPNY--------ALFITSAADKL-----------
----------------TYQPNRAS------------------------------------
--AVNPDHLSFFKFVGRVIGKAIYDGRLLDAYFTR--SFYKHILR---------------
--------RPVDYRDVEA----------------------IDPEYYKS--------LVWM
LEN--DIT-DII--
-DLTFSIETDF---------------------------FGT-KETVD-LRPD--------
GRNIP---
------------------------------VTEANKH-EYVTL-VTEQKLTTA-IKDQIN
-AFVQG---FHDVIPA--SLIQ-IFNEQ-ELELLISG-----------------------
----LPDID-----------IDDW----KNNTIYES-YSQS-------------------
------------------------SAPI-QWFWRAVR--------------------SFD
QEERAKLLQF-------ATGTSKVP------------LEG-----FA-HLQGSS--GIQK
FQIHKDFGGE------------------------KRLPSAHTXF-NQ-IDLPQY-DSYEN
LRANLF-KAINECSTGF
>Choanephora_cucurbitarum_LUGH01000166.1 .
LNVRRQYV-FEDSYHQL--------------QGRTGDEIKNGKLAVKFYD-------EEG
VDAGGVTREW---FSVL----ARQMFDPNY--------ALFITSAADKL-----------
----------------TYQPNRDS------------------------------------
--AVNPDHLSFFKFVGRVIGKAIYDGRLLDAYFTR--SFYKHILG---------------
--------RTVDYRDVEA----------------------LDPEYYKS--------LVWM
LEN--DIT-DII--
-DLTFSIETDF---------------------------FGT-KETVD-LKPN--------
GRNIP---
------------------------------VTEANKH-EYVTL-VTEQKLTTA-IKDQIN

-AFVQG---FHDVIPA--SLIQ-IFNEQ-ELELLISG-----------------------
----LPDID-----------IDDW----KNNTEYTG-YSST-------------------
------------------------SPPV-QWFWRAVR--------------------SFD
QEERAKLLQF-------ATGTSKVP------------LEG-----FS-QLQGSG--GVQK
FQIHKDFGGQ------------------------DRLPSAHTCF-NQ-VDLPQY-DSYEN
LRTNLF-KAINECSTGF
>Actinomucor_elegans_BCHK01000008.1 .
LNVRRQYV-FEDSYHQL--------------QGRTGEEIKNGKLAVRFYD-------EEG
LDAGGVTREW---FSVL----ARQMFDPNY--------ALFITSAADKL-----------
----------------TYQPNRDS------------------------------------
--AVNPDHLSFFKFVGRVIGKAIYDGRLLDAYFTR--SFYKHILG---------------
--------RTVDYRDVEA----------------------LDPEYYKS--------LVWM
LEN--DIT-DII--
-DLTFSIETDF---------------------------FGT-KETVD-LKPN--------
GRNIP---
------------------------------VTEANKR-EYVTL-VTEQKLTTA-IKDQIN
-AFVQG---FHDVIPA--SLIQ-IFNEQ-ELELLISG-----------------------
----LPDID-----------IDDW----KNNTEYEG-YTPG-------------------
------------------------SAPI-QWFWRAVR--------------------SFD
QEERAKLLQF-------ATGTSKVP------------LEG-----FS-QLQGSS--GVQK
FQIHKDYAGE------------------------NRLPSAHTXF-NQ-IDLPNY-DSYES
LRTNLF-KAISECSTG-
>Mucor_indicus_JNEK01003082.1 .
LNVRRQYV-FEDSYHQL--------------QGRTGDEIKHGKLAVRFYD-------EEG
VDAGGVTREW---FSVL----ARQMFDPNY--------ALFITSAADKL-----------
----------------TYQPNRDS------------------------------------
--AVNPDHLSFFKFVGRVIGKAIYDGRLLDAYFTR--SFYKHILG---------------
--------RTVDYRDVEA----------------------IDPEYYKS--------LVWM
LEN--DIT-DII--
-DLTFSIETDF---------------------------FGT-KETVD-LKPN--------
GRNIP---
------------------------------VVEANKH-EYVTL-VTEQKLTTA-IKDQIN
-AFVQG---FHDIIPA--SLIQ-IFNEQ-ELELLISG-----------------------
----LPDID-----------IDDW----KNNTEYQG-YTSS-------------------
------------------------SPPI-QWFWRAVR--------------------SFD
QEERAKLLQF-------ATGTSKVP------------LEG-----FS-QLQGSS--GVQK
FQIHKEFSRE------------------------NRLPSAHTXF-NQ-IDLPQY-DSYES
LRANLF-KAINECSTGF
>Parasitella_parasitica_CCXP01000598.1 .
LNVRRQYV-FEDSYHQL--------------QGRTGEEIKNGKLAVRFYD-------EEG
VDAGGVTREW---FSVL----ARQMFDPNY--------ALFITSAADKL-----------
----------------TYQPNRDS------------------------------------
--AVNPDHLSFFKFVGRVIGKAIYDGRLLDAYFTR--SFYKHILG---------------
--------RTVDYRDVEA----------------------LDPEYYKS--------LVWM
LDN--DIT-DII--
-DLTFSIETDF---------------------------FGT-KETVD-LKPD--------
GRNIP---
------------------------------VTEANKH-EYVTL-VTEQKLTTA-IKDQIN
-AFVQG---FHDVIPA--PLIQ-IFNEQ-ELELLISG-----------------------
----LPDID-----------IDDW----KNNTEYQG-YTAS-------------------
------------------------SPPI-QWFWRAVR--------------------SFD
QEERAKLLQF-------ATGTSKVP------------LEG-----FS-QLQGSS--GVQK
FQIHKDFGGD------------------------NRLPSAHTXF-NQ-VDLPQY-DSYEN
LRANLF-KAISECSTGF
>Mucor_racemosus_JNEI01005158.1 .
LNVRRQYV-FEDSYHQL--------------QGRTGEEIKNGKLAVRFYD-------EEG
VDAGGVTREW---FSVL----ARQMFDPNY--------ALFITSAADKL-----------
----------------TYQPNRDS------------------------------------

--AVNPDHLSFFKFVGRVIGKAIYDGRLLDAYFTR--SFYKHILG---------------
--------RTVDYRDVEA----------------------LDPEYYKS--------LVWM
LDN--DIT-DII--
-DLTFSIETDF---------------------------FGT-KETVD-LKPD--------
GRNIP---
------------------------------VTEANKH-EYVAL-VTEQKLTTA-IKDQIN
-AFVQG---FHDVIPA--PLIQ-IFNEQ-ELELLISG-----------------------
----LPDID-----------IDDW----KNNTEYQS-YTAS-------------------
------------------------SPPI-QWFWRAVR--------------------SFD
QEERAKLLQF-------ATGTSKVP------------LEG-----FS-QLQGSS--GVQK
FQIHKEFGGE------------------------NRLPSAHTXF-NQ-IDLPQY-DSYES
LRANLF-KAISECSTGF
>Mucor_velutinosus_JNDK01002322.1 .
LNVRRQYV-FEDSYHQL--------------QGRTFEEIKNGKLAVRFYD-------EEG
VDAGGVTREW---FSVL----ARQMFDPNY--------ALFITSAADKL-----------
----------------TYQPNRDS------------------------------------
--AVNPDHLSFFKFVGRVIGKAIYDGRLLDAYFTR--SFYKHILG---------------
--------RTVDYRDVEA----------------------LDPEYYKS--------LVWM
LEN--DIT-DII--
-DLTFSIETDF---------------------------FGT-KETVD-LKPN--------
GRNIP---
------------------------------VTEASKH-EYVTL-VTEQKLTTA-IKDQIN
-AFVQG---FHDVIPA--PLIQ-IFNEQ-ELELLISG-----------------------
----LPDID-----------IDDW----KNNTQYEG-YDAS-------------------
------------------------SPPV-QWFWRAVR--------------------SFD
QEERAKLLQF-------ATGTSKVP------------LEG-----FS-QLQGSS--GVQK
FQIHKDFGGE------------------------NRLPSAHTXF-NQ-IDLPQY-DSYEN
LRANLF-KAISECSTGF
>Mucor_irregularis_GFBC01015036.1 .
LNVRRQYV-FEDSYHQL--------------QGRTGEEIKNGKLAVRFYD-------EEG
VDAGGVTREW---FSVL----ARQMFDPNY--------ALFITSAADKL-----------
----------------TYQPNRDS------------------------------------
--AVNPDHLSFFKFVGRVIGKAIYDGRLLDAYFTR--SFYKHILG---------------
--------RTVDYRDVEA----------------------LDPEYYKS--------LVWM
LEN--DIT-DII--
-DLTFSIETDF---------------------------FGT-KETVD-LKPD--------
GRNIP---
------------------------------VTEANKH-EYVTL-VTEQKLTTA-IKDQIN
-AFVQG---FHDVIPA--PLIQ-IFNEQ-ELELLISG-----------------------
----LPDID-----------IDDW----KNNTEYQG-YTAS-------------------
------------------------SPPV-QWFWRAVR--------------------SFD
QEERAKLLQF-------ATGTSKVP------------LEG-----FS-QLQGSS--GVQK
FQIHKDFSGE------------------------NRLPSAHTCF-NQ-VDLPQY-NSYEN
LRANLF-KAINECSTGF
>Rhizomucor_variabilis_JNES01000565.1 .
LNVRRQYV-FEDSYHQL--------------QGRTFEEIKNGKLAVRFYD-------EEG
VDAGGVTREW---FSVL----ARQMFDPNY--------ALFITSAADKL-----------
----------------TYQPNRDS------------------------------------
--AVNPDHLSFFKFVGRVIGKAIYDGRLLDAYFTR--SFYKHILG---------------
--------RTVDYRDVEA----------------------LDPEYYKS--------LVWM
LEN--DIT-DII--
-DLTFSIETDF---------------------------FGT-KETVD-LKPD--------
GRNIP---
------------------------------VTEANKH-EYVTL-VTEQKLTTA-IKDQIN
-AFVQG---FHDVIPA--PLIQ-IFNEQ-ELELLISG-----------------------
----LPDID-----------IDDW----KNNTEYQG-YTAS-------------------
------------------------SPPV-QWFWRAVR--------------------SFD
QEERAKLLQF-------ATGTSKVP------------LEG-----FS-QLQGSS--GVQK

FQIHKDFSGE------------------------NRLPSAHTXF-NQ-VDLPQY-NSYEN
LRANLF-KAINECSTGF
>Mucor_ambiguus_BBKB01004600.1 .
LNVRRQYV-FEDSYHQL--------------QGRTGEEIKNGKLAVRFYD-------EEG
VDAGGVTREW---FSVL----ARQMFDPNY--------ALFITSAADKL-----------
----------------TYQPNRDS------------------------------------
--AVNPDHLSFFKFVGRVIGKAIYDGRLLDAYFTR--SFYKHILG---------------
--------RTVDYRDVEA----------------------LDPEYYKS--------LVWM
LEN--DIT-DII--
-DLTFSIETDF---------------------------FGT-KETVD-LKPD--------
GRNIP---
------------------------------VTEANKH-EYVTL-VTEQKLTTA-IKDQIN
-AFVQG---FHDVIPA--PLIQ-IFNEQ-ELELLISG-----------------------
----LPDID-----------IDDW----KNNTEYQG-YNAS-------------------
------------------------SPPV-QWFWRAVR--------------------SFD
QEERAKLLQF-------ATGTSKVP------------LEG-----FS-QLQGSS--GVQK
FQIHKDFGGE------------------------NRLPSAHTXF-NQ-VDLPQY-DCYEN
LRANLF-KAISECSTGF
>Mucor_circinelloides_AMYB01000003.1 .
LNVRRQYV-FEDSYHQL--------------QGRTGEEIKNGKLAVRFYD-------EEG
VDAGGVTREW---FSVL----ARQMFDPNY--------ALFITSAADKL-----------
----------------TYQPNRDS------------------------------------
--AVNPDHLSFFKFVGRVIGKAIYDGRLLDAYFTR--SFYKHILG---------------
--------RTVDYRDVEA----------------------LDPEYYKS--------LVWM
LEN--DIT-DII--
-DLTFSIETDF---------------------------FGT-KETVD-LKPD--------
GRNIP---
------------------------------VTEANKH-EYVTL-VTEQKLTTA-IKDQIN
-AFVQG---FHDVIPA--PLIQ-IFNEQ-ELELLISG-----------------------
----LPDID-----------IDDW----KNNTEYQG-YNAS-------------------
------------------------SAPV-QWFWRAVR--------------------SFD
QEERAKLLQF-------ATGTSKVP------------LEG-----FS-QLQGSS--GVQK
FQIHKDFGDE------------------------NRLPSAHTXF-NQ-VDLPQY-DSYEN
LRANLF-KAISECSTGF
>Umbelopsis_isabellina_BAVE01000005.1 .
LNVRRQYV-FEDSYHQL--------------QGRSGEEIKFGKLSVRFYD-------EEG
VDAGGVSREW---FSVL----ARQMFDPNY--------ALFKTSAADKL-----------
----------------TYQPNRAS------------------------------------
--FVNPDHLSFFKFVGRVIGKAIYDGRLLDAYFTR--SFYKHILG---------------
--------RQVDYRDVEA----------------------IDPEYYKS--------LVWM
LEN--DIT-DII--
-DLTFSVETDD---------------------------FGN-MKTVD-LKPN--------
GRDIP---
------------------------------VTEENKQ-EYVKL-ITEQKLTLG-IKDQIN
-AFLDG---FHDIISE--QLIS-IFNEQ-ELELLISG-----------------------
----MPDID-----------IDDW----KNNTEYQG-YSPS-------------------
------------------------SPQI-NWFWRAVR--------------------SFD
QEERAKLLQF-------ATGTSKVP------------LEG-----FA-HLQGSG--GTQK
FQIHCQY-ST------------------------DRLPSAHTCF-NQ-VDLPQY-ESYES
LRANLL-TAIRECSTGF
>Lichtheimia_ramosa_LK023315.1 .
LNVRRRYV-FQDSYHQL--------------LGKTGDEIKYGRLNVQFRD-------EDG
VDAGGVSREW---YSVL----ARQMFDPNY--------ALFITSTADKL-----------
----------------TYQPNRAS------------------------------------
--SVNPDHLSYLKFVGRVIGKAIYDGRLLDAYFTR--SFYKHILG---------------
--------RSVDYKDMAA----------------------IDHEYYKS--------LVWM
LEN--DIT-DVI--
-DLTFSLEVDD---------------------------FGT-THVID-LKPD--------

GRNLP---
------------------------------VTEDNKH-EYVSL-VTEHRLTTA-IKDQIE
-AFLQG---FHEIIPA--SLIQ-IFNEQ-ELELLISG-----------------------
----LPDID-----------IDDW----KNNTEYQGGYSSN-------------------
------------------------STQI-QWFWRAVR--------------------SFD
QEERAKLLQF-------ATGTSKVP------------LEG-----FA-HLQGSG--GVQK
FQIHKQYSSN------------------------NRLPTAHT------------------

>Lichtheimia_corymbifera_CBTN010000010.1 .
LNVRRRYV-FQDSYHQL--------------LGKTGDEIKYGRLSVQFRD-------EDG
VDAGGVSREW---YSVL----ARQMFDPNY--------ALFITSAADKL-----------
----------------TYQPNRAS------------------------------------
--SVNPDHLSYLKFVGRVIGKAIYDGRLLDAYFTR--SFYKHILG---------------
--------RSVDYKDMEA----------------------IDHEYYKS--------LVWM
LEN--DIT-DVI--
-DLTFSLEVDD---------------------------FGT-THVID-LKPD--------
GRNLP---
------------------------------VTEENKH-EYVSL-VTEHRLTTA-IKDQIE
-AFLQG---FHEVIPA--SLIQ-IFNEQ-ELELLISG-----------------------
----LPDID-----------IDDW----KNNTEYQGGYSSN-------------------
------------------------STQI-QWFWRAVR--------------------SFD
QEERAKLLQF-------ATGTSKVP------------LEG-----FA-HLQGSG--GVQK
FQIHKEYSMD------------------------NRLPTAHTXF-NQ-IDLPMY-DSYSN
LRHNLF-KAINE-----
>Mortierella_alpina_ADAG01001083.1 .
MNVRREMV-FVDSFSQL--------------QSRSGEEIKYSKLNVHFHG-------EEG
VDAGGVTREW---FQVL----ARQMFNPDY--------ALFKTSAADKL-----------
----------------TYQPNRAS------------------------------------
--WVNSDHLLFFKFIGRVIGKAIYDGRLLDAYFTR--SFYKHILG---------------
--------RPVDYRDVEA----------------------VDPEYYKS--------LVWM
LEN--DIT-DIV--
-EETFSVETDD---------------------------FGN-TKVVD-LKPN--------
GRNIP---
------------------------------VTEQNKH-EYVKY-ITEQKLTLA-IKDQIH
-AFLQG---FHEVIPA--HLIS-IFNEQ-ELELLISG-----------------------
----LPDID-----------VDEW----KNCTEYQN-YSSS-------------------
------------------------SPQI-QNFWRAVR--------------------SFD
QTERAKLLQF-------VTGTSKVP------------LGG-----FS-QLQGIS--GVQK
FQIHKDFSST------------------------KRLPSAHTXF-NQ-LDLPEY-ETYEE
LRQQLL-TAISECSTGF
>Mortierella_verticillata_AEVJ01000143.1 .
MNVRREMV-FVDSFNHW--------------QSKKGDDIKYSKLNVKFHG-------EEG
VDAGGVTREW---FQVL----ARQMFNPDY--------ALFKTSAADKL-----------
----------------TYQPNRAS------------------------------------
--WANSEHLLFFKFIGRVIGKAIYDGRLLDAYFTR--SFYKHILG---------------
--------RPVDYRDVEA----------------------VDPEYYKS--------LVWM
LEN--DIT-NIV--
-DETFSVETDD---------------------------FGT-TKTVD-LKPG--------
GRDIP---
------------------------------VTEENKH-EYVKY-ITEQKLTLA-IKDQIH
-SFLQG---FHEIIPS--SLIS-IFNEQ-ELELLISG-----------------------
----LPDID-----------VDEW----KNCTEYQN-YSPG-------------------
------------------------SPQV-QNFWRAVR--------------------SFD
QTERAKLLQF-------VTGTSKVP------------LGG-----FA-QLQGIS--GVQK
FQIHKDFSST------------------------KRLPSAHTXF-NQ-LDLPEY-ETYEE
LRQQLL-TAISECSTGF
>Basidiobolus_meristosporus_MCFE01000412.1 .
LNVRRQYV-FEDSFHQL--------------QGRGGDEVKNGKLSVRFYD-------EEG

VDAGGVTREW---FQVL----SRQMFNPDY--------ALFKTSAVDKV-----------
----------------TYQPNRAS------------------------------------
--WVNPDHLLYFKFVGRIIGKAIYDGRLLDCYFTR--SFYKHILG---------------
--------KSVDYKDVEA----------------------LDPEYFKS--------LVWM
LEN---DIT-DII--
-DLTFSIEADD---------------------------FGK-MKVID-LKPN--------
GRDIP---
------------------------------VTEENKY-EYVKL-VTEQKLTLA-IKDQID
-NFLVG---FHEIISP--QLIS-IFNEQ-ELELLISG-----------------------
----MPDID-----------VDDW----KNNTEYQG-YTTS-------------------
------------------------SPQI-QWFWRAVR--------------------SFD
QEERAKLIQF-------VTGTSKVP------------LEG-----FS-SLQGVN--GVQK
FQIHKDPSST------------------------HRLPSAHTCF-NQ-LDLPQY-ESYEQ
LRSNTL-TAIQECNTGF
>Gigaspora_margarita_GBYF01006209.1 .
LNVRRQYV-FEDSYQNL---------------QRRTGDEIKYGKLSVRFYE-------EEG
VDAGGVTREW---FQVL----ARQMFDENY--------ALFKTSAADRL-----------
----------------TYQPNRAS------------------------------------
--SANPEHLSFFKFVGRVIGKAIYDGRLLDAYFTR--SFYKHILG---------------
--------KPVDYRDVEA----------------------IDLEYYNS--------LVWM
LNN---DIT-NVV--
-DLTFSVVTND---------------------------FGE-EKVID-LKPN--------
GSNIT---
------------------------------VTDENKR-EYVML-VTEQRLTLA-IKDQIE
-NFLAG---FHEIIPA--HLIS-IFNEQ-ELELLISG-----------------------
----MPDID-----------IDDW----KNNTEYQN-YTSS-------------------
------------------------SPQI-QWFWRAVR--------------------SFS
QEERAKLLQF-------VTGTSKVP------------LEG-----FS-ALQGVH--GVQK
FQIHKDFSSP------------------------DRLPSAHTCF-NQ-LDIPEY-EDYEQ
LRSQLL-LAISEGTIG-
>Rhizophagus_irregularis_JEMT01008830.1 .
LNVRRQYV-FEDSFQNL---------------QRRTGDEIKYGKLSVRFYE-------EEG
VDAGGVTREW---FQVL----ARQMFNEDY--------ALFKTSAADRL-----------
----------------TYQPNRAS------------------------------------
--GANPEHLLFFKFVGRVIGKAIYDGRLLDAYFTR--SFYKHILG---------------
--------KPVDYRDVEA----------------------IDLEYYNS--------LVWM
LNN---DIT-GVI--
-DYTFSVETDD---------------------------FGQ-KKIID-LKEN--------
GRNIP---
------------------------------VTEENKR-EYVKL-VTEQKLTLA-IKDQIE
-QFLAG---FHEIIPA--HLIS-IFNEQ-ELELLISG-----------------------
----MPDID-----------IDDW----KNNTEYQN-YTPS-------------------
------------------------SPQI-QWFWRAVR--------------------SFD
QEERAKLLQF-------VTGTSKVP------------LEG-----FS-VLQGVH--GVQK
FQIHKDFASP------------------------DRLPSAHTXF-NQ-IDIPEY-ENYEQ
LRSQLL-LAISEGTTGF
>Entomophthora_muscae_GEMZ01017396.1 .
LNVRRQYV-FEDSFRQF---------------QGRSGQDIKYGKLNVKFHD-------EEG
VDAGGVTREW---FSVL----SRQMFNPNY--------ALFKSSANDKV-----------
----------------TYQPNRTS------------------------------------
--WVNSDHLLYFKFVGRIIGKAIYDDHLFDAYFTR--SFYKHILG---------------
--------KPVEIRDLEA----------------------IDPEFYKS--------MVWM
LEN---DIT-DVV--
-YANFCVETDD---------------------------FGR-KLVIE-LKPG--------
GQDIT---
------------------------------VTQENKF-EYVQL-VTEQKLYGA-IKDQIE
-HFLRG---FHEVIPK--ELIS-IFNEQ-ELELLISG-----------------------
----LPDID-----------IDDW----KNNTDYEG-YTTS-------------------

------------------------SPQI-QWFWRAVR---------------------SFD
QSERAKLLQF-------VTGTSKVP------------LEG-----FA-HLQGST--GVQK
FQIHRDFASV------------------------KRLPSAHTCF-NQ-LDIPMY-ESYDD
LRRQLL-LAIEEC----
>Conidiobolus_coronatus_JXYT01001333.1 .
LNVRRSHV-FEDSYHQL--------------QGRTGDEIKYGKLNVKFHD-------EEG
VDAGGVTREW---SQVL----SRQMFNPDY--------ALFKTSAVDRV-----------
----------------TYQPNRSS------------------------------------
--WVNSDHLSYFKFVGRVIGKAIYDNRLLDCYFTR--SFYKHILG---------------
--------KTVEIRDMEA----------------------ADPTFYRS--------LMWM
NEN--DIT-GVL--
-DLTFSMEDEE---------------------------FGE-KRIVD-LKPN--------
GRDIP---
------------------------------VTEENKQ-EYIHL-ITQQKLTVA-IKDQIK
-SFLTG---FNEIIPP--WLIR-IFNEQ-ELELLISG-----------------------
----LPYID-----------VDDW----KNNTEYQG-YTES-------------------
------------------------SPQI-QWFWRAVR---------------------SFD
QEERTKLLQF-------VTGTSKVP------------LEG-----FS-NLQGSG--GVQK
FQIHKDFSSI------------------------QRLPSAHTXF-NQ-LDIPLY-ENYEQ
LRSQVL-LAISECGTGF
>Conidiobolus_incongruus_JNEM01006077.1 .
LNVRRSHV-FEDSYHQL--------------QGRSGEEIKYGKLNVKFHD-------EEG
VDAGGVTREW---SQVL----SRQMFNPDY--------ALFKTSAVDRV-----------
----------------TYQPNRSS------------------------------------
--WVNNDHLSYFKFVGRIIGKAIYDNRLLDCYFTR--SFYKHILN---------------
--------KPVEIRDMEA----------------------VDPTFYRS--------LMWM
TEN--DIT-GVL--
-DLTFSMEDEE---------------------------FGE-KRIID-LKPN--------
GRDIP---
------------------------------VTEENKH-EYIHL-ITQQKLTVA-IKDQIK
-NFLIG---FHEIIPP--WLIR-IFNEQ-ELELLISG-----------------------
----LPYID-----------VDDW----KNNTEYQG-YTES-------------------
------------------------SPQI-QWFWRAVR---------------------SFD
QEERAKLLQF-------VTGTSKVP------------LEG-----FS-NLQGSG--GVQR
FQIHKDFSST------------------------QRLPSAHTXF-NQ-LDIPLY-ESYEQ
LRSQIL-LAISECGTG-
>Rhizoclosmatium_globosum_MCGO01000158.1 .
LNVRRAQV-FEDSYHQL--------------HGRSGDEIKFGKLNIRFHD-------EEG
VDAGGVTREF---FQVL----ARQMFNPDY--------ALFKPSAVDKV-----------
----------------TYQPNRSS------------------------------------
--WINPDHLLYFRFVGRIIGKAIYDGRLLDAYFTR--SFYKCMLE---------------
--------APVDWKDMEA----------------------IDPSFHKS--------LQWI
LEN--DIT-DVM--
-DLTFSTEVEE---------------------------FGV-EKIID-LKEN--------
GRNIA---
------------------------------VTDENKK-EYVSL-ITEQRLTKA-IQEQIN
-AFLGG---FHDIIPK--DLIK-IFNEQ-ELELLISG-----------------------
----LPDID-----------IDDM----KNNTEYQN-YTQS-------------------
------------------------SPQI-QWFWRAVR---------------------SFS
QEERAKLIQF-------ATGTSKVP------------LEG-----FK-ALEGSN--GIQK
FQIHKDFASK------------------------LRLPSAHTXF-NQ-IDLPEY-DCYED
LRANLL-TAISECSTGF
>Homoloaphlyctis_polyrhiza_AFSM01001127.1_R .
INVRRQYV-FEDSFHQL--------------SGRSGDELKYGKLAVRFHE-------EEG
IDAGGVTREW---FSVL----ARQMFNPDY--------ALFRPSAADRV-----------
----------------TYQPNRAS------------------------------------
--GINPDHLHYFKFVGRIIGKAIYDGRLLDAYFTR--SFYKCILG---------------
--------SQVDYKDMEA----------------------IDPEFHKS--------LEWI

```
LQN------------------------------------------------DIT-DVL--
-DLTFSTEIDD---------------------------FGR-QRVID-LKPN--------
GRNIV-------------------------------------------------------
------------------------------VTEANKV-EYVKL-ITEQRLVVA-IKDQID
-AFLKG---FHEVIPA--DLVR-IFNEQ-ELELLISG-----------------------
----MPDLD-----------IDDW----KNNTEYQN-YTAA-------------------
------------------------SPQV-QWFWRAVR--------------------SFT
QEERAKLIQF-------ATGTSKVP------------LEG-----FA-ALQGSS--GVQK
FQIHKEFSDT------------------------SRLPSAHTWF-NQ-IDLPQY-ESYEQ
LRQMLL-TAISECGTG-
>Batrachochytrium_dendrobatidis_XM_006681556.1 .
INVRRQYV-FEDSFHQL--------------SGRSGDELKYSKLAVRFHE-------EEG
IDAGGVAREW---FSVL----ARQMFNPDY--------ALFRPSAADKV-----------
----------------TYQPNRAS------------------------------------
--GVNPDHLHYFKFVGCIIGKAIYDGRLLDAYFTR--SFYKCILG---------------
--------IQVDYKDMEA----------------------IDPGFHKS--------LEWI
LQN------------------------------------------------DIE-DVL--
-DLTFSTEVDD---------------------------FGR-QRIID-LKPN--------
GRNIT-------------------------------------------------------
------------------------------VTDENKV-EYVKL-ITEQRLVVA-IKDQIH
-AFLAG---FNQVIPA--DLVR-IFNEQ-ELELLISG-----------------------
----MPDID-----------IDDW----KNNTEYQN-YTAS-------------------
------------------------SPQV-QWFWRAVR--------------------SFS
QEERAKLIQF-------ATGTSKVP------------LEG-----FK-ALEGST--GVQK
FQIHKEFSDV------------------------SRLPSAHTCF-NQ-IDLPQY-DSYEQ
LRSMLL-TAISECGTGF
>Spizellomyces_punctatus_XM_016753891.1 .
INVRRQYV-FEDSYHQL--------------QGRSGDEIKFSKLNVRFYD-------EEG
VDAGGVTREW---FSVL----ARQMFNPDY--------ALFRPSAVDKV-----------
----------------TYQPNRLS------------------------------------
--YINPDHLLYFKFVGRIIGKAIYDGRLLDAYFTR--SFYKSMIE---------------
--------VPVDYKDMEA----------------------VDPEYHKS--------LEWI
LQN------------------------------------------------DNV-EVL--
-DLTFSTEIDE---------------------------FGK-KQTID-LKPN--------
GRDIQ-------------------------------------------------------
------------------------------VTEENKH-EYVKL-VVEQRLMTA-IRAQID
-AFLSG---FHDIIPK--DLVK-IFNEQ-ELELLISG-----------------------
----MPDID-----------IDDW----KNNTEYQN-YTSS-------------------
------------------------SPQV-QWFWRAVR--------------------SFS
QEERAKLVQF-------ATGTSKVP------------LEG-----FA-QLQGAN--GVQK
FQIHKDFSSA------------------------DRLPSAHTCF-NQ-LDLPQY-ESYEQ
LRSNLL-LAISEGGTGF
>Pecoramyces_ruminatium_ASRE01028691.1 .
IAVRRQYV-FEDSFHKL--------------QGKTGNEIKYSKLNVRFVE-------EEG
VDVGGVTREW---FSAL----ARQMFNPDY--------ALFKPSAQDRV-----------
----------------TYQPNRNS------------------------------------
--WINPDHLSFFKFVGRIIGKAIYDGRALDCYFTR--SFYKHILN---------------
--------IAVDYKDIEA----------------------IDPEYFKS--------LEWI
LHN------------------------------------------------DIT-DVL--
-DLTFSLEIDE---------------------------FGK-KSIID-LKPD--------
GRNIP-------------------------------------------------------
------------------------------VTEENKV-EYVKL-VTEQRLTVA-IKKQIK
-AFLDG---FHDIIPH--SLIS-IFNEQ-ELELLISG-----------------------
----LPEID-----------IDDW----KNNTVYEN-YSSS-------------------
------------------------SPQV-QWFWRAVR--------------------SFT
QEERAKLIQF-------TTGTSKVP------------LEG-----FS-NLQGVN--GIQK
FQIHKDFGSI------------------------ERLPSAHTXF-NQ-LDIPAY-ESYEH
LRKALL-LAINECSVGF
```

>Neocallimastix_californiae_MCOG01000243.1 .
IAVRRQYV-FEDSFHKL--------------QGKTGNEIKYSKLNVRFVE-------EEG
VDVGGVTREW---FSAL----ARQMFNPDY--------ALFKPSAQDRV-----------
----------------TYQPNRNS------------------------------------
--WINPDHLSFFKFVGRIIGKAIYDGRALDCYFTR--SFYKHILN---------------
--------IDVDYKDIEA----------------------IDPEYFKS--------IEWI
LHN--DIT-DVL--
-DLTFSLEIDE---------------------------FGK-KSVID-LKPN--------
GRNIP---
------------------------------VTEENKV-EYVKL-VTEQRLTVA-IKQQIE
-AFLDG---FHDIIPH--SLIS-IFNEQ-ELELLISG-----------------------
----LPEID-----------IDDW----KNNTVYEN-YSSS-------------------
------------------------SPQV-QWFWRAVR--------------------SFT
QEERAKLIQF-------TTGTSKVP------------LEG-----FS-NLQGVN--GIQK
FQIHKDFGSI------------------------ERLPSAHTXF-NQ-LDIPAY-ESYEH
LRKALL-LAINECSVGF
>Piromyces_finnis_MCFH01000037.1 .
IAVRRQYV-FEDSFHKL--------------QGKTGNEIKYSKLNVRFAE-------EEG
VDVGGITREW---FSAL----ARQMFNPDY--------ALFKPSAVDRV-----------
----------------TYQPNRNS------------------------------------
--WINPDHLSFFKFVGRIIGKAIYDGRCLDCYFTR--SFYKHILN---------------
--------IDVDYKDIEA----------------------IDPEYFKS--------LEWI
LHN--DIT-DVL--
-DLTFSLEIDE---------------------------FGK-KSIID-LKPD--------
GRNIP---
------------------------------VTEDNKV-EYVKL-VTEQRLTVA-IKKQIE
-AFLDG---FHDIIPH--SLIS-IFNEQ-ELELLISG-----------------------
----LPDID-----------IDDW----KNNTVYEN-YSSS-------------------
------------------------SPQV-QWFWRAVR--------------------SFT
QEERAKLIQF-------TTGTSKVP------------LEG-----FS-SLQGVN--GIQK
FQIHKDFGSI------------------------ERLPSAHTWF-NQ-LDIPAY-ESYEH
LRKALL-LAINECS---
>Anaeromyces_robustus_MCFG01000004.1 .
IAVRRQYV-FEDSFHKL--------------QGKTGNEIKYGKLNVRFME-------EEG
VDVGGVTREW---FSAL----ARQMFNPDY--------ALFKPSAVDRV-----------
----------------TYQPNRNS------------------------------------
--WINPDHLSFFKFVGRIIGKAIYDGRLLDCYFTR--SFYKHILN---------------
--------IDVDYKDIEA----------------------IDPEYFKS--------LEWI
LHN--DIT-DVL--
-DLTFSLEIDE---------------------------FGK-KSIID-LKPD--------
GRNIP---
------------------------------VTEENKV-EYVKL-VTEQRLTVA-IKQQIE
-AFLNG---FHDIIPH--SLIS-IFNEQ-ELELLISG-----------------------
----LPEID-----------IDDW----KNNTVYEN-YSSS-------------------
------------------------SPQV-QWFWRAVR--------------------SFT
QEERAKLIQF-------TTGTSKVP------------LEG-----FS-NLQGVN--GIQK
FQIHKDFGSI------------------------ERLPSAHTXF-NQ-LDIPAY-ESYEH
LRKALL-LAINECSVGF
>Linderina_pennispora_MCFD01000009.1 .
LNVRRDNV-FEDSFHQF--------------AGKSGDEIKRGRLHVKFHD-------EEG
VDAGGVSREW---FQAL----ARQMFNPDY--------AMFKPSASGRV-----------
----------------TYQPNPQS------------------------------------
--WANPEHLLYFKFVGRIIGKAIVDQRVLDAYFTR--SFYKHILG---------------
--------RKVDYRDMEA----------------------IDPSYYQS--------LEWI
LQN--DIT-DVV--
-EETFSMEVDN---------------------------FGQ-QEIID-LIPD--------
GQDVL---
------------------------------VTEANKA-EYVRL-VTEQRLSRA-IKDQIA

-AFLTG---FHDLIPQ--DLIQ-IFNEQ-ELELLISG----------------------
----MPDID-----------VDDW----RNNTDYHGGYSAS-------------------
------------------------SAQI-QWFWRAVR--------------------SFD
QEERAKLLQF-------VTGTSKVP------------LEG-----FT-HLQGSQ--GVQK
FQVHKDFGSP-----------------------TRLPTAHTCF-NQ-LDLPMY-DCFET
LKANLL-VAISECSTGF
>Smittium_culicis_LSSN01004393.1 .
LNVRRQYV-FEDSFHQF--------------AGKSGNEIRGGKLNVKFHD-------EEG
VDVGGVTREW---FQVL----SRQMFNPDY--------ALFLPSASDRI-----------
----------------TYQPNPQS------------------------------------
--WANPDHLFYFKFVGRIIGKAICDQRLLDAYFTR--SFYKHMLG---------------
--------KPVDYKDLEA----------------------LDPEYSKS--------LQWI
LDN--DIT-DVV--
-DETFSVELDD---------------------------FGQ-RRVIE-LLPN--------
GSQIQ---
------------------------------VTEENKA-EYVKL-VAEQRLSLA-IKDQIK
-SFLDG---FHELVPK--DLIQ-IMNEQ-EIELLISG----------------------
----MPDID-----------VDDW----RNNTEYQGGYSPS-------------------
------------------------SPQI-IWFWRTVR--------------------SFD
QEERAKLLQF-------VTGTSKVP------------LEG-----FS-KLQGSG--GIQK
FQIHRDFSSA-----------------------ARLPSAHTCF-NQ-LDLPLY-DSYET
LRSQLL-LAISECTTGF
>Smittium_mucronatum_LSSL01002073.1 .
LNVRRQYV-FEDSFHQF--------------AGKSGKEIRGGKLNVKFHD-------EEG
VDVGGVTREW---FQVL----SRQMFNPDY--------ALFLPSASDRI-----------
----------------TYQPNPQS------------------------------------
--WANPDHLLYFKFVGRVIGKAICDQRLMDAYFTR--SFYKHILG---------------
--------KPVDYKDLEA----------------------LDPEYFKS--------LQWI
LEN--DIT-DIV--
-YETFSVEVDD---------------------------FGQ-HRIVE-LIPN--------
GSTIQ---
------------------------------VNEENKA-EYVRL-VAEQRLSLA-IKDQIQ
-AFLNG---FHELVPK--DLIQ-IMNEQ-EIELLISG----------------------
----MPDID-----------VDDW----RNNTEYNGGYSPS-------------------
------------------------SPQI-IWFWRTVR--------------------SFD
QEERAKLLQF-------VTGTSKVP------------LEG-----FS-KLQGSG--GIQK
FQIHRDFSST-----------------------ARLPSAHTCF-NQ-LDLPLY-DSYEI
LRSQLL-LAVNE-----
>Capniomyces_stellatus_LUVW01000003.1 .
LNVRRQYV-FEDSFHQF--------------AGKSGPEIRGGRLNVKFYD-------EEG
VDAGGVTREW---FQVL----SRQMFNPDY--------ALFRPSAADKI-----------
----------------TYQPNPQS------------------------------------
--WANPDHLLYFKFVGRIIGKAICDQRLFDAYFTR--SFYKHMLG---------------
--------RSVDYRDLEA----------------------VDPEYAKS--------LQWI
LDN--NIT-DVF--
-DETFSVEVDD---------------------------FGQ-HRIVD-LVPG--------
GRTIA---
------------------------------VTEENKV-EYVRL-VAEQRLTVA-IRDQID
-AFLTG---FHELVPK--DLVQ-IMNEQ-EIELLISG----------------------
----MPDID-----------VDDW----RNNADYNGGYTNS-------------------
------------------------SAQI-QWFWRAVR--------------------SFD
LEERAKLLQF-------VTGTSKVP------------LEG-----FA-KLQGSG--GHQK
FQIHKDFSSV-----------------------NRLPSAHTCF-NQ-LDLPLY-ESYEI
LRSQLL-LAISECSTGF
>Zancudomyces_culisetae_LSSK01000893.1 .
LNVRRQYV-FEDSFHQF--------------AGKSGAEIRHGKLNVKFYE-------EEG
VDAGGVTREW---FTVL----SRQMFNPDY--------ALFKPSAADKI-----------
----------------TYQPNPQS------------------------------------

--WANPDHLLYFKFVGRIIGKAICDQRLFDAYFTR--SFYKHMLG---------------
--------RPVDYKDMEA----------------------VDPEYFKS--------LQWI
LDN--DIT-DIV--
-DETFSVHVDD---------------------------FGQ-LRIVD-LIPD--------
GRNIP---
------------------------------VTQENKV-EYVRL-VSEQRLTVA-IQDQIN
-AFLEG---FHELVPK--DLIQ-IMNEQ-EVELLISG-----------------------
----LPDID-----------VDDW----RNNTEYHGGYSSS-------------------
------------------------CVQI-QWFWRAVR--------------------AFD
QEERAKLLQF-------VTGTSKVP------------LEG-----FS-KLQGSG--GYQK
FQIHKDFSSN------------------------LRLPSAHTCF-NQ-LDLPLY-ESYEV
LRDQLL-TAISECSTGF
>Cladosporium_cladosporioides_MSJH02000448 1..
LSIRRDHI-FEDTYHQL--------------QDRTGNEIRYGKLKVYFQD-------EEG
VDEGGVSREW---FSAL----AGQMFDPNY--------ALFITSAADRL-----------
----------------TYQPNRAS------------------------------------
--GVNPEHLSYFKFVGRVIGKAIHDGRVLDAYFTR--SFYKLILG---------------
--------RPVDCKDLET----------------------VDPEYYKS--------LVWM
LDN--DIT-NVI--
-DLTFSVETDD---------------------------FGT-CKIVD-LIPN--------
GRNIA---
------------------------------VTEANKH-KYVNL-IAQQKLVLA-IKPQVD
-AFLEG---FYEIIPA--NLIQ-IFNEQ-ELELLISG-----------------------
----LPDID-----------IDDW----KANTVYEG-YTIQ-------------------
------------------------SPQI-QWFWRAVR--------------------SFD
QEERAKLLQF-------ATGTSKVP------------LGG-----FS-ALQGSN--GVQK
FQIHKEFGDT------------------------NRLPSAHTXF-NQ-IDLPQY-KTYED
LRRNLF-KAISECS---
>Rhizopus_microsporus_CCYT01000585.1 .
LSIRRDHI-FEDTYHQL--------------QDRTGDEIRYGKLKVYFQD-------EEG
VDEGGVSREW---FSAL----AGQMFDPNY--------ALFITSAADRL-----------
----------------TYQPNRAS------------------------------------
--GVNPEHLSYFKFVGRVIGKAIHDGRVLDAYFTR--SFYKLILG---------------
--------RPVDCKDLET----------------------VDPEYYKS--------LVWM
LDN--DIT-NVI--
-DLTFSVETDD---------------------------FGT-CKIVD-LIPN--------
GRNIA---
------------------------------VTEANKH-KYVNL-IAQQKLVLA-IKPQVD
-AFLEG---FYEIIPA--NLIQ-IFNEQ-ELELLISG-----------------------
----LPDID-----------IDDW----KANTVYEG-YTIQ-------------------
------------------------SPQI-QWFWRAVR--------------------SFD
QEERAKLLQF-------ATGTSKVP------------LGG-----FS-ALQGSN--GVQK
FQIHKEFGDT------------------------NRLPSAHT------------------

>Rhizopus_oryzae_GDUK01004271.1 .
LSIRRDYI-FEDTYQQL--------------QDRTGNEIRYGKLKVHFQD-------EEG
VDEGGVSREW---FSAL----ARQMFDPNY--------ALFITSAADKL-----------
----------------TYLPNRAS------------------------------------
--GVNPDHLSYFKFVGRVIGKAIHDGRLLDAYFTR--SFYKLILG---------------
--------RSIDYKDLEA----------------------IDPTYYKS--------LVWM
LEN--DIT-NVI--
-DLTFSVETDD---------------------------FGT-TKTID-LKPD--------
GRNIP---
------------------------------VTEENKH-EYVYL-IAQQRLVLA-IKPQVD
-AFLEG---FHEIIPS--SLIS-IFNEQ-ELELLISG-----------------------
----LPDID-----------IDDW----KANTVYQG-YNFQ-------------------
------------------------SPQI-QWFWRAVR--------------------SFD
EEERAKLLQF-------ATGTSKVP------------LGG-----FS-ALQGSN--GLQK

FQIHKEFSDI------------------------NRLPSAHTCF-NQ-IDLPQY-QNYED
LRRNLF-KAISECSTG-
>Rhizopus_delemar_JNEA01004412.1 .
LSIRRDYI-FEDTYQQL--------------QDRTGDEIRYGKLKVHFQD-------EEG
VDEGGVSREW---FSAL----ARQMFDPNY--------ALFITSAADKL-----------
----------------TYLPNRAS------------------------------------
--GVNPDHLSYFKFVGRVIGKAIHDGRLLDAYFTR--SFYKLILG---------------
--------RSIDYKDLEA----------------------IDPTYYKS--------LVWM
LEN--DIT-NVI--
-DLTFSVETDD---------------------------FGT-TKTID-LKPD--------
GRNIP---
------------------------------VTEENKH-EYVYL-IAQQRLVLA-IKPQVD
-AFLEG---FHEIIPS--SLIS-IFNEQ-ELELLISG-----------------------
----LPDID-----------IDDW----KANTVYQG-YNFQ-------------------
------------------------SPQI-QWFWRAVR--------------------SFD
EEERAKLLQF-------ATGTSKVP------------LGG-----FS-ALQGSN--GLQK
FQIHKEFSDI------------------------NRLPSAHTXF-NQ-IDLPQY-QNYED
LRRNLF-KAISECS---
>Cokeromyces_recurvatus_JNEH01001672.1 .
LSVSRERV-FEDTYEQL--------------HPFSGKEIRNSKIAVEFMN-------EDG
IDEGGVSREW---FSVL----ARQMFDPNY--------ALFIASAADRL-----------
----------------TYQPNRAS------------------------------------
--GVSAEHLNYFKFVGRIIGKAIHDGRLLDAYFTR--SFYKLILG---------------
--------RSVDYRDVET----------------------VDPTYYKS--------LVWM
LEN--DIT-DII--
-DATFSIEVDD---------------------------FGK-RKIID-LKAN--------
GRNIA---
------------------------------VTEANKH-EYVAL-VTEQKLVLA-IKDQVN
-AFLEG---FYDIIPV--ELIQ-IFNEQ-ELELLISG-----------------------
----LPDID-----------IDDW----KANTVYVG-YTSA-------------------
------------------------SPQI-HWFWRAVR--------------------SFT
QEERAKLLQF-------TTGTSKVP------------LEG-----FS-QLQGSN--GVQK
FQIHKEFGDV------------------------NRLPSAHTXF-NQ-IDLPQY-VTYED
LRSSLF-KAMNECSTGF
>Mucor_indicus_JNEK01001199.1 .
LTVRREQV-FEDTYRRL--------------QGLSGDEIRFGKLNVQFQD-------EEG
VDAGGVAREW---FSVL----ARQMFDPNY--------ALFITSAADKL-----------
----------------TYQPNRAS------------------------------------
--GVNSEHLSYFKCVGRIIGKAIHDGRLLDAYFTR--SFYKLILG---------------
--------RSVDYKDVEA----------------------IDPTYYKS--------LVWM
LEN--DIT-DII--
-DLTFSVETDD---------------------------FGT-TKIID-LKPN--------
GRDIP---
------------------------------VTEENKH-EYVNL-VSEQRLYLA-IKDQVN
-AFIEG---FNDIIPR--ELIQ-IFNEQ-ELELLISG-----------------------
----LPDID-----------IDEW----KANTVYVG-YTLS-------------------
------------------------SPQI-QWFWRAVR--------------------SFD
QEERAKLLQF-------STGTSKVP------------LEG-----FA-QLQGSN--GVQK
FQIHKEFSDV------------------------NRLPSAHTXF-NQ-IDLPQY-LTYED
LRANLF-KAINECSTGF
>Actinomucor_elegans_BCHK01000021.1 .
LTVRRAHV-FEDTYQKL--------------QGLSGKEIKYGKLNVQFHN-------EEG
VDAGGVAREW---FSVL----ARQMFDPNY--------ALFVTSAADKL-----------
----------------TYQPNRAS------------------------------------
--GINSEHLNYFKCVGRIIGKAIHDGRLLDAYFTR--SFYKLMLG---------------
--------RSVDYRDIET----------------------VDPAYYKS--------LVWM
LDN--DIT-DII--
-DLTFSIEVDD---------------------------FGT-NKIID-LKPN--------

GRHIP---
------------------------------VTEANKH-EYVSL-ITEQRLVLA-IKDQVN
-AFLEG---FHDIIPE--QLIQ-IFNEQ-ELELLISG-----------------------
----LPDID-----------IDEW----KANTVYEG-YTLS-------------------
------------------------SPQI-QWFWRAVR--------------------SFD
QEERAKLLQF-------STGTSKVP------------LEG-----FA-QLQGSN--GVQK
FQIHKEFGDV------------------------NRLPSAHTXF-NQ-IDLPQY-PSYEE
LRNSLF-KAISECSTGF
>Choanephora_cucurbitarum_LUGH01000002.1 .
LSVRRAHV-FEDTYQKL--------------QGLTGEEIKHGKLNVSFHN-------EEG
VDAGGVAREW---FSVL----ARQMFDPNY--------ALFITSAADKL-----------
----------------TYQPNRAS------------------------------------
--GVNSEHLSYFKCVGRIIGKAIHDGRLLDAYFTR--SFYKLMLG---------------
--------RSVDYRDVEA----------------------VDPAYYKS--------LVWM
LEN--DIT-DII--
-DLTFSIEVDD---------------------------FGT-NKTID-LKPN--------
GRNIP---
------------------------------VTEANKH-EYVSL-ITEQKLVLA-IKDQVN
-AFLEG---FHDIIPP--HLIQ-IFNEQ-ELELLISG-----------------------
----LPDID-----------IDEW----KANTVYEG-YTIK-------------------
------------------------SPQI-QWFWRAVR--------------------SFD
QEERAKLLQF-------STGTSKVP------------LEG-----FA-QLQGSN--GVQK
FQIHKEFGDV------------------------NRLPSAHT------------------

>Mucor_irregularis_GFBC01017477.1 .
LNVRRAHV-FEDTYRQL--------------QGRTGKEIKHGKLNVQFHN-------EEG
VDAGGVAREW---FSVL----ARQMFDPNY--------ALFITSAADKL-----------
----------------TYQPNRAS------------------------------------
--GVNSDHLSYFKCVGRIIGKAIHDGRLLDAYFTR--SFYKLMLG---------------
--------RSVDYRDVEA----------------------VDPAYYKS--------LVWM
LDN--DIT-DII--
-DLTFSVETDD---------------------------FGT-NKIID-LKPD--------
GRNIA---
------------------------------VTESNKH-EYVTL-ITEQKLVLA-IKDQVN
-AFLEG---FHDIIPA--QLIQ-IFNEQ-ELELLISG-----------------------
----LPDID-----------IDEW----KANTVYEG-YTLS-------------------
------------------------SPQI-QWFWRAVR--------------------SFD
QEERAKLLQF-------STGTSKVP------------LEG-----FS-QLQGSN--GVQK
FQIHKEFGDV------------------------NRLPSAHTCF-NQ-IDLPQY-LSYED
LRSNLF-KAISECSTG-
>Mucor_racemosus_JNEI01001689.1 .
LSLDRANV-FEDTYQKL--------------QGLTGDEIKYGKLNVHFHG-------EEG
VDAGGVAREW---FSVL----ARQMFDPNY--------ALFITSAADKL-----------
----------------TYQPNRAS------------------------------------
--GVNSEHLSYFKCVGRIIGKAIHDGRLLDAYFTR--SFYKLMLG---------------
--------RSVDYRDVEA----------------------VDPAYYKS--------LVWM
LDN--DIT-DII--
-DLTFSVETDD---------------------------FGT-NKTID-LKPG--------
GRDIP---
------------------------------VTEENKH-EYVTL-VTEQKLVLA-IKDQVN
-TFLEG---FHDIIPA--TLIQ-IFNEQ-ELELLISG-----------------------
----LPDID-----------IDEW----KANTVYEG-YTLS-------------------
------------------------SPQI-QWFWRAVR--------------------SFD
QEERAKLLQF-------STGTSKVP------------LEG-----FA-ELQGSN--GVQK
FQIHKEFGDV------------------------NRLPSAHTXF-NQ-IDLPQY-LTYED
LRSNLF-KAISECSTGF
>Mucor_circinelloides_AOCY01000079.1 .
LSLDRANV-FEDTYRKL--------------QGLTGDEIKYGKLNVHFHG-------EEG

VDAGGVAREW---FSVL----ARQMFDPNY--------ALFITSAADKL-----------
----------------TYQPNRAS------------------------------------
--GVNSEHLSYFKCVGRIIGKAIHDGRLLDAYFTR--SFYKLMLG---------------
--------RSVDYRDVEA----------------------VDPAYYKS--------LVWM
LEN--DIT-DII--
-DLTFSVETDD---------------------------FGT-NKIID-LKPD--------
GRNIP---
------------------------------VTEENKH-EYVTL-VTEQKLVLA-IKDQVN
-AFLEG---FHDIIPA--SLIQ-IFNEQ-ELELLISG-----------------------
----LPDID-----------IDEW----KANTVYEG-YTLS-------------------
------------------------SPQI-QWFWRAVR--------------------SFD
QEERAKLLQF-------STGTSKVP------------LEG-----FA-ELQGSN--GVQK
FQIHKEFGDV------------------------NRLPSAHTXF-NQ-IDLPQY-LTYED
LRSNLF-KAISECSTGF
>Mucor_velutinosus_JNDK01002064.1 .
LSLDRANV-FEDTYRKL--------------QGLTGDEIKYGKLNVHFHG-------EEG
VDAGGVAREW---FSVL----ARQMFDPNY--------ALFITSAADKL-----------
----------------TYQPNRAS------------------------------------
--GVNSEHLSYFKCVGRIIGKAIHDGRLLDAYFTR--SFYKLMLG---------------
--------RSVDYRDVEA----------------------VDPAYYKS--------LVWM
LEN--DIT-DII--
-DLTFSVETDD---------------------------FGT-NKIID-LKPD--------
GRNIP---
------------------------------VTEENKH-EYVTL-VTEQKLVLA-IKDQVN
-AFLEG---FHDIIPA--SLIQ-IFNEQ-ELELLISG-----------------------
----LPDID-----------IDEW----KANTVYEG-YTLS-------------------
------------------------SPQI-QWFWRAVR--------------------SFD
QEERAKLLQF-------STGTSKVP------------LEG-----FA-ELQGSN--GVQK
FQIHKEFGDV------------------------NRLPSAHTXF-NQ-IDLPQY-LTYED
LRANLF-KAISECSTGF
>Mucor_ambiguus_BBKB01004437.1 .
LSLDRANV-FEDTYRKL--------------QGLTGDEIKYGKLNVHFHG-------EEG
VDAGGVAREW---FSVL----ARQMFDPNY--------ALFITSAADKL-----------
----------------TYQPNRAS------------------------------------
--GVNSEHLSYFKCVGRIIGKAIHDGRLLDAYFTR--SFYKLMLG---------------
--------RSVDYRDVEA----------------------VDPAYYKS--------LVWM
LEN--DIT-DII--
-DLTFSVETDD---------------------------FGT-NKIID-LKPD--------
GRNIP---
------------------------------VTEENKH-EYVTL-VTEQKLVLA-IKDQVN
-AFLEG---FHDIIPA--SLIQ-IFNEQ-ELELLISG-----------------------
----LPDID-----------IDEW----KANTVYEG-YTLS-------------------
------------------------SPQI-QWFWRAVR--------------------SFD
QEERAKLLQF-------STGTSKVP------------LEG-----FA-ELQGSN--GVQK
FQIHKEFGDV------------------------NRLPSAHTXF-NQ-IDLPQY-LTYED
LRANLF-KAISECSTGF
>Allomyces_macrogynus_ACDU01002042.1 .
LNLRRQNV-FEQSYQQM--------------QHLSGEELKNGKLNVRFYG-------EEG
VDAGGLTREW---FSVL----ARQIFNPDY--------ALFKTSAVDKA-----------
----------------TYQPNRAS------------------------------------
--WVNPEHLHYFKFVGRFIGKAIYDQRLLDCYFTR--SFYKHLLG---------------
--------KAVDIRDMEA----------------------VDPSYYKS--------LEWI
LEN--EIN-DIM--
-DLTFSVETDD---------------------------FGK-TKVID-LKPD--------
GRNIA---
------------------------------VTDENKH-EYVRL-VVEQRLTLA-IRDQIA
-AFTEG---FFDMVPR--DLVS-IFNEQ-ELELLISG-----------------------
----MPEID-----------VDDW----RNNTEYHH-FTAS-------------------

------------------------APVI-QWFWRAVR--------------------SFD
QEHRAKLVQY-------VTGTSKVP------------MEG-----FR-ALQGST--GVQK
FQIVRDPGGT------------------------HRLPSAHTCF-NQ-LDLPEY-ESYEK
LRTMLL-KAVDEASE--
>Catenaria_anguillulae_MCFL01000019.1 .
LNVRRQFV-FEDSYHQM--------------QHRSGDELKYARLNVTFVD-------EDG
VDAGGLTREW---YSVL----ARQIFNPGY--------ALFTAAAQDKT-----------
----------------TYQPNRAS------------------------------------
--WVNPDHLHFFRFVGRFIGKAIFDQRLLDCYFTR--SFYKHILD---------------
--------KAVDVRDMEA----------------------VDPSYFKS--------LEWI
LEN--SID-DVM--
-ELTFSVETDD---------------------------FGT-TKTID-LKEN--------
GRNIL---
------------------------------VTDENKA-EYVRL-VVEQKLTRA-IKDQLG
-AFISG---FYEMIPR--PLVS-IFNEQ-ELELLISG-----------------------
----MPEID-----------IDDW----RNNTVYHS-YTSS-------------------
------------------------SPVI-QWFWRAVR--------------------SFD
QEQRAKLVQF-------TTGTSKVP------------MEG-----FK-ALQGSG--GVNK
FTIVKDPGGT------------------------HRLPSAHTCF-NQ-LDLPEY-ESYDK
LRAMVL-KVLDEASEGF
>Sporisor_scitamineum_LK056653.1 .
LSVRRNSV-FEDSFRYF--------------SRKTGPEVKHGKLNVRFNN-------EEG
VDAGGVTREW---FQVL----ARAMFNPDY--------ALFQPCAADRT-----------
----------------TYQPNRMS------------------------------------
--YVNPDHLSFFKFVGRIIGKAIYDGRLLDAYFTR--SFYKHILG---------------
--------KPVDYRDLES----------------------IDPEYFKS--------LEWM
LNN--DIT-DIL--
-DLTFSVDDEE---------------------------FGE-TKVIE-LKPN--------
GTTIA---
------------------------------VTEANKQ-EYVRL-VTEQRLTNS-IKSQID
-AFLGG---FNEIIPS--ELIR-IFSEQ-ELELLISG-----------------------
----LPDID-----------VDAW----KNNTELHG-YSSG-------------------
------------------------DAVI-QWWWRAVR--------------------SFD
QTEKAKLLQF-------ITGTSKVP------------LEG-----FA-HLQGVQ--GTQR
FNIHKAYGA-------------------------DRLPAAHTCF-NQ-LDLPQY-ESYDK
LRSSLL-LAMNE-----
>Sporisor_reilianum_FQ311474.1 .
LSVRRNSV-FEDSFRYF--------------SRKTGPEVKHGKLNVRFNN-------EEG
IDAGGVTREW---FQVL----ARAMFNPDY--------ALFQPCAADRT-----------
----------------TYQPNRMS------------------------------------
--YVNPDHLSFFKFVGRIIGKAIYDGRLLDAYFTR--SFYKHILG---------------
--------KPVDYRDLES----------------------IDPEYFKS--------LEWM
LNN--DIT-DIL--
-DLTFSVDDEE---------------------------FGE-TKVID-LKPN--------
GSTVA---
------------------------------VTEANKQ-EYVRL-VTEQRLTNS-IRSQID
-AFLGG---FNEIIPS--ELIR-IFSEQ-ELELLISG-----------------------
----LPDID-----------VDAW----KNNTELHG-YSSG-------------------
------------------------DAVI-QWWWRAVR--------------------SFD
QTEKAKLLQF-------ITGTSKVP------------LEG-----FA-HLQGVQ--GTQR
FNIHKAYGA-------------------------DRLPAAHTCF-NQ-LDLPQY-ESYDK
LRSSLL-LAMNE-----
>Pseudozyma_hubeiensis_XM_012332066.1 .
LSVRRNSV-FEDSFRYF--------------SRKTGPEVKHGKLNVRFNN-------EEG
IDAGGVTREW---FQVL----ARAMFNPDY--------ALFQPCAADRT-----------
----------------TYQPNRMS------------------------------------
--YVNPDHLSFFKFVGRIIGKAIYDGRLLDAYFTR--SFYKHILG---------------
--------KPVDYRDLES----------------------IDPEYFKS--------LEWM

LNN--DIT-DIL--
-DLTFSVDDEE---------------------------FGE-TKVID-LKPN--------
GNTIA---
------------------------------VTEANKQ-EYVRL-VTEQRLTKS-IKSQID
-AFLGG---FNEIIPS--DLIR-IFSEQ-ELELLISG-----------------------
----LPDID-----------VDAW----KNNTELHG-YSSG-------------------
------------------------DAVI-QWWWRAVR--------------------SFD
QTEKAKLLQF-------ITGTSKVP------------LEG-----FA-HLQGVQ--GTQR
FNIHKAYGA-------------------------DRLPAAHTCF-NQ-LDLPQY-ESYEK
LRSSLL-LAMTEGGEG-
>Ustilago_maydis_XM_011391602.1 .
LSVRRNSV-FEDSFRYF--------------SRKTGPEVKHGKLNVRFTN-------EEG
IDAGGVTREW---FQVL----ARAMFNPDY--------ALFQPCAADRT-----------
----------------TYQPNRMS------------------------------------
--YVNPDHLSFFKFVGRIIGKAIYDGRLLDAYFTR--SFYKHILG---------------
--------KPVDYRDLES----------------------IDPEYFKS--------LEWM
LSN--DIT-DIL--
-DLTFSVDDEE---------------------------FGE-TKVVD-LKPN--------
GTSIS---
------------------------------VTEANKQ-EYVRL-VTEQRLTKS-IKSQID
-AFLGG---FNEIIPS--DLIR-IFSEQ-ELELLISG-----------------------
----LPDID-----------VDAW----KNNTELHG-YSSG-------------------
------------------------DAVV-QWWWRAVR--------------------SFD
QTEKAKLLQF-------ITGTSKVP------------LEG-----FA-HLQGVQ--GTQR
FNIHKAYGA-------------------------DRLPAAHTCF-NQ-LDLPQY-ESYEK
LRSSLL-LAMNE-----
>Kalmanozyma_brasiliensis_XM_016433987.1 .
LSVRRNSV-FEDSFRYF--------------SRKSGPEVKHGKLNVRFNN-------EEG
IDAGGVTREW---FQVL----ARAMFNPDY--------ALFQPCAADRT-----------
----------------TYQPNRMS------------------------------------
--YVNPDHLSFFKFVGRIIGKAIYDGRLLDAYFTR--SFYKHILG---------------
--------KPVDYRDLES----------------------IDPEYFKS--------LEWM
LNN--DIT-DIL--
-DLTFSVDDEE---------------------------FGE-TKIVE-LQPN--------
GASIA---
------------------------------VTEANKQ-EYVRL-VTEQRLTKS-IKSQIE
-AFLGG---FNEIIPS--DLIR-IFSEQ-ELELLISG-----------------------
----LPDID-----------VDAW----KNNTELHG-YSSG-------------------
------------------------DAVI-QWWWRAVR--------------------SFD
QTEKAKLLQF-------ITGTSKVP------------LEG-----FA-HLQGVQ--GTQR
FNIHKAYGA-------------------------DRLPAAHTCF-NQ-LDLPQY-ESYEK
LRSSLL-LAMNE-----
>Pseudozyma_antarctica_XM_014800822.1 .
LSVRRNSV-FEDSFRYF--------------SRKNGPEVKHGKLNVRFNN-------EEG
IDAGGVTREW---FQVL----ARAMFNPDY--------ALFQPCAADRT-----------
----------------TYQPNRMS------------------------------------
--YVNPDHLSFFKFVGRIIGKAIYDGRLLDAYFTR--SFYKHILA---------------
--------KPVDYRDLES----------------------IDPEYFKS--------LEWM
LNN--DIT-DIL--
-DLTFSVDDEE---------------------------FGE-TKIIE-LKLN--------
GANIT---
------------------------------VTESNKQ-EYVRL-VTEQRLTKS-IQSQID
-AFLTG---FNEIIPA--DLIR-IFSEQ-ELELLISG-----------------------
----LPDID-----------VDAW----KNNTELHG-YGSG-------------------
------------------------DAVI-QWWWRAVR--------------------SFD
QTEKAKLLQF-------ITGTSKVP------------LEG-----FA-HLQGVQ--GTQR
FNIHKAYGA-------------------------DRLPAAHTCF-NQ-LDLPQY-DSYEK
LRSSLL-TAMNEGGEG-

>Ustilago_hordei_CAGI01000163.1 .
LSVRRNSV-FEDSFRYF--------------SRKTGPEVKHGKLNVRFNN-------EEG
IDAGGVTREW---FQVL----ARAMFNPDY--------ALFQPCAADRT-----------
----------------TYQPNRMS------------------------------------
--YVNPDHLSFFKFVGRIIGKAIYDGRLLDAYFTR--SFYKHILG---------------
--------KPVDYRDLES----------------------IDPEYFKS--------LEWM
LNN--DIT-DIL--
-DLTFTVDDEE---------------------------FGE-TKVID-LKPN--------
GTNIA---
------------------------------VTELNKQ-EYLRL-VTEQRLTKS-IRSQID
-AFLDG---FNEIIPT--DLIR-IFSEQ-ELELLISG-----------------------
----LPDID-----------VDAW----KNNTELHG-YSSG-------------------
------------------------DAVI-QWWWRAVR--------------------SFD
QTEKAKLLQF-------ITGTSKVP------------LEG-----FA-HLQGVQ--GTQR
FNIHKAYGA-------------------------DRLPAAHTCF-NQ-LDLPQY-DSYDK
LRSLLL-IAINEGGEGF
>Ustilago_bromivora_LT558126.1 .
LSVRRNSV-FEDSFRYF--------------SRKTGPEVKHGKLNVRFNN-------EEG
IDAGGVTREW---FQVL----ARAMFNPDY--------ALFQPCAADRT-----------
----------------TYQPNRMS------------------------------------
--YVNPDHLSFFKFVGRIIGKAIYDGRLLDAYFTR--SFYKHILG---------------
--------KPVDYRDLES----------------------IDPEYFKS--------LEWM
LNN--DIT-DIL--
-DLTFTVDDEE---------------------------FGE-TKVID-LKPN--------
GTNIA---
------------------------------VTELNKQ-EYLRL-VTEQRLTKS-IRSQID
-AFLDG---FNEIIPA--DLIR-IFSEQ-ELELLISG-----------------------
----LPDID-----------VDAW----KNNTELHG-YSPG-------------------
------------------------DAVI-QWWWRAVR--------------------SFD
QTEKAKLLQF-------ITGTSKVP------------LEG-----FA-HLQGVQ--GTQR
FNIHKAYGA-------------------------DRLPAAHTCF-NQ-LDLPQY-DSYEK
LRSLLL-IAINE-----
>Melanopsichium_pennsylvanicum_HG529608.1 .
LSVRRNSV-FEDSYRYF--------------SRKNGPDVKHGKLNVRFNN-------EEG
IDAGGVTREW---FQVL----ARAMFNPDY--------ALFQPCAADRT-----------
----------------TYQPNRMS------------------------------------
--YVNPDHLSFFKFVGRIIGKAIYDGRLLDAYFTR--SFYKHILG---------------
--------KPVDYRDIES----------------------VDPEYFKS--------LEWM
LNN--DIT-DIL--
-DFTFSVDDEE---------------------------FGE-TKVIE-LKPD--------
GTNIP---
------------------------------VSEENKH-EYVRL-VTEQRLTKS-IKSQID
-AFLGG---FNEIIPA--DLIR-IFSEQ-ELELLISG-----------------------
----LPDID-----------VDAW----KNNTELHG-YGSG-------------------
------------------------DAVI-QWWWRAVR--------------------SFD
QTEKAKLLQF-------ITGTSKVP------------LEG-----FA-HLQGVQ--GTQR
FNIHKAYGA-------------------------DRLPAAHTCF-NQ-LDLPQY-DSYEK
LRSSLL-LAMNE-----
>Pseudozyma_flocculosa_XM_007879733.1 .
LSVRRQYV-FEDSFQYL--------------NRRKGPEVKHGKLNVRFYN-------EEG
IDAGGVTREW---FQVL----ARAMFNPDY--------ALFQPCAADRT-----------
----------------TYQPNRMS------------------------------------
--SVNEHHLSFFKFVGRVIGKAIYDNRLLDAYFTR--SFYKHILG---------------
--------KSVDYRDLES----------------------IDPEYFKS--------LQWM
LDN--DIT-DVL--
-DLTFSVDAEE---------------------------FGE-TKIIE-LKPD--------
GANIP---
------------------------------VTEANKV-EYVRL-VTEQRLTQS-IRSQID

-AFLEG---FNDIIPA--DLIR-IFSEQ-ELELLISG-----------------------
----LPDID-----------VDAW----KNNTELHG-YSSG-------------------
------------------------DAVI-QWWWRAVR--------------------SFD
QTEKAKLLQF-------ITGTSKVP------------LEG-----FG-HLQGVQ--GTQR
FNIHKAYGS-------------------------DRLPAAHTCF-NQ-LDLPQY-ESYEK
LRSSLL-IAMNEGGEG-
>NWGS3B_2_Meira_nashicola_BCJU01000002.1 .
ISVRRQHV-FSDSFRCL--------------QGHTGPEIKHGKLNVRFYN-------EEG
IDAGGVTREW---YSIL----ARAMFNPDY--------ALFAPCAADRT-----------
----------------TYQPNRAS------------------------------------
--AINPDHLAFFKFVGRVIGKAIYDGRLLDAYFTR--SFYKHILG---------------
--------KAVDYRDLEA----------------------IDPEYFKS--------LQWM
LEN--DIE-DVL--
-DLTFTVDNEE---------------------------FGE-TTVVE-LKPD--------
GANIP---
------------------------------VTDKNKQ-EYVRL-VTEQRLTLS-IQKQIE
-AFLNX---FHDVIPK--DLVR-IFSEQ-ELELLISG-----------------------
----LPDID-----------VVAW----KNNTELHG-YSTS-------------------
------------------------DPVV-QWFWRAVR--------------------SFD
QTDKAKLLQF-------ITGTSKVP------------LEG-----FA-HLQGVQ--GTQR
FNIHKAYGA-------------------------DRLPAAHTCF-NQ-LDLPQY-DSYEK
LRSQLL-LAMNEGGEGF
>Tilletiopsis_pallescens_BCHO01000002.1 .
LSLRRDFV-FMDSFTFF--------------ARHSGPEIKHGKLNIRFYG-------EEG
VDAGGVTREW---FQVL----ARAMFNPDY--------ALFAPCAADRT-----------
----------------TYQPNRMS------------------------------------
--SVNPDHEKFFKFVGRVIAKAIYDGRLLDAYFTR--SFYKHILG---------------
--------RAVDYKDLES----------------------VDPEYFKS--------LEWM
LQN--DIT-DVL--
-DLTFSVDADD---------------------------FGE-TKVID-LKPD--------
GRTIP---
------------------------------VTEANKL-EYVKL-VTEQRLTNS-IQKQID
-AFLEG---FHDIISP--SLIR-IFSEQ-ELELLISG-----------------------
----LPDID-----------VDSW----KGDTELHG-YSAG-------------------
------------------------DAVV-QWFWRAVR--------------------SFD
QTEKAKLLQF-------VTGTSKVP------------LEG-----FA-HLQGMQ--GTQK
FNIHKAYGN-------------------------DRLPAAHTXF-NQ-LDLPQY-DSYER
LRSQLL-LAMNEGGEGF
>Salmacisia_buchloeana_MOEQ01000025.1 .
LNVRRSEV-FGDSQMAF--------------SRYTGPEIKHGRLNVRFLG-------EEG
IDAGGVTREW---FSVL----ARAMFNPDY--------ALFTPCAADRT-----------
----------------TYQPNRLS------------------------------------
--SVDRNHLLYFKFVGRVIGKAIYDGRLLDAYFTR--SFYKHILG---------------
--------KPVDYRDMEA----------------------VDPEYYKS--------LEWI
LEN--DIT-DVL--
-DLNFTMDAED---------------------------FGE-TRVIE-LKPD--------
GASIP---
------------------------------VTEENKV-EYVRL-VTEQMILRT-LSLQID
-AFLEG---FHDIIPP--ELVR-IFTEQ-ELELLISG-----------------------
----LPDID-----------VDSW----KNNTELHG-YTSS-------------------
------------------------DPVI-QWFWRAVR--------------------SFD
QTEKAKLLQF-------ITGTSKVP------------LEG-----FG-HLQGTQ--GIQK
FNIHKAYGA-------------------------NRLPAAHTXF-NQ-LDLPQY-ESYDK
LRSQLL-ICMNEASEGF
>Tilletia_horrida_LAXH01000245.1 .
LNIRRSEV-FGDSQKFF--------------SRYTGPEIKHGRLNVRFLG-------EEG
IDAGGVTREW---FSVL----ARAMFNPDY--------ALFTPCAADRT-----------
----------------TYQPNRLS------------------------------------

--FVDSHHLIYFKFVGRVIGKAIYDGRLLDAYFTR--SFYKHILG---------------
--------KPVDYRDMEA----------------------VDPEYYKS--------LEWI
LEN--DIT-DVL--
-DLNFTMDAED---------------------------FGE-TRVIE-LKPD--------
GATMP---
------------------------------VTEENKV-EYVRL-VTEQRLTQS-IRPQID
-AFLEG---FHDIIPP--ELIR-IFTEQ-ELELLISG-----------------------
----LPDID-----------VDSW----KNNTELHG-YSSS-------------------
------------------------DAVI-QWFWRAVR--------------------SFD
QTEKAKLLQF-------ITGTSKVP------------LEG-----FG-HLQGTQ--GIQK
FNIHKAYGX--------------------------RIP--HYSF-NQ-LDLPQY-ESYEK
LRSWLL-ICMNEASEGF
>Tilletia_controversa_LWDE01000348.1 .
LNVRRTEV-FGDSQKAF--------------SRYTGPEIKHGRLNVRFLG-------EEG
IDAGGVTREW---FSVL----ARAMFNPDY--------ALFTPCAADRT-----------
----------------TYQPNRLS------------------------------------
--SVDSNHLLYFKFVGRVIGKAIYDGRLLDAYFTR--SFYKHILG---------------
--------KPVDYRDMEA----------------------VDPEYYKS--------LEWI
LEN--DIT-DVL--
-DLNFTMDAED---------------------------FGQ-TRVIE-LKPD--------
GATIP---
------------------------------VTEETKV-EYIRL-VTEQRLTHT-IRSQID
-SFLTG---FHEIIPP--ELIR-IFTEQ-ELELLISG-----------------------
----LPDID-----------VDSW----KNNTELHG-YNSS-------------------
------------------------DPVI-QWFWRAVR--------------------SFD
QTEKAKLLQF-------ITGTSKVP------------LEG-----FG-HLQGTQ--GIQK
FNIHKAYGA-------------------------DRLPAAHTXF-NQ-LDLPQY-ESYEK
LRSQLL-ICMNEAS---
>Tilletia_indica_MAPW01000241.1 .
LNVRRTEV-FGDSQKAF--------------SRYTGPEIKHGRLNVRFLG-------EEG
IDAGGVTREW---FSVL----ARAMFNPDY--------ALFTPCAADRT-----------
----------------TYQPNRLS------------------------------------
--SVDNNHLLYFKFVGRVIGKAIYDGRLLDAYFTR--SFYKX------------------
---------------MEA----------------------VDPEYYKS--------LEWI
LEN--DIT-DVL--
-DLNFTMDAED---------------------------FGQ-TRVIE-LKPD--------
GATIP---
------------------------------VTEETKV-EYIRL-VTEQRLTHS-IRSQVD
-AFLTG---FHEIIPM--ELIR-IFTEQ-ELELLISG-----------------------
----LPDID-----------VDSW----KNNTELHG-YNSS-------------------
------------------------DAVI-QWFWRAVR--------------------SFD
QTEKAKLLQF-------ITGTSKVP------------LEG-----FG-HLQGTQ--GIQK
FNIHKAYGA-------------------------DRLPAAHTXF-NQ-LDLPQY-ESYEK
LRSQLL-ICMNEASE--
>Tilletia_walkeri_LWDG01000113.1 .
LNVRRTEV-FGDSQKAF--------------SRYTGPEIKHGRLNVRFLG-------EEG
IDAGGVTREW---FSVL----ARAMFNPDY--------ALFTPCAADRT-----------
----------------TYQPNRLS------------------------------------
--SVDNNHLLYFKFVGRVIGKAIYDGRLLDAYFTR--SFYKHILG---------------
--------KPVDYRDMEA----------------------VDPEYYKS--------LEWI
LEN--DIT-DVL--
-DLNFTMDAED---------------------------FGQ-TRVIE-LKPD--------
GATIP---
------------------------------VTEETKV-EYIRL-VTEQRLTHS-IRSQVD
-AFLTG---FHEIIPM--ELIR-IFTEQ-ELELLISG-----------------------
----LPDID-----------VDSW----KNNTELHG-YNSS-------------------
------------------------DAVI-QWFWRAVR--------------------SFD
QTEKAKLLQF-------ITGTSKVP------------LEG-----FG-HLQGTQ--GIQK

FNIHKAYGA-------------------------DRLPAAHTXF-NQ-LDLPQY-ESYEK
LRSQLL-ICMNEASE--
>Tilletiaria_anomala_XM_013389925.1 .
MSIRRSEI-FYDSYRFF--------------SRYTGPEIKHGKLNVRFHG-------EEG
VDAGGVTREW---FSVL----ARAMFNPDY--------ALFAPCAADRT-----------
----------------TFQPNRMS------------------------------------
--WINEHHLSFFKFVGRIIGKAIYDGRLLDAYFSR--SFYKHILG---------------
--------KAVDYRDIEA----------------------VDPEYFKS--------LEWM
LNN--DIT-GVL--
-DLTFSVDAED---------------------------FGE-AKTIE-LKPG--------
GKDIA---
------------------------------VTEENKV-EYVKL-ITEQRLTKS-IRKQID
-AFLEG---FHDIIPV--DLIR-IFNEQ-ELELLISG-----------------------
----LPDIN-----------VDDW----KNNTELHG-YSSG-------------------
------------------------DPVI-QWFWRAVR--------------------SFD
QTQKANLLQF-------ITGTSKTP------------IEG-----FK-ALQGVQ--GNQR
FNIHKAYGE-------------------------NRLPTAHTCF-NQ-LDLPAY-ETYEK
LRSQLL-TAMVEGGTGF
>Malassezia_restricta_LFDA01000019.1 .
LNVRRQFV-FEDSYHQM--------------QHRSGDELKYARLNVRFHN-------EDG
VDAGGVTREW---FQVL----SREMFNPDY--------ALFQPCAADCT-----------
----------------TYQPNKMS------------------------------------
--SVNDMHLAFFKFIGRVIGKAIYDGRLLDAYFTR--SFYKHILG---------------
--------RKVDYRDLEA----------------------VDPEYYNS--------IQWM
LNN--DIT-DVL--
-DLTFAVEEDV---------------------------FGE-TRTIE-LKPG--------
GSSIP---
------------------------------VTESNKH-EYVRL-VTEQSLTNS-IRSQID
-AFLAG---FHEIIPP--SLIK-LFSER-ELELLISG-----------------------
----LPDID-----------VDEW----KNNTDLRG-YKSS-------------------
------------------------DPMI-QWWWRAVR--------------------SFD
QTEKAKLLQF-------ITGTSKVP------------LEG-----FA-HLQGVN--GTQR
FNIHRAYGE-------------------------DRLPAAHTCF-NQ-LDLPAY-ESYEK
LRSQLL-LAMKEGAEGF
>Malassezia_slooffiae_LFGK01000372.1 .
LTVRRAYV-FYDSFQFF--------------NRKSGPEIKHGKLNVRFQG-------EEG
VDAGGVTREW---FQVL----AREMFNPDY--------ALFAPCAADRT-----------
----------------TYQPNRMS------------------------------------
--AVNDLHLSFFKFIGRVIGKAIYDGRLLDAYFTR--SFYKHILG---------------
--------KRVDYKDLEA----------------------VDPEYYNS--------LEWM
LHN--DIE-DVL--
-ELNFAIESEE---------------------------FGE-TKVIE-LKPD--------
GANIP---
------------------------------VTDANKH-EYVRL-VTEQRLTNS-IRSQID
-AFLQG---FNEVIPP--SLIQ-IFSEH-ELELLISG-----------------------
----LPDID-----------VDAW----KNNTELYG-YSSS-------------------
------------------------DPVV-QWWWRAVR--------------------SFD
QTEKAKLLQF-------ITGTSKVP------------LEG-----FA-HLQGIQ--GTQR
FNIHKAFDS-------------------------ERLPAAHTCF-NQ-LDLPAY-DSYER
LRSQLL-IAMNEGSEGF
>Malassezia_cuniculi_LFFW01000054.1 .
LTVRRNHV-FYDSFQFF--------------SRKSGPEIKHGRLSVKFQG-------EEG
VDAGGLTREW---FQVL----AREMFNPDY--------ALFAPCAADQT-----------
----------------TYQPNRMS------------------------------------
--SVNDMHLSFFKFIGRVIGKAIYDGRLLDSYFTR--SFYKHILH---------------
--------KGVDYKDLEA----------------------VDPEYYNS--------LEWM
LHN--DIK-DVL--
-DLTFTIDSEE---------------------------FGV-TRTIE-LIPD--------

GAKKA---
------------------------------VTDENKL-EYVRL-VTEQRLTNS-IRSQID
-AFLDG---FHEIIQP--ELIQ-IFTEH-ELELLISG-----------------------
----LPDID-----------VDEW----KNNTELHG-YNSS-------------------
------------------------DPTI-QWWWRAVR--------------------SFD
QTEKAKLLQF-------ITGTSKVP------------LEG-----FS-HLQGVN--GKQR
FNIHKAFGT-------------------------DRLPAAHTCF-NQ-LDLPQY-ETYEK
LRSQLL-VAMNEGGEGF
>Malassezia_japonica_LFDB01000127.1 .
LSVRRKFV-FYDSFQYF--------------QRRSGPEIKHGKLNVRFHN-------EDG
IDAGGVTREW---FQVL----SREMFNPDY--------ALFQPCAADRT-----------
----------------TYQPNRMS------------------------------------
--SVNDLHLSFFTFIGRVIGKAIYDGRLLDAYFTR--SFYKHILG---------------
--------RAVDYKDLEA----------------------VDPEYYNS--------LEWM
LHN--DIT-DVL--
-DLTFAVEEEV---------------------------FGE-TQLVE-LRPD--------
GASIP---
------------------------------VTNENKE-EYVRL-VTEQRLTNS-IKQQID
-AFLTG---FLEVIPR--DLIQ-IFSEQ-ELELLISG-----------------------
----LPDID-----------VDAW----KNHTELQG-YSSS-------------------
------------------------DSMV-QWWWRAVR--------------------SFD
QTQKAKLLQF-------ITGTSKVP------------LEG-----FA-QLQGVH--GTQR
FNIHKAFGE-------------------------NRLPVAHTWY-NQ-LDLPQY-DSYEK
LRSQLL-LAMNEGGEGF
>Malassezia_obtusa_LFGC01001680.1 .
LSVRRKQV-FYDSFQYF--------------QRRSGPEIKHGKLNVRFHN-------EDG
VDAGGVTREW---FQVL----SREMFNPDY--------ALFQPCAADRT-----------
----------------TYQPNRMS------------------------------------
--SVNDLHLSFFTFIGRVIGKAIYDGRLLDAYFTR--SFYKHILG---------------
--------RAVDYKDLEA----------------------VDPEYYNS--------LEWM
LHN--DIT-DVL--
-DLTFAIEEEV---------------------------FGE-TTLVE-LVPN--------
GAAIP---
------------------------------VTNENKP-EYVRL-VTEQRLTKS-IQSQID
-AFLTG---FHEVIPR--DLIQ-IFSEQ-ELELLISG-----------------------
----LPDID-----------VDEW----KNHTELQG-YSSS-------------------
------------------------DPMI-QWFWRTVR--------------------SFD
QTEKAKLLQF-------ITGTSKVP------------LEG-----FA-HLQGVH--GTQR
FNIHKAFGE-------------------------GRLPVAHTCF-NQ-LDLPQY-ESYEK
LRSQLL-LAMNEGGEGF
>Malassezia_yamatoensis_LFCX01000032.1 .
LSVRRKQV-FHDSFQFF--------------QRRSGEIPKHGKLSVRFHN-------EDG
VDAGGVTREW---FQVL----SREMFNPDY--------ALFQPCAADRT-----------
----------------TYQPNRMS------------------------------------
--SINDMHLAFFTFIGRIIGKAIYDARLLDAYFTR--SFYKHMLG---------------
--------RAVDYKDLEA----------------------VDPEYYNS--------LEWM
LHN--DIT-DIL--
-ELTFAMEEEV---------------------------FGE-TSLVE-LLPD--------
GAAIP---
------------------------------VTNENKH-EYVRL-VTEQRLTKS-IQSQID
-AFLTG---FHEIIPR--ELIE-IFSEQ-ELELLISG-----------------------
----LPDID-----------VDEW----KNHTELRG-YNSS-------------------
------------------------DPMI-QWFWRAVR--------------------SFD
QTEKAKLLQF-------ITGTSKVP------------LEG-----FA-HLQGVQ--GTQR
FNIHRAYGE-------------------------GRLPAAHTCF-NQ-LDLPSY-ESYER
LRSQLL-LAMNEGGEGF
>Malassezia_furfur_LFDD01003350.1 .
LSVRRKQV-FYDSFQYF--------------QRRSGEIPKHGKLNVRFHN-------EDG

VDAGGVTREW---FQVL----SREMFNPDY--------ALFQPCAADRT-----------
----------------TYQPNRMS------------------------------------
--AVNDMHLAFFRFIGRVIGKAIYDGRLLDAYFTR--SFYKHLLG---------------
--------RAVDYKDLEA----------------------VDPEYYNS--------LEWM
LHN--DIT-DVL--
-DLTFAIEEEV---------------------------FGE-TTLVE-LVPN--------
GASIA---
------------------------------VTNENKH-EYVRL-VTEQRLTKS-IQSQID
-AFLSG---FHEVIPR--ELIE-IFTEQ-ELELLISG-----------------------
----LPDID-----------VDEW----KNHTELRG-YNSS-------------------
------------------------DPMI-QWFWRAVR--------------------SFD
QTEKAKLLQF-------ITGTSKVP------------LEG-----FS-QLHGVH--GTQR
FNIHRAYGE-------------------------GRLPVAHTCF-NQ-LDLPSY-DSYEK
MRAQLL-LAMNEGGEGF
>Malassezia_globosa_XM_001730091.1 .
LTVRRQHV-FYDSFQYF--------------NRKSGPEIKHGKLNVRFHH-------EEG
VDAGGVTREW---FQVL----SREMFNPDY--------ALFQPCAADRT-----------
----------------TYQPNRMS------------------------------------
--SVNDMHLAFFKFIGRVIGKAIYDGRLLDAYFTR--SFYKHILG---------------
--------RKVDYKDLEA----------------------VDPEYYNS--------IEWM
LHN--DIT-DVL--
-ELTFSVDEDV---------------------------FGE-TRVVE-LKPG--------
GASIP---
------------------------------VTEANKH-EYVRL-VTEQRLTNS-IRSQID
-AFLDG---FHEVIPH--SLIQ-LFSEQ-ELELLISG-----------------------
----LPDID-----------VDEW----KNHTELQG-YKSS-------------------
------------------------DPVI-QWWWRAVR--------------------SFD
QTEKAKLLQF-------ITGTSKVP------------LEG-----FA-HLQGVN--GTQR
FNIHRAYGE-------------------------DRLPAAHTCF-NQ-LDLPAY-DSYEK
LRSQLL-LAMNEGSEGF
>Malassezia_pachydermatis_XM_018134861.1 .
LTVRRQHV-FYDSFQYF--------------NRKSGPEIKHGKLNVRFHN-------EDG
VDAGGVTREW---FQVL----SREMFNPDY--------ALFQPCAADRT-----------
----------------TYQPNRMS------------------------------------
--SVNDMHLSFFKFIGRVIGKAIYDGRLLDAYFTR--SFYKHILG---------------
--------RSVDYKDLEA----------------------VDPEYYNS--------IEWM
LHN--DIT-DVL--
-DLTFTVEDEV---------------------------FGE-TQVVE-LKPD--------
GASIP---
------------------------------VTEENKH-EYVRL-VTEQRLTNS-IRSQID
-AFLDG---FHEIIPR--ELIK-LFTEQ-ELELLISG-----------------------
----LPDID-----------VDEW----KNNTELQG-YNSS-------------------
------------------------DPMI-QWWWRAVR--------------------SFD
QTEKAKLLQF-------ITGTSKVP------------LEG-----FG-QLQGVN--GTQR
FSIHRAFGE-------------------------DRLPAAHTCF-NQ-LDLPTY-DSYEK
LRSQLL-LAMNEGGEGF
>Malassezia_nana_LFGD01000008.1 .
LTVRRQHV-FYDSFQYF--------------HRKTGPEIKHGKLNVRFHN-------EEG
VDAGGVTREW---FQVL----SREMFNPDY--------ALFQPCAADRT-----------
----------------TYQPNRMS------------------------------------
--SVNDMHLSFFKFIGRVIGKAIYDGRLLDAYFTR--SFYKHMLG---------------
--------RSVDYKDLET----------------------VDPEYYNS--------IEWM
LHN--DIT-DVL--
-ELTFAVEDEV---------------------------FGE-TQVVE-LKPG--------
GASIP---
------------------------------VTEENKY-EYVRL-VTEQRLTNS-IRSQID
-AFLEG---FHEVIPR--SLIQ-LFSEQ-ELELLISG-----------------------
----LPDID-----------VDEW----KNNTELQG-YSSS-------------------

------------------------DPVI-QWWWRAVR--------------------SFD
QTEKAKLLQF-------ITGTSKVP------------LEG-----FG-HLQGVN--GTQR
FSIHRAFGE-------------------------DRLPVAHTCF-NQ-LDLPTY-ETYEK
LRSQLL-VAMNEGAEGF
>Malassezia_equina_LFFY01000113.1 .
LTVRRQHV-FYDSFQYV--------------HRKTGEIPKHGKLNVRFHN-------EEG
VDAGGVTREW---FQVL----SREMFNPDY--------ALFQPCAADRT-----------
----------------TYQPNRMS------------------------------------
--AVNDMHLSFFKFIGRVIGKAIYDGRLLDAYFTR--SFYKHMLG---------------
--------RSVDYKDLEA----------------------VDPEYYNS--------IEWM
LHN--DIT-DVL--
-ELTFAVEDEV---------------------------FGE-THVVE-LKPE--------
GASIP---
------------------------------VTEENKH-EYVRL-VTEQRLTNS-IRSQID
-AFLEG---FHEVIPR--PLIQ-LFSEQ-ELELLISG-----------------------
----LPDID-----------VDEW----KNNTELQG-YSSS-------------------
------------------------DPMI-QWWWRAVR--------------------SFD
QTQKAKLLQF-------ITGTSKVP------------LEG-----FG-HLQGVN--GTQR
FNIHRAFGE-------------------------DRLPAAHTCF-NQ-LDLPTY-DSYEK
LRSQLL-IAMNEGAEGF
>Malassezia_sympodialis_XM_018884104.1 .
LTVRRQHV-FYDSFQYF--------------HRKTGPEIKHGKLNVRFHN-------EEG
VDAGGVTREW---FQVL----SREMFNPDY--------ALFQPCAADRT-----------
----------------TYQPNRMS------------------------------------
--AVNDMHLSFFKFIGRVIGKAIYDGRLLDAYFTR--SFYKHILG---------------
--------RKVDYKDLEA----------------------VDPEYYNS--------IEWM
LHN--DIT-DVL--
-ELTFAVEDEV---------------------------FGV-TQVVE-LKPE--------
GASIP---
------------------------------VTEENKH-EYVRL-VTEQRLTNS-IRSQID
-AFLEG---FHEVIPR--PLIQ-LFSEQ-ELELLISG-----------------------
----LPDID-----------VDEW----KNNTELQG-YSSG-------------------
------------------------DPMI-QWWWRAVR--------------------SFD
QTQKAKLLQF-------ITGTSKVP------------LEG-----FA-HLQGVN--GTQR
FSIHRAFGE-------------------------DRLPAAHTCF-NQ-LDLPTY-DSYEK
LRSQLL-VAMNEGAEGF
>Malassezia_dermatis_LFFX01000019.1 .
LTVRRQHV-FYDSFQYF--------------HRKTGPEIKHGKLNVRFHN-------EEG
VDAGGVTREW---FQVL----SREMFNPDY--------ALFQPCAADRT-----------
----------------TYQPNRMS------------------------------------
--AVNDMHLSFFKFIGRVIGKAIYDGRLLDAYFTR--SFYKHMLG---------------
--------RSVDYKDLET----------------------VDPEYYNS--------IEWM
LHN--DIT-DVL--
-ELTFAVEDEV---------------------------FGE-TQVVE-LKPE--------
GASIP---
------------------------------VTEENKH-EYVRL-VTEQRLTNS-IRSQID
-AFLEG---FHEVIPR--SLIQ-LFSEQ-ELELLISG-----------------------
----LPDID-----------VDEW----KNNTELQG-YSSG-------------------
------------------------DPMI-QWWWRAVR--------------------SFD
QTQKAKLLQF-------ITGTSKVP------------LEG-----FA-HLQGVN--GTQR
FSIHRAFGE-------------------------DRLPAAHTCF-NQ-LDLPTY-DSYEK
LRSQLL-IAMNEGAEGF
>Malassezia_caprae_LFFV01000063.1 .
LTVRRQHV-FYDSFQYF--------------HRKTGPEIKHGKLNVRFHN-------EEG
VDAGGVTREW---FQVL----SREMFNPDY--------ALFQPCAADRT-----------
----------------TYQPNRMS------------------------------------
--AVNDMHLSFFKFIGRVIGKAIYDGRLLDAYFTR--SFYKHMLG---------------
--------RSVDYKDLEA----------------------VDPEYYNS--------IEWM

```
LHN------------------------------------------------DIT-DVL--
-ELTFAVEDEV---------------------------FGE-TQVVE-LKPE--------
GASIP-------------------------------------------------------
------------------------------VTEENKH-EYVRL-VTEQRLTNS-IRSQID
-AFLEG---FHEVIPR--PLIQ-LFSEQ-ELELLISG-----------------------
----LPDID-----------VDEW----KNNTELQG-YSSS-------------------
------------------------DPMI-QWWWRAVR--------------------SFD
QTQKAKLLQF-------ITGTSKVP------------LEG-----FA-HLQGVN--GTQR
FSIHRAFGE-------------------------DRLPAAHTCF-NQ-LDLPTY-DSYEK
LRSQLL-IAMNEGAEGF
>Wallemia_ichthyophaga_XM_009269350.1 .
VNVRRSHV-FADSFQYL--------------QRKSGDEIKYGKLSVKFHG-------EEG
VDAGGVAREW---FQVL----AQQMFNPNY--------ALFQPCDADRL-----------
----------------TYQPNRAS------------------------------------
--YVNEHHLDFFKFVGRIIGKAIYDGRLLDAYFTR--SFYKHMLG---------------
--------RQVDFKDLES----------------------VDLSYYNS--------LVWM
LEN------------------------------------------------SLE-GVL--
-ELTFSIEDDE---------------------------FGV-VNIID-LIPN--------
GRNIP-------------------------------------------------------
------------------------------VTDQNKK-EYVKL-VTEFRLTTA-IERQIQ
-CFLEG---FHEIIPK--DLVK-IFSEN-ELELLISG-----------------------
----LPDID-----------VDAW----KNQTDYHG-FTPS-------------------
------------------------DPVI-GWFWRVLR--------------------SFD
STQKASFLQF-------ATGSSRVP------------LEG-----FG-ALQGSQ--GTQR
FNIHKAYGAE------------------------DRLPAAHTCF-NQ-LDLGPY-SSYEA
LRRQIL-TAIHEGNTGF
>Wallemia_sebi_XM_006956023.1 .
VNVRRSQV-FSDSFQYL--------------QRKTGDEIKYGKLSVKFYG-------EEG
VDAGGVAREW---FQVL----TQQMFNPDY--------ALFQPCDADRL-----------
----------------TYQPNRAS------------------------------------
--YVNEHHLSFFKFVGRIIGKAIYDGRLLDAYFTR--SFYKHMLG---------------
--------RQVDFKDLES----------------------VDLSYYNS--------LVWM
LEN------------------------------------------------SIE-GVL--
-EPTFSVDNEE---------------------------FGV-VNVID-LIPN--------
GRNIM-------------------------------------------------------
------------------------------VTDANKK-EYVKL-NTEFRLTKA-IEKQIQ
-CFLEG---FHEIIPK--DLAK-IFSES-ELELLISG-----------------------
----LPDID-----------VDEW----KNQTDYHG-FTPS-------------------
------------------------DPIV-NWFWRVLR--------------------SFD
STQKASFLQF-------VTGSSRVP------------LEG-----FG-SLQGSQ--GTQR
FNIHKAYGEE------------------------DKLPTAHTCF-NQ-LDLGPY-SSYEA
LRKQIL-TAIHEGNTGF
>Wallemia_mellicola_AFQX01000003.1 .
VNVRRSQV-FSDSFQYL--------------QRKTGDEIKYGKLSVKFYG-------EEG
VDAGGVAREW---FQVL----TQQMFNPDY--------ALFQPCDADRL-----------
----------------TYQPNRAS------------------------------------
--YVNEHHLSFFKFVGRIIGKAIYDGRLLDAYFTR--SFYKHMLG---------------
--------RQVDFKDLES----------------------VDLSYYNS--------LVWM
LEN------------------------------------------------SIE-GVL--
-EPTFSVDNEE---------------------------FGV-VNVID-LIPN--------
GRNIM-------------------------------------------------------
------------------------------VTDANKK-EYVKLN-TEFRLTKA-IEKQIQ
-CFLEG---FHEIIPK--DLAK-IFSES-ELELLISG-----------------------
----LPDID-----------VDEW----KNQTDYHG-FTPS-------------------
------------------------DPIV-NWFWRVLR--------------------SFD
STQKASFLQF-------VTGSSRVP------------LEG-----FG-SLQGSQ--GTQR
FNIHKAYGEE------------------------DKLPTAHTCX-NQ-LDLGPY-SSYEA
LRKQIL-TAIHEGNTGF
```

>Cryptococcus_neoformans_XM_768982.1 .
LNIRRQYV-FEDSFLAL--------------QRWNGEELKYGKLSVKFRH-------EDG
VDVGGVTREW---YSVL----AQQIFDPNF--------ALFEPCAADQQ-----------
----------------TYQPNKTS------------------------------------
--WINDVHLSYFKFVGRVIGKAVYDGRLLDAYFNR--AFYKQILG---------------
--------RTVDMRDLES----------------------IDPEYHKS--------LQWM
LDN--DIT-GVI--
-DQEFTIEDDQ---------------------------FGE-KKTVE-LKEN--------
GANIP---
------------------------------VTEGNKE-EYVRL-VVSYRLDNS-IRDQIK
-SFLEG---FYDIIPQ--ELIQ-IFEPD-QLELLISG-----------------------
----ITTVD-----------VDEL----KNATQLNG-WKAT-------------------
------------------------DPEV-AWFWRALR--------------------SFS
QEERSRFLMF-------VTSSSRVP------------LGG-----FS-QLQGSS--GTQP
LQLQKLHGKE------------------------GGLPQASTCF-NL-LLLPTY-ASYEQ
LRERLQ-FAITE-TGGF
>Cryptococcus_neoformans_XM_572687.1 .
LNIRRQYV-FEDSFLAL--------------QRWNGEELKYGKLSVKFRH-------EDG
VDVGGVTREW---YSVL----AQQIFDPNF--------ALFEPCAADQQ-----------
----------------TYQPNKTS------------------------------------
--WINDVHLSYFKFVGRVIG-----GRLLDAYFNR--AFYKQILG---------------
--------RTVDMRDLES----------------------IDPEYHKS--------LQWM
LDN--DIT-GVI--
-DQEFTIEDDQ---------------------------FGE-KKTVE-LKEN--------
GANIP---
------------------------------VTEGNKE-EYVRL-VVSYRLDNS-IRDQIK
-SFLEG---FYDIIPQ--ELIQ-IFEPD-QLELLISG-----------------------
----ITTVD-----------VDEL----KNATQLNG-WKAT-------------------
------------------------DPEV-AWFWRALR--------------------SFS
QEERSRFLMF-------VTSSSRVP------------LGG-----FS-QLQGSS--GTQP
LQLQKLHGKE------------------------GGLPQASTCF-NL-LLLPTY-ASYEQ
LRERLQ-FAITE-TGGF
>Cryptococcus_neoformans_XM_012196494.1 .
LNIRRQYV-FEDSFLAL--------------QRWNGEELKYGKLSVKFRH-------EDG
VDVGGVTREW---YSVL----AQQIFDPNF--------ALFEPCAADQQ-----------
----------------TYQPNKTS------------------------------------
--WINDVHLSYFKFVGRVIGKAVYDGRLLDAYFNR--AFYKQILG---------------
--------RTVDMRDLES----------------------IDPEYHKS--------LQWM
LDN--DIT-GVI--
-DQEFTIEDDQ---------------------------FGE-KKIVE-LKEN--------
GANIP---
------------------------------VTEENKE-EYVRL-VVSYRLDNS-IRDQIK
-SFLEG---FYDIIPQ--ELIQ-IFEPD-QLELLISG-----------------------
----ITTVD-----------VDEL----KNATQLNG-WKAT-------------------
------------------------DPEV-AWFWRALR--------------------SFS
QEERSRFLMF-------VTSSSRVP------------LGG-----FS-QLQGSS--GTQP
LQLQKLHGKE------------------------GGLPQASTCF-NL-LLLPTY-ASYEQ
LRERLQ-FAITE-TGGF
>Tsuchiyaea_wingfieldii_XM_019178606.1 .
LNIRRQYV-FEDSFSAL--------------QRWSGEELKYGKLSVKFRQ-------EDG
VDAGGVTREW---YSVL----AQQIFDPNF--------ALFEPCAADQQ-----------
----------------TYQPNKMS------------------------------------
--WINDVHLSYFKFVGRVIGKAVYDGRLLDAYFNR--AFYKQILG---------------
--------RTVDMRDLES----------------------IDPEYHKS--------LQWM
LNN--DIT-GII--
-DVRFTTEDDQ---------------------------FGE-KKTVE-LKPD--------
GANIP---
------------------------------VTEENKE-EYVRL-VVSYRLDNS-IRDQIK

-AFLEG---FYDIIPH--ELIQ-IFEPD-QLELLISG-----------------------
----ITTVD-----------VDEL----KNATQLSG-WKST-------------------
------------------------DPEI-SWFWRALR--------------------IFS
QEERSRFLMF-------VTSSSRVP------------LGG-----FA-QLQGSS--GTQP
FQIQKLYGKE------------------------GGLPQASTCF-NL-LLLPTY-TSYEQ
LRERLQ-FAITE-TGGF
>Cryptococcus_amylolentus_XM_019142768.1 .
LNVRRQYV-FEDSFSAL--------------QRWSGEELKYGKLSVKFRQ-------EDG
VDAGGVTREW---YSVL----AQQIFDPNF--------ALFEPCAADQQ-----------
----------------TYQPNKMS------------------------------------
--WINDVHLSYFKFVGRVIGKAVYDGRLLDAYFNR--AFYKQILG---------------
--------RTVDMRDLES----------------------IDPEYHKS--------LQWM
LNN--DIT-GII--
-DQEFTTEDDQ---------------------------FGE-KKTVE-LKPD--------
GANIP---
------------------------------VTEDNKE-EYVRL-VVSYRLDNS-IRDQIK
-AFLEG---FYDIIPH--ELIQ-IFEPD-QLELLISG-----------------------
----ITTVD-----------VDEL----KNATXXXG-WKST-------------------
------------------------DPEI-SWFWRALR--------------------IFS
QEERSRFLMF-------VTSSSRVP------------LGG-----FA-QLQGSS--GTQP
FQIQKLYGKE------------------------GGLPQASTCF-NL-LLLPTY-TSYEQ
LRERLQ-FAITE-TGGF
>Cryptococcus_amylolentus_XM_019142767.1 .
LNVRRQYV-FEDSFSAL--------------QRWSGEELKYGKLSVKFRQ-------EDG
VDAGGVTREW---YSVL----AQQIFDPNF--------ALFEPCAADQQ-----------
----------------TYQPNKMS------------------------------------
--WINDVHLSYFKFVGRVIGKAVYDGRLLDAYFNR--AFYKQILG---------------
--------RTVDMRDLES----------------------IDPEYHKS--------LQWM
LNN--DIT-GII--
-DQEFTTEDDQ---------------------------FGE-KKTVE-LKPD--------
GANIP---
------------------------------VTEDNKE-EYVRL-VVSYRLDNS-IRDQIK
-AFLEG---FYDIIPH--ELIQ-IFEPD-QLELLISG-----------------------
----ITTVD-----------VDEL----KNATQLSG-WKST-------------------
------------------------DPEI-SWFWRALR--------------------IFS
QEERSRFLMF-------VTSSSRVP------------LGG-----FA-QLQGSS--GTQP
FQIQKLYGKE------------------------GGLPQASTCF-NL-LLLPTY-TSYEQ
LRERLQ-FAITE-TGGF
>Kwoniella_heveanensis_ASQB01000035.1 .
-----------DSFHAL--------------QRRTGDEIKYGKLSVKFYN-------EDG
VDAGGVTREW---YSVL----AQQIFDPNF--------ALFEPCAADQQ-----------
----------------TFQPNKAS------------------------------------
--SINTDHLAYFKFVGRVIGKAVYDGRLLDAYFNR--AFYKQILG---------------
--------RTVDMRDLES----------------------IDPEYHKS--------LQWM
LDN--DIT-GVI--
-DQEFTIEDDQ---------------------------FGE-KKVVE-LKPG--------
GASIP---
------------------------------VTEENKE-EYVRL-VVSYRLDNS-IKDQIK
-AFLEG---FYDIIPR--QLIQ-IFEPD-QLELLISG-----------------------
----ITTVD-----------VDEL----KNATQLSG-WKST-------------------
------------------------DPEI-QWFWRALR--------------------SFS
QEERSRFLMF-------VTSSSRVP------------LGG-----FA-QLQGSS--GIQP
FQIQKVSQSX------------------------GGLPQASTCF-NL-LLLPTY-ASYEQ
LRERLQ-FAIVE-TGG-
>Kwoniella_mangroviensis_XM_019149254.1 .
LNIRRQYV-FEDSFHAM--------------QRRTGEEIKSGKLSVKFYN-------EDG
VDAGGVTREW---YSVL----AQQIFDPNF--------ALFEPCAADQQ-----------
----------------TYQPNKAS------------------------------------

--SINGDHLAYFKFVGRVIGKAVYDGRLLDAYFNR--AFYKQILG---------------
--------KTVDMRDLES----------------------IDPEYHKS--------LQWM
LDN--DIT-GVI--
-DQEFTIEDDQ---------------------------FGE-KKIVE-LKPG--------
GSSIP---
------------------------------VTEENKE-EYVRL-VVSYRLDNS-IKEQIK
-AFLEG---FYDIIPR--QLIQ-IFEPD-QLELLISG-----------------------
----ITTVD-----------VDEL----KNATQLSG-WKAT-------------------
------------------------DPEI-SWFWRALR--------------------SFS
QEERSRFLMF-------VTSSSRVP------------LGG-----FT-QLQGSS--GTQP
FQIQKLYAKE------------------------GSLPQASTCF-NL-LLLPTY-ASYEQ
LRDKLQ-FAIVE-TGGF
>Kwoniella_dejecticola_XM_018410035.1 .
LNIRRQYV-FEDSFHAL--------------QRRSGDEIKYGKLSVKFYN-------EDG
VDAGGVTREW---YSVL----AQQIFDPNF--------ALFEPCAADQQ-----------
----------------TYQPNKAS------------------------------------
--SVNGDHLAYFKFVGRVIGKAVYDGRLLDAYFNR--AFYKQILG---------------
--------RTVDTRDLES----------------------IDPEYHKS--------LQWM
LDN--DIT-GVI--
-DVSFTIEDDQ---------------------------FGE-KKIVE-LKPD--------
GAATP---
------------------------------VTEENKE-DYVRL-VVSYRLDNS-IKDQIK
-AFLDG---FYDIIPR--QLIQ-IFEPD-QLELLISG-----------------------
----ITTVD-----------VDEL----KNATQLSG-WKSS-------------------
------------------------DPEI-SWFWRALR--------------------SFS
QEERSRFLMF-------VTSSSRVP------------LGG-----FT-QLQGSS--GTQP
FQIQKLYAKE------------------------GSLPQASTCF-NL-LLLPTY-ASYEQ
LREKLQ-FAIVE-TGGF
>Kwoniella_bestiolae_XM_019193863.1 .
LNIRRQYV-FEDSFHAM--------------QRRTGDEIKFGKLSVKFYN-------EDG
VDAGGVTREW---YSVL----AQQIFDPNF--------ALFEPCAADQQ-----------
----------------TYQPNKAS------------------------------------
--SVNGDHLAYFKFVGRVIGKAVYDGRLLDAYFNR--AFYKQILG---------------
--------RTVDMRDLES----------------------IDPEYHKS--------LQWM
LDN--DIT-GVI--
-DQEFTIEDDQ---------------------------FGE-KKIVE-LKPG--------
GSSIP---
------------------------------VTEENKE-EYVRL-VVSYRLDNS-IKDQIK
-AFLEG---FYDIIPR--QIIQ-IFEPD-QLELLISG-----------------------
----ITTVD-----------VDEL----KNATQLSG-WKAT-------------------
------------------------DPEI-SWFWRALR--------------------SFS
QEERSRFLMF-------VTSSSRVP------------LGG-----FT-QLQGSS--GIQP
FQIQKLYAKE------------------------GSLPQASTCF-NL-LLLPTY-ASYEQ
LRDKLQ-FAIVE-TGGF
>Kwoniella_pini_XM_019156662.1 .
LNIRRQYV-FEDSFHAL--------------QRRTGDEIKYGKLSVKFYN-------EDG
VDAGGVTREW---YSVL----AQQIFDPNF--------ALFEPCAADQQ-----------
----------------TYQPNKAS------------------------------------
--SVNGDHLAYFKFVGRVIGKAVYDGRLLDAYFNR--AFYKQILG---------------
--------RTVDMRDLES----------------------IDPEYHKS--------LQWM
LEN--DIT-GVI--
-DVSFTIEDDQ---------------------------FGE-KKIVE-LKPG--------
GASVP---
------------------------------VTQDNKE-EYVRL-VVSYRLDNS-IKDQIK
-AFLEG---FYDIIPR--QIIQ-IFEPD-QLELLISG-----------------------
----ITTVD-----------VDEL----KNATQLSG-WKTS-------------------
------------------------DPEI-SWFWRALR--------------------SFS
QEERSRFLMF-------VTSSSRVP------------LGG-----FT-QLQGSS--GTQP

FQIQKLYAKE------------------------GSLPQASTCF-NL-LLLPTY-ASYEQ
LRDRLQ-FAIVE-TGGF
>Tremella_mesenterica_XM_007001922.1 .
LNIRRQYV-FEDSFHAL--------------QRRKGDEIKYGKLNVRFYN-------EDG
IDAGGVTREW---YSAL----ARQIFDPNF--------ALFEPCAADDR-----------
----------------TYQPNKAS------------------------------------
--SVNHDHLAYFKFVGRVIGKAIYDGRLLDAYFSR--AFYKQILG---------------
--------RSVDIRDMES----------------------IDPEYHKS--------LQWM
LEN--DIT-GVI--
-DQEFTIEDDQ---------------------------FGE-KQVVE-LKDG--------
GASIP---
------------------------------VTEENKD-EYVRL-VVSYRLHNS-IKEQLT
-AFLDG---FYDVVPR--HLIE-IFEPD-QLELLISG-----------------------
----ITTID-----------VDEL----KNATQLSG-WKTD-------------------
------------------------DADV-AWFWRALR--------------------SYS
QEERARFLMF-------VTSSSRVP------------LGG-----FT-QLQGSS--GVQP
FQLQRLYGKD------------------------GSLPQASTCF-NL-LLLPKY-DSYEQ
LREKLL-FAITE-TGGF
>Cutaneotrichosp_oleaginosus_JZUH01000088. 1..
---------------------------------------KYGKINVKFIN-------EDG
VDAGGVTREW---YHVL----AQQIFDPDY--------ALFEPCAADNQ-----------
----------------TYQPNKHS------------------------------------
--SINPDHLSYFKFVGRVIGKAIYDGRLLDAYFNR--AFYKQILG---------------
--------RGCDIRDLEA----------------------IDPEYHKS--------LQWM
LEN--DIT-DVI--
-DQEFTIEDES---------------------------FGA-KQIVE-LKPG--------
GATIP---
------------------------------VTEANKD-EYVRL-VCAYRLENS-VKEQMK
-AFLTG---FYDIVPR--ELVQ-IFEPE-QLELLISG-----------------------
----VNTFD-----------VDEL----KNSTQISG-WKNN-------------------
------------------------DNEV-MWFWRALR--------------------SFS
QEERARFLIF-------VTASSRVP------------LGG-----FE-KLQGAS--GIQP
FQIQKLFGKP------------------------GILPQASTCF-NL-LLLPPY-ESYEQ
LRERLL-FAVTE-TEG-
>Trichosporon_oleaginosus_XM_018420928.1 .
LNVRRQYV-FQDSYSAL--------------LHRAGDEIKYGKINVKFIN-------EDG
VDAGGVTREW---YHVL----AQQIFDPDY--------ALFEPCAADNQ-----------
----------------TYQPNKHS------------------------------------
--SINPDHLSYFKFVGRVIGKAIYDGRLLDAYFNR--AFYKQILG---------------
--------RGCDIRDLEA----------------------IDPEYHKS--------LQWM
LEN--DIT-DVI--
-DQEFTIEDES---------------------------FGA-KQIVE-LKPG--------
GATIP---
------------------------------VTEANKD-EYVRL-VCAYRLENS-VKEQMK
-AFLTG---FYDIVPR--ELVQ-IFEPE-QLELLISG-----------------------
----VNTFD-----------VDEL----KNSTQISG-WKNN-------------------
------------------------DNEV-MWFWRALR--------------------SFS
QEERARFLIF-------VTASSRVP------------LGG-----FE-KLQGAS--GIQP
FQIQKLFGKP------------------------GILPQASTCF-NL-LLLPPY-ESYEQ
LRERLL-FAVTE-TEGF
>Trichosporon_cutaneum_BCKU01000003.1 .
---------------------------------------KYGKINVKFIN-------EDG
VDAGGVTREW---YHVL----AQQIFDPDY--------ALFEPCAADNQ-----------
----------------TYQPNKHS------------------------------------
--SVNPDHLSYFKFVGRVIGKAIYDGRLLDAYFNR--AFYKQILG---------------
--------RACDIRDLEA----------------------IDPEYHKS--------LQWM
LEN--DIT-DVI--
-DQEFTIDDES---------------------------FGA-KQTVE-LKPG--------

GASIP---
------------------------------VTEANKD-EYVRL-VCAYRLENS-VKEQMK
-AFLTG---FYEMIPH--ELVQ-IFEPE-QLELLISG-----------------------
----VNTFD-----------VDEL----KNSTQMAG-WKST-------------------
------------------------DVEV-SWFWRALR--------------------SFS
QEERARFLIF-------VTASSRVP------------LGG-----FE-KLQGAS--GVQP
FQIQRLYGKE------------------------GILPQASTCF-NL-LLLPKY-ESYEQ
LRERLL-FAVTE-TEG-
>Cutaneotrichosp_cutaneum_LRUG01000116.1 .
---------------------------------------KYGKINVKFIN-------EDG
VDAGGVTREW---YHVL----AQQIFDPDY--------ALFEPCAADNQ-----------
----------------TYQPNKHS------------------------------------
--SVNPDHLSYFKFVGRLIGKAIFDGRLLDAYFNR--AFYKQILG---------------
--------RRCDIRDLEA----------------------IDPEYHKS--------LQWM
LDN--DIT-DVI--
-DQEFTIEDDS---------------------------FGA-KQIVE-LKPG--------
GASIP---
------------------------------VTEANKE-EYVRL-VCAYRLENS-IKEQMN
-AFLSG---FYEIIPR--SIIQ-IFEPD-QLELLISG-----------------------
----LSTFD-----------VDEL----KNSTQMSG-WKSS-------------------
------------------------DVEI-SWFWRALR--------------------SFS
QEERSRFLIF-------VTASSRVP------------LGG-----FE-KLQGAS--GVQP
FQIQRLYGKE------------------------GILPQASTCF-NL-LLLPKF-ESYEQ
LRERLL-FAVTE-TEG-
>Cutaneotrichosp_curvatus_LDEP01000079.1 .
---------------------------------------KYGKINVKFIN-------EDG
VDAGGVTREW---YHVL----ATQIFDPNF--------ALFEPCAADKQ-----------
----------------TYQPNKHS------------------------------------
--NINGDHLSFFKFIGRLIGKAIFDGRLLDAYFNR--AFYKQILG---------------
--------RPVDIRDLES----------------------IDPEYHKS--------LQWM
LDN--DIT-DVI--
-DQEFTIEDDN---------------------------FGA-KQIVE-LKPG--------
GSQIP---
------------------------------VTEENKA-EYVRL-VCAYRLENS-IKEQMN
-AFLTG---FYDVIPR--ALIQ-IFEPD-QLEVLISG-----------------------
----MTTID-----------VDEL----KNSTQMQG-WKSS-------------------
------------------------DPEV-SWFWRALR--------------------SFS
QEERARFMIF-------VTASSRVP------------LGG-----FT-QLQGAS--GIQP
FQLQRLYGKD------------------------GILPQASTCF-NL-LLLPKY-DSYEH
LRERLL-FAVTE-TSG-
>NWGS3B_2_Trichosporon_inkin_JXYM01000005.1 .
---------------------------------------KYGKINVKFIN-------EDG
VDAGGVTREW---YHVL----ATQIFDPNF--------ALFEPCAADKQ-----------
----------------TYQPNKHS------------------------------------
--SVVDDHLSFFKFVGRVIGKAIYDGRLLDAYFSR--AFYKQILG---------------
--------RDVDMRDLES----------------------IDPEYHKS--------LQWI
LDN--DIT-DII--
-DQEFTIEDDS---------------------------FGE-KKIVE-LKEG--------
GAEIP---
------------------------------VTEENKS-EYVRL-VCAYRLENS-IRDQMK
-AFLTG---FYDIIPQ--NLIQ-IFEPD-QLELLISG-----------------------
----MTTID-----------VDEL----KNSTQMAG-WKGS-------------------
------------------------DPEI-SWFWRALR--------------------SFS
QEERSRFLMF-------VTSSSRVP------------LGG-----FS-QLQGAS--GTQP
FQIQKLYGKE------------------------GILPQASTCF-NL-LLLPKY-ASYEQ
LRERLL-FAITE-TSG-
>Trichosporon_coremiiforme_JXYL01000030.1 .
---------------------------------------KYGKINIKFIN-------EDG

VDAGGVTREW---FHVL----ATQIFDPNF--------ALFEPCAADKQ-----------
----------------TYQPNKHS------------------------------------
--SVVDDHLSFFKFVGRVIGKAIYDGRLLDAYFSR--AFYKQILG---------------
--------RDVDMRDLES----------------------IDPEYHKS--------LQWI
LDN--DIT-DVI--
-DQEFTIEDDS---------------------------FGE-TKIVE-LKEG--------
GAKIP---
------------------------------VTEENKA-EYVRL-VCAYRLENS-IRDQMK
-AFLTG---FYDIIPQ--SLIQ-IFEPD-QLELLISG-----------------------
----MTTID-----------VDEL----KNSTQMAG-WKGS-------------------
------------------------DPEI-SWFWRALR--------------------SFS
QEERSRFLMF-------VTSSSRVP------------LGG-----FS-QLQGAS--GTQP
FQIQKLYGKE------------------------GILPQASTCF-NL-LLLPKY-ASYEQ
LRERLL-FAITE-TSG-
>Trichosporon_asahii_XM_014323711.1 .
LNVRRQYV-FQDSYSAL--------------LHRSGDEVKYGKINIKFIN-------EDG
IDAGGVTREW---FHVL----ATQIFDPNF--------ALFEPCAADKQ-----------
----------------TYQPNKHS------------------------------------
--SVVDDHLSFFKFVGRVIGKAIYDGRLLDAYFSR--AFYKQILG---------------
--------RDVDMRDLES----------------------IDPEYHKS--------LQWI
LDN--DIT-DVI--
-DQEFTIEDDS---------------------------FGE-TKIVE-LKEG--------
GAKIP---
------------------------------VTEENKA-EYVRL-VCAYRLENS-IRDQMK
-AFLTG---FYDIIPQ--SLIQ-IFEPD-QLELLISG-----------------------
----MTTID-----------VDEL----KNSTQMAG-WKGS-------------------
------------------------DPEI-SWFWRALR--------------------SFS
QEERSRFLMF-------VTSSSRVP------------LGG-----FS-QLQGAS--GTQP
FQIQKLYGKE------------------------GILPQASTCF-NL-LLLPKY-ASYEQ
LRERLL-FAITE-TSGF
>Trichosporon_faecale_JXYK01000006.1 .
---INIKFIN-------EDG
VDAGGVTREW---FHVL----ATQIFDPNF--------ALFEPCAADKQ-----------
----------------TYQPNKHS------------------------------------
--SVVDDHLSFFKFVGRVIGKAIYDGRLLDAYFSR--AFYKQILG---------------
--------RDVDMRDLES----------------------IDPEYHKS--------LQWI
LDN--DIT-DVI--
-DQEFTIEDDS---------------------------FGE-TKIVE-LKEG--------
GAKIP---
------------------------------VTEENKA-EYVRL-VCAYRLENS-IRDQMK
-AFLTG---FYDIIPQ--SLIQ-IFEPD-QLELLISG-----------------------
----MTTID-----------VDEL----KNSTQMAG-WKGS-------------------
------------------------DPEI-SWFWRALR--------------------SFS
QEERSRFLMF-------VTSSSRVP------------LGG-----FS-QLQGAS--GTQP
FQIQKLYGKE------------------------GILPQASTCF-NL-LLLPKY-ASYEQ
LRERLL-FAITE-TSG-
>Naganishia_albida_LLJT01000015.1 .
---------------------------------------KYGKLNVKFHG-------EEG
VDAGGVTREW---YSAL----AQSIFNPGY--------CLFEPCAADQL-----------
----------------TYQPSHRS------------------------------------
--WVNPEHIGFFRFIGKMIGKAIYDGRLLDAYFSR--SFYKQILG---------------
--------RKVELRDLES----------------------VDPDYYKS--------LVWI
LEN--PIE-GVV--
-DVDFTLEADE---------------------------FGV-NKTVE-LKEN--------
GAQIP---
------------------------------VTEENKQ-EYVRL-VVEYRLDTA-IKDQVK
-AFLDG---FYEIIPR--GLIS-IFDPD-QLX-----------------------------
----------------------------------QG-YKPT-------------------

------------------------DAEI-TWFWRALR--------------------SFS
QEERARFLMF-------VTSSSRVP------------LGG-----FS-QLQGSS--GTQP
FQIHRVSKRX------------------------DVLPSAATCF-NE-LLLPSY-SSYED
LRAKLL-TAIQE-GA--
>Cryptococcus_albidus_LKPZ01000027.1 .
---------------------------------------KYGKLNVKFHG-------EEG
VDAGGVTREW---YAVL----AQSIFNPGY--------CLFEPCAADQL-----------
----------------TYQPSRQS------------------------------------
--WVNPEHIGFFRFIGKMIGKAIYDGRLLDAYFSR--AFYKQMLG---------------
--------RKVDIKDLES----------------------VDPEYHKS--------LVWM
LNN--DIT-NVI--
-DLDFTLEVDE---------------------------FGA-KQVVD-LKEN--------
GSQIP---
------------------------------VTEENKQ-EYVKL-VVEHRLETA-IKDQVK
-AFLDG---FYEVIPR--SLIS-IFDPD-QLXVLISG-----------------------
----ISVID-----------VDEL----KNATXYHG-WKNG-------------------
------------------------DAEI-SWFWRALR--------------------SFS
QEERARFLMF-------VTSSSRVP------------LGG-----FS-QLQGSS--GIQP
FQIHKVSGRX------------------------DVLPSSATCF-NE-LLLPSY-SSYED
LRAKLL-TAINE-G---
>Cronartium_ribicola_GBSG01010864.1 .
LNVRRPHV-FEDSFHSL--------------ARRTGDELKFGKLSVRFYD-------EEG
VDAGGVTREW---LTIL----VKQMLDPNY--------ALFTGSAADSK-----------
----------------TYQPNRAS------------------------------------
--AVNPDHLGFFTFCGRVIGKALYDGRVVDAYFTL--AFYKHLLG---------------
--------TSVGLSDLES----------------------VDPDHHRS--------LKWM
LDN--DID-GIF--
-ELTFSVEADD---------------------------FGS-TRIVD-LKPD--------
GRNIP---
------------------------------VTNENKP-EYVQL-LVQNRLTVS-IKEQIE
-AFKKG---FDEIIPR--ELVR-IFSAT-ELQLLLNG-----------------------
----LPDIN-----------VEDW----RANTELHQ-FQQS-------------------
------------------------DSTV-TWFWRAVR--------------------SFD
QEERAKLLQF-------STGSSRVP------------LEG-----FG-ALQGAQ--GPTK
FSLVNAHTK-------------------------NILPSAHTCF-NQ-IDLPSY-DSYDD
LRRMLL-IAINE-----
>Melampsora_larici_populina_XM_007418528.1 .
LNVRRPHV-FEDSFHSL--------------ARRTGDELKYGKLSVRFYD-------EEG
VDAGGVTREW---LTIL----VKQMLDPNY--------ALFTGSAADSK-----------
----------------TYQPNRAS------------------------------------
--AINPDHLGFFTFCGRVIGKALYDGRVVDAYFTL--AFYKHLLG---------------
--------TSVGLSDLES----------------------VDPDHHRS--------LKWM
LDN--DID-GIF--
-ELTFSVEADD---------------------------FGS-TRIVD-LKPD--------
GRNLP---
------------------------------VTNENKA-EYVQL-LVQNRLTLS-IKDQIE
-AFKKG---FDEIIPR--DLVR-IFSAT-ELQLLLNG-----------------------
----LPDIN-----------VEDW----RANTELHQ-FQQS-------------------
------------------------DSTV-TWFWRAVR--------------------SFD
QEERAKLLQF-------STGSSRVP------------LEG-----FG-ALQGAQ--GATK
FSLVNAHTK-------------------------NILPSAHTCF-NQ-IDLPSY-DSYDD
LRRMLL-IAINE-----
>Puccinia_striiformis_GAIS01005873.1 .
LNVRRPHV-FEDSFHSL--------------ARRTGDELKYGKLSVRFYD-------EEG
VDAGGVTREW---LTIL----VKQMLDPNY--------ALFTGSAADSK-----------
----------------TYQPNRAS------------------------------------
--AVNPDHLGFFTFCGRVIGKALYDGRVVDAYFTL--AFYKHLLG---------------
--------ISVGLSDLES----------------------VDPDHHRS--------LKWM

LDN--DID-GIF--
-ELTFSVEADD---------------------------FGS-TRIVD-LKPG--------
GQEIP---
------------------------------VTNENKP-EYVQL-LVQNRLTVS-IREQID
-AFKKG---FDEIIPR--DLVR-IFSAT-ELQLLLNG-----------------------
----LPDIN-----------VEDW----RANTELHQ-FQQS-------------------
------------------------DSTV-TWFWRAVR--------------------SFG
QEERAKLLQF-------ATGSSRVP------------LEG-----FG-ALQGAQ--GATK
FSLVNAHTK-------------------------NVLPSAHTCF-NQ-IDLPSY-DSYEE
LRKMFL-IAINE-----
>Puccinia_graminis_XM_003319784.2 .
LNVRRPHV-FEDSFHSL--------------ARRTGDELKYGKLSVRFYD-------EEG
VDAGGVTREW---LTIL----VKQMLDPNY--------ALFTGSAADSK-----------
----------------TYQPNRAS------------------------------------
--AVNPDHLGFFTFCGRVIGKALYDGRVVDAYFTL--AFYKHLLG---------------
--------IPVGLSDLES----------------------VDPDHHRS--------LKWM
LDN--DID-GIF--
-ELTFSVEADD---------------------------FGS-TRIVD-LKPG--------
GQEIP---
------------------------------VTNENKA-EYVQL-LVQNRLTVS-IREQID
-AFKKG---FDEIIPR--DLVR-IFSAT-ELQLLLNG-----------------------
----LPDIN-----------VEDW----RANTELHQ-FQQS-------------------
------------------------DSTV-TWFWRAVR--------------------SFG
QEERAKLLQF-------ATGSSRVP------------LEG-----FG-ALQGAQ--GATK
FSLVNAHTK-------------------------NVLPSAHTCF-NQ-IDLPSY-DSYEE
LRRMFL-IAINE-GSEG
>Mixia_osmundae_XM_014715414.1 .
INVRRPYV-FEDSFSQL--------------QRKTGEEIKHGKLSVRFYD-------EEG
VDVGGVTREW---FHVL----ARQMFNPGY--------ALFEPCLGDRL-----------
----------------TYHPRRTS------------------------------------
--SVVTDHLAFFKFVGRIIGKAVFDGRLLDAYFTR--SLYKQMIG---------------
--------KPVSPSDLES----------------------IDPEYYKS--------LTWM
LQN--DIT-GVM--
DDYTFSIEEDV---------------------------FGE-MKIVE-LKPN--------
GANIN---
------------------------------VTQENKH-EYVRL-VTEQRLTKS-VQAQID
-SFLAG---LWEIIPK--DLIQ-IFSDN-ELELLISG-----------------------
----LPDID-----------VDEW----RANTVYHN-LPAN-------------------
------------------------STTV-TWFWRAVR--------------------SLD
QEERAKLLQF-------VTGSSRVP------------LEG-----FG-ALQGVS--GVTK
FTIVAAHTH-------------------------DSLPSAHTCF-NQ-IDLPEY-SSYED
LRKYLL-IAITE-----
>Rhodotorula_toruloides_XM_016421769.1 .
LNVRRAHV-FEDSFHVF--------------NRRSGEEIKYGKLNVKFYD-------EEG
VDAGGVTREW---FGVL----ARQMFNPGY--------ALFQPQAADSL-----------
----------------TYQPNKSS------------------------------------
--AINENHLEYFRFVGRLIGKAIFDQRILEAHFSR--SVYKHMLG---------------
--------KPVDHRDLES----------------------IDPEYYKS--------LVWM
LEN--DIE-GII--
-DLTFSVERDE---------------------------FGV-MEVVD-LIPN--------
GRNIP---
------------------------------VTNENKH-DYVRR-IADQRLSIE-IKDQMD
-ALLKG---LYDVVSK--DLLQ-IFSER-ELELLISG-----------------------
----LPTLD-----------VDDM----RAHTDLVG-FSPS-------------------
------------------------DPVV-AWFWRAVR--------------------SFS
QEERAKLLQF-------VSGSSRVP------------LEG-----FA-ALQGMN--GVTR
FNIHKAGNN-------------------------ASLPTAHTCF-NQ-LDLPTGYESYEH
FRRHLS-LAIHE-----

>Rhodotorula_graminis_XM_018412335.1 .
LNIRRAHV-FEDSFSVF--------------QRKSGEDIKYGKLNVKFYG-------EEG
VDAGGVTREW---FGAL----ARRMFNPDY--------ALFQPQAADSL-----------
----------------TYQPNKSS------------------------------------
--AINEYHLHYFRFVGRIIGKAIHDQRILEAYFSR--SVYKHMLG---------------
--------KPIDHRDLES----------------------IDPEYYKS--------LVWM
LEN--DID-GVL--
-DLTFSTERDE---------------------------FGV-VETID-LVPN--------
GRNVA---
------------------------------VTNENKA-DYVRR-IADQRLSIE-IKDQMK
-AMLEG---LYEIVPK--ELLQ-IFSER-EVELLISG-----------------------
----LPDVD-----------VDDW----RAHCDLVG-YSPS-------------------
------------------------DPVI-GYLWRSIR--------------------SFS
QEERAKLLQF-------VTGSSRVP------------LEG-----FK-ALQGMH--GVTR
FNIHKAGAN-------------------------DSLPSAHTCF-NQ-LDLPSGYESYEQ
FRQKLL-LAITE-----
>Rhizoctonia_solani_JP285292.1 .
LNLRRPHV-FEDSFQNL--------------QRKTGEQIKYGKLSVRFYN-------EEG
VDAGGVTREW---FQIL----ARQMFNPDY--------ALFQPCAADKL-----------
----------------TFQPNRAS------------------------------------
--MVNPEHLSFFKFVGRVIGKAIYDGRLMDAHFAR--SLYRQILG---------------
--------KPVDYRDVEW----------------------VDPDYYKS--------LIWI
LEN--DPT--VL--
-ETTFTVEAEE---------------------------FGV-HKVVP-LKEN--------
GDKTV---
------------------------------VTEENKK-EFVQL-SAQYRLYTS-IKDQIE
-ALLAG---FYDIIPK--ELIS-IFNEQ-ELELLISG-----------------------
----TPDID-----------IDEW----RAATDYNG-YSPS-------------------
------------------------DPAI-VWWWRALK--------------------SFD
REERAKVLSF-------ATGTSRVP------------LEG-----FK-DLQGVQ--GTQR
FSIHRAYGES------------------------DRLPQAHTCF-NQ-IDLPQY-SSYEK
LRTQLL-LAINE-----
>Rigidoporus_microporus_GDMN01026518.1 .
LNVRRAQV-FQDSYHIF--------------IHKSGDQIKYGKLSVRFYN-------EEG
VDAGGVTREW---FQIL----ARQMFDVNY--------ALFEPCAADTQ-----------
----------------TYQPNRAS------------------------------------
--AVNPDHLSYFKFVGRVIGKAIFDGRLMDAHFAR--SLYRQLLG---------------
--------KKVDYRDVEW----------------------VDPEYYKS--------LCWI
LDN--DPT--VL--
-DLTFITEVDE---------------------------FGR-RDIIP-LKEN--------
GTSIP---
------------------------------VTLENRK-EYVQL-SAQYRLTDS-IKDQIE
-KLLEG---FYEIIPK--DLIS-IFNEQ-EVELLIAG-----------------------
----TPDID-----------VDEW----RAATEYNG-YSSS-------------------
------------------------DPVI-VWWWRALK--------------------SFS
RDERAKVLSF-------ATGTSRVP------------LGG-----FT-DLQGVQ--GVQR
FSIHKAYGQP------------------------DRLPQAHTCF-NQ-IDLPQY-TSYEM
LRQQLL-LAINE-----
>Fomitiporia_mediterranea_XM_007266601.1 .
VNVRRSRV-FEDSFHAF--------------QHKTGDQIKYGKLSVRFYA-------EEG
VDAGGVTREW---FQIL----ARQMFNPNY--------ALFEPCAADRQ-----------
----------------TYQPNRAS------------------------------------
--EINPDHLSYFKFVGRVIGKAIYDGRLMDAHFAR--SLYRMLLG---------------
--------KRVDYRDVEW----------------------VDPDYYKS--------LCWI
LEN--DPS--ML--
-DLNFITEVDE---------------------------FGR-HAVIP-LKEN--------
GASIP---
------------------------------VTMENRK-EYVQL-AAQYRLHSS-IAKQIE

-NLLAG---FYEIVPK--ELIS-IFNEQ-EVELLISG-----------------------
----TPDID-----------VDEW----RAATEYHG-YSSS-------------------
------------------------DPVI-VWWWRALK--------------------SFN
RDERAKVLSF-------ATGTTRVP------------LGG-----FG-ELQGVQ--GVQR
FSIHRAYGEP------------------------DRLPQAHTCF-NQ-IDLPEY-SSYER
LRHQLL-LAINE-----
>Phanerochaete_carnosa_XM_007397074.1 .
LNVRRQRV-FEDSFQYL--------------QRKSGEQIKYGKLSVRFYD-------EEG
VDAGGVTREW---FQIL----ARQMFDPNY--------CLFQPCAADRL-----------
----------------TYQPNKAS------------------------------------
--SINPEHLSFFKFVGRIIGKAIYDGRLLDAYFAR--SLYRQILG---------------
--------KPVDYRDVEW----------------------VDPEYYKS--------LCWI
LEN--DPT--LL--
-DLTFSVEADE---------------------------FGV-TKLIE-LKEN--------
GAHIP---
------------------------------VTNENKR-EFVQL-SANYRLYSS-IKDQIE
-ALLTG---FYEIIPK--DLIQ-IFDEK-ELELLISG-----------------------
----TPDID-----------VDEW----RAATEYNG-YTSS-------------------
------------------------DPVI-VWFWRALK--------------------SFN
REERAKVLSF-------ATGTSRVP------------LGG-----FV-DLQGVQ--GVQR
FSIHKAYGEV------------------------DRLPQAHTCF-NQ-IDLPQY-SSYEM
LRQQLL-LAIHE-----
>Irpex_lacteus_GBRE01005119.1 .
LNVRRNRV-FEDSYQYL--------------QRKTGDQIKYGKLSVRFFN-------EEG
VDAGGVTREW---FQIL----ARQMFNADY--------CLFQPCAADRL-----------
----------------TYQPNKAS------------------------------------
--SINPEHLSFFKFVGRIIGKAIYDGRLLDAYFAR--SLYRQILG---------------
--------KPVDYRDVEW----------------------IDPEYYKS--------LCWI
LEN--DPS--LL--
-ELTFSVEADE---------------------------FGV-MKLVE-LKEG--------
GATIP---
------------------------------VTQENKR-EFVQL-SAQWRLYSS-IKEQIE
-ALLSG---FYEIIPK--DLIT-IFDEK-DLELLISG-----------------------
----TPDID-----------VDEW----RAATEYNG-YTSS-------------------
------------------------DPAI-VWFWRALK--------------------SFN
REERAKVLSF-------ATGTSRVP------------LGG-----FV-DLQGVQ--GTQR
FSIHRAYGEV------------------------DRLPQAHTCF-NQ-IDLPQY-SSYEM
LRQQLL-LAINE-----
>Agaricus_bisporus_XM_007328014.1 .
LNVRRARV-FEDSFQYL--------------QRKSGDQIKYGKLSVRFYD-------EEG
VDAGGVTREW---FQIL----ARQMFDPNN--------ALFQPCAADKL-----------
----------------TYQPNKNS------------------------------------
--WVNPEHLSFFKFVGRVIGKAIYDGRLLDAYFAK--SLYRQILG---------------
--------KPVDYRDVEW----------------------VDPEYYNS--------LCWI
LEN--DPG--VL--
-ELNFSVEADE---------------------------FGV-NRIVP-LKDN--------
GEAIA---
------------------------------VTQDNKR-EFVQL-SAQYRLYSS-IKEQIE
-NLLNG---FYEIIPK--ELIA-IFNEQ-ELELLISG-----------------------
----TPDID-----------VDEW----RAATEYNG-YSSS-------------------
------------------------DPNI-VWWWRALK--------------------SFN
REERAKVLSF-------ATGTSRVP------------LSG-----FV-DLQGVQ--GVQR
FSIHRAYGES------------------------DRLPQAHTCF-NQ-IDLPQY-SSYEM
LRQQLL-LAINE-----
>Leucocoprinus_sp._GEHG01003484.1 .
LNVRRARV-FEDSFQYL--------------QRKTGDQIKYGKLSVRFYD-------EEG
VDAGGVTREW---FQIL----ARQMFDPNN--------ALFQPCAADKL-----------
----------------TYQPNKNS------------------------------------

--WVNPEHLSFFKFVGRVIGKAIYDGRLLDAYFAK--SLYRQILG---------------
--------KPVDYRDVEW----------------------VDPEYFNS--------LCWI
LEN--DPT--VL--
-ELTFSVEADE---------------------------FGI-NRIVP-LKEN--------
GETLP---
------------------------------VTQENKR-EFVQL-SAQYRLYSS-IKDQIE
-NLLSG---FSDIIPK--DLIT-IFNEQ-ELELLISG-----------------------
----TPDID-----------VDEW----RAATEYNG-YSSS-------------------
------------------------DPNI-VWWWRALK--------------------SFN
REERAKVLSF-------ATGTSRVP------------LSG-----FV-DLQGVQ--GVQR
FSIHRAYGEP------------------------DRLPQAHTCF-NQ-IDLPQY-SSYEM
LRQQLI-LAINE-----
>Moniliophthora_roreri_XM_007845352.1 .
LNVRRARV-FEDSFQQF--------------QRKTGDQIKYGKLNIRFYE-------EEG
VDAGGLTREW---FQIL----ARQMFDPNN--------ALFQPCAADKL-----------
----------------TYQPNKNS------------------------------------
--WVNPEHLSFFKFVGRVIGKAIYDGRLLDAYFAR--SLYRQILG---------------
--------KPVDYRDVEW----------------------VDPEYYNS--------LCWI
LEN--DPT--PL--
-ELTFSVEADE---------------------------FGV-NRIVP-LKEG--------
GETLP---
------------------------------VTQENKR-EFVQL-SAQYRLYSS-IKEQIE
-NLLNG---FYEIIPK--DLIT-IFNEQ-ELELLISG-----------------------
----TPDID-----------VDEW----RAATEYNG-YTSS-------------------
------------------------DPNI-VWWWRALK--------------------SFN
REERAKVLSF-------ATGTSRVP------------LGG-----FV-DLQGVQ--GVQR
FSIHRAYGES------------------------DRLPQAHTCF-NQ-IDLPQY-SSYEM
LRQQVL-LAISE-----
>Lyophyllum_decastes_BCJR01000038.1 .
--KYGKLSVRFYD-------EEG
VDAGGVTREW---FQIL----ARQMFDPNN--------ALFQPCAADKL-----------
----------------TYQPNKNS------------------------------------
--WVNPEHLTFFKFVGRVIGKAIYDGRLLDAYFAR--SLYRQLLG---------------
--------KPVDYKDVEW----------------------VDPEYYNS--------LCWI
LEN--DPT--LL--
-DLTFSVEADX---------------------------FGV-NRIVP-LKEG--------
GELIP---
------------------------------VTQENKR-EFVQLS-AQYRLYSS-IKTQIE
-SLSTG---FYEIIPK--DLIT-XFNEQ-ELELLISG-----------------------
----TPDID-----------VDEW----RAATEYNG-YTSS-------------------
------------------------DPNI-VWWWRALK--------------------SFN
RDERAKVLSF-------ATGTSRVP------------LGG-----FV-DLQGVQ--GVQR
FSIHKAYGES------------------------DRLPQAHTC-----------------

>Laccaria_bicolor_XM_001877693.1 .
LNVRRARV-FEDSFQYL--------------QRKTGDQIKHGKLSVRFYD-------EEG
VDAGGVTREW---FQIL----ARQMFDPNN--------ALFQPCAADKL-----------
----------------TYQPNKNS------------------------------------
--WVNPEHLSFFKFVGRVIGKAIYDGRLLDAYFAR--SLYRQLLG---------------
--------KPVDYKDVEW----------------------VDPEYYKS--------LCWI
LEN--DPT--VL--
-DLNFSVEADA---------------------------FGV-NQIIP-LKEG--------
GESIS---
------------------------------VTQENKR-EFVQH-SAQYRLYSS-IKDQIE
-SLSTG---FYEIIPK--DLIT-IFNEQ-ELELLISG-----------------------
----TPDID-----------VDEW----RAATEYNG-YTSS-------------------
------------------------DPNI-VWWWRALK--------------------SFN
RDERAKVLSF-------ATGTSRVP------------LSG-----FV-DLQGVQ--GVQR

```
FSIHRAYGES------------------------DRLPQAHTCF-NQ-IDLPQY-SSYEM
LRQQLL-MAINE-----
>Schizophyllum_commune_XM_003029381.1 .
LNVRRARV-FEDSFQHL--------------QRKTGDQIKYGKLSVRFYD-------EEG
VDAGGVTREW---FQIL----ARQMFDPNN--------ALFQPCAADRQ-----------
----------------TYQPNKNS------------------------------------
--WVNPEHLSFFKFVGRVIGKAIYDGRLLDAYFAK--SLYRQLLG---------------
--------KPVDYRDVEW----------------------VDPEYYNS--------LCWI
LEN------------------------------------------------DPT--PL--
-DLTFSVEADE---------------------------FGV-QRIVP-LKEG--------
GETLP-------------------------------------------------------
------------------------------VTNENKR-EFVQL-SAQYRLYSS-IKSQIE
-ALSEG---FYEIIPK--DMIT-IFNEQ-ELELLISG-----------------------
----TPDID-----------VDEW----RAATDYVG-YTSS-------------------
------------------------DPNI-VWWWRALK--------------------SFD
RDERAKVLSF-------ATGTSRVP------------LGG-----FT-ELQGVQ--GTQK
FSIHRAYGDE------------------------DRLPSAHTCF-NQ-IDLPQY-SSYEK
LRQQLL-LAISE-GATG
>Coprinopsis_cinerea_XM_001830199.2 .
LNVRRARV-FEDSFQHL--------------QRKTGDQIKYGKLNVRFYD-------EEG
VDAGGVTREW---FQIL----ARQMFDPNN--------ALFQPCAADKQ-----------
----------------TYQPNKNS------------------------------------
--WVNPEHLSFFKFVGRVIGKAIFDGRLLDAYFAR--SLYRQLLG---------------
--------KPVDYKDVEW----------------------VDPEYYKS--------LCWI
LEN------------------------------------------------DPT--VL--
-DLTFSVEADE---------------------------FGV-NRVIP-LKEG--------
GDQIP-------------------------------------------------------
------------------------------VTQENKR-EFVQL-SAQYRLYSS-IKEQIE
-NLSAG---FYEIVPK--DLIT-IFNEQ-ELELLISG-----------------------
----TPDID-----------VDEW----RAATDYVG-YTSS-------------------
------------------------DPNI-VWWWRALK--------------------SFN
RDERAKVLSF-------ATGTSRVP------------LNG-----FT-DLQGVQ--GVQR
FSIHRAYGEN------------------------DRLPQAHTCF-NQ-IDLPQY-SSYEM
LRQQLL-LAINE-----
>Coniophora_puteana_XM_007768651.1 .
LNVRRARV-FEDSFQQF--------------HSKDGERIKHAKLNVRFYD-------EEG
VDAGGVTREW---FQIL----ARQMFDPNN--------ALFQPCAADRL-----------
----------------TYQPNKNS------------------------------------
--WVNPEHLSFFKFVGRVIGKAIYDGRLLDAYFAK--SIYRQLLG---------------
--------KPVDYRDVEW----------------------VDPEYYNS--------LCWI
LEN------------------------------------------------DPT--PL--
-ELTFS--------------------------------FGR-NRIFP-LKEG--------
GEQIS-------------------------------------------------------
------------------------------VTNENKR-EFVQL-SASFRLYSS-IKEQIE
-HLVSG---FHDIIPK--DLVN-IFNEK-ELELLISG-----------------------
----TPDID-----------VDEW----RAATEYNG-YTSS-------------------
------------------------DPVI-VWWWRALK--------------------SFN
REERAKVLSF-------ATGTSRVP------------LGG-----FV-DLQGVQ--GVQR
FSIHRAYGDP------------------------DRLPQAHTCF-NQ-IDLPQY-SSYEM
LRQQLM-LAISE-----
>Trametes_sanguinea_GAKI01001196.1 .
LNVRRQRV-FEDSFQYL--------------QRKTGDQIKYGKLSVRFYD-------EEG
VDAGGVTREW---FQIL----ARQMFDPNY--------ALFQPCAVDKL-----------
----------------TYQPNRAS------------------------------------
--WVNPEHLSFFKFVGRVIGKAIYDGRLLDAYFAR--SIYKQLLG---------------
--------KPVDYKDVEW----------------------VDPEYYNS--------LVWI
LEN------------------------------------------------DPS--PL--
-DLTFSVEADE---------------------------FGV-TKIVD-LKPG--------
```

```
GASIP-------------------------------------------------------
------------------------------VTQENKK-EFVQL-SANYRLYSS-IKDQIE
-ALLAG---FYEIIPK--DLIT-IFNER-ELELLISG-----------------------
----TPDID-----------VDEW----RSATEYNG-YTSS-------------------
------------------------DPVI-VWFWRALK--------------------SFT
REERAKVLSF-------ATGTSRVP------------LGG-----FV-ELQGVQ--GTQR
FSIHKAYGDP------------------------DRLPQAHTCF-NQ-IDLPQY-SSYEM
LRQQLL-LAINE-----
>Trametes_versicolor_XM_008036066.1 .
LNVRRQRV-FEDSFQYL--------------QRKTGDQIKYGKLSVRFYD-------EEG
VDAGGVTREW---FQIL----ARQMFDPNY--------ALFQPCAVDKL-----------
----------------TYQPNRAS------------------------------------
--WVNPEHLSFFKFVGRVIGKAIYDGRLLDAYFAR--SIYKQLLG---------------
--------KPVDYKDVEW----------------------VDPEYYNS--------LVWI
LEN------------------------------------------------DPS--PL--
-DLTFSVEADE---------------------------FGV-TKLVE-LKEG--------
GATIP-------------------------------------------------------
------------------------------VTQENKK-EFVQL-SANYRLYSS-IKEQIE
-ALLTG---FYEIIPK--DLGS-IFNER-ELELLISG-----------------------
----TPDID-----------VDEW----RSATEYNG-YTGS-------------------
------------------------DPVI-VWWWRALK--------------------SFT
REERAKVLSF-------ATGTSRVP------------LGG-----FV-DLQGVQ--GTQR
FSIHKAYGDT------------------------DRLPQAHTCF-NQ-IDLPQY-SSYEM
LRQQVL-LAINE-----
>Trametes_hirsuta_CP019378.1 .
LNVRRQRV-FEDSFQYL--------------QRKTGDQIKYGKLSVRFYD-------EEG
VDAGGVTREW---FQIL----ARQMFDPNY--------ALFQPCAVDKL-----------
----------------TYQPNRAS------------------------------------
--WVNPEHLSFFKFVGRVIGKAIYDGRLLDAYFAR--SIYKQLLG---------------
--------KPVDYKDVEW----------------------VDPEYYNS--------LVWI
LEN------------------------------------------------DPS--PL--
-DLTFSVEADE-------------------------------------------------
------------------------------------------------------------
------------------------------------------------------------
------------------------FNER-ELELLISG-----------------------
----TPDID-----------VDEW----RSATEYNG-YTSS-------------------
------------------------DPVI-VWFWRALK--------------------SFT
REERAKLLSF-------ATGTSRVP------------LGG-----FV-ELQGVQ--GTQR
FSIHKAYGDA------------------------DRLPQAHTCX-NQ-IDLPQY-SSYEM
LRQQLL-LAINE-----
>Coriolopsis_gallica_GBYM01001049.1 .
---------------------------------------KYGKLSVRFYD-------EEG
VDAGGVTREW---FQIL----ARQMFDPNY--------ALFQPCAADRL-----------
----------------TYQPNRAS------------------------------------
--WVNPEHLSFFKFVGRVIGKAIYDGRLLDAYFAR--SLYRQLLG---------------
--------KQVDYKDVEW----------------------VDPEYYNS--------LCWI
LEN------------------------------------------------DPS--PL--
-DLTFSVEADE---------------------------FGV-TKLVE-LKEG--------
GASIP-------------------------------------------------------
------------------------------VTQENKK-EFVQLS-ANYRLYSS-IKDQIE
-SLVAG---FYEIIPK--DLVS-IFNEQ-ELELLISG-----------------------
----TPDID-----------VDEW----RSATEYNG-YTSS-------------------
------------------------DPVI-VWFWRALK--------------------SFN
REERAKVLSF-------ATGTSRVP------------LGG-----FV-ELQGVQ--GTQR
FSIHKAYGDT------------------------DRLPQAHTCF-NQ-IDLPQY-SSYEM
LRQQLL-LAINE-G---
>Dichomitus_squalens_XM_007366811.1 .
LNVRRQRV-FEDSFQYL--------------QRKTGEQIKYGKLSVRFYD-------EEG
```

VDAGGVTREW---FQIL----ARQMFDPNY--------ALFQPCAADKL-----------
----------------TYQPNRAS------------------------------------
--WVNPEHLSFFKFVGRVIGKAIYDGRLLDAYFAR--SLYRQLLG---------------
--------KQVDYKDVEW----------------------VDPEYYNS--------LCWI
LEN--DPS--PL--
-DLTFSVEADE---------------------------FGV-TKLVE-LKEG--------
GASIP---
------------------------------VTQENRK-EFVQL-SANYRLYSS-IKDQIE
-SLLAG---FYEIIPK--DLVS-IFNEQ-ELELLISG-----------------------
----TPDID-----------VDEW----RSATEYNG-YTSS-------------------
------------------------DPVI-VWFWRALK--------------------SFN
REERAKVLSF-------ATGTSRVP------------LGG-----FV-ELQGVQ--GTQR
FSIHKAYGDT------------------------DRLPQAHTCF-NQ-IDLPQY-SSYEM
LRQQLL-LAINE-----
>Ganoderma_lucidum_AHGX01000550.1_R .
LNVRRQRV-FEDSFQYL--------------QRKTGEQIKYGKLSVRFYD-------EEG
VDAGGVTREW---FQIL----ARQMFDPNY--------ALFQPCAADRL-----------
----------------TYQPNRAS------------------------------------
--WVNPEHLSFFKFVGRVIGKAIYDGRLLDAYFAR--SLYRQLLG---------------
--------KQVDYKDVEW----------------------VDPEYYNS--------LCWI
LEN--DPS--PL--
-DLNFSVEADE---------------------------FGV-TKLVE-LKEG--------
GATIP---
------------------------------VTQENRK-EFVQL-SANYRLYSS-IKEQIE
-SLLVG---FYEIIPK--DLVS-IXNEQ-ELELLISG-----------------------
----TPDID-----------VDEW----RSATEYNG-YTSS-------------------
------------------------DPVI-VWFWRALK--------------------SFN
REERAKVLSF-------ATGTSRVP------------LGG-----FV-ELQGVQ--GTQR
FSIHKAYGDT------------------------DRLPQAHTCX-NQ-IDLPQY-SSYEM
LRQQLL-LAIN------
>Auricularia_delicata_AFVO01000136.1 .
LNVRRARV-FEDSFQYL--------------QRRTGDQIKYGKLSVRFYD-------EEG
VDAGGVTREW---FQIL----ARQMFNPDY--------CLFQPCAADKL-----------
----------------TYQPNRAS------------------------------------
--AVNPEHLSFFKFVGRVIGKAIYDGRLLDAYFAR--SLYRQLLG---------------
--------KPVDYRDVEW----------------------VDPSYYSS--------LCWL
LEN--DPA--PL--
-DMTFSIDTDE---------------------------FGV-TKVVP-LKEN--------
GASIP---
------------------------------VTIENRR-EFVQL-AAEYRLYSS-IKDQIE
-SLLSG---FYEIIPK--DLIS-IXNEQ-EVELLISG-----------------------
----TPDID-----------VDEW----RAATEYNG-YTAS-------------------
------------------------DPVI-VWWWRALK--------------------SFS
RDERAKVLSF-------ATXTSRVP------------LGG-----FV-ELQGVQ--GVQR
FSIHKAYGGT------------------------DRLPQAHTCF-NQ-VDLPQY-SSYEM
LRTQLL-LAINE-----
>Auricularia_subglabra_XM_007339319.1 .
LNVRRARV-FEDSFQYL--------------QRRTGDQIKYGKLSVRFYD-------EEG
VDAGGVTREW---FQIL----ARQMFNPDY--------CLFQPCAADKL-----------
----------------TYQPNRAS------------------------------------
--AVNPEHLSFFKFVGRVIGKAIYDGRLLDAYFAR--SLYRQLLG---------------
--------KPVDYRDVEW----------------------VDPSYYSS--------LCWL
LEN--DPA--PL--
-DMTFSIDTDE---------------------------FGV-TKVVP-LKEN--------
GASIP---
------------------------------VTIENRR-EFVQL-AAEYRLYSS-IKDQIE
-SLLSG---FYEIIPK--DLIS-IFNEQ-EVELLISG-----------------------
----TPDID-----------VDEW----RAATEYNG-YTAS-------------------

------------------------DPVI-VWWWRALK--------------------SFS
RDERAKVLSF-------ATGTSRVP------------LGG-----FV-ELQGVQ--GVQR
FSIHKAYGGT------------------------DRLPQAHTCF-NQ-VDLPQY-SSYEM
LRTQLL-LAI-------
>Auricularia_subglabra_XM_007357728.1 .
LNVRRARV-FEDSFQYL--------------QRRTGDQVKYGKLSVRFYD-------EEG
LDAGGVTREW---FQIL----ARQMFNPDY--------CLFQPCAADKL-----------
----------------TYQPNRAS------------------------------------
--AVHPEHLSFFKFVGRVIGKALYDGRLLDAYFAR--SLYRQLLG---------------
--------KPVDYRDVEW----------------------VDPSYYSS--------LCWL
LEN--DPA--PL--
-DMTFSIDTDE---------------------------FGV-TKVVP-LKEN--------
GASIP---
------------------------------VTIDNRR-EFVQL-AAEYRLYSS-IKDQIE
-SLLGG---FYEIIPK--DLIS-IFNEQ-EVELLISG-----------------------
----TPDVD-----------VDEW----RAATEYNG-YTPS-------------------
------------------------DPVI-VWWWRALK--------------------SFS
RDERAKVLGF-------ATGTSRVP------------LGG-----FV-ELQGVQ--GVQR
FSIHKAYGGT------------------------DRLPQAHTCF-NQ-VDLPQY-PSYEM
LRAQLL-LAI-------
>Punctularia_strigosozonata_XM_007381277.1 .
LNVRRARV-FEDSFQYL--------------QRKTGDQIKYGKLSIRFYD-------EEG
VDAGGLTREW---FQIL----ARQMFNPDY--------ALFQPCVADKL-----------
----------------TYQPNRAS------------------------------------
--WVNPEHLSFFKFVGRIIGKAIYDGRLLDAYFAR--SFYRQLLG---------------
--------KPVDYRDVEW----------------------VDPEYYNS--------LCWI
LEN--DPT--GL--
-DLTFDVEADE---------------------------FGV-TKIVP-LKEN--------
GTQIH---
------------------------------VTNENKK-EFVQL-SAQYRLYTS-IKDQID
-AILAG---FYDIIPK--DLIS-IFNEQ-EVELLISG-----------------------
----TPDID-----------VDEW----RAATEYHG-YTSS-------------------
------------------------DPTI-VWWWRALK--------------------SFN
REERAKVLSF-------ATGTARVP------------LGG-----FG-DLQGVQ--GVQK
FSIHKAYGEQ------------------------DRLPQAHTCF-NQ-IDLPEY-SSYEM
LRQQLL-LAINE-GGEG
>Gloeophyllum_trabeum_XM_007866419.1 .
LNVRRSRV-FEDSVQHL--------------QRKTGDQIKYGKLSVRFYD-------EEG
VDAGGVTREW---FQIL----ARQMFDPNN--------ALFQPCAADKL-----------
----------------TYQPNSRS------------------------------------
--WINPEHLIFFKFVGRVIGKAIYDGRLLDAYFAR--SLYRQILG---------------
--------KPVDYRDVEW----------------------VDPEYYNS--------LCWI
LEN--DPS--PL--
-DLTFSVEADE---------------------------FGT-TKVIP-LKEG--------
GTSIP---
------------------------------VTQENKK-EFVQL-SAQYRLYLS-TKDQIE
-ALLAG---FYEIIPK--DLIA-IFNEQ-ELELLISG-----------------------
----TPDID-----------VDEW----RAATEYHG-YSSS-------------------
------------------------DPVI-VWWWRALK--------------------SFN
RDERAKVLSF-------ATGTARVP------------LGG-----FT-ELQGVQ--GTQR
FSIHKAYGDP------------------------DRLPQAHTCF-NQ-IDLPQY-TSYEM
LRQQLL-LAINE-----
>Heterobasidion_irregulare_XM_009548886.1 .
LNVRRQRL-FEDSFQYF--------------HRRTGEQIKYGKLSVRFYD-------EEG
VDAGGVTREW---FQIL----ARQMFDPNN--------ALFEPCAADRL-----------
----------------TYQPNKAS------------------------------------
--WVNPEHLSFFKFVGRVIGKAIYDGRLLDAYFAR--SLYRQILG---------------
--------KPVDYKDVEW----------------------IDPEYYKS--------LCWI

```
LEN------------------------------------------------DPS--LL--
-DLTFSVEGDE---------------------------FGV-TKIVD-LKEN--------
GASIP-------------------------------------------------------
------------------------------VTMANRR-EFVQL-SAQYRLYSS-IKEQLE
-HLLSG---FYEIIPK--DLVA-IFNEQ-ELELLISG-----------------------
----TPDID-----------VDEW----RAATDYNG-YTSS-------------------
------------------------DPVI-VWWWRALK--------------------SFN
RDERAKVLSF-------ATGTSRVP------------LSG-----FG-DLQGVQ--GVQR
FSIHRAYGDS------------------------DRLPQAHTCF-NQ-IDLPQY-SSYEM
LRQQLL-LAINE-----
>Stereum_hirsutum_XM_007307864.1 .
LNVRRQRL-FEDSFQYL--------------QRKTGDQIKYGKLSVRFYD-------EEG
VDAGGVTREW---FQIL----ARQMFDPNN--------ALFEPCAADKL-----------
----------------TYQPNKAS------------------------------------
--WVNPEHLSFFKFVGRVIGKAIYDGRLLDAYFAR--SLYRQILG---------------
--------KPVDYRDVEW----------------------IDPDYYKS--------LCWI
LEN------------------------------------------------DPT--AL--
-DMTFSVEGDE---------------------------FGV-MKIVP-LKEG--------
GETLP-------------------------------------------------------
------------------------------VTLENRR-EFVQL-AAQYRLYSS-IKDQIE
-NLLSG---FYDIIPK--DLVS-IFNEQ-ELELLISG-----------------------
----TPEID-----------VDEW----RAATDYNG-YNSS-------------------
------------------------DPVI-VWWWRALK--------------------SFN
RDERAKVLSF-------ATGTSRVP------------LSG-----FG-DLQGVQ--GTQR
FSIHRAYGDP------------------------DRLPQAHTCF-NQ-IDLPQY-SSYEK
LRQQLL-LAINE-----
>Boletopsis_grisea_GEZR01001879.1 .
LNVRRQRV-FEDSYQYL--------------QRKTGEQIKHGKLSIRFYD-------EEG
VDAGGVTREW---FQIL----ARQMFNPNY--------ALFQPCAADRL-----------
----------------TYQPNRAS------------------------------------
--AVNPEHLSFFKFVGRVIGKAIFDGRLLDAYFAR--SLHRQLLG---------------
--------KPVDYRDVEW----------------------IDPEYYNS--------LCWI
LEN------------------------------------------------DPS--PL--
-DLTFSVETDE---------------------------FGV-TKIVS-LKEN--------
GDKMP-------------------------------------------------------
------------------------------VTLENRK-EFVQL-SAQYRLYSS-IKDQIE
-SLLGG---FYEIIPK--DLIS-IFNEQ-EVELLISG-----------------------
----TPDID-----------VDEW----RAATDYNG-YSSS-------------------
------------------------DPVI-VWWWRALK--------------------SFN
REERAKVLSF-------ATGTSRVP------------LGG-----FV-ELQGVQ--GVQR
FSIHRAYGDQ------------------------DRLPQAHTCF-NQ-IDLPQY-SSYEM
LRQQLL-LAINE-----
>Ceriporiopsis_subvermispora_AEOV01000330.1_R .
LNVRRQRV-FEDSFQYL--------------QRKTGDQIKYGKLSVRFYE-------EEG
VDAGGVTREW---FQIL----ARQMFDPNY--------ALFQPCAADRL-----------
----------------TYQPNKAS------------------------------------
--WVNPEHLSFFKFVGRIIGKAIYDGRLLDAYFAR--SLYRQILA---------------
--------KPVDYRDVEW----------------------VDPEYYNS--------LCWI
LEN------------------------------------------------DPT--PL--
-DLTFSVEADX---------------------------FGV-TKIVD-LKPG--------
GASIP-------------------------------------------------------
------------------------------VTQENKK-EFVQL-SAQYRLYSS-IKDQIE
-SLLTG---FYEIIPK--DLIS-IVSSR-ELELLISG-----------------------
----TPDID-----------VDEW----RSATEYNG-YTSS-------------------
------------------------DPVI-VWWWRALK--------------------SFN
REERAKVLSF-------ATGTSRVP------------LGG-----FV-ELQGVQ--GVQR
FSIHKAYGDT------------------------DRLPQAHTCX-NQ-IDLPQY-SSYEM
LRQQLL-LAINE-GGEG
```

>Sparassis_latifolia_GELB01006542.1 .
LNVRRQRV-FEDSFQHL--------------QRKTGDQIKYGKLSIRFYD-------EEG
VDAGGVTREW---FQIL----ARQMFDPNY--------ALFQPCAADRL-----------
----------------TYQPNKAS------------------------------------
--WVNPEHLSFFKFVGRVIGKAIYDGRLLDAYFAR--SLYRQILG---------------
--------KPVDYRDVEW----------------------VDPEYYNS--------LCWI
LEN--DPS--AL--
-ELTFSVEADE---------------------------FGV-TKIVD-LKEN--------
GSSIP---
------------------------------VTQENKR-EFVQL-SAQYRLYSS-IKDQIE
-SLLTG---FYEIIPK--ELIS-IFNEQ-ELELLISG-----------------------
----TPDID-----------VDEW----RSATEYNG-YTSS-------------------
------------------------DPVI-VWWWRALK--------------------SFN
REERAKVLSF-------ATGTSRVP------------LGG-----FV-DLQGVQ--GVQR
FSIHRAYGDT------------------------DRLPQAHTCF-NQ-IDLPQY-SSYEM
LRQQLL-LAINE-----
>Postia_placenta_ABWF01001230.1_R .
LNVRRPRV-FEDSFQYL--------------QRKTGEQIKYGKLSVRFYD-------EEG
VDAGGVTREW---FQIL----ARQMFDPNY--------ALFQPCAADRL-----------
----------------TYQPNKAS------------------------------------
--WVNPEHLSFFKFVGRIIGKAIYDGRLLDAYFAR--SLYRLILG---------------
--------KPVDYRDVEW----------------------VDPEYYNS--------LCWI
LDN--DPS--AL--
-ELTFNVEADE---------------------------FGV-TKIVD-LKEN--------
GRSIP---
------------------------------VTQESKR-EFVQL-SAQYRLYSS-IKDQIE
-ALLAG---FYEIIPK--DLIS-IXNEQ-ELELLISG-----------------------
----TPDID-----------VDEW----RAATEYNG-YTSS-------------------
------------------------DPVI-VWFWRALK--------------------SFN
REERAKVLSF-------ATGTSRVP------------LGG-----FT-ELQGVQ--GVQR
FSIHRAYGDT------------------------DRLPQAHTCF-NQ-IDLPQY-SSYEM
LRQQLL-LAINE-----
>Fibroporia_radiculosa_XM_012325051.1 .
LNVRRPRV-FEDSFQYL--------------QRKTGDQIKYGKLSVRFYE-------EEG
VDAGGVTREW---FQIL----ARQMFDPNY--------ALFQPCAADRL-----------
----------------TYQPNKAS------------------------------------
--WVNPEHLSFFKFVGRVIGKAIYDGRLLDAYFAR--SLYRQILA---------------
--------KPVDYRDVEW----------------------VDPEYYNS--------LCWI
LDN--DPS--AL--
-ELTFSVEADE---------------------------FGV-TKIVD-LREN--------
GRSVA---
------------------------------VTQENKR-EFVQL-SAQYRLYSS-IKDQIE
-ALLTG---FYEIIPK--DLIA-IFNEQ-ELELLISG-----------------------
----TPDID-----------VDEW----RAATEYNG-YTSS-------------------
------------------------DPVI-VWWWRALK--------------------SFN
REERAKVLSF-------ATGTSRVP------------LGG-----FT-ELQGVQ--GVQR
FSIHRAYGDQ------------------------DRLPQAHTCF-NQ-IDLPQY-SSYEM
LRQQLL-LAINE-----
>Starmerella_bacillaris_MWPI01000016.1 .
IHVSRDSL-YADTYK-L-------------------YPIKD-LLNIHFNN-------EEG
EDAGGVSREF---YDLM----STE-FI-NH--------KLFRSCTPGSA-----------
----------------VYCIN---------------------------------------
----ESANLKELEFAGWLLASAMVTHKTVGIHFTT--SIYKFLLE---------------
--------RKVNINDMSV----------------------LDEEYTTS--------LRWI
LDN--DIT-DVI--
-YESFSID------------------------------------SKD-LIPG--------
GKEVE---
------------------------------VTNENKQ-DYVSK-VIEYKLVES-VRDQLE

-SLKKG---FNRVIPI--ESYN-IYDEK-ELELILCG-----------------------
----LPTID-----------VTDW----HRNTIYEG-YTVT-------------------
------------------------SPQI-KWFWSTVT--------------------SMS
TDERAKLLQF-------CTGTSRVP------------VTG-----FA-SLESTS--GFGK
FVIKKIGAST------------------------SHLPSSHTCM-NT-LNLPEY-ETAKK
LKVSLL-KAILEGS---
>Candida_apicola_LBNK01000009.1 .
LELNRDKI-LSDSFDGI--------------MGLNHSLLRC-PLNIQFVG-------EDG
VDAGGVAREW---LELI----SRELVDPKL--------SLFTTTATGSS-----------
----------------VYQINPGS------------------------------------
----RVDQLEYFTFTGRILGRAVLDLRTIGTHFSR--VIFKYILG---------------
--------LPISLNDMET----------------------VDDEYCRN--------LQWM
LEN--NVT-DII--
-YETFSVDIDK---------------------------SGE-KQTID-LIPD--------
GRNIY---
------------------------------VSEENKR-EYVDR-IVAYRLVDS-VEPQLK
-QIAKG---FNEIVPV--EYIR-DFTDQ-EMELLISG-----------------------
----VPDID-----------IDEW----KRNTMYVG-YTPT-------------------
------------------------SPQI-KWFWVVVK--------------------SFT
KEERAKLLQF-------CTGTSRVP------------AQG-----FE-LLESSE--GYGK
FSIHKERSSA------------------------DRLPSSHTCM-NQ-LMLPQY-ESYKK
LRSRLL-HAITEGN---
>2_Candida_infanticola_LWLF01000022.1 .
LKVSRSNL-FMDSMDHL--------------MKLEPFELRLGQLDVHFDG-------EEG
IDQGGLNREW---YSEM----TKYIFHPEN--------GLFEQHASGL------------
----------------RPKAVPGS------------------------------------
--------LDKFLFIGRFIGKAVVDGQLLDVVFSP--SVYKFLLK---------------
--------ETITMEDLKV----------------------IDETYYNS--------LKWM
LEN--DIT-DVI--
-DEVFAVET-----------------------------PGHPDQLID-LIPN--------
GSKIP---
------------------------------MTESNKK-EYVEL-RAAWDLYKR-VEPQLE
-QLSTG---FYELMKF--SLVS-LFNPK-ELELVIGG-----------------------
----IPKID-----------ITDW----RLNTEYEG-YSAS-------------------
------------------------SPQI-RWFWRTVK--------------------SYD
HAQRAKLLQF-------CTGSAKVP------------VDG-----FQ-HLPA------GR
FNISRDRRSP------------------------EWLPTAHTCT-NH-LCLPEY-TSYDK
LRAAVS-RAISDGAG--
>Mitosporidium_daphniae_XM_013383512.1 .
LRIRRDRL-FEDSFREI--------------MTLPATALAERPLSIAFDG-------EEG
VDAGGLTREW---FSLL----AQQMLNAGY--------ALFLPVGGGQGGSGA-------
----------------VFYPNRLS------------------------------------
--AVNPEHLDYFEFVGRIIGKAVVESQLLACHFAC--AFYKILLD---------------
--------IPLELSDLEA----------------------VDASYYRS--------LAWM
LSQA---DPE-QLE--
-ELSLFMVTELDD-------------------------FGR-PRMVD-LIPN--------
GSTIP---
------------------------------VTA-DRIEEYVSL-TVDVRLRGA-LEAQFD
-AIRRG---LFGVLSQ--EILA-IFTEK-ELELFISG-----------------------
----LALIDD----------IDDW----KRNTEYGTGYTAT-------------------
------------------------SPQV-VWFWRAVR--------------------SMS
YEERSQLLQF-------VTGSMRVP------------LGG-----FS-RLLGSN-GALQR
FSIHRDTGGA------------------------HRLPQAHTCF-NQ-LDIPEY-ESYEQ
LRRALL-VAIREGTTGF
>Schizosacch_japonicus_XM_002174952.1 .
VPVRREHV-FTDSYRVL--------------YAKDAKELLHARLSIKFEG-------EEG
VDAGGITREW---FQVV----THQMLNPNY--------ALFSPSPTDPN-----------
----------------TFHPNKTS------------------------------------

--AVNPEHLSYFRFVGRLLGKALFDERLLDCHFSN--AVYKALLN---------------
--------KPISLNDIDS----------------------LDTNYSKS--------LHWM
LEN--NIT-DVI--
-TETFSVETDT---------------------------FGV-HKVID-LVPN--------
GQSIN---
------------------------------VTEENKR-DYVHL-LTEYHLKSS-VQQQLE
-HLKAG---FSEMIDE--SLIC-IFSEK-ELELLLSG-----------------------
----LPDID-----------IDDW----KNNTEYVN-YTPA-------------------
------------------------SSNI-QWFWRALR--------------------SLG
KEDLAKLLQF-------VTGTSKVP------------LNG-----FA-GLMGTT--GLQK
FSIHKDYSTVP-----------------------GKLPQSHTCF-NQ-LDLPEY-GSYEE
LKSALL-TAIHEGSEGF
>Hanseniaspora_uvarum_APLS01000004.1 .
LKVNRSSV-FEDSYRVL--------------YSDPSSNFKTGKLNVTFEG-------EEG
VDAGGLTREW---YQII----SREMVSPDL--------SLFTPVSEDNND----------
----------------TFIPNRNS------------------------------------
--SVNPSHFSYFEFCGMIMAKAVYDSCFLDCHFIR--PIYKAIIG---------------
--------KNPGLKDMES----------------------IDAEYYKS--------VVWI
LEN--DIT-DVL--
-DMTFSIETED---------------------------SGV-YKSHD-LIKN--------
GSEIA---
------------------------------VTEENKK-SYVEK-VIDFKLKLS-FEKQLH
-CLVEG---FYSIIDK--DLIE-IFNEN-ELELLLSG-----------------------
----LPDID-----------VDDW----KNNTEYVN-YTNQ-------------------
------------------------SQEI-SLFWRCVR--------------------SFN
QVEKAKLLQF-------ITGTSKVP------------LNG-----FK-DLQGSN--GSSK
FSIHKDFGSS------------------------SRLPQAHTCF-NQ-LDLPSY-KTYDE
MKRALL-VAINEGS---
>Spathaspora_arborariae_AYLH01000137.1 .
INVRRDQV-FLDSYRSL--------------FFKSRDEFRDCKLEINFKG-------EEG
IDAGGVTREW---YQVL----SRQMFNPDY--------ALFTPVVADKT-----------
----------------TFHPNKTS------------------------------------
--YINPEHLSFFKFIGRIIGKAIYDNCYLDCHFSR--AVYKQILG---------------
--------KKTSLKDMET----------------------LDLEYFKS--------LMWM
LEN--DIT-DVI--
-TEDFSVETDE---------------------------FGE-HKIID-LIPD--------
GRNIP---
------------------------------VTEANKH-EYVKK-VVEYRLQTS-VVEQMD
-NFLIG---FHEIIPK--DLVA-IFDEQ-ELELLISG-----------------------
----LPDIN-----------VSDW----QSNTIYNN-YSPS-------------------
------------------------SLQI-QWFWRAVK--------------------SFD
NEERARLLQF-------ATGTSKVP------------LNG-----FK-ELSGAS--GTCK
FSIHRDYGSI------------------------DRLPSSHTCF-NQ-IDLPAY-ESYET
LRGSLL-MAITEGHEGF
>Spathaspora_girioi_LQMS01002369.1 .
INIRRDQV-FLDSYRSL--------------FFKSRDEFRNSKLEINFKG-------EQG
IDAGGVTREW---YQVL----SRQMFNPDY--------ALFTPVVADKT-----------
----------------TFHPNRTS------------------------------------
--YVNPEHLSFFKFIGRIIGKAIYDNCYLDCHFSR--AVYKQILA---------------
--------KKQSLKDMET----------------------LDLEYFKS--------LMWM
LEN--DIT-DVI--
-TEDFSVETND---------------------------YGE-RNIID-LIPN--------
GRNIP---
------------------------------VTEANKH-EYVKQ-VVEYRLQTS-VVEQMD
-NFLIG---FHEIIPK--DLIV-IFDEQ-ELELLISG-----------------------
----LPDIN-----------VSDW----QNHTVYNN-YSAS-------------------
------------------------SLQI-QWFWRAVK--------------------SFD
NEERAKLLQF-------ATGTSKVP------------LNG-----FK-ELSGAN--GTCK

FSIHRDYGSI------------------------DRLPSSHTCF-NQ-IDLPAY-ESYET
LRGSLL-MAITEGHEGF
>Spathaspora_passalidarum_XM_007374915.1 .
VNVRRDQV-FLDSYRSL--------------FFKSRDEFRNSKLEINFKG-------EQG
IDAGGVTREW---YQVL----SRQMFNPDY--------ALFTPVVADRT-----------
----------------TFHPNRTS------------------------------------
--YINPEHLSFFKFIGRIIGKAIYDNCYLDCHFSR--AVYKQILG---------------
--------KKQSLKDMES----------------------LDLEYTKS--------LMWM
LEN--DIT-DVI--
-TEDFSVETDD---------------------------YGE-HKIID-LIPN--------
GRNIP---
------------------------------VTEENKQ-EYVKK-VVEYRLQTS-VEEQMD
-NFLIG---FHEIIPK--DLIA-IFDEQ-ELELLISG-----------------------
----LPDIN-----------VSDW----QSNTIYNN-YSPS-------------------
------------------------SIQI-QWFWRAVK--------------------SFD
NEERARLLQF-------ATGTSKVP------------LNG-----FK-ELSGAS--GTCK
FSIHRDYGST------------------------DRLPSSHTCF-NQ-VDLPAY-ESYET
LRGSLL-MAITEGHEGF
>Spathaspora_xylofermentans_NDXA01000014.1 .
VNVRRDQV-FLDSYRAL--------------FFKAKDEFRNSQLDINFKG-------EQG
IDAGGVTREW---YQVL----SRQMFNPDY--------ALFTPVVSDKT-----------
----------------TFHPNRTS------------------------------------
--YINPEHLSFFKFIGRIIGKAIFDNCYLDCHFSR--AVYKRILG---------------
--------RPQSLKDMES----------------------LDLEYFKS--------LMWM
LEN--DIT-DVI--
-TEDFSVESDD---------------------------YGE-HKIID-LIPD--------
GRNIP---
------------------------------VTEENKH-DYVRK-VVEYKLQTS-VVEQMD
-NFLIG---FHEIIPK--DLIA-IFDEQ-ELELLISG-----------------------
----LPDID-----------VVDW----QNNTIYNN-YSPS-------------------
------------------------SVQI-QWFWRAVK--------------------SYD
NEERARLLQF-------ATGTSKVP------------LNG-----FK-ELSGAS--GTCK
FSIHRDYGST------------------------DRLPSSHTCF-NQ-IDLPAY-DTYET
LRGSLL-MAITEGHEGF
>Spathaspora_hagerdaliae_LQHL01001588.1 .
INVRREQV-FLDSYRSL--------------FFKSKDEFRNSKLEINFKG-------EQG
IDAGGVTREW---YQVL----SRQMFNADY--------ALFTPVVSDKT-----------
----------------TFHPNRTS------------------------------------
--YINPEHLSFFKFIGRIIGKAIYDNCFLDCHFSR--AVYKRILG---------------
--------RPTSLKDMES----------------------LDLEYFKS--------LMWM
LEN--DIT-DVI--
-TEDFSMETDD---------------------------YGE-HKIID-LIAN--------
GRNIP---
------------------------------VVEENKH-EYVKK-VVEYKLLTS-VSEQMD
-NFLIG---FHEIISK--DLVA-IFDEQ-ELELLISG-----------------------
----LPDID-----------VMDW----QNNTNYNN-YSPS-------------------
------------------------SIQI-QWFWRAVK--------------------SFD
NEERARLLQF-------ATGTSKVP------------LNG-----FK-ELSGAS--GTCK
FSIHRDYGSI------------------------DRLPSSHTCF-NQ-IDLPAY-ESYET
LRGSLL-MAITEGHEGF
>Spathaspora_gorwiae_LQMZ01002370.1 .
INVRREQV-FLDSYRSL--------------FFKSKDEFRNSKLEINFKG-------EQG
IDAGGVTREW---YQVL----SRQMFNPDY--------ALFTPVVSDKT-----------
----------------TFHPNRTS------------------------------------
--YINPEHLSFFKFIGRIIGKAIYDNCFLDCHFSR--AVYKRILG---------------
--------RPTSLKDMES----------------------LDLEYFKS--------LMWM
LEN--DIT-DVI--
-TEDFSVETDD---------------------------YGE-HKIID-LIAN--------

GRNIP---
------------------------------VIEENKH-EYVKK-VVEYKLLTS-VSEQMD
-NFLIG---FHEIISK--DLVA-IFDEQ-ELELLISG-----------------------
----LPDID-----------VIDW----QNNTNYNN-YSPS-------------------
------------------------SLQI-QWFWRAVK--------------------SFD
NEERARLLQF-------ATGTSKVP------------LNG-----FK-ELSGAS--GTCK
FSIHRDYGSI------------------------DRLPSSHTCF-NQ-IDLPAY-ESYET
LRGSLL-MAITEGHVGF
>Candida_maltosa_AOGT01000089.1 .
VSVRRDQV-FLDSYRGL--------------FFKGKDEFKNSKLEINFKG-------EQG
IDAGGVTREW---YQVL----SRQMFNPDY--------ALFTPVVSDET-----------
----------------TFHPNRTS------------------------------------
--YINPEHLSFFKFIGRIIGKAIYDNCFLDCHFSR--AVYKRILG---------------
--------KQQSLKDMET----------------------LDLEYFKS--------LMWM
LEN--DIT-DVI--
-TEDFSVETDD---------------------------YGE-HKIID-LIPN--------
GRNIP---
------------------------------VTEENKH-EYVQK-VVDYRLQTS-VEEQME
-NFLIG---FHEIIPK--DLVA-IFDEK-ELELLISG-----------------------
----LPDID-----------VTDW----QGNTTYNN-YSPL-------------------
------------------------SLQI-QWFWRAVK--------------------SFD
NEERARLLQF-------ATGTSKVP------------LNG-----FK-GLSGAS--GTCK
FSIHRDYGSA------------------------DRLPSSHTCF-NQ-IDLPAY-ESYET
LRGSLL-MAITEG----
>Candida_sojae_LMTL01000099.1 .
VSVRRDQV-FLDSYRAL--------------FFKPKDEFRNSKLEINFKG-------EQG
IDAGGVTREW---YQVL----SRQMFNPDY--------ALFTPVVSDET-----------
----------------TFHPNRTS------------------------------------
--YINPEHLSFFKFIGRIIGKAIYDNCFLDCHFSR--AVYKRILG---------------
--------KQQSLKDMET----------------------LDLEYFKS--------LMWM
LEN--DIT-DVI--
-TEDFSVETDD---------------------------YGE-HKIID-LIPN--------
GRNIP---
------------------------------VTEENKH-EYVQR-VVEYRLQTS-VEEQME
-NFLMG---FHEIIPK--DLVA-IFDEK-ELELLISG-----------------------
----LPDID-----------VVDW----QNNTTYNN-YSPL-------------------
------------------------SLQI-QWFWRAVK--------------------SFD
NEERARLLQF-------ATGTSKVP------------LNG-----FK-ELSGAS--GTCK
FSIHRDYGSS------------------------DRLPSSHTCF-NQ-IDLPNY-ESYET
LRGSLL-MAITEG----
>Candida_tropicalis_XM_002546955.1 .
VSVRRDQV-FLDSYRAL--------------FFKPKDEFRNAKLEINFKG-------EQG
IDAGGVTREW---YQVL----SRQMFNPDY--------ALFTPVVSDET-----------
----------------TFHPNRTS------------------------------------
--YINPEHLSFFKFIGRIIGKAIHDNCFLDCHFSR--AVYKRILG---------------
--------KQQSLKDMET----------------------LDLEYFKS--------LMWM
LEN--DIT-DVI--
-TEDFSVETDD---------------------------YGE-HKIID-LIPN--------
GRNIP---
------------------------------VTEENKH-EYVKK-VVEYRLQTS-VEEQME
-NFLMG---FHEIIPK--DLVA-IFDEK-ELELLISG-----------------------
----LPDID-----------VVDW----QNNTTYNN-YSPL-------------------
------------------------SLQI-QWFWRAVK--------------------SFD
NEERARLLQF-------ATGTSKVP------------LNG-----FK-ELSGAS--GTCK
FSIHRDYGAS------------------------DRLPSSHTCF-NQ-IDLPSY-ESYET
LRGSLL-MAITEGHEGF
>Candida_dubliniensis_XM_002418227.1 .
VSVRRDQV-FLDSYRSL--------------FFKPKDEFRNSKLEINFKG-------EQG

IDAGGVTREW---YQVL----SRQMFNPDY--------ALFTPVVSDET-----------
----------------TFHPNRTS------------------------------------
--YINPEHLSFFKFIGRIIGKAIYDNCFLDCHFSR--AVYKRILG---------------
--------KPQSLKDMET----------------------LDLEYFKS--------LMWM
LEN--DIT-DVI--
-TEDFSVETDD---------------------------YGE-HKIID-LIPN--------
GRNIP---
------------------------------VIEENKH-EYVKK-VVEYRLQTS-VEEQME
-NFLIG---FHEIIPK--DLVA-IFDEK-ELELLISG-----------------------
----LPDID-----------VSDW----QNHTTYNN-YSPL-------------------
------------------------SLQI-QWFWRAVK--------------------SFD
NEERARLLQF-------ATGTSKVP------------LNG-----FK-ELSGAS--GTCK
FSIHRDYGST------------------------DRLPSSHTCF-NQ-IDLPAY-DSYET
LRGSLL-MAITEGHEGF
>Candida_albicans_XM_710225.1 .
VSVRRDQV-FLDSYRSL--------------FFKPKDEFRNSKLEINFKG-------EQG
IDAGGVTREW---YQVL----SRQMFNPDY--------ALFTPVVSDET-----------
----------------TFHPNRTS------------------------------------
--YINPEHLSFFKFIGRIIGKAIYDNCFLDCHFSR--AVYKRILG---------------
--------KPQSLKDMET----------------------LDLEYFKS--------LMWM
LEN--DIT-DVI--
-TEDFSVETDD---------------------------YGE-HKIID-LIPN--------
GRNIP---
------------------------------VTEENKN-EYVKK-VVEYRLQTS-VEEQME
-NFLIG---FHEIIPK--DLVA-IFDEK-ELELLISG-----------------------
----LPDID-----------VSDW----QNHTSYNN-YSPL-------------------
------------------------SLQI-QWFWRAVK--------------------SFD
NEERARLLQF-------ATGTSKVP------------LNG-----FK-ELSGAS--GTCK
FSIHRDYGST------------------------DRLPSSHTCF-NQ-IDLPAY-DCYET
LRGSLL-MAITEGHEGF
>Candida_africana_GEVV02004309.1 .
VSVRRDQV-FLDSYRSL--------------FFKPKDEFRNSKLEINFKG-------EQG
IDAGGVTREW---YQVL----SRQMFNPDY--------ALFTPVVSDET-----------
----------------TFHPNRTS------------------------------------
--YINPEHLSFFKFIGRIIGKAIYDNCFLDCHFSR--AVYKRILG---------------
--------KPQSLKDMET----------------------LDLEYFKS--------LMWM
LEN--DIT-DVI--
-TEDFSVETDD---------------------------YGE-HKIID-LIPN--------
GRNIP---
------------------------------VTEENKN-EYVKK-VVEYRLQTS-VEEQME
-NFLIG---FHEIIPK--DLVA-IFDEK-ELELLISG-----------------------
----LPDID-----------VSDW----QNHTSYNN-YSPL-------------------
------------------------SLQI-QWFWRAVK--------------------SFD
NEERARLLQF-------ATGTSKVP------------LNG-----FK-ELSGAS--GTCK
FSIHRDYGST------------------------DRLPSSHTCF-NQ-IDLPAY-DCYET
LRGSLL-MAITE-----
>Lodderomyces_elongisporus_XM_001528734.1 .
VDVRREQV-FLDSYRSL--------------FFKNKDEFKNSKLEITFKG-------ELG
IDAGGVTREW---YQVL----SRQMFNPDY--------ALFTPVVSDSN-----------
----------------TFHPNRTS------------------------------------
--YINPEHLSFFKFIGRIIGKAIYDNCFLDCHFTR--AVYKRILG---------------
--------QPQSLKDMET----------------------LDLEYYKS--------LLWM
LEN--DIT-DVI--
-TETFSVETDD---------------------------YGE-HKVID-LIEN--------
GRDTP---
------------------------------VTEENKH-EYVKK-VVEYKLQTS-VEEQME
-NFLIG---FHEIIPK--ELVA-IFDEK-ELELLISG-----------------------
----LPDID-----------VHDW----QLHSTYSN-YSPS-------------------

------------------------SLQI-QWFWRAVK--------------------SFD
NEERARLLQF-------ATGTSKVP------------LNG-----FK-ELSGAS--GTSK
FSIHRDYGST------------------------DRLPSSHTCF-NQ-IDLPAY-ENYET
LRGALL-MAITEGHEGF
>Candida_orthopsilosis_XM_003866881.1 .
ISIRRDQV-FLDSYRAL--------------FFKSKDEFKNSKLEINFKG-------EQG
IDAGGVTREW---YQVL----SRQMFNPDY--------ALFLPVVSDKT-----------
----------------TFHPNRTS------------------------------------
--YVNPEHLSFFKFIGRIIGKAIYDNCFLDCHFSR--AVYKQILG---------------
--------QPQSLKDMET----------------------LDLEYYKS--------LIWM
LEN--DIT-DVI--
-TETLSVETDD---------------------------YGE-HKVID-LIPN--------
GSNIP---
------------------------------VTEENKQ-LYVKK-VVEYRLQTS-VEEQME
-NFLIG---FHEIIPK--DLVA-IFDEK-ELELLISG-----------------------
----LPDID-----------VHDW----QSHTQYVN-YSPS-------------------
------------------------SVQI-QWFWRAVK--------------------SFD
NEERARLLQF-------ATGTSKVP------------LNG-----FK-ELTGAS--GTCK
FSIHRDYGAT------------------------DRLPSSHTCF-NQ-IDLPAY-ENYET
LRGSLL-MAITEGHEGF
>Candida_metapsilosisCBZN020000072.1 .
ISIRRDQV-FLDSYRAL--------------FFKPKDEFKNSKLEINFKG-------EQG
IDAGGVTREW---YQVL----SRQMFNPDY--------ALFLPVVSDKT-----------
----------------TFHPNRTS------------------------------------
--YVNPEHLSFFKFIGRIIGKAIYDNCFLDCHFSR--AVYKRILG---------------
--------QPQSLKDMET----------------------LDLEYYKS--------LLWM
LEN--DIT-DVI--
-TETLSVETDD---------------------------YGE-HKVID-LIPN--------
GSNIP---
------------------------------VTEENKQ-LYVKK-VVEYRLQTS-VEEQME
-NFLIG---FHEIIPK--DLVA-IFDEK-ELELLISG-----------------------
----LPDID-----------VHDW----QSHTQYVN-YSPS-------------------
------------------------SVQI-QWFWRAVK--------------------SFD
NEERARLLQF-------ATGTSKVP------------LNG-----FK-ELTGAS--GTCK
FSIHRDYGAT------------------------DRLPSSHTCF-NQ-IDLPVY-ENYET
LRGSLL-MAITEGHEGF
>Candida_parapsilosis_HE605206.1 .
ISIRRDQV-FLDSYRAL--------------FFKPKDEFKNSKLEINFKG-------EQG
IDAGGVTREW---YQVL----SRQMFNPDY--------ALFLPVVSDKT-----------
----------------TFHPNRTS------------------------------------
--YVNPEHLSFFKFIGRIIGKAIYDNCFLDCHFSR--AVYKRILG---------------
--------QPQSLKDMET----------------------LDLEYYKS--------LIWM
LEN--DIT-DVI--
-TETLSVETDD---------------------------YGE-HKVID-LIRD--------
GSNIP---
------------------------------VTEENKQ-LYVKK-VVEYRLQTS-VEEQME
-NFLIG---FHEIIPK--DLVA-IFDEK-ELELLISG-----------------------
----LPDID-----------VHDW----QSHTQYVN-YSAS-------------------
------------------------SVQI-QWFWRAVK--------------------SFD
NEERARLLQF-------ATGTSKVP------------LNG-----FK-ELTGAS--GTCK
FSIHRDYGAT------------------------DRLPSSHTCF-NQ-IDLPAY-ENYET
LRGSLL-MAITE-----
>Priceomyces_haplophilus_BCIF01000003.1 .
VSIRRDQV-FLDSYRAL-------------FFKPSDEFKKAKLEISFKG-------EAG
VDAGGVTREW---YQVL----SRQMFNPDY--------ALFTPVSSDDT-----------
----------------TFHPNRTS------------------------------------
--YINPEHLSFFKFIGRIIGKAIYDGSFLDSHFSR--AVYKRILG---------------
--------IPVSLKDMET----------------------LDLEYFKS--------LMWM

LEN--DIT-DVI--
-TEDFSVETDD---------------------------YGE-HKVID-LIEN--------
GRNIP---
------------------------------VTDQNKQ-EYVRR-VIEYKLQTS-VAEQMD
-NFLMG---FHEIIPK--DLVS-IFDEQ-ELELLISG-----------------------
----LPDID-----------VADW----QNNTVHSN-YSPS-------------------
------------------------SLQI-QWFWRAVK--------------------SFD
NEERAKLLQF-------ATGTSKVP------------LNG-----FK-ELSGAD--GTCK
FSIHRDYGST------------------------DRLPSSHTCF-NQ-IDLPAY-TSYEM
LRGSLL-LAITEGHEGF
>Hyphopichia_burtonii_XM_020222480.1 .
VNIRRDQV-FLDSYRAL--------------FFKSRDEFRDSKLDINFKG-------ESG
IDAGGVTREW---YQVL----SRQMFNPDY--------ALFTPVASDET-----------
----------------TFHPNRTS------------------------------------
--YINPEHLSFFKFIGRIIGKAIYDGSYLDCHFSR--AVYKRILG---------------
--------KQVSLKDMEN----------------------LDLEYFKS--------LMWM
LEN--DIT-DVI--
-TEDFLVETDD---------------------------YGE-HKIID-LVPN--------
GRNIP---
------------------------------VTEENKN-EYVKL-VVEYRLTTS-VSEQMD
-NFISG---FHEIIPK--DLVS-IFGEQ-ELELLISG-----------------------
----LPDIN-----------VDDW----KNNTTYNN-YSPS-------------------
------------------------SIQI-QWFWRAIK--------------------SFD
NEERAKLLQF-------ATGTSKVP------------LNG-----FK-ELSGAN--GTCK
FSIHRDYGST------------------------DRLPSSHTCF-NQ-IDLPAY-ETYET
LRGSLL-LAFTEGHEGF
>Candida_homilentoma_BCGB01000004.1 .
INVRRDQV-FLDSYRAL--------------FFKSRDEFRDSKLEITFKG-------ESG
IDAGGVTREW---YQVL----SRQMFNPDY--------ALFTPVASDET-----------
----------------TFHPNRTS------------------------------------
--YINPEHLSFFKFIGRIIGKAIYDGSYLDCHFSR--AVYKRILG---------------
--------KQVSLKDMET----------------------LDLEYFKS--------LMWM
LEN--DIT-DVI--
-TEDFSVETDD---------------------------YGE-HKVID-LIPD--------
GRNIP---
------------------------------VTEANKQ-EYVKL-VVEYRLTTS-VTEQMD
-NFITG---FHEIIPK--DLVS-IFDEQ-ELELLISG-----------------------
----LPDIS-----------VDDW----KNNTTYNN-YSPS-------------------
------------------------SIEI-QWFWRAVK--------------------SFD
NEERAKLLQF-------ATGTSKVP------------LNG-----FK-ELSGAS--GTCK
FSIHRDYGST------------------------DRLPSSHTCF-NQ-IDLPAY-ETYET
LRGSLL-LAITEGHEGF
>Sugiyamaella_xylanicola_MQSX01000019.1 .
INIRRDQV-FLDSYRSL--------------FFKSKDEFRTSKLEVAFKG-------EAG
IDAGGVTREW---YQVL----SRQMFNPDY--------ALFTPVASDVT-----------
----------------TFHPNRTS------------------------------------
--YVNPEHLSFFKFIGRIIGKAIYDNCFLDCHFSR--AVYKRILG---------------
--------IPVSLKDMET----------------------LDLEYFKS--------LIWM
LEN--DIT-DII--
-TEDFSVETDD---------------------------YGE-HKIIE-LIPN--------
GSNIP---
------------------------------VTEENKN-DYVRK-VVEYRLQTS-VSEQMD
-NFLIG---FHEIIPK--DLVS-IFDEQ-ELELLISG-----------------------
----LPDID-----------VQDW----QYNTTYNN-YSAS-------------------
------------------------SVQI-QWFWRAVK--------------------SFD
NEQRARLLQF-------ATGTSKVP------------LNG-----FK-ALGGAYG-GTCK
FSIHRDYGST------------------------DRLPSSHTCF-NQ-IDLPAY-ENYET
LRGSLL-LAITEGHEGF

```
>Debaryomyces_hansenii_XM_461787.1 .
ISIRRDQV-FLDSYRAL--------------FFKPKDEFKNSKLEVNFKG-------ESG
IDAGGVTREW---YQVL----SRQMFNPDY--------ALFTPVASDET-----------
----------------TFHPNRTS------------------------------------
--YINPEHLSFFKFIGKIIGKAIFDNSFLDCHFSR--AVYKRILG---------------
--------KSVSLKDMET----------------------LDLEYFKS--------LVWM
LEN--------------------------------------------------DIT-DVI--
-TEDFSVETDD---------------------------YGE-HKIID-LIPN--------
GRDIA-------------------------------------------------------
------------------------------VTEENKH-EYVKL-VVQYRLQTS-VTEQMD
-NFLLG---FHEIISK--DLVS-IFDEQ-ELELLISG-----------------------
----LPDID-----------VLDW----QNNSTYNN-YSPS-------------------
------------------------SEQI-QWFWRAVK--------------------SFD
NEERAKLLQF-------ATGTSKVP------------LNG-----FK-ELSGAS--GTCK
FSIHRDYGTT------------------------DRLPSSHTCF-NQ-IDLPAY-ESYET
LRGSVL-LAITEGHEGF
>Debaryomyces_fabryi_XM_015609004.1 .
INIRRDQV-FLDSYRAL--------------FFKPKDEFKNSKLEVNFKG-------ESG
IDAGGVTREW---YQVL----SRQMFNPDY--------ALFTPVASDET-----------
----------------TFHPNRTS------------------------------------
--YINPEHLSFFKFIGKIIGKAIFDNSFLDCHFSR--AVYKRILG---------------
--------KSVSLKDMET----------------------LDLEYFKS--------LVWM
LEN--------------------------------------------------DIT-DVI--
-TEDFSVETDD---------------------------YGE-HKIID-LIPN--------
GRDIA-------------------------------------------------------
------------------------------VTEENKH-EYVKL-VVQYRLQTS-VTEQMD
-NFLLG---FHEIISK--DLVS-IFDEQ-ELELLISG-----------------------
----LPDID-----------VLDW----QNNTTYNN-YSPS-------------------
------------------------SEQI-QWFWRAVK--------------------SFD
NEERAKLLQF-------ATGTSKVP------------LNG-----FK-ELSGAS--GTCK
FSIHRDYGTT------------------------DRLPSSHTCF-NQ-IDLPAY-ESYET
LRGSVL-LAITEGHEGF
>Candida_tanzawaensis_XM_020210918.1 .
ISIRRDQV-FLDSYRSL--------------FFKSKEEFKNSKLEVNFKG-------ETG
VDAGGVTREW---YQVL----SRQMFNPDY--------ALFTPVASDET-----------
----------------TFHPNRTS------------------------------------
--FVNPEHLSFFKFIGRIIGKAIHDGSFLDCHFSR--AVYKRILS---------------
--------RPVSLKDMET----------------------LDLEYFKS--------LMWM
LEN--------------------------------------------------DIT-DVL--
-TEDFSVETDD---------------------------YGE-HKVID-LIPD--------
GRNIP-------------------------------------------------------
------------------------------VTEENKQ-EYVSK-VVEYRLQTS-VTEQMD
-NFLIG---FHEIIPK--DLVS-IFDEQ-ELELLISG-----------------------
----LPDID-----------VNDW----QNNCTYNN-YSPS-------------------
------------------------SLQI-QWFWRAVK--------------------SFD
NEERAKLLQF-------ATGTSKVP------------LNG-----FK-ELSGAN--GTCK
FSIHRDYGTT------------------------DRLPSSHTCF-NQ-IDLPAY-ESYET
LRGSLL-LAITEGHEGF
>Wickerhamia_fluorescens_BCGE01000001.1 .
ISVRRDQV-FLDSYRAL--------------FFKSKDEFRHSKLEINFKG-------EAG
VDAGGVTREW---YQVL----SRQMFNPDY--------ALFTPVASDET-----------
----------------TFHPNRTS------------------------------------
--YINPEHLSFFKFIGRIIGKAIYDCSFLDCHFSR--AVYKRILG---------------
--------RPVSLKDMET----------------------LDLEYFKS--------LMWM
LEN--------------------------------------------------DIT-DVI--
-TEDFSVETDD---------------------------YGE-HKIID-LIPN--------
GRNIP-------------------------------------------------------
------------------------------VSEENKQ-EYVLK-VVEYRLQTS-VAEQMD
```

```
-NFLIG---FHEIIPK--DLVA-IFDEQ-ELELLISG-----------------------
----LPDIN-----------VADW----QNNCTYNN-YSAS-------------------
------------------------SIQI-QWFWRAVK--------------------SFD
NEERAKLLQF-------ATGTSKVP------------LNG-----FK-ELSGAN--GTCK
FSIHRDYGSL------------------------DRLPSSHTCF-NQ-IDLPAY-ETYET
LRGSLL-LAITEGHEGF
>Scheffersomyces_stipitis_XM_001382813.1 .
INVRREQV-FLDSYRSL--------------FFKSKDEFRNSKLEINFKG-------ESG
VDAGGVTREW---YQVL----SRQMFNPDY--------ALFSPVASDET-----------
----------------TFHPNRTS------------------------------------
--YVNPEHLSFFKFIGRVIGKAIYDNCYLDCHFSR--AVYKRILG---------------
--------RPVSLKDMET----------------------LDLEYFKS--------LMWM
LEN------------------------------------------------DIT-DVI--
-TEDFSVETDD---------------------------YGE-HKIID-LIPN--------
GRNIP-------------------------------------------------------
------------------------------VTEENKH-DYVKK-VVEYRLQTS-VAEQMD
-NFLIG---FHEIIPK--ELVA-IFDEQ-ELELLISG-----------------------
----LPDIS-----------VIDW----QSHTTYNN-YSPS-------------------
------------------------SLQI-QWFWRAVK--------------------SFD
NEERAKLLQF-------ATGTSKVP------------LNG-----FK-ELSGAN--GTCK
FSIHRDYGLT------------------------ERLPSSHTCF-NQ-IDLPAY-ETYET
LRGSLL-LAITEGHEGF
>Scheffersomyces_shehatae_BDMO01000002.1 .
INIRREQV-FLDSYRSL--------------FFKSRDEFRDSKLEINFKG-------EAG
VDAGGVTREW---YQVL----SRQMFNPDY--------ALFTPVASDDT-----------
----------------TFHPNRTS------------------------------------
--YINPEHLSFFKFIGRIIGKAIYDNSYLDCHFSR--AVYKRLLG---------------
--------RPVSLKDMET----------------------LDLEYFKS--------LMWM
LEN------------------------------------------------DIT-DVI--
-TEDFSVETDD---------------------------YGE-HKIID-LVPN--------
GRNIP-------------------------------------------------------
------------------------------VTEENKH-DYVKK-VVEYRLQTS-VAEQMD
-NFLIG---FHEIIPK--ELVA-IFDEQ-ELELLISG-----------------------
----LPDIS-----------VQDW----QNNSVYNN-YSPS-------------------
------------------------SLQI-QWFWRAVK--------------------SFD
NEERAKLLQF-------ATGTSKVP------------LNG-----FK-ELSGAN--GTCK
FSIHRDYGST------------------------DRLPSSHTCF-NQ-IDLPAY-ETYET
LRGSLL-LAITEGHEGF
>Scheffersomyces_lignosus_BCGS01000003.1 .
INIRREQV-FLDSYRSL--------------FFKSRDEFRDSKLEINFKG-------EAG
VDAGGVTREW---YQVL----SRQMFNPDY--------ALFTPVASDET-----------
----------------TFHPNRTS------------------------------------
--YINPEHLSFFKFIGRIIGKAIYDNSYLDCHFSR--AVYKRLLG---------------
--------RPVSLKDMET----------------------LDLEYFKS--------LMWM
LEN------------------------------------------------DIT-DVI--
-TEDFSVETDD---------------------------YGE-HKIID-LVPN--------
GRNIP-------------------------------------------------------
------------------------------VTEENKH-DYVKK-VVEYRLQTS-VAEQMD
-NFLIG---FHEIIPK--DLVS-IFDEQ-ELELLISG-----------------------
----LPDIS-----------VQDW----QNNSVYNN-YSPS-------------------
------------------------SLQI-QWFWRAVK--------------------SFD
NEERAKLLQF-------ATGTSKVP------------LNG-----FK-ELSGAN--GTCK
FSIHRDYGST------------------------DRLPSSHTCF-NQ-IDLPAY-ETYET
LRGSLL-LAITEGHEGF
>Candida_tenuis_XM_006684551.1 .
INVRRDQV-FLDSYRAL--------------FFKSNDEFKNSRLDVNFKG-------ESG
IDAGGVTREW---YQVL----SRQMFNPDY--------ALFTPVSSDSN-----------
----------------TYHPNRTS------------------------------------
```

--YINPEHLSFFKFIGKIIGKAIFDGCFLDCHFSR--AVYKQILG---------------
--------RSVSFKDMEA----------------------LDLEYFKS--------LIWI
LEN--DIT-DVI--
-TEDFLVETDD---------------------------YGE-KKIID-LIPN--------
GRNIP---
------------------------------VTEDNKQ-EYVKF-VVEYRLQRS-VSEQMD
-NFLIG---FHEMIPK--DLVS-IFDEQ-ELELLISG-----------------------
----LPDID-----------VQDW----QNNTIYNN-YSPS-------------------
------------------------SLQI-QWFWRAVK--------------------SFD
NEERAKLLQF-------ATGTSRVP------------LNG-----FK-ELKGAN--DGSK
FSIHRDYGSI------------------------ERLPSSHTCF-NQ-IDLPAY-ESYET
LRGSLL-LAITEGHEGF
>Millerozyma_acaciae_BCKO01000002.1 .
ISVRRDQV-FLDSYRAL--------------FFKSKDEFKNSTLEVTFKG-------ESG
IDAGGITREW---YQVL----SRQMFNPDY--------ALFTPVASDET-----------
----------------TFHPNRTS------------------------------------
--YVNPEHLSFFKFIGKIIGKAIFDGSFLDCHFSR--AVYKRILG---------------
--------KSVSLKDMET----------------------LDLEYFRS--------LMWM
LEN--DIT-DVI--
-TEDFSVETDD---------------------------YGE-HKIID-LIPN--------
GRNIS---
------------------------------VTEENKQ-EYVKL-VVEYRLQTS-VIDQMD
-NFLMG---FHEIIPK--DLVS-IFDEQ-ELELLISG-----------------------
----LPDID-----------VIDW----QNNTEYVN-YSPS-------------------
------------------------NEQI-EWFWRAVK--------------------SFD
NEERAKLLQF-------ATGTSKVP------------LNG-----FK-ELTGSG--GSNK
FSIHRDFGKT------------------------DRLPSSHTCF-NQ-IDLPAY-ESYEA
LRGSLL-LAITEGHEGF
>Pichia_sorbitophila_FO082057.1 .
ITIRRDQV-FLDSYRAL--------------FFKSKDEFKNSTLEVNFKG-------ESG
IDAGGVTREW---YQVL----SRQMFNPDY--------ALFTPVASDET-----------
----------------TFHPNRTS------------------------------------
--YVNPEHLSFFKFIGKVIGKAIFDNCFLDCHFSR--AVYKRILG---------------
--------KPVSLKDMET----------------------LDLEYFRS--------LMWM
LEN--DIT-DVI--
-TEDFSVETDD---------------------------YGE-HKIID-LIPN--------
GRNID---
------------------------------VTEENKH-EYVKL-VVEYRLQTS-VLEQMD
-HFLQG---FHEIIPK--ELIA-IFDEQ-ELELLISG-----------------------
----LPDID-----------VTDW----QNNTVYNN-YSPS-------------------
------------------------TEQI-QWFWRAVK--------------------SFD
NEERAKLLQF-------ATGTSKVP------------LNG-----FK-ELTGSN--GTCK
FSIHRDYGST------------------------DRLPSSHTCF-NQ-IDLPAY-ESYEM
LRGALL-LAIRE-----
>Candida_carpophila_BCGK01000003.1 .
INVRRDQV-FLDSYRAL--------------FFKSKEEVRKSKLEISFKG-------ESG
VDAGGVTREW---YQVL----SRQMFNPDY--------ALFTPVASDET-----------
----------------TFHPNRTS------------------------------------
--FINPEHLSFFKFIGMIIGKAIYDSNFLDCHFSR--AVYKRLLG---------------
--------RPVSLKDMET----------------------LDNDYFKS--------LMWM
LEN--DIT-DVI--
-TEDFSVETDD---------------------------YGE-HKVID-LIEN--------
GHNIP---
------------------------------VTEENKQ-EYVKL-VVEYRLQTS-VAEQMN
-NFLAG---FHDMIPK--DLVL-IFDEQ-ELELLISG-----------------------
----LPDID-----------VSDW----KSNTEYHN-YSPS-------------------
------------------------SIQI-QWFWRAVM--------------------SFD
NEERAKLLQF-------ATGTSKVP------------LNG-----FK-ELSGSN--GICK

FSIHRDYGST------------------------DRLPSSHTCF-NQ-IDLPAY-ETYET
LRGSLL-LAITEGHEGF
>Meyerozyma_guilliermondii_XM_001485141.1 .
INVRRDQV-FLDSYRAL--------------FFKSKDEVRKSKLEISFKG-------ESG
VDAGGVTREW---YQVL----SRQMFNPDY--------ALFTPVASDET-----------
----------------TFHPNRTS------------------------------------
--FINPEHLSFFKFIGMIIGKAIYDSNFLDCHFSR--AVYKRLLG---------------
--------RPVSLKDMET----------------------LDNDYFKS--------LMWM
LEN--DIT-DVI--
-TEDFSVETDD---------------------------YGE-HKVID-LIEN--------
GHNIP---
------------------------------VTEENKQ-EYVKL-VVEYRLQTS-VAEQMN
-NFLAG---FHDMIPK--DLVL-IFDEQ-ELELLISG-----------------------
----LPDID-----------VSDW----KSNTEYHN-YSPS-------------------
------------------------SIQI-QWFWRAVM--------------------SFD
NEERAKLLQF-------ATGTSKVP------------LNG-----FK-ELSGSN--GISK
FSIHRDYGTT------------------------DRLPSSHTCF-NQ-IDLPAY-ETYET
LRGSLL-LAITEGHEGF
>Metschnikowia_bicuspidata_XM_018856760.1 .
VSIRRDQV-FLDSYRAL--------------FFKSVQDFKKAHLEINFKG-------EAG
IDAGGVTREW---YQVL----SRQMFNPDY--------ALFTPVASDEN-----------
----------------TYHPNRTS------------------------------------
--YINPEHLSFFKFIGRTIGKAIYDGCFLDCHFSR--AVYKKILD---------------
--------RSVSLKDMEN----------------------LDLEYFKS--------LMWM
LEN--DIT-DII--
-TEDFSVETDD---------------------------YGE-HKVID-LVPD--------
GRNIP---
------------------------------VDESNKH-EYVRL-VVEYRLQTS-VNEQMQ
-NFITG---FHEIIPR--DLVA-IFDEQ-ELELLISG-----------------------
----LPDID-----------VQDW----QNNTNYHN-YSAS-------------------
------------------------SEQI-QWFWRAVK--------------------SFD
NEERAKLLQF-------STGTSKVP------------LNG-----FK-DLRGAN--GVCK
FSIHRDYGAK------------------------DRLPSSHTCF-NQ-IDLPVY-ESYET
LRGSLL-LALSEGHEGF
>Metschnikowia_australis_MVNQ01000024.1 .
VSIRRDQV-FLDSYRAL--------------FFKSVQEFKKAHLEINFKG-------EAG
IDAGGVTREW---YQVL----SRQMFNPDY--------ALFTPVASDEN-----------
----------------TYHPNRTS------------------------------------
--YINPEHLSFFKFIGRTIGKAIYDGCFLDCHFSR--AVYKKILD---------------
--------RSVSLKDMEN----------------------LDLEYSKS--------LMWM
LEN--DIT-DII--
-TEDFSVETDD---------------------------YGE-HKTID-LVPN--------
GRNIP---
------------------------------VDQSNKH-EYVRL-VVEYRLQTS-VNEQMQ
-NFITG---FHEVIPR--DLVA-IFDEQ-ELELLISG-----------------------
----LPDID-----------VQDW----KNNTNYHN-YSAS-------------------
------------------------SEQI-QWFWRAVK--------------------SFD
NEERAKLLQFS-------TGTSKVP------------LNG-----FK-ELRGAN--GVCK
FSIHRDYGAK------------------------DRLPSSHTCF-NQ-IDLPVY-ESYET
LRGSLL-LALSEGHEGF
>Metschnikowia_fructicola_ANFW02000030.1 .
VSIRREQV-FLDSYRAL--------------FFKSVEEFKKAHLEINFKG-------EAG
IDAGGVTREW---YQVL----SRQMFNPDY--------ALFTAVASDEN-----------
----------------TYHPNRTS------------------------------------
--YINPEHLSFFKFIGRTIGKAIYDGCFLDCHFSR--AVYKKILD---------------
--------QSVSLKDMES----------------------LDLEYYKS--------LIWM
LEN--DIT-DII--
-TEDFSVETDD---------------------------YGE-HKIID-LVPD--------

```
GRNIP-------------------------------------------------------
------------------------------VTEENKQ-DYVRL-VVEYRLQTS-VNEQMQ
-NFITG---FHEIIPR--ELVA-IFDEQ-ELELLISG-----------------------
----LPDID-----------VQDW----QNNTNYNN-YSAS-------------------
------------------------SEQI-QWFWRAVK--------------------SFD
NEERAKLLQFS-------TGTSKVP------------LNG-----FK-DLRGAN--GVCK
FSIHRDYGAK------------------------DRLPSSHTCF-NQ-IDLPVY-ESYET
LRGSLL-LAVSEGHEGF
>Candida_auris_XM_018312725.1 .
ISIRRDQV-FLDSYRAL--------------FFKSVEEFRKAKLEINFKG-------ESG
IDAGGVTREW---YQVL----SRQMFNPDY--------ALFSAIASDET-----------
----------------TFHPNRTS------------------------------------
--YVNPEHLSFFKFIGRIIGKAIFDGSFLDCHFSR--AVYKKILD---------------
--------RPMSLKDMET----------------------LDLEYFKS--------LMWM
LDN------------------------------------------------DIT-DII--
-TEDFSVESDD---------------------------YGE-HKIID-LIPN--------
GRNVP-------------------------------------------------------
------------------------------VTEENKH-EYVRL-VVEYRLLKS-VEEQMN
-NFSTG---FHEIIPK--DLVA-IFDEQ-ELELLISG-----------------------
----LPDID-----------VGDW----QANTSYNN-YSPS-------------------
------------------------SEQI-QWFWRAVK--------------------SFD
NEERAKLLQF-------ATGTSKVP------------LNG-----FK-ELRGAS--GTCK
FSIHRDYGST------------------------DRLPSSHTCF-NQ-IDLPAY-ESYET
LRGSLL-LAITEGHEGF
>Clavispora_lusitaniae_XM_002618067.1 .
ISIRRDQV-FLDSYRGL--------------FFKSVDTFRNAVLEINFKG-------EAG
VDAGGVTREW---YQVL----SRQMFNPDY--------ALFTAVASDET-----------
----------------TFHPNRTS------------------------------------
--YINPEHLSFFKFIGRIIGKAIFDNCFLDCHFSR--AVYKKILD---------------
--------RPVSLKDMEN----------------------LDLEYFKS--------LMWM
LEN------------------------------------------------DIT-DII--
-TEDFSVETDD---------------------------YGE-HKIID-LIPN--------
GRNIP-------------------------------------------------------
------------------------------VTEENKQ-EYVRL-VVEYRLQTS-VAEQMN
-NFITG---FHEIIPR--DLVA-IFDEQ-ELELLISG-----------------------
----LPDID-----------VQDW----QNNTTYVN-YSPS-------------------
------------------------SEQI-QWFWRSVK--------------------SFD
NEERAKLLQF-------ATGTSKVP------------LNG-----FK-ELRGAN--GGCK
FSIHRDYGST------------------------DRLPSSHTCF-NQ-IDLPAY-ETYET
LRGSLL-LAITEGHEGF
>Candida_intermedia_LT635758.1 .
INVRRDQV-FLDSYRSL--------------FFKSVEEFRNSRLEVNFKG-------EAG
IDAGGVTREW---YQVL----SRQMFNPDY--------ALFTAVASDET-----------
----------------TFHPNRTS------------------------------------
--YINPEHLSFFKFIGRIIGKAIFDDCFLDCHFSR--AVYKKILD---------------
--------RPVSLKDMEN----------------------LDLEYFKS--------LMWM
LEN------------------------------------------------DIT-DII--
-TEDFSVETDD---------------------------YGE-HKIID-LVPD--------
GRNIP-------------------------------------------------------
------------------------------VTEANKQ-EYVRL-VVEYRLQTS-VIEQMN
-NFIAG---FHEIIPR--DLIA-IFDEQ-ELELLISG-----------------------
----LPDID-----------VNDW----QNNTTYVN-YSPS-------------------
------------------------SEQI-QWFWRAVK--------------------SFD
NEERAKLLQF-------ATGTSKVP------------LNG-----FK-ELRGAN--GGCK
FSIHRDYGST------------------------ERLPSSHTCF-NQ-VDLPAY-DSYET
LRGSLL-LAITE-----
>Candida_aaseri_LKAN01000058.1 .
INVRREQV-FLDSYRAL--------------FFKSLDQFRNSKLDINFKG-------ESG
```

IDAGGVTREW---YQVL----SRQMFNPDY--------ALFTPVSAN-------------
----------------TYHPNRTS------------------------------------
--YINPEHLLFFKFIGKIIGKAIYDNCFLDCHFTR--AIYKRILG---------------
--------INLSLKDMET----------------------LDLEYYKS--------LMWM
LEN--DIT-DVI--
-TEDFSVEIDD---------------------------YGE-KKIID-LIPG--------
GRNIP---
------------------------------VTEENKK-EYVQK-VVDYRLMTS-VEEQLN
-HFLQG---FHEIIPK--ELIS-IFNEQ-ELELLISG-----------------------
----LPDID-----------VDDW----QYNTVYNN-YSPS-------------------
------------------------SPQI-QWFWRAVR--------------------SFD
NEERAKLLQF-------STGTSKVP------------LNG-----FK-ELRGSN--GSSN
FSIHRDYGST------------------------DRLPSSHTCF-NQ-IDLPAY-ESYEA
LRGNLL-MAIREGYEGF
>Pachysolen_tannophilus_CAHV01000055.1 .
VNVRRSQV-FLDSYRSL--------------FFKSSEEFRNAKLDIKFRG-------EAG
VDAGGLTREW---YQVL----SRQMFNPDY--------ALFTPVASDKT-----------
----------------TFHPNRTS------------------------------------
--WVNPEHLSFFKFIGRVIGKAVCDNCYLDCHFSR--AVYKCILG---------------
--------DQLSLKDMET----------------------LDLDYYKS--------LVWM
LEN--DIT-DII--
-VETFSVETDD---------------------------YGE-HKIVD-LIPN--------
GRNIA---
------------------------------VSEENKQ-EYVRL-IVEYRLQSS-VAEQMD
-NFLQG---FHEIIPK--DLIA-IFNEQ-ELELLISG-----------------------
----LPDID-----------IDDW----KTNTTYNN-YSAS-------------------
------------------------SPQI-QWFWRAVR--------------------SFD
AEERAKLLQF-------ATGTSKVP------------LHG-----FK-ELSGVD--GVSK
FSIHRDYGST------------------------DRLPSSHTCF-NQ-IDLPEY-ESYET
LRGSLL-LAITEGREGF
>2_Nakazawaea_peltata_BCGQ01000004.1 .
VNVRRDQV-FLDSFRSL--------------FYKPTVDFKNSKLDIKFKG-------EEG
VDAGGVTREW---YQVL----SRQMFNPDY--------ALFTPVAADKT-----------
----------------TFHPNRTS------------------------------------
--WVNPEHLSFFKFIGRIIGKAVFDGNVLDCHFSR--AVYKSILG---------------
--------IPVSLKDMET----------------------LDLDYYKS--------LVWM
LEN--DIT-DII--
-VETFSVETDD---------------------------YGE-HKIID-LIPD--------
GRNIP---
------------------------------VDESNKQ-DYVHR-IVEYRLQTS-IAEQLE
-NFLLG---FHEVIPK--DLIA-IFSEQ-ELELLVSG-----------------------
----LPDID-----------VDDW----KNNTTYTN-YSAS-------------------
------------------------SPQI-QWFWRAVR--------------------SFD
VEERAKLLQF-------ATGTSKVP------------LHG-----FK-ELAGVN--GVSK
FSVHRDYGST------------------------DRLPSSHTCF-NQ-IDLPEY-DSYET
LRAALL-VAINEGREGF
>Babjeviella_inositovora_XM_019129949.1 .
VSVGRDLV-FLDSYRAL--------------FFKSKEEMKNAKLDIKFKG-------EDG
VDAGGVTREW---YLVL----SRQMFNPDY--------ALFAPVASDKT-----------
----------------TFHPNRTS------------------------------------
--WVNPEHLSFFKFIGRIIGKAIYDGFFLDCHFSR--AVYKQILG---------------
--------CPLSLKDMET----------------------LDLEYFKS--------LIWM
LEN--DIT-DII--
-IEDFSVETDD---------------------------YGE-HKVID-LIPD--------
GRNVP---
------------------------------VTQENKQ-DYVRR-VVAYRLQTS-IAEQMD
-NFLQG---FHDIIPR--DLIA-IFDEQ-ELELLISG-----------------------
----LPDID-----------VDDW----KNNSNYVN-YSGS-------------------

------------------------SPQI-QWFWRAVR--------------------SFD
VEERAKLLQF-------ATGTSKVP------------LNG-----FK-ELSGVN--GIAK
FSIHRDYGKT------------------------DRLPSSHTCF-NQ-IDLPEY-DSYEQ
LRGSLL-LATTEGHEGF
>Komagataella_pastoris_FBUC01000036.1 .
ISVSREQV-FLDSYRAL--------------FFKKVDEIKNSKLEISFKG-------EAG
VDAGGVTREW---YQVL----SRQMFNPDY--------ALFIPVASDKT-----------
----------------TFHPNRTS------------------------------------
--YINPEHLSFFKFIGRIIGKAVYDNCFLDCHFSR--AVYKRILG---------------
--------RQVTLKDMET----------------------LDLDYYKS--------LVWI
LEN--DIT-DII--
-DETFSVESDD---------------------------YGV-HTIVD-LKPN--------
GRNIL---
------------------------------VTEENKQ-EYVRL-ITEYRLQTS-VKEQMN
-NFLIG---FHEIIPK--DLVA-IFGDQ-ELELLISG-----------------------
----LPDID-----------VDDW----KANAVYEN-YSPS-------------------
------------------------SIQI-QWFWRAVR--------------------SFD
VEERAKLLQF-------ATGTSKVP------------LGG-----FK-ELTGVD--GVSK
FSIHRDYGST------------------------DRLPSSHTCF-NQ-IDLPEY-ESYET
LRGSLL-LAVTEGHEGF
>Komagataella_phaffii_XM_002492207.1 .
ISVSREQV-FLDSYRAL--------------FFKKVDEIKNSKLEISFKG-------EAG
VDAGGVTREW---YQVL----SRQMFNPDY--------ALFIPVASDKT-----------
----------------TFHPNRTS------------------------------------
--YINPEHLSFFKFIGRIIGKAVYDNCFLDCHFSR--AVYKRILG---------------
--------RQVTLKDMET----------------------LDLDYYKS--------LVWI
LEN--DIT-DII--
-DETFSVESDD---------------------------YGV-HTIVD-LKPN--------
GRNIL---
------------------------------VTEENKQ-EYVRL-ITEYRLQTS-VKEQMN
-NFLIG---FHEIIPK--DLVA-IFGDQ-ELELLISG-----------------------
----LPDID-----------VDDW----KANAVYEN-YSPS-------------------
------------------------SIQI-QWFWRAVR--------------------SFD
VEERAKLLQF-------ATGTSKVP------------LGG-----FK-ELTGVD--GVSK
FSIHRDYGST------------------------DRLPSSHTCF-NQ-IDLPEY-ESYET
LRGSLL-LAVTEGHEGF
>Sporopachydermia_quercuum_BCGN01000006.1 .
INVRRENV-FLDSYRTL--------------FYKTSEEIKNAKLDVVFRG-------EAG
VDAGGXSREW---YQVL----SRQMFNPDY--------ALFTPVSSDRT-----------
----------------TFHPNRTS------------------------------------
--WVNPEHLSFFKFIGRIIAKAIYDQRFLDCHFSR--AVYKRILG---------------
--------KSVSLKDMET----------------------LDLDYYKS--------MVWM
LEN--DIT-DII--
-VETFSIEADD---------------------------YGE-LKIID-LIPD--------
GRNIA---
------------------------------VTEGNKQ-EYVRL-MIEYKLQTS-IQEQMD
-AFLTG---FHEIIPK--ELIS-IFDEQ-ELELLISG-----------------------
----LPDID-----------VDDW----RNNTSYVN-YTPS-------------------
------------------------SPQI-QWFWRAVR--------------------SFD
VEERAKLLQF-------ATGTSKVP------------LYG-----FK-ELGGVN--GVSK
FSIHRDYGSK------------------------DRLPQSHTCF-NQ-IDLPEY-DSYET
LRGSLL-LAVTEGHEGF
>Kuraishia_capsulata_CBUD020000046.1 .
VNVRRDQV-FLDSYRSL--------------FYRPSNELKDSKLDIQFRG-------EAG
VDAGGVTREW---FQVL----SRQMFNPDY--------ALFTPVASDTT-----------
----------------TFHPNRTS------------------------------------
--YANPEHLSFFKFIGKIIGKALYDGFLLDCHFSR--AVYKRILG---------------
--------RPLTLKDVES----------------------LDPSYFKS--------LIWM

```
LEN------------------------------------------------DIT-DVI--
-EETFSVESDD---------------------------FGE-HKVID-LIEN--------
GHNIP-------------------------------------------------------
------------------------------VTEDNKK-MYVSL-IVEYRLNTS-VEQQMD
-SFLEG---FHQVIPK--ELVS-IFDDQ-ELELLISG-----------------------
----LPDID-----------VDDW----RNNTVYQN-YSPS-------------------
------------------------SPQI-QWFWRAVK--------------------SFD
VEERAKLLQF-------ATGTSKVP------------LHG-----FK-ELSGVS--GVSK
FSIHKDYGSS------------------------DRLPSSHTCF-NQ-IDLPEY-ASYDA
LRGSLL-LAITEGHEGF
>Candida_succiphila_BCGL01000006.1 .
IQVRREQV-FLDSYRAL--------------FFKSTDAIKKNKLEITFRG-------EEG
VDAGGVMREW---YQVL----ARQMFNPDY--------ALFTPVASDST-----------
----------------TFHPNRTS------------------------------------
--YINPEHLSFFKFVGIIIGKAIYDNCLLDCHFSR--AVYKRFLS---------------
--------RPVSLKDMES----------------------LDLDYYKS--------LLWM
LEN------------------------------------------------DIT-DVI--
-VENFSVETDD---------------------------YGE-HKIID-LKEN--------
GRDIP-------------------------------------------------------
------------------------------VTEENKQ-EYVRL-IVEYRLQKS-VQEQMD
-NFLQG---FHSIIPK--DLVS-IFDDQ-ELELLISG-----------------------
----LPEID-----------VDDW----RNNTVYNN-YTAS-------------------
------------------------SPQI-QWFWRAVK--------------------SFD
AEERAKLLQF-------ATGTSKVP------------LNG-----FK-DLVGSVAGPRSP
FSIHRVYGST------------------------EKLPSSHTCF-NQ-IDLPEY-ETYEK
LRSALL-FAITEGHEGF
>Ogataea_methanolica_IAAJ01012358.1 .
VSIRRDQV-FLDSYRAL--------------FFKNPATIRKSKLDIQFRG-------EQG
VDAGGLTREW---YQVL----SRQMFNPDY--------ALFSPVASDKT-----------
----------------TFHPNRTS------------------------------------
--WVNPEHLSFFKFVGIIIGKAVYDGCMLDCHFSR--AVYKLILG---------------
--------RPVSLKDIES----------------------LDLDYYKS--------LVWM
LEH------------------------------------------------DIT-DII--
-VETFSVETDD---------------------------YGE-HKIID-LKED--------
GRNIA-------------------------------------------------------
------------------------------VTDENKH-EYVRL-IVEYRLQTS-VIEQME
-NFLQG---FYQIISK--DLIA-IFDDQ-ELELLISG-----------------------
----LPDID-----------VDDW----KNNTTYQN-YSAS-------------------
------------------------SAQI-QWFWRAVK--------------------SFD
TEEKAKLLQF-------STGTSKVP------------LNG-----FK-ELTGMS--GVSK
FSIHRSYGPT------------------------DRLPSSHTCF-NQ-IDLPEY-ESYEK
LRGSLL-LAITE-----
>Candida_arabinofermentans_LWUO01000244.1 .
VNIRRDQV-FLDSFRAL--------------FFKSPDDIRKAKLDIQFRG-------EQG
VDAGGLTREW---YQVL----SRQMFNPDY--------ALFTPVASDKT-----------
----------------TFHPNRTS------------------------------------
--WVNPEHLSFFKFVGIIIGKAVYDGCMLDCHFSR--AVYKRLLG---------------
--------RPVSLKDIES----------------------LDLDYYKS--------LIWM
LEN------------------------------------------------DIT-DII--
-SETFSVETDD---------------------------YGV-HTTID-LKEN--------
GSNIA-------------------------------------------------------
------------------------------VTEENKQ-EYVRL-IVEYRLQTS-VTDQME
-NFLQG---FHQIIPK--ELIA-VFDDQ-ELELLISG-----------------------
----LPDID-----------VDDW----KNNSTYHS-YTAS-------------------
------------------------SPQI-QWFWRAVK--------------------SFD
TEEKAKLLQF-------ATGTSKVP------------LNG-----FK-ELTGTS--GVSK
FSIHRVYGAT------------------------HKLPTSHTCF-NQ-IDLPEY-ESYEK
LRGSLL-KALTEGYEGF
```

>Ogataea_polymorpha_XM_018356641.1 .
VNVKRDQV-FLDSYRAL--------------FFKSPQDIRKSKLDIQFRG-------EQG
VDAGGLTREW---YQVL----SRQMFNPDY--------ALFTPVASDKT-----------
----------------TFHPNRTS------------------------------------
--WVNPEHLSFFKFVGIIIGKAVYDGCMLDCHFSR--AVYKQILG---------------
--------RPVSLKDIES----------------------LDLDYYKS--------LIWM
LEN--DIT-DII--
-VETFSVETND---------------------------YGE-EKVID-LKPN--------
GRDIA---
------------------------------VTEENKH-EYVRL-IVEYRLKTS-VQEQMD
-NFLKG---FYEIIPK--DLIA-IFDDQ-ELELLISG-----------------------
----LPDID-----------VDDW----KNNTEYQN-YSAS-------------------
------------------------SPQV-QWFWRAVK--------------------SFD
AEEKAKLLQF-------ATGTSKVP------------LNG-----FK-ELPGMV--GVSK
FSIHRVYGST------------------------DRLPSSHTCF-NQ-IDLPEY-ESYEK
LRGSLL-LAIMEGHEGF
>Candida_boidinii_MSRZ01000055.1 .
VNVRRDQV-FLDSYRAL--------------FFKNPEEVRKAKLEIQFKG-------EQG
VDAGGLTREW---YQVL----SRQMFNPDY--------ALFTPVASDKT-----------
----------------TFHPNRTS------------------------------------
--WVNPEHLSFFKFVGTIIGKAVYDNCMLDCHFSR--AVYKRILG---------------
--------RPVSLKDMES----------------------LDLDYYKS--------LVWM
LEN--DIT-DII--
-VETFSVETDD---------------------------YGE-HKIID-LIED--------
GRNVS---
------------------------------VTEENKR-QYVKL-IVEYRLQTS-VKDQME
-NFLQG---FHSVIPK--ELVA-VFDDQ-ELELLISG-----------------------
----LPEID-----------VDDW----KNNTVYQN-YSAS-------------------
------------------------SPQV-QWFWRAVK--------------------SFD
QEEKAKLLQFS-------TGTSKVP------------LNG-----FK-ELTGMS--GVAK
FSIHRVYDNT------------------------DRLPSSHTCF-NQ-VDLPEY-ESYEK
LRNALL-LAITEGHVGF
>Candida_ethanolica_ANNA01000770.1 .
INIRRDQV-FLDSYRSI--------------FFKPREKVRKSILEINFNG-------EEG
VDAGGVTREW---YQVL----SRQIFDPNY--------ALFIPVASDKT-----------
----------------TFHPNRTS------------------------------------
--WVNPEHLSFFKFVGMIIGKAVYDGYVLDCHFSR--AVFKRILG---------------
--------KPVSLKDMES----------------------LDLDYYKS--------LVWM
LEN--DIT-DII--
-VETFSVETDD---------------------------YGE-HKIID-LIPN--------
GRDIA---
------------------------------VTQENKQ-EYVKA-IVEYRLLTS-VKEQMD
-NFLEG---FYSMIPK--DLIS-IFDEQ-ELELLVSG-----------------------
----LPDID-----------VDDW----KNNTIYTT-YLPS-------------------
------------------------SPQV-QWFWRAVK--------------------SFD
AEERAKLLQF-------VTGTSKVP------------LNG-----FK-ELSGMN--GISK
FTIHKTFGTD-------------------------RLPTAHTCF-NQ-LDLPEY-ESYSK
LRNALL-LAIREGHEGF
>Pichia_membranifaciens_XM_019161785.1 .
INIRRNQV-FLDSYRSI--------------FFKPSDKIRKSILEIHFNG-------EEG
VDAGGVTREW---YQVL----SRQMFDPNY--------ALFIPVASDKT-----------
----------------TFHPNRTS------------------------------------
--WVNPEHLSFFKFVGMIIGKAVYDGYVLDCHFSR--AVFKRMLG---------------
--------KPVSLKDMES----------------------LDLDYYKS--------LVWM
LEN--DIT-DII--
-VETFSVETDD---------------------------YGE-HKIID-LIPN--------
GKDIA---
------------------------------VTEENKQ-EYVKA-IVEYRLLTS-VKEQMD

-NFLEG---FYSMIPQ--DLVS-IFDEQ-ELELLVSG----------------------
----LPDID-----------VDDW----KNNTIYEN-YSPS-------------------
------------------------SPQV-QWFWRAVK--------------------SFD
VEERAKLLQF-------ATGTSKVP------------LNG-----FK-ELTGMN--GISK
FSIHRVYNAT-----------------------DRLPTAHTCF-NQ-IDLPEY-QNYTR
LRNALL-LALREGHEGF
>Pichia_kudriavzevii_XM_020688683.1 .
INIRRDQV-FLDSYRNM--------------FYKPTEKVKKSLLDIKFNG-------EEG
VDAGGVTREW---YQVL----SRQIFDPNY--------ALFIPVASDKS-----------
----------------TFHPNRTS------------------------------------
--WVNPEHLSYFKFVGMIIGKAVYDGYVLDCHFSR--AVFKMILS---------------
--------KQVSLKDMES----------------------LDLDYYKS--------LVWM
LEN---DIT-DII--
-VETFSVETDD---------------------------YGE-HKVID-LVPN--------
GSNVS---
------------------------------VTEENKQ-EYVKK-IVEYRLLTS-VKDQMN
-NFLEG---FYSMIPK--DLVS-IFDEQ-ELELLVSG----------------------
----LPDID-----------VDDW----RNNTIYEN-YSPS-------------------
------------------------SPQI-QWFWRAVK--------------------SFD
AEERAKLLQF-------ATGTSKVP------------LNG-----FK-ELVGMN--GISK
FSVHRVYNST-----------------------ERLPTAHTCF-NQ-IDLPEY-ESYGK
LRNALL-LAIREGEEGF
>Candida_sorboxylosa_BCGC01000007.1 .
IKIRRDQV-FLDSYRNI--------------FFKPSDKIKKSILEIHFNG-------EEG
VDAGGVTREW---FQVL----SRQIFDPNY--------ALFVPCASDKT-----------
----------------TFHPNRTS------------------------------------
--WVNPEHLSFFKFVGMIIGKAVYDGYFLDCHFSR--AVFKRLLG---------------
--------KPVSLKDMES----------------------LDLEYYKS--------LVWM
LEN---DIT-DII--
-VEDFSVEADD---------------------------YGE-HKIID-LIPD--------
GRNIS---
------------------------------VTEENKQ-LYVKS-IVEYRLLTS-VKDQMD
-NFLEG---FFSMIPK--DLVS-IFDEQ-ELELLVSG----------------------
----LPDID-----------VDDW----KNNTTYEN-YSPS-------------------
------------------------SQQV-QWFWRAVK--------------------SFD
AEERAKLLQF-------ATGTSKVP------------LDG-----FK-ELMGMN--GVSK
FSIHRVYNST-----------------------DRLPTAHTCF-NQ-IDLPEY-EDYQK
LRNFLL-LAIKEGHEGF
>Ambrosiozyma_monospora_BCIP01000022.1 .
ITISRDQV-FLDSYRAL--------------FFKPPELIRKSNLEIHFRG-------EEG
VDAGGLTREW---YQVL----ARQMFNPDY--------ALFTPVASDKT-----------
----------------TFHPNRTS------------------------------------
--WVNPEHLSFXKFVGMIIGKAVYDGFMLDCHFSR--AVFKQMLG---------------
--------RSVSLKDIES----------------------LDLDYYKS--------LVWM
LEN---DIT-DII--
-VETFSVETDD---------------------------YGE-HKIID-LKEN--------
GRDIA---
------------------------------VTEENKQ-EYVKL-IVEYRLKTS-IKDQIT
-NFLEG---FHQIIPK--DLVS-IFDDQ-ELELLISG----------------------
----LPDID-----------VDDW----KNNSNYVN-YSAS-------------------
------------------------SAQI-QWFWRAVK--------------------SFD
QEQKAKLLQF-------ATGTSKVP------------LNG-----FK-ELSGVN--GVSK
FSIHRVYGAT-----------------------DRLPSAHTCF-NQ-VDLPEY-ESYEK
LRGSLL-LAITEGHEGF
>Ambrosiozyma_kashinagacola_IAAG01003397.1 .
ITIRRDQV-FLDSYRAL--------------FFKAPELVRKSNLEIHFRG-------EEG
VDAGGLTREW---YQVL----ARQMFNPDY--------ALFTPVASDKT-----------
----------------TFHPNRTS------------------------------------

--WVNPEHLSFFKFVGMIIGKAVYDGYMLDCHFSR--AVFKQMLG---------------
--------RPVSLKDIES----------------------LDLDYYKS--------LIWM
LEN--DIT-DII--
-VETFSVETDD---------------------------YGE-HKIID-LKED--------
GRNIA---
------------------------------VTQENKQ-EYVRL-IVEYRLQTS-IKDQIT
-NFLEG---FHQIISK--DLVS-IFDDQ-ELELLISG-----------------------
----LPDID-----------VDDW----KNNTNYVN-YSAS-------------------
------------------------SAQI-QWFWRAVK--------------------SFD
QEEKAKLLQF-------STGTSKVP------------LNG-----FK-ELSGMN--GISK
FSIHRVYDST------------------------DRLPSAHTCF-NQ-IDLPEY-ESYDK
LRGSLL-LAITE-----
>Brettanomyces_naardenensis_MDSA01000001.1 .
VTIRRDQV-FLDSYRAI--------------FFKPPETVRKSNLEIHFRG-------EEG
VDAGGLTREW---YQVL----SRQIFNPDY--------ALFTPVTSDKT-----------
----------------TFHPNRTS------------------------------------
--WVNPEHLSFFKFVGMIIGKAVYDGYMLDCHFTR--AVFKRILG---------------
--------KPVSLKDMES----------------------LDPDYYKS--------LIWM
LEN--DIT-DII--
-VETFSVEADD---------------------------YGE-HKIID-LKEN--------
GRNIP---
------------------------------VTDENKQ-EYVRL-IVEYRLLDS-VKDQMD
-NFLEG---FYQIIPK--DLVA-IFDEQ-ELELLISG-----------------------
----LPDID-----------VDDW----KNNTSYVN-YSAS-------------------
------------------------SSQI-QWFWRAVK--------------------SFN
KEERAKLLQF-------ATGTSKVP------------LNG-----FK-ELSGVN--GVSK
FSIHRVYGDT------------------------DRLPSAHTCF-NQ-IDLPEY-ENYEK
LRAALL-LAVREGHEGF
>Brettanomyces_custersianus_MDVK01000013.1 .
VTVSREQV-FLDSYRAL--------------FFKPVDAVRTSVLEIHFKD-------EEG
VDAGGLTREW---YQVL----SRQMFNPGY--------ALFTPVTSDKT-----------
----------------TFHPNRAS------------------------------------
--WVNPEHLSFFKFVGMIIGKAIYDGYMLDCHFTR--AVFKSILG---------------
--------KPVSLKDMES----------------------LDPDYYKS--------LVWM
LKN--NIT-GII--
-SETFSVEADD---------------------------YGE-HKIID-LKDN--------
GRNIP---
------------------------------VTEENKQ-EYVRL-LVDYRLLTS-VKDQMD
-NFLKG---FYEIVPK--KLIS-IFDER-ELELLISG-----------------------
----LPDID-----------VDDW----KANATYVN-YSSA-------------------
------------------------SAQI-QWFWRAVK--------------------SFD
EEERAKLLQF-------VTGTSKVP------------LNG-----FK-ELSGVN--GISK
FSIHRVYCDT------------------------NRLPTAHTCF-NQ-LDLPEY-ESYGK
LRAALL-LAVKEGHEGF
>Brettanomyces_anomalus_MDSB01000008.1 .
VTIRRDQV-FLDSYRAL--------------FFKPIEKVAKSNLEIHFKG-------EEG
VDAGGLTREW---YQVL----SRQIFNPDY--------ALFTPVTSDKT-----------
----------------TFHPNRTS------------------------------------
--WINPEHLSFFKFVGMIIGKAIYDGYMLDCHFTR--AVFKRLLG---------------
--------KPVSLKDMES----------------------LDPDYYKS--------LVWM
LQN--DIT-DII--
-TETFSVEEDN---------------------------YGE-HKIID-LKEN--------
GRNVP---
------------------------------VTEKNKQ-EYVRL-IVDYRLITS-VKEQLD
-NFLQG---FYQIVPK--SLVA-IFDER-ELELLISG-----------------------
----LPDID-----------VDDW----KNNTNYVN-YSAS-------------------
------------------------SPQI-QWFWRAVK--------------------SFD
TEERAKLLQFS-------TGTSKVP------------LNG-----FK-ELSGVN--GVSK

```
FSIHRTYEST------------------------DRLPTAHTCF-NQ-IDLPAY-ESYGK
LRAALL-LAVREGHEGF
>Brettanomyces_bruxellensis_AHMD01000627.1 .
VTVRRDQV-FLDSYRAL--------------FFKPIDKVAKSSLEIHFKG-------EEG
VDAGGLTREW---YQVL----SRQIFNPDY--------ALFTPVTSDKT-----------
----------------TFHPNRTS------------------------------------
--WINPEHLSFFKFVGMIIGKAIYDGYMLDCHFTR--AVFKRLLG---------------
--------KPVSLKDMES----------------------LDPDYYKS--------LVWM
LQN------------------------------------------------NIT-DII--
-TETFSVEEDN---------------------------YGE-HKVID-LKEN--------
GRNIP-------------------------------------------------------
------------------------------VTEQNKH-EYVRL-IVDYRLITS-VKQQLD
-NFLLG---FYQVVPE--SLVS-IFDER-ELELLISG-----------------------
----LPEID-----------VDDW----KNNTNYVN-YSAS-------------------
------------------------SPQI-QWFWRAVK--------------------SFD
TEERAKLLQFS-------TGSSKVP------------LNG-----FK-ELSGVN--GISK
FSIHRTYEDT------------------------DRLPTAHTCF-NQ-IDLPAY-ENYAK
LRAALL-LAVREGHEGF
>Cyberlindnera_jadinii_XM_020214312.1 .
ISVRRKEV-FLDSYRAL--------------FFKSKDEFRNAKLEINFKG-------EAG
VDAGGVTREW---YQVL----SRQMFNPDY--------ALFLPIASDKT-----------
----------------TFHPNRTS------------------------------------
--WVNPEHLSFFKFVGRIIGKAIYDGCFLDCHFTR--DVYKSILG---------------
--------CKVSLKDVET----------------------IDLNIYKS--------LTWM
LEN------------------------------------------------DIT-DVI--
-IETFSIEADD---------------------------YGE-KKIID-LIPD--------
GRNIA-------------------------------------------------------
------------------------------VTEENKQ-QYVKK-VVEFRVQKS-VEEQVE
-NFLQG---FYEIIPK--TLIS-IFDEQ-ELELLISG-----------------------
----LPDID-----------VDDW----KNNTTYVN-YTAS-------------------
------------------------SREI-SYFWRAVR--------------------SFD
TEERAKLLQF-------ATGTSKVP------------LNG-----FK-ELEGAN--GATK
FNIHKDFGST------------------------DRLPSSHTCF-NQ-IDLPAY-DSYEK
LRKALL-LAINEGHEGF
>Cyberlindnera_fabianii_MPUK01000002.1 .
VNVSRDQV-FLDSYRSL--------------FFKPKEEFRNAKLDISFKN-------EAG
VDAGGVTREW---YQVL----SRQMFNPDY--------ALFLPVASDKT-----------
----------------TFHPNRTS------------------------------------
--WVNPEHLSFFKFIGRVIGKALYDGCFLDCHFSR--DVYKSILG---------------
--------RPVSLKDLET----------------------IDLEYYNS--------LMWM
LEN------------------------------------------------DIT-DVI--
-IETFSVETDD---------------------------YGE-VKTID-LVPG--------
GRDIS-------------------------------------------------------
------------------------------VTEENKH-EYVRL-VVEYRLQKS-VEEQVD
-NFLQG---FHEVIPK--ELIA-IFDEQ-ELELLISG-----------------------
----LPDID-----------VDDW----KNNTTYVN-YNSN-------------------
------------------------SKEV-GYFWRAVR--------------------SFD
KEERAKLLQF-------ATGTSKVP------------LNG-----FK-ELEGAS--GATK
FSIHKDFGRT------------------------DRLPQSHTCF-NQ-IDLPAY-DSYET
LRRALL-IAITEGHEGF
>Wickerhamomyces_anomalus_XM_019181627.1 .
VSIRRDQV-FLDSYRAL--------------FFKSSEEFRNAKLDISFKG-------EAG
IDAGGVTREW---YQVL----SRQMFNPDY--------ALFLPVASDKT-----------
----------------TFHPNRTS------------------------------------
--WVNPEHLSFFKFIGRVIGKAIYDNCFLDCHFSR--DVYKSILG---------------
--------RSVSLKDLET----------------------IDLEYFKS--------LMWM
LEN------------------------------------------------DIT-DII--
-IETFAVETED---------------------------YGE-VKIID-LIPD--------
```

GRNIA---
------------------------------VTEDNKQ-EYVRL-VVEYRLQKS-VEEQVS
-NFLQG---FHEIIPK--ELIS-IFDEQ-ELELLISG-----------------------
----LPDID-----------VDDW----KNNTTYVN-YSPS-------------------
------------------------SKEI-SYFWRAVR--------------------SFD
AEERAKLLQF-------ATGTSKVP------------LNG-----FK-ELGGSN--GTSK
FSIHRDFGST------------------------ERLPSSHTCF-NQ-IDLPAY-DSYET
LRGSLL-LAITEGHEGF
>Wickerhamomyces_ciferrii_XM_011275918.1 .
VSIRRDQV-FLDSYRAL--------------FFKSKDEFRNAKLDISFKG-------EAG
VDAGGVTREW---FQVL----SRQMFNADY--------ALFLPVASDKT-----------
----------------TFHPNRTS------------------------------------
--WVNPEHLSFFKFIGRIIGKAIYDNCFLDCHFSR--DVYKSILG---------------
--------RIVSLKDLET----------------------IDLEYFNS--------LMWM
LNN--DIT-DII--
-IETFAVETDD---------------------------YGA-VQTVD-LIPG--------
GRDIP---
------------------------------VTEENKQ-DYVRL-VVEYRLQKS-VQEQMD
-NFLQG---FHEIIPK--ELIS-IFDEQ-ELELLISG-----------------------
----LPDID-----------VDDW----KNNTTYVN-YSPS-------------------
------------------------SKEI-SYFWRAVR--------------------SFD
AEERAKLLQF-------ATGTSKVP------------LNG-----FK-ELGGSG--DNSK
FSIHKDFGST------------------------ERLPSSHTCF-NQ-IDLPAY-DSYET
LRGSLL-LAITEGHEGF
>Hanseniaspora_vineae_LSNF01000448.1 .
IEVTRDQV-FLDSYRAL--------------FFKPIKVFKESKLDVTFKG-------EEG
VDAGGVKREW---YQSI----SKQMVNPDY--------ALFLSASSDSN-----------
----------------TFHPNRTS------------------------------------
--WVNPEHLSFFKFIGMIIGKSIRDKCFVDCYFSR--AVYKQLLG---------------
--------IPVSLKDMES----------------------IDPDYYKS--------LVWI
LEN--DIT-DII--
-DLTFSVDTDD---------------------------YGE-HKIID-LIEN--------
GRDIA---
------------------------------VTQENKS-DYVTK-IVEYKLSKS-VSEQMS
-NLLQG---FYSIIPK--KLIT-IFNAK-ELELLVSG-----------------------
----LPDID-----------VLDW----KNNTQYVN-YTAS-------------------
------------------------SREI-GYFWRAVR--------------------SFS
KEEKAKLLQF-------VTGTSKVP------------LNG-----FK-ELAGVN--GVSK
FSIHKDYGAT------------------------DRLPQSHTCF-NQ-LDLPAY-ESYEM
LRKALL-LAISEGHEGF
>Hanseniaspora_osmophila_LPNM01000011.1 .
IEVTRDQV-FLDSYRAL--------------FFKPIKIFKESKLDITFKG-------EEG
VDAGGVKREW---YQSI----SKQMVNPDY--------ALFLSASSDSN-----------
----------------TFHPNRTS------------------------------------
--WVNPEHLSFFKFIGMIIGKSIRDRCFVDCYFSR--AVYKQLLG---------------
--------IPVSLKDMES----------------------IDPDYYKS--------LLWI
LEN--DIT-DII--
-DLTFSVDTDD---------------------------YGE-HKIID-LIEN--------
GRDIA---
------------------------------VTQENKS-DYVTK-VVEYKLSTS-VSEQMS
-NLLQG---FYSIIPK--KLIT-IFNAK-ELELLVSG-----------------------
----LPDID-----------VLDW----KNNTQYIN-YTAS-------------------
------------------------SREI-GYFWRAVR--------------------SFS
KEEKAKLLQF-------VTGTSKVP------------LNG-----FK-DLAGVN--GVSK
FSIHKDYGAT------------------------DRLPQSHTCF-NQ-LDLPAY-ESYEM
LRRALL-LAISEGHEGF
>Hanseniaspora_valbyensis_LXPE01000017.1 .
VDISRDQV-FLDSYRVL--------------FFKPIKEFINSKFNITFQG-------EQG

VDAGGVKREW---YQII----SRQMVNPDY--------ALFVPVSSDVS-----------
----------------TFHPNRTS------------------------------------
--GINPEHLSFFKFIGMILAKSIRDNCYIDSHFSR--AVYKQILG---------------
--------KNVSLKDMES----------------------IDPDYYKS--------LCWI
LEN--DIT-DIL--
-DMTFSVETDD---------------------------YGE-HKVID-LIPN--------
GSNID---
------------------------------VTEANKR-EYVER-MINYKLKDS-VKEQMD
-NLIQG---FYSVISK--ENIT-IFNEK-ELELLMNG-----------------------
----LPEID-----------VDDW----KNNTTYVN-YSTT-------------------
------------------------SKEI-NYFWRAVR--------------------SFS
KDERAKLLQF-------ITGTSKVP------------LDG-----FK-QLAGVN--GTSK
FSIHKDFGSD------------------------DRLPQSHTCF-NQ-LDLPSY-KSYED
LKRALL-IAINEGYEGF
>Saccharomyces_eubayanus_XM_018363851.1 .
ITVRREQV-FLDSYRAL--------------FFKTNDEIKNSRLEITFKG-------ESG
VDAGGLTREW---YQVL----SRQMFNPDY--------ALFLPVPSDKT-----------
----------------TFHPNRTS------------------------------------
--GINPEHLSFFKFTGMVIGKAIRDQCFLDCHFSR--EVYKDILG---------------
--------RPVSLKDMES----------------------LDPDYYKS--------LVWI
LEN--DIT-DII--
-DETFSVETDD---------------------------YGE-HKVID-LTEG--------
GKDIM---
------------------------------VTELNKQ-DYVKK-IVEYKLQTS-VKEQMD
-NFLVG---FYALISK--DLIT-IFDEQ-ELELLISG-----------------------
----LPDID-----------VDDW----KNNTTYVN-YTAT-------------------
------------------------CKEV-NYFWRAVR--------------------SFD
AEERAKLLQF-------VTGTSKVP------------LNG-----FK-ELSGVN--GVCK
FSIHRDFGSS------------------------ERLPSSHTCF-NQ-LNLPPY-ESYET
LRGSLL-LAINEGHEGF
>Saccharomyces_pastorianus_ABPO01000272.1 .
ITVRREQV-FLDSYRAL--------------FFKTNDEIKNSRLEITFKG-------ESG
VDAGGLTREW---YQVL----SRQMFNPDY--------ALFLPVPSDKT-----------
----------------TFHPNRTS------------------------------------
--GINPEHLSFFKFTGMVIGKAIRDQCFLDCHFSR--EVYKDILG---------------
--------RPVSLKDMES----------------------LDPDYYKS--------LVWI
LEN--DIT-DII--
-DETFSVETDD---------------------------YGE-HKVID-LTEG--------
GKDIM---
------------------------------VTELNKQ-DYVKK-IVEYKLQTS-VKEQMD
-NFLVG---FYALISK--DLIT-IFDEQ-ELELLISG-----------------------
----LPDID-----------VDDW----KNNTTYVN-YTAT-------------------
------------------------CKEV-NYFWRAVR--------------------SFD
AEERAKLLQF-------VTGTSKVP------------LNG-----FK-ELSGVN--GVCK
FSIHRDFGSS------------------------ERLPSSHTCF-NQ-LNLPPY-ESYET
LRGSLL-LAINEGHEGF
>Saccharomyces_bayanus_AACG02000016.1 .
ITVRREQV-FLDSYRAL--------------FFKTNDEIKNSRLEITFKG-------ESG
VDAGGLTREW---YQVL----SRQMFNPDY--------ALFLPVPSDKT-----------
----------------TFHPNRTS------------------------------------
--GINPEHLSFFKFTGMVIGKAIRDQCFLDCHFSR--EVYKNILG---------------
--------RPVSLKDMES----------------------LDPDYYKS--------LVWI
LEN--DIT-DII--
-DETFSVETDD---------------------------YGE-HKVID-LTEG--------
GKDII---
------------------------------VTELNKQ-DYVKK-IVEYKLQTS-VKEQMD
-NFLVG---FYALISK--DLIT-IFDEQ-ELELLISG-----------------------
----LPDID-----------VDDW----KNNTTYVN-YTAT-------------------

------------------------CKEV-NYFWRAVR--------------------SFD
AEERAKLLQF-------VTGTSKVP------------LNG-----FK-ELSGVN--GVCK
FSIHRDFGSS------------------------ERLPSSHTCF-NQ-LNLPPY-ESYET
LRGSLL-LAINEG----
>Saccharomyces_uvarum_JNVO01000208.1 .
ITVRREQV-FLDSYRAL--------------FFKTNDEIKNSRLEITFKG-------ESG
VDAGGLTREW---YQVL----SRQMFNPDY--------ALFLPVPSDKT-----------
----------------TFHPNRTS------------------------------------
--GINPEHLSFFKFTGMVIGKAIRDQCFLDCHFSR--EVYKNILG---------------
--------RPVSLKDMES----------------------LDPDYYKS--------LVWI
LEN--DIT-DII--
-DETFSVETDD---------------------------YGE-HKVID-LTEG--------
GKDII---
------------------------------VTELNKQ-DYVKK-IVEYKLQTS-VKEQMD
-NFLVG---FYALISK--DLIT-IFDEQ-ELELLISG-----------------------
----LPDID-----------VDDW----KNNTTYVN-YTAT-------------------
------------------------CKEV-NYFWRAVR--------------------SFD
AEERAKLLQF-------VTGTSKVP------------LNG-----FK-ELSGVN--GVCK
FSIHRDFGSS------------------------ERLPSSHTCF-NQ-LNLPPY-ESYET
LRGSLL-LAINEGHEGF
>Saccharomyces_kudriavzevii_AJIH01001693.1 .
ITVRREQV-FLDSYRAL--------------FFKTNDEIKNSRLEITFKG-------ESG
VDAGGLTREW---YQVL----SRQMFNPDY--------ALFLPVPSDKT-----------
----------------TFHPNRTS------------------------------------
--GINPEHLSFFKFTGMIIGKAIRDQCFLDCHFSR--EVYKNILG---------------
--------RPVSLKDMES----------------------LDPDYYKS--------LVWI
LEN--DIT-DII--
-EETFSVETDD---------------------------YGE-HKVID-LIQG--------
GKDIV---
------------------------------VTESNKQ-EYVKK-IVEYKLQTS-VKEQMD
-NFLVG---FYALISK--DLIT-IFDEQ-ELELLISG-----------------------
----LPDID-----------VDDW----KNNTTYVN-YTAT-------------------
------------------------CKEV-NYFWRAVR--------------------SFD
AEERAKLLQF-------VTGTSKVP------------LNG-----FK-ELSGVN--GICK
FSIHRDFGSS------------------------ERLPSSHTCF-NQ-LNLPPY-ESYET
LRGSLL-LAINEGHEGF
>Saccharomyces_mikatae_AACH01000088.1 .
ITVRREQV-FLDSYRAL--------------FFKTNDEIKNSRLEITFKG-------ESG
VDAGGVTREW---YQVL----SRQMFNPDY--------ALFLPVPSDKT-----------
----------------TFHPNRTS------------------------------------
--GINPEHLSFFKFIGMIIGKAIRDQCFLDCHFSR--EVYKNILG---------------
--------RPVSLKDMES----------------------LDPDYYKS--------LVWI
LEN--DIT-GVI--
-EETFSVETDD---------------------------YGE-HKIIE-LIEG--------
GKDII---
------------------------------VSETNKQ-DYVKK-VVEYKLQTS-VKEQMD
-NFLVG---FYALISK--DLIT-IFDEQ-ELELLISG-----------------------
----LPDID-----------VDDW----KNNTTYVN-YTST-------------------
------------------------CKEV-NYFWRAVR--------------------SFD
AEERAKLLQF-------VTGTSKVP------------LNG-----FK-ELSGVN--GVCK
FSIHRDFGSS------------------------ERLPSSHTCF-NQ-LNLPPY-ESYET
LRGSLL-LAINEGHEG-
>Saccharomyces_arboricola_ALIE01000159.1 .
ITVRREQV-FLDSYRAL--------------FFKSNDEIKNSRLEITFKG-------ESG
VDAGGVTREW---YQVL----SRQMFNPDY--------ALFLPVPSDKT-----------
----------------TFHPNRTS------------------------------------
--GINPEHLSFFKFIGMIIGKAIRDQCFLDCHFSR--EVYKNILG---------------
--------RPVSLKDMES----------------------LDPDYYKS--------LLWI

LEN--DIT-DII--
-EETFSVETDD---------------------------YGE-HKVID-LIEG--------
GKDII---
------------------------------VTEANKQ-DYVKK-IVEYKLQTS-VKEQMD
-NFLVG---FYALISK--DLIT-IFDEQ-ELELLISG-----------------------
----LPDIE-----------VDDW----KNNTTYVN-YTAT-------------------
------------------------CKEV-NYFWRAVR--------------------SFD
AEERAKLLQF-------VTGTSKVP------------LNG-----FK-ELSGVN--GVCK
FSIHRDFGSS------------------------ERLPSSHTCF-NQ-LNLPPY-ESYET
LRGSLL-LAINEGHEGF
>Saccharomyces_cerevisiae_CP004732.2 .
ITVRREQV-FLDSYRAL--------------FFKTNDEIKNSKLEITFKG-------ESG
VDAGGVTREW---YQVL----SRQMFNPDY--------ALFLPVPSDKT-----------
----------------TFHPNRTS------------------------------------
--GINPEHLSFFKFIGMIIGKAIRDQCFLDCHFSR--EVYKNILG---------------
--------RPVSLKDMES----------------------LDPDYYKS--------LVWI
LEN--DIT-DII--
-EETFSVETDD---------------------------YGE-HKVIN-LIEG--------
GKDIV---
------------------------------VTEANKQ-DYVKK-VVEYKLQTS-VKEQMD
-NFLVG---FYALISK--DLIT-IFDEQ-ELELLISG-----------------------
----LPDID-----------VDDW----KNNTTYVN-YTAT-------------------
------------------------CKEV-SYFWRAVR--------------------SFD
AEERAKLLQF-------VTGTSKVP------------LNG-----FK-ELSGVN--GVCK
FSIHRDFGSS------------------------ERLPSSHTCF-NQ-LNLPPY-ESYET
LRGSLL-LAINE-----
>Saccharomyces_paradoxus_CP020313.1 .
ITVRREQV-FLDSYRAL--------------FFKTNDEIKNSKLEITFKG-------ESG
VDAGGVTREW---YQVL----SRQMFNPDY--------ALFLPVPSDKT-----------
----------------TFHPNRTS------------------------------------
--GINPEHLSFFKFIGMIIGKAIRDQCFLDCHFSR--EVYKNILG---------------
--------RPVSLKDMES----------------------LDPDYYKS--------LVWI
LEN--NIT-DII--
-EETFSVETDD---------------------------YGE-HKVID-LIEG--------
GKDII---
------------------------------VTEANKQ-DYVKK-VVEYKLQKS-VKEQMD
-NFLVG---FYALISK--DLIK-IFDEQ-ELELLISG-----------------------
----LPDID-----------VDDW----KNNTTYVN-YTAT-------------------
------------------------CKEV-NYFWRAVR--------------------SFD
AEERAKLLQF-------VTGTSKVP------------LNG-----FK-ELSGVN--GVCK
FSIHRDFGSS------------------------ERLPSSHTCF-NQ-LNLPPY-ESYET
LRGSLL-LAINE-----
>Torulaspora_delbrueckii_XM_003678936.1 .
ITVRREQV-FLDSYRAL--------------FFKSNDDIKKSKLEITFKG-------ESG
VDAGGLTREW---YQVL----SRQMFNPDY--------ALFLPVESDKT-----------
----------------TFRPNRTS------------------------------------
--GINPEHLSFFKFIGMIIGKAIRDQCFLDCHFSR--EVYKNILG---------------
--------RPVSLKDMES----------------------LDLDYYKS--------LLWI
LEN--DIT-DVI--
-EETFSVETDD---------------------------YGE-HKIID-LIEN--------
GKNVP---
------------------------------VTEQNKQ-DYVKK-IVEYKLHIS-VKEQMD
-NFLQG---FYALIPI--ELIS-IFDEQ-ELELLVSG-----------------------
----LPDID-----------VDDW----KNNTTYVN-YTAN-------------------
------------------------CKQI-NYYWRAVR--------------------SFD
AEERAKLLQF-------VTGTSKVP------------LNG-----FK-ELSGVS--GVCK
FSIHRDYGSV------------------------DRLPSSHTCF-NQ-LNLPAY-SSYET
LRGSLL-LAINEGHEGF

```
>Nakaseomyces_delphensis_CAPT01000141.1 .
ITVRREQV-FLDSYRAL--------------FFKADQEIKNSKLDITFKG-------ESG
VDAGGVTREW---YQVL----SRQMFNPDY--------ALFLPVASDKT-----------
----------------TFRPNRTS------------------------------------
--GINPEHLSFFKFIGMIIGKAIRDQCFLDCHFSR--EVYKNILG---------------
--------KPVSLKDMES----------------------LDLDYYKS--------LVWI
LEN------------------------------------------------DIT-DII--
-EETFSVETDD---------------------------YGE-HKIID-LIEG--------
GRNIN-------------------------------------------------------
------------------------------VTEENKQ-DYVKK-IVEYKLHTS-VKEQMD
-NFLKG---FYALIPK--EIIS-IFDEQ-ELELLVSG-----------------------
----LPDID-----------VDDW----KNNTTYVN-YTAN-------------------
------------------------CKQV-NYFWRAVR--------------------SFD
AEEKAKLLQF-------VTGTSKVP------------LNG-----FK-ELSGVN--GVSK
FSVHRDYGSV------------------------ERLPSSHTCF-NQ-LNLPAY-VSYDT
LRGSLL-LAINEGHEGF
>Candida_nivariensis_CAPV01000090.1 .
ITVRRDQV-FLDSYRAL--------------FFKADQEIKNSKLDITFKG-------ESG
VDAGGVTREW---YQVL----SRQMFNPDY--------ALFLPVASDKT-----------
----------------TFRPNRTS------------------------------------
--GINPEHLSFFKFIGMIIGKAIRDQCFLDCHFSR--EVYKNILG---------------
--------KPVSLKDMES----------------------LDLDYYKS--------LVWI
LEN------------------------------------------------DIT-DII--
-EETFSVETDD---------------------------YGE-HKIID-LIEN--------
GRNIN-------------------------------------------------------
------------------------------VTEENKQ-DYVKK-IVEYKLHTS-VKEQMD
-NFLRG---FYALIPK--DIIS-IFDEQ-ELELLVSG-----------------------
----LPDID-----------VDDW----KNNSTYVN-YTAN-------------------
------------------------CKQV-NYFWRAVR--------------------SFD
AEEKAKLLQF-------VTGTSKVP------------LNG-----FK-ELSGVN--GVSK
FSIHRDYGSV------------------------ERLPSSHTCF-NQ-LNLPAY-ASYDT
LRGSLL-LAINEGHEGF
>Kazachstania_naganishii_HE978316.1 .
ITVRREQV-FLDSYRSL--------------FFKTNEEIKRSKLDITFKG-------ESG
VDAGGVTREW---YQVL----SRQMFNPDY--------ALFLPVASDVT-----------
----------------TFHPNRTS------------------------------------
--GINPEHLSFFKFIGMIIGKAIRDQCFLDCHFSR--EVYKNILG---------------
--------KPVSLKDMES----------------------LDPDYYKS--------LVWI
LEN------------------------------------------------DIT-DII--
-DETFSVETDD---------------------------YGE-HTVVD-LIEN--------
GRNIP-------------------------------------------------------
------------------------------VTEQNKQ-QYVRS-IIEFKLHLS-VKEQMD
-NFLDG---FYALIPK--DLIS-IFDEQ-EIELLISG-----------------------
----LPDID-----------VDDW----KNNTTYVN-YTST-------------------
------------------------CKQV-NYFWRAVR--------------------SFE
AEERAKLLQF-------VTGTSKVP------------LNG-----FK-ELSGVS--GVCK
FSIHRDYGST------------------------ERLPSSHTCF-NQ-LNLPAY-NSYET
LRGSLL-LSINE-----
>Candida_castellii_CAPW01000002.1 .
ITVTRDQV-FLDSYRAL--------------FFKTNEEIKHSKLEITFKN-------ESG
VDAGGVTREW---YQVL----SRQIFNPDY--------ALFLPVASDKT-----------
----------------TFHPNRTS------------------------------------
--GVNPEHLSFFKFIGMIIGKAIRDQCFLDCHFSR--DVYKNILG---------------
--------KPVSLKDMES----------------------LDLDYYKS--------LMWI
LEN------------------------------------------------DIT-DVI--
-EETFSVETDD---------------------------YGE-HKLID-LVEN--------
GQNIL-------------------------------------------------------
------------------------------VTEENKH-DYVKR-IVEYKLYTS-VKEQMD
```

-NFLVG---FYALIPK--DMIT-IFDEQ-ELELLISG-----------------------
----LPDID-----------VDDW----RNNTTYVN-YTPS-------------------
------------------------CKQV-NYFWRAVK--------------------SFD
AEERAKLLQF-------VTGTSKVP------------LNG-----FK-ELSSVN--GVCK
FSIHKDYGST------------------------NRLPSSHTCF-NQ-LNLPAY-NSYEG
LRKAVL-LAISEGHEGF
>Nakaseomyces_bacillisporus_CAPX01000126.1 .
ITVRREQV-FLDSYRSL--------------FFKSDDEFKTSKLEITFKG-------ESG
VDAGGLTREW---YQVL----SRQMFNPDY--------ALFLPVASDST-----------
----------------TFHPNRTS------------------------------------
--GINPEHLSFFKFIGMVIGKAIRDECFLDCHFSR--EVYKNILG---------------
--------KPVSLKDMES----------------------LDLDYCKS--------LVWI
LEN--DIT-DII--
-EETFSVETDD---------------------------YGE-HKIID-LIEN--------
GRNIP---
------------------------------VTEENKG-EYVKK-ITEYKLHTS-VKEQMD
-NFLQG---FYALIPK--DMIS-IFDEQ-ELELLVSG-----------------------
----LPDID-----------VDDW----KNNSTYVN-YTST-------------------
------------------------CKQV-NYFWRAVR--------------------SFD
AEERAKLLQF-------VTGTSKVP------------LNG-----FK-ELSAVN--GYCK
FSIHKDYGSI------------------------DRLPSSHTCF-NQ-LNLPAY-NSYES
LRRALI-LAINEGHEGF
>Candida_glabrata_XM_448497.1 .
IPVRRDQV-FLDSYRAL--------------FFKSNEEIKNSRLEITFKG-------ESG
VDAGGVTREW---YQVL----SRQMFNPGY--------ALFTPVASDKT-----------
----------------TFRPNRAS------------------------------------
--GVNPEHLSFFKFVGMVIGKAIRDQCFLDCHFSR--EVYKSILG---------------
--------KPVALKDMES----------------------LDLDYYKS--------LVWI
LEN--DIT-DII--
-EETFSVETDD---------------------------YGE-HKIID-LIDN--------
GRNVS---
------------------------------VTESNKQ-DYVRK-IVEYKLHTS-VKEQMD
-NFLSG---FYALIPK--DVIS-IFDEQ-ELELLISG-----------------------
----LPDID-----------VDDW----KNNTTYVN-YTES-------------------
------------------------CKQV-SYFWRAVR--------------------SFD
AEEKAKLLQF-------VTGTSKVP------------LNG-----FK-ELSGVS--GVCK
FSIHRDYGST------------------------ERLPSSHTCF-NQ-LNLPAY-ASYDT
LRGSLL-IAINEGHEGF
>Kazachstania_africana_XM_003958980.1 .
ISVNRNQV-FLDSYRAL--------------FFKADDEIKKSKLEITFKG-------ESG
VDAGGVTREW---YQVL----SRQMFNPDY--------ALFLPVASDKT-----------
----------------TFHPNRAS------------------------------------
--GINPEHLSFFKFIGMIIGKAICDQCFLDCHFSR--EVYKNILG---------------
--------KPVSLKDMES----------------------LDLDYYKS--------LVWI
LEN--DIT-DIF--
-EETFSVEVDD---------------------------YGE-HKIVD-LIEN--------
GRNIP---
------------------------------VSEANKR-EYVKS-IVEYKLHLS-VKEQMD
-NFLTG---FYTLIPK--DIIS-IFDEQ-ELELLISG-----------------------
----LPDID-----------VDDW----RNNTTYVN-YTES-------------------
------------------------CKQV-NYFWRAVR--------------------SFD
AEERAKLLQF-------ITGTSKVP------------LNG-----FK-ELSGVS--GVCK
FSIHRDYGST------------------------ERLPSSHTCF-NQ-LNLPAY-SSYET
LRGSLL-IAINE-----
>Kazachstania_saulgeensis_FXLY01000006.1 .
VSVRREQV-FLDSYRSL--------------FFKNNNEIKDSKLEITFKG-------ESG
VDAGGLTREW---YQVL----SRQMFNPDY--------ALFLPIASDKT-----------
----------------TFHPNRSS------------------------------------

--GINPEHLSFFKFIGMIIGKSIRDQCYLDCHFSR--EVYKNILG---------------
--------KPVSLKDMES----------------------IDPDYYKS--------LVWI
LEN--DIT-DII--
-EETYSLETDD---------------------------YGE-HKIID-LVEN--------
GRNIP---
------------------------------VTNDDKQ-TYVQK-IIDYKLHKS-VQEQMD
-NFLLG---FYALIPK--NLIS-IFDEQ-EIELLING-----------------------
----LPDID-----------VDDW----KNNTNYVN-FTAN-------------------
------------------------SKEV-SYFWRAVR--------------------SFD
SEERAKLLQF-------VTGTSKVP------------LNG-----FK-ALSGVG--GVCK
FSIHRDYGST------------------------ERLPSSHTCF-NQ-LILPAY-NSYDM
LRRLLL-HAITEGSEGF
>Naumovozyma_castellii_XM_003676927.1 .
ITVRRDQV-FLDSYRAL--------------FFKKDEEIKDSKLEITFKG-------ESG
VDAGGLTREW---YQVL----SRQMFNPDY--------ALFLPVASDKT-----------
----------------TFHPNRTS------------------------------------
--GINPEHLSFFKFIGMVIGKAIRDQCFLDCHFSR--EVYKNILG---------------
--------KPVSLRDMES----------------------LDPDYYKS--------LVWI
LEN--DIT-DII--
-EETFSVELDD---------------------------YGE-HKTID-LIEN--------
GANIP---
------------------------------VTEENKQ-EYVKK-IVEYKLNTS-VKEQMD
-NFLRG---FYALIPL--NLIS-IFDEQ-ELELLISG-----------------------
----LPDID-----------VDDW----KNNTNYVN-YTIS-------------------
------------------------DREI-GYFWRAVR--------------------SFD
GEERAKLLQF-------VTGTSKVP------------LNG-----FK-ELGGVN--GVCK
FSIHKDYGST------------------------ERLPSSHTCF-NQ-LNLPAY-NSYET
LRGSLL-IAINEGHEGF
>Naumovozyma_dairenensis_XM_003671556.1 .
VSVRRDQV-FLDSYRSL--------------FFKSDDEIKNSRLEITFKG-------ESG
VDAGGLTREW---YQVL----SRQMFNPDY--------ALFLPVSSDKT-----------
----------------TFHPNRTS------------------------------------
--GVNPEHLSFFKFIGMVLGKAIRDQCFLDCHFSR--DVYKNMLG---------------
--------KPVSLKDMES----------------------IDLDYYKS--------LVWI
LEN--DIT-DVI--
-EETFSVETDD---------------------------YGE-HKVID-LIEN--------
GSNVP---
------------------------------VTEENKK-DYVKK-IVEYKLHTS-VKEQMD
-NFLRG---FYSLIPK--ELVS-IFDEQ-ELELLVSG-----------------------
----LPDID-----------VDDW----KNNTNYTN-YTSN-------------------
------------------------DKQI-NYFWRAVR--------------------SFD
VEERARLLQF-------VTGTSKVP------------LNG-----FK-ELSGVE--GVCK
FAIHRDYGST------------------------DRLPSSHTCF-NQ-LNLPAY-NSYET
LRGSLL-IAINEGHEGF
>Zygosaccharomyces_rouxii_XM_002496986.1 .
ISVRREQV-FLDSYRAL--------------FFKSNEDIKKSKLEITFKG-------ESG
VDAGGLTREW---YQVL----SRQMFNPDY--------ALYLPVESDRT-----------
----------------TFRPNRTS------------------------------------
--GINPEHLSFFKFVGMVIGKAICDQCFLDCHFSR--EVYKNILG---------------
--------RPVSLKDMES----------------------LDLDYYKS--------LIWI
LEN--DIT-DII--
-EETFSLETDD---------------------------YGE-RKVVE-LIPN--------
GSEIQ---
------------------------------VTEENKQ-EYVKK-IVEYKLHLS-VKEQMD
-NFLQG---FYALIPR--DLIS-IFDEQ-ELELLISG-----------------------
----LPDVD-----------VDDW----RNNTNYVN-YTAN-------------------
------------------------CKQI-NYFWRAVR--------------------SFD
AEERAKLLQF-------VTGTSKVP------------LNG-----FK-ELTGVS--GICK

FSIHRDYCPT------------------------DRLPSSHTCF-NQ-LNLPSY-NSYDT
LRGSLL-LAINEGHEGF
>Candida_versatilis_LAVI01000262.1 .
ISVRREQV-FLDSYRAL--------------FFKSNEDIKKSKLEITFKG-------ESG
VDAGGLTREW---YQVL----SRQMFNPDY--------ALYLPVESDRT-----------
----------------TFRPNRTS------------------------------------
--GINPEHLSFFKFVGMVIGKAICDQCFLDCHFSR--EVYKNILG---------------
--------RPVSLKDMES----------------------LDLDYYKS--------LIWI
LEN--DIT-DII--
-EETFSLETDD---------------------------YGE-RKVVE-LIPN--------
GSEIQ---
------------------------------VTEENKQ-EYVKK-IVEYKLHLS-VKEQMD
-NFLQG---FYALIPR--DLIS-IFDEQ-ELELLISG-----------------------
----LPDVD-----------VDDW----RNNTNYVN-YTAN-------------------
------------------------CKQI-NYFWRAVR--------------------SFD
AEERAKLLQF-------VTGTSKVP------------LNG-----FK-ELTGVS--GICK
FSIHRDYCPT------------------------DRLPSSHTCF-NQ-LNLPSY-NSYDT
LRGSLL-LAINEGHEGF
>Zygosaccharomyces_bailii_HG316458.1_a.
ISVRRDQV-FLDSYRAL--------------FFKSNEDIKKSKLEITFKG-------ESG
VDAGGLTREW---YQVL----SRQMFNPDY--------ALFLPVESDRT-----------
----------------TFRPNRTS------------------------------------
--GINPEHLSFFKFIGMVIGKAICDQCFLDCHFSR--EVYKNILG---------------
--------RPVSLKDMES----------------------LDLDYYKS--------LIWI
LEN--DIT-DVI--
-EETFSLETDD---------------------------YGE-HKVVE-LIPN--------
GSQIQ---
------------------------------VTEENKQ-EYVKK-IVEYKLHLS-VKEQMD
-NFLQG---FYALIPI--ELIS-IFDEQ-ELELLISG-----------------------
----LPDLD-----------VDDW----RNNTTYVN-YTPN-------------------
------------------------CKQV-NYFWRAVR--------------------SFD
AEERAKLLQF-------VTGTSKVP------------LNG-----FK-ELTGVS--GICK
FSIHRDYGPT------------------------DRLPSSHTCF-NQ-LNLPSY-NSYET
LRGSLL-LAINE-----
>Zygosaccharomyces_parabailii_CP019500.1 .
ISVRRDQV-FLDSYRAL--------------FFKSNEDIKKSKLEITFKG-------ESG
VDAGGLTREW---YQVL----SRQMFNPDY--------ALFLPVESDRT-----------
----------------TFRPNRTS------------------------------------
--GINPEHLSFFKFIGMVIGKAICDQCFLDCHFSR--EVYKNILG---------------
--------RPVSLKDMES----------------------LDLDYYKS--------LIWI
LEN--DIT-NVI--
-EETFSLETDD---------------------------YGE-HKVVE-LIPN--------
GSQIQ---
------------------------------VTEENKQ-EYVKK-IVEYKLHLS-VKEQMD
-NFLQG---FYALIPI--ELIS-IFDEQ-ELELLISG-----------------------
----LPDLD-----------VDDW----RNNTTYVN-YTAS-------------------
------------------------CKQV-NYFWRAVR--------------------SFD
AEERAKLLQF-------VTGTSKVP------------LNG-----FK-ELTGVS--GICK
FSIHRDYGPT------------------------DRLPSSHTCF-NQ-LNLPSY-NSYET
LRGSLL-LAINE-----
>Vanderwaltozyma_polyspora_XM_001646709.1 .
ITVRRDQV-FLDSYRSL--------------FFKSNEEIKNSKLEIVFKG-------ESG
VDAGGLTREW---YQVL----SRQMFNPDY--------ALFIPVASDTT-----------
----------------TFRPNRTS------------------------------------
--GINPEHCSFFKFIGMIIGKAIRDQCYLDCHFSR--EVYKNILG---------------
--------KSVSLKDMES----------------------LDLDYYKS--------LIWI
IEN--DIT-DII--
-EETFSVETDD---------------------------YGE-HKIID-LIKD--------

GRNIA---
------------------------------VTEENKQ-EYVQK-IVEYKLQTS-VNEQME
-NFLQG---FYALIPK--DLIS-IFDEQ-ELELLISG-----------------------
----LPDID-----------VDDW----KSNSTYVN-YTSS-------------------
------------------------CKQI-NYFWRAVK--------------------SFD
QEERVKLLQF-------VTGTSKVP------------LNG-----FK-ELAGVN--GVCK
FSIHKDYGAI------------------------DRLPTSHTCF-NQ-LDLPAY-NSYET
LRRFLL-LAISEGYEGF
>Tetrapisispora_phaffii_XM_003687548.1 .
ITVSRDQV-FLDSYRAL--------------FFKPDEEIKKSKLEITFKG-------ESG
VDAGGLTREW---YQVL----SRQMFNPDY--------ALFIPVASDKT-----------
----------------TFRPNRTS------------------------------------
--GINPEHHSFFKFIGMIIGKAIRDQCYLDCHFSR--EVYKNILG---------------
--------KPVTLKDMES----------------------LDLDYYKS--------LNWI
LEN--DIT-DII--
-EETFSVETDD---------------------------YGE-HKIID-LIDD--------
GRNIA---
------------------------------VTNLNKQ-DYVQK-IVQYKLKTS-VNDQMD
-NFLKG---FYALIPK--ELIS-IFNEQ-ELELLISG-----------------------
----LPDID-----------VNDW----QSNTTYVN-YTST-------------------
------------------------SKQI-NYFWRAVK--------------------SFD
TEERVKLLQF-------VTGTSKVP------------LNG-----FK-ELAGVN--GLCK
FSIHKDYGPS------------------------DRLPSSHTCF-NQ-LDLPAY-DSYET
LRGSLL-LAITEGYEGF
>Tetrapisispora_blattae_XM_004180813.1 .
ITVRRDQV-FLDSYRAL--------------FFKSDEEIKKSKLEITFKG-------ESG
VDAGGLTREW---YQVL----SRQMFNPDY--------ALFIPVASEKT-----------
----------------TFRPNRTS------------------------------------
--GINPEHYSFFKFIGMVIGKAIRDKCYLDCHFSR--EVYKNILG---------------
--------KSVSLKDMES----------------------LDLEYYKS--------LIWM
LEN--DIT-GVI--
-EETFSVETDD---------------------------YGE-HKIID-LIEN--------
GRNVQ---
------------------------------VTQENKH-DYVKK-IVEYKLQTS-VSGQME
-NFLQG---FHALIPK--DLIS-IFDEQ-ELELLISG-----------------------
----LPDID-----------VDDW----KNNTTYVT-YTAS-------------------
------------------------CKQI-GYFWRAVK--------------------SFD
TEERAKLLQF-------VTGTSKVP------------LNG-----FK-ELSGVN--GICK
FSIHKDYGST------------------------DRLPSSHTCF-NQ-LDLPAY-NSYET
LRGSLL-LAINEGHEGF
>Kluyveromyces_marxianus_CP009306.1 .
ITVRRDQV-FLDSYRAL--------------FFKSNEEIKNCKLDITFKG-------EAG
VDEGGVTREW---YQVL----SRQMFNPDY--------ALFIPVGSDNT-----------
----------------KFRPNRTS------------------------------------
--GINPEHLSFFKFVGMIIGKAISDNCFLDCHFSR--EVYKNILG---------------
--------KPVSLKDMES----------------------LDLEYYKS--------LNWM
LEN--DIT-YVI--
-EETFSIDTDD---------------------------YGE-HKTID-LIPN--------
GRNIA---
------------------------------VTEENKK-DYVQK-VVEYKLQES-VKEHMA
-NLLQG---FYTIIDK--DLIS-IFDEQ-ELELLISG-----------------------
----LPDID-----------VDDW----KNNTTYVN-YTPT-------------------
------------------------CKQI-NYFWRAVR--------------------SFD
KEERAKLLQF-------VTGTSKLP------------LNG-----FK-DLSGIN--GDSK
FSIHRDYGST------------------------DRLPSSHTCF-NQ-LDLPAY-DSYEQ
LRGSLL-LAINEG----
>Kluyveromyces_lactis_XM_451611.1 .
ITVLRDQV-FLDSYRAL--------------FFKSNDEIKNCKLDITFKG-------EAG

VDEGGVTREW---YQVL----SRQMFNPDY--------ALFIPVGTDNT-----------
----------------KFRPNRTS------------------------------------
--GINPEHLSFFKFVGMIIGKAISDNCFLDCHFSR--EVYKNILG---------------
--------KPVSLKDMES----------------------LDLEYYKS--------LNWM
LEN--DIT-YVI--
-DETFSVDTDD---------------------------YGE-HKTID-LIPN--------
GRNIP---
------------------------------VTEENKK-EYVQK-IVEYKLQES-VKDHMQ
-NLLQG---FYAVIDK--DLIS-IFDEQ-ELELLISG-----------------------
----LPDID-----------VDDW----KNNTTYVN-YTPT-------------------
------------------------CKQI-NYFWRAVR--------------------SFD
KEERAKLLQF-------VTGTSKLP------------LNG-----FK-DLSGIN--GDSK
FSIHRDYGST------------------------ERLPSSHTCF-NQ-LDLPAY-DSYEQ
LRGSLL-LAINEGHEGF
>Kluyveromyces_wickerhamii_AEAV01000205.1 .
ISIRRDQV-FLDSYRAL--------------FFKSNDEIKNSKLDISFKG-------EAG
VDEGGVTREW---YQVL----SRQMFNPDY--------ALFIPVASDKT-----------
----------------TFRPNRTS------------------------------------
--SINPEHLSFFKFIGMIIGKAISDKCFLDCHFSR--EVYKNILG---------------
--------KPVSLKDMES----------------------LDLEYYKS--------LNWI
LDN--DIT-YVI--
-EETFSVDTDD---------------------------YGE-HKIID-LIEN--------
GHEIP---
------------------------------VTEQNKK-EYVQK-IVEYKLQES-VKDQMY
-NFLQG---FYAVIEK--DLIS-IFDEQ-ELELLISG-----------------------
----LPDID-----------VDDW----KNNSTYVN-YTPT-------------------
------------------------CKQI-NYFWRAVR--------------------SFD
KEERAKLLQF-------VTGTSKVP------------LNG-----FK-ELSGIH--GVSK
FSIHRDYGAT------------------------DRLPSSHTCF-NQ-LDLPAY-DSYEQ
LRGSLL-LAINEGHEGF
>Kluyveromyces_aestuarii_AEAS01000003.1 .
ISVRRDQV-FLDSYRSL--------------FFKPNDEIKNCKLDISFKG-------ESG
VDEGGVTREW---YQVL----SRQMFNPDY--------ALFIPVASDKT-----------
----------------TFRPNRTS------------------------------------
--GINPEHLSFFKFVGMIIAKAITDKCFLDCHFSR--EVYKNILG---------------
--------KTVSLKDMES----------------------LDLEYYKS--------LIWI
LEN--DIT-YVI--
-EETFSVDTDD---------------------------YGE-HKTID-LIEN--------
GHNIA---
------------------------------VTEENKK-EYVQK-VVEYKLQDS-VKDQME
-NFLQG---FYAIIPM--DLIS-IFDEQ-EIELLISG-----------------------
----LPDID-----------VDDW----KNNTTYVN-YTST-------------------
------------------------CKQI-NYFWRAVR--------------------SFD
KEERAKLLQF-------VTGTSKVP------------LNG-----FK-ELTGIH--GVSK
FSIHRDYGST------------------------ERLPSSHTCF-NQ-LDLPAY-DSYEQ
LRGSLL-LAINEGHEGF
>Lachancea_mirantina_FJUN01000018.1 .
ISVRRNEV-FLDSYRAL--------------FFKSNDEIKSSKLEITFKG-------EAG
VDAGGVTREW---YQVL----SRQMFNPDY--------ALFLPVASDKT-----------
----------------TFHPNRTS------------------------------------
--GVNPEHLSFFKFIGMIIGKAISDQCFLDCHFSR--EVYKNILG---------------
--------KPVSLKDMES----------------------LDLDYYKS--------LIWI
LEN--DIT-GII--
-EETFSVETDD---------------------------YGE-HKIID-LIPD--------
GRDIA---
------------------------------VTEDCKQ-EYVQK-IVEYKLHTS-VKEQMD
-NFLLG---FYAVIPK--ELIA-IFDEQ-ELELLISG-----------------------
----LPDID-----------VDDW----KNNTVYVN-YTAT-------------------

------------------------CKQI-SHFWRAVR--------------------SFD
TEERAKLLQF-------VTGTSKVP------------LNG-----FR-ELSGVN--GISK
FSIHRDYGST------------------------ERLPSSHTCF-NQ-LDLPAY-SSYET
LRGSLL-LAINEGHEGF
>Eremothecium_cymbalariae_XM_003645959.1 .
ISVRREHV-FLDSYRSL--------------FFKSNEDIKNSKLEIAFKG-------EAG
VDAGGVTREW---YQVL----SRQMFNPDY--------ALFLPVASDKT-----------
----------------TFRPNRTS------------------------------------
--GINPEHLSFFKFIGMIIGKAISDQCFLDCHFSR--EVYKNILG---------------
--------KPVSLKDMES----------------------LDLDYYKS--------LIWI
LEN--DIT-DII--
-EETFSVEADD---------------------------YGE-HKIID-LIEN--------
GSHVA---
------------------------------VTEENKH-DYVKK-IVEYKLQTS-VKVQMD
-NFLQG---FYAIIPK--ELIS-IFDEQ-ELELLISG-----------------------
----LPDID-----------VDDW----RNNTTYVN-YTPT-------------------
------------------------CKQI-NYFWRAVR--------------------SFD
KEERAKLLQF-------ITGTSKVP------------LNG-----FK-ELSGVN--GISK
FSIHRDYGSI------------------------DRLPSSHTCF-NQ-LDLPAY-DLYET
LRGSLL-LAINEGHVGF
>Ashbya_gossypii_NM_210515.2 .
ISVRREHV-FLDSYRSL--------------FFKSNEDIKISKLEISFKG-------EAG
VDAGGITREW---YQVL----SRQMFNPDY--------ALFIPVASDKT-----------
----------------TFRPNRTS------------------------------------
--GINPEHLSFFKFIGMIIGKAISDQCFLDCHFSR--EVYKNILG---------------
--------KPVALKDMES----------------------LDLDYYKS--------LIWI
LEN--DIT-DII--
-EETFSVETDD---------------------------YGE-HKVIE-LIEN--------
GAHVA---
------------------------------VTEQNKH-DYVKK-IVEYKLQTS-VKDQME
-NFLQG---FYAIIPK--DLIS-IFDEQ-ELELLVSG-----------------------
----LPDID-----------VDDW----KNNTIYVN-YTPT-------------------
------------------------CKQI-NYFWRAVR--------------------SFD
KEERAKLLQF-------VTGTSKVP------------LNG-----FK-ELSGVN--GISK
FSIHRDYGSI------------------------DRLPSSHTCF-NQ-LDLPAY-DSYET
LRGSLL-LAINEGHEGF
>Eremothecium_sinecaudum_XM_018133268.1 .
ISVRREHV-FLDSYRSL--------------FFKSNDDIKNSKLEITFKG-------EAG
VDAGGVTREW---YQVL----SRQMFNPDY--------ALFLPVASDKT-----------
----------------TFRPNRTS------------------------------------
--GINPEHLSFFKFIGMIIGKAISDQCFLDCHFSR--EVYKNILG---------------
--------KPVSLKDMES----------------------LDLDYYKS--------LIWI
LEN--DIT-DII--
-DETFSLETDD---------------------------YGE-HKTIE-LIEN--------
GSNVA---
------------------------------VTEANKH-DYVKK-IVEHKLQTS-VKEQMD
-NFLQG---FYAIIGK--DLIS-IFDEQ-ELELLISG-----------------------
----LPDID-----------VDDW----RNNTTYVN-YTST-------------------
------------------------CKQI-NYFWRAVR--------------------SFD
KEERAKLLQF-------ITGTSKVP------------LNG-----FK-ELSGVN--GVSK
FSIHRDYGST------------------------ERLPSSHTCF-NQ-LDLPAY-DSYET
LRGSLL-LAINEGHEGF
>Lachancea_kluyveri_AACE03000001.1 .
ITIRREQV-FLDSYRSL--------------FFKSNDEIKNSKLEITFKG-------EAG
VDAGGVTREW---YQVL----SRQMFNPDY--------ALFLPVASDKT-----------
----------------TFHPNRTS------------------------------------
--GINPEHLSFFKFIGMIIGKAINDQCFLDCHFSR--DVYKNMLG---------------
--------KPVSLKDMES----------------------LDLDYYKS--------LIWI

LEN--DIT-DII--
-EETFSVETDD---------------------------YGE-HKTID-LIEN--------
GHNVP---
------------------------------VTEENKQ-DYVKK-IVEYKLHTS-VKDQID
-NFLQG---FYAIIPK--DLIS-IFDEQ-ELELLISG-----------------------
----LPDID-----------VDDW----KNNTTYGN-YTPT-------------------
------------------------CKQI-NYFWRAVR--------------------SFD
AEERAKLLQF-------VTGTSKVP------------LNG-----FK-ELTGVN--GVSK
FSIHRDYGPT------------------------DRLPSSHTCF-NQ-LDLPAY-DSYET
LRGSLL-LAINEGHEGF
>Lachancea_cidri_FJUT01000009.1 .
ISIRRDQV-FLDSYRAL--------------FFKSNDDIKSSKLEITFKG-------EAG
VDAGGITREW---YQVL----SRQMFNPDY--------ALFLPVASDKT-----------
----------------TFHPNRTS------------------------------------
--AINPEHLSFFKFIGMIIGKAISDQCFLDCHFSR--EVYKNILS---------------
--------KPVALKDMES----------------------LDLDYYKS--------LIWI
LEN--DIT-DII--
-EETFSVETDD---------------------------YGE-HKVID-LIED--------
GRNIP---
------------------------------VTEVNKQ-EYVKK-IVEYKLHTS-VKEQMD
-NFLLG---FYAIIPK--ELIS-IFDEQ-ELELLISG-----------------------
----LPDID-----------VDDW----KNNTVYVN-YTAT-------------------
------------------------CKQI-NYFWRAVR--------------------SFD
VEERAKLLQF-------VTGTSKVP------------LNG-----FK-ELSGVN--GVSK
FSIHRDYGST------------------------DRLPSSHTCF-NQ-LDLPSY-NSYET
LRGSLL-LAINEGHEGF
>Lachancea_fermentati_FJUO01000027.1 .
ISIRRDQV-FLDSYRAL--------------FFKSNNDIKNSKLEITFKG-------EAG
VDAGGVTREW---YQVL----SRQMFNPDY--------ALFLPVASDKT-----------
----------------TFHPNRTS------------------------------------
--GINPEHLSFFKFIGMIIGKAISDQCFLDCHFSR--EVYKNILS---------------
--------KPVALKDMES----------------------LDLDYYKS--------LIWI
LEN--DIT-DII--
-EETFSVETDD---------------------------YGE-HKVID-LIED--------
GRNIP---
------------------------------VTEENKQ-DYVKK-IVEYKLHTS-VKEQMD
-NFLLG---FYAIIPK--ELIS-IFDEQ-ELELLISG-----------------------
----LPDID-----------VDDW----KNNTVYVN-YTPT-------------------
------------------------CKQI-NYFWRAVR--------------------SFD
VEERAKLLQF-------VTGTSKVP------------LNG-----FK-ELSGVN--GVSK
FSIHRDYGST------------------------ERLPSSHTCF-NQ-LDLPAY-SSYET
LRGSLL-LAINEGHEGF
>Lachancea_thermotolerans_XM_002555204.1 .
ISVRREQV-FLDSYRSL--------------FFKSNEDIKNSKLEITFKG-------EAG
VDAGGITREW---YQVL----SRQMFNPDY--------ALFLPVASDKT-----------
----------------TFHPNRTS------------------------------------
--GINPEHLSFFKFIGMIIGKAICDQCFLDCHFSR--EVYKNILS---------------
--------KPVALKDMES----------------------LDLDYYKS--------LIWI
LEN--DIT-DII--
-EETFSVETDD---------------------------YGE-HKVID-LIED--------
GHNVP---
------------------------------VTEQNKQ-EYVKK-IVEYKLHTS-VKEQMD
-NFLLG---FYAIIPK--DLVS-IFDEQ-ELELLISG-----------------------
----LPDID-----------VDDW----KNNTNYVN-YTPT-------------------
------------------------CKQI-SYFWRAVR--------------------SFD
SEERAKLLQF-------VTGTSKVP------------LSG-----FK-ELTGVN--GISK
FSIHRDYGST------------------------ERLPSSHTCF-NQ-LDLPAY-DSYET
LRGSLL-LAINEGHEGF

```
>Lachancea_quebecensis_CZLH01000076.1 .
ISVRREQV-FLDSYRSL--------------FFKSNEDIKNSKLEITFKG-------EAG
VDAGGVTREW---YQVL----SRQMFNPDY--------ALFLPVASDKT-----------
----------------TFHPNRTS------------------------------------
--GINPEHLSFFKFIGMIIGKAICDQCFLDCHFSR--EVYKNILS---------------
--------KPVALKDMES----------------------LDLDYYKS--------LIWI
LEN------------------------------------------------DIT-DII--
-EETFSVETDD---------------------------YGE-HKIID-LIED--------
GHNIP-------------------------------------------------------
------------------------------VTEENKQ-EYVKK-IVEYKLHTS-VKEQMD
-NFLLG---FYAIIPK--DLVS-IFDEQ-ELELLISG-----------------------
----LPDID-----------VDDW----KNNTNYVN-YTST-------------------
------------------------CKQI-SYFWRAVR--------------------SFD
SEERAKLLQF-------VTGTSKVP------------LSG-----FK-ELTGVN--GISK
FSIHRDYGSI------------------------DRLPSSHTCF-NQ-LDLPAY-DSYET
LRGSLL-LAINEGHEGF
>Lachancea_waltii_AADM01000155.1 .
ISVRRDQV-FLDSYRSL--------------FFKSNEDIKNSKLEITFKG-------EAG
VDAGGVTREW---YQVL----SRQMFNPDY--------ALFLPVASDKT-----------
----------------TFHPNRTS------------------------------------
--SINPEHLSFFKFIGMIIGKAICDQCFLDCHFSR--EVYKNILG---------------
--------KPVALKDMES----------------------LDLDYYKS--------LIWI
LEN------------------------------------------------DIT-DII--
-EESFSVETDD---------------------------YGE-HKVID-LIEN--------
GRNIL-------------------------------------------------------
------------------------------VTEQNKQ-EYVKK-IVEYKLHTS-VKEQMD
-NFLLG---FYAIIPK--ELVA-IFDEQ-ELELLISG-----------------------
----LPDID-----------VDDW----KNNTNYVN-YTPT-------------------
------------------------CKQI-NYFWRAVR--------------------SFD
SEERAKLLQF-------VTGTSKVP------------LSG-----FK-ELTGVN--GISK
FSIHRDYGAT------------------------DRLPSSHTCF-NQ-LDLPAY-DSYET
LRGSLL-LAVNEGHEGF
>Lachancea_nothofagi_FJUQ01000005.1 .
ISVRREQV-FLDSYRSL--------------FFKSNEDIKNSKLEITFKG-------EAG
VDAGGVTREW---YQVL----SRQMFNPDY--------ALFLPVASDKT-----------
----------------TFHPNRTS------------------------------------
--GINPEHLSFFKFIGMIIGKAICDQCFLDCHFSR--EVYKNILS---------------
--------KPVALKDMES----------------------LDLDYYKS--------LIWI
LEN------------------------------------------------DIT-DII--
-EETFSVETDD---------------------------YGE-HTIVD-LTEN--------
GRDVL-------------------------------------------------------
------------------------------VTEKNKQ-DYVKK-IVEFKLHTS-VKEQMD
-NFLLG---FYAIIPK--ELIS-VFDEQ-ELELLISG-----------------------
----LPDID-----------VDDW----KNNTNYIN-YTPT-------------------
------------------------CKQI-SYFWRAVR--------------------SFD
CEERAKLLQF-------VTGTSKVP------------LSG-----FK-ELTGVN--GISK
FSIHRDYGAT------------------------DRLPSSHTCF-NQ-LDLPAY-DSYEQ
LRGSVL-LAINEGHEGF
>2_Lachancea_dasiensis_FJUP01000011.1 .
ISVRRDQV-FLDSYRSL--------------FFKSNEDIKNSKLEITFKG-------EAG
VDAGGVTREW---YQVL----SRQMFNPDY--------ALFLPVASDKT-----------
----------------TFHPNRTS------------------------------------
--GINPEHLSFFKFIGMIIGKAICDQCYLDCHFSR--EVYKNILS---------------
--------KPVALKDMES----------------------LDLDYYKS--------LIWI
LEN------------------------------------------------DIT-DII--
-EETFSVETDD---------------------------YGE-HKTVD-LIEN--------
GRSIL-------------------------------------------------------
------------------------------VTEENKQ-EYVKK-IVEFKLHTS-VKEQMD
```

-NFLLG---FYAIIPK--DLIS-VFDEQ-ELELLISG----------------------
----LPDID-----------VDDW----KNNTNYVN-YTPT-------------------
------------------------CKQI-NYFWRAVR--------------------SFD
SEERAKLLQF-------VTGTSKVP------------LSG-----FK-ELTGVN--GISK
FSIHRDYGAT------------------------DRLPSSHTCF-NQ-LDLPAY-DSYET
LRGSLL-LAINEGHEGF
>Lachancea_lanzarotensis_CDLU01000002.1 .
ISVRRDQV-FLDSYRSL--------------FFKSNEDIKNSKLEITFKG-------EAG
VDAGGVTREW---YQVL----SRQMFNPDY--------ALFLPVASDKT-----------
----------------TFHPNRTS------------------------------------
--GINPEHLSFFKFIGMIIGKAICDQCFLDCHFSR--EVYKNILS---------------
--------KPVALKDMES----------------------LDLDYYKS--------LIWI
LEN---DIT-DII--
-EETFSVETDD---------------------------YGE-HKVVD-LIEN--------
GSGIP---
------------------------------VTERNKQ-EYVKK-IVEYKLHTS-VKEQMD
-NFLLG---FYAIIPK--DLIS-VFDEQ-ELELLISG----------------------
----LPDID-----------VDDW----KNNTNYVN-YTPT-------------------
------------------------CKQI-NYFWRAVR--------------------SFD
TEERAKLLQF-------ITGTSKVP------------LSG-----FK-ELTGVN--GISK
FSIHRDYGAT------------------------DRLPSSHTCF-NQ-LDLPAY-DSYET
LRGSLL-LALNEGHEGF
>Lachancea_meyersii_FJUM01000009.1 .
ISIRRDQV-FLDSYRSL--------------FFKSNEDIKNSKLEINFKG-------EAG
VDAGGVTREW---YQVL----SRQMFNPDY--------ALFLPVASDKT-----------
----------------TFHPNRTS------------------------------------
--GINPEHLSFFKFIGMIIGKAICDQCFLDCHFSR--EVYKNILS---------------
--------KPVALKDMES----------------------LDLDYYKS--------LIWI
LEN---DIT-DII--
-EETFSVETDD---------------------------YGE-HKTVD-LIEN--------
GRDIP---
------------------------------VTEQNKQ-DYVRK-IVEFKLHTS-VKEQMD
-NFLLG---FYAIIPK--DLIS-VFDEQ-ELELLISG----------------------
----LPDID-----------VDDW----KNNTNYVN-YTPT-------------------
------------------------CKQI-NYFWRAVR--------------------SFD
SEERAKLLQF-------ITGTSKVP------------LSG-----FK-ELTGVN--GISK
FSIHRDYGAT------------------------DRLPSSHTCF-NQ-LDLPAY-DSYET
LRGSLL-LAINEGHEGF
>Saccharomycopsis_malanga_BCGJ01000004.1 .
INVSRDRV-FLDSYRAC--------------AFKSPEEFRDAKLDISFKG-------EAG
IDAGGVTREW---YQVL----SRQIFNPDY--------ALFVPVASDKS-----------
----------------TFHPNRTS------------------------------------
--WVNSEHLLFFRFIGRVIGKAIYDNQYLDAHFSR--AVYKRLLG---------------
--------RPVSLKDMET----------------------LDLDYFKS--------LIWM
LEN---DIT-DII--
-TETFSVETDD---------------------------YGE-VKVVD-LKPD--------
GRNIP---
------------------------------VTEENKQ-EYVKL-VVEYRLQES-VKEQME
-NFLFG---FHEIIPK--DLIA-IFDEQ-ELELLISG----------------------
----LPDID-----------VDDW----KNNSVYTN-YSPS-------------------
------------------------SPAI-QWFWRAVK--------------------SFD
NEQKAKLLQF-------VTGTSKVP------------LQG-----FS-ALAGVN--GNHK
FSIHRDYGSL------------------------ERLPSAHTCF-NQ-LDLPPY-ESYEQ
LRGSLL-LAITEGHEGF
>Saccharomycopsis_fibuligera_CP012823.1 .
IKVSRDRV-FLDTYRAC--------------AFKPADVFRDSKLDITFKG-------EAG
VDAGGVTREW---YQVL----SRQIFNPDY--------ALFIPVASDRS-----------
----------------TFHPNRTS------------------------------------

--GVNSEHLLFFKFIGRIIGKAIYDSQYLDAHFSR--AVYKRLLG---------------
--------RSVSLKDMET----------------------LDLDYFKS--------LMWM
LEN--DIT-DII--
-TETFSVETDD---------------------------YGE-HKVID-LKPD--------
GSNIP---
------------------------------VTEENKQ-EYVKL-VVEYKLQES-VKEQMD
-NFLQG---FHEIIPK--DLIA-IFDEQ-ELELLISG-----------------------
----LPDID-----------VDDW----KNNSVYTN-YSPS-------------------
------------------------NTVI-QWFWRAVK--------------------SFD
KEQKAKLLQF-------VTGTSKVP------------LTG-----FA-ALAGVN--GNHK
FSIHRDYGSL------------------------ERLPSAHTCF-NQ-LDLPPY-ESYEQ
LRGSLL-LAITE-----
>Ascoidea_asiatica_BCKQ01000009.1 .
VVVSRDNV-FLDSFKVL--------------FYKNRDDLRASKLDIRFRG-------EAG
VDAGGLTREW---YQVL----SRQMFNPDY--------ALFTPVASDKT-----------
----------------TFHPNRTS------------------------------------
--GINPEHLSFFKFIGTIIGKAIFDGCFLDCHFSR--ALYKSLLG---------------
--------RKVSIKDMEN----------------------LDPEYYKS--------VEWI
LNN--DIT-DIL--
-TETFSIETDD---------------------------FGI-TKVFD-LIPN--------
GRNIP---
------------------------------LTNENKK-DYVQK-VIEFRLQTS-VKEQID
-NFLIG---FYEIIPK--ELVS-ILNEQ-ELELLISG-----------------------
----LPYID-----------VDDW----KNNTIYKN-FSPS-------------------
------------------------NAVI-QWFWRAVK--------------------SFD
KEQRAKLLQF-------VTGTSKVP------------LEG-----FG-KLSGVN--GAQK
FSIHRDYGTA------------------------DRLPSAHTCF-NQ-LDLPQY-ETYES
LRGSLL-LAITEGHEGF
>Ascoidea_rubescens_XM_020194098.1 .
VAVSRNNV-FFDSYRSI--------------FYKKRDDIRASRLQIRFKG-------EAG
LDAGGVTREW---YQVL----SRQMFNPDY--------VLFTPVASDKT-----------
----------------TFHPNRMS------------------------------------
--GINPDHLSYFKFIGTIIGKAIFDGCFLDCHFSR--ALYKSLLG---------------
--------RKVSLRDMEN----------------------LDPEYYKS--------VEWI
LNN--DIT-DVL--
-TETFSVETND---------------------------FGV-TKVYD-LMPN--------
GRNIS---
------------------------------LTNENKK-DYVEK-VVEFRLQTS-VKEQID
-NFLIG---FYEIIPK--DLVG-IFNEQ-ELELLISG-----------------------
----LPNID-----------VDDW----KNNTIYKN-YSPS-------------------
------------------------STVI-RWFWRAVK--------------------SFD
KEQRAKLLQF-------VTGTSKVP------------LEG-----FS-SLAGVD--GTQK
FAIHRVYGAT------------------------DRLPSAHTCF-NQ-LDLPQY-ESYEN
LRTALL-LAITEGHEGF
>Saprochaete_clavata_CBXB010000137.1 .
VNVRREQA-FLDSYRTL--------------YYKSPEEIKRSRLSVKFKG-------EEG
IDAGGLTREW---YQVM----SRQMFNPDY--------ALFQPVAADRT-----------
----------------TFHPNRTS------------------------------------
--GVNEDHLSYFKFVGRIIGKAIYDNKLLDCHFSR--AMYKKILG---------------
--------KPVSLKDMEN----------------------LDLDYYKS--------LQWI
LDN--DIT-DII--
-TETFSIDTDD---------------------------YGE-RKVID-LKPN--------
GRNIP---
------------------------------VTEENKA-EYVRL-VCEYRLIES-VRTQLD
-HFLEG---FHEIIPK--DLVS-IFDEQ-ELELLISG-----------------------
----LPDID-----------IDDW----RNNTQYQN-YSPS-------------------
------------------------SIQI-KWFWRAVR--------------------AMD
AEERAKLLQF-------VTGTSKVP------------LNG-----FK-ELVGMD--GVSK

FSIHRAYGSN------------------------DRLPSSHTCF-NQ-IDLPAY-DSYEM
LREALL-KAITEGGESF
>Tortispora_caseinolytica_LSKT01000007.1 .
VNVRRDQV-FLDSYKAL--------------YFKTAEEIKNSKFAVKFNG-------EEG
VDAGGVTREW---FQVL----SRQMFNPDY--------ALFIPVASDRT-----------
----------------TFHPNRLS------------------------------------
--GVNPEHLSFFKFIGRIIGKATFEERVLDCHFSR--AVYKRILG---------------
--------RPVALKDMEN----------------------LDLEYFKS--------LEWI
LNN--DIT-DII--
-SETFSLVTDD---------------------------YGD-QQIID-LIPD--------
GRNIA---
------------------------------VTESNKE-EYVKL-VVEYRLVDS-VKEQLD
-SFLAG---FYDMIPR--DLVA-IFDEQ-ELELLISG-----------------------
----LPDID-----------VDDW----RNNTEYHN-YSIS-------------------
------------------------SPQI-QWFWKMVR--------------------SFD
PEERAKLLQF-------VTGTSKVP------------LNG-----FK-ELEGMN--GVSK
FSIHRAFGGD------------------------DRLPSAHTCF-NQ-LDLPEY-KSYES
LKSNVL-LAITEGREGF
>Yarrowia_lipolytica_XM_500551.2 .
LNVRRDQV-FLDSYKSM--------------YFKSAAEIRSGKLNIHFSG-------EEG
VDAGGVTREW---YQVL----ARQMFNPDY--------ALFTPVASDTT-----------
----------------TFHPNRTS------------------------------------
--WVNPEHLSFFKFIGRIIGKAIFDQRLLDCHFSR--AVYKKILG---------------
--------RGVSLKDMET----------------------LDIEYHKS--------LVWM
LEN--DIT-DII--
-TETMSIETED---------------------------YGE-KKTID-LMPD--------
GRNIA---
------------------------------VDESNKA-EFVQR-VVEYRLITS-VEEQLE
-HFLQG---FHDIIPK--ELVS-IFNEQ-ELELLICG-----------------------
----LPEID-----------VDDW----RNNTVYTN-YSAS-------------------
------------------------SPQI-QWFWRSIR--------------------SFD
DEERAKLLQF-------VTGTSKVP------------LDG-----FK-ELEGMN--GPTK
FNIHRAYGNN------------------------ERLPSSHTCF-NQ-LDLPEY-DSYET
LRGSLL-LAITEGREGF
>Yarrowia_keelungensis_BCJD01000006.1 .
LNVRRDQV-FLDSYKSM--------------YFKSAAEIRSGKLNIHFSG-------EEG
VDAGGVTREW---YQVL----ARQMFNPDY--------ALFTPVASDTT-----------
----------------TFHPNRTS------------------------------------
--WVNPEHLSFFKFIGRIIGKAIFDQRLLDCHFSR--AVYKKILG---------------
--------RGVSLKDMET----------------------LDIEYHKS--------LVWM
LEN--DIT-DII--
-TETMSIETED---------------------------YGE-KKTID-LMPD--------
GRNIA---
------------------------------VDESNKA-EFVQR-VVEYRLITS-VEEQLE
-HFLQG---FHDIIPK--ELVS-IFNEQ-ELELLICG-----------------------
----LPEID-----------VDDW----RNNTVYTN-YSAS-------------------
------------------------SPQI-QWFWRSIR--------------------SFD
DEERAKLLQF-------VTGTSKVP------------LDG-----FK-ELEGMN--GPTK
FNIHRAYGNN------------------------ERLPSSHTCF-NQ-LDLPEY-DSYET
LRGSLL-LAITEGREGF
>Nadsonia_fulvescens_LXPB01000140.1 .
LNVRRDQV-FLDSYRSL--------------FFKK--EFKEARLNIRFQG-------EEG
VDAGGVTREW---YQVL----ARQIFNPDY--------ALFTPVASDQT-----------
----------------TFHPNRIS------------------------------------
--WVNPEHLSFFKFVGRIIGKAIYDGRVLDCHFSR--AVYKKILG---------------
--------KTVSLKDMET----------------------LDLDYYKS--------LVWM
MEN--DIT-DII--
-TETMSIDTDD---------------------------YGE-QKTID-LIPN--------

GRDIP---
------------------------------VTEENKH-EYVRL-VCEYRLITS-VQEQMD
-NLLEG---FHDIIPE--DIIA-IFDEQ-ELELLISG-----------------------
----MPDID-----------IEDW----RNNTEYRN-YTVN-------------------
------------------------SPQV-QWFWRAVR--------------------SFD
AEERAKLLQF-------STGTSKVP------------LSG-----FK-ALEGRN--GVYK
FNISRDYGNK------------------------DRLPSSHTCF-NQ-VDLPEY-DTYET
LRQALL-LAIMEGSEGF
>Galactomyces_candidum_CCBN010000016.1 .
LNVRRDQV-FLDSFKSL--------------YYKSASEIKDARLNIKFQG-------EEG
VDVGGVTREW---YQVL----SRQIFNPDY--------ALFTPVASDRT-----------
----------------TFHPNRTS------------------------------------
--SVNPEHLLFFKFIGRIIGKAIYDNKLLDCHFSR--AVYKRILG---------------
--------KSVSVKDMET----------------------LDLDYYKS--------LVWM
LEN--DIT-DII--
-TETFSIDADD---------------------------YGE-QKVID-LKPN--------
GRNIP---
------------------------------VTEENKA-EYVKL-VVEYRLVES-IKDQLN
-AFLEG---FHDIISK--DAVA-IFDEQ-ELELLISG-----------------------
----MPDID-----------LDDW----TSNTEYRN-YTAA-------------------
------------------------SPQI-KWFWRAVK--------------------SFD
AEEKAKLLQF-------ATGTSKVP------------LNG-----FK-ELLGMH--GVSK
FSIHRDYGSK------------------------DRLPSSHTCF-NQ-IDLPEY-SSYEA
LRSALL-KAITEGTEGF
>2_Blastobotrys_adeninivorans_CBZY010000004. 1..
LNVRRDQV-FLDSYKAL--------------YFKSAEEIKNSRLNVRFQG-------EEG
VDAGGVAREW---YQVL----SRQMFNPDY--------ALFTPVAADST-----------
----------------TFHPNRTS------------------------------------
--WVNPEHLSFFKFIGRIIGKAINDLKVLDCHFSR--AVYKKILG---------------
--------KAVSLKDMET----------------------LDLDYHKS--------LVWM
LEN--DIT-DII--
-TETFSIEADD---------------------------YGE-QKVID-LKPG--------
GRDIP---
------------------------------VTEENKR-EYVSL-VVDYRLLRS-VKEQMD
-HFLEG---FHDIIPK--EVVS-IFDEQ-ELELLISG-----------------------
----MPDID-----------LDDW----RNNTVYHN-YTPS-------------------
------------------------SPQV-KWFWRAVR--------------------SFD
AEEKAKLLQF-------ATGTSKVP------------LNG-----FG-SLEGMN--GVSK
FNIHRDFGSK------------------------DRLPSSHTCF-NQ-IDLPEY-DSYET
LRGSLL-MAITEGKEGF
>Sugiyamaella_lignohabitans_XM_018880641.1 .
IHVRRDQV-FLDSYKAL--------------YFKSASEIKNSRFNIRFQG-------EEG
VDAGGVTREW---YQVL----SRQIFNPDY--------ALFSPVASDST-----------
----------------TFHPNRTS------------------------------------
--WVNPEHLSFFKFIGRIIGKAIFDFKLLDCHFSR--AVYKKILG---------------
--------KPVSIKDMET----------------------LDLDYYKS--------LVWM
LEN--DIT-DII--
-TETFSIEAED---------------------------YGE-QKIID-LKPG--------
GRDIP---
------------------------------VTEENKH-EYVRL-VVDYRLIKS-VQEQLD
-HFLEG---FHDIIPK--DVVA-IFDEQ-ELELVISG-----------------------
----MPDID-----------IDDW----RNNTEYHN-YTPS-------------------
------------------------SPQI-KWFWRAVR--------------------SFD
AEEKAKLLQF-------ATGTSKVP------------LNG-----FE-KLEGMN--GVSR
FNIHRDYGSK------------------------DRLPSSHTCF-NQ-IDLPEY-ESYET
LRGALR-TAITEGREGF
>Taphrina_wiesneri_BAVU01000033.1 .
---LNIRFVG-------EEG

VDAGGVSREW---FQVL----ARQMFDPNY--------ALFVPVNADRN-----------
----------------TYHPNKTS------------------------------------
--GINPEHLLFFKFIGRIVGKALYDGRLLDCHFSR--PIYCNMLG---------------
--------KNVSLKDMET----------------------LDLEYYKS--------LVWM
ANN--DIT-DVI--
-TESFAVERED---------------------------FGE-VNVID-LIPN--------
GRNIV---
------------------------------VNEQNKQ-EYITR-VTEYRLLES-VSDQLK
-HFIVG---FNEIIPA--ELVS-IFNEQ-ELELLISG-----------------------
----LPDID-----------PDDW----KNNTEYHN-YTAG-------------------
------------------------SAQI-QWFWRAVR--------------------SFD
DEERARLLQF-------CTGSSRIP------------IEG-----FA-NLEGMH--GVQK
FNIHRDYTTG------------------------SRLPQSHTCF-NQ-LDLPEY-ENYEV
LRSSLL-TAISEGS---
>Protomyces_lactucaedebilis_MCFI01000002.1 .
LNVRRDMI-FLDSYKNL--------------HFKSGDEIKYAKLNIRFAG-------EEG
VDAGGVTREW---FQVL----ARQMFDPNY--------ALFVPVNADRN-----------
----------------TYHPNKLS------------------------------------
--GINPEHLSFFKFVGRIVGKALYDGRLLDCHFSR--PVYRKMLG---------------
--------KNVSLKDMET----------------------LDLEYYKS--------LLWM
LEN--DIT-DVI--
-TETFSVERDD---------------------------FGE-VTIID-LIPN--------
GREIP---
------------------------------VTEENKQ-EYVAR-VTQYRLLDS-VSEQLD
-HFMKG---FNDIISP--ELVS-IFNEQ-ELELLISG-----------------------
----LPDID-----------ADDW----RNNTEYHN-YTAA-------------------
------------------------SPQI-QWFWRAVR--------------------SFD
DEERARLLQF-------ATGSSRIP------------IEG-----FA-NLEGMQ--GVQK
FNIHRDYTNG------------------------DRLPQSHTCF-NQ-LDLPEY-ESYEA
LRNSLL-TAISEGN---
>Taphrina_populina_BAVX01000003.1 .
LNVRRDAV-FLDSYKEL--------------HFKRSDEIKYSKFNIRFVG-------EEG
VDAGGVSREW---FQIL----ARQMFDPNY--------ALFVPVNADRN-----------
----------------TYHPNETS------------------------------------
--GVNPEHFSFFKFIGRIIGKALYDGRLLDCHFSR--PIYCKMLG---------------
--------KKVTLKDMET----------------------LDLEYYNS--------LMWM
LDN--DIT-DVI--
-TETFSVQRDD---------------------------FGQ-IIIVD-LVPN--------
GRNVP---
------------------------------VTEENKK-EYVAL-VTNHRLLDS-VSQQLE
-HFMSG---FNEIVAP--DLIS-IFTEQ-ELELLISG-----------------------
----LPDID-----------PDDW----KNNTEYHS-YTAA-------------------
------------------------SPQV-QWFWRAVR--------------------SFD
DEERARLLQF-------CTGSSRIP------------IEG-----FA-HLEGMH--GVQR
FNIHKDPTTG------------------------VRLPQSHTCF-NQ-LDLPAY-ESYEI
LRSSLL-TAISEGNEGF
>Cladophialophora_yegresii_XM_007754313.1 .
LNVRRDQV-FLDSYKAL--------------YYKSAEEMKYGKLNIRFNG-------EEG
VDAGGVTREW---FQVL----ARGMFNPDW--------ALWQPVAADRT-----------
----------------TFHPSPLS------------------------------------
--WINGEHLLYFKFIGRIIGKALHEGRVLDCHFSR--AVYKRLLG---------------
--------KEPNLKDLES----------------------MDLDYYKS--------LVWI
LEN--DIT-DVI--
-TEDFSVIEEQ---------------------------FGV-EKVID-LIPN--------
GRNIP---
------------------------------VTEENKR-EYVNA-QVRYRLTTS-VKDQIE
-NFVKG---FHDIVPA--ELIA-IFDEQ-ELELLISG-----------------------
----LPEID-----------VDDW----KAHTEYHN-YNGS-------------------

------------------------SPQV-IWFWRIVR--------------------GMS
NEERAKLLQF-------VTGTSKVP------------LNG-----FK-DLEGMQ--GNTL
FSIHKDPSQ-------------------------SRLPTSHTCF-NQ-LDLPAY-DDYDT
LKNNLM-TAINLGADYF
>Cladophialophora_carrionii_XM_008723697.1 .
LNVRRDQV-FLDSYKAL--------------YYKSAEEMKYGKLNIRFNG-------EEG
VDAGGVTREW---FQVL----ARGMFNPDW--------ALWQPVAADRT-----------
----------------TFHPSPLS------------------------------------
--WINGEHLLYFKFIGRIIGKALHEGRVLDCHFSR--AVYKRLLG---------------
--------KEPNLKDLES----------------------MDLDYYKS--------LVWI
LEN--DIT-DVI--
-TEDFSVIEEQ---------------------------FGV-EKVID-LIPN--------
GRNIP---
------------------------------VTEENKR-EYVNA-QVRYRLTTS-VQDQIE
-NFVKG---FHDIIPA--ELIA-IFDEQ-ELELLISG-----------------------
----LPEID-----------VDDW----KAHTEYHN-YNGS-------------------
------------------------SPQV-VWFWRIVR--------------------GMS
NEERAKLLQF-------VTGTSKVP------------LNG-----FK-DLEGMQ--GNTL
FSIHKDPSQ-------------------------SRLPTSHTCF-NQ-LDLPAY-DDYDT
LKNNLM-TAINLGADYF
>Phialophora_verrucosa_MSED01000005.1 .
LNVRRDQV-FLDSYKAL--------------YYKSAEEMKYGKLNIRFNG-------EEG
VDAGGVTREW---FQVL----ARGMFNPDW--------ALWQPVAADRT-----------
----------------TFHPSPLS------------------------------------
--WINGEHLLYFKFIGRIIGKALHEGRVLDCHFSR--AVYKRLLG---------------
--------KEPNLKDLES----------------------MDLDYYKS--------LVWI
LEN--DIT-DVI--
-TEDFSVIEEQ---------------------------FGE-EKVID-LIPN--------
GRNIP---
------------------------------VTEENKR-EYVNA-QVRYRLTTS-VKDQIE
-NFVKG---FHDIIPA--ELVA-IFDEQ-ELELLISG-----------------------
----LPEID-----------VDDW----KAHTEYHN-YNGS-------------------
------------------------SPQV-IWFWRIVR--------------------GMS
NEERAKLLQF-------VTGTSKVP------------LNG-----FK-DLEGMQ--GNTL
FSIHKXPSQ-------------------------SRLPTSHTCF-NQ-LDLPAY-DDYDT
LKNNLM-TAINLGADYF
>Phialophora_americana_JYCC01000013.1 .
LNVRRDQI-FLDSYKAL--------------YYKSAEEMKYGKLNIRFNG-------EEG
VDAGGVTREW---FQVL----ARGMFNPDW--------ALWQPVAADRT-----------
----------------TFHPSPLS------------------------------------
--WINGEHLLYFKFIGRIIGKALHEGRVLDCHFSR--AVYKRILG---------------
--------KEPNLKDLES----------------------MDLDYYKS--------LVWI
LEN--DIT-DVI--
-TEDFSVIEEQ---------------------------FGE-EKVID-LIPN--------
GRNIP---
------------------------------VTEENKR-DYVNE-QVRYRLTTS-VKDQIE
-NFVKG---FHDIIPA--ELVA-IFDEQ-ELELLISG-----------------------
----LPEID-----------VDDW----KAHTEYHN-YNGS-------------------
------------------------SPQV-IWFWRIVR--------------------GMS
NEERAKLLQF-------VTGTSKVP------------LNG-----FK-DLEGMQ--GNTL
FSIHKDPSQ-------------------------SRLPTSHTCF-NQ-LDLPAY-DDYDT
LKNNLM-TAINLGADYF
>Exophiala_calicioides_BCHZ01000002.1 .
LNVRRDQV-FLDSYKAL--------------YYKSAEEMKYGKLNIRFNG-------EEG
VDAGGVTREW---FQVL----ARGMFNPDW--------ALWQPVAADRT-----------
----------------TFHPNPLS------------------------------------
--WINGEHLLYFKFIGRIIGKALHEGRVLDCHFSR--AVYKRILG---------------
--------KETSVKDLES----------------------MDLDYYKS--------LLWI

LEN--DIT-DVI--
-TEDFSVIEEQ---------------------------FGE-EKVVD-LIPD--------
GRNIP---
------------------------------VTEENKR-EYVNA-QVRYRLTTS-VREQLE
-NFVRG---FHDIIPA--ELVA-IFDEQ-ELELLISG-----------------------
----LPEID-----------VDDW----KAHTEYHN-YSAS-------------------
------------------------SPQV-TWFWRIVR--------------------GMS
NEERAKLLQF-------VTGTSKVP------------LNG-----FK-DLEGMQ--GNTL
FSIHKXPSQ-------------------------SRLPTSHTCF-NQ-LDLPAY-DDYDT
LKNNLM-TAINLGADYF
>Rhinocladiella_mackenziei_XM_013413802.1 .
LNVRRDQV-FLDSYKAL--------------YYKSAEEMKYGKLNIRFNG-------EEG
VDAGGVTREW---FQVL----ARGMFNPDW--------ALWQPVAADRT-----------
----------------TFHPNPLS------------------------------------
--WINGEHLLYFKFIGRIIGKALHEGRVLDCHFSR--AVYKRLLG---------------
--------KEPNLKDLES----------------------MDLDYYKS--------LLWI
LEN--DIT-DVI--
-TEDFSVIEEQ---------------------------FGE-EKVVD-LIEN--------
GRNIP---
------------------------------VTEENKR-DYVNA-QVRYRLTTS-VREQLE
-NFVRG---FHDIIPA--ELVA-IFDEQ-ELELLISG-----------------------
----LPEID-----------VDDW----RAHTEYHN-YNAN-------------------
------------------------SPQV-TWFWRIVR--------------------GMS
NEERAKLLQF-------VTGTSKVP------------LNG-----FK-DLEGMQ--GNTL
FSIHRDPSQ-------------------------SRLPTSHTCF-NQ-LDLPAY-SDYET
LKSNLM-TAINLGADYF
>Exophiala_alcalophila_BCHY01000004.1 .
LNVRRDQI-FLDSYKAL--------------YYKSAEEMKYGKLNIRFNG-------EEG
VDAGGVTREW---FQVL----ARGMFNPDW--------ALWQPVAADRT-----------
----------------TFHPNPLS------------------------------------
--WINGEHLLYFKFIGRIIGKALHEGRVLDCHFSR--AVYKRLLG---------------
--------KEPNLKDLES----------------------MDLDYYKS--------LVWI
LEN--DIT-DVI--
-AEDFSVIEEQ---------------------------FGQ-EKVVD-LIPD--------
GRNIP---
------------------------------VTEENKR-EYVNA-QVRYRLTTS-VREQLE
-NFTRG---FHDIIPA--ELVA-IFDEQ-ELELLISG-----------------------
----LPEID-----------VDDW----KAHTEYHN-YNAN-------------------
------------------------SPQV-TWFWRIVR--------------------GMS
NEERAKLLQF-------VTGTSKVP------------LNG-----FK-DLEGMQ--GNTL
FSIHKDPSQ-------------------------TRLPTSHTCF-NQ-LDLPAY-DDYDT
LKNSLM-TAINLGADYF
>Exophiala_aquamarina_XM_013404924.1 .
LNVRRDQV-FLDSYKAL--------------YYKSAEEMKYGKLNIRFNG-------EEG
VDAGGVTREW---FQVL----ARGMFNPDW--------ALWQPVAADRT-----------
----------------TFHPNPLS------------------------------------
--WINGEHLLYFKFIGRIIGKALHEGRVLDAHFSR--AVYKRLLG---------------
--------KEPNLKDLES----------------------MDLDYYKS--------LLWI
LEN--DIT-DVI--
-SEDFSVIEEQ---------------------------FGE-EKVVD-LIPN--------
GRNIP---
------------------------------VTDENKR-EYVNA-QVRYRLTTS-VREQLG
-DFVRG---FHDIIPA--ELVA-IFDEQ-ELELLISG-----------------------
----LPEID-----------VDDW----KANTEYHN-YTAN-------------------
------------------------SPQV-TWFWRIVR--------------------GMS
NEEKAKLLQF-------VTGTSKVP------------LNG-----FK-DLEGMQ--GNTL
FSIHKDPSQ-------------------------SRLPTSHTCF-NQ-LDLPSY-DDYDT
LKSNLM-TAINLGADYF

>Exophiala_xenobiotica_XM_013454491.1 .
LNVRRDQV-FLDSYKAL--------------YYKTADEMKYGKLNIRFNG-------EEG
VDAGGVTREW---FQVL----ARGMFNPDW--------ALWQPVAADRT-----------
----------------TFHPNPLS------------------------------------
--WINGEHLLYFKFIGRIIGKALHEGRVLDCHFSR--AVYKRLLG---------------
--------KEPNLKDLES----------------------MDLDYYKS--------LVWI
LEN--DIT-DVI--
-TEDFSVIEEQ---------------------------FGE-EKVVD-LIPN--------
GRNIP---
------------------------------VTDQNKR-EYVNA-QVRYRLTTS-VQEQLE
-SFVRG---FHDIIPA--ELVA-IFDEQ-ELELLISG-----------------------
----LPEID-----------VDDW----RGHTEYHN-YTAN-------------------
------------------------SPQI-TWFWRIVR--------------------GMS
NEERAKLLQF-------VTGTSKVP------------LNG-----FK-DLEGMQ--GNTL
FSIHKDPSQ-------------------------NRLPTSHTCF-NQ-LDLPAY-DDFDT
LKSNLM-TAINLGADYF
>Exophiala_oligosperma_XM_016404370.1 .
LNVRRDQV-FQDSYKAL--------------YYKTADEMKYGKLNIRFNG-------EEG
VDAGGVSREW---FQVL----ARGMFNPDW--------ALWQPVASDRT-----------
----------------TFHPNPLS------------------------------------
--WINGEHLLYFKFIGRIIGKALHEGRVLDAHFSR--AVYKRLLG---------------
--------KEPNLKDLES----------------------MDLDYYKS--------LVWI
LEN--DIT-DVI--
-TEDFSVIEEQ---------------------------FGE-EKVVD-LIPN--------
GRNIP---
------------------------------VTDENKR-EYVNA-QVRYRLTTS-VSEQLE
-NFVRG---FHDIIPA--ELVA-IFDEQ-ELELLISG-----------------------
----LPEVD-----------VDDW----RAHTEYHN-YTAN-------------------
------------------------SAQI-TWFWRIVR--------------------GMS
NEERAKLLQF-------VTGTSKVP------------LNG-----FK-DLEGMQ--GNTL
FSIHKDPSQ-------------------------NRLPTSHTCF-NQ-LDLPAY-DDYDT
LKSSLM-TAINLGADYF
>Exophiala_spinifera_XM_016380320.1 .
LNVRRDQV-FQDSYKAL--------------YYKTADEMKYGKLNIRFNG-------EEG
VDAGGVTREW---FQVL----ARGMFNPDW--------ALWQPVASDRT-----------
----------------TFHPNPLS------------------------------------
--WINGEHLLYFKFIGRIIGKALHEGRVLDAHFSR--AVYKRLLG---------------
--------KEPNLKDLES----------------------MDLDYYKS--------LVWI
LEN--DIT-DVI--
-TEDFSVIEEQ---------------------------FGE-EKVVD-LIPN--------
GRNIP---
------------------------------VTDENKR-EYVNA-QVRYRLTTS-VQEQLE
-NFVRG---FHDIIPA--ELVA-IFDEQ-ELELLISG-----------------------
----LPEID-----------VDDW----RAHTEYHN-YTAN-------------------
------------------------SPQI-TWFWRIVR--------------------GMS
NEERAKLLQF-------VTGTSKVP------------LNG-----FK-DLEGMQ--GNTL
FSIHKDPSQ-------------------------NRLPTSHTCF-NQ-LDLPAY-DDFDT
LKGSLM-TAINLGADYF
>Fonsecaea_nubica_LVCJ01000110.1 .
LNVRRSQV-FLDSYKAL--------------YYKSAEEMKYGKLNIRFNG-------EEG
VDAGGVTREW---FQVL----ARGMFNPDW--------ALWQPVAADRT-----------
----------------TFHPNPLS------------------------------------
--WINGEHLLYFKFIGRIIGKALHEGRVLDCHFSR--AVYKRLLG---------------
--------KEPNLKDLES----------------------MDLDYYKS--------LVWI
LEN--DIT-DVI--
-TEDFSVVEEQ---------------------------FGE-EKIVD-LVPN--------
GRNIP---
------------------------------VTEENKR-EYVNA-QVRYRLTTS-VKEQLE

-NFVKG---FHDIIPA--ELIA-IFDEQ-ELELLISG----------------------
----LPEID-----------VDDW----RAHTEYHN-YNAN-------------------
------------------------SPQV-TWFWRIVR--------------------GMS
NEERAKLLQF-------VTGTSKVP------------LNG-----FK-DLE---------

>Cladophialophora_psammophila_XM_007744712.1 .
LNVRRSQV-FLDSYKAL--------------YYKSADEMKYGKLNIRFNG-------EEG
VDAGGVTREW---FQVL----ARGMFNPDW--------ALWQPVAADRT-----------
----------------TFHPNPLS------------------------------------
--WINGEHLLYFKFIGRIIGKALHEGRVLDCHFSR--AVYKRLLG---------------
--------KEPNLKDLES----------------------MDLDYYKS--------LVWI
LEN--DIT-DVI--
-TEDFSVVEEQ---------------------------FGE-EKVVD-LIPN--------
GRNIP---
------------------------------VTEENKR-EYVNA-QVRYRLTTS-VKEQLE
-NFVKG---FHDIIPA--ELIA-IFDEQ-ELELLISG----------------------
----LPEID-----------VDDW----RAHTEYHN-YNAN-------------------
------------------------SPQV-TWFWRIVR--------------------GMS
NEERAKLLQF-------VTGTSKVP------------LNG-----FK-DLEGMQ--GNTR
FSIHKDPSQ-------------------------SRLPTSHTCF-NQ-LDLPAY-DDYDT
LKNSLM-TAINLGADYF
>Cladophialophora_bantiana_XM_016761127.1 .
LNVRRSQV-FLDSYKAL--------------YYKSADEMKYGKLNIRFNG-------EEG
VDAGGVTREW---FQVL----ARGMFNPDW--------ALWQPVAADRT-----------
----------------TFHPNPLS------------------------------------
--WINGEHLLYFKFIGRIIGKALHEGRVLDCHFSR--AVYKRLLG---------------
--------KEPNLKDLES----------------------MDLDYYKS--------LVWI
LEN--DIT-DVI--
-TEDFSVVEEQ---------------------------FGE-EKVVD-LIPN--------
GRNIP---
------------------------------VTEENKR-EYVNA-QVRYRLTTS-VKEQLE
-NFVKG---FHDIIPA--ELIA-IFDEQ-ELELLISG----------------------
----LPEID-----------VDDW----RAHTEYHN-YNAN-------------------
------------------------SPQV-TWFWRIVR--------------------GMS
NEERAKLLQF-------VTGTSKVP------------LNG-----FK-DLEGMQ--GNTR
FSIHKDPSQ-------------------------SRLPTSHTCF-NQ-LDLPAY-DDYDT
LKNSLM-TAINLGADYF
>Fonsecaea_pedrosoi_XM_013427263.1 .
LNVRRSQV-FLDSYKAL--------------YYKSAEEMKYGKLNIRFNG-------EEG
VDAGGVTREW---FQVL----ARGMFNPDW--------ALWQPVAADRT-----------
----------------TFHPNPLS------------------------------------
--WINGEHLLYFKFIGRIIGKALHEGRVLDCHFSR--AVYKRLLG---------------
--------KEPNLKDLES----------------------MDLDYYKS--------LVWI
LEN--DIT-DVI--
-TEDFSVVEEQ---------------------------FGE-EKIVD-LVPN--------
GRNIP---
------------------------------VTEENKR-EYVNA-QVRYRLTTS-VKEQLE
-NFVKG---FHDIIPA--ELIA-IFDEQ-ELELLISG----------------------
----LPEID-----------VDDW----RAHTEYHN-YNAN-------------------
------------------------SPQV-TWFWRIVR--------------------GMS
NEERAKLLQF-------VTGTSKVP------------LNG-----FK-DLEGMQ--GNTL
FSIHKDPSQ-------------------------SRLPTSHTCF-NQ-LDLPAY-DDYDT
LKNNLM-TAINLGADYF
>Fonsecaea_multimorphosa_XM_016774518.1 .
LNVRRSQV-FLDSYKAL--------------YFKSAEEMKYGKLNIRFNG-------EEG
VDAGGVTREW---FQVL----ARGMFNPDW--------ALWQPVAADRT-----------
----------------TFHPNPLS------------------------------------

--WINGEHLLYFKFIGRIIGKALHEGRVLDCHFSR--AVYKRLLG---------------
--------KEPNLKDLES----------------------MDLDYYKS--------LVWI
LEN---DIT-DVI--
-TEDFSVVEEQ---------------------------FGE-EKIVD-LIPD--------
GRNIP---
------------------------------VTEENKR-EYVNA-QVRYRLTTS-VKDQLE
-NFVKG---FHDIIPA--ELIA-IFDEQ-ELELLISG-----------------------
----LPEID-----------VDDW----KAHTEYHN-YNAN-------------------
------------------------SPQV-TWFWRIVR--------------------GMS
NEERAKLLQF-------VTGTSKVP------------LNG-----FK-DLEGMQ--GNTL
FSIHKDPSQ-------------------------SRLPTSHTCF-NQ-LDLPAY-DDYDT
LKNNLM-TAINLGADYF
>Cladophialophora_immunda_XM_016397974.1 .
LNVRRSQV-FLDSYKAL--------------YFKSAEEMKYGKLNIRFNG-------EEG
VDAGGVTREW---FQVL----ARGMFNPDW--------ALWQPVAADRT-----------
----------------TFHPNPLS------------------------------------
--WINGEHLLYFKFIGRIIGKALHEGRVLDCHFSR--AVYKRLLG---------------
--------KEPNLKDLES----------------------MDLDYYKS--------LVWI
LEN---DIT-DVI--
-TEDFSVVEEQ---------------------------FGE-EKIVD-LIPN--------
GRNIP---
------------------------------VTEENKR-EYVNA-QVRYRLTTS-VKDQLE
-NFVKG---FHDIIPA--ELIA-IFDEQ-ELELLISG-----------------------
----LPEID-----------VDDW----RAHTEYHN-YNAN-------------------
------------------------SPQV-TWFWRIVR--------------------GMS
NEERAKLLQF-------VTGTSKVP------------LNG-----FK-DLEGMQ--GNTL
FSIHKDPSQ-------------------------SRLPTSHTCF-NQ-LDLPAY-DDYDT
LKNNLM-TAINLGADYF
>Fonsecaea_erecta_XM_018838770.1 .
LNVRRSQV-FLDSYKAL--------------YFKSAEEMKYGKLNIRFNG-------EEG
VDAGGVTREW---FQVL----ARGMFNPDW--------ALWQPVAADRT-----------
----------------TFHPNPLS------------------------------------
--WINGEHLLYFKFIGRIIGKALHEGRVLDCHFSR--AVYKRLLG---------------
--------KEPNLKDLES----------------------MDLDYYKS--------LVWI
LEN---DIT-DVI--
-TEDFSVVEEQ---------------------------FGE-EKIVD-LIPN--------
GRNIP---
------------------------------VTEENKR-DYVNA-QVRYRLTTS-VKDQLE
-NFVKG---FHDIIPA--ELIA-IFDEQ-ELELLISG-----------------------
----LPEID-----------VDDW----RAHTEYHN-YTAN-------------------
------------------------SPQV-TWFWRIVR--------------------GMS
NEERAKLLQF-------VTGTSKVP------------LNG-----FK-DLEGMQ--GNTL
FSIHKDPSQ-------------------------SRLPTSHTCF-NQ-LDLPAY-DDYDT
LKNNLM-TAINLGADYF
>Exophiala_dermatitidis_XM_009159908.1 .
LNVRRDQV-FLDSYKAL--------------YYKTADEMKYGKLNIRFNG-------EEG
VDAGGVTREW---FQVL----ARGMFNPDW--------ALWQPVAADRT-----------
----------------TFHPNPLS------------------------------------
--WINGEHLLYFKFIGRIIGKALHEGRVLDCHFSR--AVYKRLLG---------------
--------KEPNLKDLES----------------------MDLDYYKS--------LVWI
LEN---DIT-DVI--
-TEDFSVIEEQ---------------------------FGE-EKVVD-LIPN--------
GRNIP---
------------------------------VTEENKR-EYVHA-QVRYRLTTS-VKDQLE
-AFVKG---FHDIIPA--ELIA-IFDEQ-ELELLISG-----------------------
----LPEID-----------VDDW----RAHTEYHN-YTAN-------------------
------------------------SPQV-TWFWRVVR--------------------NMS
NEERAKLLQF-------VTGTSKVP------------LNG-----FK-DLEGMQ--GNTL

FSIHKDPSS-------------------------DRLPTSHTCF-NQ-LDLPTY-DDYET
LKSNLM-KAINLGADYF
>Capronia_coronata_XM_007721087.1 .
LNVRRDQI-FLDSYKAL--------------YYKTAEEMKYGKLNIRFNG-------EEG
VDAGGVTREW---FQVL----ARGMFNPDW--------ALWQPVAADRT-----------
----------------TFHPNPLS------------------------------------
--WINGEHLLYFKFIGRIIGKALHEGRVLDCHFSR--AVYKRLLG---------------
--------KEPNLKDLES----------------------MDLDYYKS--------LLWI
LEN--DIT-DVI--
-TEDFSVIEEQ---------------------------FGE-EKVVD-LIPN--------
GRNIP---
------------------------------VTEENKR-EYVNA-QVRYRLTTS-VKDQLE
-AFVRG---FHDIIPA--ELIA-IFDEQ-ELELLISG-----------------------
----LPEID-----------VDDW----KAHTEYHN-YNAN-------------------
------------------------SPQV-IWFWRVVR--------------------NMS
NEERAKLLQF-------VTGTSKVP------------LNG-----FK-DLEGMQ--GNTL
FSIHKDPSQ-------------------------SRLPTSHTCF-NQ-LDLPAY-DDYET
LKNNVM-KAISLGADYF
>Capronia_epimyces_XM_007732183.1 .
LNVRRDQV-FLDSYKAL--------------YYKTAGEMKYGKLNIRFNG-------EEG
VDAGGVTREW---FQVL----ARGMFNPDW--------ALWQPVAADRT-----------
----------------TFHPNPLS------------------------------------
--WINGEHLLYFKFIGRIIGKALHEGRVLDCHFSR--AVYKRLLG---------------
--------KEPNLKDLES----------------------MDLDYYKS--------LLWI
LEN--DIT-EVI--
-TEDFSVIEEQ---------------------------FGE-EKVVD-LIPD--------
GRNIP---
------------------------------VTEENKR-EYVNA-QVRYRLTTS-VKDQLE
-AFVRG---FHDIIPA--ELIA-IFDEQ-ELELLISG-----------------------
----LPEID-----------VDDW----KAHTEYHN-YTAN-------------------
------------------------SPQV-IWFWRVVR--------------------NMS
NEERAKLLQF-------VTGTSKVP------------LNG-----FK-DLEGMQ--GNTL
FSIHKDPSQ-------------------------SRLPTSHTCF-NQ-LDLPTY-DDYET
LKNNLM-KAINLGADYF
>Exophiala_mesophila_XM_016366139.1 .
LSVRRDQV-FLDSYKAL--------------YYKSADEMKYGKLNIRFSG-------EEG
VDAGGVTREW---FQVL----ARGMFNPDW--------ALWQPVAADRT-----------
----------------TFHPNPLS------------------------------------
--WINGEHLLYFKFIGRIIGKALHEGRVLDAHFSR--AVYKRLLG---------------
--------KESNLKDLES----------------------MDLDYYKS--------LMWI
LQN--DIT-DVI--
-TEDFSVIEEQ---------------------------FGE-EKVVD-LIPD--------
GRNIP---
------------------------------VTDENKM-EYVNA-QVRYRLTTS-VKEQLE
-SFVRG---FHDIIPA--ELIA-IFDEQ-ELELLISG-----------------------
----LPEID-----------VDDW----RAHTEYHN-YTAN-------------------
------------------------SPQV-TWFWRIVR--------------------GMS
NEEKAKLLQF-------VTGTSKVP------------LNG-----FK-DLEGMT--GTTL
FSIHKDPSQ-------------------------SRLPTSHTCF-NQ-LDLPAY-DDYDT
LRSSLM-TAINLGADYF
>Exophiala_sideris_JYBR01000058.1 .
LNVRRDQI-FLDSYKAL--------------YYKTADEMKYGKLNIRFNG-------EEG
VDAGGVTREW---FQVL----ARGMFNPDW--------ALWQPVAADRT-----------
----------------TFHPNPLS------------------------------------
--WINGEHLLYFKFIGRIIGKAVHEGRVLDCHFSR--AVYKRLLG---------------
--------KEPNLKDLES----------------------MDLDYYKS--------LVWI
LEN--DIT-DVI--
-SEDFSVIEEQ---------------------------FGE-EKVVD-LIPN--------

```
GRNIP-------------------------------------------------------
------------------------------VTEENKK-EYVNA-QVRYRLTTS-VQEQLE
-NFTRG---FHDIIPA--ELIA-IFDEQ-ELELLISG-----------------------
----LPEID-----------VDDW----KAHSEYHN-YSAN-------------------
------------------------SPQI-TWFWRIVR--------------------GMS
NEERAKLLQF-------VTGTSKVP------------LNG-----FK-DLEX--------
------PSQ-------------------------TRLPTSHTCF-NQ-LDLPAY-DDYET
LKSSLM-TAINLGADYF
>Fonsecaea_multimorphosa_LVCI01000048.1 .
---------------------------------------KYGKLNIRFNG-------EEG
VDAGGVTREW---FQVL----ARGMFNPDW--------ALWQPVAADRT-----------
----------------TFHPNPLS------------------------------------
--WINGEHLLYFKFIGRIIGKALHEGRVLDCHFSR--AVYKRLLG---------------
--------KEPNLKDLES----------------------MDLDYYKS--------LVWI
LEN------------------------------------------------DIT-DVI--
-TEDFSVVEEQ---------------------------FGE-EKIVD-LIPD--------
GRNIP-------------------------------------------------------
------------------------------VTEENKR-EYVNA-QVRYRLTTS-VKDQLE
-NFVKG---FHDIIPA--ELIA-IFDEQ-ELELLISG-----------------------
----LPEID-----------VDDW----KAHTEYHN-YNAN-------------------
------------------------SPQV-TWFWRIVR--------------------GMS
NEERAKLLQF-------VTGTSKVP------------LNG-----FK-DLE---------
------------------------------------------------------------
-----------------
>Phialophora_attae_XM_018139491.1 .
LNIRRDQV-FQDSYKAL--------------YFKSPDEMKYGKLNIRFNG-------EEG
VDAGGVTREW---FQVL----ARGIFNPDW--------ALWQPVASDKT-----------
----------------TFHPNSLS------------------------------------
--WINGEHLVYFKFIGRIIGKALHESRVLDCHFSR--AVYKRMLG---------------
--------KQPNLKDLES----------------------MDLDYYKS--------LCWI
LEN------------------------------------------------DIT-DII--
-TEDFSVVEEQ---------------------------FGE-EKIVD-LIPD--------
GRNIP-------------------------------------------------------
------------------------------VTEDNKR-DYVQK-LVEYRLTGS-VSEQLE
-NFVKG---FHDIIPS--ELVS-IFDEQ-ELELLISG-----------------------
----LPEID-----------VDDW----KANTEYHN-YNAN-------------------
------------------------SPQV-TWFWRIVR--------------------SMS
NEERAKLLQF-------ITGTSKVP------------LNG-----FK-DLEGMQ--GTTR
FSIHREPNL-------------------------HRLPTSHTCF-NQ-LDLPAY-DNQES
LKTSLM-KAINLGADYF
>Cyphellophora_europaea_XM_008721609.1 .
LNVRRDQV-FQDSYKAL--------------YFKTADEMKYGKLNIRFNG-------EEG
VDAGGVTREW---FQVL----ARGIFNPDW--------ALWQPVASDKT-----------
----------------TFHPNPLS------------------------------------
--WINGEHLVYFKFIGRIIGKALHEGRVLDCHFSR--AAYKRMLG---------------
--------KQPNLKDLES----------------------MDLDYYKS--------LLWI
LEN------------------------------------------------DIT-DIL--
-TEDFSIVEEQ---------------------------FGE-EKIVD-LIPD--------
GRNIP-------------------------------------------------------
------------------------------VTEENKR-EYVQK-LVEYRLTGS-VSEQLE
-HFIRG---FHDIIPA--ELVA-IFDEQ-ELELLISG-----------------------
----LPEID-----------VDDW----KANTDYHN-YNAN-------------------
------------------------SPQI-TWFWRIVR--------------------AMS
NEERAKLLQF-------ITGTSKVP------------LNG-----FK-DLEGMQ--GTTR
FSVHREPNL-------------------------NRLPTSHTCF-NQ-LDLPAY-DNQES
LKTNIM-KAINLGADYF
>Herpotrichiellaceae_sp._AMYF01000005.1 .
LNVRRDQV-FIDSYKAL--------------YFKNAEEMKYGKLNIRFNG-------EEG
```

VDAGGVTREW---FQVL----ARGMFDPNY--------ALFQPVASDKT-----------
----------------TFHPSPLS------------------------------------
--RVNPEHLLYFKFIGRIIGKALHEGRVLDCHFSR--AVYKRILG---------------
--------KKPNLKDLES----------------------SDVDYYKS--------LVWI
LEN--DIT-DIL--
-TEEFCVIEDE---------------------------FGE-EKTID-LIPN--------
GRNIA---
------------------------------VTEENKK-EYVQA-LVEYRLTES-VKEQLD
-SFLGG---FHDIIPA--ELIS-IFNEQ-ELELLISG-----------------------
----LPEID-----------VDDW----KANTEYHN-YNPS-------------------
------------------------SQQI-VWFWRIVK--------------------AMT
NEERAKLLQF-------ITGTSKVP------------LNG-----FK-ELEGVS--GLTK
CNIHKDPST-------------------------NRLPTSHTCF-NQ-LDLPAY-ESFEI
MKQNLN-TAISLGADYF
>Schizosacch_octosporus_XM_013163476.1 .
INVRRDQV-FLDSYRAL--------------HFKDADEVKFSKLNIHFRD-------EEG
VDAGGVTREW---LQVL----ARQMFNPDY--------ALFLPVAGDAT-----------
----------------TFHPNRDS------------------------------------
--SVNPDHLSFFKFTGRIIGKSVYDGRLLDCHFSR--AVYKHMLH---------------
--------RSVSVKDIES----------------------LDPDYCKS--------LVWM
LNN--DIT-DII--
-TEEFAVEKDV---------------------------FGE-KTIVD-LIPN--------
GRNIP---
------------------------------VTELNKH-DYVNR-MVDYKLVES-VKDQLQ
-SLLEG---FSDIIPP--NLIQ-IFNEQ-ELELLISG-----------------------
----LPEID-----------NDDW----RNNTEYHG-YNVS-------------------
------------------------SPQI-QWFWRAVR--------------------SFD
EEERAKLLQF-------ATGTSKVP------------LNG-----FK-ELEGMS--GFQR
FNIHKSYGSL------------------------NRLPQSHTCF-NQ-LDLPEY-ETYEQ
LRTMLL-TAINEGSEGF
>Schizosacch_cryophilus_XM_013169236.1 .
INVRRDQV-FLDSYRAL--------------HFKDADEVKYSKLNIHFRE-------EEG
VDAGGVTREW---LQVL----ARQMFNPDY--------ALFLPVAGDAT-----------
----------------TFHPNRDS------------------------------------
--SVNPDHLSFFKFTGRIIGKSVYDGRLLDCHFSR--AVYKHMLH---------------
--------RSVSVKDIES----------------------LDPDYCKS--------LVWM
LNN--DIT-DII--
-TEEFAVEKDV---------------------------FGE-KTIVD-LIPN--------
GRNIP---
------------------------------VTELNKH-DYVNR-MVDYKLVES-VKDQLQ
-SLLEG---FSDIIPP--NLIQ-IFNEQ-ELELLISG-----------------------
----LPEID-----------IDDW----RNNTEYHG-YNVS-------------------
------------------------SPQI-QWFWRAVR--------------------SFD
EEERAKLLQF-------ATGTSKVP------------LNG-----FK-ELEGMS--GFQR
FNIHKSYGSL------------------------NRLPQSHTCF-NQ-LDLPEY-ETYEQ
LRTMLL-TAINEGSEGF
>Schizosacch_pombe_NM_001020331.1 .
ITVRRDHV-FLDSYRAL--------------HFKDADEVKFSKLNIHFRD-------EEG
VDAGGVTREW---LQVL----ARQMFNPDY--------ALFLPVTGDAT-----------
----------------TFHPNRDS------------------------------------
--SVNPDHLSFFKFTGRIIGKALYDGRLLDCHFSR--AVYKHMLH---------------
--------RSVSVKDIES----------------------LDPDYYKS--------LVWM
LNN--DIT-DII--
-TEEFAVEKDV---------------------------FGE-KTVVD-LIPN--------
GRNIP---
------------------------------VTELNKQ-NYVNR-MVDYKLRES-VKDQLK
-SLLDG---FSDIIPS--HLIQ-IFNEQ-ELELLISG-----------------------
----LPEID-----------IDDW----KNNTEYHG-YNVS-------------------

------------------------SPQV-QWFWRAVR--------------------SFD
EEERAKLLQF-------ATGTSKVP------------LNG-----FK-ELEGMS--GFQR
FNIHKSYGSL------------------------NRLPQSHTCF-NQ-LDLPEY-DTYEQ
LRSMLL-TAINEGSEGF
>Schizosacch_japonicus_XM_002173541.2 .
ITVRRDQV-FLDSYRAL--------------HFKNADEVKYSKLNIRFRD-------EEG
VDAGGVTREW---LQVL----ARQMFNPDY--------ALFLPVVGDST-----------
----------------TFHPNRDS------------------------------------
--AVNPDHLSFFKFTGRIIGKALYDGRLLDCHFSR--AVYKQILK---------------
--------CPLSLKDMES----------------------LDPDYYKS--------LVWM
LSN--NIS-DII--
-TEEFAVEKDV---------------------------FGE-RTIVD-LIEN--------
GRNIP---
------------------------------VTEENKH-QYVNL-MVNYKLKES-VKDQLQ
-SLCDG---FYDIIAP--QLVQ-IFNER-ELELLISG-----------------------
----LPEID-----------IDDW----RNNTEYHN-YTMS-------------------
------------------------SPQI-QWFWRAVR--------------------SFD
EEERAKLLQF-------TTGTSKVP------------LNG-----FK-ELEGMS--GFQR
FNIHKSYGSL------------------------QRLPQSHTCF-NQ-LDLPEY-ESYEQ
LRSMLL-TAINEGSEGF
>Saitoella_complicata_XM_019168265.1 .
LNVRRDQV-FHDSFRAL--------------YFKTADEIKYSKLNIRFHG-------EEG
VDAGGVTREW---FQVL----ARQMFNPDY--------ALFTPVSSDST-----------
----------------TFHPNQTS------------------------------------
--GVNPEHLLFFKFIGRIIGKALYDNRLLDCYFSR--AVYKRMLG---------------
--------TPVSLKDMES----------------------LDLDYYKS--------LCWI
LEN--DIT-DII--
-AETFSMEVDE---------------------------YGV-TKIID-LKPD--------
GRNIP---
------------------------------VTEENKQ-EYVKL-VVEYRLITS-VKEQLD
-NFIMG---FHDIISP--DLVK-IFNEK-ELELLISG-----------------------
----LPDID-----------IDDW----RNNTEYQN-YTAA-------------------
------------------------SPQV-QWFWRAVR--------------------SFD
DEERAKLLQF-------STGTSKVP------------LNG-----FK-ELEGMQ--GIQK
FSIHRDFASI------------------------ERLPQSHTCF-NQ-LDLPAY-ESYEQ
LRTQLL-MAISEGNVGF
>Pneumocystis_jirovecii_XM_018374176.1 .
LNVRREMI-FLDSYLAL--------------YFKSGDEMKYSKLNIRFHG-------EEG
VDAGGLTREW---YQAL----ARQMFNPDY--------ALFIPVAADRT-----------
----------------TFHPNTRS------------------------------------
--DVNQDHLSFFKFIGRIIGKALYDNRLLDSHFSR--AVYKKILG---------------
--------KPVSLKDIET----------------------LDLEYYKS--------LVWM
LEN--DIT-DVI--
-TETFSVETEN---------------------------YGA-TETVD-LIPG--------
GRSIL---
------------------------------VTEENKH-EYVKA-VIEYRLINS-VKDQLD
-NFLIG---FYDIIPP--DLIQ-IFNEQ-ELELLISG-----------------------
----LPDID-----------VDDW----RHNTEYYN-YTAS-------------------
------------------------SPQI-QWFWRAVR--------------------SFD
DEQRAKLLQF-------ATGTSKVP------------LNG-----FK-ELEGMQ--GIQK
FSIHRDPTSS------------------------DRLPQSHTCY-NQ-IDLPVY-ESYEA
LRAALL-TAINEGSEGF
>Pneumocystis_carinii_XM_018370458.1 .
LNVRREMI-FLDSYLAL--------------YFKSGDEMKYSKLNIRFHG-------EEG
VDAGGLTREW---YQAL----ARQMFNPDY--------ALFIPVAADRT-----------
----------------TFHPNRRS------------------------------------
--DVNQDHLSFFKFIGRIIGKALYDNRLLDSHFSR--AVYKKILG---------------
--------KPVSLKDIET----------------------LDLEYYKS--------LVWM

LEN--DIT-DVI--
-TETFSVETEN---------------------------YGA-TETVD-LVPG--------
GRTIL---
------------------------------VTEENKH-EYVKA-VIEYRLIDS-VKDQLD
-NFLVG---FYDIIPP--DLIQ-IFNEQ-ELELLISG-----------------------
----LPDID-----------VDDW----RHNTEYFN-YTAS-------------------
------------------------SPQI-QWFWRAVR--------------------SFD
DEQRAKLLQF-------ATGTSKVP------------LNG-----FK-ELEGMQ--GIQK
FSIHRDPTSS------------------------DRLPQSHTCY-NQ-IDLPEY-GSYEA
LRSALL-TAINEGSEGF
>Pneumocystis_murina_XM_007875791.1 .
LNVRREMI-FLDSYLAL--------------YFKSGDEMKYSKLNIRFHG-------EEG
VDAGGLTREW---YQAL----ARQMFNPDY--------ALFIPVAADRT-----------
----------------TFHPNRRS------------------------------------
--DVNQDHLSFFKFIGRIIGKALYDNRLLDSHFSR--AVYKKILG---------------
--------KPVSLKDIET----------------------LDLEYYKS--------LVWM
LEN--DIT-DVI--
-TETFSVETEN---------------------------YGA-TETVD-LVPG--------
GRSIL---
------------------------------VTEENKH-EYVKA-VIEYRLINS-VKDQLD
-NFLVG---FYDIIPP--DLIQ-IFNEQ-ELELLISG-----------------------
----LPDID-----------VDDW----RHNTEYFN-YTAS-------------------
------------------------SPQI-QWFWRAVR--------------------SFD
DEQRAKLLQF-------ATGTSKVP------------LNG-----FK-ELEGMQ--GIQK
FSIHRDPTSS------------------------DRLPQSHTCY-NQ-IDLPEY-GSYEA
LRSALL-TAINEGSEGF
>Epichloe_festucae_ADFL02000131.1_R .
LSVRRENV-FHDSFRSL--------------YFKSGDEMKFGKLNIRFHG-------EEG
VDAGGVTREW---FQVL----ARQMFDPNY--------VLFTPVSSDRT-----------
----------------TFHPHKLS------------------------------------
--AVNPEHLLFFKFIGRIIGKALYEGRLLDCFFSR--AVYKRILG---------------
--------KSVSVKDMES----------------------FDPDYYKS--------LCWM
LDN--DIT-DII--
-TETFSVEDDE---------------------------FGV-TNVVD-MIAN--------
GRDIA---
------------------------------VTEENKH-EYVRL-VVEHKLLSS-VKDQME
-SFLKG---FHEIIPA--ELIS-IFNEQ-ELELLISG-----------------------
----LPDID-----------VDDW----KSNTEYQN-YTPS-------------------
------------------------SQQI-QWFWRAVR--------------------SFD
KEERAKLLQF-------VTGTSKVP------------LNG-----FK-ELEGMN--GISR
FNIHRDYGDK------------------------GRLPSSHTCF-NX-LDVPEY-ESYDI
LRAQIL-KAITQGSEY-
>Epichloe_typhina_AFSE01000593.1_R .
LSVRRENV-FHDSFRSL--------------YFKSGDEMKFGKLNIRFHG-------EEG
VDAGGVTREW---FQVL----ARQMFDPNY--------VLFTPVSSDRT-----------
----------------TFHPHKLS------------------------------------
--AVNPEHLLFFKFIGRIIGKALYEGRLLDCFFSR--AVYKRILG---------------
--------KSVSVKDMES----------------------FDPDYYKS--------LCWM
LDN--DIT-DII--
-TETFSVEDDE---------------------------FGV-TNVVD-MIAN--------
GRDIA---
------------------------------VTEENKH-EYVRL-VVEHKLLSS-VKDQME
-SFLKG---FHEIIPA--ELIS-IFNEQ-ELELLISG-----------------------
----LPDID-----------VDDW----KSNTEYQN-YTPS-------------------
------------------------SQQI-QWFWRAVR--------------------SFD
KEERAKLLQF-------VTGTSKVP------------LNG-----FK-ELEGMN--GISR
FNIHRDYGDK------------------------GRLPSSHTCF-NX-LDVPEY-ESYDI
LRAQIL-KAITQGSEY-

>Epichloe_brachyelytri_AFRB01001099.1_R .
--VRRENV-FHDSFRSL--------------YFKSGDEMKFGKLNIRFHG-------EEG
VDAGGVTREW---FQVL----ARQMFDPNY--------VLFTPVSSDRT-----------
----------------TFHPHKLS------------------------------------
--AVNPEHLLFFKFIGRIIGKALYEGRLLDCFFSR--AVYKRILG---------------
--------KSVSVKDMES----------------------FDPDYYKS--------LCWM
LDN--DIT-DII--
-TETFSVEDDE---------------------------FGV-TNVVD-MIAN--------
GRDIA---
------------------------------VTEENKH-EYVRL-VVEHKLLSS-VKDQME
-SFLKG---FHEIIPA--ELIS-IFNEQ-ELELLISG-----------------------
----LPDID-----------VDDW----KSNTEYQN-YTPS-------------------
------------------------SQQI-QWFWRAVR--------------------SFD
KEERAKLLQF-------VTGTSKVP------------LNG-----FK-ELEGMN--GISR
FNIHRDYGDK------------------------GRLPSSHTCF-NX-LDVPEY-ESYDI
LRAQIL-KAITQGSEY-
>Epichloe_amarillans_AFRF01000001.1_R .
--VRRENV-FHDSFRSL--------------YFKSGDEMKFGKLNIRFHG-------EEG
VDAGGVTREW---FQVL----ARQMFDPNY--------VLFTPVSSDRT-----------
----------------TFHPHKLS------------------------------------
--AVNPEHLLFFKFIGRIIGKALYEGRLLDCFFSR--AVYKRILG---------------
--------KSVSVKDMES----------------------FDPDYYKS--------LCWM
LDN--DIT-DII--
-TETFSVEDDE---------------------------FGV-TNVVD-MIAN--------
GRDIA---
------------------------------VTEENKH-EYVRL-VVEHKLLSS-VKDQME
-SFLKG---FHEIIPA--ELIS-IFNEQ-ELELLISG-----------------------
----LPDID-----------VDDW----KSNTEYQN-YTPS-------------------
------------------------SQQI-QWFWRAVR--------------------SFD
KEERAKLLQF-------VTGTSKVP------------LNG-----FK-ELEGMN--GISR
FNIHRDYGDK------------------------GRLPSSHTCF-NX-LDVPEY-ESYDI
LRAQIL-KAITQGSEY-
>Epichloe_glyceriae_AFRG01000186.1_R .
LSVRRENV-FHDSFRSL--------------YFKSGDEMKFGKLNIRFHG-------EEG
VDAGGVTREW---FQVL----ARQMFDPNY--------VLFTPVSSDRT-----------
----------------TFHPHKLS------------------------------------
--AVNPEHLLFFKFIGRIIGKALYEGRLLDCFFSR--AVYKRILG---------------
--------KSVSVKDMES----------------------FDPDYYKS--------LCWM
LDN--DIT-DII--
-TETFSVEDDE---------------------------FGV-TNVID-MIAN--------
GRDIA---
------------------------------VTEENKH-EYVRL-VVEHKLLSS-VKDQME
-SFLKG---FHEIIPA--ELIS-IFNEQ-ELELLISG-----------------------
----LPDID-----------VDDW----KSNTEYQN-YTPS-------------------
------------------------SQQI-QWFWRAVR--------------------SFD
KEERAKLLQF-------VTGTSKVP------------LNG-----FK-ELEGMN--GISR
FNIHRDYGDK------------------------GRLPSSHTCF-NR-LDVPEY-ESYDI
LRAQIL-KAITQGSEY-
>Neotyphodium_gansuense_AFRE01000024.1_R .
LSVRRENV-FHDSFRSL--------------YFKSGDEMKFGKLNIRFHG-------EEG
VDAGGVTREW---FQVL----ARQMFDPNY--------VLFTPVSSDRT-----------
----------------TFHPHKLS------------------------------------
--AVNPEHLLFFKFIGRIIGKALYEGRLLDCFFSR--AVYKRILG---------------
--------KSVSVKDMES----------------------FDPDYYKS--------LCWM
LDN--DIT-DII--
-TETFSVEDDE---------------------------FGV-TNVID-MIPN--------
GRDIA---
------------------------------VVEENKH-EYVRL-VVEHKLLSS-VKDQME

-SFLKG---FHEIIPA--ELIS-IFNEQ-ELELLISG-----------------------
----LPDID-----------VDDW----KSNTEYQN-YTPS-------------------
------------------------SQQI-QWFWRAVR--------------------SFD
KEERAKLLQF-------VTGTSKVP------------LNG-----FK-ELEGMN--GISR
FNIHRDYGDK------------------------NRLPSSHTCF-NR-LDVPEY-ESYDT
LRAQIL-KAITQGSEY-
>Atkinsonella_hypoxylon_JFHB01000023.1 .
LSVRRDHV-FHDSFRSL--------------YFKSGDEMKFGKLNIRFHG-------EEG
VDAGGVTREW---FQVL----ARQMFDPNY--------VLFTPVSSDRT-----------
----------------TFHPNKLS------------------------------------
--AVNPEHLLFFKFIGRIIGKALYEGRLLDCFFSR--AVYKRILG---------------
--------KSVSVKDMES----------------------FDPDYYKS--------LCWM
LEN--DIT-DII--
-TETFSVEDDE---------------------------FGV-TNIVD-LISK--------
GRDIA---
------------------------------VTEENKY-EYVRL-VVEHKLLSS-VKDQME
-SFLKG---FHEIIPA--ELIS-IFNEQ-ELELLISG-----------------------
----LPDID-----------IDDW----KSNTEYQN-YTPS-------------------
------------------------SQQI-QWFWRAVR--------------------SFD
KEERAKLLQF-------VTGTSKVP------------LNG-----FK-ELEGMN--GISR
FNIHRDYGDK------------------------DRLPSSHTCF-NX-LDIPEY-ESYDM
LRAQLL-KAITQGSEYF
>Periglandula_ipomoeae_AFRD01000370.1 .
LSVRRDHV-FHDSFRSL--------------YFKSGDEMKFGKLNIRFHG-------EEG
VDAGGVTREW---FQVL----ARQMFDPNY--------VLFIPVSSDRT-----------
----------------TFHPNKLS------------------------------------
--AVNPEHLLFFKFIGRIIGKALYEGRLLDCFFSR--AVYKRILG---------------
--------KSVSVKDMEL----------------------FDPDYYKS--------LCWM
LDD--DIT-DII--
-TETFCVEDDE---------------------------FGV-TNVID-LVPN--------
GRDIA---
------------------------------VTEENKH-EYVRL-VVEHKLLSS-VKDQME
-SFLKG---FHEIIPA--ELIS-IFNEQ-ELELLISG-----------------------
----LPDID-----------IDDW----KSNTEYQN-YTPS-------------------
------------------------SQQI-QWFWRAVR--------------------SFD
KEERAKLLQF-------VTGTSKVP------------LNG-----FK-ELEGMN--GISR
FNIHRDYGDK------------------------DRLPSSHTCF-NX-LDIPEY-ESYDM
FRAQLL-KAITQGSEYF
>Aciculosporium_take_AFQZ01000418.1_R .
LSVRRDHV-FHDSFRSL--------------YFKSGDEMKFGKLNIRFHG-------EEG
VDAGGVTREW---FQVL----ARQMFDPNY--------VLFIPVSSDRT-----------
----------------TFHPNKLS------------------------------------
--AVNPEHLMFFKFIGRIIGKALYEGRLLDCFFSR--AVYKRILG---------------
--------KSVSVKDMES----------------------FDPDYYKS--------LCWM
LDN--DIA-DII--
-TETFSVEDDE---------------------------FGV-TNVVD-LIPS--------
GRDIS---
------------------------------VTEEDKH-EYVRL-VVEHRLLSS-VKDQME
-SFLKG---FHEIIPA--ELIS-IFNEQ-ELELLISG-----------------------
----LPDID-----------IDDW----KSNTEYQN-YTPS-------------------
------------------------SQQI-QWFWRAVR--------------------SFD
KEERAKLLQF-------VTGTSKVP------------LNG-----FK-ELEGMN--GISR
FNIHRDYGNK------------------------DRLPSSHTCF-FTELDIPEY-ESYDI
LRTQLH-KAITQGSEY-
>Claviceps_paspali_AFRC01000001.1 .
LSVRRDHV-FHDSFRSL--------------YFKSGDEMKFGKLNIRFHG-------EEG
VDAGGVTREW---FQVL----ARQMFDPNY--------VLFIPVSSDRT-----------
----------------TFHPNKLS------------------------------------

--AVNPEHLLFFKFIGRIIGKALYEGRLLDCFFSR--AVYKRILG---------------
--------KSVSVKDMES----------------------FDPDYYKS--------LCWM
LDN--EIT-DII--
-TETFSVEDDE---------------------------FGV-TNVVD-LVPG--------
GRDII---
------------------------------VTEENKH-EYVRL-VVEHKLLSS-VKDQME
-SFLRG---XHEIIPA--ELIS-IFNEQ-ELELLISG-----------------------
----LPDID-----------VDDW----KSNTEYQN-YNPS-------------------
------------------------SPQI-QWFWRAVR--------------------SFD
KEERAKLLQF-------VTGTSKVP------------LNG-----FK-ELEGMN--GISR
FNIHRDYGDK------------------------NRLPTSHTCF-NX-LDIPEY-ESYDM
LRAQLH-KAITQGSEYF
>2_Ustilaginoidea_virens_BBTG02000005.1 .
LSVRRDHV-FHDSFRSL--------------YYKSGDEMKFGKLNIRFHG-------EEG
VDAGGVTREW---FQVL----ARQMFDPNY--------VLFIPVSSDRT-----------
----------------TFHPNKLS------------------------------------
--AVNPEHLLFFKFIGRIIGKALYEGRLLDCFFSR--AVYKRILG---------------
--------KSVSVKDMES----------------------FDPDYYKS--------LCWM
LDN--DIT-DII--
-TETFSVEDDE---------------------------FGV-TKVVD-LVPS--------
GREMV---
------------------------------VTEENKH-DYVRX--------------QME
-SFLKG---FHEIIPS--DLIS-IFNEQ-ELELLISG-----------------------
----LPDID-----------IDDW----KSNTEYQN-YSPS-------------------
------------------------SQQM-QWFWRAVR--------------------SFD
KEERAKLLQF-------VTGTSKVP------------LNG-----FK-ELEGMN--GVSR
FNIHRDYGDK------------------------DRLPSSHTCF-NX-LDIPEY-ESYDM
LRAQLH-KAITQGSEYF
>Pochonia_chlamydosporia_XM_018293197.1 .
LSVRRDHV-FHDSFRSL--------------YFKSGDEMKFGKLNIRFHG-------EEG
VDAGGVTREW---FQVL----ARQMFDPNY--------ALFTPVSSDRT-----------
----------------TFHPNKLS------------------------------------
--GINPEHLMFFKFIGRIIGKALYEGRLLDCFFSR--AVYKRILG---------------
--------KSVSVKDMES----------------------FDPDYYKS--------LCWM
LDN--DIT-DII--
-TETFSVEDDE---------------------------FGV-TNVVD-LIPN--------
GRDVG---
------------------------------VTEDNKH-EYVRL-VVEHKLLSS-VKEQME
-KFLQG--------KS--FLIS-IFNEQ-ELELLISG-----------------------
----LPDID-----------IDDW----KSNTEYQN-YTPS-------------------
------------------------SQQI-QWFWRAVR--------------------SFD
KEERAKLLQF-------VTGTSKVP------------LNG-----FK-ELEGMN--GISR
FNIHRDYGDK------------------------DRLPSSHTCF-N--------------

>Metarhizium_anisopliae_ADNJ01000348.1 .
--VRRDHV-FHDSFRSL--------------YFKSGDEMKFGKLNIRFHG-------EEG
VDAGGVTREW---FQVL----ARQMFDPNY--------ALFTPVSSDRT-----------
----------------TFHPNKLS------------------------------------
--GINPEHLMFFKFIGRIIGKALYEGRLLDCFFSR--AVYKRILG---------------
--------KSVSVKDMES----------------------FDPDYYKS--------LCWM
LDN--DIT-DII--
-TETFSVEDDE---------------------------FGV-TNVFD-LVPN--------
GRDVA---
------------------------------VTEDNKH-EYVRL-VVEHKLX---------
-----G---FHDIIPA--ELIS-IFNEQ-ELELLISG-----------------------
----LPDID-----------IDDW----KSNTEYQN-YTPS-------------------
------------------------SQQI-QWFWRAVR--------------------SFD
KEERAKLLQF-------VTGTSKVP------------LNG-----FK-ELEGMN--GISR

FNIHRDYGDK------------------------DRLPSSHTCF-NX-LDLPEY-ESYDM
LRAQLH-KAITQGSEY-
>Metarhizium_acridum_XM_007813798.1 .
LSVRRDHV-FHDSFRSL--------------YFKSGDEMKFGKLNIRFHG-------EEG
VDAGGVTREW---FQVL----ARQMFDPNY--------ALFTPVSSDRT-----------
----------------TFHPNKLS------------------------------------
--GINPEHLMFFKFIGRIIGKALYEGRLLDCFFSR--AVYKRILG---------------
--------KSVSVKDMES----------------------FDPDYYKS--------LCWM
LDN--DIT-DII--
-TETFSVEDDE---------------------------FGV-TNVFD-LVPN--------
GRDVA---
------------------------------VTEDNKH-EYVRL-VVEHKLLSS-VKEQME
-KFLQG---FHDIIPA--ELIS-IFNEQ-ELELLISG-----------------------
----LPDID-----------IDDW----KSNTEYQN-YTPS-------------------
------------------------SQQI-QWFWRAVR--------------------SFD
KEERAKLLQF-------VTGTSKVP------------LNG-----FK-ELEGMN--GISR
FNIHRDYGDK------------------------DRLPSSHTCF-NQ-LDLPEY-ESYDM
LRAQLH-KAITQGSEYF
>Metarhizium_robertsii_XM_007819853.2 .
LSVRRDHV-FHDSFRSL--------------YFKSGDEMKFGKLNIRFHG-------EEG
VDAGGVTREW---FQVL----ARQMFDPNY--------ALFTPVSSDRT-----------
----------------TFHPNKLS------------------------------------
--GINPEHLMFFKFIGRIIGKALYEGRLLDCFFSR--AVYKRILG---------------
--------KSVSVKDMES----------------------FDPDYYKS--------LCWM
LDN--DIT-DII--
-TETFSVEDDE---------------------------FGV-TNVFD-LVPN--------
GRDVA---
------------------------------VTEDNKH-EYVRL-VVEHKLLSS-VKEQME
-KFLQG---FHDIIPA--ELIS-IFNEQ-ELELLISG-----------------------
----LPDID-----------IDDW----KSNTEYQN-YTPS-------------------
------------------------SQQI-QWFWRAVR--------------------SFD
KEERAKLLQF-------VTGTSKVP------------LNG-----FK-ELEGMN--GISR
FNIHRDYGDK------------------------DRLPSSHTCF-NQ-LDLPEY-ESYDM
LRAQLH-KAITQGSEYF
>Metarhizium_brunneum_XM_014691392.1 .
LSVRRDHV-FHDSFRSL--------------YFKSGDEMKFGKLNIRFHG-------EEG
VDAGGVTREW---FQVL----ARQMFDPNY--------ALFTPVSSDRT-----------
----------------TFHPNKLS------------------------------------
--GINPEHLMFFKFIGRIIGKALYEGRLLDCFFSR--AVYKRILG---------------
--------KSVSVKDMES----------------------FDPDYYKS--------LCWM
LDN--DIT-DII--
-TETFSVEDDE---------------------------FGV-TNVFD-LVPN--------
GRDVA---
------------------------------VTEDNKH-EYVRL-VVEHKLLSS-VKEQME
-KFLQG---FHDIIPA--ELIS-IFNEQ-ELELLISG-----------------------
----LPDID-----------IDDW----KSNSEYQN-YTPS-------------------
------------------------SQQI-QWFWRAVR--------------------SFD
KEERAKLLQF-------VTGTSKVP------------LNG-----FK-ELEGMN--GISR
FNIHRDYGDK------------------------DRLPSSHTCF-NQ-LDLPEY-ESYDM
LRAQLH-KAITQGSEYF
>Metarhizium_majus_XM_014727471.1 .
LSVRRDHV-FHDSFRSL--------------YFKSGDEMKFGKLNIRFHG-------EEG
VDAGGVTREW---FQVL----ARQMFDPNY--------ALFTPVSSDRT-----------
----------------TFHPNKLS------------------------------------
--GINPEHLMFFKFIGRIIGKALYEGRLLDCFFSR--AVYKRILG---------------
--------KSVSVKDMES----------------------FDPDYYKS--------LCWM
LDN--DIT-DII--
-TETFSVEDDE---------------------------FGV-TNVFD-LVPN--------

```
GRDVA-------------------------------------------------------
------------------------------VTEDNKH-EYVRL-VVEHKLLSS-VKEQME
-KFLQG---FHDIVPA--ELIS-IFNEQ-ELELLISG-----------------------
----LPDID-----------IDDW----KSNTEYQN-YTPS-------------------
------------------------SQQI-QWFWRAVR--------------------SFD
KEERAKLLQF-------VTGTSKVP------------LNG-----FK-ELEGMN--GISR
FNIHRDYGDK------------------------DRLPSSHTCF-NQ-LDLPEY-ESYDM
LRAQLH-KAITQGSEYF
>Purpureocillium_lilacinum_XM_018324184.1 .
LSVRREQV-FRDSFKAL--------------YFKSGDEMKFGKLNIRFHG-------EEG
VDAGGVTREW---FQVL----ARQMFDPNY--------ALFIPVSSDRT-----------
----------------TFHPNKLS------------------------------------
--GVNEAHLMFFKFIGRIIGKALYEGRLLDCFFSR--AVYKRILG---------------
--------KSVSVKDMES----------------------FDPDYYKS--------LCWM
LDN------------------------------------------------DIT-DII--
-TETFSVEDDE---------------------------FGV-TNVVD-LVPN--------
GREIS-------------------------------------------------------
------------------------------VTEENKQ-EYVRL-VVEHKLLSS-VKEQME
-NFLKG---FHEIIPA--NLIS-IFSEQ-ELELLISG-----------------------
----LPDID-----------IDDW----KSNTEYQN-YTPS-------------------
------------------------SQQI-QWFWRAVR--------------------SLD
KEERAKLLQF-------VTGTSKVP------------LNG-----FK-ELEGMN--GVNR
FNIHRDYGSK------------------------DRLPSSHTCF-NQ-LDLPEY-ETYDM
LRSQLV-KAITQGSEYF
>Ophiocord_unilateralis_LAZP01000068.1 .
LSVRRDHV-FHDSFKSL--------------YFKSGDEMKFGKLNIRFHG-------EEG
VDAGGVTREW---FQVL----ARQMFDPNY--------ALFIPVSSDRT-----------
----------------TFHPNKLS------------------------------------
--GINDEHLMFFKFIGRVIGKALYEGRLLDCFFSR--AVYKRILG---------------
--------KSVSVKDMES----------------------FDPDYYKS--------LCWM
LDN------------------------------------------------EIT-DII--
-TETFSVENDE---------------------------FGV-TTVVD-LVPN--------
GRENS-------------------------------------------------------
------------------------------VSEENKH-DYVRL-VVEHKLLAS-VKDQME
-HFLKG---FHEIIPA--KLIS-IFNEQ-ELELLISG-----------------------
----LPDID-----------IDDW----KSNTEYQN-YTPS-------------------
------------------------SQQI-QWFWRAVR--------------------SFD
KEERAKLLQF-------VTGTSKVP------------LNG-----FK-ELEGMN--GVNR
FNIHRDYGNK------------------------DRLPSSHTCF-NX-LDIPEY-ETYDM
LRSQLH-KAITQGSEYF
>Ophiocord_sinensis_GCQL01008690.1 .
LSVRRDHV-FHDSFKSL--------------YFKSGDEMKFGKLNIRFHG-------EEG
VDAGGVTREW---FQVL----ARQMFDPNY--------ALFIPVSSDRT-----------
----------------TFHPNKLS------------------------------------
--GINDEHLMFFKFIGRIIGKALYEGRLLDCFFSR--AVYKRILG---------------
--------KSVSVKDMES----------------------FDPDYYKS--------LCWM
LDN------------------------------------------------DIT-DII--
-TETFSIEDDA---------------------------FGV-TTVVD-LVLN--------
GREIL-------------------------------------------------------
------------------------------VTEDNKH-DYLRL-VVEHKLLSS-VKDQME
-HFLKG---FHEIVPA--KLIS-IFNEQ-ELELLISG-----------------------
----LPDID-----------IDDW----KSNTEYQN-YNPS-------------------
------------------------TQQI-QWFWRAVR--------------------SFD
KEERAKLLQF-------VTGTSKVP------------LNG-----FK-ELEGMN--GINR
FNIHRDYGNK------------------------DRLPSSHTCF-NQ-LDLPEY-ETYDM
LRCQLL-KAITQ-----
>Albophoma_yamanashiensis_BCKH01000004.1 .
LSVRRDHV-FHDSFKSL--------------YFKSGDEMKFGKLNIRFHG-------EEG
```

VDAGGVTREW---FQVL----ARQMFDPNY--------ALFIPVSSDRT-----------
----------------TFHPNKLS------------------------------------
--GINDEHLMFFKFIGRIIGKALYEGRLLDCFFSR--AVYKRILG---------------
--------KSVSVKDMES----------------------FDPDYYKS--------LCWM
LEN--DIT-DII--
-TETFSVEDDE---------------------------FGV-TNVVD-LISN--------
GREIS---
------------------------------VTEENKH-EYVRL-VVEHKLLAS-VKDQME
-NFLKG---FHEIIPA--KLIS-IFNEQ-ELELLISG-----------------------
----LPDID-----------IDDW----KSNTEYQN-YTPS-------------------
------------------------SQQI-QWFWRAVR--------------------SFD
KEERAKLLQF-------VTGTSKVP------------LNG-----FK-ELEGMN--GVNR
FNIHRDYGNK------------------------DRLPSSHTCF-NX-LDLPEY-ESYDA
LRSQIH-KAITQGSEYF
>Tolypocladium_inflatum_AOHE01000160.1 .
LSVRRDHV-FHDSFKSL--------------YFKSGDEMKFGKLNIRFHG-------EEG
VDAGGVTREW---FQVL----ARQMFDPNY--------ALFIPVSSDRT-----------
----------------TFHPNKLS------------------------------------
--GINDEHLMFFKFIGRTIGKALYEGRLLDCFFSR--AVYKRILG---------------
--------KSVSVKDMES----------------------FDPDYYKS--------LCWM
LEN--DIT-DII--
-TETFSVEDDE---------------------------FGV-TNVVD-LIPN--------
GREIS---
------------------------------VTEENKH-EYVRL-VVEHKLLSS-VKDQME
-NFLKG---FHEIVPA--KLIS-IFNEQ-ELELLISG-----------------------
----LPDID-----------IDDW----KSNTEYQN-YTPS-------------------
------------------------SPQI-QWFWRALR--------------------SFD
KEERAKLLQF-------VTGTSKVP------------LNG-----FK-ELEGMN--GVNR
FNIHRDYGNK------------------------DRLPSSHTCF-NX-LDLPEY-ESYDA
LRSQIH-KAITQGSEYF
>Trichoderma_atroviride_XM_014089110.1 .
LSVRREHV-FHDSFKWL--------------CFKSGEEMKYGKLNIRFNG-------EEG
VDAGGVTREW---FQVL----ARQMFDPNY--------ALFIPVSSDRT-----------
----------------TFHPNKLS------------------------------------
--GINDEHLRFFKFIGRIIGKALYEGRLLDCFFSR--AVYKRILG---------------
--------KSVSVKDMES----------------------FDPDYYKS--------LCWM
LEN--DIT-DII--
-TETFSVEDDE---------------------------FGV-TKIVD-LIPN--------
GREVA---
------------------------------VTEDNKH-EYVRV-VVEHKLLSS-VKEQME
-NFLMG---FHDIIPA--ELIS-IFNEQ-ELELLISG-----------------------
----LPDID-----------IDDW----KANTEYHN-YNPS-------------------
------------------------SPQI-QWFWRAVR--------------------SFD
KEELAKLLQF-------VTGTSKVP------------LNG-----FK-ELEGMN--GVNR
FNIHRDYGNK------------------------DRLPSTHTCF-NQ-LDLPEY-DSYDV
LRSQII-KAITQGSDYF
>Trichoderma_gamsii_XM_018808156.1 .
LSVRREHV-FHDSFKWL--------------CFKSGEEMKYGKLNIRFNG-------EEG
VDAGGVTREW---FQVL----ARQMFDPNY--------ALFIPVSSDRT-----------
----------------TFHPNKLS------------------------------------
--GINDEHLRFFKFIGRIIGKALYEGRLLDCFFSR--AVYKRILG---------------
--------KSVSVKDMES----------------------FDPDYYKS--------LCWM
LEN--DIT-DII--
-TETFSVEDDE---------------------------FGV-TKIVD-LIPN--------
GREVA---
------------------------------VTEDNKH-EYVRV-VVEHKLLSS-VKEQME
-NFLMG---FHDIIPA--ELIS-IFNEQ-ELELLISG-----------------------
----LPDID-----------IDDW----KANTEYHN-YNPS-------------------

------------------------SPQI-QWFWRAVR--------------------SFD
KEELAKLLQF-------VTGTSKVP------------LNG-----FK-ELEGMN--GVNR
FNIHRDYGNK------------------------DRLPSTHTCF-NQ-LDLPEY-DSYDV
LRSQII-KAITQGSDYF
>Trichoderma_virens_XM_014095481.1 .
ISVRREHV-FHDSFKWL--------------CFKSGDEMKYGKLNIRFNG-------EEG
VDAGGVTREW---FQVL----ARQMFDPNY--------ALFIPVSSDRT-----------
----------------TFHPNKLS------------------------------------
--GINDEHLRFFSFIGRIIGKALYEGRLLDCFFSR--AVYKRILG---------------
--------KSVSVKDMES----------------------FDPDYYKS--------LCWM
LEN--DIT-DII--
-TETFSVEDDE---------------------------FGV-TKIVD-LVPN--------
GREIA---
------------------------------VTEENKH-EYVRV-VVEHKLLSS-VKDQME
-NFLSG---FHDIIPA--ELIS-IFNEQ-ELELLISG-----------------------
----LPDID-----------IDDW----KANTEYHN-YSPS-------------------
------------------------SPQI-QWFWRAVR--------------------SFD
KEELAKLLQF-------VTGTSKVP------------LNG-----FK-ELEGMN--GVNR
FNIHRDYGNK------------------------DRLPSTHTCF-NQ-LDLPEY-DSYDI
LRSQII-KAITQGSDYF
>Trichoderma_reesei_XM_006962994.1 .
ISVRREHV-FHDSFKWL--------------CFKSADEMKYGKLNIRFNG-------EEG
VDAGGVTREW---FQVL----ARQMFDPNY--------ALFIPVSSDRT-----------
----------------TFHPNKLS------------------------------------
--GVNDEHLRFFKFIGRIIGKALYEGRLLDCFFSR--AVYKRILG---------------
--------KSVSVKDMES----------------------FDPDYYKS--------LCWM
LEN--DIT-DII--
-TETFSVEDDE---------------------------FGV-TKIVD-LIPN--------
GREIA---
------------------------------VTEENKH-EYVRV-VVEHKLLSS-VKDQME
-NFLSG---FHDIIPA--ELIS-IFNEQ-ELELLISG-----------------------
----LPDID-----------IDDW----KANTEYQN-YSPS-------------------
------------------------SPQI-QWFWRAVR--------------------SFD
KEELAKLLQF-------VTGTSKVP------------LNG-----FK-ELEGMN--GINR
FNIHRDYGNK------------------------DRLPSTHTCF-NQ-LDLPEY-DSYDI
LRSQIL-KAITQGSDYF
>Trichoderma_parareesei_LFMI01000417.1 .
ISVRREHV-FHDSFKWL--------------CFKSADEMKYGKLNIRFNG-------EEG
VDAGGVTREW---FQVL----ARQMFDPNY--------ALFIPVSSDRT-----------
----------------TFHPNKLS------------------------------------
--GVNDEHLRFFKFIGRIIGKALYEGRLLDCFFSR--AVYKRILG---------------
--------KSVSVKDMES----------------------FDPDYYKS--------LCWM
LEN--DIT-DII--
-TETFSVEDDE---------------------------FGV-TKIVD-LIPN--------
GREIA---
------------------------------VTEENKH-EYVRV-VVEHKLLSS-VKDQME
-NFLSG---FHDIIPA--ELIS-IFNEQ-ELELLISG-----------------------
----LPDID-----------IDDW----KANTEYQN-YSPS-------------------
------------------------SPQI-QWFWRAVR--------------------SFD
KEELAKLLQF-------VTGTSKVP------------LNG-----FK-ELEGMN--GINR
FNIHRDYGNK------------------------DRLPSTHTCF-NX-LDLPEY-DSYDI
LRSQIL-KAITQGSDYF
>Isaria_fumosorosea_XM_018851752.1 .
LKVRRESV-YRDSFANL--------------YYKDKDEIKYGKLNIRFDG-------EEG
VDAGGVTREW---FQVL----ARQMFDPNN--------ALFIPVSSDRT-----------
----------------TFHPNKLS----------------------------KWHLNDGT
DGNGESTDSIHFKFIGRIIGKALYEGRLLDCFFSR--AVYKRILG---------------
--------KSVSVKDMES----------------------FDPDYYKS--------LCWM

LEN--DIT-DII--
-TETFSEEEDE---------------------------FGV-TKIVD-LVPN--------
GREIP---
------------------------------VTEENKQ-EYVRL-VVEHRLLTS-VKDQME
-SFLNG---FHEIIPA--ELIS-IFNEQ-ELELLISG-----------------------
----LPDID-----------IDDW----RSNTEYHN-YTPS-------------------
------------------------SQQV-QWFWRAVR--------------------SFD
KEELAKLLQF-------VTGTSKVP------------LNG-----FK-ELEGMN--GISR
FNIHRDYGDK------------------------DRLPTSHTCF-NQ-LDLPEY-DSYDT
LRAQLY-KAITAGNEYF
>Cordyceps_cicadae_AEIW01002045.1 .
LKVRRESV-YRDSFTNL--------------YYKDKDEIKYGKLNIRFDG-------EEG
VDAGGVTREW---FQVL----ARQMFDPNN--------ALFIPVSSDRT-----------
----------------TFHPNKLS----------------------------KWHLNDGT
DGNGESTDNIHFKFIGRIIGKALYEGRLLDCFFSR--AVYKRILG---------------
--------KSVSVKDMES----------------------FDPDYYKS--------LCWM
LEN--DIT-DII--
-TETFSEEEDE---------------------------FGV-TKIVD-LVPN--------
GREIP---
------------------------------VTEENKQ-EYVRL-VVEHRLLTS-VKDQME
-SFLNG---FHEIIPA--ELIS-IFNEQ-ELELLISG-----------------------
----LPDID-----------IDDW----RSNTEYHN-YTPS-------------------
------------------------SQQV-QWFWRAVR--------------------SFD
KEELAKLLQF-------VTGTSKVP------------LNG-----FK-ELEGMN--GISR
FNIHRDYGDK------------------------DRLPTSHTCF-NX-LDLPEY-DSYDV
LRAQLH-KAITAGNEYF
>Lecanicillium_fungicola_FWCC01000068.1 .
LSVRRESV-YRDSFANL--------------YYKSGDEIKYGKLNIRFGG-------EEG
VDAGGVTREW---FQVL----ARQMFDPNN--------ALFIPVSSDRT-----------
----------------TFHPNKLS----------------------------KFHLDDES
SDGAESADSIHFKFIGRIIGKALYEGRLLDCFFSR--AVYKRILG---------------
--------KTVSVKDMES----------------------FDPDYYKS--------LCWM
LEN--DIT-DII--
-TETFSEEEDE---------------------------FGV-TKTVD-LVPN--------
GRDIP---
------------------------------VTEDNKQ-DYVRL-VVEHRLLTS-VKDQME
-SFLKG---FHEIIPA--ELIS-IFNEQ-ELELLISG-----------------------
----LPDID-----------IDDW----RSNTEYHN-YTPS-------------------
------------------------SQQV-QWFWRAVR--------------------SFD
KEELAKLLQF-------VTGTSKVP------------LNG-----FK-ELEGMN--GISR
FNIHRDYGAK------------------------DRLPTSHTCF-NX-LDLPEY-DSYDT
LRAQLY-KAITAGNEYF
>Cordyceps_confragosa_AZHF01000002.1 .
LSVRRESV-YRDSFANL--------------YYKSGDEIKYGKLNIRFGG-------EEG
VDAGGVTREW---FQVL----ARQMFDPNN--------ALFIPVSSDRT-----------
----------------TFHPNKLS----------------------------KFHLNE-E
SNAGESTDSIHFKFIGRIIGKALYEGRLLDCFFSR--AVYKRILG---------------
--------KTVSVKDMES----------------------FDPDYYKS--------LCWM
LEN--DIT-DII--
-TETFSEEEDE---------------------------FGV-TKIVD-LVPN--------
GREIP---
------------------------------VTEENKQ-DYVRL-VVEHRLLTS-VKDQME
-SFLKG---FHEIIPA--ELIS-IFNEQ-ELELLISG-----------------------
----LPDID-----------IDDW----RSNSEYHN-YTPS-------------------
------------------------SQQV-QWFWRAVR--------------------SFD
KEELAKLLQF-------VTGTSKVP------------LNG-----FK-ELEGMN--GVSR
FNIHRDYGDK------------------------DRLPTSHTCF-NX-LDLPEY-DSYDT
LRAQLY-KAITAGNEYF

>Cordyceps_militaris_XM_006670326.1 .
LSVRRESV-YRDSFANL--------------YYKSGDEIKYGKLNIRFGG-------EEG
VDAGGITREW---FQVL----ARQMFDPNN--------ALFIPVSSDRT-----------
----------------TFHPNKLS----------------------------KFHLNE-D
SNAGESTDSIHFKFIGRIIGKALYEGRLLDCFFSR--AVYKRILG---------------
--------KSVSVKDMES----------------------FDPDYYKS--------LCWM
LEN--DIS-DII--
-TETFSEEEDE---------------------------FGV-TRIVD-LVPN--------
GREIP---
------------------------------VTDDNKQ-EYVRL-VVEHRLLTS-VKDQME
-SFLKG---FHEIIPA--ELIS-IFNEQ-ELELLISG-----------------------
----LPDID-----------TDDW----RSNTEYHN-YSPS-------------------
------------------------SQQV-QWFWRAVR---------------------SFD
KEELAKLLQF-------VTGTSKVP------------LNG-----FK-ELEGMN--GISR
FNIHRDYGDK------------------------DRLPTSHTCF-NQ-LDLPEY-DSYDV
LRAQLY-KAITAGNEYF
>Paecilomyces_hepiali_LNDK02000103.1 .
LSVRRESV-YRDSFANL--------------YYKSGDEIKYGKLNIRFGG-------EEG
VDAGGVTREW---FQVL----ARQMFDPNN--------ALFIPVSSDRT-----------
----------------TFHPNKLS----------------------------KFHLNE-D
SNAGESTDSIHFKFIGRIIGKALYEGRLLDCFFSR--AVYKRILG---------------
--------KSVSVKDMES----------------------FDPDYYKS--------LCWM
LEN--DIT-DII--
-TETFSEEEDE---------------------------FGV-TKIVD-LVPN--------
GREIP---
------------------------------VTEDNKQ-EYVRL-VVEHRLLTS-VKDQME
-SFLKG---FHEIIPA--ELIS-IFNEQ-ELELLISG-----------------------
----LPDID-----------IDDW----RSNTEYHN-YTPS-------------------
------------------------SQQV-QWFWRAIR---------------------SFD
KEELAKLLQF-------VTGTSKVP------------LNG-----FK-ELEGMN--GISR
FNIHRDYGDK------------------------DRLPTSHTCF-NX-LDLPEY-DSYDT
LRAQLY-KAITAGNEYF
>Isaria_farinosa_JMNC01001003.1 .
LSVRRESV-YRDSFANL--------------YYKSGDEIKYGKLNIRFGG-------EEG
VDAGGVTREW---FQVL----ARQMFDPNN--------ALFIPVSSDRT-----------
----------------TFHPNKLS----------------------------KFHLNE-D
SNAGESTDSIHFKFIGRIIGKALYEGRLLDCFFSR--AVYKRILG---------------
--------KSVSVKDMES----------------------FDPDYYKS--------LCWM
LEN--DIT-DII--
-TETFSEEEDE---------------------------FGV-TKIVD-LVPN--------
GREIP---
------------------------------VTEENKQ-EYVRL-VVEHRLLTS-VKDQME
-SFLKG---FHEIIPA--ELIS-IFNEQ-ELELLISG-----------------------
----LPDID-----------IDDW----RSNTEYHN-YTPS-------------------
------------------------SQQV-QWFWRAVR---------------------SFD
KEELAKLLQF-------VTGTSKVP------------LNG-----FK-ELEGMN--GISR
FNIHRDYGDK------------------------DRLPTSHTCF-NX-LDIPEY-DSYDT
LRAQLY-KAITAGNEYF
>Beauveria_bassiana_XM_008600615.1 .
LSVRRESV-YRDSFANL--------------YYKSGDEIKYGKLNIRFGG-------EEG
VDAGGVTREW---FQVL----ARQMFDPNN--------ALFIPVSSDRT-----------
----------------TFHPNKLS----------------------------KFHLNE-D
SNAGESTDSIHFKFIGRIIGKALYEGRLLDCFFSR--AVYKRILG---------------
--------KSVSVKDMES----------------------FDPDYYKS--------LCWM
LEN--DIT-DII--
-TETFSEEEDE---------------------------FGV-TKIVD-LVPN--------
GREIP---
------------------------------VTEENKQ-EYVRL-VVEHRLLTS-VKDQME

-SFLKG---FHEIIPA--ELIS-IFNEQ-ELELLISG-----------------------
----LPDID-----------IDDW----RSNAEYHN-YTPS-------------------
------------------------SQQV-QWFWRAVR--------------------SFD
KEELAKLLQF-------VTGTSKVP------------LNG-----FK-ELEGMN--GISR
FNIHRDYGDK------------------------DRLPTSHTCF-NQ-LDLPEY-DSYDT
LRAQLY-KAITAGNEYF
>Cordyceps_brongniartii_AZHA01000001.1 .
LSVRRESV-YRDSFANL--------------YYKSGDEIKYGKLNIRFGG-------EEG
VDAGGVTREW---FQVL----ARQMFDPNN--------ALFIPVSSDRT-----------
----------------TFHPNKLS----------------------------KFHLNE-D
SNAGESTDSIHFKFIGRIIGKALYEGRLLDCFFSR--AVYKRILG---------------
--------KSVSVKDMES----------------------FDPDYYKS--------LCWM
LEN--DIT-DII--
-TETFSEEEDE---------------------------FGV-TKIVD-LVPN--------
GREIP---
------------------------------VTEENKQ-EYVRL-VVEHRLLTS-VKDQME
-SFLKG---FHEIIPA--ELIS-IFNEQ-ELELLISG-----------------------
----LPDID-----------IDDW----RSNAEYHN-YTPS-------------------
------------------------SQQV-QWFWRAVR--------------------SFD
KEELAKLLQF-------VTGTSKVP------------LNG-----FK-ELEGMN--GISR
FNIHRDYGDK------------------------DRLPTSHTCF-NX-LDLPEY-DSYDT
LRAQLY-KAITAGNEYF
>Claviceps_fusiformis_AFRA01000582.1_R .
LSVRRDHV-FHDSFRSL--------------YFKSGDEMKFGKLNIKFHG-------EEG
VDAGGVTREW---FQVL----ARQMFDPNY--------VLFTPVSSDRT-----------
----------------TFHPNKLS------------------------------------
--AVNPEHLLFFKFIGRIIGKALYEGRLLDCFFSR--AVYKRILG---------------
--------KSVSVKDMES----------------------FDPDYYKS--------LCWM
LNN--DIA-DII--
-TETFSVEDDE---------------------------FGV-TNIVD-LIPN--------
GREVA---
------------------------------VTEENKH-EYVRL-VVEHRLLAS-VKDQME
-SFLIG---FHEIIPA--ELIS-IFNEQ-ELELLISG-----------------------
----LPDID-----------VDDW----KSNTEYQN-YTPS-------------------
------------------------SQQI-QWFWRAVR--------------------SFD
KEERAKLLQF-------VTGTSKVP------------LNG-----FK-ELEGMN--GVSR
FNIHRDYGDK------------------------DRLPSSHTCF-NR-------------

>Stachybotrys_chlorohalonata_APWP01004461. 1..
IGVRRENV-FHDSFRSL--------------FFLSGDELKYGKLNIRFHG-------EEG
VDAGGVTREW---FQVL----ARQMFDPNY--------VLFTPVSSDRT-----------
----------------TFHPNKLS------------------------------------
--AINDEHLAFFKFIGRVIGKALYEGRVLDCFFSR--AVYKRILG---------------
--------KSVSVKDMES----------------------FDPDYYKS--------LCWM
LDN--DIT-DII--
-TESFSVEDDA---------------------------FGA-TKVVD-LIPN--------
GREVP---
------------------------------VTEDNKH-EYVRL-VVEHKLLSS-VKEQME
-NFLFG---FHEIIPA--ELIS-IFDEQ-ELELLISG-----------------------
----LPDID-----------VDDW----KSNTEYHN-YSPS-------------------
------------------------SQQI-QWFWRAVR--------------------SFD
KEERAKLLQF-------VTGTSKVP------------LNG-----FK-ELEGMN--GVNR
FNIHRDYGKK------------------------DRLPTSHTCF-NX-LDIPEY-DSYDV
LRSQIM-KAITAGSDYF
>Stachybotrys_chartarum_AQPQ01002140.1 .
IGVRRENV-FHDSFRSL--------------FFLSGDELKYGKLNIRFHG-------EEG
VDAGGVTREW---FQVL----ARQMFDPNY--------VLFTPVSSDRT-----------
----------------TFHPNKLS------------------------------------

--AINDEHLAFFKFIGRVIGKALYEGRVLDCFFSR--AVYKRILG---------------
--------KSVSVKDMES----------------------FDPDYYKS--------LCWM
LDN--DIT-DII--
-TESFSVEDDA---------------------------FGA-TKVVD-LIPN--------
GREVP---
------------------------------VTEDNKH-EYVRL-VVEHKLLSS-VKEQME
-NFLFG---FHEIIPA--ELIS-IFDEQ-ELELLISG-----------------------
----LPDID-----------VDDW----KSNTEYHN-YSPS-------------------
------------------------SQQI-QWFWRAVR--------------------SFD
KEERAKLLQF-------VTGTSKVP------------LNG-----FK-ELEGMN--GVNR
FNIHRDYGKK------------------------DRLPTSHTCF-NX-LDIPEY-DSYDV
LRSQIM-KAITAGSDYF
>Fusarium_poae_LYXU01000001.1 .
---LNIRFHG-------EEG
VDAGGVTREW---FQVL----SRQMFDPNY--------VLFTPVSSDRT-----------
----------------TFHPNKLS------------------------------------
--GINDEHLLFFKFIGRIIGKALYEGRVLDCYFSR--ALYKRILG---------------
--------KSVSVKDMES----------------------FDPDYYKS--------LCWM
LNN--DIT-DII--
-TETFSVEDDE---------------------------FGV-TNVVD-LIPN--------
GREIA---
------------------------------VTEENKH-DYVRL-VVEHKLLSS-VKEQMA
-HFLQG-----XIIPA--ELIS-IFNEQ-ELELLISG-----------------------
----LPDID-----------IDDW----KSNTEYHN-YTPS-------------------
------------------------SQQI-QWFWRALR--------------------SFD
KEERAKMLQF-------VTGTSKVP------------LNG-----FK-ELEGMN--GVNR
FNIHRDYGNK------------------------NRLPSSHTCF-N--------------

>Calonectria_pseudoreteaudii_MOCD01000065. 1..
LSVRREHV-FHDSFKSL--------------YFKLGDEMKFGKLNIRFHG-------EEG
VDAGGVTREW---FQVL----SRQMFDPNY--------VLFTPVSSDRT-----------
----------------TFHPNKLS------------------------------------
--GINDEHLMFFKFIGRIIGKALYEGRVLDCYFSR--AVYKRILG---------------
--------KSVSVKDMES----------------------FDPDYYKS--------LCWM
LDN--DIT-DII--
-TETFSVEDDE---------------------------FGV-TNVVD-LIPN--------
GREIA---
------------------------------VTEENKH-DYVRL-VVEHKLLSS-VKEQME
-SFLRG---FHEIIPA--ELIA-IFNEQ-ELELLISG-----------------------
----LPDID-----------IDDW----KSNTEYHN-YSPS-------------------
------------------------SQQI-QWFWRALR--------------------SFD
KEERAKLLQF-------VTGTSKVP------------LNG-----FK-ELEGMN--GVNR
FNIHRDYGNK------------------------DRLPSSHTCF-NX-LDLPEY-ESYDV
LRSQIM-KAITAGSEYF
>Calonectria_pseudonaviculata_JYJY01000116 1..
LSVRREHV-FHDSFKSL--------------YFKSGDEMKFGKLNIRFHG-------EEG
VDAGGVTREW---FQVL----SRQMFDPNY--------VLFTPVSSDRT-----------
----------------TFHPNKLS------------------------------------
--GINDEHLMFFKFIGRIIGKALYEGRVLDCYFSR--AVYKRILG---------------
--------KSVSVKDMES----------------------FDPDYYKS--------LCWM
LDN--DIT-DII--
-TETFSVEDDE---------------------------FGV-TNVVD-LIPN--------
GREIA---
------------------------------VTEENKH-DYVRL-VVEHKLLSS-VKEQME
-SFLKG---FHEIIPA--ELIA-IFNEQ-ELELLISG-----------------------
----LPDID-----------VDDW----KSNTEYHN-YSPS-------------------
------------------------SQQI-QWFWRALR--------------------SFD
KEERAKLLQF-------VTGTSKVP------------LNG-----FK-ELEGMN--GVNR

FNIHRDYGNK------------------------DRLPSSHTCF-NX-LDLPEY-ESYDV
LRSQIM-KAITAGSEYF
>Ilyonectria_destructans_MPHF01000044.1 .
LSVRREHV-FHDSFKSL--------------YFKSGDEMKFGKLNIRFHG-------EEG
VDAGGVTREW---FQVL----SRQMFDANY--------VLFTPVSSDRT-----------
----------------TFHPNKLS------------------------------------
--GINDEHLMFFKFIGRIIGKALYEGRVLDCYFSR--AVYKRILG---------------
--------KSVSVKDMES----------------------FDPDYYKS--------LCWM
LEN--DIT-DII--
-TETFSVEDDE---------------------------FGV-TNVVD-LIPN--------
GREVA---
------------------------------VTEENKH-DYVRL-VVEHKLLSS-VKEQME
-SFLRG---FHEIIPA--ELIA-IFNEQ-ELELLISG-----------------------
----LPDID-----------VDDW----KSNTEYHN-YNPS-------------------
------------------------SQQI-QWFWRALR--------------------SFD
KEERAKLLQF-------VTGTSKVP------------LNG-----FK-ELEGMN--GVNR
FNIHRDYGNK------------------------DRLPSSHTCF-NX-LDLPEY-ESYDA
LRSQIM-KAITAGSEYF
>Neonectria_ditissima_LKCW01000265.1 .
LSVRREHV-FHDSFKSL--------------YFKSGDEMKFGKLNIRFHG-------EEG
VDAGGVTREW---FQVL----SRQMFDANY--------VLFTPVSSDRT-----------
----------------TFHPNKLS------------------------------------
--GINDEHLMFFKFIGRIIGKALYEGRLLDCYFSR--AVYKRILG---------------
--------KSVSVKDMES----------------------FDPDYYKS--------LCWM
LDN--DIT-DII--
-TETFSVEDDE---------------------------FGV-TNVVD-LIEN--------
GREIA---
------------------------------VTEENKH-DYVRL-VVEHKLLSS-VKEQME
-SFLRG---FHEIIPS--ELIA-IFNEQ-ELELLISG-----------------------
----LPDID-----------VDDW----KSNTEYHN-YNPS-------------------
------------------------SQQI-QWFWRALR--------------------SFD
KEERAKLLQF-------VTGTSKVP------------LNG-----FK-ELEGMN--GVNR
FNIHRDYGNK------------------------DRLPSSHTCF-NX-LDLPEY-ESYDA
LRSQIM-KAITAGSEYF
>Dactylonectria_macrodidyma_JYGD01000056.1 .
LSVRREHV-FHDSFKSL--------------YFKSGDEMKFGKLNIRFHG-------EEG
VDAGGVTREW---FQVL----SRQMFDPNY--------VLFTPVSSDRT-----------
----------------TFHPNKLS------------------------------------
--GINDEHLMFFKFIGRIIGKALYEGRLLDCYFSR--AVYKRILG---------------
--------KSVSVKDMES----------------------FDPDYYKS--------LCWM
LDN--DIT-DII--
-TETFSVEDDE---------------------------FGA-TTVVD-LIPN--------
GREVA---
------------------------------VTEENKG-EYVRL-VVEHKLLSS-VKEQME
-SFLRG---FHEIIPA--ELIA-IFNEQ-ELELLISG-----------------------
----LPDID-----------IDDW----KSNTEYHN-YNPS-------------------
------------------------SQQI-QWFWRALR--------------------SFD
KEERAKLLQF-------VTGTSKVP------------LNG-----FK-ELEGMN--GVNR
FNIHRDYGNK------------------------DRLPSSHTCF-NX-LDLPEY-ESYDA
LRSQIM-KAITAGSEYF
>Fusarium_meridionale_LHUA01000031.1 .
LSVRREHV-FHDSFKHL--------------YFKSGDEMKFGKLNIRFHG-------EEG
VDAGGVTREW---FQVL----SRQMFDPNY--------VLFTPVSSDRT-----------
----------------TFHPNKLS------------------------------------
--GINDEHLLFFKFIGRIIGKALYEGRVLDCYFSR--ALYKRILG---------------
--------KSVSVKDMES----------------------FDPDYYKS--------LCWM
LDN--DIT-DII--
-TETFSVEDDE---------------------------FGV-TNVVD-LIPN--------

GREIA---
------------------------------VTEENKH-DYVRL-VVEHKLLSS-VKEQMA
-HFLQG---FHDIIPA--ELIS-IFNEQ-ELELLISG-----------------------
----LPDID-----------IDDW----KSNTEYHN-YTPS-------------------
------------------------SQQI-QWFWRALR--------------------SFD
KEERAKMLQF-------VTGTSKVP------------LNG-----FK-ELEGMN--GVNR
FNIHRDYGNK------------------------NRLPSSHTCF-NX-LDLPEY-ESYDH
LRSQVM-KAITAGSEYF
>Fusarium_graminearum_XM_011318023.1 .
LSVRREHV-FHDSFKHL--------------YFKSGDEMKFGKLNIRFHG-------EEG
VDAGGVTREW---FQVL----SRQMFDPNY--------VLFTPVSSDRT-----------
----------------TFHPNKLS------------------------------------
--GINDEHLLFFKFIGRIIGKALYEGRVLDCYFSR--ALYKRILG---------------
--------KSVSVKDMES----------------------FDPDYYKS--------LCWM
LDN--DIT-DII--
-TETFSVEDDE---------------------------FGV-TNVVD-LIPN--------
GREIA---
------------------------------VTEENKH-DYVRL-VVEHKLLSS-VKEQMA
-HFLQG---FHDIIPA--ELIS-IFNEQ-ELELLISG-----------------------
----LPDID-----------IDDW----KSNTEYHN-YTPS-------------------
------------------------SQQI-QWFWRALR--------------------SFD
KEERAKMLQF-------VTGTSKVP------------LNG-----FK-ELEGMN--GVNR
FNIHRDYGNK------------------------NRLPSSHTCF-NQ-LDLPEY-ESYDH
LRSQVM-KAITAGSEYF
>Fusarium_pseudograminearum_CBMD010000084. 1..
LSVRREHV-FHDSFKHL--------------YFKSGDEMKFGKLNIRFHG-------EEG
VDAGGVTREW---FQVL----SRQMFDPNY--------VLFTPVSSDRT-----------
----------------TFHPNKLS------------------------------------
--GINDEHLLFFKFIGRIIGKALYEGRVLDCYFSR--ALYKRILG---------------
--------KSVSVKDMES----------------------FDPDYYKS--------LCWM
LDN--DIT-DII--
-TETFSVEDDE---------------------------FGV-TNVVD-LIPN--------
GREIA---
------------------------------VTEENKH-DYVRL-VVEHKLLSS-VKEQMA
-HFLQG---FHDIIPA--ELIS-IFNEQ-ELELLISG-----------------------
----LPDID-----------IDDW----KSNTEYHN-YTPS-------------------
------------------------SQQI-QWFWRALR--------------------SFD
KEERAKMLQF-------VTGTSKVP------------LNG-----FK-ELEGMN--GVNR
FNIHRDYGNK------------------------NRLPSSHTCF-NX-LDLPEY-ESYDH
LRSQVM-KAITAGSEYF
>Fusarium_langsethiae_JXCE01000015.1 .
LSVRREHV-FHDSFKHL--------------YFKSGDEMKFGKLNIRFHG-------EEG
VDAGGVTREW---FQVL----SRQMFDPNY--------VLFTPVSSDRT-----------
----------------TFHPNKLS------------------------------------
--GINDEHLLFFKFIGRIIGKALYEGRVLDCYFSR--ALYKRILG---------------
--------KSVSVKDMES----------------------FDPDYYKS--------LCWM
LDN--DIT-DII--
-TETFSVEDDE---------------------------FGV-TNVVD-LIPN--------
GREIA---
------------------------------VTEENKH-DYVRL-VVEHKLLSS-VKEQMA
-HFLQG---FHDIIPA--ELIS-IFNEQ-ELELLISG-----------------------
----LPDID-----------IDDW----KSNTEYHN-YTPS-------------------
------------------------SQQI-QWFWRALR--------------------SFD
KEERAKMLQF-------VTGTSKVP------------LNG-----FK-ELEGMN--GVNR
FNIHRDYGNK------------------------NRLPSSHTCF-NX-LDLPEY-ESYDH
LRSQIM-KAITAGSEYF
>Fusarium_culmorum_LT598659.1_a.
LSVRREHV-FHDSFKHL--------------YFKSGDEMKFGKLNIRFHG-------EEG

VDAGGVTREW---FQVL----SRQMFDPNY--------VLFTPVSSDRT-----------
----------------TFHPNKLS------------------------------------
--GINDEHLLFFKFIGRIIGKALYEGRVLDCYFSR--ALYKRILG---------------
--------KSVSVKDMES----------------------FDPDYYKS--------LCWM
LDN---DIT-DII--
-TETFSVEDDE---------------------------FGV-TNVVD-LIPN--------
GREIA---
-----------------------------VTEENKH-DYVRL-VVEHKLLSS-VKEQMA
-HFLQG---FHDIIPA--ELIS-IFNEQ-ELELLISG-----------------------
----LPDID-----------IDDW----KSNTEYHN-YTPS-------------------
------------------------SQQI-QWFWRALR--------------------SFD
KEERAKMLQF-------VTGTSKVP------------LNG-----FK-ELEGMN--GVNR
FNIHRDYGNK------------------------NRLPSSHTCF-NX-LDLPEY-ESYDH
LRSQIM-KAITAGSEY-
>Fusarium_praegraminearum_LXHY01000027.1 .
LSVRREHV-FHDSFKHL--------------YFKSGDEMKFGKLNIRFHG-------EEG
VDAGGVTREW---FQVL----SRQMFDPNY--------VLFTPVSSDRT-----------
----------------TFHPNKLS------------------------------------
--GINDEHLLFFKFIGRIIGKALYEGRVLDCYFSR--ALYKRILG---------------
--------KSVSVKDMES----------------------FDPDYYKS--------LCWM
LEN---DIT-DII--
-TETFSVEDDE---------------------------FGV-TNVVD-LIPN--------
GREIA---
-----------------------------VTEENKH-DYVRL-VVEHKLLSS-VKEQMA
-HFLQG---FHDIIPA--ELIS-IFNEQ-ELELLISG-----------------------
----LPDID-----------IDDW----KSNTEYHN-YTPS-------------------
------------------------SQQI-QWFWRALR--------------------SFD
KEERAKMLQF-------VTGTSKVP------------LNG-----FK-ELEGMN--GVNR
FNIHRDYGNK------------------------NRLPSSHTCF-NX-LDLPEY-ESYDH
LRSQVM-KAITAGSEYF
>Nectria_haematococca_XM_003054392.1 .
LSVRREQV-FHDSFKSL--------------YFKSGDEMKFGKLNIRFHG-------EEG
VDAGGVTREW---FQVL----SRQMFDPNY--------VLFIPVSSDRT-----------
----------------TFHPNKLS------------------------------------
--GINDEHLMFFKFIGRIIGKALYEGRVLDCYFSR--AVYKRILG---------------
--------KSVSVKDMES----------------------FDPDYYKS--------LCWM
LDN---DIT-DII--
-TETFSVEDDE---------------------------FGV-TNVVD-LIPN--------
GREIA---
-----------------------------VTEENKH-DYVRL-VVEHKLLSS-VKEQMA
-HFLQG---FHDIIPA--ELIS-IFNEQ-ELELLISG-----------------------
----LPDID-----------IDDW----KSNTEYHN-YNPS-------------------
------------------------SQQI-QWFWRALR--------------------SFD
KEERAKLLQF-------VTGTSKVP------------LNG-----FK-ELEGMN--GVNR
FNIHRDYGNK------------------------DRLPSSHTCF-NQ-LDLPEY-ESYDQ
LRSQII-KAITAGSEYF
>Fusarium_virguliforme_GBJV01000293.1 .
LSVRREQV-FHDSFKSL--------------YFKSGDEMKFGKLNIRFHG-------EEG
VDAGGVTREW---FQVL----ARQMFDANY--------VLFTPVSSDRT-----------
----------------TFHPNKLS------------------------------------
--DINDEHLMFFKFIGRIIGKALYEGRVLDCYFSR--AVYKRILG---------------
--------RSVSVKDMES----------------------FDPDYYKS--------LCWM
LEN---DIT-DII--
-TETFSVEEES---------------------------FGV-FKVVD-LAPN--------
GREIA---
-----------------------------VTEENKH-DYVRL-VVEHKLLSS-VKEQMA
-HFLQG---FHDIIPA--ELIS-IFNEQ-ELELLISG-----------------------
----LPDID-----------IDDW----KSNTEYHN-YNPS-------------------

------------------------SQQI-QWFWRALR--------------------SFD
KEERAKLLQF-------VTGTSKVP------------LNG-----FK-ELEGMN--GVNR
FNIHRDYGNK------------------------DRLPSSHTCF-NQ-LDLPEY-ESYDH
LRSQIL-KAITAGSEY-
>Fusarium_tucumaniae_MAEF01005620.1 .
LSVRREQV-FHDSFKSL--------------YFKSGDEMKFGKLNIRFHG-------EEG
VDAGGVTREW---FQVL----ARQMFDANY--------VLFTPVSSDRT-----------
----------------TFHPNKLS------------------------------------
--DINDEHLMFFKFIGRIIGKALYEGRVLDCYFSR--AVYKRILG---------------
--------RSVSVKDMES----------------------FDPDYYKS--------LCWM
LEN--DIT-DII--
-TETFSVEEES---------------------------FGV-FKVVD-LAPN--------
GREIA---
------------------------------VTEENKH-DYVRL-VVEHKLLSS-VKEQMA
-HFLQG---FHDIIPA--ELIS-IFNEQ-ELELLISG-----------------------
----LPDID-----------IDDW----KSNTEYHN-YNPS-------------------
------------------------SQQI-QWFWRALR--------------------SFD
KEERAKLLQF-------VTGTSKVP------------LNG-----FK-ELEGMN--GVNR
FNIHRDYGNK------------------------DRLPSSHTCF-NX-LDLPEY-ESYDH
LRSQIL-KAITAGSEYF
>Fusarium_azukicola_MAEG01002667.1 .
LSVRREQV-FHDSFKSL--------------YFKSGDEMKFGKLNIRFHG-------EEG
VDAGGVTREW---FQVL----ARQMFDANY--------VLFTPVSSDRT-----------
----------------TFHPNKLS------------------------------------
--DINDEHLMFFKFIGRIIGKALYEGRVLDCYFSR--AVYKRILG---------------
--------RSVSVKDMES----------------------FDPDYYKS--------LCWM
LEN--DIT-DII--
-TETFSVEEES---------------------------FGV-FKVVD-LAPN--------
GREIA---
------------------------------VTEENKH-DYVRL-VVEHKLLSS-VKEQMA
-HFLQG---FHDIIPA--ELIS-IFNEQ-ELELLISG-----------------------
----LPDID-----------IDDW----KSNTEYHN-YNPS-------------------
------------------------SQQI-QWFWRALR--------------------SFD
KEERAKLLQF-------VTGTSKVP------------LNG-----FK-ELEGMN--GVNR
FNIHRDYGNK------------------------DRLPSSHTCF-NX-LDLPEY-ESYDH
LRSQIL-KAITAGSEYF
>Fusarium_verticillioides_XM_018887114.1 .
LSVRREQV-FHDSFKSL--------------YFKSGDEMKFGKLNIRFHG-------EEG
VDAGGVTREW---FQVL----SRQMFDPNY--------VLFTPVSSDRT-----------
----------------TFHPNKLS------------------------------------
--GINDEHLMFFKFIGRIIGKALYEGRVLDCYFSR--AVYKRILG---------------
--------KSVSVKDMES----------------------FDPDYYKS--------LCWM
LDN--DIT-DII--
-TETFSVENDE---------------------------FGA-TTVVD-LIPN--------
GREIA---
------------------------------VTEENKH-DYVRL-VVEHKLLSS-VKEQMA
-HFLQG---FHDIIPA--ELIS-IFNEQ-ELELLISG-----------------------
----LPDID-----------IDDW----KSNTEYHN-YTPS-------------------
------------------------SQQI-QWFWRALR--------------------SFD
KEERAKLLQF-------VTGTSKVP------------LNG-----FK-ELEGMN--GVNR
FNIHRDYGNK------------------------DRLPSSHTCF-NQ-LDLPEY-ESYDH
LRSQIM-KAITAGSEYF
>Fusarium_oxysporum_XM_018377625.1 .
LSVRREQV-FHDSFKSL--------------YFKSGDEMKFGKLNIRFHG-------EEG
VDAGGVTREW---FQVL----SRQMFDPNY--------VLFTPVSSDRT-----------
----------------TFHPNKLS------------------------------------
--GINDEHLMFFKFIGRIIGKALYEGRVLDCYFSR--AVYKRILG---------------
--------KSVSVKDMES----------------------FDPDYYKS--------LCWM

LDN--DIT-DII--
-TETFSVENDE---------------------------FGA-TTVVD-LIPN--------
GREIA---
------------------------------VTEENKH-DYVRL-VVEHKLLSS-VKEQMA
-HFLQG---FHDIIPA--ELIS-IFNEQ-ELELLISG-----------------------
----LPDID-----------IDDW----KSNTEYHN-YTPS-------------------
------------------------SQQI-QWFWRALR--------------------SFD
KEERAKLLQF-------VTGTSKVP------------LNG-----FK-ELEGMN--GVNR
FNIHRDYGNK------------------------DRLPSSHTCF-NQ-LDLPEY-ESYDH
LRSQIM-KAITAGSEYF
>Fusarium_mangiferae_FCQH01000001.1 .
LSVRREQV-FHDSFKSL--------------YFKSGDEMKFGKLNIRFHG-------EEG
VDAGGVTREW---FQVL----SRQMFDPNY--------VLFTPVSSDRT-----------
----------------TFHPNKLS------------------------------------
--GINDEHLMFFKFIGRIIGKALYEGRVLDCYFSR--AVYKRILG---------------
--------KSVSVKDMES----------------------FDPDYYKS--------LCWM
LEN--DIT-DII--
-TETFSVENDE---------------------------FGA-TTVVD-LIPN--------
GREIA---
------------------------------VTEENKH-DYVRL-VVEHKLLSS-VKEQMA
-HFLQG---XHDIIPA--ELIS-IFNEQ-ELELLISG-----------------------
----LPDID-----------IDDW----KSNTEYHN-YTPS-------------------
------------------------SQQI-QWFWRALR--------------------SFD
KEERAKLLQF-------VTGTSKVP------------LNG-----FK-ELEGMN--GVNR
FNIHRDYGNK------------------------DRLPSSHTCF-NX-LDLPEY-ESYDH
LRSQIM-KAITAGSEYF
>Fusarium_fujikuroi_MBPS01000015.1 .
LSVRREQV-FHDSFKSL--------------YFKSGDEMKFGKLNIRFHG-------EEG
VDAGGVTREW---FQVL----SRQMFDPNY--------VLFTPVSSDRT-----------
----------------TFHPNKLS------------------------------------
--GINDEHLMFFKFIGRIIGKALYEGRVLDCYFSR--AVYKRILG---------------
--------KSVSVKDMES----------------------FDPDYYKS--------LCWM
LEN--DIT-DII--
-TETFSVENDE---------------------------FGA-TTVVD-LIPN--------
GREIA---
------------------------------VTEENKH-DYVRL-VVEHKLLSS-VKEQMA
-HFLQG---FHDIIPA--ELIS-IFNEQ-ELELLISG-----------------------
----LPDID-----------IDDW----KSNTEYHN-YTPS-------------------
------------------------SQQI-QWFWRALR--------------------SFD
KEERAKLLQF-------VTGTSKVP------------LNG-----FK-ELEGMN--GVNR
FNIHRDYGNK------------------------DRLPSSHTCF-NX-LDLPEY-ESYDH
LRSQIM-KAITAGSEYF
>Neurospora_crassa_XM_958337.2 .
LQVRREHV-FHDSFRSL--------------YYKKADELKFGKLNIRFQG-------EEG
VDAGGVTREW---FQVL----SRQMFDPNY--------VLFVPVSSDRT-----------
----------------TFHPNKLS------------------------------------
--PINDEHLPFFKFIGRIIGKALYEGRLLECYFSR--AVYKRILG---------------
--------KPVSVKDMES----------------------FDPDYYKS--------LVWM
LEN--DIT-DII--
-TETFSVEDDV---------------------------FGE-VKVVD-LIEN--------
GRNIP---
------------------------------VTEENKH-EYVRL-IVEHKLITS-VKDQMK
-AFLTG---FHEIIPE--ELIA-IFNEQ-ELELLISG-----------------------
----LPDID-----------IDDW----KANTEYHN-YSAG-------------------
------------------------APQI-QWFWRAVR--------------------SFD
KEELAKLLQF-------VTGTSKVP------------LNG-----FK-ELEGMN--GVSR
FNIHRDYGSK------------------------DRLPSSHTCF-NQ-LDLPEY-ENYET
LRSQLL-KAITAGSDYF

```
>Neurospora_tetrasperma_XM_009853587.1 .
LQVRREHV-FHDSFRSL--------------YYKKADELKFGKLNIRFQG-------EEG
VDAGGVTREW---FQVL----SRQMFDPNY--------VLFVPVSSDRT-----------
----------------TFHPNKLS------------------------------------
--PINDEHLPFFKFIGRIIGKALYEGRLLECYFSR--AVYKRILG---------------
--------KPVSVKDMES----------------------FDPDYYKS--------LVWM
LEN------------------------------------------------DIT-DII--
-TETFSVEDDV---------------------------FGE-VKVVD-LIEN--------
GRNIP-------------------------------------------------------
------------------------------VTEENKH-EYVRL-IVEHKLITS-VKDQMK
-AFLTG---FHEIIPE--ELIA-IFNEQ-ELELLISG-----------------------
----LPDID-----------IDDW----KANTEYHN-YSAG-------------------
------------------------APQI-QWFWRAVR--------------------SFD
KEELAKLLQF-------VTGTSKVP------------LNG-----FK-ELEGMN--GVSR
FNIHRDYGSK------------------------DRLPSSHTCF-NQ-LDLPEY-ENYET
LRSQLL-KAITAGSDYF
>Neurospora_africana_CAPO020007926.1 .
LQVRREHV-FHDSFRSL--------------YYKKADELKFGKLNIRFQG-------EEG
VDAGGVTREW---FQVL----SRQMFDPNY--------VLFVPVSSDRT-----------
----------------TFHPNKLS------------------------------------
--PINDEHLPFFKFIGRIIGKALYEGRLLECYFSR--AVYKRILG---------------
--------KPVSVKDMES----------------------FDPDYYKS--------LVWM
LEN------------------------------------------------DIT-DII--
-TETFSVEDDV---------------------------FGE-VKVVD-LIEN--------
GRNIP-------------------------------------------------------
------------------------------VTEENKH-EYVRL-IVEHKLITS-VKDQMK
-AFLTG---FHEIIPE--ELIA-IFNEQ-ELELLISG-----------------------
----LPDID-----------IDDW----KANTEYHN-YSAG-------------------
------------------------APQI-QWFWRAVR--------------------SFD
KEELAKLLQF-------VTGTSKVP------------LNG-----FK-ELEGMN--GVSR
FNIHRDYGSK------------------------DRLPSSHTCF-NX-LDLPEY-ESYET
LRSQLL-KAITAGSDYF
>Sordaria_macrospora_XM_003345562.1 .
LQVRREHV-FHDSFRSL--------------YYKKAEELKFGKLNIRFQG-------EEG
VDAGGVTREW---FQVL----SRQMFDPNY--------VLFVPVSSDRT-----------
----------------TFHPNKLS------------------------------------
--PINDEHLPFFKFIGRIIGKALYEGRLLDCYFSR--AVYKRILG---------------
--------KPVSVKDMES----------------------FDPDYYKS--------LVWM
LEN------------------------------------------------DIT-DII--
-TETFSVEDDV---------------------------FGE-VKVVD-LIEN--------
GRNIP-------------------------------------------------------
------------------------------VTEENKH-EYVRL-IVEHKLITS-VKDQMK
-AFLTG---FHEIIPE--ELIA-IFNEQ-ELELLISG-----------------------
----LPDID-----------IDDW----KANTEYHN-YSAG-------------------
------------------------APQI-QWFWRAVR--------------------SFD
KEELAKLLQF-------VTGTSKVP------------LNG-----FK-ELEGMN--GVSR
FNIHRDYGSK------------------------DRLPSSHTCF-NQ-LDLPEY-ESYET
LRSQLL-KAITAGSDYF
>Chaetomium_thermophilum_XM_006691009.1 .
LSVRRDHV-FHDSFKSL--------------YFKSGPEMKYGKLNIRFHG-------EEG
VDAGGVTREW---FQVL----ARQMFDPNY--------ALFEPVSADRT-----------
----------------TFHPNKLS------------------------------------
--GINPEHLLFFKFIGRIIGKALYEGRLLDCYFSR--AVYKRILG---------------
--------KPVSVKDMES----------------------FDPEYYKS--------LIWM
LEN------------------------------------------------DIT-DVI--
-TETFSIEDEE---------------------------FGV-KKVVD-LIEN--------
GRNIP-------------------------------------------------------
------------------------------VTEENKH-EYVRL-IVEHKLLTS-VKEQME
```

-HFLKG---FHDIIPA--DLIS-IFNEQ-ELELLISG-----------------------
----LPDID-----------IDDW----KSNTEYHN-YTAA-------------------
------------------------SPQI-QWFWRAVR--------------------SFD
KEEQAKLLQF-------VTGTSKVP------------LNG-----FK-ELEGMN--GVNR
FNIHRDYGSK------------------------DRLPSSHTCF-NQ-LDLPEY-DSYET
LRSQLL-KAITAGSDYF
>Chaetomium_globosum_XM_001228090.1 .
LSVRRDQV-FHDSFKSL--------------YFKSGPEMKFGKLNIRFHG-------EEG
VDAGGVTREW---FQVL----ARQMFDPNY--------ALFIPVSSDRT-----------
----------------TFHPNKLS------------------------------------
--GINDEHLMFFKFIGRIIGKALYEGRLLDCYFSR--AVYKRILG---------------
--------KPVSVKDMES----------------------FDPDYYKS--------LVWM
LEN--DIT-DII--
-VETFSVEDDE---------------------------FGV-TKVVD-LIEN--------
GRNIP---
------------------------------VTEDNKH-EYVRL-IVEHKLLSS-VKEQME
-NFLKG---FHDIIPE--DLIA-IFTEQ-ELELLISG-----------------------
----LPDID-----------VDDW----KSNTEYHN-YTAA-------------------
------------------------SQQI-QWFWRG-------------------------
--ELEGMN--GVNR
FNIHRDYGNK------------------------ERLPSSHTCF-NQ-LDLPEY-ESYDI
LRSQLL-KAITAGNDYF
>Chaetomium_cochliodes_LSBY01000204.1 .
LSVRRDQV-FHDSFKSL--------------YFKSGPEMKFGKLNIRFHG-------EEG
VDAGGVTREW---FQVL----ARQMFDPNY--------ALFIPVSSDRT-----------
----------------TFHPNKLS------------------------------------
--GINDEHLMFFKFIGRIIGKALYEGRLLDCYFSR--AVYKRILG---------------
--------KPVSVKDMES----------------------FDPDYYKS--------LVWM
LEN--DIT-DII--
-VETFSVEDDE---------------------------FGV-TKVVD-LIEN--------
GRNIP---
------------------------------VTEDNKH-EYVRL-IVEHKLLTS-VKEQME
-NFLKG---FHDIIPE--DLIA-IFTEQ-ELELLISG-----------------------
----LPDID-----------VDDW----KSNTEYHN-YTAA-------------------
------------------------SQQI-QWFWRAVR--------------------SFD
KEERAKLLQF-------VTGTSKVP------------LNG-----FK-ELEGMN--GVNR
FNIHRDYGNK------------------------ERLPSSHTCF-NX-LDLPEY-ESYDI
LRSQVL-KAITAGNDYF
>Thielavia_terrestris_XM_003657767.1 .
LSVRREHV-FHDSFKSL--------------YFKSGPEMKFGKLNIRFHG-------EEG
VDAGGVTREW---FQVL----ARQMFDPNY--------ALFIPVSSDRT-----------
----------------TFHPNKLS------------------------------------
--GINDEHLMFFKFIGRIIGKALYEGRLLDCYFSR--AVYKRILG---------------
--------KPVSVKDMES----------------------FDPDYYKS--------LVWM
LEN--DIT-DII--
-TETFSVEDDE---------------------------FGV-TKVVD-LIEN--------
GRNIP---
------------------------------VTEENKH-EYVRL-IVEHKLLTS-VKDQME
-HFLKG---FHDIIPE--DLIA-IFTEQ-ELELLISG-----------------------
----LPDID-----------VDDW----KSNTEYHN-YTAA-------------------
------------------------SQQI-QWFWRAVR--------------------SFD
KEERAKLLQF-------VTGTSKVP------------LNG-----FK-ELEGMN--GINR
FNIHRDYGNK------------------------DRLPSSHTCF-NQ-LDLPEY-ESYDI
LRSQLL-KAITAGSDYF
>Madurella_mycetomatis_LCTW02000008.1 .
LSVRREHV-FHDSFRSL--------------YFKSGPEMKFGRLNIRFHG-------EEG
VDAGGVTREW---FQVL----ARQMFDPNY--------ALFIPVSSDRT-----------
----------------TFHPNKLS------------------------------------

--GINDEHLMFFKFIGRIIGKALYEGRLLDCYFSR--AVYKRILG---------------
--------KPVSVKDMES----------------------FDPDYYKS--------LVWM
LEN--DIT-DII--
-VETFSVEDDE---------------------------FGV-TKVVD-LIEN--------
GRNIP---
------------------------------VTEENKH-EYVRL-IVEHKLLTS-VKEQME
-NFLKG---FHDIIPE--DLIA-IFNEQ-ELELLISG-----------------------
----LPDID-----------VDDW----KSNTEYHN-YTAA-------------------
------------------------SQQI-QWFWRAVR--------------------SFD
KEERAKLLQF-------VTGTSKVP------------LNG-----FK-ELEGMN--GVSR
FNIHRDYGNK------------------------ERLPSSHTCF-NX-LDLPEY-ESYDT
LRSQLL-KAITAGSDYF
>Podospora_anserina_XM_001909532.1 .
LSVRREHV-FHDSFKSL--------------YFKTGDEMKFGKLNIRFHG-------EEG
VDAGGVTREW---FQVL----ARQMFDPNY--------ALFIPVSSDRT-----------
----------------TFHPNQLS------------------------------------
--SINEEHLMFFKFIGRIIGKALYEGRLLDCYFSR--AVYKRILG---------------
--------KPVSVKDMES----------------------FDPNYYKS--------LVWI
LEN--DIT-DII--
-TETFSVEDDE---------------------------FGV-TKTVD-LIPD--------
GRNIP---
------------------------------VTEENKS-EYVRL-IVEHKLLTS-VKDQME
-HFLKG---FHDIIPE--ELIA-IFNEQ-ELELLISG-----------------------
----LPDID-----------VDDW----KSNTEYHN-YTAA-------------------
------------------------SQQI-QWFWRAIR--------------------SFD
KEERAKLLQF-------VTGTSKVP------------LNG-----FK-ELEGMN--GVSR
FNIHRDYGNK------------------------DRLPSSHTCF-NQ-LDLPEY-ESYDT
LRSQIL-KAITAGSDYF
>Myceliophthora_thermophila_XM_003666911.1 .
LSVRRDQV-FHDSFKSL--------------YFKSGPEMKFGKLNIRFQG-------EEG
VDAGGVTREW---FQVL----SRQMFDPNY--------ALFIPVSSDRT-----------
----------------TFHPNKLS------------------------------------
--GVNDEHLMFFKFIGRIIGKALYEGRVLDCYFSR--AVYKRILG---------------
--------KPVSVKDMES----------------------FDPDYYKS--------LVWM
LEN--DIT-DII--
-TETFSVEDDE---------------------------FGV-TKVVD-LIEN--------
GRNIP---
------------------------------VTEENKH-EYVRL-IVEHKLLTS-VKDQME
-NFLKG---FHDIIPE--DLIS-IFTEQ-ELELLISG-----------------------
----LPEID-----------VDDW----KANTEYQN-YTPA-------------------
------------------------SQQI-QWFWRAVR--------------------SFD
KEERAKLLQF-------VTGTSKVP------------LNG-----FK-ELEGMN--GINR
FNIHRDYGNK------------------------DRLPSSHTCF-NQ-LDLPEY-ESYEV
LRSQLL-KAITAGSDYF
>Magnaporthe_oryzae_XM_003716674.1 .
LSVRRDHV-FHDSFKSL--------------YFKKGEEMKYGKLNIRFHG-------EEG
VDAGGVTREW---FQVL----SRQMFDPNY--------VLFTPVSSDRT-----------
----------------TFHPNKLS------------------------------------
--SINDEHLMFFKFIGRIIGKALYEGRVLDCYFSR--AVYKRILG---------------
--------KPVSVKDMES----------------------FDPEYYKS--------LVWM
LEN--DIT-DII--
-TETFAVEDDA---------------------------FGV-TKTVD-LCEN--------
GRNIP---
------------------------------VTEDNKH-EYVRL-VVEHKLLAS-VKDQMA
-EFLQG---FHDIIPA--ELIA-IFNEQ-ELELLISG-----------------------
----LPDID-----------VDDW----KAHTEYHN-YQPS-------------------
------------------------SQQI-QWFWRAVR--------------------SFD
KEERAKLLQF-------VTGTSKVP------------LNG-----FK-ELEGMN--GVSR

FNIHRDYGRP------------------------DRLPSSHTCF-NQ-LDLPEY-ESYDV
LRKQIL-KAITAGSDYF
>Falciphora_oryzae_JNVV01000011.1 .
LSVRRDHV-FHDSFKSL--------------YFKKGEEMKYGKLNIRFHG-------EEG
VDAGGVTREW---FQVL----SRQMFDPNY--------VLFIPVSSDRT-----------
----------------TFHPNKLS------------------------------------
--SINDEHLMFFKFIGRVIGKALYEGRVLDCYFSR--AVYKRILG---------------
--------KPVSVKDMES----------------------FDPEYYKS--------LVWM
LEN--DIT-DII--
-TETFAVEDDA---------------------------FGM-TKTVD-LCEN--------
GRNIP---
------------------------------VTEDNKH-DYVRL-VVEHKLLAS-VKDQMA
-EFLTG---FHDIIPA--ELIA-IFNEQ-ELELLISG-----------------------
----LPDID-----------VDDW----KSHTEYHN-YTPS-------------------
------------------------SQQI-QWFWRAVR--------------------SFD
KEERAKLLQF-------VTGTSKVP------------LNG-----FK-ELEGMN--GVSR
FNIHRDYGNK------------------------ERLPSSHTCF-NX-LDLPEY-ESYEI
LRAQIM-KAITAGSDYF
>Magnaporthiopsis_poae_ADBL01000164.1 .
LSVRRDHV-FHDSFKSL--------------YFKKGEEMKYGKLNIRFHG-------EEG
VDAGGVTREW---FQVL----SRQMFDPNY--------VLFIPVSSDRT-----------
----------------TFHPNKLS------------------------------------
--SINDEHLMFFKFIGRIIGKALYEGRVLDCYFSR--AVYKRILG---------------
--------KPVSVKDMES----------------------FDPEYYKS--------LVWM
LEN--DIT-DII--
-TETFAVEDDA---------------------------FGA-TETVD-LCEN--------
GRHIP---
------------------------------VTEDNKH-DYVRL-VVEHKLLAS-VKDQMA
-EFLTG---FHDIIPA--ELIA-IFNEQ-ELELLISG-----------------------
----LPDID-----------VDDW----KSHTEYHN-YTPS-------------------
------------------------SQQI-QWFWRAVR--------------------SFD
KEERAKLLQF-------VTGTSKVP------------LNG-----FK-ELEGMN--GVSR
FNIHRDYGNK------------------------ERLPSSHTCF-NX-LDLPEY-ESYEI
LRAQIM-KAITAGSDYF
>Gaeumannomyces_tritici_ADBI01000923.1 .
LSVRRDHV-FHDSFKSL--------------YFKKGEEMKYGKLNIRFHG-------EEG
VDAGGVTREW---FQVL----SRQMFDPNY--------ALFIPVSSDRT-----------
----------------TFHPNKLS------------------------------------
--SINDEHLMFFKFIGRIIGKALYEGRVLDCYFSR--AVYKRILG---------------
--------KPVSVKDMES----------------------FDPEYYKS--------LVWM
LEN--DIT-DII--
-TETFAVEEDA---------------------------FGA-TETVD-LCEN--------
GRNIP---
------------------------------VTEDNKH-DYVRL-VVEHKLLAS-VKDQMA
-EFLTG---FHDIIPA--ELIA-IFNEQ-ELELLISG-----------------------
----LPDID-----------VDDW----KSHTEYHN-YTPS-------------------
------------------------SQQI-QWFWRAVR--------------------SFD
KEERAKLLQF-------VTGTSKVP------------LNG-----FK-ELEGMN--GVSR
FNIHRDYGNK------------------------ERLPSSHTCF-NX-LDLPEY-ESYEI
LRAQVM-KAITAGSDYF
>Gaeumannomyces_graminis_XM_009229095.1 .
LSVRRDHV-FHDSFKSL--------------YFKKGEEMKYGKLNIRFHG-------EEG
VDAGGVTREW---FQVL----SRQMFDPNY--------ALFIPVSSDRT-----------
----------------TFHPNKLS------------------------------------
--SINDEHLMFFKFIGRIIGKALYEGRVLDCYFSR--AVYKRILG---------------
--------KPVSVKDMES----------------------FDPEYYKS--------LVWM
LEN--DIT-DII--
-TETFAVEEDA---------------------------FGA-TETVD-LCEN--------

```
GRNIP-------------------------------------------------------
------------------------------VTEDNKH-DYVRL-VVEHKLLAS-VKDQMA
-EFLTG---FHDIIPA--ELIA-IFNEQ-ELELLISG-----------------------
----LPDID-----------VDDW----KSHTEYHN-YTPS-------------------
------------------------SQQI-QWFWRAVR--------------------SFD
KEERAKLLQF-------VTGTSKVP------------LNG-----FK-ELEGMN--GVSR
FNIHRDYGNK------------------------ERLPSSHTCF-NQ-LDLPEY-ESYEI
LRAQVM-KAITAGSDYF
>Coniochaeta_ligniaria_MNPN01000083.1 .
LSVRRNNV-FHDSFKSL--------------YYKSGDEMKFGKLNIRFHG-------EEG
VDAGGVTREW---FQAL----SRQMFDPNY--------ALFTPVSSDRT-----------
----------------TFHPNKLS------------------------------------
--SINEEHLLFFKFIGRIIGKALYEGRVLDCYFSR--AVYKRILG---------------
--------KPVSVKDMES----------------------FDPDYYKS--------LVWM
LEN------------------------------------------------DIT-DAI--
-FQTFSVEDEE---------------------------FGV-THVVD-LVPN--------
GRNIP-------------------------------------------------------
------------------------------VTEENKH-DYVRL-VVEHKLLSS-VQEQME
-HFLKG---FHDIIPA--ELIA-IFNEQ-ELELLISG-----------------------
----LPDID-----------VDDW----KANTEYQN-YTAA-------------------
------------------------SQQI-QWFWRAVR--------------------SFD
KEERAKLLQF-------VTGTSKVP------------LNG-----FK-ELEGMN--GVSR
FNIHRDYGDT------------------------KRLPSSHTCF-NX-LDLPEY-ESYDV
LRSRIL-KAITAGSDYF
>Leptographium_lundbergii_LDEF01000012.1 .
LSVRRDLV-FHDSFRSL--------------YFKTGDEMKYGKLNIRFYG-------EEG
VDAGGVTREW---FQVL----ARQMFDANY--------ALFIPVSSDRT-----------
----------------TFHPNKLS------------------------------------
--GINDEHLMFFKFIGRIIGKALYEGRLLECYFSR--AVYKRILG---------------
--------KPVSVKDMES----------------------FDPEYYKS--------LVWM
LEN------------------------------------------------DIT-DVI--
-TETFSVVDDE---------------------------FGV-TTVKD-LIDG--------
GRDVA-------------------------------------------------------
------------------------------VTDENKH-DYVRL-VVEHKLLVS-VKDQME
-HFLKG---FHDIIPA--GLIS-IFTEQ-ELELLISG-----------------------
----LPDID-----------IDDW----RSHTEYHN-YNAA-------------------
------------------------SPQI-QWFWRAVR--------------------SFD
KEERAKLLQF-------VTGTSKVP------------LNG-----FK-ELEGMN--GVNR
FNIHRDYGNK------------------------DRLPSSHTCF-NX-LDLPEY-DSYEI
MRSQLI-KAITTGNDYF
>Grosmannia_clavigera_XM_014312569.1 .
LSVRRDQV-FHDSFRSL--------------YFKTGDEMKYGKLNIRFYG-------EEG
VDAGGVTREW---FQVL----ARQMFDANY--------ALFIPVSSDRT-----------
----------------TFHPNKLS------------------------------------
--GINDEHLMFFKFIGRIIGKALYEGRLLECYFSR--AVYKRILG---------------
--------KPVSVKDMES----------------------FDPEYYKS--------LVWM
LEN------------------------------------------------DIT-DVI--
-TETFSVVDDE---------------------------FGV-TTVKD-LIDG--------
GRDVA-------------------------------------------------------
------------------------------VTEDNKH-DYVRL-VVEHKLLVS-VKDQME
-DFLRG---FHDIIPA--ELIS-IFTEQ-ELELLISG-----------------------
----LPDID-----------IDDW----RSHTEYHN-YNAA-------------------
------------------------SPQI-QWFWRAVR--------------------SFD
KEERAKLLQF-------VTGTSKVP------------LNG-----FK-ELEGMN--GVNR
FNIHRDYGNK------------------------DRLPSSHTCF-NQ-LDLPEY-DSYEI
MRSQLI-KAITAGNDYF
>Leptographium_procerum_JRUC01001665.1 .
LSVRRDQV-FHDSFRSL--------------YFKTGDEMKYGKLNIRFYG-------EEG
```

VDAGGVTREW---FQVL----ARQMFDANY--------VLFIPVSSDRT-----------
----------------TFHPNKLS------------------------------------
--GINDEHLMFFKFIGRIIGKALYEGRLLECYFSR--AVYKRILG---------------
--------KSVSVKDMES----------------------FDPEYYKS--------LVWM
LEN--DIT-DVI--
-TETFSVVDDE---------------------------FGV-TTVKD-LIEN--------
GRDVA---
------------------------------VTEENKH-DYVRL-VVEHKLLAS-VKDQME
-NFLKG---FHDIIPA--ELIS-IFTEQ-ELELLISG-----------------------
----LPDID-----------IDDW----RSHTEYHN-YNAA-------------------
------------------------SPQI-QWFWRAVR--------------------SFD
KEERAKLLQF-------VTGTSKVP------------LNG-----FK-ELEGMN--GVNR
FNIHRDYGNK------------------------DRLPSSHTCF-NX-LDLPEY-DSYEV
MRSQLV-KAITAGNDYF
>3_Raffaelea_quercivora_BCFZ01000003.1 .
LSVRREQV-FHDSFRSL--------------YFKTGDEMKYGKLNIRFYG-------EEG
VDAGGVTREW---FQVL----ARQMFDPNY--------ALFIPVSSDRT-----------
----------------TFHPNKLS------------------------------------
--GINDEHLMFFKFIGRIIGKALYEGRLLECYFSR--AVYKRILG---------------
--------KPVSVKDMES----------------------FDPEYYKS--------LVWM
LEN--DIT-DVI--
-TETFSVVDDE---------------------------FGV-TTVKD-LIEN--------
GRDVA---
------------------------------VTEENKH-DYVRL-VVEHKLLKS-VKEQME
-YFLRG---FHDIIPA--ELIS-IFTEQ-ELELLISG-----------------------
----LPDID-----------VDDW----RSHTEYQN-YSAA-------------------
------------------------SPQI-QWFWRAVR--------------------SFD
KEERAKLLQF-------VTGTSKVP------------LNG-----FK-ELEGMN--GVNR
FNIHRDYGNK------------------------DRLPSSHTCF-NX-LDLPEY-DSYEA
MRSQLL-KAITAGNEYF
>Grosmannia_penicillata_MLJV01000034.1 .
LSVRRERV-FHDSFRSL--------------YYKSGDEMKYGKLNIRFSG-------EEG
VDAGGVTREW---FQVL----ARQMFDPNY--------ALFIPVSSDRT-----------
----------------TFHPNKLS------------------------------------
--GINDEHLRFFKFIGRIIGKALYEGRLLECYFSR--AVYKRILG---------------
--------KPVSVKDMES----------------------FDPEYYKS--------LVWM
LEN--DIT-DVI--
-TETFAVEDDA---------------------------FGV-TTVVD-LIEN--------
GRDVP---
------------------------------VTEENKH-DYVRL-VVEHKLLAS-VQEQME
-NFLKG---FHDIIPA--ELIS-IFNEQ-ELELLISG-----------------------
----LPDID-----------IDDW----RGHTEYHN-YSAS-------------------
------------------------SAQI-QWFWRAVR--------------------SFD
KEERAKLLQF-------VTGTSKVP------------LNG-----FK-ELEGMN--GVNR
FNIHRDYGNK------------------------DRLPSSHTCF-NX-LDLPEY-DSYEA
MRSQLL-KAITAGNEYF
>Sporothrix_pallida_JNEX02000053.1 .
LSVRRDQV-FHDSFRSL--------------YFKSGDEMKYGKLNIRFHG-------EEG
VDAGGVTREW---FQVL----SRQMFDPNY--------ALFIPVSSDRT-----------
----------------TFHPNKLS------------------------------------
--GINDEHLLFFKFIGRIIGKALYEGRLLECYFSR--AVYKRILG---------------
--------KPVSVKDMES----------------------FDPEYYKS--------LVWM
LEN--DIT-DVI--
-TETFSVVDDE---------------------------FGA-TKVVD-LIPN--------
GRDVG---
------------------------------VTEENKH-DYVRL-VVEHKLLAS-VKDQMG
-EFLKG---FHDIIPA--ELIS-IFTEQ-ELELLISG-----------------------
----LPDID-----------IDDW----RGNTEYHN-YSGG-------------------

------------------------APQI-QWFWRAVR--------------------SFD
KEERAKLLQF-------VTGTSKVP------------LNG-----FK-ELEGMN--GINR
FNIHRDYGQK------------------------DRLPSSHTCF-NQ-LDLPEY-ESYDA
MRSQLL-KAITAGNEYF
>Sporothrix_globosa_LVYX01000006.1 .
LSVRRDQV-FHDSFRSL--------------YFKSGDEMKYGKLNIRFHG-------EEG
VDAGGVTREW---FQVL----SRQMFDPNY--------ALFIPVSSDRT-----------
----------------TFHPNKLS------------------------------------
--GINDEHLMFFKFIGRIIGKALYEGRLLECYFSR--AVYKRILG---------------
--------KPVSVKDMES----------------------FDPEYYKS--------LVWM
LEN--DIT-DVI--
-TETFSVVDDE---------------------------FGA-TKVVD-LILN--------
GRDVA---
------------------------------VTEENKH-DYVRL-VVEHKLLAS-VKDQMS
-EFLKG---FHDIIPA--ELIS-IFTEQ-ELELLISG-----------------------
----LPDID-----------IDDW----RGNTEYHN-YNSA-------------------
------------------------APQI-QWFWRAVR--------------------SFD
KEERAKLLQF-------VTGTSKVP------------LNG-----FK-ELEGMN--GINR
FNIHRDYGDK------------------------DRLPSSHTCF-N--------------

>Sporothrix_schenckii_XM_016734862.1 .
LSVRRDQV-FHDSFRSL--------------YFKSGDEMKYGKLNIRFHG-------EEG
VDAGGVTREW---FQVL----SRQMFDPNY--------ALFIPVSSDRT-----------
----------------TFHPNKLS------------------------------------
--GINDEHLMFFKFIGRIIGKALYEGRLLECYFSR--AVYKRILG---------------
--------KPVSVKDMES----------------------FDPEYYKS--------LVWM
LEN--DIT-DVI--
-TETFSVVDDE---------------------------FGA-TKVVD-LVPN--------
GRDVA---
------------------------------VTEENKH-DYVRL-VVEHKLLAS-VKDQMS
-EFLKG---FHDIIPA--ELIS-IFTEQ-ELELLISG-----------------------
----LPDID-----------IDDW----RGNTEYHN-YNSA-------------------
------------------------APQI-QWFWRAVR--------------------SFD
KEERAKLLQF-------VTGTSKVP------------LNG-----FK-ELEGMN--GINR
FNIHRDYGDK------------------------DRLPSSHTCF-NQ-LDLPEY-ETYDA
MRSQLL-KAITAGNEYF
>Sporothrix_brasiliensis_AWTV01000011.1 .
LSVRRDQV-FHDSFRSL--------------YFKSGDEMKYGKLNIRFHG-------EEG
VDAGGVTREW---FQVL----SRQMFDPNY--------ALFIPVSSDRT-----------
----------------TFHPNKLS------------------------------------
--GINDEHLMFFKFIGRIIGKALYEGRLLECYFSR--AVYKRILG---------------
--------KPVSVKDMES----------------------FDPEYYKS--------LVWM
LEN--DIT-DVI--
-TETFSVVDDE---------------------------FGA-TKVVD-LIPN--------
GRDVA---
------------------------------VTEENKH-DYVRL-VVEHKLLAS-VKDQMS
-EFLKG---FHDIIPA--ELIS-IFTEQ-ELELLISG-----------------------
----LPDID-----------IDDW----RGNTEYHN-YNSA-------------------
------------------------APQI-QWFWRAVR--------------------SFD
KEERAKLLQF-------VTGTSKVP------------LNG-----FK-ELEGMN--GINR
FNIHRDYGDK------------------------DRLPSSHTCF-NX-LDLPEY-ETYDA
MRSQLL-KAITAGNEYF
>Ophiostoma_novo-ulmi_AMZD01000118.1 .
LSVRRDLV-FHDSFRSL--------------YFKSGDEMKYGKLNIRFHG-------EEG
VDAGGVTREW---FQVL----SRQMFDPNN--------ALFIPVSSDRT-----------
----------------TFHPNKSS------------------------------------
--WINDEHLLFFKFIGRIIGKALYEGRLLECYFSR--AVYKRLLG---------------
--------KPVSVKDMES----------------------FDPEYYKS--------LVWM

LEN--DIT-DVI--
-TETFSVINSA---------------------------FGA-DQEMD-LIEN--------
GRNVP---
------------------------------VTEENKH-EYVRL-VVEFKLVKS-VEEQMG
-EFLKG---FHEIIPA--ELIQ-IFTEQ-ELELLISG-----------------------
----LPDID-----------IDDW----RANTEYHN-YSSG-------------------
------------------------APQI-QWFWRAVR--------------------SFD
KEERAKLLQF-------VTGTSKVP------------LNG-----FK-ELEGMN--GINR
FNIHRDYGNN------------------------NRLPSSHTCF-NQ-LDLPEY-ESYDS
MRSQLL-KAITTGNEYF
>Corollospora_maritima_GDFX01000067.1 .
LSVRRDLV-FHDSFRSL--------------YFKTGDEMKYGKLNIRFHG-------EEG
VDAGGVTREW---FQVL----SRQMFNPNY--------ALFIPVSSDRT-----------
----------------TFHPNKLS------------------------------------
--GINDEHLAFFKFIGRIIGKALYEGRVLECYFSR--AVYKRILG---------------
--------KSVSVKDMES----------------------FDPDYYKS--------LVWM
LDN--DIT-DII--
-TETFSVEDDE---------------------------FGV-TKVVD-LIEN--------
GRNVT---
------------------------------VTEENKN-EYVRL-VVEHKLLSS-VKEQME
-HFLTG---FHDIIPS--ELIS-IFNEQ-ELELLISG-----------------------
----LPDID-----------VDDW----KGNTEYHN-YSAS-------------------
------------------------SPQI-QWFWRAVR--------------------SFD
KEERAKLLQF-------VTGTSKVP------------LNG-----FK-ELEGMN--GINR
FNIHRDYGNK------------------------ERLPSSHTCF-NQ-LDLPEY-DSYDI
LRKQVL-KAI-------
>Gliomastix_tumulicola_BCHX01000001.1 .
LSVRREQV-FHDSFRSL--------------YFKTGDEMKFGKLNIRFHN-------EEG
VDAGGVTREW---FQVL----SRQMFDANY--------ALFIPVSSDRT-----------
----------------TFHPNKLS------------------------------------
--GINDEHLSFFKFIGRVIGKALYEGRVLDCYFSR--AVYKRILS---------------
--------KSVSVKDMES----------------------FDPDYYKS--------LCWM
LEN--DIT-DII--
-TETFSVEDEE---------------------------FGV-TNIVD-LIPN--------
GRDVP---
------------------------------VTDENKH-EYVRL-VVEHKLLSS-VKEQME
-HFLKG---FHDIIPA--ELIS-IFNEQ-ELELLISG-----------------------
----LPEID-----------VDDW----KRNTEYQN-YNAT-------------------
------------------------SAQI-QWFWRAVR--------------------SFD
KEERAKLLQF-------VTGTSKVP------------LNG-----FK-ELEGMN--GVNR
FNIHRDYGNK------------------------DRLPSSHTCF-NX-LDLPEY-ENYDA
LRSQLI-KAITAGSEYF
>Colletotrichum_fioriniae_XM_007598489.1 .
LSVRREHV-FHDSFKSL--------------YFKTGDEMKYGKLNIRFHN-------EEG
VDAGGVTREW---FQVL----SRQMFDANY--------ALFIPVSSDRT-----------
----------------TFHPNKLS------------------------------------
--GINDEHLMFFKFIGRIIGKALYEGRVLDCYFSR--AVYKRILG---------------
--------KSVSVKDMES----------------------FDPDYYKS--------LVWM
LDN--DIT-DII--
-TETFSVEDDE---------------------------FGV-TRTVD-LCPG--------
GRDIA---
------------------------------VTEENKH-DYVRL-VVEHKLLSS-VKEQME
-HFLKG---FHDIIPA--DLIS-IFNEQ-ELELLISG-----------------------
----LPDID-----------VDDW----KSNTEYHN-YTPS-------------------
------------------------SPQI-QWFWRAIR--------------------SFD
KEERAKLLQF-------VTGTSKVP------------LNG-----FK-ELEGMN--GINR
FNIHRDYGNK------------------------ERLPSSHTCF-NQ-LDLPEY-ENYEM
LRQQLM-KAITAGSDYF

>Colletotrichum_higginsianum_XM_018305377.1 .
LSVRREHV-FHDSFKSL--------------YFKTGDEMKYGKLNIRFHN-------EEG
VDAGGVTREW---FQVL----SRQMFDANY--------ALFIPVSSDRT-----------
----------------TFHPNKLS------------------------------------
--GINDEHLMFFKFIGRIIGKALYEGRVLDCYFSR--AVYKRILG---------------
--------KSVSVKDMES----------------------FDPDYYKS--------LVWM
LDN--DIT-DII--
-TETFSVEDDE---------------------------FGV-TRTVD-LCPN--------
GRDIA---
------------------------------VTEENKH-DYVRL-VVEHKLLSS-VKEQME
-HFLKG---FHDIIPA--DLIS-IFNEQ-ELELLISG----------------------
----LPDID-----------VDDW----KSNTEYHN-YTPS-------------------
------------------------SPQI-QWFWRAIR--------------------SFD
KEERAKLLQF-------VTGTSKVP------------LNG-----FK-ELEGMN--GVNR
FNIHRDYGNK------------------------ERLPSSHTCF-NQ-LDLPEY-ESYEM
LRQQLM-KAITAGSDYF
>Colletotrichum_graminicola_XM_008097467.1 .
LAVRREHV-FHDSFKHL--------------YYKTGDEMKYGKLNIRFHN-------EEG
VDAGGVTREW---FQVL----SRQMFDANY--------ALFIPVSSDRT-----------
----------------TFHPNKLS------------------------------------
--GINDEHLMFFKFIGRIIGKALYEGRVLDCYFSR--AVYKRILG---------------
--------KSVSVKDMES----------------------FDPDYYKS--------LVWM
LDN--DIT-DII--
-TETFSVEDDE---------------------------FGV-TRTVD-LCPN--------
GRDIA---
------------------------------VTEENKH-DYVRL-VVEHKLLSS-VKEQME
-HFLKG---FHDIIPA--DLIS-IFNEQ-ELELLISG----------------------
----LPDID-----------VDDW----KSNTEYHN-YTPS-------------------
------------------------SPQI-QWFWRAVR--------------------SFD
KEERAKLLQF-------VTGTSKVP------------LNG-----FK-ELEGMN--GVNR
FNIHRDYGNK------------------------ERLPSSHTCF-NQ-LDLPEY-ESYEM
LRQQLM-KAITAGSDYF
>Colletotrichum_gloeosporioides_XM_007279160.1 .
LSVRREHV-FHDSFKSL--------------YFKTGDEMKYGKLNIRFHN-------EEG
VDAGGVTREW---FQVL----SRQMFDANY--------ALFTPVSSDRT-----------
----------------TFHPNSLS------------------------------------
--GINDEHLMFFKFIGRIIGKALYEGRVLDCYFSR--AVYKRILG---------------
--------KSVSVKDMES----------------------FDPDYYKS--------LVWM
LDN--DIT-DII--
-TETFSVEDDE---------------------------FGV-TRTID-LCPN--------
GRDIA---
------------------------------VTEENKH-DYVRL-VVEHKLLSS-VREQME
-HFLKG---FHDIIPA--DLIS-IFNEQ-ELELLISG----------------------
----LPDID-----------VDDW----KSNTEYHN-YTPS-------------------
------------------------SQQI-QWFWRAIR--------------------SFD
KEERAKLLQF-------VTGTSKVP------------LNG-----FK-ELEGMN--GINR
FNIHRDYGNK------------------------ERLPSSHTCF-NQ-LDLPEY-ESYET
LRAQVM-KAITAGSDYF
>2_Colletotrichum_acutatum_LUXP01000006.1 .
LSVRREHV-FHDSFKSL--------------YFKTGDEMKYGKLNIRFHN-------EEG
VDAGGVTREW---FQVL----SRQMFDANY--------ALFIPVSSDRT-----------
----------------TFHPNKLS------------------------------------
--GINDEHLMFFKFIGRIIGKALYEGRVLDCYFSR--AVYKRILG---------------
--------KSVSVKDMES----------------------FDPDYYKS--------LVWM
LDN--DIT-DII--
-TETFSVEDDE---------------------------FGV-TRTVD-LCPG--------
GRDIA---
------------------------------VTEENKH-DYVRL-VVX-------------

-----G---FHDIIPA--DLIS-IFNEQ-ELELLISG-----------------------
----LPDID-----------VDDW----KSNTEYHN-YTPS-------------------
------------------------SPQI-QWFWRAIR--------------------SFD
KEERAKLLQF-------VTGTSKVP------------LNG-----FK-ELEGMN--GINR
FNIHRDYGNK------------------------ERLPSSHTCF-N--------------

>Aureobasidium_pullulans_MSJF01000343.1 .
LSVRREHV-FHDSFKSL--------------YFKSGDEMKYGKLNIRFHG-------EEG
VDAGGVTREW---FQVL----SRQMFDPNY--------ALFIPVSSDRT-----------
----------------TFHPNKLS------------------------------------
--GINDEHLRFFKFIGRIIGKALYEGRLLDCYFSR--AVYKRILG---------------
--------KSVSVKDMES----------------------FDPDYYKS--------LVWM
LDN--DIT-EII--
-TETFSLEDDE---------------------------FGR-TNTVD-LIPN--------
GRNIP---
------------------------------VTEENKH-EYVRL-VVEHKLLNS-VKEQME
-HFLKG---FHDIIPA--DLIS-IFNEQ-ELELLISG-----------------------
----LPDID-----------VDDW----KSNTEYHN-YTAS-------------------
------------------------SPQI-QWFWRAVR--------------------SFD
KEERAKLLQF-------VTGTSKVP------------LNG-----FK-ELEGMN--GINR
FNIHRDYGNK------------------------SRLPSSHTCF-NX-LDLPEY-ESYDM
LRKQLL-KAITAGSDYF
>Scedosporium_apiospermum_XM_016783569.1 .
LSVRREHV-FHDSFKSL--------------YFKSGDEMKYGKLNIRFHG-------EEG
VDAGGVTREW---FQVL----SRQMFDPNY--------ALFIPVSSDRT-----------
----------------TFHPNKLS------------------------------------
--GINDEHLRFFKFIGRIIGKALYEGRLLDCYFSR--AVYKRILG---------------
--------KSVSVKDMES----------------------FDPDYYKS--------LVWM
LDN--DIT-EII--
-TETFSLEDDE---------------------------FGR-TNTVD-LIPN--------
GRNIP---
------------------------------VTEENKH-EYVRL-VVEHKLLNS-VKEQME
-HFLKG---FHDIIPA--DLIS-IFNEQ-ELELLISG-----------------------
----LPDID-----------VDDW----KSNTEYHN-YTAS-------------------
------------------------SPQI-QWFWRAVR--------------------SFD
KEERAKLLQF-------VTGTSKVP------------LNG-----FK-ELEGMN--GINR
FNIHRDYGNK------------------------TRLPSSHTCF-NQ-LDLPEY-ESYDM
LRKQLL-KAITAGSDYF
>Verticillium_longisporum_CVQH01020452.1 .
LAVRREHV-FHDSFRSL--------------YFKSGDEMKYGKLNIRFHG-------EEG
VDAGGVTREW---FQVL----ARQMFDANY--------ALFIPVSSDRT-----------
----------------TFHPNKLS------------------------------------
--GINDMHLMYFKFVGRIIGKALYEGRLLDCYFSR--AVYKRILG---------------
--------KSVSVKDMES----------------------FDPDYYKS--------LVWM
LEN--DIT-DII--
-TETFSVEDDE---------------------------FGV-TTIVD-LCPD--------
GRNIA---
------------------------------VTEENKN-DYVRL-VVEHKLLSS-VKEQME
-HFLKG---FHEIIPS--ELIR-IFNEQ-ELELLISG-----------------------
----LPDID-----------IDDW----KSNTEYHN-YTPS-------------------
------------------------SQQI-QWFWRAIR--------------------SFD
KEELAKLLQF-------VTGTSKVP------------LNG-----FK-ELEGMN--GVSR
FNIHRDYGNK------------------------ERLPSSHTCF-NX-LDLPEY-ESYET
LRSQLM-KAITAGSDYF
>Verticillium_dahliae_XM_009655812.1 .
LAVRREHV-FHDSFRSL--------------YFKSGDEMKYGKLNIRFHG-------EEG
VDAGGVTREW---FQVL----ARQMFDANY--------ALFIPVSSDRT-----------
----------------TFHPNKLS------------------------------------

--GINDMHLMYFKFVGRIIGKALYEGRLLDCYFSR--AVYKRILG---------------
--------KSVSVKDMES----------------------FDPDYYKS--------LVWM
LEN---DIT-DII--
-TETFSVEDDE----------------------------FGV-TTIVD-LCPD--------
GRNIA---
------------------------------VTEENKN-DYVRL-VVEHKLLSS-VKEQME
-HFLKG---FHEIIPS--ELIR-IFNEQ-ELELLISG-----------------------
----LPDID-----------IDDW----KSNTEYHN-YTPS-------------------
-------------------------SQQI-QWFWRAIR--------------------SFD
KEELAKLLQF-------VTGTSKVP------------LNG-----FK-ELEGMN--GVSR
FNIHRDYGNK------------------------ERLPSSHTCF-NQ-LDLPE--ESYET
LRSQLM-KAITAGSDYF
>Verticillium_tricorpus_JPET01000007.1 .
LAVRREHV-FHDSFRSL--------------YFKSGDEMKYGKLNIRFHG-------EEG
VDAGGVTREW---FQVL----SRQMFDANY--------ALFIPVSSDRT-----------
----------------TFHPNKLS------------------------------------
--GINDMHLMYFKFVGRIIGKALYEGRLLDCYFSR--AVYKRILG---------------
--------KSVSVKDMES----------------------FDPDYYKS--------LVWM
LEN---DIT-DII--
-TETFSVEDDE----------------------------FGV-TTIVD-LCPD--------
GRNIA---
------------------------------VTEENKN-DYVRL-VVEHKLLSS-VKEQME
-HFLKG---FHEIIPS--ELIS-IFNEQ-ELELLISG-----------------------
----LPDID-----------IDDW----KSNTEYHN-YTPS-------------------
-------------------------SQQI-QWFWRAVR--------------------SFD
KEELAKLLQF-------VTGTSKVP------------LNG-----FK-ELEGMN--GVSR
FNIHRDYGNK------------------------ERLPSSHTCF-NX-IDLPEY-ESYET
LRAQLM-KAITAGSDYF
>Acremonium_furcatum_BCIA01000004.1 .
LAVRRDQV-FHDSFRSL--------------YFKSGDEMKYGKLNIRFHG-------EEG
VDAGGVTREW---FQVL----ARQMFDANY--------ALFIPVSSDRT-----------
----------------TFHPNKLS------------------------------------
--GINDMHLMYFKFVGRIIGKALYEGRVLDCYFSR--AVYKRILG---------------
--------KKVSVKDMES----------------------FDPDYYKS--------LVWM
LEN---DIT-DII--
-TETFSVEDDE----------------------------FGV-TTIVD-LCPD--------
GRNIP---
------------------------------VTDENKH-EYVRL-VVEHKLLSS-VKEQME
-HFLKG---FHEIIPA--ELIS-IFNEQ-ELELLISG-----------------------
----LPDID-----------IDDW----KSNTEYHN-YNPS-------------------
-------------------------SQQI-QWFWRAIR--------------------SFD
KEELAKLLQF-------VTGTSKVP------------LNG-----FK-ELEGMN--GINR
FNIHRDYGNK------------------------ERLPSSHTCF-NX-LDLPEY-DSYDT
LRSQVL-KAITAGSDYF
>Termitomyces_clypeatus_GAGE01008105.1 .
LSVRREHV-FHDSFKSL--------------YFKSGDEMKFGKLNIRFHG-------EEG
VDAGGVTREW---FQVL----SRQMFDPNY--------ALFVPVSSDRT-----------
----------------TFHPNKLS------------------------------------
--GINDEHLMFFKFIGRVIGKALYEGRLLDCYFSR--AVYKRILS---------------
--------KPVSVKDMES----------------------FDPEYYKS--------LVWM
LEN---DIT-DII--
-TESFAVEDDE----------------------------FGV-KTIED-LIPN--------
GRDIP---
------------------------------VTEENKN-DYVRL-VVEHKLLKS-VKDQME
-HFLKG---FHEIIPA--ELIA-IFNEQ-ELELLISG-----------------------
----LPDID-----------VDDW----KSNTEYHN-YTPS-------------------
-------------------------SQQI-QWFWRAVR--------------------SFD
KEELAKLLQF-------VTGTSKVP------------LNG-----FK-ELEGMN--GINR

FNIHRDYGNK------------------------ERLPSSHTCF-NQ-LDLPEY-ESYDT
LRAQVI-KAITAGSDYF
>Arthrinium_malaysianum_GEGR01018075.1 .
LSVRREHV-FHDSFKSL--------------YFKSGDEMKFGKLNIRFHG-------EEG
VDAGGVTREW---FQVL----SRQMFDPNY--------ALFVPVSSDRT-----------
----------------TFHPNKLS------------------------------------
--GINDEHLMFFKFIGRVIGKALYEGRLLDCYFSR--AVYKRILS---------------
--------KPVSVKDMES----------------------FDPEYYKS--------LVWM
LEN--DIT-DII--
-TESFAVEDDE---------------------------FGV-KTIED-LIPN--------
GRDIP---
------------------------------VTEENKN-DYVRL-VVEHKLLKS-VKDQME
-HFLKG---FHEIIPA--ELIA-IFNEQ-ELELLISG-----------------------
----LPDID-----------VDDW----KSNTEYHN-YTPS-------------------
------------------------SQQI-QWFWRAVR--------------------SFD
KEELAKLLQF-------VTGTSKVP------------LNG-----FK-ELEGMN--GINR
FNIHRDYGNK------------------------ERLPSSHTCF-NQ-LDLPEY-ESYDT
LRAQVI-KAITAGSDYF
>Eutypa_lata_XM_007797372.1 .
LQVRREHV-FHDSFKSL--------------YFKSGDEMKFGKLSIRFHG-------EEG
VDAGGVTREW---FQVL----SRQMFDPNY--------ALFVPVSSDRT-----------
----------------TFHPNKLS------------------------------------
--GINDEHLMFFKFIGRVIGKALYEGRVLDCYFSR--AVYKRILG---------------
--------KPVSVKDMES----------------------FDPDYYKS--------LVWM
LEN--DIT-DII--
-TESFSVEDDV---------------------------FGV-TRIED-LCED--------
GRNVP---
------------------------------VTEDNKH-EYVRL-VVEHKLLSS-VKEQME
-NFLKG---FHEIIPA--ELIS-IFNEQ-ELELLISG-----------------------
----LPDID-----------VDDW----KSNTEYHN-YTPS-------------------
------------------------SPQI-QWFWRAVR--------------------SLD
KEELAKLLQF-------VTGTSKVP------------LNG-----FK-ELEGMN--GVNR
FNIHRDYGNK------------------------DRLPSSHTCF-NQ-LDLPEY-ESYDA
LRRQLL-KAITAGSDYF
>Daldinia_eschscholzii_AIID01006985.1 .
LQVRREHV-FHDSFKSL--------------YFKTGDEMKFGKLSIRFHG-------EEG
VDAGGVTREW---FQVL----ARQMFDANY--------ALFVPVSSDRT-----------
----------------TFHPNKLS------------------------------------
--GINDEHLMFFKFIGRIIGKALYEGRLLDCYFSR--AVYKRILG---------------
--------KPVSVKDMES----------------------FDPDYYKS--------LSWM
LNN--DIT-DII--
-TETFSVEDDE---------------------------FGV-TKIHD-LIEN--------
GRNVP---
------------------------------VTEENKH-EYVRL-VVEHKLLTS-VKDQME
-NFLKG---FHEIIPA--ELIS-IFNEQ-ELELLISG-----------------------
----LPDVD-----------IDDW----KSNTEYHN-YSPS-------------------
------------------------SPQI-QWFWRAVR--------------------SFD
KEERAKLLQF-------VTGTSKVP------------LNG-----FK-ELEGMN--GVNR
FNIHRDYGNK------------------------DRLPSSHTCF-NX-LDLPEY-ESYDI
LRKQVL-KAITAGSDYF
>Togninia_minima_XM_007917676.1 .
LSVRREHV-FHDSFKSL--------------YFKSGDEMKFGKLNIRFHG-------EEG
VDAGGVTREW---FQVL----SRQMFDPNY--------ALFIPVSSDRT-----------
----------------TFHPNKLS------------------------------------
--GINDEHLMFFKFIGRIIGKALYEGRVLDCYFSR--AVYKRILG---------------
--------KQVSVKDMES----------------------FDPDYYKS--------LVWM
LEN--DIT-DII--
-TETFSVEDDE---------------------------FGV-TKIVD-LCEN--------

GRNIP---
------------------------------VTEENKH-EYVRL-VVDHKLLSS-VKDQME
-NFLKG---FHDIIPS--ELIA-IFNEQ-ELELLISG-----------------------
----LPDID-----------VDDW----KSNAEYHN-YTAA-------------------
------------------------SQQI-QWFWRAVR--------------------SFD
KEERAKLLQF-------VTGTSKVP------------LNG-----FK-ELEGMN--GVNR
FNIHRDYGNK------------------------DRLPSSHTCF-NQ-LDLPEY-ESYDI
LRAQLM-KAITAGSDYF
>Pseudomassariella_vexata_MCFJ01000007.1 .
LSVRREHV-FHDSFKSL--------------YFKSGDEMKFGKLNIRFHG-------EEG
VDAGGVTREW---FQVL----ARQMFDPNY--------ALFVPVSSDRT-----------
----------------TFHPNKLS------------------------------------
--GINDEHLMFFKFIGRIIGKSLYEGRVLDCYFSR--AVYKRILG---------------
--------KPVSVKDMES----------------------FDPDYYKS--------LVWM
LEN--DIT-DII--
-TETLSVEDDE---------------------------FGV-TRIED-LCEN--------
GRNIP---
------------------------------VTEDNKH-DYVRL-VVEHKLLSS-VKEQME
-HFLKG---FHEIIPA--ELIA-IFNEQ-ELELLISG-----------------------
----LPDID-----------VDDW----KSNTEYHN-YTPS-------------------
------------------------SQQI-QWFWRALR--------------------SFD
KEERAKLLQF-------VTGTSKVP------------LNG-----FK-ELEGMN--GINR
FNIHRDYGNK------------------------DRLPSSHTCF-NX-LDLPEY-ESYDT
LRQQVL-KAITAGSDYF
>Reticulascus_tulasneorum_LSAY01000002.1 .
LSVRREHV-FHDSFKSL--------------YFKSGDEMKFGKLNIRFHG-------EEG
VDAGGVTREW---FQVL----SRQMFDANY--------ALFIPVSSDRT-----------
----------------TFHPNKLS------------------------------------
--GINDEHLMFFKFIGRIIGKALYEGRVLDCYFSR--AVYKRILG---------------
--------KSVSVKDMES----------------------FDPDYYKS--------LVWM
LDN--DIT-DII--
-TETFSVEDDE---------------------------FGV-TRIVD-LCPN--------
GREIA---
------------------------------VTEDNKH-EYVRL-VVEHKLLSS-VKDQME
-HFLKG---FHDIIPA--DLIA-IFNEQ-ELELLISG-----------------------
----LPDID-----------VDDW----KSNTEYHN-YNPS-------------------
------------------------SQQI-QWFWRAVR--------------------SFD
KEERAKLLQF-------VTGTSKVP------------LNG-----FK-ELEGMN--GINR
FNIHRDYGNK------------------------ERLPSSHTCF-NX-LDLPEY-ENYDA
LRAQVL-KAITAGSDYF
>2_Microdochium_bolleyi_LSSP01000005.1 .
LQVRRDQV-FQDSFKSL--------------YFKSGDEMKFGKLNIRFHG-------EEG
VDAGGVTREW---FQVL----SRQMFDPNY--------ALFVPVSSDRT-----------
----------------TFHPNKLS------------------------------------
--GINDEHLMYFKFIGRVIGKALYEGRVLDCYFSR--AVYKRILG---------------
--------KQVSVKDMES----------------------FDPDYYKS--------LVWM
LEN--DIT-DII--
-TETFSVETDE---------------------------FGV-TRVED-LREN--------
GRNIP---
------------------------------VTEENKQ-EYVRL-VVEHKLLSS-VRDQME
-QFLKG---FHEIIPA--DLIS-IFNEQ-ELELLISG-----------------------
----LPDID-----------VDDW----KGNTEYHN-YSPS-------------------
------------------------SQQI-QWFWRALR--------------------SFD
KEELAKLLQF-------VTGTSKVP------------LNG-----FK-ELEGMN--GVNR
FNIHRDYGNK------------------------DRLPSSHTCF-NR-KTLP--------

>Ceratocystis_adiposa_LXGU01000053.1 .
ISVRRDMV-FYDSYRSL--------------YFKTGDEMKYGKLNIRFQG-------EDG

VDAGGVTREW---FQVL----SRQMFDANY--------CLFTPVSSDRT-----------
----------------TFHPNRLS------------------------------------
--GVNDQHLNFFKFVGRVIGKALYEGRVLDCHFSR--AVYKRILG---------------
--------KSVSVKDMES----------------------FDPDYYKS--------LIWM
LDN--DIT-DIL--
-TDTFSVEDEE--------------------------FGV-TKVVD-LVEN--------
GRNIE---
-----------------------------VTEENKR-DYVRL-VVEYKLLSS-VKEQME
-HFLAG---FHDIIPS--ELIS-IFNEQ-ELELLISG-----------------------
----LPDVD-----------VDDW----KAHTEYHN-YTPA-------------------
------------------------SPQI-QWFWRAVR--------------------SFD
KEEKAKLLQF-------VTGTSKVP------------LNG-----FK-ELEGMN--GVSR
FNIHRDYGNK------------------------DRLPSSHTCF-NX-LDVPEY-ESYDV
LRAQLL-KAITAGNDYF
>Huntiella_bhutanensis_MJMS01000086.1 .
VSVRRDMV-FYDSYRSL---------------YFKKGDEMKYGKLNIRFQG-------EEG
VDAGGVTREW---FQVL----SRQMFNADY--------ALFSPVSSDRT-----------
----------------TFHPNKLS------------------------------------
--AVNDQHLLYFKFVGRIIGKALYEGRVLDCHFSR--AVYKRILG---------------
--------KSVSVKDMES----------------------FDPDYYKS--------LVWM
LNN--DIT-DII--
-VETFSVEDEE--------------------------FGV-TTIVD-LIEN--------
GREVA---
-----------------------------VTEENKR-EYVKL-VVEHKLLSS-VKEQLE
-SFLAG---FHDIIPA--NLIS-IFNEQ-ELELLISG-----------------------
----LPDVD-----------VDDW----KAHTEYHN-YTAA-------------------
------------------------SPQI-QWFWRAVR--------------------SFD
KEEKAKLLQF-------VTGTSKVP------------LNG-----FK-ELEGMN--GVSR
FNIHRDYGDS------------------------DRLPSSHTCF-NQ-LDIPEY-ESYEV
LRTRLL-KAITAGNDYF
>Huntiella_omanensis_JSUI01006128.1 .
VSVRRDMV-FYDSYRSL---------------YFKKGDEMKYGKLNIRFQG-------EEG
VDAGGVTREW---FQVL----SRQMFNADY--------ALFSPVSSDRT-----------
----------------TFHPNKLS------------------------------------
--AVNDQHLLYFKFVGRIIGKALYEGRVLDCHFSR--AVYKRILG---------------
--------KSVSVKDMES----------------------FDPDYYKS--------LVWM
LNN--DIT-DII--
-VETFSVEDEE--------------------------FGV-TRIVD-LIEN--------
GREVA---
-----------------------------VTEENKR-EYVKL-VVEHKLLSS-VKEQLE
-SFLAG---FHDIIPA--NLIS-IFNEQ-ELELLISG-----------------------
----LPDVD-----------VDDW----KAHTEYHN-YTAA-------------------
------------------------SPQI-QWFWRAVR--------------------SFD
KEEKAKLLQF-------VTGTSKVP------------LNG-----FK-ELEGMN--GVSR
FNIHRDYGDS------------------------DRLPSSHTCF-NQ-LDIPEY-ESYEV
LRTRLL-KAITAGNDYF
>Huntiella_moniliformis_JMSH01000088.1 .
VSVRRDMV-FYDSYRSL---------------YFKKGDEMKYGKLNIRFQG-------EEG
VDAGGVTREW---FQVL----SRQMFNADY--------ALFSPVSSDRT-----------
----------------TFHPNKLS------------------------------------
--AVNDQHLLYFKFVGRIIGKALYEGRVLDCHFSR--AVYKRILG---------------
--------KSVSVKDMES----------------------FDPDYYKS--------LVWM
LNN--DIT-DII--
-VETFSVEDEE--------------------------FGV-TKIVD-LIEN--------
GREVA---
-----------------------------VTEENKR-EYVKL-VVEHKLLSS-VKEQLE
-SFLAG---FHDIIPA--NLIS-IFNEQ-ELELLISG-----------------------
----LPDVD-----------VDDW----KAHTEYHN-YTAA-------------------

------------------------SPQI-QWFWRAVR--------------------SFD
KEEKAKLLQF-------VTGTSKVP------------LNG-----FK-ELEGMN--GVSR
FNIHRDYGDS------------------------DRLPSSHTCF-NQ-LDIPEY-ESYEA
LRTRLL-KAITAGNDYF
>Endoconidiophora_laricicola_LXGT01000170. 1..
LNVRRDRV-FHDSYRAL--------------YYKNGGEMKYGKLNIRFNG-------EEG
VDAGGVTREW---FQVL----SRQMFDANY--------ALFTPVSSDRT-----------
----------------TFHPNKLS------------------------------------
--AINDQHLAYFKFVGRIIGKALYEGRVLDCHFSR--AVYKRILG---------------
--------KSVSVKDMES----------------------FDPDYYKS--------LLWM
LDN--NIT-DII--
-VDTFSIEDEE---------------------------FGV-TKTVD-LVEG--------
GRDIA---
------------------------------VTEENKK-DYVRL-VVEHKLLAS-VKEQME
-YFLKG---FHDIIPA--EIIS-IFNEQ-ELELLISG-----------------------
----LPDVD-----------VDDW----KAHTEYHN-YTAA-------------------
------------------------SQQI-QWFWRAVR--------------------SFD
KEERAKLLQF-------VTGTSKVP------------LNG-----FK-ELEGMN--GVSR
FNIHRDYGNK------------------------NRLPSSHTCF-NX-LDIPEY-ESYET
LRQQLI-KAITAGNDYF
>Endoconidiophora_polonica_LXKZ01000477.1 .
LNVRRDRV-FHDSYRAL--------------YYKNGSEMKYGKLNIRFNG-------EEG
VDAGGVTREW---FQVL----SRQMFDANY--------ALFTPVSSDRT-----------
----------------TFHPNKLS------------------------------------
--AINDQHLAYFKFVGRIIGKALYEGRVLDCHFSR--AVYKRILG---------------
--------KSVSVKDMES----------------------FDPDYYKS--------LLWM
LDN--NIT-DII--
-VDTFSIEDEE---------------------------FGV-TNTVD-LVEG--------
GRDIA---
------------------------------VTEENKK-DYVRL-VVEHKLLAS-VKEQME
-YFLKG---FHDIIPA--EIIS-IFNEQ-ELELLISG-----------------------
----LPDVD-----------VDDW----KAHTEYHN-YTAA-------------------
------------------------SQQI-QWFWRAVR--------------------SFD
KEERAKLLQF-------VTGTSKVP------------LNG-----FK-ELEGMN--GVSR
FNIHRDYGNK------------------------NRLPSSHTCF-NX-LDIPEY-ESYET
LRQQLI-KAITAGNDYF
>Davidsoniella_virescens_LJZU01000147.1 .
LNVRRDRV-FHDSYRAL--------------YFKNGNEMKYGKLNIRFNG-------EEG
VDAGGVTREW---FQVL----SRQMFDANY--------ALFSPVSSDRT-----------
----------------TFHPNKLS------------------------------------
--AINDQHLAYFKFVGRIIGKALYEGRVLDCHFSR--AVYKRILG---------------
--------KPVSVKDMES----------------------FDPDYYKS--------LLWM
LDN--NIT-DII--
-VDTFSIEDEE---------------------------FGV-TNIVD-LVEG--------
GRDIA---
------------------------------VTEENKK-DYVRL-VVEHKLLAS-VKDQME
-NFLKG---FHDIIPS--ELIS-IFNEQ-ELELLISG-----------------------
----LPEVD-----------VDDW----KAHTEYHN-YTAA-------------------
------------------------SQQI-QWFWRAVR--------------------SFD
KEERAKLLQF-------VTGTSKVP------------LNG-----FK-ELEGMN--GVSR
FNIHRDYGNK------------------------NRLPSSHTCF-NX-LDIPEY-ESYEI
LRQQLI-KAITAGNDYF
>Ceratocystis_albifundus_JSSU01001359.1 .
LSVRRDMV-FHDSYRSL--------------YFKTGNEMKYGKLNIRFHG-------EEG
VDAGGVTREW---FQVL----SRQMFDANY--------ALFTPVSSDRT-----------
----------------TFHPNKLS------------------------------------
--AVNDQHLTYFKFVGRIIGKALYEGRLLDCHFSR--AVYKRILG---------------
--------KSVSVKDMES----------------------FDPDYYKS--------LLWM

LDN--DIT-DII--
-VETFSVEDEE---------------------------FGA-TTIVD-LIPD--------
GRNIA---
------------------------------VTEENKK-EYVRL-VVEHKLLSS-VKEQME
-NFLKG---FHDIIPA--ELIS-IFNEQ-ELELLISG-----------------------
----LPDVD-----------IDDW----KTHTEYHN-YTAA-------------------
------------------------SPQI-QWFWRALR--------------------SFD
KEERAKMLQF-------VTGTSKVP------------LNG-----FK-ELEGMN--GVSR
FNIHRDYGNK------------------------DRLPSSHTCF-NX-LDLPEY-ESYEV
LRKQLI-KAITAGNDYF
>Ceratocystis_harringtonii_MKGM01000177.1 .
LSVRRDMV-FHDSYRSL--------------YFKSGNEMKYGKLNIRFHG-------EEG
VDAGGVTREW---FQVL----SRQMFDANY--------ALFTPVSSDRT-----------
----------------TFHPNKLS------------------------------------
--AVNDQHLTYFKFVGRIIGKALYEGRLLDCHFSR--AVYKRILG---------------
--------KSVSVKDMES----------------------FDPDYYKS--------LLWM
LDN--DIT-DII--
-VETFSVEDEE---------------------------FGA-TTIVD-LIPD--------
GRNIA---
------------------------------VTEENKK-EYVRL-VVEHKLLSS-VKEQME
-NFLKG---FHDIIPA--ELIS-IFNEQ-ELELLISG-----------------------
----LPDVD-----------VDDW----KTHTEYHN-YTAA-------------------
------------------------SPQI-QWFWRALR--------------------SFD
KEERAKLLQF-------VTGTSKVP------------LNG-----FK-ELEGMN--GVSR
FNIHRDYGNK------------------------DRLPSSHTCF-NX-LDLPEY-ESYEV
LRKQLI-KAITAGNDYF
>Chalaropsis_thielavioides_BCGU01000008.1 .
LSVRRDMV-FHDSYRSL--------------YFKSGDEMKYGKLNIRFHG-------EEG
VDAGGVTREW---FQVL----SRQMFDANY--------ALFIPVSSDRT-----------
----------------TFHPNKLS------------------------------------
--AVNDQHLTYFKFVGRIIGKALYEGRLLDCHFSR--AVYKRILG---------------
--------KSVSVKDMES----------------------FDPDYYKS--------LLWM
LDN--DIT-DII--
-VETFSVEDEE---------------------------FGA-TTIVD-LIPD--------
GRNIV---
------------------------------VTEDNKK-EYVRL-VVEHKLLSS-VKEQME
-HFLKG---FHDIIPA--ELIS-IFNEQ-ELELLISG-----------------------
----LPDVD-----------IDDW----KTHTEYHN-YTAA-------------------
------------------------SPQI-QWFWRALR--------------------SFD
KEERAKLLQF-------VTGTSKVP------------LNG-----FK-ELEGMN--GVSR
FNIHRDYGNK------------------------DRLPSSHTCF-N--------------

>Ceratocystis_fagacearum_MKGJ01000539.1 .
ISVRRDMV-FHDSYRSL--------------YFKSGDEMKYGKLNIRFQG-------EDG
VDAGGITREW---FQVL----SRQMFDANY--------ALFTPVSSDRT-----------
----------------TFHPNKLS------------------------------------
--AVNDQHLLYFKFVGRIIGKALYEGRVLDCHFSR--AVYKRILG---------------
--------KSVSVKDMES----------------------FDPDYYKS--------LLWM
LNN--DIT-DII--
-VEMFSIEDEE---------------------------FGV-TTIVD-LVEN--------
GRDIA---
------------------------------VTEENKR-DYVRL-VVEHKLLSS-VKEQME
-HFLKG---FHEIIPA--ELIS-IFNEQ-ELELLISG-----------------------
----LPDVD-----------IDDW----KAHTEYHN-YTVA-------------------
------------------------SPQI-QWFWRAVR--------------------SFD
KEEKAKLLQF-------VTGTSKVP------------LNG-----FK-ELEGMN--GVSR
FNIHRDYGNK------------------------DRLPSSHTCF-NX-LDIPEY-ENYEV
LRTQLL-KAITAGNDYF

>Thielaviopsis_punctulata_LAEV01000882.1 .
LSVRRNMV-FHDSYRAL--------------YFKSGEEMKYGKLNIRFHG-------EEG
VDAGGVTREW---FQVL----SRQMFDANY--------ALFTPVSSDRT-----------
----------------TFHPNKLS------------------------------------
--AVNDQHLLYFKFVGRIIGKALYEGRVLDCHFSR--AVYKRILG---------------
--------KSVSVKDMES----------------------FDPDYYKS--------LLWM
LDN--DIT-DII--
-VETFSVEDEE---------------------------FGV-TTIVD-LVPD--------
GRNIA---
------------------------------VTEENKK-DYVRL-VVEHKLLSS-VKEQME
-HFLKG---FHDIIPA--ELIS-IFNEQ-ELELLISG-----------------------
----LPDVD-----------IDDW----KAYTEYHN-YTAA-------------------
------------------------SQQI-QWFWRAVR--------------------SFD
KEELAKLLQF-------VTGTSKVP------------LNG-----FK-ELEGMN--GVSR
FNIHRDYGNK------------------------SRLPSSHTCF-NQ-LDLPEY-ESYEV
LRTQLL-KAITAGNDY-
>Thielaviopsis_musarum_LKBB01000165.1 .
LSVRRNMV-FHDSYRAL--------------YFKNGDEMKYGKLNIRFHG-------EEG
VDAGGVTREW---FQVL----SRQMFDANY--------ALFTPVSSDRT-----------
----------------TFHPNKLS------------------------------------
--AVNDQHLLYFKFVGRIIGKALYEGRVLDCHFSR--AVYKRILG---------------
--------KSVSVKDMES----------------------FDPDYYKS--------LLWM
LDN--DIT-DII--
-VETFSVEDEE---------------------------FGV-TTIVD-LVPD--------
GRNIA---
------------------------------VTEENKK-DYVRL-VVEHKLLSS-VKEQME
-HFLKG---FHDIIPA--ELIS-IFNEQ-ELELLISG-----------------------
----LPDVD-----------IDDW----KAHTEYHN-YTAA-------------------
------------------------SQQI-QWFWRAVR--------------------SFD
KEELAKLLQF-------VTGTSKVP------------LNG-----FK-ELEGMN--GVSR
FNIHRDYGNK------------------------SRLPSSHTCF-NX-LDLPEY-ESYEV
LRKQLM-KAITAGNDY-
>Thielaviopsis_paradoxa_BCFY01000036.1 .
LSVRRNMV-FHDSYRAL--------------YFKNGDEMKYGKLNIRFHG-------EEG
VDAGGVTREW---FQVL----SRQMFDANY--------ALFTPVSSDRT-----------
----------------TFHPNKLS------------------------------------
--AVNDQHLLYFKFVGRIIGKALYEGRVLDCHFSR--AVYKRILG---------------
--------KSVSVKDMES----------------------FDPDYYKS--------LLWM
LDN--DIT-DII--
-VETFSVEDEE---------------------------FGV-TTIVD-LVPD--------
GRNIA---
------------------------------VTEENKK-DYVRL-VVEHKLLSS-VKEQME
-HFLKG---FHDIIPA--ELIS-IFNEQ-ELELLISG-----------------------
----LPDVD-----------IDDW----KAHTEYHN-YTAA-------------------
------------------------SQQI-QWFWRAVR--------------------SFD
KEELAKLLQF-------VTGTSKVP------------LNG-----FK-ELEGMN--GVSR
FNIHRDYGNK------------------------SRLPSSHTCF-NX-LDLPEY-ESYEV
LRKQLM-KAITAGNDYF
>Chrysoporthe_cubensis_LJCY01000251.1 .
VGVRRDSV-FHDSYRNL--------------AFKSDEEVKYGKLNVRFHG-------EEG
VDAGGVTREW---FQVL----TRAMFDPNY--------VLFTPVSSDRT-----------
----------------TFHPNKSS------------------------------------
--SINDEHLNFFKFIGRIIGKAVYEGRLLDCYFSR--AVYKRILG---------------
--------KQVSVKDMES----------------------FDPDFYRN--------HSWM
LEN--DIT-GTV--
-YETFSVEEDE---------------------------FGA-KVTVD-LIEN--------
GRNID---
------------------------------VTNENKH-EYVRL-IVEHKLLSS-VKEQMD

-YFLKG---FHGIIPA--DLIS-IFNEQ-ELELLISG-----------------------
----LPDID-----------VDDW----KSNTEYTN-YTAA-------------------
------------------------SQQI-QWFWRAVR--------------------AFD
KEERAKLLQF-------VTGTSKVP------------LNG-----FK-ELEGMN--GIQR
FNIHRDYGNK------------------------DRLPSSHTCF-NX-LDLPEY-ESYDA
LRSQLL-KAITAGSDYF
>Valsa_mali_JUIZ01002076.1 .
VNVRRDNV-FHDSYRTL--------------AFKSDDEIKYGKLNVRFHG-------EEG
VDAGGVTREW---FAVL----TRAMFNPDY--------ALFTPVSADRT-----------
----------------TFHPNKAS------------------------------------
--HINEEHLHFFKFIGRIIGKAVYEGRLLECYFSR--ALYKRILG---------------
--------KPVSVKDMES----------------------FDPDYYKS--------LVWM
LEN--DIT-GAV--
-IETFSVEEEE---------------------------FGA-SKTVD-LIEN--------
GSNIE---
------------------------------VTNDNKH-EYVRL-IVEHKLLSS-VKVQME
-HFLQG---FHGVIPA--DLIA-IFNEQ-ELELLISG-----------------------
----LPDID-----------VDDW----KGNTEYHN-YTAA-------------------
------------------------SQQI-QWFWRALR--------------------SFD
KEERAKLLQF-------VTGTSKVP------------LNG-----FK-ELEGMN--GINR
FNIHRDYGNK------------------------DRLPSSHTCF-NX-LDLPEY-ESYEA
LRTQVL-KAITAGSDYF
>Diaporthe_helianthi_MAVT01009999.1 .
VNVRRDNV-FHDSYRAL--------------AFKSDEEVKHGKLNVRFHG-------EEG
VDAGGVTREW---FQVL----TRAMFNPDY--------ILFTPVSADRT-----------
----------------TFHPNKSS------------------------------------
--HYNEEHLAFFKFIGRIIGKAVYEGRLLDCYFSR--AVYKRILG---------------
--------KPVSVKDMES----------------------FDPDYYKS--------LVWM
LEN--DIT-NTV--
-IETFSVEEDE---------------------------FGA-SKVVD-LIEN--------
GRNIE---
------------------------------VTNENKH-EYVRL-IVEHKLLSS-VKIQME
-HFLQG---FHGVIPA--DLIA-IFNEQ-ELELLISG-----------------------
----LPDID-----------VDDW----KSNTEYHN-YTAA-------------------
------------------------SQQI-QWFWRAVR--------------------SFD
KEEQAKLLQF-------VTGTSKVP------------LNG-----FK-ELEGMN--GVNR
FNIHRDYGNK------------------------DRLPSSHTCF-N--------------

>Diaporthe_longicolla_AYRD02012323.1 .
VNVRRDNV-FHDSYRAL--------------AFKSDEEVKHGKLNVRFHG-------EEG
VDAGGVTREW---FQVL----TRAMFNPDY--------ILFTPVSADRT-----------
----------------TFHPNKSS------------------------------------
--HYNEEHLAFFKFIGRIIGKAVYEGRLLDCYFSR--AVYKRILG---------------
--------KPVSVKDMES----------------------FDPDYYKS--------LVWM
LEN--DIT-NTV--
-IETFSVEEDE---------------------------FGA-SKVVD-LIEN--------
GRNIE---
------------------------------VTNENKH-EYVRL-IVEHKLLSS-VKVQME
-HFLQG---FHGVIPA--DLIA-IFNEQ-ELELLISG-----------------------
----LPDID-----------VDDW----KSNTEYHN-YTAA-------------------
------------------------SQQI-QWFWRAVR--------------------SFD
KEEQAKLLQF-------VTGTSKVP------------LNG-----FK-ELEGMN--GVNR
FNIHRDYGNK------------------------DRLPSSHTCF-NX-LDLPEY-ESYEA
LRSQVI-KAITTGGDYF
>Diaporthe_ampelina_LCUC01000455.1 .
VNVRRDNV-FHDSYRAL--------------AFKSDDEVKHGKLNVRFHG-------EEG
VDAGGVTREW---FQVL----TRAMFNPDY--------ILFTPVSADRT-----------
----------------TFHPNKSS------------------------------------

--HYNEEHLAFFKFIGRIIGKAVYEGRLLDCYFSR--AVYKRILG---------------
--------KPVSVKDMES----------------------FDPDYYKS--------LVWM
LEN--DIT-GTV--
-IESFSVEEDE---------------------------FGA-SKVVD-LIEN--------
GRDIE---
------------------------------VTNENKH-EYVRL-IVEHKLLSS-VKIQME
-HFLQG---FHGVIPA--DLIA-IFNEQ-ELELLISG-----------------------
----LPDID-----------VDDW----KSNTEYHN-YTAA-------------------
------------------------SQQI-QWFWRAVR--------------------SFD
KEEQAKLLQF-------VTGTSKVP------------LNG-----FK-ELEGMN--GVNR
FNIHRDYGNK------------------------DRLPSSHTCF-NX-LDLPEY-ESYEA
LRSQVI-KAITTGGDYF
>Diaporthe_aspalathi_LJJS01000760.1 .
VNVRRDNV-FHDSYRAL--------------AFKSDDEVKHGKLNVRFHG-------EEG
VDAGGVTREW---FQVL----TRAMFNPDY--------ILFTPVSADRT-----------
----------------TFHPNKSS------------------------------------
--HFNEEHLAFFKFIGRIIGKAVYEGRLLDCYFSR--AVYKRILR---------------
--------KPVSVKDMES----------------------FDPDYYKS--------LAWM
LEN--DIT-GTV--
-IESFSVEEDE---------------------------FGA-NKVVD-LIEN--------
GRNIE---
------------------------------VTNENKH-EYVRL-IVEHKLLSS-VKVQME
-HFLQG---FHGVIPA--DLIA-IFNEQ-ELELLISG-----------------------
----LPDID-----------VDDW----KSNTEYHN-YTAA-------------------
------------------------SQQI-QWFWRAVR--------------------SFD
KEEQAKLLQF-------VTGTSKVP------------LNG-----FK-ELEGMN--GVNR
FNIHRDYGNK------------------------DRLPSSHTCF-NX-LDLPEY-ESYEA
LRSQVI-KAITTGGDYF
>Mycosphaerella_eumusae_GDIK01005317.1 .
LNIRRDQV-FLDSFKSL--------------YYKSPSEIKYGKLNIRFYG-------EEG
IDAGGVSREW---FAAM----ARQMFNPDY--------ALFNPVAADRT-----------
----------------TFHPNSLS------------------------------------
--EINPEHLMFFKFIGRVIGKALYENRVLDCHFSR--AVYRKILG---------------
--------KSVSLKDMES----------------------LDLDYYKS--------LVWI
LEN--DIT-DVT--
-FETFSVDVDK---------------------------FGV-TETID-LIPN--------
GRNIA---
------------------------------VTEENKQ-EYVRL-VVDYRLIKS-VQGQLD
-NFLEG---FHDIIPA--ELVS-IFNEQ-ELELLISG-----------------------
----LPDID-----------VDDW----KNNTEYHN-YQST-------------------
------------------------SPQV-QWFWRAVR--------------------SFD
KEEKAKLLQF-------VTGTSKVP------------LNG-----FK-ELEGMN--GFSK
FNIHRDFSSK------------------------EKLPSSHTCF-NQ-LDLPEY-ESYEH
LRHQLY-TAITAGSEYF
>Mycosphaerella_eumusae_LFZN01000026.1 .
LNIRRDQV-FLDSFKSL--------------YYKSPSEIKYGKLNIRFYG-------EEG
IDAGGVSREW---FAAM----ARQMFNPDY--------ALFNPVAADRT-----------
----------------TFHPNSLS------------------------------------
--EINPEHLMFFKFIGRVIGKALYENRVLDCHFSR--AVYRKILG---------------
--------KSVSLKDMES----------------------LDLDYYKS--------LVWI
LEN--DIT-DVT--
-FETFSVDVDK---------------------------FGV-TETID-LIPN--------
GRNIA---
------------------------------VTEENKQ-EYVRL-VVDYRLIKS-VQGQLD
-NFLEG---FHDIIPA--ELVS-IFNEQ-ELELLISG-----------------------
----LPDID-----------VDDW----KNNTEYHN-YQST-------------------
------------------------SPQV-QWFWRAVR--------------------SFD
KEEKAKLLQF-------VTGTSKVP------------LNG-----FK-ELEGMN--GFSK

FNIHRDFSSK------------------------EKLPSSHTCF-NX-LDLPEY-ESYEH
LRHQLY-TAITAGSEYF
>Pseudocerc_musae_GDIN01009256.1 .
LNIRRDQV-FLDSFKSL--------------YYKSPSEIKYGKLNIRFHG-------EEG
IDAGGVSREW---FAAM----ARQMFNPDY--------ALFNPVAADRT-----------
----------------TFHPNSLS------------------------------------
--EINPEHLMFFKFIGRVIGKALYENRVLDCHFSR--AVYRKILG---------------
--------KSVSLKDMES----------------------LDLDYYKS--------LVWI
MEN--DIT-DVT--
-FETFSVDVDK---------------------------FGV-TETID-LIPN--------
GRNIA---
------------------------------VTEENKQ-EYVRL-VVDYRLIKS-VQGQLD
-NFLEG---FHDIIPA--ELVS-IFNEQ-ELELLISG-----------------------
----LPDID-----------VDDW----KNNTEYHN-YQST-------------------
------------------------SPQV-QWFWRAVR--------------------SFD
KEEKAKLLQF-------VTGTSKVP------------LNG-----FK-ELEGMN--GFSK
FNIHRDFSSK------------------------DKLPSSHTCF-NQ-LDLPEY-ESYEH
LRHQLY-TAITAGSEYF
>Pseudocerc_fijiensis_XM_007922474.1 .
LNIRRDQV-FLDSFKSL--------------YYKSPSEIKYGKLNIRFHG-------EEG
IDAGGVSREW---FAAM----ARQMFNPDY--------ALFNPVAADRT-----------
----------------TFHPNSLS------------------------------------
--EINPEHLMFFKFIGRVIGKALYENRVLDCHFSR--AVYRKILG---------------
--------KSVSLKDMES----------------------LDLDYYKS--------LVWI
LEN--DIT-DVT--
-FETFSVDVDK---------------------------FGV-TETID-LIPN--------
GRNIA---
------------------------------VTEENKQ-EYVRL-VVDYRLIKS-VQGQLD
-NFLEG---FHDIIPA--ELVS-IFNEQ-ELELLISG-----------------------
----LPDID-----------VDDW----KNNTEYHN-YQQT-------------------
------------------------SPQV-QWFWRAVR--------------------SFD
KEEKAKLLQF-------VTGTSKVP------------LNG-----FK-ELEGMN--GFSK
FNIHRDFSSK------------------------EKLPSSHTCF-NQ-LDLPEY-ESYEH
LRHQLY-TAITAGSEYF
>Pseudocerc_pini_densiflorae_AWYD02003323. 1..
LNIRRDQV-FLDSFKSL--------------YYKSPSEIKYGKLNIRFHG-------EEG
IDAGGVSREW---FAAM----ARQMFNPDY--------ALFNPVAADRT-----------
----------------TFHPNSLS------------------------------------
--EINPEHLMFFKFIGRVIGKALYENRVLDCHFSR--AVYRKILG---------------
--------KSVSLKDMES----------------------LDLDYYKS--------LVWI
LEN--DIT-DVT--
-FETFSVDVDK---------------------------FGV-TETID-LIPN--------
GRNIA---
------------------------------VTEENKQ-EYVRL-VVDYRLIKS-VQGQLD
-NFLEG---FHDIIPA--ELVS-IFNEQ-ELELLISG-----------------------
----LPDID-----------VDDW----KNNTEYHN-YQST-------------------
------------------------SPQV-QWFWRAVR--------------------SFD
KEEKAKLLQF-------VTGTSKVP------------LNG-----FK-ELEGMN--GFSK
FNIHRDFSSK------------------------EKLPSSHTCF-NX-LDLPEY-DTYEH
LRHQLY-TAITAGSEYF
>Phaeocryptopus_gaeumannii_MWSP01000387.1 .
LSVRRDQV-FLDSFKSL--------------YYKSGDEIKYGKLNIRFHG-------EEG
IDAGGVSREW---FAAM----ARQMFNPDY--------ALFNPVAADRT-----------
----------------TFHPNTLS------------------------------------
--GINQEHLMFFKFIGRIIGKALYENRVLDCHFSR--AVYRKILG---------------
--------KSVSLKDMET----------------------LDLDYYKS--------LVWI
LEN--DIT-DVT--
-FETFSVDVDR---------------------------FGV-TETID-LMDN--------

GRNIA---
------------------------------VTEDSKR-EYVRL-VVEYRLIKS-VQEQLD
-HFLQG---FHEIIPA--ELVA-IFTEQ-ELELLISG-----------------------
----LPDID-----------VDDW----KNNTDYTN-YQPN-------------------
------------------------SPQV-QWFWRAVR--------------------SFD
KEEKAKLLQF-------VTGTSKVP------------LNG-----FK-ELEGMN--GFSK
FNIHRDYSNK------------------------EKLPSSHTCF-NX-LDLPEY-ESYEH
LRQQLY-TAITAGSEYF
>Cercospora_sojina_AHPQ01000122.1 .
LNVRRDQV-FHDSFKSL--------------YYKSPNEIKYGKLNIRFHG-------EEG
IDAGGVSREW---FAAM----ARQMFNPDY--------ALFNPVAADRT-----------
----------------TFHPNTLS------------------------------------
--TVNPEHLLFFKFIGRIIGKALYENRVLDCHFSR--AVYRKILG---------------
--------KSVSLKDMET----------------------LDLDYYKS--------LVWI
LEN--DIT-DIT--
-FETFSVDIDR---------------------------FGS-TETVD-LVEN--------
GREIP---
------------------------------VTEENKH-EYVRH-VVEYRLIKS-VQAQLD
-EFLTG---FHDIIPP--ELIS-IFNEQ-ELELLISG-----------------------
----LPDID-----------VDDW----KNNTEYTN-YTPT-------------------
------------------------SPQV-QWFWRAVR--------------------SFD
KEEKAKLLQF-------VTGTSKVP------------LNG-----FK-ELEGMN--GFSR
FNIHRDYSSK------------------------EKLPSSHTCF-NX-LDLPEY-DSYEH
LRHQLY-TAITAGSEYF
>Cercospora_canescens_ANSM01000452.1 .
LNVRRDQV-FLDSFKSL--------------YYKSGNEIKYGKLNIRFHG-------EEG
IDAGGVSREW---FAAM----ARQMFNPDY--------ALFNPVAADRT-----------
----------------TFHPNTLS------------------------------------
--SINGEHLLFFKFIGRIIGKALYENRVLDCHFSR--AVYRKILG---------------
--------KSVSLKDMET----------------------LDLDYYKS--------LVWI
LEN--DIT-DIT--
-FETFSVDIDR---------------------------FGV-TETVD-LIEN--------
GREIP---
------------------------------VTEENKH-EYVRH-VVEYRLIKS-VQAQLD
-NFLSG---FHEIIPP--ELIS-IFNEQ-ELELLISG-----------------------
----LPDID-----------VDDW----KNNTEYTN-YTPT-------------------
------------------------SPQV-QWFWRAVR--------------------SFD
KEEKAKLLQF-------VTGTSKVP------------LNG-----FK-ELEGMN--GFSR
FNIHRDYSSK------------------------EKLPSSHTCF-NX-LDLPEY-DSYEH
LRHQLY-TAITAGSEYF
>Sphaerulina_populicola_AIDU01000338.1 .
LNIRRNQV-FLDSFKSL--------------YYKTGNEIKYGKLNIRFHG-------EEG
VDAGGVSREW---FAAM----ARQMFNPDY--------GLFNPVAADRT-----------
----------------TFHPNTHS------------------------------------
--GVNDEHFMFFKFIGRIIGKALYENRVLDCHFSR--AVYRKILG---------------
--------KSVSLKDMES----------------------LDLDYYKS--------LVWI
LEN--DIT-DVT--
-FETFSIDLDK---------------------------FGV-TETID-LIEG--------
GRDIS---
------------------------------VTEENKH-EYVRH-VVEYRLIKS-VQEQLD
-NFLQG---FHEVIPA--ELIS-IFNEQ-ELELLISG-----------------------
----LPDID-----------VDDW----KNNTEYTN-YTPT-------------------
------------------------SPQV-QWFWRAVR--------------------SFD
KEEKAKLLQF-------VTGTSKVP------------LNG-----FK-ELEGMN--GFSR
FAIHRDYSNK------------------------ERLPSSHTCF-NX-LDLPEY-DSYEH
LRHQLY-TAITAGSEYF
>Sphaerulina_musiva_XM_016903118.1 .
LNIRRNQV-FLDSFKSL--------------YYKTGNEIKYGKLNIRFHG-------EEG

```
VDAGGVSREW---FAAM----ARQMFNPDY--------GLFNPVAADRT-----------
----------------TFHPNTHS------------------------------------
--GVNDEHFMFFKFIGRIIGKALYENRVLDCHFSR--AVYRKILG---------------
--------KSVSLKDMES----------------------LDLDYYKS--------LVWI
LEN---------------------------------------------DIT-DVT--
-FETFSIDLDK---------------------------FGV-TETID-LIEG--------
GRDIP-------------------------------------------------------
-----------------------------VTEENKH-EYVRH-VVEYRLIKS-VQEQLD
-NFLQG---FHEVIPA--ELIS-IFNEQ-ELELLISG-----------------------
----LPDID-----------VDDW----KNNTEYTN-YTPT-------------------
------------------------SPQV-QWFWRAVR--------------------SFD
KEEKAKLLQF-------VTGTSKVP------------LNG-----FK-ELEGMN--GFSR
FAIHRDYSNK------------------------EKLPSSHTCF-NQ-LDLPEY-DSYEH
LRHQLY-TAITAGSEYF
>Ramularia_endophylla_MWSQ01004189.1 .
LSLRRSEV-FLDSFKSL---------------YYKSANEIKYGKLNIRFHN-------EEG
VDAGGVSREW---FAAM----ARQMFNPNY--------ALFTPVASDRT-----------
----------------TFHPNTMS------------------------------------
--GVNNEHLLFFKFVGRIIGKALYENRVLDCHFSR--AVYRRILG---------------
--------KGVSLKDMET----------------------LDLDYYKS--------LVWI
LEN---------------------------------------------DIT-DIT--
-FETFSVDIDK---------------------------FGV-TETID-LIPN--------
GRNIP-------------------------------------------------------
-----------------------------VTEDNKH-DYVRC-VVDYRLVTS-VKDQLD
-EFLKG---FHEIIPA--ELVA-IFNEQ-ELELLISG-----------------------
----LPEID-----------VDDW----KNNTEYTN-YQPT-------------------
------------------------SPQI-LWFWRAVK--------------------SFD
KEEKAKLLQF-------ITGTSKVP------------LNG-----FK-ELEGMN--GFSK
FSIHRDYGNK------------------------ERLPSSHTCF-NX-LDLPEY-ESYEA
LRHQLY-TAITAGSEYF
>Ramularia_collo-cygni_CZLF01000441.1 .
LSLRRSEV-FLDSFKSL---------------YYKTANEIKYGKLNIRFHN-------EEG
VDAGGVSREW---FAAM----ARQMFDANY--------ALFTPVASDRT-----------
----------------TFHPNTMS------------------------------------
--GINGEHLMFFKFVGRIIGKALYENRVLDCHFSR--AVYRRILG---------------
--------KSVSLKDMES----------------------LDLDYYKS--------LVWI
LEN---------------------------------------------DIT-DVT--
-FDTFSTDVDK---------------------------FGL-KETID-LIPN--------
GRNIP-------------------------------------------------------
-----------------------------VTEENKH-EYVRH-VVDYRLVTS-VKDQLD
-NFLEG---FHEIIPI--ELVS-IFNEQ-ELELLISG-----------------------
----LPEID-----------VDDW----KNNTEYTN-YQPT-------------------
------------------------SPQI-MWFWRAVK--------------------SFD
KEEKAKLLQF-------ITGTSKVP------------LNG-----FK-ELEGMN--GFSK
FSIHRDYGNK------------------------DRLPSSHTCF-NX-LDLPEY-ESYEA
LRHQLY-LAITAGSEYF
>Zymoseptoria_passerinii_AFIY01000099.1_R .
LSVRRDQV-FLDSFKSL---------------YYKSGNEIKYGKLNIRFIG-------EEG
VDAGGVSREW---FAAM----ARQMFNPDY--------ALFNPVASDRT-----------
----------------TFHPNTLS------------------------------------
--EVNPEHLLFFKFIGRIIGKALYENRVLDCHFSR--AVYRXILG---------------
--------KSVSLKDMET----------------------LDLEYYKS--------LVWM
LEN---------------------------------------------DIT-DVA--
-FETFSVDVDK---------------------------FGV-TETVD-LIPN--------
GRNVP-------------------------------------------------------
-----------------------------VTEVNKH-EYVRH-VVDYRLVTS-VKNQLD
-NFLQG---FHEIIPA--ELVS-IFNEQ-ELELLVSG-----------------------
----LPDID-----------VDDW----KNNTDYTN-YQPT-------------------
```

------------------------SPQV-QWFWRAVR---------------------SFD
KEEKAKLLQF-------VTGTSKVP------------LNG-----FK-ELEGMN--GFSK
FSIHRDYSNK------------------------ERLPSSHTCF-NX-LDLPEY-ESYEA
LRHQLY-TAITAGSEY-
>Zymoseptoria_tritici_LT853692.1_a.
LSIRRDQV-FLDSFKSL--------------YYKSGNEIKYGKLNIRFIG-------EEG
VDAGGVSREW---FAAM----ARQMFNPDY--------ALFNPVASDRT-----------
----------------TFHPNTLS------------------------------------
--EVNPEHLMFFKFIGRIIGKALYENRVLDCHFSR--AVYRRILG---------------
--------KSVSLKDMET----------------------LDLDYYKS--------LVWI
LEN--DIT-DVT--
-FETFSVDVDK---------------------------FGV-TETVD-LIPN--------
GRNIP---
------------------------------VTEENKH-EYVRH-VVDYRLVTS-VKNQLD
-NFLQG---FHEIIPA--ELVS-IFNEQ-ELELLISG-----------------------
----LPDID-----------VDDW----KNNTDYTN-YQPT-------------------
------------------------SPQI-QWFWRAVR---------------------SFD
KEEKAKLLQF-------VTGTSKVP------------LNG-----FK-ELEGMN--GFSK
FNIHRDYSNK------------------------ERLPSSHTCF-NX-LDLPEY-ESYEA
LRHQLY-TAITAGSEYF
>Zymoseptoria_pseudotritici_AFIQ01000807.1 .
LSIRRDQV-FLDSFKSL--------------YYKSGNEIKYGKLNIRFIG-------EEG
VDAGGVSREW---FAAM----ARQMFNPDY--------ALFNPVASDRT-----------
----------------TFHPNTLS------------------------------------
--EVNPEHLMFFKFIGRIIGKALYENRVLDCHFSR--AVYRRILG---------------
--------KSVSLKDMET----------------------LDLDYYKS--------LVWI
LEN--DIT-DVT--
-FETFSVDVDK---------------------------FGV-TETVD-LIPN--------
GRNIP---
------------------------------VTEENKH-EYVRH-VVDYRLVTS-VKNQLD
-NFLQG---FHEIIPA--ELVS-IFNEQ-ELELLISG-----------------------
----LPDID-----------VDDW----KNNTDYTN-YQPT-------------------
------------------------SPQI-QWFWRAVR---------------------SFD
KEEKAKLLQF-------VTGTSKVP------------LNG-----FK-ELEGMN--GFSK
FNIHRDYSNK------------------------ERLPSSHTCF-NX-LDLPEY-ESYEA
LRHQLY-TAITAGSEYF
>Zymoseptoria_brevis_LAFY01000332.1 .
LSIRRDQV-FLDSFKSL--------------YYKSGNEIKYGKLNIRFIG-------EEG
VDAGGVSREW---FAAM----ARQMFNPDY--------ALFNPVASDRT-----------
----------------TFHPNTLS------------------------------------
--EVNPEHLMFFKFIGRIIGKALYENRVLDCHFSR--AVYRRILG---------------
--------KSVSLKDMET----------------------LDLDYYKS--------LVWI
LEN--DIT-DVT--
-FETFSVDVDK---------------------------FGV-TETVD-LIPN--------
GRNIP---
------------------------------VTEENKH-EYVRH-VVDYRLVTS-VKNQLD
-NFLQG---FHEIIPA--ELVS-IFNEQ-ELELLISG-----------------------
----LPDID-----------VDDW----KNNTDYTN-YQPT-------------------
------------------------SPQI-QWFWRAVR---------------------SFD
KEEKAKLLQF-------VTGTSKVP------------LNG-----FK-ELEGMN--GFSK
FNIHRDYSNK------------------------ERLPSSHTCF-NX-LDLPEY-ESYEA
LRHQLY-TAITAGSEYF
>Mycosphaerella_graminicola_XM_003857457.1 .
LSIRRDQV-FLDSFKSL--------------YYKSGNEIKYGKLNIRFIG-------EEG
VDAGGVSREW---FAAM----ARQMFNPDY--------ALFNPVASDRT-----------
----------------TFHPNTLS------------------------------------
--EVNPEHLMFFKFIGRIIGKALYENRVLDCHFSR--AVYRRILG---------------
--------KSVSLKDMET----------------------LDLDYYKS--------LVWI

LEN--DIT-DVT--
-FETFSVDVDK---------------------------FGV-TETVD-LISN--------
GRNIP---
------------------------------VTEENKH-EYVRH-VVDYRLVTS-VKNQLD
-NFLQG---FHEIIPA--ELVS-IFNEQ-ELELLISG-----------------------
----LPDID-----------VDDW----KNNTDYTN-YQPT-------------------
------------------------SPQI-QWFWRAVR--------------------SFD
KEEKAKLLQF-------VTGTSKVP------------LNG-----FK-ELEGMN--GFSK
FNIHRDYSNK------------------------ERLPSSHTCF-NQ-LDLPEY-ESYEA
LRHQLY-TAITAGSEYF
>Zymoseptoria_ardabiliae_AFIU01000685.1 .
LSIRRDQV-FLDSFKSL--------------YYKSGNEIKYGKLNIRFIG-------EEG
VDAGGVSREW---FAAM----ARQMFNPDY--------ALFNPVASDRT-----------
----------------TFHPNTLS------------------------------------
--EVNPEHLMFFKFIGRIIGKALYENRVLDCHFSR--AVYRRILG---------------
--------KSVSLKDMET----------------------LDLDYYKS--------LVWI
LEN--DIT-DVT--
-FETFSVDVDK---------------------------FGV-TETVD-LIPN--------
GRNIP---
------------------------------VTEENKH-EYVRH-VVDYRLVTS-VKNQLD
-NFLQG---FHEIIPA--ELVS-IFNEQ-ELELLISG-----------------------
----LPDID-----------VDDW----KNNTDYTN-YQPT-------------------
------------------------SPQI-QWFWRAVR--------------------SFD
KEEKAKLLQF-------VTGTSKVP------------LNG-----FK-ELEGMN--GFSK
FNIHRDYSNK------------------------ERLPSSHTCF-NX-LDLPEY-ESYEA
LRHQLY-TAITAGSEYF
>Mycosphaerella_laricina_AWYE02000733.1 .
LSIRRDQV-FLDSFKSL--------------YYKTGNEIKYGKLNIRFHG-------EEG
IDAGGVSREW---FAAM----ARQMFNPDY--------ALFNPVASDRT-----------
----------------TFHPNNLS------------------------------------
--EVNPEHLLFFKFIGRIIGKALYENRVLDCHFSR--AIYRKMLG---------------
--------KNVTLKDMET----------------------LDLDYYKS--------LVWI
LEN--DIT-DVT--
-FETFSVDVDK---------------------------FGV-TETID-LIPG--------
GRDIS---
------------------------------VTEENKQ-EYVRL-VVEHRLIKS-VQQQID
-HFLEG---FHEIIPA--DMVA-IFTEQ-ELELLISG-----------------------
----LPDID-----------VDDW----KNNTDYTN-YQPT-------------------
------------------------SPQI-QWFWRAVR--------------------SFD
KEEKAKLLQF-------VTGTSKVP------------LNG-----FK-ELEGMN--GFHK
FNIHRDYSSK------------------------EKLPSSHTCF-NX-LDLPEY-ESYEH
LRQQLY-TAITAGGEYF
>Mycosphaerella_arachidis_LIHB01000013.1 .
LSVRRDQV-FLDSFKSL--------------YYKTGNEIKYGKLNIRFHG-------EEG
IDAGGVSREW---FAAM----ARQMFNPDF--------ALFIPVASDRT-----------
----------------TFHPNNLS------------------------------------
--EVNPEHLLFFKFIGRIIGKALYENRVLDCHFSR--AVYRKILG---------------
--------KNVSLKDMET----------------------LDLDYYKS--------LVWI
LEN--DIT-DVA--
-FETFSVDVDK---------------------------FGV-TETID-LIPG--------
GRDIS---
------------------------------VTEQNKH-DYVRL-VVEHRLIKS-VQQQID
-HFLEG---FHEIIPA--ELIS-IFNEQ-ELELLISG-----------------------
----LPDID-----------VDDW----KNNTDYTN-YQPT-------------------
------------------------SPQI-QWFWRAIR--------------------SFD
KEEQAKLLQF-------VTGTSKVP------------LNG-----FK-ELEGMN--GFAK
FNIHRDYSNK------------------------EKLPSSHTCF-NX-LDLPEY-ESYEH
LRQQLY-TAITAGSEYF

>Passalora_fulva_AMRR01003002.1 .
LSVRRDQV-FLDSFKSL--------------YYKTGNEIKYGKLNIRFHG-------EEG
IDAGGVSREW---FAAM----ARQMFNADY--------ALFNPVASDRT-----------
----------------TFHPNNLS------------------------------------
--EVNPEHLLFFKFIGRIIGKALYENRVLDCHFSR--AVYRKMLG---------------
--------KNVSLKDMET----------------------LDLDYYKS--------LVWI
LEN--DIT-DVA--
-FETFSVDVDK---------------------------FGV-TETID-LVPG--------
GRDIS---
------------------------------VTEDNKQ-DYVRL-VVEHRLIKS-VQQQID
-HFLEG---FHEIIPA--ELIS-IFNEQ-ELELLISG-----------------------
----LPDID-----------VDDW----KNNTDYTN-YQPT-------------------
------------------------SPQI-QWFWRAVR--------------------SFD
KEEKAKLLQF-------VTGTSKVP------------LNG-----FK-ELEGMN--GFAK
FNIHRDYSNK------------------------EKLPSSHTCF-NX-LDLPEY-ESYEH
LRQQLY-TAITAGSEYF
>Dothistroma_pini_MWSO01000950.1 .
LSVRRDQV-FLDSFKSL--------------YYKTGNEIKYGKLNIRFHG-------EEG
IDAGGVSREW---FAAM----ARQMFNPDY--------ALFNPVASDRT-----------
----------------TFHPNNLS------------------------------------
--EVNPEHLLFFKFIGRIIGKALYENRVLDCHFSR--AVYRKILG---------------
--------KNVSLKDMET----------------------LDLDYYKS--------LVWI
LEN--DIT-DVA--
-FETFSVDVDK---------------------------FGV-TETID-LVPG--------
GRDIS---
------------------------------VTEENKH-DYVRL-VVEHRLIKS-VQQQID
-HFLEG---FHEIIPA--ELIS-IFNEQ-ELELLISG-----------------------
----LPDID-----------ADDW----KNNTDYTN-YQPT-------------------
------------------------SPQI-QWFWRAVR--------------------SFD
KEEKAKLLQF-------VTGTSKVP------------LNG-----FK-ELEGMN--GFAK
FNIHRDYSNK------------------------EKLPSSHTCF-NX-LDLPEY-ESYEH
LRQQLY-TAITAGSEYF
>Dothistroma_septosporum_AIEN01000008.1 .
LSVRRDQV-FLDSFKSL--------------YYKTGNEIKYGKLNIRFHG-------EEG
IDAGGVSREW---FAAM----ARQMFNPDY--------ALFNPVASDRT-----------
----------------TFHPNNLS------------------------------------
--EVNPEHLLFFKFIGRIIGKALYENRVLDCHFSR--AVYRKILG---------------
--------KNVSLKDMET----------------------LDLDYYKS--------LVWI
LEN--DIT-DVA--
-FETFSVDVDK---------------------------FGV-TETID-LVPG--------
GRDIS---
------------------------------VTEENKH-DYVRL-VVEHRLIKS-VQQQID
-HFLEG---FHEIIPA--ELIS-IFNEQ-ELELLISG-----------------------
----LPDID-----------ADDW----KNNTDYTN-YQPT-------------------
------------------------SPQI-QWFWRAVR--------------------SFD
KEEKAKLLQF-------VTGTSKVP------------LNG-----FK-ELEGMN--GFAK
FNIHRDYSNK------------------------EKLPSSHTCF-NX-LDLPEY-ESYEH
LRQQLY-TAITAGSEYF
>Hortaea_werneckii_AIJO01004832.1 .
LNIRRDNV-FMDSYKSL--------------YFKSAEEIKYGKLNIRFHG-------EEG
IDAGGVSREW---FGSM----ARQMFNPDY--------ALFNPVASDRT-----------
----------------TFHPNPHS------------------------------------
--DVNEEHLSFFKFIGRIIGKALYEGRLLDCHFSR--AVYRRILG---------------
--------RTVSLKDMES----------------------LDLDYYRS--------LVWI
LEN--DIT-DVT--
-FETFSVDVDR---------------------------FGA-EETVD-LIPD--------
GRNIP---
------------------------------VTEENKQ-QYVQL-VVEHRLITS-VQQQLD

-KFLEG---FHDIIPQ--ELVS-IFNEQ-ELELLISG-----------------------
----LPEID-----------LDDW----KNNTEYHN-YQAT-------------------
------------------------SPQI-QWFWRAVR--------------------SFD
KEEKAKLLQF-------ITGTSKVP------------LNG-----FK-ELEGMN--GISR
FNIHRDYSSK------------------------EKLPSSHTCF-NQ-LDLPEY-ESYEH
LRQQLY-TAITAGNEYF
>Baudoinia_panamericana_XM_007679448.1 .
LSIRRDQV-FLDSFKSL--------------YHKRGDEIKYGKLNIRFHG-------EEG
IDAGGVSREW---FAAM----ARQMFNPDY--------ALFNPVASDRT-----------
----------------TFHPNPLS------------------------------------
--DINNEHLMFFKFIGRIIGKALYENRVLDCHFSR--AVYRRILG---------------
--------KSVSLKDMES----------------------LDLDYYKS--------LVWI
LEN--DIT-DVF--
-DETFSIDVDR---------------------------FGA-IETVD-LIEN--------
GRNIP---
------------------------------VTEENKQ-EYVQL-VVEHRLIKS-VGPQLE
-NFLDG---FHDIIPK--ELVA-IFNEQ-ELELLISG-----------------------
----LPDID-----------VDDW----KNNTEYHN-YQAT-------------------
------------------------SSQV-QWFWRAVR--------------------SFD
KEEKAKLLQF-------VTGTSKVP------------LNG-----FK-ELEGMN--GFAK
FNIHRDYSSK------------------------EKLPSSHTCF-NQ-LDLPEY-ESYEH
LRQQLY-TAITAGSEYF
>Acidomyces_richmondensis_JPDO01000302.1 .
LSVRRDYV-FLDSFKSL--------------YYKTPDEIKYGKLNVRFHG-------EEG
IDAGGVSREW---FAAM----SRQMFNPDY--------ALFRPVASDRT-----------
----------------TFHPNENS------------------------------------
--EVNSEHLLFFKFIGRIIGKALYENRLLDCHFSR--AVYRKLLG---------------
--------KTVSLKDMES----------------------LDLQDYKA--------LVWM
LEN--PID-GIL--
-YHTFAYSYDH---------------------------FGE-TQTKD-LIPN--------
GRNID---
------------------------------VTDENKQ-EYVQK-VVEFRLIGS-VEEQLD
-KFLEG---FHEIIPR--ELVS-MFNEQ-ELELLISG-----------------------
----LPDID-----------VDDW----KNNTEYHN-YQPT-------------------
------------------------SPQI-QWFWRAVK--------------------SFD
KEEKAKLLQF-------VTGTSKVP------------LNG-----FK-ELEGMN--GFAK
FNIHRDYSSK------------------------EKLPTSHTCF-NQ-LDLPEY-ESYEQ
LRHQLY-TAITAGAEYF
>Rachicladosp_antarcticum_NAJO01000012.1 .
LNVRRDQV-FMDSYKAL--------------YYKSPNEIKHGKLNIRFNG-------EEG
IDAGGVSREW---FAVM----ARQMFNPDY--------ALFNPVASDRT-----------
----------------TFHPNRLS------------------------------------
--DINTEHLSFFKFIGRVIGKALYENRVLDCHFSR--AVYRRILG---------------
--------KSVSLKDMET----------------------LDLDYYRS--------LVWI
LEN--DIT-DVT--
-FETFSVEVDR---------------------------FGV-VTTDD-LIPN--------
GRDIA---
------------------------------VTEDNKQ-EYVRL-VVEYRLIKS-VEGQLD
-AFLGG---FHDIIPA--ELVS-IFNEQ-ELELLISG-----------------------
----LPEID-----------VDDW----KNNTEYHN-YQAT-------------------
------------------------SPQI-QWLWRAIK--------------------SFD
PEERAKLLQF-------VTGTSKVP------------LNG-----FK-ELEGMN--GFAK
FNIHRDYSSK------------------------EKLPTSHTCF-NX-LDLPEY-ESYEH
LRQQLY-TAITAGSEYF
>Peltaster_fructicola_LJAO01000002.1 .
LNVRRDEV-FMDSYKAL--------------FFKSGEEIKNGKLNIRFNG-------EEG
IDAGGVSREW---FAVM----ARQMFNPDF--------ALFNPVASDRT-----------
----------------TFHPNRLS------------------------------------

--DINEEHLPFFKFIGRVIGKALYEGRVLDCHFSR--AVYRRILG---------------
--------KSVSLKDMES----------------------LDLEYYKS--------LVWI
LEN--DIT-DIA--
-FETFSVEVDR---------------------------FGE-IEIVD-LIEN--------
GRNIA---
------------------------------VTEDNKQ-EYVRL-VVEHRLIKS-VEEQLN
-SFLKG---FHDIIPA--ELIS-IFNEQ-ELELLISG-----------------------
----LPEID-----------VDDW----KNNTEYHN-YQAT-------------------
------------------------SAQI-QWFWRAVK--------------------SFD
PEERAKLLQF-------VTGTSKVP------------LNG-----FK-ELEGMN--GFAR
FNIHRDYSSK------------------------EKLPTSHTCF-NX-LDLPEY-ESYEH
LRQQLY-TAITAGSEYF
>Preussia_sp._LJJI01000331.1 .
LAVRRDHV-FLDSYKAL--------------YYKSGEEIKNGKLNIRFNG-------EEG
IDAGGVSREW---FAVM----ARQMFNPDY--------ALFNPVASDRT-----------
----------------TFHPNKLS------------------------------------
--GINDEHLSFFKFIGRVIGKALYEGRLLDAHFSR--AVYRRMLG---------------
--------KSVSLKDMET----------------------LDLDYYRS--------LVWI
LEN--DIT-DVT--
-FETFSAEVEA---------------------------FGV-TETVD-LVPG--------
GRDIA---
------------------------------VTEENKQ-EYVRL-VVEHRLIKS-VEAQLD
-AFLGG---FHDIVPA--ELVS-IFNEQ-ELELLISG-----------------------
----LPDID-----------VDDW----KNTTEYHN-YQST-------------------
------------------------SPQI-QWFWRAVK--------------------SFD
EEERAKLLQF-------VTGTSKVP------------LNG-----FK-ELEGMN--GFAK
FNIHRDYSSK------------------------DKLPTSHTCF-NQ-LDLPEY-ESYEV
LRQQLY-TAVTAGNEYF
>Cladosporium_sphaerospermum_MSJI02000704. 1..
LAVRRDHV-FLDSYKAL--------------YYKSGEEIKNGKLNIRFNG-------EEG
IDAGGVSREW---FAVM----ARQMFNPDY--------ALFNPVASDRT-----------
----------------TFHPNKLS------------------------------------
--GINEEHLSFFKFIGRVIGKALYEGRLLDAHFSR--AVYRRMLG---------------
--------KSVSLKDMET----------------------LDLDYYRS--------LVWI
LEN--DIT-DVT--
-FETFSAEIET---------------------------FGV-TETVD-LVPG--------
GRDIP---
------------------------------VTEENKQ-EYVRL-VVEHKLVKS-VEDQLN
-HFLGG---FHDIVPA--ELVS-IFNEQ-ELELLISG-----------------------
----LPDID-----------VDDW----KNTTEYHN-YTAT-------------------
------------------------SPQI-QWFWRAVK--------------------SFD
EEERAKLLQF-------VTGTSKVP------------LNG-----FK-ELEGMN--GFAK
FNIHRDYSSK------------------------DKLPTSHTCF-NQ-LDLPEY-DSYEQ
LRQQLY-TAVTAGNEYF
>Cladosporium_sphaerospermum_AIIA02000267. 1..
LQVSRDRV-FLDSYKSL--------------YYKSGEEIKNGKLNIRFIG-------EEG
IDAGGVSREW---FAVM----ARQMFNPDY--------ALFNPVASDRT-----------
----------------TFHPNKLS------------------------------------
--GINEEHLSFFKFIGRIIGKALYEGRLLDAHFSR--AVYRRMLG---------------
--------KSVSLKDMET----------------------LDLDYYRS--------LVWI
LEN--DIT-DVT--
-FETFSAEADT---------------------------FGV-TETID-LIPD--------
GRNIP---
------------------------------VTEENKQ-EYVRL-VVEHKLVKS-VEDQLN
-HFLSG---FHDIVPA--ELVS-IFNEQ-ELELLISG-----------------------
----LPDID-----------VDDW----KNTTEYHN-YTAT-------------------
------------------------SPQI-QWFWRAVK--------------------SFD
EEERAKLLQF-------VTGTSKVP------------LNG-----FK-ELEGMN--GFAK

```
FNIHRDYSSK------------------------DKLPTSHTCF-NQ-LDLPEY-ESYEQ
LRQQLY-TAVTAGNEYF
>Aureobasidium_subglaciale_XM_013486206.1 .
LNIRRDQI-FMDSFKNL--------------YYKSPNEIKYGKLNIRFHG-------EEG
VDAGGVTREW---FAAL----SRQMFNPDY--------ALFNPVASDRT-----------
----------------TFHPNPMS------------------------------------
--DINPEHLTFFKFVGRIIGKALYEGRVLDCHFSR--AVYRRILG---------------
--------KPVSLKDMET----------------------LDLDYYKS--------LCWI
LEN------------------------------------------------DIT-DVT--
-FETFSVEVDR---------------------------FGE-TETVD-LIEN--------
GRDIP-------------------------------------------------------
------------------------------VTEENKQ-EYVRL-VVDHRLIKS-VEEQLE
-HFLKG---FHEIIPA--ELVA-IFNEQ-ELELLISG-----------------------
----LPDID-----------VDDW----KGNTEYHN-YQQT-------------------
------------------------SPQI-QWFWRAVR--------------------SFD
KEEKAKLLQF-------VTGTSKVP------------LNG-----FK-ELEGMN--GFTK
FNIHRDYGSK------------------------DRLPSSHTCF-NQ-IDLPEY-ETYEQ
LRHQMY-TAMTQGSEYF
>Aureobasidium_pullulans_AMCU01000026.1 .
LNIRRDQI-FLDSFKNL--------------YFKTANEIKYGKLNIRFHG-------EEG
VDAGGVTREW---FAAL----SRQMFNPDY--------ALFNPVASDRT-----------
----------------TFHPNPMS------------------------------------
--GINDEHLTFFKFVGRIIGKAMYEGRVLDCHFSR--AVYRRILG---------------
--------KPVSLKDMET----------------------LDLDYYKS--------LCWI
LEN------------------------------------------------DIT-DIT--
-FETFSVEVDR---------------------------FGE-TEIVD-LIEN--------
GRDIP-------------------------------------------------------
------------------------------VTEENKQ-EYVRL-VVEHRLIKS-VEAQLE
-HFLKG---RSXIIPA--ELVS-IFNEQ-ELELLISG-----------------------
----LPDID-----------VDDW----KNNTEYHN-YQQT-------------------
------------------------SPQI-QWFWRAVR--------------------SFD
KEEKAKLLQF-------VTGTSKVP------------LNG-----FK-ELEGMN--GFTK
FNIHRDYGSK------------------------DRLPSSHTCF-N--------------
-----------------
>Aureobasidium_melanogenum_MWII01000016.1 .
LNIRRDQI-FLDSFKNL--------------YFKTPNEIKYGKLNIRFHG-------EEG
VDAGGVTREW---FAAL----SRQMFNPDY--------ALFNPVASDRT-----------
----------------TFHPNPMS------------------------------------
--NINDEHLTFFKFVGRIIGKAMYEGRVLDCHFSR--AVYRRILG---------------
--------KPVSLKDMET----------------------LDLDYYKS--------LCWI
LEN------------------------------------------------DIT-DVT--
-FETFSVEVDR---------------------------FGE-TEVVD-LIEN--------
GRDIP-------------------------------------------------------
------------------------------VTEENKQ-EYVRL-VVEHRLIKS-VEEQLE
-NFLKG---FHEIIPA--ELVS-IFNEQ-ELELLISG-----------------------
----LPDID-----------VDDW----KNNTEYHN-YQQT-------------------
------------------------SPQI-QWFWRAVR--------------------SFD
KEEKAKLLQF-------VTGTSKVP------------LNG-----FK-ELEGMN--GFTK
FNIHRDYGSK------------------------DRLPSSHTCF-NX-LDLPEY-DTYEQ
LRQQMY-TAMTQGSEYF
>Aureobasidium_namibiae_XM_013568371.1 .
LNIRREQI-FLDSFKNL--------------YFKTPNEIKYGKLNIRFHG-------EEG
VDAGGVTREW---FAAL----SRQMFNPDY--------ALFNPVASDRT-----------
----------------TFHPNPMS------------------------------------
--GINDEHLTFFKFVGRIIGKAMYEGRVLDCHFSR--AVYRRILG---------------
--------KPVSLKDMET----------------------LDLDYYKS--------LCWI
LEN------------------------------------------------DIT-DVT--
-FETFSVEVDR---------------------------FGE-TETVD-LIEN--------
```

```
GRDIP-------------------------------------------------------
------------------------------VTEENKQ-EYVRL-VVEHRLIKS-VEEQLE
-HFLKG---FHEIIPA--ELVS-IFNEQ-ELELLISG-----------------------
----LPDID-----------VDDW----KNNAEYHN-YQQT-------------------
------------------------SPQI-QWFWRAVR--------------------SFD
KEEKAKLLQF-------VTGTSKVP------------LNG-----FK-ELEGMN--GFTK
FNIHRDYGSK------------------------DRLPSSHTCF-NQ-LDLPEY-ETYEQ
LRHQMY-TAMTQGSEYF
>Leptoxyphium_fumago_LSHF01000115.1 .
LNIRRDSV-FLDSFKSL--------------YFKSGNEIKYGKLNIRFHG-------EEG
IDAGGVTREW---FGAM----ARQMFNPDY--------ALFNPVASDRT-----------
----------------TFHPNPLS------------------------------------
--DINEQHLMFFKFIGRIIGKALYEGRLLDCHFSR--AVYKRILS---------------
--------RPVSLKDMET----------------------LDLDFYKS--------LDWM
LNN------------------------------------------------DIT-GIT--
-FETFSTEVDR---------------------------FGE-TKVID-LKPD--------
GRNIE-------------------------------------------------------
------------------------------VTDENKH-EYVRL-ITQNRLIDS-VREQLD
-NFLTG---FYEIIPK--ELVA-IFNEQ-ELELLISG-----------------------
----LPDID-----------SDDW----KNNTEYHN-YQAT-------------------
------------------------SPQI-QWFWRAVR--------------------SFD
KEEKAKLLQF-------VTGTSKVP------------LNG-----FK-ELEGMN--GPTK
FNIHRDYSKG------------------------EKLPTSHTCF-NQ-LDLPEY-ETYEQ
LRQQLY-TAITAGSDYF
>Ascosphaera_apis_AARE01000189.1 .
LSVRRDRV-FHDSFRSL--------------YYKSPNEVKYGKLNIKFHG-------EEG
IDAGGVAREW---FHVL----ARSMFNPDF--------ALFIPVASDRT-----------
----------------TFHPNRLS------------------------------------
--GINEEHLAFFKFIGRIISKALYENRVLDCHFSR--AVYKRILG---------------
--------KDVSIKDMET----------------------LDLDYYKS--------LQWM
LEN------------------------------------------------DIT-DII--
-TETFSVEADS---------------------------FGE-TQIID-LIPN--------
GRNIP-------------------------------------------------------
------------------------------VTQENKE-EYVQK-VVNYRLVGS-VEEQLD
-NFLKG---FHEIVPR--ELIS-IFNEQ-ELELLISG-----------------------
----LPEID-----------IDDW----KANTDYHN-YTST-------------------
------------------------SPTI-QWFWRAVR--------------------SFD
KEERAKLLQF-------VTGTSKVP------------LNG-----FK-ELEGMN--GFTK
FNIHRDYGSK------------------------DRLPSSHTCF-NX-LDLPEY-DTYED
LRQRLY-TAMTTGSGYF
>Ciborinia_camelliae_LGKQ01000067.1 .
LSVRREQV-FHDSFKSL--------------YFQTPDQMKYGKLSIRFHG-------EEG
VDAGGVTREW---FQVL----SRQMFDPGY--------ALFIPVSSDRT-----------
----------------TFHPNQLS------------------------------------
--SINEEHLMFFKFIGRIIGKALYEGRVLDCHFSR--AVYKRILG---------------
--------KAVSVKDMES----------------------LDPDYYKS--------LIWM
LEN------------------------------------------------DIT-DII--
-TETFSVDNDK---------------------------FGV-VETID-FIED--------
GRNVA-------------------------------------------------------
------------------------------VTEENKH-EYVRL-MVEWKLTGS-VKAQLD
-EFLKG---FHDIIPA--ELVS-IFNEQ-ELELLISG-----------------------
----LPEID-----------VDDW----KSNTEYHN-YSAS-------------------
------------------------SPQI-QWFWRAIR--------------------SYD
KEERAKLLQF-------VTGTSKVP------------LNG-----FK-ELEGMN--GFSR
FNIHRDYGNK------------------------ERLPSSHTCF-NX-LDLPEY-ESYET
LRAQVL-TAITAGSEYF
>Monilinia_aucupariae_NGKF01000008.1 .
LSVRREQV-FHDSFKSL--------------YFQTPDQMKYGKLSIRFHG-------EEG
```

VDAGGVTREW---FQVL----SRQMFDPGY--------ALFIPVSSDRT-----------
----------------TFHPNQLS------------------------------------
--SINEEHLMFFKFIGRIIGKALYEGRVLDCHFSR--AVYKRILG---------------
--------KAVSVKDMES----------------------LDPDYYKS--------LIWM
LEN--DIT-DII--
-TETFSVDNDK---------------------------FGV-VETID-FIED--------
GRNVA---
------------------------------VTEENKH-EYVRL-MVEWKLTGS-VKAQLD
-EFLKG---FHDIIPA--ELVS-IFNEQ-ELELLISG-----------------------
----LPEID-----------VDDW----KSNTEYHN-YSAS-------------------
------------------------SPQI-QWFWRAIR--------------------SYD
KEERAKLLQF-------VTGTSKVP------------LNG-----FK-ELEGMN--GFSR
FNIHRDYGNK------------------------ERLPSSHTCF-NX-LDLPEY-ESYET
LRAQVL-TAITAGSEYF
>Myriosclerotinia_curreyana_NGKJ01000108.1 .
LSVRREQV-FHDSFKSL--------------YFQTPDQMKYGKLSIRFHG-------EEG
VDAGGVTREW---FQVL----SRQMFDPGY--------ALFIPVSSDRT-----------
----------------TFHPNQLS------------------------------------
--SINEEHLMFFKFIGRIIGKALYEGRVLDCHFSR--AVYKRILG---------------
--------KAVSVKDMES----------------------LDPDYYKS--------LIWM
LEN--DIT-DII--
-TETFSVDNDK---------------------------FGV-VETID-FIEN--------
GRNVA---
------------------------------VTEENKH-EYVRL-MVEWKLTGS-VKAQLD
-EFLKG---FHDIIPA--ELVS-IFNEQ-ELELLISG-----------------------
----LPEID-----------VDDW----KSNTEYHN-YSAS-------------------
------------------------SPQI-QWFWRAIR--------------------SYD
KEERAKLLQF-------VTGTSKVP------------LNG-----FK-ELEGMN--GFSR
FNIHRDYGNK------------------------ERLPSSHTCF-NX-LDLPEY-ESYET
LRAQVL-TAITAGSEYF
>Monilinia_fructicola_NGKE01000026.1 .
LSVRREQV-FHDSFKSL--------------YFQTPDQMKYGKLSIRFHG-------EEG
VDAGGVTREW---FQVL----SRQMFDPGY--------ALFIPVSSDRT-----------
----------------TFHPNQLS------------------------------------
--SINEEHLMFFKFIGRIIGKALYEGRVLDCHFSR--AVYKRILG---------------
--------KAVSVKDMES----------------------LDPDYYKS--------LIWM
LEN--DIT-DII--
-TETFSVDNDK---------------------------FGV-VETID-FIED--------
GRNVA---
------------------------------VTEENKH-EYVRL-MVEWKLTGS-VKAQLD
-EFLKG---FHDIIPA--ELVS-IFNEQ-ELELLISG-----------------------
----LPEID-----------VDDW----KSNTEYHN-YSAS-------------------
------------------------SPQI-QWFWRAIR--------------------SFD
KEERAKLLQF-------VTGTSKVP------------LNG-----FK-ELEGMN--GFSR
FNIHRDYGNK------------------------ERLPSSHTCF-NX-LDLPEY-ESYET
LRAQVL-TAITAGSEYF
>Botrytis_cinerea_CP009808.1 .
LSVRREQV-FHDSFKSL--------------YFQTPDQMKYGKLSIRFHG-------EEG
VDAGGVTREW---FQVL----SRQMFDPGY--------ALFIPVSSDRT-----------
----------------TFHPNQLS------------------------------------
--SINEEHLMFFKFIGRIIGKALYEGRVLDCHFSR--AVYKRILG---------------
--------KAVSVKDMES----------------------LDPDYYKS--------LIWM
LEN--DIT-DII--
-TETFSVDNDK---------------------------FGV-VETID-FIED--------
GRNVP---
------------------------------VTEENKH-EYVRL-MVEWKLTGS-VKAQLD
-EFLKG---FHDIIPA--ELVS-IFNEQ-ELELLISG-----------------------
----LPEID-----------VDDW----KSNTEYHN-YSAS-------------------

------------------------SPQI-QWFWRAIR--------------------SFD
KEERAKLLQF-------VTGTSKVP------------LNG-----FK-ELEGMN--GFSR
FNIHRDYGNK------------------------ERLPSSHTCF-NX-LDLPEY-ESYET
LRAQVL-TAITAGSEYF
>Botryotinia_fuckeliana_XM_001557601.1 .
LSVRREQV-FHDSFKSL--------------YFQTPDQMKYGKLSIRFHG-------EEG
VDAGGVTREW---FQVL----SRQMFDPGY--------ALFIPVSSDRT-----------
----------------TFHPNQLS------------------------------------
--SINEEHLMFFKFIGRIIGKALYEGRVLDCHFSR--AVYKRILG---------------
--------KAVSVKDMES----------------------LDPDYYKS--------LIWM
LEN--DIT-DII--
-TETFSVDNDK---------------------------FGV-VETID-FIED--------
GRNVP---
------------------------------VTEENKH-EYVRL-MVEWKLTGS-VKAQLD
-EFLKG---FHDIIPA--ELVS-IFNEQ-ELELLISG-----------------------
----LPEID-----------VDDW----KSNTEYHN-YSAS-------------------
------------------------SPQI-QWFWRAIR--------------------SFD
KEERAKLLQF-------VTGTSKVP------------LNG-----FK-ELEGMN--GFSR
FNIHRDYGNK------------------------ERLPSSHTCF-NQ-LDLPEY-ESYET
LRAQVL-TAITAGSEYF
>Botrytis_paeoniae_LBGX01000555.1 .
LSVRREQV-FHDSFKSL--------------YFQTPDQMKYGKLSIRFHG-------EEG
VDAGGVTREW---FQVL----SRQMFDPGY--------ALFIPVSSDRT-----------
----------------TFHPNQLS------------------------------------
--SINEEHLMFFKFIGRIIGKALYEGRVLDCHFSR--AVYKRILG---------------
--------KAVSVKDMES----------------------LDPDYYKS--------LIWM
LEN--DIT-DII--
-TETFSVDNDK---------------------------FGV-VETID-FIED--------
GRNVP---
------------------------------VTEENKH-EYVRL-MVEWKLTGS-VKAQLD
-EFLKG---FHDIIPA--ELVS-IFNEQ-ELELLISG-----------------------
----LPEID-----------VDDW----KSNTEYHN-YSAS-------------------
------------------------SPQI-QWFWRAIR--------------------SFD
KEERAKLLQF-------VTGTSKVP------------LNG-----FK-ELEGMN--GFSR
FNIHRDYGNK------------------------ERLPSSHTCF-NX-LDLPEY-ESYET
LRAQVL-TAITAGSEYF
>Sclerotium_cepivorum_NGKD01000027.1 .
LSVRREQV-FHDSFKSL--------------YFQTPDQMKYGKLSIRFHG-------EEG
VDAGGVTREW---FQVL----SRQMFDPGY--------ALFIPVSSDRT-----------
----------------TFHPNQLS------------------------------------
--SINEEHLMFFKFIGRIIGKALYEGRVLDCHFSR--AVYKRILG---------------
--------KAVSVKDMES----------------------LDPDYYKS--------LIWM
LEN--DIT-DII--
-TETFSVDNDK---------------------------FGV-VETID-FIEN--------
GRNVA---
------------------------------VTEENKH-EYVRL-MVEWKLTGS-VKAQLD
-EFLKG---FHDIIPA--ELVS-IFNEQ-ELELLISG-----------------------
----LPEID-----------VDDW----KSNTEYHN-YSAS-------------------
------------------------SPQI-QWFWRAVR--------------------SYD
KEERAKLLQF-------VTGTSKVP------------LNG-----FK-ELEGMN--GFSR
FNIHRDYGNK------------------------ERLPSSHTCF-NX-LDLPEY-ESYET
LRAQVL-TAITAGSEYF
>Sclerotinia_sclerotiorum_XM_001588216.1 .
LSVRREQV-FHDSFKSL--------------YFQTPDQMKYGKLSIRFHG-------EEG
VDAGGVTREW---FQVL----SRQMFDPGY--------ALFIPVSSDRT-----------
----------------TFHPNQLS------------------------------------
--SINEEHLMFFKFIGRIIGKALYEGRVLDCHFSR--AVYKRILG---------------
--------KAVSVKDMES----------------------LDPDYYKS--------LIWM

LEN--DIT-DII--
-TETFSVDNDK---------------------------FGV-VETID-FIEN--------
GRNVA---
------------------------------VTEENKH-EYVRL-MVEWKLTGS-VKAQLD
-EFLKG---FHDIIPA--ELVS-IFNEQ-ELELLISG-----------------------
----LPEID-----------VDDW----KSNTEYHN-YSAS-------------------
------------------------SPQI-QWFWRAVR--------------------SYD
KEERAKLLQF-------VTGTSKVP------------LNG-----FK-ELEGMN--GFSR
FNIHRDYGNK------------------------ERLPSSHTCF-NQ-LDLPEY-ESYET
LRAQVL-TAITAGSEYF
>Sclerotinia_glacialis_NGKH01000022.1 .
LSVRREQV-FHDSFKSL--------------YFQTPDQMKYGKLSIRFHG-------EEG
VDAGGVTREW---FQVL----SRQMFDPGY--------ALFIPVSSDRT-----------
----------------TFHPNQLS------------------------------------
--SINEEHLMFFKFIGRIIGKALYEGRVLDCHFSR--AVYKRILG---------------
--------KAVSVKDMES----------------------LDPDYYKS--------LIWM
LEN--DIT-DII--
-TETFSVDNDK---------------------------FGV-VETID-FIEN--------
GRNVA---
------------------------------VTEENKH-EYVRL-MVEWKLTGS-VKAQLD
-EFLKG---FHDIIPA--ELVS-IFNEQ-ELELLISG-----------------------
----LPEID-----------VDDW----KSNTEYHN-YSAS-------------------
------------------------SPQI-QWFWRAVR--------------------SYD
KEERAKLLQF-------VTGTSKVP------------LNG-----FK-ELEGMN--GFSR
FNIHRDYGNK------------------------ERLPSSHTCF-NX-LDLPEY-ESYET
LRAQVL-TAITAGSEYF
>Sclerotinia_borealis_AYSA01000167.1 .
LSVRREQV-FHDSFKSL--------------YFQTPDQMKYGKLSIRFHG-------EEG
VDAGGVTREW---FQVL----SRQMFDPGY--------ALFIPVSSDRT-----------
----------------TFHPNQLS------------------------------------
--SINEEHLMFFKFIGRIIGKALYEGRVLDCHFSR--AVYKRILG---------------
--------KAVSVKDMES----------------------LDPDYYKS--------LIWM
LEN--DIT-DII--
-TETFSVDNDK---------------------------FGV-VETID-FKEN--------
GRNIA---
------------------------------VTEENKH-EYVRL-MVEWRLTGS-VKAQLD
-EFLKG---FHDIIPA--ELVA-IFNEQ-ELELLISG-----------------------
----LPEID-----------VDDW----KSNTEYHN-YSAS-------------------
------------------------SPQI-QWFWRAIR--------------------SYD
KEERAKLLQF-------VTGTSKVP------------LNG-----FK-ELEGMN--GFSR
FNIHRDYGNK------------------------ERLPSSHTCF-NX-LDLPEY-ESYET
LRTQVL-TAITAGSEYF
>Rutstroemia_echinophila_JWJA01002412.1 .
LSVRREQV-FHDSFKSL--------------YFQTPDQMKYGKLSIRFHG-------EEG
VDAGGVTREW---FQVL----SRQMFDPGY--------ALFIPVSSDRT-----------
----------------TFHPNQLS------------------------------------
--SINEEHLMFFKFIGRIIGKALYEGRVLDCHFSR--AVYKRILG---------------
--------KAVSVKDMES----------------------LDPDYYKS--------LIWM
LEN--DIT-DII--
-TETFSVDNDK---------------------------FGV-LETID-FIEG--------
GRNIP---
------------------------------VTEENKH-EYVRL-MVEWKLTGS-VKDQLD
-EFLKG---FHDIIPA--ELVA-IFNEQ-ELELLISG-----------------------
----LPEID-----------VDDW----KSNTEYHN-YSAS-------------------
------------------------SSQI-QWFWRAVR--------------------SFD
KEERAKLLQF-------VTGTSKVP------------LNG-----FK-ELEGMN--GFSR
FNIHRDYGNK------------------------ERLPSSHTCF-NX-LDLPEY-ESYDT
LRTQVL-TAITAGSEYF

>Sclerotinia_homoeocarpa_JU092177.1 .
LSVRREQV-FHDSFKSL--------------YFQTPDQMKYGKLSIRFHG-------EEG
VDAGGVTREW---FQVL----SRQMFDPGY--------ALFIPVSSDRT-----------
----------------TFHPNQLS------------------------------------
--GVNEEHLMFFKFIGRIIGKALYEGRVLDCHFSR--AVYKRILG---------------
--------KSVSVKDMES----------------------LDPDYYKS--------LVWM
LEN--DIT-DII--
-TETFSVDNDK---------------------------FGV-VETID-FIEN--------
GRNIP---
------------------------------VTQENKH-EYVRL-MVEWRLTGS-VKEQLD
-EFLKG---FHDIIPA--ELVA-IFNEQ-ELELLISG-----------------------
----LPEID-----------VDDW----KSNTEYHN-YSAS-------------------
------------------------SPQI-QWFWRAVR--------------------SFD
KEERAKLLQF-------VTGTSKVP------------LNG-----FK-ELEGMN--GFSR
FNIHRDYGNK------------------------ERLPSSHTCF-NQ-LDLPEY-ENYET
LRTQVL-TAITAGSEYF
>Glarea_lozoyensis_XM_008083787.1 .
LSVRRDQV-FHDSFKSL--------------YFQTGDQMKYGKLSIRFHG-------EEG
VDAGGVTREW---FQVL----SRQMFDPGY--------ALFIPVSSDRT-----------
----------------TFHPNQLS------------------------------------
--SINEEHLMFFKFIGRIIGKALYEGRVLDCHFSR--AVYKRILG---------------
--------KPVSVKDMES----------------------LDPDYYKS--------LVWM
LEN--DIT-DII--
-TETFSVDNDK---------------------------FGV-VETID-FIEN--------
GRNIP---
------------------------------VTEENKH-EYVRL-MVEWRLTGS-VKEQLD
-EFLKG---FHDIIPA--ELIA-IFNEQ-ELELLISG-----------------------
----LPEID-----------VDDW----KSNTEYHN-YSAS-------------------
------------------------SPQI-QWFWRAIR--------------------SYD
KEERAKLLQF-------VTGTSKVP------------LNG-----FK-ELEGMN--GFSR
FNIHRDYGNK------------------------DRLPSSHTCF-NQ-LDLPEY-ESYET
LRQQVL-TAITAGSEYF
>Hymenoscyphus_fraxineus_LLCC01000496.1 .
LSVRRDQV-FHDSYRSL--------------FFQTGDQMKYGKLSIRFHG-------EEG
VDAGGVTREW---FQVL----SRQMFDPGY--------ALFIPVSSDRT-----------
----------------TFHPNQLS------------------------------------
--SVNEEHLNFFKFIGRIIGKALYEGRVLDCHFSR--AVYKRILG---------------
--------RPVSVKDMES----------------------LDPEYYKS--------LVWM
LEN--DIT-DVI--
-TETFSVDNDK---------------------------FGV-VETID-FIEN--------
GRNIP---
------------------------------VTEENKH-EYIRL-MVEWKLTGS-VKDQLD
-NFLKG---FHDIIPA--ELIA-IFNEQ-ELELLISG-----------------------
----LPEID-----------VDDW----KSNTEYHN-YSAS-------------------
------------------------SPQI-QWFWRAIR--------------------SYD
KEERAKLLQF-------VTGTSKVP------------LNG-----FR-ELEGMN--GFSR
FNIHRDYGNK------------------------DRLPSSHTCF-N--------------

>Hymenoscyphus_salicellus_LLCD01000003.1 .
LSVRRDQV-FHDSFKSL--------------YFQTGDQMKYGKLSIRFHG-------EEG
VDAGGVTREW---FQVL----SRQMFDPGY--------ALFIPVSSDRT-----------
----------------TFHPNQLS------------------------------------
--SINEEHLMFFKFIGRIIGKALYEGRVLDCHFSR--AVYKRILG---------------
--------RPVSVKDMES----------------------LDPEYYKS--------LVWM
LEN--DIT-DVI--
-TETFSVDNDK---------------------------FGV-VETID-FIEN--------
GRNVP---
------------------------------VTEENKH-EYIRL-MVEWKLTGS-VKGQLD

-DFLKG---FHDIIPA--ELIA-IFNEQ-ELELLISG----------------------
----LPEID-----------VDDW----KSNTEYHN-YSAS------------------
------------------------SPQI-QWFWRAIR--------------------SYD
KEERAKLLQF-------VTGTSKVP------------LNG-----FK-ELEGMN--GFSR
FNIHRDYGNK------------------------DRLPSSHTCF-NX-LDLPEY-ESYEQ
LRQQVL-TAITAGSEYF
>Ascocoryne_sarcoides_AIAA01000120.1 .
LSVRRDQV-FHDSFKSL--------------YFQTGDQMKYGKLSIRFHG-------EEG
VDAGGVTREW---FQVL----SRQMFDPGY--------ALFIPVSSDRT-----------
----------------TFHPNQLS------------------------------------
--SVNEEHLMFFKFIGRIIGKALYEGRVLDCHFSR--AVYKRILG---------------
--------KAVSVKDMES----------------------LDPDYYKS--------LVWM
LEN---DIT-DII--
-TETFSVDNDK---------------------------FGV-VETID-FIPN--------
GRNIA---
------------------------------VTEENKQ-EYVRL-MVEWKLTGS-VKEQLD
-EFLKG---FHDIISA--DLIS-IFNEQ-ELELLISG----------------------
----LPEID-----------VDDW----KSNTEYHQ-YTAS------------------
------------------------SPQI-QWFWRAVR--------------------SFD
KEERAKLLQF-------VTGTSKVP------------LNG-----FK-ELEGMN--GFSR
FNIHRDYGDK------------------------DRLPSSHTCF-NX-LDLPEY-ESYES
LRQQVH-TAITVGSEYF
>Phialocephala_scopiformis_XM_018221312.1 .
LSVRRDQV-FHDSFKSL--------------YFQTGDQMKYGKLSIRFHG-------EEG
VDAGGVTREW---FQVL----SRQMFDPGY--------ALFIPVSSDRT-----------
----------------TFHPNQLS------------------------------------
--SVNEEHLMFFKFIGRIIGKALYEGRVLDCHFSR--AVYKRILG---------------
--------KAVSVKDMES----------------------LDPDYYKS--------LIWM
LEN---DIT-DII--
-TETFSVDNDK---------------------------FGV-VETID-FIPD--------
GRNIP---
------------------------------VTEENKQ-EYVRL-MVEWKLTGS-VKEQLD
-EFLKG---FHDIIPA--PLVA-IFNEQ-ELELLISG----------------------
----LPEID-----------VDDW----KSNTEYHN-YSAS------------------
------------------------SPQI-QWFWRAVR--------------------SYD
KEERAKLLQF-------VTGTSKVP------------LNG-----FK-ELEGMN--GFSR
FNIHRDYGNK------------------------DRLPSSHTCF-NQ-LDLPEY-ESYEA
LRQQVL-TAITAGSEYF
>Phialocephala_subalpina_FJOG01000003.1 .
LSVRRDQV-FHDSFKSL--------------YFQTGDQMKYGKLSIRFHG-------EEG
VDAGGVTREW---FQVL----SRQMFDPGY--------ALFIPVSSDRT-----------
----------------TFHPNQLS------------------------------------
--SINEEHLMFFKFIGRIIGKALYEGRVLDCHFSR--AVYKRILG---------------
--------KAVSVKDMES----------------------LDPDYYKS--------LVWM
LEN---DIT-DII--
-TETFSVDNDK---------------------------FGV-VETID-FIPN--------
GRNIP---
------------------------------VTEENKQ-EYVRL-MVEWKLTGS-VKEQLD
-EFLKG---FHDIIPA--DLVA-IFNEQ-ELELLISG----------------------
----LPEID-----------VDDW----KGNTEYHN-YTAS------------------
------------------------SPQI-QWFWRAVR--------------------SFD
KEERAKLLQF-------VTGTSKVP------------LNG-----FQ-ELEGMN--GFSR
FNIHRDYGNK------------------------DRLPSSHTCF-NX-LDLPEY-ESYEV
LRQQVL-TAITAGSEYF
>Amorphotheca_resinae_JZSE01000224.1 .
LSVRRDQV-FHDSFKAL--------------YFQTADQMKFGKLSIRFHG-------EEG
VDAGGVTREW---FQVL----SRQMFDPGY--------ALFIPVSSDRT-----------
----------------TFHPNQLS------------------------------------

--GVNEEHLMFFKFIGRIIGKALYEGRVLDCHFSR--AVYKRILG---------------
--------KAVSVKDMES----------------------LDPDYYKS--------LVWM
LEN--DIT-DII--
-TETFSVDNDK---------------------------FGV-VETID-FIPN--------
GRNIA---
------------------------------VTEENKH-EYVRL-MVEWRLTGS-VKEQLD
-EFLKG-----XIIPA--ELIA-IFNEQ-ELELLISG-----------------------
----LPEID-----------VDDW----KSNTEYHN-YSAS-------------------
------------------------SPQI-QWFWRAVR--------------------SFD
KEERAKLLQF-------VTGTSKVP------------LNG-----FK-ELEGMN--GFSR
FNIHRDYGRK------------------------ERLPSSHTCF-NX-LDLPEY-ESYEV
LRQQVL-TAITAGSQYF
>Erysiphe_pisiCACM01012766.1 .
LSVRRDLV-FHDSFKSL--------------YFQSGDQMKYGKLSIRFHG-------EEG
VDAGGVTREW---FQVL----SRQMFDPGY--------ALFIPVSSDRT-----------
----------------TFHPNQLS------------------------------------
--SVNEEHLMFFKFIGRIIGKALYEGRVLDCHFSR--AVYKRILG---------------
--------KAVSLKDMES----------------------LDPDYYKS--------LIWM
LEN--DIT-DIM--
-TETFSVDSDK---------------------------FGV-NETID-FIPN--------
GRNIN---
------------------------------VTEENKH-EYVRL-MVEWRLTGS-VKEQLD
-EFLKG---FHDIVPA--DLVS-IFNEQ-ELELLISG-----------------------
----LPEID-----------VDDW----KGNTEYHN-YTAS-------------------
------------------------SPQI-QWFWRAVR--------------------SFD
KEERAKLLQF-------VTGTSKVP------------LNG-----FK-ELEGMN--GFSR
FNIHRDYGNK------------------------NRLPSSHTCF-NX-LDLPEY-ENYEI
LRQQLF-TAITAGSEYF
>Blumeria_graminis_ANZE01016071.1 .
LSVRRDLV-FHDSFKSL--------------YFQSGDQMKYGKLSIRFHG-------EEG
VDAGGVTREW---FQVL----SRQMFDPGY--------ALFIPVSSDRT-----------
----------------TFHPNQLS------------------------------------
--SVNEEHLMFFKFIGRIIGKALYEGRVLDCHFSR--AVYKRILG---------------
--------KSVSVKDMES----------------------LDPDYYKS--------LVWM
LEN--DIT-DII--
-TETFSVDNDK---------------------------FGV-NETID-FIPN--------
GRNIP---
------------------------------VTEENKH-EYVRL-MVEWKLTGS-VKEQLD
-EFLKG---FHDIIPA--DLVA-IFNEQ-ELELLISG-----------------------
----LPEID-----------VDDW----KGNTEYHN-YSTS-------------------
------------------------SAQI-QWFWRAVR--------------------SFD
KEERAKLLQF-------VTGTSKVP------------LNG-----FK-ELEGMN--GFSR
FNIHRDYGNK------------------------DRLPSSHTCF-NX-LDIPEY-ESYEI
LRQQLL-TAITAGSEYF
>Podosphaera_xanthii_GEUO01003016.1 .
LSVRRDLV-FHDSFKSL--------------YFQSGDQMKYGKLSIRFHG-------EEG
VDAGGVTREW---FQVL----SRQMFDPGY--------ALFIPVSSDRT-----------
----------------TFHPNQLS------------------------------------
--SVNEEHLMFFKFIGRIIGKALYEGRVLDCHFSR--AVYKRILG---------------
--------KSVSVKDMES----------------------LDPDYYKS--------LIWM
LEN--DIT-DII--
-TETFSIDSDK---------------------------FGV-NETID-FIPN--------
GRNIA---
------------------------------VTEENKH-EYVRL-MVEWKLTGS-VKEQLD
-EFLKG---FHDIIPA--DLVS-IFNEQ-ELELLISG-----------------------
----LPEID-----------VDDW----KGNTEYHN-YSTS-------------------
------------------------SIQI-QWFWRAVR--------------------SFD
KEERAKLLQF-------VTGTSKVP------------LNG-----FK-ELEGMN--GFSR

FNIHRDYGNK------------------------NRLPSSHTCF-NQ-LDIPEY-ESYEI
LRQQLL-TAITAGSEYF
>Marssonina_brunnea_XM_007290475.1 .
LSVRRDQV-FHDSFKSL--------------YFQTGDQMKFGKLSIRFHG-------EEG
VDAGGVTREW---FQVL----SRQMFDPGY--------ALFVPVSSDRT-----------
----------------TFHPNHSS------------------------------------
--SINEEHLMFFKFIGRIIGKALYEGRVLDCHFSR--AVYKRILG---------------
--------KAVSVKDMES----------------------LDLDYYKS--------LVWM
LEN---DIT-DII--
-TETFSTEQDK---------------------------FGV-TETID-FIPN--------
GRNIP---
------------------------------VTEENKH-EYVRL-MTEWRLTGS-VKEQLD
-EFLKG---FHDIIPA--ELVA-IFNEQ-ELELLISG-----------------------
----LPEID-----------VDDW----KSNTEYHN-YTAS-------------------
------------------------SPQI-QWFWRAIR--------------------SFD
KEERAKLLQF-------VTGTSKVP------------LNG-----FK-ELEGMN--GFSR
FNIHRDYGSK------------------------DRLPSSHTCF-NQ-LDLPEY-ESYES
LRNQVL-TAITAGSEYF
>Pseudogymnoascus_destructans_XM_012888536.1 .
LSVRRDQV-FHDSFKSL--------------YFKSGDEMKFGKLSIRFHG-------EEG
VDAGGVTREW---FQVL----SRQMFDPGY--------ALFIPVSSDRT-----------
----------------TFHPNLTS------------------------------------
--SINPEHLMFFKFIGRVIGKALYEGRVLDCHFSR--AVYKRILG---------------
--------KAVSVKDMES----------------------LDPEYYKS--------VVWM
LEN---DIT-DII--
-SESFSVDNDK---------------------------FGV-VETVD-LIEN--------
GRNVP---
------------------------------VTEENKH-EYVRL-MVEFRLTGS-VQEQLD
-NFLKG---FHEIIPA--ELVA-IFNEQ-ELELLISG-----------------------
----LPEID-----------VDDW----KATTEYHN-YTAS-------------------
------------------------SPQI-QWFWRAIR--------------------SFD
KEERAKMLQF-------VTGTSKVP------------LNG-----FK-ELEGMN--GFSR
FNIHRDYGNK------------------------DRLPSSHTCF-NQ-LDLPEY-ESYEM
LRQQVL-TAITTGSEYF
>Pseudogymnoascus_verrucosus_XM_018274970.1 .
LSVRRDQV-FHDSFKSL--------------YFKSGDEMKFGKLSIRFHG-------EEG
VDAGGVTREW---FQVL----SRQMFDPGY--------ALFIPVSSDRT-----------
----------------TFHPNLTS------------------------------------
--SINPEHLMFFKFIGRVIGKALYEGRVLDCHFSR--AVYKRILG---------------
--------KAVSVKDMES----------------------LDPDYYKS--------VVWM
LEN---DIT-DII--
-SESFSVDNDK---------------------------FGV-VETVD-LIEN--------
GRNVP---
------------------------------VTEENKH-EYVRL-MVEFKLTGS-VQEQLD
-NFLKG---FHEIIPA--ELVA-IFNEQ-ELELLISG-----------------------
----LPEID-----------VDDW----KATTEYHN-YTAS-------------------
------------------------SPQI-QWFWRAIR--------------------SFD
KEERAKMLQF-------VTGTSKVP------------LNG-----FK-ELEGMN--GFSR
FNIHRDYGNK------------------------DRLPTSHTCF-NQ-LDLPEY-ESYEM
LRQQVL-TAITTGSEYF
>Dactylellina_haptotyla_XM_011109578.1 .
LHVRRDQV-FMDSYRSL--------------HYKRGDEIKYSKLNIRFQG-------EEG
VDAGGVTREW---FQVL----ARQMFNPNW--------ALFSPVASDRT-----------
----------------TFHPNRMS------------------------------------
--GINAEHLSFFKFIGRIIGKALYEGRVLDCHFSR--AMYKRILG---------------
--------QNVSLKDMEN----------------------LDLDYYKS--------LLWM
LNN---EIQ-DIL--
-LLTFSIETDE---------------------------FGV-EKVLD-LVPN--------

GSNIP---
------------------------------VTDENKE-EYVKK-VTEFRLIQS-VKDQMD
-WFLQG---FHDVVPA--ELIA-IFNEQ-ELELLISG-----------------------
----LPDID-----------VDDW----RNNTEYHN-YSAS-------------------
------------------------SPQV-QWFWRAVR---------------------SFD
KEERAKLLQF-------VTGTSKVP------------LNG-----FK-ELEGMN--GYSK
FNIHRDYGNK------------------------DRLPSSHTCF-NQ-IDLPEY-ESYEL
LRKNLL-TAITAGAEYF
>Arthrobotrys_oligospora_XM_011124945.1 .
LNVRRDQV-FMDSYRSM--------------HYKRGDEIKYSKLNIRFQG-------EEG
VDAGGVTREW---FQVL----ARQMFNPGY--------ALFLPVASDRT-----------
----------------TFHPNRMS------------------------------------
--SVNSEHLSFFKFIGRIIGKALYEGRVLDCHFSR--AMYKRILG---------------
--------QNVSLKDMEN----------------------LDLEYYKS--------LQWM
LKN--TIE-DVL--
-LLSFSVDTDD---------------------------FGV-ERVLD-LVPN--------
GSNIP---
------------------------------VTDENKE-DYVKK-VTQFRLVGS-VKEQMD
-HFLQG---FHDIVPA--ELIA-IFNEQ-ELELLISG-----------------------
----LPDID-----------VDDW----RNNTEYQN-YTAS-------------------
------------------------SPQV-QWFWRAVR---------------------SFD
KEERAKLLQF-------VTGTSKVP------------LNG-----FK-ELEGMN--GYSK
FNIHRDYGNK------------------------DRLPSSHTCF-NQ-IDLPEY-ETYEH
LRKNLL-TAITAGAEYF
>Drechslerella_stenobrocha_ASQI01000062.1 .
LHVRRDQV-FMDSYRSL--------------HYKRGDEIKYSKLNIRFQG-------EEG
VDAGGVTREW---FQVL----ARQMFNPDY--------ALFLPVASDRT-----------
----------------TFHPNRMS------------------------------------
--GINSEHLSFFKFIGRIIAKALYEGRVLDCHFSR--AVYKRILG---------------
--------QNVSLKDMEN----------------------LDLEYYKS--------LQWM
LEN--DIT-DIL--
-LLNFNVETDE---------------------------FGV-EKIVD-LIPD--------
GRKVT---
------------------------------VTQENKH-EYVKL-VTEFRLLDS-VKEQMD
-SFLQG-----XIVPA--ELIA-IFNEQ-ELELLISG-----------------------
----LPDID-----------VDDW----RNNTEYHN-YSAS-------------------
------------------------SPQV-QWFWRAVR---------------------SFD
KEERAKLLQF-------VTGTSKVP------------LNG-----FK-ELEGMN--GYSK
FNIHRDYGNK------------------------DRLPSSHTCF-N--------------

>Tuber_melanosporum_XM_002835388.1 .
LNVRRDQV-FLDSYKSM--------------YYKNGDEIKYAKLSIRFHG-------EEG
VDAGGVTREW---FQVM----ARQMFNPDY--------ALFIPVASDRT-----------
----------------TFHPSRMS------------------------------------
--GVNPEHLSFFKFIGRIIGKALYEGRVLDCHFSR--AVYKRILG---------------
--------KSVSLKDMET----------------------LDLDYYKS--------LVWM
LEN--DIT-DII--
-TETFSVETDD---------------------------FGD-KKIID-LVPD--------
GRNVP---
------------------------------VTDDNKH-EYVRL-LVEYRLLTS-VQEQME
-NFLVG---FHDIVPA--ELIS-IFNEQ-ELELLISG-----------------------
----LPEID-----------VDDW----RNNTEYHN-YSAS-------------------
------------------------SPQI-QWFWRAVR---------------------SFD
KEERAKLLQF-------VTGTSKVP------------LNG-----FK-ELEGMN--GFSK
FNIHRDYGSK------------------------DRLPSSHTCF-NQ-IDLPEY-ESYES
LRQNIL-TAITQGAEYF
>Venturia_carpophila_MECS01000068.1 .
LSVRRDQV-FLDSFKSL--------------YYKKADEFKYGKLSIRFSG-------EEG

```
VDAGGVSREW---FQVL----TKQMFDPNY--------ALFNPVASDRT-----------
----------------TFHPNPQS------------------------------------
--DINEQHLMFFKFIGRIIGKSLYEGRVLDCHFSR--AVYKRILG---------------
--------KAISIKDMES----------------------LDLEYYKS--------LQWI
MEN------------------------------------------------DIS-DVI--
-EETFSVETER---------------------------FGE-HKVED-LIEN--------
GRDIP-------------------------------------------------------
------------------------------VTEANKM-DYVRAN-VEHKLIGS-VKEQLD
-SFLQG---FHDIVQA--DLIS-IFNEQ-ELELLISG-----------------------
----LPDID-----------IDDW----KNNTEYHN-YQAS-------------------
------------------------SPQI-QWFWRAVR--------------------SFE
KEERAKLLQF-------VTGTSKVP------------LNG-----FK-ELEGMN--GFSR
FNIHRDYGSK------------------------ERLPSSHTCF-NQ-LDLPEY-ESYED
LRKALY-TAMTAGSEYF
>Venturia_pyrina_JEMP01000127.1 .
LSVRRDQV-FLDSFKSL--------------YYKKADEFKYGKLSIRFSG-------EEG
VDAGGVSREW---FQVL----TKQMFDPNY--------ALFNPVASDRT-----------
----------------TFHPNSQS------------------------------------
--GINEQHLMFFKFIGRIIGKSLYEGRVLDCHFSR--AVYKRILG---------------
--------KAISIKDMES----------------------LDLEYYKS--------LQWI
LEN------------------------------------------------DIS-GIL--
-EETFSVETEA---------------------------FGE-HKVED-LIEN--------
GRDIP-------------------------------------------------------
------------------------------VTEANKM-DYVRAN-VEHKLIGS-VKDQLD
-SFLQG------X-----------------------------------------------
---------------------DDW----KNNSEYHN-YQAS-------------------
------------------------SPQI-QWFWRAVR--------------------SFE
KEERAKLLQF-------VTGTSKVP------------LNG-----FK-ELEGMN--GFSR
FNIHRDYGSK------------------------DRLPSSHTCF-NQ-LDLPEY-ESYED
LRKALY-TAMTAGS---
>Ochroconis_constricta_AZYM01000269.1 .
LSVRRDQV-FLDSFKHL--------------YYKKPEEFKYGKLSIRFAG-------EEG
VDAGGVTREW---FQAL----TKQMFDPNY--------ALFVPVAADRT-----------
----------------TFHPNSES------------------------------------
--AINEQHLTFFKFIGRIIGKALYEGRVLDCHFSR--AVYKRILG---------------
--------RSISIKDMES----------------------LDNDYAKN--------LQWI
LDN------------------------------------------------DIT-DLI--
-TETFSVSTDV---------------------------FGE-EKTVD-LIPG--------
GRDIE-------------------------------------------------------
------------------------------VTEENKH-DYVQR-VVEYKLIGS-VKEQME
-HFLQG-----XIVPA--DLVS-IFNEQ-ELELLISG-----------------------
----LPDID-----------VEDW----RNNTEYHN-YNAS-------------------
------------------------SPQI-QWYWRAVR--------------------SFD
KEERAKLLQF-------VTGTSKVP------------LNG-----FK-ELEGMN--GFSR
FNIHRDYGAK------------------------DRLPSSHTCF-NQ-LDLPEY-DSYED
LRKALY-TAITTGS---
>Verruconis_gallopava_XM_016355991.1 .
LHVRRDQV-FLDSFKHL--------------CYRKPEEIKYGKLNIRFAG-------EEG
VDAGGVTREW---FQAI----TKQMFDPNY--------ALFVPVAADRT-----------
----------------TFHPNPAS------------------------------------
--SINEQHLTFFKFIGRIIGKALYEGRVLDCHFSR--AVYKRILG---------------
--------RTISIKDMES----------------------LDNDYAKN--------LQWI
LDN------------------------------------------------DIT-DLI--
-TETFSVSADV---------------------------FGE-EKIVD-LIPN--------
GRNIE-------------------------------------------------------
------------------------------VTEENKH-DYVQR-VVEYKLIGS-VKEQME
-HFLQG---FHDIVPA--DLVS-IFNEQ-ELELLISG-----------------------
----LPDID-----------VEDW----RNNTEYHN-YTAS-------------------
```

------------------------SPQV-QWFWRAVR--------------------SFD
KEERAKLLQF-------VTGTSKVP------------LNG-----FK-ELEGMN--GFSR
FNIHRDYGNK------------------------DRLPSSHTCF-NQ-LDLPEY-DSYED
LRKALY-TAITTGSEYF
>Alternaria_alternata_XM_018528864.1 .
LSVRRDQV-FLDSFKSL--------------YFKSADEMKFGKLSIRFHG-------EEG
VDAGGVTREW---FQSI----SRQMFNADY--------ALFVPVASDRT-----------
----------------TFHPNRLS------------------------------------
--SINPEHLMFFKFIGRIIGKALYEGRVLDCHFSR--AVYKQIMG---------------
--------KQVSLKDMET----------------------LDLEYYKS--------LEWM
IHN--EIT-DII--
-TETFSVEVEA---------------------------FGE-MQTVD-LIEN--------
GRNIP---
------------------------------VTEDNKH-EYVRL-ITEHRLVGA-VQEQLE
-NFLKG---FHDIVPA--ELVS-IFSEQ-ELELLISG-----------------------
----LPDIN-----------VDDW----KNNTEYHN-YTAA-------------------
------------------------SPQI-QWFWRAVR--------------------TFE
KEEQAKLLQF-------VTGTSKVP------------LNG-----FK-ELEGMN--GFSK
FNIHRDYGSK------------------------DRLPSSHTCF-NQ-LDLPEY-ETYED
LRKALY-TAMTAGGEYF
>Alternaria_arborescens_AIIC01000057.1 .
LSVRRDQV-FLDSFKSL--------------YFKSADEMKFGKLSIRFHG-------EEG
VDAGGVTREW---FQSI----SRQMFNADY--------ALFVPVASDRT-----------
----------------TFHPNRLS------------------------------------
--SINPEHLMFFKFIGRIIGKALYEGRVLDCHFSR--AVYKQIMG---------------
--------KQVSLKDMET----------------------LDLEYYKS--------LEWM
IHN--EIT-DII--
-TETFSVEVEA---------------------------FGE-MQTVD-LIEN--------
GRNIP---
------------------------------VTEDNKH-EYVRL-ITEHRLVGA-VQEQLE
-NFLKG---FHDIVPA--ELVS-IFSEQ-ELELLISG-----------------------
----LPDIN-----------VDDW----KNNTEYHN-YTAA-------------------
------------------------SPQI-QWFWRAVR--------------------TFE
KEEQAKLLQF-------VTGTSKVP------------LNG-----FK-ELEGMN--GFSK
FNIHRDYGSK------------------------DRLPSSHTCF-NX-LDLPEY-ETYED
LRKALY-TAMTAGGEYF
>Alternaria_brassicicola_ACIW01002098.1 .
LSVRRDQV-FLDSFKSL--------------YFKSADEMKFGKLSIRFHG-------EEG
VDAGGVTREW---FQSI----SRQMFNADY--------ALFVPVASDRT-----------
----------------TFHPNRLS------------------------------------
--SINPEHLMFFKFIGRIIGKALYEGRVLDCHFSR--AVYKQIMG---------------
--------KQVSLKDMET----------------------LDLEYYKS--------LEWM
IHN--EIT-DII--
-TETFSVEVEA---------------------------FGE-MQTVD-LIEN--------
GRNIP---
------------------------------VTEDNKH-EYVRL-ITEHRLVGA-VQEQLE
-NFLKG---FHDIVPA--ELVS-IFSEQ-ELELLISG-----------------------
----LPDIN-----------VDDW----KNNTEYHN-YTAA-------------------
------------------------SPQI-QWFWRAVR--------------------TFE
KEEQAKLLQF-------VTGTSKVP------------LNG-----FK-ELEGMN--GFSK
FNIHRDYGSK------------------------DRLPSSHTCF-NX-LDLPEY-ETYED
LRKALY-TAMTAGGEYF
>Alternaria_consortialis_BCGG01000008.1 .
LSVRRDQV-FLDSFKSL--------------YFKSADEMKFGKLSIRFHG-------EEG
VDAGGVTREW---FQSI----SRQMFNADY--------ALFVPVASDRT-----------
----------------TFHPNRLS------------------------------------
--SINPEHLMFFKFIGRIIGKALYEGRVLDCHFSR--AVYKQIMG---------------
--------KQVSLKDMET----------------------LDLEYYKS--------LEWM

IHN---EIT-DII--
-TETFSVEVEA---------------------------FGE-MQTVD-LIEN--------
GRNIP---
------------------------------VTEDNKH-EYVRL-ITEHRLVGA-VQEQLE
-NFLKG---FHDIVPA--ELVS-IFSEQ-ELELLISG-----------------------
----LPDIN-----------VDDW----KNNTEYHN-YTAA-------------------
------------------------SPQI-QWFWRAVR--------------------TFE
KEEQAKLLQF-------VTGTSKVP------------LNG-----FK-ELEGMN--GFSK
FNIHRDYGNK------------------------DRLPSSHTCF-NX-LDLPEY-ETYED
LRKALY-TAMTAGGEYF
>Pyrenophora_tritici_repentis_XM_001935731.1 .
LSVRRDQV-FLDSFKSL--------------YFKSADEMKYGKLSIRFHG-------EEG
VDAGGVTREW---FQSI----SRQMFNADY--------ALFVPVASDRT-----------
----------------TFHPNRLS------------------------------------
--SINPEHLMFFKFIGRIIGKALYEGRVLDCHFSR--AVYKQIMG---------------
--------KQVSLKDMET----------------------LDLEYYKS--------LEWM
IHN---EIT-DII--
-TETFSVEVEA---------------------------FGE-MQTVD-LIEN--------
GRNIP---
------------------------------VTEDNKH-EYVRL-ITEHRLTGA-VHEQLE
-NFLKG---FHDIVPA--ELVS-IFSEQ-ELELLISG-----------------------
----LPDIN-----------VDDW----KNNTEYHN-YTAA-------------------
------------------------SPQI-QWFWRAVR--------------------TFE
KEEQAKLLQF-------VTGTSKVP------------LNG-----FK-ELEGMN--GFSK
FNIHRDYGSK------------------------DRLPSSHTCF-NQ-LDLPEY-ETYED
LRKALY-TAMTAGGEYF
>Pyrenophora_seminiperda_ATLS01000644.1 .
LSVRRDQV-FLDSFKSL--------------YFKTAEEMKYGKLSIRFHG-------EEG
VDAGGVTREW---FQSI----SRQMFNADY--------ALFVPVASDRT-----------
----------------TFHPNRLS------------------------------------
--SINPEHLMFFKFIGRIIGKALYEGRVLDCHFSR--AVYKQIMG---------------
--------KQVSLKDMET----------------------LDLEYYKS--------LEWM
IHN---EIT-DII--
-TETFSVEVEA---------------------------FGE-MQTVD-LIEN--------
GRNIP---
------------------------------VTEDNKH-EYVRL-ITEHRLTGA-VQEQLE
-NFLKG---FHDIVPA--ELVS-IFSEQ-ELELLISG-----------------------
----LPDIN-----------VDDW----KNNTEYHN-YTAA-------------------
------------------------SPQI-QWFWRAVR--------------------TFE
KEEQAKLLQF-------VTGTSKVP------------LNG-----FK-ELEGMN--GFSK
FNIHRDYGSK------------------------DRLPSSHTCF-NX-LDLPEY-ETYED
LRKALY-TAMTAGGEYF
>Pyrenophora_teres_XM_003296520.1 .
LSVRRDQV-FLDSFKSL--------------YFKSADEMKYGKLSIRFHG-------EEG
VDAGGVTREW---FQSI----SRQMFNADY--------ALFVPVASDRT-----------
----------------TFHPNRLS------------------------------------
--SINPEHLMFFKFIGRIIGKALYEGRVLDCHFSR--AVYKQIMG---------------
--------KQVSLKDMET----------------------LDLEYYKS--------LEWM
IHN---DIT-DII--
-TETFSVEVEA---------------------------FGE-MQTVD-LIEN--------
GRNIP---
------------------------------VTEDNKH-EYVRL-ITEHRLVGA-VQEQLE
-NFLKG---FHDIVPA--ELVS-IFSEQ-ELELLISG-----------------------
----LPDIN-----------VDDW----KNNTEYHN-YTAA-------------------
------------------------SPQI-QWFWRAVR--------------------TFE
KEEQAKLLQF-------VTGTSKVP------------LNG-----FK-ELEGMN--GFSK
FNIHRDYGSK------------------------DRLPSSHTCF-NQ-LDLPEY-ETYED
LRKALY-TAMTAGGEYF

>Setosphaeria_turcica_XM_008023317.1 .
LSVRRDQV-FLDSFKSL--------------YFKSADEMKYGKLSIRFHG-------EEG
VDAGGVTREW---FQSI----SRQMFNPDY--------ALFVPVASDRT-----------
----------------TFHPNRLS------------------------------------
--SINPEHLMFFKFIGRIIGKALYEGRVLDCHFSR--AVYKQIMG---------------
--------KQVSLKDMET----------------------LDLEYYKS--------LEWM
IHN--EIT-DII--
-TETFSVEVEA---------------------------FGE-MQTVD-LIEN--------
GRNIP---
------------------------------VTEDNKH-EYVRL-ITEHRLLGA-VQEQLE
-NFLKG---FHDIVPA--ELVS-IFSEQ-ELELLISG-----------------------
----LPDIN-----------VDDW----KNNTEYHN-YTAA-------------------
------------------------SPQI-QWFWRAVR--------------------TFE
KEEQAKLLQF-------VTGTSKVP------------LNG-----FK-ELEGMN--GFSK
FNIHRDYGSK------------------------DRLPSSHTCF-NQ-LDLPEY-ETYED
LRKALY-TAMTAGGEYF
>Setosphaeria_turcica_AIHT01000979.1 .
LSVRRDQV-FLDSFKSL--------------YFKSADEMKYGKLSIRFHG-------EEG
VDAGGVTREW---FQSI----SRQMFNPDY--------ALFVPVASDRT-----------
----------------TFHPNRLS------------------------------------
--SINPEHLMFFKFIGRIIGKALYEGRVLDCHFSR--AVYKQIMG---------------
--------KQVSLKDMET----------------------LDLEYYKS--------LEWM
IHN--EIT-DII--
-TETFSVEVEA---------------------------FGE-MQTVD-LIEN--------
GRNIP---
------------------------------VTEDNKH-EYVRL-ITEHRLLGA-VQEQLE
-NFLKG---FHDIVPA--ELVS-IFSEQ-ELELLISG-----------------------
----LPDIN-----------VDDW----KNNTEYHN-YTAA-------------------
------------------------SPQI-QWFWRAVR--------------------TFE
KEEQAKLLQF-------VTGTSKVP------------LNG-----FK-ELEGMN--GFSK
FNIHRDYGSK------------------------DRLPSSHTCF-NX-LDLPEY-ETYED
LRKALY-TAMTAGGEYF
>Stemphylium_lycopersici_LGLR01000196.1 .
LSVRRDQV-FLDSFKSL--------------YFKSADEMKYGKLSIRFHG-------EEG
VDAGGVTREW---FQSI----SRQMFNPDY--------ALFVPVASDRT-----------
----------------TFHPNRLS------------------------------------
--SINPEHLMFFKFIGRIIGKALYEGRVLDCHFSR--AVYKQIMG---------------
--------KQVSLKDMET----------------------LDLEYYKS--------LEWM
IHN--EIT-DII--
-TETFSVEVEA---------------------------FGE-MQTVD-LIEN--------
GRNIP---
------------------------------VTEDNKH-EYVRL-ITEHRLLGA-VQEQLE
-NFLKG---FHDIVPA--ELVS-IFSEQ-ELELLISG-----------------------
----LPDIN-----------VDDW----KNNTEYHN-YTAA-------------------
------------------------SPQI-QWFWRAVR--------------------TFE
KEEQAKLLQF-------VTGTSKVP------------LNG-----FK-ELEGMN--GFSK
FNIHRDYGSK------------------------DRLPSSHTCF-NX-LDLPEY-ETYED
LRKALY-TAMTAGGEYF
>Bipolaris_oryzae_LNFW01000192.1 .
LSVRRDQV-FLDSFKSL--------------YFKSADEMKYGKLSIRFHG-------EEG
VDAGGVTREW---FQSI----SRQMFNPDY--------ALFVPVASDRT-----------
----------------TFHPNRLS------------------------------------
--SINPEHLMFFKFIGRIIGKALYEGRVLDCHFSR--AVYKQIMG---------------
--------KQVSLKDMET----------------------LDLEYYKS--------LEWM
IHN--EIT-DII--
-TETFSVEVEA---------------------------FGE-MQTVD-LIEN--------
GRNIP---
------------------------------VTEDNKH-EYVRL-ITEHRLLGA-VQEQLD

```
-HFLKG---FHDIVPA--ELVS-IFSEQ-ELELLISG-----------------------
----LPDIN-----------VDDW----KNNTEYHN-YTAA-------------------
------------------------SPQI-QWFWRAVR--------------------TFE
KEEQAKLLQF-------VTGTSKVP------------LNG-----FK-ELEGMN--GFSK
FNIHRDYGSK------------------------DRLPSSHTCF-NX-LDLPEY-ETYED
LRKALY-TAMTAGGEYF
>Bipolaris_zeicola_AMCN01000037.1 .
LSVRRDQV-FLDSFKSL--------------YFKSADEMKYGKLSIRFHG-------EEG
VDAGGVTREW---FQSI----SRQMFNPDY--------ALFVPVASDRT-----------
----------------TFHPNRLS------------------------------------
--SINPEHLMFFKFIGRIIGKALYEGRVLDCHFSR--AVYKQIMG---------------
--------KQVSLKDMET----------------------LDLEYYKS--------LEWM
IHN------------------------------------------------EIT-DII--
-TETFSVEVEA---------------------------FGE-MQTVD-LIEN--------
GRNIP-------------------------------------------------------
------------------------------VTEDNKH-EYVRL-ITEHRLLGA-VQEQLD
-HFLKG---FHDIVPA--ELVS-IFSEQ-ELELLISG-----------------------
----LPDIN-----------VDDW----KNNTEYHN-YTAA-------------------
------------------------SPQI-QWFWRAVR--------------------TFE
KEEQAKLLQF-------VTGTSKVP------------LNG-----FK-ELEGMN--GFSK
FNIHRDYGSK------------------------DRLPSSHTCF-NX-LDLPEY-ETYED
LRKALY-TAMTAGGEYF
>Cochliobolus_sativus_XM_007696502.1 .
LSVRRDQV-FLDSFKSL--------------YFKSADEMKYGKLSIRFHG-------EEG
VDAGGVTREW---FQSI----SRQMFNPDY--------ALFVPVASDRT-----------
----------------TFHPNRLS------------------------------------
--SINPEHLMFFKFIGRIIGKALYEGRVLDCHFSR--AVYKQIMG---------------
--------KQVSLKDMET----------------------LDLEYYKS--------LEWM
IHN------------------------------------------------EIT-DII--
-TETFSVEVEA---------------------------FGE-MQTVD-LIEN--------
GRNIP-------------------------------------------------------
------------------------------VTEDNKH-EYVRL-ITEHRLLGA-VQEQLD
-HFLKG---FHDIVPA--ELVS-IFSEQ-ELELLISG-----------------------
----LPDIN-----------VDDW----KNNTEYHN-YTAA-------------------
------------------------SPQI-QWFWRAVR--------------------TFE
KEEQAKLLQF-------VTGTSKVP------------LNG-----FK-ELEGMN--GFSK
FNIHRDYGSK------------------------DRLPSSHTCF-NQ-LDLPEY-ETYED
LRKALY-TAMTAGGEYF
>Bipolaris_sorokiniana_AEIN01000013.1 .
LSVRRDQV-FLDSFKSL--------------YFKSADEMKYGKLSIRFHG-------EEG
VDAGGVTREW---FQSI----SRQMFNPDY--------ALFVPVASDRT-----------
----------------TFHPNRLS------------------------------------
--SINPEHLMFFKFIGRIIGKALYEGRVLDCHFSR--AVYKQIMG---------------
--------KQVSLKDMET----------------------LDLEYYKS--------LEWM
IHN------------------------------------------------EIT-DII--
-TETFSVEVEA---------------------------FGE-MQTVD-LIEN--------
GRNIP-------------------------------------------------------
------------------------------VTEDNKH-EYVRL-ITEHRLLGA-VQEQLD
-HFLKG---FHDIVPA--ELVS-IFSEQ-ELELLISG-----------------------
----LPDIN-----------VDDW----KNNTEYHN-YTAA-------------------
------------------------SPQI-QWFWRAVR--------------------TFE
KEEQAKLLQF-------VTGTSKVP------------LNG-----FK-ELEGMN--GFSK
FNIHRDYGSK------------------------DRLPSSHTCF-NX-LDLPEY-ETYED
LRKALY-TAMTAGGEYF
>Curvularia_papendorfii_JXCC01000131.1 .
LSVRRDQV-FLDSFKSL--------------YFKSADEMKYGKLSIRFHG-------EEG
VDAGGVTREW---FQSI----SRQMFNPDY--------ALFVPVASDRT-----------
----------------TFHPNRLS------------------------------------
```

--SINPEHLMFFKFIGRIIGKALYEGRVLDCHFSR--AVYKQIMG---------------
--------KQVSLKDMET----------------------LDLEYYKS--------LEWM
IHN--DIT-DII--
-TETFSVEVEA---------------------------FGE-MQTVD-LIEN--------
GRNIP---
------------------------------VTEDNKH-EYVRL-VTEHRLLGA-VQEQLE
-HFLKG---FHDIVPA--ELVS-IFSEQ-ELELLISG-----------------------
----LPDIN-----------VDDW----KNNTEYHN-YTAA-------------------
------------------------SPQI-QWFWRAVR--------------------TFE
KEEQAKLLQF-------VTGTSKVP------------LNG-----FK-ELEGMN--GFSK
FNIHRDYGSK------------------------DRLPSSHTCF-NX-LDLPEY-ETYED
LRKALY-TAMTAGGEYF
>Leptosphaeria_maculans_XM_003836829.1 .
LSVRRDQV-FLDSFKSL--------------YFKSADEMKYGKLSIRFHG-------EEG
VDAGGVTREW---FQVI----SRQMFNADY--------ALFVPVASDRT-----------
----------------TFHPNRLS------------------------------------
--SINPEHLMFFKFIGRIIGKALYEGRVLDCHFSR--AVYKQIMG---------------
--------KQVNLKDMET----------------------LDLEYYKS--------LEWM
LHN--DIT-DII--
-TETFSVEVEA---------------------------FGE-MQVVD-LIEN--------
GRNIP---
------------------------------VTEDNKQ-EYIRL-ITEHRLTGA-VQEQLE
-HFLKG---FHDIVPA--ELVS-IFSEQ-ELELLISG-----------------------
----LPDIN-----------VDDW----KNNTEYHN-YTAA-------------------
------------------------SPQI-QWFWRAIR--------------------TFE
KEEQAKLLQF-------ITGTSKVP------------LNG-----FK-ELEGMN--GFSK
FNIHRDYGSK------------------------DRLPSSHTCF-NQ-LDLPEY-ETYED
LRKALY-TAMTAGGEYF
>Leptosphaeria_biglobosa_FO905658.1 .
LSVRRDQV-FLDSFKSL--------------YFKSADEMKYGKLSIRFHG-------EEG
VDAGGVTREW---FQVI----SRQMFNADY--------ALFVPVASDRT-----------
----------------TFHPNRLS------------------------------------
--SINPEHLMFFKFIGRIIGKALYEGRVLDCHFSR--AVYKQIMG---------------
--------KQVNLKDMET----------------------LDLEYYKS--------LEWM
LHN--DIT-DII--
-TETFSVEVEA---------------------------FGE-MQVVD-LIEN--------
GRNIP---
------------------------------VTEDNKQ-EYIRL-ITEHRLTGA-VQEQLE
-HFLKG---FHDIVPA--ELVS-IFSEQ-ELELLISG-----------------------
----LPDIN-----------VDDW----KNNTEYHN-YTAA-------------------
------------------------SPQI-QWFWRAIR--------------------TFE
KEEQAKLLQF-------ITGTSKVP------------LNG-----FK-ELEGMN--GFSK
FNIHRDYGSK------------------------DRLPSSHTCF-NX-LDLPEY-ESYED
LRKALY-TAMTAGGEYF
>Pyrenochaeta_lycopersici_GAJI01025518.1 .
LSVRRDQV-FLDSFKSL--------------YFKSADEMKFGKLSIRFHG-------EEG
VDAGGVTREW---FQVI----SRQMFNADY--------ALFVPVASDRT-----------
----------------TFHPNRLS------------------------------------
--SINPEHLMFFKFIGRIIGKALYEGRVLDCHFSR--AVYKQIMS---------------
--------KQVNLKDMET----------------------LDLEYYKS--------LEWM
LHN--DIT-DII--
-TETFSVEVEA---------------------------FGE-MQVVN-LIEN--------
GQNIP---
------------------------------VTEDNKH-EYIRL-ITEHRLTGA-VQEQLE
-HFLKG---FHDIVPA--ELVS-IFSEQ-ELELLISG-----------------------
----LPDIN-----------VDDW----KNNTEYHN-YTAA-------------------
------------------------SPQI-QWFWRAVR--------------------TFE
KEEQAKLLQF-------VTGTSKVP------------LNG-----FK-ELEGMN--GFSK

FNIHRDYGSK------------------------DRLPSSHTCF-NQ-LDLPEY-TSYED
LRKALY-TAMTAGGEYF
>Paraphoma_sp._BCLK01000308.1 .
LAVRRDQV-FLDSFKSL--------------YFKSADEMKYGKLSIRFHG-------EEG
VDAGGVTREW---FQVI----ARQMFNADY--------ALFVPVASDRT-----------
----------------TFHPNRLS------------------------------------
--SINPEHLMFFKFIGRIIGKALYEGRVLDCHFSR--AVYKQIMG---------------
--------KQVNLKDMET----------------------LDLEYYKS--------LEWM
LNN--DIT-DII--
-TETFSVEVEA---------------------------FGE-MQVVD-LIEN--------
GREIP---
------------------------------VTEENKQ-EYIRL-ITEHRLTGS-VKEQLE
-NFLKG---FHDIVPA--ELVN-IFSEQ-ELELLISG-----------------------
----LPDIN-----------VDDW----KNNTEYHN-YTAA-------------------
------------------------SPQI-QWFWRAVR--------------------TFE
KEEQAKLLQF-------VTGTSKVP------------LNG-----FK-ELEGMN--GFSK
FNIHRDYGSK------------------------DRLPSSHTCF-NX-LDLPEY-ETYED
LRKALY-TAMTAGGEYF
>Pleosporales_sp._AJMS01007776.1 .
LAVRRDQV-FLDSFKSL--------------YFKSADEMKYGKLSIRFHG-------EEG
VDAGGVTREW---FQVI----SRQMFNADY--------ALFVPVASDRT-----------
----------------TFHPNRLS------------------------------------
--SINPEHLMFFKFIGRIIGKALYEGRVLDCHFSR--AVYKQIMG---------------
--------KQVNLKDMET----------------------LDLEYYKS--------LEWM
LNN--DIT-DII--
-TETFSVEVEA---------------------------FGE-MQVVD-LIEN--------
GREIP---
------------------------------VTEDNKH-EYIRL-ITEHRLTGS-VKEQLE
-NFLKG---FHDIVPA--ELVS-IFSEQ-ELELLISG-----------------------
----LPDIN-----------VDDW----KNNTEYHN-YTAA-------------------
------------------------SPQI-QWFWRAVR--------------------TFE
KEEQAKLLQF-------VTGTSKVP------------LNG-----FK-ELEGMN--GFSK
FNIHRDYGSK------------------------DRLPSSHTCF-NX-LDLPEY-ESYED
LRKALY-TAMTAGGEYF
>Stagonospora_sp._LXTA01000114.1 .
LAVRRDQV-FLDSFKSL--------------YFKSADEMKFGKLSIRFHG-------EEG
VDAGGVTREW---FQVI----SRQMFNADY--------ALFVPVASDRT-----------
----------------TFHPNRLS------------------------------------
--SINPEHLMFFKFIGRIIGKALYEGRVLDCHFSR--AVYKQIMG---------------
--------KQVNLKDMET----------------------LDLEYYKS--------LEWM
LNN--DIT-DII--
-TETFSVEVEA---------------------------FGE-MQIVD-LIEN--------
GRDIP---
------------------------------VTEENKH-EYIRL-ITEHRLTGS-VKEQLE
-NFLKG---FHDIVPA--ELVS-IFSEQ-ELELLISG-----------------------
----LPDIN-----------VDDW----KNNTEYHN-YTAA-------------------
------------------------SPQI-QWFWRAVR--------------------TFE
KEEQAKLLQF-------VTGTSKVP------------LNG-----FK-ELEGMN--GFSK
FNIHRDYGSK------------------------DRLPSSHTCF-NX-LDLPEY-ESYED
LRKALY-TAMTAGGEYF
>Hymenoscyphus_laetus_LLCA01000038.1 .
LAVRRDQV-FLDSFKSL--------------YFKSADEMKYGKLSIRFHG-------EEG
VDAGGVTREW---FQVI----SRQMFNADY--------ALFVPVASDRT-----------
----------------TFHPNRLS------------------------------------
--SINPEHLMFFKFIGRIIGKALYEGRVLDCHFSR--AVYKQIMG---------------
--------KQVNLKDMET----------------------LDLEYYKS--------LEWM
LNN--DIT-DII--
-TETFSVEVEA---------------------------FGE-MQVVD-LIDN--------

GREIP---
------------------------------VTEDNKH-EYIRL-ITEHRLTGS-VREQLD
-HFLKG---FHDIVPS--TLVS-IFSEQ-ELELLISG-----------------------
----LPDIN-----------VDDW----KNNTEYHN-YTAA-------------------
------------------------SPQI-QWFWRAVR--------------------TFE
KEEQAKLLQF-------VTGTSKVP------------LNG-----FK-ELEGMN--GFSK
FNIHRDFGNK------------------------DRLPSSHTCF-NX-LDLPEY-ETYED
LRKALY-TAMTAGGEYF
>Parastagonospora_nodorum_AAGI01000367.1 .
LAVRRDQV-FLDSFKSL--------------YFKSADEMKYGKLSIRFHG-------EEG
VDAGGVTREW---FQVI----SRQMFNADY--------ALFVPVASDRT-----------
----------------TFHPNRLS------------------------------------
--SINPEHLMFFKFIGRIIGKALYEGRVLDCHFSR--AVYKQIMG---------------
--------KQVNLKDMET----------------------LDLEYYKS--------LEWM
LNN--DIT-DII--
-TETFSVEVEA---------------------------FGE-MQVVD-LIDN--------
GREIP---
------------------------------VTEDNKH-EYIRL-ITEHRLTGS-VREQLD
-HFLKG---FHDIVPS--TLVS-IFSEQ-ELELLISG-----------------------
----LPDIN-----------VDDW----KNNTEYHN-YTAA-------------------
------------------------SPQI-QWFWRAVR--------------------TFE
KEEQAKLLQF-------VTGTSKVP------------LNG-----FK-ELEGMN--GFSK
FNIHRDFGSK------------------------DRLPSSHTCF-NX-LDLPEY-ETYED
LRKALY-TAMTAGGEYF
>Phaeosphaeria_nodorum_XM_001805745.1 .
LAVRRDQV-FLDSFKSL--------------YFKSADEMKYGKLSIRFHG-------EEG
VDAGGVTREW---FQVI----SRQMFNADY--------ALFVPVASDRT-----------
----------------TFHPNRLS------------------------------------
--SINPEHLMFFKFIGRIIGKALYEGRVLDCHFSR--AVYKQIMG---------------
--------KQVNLKDMET----------------------LDLEYYKS--------LEWM
LNN--DIT-DII--
-TETFSVEVEA---------------------------FGE-MQVVD-LIDN--------
GREIP---
------------------------------VTEDNKH-EYIRL-ITEHRLTGS-VREQLD
-HFLKG---FHDIVPS--TLVS-IFSEQ-ELELLISG-----------------------
----LPDIN-----------VDDW----KNNTEYHN-YTAA-------------------
------------------------SPQI-QWFWRAVR--------------------TFE
KEEQAKLLQF-------VTGTSKVP------------LNG-----FK-ELEGMN--GFSK
FNIHRDFGSK------------------------DRLPSSHTCF-NQ-LDLPEY-ETYED
LRKALY-TAMTAGGEYF
>Didymella_rabiei_GDJN01004599.1 .
LSVRRDQV-FLDSFKSL--------------YFKTAEEMKYGKLSIRFHG-------EEG
VDAGGVTREW---FQVI----ARQMFNADY--------ALFVPVASDRT-----------
----------------TFHPNRLS------------------------------------
--SINPEHLMFFKFIGRIIGKALYENRVLDCHFSR--AVYKQIMS---------------
--------KQVNLKDMET----------------------LDLEYYKS--------LEWM
LHN--DIT-DII--
-TETFSTEVEA---------------------------FGE-MQTVD-LIED--------
GRNIP---
------------------------------VTEENKH-EYIRL-ITEYRLTGA-VQEQLQ
-EFLRG---FHDIVPA--ELVS-IFSEQ-ELELLISG-----------------------
----LPDIN-----------VDDW----KNNTEYHN-YTAA-------------------
------------------------SPQI-QWFWRAVR--------------------TFE
KEEQAKLLQF-------VTGTSKVP------------LNG-----FK-ELEGMN--GFSK
FNIHRDYGSK------------------------DRLPSSHTCF-NQ-LDLPEY-ESYED
LRKALY-TAMTAGGEYF
>Ascochyta_rabiei_JYNV01000244.1 .
LSVRRDQV-FLDSFKSL--------------YFKTAEEMKYGKLSIRFHG-------EEG

VDAGGVTREW---FQVI----ARQMFNADY--------ALFVPVASDRT-----------
----------------TFHPNRLS------------------------------------
--SINPEHLMFFKFIGRIIGKALYENRVLDCHFSR--AVYKQIMS---------------
--------KQVNLKDMET----------------------LDLEYYKS--------LEWM
LHN--DIT-DII--
-TETFSTEVEA---------------------------FGE-MQTVD-LIED--------
GRNIP---
------------------------------VTEENKH-EYIRL-ITEYRLTGA-VQEQLQ
-EFLRG---FHDIVPA--ELVS-IFSEQ-ELELLISG-----------------------
----LPDIN-----------VDDW----KNNTEYHN-YTAA-------------------
------------------------SPQI-QWFWRAVR--------------------TFE
KEEQAKLLQF-------VTGTSKVP------------LNG-----FK-ELEGMN--GFSK
FNIHRDYGSK------------------------DRLPSSHTCF-NX-LDLPEY-ESYED
LRKALY-TAMTAGGEYF
>Epicoccum_nigrum_NCTX01000186.1 .
LSVRRDQV-FLDSFKSL--------------YFKSAEEMKYGKLSIRFHG-------EEG
VDAGGVTREW---FQAI----SRQMFNADY--------ALFVPVASDRT-----------
----------------TFHPNRLS------------------------------------
--SINPEHLMFFKFIGRIIGKALYEGRVLDCHFSR--AVYKQIMN---------------
--------KQVNLKDMET----------------------LDLEYYKS--------LEWM
LHN--DIT-DVI--
-TETFSTEVEA---------------------------FGE-MQTVD-LIEN--------
GRNIP---
------------------------------VTEDNKH-EYIRL-ITEYRLTGA-VEEQLK
-EFLRG---FHDIVPA--DLVS-IFSEQ-ELELLISG-----------------------
----LPDIN-----------VDDW----KNNTEYHN-YTAA-------------------
------------------------SPQI-QWFWRAVR--------------------TFE
KEEQAKLLQF-------VTGTSKVP------------LNG-----FK-ELEGMN--GFSK
FNIHRDYGSK------------------------DRLPSSHTCF-NX-LDLPEY-ESYED
LRKALY-TAMTVGGEYF
>Epicoccum_sorghinum_MIEO01000390.1 .
LSVRRDQV-FLDSFKSL--------------YFKSAEEMKYGKLSIRFHG-------EEG
VDAGGVTREW---FQVI----ARQMFNADY--------ALFVPVASDRT-----------
----------------TFHPNRLS------------------------------------
--SINPEHLMFFKFIGRIIGKALYEGRVLDCHFSR--AVYKQIMN---------------
--------KQVNLKDMET----------------------LDLEYYKS--------LEWM
LHN--DIT-DVI--
-TETFSTEVEA---------------------------FGE-MQTVD-LIEN--------
GRNIP---
------------------------------VTEDNKH-EYIRL-ITEYRLTGA-VEEQLK
-EFLRG---FHDIVPA--ELVS-IFSEQ-ELELLISG-----------------------
----LPDIN-----------VDDW----KNNTEYHN-YTAA-------------------
------------------------SPQI-QWFWRAVR--------------------TFE
KEEQAKLLQF-------VTGTSKVP------------LNG-----FK-ELEGMN--GFSK
FNIHRDYGSK------------------------DRLPSSHTCF-NX-LDLPEY-ESYED
LRKALY-TAMTAGGEYF
>Stagonosporopsis_tanaceti_JUDZ01009623.1 .
LSVRRDQV-FLDSFKSL--------------YFKSAEEMKYGKLSIRFHG-------EEG
VDAGGVTREW---FQVI----ARQMFNADY--------ALFVPVASDRT-----------
----------------TFHPNRLS------------------------------------
--SINPEHLMFFKFIGRIIGKALYEGRVLDCHFSR--AVYKQIMS---------------
--------KQVNLKDMET----------------------LDLEYYKS--------LEWM
LHN--DIT-DII--
-TETFSTEVEA---------------------------FGE-MQTVD-LIEN--------
GRNIP---
------------------------------VTEDNKH-EYIRL-ITEYRLTGA-VEEQLK
-EFLRG---FHDIVPA--ELVS-IFSEQ-ELELLISG-----------------------
----LPDIN-----------VDDW----KNNTEYHN-YTAA-------------------

------------------------SPQI-QWFWRAVR--------------------TFE
KEEQAKLLQF-------VTGTSKVP------------LNG-----FK-ELEGMN--GFSK
FNIHRDYGSK------------------------DRLPSSHTCF-NX-LDLPEY-ESYED
LRKALY-LAMTAGGEYF
>Phoma_herbarum_BCGR01000005.1 .
LSVRRDQV-FLDSFKSL--------------YFKSAEEMKYGKLSIRFHG-------EEG
VDAGGVTREW---FQVI----ARQMFNADY--------ALFVPVASDRT-----------
----------------TFHPNRLS------------------------------------
--SINPEHLMFFKFIGRIIGKALYEGRVLDCHFSR--AVYKQIMS---------------
--------KQVNLKDMET----------------------LDLEYYKS--------LEWM
LHN--DIT-DII--
-TETFSTEVEA---------------------------FGE-MQTVD-LIEN--------
GRNIP---
------------------------------VTEDNKH-EYIRL-ITEYRLTGA-VEEQLK
-EFLRG---FHDIVPA--ELVS-IFSEQ-ELELLISG-----------------------
----LPDIN-----------VDDW----KNNTEYHN-YTAA-------------------
------------------------SPQI-QWFWRAVR--------------------TFE
KEEQAKLLQF-------VTGTSKVP------------LNG-----FK-ELEGMN--GFSK
FNIHRDYGSK------------------------DRLPSSHTCF-NX-LDLPEY-ESYED
LRKALY-TAMTAGGEYF
>Corynespora_cassiicola_FWCK01000061.1 .
LAVRRDQV-FLDSFKSL--------------YFKNADEMKYGKLSIRFHG-------EEG
VDAGGVTREW---FQVI----ARQMFNPDY--------ALFVPVASDRT-----------
----------------TFHPNRLS------------------------------------
--SINPEHLMFFKFIGRIIGKALYEGRVLDCHFSR--AVYKRILG---------------
--------RPVALKDMET----------------------LDLDYYKS--------LQWM
LEN--DIT-DII--
-TETFSVEVEA---------------------------FGE-IVTAD-LIEN--------
GRNIP---
------------------------------VTEENKH-EYVRL-VTEHRLTGA-VQEQLD
-EFLSG---FHDIIPA--ELVS-IFSEQ-ELELLISG-----------------------
----LPDIN-----------VDDW----KNNTEYHN-YTAA-------------------
------------------------SPQI-QWFWRAVR--------------------SFE
KEEQAKLLQF-------VTGTSKVP------------LNG-----FK-ELEGMN--GFSR
FNIHRDYGSK------------------------ERLPSSHTCF-NQ-LDLPEY-ESYED
LRKQLY-TAMTAGGEYF
>Glonium_stellatum_LKAO01001460.1 .
LAVRRDQV-FLDSFKSL--------------YFKTADEMKYGKLSIRFHG-------EEG
VDAGGVTREW---FQVL----SRQMFNPDY--------ALFVPVASDRT-----------
----------------TFHPNRLS------------------------------------
--AINQEHLMFFKFIGRIIGKALYEGRVLDCHFSR--AVYKRILG---------------
--------KPVSLKDMET----------------------LDLEYYKS--------LLWM
LEN--DIT-DII--
-TETFSTDVEA---------------------------FGE-TQTVD-LIEN--------
GRNIP---
------------------------------VTEENKH-EYVRL-VTEHRLTGA-VQEQLE
-HFLKG---XMTLVPA--ELIS-IFSEQ-ELELLISG-----------------------
----LPDID-----------VEDW----KNNTEYHN-YTAG-------------------
------------------------SPQI-QWFWRAVR--------------------SFD
KEERAKLLQF-------VTGTSKVP------------LNG-----FK-ELEGMN--GFSR
FNIHRDYGSK------------------------DRLPSSHTCF-NX-LDLPEY-DTYEA
LRQQLY-TAMTAGSEYF
>Cenococcum_geophilum_LKKR01000150.1 .
LAVRRDQV-FLDSFKSL--------------YFKTADEMKYGKLSIRFHG-------EEG
VDAGGVTREW---FQVL----SRQMFNPDY--------ALFVPVASDRT-----------
----------------TFHPNRLS------------------------------------
--AINQEHLMFFKFIGRIIGKALYEGRVLDCHFSR--AVYKRILG---------------
--------KPVSLKDMET----------------------LDLEYYKS--------LLWM

```
LEN------------------------------------------------DIT-DII--
-TETFSTDVEA---------------------------FGE-TQTVD-LIEN--------
GRNIP-------------------------------------------------------
------------------------------VTEENKH-EYVRL-VTEHRLTGA-VQEQLE
-HFLKG---FHDIVPA--ELIS-IFSEQ-ELELLISG-----------------------
----LPDID-----------VEDW----KNNTEYHN-YTAG-------------------
------------------------SPQI-QWFWRAVR--------------------SFD
KEERAKLLQF-------VTGTSKVP------------LNG-----FK-ELEGMN--GFSR
FNIHRDYGSK------------------------DRLPSSHTCF-NX-LDLPEY-DTYEA
LRQQLY-TAMTAGSEYF
>Lepidopterella_palustris_LKAR01000230.1 .
LAVRRDQV-FLDSFKSL--------------YFKTADEMKYGKLSIRFHG-------EEG
VDAGGVTREW---FQVL----SRQMFNPDY--------ALFVPVASDRT-----------
----------------TFHPNRLS------------------------------------
--AINQEHLMFFKFIGRIIGKALYEGRVLDCHFSR--AVYKRILG---------------
--------KSVSLKDMET----------------------LDLEYYKS--------LLWM
MEN------------------------------------------------DIT-DII--
-TETFSVDVEA---------------------------FGE-TQTVD-LIEN--------
GRNVP-------------------------------------------------------
------------------------------VTEENKH-EYVRL-VTEHRLTGS-VQDQLE
-HFLKG---FHDIVPA--ELVS-IFSEQ-ELELLISG-----------------------
----LPDID-----------VDDW----KNNTEYHN-YTAA-------------------
------------------------SPQI-QWLWRAVR--------------------SFD
KEERAKLLQF-------VTGTSKVP------------LNG-----FK-ELEGMN--GFSR
FNIHRDYGSK------------------------DRLPSSHTCF-NX-LDLPEY-DTYET
LRQQLY-TAMTAGSEYF
>Hysterium_pulicare_AJFK01001090.1 .
LAVRRDQV-FLDSFKSL--------------YFKTADEMKYGKLSIRFHG-------EEG
VDAGGVTREW---FQVL----ARQMFNPDY--------ALFVPVASDRT-----------
----------------TFHPNRLS------------------------------------
--EINQEHLMFFKFIGRIIGKALYEGRVLDCHFSR--AVYKQILG---------------
--------KPVNLKDMET----------------------LDLEYYKS--------LQWM
LEN------------------------------------------------DIT-EII--
-TETFSVDVEA---------------------------FGE-TRTVD-LIEN--------
GRNIP-------------------------------------------------------
------------------------------VTEDNKH-EYVRL-VTEHRLTGS-VQEQLD
-NFLKG---FHDIVPA--ELVS-IFSEQ-ELELLISG-----------------------
----LPDIN-----------VDDW----KNNTEYHN-YTVA-------------------
------------------------SPQI-QWFWRAVR--------------------SFD
KEERAKLLQF-------VTGTSKVP------------LNG-----FK-ELEGMN--GFSR
FNIHRDYGNK------------------------DRLPSSHTCF-NX-LDLPEY-ETYEA
LRQQLY-TAMTAGSEYF
>Beverwykella_pulmonaria_BCHH01000002.1 .
LAVRRDQV-FLDSFKSL--------------YFKTPDEMKYGKLSIRFHG-------EEG
VDAGGVTREW---FQVI----ARQMFNADY--------ALFVPVASDRT-----------
----------------TFHPNRLS------------------------------------
--SINPEHLMFFSFIGRIIGKALYEGRVLDCHFSR--AVYKRMLG---------------
--------KPVNLKDMET----------------------LDLEYHKS--------LQWM
LSN------------------------------------------------DIT-DII--
-TEAFSVDVEA---------------------------FGE-TQVVD-LIEN--------
GRNIP-------------------------------------------------------
------------------------------VTEENKH-EYVRL-VCEHRLTGA-VQDQLE
-HFLTG---FHDIVPK--ELIS-IFSEQ-ELELLISG-----------------------
----LPDIN-----------VDDW----KNNTEYHN-YTAA-------------------
------------------------SPQI-QFFWRAVR--------------------SFD
KEEQAKLLQF-------VTGTSKVP------------LNG-----FK-ELEGMN--GFSR
FNIHRDYGSK------------------------DRLPSSHTCF-NX-LDLPEY-ETYED
LRKQLH-TAITAGEGYF
```

>Clohesyomyces_aquaticus_MCFA01000200.1 .
LSVRRDQV-FLDSFKSL--------------YFKTADEMKYGKLSIRFHG-------EEG
VDAGGVTREW---FQVI----ARQMFNPDY--------ALFVPVAADRT-----------
----------------TFHPNRLS------------------------------------
--AINPEHLMFFKFIGRIIGKALYEGRVLDCHFSR--AVYKRILS---------------
--------RPINLKDMES----------------------LDLEYYKS--------LNWM
LEN--SIE-GVM--
-TETFSVDVEA---------------------------FGE-VQTVD-LIEN--------
GRNIP---
------------------------------LTDENKH-EYARL-VTEHRLTGS-VQEQLE
-HFLKG---FHDIVPA--ELIS-IFSEQ-ELELLISG-----------------------
----LPDVN-----------VDDW----KNNTEYHN-YTAA-------------------
------------------------SPQI-QWFWRAVR--------------------SFD
KEERAKLLQF-------VTGTSKVP------------LNG-----FR-ELEGMN--GFSR
FNIHRDYGSK------------------------ERLPSSHTCF-NX-LDLPEY-DTYED
LRKQLY-TAMTAGGEYF
>Rhytidhysteron_rufulum_AJFL01000340.1 .
LAVRRDQV-FLDSFKSL--------------YFKTSDEMKYGKLNIRFHG-------EEG
VDAGGVTREW---FQVL----ARQMFNPDY--------ALFVPVASDRT-----------
----------------TFHPNRLS------------------------------------
--AINQEHLMFFKFIGRIIGKALYEGRVLDCHFSR--AVYKRILG---------------
--------RPVNLKDMET----------------------LDLEYYKS--------LQWM
LEN--DIT-DII--
-TETFSVDVEA---------------------------FGE-TQTVD-LIEN--------
GRNIP---
------------------------------VTEENKH-EYVRL-VTEHRLTGA-VQEQLE
-NFLKG---FHDIVPA--ELVS-IFSEQ-ELELLISG-----------------------
----LPDIN-----------IDDW----KNNTEYHN-YTAA-------------------
------------------------SPQI-QWFWRAVR--------------------SFD
KEERAKLLQF-------VTGTSKVP------------LNG-----FK-ELEGMN--GFSR
FNIHRDYGSK------------------------DRLPSSHTCF-NQ-LDLPEY-ESYES
LRQQLY-TAMTAGSEYF
>Biatriospora_mackinnonii_JGVQ01000269.1 .
LAVRRDQV-FLDSFKSL--------------YFKTADEMKYGKLSIRFHG-------EEG
VDAGGVTREW---FQVI----ARQMFNPDY--------ALFVPVASDRT-----------
----------------TFHPNRLS------------------------------------
--AINPEHLMFFKFIGRIIGKALYEGRVLDCHFSR--AVYKRILG---------------
--------RPVNLKDMET----------------------LDLEYYKS--------LQWM
LEN--DIT-DII--
-TETFSVDVEA---------------------------FGE-TQTVD-LIEN--------
GQNVP---
------------------------------VTEENKH-EYVRL-VTEHRLTGA-VHEQLE
-HFLKG---FHDIVPA--ELVS-IFSEQ-ELELLISG-----------------------
----LPDIN-----------VDDW----KNNTEYHN-YTAA-------------------
------------------------SPQI-QWFWRAVR--------------------SFD
KEERAKLLQF-------VTGTSKVP------------LNG-----FK-ELEGMN--GFSR
FNIHRDYGSK------------------------DRLPSSHTCF-NX-LDLPEY-ETYED
LRKQLY-TAMTAGSEYF
>Helminthosporium_solani_AWWW01000776.1 .
LAIRRDQV-FMDSFKSL--------------YFKTADEMKYGKLSIRFHG-------EEG
VDAGGVTREW---FQVI----ARQMFDPNY--------ALFIPVASDRT-----------
----------------TFHPNRLS------------------------------------
--GVNQEHLMFFKFIGRIIGKALYEGRVLDCHFSR--AVYKRILG---------------
--------RPVNLKDMET----------------------LDLEYYKS--------LLWM
LEN--DIT-DII--
-TETFSTDVEA---------------------------FGE-TEVVD-LIEN--------
GRNIP---
------------------------------VTEDNKH-EYVRL-VTEHRLTGA-VQEQLE

-HFLRG---FHDIIPA--ELIS-IFSEQ-ELELLISG-----------------------
----LPDIN-----------IEDW----KANTDYHN-YTQN-------------------
------------------------SPQI-HWFWRAVR--------------------SFD
KEEQAKLLQF-------VTGTSKVP------------LNG-----FK-ELEGMN--GFSK
FNIHRDYGSK------------------------DRLPSSHTCF-NX-LDLPEY-EDYDA
LKKALY-TAMTAGGEYF
>Paraphaeosphaeria_sporulosa_XM_018184806.1 .
LAVRRDQV-FLDSFKSL--------------YFKTADEMKYGKLSIRFHG-------EEG
VDAGGVTREW---FQVI----ARQMFNPDY--------ALFIPVASDRT-----------
----------------TFHPNRLS------------------------------------
--GINPEHLMFFKFIGRVIGKALYEGRVLDCHFSR--AVYKRILS---------------
--------RPVNVKDMES----------------------LDEEYYKS--------LLWI
LNN--DPT-DVI--
-FETFSVETER---------------------------FGE-TEIVD-LMEN--------
GRNIP---
------------------------------LSEENKH-DYVRL-VTEYRLTGA-VQEQLE
-HFLKG---FHDIIPS--DLVS-IFSEQ-ELELLISG-----------------------
----LPDIN-----------IEDW----KANTEYSN-YTQN-------------------
------------------------SPQI-HWFWRAVR--------------------SFD
KEEQAKLLQF-------VTGTSKVP------------LNG-----FK-ELEGMN--GFSK
FNIHRDYGSK------------------------DRLPSSHTCF-NQ-LDLPEY-DDYES
LKKALY-TAMTAGGEYF
>Umbilicaria_muehlenbergii_JFDN01000110.1 .
LSVRRDQV-FLDSFKSL--------------YFKSPDEMKYGKLSIRFHG-------EEG
VDAGGVTREW---FQVL----SRQMFNPDY--------ALFVPVAADRT-----------
----------------TFHPNKLS------------------------------------
--TINPEHLYFFKFIGRVIGKALYEGRALDCHFSR--AVYKRILG---------------
--------KPVSIKDMET----------------------LDLDYYKS--------LLWM
LEN--DIT-DII--
-TENFCDETEA---------------------------FGD-TQIVD-LVEN--------
GRNIP---
------------------------------VTEENKQ-EYVQL-LVEYRLTGS-VQAQLE
-EFLKG---FHDIVPA--ELIS-IFNEQ-ELELLISG-----------------------
----LPDID-----------VDDW----KNNTEYHN-YSAS-------------------
------------------------SPQI-QWFWRAVR--------------------SFD
KEERAKLLQF-------VTGTSKVP------------LNG-----FK-ELEGMN--GFSR
FNIHRDYGNK------------------------DRLPSSHTCF-NX-LDLPEY-ESYES
LRQQVY-TAMTAGSEYF
>Umbilicaria_pustulata_JYIL01001429.1 .
LSVRRDQV-FLDSFKSL--------------YFKSPDEMKYGKLSIRFHG-------EEG
VDAGGVTREW---FQVL----SRQMFNPDY--------ALFVPVAADRT-----------
----------------TFHPNKLS------------------------------------
-TINSEHHLYFFKFIGRVIGKALYEGRALDCHFSR--AVYKRILG---------------
--------KPVSIKDMET----------------------LDLDYYKS--------LLWM
LEN--DIT-DII--
-TENFCDETEA---------------------------FGD-TQIVD-LIEN--------
GRNIP---
------------------------------VTEENKQ-EYVQL-LVEYRLTGS-VQAQLE
-EFLKG---FHDIVPA--ELIS-IFNEQ-ELELLISG-----------------------
----LPDID-----------FDDW----KNNTEYHN-YSAS-------------------
------------------------SPQI-QWFWRAVR--------------------SFD
KEERAKLLQF-------VTGTSKVP------------LNG-----FK-ELEGMN--GFSR
FNIHRDYGNK------------------------DRLPSSHTCF-NX-LDLPEY-ESYES
LRQQVY-TAMTAGSEYF
>Gyalolechia_flavorubescens_AUPK01000014.1 .
LNVRRDQV-FLDSYKFM--------------HFKSGDEIKYGKLSIRFHG-------EEG
VDAGGVSREW---FQVL----SRQMFNPDY--------ALFVPVASDRT-----------
----------------TFHPNKLS------------------------------------

```
--KVNEEHLDFFKFIGRIIGKALYEGRALDCHFSR--AVYKRILG---------------
--------NSVSIKDMET----------------------LDLDYYKS--------LLWM
LEN-------------------------------------------------DIT-EII--
-TETFSIETDD---------------------------FGV-TEIVD-LIEN--------
GRNIP-------------------------------------------------------
------------------------------VTEENKH-EYVQR-VVEYRLTGS-VQSQLE
-KFLGG---FHDIVSP--ELIA-IFNEQ-ELELLISG-----------------------
----LPDID-----------VDDW----KNNSEYHN-YSAS-------------------
------------------------SSQI-QWFWRAVR--------------------SFD
KEERAKLLQF-------VTGTSKVP------------LNG-----FS-QLEGMN--GVSR
FNIHRDYGNK------------------------DRLPSSHTCF-NX-LDLPEY-ESYEQ
LRQQVY-TAMTAGNQYF
>Xylona_heveae_XM_018335429.1 .
LQVRRDQV-FLDSFKSL--------------YFKTGEEMKYGKLNIRFHR-------EEG
VDAGGVTREW---FQVL----SRQMFNPDY--------ALFTPVAADRT-----------
----------------TFHPNRLS------------------------------------
--SVNQEHLMFFKFIGRIIGKALYEGRVLDCHFSR--AVYKRILG---------------
--------KPVSIKDMET----------------------LDLDYYKS--------LVWM
LEN-------------------------------------------------DIT-DII--
-TETFSVETDA---------------------------FGE-SQTID-LIEN--------
GRNIP-------------------------------------------------------
------------------------------VTEENKH-EYVRL-VVEYRMTGS-VREQLE
-HFLKG---FHDIVPA--ELIS-IFNEQ-ELELLISG-----------------------
----LPDID-----------VDDW----KNNTEYHN-YSAS-------------------
------------------------SPQI-QWFWRAVR--------------------SFD
KEERAKLLQF-------VTGTSKVP------------LNG-----FK-ELEGMN--GFSR
FNIHRDYGSK------------------------DRLPSSHTCF-NQ-LDLPEY-ESYEA
LRKQLY-KAMTVGSSYF
>Symbiotaphrina_buchneri_BCIG01000021.1 .
LQVRRDQV-FLDSFKSL--------------YFKTGDEMKYGKLNIRFHG-------EEG
VDAGGVTREW---FQVL----SRQMFNPDY--------ALFIPVAADRT-----------
----------------TFHPNRLS------------------------------------
--SVNQEHLLFFKFIGRVIGKALYEGRVLDCHFSR--AVYKRILG---------------
--------KPVSIKDMET----------------------LDLDYYKS--------LIWM
LEN-------------------------------------------------DIT-DII--
-TETFSVETEA---------------------------FGE-IQIID-LIEN--------
GRNIP-------------------------------------------------------
------------------------------VTEENKH-EYVRL-VVEYRLTGS-VQEQLE
-NFLRG---FHDIIPA--ELIA-IFNEQ-ELELLISG-----------------------
----LPDID-----------VDDW----KNNTEYHN-YSAS-------------------
------------------------SPQI-QWFWRAVR--------------------SFD
KEERAKLLQF-------VTGTSKVP------------LNG-----FK-ELEGMH--GFSR
FNIHRDYGNK------------------------DRLPSSHTCF-NX-LDLPEY-ESYEV
LRERLY-TAITAGSEYF
>Coniosporium_apollinis_XM_007781852.1 .
LAVRRDQV-FLDSFKSL--------------HFRSGDEMKFGKLSIRFQG-------EEG
VDAGGVTREW---FQVL----SRQMFNPDY--------ALFIPVASDRT-----------
----------------TFHPNRLS------------------------------------
--SVNQEHLMFFKFIGRIIGKALYENRVLDCHFSR--AVYKCILG---------------
--------KPVSIKDMET----------------------LDLEYYKS--------LLWI
LEN-------------------------------------------------DIT-DII--
-TETFSVETDD---------------------------FGE-KQIID-LVEN--------
GRNIP-------------------------------------------------------
------------------------------VTDENKH-EYVRL-VVEYKLTGS-VKDQLE
-NFLRG---FHDIVPA--ELVS-IFDEQ-ELELLISG-----------------------
----LPEID-----------IDDW----KNNTEYHN-YTAA-------------------
------------------------SPQI-QWFWRAVR--------------------SFD
QEEQAKLLQF-------VTGTSKVP------------LNG-----FK-ELEGMN--GFSR
```

FNIHRDYGNK------------------------DRLPSSHTCF-NQ-LDLPEY-ESYEQ
LRQQVY-TAMTAGSEYF
>Phyllosticta_capitalensis_LOEO01000812.1 .
LSVRRDQV-FLDSFKSL--------------YFKTGDEMKYGKLSIRFHG-------EEG
VDAGGVTREW---FQVL----SRQMFNPDY--------ALFIPVASDRT-----------
----------------TFHPNRLS------------------------------------
--SINAEHLLFFKFIGRIIGKALYEGRVLDCHFSR--AVYKRILG---------------
--------RPVSIKDMET----------------------LDLDYYKS--------LLWM
LEN--DIT-DVI--
-TETFSVDVEE---------------------------FGV-TRTVD-LIEN--------
GANIP---
------------------------------VTEENKH-EYVRL-MVEHKLTGS-VEEQLN
-EFLKGKS-FLSIVPP--ELIS-IFNEQ-ELELLISG-----------------------
----LPEID-----------VDDW----KNNTEYHN-YTPA-------------------
------------------------SPQI-QWFWRAVR--------------------SFD
KEERAKLLQF-------VTGTSKVP------------LNG-----FK-ELEGMN--GFSK
FNIHRDFGSK------------------------DRLPSSHTCF-NQ-LDLPEY-ESYEQ
LRQQVY-TAMTAGSEYF
>Phyllosticta_citricarpa_AOTE01001096.1 .
LSVRRDQV-FLDSFKSL--------------YFKSGDEMKYGKLSIRFHG-------EEG
VDAGGVTREW---FQVL----SRQMFNPDY--------ALFIPVASDRT-----------
----------------TFHPNRLS------------------------------------
--SINQEHLLFFKFIGRIIGKALYEGRVLDCHFSR--AVYKRILG---------------
--------RPVSIKDMET----------------------LDLDYYKS--------LLWM
LEN--DIT-DVI--
-TETFSVDVEE---------------------------FGT-TRTVD-LIEN--------
GRNIP---
------------------------------VTEENKH-EYVRL-MVEYRLTGS-VEEQLR
-EFLKX---FHDIVPP--ELIS-IFNEQ-ELELLISG-----------------------
----LPEID-----------VDDW----KNNTEYHN-YTPA-------------------
------------------------SPQI-QWFWRAVR--------------------SFD
KEERAKLLQF-------VTGTSKVP------------LNG-----FK-ELEGMN--GFSK
FNIHRDFGSK------------------------DRLPSSHTCF-NQ-LDLPEY-ESYEQ
LRQQVY-TAMTAGS---
>Diplodia_corticola_XM_020274308.1 .
LSVRREHV-FLDSFKSL--------------YFKSGDEMKYGKLSIRFHG-------EEG
VDAGGVTREW---FQVL----ARQMFNPDY--------ALFIPVASDRT-----------
----------------TFHPNRLS------------------------------------
--SINQEHLLFFKFIGRIIGKALYEGRVLDCHFSR--AVYKRILG---------------
--------KPVSIKDMET----------------------LDLDYYKS--------LLWM
LEN--DIT-DII--
-TETFSVEAEE---------------------------FGV-TQTID-LIPN--------
GRNIP---
------------------------------VTEENKQ-EYVRL-MVEYRLTGS-VQDQLT
-EFLKG---FHDIVPA--ELIS-IFNEQ-ELELLISG-----------------------
----LPEID-----------VDDW----KNNAEYHN-YTAA-------------------
------------------------SPQI-QWFWRAVR--------------------AFD
KEERAKLLQF-------VTGTSKVP------------LNG-----FK-ELEGMN--GFSR
FNIHRDYGSK------------------------DRLPSSHTCF-NQ-LDLPEY-ETYEA
LRQQLH-TAITAGSEYF
>Diplodia_sapinea_JHUM01000878.1 .
LAVRREHV-FLDSFKSL--------------YFKTGDEMKYGKLSIRFHG-------EEG
VDAGGVTREW---FQVL----ARQMFNPDY--------ALFIPVASDRT-----------
----------------TFHPNRLS------------------------------------
--AINQEHLLFFKFIGRIIGKALYEGRVLDCHFSR--AVYKRILG---------------
--------KPVSIKDMET----------------------LDLDYYKS--------LLWM
LEN--DIT-DII--
-TETFSVEAEE---------------------------FGV-TQTID-LIPN--------

GRNIP---
------------------------------VTEENKQ-EYVRL-MVEYKLTGS-VQDQLT
-EFLKG---FHDIVPA--ELIS-IFNEQ-ELELLISG-----------------------
----LPEID-----------VDDW----KNNAEYHN-YTAA--------------------
------------------------SPQI-QWFWRAVR---------------------AFD
KEERAKLLQF-------VTGTSKVP------------LNG-----FK-ELEGMN--GFSR
FNIHRDYGSK------------------------DRLPSSHTCF-NQ-LDLPEY-ETYEA
LRQQLH-TAITAGSEYF
>Macrophomina_phaseolina_LFIX01001429.1 .
LSVRREHV-FLDSFKSL--------------YFKTGDEMKYGKLSIRFHG-------EEG
VDAGGVTREW---FQVL----ARQMFNPDY--------ALFIPVASDRT-----------
----------------TFHPNRLS------------------------------------
--AINQEHLLFFKFIGRIIGKALYEGRVLDCHFSR--AVYKRILG---------------
--------KPVSIKDMET----------------------LDLDYYKS--------LLWM
LEN--DIT-DII--
-TETFSVESEE---------------------------FGV-TQTID-LIPN--------
GRNIP---
------------------------------VTDDNKQ-EYVRL-MVEYKLTGS-VQDQLT
-EFLKG---FHDIVPA--ELIS-IFNEQ-ELELLISG-----------------------
----LPEID-----------VDDW----KNNTEYHN-YTAA--------------------
------------------------SPQI-QWFWRAVR---------------------SFD
KEERAKLLQF-------VTGTSKVP------------LNG-----FK-ELEGMN--GFSR
FNIHRDYGSK------------------------DRLPSSHTCF-NQ-LDLPEY-ESYEA
LRQQLY-TAITAGSEY-
>Botryosphaeria_dothidea_MDSR01000023.1 .
LSVRREHV-FLDSFKSL--------------YFKTGDEMKYGKLSIRFHG-------EEG
VDAGGVTREW---FQVL----ARQMFNPDY--------ALFIPVASDRT-----------
----------------TFHPNRLS------------------------------------
--AINQEHLLFFKFIGRIIGKALYEGRVLDCHFSR--AVYKRILG---------------
--------KPVSIKDMET----------------------LDLDYYKS--------LLWM
LEN--DIT-DII--
-TETLSVEAEE---------------------------FGV-TQTID-LIPN--------
GRNIP---
------------------------------VTEENKQ-EYVRL-MVEHRLTGS-VQDQLS
-EFLKG---FHDIVPA--ELIS-IFNEQ-ELELLISG-----------------------
----LPEID-----------VDDW----KNNTEYHN-YTAA--------------------
------------------------SPQI-QWFWRAVR---------------------SFD
KEERAKLLQF-------VTGTSKVP------------LNG-----FK-ELEGMN--GFSR
FNIHRDYGSK------------------------DRLPSSHTCF-NQ-LDLPEY-ESYEA
LRQQLY-TAITAGSEYF
>Lasiodiplodia_theobromae_MDYX01000009.1 .
LSVRREHV-FLDSFKSL--------------YFKTGDEMKYGKLSIRFHG-------EEG
VDAGGVTREW---FQVL----ARQMFNPDY--------ALFIPVASDRT-----------
----------------TFHPNRLS------------------------------------
--AINQEHLLFFKFIGRIIGKALYEGRVLDCHFSR--AVYKRILG---------------
--------KPVSIKDMET----------------------LDLDYYKS--------LLWM
LEN--DIT-DII--
-TETFSVEAEE---------------------------FGV-TQTID-LIPN--------
GRNIP---
------------------------------VTEDNKQ-EYVRL-MVEYRLTGS-VQDQLS
-EFLKG---FHDIVPA--ELIS-IFNEQ-ELELLISG-----------------------
----LPEID-----------VDDW----KNNTEYHN-YTAA--------------------
------------------------SPQI-QWFWRAVR---------------------SFD
KEERAKLLQF-------VTGTSKVP------------LNG-----FK-ELEGMN--GFSR
FNIHRDYGSK------------------------DRLPSSHTCF-NQ-LDLPEY-ESYEA
LRQQLY-TAMTAGSEYF
>Neofusicoccum_parvum_AORE01001876.1 .
LSVRREHV-FLDSFKSL--------------YFKTGDEMKYGKLSIRFHG-------EEG

VDAGGVTREW---FQVL----ARQMFNPDY--------ALFIPVASDRT-----------
----------------TFHPNRLS------------------------------------
--AINQEHLLFFKFIGRIIGKALYEGRVLDCHFSR--AVYKRILG---------------
--------KPVSIKDMET----------------------LDLDYYKS--------ILWM
LEN--DIT-DII--
-TETFSVEAEE---------------------------FGV-TQTID-LIPN--------
GRNIP---
------------------------------VTEDNKQ-EYVRL-MVEYRLTGS-VQDQLS
-EFLKG---FHDIVPA--ELIS-IFNEQ-ELELLISG-----------------------
----LPEID-----------VDDW----KNNTEYHN-YTAA-------------------
------------------------SPQI-QWFWRAVR--------------------SFD
KEERAKLLQF-------VTGTSKVP------------LNG-----FK-ELEGMN--GFSR
FNIHRDYGSK------------------------DRLPSSHTCF-NQ-LDLPEY-ESYEA
LRQQLY-TAMTAGSEYF
>Phaeomoniella_chlamydospora_JACF01000296. 1..
LSVRRDQV-FLDSFKSL--------------YFKSAEEMKYGKLSIRFHG-------EEG
VDAGGVTREW---FQVL----ARGMFNPDY--------ALFIPVASDRT-----------
----------------TFHPNRLS------------------------------------
--GVNNEHLMFFKFIGRIIGKALYEGRVLDCHFSR--AVYKCILG---------------
--------KSVSIKDMET----------------------LDLDYYKS--------LVWM
LEN--DIT-DII--
-TETFSVETDD---------------------------FGE-AKTID-LIEN--------
GHNIP---
------------------------------VTEENKQ-EYVQL-VVEYRLTGS-VNEQLE
-HFLKG---FHDIIPA--ELIS-IFNEQ-ELELLISG-----------------------
----LPDID-----------VDDW----RANTEYHN-YSAS-------------------
------------------------SQQI-QWFWRAVR--------------------SFD
KEEQAKLLQF-------VTGTSKVP------------LNG-----FK-ELEGMN--GFSR
FNIHKDYGNK------------------------DRLPSSHTCF-NX-LDLPEY-DSYDT
LRQRLH-MAMTTGSEYF
>Endocarpon_pusillum_XM_007801947.1 .
LHVRRDQC-FLDSFKAL--------------YFNSADEMKYGKLSIRFNG-------EEG
VDAGGVTREW---FQVL----ARGMFNPNY--------ALFIPVASDRT-----------
----------------TFHPNRLS------------------------------------
--GVNAEHLLFFKFIGRIIGKALYESRVLDCHFSR--AVYKKILG---------------
--------KTVSIKDMET----------------------LDLDYYKS--------LVWM
LEN--DIT-DII--
-TETFSIESDD---------------------------FGE-SQIID-LIED--------
GRNIP---
------------------------------VTEENKQ-EYVQL-LVEYRLTGS-VQEQLE
-NFLKG---FHDIIPA--ELVS-IFTEQ-ELELLISG-----------------------
----LPDID-----------VDDW----KANTEYHN-YSAS-------------------
------------------------SPQI-QWFWRAVR--------------------SFD
KEEQAKLLQF-------VTGTSKVP------------LNG-----FK-ELEGMN--GFSR
FNIHKDYGNK------------------------DRLPSSHTCF-NQ-LDLPEY-ENYET
LRQRLL-TAMLQGSEYF
>Ajellomyces_dermatitidis_XM_002626298.1 .
LSVRRDQV-FLDSFKSL--------------YFKTANEMKYGKLNIRFHG-------EEG
VDAGGVTREW---FQVL----ARGMFNPNY--------ALFIPVASDRT-----------
----------------TFHPNRLS------------------------------------
--GVNQEHLMFFKFIGRIIGKALYEGRVLDCHFSR--AVYKRILG---------------
--------KSVSIKDMET----------------------LDLDYYKS--------LLWM
LEN--DIT-DIL--
-TENFSVESDD---------------------------FGE-KQTID-LVEN--------
GRNIP---
------------------------------VTQENKE-EYVQR-VVEYRLVGS-VKDQLD
-NFLKG---FHDIIPA--DLIA-IFNEQ-ELELLISG-----------------------
----LPEID-----------VDDW----KNNSEYHN-YSAS-------------------

------------------------SPQI-QWFWRAVR--------------------SFD
KEERAKLLQF-------VTGTSKVP------------LNG-----FK-ELEGMN--GFSK
FNIHRDYGNK------------------------DRLPSSHTCF-NQ-LDLPEY-DSYET
LRQRLY-TAMTAGSEYF
>Emergomyces_orientalis_MOWL01000068.1 .
LSVRRDQV-FLDSFKSL--------------YFKTANEMKYGKLNIRFHG-------EEG
VDAGGVTREW---FQVL----ARGMFNPNY--------ALFIPVASDRT-----------
----------------TFHPNRLS------------------------------------
--GVNQEHLMFFKFIGRIIGKALYEGRVLDCHFSR--AVYKRILG---------------
--------KSVSIKDMET----------------------LDLDYYKS--------LLWM
LEN--DIT-DIL--
-TENFSVESDD---------------------------FGE-KQTID-LVEN--------
GRNIP---
------------------------------VTQENKE-EYVQR-VVEYRLVGS-VKDQLD
-NFLKG---FHDIIPA--DLIA-IFNEQ-ELELLISG-----------------------
----LPEID-----------VDDW----KNNSEYHN-YSAS-------------------
------------------------SPQI-QWFWRAVR--------------------SFD
KEERAKLLQF-------VTGTSKVP------------LNG-----FK-ELEGMN--GFSK
FNIHRDYGNK------------------------DRLPSSHTCF-NX-LDLPEY-DSYET
LRQRLY-TAMTAGSEYF
>Blastomyces_dermatitidis_ACBT01000467.1 .
LSVRRDQV-FLDSFKSL--------------YFKTANEMKYGKLNIRFHG-------EEG
VDAGGVTREW---FQVL----ARGMFNPNY--------ALFIPVASDRT-----------
----------------TFHPNRLS------------------------------------
--GVNQEHLMFFKFIGRIIGKALYEGRVLDCHFSR--AVYKRILG---------------
--------KSVSIKDMET----------------------LDLDYYKS--------LLWM
LEN--DIT-DIL--
-TENFSVESDD---------------------------FGE-KQTID-LVEN--------
GRNIP---
------------------------------VTQENKE-EYVQR-VVEYRLVGS-VKDQLD
-NFLKG---FHDIIPA--DLIA-IFNEQ-ELELLISG-----------------------
----LPEID-----------VDDW----KNNSEYHN-YSAS-------------------
------------------------SPQI-QWFWRAVR--------------------SFD
KEERAKLLQF-------VTGTSKVP------------LNG-----FK-ELEGMN--GFSK
FNIHRDYGNK------------------------DRLPSSHTCF-NX-LDLPEY-DSYET
LRQRLY-TAMTAGSEYF
>Blastomyces_percursus_LGTZ01000977.1 .
LSVRRDQV-FLDSFKSL--------------YFKTANEMKYGKLNIRFHG-------EEG
VDAGGVTREW---FQVL----ARGMFNPNY--------ALFIPVASDRT-----------
----------------TFHPNRLS------------------------------------
--GVNQEHLMFFKFIGRIIGKALYEGRVLDCHFSR--AVYKRILG---------------
--------KSVSIKDMET----------------------LDLDYYKS--------LLWM
LEN--DIT-DIL--
-TENFSVESDD---------------------------FGE-KQTID-LVEN--------
GRNIP---
------------------------------VTQENKE-EYVQR-VVEYRLVGS-VKDQLD
-NFLRG---FHDIIPA--DLIA-IFNEQ-ELELLISG-----------------------
----LPEID-----------VDDW----KNNSEYHN-YSAS-------------------
------------------------SPQI-QWFWRAVR--------------------SFD
KEERAKLLQF-------VTGTSKVP------------LNG-----FK-ELEGMN--GFSK
FNIHRDYGNK------------------------DRLPSSHTCF-NX-LDLPEY-DSYET
LRQRLY-TAMTAGSEYF
>Emergomyces_pasteuriana_LGRN01000071.1 .
LSVRRDQV-FLDSFKSL--------------YFKTANEMKYGKLNIRFHG-------EEG
VDAGGVTREW---FQVL----ARGMFNPNY--------ALFIPVASDRT-----------
----------------TFHPNRLS------------------------------------
--GVNQEHLMFFKFIGRIIGKALYEGRVLDCHFSR--AVYKRILG---------------
--------KSVSIKDMET----------------------LDLDYYKS--------LLWM

LEN--DIT-DIL--
-TENFSVESDD---------------------------FGE-KQIID-LVEN--------
GRNIP---
------------------------------VTQENKE-EYVQR-VVEYRLVGS-VKDQLD
-NFLKG---FHDIIPA--DLIS-IFNEQ-ELELLISG-----------------------
----LPEID-----------VDDW----KNNSEYHN-YSAS-------------------
------------------------SPQI-QWFWRAVR--------------------SFD
KEERAKLLQF-------VTGTSKVP------------LNG-----FK-ELEGMN--GFSK
FNIHRDYGNK------------------------DRLPSSHTCF-NX-LDLPEY-ESYET
LRQRLY-TAMTAGSEYF
>Paracoccidioides_lutzii_XM_002795110.2 .
LSVRREQV-FLDSFKSL--------------YFKSADEMKYGKLNIRFHG-------EEG
VDAGGVTREW---FQVL----ARGMFNPNY--------ALFIPVASDRT-----------
----------------TFHPNRLS------------------------------------
--GVNQEHLMFFKFIGRIIGKALYEGRVLDCHFSR--AVYKRILG---------------
--------KSVSIKDMET----------------------LDLDYYKS--------LLWM
LEN--DIT-DIL--
-TENFSVESDD---------------------------FGE-KQIID-LVDN--------
GRNIP---
------------------------------VTQENKE-EYVQR-VVEYRLVGS-VKDQLD
-NFLKG---FHDIIPA--DLIA-IFNEQ-ELELLISG-----------------------
----LPEID-----------VDDW----KNNSEYHN-YSAS-------------------
------------------------SPQI-QWFWRAVR--------------------SFD
KEERAKLLQF-------VTGTSKVP------------LNG-----FK-ELEGMN--GFSK
FNIHRDYGHK------------------------DRLPSSHTCF-NQ-LDLPEY-DNYET
LRQRLY-TAMTAGSEYF
>Byssoonygena_ceratinophila_GDRB01010003.1 .
LSVRRDQV-FLDSFKSL--------------YFKSAEEMKYGKLSVRFHG-------EEG
VDAGGVTREW---FQVL----ARGMFNPNY--------ALFIPVASDRT-----------
----------------TFHPNRLS------------------------------------
--GVNQEHLMFFKFIGRIIGKALYEGRVLDCHFSR--AVYKRILG---------------
--------KSVSIKDMET----------------------LDLDYYKS--------LLWM
LEN--DIT-DIL--
-TENFSVEVED---------------------------FGE-KQVID-LVEN--------
GRNIP---
------------------------------VTQENKE-EYVQL-VVEHRLVGS-VKEQLD
-NFLKG---FHDIIPA--DLIA-IFNEQ-ELELLISG-----------------------
----LPEID-----------VDDW----KNNTDYHN-YSAS-------------------
------------------------SPQI-QWFWRAVR--------------------SFD
KEERAKLLQF-------VTGTSKVP------------LNG-----FR-ELEGMN--GFSK
FNIHRDYGNK------------------------DRLPSSHTCF-NX-LDLPEY-DSYET
LRQRLY-IAITAGSEYF
>Amauroascus_niger_GDRA01002723.1 .
LSVRRDQV-FLDSFKSL--------------YFKSADEMKYGKLNIRFHG-------EEG
VDAGGVTREW---FQVL----ARGMFNPNY--------ALFIPVASDRT-----------
----------------TFHPNRLS------------------------------------
--GVNQEHLMFFKFIGRIIGKALYEGRVLDCHFSR--AVYKRILS---------------
--------KTVSIKDMET----------------------LDLDYYKS--------LLWM
LEN--DIT-DIL--
-TENFSVEVEA---------------------------FGE-KQVID-LIEN--------
GRNIP---
------------------------------VTQENKE-EYVQL-VVEHRLVGS-VKEQLD
-NFLRG---FHDIIPA--DLIS-IFNEQ-ELELLISG-----------------------
----LPEID-----------VDDW----KNNTDYHN-YSAS-------------------
------------------------SPQI-QWFWRAVR--------------------SFD
KEERAKLLQF-------VTGTSKVP------------LNG-----FR-ELEGMN--GFSK
FNIHRDYGNK------------------------DRLPSSHTCF-NX-LDLPEY-DSYET
LRQRVY-TAITAGSEYF

>Uncinocarpus_reesii_XM_002584380.1 .
LSVRRDQV-FLDSFKSL--------------YFKSADEMKYGKLNIRFHG-------EEG
VDAGGVTREW---FQVL----ARGMFNPNY--------ALFIPVASDRT-----------
----------------TFHPNRLS------------------------------------
--GVNQEHLMFFKFVGRIIGKALYEGRVLDCHFSR--AVYKRILG---------------
--------KTVSIKDMET----------------------LDLDYYKS--------LLWM
LEN--DIT-DIL--
-TENFSVEVED---------------------------FGE-KQVID-LIEN--------
GRNIP---
------------------------------VTQENKE-EYVQL-VVEHRLVGS-VKEQLD
-NFLKG---FHDIIPA--DLIS-IFNEQ-ELELLISG-----------------------
----LPEID-----------VDDW----KNNTEYQN-YSAS-------------------
------------------------SPQI-QWFWRAVR--------------------SFD
KEERAKLLQF-------VTGTSKVP------------LNG-----FR-ELEGMN--GFSK
FNIHRDYGNK------------------------DRLPSSHTCF-NQ-LDLPEY-DSYET
LRQRLY-VAMTAGSEYF
>Amauroascus_mutatus_GDQZ01007544.1 .
LSVRRDQV-FLDSFKSL--------------YFKTADEMKYGKLNIRFHG-------EEG
VDAGGVTREW---FQVL----ARGMFNPNY--------ALFIPVASDRT-----------
----------------TFHPNRLS------------------------------------
--GVNQEHLMFFKFIGRIIGKALYEGRVLDCHFSR--AVYKRILG---------------
--------KSVSIKDMET----------------------LDLDYYKS--------LLWM
LEN--DIT-DIL--
-TENFSVEVED---------------------------FGE-KQVID-LIEN--------
GRNIP---
------------------------------VTQENKE-EYVQL-VVEHRLVGS-VKEQLD
-NFLKG---FHDIIPA--DLIS-IFNEQ-ELELLISG-----------------------
----LPEID-----------VDDW----KNNTDYHN-YSAS-------------------
------------------------SPQI-QWFWRAVR--------------------SFD
KEERAKLLQF-------VTGTSKVP------------LNG-----FR-ELEGMN--GFSK
FNIHRDYGNK------------------------DRLPSSHTCF-NQ-LDLPEY-ESYET
LRQRLY-TAMTAGSEYF
>Chrysosporium_queenslandicum_GDRC01000238.1 .
LSVRRDQV-FLDSFKSL--------------YFKTADEMKYGKLNVRFHG-------EEG
VDAGGVTREW---FQVL----ARGMFNPNY--------ALFIPVASDRT-----------
----------------TFHPNRLS------------------------------------
--GVNQEHLMFFKFIGRIIGKALYEGRVLDCHFSR--AVYKRILG---------------
--------KSVSIKDMET----------------------LDLDYYKS--------LLWM
LEN--DIT-DIL--
-TENFSVEVEA---------------------------FGE-KQVID-LVEN--------
GRNIP---
------------------------------VTQENKE-EYVQL-VVEHRLVGS-VKEQLD
-NFLKG---FHDIIPA--DLIS-IFNEQ-ELELLISG-----------------------
----LPEID-----------VDDW----KNNTDYHN-YSAS-------------------
------------------------SPQI-QWFWRAVR--------------------SFD
KEERAKLLQF-------VTGTSKVP------------LNG-----FR-ELEGMN--GFSK
FNIHRDYGNK------------------------DRLPSSHTCF-NQ-LDLPEY-DSYET
LRQRLY-TAMTAGSEYF
>Coccidioides_posadasii_XM_003065690.1 .
LSVRRDQV-FLDSFKSL--------------YFKTADEMKYGKLSVRFHG-------EEG
VDAGGVTREW---FQVL----ARGMFNPNY--------ALFIPVASDRT-----------
----------------TFHPNRLS------------------------------------
--GVNQEHLMFFKFIGRIIGKAIYEGRVLDCHFSR--AVYKRILG---------------
--------KSVSIKDMET----------------------LDLDYYKS--------LLWM
LEN--DIT-DIL--
-TENFSVEVEA---------------------------FGE-KQVID-LVEN--------
GRNIP---
------------------------------VTQENKE-EYVQL-VVEHRLVGS-VKEQLD

```
-NFLKG---FHDIIPA--DLIS-IFNEQ-ELELLISG-----------------------
----LPEID-----------VDDW----KNNTDYHN-YSAS-------------------
------------------------SPQI-QWFWRAVR---------------------SFD
KEERAKLLQF-------VTGTSKVP------------LNG-----FR-ELEGMN--GFSK
FNIHRDYGNK------------------------DRLPSSHTCF-NQ-LDLPEY-DSYET
LRQRLY-TAMTAGSEYF
>Coccidioides_immitis_XM_001247708.2 .
LSVRRDQV-FLDSFKSL--------------YFKTADEMKYGKLSVRFHG-------EEG
VDAGGVTREW---FQVL----ARGMFNPNY--------ALFIPVASDRT-----------
----------------TFHPNRLS------------------------------------
--GVNQEHLMFFKFIGRIIGKAIYEGRVLDCHFSR--AVYKRILG---------------
--------KSVSIKDMET----------------------LDLDYYKS--------LLWM
LEN------------------------------------------------DIT-DIL--
-TENFSVEVEA---------------------------FGE-KQVID-LVEN--------
GRNIP-------------------------------------------------------
------------------------------VTQENKE-EYVQL-VVEHRLVGS-VKEQLD
-NFLKG---FHDIIPA--DLIS-IFNEQ-ELELLISG-----------------------
----LPEID-----------VDDW----KNNTDYHN-YSAS-------------------
------------------------SPQI-QWFWRAVR---------------------SFD
KEERAKLLQF-------VTGTSKVP------------LNG-----FR-ELEGMN--GFSK
FNIHRDYGNK------------------------DRLPSSHTCF-NQ-LDLPEY-ESYET
LRQRLY-TAMTAGSEYF
>Onygena_corvina_JWPT01000072.1 .
LSVRRDQV-FLDSFKSL--------------YFKTAEEMKYGKLSIRFHG-------EEG
VDAGGVSREW---FQVL----ARGMFNPDY--------ALFIPVASDRT-----------
----------------TFHPNRLS------------------------------------
--GVNQEHLMFFKFIGRIIGKALYEGRVLDCHFSR--AVYKRILG---------------
--------KSVSIKDMET----------------------LDLGYYRS--------LLWM
LAN------------------------------------------------DIT-DVL--
-TENFSVEVQD---------------------------FGE-SRIID-LVEN--------
GQNIP-------------------------------------------------------
------------------------------VTQENKE-EYVQR-LVEHRLTGS-VKEQLD
-HFLRG---FHDIIPA--DLIA-IFNEQ-ELELLISG-----------------------
----LPEID-----------VTDW----INNTEYQN-YSAS-------------------
------------------------SPQI-QWFWRAVR---------------------SFD
KEERAKLLQF-------VTGTSKVP------------LNG-----FR-ELEGMN--GFSK
FNIHRDYGNK------------------------NRLPSSHTCF-NX-LDLPEY-DSYET
LRKSLY-TAMTAGGEYF
>Arthroderma_otae_XM_002844412.1 .
LSVRRDQV-FLDSFKSL--------------YFKTADEMKYGKLSIRFHG-------EEG
VDAGGVSREW---FQVL----ARGMFNPDY--------ALFIPVASDRT-----------
----------------TFHPNRLS------------------------------------
--GVNQEHLMFFKFIGRIIGKALYEGRVLDCHFSR--AVYKRILG---------------
--------KSVSIKDMET----------------------LDLDYYKS--------LLWM
LEN------------------------------------------------DIT-DIL--
-TENFSVEVED---------------------------FGE-TRVID-LVEN--------
GRNIP-------------------------------------------------------
------------------------------VTQENKE-EYVQH-VVEHRLTGS-VKEQLD
-HFLRG---FHDIIPA--DLIS-IFNEQ-ELELLISG-----------------------
----LPEID-----------VNDW----KNNTEYHN-YSAS-------------------
------------------------SPQI-QWFWRAVR---------------------SFD
KEERAKLLQF-------VTGTSKVP------------LNG-----FR-ELEGMN--GFSK
FNIHRDYGSN------------------------DRLPSSHTCF-N--------------
-----------------
>Trichophyton_equinum_ABWI01000693.1 .
LSVRRDQV-FLDSFKSL--------------YFKTADEMKYGKLSIRFHG-------EEG
VDAGGVSREW---FQVL----ARGMFNPDY--------ALFIPVASDRT-----------
----------------TFHPNRLS------------------------------------
```

```
--GVNQEHLMFFKFIGRIIGKALYEGRVLDCHFSR--AVYKRILG---------------
--------KSVSIKDMET----------------------LDLDYYKS--------LLWM
LEN------------------------------------------------DIT-DIL--
-TENFSVEVED---------------------------FGE-TRVID-LVEN--------
GRNIP-------------------------------------------------------
------------------------------VTQENKE-EYVQH-VVEHRLTGS-VKEQLD
-HFLRG---FHDIIPA--DLIS-IFNEQ-ELELLISG-----------------------
----LPEID-----------VNDW----KNNTEYHN-YSAS-------------------
------------------------SPQI-QWFWRAVR--------------------SFD
KEERAKLLQF-------VTGTSKVP------------LNG-----FR-ELEGMN--GFSK
FNIHRDYGSK------------------------DRLPSSHTCF-NX-LDLPEY-DSYES
LRKCLY-TAMTAGSEYF
>Trichophyton_interdigitale_AOKS01000085.1 .
LSVRRDQV-FLDSFKSL--------------YFKTADEMKYGKLSIRFHG-------EEG
VDAGGVSREW---FQVL----ARGMFNPDY--------ALFIPVASDRT-----------
----------------TFHPNRLS------------------------------------
--GVNQEHLMFFKFIGRIIGKALYEGRVLDCHFSR--AVYKRILG---------------
--------KSVSIKDMET----------------------LDLDYYKS--------LLWM
LEN------------------------------------------------DIT-DIL--
-TENFSVEVED---------------------------FGE-TRVID-LVEN--------
GRNIP-------------------------------------------------------
------------------------------VTQENKE-EYVQH-VVEHRLTGS-VKEQLD
-HFLRG---FHDIIPA--DLIS-IFNEQ-ELELLISG-----------------------
----LPEID-----------VNDW----KNNTEYHN-YSAS-------------------
------------------------SPQI-QWFWRAVR--------------------SFD
KEERAKLLQF-------VTGTSKVP------------LNG-----FR-ELEGMN--GFSK
FNIHRDYGSK------------------------DRLPSSHTCF-NX-LDLPEY-DSYES
LRKCLY-TAMTAGSEYF
>Trichophyton_verrucosum_XM_003020858.1 .
LSVRRDQV-FLDSFKSL--------------YFKTADEMKYGKLSIRFHG-------EEG
VDAGGVSREW---FQVL----ARGMFNPDY--------ALFIPVASDRT-----------
----------------TFHPNRLS------------------------------------
--GVNQEHLMFFKFIGRIIGKALYEGRVLDCHFSR--AVYKRILG---------------
--------KSVSIKDMET----------------------LDLDYYKS--------LLWM
LEN------------------------------------------------DIT-DIL--
-TENFSVEVED---------------------------FGE-TRVID-LVEN--------
GRNIP-------------------------------------------------------
------------------------------VTQENKE-EYVQH-VVEHRLTGS-VKEQLD
-HFLRG---FHDIIPA--DLIS-IFNEQ-ELELLISG-----------------------
----LPEID-----------VNDW----KNNTEYHN-YSAS-------------------
------------------------SPQI-QWFWRAVR--------------------SFD
KEERAKLLQF-------VTGTSKVP------------LNG-----FR-ELEGMN--GFSK
FNIHRDYGSK------------------------DRLPSSHTCF-NQ-LDLPEY-DSYES
LRKCLY-TAMTAGSEYF
>Nannizzia_gypsea_XM_003170838.1 .
LSVRRDQV-FLDSFKSL--------------YFKTADEMKYGKLSIRFHG-------EEG
VDAGGVSREW---FQVL----ARGMFNPDY--------ALFIPVASDRT-----------
----------------TFHPNRLS------------------------------------
--GVNQEHLMFFKFIGRIIGKALYEGRVLDCHFSR--AVYKRILG---------------
--------KSVSIKDMET----------------------LDLDYYKS--------LLWM
LEN------------------------------------------------DIT-DIL--
-TENFSVEVED---------------------------FGE-TRVID-LVEN--------
GRNIP-------------------------------------------------------
------------------------------VTQENKE-EYVQH-VVEHRLTGS-VKEQLD
-HFLRG---FHDIIPA--DLIS-IFNEQ-ELELLISG-----------------------
----LPEID-----------VNDW----KNNSEYHN-YSAS-------------------
------------------------SPQI-QWFWRAVR--------------------SFD
KEERAKLLQF-------VTGTSKVP------------LNG-----FR-ELEGMN--GFSK
```

FNIHRDYGSK------------------------DRLPSSHTCF-NX-LDLPEY-DSYES
LRKCLY-TAMTAGSEYF
>Trichophyton_rubrum_XM_003232284.1 .
LSVRRDQV-FLDSFKSL--------------YFKTADEMKYGKLSIRFHG-------EEG
VDAGGVSREW---FQVL----ARGMFNPDY--------ALFIPVASDRT-----------
----------------TFHPNRLS------------------------------------
--GVNQEHLMFFKFIGRIIGKALYEGRVLDCHFSR--AVYKRILG---------------
--------KSVSIKDMET----------------------LDLDYYKS--------LLWM
LEN--DIT-DIL--
-TENFSVEVED---------------------------FGE-TRVID-LVEN--------
GRNIP---
------------------------------VTQENKE-EYVQH-VVEHRLTGS-VKEQLD
-HFLRG---FHDIIPA--DLIS-IFNEQ-ELELLISG-----------------------
----LPEID-----------VNDW----KNNTEYHN-YSAS-------------------
------------------------SPQI-QWFWRAVR--------------------SFD
KEERAKLLQF-------VTGTSKVP------------LNG-----FR-ELEGMN--GFSK
FNIHRDYGSK------------------------DRLPSSHTCF-NQ-LDLPEY-DSYES
LRKCLY-TAMTAGSEYF
>Emmonsia_parva_LDEV01000750.1 .
LSVRRDQV-FLDSFKSL--------------YFKTANEMKYGKLNIRFHG-------EEG
VDAGGVTREW---FQVL----ARGMFNPNY--------ALFIPVASDRT-----------
----------------TFHPNRLS------------------------------------
--GVNQEHLMFFKFIGRIIGKALYEGRVLDCHFSR--AVYKRILG---------------
--------KSVSIKDMET----------------------LDLEYYKS--------LVWM
LEN--DIT-DIM--
-TEDFSVESDD---------------------------FGE-KQTID-LVEN--------
GRNIP---
------------------------------VTQENKE-EYIQR-VVEYRLVSS-VKDQLD
-NFLKG---FHDIIPA--DLIA-IFNEQ-ELELLISG-----------------------
----LPEIN-----------IDDW----KANTEYIN-YSAS-------------------
------------------------SNQI-VWFWCAVE--------------------SFD
KEERAKLLQF-------VTGTSKVP------------LNG-----FK-ELEGMN--GFTK
FNIHRDFSDDQ-----------------------DRLPTSHTCF-NX-LDLPKY-KSYEI
LRQRLH-TAITAGNEYF
>Aspergillus_ruber_AWRT01000013.1 .
LSVRRDQV-FLDSFKSL--------------YFKTADELKYGKLNVRFIG-------EEG
VDAGGVTREW---FQVL----ARGMFNPNY--------ALFIPVAADST-----------
----------------TFHPNRLS------------------------------------
--GVNSEHLMFFKFIGRIIGKALYEGRVLDCHFSR--AVYKCILG---------------
--------RNVSIKDMET----------------------LDLDYYKS--------LLWM
LEN--DIT-DII--
-TETFSVETDD---------------------------FGE-KQVID-LIEN--------
GHNIP---
------------------------------VTQENKE-EYVQR-VVDYRLLRS-VNEQLD
-NFLKGM--FTFCYPP--ELIS-IFTEQ-ELELLISG-----------------------
----LPEIE-----------VDDW----KANTEYHN-YSAS-------------------
------------------------SPQI-QWFWRAVR--------------------SFD
KEERAKLLQF-------VTGTSKVP------------LNG-----FK-ELEGMN--GINR
FNIHRDYGDK------------------------ERLPSSHTCF-N--------------

>Aspergillus_glaucus_LSTL01000002.1 .
LSVRRDQV-FLDSFKSL--------------YFKTADELKYGKLNVRFIG-------EEG
VDAGGVTREW---FQVL----ARGMFNPNY--------ALFIPVAADST-----------
----------------TFHPNRLS------------------------------------
--GVNSEHLMFFKFIGRIIGKALYEGRVLDCHFSR--AVYKCILG---------------
--------RNVSIKDMET----------------------LDLDYYKS--------LLWM
LEN--DIT-DII--
-TETFAVETDD---------------------------FGE-KQVID-LIEN--------

GHNIP---
------------------------------VTQENKE-EYVQR-VVDYRLLRS-VNEQLD
-NFLKGM--FTFCYPP--DLIS-IFTEQ-ELELLISG-----------------------
----LPEIE-----------VDDW----KANTEYHN-YSAS-------------------
------------------------SPQI-QWFWRAVR---------------------SFD
KEERAKLLQF-------VTGTSKVP------------LNG-----FK-ELEGMN--GINR
FNIHRDYGDK------------------------ERLPSSHTCF-N--------------

>Aspergillus_cristatus_JXNT01000001.1 .
LSVRRDQV-FLDSFKSL--------------YFKTADELKYGKLNVRFIG-------EEG
VDAGGVTREW---FQVL----ARGMFNPNY--------ALFIPVAADST-----------
----------------TFHPNRLS------------------------------------
--GVNSEHLMFFKFIGRIIGKALYEGRVLDCHFSR--AVYKCILG---------------
--------RNVSIKDMET----------------------LDLDYYKS--------LLWM
LEN--DIT-DII--
-TETFSVETDD---------------------------FGE-KQVID-LIEN--------
GRNIP---
------------------------------VTQENKE-EYVQR-VVDYRLLRS-VNEQLD
-NFLKG---FHEIIPP--DLIS-IFTEQ-ELELLISG-----------------------
----LPEID-----------VDDW----KANTEYHN-YSAS-------------------
------------------------SAQI-QWFWRAVR---------------------SFD
KEERAKLLQF-------VTGTSKVP------------LNG-----FK-ELEGMN--GINR
FNIHRDYGDK------------------------ERLPSSHTCF-N--------------

>Aspergillus_chevalieri_BCIE01000001.1 .
LSVRRDQV-FLDSFKSL--------------YFKTADELKYGKLNVRFIG-------EEG
VDAGGVTREW---FQVL----ARGMFNPNY--------ALFIPVAADST-----------
----------------TFHPNRLS------------------------------------
--GVNSEHLMFFKFIGRIIGKALYEGRVLDCHFSR--AVYKCILG---------------
--------RSVSIKDMET----------------------LDLDYYKS--------LLWM
LEN--DIT-DII--
-TETFSVETDD---------------------------FGE-KQVID-LIEN--------
GRNIP---
------------------------------VTQENKE-EYVQR-VVDYRLLRS-VNEQLD
-NFLKG---FHEIIPP--DLIS-IFTEQ-ELELLISG-----------------------
----LPEID-----------VDDW----KANTEYHN-YSAS-------------------
------------------------SAQI-QWFWRAVR---------------------SFD
KEERAKLLQF-------VTGTSKVP------------LNG-----FK-ELEGMN--GINR
FNIHRDYGDK------------------------ERLPSSHTCF-N--------------

>Xeromyces_bisporus_CCCX01000492.1 .
LSVRRDQV-FLDSFKSL--------------YFKTADELKYGRLNVRFIG-------EEG
VDAGGVTREW---FHVL----ARGMFNPNY--------ALFIPVASDRT-----------
----------------TFHPSRLS------------------------------------
--GINSEHLMFFRFIGRIIGKALYEGRVLDCHFSR--AVYKNLLG---------------
--------RAVSIKDMET----------------------LDLDYYKS--------LLWM
LEN--DIT-DII--
-TETFALETDD---------------------------FGE-RQVID-LVPD--------
GRNIP---
------------------------------VTQENKE-EYVQR-VVDYRLVRS-VREQLD
-SFLKG-----XIIPP--ELIS-IFNEQ-ELELLISG-----------------------
----LPEID-----------VDDW----KSNTELHN-YSAS-------------------
------------------------SPQI-QWFWRAVR---------------------SFD
KEERAKLLQF-------VTGTSKVP------------LNG-----FK-ELEGMN--GVSR
FNIHRDYGSK------------------------DRLPTTHTCF-NX-LDMPEY-DSYET
LRQRLI-VAMTAGS---
>Aspergillus_nidulans_XM_654478.1 .
LAVRRDQV-FLDSFRAL--------------YFKSAEELKHGKLNVRFHG-------EEG

VDAGGVTREW---FQVL----ARGMFNPDY--------ALFIPVAADRT-----------
----------------TFHPNRLS------------------------------------
--GVNPEHLMFFKFIGRIIGKALYEGRVLDCHFSR--AVYKCILG---------------
--------RNVSIKDMET----------------------LDLDYYKS--------LLWM
LEN---DIT-DII--
-TETFAVETDD--------------------------FGE-KQTID-LIEN--------
GRNIP---
-----------------------------VTQENKE-EYVQK-VVDYRLVAS-VREQLD
-NFLKG---FHEIIPP--ELIS-IFNEQ-ELELLISG-----------------------
----LPEID-----------VDDW----KANTEYHN-YSAS-------------------
------------------------SPQI-QWFWRAVR--------------------SFD
KEERAKLLQF-------VTGTSKVP------------LNG-----FK-ELEGMN--GVSR
FNIHRDYGNK------------------------DRLPSSHTCF-NQ-LDLPEY-DSYET
LRQRLY-IAMTTGSEYF
>Aspergillus_versicolor_MRBN01000030.1 .
LAVRRDQV-FLDSFRSL--------------YFKSADELKHGKLNVRFHG-------EEG
VDAGGVTREW---FQVL----ARGMFNPDY--------ALFIPVAADRT-----------
----------------TFHPNRLS------------------------------------
--AINPEHLMFFKFIGRIIGKALYEGRVLDCHFSR--AVYKCILG---------------
--------RNVSIKDMET----------------------LDLDYYKS--------LLWM
LEN---DIT-DII--
-TETFALETDA--------------------------FGE-KQVID-LIEN--------
GHNIP---
-----------------------------VTQENKE-EYVQK-VVDYRLVVS-VQEQLD
-NFLKG---FHEIIPP--ELIS-IFNEQ-ELELLISG-----------------------
----LPEVE-----------VDDW----KAHTEYHN-YSAS-------------------
------------------------SPQI-QWFWRAVR--------------------SFD
KEERAKLLQF-------VTGTSKVP------------LNG-----FK-ELEGMN--GVSR
FNIHRDYGNK------------------------DRLPSSHTCF-NX-LDLPEY-DSYET
LRQRLH-TAITTGSEYF
>Aspergillus_sydowii_MRCH01000050.1 .
LAVRRDQV-FLDSFRSL--------------YFKSADELKHGKLNVRFHG-------EEG
VDAGGVTREW---FQVL----ARGMFNPDY--------ALFIPVAADRT-----------
----------------TFHPNRLS------------------------------------
--AINPEHLMFFKFIGRIIGKALYEGRVLDCHFSR--AVYKCILG---------------
--------RNVSIKDMET----------------------LDLDYYKS--------LLWM
LEN---DIT-DII--
-TETFALETDA--------------------------FGE-KQVID-LIEN--------
GRNIP---
-----------------------------VTQENKE-EYVQK-VVDYRLVVS-VQEQLD
-NFLKG---FHEIIPP--ELIS-IFNEQ-ELELLISG-----------------------
----LPEVE-----------VDDW----KAHTEYHN-YSAS-------------------
------------------------SPQI-QWFWRAVR--------------------SFD
KEERAKLLQF-------VTGTSKVP------------LNG-----FK-ELEGMN--GVSR
FNIHRDYGNK------------------------DRLPSSHTCF-NX-LDLPEY-DSYET
LRQRLH-TAITTGSEYF
>Aspergillus_calidoustus_CDMC01000005.1 .
LAVRRDQV-FLDSFKSL--------------YFKNADELKYGKLNVRFHG-------EEG
VDAGGVTREW---FQVL----ARGMFNPDY--------ALFIPVAADRT-----------
----------------TFHPNRLS------------------------------------
--GVNPEHLMFFKFIGRIIGKALYEGRVLDCHFSR--AVYKCILG---------------
--------RNVSIKDMET----------------------LDLDYYKS--------LLWM
LEN---DIT-DII--
-TETFAVETDD--------------------------FGE-KQVID-LIEN--------
GRNIP---
-----------------------------VTQENKE-EYVQK-VVDYRLVVS-VREQLD
-NFLIGIG-FHEIIPP--ELIS-IFNEQ-ELELLISG-----------------------
----LPEIE-----------VDDW----KANTEYHN-YSAS-------------------

```
------------------------SPQI-QWFWRAVR--------------------SFD
KEERAKLLQF-------VTGTSKVP------------LNG-----FK-ELEGMN--GVSR
FNIHRDYGNK------------------------DRLPSSHTCF-N--------------
-----------------
>Aspergillus_ustus_JOMC01000110.1 .
LAVRRDQV-FLDSFKSL--------------YFKNADELKYGKLNVRFHG-------EEG
VDAGGVTREW---FQVL----ARGMFNPDY--------ALFIPVAADRT-----------
----------------TFHPNRLS------------------------------------
--GVNPEHLMFFKFIGRIIGKALYEGRVLDCHFSR--AVYKCILS---------------
--------RNVSIKDMET----------------------LDLDYYKS--------LLWM
LEN------------------------------------------------DIT-DII--
-TETFAVETDD---------------------------FGE-KQVID-LIEN--------
GRNIP-------------------------------------------------------
------------------------------VTQENKE-EYVQK-VVDYRLVVS-VREQLD
-NFLKG---FHEIIPP--ELIS-IFNEQ-ELELLISG-----------------------
----LPEIE-----------VDDW----KANTEYHN-YSAS-------------------
------------------------SPQI-QWFWRAVR--------------------SFD
KEERAKLLQF-------VTGTSKVP------------LNG-----FK-ELEGMN--GVSR
FNIHRDYGNK------------------------DRLPSSHTCF-NX-LDLPEY-DSYET
LRQRLY-IAMTTGSEYF
>Cladosporium_cladosporioides_MSJH02000812 1..
LSVRRDQV-FLDSFKSL--------------YFKTADEMKYGKLSVRFHG-------EEG
VDAGGVTREW---FQVL----ARGMFNPNY--------ALFIPVASDRT-----------
----------------TFHPNRLS------------------------------------
--GVNSEHLMFFKFIGRIIGKALYEGRVLDCHFSR--AVYKRILG---------------
--------KSVSLKDMET----------------------LDLDYYKS--------LVWM
LEN------------------------------------------------DIT-DII--
-TETFAIETDD---------------------------FGE-TQIID-LIPN--------
GRNIP-------------------------------------------------------
------------------------------VTQENKE-EYVQR-VVEYRLIDS-VKEQLD
-NFLKG---FHDIIPA--DLIS-IFNEQ-ELELLISG-----------------------
----LPEID-----------VDDW----KNNTEYHN-YSAS-------------------
------------------------SPQI-QWFWRAVR--------------------SFD
KEERAKLLQF-------VTGTSKVP------------LNG-----FK-ELEGMN--GFSK
FNIHRDYGNK------------------------DRLPSSHTCF-NX-MDLPEY-ESYED
LRSRLY-TAMTAGSEYF
>Byssochlamys_spectabilis_BAUL01000093.1 .
LSVRRDQV-FLDSFKSL--------------YFKTADEMKYGKLSIRFHG-------EEG
VDAGGVTREW---FQVL----ARGMFNPNY--------ALFIPVASDRT-----------
----------------TFHPNRLS------------------------------------
--GVNSEHLMFFKFIGRIIGKALYEGRVLDCHFSR--AVYKRILG---------------
--------KSVSLKDMET----------------------LDLDYYKS--------LVWM
LEN------------------------------------------------DIT-DII--
-TETFAIETDD---------------------------FGE-TQVID-LIPN--------
GRNIP-------------------------------------------------------
------------------------------VTQENKE-EYVQR-VVEYRLIDS-VKEQLD
-NFLKG---FHDIIPA--DLIS-IFNEQ-ELELLISG-----------------------
----LPEID-----------VDDW----KNNTEYHN-YSAS-------------------
------------------------SPQI-QWFWRAVR--------------------SFD
KEERAKLLQF-------VTGTSKVP------------LNG-----FK-ELEGMN--GFSK
FNIHRDYGNK------------------------DRLPSSHTCF-NX-LDLPEY-ESYED
LRSRLY-TAMTAGSEYF
>Thermomyces_lanuginosus_ANHP01000232.1 .
LSVRRDQV-FLDSFKSL--------------YFKTADEVKYGKLNVRFHG-------EEG
IDAGGVTREW---FQVL----ARGMFNPNY--------ALFIPVASDRT-----------
----------------TFHPNRLS------------------------------------
--GVNSEHLMFFKFIGRIIGKALYEGRVLDCHFSR--AVYKSILG---------------
--------KPVSIKDMET----------------------LDLDYYKS--------LVWM
```

```
LEN------------------------------------------------DIT-DII--
-TETFAIETDD---------------------------FGE-KQIID-LIPN--------
GRNIP-------------------------------------------------------
------------------------------VTQENKE-EYVQR-VVEYKLVGS-VKEQLE
-HFLKG---FHDIIPA--DLIS-IFNEQ-ELELLISG-----------------------
----LPEID-----------VDDW----RNNTEYHN-YSPS-------------------
------------------------SPQI-QWFWRAVR--------------------SFD
KEERAKLLQF-------VTGTSKVP------------LNG-----FK-ELEGMN--GFSK
FNIHRDYGDK------------------------DRLPSSHTCF-NX-LDLPEY-ESYEE
LRSRLY-TAMTWGSEYF
>Rasamsonia_emersonii_XM_013471677.1 .
LSVRRDQV-FLDSFKSL--------------YFKTAEEVKYGKLNVRFHG-------EEG
VDAGGVTREW---FQVL----ARGMFNPNY--------ALFIPVASDRT-----------
----------------TFHPNRLS------------------------------------
--GVNSEHLMFFKFIGRIIGKALYEGRVLDCHFSR--AVYKRILG---------------
--------KPVSLKDMET----------------------LDLDYYKS--------LVWM
LEN------------------------------------------------DIT-DII--
-TETFSVETDD---------------------------FGE-KQIID-LIPN--------
GRNIP-------------------------------------------------------
------------------------------VTQENKE-EYVQR-VVEYRLVGS-VKEQLD
-NFLKDRKGFHDIIPP--DLIS-IFNEQ-ELELLISG-----------------------
----LPEID-----------VDDW----KNNTEYHN-YSPS-------------------
------------------------SPQI-QWFWRAVR--------------------SFD
KEERAKLLQF-------VTGTSKVP------------LNG-----FA-ELEGMN--GFSK
FNIHRDYGNK------------------------DRLPSSHTCF-NQ-LDLPEY-ESYEE
LRSRLY-TAMTAGSEYF
>Talaromyces_stipitatus_XM_002341784.1 .
LSVRRDQV-FLDSFKSL--------------YFKTAEEVKYGKLNIRFHG-------EEG
VDAGGVTREW---FQVL----ARGMFNPNY--------ALFIPVASDRT-----------
----------------TFHPNRLS------------------------------------
--GVNTEHLMFFKFIGRIIGKALYEGRVLDCHFSR--AVYKSILG---------------
--------KSVSIKDMET----------------------LDLDYYKS--------LLWM
LEN------------------------------------------------DIT-DII--
-TETFAIETDD---------------------------FGE-TQVID-LIPN--------
GRNIP-------------------------------------------------------
------------------------------VTQENKE-EYVQR-VVEYKLVGS-VKEQLE
-NFLKG---FHDIIQP--DLIS-IFNEQ-ELELLISG-----------------------
----LPEID-----------VDDW----KNNTEYHN-YSAS-------------------
------------------------SQQI-QWFWRAVR--------------------SFD
KEERAKLLQF-------VTGTSKVP------------LNG-----FK-ELEGMN--GFSR
FNIHRDYGNK------------------------DRLPSSHTCF-NQ-LDLPEY-ESYED
LRSRLY-KAMTAGGDYF
>Talaromyces_verruculosus_LHCL01000050.1 .
LSVRRDQV-FLDSFKSL--------------YFKTAEEVKYGKLNIRFHG-------EEG
VDAGGVTREW---FQVL----ARGMFNPNY--------ALFIPVASDRT-----------
----------------TFHPNRLS------------------------------------
--GVNTEHLMFFKFIGRIIGKALYEGRVLDCHFSR--AVYKSILG---------------
--------KSVSIKDMET----------------------LDLDYYKS--------LLWM
LEN------------------------------------------------DIT-DII--
-TETFAIETDD---------------------------FGE-TQVID-LIPN--------
GRNIP-------------------------------------------------------
------------------------------VTQENKE-EYVQR-VVEYKLAGS-VKEQLE
-NFLKG---MSXIIPP--DLIS-IFNEQ-ELELLISG-----------------------
----LPEID-----------VDDW----KNNTEYHN-YSAS-------------------
------------------------SQQI-QWFWRAVR--------------------SFD
KEERAKLLQF-------VTGTSKVP------------LNG-----FK-ELEGMN--GFSR
FNIHRDYGNK------------------------ERLPSSHTCF-NX-LDLPEY-ESYED
LRSRLY-KAMTAGGDYF
```

>Talaromyces_amestolkiae_MIKG01000002.1 .
LSVRRDQV-FLDSFKSL--------------YFKTAEEVKYGKLNIRFHG-------EEG
VDAGGVTREW---FQVL----ARGMFNPNY--------ALFIPVASDRT-----------
----------------TFHPNRLS------------------------------------
--GVNTEHLMFFKFIGRIIGKALYEGRVLDCHFSR--AVYKSILG---------------
--------KSVSIKDMET----------------------LDLDYYKS--------LLWM
LEN--DIT-DII--
-TETFAIETDD---------------------------FGE-TQVID-LIPN--------
GRNIP---
------------------------------VTQENKE-EYVQR-VVEYKLAGS-VKEQLE
-NFLKG---FHDIIPP--DLIS-IFNEQ-ELELLISG-----------------------
----LPEID-----------VDDW----KNNTEYHN-YSAS-------------------
------------------------SQQI-QWFWRAVR--------------------SFD
KEERAKLLQF-------VTGTSKVP------------LNG-----FK-ELEGMN--GFSR
FNIHRDYGNK------------------------ERLPSSHTCF-NX-LDLPEY-ESYED
LRSRLY-KAMTAGGDYF
>Penicillium_marneffei_XM_002151180.1 .
LSVRRDQV-FLDSFKSL--------------YFKTAEEVKYGKLNIRFHG-------EEG
VDAGGVTREW---FQVL----ARGMFNPNY--------ALFIPVASDRT-----------
----------------TFHPNRLS------------------------------------
--GVNTEHLMFFKFIGRIIGKALYEGRVLDCHFSR--AVYKSILG---------------
--------KSVSIKDMET----------------------LDLDYYKS--------LLWM
LEN--DIT-DII--
-TETFAIETDD---------------------------FGE-TQVID-LIPN--------
GRNIP---
------------------------------VTQENKE-EYVQR-VVEYKLAGS-VKEQLE
-NFLKG---FHDIIPP--DLIS-IFNEQ-ELELLISG-----------------------
----LPEID-----------VDDW----KNNTEYHN-YSAS-------------------
------------------------SQQI-QWFWRAVR--------------------SFD
KEERAKLLQF-------VTGTSKVP------------LNG-----FK-ELEGMN--GFSR
FNIHRDYGNK------------------------ERLPSSHTCF-NQ-LDLPEY-ESYED
LRSRLY-KAMTAGGDYF
>Aspergillus_sojae_BACA01000963.1 .
LSVRRDQV-FLDSFKSL--------------YFKSADELKYGKLNVRFHG-------EEG
VDAGGVTREW---FQVL----ARGMFNPNY--------ALFIPVAADRT-----------
----------------TFHPNRLS------------------------------------
--GVNSEHLMFFKFIGRIIGKALYEGRVLDCHFSR--AVYKCILG---------------
--------RSVSIKDMET----------------------LDLDYYKS--------LLWM
LEN--DIT-DII--
-TETFAVETDD---------------------------FGE-KQVID-LVEN--------
GSNIP---
------------------------------VTQENKE-EYVQR-VVDYRLVRS-VKEQLD
-NFLKG---FHEIIPA--DLIS-IFNEQ-ELELLISG-----------------------
----LPEID-----------VDDW----KTNTEYHN-YSAS-------------------
------------------------SPQI-QWFWRAVR--------------------SFD
KEERAKLLQF-------VTGTSKVP------------LNG-----FK-ELEGMN--GVSR
FNIHRDYGNK------------------------DRLPSAHTCF-NX-LDLPEY-DSYET
LRQRLY-TAMTAGSEYF
>Aspergillus_flavus_XM_002374150.1 .
LSVRRDQV-FLDSFKSL--------------YFKSADELKYGKLNVRFHG-------EEG
VDAGGVTREW---FQVL----ARGMFNPNY--------ALFIPVAADRT-----------
----------------TFHPNRLS------------------------------------
--GVNSEHLMFFKFIGRIIGKALYEGRVLDCHFSR--AVYKCILG---------------
--------RSVSIKDMET----------------------LDLDYYKS--------LLWM
LEN--DIT-DII--
-TETFAVETDD---------------------------FGE-KQVID-LVEN--------
GSNIP---
------------------------------VTQENKE-EYVQR-VVDYRLVRS-VKEQLD

-NFLKG---FHEIIPA--DLIS-IFNEQ-ELELLISG-----------------------
----LPEID-----------VDDW----KANTEYHN-YSAS-------------------
------------------------SPQI-QWFWRAVR--------------------SFD
KEERAKLLQF-------VTGTSKVP------------LNG-----FK-ELEGMN--GVSR
FNIHRDYGNK------------------------DRLPSAHTCF-NQ-LDLPEY-DSYET
LRQRLY-TAMTAGSEYF
>Aspergillus_westerdijkiae_LKBE01000025.1 .
LSVRRDQV-FLDSFKSL--------------YFKTADELKHGKLNVRFHG-------EEG
VDAGGVTREW---FQVL----ARGMFNPNY--------ALFIPVAADRT-----------
----------------TFHPNRLS------------------------------------
--GVNSEHLMFFKFIGRIIGKALYEGRVLDCHFSR--AVYKCILG---------------
--------RSVSIKDMET----------------------LDLDYYKS--------LLWM
LEN--DIT-DII--
-TETFAVETDD---------------------------FGE-KQVID-LIEN--------
GSNIP---
------------------------------VTQENKE-EYVQR-VVDYRLVRS-VKEQLD
-NFLKG---X--
--------E-----------VDDW----KVNTEYHN-YSAS-------------------
------------------------SPQI-QWFWRAVR--------------------SFD
KEERAKLLQF-------VTGTSKVP------------LNG-----FK-ELEGMN--GVSR
FNIHRDYGNK------------------------DRLPSSHTCF-NX-LDLPEY-DSYET
LRQRLY-TAMTAGS---
>Aspergillus_nomius_XM_015550680.1 .
LSVRRDQV-FLDSFKSL--------------YFKTADELKYGKLNVRFHG-------EEG
VDAGGVTREW---FQVL----ARGMFNPNY--------ALFIPVAADRT-----------
----------------TFHPNRLS------------------------------------
--GVNSEHLMFFKFIGRIIGKALYEGRVLDCHFSR--AVYKCILG---------------
--------RSVSIKDMET----------------------LDLDYYKS--------LLWM
LEN--DIT-DII--
-TETFAVETDD---------------------------FGE-KQVID-LIEN--------
GSNIP---
------------------------------VTQENKE-EYVQR-VVDYRLVRS-VKEQLD
-NFLKG---FHEIIPA--DLIS-IFNEQ-ELELLISG-----------------------
----LPEID-----------VDDW----KANTEYHN-YSAS-------------------
------------------------SPQI-QWFWRAVR--------------------SFD
KEERAKLLQF-------VTGTSKVP------------LNG-----FK-ELEGMN--GVSR
FNIHRDYGNK------------------------DRLPSAHTCF-NQ-LDLPEY-ESYET
LRQRLY-TAMTAGSEYF
>Aspergillus_parasiticus_LOAP01000398.1 .
LSVRRDQV-FLDSFKSL--------------YFKSADELKYGKLNVRFHG-------EEG
VDAGGVTREW---FQVL----ARGMFNPNY--------ALFIPDAPDRT-----------
----------------TFHPNRLS------------------------------------
--GVNSEHLMFFKFIGRIIGKALYEGRVLDCHFSR--AVYKCILG---------------
--------RSVSIKDMET----------------------LDLDYYKS--------LLWM
LEN--DIT-DII--
-TETFAVETDD---------------------------FGE-KQAID-LVEN--------
GSNIP---
------------------------------VTQENKE-EYVQR-VVDYRLVRS-VKEQLD
-NFLKG---FHEIIPA--DLIS-IFNEQ-ELELLISG-----------------------
----LPEID-----------VDDW----KTNTEYHN-YSAS-------------------
------------------------SPQI-QWFWRAVR--------------------SFD
KEERAKLLQF-------VTGTSKVP------------LNG-----FK-ELEGMN--GVSR
FNIHRDYGNK------------------------DRLPSAHTCF-NX-LDLPEY-DSYET
LRQRLY-TAMTAGSEY-
>Aspergillus_oryzae_AP007155.1_a.
LSVRRDQV-FLDSFKSL--------------YFKSADELKYGKLNVRFHG-------EEG
VDAGGVTREW---FQVL----ARGMFNPNY--------ALFIPVAADRT-----------
----------------TFHPNRLS------------------------------------

--GVNSEHLMFFKFIGRIIGKALYEGRVLDCHFSR--AVYKCILG---------------
--------RSVSIKDMET----------------------LDLDYYKS--------LLWM
LEN--DIT-DII--
-TETFAVETDD---------------------------FGE-KQVID-LVEN--------
GSNIP---
------------------------------VTQENKE-EYVQR-VVDYRLVRS-VKEQLD
-NFLKG---FHEIIPA--DLIS-IFNEQ-ELELLISG-----------------------
----LPEID-----------VDDW----KANTEYHN-YSAS-------------------
------------------------SPQI-QWFWRAVR--------------------SFD
KEERAKLLQF-------VTGTSKVP------------LNG-----FK-ELEGMN--GVSR
FNIHRDYGNK------------------------DRLPSAHTCF-N--------------

>Aspergillus_terreus_XM_001213169.1 .
LSVRRDQV-FLDSFKSL--------------YFKTADELKYGKLNVRFHG-------EEG
VDAGGVTREW---FQVL----ARGMFNPNY--------ALFIPVAADRT-----------
----------------TFHPNRLS------------------------------------
--GVNSEHLMFFKFIGRIIGKALYEGRVLDCHFSR--AVYKCILG---------------
--------RSVSIKDMET----------------------LDLDYYKS--------LLWM
LEN--DIT-DII--
-TETFAVETDD---------------------------FGE-KQVID-LVEN--------
GRNIP---
------------------------------VTQENKE-EYVQR-VVDYRLLRS-VKEQLD
-NFLKG---FHEIIPP--DLIS-IFNEQ-ELELLISG-----------------------
----LPEID-----------VDDW----KVNTEYHN-YSAS-------------------
------------------------SPQI-QWFWRAVR--------------------SFD
KEERAKLLQF-------VTGTSKVP------------LNG-----FK-ELEGMN--GVSR
FNIHRDYGNK------------------------DRLPSSHTCF-NQ-LDLPEY-ESYET
LRQRLY-TAMTAGSEYF
>Aspergillus_clavatus_XM_001271948.1 .
LSVRRDQV-FLDSFKSL--------------YFKSADELKYGKLNVRFHG-------EEG
VDAGGVTREW---FQVL----ARGMFNPNY--------ALFIPVAADRT-----------
----------------TFHPNRLS------------------------------------
--GVNSEHLMFFKFIGRIIGKALYEGRVLDCHFSR--AVYKCILS---------------
--------RSVSIKDMET----------------------LDLDYYKS--------LLWM
LEN--DIT-DII--
-TETFAVETDD---------------------------FGE-KQVID-LIEN--------
GSNIP---
------------------------------VTQENKE-EYVQR-VVDYRLVKS-VKDQLD
-NFLNG---FHEIIPP--DLIS-IFNEQ-ELELLISG-----------------------
----LPEID-----------VDDW----KANTEYHN-YSAS-------------------
------------------------SPQI-QWFWRAVR--------------------SFD
KEERAKLLQF-------VTGTSKVP------------LNG-----FK-ELEGMN--GVSK
FNIHRDYGNK------------------------DRLPSSHTCF-NQ-LDLPEY-ENYET
LRQRLY-TAITAGSEYF
>Aspergillus_lentulus_BCLY01000009.1 .
LSVRRDQV-FLDSFKSL--------------YFKSADELKYGKLNVRFHG-------EEG
VDAGGVTREW---FQVL----ARGMFNPNY--------ALFIPVAADRT-----------
----------------TFHPNRLS------------------------------------
--GVNSEHLMFFKFIGRIIGKALYEGRVLDCHFSR--AVYKCILG---------------
--------RSVSIKDMET----------------------LDLDYYKS--------LLWM
LEN--DIT-DII--
-TETFAVETDD---------------------------FGE-KQVID-LIEN--------
GRNIP---
------------------------------VTQENKE-EYVQR-VVDYRLVKS-VKDQLD
-NFLKG---FHEIIPP--DLIS-IFNEQ-ELELLISG-----------------------
----LPEID-----------VDDW----KANTEYHN-YSAS-------------------
------------------------SPQI-QWFWRAVR--------------------SFD
KEERAKLLQF-------VTGTSKVP------------LNG-----FK-ELEGMN--GVSK

FNIHRDYGNK------------------------DRLPSSHTCF-NX-LDLPEY-DSYET
LRQRLY-TAMTAGSDYF
>Aspergillus_fumigatus_XM_746656.1 .
LSVRRDQV-FLDSFKSL--------------YFKTADELKYGKLNVRFHG-------EEG
VDAGGVTREW---FQVL----ARGMFNPNY--------ALFIPVAADRT-----------
----------------TFHPNRLS------------------------------------
--GVNSEHLMFFKFIGRIIGKALYEGRVLDCHFSR--AVYKCILG---------------
--------RSVSIKDMET---------------------LDLDYYKS--------LLWM
LEN---DIT-DII--
-TETFAVETDD---------------------------FGE-KQVID-LIEN--------
GRNIP---
------------------------------VTQENKE-EYVQR-VVDYRLVKS-VKDQLD
-NFLKG---FHDIIPP--DLIS-IFNEQ-ELELLISG-----------------------
----LPEID-----------VDDW----KANTEYHN-YSAS-------------------
------------------------SPQI-QWFWRAVR--------------------SFD
KEERAKLLQF-------VTGTSKVP------------LNG-----FK-ELEGMN--GVSK
FNIHRDYGNK------------------------DRLPSSHTCF-NQ-LDLPEY-DSYET
LRQRLY-TAMTAGSDYF
>Neosartorya_fischeri_XM_001266939.1 .
LSVRRDQV-FLDSFKSL--------------YFKTADELKYGKLNVRFHG-------EEG
VDAGGVTREW---FQVL----ARGMFNPNY--------ALFIPVAADRT-----------
----------------TFHPNRLS------------------------------------
--GVNSEHLMFFKFIGRIIGKALYEGRVLDCHFSR--AVYKCILG---------------
--------RSVSIKDMET---------------------LDLDYYKS--------LLWM
LEN---DIT-DII--
-TETFAVETDD---------------------------FGE-KQVID-LIEN--------
GRNIP---
------------------------------VTQENKE-EYVQR-VVDYRLVKS-VKDQLD
-NFLKG---FHEIIPP--DLIS-IFNEQ-ELELLISG-----------------------
----LPEID-----------VDDW----KANTEYHN-YSAS-------------------
------------------------SPQI-QWFWRAVR--------------------SFD
KEERAKLLQF-------VTGTSKVP------------LNG-----FK-ELEGMN--GVSK
FNIHRDYGNK------------------------DRLPSSHTCF-NQ-LDLPEY-DSYET
LRQRLY-TAMTAGSDYF
>Aspergillus_rambellii_JZBS01001556.1 .
LSVRRDQV-FLDSFKSL--------------YFKSAEELKYGKLNVRFHG-------EEG
VDAGGVTREW---FQVL----ARGMFNPNY--------ALFIPVAADRT-----------
----------------TFHPNRLS------------------------------------
--GVNSEHLMFFKFIGRIIGKALYEGRVLDCHFSR--AVYKCILG---------------
--------RNVSIKDMET---------------------LDLDYYKS--------LLWM
LEN---DIT-DII--
-TETFAVETDD---------------------------FGE-KQVID-LVEN--------
GRNIP---
------------------------------VTQENKE-EYVQR-VVDYRLVVS-VKEQLD
-NFLKG---FHEIIPP--ELIS-IFNEQ-ELELLISG-----------------------
----LPEID-----------VDDW----KANAEYHN-YSAS-------------------
------------------------SPQI-QWFWRAVR--------------------SFD
KEERAKLLQF-------VTGTSKVP------------LNG-----FK-ELEGMN--GVSR
FNIHRDYGNK------------------------DRLPSSHTCF-NX-LDLPEY-DSYET
LRQRLY-TAITTGSEYF
>Aspergillus_aculeatus_XM_020200648.1 .
LSVRRDQV-FLDSFKSL--------------YFKTADELKFGKLNVRFHG-------EEG
VDAGGVTREW---FQVL----ARGMFNPNY--------ALFIPVASDRT-----------
----------------TFHPNRLS------------------------------------
--GVNSEHLMFFKFIGRIIGKALYEGRVLDCHFSR--AVYKCILS---------------
--------RSVSIKDMET---------------------LDLDYYKS--------LLWM
LEN---DIT-DII--
-TETFAVETDD---------------------------FGE-KQVID-LIEN--------

GSNIP---
------------------------------VTEENKE-EYVQR-VVDYRLIGS-VKEQLD
-NFLKG---FHEIIPA--DLIS-IFNEQ-ELELLISG-----------------------
----LPEID-----------VDDW----KVNTEYHN-YSAS-------------------
------------------------SPQI-QWFWRAVR--------------------SFD
KEERAKLLQF-------VTGTSKVP------------LNG-----FK-ELEGMN--GVSR
FNIHRDYGNK------------------------DRLPSSHTCF-NQ-LDLPEY-ESYET
LRQRLY-TAMTAGSEYF
>Aspergillus_carbonarius_AHIG01000110.1 .
LSVRRDQI-FLDSFKSL--------------YFKTADELKYGKLNVRFHG-------EEG
VDAGGVTREW---FQVL----ARGMFNPNY--------ALFIPVASDRT-----------
----------------TFHPNRLS------------------------------------
--GVNAEHLMFFKFIGRIIGKALYEGRVLDCHFSR--AVYKCILG---------------
--------RSVSIKDMET----------------------LDLDYYKS--------LLWM
LEN--DIT-DII--
-TETFAVETDD---------------------------FGE-KQVID-LTEN--------
GRNIP---
------------------------------VTEENKE-EYIQK-VVDYRLVGS-VKEQLD
-NFLKG---FHEIIPS--DLIS-IFNEQ-ELELLISG-----------------------
----LPEIE-----------VDDW----KVNTEYHN-YSAS-------------------
------------------------SPQI-QWFWRAVR--------------------SFD
KEERAKLLQF-------VTGTSKVP------------LNG-----FK-ELEGMN--GVSR
FNIHRDYGNK------------------------DRLPSSHTCF-NX-LDLPEY-ESYET
LRQRLY-TAMTAGSEYF
>Aspergillus_tubingensis_LJXU01000047.1 .
LSVRRDQV-FLDSFKSL--------------YFKTADELKYGKLNVRFHG-------EEG
VDAGGVTREW---FQVL----ARGMFNPNY--------ALFIPVASDRT-----------
----------------TFHPNRLS------------------------------------
--GVNSEHLMFFKFIGRIIGKALYEGRVLDCHFSR--AVYKCILG---------------
--------RSVSIKDMET----------------------LDLDYYKS--------LLWM
LEN--DIT-DII--
-TETFAVETDD---------------------------FGE-KQVID-LIEN--------
GRNIP---
------------------------------VTEENKE-EYIQR-VVDYRLVGS-VKEQLD
-NFLKG---FHEIIPS--DLIS-IFNEQ-ELELLISG-----------------------
----LPEIE-----------VDDW----KVNTEYHN-YSAS-------------------
------------------------SPQI-QWFWRAVR--------------------SFD
KEERAKLLQF-------VTGTSKVP------------LNG-----FK-ELEGMN--GVSR
FNIHRDYGNK------------------------DRLPSSHTCF-NX-LDLPEY-ESYET
LRQRLY-TAMTAGSEYF
>Aspergillus_niger_XM_001401916.2 .
LSVRRDQV-FLDSFKSL--------------YFKTADELKYGKLNVRFHG-------EEG
VDAGGVTREW---FQVL----ARGMFNPNY--------ALFIPVASDRT-----------
----------------TFHPNRLS------------------------------------
--GVNSEHLMFFKFIGRIIGKALYEGRVLDCHFSR--AVYKCILG---------------
--------RSVSIKDMET----------------------LDLDYYKS--------LLWM
LEN--DIT-DII--
-TETFAVETDD---------------------------FGE-KQVID-LIEN--------
GRNIP---
------------------------------VTEENKE-EYIQR-VVDYRLVGS-VKEQLD
-NFLKG---FHEIIPS--DLIS-IFNEQ-ELELLISG-----------------------
----LPEIE-----------VDDW----KVNTEYHN-YSAS-------------------
------------------------SPQI-QWFWRAVR--------------------SFD
KEERAKLLQF-------VTGTSKVP------------LNG-----FK-ELEGMN--GVSR
FNIHRDYGNK------------------------DRLPSSHTCF-NQ-LDLPEY-ESYET
LRQRLY-TAMTAGSEYF
>Aspergillus_kawachii_BACL01000033.1 .
LSVRRDQV-FLDSFKSL--------------YFKTADELKYGKLNVRFHG-------EEG

VDAGGVTREW---FQVL----ARGMFNPNY--------ALFIPVASDRT-----------
----------------TFHPNRLS------------------------------------
--GVNSEHLMFFKFIGRIIGKALYEGRVLDCHFSR--AVYKCILG---------------
--------RSVSIKDMET----------------------LDLDYYKS--------LLWM
LEN--DIT-DII--
-TETFAVETDD---------------------------FGE-KQVID-LIEN--------
GRNIP---
------------------------------VTEENKE-EYIQR-VVDYRLVGS-VKEQLD
-NFLKG---FHEIIPS--DLIS-IFNEQ-ELELLISG-----------------------
----LPEIE-----------VDDW----KNVTEYHN-YSAS-------------------
------------------------SPQI-QWFWRAVR--------------------SFD
KEERAKLLQF-------VTGTSKVP------------LNG-----FK-ELEGMN--GVSR
FNIHRDYGNK------------------------DRLPSSHTCF-NX-LDLPEY-ESYET
LRQRLY-TAMTAGSEYF
>Thermoascus_crustaceus_BCIC01000007.1 .
LSVRRDQV-FLDSFKSL---------------YFKTAEEMKYGKLSIRFHG-------EEG
VDAGGVTREW---FQVL----ARGMFNPNY--------ALFIPVASDRT-----------
----------------TFHPNRLS------------------------------------
--GVNSEHLMFFKFIGRIIGKALYEGRVLDCHFSR--AVYKRILG---------------
--------KSVSIKDMET----------------------LDLDYYKS--------LLWM
LEN--DIT-DII--
-TETFAVETDD---------------------------FGE-KQVID-LVEN--------
GRNIP---
------------------------------VTQENKE-EYVQR-VVEYRLVGS-VKEQLD
-NFLKG---KSXIIPP--DLIS-IFNEQ-ELELLISG-----------------------
----LPEID-----------VDDW----KNNTEYHN-YSAS-------------------
------------------------SPQI-QWFWRAVR--------------------SFD
KEERAKLLQF-------VTGTSKVP------------LNG-----FK-ELEGMN--GFSK
FNIHRDYGNK------------------------DRLPSSHTCF-NX-LDLPEY-ESYEV
LRQRLY-TAMTAGS---
>Talaromyces_leycettanus_JSYV01000274.1 .
LSVRRDQV-FLDSFKSL---------------YFKTADEMKYGKLNIRFHG-------EEG
VDAGGVTREW---FQVL----ARGMFNPNY--------ALFIPVAADRT-----------
----------------TFHPNRLS------------------------------------
--GVNSEHLMFFKFIGRIIGKALYEGRVLDCHFSR--AVYKCILG---------------
--------KSVSIKDMET----------------------LDLDYYKS--------LLWM
LEN--DIT-DII--
-TETFSVETDA---------------------------FGE-KQVID-LVEN--------
GRNIP---
------------------------------VTQENKE-EYVQR-VVEYRLVGS-VKEQLD
-NFLKG---FHEIIPP--DLIS-IFNEQ-ELELLISG-----------------------
----LPEID-----------VDDW----KNNTEYHN-YSAS-------------------
------------------------SPQI-QWFWRAVR--------------------SFD
KEERAKLLQF-------VTGTSKVP------------LNG-----FK-ELEGMN--GVNK
FNIHRDYGNK------------------------DRLPSAHTCF-NX-LDLPEY-ESYED
LRQRLY-TAMTAGSEYF
>Penicilliopsis_zonata_MRBM01000155.1 .
LSVRRDQV-FLDSFKSL---------------YFKTADEMKYGKLNIRFHG-------EEG
VDAGGVTREW---FQVL----ARGMFNPNY--------ALFIPVAADRT-----------
----------------TFHPNRLS------------------------------------
--GVNSEHLMFFKFIGRIIGKALYEGRVLDCHFSR--AVYKSILG---------------
--------RSVSIKDMET----------------------LDLDYYKS--------LLWM
LEN--DIT-DII--
-TETFSLETDA---------------------------FGE-KQVID-LVEN--------
GHNIP---
------------------------------VTQENKE-EYIQR-VVEYRLVGS-VKEQLD
-NFLKG---FHEIIPP--DLIS-IFNEQ-ELELLISG-----------------------
----LPEIE-----------VDDW----KNNTEYHN-YSAS-------------------

------------------------SPQI-QWFWRAVR--------------------SFD
KEERAKLLQF-------VTGTSKVP------------LNG-----FK-ELEGMN--GVSK
FNIHRDYGNK------------------------DRLPSSHTCF-NX-LDLPEY-ESYET
LRQRLY-TAMTAGS---
>Talaromyces_purpureogenus_LIAB01000659.1 .
LSVRRDQV-FLDSFKSL--------------YFKSADELKYGKLNVRFHG-------EEG
VDAGGVTREW---FQVL----ARGMFNPNY--------ALFIPVAADRT-----------
----------------TFHPNRLS------------------------------------
--GVNSEHLMFFKFIGRIIGKALYEGRVLDCHFSR--AVYKCILG---------------
--------RTVSIKDMET----------------------LDLDYYKS--------LLWM
LEN--DIT-DII--
-TENFAIETDA---------------------------FGE-KQVID-LIPD--------
GCNIP---
------------------------------VTQENKE-EYIQR-VVEYRLVES-VREQLD
-NFLKG---FHEIIPP--DLIS-IFNEQ-ELELLISG-----------------------
----LPDID-----------VDEW----KNNTEYHN-YSAS-------------------
------------------------SSQI-QWFWRAVR--------------------SFD
KEERAKLLQF-------VTGTSKVP------------LNG-----FK-ELEGMN--GVSK
FNIHRDYGNK------------------------DRLPSAHTCF-NX-LDLPEY-ESYED
LRQRLY-TAVTAGSEYF
>Penicillium_subrubescens_MNBE01000120.1 .
LSVRRDQV-FLDSFKSL--------------YFKSADELKYGKLNVRFHG-------EEG
VDAGGVTREW---FQVL----ARGMFNPNY--------ALFIPVAADRT-----------
----------------TFHPNRLS------------------------------------
--GVNSEHLMFFKFIGRIIGKALYEGRVLDCHFSR--AVYKCILG---------------
--------RTVSIKDMET----------------------LDLDYYKS--------LLWM
LEN--DIT-DII--
-TENFAIETDA---------------------------FGE-KQVID-LIPD--------
GRNIP---
------------------------------VTQENKE-EYIQR-VVEYRLVES-VREQLD
-NFLKG---FHEIIPP--DLIS-IFNEQ-ELELLISG-----------------------
----LPDID-----------VDEW----KNNTEYHN-YSAS-------------------
------------------------SSQI-QWFWRAVR--------------------SFD
KEERAKLLQF-------VTGTSKVP------------LNG-----FK-ELEGMN--GVSK
FNIHRDYGNK------------------------DRLPSAHTCF-NX-LDLPEY-ESYED
LRQRLY-TAVTAGSEYF
>Penicillium_janthinellum_GBSP01007413.1 .
LSVRRDQV-FLDSFKSL--------------YFKTADELKYGKLNVRFHG-------EEG
VDAGGVTREW---FQVL----ARGMFNPNY--------ALFIPVAADRT-----------
----------------TFHPNRLS------------------------------------
--GVNSEHLMFFKFIGRIIGKALYEGRVLDCHFSR--AVYKCILG---------------
--------RTVSIKDMET----------------------LDLDYYKS--------LLWM
LEN--DIT-DII--
-TETFAIETDA---------------------------FGE-KQVID-LIPD--------
GRNIP---
------------------------------VTQENKE-EYIQR-VVEYRLVES-VREQLD
-NFLKG---FHEIIPP--DLIS-IFNEQ-ELELLISG-----------------------
----LPDID-----------VDEW----KINTEYHN-YSAS-------------------
------------------------SSQI-QWFWRAVR--------------------SFD
KEERAKLLQF-------VTGTSKVP------------LNG-----FK-ELEGMN--GVSK
FNIHRDYGNK------------------------DRLPSAHTCF-NQ-LDLPEY-ESYED
LRQRLY-TAVTAGSEYF
>Penicillium_oxalicum_AGII01000285.1 .
LSVRRDQV-FLDSFKSL--------------YFKSADELKYGKLNVRFHG-------EEG
VDAGGVTREW---FQVL----ARGMFNPNY--------ALFIPVAADRT-----------
----------------TFHPNRLS------------------------------------
--GVNSEHLMFFKFIGRIIGKALYEGRVLDCHFSR--AVYKCILG---------------
--------RTVSIKDMET----------------------LDLDYYKS--------LLWM

LEN--DIT-DII--
-TETFAIETDA---------------------------FGE-KQVID-LIPD--------
GRNIP---
------------------------------VTQENKE-EYIQR-VVEYRLVVS-VREQLD
-NFLKG---FHEIIPP--DLIS-IFNEQ-ELELLISG-----------------------
----LPEID-----------VDEW----KNNTEYHN-YSAS-------------------
------------------------SPQI-QWFWRAVR--------------------SFD
KEERAKLLQF-------VTGTSKVP------------LNG-----FK-ELEGMN--GVSK
FNIHRDYGNK------------------------DRLPSAHTCF-NX-LDLPEY-ESYED
LRQRLY-TAMTAGSEYF
>Penicillium_decumbens_MDYL01000033.1 .
LSVRRDQV-FLDSFKSL--------------YFKSAEELKFGKLNVRFHG-------EEG
VDAGGVTREW---FQVL----ARGMFNPNY--------ALFIPVAADRT-----------
----------------TFHPNRLS------------------------------------
--GVNSEHLMFFKFIGRIIGKALYEGRVLDCHFSR--AVYKSILG---------------
--------RSVSIKDMET----------------------LDLEYYKS--------LLWM
LEN--DIT-DII--
-TETFAIETDA---------------------------FGE-TQVID-LIPD--------
GRNVP---
------------------------------VTQENKE-EYIQR-VVEYRLVES-VREQLD
-SFLKG---FHEIIPP--DLIS-IFNEQ-ELELLISG-----------------------
----LPEID-----------VDEW----KNNTEYHN-YSAS-------------------
------------------------SSQI-QWFWRAVR--------------------SFD
KEERAKLLQF-------VTGTSKVP------------LNG-----FK-ELEGMN--GVSK
FNIHRDYGSK------------------------DRLPSSHTCF-NX-LDLPEY-ESYED
LRQRLY-TAMTTGSGYF
>Penicillium_antarcticum_MDYN01000005.1 .
LSVRRDQV-FLDSFKSL--------------YFKSADELKYGKLNVRFHG-------EEG
VDAGGVTREW---FQVL----ARGMFNPNY--------ALFIPVASDRT-----------
----------------TFHPNRLS------------------------------------
--GVNSEHLMFFKFIGRIIGKALYEGRVLDCHFSR--AVYKNILS---------------
--------RSVSIKDMET----------------------LDLDYYKS--------LLWM
LEN--DIT-DII--
-TETFAIETDD---------------------------FGE-KQVID-LIPG--------
GRDIP---
------------------------------VTQENKE-QYVQR-VVEYRLVES-VREQLD
-NFLKG---FHEIIPR--DLIS-IFNEQ-ELELLISG-----------------------
----LPEID-----------VDEW----KNNTEYHN-YSAS-------------------
------------------------SSQI-QWFWRAVR--------------------SFD
KEERAKLLQF-------VTGTSKVP------------LNG-----FK-ELEGMN--GVSK
FNIHRDYGNK------------------------DRLPSSHTCF-NX-LDLPEY-ESYED
LRQRLY-TAMTAGSEYF
>Penicillium_capsulatum_JPLQ01000028.1 .
LSVRRDQV-FLDSFKSL--------------YFKSADELKYGKLNVRFHG-------EEG
VDAGGVTREW---FQVL----ARGMFNPNY--------ALFIPVASDRT-----------
----------------TFHPNRLS------------------------------------
--GVNSEHLMFFKFIGRIIGKALYEGRVLDCHFSR--AVYKNILS---------------
--------RSVSIKDMET----------------------LDLDYYKS--------LLWM
LEN--DIT-DII--
-TETFAIETDD---------------------------FGE-KQVID-LIPG--------
GRDIP---
------------------------------VTQENKE-QYVQR-VVEYRLVES-VREQLD
-NFLKG---FHEIIPR--DLIS-IFNEQ-ELELLISG-----------------------
----LPEID-----------VDEW----KNNTEYHN-YSAS-------------------
------------------------SSQI-QWFWRAVR--------------------SFD
KEERAKLLQF-------VTGTSKVP------------LNG-----FK-ELEGMN--GVSK
FNIHRDYGNK------------------------DRLPSSHTCF-NX-LDLPEY-ESYED
LRQRLY-TAMTAGSEYF

```
>Penicillium_raistrickii_GFHR01008368.1 .
LSVRRAEV-FLDSFKSL--------------YFKSADELKYGKLNVRFHG-------EEG
VDAGGVTREW---FQVL----ARGMFNPNY--------ALFIPVAADRT-----------
----------------TFHPNRLS------------------------------------
--GVNSEHLMFFKFIGRIIGKALYEGRVLDCHFSR--AVYKNILG---------------
--------RSVSIKDMET----------------------LDLEYYKS--------LLWM
LEN------------------------------------------------DIT-DII--
-TETFAIETDD--------------------------FGE-KQVID-LKPN--------
GRDIP-------------------------------------------------------
------------------------------VTQENKE-EYVQR-VVEYRLVES-VREQLD
-NFLKG---FHEIIPP--DLIS-IFNEQ-ELELLISG-----------------------
----LPEID-----------VDEW----KNHTEYHN-YSAS-------------------
------------------------SSQI-QWFWRAVR--------------------SFD
KEERAKLLQF-------VTGTSKVP------------LNG-----FK-ELEGMN--GVSK
FNIHRDYGNK------------------------DRLPSSHTCF-NQ-LDLPEY-ESYED
LRARLY-TAMTAGSEYF
>Penicillium_nalgiovense_CBXQ010000187.1 .
LSVRRSEV-FLDSFKSL--------------YFKSADELKYGKLNVRFHG-------EEG
VDAGGVTREW---FQVL----ARGMFNPNY--------ALFIPVAADRT-----------
----------------TFHPNRLS------------------------------------
--GVNSEHLMFFKFIGRIIGKALYEGRVLDCHFSR--AVYKNILG---------------
--------RSVSIKDMET----------------------LDLDYYKS--------LLWM
LEN------------------------------------------------DIT-DII--
-TETFAIETDD--------------------------FGE-KQVID-LKPG--------
GRDIP-------------------------------------------------------
------------------------------VTQENKE-EYVQR-VVEYRLVES-VREQLD
-NFLKG---FHEIIPP--ELIS-IFNEQ-ELELLISG-----------------------
----LPEID-----------VDEW----KNNTEYHN-YSAS-------------------
------------------------SSQI-QWFWRAVR--------------------SFD
KEERAKLLQF-------VTGTSKVP------------LNG-----FK-ELEGMN--GVSK
FNIHRDYGHK------------------------DRLPSSHTCF-NX-LDLPEY-ESYED
LRQRLY-TAVTTGSEYF
>Penicillium_chrysogenum_XM_002564676.1 .
LSVRRSEV-FLDSFKSL--------------YFKSADELKYGKLNVRFHG-------EEG
VDAGGVTREW---FQVL----ARGMFNPNY--------ALFIPVAADRT-----------
----------------TFHPNRLS------------------------------------
--GVNSEHLMFFKFIGRIIGKALYEGRVLDCHFSR--AVYKNILG---------------
--------RSVSIKDMET----------------------LDLDYYKS--------LLWM
LEN------------------------------------------------DIT-DII--
-TETFAIETDD--------------------------FGE-KQVID-LKPG--------
GRDIP-------------------------------------------------------
------------------------------VTQENKE-EYVQR-VVEYRLVES-VREQLD
-NFLKG---FHEIIPP--ELIS-IFNEQ-ELELLISG-----------------------
----LPEID-----------VDEW----KNNTEYHN-YSAS-------------------
------------------------SSQI-QWFWRAVR--------------------SFD
KEERAKLLQF-------VTGTSKVP------------LNG-----FK-ELEGMN--GVSK
FNIHRDYGHK------------------------DRLPSSHTCF-NQ-LDLPEY-ESYED
LRQRLY-TAVTTGSEYF
>Penicillium_coprophilum_MDDG01000003.1 .
LSVRRAEV-FLDSFKSL--------------YFKSADELKYGKLNVRFHG-------EEG
VDAGGVTREW---FQVL----ARGMFNPNY--------ALFIPVAADRT-----------
----------------TFHPNRLS------------------------------------
--GVNSEHLMFFKFIGRIIGKALYEGRVLDCHFSR--AVYKNILG---------------
--------RAVSIKDMET----------------------LDLDYYKS--------LLWM
LEN------------------------------------------------DIT-DII--
-TETFAIETDA--------------------------FGE-KQVID-LKPG--------
GRDIP-------------------------------------------------------
------------------------------VTQENKE-EYVQR-VVEYRLVES-VREQLD
```

-NFLKG---FHEIIPP--ELIS-IFNEQ-ELELLISG-----------------------
----LPEID-----------VDEW----KNNTEYHN-YSAS-------------------
------------------------SSQI-QWFWRAVR--------------------SFD
KEERAKLLQF-------VTGTSKVP------------LNG-----FK-ELEGMN--GVSK
FNIHRDYGHK------------------------DRLPSSHTCF-NX-LDLPEY-ESYED
LRQRLY-TAVTTGSEYF
>Penicillium_digitatum_XM_014680434.1 .
LSVRRAEV-FLDSFKSL--------------YFKSADELKYGKLNVRFHG-------EEG
VDAGGVTREW---FQVL----ARGMFNPNY--------ALFIPVAADRT-----------
----------------TFHPNRLS------------------------------------
--GVNSEHLMFFKFIGRIIGKALYEGRVLDCHFSR--AVYKNILG---------------
--------RSVSIKDMET----------------------LDLDYYKS--------LLWM
LEN--DIT-DII--
-TETFSIETDD---------------------------FGE-KQVID-LKPG--------
GHDIP---
------------------------------VTQENKE-EYVQR-VVEYRLVGS-VREQLD
-NFLKG---FHEIIPP--ELIS-IFNEQ-ELELLISG-----------------------
----LPEID-----------VDEW----KNHTEYHN-YSAS-------------------
------------------------SSQI-QWFWRAVR--------------------SFD
KEERAKLLQF-------VTGTSKVP------------LNG-----FK-ELEGMN--GVSK
FNIHRDYGHK------------------------DRLPSSHTCF-NQ-LDLPEY-ESYED
LRQRLY-TAVTAGSEYF
>Penicillium_vulpinum_MDYP01000005.1 .
LSVRRAEV-FLDSFKSL--------------YFKSADELKYGKLNVRFHG-------EEG
VDAGGVTREW---FQVL----ARGMFNPNY--------ALFIPVAADRT-----------
----------------TFHPNRLS------------------------------------
--GVNSEHLMFFKFIGRIIGKALYEGRVLDCHFSR--AVYKNILG---------------
--------RSVSIKDMET----------------------LDLDYYKS--------LLWM
LEN--DIT-DII--
-TETFAIETDD---------------------------FGE-KQVID-LKPG--------
GHDIP---
------------------------------VTQENKE-EYVQR-VVEYRLVES-VREQLD
-NFLKG---FHEIIPP--ELIS-IFNEQ-ELELLISG-----------------------
----LPDID-----------VDEW----KNNTEYHN-YSAS-------------------
------------------------SSQI-QWFWRAVR--------------------SFD
KEERAKLLQF-------VTGTSKVP------------LNG-----FK-ELEGMN--GVSK
FNIHRDYGNK------------------------DRLPSSHTCF-NX-LDLPEY-ESYED
LRQRLY-TAVTAGSEYF
>Penicillium_griseofulvum_LWCZ01000553.1 .
LSVRRAEV-FLDSFKSL--------------YFKSADELKYGKLNVRFHG-------EEG
VDAGGVTREW---FQVL----ARGMFNPNY--------ALFIPVAADRT-----------
----------------TFHPNRLS------------------------------------
--GVNSEHLMFFKFIGRIIGKALYEGRVLDCHFSR--AVYKNILG---------------
--------RAVSIKDMET----------------------LDLDYYKS--------LLWM
LEN--DIT-DII--
-TETFSIETDD---------------------------FGE-KQVID-LKPG--------
GHDIP---
------------------------------VTQENKE-EYVQR-VVEYRLVES-VREQLD
-NFLKG---FHEIIPP--ELIS-IFNEQ-ELELLISG-----------------------
----LPEID-----------VDEW----KNNTEYHN-YSAS-------------------
------------------------SSQI-QWFWRAVR--------------------SFD
KEERAKLLQF-------VTGTSKVP------------LNG-----FK-ELEGMN--GVSK
FNIHRDYGHK------------------------DRLPSSHTCF-NX-LDLPEY-ESYED
LRQRLY-TAVTAGSEYF
>Penicillium_italicum_JMDK01000789.1 .
LSVRRAEV-FLDSFKSL--------------YFKSADELKYGKLNVRFHG-------EEG
VDAGGVTREW---FQVL----ARGMFNPNY--------ALFIPVAADRT-----------
----------------TFHPNRLS------------------------------------

--GVNSEHLMFFKFIGRIIGKALYEGRVLDCHFSR--AVYKNILG---------------
--------RSVSIKDMET----------------------LDLDYYKS--------LLWM
LEN--DIT-DII--
-TETFSIETDD---------------------------FGE-KQVID-LKPG--------
GHDIP---
------------------------------VTQENKE-EYVQR-VVEYRLVES-VREQLD
-NFLKG---FHEIIPP--ELIS-IFNEQ-ELELLISG-----------------------
----LPEID-----------VDEW----KNNTEYHN-YSAS-------------------
------------------------SSQI-QWFWRAVR--------------------SFD
KEERAKLLQF-------VTGTSKVP------------LNG-----FK-ELEGMN--GVNK
FNIHRDYGHK------------------------DRLPSSHTCF-NX-LDLPEY-ESYED
LRQRLY-TAVTAGSEYF
>Penicillium_polonicum_MDYM01000006.1 .
LSVRRAEV-FLDSFKSL--------------YFKSADELKYGKLNVRFHG-------EEG
VDAGGVTREW---FQVL----ARGMFNPNY--------ALFIPVAADRT-----------
----------------TFHPNRLS------------------------------------
--GVNSEHLMFFKFIGRIIGKALYEGRVLDCHFSR--AVYKNILG---------------
--------RSVSIKDMET----------------------LDLDYYKS--------LLWM
LEN--DIT-DII--
-TETFSIETDD---------------------------FGE-KQVID-LKPG--------
GHDIP---
------------------------------VTQENKE-EYVQR-VVEYRLVES-VREQLD
-NFLKG---FHEIIPP--ELIS-IFNEQ-ELELLISG-----------------------
----LPEID-----------VDEW----KNNTEYHN-YSAS-------------------
------------------------SSQI-QWFWRAVR--------------------SFD
KEERAKLLQF-------VTGTSKVP------------LNG-----FK-ELEGMN--GVSK
FNIHRDYGHK------------------------DRLPSSHTCF-NX-LDLPEY-ESYED
LRQRLY-TAVTTGSEYF
>Penicillium_freii_LLXE01000097.1 .
LSVRRAEV-FLDSFKSL--------------YFKSADELKYGKLNVRFHG-------EEG
VDAGGVTREW---FQVL----ARGMFNPNY--------ALFIPVAADRT-----------
----------------TFHPNRLS------------------------------------
--GVNSEHLMFFKFIGRIIGKALYEGRVLDCHFSR--AVYKNILG---------------
--------RSVSIKDMET----------------------LDLDYYKS--------LLWM
LEN--DIT-DII--
-TETFSIETDD---------------------------FGE-KQVID-LKPG--------
GHDIP---
------------------------------VTQENKE-EYVQR-VVEYRLVES-VREQLD
-NFLKG---FHEIIPP--ELIS-IFNEQ-ELELLISG-----------------------
----LPEID-----------VDEW----KNNTEYHN-YSAS-------------------
------------------------SSQI-QWFWRAVR--------------------SFD
KEERAKLLQF-------VTGTSKVP------------LNG-----FK-ELEGMN--GVSK
FNIHRDYGHK------------------------DRLPSSHTCF-NX-LDLPEY-ESYED
LRQRLY-TAVTTGSEYF
>Penicillium_solitum_MJCB01000425.1 .
LSVRRAEV-FLDSFKSL--------------YFKSADELKYGKLNVRFHG-------EEG
VDAGGVTREW---FQVL----ARGMFNPNY--------ALFIPVAADRT-----------
----------------TFHPNRLS------------------------------------
--GVNSEHLMFFKFIGRIIGKALYEGRVLDCHFSR--AVYKNILG---------------
--------RSVSIKDMET----------------------LDLDYYKS--------LLWM
LEN--DIT-DII--
-TETFSIETDD---------------------------FGE-KQVID-LKPG--------
GHDIP---
------------------------------VTQENKE-EYVQR-VVEYRLVES-VREQLD
-NFLKG---FHEIIPP--ELIS-IFNEQ-ELELLISG-----------------------
----LPEID-----------VDEW----KNNTEYHN-YSAS-------------------
------------------------SSQI-QWFWRAVR--------------------SFD
KEERAKLLQF-------VTGTSKVP------------LNG-----FK-ELEGMN--GVSK

FNIHRDYGHK------------------------DRLPSSHTCF-NX-LDLPEY-ESYED
LRQRLY-TAVTTGSEYF
>Penicillium_camemberti_CBVV010000570.1 .
LSVRRAEV-FLDSFKSL--------------YFKSADELKYGKLNVRFHG-------EEG
VDAGGVTREW---FQVL----ARGMFNPNY--------ALFIPVAADRT-----------
----------------TFHPNRLS------------------------------------
--GVNSEHLMFFKFIGRIIGKALYEGRVLDCHFSR--AVYKNILG---------------
--------RSVSIKDMET----------------------LDLDYYKS--------LLWM
LEN--DIT-DII--
-TETFSIETDD---------------------------FGE-KQVID-LKPG--------
GHDIP---
------------------------------VTQENKE-EYVQR-VVEYRLVES-VREQLD
-NFLKG---FHEIIPP--ELIS-IFNEQ-ELELLISG-----------------------
----LPEID-----------VDEW----KNNTEYHN-YSAS-------------------
------------------------SSQI-QWFWRAVR--------------------SFD
KEERAKLLQF-------VTGTSKVP------------LNG-----FK-ELEGMN--GVSK
FNIHRDYGHK------------------------DRLPSSHTCF-NX-LDLPEY-ESYED
LRQRLY-TAVTTGSEYF
>Penicillium_expansum_XM_016738102.1 .
LSVRRAEV-FLDSFKSL--------------YFKSADELKYGKLNVRFHG-------EEG
VDAGGVTREW---FQVL----ARGMFNPNY--------ALFIPVAADRT-----------
----------------TFHPNRLS------------------------------------
--GVNSEHLMFFKFIGRIIGKALYEGRVLDCHFSR--AVYKNILG---------------
--------RSVSIKDMET----------------------LDLDYYKS--------LLWM
LEN--DIT-DII--
-TETFSIETDD---------------------------FGE-KQVID-LKPG--------
GHDIP---
------------------------------VTQENKE-EYVQR-VVEYRLVES-VREQLD
-NFLKG---FHEIIPP--ELIS-IFNEQ-ELELLISG-----------------------
----LPEID-----------VDEW----KNNTEYHN-YSAS-------------------
------------------------SSQI-QWFWRAVR--------------------SFD
KEERAKLLQF-------VTGTSKVP------------LNG-----FK-ELEGMN--GVSK
FNIHRDYGHK------------------------DRLPSSHTCF-NQ-LDLPEY-ESYED
LRQRLY-TAVTAGSEYF
>Penicillium_carneum_CBXS010000410.1 .
LSVRRAEV-FLDSFKSL--------------YFKSADELKYGKLNVRFHG-------EEG
VDAGGVTREW---FQVL----ARGMFNPNY--------ALFIPVAADRT-----------
----------------TFHPNRLS------------------------------------
--GVNSEHLMFFKFIGRIIGKALYEGRVLDCHFSR--AVYKNILG---------------
--------RSVSIKDMET----------------------LDLDYYKS--------LLWM
LEN--DIT-DII--
-TETXXIETDD---------------------------FGE-KQVID-LKPG--------
GHDIP---
------------------------------VTQENKE-EYVQR-VVEYRLVES-VREQLD
-NFLKG---FHEIIPP--ELIS-IFNEQ-ELELLISG-----------------------
----LPEID-----------VDEW----KNNTEYHN-YSAS-------------------
------------------------SSQI-QWFWRAVR--------------------SFD
KEERAKLLQF-------VTGTSKVP------------LNG-----FK-ELEGMN--GVSK
FNIHRDYGHK------------------------DRLPSSHTCF-NX-LDLPEY-ESYED
LRQRLY-TAVTXGSEYF
>Penicillium_nordicum_LHQQ01000043.1 .
LSVRRAEV-FLDSFKSL--------------YFKSADELKYGKLNVRFHG-------EEG
VDAGGVTREW---FQVL----ARGMFNPNY--------ALFIPVAADRT-----------
----------------TFHPNRLS------------------------------------
--GVNSEHLMFFKFIGRIIGKALYEGRVLDCHFSR--AVYKNILG---------------
--------RSVSIKDMET----------------------LDLDYYKS--------LLWM
LEN--DIT-DII--
-TETFAIETDD---------------------------FGE-KQVID-LKPG--------

GHDIP---
------------------------------VTQENKE-EYVQR-VVEYRLVES-VREQLD
-NFLKG---FHEIIPP--ELIS-IFNEQ-ELELLISG-----------------------
----LPEID-----------VDEW----KNNTEYHN-YSAS-------------------
------------------------SSQI-QWFWRAVR--------------------SFD
KEERAKLLQF-------VTGTSKVP------------LNG-----FK-ELEGMN--GVSK
FNIHRDYGHK------------------------DRLPSSHTCF-NX-LDLPEY-ESYED
LRQRLY-TAVTAGSEYF
>Penicillium_verrucosum_LAKW01001866.1 .
LSVRRAEV-FLDSFKSL--------------YFKSADELKYGKLNVRFHG-------EEG
VDAGGVTREW---FQVL----ARGMFNPNY--------ALFIPVAADRT-----------
----------------TFHPNRLS------------------------------------
--GVNSEHLMFFKFIGRIIGKALYEGRVLDCHFSR--AVYKNILG---------------
--------RSVSIKDMET----------------------LDLDYYKS--------LLWM
LEN--DIT-DII--
-TETFAIETDD---------------------------FGE-KQVID-LKPG--------
GHDIP---
------------------------------VTQENKE-EYVQR-VVEYRLVES-VREQLD
-NFLKG---FHEIIPP--ELIS-IFNEQ-ELELLISG-----------------------
----LPEID-----------VDEW----KNNTEYHN-YSAS-------------------
------------------------SSQI-QWFWRAVR--------------------SFD
KEERAKLLQF-------VTGTSKVP------------LNG-----FK-ELEGMN--GVSK
FNIHRDYGHK------------------------DRLPSSHTCF-NX-LDLPEY-ESYED
LRQRLY-TAVTAGSEYF
>Entomophthora_muscae_GENB01017218.1 .
LKVQREKI-LESSMKST--------------KNFSVSDW-CGNFEVTFQG-------EQG
IDWGGLRREW---FELI----CSSLFDPRG--------GLFCSFHDKHQA----------
----------------LVHPNPHR------------------------------------
---PSHLKLKHFEFAGKIVGKCLYESXLVRARFTR--SFLAQLIG---------------
------LRVHYKYFEQDD----------------------PDLYLSK---------IKYI
LDTDL--DTT-DSL--
--ELYFVEEIYDGS------------------------GQL-NKTLE-LIPN--------
GSKVR---
------------------------------VTNATKN-QYLDA-LAQQRLCNS-VKDEVD
-SFLKG---LNGIIPD--NLLS-IFDEN-ELELLMCG-----------------------
----TGEYS-----------IADF----KAHHIANG-NSSEF------------------
------------------------RRVL-AWFWAGVS--------------------NFS
QTEMARLLQF-------TTGCSQLP------------PGG-----FQ-ELNPQF--QITA
APTF------------------------------GNLPTAHTCF-NQ-LCLPDY-ESYEQ
FEKALL-LAISEGTEGF
>Mitosporidium_daphniae_XM_013383569.1 .
IKVRRDDI-FRSSFTAF--------KRILSIPKEHSALLHIHSFEVEFLN-------EEG
VDAGGLSREW---ATLM----AAQLQVSEL--------SIFNSYKE--------------
----------------GFYFRRDG------------------------------------
------KNKEYAKFCGAFLGLAISKELTLDCRFSD--LFYRILTS---------------
----DPKNIPLLFKDMEL----------------------IDTDLHRGFSNSDSN-LLND
LENFYKDGDDKKKL-------------------------------------DFF-KAC--
--KRYLIVSPD---------------------------EKI-GAHLP-RDKD--------
GETFTSI---
------------------------------VLTDDDL-NSLRD-ELTHEIYYSALKDTIE
-RFLDG---VHIFIPP--EELQ-LFSIE-GLRKKVEG-----------------------
---EFSEINDKA--------FEKW----KAITVWKY-LTNGKTGIEEETWKNGL------
------------------------KIQK-DWFWEIIQ--------------------KFS
EKEKRDLLQF-------WTGSRNIP------------KT------L--EVNHG---GTV-
----------------------------------NYFPSSSTCL-LS-LNLPYY--KKEK
VAVDGI-ETIKGAKEIM
>Mitosporidium_daphniae_XM_013383402.1 .
FDRIRTNM-TRQEFRPK--------KFCIKSSFKAFKGVLRTEGNTTPLLH------AKG

IDLDGFSREW---ASLM----AIQLQVSEL--------SIFNSYKE--------------
-----------------GFYFRRDG-------------------------------------
------KNKEYAKFCGAFLGLAISKELTLDCRFSG--LFYHILTS---------------
----DPKNIQLSLEDMKL----------------------IDTTLYTGFSNSDSN-LLNE
LEDFYKDGDDKKKL--DFF-KTF--
--KRHLIASSD----------------------------EKI-GTHLP-REEN--------
GEISRSI---
-------------------------------VLTNDEF-NALKD-ELTHEIFYSALEDTIK
-PFLDG---LNMFIPP--EKLQ-QFRIE-DLRKKVEG----------------------
---EFSEINDKA--------FEKW----KAITVWTD-CTNSGIGIKKKTWKKKL------
-------------------------KTQK-DWFWEIVY--------------------EFS
EKEKRDLVQF-------WTGSGNIP------------EN------L--EVIHG---GTV-
-------------------------------------NCLPSSHTCV-FT-LELPYY--KNEV
KVSVDI-ETIKGAKEIM
>Zygosaccharomyces_bailii_HG316454.1 .
ATISREHM-LEDACNAF----------------NSIGERFKA-KLAVTFVNEFGP---EVG
IDGGGITKEF---LTSV----SDEGFNSDKY-------GLFQTNDNY-------------
-----------------ELYPATNL-------------------------------------
----NPQQLRYLWFLGKVLGKCLYDHVLIDVTFAD--FFLKKLLN---------------
----YSTRFTSSIDDMCS----------------------LDPTLYSN--------LVKL
LSMSA---EEV-DALE-
--LSFEVSSE-----------------------------TG----PIE-LVPH--------
GSQLR---
-------------------------------VQKDNVL-FYIVK-VADYKLNQS-LFKQVF
-NFHGG---ISMIIAP--HWME-MFNSV-ELQMLISG----------------------
---KGKDID-----------LQDL----KDNTEYGG-FLPT-------------------
-------------------------DTTI-VHFWQILA--------------------EFN
REERFSFIKF-------VTSVPRAP------------LQG-----FR-SLEPKF--GIRN
AGSEL-----------------------------DRLPTASTCV-NL-LKLPDY-QNKEL
LRQKLL-YAINS-GARF
>Zygosaccharomyces_parabailii_CP019490.1 .
ATISREHM-LEDACNAF----------------NSIGERFKA-KLAVTFVNEFGP---EVG
IDGGGITKEF---LTSV----SDEGFNSDKY-------GLFQTNDNY-------------
-----------------ELYPATNL-------------------------------------
----NPQQLRYLWFLGKVLGKCLYDHVLIDVTFAD--FFLKKLLN---------------
----YSTRFTSSIDDMCS----------------------LDPTLYSN--------LVKL
LSMSA---EEI-DALE-
--LSFEVSSE-----------------------------TG----PVE-LVPH--------
GSQLR---
-------------------------------VQKDNVL-FYIVK-VADYKLNQS-LFKQVF
-NFHGG---ISMIIAP--HWME-MFNSV-ELQMLISG----------------------
---KGKDID-----------LQDL----KDNTEYGG-FLPT-------------------
-------------------------DTTI-VHFWQILA--------------------EFN
REERFSFIKF-------VTSVPRAP------------LQG-----FR-SLEPKF--GIRN
AGSEL-----------------------------DRLPTASTCV-NL-LKLPDY-QNKEL
LRQKLL-YAINS-GARF
>Zygosaccharomyces_rouxii_XM_002494634.1 .
ATISREHM-LEDACNAF----------------NSIGERFKA-KLAVTFVNEFGP---EVG
IDGGGITKEF---LTSV----SDEGFNSDKY-------GLFQSNDNY-------------
-----------------ELYPSTSV-------------------------------------
----DSQQLRYLWFLGKVLGKCLYDHVLIDVTFAD--FFLKKLLN---------------
----YSTRFTSSIDDMCS----------------------LDPTLYSN--------LVKL
LSMSA---EEI-ASLD-
--LTFEITTD-----------------------------NG----PVE-LIPN--------
GSKTR---
-------------------------------VQKETVL-YYIVK-VSDYKLNRS-LFKQIF
-NFHGG---MSMIIAP--HWME-MFNSV-ELQMLISG----------------------
---KGKDVD-----------LQDL----KNNTEYGG-FSQT-------------------

------------------------DPTI-RHFWQILE--------------------EFE
REERFSFIKF-------VTSVPRAP------------LQG-----FK-SLEPKF--GIRN
AGSEL-----------------------------ERLPTASTCV-NL-LKLPDY-RDKEL
LKKKLL-YAINS-GARF
>Torulaspora_delbrueckii_XM_003682796.1 .
ATISRENM-LEDACNAF---------------NSIGERFKA-KLAVTFVNEFGP---EAG
IDGGGITKEF---LTSV----AEEGFSNTRH-------ELFQTNDQH-------------
----------------ELYPTTAV------------------------------------
----NSQKLRYLWFLGKILGKCLYDHVLIDVTFAD--FFLKKMLN---------------
----PSSRFTSSFDDLHS----------------------LDAILYSN--------LVKL
LSMTA--EQL-EELD-
--LSFEINSP----------------------------NGT-GSVVE-LIPN--------
GSKVK---
------------------------------VRKDNVL-LYIIK-VADFKLNKT-LYRQTQ
-NFHGG---MSMIIAP--HWME-MFNSV-ELQMLISG-----------------------
---GGKDID-----------LVDL----KNNTEYGG-FLEN-------------------
------------------------DKTI-NDFWTILQ--------------------EFK
PEERLNFIKF-------VTSVPRAP------------LQG-----FQ-SLEPKF--GIRN
AGRDL-----------------------------ERLPTASTCV-NL-LKLPDY-QDKEL
LRDKLL-YSINS-GARF
>Vanderwaltozyma_polyspora_XM_001645346.1 .
ITISREHV-LEDAYESF---------------NQIGERIKS-KLSVTFVNEFGP---EAG
IDGGGITKEF---LTSV----SEEGFKNEKY-------KLFLTNDKH-------------
----------------ELYPNLNL------------------------------------
----NPVQLKYLWFLGKIVGKCLYDRVLIDVKFTD--FFVKKLLN---------------
----YSNHFSSSFDDLSS----------------------LDSSLYQN--------LVKL
LTMSA--AEI-EDLD-
--LTFELTDE----------------------------SDV-SKVVE-LLPN--------
GSSIK---
------------------------------VNKGNVL-QYIFL-ISDFKLNKS-QFRQVS
-YFHGG---MSMIIAP--HWME-MFNSV-ELQMLISG-----------------------
---GDRDID-----------LNDL----KQNTVYGG-FEEG-------------------
------------------------DVTV-KNFWEILK--------------------EFE
PEERLNFIKF-------VTSVPRGP------------LQG-----FG-SLNPKF--GIRN
SGSDS-----------------------------DRLPTASTCV-NL-LKLPDY-KDKKV
MKEKLL-YAINS-GARF
>Naumovozyma_castellii_XM_003675982.1 .
AVISREHV-LEDAFNSY---------------NKIGERFKS-RLSVTFVNEFGP---EAG
IDGGGITKEF---LTSV----SEEGFKKSEY-------KLFETNDQY-------------
----------------ELYPSTDV------------------------------------
----TPEKLKYLWFMGKIVGKCLYEHVLIDVSFAN--FFLKKLLN---------------
----YSNNFQLSFDDLSS----------------------LDSSLYMN--------LTKL
LAMTD--EEI-QSLG-
--LTFEMTS-----------------------------STK-DKPIE-LIPN--------
GSHIP---
------------------------------VTKENVL-QYLTK-VSNYKLNVS-MFRQCV
-SFHGG---LSTMILP--TWVE-MFNSI-ELQMLISG-----------------------
---GGKDID-----------LDDL----KNNVEYGG-FSSD-------------------
------------------------DKTI-VALWEIMA--------------------ECN
TEERLAFIKF-------VTSVPQAP------------LQG-----FQ-ALEPRF--GIRN
AGTEL-----------------------------DRLPTASTCV-NL-LKLPDY-QDKEL
LREKLL-YAIKS-GARF
>Nakaseomyces_bacillisporus_CAPX01000169.1 .
ATISREHV-LEDAYNSF---------------SGIGERFKS-KLSVTFVNEFGP---EAG
IDGGGITKEF---LTSV----SEEGFTNEKY-------HLFNNNDHY-------------
----------------ELYPSSNL------------------------------------
----NSQQLKYMWFLGKIVGKCLYDHVLIDVRFAD--FFLKKLLN---------------
----FSNKFASTFDDLQS----------------------LDSVLYLN--------LFKL

```
LKMAS----------------------------------------------SEL-ESLD-
--LTFEINESHPI-------------------------TSE-VRTYP-LIED--------
GSSIK-------------------------------------------------------
------------------------------VTQQNVL-LYVMR-VVEFKLNKS-LFKPVH
-SFHSG---LSMIVAP--HWME-IFSSY-ELQMLISG-----------------------
---EGKDID-----------LNDL----QTNTEYGG-FTPE-------------------
------------------------DKTI-RYFWEILE--------------------EFK
PEQRLMFLKF-------VTSVPQAP------------LQG-----FQ-VLEPKF--GIRN
AGPEY-----------------------------DRLPTASTCV-NL-LKLPDY-KNKKL
LRDKLL-YAINS-GAGF
>Candida_castellii_CAPW01000081.1 .
AVISRESV-LEDAFNAF---------------NAIGERLKA-KLAVTFVNEFGR---EAG
IDGGGITKEF---LTSV----TEEGFSEERY-------GLFEHNDNY-------------
----------------ELYPKSHL------------------------------------
----SSQELKYMFFLGKVIGKCLYDRVLVDVTFAE--FFLKKLLN---------------
----FGQRFASGFDDLQS----------------------MDFALYSN--------LAKL
LNMTD----------------------------------------------EEL-RSLD-
--LTFEVSDYNPV-------------------------DKT-VYTVE-LFKG--------
GKHET-------------------------------------------------------
------------------------------VNKANVL-LYVMK-ISDFKLNRS-LFKAVY
-AFYTG---FSLIIAP--HWVW-LFDSK-ELQMLISG-----------------------
---EGKDID-----------LKNL----RENTEYGG-FEGN-------------------
------------------------DLTV-KLFWEILE--------------------EFT
SEERLSFLKF-------VTSVPQAP------------LQG-----FQ-VLEPKF--GIKN
DGPDV-----------------------------DRLPTASTCV-NL-LKLPDY-RDKEL
LRQKLL-YAINA-GARF
>Candida_glabrata_XM_446308.1 .
VNISRDNV-LEDAYSSF---------------NPAGERFKG-KLSVTFTNEFGP---EAG
IDGGGITKEF---LTSV----TEEGFKNDKY-------KLFDTNQNY-------------
----------------ELYPSSEV------------------------------------
----NATKMKYYYFLGKVLGKCIYDRVLIDIHFTD--FLLKKLLN---------------
----SSNHYISYFDDLQS----------------------LDESLYQN--------LVKL
LAMSA----------------------------------------------SDL-ESLE-
--LHFEVDDLS---------------------------TGK-NRVTE-LIPN--------
GSKTV-------------------------------------------------------
------------------------------VTKNNVL-QYLVL-IADFKLNRS-LNKQVA
-ALHRG---LSVMIAP--HWFE-MFNSS-ELQKLISG-----------------------
---EGKDIN-----------LEDL----KSNTIYGG-FSDT-------------------
------------------------SLTI-QYFWQILE--------------------EFE
PQQRLDLLKF-------VTSVPQAP------------LQG-----FG-ALEPKF--GIRN
SGPER-----------------------------DRLPTAATCV-NL-LKLPDY-ADKEL
LRQKLL-YAINA-GAGF
>Nakaseomyces_delphensis_CAPT01000061.1 .
ANISRENI-LEDAYNSF---------------NVVGERFKA-KLSVTFTNEFGP---EAG
IDGGGITKEF---LTSV----SEEALKSGKY-------SLFQTNDKY-------------
----------------ELYPSTQI------------------------------------
----TPTKLKYLYFLGKVVGKCIYDRVLIDVQFAD--FFLKKILN---------------
----YSNHYVSLFDDLQS----------------------LDSSLYHN--------LVKL
LAMDA----------------------------------------------DEL-ATID-
--LRFELTDTS---------------------------NGA-HRIIE-LVPN--------
GSNIR-------------------------------------------------------
------------------------------VNKENVL-KYLVL-IADYKLNKT-LFKQTS
-AFYCG---LRVMIAP--HWME-MFNSG-ELQMLISG-----------------------
---GGKDVD-----------LKDL----MDNTEYGG-FLPG-------------------
------------------------DATI-QYFWEVLA--------------------EFE
PEQRLSFLKF-------VTSVPQAP------------LQG-----FA-TLEPKF--GVRN
TGPDI-----------------------------TRLPTASTCV-NL-LKLPDY-KDKKL
LREKLL-YAINA-GAGF
```

```
>Candida_bracarensis_CAPU01000182.1 .
ANISRENI-LEDAYNSF---------------NVVGERFKA-KLSVTFTNEFGP---EAG
IDGGGITKEF---LTSV----SEEAIKSEKY-------SLFQTNDKY-------------
----------------ELYPSTQI------------------------------------
----NPTKLKYLYFLGKIVGKCIYDRVLIDVQFAD--FFLKKLLN---------------
----YSSHYTSLFDDLQS----------------------LDSSLYQN--------LVKL
LTMEA----------------------------------------------EEL-EAID-
--LRFELTDTS---------------------------SGQ-NKTIE-LVPN--------
GSNIK-------------------------------------------------------
------------------------------VKKENVL-KYLVL-IADFKLNKT-LFKQTS
-AFHRG---LSVMIAP--HWME-LFNSG-ELQMLISG-----------------------
---GGKDVD-----------LTDL----KNNTEYGG-FLPD-------------------
------------------------DPTI-TYFWEILE--------------------EFE
PEQRLSFLKF-------VTSVPQAP------------LQG-----FA-TLEPRF--GIRN
TGSDI-----------------------------TRLPTASTCV-NL-LKLPDY-KTKEL
LRQKLL-YAINA-GAGF
>Saccharomyces_paradoxus_CP020316.1 .
AIISRENV-LEDAFNAF---------------NSIGERFKA-SLDVTFINEFGE---EAG
IDGGGITKEF---LTTV----SDEGFKDPKH-------ELFRTNDRY-------------
----------------ELYPSIVY------------------------------------
----DTLKLKYIWFLGKVVGKCLYEHVLIDVSFAD--FFLKKLLN---------------
----YSNGFLSSFSDLGS----------------------YDSVLYSN--------LIKL
LNMTT----------------------------------------------DEI-KSLD-
--LTFEIDEPES----------------------------P-AKVVD-LIPN--------
GSKTY-------------------------------------------------------
------------------------------VTKGNVL-LYVTK-VTDYKLNKR-CFKPVS
-AFHGG---LSVIIAP--HWME-MFNSI-ELQMLISG-----------------------
---ERDNID-----------LDDL----KSNTEYGG-YTEE-------------------
------------------------DQTI-VDFWEVLN--------------------EFK
FEEKLNFLKF-------VTSVPQAP------------LQG-----FK-ALDPKF--GIRN
AGTEK-----------------------------YRLPTASTCV-NL-LKLPDY-RNKKI
LREKLL-YAINS-GARF
>Saccharomyces_cerevisiae_NM_001181006.1 .
AIISRDNV-LEDAFNAF---------------NSIGERFKA-SLDVTFINEFGE---EAG
IDGGGITKEF---LTTV----SDEGFKDPKH-------ELFRTNDRY-------------
----------------ELYPSVVY------------------------------------
----DATKLKYIWFLGKVVGKCLYEHVLIDVSFAD--FFLKKLLN---------------
----YSNGFLSSFSDLGS----------------------YDSVLYNN--------LIKL
LNMTT----------------------------------------------DEI-KSLD-
--LTFEIDEPES----------------------------S-AKVVD-LIPN--------
GSKTY-------------------------------------------------------
------------------------------VTKDNVL-LYVTK-VTDYKLNKR-CFKPVS
-AFHGG---LSVIIAP--HWME-MFNSI-ELQMLISG-----------------------
---ERDNID-----------LDDL----KSNTEYGG-YKEE-------------------
------------------------DQTI-VDFWEVLN--------------------EFK
FEEKLNFLKF-------VTSVPQAP------------LQG-----FK-ALDPKF--GIRN
AGTEK-----------------------------YRLPTASTCV-NL-LKLPDY-RNKTI
LREKLL-YAINS-GARF
>Saccharomyces_mikatae_AACH01001864.1 .
AIISRDNV-LEDAFNAF---------------NSIGERFKA-PLDVTFINEFGE---EAG
IDGGGITKEF---LTNV----SDEGFKDPKH-------ELFQTNDRY-------------
----------------ELYPSVVY------------------------------------
----DSKKLKFIWFLGKVVGKCLYEHVLIDVSFAD--FFLKKLLN---------------
----CSNGFLSSFPDLGS----------------------YDSVLYSN--------LIKL
LNMTV----------------------------------------------DEI-RSLD-
--LTFEIDEPES----------------------------P-AKVID-LIPN--------
GSKTY-------------------------------------------------------
------------------------------VTKDNVL-LYITK-VTDYKLNKR-CFKPVS
```

-AFHGG---LSAIIAP--HWME-MFNSI-ELQMLISG-----------------------
---ERDNID-----------LDDL----KCNTEYGG-YTED-------------------
------------------------DQTV-VDFWEVLN--------------------EFK
YEDKLNFLKF-------VTSVPQAP------------LQG-----FK-ALDPKF--GVRN
AGTEK-----------------------------YRLPTASTCV-NL-LKLPDY-RNKRI
LREKLL-YAINS-GARF
>Saccharomyces_kudriavzevii_AJIH01002428.1 .
AVISRDSV-VEDAFNAF---------------NSIGERFKA-SLDVTFVNEFGE---EAG
IDGGGITKEF---LTNV----SEEGFKDPKH-------GLFQTNDRY-------------
----------------ELYPSVIY------------------------------------
----DTSKLKYIWFLGKIVGKCLYEHVLIDVSFAD--FFLKKLLN---------------
----HSNGFLSSFSDLGS----------------------YDSVLYSN--------LVKL
LNMTA---DXI-KSLE-
--LTFEIDVPES----------------------------P-AKAID-LIPN--------
GSKIY---
------------------------------VTKDNVL-LYITK-VTDYKLNKR-CVKPVS
-AFHGG---LSVIIAP--HWME-MFNSI-ELQMLISG-----------------------
---ERDNID-----------LDDL----KSNTEYGG-YTEA-------------------
------------------------DQTI-VDFWEVLN--------------------DFK
FEEKLNFLKF-------VTSVPQAP------------LQG-----FK-ALDPKF--GIRN
AGTEK-----------------------------YRLPTASTCV-NL-LKLPDY-RNKKI
LKEKLL-YAINS-GARF
>Saccharomyces_arboricola_ALIE01000079.1 .
AVISRDNV-LEDAFNAF---------------NSIGERFKA-SLDVTFVNEFGE---EAG
IDGGGITKEF---LTNV----SEEGFKDPKH-------ELFQTNDRY-------------
----------------DLYPSVIY------------------------------------
----DTSKLKYMWFLGKIVGKCLYEHVLIDVSFAD--FFLKKLLN---------------
----YSNGFLSSFSDLGS----------------------YDSVLYSN--------LVKL
LNMTA---DQI-QSLD-
--LTFEIDEPEI----------------------------P-AKVID-LIPN--------
GSKTY---
------------------------------VTKDNVL-LYITK-VTDYKLNKR-CFKPVS
-AFHGG---LSVIIAP--HWME-MFNSI-ELQMLISG-----------------------
---ERDNID-----------LDDL----KSNTEYGG-YTAE-------------------
------------------------DQTI-VDFWEVLN--------------------EFE
LEEKLNFLKF-------VTSVPQPP------------LQG-----FK-ALDPKF--GVRN
AGTEK-----------------------------YRLPTASTCV-NL-LKLPDY-RDKRI
LKEKLL-YAINS-GARF
>Saccharomyces_eubayanus_XM_018365000.1 .
AIISRENV-LDDAFNAF---------------NSIGERFKA-SLDVTFVNEFGE---EAG
IDGGGITKEF---LTNV----TEEGFKDSKH-------ELFQTNDHY-------------
----------------DLYPSVVY------------------------------------
----DASKLKYIWFLGKIVGKCLYEHVLIDVSFSD--FFLKKLLN---------------
----YSNGFLSSFSDLGS----------------------YDSVLYSN--------LVKL
LNMSA---EEI-KSLD-
--LTFEIDEPNS----------------------------P-AKVVD-LIPN--------
GSKTY---
------------------------------VTKDNVL-LYITK-VTDYKLNRR-CFKPVS
-AFHGG---LSVIIAP--HWME-MFNSI-ELQMLISG-----------------------
---ERDNID-----------LGDL----KANTEYGG-YTED-------------------
------------------------DQTI-VDFWEVLN--------------------TFK
FEEKLSFLKF-------VTSVPQAP------------LQG-----FK-ALDPKF--GIRN
AGTEK-----------------------------FRLPTASTCV-NL-LKLPDY-RNKKV
LKEKLL-YAINS-GARF
>Saccharomyces_pastorianus_LOQJ01003216.1 .
AIISRENV-LDDAFNAF---------------NSIGERFKA-SLDVTFVNEFGE---EAG
IDGGGITKEF---LTNV----TEEGFKDSKH-------ELFQTNDHY-------------
----------------DLYPSVVY------------------------------------

----DASKLKYIWFLGKIVGKCLYEHVLIDVSFSD--FFLKKLLN---------------
----YSNGFLSSFSDLGS----------------------YDSVLYSN--------LVKL
LNMSA---EEI-KSLD-
--LTFEIDEPNS----------------------------P-AKVVD-LIPN--------
GSKTY---
------------------------------VTKDNVL-LYITK-VTDYKLNRR-CFKPVS
-AFHGG---LSVIIAP--HWME-MFNSI-ELQMLISG-----------------------
---ERDNID-----------LGDL----KANTEYGG-YTED-------------------
------------------------DQTI-VDFWEVLN--------------------TFK
FEEKLSFLKF-------VTSVPQAP------------LQG-----FK-ALDPKF--GIRN
AGTEK------------------------------FRLPTASTCV-NL-LKLPDY-RNKKV
LKEKLL-YAINS-GARF
>Saccharomyces_bayanus_AACG02000003.1 .
AIISRENV-LDDAFNAF---------------NSIGERFKA-SLDVTFVNEFGE---EAG
IDGGGITKEF---LTNV----TEEGFKDPKH-------ELFQTNDHY-------------
----------------DLYPSVVY------------------------------------
----DASKLKYIWFLGKIVGKCLYEHVLIDVSFAD--FFLKKLLN---------------
----YSNGFLSSFSDLGS----------------------YDSVLYSN--------LVKL
LNMSA---DEI-KSLD-
--LTFEIDEPNS----------------------------P-AKVVD-LIPN--------
GSKTY---
------------------------------VTKDNVL-LYITK-VTDYKLNRR-CFKPVS
-AFHGG---LSVIIAP--HWME-MFNSI-ELQMLISG-----------------------
---ERDNID-----------LSDL----KSNTEYGG-YTAD-------------------
------------------------DQTI-VDFWEVLN--------------------EFE
FEEKLSFLKF-------VTSVPQAP------------LQG-----FK-ALDPKF--GIRN
AGTEK------------------------------YRLPTASTCV-NL-LKLPDY-RNKKL
LREKLL-YAINS-GARF
>Saccharomyces_uvarum_JNVO01000054.1 .
AIISRENV-LDDAFNAF---------------NSIGERFKA-SLDVTFVNEFGE---EAG
IDGGGITKEF---LTNV----TEEGFKDPKH-------ELFQTNDHY-------------
----------------DLYPSVVY------------------------------------
----DASKLKYIWFLGKIVGKCLYEHVLIDVSFAD--FFLKKLLN---------------
----YSNGFLSSFSDLGS----------------------YDSVLYSN--------LVKL
LNMSA---DEI-KSLD-
--LTFEIDEPNS----------------------------P-AKVVD-LIPN--------
GSKTY---
------------------------------VTKDNVL-LYITK-VTDYKLNRR-CFKPVS
-AFHGG---LSVIIAP--HWME-MFNSI-ELQMLISG-----------------------
---ERDNID-----------LSDL----KSNTEYGG-YTAD-------------------
------------------------DQTI-VDFWEVLN--------------------EFE
FEEKLSFLKF-------VTSVPQAP------------LQG-----FK-ALDPKF--GIRN
AGTEK------------------------------YRLPTASTCV-NL-LKLPDY-RNKKL
LREKLL-YAINS-GARF
>Kazachstania_africana_XM_003955862.1 .
AVISREHI-LEDAMKSF---------------NGIGDKFKS-KLSVTFVNEFGP---EEG
IDGGGVTKEF---LTSV----SEEGFTDPKY-------DLFQTNTDY-------------
----------------DIYPKPTR------------------------------------
----DLAKLNKLEFLGKIVGKCIYERVLIDVTFAD--FFLKKLLN---------------
----YGDHFRSSFDDLSS----------------------LDFDLYAN--------LTKL
LLMSS---EEI-DSLD-
--LYFELTDMTN----------------------------A-SRTIE-LIPN--------
GSHVK---
------------------------------VTKRTLL-QYILK-IAEYKLDRT-LFEPVY
-FFHRG---VSSLIGP--MWLE-LFNSI-ELQMLISG-----------------------
---GHKNVD-----------LKDL----RENTEYGG-YIEN-------------------
------------------------DDTI-KYFWEILE--------------------EFS
MDERSKFLKF-------VTSVPQAP------------LQG-----FG-ALEPKF--GIRN

AGFDL-----------------------------DRLPTASTCV-NL-LKLPDY-GNKEL
LKEKLL-YSINS-GARF
>Kazachstania_saulgeensis_FXLY01000003.1 .
STISRDNI-LEDAMEAY---------------NNKGDLFKS-SLSVKFINEFGP---EEG
IDGGGVTKEF---LTSV----AEEGFKDAKY-------GLFTTNEQY-------------
----------------ELYPHPTG------------------------------------
----DIMALERLSFMGKIVGKCLYDHVLIDIDFAR--FFLKKLLN---------------
----YSNMFRSTFDDLNS----------------------LDSTLYRT--------LTKL
LAIDNT---KEI-DSLD-
--LYFEINDS------------------------------K-GNIIE-LIPN--------
GSKTK---
------------------------------VNKHNIL-KYVLK-IAEYKLDNS-LLEAVT
-AFHRG---LSVMIPP--IWLE-MFNPE-EFEMLISG-----------------------
---GKKDFD-----------IVDL----QNNTEYGG-YKED-------------------
------------------------DITI-RNFWDIIN--------------------DMT
SEERCKFLKF-------VTSVPQAP------------LQG-----FE-SLDPKF--GIRN
AGNDL-----------------------------TRLPTASTCV-NL-LKLPDY-VDKET
MRSKLL-YSINS-GARF
>Tetrapisispora_phaffii_XM_003684998.1 .
VSISRENL-LEDAYNAY---------------NNVGEQLKS-KISVNFVNEFGP---EAG
IDGGGISKEL---LTSV----TEEGFNSDKY-------GLFRTNDNY-------------
----------------ELYPNPNV------------------------------------
----TLSKLNLILFLGKIVGKCLYDRVLIDVKFTE--FFMKKILN---------------
----YSNKLISSFDDLSS----------------------LDNVLYQN--------LVKL
LNMDS--NEV-EALE-
--LTFEIADENN----------------------------R-QKVIE-LVPN--------
GSTKK---
------------------------------VTKSNIL-QYIFA-IADLKLNRQ-LYLPCL
-YFMKG---MNMIIAP--HWME-IFNPV-ELQMLISG-----------------------
---GDRDID-----------LEDL----RENTIYGG-FEQN-------------------
------------------------DRTI-QDFWQTLS--------------------DFE
PSERVKFIKF-------VTSVPRGP------------LQG-----FG-SLTPKF--GIRN
SGTDK-----------------------------DRLPTASTCV-NL-LKLPNY-QDKAL
LKQKLL-YAINS-GARF
>Saccharomycetaceae_sp._CP006025.1 .
AIISRNNI-LEDAYEAY---------------NPLGEDFKD-QLAVTFVNEFGP---EAG
IDGGGITKEF---LTSV----SDEGFNKDKY-------NLFKANDAY-------------
----------------EIYPSDAT------------------------------------
----SPQQLQYLHFLGKVLGKCLYEKVLIDVHFAD--FFLHKLLS---------------
----TSKNMACSFDNLPS----------------------FDRELYDN--------LNKL
FDMST--AEL-QLLD-
--LRMEIVDEN-----------------------------T-KKIVE-LVPG--------
GSHIQ---
------------------------------VTATNVR-QYALA-VASYRMNAQ-LARATF
-YFQTG---MAVIIPP--HWIA-MFSSS-ELSKLISG-----------------------
---GGRDID-----------LADL----RANVTYGG-YVES-------------------
------------------------DPTI-QHLWQLLV--------------------EFT
PEQRCKFIKF-------ATSVPRAP------------LLG-----FQ-MLNPKF--GIRN
AGPDY-----------------------------NRLPTASTCV-NL-LKLPDY-QDKDL
LREKLL-YAINS-EARF
>Ashbya_gossypii_NM_209169.1 .
AIISRNNI-LEDAYEAY---------------NPLGEDFKD-QLAVTFINEFGP---EAG
IDGGGITKEF---LTSV----SDEGFNKDKY-------RLFKANSVY-------------
----------------ELYPSDAA------------------------------------
---TSPQQLQYLHFLGKVLGKCLYEKVLIDVHFAD--FFLHKLLS---------------
----TSKNMACSFDNLPS----------------------FDRELYDN--------LNKL
FDMST--AEL-QLLD-
--LRMEIVDEN-----------------------------T-MKIVE-LVPG--------

GSQIP---
------------------------------VTTTNVR-QYALA-VASYRMNSQ-LARATF
-YFQTG---MAVIIPP--HWIA-IFSSS-ELSKLISG-----------------------
---GGRDID-----------LADL----RANVTYGG-YVES-------------------
------------------------DPTI-QHLWQLLL--------------------EFT
PEQRCKFIKF-------ATSVPRAP------------LLG-----FQ-MLNPKF--GIRN
AGTDY-----------------------------NRLPTASTCV-NL-LKLPDY-QDKDL
LREKLL-YAINS-EARF
>Eremothecium_cymbalariae_XM_003645631.1 .
ATISRSRI-LEDAYEAF---------------NPIGEAFKE-QLAVTFVNEFGP---EVG
IDGGGITKEF---LTSI----SDEGFNKDKY-------GLFESNSSY-------------
----------------ELYPSDTA------------------------------------
---TSPLQLKYLQFLGKILGKCLYEKVLIDVNFAD--FFLHKLLN---------------
----TSKNMVCSFDNLSS----------------------FDQELYDN--------LNKL
FDMTN--DEL-LQLD-
--LRMELFDNA-----------------------------T-KKFVE-LIPG--------
GSDIP---
------------------------------VSVTNVR-QYAMA-IASYKINAK-LYKPTL
-FFQTG---MSTIIPP--HWIS-IFSSR-EISNLISG-----------------------
---GGKDID-----------LSDL----KANVTYGG-YVET-------------------
------------------------DKTI-QDLWQLLI--------------------EFT
PEERCKFIKF-------ATSVPRAP------------LLG-----FQ-MLNPNF--GIHN
AGRDG-----------------------------NRLPTASTCV-NL-LKLPDY-QDKNL
LREKLL-YAINA-EARF
>Eremothecium_coryli_AZAH01000001.1 .
ATISRQNV-LEDAYVAF---------------NPIGEAFKE-QLSVTFVNEFGP---EAG
IDGGGITKEF---LTSV----SDEGFNNDKY-------GLFKSNSNY-------------
----------------ELYPSDEA------------------------------------
---TTPQQLQYLRFMGKILGRCLYENVLIDVSFAD--FFLHKLLN---------------
----TSKNMVCSFDNLSS----------------------YDKELYDN--------LNKL
FDMSN--DEL-TQLD-
--LTMEIFDDS-----------------------------S-KKLVT-LIPD--------
GANIP---
------------------------------VTQTNVR-QYAMA-VASYKLNSK-LHKPTS
-YFQMG---MSVIIPP--HWIS-MFSST-ELNNLISG-----------------------
---DARDID-----------LEDL----RANVSYGG-YLES-------------------
------------------------DPTI-QNLWLILS--------------------EFS
PAERCKFIKF-------TTSVPRAP------------LLG-----FK-MLNPKF--GIRN
AGSDL-----------------------------LRLPTASTCV-NL-LKLPDY-QDKEL
LREKLL-YALNS-EARF
>Eremothecium_sinecaudum_XM_018131064.1 .
ATISRENV-LEDAFAAY---------------NPIGEAFKE-QLAVTFVNEFGP---EAG
IDGGGITKEF---LTSV----SDQGFNNDKY-------GIFKTNDRY-------------
----------------ELYPSEEA------------------------------------
---VTQQQLQYLHFLGKVLGRCLYEKVLIDVHFAD--FFLHKLLN---------------
----ASKNMVCSFDNLSS----------------------YDKELHDN--------LNKL
FVMSK--EEL-LQLD-
--LRMEIVDDS-----------------------------T-KNLVQ-LVPN--------
GSKVP---
------------------------------VTDTNVR-QYAMA-IASYKMNSK-LYRPTL
-YFQMG---LSVIIPS--HWIE-MFSST-ELSNLIAG-----------------------
---EARDID-----------LADL----KSNVSYGD-YLDT-------------------
------------------------DPTI-RYLWEILA--------------------EFS
PEERCKFIKF-------TTSVPRAP------------LLG-----FQ-MLNPKF--GIRN
AGSDA-----------------------------TRLPTASTCV-NL-LKLPDY-QNKEL
LRQKLL-YALNS-ESRF
>2_Lachancea_kluyveri_AACE03000003.1 .
ATISRENV-LEDAYNSF---------------NPIGESFKA-KLGVTFINEFGQ---EAG

IDGGGITKEF---LTSV----SDEGFKNEKY-------RLFVENSEH-------------
----------------ELYPSSRI------------------------------------
---KSPLHLKYLWFMGKVLGKCLYEQVLIDVTFAD--FFMKKILK---------------
----NEQNYHSSFDDLAS----------------------LDQELYSN--------LVKL
LDMRQ--DEI-DALE-
--LHFEITDDD-----------------------------T-KTSVE-LVPN--------
GSDKR---
------------------------------VTKANIF-QYVLC-VADYKLNRK-LYRPTL
-YFQRG---LSAMIPS--HWIE-IFNSV-ELQMLISG-----------------------
---GSKDID-----------LEDL----KASTQYGG-YLEN-------------------
------------------------DKTV-VHFWEILA--------------------EFT
PQQRFKFIKF-------VTSVPRAP------------LQG-----FA-ALNPHF--GIRN
AGREI-----------------------------DRLPTASTCV-NL-LKLPDY-KDKNL
LKQKLL-YAINA-EARF
>Lachancea_mirantina_FJUN01000011.1 .
ATISREHT-LEDAFKAF---------------NPIGESLKS-RLAVTFVSEFGP---EAG
IDGGGITKEF---LTSV----TEEGFKADTM-------ELFKANEKH-------------
----------------EIYPSPKI------------------------------------
---NSKQHFNYLFFMGKILGKCLYDHVLIDVAFAD--FLLKKMLS---------------
----SQNSFQSSFDDLAS----------------------LDSTLYRN--------LVKL
LAMDT--KEL-ELLD-
--LRFEVTDDE-----------------------------S-KQAVE-LINR--------
GSQIP---
------------------------------VTRSNVL-EYVLS-IAEYKLNRK-SRIPTR
-YFLGG---LTVMIPA--HWIQ-MFNSV-ELQMLISG-----------------------
---GGKDIN-----------LADL----RANTEYGD-YVDG-------------------
------------------------DITI-RHFWDILR--------------------EFS
PEQRLKFVKF-------VTSVPRAP------------LQG-----FG-SLDPHF--GIRN
AGRDI-----------------------------ARLPTASTCV-NL-LKLPDY-RDKDL
LRQKLL-YALTS-DARF
>Lachancea_cidri_FJUT01000025.1 .
ATISRENP-LDDAFEAF---------------NPIGESFKA-KLSVTFVNEFGP---EAG
IDGGGITKEF---LTSV----SDEGFKDGKY-------GLFKQNEKH-------------
----------------EIYPSSKI------------------------------------
---DSSKHLNYLWFMGKVLGKCLYEHVLIDVSFTD--FFLKKLLN---------------
----SQNNFSSSFDDLAS----------------------LDSTLYSN--------LVKL
LGMSA--AEL-EALD-
--LHFEVTDDD-----------------------------T-LLDVN-LIPN--------
GSHTV---
------------------------------VTKNNAL-QYVLS-VADYKLNRK-LKKATR
-YFCGG---LSMMIPF--HWIG-MFNSV-ELQMLISG-----------------------
---GGKDID-----------FKDL----RSNTEYGD-YTER-------------------
------------------------DETI-INFWQILE--------------------ECT
SDQRLKFVKF-------VTSVPRAP------------LQG-----FG-SLVPRF--GIRN
AGNDV-----------------------------ARLPTSSTCV-NL-LKLPDY-RDKEL
LRQKLL-YAINA-EARF
>Lachancea_fermentati_FJUO01000015.1 .
ATISRESA-LDDAFKAF---------------NPIGESFKA-RLSVTFVNEFGP---EAG
IDGGGITKEF---LTSV----SDEGFKDVKY-------HLFKHNERH-------------
----------------EIYPSGKI------------------------------------
---DSPQHLKYLWFMGKVLGKCLYEHVLIDVSFTD--FFLKKILN---------------
----SQNQFYSSFDDLAS----------------------LDATLYAN--------LVKL
LEMST--AEL-RALD-
--LHFEVTDED-----------------------------S-LRNVE-LILN--------
GSNTK---
------------------------------VDKSNVL-QYVLS-VADFKLNRK-LNKATR
-YFCGG---LSVMIPL--HWVG-MFNSV-ELQMLISG-----------------------
---GGKDID-----------LNDL----HANTEYGD-YSEQ-------------------

------------------------DKTI-QYFWRILE--------------------EFT
PEQRLKFVKF-------VTSVPRAP------------LQG-----FG-SLVPRF--GIRN
AGSDI-----------------------------GRLPTASTCV-NL-LKLPNY-KDKHL
LKDKLL-YAINA-EARF
>Lachancea_thermotolerans_XM_002554575.1 .
ATISREHL-LEDAYNAY---------------NPIGESFKS-KLSITFVNEFGP---EAG
IDGGGITKEF---LTSV----TGEAFKIDKH-------KLFRSNNNH-------------
----------------EIYPTSNL------------------------------------
---TSAQSVKFLWFLGKVLGKCLYDHVLINVTFAD--FFLKKLLN---------------
-----VNNANSSFDDLAS----------------------LDSELYSN--------LAKL
SEMNA--EQL-QSLG-
--LRFEVTDED-----------------------------T-LEAVD-LMPS--------
CPASF---
------------------------------VTKSNVL-QYLLA-VADYKLNRK-LKTGSR
-AFAGG---LYTMIPP--HWIE-MFNSV-ELQMLISG-----------------------
---GDKDID-----------LADL----RKHTEYGD-YSEQ-------------------
------------------------HQTV-RDFWEILE--------------------ECT
AEERLKFIKF-------VTSVPRAP------------LQG-----FG-SLNPLF--GIRN
AGRET-----------------------------ARLPTASTCV-NL-LKLPDY-RNKQL
LKTKLL-YAINA-EARF
>Lachancea_quebecensis_CZLH01000067.1 .
ATISREHL-LEDAYNAY---------------NPIGESFKS-KLSITFVNEFGP---EAG
IDGGGITKEF---LTSV----TDEAFKIDKH-------KLFRSNSNH-------------
----------------EIYPASKL------------------------------------
---LSAQSVKYLWFLGKVLGKCLYDHVLIDVTFAD--FFLKKLLN---------------
-----INNAKSSFDDLAS----------------------LDSELYSN--------LAKL
SEMNA--EQL-QSLG-
--LRFEATDED-----------------------------T-LETVN-LMPN--------
-RPAS---
-----------------------------IVTKSNVL-QYLLA-VADYKLNRK-LKTGSR
-AFTGG---LYTMIPP--HWVE-MFNSV-ELQMLISG-----------------------
---GDKDID-----------VADL----RKHTEYGD-YTDQ-------------------
------------------------HQTV-HDFWEILE--------------------ECT
AEERLKFIKF-------VTSVPRAP------------LQG-----FG-SLNPLF--GIRN
AGRET-----------------------------ARLPTASTCV-NL-LKLPDY-RNKQL
LKTKLL-YAINA-EARF
>Lachancea_waltii_AADM01000007.1 .
ATISRENL-LEDAFAAY---------------NPIGESFKS-KLSITFVNEFGP---EAG
IDGGGITKEF---LTSI----TDKAFKDDKY-------QLFKHNKNH-------------
----------------EIYPASNL------------------------------------
---NDSANVNYVWFLGKVLGKCLYDHVLVDVTFAD--FFLKKLLN---------------
-----FNNSNSSFDDLAS----------------------LDPDLYSN--------LAKL
LVMNS--DEL-QALG-
--LLFEITDED-----------------------------T-HRTVE-LVPN--------
GSSLR---
------------------------------VDKSNVL-HFLLA-VSDFKLNKK-LRVGTR
-AFTGG---LYTMIPP--HWIE-MFNSA-ELQMLISG-----------------------
---GGKDID-----------LEDL----QNNTEYGD-YTYK-------------------
------------------------HQTI-KDFWEILA--------------------ECS
PEDRLKFVKF-------VTSVPRAP------------LQG-----FG-SLSPRF--GIRN
AGQDT-----------------------------SRLPTASTCV-NL-LKLPDY-RNKEI
LRAKLL-YAINA-EARF
>Lachancea_dasiensis_FJUP01000022.1 .
ATISREHL-LEDAFNAY---------------NPIGENFKS-KLSVTFVSEFGP---EAG
IDGGGITKEF---LTSV----SDQGFKDEKY-------HLFEENEHH-------------
----------------EIYPSASI------------------------------------
---HSSKHLKYLWFLGKVLGKCLYDHVLIDVTFAD--FFLKKLLN---------------
-----VNQMNSSFDDLAS----------------------FDASLYTN--------LARL

```
IKMNS----------------------------------------------SEL-QALG-
--LRFEITDNE-----------------------------S-LQTVD-LIPS--------
GADTA-------------------------------------------------------
------------------------------VTKTNVL-QYLLA-VADYKLNRK-LRLGTR
-SFTGG---LYTIVPP--HWLE-MFSSI-ELQMLISG-----------------------
---GGKDID-----------LTDL----HKHTEYGD-YSEQ-------------------
------------------------DQTI-KDFWSILA--------------------DFD
SQDRLKFVKF-------VTSVPRAP------------LQG-----FR-ALNPLF--GIRN
AGSDV-----------------------------TRLPTASTCV-NL-LKLPDY-QNREL
LKTKLL-YAITA-EARF
>Lachancea_nothofagi_FJUQ01000033.1 .
ATISREHL-LEDAFNAY---------------NPIGESFKS-KLSVTFVNQFGP---EAG
IDGGGITKEF---LTSV----SDEGFKQDQY-------HLFKENHDH-------------
----------------ELYPSDTL------------------------------------
---TSAQHVKYLWFLGKVLGKCLYDHVLIDVNFAD--FFLKKLLN---------------
-----VNQSNSSFDDLAS----------------------FDSALYSN--------LVKL
LGMSA----------------------------------------------DEL-QNLG-
--LRFEITDEA-----------------------------T-LQTVD-LVPN--------
GSDII-------------------------------------------------------
------------------------------VTKSNTL-QYLFA-VADFKLNRK-LRLGTR
-SFTGG---LYTMIPP--HWLE-MFNSI-ELQMLISG-----------------------
---GGKDID-----------LSDL----HRHSEYGD-FSEQ-------------------
------------------------DQTI-RDFWTILA--------------------EFE
PQDRFKFVKF-------VTSVPRAP------------LQG-----FG-SLNPLF--GIRN
AGPDA-----------------------------SRLPTASTCV-NL-LKLPDY-RNREV
LRNKLL-YAINA-EARF
>Lachancea_meyersii_FJUM01000012.1 .
ATISRENL-LEDAFEAF---------------NPIGESFKS-KLSVAFVNEFGP---EAG
IDGGGITKEF---LTSV----SDEGFKTDKH-------KLFEGNENH-------------
----------------EIYPSGNL------------------------------------
---NSAQSLRYMWFLGKVLGKCLYDHVLIDVTFAD--FFLKKLLN---------------
-----VNNSNSSFDDLSS----------------------LDSTLYSN--------LVKL
LNMTP----------------------------------------------WDL-QSLG-
--LRFEVTDEE-----------------------------T-LQTVE-LVPN--------
GSQKA-------------------------------------------------------
------------------------------VDKSNTL-QYLLA-TADYKMNRK-LRLGTR
-AFTGG---LYTMIPP--HWLE-MFNSI-ELQMLISG-----------------------
---GGKDID-----------LADL----RKHTEYGE-FSDD-------------------
------------------------DQTV-QDFWAIMA--------------------EFG
PEDRLNLVKF-------VTSVPRAP------------LQG-----FG-SLNPLF--GIRN
AGHDV-----------------------------SRLPTASTCV-NL-LKLPNY-QNREL
LKNKLL-YAINA-EARF
>Lachancea_lanzarotensis_CDLU01000007.1 .
ATISRENV-LEDAYNAY---------------NPIGESFKS-KLSVTFVNEFGP---EAG
IDGGGITKEF---LTSV----SDEGFKTDRH-------KLFEHNDSH-------------
----------------EIYPSGKL------------------------------------
---KSAQSLRYLWFLGKVLGKCLYDHVLVDVTFAD--FFLKKVLN---------------
-----VNSSNSSFDDLSS----------------------FDSDLYAN--------VVKL
LGMSP----------------------------------------------SEL-QALG-
--LRFEVTDEE-----------------------------T-FQTVE-LVPN--------
GSNKV-------------------------------------------------------
------------------------------VDKTNTL-QYLLA-LADYKLNRK-LRLGTR
-SFTGG---LYTMIPP--HWLE-MFNSV-ELQMLISG-----------------------
---GGKDID-----------LSDL----RQHTEYGE-FSDE-------------------
------------------------DRTI-QDFWSIMA--------------------EFQ
PQERLNLVKF-------VTSVPRAP------------LQG-----FS-SLNPLF--GIRN
AGQDI-----------------------------SRLPTASTCV-NL-LKLPDY-QDRQT
LKNKLL-YAINA-EARF
```

>Tetrapisispora_blattae_XM_004178993.1 .
ATISRKNL-LKDAFESF---------------NKIGERFKG-KLSVQFKNEFDEI--EVG
IDGGGISKEF---LTSL----CAESFNGDS--------RLFQNNEQY-------------
----------------EIYPKVEG------------------------------------
---IKSEDMRYYWFLGKVIGKCLYEHTLVDVQFAP--FFLRKLLN---------------
----YKNQFKSNIDDLNN----------------------YDPTIYEN--------LMKL
IHMNE--EEL-MTMD-
--LSFKK--N--------
GEDEK---
------------------------------VNKRNVI-RYLME-YCNRKLNEE-LNRQTR
-AFHGG---LSVIIAP--HWMD-MFNSI-ELQELISG----------------------
---DGNDID-----------LMDL----KNNINYGI-YDQK-------------------
------------------------DLTI-QYLWEVLE--------------------EMD
SKERLKFVKF-------VTSIPRAP------------LQG-----FG-SLNPKF--GIHN
AGDNT-----------------------------NLLPTASTCV-NL-LKLPNY-RNKEL
LRSKLL-YAINA-GAGF
>Naumovozyma_dairenensis_XM_003671696.1 .
GTISRDSI-LEDAYNSF---------------GDIGEGMKM-KLDVTFVNEFGP---EAG
IDGGGITKEM---LTCV----TDEGFKDPEK-------ELFQTTGTY-------------
----------------KLYPMIAK------------------------------------
-NGITREKGKYFEFMGAILGKCLYDHVLIDVQFAN--FFLQKLLN---------------
----CSNNFLVGFDDLVD----------------------LDPVLYRN--------LLKL
VSMSA--TEL-KELD-
--LNFEI-TSAT----------------------------G-DGTVE-LIPN--------
GANVK---
------------------------------VSKENVL-LYLTK-VSDYKLNIS-IAIQTQ
-RFHDG---LMSIIKP--LWME-LFNAR-ELQMLISG----------------------
---AGKDID-----------LKNL----KENTVYGG-YVET-------------------
------------------------DLTI-RYFWEILE--------------------EFT
TEQRFEFVKF-------VTSVPQAP------------LRG-----FQ-TLEPLF--GIRN
AGSGDL----------------------------GRLPTASTCV-NL-LKLPDY-QNKTI
LKEKLL-YAIEA-GAGF
>Hanseniaspora_osmophila_LPNM01000005.1 .
ATISRDNV-LLDSFNAF---------------DSLGEKFKG-KLSLTFTNEFGE---EAG
IDGGGLTKEF---LTSV----TANGFLEHP--------ALFAENIDH-------------
----------------EIFCKPYN------------------------------------
--NISLENYRLIRFMGKCLGKCLYENVLIDVQFAP--FFLNKLLK---------------
-CDRFAGGYKSSFDELSS----------------------MDSVLYKN--------LMQL
WTMSA--KEL-ESMG-
--LSFEITDDE---------------------------NPY-GSMIE-LVPN--------
GAQII---
------------------------------VDRTNVL-DYMNK-VADYKMNSKRMLASMK
-AFIEG---FNTIIPQ--HWIG-LFTPL-ELQKLISG----------------------
---GESHIN-----------ISDL----RNHTEYGG-YLPT-------------------
------------------------DETI-VDLWDVLEN-------------------ELS
DEEKAKFVKF-------VTSVPKAP------------LSG-----FK-ALQPLF--GIRN
AGREL-----------------------------GKLPTSSTCV-NL-LKLPDY-RDKKL
LAAKLR-KSILS-ESGF
>Hanseniaspora_vineae_JFAV02000080.1 .
ATISRNNV-LLDSFNAF---------------DFLGEKFKG-KLSLTFTNEFGE---EAG
IDGGGLTKEF---LTSV----TANGFLEHP--------ELFTENVDH-------------
----------------EVFCKPYN------------------------------------
--KITQNNYKLIRFMGKCLGKCLYENVLIDVQFAP--FFLNKLLK---------------
-CDRFAGGYKSSLDELSS----------------------MDSILYKN--------LMQL
WTMRT--DQL-ENMG-
--LAFEITDDE---------------------------NPY-GPMIE-LVPN--------
GSHIP---
------------------------------VDRSNVL-DYMNK-VADYKMNSKRMLTSMK

-AFIEG---FNTIIPQ--HWIG-LFTPL-ELQKLISG-----------------------
---GESHID-----------ISDL----REHTEYGG-YLAT-------------------
------------------------DQTI-VDLWDVLEN-------------------ELS
NEERAKFVKF-------VTSVPKAP------------LTG-----FK-ALQPLF--GIRN
AGREI-----------------------------GKLPTSSTCV-NL-LKLPDY-RDKKL
LAAKLR-KSIHS-ESGF
>Kazachstania_naganishii_HE978322.1 .
ATISREHV-LEDAMNSF----------------SRGSDLKS-KLSVTFVNQFGV---EEG
IDGGGVTKEF---LTSV----IEEGFKDEDDKY-----RLFTTNDQH-------------
----------------ELYPVPTY------------------------------------
----DHERLRAVEFLGRIVGKCLYDHILVDLTFAD--FFLKCLLT---------------
------GSEHFSIDDLKS----------------------LDSSLHSS--------LVKL
LNMPG--PEI-DRLE-
--LCFEATDMF---------------------------DP--HLTRD-LVPN--------
GSRVP---
------------------------------VTRGNVL-EYVWR-LTQYKLRSI-AKPATM
-RFREG---LAGLIPH--HWFA-MFNTV-DLEMLISG-----------------------
---GRRQID-----------LEDL----QRNTEYGG-YEAA-------------------
------------------------DETV-RDFWAVLG--------------------EFD
EPERRKFLKF-------VTSVPQAP------------LKG-----FS-ALEPKF--GIRN
SGQEDR----------------------------DRLPTASTCV-NL-LKLPDY-RDRRH
LKQKLL-YSINS-GARF
>Kluyveromyces_aestuarii_AEAS01000169.1 .
ATIRRAYP-LEDAYSAF---------------GSFGEGFKE-KLGIQFVNEFGP---EAG
IDGGGITKEF---LQTL----IEEAFNKNSY-------QLFSTTPFH-------------
----------------KLYPSNSI------------------------------------
----SSENLKYISFMGKVLGKCLYDHILVDVDLAD--FFLKKILN---------------
----VENNMNVPFNDLYS----------------------LDPEYYKN--------LMKL
LEMSK--EEL-QSMD-
--LFFEI-DST---------------------------AG---HRVP-LIND--------
GFSIK---
------------------------------VNRDNVF-EYITR-VAHYKLNLQ-LYSVTS
-RFVSG---LSFIIPP--HWLR-MFTSY-ELKTLISG-----------------------
---SERELD-----------VDDL----KANTEYGG-YTET-------------------
------------------------NLTI-KYFWQVLE--------------------SFD
AEEKRKFLKF-------VFSVPTAP------------LQG-----FH-SLNPLF--GIHN
AGRDK-----------------------------QRLPTASTCV-NL-LKLPDY-QDYEI
LKTKLL-ASINS-NSRF
>Kluyveromyces_wickerhamii_AEAV01000173.1 .
ATIRREHA-LEDAYEAF---------------ASIGEGFKE-KLGIQFVNDFGP---EAG
IDGGGITKEF---LQTL----IQEGFIKNSF-------ELFDTTPFN-------------
----------------KLYPSKNV------------------------------------
----SSQNLKYISFMGKVLGKCLYEHILVDVELSD--FFLKKILN---------------
----VENDMNVPFNDLYS----------------------LDPEYYRN--------LMKL
LEMSK--EEL-EFMD-
--LYFEVPDTT---------------------------DG---KRIP-LIKD--------
GLLTK---
------------------------------VNPQNVF-EYITR-VSHYKLNLQ-LYTVTS
-RFISG---LSYMIPP--HWLR-MFTSY-ELKTLISG-----------------------
---SEKDFD-----------LEDL----KKHTQYGG-YTEN-------------------
------------------------SLTI-QYFWKALG--------------------SFT
MEERRKFLKF-------VFSVPTAP------------LKG-----FH-SLNPLF--GIRN
AGEET-----------------------------DRLPTASTCV-NL-LKLPDY-KDYKT
LREKLL-TSINS-DSRF
>Kluyveromyces_lactis_XM_454612.1 .
STIRREHA-LEDAYNAY---------------GAVGEMFKE-KLGIQFVNEFGP---EAG
IDGGGITKEF---LQTL----VQEGFINQPF-------KLFDTTSFN-------------
----------------KLYPSKNV------------------------------------

----SPQNLKYISFMGKILGKCLYENILVDVELAD--FFLKKILN---------------
----VENNMNVPFNDLYS----------------------LDPEYYRN--------LMKL
LEMSE--NEL-AYMD-
--LYFEVPDAP---------------------------NG---KRIP-LIKD--------
GLSTK---
------------------------------VTQKNVF-EYITR-ISHYKLNLQ-LYTVTS
-RFITG---LSYMIPA--HWLR-MFTSY-ELKTLISG-----------------------
---SEKDFD-----------LEDL----KKNTSYGG-FSES-------------------
------------------------SVTI-QHFWQVLS--------------------SFT
PEERRKFLKF-------VFSVPTAP------------LKG-----FH-SLNPLF--GIRN
AGDET-----------------------------DRLPTASTCI-NL-LKLPDY-QNYHT
LREKLL-TAINS-DSRF
>Kluyveromyces_marxianus_AP014604.1 .
ATIRRDHA-LEDAYNAY---------------GSIGEGFKE-KLSIQFINEFGP---EAG
IDGGGITKEF---LQTL----IQEAFIKGSF-------GLFEVTSFH-------------
----------------KLYPSKDV------------------------------------
----SPQNLKYISFMGKVLGKCLYENILVDVEFAD--FFLKKILN---------------
----VENNMNVPFNDLYS----------------------LDPEYYKN--------LMKL
LEMSE--DEL-NYMD-
--LYFEVPDMP---------------------------NG---RRIP-LIKD--------
GLSVK---
------------------------------VTQKNVF-EYITR-ISHYKLNLQ-LYTVTS
-RFISG---LSYMIPP--HWLR-MFTSY-ELKTLISG-----------------------
---SEKDFD-----------LEDL----RKNTEYGG-FTDD-------------------
------------------------SITI-QHFWMALS--------------------SFT
PEERRKFLKF-------VFSVPTAP------------LKG-----FH-SLNPLF--GIRN
AGDET-----------------------------NRLPTASTCI-NL-LKLPDY-KDYNT
LREKLL-TAINS-DSRF
>Nadsonia_fulvescens_LXPB01000160.1 .
ADVRREFL-LEDAYEGF---------------NKLGPDLKS-AIGVTFYNDYGP---EAG
IDGGGITKEF---LTSV----CKEAFNPSHPL------KLFASTAGH-------------
----------------LLYPNPAI--------------------GSPI-SRAMNDNSVSE
RITYRRRQLSYIEFLGRIIGKCLYEGILVDVEFAP--FFLQKWTN---------------
RNVISNSNIKSSFDDLYW----------------------LDPELYNG--------LIKV
HKYPG--DVEHDLG--
--LNFTTMHIDD--------------------------RGQ-PISID-LKRGGG------
GHSLA---
------------------------------VTNANRL-EYIHA-VANFKLNTV-LQVQTN
-AFITG---MSDLISA--NWLS-MFNAP-ELQMLVSG-----------------------
---GSAGID-----------IADL----RRHTLYGG-YLET-------------------
------------------------DETV-SHFWEVLE--------------------EFT
DVERRKLIKF-------VTSVARAP------------LLG-----FE-VLRPLF--AIRN
AGSDK-----------------------------ERLPTASTCV-NL-LKLPDY-KSKSL
LRKKLL-YAINA-EAGF
>Sugiyamaella_lignohabitans_CP014501.1_a.
ADIRRENL-FEDAYRNF---------------ASAGPSFKF-PIGVTFLSQGMP---ESG
IDGGGLTKEF---LTSV----CQEGFYENDL-------DLFHSTKDH-------------
----------------LLYPNPVF--------------------------GVSRKF-SNL
SDEELEYSLSRIEFLGQIVGKCLYSEILVDVEFAP--FFLLKWAG---------------
------KVARNSFDDLYS----------------------LDPELYEN--------LVKV
HKYPG--NVEEDLN--
--LNFVTVQETG--------------------------HGA-HRSIE-LRPN--------
GEDTP---
------------------------------VTNSNRL-EYIHS-IANYKLNSV-LALQTN
-RFLRG---MSKVISL--NWLS-MFNAK-ELQMLISG-----------------------
---GNSKID-----------LTDL----KTNTVYGG-YLDH-------------------
------------------------DPTV-EYFWQVVE--------------------ELD
DSDKRDFIKF-------VTSVPKAP------------LLG-----FS-QLNPKF--AIRN

AGSDR-----------------------------ERLPTSSTCV-NL-LKLPDY-KNKQL
LKEKLI-YAIRS-GAGF
>Galactomyces_candidum_CCBN010000003.1 .
ANIRRSNL-LEDAFEGF---------------DKLSEGFKS-KIAVTFFNEYGP---EMG
IDGGGIMKEF---LTSI----CSEAFQPTE--------XLFSVNKDH-------------
----------------LLYPNPIF--------------------------GVNSKY-SAL
TPEEKKEGLRYIKFLGKIIGKCLYEGILVDVEFSL--FFLQKLSG---------------
-------IIDNSFDDMYS----------------------LDPEVYNS--------LIKL
YNYPG--DVE-ELS--
--LDFTISQKVG--------------------------HGK-YVTIP-LVPD--------
GQSIA---
------------------------------VTNNNRL-RYIHA-VSQYKLNTV-LTPQTR
-SFLSG---MSTLISL--DWFS-MFNGP-ELQMLISG-----------------------
---GSSRIN-----------ISDL----KAHTLIYD-FPAN-------------------
------------------------DTTI-SDLWDILEN-------------------DFT
EEERRLFIKF-------VTSVPKAP------------LLG-----FG-ALVPNF--AIRR
ASNDQ-----------------------------NRLPTASTCV-NL-LKLPHY-TSRAV
LKKKLL-DSIHA-EAGF
>2_Sporopachydermia_quercuum_BCGN01000004.1 .
AIIHRNSV-LADAYESL---------------GHLGSKLKK-NISVTFYDQFGNE--EAG
IDGGGLTKEF---LTSV----VKEGFDPTGP-------ELFAETSDN-------------
----------------LIYPNPMT--------------------------GITDAYLTPG
QIEVRNEQLACIRFLGTIIGKCLYEGILVDVEFAS--FFLQKWAS---------------
-------RSKLLFEDLYS----------------------LDPELYNN--------LLKL
LRYSD--DVENDIS--
--LNFTVTQELE--------------------------NNR-TISIE-LIPN--------
GKNIP---
------------------------------VTNTNRL-NYVHA-IANYKLNFL-MNLPSN
-AFVKG---LSEIISP--LWLG-MFNPR-ELQILVSG-----------------------
---GEAEID-----------IADL----KRNVEYDATYQIEAQDK---------------
------------------------TSTI-SLLWEVLE--------------------EMS
NAERKEVIKF-------ITSVPRAP------------LLG-----FS-SLKPRI--GIRC
AGNDK-----------------------------SRLPTASTCF-NL-LKLPDY-RDKKL
IREKLL-YSIHA-GAGF
>Alloascoidea_hylecoeti_BCKZ01000012.1 .
GTIRRNNV-FGDAYESF---------------HDLGADFKH-NIRVTFLNEYGP---EAG
IDGGGLTKEF---LTSV----CNDGFSPST--------GLFVTTDDH-------------
----------------LLYPNPIL--------------------------GVPDKYLTEE
DKKIRRKKLQYIKFLGSIIGKCLYEGVLVPVDFAS--FFLQKWFS---------------
-----KNQIRNSFDDLYS----------------------LDPDLHSG--------LAKL
RRYSD--NVE-DLA--
--LNFTIVQEVLYGD-----------------------STR-QVTID-LQPN--------
GSNIA---
------------------------------VTNXNRL-EYVHA-VANFKLNSV-LHLQSQ
-AFLQG---MTSFISP--LWLG-MFNPN-ELQMLVSG-----------------------
---GSSSIN-----------IDDL----RKHTVYGG-YLEN-------------------
------------------------EITV-KYFWEVLA--------------------EMT
PTELSKLIKF-------VTSVPRAP------------LLG-----FG-ELKPLF--SLRN
SGSDIN-----------------------------RLPTASTCV-NL-LKLPDY-KDKKI
LREKLL-YSISA-EAGF
>Blastobotrys_adeninivorans_CBZY010000005. 1..
IRVRRGHI-FEDAFENV-----------------DKIDLKS-QLRVEFYNEYGV---EAG
IDGGGLTKEF---LISA----CAEGFDSSE--------GLFTTTQNH-------------
----------------LLYPEPVL--------------------------GVVSEI-SKI
PSDERALMLSKLNLLGKLVGKCLYEGILVDAEFAP--FFLQKLLG---------------
--------IKNSFDDLYT----------------------LDPEIYDS--------LVKL
RKYSG--NVE-DLN--
--LTFSIDQDMG--------------------------NGK-HASLE-LIRG--------

GDNIT---
------------------------------VTKANRL-EYIHA-IANFKLNLV-LSPQAN
-AFFSG---MSTVISP--HWLK-MFNAV-ELQMLISG-----------------------
---GHSKID-----------VNDL----RSNTIYGG-FTED-------------------
------------------------DPTV-KLFWEVLE--------------------EMS
EEDRCLLLKF-------VTSAPKAP------------LGG-----FR-SLNPKF--ALRN
AGAREV----------------------------DRLPTASTCV-NL-LKLPDY-RDKEA
LREKLL-YSIRS-GTGF
>Saprochaete_clavata_CBXB010000007.1 .
IRIRRNYL-LEDAFNAF---------------NALGSDFKQ-PISVLFENEHGV---EAG
IGPG-VTKEF---LIQV----SQEGFKPSE--------XLFSVTKDN-------------
----------------LLYPNPLL--------------------------GIPSKY-SNL
TKEERSDGLTYMMFLGKIIGKCLFENILVDVEFAP--FFLQKWTL---------------
------PNFKNSFNDLYS----------------------LDPEIYDN--------IIKL
CNYPG--NVETDFM--
--LDFTIDQNVG--------------------------HGK-QATIE-LIPG--------
GSKIP---
------------------------------VTNSNRL-EYAHL-VANFKLNAS-LYYQTL
-MFYRG---LSMIIPP--RWLN-MFNAR-ELQMLIAG-----------------------
---RPSVFD-----------VDDL----RANTVLAG-YTES-------------------
------------------------DITV-QYFWEVLR--------------------ELT
DEQKSQFLKF-------VTSAPKAP------------LLG-----FR-VLTPKF--AIRN
AGADI-----------------------------NRLPTSSTCI-NL-IKLPDY-RNKQL
LKRKLL-ESIDN-GIGF
>Ogataea_polymorpha_XM_018355363.1 .
GVVSRENV-LFDAFEQF--------------GELRGQQFKL-PLSVEFINQFGEK--EAG
IDGGGLTKEL---LTSI----VDSAFYVSNSNADKRGGMRFFNATSNF------------
----------------QLYPNPDY----------------------------------FL
NSEEKTFHLQMLRFLGMVIGKCLYENVLTDIQFAP--FFLSRWTT---------------
---TPSFQQRISFDDLKI----------------------YDSELHEN--------LSKL
LKLTE--HEI-DELD-
--LNFTISEKLQD-------------------------SPE-VVTID-LLPG--------
GRKMK---
------------------------------VNQQNKL-QYVYC-VAKFKMDQS-IAIQSK
-YFIEG---LSQIIKP--RWLL-FFNAY-ELAKLISG-----------------------
---GEKEID-----------VEDL----RRNTALGG-YTMH-------------------
------------------------DPTI-VYLFELLE--------------------EFD
NDLRSKFLKF-------VTSSSREP------------LLG-----FK-ELNPKF--GIRN
SGPDT-----------------------------SRLPTASTCV-NL-LKLPDY-RDKKL
LKEKLL-YSINS-HAGF
>Ogataea_polymorpha_XM_018356855.1 .
GVVSRENV-LFDAFEQF--------------GELRGQQFKL-PLSVEFINQFGEK--EAG
IDGGGLTKEL---LTSI----VDSAFYVSNSNADKRGGMRFFNATSNF------------
----------------QLYPNPDY-------------------FLKLK-LQQQNPS-YDF
SSEEKTFHLQMLRFLGMVIGKCLYENVLTDIQFAP--FFLSRWTT---------------
---TPSFQQRISFDDLKI----------------------YDSELHEN--------LSKL
LKLTE--HEI-DELD-
--LNFTISEKLQD-------------------------SPE-VVTID-LLPG--------
GRKMK---
------------------------------VNQQNKL-QYVYC-VAKFKMDQS-IAIQSK
-YFIEG---LSQIIKP--RWLL-FFNAY-ELAKLISG-----------------------
---GEKEID-----------VEDL----RRNTALGG-YTMH-------------------
------------------------DPTI-VYLFELLE--------------------EFD
NDLRSKFLKF-------VTSSSREP------------LLG-----FK-ELNPKF--GIRN
SGPDT-----------------------------SRLPTASTCV-NL-LKLPDY-RDKKL
LKEKLL-YSINS-HAGF
>Ogataea_parapolymorpha_XM_014079020.1 .
GVVSRENV-LFDAFEQF--------------GELRGQQFKL-PLSVEFINQFGEK--EAG

IDGGGLTKEL---LTSI----VDSAFYVSNSNADKNGGMRFFNATSNF------------
----------------QLYPNPDY-------------------FLKLK-LQQQNPS-YDF
SSEEKTFHLQMLRFLGMVIGKCLYENVLTDIQFAP--FFLSRWTN---------------
---TPSFQQRISFDDLKI----------------------YDSELHEN--------LSKL
LKLTE--HEI-DDLD-
--LNFTISEKLQD-------------------------SSE-VVTID-LLPG--------
GRKMK---
------------------------------VNQQNKL-QYVYC-VAKFKMDQS-IAIQSK
-YFIEG---LSQIIKP--KWLS-FFNAY-ELAKLISG-----------------------
---GEKEID-----------IEDL----RRNTALGG-YTMH-------------------
------------------------DQTI-VYLFELLE--------------------EFD
NDLRSKFLKF-------VTSSSREP------------LLG-----FK-ELNPKF--GIRN
SGTDT-----------------------------SRLPTASTCV-NL-LKLPDY-RNKKL
LKEKLL-YSINS-HAGF
>Ogataea_methanolica_IAAJ01004704.1 .
GVVSRENI-LFDAFEAF--------------GDATGDQFKA-PLSVEFLNKFGEK--EAG
IDGGGLTKEL---LTSL----IDNAFLVSSSNESQNGGLKYFTTTSNY------------
----------------QLYPNPDY-------------------FLKLK-YQQQNPSLDYF
DSEERIFQLQLMRFLGMVIGKCIYEDILIDISFAP--FFLSKWSS-GNNSTSIRNLLGFK
QQQGESQQYRNSFDDLKT----------------------FDSELYNN--------LIKL
LRMSE--DEL-DLLD-
--LTFTISEKLPDTN-----------------------ENI-VLNLN-LIPN--------
GSQIK---
------------------------------VTQQNRL-QYIYS-VAKFKLDQS-IATQST
-YFLDG---LFEIIKP--RWLS-LFNPY-ELTKLISG-----------------------
---GEKEID-----------IKDL----RSNVVYGG-YLNT-------------------
------------------------DQTV-LDLFEILEG--------------------FD
NSEKSKFLKF-------VTSSSKQP------------LLG-----FK-ELNPKF--GIRN
SGSDV-----------------------------SRLPTASTCV-NL-LKLPDY-KDKEL
LKRKLL-YSINA-EAGF
>Ambrosiozyma_kashinagacola_IAAG01005665.1 .
GTVARENL-LFDAFEAF--------------GQASSDRFKN-VLHVEFLNKLGER--ESG
IDGGGLTKEL---LTGI----VNTCFIDSKANEAQNGGLKFFDKTSHY------------
----------------ELYPDPTY-------------------FLKLK-YHQQHPEKKFY
PYEDRNFYLQLMKFLGMIIGKCLYSNVLVDVAFAP--FFLGRWSM---EPSQPGKHMLGA
GGRDSQNNYRSSFDDLKG----------------------FDPELYEN--------LIKL
LSLSD--EEL-SELD-
--SYFTITESLEVDG-----------------------EQV-PITLP-LVKG--------
GEHIK---
------------------------------VNSQNKL-QYIYS-IAKFKLDQR-IMIQSN
-FFLEG---VFKLIDP--KWLS-LFNPY-ELQKLISG-----------------------
---GEKEFD-----------PEDL----RANTVLYR-YNDN-------------------
------------------------DPTI-KYFFEVLK--------------------EFN
KEQKGKFLKF-------ATSSSKQP------------LLG-----FK-ELDPKF--AIQN
SSSDK-----------------------------TRLPSASTCF-NL-LKLPDY-NDKET
LREKLT-YAMNS-EAGF
>Nakazawaea_peltata_BCGQ01000003.1 .
ATISRENL-FFDAFELF---------------GDVPSSQFKKKLDVTFVNQFGP---ESG
IDGGGLTKEF---LTSV----CLEGFX---------------------------------

---------RMMKFLGRVIGKCLYENVLIDISFAP--FFLSRWCS----AEKSGSAGATT
GVYGAQSSQRASFDDLKS----------------------LDSSLYHS--------LLKL
MEMSD--EEL-ASLD-
--LNFTISEKLAH-------------------------ENT-IVTVD-LVPN--------
GSQIV---
------------------------------LNSSNRL-HYIYA-MANFKLSRS-IDLQTR
-QFLSG---LYEIINP--LWLN-LFSYS-ELLVLVSG-----------------------
---GDKPID-----------LNDL----KRNTVYGG-YLET-------------------

------------------------DLTV-KLFWEVME--------------------EFS
AEERSKVVKF-------VTSSPKAP------------LLG-----FK-ELVPKF--AIRD
ALDT------------------------------QRLPTASTCV-NL-LKLPDY-RDKKV
LKDKLL-YAINA-GSGF
>Ascoidea_asiatica_BCKQ01000017.1 .
AVIHRETL-LQDAFNSF---------------YKYSSKFKA-PSSIKFLDEDGEP--EAG
IDGGGITKEF---LISV----IEKGFTDK---------NLFIETENF-------------
----------------ELYPNPNI-----------------------------YFNLKCD
KNKKVIKNLQMIEFLGMIIGKCIYEKILVNIKFSS--FFLNKWNP---------------
----NSKFLRSSFNDLSS----------------------LDPELYNG--------LNKL
LYLSD--NEI-NELG-
--FTFSIDEKIYKDFSNKE-------------------NSR-IITIN-LIPN--------
GSKIK---
------------------------------LNSSNKI-QYISS-LSNFKLNTS-IRLQMS
-YFVKG---LNAILPS--KWLN-MFNAY-ELQMLVSG-----------------------
--GNDTKID-----------IEDL----KENVIFGGGYKPE-------------------
------------------------DLTI-KYFWEVIS--------------------EFS
IENQQNLLKF-------VTSVPRPP------------LLG-----FG-EYNPKF--GISK
GGFDS-----------------------------SRLPTASTCV-NL-LKLPDY-RNKEI
LKEKLL-FSINS-KAGF
>Babjeviella_inositovora_XM_019131551.1 .
GNIRRQHI-IEDAYAEF---------------GKLGDGFKE-QLSITFYNEYDGDKPEAG
IDGGGITKEF---LTSI----VRELSSSES--------GYFQETPSRG------------
----------------QLYPNPDI----------------------------YYKLRSGV
EKPEQLHRIEVFKFLGNVIGKCLYETVLIDMTFSP--FFMSKWQN---------------
SSTRNVAQSRSTFNDLRD----------------------LDSELHHN--------LVKL
LQLTG--EDL-ASLD-
--LNFTINEVV---------------------------NGE-VKTFD-LIAQ--------
GSSIR---
------------------------------VDEHNRL-QYLQA-VADFKLNRS-LSFQTV
-SFVEG---LYEMIPQ--QWLN-MFNPF-ELQMLLSG-----------------------
---GEKDIN-----------VDDL----KQNVQYGG-YDLDI------------------
------------------------DLTI-RDFWEIFEH-------------------EMT
PVERFKLIKF-------VTSVPRAP------------LLG-----FA-SLTPRF--GIRN
AGFQDR----------------------------ERLPTASTCV-NL-LKLPDY-RDKKV
LKEKLL-YAINA-EAGF
>Saccharomycopsis_fibuligera_CP012824.1 .
AEVRRDHL-LEDSFNAF---------------NKLTRNFKA-RLQVTFIDQYGKP--EAG
IDGGGITKEF---LTSV----VNDGFNNPAS-------GLFRQNQNF-------------
----------------QLYPNPDI--------------------YLNL-KHNPAVYGNKD
VKQAEYKKLQKLQFLGKVIGKCLYEGILVDVNFVP--SFLKKFKT---------------
------IGKVSTFDELAD----------------------VDPELYVG--------LSKL
LKLDD--SFF-KLSE-
--MNFTLTEKLTYTIPEKSTVNVYTGEEETELAQT---RSK-LITID-LIPN--------
GSNIA---
------------------------------VTNANKL-NYINA-IANFKLNKS-LFPQID
-SFLKG---LFQIIPS--EWLN-MFNPA-ELQMLVSG-----------------------
---GEKDID-----------IDDF----KANVEYGG-FLEN-------------------
------------------------DPTV-VYFWECVA--------------------EMK
PEERFKLVKF-------VTSVPRAP------------LLG-----FG-DLNPRF--GIRN
AGYST-----------------------------EWLPTASTCV-NL-LKLPDY-RDKQI
LKEKLL-YVINS-EAGF
>Saccharomycopsis_malanga_BCGJ01000001.1 .
AEIRREHL-LDDSFAAF---------------HKLGDSFKF-PMSVTFIDKYGKP--EAG
IDGGGITKEF---LTSV----VKEGFDPHT--------GLFVANTNY-------------
----------------QLYPNPNI--------------------YLNL---KYNAEMPSS
LKRAEVDKLQKLEFLGKVIGKCLYEGILVDVNFVP--LFLKKWKS---------------
------IGKLSTFDELND----------------------VDPELYSG--------LSKL

LKLND--DFF-KSSD-
--MSFTLTSKITYTIPSKTTVDLTADESVMSVDEQV--EEK-IVNID-LVHN--------
GSNIP---
------------------------------VTNFNKL-NYINA-VANFKLNKS-IYPQIE
-SFLKG---LFQMIPS--DWLN-MFNPT-ELQMLVSG-----------------------
---GEKDID-----------IQDF----QQNVEYGG-FLET-------------------
------------------------DLTV-RYFWECVN--------------------EMS
PTERFKLIKF-------VTSVPRAP------------LLG-----FS-DLNPKF--GIRN
AGSQT-----------------------------NRLPTASTCV-NL-LKLPDY-KDKQL
LKAKLL-YVINS-EAGF
>Cyberlindnera_fabianii_MPUK01000003.1 .
AEIRREHL-LNDAYESF---------------GKMGGTFKN-QLAIRFINKHGT---EDG
VDGGGLTKEF---LTGV----VKEGFN-----------GLFIENYNH-------------
----------------ELYPDPEI----------------------------GIKYQYRV
DSAEQLLKLNHTNFMGKVIGKCLYDRVLVDACFAP--FFLTKLNR---------------
-------DFRNSFDDLNS----------------------LDPDLYAN--------LVKL
LSMSE--EEL-AALA-
--LTFSVDERV---------------------------GNK-SITIN-LIPN--------
GSMVP---
------------------------------VTTSNRL-KFIHE-MSDYKLNKV-INTQSN
-SFLTG---LYEIVSK--DWLA-MFNPY-ELQILISG-----------------------
----ETDIN-----------ITDL----KTHTVYGG-FSEQ-------------------
------------------------DKTI-TDFWEVVE--------------------EMT
NEEKSLLVKF-------ATSVPRAP------------LLG-----FK-ALNPQF--GITN
AGGLKEHS--------------------------DSLPTASTCV-NL-LKLPNY-QNKEK
LRQKLL-YAIKA-EAGF
>Cyberlindnera_jadinii_XM_020215913.1 .
ATIRREHL-LDDAFENF---------------GKLGEQFKT-KLGIEFVNQYGR---EEG
IDGGGITKEF---LTSV----VREGFRE----------PLFVENDHH-------------
----------------ELYPNPQI----------------------------GLRYRNRI
DSSKQLEHLSYLNFMGKVLGKCLYDRVLVDVAFAN--FFLTKFNS---------------
-------GYKTSFDDLES----------------------LDSELYSN--------LTKL
LSLTD--DEL-SNLG-
--LTFSLDELV---------------------------HDR-HITFD-LIPK--------
GSTIS---
------------------------------VTSANRL-KFIHE-VSNYKLNKT-VSLQCN
-SFLNG---LYEMISK--EWLA-MFNPY-ELQMLISG-----------------------
----ETDVN-----------IEDL----KENCVYGG-YSES-------------------
------------------------DQTI-QDLWEIVA--------------------EMT
SADRFQFVKF-------VTSVPRAP------------LLG-----FK-ALVPNF--GIRN
TGSDI-----------------------------DRLPTSSTCV-NL-LRLPNY-RNKQV
LKEKLL-YAINA-EAGF
>Wickerhamomyces_anomalus_XM_019181629.1 .
ATIRREHM-LEDAYDSF---------------NKLGETFKS-KLGINFINEHGE---EVG
VDGGGLTKEF---LTGV----VKEGFK-----------DLFVENDNH-------------
----------------ELYPNPQI----------------------------GLKYQYRI
DSEEQLKSLNYTNFLGKVIGKCLYERVLVDISFSS--FFLTKFNT---------------
-------GYKNSFDDLRS----------------------LDGELYTN--------LVKL
LSMTD--EEL-EGLN-
--LTFSIDEKL---------------------------DNR-NISID-LVPN--------
GSITP---
------------------------------VTSSNKL-KFVHE-VANFKLNRL-INIQCN
-SFLNG---LFEIISK--EWLA-MFNPY-ELQMLISG-----------------------
----EKDIN-----------VQDL----KEYVVYGG-YTLN-------------------
------------------------DKTI-RDFWEIVH--------------------EMT
NTERFQLVKF-------VTSVPRAP------------LLG-----FK-ALVPRF--GIKN
GGDEP-----------------------------ERLPTSSTCV-NL-LKLPNY-RNKQL
LKEKLL-YAINA-EAGF

>Wickerhamomyces_ciferrii_XM_011279367.1 .
ANIRRGHM-LEDAYESF---------------NTLGEAFKG-PLKINFTNEHGQV--EQG
IDGGGLTKEF---LTST----VKEGFK-----------DLFVENDSH-------------
----------------ELYPNPQI----------------------------GLKYQYRV
ESEEQLKSLNYLNFLGRIIGKCLYERVLVDINFSP--FFLTKFNT---------------
-------GYKNSFDDLRS----------------------LDKDIYSN--------LVKL
LNMND--EEL-ESMN-
--LTFTIDEQI---------------------------NNK-NVSID-LIPN--------
GSITQ---
------------------------------VNSSNKL-KFIHE-VSNFKLNKL-INIQSN
-SFLNG---LFGMISK--EWLA-MFNPY-EVQMLISG-----------------------
----EKDVD-----------INDL----KENVHYGN-CEPT-------------------
------------------------DLTV-KYFWEVVE--------------------EMS
NTDRFQLVKF-------VTSVPRAP------------LLG-----FK-ALNPRL--GIFL
DKSLGHR---------------------------DALPSSATCT-NM-LRLPDY-KDKKT
LREKLI-YAINS-EAGF
>Lodderomyces_elongisporus_XM_001523653.1 .
GNIRREHV-LEDAFAAF---------------ASTGSQFKQ-RIRVHYVNQHGP---EAG
IDGGGITKEL---LTGV----TTEGFDPEN--------GLFQESSDH-------------
----------------RIYPTTDX----------------------------GSGSGSES
DFELMKLKLDYLRFMGMCVGKCMYEGILIDASFAP--FFLNKWCH---------------
------DSFKNTVDDLKY----------------------MDVELYNN--------TIKL
LDMSS--TEL-DALD-
--LTFFINERISG-------------------------NTK-IVQYD-LLPN--------
GSKIK---
------------------------------VDRSNVH-LYNHK-FADFKLNKS-LAQQTK
-HFLSG---VQSIIPL--RWLT-LFDYH-ELQMLISG-----------------------
---GEKAID-----------VDDW----RRNAVTNG-FEFD-------------------
------------------------DTTL-NIFWEVVA--------------------EMS
SEERRKLLAF-------VTSSPRPP------------LLG-----FD-QLNPKF--GIQN
KGAEL-----------------------------NVLPSAATCV-NL-LKLPDY-RDKNV
LKEKLL-YAINS-AAGF
>2_Yamadazyma_sp._MVNT01000007.1 .
VHARRDHL-LEDGFEYL---------------GQSGAQLKH-GLRVTFFNEYGQ---EAG
VDGGGITKEF---LSGV----VKEGFNPNGKY------KIFKETTEH-------------
----------------MLYPNDEI----------------------------FVKYYNKI
DFEEQLKNLQYLKFLGNIVGKCIYELVLIDVAFAP--FFLSKWCN---------------
----SKNAMKNSINDLNY----------------------LDPQLFTN--------LLKL
DRMQA--SEI-EALE-
--LNFTVNAQA---------------------------DGK-IFPFD-LMKG--------
GESVK---
------------------------------VTAANRM-NYIHQ-MANFLLNVS-LFIQSK
-HFLEG---VFEVINS--NWLK-MFDFN-ELQMLISG-----------------------
---SKAKLD-----------MENW----KQNVEYGG-YLQQ-------------------
------------------------DITI-QHFWEVVE--------------------EMS
SEEQSKLLKF-------VTSVSRAP------------LLG-----FL-ELTPKF--GIRN
SGRSS-----------------------------ERLPTASTCV-NL-LKLPDY-QDKHL
LKEKLL-YAIDM-DAGF
>Candida_tenuis_XM_006684866.1 .
VNARREHL-LEDGFKYL---------------GQSGPQLKH-GIQVTFFNEYGQ---EAG
VDGGGITKEF---LSGV----VLEGFDPNGQR------RLFKETSEH-------------
----------------QLYPNDEI----------------------------FTKYYNKI
DFEQQLEQLQYLRFLGNIVGKCLYDQVLIDVSFAP--FFLNKWCN---------------
----AKNMMKNSINDLNY----------------------LDPELFKN--------LVKF
TKMSG--PDI-EDLD-
--LNFTVNTRA---------------------------DGK-QFKFD-LVKN--------
GEAIK---
------------------------------VTAANRL-NYIHQ-MANFLLNTS-LHIQSK

-HFLQG---VFAVVNS--NWLK-MFDFN-ELQMLISG-----------------------
---SKAKLD-----------IANW----KQNVEYGG-YLES-------------------
------------------------ESTI-QYFWQVVE--------------------EMT
SEEQSKLVKF-------VTSVSRAP------------LLG-----FQ-SLNPKF--GIRN
SGRST-----------------------------DRLPTAATCV-NL-LKLPDY-QDKEV
LRQKLI-YAINV-DAGF
>Clavispora_lusitaniae_XM_002618273.1 .
ADIHRETI-MEDAYEQF---------------HKVGSNFKN-KIQVTFYNEHGP---EAG
IDGGGLTKEL---LTSV----VSEGFDPKFDL------HLFKETEANN------------
----------------ELYPNDDI----------------------------YLKIAKRI
DLPKQQQRLNYVKFLGMIVGKCLYESVLIDIGFAP--FFLAKWKV---------------
----AQSSQNCSINDLSY----------------------LDRQLYLN--------LSKL
LTMSA--DDI-NALD-
--LTFVIDENV---------------------------DSK-ILQYD-LAPPY-------
GAMTS---
------------------------------VSKSNRL-QYIHL-VANFKLNKS-LHVQTK
-YFLEG---LFEIIDA--EWLN-IFDPF-ELQMLISG-----------------------
----GNDID-----------VQDW----KENVHYGG-YFDD-------------------
------------------------DLTI-VLFWEVVE--------------------EMS
PQERCDLIKF-------VTSVSRAP------------LLG-----FK-ALTPHF--GIHN
SGSP------------------------------DRLPTASTCV-NL-LKLPDY-KDKTL
IREKLL-YASKA-NSGF
>Candida_intermedia_LT635764.1 .
ADIYRENL-LESSFERF---------------NKVGAQFKN-KLQITFHNEHGV---EAG
IDGGGLTKEF---LTSV----VTDAFSTNNAL------KLFRETEADN------------
----------------DLYPNHDI----------------------------YLKILKRI
DLPEQQIRLQYMRFLGMIVGKCLYEHVLIDVAFAP--FFLAKWRV---------------
----AQNSMKNSINDLKF----------------------LDRDLYQN--------LNKL
LEMSE--EQI-QQLD-
--LDFTINEHV---------------------------DSD-TLQYD-LQPPH-------
GETIK---
------------------------------VTSSNRL-NYIHQ-IANFKLNQS-LHIQTK
-YFLEG---LFELINA--TWLN-MFDPY-ELQMLISG-----------------------
----GNDVN-----------IKDW----KENVLYGG-YFDD-------------------
------------------------DVTV-VLFWQVVE--------------------EMS
PQERCDLIKF-------VTSVSRAP------------LLG-----FG-ALSPKF--GIHN
SGPS------------------------------NRLPTASTCV-NL-LKLPDY-KDKDV
IRQKLL-YSISA-NSGF
>Metschnikowia_bicuspidata_XM_018855152.1 .
ADINRESL-IEDAFENF---------------FRLGKNFKN-PLLVTFYNEHGP---EAG
IDGGGLTKEF---LTSV----VQEGFNPRSDI------SLFKESSSC-------------
----------------SLYPNDDI----------------------------FWNTINNI
QLPTQRKRLMYTKFLGMIIGKCLYEEILIDITFVP--SFLSKWRV---------------
----AQNNLKNSIDDLVF----------------------LDPELYTN--------LNKL
LEMNE--GQL-ESLD-
--LNFTIDEVI---------------------------GNS-SYLFD-LLPPN-------
GAQET---
------------------------------VTQSNRL-SYIHK-VSHFKLNQS-LHLQTK
-HFLEG---LFQLIKP--SWLN-MVDPL-ELQMLISG-----------------------
----GHDID-----------LEDW----KFNVRYGG-YFDD-------------------
------------------------DLTI-KLFWQVVE--------------------EMT
AQERCDLVKF-------VTSVARAP------------LLG-----FG-AMYPKF--AINN
AGSP------------------------------DRLPTASTCV-NL-LKLPDY-KDKVL
IRQKLL-YSVYA-NSGF
>Candida_auris_XM_018313795.1 .
ADISRNAV-LEDAFKNF---------------FPLGHKFKE-RISVTFHSEHGP---EAG
IDGGGITKEL---LTSV----AMEGFNPNLKH------ALFKETPSHN------------
----------------ELYPNDDI----------------------------YMSVHQQI

```
NLKRQQERLLYIQFLGMVIGKCFYENVLVDIEFAP--FFLTKWTS---------------
----AHSASRSSVNDLQY----------------------LDVDLYNN--------LMKL
LEMND-----------------------------------------------KEI-RALD-
--LNFTINEVV----------------------------DGK-VVRFD-LQPPH-------
GESVS-------------------------------------------------------
------------------------------VTSTNRL-NYIHE-VANFKLNQC-LYLPSR
-HFLEG---LHKIIDR--KWLN-MFDPF-ELQKLISG-----------------------
---GESNVN-----------IEDW----KNNVSYGG-YFDD-------------------
------------------------DITI-RLFWEVVE--------------------EMS
PKERCRLIKF-------VTSVSKAP------------LLG-----FG-SLTPKF--GIRN
SGRYP------------------------------DRLPTASTCV-NL-LKLPDY-QDKEL
IRQKLL-YAINV-DSGF
>Candida_dubliniensis_XM_002419683.1 .
AEINRDSI-LEDAFNAY---------------HKEGANFKN-RLQVEFFNEYGR---EAG
IDGGGITKEF---LTCI----VKEGFNPDNAY------ELFKETGDN-------------
----------------QLYPNDKI-----------------------------FEILYIGM
DKEFQQIKLDYIRFLGMIIGKCLYENVLIDVWFAP--FFLNKWCN---------------
------DAMKNSINDLSY----------------------LDNELFKN--------LMKL
TKMTN-----------------------------------------------DEL-KMLE-
--LTFSINLKI----------------------------DNK-SYTVE-LLPN--------
GAVIE-------------------------------------------------------
------------------------------VDLSNIL-SYIHQ-FANYKLNQS-LKIQTK
-YFLEG---LYSIISK--SWLS-MFDCF-ELQMLISG-----------------------
---GKDDIN-----------IDDW----KNNVEYGG-YSDS-------------------
------------------------DPAV-IMFWEIVE--------------------EMS
PHERCKLIKF-------VTSVSRAP------------LLG-----FG-SLVPKF--GIRN
SGTDS------------------------------VRLPTASTCV-NL-LKLPNY-QDKKT
MKEKLL-YAINT-EARF
>Candida_albicans_XM_704929.2 .
AKINRDSI-LEDAYNAY---------------HRQGANFKN-RLQVEFFNQYGK---EAG
IDGGGITKEF---LTCV----VKEGFNPDNAF------ELFKETGDN-------------
----------------QLYPNDKI-----------------------------FEILYVGM
DREFQQIKLDYIRFLGMIVGKCLYENVLIDVSFAP--FFLNKWCN---------------
------DGMKNSINDLSY----------------------LDNELFKN--------LMKL
TKMTN-----------------------------------------------DEL-KQLE-
--LTFSINLKI----------------------------DNK-SYNLD-LLPN--------
GANVE-------------------------------------------------------
------------------------------VDLSNIL-NYIHQ-LANYKLNQS-LKIQTK
-YFLEG---LYSMISK--SWLS-MFDCF-ELQMLISG-----------------------
---GKDDIN-----------IDDW----KNNVEYGG-YLDD-------------------
------------------------DPAV-IMFWEIVE--------------------EMT
PQERCKLIKF-------VTSVSRAP------------LLG-----FG-SLAPKF--GIRN
SGNDS------------------------------VRLPTASTCV-NL-LKLPNY-RDKKT
MREKLL-YAINT-EAGF
>Candida_tropicalis_XM_002545253.1 .
AKINRDTI-LEDAFHAF---------------HRQGSNFKN-RIQVEFFNEYGR---EIG
IDGGGITKEF---LTSV----VKEGFNPDNAY------ELFKETLTDN------------
----------------QIYPNDKI-----------------------------YEIMHAGM
DQEFQQEKLEYIRFLGMIIGKCLYEHVLIDVSFAP--FFLNKWCN---------------
------DSMKNSINDLSY----------------------LDHELFVN--------LMKL
TKMSS-----------------------------------------------EEL-ESLD-
--LTFSVDILL----------------------------DGK-GYTFD-LLPN--------
GRNVK-------------------------------------------------------
------------------------------VDSTNIL-NYMHQ-MANFKLNQS-LKLQTK
-YFLEG---LYSMISS--SWLS-MFDCF-ELQMLISG-----------------------
---GKSDIN-----------IEDW----KNNVEYGG-YSDD-------------------
------------------------DPSI-KMFWEIVE--------------------EMT
PEERFKLIKF-------VTSVRRAP------------LLG-----FG-ALYPKF--GIRN
```

SGQGL-----------------------------LRLPTASTCV-NL-LKLPDY-KDKKV
MKEKLL-YAINT-DAGF
>Candida_parapsilosis_CABE01000024.1 .
AEIRRQNL-LQDAFEAF---------------SKMGNLFKN-RIQVEFFNEYGP---EAG
IDGGGITKEF---LTSV----VKEGFDPVQ--------GLFKETRDN-------------
----------------QIYPNEEI----------------------------CLKMKLGH
DVYDARGKLEYIQFMGMCVGKCLYENVLIDVSFAP--FFINKWCM---------------
------DGFKNTINDLSY----------------------FDAELFKN--------LIKL
SHMTD--VEL-RNLD-
--LSFAVDEKV---------------------------DGA-THSFE-LIPD--------
GKSIS---
------------------------------VAKPNIQ-FYLHK-FADFKLNQS-LLPQSK
-AFLSG---LFSIIPR--KWFM-MFDYY-ELQMLISG-----------------------
---GKKDVD-----------VTDW----QNNVEYGG-YLPQ-------------------
------------------------DISV-RYFWEIVY--------------------EMT
PEEKSKLIKF-------VTSVSRAP------------LLG-----FA-VLNPKF--GIRN
SGRDV-----------------------------TRLPTASTCV-NL-LKLPDY-QNKEI
MREKLL-YAINT-ESGF
>Candida_orthopsilosis_XM_021357152.1 .
AEIRRQNL-LQDAFEAF---------------SKMGNLFKN-RIQVEFFNEYGP---EAG
IDGGGITKEF---LTSV----VKEGFDPVQ--------GLFKETGDN-------------
----------------QIYPNEEI----------------------------CLKMKLGQ
DVYDARGKLEYIQFMGMCVGKCLYENVLIDVSFAP--FFINKWCM---------------
------DGFKNTVNDLSY----------------------FDGELFTN--------LIKL
THMSN--EEL-QHLD-
--LVFAINEKV---------------------------DGE-TYNFE-LIPN--------
GKSIE---
------------------------------VNKLNVQ-YYLHK-FADFKLNQL-LLPQTK
-AFLSG---LFSIIPR--KWFM-MFDYY-ELQMLISG-----------------------
---GKKDVD-----------VNDW----QNNVEYGG-YLPH-------------------
------------------------DISI-RYFWEIVN--------------------EMS
PQEKSKLIKF-------VTSVSRAP------------LLG-----FG-VLNPKF--GIRN
SGRDV-----------------------------TRLPTASTCV-NL-LKLPDY-QNKQI
MKEKLL-YAINT-ESG-
>Meyerozyma_caribbica_BADS01000003.1 .
ADIRREFI-LEDAFNNF---------------NNIGQNFKN-PLSVTFFNEYGQ---EAG
IDGGGITKEF---LTSV----VQEGFLPGGQF------DLFKETATNN------------
----------------QLYPNDEI----------------------------FKKLRMNY
NVPEQKLKLEYLRFLGAIVGKCLYENVLIDVSFAP--FFINKWSN---------------
----VAHLMKSSINDLRS----------------------LDEELFSN--------LLKL
NSMSD--QEL-VELD-
--LNFMIEEQV---------------------------SGK-KFIYD-LMPPN-------
GRNTQ---
------------------------------VNHNNRL-NYIHQ-VSNFKLNQS-LHLQTK
-YFVEG---LFEMISA--AWLT-MFDPI-ELQMLISG-----------------------
---GEQDIN-----------IDDW----KDNVEYGG-YFDD-------------------
------------------------DLTI-VYFWEVVK--------------------EMS
PQERFKLVKF-------VTSVSRAP------------LLG-----FQ-SLTPKF--GIRN
SGREI-----------------------------DRLPTASTCV-NL-LKLPDY-QDKEL
IRSKLL-YAINM-GAGF
>Meyerozyma_guilliermondii_XM_001484729.1 .
ADIRREFI-LEDAFNNF---------------NNIGQNFKN-PLSVTFFNEYGQ---EAG
IDGGGITKEF---LTSV----VQEGFLPGGQF------DLFKETATNN------------
----------------QLYPNDEI----------------------------FKKLRMNY
NVPEQKLKLEYLRFLGAIVGKCLYENVLIDVSFAP--FFINKWSN---------------
----VAHLMKSSINDLRS----------------------LDEELFSN--------LLKL
NSMSD--QEL-VELD-
--LNFMIEEQV---------------------------SGK-KFIYD-LMPPN-------

GRNTQ---
------------------------------VNHNNRL-NYIHQ-VSNFKLNQS-LHLQTK
-YFVEG---LFEMISA--AWLT-MFDPI-ELQMLISG-----------------------
---GEQDIN-----------IDDW----KDNVEYGG-YFDD-------------------
------------------------DLTI-VYFWEVVK--------------------EMS
PQERFKLVKF-------VTSVSRAP------------LLG-----FQ-SLTPKF--GIRN
SGREI-----------------------------DRLPTASTCV-NL-LKLPDY-QDKEL
IRSKLL-YAINM-GAGF
>2_Candida_carpophila_BCGK01000003.1 .
ADIRREFI-LEDAFDNF---------------NNIGQNFKN-PLSVTFFNEYGQ---EAG
IDGGGITKEF---LTSV----VQEGFLPGGQF------DLFKETATNN------------
----------------QLYPNDEI----------------------------FKKLRMNY
DAPEQKLKLDYLRFLGAIVGKCLYENVLIDVSFAP--FFINKWSN---------------
----VAHLMKSSINDLRS----------------------LDEELFNN--------LLKL
NSMSD--QEL-AELD-
--LNFVIEEQV---------------------------SGK-KFVYD-LMPPN-------
GRNTQ---
------------------------------VNHNNRL-NYIHQ-VSNFKLNQS-LHLQTK
-YFVEG---LFEMISA--AWLT-MFDPI-ELQMLISG-----------------------
---GEQDIN-----------INDW----KDNVEYGG-YFDD-------------------
------------------------DLTV-QYFWEVVK--------------------EMT
PQERFKLVKF-------VTSVSRAP------------LLG-----FQ-SLTPKF--GIRN
SGREI-----------------------------DRLPTASTCV-NL-LKLPDY-QDKEL
IRSKLL-YAINM-GAGF
>Debaryomyces_hansenii_XM_462492.1 .
ADIRRESL-FEDAFNNF---------------HKCGSDFKH-TLSVTLFNEQGGQ--EAG
IDGGGITKEF---LTSV----AMEGFQPHGKF------ELFKETAEN-------------
----------------QLYPSDEI----------------------------YKMLSKKI
EVKEQQQKLLYLRFLGNIIGKCFYENVLIDLSFAP--FFLNKWCS---------------
-FNRGQNSMKNSINDLNY----------------------LDRELFTN--------LMKL
TSMDS--NEL-EQLD-
--LNFTIDEKIN--------------------------DFN-CTFDL-LPPN--------
GAEIK---
------------------------------VDQSNRL-NYIHQ-ISNFRLNQS-LHVQTK
-FFLNG---LFEIISS--SWLS-MFDAS-ELQMLISG-----------------------
---GENDVN-----------IQDW----KENVEYGG-YFDD-------------------
------------------------DTTV-VYFWEVIS--------------------EMS
PEERFKLIKF-------VTSVSRAP------------LLG-----FG-SLNPKF--GIRN
AGRSI-----------------------------DRLPTASTCV-NL-LKLPDY-QDKEL
VRSKLL-YAINT-EARF
>Debaryomyces_fabryi_XM_015611082.1 .
ADIRRESL-FEDAFNSF---------------HKCGSDFKH-TLSVTLFNEQGGQ--EAG
IDGGGITKEF---LTSV----AMEGFHPQGKF------ELFKETAEN-------------
----------------QLYPSDEI----------------------------YKMLSKKL
NIQEQQQKLMYLRFLGNIIGKCFYENVLIDLSFAP--FFLNKWCS---------------
-FNRGQNSMKNSINDLNY----------------------LDRELFKN--------LMKL
TSMDS--KEL-EQLD-
--LNFTIDEKIN--------------------------DLN-CTFDL-LPPN--------
GAEIK---
------------------------------VDQSNIL-NYIHQ-ISNFRLNQS-LHVQTK
-FFLNG---LFEIISS--SWLS-MFDAY-ELQMLISG-----------------------
---GENDVN-----------IQDW----KENVEYGG-YFDD-------------------
------------------------DITV-VYFWEVIS--------------------EMN
PEERFKLIKF-------VTSVSRAP------------LLG-----FG-SLNPKF--GIRN
AGRSI-----------------------------DRLPTASTCV-NL-LKLPDY-QDKEL
VRSKLL-YAINT-EARF
>2_Millerozyma_acaciae_BCKO01000002.1 .
ANIRREHL-LEDAFNSF---------------HKYGSGFKN-QLSVTLFNNEGGQ--EAG

IDGGGITKEF---LTSV----AMEGFSPGGKF------ALFKETDDH-------------
----------------QLYPNDEI----------------------------SKKLHKGV
DVNEQRQKLQYLQFLGNIIGKCFHENVLIDISFAP--FFLNKWCS---------------
-YKGLGGSMKNSINDLSY----------------------LDREIYTN--------LMKL
TSMDA--KEL-KDLD-
--LTFTIDEKI---------------------------DNV-DYTFD-LNPPR-------
GESTK---
------------------------------VDMSNRL-NYVHQ-ISNFKLNQS-LFIQTK
-YFLTG---LFQIVSS--RWLS-MFDSR-ELQMLISG-----------------------
---EESDVN-----------IQDW----KQNVEYGG-YFDD-------------------
------------------------DITV-VYFWEVVS--------------------EMT
AEERCKLIKF-------VTSVSRAP------------LLG-----FG-SLNPKF--GIRN
AGFRP-----------------------------EWLPTASTCV-NL-LKLPDY-KDKEL
IRSKLL-YSINT-EARF
>Millerozyma_farinosa_FO082058.1 .
ADIRRESL-FEDAFNNF---------------HKCGLDFKF-PLNVTLFNEQGGQ--EAG
IDGGGITKEF---LTSV----VFEGFDPYDKY------KFFKENESH-------------
----------------QLYPNDDI----------------------------QKKLSHHI
DVEEQRMNLLYIQFLGSVIGKCLYENVLIDISFAP--FFLNKWVS---------------
-HNGAYRGMKNSINDLSY----------------------LDEQLFNN--------LIKL
TNMSD--EEL-KALD-
--LNFTIDEIIG--------------------------GKK-YTEDL-MPPN--------
GEQIQ---
------------------------------VASNNRL-NYIHQ-ISNYKLNKL-MYVQTK
-YFLSG---LYEIISS--NWLS-MFDSY-ELQMLISG-----------------------
---GESDVN-----------IQDW----KQNVEYGG-YFDD-------------------
------------------------DITI-IYFWEVIC--------------------EMS
AEERFKLLKF-------VTSVSRAP------------LLG-----FS-SLNPKF--GIRN
AGRCL-----------------------------DRLPTASTCV-NL-LKLPDY-QDKEV
MRAKLL-YAINT-EARF
>Hyphopichia_burtonii_XM_020219730.1 .
ANIRREFL-LEDAFENF---------------HKLGSQFKN-KLSVTFFNEYGGQ--EAG
IDGGGITKEF---LTSV----VLEAFKPSNKF------GLFKETPSDY------------
----------------QLYPNDDI----------------------------YKKLYKKI
DVDIQREKLFYLKFLGSIIGKCFYENVLVDISFAP--FFLNKWCN---------------
----VNNSMKNSINDLNY----------------------LDNELFKN--------LMKL
LSMTS--DEL-KELD-
--LNFTIEEKI---------------------------DGL-DYKFD-LLPPN-------
GETTL---
------------------------------VNTSNEL-NYIHQ-VSNFKLNQS-LHIQSK
-FFLDG---LFEIINA--NWLS-MFDSF-ELQMLISG-----------------------
---GENDIN-----------IQDW----KENVEYGG-FFDD-------------------
------------------------DITI-INFWQVVS--------------------EMS
PQERFKLIKF-------VTSVSRAP------------LLG-----FG-ALSPKF--GIRN
SGRSI-----------------------------ERLPTASTCV-NL-LKLPDY-QDKEL
IRTKLL-YAINT-DSRF
>Spathaspora_passalidarum_XM_007376682.1 .
ADIRRENL-LVDAFNAF---------------NKSGASFKN-RIQVVFYNEYGQ---EAG
IDGGGITKEF---LTSV----VKEGFNPENEL------KLFKETLSHN------------
----------------QLYPNNDI----------------------------YDKLHLGL
EMEAQQVRLQYLRFLGMVVGKCFYENVLIDISFAP--FFLNKWCN---------------
--------MKNSINDLNY----------------------LDSELFVN--------LMKL
TKMTN--EEL-EALD-
--LNFTIYEKV---------------------------NQK-TYLFD-LAPN--------
GQSTQ---
------------------------------VNSSNIL-QYIHQ-LSNFKLNQS-LTIQTR
-SFLQG---LFLIISS--NWLN-MFDCF-ELQMLISG-----------------------
---EPTDVN-----------INDW----KLNVEYGG-YLEN-------------------

------------------------DLTI-KYFWQVVE--------------------EMT
KEQRFKLIKF-------VTSVGRVP------------LLG-----FG-SLNPKF--GLRN
SGRDG-----------------------------DRLPTASTCV-NL-LKLPDY-QNKQI
LKEKLL-YAISI-DSGF
>Spathaspora_hagerdaliae_LQHL01001400.1 .
AKIHRQNL-LVDAYNSF---------------AKTGVNFKN-RIQVMFFNEYGP---EAG
IDGGGITKEF---LTCV----VQEGFSPDNKL------QLFKETYSHN------------
----------------QVYPNEEI----------------------------YQKLTMGI
DHELQLEKLLYLRFLGMIIGKCFYENVLVDISFAP--FFLNKWCR---------------
------DNMKNSIDDLKY----------------------LDQELFDN--------LMKL
IKMND---EEL-KLLD-
--LNFIIDEKVMINGTG---------------------HIL-PVQFD-LLPPH-------
GANIQ---
------------------------------VNSSNIL-NYIHQ-ISNFKLNQS-LRIQTK
-YFLEG---LYLMINP--NWLS-MFDGF-ELQMLISG-----------------------
---GENDVN-----------IEDW----KSNVEYGG-YLPN-------------------
------------------------DKTI-DMFWEIVE--------------------EMS
SGERFKLIKF-------VTSVSRAP------------LLG-----FG-SLSPKF--GIRN
SXGREI----------------------------TRLPTASTCV-NL-LKLPDY-QNKEI
MREKLL-YAISV-ESGF
>Candida_tanzawaensis_XM_020210289.1 .
GDIRREFL-LEDAFKSF---------------YEVGSNFKN-RIQVVFYNEYGGK--EAG
IDGGGITKEF---LTSV----VQEGFNPSHEL------KLFNETSEH-------------
----------------EIYPNNEI----------------------------GLNLTKKI
DTEYQHSRLKYLKFLGMVIGKCFYENVLVDVSFAP--FFINKWCN---------------
------ENLKNSINDLRS----------------------LDNELFQN--------LMKL
ERMNA---EEL-KELD-
--LNFAVNENL---------------------------NGK-SHVFE-ISKN--------
GESLP---
------------------------------VTEKNKL-NYIHQ-ISNFKLNVS-LYIQTK
-FFIEG---LEEIISP--NWLK-MFDSF-ELQMLISG-----------------------
---GESDVN-----------IDDW----KDNVEYGG-FFDD-------------------
------------------------DLTI-RYFWEVVR--------------------EMN
SEERFKLIKF-------VTSVSRAP------------LLG-----FG-SLSPKF--GIRN
SGRDI-----------------------------ERLPTASTCV-NL-LKLPDY-QNKKL
IREKLL-YAINE-DARF
>2_Wickerhamia_fluorescens_BCGE01000002.1 .
ADIRREHL-LHDAFQLF---------------HKSGSHFKN-QLQVSFYNKYGGQ--EAG
IDGGGITKEF---LTSV----VGEGFNPSNEM------HLFKETSNN-------------
----------------QIYPDDMI----------------------------FKKLSKKI
EVAEQQEKLLYLKFLGSVIGKCFYENVLIDISFAP--FFLNKWCH---------------
------DNMKNSIDDLGF----------------------LDQELFNN--------LMKL
VKMSD---TEL-EELD-
--LNFSLNETI---------------------------NGH-FFVFD-IAPA--------
GESIK---
------------------------------LNAANRL-NYIHL-TSNYKLNQS-LHIQTK
-FFLEG---LYEIISS--NWLS-MFDSS-ELQMLISG-----------------------
---GESDVN-----------IKDW----MENVEYGG-FFDD-------------------
------------------------DLTI-KYFWEVIA--------------------EMT
PKERFRLIKF-------VTSVSRAP------------LLG-----FG-ALSPKF--GIRN
SGRDV-----------------------------ERLPTASTCV-NL-LKLPDY-KDKNL
IRNKLV-YAINV-DAGF
>Scheffersomyces_stipitis_CP000498.1 .
ANIRREFL-LEDAYNSF---------------HKAGSNFKN-RIQVSFFNEYGP---EAG
IDGGGITKEF---LTSV----VREGFDPSNEL------ELFKETISDN------------
----------------QIYPNDDI----------------------------HKSITVGD
DPQLQQKKLLYLKFLGSIVGKCLYENVLIDVSFAP--FFLNKWCN---------------
------DNMKNSINDLNY----------------------LDHELFMG--------LMKL

VKMPE--QEL-DSLD-
--LNFTVNETL---------------------------KGK-NYVFD-LLPPN-------
GENTK---
------------------------------LNLSNKL-SYIHQ-ISNFKLNQS-LHIQTK
-YFIEG---LFGLISS--SWLS-MFDSF-ELQMLISG-----------------------
---GQNDIN-----------ILDW----KNNVEYGG-YLDS-------------------
------------------------DITV-RYFWEVVA--------------------EMT
PDERFALIKF-------VTSVSRAP------------LLG-----FG-SLNPKF--GIRN
SGSDT-----------------------------SRLPTASTCV-NL-LKLPDY-RNKEL
IRSKLL-YAIEA-EAGF
>Scheffersomyces_shehatae_BDMP01000003.1 .
ADIRREHL-LEDAFNSF---------------HNSGSNFKQ-VLQVSFFNEYGQ---EAG
IDGGGITKEF---LTSV----VTEGFSPSNDL------HLFKETLSDN------------
----------------QIYPNDDI----------------------------YKSINVGI
DLELQQEKLNYLKFLGNVVGKCFYENVLIDVSFAP--FFLNKWCN---------------
------DNMKNSINDLNY----------------------LDHELFMG--------LMKL
LKMPE--AEL-NDLD-
--LNFTINEKV---------------------------NGK-SFLFD-LLPPN-------
GENTK---
------------------------------LSLSNKL-SYIHQ-ISNFKLNQS-LHIQTK
-YFLEG---LYEIISS--SWLS-MFDSF-ELQMLISG-----------------------
---GESDVN-----------IVDW----KENVEYGG-YFDD-------------------
------------------------DITI-KYFWEVIA--------------------EMS
AEERFKLIKF-------VTSVSRAP------------LLG-----FG-SLSPRF--GIRN
SGRDV-----------------------------ARLPTASTCV-NL-LKLPDY-QDKEL
IRSKLL-YAIYT-EARF
>Pichia_membranifaciens_XM_019162646.1 .
GVISRNNM-LFDSYNSF--------------GNLTGTEFKR-QFSVQFVNEFGET--EAG
IDGGGLTKEL---LTTL----VASTFVPSE--------ENRKNNKGL-------------
----------------QLFKEAIS------------------------------------
YACTNDEYLKISRFLGMVIGKCLYDNVLLDISFTS--FFLNTCAT---------------
--LGSKYFKN--------LIGE
KISTDP---EKF-ESMS-
--LLFTVDDIFYDEDG----------------------NLH-HVEVP-LIPPQKKSPDSA
PEPVP---
------------------------------VTVNNKM-QFVRL-MTSFKLSKQ-TDLVMK
-YFVEG---LFQVIKP--YWLL-LFNPF-ELQTLISG-----------------------
---DDDAID-----------IDDL----QRNVEYGG-FLES-------------------
------------------------DQTV-HDLFTILR--------------------EFD
RTDRGKFVKF-------VTSSPKQP------------LLG-----FK-ELNPKF--GIRN
SGSDT-----------------------------SRLPTASTCV-NL-LKIPDY-RDKEL
LREKLL-YSINS-KAGF
>Pichia_kudriavzevii_XM_020688198.1 .
GVISRDNI-LFDAYKYY--------------GKTRGKDFKR-PFAVQFINHFGEA--EAG
IDGGGLTKEL---LTSV----VSCAMTPSESNRQANRGLEFFRIGTDY------------
----------------HLYFNPEF--------------------YFKL-YYEREQHSKVP
YACSNEEYLHMCHFLGMVIGKCLYSNILLDVSFTS--FFLITCAK-----MGGQYFRNLV
GDKVDFIGYSVSLDELKN----------------------IDEALYQS--------VNYI
LKQTEE---SKF-KSMG-
--IQFSVDDEFYDI------------------------NGK-NYHVS-IPLLRNKD----
GSVVE---
------------------------------VTNGNKM-QFARM-LASFKLSKQ-NKLEMK
-SFVDG---LFQVIRP--HWLL-LFNPI-ELQTLISG-----------------------
----DDEID-----------IEDL----RRNVVYGGGYTEE-------------------
------------------------DQTI-KDLFEILH--------------------EFN
HENRSKFIKY-------VTSSSKQP------------LLG-----FK-ELDPHF--GIFN
AGADT-----------------------------NRLPTASTCV-NL-LKLPNY-KDKDQ
LRRKLL-YAITS-NAGF

>Komagataella_phaffii_XM_002491005.1 .
GTISRDNI-IEDGFALL--------------SKLTGEQLKN-YLGIQFTNQFGA---EAG
IDGGGLTKEF---LTET----VKKGFATTNESGVEDL-MLFKHSPRN-------------
----------------EIYPNPAL-------------------------------YLKRT
KGVDVHKDLEMIRYLGSIVGKCLYEKILVDLPFSP--FFLTRWSV---------------
-----NQPYKTMFDDLKY----------------------YDEDLFRN--------LNKL
LSLNS--DQV-DALE-
--LNFTLTERFYYNG-----------------------AFK-TTTIM-LLPN--------
GANIP---
------------------------------VTTANRL-QYVFA-VSNFKLYTS-LRLQTD
-YFLQG---LFKVIPS--QWLE-VFNPM-ELQILVSG-----------------------
---RDRGID-----------ISDL----RKNTVFSE-YTEN-------------------
------------------------DQTI-IDLFDILEH-------------------DFS
NEERSKFIRF-------VTSSSKAP------------LLG-----FS-QLNPKF--GVRN
IGPFS-----------------------------DRLPSAFTCF-NQ-LKLPDY-GNRKV
LKEKLL-YAISS-ESGF
>Komagataella_pastoris_CP014587.1 .
GTISRDNI-IEDGFALL--------------SRLTGEQLKN-HLGIQFTNQFGA---EAG
IDGGGLTKEF---LTET----VKKGFATTNESGVEDL-MLFKHSPRN-------------
----------------EIYPNPAL-------------------------------YLKRS
KGINVHKDLEMIRYLGSIVGKCLYEKILVDLPFSP--FFLTRWSV---------------
-----NQPYKTMFDDLKY----------------------YDEDLFRN--------LNKL
LSLSS--AQV-DALE-
--LNFTLTERFYHNG-----------------------AFK-TTTIM-LLPN--------
GATIP---
------------------------------VTTANRL-QYVFA-VSNFKLYTS-LRLQTD
-YFLQG---LFKVIPS--QWLE-VFNPM-ELQILVSG-----------------------
---RDRGID-----------ISDL----KKNTVFSE-YSEN-------------------
------------------------DQTI-IDLFDILEH-------------------DFS
NEERSQFIRF-------VTSSSKAP------------LLG-----FS-QLNPKF--GVRN
IGPFS-----------------------------DRLPSAFTCF-NQ-LKLPNY-GNRKV
LKEKLL-YAISS-ESGF
>Tortispora_caseinolytica_LSKT01000059.1 .
IPINRDDV-IGSGIDAI---------------TLLGNDFRK-HTKIEFHNAHGI---EAG
IDGGGLTKEF---ITLA----CKELFSPEL--------GLFVESPLH-------------
----------------FLYPNPTA------------------------------------
---TTPEQLKMLHFCGMLIGKAVRSQVLLEVQLAQ--FFMRKWRN---------------
------YAVENSTDDLID----------------------LDPEFYRS--------LMNL
KNYEG--DID-ELH--
--MPYSVVNA----------------------------EGR-EIKLS-STPE--------
DQ--
-----------------------------FVTKKNVA-WYIFQ-VSNYKLNTE-LRAQTN
-AFLSG---LGKVLPI--SWLS-MFNAA-ELQELVCG-----------------------
---TMKPVS-----------IEDL----KNNTVYSV-YDES-------------------
------------------------SPTI-KAFWEVLE--------------------EMS
EEDKAKFLLF-------VTSSSRPP------------LQG-----FK-QLNPKF--SIRH
AGNEI-----------------------------YRLPSASTCV-NL-LKLPNY-QSKEL
LRERLM-YAIQS-AKG-
>2_Yarrowia_keelungensis_BCJD01000001.1 .
APVIRGRE-FESAYEKL---------------YSYGSELKK-PLSIRFFNDFGP---EAG
IDGGGLTKEF---LTSV----TDDAFNPSR--------GLFAATENH-------------
----------------LLYPNPAT------------------------------------
------HDTKQLAFLGNLVGKGLYENILIEHGFAS--FFLQKWTQ---------------
------GSMRSSIDDLYS----------------------LDPNLYDS--------LASL
KQIYAT---QGDADLG--
--LTFSIDYEDA--------------------------QGN-VRTRD-LIRN--------
GSEVP---
------------------------------VTKSNYL-RYIYE-VANFKLNTS-IRTQTD

-AFLGG---LYQLIDP--SWVS-MFNAD-ELQMLISG-----------------------
---GHANVD-----------VWDL----KTHTNYGG-YLDT-------------------
------------------------DQTV-KDFWTVFE--------------------SFE
EEDKRLLLKF-------VTSVSKAP------------LQG-----FK-ALNPSF--AIRN
AGRQV-----------------------------DRFPTASTCV-NL-LKLPDY-QDIDQ
LRKKLL-YAIRS-HAGF
>Yarrowia_lipolytica_XM_500283.1 .
APVIRGRE-FESAYEKL---------------YSYGSELKK-PLSIRFFNDFGP---EAG
IDGGGLTKEF---LTSV----TDDAFNTSR--------GLFAATDNH-------------
----------------LLYPNPTT------------------------------------
------HDTKQLAFLGNLVGKGLYENILIEHGFAS--FFLQKWTQ---------------
------GSMRSSIDDLYS----------------------LDPNLYES--------LASL
KQIYAT---QGDADLG--
--LTFSIDYADS--------------------------EGN-VRTRD-LIRN--------
GSEVP---
------------------------------VTKANYL-RYIYE-VANFKLNTS-IRTQTD
-AFLGG---LYQLIDP--SWVS-MFNAD-ELQMLISG-----------------------
---GHANVD-----------VWDL----KTHTNYGG-YLDT-------------------
------------------------DQTI-KDFWTVFE--------------------SFE
EEDKRLLLKF-------VTSVSKAP------------LQG-----FK-ALNPSF--AIRN
AGRQV-----------------------------DRFPTASTCV-NL-LKLPDY-QDIDQ
LRKKLL-YAIRS-HAGF
>Yarrowia_deformans_BCIW01000011.1 .
APVIRGRE-FESAYEKL---------------YSYGSELKK-PLSIRFFNDFGP---EAG
IDGGGLTKEF---LTSV----TDDAFNPSR--------GLFAATDNH-------------
----------------LLYPNPTT------------------------------------
------HDTKQLAFLGNLVGKGLYENILIEHGFAS--FFLQKWTQ---------------
------GSMRSSIDDLYS----------------------LDPNLYES--------LASL
KQIYAT---QGDADLG--
--LTFSIDYADS--------------------------NGN-VRTRD-LIRN--------
GSEVP---
------------------------------VTKANYL-RYIYE-VANFKLNTS-IRTQTD
-AFLGG---LYQLIDP--SWVS-MFNAD-ELQMLISG-----------------------
---GHANVD-----------VWDL----KTHTNYGG-YLDS-------------------
------------------------DQTV-KDFWTVFE--------------------SLE
EEDKRLFLKF-------VTSVSKAP------------LQG-----FK-ALNPSF--AIRN
AGRQV-----------------------------DRFPTASTCV-NL-LKLPDY-QDIDQ
LRKKLL-YAIRS-HAGF
>Starmerella_bacillaris_MWSF01000009.1 .
--IDRDNL-LHSARKLF---------------LDLGSSTKN--MQISFTSNDSA---EVG
IDGGGLTRELLIDLIPL----TLSPNTSDVRGLNFDGHPMFVENEHH-------------
----------------LLLPNPIF---------------------------------MEV
SKYRTKEVAENFMFLGQIVGKCIYDGVLLDFEFAP--QFLAHWTC---------------
-------MATSKFDDLKT----------------------IDPMLYKN--------LRSI
CSMSA--DEV-DSL--
-GLDFTVTGC------------------------------Q-NQTIE-LREG--------
GSKIA---
------------------------------VTEHNKF-EYAME-LARYKMSTC-IKRQTV
-SFLKG---LRQIISE--TCFN-MFNPD-ELNVILSG-----------------------
---SSRDLD-----------IDDL----REHSVCSG-FAPG-------------------
------------------------STYV-DSLWEIVK--------------------SFT
PLQKRKFLKF-------VTSVPRGP------------LLG-----FR-MLEPNF--GVRR
AGEND-----------------------------SHFPTASTCV-NL-LAIPEY-SSLEL
LRDRL------------
>Candida_infanticola_LWLF01000001.1 .
--IRREAL-TEDAER-V--------------FAALGPNLRN--LRVQLVSNGEP---EAG
IDGGGLTKEV---LMSI----TDEMFKNPTSAR-----PLFVTNESH-------------
----------------LLFPNPIN----------------------------------ST

PGRESAEALKRYRFLGQVIAKCLYEGVLLDVEFSR--FFLQKWAG---------------
-----DTAVKNSFDDLYY----------------------LDPELYSS--------LVAL
YNYEG--DVE-ELS--
--LDFTTANS-----------------------------GG--SSIN-LRPN--------
GASIA---
------------------------------VTNANRL-EYIHA-IANYRLNTS-IASQTR
-AFLNG---MVDLIPL--HWLR-MFNPH-EIQTLISG-----------------------
---GTTSVD-----------IDDL----ERNTIVNG-FPAN-------------------
------------------------SQTI-KNFWKVVR--------------------EMT
PEQQHKLLQF-------VTSVPKGP------------LLG-----FS-NLSPKF--GIRC
ATTGPSPVFDF----------YGNNQDNGVVRDDGRLPTASTCV-NL-LKLPDY-SSIDI
LREKLL-FVIES-NAGF
>Candida_versatilis_BCJV01000003.1 .
--INRETL-VDDAET-V--------------FAELGPRIKD-DLAVRLVSNGVP---EAG
IDGGGLTKEV---LTAI----AAEAFTPQS--------PYWCETPSH-------------
----------------TLIPNPIW--------------------------------SERP
WPNGIEAARAKYRFLGQVVGKCLYENLLIDVEFSY--VFLMKWAG---------------
-----ASATRASFDDLYL----------------------LDLDLYNS--------LVSL
LHLSPS---DIE-ALD--
--LDFTIQGP-----------------------------GS--TVVE-LKPH--------
GAQVQ---
------------------------------LTDSNKL-EYARC-VANYKLNNE-MGPQTN
-WFLQG---FGEIINL--AWLQ-MFNGS-EIQMLISG-----------------------
---GHQDID-----------IEDL----RAHAEFSG-WAFD-------------------
------------------------SPTL-ENLWHVLH--------------------EFN
SDQRKAFLKF-------VTSVPRGP------------LLG-----FK-HLTPRF--GIRC
AGRSD-----------------------------ERLPTSSTCV-NL-LKIPEY-SSKEI
LRNKLL-YSIES-GAGF
>Rhizopus_oryzae_GDUK01021469.1 .
ITVKRGQV-LLDGYQQL--------------SSLPTSAWKG-KIRVNFINELGME--EAG
IDRGGPFKEF---ITTL----ISEAFKPNY--------GLFEATSQN-------------
----------------SFYPSPSS------------------------------------
-AVHGKNHIQLFEFIGKAIGKAVCEGILLDVQFAS--FLLAKLLG---------------
--------RNVFLEELKE----------------------LDEDVWKN--------LIYV
KNYEG--DVE-VLG--
--LTFEVDEDV---------------------------FGK-IESHE-LKYR--------
GKHVP---
------------------------------VVNSNRI-EYVYL-MADYKLNQR-AKNQTN
-AFIQG---FKTVVSE--SWIR-LFSPP-ELQRVLTG-----------------------
---EDKDFD-----------VLDM----RKHTVYEDGYFDE-------------------
------------------------HPVI-RSFWQIVE--------------------QFT
FEEKKTLLKF-------VTGCSKPP------------LGG-----FS-YLQPPF--TIRM
VSTELDGPASI----------RMIKSVLKMNTKSGRLPTSSTCF-NL-LKLPAY-TRKAQ
LKEKLS-YSINS-NTGF
>Mucor_irregularis_GFBC01021656.1 .
ITVRRGQV-LEDGFQGL--------------SGLSPSAWKG-TIRVAFVNEVGAT--EAG
IDQGGPFKDF---LTMM----ISEAFEPNY--------GLFSSTKTN-------------
----------------SFYPSATS------------------------------------
-IVHGRNHILLFEFIGKAIGKALYEGILLDVKFAG--FLLARLLG---------------
--------RNVFLEELKE----------------------LDEEVWKS--------LTFI
KHYEG--DVE-DLG--
--LTFEADENN---------------------------FGK-VQSHE-LKYR--------
GKSTA---
------------------------------VTDGNKI-EYVYL-MADYKLNQR-AKEQTK
-AFIHG---FRSIISE--NWIK-LFSPP-ELQRVLSG-----------------------
---EDTDFD-----------ISDL----RKHTEYQDGYFDL-------------------
------------------------HPVI-RLLWQIVG--------------------ELS
STEKRAFLKF-------ATGCPKPP------------LGG-----FE-YLQPPF--TVRM

VSTDASEGPK------------KSFFKMSIGNKSGRLPTSSTCF-NL-LKLPAY-TKKSL
LKEKLQ-YAINA-NTGF
>Phycomyces_blakesleeanus_XM_018428618.1 .
ITVRRNFV-LEDGLRGL--------------SNLSPTAWKG-TIRVSFVNELGIE--EAG
IDQGGPFKDF---ISLL----VAEVFKPSC--------ELFAATPKTN------------
----------------LFYPAASS------------------------------------
-HIIGASHVAYFELIGKIIGKAVYEGILIDAQFAS--FLLSKLLG---------------
--------RNVFLEELRE----------------------LDEDIWRN--------LTFV
KHQD---NIE-DLG--
--LTFATDEQI---------------------------NGK-VVTHE-LKFL--------
GSQTA---
------------------------------VTDSNKV-EYVYL-MADYKLNQQ-AKEQTK
-AFING---FRSVIFD--GWIK-VFSPP-ELQRVISG-----------------------
---EDTDFD-----------VHDL----RRHTDYQNGYFDQ-------------------
------------------------HPVI-KLMWQIVD--------------------GLT
SQEKRAFLKF-------VTGCPKPP------------LGG-----FD-YLQPPF--TIRM
VSPDKDQQSMEG-----LGIVKSFFKINGLQNKGGRLPTSSTCF-NL-LKLPGK------
-KIKHL-YHIEP-SK--
>Gigaspora_margarita_GBYF01078893.1 .
IKIRRDHV-LDDGYKQL--------------GRLTATQIKG-QIRVMFVNETGVD--EIG
VDQGGPFKEF---ITQL----IAEAFDPKC--------GLFAVTPDG-------------
----------------SLYPNPHS------------------------------------
----AHTKLPLYTFLGRMIARAMKEKILLDSQFAE--FFLSKISG---------------
--------RAVFLEDLIG----------------------WNNELWKN--------LIFL
KRYEG--NVE-DLG--
--LYFVVDEEV---------------------------NGR-IISKE-LRAG--------
GSHMA---
------------------------------VTNYNRI-QYIYL-MADYKLNKQ-IKEQTE
-AFISG---FQSMMPI--NRLR-MFSPP-ELRRVMSG-----------------------
---EDVDWD-----------VSDL----RQHTTYQSGYFDQ-------------------
------------------------HRTI-RNLWSILE--------------------EFD
SKNKNAFLKF-------VTSCSRPP------------LGG-----FK-YLQPQF--TIRM
VSMEYDDSESRPV---LAPVKAFFSFGNSKSAAKGRLPTSSTCF-NL-LKLPAY-PQKSI
LKEKLK-YAIHS-NTG-
>Spizellomyces_punctatus_XM_016754283.1 .
VVVHRNAV-LEDGYRQL--------------ARVPPQQLKQ-AIRVKFVNELGLE--EAG
IDQSGIFKEF---LEEM----CKRAFSTNL--------NLFHTTPDG-------------
----------------YVTPSPTS------------------------------------
--FIHEEHLQLLEFVGKIFGKALYEGIAIDIPFAN--FIYAKLLG---------------
--------RYNYLDELPS----------------------LDPQLYKN--------LTFL
KRYEG--DSE-DLG--
--LTFTIDQDI---------------------------FGN-VVSKE-IKPG--------
GAAIS---
------------------------------VTNENKF-EYVHL-MADYRLNRE-CRDQFK
-ALIRG---FRSIISE--KWLG-FFSPT-ELQKLMCG-----------------------
---ENVEFD-----------VKDL----RAHVRYEGGYFDQ-------------------
------------------------HKTI-RSLWQVVS--------------------DFG
PKDKAAFLKF-------VTSCSKPP------------VGG-----FQ-YLNPPF--TIRY
VAESDEGGTQTENPVVAGARLIGSAFGITLGKDMNRLPTASTCF-NM-LKLPAY-KKKST
LKEKLL-YAINS-GAGF
>Batrachochytrium_dendrobatidis_XM_006674966.1 .
IKVFRQRL-LEDGFSQL--------------RKFTCSQLKQ-TVRVKFVDQFGSE--EIG
IDQNGIFKEF---MEDI----CKQAFSTEF--------GLFKTTLDG-------------
----------------NVTPFLNS------------------------------------
--VVHDEHLQLFEFIGRMMGKALYEGIVIDIPFAL--YIYAKLLG---------------
--------RYNYMDDLPS----------------------LDPELYRS--------LTFI
KDYEG--DCS-DLG--
--LDFTISQDI---------------------------FGH-VTNVE-IKPG--------

GAHIP---
------------------------------LTNDNVF-EYIHL-MADFRLNQE-CKDQFQ
-AFICG---FRSIIQD--QWLH-IFSPM-ELQWLMSG-----------------------
---ENSKLD-----------IKDL----RMHTRYEGGYFDL-------------------
------------------------HRTI-RNFWAVLG--------------------EFT
PKDQSAFLKY-------VTSCSKVR----------FFIEGISE--TK-MIKSTF--WNA-
----------------------------------DRLPTARTCF-NL-LKLPSY-SKKST
LYKKLL-YAIQY-GAGF
>Rozella_allomycis_ATJD01000205.1 .
INIQRGNE-FESGFQQM---------------NSLGHKWKQ-KIRISFLDEFQQV--EAG
VDGGGLYKEF---LNNL----LKIALSPNY--------GLFKVNHLG-------------
----------------QVYPNNNS----------------------------FSSHGIKL
KFITIDDYLTLFSFLGRVIGKALCDRILIDVPFVR--FFIAKWIG--------------K
HISCNKFYIKCLVDDLAL----------------------LDQDLYKN--------LLFL
RDYKG--NVE-DLN--
--LTFSIADDGKLYLRLIKD------------------LNF-NSSKN-LIPN--------
GSEIS---
------------------------------VTNDNRL-NYIYL-LANEKLNNQ-IRHQTL
-AFLDG---LSNLVSL--EWLK-IFTQS-ELQLLLSG-----------------------
---TTTPID-----------LNDW----KKHCVYQGDYSQLGHE----------------
------------------------DPTI-KLFWEIIS--------------------EMS
EVEKRKVLKF-------VTSCPRPP------------LLG-----FS-QLEPKF--GIQS
TGTD------------------------------DRLPSSSTCF-NL-LKLPRY-TSKSV
LKGKLL-YAINS-EAG-
>Entomophthora_muscae_GENB01019891.1 .
LTVRRTHL-YEDAYDKL--------------RPENEPDLRL-KFRVQFVSSLGLD--EAG
IDGGGVFREF---LSEL----IKTAFDPNR--------GFFMVTTDN-------------
----------------KLYPNPNV------------------------------------
-GDLVPDFEKHYYFIGRILGKAIYENLLVELPLAE--FFLTKLAG---------------
------KYADVDIHQLAS----------------------LDPELYKN--------LLYL
KDYEG--DVS-ELN--
--LDFTVASSS---------------------------LGQ-TQVIE-LKHQ--------
GQTIP---
------------------------------VTNSNRI-EYLQL-MADYKLNVQ-IRQHCI
-AFRKG---LSNVLPV--EWLY-MFSNK-ELQILISG-----------------------
---AEIPID-----------LEDL----KKHCKYGGEYSQE-------------------
------------------------HPSI-IAFWSALE--------------------SFD
DLQKRQLLKF-------VTSCSRPP------------LLG-----FK-DLDPPF--FIQN
AGDM------------------------------ERLPTASTCT-NL-LKLPPF-KTIEQ
MREKLL-YAIQS-GVGF
>Trichosporon_veenhuisii_BCKJ01000001.1 .
AEIRRTHL-AEDGFRQL---------------NRLGPSLKG-MVQITFIDQWGNE--EAG
IDGGGLFKEF---LTAL----SKEAFDTAR--------GLWLANYNN-------------
----------------QLYPNPHS------------------------------------
-YSKESHQLEWYSFIGRVLGKALYEDILVDVSFAG--FFVAKWLG---------------
--------RQSYLDDLAS----------------------LDKDLYRG--------LISL
KNYAG--NPE-DLS--
--LNFTVTEDD---------------------------FGV-ARSVD-LVPG--------
GSDIA---
------------------------------VTSENKH-EYIQL-VCKYKLDRQ-FAEQSR
-AFFSG---LSDIIDP--KWLR-MFDQQ-ELAQLLGG-----------------------
---EEAPID-----------IADL----RKHTTVSG-FDD--------------------
------------------------GNTP-AMFWRVVA--------------------GFD
QEQRRALVRF-------VTSCSRPP------------LLG-----FG-ALNPGF--AIRN
SGTDT-----------------------------ARLPTASSCA-NL-LKLPDY-KDEQL
LRSKLL-KAINS-GAGF
>Cutaneotrichosp_cutaneum_LTAL01000394.1 .
AEIRRTHL-AEDGFRQL---------------NRLGPSLKG-MVQITFIDQWGNE--EAG

```
IDGGGLFKEF---LTAL----SKEAFDTAR--------GLWLANYNN-------------
----------------ELYPNPHS------------------------------------
-YSTESHQLQWYSFIGRVLGKALYEDILVDVSFAG--FFVAKWLG---------------
--------RQSYLDDLAS----------------------LDKDLYRG--------LISL
KNYAG----------------------------------------------NPE-DLS--
--LNFTVTEDD---------------------------FGV-ARSVD-LVPG--------
GSDIA-------------------------------------------------------
------------------------------VTAENKH-EYIQL-VCKYKLDRQ-FAEQSR
-AFFSG---LSDIIDP--KWLR-MFDQQ-ELAQLLGG-----------------------
---EEVPID-----------IADL----RKHTTVSG-FDD--------------------
------------------------GNTP-AMFWRVVN--------------------GFN
QEQRRALVRF-------VTSCSRPP------------LLG-----FG-SLNPGF--AIRN
SGTDT-----------------------------ARLPTASSCA-NL-LKLPDY-KDEQL
LRSKLL-KAINS-GAGF
>Trichosporon_gracile_BCJO01000003.1 .
AEIRRSHL-AEDGFRQL---------------NRLGPALKG-MVQITFIDQWGNE--EPG
IDGGGLFKEF---LTAL----SKEAFDTER--------GLWLANYNN-------------
----------------ELFPNPHK------------------------------------
-YATEPHQLEWYSFIGRVLGKALYEDILVDVTFAG--FFVAKWLG---------------
--------RQSYLDDLAS----------------------LDKDLYRG--------LISL
KNYPG----------------------------------------------NPE-DLS--
--LNFTITEDD---------------------------FGV-ARSVD-LVPG--------
GSDIA-------------------------------------------------------
------------------------------VTNENKH-EYIQL-VCKYKLDRQ-FFQQSR
-AFFSG---LSDIIDP--KWLR-MFDQQ-ELAQLLGG-----------------------
---EAVPID-----------ISDL----RKHTTVSG-FDD--------------------
------------------------GYTT-QLFWHVVE--------------------SFD
EEQRRALLRF-------VTSCSRPP------------LLG-----FG-YLNPGF--AIRN
SGNDT-----------------------------ARLPTASSCA-NL-LKLPDY-KDEKL
LRTKLL-QAINS-GAGF
>Trichosporon_montevideense_BCFV01000001.1 .
--IRRSHL-SEDGFRGL---------------NRLGPSLKG-MIQIQFIDKWGGE--EAG
IDGGGLFKEF---LTQL----SKEAFDTEH--------GLWLANYNN-------------
----------------ELYPNPHN------------------------------------
-YAKEEHQLQWYSFLGRMLGKALYEDILVDVSFAG--FFVAKWLG---------------
--------RQSYLDDLAS----------------------LDRDLYRG--------LISL
KNYPG----------------------------------------------NPE-DLS--
--LNFTMTEDD---------------------------FGV-ARSVD-LVPG--------
GSDIA-------------------------------------------------------
------------------------------VTAENRH-EYIQL-VCRYKLDRQ-FAEQSR
-AFFSG---LSDIIDP--KWLR-MFDQQ-ELAQLLGG-----------------------
---EEVPID-----------IADL----RKHTQVSG-YPD--------------------
------------------------AQTP-QLFWRVVE--------------------GFN
DEQRRALIRF-------VTSCSRPP------------LLG-----FA-YLNPGF--AIRN
AGSDM-----------------------------ARLPTASSCA-NL-LKLPDY-KDEEL
LRRKLL-QAINS-GAGF
>Trichosporon_porosum_BCJG01000002.1 .
ATIRRSDL-AHDGFRQL---------------NDVGPLLKG-TIQITFVDQWGQE--EAG
IDGGGLFKEF---LTSL----NKEAFDTER--------GLWLTNYNN-------------
----------------ELYPNPHQ------------------------------------
-YATEPHQLAWYGFIGRVLGKALYEDILVDVSFAG--FFLAKWLG---------------
--------RQSYLDDLAS----------------------LDRDLYRG--------LILL
KNYP-----------------------------------------------EPE-ELS--
--LNFTVTEED---------------------------FGV-ARSVD-LVPG--------
GSDIA-------------------------------------------------------
------------------------------VTADNRH-EYIQL-VCRYKLDKQ-FSLQSR
-SFFAG---LSDLIDP--KWLR-MFDQT-ELAQLLGG-----------------------
---EETPID-----------LVDL----REHTNVTG-FES--------------------
```

------------------------GYTP-QLFWRVVS--------------------TFT
QEQQRALLRF-------VTSCSRPP------------LLG-----FK-HLNPGF--AIRN
SGADT-----------------------------ERLPTASSCA-NL-LKLPDY-KDEKL
LRTKLL-QAITS-GAGF
>NWGS3B_2_Trichosporon_gamsii_BCJN01000003.1 .
ATIRRSDL-AHDGFRQL---------------NDLGPMLKG-TLHIHFVDQFGLE--EAG
IDGGGLFKEF---LTSL----SKEAFDTER--------GLWLTNYNN-------------
----------------ELYPNPHS------------------------------------
-YATEPHQLEWYGFIGRVLGKALYEDILVDVKFAG--FFLAKWLS---------------
--------RQSYLDDLAS----------------------LDRDLYRG--------LIML
KNYPG--DPE-DLS--
--LNFTITEDD---------------------------FGV-ARSVD-LVPG--------
GSEIA---
------------------------------VTAENRH-EYIQL-VCKYKLDRQ-FAQQSR
-AFFSG---LSDLIDP--KWLR-MFDQT-ELAQLLGG-----------------------
---EQTPID-----------LADL----REHTSILG-YDD--------------------
------------------------GYTP-QLFWRVVA--------------------GFT
QEQQRALLRF-------VTSCSRPP------------LLG-----FK-HLNPGF--AIRN
GGHDK-----------------------------ERLPTASSCV-NL-LKLPDY-RDEAV
LRQKLL-QAITS-GAGF
>Cutaneotrichosp_curvatus_LDEP01000114.1 .
VTIRRTDL-AEDGFRQI---------------NSLGSHLKG-TLEITFIDQWGNE--EPG
IDGGGLFKEF---LHDL----SKEAFDSER--------GLWLANANN-------------
----------------ELYPNPHS------------------------------------
-YATEPHQLAWYGFIGRLLGKALYADILVDVKFAG--FFLAKWLG---------------
--------RQSYLDDLAS----------------------LDKELYRG--------LIQL
KNYP--NPE-DLS--
--LNFTIAEDE---------------------------FGV-TRSVD-LVPG--------
GSEIA---
------------------------------VTAANRH-EYIHL-VCKYKLDRQ-FALQSQ
-AFFAG---LSDIIDP--RWLR-MFDQN-ELAQLLGG-----------------------
---EETPID-----------IQDL----RANTSISG-FGG--------------------
------------------------SATP-QMFWNVVN--------------------SFN
QQQRRDLLRF-------VTSCSRPP------------LLG-----FK-HLNPPF--GVRN
SGDDR-----------------------------DRLPTASACA-NL-LKLPDY-KDERV
LRAKLL-QSINS-GAGF
>Cutaneotrichosp_cutaneum_LRUG01000069.1 .
VAIRRTHL-AEDGFRQL---------------NSLGSGLKN-TIEITFIDQWGNE--EAG
IDGGGLFKEF---LHNL----SKEAFDTER--------GLWLANLRN-------------
----------------ELYPNPHS------------------------------------
-YATEPHQLAWYSFIGRLLGKALYANMLVDVTFAG--FFLAKWLG---------------
--------RQSYVDDLAS----------------------LDRELYRG--------LIQL
KNYP--NPE-ELS--
--LTFSVTEDE---------------------------FGV-AKSID-LVPG--------
GSELP---
------------------------------VTAENRH-EYIAL-VCKYKLDRQ-FALQSE
-AFFAG---LSDLIDP--RWLR-MFDQN-ELAQLLGG-----------------------
---EETPID-----------ILDL----RKHTTVMG-FDD--------------------
------------------------ERTP-QMFWRVVN--------------------GFT
EEQKRQLLTF-------VTSCSRPP------------LLG-----FA-QLNPPF--GVRN
GGADK-----------------------------ERLPTASSCA-NL-LKLPDY-KDEHM
LRAKLL-QAITS-GAGF
>Cutaneotrichosp_oleaginosus_JZUH01000040. 1..
VSIRRSHL-AEDGFRQL---------------NSLGPGLKN-IVEITFIDQWGNE--EAG
IDGGGLFKEF---LHNL----SKEAFDTER--------GLWLANLRN-------------
----------------ELYPNPHS------------------------------------
-YAKESHQLEWYSFIGRLLGKALYANMLVDVTFAG--FFLAKWLG---------------
--------RQSYVDDLAS----------------------LDQELYRG--------LIQL

```
KNYP-----------------------------------------------NPE-ELS--
--LTFSVTENE---------------------------FGV-AKNVD-LIPG--------
GSEIP-------------------------------------------------------
------------------------------VTAENRH-EYIAL-VCKYKLDWQ-FAAQSE
-AFFAG---LSDLIDP--RWLR-MFDQN-ELAQLLGG-----------------------
---EETPID-----------IMDL----RKHTTVTG-FED--------------------
------------------------ERTP-AMFWRVVD--------------------SFS
EEQKRQLLTF-------VTSCSRPP------------LLG-----FA-QLNPPF--GVRN
GGSDK-----------------------------SRLPTASSCA-NL-LKLPDY-RDEAM
LRSKLL-QAITS-GAGF
>Trichosporon_oleaginosus_XM_018422820.1 .
VSIRRSHL-AEDGFRQL---------------NSLGPGLKN-IVEITFIDQWGNE--EAG
IDGGGLFKEF---LHNL----SKEAFDTER--------GLWLANLRN-------------
----------------ELYPNPHS------------------------------------
-YAKESHQLEWYSFIGRLLGKALYANMLVDVTFAG--FFLAKWLG---------------
--------RQSYVDDLAS----------------------LDQELYRG--------LIQL
KNYP-----------------------------------------------NPE-ELS--
--LTFSVTENE---------------------------FGV-AKNVD-LIPG--------
GSEIP-------------------------------------------------------
------------------------------VTAENRH-EYIAL-VCKYKLDWQ-FAAQSE
-AFFAG---LSDLIDP--RWLR-MFDQN-ELAQLLGG-----------------------
---EETPID-----------IMDL----RKHTTVTG-FED--------------------
------------------------ERTP-AMFWRVVD--------------------SFS
EEQKRQLLTF-------VTSCSRPP------------LLG-----FA-QLNPPF--GVRN
GGSDK-----------------------------SRLPTASSCA-NL-LKLPDY-RDEAM
LRSKLL-QAITS-GAGF
>Trichosporon_asahii_XM_014321649.1 .
AQIRRTSV-AHDGFRQL----------------NRPGALRG-MVQIQFVNEHGLP--EAG
IDGGGLFKEF---LTCL----VKEAFDVER--------GLWLANDEN-------------
----------------ELYPNPHG------------------------------------
-YATEPHQLEWY----------------------------TFWLG---------------
--------RQSYLDDLSS----------------------LDAELYRG--------LISV
KNYP-----------------------------------------------KPE-ELS--
--LSFTVTEDD---------------------------FGV-ARSVD-LVPG--------
GSEIP-------------------------------------------------------
------------------------------VTAENRA-EYIQL-MCQYKLGRQ-FAAQSR
-AFFSG---LAEMIDP--RWLR-IFDQA-ELAQLLGG-----------------------
---EHADID-----------LADL----KRHTHFSG-FAPD-------------------
------------------------SSTP-TAFWRVLE--------------------SLS
QEQRGKLVKF-------VTSCSRPP------------LLG-----FK-YLNPEF--AVRN
AGSDT-----------------------------QRLPTASSCA-NL-LKLPDY-KDERL
LREKLL-QAIES-GAGF
>Tremella_mesenterica_XM_007002417.1 .
ATIRRDHL-AQDGFDQL---------------NELGSALKG-TIVIKFVDQFGQR--EAG
VDGGGLFKEF---LTNL----SKEVFDTER--------GLWLATDQN-------------
----------------ALYPNPHS------------------------------------
-YAVEPHQLQWYGFIGRMLGKAIYEGILVDVSFAP--FFLAKWLG---------------
--------KQSYLDDLSS----------------------LDPELYKG--------LIIL
KNYP-----------------------------------------------KPE-DLA--
--LNFTVTQQE---------------------------FGT-TQTVD-LVPG--------
GSEIP-------------------------------------------------------
------------------------------VTAENRH-EYIQL-VCKYKLDQQ-ISAQSK
-AFFNG---LAEVIDQ--KWLR-MFDQH-ELQQLIGG-----------------------
---EETPID-----------IDDL----RENSNITG-FIH--------------------
------------------------NETV-PLFWKVVR--------------------QFN
EEERRALLKF-------VTSCERPP------------LLG-----FA-NLNPKF--GIHS
SGTNT-----------------------------DRLPSASSCF-NL-LKLPEY-TDERV
LRQKLL-QAITS-GAGF
```

```
>Kwoniella_bestiolae_XM_019190329.1 .
AKIRRDRI-SQDGFDEL---------------GNLGPALKS-RIEITFVDQHGMT--EAG
IDGGGLFKEF---LTNL----SKEVFDTNR--------GLWLATDQN-------------
----------------ELYPNPHS------------------------------------
-YATEPHQLEWYRFIGQVLGKAMYEGMLVDVSFAD--FFLAKWLG---------------
--------RQSYLDDLNS----------------------LDKELYKG--------LIIL
KNYP-----------------------------------------------KPE-ELA--
--LNFAITEDE---------------------------FGV-KRTID-LVPN--------
GSEIA-------------------------------------------------------
------------------------------VNAENRH-EYIQL-VCKYKLDRQ-ISAQSR
-AFFNG---LSDLIDA--KWLR-MFDQQ-ELQQLIGG-----------------------
---EETLID-----------IDDL----RAHCSVDG-FPN--------------------
------------------------DITP-RLFWKVVK-------------------G-FS
HEQRRALLRF-------VTSCSRPP------------LLG-----FG-YLYPKF--GVKF
NGNDT-----------------------------SRLPSASACF-NL-LKLPGY-TTEQS
LRAKLL-QAITS-GAGF
>Kwoniella_mangroviensis_XM_019145259.1 .
AKIRRSHI-AQDGFDEL---------------GNLGPALKS-RIEITFVDQHGMT--EAG
IDGGGLFKEF---LTNL----SKEVFDTNR--------GLWLATDQN-------------
----------------ELYPNPHS------------------------------------
-YATEPHQLEWYRFIGQVLGKAMYEGMLVDVSFAD--FFLAKWLG---------------
--------RQSYLDDLNS----------------------LDKELYKG--------LIIL
KNYP-----------------------------------------------KPE-ELA--
--LNFAITEDD---------------------------FGV-KRTLN-LVPN--------
GSEIA-------------------------------------------------------
------------------------------VTAENRH-EYIQL-VCKYKLDRQ-ISAQSR
-AFFNG---LSDLIDA--KWLR-MFDQQ-ELQQLIGG-----------------------
---EETLID-----------IDDL----RAHCSVDG-FPN--------------------
------------------------DTTP-RLFWKVVK-------------------G-FT
QEQRRALLRF-------VTSCSRPP------------LLG-----FG-YLYPKF--GVKF
NGNDT-----------------------------SRLPSASACF-NL-LKLPGY-TTEQS
LRTKLL-QAITS-GAGF
>Kwoniella_pini_XM_019158673.1 .
AKIRRTHI-SQDGFDEL---------------GELGPALKS-RIEITFVDQYGLT--EAG
IDGGGLFKEF---LTNL----SKEVFDTNR--------GLWLATDQN-------------
----------------ELYPNPHS------------------------------------
-YATEPHQLAWYRFIGQILGKAMYEGILVDVSFAD--FFLAKWLG---------------
--------RQSYLDDLSS----------------------LDKELYKG--------LIIL
KNYP-----------------------------------------------KPE-ELA--
--LNFAITEDD---------------------------LGV-KRTID-LVPN--------
GSEIA-------------------------------------------------------
------------------------------VTSENRH-EYIQL-VCKYKLDRQ-ISAQSR
-AFFNG---LSDLIDA--KWLR-MFDQQ-ELQQLIGG-----------------------
---EETLID-----------IDDL----RAHCSVDG-FPN--------------------
------------------------DVTP-RLFWKVVK-------------------G-FT
HEQRRALLRF-------VTSCSRPP------------LLG-----FG-YLYPKF--GVKY
NGGNT-----------------------------DRLPSASACF-NL-LKLPGY-TTEQA
LRTKLL-QAITS-GAGF
>Kwoniella_dejecticola_XM_018405120.1 .
AKIRRTHI-SQDGFDEL---------------GELGPALKS-RIEITFVDQYGLT--EAG
IDGGGLFKEF---LTNL----SKEVFDTNR--------GLWLATDQN-------------
----------------ELYPNPHS------------------------------------
-YATESHQLAWYRFIGQILGKAMYEGMLVDVSFAG--FFLAKWLG---------------
--------RQSYLDDLNS----------------------LDKDLYKG--------LIIL
KNYP-----------------------------------------------KPE-ELA--
--LNFAMTEDD---------------------------FGV-KRNID-LIHN--------
GSEVA-------------------------------------------------------
------------------------------VTAENRH-EYIQL-VCKYKLDRQ-IYAQSR
```

-AFFNG---LSDLIDP--KWLR-MFDQQ-ELQQLIGG-----------------------
---EETLID-----------IDDL----RAHCHVDG-FPN--------------------
------------------------DITP-RLFWKVVK-------------------G-FS
HEERRALLRF-------VTSCSRPP------------LLG-----FG-YLYPKF--GVKF
NGPDL-----------------------------DRLPTASACF-NL-LKLPGY-T----

>Cryptococcus_amylolentus_XM_019137396.1 .
AEIRRDHV-AQDGFDGL---------------ASAGPALKG-RVDITFVDQHGLT--EAG
IDGGGLYKEF---LTML----TKEVFDSNR--------GLWLVTNQN-------------
----------------ELYPNPHD------------------------------------
-FASESFNLSWYRFIGQMLGKAIYDGILVDATFAG--FFLAKWLG---------------
--------RQSSLDDLAS----------------------LDKSLYKG--------LIIL
KNDP---KPE-DMA--
--LTFSMTEED---------------------------FGV-QKQVD-LIPG--------
GSDIP---
------------------------------VTADNRH-EYIQL-VCRYKLDKQ-IAAQSK
-AFFLG---LSDLIDA--KWLR-MFDQQ-ELQQLIGG-----------------------
---EETPID-----------LKDL----RAHCHVDG-FPN--------------------
------------------------DTTP-TLFWKVVG--------------------SFT
EEQKRDLLRF-------VTSCSRPP------------LLG-----FS-QLYPKF--AVKF
NGSDM-----------------------------DRLPTASACF-NL-LKLPGY-TNEAT
LRSKLL-QAITS-GAGF
>Cryptococcus_amylolentus_XM_019137397.1 .
AEIRRDHV-AQDGFDGL---------------ASAGPALKG-RVDITFVDQHGLT--EAG
IDGGGLYKEF---LTML----TKEVFDSNR--------GLWLVTNQN-------------
----------------ELYPNPHD------------------------------------
-FASESFNLSWYRFIGQMLGKAIYDGILVDATFAG--FFLAKWLG---------------
--------RQSSLDDLAS----------------------LDKSLYKG--------LIIL
KNDP---KPE-DMA--
--LTFSMTEED---------------------------FGV-QKQVD-LIPG--------
GSDIP---
------------------------------VTADNRH-EYIQL-VCRYKLDKQ-IAAQSK
-AFFLG---LSDLIDA--KWLR-MFDQQ-ELQQLIGG-----------------------
---EETPID-----------LKDL----RAHCHVDG-FPN--------------------
------------------------DTTP-TLFWKVVG--------------------SFT
EEQKRDLLRF-------VTSCSRPP------------LLG-----FS-QLYPKF--AVKF
NGSDM-----------------------------DRLPTASACF-NL-LKLPGY-T----

>Tsuchiyaea_wingfieldii_XM_019172565.1 .
AKIRRDHV-AQDGFDEL---------------ASAGPALKG-RVDITFVDQHGIT--EAG
IDGGGLYKEF---LTML----TKEVFDSNR--------GLWLVTNQN-------------
----------------ELYPNPHH------------------------------------
-FASESFNLSWYRFIGQMLGKAIYDGILVDATFAG--FFLAKWLG---------------
--------RQSYLDDLAS----------------------LDKSLYKG--------LIIL
KNDP---KPE-DMA--
--LTFSMTEED---------------------------FGV-QKQVD-LIPG--------
GSDIP---
------------------------------VTADNRH-EYIQL-VCKYKLDKQ-IAAQSK
-AFFLG---LSDLIDA--KWLR-MFDQQ-ELQQLIGG-----------------------
---EETPID-----------LKDL----RAHCHVDG-FPN--------------------
------------------------DTTP-TLFWKVVG--------------------SFT
EEQKRDLLRF-------VTSCSRPP------------LLG-----FS-QLYPAF--AVKF
NGLDM-----------------------------DRLPTASACF-NL-LKLPGY-TNEAT
LRSKLL-QAITS-GAGF
>Cryptococcus_gattii_XM_003193988.1 .
AKIRRDHV-AQDGFDEL---------------SNLGPALKG-RVDITFVDKYGIT--EAG
IDGGGLYKEF---LTIL----SKEVFDSNR--------GLWLTTDQN-------------
----------------ELYPNPHS------------------------------------

-YASESHNLSWYRFIGQVLGKAIYDGILVDVTFAA--FFLAKWLG---------------
--------RQSYLDDLAS----------------------LDKDLYKG--------LIIL
KNDS---KPE-DMA--
--LTFSTTIEE----------------------------FGV-QRQID-LIPG--------
GSEIP---
------------------------------VTAENRH-EYIQL-VCKYKLDKQ-IAAQSK
-AFFIG---LSDLLDS--KWLR-MFDQQ-ELQQLIGG-----------------------
---EEKPID-----------LKDL----KAHCNFDG-FPN--------------------
------------------------DVTP-ALFWKVIQ-------------------G-FT
EEQKRALLRF-------VTSCSRPP------------LLG-----FS-QLNPQF--GVRF
NGGDM------------------------------DRLPSASACF-NL-LKLPGY-TTEAT
LRAKLL-QAINS-GAGF
>Cryptococcus_neoformans_XM_012196594.1 .
AKIRRDHV-AQDGFDEL---------------SNLGPALKG-RVDITFVDQYGIT--EAG
IDGGGLYKEF---LTIL----SKEVFDSNR--------GLWLVTDQN-------------
----------------ELYPNPHS------------------------------------
-YASESHNLSWYRFIGQVLGKAIYDGILVDVTFAA--FFLAKWLG---------------
--------RQSYLDDLAS----------------------LDKDLYKG--------LIIL
KNDP---KPE-DMA--
--LTFSTTIEE----------------------------FGV-QRQID-LVPG--------
GSDIP---
------------------------------VTAENRH-EYIQL-VCKYKLDKQ-IAAQSK
-AFFIG---LSDLLDA--KWLR-MFDQQ-ELQQLIGG-----------------------
---EEKPID-----------LKDL----KAHCNFDG-FPN--------------------
------------------------DVTP-ALFWKVVQ--------------------EFT
EEQKRALLRF-------VTSCSRPP------------LLG-----FS-QLNPQF--GVRF
NGGDM------------------------------DRLPSASACF-NL-LKLPGY-TTEAT
LRAKLL-QAINS-GAGF
>Cryptococcus_neoformans_XM_768105.1 .
AKIRRDHV-AQDGFDEL---------------SNLGPALKG-RVDITFVDQYGIT--EAG
IDGGGLYKEF---LTIL----SKEVFDSNR--------GLWLVTDQN-------------
----------------ELYPNPHS------------------------------------
-YASESHNLSWYRFIGQVLGKAIYDGILVDVTFAA--FFLAKWLG---------------
--------RQSYLDDLAS----------------------LDKDLYKG--------LIIL
KNDP---KPE-DMA--
--LTFSTTIEE----------------------------FGV-QRQID-LVPG--------
GSDIP---
------------------------------VTAENRH-EYIQH-VCKYKLDKQ-IAAQSK
-AFFIG---LSDLLDA--KWLR-MFDQQ-ELQQLIGG-----------------------
---EEKPID-----------LKDL----KAHCNFDG-FPN--------------------
------------------------DVTP-ALFWKVVQ-------------------G-FT
EEQKRALLRF-------VTSCSRPP------------LLG-----FS-QLNPQF--GVRF
NGGDM------------------------------DRLPSASACF-NL-LKLPGY-TTEAT
LRAKLL-QAINS-GAGF
>NWGS3B_2_Cryptococcus_albidus_BCHV01000003.1 .
ATIRRGHI-SEDGFSEL---------------YNLGPLMKG-TIQIRFIDEWGQE--EAG
IDGGGLFKEF---LTSL----SKEAFDTDR--------GLWLATSQN-------------
----------------ELYPNPHS------------------------------------
-YAKEGXQLHWYFXIGRVLGKALYEGVLVDVSFAG--FFVAKWLG---------------
--------RQSYLDDLAS----------------------LDPELYKG--------LVQL
KNYPG--NPE-DLS--
--LNFTVAEEE----------------------------FGV-TRSID-LVPG--------
GSDIA---
------------------------------VTRVNRX-EDIQL-VCDYKLNRQ-IAPQCE
-AFFSG---LSDIIDP--KWLR-MYDQT-ELQTLIGG-----------------------
---TLSPVD-----------VDDL----ERNCAMPD-GTD--------------------
------------------------DVTI-RLFWKVVR--------------------SFN
QTELKALLKF-------VTSTPNPP------------LLG-----FK-YLNPNF--GIRL

AGSDT-----------------------------TRLPSASACA-NL-LKLPRY-LDERT
LRTKLL-QAINS-NAGF
>Naganishia_albida_LLJT01000019.1 .
ATIRRGHI-SEDGFSEL---------------YNLGPLMKG-TIQIRFIDEQGQE--EAG
IDGGGLFKEF---LTSL----SKEAFDTDR--------GLWLATSQN-------------
----------------ELYPNPHS------------------------------------
-YAKEGXQLHWYCXIGRVLGKALYEGVLVDVSFAG--FFVAKWLG---------------
--------RQSYLDDLAS----------------------LDPELYKG--------LVQL
KNYPG--NPE-DLS--
--LNFTVAEEE---------------------------FGV-TRSID-LVPG--------
GSDIA---
------------------------------VTRVNRX-EDIQL-VCDYKLNRQ-IAPQCE
-AFFSG---LSDIIDP--KWLR-MYDQT-ELQTLIGG-----------------------
---TLSPVD-----------VDDL----ERNCAMPD-GTD--------------------
------------------------DVTI-RLFWKVVR--------------------SFN
QTELKALLKF-------VTSTPNPP------------LLG-----FK-YLNPNF--GIRL
AGSDT-----------------------------TRLPSASACA-NL-LKLPRY-LDERT
LRTKLL-QAINS-NAGF
>Rhodotorula_graminis_XM_018413525.1 .
AVVRRGNHLAEDAFKQL---------------NGLGAELKK-RVEIVFVDEHGIE--ESG
IDGGGLFKEL---LTSL----SKEVFNTDR--------GLWLATSQN-------------
----------------ELYPNPHT------------------------------------
-YAREPNQLAWFAFVGRILGKALYQGILVNVKFAG--FFLSKWLG---------------
--------RQAYLDDLAS----------------------LDPELYRG--------LVSL
KNYSG--NVE-DLS--
--LNFTITEED---------------------------FGV-TRTID-LIPR--------
GSEIA---
------------------------------VTNENRL-QYIVL-VSNYRLNVQ-IAPQCR
-AFFSG---LSEMVNP--RWLR-LFSQS-ELAVLVGG-----------------------
---TEDPID-----------IDDL----RAHTVYSG-WSADEN-----------------
------------------------TPTI-RAFWDVVS--------------------SFG
KEDRAKLVRF-------VTACERPP------------LLG-----FG-QLNPLF--AIRK
AGDNQ-----------------------------SRLPTSSTCI-NL-LRLEEY-SDPAN
LREKLL-YAINS-GAGF
>Rhodosporidium_toruloides_GEEN01005922.1 .
AVVRRTHL-AEDAYTHM---------------NGLGPELKK-RIEIVFIDEHGME--ESG
IDGGGLFKEL---LTSL----SKEVFDTNR--------GLWLATSEQ-------------
----------------EIYPNPHA------------------------------------
-YAKESTQLSWFTFVGRILGKAIYSGILVNVKFAN--FFLAKWLG---------------
--------RQSYLDDLAS----------------------LDPELYNG--------LLKL
KNYPG--NVEEDLA--
--LNFTITEED---------------------------FGV-SRSID-LIPG--------
GSEIP---
------------------------------VTNDNRM-QYIVL-VSNYRLNVQ-IAPQCR
-AFYQG---LFEIVNP--RWLH-MFNQS-ELAILVGG-----------------------
---TEEVID-----------IDDL----KANTVYSG-FAEEEN-----------------
------------------------TPTI-RAFWDVVE--------------------SFD
KDQRAKLVKF-------VTACERPP------------LLG-----FG-QLNPKF--AIRN
AGGDQ-----------------------------TRLPTSATCV-NL-LKLPDY-QDPTN
LREKLI-YAINS-GAGF
>Rhodotorula_toruloides_XM_016421121.1 .
AVVRRTHL-AEDAYTHM---------------NGLGPELKK-RIEIVFIDEHGME--ESG
IDGGGLFKEL---LTSL----SKEVFDTNR--------GLWLATSEQ-------------
----------------EIYPNPHA------------------------------------
-YAKESTQLSWFTFVGRILGKAIYSGILVNVKFAN--FFLAKWLG---------------
--------RQSYLDDLAS----------------------LDPELYNG--------LLKL
KNYPG--NVEEDLA--
--LNFTITEED---------------------------FGV-SRSID-LIPG--------

```
GSEIP-------------------------------------------------------
------------------------------VTNDNRM-QYIVL-VSNYRLNVQ-IAPQCR
-AFYQG---LFEIINP--RWLH-MFNQS-ELAVLVGG-----------------------
---TEEAID-----------IEDL----KANTVYSG-FAEEEN-----------------
------------------------TPTI-RAFWDVVE--------------------SFD
KDQRAKLVKF-------VTSCERPP------------LLG-----FG-QLNPKF--AIRN
AGGDQ-----------------------------TRLPTSATCV-NL-LKLPDY-QDPNN
LREKLI-YAINS-GAGF
>Puccinia_graminis_XM_003322600.1 .
ASIRRQFV-SEDGFTHL---------------NALGPRLKE-TIEIKFIDPYGLE--EAG
IDGGGVFKEF---LTSL----TKEVFDVNK--------GLWLVNKNQ-------------
----------------EIYPNPHS------------------------------------
-YSRQPLSLEWYKFLGRVLGKALYEGILIDIDFAD--FFLNKWLG---------------
--------KQSYLDDLAS----------------------LDPELYQG--------LIFL
KHYKG----------------------------------------------DVEADLS--
--LNFTVTNNE---------------------------FDA-SETIE-LIPE--------
GSKTS-------------------------------------------------------
------------------------------VTAQNRI-NYIYL-MSNYKLNIQ-LESQCA
-AFFKG---LNDIIEL--KWLR-MFNQL-ELKVLVGG-----------------------
--LDGQDLD-----------IDDM----QQWTVYGG-WDES-------------------
------------------------HPTI-RIFWKVLR--------------------EFD
NLTKRKLLRF-------VTSCARPP------------LLG-----FK-ELRPSF--AIRS
SGVDR-----------------------------SRLPSASTCV-NL-LKLPEY-QTEAE
LREKVL-YAINA-GAGF
>Puccinia_striiformis_AEEW01008792.1 .
ASIRREFV-SEDGFTHL---------------NNLGFKLKE-TIEIKFIDSYGLE--EAG
IDGGGVFKEF---LTSL----TKEIFDINK--------GLWLVNKNM-------------
----------------EIYPNPHS------------------------------------
-YSKSPVSLEWFTFLGRVIGKALYEGILIDIDFAD--FFLNKWLX---------------
----------SYLDDLGS----------------------LDPELYQG--------LIFL
KHYKG----------------------------------------------DIETDLS--
--LNFTITNNE---------------------------FDE-STTIE-LVPG--------
GSSKS-------------------------------------------------------
------------------------------VTVQNRI-NYIDL-ISNYKLNIQ-IEEQCN
-AFFKG---LNDLIDL--KWLR-MFNQI-ELKSLVGG-----------------------
--LDGQDLD-----------IDDM----QANTVYGG-WDES-------------------
------------------------HPTI-RXFWKVIR--------------------KFD
NSNQRKLLKF-------VTSCGRPP------------LLG-----FK-ELRPSF--AIRS
SGTDR-----------------------------SRLPSASTCV-NL-LKLPEY-LNEIE
LENKLV-YAINS-GAGF
>Cronartium_ribicola_GBSG01010958.1 .
AVIRREHI-AEDGFLHL---------------NALGSRLKE-PIEIKFIDQHGLE--EAG
IDGGGVFKEF---LTNL----TKLIFDSNK--------GLWLVNANM-------------
----------------ELYPNPHS------------------------------------
-YSRESLSLEWYAFLGRVLGKALYEGILVDVEFAD--FFLNKWLG---------------
--------KQSYLDDLAS----------------------LDPELYQG--------LLFL
KNYGG----------------------------------------------DVESDLS--
--LNFTISSQE---------------------------FGI-SKTIE-LLPN--------
GAQIP-------------------------------------------------------
------------------------------VTSKNRI-NYIYL-ISNYKLNSQ-FEIQCS
-AFFKG---LNDIVEA--KWLR-MFNTV-ELKVLVGG-----------------------
--LDGQELD-----------FDDL----QRCTVYGG-WDEA-------------------
------------------------HETI-RMFWRVVK--------------------RFD
NPTKAKLLKF-------VTSCARPP------------LLG-----FK-ELRPSF--AIRC
AGQDE-----------------------------SRLPSASTCV-NL-LKLPEY-KTEIG
LEEKLV-YAINA-GAGF
>Melampsora_larici_populina_XM_007412061.1 .
AVIRRDRI-SEDGFLHL---------------NALGPRLKD-PIEIKFIDQYGLE--EAG
```

```
IDGGGVFKEF---LTSL----TKDIFDTNK--------GLWLVNQNQ-------------
----------------ELYPNPHT------------------------------------
-FSQEALSLEWYGFLGRVLGKALYEGILIDVEFAD--FFLNKWLG---------------
--------KQSYLDDLAS----------------------LDPELYQG--------LLFL
KNYKG----------------------------------------------DVEADLS--
--LNFTITSNE---------------------------FDQ-STTIE-LIPN--------
GSKIS-------------------------------------------------------
------------------------------VTARNRI-NYIYL-ISNYKLNIQ-FEAQCA
-AFFRG---LNDIVEL--KWLR-MFNQV-EIKALIGG-----------------------
--LDGQDLD-----------FGDL----ERCTVYGG-WNES-------------------
------------------------HETI-RIFWRVVK--------------------RFD
NATKRKLLKF-------VTSCSRPP------------LLG-----FK-ELRPAF--AIRS
SGVDE-----------------------------SRLPSASTCV-NL-LKLPEY-KSETQ
LEEKLV-YAINS-GAGF
>Melampsora_abietis_canadensis_MWRH0100882 .
AVIRRDRI-SEDGFLHL----------------NALGPKMKD-PIEIKFIDQYGLE--EAG
IDGGGVFKEF---LTSL----TKDIFDTNK--------GLWLVNQNQ-------------
----------------ELYPNPHT------------------------------------
-FSQEPLSLEWYGFLGRVLGKALYEGILIDVEFAD--FFLNKWLG---------------
--------XQSYLDDLAS----------------------LDPELYQG--------LLFL
KNYKG----------------------------------------------DVEADLS--
--LNFTITSNE---------------------------FDQ-SKTIE-LIPN--------
GSKIS-------------------------------------------------------
------------------------------VTARNRIN-YIYL-ISNYKLNIQ-FEAQCA
-AFFKG---LNDIVEL--KWLR-MFNQV-EIKALIGG-----------------------
--LDGQDLD-----------FGDL----ESCTVYGG-WDES-------------------
------------------------HETI-XQFWRVVK--------------------RFD
NSTKRKLLKF-------VTSCSRPP------------LLG-----FK-ELRPAF--AIRS
SGVDE-----------------------------SRLPSASTCV-NL-LKLPEY-KSEAQ
LEEKLV-YAINA-GAGF
>Melampsora_occidentalis_MWRL01017619.1 .
AVIRRDRI-SEDGFLHL----------------NALGPKMKD-PIEIKFIDQYGLE--EAG
IDGGGVFKEF---LTSL----TKDIFDTNK--------GLWLVNQNQ-------------
----------------ELYPNPHT------------------------------------
-FSQEPLSLEWYGFLGRVLGKALYEGILIDVEFAD--FFLNKWLG---------------
--------KQSYLDDLAS----------------------LDPELYQG--------LLFL
KNYKG----------------------------------------------DVEADLS--
--LNFTITSNE---------------------------FDQ-SKTIE-LIPN--------
GSKIS-------------------------------------------------------
------------------------------VTARNRI-NYIYL-ISNYKLNIQ-FEAQCA
-AFFKG---LNDIVEL--KWLR-MFNQV-EIKALIGG-----------------------
--LDGQDLD-----------FGDL----ESCTVYGG-WDES-------------------
------------------------HETI-XQFWRVVK--------------------RFD
NSTKRKLLKF-------VTSCSRPP------------LLG-----FR-ELRPAF--AIRS
SGVDE-----------------------------SRLPSASTCV-NL-LKLPEY-KSEAQ
LEEKLV-YAINA-GAGF
>Melampsora_medusae_MWRK01009508.1 .
AVIRRDRI-SEDGFLHL----------------NALGPKMKD-PIEIKFIDQYGLE--EAG
IDGGGVFKEF---LTSL----TKDIFDTNK--------GLWLVNQNQ-------------
----------------ELYPNPHT------------------------------------
-FSQEPLSLEWYGFLGRVLGKALYEGILIDVEFAD--FFLNKWLG---------------
--------KQSYLDDLAS----------------------LDPELYQG--------LLFL
KNYKG----------------------------------------------DVEADLS--
--LNFTITSNE---------------------------FDQ-SKTIE-LIPN--------
GSKIS-------------------------------------------------------
------------------------------VTARNRI-NYIYL-ISNYKLNIQ-FEAQCA
-AFFKG---LNDIVEL--KWLR-MFNQV-EIKALIGG-----------------------
--LDGQDLD-----------FGDL----ESCTVYGG-WDES-------------------
```

------------------------HETI-XQFWRVVK--------------------RFD
NSTKRKLLKF-------VTSCSRPP------------LLG-----FR-ELRPAF--AIRS
SGVDE-----------------------------SRLPSASTCV-NL-LKLPEY-KSEAQ
LEEKLV-YAINA-GAGF
>Mixia_osmundae_XM_014713063.1 .
AIIRRDHV-ADDGFAHL---------------AKLGKELKH-RMQITFVDVFGEE--EAG
IDGGGLTKEF---LTSL----GKEVFDTDR--------GLWRANAKQ-------------
----------------ELYPSPSL------------------------------------
-YARGSDQLAWFRFLGQITGKALYDGILVNTPFAD--FFLAKWLG---------------
--------RSNFLDDLAS----------------------LDPELYQG--------LVFL
KNYAG--NVEKDLA--
--LTFSVTDEE---------------------------FGE-SRTID-LIPD--------
GSNVA---
------------------------------VTASNRI-RYITL-VSHYRLNYQ-IREQSE
-AFTAG---LFDIIEP--RWLR-LFNQS-ELRLICSG-----------------------
---TNSEID-----------LEDL----RRNTVYGR-LDEG-------------------
------------------------GDLV-RWFWSTVK--------------------AFS
QAEKEALLSF-------VTSCPRPP------------LLG-----FK-ELRPSF--CIQA
GENDP-----------------------------GRLPTASTCV-NL-LKLPAY-TSPDT
LRQKLL-TAIQS-KAGF
>Meira_nashicola_BCJU01000003.1 .
ITIRRGSV-AEDGFTQL---------------NGLGENLKH-RIAIRFVDQFGIE--ESG
IDGGGLFKEF---LTSL----IKEAFDTDR--------GLWSATSDQ-------------
----------------QLYPNPHS------------------------------------
-YAKQDDNLQWYKFLGRVLGKALYQGILVDINFAS--FFLSKWLG---------------
----RGHGYLDDLSGLES----------------------LDKEVYRN--------LLWL
KHYNG--DVEKDLT--
--LDFTVADEE---------------------------FGE-KRIHE-LVPN--------
GRNIP---
------------------------------VTRENRL-EYIYR-VSHYRLNEQ-IRQQSE
-AFLSG---LTDLIDL--RWLR-MLNRD-ELRILISG-----------------------
---TEGDID-----------LKDL----QENTVLGG-YHEK-------------------
------------------------DQIM-DFFWRSLE--------------------SFT
HAQRKAFLKF-------ATSCPSPP------------LLG-----FQ-QLNPKF--GIRH
AGDDT-----------------------------SRLPTASTCV-NL-LKLPRY-TSFEQ
CREKLL-YAIES-GAGF
>Malassezia_caprae_LFFV01000169.1 .
ATIRRGHI-AEDGMAQL---------------YALGPRLKE-PLEIVFIDQWGQP--EAG
IDGGGVFKEF---LTSI----VHEVFDTDR--------GLWCTNDRH-------------
----------------ELYPSPYS------------------------------------
-YAQADEQLVWYTFIGRILGKALYEGILVDVKFAG--FFLNKWLG---------------
-----LQGYVDELASLES----------------------LDKDLYRG--------LIAL
KNYTG--DVENDFS--
--LNFTVSKDE---------------------------FGE-QTVTE-LIPG--------
GSDIP---
------------------------------VTRENRL-SYIYH-MTRYRLSVQ-IEAQCR
-AFFGG---LSDLIDP--RWLK-LLNRE-ELRILLCG-----------------------
---TESPID-----------LEDL----RRHTVYGG-YHEK-------------------
------------------------DLSV-QYFWEALA--------------------LLD
QSSLKAFLRF-------VTSSPNPP------------LLG-----FG-ELNPKF--AIRH
AGDDA-----------------------------TRLPTASTCV-NL-LKLPAY-SSREQ
CLEKLK-YAIHA-EAGF
>Malassezia_dermatis_LFFX01000072.1 .
ATIRRGHV-AEDGMAQL---------------YALGSRLKE-PLEIVFIDQWGQP--EAG
IDGGGVFKEF---LTSI----VHEVFNTDR--------GLWCANDRH-------------
----------------ELYPSPYS------------------------------------
-YAQADEQLVWYTFIGRILGKALYEGILVDIKFAG--FFLNKWLG---------------
-----LHGYVDEIAGLES----------------------LDKDLYRG--------LIAL

KNYTG--DVENDFS--
--LNFTVSKDE---------------------------FGE-QTVSE-LIPG--------
GSDIP---
------------------------------VTRENRL-SYIYH-MTRYRLSVQ-IDAQCR
-AFFGG---LSDLIDP--RWLK-LLNRE-ELRILLCG-----------------------
---TESPID-----------LQDL----RRHTVYGG-YHEK-------------------
------------------------DLVV-QYFWEALA--------------------MMD
QSSLKAFLRF-------VTSSPNPP------------LLG-----FG-ELNPKF--AIRH
AGDDP-----------------------------TRLPTASTCV-NL-LKLPAY-LTREQ
CHEKLM-YAIHA-EAGF
>Malassezia_sympodialis_LT671798.1 .
ATIRRDHI-AEDGMAQL---------------YALGPRLKE-PLEIVFIDAWGQP--EAG
IDGGGVFKEF---LTSI----VHEVFNTDR--------GLWCTNDRH-------------
----------------ELYPSPYS------------------------------------
-YAQAEEQLVWYTFIGRILGKALYEGILVDVKFAG--FFLNKWLG---------------
-----LQGYVDELASLES----------------------LDKDLYRG--------LIAL
KNYNG--DVENDFS--
--LNFTVSKDE---------------------------FGE-QTVLE-LIPG--------
GSEIP---
------------------------------VTRENRL-SYIYH-MTRYRLTVQ-IEAQCR
-AFFSG---LSDLIDP--RWLK-LLNRE-ELRILLCG-----------------------
---TESPID-----------LDDL----RHHTVYGG-YHEK-------------------
------------------------DVAV-QYFWEALA--------------------MLD
QPSLKAFLRF-------VTSSPNPP------------LLG-----FG-ELNPKF--AIRH
AGDDP-----------------------------TRLPTASTCV-NL-LKLPAY-LSREQ
CLEKLK-YAIHA-EAG-
>Malassezia_equina_LFFY01000025.1 .
ATIRRGHI-AEDGMAQL---------------YALGPRLKE-PLEIVFVDQWGQQ--EAG
IDGGGVFKEF---LTSI----VREVFDTDR--------GLWCTNDCH-------------
----------------ELYPSPFS------------------------------------
-YAQAKEQLIWYTFVGRILGKALYEGILVDVKFAV--FFLNKWLG---------------
-----LQGYVDELASLES----------------------LDKDLYRG--------LIAL
KNYTG--DVENDFA--
--LNFTVSKDE---------------------------FGQ-QSVTE-LIPG--------
GADIP---
------------------------------VTRENRL-SYIYH-MTRYRLSVQ-IEPQCR
-AFFRG---LSDLIDP--RWLR-LLNRE-ELRILLCG-----------------------
---TESPID-----------LEDL----RRNTVYGG-YHEK-------------------
------------------------DLAV-QYFWEALS--------------------LLD
QPSLKAFLRF-------VTSSPNPP------------LLG-----FG-ELNPKF--AIRH
AGDDA-----------------------------TRLPTASTCV-NL-LKLPAY-SSREQ
CLEKLR-YAIHA-EAGF
>Malassezia_nana_LFGD01000075.1 .
ATIRRDHI-AEDGMTQL---------------YALGPRLKE-PLEIVFVDKWGQP--EAG
IDGGGVFKEF---LTSI----VHEVFDTDR--------GLWCTNHRH-------------
----------------ELYPSPYK------------------------------------
-YAQEEEQLVWYTFIGRILGKALYEGILVDVKFAG--FFLNKWLG---------------
-----HQGYVDEVASLES----------------------LDKDLYRG--------LIAL
KNYTG--DVENDFA--
--LNFTVTKEE---------------------------YGQ-QTVVE-LIPG--------
GADIP---
------------------------------VTRENRL-SYIYH-MTRYRLSVQ-IEPQCR
-AFFAG---LSDLIEP--RWIK-MLDRE-ELRILLCG-----------------------
---TESPID-----------LEDL----RRNTVYGG-YHEK-------------------
------------------------DLAV-QYFWEALS--------------------MLD
QASLKAFLRF-------VTSSPNPP------------LMG-----FS-ELNPKF--AIRH
SSDDT-----------------------------TRLPTASTCV-NL-LKLPAY-SSREQ
CLEKLR-YAIHA-GAGF

>Malassezia_pachydermatis_XM_018136617.1 .
ATIRREHI-AEDGMAQL---------------NALGPHLKE-PLEIAFIDQWGMQ--EAG
IDGGGVFKEF---LTSM----VRTVFDTDR--------GLWCANERQ-------------
----------------ELYPNPHS------------------------------------
-YARAEEQLMWYVFLGRILGKALYEGILVDVKFAG--FFLSKWLG---------------
-----QQGYVDDVASLES----------------------LDSELYRG--------LITL
KNYSG--DVENDFA--
--LNFTVVNEE---------------------------FGV-RETVP-LISN--------
GAEIP---
------------------------------VTRENRL-SYIYH-MTRYRLSTQ-IEPQCR
-AFFHG---LSELIEP--RWLR-LFNRE-ELRVLVCG-----------------------
---TEDPID-----------VDDL----QQNTVYGG-YHEK-------------------
------------------------DMAI-QYFWEALR--------------------SLD
QPSRKAFLRF-------VTSSPNPP------------LLG-----FS-ELHPKF--AIRH
AGDDV-----------------------------TRLPTASTCV-NL-LKLPAY-ESTQQ
CLEKLR-YAIDA-GAGF
>Malassezia_restricta_LFCZ01000061.1 .
ATIRRGHV-AEDGIAQL---------------NGLGSNLKE-PLEIMFVDQWGMP--EAG
IDGSGLFKEF---LVSM----IQEVFDTDR--------GLWCSNEIH-------------
----------------ELYPNPHS------------------------------------
-YAHASEQLIWYLFMGRILGKALYEGILVDVKFAD--FFLSKWLG---------------
-----QQSYIDDLASLES----------------------LDSELYRG--------LIAL
KNYSG--NVEGDFA--
--LNFTVADDE---------------------------FGI-RTSRE-LVPG--------
GTDIP---
------------------------------VTRENRL-SYIYL-ITRYRLSTQ-IEDQSR
-AFLQG---LTELINP--RWLR-LFNTE-ELRVLVTG-----------------------
---ADTPID-----------VEDL----RRNTVYGG-YHEK-------------------
------------------------DMAV-QYFWEALS--------------------SLD
QASLKAFLRF-------VTSSPNPP------------LLG-----FS-ELNPKF--AIRH
AGDDI-----------------------------TRLPTASTCV-NL-LKLPAY-NSTAQ
CLEKVK-YAIYS-GAG-
>Malassezia_globosa_XM_001730276.1 .
ATIRRGHI-AEDGMAKL---------------NGLGPSLKE-PLEIVFIDQWGMP--EAG
IDGSGLFKEF---LVSI----IREVFDTDR--------GLWCANERQ-------------
----------------ELYPNPHS------------------------------------
-YAHGEEQLTWYTFLGRILGKALYEGILVDVKFAD--FFLTKWLG---------------
-----PKGYIDDLASLES----------------------LDSDLYRG--------LIAL
KNYTG--NVENDFA--
--LNFTVTDEE---------------------------FGV-RMNRE-LIPG--------
GNDVP---
------------------------------VTRENRL-SYIYC-MTRYRLSTQ-IEDQCN
-AFFQG---LSEMIDP--RWLR-LFNRE-ELRVLVSG-----------------------
---AESPID-----------VDDL----RRNTIYGG-YHEK-------------------
------------------------DMAV-QYFWEALS--------------------HFD
QSSLKAFLRF-------VTSSPNPP------------LLG-----FG-ELNPKF--AIRH
AGDDV-----------------------------TRLPTASTCV-NL-LKLPAY-ESVAQ
CADKLR-YAIYA-GAGF
>Malassezia_slooffiae_LFGK01000044.1 .
VTVRRGHI-AEDGMAQL---------------NGLGPHLKE-PIEIVFVDQFGQE--EAG
IDGGGVFKEF---LTSL----VREVFDTDR--------GLWRANQRQ-------------
----------------ELYPNPHS------------------------------------
-YAKAPEQLVWYTFLGRILGKALYEGILVDVKFAS--FFLSKWLG---------------
-----HQTYLDDLASLDS----------------------LDGELYRG--------LIYL
KNYQG--DVEADLA--
--LSFTVADEE---------------------------FGV-SHTTE-LIPG--------
GAEVP---
------------------------------VTRENRL-SYIYL-VSRYRLSKQ-IEPQCA

```
-AFFRG---LSEMIHP--RWLR-LLNRE-ELRVLVSG-----------------------
---TEAPID-----------IADL----RANTVYGG-YHEK-------------------
------------------------DLAV-DYFWQALE--------------------QLD
QPSRKAFLRF-------VTSSPNPP------------LLG-----FA-ELNPKF--AIRH
AGDDA-----------------------------HRLPTASTCV-NL-LKLPAY-TSTEQ
CLERLR-YAIHS-GAGF
>Malassezia_furfur_LFGI01000985.1 .
ATVRRAAI-AEDGMAQL---------------NALGPRLKE-PIEIVFIDQFGQP--EAG
IDGGGVFKEF---LTSL----VRQVFDTDR--------GLWRANARN-------------
----------------ELYPNPHS------------------------------------
-YARQPEQLGWYTFLGRILGKALYEGILVDVPFAS--FFLGKWLG---------------
-----HQSYLDDVASLQS----------------------LDADLYRG--------LIQL
KNYTG-----------------------------------------------DVENDFA--
--LNFTVSDEE---------------------------FGV-THTTE-LVPG--------
GAEIP-------------------------------------------------------
------------------------------VTNENRL-SYIYH-MSRYRLSTQ-IEPQCA
-AFLRG---LTELIEP--RWLR-VLSRD-ELRVLVSG-----------------------
---TEAPVD-----------VADL----RAHTVYGG-YHEK-------------------
------------------------DLAV-SYFWEALE--------------------SLD
PASRRAFLRF-------VTSSPNPP------------LLG-----FG-ELHPQF--AIRH
AGDDV-----------------------------SRLPTASTCV-NL-LKLPAY-TSREQ
CLEKLR-YAIHS-EAGF
>Malassezia_yamatoensis_LFCX01000042.1 .
ATVRRESI-AEDGMAQL---------------NGLAARLKE-PVEIVFIDQFGQP--EAG
IDGGGVFKEF---LTSL----VRQVFDTDR--------GLWRANEHQ-------------
----------------ELYPNPHS------------------------------------
-YARQPEQLEWYTFLGRILGKALYEGILVDVKLAS--FFLGKWLG---------------
-----QQSYLDDVASLQS----------------------LDADLYRG--------LIQL
KNYPG-----------------------------------------------DVENDFA--
--LNFTVTDEE---------------------------FGV-THTTE-LIPN--------
GADIP-------------------------------------------------------
------------------------------VTRDNRL-SYIYH-VSRYRLSKQ-IEPQCR
-AFLHG---LSELIEP--RWLR-VLGRE-ELRVLISG-----------------------
---TEAPID-----------LQDL----RSHTVYGG-YHEK-------------------
------------------------DMAV-TYFWEALE--------------------KLD
PDSRKAFLRF-------VTSSPNPP------------LLG-----FA-ELNPQF--AIRH
AGDDV-----------------------------SRLPTASTCV-NL-LKLPSY-TSTQQ
CLEKLR-YAIHS-DAGF
>Malassezia_japonica_LFDB01000134.1 .
ATVRRESI-ATDGMAQL---------------NSLGPRLKE-PVEIVFIDQFGQP--EAG
IDGGGVFKEF---LTTL----TRQVFDTDR--------GLWRTNERQ-------------
----------------ELYPNPHS------------------------------------
-YARQPDQLEWYTFLGRILGKALYDGILVDVKLAS--FFLGKWLG---------------
-----HQGYLDDLASLHS----------------------LDAALYRG--------LIQL
KNYTG-----------------------------------------------DVENDFA--
--LNFTVSDEE---------------------------FGV-THTTE-LIPG--------
GADIP-------------------------------------------------------
------------------------------VTKENRL-SYIYH-VSRYRLSKQ-IEPQCR
-AFFQG---LSELIDP--RWLR-LLSRD-ELGVLVSG-----------------------
---TDSPID-----------LADL----RAHTVYGG-FHEK-------------------
------------------------DLAV-TYFWEALE--------------------SMD
QASRKAFLRF-------VTSSPNPP------------LLG-----FS-ELNPQF--AIRN
AGDDV-----------------------------TRLPTASTCV-NL-LKLPAY-ASRAQ
CLEKLL-YAVHS-EAGF
>Malassezia_cuniculi_LFFW01000030.1 .
ATVRRNSV-ASDGMVQL---------------GFLGARLKE-PVKITFIDEFGQE--EAG
IDGGGVFKEF---LTAL----VREAFDTDR--------GLWCANAAQ-------------
----------------ELYPNPHS------------------------------------
```

```
-YARGEEQLAWYTFLGRIIGKALYEGILVNAQFAR--FFLSKWLG---------------
-----QKSYLDDLASLDS----------------------LDPVLYRG--------LLYL
KNYTG----------------------------------------------DVESDLA--
--LNFTVADDE---------------------------FGV-NQTTE-LVPG--------
GADLP-------------------------------------------------------
------------------------------VTRENRL-SYIYL-VTRYRLWRQ-IEPQST
-AFFRG---LADIIEP--RWLR-MFDPE-ELRVLVSG-----------------------
---ADRDID-----------LADL----RANTVYGG-YHEK-------------------
------------------------DPSV-QYFWEALE--------------------TMD
SASRRAFLRF-------VTSSPNPP------------LLG-----FG-ELNPKF--AIRH
AGNDT-----------------------------TRLPTASTCV-NL-LKLPEY-SSREQ
CLEKLR-YAIDS-AAGF
>Pseudozyma_flocculosa_XM_007883821.1 .
IKVRRGMI-AEDGFAQM---------------NPLGPRLKE-RLEIAFIDQWGHE--EAG
IDGGGVYKEF---LTSL----VREAFDTNR--------GLWKATDAQ-------------
----------------ELYPNPHS------------------------------------
-YAKTPDQLEWYTFLGRVLGKALYEGILVDVKFAS--FFLSKWLG---------------
-----KQGYLDDLASLDS----------------------LDKELYRG--------LIYL
KNYGG----------------------------------------------DVEADLA--
--LNFTVTDDE---------------------------FGV-SKTTE-LVPG--------
GSEVP-------------------------------------------------------
------------------------------VTRENRL-EYIYR-VSHYRLSVQ-IAEQCR
-AFFLG---LSEMIDP--RWLR-MMNRE-ELRVLVCG-----------------------
---TEEPID-----------VADL----RANTVYGG-YHER-------------------
------------------------DVAV-DYFWQALE--------------------SFD
QPQRKAFLKF-------VTSSPNPP------------LLG-----FA-ELNPKF--SIRN
AGPDA-----------------------------TRLPTASTCV-NL-LKLPEY-NDAET
CRQKLV-YAIQS-GAGF
>Tilletiaria_anomala_XM_013389211.1 .
IKVRRGHV-ADDGFAQL---------------NLHGDMLKH-PIEIAFIDEWGQE--EAG
IDGGGLFKEF---LTSL----VKEVFDTDR--------GLWCATNEQ-------------
----------------EIYPNPHS------------------------------------
-YAQTPESLEWYTFLGRILGKAMYEGILVDVKFAS--FFLSKWLG---------------
-----KQSYLDDLSSLGS----------------------LDKELYKG--------LIYL
KNYPG----------------------------------------------DVEADLA--
--LNFTVTDEE---------------------------FGV-QHTTE-LVPG--------
GSSIA-------------------------------------------------------
------------------------------VTRENRL-SYIYR-VSHYRLTAQ-IARQCR
-AFFNG---LSEMIDP--RWLR-MMNRE-ELRVIVSG-----------------------
---ADQPID-----------IDDL----KANTVLAG-YHEK-------------------
------------------------DLAV-EYFWNALA--------------------TFD
DTARRALLKF-------VTSCPSPP------------LLG-----FS-QLNPKF--AIQH
SSDDS-----------------------------SRLPTASTCI-NL-LKLPRY-TSQGQ
CLEKLR-YAIQA-NSGF
>Pseudozyma_tsukubaensis_MAIP01000001.1 .
VKIRRGHV-AQDGFASL---------------APLGADLKK-PLEILFVDQFGNV--EAG
IDGGGLFKEF---LTSL----VLEAFDTDK--------GLWKATDAQ-------------
----------------ELYPNPHT------------------------------------
-YATSGDQLVFYEFLGRIIGKALYDGILVNTKFAG--FFLSKMLG---------------
-----KQSYLDDLGSIDS----------------------LDKELYKG--------LISL
KNYHG----------------------------------------------NVE-DLS--
--LNFTVTDEE---------------------------FGV-SLTRE-LIPG--------
GANIP-------------------------------------------------------
------------------------------VTNLNRM-DYIYR-ISHYRLSTQ-IQAQCS
-AFFTG---LADIINP--RWLR-NFNRE-ELSILISG-----------------------
---TDDPVD-----------IEDL----RKNTVLAG-YHEQ-------------------
------------------------DLTV-QSFWKALE--------------------GFD
QKMRKAFLKF-------VTSSSNPP------------LLG-----FG-QLNPKF--GLRK
```

```
AGDDK-----------------------------SRLPTASTCF-NM-LKLPDY-EDEAT
CREKLR-YAITS-EAGF
>Melanopsichium_pennsylvanicum_HG529499.1 .
VKIRRGHV-APDGFASL---------------AALGPELKK-PLEIVFVDKFGQP--EPG
IDGGGLFKEF---LTSL----VREAFDTNR--------GLWKATDAQ-------------
----------------ELYPNPHT------------------------------------
-YAQSGEQLDWYEFLGRVIGKALYEGILVDAKFAG--FFLSKMLG---------------
-----KQSFLDDLGSIDS----------------------LDKELYKG--------LISL
KNYQG----------------------------------------------NVE-DLS--
--LNFTVTDEE---------------------------FGV-SMTRE-LVPD--------
GANVP-------------------------------------------------------
------------------------------VTNLNRM-EYIYR-ISHYRLSTQ-IQHQCN
-SFFNG---LADMINP--RWLR-NFNRE-ELSVLISG-----------------------
---TDDPVD-----------IADL----RKHTVLGG-YHEQ-------------------
------------------------DLTV-QHFWRALE--------------------SFD
QPMRKAFLKF-------VTSSPNPP------------LLG-----FS-QLNPMF--AIRK
AGEDK-----------------------------TRLPTASTCV-NM-LKLPDY-EDEQT
CRDKLK-YAIMS-EAGF
>Pseudozyma_antarctica_XM_014804064.1 .
VKIRRSHV-AQDGFAAL---------------APLGAEIKK-PLEIVFVDQFGNN--EAG
IDGGGLFKEF---LTSL----VREAFDTNR--------GLWKATGAQ-------------
----------------ELYPNPHT------------------------------------
-YATSGDQLEWYGFLGRVIGKALYEGILVDAKFAG--FFLSKMLG---------------
-----KQSFLDDLGSIDS----------------------LDKELYKG--------LISL
KNYQG----------------------------------------------NVE-ELA--
--LNFTVTDEE---------------------------FGV-SLTRE-LVPG--------
GANIP-------------------------------------------------------
------------------------------VTNLNRM-EYIYR-ISHYRLSTQ-IQHQCA
-AFFTG---LADIINP--RWLR-NFNHE-ELSILISG-----------------------
---SDDPVD-----------IDDL----RKHTVLGG-YHEK-------------------
------------------------DLTV-EHFWHVLE--------------------GFD
QPMRKAFLKF-------VTSSPKPP------------LLG-----FS-QLNPMF--AIRK
AGDDT-----------------------------SRLPTASTCV-NM-LKLPDY-ADEAT
CREKLL-YAIQS-EAGF
>NWGS3B_2_Moesziomyces_aphidis_AWNI01000008.1 .
VKIRRGHV-AQDGFAAL---------------APLGAEIKK-PLEIVFVDQFGNN--EAG
IDGGGLFKEF---LTSL----VREAFDTNR--------GLWKATDAQ-------------
----------------ELYPNPHT------------------------------------
-YATSGDQLEWYGFLGRVIGKALYEGILVDAKFAG--FFLSKMLG---------------
-----KQSFLDDLGSIDS----------------------LDKELYKG--------LISL
KNYQG----------------------------------------------NVE-DLA--
--LNFTVTDEE---------------------------FGV-SLTRE-LVPG--------
GANIP-------------------------------------------------------
------------------------------VTNLNRI-EYIYR-ISHYRLSTQ-IQHQCN
-AFFTG---LADIINP--RWLR-NFNHE-ELSILISG-----------------------
---SDDPVD-----------IDDL----RKHTVLGG-YHDK-------------------
------------------------DLTV-EHFWHVLE--------------------GFD
QPMRKAFLKF-------VTSSPKPP------------LLG-----FS-QLNPMF--AIRK
AGDDT-----------------------------SRLPTASTCV-NM-LKLPDY-ADEAT
CREKLL-YAIQS-EAGF
>Ustilago_maydis_XM_011388361.1 .
VKIRRDHV-AQDGFVSL---------------FPLGAELKK-PLEIIFVDQFGQP--EAG
IDGGGLFKEF---LTSL----VREAFDTNR--------GLWKATDAQ-------------
----------------ELYPNPHT------------------------------------
-YATSSDQLEWYEFLGRVIGKALYEGILVDAKFAG--FFLSKMLG---------------
-----KQSYLDDLGSIDS----------------------LDKELYKG--------LISL
KNYQG----------------------------------------------NVE-DLS--
--LNFTVTDEE---------------------------FGV-SMTRE-LVPG--------
```

GANIP---
------------------------------VTNLNRM-EYIFR-ISHYRLSTQ-IQHQCT
-AFFNG---LADIVNP--RWLR-NFNRE-ELSILISG-----------------------
---TEDPVD-----------IDDL----RKHTVLGG-YHEA-------------------
------------------------DLTV-QHFWKVLE--------------------GFD
QRMRKAFLKF-------VTSSPNPP------------LLG-----FS-QLNPLF--AIRK
AGDDT-----------------------------SRLPTASTCV-NM-LKLPDY-ADEQT
CEDKLR-YAIQS-EAGF
>Pseudozyma_hubeiensis_XM_012333661.1 .
VKIRREHV-AQDGFASL---------------APLGADIKK-PLEIIFVDQFGQP--EAG
IDGGGLFKEF---LTSL----VREAFDTNR--------GLWKATDAQ-------------
----------------ELYPNPHT------------------------------------
-YATSGDQLEWYEFLGRVIGKALYEGILVDAKFAG--FFLSKMLG---------------
-----KQSYLDDLGSIDT----------------------LDKELYKG--------LISL
KNYQG--NVE-DLS--
--LNFTVTDEE---------------------------FGV-SMTRE-LVPG--------
GANIP---
------------------------------VTNLNRM-EYIFR-ISHYRLSTQ-IQHQCN
-AFFNG---LADIINP--RWLR-NFNRE-ELSILISG-----------------------
---TDDPVD-----------IADL----RQNTVLGG-YHEA-------------------
------------------------DLTV-QHFWKVLE--------------------DFD
QPMRKAFLKF-------VTSSPNPP------------LLG-----FS-QLNPRF--GIRK
AGDDK-----------------------------SRLPTASTCV-NM-LKLPDY-ADEQT
CREKLR-YAIQS-EAGF
>Ustilago_vetiveriae_MAIM01000045.1 .
VKIRRDHV-AQDGFVSL---------------APLGAELKK-PLEIVFVDQFGQP--EAG
IDGGGLFKEF---LTSL----VREAFDTNR--------GLWRATDAQ-------------
----------------ELYPNPHT------------------------------------
-YATSADQLEWYEFLGRVIGKALYEGILVDAKFAG--FFLSKMLG---------------
-----KQSYLDDLGSIDS----------------------LDKELYKG--------LLSL
KKYQG--NVE-DLS--
--LNFTVTDEE---------------------------FGV-SMTRE-LVPG--------
GANIP---
------------------------------VTNLNRM-EYIFR-ISHYRLSTQ-IQHQCN
-AFFNG---LADIINP--RWLR-NFNRE-ELSILISG-----------------------
---TDDPVD-----------IEDL----RKHTVLGG-YHEA-------------------
------------------------DLTV-EHFWRVLE--------------------SFD
QSMRKAFLKF-------VTSSPNPP------------LLG-----FS-QLNPLF--AIRK
AGDDK-----------------------------SRLPTASTCV-NM-LKLPDY-SDEET
CREKLR-YAIQS-EAGF
>Sporisor_reilianum_FQ311430.1_a.
VQIRRGHV-AQDGFVHL---------------APLGSELKK-PLEIVFIDQFGQP--EAG
IDGGGLFKEF---LTSL----VREAFDTNR--------GLWKATDAQ-------------
----------------ELYPNPHT------------------------------------
-YATSGEQLEWYEFLGRVIGKALYEGILVDAKFAG--FFLSKMLG---------------
-----KQSFLDDLGLIDT----------------------LDKELYKG--------LISL
KNYQG--NVE-DLA--
--LNFTVTDEE---------------------------FGV-SMTRE-LIPG--------
GANVP---
------------------------------VTNLNRM-EYIYR-ISHYRLSTQ-IQHQCN
-AFFNG---LADMINP--RWLR-NFNRE-ELSVLISG-----------------------
---TDEPVD-----------LEDL----RKHTVLGG-YHEQ-------------------
------------------------DVTV-QHFWKVLE--------------------GFD
QPMRKAFLKF-------VTSSPNPP------------LLG-----FS-QLNPLF--AIRK
AGDDK-----------------------------SRLPTASTCV-NL-LKLPDY-ADEQT
CRDKLK-YAIQS-EAGF
>Sporisor_scitamineum_LK056664.1 .
VQIRRGHV-AQDGFARL---------------APLGANLKK-PLEIVFIDQFGRP--EAG

IDGGGLFKEF---LTSL----VREAFDTNR--------GLWKATDAQ-------------
----------------ELYPNPHT------------------------------------
-YATSGEQLEWYEFLGRVIGKALYEGILVDAKFAG--FFLSKMLG---------------
-----KQSFLDDLGSIDT----------------------LDKELYKG--------LISL
KNYQG--NVE-DLS--
--LNFTVTDEE---------------------------FGV-SMTCE-LIPG--------
GANIP---
-----------------------------VTNLNRM-EYIYR-ISHYRLSTQ-IQHQCN
-AFFNG---LADMINP--RWLR-NFNRE-ELSVLISG-----------------------
---TDDPVD-----------LEDL----RKHTVLGG-YHEQ-------------------
------------------------DLTV-QHFWKVLE--------------------GFD
QPMRKAFLKF-------VTSSPNPP------------LLG-----FG-QLNPLF--AIRK
AGDDK-----------------------------SRLPTASTCV-NL-LKLPDY-EDEQT
CRDKLK-YAIQS-EAGF
>Sporisor_iseilematis_ciliati_MJEU01000005 1..
VQIRRGHV-AQDGFVHL----------------APLGADLKK-PLEIVFIDQFGEP--EAG
IDGGGLFKEF---LTSL----VREAFDTNR--------GLWKATDAQ-------------
----------------ELYPNPHT------------------------------------
-YATSGEQLEWYEFLGRVIGKALYEGILIDAKFAG--FFLSKMLG---------------
-----KQSFLDDLGSIDS----------------------LDKELYRG--------LISL
KNYQG--NVE-DLS--
--LNFTVVDEE---------------------------FGV-SMTRE-LIPG--------
GANIP---
-----------------------------VTNLNRM-EYIYR-ISHYRLSTQ-IQHQCN
-AFFNG---LADIISP--RWLR-NFNRE-ELSVLISG-----------------------
---TDEPVD-----------LADL----RKHTVLGG-YHEQ-------------------
------------------------DVTV-QNFWKVLE--------------------GFD
QPMRKAFLKF-------VTSSPNPP------------LLG-----FS-RLNPLF--AIRK
AGDDK-----------------------------TRLPTASTCV-NL-LKLPDY-ADEQT
CREKLK-YAIQS-EAGF
>Kalmanozyma_brasiliensis_XM_016436064.1 .
VNIRRDHV-AQDGFASL----------------APLGADLKK-PLEIVFIDKFGQP--EAG
IDGGGLFKEF---LTSL----VREAFDTNR--------GLWKATDAQ-------------
----------------ELYPNPHT------------------------------------
-YATSGEQLEWYEFLGRVIGKALYEGILIDAKFAG--FFLSKALG---------------
-----KQSFLDDLGSIDT----------------------LDKELYKG--------LISL
KNYQG--NVE-DLS--
--LNFTVTDEE---------------------------FGV-SMTRE-LVPG--------
GANVA---
-----------------------------VTNLNRM-EYIYR-ISHYRLSTQ-IQHQCN
-AFFNG---LADIINP--RWLR-NFNRE-ELSVLISG-----------------------
---TEEPVD-----------IADL----RKHTVLGG-YHEQ-------------------
------------------------DLTV-QHFWNVLE--------------------GFD
QPMRKAFLKF-------VTSSPNPP------------LLG-----FS-QLNPLF--AIRK
AGEDK-----------------------------SRLPTASTCV-NM-LKLPDY-SDEQT
CREKLR-YAIQS-EAGF
>Ustilago_trichophora_LVYE01000041.1 .
VKIRREHV-AQDGFASL----------------APLGAELKK-PLEILFIDKFGQV--EAG
IDGGGLFKEF---LTSL----VREAFDTNR--------GLWKATDAQ-------------
----------------ELYPNPHT------------------------------------
-YATSGDQLEWYEFLGRVIGKALYEGILVDAKFAG--FFLSKMLG---------------
-----KQSYLDDLGSLDT----------------------LDKELYKG--------LISL
KNYQG--NVE-DLS--
--LNFTVTDEE---------------------------FGV-SMTRE-LIPG--------
GANVP---
-----------------------------VTNLNRM-EYIYR-ISHYRLSTQ-IQHQCN
-AFFTG---LADIIDP--RWLR-NFNRE-ELSVLISG-----------------------
---TDEPVD-----------IADL----RKHTVLGG-YHEK-------------------

------------------------DLTV-QNLWKVLE--------------------SFD
QPMRKAFLKF-------VTSSPNPP------------LLG-----FS-QLNPLF--GIRK
AGEDK-----------------------------SRLPTASTCV-NM-LKLPDY-EDEDT
CRDKLR-YAILS-EAG-
>Ustilago_bromivora_LT558118.1 .
VRIRREHV-AQDGFVNL---------------APLGAEIKK-PLEIQFIDKFGQV--EAG
IDGGGLFKEF---LTAL----VREAFDTNR--------GLWRATDAQ-------------
----------------ELYPNPHT------------------------------------
-YATSGDQLEWYTFLGRVIGKALYEGILVDAKFAG--FFLSKMLG---------------
-----KQSYLDDLGSIDS----------------------LDKELYKG--------LISL
KNYQG--NVE-DLS--
--LNFTVTDEE---------------------------FGV-SMTRE-LIPG--------
GANIP---
------------------------------VTNLNRM-EYIYR-ISHYRLSTQ-IQHQCN
-AFFTG---LADIINP--RWLR-NFNRE-ELSVLISG-----------------------
---TDEPVD-----------IEDL----RKHTVLGG-YHEQ-------------------
------------------------DLTV-QHFWKVLE--------------------DFD
QPMRKAFLKF-------VTSSPNPP------------LLG-----FS-QLNPLF--AIRK
AGDDT-----------------------------SRLPTASTCV-NM-LKLPDY-ADEKT
CREKLR-YAIQS-EAG-
>Ustilago_hordei_CAGI01000189.1 .
VRIRREHV-AQDGFVNL---------------APLGAEIKK-PLEIQFIDKFGQV--EAG
IDGGGLFKEF---LTAL----VREAFDTNR--------GLWRATDAQ-------------
----------------ELYPNPHT------------------------------------
-YAISGDQLEWYTFLGRVIGKALYEGILVDAKFAG--FFLSKMLG---------------
-----KQSYLDDLGSIDS----------------------LDKELYKG--------LISL
KNYQG--NVE-DLS--
--LNFTVTDEE---------------------------FGV-SMTRE-LIPE--------
GANIP---
------------------------------VTNLNRM-EYIYR-ISHYRLSTQ-IQHQCN
-AFFTG---LADIINP--RWLR-NFNRE-ELSVLISG-----------------------
---TDEPVD-----------IEDL----RKHTVLGG-YHEQ-------------------
------------------------DLTV-QHFWKVLE--------------------DFD
QPMRKAFLKF-------VTSSPNPP------------LLG-----FS-QLNPLF--AIRK
AGDDT-----------------------------SRLPTASTCV-NM-LKLPDY-ADEKT
CKEKLR-YAIQS-EAGF
>Ustilago_xerochloae_MAIN01000022.1 .
-----DHV-AQDGFVNL---------------APLGSEIKK-PLEIVFIDKFGQI--EAG
IDGGGLFKEF---LTAL----VREAFDTNR--------GLWRATDSQ-------------
----------------ELYPNPHT------------------------------------
-YATSGDQLEWYTFLGRVLGKALYEGILVDAKFAG--FFLSKMLG---------------
-----KQSYLDDLGSIDS----------------------LDKELYKG--------LISL
KNYQG--NVE-DLS--
--LNFTVTDEE---------------------------FGV-SMTRE-LIPG--------
GANIP---
------------------------------VTNLNRM-EYIYR-ISHYRLSTQ-IQHQCN
-AFFTG---LADIINP--RWLR-NFNRE-ELSVLISG-----------------------
---TDEPVD-----------IEDL----RKHTVLGG-YHEQ-------------------
------------------------DLTV-QHFWKVLE--------------------DFD
QPMRKAFLKF-------VTSSPNPP------------LLG-----FS-QLNPLF--AIRR
AGDDT-----------------------------SRLPTASTCV-NM-LKLPAY-SDEKT
CREKLR-YAIQS-EAGF
>Ustilago_cynodontis_LZZZ01000003.1 .
-----DHV-AQDGFVNL---------------APLGSEIKK-PLEIVFIDKFGQI--EAG
IDGGGLFKEF---LTAL----VREAFDTNR--------GLWRATDSQ-------------
----------------ELYPNPHT------------------------------------
-YATSGDQLEWYTFLGRVLGKALYEGILVDAKFAG--FFLSKMLG---------------
-----KQSYLDDLGSIDS----------------------LDKELYKG--------LISL

KNYQG--NVE-DLS--
--LNFTVTDEE---------------------------FGV-SMTRE-LIPG--------
GANIP---
------------------------------VTNLNRM-EYIYR-ISHYRLSTQ-IQHQCN
-AFFTG---LADIINP--RWLR-NFNRE-ELSVLISG-----------------------
---TDEPVD-----------IEDL----RKHTVLGG-YHEQ-------------------
------------------------DLTV-QHFWKVLE--------------------DFD
QPMRKAFLKF-------VTSSPNPP------------LLG-----FS-QLNPLF--AIRR
AGDDT-----------------------------SRLPTASTCV-NM-LKLPDY-ADEKT
CREKLR-YAIQS-EAGF
>Wallemia_sebi_XM_006957417.1 .
ADIRRDNV-AEDGFNQL---------------NIPGDQLKG-KVFIRFFDQWGIP--EVG
VDGGGLFKEF---LTSL----AKEVFDTDR--------GLWLSSEKR-------------
----------------LLYPNPHS------------------------------------
-YAKEPSQLDWYRFLGKIIGKALYSGILLDVGFAE--FFLAKWNG---------------
--------RQSHLDDLAS----------------------LDPQLYDG--------LLYL
KNYPG--DPQ-DLA--
--LTFAASEEE---------------------------FGA-TRTID-LIPN--------
GQNIP---
------------------------------VTRDNKI-QYIHL-ISHYRLNTQ-IEPQCK
-AFFQG---LSSIIDN--KWLR-MFDQQ-ELGVLIGG-----------------------
---AESDID-----------LDDL----KIHTVYNN-YSEA-------------------
------------------------DEVI-INFWSVVR--------------------SFN
QRQREQLLSF-------VTSCPRAP------------LLG-----FR-ELRPFF--AISK
ASDDD-----------------------------SWLPTASTCV-NM-LKLPAY-SNKQK
LKEKLL-AAITS-GARF
>Wallemia_ichthyophaga_XM_009268669.1 .
ADIRRDNV-AEDGFNQL---------------NIPGDQLKG-KVFIRFFDQWGNL--EAG
VDGGGLFKEF---LTSL----AKEVFDTDR--------GLWLSNEKR-------------
----------------LLYPNPHS------------------------------------
-YAKEPSQLDWYRFLGKIIGKALYSGILLDIGFAE--FFLAKWNG---------------
--------RQSHLDDLAS----------------------LDPQLYDG--------LLYL
KNYPG--DSQ-DLA--
--LTFAASEEE---------------------------FGV-TRTMD-LIPN--------
GQNIT---
------------------------------VTRDNKI-QYIHL-VSHYRLNTQ-IEPQCN
-AFFQG---LSSIIDN--KWLR-MFDQQ-ELGVLIGG-----------------------
---AESDID-----------LDDL----KKHTVYNN-YSEA-------------------
------------------------DEVV-INFWSVVR--------------------SFN
QREKELLLSF-------VTSCPRAP------------LLG-----FR-ELRPFF--AISK
ASEDD-----------------------------SWLPTASTCV-NL-LKLPAY-SNKEK
LRDKLL-AAITS-GSRF
>Rigidoporus_microporus_GDMN01016353.1 .
VTIRRDRV-AKDGFDKL-----------------GQANLKQ-RLRIQFIDKFGNE--EAG
IDGGGLFKEF---FTSL----CKEVFHPDR--------GLWVTNEKN-------------
----------------EVYPSPLS------------------------------------
-YAKEPHSLDWYRFIGRIVGKALYESILIDVPFAG--FFLAKWLK---------------
--------KQSYLDDLES----------------------LDKELYQG--------LLFV
KHHPE--NLE-DLA--
--LTFTIAEEE---------------------------YGV-TESVD-LIPN--------
GSNIA---
------------------------------VTSENRL-QYITL-VCYHRLTKQ-IRPQSE
-AFFQG---LSEIISP--KWLR-MFNQQ-EMQILIGG-----------------------
---VDTAID-----------IEDL----KKNTVYSGLYNEE-------------------
------------------------EPTI-QMFWGVVS--------------------EFN
QKQKQALLRF-------VTSCGRPP------------LLG-----FK-ELSPSF--SIRD
SGSDQ-----------------------------TRLPTSSTCV-NL-LKLPRY-QSIHT
LREKLL-KAVNS-GAGF

>Coriolopsis_gallica_GBYM01004111.1 .
VVIRRDHI-AQDGFDKL-----------------GELDLKA-PIAITFIDQFGEQ--EQG
IDGGGVFKEF---LTSL----SREVFHTDR--------GLWMENQNH-------------
----------------ELYPASHS------------------------------------
-LVMEPHSLAWYKFIGRILGKALYEGILVDVAFAG--FFLAKWLG---------------
--------KQSFLDDLAS----------------------LDPEFYHN--------LIYL
KHQDLR---KLETDGKGG
LGLDFTILLDD---------------------------FGR-NETVE-LIPG--------
GSEIP---
------------------------------VTEENKL-QYIYL-VAHYKLTKQ-IKKQSA
-AFFEG---LSDMIEP--RWLR-MFNQQ-ELQILLGG-----------------------
---VNTPID-----------LDDL----RKHTNYGGLYDDH-------------------
------------------------HPVI-QMFWEVVN--------------------TFT
QEERQKLLAF-------ATSCSRPP------------LLG-----FK-ELNPNF--CIRD
SSDDQ-----------------------------ERLPSSSTCV-NL-LKLP--------

>Moniliophthora_roreri_XM_007852604.1 .
VEIRRTHI-AQDGFDKL----------------NQDVDLKK-GVEIVFMDRWGEE--EAG
IDGGGVFKEF---LTSL----SKEVFDTDR--------GLWQVNRER-------------
----------------ELYPATGS------------------------------------
-LAIETHSLNWYRFIGRILGKAMYDGILVDVAFAN--FFLGKWLS---------------
-------RTHTSLDDLAS----------------------LDPDLYNG--------LVFL
KHYPKE---KVE-DLS--
--LTFVIGVEE---------------------------YGL-QQTIP-LIPN--------
GASIP---
------------------------------VTASNRL-QYITL-VSHFKLSKQ-IKKQSE
-AFWQG---VSDLVQA--RWVR-MFNPH-ELQILLGG-----------------------
---VNTPID-----------IEDL----RNNTNYGGVYDER-------------------
------------------------EQTV-GMFWDVVN--------------------SFS
EDQKRALLRF-------VTSCSRPP------------LLG-----FK-ELNPHF--AIRD
AGNDQ-----------------------------HRLPTSSTCV-NL-LKLPKY-QDKQT
LRAKLV-QAINA-GAGF
>Auricularia_subglabra_XM_007356987.1 .
IVIRRDRIAEADGFDHL---------------NDLGPALKG-SLSIVFVDQFGQE--EAG
IDGGGVFKEF---LTSL----SKEVFNADR--------GLWLTTSQH-------------
----------------ELYPNPMS------------------------------------
-IATEPHHLNWYRFVGRILGKALYQGILVEVAFAS--FFLAKWLS---------------
--------KQSFLDDLAS----------------------LDRELYNG--------LIFL
KHYQG--NLE-DLA--
--LNFTINEDD---------------------------FGV-TRTID-LIPN--------
GSNIP---
------------------------------VTRENRL-QYIHL-VCHHRLSRQ-IRKQST
-AFFEG---LSEIIDP--KWLR-MFNQQ-ELQILVAG-----------------------
---VNADVD-----------IDDL----RHNTNYGGVYDDN-------------------
------------------------EETI-VAFWKVVK--------------------SFD
ATQKRQLLRF-------VTSCSRPP------------LLG-----FK-ELVPLF--CIRD
SGNDQ-----------------------------TRLPTASTCV-NL-LKLPRY-KNEHV
LRQKLL-QAISS-NAGF
>Fomitiporia_mediterranea_XM_007271664.1 .
VVVHRGNV-AADGFDKL-----------------ANMNLKG-LIEIQFVDQFGNT--EAG
IDGGGVFKEF---FTSL----CKEVFDTDR--------GLWLATKQN-------------
----------------ELYPNPHS------------------------------------
-YAKEPHSLEWYRFIGRILGKALYEGILVDVAFAG--FFLAKWLG---------------
--------KQSYLDDLAS----------------------LDPELYQG--------LLFL
KNYDD--NPQ-DLS--
--LTFAVSEEE---------------------------FGA-AKTRE-LIPN--------
GSNTP---
------------------------------VTKENRL-PYIYL-MCHYHLSKK-IRSQSE

```
-AFFEG---LSEMIDP--KWIR-MFNQQ-ELQILIGG-----------------------
---VNAPID-----------LDDL----RKNTNYGG-FKDD-------------------
------------------------DPVI-EAFWRVVS--------------------SFD
QEQRRMLLRF-------VTSCSRPP------------LLG-----FK-ELQPLF--SIRR
ASEDQ-----------------------------SRLPTASTCV-NL-LKLPAY-QDERV
MKSKLL-QAISS-GAGF
>Boletopsis_grisea_GEZR01002480.1 .
ISVRRDKI-SQDGFDKL-----------------QDVDMKG-PLEIVFIDQFGSE--EAG
IDGGGVFKEF---LTSL----AKEVFDSDR--------GLWLATNQN-------------
----------------ELYPNPHS------------------------------------
-YARDLYSMNWYRFVGRVLGKALYESILVDVAFAG--FFLAKWLG---------------
--------KHSFLDDLAS----------------------LDPELYQG--------LLFL
KHYSG----------------------------------------------DPE-DLV--
-LN-FTVTQEE---------------------------FGV-AKSIE-LIPN--------
GSNIP-------------------------------------------------------
------------------------------VTKENKL-QYIFL-VSHFKLTKQ-IKLQSE
-AFFEG---LSDVIDP--KWLR-MFNQQ-ELQVLLGG-----------------------
---VNSPID-----------VADL----RKYTNYGGLYSDQ-------------------
------------------------EDTI-QKFWNVVN--------------------AFD
QDQRRALLRFS-------TGCGRPP------------LLG-----FK-ELRPKF--AIRD
AGDDA-----------------------------ERLPTASTCM-NL-LKLPRY-TSEKV
LRGKLL-QAIYS-GAGF
>Agaricus_bisporus_XM_006460489.1 .
VQVRRGTV-AQDGFDRL-----------------AEADLKQ-PIEITFIDQFGQE--EAG
IDGGGVFKEF---LTSL----SKEVFDTDR--------GLWLANKKN-------------
----------------ELYPNPHS------------------------------------
-YATEPHSLNWYRFIGRMLGKAMYDGILIDVAFAG--FFLAKWLG---------------
--------KQSFLDDLAS----------------------LDPELYNG--------LIFL
KHYTG----------------------------------------------NPE-DLS--
--LNFTVAVDE---------------------------FGV-TKMMD-LMKY--------
GSETA-------------------------------------------------------
------------------------------VTKENKL-MYIYL-ISHYRLSKQ-IKLQSN
-AFFEG---LSEMIDP--KWLR-MFNQQ-EVQILLGG-----------------------
---VNSPID-----------IEDL----KMCTNYGGLYDSG-------------------
------------------------HLTI-RMFWKVVS--------------------SFT
EEQKRALLRF-------VTSCSRPP------------LLG-----FK-ELVPNF--SIRD
AGSDE-----------------------------LRLPTASTCV-NL-LKLPRY-TTEKA
LRNKLV-QAITS-NAGF
>Agaricus_bisporus_XM_007333265.1 .
VQVRRGTV-AQDGFDRL-----------------AEADLKQ-PIEITFIDQFGQE--EAG
IDGGGVFKEF---LTSL----SKEVFDTDR--------GLWLANKKN-------------
----------------ELYPNPHS------------------------------------
-YATEPHSLNWYRFIGRMLGKAMYDGILIDVAFAG--FFLAKWLG---------------
--------KQSFLDDLAS----------------------LDPELYNG--------LIFL
KHYTG----------------------------------------------NPE-DLS--
--LNFTVAVDE---------------------------FGV-TKMMD-LMKY--------
GSETA-------------------------------------------------------
------------------------------VTKENKL-MYIYL-ISHYRLSKQ-IKLQSN
-AFFEG---LSEMIDP--KWLR-MFNQQ-EVQILLGG-----------------------
---VNSPID-----------IEDL----KICTNYGGLYDSG-------------------
------------------------HLTI-RMFWKVVS--------------------SFT
EEQKRALLRF-------VTSCSRPP------------LLG-----FK-ELVPNF--SIRD
AGSDE-----------------------------LRLPTASTCV-NL-LKLPRY-TTEKA
LRNKLV-QAITS-NAGF
>Agaricus_subrufescens_GBEJ01009466.1 .
IQVRRDTV-AQDGFDKL-----------------AEADLKQ-PIEITFIDQFGQE--EAG
IDGGGVFKEF---LTSL----SKQVFDTDR--------GLWLANKKN-------------
----------------ELYPNPHS------------------------------------
```

-YATEPHSLNWYRFIGRILGKAMYDGILIDVAFAG--FFLAKWLG---------------
--------KQSFLDDLAS----------------------LDPELYNG--------LIFL
KHSTG--NPE-ELS--
--LNFTVAVDE---------------------------FGV-TKTID-LIKN--------
GSETP---
------------------------------VTKENKL-TYIYL-ISHYRLSKQ-IKLQSN
-AFFEG---LSEMIDP--KWLR-MFNQQ-EVQILLGG-----------------------
---VNSPID-----------LDDL----KRYTNYGGLYDTG-------------------
------------------------NATI-RLFWKVVG--------------------SFT
EEQKRALLRF-------VTSCSRPP------------LLG-----FK-ELVPNF--SIRD
AGSDE-----------------------------LRLPTASTCV-NL-LKLPRY-TTERA
LKSKLV-QAITS-NAGF
>Leucocoprinus_gongylophorus_HAAN01000619.1 .
VKVRREMV-SQDGFDRL-----------------XDADLKQ-PIEITFIDRFGQE--EAG
IDGGGVFKEF---LTSL----SKEVFDTDR--------GLWLANKKN-------------
----------------ELYPNPHS------------------------------------
-YATESYSLNWYRFIGRILGKAMYEGILIDVAFAG--FFLSKWLG---------------
--------KQSFLDDLAS----------------------LDPELYNG--------LIFL
KHYTG--NAE-DLS--
--LNFTVAVDE---------------------------YGI-TKSID-LMPN--------
GSETP---
------------------------------VTKENRL-TYIYM-ISHYRLSKQ-IRLQSN
-AFFEG---LSEMIDP--KWLR-MFNQQ-EVQILLGG-----------------------
---VNTPID-----------VQDL----KNNTNYGGLFDAR-------------------
------------------------HPTI-LLFWKVVS--------------------SFS
EDQKRLLLRF-------VSSCSRPP------------LLG-----FK-ELVPNF--SIRD
AGSDQ-----------------------------LRLPTASTCV-NL-LKLPRY-TMEQT
LRAKLL-QAITS-NAGF
>Hypsizygus_marmoreus_GBCL01016336.1 .
VQVRRGSV-AQDGFDRL-----------------ADADLKA-PVEITFVDQFGAE--EAG
IDGGGVFKEF---FTSL----CKEVFDTDR--------GLWLANKKN-------------
----------------ELYPNPHA------------------------------------
-YATEGHSLNWYRFIGRILGKAMYEGILVDVAFAG--FFLAKWLG---------------
--------KQSFLDDLAS----------------------LDPDLYNG--------LIFL
KHYTG--PVE-ELS--
--LNFTVAIDE---------------------------FGV-TKTID-LIPD--------
GSNVA---
------------------------------VTKDNRL-QYIQL-VSHYRLSKQ-IKLQSE
-AFFEG---LSEMIDP--KWIR-MFNQQ-EIQILLGG-----------------------
---VNAPID-----------FDDL----RANTNYGGLYDDN-------------------
------------------------EETI-VAFWNVVN--------------------SFN
QEQRMALLRF-------VTSCSRPP------------LLG-----FK-ELVPNF--SIRD
AGSDQ-----------------------------LRLPTSSTCV-NL-LKLPRY-KSEKV
LRAKLL-QAINA-NAGF
>Laccaria_bicolor_XM_001885008.1 .
VQVRRGMV-AQDGFDRL-----------------GEVDLKA-PIEITFIDQFGQE--EAG
IDGGGVFKEF---FTSL----CKEVFDTDR--------GLWLENKKN-------------
----------------ELYPNPHA------------------------------------
-YATEAHSLNWYRFIGRILGKAMYEGILVDVAFAG--FFLAKWLG---------------
--------KQSFLDDLVS----------------------LDPDLYNG--------LIFL
KHYTG--NTD-DLS--
--LNFTIAVDE---------------------------IGV-TKIID-LIPN--------
GSNIA---
------------------------------VTKENRL-KYIYL-VSHYRLTKQ-IRRQSD
-AFFEG---LSEMIDP--KWLR-MFNQQ-EVQILIGG-----------------------
---VNSLID-----------LDDL----RRHTNYGGLYDDK-------------------
------------------------HENI-VDFWNVVN--------------------TFD
QDQRRALLRF-------VTSCSRPP------------LLG-----FK-ELVPNF--SIRD

AGADP-----------------------------QRLPTASTCI-NL-LKLPRY-PNARI
LKSKLL-QAISS-GAGF
>Coprinopsis_cinerea_XM_001840013.2 .
IRVRRGRV-AQDGFDRL-----------------SEVDLKK-PVEITFVDQWGQE--EAG
IDGGGVFKEF---FTDL----CKEVFDTDR--------GLWLANKKN-------------
----------------ELYPNPHS------------------------------------
-YATEQHSLNWYRFIGRVLGKAMYEGILVDVAFAS--FFLAXWLG---------------
--------KQSFLDDLAS----------------------LDPELYKG--------LIFL
KHYKG--NPE-DLA--
--LNFTIAVDE---------------------------LGV-TKDVE-LVPG--------
GSNIT---
------------------------------VTKANRL-DYITL-VSHYRLKKQ-IKKQSD
-AFFDG---LSDMIDP--RWLR-MFNQQ-EVQILIGG-----------------------
---VNSPID-----------LDDL----RQHTNYGGLYDDR-------------------
------------------------HPVI-VAFWKVVN--------------------SFD
QEQRRALLRF-------VTSCSRPP------------LLG-----FK-ELVPHF--SIRD
AGTDE-----------------------------LRLPTST-------------------

>Punctularia_strigosozonata_XM_007380595.1 .
AVVRRGQV-AKDGFDRL-----------------YDADLKL-PIEITFIDQFGQE--EAG
IDGGGVFKEF---FTSL----CKEVFDTDR--------GLWLVNKDN-------------
----------------ELYPNPHS------------------------------------
-YATESHSLNWYRFIGRILGKAMYDGILVDVAFAP--FFLAKWLG---------------
--------KQSFLDDLAS----------------------LDPELYNG--------LIFL
KNSTE--NPE-ELS--
--LNFTVAVEE---------------------------FGV-MKTLD-LIPN--------
GSNVP---
------------------------------VTRENKL-QYIYL-VSNYRLNRQ-IKRQSE
-AFFEG---LSEMIDP--KWLR-MFNQQ-EVQILISG-----------------------
---VNSPID-----------IDDL----RNHTVYGGLYDAG-------------------
------------------------HETI-QLFWKVVH--------------------SLD
HNQRRDLLRF-------VTSCSRPP------------LLG-----FK-ELRPNF--AIRD
AGGDE-----------------------------ARLPTASTCV-NL-LKLPRY-KTEAI
LRVKLL-QAINA-NAGF
>Stereum_hirsutum_XM_007311441.1 .
VTVRRGHV-AEDGFNRL-----------------AEADLRM-PVEITFVDQFGEV--ESG
IDGGGVFKEF---FTEL----CKEAFDTNR--------GLWLANQQN-------------
----------------ELYPNPHS------------------------------------
-YAREAHNLNWYRFIGRILGKALYEGILVDVAFAG--FFLAKWLG---------------
--------KQSFLDDLAS----------------------LDPDLYHG--------LIFL
KHYTG--NPE-ELS--
--LNFTVAEEE---------------------------FGV-TNTID-LCPN--------
GSNIP---
------------------------------VTRDNRL-EYIYR-MSHYRLTKQ-IRKQTE
-AFFDG---LSDVIDQ--KWLR-MFNQQ-ELQVLLGG-----------------------
---VNAPID-----------VDDL----RSHTNYGGLYDDN-------------------
------------------------EPTI-VAFWNVLK--------------------TFD
HDQRRAVLRF-------VTSVGRPP------------LLG-----FK-ELHPNF--SIRD
AGSDQ-----------------------------TRLPTSSTCV-NL-LKLPRY-KDEKT
LRDKLL-QAAFS-GAGF
>Gloeophyllum_trabeum_XM_007872379.1 .
VSVRRDHI-AEDGFDRL-----------------GDADLKA-PIEIVFIDQFGQE--EAG
IDGGGVFKEF---LTSL----CKEVFDRDR--------GLWLANKKN-------------
----------------ELYPNPHS------------------------------------
-YAKEPHSLNWYRFIGRVLGKALYEGILVDVAFAG--FFLAKWLG---------------
--------KQSFLDDLAS----------------------LDPDLYNG--------LIFL
KHYNG--NPE-DLS--
--LTFTVDEED---------------------------LGV-TKTTE-LIPN--------

```
GSNIP-------------------------------------------------------
------------------------------VTRENRL-QYITL-VSHYRLTKQ-IRLQSE
-AFFEG---LSQMIDT--KWLR-MFNQQ-ELQILLGG-----------------------
---VDTPID-----------LDDL----RSHTNYGGLYDDN-------------------
------------------------HPTI-VMFWNVVN--------------------TFN
QDQRKALLRF-------VTSCSRPP------------LLG-----FK-ELVPNF--AIRD
AGSDE-----------------------------ARLPTSSTCV-NL-LKLPRY-QSEKT
LRDKLL-QAIYS-GAGF
>Coniophora_puteana_XM_007777017.1 .
VAIRRGHI-SQDGFDRL-----------------GEMDLRE-PVSITFIDQFGNE--EAG
IDGGGVFKEF---FTSL----CKEVFDTDR--------GLWLANKKN-------------
----------------ELYPNPIS------------------------------------
-YATESHSLNWYRFIGRIIAKALYEGILVEVAFAG--FFLAKWLG---------------
--------KQSLLDDLAS----------------------LDPDLHQG--------LIFL
KNYNG----------------------------------------------DVG-DLS--
--LNFTIAIDE---------------------------FGA-NKTID-LIPN--------
GSNVA-------------------------------------------------------
------------------------------VTKENRL-QYIYL-VSHYRLSRQ-IRLQSD
-AFFEG---LSEIIDD--KWLK-MFNQQ-ELQILLGG-----------------------
---VNAPID-----------LQDL----RANTQYGGLYDDH-------------------
------------------------ASTI-EAFWKVLE--------------------SFN
QEQRRAFLRF-------VTSCSRPP------------LLG-----FK-ELRPQF--AIRD
SSNDQ-----------------------------NWLPTASTCV-NL-LKLPRY-ESESV
LRDKLL-QAIYS-GAGF
>Serpula_lacrymans_XM_007323942.1 .
ITVRRGHI-AEDGFDRL-----------------SEANLKA-PIRIEFIDQFGEP--EAG
IDGGGVFKEF---FTSL----CKEVFDTNR--------GLWLATKKN-------------
----------------ELYPNPHS------------------------------------
-YATEPHSLNWYRFIGRILGKALYEGILVEVAFAG--FFLAKWLG---------------
--------KQSFLDDLAS----------------------LDPDLYQG--------LIFL
KNYTG----------------------------------------------NPE-DLS--
--LNFTAAVEE---------------------------FGV-AKTVD-LIPD--------
GSNVA-------------------------------------------------------
------------------------------VTRENRL-QYIYL-VSHYRLSKQ-IKQQSD
-AFFEG---LSEMIDP--KWLR-MFNQQ-GLQILLGG-----------------------
---VDSPID-----------LDDL----RENTSYGGLFDDK-------------------
------------------------DATI-EIFWKVVN--------------------SFD
QEQRRAFLRF-------VTSCSRPP------------LLG-----FK-ELAPNF--AIRD
SGSDE-----------------------------KRLPTSSTCV-NL-LKLPRY-QSEAV
LRQKLL-QAITS-GAGF
>Armillaria_ostoyae_GAHM01002288.1 .
VTIRRDHI-AEDGFDKL-----------------GDANLKG-HVQITFIDKFGEP--EAG
IDGGGVFKEF---FTSL----CKEVFDTDR--------GLWLANNNN-------------
----------------ELYPNPHG------------------------------------
-YAKEGHSLSWYRFIGRILGKAIYEGILVDVAFAG--FFLAKWLG---------------
--------RQNLLDDLAS----------------------LDPELYNG--------LMFL
KHYTG----------------------------------------------NPE-DLS--
--LNFTVAIEE---------------------------FGV-TKTID-LIPE--------
GSTTA-------------------------------------------------------
------------------------------VTRENRL-QYIYF-VSHYRLSKQ-IKLQSE
-AFFEG---LSETIDP--KWLR-MFNQQ-EVQILIGG-----------------------
---VNSPID-----------LEDL----RENTNYGGLYDND-------------------
------------------------EPTI-VAFWKVVE--------------------TFD
QDQRRALLRF-------VTSCSRPP------------LLG-----FK-ELVPKF--GIRD
SGSDE-----------------------------HRLPTASTCF-NL-LKLPRY-QSEKV
LKDKLL-RAITS-GAGF
>Ganoderma_lucidum_AHGX01000115.1_R .
VVVRRDHI-AQDGFDKL-----------------GEVDLRA-PIAITFVDQWGN---EAG
```

IDGGGVFKEF---LTSL----TREVFDTDR--------GLWLANKKN-------------
----------------ELYPAX---------------------------------------
--------LAWYRFIGRILGKALYEGILIDVAFAG--FFLAKWLD---------------
--------KQSYLDDLAS----------------------LDPELYQG--------LMQL
KHYTG--DPE-ELS--
--LNFTVAQEE---------------------------FGE-TRTID-LLPN--------
GSNIA---
------------------------------VTRDNRL-QYMYL-VAHYRLTKQ-IKKQTA
-AFFDG---LSEIIDP--KWLR-MFNQQ-ELQILLGG-----------------------
---VNSPID-----------LDDL----RANTQYGGLYDDH-------------------
------------------------QHTI-TMFWEVVG--------------------SFT
QEDRRKLLRF-------ATSCSRPP------------LXG-----FK-ELNPHF--AIRD
ATDDQ-----------------------------SRLPTASTCV-NL-LKVPRY-TNKRT
LKEKLL-QAINS-NAGF
>Dichomitus_squalens_XM_007370525.1 .
IVIRRDHI-AQDGFDRL------------------ADADLRA-PIAITFIDQFGNE--EAG
IDGGGVFKEF---LTSL----TREVFDTDR--------GLWLATKKN-------------
----------------ELYPAPHS------------------------------------
-YATEPHSLAWYRFIGRILGKALYEGILVDIAFAG--FFLAKWLD---------------
--------KQSWLDDLAS----------------------LDPELYQG--------LMQL
KHYTG--DPE-ELS--
--LNFTVAQEE---------------------------FGV-TKSID-LIPN--------
GSNIP---
------------------------------VTRENRL-QYMYL-VAHYKLTKQ-IKKQST
-AFFEG---LSEIIDP--KWLR-MFNQQ-ELQILLGG-----------------------
---VDSPID-----------VEDL----RANTQYGGLYDDH-------------------
------------------------HLTI-QLFWEVVN--------------------ELS
HEDRRKLLRF-------ATSCSRPP------------LLG-----FK-ELNPHF--AIRD
ATDDQ-----------------------------TRLPTASTCV-NL-LKLPRY-TDRRV
LKEKLV-QAINS-NAGF
>Trametes_versicolor_XM_008047415.1 .
VTVRRTNI-AQDGFDRL------------------GDVDLKT-PIAITFMDQFGNE--EAG
IDGGGVFKEF---LTSL----CKEVFDSDR--------GLWLANKKN-------------
----------------ELYPAPHS------------------------------------
-YATEAHSLAWYRFIGRILGKALYDGILIDVAFAG--FFLAKWLG---------------
--------KQSWLDDLAS----------------------LDPELYSG--------LIFL
KHYEG--DPE-ELS--
--LNFTVSQEE---------------------------FGV-MKTAD-LIPN--------
GSNIP---
------------------------------VTRENRL-QYIYL-VAHHRLTKQ-IKKQST
-AFFEG---LSEIIDP--KWLR-MFNQQ-ELQILLGG-----------------------
---VNAPID-----------LDDL----RSHTQYGGLYDDH-------------------
------------------------EHTI-EIFWSVVK--------------------TFN
QDERRKLLRF-------VTSCSRPP------------LLG-----FK-ELNPNF--AIRD
ATGDE-----------------------------TRLPTASTCV-NL-LKLPRY-KSPQV
LREKLV-QAINS-NAGF
>Irpex_lacteus_GBRE01004950.1 .
VTVRRGSI-AQDGFDRL------------------DGYDLRS-SIAITFIDQFGQE--EAG
IDGGGVFKEF---LTSL----CKEVFDTDR--------GLWLANKEH-------------
----------------ELYPNPHS------------------------------------
-YATEPHSLAWYRFIGRILGKALYDGILVDVAFAG--FFLAKWLG---------------
--------KQSFLDDLAT----------------------LDPELYNG--------LIFL
KHYDG--NPE-DLS--
--LNFTVALEE---------------------------FGV-TTTVP-LIPN--------
GDKIA---
------------------------------VTKENRL-QYIYL-VSNYKLNRQ-IKKQSD
-AFFEG---LSEIIDP--KWLR-MFNQQ-ELQILLGG-----------------------
---VNSPID-----------LEDL----RAHTQYGGLYNDQ-------------------

------------------------EPTI-QVFWRVVN--------------------SFN
HEQRRALLRF-------ATSCSRPP------------LLG-----FK-ELYPNF--AIRD
AGSDE-----------------------------SRLPTASTCV-NL-LKLPRY-TSERT
LRQKLV-QAIQS-NAGF
>Phanerochaete_carnosa_XM_007398460.1 .
VTVRRGNI-AQDGFNRL-----------------GDVDLKG-PIAITFIDQFGQE--EAG
IDGGGVFKEF---LTEL----CKEVFDTDR--------GLWLASKKN-------------
----------------ELYPNPHS------------------------------------
-YATESGSLAWYRFIGRILGKALYEGILVDVAFAG--FFLAKWLG---------------
--------KQSFLDDLAS----------------------LDPELYQG--------LIFL
KHYTG--NPE-DLS--
--LTFAVTSEE---------------------------FDV-TRTIP-LIPN--------
GDQIP---
------------------------------VTRENRL-QYIYL-ISHYRLNKQ-IKRQSD
-AFFEG---LSEMIDP--KWLR-MFNQQ-ELQILLGG-----------------------
---VNSPID-----------LDDL----RTNTQYGGLFNDH-------------------
------------------------ESTI-EMFWRVVN--------------------TFN
QDQRRALLRF-------ATSCSRPP------------LLG-----FK-ELVPNF--AVRD
AGSDE-----------------------------NRLPTASTCV-NL-LKLPRY-TSERM
LREKLL-QAIQS-NAGF
>Sparassis_latifolia_GELB01007887.1 .
VTVRRGMI-AQDGFDHL-----------------QDVDLKS-PIAISFIDQFGQE--EAG
IDGGGVFKEF---LTSL----CKEVFDTDR--------GLWLANQKH-------------
----------------ELYPNPHS------------------------------------
-YATETHSLNWYRFIGRILGKALYEGILIDVAFAG--FFLAKWLG---------------
--------KQSFLDDLAS----------------------LDPELYQG--------LIFL
KHYTG--NPE-DLS--
--LNFTVAEEE---------------------------FGV-AKAME-LIPD--------
GSNIP---
------------------------------VTRENRL-QYIYL-VSHYRLTKQ-IKLQSE
-AFFEG---LSEMIDS--KWLR-MFNQQ-ELQILLGG-----------------------
---VNSPID-----------LEDL----REHTHYGGLYDDH-------------------
------------------------EPTI-QVFWRVVD--------------------SFD
QEQRRKLLRF-------ATSCSRPP------------LLG-----FR-ELVPNF--SVRD
AGSDE-----------------------------NRLPTASTCV-NL-LKLPRY-KSEIV
LRDKLL-QAINA-NAGF
>Fibroporia_radiculosa_XM_012329328.1 .
VTVRRSSL-AQDGFDKL-----------------QDVDLHV-PIAITFIDQFGNE--EAG
IDGGGVFKEF---LTSL----CKEVFDTDR--------GLWLANKKN-------------
----------------ELYPAPHS------------------------------------
-LATERHSLNWYRFIGRILGKALYEGILIDVAFAG--FFLAKWLG---------------
--------KQSFLDDLAS----------------------LDPDLYNG--------LLFL
KHYTG--DPE-DLS--
--LNFTAAEEE---------------------------FGV-ARTVE-LIPN--------
GSNIA---
------------------------------VSKENRL-QYIYL-VSHYRLSKQ-IKRQSE
-AFFEG---LSEMIDP--KWLR-MFNQQ-ELQILLGG-----------------------
---VNAPVD-----------LENL----RQHTNYGGLYDDH-------------------
------------------------ETTI-ELFWKVVN--------------------SFD
HEQRRGLLRF-------ATSCSRPP------------LLG-----FE-ALVPNF--AIRD
AGADE-----------------------------NRLPTASTCV-NL-LKLPRY-QNETV
LREKLL-QAINS-NAGF
>Mortierella_alpina_ADAG01001035.1 .
---------------CL---------------LVIGPKLKG-RIAISFIDQYGIP--EAG
IDGGGVFKEF---LTSL----VLQAFDTNY--------GLFLSTSDQ-------------
----------------LLYPNPHR------------------------------------
-FAQEMTQLKHYEFLGRILGKALYEGILIDAAFAG--FFLGKCLG---------------
--------QVNYLDDLPS----------------------LDPDLYKG--------LMFL

KNYEG--NVE-DLS--
--LYFTVDDEG---------------------------KGX-TITRE-LIPN--------
GGNTL---
------------------------------VTRANRI-RYIYL-TAHYRLNTQ-IDRQCR
-AFFRG---LSDLIDP--KWLW-MFNQQ-ELQVMLGG-----------------------
---AQTAIS-----------LADL----EKNVVYSN-FSRT-------------------
------------------------HPTI-EHFWSVVQ--------------------SMK
EEDRRLLIKF-------ITSCARPP------------LLG-----FA-ELNPRL--CIRN
AGQEE-----------------------------DRLPTSSTCM-NL-LKLPAF-TSRER
LKEKLM-YAIHS-QAGF
>Mortierella_verticillata_AEVJ01000465.1 .
----------------------------------IGRKLKG-RIAIEFIDQHGIS--EAG
IDGGGVFKEF---LTSL----VHQAFDTNY--------GLFLNTSDQ-------------
----------------LLFPNPHA------------------------------------
-FAREATQLKHYEFLGRILGKALYEGILIDAAFAG--FFLGKCLG---------------
--------QVNYLDDLPS----------------------LDPELYRG--------LMFL
KNYEG--DVE-DLS--
--LFFTVDDEG---------------------------XGT-KITRE-LVPN--------
GSNIA---
------------------------------VTRQNRI-RYIYM-TAHYRLNTQ-INRQCK
-AFFRG---LSDLIDP--KWLW-MFNEQ-ELQVMLGG-----------------------
---AQIEIS-----------LEDL----KKNVVYSN-FEVG-------------------
------------------------DPTI-QFFWSVVQ--------------------EMA
EEDRRLLIKF-------ITSCARPP------------LLG-----FA-ELNPKL--CIRH
AGVEE-----------------------------SRLPTSSTCM-NL-LKLPAF-STRQR
LKEKLM-YAIHS-EAGF
>Spizellomyces_punctatus_XM_016755140.1 .
VTIRRQYV-FKDGYTHL---------------NALGSRLKN-RVAITFISEQGLV--EAG
IDGGGVFKEF---LTTL----CRQAFDLNY--------GLFQATTDQ-------------
----------------LLYPSPHS------------------------------------
-YATQETQLKHLEFLGRILGKALYEGILVDVGFAN--FFLAKWLG---------------
--------RTSYLDDLPS----------------------LDPDLYQG--------LLFL
KNYQG--DVR-DLG--
--LTFSVDDTE---------------------------FGA-QKTIE-LIPN--------
GSTIP---
------------------------------VTNTNRI-KYIYL-MAHWKLNTR-IERQCK
-AFFGG---LVDLIDP--SWVR-MFNQQ-ELQILLSG-----------------------
---TPTPIS-----------LTSL----REHTTYAGGYTST-------------------
------------------------HPTI-LLFWTVLE--------------------QFD
EEYRRGLLRF-------VTSCAREP------------LLG-----FG-ELRPGF--CIRF
AGDEE-----------------------------DRLPTASTCV-NL-LKLPAY-KSLEV
MR---------------
>Batrachochytrium_dendrobatidis_ADAR01000059.1 .
--IRRSSV-FEDGFDHL---------------NSLGSGLKG-RVSITFIDEHGLV--EAG
IDGGGVFKEF---LTAC----LKQAFNSNY--------GLFETTKDQ-------------
----------------LLFPSX--------------------------------------
------SQLQLMEFLGRIIGKALYDGVLLDSAFAT--FFLAKWLG---------------
--------XYNTVDDLPS----------------------LDTEFYNG--------LLFL
KKYTG--DVEKDLA--
--LNFTVSENE---------------------------FGV-PKIVE-LIPN--------
GANIS---
------------------------------VTKENRI-RYIYL-VANYRLNTK-IAKQCQ
-AFFHG---LSDLIHP--SWLK-LFDEX-ELQVLLGG-----------------------
---SAVPID-----------LDDL----RQNTVYSGVYDDK-------------------
------------------------HPTI-VMFWEVLQ--------------------DFD
EDQRRKLVKY-------VTSCSRPP------------ILG-----FC-ELYPLF--SIRD
SSTDQ-----------------------------ARLATASTCV-NL-LKLPRY-ESKDI
LRQKLI-YAIDS-DAGF

```
>Homoloaphlyctis_polyrhiza_AFSM01002710.1_R .
VTIRRDSI-FRDGFDQL---------------NGLKRQLKG-RIAISFIDTHGLP--EAG
IDGGGVFKEF---LTSX---------DSSY--------GLFQTTNNQ-------------
----------------LLYPSASE------------------------------------
-YATQDSQLRLMEFLGRIIGKALYEGVLIEAAFAN--FFLSKWLG---------------
--------XGIIVDDLPG----------------------LDQELYNG--------LLFL
KNYAG----------------------------------------------SVEADLG--
--LNFTVSENE---------------------------FGV-TRNVE-LIPD--------
GANVP-------------------------------------------------------
------------------------------VTNENRI-RYVYF-TANYKLNTK-IARQCQ
-AFFRG---LSDLIDP--SWLK-LFDEV-QLQILLGG-----------------------
---NAVPID-----------IADL----RRNTVYNGVYSHD-------------------
------------------------HPTI-EMFWRVVE--------------------QMD
DAERRMLVQY-------ITSCSRPP------------LLG-----FG-ELQPKL--CIRD
SGSDE-----------------------------DRLPTASTCV-NL-LKLPIY-KSEAT
LQRKLQ-YAIHA-EAGF
>Gigaspora_margarita_GBYF01053735.1 .
VTIRRSKI-FDDGFTHL---------------NAMGPRLKN-PVAIQFIDEFGIQ--EAG
IDGGGLFKEF---LTSL----TRIAFDAEY--------GLFLSTNEQ-------------
----------------LLYPNPHS------------------------------------
-YARNETQLHHYEFLGRILGKALYEGILVDAAFAG--FFLSKWLG---------------
--------ERSYLDDLPS----------------------VDPELYQG--------LVYL
KNYKG----------------------------------------------DVESDLS--
--LNFTVVDNE---------------------------FGE-SRTIE-LITG--------
GSDIP-------------------------------------------------------
------------------------------VTKHNRI-NYIYK-MVNYRLNVQ-IGLQCQ
-AFFRG---LIDLIEP--KWLK-YFEQQ-ELQILVGG-----------------------
---AYIPIN-----------IADL----QQNTVYSD-YKDD-------------------
------------------------DPVI-INFWKVVS--------------------EFS
EEQKQKLIKF-------VTSCSRPP------------LLG-----FK-ELNPKF--SIRR
AGTG------------------------------ARLPSSSTCV-NL-LKLPAY-PDENT
LRQKLS-YAINA-DVGF
>Absidia_caerulea_GFAW01016702.1 .
ARIRRDHV-FEDGFNQL---------------YKLGPDLKK-RIAILFIDSFGME--ESG
IDGGGVFKEF---LTSL----GHEAFDTDY--------GLFASTPEQ-------------
----------------LLYPNPGS------------------------------------
-FATEASQLAFFEFLGLIIGKALYEGILLDVAFAE--FFLKKCLG---------------
--------KTNYLDDLPS----------------------LDPELYKG--------LIQV
KNFNG----------------------------------------------NVE-DLC--
--LDFTLTENG---------------------------NQ--SRSIN-LIPN--------
GSNIQ-------------------------------------------------------
------------------------------VTNENRI-RYVYL-VANYRLNIQ-IARQCK
-AFFSG---LSTIVDT--KWLR-MFNQQ-ELQVMLGG-----------------------
---ASIPID-----------LQDL----RRHCVYAD-YQDT-------------------
------------------------DPTI-KYFWQALS--------------------SFT
NDERMKFVKF-------VTSCSRPP------------VLG-----FK-ELQPQL--CIRR
AGTDE-----------------------------SRLPTSSTCV-NL-LKLPAF-TTYQG
LREKLL-YAINA-DAGF
>Phycomyces_blakesleeanus_XM_018427911.1 .
VTIHRNNV-LEDGFTQL---------------YPLGANLKK-RIAISFVDEFGLL--EAG
IDGGGVFKEF---LTCL----GHEAFDTNY--------GLFLATPDQ-------------
----------------LLYPNPSA----------------------------------LI
SNESLAEKLVFYEFLGLIIGKALYEGILLDVAFAE--FFLKRCLG---------------
--------KVNYLDDLPS----------------------LDPELYKG--------LIEV
KNFQG----------------------------------------------NVE-DLC--
--LDFSLAETA---------------------------DGK-SKVIE-LIPG--------
GSDIA-------------------------------------------------------
------------------------------VTEKNRI-RYVHL-VANYRLNVQ-IAKQCK
```

-AFFRG---LSTIVDI--KWLR-MFNER-ELQILLGG-----------------------
---ASVPID-----------IEDL----RRHTVYAG-YREN-------------------
------------------------DPTV-KDLWKALE--------------------SFD
NTQRMKFVKF-------VTSCSRPP------------LLG-----FK-ELRPQL--CIRE
AGSDS-----------------------------GRLPTSSTCV-NL-LKLPKF-ASYNI
LRQKLL-YAINA-DAGF
>Rhizopus_oryzae_GDUK01016278.1 .
ATIRRNHI-FEDGFNAF---------------HKLESALKD-KVAISFVDEFGLE--EAG
IDGGGVFKEF---LTGL----SHDAFNVNY--------GLFVATPEQ-------------
----------------LLYPNPNS------------------------------------
-FATEPLQLEYFRFLGLIIGKAVYEGILLDIPFAS--FFLKKCLG---------------
--------KVNYLDDLSS----------------------LDPELYRG--------LLTL
KNYDG--NVE-DLS--
--LDFTITHDE---------------------------LGK-SKTVE-LIPN--------
GSQIA---
------------------------------VTNQNRI-QYIYL-VANYRLNIQ-IAKQCR
-AFFKG---LSTIVDI--KWLR-MFNEQ-ELQVLLGG-----------------------
---ASIPID-----------LDDL----RANTVLAG-YMEH-------------------
------------------------DATV-QNFWKVLE--------------------SFD
NTLRMKFVKF-------VTSCSRPP------------LLG-----FK-ELVPKF--CIRN
AGVDD-----------------------------ERLPTSSTCV-NL-LKLPNF-SSFER
LKE--------------
>Mucor_indicus_JNEK01001778.1 .
ITVRRDHI-FEDGFNSL---------------HSLGAELKN-KVAISFVDEFGLE--EAG
IDGGGVFKEF---LTGL----SKEAFDKNY--------GLFTTTPEQ-------------
----------------LLYPNPTL---------------------QAT-ERKSKDGFWSC
RCYVLAEQLEHFQFLGLIIGKAVYEGILLDVAFAE--FFLKKCLG---------------
--------QVNXVDDLSS----------------------LDPDLYKG--------LLAL
KDLEG--NIE-DLS--
--LDFTSTHIE---------------------------NGR-SITVD-LIPQ--------
GSNVP---
------------------------------VTNQNRI-QYIYS-MANYRLNTQ-IAKQCR
-AFFRG---LSTIIDI--KWLR-MFNQQ-ELQILLGG-----------------------
---ASVPID-----------LNDL----RQHTVLAG-YMDH-------------------
------------------------DPTV-KNFWKVME--------------------SFD
NDLRMKFVKF-------VTSCSRPP------------LLG-----FK-ELVPQF--CLRN
AGADD-----------------------------ERLPTSSTCV-NL-LKLPNY-SSYRI
LKE--------------
>Mucor_irregularis_GFBC01025636.1 .
ATVRRDHI-FEDGFASL---------------NGLDVELKN-KVAISFVDQWGLE--EAG
IDGGGVFKEF---LTCL----SLEAFNTNY--------GLFVATPDQ-------------
----------------LLYPNPSS------------------------------------
-FATEPRQLEYFRFLGLVIGKAVYEGILLDVPFAE--FFLKKCLG---------------
--------KVNYLDDLAS----------------------LDPELYKG--------LLSL
KNYDG--NVE-DLS--
--LDFTSTHLE---------------------------NGK-SVVVN-LIPN--------
GSNIP---
------------------------------VTNQNRI-QYIYL-IANYRLNVQ-IAKQCR
-AFFRG---LSTIVDI--KWLR-MFNQQ-ELQILLGG-----------------------
---ASVPID-----------LADL----RAHTVLGG-YMEH-------------------
------------------------DATV-KNFWKALE--------------------SFD
NPLRMKFVKF-------VTSCSRPP------------LLG-----FK-ELVPKF--SLRN
AGVDD-----------------------------ERFPTSSTCV-NL-LKLPNY-SSYET
LREKLL-YAINA-DAGF
>Parasitella_parasitica_CCXP01001298.1 .
ATVRRDHM-FEDGFDQL---------------NSLGVELKN-KVAISFVDEFGLE--EAG
IDGGGVFKEF---LTGL----GKEAFNIDY--------GLFTATPEQ-------------
----------------LLYPNPSS-----------------YATERKF-FRLTGAVESDI

RFSFVAQQLEYFKFLGLIIGKAVYEGILLDVAFAE--FFLKKCLG---------------
--------KVNXVDDLSS----------------------LDPELYKG--------LSAV
KNYNG---NVE-DLS--
--LDFTSTHME---------------------------NGR-SINVE-LIPG--------
GSNIP---
------------------------------VTNQNRI-QYIYS-IANYRLNIQ-IAKQCR
-AFFKG---LSTIIDN--KWLR-MFNQQ-ELQIVLGG-----------------------
---ASVPID-----------LDDL----RSHTVLAG-FMEH-------------------
------------------------DATV-KNFWKALE--------------------SFD
NNLRMKFVKF-------VTSCSRPP------------LLG-----FK-ELVPQF--CLRN
AGVDE-----------------------------ERLPTSSTCV-NL-LKLPNY-PTYQI
LREXLL-YAITA-DAGF
>Pneumocystis_carinii_XM_018371944.1 .
VTIRRNNI-FNDGFESL---------------YTIGKDIKK-PINIVFIDQYGLP--EVG
IDGGGITKEF---LTCI----CKQALDVNF--------GLFHETSEH-------------
----------------MLYPNPHS------------------------------------
-YACESSQLQCFEFLGKLIGKCIYESVLLDVTFAP--FFLTKILG---------------
--------KKSYLDDLVI----------------------LDFELYKG--------LMFL
KRYTG---DVQNDFS--
--LNFTIIEQE---------------------------FGK-TTTIE-LIPG--------
GSDIS---
------------------------------VTNTNRL-QYIYT-MADYRLNKV-ISKQSY
-AFAKG---LFDIIDI--KWLS-MFSSQ-ELQKIIGG-----------------------
---SSLPID-----------IDDL----RNNSVYGG-FHDN-------------------
------------------------DPTI-ELFWSVLY--------------------EFS
VFERQTFLKF-------VTSVSRPP------------LLG-----FK-DLKPLF--CIRD
GGDDT-----------------------------NRLPTASTCI-NL-LILPRY-NDKAT
MKR--------------
>Pneumocystis_murina_XM_007876866.1 .
VTIRRNNI-FDDGFESL---------------YTIGKDIKK-PINIVFIDRYGLP--EVG
IDGGGITKEF---LTCI----CKQALDVNF--------GLFYETSEH-------------
----------------LLYPNPHS------------------------------------
-YACESSQLQCFEFLGRLIGKCIYESVLLDVTFAP--FFLTRILG---------------
--------KKSYLDDLVI----------------------LDFELYRG--------LMLL
KQYTG---DVQNDFS--
--LNFTIIEQE---------------------------FGK-TTTIE-LIPG--------
GSDIS---
------------------------------VTNTNRL-QYIYT-MADYRLNKV-ISKQSC
-AFAKG---LFDIIDI--KWLS-MFSSQ-ELQKIIGG-----------------------
---SSLPID-----------IDDL----RSNSVYGG-FHDD-------------------
------------------------DPTI-ELFWSVLR--------------------EFS
VFERQAFLKF-------VTSVSRPP------------LLG-----FK-DLKPLF--CIRD
GGNDT-----------------------------NRLPTASTCV-NL-LILPRY-SDRAT
MKSKLL-YAINF-CAGF
>Pneumocystis_jirovecii_XM_018375020.1 .
VTIRRDNI-FDDGFATL---------------YSIGKDIKK-PISIIFVDRYGLP--EVG
IDGGGITKEF---LVSI----CKQAFDIDF--------GLFCETHEH-------------
----------------LLYPNPHS------------------------------------
-YAREPAQLLCFEFLGRLVGKCIYETILLDVTFAP--FFLMKLLG---------------
--------KMSHLDDLFV----------------------LDPELYKG--------LIFL
KRYTG---DVENDLF--
--LNFTVIEQE---------------------------FGK-SITVE-LVAD--------
GSNVS---
------------------------------VTKKNRL-KYIYT-LADYRLNKV-ILKQSN
-AFLKG---LSEIINI--KWLS-MFSCQ-ELQNLIGG-----------------------
---SSVPID-----------INDL----RNNSVYGG-FQDD-------------------
------------------------DPTI-ELFWSVVS--------------------EFS
DIERRLLLKF-------VTSVSRPP------------LLG-----FK-DLRPLF--CIRN

GGDDI-----------------------------NRLPTASTCV-NL-LILPRY-SDRTI
MKSKLL-YAINF-GAGF
>Schizosacch_cryophilus_XM_013169100.1 .
AVIRRNRI-FDDGFDAF---------------YNLGKMFKG-SIRITFVDEHGVV--EEG
IDGGGLTKEF---LTSI----CKTVFDINY--------GLFSETKAH-------------
----------------LLYPNTHA------------------------------------
-YAQDIERLRCYEFLGMLIGKCIYEGIQIDAAFAP--FFVSKWLG---------------
--------HPSYFDDLTS----------------------LDNNLYEG--------LIFL
KHYDG--DVENDLA--
--LNFTVVHEE---------------------------FGV-RNVIE-LIPN--------
GGNVA---
------------------------------VTNDNRL-QYIHL-ISNYYLNAR-LSKQCR
-AFTNG---FTQIIDP--HWLA-MFHEN-EIQVLVGG-----------------------
---DPVPID-----------VSDL----KKHTVYAGGYELN-------------------
------------------------SPTI-QLFWEVLR--------------------ELD
EEDKRNFVKF-------VTSVARPP------------ILG-----FK-ALMPSF--CIRT
NGEDE-----------------------------SRLPTASTCV-NL-LKLPVY-TSKKT
LKEKLI-VAVRS-GGGF
>Schizosacch_octosporus_XM_013160858.1 .
AVIRRNRI-FDDGFDAF---------------FNLGKMFKG-TIRITFVDEHGVV--EEG
IDGGGLTKEF---LTSI----CKTVFDINY--------GLFSETKAH-------------
----------------LLYPNTHG------------------------------------
-YAQDIERLRCYEFLGMLIGKCIYEGIQIDAAFAA--FFVSKWLG---------------
--------HPSYFDDLTS----------------------LDNNLYEG--------LIFL
KNYDG--DVENDLA--
--LNFTVVHEE---------------------------FGV-RNVIE-LIPN--------
GGNVA---
------------------------------VTNDNRL-QYIHL-ISNYYLNAR-LSKQCR
-AFTNG---FTQIIDP--HWLA-MFHEN-EIQVLVGG-----------------------
---DPVPID-----------VNDL----KKNTVYAGGYELK-------------------
------------------------SPTI-QMFWEVLR--------------------ELD
EEDKRNFVKF-------VTSVARPP------------ILG-----FK-ALMPAF--CIRT
NGEDE-----------------------------SRLPTASTCV-NL-LKLPIY-TSKQT
LKEKLL-VSVRS-GGGF
>Schizosacch_pombe_NM_001018810.2 .
AVIRRNRI-FDDGFDAF---------------YNFGKLLKG-PIRITFVDEHGVV--EEG
IDGGGLTKEF---LTSI----CKTVFDINY--------GLFSETKAH-------------
----------------LLYPNTHA------------------------------------
-YAQDVERLRCYEFLGMLIGKCIYEGIQIDAAFAS--FFVAKWLG---------------
--------HPSYFDDLTS----------------------LDPNLYEG--------LVFL
KNYDG--DVENDMA--
--LNFTVVHEE---------------------------FGV-RNVID-LIPN--------
GSNIS---
------------------------------VTNENRL-QYIHL-VSNYYLNAR-LSRQCR
-AFTNG---FTQIIDP--HWLA-MFHES-EIQILVGG-----------------------
---DPVPID-----------IDDL----RRHTVYAGGYEPN-------------------
------------------------SPTI-VLFWEVLR--------------------EFE
EEDKRSFVKF-------VTSVARPP------------ILG-----FK-ALMPSF--CIRV
NGEDE-----------------------------TRLPTASTCV-NL-LKLPMY-STKQT
LRDKLL-TAVRS-GVGF
>Schizosacch_japonicus_XM_002175501.2 .
VIIRRNRI-FDDGYDAF---------------HTFGPLFKG-PIKITFVDEHGVV--EEG
IDGGGLTKEF---LTSI----CRTVFDTNY--------GLFSETFAN-------------
----------------LLYPSTKA------------------------------------
-YAQDPERLHYYEFLGMLIGKCIYEGIQIDAAFAP--FFIAKWLG---------------
--------HPSHIDDLAA----------------------LDLKLYEG--------LAFL
KHYKG--DVENDLS--
--LSFTVVHEE---------------------------FGV-KEEID-LIPN--------

GKNIS---
------------------------------VTEANRL-QYIHL-VSNYYLNAR-LSKQSK
-AFTQG---FTQLINP--HWLA-MFHEN-ELQLLVGG-----------------------
---APVSID-----------VDDL----RKHTVYAGEFDEN-------------------
------------------------SPTI-LFFWQVLR--------------------EFS
EDDKRALLKF-------VTSVARPP------------ILG-----FK-ELNPLF--CIRS
NGNDE-----------------------------TRLPTASTCV-NL-LKLPVY-GSKQI
LRDKLS-IAIQA-NTGF
>Saitoella_complicata_XM_019170150.1 .
ADIRRDRV-FEDAFNQI---------------GSMGPRLKG-PLSVTFFDSYGMA--EAG
IDGGGVTKEF---LTAV----CRQAFDANY--------GLFKETKEN-------------
----------------LLYPNPHA------------------------------------
-FSREKTQLAYYEFLGKIIGKCLYEGILVDVAFAP--FFLLKLVG---------------
--------RSSYLDDLPG----------------------LDPELYQG--------LVFL
KQYTG--DVESDLA--
--LNFTVTNND---------------------------MGA-AKQVE-LLPG--------
GADIA---
------------------------------VTRANRL-QYIYL-VSNYRLNTQ-LAHQSR
-AFVRG---LSEMVDK--KWLA-MFDQA-ELQTLIGG-----------------------
---APVPIN-----------VADL----KRNTDYGG-YLEK-------------------
------------------------DDTI-KYFWEVME--------------------EFA
DCDRRELVKF-------VTSVARPP------------LLG-----FK-ELNPKF--GIRD
AGGDE-----------------------------SRLPTASTCV-NL-LKLPRY-RSKQV
LKEKML-YAIRS-GAGF
>Taphrina_populina_BAVX01000010.1 .
VVCRRGNE-FLDGFEQL---------------WPLGGKLKE-NLAVDFKDVHGMP--EAG
IDGGGLTKEF---LTAL----CKQAFRPES--------GLFVETEGH-------------
----------------LLYPNPNP------------------------------------
-MFKSPSHLQRYEFLGRVIAKCIYEGILLDVEFAP--FFVLKWIH---------------
--------KSVYLDDLKA----------------------LDVSLYNG--------LVYL
KQHQG--EVENELA--
--LEFMIDEEF---------------------------NGR-RYRIP-IGES--------
KQRNR---
-----------------------------IVTSENRA-EYIEE-VCKYKLSTR-IEKQTS
-AFVEG---MSQILDL--RWFG-MFETS-EMQALVGG-----------------------
---NPVPID-----------VDNL----KLHTVYAG-YHDS-------------------
------------------------DNTV-RIFWDVLR--------------------EFS
NEERRKFVKF-------VTSTPRPP------------LQG-----FS-VLEPKF--ALHN
AGADE-----------------------------QRLPTASTCV-NL-LKLPAY-STHDQ
MRLKLR-LSINA-EAGF
>Taphrina_deformans_BAVV01000055.1 .
VVCRREHE-FEDGFGAL---------------WQIGGGIKG-PLSVDYRDIHGMQ--EAG
IDGGGLTKEF---LTAV----CKQAFHPSF--------GLFIETPER-------------
----------------SLYPNPAS------------------------------------
-SSRTPEKLQQYEFLGRIIAKCIYAGILVDVTFAP--FFLLNWVG---------------
--------KSAYLDDLQA----------------------MDKDLYTG--------LIQL
KNYTG--DVENDLS--
--LDFTVNEQKG--------------------------SGR-KATVN-LLPD--------
GSQVP---
------------------------------VTRANRL-QYIHL-ICHYKLNAR-LSKQSK
-AFVRG---MADVLDL--RWLG-MFNQT-EMQALVGG-----------------------
---APVPID-----------IDDL----RRNTVYGG-FDEQ-------------------
------------------------DPAV-RIFWDVVR--------------------EFD
NEERRKLVKF-------VTSTPRPP------------LLG-----FK-ELNPKF--SLRN
AGSDV-----------------------------QRLPTASTCV-NL-LKLPNY-TTHEQ
MRRKLR-LSITA-EAGF
>Taphrina_flavorubra_BAVW01000018.1 .
VVCRRDHE-FEDGFEAL---------------WPTGGGIKG-PLSVDYRDVHGMK--EAG

IDGGGLTKEF---LTAV----CKQAFHPSF--------GLFIETPER-------------
----------------SLYPNPAP------------------------------------
-ISKTPQKLQQYEFLGRIIAKCIYAGILVDVTFAP--FFLLNWVG---------------
--------KSAYLDDLQA----------------------LDKDLYTG--------LIQL
KNYTG--DVENDLS--
--LDFTVNEQDGQ-------------------------GGV-QSTVN-LVPQ--------
GNQVP---
------------------------------VTRANRL-QYIHL-VCHYKLNAR-VSRQSK
-AFVQG---MSEVLDL--RWLG-MFNTN-EMQALVGG-----------------------
---APVPID-----------IDDL----RRNTVYGG-FDER-------------------
------------------------DDAV-RIFWDVVR--------------------EFD
NEERRLLVKF-------VTSTPRPP------------LLG-----FK-ELVPKF--SLRN
AGSDV-----------------------------QRLPTASTCG-D--------------

>Taphrina_wiesneri_BAVU01000078.1 .
VVCRREHE-FEDGFEAL---------------WPVGGGIKG-PLSVDYRDVHGLQ--EAG
IDGGGLTKEF---LTAV----CKQAFHPSF--------GLFIETPER-------------
----------------SLYPNPAL------------------------------------
-TSRTPQKLQQYEFLGRIIAKCIYAGILVDVTFAP--FFLLNWVG---------------
--------KSAYLDDLQA----------------------LDKDLYTG--------LIQL
KNYTG--DVENDLS--
--LDFTVNEEEE--------------------------GGR-QVTIN-LLPQ--------
GNEIP---
------------------------------VTRANRL-QYIHL-MCHYKLNAR-LSRQSK
-AFVQG---MSSVLDL--RWLG-MFNQT-EMQALVGG-----------------------
---SPVPID-----------IEDL----RRNTVYGG-FDEQ-------------------
------------------------DAAV-GIFWDVVR--------------------GFD
NDERRKLVKF-------VTSTPRPP------------LLG-----FK-ELNPKF--SLRN
AGSDV-----------------------------QRLPTASTCV-NL-LKLPNY-TTHEQ
MRKKLR-LSITA-EAGF
>Entomophthora_muscae_GEND01016789.1 .
VNIRRDNV-FEDGFAQL---------------NPLGSGLKG-RVSITMVSSLGMP--EAG
IDGGGVFKEF---LNAL----IKQAFAPET--------DLFLTTPAE-------------
----------------LLYPSPHR------------------------------------
-ANREHPRLRQYEFLGRMVGKALFEGILIDASFAR--FFLSKWLG---------------
--------RRSFLDDLPS----------------------LDDELYAG--------LMSL
LRYDG--DPE-DMA--
--LNFTLVDREP--------------------------SG--PVNVE-LVRG--------
GEKLP---
------------------------------VTAANKF-EYXRL-VADYRLNIQ-LQRQSR
-AFLGG---LIDILPP--RWLQ-MFDPA-ELQMVISG-----------------------
---GRQPID-----------VQEL----QTHITYSGVYHAE-------------------
------------------------HPTI-VAFWSVVH--------------------GLE
TDTLALLIKF-------ITSCSRPP------------LQG-----FK-ALSPPM--CIRD
SGRDD-----------------------------GRLPTASTCV-NL-LKLPIY-SSPEA
LKNKLL-YAIHS-ESGF
>Linderina_pennispora_MCFD01000007.1 .
---------------------------------------KR-RMHIEFIDRYGMP--EAG
IDGGGVFKEF---LTSL----VREAFDPKL--------GLFNTTPQN-------------
----------------NLYPSPSA----------------------------------LD
DAEQRILTLDKLRFLGAVIGKALYEGVLVDAPFAL--FFLIRWMN---------------
--------QLPGFNDLPT----------------------LDEDLYRG--------LVNL
KNYPVG---DAT-XFG--
--LDFTITGSTR--------------------------NGK-TRTVP-LVPR--------
GDRLK---
------------------------------VTSQNRL-LYVDL-VAQYRLVKQ-IDAPVN
-AFVAG---LHSVIPP--VWLTLLFASPLELSHLLCG-----------------------
---NANAID-----------IDDW----KRNTVYEGAYKAAATK----------------

------------------------HPTI-VNFWSVVE-------------------HDLT
ERQRSELCRF-------ATSCERPP------------LLG-----FG-ELTPRF--XLRP
SEEQN-----------------------------ARLPSASTCV-NL-LKLPIY-SSRGV
LREKLV-TAIES-GAG-
>Neosartorya_fischeri_XM_001265242.1 .
ANIRRESV-FEDAFDQF---------------YELGEGLKE-PIQISFIDKFNTV--EAG
IDGGGVTKEF---LTSV----TNEAFKSGSEP------KLFEENDQH-------------
----------------LLYPNPAA----------------VEQRREVL-RQLGFVENSPE
WNEKVRDLLRRYEFLGRIIGKCLYEGILVDVNFAS--FFLLKWAL-----------TGGA
GSAQRETAYRANLNDLKD----------------------LDQGLYQG--------LLQL
KNYTG--DVE-DFA--
--LNFTVTDTIPLPNG----------------------GTR-TVTRD-LKSN--------
GSDIP---
------------------------------VTNQNRL-VYISY-IARYRLQVQ-PALQTN
-AFLQG---LGHIIQP--SWLS-MFNQS-ELQTLVSG-----------------------
---ESGDID-----------VADL----RRNTLYGGVYTIGDDKEE--------------
------------------------HPTV-KLFWQVME--------------------EMS
NEERQKVLRF-------VTSTPRAP------------LLG-----FS-HLNPRF--SIRD
SSEDQ-----------------------------DRLPSTSTCV-NL-LKLPRY-TNAKV
LREKLL-YAINS-GAGF
>Aspergillus_fumigatus_XM_745075.1 .
ANIRRESV-FEDAFDQF---------------YELGEGLKE-PIQISFIDKFNTV--EAG
IDGGGVTKEF---LTSV----TNEAFKSGSEP------KLFEENDQH-------------
----------------LLYPNPAA----------------VEQRREVL-RQLGFVENSPE
WNEQVRDLLRRYEFLGRIIGKCLYEGILVDVNFAP--FFLLKWAL-----------TGGA
GSAQRETAYRANLNDLKD----------------------LDQGLYQG--------LLQL
KNYTG--DVE-DFA--
--LNFTVTDTIPLPNG----------------------GTR-TVTQD-LKSN--------
GSDIP---
------------------------------VTNQNRL-VYISY-IARYRLQVQ-PALQTN
-AFLQG---LGHIIQP--SWLS-MFNQS-ELQTLVSG-----------------------
---ESGDID-----------VADL----RRNTLYGGVYTIGDDKEE--------------
------------------------HPTV-KLFWQVME--------------------EMS
NEERQKVLRF-------VTSTPRAP------------LLG-----FS-HLNPRF--SIRD
SSEDQ-----------------------------DRLPSTSTCV-NL-LKLPRY-TNAKV
LREKLL-YAINS-GAGF
>Aspergillus_clavatus_XM_001269718.1 .
ANIRRESV-FEDAFEQF---------------YELGEGLKE-PIQISFIDKFDTV--EAG
IDGGGVTKEF---LTSV----TTEAFKAGSGP------KLFEENDQH-------------
----------------LLYPNPAA----------------VEQRREAL-RQLGFVENSPE
WNERVRDLLRRYEFLGRIIGKCLYEGILVDVNFAP--FFLLKWAL-----------TGGV
GSAQRETAYRANLNDLKD----------------------LDQGLYQG--------LLQL
KNYPG--DVE-DFA--
--LNFTVSDTIPLPDG----------------------GSR-TVTRD-LKSH--------
GSDIA---
------------------------------VTNQNRL-VYISY-IARYRLQVQ-PALQTH
-AFLQG---LGHIIQP--SWLS-MFNQA-ELQTLVSG-----------------------
---ESGDID-----------VADL----RRNTLYGGVYTIGDDKEE--------------
------------------------HPTV-KLFWEVME--------------------KMS
NEERQKVLRF-------VTSTPRAP------------LLG-----FS-HLNPRF--SIRD
SSEDQ-----------------------------ERLPSTSTCV-NL-LKLPRY-SSAKV
LREKLL-YAINS-GAGF
>Aspergillus_niger_XM_001388703.2 .
ANIRRESI-FKDAFDEF---------------YELGDGLKE-PIQITFIDKFNTT--EAG
IDGGGVTKEF---LTSV----TSEAFKSTSDL------NLFEENDQH-------------
----------------LLYPNPAA----------------VEQRRELL-RQLGFVENTAE
WNENVRDLLRRYEFLGRIIGKCLYEGILVDVNFAP--FFLLKWAL-----------TGGT
GSAQRETAYRANLNDLKD----------------------LDQGLYQG--------LLQL

KNYPG--DVE-DFS--
--LNFTVTDTIPLPDG----------------------GNR-TTTRD-LKSH--------
GSDIP---
------------------------------VTNQNRL-VYISY-IARYRLQVQ-PALQTN
-AFLQG---LGQIIQP--SWLS-MFNQT-ELQTLVSG-----------------------
---DSGDID-----------VADL----RRNTLYGGVYTIGDDKEE--------------
------------------------HPTV-KLFWEVME--------------------KMS
NEERQKVLRF-------VTSTPRAP------------LLG-----FS-HLNPRF--SIRD
SSEDQ-----------------------------ERLPSTSTCV-NL-LKLPRY-TNANI
LREKLL-YAVNS-GAGF
>Aspergillus_nidulans_XM_656511.1 .
ANIRRESL-FDDAFDQF---------------YELGEGLKE-PIQITFIDKFNAP--EAG
IDGGGVTKEF---LTSV----TNEAFKSTVGL------RLFEENDQH-------------
----------------LLYPSPVA----------------VEQRKENL-RQLGLKENTPE
WNDQVRDLLRRYEFLGRVIGKCLYEGILVDVSFAP--FFLLKWAL-----------TGGT
GSAQRETAYRANLNDLKD----------------------LDSGLYQG--------LLQL
KNYPG--DVE-DFS--
--LNFTVTDTIPLPDG----------------------GSR-TITRD-LKSN--------
GSDTA---
------------------------------VTNQNRL-VYISY-IARYRLQVQ-PALQTN
-AFLQG---LGQIIQP--SWLS-MFNQS-ELQTLVSG-----------------------
---ESGDID-----------VQDL----RRNTLYGGVYTIGDDKEE--------------
------------------------HPTI-KLFWEVLE--------------------QMT
NEERRKVLRF-------VTSTPRAP------------LLG-----FS-HLNPRF--SIRD
SSEDD-----------------------------QRLPSTSTCV-NL-LKLPRY-KSAST
LREKLL-YAVNS-GAGF
>Aspergillus_aculeatus_XM_020204566.1 .
ANIRRDSV-FEDAFEEF---------------YELGEGLKE-PIQITFIDKWNTA--EAG
IDGGGVTKEF---LTSV----TSEAFKSRNEM------SLFEENDQH-------------
----------------LLYPNPTA----------------VEQRKELL-RQVGFVENSPD
WNEKVRELLRRYEFLGRIIGKCMYEGILVDVNFAP--FFLLKWAL-----------TGGA
GSAQRESAYRANLNDLKD----------------------LDQGLYQG--------LLQL
KNYPG--DVE-DFS--
--LNFTITDTIPLPGS----------------------GSR-TITRD-LKSQ--------
GSDIP---
------------------------------VTNQNRL-VYISY-IARYRLQVQ-PALQTN
-AFLQG---LGQIIQP--SWLS-MFNQT-ELQTLVSG-----------------------
---DRGDID-----------VADL----RRNTLYGGVYTIGDDNEE--------------
------------------------HPTI-KLFWEVME--------------------KMT
NEQRQNVLRF-------VTSTPRAP------------LLG-----FS-HLNPRF--SIRD
SSEDQ-----------------------------ERLPSTSTCV-NL-LKLPRY-SNADV
LREKLL-YAVNS-GAGF
>Aspergillus_terreus_XM_001212966.1 .
ANIRRESV-FEDAFDQF---------------YELGEGLKE-PIQITFIDKFNTA--EAG
IDGGGVTKEF---LTSV----TNEAFRSTNDS------NLFEENDQH-------------
----------------LLYPNPAA----------------VERQRHLL-RQLGFTEGSPE
WNDGIRELLRRYEFLGRVIGKCLYEGILVDVNFAP--FFLLKWAL-----------TGGT
GSAQRETAYRANLNDLKD----------------------LDQGLYQG--------LLQL
KNYPG--DVE-DFA--
--LNFTVTDTIPLAES----------------------ESR-TVTRD-LKPH--------
GSEIP---
------------------------------VTNQNRL-VYISY-IARYRLQVQ-PALQTN
-AFLQG---LGQIIQP--SWLS-MFNQS-ELQTLVRG-----------------------
---ESGDVD-----------VADL----RRNTLYGGVYSIGDDGKE--------------
------------------------HPTV-QLFWEVME--------------------KMT
NEERQKVLRF-------VTSTPRAP------------LLG-----FS-HLNPRF--SIRD
SSDDQ-----------------------------ERLPSTSTCV-NL-LKLPRY-TDANT
LREKLL-YAVNS-GAGF

```
>Aspergillus_nomius_XM_015549578.1 .
ANIRRERV-FEDAFDQF---------------YELGESLKE-PIQITFIDKFNTP--EAG
IDGGGVTKEF---LTSV----TNEAFKSISDL------NLFEENEQH-------------
----------------LLYPNPAA----------------VEHRRELL-RQGGLAENGPD
WNDNIRDLLRRYEFLGRVIGKCLYEGILVDVNFAP--FFLLKWAL-----------TGGT
GSAQKETAYRANLNDLKD----------------------LDQGLYQG--------LLQL
KNYPG----------------------------------------------DVE-DFS--
--LNFTVTDIIPLSNG----------------------RSR-TITRD-LKPH--------
GSDIP-------------------------------------------------------
------------------------------VTNQNRL-VYISY-IARYRLQAQ-PALQTN
-AFLQG---LGQIIQP--SWLS-MFNQS-ELQTLVSG-----------------------
---ESGDID-----------VSDL----RRNTQYGGVYTIGDDREE--------------
------------------------HPTI-QLFWEVMH--------------------KMT
NEERQKVLRF-------VTSTPRAP------------LLG-----FS-HLNPRF--SIRD
SSEDQ-----------------------------ERLPSTSTCV-NL-LKLPRY-TNADI
LREKLL-YAINS-GAGF
>Aspergillus_sojae_BACA01000970.1 .
ANIRRESV-FKDAFDQF---------------YELGEGLKE-PIQITFIDKFNTP--EAG
IDGGGVTKEF---LTSV----TNEAFKSISDL------NLFEENEQH-------------
----------------LLYPNPAA----------------VEYRRELL-RQVGLAENSPD
WNDNIRDLLRRYEFLGRVIGKCLYEGILVDVNFAP--FFLLKWAL-----------TGGT
GSAQKETAYRANLNDLKD----------------------LDQGLYQG--------LVX-
------------------------------------------------------------
--LNFTVTDIIPLSDG----------------------RSR-TITRD-LKPH--------
GSDIP-------------------------------------------------------
------------------------------VTNQNRL-VYISY-IARYRLQAQ-PALQTN
-AFLQG---LGQIIQP--SWLS-MFNQS-ELQTLVSG-----------------------
---ESGDID-----------VSDL----RRNTQYGGVYTIGDNREE--------------
------------------------HPTI-RLFWEVMH--------------------KMT
NEERQKVLRF-------VTSTPRAP------------LLG-----FS-HLNPRF--SIRD
SSEDQ-----------------------------ERLPSTSTCV-NL-LKLPRY-TNANT
LREKLL-YAINS-GAGF
>Aspergillus_flavus_XM_002374361.1 .
ANIRRESV-FKDAFDQF---------------YELGEGLKE-PIQITFIDKFNTP--EAG
IDGGGVTKEF---LTSV----TNEAFKSISDL------NLFEENEQH-------------
----------------LLYPNPAA----------------VEHRRELL-RQVGLAENSPD
WNDNIRDLLRRYEFLGRVIGKCLYEGILVDVNFAP--FFLLKWAL-----------TGGT
GSAQKETAYRANLNDLKD----------------------LDQGLYQG--------LLQL
KNYPG----------------------------------------------DVE-DFS--
--LNFTVTDIIPLSDG----------------------RSR-TITRD-LKPH--------
GSDIP-------------------------------------------------------
------------------------------VTNQNRL-VYISY-IARYRLQAQ-PALQTN
-AFLQG---LGQIIQP--SWLS-MFNQS-ELQTLVSG-----------------------
---ESGDID-----------VSDL----RRNTQYGGVYTIGDDREE--------------
------------------------HPTI-QLFWEVMH--------------------KMT
NEERQKVLRF-------VTSTPRAP------------LLG-----FS-HLNPRF--SIRD
SSEDQ-----------------------------ERLPSTSTCV-NL-LKLPRY-TNANT
LREKLL-YAINS-GAGF
>Aspergillus_oryzae_XM_001819992.2 .
ANIRRESV-FKDAFDQF---------------YELGEGLKE-PIQITFIDKFNTP--EAG
IDGGGVTKEF---LTSV----TNEAFKSISDL------NLFEENEQH-------------
----------------LLYPNPAA----------------VEHRRELL-RQVGLAENSPD
WNDNIRDLLRRYEFLGRVIGKCLYEGILVDVNFAP--FFLLKWAL-----------TGGT
GSAQKETAYRANLNDLKD----------------------LDQGLYQG--------LLQL
KNYPG----------------------------------------------DVE-DFS--
--LNFTVTDIIPLSDG----------------------RSR-TITRD-LKPH--------
GSDIP-------------------------------------------------------
------------------------------VTNQNRL-VYISY-IARYRLQAQ-PALQTN
```

-AFLQG---LGQIIQP--SWLS-MFNQS-ELQTLVSG-----------------------
---ESGDID-----------VSDL----RRNTQYGGVYTIGDDREE--------------
------------------------HPTI-QLFWEVMH--------------------KMT
NEERQKVLRF-------VTSTPRAP------------LLG-----FS-HLNPRF--SIRD
SSEDQ-----------------------------ERLPSTSTCV-NL-LKLPRY-TNANT
LREKLL-YAINS-GAGF
>Rasamsonia_emersonii_XM_013470489.1 .
ANIRRDNV-FEDAFDQF---------------YDLGDGLKE-PIQISFIDKFGTV--EAG
IDGGGVTKEF---LTSV----TNEAFKSLDGL------NLFVENDQN-------------
----------------LLYPNPAA----------------VEQRKELL-REMGFVEGSPE
WNEQVRDLLRRYEFLGRVIGKCLYEGILVDVHFAG--FFLLKWAL-----------TGGL
SSAQKESAYRANLNDLRE----------------------LDEGLYQG--------LLQL
KNYPG--DVE-DFA--
--LNFTVTDTVPMPDG----------------------TTR-TITRE-LKPN--------
GSNIP---
------------------------------VTNQNRL-VYISY-IARHRLQVQ-PFYQTN
-AFLQG---LGQIIQP--SWLS-MFNQS-ELQTLVGG-----------------------
---DSGEID-----------VADL----RRNTLYGGVYSIGDDKEE--------------
------------------------HPTV-KLFWEVME--------------------GMT
NEERQKVLKF-------VTSTPRAP------------LLG-----FS-HLNPRF--SIRD
SSDDE-----------------------------ERLPSTSTCV-NL-LKLPRY-SNARI
LKEKLL-YAINA-GAGF
>Talaromyces_atroroseus_XM_020267709.1 .
ASIRRESV-FEDAFDQF---------------YELGEGLKE-PIQISFIDQFGSV--EAG
IDGGGVTKEF---LTSI----TNEAFMSTSGL------NLFIENDQN-------------
----------------LLYPNPAA----------------VEQRREVL-RELGLDENSLQ
FSDGVRDLLKRYEFLGRVIGKCLYEGILVDVHFAG--FFLLKWAL-----------TGGS
TSARRESAYRANLNDLRD----------------------LDEGLYQG--------LLQL
KNYTG--DVE-DFS--
--LNFTITDTIPIPGS----------------------KSR-IVTKE-LKPG--------
GANIA---
------------------------------VTNQNRL-VYISY-IARHRLQIQ-PAPQTT
-AFLKG---LGDIIQP--SWLS-MFNQS-ELQTLVGG-----------------------
---DAGEIN-----------VADL----RRNTLYGGVYTIGDDNEE--------------
------------------------HPTV-QLFWQVME--------------------ELS
NEERQRVLKF-------VTSTPRAP------------LLG-----FS-HLNPRF--SIRD
SSDDQ-----------------------------ERLPSTSTCV-NL-LKLPRY-STAQI
LRQKLL-YAVNS-GAGF
>Talaromyces_stipitatus_XM_002483937.1 .
ASIHRDSV-FDDAFEQF---------------YDLGEGLKE-PIQISFIDKFGAT--EAG
IDGGGVTKEF---LTSI----TNEAFMPSNGL------DLFVENDQN-------------
----------------LLYPNPVA----------------VEQRKEVL-RQMGCKEGSPE
FVDGVRDLLKRYEFLGRVIGKCLYEGILVDVHFAG--FFLLKWAL-----------TGGS
TSARRESAYRANLNDLRD----------------------LDEGLYQG--------LLQL
KNYPG--DVE-DFA--
--LNFTVTDTIPVPGS----------------------KPR-IITKE-LKPG--------
GANIS---
------------------------------VTNQNRL-VYISY-IARHRLQNQ-PAPQTN
-AFLKG---LGDIIQP--SWLS-MFNQS-ELQTLVGG-----------------------
---DAGEID-----------VADL----RRNTLYGGVYTIGDDNQE--------------
------------------------HPTI-QLFWQVMQ--------------------ELS
NEDRQKVLKF-------VTSTPRAP------------LLG-----FS-HLNPRF--SIRD
SSDDQ-----------------------------DRLPSTSTCV-NL-LKLPRY-SSAEV
LRQKLL-YAVNS-GAGF
>Penicillium_marneffei_XM_002150245.1 .
ASIHRESV-FEDAFDQF---------------YELGEGLKE-PIQISFIDKFGTT--EAG
IDGGGVTKEF---LTSI----TNEAFMPSNGL------DLFVENDQN-------------
----------------LLYPNPTA----------------VEQRKEIL-RQIGCTEGSPQ

FVDGVRDLLKRYEFLGRVVGKCLYEGILVDVHFAG--FFLLKWAL-----------TGGS
TSARRESAYRANLNDLRD----------------------LDEGLYQG--------LLQL
KNYPG--DVE-DFS--
--LNFTVTDTIPIPGS----------------------KPR-IITKD-LKPG--------
GANIA---
------------------------------VTNQNRL-VYISY-IARHRLQNQ-PAPQTN
-AFLKG---LGEIIQP--SWLS-MFNQS-ELQTLVGG-----------------------
---DAGEID-----------VADL----RRNTLYGGVYVIGDDNQE--------------
------------------------HPTI-QLFWQVMQ--------------------ELS
NEERQKVLKF-------VTSTPRAP------------LLG-----FS-HLNPRF--SIRD
SSDDQ------------------------------DRLPSTSTCV-NL-LKLPRY-SNAHT
LRQKLL-YAVNS-GAGF
>Talaromyces_amestolkiae_MIKG01000029.1 .
ASIHRESV-FDDAFDQF---------------YELGEGLKE-PIQISFIDKFGAT--EAG
IDGGGVTKEF---LTSI----TNEAFMPSNGPSNGL--DLFVENDQN-------------
----------------LLYPNPSA----------------VEQRKEVM-RQLGITEGSPQ
FVDGVRDLLKRYEFLGRVVGKCLYEGILVDVQFAG--FFLLKWAL-----------TGGS
TSARRESAYRANLNDLRD----------------------LDEGLYQG--------LVCL
XNYPG--DVE-DFS--
--LNFTVTDTIPIPGS----------------------KSR-IITKE-LKPG--------
GANIA---
------------------------------VTNQNRL-VYISY-IARHRLQNQ-PAPQTN
-AFLKG---LGDIIQP--SWLS-MFNQS-ELQTLVGG-----------------------
---DAGEID-----------VADL----RRNTLYGGVYTIGDDNQE--------------
------------------------HPTI-QLFWQVMQ--------------------DLS
NEERQKVLKF-------VTSTPRAP------------LLG-----FS-HLNPRF--SIRD
SSDDQ------------------------------DRLPSTSTCV-NL-LKLPRY-STAHI
LRQKLL-YAVNS-GAGF
>Penicillium_sclerotiorum_MJCA01000199.1 .
AEIRRDHI-FSDALEQF---------------YSLGDGLKE-PIQISFIDRFGSR--EAG
IDGGGVTKEF---LTSI----TNEAFQTSWG-------SMFAENDQH-------------
----------------LLYPNPTL----------------IEQCKEQL-RDLEMEEGGSP
WKDSIRMVLQRYEFLGRVIGKCLYEGILVDVHFAP--FFLLKWAL-----------TGGT
GSALKESSYRANINDLKD----------------------LDAGLYQG--------LVXL
KNYPG--NVE-DFG--
--LDFTVNDVIPLGDK----------------------PSR-TKTKE-LRPD--------
GSNIS---
------------------------------VTNQNRL-VYISY-IARYRLQVQ-PLQQTN
-AFLMG---LGQIIQP--SWLS-MFNQS-ELQTLVSG-----------------------
---EASSID-----------VEDL----RRNTLYGGIYVIGDDQQE--------------
------------------------HPTI-RLFWQVLH--------------------AMS
AEERQMVVRF-------VTSTPRAP------------LLG-----FS-HLNPRF--SIRD
SSTDE------------------------------ERLPTTSTCV-NL-LKLPRY-SSAET
LRTKLL-YAVSS-GAGF
>Penicillium_decumbens_MDYL01000008.1 .
ADIRREHV-FDDALSQF---------------YQLGDGLKE-PIQISFIDQFGSL--EAG
IDGGGVTKEF---LTSV----TSEAFKAESDF------SMFAENDQH-------------
----------------LLYPNPTA----------------LEQFREQL-LENGVSKSSPE
WQAGIRDLLRRYEFLGRVIGKCLYEGILVDVNFAP--FFLLKWAL-----------TGGT
GSAMKESSYRANLNDLKD----------------------LDAGLYQG--------LVXL
KNYSG--NVE-DFS--
--LDFTVTDTIPLPGS----------------------RRR-TVTKE-LRPG--------
GSSIP---
------------------------------VTNQNRL-VYISY-IARYRLQLQ-PLYQTN
-AFLQG---LGQMIQP--AWLS-MFNQA-ELQTLVSG-----------------------
---ENADID-----------VEDL----RRNTHCGGVYAVGDDNQE--------------
------------------------HPTI-QMFWQVMH--------------------EMS
NEERRKVVRF-------VTSTPRAP------------LLG-----FS-HLNPRF--SIRD

SGEEE-----------------------------DRLPSTSTCV-NL-LKLPQY-SSVEL
LRSKLL-YAVNS-GAGF
>Penicillium_janthinellum_GBSQ01009896.1 .
AEIRRENV-FEDAFDQF---------------YSLGEALKE-PIQISFIDRFGAM--EAG
IDGGGVTKEF---LTSI----TSEAFQTESDF------SMFVENDQH-------------
----------------LLYPNPTA----------------VEQCKERL-REAGVNESSLK
WSESIRDLLRRYEFLGRVIGKCLYEGILVEVNFAP--FFLLKWAL-----------TGGT
GSAMKESSYRANLNDLKD----------------------LDQGLYQG--------LLQL
KNYPG--DVE-DFS--
--LDFTVTDTIPMPGS----------------------ANR-TTTRE-LRYN--------
GSKTP---
------------------------------VTNQNRL-VYISY-IARYRLQVQ-PAMQTN
-AFLQG---LGQIIQP--AWLS-MFNQS-ELQTLISG-----------------------
---ETGDID-----------VEDL----RRNTQYGGVYTIGDDNQE--------------
------------------------HPTI-QLFWKVMH--------------------EMT
NEERQKVIRF-------VTSTPRAP------------LLG-----FS-HLNPRF--SIRD
SSDDQ-----------------------------ERLPSTSTCV-NL-LKLPRY-ATAET
LRSKLL-YAVSS-GAGF
>Penicillium_raistrickii_GFHR01000007.1 .
ADIRRGSV-FEDAFSQF---------------YPLGDGLKE-PIQISFIDQFGAM--EAG
IDGGGVTKEF---LTSV----TSQAFKTDDHE------SMFAENDHH-------------
----------------LLYPHPAA----------------VDQRKAIL-NEAGLSSSTIE
WQDEVRGLLRRYEFLGRVIGKCLYEGILVDVNFAP--FFLLKWAL-----------TGGS
RSAVKESSYRANLNDLKD----------------------LDEGLYQG--------LLQL
KNYPG--NVE-DFS--
--LDFTVNNTIPMPSG----------------------RNR-TATVE-LIPN--------
GSQTA---
------------------------------VTNKNRL-VYISY-MARYRLQLQ-PAMQTN
-AFLQG---LGQIIQP--AWLS-MFNQS-ELQTLVSG-----------------------
---DKADID-----------VEDL----RRNTLYGGVYVIGDDNME--------------
------------------------HPTI-ALFWQVMH--------------------QMN
NEERQKVIRF-------VTSTPRAP------------LLG-----FS-HLHPRF--SIRD
SSEDQ-----------------------------DRLPSTSTCV-NL-LKLPRY-SDAET
LRAKLL-YAVSS-GAGF
>Penicillium_chrysogenum_XM_002561175.1 .
ADIRRESV-FEDAFSQY---------------YGLGDGLKE-PIQISFIDQFGAM--EAG
IDGGGVTKEF---LTSV----TAEAFKTDDYE------SMFAENDHH-------------
----------------LLYPSPVA----------------VDQLKKVL-SEAGLTSSSPE
WQNDVRGLLRRYEFLGRIIGKCLYEGILVDVNFAP--FFLLKWAL-----------TGGS
RSAVKESSYRANLNDLKD----------------------LDEGLYQG--------LLQL
KNYPG--DVE-DFG--
--LDFTINNVIRMPGG----------------------QNR-TVTAE-LKPS--------
GSQTP---
------------------------------VTSKNRL-VYISY-VARYRLQLQ-PALQTN
-AFLQG---LGQIIQP--AWLS-MFNQS-ELQTLVSG-----------------------
---DKADID-----------VEDL----RRNTLYGGVYVIGDDNLE--------------
------------------------HPTI-ALFWQVMH--------------------EMT
NEERQKVIRF-------VTSTPRAP------------LLG-----FS-HLRPHF--SIRD
SSDDQ-----------------------------ERLPSTSTCV-NL-LKLPRY-SDADT
LRSKLL-YAVSS-GAGF
>Penicillium_expansum_XM_016744442.1 .
ADIRRESV-FEDAFSQY---------------YGLGDGLKE-PIQISFIDQFGAM--EAG
IDGGGVTKEF---LTSI----TSQAFKTDDHE------SMFAENDHH-------------
----------------LLYPSPTA----------------VDQLKKVL-SEAGLTSSSLE
WQDDVRGLLRRYEFLGRIIGKCLYEGILVDVNFAP--FFLLKWAL-----------TGGS
RSAVKESSYRANLNDLKD----------------------LDEGLYQG--------LLQL
KNYPG--NVE-DFG--
--LDFTINNVIRMPSG----------------------ANR-TVTAE-LKPN--------

GSQTA---
------------------------------VTNKNRL-VYISY-VARYRLQLQ-PALQTN
-AFLQG---LGQIIQP--AWLS-MFNQS-ELQTLVSG------------------------
---DNADID-----------VEDL----RRNTLYGGVYVIGDDNLE--------------
------------------------HPTI-ALFWQVMH--------------------KMN
NEERQKVIRF-------VTSTPRAP------------LLG-----FS-HLRPNF--SIRD
SSEDQ-----------------------------ERLPST--------------------

>Penicillium_digitatum_XM_014681767.1 .
ADIRRESV-FEDAFSQY---------------YGLGDGLKE-PIQISFIDQFGAM--EAG
IDGGGVTKEF---LTSI----TSQAFKTDDYE------SMFAENDHH-------------
----------------LLYPSPIA----------------VDQLKKVL-SEAGLTNSSPE
WQDDVRGLLRRYEFLGRIIGKCLYEGILVDVDFAP--FFLLKWAL-----------TGGS
RSAVKESSYRANLNDLKD----------------------LDEGLYQG--------LLQL
KNYPG--NVE-DFG--
--LDFTINNKIRMPSG----------------------ANR-TVTAE-LKPN--------
GSQTA---
------------------------------VTNKNRL-VYISY-VARYRLQLQ-PALQTN
-AFLQG---LGQIVQP--AWLS-MFNQS-ELQTLVSG------------------------
---DKADID-----------VEDL----RRNTLYGGVYVIGDDNLE--------------
------------------------HPTI-ALFWQVMH--------------------EMT
NEERQKVIRF-------VTSTPRAP------------LLG-----FS-HLRPNF--SIRD
SSEDQ-----------------------------ERLPSTSTCV-NL-LKLPRY-SDADT
LRSKLL-YAVSS-GAGF
>Ajellomyces_dermatitidis_XM_002625976.1 .
ANIRRESL-FQDAFEQF---------------YELGDGLKE-PIQISFIDKFGTP--EAG
IDGGGVTKEF---LTSV----INDAFNPSGAS------SLFIENDQH-------------
----------------LLYPNPTL----------------IEQRKAEL-RRQGIPDRSVT
MNAEVKELLKRYEFLGRIIGKCLYEGILVDVSFAG--FFLLKWAL-----------TGGT
SSARKESSYRANLNDVRD----------------------LDESLYQG--------LLQL
KNYPG--DVE-DFS--
--LNFTLTDTIDIPIEKPDGTTET--------------ITE-SITRD-LKPH--------
GSEIP---
------------------------------VTNQNRL-VYISY-IARHRLQAQ-PYLQTN
-AFLQG---LGTIIQP--SWLS-MFNQS-ELQTLIGG------------------------
---DAGEID-----------VADL----RRNTVYSGVYVIGDDNQE--------------
------------------------HPTI-KLFWEVLH--------------------AMT
NADRQKVLKF-------VTSTPRAP------------LLG-----FS-HLNPRF--SIRD
SSADE-----------------------------ERLPSTSTCA-NL-LKLPRY-TRRDT
LREKLM-YAINA-GAGF
>Ajellomyces_capsulatus_XM_001538372.1 .
-----ESL-FQDAFDKF---------------YELGDGLKE-PIQISFIDKFGTP--EAG
IDGGGVTKEF---LTSV----ISDAFNPSGPS------SLFIENDQH-------------
----------------LLYPNPTL----------------IEQRKAQL-RRQGVPERSPT
MNAEVKEILKRYEFLGRIIGKCLYEGILVDVSFAG--FFLLKWAL-----------TGGT
SSARKESSYRANLNEVRD----------------------LDEGLYQG--------LLQL
KNYPG--DVE-DFA--
--LNFTVTDTIAVPIEKPDGTLET--------------ITE-NITKD-LKPH--------
GSEIP---
------------------------------VTNQNRL-VYISY-IARHRLQAQ-PYLQTN
-AFLQG---LGTIIQP--SWLS-MFNQS-ELQTLVGG------------------------
---DTGEID-----------VADL----RRNTVYSGSYVIGDDNQE--------------
------------------------HPTI-KLFWEVLQ--------------------TMT
NADRQKLLKF-------VTSTPRAP------------LLG-----FS-HLNPRF--SIRD
SSADE-----------------------------ERLPSTSTCA-NL-LKLPRY-TRLDT
LREKLM-YAINA-GAGF
>Paracoccidioides_brasiliensis_XM_010765021.1 .
ATINRESL-FQDAFEQF---------------YDLGDGLKE-PIQISFIDKFGTQ--EAG

IDGGGVTKEF---LTSV----IGDAFNPSGDS------SLFIENDQH-------------
----------------LLYPNPTI----------------VEQRKAQL-RHQGVKERSVE
WNREIKDILKRYEFLGRIIGKCLYEGILVDVSFAG--FFLLKWAL-----------TGGT
SSARKESSYRANLNDVRD---------------------LDESLYQG---------LLQL
KNYPG--DVE-DFS--
--LNFTVTDTVTVPLTTPTGKPYT--------------TTQ-STTKE-LKPH--------
GSDIP---
------------------------------VTNQNRL-VYISY-IARHRLQAQ-PYLQTN
-AFLQG---LGTIIQP--SWLS-MFNQS-ELQTLVGG-----------------------
---EAGEID-----------VSDL----RRNTVYSGVYIVGDDGQE--------------
------------------------HPTI-KLFWEVME--------------------AMT
NEERRKVLKF-------VTSTPRAP------------LLG-----FS-HLNPRF--SIRD
SSVDE-----------------------------ERLPSTSTCA-NL-LKLPRY-TRRET
LREKLM-YAINS-GAGF
>Paracoccidioides_lutzii_XM_002797661.1 .
ATINRESL-FQDAFEQF---------------YDLGDGLKE-PIQISFIDKFGTQ--EAG
IDGGGVTKEF---LTSV----IGDAFNPSGDS------SLFIENDQH-------------
----------------LLYPNPTI----------------VEQRKAQL-RHHGVKERSVE
WNREIKDILKRYEFLGRIIGKCLYEGILVDVSFAG--FFLLKWAL-----------TGGT
SSARKESSYRANLNDVRD---------------------LDESLYQG---------LLQL
KNYPG--DVE-DFS--
--LNFTVTDTVTVPLTTPTGKPYT--------------TTQ-STTKE-LKPH--------
GSDIP---
------------------------------VTNQNRL-VYISY-IARHRLQAQ-PYLQTN
-AFLQG---LGTIIQP--SWLS-MFNQS-ELQTLVGG-----------------------
---EAGEID-----------VSDL----RRNTVYSGVYIVGDDGHE--------------
------------------------HPTI-KLFWEVME--------------------EMT
NEERRKVLKF-------VTSTPRAP------------LLG-----FS-HLNPRF--SIRD
SSADE-----------------------------ERLPSTSTCA-NL-LKLPRY-SRRET
LRQKLM-YAINS-GAGF
>Trichophyton_rubrum_XM_003234252.1 .
ADIRRENL-FEDAFEQF---------------YPLGEAFKE-PIQITFIDQFDTV--EAG
IDGGGVTKEF---LTSI----INDAFNPSGTL------SMFTENDEH-------------
----------------LLYPNPTS----------------VEQQKAQL-REAGIIERSPE
WNEHIRELLKRYEFLGRIIGKCLYEGILVDVSFAN--FFLLKWAL-----------TGGT
GSASRESAYRANLNDVQD---------------------LDKSLYQG---------LLQL
KNYPG--NVE-DFA--
--LNFTVTDTVSVPDEKGKE------------------KTQ-TITRE-LKPG--------
GSNIP---
------------------------------VTNQNRL-VYISY-IARHRLQLQ-PYLQTN
-AFLQG---LGQIIQP--SWLS-MFNQG-ELQRLVGG-----------------------
---DAIEID-----------VNDL----RRNTVYSGIYVLGDDQQD--------------
------------------------HPTI-KLFWEVLE--------------------AMP
NKDRQKVLKF-------VTSTPRAP------------LLG-----FS-HLNPRF--SIRD
SSSDE-----------------------------ERLPSASTCA-NL-LKLPRY-TNANT
LREKLM-YAVNS-GAGF
>Trichophyton_verrucosum_XM_003020558.1 .
ADIRRENL-FEDAFEQF---------------YPLGEAFKE-PIQITFIDQFDTV--EAG
IDGGGVTKEF---LTSI----INDAFNPSGTL------SMFTENDEH-------------
----------------LLYPNPTS----------------VEQQKAQL-REAGIIERSPE
WNEHIRELLKRYEFLGRIIGKCLYEGILVDVSFAN--FFLLKWAL-----------TGGT
GSASRESAYRANLNDVQD---------------------LDKSLYQG---------LLQL
KNYPG--NVE-DFA--
--LNFTVTDTVSVPDEKGKE------------------KTQ-TITRE-LKPG--------
GSNIP---
------------------------------VTNQNRL-VYISY-IARHRLQLQ-PYLQTN
-AFLQG---LGQIIQP--SWLS-MFNQG-ELQRLVGG-----------------------
---DAIEID-----------VNDL----RRNTVYSGIYVLGDDQQD--------------

------------------------HPTI-KLFWEVLE--------------------AMP
NKDRQKVLKF-------VTSTPRAP------------LLG-----FS-HLNPRF--SIRD
SSSDE-----------------------------ERLPSASTCA-NL-LKLPRY-TNANT
LREKLM-YAVNS-GAGF
>Arthroderma_benhamiae_XM_003012442.1 .
ADIRRENL-FEDAFEQF---------------YPLGEAFKE-PIQITFIDQFDTV--EAG
IDGGGVTKEF---LTSI----INDAFNPSGTL------SMFTENDEH-------------
----------------LLYPNPTS----------------VEQQKAQL-REAGIIERSPE
WNEHIRELLKRYEFLGRIIGKCLYEGILVDVSFAN--FFLLKWAL-----------TGGT
GSASRESAYRANLNDVQD----------------------LDKSLYQG--------LLQL
KNYPG--NVE-DFA--
--LNFTVTDTVSVPDDKGKE------------------KTQ-TITRE-LKPG--------
GSNIP---
------------------------------VTNQNRL-VYISY-IARHRLQLQ-PYLQTN
-AFLQG---LGQIIQP--SWLS-MFNQG-ELQRLVGG-----------------------
---DAIEID-----------VNDL----RRNTVYSGIYVLGDDQQD--------------
------------------------HPTI-KLFWEVLE--------------------AMP
NKDRQKVLKF-------VTSTPRAP------------LLG-----FS-HLNPRF--SIRD
SSSDE-----------------------------ERLPSASTCA-NL-LKLPRY-TSANT
LREKLM-YAVNS-GAGF
>Nannizzia_gypsea_XM_003175498.1 .
ADIRRDNL-FEDAFEQF---------------YPLGEAFKE-PIQITFIDRFDTV--EAG
IDGGGVTKEF---LMSI----INDAFNPSGTL------SMFTENDEH-------------
----------------LLYPNPTS----------------VEQQKAQL-HEAGIIERSTE
WNEHIRELLKRYEFLGRIIGKCLYEGILVDVSFAN--FFLLKWAL-----------TGGT
GSASRESAYRANLNDVQD----------------------LDKSLYQG--------LLQL
KNYPG--NVE-DFA--
--LNFTVTDTVSVPDEKGKE------------------KTQ-TITRE-LKPG--------
GSNIP---
------------------------------VTNQNRL-VYISY-IARHRLQLQ-PYLQTN
-AFLQG---LGQIIQP--SWLS-MFNQG-ELQRLVGG-----------------------
---DAIEID-----------VNDL----RRNTVYSGVYVLGDDQQD--------------
------------------------HPTI-KLFWEVLE--------------------AMP
NKDRQKVLKF-------VTSTPRAP------------LLG-----FS-HLNPRF--SIRD
SSSDE-----------------------------ERLPSASTCA-NL-LKLPRY-TNADT
LREKLM-YAVNS-GAGF
>Arthroderma_otae_XM_002847911.1 .
ADIRRENL-FEDAFEQF---------------YPLGEAFKE-PIQITFIDKFDTV--EAG
IDGGGVTKEF---LTSI----INDAFNPSGTL------SMFAENDEH-------------
----------------LLYPNPAS----------------VEQQKSQL-REAGIAERSPE
WNEHIRELLKRYEFLGRIIGKCLYEGILVDVSFAG--FFLLKWAL-----------TGGT
GSASRESAYRANLNDVQD----------------------LDKSLYQG--------LLQL
KNYPG--NVE-DFA--
--LNFTVTDTVSLPDERGKE------------------KTQ-TITRE-LKPG--------
GSNIP---
------------------------------VTNQNRL-VYISY-IARHRLQLQ-PYLQTQ
-AFLQG---LGQIIQP--SWLS-MFNQG-ELQRLVGG-----------------------
---DAVEID-----------VNDL----RRNTVYSGVYVLGDDQQD--------------
------------------------HPTI-KLFWEVLE--------------------AMP
NTDRQKVLKF-------VTSTPRAP------------LLG-----FS-HLNPRF--SIRD
SSSDE-----------------------------ERLPSASTCA-NL-LKLPLY-TNAHT
LREKLI-MIPSS-----
>Uncinocarpus_reesii_XM_002583545.1 .
ANIRRENL-VEDAFEQF---------------YPLGEGLKE-PIQITFIDKFDTV--EAG
IDGGGVTKEF---LTSV----ISDAFDPSGTL------SLFSENDQH-------------
----------------LLFPNPTA----------------VEQRKALL-RQAGVPERSVE
WNQQIRELLKRFEFLGRIIGKCLYEGILVDVNFAG--FFLLKWAL-----------TGGS
SSASKESAYRANLNDVRD----------------------LDESLYQG--------LLQL

```
KNYPG----------------------------------------------DVE-DFS--
--LNFTVTDTVTVPGTGIDDPE----------------KTQ-SITRD-LKPN--------
GSNIA-------------------------------------------------------
------------------------------VTNQNRL-VYISY-IARHRLQAQ-PYLQTN
-AFLQG---VGQIIQP--SWLS-MFNQG-ELQRLIGG-----------------------
---DAGEVD-----------VADL----RRNTLYSGVYSLGEDMEE--------------
------------------------HLTV-KLFWQVME--------------------SMS
NADRQKVLKF-------VTSTPRAP------------LLG-----FS-HLNPRF--SIRD
SSSDE-----------------------------ERLPSTSTCA-NL-LKLPRY-TSAKT
LEQKLM-YAINS-GAGF
>Chrysosporium_queenslandicum_GDRC01009890.1 .
ANIRRQNL-FEDAYEQF---------------YPLGEGLKE-PIQITFIDKFDTI--EAG
IDGGGVTKEF---LTSI----ISDAFDPSGVS------SLFSENDQH-------------
----------------LLYPNPTA----------------IEQRRAQL-RQAGIPERSAE
WNQQIRELLKRYEFLGRIIGKCLYEGILVDVNFAG--FFLLKWAL-----------TGGS
SSASKESAYRANLNDVRD----------------------LDESLYQG--------LLQL
KNYPG----------------------------------------------DVE-DFS--
--LNFTVTDTVTVPGAGPDDPE----------------RTE-TITRE-LKPN--------
GSNIA-------------------------------------------------------
------------------------------VTNQNRL-VYISY-IARHRLQAQ-PYLQTN
-AFLQG---VGQIIQP--SWLS-MFNQG-ELQRLIGG-----------------------
---DSGEID-----------VADL----RSNTVYSGVYALGDDMEE--------------
------------------------HLTI-KLFWQVLE--------------------AMS
NADRQKVLKF-------VTSTPRAP------------LLG-----FS-HLNPPF--SIRD
SSSDE-----------------------------ERLPSTSTCA-NL-LKLPRY-TNAKT
LHEKLM-YAINS-GAGF
>Byssoonygena_ceratinophila_GDRB01009277.1 .
ANIRRENL-FEDAFEQF---------------YPLGEGLKE-PIQITFIDKFDTI--EAG
IDGGGVTKEF---LTSI----ISDAFDPSDIL------SLFLENDQH-------------
----------------LLYPNPTT----------------VEQRKAQL-RQDGIPERSAE
WNQQIRELLKRYEFLGRIIGKCLYEGILVDVNFAG--FFLLKWAL-----------TGGS
SSASKESAYRANLNDVRD----------------------LDESLYQG--------LLQL
KNYPG----------------------------------------------DVE-DFS--
--LNFTVSDTVTIPGAGPDDPE----------------ITQ-TFTRE-LKPN--------
GSNIA-------------------------------------------------------
------------------------------VTNQNRL-VYISY-IARHRLQAQ-PYLQTN
-AFLQG---VGQIIQP--SWLS-MFNQG-ELQRLVGG-----------------------
---DSGEID-----------VADL----RRNTVYSGVYVIGDDMEE--------------
------------------------HLTV-KLFWQTME--------------------SMS
NADRQKVLKF-------VTSTPRAP------------LLG-----FS-HLNPRF--SIRD
SSSDE-----------------------------ERLPSASTCA-NL-LKLPRY-TSAKT
LQEKLM-YAINS-GAGF
>Amauroascus_mutatus_GDQZ01003362.1 .
ANIRRENL-FEDAYEQF---------------YPLGVGLKE-PIQITFIDKFDTV--EAG
IDGGGVTKEF---LTSI----INDAFDPSSIL------SFFSENDQH-------------
----------------LLYPNPTA----------------VEQRRALL-RQAGVLERSAE
WNLQIRELLKHYEFLGRIIGKCLYEGILVDVNFAG--FFLLKWAL-----------TGGS
SSASKESAYRANINDIRD----------------------LDESLYQG--------LLQL
KNYPG----------------------------------------------NVE-DFS--
--LNFTVTDTITIPGTAGPDDPE---------------KTQ-TVTRE-LKPD--------
GSNIT-------------------------------------------------------
------------------------------VTNQNRL-VYISY-IARHRLQAQ-PFLQTN
-AFLQG---VGQIIQP--SWLS-MFNQG-ELQRLVGG-----------------------
---DSGEID-----------VADL----RRNTVYGGVYTLGDDMEE--------------
------------------------HLTV-KLFWQVME--------------------SLS
NTDRQKVLKF-------VTSTPRAP------------LLG-----FS-HLIPRF--SIRD
SSSDE-----------------------------ERLPSTSTCA-NL-LKLPRY-TNAKT
LQKKLM-YAVNS-GAGF
```

>Amauroascus_niger_GDRA01006611.1 .
ANIRRENL-FEDAFEQF---------------YALGEGLKE-PIQITFIDKFDTV--EAG
IDGGGVTKEF---LTSI----INDAFDPSGVL------SLFSENDQH-------------
----------------LLYPNPAA----------------VEQRRALL-RQAGIPERSAE
WNQQIRELLKRYEFLGRIIGKCLYEGILVDANFAG--FFLLKWAL-----------TGGS
SSASKESAYRANLNDVRD----------------------LDESLYQG--------LLQL
KNYPG--EVE-DFS--
--LNFTVTDTVTVPGADPDVPE----------------KTQ-TITRE-LKPD--------
GSNIA---
------------------------------VTNQNRL-VYISY-IARHRLQAQ-PYLQTN
-AFLQG---VGQIIQP--SWLS-MFNQG-ELQRLVGG-----------------------
---DSGEID-----------VADW----RRNTMYSGVYAIGDDMED--------------
------------------------HLTV-KLFWQVME--------------------SMT
NVDRQKVLKF-------VTSTPRAP------------LLG-----FS-HLNPRF--SIRD
SSSDE-----------------------------ERLPSASTCA-NL-LKLPRY-TSANV
LHEKLM-YAINS-GAGF
>Coccidioides_immitis_XM_001245550.2 .
ANIRRENL-FEDAFEQF---------------YPLGEGLKE-PIQITFIDKFDTV--EAG
IDGGGVTKEF---LTSI----INDAFDPSGVL------SLFSENDQH-------------
----------------LLYPNPTA----------------VEQRKALL-RQAGVPERSAE
WNQQIRELLKRYEFLGRVIGKCLYEGILVDVNFAG--FFLLKWAL-----------TGGS
SLASKESAYRANLNDIRD----------------------LDESLYQG--------LLQL
KNYPG--NVE-DFS--
--LNFTVTDTVTVPGTGPDDPE----------------KAQ-TITRE-LKPN--------
GSNIA---
------------------------------VTNQNRL-VYISY-IARHRLQAQ-PYLQTN
-AFLQG---VGQIIQP--SWLS-MFNQG-ELQRLVGG-----------------------
---DSGEID-----------VADL----RRNTVYSGVYTLGDDMEE--------------
------------------------HMTV-KLFWQVME--------------------SMP
NTDRQKVLKF-------VTSTPRAP------------LLG-----FS-HLNPRF--SIRD
SSSDE-----------------------------ERLPSTSTCA-NL-LKLPRY-TSART
LHEKLM-YAINS-GAGF
>Coccidioides_posadasii_ABIW01001021.1 .

-DGGGVTKEF---LTSI----INDAFDPSGVL------SLFSENDQH-------------
----------------LLYPNPTA----------------VEQRKALL-RQAGVPERSAE
WNQQIRELLKRYEFLGRVIGKCLYEGILVDVNFAG--FFLLKWAL-----------TGGS
SLASKESAYRANLNDIRD----------------------LDESLYQG--------LXQL
KNYPG--NVE-DFS--
--LNFTVTDTVTVPGTGPDDPE----------------KAQ-TITRE-LKPN--------
GSNIA---
------------------------------VTNQNRL-VYISY-IARHRLQAQ-PYLQTN
-AFLQG---VGQIIQP--SWLS-MFNQG-ELQRLVGG-----------------------
---DSGEID-----------VADL----RRNTVYSGVYTLGDDMEE--------------
------------------------HMTV-KLFWQVME--------------------SMP
NTDRQKVLKF-------VTSTPRAP------------LLG-----FS-HLNPRF--SIRD
SSSDE-----------------------------ERLPSTSTCA-NL-LKLPRY-TSART
LHEKLM-YAINS-GAGF
>Xylona_heveae_XM_018330147.1 .
AKIRRESV-FEDAYEQF---------------YELGEGLKE-PIQITFVDKFDTV--EAG
IDGGGVTKEF---LTSV----TNEAFSPDSDL------NLFVENDQS-------------
----------------LLYPNPAA----------------VDERREWL-REAGVREGSRD
WNEQIRDLLRRYEFLGRVIGKCLYEGILVDISFAP--FFLLKWAL-----------TGGS
GSAARESGYRANLNDLRD----------------------LDESLYQG--------LLQL
KNYPG--NVE-DFA--
--LNFTVTDNITTPRKAPDGTMEG--------------PIR-TTTRD-LRPG--------
GSDIP---
------------------------------VTNENRL-VYISY-MARHRLQVQ-PHLQTS

-AFLRG---LGEIIPP--SWLS-MFNQS-ELQTLVGG----------------------
---ASAEID-----------VFDL----RRNTLYGGVYTIGDDNLE--------------
------------------------HPSV-QLFWQVMQ--------------------SLS
DAERRKVLKF-------VTSTPRAP------------LLG-----FG-QLNPRF--SIRD
AGDDQ-----------------------------VRLPSTSTCV-NL-LKLPRY-SDADT
LREKLL-YAVNS-GAGF
>Verruconis_gallopava_XM_016361626.1 .
ATIRRGHE-FEDAYEQF---------------YSLGEGLKE-PIQITFMDRFGQQ--EAG
IDGGGVTKEF---LTSV----TNQAFTPSSDGGL----DLFKENQQR-------------
----------------LLFPNPTA----------------VEEQKEIL-RQAGIQPRTPE
YVRLMTELMQRYEFLGRIVGKCLYEGILVDINFAP--FFLLKWAL-----------TGGD
GAAPRESGYRANLNDLRD----------------------IDPDLYRG--------LIQL
KNYPG---NVEEDLA--
--LDFTIADTFVVDHRTG--------------------EEK-VIMRE-LRPN--------
GANIH---
------------------------------VTNENRL-VYISC-VARHRLVTQ-PYAQTN
-AFLKG---LSSMISP--SWLS-MFNQS-ELQMLIAG----------------------
---TSSSID-----------IADL----RRNTLYGGVYVIGDDGRE--------------
------------------------HPTV-QLFWSVME--------------------SLS
DEDRRKVLKF-------VTSTPRAP------------LLG-----FA-SLNPRF--SLRD
SGDDE-----------------------------SRLPSTSTCV-NL-LKLPRY-KTAEV
LKEKLL-YAVNS-GAGF
>Neofusicoccum_parvum_XM_007581273.1 .
AKVRRKHE-FDDAFEQF---------------FKLGEALKE-PIQITFVDQFDTV--EAG
IDGGGVTKEF---LTSV----SSQAFAPSENGI-----DLFVENEAH-------------
----------------LLYPNPSA----------------VEHQKEIL-REAGVRENSKL
WKDTVQELLERYEFLGRIVGKCLYEGILVDINFAP--FFLLKWAL-----------TGGT
NSAAKESGYRANLNDLRD----------------------LDEDLYQG--------LLTL
KNYPG---NVEEDFS--
--LNFTITDVIRTDTSTG--------------------QQI-TRTKD-LVSD--------
GSNMP---
------------------------------VTNENRL-VYISY-VARHRLQVQ-PAPQTA
-AFLRG---LSTMISP--SWLS-MFNQS-ESQTLIGG----------------------
---TSSSID-----------VADL----RAHTQYGGLYVIGDDRLE--------------
------------------------HPSV-QLFWRVME--------------------SLS
DDERRAVLKF-------VTSTPRAP------------LLG-----FS-SLNPRF--SIRD
AGHDV-----------------------------TRLPSTSTCV-NL-LKLPLY-KDERT
LREKLL-YAVNS-GAGF
>Coniosporium_apollinis_XM_007783990.1 .
AKIRRGHE-FEDAFEQF---------------YPLKEGLKE-PIQITFVDQFDTV--EAG
IDGGGVTKEF---LTSV----TNQAFNPSSSEDDEL--PLFVENEHH-------------
----------------LLYPNPSA----------------IDAQKDLL-HQLGYRDGSPE
YREQLRGLRQRYEFLGRVVGKCLYEGILVDIHFAG--FFLLKWAL-----------TGGH
GSAPKESGYMSNLNDLRD----------------------LDEGLYQG--------LLQL
KNYAG---NVE-DFA--
--LNFTITDTVSTDPATG--------------------ATK-TLTRN-LIPN--------
GAAVP---
------------------------------VTNQNRI-PYISL-VARHRLVTQ-PAAQTA
-AFLKG---LADMVQP--SWLR-MFNQS-ELQTLLAG----------------------
---TSSPLD-----------IADL----RRHTLYGGVYALGDDRAE--------------
------------------------HPSI-RAFWRVMA--------------------SLS
DEERRAVLKF-------VTSSPRAP------------LLG-----FG-ALNPRF--SIRD
AGDEQ-----------------------------SRLPSTSTCV-NL-LKLPRY-RDEQT
LRERLL-YAVFS-GAGF
>Paraphaeosphaeria_sporulosa_XM_018180666.1 .
ARVRRGAE-FDDAFAQF---------------YDLGEGLKE-PIQITFVDQFDQA--EAG
IDGGGVTKEF---LTSV----TNTAFTPTEDNI-----DMFIENNQH-------------
----------------LLYPNPSA----------------IEELKEALRSKAGLSDGSAE

FRQQVSESLQRYEFLGRIIGKCMYEGILVDVNFAP--FFLRKWAL-----------TGGT
GSAPNETGYRPTLNDIRD---------------------LDEELYQG--------LHKL
KTYDG---DVE-DFG--
--LNFTITDTVVTDHATK--------------------KTK-AVTKE-LRPD--------
GANTP--
------------------------------VTNQNRL-VYISY-VARHRLQNQ-PYLQTS
-AFLRG---LSAMIQP--SWLS-MFNAP-ELQTLIGG-----------------------
---TSSSID-----------IDDL----RRNTMYGGTYVIGDDGLE--------------
------------------------HPTV-QLFWKTMK--------------------DLS
DGERRAVLKF-------VTSTPRAP------------LLG-----FK-TLNPRF--SIRD
AGSDQ-----------------------------ERLPSTSTCV-NL-LKLPMY-RDEGV
LREKLL-YSVFS-GAGF
>Pyrenochaeta_lycopersici_GAJI01014483.1 .
ANIRRENE-FEDAFEQF---------------FPLGQGLKE-PIQITFMDQFGAP--EAG
IDGGGVTKEF---LTSV----TNRAFMPTEYI------DMFIENDQH-------------
----------------LLYPNPVA----------------VEEHKEAL-RQTGYLENSPE
FRDQISRLLQRYEFLGRIIGKCLYEGILVDVNFAP--FFLRKWAL-----------TDGA
GSTSNESGYRPTLNDLRD---------------------LDEELYQG--------LHKL
KTYPG---DVE-DFS--
--LNFSVTDTIVVDHTTEPK------------------KTK-AITKE-LMPN--------
GSDTP--
------------------------------VTNQNRL-VYISY-MARHRLQSQ-PFLQTT
-AFLRG---LSTMIQP--SWLS-MFNQS-ELQTLISG-----------------------
---TRTSID-----------VEDL----RRNTMYGGTYVIGDDGLE--------------
------------------------HPTI-RLFWKILT--------------------DMS
DEERRAVLKF-------VTSTPRAP------------LLG-----FR-TLNPRF--SIRD
AGSDQ-----------------------------DRLPSTSTCV-NL-LKLPMY-RDEEV
LKEKLL-YSVFS-GAGF
>Parastagonospora_nodorum_AAGI01000140.1 .
AKIRRENE-FEDAFEQF---------------YPLGAGLKE-PIQITFMDQFGSP--EAG
IDGGGVTKEF---LTSV----TDRAFMPSEEL------DMFVENDQH-------------
----------------LLYPNPTA----------------VEELKAAM-RQCNLNERTAE
FRTQISDLLQRYEFLGRIIGKCLYEGILVDVNFAP--FFLRKWAL-----------TGGE
GHAPNESSYRPTLNDLRD---------------------LDEELYQG--------LHKL
KTYPG---NVE-DFG--
--LNFTVTDILTIDPTSSPK------------------KTL-AVEKE-LKPD--------
GANTP--
------------------------------VTNQNRL-VYISY-MARHRLQNQ-PYLQTS
-AFLRG---LRQMVQP--SWLS-MFNHS-ELQTLVSG-----------------------
---TRTSID-----------VEDL----RRNTIYGGTYVIGTDGLE--------------
------------------------HPTI-SLFWKIMK--------------------ELS
DDERRAVLKF-------VTSTPRAP------------LLG-----FG-TLNPRF--SIRD
AGSDQ-----------------------------QRLPSTSTCV-NL-LKLPMY-RDEGV
MKEKLL-YSAFS-GAGF
>Hymenoscyphus_laetus_LLCA01000004.1 .
AKIRRENE-FEDAFEQF---------------YPLGAGLKE-PIQITFMDQFGSP--EAG
IDGGGVTKEF---LTSV----TDRAFMPSDEI------DMFVENDQH-------------
----------------LLYPNPTA----------------VEEIKTAM-RRCGLNERTAE
FRTQISDMLQRYEFLGRIIGKCLYEGILVDVNFAP--FFLRKWAL-----------TGGE
GHAPNESSYRPTLNDLRD---------------------LDEELYQG--------LHKL
KTYAG---NVE-DFG--
--LNFTVTDTIVIDHDSSPK------------------KTV-AVEKE-LKPD--------
GANTP--
------------------------------VTNQNRL-VYISY-MARHRLQNQ-PYLQTS
-AFLRG---LRQMVQP--SWLS-MFNHS-ELQTLVSG-----------------------
---TRTSID-----------VEDL----RRNTIYGGTYVIGTDGLE--------------
------------------------HPTI-SLFWKIMK--------------------ELS
DDERRAVLKF-------VTSTPRAP------------LLG-----FG-TLNPRF--SIRD

```
AGSDQ-----------------------------QRLPSTSTCV-NL-LKLPMY-RDEKV
MKEKLL-YSVFS-GAGF
>Stagonospora_sp._LXTA01000084.1 .
AKIQRENE-FEDAFEQF---------------YPLGAGLKE-PIQITFVDQFGSP--EAG
IDGGGVTKEF---LTSV----TDRAFMPTDSI------DMFIENDQH-------------
----------------LLYPNPTA----------------IEELKAAL-RQAGLKDNSAD
FRSQISSFLQRYEFLGRIIGKCLYEGILVDVNFAP--FFLRKWAL-----------TGGE
GHAPNESGYRPTLNDLRD----------------------LDEELYQG--------LHKL
KTYPG----------------------------------------------DVE-DFS--
--LNFTVTDNVIIDHSTNPK------------------KTK-AVEKE-LKPD--------
GANTS-------------------------------------------------------
------------------------------VTNQNRL-VYISY-MARHRLQNQ-PYAQTN
-AFLRG---LRTMVQP--SWLS-MFNHS-ELQTLVSG-----------------------
---TRTNID-----------IEDL----RRNTIYGGTYVIGTDGLE--------------
------------------------HPTI-ALFWKILK--------------------DMS
DDERRAVLKF-------VTSTPRAP------------LLG-----FG-TLNPRF--SIRD
AGSDQ-----------------------------QRLPSTSTCV-NL-LKLPMY-RDEEL
MKEKLL-YSVFS-GAGF
>Paraphoma_sp._BCLK01000257.1 .
AKIRRENE-FEDAFDQF---------------FPLGAGLKE-PIQITFLDQFGAQ--EAG
IDGGGVTKEF---LTSV----TNRAFMPTDFI------DMFIENDQH-------------
----------------LLYPNPTA----------------IEELKAAL-RQDGLADNSPE
FRLHVNEMLHRYEFLGRIIGKCLYEGILVDVNFAP--FFLRKWAL-----------TGGE
GSAPNETGYRPTLNDLRD----------------------LDEELYQG--------LHKL
KTYPG----------------------------------------------DVE-DFG--
--LSFTVTDTVIVDHTTSPK------------------KTK-AIDRE-LKPD--------
GANTP-------------------------------------------------------
------------------------------VTNQNRL-VYISY-MARHRLQNQ-PFQQTA
-AFLRG---LGQMVQP--SWLS-MFNHS-ELQTLVSG-----------------------
---TRNNID-----------VEDL----RRNTAYGGTYVIGDDGLE--------------
------------------------HPTV-QLFWKIMK--------------------EIS
DDERRAVLKF-------VTSTPRAP------------LLG-----FG-TLNPRF--SIRD
AGSDQ-----------------------------ERLPSTSTCV-NL-LKLPMY-REESV
LKEKLL-YSVFS-GAGF
>Leptosphaeria_maculans_XM_003834964.1 .
ARIRRENE-FEDAFEQF---------------YPLGSGLKE-PISITFMDQFGEP--EAG
IDGGGVTKEF---LTSV----TDRAFMPTNFI------DMFVENDQH-------------
----------------LLYPNPAA----------------VDEHKEAL-RQAGLRENSPE
SRLQISELLQKYEFLGRIIGKCLYEGILVDVNFAP--FFLRKWAL-----------TGGA
GSAPNETGYRPTLNDLRD----------------------LDEELYQG--------LHKL
KTYPG----------------------------------------------DVE-DFS--
--LNFTVTDNITIDHTSNPK------------------KTK-AVTKE-LKPD--------
GANTP-------------------------------------------------------
------------------------------VTNQNRL-VYISY-MARHRLQNQ-PYLQTT
-AFLRG---LGTMIQP--SWLS-MFNHS-ELQTLISG-----------------------
---TRTSID-----------VEDL----RRNTVYGGLYLIGNQGLE--------------
------------------------HPTI-QILWKVMK--------------------EMS
DDERRAVLKF-------VTSTPRAP------------LLG-----FG-TLNPRF--SIRD
AGSDQ-----------------------------ERLPSTSTCV-NL-LKLPMY-RDEKT
LKEKLL-YSIFS-GAGF
>Alternaria_alternata_XM_018535643.1 .
ARIRRENE-FDDAFEQF---------------YELGQGLKE-PIQITFMDQFGAP--EAG
IDGGGVTKEF---LTSV----TSRAFMPTDYI------DMFVENDQH-------------
----------------LLYPNPAA----------------LEEHKEAL-RQAGIREGSQE
YRAQITELLQRYEFLGRIIGKCLYEGILVDVNFAP--FFLRKWAL-----------TGGT
GSAPNESGYRPTLNDLRD----------------------LDEELYQG--------LHKL
KTYPG----------------------------------------------DVE-DFS--
--LNFTVTDTVVVDHTTSPK------------------KTK-AITKD-LKPD--------
```

GSNTP---
------------------------------VTNQNRL-VYISY-MARHRLQNQ-PYAQTA
-AFLRG---LSTMIQP--SWLS-MFNAS-ELQTLISG-----------------------
---TRTQID-----------IDDL----RRNTIYGGTYVIGDDGLE--------------
------------------------HPTI-QLLWKVMK--------------------EMS
DDERRAVLKF-------VTSTPRAP------------LLG-----FG-TLNPRF--SIRD
AGRDQ-----------------------------SRLPSTSTCV-NL-LKLPMY-EDEET
LKEKLL-YSVFS-GAGF
>Alternaria_arborescens_AIIC01000032.1 .
ARIRRENE-FDDAFEQF---------------YELGQGLKE-PIQITFMDQFGAP--EAG
IDGGGVTKEF---LTSV----TSRAFMPTDYI------DMFVENDQH-------------
----------------LLYPNPAA----------------LEEHKEAL-RQAGIREGSQE
YRAQITELLQRYEFLGRIIGKCLYEGILVDVNFAP--FFLRKWAL-----------TGGT
GSAPNESGYRPTLNDLRD----------------------LDEELYQG--------LHKL
KTYPG--DVE-DFS--
--LNFTVTDTVVVDHTTSPK------------------KTK-AITKD-LKAD--------
GSNTP---
------------------------------VTNQNRL-VYISY-MARHRLQNQ-PYAQTA
-AFLRG---LSTMIQP--SWLS-MFNAS-ELQTLISG-----------------------
---TRTQID-----------IDDL----RRNTLYGGTYVIGDDGLE--------------
------------------------HPTI-QLFWKCMV--------------------EMS
DDERRAVLKF-------VTSTPRAP------------LLG-----FG-TLNPRF--SIRD
AGRDQ-----------------------------SRLPSTSTCV-NL-LKLPMY-EDEET
LKEKLL-YSVFS-GAGF
>Bipolaris_maydis_XM_014225563.1 .
ARIRRENE-FDDAFDQF---------------YDLGQGLKE-PIQITFLDQFGAQ--EAG
IDGGGVTKEF---LTSV----TNRAFMPTDYI------DMFIENDQH-------------
----------------LLYPNPAA----------------LEEHKEAL-RQAGIREGSQE
YRAQITELLQRYEFLGRIIGKCMYEGILIDVNFAP--FFLRKWAL-----------TGGA
GSAPNESGYRPTLNDLRD----------------------LDEELYQG--------LHKL
KTYPG--DVE-DFS--
--LNFTVTDTIVVDHTTTPK------------------KTK-AITKD-LKPD--------
GSNTP---
------------------------------VTNQNRL-VYISY-MARHRLQNQ-PYAQTA
-AFLRG---LSTMIQP--SWLS-MFNQS-ELQTLISG-----------------------
---TRTSID-----------VEDL----RRNTIYGGTYVIGDDGLE--------------
------------------------HPTI-QLLWKVMK--------------------EMS
DDERRAVLKF-------VTSTPRAP------------LLG-----FA-TLNPRF--SIRD
AGSDQ-----------------------------ERLPSTSTCV-NL-LKLPMY-RDEAT
LKEKLL-YSVFS-GAGF
>Cochliobolus_sativus_XM_007697172.1 .
ARIRRENE-FDDAFDQF---------------YDLGQGLKE-PIQITFLDQFGSQ--EAG
IDGGGVTKEF---LTSV----TNRAFMPTDYI------DMFIENDHH-------------
----------------LLYPNPAA----------------LEEHKEAL-RQAGIREGSQE
YRAQITELLQRYEFLGRIIGKCMYEGILIDINFAP--FFLRKWAL-----------TGGA
GSAPNESGYRPTLNDLRD----------------------LDEELYQG--------LHKL
KTYPG--DVE-DFS--
--LNFTVTDTIVVDHTTTPK------------------KTK-AITKD-LKPD--------
GSNTP---
------------------------------VTNQNRL-VYISY-MARHRLQNQ-PYAQTA
-AFLRG---LSTMIQP--SWLS-MFNQS-ELQTLISG-----------------------
---TRTSID-----------VEDL----RRNTIYGGTYVIGDDGLE--------------
------------------------HPTI-QLLWKVMK--------------------EMS
DDERRAVLKF-------VTSTPRAP------------LLG-----FA-TLNPRF--SIRD
AGSDQ-----------------------------ERLPSTSTCV-NL-LKLPMY-RDEAT
LKEKLL-YSVFS-GAGF
>Pyrenophora_seminiperda_ATLS01000654.1 .
ARIRRENE-FDDAFEQF---------------YELGQGLKE-PIQITFMDQFGAP--EAG

IDGGGVTKEF---LTSV----TNRAFMPTDYI------DMFVENDQH-------------
----------------LLYPNSAA----------------LEEHKEAL-RQAGLRENSPE
YRAQINELLQRYEFLGRIIGKCLYEGILVDVNFAP--FFLRKWAL-----------TGGA
GSAPNESGYRPTLNDLRD----------------------LDEELYQG--------LHKL
KTYPG---DVE-DFS--
--LNFTVTDTVVVDHTTSPK------------------KTK-AITKE-LKHD--------
GSNTP---
-----------------------------VTNQNRL-VYISY-MARHRLQNQ-PYRQTT
-AFLRG---LSTMIQP--SWLS-MFNQS-ELQTLISG-----------------------
---TRTSID-----------IEDL----RRNTIYGGTYVIGDDGLE--------------
------------------------HPTI-QILWKVMK--------------------EMS
DDERRAVLKF-------VTSTPRAP------------LLG-----FG-TLNPRF--SIRD
AGGDQ-----------------------------ERLPSTSTCV-NL-LKLPMY-RDEKT
LKEKLL-YSVFS-GAGF
>Pyrenophora_triticirepentis_XM_001931824.1 .
ARIRRENE-FDDAFEQF---------------YELGQGLKE-PIQITFMDQFGAA--EAG
IDGGGVTKEF---LTSV----TNRAFMPTDYI------DMFVENDQH-------------
----------------LLYPNPAA----------------LEEHKEAL-RQAGLRENSPE
YRAQVTELLQRYEFLGRIIGKCLYEGILVDVNFAP--FFLRKWAL-----------TGGA
GSAPNESGYRPTLNDLRD----------------------LDEELYQG--------LHKL
KTYPG---DVE-DFS--
--LNFTVTDTVVVDHATSPK------------------KTK-AITKE-LKPD--------
GSNTP---
-----------------------------VTNQNRL-VYISY-MARHRLQNQ-PYAQTT
-AFLRG---LSTMIQP--SWLS-MFNQS-ELQTLISG-----------------------
---TRTSID-----------VEDL----RRNTIYGGTYVIGDDGQE--------------
------------------------HPTI-QILWKVMK--------------------EMS
DDERRAVLKF-------VTSTPRAP------------LLG-----FG-TLNPRF--SIRD
AGSDQ-----------------------------ERLPSTSTCV-NL-LKLPMY-RDEKT
LKEKLL-YSVFS-GAGF
>Pyrenophora_teres_XM_003298629.1 .
ARIRRENE-FDDAFEQF---------------YELGQGLKE-PIQITFMDQFGAA--EAG
IDGGGVTKEF---LTSV----TNRAFMPTDYI------DMFVENDQH-------------
----------------LLYPNPAA----------------LEEHKEAL-RQAGLRENSPE
YRAQVTELLQRYEFLGRIIGKCLYEGILVDVNFAP--FFLRKWAL-----------TGGT
GSAPNESGYRPTLNDLRD----------------------LDEELYQG--------LHKL
KTYPG---DVE-DFS--
--LNFTVTDTVVVDHTISPK------------------KTK-AITKE-LKPD--------
GSNTP---
-----------------------------VTNQNRL-VYISY-MARHRLQNQ-PYAQTT
-AFLRG---LSTMIQP--SWLS-MFNQS-ELQTLISG-----------------------
---TRTSID-----------VEDL----RRNTIYGGTYVIGDDGQE--------------
------------------------HPTI-QILWKVMK--------------------EMS
DDERRAVLKF-------VTSTPRAP------------LLG-----FG-TLNPRF--SIRD
AGSDQ-----------------------------ERLPSTSTCV-NL-LKLPMY-RDEKT
LKEKLL-YSVFS-GAGF
>Ramalina_peruviana_MSTJ01000002.1 .
ATVRREQI-FDDAYKQF---------------YDLKEGLKE-PIQIRFVDKFGTV--EEG
IDGGGVTKEF---LTSI----TNEAFGATNGLE-----SLFIENDQH-------------
----------------LLFPNPSA----------------VEERKETL-RQSMLTEGSPD
WNETIRDLLRRYEFLGRIVGKCLYEGILIDIHFAP--FFLLKWAL-----------TGGS
GSASKESGYRANLNDLRD----------------------LDESLYQG--------LLQL
KNYAG---DVETDFS--
--LNFAVTDTLDLSPT----------------------QTK-TITRE-LRPG--------
GENIP---
-----------------------------VTNENRP-VYIAS-IARYRLQQQ-PHQQTS
-AFLRG---LGTIISP--SWLS-MFNQS-ELQTLIGG-----------------------
---SGAEVS-----------IADL----RQNTQYGGIYVIGDDGME--------------

------------------------HPSI-QLFWKVMES--------------------LD
MTDVAKVLKF-------VTSTPRAP------------LLG-----FG-NLTPRF--SIRD
SGPDQ-----------------------------TRLPSTSTCV-NL-LKLPIY-RDEKV
LKDRLL-YSINA-GAGF
>Gyalolechia_flavorubescens_AUPK01000019.1 .
ARVRRESI-FDDAFKQY---------------YELGEGLKE-PIQISFVDKFDTV--EEG
IDGGGVTKEF---LTSV----TNEAFNSMSGL------DMFVENDQH-------------
----------------LLYPNPTA----------------VEERKELL-RQAGEAEGSQF
YNESVRDLLRRFEFMGRIIGKCLYEGILVDIHFAP--FFLLKWSL-----------TGGH
AAATRESSYRANLNDLRD----------------------LDEALYQG--------LLQL
KNYKP------------------------------------NSNDENSTIE-SLA--
--LTFEVTDTLFPADRPSSHPNQ---------------NPI-PRTVE-LRPN--------
GSNIP---
-----------------------------VDNANRL-VYISY-IARHRLSIQ-PWHQTS
-AFLKG---LGQIISP--AWLS-MFNQH-ELQTLVSG----------------------
---SAASID-----------IADL----RANTQYGGLYVVGDDGVE--------------
------------------------HPTI-QLFWDVVS--------------------QFS
NEDKERLVGF-------VTSTPRAP------------LLG-----FG-NLNPRF--SIRD
GGRDE-----------------------------GRLPTTSTCV-NL-LKLPIF-TDGEV
LRERLL-YSIRA-GAGF
>Phaeomoniella_chlamydospora_JACF01000323. 1..
ADIKRNAT-FESAFDQF---------------YTLGDGLKE-PIQISFIDQFGSV--EAG
IDGGGVTKEF---LTSV----TKEAFDPHGDL------GLFTENDQH-------------
----------------LLYPTPGS----------------LEERKELL-RQAEFREGSPD
WREGIAELLRRYEFLGRIIGKCLYEGILVDVNFAG--FFLLKWAL-----------TGGT
TSASNESAYKPNLNDLRD----------------------LDEGLYQG--------LLQL
KNYAG---NVE-DFS--
--LHFSITDTFALPEVDTVR------------------RTK-NIDHD-LIPN--------
GSSIP---
-----------------------------VTNQNRP-HYISL-VTRHRLASQ-QYYQTK
-AFLQG---LGQMISS--SWLS-MFNQH-ELQTLVSG----------------------
--DTSGEID-----------VEDL----RAHTQYGGLYVIGDDGLE--------------
------------------------HPNI-QMFWDVMR--------------------SFS
HEDRRKVLKF-------VTSTPRAP------------LGG-----FS-HLNPRF--SIRD
SGTSEGE---------------------------VRLPSTSTCV-NL-LKLPRY-RTKDQ
MKEKLK-YSIES-GAGF
>Phialocephala_scopiformis_XM_018211566.1 .
AKIRRDQV-FEDAYEQF---------------FELGEGLKE-PIQITFVDQFDTV--EAG
IDGGGVTKEF---LTSV----TNEAFRAEDGP------NLFITNDQN-------------
----------------LLYPNPAA----------------LDERQELL-KQAGLRESSTE
WRENIHELLRHYEFLGRIVGKCLYEGILVDIGFAG--FFLLKWAA--------------A
GATGAESGYRPNINDLRD----------------------LDEGLYQG--------LLKL
KNYSG---NVE-DFS--
--LDFTITDTISLPGA----------------------PTQ-TIERD-LMPN--------
GSKTA---
-----------------------------VTNENRL-LYVSC-VARHRLQKQ-PYRQTQ
-AFLKG---LSEIINP--SWLS-MFNQS-ELQTLIGG----------------------
---DSSEID-----------VADL----RRNTLYGGVYVIGDDGQE--------------
------------------------HPTV-KLFWKVMK--------------------ALD
DSDRRKVLKY-------VTSTPRAP------------LLG-----FS-QLNPRF--SIRD
AGSDE-----------------------------TRLPSTSTCV-NL-LKLPRY-STEDI
LRQKLL-YAVNS-GAGF
>Phialocephala_subalpina_FJOG01000009.1 .
AKIRRDQI-FEDAYEQF---------------YELGEGLKE-PIQITFVDQFDTI--EAG
IDGGGVTKEF---LTSV----TNEAFRSEDGP------NLFITNDQN-------------
----------------LLYPNPGA----------------VDERQELL-KQAGLRESSSE
WRENITELLKHYEFLGRIVGKCLYEGILVDIGFAG--FFLLKWAT-------------FK
AAAGSESGYRANINDLRD----------------------LDEGLYQG--------LLKL

KSFPG--NVE-DFG--
--LDFTITDTISLPGA----------------------PTQ-TIERE-LLPN--------
GSNMS---
------------------------------VTNENRL-LYLAS-VARHRLLVQ-PQRQTR
-AFLKG---LSEIVNP--SWLS-MFNQA-ELQTLIGG-----------------------
---DSSEID-----------VEDL----RQNTLYGGIYVIGDDNQE--------------
------------------------HPTI-QLFWKVMK--------------------ALD
DSDRRKVLKY-------VTSTPRAP------------LLG-----FS-QLNPRF--SIRD
ASSDE-----------------------------TRLPSTSTCV-NL-LKLPRY-STEAI
LREKLL-YAVNS-GAGF
>Marssonina_brunnea_XM_007289005.1 .
AKIRRDQI-FEDAYKQF---------------YQLGQGLKE-PIQITFVDQFDTV--EAG
IDGGGVTKEF---LTSI----TNEAFLSEEASP-----RLFIANDQN-------------
----------------LLYPNPAT----------------VDERKELL-KAAGLRETSPV
WKESMNDLLKRYEFLGRIVGKCLYEGILVNIGFAG--FFLLKWAT---------------
--AGSESGYRANINDLRD----------------------LDEGLYQG--------LLKL
KNYPG--NVE-DFS--
--LDFTITDSISSDGG----------------------HEK-IITKD-LMPN--------
GSNIP---
------------------------------VTDKNRP-VYVSY-VARHRLQVQ-PFRQTQ
-AFLRG---LTEIINP--TWLS-MFNQS-ELQTLIGG-----------------------
---DSSEVN-----------VEDL----RRNTQYGGVYQIGDDGQE--------------
------------------------HSTI-KIFWKVLK--------------------ELD
DSDRRKVLKY-------VTSTPRAP------------LLG-----FS-QLNPRF--SIRD
AGRDE-----------------------------ERLPSTSTCV-NL-LKLPMY-TSEAR
LRTKLL-YAVNS-GAGF
>Cairneyella_variabilis_AYLM01000623.1 .
AQISRHQV-FEDAYKQF---------------YKLGEGLKE-PIQITFVDQFGTT--EAG
IDGGGVTKEF---LTSI----TNEAFRAEEAGP-----NLFVANDQN-------------
----------------LLYPNPAA----------------IDARKEIM-SQFGMKEGSDE
WRLTISELLKRFEFLGRVIGKCLYEGILVDIGFAG--FFLLKWAA--------------H
GATGSESGYRANINDLRD----------------------LDESLYQG--------LLKL
KNYPG--NVE-DFG--
--LDFTITDTISLPGE----------------------PTK-TITRE-LMPN--------
GSNVP---
------------------------------VTNENRP-LYVSY-MARHRLQVQ-PYQQTQ
-AFLRG---LSQIIKP--SWLS-MFNQS-ELQTLIGG-----------------------
---DSSEID-----------VDDL----RANTQYGGVYQIGDDGME--------------
------------------------HDTV-RLFWKVMK--------------------ALD
DSDRRKVLKY-------VTSTPRAP------------LLG-----FS-QLNPRF--SIRD
AGSDE-----------------------------SRLPSTSTCV-NL-LKLPKY-STERT
LREKLL-YAVNS-GAGF
>Hymenoscyphus_scutula_LKTO01000213.1 .
AKIRRDQV-FEDAYEQF---------------FELGEGLKE-PIQITFVDQFDTV--EAG
IDGGGVTKEF---LTSV----TNEAFRGEDGP------KWFISNDQN-------------
----------------LLYPNPSA----------------VDERREWL-RKIGLVEGSLE
WGENIAEILRRYEFLGRVVGKCLYEGILIDIGFAG--FFLLKWAA--------------A
GAAGSESGYRANINDLRD----------------------LDESLYQG--------LLKL
KNYPG--DVE-DFS--
--LDFTITDTISPPGE----------------------PTQ-TITRD-LKPN--------
GSNIP---
------------------------------VTNENRT-LYISY-VARHRLQVQ-PYQQTQ
-AFLKG---LGEIIKP--TWLS-MFNQS-ELQTLVGG-----------------------
---DSSEID-----------VEDL----RENTQYGGVYQIGDDGLE--------------
------------------------HSTI-QMFWQVMK--------------------NLD
DSDRRKVLKY-------VTSTPRAP------------LLG-----FS-QLNPRF--SIRD
AGRDE-----------------------------TRLPSTSTCV-NL-LKLPMY-TTIGV
LKQKLL-YAVNS-GAGF

```
>Hymenoscyphus_repandus_LLCE01000039.1 .
AKIRRDQV-FEDAYEEF---------------YQLGEGLKE-PIQITFVDKFDTV--EAG
IDGGGVTKEF---LTSV----TNEAFRSEGGP------NWFVANDNN-------------
----------------LLYPNPSA----------------VDERREQM-KKVELFEGSTE
WSDTLTEILKRYEFLGRIVGKCLYEGILIDIGFAG--FFLLKWAR--------------S
ASAGSESGYRANINDLRD----------------------LDEGLYQG--------LLKL
KNYSG----------------------------------------------NVE-DFG--
--LDFTITDTISSPNE----------------------PTA-TITRD-LMPS--------
GSSMP-------------------------------------------------------
------------------------------VTNENRP-LYVYR-VAQHRLQLQ-PYQQTQ
-AFLKG---LGEIIKP--SWLS-MFNQS-ELQTLVGG-----------------------
---DSSGID-----------VDDL----RQNTLYGGVYQIGDDGLE--------------
------------------------HPTI-QLFWQVLK--------------------NLE
DIDRRKVLKY-------VTSTPRAP------------LLG-----FS-QLNPRF--SIRD
AGSDE-----------------------------SRLPSTSTCV-NL-LKLPRY-TSANV
LKQKLL-YAVNS-GAGF
>Glarea_lozoyensis_XM_008080864.1 .
AKIRRGSL-FEDAFQQF---------------YDLREGLKE-PIQITFVDKFDTA--EAG
IDGGGVTKEF---LTSV----TNEAFKEGH--------KWFVANDQN-------------
----------------LLYPNPVV----------------IDEIRQRL-KQFGYEEGSPE
WQDATQDALKRFEFLGRIVGKCLYEGILVDIGFAG--FFLLKWAA--------------S
GGGAVESGYRANINDLRD----------------------MDESLYQG--------LLKL
KNYPG----------------------------------------------NVE-DFS--
--LDFTIVDEVSLEGE----------------------PSE-TIVKE-LMPN--------
GSNIA-------------------------------------------------------
------------------------------VTNENRL-LYVSY-VARHRLQLQ-PYRQTQ
-AFLKG---LGEIIKP--SWLS-MFNQS-ELQTLVGG-----------------------
---DASEID-----------VEDL----RLNTQYGGVYQIGDDGQE--------------
------------------------HDTI-KLFWHVLK--------------------HLD
DEDRRKVLKY-------VTSTPRAP------------LLG-----FS-QLNPRF--SIRD
AGGDE-----------------------------SRLPSTSTCV-NL-LKLPRY-STASV
LKSKLL-YAVNS-GAGF
>Sclerotinia_homoeocarpa_JW827358.1 .
AKIHRDRV-FEDAYKAF---------------YELGEGLKE-PIQITFVDQFDTQ--EAG
IDGGGVTKEF---LTSI----TNEAFKSENGA------NLFITNAQN-------------
----------------LLYPNPAA----------------LDERKALL-KEAGLTEDTPE
YAENINDLLRQYEFLGRVIGKCLYEGILVDISFAG--FFLLKWAH---------------
-SYAGQEAYRPNINDLRD----------------------LDEELYQG--------LLKL
KNYPG----------------------------------------------NVE-DFG--
--LDFTIVDTISLPGS----------------------PVQ-TITRD-LIDN--------
GSSIP-------------------------------------------------------
------------------------------VTNENRT-LYIAY-VARHRLQRQ-PRYQTA
-AFLKG---LGQIINP--SWLS-MFNQS-ELQTLIGG-----------------------
---ESSQID-----------IDDL----RRNTMYGGVYEIGDDGLE--------------
------------------------HPTI-QMFWHVMK--------------------ELD
DEDRRKVLKY-------VTSTPRAP------------LLG-----FS-QLNPRF--SIRD
SSGDE-----------------------------ERLPSTSTCV-NL-LKLPRY-GSVRK
LREKLL-YAVNS-GAGF
>Pseudogymnoascus_destructans_XM_012885741.1 .
ARIRRDNV-LEDAFGQF---------------FSLGEGLKE-PIQITFVDKFDNV--EAG
IDGGGVTKEF---LTTV----TNEAFRSEDGP------GYFVTNDQN-------------
----------------LLYPNPAA----------------VDQCKDVL-RHQGWEESDTE
WNESITQLLKRYEFLGRVVGKCLYEGILVDIGFAG--FFLLKWAS------------TAT
ADSASELRYQANINDLRD----------------------LDETLYQG--------LLQL
KNYTG----------------------------------------------NAQ-DFA--
--LDFTIDDVVSLANE----------------------PVR-TITRE-LIPN--------
GANKP-------------------------------------------------------
------------------------------VNNENRV-LYISL-VARHRLHSQ-PYHQTR
```

-AFLRG---LSQIIQP--SWLS-MFNQT-ELQTLIGG-----------------------
---DMAEID-----------VDDL----RRNTEYSGVYVIGDDGLE--------------
------------------------HPTI-NLFWQVMK--------------------QLD
DQDRRKVLKY-------VTSTPRSP------------LLG-----FG-QLNPKF--SIRD
AGGDE-----------------------------QRLPSASTCI-NL-LKLPRY-TSAKT
MSEKLL-YAVNS-GAGF
>Pseudogymnoascus_verrucosus_XM_018277696.1 .
ARIRRDNV-LEDAFGQF---------------FSLGEGLKE-PIQITFVDKFDNV--EAG
IDGGGVTKEF---LTTV----TNEAFRSVDGP------GYFVTNDQN-------------
----------------LLYPNPAA----------------VDQCKDVL-RHQGWQESDAE
WNDSITQLLKRYEFLGRVVGKCLYEGILVDIGFSG--FFLLKWAS------------TAN
ADSASELRYQANINDLRD----------------------LDETLYQG--------LLQL
KNYTG--NAE-DFA--
--LDFTIDDVVSLANE----------------------PVR-TITRE-LIPN--------
GANKP---
------------------------------VNNENRV-LYISL-VARHRLHSQ-PYHQTR
-AFLRG---LSQIIQP--SWLS-MFNQT-ELQTLIGG-----------------------
---DMAEID-----------VDDL----RRNTEYSGVYVIGDDGLE--------------
------------------------HPTI-NLFWQVMK--------------------QLD
DQDRRKVLKY-------VTSTPRSP------------LLG-----FG-QLNPKF--SIRD
AGGDE-----------------------------QRLPSASTCI-NL-LKLPRY-TSAKT
MSEKLL-YAVNS-GAGF
>Blumeria_graminis_CAUH01003100.1 .
AKIRRSNV-FEDAYHQF---------------YGLGEGIKE-PIQITFVDQFDAA--EAG
IDGGGVTKEF---LTSI----SNEAFCLKNDP------RLFITNDQN-------------
----------------LLYPNPSV----------------LDEARESL-RLEGHQESSLY
WKERINEILSQFVFLGRIVGKCLYEGILIDMEFAG--FFLLKWAT--------------S
GLMGSDLRYRANIDHLRD----------------------LDESLYQG--------LLKL
KNYHG--NVE-DFF--
--LDFTITDSITLKNG----------------------VDK-TITRE-LIPN--------
GSQVP---
------------------------------VNNENRP-LYVSY-VARHRLQVQ-PHQQTQ
-AFLKG---LTDVINP--TWIS-MFNQS-ELQRLISG-----------------------
---DSSEID-----------IDDL----RRNTVYSGVYEIGDDGEE--------------
------------------------HDTI-KIFWKVFK--------------------SFD
DSDRRKVLKY-------VTSTPRAP------------LLG-----FS-QLNPLF--TIRD
SGNDE-----------------------------ERLPSTSTCV-NL-LKLPRY-ANESN
LKEKLL-YAVHS-GAGF
>Erysiphe_pisiCACN01002656.1 .
AKIRRNNM-FEDAYHHF---------------YSLGDELKE-PIRINFVDQFDTV--EAG
IDGGGVTKEF---LTSV----ITEALCNKTSP------KFFVTNDQN-------------
----------------LLYPNPTA----------------LDEERENL-RREGHVESSPY
WKERIKDLLQQFEFLGRIIGKCLYEGILIDIGFAS--FFLLKWAA---------------
----SGMTYRANINDIRD----------------------LDEKLYEG--------LLQL
KNYNG--NVE-EFS--
--LDFTTTDTFLAKSG----------------------QTI-AITRD-LIPN--------
GSTMS---
------------------------------VTNENRP-LYVSY-VARHRLQVQ-PYQQTQ
-AFLKG---LSDVINP--AWIS-MFNRS-ELQILISG-----------------------
---DSSEID-----------VEDL----RRNTLYGGVYQIGDDGEE--------------
------------------------HETI-KYFWKVMK--------------------ALD
DSDRRKVLKF-------ITSSPRAP------------LLG-----FS-KLNPLF--SIRD
SGSDE-----------------------------DRLPSTSTCV-NL-LKLPRY-SNEDI
LKHKLL-YAVNS-GAGF
>Sclerotinia_glacialis_NGKH01000004.1 .
AQIRRQNV-FEDAFEAF---------------YPLAEGLKE-PIQITFIDSFGAV--EAG
IDGGGVTKEF---LTSV----TNEAFRGPLDYGGSP--SLFVTNDQN-------------
----------------LLYPNPTA----------------TEQDYEFL-RSFGINETIPG

```
WKSYSVPLRNRMQFLGRVVGKCLYEGILVDITFAG--FFLLKWSA---------------
AYSGPEAASRANLNDLRD----------------------LDEGLYQG--------LLKL
KNHPG-----------------------------------------------NVE-DFS--
--LDFTITETVPSPILGG--------------------RPK-IITRD-LMPN--------
GSNIP-------------------------------------------------------
------------------------------VTNENRL-LYIAY-VVRHRLHIQ-PYELTQ
-AFLRG---LGQIINP--SWLS-MFNQV-ELQTLISG-----------------------
---ETSEIN-----------IDDL----RKNTRYGGVYEIGDDGLE--------------
------------------------HPTI-MMFWQVMK--------------------EFE
DEDRRKVLKY-------VTSTPRAP------------LLG-----FS-SLNPLF--SIRD
GSADE-----------------------------ERLPSASTCV-NL-LKLPRY-QSKEK
LREKLL-YAINA-GAGF
>Sclerotium_cepivorum_NGKD01000001.1 .
AQIRRQNV-FEDAFEAF---------------YPLAEGLKE-PIQITFIDSFGAV--EAG
IDGGGVTKEF---LTSV----TNEAFRGPLDYGSCP--SLFVTNDQN-------------
----------------LLYPNPTA----------------TEQDYEFL-RSNGINETSPE
WRAYSVQLRNRMQFLGRVVGKCLYEGILIDINFAG--FFLLKWSA---------------
AYSGPEAASRGNLNDLRD----------------------LDEGLYQG--------LLKL
KNYPG-----------------------------------------------NVE-DFS--
--LDFTITDTVPSPLPGG--------------------RPK-IITRD-LMPN--------
GSNIP-------------------------------------------------------
------------------------------VTNENRL-LYIAY-VVRHRLHIQ-PYELTQ
-AFLRG---LGQIINP--SWLS-MFNQV-ELQTLISG-----------------------
---ETSEIN-----------IDDL----RRNTRYGGVYQIGDDGLE--------------
------------------------HPTI-MMFWQVME--------------------EFE
DEDRRKVLKY-------VTSTPRAP------------LLG-----FS-SLNPLF--SIRD
GSADE-----------------------------ERLPSASTCV-NL-LKLPRY-QSKEK
LREKLL-YAVNA-GAGF
>Sclerotinia_sclerotiorum_XM_001598229.1 .
ARIRRESV-FEDAFEAF---------------YPLSEGLKE-PIQITFIDSFGTA--EAG
IDGGGVTKEF---LTSA----TNEAFRGPLGYGSRT--SLFVTNDQN-------------
----------------LLYPNPTA----------------TEQDYEFL-RSNGITETNPD
WKAHSAQLRNRMQFLGRIVGKCLYEGILVDINFAG--FFLLKWSA---------------
AFSGSEATSRGNLNDLRD----------------------LDEGLYQG--------LLKL
KNYPG-----------------------------------------------NVE-DFS--
--LDFTITDTIPSHIPGG--------------------RPK-IITRD-LMPN--------
GSNIP-------------------------------------------------------
------------------------------VTNENRL-LYIAY-VVRHRLHIQ-PYELTQ
-AFLRG---LGQIINP--SWLS-MFNQV-ELQTLISG-----------------------
---ETSEIN-----------IDDL----RRNTRYGGVYEIGDDGLE--------------
------------------------HPTV-MMFWQVMK--------------------EFE
DEDRRKVLKY-------VTSTPRAP------------LLG-----FS-SLVPRF--SIRD
GSLDE-----------------------------KRLPSASTCV-NL-LKLPRY-QSKEK
LKEKLL-YAINA-GAGF
>Botrytis_paeoniae_LBGX01000139.1 .
ASIRRESV-FEDAFEAF---------------FPLSEGLKE-PIQITFVDSFGAI--EAG
IDGGGVTKEF---LTSV----TNEAFRSPLDYGSNP--SLFVTNDQN-------------
----------------LLYPNPTA----------------IEQDHEFL-RSIGINETSPD
WRTHSVQLRNRMHFLGRIVGKCLYEGILIDINFAG--FFLLKWSA---------------
ANSGPEAASRTNLNDLRD----------------------LDEGLYQG--------LLKL
KNYPG-----------------------------------------------NVE-DFS--
--LDFTITDTIPNGMPDG--------------------RPK-VITRD-LMPN--------
GSNIP-------------------------------------------------------
------------------------------VTNENRL-LYIAY-VVRHRLHIQ-PYELTQ
-AFLRG---LGQIINP--SWLS-MFNQV-ELQTLISG-----------------------
---ETSDIN-----------INDL----RENTQYGGVYQIGDDGRE--------------
------------------------HPTI-MMFWQVME--------------------ELK
DEDRRKVLKY-------VTSTPRAP------------LLG-----FG-SLNPLF--SIRD
```

SSSDE-----------------------------DRLPSASTCV-NL-LKLPRY-QSKEK
LREKLL-YAVNA-GAGF
>Botrytis_cinerea_AORW01000050.1 .
ASIRRESV-FEDAFEAF---------------FPLSEGLKE-PIQITFVDSFGAI--EAG
IDGGGVTKEF---LTSV----TNEAFRGPLDYGNNP--SLFVTNDQN-------------
----------------LLYPNPTA----------------MEQDYAFL-RSVGINETSPG
WKTHSVQLRNRMHFLGRIVGKCLYEGILIDINFAG--FFLLKWSA---------------
ANSGPEAASRSNLNDLRD----------------------LDEGLYQG--------LLKL
KNYPG--NVE-DFS--
--LDFTITDQIPNGMPDG--------------------RAK-VITRD-LMPN--------
GSNIP---
------------------------------VTNENRL-LYIAY-VVRHRLHIQ-PYELTQ
-AFLRG---LGQIINP--SWLS-MFNQV-ELQTLISG-----------------------
---ETSDIN-----------INDL----RKNTQYGGVYQIGYDGRE--------------
------------------------HPTI-RMFWQVME--------------------EFK
DEDRRKVLKY-------VTSTPRAP------------LLG-----FA-SLNPQF--SIRD
SSSDE-----------------------------DRLPSASTCV-NL-LKLPRY-QSKEK
LREKLL-YAVNA-GAGF
>Monilinia_aucupariae_NGKF01000028.1 .
AQIRRDNV-FEDAFDTF---------------NSLGEGLKE-PIQITFVDSFGSI--EAG
IDGGGVTKEF---LTSV----TNEAFHGPLKFGSDA--TLFVTNDQN-------------
----------------LLYPNPVA----------------VEQDYENL-RERGVDETDPN
WRAYGMQLRSRMNFLGRIVGKCLYEGILIDINFAG--FFLLKWSA---------------
AYSGSEAAGRANLNDLRD----------------------LDEGLYQG--------LLKL
KNYPG--NVE-DFS--
--LDFTITDTIPISKPGV--------------------RPK-IITRE-LIPN--------
GSNIP---
------------------------------VTNENRL-LYIAY-VVRHRLHIQ-PYDLTQ
-AFLRG---LGQIINP--SWLS-MFNQA-ELQTLISG-----------------------
---ETSEID-----------VDDL----RNHTQYGGVYSIGDDGLE--------------
------------------------HPTV-VMFWEVME--------------------ELE
DEDRRKVLKY-------VTSTPRAP------------LLG-----FA-QLNPLF--SIRD
SSADE-----------------------------ERLPSASTCV-NL-LKLPRY-QSKEK
LREKLL-YAVRA-GAGF
>Monilinia_fructicola_NGKE01000003.1 .
AQIRRDNV-FEDAFATF---------------NSLGEGLKE-PIQITFVDSFGSV--EAG
IDGGGVTKEF---LTSV----TNEAFHGPLKFGSDA--TLFVTNDQN-------------
----------------LLYPNPTA----------------VEQDYETL-RENGVDETDPN
WRAYGMQLRNRMNFLGRIVGKCLYEGILIDINFAG--FFLLKWSA---------------
AYSGSEAAGRANLNDLRD----------------------LDEGLYQG--------LLKL
KNYPG--NVE-DFS--
--LDFTITDTIPINKPDV--------------------RPK-IITRD-LIPN--------
GSNIP---
------------------------------VTNENRL-LYIAY-VVRHRLHIQ-PYELTQ
-AFLRG---LGQIINP--SWLS-MFNQA-ELQTLISG-----------------------
---ETSEID-----------VNDL----RENTQYGGVYSIGDDGLE--------------
------------------------HPTV-VMFWEVME--------------------ELE
DEDRRRVLKY-------VTSTPRAP------------LLG-----FS-QLNPLF--SIRD
SSADE-----------------------------ERLPSASTCV-NL-LKLPRY-QSKER
LREKLL-YAVRA-GAGF
>Ciborinia_camelliae_LGKQ01000453.1 .
AQIRRESV-FEDAFRNF---------------YSLAEGLKE-PIQITFVDSFGAI--EAG
IDGGGVTKEF---LTSV----TNEAFRGPLDFGSGP--SLFVTNDQN-------------
----------------LLYPNPTA----------------IEQDYEFL-RSSGVDETNPE
WRTYSSQLRNRMQFLGRIVGKCLYEGILIDINFAG--FFLLKWSA---------------
AYSGPEAASRANLNDLRD----------------------LDEGLYQG--------LLKL
KNYPG--NVE-DFS--
--LDFTITDTIPVL--DG--------------------RPK-IITRD-LMPN--------

GSNIP---
------------------------------VTNENRL-LYIAY-VVRHRLQIQ-PYDVTQ
-AFLRG---LGQIINP--SWLS-MFNQA-ELQTLISG-----------------------
---ETSEIN-----------INDL----RENTQYGGLYQVGDDGVE--------------
------------------------HPTI-VMFWEVME--------------------ELE
DEDRRKVLKY-------VTSTPRAP------------LLG-----FA-QLNPLF--SIRD
SSVDE-----------------------------ERLPSASTCV-NL-LKLPRY-QTKER
LRTKLL-YAVNA-GAGF
>Sclerotinia_borealis_AYSA01000316.1 .
AQIRRENV-FEDAFRAF---------------YPLADGLKE-PIQITFIDSFGAV--EAG
IDGGGVTKEF---LTSV----TNEAFRAPLDSGSGP--SLFVTNDQN-------------
----------------LLYPNPTA----------------VQQDHEFL-RSNEIDETNPD
WKAYSVQLRNRMHFLGRIVGKCLYEGILIDINFAG--FFLLKWSA---------------
AYSGPEAASRANLNDLRD----------------------LDEGLYQG--------LLKL
KNYAG--NVE-DFS--
--LDFTIIDTIPNSIPGG--------------------RPK-IITRD-LMPN--------
GSNIT---
------------------------------VTNENRL-LYIAY-VVRHRLQIQ-PYEVTQ
-AFLRG---LGQIINP--SWLS-MFNQA-ELQTLIAG-----------------------
---ETSEID-----------IDDL----RDNTQYGGVYQIGDDGLE--------------
------------------------HPTI-VMFWEVME--------------------EFP
DEDKRKVLKY-------VTSTPRAP------------LLG-----FA-QLNPPF--SIRD
SSGDE-----------------------------ERLPSASTCV-NL-LKLPRY-QSRER
LREKLL-YAVNA-GAGF
>Myriosclerotinia_curreyana_NGKJ01000034.1 .
AQISRDNV-FEDAFASF---------------NSLAEGLKE-PIQITFVDSFGAV--EAG
IDGGGVTKEF---LTSV----TNEAFRGPLKSGGGQ--FLFVANDQN-------------
----------------LLYPNPAA----------------VEQEHEFL-RSSGIDKTNPD
WGLYSSQTRNRMNFLGRIVGKCLYEGILIDINFAG--FFLLKWSA---------------
AYSGSEGTSRANLNDLRD----------------------LDEGLYQG--------LLKL
KNYPG--NVE-EFS--
--LDFTITDTIPSTTPDG--------------------RPK-IITRD-LIPN--------
GSNVP---
------------------------------VTNENRL-LYIAY-VVRHRLQIQ-PYEVTQ
-AFLRG---LGQIINP--SWLS-MFNQA-ELQTLISG-----------------------
---ETSEID-----------INDL----RNNTQYGGVYQIGDDGLE--------------
------------------------HPTI-MMFWEVME--------------------EFA
DEERRKVLKY-------VTSTPRAP------------LLG-----FA-QLNPPF--SIRD
SSGDE-----------------------------ERLPSASTCV-NL-LKLPRY-STRGR
LREKLL-YAVNA-GAGF
>Myriosclerotinia_scirpicola_NGKG01000003. 1..
AQISRDNV-FEDAFASF---------------NSLAEGFKE-PIQITFVDSFGAV--EAG
IDGGGVTKEF---LTSV----TNEAFRGPLKSGSGQ--HLFVANDQN-------------
----------------LLYPNPAA----------------VEQEHEFL-RSSGIDRTNPD
WGLYSSQTRNRMNFLGRIVGKCLYEGILIDINFAG--FFLLKWSA---------------
AYSGSEGTSRANLNDLRD----------------------LDEGLYQG--------LLKL
KNYPG--NVE-EFS--
--LDFTITDTIPSTTPGG--------------------RPK-IITRD-LIPN--------
GSNIP---
------------------------------VTNENRL-LYIAY-VVRHRLQIQ-PYEVTQ
-AFLRG---LGQIINP--SWLS-MFNQA-ELQTLISG-----------------------
---ETSEID-----------INDL----RNNTQYGGVYQIGDDGLE--------------
------------------------HPTI-MMFWEVME--------------------EFE
DEDRRKVLKY-------VTSTPRAP------------LLG-----FA-QLNPLF--SIRD
SSGDE-----------------------------ERLPSASTCV-NL-LKLPRY-STRER
LREKLL-YAVNA-GAGF
>Myriosclerotinia_duriaeana_NGKI01000001.1 .
AQISRDNV-FEDAFASF---------------NSLSEGLKE-PIQITFVDSFGAV--EAG

IDGGGVTKEF---LTSV----TNEAFRGPLKSGSGQ--FLFVANDQN-------------
----------------LLYPNPAA----------------VEQEHEFL-RSSGIDKTNPE
WGLYSSQTRNRMNFLGRIVGKCLYEGILIDINFAG--FFLLKWSA---------------
AYSGSEGTSRANLNDLRD----------------------LDEGLYQG--------LLKL
KNYPG--NVE-EFS--
--LDFTITDTIPSTTPGG--------------------RPK-IITRD-LIPN--------
GSNIP---
------------------------------VTNENRL-LYIAY-VVRHRLQIQ-PYEVTQ
-AFLRG---LGQIINP--SWLS-MFNQA-ELQTLISG-----------------------
---ETSEID-----------INDL----RNNTQYGGVYQIGDDGLE--------------
------------------------HPTI-MMFWEVME--------------------EFE
DEDRRKVLKY-------VTSTPRAP------------LLG-----FA-QLNPPF--SIRD
SSGDE-----------------------------ERLPSASTCV-NL-LKLPRY-STRER
LREKLL-YAVNA-GAGF
>Aureobasidium_pullulans_AYEO01000003.1 .
ANVRRKHE-FEDAYKQF---------------FSLGDGLKE-PIQITFLDEFGLQ--EAG
IDGGGVTKEF---LTSI----TTEAFNPEK--------GLFMENSQH-------------
----------------SLYPDPTS----------------VEALKEML-RLEGYPDDTQE
HRESRQDLLQRYEFLGRVVGKCLYEGILIDINFAS--FFLTKWAL-----------FGGR
NSASRESSYRASINDLRD----------------------LDEELYQG--------LLSL
KYYSG--DVA-EWG--
--TYFAINDTIPLPNG----------------------HTK-TIEHE-LVPN--------
GANKL---
------------------------------VNRENRL-LYISA-VARYRLSLQ-SKPQTD
-AFLGG---LSSIIQP--SWLN-MFNQK-ELQTLIGG-----------------------
---AASSID-----------ISDL----RANTEYNGVYVIGDDGAE--------------
------------------------HPSV-QLFWQVMQ--------------------DLP
DADRRKVLKF-------VTSTPRAP------------LLG-----FG-SLNPRF--TIRD
SGEDE-----------------------------HRFPTASTCV-NL-LKLPRF-RSQQA
LRDKLL-YAVNS-GAGF
>Aureobasidium_subglaciale_XM_013493559.1 .
ASIRRKNE-FEDAFSQF---------------YSLGDGLKE-PIQITFLDEFGLQ--EAG
IDGGGVTKEF---LTSI----TSEAFNPEK--------GLFMENSQH-------------
----------------SLYPDPTS----------------VEAHKEML-RQEGYTNDTEE
YRESMKDLLNRYEFLGRVVGKCLYEGILIDINFAS--FFLTKWAL-----------FGGR
GAASKESGYRASINDLRD----------------------LDEELYQG--------LQTL
KHHPG--EVG-EWG--
--TYFAINDTIPLPNG----------------------QTK-TVEFE-LIPN--------
GANIL---
------------------------------VTRENRL-LYISH-VARYRLSRQ-SKLQTD
-AFLTG---LSSIIQP--SWLN-MFNQK-ELQTLVGG-----------------------
---AASSIN-----------IPDL----RANTQYSGVYVIGDDGQE--------------
------------------------HPSV-QLFWQLML--------------------DLP
DIERRKVLKF-------VTSTPRAP------------LLG-----FG-SLNPRF--TIRD
SGDDE-----------------------------HRFPTASTCV-NL-LKLPRF-RSKEA
LRDKLL-YAVNS-GAGF
>Aureobasidium_namibiae_XM_013567727.1 .
ASIRRKNE-FEDAFKQF---------------YSLGEKLKE-PIQITFLDEFGLQ--EAG
IDGGGVTKEF---LTSV----TTEAFNPEK--------LYFTENSQH-------------
----------------SLYPNPTS----------------VEVHKEML-RMTGFPDDTEE
HREVIRDLLQRYEFLGRVVGKCLYEGILIDINFAS--FFLSKWAL-----------FGGT
NNASRESAYKASVNELRD----------------------LDEELYQG--------LMSL
KHYPG--DVA-EWG--
--TYFAVNNTITLPNG----------------------QTK-TMEHE-LIPN--------
GANTL---
------------------------------VNRGNRL-VYIAK-VAQYRLSVQ-SHLQTT
-AFLTG---LSAIIQP--SWLN-MFNQK-ELQTLIGG-----------------------
---AASNID-----------VSDL----RANTHYNGVYEIGTDGQE--------------

```
------------------------HPSI-QLFWQIML--------------------ELP
DADRRKVLKF-------VTSTPRAP------------LLG-----FG-SLNPRF--TIRD
SGDDE-----------------------------KRFPTASTCV-NL-LKLPRF-KSKEA
LRDKLL-YAVNS-GAGF
>Aureobasidium_pullulans_AMCU01000119.1 .
ASIRRKNE-FEDAFRQF---------------YSLGDGLKE-PIQITFLDEFGLQ--EAG
IDGGGVTKEF---LTSI----TTEAFNPEK--------LLFTENSQH-------------
----------------SLYPNPTS----------------VEAHKEML-RQSGYPDDMEE
HRESVKDLLQRYEFLGRVVGKCLYEGILIDINFAA--FFLTKWAL-----------FGGQ
GAAKRESGYRASVNDLRD----------------------LDEELYQG--------LMSL
KHYPG----------------------------------------------EVA-DWG--
--TYFAVNSTVTLPNG----------------------QSK-TIEHE-LISN--------
GADTL-------------------------------------------------------
------------------------------VDSRNRL-IYISK-VANYRLSLQ-SKLQTD
-AFLTG---LSSIIQP--SWLN-MFNQQ-ELQTLIGG-----------------------
---AASSID-----------IADL----RANTYYNGVYQIGDDGQE--------------
------------------------HPSV-QLFWQTML--------------------ELP
DAEKRKVLKF-------VTSTPRAP------------LLG-----FG-SLNPRF--TIRD
SGSDE-----------------------------HRFPTASTCI-NL-LKLPRY-TSKEM
LKEKLL-YAVNS-GAGF
>Aureobasidium_melanogenum_AYEN01000051.1 .
ASIRRKNE-FEDAFRQF---------------YSLGDGLKE-PIQITFLDEFGLQ--EAG
IDGGGVTKEF---LTSI----TTEAFNPEK--------LLFTENSQH-------------
----------------SLYPNPNS----------------VEAHKEML-RQSGYPDDMEE
HRESVKDLLQRYEFLGRVVGKCLYEGILIDINFAA--FFLTKWAL-----------FGGR
GAAKRESGYRASVNDLRD----------------------LDEELYQG--------LMSL
KHYPG----------------------------------------------EVA-DWG--
--TYFAVNSTVTLPNG----------------------QSK-TIEYE-LIPN--------
GTDTL-------------------------------------------------------
------------------------------VDSRNRL-IYISK-VANYRLSLQ-SKRQTD
-AFLTG---LSSIIQP--SWLN-MFNQQ-ELQTLIGG-----------------------
---AASSID-----------IADL----RANTYYNGVYQIGDDGQE--------------
------------------------HPSV-QLFWQTML--------------------ELP
DAEKRKVLKF-------VTSTPRAP------------LLG-----FG-SLNPRF--TIRD
SGDDE-----------------------------HRFPTASTCI-NL-LKLPRY-TSKEM
LKEKLL-YAVNS-GAGF
>Endocarpon_pusillum_JFDM01000015.1 .
ANIRRQHI-FSSAFDQF---------------NKLGDGLKE-PVQITFIDQFGSE--EAG
IDGGGVTKEF---LTSI----TKETFDPLNEE------QLFVANEQN-------------
----------------LLYPNPGK----------------LDAYIYLL-RAAGMRDKTAE
MDAAVKELLARYRFLGRIIGKCLYEGFLIDVSFAG--YFLLRWAL-----------TGGT
TQAAKESGYRPTIDDLRE----------------------LDEDLYQG--------ILKL
KHYDG----------------------------------------------DVEADFG--
--LNFTVVDTFEVPAPTPSDPHNT--------------ETK-SITRP-LISS--------
GADIP-------------------------------------------------------
------------------------------VTNINRP-QYISA-LVNHRLRTQ-SLFQTN
-AFLLG---LGEIIQP--MWLS-MFNQS-ELQRLIGG-----------------------
---DSREID-----------ISDL----RTNTVYSGLYVLGDNDEE--------------
------------------------HPTI-KLFWDVLR--------------------QMD
DQDRRKVLKF-------VTSTPRAP------------LLG-----FG-HLNPRF--SIRD
SGAGDE----------------------------GRLPSTSTCV-NL-LKLPRY-SNARV
LREKLL-YAVNS-GAGF
>Cyphellophora_europaea_XM_008721584.1 .
AEIRRESV-FEDAYESY---------------YPLGDKLKE-PIQISFIDKFGAP--EAG
IDGGGVTKEF---LTSV----TSEALDPNRSM------AMFSENAQH-------------
----------------YLYPNPVI----------------WQETQENL-RRMGLKEGSEA
RNHHLREFLRRYEFLGRVIGKCLYEGILIDINFAG--FFLLKWAL-----------TGGS
TVATKETGYRASINDLRE----------------------YDEELYQG--------LLKL
```

KYYPG---DVEADFG--
--LNFTVNDTLHLTDSDGKE------------------TEV-VKTIK-LYPD--------
GANKV---
------------------------------VDNVWRH-IYIDR-MVRYRLQDQ-PERVTS
-AFLQG---LGQIVSP--SWLA-MFNQK-ELQKLVGG----------------------
---DNTDLD-----------IADL----RRNTQYGGLYVIGDDGLE--------------
------------------------HPTV-ELFWKAMQ--------------------EMD
DADRRRVLKF-------VTSTPRAP------------LLG-----FS-NLNPKF--SIRD
SSADQ-----------------------------ERLPSTSTCV-NL-LKLPRY-GDLQT
MKDKLL-YAANS-GAGF
>Phialophora_attae_XM_018147004.1 .
AEIRREHV-FEDAYDIY---------------YQLGDKLKE-PIQISFIDKFGTP--EAG
IDGGGVTKEF---LTSV----TSEALDPSGPS------PMFTENAEH-------------
----------------FLYPNPLI----------------WQEMQESL-RREGTRDGSEG
WNAVLRDYLRRYEFLGRVIGKCLYEGILIDVNFAG--FFLLKWAL-----------TGGS
TVATNESAYRASINDLRE----------------------YDEELYQG--------LLKL
KYYPG---NVEADFG--
--LNFTVNDTLNVQDEMGKI------------------TSV-VQTVN-LYPD--------
GGNKA---
------------------------------VDNVWRH-LYIDR-IVKYRLQDQ-PEKVTN
-AFLRG---LGQIISP--TWLA-MFNQK-ELQKLVGG----------------------
---DSSELD-----------IADL----RRNTQYGGIYVIGDDGLE--------------
------------------------HPTV-ELFWKAMQ--------------------EMD
DGDRRKVLKF-------VTSTPRAP------------LLG-----FG-NLNPKF--SIRD
SSTDQ-----------------------------SRLPSTSTCV-NL-LKLPRY-GDLST
MKDKLL-YAANA-GAGF
>Herpotrichiellaceae_sp._AMYF01000100.1 .
ADIHRGSV-FEDAFESF---------------YPLGEELKE-PIQISFIDQFGQP--EAG
IDGGGVTKEF---LMSV----TSEAFDPQSEM------PLFEENEQR-------------
----------------YLYPKPTT----------------LQQAKYVL-VKDGNKPGTEG
YNAAMREILQRYEFLGRIVGKCMYEGILIDVNFAG--FFLLKWAL-----------TGGT
TSATSESAFRASINDLRE----------------------FDEQLYQG--------LLKL
KNYSG---DVEADFG--
--LNFTVSDTFDVEG-----------------------RTI-SRTTD-LIPG--------
GSHES---
------------------------------VTNKNRL-KYIDR-IVRYRLQEQ-PRLITN
-AFLKG---LGQIISP--MWLA-MFNQK-ELQKLVGG----------------------
---DNQELD-----------ILDL----RRNTQYGGIYVIGDDGLE--------------
------------------------HPTV-AMFWKVLQ--------------------QMS
DADRRKVLKF-------VTSTPRAP------------LLG-----FS-HLNPKF--SIRD
SSEDQ-----------------------------TRLPSTSTCV-NL-LKLPRY-SDERT
MREKLL-YAANA-GAGF
>Exophiala_aquamarina_XM_013404842.1 .
AEIHRESV-FEDAYDAF---------------YPLGEALKE-PIQITFIDQFGAP--EAG
IDGGGVTKEF---LMSV----TSEAFDPDSSL------SAFKENNQR-------------
----------------YLFPNPLI----------------YEETVRFL-VRMGRSPNTEG
YTRPMNEFLRRFQFLGRVVGKCLYEGILIDVTFAG--FFLLKWAL-----------TGGS
TVGSNETAYRPSINDVRE----------------------YDEELYQG--------LLKL
KNYPG---DVESDFA--
--LDFTVTDTFNVLDVDGKQ------------------IPE-TITTE-LRPN--------
GANIA---
------------------------------VTNINRF-DYINR-IVRRKLLEQ-PKMVTD
-AFLSG---LGQIIQP--SWLA-MFNQK-ELQKLVGG----------------------
---DSSELD-----------IADL----RKHTQYGGLYQIGDDGLE--------------
------------------------HPTV-QLFWEALQ--------------------EMD
NEDRRKVLKF-------VTSTPRAP------------LLG-----FS-HLNPRF--SIRD
SSEDQ-----------------------------ERLPSTSTCV-NL-LKLPRY-GDIKT
MKDKLL-YAVNS-GAGF

```
>Exophiala_mesophila_XM_016365929.1 .
AEIHRESI-FEDALDQF---------------YDLGEALKE-PIQITFIDKFNTP--EAG
IDGGGVTKEF---LMSV----TKDALDPNNPA------AMFCETPQK-------------
----------------SLYPSPLL----------------YDELAKYY-ASVGHAP---S
TDDSMTNWMRRFQFIGRIIGKCLYEGILIDVTFAG--FFLLKWAL-----------TGGS
TVGSNESAYRPTINDLRE----------------------YDEELYQG--------LLKL
KNYPG----------------------------------------------DVEADFG--
--LDFTLTDTLTILGKEPK-------------------RFE-TITTE-LKPN--------
GANIP-------------------------------------------------------
------------------------------VTNANRL-EYINR-VVQRKLVGQ-PKTVTG
-HFLAG---LGQIIQP--SWLA-MFNQK-ELQKLVGG-----------------------
---DSSELD-----------IADL----RNNTQYGGLYVVGDDGQE--------------
------------------------HPTV-QLFWKALQ--------------------DMD
DDDRRKVLKF-------VTSTPRAP------------LLG-----FS-HLNPKF--SIRD
SSEDQ-----------------------------TRLPSTSTCV-NL-LKLPRY-GDYKT
MKEKLL-YAVNS-GAGF
>Exophiala_spinifera_XM_016381802.1 .
AEIHRDSV-VQDAFDAF---------------FPLGEGLKE-PIQITFIDKFGAP--EAG
IDGGGVTKEF---LMSV----TSEALQPEDDF------SMFAESPQH-------------
----------------AIYPNPKL----------------IPTLTNYMRKHGILPGTDGV
YESKVKRMLHQMQFIGRIVGKCLYEGILIDVNFAG--FFLLKWAL-----------TGGT
TVGSNESAYRATINDLRD----------------------YDEELYHG--------LLKL
KNYPG----------------------------------------------DVEADFS--
--LDFTVTDMMHDCDENGAD------------------VSR-PHTIE-LIPN--------
GANTP-------------------------------------------------------
------------------------------VTNTNRL-VYIDR-VVRYRLQQQ-PKAVTD
-AFLKG---LGQIIQP--MWLA-MFNQK-ELQKLVGG-----------------------
---DNTELD-----------IADL----RRNTMYGGVYAIGDDGQE--------------
------------------------HPTV-QLFWKALQ--------------------EMD
DDDRRKVLKF-------VTSTPRAP------------LLG-----FS-HLNPKF--SIRD
SSDDQ-----------------------------ERLPSTSTCV-NL-LKLPRY-EDIRT
MREKLL-YAVNS-GAGF
>Exophiala_oligosperma_XM_016408033.1 .
AEIHRESV-VDDAFQAF---------------FELGEGLKE-PIQITFIDKFGTP--EAG
IDGGGVTKEF---LMSV----TSEALKPENDF------SMFAESPQH-------------
----------------AIFPNWKI----------------IPTLTGYMKRIGMTPGTDGE
FESKVKRLLQQVQFVGRVVGKCLYEGILIDVNFAG--FFLLKWAL-----------TGGT
TVGSNESAYRATINDLRD----------------------YDEELYNG--------LLKL
KNYPG----------------------------------------------DVEADFS--
--LDFTVTDMMLGCDEQGNE------------------IPV-PHTIE-LIPN--------
GANTP-------------------------------------------------------
------------------------------VTNTNRL-VYIDR-VVRFRLQQQ-PKTMTD
-AFLKG---LGEIISP--MWLA-MFNQK-ELQKLVGG-----------------------
---DNTELD-----------IADL----RRNTQYGGVYTIGDDGLE--------------
------------------------HPTV-QLFWKALQ--------------------EMD
DDDRRKVLKF-------VTSTPRAP------------LLG-----FS-HLNPRF--SIRD
SSGDQ-----------------------------ERLPSTSTCV-NL-LKLPRY-GDIET
MKEKLL-YAVNS-GAGF
>Fonsecaea_monophora_LVKK01000048.1 .
AQIHRDKV-FTDAYEAF---------------YGLGEGLKE-PIQITFIDQFGTP--EAG
IDGGGVTKEF---LISV----TNEAFDPDAKL------PMFKENAQR-------------
----------------YLYPNPTI----------------YQETAEYL-RVAGVRWGTQG
MAFPMAEFLRRYQFLGRIVGKCLYEGILIDVTFAG--FFLLKWAL-----------TGGT
TVGSNESAYRATINDLRE----------------------YDEELYQG--------LLKL
KNYTG----------------------------------------------DVETDFS--
--LDFTVTDTITIEDDDGKK------------------VHK-TFVTE-LLPN--------
GANTP-------------------------------------------------------
------------------------------VTNANRL-LYIDR-IVRYRLQQQ-PKAVTD
```

-AFLRG---LGQIIQP--MWLA-MFNQK-ELQKLVGG----------------------
---DNKELD-----------IADL----RRNTQYGGVYAIGDDGLE--------------
------------------------HPTI-QLFWKALQ--------------------EMS
DEDRRKVLKF-------VTSTPRAP------------LLG-----FS-HLNPKF--SIRD
SSGDQ-----------------------------DRLPSTSTCV-NL-LKLPRY-EDLKT
MKEKIL-YAVNS-GAG-
>Fonsecaea_pedrosoi_XM_013425462.1 .
AQIHRDKV-FTDAYEAF---------------YGLGEGLKE-PIQITFIDQFGTP--EAG
IDGGGVTKEF---LISV----TNEAFDPDAKL------PMFKENAQR-------------
----------------YLYPNPTI----------------YQETAEYL-RVAGVRWGTQG
MAFPMAEFLRRYQFLGRIVGKCLYEGILIDVTFAG--FFLLKWAL-----------TGGT
TVGSNESAYRATINDLRE----------------------YDEELYQG--------LLKL
KNYTG---DVETDFS--
--LDFTVTDTITIEDDDGKK------------------VHK-TFVTE-LLPN--------
GANTP---
------------------------------VTNANRL-LYIDR-IVRYRLQQQ-PKAVTD
-AFLRG---LGQIIQP--MWLA-MFNQK-ELQKLVGG----------------------
---DNKELD-----------IADL----RRNTQYGGVYAIGDDGLE--------------
------------------------HPTI-QLFWKALQ--------------------EMS
DEDRRKVLKF-------VTSTPRAP------------LLG-----FS-HLNPKF--SIRD
SSGDQ-----------------------------DRLPSTSTCV-NL-LKLPRY-EDLKT
MKEKIL-YAVNS-GAGF
>Fonsecaea_nubica_LVCJ01000042.1 .
AQIRRDKV-FTDAYEAF---------------YGLGEGLKE-PIQITFIDQFGTP--EAG
IDGGGVTKEF---LISV----TNEAFDPDANL------PMFKENAQR-------------
----------------YLYPNPTI----------------YQETAEYL-RVAGVRWGTQG
MAFPMAEFLRRYQFLGRIVGKCLYEGILIDVTFAG--FFLLKWAL-----------TGGT
TVGSNESAYRATINDLRE----------------------YDEELYQG--------LLKL
KNYTG---DVETDFS--
--LDFTVTDTITIEDDDGKK------------------VHK-TFVTE-LLPN--------
GANTP---
------------------------------VTNANRL-LYIDR-IVRYRLQQQ-PKAVTD
-AFLRG---LGQIIQP--MWLA-MFNQK-ELQKLVGG----------------------
---DNKELD-----------IADL----RRNTQYGGVYAIGDDGLE--------------
------------------------HPTI-QLFWKALQ--------------------EMS
DEDRRKVLKF-------VTSTPRAP------------LLG-----FS-HLNPKF--SIRD
SSGDQ-----------------------------DRLPSTSTCV-NL-LKLPRY-EDLKT
MKEKIL-YAVNS-GAG-
>Fonsecaea_multimorphosa_XM_016777430.1 .
AQIRRGEV-FMDAYEAF---------------YGLGEGLKE-PIQITFIDQFGTP--EAG
IDGGGVTKEF---LISV----TSEAFDPDAKL------PMFKENAQR-------------
----------------YLFPNPTI----------------YQETAEYQ-RLLGVPWGTQE
MAFAMAELLRHYQFLGRIVGKCLYEGILIDVYFAG--FFLLKWAL-----------TGGT
TVGSNESAYRATINDLRE----------------------YDEELYQG--------LLKL
KNYSG---DVETDFS--
--LDFTVTDEITIDDDDGTK------------------VSK-TITTE-LLPN--------
GANTP---
------------------------------VTNANRL-LYIDR-IVRYRLQQQ-PKAVTD
-AFLKG---LGQIIQP--MWLA-MFNQK-ELQKLVGG----------------------
---DNTELD-----------IPDL----RRNTQYGGVYAIGDDGRE--------------
------------------------HPTI-QLFWKALQ--------------------EMS
DEDRRKVLKF-------VTSTPRAP------------LLG-----FS-HLTPKF--SIRD
SSGDQ-----------------------------DRLPSTSTCV-NL-LKLPRY-EDLKT
MKEKLL-YAVNA-GAGF
>Cladophialophora_immunda_XM_016396178.1 .
AQIRRSSV-FADAFEAF---------------YGLGEGLKE-PIQITFIDQFGAP--EAG
IDGGGVTKEF---LISV----TNEAFDPDANL------PMFKENAQR-------------
----------------YLYPNPMI----------------YQETAEYL-KVAASRGDTEG

MAISMADLIRRYQFLGRVVGKCLYEGILIDVNFAG--FFLLKWAL-----------TGGT
TVGSNESAYRATINDLRE----------------------YDEELYQG--------LLKL
KNYVG---DVETDFS--
--LDFTVADTITIEADDGTE------------------VHK-TVTTE-LRPN--------
GANIP---
------------------------------VTNTNRL-LYIDR-IVRYRLQQQ-PKAVTD
-AFLRG---LGQIIQP--MWLA-MFNQK-ELQKLVGG-----------------------
---DNTELD-----------IADL----RRNTQYGGVYSIGDDGRE--------------
------------------------HPTI-QLFWKVLQ--------------------DMS
DEDRRKVLKF-------VTSTPRAP------------LLG-----FS-HLNPKF--SIRD
SSGDQ-----------------------------DRLPSTSTCV-NL-LKLPRY-EDLKT
MKEKLL-YAVNS-GAGF
>Fonsecaea_erecta_XM_018841367.1 .
AEISRGRV-FMDAFEAF---------------YELGDGLKE-PIQITFIDQFGAP--EAG
IDGGGVTKEF---LISV----TNEAFDPNPKM------SMFKENAQR-------------
----------------YLFPNPMI----------------YQETAEYL-KMPGLKWNVDG
VSSPLVELLRRYQFLGRIVGKCLYEGILIDVNFAG--FFLLKWAL-----------TGGT
TVGSNESAYRATINDVRE----------------------FDEELYQG--------LLKL
KNYPG---DVETDFS--
--LDFTVTDTVTYENEDGTE------------------AHK-TFTTE-LLPN--------
GANIP---
------------------------------VTNTNRL-LYIDR-IVRYRLQQQ-PKAVTD
-AFLKG---LGQIIQP--MWLA-MFNQK-ELQKLVGG-----------------------
---DNTELD-----------IADL----RRNTQYGGVYAIGDDGQE--------------
------------------------HPTI-QLFWKALQ--------------------EMS
DEDRRKVLKF-------VTSTPRAP------------LLG-----FS-HLNPKF--SIRD
SSGDQ-----------------------------DRLPSTSTCV-NL-LKLPRY-EDLKT
MKEKLL-YAVNS-GAGF
>Cladophialophora_psammophila_XM_007752628.1 .
AEIRRTSV-FEDAYESF---------------YKLGEGLKE-PIQITFIDRFGAP--EAG
IDGGGVTKEF---LISV----TSEAFDPDSKP------AMFKENAQR-------------
----------------YLYPNPTI----------------YQETAEHL-RLAGLKGGSQE
TAFAMAELLHRFQFLGRIVGKCLYEGILIDVTFAG--FFLLKWAL-----------TGGT
TVGSNESAYRATINDLRE----------------------YDEELYQG--------LLKL
KNYTG---DVETDFA--
--LDFTVTDTLTVEDEQGKT------------------IHK-TSTRE-LLPN--------
GANTP---
------------------------------VTNTNRL-LYIDR-IVRYRLQQQ-PKAVTD
-AFLRG---LGEIIQP--MWLA-MFNQK-ELQKLVGG-----------------------
---DNTELD-----------IADL----RRNTQYGGVYTIGDDGLE--------------
------------------------HATV-QLFWQALQ--------------------EMS
DEDRRKVLKF-------VTSTPRAP------------LLG-----FS-HLNPKF--SIRD
SSGDQ-----------------------------ERLPSTSTCV-NL-LKLPRY-GDLKT
MKEKLL-YAVNS-GAGF
>Cladophialophora_bantiana_XM_016770025.1 .
AEIRRTSV-FEDAYESF---------------YMLGEGLKE-PIQITFIDRFGAP--EAG
IDGGGVTKEF---LISV----TSEAFDPDSKP------AMFKENAQR-------------
----------------YLYPNPTI----------------YQETAEHL-RLSGLKGGSQE
TAFAMAELLHRFQFLGRIVGKCLYEGILIDVTFAG--FFLLKWAL-----------TGGT
TVGSNESAYRATINDLRE----------------------YDEELYQG--------LLKL
KNYTG---DVETDFA--
--LDFTVTDTLAVEDEQGKT------------------IHK-TSTRE-LLPN--------
GANTP---
------------------------------VTNTNRL-LYIDR-IVRYRLQQQ-PKAVTD
-AFLRG---LGEIIQP--MWLA-MFNQK-ELQKLVGG-----------------------
---DNTELD-----------IADL----RRNTQYGGVYTIGDDGLE--------------
------------------------HATV-QLFWQALQ--------------------EMS
DEDRRKVLKF-------VTSTPRAP------------LLG-----FS-HLNPKF--SIRD

```
SSGDQ-----------------------------DRLPSTSTCV-NL-LKLPRY-GDLKT
MKEKLL-YAVNS-GAGF
>Exophiala_dermatitidis_XM_009162833.1 .
AEIHRDSV-FEDAYEAF---------------YPLGEGLKE-PIQITFIDKFGNP--EAG
IDGGGVTKEF---LMSV----TAEAFDPEASL------SMFKQNKQG-------------
----------------YLYPNPLI----------------YQETAEYL-KHAGRKPGTEG
FDYPMAEFLRRFQFVGRVVGKCLYEGILVDVNFAG--FFLLKWAL-----------TGGT
TVGSNESAYRATINDVRE----------------------YDEELYNG--------LLKL
KNYPG----------------------------------------------DVEADFA--
--LDFTVSDTITIIDERGKE------------------ITK-TITTE-LMPN--------
GANTP-------------------------------------------------------
------------------------------VTNTNRL-LYIDR-IVRYRLQQQ-PKAVTD
-AFLKG---LGQIIQP--MWLA-MFNQK-ELQKLVGG-----------------------
---DNSQLD-----------IDDL----RRNTQYGGLYVIGDDGLE--------------
------------------------HPTV-QLFWKAMY--------------------EFS
DEDRRKVLKF-------VTSSPRAP------------LLG-----FS-HLHPKF--SIRD
ASEDQ-----------------------------TRLPSASTCV-NL-LKLPRY-EDLKT
MKEKLL-YAVNA-GAGF
>Capronia_coronata_XM_007729688.1 .
AEIHRDSV-FEDAYEAF---------------YPLGEALKE-PIQITFIDKFGSP--EAG
IDGGGVTKEF---LLSV----TTEAFDPEASL------SMFKQNKQG-------------
----------------YLYPNPLI----------------YQETAELL-KHAGRKPGTEG
FDYPMSEFLRRFQFLGRIVGKCLYEGILIDVNFAG--FFLLKWAL-----------TGGT
TVGSNESAYRATINDVRE----------------------YDEELYSG--------LLKL
KNYPG----------------------------------------------DVETDFA--
--LDFTITDTITIMDAHGKE------------------ITR-TITTE-LMPN--------
GANTP-------------------------------------------------------
------------------------------VTNTNRL-LYIDR-IVRYRLQQQ-PKAVTD
-AFLKG---LGQIIQP--MWLA-MFNQK-ELQKLVGG-----------------------
---DNSELD-----------INDL----RRNTQYGGVYAIGDDGQE--------------
------------------------HPSV-QLFWKAMQ--------------------EFS
DEDRRKVLKF-------VTSSSRAP------------LLG-----FS-HLNPKF--SIRD
SSEDQ-----------------------------TRLPSASTCV-NL-LKLPRY-GDLKT
MKEKLL-YAVNA-GAGF
>Capronia_epimyces_XM_007736463.1 .
AEIHRDSV-FEDAYEAF---------------YPLGEALKE-PIQITFIDKFNSP--EAG
IDGGGVTKEF---LMSV----TSEALDPEAKL------SMFKQNRQG-------------
----------------YLYPNPLV----------------YQETAEYL-KHLGMMPGTGG
FDHPMAEFLRHFQFLGRIVGKCLYEGILIDVNFAG--FFLLKWAL-----------TGGT
TVGSNESAYRATINDVRE----------------------FDEELYNG--------LLKL
KNYPG----------------------------------------------NVEADFA--
--LDFTVTDTVSVGKENGKE------------------ITR-TITTE-LIPN--------
GANTP-------------------------------------------------------
------------------------------VTNSNRP-LYIDR-IVRYRLQQQ-PKAVTD
-AFLKG---LGQIIQP--MWLA-MFNQK-ELQKLVGG-----------------------
---DNSELD-----------VDDL----RRNTQYGGVYTIGDDGRE--------------
------------------------HPTV-QLFWQAMQ--------------------ECS
DEDRRKVLKF-------VTSSSRAP------------LLG-----FS-HLNPKF--SIRD
SSDDQ-----------------------------TRLPSASTCV-NL-LKLPRY-GELET
VKEKLL-YAVNA-GAGF
>2_Exophiala_spinifera_BCHD01000007.1 .
AEIHRENV-FEDAYDAF---------------FQLGEGLKE-PIQITFIDKFGAP--EAG
IDGGGVTKEF---LMSV----TSEAFDPNASL------SMFKENPQH-------------
----------------SLYPNPLI----------------YQETAEYL-KRVGRKPGTDG
FDKPLTEFLRRFQFLGRIVGKCLYEGILIDVTFAG--FFLLKWAL-----------TGGT
TVGSNESAYHATINDVRD----------------------YDEELYQG--------LLKL
KNYPG----------------------------------------------DVEADFA--
--LDFTVTDTLNIMDEYGKE------------------TPH-TITTE-LIPN--------
```

GANTP---
------------------------------VTNTNRP-LYIDR-IVRYKLQQQ-PKAVTD
-AFLQG---LGQIIQP--MWLA-MFNQK-ELQKLVGG-----------------------
---DNTELD-----------IADL----RRNTQYGGVYSIGDDGLE--------------
-------------------------HPTV-RLFWKALQ--------------------EMS
DDDRRKVLKF-------VTSTPRAP------------LLG-----FS-HLNPKF--SIRD
SSDDQ-----------------------------ERLPSTSTCV-NL-LKLPRY-GDIRT
MKEKLL-YAVNA-GAGF
>Exophiala_xenobiotica_XM_013464526.1 .
AEIHRENV-FEDAYDAF----------------FQLGEGLKE-PIQITFIDKFGAP--EAG
IDGGGVTKEF---LMCV----TSEAFDPNASL------SMFKENPQH-------------
----------------SLYPNPLI----------------YQETAEYL-KRVGRKPGTDG
FDKPLTEFLRRFQFLGRIVGKCLYEGILIDVTFAG--FFLLKWAL-----------TGGT
TVGSNESAYHATINDVRD---------------------YDEELYQG--------LLKL
KNYPG---DVEADFA--
--LDFTVTDTLNIMDEYGKE------------------TPH-TITTE-LIPN--------
GANTP---
------------------------------VTNTNRP-LYIDR-IVRYKLQQQ-PKAVTD
-AFLQG---LGQIIQP--MWLA-MFNQK-ELQKLVGG-----------------------
---DNTELD-----------IADL----RRNTQYGGVYSIGDDGLE--------------
-------------------------HPTV-RLFWKALQ--------------------EMS
DDDRRKVLKF-------VTSTPRAP------------LLG-----FS-HLNPKF--SIRD
SSDDQ-----------------------------ERLPSTSTCV-NL-LKLPRY-GDIRT
MKEKLL-YAVNA-GAGF
>Exophiala_sideris_JYBR01000048.1 .
AEIHRESV-FEDAYGAF---------------YELGEGLKE-PIQITFIDKFGSP--EAG
IDGGGVTKEF---LMSV----TSEAFDPDADL------HMFVENAQR-------------
----------------YLYPSPLI----------------YQETAEYL-RRNGLKSGTEG
FDKSMTELLRRFQFLGRVVGKCLYEGILIDVNFAG--FFLLKWAL-----------TGGT
TVGSNESAYRATINDVRE---------------------YDEELYQG--------LLKL
KNYPG---DVETDFA--
--LDFTVTDTLTTMDQYGKE------------------ITR-TVTTE-LIPN--------
GSKTP---
------------------------------VTNTNRL-LYIDR-VVRYKLQQQ-PKAVTD
-AFLKG---LGQVVQP--MWLA-MFNQK-ELQKLVGG-----------------------
---DSTELD-----------IADL----RRNTQYGGVYSIGDDGLE--------------
-------------------------HPTV-QLFWKALQ--------------------EMD
EGDRRKVLKF-------VTSTPRAP------------LLG-----FS-HLNPKF--SIRD
SSDDQ-----------------------------DRLPSTSTCV-NL-LKLPRY-GDLRT
MREKLL-YAVNA-GAGF
>Rhinocladiella_mackenziei_XM_013415796.1 .
AEIHRESV-FEDAYEAF----------------YPLGEGLKE-PIQITFIDKFGAP--EAG
IDGGGVTKEF---LMSV----TSEAFDPNAGM------NMFKENAQR-------------
----------------YLYPNPLI----------------YQETAEYF-KRAGRKPHTEN
FDSPMAEFLRRFQFLGRVVGKCLYEGILIDVNFAG--FFLLKWAL-----------TGGT
TVGSNESAYRATINDVRE---------------------YDEELYHG--------LLKL
KNYPG---DVEADFS--
--LDFTVTDTLTVGNENGKE------------------VTH-TVTTE-LMPN--------
GSNIP---
------------------------------VTNTNRL-LYIDR-VVRYKLQQQ-PKAVTD
-AFLKG---LGQIIQP--MWLA-MFNQK-ELQKLVGG-----------------------
---DNSELD-----------IADL----RRNTQYGGLYTIGDDGLE--------------
-------------------------HPTI-QLFWKALQ--------------------DMD
DEDRRKVLKF-------VTSTPRAP------------LLG-----FS-HLNPKF--SIRD
SSEDQ-----------------------------ERLPSTSTCV-NL-LKLPRY-GDIKT
LKEKLL-YAVNS-GAGF
>2_Exophiala_alcalophila_BCHY01000001.1 .
AEIRRESV-FEDAYEAF---------------YGLGEGLKE-PIQITFIDKFGAP--EAG

IDGGGVTKEF---LMSV----TSEAFDPDASL------AMFKENAQR-------------
----------------YLYPNPLI----------------YQETAEYL-KQAGRKPNSEG
FDYPMSEFLRHFQFLGRVVGKCLYEGILIDVSFAG--FFLLKWAL-----------TGGT
TVGSNESAYRATINDLRE----------------------YDEELYHG--------LLKL
KNYPG--DVESDFG--
--LDFTVTDTLTMVDEHGVE------------------THR-TITTE-LMRD--------
GSNIP---
------------------------------VTNTNRL-LYIDR-MVRYRLQQQ-PKAVTD
-AFLKG---LGHIVQP--MWLA-MFNQK-ELQKLVGG-----------------------
---DNSELD-----------IADL----RRNTQYGGVYTIGDDGLE--------------
------------------------HPTV-QMFWKALQ--------------------DMD
DEDRRKVLKF-------VTSTPRAP------------LLG-----FS-HLNPKF--SIRD
SSEDQ-----------------------------ARLPSTSTCV-NL-LKLPRY-GDIKT
MREKLL-YAVNS-GAGF
>Cladophialophora_carrionii_XM_008733655.1 .
AEIRREKV-FEDAYEAF---------------YGLGEGLKE-PIQITFIDKFGAP--EAG
IDGGGVTKEF---LMSV----TSEAFDPDASL------AMFKENAQR-------------
----------------YLYPNPLI----------------YQETAAYL-KRAGRKPGTEG
FDFPMAEFLRRFQFLGRVVGKCLYEGILIDVSFAG--FFLLKWAL-----------TGGT
TVGSNESAYRATINDLRE----------------------YDEELYQG--------LLKL
KNYPG--DVENDFG--
--LDFTVTDTITIEDERHNE------------------VHQ-TITTE-LITN--------
GANTP---
------------------------------VTNTNRL-VYIDR-IVRYRLQQQ-PKAVTD
-AFLKG---LGQIIQP--MWLA-MFNQK-ELQKLVGG-----------------------
---DNSELD-----------IADL----RRNTQYGGLYVIGDDGQE--------------
------------------------HPTV-QLFWKALQ--------------------EMS
DSDRRKVLKF-------VTSTPRAP------------LLG-----FS-HLNPRF--SIRD
SSEDQ-----------------------------ERLPSTSTCV-NL-LKLPRY-GDIKT
MKEKLL-YAVNS-GAGF
>Cladophialophora_yegresii_XM_007763387.1 .
AEIRREHV-FEDAYEAF---------------YGLGEGLKE-PIQITFIDKFGAP--EAG
IDGGGVTKEF---LMSV----TSEAFDPDASL------PMFKENAQR-------------
----------------YLYPNPLI----------------YQETAAYL-KRAGRKPGTEG
FDFQMAEFLRRFQFLGRVVGKCLYEGILIDVSFAG--FFLLKWAL-----------TGGT
TVGSNESAYRATINDLRE----------------------YDEELYQG--------LLKL
KNYPG--DVENDFG--
--LDFTVTDTITIEDERGND------------------VHQ-TNTTE-LIAN--------
GANTP---
------------------------------VTNTNRL-VYIDR-IVRYRLQQQ-PRAVTD
-AFLRG---LGQIIQP--MWLA-MFNQK-ELQKLVGG-----------------------
---DNSELD-----------IADL----RRNTQYGGLYVIGDDGQE--------------
------------------------HPTV-QLFWKALQ--------------------DMS
DSDRRKVLKF-------VTSTPRAP------------LLG-----FS-HLNPRF--SIRD
SSEDQ-----------------------------ERLPSTSTCV-NL-LKLPRY-ADIKT
MKEKLL-YAVNS-GAGF
>2_Phialophora_verrucosa_MSED01000007.1 .
AEIRRESV-FEDAYEAF---------------YGLGEGLKE-PIQITFIDKFGAP--EAG
IDGGGVTKEF---LMSV----TSEAFDPDASL------AMFKENAQR-------------
----------------YLYPNPLI----------------YQETAEYL-KRAGRKPGTEG
FDYPMAEFLRRFQFLGRVVGKCLYEGILIDVSFAG--FFLLKWAL-----------TGGT
TVGSNESAYRATINDLRE----------------------FDEELYQG--------LLKL
KNYPG--DVENDFG--
--LDFTVTDTITIEDERHNE------------------VHQ-TVTTE-LIAN--------
GASTP---
------------------------------VTNTNRL-VYIDR-IVRYRLQQQ-PKAVTD
-AFLRG---LGQIIQP--MWLA-MFNQK-ELQKLVGG-----------------------
---DNTELD-----------IADL----RRNTQYGGLYVIGDDGQE--------------

------------------------HPTV-QLFWKALQ--------------------DMS
DSDRRKVLKF-------VTSTPRAP------------LLG-----FS-HLNPKF--SIRD
SSEDQ-----------------------------ERLPSTSTCV-NL-LKLPRY-GDIKT
MKEKLL-YAVNS-GAGF
>Phialophora_americana_JYCC01000057.1 .
AEIRRQSV-FEDAYEAF---------------YGLGEGLKE-PIQITFIDKFGTA--EAG
IDGGGVTKEF---LMSV----TSEAFDPDASL------AMFKENAQR-------------
----------------YLYPNPLI----------------YQETAEYL-KRAGRKPGTEG
FDYPMAEFLRRFQFLGRVVGKCLYEGILIDVTFAG--FFLLKWAL-----------TGGT
TVGSNESAYRATINDLRE----------------------YDEELYQG--------LLKL
KNYPG--DVENDFG--
--LDFTVTDTITIEDERHNE------------------VHH-TITTE-LIAN--------
GANTP---
------------------------------VTNTNRL-VYIDR-IVRYRLQQQ-PKAVTD
-AFLKG---LGQIVQP--MWLA-MFNQK-ELQKLVGG-----------------------
---DNTELD-----------IADL----RRNTQYGGLYVIGDDGQE--------------
------------------------HPTV-QLFWKALQ--------------------DMS
DSDRRKVLKF-------VTSTPRAP------------LLG-----FS-HLNPKF--SIRD
SSEDQ-----------------------------ERLPSTSTCV-NL-LKLPRY-GDIKT
MKEKLL-YAVNS-GAGF
>Mycosphaerella_sp._MWSN01000167.1 .
ATIRRRHL-FYDAFEHF---------------YKIGADLKE-PIQITFVDKWNMK--EAG
IDGGGVTKEF---LTSV----INEAMDPQCNKEWP---PFFTANDEH-------------
----------------LLHPNPVA----------------FEVCRLRL-RGGD---QDEV
VREEFSMLSQQYEFLGRIIGKCLYEGILVDVSFAG--FFLKKWAL-----------TGGA
GSSANDNHYRPNINDLRE----------------------LDEGLYRG--------LLAV
KNAE---NAE-DLG--
--LTFSVTDEVIYDCKRAPDIWPDENVRESPQPAPEDLERE-VYEVD-LMPN--------
GSNIP---
------------------------------VTNQNRL-AYINA-VASYRLKGQ-SHRQTR
-AFLRG---LSDIIQP--SWLS-MFNQS-ELQTLISG-----------------------
---SGAGID-----------IADL----RRNTQYGGTYVIGNDGQE--------------
------------------------HPTI-QLFWKVFS--------------------TID
DADRRKVLKF-------VTSTPRGP------------LLG-----FS-NLNPRF--SIRD
SGPDE-----------------------------NRYPTTSTCV-NL-LKLPMY-KSEGV
LRERLL-AAVRS-GAGF
>Sphaerulina_musiva_XM_016906288.1 .
ATIRRRHL-FYDAFEHF---------------YKIGADLKE-PIQITFVDKWNMK--EAG
IDGGGVTKEF---LTSV----INEAMDPQCNKEWP---PFFTANDEH-------------
----------------LLHPNPVA----------------FEVCRLRL-RGGD---QDEV
VREEFSMLSQQYEFLGRIIGKCLYEGILVDVSFAG--FFLKKWAL-----------TGGA
GSSANDNHYRPNINDLRE----------------------LDEGLYRG--------LLAV
KNAE---NAE-DLG--
--LTFSVTDEVIYDCKRAPDIWPDENVRESPQPAPEDLERE-VYEVD-LMPN--------
GSNIP---
------------------------------VTNQNRL-AYINA-VASYRLKGQ-SHRQTR
-AFLRG---LSDIIQS--SWLS-MFNQS-ELQTLISG-----------------------
---SGAGID-----------IADL----RRNTQYGGTYVIGNDGQE--------------
------------------------HPTI-QLFWKVFS--------------------TID
DADRRKVLKF-------VTSTPRGP------------LLG-----FS-HLNPRF--SIRD
SGPDE-----------------------------NRYPTTSTCV-NL-LKLPMY-KSEGV
LRERLL-AAVRS-GAGF
>Sphaerulina_populicola_AIDU01001263.1 .
ATIRRRHL-FYDAFEHF---------------YKIGADLKE-PIQITFVDKWNMK--EAG
IDGGGVTKEF---LTSV----INEAMDPQCNKEWP---PFFTANDEH-------------
----------------LLHPNPVA----------------FEVCRLRL-PGGD---QDEV
VREEFSMLSQQYEFLGRIIGKCLYEGILVDVSFAG--FFLKKWAL-----------TGGA
GSSANDNHYRPNINDLRE----------------------LDEGLYRG--------LLAV

KNAE---NAE-DLG--
--LTFSVTDEVIYDCKRAPDIWPDENVRESPQPEPEDLERE-VYEVD-LMPN--------
GSNIP---
------------------------------VTNQNRL-AYINA-VASYRLKGQ-SHRQTR
-AFLRG---LSDIIQP--SWLS-MFNQS-ELQTLISG-----------------------
---SGAGID-----------IADL----RRNTQYGGTYVIGNDGQE--------------
------------------------HPTI-QLFWKVFS--------------------TID
DADRRRVLKF-------VTSTPRGP------------LLG-----FS-HLNPRF--SIRD
SGPDE-----------------------------NRYPTTSTCV-NL-LKLPMY-KSEGV
LRERLL-AAVRS-GAGF
>Cladosporium_sphaerospermum_AIIA01014291.1 .
AQIKRKSE-LEDAFQAF---------------YELGADLKE-PIQITFLDEFGLQ--EAG
IDGGGVTKEF---LTSV----INQAFNPANGGLE----KYFIENEKH-------------
----------------LLFPNPTA----------------MDDAIAIL-REAG--LKDGH
MKPFLREVFQKYEFLGRIIGKCLYEGILVDVSFAG--FFLKKWAL-----------TGGH
GSAPSESGYRANINDLRD----------------------FDEALYKG--------LLDV
KSAP---DAS-AYG--
--LTFTVDDLVGSRD-----------------------KKQ-VVERE-LIAG--------
GADTP---
------------------------------VTNENRL-IYLNR-MSWYRLQGQ-SAPQTN
-AFLKG---LSSIIQP--TWLS-MFNQA-ELQTLIGG-----------------------
---AQAGID-----------VGDL----RRNTQYGGVYVIGDDGQE--------------
------------------------HPSV-RLFWRVME--------------------GLP
DEDRRKVLKF-------VTSTPRGP------------LLG-----FS-NLNPRF--SIRD
SSSDE-----------------------------GRIPSASTCV-NL-LKLPMY-KSEDV
LREKLL-YAVNS-GAGF
>2_Rachicladosp_antarcticum_NAJO01000003.1 .
AQIKRSSE-FEDAFDAF---------------YELGADLKE-PIQITFMDEFGLQ--EAG
IDGGGVTKEF---LTSV----VAQAFVDPNRTGAD---RYFIENENH-------------
----------------LLYPNPTA----------------LEDYRVTL-ENAG--LRHSE
VASLRRDMLRKYEFLGRIIGKCLYEGILVDVSFAG--FFLKKWAL-----------TGGH
GSAPGESGYRASINDLRD----------------------FDQSLYAG--------LLAL
RTAP---DAS-DFG--
--LTFTVDDLIGPPG-----------------------AQR-PKSSP-LIPG--------
GTDIA---
------------------------------VTNENRL-LYLNK-MSWYRLQGQ-SAAQTN
-AFLKG---LATIVQP--SWLS-MFNQA-ELQTLIGG-----------------------
---ASAGIN-----------VPDL----RANTLYGGTYVIGDDGRE--------------
------------------------HPTV-RVFWKVMT--------------------SLT
DAEKSKVLKF-------VTSTPRGP------------LLG-----FS-SLNPRF--SIRD
SGSDE-----------------------------SRYPSASTCV-NL-LKLPMY-RSEKI
LREKLL-GAVNS-GAGF
>Baudoinia_panamericana_XM_007676758.1 .
AKIRRKNE-FADAFEQF---------------YDLGGDLKE-PIQITFVDEFDIP--EAG
IDGGGVTKEF---LTSV----ITQAFGPDA--------DFFSETETH-------------
----------------FLHPKPTA----------------IEDLKVRL-RNMG--FKEHS
MRGEVKSLLQQYEFLGRIIGKCLYEGILVDVSFAG--FFLKKWAL-----------TGGM
HSAPMETGYRANINDLKE----------------------LDEGLYRG--------LLAL
KNAPAE---QVE-DFG--
--LTFTIDDLVGPDD-----------------------DRH-VVERE-LVPG--------
GANTS---
------------------------------VTAENRL-IYINR-MSWYRLQGQ-SAQQTN
-AFLKG---ISSIVQP--SWLS-MFNQT-ELQTLIGG-----------------------
---AAANID-----------VADL----RRNTLYGGTYVIGDDGRE--------------
------------------------HPSI-QLFWKVMA--------------------QLP
DEDRRKVLKF-------VTSTPRGP------------LLG-----FG-QLNPRF--SIRD
SGSDE-----------------------------NRFPTTSTCV-NL-LKLPMY-KSERM
LRDKLL-AAVNS-GAGF

>Acidomyces_richmondensis_JPDO01000840.1 .
AKVRRKNE-FQDAFDQF---------------YDLGAELKE-PIQITFVDEFDIP--EAG
IDGGGVTKEF---LTSV----IAQAFDPSNENIGX---QFFVENDKH-------------
----------------LLYPNPTA----------------IEDLKVRC-ERSG----MSK
TSAAVKSLLQQYEFLGRIIGKCLYEGILVDVSFAG--FFLKKWAL-----------FGGP
GSAPGENGYRSNINDLRE----------------------LDEGLYRG--------LLAL
KNAPQE---EVE-NFG--
--LTFTVDDAVGPKG-----------------------EKK-ILETE-LVPG--------
GANIP---
------------------------------VTAENRL-IYINR-MARYRLQDQ-SQKQTR
-AFLEG---LSSIIQP--SWLS-MFNQS-ELQTLIGG-----------------------
---AAAEID-----------VEDL----RRNTLYGGTYVIGDDGQE--------------
------------------------HPSV-RMFWNVMH--------------------TLP
DEDRRKVLKF-------VTSTPRGP------------LLG-----FG-QLNPRF--SIRD
SGSDE-----------------------------NRYPTTSTCV-NL-LKLPMY-KSEKT
LKEKLL-AAVTS-GAGF
>Pseudocerc_musae_GDIN01003251.1 .
AKVKRTQE-FQDAYEQF---------------YDLGADLKE-PIQITFLDQWDMP--EAG
IDGGGVTKEF---LTSV----ISQAFDQDPTASGH---RFFVENEEH-------------
----------------LLYPNPTI----------------FEDLKLQH-QLYGFSERSPE
VAEACRDLGRQYEFLGRIIGKCLYEGILVDVSFAG--FFLKKWAL-----------TGGA
GSARSESHYRPNINDLRE----------------------LDEGLYRG--------LLQV
KNAD---NAE-ELG--
--MTFSVNDEVGPPD-----------------------GKR-VVEVD-LVPH--------
GGNIA---
------------------------------VTNENRL-QYINQ-IAAYRLARQ-AFQQTR
-MFLRG---LGDIIQP--SWLS-MFNQS-ELQTLIGG-----------------------
---AAAGID-----------VNDL----RRNTQYGGTYVIGNDGME--------------
------------------------HPSV-QMFWNVMQ--------------------KME
DQDRRKVLKF-------VTSTPRGP------------LLG-----FD-HLNPRF--SIRD
SGSDE-----------------------------NRYPTTSTCV-NL-LKLPMY-KSEAV
LREKLL-AAVNS-GAGF
>Pseudocerc_pini_densiflorae_AWYD02002152. 1..
AKVKRTQE-FQDAYEQF---------------YELGADLKE-PIQITFLDQWDMP--EAG
IDGGGVTKEF---LTSV----ISQAFDQDPTASGH---RFFVENEEH-------------
----------------LLYPNPTI----------------FEDLKLQH-QLYGFSERSPE
VAEACRDLGRQYEFLGRIIGKCLYEGILVDVSFAG--FFLKKWAL-----------TGGT
GSARSESHYRPNINDLRE----------------------LDEGLYRG--------LLQV
KNAD---NAE-ELG--
--MTFSVNDEVGPPD-----------------------SKR-IVEVD-LVPH--------
GGNIA---
------------------------------VTNENRL-RYINQ-IAAYRLARQ-AFQQTR
-MFLRG---LGDIIQP--SWLS-MFNQS-ELQTLIGG-----------------------
---AAAGID-----------VNDL----RRNTQYGGTYVIGNDGME--------------
------------------------HPSV-QMFWNVMQ--------------------KME
DQDRRKVLKF-------VTSTPRGP------------LLG-----FG-HLNPRF--SIRD
SGSDE-----------------------------NRYPTTSTCV-NL-LKLPMY-KSEAV
LKEKLL-AAVNS-GAGF
>Mycosphaerella_eumusae_GDIK01019526.1 .
AKVKRTQE-FQDAYEQF---------------YELGADLKE-PIQITFLDQWDMP--EAG
IDGGGVTKEF---LTSV----ISQAFDQDPESSGH---RFFVENEEH-------------
----------------LLYPNPTI----------------FEDLKLEY-QLLGFSERSPE
VAEACRDLGRQYEFLGRIIGKCLYEGILVDVSFAG--FFLKKWAL-----------TGGT
GSARSESHYRPNINDLRE----------------------LDEGLYRG--------LLQV
KNAD---NAE-ELG--
--MTFSVNDEVGPPD-----------------------GKR-VVEVD-LVPH--------
GGNVA---
------------------------------VTNENRL-QYINQ-IAAYRLARQ-AFQQTR

-MFLRG---LGDIIQP--SWLS-MFNQS-ELQTLIGG----------------------
---AAAGID-----------VNDL----RRNTQYGGTYVIGNDGME--------------
------------------------HPSV-QMFWNVMQ--------------------KME
DQDRRKVLKF-------VTSTPRGP------------LLG-----FS-HLNPRF--SIRD
SGSDE-----------------------------NRFPTTSTCV-NL-LKLPMY-KSETV
LREKLL-AAVNS-GAGF
>Pseudocerc_fijiensis_XM_007926371.1 .
AKVKRTQE-FQDAYEQF---------------YELGADLKE-PIQITFLDQWDMP--EAG
IDGGGVTKEF---LTSV----ISQAFDQDPKASGH---RFFVENDQH-------------
----------------LLYPNPTI----------------FEDLKLQH-QLYGFSERSPE
VAEACRDLGRQYEFLGRIIGKCLYEGILVDVSFAG--FFLKKWAL-----------TGGT
GSARSESHYRPNINDLRE----------------------LDEGLYRG--------LLQV
KNAD--NAE-ELG--
--MTFSVTDEVGPPD----------------------GKR-VVEVD-LVPH--------
GGNIA---
------------------------------VTNENRL-QYINL-IAAYRLARQ-ASQQTR
-TFLRG---LSDIIQP--SWLS-MFNQS-ELQILIGG----------------------
---AAAGID-----------VNDL----RRNTQYGGTYVIGNDGME--------------
------------------------HPSV-QMFWNVMQ--------------------KME
DQDRRKVLKF-------VTSTPRGP------------LLG-----FG-HLNPRF--SIRD
SGSDE-----------------------------NRYPTTSTCV-NL-LKLPMY-KSEAV
LREKLL-AAVNS-GAGF
>Phaeocryptopus_gaeumannii_MWSP01001094.1 .
AKIKRTQE-FQDAYDQF---------------YNLGAALKE-PIQITFVDQWDMP--EAG
IDGGGVTKEP---LTSV----INQAFDQEAAHLE----KFFVENEQH-------------
----------------LLYPNPTS----------------FEDLKIKY-QQIGFSPQTPE
SKAAQTELYKQYQFLGRAIGKCLYEGILVDVSFAG--FFLKQWAL-----------TGGS
QSARSESHYKADLNDLRE----------------------LDEGLYRG--------LLQL
KNAD--NAE-ELG--
--MTFSVNEALGPEG----------------------KKQ-IREVD-LMTN--------
GSDTP---
------------------------------ITNQNRL-IYIQQ-IARYKLQAQ-AAKQIR
-AFISG---LSDIIQP--SWLS-MFNQM-ELQTLIGG----------------------
---SGAGID-----------VQDL----RRNTLYGGTYVIGDDGRE--------------
------------------------HPSI-ELFWNVMK--------------------DME
DVDRRKVLKF-------VTSTPRGP------------LLG-----FD-HLNPRF--SIRD
SGSDE-----------------------------NRYPTTSTCV-NL-LKLPMY-KSEKT
LREKLL-AAVNS-GAGF
>Cercospora_sojina_AHPQ01000313.1 .
AKIKRTQE-FEDAFEQF---------------YELGPDLKE-PIQITFVDQWDMP--EAG
IDGGGVTKEF---LTSV----INQAFDQKEDNEQT---KYFVENEEH-------------
----------------LLYPNPTA----------------FEELKLIL-KSYGYDENTQQ
TREVLREYFKKYEFLGRIIGKCLYEGILVDVSFAG--FFLKKWAL-----------TGGA
GSSASDNHYRPNINDLRE----------------------LDEGLYRG--------LLQV
KNAE--NVE-ELG--
--MTFSVNDAITINMDGTA-------------------ETK-IVEVD-LVPD--------
GSNTP---
------------------------------VTNQNRL-AYINA-IASYRLKGQ-AQRQTR
-AFLRG---LSDIIQP--SWLS-MFNQS-ELQTLIGG----------------------
---SGAGID-----------INDL----RRNTLYGGTYVIGNDGLE--------------
------------------------HPSI-QMFWKVFS--------------------SLP
DEDRRKVLKF-------VTSTPRGP------------LLG-----FS-HLNPKF--SIRD
SGSDE-----------------------------NRFPTTSTCV-NL-LKLPMY-KSEKV
LREKLL-AAVNS-GAGF
>Zymoseptoria_passerinii_AFIY01000240.1 .
AKIKRTQE-FQDAYEQF---------------YDLGPDLKE-PIQITFVDQWDMP--EAG
IDGGGVTKEF---LTSV----ISQAFDSNADKME----QFFVENDQH-------------
----------------LLHPNPTA----------------FESLKHRL-ISAGHRPDSDE

VRTPIRELYRQYEFLGRIIGKCLYEGILVDVSFAG--FFLKKWAL-----------TGGT
GSAPSESHYRANINDLRE---------------------LDESLYKG--------LLQV
KYAP---DAS-DLG--
--MTFSVNDIVTSSVDA---------------------KPH-VLEVD-LIPN--------
GANTP--
------------------------------VTNENRL-LYINA-LSRYRLQTQ-STVQTR
-AFLRG---LADMIQP--SWLS-MFNQS-ELQTLIGG-----------------------
---ASVGID-----------VQDL----RRNTLYGGTYVLGTDGQE--------------
------------------------HPSI-QHFWRVMD--------------------SLP
DADRRKVLKF-------VTSTPRGP------------LLG-----FA-HLNPRF--SIRD
SGSDE-----------------------------NRFPTTSTCV-NL-LKLPMY-GSEAR
LRRMLL-AAVNS-GAGF
>Zymoseptoria_ardabiliae_GCJX01009489.1 .
AKIKRTQE-FQDAYEQF----------------YELGADLKE-PIQITFVDQWDMP--EAG
IDGGGVTKEF---LTSI----ISQAFDPDANNME----QFFVENDQH-------------
-----------------LLHPNPTA----------------FETLKYRL-ISAGHRPDSDE
VRTQIRELYRQYEFLGRIIGKCLYEGILVDVSFAG--FFLKKWAL-----------TGGT
GSAPSESHYRANINDLRE---------------------LDESLYRG--------LLQV
KYAS---DAS-DLG--
--MTFSVNDIVTSPPPS---------------------KPH-VLEVD-LIPN--------
GANTP--
------------------------------VTNENRL-LYINA-LSRYRLQTQ-STAQSR
-AFLRG---LGDMIQP--SWLS-MFNQS-ELQTLIGG-----------------------
---ASAGID-----------VQDL----RRNTLYGGTYVIGTDGLE--------------
------------------------HPSI-QHFWRVME--------------------TLP
DEDRRKVLKF-------VTSTPRGP------------LLG-----FG-HLNPRF--SIRD
SGRDE-----------------------------NRFPTTSTCV-NL-LKLPMY-GSEER
LRRMLL-AAVNS-GAGF
>Mycosphaerella_graminicola_AFIV01000278.1 .
AKIKRTQE-FQDAYEQF----------------YELGADLKE-PIQITFVDQWDMP--EAG
IDGGGVTKEF---LTSI----ISQAFDPDANNME----QFFVENDQH-------------
-----------------LLHPNPTA----------------FETLKYRL-ISAGHRPDSDE
VRTQIRELYRQYEFLGRIIGKCLYEGILVDVSFAG--FFLKKWAL-----------TGGT
GSAPSESHYRANINDLRE---------------------LDESLYRG--------LLQV
KYAS---DAS-DLG--
--MTFSVNDIVTSPPPS---------------------KPH-VLEVD-LIPN--------
GANTP--
------------------------------VTNENRL-LYINA-LSRYRLQTQ-STAQSR
-AFLRG---LGDMIQP--SWLS-MFNQS-ELQTLIGG-----------------------
---ASAGID-----------VQDL----RRNTLYGGTYVIGTDGLE--------------
------------------------HPSI-QHFWRVME--------------------TLP
DEDRRKVLKF-------VTSTPRGP------------LLG-----FG-HLNPRF--SIRD
SGRDE-----------------------------NRFPTTSTCV-NL-LKLPMY-GSEER
LRRMLL-AAVNS-GAGF
>Zymoseptoria_tritici_GCJU01013243.1 .
AKIKRTQE-FQDAYEQF----------------YELGADLKE-PIQITFVDQWDMP--EAG
IDGGGVTKEF---LTSV----ISQAFDPDANNME----QFFVENDQH-------------
-----------------LLHPNPTA----------------FETLKYRL-VSAGHRPDSDE
VRTQMRELYRQYEFLGRIIGKCLYEGILVDVSFAG--FFLKKWAL-----------TGGT
GSAPSESHYRANINDLRE---------------------LDESLYKG--------LLQV
KYAS---DAS-DLG--
--MTFSVNDIVTSSPSS---------------------KPH-VLEVD-LIPN--------
GANTP--
------------------------------VTNENRL-LYINA-LSRYRLQTQ-STAQTR
-AFLRG---LGDMIQP--SWLS-MFNQS-ELQTLIGG-----------------------
---ASAGID-----------VQDL----RRNTLYGGTYVIGTDGLE--------------
------------------------HPSI-QHFWRVME--------------------TFP
DEDRRKVLKF-------VTSTPRGP------------LLG-----FG-HLNPRF--SIRD

SGRDE-----------------------------NRFPTTSTCV-NL-LKLPMY-GSEER
LRRMLL-AAVNS-GAGF
>Zymoseptoria_brevis_GCVP01008566.1 .
AKIKRTRE-FQDAYEQF---------------YELGADLKE-PIQITFVDQWDMP--EAG
IDGGGVTKEF---LTSV----ISQAFDPDATNME----QFFVENDQH-------------
----------------LLHPNPTA----------------FETLKYRL-ISAGHRPDSDE
VRTQIRELYRQYEFLGRIIGKCLYEGILVDVSFAG--FFLKKWAL-----------TGGT
GSAPSESHYRANINDLRE----------------------LDESLYKG--------LLQV
KYAS---DAS-DLG--
--MTFSVNDIVTSSTSS---------------------KPH-VLEVD-LIPN--------
GANTP---
------------------------------VTNENRL-LYINA-LSRYRLQTQ-STAQTR
-AFLRG---LGDMIQP--SWLS-MFNQS-ELQTLIGG-----------------------
---ASAGID-----------VQDL----RRNTLYGGTYVIGTDGLE--------------
------------------------HPSI-QHFWRVME--------------------TFP
DEDRRKVLKF-------VTSTPRGP------------LLG-----FG-HLNPRF--SIRD
SGRDE-----------------------------NRFPTTSTCV-NL-LKLPMY-GSEER
LRRMLL-AAVNS-GAGF
>Zymoseptoria_pseudotritici_GCJY01009432.1 .
AKIKRTQE-FQDAYEQF---------------YELGADLKE-PIQITFVDQWDMP--EAG
IDGGGVTKEF---LTSV----ISQAFDPDADNME----QFFVENDQH-------------
----------------LLHPNPTA----------------FETLKYRL-ISAGHRPDSDE
VRTQIRELYRQYEFLGRIIGKCLYEGILVDVSFAG--FFLKKWAL-----------TGGT
GSAPSESHYRANINDLRE----------------------LDESLYKG--------LLQV
KYAS---DAS-DLG--
--MTFSVNDIVTSSPSS---------------------KPH-VLEVD-LIPN--------
GANTP---
------------------------------VTNENRL-LYINA-LSRYRLQTQ-STAQTR
-AFLRG---LGDMIQP--SWLS-MFNQS-ELQTLIGG-----------------------
---ASAGID-----------VQDL----RRNTLYGGTYVIGTDGLE--------------
------------------------HPSI-QHFWRVME--------------------TFP
DEDRRKVLKF-------VTSTPRGP------------LLG-----FG-HLNPRF--SIRD
SGRDE-----------------------------NRFPTTSTCV-NL-LKLPMY-GSEER
LRRMLL-AAVNS-GAGF
>Tuber_melanosporum_XM_002841937.1 .
ATIRRNRV-FEDAYEQF---------------WPLGEGLKE-PIQITFIDRFGVE--EAG
IDGGGVTKEF---LTGV----CSDAFTPSK--------NLFLENEQH-------------
----------------LLYPNPTS----------------IEELKEEL-IKTRGPYQEVI
GDEV-KPLSKRYEFLGRVLGKCLYEGILVDVAFAP--FFLLKWSQ---------------
QASDSRASMAIGVNDLRD----------------------LDEGLYRG--------LVKL
KNYTGD---VES-DFG--
--LNFTISSRIPR-------------------------HDK-TITVE-LKPG--------
GETIP---
------------------------------VTNANKL-EYVHL-VSRYRLSVQ-AHIQTS
-AFLKG---LSSIINP--SWLS-MFNQS-ELQTLVGG-----------------------
--DINTPID-----------VEDL----RRNTIYGGVYQLGEDGIE--------------
------------------------HESI-RLFWEVMR--------------------SLG
DEERRKVLKF-------VTSVARAP------------LLG-----FG-VLRPRF--SIRD
AGEDQ-----------------------------GRLCSASTCV-NL-LKLPRY-RDPRI
LREKLL-YSVNS-NAGF
>Dactylellina_haptotyla_XM_011113290.1 .
GVIRRDHV-FEDAYDEF---------------YALGDGFKE-PIQITFVDSFGAE--EPG
IDGGGILKEF---LSSI----VQESYRNDPAWGV----PFFSETEGH-------------
----------------LLYPNPTL-------------LDQISWGLYKQ-NQRGFHDIKSE
VSHEVSELLKRFEFLGRIIGKCMYEGILVDVAFAD--FFLLKWSK---------------
-VSLGNEDLGLGVDDLKS----------------------LDRSLWKG--------LQSL
KYYD---DHTIENLE-
--LTFTTENIFQLPDSLALNGV----------------SKK-ITLVD-LKPG--------

GSQLP---
------------------------------VTSSNRL-EYIHL-LSRYKLVTQ-GKLQTN
-AFLKG---LSSIISP--RWLS-MFNQS-ELQTLVGG-----------------------
---NESPLD-----------ISDL----RRNTVYGGIYTIGDDNQE--------------
------------------------HPTI-EMFWSVME--------------------RLE
ESDKRAVLKF-------VTSVSRAP------------LLG-----FG-SLNPKF--SIRD
AGSDP-----------------------------TRLPSTSTCV-NL-LKLPRY-QSEHV
LEEKLL-YAANA-GAGF
>Endoconidiophora_laricicola_LXGT01000069. 1..
VTIKREHV-LESAFEEM---------------WKMGAAIKN-PLSISFVDRFGAN--EAG
IDGGGVTKEF---LISV----INEAFGKS---------GLFTTNTQN-------------
----------------QYYPDPAP----------------YAWLEAHA-KKSASPEETEE
RCKSMQNLLDRYEFIGRLLGKCIYEGLLIDVSFAP--FFILKWTL---------------
-GLGGENGYKGNVNDLRD----------------------LDEQTYEG--------LVAL
KNAP---DAS-ELG--
--LDFSVNDRAAMKGQ----------------------KTI-YLSRL-LVPG--------
GDQLA---
------------------------------VTNENRL-LYASY-FARHRLVLQ-SQKISH
-AFLQG---LWTVIEP--TWIS-MFNQW-ELQRVVGG-----------------------
---DSSKVD-----------LSDW----RANTTYSGIYVIGDDGLE--------------
------------------------HPVI-QMFWEVME--------------------EFS
EQERRLVLQY-------ATSVPRAP------------LLG-----FS-KLTPRF--AIRD
SKSVEGL---------------------------DRLPSASTCM-NL-LKLPPY-KSKDV
MRNKLR-YAINC-GAGF
>2_Endoconidiophora_polonica_LXKZ01000184.1 .
VTIKREHV-LESAFEEM---------------WKMGAAIKN-PLSISFVDRFGAN--EAG
IDGGGVTKEF---LISV----INDAFGKS---------GLFTTNTQN-------------
----------------QYYPDPAP----------------YAWLVAHA-KKSASPEEMEE
RCKSMQNLLDRYEFIGRLLGKCIYEGLLIDVSFAP--FFILKWTL---------------
-GLGGENGYKGNVNDLRD----------------------LDEQTYEG--------LVAL
KNAP---DAS-ELG--
--LDFSVNDRAAMNGQ----------------------KTI-YLSRN-LVPG--------
GDQLA---
------------------------------VTNENRL-LYASY-FARHRLVLQ-SQKISH
-AFLQG---LWTVIEP--TWIS-MFNQW-ELQRVVGG-----------------------
---DSSKVD-----------LSDW----RANTTYSGIYVIGDDGLE--------------
------------------------HPVI-QMFWEVME--------------------EFS
EQERRLVLQY-------ATSVPRAP------------LLG-----FS-QLTPRF--AIRD
SKSVEGL---------------------------DRLPSASTCM-NL-LKLPPY-KSKDV
MRNKLR-YAINC-GAGF
>Davidsoniella_virescens_LJZU01000206.1 .
VKIRREEA-LDSAFKEM---------------WNMRGGIKN-PLSISFVNRFGVN--EAG
IDGGGVTKEF---LISV----IKEAFADS---------GLFTTNTQN-------------
----------------QYYPDPAP----------------YDWLMELA-KEAESPEELDK
RCKHIQDLLERYEFIGRLLGKCIYEGILIDVSFAP--FFILKWTM---------------
-GLEGENGYKGNVNDLRD----------------------LDEQTYEG--------LVML
KNAA---DAS-ELG--
--LDFSVNDKAAMKDQ----------------------KAV-YLSRN-LIPG--------
GDQIP---
------------------------------VTNENRL-LYSSY-FARHRLVMQ-SQKISQ
-AFLRG---LWIVIEP--TWLS-MFNQW-EIQRVVGG-----------------------
---DSAKVD-----------LRDW----RASTVYSGIYVIGDDGLE--------------
------------------------HPVI-QMFWEVME--------------------EFS
EQERRLVLQF-------ATSVPRAP------------LLG-----FS-QLTPRF--AIRD
SKSSEDL---------------------------DRLPSASTCM-NL-LKLPPY-KSKDV
MRSKLS-YAINC-GAGF
>2_Chalaropsis_thielavioides_BCGU01000009.1 .
VQIERQRL-FESAFDEL---------------WEVGAEIKK-PLHIIFVDQFGAT--EAG

IDGGGVTKEF---LISV----TSEAFGGS---------GLFTTNSQN-------------
----------------QYYPDPSQ----------------YDWFLQKS-RTASTRQEKEE
CFSKMQGLLERYEFLGRVLGKCIYEGILVDASFAP--FFLLKWIM---------------
-GVNGATGYKGNINDLRD----------------------LDEQTYQG--------LVML
KNAR---DAS-EME--
--LDFTVDDKAEIPGQ----------------------VPV-YITRN-LFPN--------
GDQIT---
------------------------------VTNENRL-LYTSY-FSRHRLVVQ-SQQISA
-AFLRG---LWDIIAP--SWIS-MFNQW-ELQRLVGG-----------------------
---DSAPVD-----------LDDW----RANTHYGGIYVIGNDNEE--------------
------------------------HPVI-KWFWEIMH--------------------EFD
EEQRRQVLQY-------ATSVPRAP------------LLG-----FS-QLNPRF--AIRD
SQSSTDV---------------------------ERLPSASTCM-NL-LKLPPY-KSKTV
MREKLL-YAIQS-GAGF
>Ceratocystis_platani_LBBL01000072.1 .
VRIKRQEL-FESAFDEL---------------WQLGAGLKN-PLHITFVDQFGAT--EAG
IDGGGVTKEF---LISL----TSEAFGGS---------RLFTTNSQN-------------
----------------QYYPDPSQ----------------YDWYYQLS-RKASTTEEKQE
CFAKMKGILNRYEFLGRILGKCIYEGILVDASFAP--FFLLKWIM---------------
-GISGATGYKGNVNDLRD----------------------LDEQTYQG--------LVML
KNAR---DAS-EME--
--LDFTVDDKAEIPGT----------------------API-YITRN-LIPN--------
GDQVM---
------------------------------VTNENRL-LYTSY-FSRHRLVVQ-SQQISA
-AFLRG---LWDIIAP--SWIS-MFNQW-ELQRLVGG-----------------------
---DSAPVN-----------LDDW----RANTYYGGIYVIGDDGEE--------------
------------------------HPVI-KWFWEVMY--------------------EFD
EGQRRQVLQY-------ATSVPRAP------------LLG-----FS-QLNPRF--AIRD
SRSSSDV---------------------------ERLPSASTCM-NL-LKLPPY-KSKDV
MRQKLL-YAIQS-GAGF
>Ceratocystis_albifundus_JSSU01001334.1 .
VRIKRQEL-FESAFDEL---------------WQLGAGLKN-PIHITFVDHFGAT--EAG
IDGGGVTKEF---LISL----TSEAFGGS---------RLFATNSHN-------------
----------------QYYPDPSQ----------------YDWYYQLS-KKASTTEEKQE
CFAKMKGILNRYELLGRILGKCIYEGILVDASFAP--FFLLKWIM---------------
-GISEATGYKGNVNDLRD----------------------LDEQTYQG--------LVML
KNAR---DAS-EME--
--LDFTVDDKAEIPGT----------------------AAI-YITRN-LIPN--------
GDQVM---
------------------------------VTNENRL-LYTSY-FSRHRLVVQ-SQRISE
-AFLRG---LWDIIAP--SWIS-MFNQW-ELQRLVGG-----------------------
---DSAPVN-----------LDDW----RANTKYSGIYVIGDDGEE--------------
------------------------HPVI-KWFWELMY--------------------EFD
EGQRRQVLQY-------VTSVPRAP------------LLG-----FS-QLNPLF--AIRD
SLSSSLSSSKV-----------------------ERLPSASTCM-NL-LKLPPY-KSKDV
MREKLL-YAIQS-GAGF
>Graphilbum_fragrans_LLKO01000026.1 .
AQVVRGST-FEDSMKQF---------------YKLGEGLKE-PIQISFVDQFGSP--EAG
IDGGGVTKEF---LMSA----TAEMLTPPS--------TLFAANADN-------------
----------------VMYPNPLP----------------HAQLSHAL-MQAKTEEEKQS
AQEQLGHLLKQYDYLGRLVGKCMYEGILLDVVFAN--FFLQKWTQ---------------
--VGMDTMYRSNINDVRE----------------------LDEELYQG--------LMHL
KNYPG--DVR-ELE--
--LDFTIDDQVSLPGQ----------------------PLK-MVTSD-LMPN--------
GSNVQ---
------------------------------VTNENRP-LYISY-VARHRLSAQ-SHYQTR
-AFLQG---LSLIIKP--EWLA-MFNQS-ELGRLVGG-----------------------
---DLAAID-----------VDDL----RANTMYAGLYVIGDDQQE--------------

------------------------HETV-KMFWKVVG--------------------ELG
DEDRRLLLKY-------VTSTSRAP------------LLG-----FR-SLLPLF--SIRD
GSTDD-----------------------------TRLPSASTCV-NL-LKLPVY-HDIDT
LREKLL-MAIQS-GAGF
>Podospora_anserina_XM_001905867.1 .
ALIRRGSL-FRDAQGSL---------------YPLEEAIKE-PVQITFLDQWGMQ--EAG
IDGGGVTKEF---LTSV----IAEMLSDT---------SLFVANSKN-------------
----------------AYYPNPLI----------------VEQWLALA-RKRGLSESE--
---TRTKLLRQYEFAGRLIGKCMYEGILINVVFAG--FFLLKWTT---------------
-----ADTKQASLNDLRE----------------------LDEELYRG--------LLFL
KNNED--QVD-DMG--
--LNFALDVDISTPEDK---------------------QPN-IVSRP-LCPN--------
GNNIP---
------------------------------VTKGNRL-KYIVQ-LAKYKLALQ-PFAQTQ
-AFLKG---LKMVIEP--GWLS-MFNQN-ELQRLVGG-----------------------
---DSGAID-----------VDDL----RRNTVYSGPYQIGDDGQE--------------
------------------------HETV-KLFWEVME--------------------EFG
DEERREVLQY-------VTSTPRAP------------LLG-----FS-QLFPRF--TIGY
GGQDE-----------------------------DRLPSASTCI-NL-LKLPRY-SRKGV
LREKLL-YAVKS-GAGF
>Scedosporium_apiospermum_XM_016789819.1 .
ADIRREHV-LDDAFKQF---------------YPLAEGLKE-PIYITFYDRFGEV--EAG
IDGGGVTKEF---LMSA----IQDAFAEDS--------SWFTRNAEG-------------
----------------LYYPNPSA----------------MDWFREKRARARTRASEREA
LENESRRVIQQYEFLGRLVGKCIYEGILIDIAFAG--FFLMKWRA---------------
--AAELNRYRGTVNDLRD----------------------LDEELYKG--------LMAL
KNEPG--DVS-GWD--
--MYFTIDDEASFGASR---------------------DKF-TVTRN-LIKD--------
GDVTK---
------------------------------VTNENRL-LFISS-VAKHRLVIQ-PALQTN
-AFLRG---LRSIIDP--SWLS-MFNRV-ELQRLVGG-----------------------
---DSRAID-----------VEDL----RRNTVYGGLYVIGDDGME--------------
------------------------HETV-RLFWRVMT--------------------KFS
DEQRREVLKF-------VTSTPRAP------------LLG-----FA-QLSPLF--SIRD
GGSAE-----------------------------DRLPSASTCI-NL-LKLPLY-SSEET
LREKLL-LAVES-GAGF
>Aureobasidium_pullulans_MSJF01000790.1 .
ADIRREHV-LDDAFKQF---------------YPLAEGLKE-PIYITFYDRFGEV--EAG
IDGGGVTKEF---LMSA----IQDAFAEDS--------SWFTRNAEG-------------
----------------LYYPNPSA----------------MDWFREKRARA-TRAPEREA
LENESRRVIQQYEFLGRLVGKCIYEGILIDIAFAG--FFLMKWRA---------------
--AAELNRYRGTVNDLRD----------------------LDEELYKG--------LMAL
KNEPG--DVS-GWD--
--MYFTIDDEASFGPSR---------------------EKF-TVTRN-LIKD--------
GDVTQ---
------------------------------VTNENRL-LFISS-VAKHRLVIQ-PALQTN
-AFLRG---LRSIIDP--SWLS-MFNRV-ELQRLVGG-----------------------
---DSRAID-----------VEDL----RRNTIYGGLYVIGDDGME--------------
------------------------HETV-RLFWRVMT--------------------KFT
DEQRREVLKF-------VTSTPRAP------------LLG-----FA-QLSPLF--SIRD
GGSAE-----------------------------DRLPSASTCI-NL-LKLPLY-SSEET
LREKLL-LAVES-GAGF
>Scedosporium_aurantiacum_JUDQ01000129.1 .
ADIRREHV-LEDAFKQF---------------YPLAEGLKE-PIYITFYDRFGEV--EAG
IDGGGVTKEF---LMSA----IQDVFAEDS--------SWFTRNAEG-------------
----------------LYYPNPSA----------------MDWFREKRARA-TQASEREE
LENESRRVIQKYEFLGRLVGKCIYEGILIDIAFAG--FFLMKWRA---------------
--AAELNRYRGTVNDLRD----------------------LDEELYKG--------LMAL

KNEPG--DVS-GWD--
--MYFTIDDEASFGADK---------------------DKF-TVTRN-LIKD--------
GDVTR---
------------------------------VTNENRL-LFISS-VAKHRLVIQ-PALQTN
-AFLRG---LRSIIDP--SWLS-MFNRV-ELQRLVGG-----------------------
---DSRAID-----------VEDL----RRNTVYGGLYVIGDDGVE--------------
------------------------HETV-RLFWKVMT--------------------KFS
DEQRREVLKF-------VTSTPRAP------------LLG-----FS-QLSPLF--SIRD
GGSAQ-----------------------------DRLPSASTCI-NL-LKLPLY-SSEET
LREKLL-LAVES-GAGF
>Corollospora_maritima_GDFX01003041.1 .
--IQRGRV-FEDAYSEF---------------YTLADGLKE-PIYITFLNHFGQV--EAG
IDGGGVTKEF---LISV----ISEAFGDDQ--------PWFTRNAQG-------------
----------------KYYPNPGI----------------NGWHWEMRKR---PGADDVA
LATQQMQALQKLEFLGRLVGKCLYEGILIDVTFAG--FFVMRWRA---------------
---EGDKEYKCTINDLRD----------------------ADEDLYKG--------LIRL
KNAAD--DVS-EWG--
--MYFTITDELRFGKDR---------------------ETI-HITRN-LLPE--------
GDMVP---
------------------------------VTNENRL-LFIAL-TAQHRLVTQ-PAAETK
-AFVRG---LQTIIDP--SWLS-MFNQT-ELQRLVGG-----------------------
---DSKEID-----------LDDL----KRNTVYSGLYVVGNDGML--------------
------------------------HPTV-QLFWKVME--------------------DFE
DDQRRDVLKF-------VTSTPRAP------------LLG-----FS-QLNPQF--AIRD
SQDDE-----------------------------TRLPSASTCI-NL-LKLPMY-TSEAT
MREKLL-LAISS-GAGF
>2_Knoxdaviesia_capensis_LNGK01000010.1 .
AQVRRGNV-FADSFKRF---------------WDLGDGLKE-PISITFVDQFGNI--EAG
IDGGGVTKEF---LYSS----ATEVLAEYG---------WFAHNQDN-------------
----------------CFYPNPAQ----------------YLYLTERL-RKVSDPTKSER
FAKERVNLLNMHEFLGRIIGKCMYEGILLDISFAP--FFLLKWTK---------------
PEKGQGGAYGGTINDLRD----------------------MDEDTYQG--------LVKL
KNEA---DSS-LAG--
--LDFTIDDEATYPQE----------------------KPM-VLTRK-LMND--------
GAKIP---
------------------------------VTNENRL-LYISY-FARHRLIHQ-PAAVTA
-AFLRG---LWSIIPP--SWLS-MFNQS-ELQRLVSG-----------------------
--DVAANID-----------IDDL----RRNTVYGGLYVIGDDGEE--------------
------------------------HPTV-QLFWKVMES--------------------LD
AESRGLVLKY-------VTSVPRAP------------LLG-----FS-KLAPHF--SIRD
GGTDE-----------------------------ERLPSASTCV-NL-LKLPRY-TTEEK
LREKLL-FAIQS-GAGF
>Knoxdaviesia_proteae_LNGL01000042.1 .
AQVRRGNV-FADSFKRF---------------WDLGDGLKE-PISITFVDQFGNI--EAG
IDGGGVTKEF---LYSS----ATEVLAEYG---------WFAHNQDN-------------
----------------CFYPNPAQ----------------YLYLTERL-RKVSDPTKSER
FAKERVNLLNMHEFLGRIIGKCMYEGILLDISFAP--FFLLKWTK---------------
PEKGQGGAYGGTINDLRD----------------------MDEDTYQG--------LVKL
KNEA---DSS-LAG--
--LDFTIDDEATYPQE----------------------KPM-VLTRK-LMND--------
GAKIS---
------------------------------VTNENRL-LYISY-FARHRLIHQ-PAAVTA
-AFLRG---LWSIIPP--SWLS-MFNQS-ELQRLVSG-----------------------
--DVAANID-----------VDDL----RRNTVYGGLYVIGDDGEE--------------
------------------------HPTV-QLFWKVMES--------------------LD
AESRGLVLKF-------VTSVPRAP------------LLG-----FS-KLAPHF--SIRD
GGTDE-----------------------------ERLPSASTCV-NL-LKLPRY-TTEEK
LREKLL-FAIQS-GAGF

>Sporothrix_brasiliensis_AWTV01000004.1 .
-RIHRDAI-FESALQDF---------------WELGEGLKE-PIQITFIDEYGNE--EAG
IDGGGVTKEF---LTCA----LMQVLSQQD--------EIFSANKEN-------------
----------------AIYPNPTR----------------LDQLKVRL-QKHGLTEGTEG
WDDRIAYLMRTYEFIGRMIGKCLYEGILIDFVFAG--FFLSKWAL---------------
--SATDSVYRANINDLRE----------------------MDEQLYEG--------MLKL
KNYDG--DVA-DLD--
--LDFTIEDQISSNNE----------------------RLQ-TETRN-LVPN--------
GDKLR---
------------------------------VTNENRP-LYISY-VARHRLMAQ-PYLVTK
-AFLRG---LGTIIDP--VWLR-MFNQN-ELQRLVGG----------------------
---DSSEID-----------VDDL----RRNTIYGGLYVIGDDHEE--------------
------------------------HPTV-KLFWDVVQ--------------------SLP
DDDRRLLLKY-------VTSTPRAP------------LLG-----FS-QLSPKF--SIRD
GGNDE-----------------------------ARLPSTSTCV-NL-LKLPRY-STEET
LRDKLL-YAIRS-NARF
>Sporothrix_schenckii_XM_016730721.1 .
GRIHRDAI-FESALQDF---------------WELGEGLKE-PIQITFIDEYGNE--EAG
IDGGGVTKEF---LTCA----LMQVLSQQD--------EIFSANKEN-------------
----------------AIYPNPTK----------------LDQLKMRL-QKHGLTEGTEG
WDDRVANLMRTYEFIGRMIGKCLYEGILIDFVFAG--FFLSKWAL---------------
--SATDSVYRANINDLRE----------------------MDEQLYEG--------MLKL
KNYDG--DVA-DLD--
--LDFTIEDQISSNNE----------------------RLQ-TETRN-LVPN--------
GDKLR---
------------------------------VTNENRP-LYISY-VARHRLMAQ-PYLVTK
-AFLRG---LGTIIDP--VWLR-MFNQN-ELQRLVGG----------------------
---DSSEID-----------VDDL----RRNTIYGGLYVIGDDHEE--------------
------------------------HPTV-KLFWDVVQ--------------------SLP
DDDRRLLLKY-------VTSTPRAP------------LLG-----FS-QLSPKF--SIRD
GGNDE-----------------------------ARLPSTSTCV-NL-LKLPRY-STEET
LRDKLL-YAIRS-NARF
>Sporothrix_globosa_LVYW01000003.1 .
-RIHRDAI-FESALQDF---------------WELGEGLKE-PIQITFIDEYGNE--EAG
IDGGGVTKEF---LTCA----LMQVLSQQD--------EIFSANKEN-------------
----------------AIYPNPTR----------------LDQLKVWM-PKHGLTEGTEG
WDDRVAYLLRTYEFIGRMIGKCLYEGILIDFVFAG--FFLSKWTL---------------
--SATDSVYRANINDLRE----------------------MDEQLYEG--------MLKL
KNYDG--DVA-DLD--
--LDFTIEDQVSSNNE----------------------RLQ-TETRN-LVPN--------
GDKLR---
------------------------------VTNENRP-LYISY-VARHRLMAQ-PYLVTK
-AFLRG---LGTIIDP--VWLR-MFNQN-ELQRLVGG----------------------
---DSSEID-----------VDDL----RRNTIYGGLYVIGDDHEE--------------
------------------------HPTV-KLFWDVVQ--------------------SLP
DDDRRLLLKY-------VTSTPRAP------------LLG-----FS-QLSPKF--SIRD
GGNDE-----------------------------ARLPSTSTCV-NL-LKLPRY-STEET
LRDKLL-YAIRS-NARF
>Sporothrix_pallida_JNEX02000003.1 .
ARIRRDAI-FDSAFQQF---------------WELGEGLKE-PIQITFIDEYGNE--EAG
IDGGGVTKEF---LTCA----LMQVLSPQD--------EIFVSNKEN-------------
----------------AIYPNPLK----------------LGQLKATL-QKHAIMEGTEE
YTEQVQSLMRHYEFIGRMIGKCLYEGILIDFVFTG--FFLLKWAL---------------
--SATDTPYRANINDLRE----------------------MDEELYQG--------MLQL
KNYDG--DVA-DMD--
--IDFTIADQVSRTNE----------------------PFK-AEVHN-LVPN--------
GDKLR---
------------------------------VTNENRP-LYISY-VARHRLMLQ-PHYVTN

-AFLRG---LGTIIDP--MWLR-MFNQN-ELQRLVGG----------------------
---DSSEID-----------VEDL----RRNTIYGGLYEIGDDHQE--------------
------------------------HPTV-KLFWDVVQ--------------------SLP
DDDRRLLLKY-------VTSTPRAP------------LLG-----FS-QLSPKF--SIRD
AGNDE-----------------------------ARLPSTSTCV-NL-LKLPRY-STEET
LRDKLL-YAIRS-NARF
>Sporothrix_insectorum_AZHD01000001.1 .
ARIRRDAV-FETAYDEF---------------WELGEGLKE-PIQITFIDEYGNE--EAG
IDGGGVTKEF---LTCA----LMQVLSPES--------EVFSSNKEN-------------
----------------AIFPNPTK----------------LDQFKQYL-LKAGVAEGSDE
WDDEVVDLMRQYEFIGRMIGKCLYEGILIDFVFAG--FFLLKWSL---------------
--STTDSAYRASLNDLRE----------------------MDEELYQG--------MLRL
KNYDG---DVA-ELD--
--LDFTIEDQISSNNES---------------------PLR-TETRN-LIPN--------
GDKIR---
------------------------------VTNENRP-LYISY-VARHRLMAQ-PYLVTK
-AFLRG---LGTIIDP--VWLR-MFNQN-ELQRLVGG----------------------
---DSSEID-----------VDDL----RRNTIYAGVYEIGDDHQE--------------
------------------------HPTV-KLFWDVVQ--------------------SLP
DEDRRLLLKY-------VTSTPRAP------------LLG-----FS-QLSPKF--SIRD
GGGDE-----------------------------TRLPSTSTCV-NL-LKLPRY-SKKET
LRSKLL-YAIRS-NARF
>Ophiostoma_novo-ulmi_AMZD01000064.1 .
AEIRRNAV-FESAFEQF---------------WELGDGLKE-PIQITFIDEYGEQ--EAG
I-GGGVTKEF---LTNA----LTQVLSPEE--------ELFQCNKEN-------------
----------------ATFPNPMS----------------LDQLKHRL-RNEGEMEGTVE
WDSAVDELKKRYEFIGRMIGKCMYEGILIDFVFAG--FFLLKWTL-------------SA
TSTDSSTGYRASVNDLRE----------------------MDEALYQG--------MMKL
KNYDGV---DVS-DLD--
--IDFTIEDQVSGNKDE---------------------RFE-TETRN-LIPN--------
GANVR---
------------------------------VTNQNRP-LYISY-VARHRLLAQ-PYIVTR
-AFLKG---LNAIIDP--AWLR-MFNQN-ELQRLVGG----------------------
---DSSEID-----------VDDM----RRNTVYSGVYEIGDDNEE--------------
------------------------HPTI-KIFWKVVA--------------------SMD
DKDRRLLLKY-------ATSTPRAP------------LLG-----FS-QLSPRF--SIRF
GGSDE-----------------------------TRLPSASTCM-NL-LKLPMY-TTEET
LREKLI-YAIRA-DVGF
>Ophiostoma_piceae_AQHS01000272.1 .
AEIRRDAA-FESAFEQF---------------WDLGEGLKE-PIQITFIDEYGQQ--EAG
I-GGGVTKEF---LTNA----LTQVLSPEE--------ELFQCNKEN-------------
----------------ATFPNPMS----------------LDQLKHRL-RNEGEMEGTVE
WEDAVDELKRRYEFIGRMIGKCMYEGILIDFVFAG--FFLLKWTL-------------SA
TSTDNSTGYRASVNDLRE----------------------LDEDLYQG--------MMKL
KNYNGA---DVS-DLD--
--IDFTIEDQVSGNKDE---------------------RLE-TVTRN-LIPN--------
GPNVR---
------------------------------VTNQNRP-LYISY-VARHRLLAQ-PYTVTR
-AFLKG---LGAIIDP--AWLR-MFNQN-ELQRLVGG----------------------
---DSSEID-----------VDDM----RRNTVYSGVYEIGDDLEE--------------
------------------------HPTI-KIFWRVVQ--------------------SMD
DKDRRLLLKY-------ATSTPRAP------------LLG-----FA-QLSPRF--SIRF
GGSDE-----------------------------TRLPSASTCM-NL-LKLPMY-TTEKT
LREKLI-YAIRA-DVGF
>Ceratocystiopsis_minuta_LZPB01000206.1 .
ARIQRNAM-FENAYTEF---------------WELGEGLKE-PIQITFLDEHGNE--EAG
IDGGGVTKEF---LTCA----LTDALSEEY--------ELFETNEEN-------------
----------------ALFPNPML----------------MDQTKDEM-RRAGVQEGSGA

WTETIGFELKQYEFLGRMIGKSMYEGILLDYVFAG--FFLRKWTQ---------------
-AATDGGGYRASINDLRE----------------------MDEGLYQG--------LLQL
KNYAGA---DVA-ELD--
--LDFTVEDQVSKRGQ----------------------AVR-TVTRP-LVPH--------
GERVR---
------------------------------VTNENRP-LYVSY-VARHRLQAQ-PHMVTQ
-AFLRG---LWLIIDP--LWLR-MFNQS-ELQRLVGG-----------------------
---DSSPVD-----------VEDL----RRHTQYSGVYAVGDDGAE--------------
------------------------HPTV-ALFWAAVR--------------------GLA
DADRRLLLKY-------VTSTPRAP------------LLG-----FA-QLRPAF--CIRD
GGGDE-----------------------------TRLPSASTCV-NL-LKLPRY-TTLET
LRDKLL-YAIRA-NARF
>Grosmannia_penicillata_MLJV01000030.1 .
ARIRRDAV-FDTAMAQF---------------FPLGEGLKE-PIQITFLDEYGNE--EAG
IDGGGVTKEF---LMSV----TKDVFTPAQGMV-----PRFESNKES-------------
----------------ALYPNPLA----------------LDQLKNSM-RRSRISEGSAD
WHGQISEMLREYEFIGRIVGKCLYEGILLDVVFAG--FFLLKWAM---------------
--STTDSAYRASINDLRE----------------------MDEGLYQG--------MLSL
KNYDG--NVA-DMD--
--LNFTLEDQVTLGDE----------------------PLR-TETRR-LMPN--------
GENIP---
------------------------------VTNENRL-LYISY-ATRHRLVAQ-PYQVTK
-AFLRG---LGTIIDP--AWLR-MFNQN-ELQRLVGG-----------------------
---DSSEID-----------VADL----RKHTVYGGVYAIGDDGEE--------------
------------------------HPSV-KLFWRVME--------------------ALP
DEDRRLLLKF-------VTSTPRAP------------LLG-----FS-QLKPYF--SIRD
GGSDE-----------------------------TRLPSASTCI-NL-LKLPRY-SNMET
MRKKLL-YAVRS-NSRF
>Raffaelea_quercivora_BCFZ01000003.1 .
ARIRRNLL-FETAYQQF---------------FPLGEGLKE-PIQITFLDEYGSE--EAG
IDGGGVTKEF---LMSI----TKEMFTTEVGHL-----RQFVANKEN-------------
----------------ALYPNPLA----------------LDQAKTWC-REMH--IEGQN
HQDHIASILSQYEFTGRIVGKCMYEGILLDAVFAG--FFLLKWSL---------------
--STSDSAYRASINDLRE----------------------MDEDLYQG--------MLRL
KNFRG--DVS-AMD--
--LNFTLEDQVSLDGM----------------------PLR-TETRK-LIPN--------
GDNIL---
------------------------------VTNENRP-LYISY-AARHRLVAQ-PYQVTK
-AFLRG---LGMIIDP--AWLR-MFNQN-ELQRLVGG-----------------------
---DSSEID-----------VADL----RQNTVYAGVYEIGLDGQE--------------
------------------------HPSV-QLFWKVLES--------------------LP
DEDRRLVLKF-------VTSTPRAP------------LLG-----FS-QLKPCF--SIRD
GGPDE-----------------------------TRLPSASTCI-NL-LKLPRY-SNLET
MRSKLL-YAVRS-NSRF
>Leptographium_procerum_JRUC01000263.1 .
ARIKRNSV-YDTAYEQF---------------NELGEGLKE-PIQITFLDEYGSE--EAG
IDGGGVTKEF---LMSV----TKEIFTTKIGNV-----MQFQSNKDG-------------
----------------ALYPNPIA----------------LDQVKTWC-DTNG----LGG
QNGNIAAMLSRFEFTGRLVGKCMYEGILLDVVFAG--FFLLKWSL---------------
--STSESAYRASINDLRE----------------------MDEELYQG--------MLRL
KNYKG--DVS-ELD--
--VNFTLEDQVSMDGM----------------------PLH-TATRN-LIPD--------
GENTA---
------------------------------VTNENRP-LYISY-VARHRLAAQ-PYQVTR
-AFLRG---LETIIDP--AWLR-MFNQN-ELQRLVGG-----------------------
---DSSEID-----------VQDL----RQNTVYGGVYEIGDDGQE--------------
------------------------HPSV-KLFWEVME--------------------SLP
DEDRRLLLKF-------VTSTPRAP------------LLG-----FS-QLKPAF--SIRD

GGHDE-----------------------------TRLPSASTCI-NL-LKLPRY-SSLET
MRSKVL-YAVRS-NSRF
>Leptographium_lundbergii_LDEF01000010.1 .
ARISRNMV-YGSAFEQF---------------YPLGEGLKE-PIQITFLDEYGSE--EAG
IDGGGVTKEF---LMSA----TKEVFTSKIGLV-----MQFQSNKDN-------------
----------------ALYPNPLA----------------LDQAKSWC-RNNEQ-LGVYQ
HRELMTAMLNQFEFTGRLVGKCMYEGILLDVVFAG--FFLLKWSL---------------
--STKDSAYRASVNDLRE----------------------MDEELYQG--------MLRL
KNYKG--DVS-GMD--
--VNFTLEDQVSSDGT----------------------PLR-TETRN-LVPD--------
GENIT---
------------------------------VTNENRP-LYISY-VARHRLVAQ-PYQVTK
-AFLRG---LEAIIDP--AWLR-MFNQN-ELQRLVGG-----------------------
---DSSEID-----------VQDL----RQNTVYGGVYEIGSDGQE--------------
------------------------HPSV-KLFWDVVG--------------------SLP
DEDRRLLLKF-------VTSTPRAP------------LLG-----FS-QLKPAF--SIRD
AGADE-----------------------------TRLPSASTCI-NL-LKLPRY-STLKT
MRDKVL-YAVRS-NSRF
>Grosmannia_clavigera_XM_014318740.1 .
ARIRRNLV-YGSAYEQF---------------YPLGEGLKE-PIQITFLDEYGSE--EAG
IDGGGVTKEF---LMSV----TKEIFTSKIGNV-----MQFQSNKDN-------------
----------------ALYPNPLA----------------LDQAKSWC-HANG--FQGAE
QRDHIAAMLSQFEFTGRLVGKCMYEGILLDVVFAG--FFLLKWSL---------------
--STKDSAYRASVNDLRE----------------------MDEELYQG--------MLRL
KNFKG--DVS-EMD--
--VNFTLEDQVSMDGL----------------------PLR-TETRN-LVPD--------
GEDIT---
------------------------------VTNENRP-LYISY-VARHRLVAQ-PYQVTK
-AFLHG---LETIIDP--AWLR-MFNQN-ELQRLVGG-----------------------
---DSSEID-----------VQDL----RQNTVYGGVYEMGDDGQE--------------
------------------------HPSV-KLFWDVVG--------------------SLP
DEDRRLLLKF-------VTSTPRAP------------LLG-----FS-QLKPAF--SIRD
AGGDE-----------------------------TRLPSASTCI-NL-LKLPRY-SSLKE
LGASAT-----------
>Chaetomium_globosum_XM_001226851.1 .
AQIQRGRM-FDDALESF---------------WGLQDGLKE-PIQITFVDEFGMP--EAG
IDGGGVTKEF---LTSV----TTEAFTPNE--------GLFIANSKN-------------
----------------SYYPNPCS----------------IDQSKNAL-QEAQIPENSEE
WAETITSRLRQYEFLGRVIGKCLYEGILIDVSFAG--FFLLKWAT---------------
-SAGASDTYRANINDLRE----------------------LDEELYQG--------MLRL
KNYPG--NVG-DLS--
--LDFTITDQISLPGE----------------------PFR-TTTRD-LVPN--------
GENVV---
------------------------------VTNENRL-LYISY-VARHRLIIQ-PYAQTR
-AFLRG---LGMIIDP--SWLS-MFNQN-ELQRLVGG-----------------------
---DSSEID-----------VEDL----RKHTAYSGVYEIGDDGQE--------------
------------------------HPTV-KLFWEVMY--------------------QLE
DRERRDVLKY-------VTSTPRAP------------LLG-----FS-QLSPPF--SIRD
GGRDQ-----------------------------ERLPSASTCV-NL-LKLPRY-DDAET
LKRKLL-YAVTS-GAGF
>Chaetomium_cochliodes_LSBY01001272.1 .
AQIQRGRI-FNDALESF---------------WDLQEGLKE-PIQITFVDEFGMP--EAG
IDGGGVTKEF---LTSV----TTEAFTPTE--------GLFIANSKN-------------
----------------SYYPNPCG----------------MDQSKNAL-REAQIPENSEE
WSETITSRLRQYEFLGRVIGKCLYEGILIDISFAG--FFLLKWAT---------------
-SAGAADTYRANINDLRE----------------------LDEELYQG--------MLRL
KNYPG--DVG-DLS--
--LDFTVTDQISLPGE----------------------PFR-TTTRN-LVPN--------

GENVA---
------------------------------VTNENRP-LYISY-VARHRLIIQ-PYAQTR
-AFLRG---LGMIIDP--GWLS-MFNQN-ELQRLVGG-----------------------
---DSSEID-----------VEDL----RRHTAYSGVYAIGDDGEE--------------
------------------------HPTV-KLFWEVMR--------------------ELE
DHERRDVLKY-------VTSTPRAP------------LLG-----FS-QLSPPF--SIRD
GGRDQ-----------------------------DRLPSASTCV-NL-LKLPQY-ETAET
LKRKLL-YAVTS-GAGF
>Thielavia_terrestris_XM_003650613.1 .
AQIQRGRV-FRDALESF---------------WDLKEGLKE-PIQITFVDEFGMT--EAG
IDGGGVTKEF---LTSV----TTEAFAPER--------RLFVANSKN-------------
----------------AYYPNPSD----------------MDQLKHYL-REAHVAEGSEE
WTEAIVYRLREYEFLGRIIGKCMYEGILIDIVFAG--FFLLKWAS---------------
-PAGAPDTYRANINDLRE----------------------LDEELYQG--------MLRL
KNYPG--DVR-DLS--
--LDFTITDQISRPSE----------------------PVR-TVTRN-LVPD--------
GENVA---
------------------------------VTNENRL-HYIIR-VAHHRLVVQ-PYAQTR
-AFLRG---LGTIIDP--AWLS-MFNQS-ELQRLVGG-----------------------
---DSSEID-----------VEDM----RRHTVYSGAYSIGDDGQE--------------
------------------------HPTV-RLFWEVMH--------------------GLE
DRERRDVLKY-------VTSTPRAP------------LLG-----FA-QLSPPF--SIRD
GGLDQ-----------------------------DRLPSASTCV-NL-LKLPQY-TSAAV
LKRKLL-YAVTS-GAGF
>Myceliophthora_thermophila_XM_003664246.1 .
AQIQRGRI-FQDALESF---------------WDLQEGLKE-PIQITFVDEFGMP--EAG
IDGGGVTKEF---LTSV----TTEAFAPSE--------RLFVANSNN-------------
----------------SYYPNPCD----------------MDQTKNML-REAQVSEGSEE
WAEAITYRLRQYEFLGRVIGKCMYEGILIDIVFAG--FFLLKWAT---------------
-SAGAGDTYRANINDLRE----------------------LDEELYQG--------MLRL
KNYPG--DVR-DLD--
--LDFTITDQVSLPDE----------------------PLR-TATRN-LIPN--------
GESVP---
------------------------------VTNENRP-LYISY-VARHRLVVQ-PYAQTR
-AFLRG---LGMIIDP--GWLS-MFNQN-ELQRLVGG-----------------------
---DSSEID-----------VEDL----RRHTVYSGVYAIGDDGEE--------------
------------------------HPTV-KMFWEVMH--------------------GLE
DRERRDVLKY-------VTSTPRAP------------LLG-----FG-QLSPPF--SIRD
GGTDQ-----------------------------ERLPSASTCV-NL-LKLPQY-RSAAV
LKKKLL-YAVTS-GAGF
>Chaetomium_thermophilum_ADUW01000032.1 .
AQVQRGRA-FADALDQF---------------WDLKEGLKE-PIQITFVDSFGMQ--EAG
IDGGGVTKEF---LLSV----TTEAFTQDQ--------KLFVTNSQN-------------
----------------AFYPNPCA----------------LDQEKHRL-QEAMVPENSDL
WKETITYLLRQYEFLGRIIGKCLYEGILIDISFAG--FFLLKWTS---------------
-AADSAETYRANINDLRE----------------------LDEELYQG--------MLQL
KNYPG--DVA-DLG--
--LDFTITDRVSLPDEH---------------------PVR-TVTRP-LIPD--------
GEKIP---
------------------------------VTNENRP-LYISY-VARHRLVVQ-PYAQTR
-AFLRG---LSMIIDP--AWLS-MFNQA-ELQRLVGG-----------------------
---DSAGID-----------VDDL----RRHTVYSGVYEIGDDGQE--------------
------------------------HPTV-KLFWEVMT--------------------ELS
DDEKRDVLKY-------VTSSPRAP------------LLG-----FS-QLNPPF--SIRD
GGLDE-----------------------------ERLPSASTCV-NL-LKLPQY-KTKEG
LKKKLL-YAVKS-GAGF
>Coniochaeta_ligniaria_MNPN01000105.1 .
AKVKRGQV-FQDALKEF---------------YQLGDGLKE-PIQITFVDQFDTV--EAG

IDGGGVTKEF---LMSV----TKEAFTDQH--------SLMVANNKN-------------
----------------AFYPNPCA----------------LDQRLEVL-RQAGVRKGSAE
WHESVNELLREYEFLGRIVGKCLYEGILIDIVFAP--FFLLKWAT---------------
--SGSETAYRANINDLRE----------------------MDDELYQG--------MLNL
KNYPG--DVS-ELG--
--LDFTINDQISYPGE----------------------PVR-TVTRN-LVPD--------
GENML---
------------------------------VTNENRP-LYISY-VARHRLAVQ-PHAQTK
-AFLRG---LGSIIDP--GWLS-MFNQN-ELQRLVGG-----------------------
---DSSEID-----------VEDL----RANTQYSGVYQIGDDGQE--------------
------------------------HPTV-QMFWEVMH--------------------GLE
DHERRDVLKY-------VTSTPRAP------------LLG-----FS-QLRPQF--TIRD
GGSDQ-----------------------------ERLPSASTCV-NL-LKLPQY-QSAKT
LKSKLL-YAVTS-GAGF
>2_Chrysoporthe_cubensis_LJCY01000118.1 .
AKVRRGKV-FQDAFAQF---------------YELRDGLKE-PIQITFVDQFDIP--EAG
IDGGGVTKEF---LTSV----TKEAFTDGL--------SLFVANSKS-------------
----------------ALYPNPCA----------------LDQRQEKL-RESGLGPDSEG
WREDISDLLAQYEFLGRIIGKCLYEGILIDVVFAG--FFLLKWAA---------------
--AASDAGYRANINDLRE----------------------FDEELYNG--------MMRL
KNYPG--DVS-ELG--
--LDFTIEDQVSLPNE----------------------TMR-IITRH-LMPN--------
GDKIT---
------------------------------VSNENKP-LYVNY-VARHRLVVQ-PFAVTR
-AFLTG---LGTIIEP--AWLS-MFNQS-ELQRLVGG-----------------------
---DSTDID-----------IEDL----RRNTVYSGLYEIGDDGLE--------------
------------------------HPTI-QMFWEVMH--------------------SLE
DSQRRDVLKY-------VTSTPRAP------------LLG-----FS-QLSPRF--SIRD
GGRDT-----------------------------TRLPSASTCV-NL-LKLPTY-DSVQL
LRDKLL-YAVSS-GAGF
>Togninia_minima_XM_007913365.1 .
AKIRRGKV-FQDAFEQF---------------YDLREGLKE-PIQITFVDQFDTP--EAG
IDGGGVTKEF---LTSV----TKEAFTDEL--------SLFVANSRN-------------
----------------ALYPNPAA----------------LDQRQDKL-RQAGLTQESPE
WRESITELLDQYEFLGRIIGKCMYEGILIDVVFAG--FFLLKWSA---------------
--AASDSGYRANINDLKE----------------------LDEELYQG--------MLSL
KNYPG--DIS-DMG--
--ADFTIEDQISMPGE----------------------PVR-TVTRN-LIPN--------
GDQVS---
------------------------------VTNENRP-LYISY-MAHHRLVAQ-PFAVTR
-AFLKG---LGSIITP--SWLS-MFNQS-ELQRLVGG-----------------------
---DSSEID-----------VEDL----RRNTVYSGLYVIGDDGEE--------------
------------------------HPTV-SMFWDVMH--------------------SLK
DEERREVLKY-------VTSTPRAP------------LLG-----FG-QLSPRF--SIRD
GGDDQ-----------------------------DRLPSASTCV-NL-LKLPKY-KSLSQ
LKSKLL-YAVKS-NAGF
>Sordaria_macrospora_XM_003353079.1 .
AKIRRGRV-FEDAMKQL---------------WALGEGLKE-PIQVTFEDEFGMQ--EAG
IDGGGVTKEF---LDSV----TTEAFTHT---------ELFVTNSKN-------------
----------------AYYPNPTL----------------IDRIRH------MWTGEPEE
TQAAVERTLKEYEFLGRVIGKCMYEGILIDIVFAG--FFLLKWAA--ANDTPGGGSNSPA
-SASATGGYRANINDLRE----------------------LDEELYQG--------MLKL
KNYTG--DIQTDLA--
--LDFTITDPINIPGE----------------------PTR-TIIRP-LIPN--------
GESIP---
------------------------------VTNENRP-LYISY-VARHRLVRQ-PYPQTR
-AFLRG---LGSIIDP--SWLS-MFNQL-ELQRLVGG-----------------------
---DSSEID-----------VEDL----RRNTYYNGVYEIGDDGEE--------------

------------------------HETI-QIFWEVMH--------------------ELR
DEERREVLKY-------VTSTPRAP------------LLG-----FG-QLRPRF--TIRD
AGRDQ-----------------------------ERLPSASTCV-NL-LKLPQY-RSKRR
LKEKLL-YAVKS-GAGF
>Neurospora_tetrasperma_XM_009855813.1 .
ARIRRGRV-FEDAMKQL---------------WELGEGLKE-PIQVTFEDEFGMQ--EAG
IDGGGVTKEF---LDSV----TTEAFTHT---------ELFVTNSKN-------------
----------------AYYPNPTL----------------IDRIRN------MRVGEPEE
TQRAVEQVLKMYEFLGRIIGKCMYEGILIDIVFAG--FFLLKWAT--ANDTPGGSSTSLA
-SSSTTAGYRANINDLRE----------------------LDEELYQG--------MLSL
KNYTG--DVQTDLA--
--LDFTITDPINSPGE----------------------PTR-TIIRP-LIPH--------
GDSTP---
------------------------------VTNENRP-LYISY-VARHRLVRQ-PYPQTR
-AFLRG---LGSVIDP--SWLS-MFNQL-ELQRLVGG-----------------------
---DSSEID-----------VEDL----RRNTHYNGVYQIGLDGEE--------------
------------------------HPTI-QIFWEVMH--------------------ELK
DEERREVLKY-------VTSTPRAP------------LLG-----FG-QLRPYF--TIRD
AGQDE-----------------------------DRLPSASTCV-NL-LKLPQY-RSKGR
LKEKLL-YAVKS-GAGF
>Neurospora_crassa_XM_955333.2 .
ARIRRGRV-FEDAMKQL---------------WELGEGLKE-PIQVTFEDEFGMQ--EAG
IDGGGVTKEF---LDSV----TTEAFTHT---------ELFVTNSKN-------------
----------------AYYPNPTL----------------IDRIRN------MRVGEPEE
TQRAVEQVLKMYEFLGRIIGKCMYEGILIDIVFAG--FFLLKWAT--ANDTPGGGSNPLA
SSSSTTAGYRANINDLRE----------------------LDEELYQG--------MLSL
KNYTG--DVQTDLA--
--LDFTITDPINSPGE----------------------PTR-TIIRP-LIPH--------
GDSTP---
------------------------------VTNENRP-LYISY-VARHRLVRQ-PYPQTR
-AFLRG---LGSVIDP--SWLS-MFNQL-ELQRLVGG-----------------------
---DSSEID-----------VEDL----RRNTHYNGVYQIGLDGEE--------------
------------------------HPTI-QIFWEVMH--------------------ELK
DEERREVLKY-------VTSTPRAP------------LLG-----FG-QLRPYF--TIRD
AGQDE-----------------------------DRLPSASTCV-NL-LKLPQY-RSKGR
LKEKLL-YAVKS-GAGF
>Magnaporthe_oryzae_XM_003712191.1 .
AKISRGSM-FDDAFEQF---------------WGLGQGLKE-PIQITFVDQFDTV--EAG
IDGGGVTKEF---LISV----TTEAFSSKH--------NLFISTKEN-------------
----------------ALYPNPSP----------------LDQLQDSM-RRSGISEGGDE
WKLNIRKLLAHYEFLGRIVGKCMYEGILVDIAFAG--FFLLKWSA---------------
--AATDHGYRANVNDLRE----------------------MDEELYQG--------LLRL
KSMD---DVS-QLG--
--LDFTVTDQVSLAGD----------------------KTV-TMTRE-LIPN--------
GKNIT---
------------------------------VTNENRP-LFISY-MARHRLVTQ-SYQQTQ
-AFLRG---LGTIVSP--SWLS-MFNQT-ELQWLVGG-----------------------
---EQSEID-----------VEDL----RRHTTYNGVYTIGDDGEE--------------
------------------------HPTV-KLFWQAMY--------------------EFQ
DSERRDVLKF-------VTSTPRAP------------LLG-----FS-QLHPSF--TIRD
GGLDL-----------------------------TRLPSASTCV-NL-MKLPQY-QTLKQ
LKTKLK-YAISS-NAGF
>Magnaporthe_grisea_LOFB01000499.1 .
AKISRGSM-FDDAFEQF---------------WGLGQGLKE-PIQITFVDQFDTV--EAG
IDGGGVTKEF---LISV----TTEAFSSKH--------NLFISTKEN-------------
----------------ALYPNPSS----------------LDQLQDSM-RRSGIPEGGDE
WKLNIRKLLAHYEFLGRIVGKCMYEGILVDIAFAG--FFLLKWSA---------------
--AATDHGYRANVNDLRE----------------------MDEELYQG--------LLRL

KSMD---DVS-QLG--
--LDFTVTDQVSLAGD----------------------KTV-TMTRE-LIPN--------
GKNIT---
------------------------------VTNENRP-LFISY-MARHRLVTQ-SYQQTQ
-AFLRG---LGTIVSP--SWLS-MFNQT-ELQWLVGG-----------------------
---EQSEID-----------VDDL----RRHTTYNGVYTIGDDGEE--------------
------------------------HPTV-KLFWQAMY--------------------EFQ
DSERRDVLKF-------VTSTPRAP------------LLG-----FS-QLLPSF--TIRD
GGLDL-----------------------------TRLPSASTCV-NL-MKLPQY-QTLEQ
LKTKLK-YAISS-NAGF
>Magnaporthiopsis_poae_ADBL01001770.1 .
AKIKRGNV-FDDAFEQF---------------YPLGDGLKE-PIQITFVDQFDTV--EAG
IDGGGVTKEF---LTSI----TMEAFGGSQH-------NLFVANKSN-------------
----------------AMYPNPSS----------------LDQLQESL-KRLGFLEGSEE
WTLQMRTLLEQYEFLGRIVGKCMYEGILIDIAFAG--FFLLKWVS---------------
--AATDLGYRASINDVRE----------------------MDEELYQG--------LLRL
KNMQS--DVS-ELD--
--LDFTINDQVSPLGE----------------------ETM-TITRN-LVPN--------
GKDVA---
------------------------------VTNENRP-LYISY-IARHRLVAQ-PLQQTR
-AFLRG---LGSIIDP--AWLS-MFNQS-ELQRLVGG-----------------------
---DSSEID-----------LEDL----RRHTVYSGVYVVGDDGKN--------------
------------------------HPTV-DLFWQAMH--------------------EFQ
DSERRAVLKY-------VTSTPRAP------------LLG-----FS-QLKPMF--SIRD
GGADP-----------------------------KRLPSASTCV-NL-LKLPRY-PTLEI
LKNKLR-YAISS-NAGF
>Falciphora_oryzae_JNVV01000001.1 .
AKIKRGNV-FDDAYDQF---------------YSLGDGLKE-PIQITFVDQFDTI--EAG
IDGGGVTKEF---LTSI----TMEAFTSSQH-------NLFVANKSN-------------
----------------AMYPNPSS----------------LDQLQESL-KKMGFLEGSDE
WSLQVQTLLEQYEFLGRIVGKCMYEGILIDIAFAG--FFLLKWVS---------------
--AVTDLGYRASINDVRE----------------------MDEELYQG--------LLRL
KNMQS--DVS-ELE--
--LDFTINDQVSPLGE----------------------ETM-TITRN-LVPN--------
GKDVA---
------------------------------VTNENRP-LYISY-VARHRLVAQ-PHQQTR
-AFLRG---LGSIIDP--AWLS-MFNQS-ELQRLVGG-----------------------
---DSSEID-----------LEDL----RKNTIYSGVYEVGDDGKD--------------
------------------------HPTI-DLFWQAMH--------------------EFQ
DSERRDVLKY-------VTSTPRAP------------LLG-----FS-QLRPLF--SIRD
GGGDP-----------------------------ERLPSAATCI-NL-LKLPRY-PTLEI
LKNKLR-YAVSS-NAGF
>Gaeumannomyces_graminis_XM_009218299.1 .
AKIKRGNV-FDDAYDQF---------------YPLGDGLKE-PIQITFVDQFDTI--EAG
IDGGGVTKEF---LTSI----TTEAFGSSQH-------NLFVANGSN-------------
----------------AMYPNPSS----------------LDQLQESL-RDMGFLEGSDE
WTLQVQTLLEQYEFLGRIVGKCMYEGILIDIAFAG--FFLLKWVS---------------
--AATDLGYRASINDVRE----------------------MDEELYQG--------LLRL
KNMQS--DVS-ELE--
--LDFTINDQVSATGE----------------------ETV-TITRD-LMPN--------
GKDVA---
------------------------------VTNENRP-LYISY-VARHRLVAQ-PYQQTR
-AFLRG---LGSIIDP--AWLC-MFNQS-ELQRLVGG-----------------------
---DSSEID-----------LEDL----RKHTVYSGVYEVGDDGKD--------------
------------------------HPTI-DLFWQAMH--------------------EFQ
DSERRDVLKY-------VTSTPRAP------------LLG-----FF-QLKPLF--SIRD
GGGDP-----------------------------ERLPSAATCI-NL-LKLPRY-PTLEI
LKNKLR-YAVSS-NAGF

>Verticillium_alboatrum_XM_003001829.1 .
--IKRGQE-FDDAFEQF---------------YELGESLKE-PVQIQFIDSFGDV--EAG
IDGGGVAKEF---LLSA----INQVFMNQSED------RYFVENNQK-------------
----------------LWYPNPII----------------FDKFREEA-EEMGLPEA--E
KKDILTGLSQRYEFLGRLVGKCMYEDILIDVNFAG--FFLLKWAA---------------
SGLTGEESYRGNVNDLRD----------------------LDASLYQG--------LVHL
KNLPAD---EVE-SLS--
--LDFTVDDQISADGE----------------------TVR-SLTRE-LIPG--------
RGSTP---
------------------------------VTKDNRL-LYISY-MARHRLVVQ-PAQQTM
-AFLRG---LRAIVAP--TWLS-MFNQT-ELQRLVGG-----------------------
---DSSEIS-----------IDDL----RDNTNYSGLYVIGDDGEE--------------
------------------------HPTI-QLFWKVVR--------------------GFT
DAQRRDLLKY-------VTSTPRAP------------LLG-----FG-SLNPLF--SIRD
GGTDE-----------------------------ARLPSASTCV-NL-LKLPRY-TDEET
LRRKLV-LAISS-GAGF
>Colletotrichum_orbiculare_AMCV01003741.1 .
-KIRRGQV-FQDAFEQF---------------FELGEGLKE-PIQIQFVDQFDTV--EAG
IDGGGVTKEF---LTSV----TTEAFSEMDGI------SLFTANSQG-------------
----------------LLYPNPTA----------------MDELREAL-SKAGIPGNSTD
WQEQVQDLLRQFEFLGRIVGKCMYEGILIDIAFAG--FFLLKWIS---------------
-GQTGENSYRGNVNDLRD----------------------LDEELYQG--------MLRL
KNYSG--NVA-DLS--
--LDFTITDQVSLPDE----------------------PVR-TVTRN-LIPN--------
GENVA---
------------------------------VTNDNRL-LYISY-VARHRLVAQ-PAQQTA
-AFLRG---LRSIIAP--SWLS-MFNQN-ELQRLVGG-----------------------
---DSSEID-----------LEDL----RRNTVYSGLYEIGDDGVE--------------
------------------------HPTI-QLFWRVMV--------------------GFS
DGERRDVLKY-------VTSTPRAP------------LLG-----FS-QLSPHF--SIRD
GGEDQ-----------------------------ARLPSTSTCV-NL-LKLPRY-KDQET
LRKKLL-YAVSS-GAGF
>Colletotrichum_lindemuthianum_MASP0200026 .
-KIRRGQV-FQDAFEQF---------------FELGEGLKE-PIQIQFVDQFDTV--EAG
IDGGGVTKEF---LTSV----TTEAFSEMDGI------SLFTANSQG-------------
----------------LLYPNPTA----------------MDELREAL-SKAGIPGDSAD
WQEQVQDLLRQFEFLGRIVGKCMYEGILIDIAFAG--FFLLKWIS---------------
-GQTGENSYRGNVNDLRD----------------------LDEELYQG--------MLRL
KNYSG--NVA-DLS--
--LDFTITDQVSLPGE----------------------PVR-TVTRN-LIPN--------
GENVA---
------------------------------VTNDNRL-LYISY-VARHRLVAQ-PAQQTA
-AFLRG---LRSIIAP--SWLS-MFNQN-ELQRLVGG-----------------------
---DSSEID-----------LEDL----RRNTVYSGLYEIGDDGLE--------------
------------------------HPTI-QLFWRVMA--------------------GFS
DGERRDVLKY-------VTSTPRAP------------LLG-----FS-QLSPHF--SIRD
GGEDQ-----------------------------ARLPSTSTCV-NL-LKLPRY-KDQET
LRKKLL-YAVSS-GAGF
>Colletotrichum_gloeosporioides_XM_007276953.1 .
-KIRRGQV-FRDAFEQF---------------YELGEGLKE-PIQIQFVDQFDTI--EAG
IDGGGVTKEF---LTSV----TTEAFSKMEGI------SLFTANSQG-------------
----------------LLYPNPTA----------------MDELKEVM-RRAGYPEYSAD
WQDQVQDLLRQFEFLGRIVGKCMYEGILIDIAFAG--FFLLKWTS---------------
-GQTGENSYRGNVNDLRD----------------------LDEELYQG--------MLHL
KNLTS--NVA-DLA--
--LDFTITDQVSLPGE----------------------PVR-TVSRN-LIPN--------
GENVP---
------------------------------VTNDNRL-LYISY-VARHRLVAQ-PAQQTA

-AFLRG---LRSIIAP--SWLS-MFNQN-EIQRLVGG----------------------
---DSSEID-----------IDDL----RQNTIYSGLYEVGDDGLE--------------
------------------------HPTV-QLFWKVMA--------------------GFS
DKERRDVLKY-------VTSTPRAP------------LLG-----FS-QLSPRF--SIRD
GGEDQ------------------------------ERLPSTSTCV-NL-LKLPRY-KDEET
LRKKLL-YAVSS-GAGF
>Colletotrichum_tofieldiae_LFHP01000533.1 .
-KIRRGQV-FQDAFEQF---------------YELGEGLKE-PIQIQFVDQFDTV--EAG
IDGGGVTKEF---LTSV----TTEAFGQMDGI------SLFTANSQG-------------
----------------LLYPNPTA----------------MDELKEAL-RRAGVPERTAE
WQEQVQDLLRQFEFLGRIVGKCMYEGILVDIAFAG--FFLLKWIS---------------
-GQTGENSYRGNVNDLRD----------------------LDEELYQG--------MLRL
KNYNE--NVA-DLA--
--LDFTINDQVSLPGE----------------------PVR-TITRN-LIPN--------
GENVT--
------------------------------VTNDNRL-LYISY-IARHRLVAQ-PAQQTA
-AFLRG---LRSIIAP--SWLS-MFNQN-ELQRLVGG----------------------
---DSSEID-----------IEDL----RRNTIYSGLYEVGDDGLE--------------
------------------------HPTV-QLFWKVMA--------------------NFS
DRERRDVLKY-------VTSTPRAP------------LLG-----FS-QLSPRF--SIRD
GGEDQ------------------------------ERLPSTSTCV-NL-LKLPRY-KDQET
LRKKLL-YAVSS-GAGF
>Colletotrichum_incanum_JTLR01001925.1 .
-KIRRGQV-FHDAFEQF---------------YELGEGLKE-PIQIQFVDQFDTV--EAG
IDGGGVTKEF---LTSV----TAEAFGQMDGI------SLFTANNQG-------------
----------------LLYPNPTA----------------MDELKEAL-RRAGVPERTAE
WQDQVQDLLRQFEFLGRIVGKCMYEGILVDIAFAG--FFLLKWIS---------------
-GQTGENSYRGNVNDLRD----------------------LDEELYQG--------MLRL
KNYNE--NVA-DLA--
--LDFTINDQVSLPGE----------------------PVR-TITRN-LIPN--------
GENVT--
------------------------------VTNDNRL-LYISY-VARHRLVAQ-PAQQTA
-AFLRG---LRSIIAP--SWLS-MFNQN-ELQRLVGG----------------------
---DSSEID-----------IEDL----RRNTIYSGLYEVGDDGLE--------------
------------------------HPTV-QLFWKVMA--------------------NFS
DRERRDVLKY-------VTSTPRAP------------LLG-----FS-QLSPRF--SIRD
GGEDQ------------------------------ERLPSTSTCV-NL-LKLPRY-KDQET
LRKKLL-YAVSS-GAGF
>Colletotrichum_higginsianum_XM_018309039.1 .
-KIRRGQV-FQDAFEQF---------------YELGEGLKE-PIQIQFVDQFDTV--EAG
IDGGGVTKEF---LTSV----TTEAFGQMDGI------SLFSANSQG-------------
----------------LLYPNPTA----------------MDELREAL-RRAGVPERTAE
WHEQVQDLLRQFEFLGRIVGKCMYEGILVDIAFAG--FFLLKWIS---------------
-GQTGENSYRGNVNDLRD----------------------LDEELYQG--------MLRL
KNYNE--NVA-DLA--
--LDFTINDQVSLPGE----------------------PTR-TISRN-LIPN--------
GENVT--
------------------------------VTNDNRL-LYISY-VARHRLVAQ-PAQQTA
-AFLRG---LRSIIAP--SWLS-MFNQN-ELQRLVGG----------------------
---DSSEID-----------IEDL----RRNTVYSGLYEVGDDGLE--------------
------------------------HPTV-QLFWKVMA--------------------NFS
DRERRDVLKY-------VTSTPRAP------------LLG-----FS-QLSPRF--SIRD
GGEDQ------------------------------ERLPSTSTCV-NL-LKLPRY-KNQET
LRKKLL-YAVSS-GAGF
>Colletotrichum_graminicola_XM_008099391.1 .
GKIRRGQV-FQDAFEQF---------------YELGEGLKE-PIQIQFVDQFDTV--EAG
IDGGGVTKEF---LTSV----TTEAFGQRDGI------SLFTANSQG-------------
----------------LLYPNPTA----------------MDELREAL-RRAGVPERSAE

WQDEVQDLLRKFEFLGRIIGKCMYEGILVDIAFAG--FFLLKWIS---------------
-GQTGENSYRGNVNDLRD----------------------MDEEFYQG--------MLRL
KNYKE---NVA-DLA--
--LDFTINDQVSLPGE----------------------PVR-TMTRN-LIPN--------
GENIT---
------------------------------VTNDNRL-LYISY-VARHRLVAQ-PAQQTA
-AFLRG---LRSIIAP--SWLS-MFNQN-ELQRLVGG-----------------------
---DSSEID-----------IEDL----RRNTIYSGLYEVGDDGLE--------------
------------------------HPTV-QLFWKVMA--------------------EFS
DRERRDVLKY-------VTSTPRAP------------LLG-----FS-QLSPRF--SIRD
GGEDQ-----------------------------ERLPSTSTCV-NL-LKLPRY-KKQET
LRQKLL-YAVSS-GAGF
>Colletotrichum_sublineola_JMSE01000096.1 .
-KIRRGQV-FQDAFEQF---------------YELGEGLKE-PIQIQFVDQFDTV--EAG
IDGGGVTKEF---LTSV----TTEAFGQMDGI------SLFTANSQG-------------
----------------LLYPNPTA----------------MDELREAL-RRAGVPERSAE
WQDAVQDLLRKFEFLGRIIGKCMYEGILVDIAFAG--FFLLKWIS---------------
-GQTGENSYRGNVNDLRD----------------------MDEELYQG--------MLRL
KNYKE---NVA-DLA--
--LDFTINDQVSLPGE----------------------PVR-TITRN-LIPN--------
GENVT---
------------------------------VTNDNRL-LYISY-IARHRLVAQ-PAQQTA
-AFLRG---LRSIIAP--SWLS-MFNQN-ELQRLVGG-----------------------
---DSSEID-----------LEDL----RRNTIYSGLYEVGDDGVE--------------
------------------------HPTI-QLFWKVMA--------------------DFS
DRERRDVLKY-------VTSTPRAP------------LLG-----FS-QLSPRF--SIRD
GGEDQ-----------------------------ERLPSTSTCV-NL-LKLPRY-KKQET
LRQKLL-YAVSS-GAGF
>Colletotrichum_orchidophilum_MJBS01000029 1..
-KIRRGQV-FQDAFEQF---------------YELGEGLKE-PIQIQFVDQFDTV--EAG
IDGGGVTKEF---LTSV----TTEAFGPRNGI------SLFTANSKG-------------
----------------LLYPNPTA----------------MDELRQVL-KRAQVPERTAE
WQDQVQGLLRQFEFLGRIVGKCMYEGILVDIAFAG--FFLLKWIS---------------
-GQTGDNSYRGNVNDLRD----------------------LDEELYQG--------MLRL
KNHNG---DVV-DWA--
--LDFTLNDLVSLPGE----------------------PVR-TVTRN-LIPN--------
GENVT---
------------------------------VTNDNRL-LYISY-VARHRLVAQ-PALQTA
-AFLRG---LRSIIAP--AWLS-MFNQN-ELQRLVGG-----------------------
---DSSEID-----------IEDL----RQNTVYSGLYEVGDDGLE--------------
------------------------HTTV-QLFWKVMA--------------------NFS
DRERRDVLKY-------VTSTPRAP------------LLG-----FS-QLSPRF--SIRD
GGEDQ-----------------------------ERLPSTSTCV-NL-LKLPRY-KDRET
LRKKLL-YAVSS-GAGF
>Colletotrichum_salicis_JFFI01002688.1 .
-KIRRGQV-FKDAFEQF---------------YELGEGLKE-PIQIQFVDQFDTV--EAG
IDGGGVTKEF---LTSV----TTEAFGPRDGI------SLFTANSKG-------------
----------------LLYPNPTA----------------MDELRQVL-KRADVPERTTE
WQEQVQGLLRQFEFLGRIVGKCMYEGILVDIAFAG--FFLLKWIS---------------
-GQIGENSYRGNVNDLRD----------------------LDEELYQG--------MLRL
KNHNG---NVA-DWA--
--LDFTLNDQVSLPGE----------------------PVR-TVTRN-LIPN--------
GENVT---
------------------------------VINDNRL-LYISY-VARHRLVAQ-PAQQTA
-AFLRG---LRSIIAP--AWLS-MFNQN-ELQRLVGG-----------------------
---DSSEID-----------IEDL----RQNTVYSGLYEVGDDGLE--------------
------------------------HPTV-QLFWKVMA--------------------NFS
DRERRDVLKY-------VTSTPRAP------------LLG-----FS-QLSPRF--SIRD

GGEDQ-----------------------------ERLPSTSTCV-NL-LKLPRY-KDQET
LRKKLL-YAVSS-GAGF
>Colletotrichum_godetiae_LZRM01001365.1 .
-KIRRGQV-FKDAFEQF---------------YELGEGLKE-PIQIQFVDQFDTV--EAG
IDGGGVTKEF---LTSV----TTEAFGPRDGI------SLFTANSKG-------------
----------------LLYPNPTA----------------MDELRQVL-KRAEVPERTTE
WQEQVQGLLRQFEFLGRIVGKCMYEGILVDIAFAG--FFLLKWIS---------------
-GQTGENSYRGNVNDLRD----------------------LDEELYQG--------MLRL
KNHNG--DVA-DWA--
--LDFTLNDQVSLPGE----------------------PVR-TVTRN-LIPN--------
GENVT---
------------------------------VTNDNRL-LYISY-VARHRLVAQ-PAQQTA
-AFLRG---LRSIIAP--AWLS-MFNQN-ELQRLVGG-----------------------
---DSSEID-----------IEDL----RQNTVYSGLYEVGDDGLE--------------
------------------------HPTV-QLFWKVMA--------------------NFS
DRERRDVLKY-------VTSTPRAP------------LLG-----FS-QLSPRF--SIRD
GGEDQ-----------------------------ERLPSTSTCV-NL-LKLPRY-KNQET
LRKKLL-YAVSS-GAGF
>Colletotrichum_nymphaeae_JEMN01000151.1 .
-KIRRGQV-FKDAFEQF---------------YELGEGLKE-PIQIQFVDQFDTV--EAG
IDGGGVTKEF---LTSV----TTEAFGPRDGI------SLFTANSKG-------------
----------------LLYPNPTA----------------MDELRQVL-KRAGVPERSAD
WQDQVQSLLRQFEFLGRIVGKCMYEGILVDIAFAG--FFLLKWIS---------------
-GQTGENSYRGNVNDLRD----------------------LDEELYQG--------MLRL
KNHHG--NVA-DWA--
--LDFTLNDQVSLPGE----------------------PVR-TVTRN-LIPN--------
GENVT---
------------------------------VTNDNRL-LYISY-VARHRLVAQ-PAQQTA
-AFLRG---LRSIIAP--AWLS-MFNQN-ELQRLVGG-----------------------
---DSSEID-----------IEDL----RQNTVYSGLYEIGDDGLE--------------
------------------------HPTV-QLFWKVMT--------------------SFS
DRERRDVLKY-------VTSTPRAP------------LLG-----FS-QLSPRF--SIRD
GGEDQ-----------------------------ERLPSTSTCV-NL-LKLPRY-KNQET
LRQKLL-YAVSS-GAGF
>Colletotrichum_fioriniae_XM_007595910.1 .
GKIRRGQV-FKDAFEQF---------------YELGEGLKE-PIQIQFVDQFDTV--EAG
IDGGGVTKEF---LTSV----TTEAFGPRDGI------SLFAANSKG-------------
----------------LLYPNPTA----------------MDELKQIL-KRGGVPERTAE
WQDQVQSLLRQFEFLGRIVGKCMYEGILVDIAFAG--FFLLKWIS---------------
-GQTGENSYRGNVNDLRD----------------------LDEELYQG--------MLRL
KNHTG--NVA-DWA--
--LDFTLNDQVSLPGE----------------------PVR-TVTRN-LIPN--------
GENVT---
------------------------------VTNDNRL-LYISY-VARHRLVAQ-PAQQTA
-AFLRG---LRSIIAP--AWLS-MFNQN-ELQRLVGG-----------------------
---DSSEID-----------IEDL----RQNTVYSGLYEIGDDGLE--------------
------------------------HPTV-QLFWKVMA--------------------NFS
DRERRDVLKY-------VTSTPRAP------------LLG-----FS-QLSPRF--SIRD
GGEDQ-----------------------------ERLPSTSTCV-NL-LKLPRY-KNQET
LRQKLL-YAVSS-GAGF
>Colletotrichum_acutatum_LVCK01001067.1 .
-KIRRGQV-FKDAFEQF---------------YELGEGLKE-PIQIQFVDQFDTV--EAG
IDGGGVTKEF---LTSV----TTEAFGPRDGI------SLFTANSKG-------------
----------------LLYPNPTA----------------MDELRQVL-KRAGVPERSAE
WQDQVQSLLRQFEFLGRIVGKCMYEGILVDIAFAG--FFLLKWIS---------------
-GQTGENSYRGNVNDLRD----------------------LDEELYQG--------MLRL
KNHNG--NVA-DWA--
--LDFTLNDQVSLPGE----------------------PVR-TVTRN-LIPN--------

GENVT---
------------------------------VTNDNRL-LYISY-VARHRLVAQ-PAQQTA
-AFLRG---LRSIIAP--AWLS-MFNQN-ELQRLVGG-----------------------
---DSSEID-----------IEDL----RQNTVYSGLYEIGDDGLE--------------
------------------------HPTV-QLFWKVMA--------------------NFS
DRERRDVLKY-------VTSTPRAP------------LLG-----FS-QLSPRF--SIRD
GGEDQ-----------------------------ERLPSTSTCV-NL-LKLPRY-KDQET
LRQKLL-YAVSS-GAGF
>Colletotrichum_simmondsii_JFBX01000271.1 .
-KIRRGQV-FKDAFEQF---------------YELGEGLKE-PIQIQFVDQFDTV--EAG
IDGGGVTKEF---LTSV----TTEAFGPRDGI------SLFTANSKG-------------
----------------LLYPNPTA----------------MDELRQVL-KRAGVPERSAE
WQDQVQSLLRQFEFLGRIVGKCMYEGILVDIAFAG--FFLLKWIS---------------
-GQTGENSYRGNVNDLRD----------------------LDEELYQG--------MLRL
KNHHG--NVA-DWA--
--LDFTLNDQVSLPGE----------------------PVR-TVTRN-LIPN--------
GENVT---
------------------------------VTNDNRL-LYISY-VARHRLVAQ-PAQQTA
-AFLRG---LRSIIAP--AWLS-MFNQN-ELQRLVGG-----------------------
---DSSEID-----------IEDL----RQNTVYSGLYEIGDDGLE--------------
------------------------HPTV-QLFWKVMA--------------------NFS
DRERRDVLKY-------VTSTPRAP------------LLG-----FS-QLSPRF--SIRD
GGEDQ-----------------------------ERLPSTSTCV-NL-LKLPRY-KNQET
LRQKLL-YAVSS-GAGF
>Pseudomassariella_vexata_MCFJ01000009.1 .
ARIKRGQV-FSDAFNQF---------------YNLGEGLKE-PIQITFIDQFDQQ--EAG
IDGGGVTKEF---LTSI----IDEAFYTSH--------RLFTANSQN-------------
----------------LLYPNPTA----------------FDEIKEMA-ISRNCAEGSPD
YLKVLQDLGKNYEFLGRIVGKCMYEGILIDVAFAN--FFLQKWSS---------------
SGISSSTESKANLYDLRE----------------------LDPELYKG--------LVSL
KNYTG--DVA-DLS--
--LDFAVTDQYVHPITK---------------------QVK-TITRP-LRKD--------
GENIP---
------------------------------VTNKDRP-LYIAA-VVHYRLVQQ-LIRQNR
-AFLRG---LGSMINP--SWLS-MFNPL-ELQRLVGG-----------------------
---DSSEID-----------IDDL----RRHTEYSGVYTIGDDGLE--------------
------------------------HPTV-QLFWQVMH--------------------SLK
DEERRDVLKY-------VTSTPRAP------------LLG-----FA-QLSPAF--SIRD
SGDDE-----------------------------ERLPSTSTCV-NL-LKLPRY-STAPR
LKEKLL-YAIQS-GAGF
>Daldinia_eschscholzii_AIID01010754.1 .
ARVKRGQV-FDDAYEQF---------------YPLGDGLKE-PIQITFVDQFDQP--EAG
IDGGGVTKEF---LISV----TDEAFNPSSGR------GLFATNSQN-------------
----------------LLYPNPST----------------FDNHAELL-RSYGYTENDAR
WKEAMIDLAKRYEFLGRVVGKCMYEGILIDKAFAG--FFLLKWSA---------------
SGHGSLNDYRANLNDLRD----------------------LDPELYQG--------LISL
KNYSG--KVV-DLA--
--LDFTITDEVTTVDG----------------------NTL-TITRP-LCKN--------
GENIP---
------------------------------VTNTNRP-LYISY-VVNHRLVAQ-PYRQTR
-AFLKG---LGSIINP--AWLS-MFNQT-ELQRLVGG-----------------------
---DSSEID-----------VEDL----RRNTQYSGLYTVGDDGME--------------
------------------------HPTV-QMFWQVMR--------------------EFQ
DSQRREVLKY-------VTSTPRAP------------LLG-----FS-QLSPHF--SIRD
GGTDE-----------------------------ERLPSTSTCV-NL-LKLPRY-SSKET
LRSKLL-YAIQS-GAGF
>Pestalotiopsis_fici_XM_007838523.1 .
ATIKRGQL-FSDAYSQF---------------YNLGEALKE-PIQITFVDKFDQP--EAG

IDGGGVTKEF---LTSV----TTEAFTPKDHL------GMFVTNSQN-------------
----------------LLYPNPTV----------------FDELVESL-RWYGIPENSAE
WNSRLQDLTQKFEFLGRVVGKCMYEGILIDLSFAP--FFLLKWSS---------------
----SGRDVRASLNELRD----------------------LDPELYRG--------LIQL
KNYPG--DVG-DFS--
--LDFTINDRVTNPVTN---------------------KVR-TITRP-LRKG--------
NENAP---
------------------------------VTNKDRP-LYISY-VVTHRLVAQ-PYRQTK
-AFLRG---LNSIIDP--TWLQ-MFNQS-ELQRLVGG-----------------------
---DSSEID-----------VEDL----RSNTVYSGVYVIGDDGEE--------------
------------------------HPTV-KMFWDVMH--------------------ELE
DSERREVLKY-------VTSTPRAP------------LLG-----FG-QLSPAF--SIRD
NGSDE-----------------------------NRLPSAATCI-NL-LKLPLY-RSRAT
LKEKLL-YAVES-GAGF
>Rosellinia_necatrix_BBSO02000584.1 .
ATISRGSV-FDDAFKQF---------------YTLGEGLKE-PIQITFVDQFNQP--EAG
IDGGGVTKEF---LISV----TNDAFRPERTDGH----TYFICNDQN-------------
----------------LLYPNPSI----------------FDDMVAKL-RHRGTLDTSMD
WKNDMGDIVRRYEFLGRIVGKCMYEGILIDISFAS--FFLLKWAS---------------
SGQSTSTDYRANLNDLRD----------------------LDPELYQG--------LTSL
KNYPG--DVA-DLS--
--LDFTIADSVWDSSG----------------------QRR-TITRP-LKKD--------
GANIP---
------------------------------VTNKDRP-LYISY-VANHRLVAQ-QYRQTK
-AFLRG---LGTIINP--SWLS-MFNQS-ELQRLIGG-----------------------
---DSSEID-----------VEDL----RQNTSYSGLYVIGDDGEE--------------
------------------------HPTI-RFFWDVMR--------------------KLK
DSERRDVLKY-------VTSTPRAP------------LLG-----FS-QLKPQF--TIRD
GGNDE-----------------------------ERLPSTSTCV-NL-LKLPVY-KSAAV
LKAKLL-YAVQS-GAGF
>2_Didymobotryum_rigidum_BCKI01000004.1 .
AQVKRGQV-FDDAYKHF---------------YSLGEGLKE-PIQITFVDQFGAQ--EAG
IDGGGVTKEF---LISV----TNEAFQPGLDGH-----GFFVTNSQN-------------
----------------LLYPNPSA----------------FDSQAELL-RYHSFKEGSTE
WRNGMADLAKRYEFLGRVVGKCMYEGILIDISFAG--FFLLKWSS---------------
SGQSAAAAYRASLNDLRD----------------------LDPELYQG--------LISL
KNYPG--DVN-DLS--
--LDFTITDQFTNPDH----------------------EVR-TITRP-LRKD--------
GENIP---
------------------------------VTNKDRP-LYISY-VANHRLVAQ-QYKQTQ
-AFLRG---LGSIISP--SWLS-MFNQS-ELQRLVGG-----------------------
---DSSEID-----------VEDL----RRNTSYSGLYVIGDDGEE--------------
------------------------HPTI-QLFWQVMK--------------------ELQ
DSERRDVLKY-------VTSTPRAP------------LLG-----FS-QLSPQF--SIRD
AGTDE-----------------------------ERLPSTSTCV-NL-LKLPRY-TNART
LKSKLL-YAVQS-GAGF
>Eutypa_lata_XM_007797295.1 .
ARVKRGVV-FDDAFDQF---------------YTLGEGLKE-PIQITFVDQFDQP--EAG
IDGGGVTKEF---LTSI----ADEAFNPSPEGH-----SFFVANKQN-------------
----------------LLYPNPTA----------------FDERAELL-RFHGLKEGTAG
WGNGFQDLAKRYEFLGRIVGKCMYEGILIDIAFAP--FFLLKWSS---------------
SGQSTPADYRANLNDLRD----------------------LDPELYQG--------LKNL
KNYPG--DVG-DLS--
--LNFAITDQIRTPDG----------------------GVR-TVTRP-LRKD--------
GENIT---
------------------------------VTNKDRP-LYISY-VASHRLIVQ-PYRQTK
-AFLRG---LGSIINP--SWLS-MFNQS-ELQRLVGG-----------------------
---DSSEID-----------VEDL----RHNTIYSGLYAIGDDGME--------------

------------------------HPTV-QMFWQVMH--------------------SLQ
DEERREVLKY-------VTSTPRAP------------LLG-----FS-QLSPHF--SIRD
AGTDE-----------------------------ERLPSTSTCV-NL-LKLPRY-TNIET
LKSKLL-YAIKS-GAGF
>Geosmithia_morbida_FNXU01000002.1 .
ANIRRGQL-FEDAFENF---------------YKIGDGLKD-PISITFIDQFGTP--EAG
IDGGGVTKEF---LTSV----TAEAFSPGKDGI-----QMFTSSEKG-------------
----------------LLYPEPAA----------------VDQLREVL-HSEGLTESEPQ
WREAMTTLLRHYEFLGRIVGKCLYEEILVNLSFAG--FFLLQWPS---------------
TGPGQSSTYKGSVNDLRD----------------------MDEDLYKG--------MVRL
KNYAG--DVS-DLG--
--LDFSINDEISLPNQ----------------------PVK-TVTRN-LVPN--------
GENVP---
------------------------------VTNDNRL-LYISY-VARHRLVNQ-PALQTS
-AFLRG---LRQIIRP--SWLS-MFNQS-ELQRLVGG-----------------------
---DSSEID-----------LEDL----RRNTMYSGLYVIGDDNKE--------------
------------------------HRSI-ELFWKVMR--------------------GFT
DQEKREVLRF-------VTSTPRAP------------LLG-----FS-QLRPLF--TIRD
SVSGL-----------------------------DKLPTASTCV-NL-LKLPPY-RTEEV
MREKLL-YAITS-GAGF
>Beauveria_bassiana_ADAH01000358.1 .
AQIRRGQL-FEDAFEEL---------------YKIGDGLKD-AVQITFVDQFDQP--EAG
IDGGGVTKEF---LISV----TSEAFGDQGGSL-----NMFTSSPDG-------------
----------------LLYPNPTA----------------VDTIKETM-REGGLNELSPE
WRRILNEQLKRYEFLGRIVGKCMYEGILVDLNFAG--FFLLKWPS---------------
SGPKEENNYKGSINDLRD----------------------MDEELYKG--------MLRL
KNYPG--DVS-ELG--
--IDFTVTDQVSMPGA----------------------PIK-TVTKL-LVAN--------
GDQIS---
------------------------------VTNDNRP-LYISY-MARHRLVYQ-PAVQTA
-AFLRG---LRAIIRP--SWLS-MFNQS-ELQRLVGG-----------------------
---DSSEID-----------IEDL----RQNTVYGGLYEVGDDKRN--------------
------------------------HPTI-DLFWKVVG--------------------GFT
DEQRRDLLKY-------VSSTPRAP------------LLG-----FS-QLRPHF--SIRD
AGADE-----------------------------ERLPSTSTCV-NL-LKLPRY-KTEAT
LREKLL-YAITS-GAGF
>Lecanicillium_fungicola_FWCC01000676.1 .
AQIRRGQL-FEDAFEEL---------------YKVGDGLKD-PIQITFVDQFGSP--EAG
IDGGGVTKEF---LISV----TTEAFGDQDNSL-----NMFTSSPDG-------------
----------------LLYPNPTS----------------IDVVKETM-REGKLSESAPD
WRRILNDQLKRYEFLGRIVGKCMYEGILVDLNFAG--FFLLKWPS---------------
SGPKEENNYKGSINDLRD----------------------MDEELYKG--------MLRL
KNYPG--DVS-ELG--
--IDFTVTDQVSMPGA----------------------PIK-TVTKL-LVAN--------
GDQVY---
------------------------------VTNDNRP-LYISY-MARHRLVVQ-SAVQTA
-AFLRG---LRAIIRP--SWLS-MFNQS-ELQRLVGG-----------------------
---DSSEID-----------IDDL----RKNTVYSGLYEVGDDNQE--------------
------------------------HPTI-KMFWRVLR--------------------SFT
DAQRRDLLKY-------VSSTPRAP------------LLG-----FS-QLRPRF--SIRD
AGTDQ-----------------------------DRLPSTSTCV-NL-LKLPRY-TNERT
LRDKLL-YAISS-GAGF
>Isaria_fumosorosea_XM_018850018.1 .
AQIRRGQL-FEDAFEEL---------------YKIGDGLKD-PIAITFVDQFDEP--EAG
IDGGGVTKEF---LISV----ISEALSGHEDGI-----NMFTASPSG-------------
----------------LLYPNPIA----------------IDAIKETM-RVGHVNESFPE
WRQMLNSQLRRYEFLGRLVGKCMYEGILVDVNFAG--FFLLKWPS---------------
AGPDDENNYKGSINDLRD----------------------LDEELYNG--------MLRL

KNYPG--DVS-ELD--
--ISFTVSDQVSMPKA----------------------PIK-TVTKL-LVAN--------
GDQVY---
------------------------------VTNDNRP-LYISY-MARHRLVVQ-SAPQTA
-AFLRG---LRAIIRP--SWLS-MFNQS-ELQRLVGG-----------------------
---DSSEID-----------LEDL----RKNTVYSGLYELGDDKEE--------------
------------------------HPTI-KIFWKVMH--------------------EFT
DEKRRNLLKY-------VSSTPRAP------------LLG-----FS-QLRPLF--CIRS
GGNDE-----------------------------ERLPSASTCI-NL-LKLPVY-KTEER
LREKLL-YAITS-GAGF
>Cordyceps_militaris_AEVU01000229.1 .
AQIRRGQL-FEDAFEEL---------------YKVGDGLKD-PIQITFVDQFNQT--EAG
IDGGGVTKEF---LTSV----ISEAFGGNEGMI-----SMFTSSPSG-------------
----------------LLYPNPIA----------------IDTTKEMM-REGKLDESSRE
WHRIIREQLKRYEFLGRMLGKCIYEGILVDLSFAG--FFLLQWPS---------------
SGSDEKNHYKGSINDLRD----------------------MDEELYAG--------MLRL
KNHPG--DVS-ELG--
--IDFTVTDQVSMPGA----------------------PIK-TVTKL-LVAN--------
GDQVY---
------------------------------VTNDNRP-LYMSY-MARHRLVVQ-PALQTA
-AFLHG---LRSIIRP--SWLS-MFNQT-ELQHLVGG-----------------------
---DSSEID-----------IDDL----RRNTVCSGLYEMGDDKKE--------------
------------------------HPTI-RMFWKVLR--------------------SFT
DAQRRDLLKY-------VSSTPRAP------------LLG-----FS-QLRPLF--SIRD
AGDDE-----------------------------ERLPSASTCV-NL-LKLPRY-RREET
LRSKLL-YAITS-GAGF
>Torrubiella_hemipterigena_CDHN01000003.1 .
-QIRRGHL-FEDAYKQF---------------YKIGDSLKD-PIQITFVDQFGAP--EAG
IDGGGVTKEF---LMGV----TSEAFSGREGEL-----SMFASSSSG-------------
----------------LLYPNPTV----------------NDVLRDEM-REMGKTERDEE
WRHALFEQLKMYEFLGRVVGKCLYEGILVDLAFAG--FFLLKWTA---------------
SGSTDESSYKGSINDLVD----------------------MDEELYNG--------MLRL
KNYPG--DVG-ELS--
--LDFTVTDQVSHPSR----------------------PVK-TITRK-LIAN--------
GDQIP---
------------------------------VTNDNRL-LYISY-VARHRLVVQ-PAAQTA
-AFLRG---LRAIIRP--SWLS-MFNQS-ELQHLVGG-----------------------
---DSSEID-----------IEDL----RKNTVYSGLYEIGDDNKE--------------
------------------------HPTI-ELFWKVVR--------------------GFT
DAQRRDLLKY-------VSSTPRAP------------LLG-----FS-QLRPKF--SIRD
ASRDE-----------------------------TRLPSTSTCV-NL-LKLPLY-TTETV
LREKLL-YAIQS-GAGF
>2_Gliomastix_tumulicola_BCHX01000004.1 .
AHIRRDRL-FEDAYDNF---------------YKLGEGLKD-SINITFVDQFGSP--EAG
IDGGGVTKEF---LTSV----TTEAFGGSDDTL-----RMFASSETG-------------
----------------LLYPNPIA----------------VDNSREVL-RRQGLTEGSVE
WRDLMGALLKRYEFLGRVVGKCLYEGILVDLAFAG--FFLLQWPS---------------
TGSTEGNTYKGSVNDLSD----------------------MDEDLYKG--------MLRL
KNYPG--DVS-ALG--
--IDFTITDQVSLPHE----------------------PPK-TITRN-LIPN--------
GDKIA---
------------------------------VTNDNRL-LYISY-VARHRLVLQ-PALQTA
-AFLRG---LREIIRP--SWLS-MFNQS-ELQRLVGG-----------------------
---DSSEID-----------IEDL----RRNTVYGGLYEIGDDGEE--------------
------------------------HPTV-KLFWKVMG--------------------TFN
DSQRRDVLKY-------VSSTPRAP------------LLG-----FS-QLRPKF--SIRD
AGSNE-----------------------------QRLPSTSTCV-NL-LKLPLY-KSEAA
LREKLL-YAISS-GAGF

```
>Acremonium_chrysogenum_JPKY01000020.1 .
--IRRGML-FEDAFKAF---------------HPLGEGLKD-TISITFVDQFGTP--EAG
IDGGGVTKEF---LTSV----TAEAFSHDEGNL-----RMFTSSETN-------------
----------------LLFPDPVA----------------VDGLRESM-RGLGLSEADQE
WRENISKLLKRFEFLGRIVGKCLYEGILLDLAFAG--FFLLQWPS---------------
ASSGESNTYKGSVNDLRD----------------------MDEELYKG--------MLRL
KNYPG----------------------------------------------DVS-EMG--
--IDFTINDQVSPPGQ----------------------PLK-TVTRN-LIPN--------
GDKTL-------------------------------------------------------
------------------------------VTNDNRL-LYISY-VARHRLVLQ-PALQTS
-AFLRG---LRQIIRP--SWLS-MFNQI-ELQRLVGG-----------------------
---DSSEID-----------VDDL----RRNTVYGGVYVIGDDNEE--------------
------------------------HPSI-KLFWRVMK--------------------SLT
DAQRRDVLKY-------VTSTPRAP------------LLG-----FS-QLKPKF--SIRD
AGRDE-----------------------------TRLPSTSTCV-NL-LKLPQY-TSMAV
MREKLL-YAITS-GAGF
>Trichoderma_gamsii_XM_018810464.1 .
-KIRRGQL-FEDAFEQF---------------YQLGEGLKD-PIQITFVDQFDTP--EAG
IDGGGVTKEF---LTSV----TSEAFGNQLGGL-----GMFTSSDKG-------------
----------------LLYPNPMA----------------LDILRESL-RQQGLTESDHE
WREAISELFKRYEFLGRIVGKCMYEGILVDLAFAG--FFLLKWPS---------------
PNRKEENSYKGSVNDLQD----------------------MDEELYKG--------LLNL
KNYPG----------------------------------------------DVS-ALG--
--FDFTITDQVSAPGE----------------------PVK-TVSRK-LVPN--------
GDEVP-------------------------------------------------------
------------------------------VTNDNRL-LYISY-AARHRLVVQ-PAPQTM
-AFLRG---LREIIRP--SWLS-MFNQS-ELQRLVGG-----------------------
---DSMEID-----------VEDL----RRNTVYSGLYAVGDDGED--------------
------------------------HPTI-KMFWNVMN--------------------GFT
DAQRRDVLKY-------VSSTPRAP------------LLG-----FA-QLNPKF--AIRD
GGSDQ-----------------------------ERLPST--------------------
-----------------
>Trichoderma_hamatum_ANCB02000256.1 .
AKIRRGQL-FEDAFEQF---------------YQLGEGLKD-PIQITFVDQFDTP--EAG
IDGGGVTKEF---LTSV----TSEAFGNQLGGL-----GMFTSSDKG-------------
----------------LLYPNPMA----------------LDILRESL-RQQGLTESDHE
WREAISELFKRYEFLGRIVGKCMYEGILVDLAFAG--FFLLKWPS---------------
PNRKEENSYKGSVNDLQD----------------------MDEELYKG--------LLNL
KNYAG----------------------------------------------DVS-ALG--
--FDFTITDQVSAPGE----------------------PVK-TVSRK-LVPN--------
GDEVP-------------------------------------------------------
------------------------------VTNDNRL-LYISY-AARHRLVLQ-PAPQTM
-AFLRG---LREIIRP--SWLS-MFNQS-ELQRLVGG-----------------------
---DSMAID-----------VEDL----RRNTIYSGLYAVGDDGED--------------
------------------------HPTI-KMFWNVMN--------------------GFT
DAQRRDVLKY-------VSSTPRAP------------LLG-----FS-QLNPKF--AIRD
GGSDQ-----------------------------ERLPSTSTCV-NL-LKLPVY-KSEST
LRTKLL-YAISS-GAGF
>Trichoderma_asperellum_JNNP01001397.1 .
AKIRRGQL-FEDAFEQF---------------YQLGEGLKD-PIQITFVDQFDTP--EAG
IDGGGVTKEF---LTSV----TSEAFGNQLGGL-----GMFTSSDKG-------------
----------------LLYPNPMA----------------LDILRESL-RQQGLTESDHE
WRETISELFRRYEFLGRIVGKCMYEGILVDLAFAG--FFLLKWPS---------------
PNRKEENSYKGSVNDLQD----------------------MDEELYKG--------LLNL
KNYPG----------------------------------------------DVS-ALG--
--FDFTITDQVSAPGE----------------------PVK-TVSRK-LVPN--------
GDEVS-------------------------------------------------------
------------------------------VTNDNRL-LYISY-AARHRLVLQ-PAPQTM
```

```
-AFLRG---LREIIRP--SWLS-MFNQS-ELQRLVGG-----------------------
---DSMAID-----------VEDL----RRNTIYSGLYAIGDDGEE--------------
------------------------HPTI-KMFWNVMN--------------------GFT
DAQRRDVLKY-------VSSTPRAP------------LLG-----FS-QLNPKF--AIRD
GGSDQ------------------------------ERLPSTSTCV-NL-LKLPVY-KSEST
LRTKLL-YAISS-GAGF
>Trichoderma_atroviride_XM_014087019.1 .
AKIRRGQL-FEDAYEQF---------------YQLGEGLKD-PIQITFVDQFDTP--EAG
IDGGGVTKEF---LTSV----TSEAFANQLGGL-----GMFTSSDKG-------------
----------------LLYPNPMA----------------LDILRESL-RQQGLTESDHE
WRETISELFKRYEFLGRIVGKCMYEGILVDLAFAG--FFLLKWPS---------------
PNRKEENSYKGSVNDLQD----------------------MDEELYKG--------LLNL
KNYPG-------------------------------------------------DVS-ALG--
--FDFTITDQVSAPGE----------------------PVK-TVSRK-LVPN--------
GDEVP-------------------------------------------------------
------------------------------VTNDNRL-LYISY-AARHRLVLQ-PAPQTM
-AFLRG---LREIIRP--SWLS-MFNQS-ELQRLVGG-----------------------
---DSMEID-----------VEDL----RRNTVYSGLYAVGDDGEE--------------
------------------------HPTI-KMFWNVMN--------------------GFT
DAQRRDVLKY-------VSSTPRAP------------LLG-----FA-QLNPKF--AIRD
GGSDQ------------------------------ERLPSTSTCV-NL-LKLPVY-KSEST
LRTKLL-YAISS-GAGF
>Trichoderma_virens_ABDF01000236.1 .
AKIRRGQL-FEDAFEQF---------------YQLGEGLKD-PIQITFVDQFDTP--EAG
IDGGGVTKEF---LTSV----TSEAFGNGAGGL-----GMFTSSGKE-------------
----------------LLYPNPTA----------------MDVLRESL-RKQGLTESDPE
WREAMSGLLRRYEFLGRIVGKCMYEGILVDLAFAG--FFLLKWPS---------------
PNRKEENNYKGSVNDLQD----------------------MDEELYRG--------LLNL
KNYSG-------------------------------------------------DVS-ALG--
--LDFTITDQISAPGE----------------------PVE-IVTRK-LVPG--------
GDEMP-------------------------------------------------------
------------------------------VTNDNRL-LYISY-AARHRLVVQ-PAPQTT
-SFLRG---LREIIRP--SWLS-MFNQS-ELQRLVGG-----------------------
---DSMAID-----------VENL----RQNTVYSGLYAIGDDGEE--------------
------------------------HPTI-KMFWNVMN--------------------SLT
DAQRRDVLKY-------VTSTPRAP------------LLG-----FA-QLNPKF--AIRD
GSSDQ------------------------------ERLPSASTCV-NL-LKLPIY-KSEST
LRKKLL-YAITA-GAGF
>Trichoderma_reesei_XM_006966642.1 .
AKIRRGQL-FDDAFEQF---------------YQLGDGLKD-PIQITFVDQFGTP--EAG
IDGGGVTKEF---LTSV----TAEAFGNEHGGF-----GLFTRSRKE-------------
----------------LLYPNPTA----------------MDVLRESL-RKQGLVESDPE
WREAVSDLLRRYEFLGRIVGKCMYEGILVDLAFAG--FFLLKWPS---------------
PSRKEENSYKGSVNDLQD----------------------MDEELYNG--------LLRL
KNYPG-------------------------------------------------DVS-ELG--
--LDFTVTDQISAPGE----------------------PVK-IVTRK-LVPN--------
GDEIP-------------------------------------------------------
------------------------------VTNDNRL-LYISY-AARHRLVVQ-PAPQTN
-AFLRG---LREIIRP--SWLS-MFNQS-ELQRLVGG-----------------------
---DSMEID-----------VEDL----RRNTVYSGLYAIGDDGEE--------------
------------------------HETI-KMFWNVMR--------------------GFT
DAQRRDVLKY-------VTSTPRAP------------LLG-----FS-QLNPKF--AIRD
GSSDQ------------------------------ERLPSTSTCV-NL-LKLPVY-KSEET
LRQKLL-YAISS-GAGF
>Trichoderma_parareesei_LFMI01000823.1 .
AKIRRGQL-FDDAFEQF---------------YQLGDGLKD-PIQITFVDQFGTP--EAG
IDGGGVTKEF---LTSV----TAEAFGNEHGGF-----GLFTRSRKE-------------
----------------LLYPNPTA----------------MDVLRESL-RKQGLVESDPE
```

WREAVSDLLRRYEFLGRIVGKCMYEGILVDLAFAG--FFLLKWPS---------------
PSRKEENSYKGSVNDLQD----------------------MDEELYNG--------LLRL
KNYPG--DVS-ELG--
--LDFTVTDQISAPGE----------------------PVK-IVTRK-LVPN--------
GDEIP---
------------------------------VTNDNRL-LYISY-AARHRLVVQ-PAPQTN
-AFLRG---LREIIRP--SWLS-MFNQS-ELQRLVGG-----------------------
---DSMAID-----------VEDL----RRNTVYSGLYAIGDDGEE--------------
------------------------HETI-KMFWNVMR--------------------GFT
DAQRRDVLKY-------VTSTPRAP------------LLG-----FS-QLNPKF--AIRD
GSSDQ-----------------------------ERLPSTSTCV-NL-LKLPVY-KSEET
LRQKLL-YAISS-GAGF
>Trichoderma_longibrachiatum_ANBJ01000017. 1..
AKIRRGQL-FDDAFEQF---------------YQLGDGLKD-PIQITFVDQFGTP--EAG
IDGGGVTKEF---LTSV----TAEAFGNEHGGF-----SMFTRSRKE-------------
----------------LLYPNPTA----------------MDVLRESL-RKQGLVETDPE
WREAISDLLRRYEFLGRIVGKCMYEGILVDLAFAG--FFLLKWPS---------------
PSRKEENSYKGSVNDLQD----------------------MDEELYNG--------LLRL
KNYPG--DVS-ELG--
--LDFTVTDQISAPGE----------------------PVK-IVTRK-LVPN--------
GDEIP---
------------------------------VTNDNRL-LYISY-AARHRLVVQ-PAPQTN
-AFLRG---LREIIRP--SWLS-MFNQS-ELQRLVGG-----------------------
---DSMAID-----------VEDL----RRNTVYSGLYAIGDDGEE--------------
------------------------HETI-KMFWNVMR--------------------GFT
DAQRRDVLKY-------VTSTPRAP------------LLG-----FS-QLNPKF--AIRD
GSSDQ-----------------------------ERLPSTSTCV-NL-LKLPVY-KSEET
LRKKLL-YAISS-GAGF
>Epichloe_amarillans_AFRF01000010.1 .
AKIKRGQL-FEDAFNQF---------------YELEDNFKD-TIQITFVDQFDTP--EAG
IDGGGVTKEF---LIGV----TSEAFANDENSL-----GMFSSNEQG-------------
----------------LLFPNPTA----------------ADTLRESL-RRGGLSEHDPE
WRAAVTGLLQRFEFLGRIVGKCLYDGILVDLAFAG--FFLLKWPS---------------
SGPRDENTYKGSINDLRD----------------------MDEELYKG--------LLRL
KNYSG--DVS-ELG--
--FDFTVTDKISPPGQ----------------------PVQ-TVTRK-LIPQ--------
GDSVP---
------------------------------VTNDNRL-LYISY-AARHRLVIQ-PKLQTS
-AFLTG---LRSIIHP--SWLS-MFNQS-ELQRLVGG-----------------------
---DSSEID-----------IGDL----RRNTVYSGLYAVGDDNLE--------------
------------------------HPTI-VLFWKVMA--------------------GFT
DSQRRDVLKY-------VSSTPRAP------------LLG-----FS-QLRPKF--SIRD
GGTDE-----------------------------DRLPSTSTCV-NL-LKLPRY-TSEAV
LREKLL-YAITS-GAGF
>Epichloe_baconii_JFGY01000236.1 .
AKIKRGQL-FEDAFNQF---------------YELGDNFKD-TIQITFVDQFDTP--EAG
IDGGGVTKEF---LIGV----TSEAFANDENSL-----GMFSSNEQG-------------
----------------LLFPNPTA----------------ADTLRESL-RQGGLSEHDPE
WRAAVTGLLQRFEFLGRIVGKCLYDGILVDLAFAG--FFLLKWPS---------------
SGPRDENTYKGSINDLRD----------------------MDEELYKG--------LLRL
KNYSG--DVS-ELG--
--FDFTVTDKISPPGQ----------------------PVQ-TVTRK-LIPQ--------
GDSVP---
------------------------------VTNDNRL-LYISY-AARHRLVIQ-PKLQTS
-AFLTG---LRSIIHP--SWLS-MFNQS-ELQRLVGG-----------------------
---DSSEID-----------IDDL----RRNTVYSGLYAVGDDNLE--------------
------------------------HPTI-VLFWKVMA--------------------GFT
DSQRRDVLKY-------VSSTPRAP------------LLG-----FS-QLRPKF--SIRD

```
GGTDE-----------------------------DRLPSTSTCV-NL-LKLPRY-TSEAV
LREKLL-YAITS-GAGF
>Epichloe_festucae_EU515145.1 .
AKIKRGQL-FEDAFNQF---------------YELGQNFKD-TIQITFVDQFDTP--EAG
IDGGGVTKEF---LIGV----TSEAFANDENSL-----GMFSSNEQG-------------
----------------LLFPNPTA----------------ADTLRESL-RQGGLSEHDPE
WRAAVTGLLQRFEFLGRIVGKCLYDGILVDLAFAG--FFLLKWPS---------------
SGPRDENTYKGSINDLRD----------------------MDEELYKG--------LLRL
KNYSG----------------------------------------------DVS-ELG--
--FDFTVTDKISPPGQ----------------------PVQ-TVTRK-LIPQ--------
GDSVP-------------------------------------------------------
------------------------------VTNDNRL-LYISY-AARHRLVIQ-PKLQTS
-AFLTG---LRSIIRP--SWLS-MFNQS-ELQRLVGG-----------------------
---DSSEID-----------IDDL----RRNTVYSGLYAVGDDNLE--------------
------------------------HPTI-VLFWKVMA--------------------SFT
DSQRRDVLKY-------VSSTPRAP------------LLG-----FS-QLRPKF--SIRD
GGTDE-----------------------------DRLPSTSTCV-NL-LKLPRY-TSEAV
LREKLL-YAITS-GAG-
>Epichloe_typhina_AFSE01000649.1 .
AKIKRGQL-FEDAFNQF---------------YELGDDFKD-TIQITFVDQFDTP--EAG
IDGGGVTKEF---LIGV----TSEAFANDENSL-----GMFSSNEQG-------------
----------------LLFPNPTA----------------ADTLRESL-RQGGLSEHDPE
WRAAVTGLLQRFEFLGRIVGKCLYDGILVDLAFAG--FFLLKWPS---------------
SGPRDENTYKGSINDLRD----------------------MDEELYKG--------LLRL
KNYSG----------------------------------------------DVS-ELG--
--FDFTVTDKISPPGQ----------------------PVQ-TVTRK-LIPQ--------
GDSVP-------------------------------------------------------
------------------------------VTNDNRL-LYISY-AARHRLVIQ-PKLQTS
-AFLTG---LRSIIRP--SWLS-MFNQS-ELQRLVGG-----------------------
---DSSEID-----------IDDL----RRNTVYSGLYAVGDDNLE--------------
------------------------HPTI-VLFWKVMA--------------------GFT
DSQRRDVLKY-------VSSTPRAP------------LLG-----FS-QLRPKF--SIRD
GGTDE-----------------------------DRLPSTSTCV-NL-LKLPRY-TSEAV
LREKLL-YAITS-GAGF
>Epichloe_brachyelytri_AFRB01000256.1 .
AKIKRGQL-FEDAFNQF---------------YEIGDDFKD-TIQITFVDQFDTP--EAG
IDGGGVTKEF---LIGV----TSEAFANDENSL-----GMFSSNEQG-------------
----------------LLFPNPTA----------------ADTLRESL-RQGGLSEHDPE
WRAAVTGLLQRFEFLGRIVGKCLYDGILVDLAFAG--FFLLKWPS---------------
SGPRDENTYKGSINDLRD----------------------MDEELYKG--------LLRL
KNYSG----------------------------------------------DVS-ELG--
--FDFTVTDKISPPGQ----------------------PVQ-TVTRK-LIPQ--------
GDSVP-------------------------------------------------------
------------------------------VTNDNRL-LYISY-AARHRLVIQ-PKLQTS
-AFLTG---LRSIIRP--SWLS-MFNQS-ELQRLVGG-----------------------
---DSSEID-----------IDDL----RRNTVYSGLYAVGDDNLE--------------
------------------------HPTI-VLFWKVMA--------------------GFT
DSQRRDVLKY-------VSSTPRAP------------LLG-----FS-QLRPKF--SIRD
GGTDE-----------------------------DRLPSTSTCV-NL-LKLPRY-TSEAV
LREKLL-YAITS-GAGF
>Epichloe_aotearoae_JFGX01000069.1 .
AKIKRGQL-FEDAFNQF---------------YELGDDFKD-TIQITFVDQFDTP--EAG
IDGGGVTKEF---LIGV----TSEAFANDENSL-----GMFSSNEQG-------------
----------------LLFPNPTA----------------ADTLRESL-RQGGLSEHDPE
WRAAVTGLLQRFEFLGRIVGKCLYDGILVDLAFAG--FFLLKWSS---------------
SGPRDENTYKGSINDLRD----------------------MDEELYKG--------LLRL
KNYSG----------------------------------------------DVS-ELG--
--FDFTVTDKISPPGQ----------------------PVQ-TVTRK-LIPQ--------
```

GDSVP---
------------------------------VTNDNRL-LYISY-AARHRLVIQ-PKLQTS
-AFLTG---LRSIIRP--SWLS-MFNQS-ELQRLVGG-----------------------
---DSSEID-----------IDDL----RRNTVYSGLYAVGDDNLE--------------
------------------------HPTI-VLFWKVMA--------------------GFT
DSQRRDVLKY-------VSSTPRAP------------LLG-----FS-QLRPKF--SIRD
GGTDE-----------------------------DRLPSTSTCV-NL-LKLPRY-TSEAV
LREKLL-YAITS-GAGF
>Epichloe_uncinata_LELE01000364.1 .
AKIKRGQL-FGDAFNQF---------------YELGDDFKD-TIQITFVDQFDTP--EAG
IDGGGVTKEF---LIGV----TSEAFANDENSL-----GMFSSNEQG-------------
----------------LLFPNPTA----------------ADTLRESL-GQGGLSEHDPE
WRAAVMGLLQRFEFLGRIIGKCLYDGILVDLAFAG--FFLLKWPS---------------
SGPRDENTYKGSINDLRD----------------------MDEELYKG--------LLRL
KNYSG--DVS-ELG--
--FDFTVTDKISPPGQ----------------------PVQ-TVTRK-LIPQ--------
GDSVP---
------------------------------VTNDNRL-LYISY-AARHRLVIQ-PKLQTS
-AFLTG---LRSIIRP--SWLS-MFNQS-ELQRLVGG-----------------------
---DSSEID-----------IDDL----RRNTVYSGLYAVGDDNLE--------------
------------------------HPTI-VLFWKVMA--------------------GFT
DSQRRDVLKY-------VSSTPRAP------------LLG-----FS-QLRPKF--SIRD
GGTDE-----------------------------DRLPSTSTCV-NL-LKLPRY-TSEAV
LREKLL-YAITS-GAGF
>Epichloe_glyceriae_AFRG01000789.1 .
AKIKRGQL-FEDAFNQF---------------YELGDDFKD-TIQITFVDQFDTP--EAG
IDGGGVTKEF---LIGV----TSEAFADDENSL-----GMFSSNEQG-------------
----------------LLFPNPTA----------------ADTLRESL-RQGGLSEHDPE
WRAAVTGLLQRFEFLGRIIGKCLYDGILVDLAFAG--FFLLKWPS---------------
SGPRDENTYKGSINDLWD----------------------MDEELHKG--------LLRL
KNYSG--DVS-ELG--
--FDFTVTDKISPPGQ----------------------PVQ-TVTRK-LIPQ--------
GDSVP---
------------------------------VTNDNRL-LYLSY-AARHRLVIQ-PRLQTS
-AFLTG---LRSIIRP--SWLS-MFNQS-ELQRLVGG-----------------------
---DSSEID-----------IGDL----RRNTVYSGLYAVGDDNLE--------------
------------------------HPTI-VLFWKVMA--------------------GFT
DSQRRDVLKY-------VSSTPRAP------------LLG-----FS-QLRPKF--SIRD
GGTDE-----------------------------DRLPSTSTCV-NL-LKLPRY-TSEAV
LREKLL-YAITS-GAGF
>Epichloe_bromicola_LBNH01000184.1 .
AKIKRGQL-FEDAFNQF---------------YELGDDFKD-TIQITFVDQFDTP--EAG
IDGGGVTKEF---LIGV----TSEAFANDENSL-----GMFSSNEQG-------------
----------------LLFPNPTA----------------ADTLRESL-RQGGLSEHDPE
WRAAVTGLLQRFEFLGRIIGKCLYDGILVDLAFAG--FFLLKWPS---------------
SGPRDENTYKGSINDLRD----------------------MDEELYKG--------LLRL
KNYSG--DVS-ELG--
--FDFTVTDKISPPGQ----------------------PVQ-TVTRK-LIPH--------
GDSVP---
------------------------------VTNDNRL-LYISY-AARHRLVIQ-PKLQTS
-AFLTG---LRSIIRP--SWLS-MFNQS-ELQRLVGG-----------------------
---DSSEID-----------INDL----RRNTVYSGLYAVGDDNLE--------------
------------------------HPTI-VLFWKVMA--------------------GFT
DSQRRDVLKY-------VSSTPRAP------------LLG-----FS-QLRPKF--SIRD
GGTDE-----------------------------DRLPSTSTCV-NL-LKLPRY-TSEAV
LREKLL-YAITS-GAGF
>Epichloe_elymi_AMDJ01000110.1 .
AKIKRGQL-FEDAFNQF---------------YELGDDFKD-TIQITFVDQFDTP--EAG

```
IDGGGVTKEF---LIGV----TSEAFANDENSL-----GMFSSNEQG-------------
----------------LLFPNPIA----------------ADTLRESL-RQGGLREHDPE
WRAAVTGLLQRFEFLGRIVGKCLYDGILVDLAFAG--FFLLKWPS---------------
SGPRYENTYKGSINDLRD----------------------MDEELYKG--------LLRL
KNYSG----------------------------------------------DVS-ELG--
--FDFTVTDKISPPGQ----------------------PVQ-TVTRK-LIPQ--------
GDSVP-------------------------------------------------------
------------------------------VTNDNRL-LYISY-AARHRLVIQ-PKLQTF
-AFLTG---LRSIIRP--SWLS-MFNQS-ELQRLVGG-----------------------
---DSSEID-----------IDDL----RRNTVYSGLYAVGDDNLE--------------
------------------------HPTI-VLFWKVMA--------------------GFT
DSQRRDVLKY-------VSSTPRAP------------LLG-----FS-QLRPKF--SIRD
SGTDE-----------------------------DRLPSTSTCV-NL-LKLPRY-TSEAV
LREKLL-YAITF-GAGF
>Epichloe_gansuensis_AMDK01000394.1 .
AKIKRGQL-FEDAFNQF---------------YELGDGFKD-TIQITFVDQFDTP--EAG
IDGGGVTKEF---LIGV----TSEAFANDENSL-----GMFSSNEQG-------------
----------------LLFPNPTA----------------ADTFCESL-RQRGLSEHDPE
WRAAVTGFLQRFEFLGRIVGKCLYDGILVDLAFAG--FFLLKWPS---------------
SGPKDENTYKGSINDLRD----------------------MDEELYKG--------LLRL
KNYSG----------------------------------------------DVS-ELG--
--FDFTVTDKISPPGQ----------------------PVQ-TVTRK-LIPQ--------
GDSVP-------------------------------------------------------
------------------------------VTNDNRL-LYISY-AARHRLVIQ-PKLQTS
-AFLRG---LRSIIRP--SWLS-MFNQS-ELQRLVGG-----------------------
---DSSEID-----------IDDL----RRNTVYSGLYALGDDNLE--------------
------------------------HPTI-VLFWKVMA--------------------GFT
DSQRRDVLKY-------VSSTPRAP------------LLG-----FS-QLRPKF--SIRD
GGTDE-----------------------------DRLPSTSTCV-NL-LKLPRY-TSEAV
LREKLL-YAITS-GAGF
>Periglandula_ipomoeae_AFRD01000158.1 .
AKIKRGQL-FEDAFNQF---------------YEIGDGFKD-TIQITFVDQFDTP--EAG
IDGGGVTKEF---LIGV----TSEAFGNSEGGL-----GMFSYNEQG-------------
----------------LLFPNPTA----------------ADTFRESL-RQRGLSENDQE
WRDAMTGFLKRFEFLGRIVGKCLYEGILVDLAFAG--FFLLKWPS---------------
SGPKEENSYKGSINDLRD----------------------MDEDLYKG--------LLRL
KNYSG----------------------------------------------DVS-ELG--
--FDFTITDQISPPGQ----------------------PVR-TVTRK-LIPQ--------
GDSVP-------------------------------------------------------
------------------------------VTNDNRL-LYISY-AARHRLVVQ-PSLQIS
-AFLRG---LRSIIRP--SWLS-MFNQS-ELQRLVGG-----------------------
---DSSEID-----------LEDL----RRNTVYSGLYVIGDDNLE--------------
------------------------HPTI-QLFWKVMA--------------------GFT
DSQRRDVLKY-------VSSTPRAP------------LLG-----FS-QLRPKF--SIRD
GGTDE-----------------------------DRLPSTSTCV-NL-LKLPRY-TTEAV
LREKLL-YAVMS-GAGF
>Atkinsonella_hypoxylon_JFHB01000037.1 .
ARIKRGQL-FEDAYNQF---------------YEIGDGFKD-AIQITFVDQFGTP--EAG
IDGGGVTKEF---LIGV----TCEAFGNAENSL-----GMFSSNEQG-------------
----------------LLFPNPTA----------------ADTFRESL-RQKGLSENDPE
WREAMTGFLKRYEFLGRIVGKCLYEGILVDLAFAG--FFLLKWPS---------------
SGPKDENTYKGSINDLRD----------------------MDEELYKG--------LLRL
KNYPG----------------------------------------------DVS-ELG--
--FDFTITDQISPPGQ----------------------SIR-TVTRR-LIPQ--------
GDSVL-------------------------------------------------------
------------------------------VTNDNRL-LYISY-AARHRLVVQ-PMLQTA
-AFLRG---LRSIIRP--SWLS-MFNQS-ELQRLVGG-----------------------
---DSSEID-----------IEDL----RRNTVYSGLYTIGDDNIE--------------
```

```
------------------------HPTI-QLFWKVMA--------------------GFT
DSQRRDVLKY-------VSSTPRAP------------LLG-----FS-ELRPKF--SIRD
GGTDE-----------------------------DRLPSTSTCV-NL-LKLPRY-RSETV
LREKLL-YAITS-GAGF
>Claviceps_fusiformis_AFRA01001486.1 .
--IKRGQL-FEDAFKQF---------------YGLGEGFKD-TIQITFVDQFDTP--EAG
IDGGGVTKEF---LIGV----TTEAFRSGDNSL-----GMFSSNEQG-------------
----------------LLFPSPTA----------------ADAFRECL-RREGLNEDDPK
WRDAMTGFLKRFEFLGRIVGKCLYDGILVNLAFAG--FFLLKWPS---------------
TGPKEENTYKGSINDLRD----------------------MDEELYKG--------LLRL
KNYPG----------------------------------------------DVS-ELG--
--FDFTVTDQISPVGQ----------------------PVQ-TITRK-LLPH--------
GDSIP-------------------------------------------------------
------------------------------VTNDNRL-LYISY-AARHRLVVQ-PMLQTA
-AFLRG---LRTIVRP--SWLS-MFNQS-ELQRLVGG-----------------------
---DSTEID-----------LDDL----RRNTVYSGLYAVGDDNLD--------------
------------------------HPTI-VLFWKVMA--------------------GFT
DSQRRDFLKY-------VSSTPRAP------------LLG-----FS-QLRPQF--SIRD
GGRDE-----------------------------KRLPSTSTCV-NL-LKLPMY-TSEAV
MREKLL-YAITS-GAGF
>Aciculosporium_take_AFQZ01000918.1 .
AKIKRGQL-FEDAFKQF---------------YEIGDGFKD-TIQITFIDQFNTP--EAG
IDGGGVTKEF---LIGV----TSEAFGDGEDSL-----GMFSSNEQG-------------
----------------LLFPNPTA----------------ADTFRESL-RHKGLDEHDPE
WRDAMAGFLKRFEFLGRIVGKCLYEGILVNLSFAG--FFLLKWSS---------------
AGPKNENTYKGSINDLRD----------------------MDEELYKG--------LLRL
KNYPG----------------------------------------------DVS-ELG--
--FDFTVTDQISPPGQ----------------------PVR-TVTRK-LILH--------
GDTVP-------------------------------------------------------
------------------------------VTNDNRL-LYISY-AARHRLVVQ-PMLQTS
-AFLKG---LRSMIRP--SWLS-MFNQS-ELQRLVGG-----------------------
---DSSEID-----------LDDL----RRNTVYSGLYAVGNDGLE--------------
------------------------HHTI-VLFWKVMA--------------------GFT
DSQRRDVLKY-------VSSTPRAP------------LLG-----FS-QLRPKF--SIRD
GGADE-----------------------------NRLPSTSTCV-NL-LKLPLY-TSEAV
LREKLL-YAVTS-GAGF
>Claviceps_paspali_AFRC01000467.1 .
AKIKRGQL-FEDAFNQF---------------YGIGEGFKD-TIQITFVDQFNAP--EAG
IDGGGVTKEF---LIGV----TSEAFGNGENSM-----GMFSSNEQG-------------
----------------LLFPNPIA----------------ADTFRESL-RRKGMEEHDPQ
WRYAMTDFLKRFEFLGRIVGKCLYEGILVDLAFAG--FFLLKWPS---------------
AGPKDENTYKGSIDDLRD----------------------MDEELYKG--------LLRL
KNYPG----------------------------------------------DVS-ELG--
--FDFTVTDQISSPDE----------------------PMK-TVTRK-LIPQ--------
GDSVL-------------------------------------------------------
------------------------------VTNDNRL-LYVSY-AARHRLVSQ-PMLQTS
-AFLRG---LRSIIRP--SWLS-MFNQS-ELQRLVGG-----------------------
---DSSEID-----------LDDL----RSNTVYSGLYAIGDDNLE--------------
------------------------HPTI-ILFWKVLA--------------------SFT
DGQRRDLLKY-------VSSSPRAP------------LLG-----FS-QLRPKF--SIRD
AGMDQ-----------------------------KRLPSTSTCV-NL-LKLPVY-SSETV
LREKLL-YAVTS-GAGF
>Ustilaginoidea_virens_BBTG02000012.1 .
AEIKRGQL-FEDAFKQF---------------YRLGDKLKG-GIQITFVDQFGTP--EAG
IDGGGMTKEF---LIGL----TTEAFGNGDGGS-----GMFSSNEQG-------------
----------------LLFPNPTA----------------VDTVREAL-RQDGLSNNDTG
RKEALSGLLKRFEFLGRIVGKCLYEGILVDLAFAG--FFLLKWQS---------------
VGPKGETTYKGSINDLRD----------------------MDEGLYKG--------LLSL
```

```
KNYAG----------------------------------------------DVS-ELG--
--FDFTITDQVSPPGE----------------------PVR-TVTRR-LIPN--------
GDQTP-------------------------------------------------------
------------------------------VTNDNRL-LYISY-AARHRLVIQ-PALQTA
-AFLRG---LRCIICP--WWLS-MFNQS-ELQRLVGG-----------------------
---DSSEID-----------IEDL----RRNTVYSGLYEVGDDNEE--------------
------------------------HPTV-KLFWKVLD--------------------GFT
DGQRRAFLKY-------VSSTPRAP------------LLG-----FS-QLRPNF--SIRD
GGTDE-----------------------------DRLPSTSTCV-NL-LKLPRY-TSEAT
LREKLL-YAVTS-GAGF
>2_Metarhizium_rileyi_AZHC01000001.1 .
AKIRRGRL-FEDAFSQF---------------YPIGERLKD-TIQITFVDQFGTP--EAG
IDGGGVTKEF---LTSI----TSEAFGNFEDGE-----ALFSSNEQG-------------
----------------LLFPNPTA----------------VDALREKL-REGGVQEDDED
YSDVVSDFLRRYEFIGRIVGKCLYEGILVDLAFAG--FFLLKWPS---------------
AGPKNENTYKGSINDLRD----------------------MDQELYKG--------LLRL
KNYYG----------------------------------------------DVS-ELG--
--FDFTVTDQVSLPGD----------------------PVK-TITRK-LIPN--------
GDRIP-------------------------------------------------------
------------------------------VTNDNRL-LYISY-AARHRLVIQ-PALQTA
-AFLRG---LRSIIRP--SWLS-MFNQS-ELQRLVGG-----------------------
---DSSEID-----------IDDL----RRNTVYSGLYEIGDDNEE--------------
------------------------HPTI-KLFWKVME--------------------GFT
DIQRRDVLKY-------VSSTPRAP------------LLG-----FA-QLRPKF--SIRD
GGTDE-----------------------------ERLPSTSTCV-NL-LKLPRY-TSEIV
LREKLL-YAVTS-GAGF
>Pochonia_chlamydosporia_XM_018281275.1 .
-KIRRGQL-FEDAFKQF---------------YQIGDGLKD-PIQITFVDQFDTP--EAG
IDGGGVTKEF---LISV----TSEAFGNSEKGG-----GMFSSNEQR-------------
----------------LLFPNPTA----------------VDTLRDFLQRRRGLNENDEE
YRDSVNDYLKRFEFLGRVIGKCLYEGILVDLAFAG--FFLLKWPS---------------
AGPKDENAYKGSINDLRD----------------------MDKELYNG--------LLTL
KNYSG----------------------------------------------DVS-ELA--
--FDFTVTDQVSPPDE----------------------PVR-TVTRK-LIPN--------
GDQIP-------------------------------------------------------
------------------------------VTNDNRL-LYISY-AARHRLVVQ-PAHQTA
-AFLRG---LRSIIRP--SWLS-MFNQS-ELQRLVGG-----------------------
---DSSEID-----------IDDL----RRNTVYSGLYEIGDDNEE--------------
------------------------HPTI-KLFWKVME--------------------GFT
DKQRRDVLKY-------VSSTPRAP------------LLG-----FS-QLRPKF--SIRD
GGTDE-----------------------------ERLPSTSTCV-NL-LKLPRY-TSESA
LRKKLL-YAVTS-GAGF
>Metarhizium_album_AZHE01000001.1 .
AKIRRGQL-FEDAFNQF---------------YQINDGLKD-TIQITFVDQFGTP--EAG
IDGGGVTKEF---LIGV----TSEAFGNKEDGT-----GMFSSNEQG-------------
----------------LLFPNPTA----------------VDTRRELV-SRQGLKDDDED
HRDEVNDLLRRFEFLGRIVGKCLYEGILVDLAFAG--FFLLKWPS---------------
AGPNDENTYKGSINDLRD----------------------MDEELYLG--------LLRL
KNYRG----------------------------------------------DVS-ELG--
--FDFTVTDQVSPPGA----------------------PVK-TITRK-LIQN--------
GDQIP-------------------------------------------------------
------------------------------VTNDNRL-LYISY-AARHRLVVQ-PALQTA
-AFLRG---LRSIIPP--SWLS-MFNQS-ELQRLVGG-----------------------
---DSSEID-----------IDDL----RRNTVYSGLYEVGDDNEE--------------
------------------------HPTI-KSFWRVME--------------------GFT
DAQRRDVLKY-------VSSTPRAP------------LLG-----FS-QLRPKF--SIRD
GGTDE-----------------------------KRLPSTSTCV-NL-LKLPVY-TSEEA
LREKLL-YAVTS-GAGF
```

>Metarhizium_acridum_XM_007811632.1 .
AKIRRGQL-FEDAFNQF---------------YQIGDGLKD-TIQITFVDQFDTP--EAG
IDGGGVTKEF---LIGV----TSEAFGNTEDGA-----GMFSSNEQG-------------
----------------LLFPNPTA----------------VDTLRESL-CRQGLKQDDEE
YRDKVNALLRRFEFLGRIVGKCLYEGILVDLAFAG--FFLLKWPS---------------
AGPKDENTYKGNINDLRD----------------------MDEELYKG--------LLRL
KNYPG--DVS-ELG--
--FDFTVTDQVSPPGA----------------------PVK-TITRK-LIQN--------
GDEIL---
------------------------------VTNDNRL-LYISY-AARHRLVVQ-PALQTA
-AFLRG---LRSIIPP--SWLS-MFNQS-ELQRLVGG-----------------------
---DSSEID-----------IEDL----RRNTVYSGLYEIGDDNEE--------------
------------------------HPTI-KLFWKVME--------------------GFT
DIQRRDVLKY-------VSSTPRAP------------LLG-----FA-QLRPKF--SIRD
GGTDE-----------------------------KRLPSTSTCV-NL-LKLPIY-TSERA
LREKLL-YAVTS-GAGF
>Metarhizium_brunneum_XM_014691755.1 .
AKIRRGRL-FEDAFNQF---------------YQIGDGLKD-TIQITFVDQFDTP--EAG
IDGGGVTKEF---LIGV----TSEAFGNTEGGA-----GMFSSNEQG-------------
----------------LLFPNPTA----------------VDTLRESL-WRRGLKQDDDE
YRDKVNALLRRFEFLGRIIGKCLYEGILVDLAFAG--FFLLKWPS---------------
AGPKDENTYKGNINDLRD----------------------MDEELYKG--------LLRL
KNYPG--DVS-ELG--
--FDFTVTDQVSPPGA----------------------PVK-TITRK-LIQN--------
GDEIL---
------------------------------VTNDNRL-LYISY-AARHRLVVQ-PALQTT
-AFLRG---LRSIIPP--SWLS-MFNQS-ELQRLVGG-----------------------
---DSSEID-----------IEDL----RRNTVYSGLYEIGDDNEE--------------
------------------------HPTI-KLFWKVMG--------------------GFT
DTQRRDVLKY-------VSSTPRAP------------LLG-----FA-QLRPKF--SIRD
GGTDE-----------------------------ERLPSTSTCV-NL-LKLPIY-TSEEA
LREKLL-YAVTS-GAGF
>Metarhizium_anisopliae_ADNJ01000355.1 .
AKIRRGRL-FEDAFNQF---------------YQIGDGLKD-TIQITFVDQFDTP--EAG
IDGGGVTKEF---LIGV----TSEAFGNTEGGA-----GMFSSNEQG-------------
----------------LLFPNPTA----------------VDTLRESL-WRRGLKQDDDE
YRDKVNALLRRFEFLGRIIGKCLYEGILVDLAFAG--FFLLKWPS---------------
AGPKDENAYKGNINDLRD----------------------MDEELYKG--------LLRL
KNYPG--DVS-ELC--
--FDFTVTDQVSPPGA----------------------PVK-TITRK-LIQN--------
GDEIL---
------------------------------VTNDNRL-LYISY-AARHRLVVQ-PALQTT
-AFLRG---LRSIIPP--SWLS-MFNQS-ELQRLVGG-----------------------
---DSSEID-----------IEDL----RRNTVYSGLYEIGDDNEE--------------
------------------------HPTI-KLFWKVMG--------------------GFT
DIQRRDVLKY-------VSSTPRAP------------LLG-----FA-QLRPKF--SIRD
GGTDE-----------------------------ERLPSTSTCV-NL-LKLPMY-TSEEA
LREKLL-YAVTS-GAGF
>Metarhizium_robertsii_XM_007820221.1 .
AKIRRGRL-FEDAFNQF---------------YQIGDGLKD-TIQITFVDQFDTP--EAG
IDGGGVTKEF---LIGV----TSEAFGNTEGGA-----GMFSSNEQG-------------
----------------LLFPNPTA----------------VDTLRESL-WRRGLKQDDDE
YRDKVNALLRRFEFLGRIIGKCLYEGILVDLAFAG--FFLLKWPS---------------
AGPKDENAYKGNINDLRD----------------------MDEELYKG--------LLRL
KNYPG--DVS-ELC--
--FDFTVTDQVSPPGA----------------------PVK-TITRK-LIQN--------
GDEIL---
------------------------------VTNDNRL-LYISY-AARHRLVVQ-PALQTT

```
-AFLRG---LRSIIPP--SWLS-MFNQS-ELQRLVGG-----------------------
---DSSEID-----------IEDL----RRNTVYSGLYEIGDDNEE--------------
------------------------HPTI-KLFWKVMG--------------------GFT
DIQRRDVLKY-------VSSTPRAP------------LLG-----FA-QLRPKF--SIRD
GGTDE-----------------------------ERLPSTSTCV-NL-LKLPMY-TSEEA
LREKLL-YAVTS-GAGF
>2_Metarhizium_guizhouense_AZNH01000001.1 .
AKIRRGRL-FEDAFNQF---------------YQIGDGLKD-TIQITFVDQFDTP--EAG
IDGGGVTKEF---LIGV----TSEAFGNTEGGA-----GMFSSNEQG-------------
----------------LLFPNPTA----------------VDTLRESL-WRRGLKQDDDE
YRDKVNALLRRFEFLGRIIGKCLYEGILVDLAFAG--FFLLKWPS---------------
AGPKDENTYKGNINDLRD----------------------MDEELYKG--------LLRL
KNYPG-------------------------------------------------DVS-ELG--
--FDFTVTDQVSPPGA----------------------PVK-TVTRK-LIQN--------
GDEIL-------------------------------------------------------
------------------------------VTNDNRL-LYISY-AARHRLVVQ-PALQTT
-AFLRG---LRSIIPP--SWLS-MFNQS-ELQRLVGG-----------------------
---DSSEID-----------IEDL----RRNTVYSGLYEIGDDNEE--------------
------------------------HPTI-KLFWKVMG--------------------GFT
DIQRRDVLKY-------VSSTPRAP------------LLG-----FA-QLRPKF--SIRD
GGTDE-----------------------------ERLPSTSTCV-NL-LKLPMY-TSEEA
LREKLL-YAVTS-GAGF
>Metarhizium_majus_XM_014727081.1 .
AKIRRGRL-FDDAFNQF---------------YQIGDGLKD-TIQITFVDQFDTP--EAG
IDGGGVTKEF---LIGV----TSEAFGNTEGGA-----GMFSSNEQG-------------
----------------LLFPNPTA----------------VDTLRESL-WRRGLKQDDDE
YRDKVNALLRRFEFLGRIIGKCLYEGILVDLAFAG--FFLLKWPS---------------
AGPKDENTYKGNINDLRD----------------------MDEELYKG--------LLRL
KNYPG-------------------------------------------------DVS-ELG--
--FDFTVTDQVSPPGA----------------------PVK-TITRK-LIQN--------
GDEIL-------------------------------------------------------
------------------------------VTNDNRL-LYISY-AARHRLVVQ-PALQTT
-AFLRG---LRSIIPP--SWLS-MFNQS-ELQRLVGG-----------------------
---DSSEID-----------IEDL----RRNTVYSGLYEIGDDNEE--------------
------------------------HPTI-KLFWKVMG--------------------GFT
DIQRRDVLKY-------VSSTPRAP------------LLG-----FA-QLRPKF--SIRD
GGTDE-----------------------------ERLPSTSTCV-NL-LKLPMY-TSEEA
LREKLL-YAVTS-GAGF
>2_Metarhizium_anisopliae_AZNF01000004.1 .
AKIRRGRL-FEDAFNQF---------------YQIGDGLKD-TIQITFVDQFDTP--EAG
IDGGGVTKEF---LIGV----TSEAFGNTEGGA-----GMFSSNEQG-------------
----------------LLFPNPTA----------------VDTLRESL-WRRGLKQDDDE
YRDKVNALLRRFEFLGRIIGKCLYEGILVDLAFAG--FFLLKWPS---------------
AGPKDENTYKGNINDLRD----------------------MDEELYKG--------LLRL
KNYPG-------------------------------------------------DVS-ELG--
--FDFTVTDQVSPPGA----------------------PVK-TITRK-LIQN--------
GDEIL-------------------------------------------------------
------------------------------VTNDNRL-LYISY-AARHRLVVQ-PALQTT
-AFLRG---LRSIIPP--SWLS-MFNQS-ELQRLVGG-----------------------
---DSSEID-----------IEDL----RRNTVYSGLYEIGDDNEE--------------
------------------------HPTI-KLFWKVMG--------------------GFT
DIQRRDVLKY-------VSSTPRAP------------LLG-----FA-QLRPKF--SIRD
GGTDE-----------------------------ERLPSTSTCV-NL-LKLPMY-TSEEA
LREKLL-YAVTS-GAGF
>Escovopsis_weberi_LGSR01000019.1 .
-EIRRGKL-FEDAFRAF---------------YPLGAGFKD-PIQITFFDQFNTP--EAG
IDGGGVTKEF---LISV----TAEAFGNV---------GMFTSNEKG-------------
----------------LLYPNPVT----------------VDALREKM-RRRGLSELSDA
```

WRDSMSNLLKKYEFLGRIIGKCMYEGILVDLAFAG--FFLLKWPS---------------
PGPKEENSYKGSINDLQD----------------------MDEELYKG--------LLRL
KNYTG--DVS-ELG--
--FDFTVTDQISAQGEQ---------------------PIK-TISRK-LIAN--------
GDEVP---
------------------------------VTNDNRL-LYISY-TARHRLVVQ-PALQTA
-AFLRG---LRDIIRP--SWLS-MFNQS-ELQRLVGG-----------------------
---DSLDID-----------IDDL----RRNTMYSGVYIIGDDGLE--------------
------------------------HPTI-QMFWNVMA--------------------GLT
DAQRRDVLKY-------VSSTPRAP------------LLG-----FS-QLRPKF--CIRD
AGSDE-----------------------------DRLPSTSTCI-NL-LKLPIY-KKEST
LREKLL-YAVNS-GAGF
>Albophoma_yamanashiensis_BCKH01000007.1 .
ARIRRGQL-FQDAYQQF---------------YQLGEGLKD-PIQITFVDQFDTP--EAG
IDGGGVTKEF---LTSV----TSEAFGNAEGDQ-----GMFSSNEQG-------------
----------------LLFPNPTA----------------ADTMRESL-RQYGLAEDSAD
WRDAMASLLKRFEFLGRIVGKCMYEGILVDLAFAG--FFLLKWPS---------------
AGPKEENSYKGSINDLRD----------------------MDEDLYKG--------MLRL
KNYPG--NVT-GLG--
--FDFTVEDQVSPPGQ----------------------PVK-TITRK-LIPN--------
GDETP---
------------------------------VTNDNRL-LYISY-MARHRLVVQ-PSLQTA
-AFLRG---LRAIIRP--SWLS-MFNQS-ELQRLVGG-----------------------
---DSSEIN-----------IEDL----RRNTVYSGLYEIGDDGEE--------------
------------------------HPTI-KLFWKVMG--------------------DFT
DAQRRDVLKY-------VSSTPRAP------------LLG-----FS-QLRPMF--SIRD
GGTDE-----------------------------ERLPSTSTCV-NL-LKLPRY-TSEAT
LREKLL-YAVTS-GAGF
>Tolypocladium_inflatum_AOHE01000056.1 .
ARIRRGQL-FQDAYKQF---------------YQLGEGFKD-PIQITFVDQFDTP--EAG
IDGGGVTKEF---LTSV----TSEAFGIEEGDE-----GMFTSNEQG-------------
----------------LLFPNPTA----------------ADTVRESL-RQYGLSEDSAD
WKGAMASLLKRFEFLGRIVGKCMYEGILVDLAFAG--FFLLKWPS---------------
AGPKEENSYKGSINDLRD----------------------MDEDLYKG--------MLRL
KNYPG--NVT-DLG--
--FDFTVEDQVSPPGR----------------------PVK-TITRK-LIPN--------
GDETP---
------------------------------VTNDNRL-LYISY-MARHRLVVQ-PSLQTA
-AFLRG---LRAIIRP--SWLS-MFNQS-ELQRLVGG-----------------------
---DSSEID-----------LEDL----RQHTVYSGLYEIGDDREE--------------
------------------------HPTI-KLFWKVMG--------------------GFT
DAQRRDVLKY-------VSSTPRAP------------LLG-----FS-QLRPKF--SIRD
GGTDE-----------------------------ERLPSTSTCV-NL-LKLPRY-TSEST
LREKLL-YAVTS-GAGF
>Tolypocladium_ophioglossoides_LFRF0100001 .
ARIRRGQL-FKDAYKQF---------------YQLGEGLKD-PIQITFVDQFDTP--EAG
IDGGGVTKEF---LTSV----TSEAFGNEEGGE-----GMFTSNKQG-------------
----------------LLFPNPTA----------------ADTVRESL-RQYGLTEDSAD
WRDAMTGLLKRFEFLGRIVGKCMYEGILVDLAFAG--FFLLKWPS---------------
TGPKEENSSKGSINNLRD----------------------MDEELYKG--------MLRL
KSYPG--NVA-DLG--
--FDFTVEDQVSQPGQ----------------------PVK-TVTRK-LIPN--------
GDETS---
------------------------------VTNDNRL-LYISY-MARHRLVVQ-PSLQTA
-AFLRG---LRAIIRP--SWLS-MFNQS-ELQRLVGG-----------------------
---DSSEID-----------LEDL----RRNTVYSGLYEIGDDGEE--------------
------------------------HSTI-KLFWRVMG--------------------GFT
DSQRRDVLRY-------VSSTPRAP------------LLG-----FS-QLRPKF--SIRD

```
GGTDE-----------------------------ERLPSTSTCV-NL-LKLPRY-TSEST
LREKLL-YAVTS-GAGF
>Purpureocillium_lilacinum_XM_018319819.1 .
AKIRRGQL-FDDAYEQF---------------YQLGDGLKD-PIQITFVDQFGTP--EAG
IDGGGVTKEF---LTSV----TSEAFQSEL--------GMFTSNEQG-------------
----------------LLYPNPTA----------------ADTRRELL-RRYGLKEDDDE
WKEAMTSLFKRFEFLGRIVGKCMYEGILVDLAFAG--FFLLKWPS---------------
TGPKEENTYKGSINDLRD----------------------MDAELYQG--------MLTL
KNYPG----------------------------------------------DVS-DLG--
--FDFTVEDQVSPPGQ----------------------PVK-TITRK-LIAN--------
GDQTP-------------------------------------------------------
------------------------------VTNDNRL-LYISY-MARHRLVVQ-PSLQTA
-AFLRG---LRAIIKP--SWLS-MFNQS-ELQRLVGG-----------------------
---DSSEID-----------IADL----RRNTVYSGLYEIGDDNEE--------------
------------------------HPTI-KLFWKVME--------------------GFT
DSQRRDVLKY-------VSSTPRAP------------LLG-----FS-QLRPKF--SIRD
GGTDE-----------------------------QRLPSTSTCV-NL-LKLPRY-SKEET
LRERLL-YAVTS-GAGF
>Ophiocord_unilateralis_LAZP01000728.1 .
AKIRRGKL-FEDAYKHF---------------YQLGDGFKD-TIQITFVDRFNTP--EAG
IDGGGVTKEF---LTSV----TSEAFGAQNSAG-----GMFVNNEQG-------------
----------------LLFPNPTA----------------TDTLRESL-RGRGWTEKDAE
WSEAMASLFKRFEFLGRIVGKCLYEGILVDLAFAG--FFLLKWPS---------------
AGPKLENTYKGSINDLRD----------------------MDEDLYKG--------MLRL
KNYTG----------------------------------------------DVL-GLG--
--FDFTVEDQVSAPGE----------------------PMK-TVTRK-LILN--------
GDQTP-------------------------------------------------------
------------------------------VDNDNRL-LYISY-MARHRLVVQ-PAAQTA
-AFLRG---LHAIISP--SWLS-MFNQS-ELQRLVGG-----------------------
---DSSEID-----------LADL----RKNTVYSGLYEIGDDKEE--------------
------------------------HPTI-KLFWKVME--------------------GFA
DTQRRDVLKY-------VSSTPRAP------------LLG-----FS-QLRPKF--SIRD
GGTDE-----------------------------ERLPSTSTCV-NL-LKLPRY-TREET
LREKLL-YAVTS-GAGF
>Ophiocord_polyrhachis_furcata_LKCN0100000 .
AKIRRGQI-FEDAYEHF---------------YQLGEGLKD-PIQITFVDRFDTQ--EAG
IDGGGVTKEF---LTSV----TSEAFATQNGAM-----GMFVSNEQG-------------
----------------LLFPNPTA----------------TDTLRESL-KSQGWTEKDPE
WKDAMTSLFRRFEFLGRIVGKCLYEGILVDLAFAG--FFLLKWPS---------------
VGPKLENTYKGSINDLRD----------------------MDEDLYKG--------MLRL
KNYPG----------------------------------------------DVL-GLG--
--FDFTVEDQVSAPGE----------------------PMK-TITRR-LILH--------
GDQTS-------------------------------------------------------
------------------------------VDNDNRL-LYISY-MARHRLVVQ-PAPQTA
-AFLRG---LHAIIRP--SWLS-MFNQS-ELQRLVGG-----------------------
---DSSEID-----------VADL----RRNTVYSGLYEIGDDQEE--------------
------------------------HPTI-KLFWKVME--------------------GFA
DSQRRDVLKY-------VSSTPRAP------------LLG-----FS-QLRPKF--SIRD
GGTDE-----------------------------ERLPSTSTCV-NL-LKLPRY-TQEER
LREKLL-YAVTS-GAGF
>Hirsutella_thompsonii_APKU01000246.1 .
ARIRRGDL-FFDAYKAF---------------YQLGDGLKD-PITITFVDQFDTP--EAG
IDGGGVTKEF---LTSV----TAEAFAECKDRNEDF--DMFVSNEQG-------------
----------------LLFPNPIA----------------ADVLRDGL-RARGIAEHDAK
WGEAFAQMCKYYEFLGRIVGKCMYEGILVDLAFAG--FFLLKWPS---------------
AGPKEENNYKGNINDLRE----------------------MDPEFYKG--------MMRL
KNYSG----------------------------------------------DVS-ELG--
--FDFTVEDQVSPLWQ----------------------PTR-TITRK-LIAH--------
```

GDETP---
------------------------------VNNDNRL-LYISY-MARHRLVVQ-PALQTT
-AFLRG---LHAMIRP--SWLS-MFNQS-ELQRLVGG-----------------------
---DSSEID-----------IDDL----RRNTVYSGVYEIGDDGRE--------------
------------------------HPTI-RLFWAVMD--------------------SFT
DAQRRDVLKY-------VSSTPRAP------------LLG-----FS-QLKPKF--SIRD
AGTDE-----------------------------TRLPSTSTCV-NL-LKLPRY-TREET
LREKLL-YAVTS-GAGF
>Hirsutella_minnesotensis_JPUM01000438.1 .
ARIRRGNL-FKDAMAQF---------------YPLGDGLKD-PIQITFVDQFDTP--EAG
IDGGGVTKEF---LTSV----TAEIFATSDNEL-----TMFASNEQG-------------
----------------LLFPSPTA----------------ADVLREDL-RTRGWTERDTE
WRESLAKLCKTYEFLGRVVGKCMYEGILVDLAFAG--FFLLKWPS---------------
AGPKEENNYKGSINDLRE----------------------MDEELYKG--------MLRL
KNYPG--DVS-ELG--
--FDFTVEDQVSHPGR----------------------PVK-TITRK-LIAH--------
GDETT---
------------------------------VNNDNRL-LYISY-MARHRLVVQ-PALQTA
-AFLRG---LRAIIHP--SWLS-MFNQS-ELQRLVGG-----------------------
---DSSEID-----------IEDL----RRNTIYSGVYEIGDDQQE--------------
------------------------HPTI-QLFWRVMR--------------------SFS
DAQRRDVLKY-------VSSTPRAP------------LLG-----FS-QLNPKF--SIRD
AGTDE-----------------------------SRLPSTSTCV-NL-LKLPRY-KSEET
LREKLL-YAVTS-GAGF
>Nilaparvata_lugens_JRMI01000849.1 .
-RIRRGHI-FEDAFRQF---------------YQLHDGLKD-PIQIEFVDQFGTQ--EAG
IDGGGMTKEF---LLSI----TMEAFGKSDKEP-----GLFVSNEQG-------------
----------------LFFPNPTM----------------VDSLRQRL-KSRGLNENDAE
WRVAMSDLLKRYEFLGCIVGKCMYEGILVDLAFAG--FFLLKWPS---------------
SGSGDENSYKGSIDDLRD----------------------MDEGLYNG--------MLRL
KNYAG--DVS-SLG--
--FDFTVEDQISPPGQ----------------------PMK-TVTRK-LIVN--------
GDGTT---
------------------------------VNNDNRL-LYISY-MARHRLVVQ-PAMQTA
-AFLRG---LRAIIRP--SWLS-MFNQS-ELQRLVGG-----------------------
---DSTEID-----------IDDL----RRNTHYSGLYEIGDDQQE--------------
------------------------HPTI-DLFWKVML--------------------SFT
DAQRRDVVKY-------VSSTPRAP------------LLG-----FS-QLKPKF--TIRD
GGTDE-----------------------------TRLPSTSTCA-NL-LKLPRY-TEEAT
LRKKLL-QAVTS-GAGF
>Cerataphis_brasiliensis_AOFP01005370.1 .
-QIRRGHV-FEDAFRQF---------------YQLHDDLKD-PIQITFVDQFGTQ--EAG
IDGGGMTKEF---LMSI----TMEAFGTTDKEP-----GLFVSNEQG-------------
----------------LLFPNPTM----------------VDSLRQWL-KSRGLNENDAE
WREAMLDLLKRYEFLGRIVGKCMYEGILVDLAFAG--FFLLKWPS---------------
SGSGDENSYKGSIDDLRD----------------------MDEGLYNG--------MLRL
KNYAG--DVS-SLG--
--FDFTVEDQISPPGQ----------------------PIK-TVTRK-LIVN--------
GDETT---
------------------------------VNNDNRL-LYISY-MARHRLVLQ-PAMQTA
-AFLRG---LRAIIRP--SWLS-MFNQS-ELQRLVGG-----------------------
---DSTEID-----------IDDL----RRNTHYGGLYEIGDDQQE--------------
------------------------HPTI-DLFWKVML--------------------SFT
DAQRRDVVKY-------VSSTPRAP------------LLG-----FS-QLNPKF--TIRD
SGTDE-----------------------------TRLPSTSTCA-NL-LKLPRY-TEEAT
LRKKLL-QAVTS-GAGF
>Calonectria_pseudoreteaudii_MOCD01000206. 1..
ARIRRQNL-FEDAYKGF---------------YELGEGFKD-PIQITFVDQFGAP--EAG

```
IDGGGVTKEF---LISA----TSEAFGNQEGGA-----GMFTSNEKG-------------
----------------LLYPDPTA----------------VDVLRQSL-KAGGLTESDPE
WKEHMASLLKRFEFLGRIVGKCMYEGILVDLAFAG--FFLLKWAS---------------
AGPNDENSYKGSVNDLRD----------------------MDEDLHKG--------MLRL
KNYPG----------------------------------------------DIS-ELS--
--IDFTVTDQVSQAGQ----------------------PVK-TITRN-LIAN--------
GDQVH-------------------------------------------------------
------------------------------VTNDNRL-LYISY-IARHRLVVQ-PSLQTA
-AFLRG---LRAIIRP--SWLS-MFNQS-ELQRLVGG-----------------------
---DSSEID-----------IEDL----RQNTIYSGLYEIGDDGQE--------------
------------------------HDTI-KLFWKVMQ--------------------GFS
DAQRRDVLKY-------VSSTPRAP------------LLG-----FS-QLKPKF--SIRD
GGTDE-----------------------------DRLPSTSTCV-NL-LKLPRY-TTEGK
LREKLL-YAITS-GAGF
>Calonectria_pseudonaviculata_JYJY01000256 1..
ARIRRQKL-FEDAYKQF---------------YELGEGLKD-PIQITFVDQFGAA--EAG
IDGGGVTKEF---LISV----TSEAFGNLDKGP-----GMFTSNEKG-------------
----------------LLYPDPSA----------------VDVVRESL-KAGGITESDPE
WKGHMSSLLKRFEFLGRIVGKCIYEGILVDLAFAG--FFLLKWAS---------------
VGPNDENNYKGSVNDLRD----------------------MDEDLYKG--------MLRL
KNYPG----------------------------------------------DIS-ELG--
--IDFTVADQVSQLGE----------------------PVK-TMTRN-LIAN--------
GDQVH-------------------------------------------------------
------------------------------VTNDNRL-LYISY-IARHRLVVQ-PSLQTA
-AFLRG---LRAIIRP--SWLS-MFNQS-ELQRLVGG-----------------------
---DSSEID-----------IEDL----RQNTIYSGLYEIGDDGQE--------------
------------------------HDTI-KLFWKVME--------------------GFT
DAQRRDVLKY-------VSSTPRAP------------LLG-----FS-QLKPKF--SIRD
GGTDE-----------------------------ERLPSTSTCV-NL-LKLPRY-TSEET
LREKLL-YAITS-GAGF
>Ilyonectria_destructans_MPHF01000069.1 .
AQIRRKRL-FEDAYKQF---------------YEIGEGLKD-PIQITFVDQFGTP--EAG
IDGGGVTKEF---LISV----TSEAFGDEDKGL-----GMFTSNEKG-------------
----------------LLYPDPTA----------------ADALRESL-RESGTTESDPE
WKGHMASLLKRFEFLGRIVGKCMYEGILVDLAFGG--FFLLKWAS---------------
SGPNDENSYKGSVNDLRD----------------------MDEELYNG--------MLRV
KNYPG----------------------------------------------DIS-ELG--
--IDFTVTDQISQTGQ----------------------PVK-TMTRK-LIAN--------
GDQVQ-------------------------------------------------------
------------------------------VTNDNRL-LYISY-IARHRLVTQ-PSLQTA
-AFLRG---LRAIIRP--SWLS-MFNQS-ELQRLVGG-----------------------
---DSSEID-----------IEDL----RRNTIYSGLYEVGDDGQE--------------
------------------------HDTI-RIFWKVMQ--------------------GFT
DTQRRDVLKY-------VSSTPRAP------------LLG-----FS-QLKPKF--SIRD
GGTDE-----------------------------ERLPSTSTCV-NL-LKLPRY-TSEAV
LREKLL-YAVTS-GAGF
>Nectria_haematococca_XM_003052569.1 .
AQIRRKRL-FEDAYKQF---------------YELGEGFKD-PIQITFVDQFGTA--EAG
IDGGGVTKEF---LISV----TSEAFGNNEGA------GMFASNEKG-------------
----------------LLFPDPTA----------------MDVLRESL-RESGLSEEDPE
FREFVTSQLRRFEFLGRIVGKCMYEGILVDLAFAG--FFLLKWAS---------------
TGPNDENNYKGSVNDLRD----------------------MDEDLYNG--------MLRL
KNYSG----------------------------------------------DIS-ELG--
--IDFTIEDQVSQPGQ----------------------PVK-TITKK-LIAN--------
GDQVH-------------------------------------------------------
------------------------------VTNDNRL-LYISY-VARHRLVVQ-PSLQTA
-AFLRG---LRGIIRP--SWLS-MFNQS-ELQRLVGG-----------------------
---DSSEID-----------IDDL----RRNTVYSGLYEIGDDGQE--------------
```

------------------------HDTI-KLFWKVMR--------------------GFT
DSQRRSVLKY-------VSSTPRAP------------LLG-----FS-QLNPKF--SIRD
GGTDE-----------------------------DRLPSTSTCV-NL-LKLPRY-KTEAV
LREKLL-YAVTS-GAGF
>Fusarium_acuminatum_CBMG010000129.1 .
AEISRERL-FDDAYDQF---------------YEIGEGLKD-PIQITFVDQFGTA--EAG
IDGGGVTKEF---LISV----TSEAFSAQHGK------GMFTSNEKG-------------
----------------LLFPDPTS----------------LDVLRDTL-RMDGLSGTDDE
YRETVTRVLRRFEFLGRIVGKCMYEGILVDLAFAG--FFLLKWAS---------------
TGPNDENNYKGSVNDLRD----------------------MDEELYNG--------MLRL
KNYPG--DVS-ELG--
--IDFTIEDQVSKPGD----------------------PVK-TITKK-LIAN--------
GDQVY---
------------------------------VTNDNRL-LYISY-VARHRLVVQ-PAPQTL
-AFLRG---LRSIIRP--TWLS-MFNQS-ELQRLVGG-----------------------
---DSSEID-----------IDDL----RRNTIYSGLYEIGDDGLE--------------
------------------------HQTI-RLFWKVMR--------------------GFT
DAQRRDVLKY-------VSSTPRAP------------LLG-----FS-QLRPKF--SIRD
GGTDE-----------------------------ERLPSTSTCV-NL-LKLPRY-TTEAT
LRKKLL-YAVQS-GAGF
>Fusarium_avenaceum_JPYM01000006.1 .
AEISRERL-FDDAYDQF---------------YEIGEGLKD-PIQITFVDQFGTA--EAG
IDGGGVTKEF---LISV----TSEAFSGQHGK------GMFTSNEKG-------------
----------------LLFPDPTS----------------LDVLRDTL-RMDGLSGTDDE
YRETVTNVLRRFEFLGRIVGKCMYEGILVDLAFAG--FFLLKWAS---------------
TGPNDENNYKGSVNDLRD----------------------MDEELYNG--------MLRL
KNYPG--DIS-ELG--
--IDFTIEDQVSKPGD----------------------PVK-TITKK-LIAN--------
GDQVY---
------------------------------VTNDNRL-LYISY-VARHRLVVQ-PAPQTL
-AFLRG---LRSIIRP--TWLS-MFNQS-ELQRLVGG-----------------------
---DSSEID-----------IDDL----RRNTIYSGLYEIGDDGLE--------------
------------------------HQTI-RLFWKVMR--------------------GFT
DAQRRDVLKY-------VSSTPRAP------------LLG-----FS-QLRPKF--SIRD
GGTDE-----------------------------ERLPSTSTCV-NL-LKLPRY-TTEAT
LRKKLL-YAVQS-GAGF
>Fusarium_graminearum_XM_011322050.1 .
AQISRKRL-FEDAFNQF---------------YEMGDGLKD-PIQITFVDQFGAQ--EAG
IDGGGVTKEF---LTSV----TTEAFGSNSGK------GMFASSENG-------------
----------------LLFPDPIA----------------LDVVREEL-KMRGYSETDNE
YRDCIANLLKRMEFLGRIVGKCMYEGILVDLAFAG--FFLLKWAS---------------
TGPNDEKNYKGSVNDLRD----------------------MDEGLYNG--------MLRL
KNYPG--DVS-DLG--
--IDFTIADQISPPGD----------------------PVK-TVTKK-LIAN--------
GDQTY---
------------------------------VTNDNRL-LYISY-VARHRLIVQ-PSIQTT
-AFLSG---LRSIIRP--NWLS-MFNQS-ELQRLVGG-----------------------
---DSSEID-----------IEDL----RQHTVYGGLYEIGDDGQE--------------
------------------------HDTI-KIFWKVMR--------------------SFT
DAQRRDVLKY-------VSSTPRAP------------LLG-----FS-QLRPKF--SIRD
GGSDE-----------------------------ERLPSASTCV-NL-LKLPIY-TSEAT
LREKML-YAIQS-GAGF
>Fusarium_culmorum_LT598660.1 .
AQISRKRL-FEDAFNQF---------------YEMGDSLKD-PIQITFVDQFGAQ--EAG
IDGGGVTKEF---LTSV----TTEAFGSNSGK------GMFASSENG-------------
----------------LLFPDPIA----------------LDVVREEL-KMRGYSETDNE
YRDCIANLLKRMEFLGRIVGKCMYEGILVDLAFAG--FFLLKWAS---------------
TGPNDEKNYKGSVNDLRD----------------------MDEGLYNG--------MLRL

```
KNYPG----------------------------------------------DVS-DLG--
--IDFTIADQISPPGD----------------------PVK-TVTKK-LIAN--------
GDQTY-------------------------------------------------------
------------------------------VTNDNRL-LYISY-VARHRLIVQ-PSIQTT
-AFLSG---LRSIIRP--NWLS-MFNQS-ELQRLVGG-----------------------
---DSSEID-----------IEDL----RQHTVYGGLYEIGDDGQE--------------
------------------------HDTI-KIFWKVMR--------------------SFT
DAQRRDVLKY-------VSSTPRAP------------LLG-----FS-QLRPKF--SIRD
GGSDE-----------------------------ERLPSASTCV-NL-LKLPIY-TSEAT
LREKML-YAIQS-GAG-
>Fusarium_pseudograminearum_XM_009258150.1 .
AQISRKRL-FEDAYNQF---------------YEIGDGLKD-PIQITFVDQFGAQ--EAG
IDGGGVTKEF---LISV----TTEAFGSNNGK------GMFASSEKG-------------
----------------LLFPDPIA----------------LDVVREDL-KMRGYSETDNE
YRDCIANLLKRMEFLGRIVGKCMYEGILVDLAFAG--FFLLKWAS---------------
TGPSDEKNYKGSVNDLRD----------------------MDEGLYNG--------MLRL
KNYPG----------------------------------------------DVS-DLG--
--IDFTIADQISPPGD----------------------PVK-TATKK-LIAN--------
GDQTY-------------------------------------------------------
------------------------------VTNDNRL-LYLSY-VARHRLIVQ-PSIQTT
-AFLSG---LRSIIRP--NWLS-MFNQS-ELQRLVGG-----------------------
---DSSEID-----------IEDL----RQHTVYGGLYEIGDDGQE--------------
------------------------HDTI-KIFWKVMR--------------------SFT
DAQRRDVLKY-------VSSTPRAP------------LLG-----FS-QLRPKF--SIRD
GGSDE-----------------------------ERLPSASTCV-NL-LKLPIY-TSEAT
LREKML-YAIQS-GAGF
>Fusarium_langsethiae_JXCE01000006.1 .
AQISRNQL-FEDAFNQF---------------YELGEGLKD-PIQITFVDQFGAQ--EAG
IDGGGVTKEF---LISV----TTEAFGSNHGK------GMFASNEKG-------------
----------------LLFPDPIA----------------LDVVREQL-KMRGYSETDKE
YRDCVANLLKRMEFLGRIVGKCMYEGILVDLAFAG--FFLLKWAS---------------
TGPNDEKNYKGSVNDLRD----------------------MDEGLYNG--------MLRL
KNYPG----------------------------------------------DVS-ELG--
--IDFTIADQISPPGE----------------------PVK-TVTKK-LIAN--------
GDQTY-------------------------------------------------------
------------------------------VTNDNRL-LYISY-VARHRLIVQ-PSLQTT
-AFLRG---LRSIIRP--NWLS-MFNQS-ELQRLVGG-----------------------
---DSSEID-----------IEDL----RRNTVYSGLYEIGDDGQE--------------
------------------------HDTI-KLFWKVMR--------------------SFT
DAQRRDVLKY-------VSSTPRAP------------LLG-----FS-QLRPKF--SIRD
GGTDE-----------------------------ERLPSTSTCV-NL-LKLPKY-TSEAT
LREKLL-YAIQS-GAGF
>Fusarium_poae_LYXU01000002.1 .
AQISRKQL-FEDAYKQF---------------YEIGEGLKD-PIQITFVDRFGAQ--EAG
IDGGGVTKEF---LISV----TTEAFGSDHGK------GMFTSNEKG-------------
----------------LLFPDPIA----------------LDVVREKL-KMSGYSETDKE
YRDCIANLLKRMEFLGRIVGKCMYEGILVDLAFAG--FFLLKWAS---------------
TGPNDEKNYKGSVNDLRD----------------------MDEELYNG--------MLRL
KNYSG----------------------------------------------DVS-ELG--
--IDFTIADQISPPGD----------------------PVK-TVTKK-LIAN--------
GDQTY-------------------------------------------------------
------------------------------VTNDNRL-LYISY-VARHRLIVQ-PSLQTT
-AFLHG---LRSIIRP--NWLS-MFNQS-ELQRLVGG-----------------------
---DSSEID-----------IEDL----RQNTVYSGLYEVGDDGEE--------------
------------------------HDTI-KIFWKVMR--------------------SFT
DAQRRDVLKY-------VSSTPRAP------------LLG-----FS-QLKPKF--SIRD
GGTDE-----------------------------ERLPSTSTCV-NL-LKLPKY-TSEAT
LREKLL-YAVQS-GAGF
```

```
>Fusarium_sambucinum_LSRD01000010.1 .
AQISRKQL-FEDAYKQF---------------YEIGDGLKD-PIQITFVDQFGTQ--EAG
IDGGGVTKEF---LISV----TTEAFGSDHGK------GMFTSNEKG-------------
----------------LLFPDPIA----------------LDVVREKL-KMSGYSETDKE
YRDCIGNILKRMEFLGRIVGKCMYEGILVDLAFAG--FFLLKWAS---------------
TGPNDEKNYKGSVNDLRD----------------------MDEELYKG--------MLRL
KNYSG----------------------------------------------DVS-ELG--
--IDFTIADQISPPGD----------------------PVK-TVAKK-LIAN--------
GDQTY-------------------------------------------------------
------------------------------VTNDNRL-LYISY-VARHRLIVQ-PSLQTT
-AFLHG---LRSIIRP--NWLS-MFNQS-ELQRLVGG-----------------------
---DSSEID-----------IEDL----RKHTIYSGIYEIGDDGEE--------------
------------------------HDTI-KIFWKVMR--------------------SFT
DAQRRDVLKY-------VSSTPRAP------------LLG-----FS-QLRPKF--SIRD
GGSDE-----------------------------ERLPSTSTCV-NL-LKLPKY-TSEAT
LREKLL-YAVQS-GAGF
>Fusarium_equiseti_CBMI010000460.1 .
AQISRKRL-FEDAYKQF---------------YEIGEGLKD-PIQITFVDQFGAQ--EAG
IDGGGVTKEF---LISV----TTEAFGAEHGK------GMFTSNEKG-------------
----------------LLFPDPTA----------------LDVAREQL-KSRGLSETDSE
YRDSIASLLRRFEFLGRIVGKCMYEGILVDLSFAG--FFLLKWAS---------------
TGPTDEKNYKGSVNDLRD----------------------MDMELYNG--------MLQL
KNYTG----------------------------------------------DVS-ELG--
--IDFTIADQVSAPGE----------------------PVK-TVTKR-LIAN--------
GDQTY-------------------------------------------------------
------------------------------VTNDNRL-LYISY-VARHRLVVQ-PSPQTT
-AFLRG---LRSIIRP--TWLS-MFNQS-ELQRLVGG-----------------------
---DSSEID-----------IEDL----RRNTIYSGLYEIGDDGEE--------------
------------------------HDTI-KLFWKVMR--------------------SFT
DAQRRDVLKY-------VSSTPRAP------------LLG-----FS-QLRPQF--SIRD
GGTDE-----------------------------ERLPSTSTCV-NL-LKLPRY-TSEKT
LREKLL-YAVQS-GAGF
>Fusarium_praegraminearum_LXHY01000029.1 .
AQISRERL-FEDAYSQF---------------YQIGDGLKD-PIQITFVDRFGAP--EAG
IDGGGVTKEF---LISV----TTEAFSPKNGK------GMFASSEEG-------------
----------------LLFPDPIA----------------LDVTREEL-KMHGYSETDNE
YRECIAKLLTRMEFIGRIVGKCMYEGILVDLAFAG--FFLLKWAS---------------
TGPNDEKNYKGSVNDLRD----------------------MDKGLYDG--------MLQL
KNYPG----------------------------------------------DVS-DLG--
--IDFTIADQISSPGD----------------------PVK-TVTKK-LIAN--------
GDQTY-------------------------------------------------------
------------------------------VTNDNRL-LYISY-VARHRLIVQ-PSIQTT
-AFLSG---LRSIIRP--SWLS-MFNQS-ELQRLVGG-----------------------
---DSSEID-----------IEDL----RENTIYGGIYQIGDDGQE--------------
------------------------HDTV-KIFWKVMR--------------------SFT
DSQRRDVLKY-------VSSTPRAP------------LLG-----FS-QLRPKF--SIRD
GGSDE-----------------------------ERLPSTSTCV-NL-LKLPVY-TSEAT
LREKLL-YAVQS-GAGF
>Fusarium_oxysporum_XM_018380830.1 .
AQISRNSL-FDDAYKQF---------------YEIGEGLKD-PIQITFVDQFGTA--EAG
IDGGGVTKEF---LISV----TTEAFGAEGGR------GMFTSNEKG-------------
----------------LLFPDPTA----------------LDVIREEL-RHAGMTEADST
FRDMISDLLKRYEFLGRIVGKCMYEGILVDLAFAG--FFLLKWTS---------------
TGPNDENTYKGSVNDLRD----------------------MDEDLYRG--------MLRL
KNYPG----------------------------------------------DVS-ELG--
--IDFTIEDQVSDPKN----------------------PVK-TVTRK-LIAN--------
GDQIH-------------------------------------------------------
------------------------------VTNDNRL-LYISY-VARHRLVVQ-PAPQTS
```

-AFLRG---LRSIIRP--SWLS-MFNQS-ELQRLVGG-----------------------
---DSSEID-----------IEDL----RNHTIYSGLYQIGDDGEE--------------
------------------------HETI-KLFWKVMR--------------------SFT
DDQRRAVLKY-------VSSTPRAP------------LLG-----FS-QLRPLF--SIRD
GGTDE-----------------------------ERLPSTSTCV-NL-LKLPRY-TSEAT
LREKLL-YAVQS-GAGF
>Fusarium_verticillioides_XM_018889203.1 .
AQISRESL-FDDAYKQF---------------YEIGDDLKG-PIQITFVDQFGAA--EAG
IDGGGVTKEF---LISV----ATEAFSAEGGR------GMFTSNEKG-------------
----------------LLYPDPTA----------------LDVIREEL-RHAGMTEADST
FRDMISDLLRRYEFLGRIIGKCMYEGILVDLVFAG--FFLLKWTS---------------
TGPNDENTYKGSVNDLRD----------------------MDEDLYRG--------MLRL
KNHPG---DVS-EMG--
--VDFTIEDQVSDPKD----------------------SVK-TVTRK-LIAN--------
GDQIH---
------------------------------VTNDNRL-LYISY-VARHRLVVQ-PAPQTS
-AFLRG---LRSIIRP--SWLS-MFNQS-ELQRLVGG-----------------------
---DSSEID-----------VEDL----RKHTIYGGLYQIGDDGEE--------------
------------------------HDTI-KLFWKVMH--------------------SFT
DDQRRAVLKY-------VSSTPRAP------------LLG-----FS-QLKPLF--SIRD
GGTDE-----------------------------ERLPSTSTCV-NL-LKLPRY-TTEAT
LREKLL-YAIQS-GAGF
>Fusarium_nygamai_LBNR01000003.1 .
AQISRKSL-FDDAYKQF---------------YEIGDDLKG-PIQITFVDQFGAA--EAG
IDGGGVTKEF---LISV----ATEAFSAEGGR------GMFTSNEKG-------------
----------------LLYPDPTA----------------LDVIREEL-RHAGMTEADST
FRDMMSDLLRRYEFLGRIIGKCMYEGILVDLVFAG--FFLLKWTS---------------
TGPNDENTYKGSVNDLRD----------------------MDEDLYRG--------MLRL
KNYPG---DVS-ELG--
--IDFTIEDQVSDPKD----------------------PVK-TVTRK-LIAN--------
GDQIH---
------------------------------VTNDNRL-LYISY-VARHRLVVQ-PAPQTS
-AFLRG---LRSIIRP--SWLS-MFNQS-ELQRLVGG-----------------------
---DSSEID-----------VEDL----RKHTIYGGLYQIGDDGEE--------------
------------------------HDTI-KLFWKVMH--------------------SFT
DEQRRAVLKY-------VSSTPRAP------------LLG-----FS-QLKPLF--SIRD
GGTDE-----------------------------ERLPSTSTCV-NL-LKLPRY-TTEAT
LREKLL-YAIQS-GAGF
>Fusarium_fujikuroi_HF679028.1 .
-QISRKSL-FDDAYKQF---------------YEIGDDLKG-PIQITFVDQFGAA--EAG
IDGGGVTKEF---LISV----STEAFGAEGGR------GMFTSNEKG-------------
----------------LLYPDPTA----------------LDVIRQEL-RHAGMTEANST
FRDMISDLLKRYEFLGRIVGKCMYEGILVDLTFAG--FFLLKWTS---------------
TGPNDENTYKGSVNDLRD----------------------MDEDLYRG--------MLRL
KNYPG---DVS-ELG--
--IDFTIEDQVSDPKD----------------------PVK-TVTRK-LIAN--------
GDQIH---
------------------------------VTNDNRL-LYISY-VARHRLVVQ-PAPQTS
-AFLRG---LRSIIRP--SWLS-MFNQS-ELQRLVGG-----------------------
---DSSEID-----------IEDL----RKHTIYGGLYQIGDDGEE--------------
------------------------HETI-KLFWKVMH--------------------SFT
DDQRRAVLKY-------VSSTPRAP------------LLG-----FS-QLKPLF--SIRD
GGTDE-----------------------------ERLPSTSTCV-NL-LKLPRY-TSEAT
LREKLL-YAIQS-GAGF
>Fusarium_mangiferae_FCQH01000004.1 .
AQISRKSL-FDDAYKQF---------------YEIGDDLKG-PIQITFVDQFGAA--EAG
IDGGGVTKEF---LISV----STEAFGAEGGR------GMFTSNEKG-------------
----------------LLYPDPTA----------------LDVIREEL-RHAGMTEANST

FRDMISDLLKRYEFLGRIVGKCMYEGILVDLTFAG--FFLLKWTS---------------
TGPNDENTYKGSVNDLRD----------------------MDEDLYRG--------MLRL
KNYPG--DVS-ELG--
--IDFTIEDQVSDPKD----------------------PVK-TVTRK-LIAN--------
GDQIH---
------------------------------VTNDNRL-LYISY-VARHRLVVQ-PAPQTS
-AFLRG---LRSIIRP--SWLS-MFNQS-ELQRLVGG-----------------------
---DSSEID-----------IEDL----RKHTIYGGLYQIGDDGEE--------------
------------------------HETI-KLFWKVMH--------------------SFT
DDQRRAVLKY-------VSSTPRAP------------LLG-----FS-QLKPLF--SIRD
GGTDE-----------------------------ERLPSTSTCV-NL-LKLPRY-TSEAT
LREKLL-YAIQS-GAGF
>Fusarium_pininemorale_NFZR01000006.1 .
AQISRKSL-FDDAYKQF---------------YEIGDDLKG-PIQITFVDQFGAA--EAG
IDGGGVTKEF---LISV----ATEAFSAEGGR------GMFTSNEKG-------------
----------------LLYPDPTA----------------LDVIREEL-RHAGMTEADST
FRDMISDLLRRYEFLGRIVGKCMYEGILVDLAFAG--FFLLKWTS---------------
TGPNDENTYKGSVNDLRD----------------------MDEDLYRG--------MLRL
KNYPG--DVS-ELG--
--IDFTIEDQVSDPKD----------------------PVK-TVTRK-LIAN--------
GDQIH---
------------------------------VTNDNRL-LYISY-VARHRLVVQ-PAPQTS
-AFLRG---LRSIIRP--SWLS-MFNQS-ELQRLVGG-----------------------
---DSSEID-----------IEDL----RKHTIYGGLYQIGDDGEE--------------
------------------------HETI-KLFWKVMH--------------------SFT
DDQRRAVIKY-------VSSTPRAP------------LLG-----FS-QLKPLF--SIRD
GGTDE-----------------------------ERLPSTSTCV-NL-LKLPRY-TSEAT
LREKLL-YAIQS-GAGF
>Fusarium_temperatum_LJGR01000006.1 .
AQISRKSL-FDDAYKQF---------------YEIGDDLKG-PIQITFVDQFGAA--EAG
IDGGGVTKEF---LISV----ATEAFSAEGGR------GMFTSNEKG-------------
----------------LLYPDPTA----------------LDVIREEL-RHAGMTEADST
FRDMISDLLRRYEFLGRIVGKCMYEGILVDLAFAG--FFLLKWTS---------------
TGPNDENTYKGSVNDLRD----------------------MDEDLYRG--------MLRL
KNYPG--DVS-ELG--
--IDFTIEDQVSDPED----------------------PVK-TVTRK-LIAN--------
GDQIH---
------------------------------VTNDNRL-LYISY-VARHRLVVQ-PAPQTS
-AFLRG---LRSIIRP--SWLS-MFNQS-ELQRLVGG-----------------------
---DSSEID-----------IEDL----RKHTIYGGLYQIGDDGEE--------------
------------------------HETI-KLFWKVMH--------------------SFT
DDQRRAVVKY-------VSSTPRAP------------LLG-----FS-QLKPLF--SIRD
GGTDE-----------------------------ERLPSTSTCV-NL-LKLPRY-TSEAT
LREKLL-YAIQS-GAGF
>Fusarium_circinatum_JRVE01000119.1 .
AQISRKSL-FDDAYKQF---------------YEIGDDLKG-PIQITFVDQFGAA--EAG
IDGGGVTKEF---LISV----ATEAFSAEGGR------GMFTSNEKG-------------
----------------LLYPDPTA----------------LDVIREEL-RHAGMTEADST
FRDMISDLLRRYEFLGRIVGKCMYEGILVDLAFAG--FFLLKWTS---------------
TGPNDENTYKGSVNDLRD----------------------MDEDLYRG--------MLRL
KNYPG--DVS-ELG--
--IDFTIEDQVSDPKD----------------------PVK-TVTRK-LIAN--------
GDQIH---
------------------------------VTNDNRL-LYISY-VARHRLVVQ-PAPQTS
-AFLRG---LRSIIRP--SWLS-MFNQS-ELQRLVGG-----------------------
---DSSEID-----------IEDL----RKHTIYGGLYQIGDDGEE--------------
------------------------HETI-KLFWKVMH--------------------SFT
DDQRRAVVKY-------VSSTPRAP------------LLG-----FS-QLKPLF--SIRD

GGTDE-----------------------------ERLPSTSTCV-NL-LKLPRY-TSEAT
LREKLL-YAIQS-GAGF
>Encephalitozoon_romaleae_XM_009266036.1 .
ITVNRETV-YRDSIEIF--------------KKMNVSNARK-QLRTTFKN-------EEG
VDSGGIRKEY---FQLL----SQEIKEDG---------RLFEHTEN--------------
----------------RIWIRPHE------------------------------------
------GDEEEYEAIGRIIAIALYNNVVLNIPFPS--LFFKKLLG---------------
--------RRPSLDDLRE----------------------ISSGIATS--------LRNL
RKLSK--DEI-NELD-
--LRFVAEHNV---------------------------DGT-TKTYP-LVKN--------
GKNIR---
------------------------------LTSENMR-LFIEK-YIEFHTDTL-IKPQFE
-AIRKG---FYSIIDK--DKMM-YLDPK-ELEKIMMG-----------------------
----SNTFD-----------IKAI----RSSTTYSG-FKED-------------------
------------------------SPII-IYFWEIFE--------------------GFN
RKKRKKLLQF-------ITGNDRIP------------VSG-----PA-SLKLVI--MRNG
CDT-------------------------------DRLPSSQTCF-NT-LLLPEY-SSKNK
LEKKLE-TALEL-TAGF
>Encephalitozoon_hellem_XM_003887026.1 .
ITVNRETV-YRDSIEIF--------------KRMNPLNARK-QLRTTFRN-------EEG
VDSGGIRKEY---FQLL----SQEIKEDG---------RLFEHTEN--------------
----------------RIWIRPHE------------------------------------
------GDEEEYEAIGRIIAIALYNNVVLNIPFPS--LFFKKLLG---------------
--------RRPTLDDLRE----------------------ISSGIATS--------LRNL
RRLSK--DEI-NGLD-
--LRFVAEHNV---------------------------DGA-TRVYP-LVKN--------
GKNIR---
------------------------------LTSENMR-LFIEK-YIEFHTDTL-VKPQFE
-AIQKG---FYSIIDR--DKMM-YLDPK-ELEKIMMG-----------------------
----SNTFD-----------IKAI----RSSATYSG-YRED-------------------
------------------------SPII-LYFWEIFE--------------------GFN
RKKRKKLLQF-------ITGNDRIP------------VSG-----PA-SLKLVI--MKNG
CDT-------------------------------DRLPSSQTCF-NT-LLLPEY-SSKSK
LEKKLE-TALEL-TAGF
>Encephalitozoon_intestinalis_XM_003072647.1 .
ITVNRETV-YRDSIEIF--------------KKMNVLDAKK-QLRTTFKN-------EEG
VDSGGIRKEY---FQLL----SQEIKEDE---------RLFEHTEN--------------
----------------RIWIRPHK------------------------------------
------GDEEEYEAIGRIIAIAFYNNIVLNIPFPS--LFFKKLLD---------------
--------RKPTLDDLRE----------------------ISSGIATS--------LRNL
KRLSK--DEI-NGLD-
--LRFVAEHIV---------------------------DGT-TRIYP-LVKN--------
GKNIK---
------------------------------LTSENMK-LFIEK-YIEFHTDTL-IKPQFE
-AIRRG---FYSIIDR--DKMV-YLDPK-ELEKIMMG-----------------------
----SNTFD-----------IKAI----RSTTTYSG-FQED-------------------
------------------------SPII-TYFWEIFE--------------------ALN
RKKKKKLLQF-------ITGNDRIP------------VSG-----SA-SLKLII--MKNG
CDT-------------------------------DRLPSSQTCF-NT-LLLPEY-SSKRK
LEKKLE-TALEL-TAGF
>Encephalitozoon_cuniculi_NM_001041082.1 .
ITVSRETV-YRDSIEIF--------------KKINVLDARK-QLRITFRN-------EEG
VDSGGIRKEY---FQLL----SQEIKEDE---------RLFEHTEN--------------
----------------RIWIRPHE------------------------------------
------GDGEGYEAIGRIIAIALYNNVVLNIPFPS--LFFKKLLD---------------
--------RRPTLDDLRE----------------------ISSGIATS--------LRNL
RRLSR--DEV-DSLD-
--LRFVVEHSV---------------------------DGI-PRSYP-LVKN--------

GEDIK---
------------------------------LTSENMR-LFIEK-YVEFHTDAL-IKPQFE
-SIKRG---FYSIIDK--DKLA-YLDPK-ELEKIMMG-----------------------
----SNTFD-----------IKAI----RSTTTYSG-FRED-------------------
------------------------SPII-VYFWEIFE--------------------AFN
RKKRKKLLQF-------ITGNDRIP------------VSG-----PA-SLKLVI--MRNG
CDT-------------------------------DRLPSSQTCF-NT-LLLPEY-SSKDK
LEGKLE-TALEL-TAGF
>Ordospora_colligata_XM_014708456.1 .
VTVDRETV-YRDSIEIF--------------KHLNPQDARK-QLRTTFRN-------EEG
VDSGGIRKEY---FQLL----SHEIREDN---------KLFEHVEN--------------
----------------KIWIKAGA------------------------------------
------KDMCEYEAVGRIIAIALYNNIVLSIPFPR--LFFKKLLE---------------
--------KKPTIEDLEE----------------------ISPDVSMS--------LKKL
RMLSK--EEI-DFLD-
--LRFVSEYYE---------------------------EGN-VLIHA-LVEN--------
GENIR---
------------------------------LTEDNIG-LFIEK-YSEFYTDVL-VRPQFE
-ALKKG---FYSVMEK--CKLV-YLNPK-ELEKIMMG-----------------------
----SNGFD-----------IKEI----RSATTYSG-FTEE-------------------
------------------------SPVI-VHFWEIFE--------------------AFD
KKKRKKLLQF-------ITGNDRIP------------VSG-----SV-SLKLVI--MRNG
CDT-------------------------------DRLPSSQTCF-NT-LLLPEY-SSKDK
LENKLE-TALEL-TAGF
>Nosema_bombycis_ACJZ01001259.1 .
ITVSRDNI-YKESMDVV--------------RCLNIEDVRK-QLRITFKC-------EEG
VDSGGIRKEY---FQLL----SHEIKHDE---------RLFYNNES--------------
----------------TIWLRNDC------------------------------------
------LDMSAYMTIGKILGIALYNNVLLNIPFPS--VFFKKLLG---------------
--------KKTTFYDLKE----------------------IDPELFCS--------LTNL
KKLSEE---DIA-GLG--
--LTFTINYTSS--------------------------KNK-TKTYN-LTED--------
KSEKS---
------------------------------VTKSNIN-SFVNR-YADFYINRS-IYRQFE
-AIKKG---FLFVIER--ETLF-YLDHK-ELEKIIMG-----------------------
----SNDFD-----------VHAL----RSTASYTD-YESD-------------------
------------------------SQII-SWFWAIFE--------------------SYG
KKMRKKLLQF-------ITGNDRIP------------VAG-----AA-SVKLII--MKNG
CDT-------------------------------DRLPSSQTCF-NT-LLLPEY-SSKKK
LESKLR-TALEM-TAG-
>Nosema_apis_ANPH01000315.1 .
ITITRENI-YSDSLKIL--------------NYIKQEDVHK-QLRITFKN-------EEG
VDSGGIRKEF---FQLL----SQIIKNDA---------NLFNIYKN--------------
----------------IIWFKNIR------------------------------------
-------SLDSYYCIGKLLGIALYNNVILNLPFPS--FFFKKLLN---------------
--------KKTTFSDLKE----------------------IDLELYNS--------LNNM
KKLNEK---EID-DLE--
--LTFTISYKI---------------------------DEK-IITHN-LNEN--------
NVEVK---
------------------------------VTKQNLH-LFINK-YADYILNKL-INKQFD
-SIKEG---FYYVIRR--DILV-YLDAK-ELEKIIIG-----------------------
----SNEFD-----------INEI----KSTTIYNG-YLVN-------------------
------------------------SDII-KWFWEIFE--------------------AYS
KKMKKKLLQF-------ITGNDRIP------------ISG-----SA-SLNLVI--MKNG
CDT-------------------------------DRLPSSQTCF-NT-LLLPEY-SSKDK
LQKKMR-TALEL-TAG-
>Nosema_ceranae_XM_002995198.1 .
ITVNRENI-YSESLKIL--------------LKIRFEDMHK-QLRITFKN-------EEG

VDSGGIRKEY---FQLL----SHEIKHDN---------SLFIIAEN--------------
----------------IIWLKNDC------------------------------------
------LDFDKYYCIGKILGIALYNNVVLNIPFPS--FFFKKLLN---------------
--------KKTTFNDLKE----------------------IDHSLYLS--------LSKL
KKLSAK---EIE-NLE--
--LTFTVIYTTT--------------------------TGE-VKSYN-LDKR--------
NREIK---
------------------------------VTKANLH-SFINK-YSDFILNKL-IKKQFN
-AIKEG---FYFVINK--NILT-PVNSK-ELEKIILG-----------------------
----SNNLN-----------VDEI----KSTTSYSG-YKND-------------------
------------------------STII-KYFWEIFE--------------------SYS
KKMKKKLIQF-------ITGHDRIP------------IAG-----AG-SLKLVI--MKNG
CDT-------------------------------ERLPSSQTCF-NT-LLLPEY-SSKEK
LEGKLR-TALEM-TAGF
>Hamiltosporidium_tvaerminnensis_ACSZ01001332. .
ITVGRSTI-YRDTMNIL--------------KNLDDSEMKK-QLKITFDN-------EEG
VDSGGIRKEY---FQLL----SEEITNDH---------GLFKIKNN--------------
----------------YLWFKITD------------------------------------
---RASDNLREYETIGKLIGIALYNDVVLNIPFPS--VFFKKLLS---------------
--------KRTNTSDLQQ----------------------ICPDIFQS--------LYNL
KKCQKE---ELK-ELE--
--QTFEIAYST---------------------------NDI-EITHF-LVEN--------
GNKVM---
------------------------------VDCENVN-EFIEK-YADFILNES-ISAEFE
-SLKKG---FFSIVKH--STVS-YLHSK-ELEKIIVG-----------------------
----TTFID-----------VEAI----HKHALNTG-FDEN-------------------
------------------------SSTL-QNFWEIFT--------------------EFD
SENRKKLLQF-------ITGNDRIP------------VSG-----PE-SLKLVI--MKNG
CDT-------------------------------DRLPSSQTCF-NT-LLLPEY-SSKEK
LKSKLL-SAIHM-TKGF
>Anncaliia_algerae_AOMW02000904.1 .
IKINRSTL-YAQTLKLL--------------RKTPNFDLRK-QVKVIFNN-------EEG
EDQGGIKKEY---FQLL----SEEIREEK---------RLFINKND--------------
----------------ILWFKLDG------------------------------------
-----SSQRPEYESIGKIIGIALYNNVVLNLPFPT--LFFKRLLN---------------
--------KETELEDLKE----------------------IDEPIFNS--------LKNL
LKMSEE---EIK-CSG--
--QTFCISYQS---------------------------GSK-IIEKR-LIKN--------
GDMVP---
------------------------------VTSENIG-LFVTR-YYKFLTSES-VEPFMK
-SIRRG---FLSVIQP--TSFE-FLHAR-ELEKIIVG-----------------------
----SKIIN-----------ITLL----KDTCIYRG-YDPD-------------------
------------------------DPIP-IWFWEIVS--------------------SYD
EEKKKKLLQF-------ITGNDRIP------------VTG------S-VLRIVF--MKNG
CDT-------------------------------DRLPSAQTCF-NI-LMIPEY-NSKEK
LENKIN-MAINY-SKG-
>Vavraia_culicis_XM_008075624.1 .
ITVNRNTL-YKDVFKIF--------------SKTNFDDIRK-EIKITFKG-------EEG
VDSGGITKEF---FQLV----SEELIHDS---------FLFTIQNN--------------
----------------LLWFTEGK------------------------------------
-------KNSRYEVVGRLIGVALYNDVVLNLPFPT--FLFKIFLN---------------
--------KKVELDDLME----------------------IEPEICHS--------LKKI
LQMNDE---ELC-VLE--
--QCFSITR-------------------------------N-TKSIE-LIPR--------
GKNVV---
------------------------------LTEKNKE-LFVEK-YVEYLTYSE-MKENIE
-NIKKG---FFHVIKL--RTIS-FLQPC-ELEKIIVG-----------------------
----SNVIN-----------VQLL----KKTAVYSG-FTED-------------------

------------------------SLVV-KTFWKIFE--------------------EYS
LEDKKKLLMF-------ITGNERVP------------VTC-----TE-NWKLII--VRNG
CDT-------------------------------DRLPSSQTCF-NT-LLLPEY-SSEEK
LRNKLG-IAISM-TKGF
>Spraguea_lophii_ATCN01000180.1 .
ITVGRDSL-YEDTIDFI-----------MKQSTGKNNQLRK-QIKVIFKG-------EEG
VDSGGMTKEF---FQLL----SEEIINDD---------RLFNMKNN--------------
----------------FLSIKRQN----------------------------NNKKEGLK
TAQNSYTIKQLYNAVGKILGIALYNDVVLNIPFPT--FLFKKILN---------------
--------KSVRMEDLEE----------------------IDPMVYIS--------LMNL
LKVK---NID-ELN--
--LNFEITVESDTTEDHY--------------------KKD-YYSVE-LIEN--------
GKNIP---
------------------------------VTNKNIN-KFISL-YVNYLTYES-IKEPLE
-QIKNG---FFDIIKH--STVL-YLQPI-ELERIIVG-----------------------
-----TYFN-----------LDSI----KENAVYNG-YTKN-------------------
------------------------STIV-KHFWSIFE--------------------NYS
SDNKNKLLKF-------ITGHDRIP------------ISN------N-PFTIVI--MKNG
CDT-------------------------------DRLPSSQTCF-NT-FLLPEY-NS---

>Vittaforma_corneae_XM_007603967.1 .
INIRRDRL-YIDTLKII--------------LEVDGIDIKK-QLKVKFLG-------EEG
VDSGGIKKEY---FLLL----GHEIENDT---------SLFTQTNN--------------
----------------RIWFRKGV------------------------------------
-------DLQLLNTIGKIVGIALYNDVVLNVPFPS--LLFKKLLH---------------
--------IPLDFDDLEE----------------------IEPEIYNS--------LKNL
QRCSPE---DFQ-FLD--
--QNFTADFEV---------------------------LGR-RINYE-LIED--------
GSGIK---
------------------------------VNKKNLE-EFTQL-YWSFLMEGI-IEDEFK
-AFSDG---FYSIINF--ENVK-KFQPH-ELEKILMG-----------------------
----VDDLD-----------FELI----KKTTTYNG-YEPK-------------------
------------------------DEII-EHFWEFFY--------------------EMC
SRKKKRLIQF-------ITGNDRLP------------VGG-----ST-ALNLII--MKNG
CDT-------------------------------DRLPSSQTCF-NT-LLLPEY-SSREK
LRDKLG-KAIGL-TAGF
>Enterocytozoon_bieneusi_XM_002649889.1 .
LAIRREYI-YEDALKIL--------------KNTSNRDLRK-QLKISFIN-------EDG
VDSGGIIKEF---FQLL----SNAIKTDK---------ELFIEKNS--------------
----------------TLWLKPGA------------------------------------
-------DLDKMKIIGKIIGIALYNNVILNIPFNT--LLFKKFIN---------------
--------KPILFEDLNY----------------------IEPEIYMS--------LCNM
EKLSTT---EFN-NLG--
--LTFEIEYEE---------------------------NET-IKKEL-LLKN--------
GHLIK---
------------------------------VTKNNFY-MFKEL-YASLYIDKM-IFNEFT
-EVLDG---FYSVIIG--SSIE-GFNYY-ELEKIVVG-----------------------
----IDQLD-----------IDKI----KKTCIYQG-YTSN-------------------
------------------------ATIV-KWFWSIVE--------------------DYN
LDMQKKLLKF-------ITGNDRLP------------IGG-----PD-ALNLTI--MKNG
CDT-------------------------------NRLPSSQTCF-NT-ILLPEY-SSYDK
LMEKMQ-KALDM-TAGF
>Enterospora_canceri_LWDP01000001.1 .
VTINRKNL-YKDTLRIL--------------KTTPNWDLKK-QLKINFKD-------EEG
VDSGGIIKEF---FQLL----SEELAADD---------SLFVEKNS--------------
----------------TLWLKPGA------------------------------------
-------DLTKMKLLGKIIGIALYNNVILNLPLTP--LIFKKFFS---------------
--------TPIGFDDLSL----------------------IEPEIHNT--------LCNM

```
EKMTSE---------------------------------------------EFE-SLG--
--MNFVVDCEM---------------------------EGE-TVRKH-LVKN--------
AENTR-------------------------------------------------------
------------------------------LSKTNFL-LFRDL-YSKYYIDEM-VSNEFT
-EVLDG---FYSVIIG--HSID-GFNYV-ELEKIVVG-----------------------
----CGRID-----------YDQI----KKHCIYQG-YTED-------------------
------------------------ALVV-RHFWTIIG--------------------SFS
KEMKRKLLKF-------ITGNDRLP------------IGR-----AE-ALKFTI--MKNG
CDT-------------------------------DRLPSAQTCF-NT-ILLPEY-ATYDK
LHDKLV-RAVEM-TAG-
>Epichloe_amarillans_AFRF01000047.1 .
LSISRSHV-LRDSLDQL--------------WRRQERELLR-PLKVHLGEGGGG---EEG
FDSGGVQQEF---FRMA----IAECLDPEY--------GAFTVDSRTR------------
----------------MAWFVPGS------------------------------------
-----LVEEWKFEMIGLLMSLAVYNGLTLPITFPK--ALYRKLLG---------------
-------HPVEELYHIAD----------------------GWPELASG--------LTAL
QEWDEENG-------------------------------------------SVE-DIFAR
-TYEFSISA-----------------------------FGG-EVTLP-MTKK--------
GSFWPQGIPEPPAKR---------------------------------------------
-----------------SLAAAADC-DAPLVTNENRD-EYISD-YVRYLTDVS-IRPQFR
-AFERG---FRACLEA--KSLS-LLTPS-ILQSVVEG-----------------------
----VQEID-----------TSEL----RRHTRYVG-WDSS-------------------
------------------------HRTV-KDFWSIVK--------------------RYD
DSMKRKLLEF-------VTASDRVP------------VGG-----MK-NVQFVI--QKNG
EEEADG----------------------------GHLPTAYTCY-GT-LLLPEY-RDKEA
LRERLG-MALEN-AQGF
>Epichloe_brachyelytri_AFRB01000936.1 .
LSISRSHV-LRDSLDQL--------------WRRQERELLR-PLKVHLGEGGGG---EEG
FDSGGVQQEF---FRMA----IAECLDPEY--------GAFTVDSRTR------------
----------------MAWFVPGS------------------------------------
-----LVEEWKFEMIGLLMSLAVYNGLTLPITFPK--ALYRKLLG---------------
-------HPVGELYHIAD----------------------GWPELASG--------LTAL
QEWDEENG-------------------------------------------SVE-DIFAR
-TYEFSISA-----------------------------FGG-EVTLP-MTKK--------
GSFWPQGIPEPPAKR---------------------------------------------
-----------------SLAAAADC-DAPLVTNENRD-EYISD-YVRYLTDVS-IRPQFR
-AFERG---FRACLEA--KSLS-LLTPS-ILQSVVEG-----------------------
----VQEID-----------TSEL----RRHTRYVG-WDSS-------------------
------------------------HCTV-KDFWSIVK--------------------RYD
DSMKRKLLEF-------VTASDRVP------------VGG-----MK-NVQFVI--QKNG
EEEADG----------------------------GHLPTAYTCY-GT-LLLPEY-RDKEA
LRERLG-MALEN-AQGF
>Epichloe_glyceriae_AFRG01000060.1 .
LSISRSHV-LRDSLDQL--------------WRRQERELLR-PLKVHLGEGGGG---EEG
FDSGGVQQEF---FRMA----IAECLDPEY--------GAFTVDSRTR------------
----------------MAWFVPGS------------------------------------
-----LVEEWKFEMIGLLMSLAVYNGLTLPITFPK--ALYRKLLG---------------
-------HPVEELYHIAD----------------------GWPELASG--------LTAL
QEWDEENG-------------------------------------------SVE-DIFAR
-TYEFSISA-----------------------------FGG-EVTLP-MTKK--------
GSFWPQGIPEPPAKR---------------------------------------------
-----------------SLAAAADC-DAPLVTNENRD-EYVSD-YVRYLTDVS-IRPQFR
-AFERG---FRACLEA--KSLS-LLTPS-ILQSVVEG-----------------------
----VQEID-----------TSEL----RRHTRYVG-WDSS-------------------
------------------------HRTV-KDFWSIVK--------------------RYD
DSMKRKLLEF-------VTASDRVP------------VGG-----MK-NVQFVI--QKNG
EEEADG----------------------------GHLPTAYTCY-GT-LLLPEY-RDKEA
LRERLG-MALEN-AQGF
```

```
>Neotyphodium_gansuense_AFRE01000383.1 .
LSISRSHV-LRDSLDQL--------------WRRQERELLR-PLKVHLGEGGGG---EEG
FDSGGVQQEF---FRMA----IAECLDPEY--------GAFTVDSRTR------------
----------------MAWFVPGS------------------------------------
-----LVEEWKFEMIGLLMSLAVYNGLTLPITFPK--ALYRKLLG---------------
-------HPVEELYHIAD----------------------GWPELASG--------LTTL
QEWDEENG----------------------------------------SVE-DIFAR
-TYEFSISA-----------------------------FGG-EVTLP-MTKK--------
GAYWPQGIPEPPAKK---------------------------------------------
-----------------SLAAAADC-DAPLVTNENRD-EYVSD-YIRYLTDIS-IRPQFR
-AFKRG---FRACLEA--KSLS-LLTPS-ILQSVVEG-----------------------
----VQEID-----------TSEL----RRHARYVG-WDPS-------------------
------------------------HRTV-KDFWSIVK--------------------RYD
DSMKRKLLEF-------VTASDRVP------------VGG-----MK-NVQFVI--QKNG
EEEADG----------------------------GHLPTAYTCY-GT-LLLPEY-RDKEV
LRERLG-MALEN-AQGF
>Claviceps_fusiformis_AFRA01000021.1 .
LSISRSHV-LRDAFDQL--------------WRRQERELLR-PLKVHLGEGGGG---EEG
FDSGGVQQEF---FRMA----IAECLDPEY--------GAFTVDSRTR------------
----------------MTWFVPGS------------------------------------
-----LVEEWKFEMIGLLMSLAVYNGLTLPITFPK--AFYRKLLG---------------
-------QPVEELYHIAD----------------------GWPELASG--------LTAL
QEWDEEDG----------------------------------------SIE-DVFAR
-TYEFSISA-----------------------------FGG-EVTLP-MTKR--------
DSVWPQGIPKPPAKS---------------------------------------------
-----------------SLAAVADS-DAPLVTNENRD-EYVSD-YVRYLTDVS-IRPQFR
-AFERG---FRACLDA--KSLS-LLTPA-ILQSLVEG-----------------------
----VQEID-----------ISEL----RRHARYVG-WDSS-------------------
------------------------HRTV-KDFWSIVK--------------------RFD
DSMKRKLLEF-------VTASDRVP------------VGG-----MK-NVQFVI--QKNG
EEEGDG----------------------------GHLPSAYTCY-GT-LLLPEY-RDKEA
LRERLN-MALEN-AQGF
>Claviceps_paspali_AFRC01000310.1 .
LSISRGRV-LRDAFDQL--------------WRRQERELLR-PLKVHLGEGGGG---EEG
FDSGGVQQEF---FRLA----IAECLDPDY--------GAFTVDGRTR------------
----------------MAWFVPGS------------------------------------
-----LVDEWKFEMIGLLMSLAVYNGLTLPITFPK--AFYRKLLG---------------
-------QPVEEVYHIAD----------------------GWPELASG--------LTAL
QEWDEENG----------------------------------------SVE-DVFAR
-TYEFSVSA-----------------------------FGG-QVTLP-MTKT--------
DSFWPQGIPVHGAKS---------------------------------------------
-----------------SVAAAADS-DAPLVTNENRD-EYVSD-YVRYLTDVS-VRPQFR
-ALERG---FRACLDG--KSLS-LLTPA-ILQSVVEG-----------------------
----VQEID-----------VSEL----RRHARYVG-WDST-------------------
------------------------HRTV-KDFWSIVK--------------------RFD
DSMKRKLLEF-------VTASDRVP------------VGG-----MK-NVQFVI--QKNG
EEAGDG----------------------------GHLPTAYTCY-GT-LLLPEY-RDREA
LRERLG-MALEN-AQ--
>Aciculosporium_take_AFQZ01000717.1 .
LSISRSHV-LRDAFDQL--------------WRRQERELLR-PLKVHLGEGAGG---EEG
FDSGGVQQEF---FRMA----IAECLDPDY--------GAFTVDSRTR------------
----------------MAWFVPGS------------------------------------
-----LVDEWKFEMIGLLMSLAVYNGLTLPITFPK--ALYRKLLG---------------
-------QPVEELYHIAD----------------------GWPELASG--------LTAL
QEWDEEDG----------------------------------------SVE-DIFAR
-TYEFSISA-----------------------------FGS-DVTLP-MTKT--------
DSAWPQGIPELPAKS---------------------------------------------
-----------------SLASVADN-DAPLVTNENRD-EYASD-YVRYLTDVS-IRPQFR
```

-AFERG---FRACLDA--KSLT-LVTPA-ILQSVVEG-----------------------
----VQEID-----------IAEL----RRHARYVG-WDSS-------------------
------------------------HRTV-KDFWSIVK--------------------RYD
DSMKRKLLEF-------VTASDRVP------------VGG-----MK-NVQFVI--QKNG
EEEGDG----------------------------GHLPTAYTCY-GT-LLLPEY-RDKEA
LRERLG-MALQN-AQGF
>Periglandula_ipomoeae_AFRD01000030.1 .
LAISRNHV-LRDAFDQL--------------WRRQERELLR-PLKVHLGEGGGG---EEG
FDSGGVQQEF---FRMA----IAEALDPEY--------GAFTVDSRTR------------
----------------MAWFVPGS------------------------------------
-----LVEEWKFEMIGLLMSLAVYNGLTLPITFPK--AFYRKLLG---------------
-------QPVQELYHIAD----------------------GWPELASG--------LTAL
QEWDEADG--SVE-DIFAR
-TYEFSVPA-----------------------------FGT-EVTRR-MVKS--------
TPYWPQGIPKPPAKS---
-----------------SLAAAADD-DAPLVTNENRD-EYVSD-YVRYLTDIS-VRPQFQ
-AFERG---FRACLDG--KSLS-LLTPS-ILQSIVEG-----------------------
----VQEID-----------ISEL----RRHARYVG-WDSS-------------------
------------------------HRTV-KDFWSIVK--------------------RYD
DTMKRKLLEF-------VTASDRVP------------VGG-----MK-NAQFVI--QKNG
EEEGDG----------------------------GHLPTAYTCY-GI-LLLPEY-RDKEA
LRERLG-MALEN-AQGF
>Pochonia_chlamydosporia_XM_018281520.1 .
LAISREHV-LRDAFDQL--------------WRRQERELLR-PLKVHLGEGSGG---EEG
FDSGGVQQEF---FRMA----IAECLDPKY--------GAFTVDDRTR------------
----------------MAWFVPGS------------------------------------
-----LVEEWKFEMVGLLMSLAVYNGLTLPITFPK--ALYRKLLG---------------
-------QPVEELYHIAD----------------------GWPELANG--------LTAL
QEWDEKDG--LVE-DVFAR
-TYEFSVGA-----------------------------FGS-DVTLP-MTKE--------
NSSWPQGIPTPPTRS---
-----------------GL-PASDD-EAPLVTNENRD-EYVSD-YIRYLTDVS-IQPQFR
-AFERG---FRACLDV--KSLT-LLTPS-ILQSVVEG-----------------------
----VQDID-----------ISEL----RRYTRYVG-WDSS-------------------
------------------------HHTV-KDFWSIVK--------------------RYD
DRMKRKLLEF-------VTASDRVP------------VGG-----MR-NLQFVV--QKNG
EEEGSG----------------------------GHLPTAYTCY-GT-LLLPEY-KDKEV
LRERLG-MALEN-AQGF
>Metarhizium_acridum_ADNI01000440.1 .
LAISRDHV-LRDAFDQL--------------WRRQERELLR-PLKIHLGEGAGG---EEG
FDSGGVQQEF---FRMA----MAECLDPNY--------GAFTVDDRTR------------
----------------MAWFVPGS------------------------------------
-----LVETWKFEMIGLLVSLAVYNGLTLPITFPK--ALYRKLLG---------------
-------QPVEELYHIAD----------------------GWPDLASG--------LTAL
QEWDEKDG--LVE-DVFAR
-TYEFSVGA-----------------------------FGA-DVTLP-MTKD--------
KSSWPQGIPTPPTRN---
-----------------SP-AALED-EAPLVTSENRD-EYVSD-YVRYLTDVS-IRPQFR
-AFERG---FRACLDA--KSLT-LLTPS-LLQSIVEG-----------------------
----VQEID-----------ISEL----RRYARYVG-WDSS-------------------
------------------------HHTV-KDFWSIVK--------------------RYD
DRMKRKLLEF-------VTASDRLP------------VGG-----MR-NVQFTV--QKNG
EEEGSG----------------------------GHLPTAYTCY-GT-LLLPEY-NDKEV
LRERLG-MALEN-AQGF
>Metarhizium_brunneum_XM_014691497.1 .
LAISRDHV-LRDAFDQL--------------WRRQERELLR-PLKIHLGEGAGG---EQG
FDSGGVQQEF---FRMA----MAECLDPNY--------GAFTVDDRTR------------
----------------MAWFVPGS------------------------------------

-----LVEIWKFEMMGLLVSLAVYNGLTLPITFPK--ALYRKLLG---------------
-------QPVEELYHIAD----------------------GWPDLANG--------LTAL
QEWDEKDG--LVE-DVFAR
-TYEFSVAA-----------------------------FGA-DVTLP-MSKD--------
KSSWPQGIPTPPARN---
-----------------SP-AALED-EAPLVTSENRD-EYVSD-YVRYLTDVS-IRPQFQ
-AFERG---FRACLDA--KSLT-LLTPS-LLQSIVEG-----------------------
----VQEID-----------VSEL----RRYARYVG-WDSS-------------------
------------------------HHTV-KDFWSIVK--------------------RYD
DRMKRKLLEF-------VTASDRLP------------VGG-----MR-NVQFTV--QKNG
EEEGSG----------------------------GHLPTAYTCY-GT-LLLPEY-NDKEV
LRERLG-MALEN-AQGF
>Metarhizium_majus_XM_014727365.1 .
LAISRDHV-LRDAFDQL--------------WRRQERELLR-PLKIHLGEGAGG---EQG
FDSGGVQQEF---FRMA----IAECLDPNY--------GAFTVDDRTR------------
----------------MAWFVPGS------------------------------------
-----LVEIWKFEMIGLLVSLAVYNGLTLPITFPK--ALYRKLLG---------------
-------QPVEELYHIAD----------------------GWPDLANG--------LTAL
QEWDEKDG--LVE-DVFAR
-TYEFSVAA-----------------------------FGA-DVTLP-MSKD--------
KSSWPQGIPTPLARN---
-----------------SP-AALED-EAPLVTSENRD-EYVSD-YVRYLTDVS-IRPQFQ
-AFERG---FRACLDA--KSLT-LLTPS-LLQSIVEG-----------------------
----VQEID-----------ISEL----RRYARYVG-WDSS-------------------
------------------------HHTV-KDFWSIVK--------------------RYD
DRMKRKLLEF-------VTASDRLP------------VGG-----MR-NVQFTV--QKNG
EEEGSG----------------------------GHLPTAYTCY-GT-LLLPEY-NDKEV
LRERLG-MALEN-AQGF
>Metarhizium_anisopliae_ADNJ01000349.1 .
LAISRDHV-LRDAFDQL--------------WRRQERELLR-PLKIHLGEGAGG---EQG
FDSGGVQQEF---FRMA----IAECLDPNY--------GAFTVDDRTR------------
----------------MAWFVPGS------------------------------------
-----LVEIWKFEMIGLLVSLAVYNGLTLPITFPK--ALYRKLLG---------------
-------QPVEELYHIAD----------------------GWPDLANG--------LTAL
QEWDEKDG--LVE-DVFAR
-TYEFSVAA-----------------------------FGA-DVTLP-MSKD--------
KSSWPQGIPTPVARN---
-----------------SP-AALED-EAPLVTSENRD-EYVSD-YVRYLTDVS-IRPQFQ
-AFERG---FRACLDA--KSLT-LLTPS-LLQSIVEG-----------------------
----VQEID-----------VSEL----RRYARYVG-WDSS-------------------
------------------------HHTV-KDFWSIVK--------------------RYD
DRMKRKLLEF-------VTASDRLP------------VGG-----MR-NVQFTV--QKNG
EEEGSG----------------------------GHLPTAYTCY-GT-LLLPEY-NDKEV
LRERLG-MALEN-AQGF
>Purpureocillium_lilacinum_XM_018321250.1 .
LSIGRDSV-VRDAFDQL--------------WRRQERELLR-PLKIHLGENGG----EEG
FDSGGVQQEF---FRMA----IAECLDPRF--------GAFTVDERTR------------
----------------MAWFVPGS------------------------------------
-----VVEDWKFEMMGLLVSLAIYNGLTLPVTFPK--ALYRKLLG---------------
-------EPVEQLYHISD----------------------GWPELAGG--------LTTL
LEWDEKDG--LIE-DVFAR
-TYEFSVAA-----------------------------FGR-DVTRE-MSKT--------
KSSWPQGLSGLQRND---
-----------------T--ASEDD-EAPMVTNANRD-DYVSD-YIRYLTDVS-VRPQFR
-AFQRG---FRACLDA--KSLS-LLTPP-ILQSIVEG-----------------------
----IQDID-----------ISDL----RRYARYVG-WDSS-------------------
------------------------HQTI-RDFWSIVK--------------------RYD
DRMKRKLLEF-------VTASDRVP------------VGG-----MR-NLQFVV--QKNG

EEDGEG----------------------------GHLPTAYTCY-GT-LLLPQY-RDKEV
LRERLA-MALEN-AQGF
>Ophiocord_sinensis_GCQL01008447.1 .
LQVSRENV-VRDAFDQL--------------WRRQERELLR-PLKIHLGEDGG----EEG
FDSGGVQQEF---FRLA----ISKCLDPDY--------GAFTIDERTR------------
----------------AAWFAPGS------------------------------------
-----VVEEWKFEMIGLLISLAVFNGLTLPVTFPK--ALYRKLLG---------------
-------QPVEQLYHIAD---------------------GWPELASG--------LTTL
LEWDEKDG--QVG-DVFAR
-TYEFSVAL-----------------------------FGG-DVTRQ-MSKE--------
DSSWPQGLPCKQHVA---
---------------------AKAD-EAPLVTNANRD-EYVSD-YVRYLTDVS-VRPQYL
-AFERG---FRACLGS--KALT-LLTPP-ILQSMVEG-----------------------
----VQTID-----------VSEL----RRYTRYVG-WDSS-------------------
------------------------HRTI-KDFWSIVR--------------------KFD
DRMKRKLLEF-------VTASDRVP------------VGG-----MA-NLHFAV--QRNG
DEEGEG----------------------------GHLPTAYTCY-GI-LLLPEY-RDKEV
LRERLC-MALEN-AQGF
>Togninia_minima_XM_007913387.1 .
LDISRDNV-ITDAFDQL--------------WRREERELMR-PLKVHLGEEGG----EEG
FDSGGVQQEF---FRMA----IAEALDPKY--------GAFTIDDRTR------------
----------------MVWFQPGS------------------------------------
-----LEAEWKFELVGLLISLAVYNGLTLPITFPK--ALYRKLLG---------------
-------EPVTELHHIAD---------------------GWPDLASG--------LTTL
LEWDEKDG--LVE-DVFAR
-TYEFSVSM-----------------------------FDE-HISRE-MNSD--------
NPEWPQFPKSVAESS---
--------------SSTSPLSAANPPDAPLVTGENRN-AYVSD-YIHYLTDVS-VKPQYE
-AFARG---FRTCLHP--KSLT-LLTSN-LLQSIVEG-----------------------
----VQDID-----------IADL----RRYTRYVG-WDST-------------------
------------------------HRTI-KDFWSIVK--------------------RYD
DRMKRKLLEF-------VTASDRVP------------VGG-----MK-NLQFVV--QRNG
E-EGDG----------------------------GHLPTAYTCY-GT-LLLPEY-HDKEI
LRERLS-MALEN-AQGF
>Verticillium_dahliae_CP010981.1 .
--ISRDGV-VRDAFDQL--------------WRREARELLR-PLKVHLGEEGG----EEG
FDSGGVQQEF---FRLA----IAECLDPDF--------GAFTVDERTR------------
----------------MAWFVPGS------------------------------------
-----IVPVWKFELLGILVSLAVYNGLTLPITFPK--AMYRKLLG---------------
-------EPVDELHHIAD---------------------GWPDLASG--------LTTL
LEWDEKDG--AVE-DVFAR
-TYEFSVSM-----------------------------FDQ-PISRE-MNSE--------
NPNWPQLKAKSESLA---
---------------------AQNPADAPLVTDENRN-AYVSD-YIRYLTDVS-VRPQFE
-AFLRG---FRACLHP--KSLG-LLTPP-LLQSVVEG-----------------------
----VQDID-----------IAEL----RRYTRYVG-WDAS-------------------
------------------------HRAV-HDFWSIVK--------------------RYD
DTMKRRLLEF-------VTASDRVP------------VGG-----MR-NLQFVV--QKNG
EEEGEG----------------------------GHLPTAYTCY-GT-LLLPEY-ESKDV
LRQRLA-MALEN-SKGF
>Verticillium_albo_atrum_XM_003001607.1 .
LDISRDGV-VRDAFDQL--------------WRREARELMR-PLKVHLGEEGG----EEG
FDSGGVQQEF---FRLA----IAECLDPDF--------GAFTVDERTR------------
----------------MAWFVPGS------------------------------------
-----IVPVWKFELLGILVSLAVYNGLTLPITFPK--AMYRKLLG---------------
-------EPVDELHHIAD---------------------GWPDLASG--------LTTL

-----QSGTRRTGLL---
----------------RMFAVCAEPSDAPLVTNEX-------------------------
---LRG---FRACLHP--KSLG-LLTPP-LLQSVVEG-----------------------
----VQDID-----------IAEL----RRYTRYVG-WDAS-------------------
------------------------HRAV-HDFWSIVK--------------------RYD
DTMKRRLLEF-------VTASDRVP------------VGG-----MR-NLQFVV--QKNG
EEEGEG----------------------------GHLPTAYTCY-GT-LLLPEY-ESKDV
LRQRLA-MALEN-SKGF
>Colletotrichum_gloeosporioides_XM_007275881.1 .
----RKHV-IRDAFDQL--------------WQREDRELHR-PLKIHLGEEGG----EEG
FDSGGVQQEF---FRLA----VAECLNPDY--------GAFTVDERTR------------
----------------MAWFVPGS------------------------------------
-----IVPVWKFEILGLLMSLAVYNGLTLPITFPK--VLYRKLLG---------------
-------EPVDELYHIAD----------------------GWPDLAMG--------LTTL
LEWDESNG---AVE-DIFAR
-TYEFSVSM-----------------------------FGQ-PVSRE-MDAK--------
ASSWPQFDATARKSL---
--------------------TSENPEDAPLVTNENRN-AYVSD-YIRYLTDVS-VRPQYK
-AFERG---FRACLHP--KALQ-LLTPS-LLQSLVEG-----------------------
----VQEID-----------VSEL----RRYARYVG-WDNS-------------------
------------------------HRSV-RDFWSIVK--------------------RYD
EKMKRKLLEF-------VTASDRVP------------VGG-----MK-NLQFVV--QRNG
EEEGEG----------------------------GHLPTAYTCY-GT-LLLPEY-KDKEV
LRERLS-MALEN-AQGF
>Colletotrichum_fioriniae_XM_007595870.1 .
LEISRKNV-IRDAFDQL--------------WRREARELHR-PLKIHLGEEGG----EEG
FDSGGVQQEF---FRLA----VAECLNPDH--------GAFTVDDRTR------------
----------------MTWFVPGS------------------------------------
-----VVPAWKFEILGLLVSLAVYNGLTLPITFPK--ALYRKLLG---------------
-------EPVDELHHIAD----------------------GWPDLAMG--------LTTL
LEWDEKQG---LVE-DVFAR
-TYEFSVSM-----------------------------FGQ-PISRE-MDGK--------
ASSWPQFEATAKTSL---
--------------------VDGNPEDAPLVTNENRN-VYVSD-YIKYLTDVS-VRPQYE
-AFERG---FRACLHP--KALG-LLTPP-LLQSLVEG-----------------------
----VQEID-----------ISEL----RRYTRYVG-WDAT-------------------
------------------------HRGV-RDFWSIVK--------------------RYD
EKMKRRLLEF-------VTASDRVP------------VGG-----MK-NMQFVV--QRNG
EEEGEG----------------------------GHLPTAYTCY-GT-LLLPEY-KDKDV
LRERLS-MALEN-AQGF
>Colletotrichum_higginsianum_XM_018303102.1 .
LEISRKNV-IRDAFDQL--------------WQREDRELHR-PLKIHLGEDAG----EEG
FDSGGVQQEF---FRLA----VAECLNPDY--------GAFTVDDRTR------------
----------------MAWFVPGS------------------------------------
-----VVPAWKFEILGLLVSLAVYNGLTLPITFPK--ALYRKLLG---------------
-------EPVNELHHIAD----------------------GWPDLAMG--------LTTL
LEWDERQG---LVE-DVFAR
-TYEFSVSM-----------------------------FGQ-PISRE-MNDQ--------
ARSWPQFEATRGKSP---
--------------------VDENPEDAPLVTNENRN-AYVSD-YIKYLTDIS-VRPQYE
-AFERG---FRACLHP--KALR-LLTPP-LLQSLVEG-----------------------
----VQEID-----------ISEL----RRYTRYVG-WDAS-------------------
------------------------HRGV-RDFWSIVK--------------------RYD
EKMKRRLLEF-------VTASDRVP------------VGG-----MK-NLQFVV--QRNG
EEEGEG----------------------------GHLPTAYTCY-GT-LLLPEY-KDKDV
LRERLA-MALEN-AQGF
>Colletotrichum_graminicola_XM_008096146.1 .
LEISRKNV-IRDAFDQL--------------WRREDRELHR-PLKIHLGEDAG----EEG

FDSGGVQQEF---FRLA----VAECLNPDY--------GAFTVDDRTR------------
----------------MAWFVPGS------------------------------------
-----IVPSWKFEILGLLVSLAVYNGLTLPITFPR--ALYRKLLG---------------
-------EPVDELHHIAD----------------------GWPDLAMG--------LTTL
LEWDERQG---LVE-DVFAR
-TYEFSVSM-----------------------------FGQ-PISRE-MNDQ--------
TRSWPQFEVAAGKSL---
--------------------ADENPKDAPLVTNENRN-AYVSD-YIKYLTDVS-VRPQYE
-AFERG---FRACLHH--KALQ-LLTPP-LLQSLVEG-----------------------
----VQEID-----------ISEL----RRYTRYVG-WDAS-------------------
------------------------HRGV-RDFWSIVK--------------------RYD
EKMKRRLLEF-------VTASDRVP------------VGG-----MK-NLQFVV--QRNG
EEEGER----------------------------GHLPTAYTCY-GT-LLLPEY-KDKDV
LRERLA-MALEN-AQGF
>Trichoderma_reesei_XM_006968711.1 .
LDIGRDNV-LRDAFDQL--------------WRRERRELLR-PLKVHLGEQSG----EEG
FDSGGVQQEF---FRVA----IAEALDPKY--------GAFTIDERTR------------
----------------MAWFVPGS------------------------------------
-----VVEEWKFELIGVIVSLAVYNGLTLPVTFPK--ALYRKLLG---------------
-------EPVEELYHIAD----------------------GWPDLASG--------LMSL
LEWNEKDG---AVE-DVFAR
-TYEFSVPN-----------------------------IGG-NVTRE-MTKD--------
MAQWPQNLDWKMRIT---
-----------------VPEETEGDHEAPLVTNENRD-QYVSD-YIRYLTDVS-IRPQYE
-AFERG---FHSCLDH--KSLS-LLSPQ-ILQSLVEG-----------------------
----VQEID-----------IAEL----RRYTRYVG-WDAS-------------------
------------------------HHTV-RDFWSIVK--------------------RYD
DRMKRKLLEF-------VTASDRVP------------VGG-----MR-NLQFVV--QRNG
DGEDGG----------------------------NRLPTAYTCY-GT-LLLPDY-RDKEM
LRQRLT-MALEN-ASGF
>Trichoderma_virens_XM_014105647.1 .
LDIGRENV-LRDAFDQL--------------WRRERRELLR-PLKVHLGEQSG----EEG
FDSGGVQQEF---FRVA----IAECMDPKY--------GAFSIDDRTR------------
----------------MAWFVPGS------------------------------------
-----VVEEWKFELMGVLVSLAVYNGLTLPVTFPR--ALYRKLLG---------------
-------EPVEELYHIAD----------------------GWPALASG--------LMSL
LEWNEKDG---QVE-DIFAR
-TYEFSVSN-----------------------------IGG-NVTRE-MTKD--------
VVQWPQNLDWKMRIN---
-----------------VPEESVDDREAPLVTNDNRD-QYVSD-YIRYLTDVS-VRPQYE
-AFERG---FNSCLEY--KSLS-LLSPQ-ILQSLVEG-----------------------
----VQEID-----------ISEL----RRYTRYVG-WDAS-------------------
------------------------HHTV-RDFWSIVK--------------------RYD
DRMKRKLLEF-------VTASDRVP------------VGG-----MR-NLQFVV--QRNG
DGEDSG----------------------------NRLPTAYTCY-GT-LLLPDY-RDKDM
LRQRLT-MALEN-AQGF
>Trichoderma_gamsii_XM_018801231.1 .
LDISRENV-LRDAFDQL--------------WRRQRRELLR-PLKVHLGELSG----EEG
FDSGGVQQEF---FRMA----IAEFMDPKY--------GAFTIDDRTR------------
----------------MAWFVPGS------------------------------------
-----VVEEWKFELMGVLVSLAVYNGLTLPVTFPK--ALYRKLLG---------------
-------EPVEELYHIAD----------------------GWPALASG--------LMSL
LEWNEADG---AVE-DVFAR
-TYEFSVPN-----------------------------IGG-NVTRE-MTKD--------
MVQWPQNLDWKMRIV---
-----------------LPEESVDDTEAPLVTNDNRD-QYVSD-YIRYLTDMS-VRPQYE
-AFERG---FKSCLSD--KSLS-LLSPQ-ILQSVVEG-----------------------
----VQEID-----------ISDL----RRYTRYVG-WDAS-------------------

------------------------HHTI-RDFWSIVK--------------------RYD
DRMKRKLLEF-------VTASDRVP------------VGG-----MR-NLQFVI--QRNG
EGEGSG----------------------------SRLPTAYTCY-GT-LLLPEY-RDKDL
LRQRLA-MALEN-AQGF
>Trichoderma_atroviride_XM_014087526.1 .
LDISRENV-LRDAFDQL--------------WRRQRRELLR-PLKVHLGELSG----EEG
FDSGGVQQEF---FRMA----IAECMDPKY--------GAFTIDDRTR------------
----------------MAWFVPGS------------------------------------
-----VVEEWKFELMGVLVSLAVYNGLTLPVTFPK--ALYRKLLG---------------
-------EPVEELYHIAD----------------------GWPALASG--------LMSL
LEWNEADG--AVE-DVFAR
-TYEFSVPN-----------------------------IGG-NVTRE-MTKD--------
MVQWPQNLDWKMRIV---
-----------------LPEDSIDDTEAPLVTNDNRD-QYVSD-YIRYLTDMS-VRPQYE
-AFERG---FKSCLSD--KSLS-LLSPQ-ILQSVVEG----------------------
----VQEID-----------ISEL----RRYTRYVG-WDAS-------------------
------------------------HHTI-RDFWSIVK--------------------RYD
DKMKRKLLEF-------VTASDRVP------------VGG-----MR-NLQFVV--QRNG
EGEGSG----------------------------SRLPTAYTCY-GT-LLLPEY-RDKDL
LRQRLA-MALEN-AQGF
>Nectria_haematococca_XM_003046791.1 .
LEISRKNV-ARDAFDQL--------------WRREERELLR-PLKVHLGEEGG----EEG
FDSGGVQQEF---FRLA----IAECLDPAY--------GAFTVDDRTR------------
----------------MAWFAPGS------------------------------------
-----LTEDWKYELVGLLMSLALFNGLTLPITFPR--ALYRKLLG---------------
-------KPVAELHHIAD----------------------GWPDLASG--------LTTL
LEWDEKDG--LVE-DIFAR
-TYEFSVSA-----------------------------LGT-NITRE-MSAD--------
KSVVWPRAAANSDDV---
-----------------APPQTSNPDDAPLVTNENRD-DYVTH-YIRYLTDIS-IRRQYI
-AFERG---FNACLDK--KSLS-LLSPS-TLQSLVEG----------------------
----IQDID-----------ISEL----KRYARYVG-WDAS-------------------
------------------------HHTV-KDFWSVVR--------------------RYD
DRMKRKLLEF-------VTSSDRVP------------VGG-----IK-NLQFVI--QKNG
EEEGDG----------------------------GHLPTAYTCY-GT-LLLPEY-RDKDV
LRERLG-MALEN-AQGF
>Fusarium_culmorum_LT598661.1 .
LEVGRENV-ARDAFDQL--------------WRRERRELLR-PLKVHLGENSG----EEG
FDSGGVQQEF---FRLA----IAECLDPGF--------GAFTVDERTR------------
----------------MAWFAPGS------------------------------------
-----LTEDWKYELVGLLMSLALYNGLTLPVTFPR--ALYRKLLG---------------
-------NPVEELHHIAD----------------------GWPDLASG--------LTTL
LEWDEKNG--LIE-DIFAR
-TYEFSVSS-----------------------------LGT-IVTRE-MRAG--------
GNTIWPNAASSSTDI---
-----------------EPPHMENADDAPLVTNDNRD-DYIVD-YIRYLTDVS-IRRQYL
-AFERG---FASCLDK--KSLS-LLCPS-TLQSLVEG----------------------
----VQEID-----------IGEL----KRYARYVG-WDTS-------------------
------------------------HRTI-KDFWSIVK--------------------RYD
EGMKQRLLEF-------VTSSDRVP------------VGG-----MK-NLQFVI--QKNG
EEDGTG----------------------------GHLPTAYTCY-GT-LLLPEY-RDKEV
LRERLG-MALQN-AQGF
>Fusarium_graminearum_HG970334.1 .
LEVGRENV-ARDAFDQL--------------WRRERRELLR-PLKVHLGENSG----EEG
FDSGGVQQEF---FRLA----IAECLDPGF--------GAFTVDERTR------------
----------------MAWFAPGS------------------------------------
-----LTEDWKYELVGLLMSLALYNGLTLPVTFPR--ALYRKLLG---------------
-------NPVEELHHIAD----------------------GWPDLASG--------LTTL

LEWDEKNG---LIE-DIFAR
-TYEFSVSS-----------------------------LGT-IVTRE-MRAG--------
GSTIWPNAASSSTDI---
-----------------EPPHMENADDAPLVTNDNRD-DYIVD-YIRYLTDVS-IRRQYL
-AFERG---FASCLDK--KSLS-LLCPS-TLQSLVEG-----------------------
----VQEID-----------IGEL----KRYARYVG-WDTS-------------------
------------------------HRTI-KDFWSIVK--------------------RYD
EGMKQRLLEF-------VTSSDRVP------------VGG-----MK-NLQFVI--QKNG
EEDGTG----------------------------GHLPTAYTCY-GT-LLLPEY-RDKEV
LRERLG-MALQN-AQGF
>Fusarium_pseudograminearum_XM_009265452.1 .
LEVGRENV-ARDAFDQL--------------WRRERRELLR-PLKVHLGENSG----EEG
FDSGGVQQEF---FRLA----IAECLDPGF--------GVFTVDERTR------------
----------------MAWFAPGS------------------------------------
-----LTEDWKYELVGLLMSLALYNGLTLPVTFPR--ALYRKLLG---------------
-------NPVEELHHIAD----------------------GWPDLASG--------LTTL
LEWDEKNG---LIE-DIFAR
-TYEFSVSS-----------------------------LGT-IVTRE-MRAG--------
GSTVWPHAASSSTDI---
-----------------EPPHMENADDAPLVTNDNRD-DYIID-YIRYLTDVS-IRRQYL
-AFERG---FASCLDK--KSLS-LLCPS-TLQSLVEG-----------------------
----VQEID-----------IGEL----KRYARYVG-WDTS-------------------
------------------------HRTI-KDFWSIVK--------------------RYD
EGMKQRLLEF-------VTSSDRVP------------VGG-----MK-NLQFVI--QKNG
EEDGTG----------------------------GHLPTAYTCY-GT-LLLPEY-RDKEV
LRERLG-MALQN-AQGF
>Fusarium_fujikuroi_HF679024.1 .
LEIGRSNV-ARDAFDQL--------------WRREKRELLH-PLKVHLGENSG----EEG
FDSGGVQQEF---FRLA----IAECLDPQY--------GAFTVDERTR------------
----------------MAWFTPGS------------------------------------
-----LTEDWKYELVGLLMSLALYNGLTLPITFPR--ALYRKLLG---------------
-------KPVEELHHIAD----------------------GWPDLASG--------LTTL
LEWDEKDG---LVE-DIFAR
-TYEFSVES-----------------------------LGT-VVTRD-MRMD--------
EQADWPKAASSSCDI---
-----------------APPHMDNPDDAPLVTNENRD-DYIID-YIRHLTDVS-IRRQYL
-AFEQG---FNSCLDR--KSLS-LLSPS-TLQSLVEG-----------------------
----VQEID-----------ISEL----KRYARYVG-WDAS-------------------
------------------------HRTI-KDFWSIVK--------------------RYD
ERMKQRLLEF-------VTSSDRVP------------VGG-----MK-NLQFVI--QKNG
EEDGAG----------------------------GHLPTAYTCY-GT-LLLPEY-KDKEV
LRERLG-MALEN-AQGF
>Fusarium_oxysporum_XM_018386967.1 .
LEIGRSNV-ARDAFDQL--------------WRREKRELLR-PLKVHLGENSG----EEG
FDSGGVQQEF---FRLA----IAECLDPQY--------GAFTVDERTR------------
----------------MAWFTPGS------------------------------------
-----LTEDWKYELVGLLMSLALYNGLTLPITFPK--ALYRKLLG---------------
-------KPVEELHHIAD----------------------GWPDLASG--------LTTL
LEWDEKDG---LVE-DIFAR
-TYEFSVES-----------------------------LGT-VVTRD-MRMG--------
EQASWPKAASSSCDI---
-----------------APPHMNNPDDAPLVTNENRD-DYIID-YIRHLTDVS-IRRQYL
-AFEQG---FNSCLDK--KSLS-LLSPS-TLQSLVEG-----------------------
----VQEID-----------IIEL----KRYARYVG-WDAS-------------------
------------------------HRTI-KDFWSIVK--------------------RYD
ERMKQRLLEF-------VTSSDRVP------------VGG-----MK-NLQFVI--QKNG
EEDGTG----------------------------GHLPTAYTCY-GT-LLLPEY-RDKEV
LRERLG-MALEN-AQGF

>Fusarium_verticillioides_XM_018894887.1 .
LEIGRSNV-ARDAFDQL--------------WRREKRELLR-PLKVHLGENSG----EEG
FDSGGVQQEF---FRLA----IAECLDPQY--------GAFTIDERTR------------
----------------MAWFTPGS------------------------------------
-----LTEDWKYELVGLLMSLALYNGLTLPITFPK--ALYRKLLG---------------
-------KPVEELHHIAD----------------------GWPDLASG--------LTTL
LEWDEKDG---LVE-DIFAR
-TYEFSVES-----------------------------LGT-IVTRD-MRMS--------
EQAGWPKAASSSCDI---
-----------------APPHMDNPDDAPLVTNENRD-DYIID-YIRHLTDVS-IRRQYL
-AFEQG---FNSCLDR--KSLS-LLSPS-TLQSLVEG-----------------------
----VQEID-----------IVEL----KRYARYVG-WDAS-------------------
------------------------HRTI-KDFWSIVK--------------------RYD
ERMKQRLLEF-------VTSSDRVP------------VGG-----MK-NLQFVI--QKNG
EEDGAG----------------------------GHLPTAYTCY-GT-LLLPEY-RDKEV
LRERLG-MALEN-AQGF
>Isaria_fumosorosea_XM_018847067.1 .
LDIGRATV-LRDAFDQL--------------WRRQRRELLR-PLKIHLGEDTG----EQG
FDSGGVQQEF---FRLA----IAECLDPVY--------GAFTVDDRSH------------
----------------MAWFKPQS------------------------------------
-----TVEDWKFELMGVLLSLAVYNGVTLPVNFPT--AFYRRLLK---------------
-------EPVDEIKHIED----------------------GWPALASG--------LTQL
LEWDESKG---LIM-DIFAR
-TYEFTMPG-----------------------------IGK-ELTIH-MLDD--------
KQKEEPGSPISIDAD---
------------------------VPEAEYVTVENRD-DYVKD-YISYLTDVS-VRPQLE
-SFRRG---FLACLSE--KALT-LLTPE-LLQSIVEG-----------------------
----ERNID-----------IAGL----RRCTRYVG-WDAS-------------------
------------------------HQTI-RDFWSIVK--------------------RYD
NQMKRKLLQF-------VTASDRVP------------VSG-----IE-NIQFTI--QKNG
ESAKD-----------------------------GRLPTAYTCY-GN-LLLPEF-QDKEV
LRERLA-MALEN-AQGF
>Cordyceps_militaris_XM_006668058.1 .
LDIGRTTV-LRDAFDQL--------------WRRQKRELLR-PLKIHLGEDAG----EEG
FDSGGVQQEF---FRLA----VAQCLDPVY--------GAFTVDERNH------------
----------------MAWFKPQP------------------------------------
-----AVEDWKFELMGLLLSLAVYNGLTLPVNFPK--AFYRKLLK---------------
-------QPVDQLYHIAD----------------------GWPELTNG--------LTKL
LEWDEKDG---LIE-DIFAR
-TYEFSVPG-----------------------------VGK-DVTIH-MLED--------
KHKEQPGSPVSADTD---
------------------------VPEAPYVTNENRD-EYVND-YIRYLTDYS-VRRQFD
-SFRRG---FVACLPE--QSLT-LLTPS-ILQSVVEG-----------------------
----VQEID-----------ISEL----RRYARYVG-WDTS-------------------
------------------------HHTV-RDFWSIVK--------------------RYD
DRMKRKLLEF-------VTASDRVP------------VSG-----LK-NVQFVI--QKNG
EENEG-----------------------------GHLPTAYTCY-GT-LLLPEY-RDKEV
LRERLG-MALEN-AQGF
>Beauveria_bassiana_XM_008600959.1 .
LDIGRTSV-LRDAFDQL--------------WRRQKRELLR-PLKIHLGEDAG----EEG
FDSGGVQQEF---FRLA----IAECLDPVY--------GAFTVDDRNH------------
----------------MAWFKPHS------------------------------------
-----AVEDWKFELMGLLLSLAVYNGLTLPVNFPK--AFYRKLLK---------------
-------QPVDQLYHIAD----------------------GWPELTSG--------LTTL
LEWDEKDG---LIE-DIFAR
-TYEFSVPG-----------------------------IGK-DLTIH-MLED--------
KHKEQPGSPISADAD---
------------------------MSEAPYVTNENRD-DFVAD-YIRYLTDYS-VHRQFD

-CFRRG---FVACLPE--KSLT-LLTPS-ILQSVVEG-----------------------
----VQEID-----------ISEL----RRYTRYVG-WDPS-------------------
------------------------HHTI-RDFWSIVK--------------------RYD
DRMKRKLLEF-------VTASDRVP------------VSG-----LK-NVQFII--QKNG
EEGEG-----------------------------GHLPTAYTCY-GT-LLLPEY-RDKEV
LRERLA-MALEN-AQGF
>Grosmannia_clavigera_ACYC01000528.1 .
LDIGRTSV-LQDAFDQL--------------WRREERELLR-PLKIHLGEEGG----EEG
LDSGGVQQEF---FRMA----IAEALDPAY--------GAFTVDSRTR------------
----------------MAWFSPTT------------------------------------
-----LEEEWKFELVGILVSLALYNGVSLPVTFPK--ALYRKLLG---------------
-------EPVTELHHISD----------------------GWPDLANG--------LTAL
LEWNEQDG---AVE-DIFVR
-TYEFSVDM-----------------------------FGQ-PISRV-MDAS--------
GSWPKIRENVTGTRA---R
SKKNKEASDSGYGPTASTLLEANPGDEAPMVTNANRN-AYVTD-YIHYLTDVS-VRRQFE
-GFSRG---FRACLHA--KSLR-LLTPA-LLQSMVEG-----------------------
----EQEID-----------VSEL----RRQARYVG-WDAS-------------------
------------------------HRTV-RDFWSVVS--------------------RYD
DVMKRKLLEF-------VTASDRVP------------VGG-----IK-NLHFVI--QRNG
EESGTG----------------------------GHLPTAYTCY-GI-LLLPVY-RDRAV
LQERLA-MALEN-AQGF
>Scedosporium_apiospermum_XM_016790789.1 .
LNVSRKTP-VRDAFDQL--------------WQREERELLR-PLKVHLGEDNG----EEG
FDLGGVQQEF---FKLA----FAEIFDPRY--------GAFQVDDRTR------------
----------------MAWFAPGS------------------------------------
-----LVEPWKFELIGVLFSLAIYNGLTLPVTLPK--ALYRKLLG---------------
-------EPVNELHHIED----------------------GWPDLANG--------LTTL
LEWDEKDG---SVE-DVFAR
-TYEFSVQV-----------------------------FDM-PISRN-MQGP--------
PMSWPRAAVPAESDT---
-----------------PAAFEENPEDAPMVTYDNRN-AYVSD-YIEYLTHVS-VLPQYT
-AFERG---FKACLNP--DSLN-ILNPT-ILQSIVEG-----------------------
----TQEID-----------LAEL----RRYTRYNG-WDAS-------------------
------------------------HQTI-RDFWSIVK--------------------RYD
EPMKRRLLEF-------VTASDRVP------------VGG-----MQ-NIQFVI--QRNG
EEGPL-----------------------------GRLPTAYTCY-GT-LLLPEY-QDKEV
LRERLG-MALEN-AQGF
>Corollospora_maritima_GDFX01004992.1 .
LSIRRKSV-VQDAFDQL--------------WYREERELLR-PLKVHLGEDSG----EEG
FDLGGVQLEF---FKLA----FAEIFDASY--------GAFSIDGRTR------------
----------------MAWFVPGS------------------------------------
-----VVELWKFELIGLLFSLAVYNGLTLPITLPL--ALYRKLLG---------------
-------QPVTELHHIAD----------------------GWPEYANG--------LTQL
LEWDERDG---AVE-DIFAL
-EYQFTTNA-----------------------------FGQ-VIARD-MTKA--------
KDEPWPSAAAESGPA---
-------------------PSEGNPEEAPMVTNENRS-AYVSD-FIEYLTDVS-VRPQFE
-AFQRG---FRTCLLA--RSLD-LLTPP-IIQSIVEG-----------------------
----SQEID-----------VNEL----RRYTRYNG-WDAS-------------------
------------------------HRTI-RDFWSVVK--------------------RYD
EGMKRKLLEF-------VTASDRVP------------VGG-----MK-NVAFVI--QKNG
EEGPL-----------------------------GRLPTAYTCY-GT-LLLPEY-SDREV
LKERLA-MALEN-ARGF
>Eutypa_lata_XM_007801455.1 .
LSIGRETV-LEDAFDQL--------------WRREEREIMR-PLKVHLGEDSG----EEG
FDSGGVQQEF---FRLA----IAEALNPDY--------GAFTVDDRTR------------
----------------MTWFQPGS------------------------------------

-----LQPEWKFELLGLLMSLAVYNGLTLPITFPK--AFYQKLLG---------------
-------EPVTELHHIAD----------------------GWPDLANG--------LTTL
LEWNERDG--SVE-DIFSL
-TYEFSASM------------------------------FGQ-PVSRK-MQPA--------
EPEWDNGNSNDSSDN---
-GDEHHWPQLAGRLSAEPSPSSSNPTDAPAVTSYNRD-AYVSD-YIRYLTTVS-VAPQFA
-ALSRG---FRRCLHP--KSLT-LLTPP-LLQSLVEG-----------------------
--ETAEDID-----------VSEL----RRAARYVG-WDAG-------------------
-------------------------HRTV-RDFWAVVR--------------------RYD
PHMRRRLLEF-------VTASDRVP------------VGG-----VG-NIQFVI--QRNG
EELGDK-----------------------------GHLPTAYTCY-GT-LLLPEY-RDRDV
LKERLS-MALEN-ARGF
>Pestalotiopsis_fici_XM_007838729.1 .
LDIGRKTI-LRDAFDQL--------------WRRQERELLR-PLKVHLGEDAG----EEG
FDSGGVQQEF---FRLA----LAEALNPDY--------GGFTVDDRTR------------
-----------------MTWFQPGS------------------------------------
-----LQPEWKFELIGLLVSIAVFNGLTLPITFPK--ALYMKILG---------------
-------EPVTELHHISD----------------------GWPDLANG--------LTSL
LEWDEADG--TVD-DIFAR
-TYEFSVNM------------------------------LEQ-PVSRE-MDSS--------
KSITWPQFSGASVAT---
--------------------LASENPEDAPLVTADNRE-AYVSD-YIRYLTDVS-VAPQYE
-AFARG---FRTCLLP--KSLQ-LLTPQ-LLQSIVEG-----------------------
----VQEID-----------INEL----RRAARYEG-WDAT-------------------
-------------------------HRTI-RDFWSIVK--------------------RYD
ENKKRKLLEF-------VTASDRVP------------VGG-----MR-NIKFVI--QKNG
AAEGKH-----------------------------GRLPSAYTCY-GI-LLLPEY-QDKET
LRERLG-FALEN-TQGF
>Arthrinium_malaysianum_GEGW01006810.1 .
LDIGRHTV-IRDAFDQL--------------WRREERELMR-PLKVHLGEDAG----EEG
FDSGGVQQEF---FRLA----IAEALNPDF--------GAFAIDDRTR------------
-----------------MTWFQPGS------------------------------------
-----LQPDWKFELIGLLISLALHNGLTLPVTFPK--ALYKRLLG---------------
-------EPVTELHHIAD----------------------GWPDLANG--------LTTL
LEWDEKDG--AVE-DVFAR
-TYEFSISM------------------------------FGQ-PVSRE-MDSS--------
STAEWPQFSNSLPLS---
----------------------SENPEDAPLVTGDNRE-AYVSD-YIRYLTDVS-VAPQFA
-AFARG---FRTCPHP--KSLR-LLNAP-LLQSLVEG-----------------------
----QQDID-----------IAEL----KRAARYVG-WDAS-------------------
-------------------------HRTV-RDFWSVVR--------------------RYD
DGMRRKLLEF-------VTASDRVP------------VGG-----MR-NIQFVV--QRNG
EEEGDG-----------------------------GHLPTAYTCY-GT-LLLPEY-RDKEA
LAERLR-MALEN-AQGF
>Daldinia_eschscholzii_AIID01011141.1 .
LDISRKNV-LRDAFDQL--------------WRRQERELLR-PLKVHLGEDSG----EEG
FDSGGVQQEF---FRLA----VAEALNPTY--------GAFVIDDRTR------------
-----------------MTWFQPGS------------------------------------
-----VQPDWKFELIGLLISLAVYNGLTLPITFPK--ALYRKLLG---------------
-------EPVTELHHIAD----------------------GWPDLANG--------LTNV
LEWNEKDG--SVE-DVFVL
-TYEFSTSM------------------------------FGQ-PVSRE-MQSS--------
NSISWPQFSSHPDST---
-------------------PLSASNPDSAPLVTGDNRN-AYVSD-YIRYLTDVS-VAPQFD
-AFARG---FRACLHP--KSLQ-LLTPS-LLQNLVEG-----------------------
----TQEID-----------VGEL----RRAARYVG-WDVS-------------------
-------------------------HRTV-RDFWSIVK--------------------RYD
DRMRRKLLEF-------VTASDRVP------------VGG-----MA-NIQFVV--QKNG

EETGEN----------------------------GHLPTAYTCY-GT-LLLPEY-RDKEV
LRERLT-MALEN-AQGF
>Chrysoporthe_austroafricana_JYIP01000752. 1..
LDISRKTV-LRDAFDQL--------------WRREERELLR-PLKVHLGEDGG----EEG
FDSGGVQQEF---FRLA----IAEALDPAF--------GAFTVDERNR------------
----------------MTWFQPGS------------------------------------
-----LEPDWKFELVGLLIGLAVYNGLTLPVSFPK--ALYRKLLG---------------
-------EPVTDIHHIND----------------------GWPQIASS--------LTEL
LEWDESNG---AVE-DIFAV
-TYEFSTNM-----------------------------FNE-HVSRE-MRSITP------
GAQPDPEEWPQFPSR---
------------AGDGPSSLSADNPADAPYVTNANRN-AYVSD-YIRYMTDVS-VRPQYD
-AFAKG---FRSILHP--KSLT-LLTPE-LLQNLVEG-----------------------
----AQEID-----------ISEL----RRAARYVG-YTQS-------------------
------------------------SRAI-KDFWAIVK--------------------RYD
DRMKRKLLEF-------VTASDRVP------------VGG-----VK-NIQFVV--QKNG
EEDGEGG----------------------------HLPTAYTCY-GT-LLLPEY-RDREV
LRERLG-MALEN-AQGF
>Chrysoporthe_cubensis_LJCY01000183.1 .
LDISRKTV-LRDAFDQL--------------WRREERELLR-PLKVHLGEDGG----EEG
FDSGGVQQEF---FRLA----IAEALDPAF--------GAFTVDERNR------------
----------------MTWFQPGS------------------------------------
-----LEPDWKFELVGLLIGLAVYNGLTLPVSFPK--ALYRKLLG---------------
-------EPVTDIHHIND----------------------GWPQIASS--------LTEL
LEWDESNG---AVE-DIFAV
-TYEFSTNM-----------------------------FNE-HVSRE-MRSITP------
GAQPDPEEWPQFPSR---
------------AGDGPSSLSADNPADAPYVTNANRN-AYVSD-YIRYMTDVS-VRPQYD
-AFAKG---FRSILHP--KSLT-LLTPE-LLQNLVEG-----------------------
----AQEID-----------ISEL----RRAARYVG-YTQS-------------------
------------------------SRAI-KDFWAIVK--------------------RYD
DRMKRKLLEF-------VTASDRVP------------VGG-----VK-NIQFVV--QKNG
EEDGEGG----------------------------HLPTAYTCY-GT-LLLPEY-RDREV
LRERLG-MALEN-TQGF
>Magnaporthe_oryzae_XM_003709264.1 .
LSISRERV-LEDTFDQL--------------WRREERELLR-PLKVRLGEGNG----EEG
FDSGGVQQEF---FRMV----MAEALNPDY--------GLFTVDERTK------------
----------------MTWFHPGS------------------------------------
-----PEPDWKFELIGLLFSLAVYNGLTLPVTFPE--ALYCKLLG---------------
-------EPVTDLHHISD----------------------GWPELASG--------LTTL
LDWDESAG---SVE-DVFSL
-TYEFSVSM-----------------------------FGQ-PVSRQ-MESD--------
QDAQWPCFPSKYAFG---
--GTALSSSWSSGTAPAPPLHEGNPSDAPAVTRDNRN-AYVAD-YVRYLTDVS-VRPQFE
-AFARG---FRTCLGT--KPLT-LLAPR-LLRSIVEG-----------------------
----VQDID-----------VAEL----RRHARYVG-WDAS-------------------
------------------------HRTV-RDFWSVVR--------------------KFD
DSMRRKLLEF-------VTASDRVP------------VGG-----LK-NLQFVI--QRNG
EED-EG----------------------------GHLPTAYTCY-GI-LLLPQY-RDKEV
LRERLC-MALGN-AQGF
>Gaeumannomyces_graminis_ADBI01000011.1 .
LSIRRDSV-LMDTFDQL--------------WRREERELMR-PLKVKLGEETG----EEG
FDSGGVQQEF---FRMA----MAEALDPDF--------GLFTVDERTR------------
----------------MTWFNPAS------------------------------------
-----PEPDFKFELIGLLFSLAVYNGLTLPVTFPE--ALYCKLLG---------------
-------EPVTDLHHISD----------------------GWPDLASG--------LTTL
LDWDEKDG---AVE-DVFSR
-TYEFSISV-----------------------------FGE-PVSRE-MKSG--------

GEARWPSFPSKHTFG---
---------GVSSASASVPLQDGNPQDAPLVTRENRN-AYVAD-YIRYLTDVS-VRPQFE
-AFARG---FRTCLGA--KALT-LLTPR-LLRSVVEG-----------------------
----VQEIE-----------VSEL----RRHARYVG-WDAS-------------------
------------------------HRTV-RDFWSIVK--------------------KFD
DGMRRKLLEF-------VTASDRVP------------VGG-----MK-NLQFVV--QRNG
EEEGDG----------------------------GHLPTAYTCY-GT-LLLPQY-RDKEV
LRERLC-MALEN-AQGF
>Neurospora_crassa_XM_952546.1 .
LEIRRNRV-IRDAFDQL--------------WRREAREIMR-PLKIHLGERAG----EEG
FDSGGVQQEF---FRLA----MAEALNPDY--------GAFTVDERTK------------
----------------MTWFQPGS------------------------------------
-----VVEEWKFELIGLLMSLAVYNGLTLPVTFPK--ALYAKLLG---------------
-------EPVTDLDHIAD----------------------GWPDLVSG--------LTML
LDWDEKEMGG---SVE-DVFAR
-TYEFSVES-----------------------------FGE-QVTKL-MTPP--------
PPPISSRSGKSAAET--------------------------------------DAFSREE
PWPQFSPAFRRLSDASSSASALPLEEEAPLVTAANRE-QFVSD-YIRYLTDVS-VRPQFE
-AFARG---FRTCLHP--KSLS-LLTPS-LLQSVVEG-----------------------
----VQEID-----------IAEL----QRYTRYVD-WDAS-------------------
------------------------HRTV-RDFWSIVK--------------------KYD
DKMKRRLLEF-------VTASDRLP------------VGG-----VK-NLVFTL--QRNG
KX---------------------------------RLPTAYTCY-GI-LLLPEY-KDKEM
LRERLA-MALEN-AQGF
>Neurospora_tetrasperma_AFCY01000172.1 .
LEIRRNRV-IRDAFDQL--------------WRREEREIMR-PLKIHLGERAG----EEG
FDSGGVQQEF---FRLA----MAEALNPDY--------GAFTVDERTK------------
----------------MTWFQPGS------------------------------------
-----VVEEWKFELIGLLMSLAVYNGLTLPVTFPK--ALYAKLLG---------------
-------EPVTDLDHIAD----------------------GWPDLVSG--------LTML
LDWDEKEMGG---SVE-DVFAR
-TYEFSVES-----------------------------FGE-QVTKL-MTPP--------
PPPISSRSGKSAAET--------------------------------------DAFSREE
PWPQFSPAFRRLSDASSSASALPLEEEAPLVTAANRE-QFVSD-YIRYLTDVS-VRPQFE
-AFARG---FRTCLHP--KSLS-LLTPS-LLQSLVEG-----------------------
----VQEID-----------IAEL----QRYTRYVD-WDAS-------------------
------------------------HRTV-RDFWSIVK--------------------KYD
DKMKRRLLEF-------VTASDRLP------------VGG-----VK-NLVFTL--QRNG
KX---------------------------------RLPTAYTCY-GI-LLLPEY-KDKEM
LRERLA-MALEN-AQGF
>Sordaria_macrospora_XM_003350146.1 .
LEVRRNRV-IRDAFDQL--------------WRREERELMR-PLKIHLGEHAG----EEG
FDSGGVQQEF---FRLA----MAEALNPDY--------GAFTVDERTK------------
----------------MTWFQPGS------------------------------------
-----AVEEWKFELIGLLMSLAIYNGLTLPVTFPK--ALYAKLLG---------------
-------EPVTDLDHIAD----------------------GWPDLVSG--------LTML
LDWDEKEMGG---SVE-DVFSR
-TYEFSVES-----------------------------FGE-QVTKL-MTPP--------
PPPICSRGGKLAAET--------------------------------------DAFSREE
PWPQFSPAFRRLSDASSTASTVRSEEEAPLVTAANRE-QFVSD-YIRYLTDVS-IRPQFE
-AFARG---FCTCLHP--KSLS-LLTPS-LLQSLVEG-----------------------
----VQEID-----------INEL----QRYTRYVD-WDAS-------------------
------------------------HRTV-RDFWSIVK--------------------KYD
DKMKRRLLEF-------VTASDRLP------------VGG-----VK-NLVFTL--QRNG
KX----------------------------------LPTAYTCY-GI-LLLPEY-RDKEM
LRERLA-MALEN-AQGF
>Chaetomium_thermophilum_XM_006694752.1 .
LQIRRNHV-IEDAFDQL--------------WRREERELLR-PLKVHLGEADG----EEG

```
FDSGGVQQEF---FRLA----IAEALNPDY--------GAFTVDERTR------------
----------------MTWFVPGS------------------------------------
-----MVDEWKFELVGLLVSLAVYNGLTLPVTFPR--ALYRKLLG---------------
-------EPVTELHHIAD----------------------GWPDLASG--------LTAL
LEWDEKDG-------------------------------------------LVE-DVFAR
-TYEFSVSV-----------------------------MGQ-HITRE-MKPP--------
GRDDEWPQFPKTAHQ---------------------------------------------
-----------SSSRPAPHEGNPPEDSTPLVTAANRN-AYVSD-YIRYLTDVS-VRPQFE
-AFARG---FRTCLHP--KSLS-LLTPS-LLQSLVEG-----------------------
----VQEID-----------ISEL----RRYARYVG-WDAS-------------------
------------------------HRTV-RDFWSIVK--------------------KYD
NNMKRKLLEF-------VTASDRLP------------VGG-----IK-NLMFVV--QRNG
EENDPA----------------------------GRLPTSYTCY-GT-LLLPEY-KDKEM
LRERLA-MALEN-AQGF
>Thielavia_terrestris_CP003009.1 .
LDISRDAV-LQDAFDQL--------------WRREERELLR-PLKVRLGESSG----EEG
FDSGGVQQEF---FRMA----IAEALNPDY--------GAFTVDERTR------------
----------------MAWFLPGS------------------------------------
-----LEDEWKFELIGLLVSLAVYNGLTLPVTFPK--ALYRKLLG---------------
-------EPVTELHHIAD----------------------GWPDLASG--------LTSL
LEWDEKDG-------------------------------------------LVE-DVFAR
-TYEFSVST-----------------------------FGQ-VVTRE-MKPL--------
SDDHHRRRSHQQDEA---------------------------------------------
----WPQFSKTRSGSPAPPRGNPDGDDAPLVTSANRN-AYVSD-YIRYLTDVS-VRPQYE
-AFARG---FHTCLHP--KSLT-LLTPS-LLQSVVEG-----------------------
----VQEID-----------IAEL----KRYVRYIG-WDAS-------------------
------------------------HRTV-RDFWSIVK--------------------RYD
DNMKRKLLEF-------VTASDRLP------------VGG-----PK-NLMFIV--QRNG
EEDDPA----------------------------GRLPTSYTCY-GT-LLLPEY-KDKEV
LRERLA-MALEN-AQGF
>Chaetomium_globosum_XM_001227135.1 .
LDIRRGTV-LQDALDQL--------------WRREERELLK-PLKVHLGEATG----EEG
FDSGGVQQEF---FRLA----IGEALNPDH--------GAFTVDERTR------------
----------------MTWFLPGS------------------------------------
-----MEDEWKFELIGLLVSLAVYNGLTLPVTFPK--AFYRKLLG---------------
-------EPVTELHHIAD----------------------GWPDLASG--------LTSL
LEWDEKDG-------------------------------------------AVE-DVFAR
-TYEFSVSA-----------------------------FGQ-HITRE-MKTP--------
SNNTHHHHHARKDEP---------------------------------------------
-WPQFAKTASQSSSRPPPHHGNPAEDEAPLVTSANRN-AYVSD-YIRYLTDVS-VRPQYE
-AFARG---FHACLHP--KSLS-LLTPS-LLQSVVEG-----------------------
----VQEID-----------IAEL----KRHARYVG-WDAS-------------------
------------------------HRTV-KDFWSVVK--------------------RYD
DAMKRKLLEF-------VTASDRVP------------VGG-----VK-NLMFII--QRNG
EEEDPA----------------------------GRLPTSYTCY-GT-LLLPEY-RDKEM
LRQRLG-MALEN-AQGF
>Myceliophthora_thermophila_CP003005.1 .
LDIRRSTV-LEDAFDQL--------------WRREERELLR-PLKVHLGESTG----EEG
FDSGGVQQEF---FRLA----IAQALNPDY--------GAFTVDERTR------------
----------------MAWFLPGS------------------------------------
-----MEDEWKFELIGLLVSLAVYNGLTLPVTFPK--ALYRKLLG---------------
-------EPVTELHHIAD----------------------GWPDLASG--------LTSL
LEWDEKDG-------------------------------------------AVE-DVFAR
-TYEFSVSA-----------------------------FGQ-HITRE-MKPL--------
SDSLHQHGRDSEDEA---------------------------------------------
-WPQFAKTLSQSSSQPAPHQGNPAEDEAPLVTGANRN-AYVSD-YIRYLTDVS-VRPQYE
-AFARG---FHTCLHP--KSLS-LLTPS-LLQSVVEG-----------------------
----VQEID-----------IAEL----KRYARYVG-WDAS-------------------
```

------------------------HRTV-KDFWSVVK--------------------RYD
ENMKRKLLEF-------VTASDRVP------------VGG-----VK-NLMFII--QRNG
EEEDPA----------------------------GRLPTSYTCY-GT-LLLPEY-KDKEM
LRERLG-MALEN-AQGF
>Podospora_anserina_CU633895.1 .
LDIRREAV-IEDAFNQL--------------WRREERELLR-PLKVRLGESTG----EEG
FDLGGVQQEF---FRLA----LAEALNPDY--------GAFTVDERSR------------
----------------MAWFVPGS------------------------------------
-----LEDEWKFELIGLLVSLAVYNGLTLPVTFPK--ALYRKLLG---------------
-------KPVDKLRHIAD----------------------GWPDIASS--------LTEV
EQWDDNGSKG---KLE-DLCI-
-TYDFSTSA-----------------------------FGH-HVIRE-MRPS--------
ASGGDEEGGDWDTHT--PSE
EEWPQFSKTASHASCNPAPHEYNPEEESKPVTIHNRD-EYIAD-YIRYLTTVS-VRPQFE
-AFARG---FRTCLQP--KSLS-LLTPS-LLQSLVEG-----------------------
----VQEID-----------ISEL----KRYAKYTG-YDEH-------------------
------------------------HRTI-KDFWSIVK--------------------RYD
EDMKRKLLEF-------VTASDRVP------------VGG-----MK-YVVFNI--QKNG
VENDELGPGGTGNR--------------------GRLPTSYTCY-ST-LLLPEY-KDKET
LRERLG-MALEN-ARGF
>Pseudogymnoascus_verrucosus_XM_018274453.1 .
LQIRRSHV-LVDAFDKL--------------WRREERELLR-PLKIKLGEEAG----EEG
SDSGGVQQEF---FRLA----IAEALNPDY--------GAFTIDERTK------------
----------------MIWFQASS------------------------------------
-----PEPLWKFELIGLLVGLAIYNGLTLPVTFPK--VLYRKLLN---------------
-------QPVTELHHIED----------------------GWPELAAG--------LTSL
LEWDETNG---LVS-DVFCR
-TYEFSANV-----------------------------FGA-PVSID-MSAP--------
NPSPWPQFASLSHTK---
----------------ISSELASEAAEAPMVDASNRN-DYVSD-YISHLATHS-VAPQFG
-AFQRG---FLTCLEP--RSLA-LFTPS-LLQSTVEG-----------------------
----VQEID-----------IAEL----RRYARYVG-WDAD-------------------
------------------------HRAI-KDFWSVVR--------------------RFG
TEEKKRLLEF-------VTASDRVP------------VGG-----MR-NLVFVV--QRNG
EGEEGDVAEE-EGEGTEGHVGR------------GRLPTSYTCY-GT-LLLPEY-KDRET
LRRKLS-MALEN-AKGF
>Pseudogymnoascus_destructans_XM_012884926.1 .
LQIRRSHV-LVDAFDKL--------------WRREERELLR-PLKIKLGEEAG----EEG
SDSGGVQQEF---FRLA----IAEALNPDY--------GAFTIDERTK------------
----------------MIWFQASS------------------------------------
-----PEPLWKFELIGLLVGLAIYNGLTLPVTFPK--VLYRKLLN---------------
-------QPVTELHHIED----------------------GWPELAAG--------LTSL
LEWDEKDG---LVS-DVFCR
-TYEFSANV-----------------------------FGA-PVSID-MSAP--------
SPSPWPQFASLSHTK---
----------------ISSELASEAAEAPMVDASNRN-DYVSD-YISHLATLS-VAPQFG
-AFQRG---FLTCLEP--RSLA-LFTPS-LLQSTVEG-----------------------
----VQDID-----------VAEL----RRYARYVG-WDAD-------------------
------------------------HRAI-KDFWSVVR--------------------RFG
TEEKKRLLEF-------VTASDRVP------------VGG-----MR-NLVFVV--QRNG
EGEEGDVAEEEEGEGTEGHVGR------------GRLPTSYTCY-GT-LLLPEY-KDRET
LRRKLS-MALEN-AKGF
>Sclerotinia_homoeocarpa_JW826167.1 .
LSIRRDHV-LLDTFNSI--------------WRREERELMR-PLKIRLGEEQG----EEG
FDLGGIQQEF---FRLA----IAEALNPDY--------GTFTIDPRTK------------
----------------MTWFQPGS------------------------------------
-----PEPLWKFELIGLIMSLAVYNGLTLPVTFPK--ALYRKLLG---------------
-------ESITELHHISD----------------------GWPDLANG--------LTSL

LEWDEKDG---SVE-DVFCR
-TYEFTVEQ-----------------------------FGQ-PISRD-MSCS--------
TQWPQFAETASHSNP---
-------------------------ADAPLVTHENRN-SYVSD-YIQWLTDIS-VRPQFD
-AFKRG---FFTCVDP--RSIT-LFDPD-TLQSLIEG-----------------------
----VQDID-----------INEM----RRNARYTG-WSAG-------------------
------------------------HRTV-KDFWAIVK--------------------KFD
LGQKRKLLEF-------VTASDRVP------------VGG-----MR-ALKFDL--QKNG
VDD-------------------------------DHLPSSYTCF-GV-LLLPEY-SSREV
LREKLE-MALEN-SK--
>Marssonina_brunnea_XM_007295961.1 .
LEIRRSHV-LLDSFNAL--------------WRREERELMR-PLKIRLGEEGG----EEG
LDSGGVQQEF---FRLA----IAEALDPDY--------GTFTIDSRTK------------
----------------MTWFQPGS------------------------------------
-----PEPLWKFELIGMVVSLAVYNGLTLPVTFPK--ALYRKLLD---------------
-------EEVTELHHIAD----------------------GWPELANG--------LTTL
LEWDEKDG---LVE-DIFAR
-TYEFSIQQ-----------------------------FGQ-SISRE-MGTA--------
STHWPQFADLQTSSN---
------------------------PTDAPSVTNQNRN-SFVSD-YIRWLTDIS-IQPQFD
-AFKAG---FYACLDR--RAIT-LFSPE-TLQSVVEG-----------------------
----VQEID-----------ISEM----RRAARYVG-WEGDAS-----------------
------------------------HRSV-RDFWSIVK--------------------RYD
LSQKRKLLEF-------VTASDRVP------------VGG-----MR-NLQFTL--QRNG
VDD-------------------------------GHLPSSYTCY-GI-LLLPEY-SSKEV
LREKLA-MALEN-SKGF
>Glarea_lozoyensis_XM_008088520.1 .
LEIGRQSV-LADTFNAI--------------WRREEREMMR-PLKIRLGENGG----EEG
MDSGGVQQEF---FRLA----IAEALDPDY--------GTFTIDSRTK------------
----------------MTWFQPGS------------------------------------
-----PEPLWKFELIGTIISLAVYNGLTLPVTFPK--ALYRKLLG---------------
-------EDVTELHHVAD----------------------GWPDLANG--------LTSL
LEWDEKNG---LVE-DIFAR
-TYEFSVEQ-----------------------------FGQ-AVSRE-MDET--------
VLWPQFSDLHTSANP---
-------------------------EDAPMVTNENRN-NYVSD-YIRWLTDIS-ISPQFE
-AFKTG---FFTCIDR--RSLS-VFDPE-TLQSVVEG-----------------------
----IQDID-----------ISEM----RRTTRYIG-WDSS-------------------
------------------------HRSV-RDFWSIVR--------------------KYD
LEHKRKLLEF-------VTASDRVP------------VGG-----MR-NLQFTL--QRNG
VDD-------------------------------GHLPSSYTCY-GI-LLLPEY-SSKDV
LREKLA-MALEN-SKGF
>Phialocephala_scopiformis_XM_018211025.1 .
LEIRRTNV-LVDTFNSI--------------WRREERELMR-PLKIRLGEEGG----EEG
LDSGGVQQEF---FRLA----IAEALDPDY--------GTFTIDARTK------------
----------------MTWFQPGS------------------------------------
-----PEPLWKFELIGMIISLAVYNGLTLPVTFPK--ALYRKLLG---------------
-------ESVDELHHIAD----------------------GWPDLTNG--------LTQL
LEWDEKEG---LVE-DVFAR
-TYEFSVEQ-----------------------------FGE-PVSRE-MGKT--------
DHWPQFSESSETAES---
-------------------------AEAPMVTHENRN-SYVSD-YIRWLTDIS-VQPQFD
-AFKRG---FFTCVDR--RSIT-LFDPD-TLQSVVEG-----------------------
----IQEID-----------ISEM----RRTTRYIG-WDAS-------------------
------------------------HHSV-RDFWSIVK--------------------RYD
LEQKRKLLEF-------VTASDRVP------------VGG-----MR-NLQFTL--QRNG
VDD-------------------------------GHLPSSYTCY-GI-LLLPEY-SSKEV
LREKLA-MALEN-SKGF

>Sclerotinia_sclerotiorum_XM_001585574.1 .
IVVSRENL-LSDAFNVL--------------WRREERELMR-PLKVSLGGEIG----EEG
VDMGGVQQEF---FRIA----MTEALNPDY--------GVFTIDGTTK------------
----------------MTWFQPGS------------------------------------
-----PEPLWKFELIGTIMSLAVYNGMTLPITFPK--AFYRKLQG---------------
-------ESITELHHISD----------------------GWPELAKG--------LTDL
LEWDENKG---AVE-DIFCR
-TYEFSQFQ-----------------------------FGK-PVSRE-MSTS--------
SQWPKYSDVNIGESN---
--------------------EDPGEVEAPLVTNENRN-NYVSD-YINWLTNIS-IQPQFE
-AFKKG---FFACLDP--RSIS-IFDSD-TLQSLVEG-----------------------
----VQEID-----------IVEM----QRGTQYIG-YEAG-------------------
------------------------DRVI-QDFWSVVK--------------------EYD
LEKKKKLLEF-------TTASDRVP------------MGG-----MR-NFQLRI--MKNG
ATD-------------------------------EQLPSSYTCF-QN-LLLPNY-SSRQI
LKERFD-IALEH-SKGF
>Botryotinia_fuckeliana_XM_001546987.1 .
IEVSRENL-LSDTFDVL--------------WRREGRELMR-PLKVSFGGEHG----EEG
LDYGGVQQEF---FRIA----MTEAINPDY--------GVFTIDNKTK------------
----------------MTWFQPGS------------------------------------
-----PEPLWKFELIGTIISLAVYNGMTLPITFPK--AFYRKLQG---------------
-------ESITELHHISD----------------------GWPELAKG--------LTDL
LEWDESKG---AVE-EIFCR
-TYEFSHFQ-----------------------------FGK-LVSRE-MVSS--------
SQWPKFSDLNVGEDF---
--------------------EDFDSTEVPLVTNENRN-SYVSD-YINWLTNVS-IQPQFE
-AFKKG---FFACLDP--RSLN-LFDAE-TLQSLVEG-----------------------
----VQEID-----------IIEM----QRGARYTG-YEPT-------------------
------------------------DRVI-QDFWSVVK--------------------EYD
LEKKKKLLEF-------TTSSDRVP------------MGG-----MK-NFQLTI--HRNG
AG--------------------------------NQLPTTSTCF-QI-LMLPNY-ASREI
LKERFD-VALEH-SSGF
>Tuber_melanosporum_XM_002837154.1 .
VEVRRETV-LQDALNQV--------------FGREIRELKR-PLKVRFA-NEG----EEG
VDHGGVQQDF---FIVA----IREALRSDY--------GLFTTDEQTR------------
----------------MNWFSVTP------------------------------------
-----IEPIHKYELLGLLVGLAVYNGVTLPITFPK--ILYKKLLG---------------
-------GKAEGLEDIED----------------------GWCQLAKG--------FKQL
LEWNDG---DVG-DVFLR
-TYDFSYX--

------------------------------VTNENRE-AFVRD-YISWLTDRS-IRRQYQ
-AFEKG---FFAVIDR--KSLS-LFTPS-NFQSLTEG-----------------------
----IQDID-----------ISEL----EKAARYEDGYSPT-------------------
------------------------HRVI-KDFWAIVR--------------------GFS
AERRRQLLEF-------VTASGRVP------------VNG-----IS-SIMFVI--QRNG
PDS-------------------------------DRVPTSLTCF-GR-LLLPEY-SRRAK
LRDKLK-LALEN-GKGF
>Cladophialophora_immunda_XM_016397642.1 .
LTIRRHDV-LNDAIDQI--------------WRRQRRELMR-PLRVRLGKDEG----EDG
LDHGGVQQEF---FRVV----FGEALRPEY--------SMFTIDSTTR------------
----------------MAWFQPGS------------------------------------
-----FEPLYRFEALGILMSMAIYNGITLPITMPL--AFYRKLLG---------------
-------LKVKKLDHIAD----------------------GWPELTKG--------LTAL
LDWSDG---DVG-EVIAR
-TYEFSYEL-----------------------------CGT-TATVD-MQKV--------
GRAAL---
------------------------------VTNANRE-QYVKD-YILWLVHKS-IEPQYE

-AFAKG---FYTCLDR--TALS-IFNPE-SLKALIEG----------------------
----YPEID-----------IDEL----QMTTTYDD-YTPD-------------------
------------------------SPTI-VDFWHIVR--------------------SMS
THQHRQLLEF-------VTASDRVP------------VNG-----MQ-SVTFIV--QKNG
EED-------------------------------DRLPSSSTCY-GR-LLLPQY-SSRAV
MEEKLT-KAIEN-CVGF
>Fonsecaea_multimorphosa_XM_016778618.1 .
LTIRRHDV-LNDAIDQI--------------WRRQRRELMR-PLRVRLGKDEG----EDG
LDHGGVQQEF---FRVV----FGEALRPEY--------SMFTVDSTTR------------
----------------MAWFQPGS------------------------------------
-----FEPLYRFEALGILMSMAIYNGITLPITMPL--AFYRKLLG---------------
-------LKVKKLDHIAD----------------------GWPELTKG--------LKAL
LEWSDG---DVG-EVIAR
-TYEFSYEL-----------------------------CGT-TVTVD-MQKV--------
GRHX--
------------------------------VTNANRE-QYVKD-YILWLTHKS-IEPQYE
-AFARG---FYTCLDR--TALS-IFNPE-SLKALIEG----------------------
----YPEID-----------IDEL----QMTATYDE-YTPD-------------------
------------------------SPTI-VDFWHIVR--------------------SMS
TMQHRQLLEF-------VTASDRVP------------VNG-----MQ-SVTFIV--QKNG
EED-------------------------------DRLPSSSTCY-GR-LLLPQY-SSRKV
MEEKLT-KAIEN-CVGF
>Fonsecaea_pedrosoi_XM_013427022.1 .
LTIRRNDI-LNDAIDQI--------------WRRQRRELMR-PLRVRLGKDEG----EDG
LDHGGVQQEF---FRAV----FGEALRPEY--------SMFTVDSTTR------------
----------------MAWFQPGS------------------------------------
-----FEPLYRFEALGILMSIAIYNGLTLPITMPL--AFYRKLLG---------------
-------LKVKKLDHIVD----------------------GWPELARG--------LKAL
LDWSDG---DVG-DVIAR
-TYEFSYEL-----------------------------CGT-TVDSD-MLEN--------
SGQEAAL---
------------------------------VTNANRE-QYVKD-YILWLTHKS-IEPQYE
-AFARG---FYTCLDR--TALS-IFNPE-SLKSLIEG----------------------
----YPDID-----------IDEL----QMTTTYDE-YTPD-------------------
------------------------HPTI-LDFWHIVR--------------------SMS
THQHRQLLEF-------VTASDRVP------------VNG-----MQ-SVTFIV--QKNG
EED-------------------------------ARLPSSSTCY-GR-LLLPQY-SSRAV
MEEKLT-KAIEN-CVGF
>Fonsecaea_erecta_XM_018838988.1 .
LTIRRHDI-LNDAIDQI--------------WQRQRRELMR-PLRVRLGKDEG----EDG
LDHGGVQQEF---FRIV----FGDALKPDY--------SMFTVDSTTR------------
----------------MTWFQPGS------------------------------------
-----FEPLYRFEALGILMSMAIYNGITLPLTMPL--AFYRKLLG---------------
-------LKVKKLDHIAD----------------------GWPDLTKG--------LKAL
LEWSDG---DVG-EVIAR
-TYEFSYEL-----------------------------CGV-SIDSD-MLEN--------
SGQEASL---
------------------------------VTNANRE-QYVKD-YILWLTHKS-IEPQYE
-AFARG---FYTCLDR--TALS-IFNPE-SLKALVEG----------------------
----YHEID-----------IDEL----QKTATYDD-YKPD-------------------
------------------------SATI-EDFWHIVR--------------------SMS
TQQHRQLLEF-------VTASDRVP------------VNG-----MQ-SVTFII--QKNG
EED-------------------------------ERLPSSSTCY-GR-LLLPKY-SSRTV
MKEKLT-KAIEN-CVGF
>Cladophialophora_psammophila_XM_007744445.1 .
LTIRRQDV-LNDAIDQI--------------WRRQRRELMC-PLRVRLGKDEG----EDG
LDHGGVQQEF---FRVV----FGEALRPEY--------SMFTVDDTTR------------
----------------MTWFQPGS------------------------------------

-----FEPLYRFEALGILMSMAIYNGITLPITMPL--AFYRKLLG---------------
-------LKVKKLEHIAD----------------------GWPELTKG--------LKSL
LDWSEG---DVG-EVIAR
-TYEFSYEL-----------------------------CGS-TVTVD-MQKV--------
GRHX--
------------------------------VTNANRE-QYVKD-YILWLTHKS-IEPQYE
-AFARG---FYTCLDR--TALS-IFNPE-SLKALIEG-----------------------
----YPDID-----------IDEL----QMTTTYDE-YTSE-------------------
------------------------SPTI-VDFWHVVR--------------------SMS
THQHRQLLEF-------VTASDRVP------------VNG-----MR-SLTFIV--QKNG
EED-------------------------------NRLPSSSTCY-GR-LLLPQY-SSRAV
MEEKLT-KAIEN-CVGF
>Cladophialophora_bantiana_XM_016760905.1 .
LTIRRQDV-LNDAIDQI--------------WRRQRRELMC-PLRVRLGKDEG----EDG
LDHGGVQQEF---FRVV----FGEALRPEY--------SMFTVDHTTR------------
----------------MTWFQPGS------------------------------------
-----FEPLYRFEALGILMSMAIYNGITLPITMPL--AFYRKLLG---------------
-------LKVKKLEHIAD----------------------GWPELTKG--------LKTL
LDWSDG---DVG-EVIAR
-TYEFSYEL-----------------------------CGS-TVDSD-MLEN--------
SGQEAAL---
------------------------------VTNANRE-QYVKD-YILWLTHKS-IEPQYE
-AFARG---FYTCLDR--TALS-IFNPE-SLKALIEG-----------------------
----CPDID-----------IDEL----QMTTTYDE-YTSD-------------------
------------------------SPTI-VDFWHVVR--------------------SMS
THQHRQLLEF-------VTASDRVP------------VNG-----MR-SLTFIV--QKNG
EED-------------------------------NRLPSSSTCY-GR-LLLPQY-SSRAV
LEEKLT-KAIEN-CVGF
>Exophiala_dermatitidis_XM_009160136.1 .
LTIRRDNV-LDDAINQI--------------WRRQRRELMR-PLRVRLGKDEG----EDG
LDHGGVQQEF---FRVV----FAEAFRPEY--------GMFTVDARTR------------
----------------MTWFQPGS------------------------------------
-----FEPLYRFEALGVLMSIAVYNGITLPITFPL--AFYRKLLG---------------
-------LKVKTLEHIAD----------------------GWPDLARG--------LQAL
LX--

------------------------------VTNANRE-QYVKD-YVFWLTHKS-IEPQYE
-AFARG---FYTCLDR--TALS-IFTPE-TLKSVIEG-----------------------
----YQEIN-----------IDEL----ERTVTYDDGYSRT-------------------
------------------------SPTI-VDFWHVVR--------------------SFS
QEQHRQLLEF-------VTASDRVP------------VNG-----LA-NVQFII--QKNG
DDD-------------------------------ARLPSSSTCY-GR-LLLPQY-SSCQV
LEEKLS-KAIEN-SVGF
>Capronia_coronata_XM_007721403.1 .
LTIRRDDV-LNDAINQI--------------WRRQRRELMR-PLRVRLGKDEG----EDG
LDHGGVQQEF---FRVV----FAEAFRPDY--------GMFTVDSTTR------------
----------------MTWFQPGS------------------------------------
-----FEPLYRFEALGVLMSIAIYNGITLPITFPL--AFYRKLLG---------------
-------LKVKTLEHISD----------------------GWPDLTRG--------LRSL
LEWTDG---DVG-DVIAR
-TYEFSYDL-----------------------------CGS-TVSVD-MX----------

------------------------------VTNANRD-QYVKD-YILWLTHKS-IEPQFE
-AFAKG---FYTCLDR--TALS-IFTPE-ALKAVIEG-----------------------
----HPEIN-----------IDEL----ERTATYDD-YERH-------------------
------------------------SPTI-VNFWHVVR--------------------NFS
PDQHRQLLEF-------VTASDRVP------------VNG-----LA-SIQFIV--QKNG

DDD-------------------------------SRLPSSSTCY-GR-LLLPQY-SSREI
LEEKLA-KAIEN-SVGF
>Rhinocladiella_mackenziei_XM_013417582.1 .
LTIRRDNV-LNDAINQI--------------WRRQRRELMR-PLRVRLGKDEG----EDG
LDHGGVQQEF---FRVV----FAEALRPDY--------GMFTVDSTTR------------
----------------MTWFQPGS------------------------------------
-----FEQLYRFEALGILMSIAVYNGITLPVTLPL--AFYRKILG---------------
-------LKVKKLEHIAD----------------------GWPDLTRG--------LRAL
LEWADG---DVG-DVIAR
-TYEFSYDL-----------------------------CGS-TVTVD-MQKI--------
GRDX--
------------------------------VTNANRT-QYVKD-YILWLTHKS-IEPQYE
-AFAKG---FYTCLDR--TALS-IFTPE-ALKSVVEG-----------------------
----YPDID-----------IDEL----ERTITYDE-YDKE-------------------
------------------------SPTI-VDFWHVVR--------------------SFS
PEQHRQLLEF-------VTASDRVP------------VNG-----IK-SIQFIV--QKNG
DDD-------------------------------NRLPSSSTCY-GR-LLLPQY-SSRQV
LEEKLT-KAIEN-CVGF
>Capronia_epimyces_XM_007732506.1 .
LTIRRDDI-LNDAINQI--------------WRRQRRELMR-PLRVRLGKDEG----EEG
LDHGGVQQEF---FRVV----FAEAFRPDY--------GMFVVDSSTR------------
----------------MTWFQPGS------------------------------------
-----FEPLYRFEAMGILMSIAVYNGITVPVTFPL--AFYRKLLG---------------
-------LKVKTLDHISD----------------------GWPDLTRG--------LRAL
LEWDDG---DVG-DVIAR
-TYEFSYDL-----------------------------CGS-AVTVD-MQKF--------
GRDX--
------------------------------VTNANRE-QYVKD-YVLWLTHKS-IEPQYE
-AFAKG---FYTCLDR--TALS-VFTPE-ALKLVVEG-----------------------
----YPQIN-----------IDEL----ERTATYDD-YDRE-------------------
------------------------SPTI-VDFWHVVR--------------------SFS
PEQHKQLLEF-------VTASDRVP------------VNG-----LS-SIQFIV--QKNG
DDD-------------------------------SRLPSSSTCY-GR-LLLPQY-SSRHV
LEEKLA-KAIEN-CVGF
>Exophiala_aquamarina_XM_013406716.1 .
LTVRRDDI-LNDAINQI--------------WRREPQELMR-PLRVRLGKDEG----EDG
LDHGGVQQEF---FRVV----FAEALKPDY--------GMFAIDSTTR------------
----------------MTWFQPGS------------------------------------
-----FEPLYRFEALGILMSLAVYNGLTLPVTFPI--AFYRKLLD---------------
-------LKVKKLEHIVD----------------------GWPDLSRG--------LQTL
LDWTEG---DVG-DVIAR
-TYEFSYEL-----------------------------CGS-TVTVD-IYKV--------
GRDX--
------------------------------VTNENRV-QYVKD-YILWLTHKS-ISEQYE
-AFARG---FYTCLDR--TALS-IFTPE-ALKFVIEG-----------------------
----HPEIE-----------IDEL----ERVTTYDD-YTRE-------------------
------------------------SQTI-IDFWQVVR--------------------NMS
PDQHRQLLEF-------VTASDRVP------------VNG-----MS-SVVFIV--QKNG
DED-------------------------------TRLPSSSTCY-GR-LLLPQY-SSREI
LKEKLS-KAIEN-CIGF
>Cladophialophora_yegresii_XM_007754427.1 .
LTIRRDDI-LNDAINQI--------------WRRQRQEIMR-PLRVRIGKDEG----EDG
LDHGGVQQEF---FRLV----FSEAFRPDY--------GMFTVDDMTR------------
----------------MAWLQPGS------------------------------------
-----LEPLYRFEALGILMSLAIYNGITIPITMPL--AFYRKLLG---------------
-------LKVKKLDHIAD----------------------GWPELVRG--------LRTM
LGWADG---DVG-DVIAR
-TYEFSYEF-----------------------------CGS-TVTVD-MQKF--------

GRDX--
------------------------------VTNMNRA-QYVKD-YIHWLTHKS-VEPQYE
-AFARG---FYTCLDR--TALS-IFTPE-ALKVLVEG-----------------------
----YADID-----------IDEL----QHTATYDE-YAAT-------------------
------------------------DPTI-LDFWSVVR--------------------NMS
PEQHKQLLEF-------VTASDRVP------------VNG-----MK-SVTFII--QKNG
EED-------------------------------TRLPSSSTCY-GR-LLLPQY-SNKKT
LEEKLT-KAIEN-SAGF
>Cladophialophora_carrionii_XM_008723824.1 .
LTIRRDDI-LNDAINQI--------------WRRQRQELMR-PLRVRIGKDEG----EDG
LDHGGVQQEF---FRLV----FSEAFRPDY--------GMFTVDNITR------------
----------------MAWFQPGS------------------------------------
-----LEPLYRFEALGILMSLAIYNGITIPITMPL--AFYRKLLG---------------
-------LKVKKLEHVAD----------------------GWPELVRG--------LRTM
LEWSHG---DVG-DVIAR
-TYEFSYEF-----------------------------CGS-SVTVD-MQKF--------
GRDX--
------------------------------VTNANRA-QYVKD-YILWLTHKS-VEPQYE
-AFARG---FYTCLDR--TALS-IFTPE-ALKVLIEG-----------------------
----YADID-----------IDEL----EHTATYDD-YTAT-------------------
------------------------DPTI-LDFWSVVR--------------------SMS
PEQHKQLLEF-------VTASDRVP------------VNG-----MK-SVTFIV--QKNG
EED-------------------------------TRLPSSSTCY-GR-LLLPQY-SNKKI
LEEKLT-KAIEN-SAGF
>Exophiala_mesophila_XM_016372735.1 .
LTIRRDDI-LKDAIDQI--------------WHRERMELLR-PLRVRLGQNEG----EDG
LDHGGVQQEF---FRVV----FAEALDKNL--------GMFTIDDTTR------------
----------------MAWFQPGS------------------------------------
-----LEPLYSFEALGVLMSVAVYNGITIPVTFPL--AFYRKLLN---------------
-------LKAKSLDHISD----------------------GWPELTKG--------LRTL
LEWSDG---DVG-DVIAR
-TYEFSYEV-----------------------------YGS-AVTID-LSRV--------
GRX---
------------------------------VTNANRE-QYVLD-YIYWLVHKS-VAPQFT
-AFARG---FYTCLDR--NALS-IFTPE-ALKLVIEG-----------------------
----HSKID-----------INEL----ERTATYDD-FERD-------------------
------------------------SEYM-HWFWAIVR--------------------NMS
PEQHKHLLEF-------VTASDRVP------------VNG-----LG-SVTFVI--QKNG
DGDVD-----------------------------GRLPSSSTCY-GR-LLLPVY-RQRAV
LEEKLT-KAIEN-HVGF
>Exophiala_xenobiotica_XM_013454952.1 .
LTIRRDNV-LNDAISQI--------------WRRQRMELMR-PLRVRLGKDEG----EDG
VDYGGVQQEF---FRVV----FAEAFDPDY--------GMFTIDSATR------------
----------------MSWFQPGS------------------------------------
-----FEPLYRFEALGILMSIAIYNGITLPVTFPL--AFYRKLLG---------------
-------LKVKKLDHIAD----------------------GWPDLTRG--------LKSL
LEWSEG---DVG-DVIAR
-TYEFSYEL-----------------------------CGN-TVSVD-MQKI--------
GRDX--
------------------------------VTNANRE-QYVKD-YILWLTHKS-IQPQYE
-AFAKG---FYTCLDR--TALS-IFTPE-ALKAVIEG-----------------------
----HPEID-----------IDEL----ERTVTYEG-YERN-------------------
------------------------SDFI-RDFWHVVR--------------------SFS
PEQHRQLLEF-------VTASDRVP------------VNG-----LS-SVQFVV--QRNG
DDDD------------------------------ARLPSSSTCY-GR-LLLPMY-SSREV
MKEKLG-RAIEN-SVGF
>Exophiala_spinifera_XM_016380070.1 .
LTVRRDNL-LNDAIDQI--------------WRRQRMEIMR-PLRVRVGKDEG----EDG

VDYGGVQQEF---FRLV----FAEAFDPDY--------GMFTVDSSTR------------
----------------TAWFQPGS------------------------------------
-----FEPLYRFEALGILMSLAIYNGITLPVTFPV--ALYRKLLS---------------
-------LKVKKLDHIAD----------------------GWPELARG--------LKTL
LEWSDG--DVG-DVIAR
-TYEFSYEL-----------------------------CGS-TVTVD-MLKI--------
GRX---
------------------------------VTNANRE-QYVKD-YILWLTHKS-VQLQYE
-AFAKG---FYTCLDR--TALS-IFTPE-ALKTVVEG-----------------------
----HPEID-----------INEL----EHTVTYEG-YDRD-------------------
------------------------SEYI-QDFWRVVR--------------------DFS
PEQHKQLLEF-------VTASDRVP------------VNG-----LG-SVQFVI--QRNG
DDED------------------------------SRLPSSSTCY-GR-LLLPMY-SSREI
LRDKLS-KAIEN-SVGF
>Exophiala_oligosperma_XM_016403876.1 .
LTVRRDNL-LNDAINQI--------------WRRQRVEIMR-PLRVRVGKDEG----EDG
VDYGGVQQEF---FRLV----FAEAFDPNY--------GMFTIDGSTR------------
----------------TAWFQPGS------------------------------------
-----FEPLYRFEALGILMSLAIYNGITLPVTFPV--ALYRKLLG---------------
-------LKVKKLDHIAD----------------------GWPELTRG--------LKTL
LEWSDG--DVG-DVIAR
-TYEFSYGL-----------------------------CGS-TVTVD-MLKI--------
GRX---
------------------------------VTNANRE-QYVKD-YILWLTHKS-VQPQYE
-AFARG---FFTCLDR--TALS-IFTAE-ALKTVVEG-----------------------
----HPQID-----------INEL----EDTVTYEG-YERE-------------------
------------------------SEYI-RGFWHVVR--------------------DFS
PEQHKQLLEF-------VTASDRVP------------VNG-----LR-SVQFVI--QRNG
DDED------------------------------SRLPSSSTCY-GR-LLLPMY-SSKEI
LRDKLS-KAIEN-SVGF
>Talaromyces_stipitatus_XM_002340463.1 .
ISVRREYV-LEDAFNQI--------------WRRERRELLK-PLKVKLGTDLG----EEG
VDLGGVQQEF---MTMA----FAEAVNPEH--------GMFTVDPTTR------------
----------------MTWFQPCS------------------------------------
-----PESLDHFELVGILMSLAVYNGLTLPVTFPL--AFYRKLLD---------------
-------LKIKLTEDIRD----------------------GWPDLAKG--------LESL
LTWSEG--DVG-DVFVR
-SYEFSFEA-----------------------------FGS-VRTVN-MEQI--------
ERDAEWPILERVPSRERRRSSVSNYSVEVQGHKDSGEV------------SEGHVDSDIG
SFKTGILKGADYNTALNTFPIQEDIEEASLVTNENRE-QYVKD-YIFWLTDKS-IRPQYE
-AFARG---FFTCIDR--SSLA-LFTPE-ILQTVIEG-----------------------
----TQEID-----------VDEL----QRNATYDGGWDCD-------------------
------------------------HPLI-VAFWDIVK--------------------KYS
REQKARLLEF-------VTASDRVP------------VSG-----IS-SINFVI--QRNG
SGN-------------------------------DRLPTSYTCY-GR-LLLPEY-TDAKA
LQLNLD-MALKN-SRGF
>Penicillium_marneffei_XM_002144851.1 .
ISVRREHI-LQDAFNQI--------------WRRERRELLR-PLKVKMGTDLG----EEG
VDLGGVQQEF---MTLA----FAEALNPEH--------GMFTVDPITR------------
----------------MAWFQPCS------------------------------------
-----PESLEHFELVGILMSLAVYNGLTIPVTFPV--ALYRKLLD---------------
-------LRIKRTEHIRD----------------------GWPDLAKG--------LESL
LTWNDG--DVE-DVFVR
-SYEFSFEA-----------------------------FGS-VX----------------

------------------------------VTNANRE-QYVKD-YLFWLTDKS-IRPQYE
-AFARG---FFTCIDR--SSLA-LFTPE-ILQTVIEG-----------------------
----TQEID-----------IEEL----QRHATYDGGWDGK-------------------

------------------------HPLI-IAFWNIVK--------------------KYS
PEQKARLLEF-------VTASDRVP------------VSG-----IS-SINFVI--QRNG
SGN-------------------------------DRLPTSYTCY-GR-LLLPEY-TNVTA
LGLNLD-MALKN-SRGF
>Arthroderma_benhamiae_ABSU01000011.1 .
ISVRRDHA-LEDALNQL--------------WRRERRELLR-PLKVRMGMDDG----EEG
VDQGGVQQEF---FRIA----MLEAMNPAF--------GMFTVDSRSG------------
----------------TYYFQPCS------------------------------------
-----FEPLYKFELLGLLVSLAIYNGVTLPVDFPI--ALYRRLLG---------------
-------LKVKTTDHIRV----------------------GWPELAKG--------LDEL
LTWEDG---DVG-DIFMR
-TYEFSFSA-----------------------------FGT-VITVD-MERT--------
DKNEPWPAPERYAQWEKTKQKLNDQRVATRRLSNQNGPDGSNSVSMLKEYSTDSRQHPRD
THVIGILKGA-PSRLPRHQVSTEPQPGASTVTNANRK-QFVKD-YIFWLTDKS-VRPQYE
-AFQRG---FFTCLDR--TALS-IFNPE-ALKTVLEG-----------------------
----IQEID-----------LAEL----QRHARYEGGWDGN---------------------
------------------------HPIV-KGFWRVVL--------------------RYP
LEKRRRLLEF-------VTASDRVP------------VNG-----IG-SILFVI--QRNG
VGX-------------------------------RRLPTSLTCF-GR-LLLPEY-TSTTV
LEEKLE-RALNN-ARGF
>Trichophyton_verrucosum_ACYE01000217.1 .
ISVRRDHA-LEDALNQL--------------WRRERRELLR-PLKVRMGMDDG----EEG
VDQGGVQQEF---FRIA----MLEAMNPAF--------GMFTVDSRSG------------
----------------TYYFQPCS------------------------------------
-----FEPLYKFELLGLLVSLAIYNGVTLPVDFPI--ALYRRLLG---------------
-------LKVKTTDHIRV----------------------GWPELAKG--------LDEL
LAWEDG---DVG-DIFMR
-TYEFSFSA-----------------------------FGT-VITVD-MERT--------
DKNEPWPAPERYAQWEKTKQKLNDQRVATRRLSNQNGPDGSNSVSMLKEYSTDSRQHPRD
THVIGILKGA-PSRLPRHQVSTEPQPGASTVTNANRK-QFVKD-YIFWLTDKS-VRPQYE
-AFQRG---FFTCLDR--TALS-IFNPE-ALKTVLEG-----------------------
----IQEID-----------LAEL----QRHARYEGGWDGN---------------------
------------------------HPIV-KGFWRAVL--------------------RYP
LEKRRRLLEF-------VTASDRVP------------VNG-----IG-SILFVI--QRNG
VGX-------------------------------RRLPTSLTCF-GR-LLLPEY-TSTTV
LEEKLE-RALNN-ARGF
>Trichophyton_equinum_ABWI01001063.1_R .
ISVRRDHA-LEDALNQL--------------WRRERRELLR-PLKVRMGMDDG----EEG
VDQGGVQQEF---FRIA----MLEAMNPAF--------GMFTVDSRSG------------
----------------TYYFQPCS------------------------------------
-----FEPLYKFELLGLLVSLAIYNGVTLPVDFPI--ALYRRLLG---------------
-------LKVKTTDHIRV----------------------GWPELAKG--------LDEL
LAWEDG---DVG-DIFMR
-TYEFSFNA-----------------------------FGT-VITVD-MERT--------
DKNEPWPAPERYTQWEKTKQKLNDQRVATRRLSNQNGHDGSNSVSMLKEYSTDSRQHPRD
THVIGILKGA-PSRLPRHQVSTEPQPEASTVTNANRK-QFVKD-YIFWLTDKS-VRPQYE
-AFQRG---FFTCLDR--TALS-IFNPE-ALKTVLEG-----------------------
----IQEID-----------LAEL----QRHARYEGGWDGN---------------------
------------------------HPIV-KGFWRVVL--------------------RYP
LEKRRRLLEF-------VTASDRVP------------VNG-----IG-SILFVI--QRNG
VGDSVC----------------------------SRLPTSLTCF-GR-LLLPEY-TSTTV
LEEKLE-RALNN-ARGF
>Trichophyton_rubrum_XM_003235732.1 .
ISVRRDHA-LEDALNQL--------------WRRERRELLR-PLKVRMGMDDG----EEG
VDQGGVQQEF---FRIA----MLEAMNPAF--------GMFTIDSRSG------------
----------------TYYFQPCS------------------------------------
-----FEPLYKFELLGLLVSLAIYNGVTLPVDFPI--ALYRRLLG---------------
-------LKVKTTDHIRV----------------------GWPELAKG--------LDEL

LTWEDG---DVG-DIFMR
-TYEFSFNA-----------------------------FGT-VVTVD-MERT--------
DKNEPWPAPERYAQWEKTKQKLNDQRVATRRLSHQNGPDWSNSVSMLKEYSTDSRQHPRD
THVIGILKGA-PSRLPRHQVSTEPQPEASTVTNANRR-QFVKD-YIFWLTDKS-VRPQYE
-AFQRG---FFTCLDR--TALS-IFNPE-ALKTVLEG-----------------------
----IQEID-----------LAEL----QRHARYEGGWDGN-------------------
------------------------HPIV-KGFWRVVL--------------------RYP
LEKRRRLLEF-------VTASDRVP------------VNG-----IG-SILFVI--QRNG
VGD-------------------------------SRLPTSLTCF-GR-LLLPEY-TSATV
LEEKLE-RALNN-ARGF
>Arthroderma_gypseum_XM_003169108.1 .
ISVRRDHA-LEDALDQL--------------WRRERRELLR-PLKVRMGMDDG----EEG
VDQGGVQQEF---FRIA----MLEAMNPAF--------GMFTIDSRSG------------
----------------MYYFQPCS------------------------------------
-----FEPLYKFELLGLLVSLAIYNGVTLPVDFPI--ALYRRLLG---------------
-------LKVKNTDHIRV----------------------GWPELAKG--------LDEL
LAWEDG---DVG-DIFMR
-TYEFSFNA-----------------------------FGT-VVTVD-MERT--------
DKNEPWPAPERYAQWEKTKQKLNDHRVATRRLSNQNTSESDNTVSMLKEYSTESRQSPRD
THVIGILKGA-PSRLPRHQVSTEPQPEASTVTNANRK-QFVKD-YIFWLTDKS-VRPQYE
-AFQRG---FFTCLDR--TALS-IFNPE-ALKTVLEG-----------------------
----IQEID-----------LVEL----QRHTRYEGGWDGN-------------------
------------------------HPIV-KGFWRVVL--------------------RYP
LEKRRQLLEF-------VTASDRVP------------VNG-----IG-SILFVI--QRNG
VGD-------------------------------SRLPTSLTCF-GR-LLLPEY-TSVAV
LEEKLE-KALDN-ARGF
>Arthroderma_otae_XM_002842676.1 .
ISVRREHA-LVDALDQL--------------WRRERRELLR-PLKVRMGMDDG----EEG
VDQGGVQQEF---FRIA----ISEALDPAF--------GMFTVDSRSG------------
----------------MYYFQPCS------------------------------------
-----FEPLYKFELLGLLVSLALYNGVTLPVDFPI--ALYRRLLG---------------
-------LKVKNTDHIRV----------------------GWPDLAKG--------LDDL
LTWEDG---DVG-DVFMR
-TYEFSFNA-----------------------------FGT-VVTVD-MERT--------
DRNEPWPAPERYAQWEKTKQKLKDQRVATKRFTSHRVPDANSNISMLKEYSSDSRQPPRD
TQVIGILKGA-PSRLPRYQVATEPQPEASIVTNDNRK-QFVKD-YIFWLTDKS-VRPQYE
-AFQRG---FFTCLDR--TALS-IFNPE-ALKMVLEG-----------------------
----IQEID-----------LSEL----RKHARYEGGWDAN-------------------
------------------------HPLV-KGFWRIVM--------------------RYS
HEKRGRLLEF-------VTASDRVP------------VNG-----IP-SIMFVI--QRNG
VGD-------------------------------SRLPTSLTCF-GR-LLLPEY-SSTAV
LEEKLD-KALEN-ARGF
>Xylona_heveae_XM_018331202.1 .
LEVRRDEV-LLDALNQL--------------WRREKRELMR-PLKVRMGMGEG----EEG
VDHGGVQQEF---FRLV----ISEALNSDY--------GTFTVDSRTR------------
----------------MTWFRPCS------------------------------------
-----LEPLYKFELLGLLFSIAIYNGLTLPITFPK--ALYRKLLG---------------
-------QQSTKIEHIQD----------------------GWPELAKG--------LTEL
LEWNEG---DVG-DIFMR
-TYDFSFDA-----------------------------FGK-VMNVD-MLKF--------
DRNSEWPPTNSPSKK-------------------------------------GKKRATNF
EPDHSRTEGA---VLDKLVSHEPSEDEAELVTNSNRE-QFVKD-YIFWLTDKS-IRPQYE
-AFARG---FFVCLDR--KAIS-MFSPE-AFQSIVEG-----------------------
----VQGID-----------IDAL----ERTARYEDGYSSG-------------------
------------------------HPLI-QQFWQIVR--------------------QFS
PVEKRHLLEF-------VTASDRVP------------VNG-----VE-SILFVI--QRNG
PDS-------------------------------DRVPTSLTCF-GR-LLLPEY-SSKAK
LEEKLR-LALEN-GKGF

```
>Ascosphaera_apis_AARE01004584.1 .
---RRDKA-VTDALDQL--------------WRREKRELLR-PLKVTMGVQEG----EEG
VDHGGVQQEF---FRTV----MAEILNPDY--------GMFTVDPVTY------------
----------------STWFQPGS------------------------------------
-----LEPLYKFELLGLLVSLAVYNGLTLPVNFPI--ALYMKLLD---------------
-------EKVRSIDHIRN----------------------GWPELAKG--------FEDL
LAWSEG---------------------------------------------DVG-DIFMR
-TYEFSFNA-----------------------------FGT-VINVD-MEKV--------
GRNEPX------------------------------------------------------
------------------------------VTNENRE-QFVKD-YIFWLTDKS-IRPQYD
-AFARG---FYACLDR--TALS-LFTPE-TLKRLVEG-----------------------
----SQHID-----------IAEL----EKHTKYHDGYYPE-------------------
------------------------HPAI-SMFWDIVR--------------------QFT
PEKTALLLEF-------VTASDRVP------------VNG-----IS-SIAFII--QRAG
NSD-------------------------------XRLPTSMTCF-GR-LLLPEY-SSREI
MKQKLE-KALEN-TKGF
>Uncinocarpus_reesii_XM_002541545.1 .
LVVRRDNA-LMDAFNQV--------------WRREKRELMR-PLRVSMGMDEG----EEG
LDHGGVQQEF---FRVV----MAEALDPAY--------GMFTLEGRDH------------
----------------ISWFQPCS------------------------------------
-----FEPLYKFELLGLLMSLAVFNGLTLPVNFPL--ALYMKLLD---------------
-------RKVKKLEHIKN----------------------GWDDLERG--------LGEL
LSWSDG---------------------------------------------DVG-DIFMR
-TYEFSFDA-----------------------------FGK-IVTVD-MTTV--------
DRNDPWPSAERGVTWNRTRFKSFAASSDSDATENFDLPE--HGMSMLQDSAGDHRSNRKD
GHLSSILKGS-SSKRPERRVPSPPEHEAPLVTNANRE-QFVRD-YIFWLTDKS-IRPQYE
-AFARG---FYTCLDR--TALS-LFTPE-ALKTVIEG-----------------------
----IQEID-----------MDEL----EKHARYEG-FQPQ-------------------
------------------------DQFI-KHFWAVVK--------------------SYT
QEKRSQLLEF-------VTASDRVP------------VNG-----IS-SILFVI--MKNG
SGD-------------------------------ERLPTSSTCF-GR-LLLPEY-SSRAV
LEEKLD-KALEN-SKGF
>Amauroascus_mutatus_GDQZ01004484.1 .
LTVRRDEA-LMDALDQL--------------WRREKRELMR-PLRVQMGMDEG----EEG
LDHGGVQQEF---FRVV----MAEALDPAY--------GMFTLDSRNH------------
----------------LSWFQPCS------------------------------------
-----FEPLYKFELVGLLMSLAVYNGLTLPVNFPV--ALYMKLLG---------------
-------RKVKKLDHIKS----------------------GWDDLRKG--------LEEL
LSWDDG---------------------------------------------DVG-DVFMR
-TYEYSFEA-----------------------------FGK-VVTVD-MTTV--------
DRNDPWPSAERNVTWNKSPLKPFLSPSDNDDTESSDPPD--YCISMLQDTPVDGRTNRRD
GRLSGILKGS-SSRPRERRVPSPPEHEAPLVSNANRE-QFVKD-YIFWLTDKS-IRPQYE
-AFARG---FYTCLDR--TALS-IFTPE-ALMTVVEG-----------------------
----IQEID-----------LDEL----ERHARYDG-FDPQ-------------------
------------------------GRVI-RDFWDIVK--------------------GYS
QKKRSQLLEF-------VTASDRVP------------VNG-----IA-TILFVI--MKNG
VGD-------------------------------ERLPTSSTCF-GR-LLLPEY-SSRKV
LEEKLE-KAIEN-CKGF
>Chrysosporium_queenslandicum_GDRC01010421.1 .
LVVGRDTV-LMDAFDQL--------------WRREKRELMR-PLRVQMGMDEG----EEG
VDHGGVQQEF---FRVV----MAQALDPVY--------GMFTVDGGDH------------
----------------ISWFKPGS------------------------------------
-----LEPLYRFELVGLLMSLAVYNGLTLPVNFPI--AFYMKLLG---------------
-------RKVKKLDHIKR----------------------GWDNLAKG--------LGEL
LSWSDG---------------------------------------------DVG-DVFMR
-TYEFSFDA-----------------------------CGK-VVSVD-MTAV--------
DRNAPWPSTERNVTWNKARFGSVASSSDSDNTETLESHN--HGTSATQDTANEKPSTHRE
GRLSGILKGP-SSRPRERCVPSLPEHEAPLVTNANRE-QFVKD-YIFWLTDKS-VRPQYE
```

-AFARG---FYTCLDR--TALS-IFTPE-ALKTVVEG-----------------------
----IQEID-----------IEEL----ERHARYEG-YDRD-------------------
------------------------DRII-KEFWDIVK--------------------RYS
QEKRSQLLEF-------VTASDRVP------------VNG-----IS-TVLFVI--LRNG
SGD--------------------------------QRLPTSSTCF-GR-LLLPEY-SCREV
LEAKLD-KALEN-SKGF
>Coccidioides_immitis_XM_001247392.1 .
LIVRRDNV-LTDTLNQL--------------WRREKQELMR-PLRVQMGMDEG----EEG
ADQGGVQQEL---FRVV----MAEILDPAY--------GMFTLSDRDH------------
----------------TSWFQPCS------------------------------------
-----FEPLYKFELAGLLMSLAIYNGITLPVNFPV--AFYMKLLD---------------
-------FEVKKLDDIRS----------------------GWEDLARG--------LSEL
LSWSDG--DVG-DIFLR
-TYEFSFDA-----------------------------FGK-VVTVD-MTTV--------
DRSDAWPPAERNVTWRKERLRSFASSSDNDDADNLDSLD--NGESMLQDTPSDSRSNRRD
GRLSGILKGS-SSKLRERRVPSPPEHEAPLVTNANRE-QFVND-YIFWLTDKS-IRPQYE
-AFARG---FYTCLDR--TALS-IFTPQ-ALKSVVEG-----------------------
----IQEID-----------MDEL----EKHTRYEG-YNPE-------------------
------------------------DRVI-RDFWDVVK--------------------TYP
QEKRSRLLEF-------VTASDRVP------------VKG-----VS-SLLFII--MKNG
VGD--------------------------------ERLPTSGTCF-GR-LLLPEY-SSRQA
LEENFD-RALEY-CKGF
>Coccidioides_posadasii_XM_003065946.1 .
LIVRRDNV-LTDTLNQL--------------WRREKQELMR-PLRVQMGMDEG----EEG
ADQGGVQQEL---FRVV----MAEILDPAY--------GMFTLSDRDH------------
----------------MSWFQPCS------------------------------------
-----FEPLYKFELAGLLMSLAIYNGITLPVNFPV--AFYMKLLD---------------
-------FEVKKLDDIRS----------------------GWEDLARG--------LSEL
LSWSDG--DVG-DIFLR
-TYEFSFDA-----------------------------FGK-VVTVD-MTAV--------
DRSDAWPPAERNVTWRKERLRSFASSSDNDDADNLDSLD--NGESMLQDTPSDSRSNRRD
GRLSGILKGS-SSKLRERRVPSPPEHEAPLVTNANRE-QFVND-YIFWLTDKS-IRPQYE
-AFARG---FYTCLDR--TALS-IFTPQ-ALKSVVEG-----------------------
----IQEID-----------MDEL----EKHTRYEG-YNPE-------------------
------------------------DRVI-RDFWDVVK--------------------TYP
QEKRSRLLEF-------VTASDRVP------------VKG-----VS-SLLFII--MKNG
VGD--------------------------------ERLPTSGTCF-GR-LLLPEY-SSRQA
LEENFD-RALEY-CKGF
>Ajellomyces_dermatitidis_XM_002624975.1 .
LVVRRDNV-LTDALNQL--------------WRRERRELMR-PLKVQMGMDEG----EEG
IDHGGVQQEF---FRVA----LGEALDPSY--------GMFTMDMRTR------------
----------------MSWFQPCS------------------------------------
-----MEPLYKFELLGLLMSLAVYNGLTLPVNFPV--ALYKKLLG---------------
-------LRVKNLDDIRV----------------------GWPELAKG--------LDDL
LSWDDG--DVG-DIFMR
-TYEFSFDA-----------------------------FGT-FVSVD-MEKV--------
DRNEPWPAPER-SAWERKKSGSYYRQSWSGKTSQPGSFD--DNTSMLRESADENSTSKRD
GVLSGILKGA-SSRTY-KATPPSPPQEAALVTNANRE-RFVKD-YIFWLTDKS-VRPQYE
-AFARG---FYTCLDR--TALS-IFTPE-ALKTVIEG-----------------------
----IQEID-----------IEEL----EHNARYEGGFEPN-------------------
------------------------HRVI-SDFWSIVK--------------------RYP
QTRKRQLLEF-------VTASDRVP------------VNG-----IS-SIMFVI--QRNG
TGD--------------------------------NRLPTSLTCF-GR-LLLPEY-SNRAI
LEEKLE-KALEN-ARGF
>Ajellomyces_capsulatus_XM_001538213.1 .
LVVRRDNV-LTDALDQL--------------WRRERRELMR-PLKVQMGMDEG----EEG
IDHGGVQQEF---FRVA----FGEALDPSY--------GMFTMDMRTR------------
----------------ISWFQPCS------------------------------------

-----MEPLYKFELLGLLTSLAVYNGLTLPVNFPV--ALYKKLLG---------------
-------LKVKTLDDIEV----------------------GWPELAKG--------LSDL
LSWDDG---DVG-DIFMR
-TYEFSFEA-----------------------------FGN-FVSVD-MEKV--------
DRTEPWPATER-PTWERKKRGSYSRQSWSGKNSPRVALD--DNISMLCECADESSTLKKD
GTLSGILKGA-SSRTY-KATRP-PPQEAALVTNANRE-RFVKD-YIFWLTDKS-IRPQYE
-AFARG---FYTCLDR--TALS-IFTPE-ALKTVIEG----------------------
----IQEIN-----------IEEL----EHHTRYEGGFEPG-------------------
------------------------HRVI-RDFWVIVK--------------------GYP
QTRKRQLLEF-------VTASDRVP------------VNG-----IS-SIMFVI--QRNG
TGD-------------------------------SRLPTSLTCF-GR-LLLPDY-SSRDI
LEEKLE-KALEN-ARGF
>Paracoccidioides_brasiliensis_XM_002797286.1 .
LVVRRDNV-LTDALDQL--------------WRRERRELMR-PLKVQMGMDEG----EEG
IDHGGVQQEF---FRVA----LGEALDPSY--------GLFTMDIRTR------------
----------------ISWFQPLS------------------------------------
-----MEPLYKFELLGLLMSLAIYNGLTLPVNFPI--ALYRKLLG---------------
-------LRVKTLDDIRV----------------------GWSELAKG--------LEEL
LSWDDC---DVG-DIFMR
-TYEFSFEA-----------------------------FGN-FVNVD-MEKV--------
DRNEPWPAPER-LAWEKRKPGSYNRRSSSGKSGPLSQRD--NDVSMLHDTTDEGPSYRRD
GVLSGILKGS-SSKIN-RAAPPSPPQEAALVTNENRE-RFVKD-YIFWLTDKS-IRPQYE
-AFARG---FYTCLDR--TALS-LFTPE-ALKLVVEG----------------------
----IQEIN-----------IREL----EYHTRYEGGFEPD-------------------
------------------------HRVI-RDFWDIVY--------------------RYS
SMRKRQLLEF-------VTASDRVP------------VNG-----IS-SIMFVI--QKNG
TGD-------------------------------NRLPTSLTCF-GR-LLLPEY-SSRDV
LEEKLS-KALEN-ARGF
>Penicillium_expansum_XM_016737707.1 .
LVVRRDNI-LSDALSQL--------------WRREKRELMR-PLKVQMGMDEG----EEG
LDHGGVQQEF---FRLL----MGQAFDPSY--------GMFTVDTRHR------------
----------------MSWFQPCS------------------------------------
-----LEPLYKFELIGLLMSIAIYNGLTLPVNLPT--AFYRKMLG---------------
-------LKVKHLDHIRD----------------------GWPELSQG--------LDTL
LAWKDG---DVG-DIFTR
-TYEFSFEA-----------------------------FGS-IETID-MQKV--------
DRDAAX--
------------------------------VTNKNRH-QFVKD-YIFWLTDKS-IRPQFE
-AFQRG---FNTCLDR--SALS-IFSPE-ALKTVVEG----------------------
----IQSID-----------VEEL----ENHTRYEGGFGPD-------------------
------------------------HRVI-RDFWDIIQ--------------------EYP
NEKRAQLLEF-------VTASDRVP------------VNG-----IS-SIMFVI--QKNG
VGD-------------------------------LRLPTSLTCF-GR-LLLPEY-SSREA
LAKKLD-KALEN-AQGF
>Penicillium_chrysogenum_XM_002557878.1 .
LVIRRDDI-LSDALSQL--------------WRREKRELMR-PLKVQMGMDEG----EEG
LDHGGVQQEF---FRLL----MGQAFDPSY--------GMFTVDTRHR------------
----------------VSWFQPCS------------------------------------
-----LEPLYKFELIGLLMSIAIYNGLTLPVNLPT--AFYRKMLG---------------
-------LKVKHLDHIRD----------------------GWPELSQG--------LDTL
LAWKDG---DVG-DIFTR
-TYEFSFEA-----------------------------FGS-IETID-MQKV--------
SRDAAWPLASKTKVTAPAPMAG--SSAWIDVPTYCDPV---ISRSPTPTAVEEAADVMSC
EPASDPTAKP-MPESISLQSPLPPAEEAALVTNKNRH-QFVKD-YIFWLTDKS-IRPQFE
-AFQRG---FNTCLDR--SALS-IFSPE-ALKTVVEG----------------------
----IQSID-----------VEEL----ENHTRYEGGFGPD-------------------
------------------------HRVI-RDFWDIIR--------------------EYP
NEKRAQLLEF-------VTASDRVP------------VNG-----IS-SIMFVI--QKNG

VGD-------------------------------LRLPTSLTCF-GR-LLLPEY-SSKTT
LAQKLD-KALEN-AQGF
>Penicillium_digitatum_XM_014676664.1 .
LVIRRDNI-LSDALSQL--------------WRREKRELMR-PLKVQMGMDEG----EEG
LDHGGVQQEF---FRLL----MGQAFDPSY--------GMFTVDTPHR------------
----------------MSWFQPCS------------------------------------
-----LEPLYKFELIGLLMSIAIYNGLTLPVNLPT--AFYRKMLG---------------
-------LKVKHLDHIRD----------------------GWPELSQG--------LDTL
VAWKDG---DVG-DVFTR
-TYEFSFET-----------------------------FGS-IETVD-MQKV--------
DRDAAX--
------------------------------VTNTNRH-QYVKD-YIFWLTDKS-IRPQFE
-AFQRG---FNTCLDR--SALS-IFSPE-ALKTVVEG-----------------------
----IQSID-----------VEEL----ENHARYEGGFGPD-------------------
------------------------HRVI-RDFWDIIQ--------------------EYP
NEKRAQLLEF-------VTASDRVP------------VNG-----IS-SLMFVI--QKNG
VGD-------------------------------LRLPTSLTCF-GR-LLLPEY-SSREA
LATKLD-KALEN-AQGF
>Penicillium_raistrickii_GFHR01004735.1 .
IVVRRDNI-LSDALSQL--------------WRREKRELMR-PLKVQMGMDEG----EEG
LDHGGVQQEF---FRIL----MGQALDPSY--------GMFTVDTRHR------------
----------------VSWFQPCS------------------------------------
-----LEPLYKFELIGLLMSIAIFNGLTLPVNFPI--AFYRKLLG---------------
-------LKVKNLEHIRE----------------------GWPELSQG--------LDML
LTWKDG---DVG-DIFTR
-TYEFSFEA-----------------------------FGS-IETID-MHKV--------
GRDAAX--
------------------------------VTNQNRH-QFVKD-YIFWLTDKS-IRPQFE
-AFQRG---FNTCLDR--SALS-IFSPE-ALKTVVEG-----------------------
----IQTID-----------VDEL----ENHTRYEGGFGPN-------------------
------------------------HRVI-RDFWSVVQ--------------------GYP
NEKRAQLLEF-------VTASDRVP------------VNG-----IS-SIMFVI--QKNG
VGD-------------------------------LRLPTSLTCF-GR-LLLPEY-SSKEA
LAEKLD-KALDN-AQGF
>Aspergillus_fumigatus_XM_744483.1 .
LMVRRDNV-LTDALNQL--------------WRRERRELMR-PLKVQMGMDEG----EEG
LDHGGVQQEF---FRVL----MAEALNPSY--------GMFTMDARTR------------
----------------ISWFQPCS------------------------------------
-----LEPLYKFELLGLLMSLAIYNGLTLPVNFPV--AFYRKLLG---------------
-------LKVKHLEHIRD----------------------GWPELTKG--------LELL
LSWQDG---DVG-DVFMR
-TYEFTFEV-----------------------------FGA-VETVD-MEKV--------
GRDAVWPLRARDRSKSSEL-----ATNWSELSQHADPA----DLSPPCSMSADHVDFL--
--ECGSSHRS-KSERLDYCVPTPPVEEASLVTNDNRA-QFVKD-YIFWLTDKS-IRPQFE
-AFARG---FYTCLDR--AALS-IFTPE-AFKTVVEG-----------------------
----IQEID-----------LGEL----ERHARYEGGFGPH-------------------
------------------------HRVI-QDFWSIVK--------------------SFS
QEKKAQLLEF-------VTASDRVP------------VNG-----IA-SIMFVI--QRNG
VGD-------------------------------ARLPTSLTCF-GR-LLLPEY-SSKSV
LEEKLN-KALEN-ARGF
>Neosartorya_fischeri_XM_001260073.1 .
LMVRRDNV-LTDALNQL--------------WRRERRELMR-PLKVQMGMDEG----EEG
LDHGGVQQEF---FRVL----MAQALDPSY--------GMFTMDARTR------------
----------------ISWFQPCS------------------------------------
-----LEPLYKFELLGLLMSLAIYNGLTLPVNFPV--AFYRKLLG---------------
-------LKVKHLEHIRD----------------------GWPELAKG--------LELL
LSWQDG---DVG-DVFMR
-TYEFTFEA-----------------------------FGA-VETVD-MEKV--------

```
DRDAVWPLRARDRGKSSEL-----ATNWSELSQHADPA----DLSPPCSMAADHADFL--
--ECGSSHRS-TSERLDHCVPTPPVEEASLVTNENRA-QFVKD-YIFWLTDKS-IRPQFE
-AFARG---FYTCLDR--AALS-IFTPE-AFKTVVEG-----------------------
----IQEID-----------LEEL----ERHARYEGGFGPH-------------------
------------------------HRVI-RDFWSIVK--------------------GFS
EEKKAQLLEF-------VTASDRVP------------VNG-----IA-SIMFVI--QRNG
VGD-------------------------------ARLPTSLTCF-GR-LLLPEY-SSKSV
LEEKLN-KALEN-ARGF
>Aspergillus_clavatus_XM_001272744.1 .
LMVRRDNV-LTDALNQL--------------WRREKRELLR-PLKVQMGMDEG----EEG
LDHGGVQQEF---FRVV----MAAALDPSY--------GMFTMDERTR------------
----------------VSWFQPCS------------------------------------
-----LEPLYKFELFGLLMSLAIYNGVTLPVNFPI--AFYRKLLG---------------
-------LKVKHLDHIQD----------------------GWPELSKG--------LGDL
LTWQDG---------------------------------------------DVG-DVFMR
-TYEFSFEA-----------------------------FGS-VETVD-MEKV--------
DKDAVWPLPTGIHRKPFILP----SKSWSELSDFAKTADM--DLSPPCSMAADDADDS--
--SPDLSHRD-IKDHAEHSVPTPPVEEASLVTNKNRA-QFVKD-YIFWLTDQS-IRTQFD
-AFTRG---FYTCLDR--AALS-IFTPE-AFKTVVEG-----------------------
----IQEID-----------IKEL----ERHARYEGGFGPS-------------------
------------------------HPTI-RGFWSIVR--------------------GYS
AEKRARLLEF-------VTASDRVP------------VNG-----IA-SIMFVI--QKNG
VGD-------------------------------ARLPTSLTCF-GR-LLLPEY-SSKKV
LEEKLD-KALEN-ARGF
>Penicillium_janthinellum_GBSP01007184.1 .
LVVRRDSV-LTDALNQL--------------WRREKRELMR-PLKVQMGMDEG----EEG
LDHGGVQQEF---FRVL----MGEALDPSY--------GMFTMDSRHH------------
----------------ISWFQPCS------------------------------------
-----WEPLYKFELLGLLMSIAVYNGITLPVNFPV--AFYRKLLG---------------
-------LKVKHLDHIRD----------------------GWPELSQG--------LDSL
LTWDDG---------------------------------------------DVG-DIFMR
-TYEFSFES-----------------------------AGK-VETVD-MX----------
------------------------------------------------------------
------------------------------VTNQNRN-QFVKD-YIFWLTDKS-IRPQFE
-AFLRG---FHTCLDR--TALS-IFTPE-ALKTVVEG-----------------------
----IQEID-----------ITQL----EHHARYEGGFGPG-------------------
------------------------HRVI-RDFWSLVH--------------------QYP
PEKRAQLLEF-------ITASDRIP------------VNG-----IS-SIMFVI--QKNG
VGX-------------------------------QRLPTSLTCF-GR-LLLPEY-SSKEA
LAEKLD-KALEN-ARGF
>Aspergillus_nidulans_XM_654386.1 .
LVIRRDNI-LTDALNQL--------------WRRERRELMR-PLKVQMGMDEG----EEG
LDHGGVQQEF---FRLL----MAEALDPSY--------GMFTTDSRTR------------
----------------CSWFQPCS------------------------------------
-----WEPLYKFEILGLLMSLAVYNGLTLPVNFPL--AFYRKLLG---------------
-------LKVKHLDHIRD----------------------GWPELTRG--------LEEL
LRWNEG---------------------------------------------DVA-DIFMR
-TYEFGFEA-----------------------------FGR-VETVD-MEKV--------
DRDAPWPNSLPPQSGRSVRY----SPSWSDVGRYTDLA----SLSPPSSMAAETAD----
--SQDDTAKS-AISLPPLPSPTSPAEEASLVTNENRG-QFVKD-YIFWLTDKS-IRPQFE
-AFAQG---FYTCLDR--TSLS-IFTPE-ALKTVVEG-----------------------
----IQEID-----------IAEL----ERHARYEGGFGAG-------------------
------------------------HRVI-QDFWSIAH--------------------QFS
AEKKAQLLEF-------VTASDRVP------------VNG-----IS-SIMFVI--QKNG
VGD-------------------------------ARLPTSLTCF-GR-LLLPEY-SSKSV
LEEKLN-KALEN-ARGF
>Aspergillus_flavus_XM_002375053.1 .
LVVRRDNL-LPDALNQL--------------WRRERRELMR-PLKVQMGMDEG----EEG
```

LDHGGVQQEF---FRVL----MAEALDQSY--------GMFATDSRTR------------
----------------VSWFQPCS------------------------------------
-----LEPLYKFELLGLLMSLAVYNGLTLPINFPT--AFYRKLLG---------------
-------LKVKHLDHIRD----------------------GWPELTKG--------LEQL
LTWEDG---DVE-DVFMR
-TYEFSFEA-----------------------------FGA-IETVD-MQKV--------
DRDAPWPLPSALARSGGGNRSLG-SPTWSEVRRYTDCV----NLSPPSSMGAEATE----
--SFADMAKS-LDGSVVMQSPTPPAEEASFVTNRNRS-QFVRD-YIFWLTDKS-IRPQFE
-AFAQG---FYTCLDR--SALS-IFTPE-ALKTVVEG-----------------------
----IQEIN-----------MDEL----EHHARYEGGFGPS-------------------
------------------------HRTI-RDFWSIAR--------------------RFS
VEKKAQLLEF-------VTASDRVP------------VNG-----IA-SIMFVI--QKNG
VGD-------------------------------ARLPTSLTCF-GR-LLLPEY-SSRSV
LEDKLN-KALEN-ARGF
>Aspergillus_oryzae_AP007155.1 .
LVVRRDNL-LPDALNQL--------------WRRERRELMR-PLKVQMGMDEG----EEG
LDHGGVQQEF---FRVL----MAEALDQSY--------GMFATDSRTR------------
----------------VSWFQPCS------------------------------------
-----LEPLYKFELLGLLMSLAVYNGLTLPINFPT--AFYRKLLG---------------
-------LKVKHLDHIRD----------------------GWPELTKG--------LEQL
LTWEDG---DVE-DVFMR
-TYEFSFEA-----------------------------FGA-IETVD-MQKV--------
DRDAPWPLPSALARSGGGNRSLG-SPTWSEVRRYTDCV----NLSPPSSMGAEATE----
--SFADMAKS-LDGSVVMQSPTPPAEEASFVTNRNRS-QFVRD-YIFWLTDKS-IRPQFE
-AFAQG---FYTCLDR--SALS-IFTPE-ALKTVVEG-----------------------
----IQEIN-----------MDEL----EHHARYEGGFGPS-------------------
------------------------HRTI-RDFWSIAR--------------------RFS
VEKKAQLLEF-------VTASDRVP------------VNG-----IA-SIMFVI--QKNG
VGX-------------------------------FRLPTSLTCF-GR-LLLPEY-SSRSV
LEDKLN-KALEN-ARGF
>Aspergillus_sojae_BACA01000986.1 .
LVVRRDNL-LPDALNQL--------------WRRERRELMR-PLKVQMGMDEG----EEG
LDHGGVQQEF---FRVL----MAEALDQSY--------GMFATDSRTR------------
----------------VSWFQPCS------------------------------------
-----LEPLYKFELLGLLMSLAVYNGLTLPINFPT--AFYRKLLG---------------
-------LKVKHLDHIRD----------------------GWPELTKG--------LEQL
LTWEDG---DVE-DVFMR
-TYEFSFEA-----------------------------FGT-IETVD-MQKV--------
DKDAPWPLPSALARPGGGNRSLG-SPPWSEVRRYTDCV----NLSPPSSMGAEATE----
--SFADMAKS-LDGSVVMQSPTPPAEEASFVTNRNRT-QFVRD-YIFWLTDKS-IRPQFE
-AFAQG---FYTCLDR--SALS-IFTPE-ALKTVVEG-----------------------
----IQEID-----------MDEL----EHHARYEGGFGPS-------------------
------------------------HRTI-RDFWSIAR--------------------QFS
VEKKAQLLEF-------VTASDRVP------------VNG-----IA-SIMFVI--QKNG
VGX-------------------------------FRLPTSLTCF-GR-LLLPEY-SSRSV
LEDKLN-KALEN-ARGF
>Aspergillus_nomius_XM_015549948.1 .
LKVRRDNL-LTDALNQL--------------WRRERRELMR-PLKVQMGMDEG----EEG
LDHGGVQQEF---FRVL----MAEALDQSY--------GMFATDSRTR------------
----------------VSWFQPCS------------------------------------
-----LEPLYKYELLGLLVSLAVYNGLTLPINFPT--AFYRKLLG---------------
-------LKVKHLDHIRD----------------------GWPELTKG--------LEQL
LTWENG---DVE-DVFMR
-TYEFSFEA-----------------------------FGT-IETVD-MQKV--------
DKDAPWPLPSALARSGGGNRSLG-SPPWSEVRRYTDCV----NLSPPSSMGAEATE----
--SFADMAKS-LDGSVVMQSPTPPAEEASFVTNQNRS-QFVRD-YIFWLTDKS-IRPQFE
-AFAQG---FYTCLDR--SALS-IFTPE-ALKTVVEG-----------------------
----IQEID-----------VDEL----EHHARYEGGFGPS-------------------

```
------------------------HRSI-RDFWSIVR--------------------RFP
AEKKAQLLEF-------VTASDRVP------------VNG-----IA-SIMFVI--QKNG
VGD-------------------------------ARLPTSLTCF-GR-LLLPEY-SSKIV
LEDKLN-KALEN-ARGF
>Aspergillus_terreus_XM_001215245.1 .
LVVRRDNV-LTDALNQL--------------WRRERRELMR-PLKVQMGMDEG----EEG
LDHGGVQQEF---FRVL----MAEALDPGY--------GMFTMDQRTR------------
----------------ISWFQPRS------------------------------------
-----LEPLYKFELLGLFMSLAVYNGVTLPVNFPT--AFYRKLLG---------------
-------LKVKHLDHIRD----------------------GWPELTKG--------LEGL
LTWHDG---------------------------------------------DVG-DVFMR
-TYEFSFEA-----------------------------FGK-IETVD-MQQV--------
DRDAAWPSPLVRSRAGSPLR----PSPWPEVHRQTDNA----SLSPPLSTAGEVPE----
--SPDDIAKS-LNGSVPIQSFTPPVEEAPLVTNRNRT-QFVKD-YIFWLTDKS-IRPQFE
-AFAQG---FYTCLDR--TSLS-IFTPE-ALKTVVEG-----------------------
----IQEID-----------MLEL----ERHARYDGGFGPS-------------------
------------------------HRVI-RDFWSIAR--------------------KFS
PEKKAQLLEF-------VTASDRVP------------VNG-----IS-SIMFVI--QKNG
VGD-------------------------------AVS-----------------------
-----------------
>Aspergillus_aculeatus_XM_020204775.1 .
LTVRRDSV-LTDALNQL--------------WRRERRELMR-PLKVQMGMDEG----EEG
LDHGGVQQEF---FRVL----MAEALDPAY--------GMFTLDGRTR------------
----------------ISWFQPCS------------------------------------
-----LEPLYKFELLGLLMSLAVYNGLTLPVNFPT--AFYRKLLG---------------
-------LKVKQLDHIQD----------------------GWPELTKG--------LEDL
LAWEEG---------------------------------------------DVG-DVFLR
-TYEFSYEV-----------------------------FGN-VETVD-MX----------
------------------------------------------------------------
-----------------------PAEEACLVTNANRG-QFVKD-YIFWLTDKS-VRPQFE
-AFAKG---FYTCLDR--TALS-IFTAE-SFKTVVEG-----------------------
----IETID-----------MREL----ERHARYEGGFSAS-------------------
------------------------HRVI-RDFWSIVR--------------------RYP
AEKRAQLLEY-------VTASDRVP------------VNG-----IA-SIMFVI--QKNG
VGD-------------------------------ARLPTSLTCF-GR-LLLPEY-SSRSV
LEEKLS-KALEN-ARGF
>Aspergillus_kawachii_BACL01000058.1 .
LMVRRDNI-LMDALNQL--------------WRRERRELMR-PLKVQMGMDEG----EEG
LDHGGVQQEF---FRVL----MAEALDPSF--------GMFTTDVRTR------------
----------------ISWFQPCS------------------------------------
-----WEPLYKFELLGLLMSLAVYNGLTLPVNFPT--AFYRKLLG---------------
-------LKVKHLEHIQD----------------------GWPELSKG--------LGDL
LTWEDG---------------------------------------------DVG-DIFMR
-TYEFSFEA-----------------------------FGA-VETVD-MQKV--------
DKDAPWPVSSMPSRARSGSRSLGYSPPWSEVRHFADHA----DLSPPSSMAAETAG----
--SYGEATKP-IDTLVPAQSPTPPADEASLVTNSNRA-QFVKD-YIFWLTDKS-VRPQFE
-AFAQG---FYTCLDR--TALS-IFTPE-ALKTVVEG-----------------------
----IQTID-----------IREL----ERHARYEGGFDPS-------------------
------------------------HRVI-RDFWSVVR--------------------RYP
AEKKAQLLEF-------VTASDRVP------------VNG-----IA-SIMFVI--QKNG
X----------------------------------RLPTSLTCF-GR-LLLPEY-SSRSV
LEEKLS-KALEN-ARGF
>Aspergillus_niger_XM_001394283.2 .
LMVRRDNI-LRDALNQL--------------WRRERRELMR-PLKVQMGMDEG----EEG
LDHGGVQQEF---FRVL----MAEALDPSF--------GMFTTDARTR------------
----------------VSWFQPCS------------------------------------
-----WEPLYKFELLGLLMSLAVYNGLTLPVNFPT--AFYRKLLG---------------
-------LKVKHLEHIQD----------------------GWPELTKG--------LGDL
```

LTWEDG---DVG-DIFMR
-TYEFSFEA-----------------------------FGV-VETVD-MX----------

----------------------PPADEASLVTNSNRA-QFVKD-YIFWLTDKS-VRPQFE
-AFAQG---FYTCLDR--TALS-IFTPE-ALKTVVEG-----------------------
----IQTID-----------IREL----ERHARYEGGFDPS-------------------
------------------------HRVI-RDFWSVVR--------------------RYP
AEKKAQLLEF-------VTASDRVP------------VNG-----IA-SIMFVI--QKNG
VGD-------------------------------ARLPTSLTCF-GR-LLLPEY-SSRSV
LEEKLS-KALEN-ARGF
>Diplodia_corticola_XM_020269417.1 .
LDVRRSHV-LEDTFDQL--------------WGLEKRQLLL-PLKVRVGKLEG----EQG
VDQGGVAQEF---FRVA----LAEAFNPDN--------GMFTVDPVTH------------
----------------VTWFQPFS------------------------------------
-----LEPLSRFELIGLLFSIAIYNGITLPVTLPG--ALYLKLIG---------------
--------KTVRPHHISE----------------------GWPDLVKS--------FQFL
LSCED--DVG-DILSR
-GYEFSFEA-----------------------------RGL-IVNIN-MDAV------KS
EFDVPSPYTMLDDSE---
------LDVTPSTSNPTPIVNWPPGTEAPLVTNANRE-RFVCD-YVAWLTDKS-IRPQYE
-AFARG---FHRCIPE--DHLQ-LLRPS-LLQHIVEG-----------------------
----STEID-----------THAL----ERVARYDGGFDAQ-------------------
------------------------HPFV-RTFWEVVH--------------------AMS
QEQKRKLLEF-------VTASDRVP------------VAG-----VE-SVVFWV--QRNG
PDG-------------------------------EGLPTSSTCF-GR-LLLPEY-DGKEK
LEKKLA-IALEN-SRGF
>Coniosporium_apollinis_XM_007785218.1 .
LDVRRSEV-LRDALDQL--------------WGREKRELLR-PLKVRMGASEG----EEG
VDHGGVSQEF---FQVA----LAEAFDPDH--------GLFTVDPITR------------
----------------MTWFQPAS------------------------------------
-----LEPLYKFELIGLLFSLAIYNGITLPVTFPL--AFYKLILH---------------
-------EPIDSIDAIRD----------------------GWPELAKG--------LEAL
LSWDDG---DVG-DVFSR
-TYEFSFET-----------------------------YGN-ISPTS-LVG---------
SMSPVA--
--------------------------EAPLVTNANRH-QYVSD-YIHWLTEKS-ISPQFS
-AFRGG---LSALIDG--NALT-LLDAP-ALRHLIEG-----------------------
----TQTLD-----------TRAL----EAVTLYADDYDAA-------------------
------------------------HPTI-RAFWEVVH--------------------AWS
QSRQRQLLEF-------VTASERVP------------VNG-----VG-SIRFEI--CRIG
ES--------------------------------EMLPQAATCF-GK-LLLPEY-PSKEV
LERKLG-VAVGE-CRGF
>Paraphaeosphaeria_sporulosa_XM_018177052.1 .
LDVTRHNP-LEETLDQL--------------WGQEKRKLLK-PLKVRIGILEG----EVG
LDQGGVTYEF---FRLI----LNEAFEPEN--------GMFTVDAENG------------
----------------MTWFQPAS------------------------------------
-----LEPLWKFEMVGVLLSLAIYNGITLPVTFPV--ALYDYVLS---------------
--EEHNSEDMMAVDFIAD----------------------GWPTLAKS--------FREF
LACPG--EVA-DVFMR
-DYTFSFSV-----------------------------FGQ-NIDVD-MQA---------
FRNRTWPDSTCSLESSSPSADMPPCR----------------------------------
-----------SLHDASWRRPRDVRTNPLTVTNEDRE-QYVCD-YVEWLTYRS-VERQLE
-AFTRG---FHTCLHR--TSLS-FFTPS-MLRSLVEG-----------------------
----SPTIS-----------IPLL---RTQAVRYEAPYHAT-------------------
------------------------HPTI-QDFWAVVE--------------------GYD
DEERNALLEF-------VTANERIP------------ITG-----YD-SVKFEI--SRSG
GDT-------------------------------ESLPTSSTCF-GK-LYLPEY-KDREK
LGKKLG-IAIRN-SKGF

```
>Leptosphaeria_maculans_XM_003843825.1 .
LTVSRENA-LEDTLDML--------------WGQEKRMLLK-PLKVKIGTEDTG---DYG
TDLGGVTNEY---FSLV----LGEAFRPEN--------GMFTIDPQTR------------
----------------MTWFQPGS------------------------------------
-----LEAPWKFEMIGLLFSIAVYNGVTLPVTFPL--AFYHMMLS-------------PV
DSSKEDLVKISTVDFIKD----------------------GWPDLAKS--------FGEL
LSWSDG--------------------------------------------DVG-EVIMR
-DAVFSYEV-----------------------------FGH-RIDHN-MAY---------
PFGHDPLEADS-------------------------------------------------
--------------------------EPEVVTNANRE-EFVGK-YIYFLTYAS-VETQLV
-AFKKG---FLTCLQP--KSLR-YFSPA-FLRKLVEG-----------------------
----SHDIS-----------MTAL----RRVVDYDEGYTPT-------------------
------------------------SATV-RAFWKVAE--------------------SYD
QEDARRLLEF-------VTASNRIP------------VTG-----YE-NVMFKI--SKVG
GQP-------------------------------HALPSSSTCF-GR-LYLPDY-ADVEV
LRQKLG-LAITH-SRGF
>Phaeosphaeria_nodorum_XM_001796840.1 .
LNVSREKP-LKDTLDQL--------------WGLEKRMLLK-PLKVQMGAEEG----ELG
QDHGGVTYEF---FRVV----LSEAFKPDH--------GMFTLDPQTR------------
----------------MTWFQPGT------------------------------------
-----LEPVWKFEMLGMIFSLAVYNGITLPVTFPH--AFYQFLLH-----------GVAP
LRATNLHNIRDCLDLVAD----------------------GWPELAKS--------FGQL
LSFEG---------------------------------------------DVA-DIFMR
-EYVFSYDV-----------------------------FGH-RVDHD-MDE---------
PYTQPGSPPLTSTPK---------------------------------------------
--------------------------EPKMVTNKNRA-QFIWD-YLNHLTYLS-VQPQLH
-AFKQG---FYACLNT--KAIA-LFTPY-TLRHLVEG-----------------------
----EQHIS-----------IPAL----RRCARYEDGYSAT-------------------
------------------------HPTI-ITFWRIVE--------------------QYS
QDDCRKLLEF-------VTASDRVP------------VTG-----YE-GITFHI--KRVG
DG--------------------------------DMLPTSSTCF-GR-LYLPEY-ETEEK
MGSKLL-LAIQN-SKGF
>Pyrenochaeta_lycopersici_GAJI01015873.1 .
LDVSREDP-LKDTLDQL--------------WGQERRMLLK-PLKVKMGQQEG----EVG
LDHGGVTYEF---FRVV----LSKAFKPDN--------GMFTIDPQTR------------
----------------MTWFQPRT------------------------------------
-----LEPDWKFEMLGILFSLALYNGITLPVTFPI--ALYDFLLP-------------PD
IPFRNRPHNRGTPDYIKD----------------------GWPGLAKA--------FGDL
LAWSDG--------------------------------------------DVG-DVMMR
-EYSFSYEV-----------------------------FGL-RFDHN-MKD---------
SFDYRAPAHTPAMSPPLAEM----------------------------------------
--------------------------ETPLVTNENRE-QFVKD-YIQYLTYAS-VAPQLW
-AFFKG---FQTCINP--KSLH-FFTPC-SLRNLVEG-----------------------
----TQHID-----------VKDL----RRCARYEEGYSAA-------------------
------------------------HHTI-VDFWAIVE--------------------GFT
QENCRHLLEF-------VTASDRVP------------VTG-----YE-SITFNI--VRIG
GEP-------------------------------DSLPSSSTCF-GK-LYLPEY-SDRQT
MRRKLE-LAIQN-SQGF
>Pyrenophora_teres_XM_003298747.1 .
LDVSRENP-LKDTLDQL--------------WGQDKRMLLK-PLKVKMGHEEG----EVG
QDHGGVTYEF---FRVV----LGEAFQPEN--------GMFTIDTETR------------
----------------MMWFQPYS------------------------------------
-----LEPCWKFEMLGILFSLAVYNGITLPVTFPL--AFYNYLWT-------------NG
DPFSKPMSLTDRISYIAD----------------------GWSTLAKS--------FEQL
LTWPDP--------------------------------------------NVE-DIFSL
-DYVFPYQV-----------------------------YGQ-HHAHN-LAH---------
PFTHSTSPPPSDNPPNP-------------------------------------------
--------------------------DPAPVTNTNRE-SYIRD-YITALTHTS-ISPQLS
```

-SFNKG---FKTCIST--PSLS-LFTPS-SLRNLIEG----------------------
----NTHIS-----------LTDL----KRCARYEDGYTPT-------------------
------------------------HSTI-RMFWDIVE--------------------RYD
QDDARRLLEF-------VTASDRVP------------VTG-----YE-SITFAI--HKIG
GAP-------------------------------MALPSSSTCF-GK-LYLPEY-ESRKG
MEGKLR-LAIRN-SRGF
>Pyrenophora_tritici_repentis_XM_001931912.1 .
LDVSRENP-LKDTLDQL--------------WGQDKRMLLK-PLKVRMGREEG----EVG
QDHGGVTYEF---FRVV----LGEAFQPEN--------GMFTIDPETR------------
----------------MMWFQPYS------------------------------------
-----LEPCWKFEMLGILFSLAVYNGITLPVTFPL--AFYNYLWT-------------NG
YTVSNPMTLTERISFISD----------------------GWPTLAKG--------FEQL
LTWPDP--NVE-DIFSL
-DSVFPYQV-----------------------------YGQ-RYAHN-LAH---------
FFTHSATPPPEDNLPNL---
--------------------------DAAPVTNENRE-EYIDN-YITTLTHAS-ISPQLS
-AFNKG---FRTCISP--PSLS-LFTPT-SLRNLIEG----------------------
----NTHIS-----------LTDL----KRCVRYEDGYTPT-------------------
------------------------HSTI-RMFWDIVE--------------------RYD
QDDARRLLEF-------VTASDRVP------------VTG-----YE-SITFAI--HKIG
GAP-------------------------------RSLPSSSTCF-GK-LYLPEY-ESREG
MEGKLG-LAIRN-SRGF
>Alternaria_arborescens_AIIC01000035.1_R .
LDVSREEP-LKDTLDQL--------------WGQDKRMLLK-PLKVKMGHKEG----EVG
LDHGGVTYEF---FRVV----LSEAFMPEN--------GMFTIDPQTR------------
----------------MTWFQPQS------------------------------------
-----LEPKWKFEMLGILFSLAVYNGITLPITFPL--AFYDCLQN-------------VG
NPLCKRVVDYDALGYIRD----------------------GWPSLAEG--------FQKL
LSWRDG--DVS-DVFMR
-EYIFSYEA-----------------------------FGQ-IVNQN-LSHE--------
SASITTPPEPSQE---
--------------------------EPDLVTNTNRE-QYVRA-YVRALTYDS-IAPQLT
-SFIRG---FLTCINA--KSLQ-LFTPS-TLRHLIEG----------------------
----TQHIS-----------ISDL----KRCAKYEDGYTAS-------------------
------------------------HSNI-RAFWDVVE--------------------DYS
QEDLRHLLEF-------VTASDRVP------------VTG-----YE-SITFHI--VRIA
GAES------------------------------GGLPSSSTCF-GK-LYLPDY-QDKKI
LEKKLG-LAIRN-SKGF
>Alternaria_alternata_XM_018528582.1 .
LDVSREEP-LKDTLDQL--------------WGQDKRMLLK-PLKVKMGHKEG----EVG
LDHGGVTYEF---FRVV----LSEAFRPEN--------GMFTIDSQTR------------
----------------MTWFQPQS------------------------------------
-----LEPKWKFEMLGILFSLAVYNGITLPITFPL--AFYDCLQS-------------VG
NPLCKRVADYDALGYIRD----------------------GWPSLAEG--------FQKL
LSWRDG--DVS-DVFMR
-EYIFSYEA-----------------------------FGQ-IVNQN-LSHE--------
SASITTPPEPSQE---
--------------------------EPDLVTNINRE-QYVRA-YVRALTYDS-IAPQLT
-SFIRG---FLTCINA--KSLQ-LFTPS-TLRHLIEG----------------------
----TQHIS-----------ISDL----KRCAKYEDGYTAS-------------------
------------------------HSNI-RAFWDVVE--------------------DYS
QEDLRHLLEF-------VTASDRVP------------VTG-----YE-SITFHI--VRIA
GAES------------------------------GGLPSSSTCF-GK-LYLPDY-QDKKI
LEKKLG-LAIRN-SKGF
>Alternaria_brassicicola_ACIW01002892.1_R .
LDVSREEP-LRDTLDQL--------------WGQDKRMLLK-PLKVKMGHKEG----EVG
LDHGGVTYEF---FRVV----LSEAFRPEN--------GMFTIDPQTR------------
----------------MTWFQPQS------------------------------------

-----LEPHWKFEILGILFSLAVYNGITLPVTFPL--AFYDCLQT-------------VG
GPRCKCVANYDALDYITD----------------------GWPKLAEG--------FREL
LSWRDG---DVQ-DVFMR
-EYVFSYEV-----------------------------FGQ-RVDQN-LDHK--------
PANVTTSSEPSPD---
--------------------------EPALVTNENRE-QYVRD-YVRALTYDS-VAPQLT
-SFTKG---FLTCINA--KSLQ-LFTPP-TLRHLIEG-----------------------
----TQHIS-----------MSDL----KRCAKYEDGYNAI-------------------
------------------------HSNI-RAFWDVVE--------------------RYS
QEDCRHLLEF-------VTASDRVP------------VTG-----YE-SITFHI--VKIA
GAP-------------------------------TALPSSSTCF-GK-LYLPDY-GDKEV
LARKLG-LAIRN-SKGF
>Setosphaeria_turcica_XM_008022320.1 .
LDVSREDP-LKDTLDQL--------------WGQDKRVLLK-PLKVKMGHDEG----EIG
VDHGGVTYEF---FRVI----LGEAFKPQH--------GMFTIDSQTR------------
----------------MTWFQPKS------------------------------------
-----LEPLWKFEMLGILFSLAVYNGITLPVTFPL--AFYEYLQT-------------TG
NPCCTCSTDYDAVEFIRD----------------------GWPTLAES--------FEQL
LLWNNG---DVE-DIFMR
-EYVFSYEV-----------------------------FGR-RIDHN-LKHP--------
FATPSTTSSAPQDETPSE--
--------------------------EPAMVTNANRD-HYVRD-YVRALTHDS-VAPQLS
-SFLQG---FLTCINA--KSLH-LFTPT-TLRALIEG-----------------------
----TQHIS-----------ISDL----KRCVKYEDGYSPT-------------------
------------------------HPTI-QFFWSTVE--------------------QYS
QQDRRHLLEF-------VTASDRVP------------VTG-----YE-GITFYI--SKIG
GAP-------------------------------HALPSSSTCF-GK-LYLPEY-REREV
MEKKVL-LAIRS-SRGF
>Bipolaris_oryzae_XM_007686146.1 .
LDVSREDP-LKDTLDQL--------------WGQDKRMLLK-PLKVKMGHGEG----EIG
VDHGGVTYEF---FRVI----LSEAFKPQH--------GMFTIDPQTR------------
----------------MTWFQPKS------------------------------------
-----LEPLWKFEMLGILFSLAVYNGITLPVTFPL--AFYEYLQT-------------NG
SSRCTHATEQDALEYIRD----------------------GWPALADS--------FQEL
LSWSEG---DVE-DIFMR
-EYVFSYEV-----------------------------YGQ-RIDHN-LHHP--------
FNNSSPNE--
--------------------------EPAMVTNQNRT-QYIRD-YIRALTHDS-VAPQLS
-SFLKG---FLSCIKL--ASLH-LFTPT-TLRALVEG-----------------------
----SQYIS-----------IAAL----KRCAKYEEGYTAT-------------------
------------------------SPTI-QSFWDIVE--------------------RYS
QEDRRHLLEF-------VTASDRVP------------VTG-----YE-SITFHI--ARIG
GAP-------------------------------HALPSSSTCF-GK-LYLPEY-RDRGV
MEGKML-LAIRS-SKGF
>Bipolaris_maydis_XM_014225376.1 .
LDVSREDP-LKDTLDQL--------------WGQDKRMLLK-PLKVKMGYGEG----EIG
VDHGGVTYEF---FRVI----LSEAFKPQH--------GMFTIDPQTR------------
----------------MTWFQPKS------------------------------------
-----LEPLWKFEMLGILFSLAVYNGITLPVTFPL--ALYEYLQT-------------SG
SSRHTHAVDKDALEYIRD----------------------GWPALADS--------FQEL
LSWSDG---DVE-DVFMR
-EYVFSYEV-----------------------------YGQ-RIDHN-LKHP--------
FGNESPDE--
--------------------------EPDMVTNENRT-QYIRD-YIRALTHDS-VAPQLQ
-SFLKG---FLSCIKP--KSLH-LFTPT-TLRALVEG-----------------------
----TQHIS-----------IADL----KRCAKYEEGYSVT-------------------
------------------------NPTI-QTFWEIVE--------------------KYS
QEDCRHLLEF-------VTASDRVP------------VTG-----YE-SITFHI--ARIG

GAP-------------------------------HALPSSSTCF-GK-LYLPEY-RDRGV
MEAKMM-LAIRS-SKGF
>Cochliobolus_sativus_XM_007697738.1 .
LDVSREDP-LKDTLDQL--------------WGQDKRMLLK-PLKVKMGHGEG----EIG
VDHGGVTYEF---FRVI----LSEAFKPQH--------GMFTIDPQTR------------
----------------MTWFQPKS------------------------------------
-----LEPLWKFEMLGILFSLAVYNGITLPVTFPL--AFYEYLQT-------------NG
SPRYTHAVEQDALEYIRD----------------------GWPALADS--------FQEL
LSWSDS---DVE-DVFMR
-EYVFSYEL-----------------------------YGQ-RIDHN-LKHP--------
FSNSNPSE--
--------------------------EPDMITNENRT-QYIRD-YIRALTHDS-VAPQLA
-SFLKG---FLSCIKP--KSLH-LFTPA-TLRALVEG-----------------------
----TQHIS-----------IADL----KRCAKYEEGYSTT-------------------
------------------------NPTI-QTFWEIVE--------------------KYS
QEDCRHLLEF-------VTASDRVP------------VTG-----YE-SITFHI--ARIG
GAP-------------------------------HALPSSSTCF-GK-LYLPEY-RDRGV
MEGKML-LAIRS-SKGF
>Bipolaris_zeicola_XM_007710746.1 .
LDVSREDP-LKDTLDQL--------------WGQDKRMLLK-PLKVKMGHGEG----EIG
VDHGGVTYEF---FRVI----LSEAFKPQH--------GMFTIDPQTR------------
----------------MTWFQPKS------------------------------------
-----LEPLWKFEMLGILFSLAVYNGITLPVTFPL--AFYEYLQT-------------NG
SPPYIHAVEQDALEYIRD----------------------GWPGLADS--------FEEL
LSWSDG---DVE-DIFTR
-EYVFSYEV-----------------------------YGQ-RIDHN-LHHP--------
FNNSSPTE--
--------------------------APDMVTNENRT-QYVCD-YIRALTHDS-VAPQLH
-SFLKG---FLSCIKP--KSLH-LFTPT-TLRALVEG-----------------------
----TQHIS-----------IADL----KSCAKYEEGYSAT-------------------
------------------------SPTI-QTFWDIVE--------------------KYS
QEDCRHLLEF-------VTASDRVP------------VTG-----YE-SITFHI--ARIG
GAP-------------------------------HALPSSSTCF-GK-LYLPEY-RDRGV
MEGKML-LAIRS-SKGF
>Bipolaris_victoriae_XM_014701678.1 .
LDVSREDP-LKDTLDQL--------------WGQDKRMLLK-PLKVKMGHGEG----EIG
VDHGGVTYEF---FRVI----LSEAFKPQH--------GMFTIDPQTR------------
----------------MTWFQPKS------------------------------------
-----LEPLWKFEMLGILFSLAVYNGITLPVTFPL--AFYEYLQT-------------NG
SPPYIHAVEQDALEYIRD----------------------GWPGLADS--------FEEL
LSWSDG---DVE-DIFTR
-EYVFSYEV-----------------------------YGQ-RIDHN-LHHP--------
FNNSSPTE--
--------------------------EPDMVTNENRT-QYVRD-YIRALTHDS-VASQLH
-SFLKG---FLSCIKP--KSLH-LFTPT-TLRALVEG-----------------------
----TQHIS-----------IADL----KRCAKYEEGYSAT-------------------
------------------------SPTI-QTFWDIVE--------------------KYS
QEDCRHLLEF-------VTASDRVP------------VTG-----YE-SITFHI--ARIG
GAP-------------------------------HALPSSSTCF-GK-LYLPEY-RDRGV
MEGKML-LAIRS-SKGF
>Verruconis_gallopava_XM_016357835.1 .
LDIERQSI-LESTFDQL--------------WGREHRELLK-PLKIVMRMDGKD---EDA
ADHGGVSQEF---FRLV----LAKAFEPEY--------GLFVVTEEQTK-----------
----------------MNWFKPLS------------------------------------
-----LEPLQTYELLGLLFGMAVYNGITLPVNLPF--AMYQVLCG---------------
-------RGPFEQFVLQD----------------------AWPSLHKS--------FQQL
TDWREEDG---DVE-EIFAR
-DYAFSFEA-----------------------------NGQ-TYTID-MLND--------

DSTLVDRLFYSEDS--
----------------EQSIFTDMRRSLTPVTYSNRI-DFISD-YKKWLVFNS-VRPQLE
-AFVKG---FRHIISI--TFTS-FITSP-TLQRFVEG-----------------------
----TRQIDASLLKAATSYAITSP----ETEPSYSDEYSSA-------------------
------------------------HPTI-QHFWSVVM--------------------NYD
QVQLQKLLTF-------VTASDRVP------------VQG-----YR-AIGFSI--EKHG
DV--------------------------------NMLPTSSTCF-GK-LFLPPY-ESEEA
VSRMLG-IAIEEGTSGF
>Baudoinia_panamericana_XM_007674977.1 .
LQVSRTNI-LQDTFDQL--------------WQRRASELKR-PLRVRLGEVDAL---DIG
QDLGGVQIEF---FNLL----CRELFAEAA--------QIFTTNVATG------------
----------------LSYFRPGS------------------------------------
-----LQPLYMFETVGLLLALAIYNGITIPVSFPR--AFYNILINGQSSNIP-LAPRNHI
RLASRSNPMDAIKDGIDD----------------------GWPQEHSS--------LIYI
LQQ--QDT-DLGL-
-EWSFPMEA-----------------------------NGY-RLYVT-RLQ---------
GDRLDHDDEVVGVD--HS
GPTDGAREVHGGVETLDWPGWRFADAPSRPVGRDQLG-DYVNT-YAWWLAYGC-VRPQWQ
-AFMRG---FYRGIDR--EDLA-LFTPA-ELKSVVEG-----------------------
----STHLD-----------IDEL----RRAARHEG-FDDD-------------------
------------------------LTYI-DTFWDIAS--------------------RWP
EETQKHLLKF-------VTAAERVP------------VGG-----AS-SITFIV--RPGV
AEDP------------------------------EALPTSSTCF-GT-LTLPIY-PSAEV
LERKLL-LAIEYGMEGF
>Pseudocerc_musae_GDIN01001970.1 .
LSVARTNV-LQDTFDQL--------------WQRRKSELRR-PLRVRLGQTDEL---EVG
HDLGGVQIEF---FNLV----CREILNEDL--------QMFTTDPKTG------------
----------------LSDFQPCS------------------------------------
-----LQPLHLFSLSGVLFALAVYNGITLPIRFPQ--VYYHILLS---------------
SLYPDNATVKLGVEALED----------------------GWPSIATS--------LKSI
LNE--EVH-GL---
-DYEFPLEA-----------------------------NGL-RLSV--LPPEKHEE----
GQCAFDLKVVDATPM-------------------------------THHGSSVRSFDQTR
ATLGTNFDQEEFSQGFPGWNVIKAAEEPMAVTSKNKN-DYVKT-YVDWLCYNS-VRPQFE
-AFLKG---FHDSQLFPEEALS-ILGPS-LLKSYIEG-----------------------
----NDIFD-----------LNDL----KAATRYDG-YDAK-------------------
------------------------SKYI-QTFWRVVG--------------------AWP
EEKQKQLLKF-------VTAAERIP------------ITG-----VK-NLVFII--KKAN
VENL------------------------------ENLPTSSTCF-GT-LMLPRY-GNADI
LREKLE-LALK------
>Sphaerulina_musiva_XM_016901028.1 .
LYVSRSNL-LKDTFDQL--------------WQRRKSELRR-PLRVRMGA-DEL---DIG
HDLGGVQVEF---FNIV----CKHIFSEEA--------QMFTTDPQTG------------
----------------LSYFTAGS------------------------------------
-----LQPLYMFELFGLLLALAIYNGITLPVRLPR--VLYHVLCD---------------
------VELPQPLAMIED----------------------AWPTLARS--------LRSL
LGE--FVE-DL---
-EYAFPLEA-----------------------------NGL-RLSCL-HVGNRHALLT--
GGRSELCIHTASLSS---SA
CGAMPDITEMEHAWPGWKLTMSTSAAEPQAVTPEHVE-DYINA-YSQWLCFDS-VLPQLE
-AFIHG---FRSSALIDSQTLS-LLGPS-RLQAYVEG-----------------------
----MDMLN-----------IHDL----KAAVRYDG-YVPT-------------------
------------------------QKYI-LAFWRVIA--------------------SWP
PEKQKLLLKF-------VTATERIP------------ISG-----AS-SLTFII--KRAY
PETL------------------------------QALPTSSTCF-GT-LMLPRY-PSTEI
MAEKLS-LALKFGAEGF
>Zymoseptoria_passerinii_AFIY01000172.1 .
LSVSRKNI-LQDAFDQL--------------WQRKKSEFRR-PLRVRLGEVDEL---EVG

HDLGGVQIEF---FNLV----CRELFSEQX--------ELFATDSTTG------------
----------------CSYFRPGS------------------------------------
-----LQPLYMFELVGLLIALAIYNGITLPVNLPW--VFYYFLCH--------------T
VKGPGLLRDHTTPEKIAD----------------------CWPATARS--------LRSI
LDE--DID-DLEL-
-EYSFPLEA-----------------------------NGL-RLAA--LLPLEQVT----
NGKMTMSIISAAPLS--------------------------------NRRDPATAVNGQV
SYTPPSLDYWAVQDDWPGWVIEKYSNTPQNVTSANKL-DYVDD-HIRWLTFSS-VRPQLA
-AFLKG---FHSAGLFEALTLR-LFGTD-RLKAYVEG-----------------------
----SDTLD-----------IDQL----RAATHYEG-YEAK-------------------
------------------------SKYI-NSFWDILR--------------------SWP
QEKQKQLLKF-------VTAAERIP------------ITG-----AS-QLTFVI--KRGY
PENL------------------------------NNLPTSSTCF-GT-LILPKY-ANVDT
LEEKLS-KAVEYGAVGF
>Mycosphaerella_graminicola_AFIU01000081.1 .
VSVSRNNI-LQDAFDQL--------------WQRRKSELRR-PLRVRLGEVDEL---EVG
HDLGGVQIEF---FNLV----CREVFSEQX--------ELFTTDSTTG------------
----------------CSYFRPGS------------------------------------
-----LQPLYMFELIGLLMALAIYNGVTLPVNLPW--VFYYFLAH--------------N
GEGRGSPRDHITLEKIVD----------------------CWPTTARS--------FRSI
LDN--DVD-GLEL-
-EYSFPLEA-----------------------------NGL-RLAA--HLPSSHSI----
GGKFEMSIMNAAPLS--------------------------------HQDESATAAEYRE
APKTPILDYRALLNEWPGWHLKSTTRNPKTVNSANKD-NYVQD-HIKWLTCYS-VLPQLT
-AFLKG---FHSAGLFEPLTLQ-LFGIE-ALKAYVEG-----------------------
----SDTLD-----------INDL----RAATRYDG-YDAK-------------------
------------------------SRYI-TSFWDIVT--------------------SWS
QEKQKQLLKF-------VTAAERIP------------ITG-----AS-QLTFVI--KRGH
PESV------------------------------NKLPTSSTCF-GT-LMLPKY-ASVET
LKEKLS-KAVEYGAVGF
>Saitoella_complicata_XM_019171883.1 .
LDIRRTHL-VHDAFAGI--------------SGRKTWELKR-PLRVRFVGGG-----EDG
VDQGGLQKEF---FASL----CHVVFDEDY--------GLFVTDADSQ------------
----------------LVWFNPAA------------------------------------
-----RDEVRTYELVGMLVGMAVYNGVILPLSFPK--MMYKKILG---------------
--------EEISLTDFAE----------------------WCPGVMRG--------MLTM
LEWEDG---DVE-EVFSL
-SYDYSWKH-----------------------------WDG-SVNTVCMKEGHE------
PGTIP---
------------------------------VTNANRA-DYAYD-YLAYTVHKS-IERQWE
-AFSRG---LAVCTNA--RMFT-LFSPT-ELQSVFQG-----------------------
----EQDLN-----------IDEW----RAITQYTDGYSPT-------------------
------------------------HRTI-RAFWSIVS--------------------ALP
PRLQRQLLVF-------VTASDRLP------------PGG-----MG-KLTFVI--QRNG
PDS-------------------------------DRLPSAQTCF-CR-LLLPEY-EGRRK
LKKMLM-TAVE------
>Homoloaphlyctis_polyrhiza_AFSM01007427.1_R .
LEVRRERL-VDDVLDQV-----------XKQIRKKESDLKK-PFKVRFVGGG-----EEG
QDQGGVQKEF---FQVI----VNKLLDPGY--------GMFVYMEETR------------
----------------SSWISGVS------------------------------------
-----LEPERQFELVGIIIGLALYNGVMLGIRFPM--LIYKKLLX---------------
--LGRG--------LQQL
LDWSDG---DVA-DVFF-
--RNFEISYEV---------------------------YGQ-VKTYP-LIAK--------
GEDIP---
------------------------------VTNENRA-EYVRL-YVEHYTDVS-IRKQFK
-AFQRG---FHKVVGG--QALK-MCKAE-ELELLICG-----------------------
--NTTTEMD-----------FGEL----QRCAEYDG-FEPDH------------------

```
------------------------QLIV-ACFWEIVH--------------------AMD
LEQKKQLLNF-------VTASDRVP------------LNG-----LG-KLMFLI--QRNG
PDT-------------------------------DRLPTALTCF-GR-LLLPEY-SSKEK
LQNRLQ-TAFEN-ATGF
>Batrachochytrium_dendrobatidis_XM_006681554.1 .
-------------------------------------DLKK-PVKVRFVGGG-----EEG
QDQGGVQKEF---FQAI----VSQLLDPGY--------GMFVYEEETR------------
----------------SSWINGAS------------------------------------
-----LEPERQFELVGIIIGLALYNGVMLGLRFPL--LLYKKLVD---------------
--------VRPTFQDFRD----------------------AFPTLGRG--------LQSL
LDWSDG---------------------------------------------DVS-DIFL-
--RNFEISYEV---------------------------YGQ-VKTYP-LVRN--------
GEDIP-------------------------------------------------------
------------------------------VTNDNRV-QYVEL-YVQHYTNES-IKRQFQ
-AFCRG---FHKVVGG--KVLK-MCRPE-ELELLICG-----------------------
--NTTAEID-----------FTEL----EHTAEYDG-FLPH-------------------
------------------------DEMV-VWFWEIMH--------------------KMD
LDQKRKLLNF-------VTASDRVP------------LSG-----FS-SLTFVV--QRNG
PDT-------------------------------DRLPTALTCF-GR-LLLPEY-ASKEK
LFDRLT-TAIEN-ATGF
>Spizellomyces_punctatus_XM_016749649.1 .
LEIRRQHL-VQDALDQI---------------RKKGADLKK-PLKVRFIGGG-----EEG
MDQGGVQKEF---FQVI----VSMLLDPAY--------GMFLYDQETR------------
----------------YCWINGAS------------------------------------
-----LESEKEFELVGTIVGLALYNGVILDVNFPK--VLYKKLLD---------------
--------EAPTLEDVKD----------------------AWPTLGRG--------LQQL
LEWSDG---------------------------------------------DVG-DVFL-
--RTFEISYDI---------------------------YGQ-VKHFP-LVEG--------
GEDIL-------------------------------------------------------
------------------------------VTNDNRK-EYVDL-YIHHYVVES-VRRQFS
-AFRRG---FHKVCGG--NALK-MCRAS-ELELMICG-----------------------
--TSTTDLD-----------FTEL----EQGAQYDDGYGPD-------------------
------------------------HEVI-QWFWEIVHS-------------------DME
LEQKKKLLNF-------VTASDRVP------------LKG-----LG-GLTFVI--QRNG
PDT-------------------------------DRLPTALTCF-GR-LLLPEY-SDKDK
LRDRLV-TAIEN-AKGF
>Basidiobolus_meristosporus_MCFE01000127.1 .
LEIRRDFF-IEDTLTQI---------------EAKARNLKK-PLKIRFVNGG-----EEG
IDQGGVQKEF---FQML----VAKLLDPVF--------GMFVYDEKTR------------
----------------YSWINGAS------------------------------------
-----KGEGKYFELVGVILGLALYNGVIIGANFPK--LIYKKILG---------------
--------KPVVLDDIKE----------------------GFPELGRG--------LEQM
LEWEDG---------------------------------------------DVY-DVFM-
--REFEIAYDE---------------------------SGA-MTTYP-LIPD--------
GSNIP-------------------------------------------------------
------------------------------VTNQNRE-EYVQL-YIDHFANKY-VSKQFS
-ALRDG---FLRVCGG--TAIS-MLRPE-ELELLLCG-----------------------
----NSEFN-----------LDEL----ERGAQYDDGYSPE-------------------
------------------------HYVI-RNFWSIVH--------------------ELS
HDQQKKLLIF-------VTASDRVP------------VKG-----LG-TVTFVI--QRNG
PDS-------------------------------ERLPTALTCF-GR-LLLPEY-SNRQK
LKKCLL-TAIEN-ARG-
>Basidiobolus_heterosporus_JNET01031885.1 .
LEIRREFF-IEDTLTQV---------------EAKAKNLKK-PLKIRFVNGG-----EEG
IDQGGVQKEF---FQML----VAKLLDPVF--------GMFVYDDKTR------------
----------------YSWINGAS------------------------------------
-----KESEKYFELVGVILGLALYNGVIIGANFPK--LMYKKILG---------------
--------KPITLDDIKE----------------------GFPELGRG--------LEQM
```

LTWKDG---DVY-DIFM-
--REFEIAYEE---------------------------DGI-MTTYP-LIPD--------
GMNIP---
------------------------------VTNHNRD-EYVQL-YIDHFANKY-VSKQFS
-ALRDG---FLRVCGG--TAMS-MLRPE-ELELLLCG-----------------------
----NSEFN-----------LDDL----ERGAQYDDGYSPE-------------------
------------------------HYVI-RNFWSIVH--------------------ELT
NEQQKKLLIF-------VTASDRIP------------VKG-----LG-TVTFVI--QRNG
PDS-------------------------------ERLPTALTCF-GR-LLLPEY-SNRQK
LKKCLL-TAIEN-ARG-
>Allomyces_macrogynus_ACDU01003958.1 .
LAVRRTHL-VADTWAQL---------------AAKPGEWKK-PLKVKFAN-------EDG
MDQGGVQKEF---FQVL----LAQLMDPLY--------GLFTYDESTR------------
----------------YSWLNAAS------------------------------------
-----LEPVRQFELVGIVLGLALYNGVIVDVRFPR--LLYRRLLG---------------
--------DAPTLDDVKC----------------------TWPDLGRG--------LQQL
LDWDDG---DVE-DVFM-
--RSFDVSVAA---------------------------YGA-VRTVE-LKPG--------
GSNLP---
------------------------------VTNANRH-EYVAL-YVDWVCRKS-VEPQFR
-ALRRG---FLKVCGG--YALG-LCRPE-EVEQLLCG-----------------------
---QEVDLD-----------MSAL----EKGCGYDDGYHAA-------------------
------------------------HPTI-RDFWSVVH--------------------AMD
LPHKKMLLEF-------VTASDRVP------------LKG-----LG-SLTFVV--QRNG
PDC-------------------------------DRLPTALTCF-GR-LLLPEY-AGKEK
MKRFLV-TAIEN-AKGF
>Absidia_glauca_FKIZ01006888.1 .
LEIRRQHF-VHDTIQQV---------------TKKWSDLKK-PLKVKFVDGG-----EEG
VDQGGVQKEF---FGVL----FEHLLDKDM--------GLFVCDPDTR------------
----------------LYWFRPCL------------------------------------
-----TPDVHSYEMFGVLLGLAIYNGVILNLAFPQ--VVWKILCMTTE---------NQV
DD-----ETAYTLDDLKQ----------------------GWPALAHG--------LEQL
LLWDEEDQGM---AVE-DVFA-
--QTYELSFAT---------------------------FEA-GLQTIPLVPH--------
GDTIP---
------------------------------VTSKNRG-QYVRD-YCTYFMYLQ-QREAIL
-ALRRG---MWCVIGS--EALESVVFPQ-ELEMITCG---------------------WE
QQGSASSLD-----------MKEL----EKVTEYDDGYHRD-------------------
------------------------HPVI-QHFWSIVHR-------------------DLT
PVQKRKLLYF-------VTASDRVP------------VGG-----LK-ELTFVV--QRNG
PDS-------------------------------DRLPTALTCF-SR-LLLPEY-ANYDK
LRDRLI-TSIEN-AKG-
>Phycomyces_blakesleeanus_XM_018428460.1 .
LEIKRISF-VEDAWDQV---------------SRKWTDIKK-PLKVRFVEGG-----EEG
MDQGGVQKEF---FGVL----FEKLLSAEL--------GLFEMDPESR------------
----------------LYWIRPYL------------------------------------
----DTDTVRHYEMTGVMIGLAIYNGIMINLPFPD--IFWKVLVAPTE---------QEV
DAQADN-HSLFTLSDLNT----------------------DWPSLASG--------LEQL
LQWPD--EVQ-DVFD-
--RNYEISIQV---------------------------FGQ-GIITVPLIPG--------
GESIP---
------------------------------VTNNNRE-AFVQD-YCTYFMYRA-QRDAIL
-ALRRG---VRSVIGS--RALD-LFTAA-EIQVVACG---------------------LR
QGPGSQDLC-----------MEDL----ESVTDYDDGYHAD-------------------
------------------------HPTI-RQFWSVVHN-------------------SLS
PDQKRELLLF-------VTASDRVP------------IGG-----LK-ELSFII--QRNG
PDS-------------------------------DRLPTALTCF-SR-LLLPEY-SSEEK
LEERLV-TAIEN-AKGF

>Mucor_irregularis_GFBC01014995.1 .
LEIRRENF-VADTFDQM---------------SRKWNDLKK-PLKVKFIEGG-----EEG
MDQGGVQKEF---FGVL----FEKLVAPDV--------GLFSQDESTR------------
----------------LCWIRPVL------------------------------------
-----DHDTRLYEMVGVMMGLSIYNGVIMNLQFPK--ILWKIFVMPNE---------ALI
DVMADR-YQLFTLEDLEE----------------------GWPDLGSG--------LRQL
LDWEDG---EVE-DVFC-
--RDYEISMDV---------------------------FGQ-GVVTKSLMPNES------
DVVVP---
------------------------------VTNENRE-QYVKD-YCKYFMYTA-QKEQIL
-ALRRG---MWSVIGS--RALN-LCTYE-ELEMVACG---------------------LR
QGPDALDLN-----------MAEL----ESIAEYDDGYNVD-------------------
------------------------HPTI-RQFWSVVQH-------------------DLT
NDQKKQLLLF-------VTASDRVP------------VGG-----LK-ELSFYI--QRNG
PDS-------------------------------DRLPTALTCF-SR-LLLPEY-ATKKK
LRDRLI-TAIEN-TKGF
>Rhizopus_stolonifer_JNDS01003412.1 .
LEIRREHF-VKDTLNQV---------------SLKWCDLKK-PLKIKFVQGG-----EEG
MDQGGVQKEF---FGVL----FEKLVSPDL--------GLFSLDESTR------------
----------------LGWIRPVS------------------------------------
-----GSDKRTFEMVGVMMGLAIYNGVMISIQFPK--VLWKVLAMPSE---------ALV
DALSER-HQLFTLQDLEE----------------------GWPTLGHG--------LRQL
LEWTDG---DVE-DIFC-
--RDYDISLEI---------------------------FGQ-GIVTLPLMSN--------
--PVP---
------------------------------VTNANRE-AYVRD-YCTYFMYTA-QREHIL
-SLRRG---LWSVIGS--RALH-LCTAE-ELEMVACG---------------------QR
QGPDAIELD-----------MTEL----EAVAEYDE-YTVD-------------------
------------------------HATI-RQFWSVVHH-------------------DLT
TEQKKQLLLF-------VTASDRVP------------VGG-----LK-ELSFFI--QRNG
PDSDRQILILLKYILILY----------------NRLPTALTCF-SR-LLLPEY-SSRRK
LCDRLI-TAIEN-TKG-
>Rhizopus_oryzae_GDUK01009237.1 .
LEIRREHF-VEDTFQQV---------------SRKWSDLRK-PLKVKFIEGG-----EEG
MDQGGVQKEF---FGVL----FEKLVSSEL--------GLFSQDESTR------------
----------------LCWIRPVS------------------------------------
-----NLDRRTYEMVGVMMGLAIYNGVMMNLQFPK--VLWKSLVMPSE---------AML
EAVAER-HHLFTLDDLEE----------------------GWPALGQG--------LKQL
LDWQDG---DVE-DVFC-
--RDYEISLEV---------------------------FGQ-GVVTQPLMPS--------
-PVVP---
------------------------------VTNANRE-AYVRD-YCTYFMYTA-QKEQIL
-ALRRG---LWSVIGS--RALH-LCTAD-ELEMVACG---------------------QR
QGPDAIELN-----------MADL----ESVAEYDE-YTSD-------------------
------------------------HPTI-RQFWSVIHH-------------------DLT
AEQKRQLLLF-------VTASDRVP------------VGG-----LK-ELSFYI--QRNG
PDS-------------------------------DRLPTALTCF-SR-LLLPEY-SSRRK
LRDRLI-TAIEN-TKGF
>Rhizopus_microsporus_JNEJ01003152.1 .
LEIRRESF-VEDTLQQV---------------SRKWSDLKK-PLKIKFVEGG-----EEG
MDQGGVQKEF---FGVL----FEKLISPEL--------GLFCQDESTR------------
----------------LCWIRPVV------------------------------------
-----NPDIRMYELVGVMMGLSIYNGVMMNLQFPR--VFWKVLVMPSE---------ALV
DALAERQQQLFTLEDLEE----------------------GWPTLGQG--------LRQL
LEWQDG---DVE-DIFC-
--RDYEISYEV---------------------------FGR-GVITQSLMRHPD------
DPVVP---
------------------------------VTNANRE-AYVKD-YCTYFMYTA-QREQIL

-ALRRG---MWSVIGS--RALY-LCTAE-ELEMVACG---------------------QR
QGPDAIELN-----------MSEL----EAVAEYDE-YTAD-------------------
------------------------HPTI-RQFWSVVHH-------------------DLT
AEQKRQLLLF-------VTASDRVP------------VGG-----LK-ELTFYI--QRNG
PDSDRYVYNHSLTSSLIF----------------NRLPTALTCF-SR-LLLPEY-SCRSK
LRDRLI-TAIEN-TKGF
>Allomyces_macrogynus_ACDU01000604.1 .
LSVRRDHV-VRDTLVQL--------------ASVPAAELRK-QLKVQFVD-------EVA
VDAGGVQKEW---LHLV----MRKVVAPEY--------GMFVPVDNNR------------
----------------GVWFRPWV------------------------------------
GTPAPKEVLEEYELIGKLVGLAVFHSVXINVPFPL--ALYKKLAN---------------
--------ERVTLADLTE----------------------IDPDLGKG--------LADL
AAYAND---DLE-DVYC-
--RNFTVDVPMQ-------------------------PGL-TVTVP-LVPD--------
GANVA---
------------------------------LTRANRM-EYIAQ-AVEFYLHRA-VEVPFR
-AFRRG---FEMVTRA--VRVX-LFTAS-EMQALIAG-----------------------
----DETLD-----------FHAL----EQRTTYDG-FTLD-------------------
------------------------SPVV-QXFWSIVH--------------------DLD
LVQHRQLLQF-------VTGSDRAP------------VGG-----LA-HLPFVL--VRAG
TDS-------------------------------VRLPSAHVCF-NA-LLLPEY-AERDM
LRHKLL-AAIAN-GEGF
>Entomophthora_muscae_GEND01029970.1 .
LNVTRENL-VQDTIREI--------------AQYNQSDLKK-PLKIKFCG-------EEA
EDAGGVRKEF---FMLL----LKDLIDPKY--------GMFKEYEDSR------------
----------------VVWFADVT------------------------------------
-----FETETMYFLIGVICGLAIYNFTIINLPFPL--ALYKKLLD---------------
--------EDVDLTDLQE----------------------LSPTLANS--------MQQL
LNYDGD---DFE-ETFD-
--LHFEISRDI---------------------------FGE-SKTEP-LKTN--------
GENIV---
------------------------------VTKENRQ-EFVDL-YVDFIFNKA-VNDQFK
-AFQSG---FMKVCSG--RVLK-IFRPE-ELMAMVVG-----------------------
----NEEYD-----------WKAL----ELNCEYKNGYTSS-------------------
------------------------DETI-KWFWEVFH--------------------NLP
LEEKKKFLLY-------LTGSDRIP------------IQG-----MK-AIHLCI--QPTN
DD--------------------------------KCLPVAHTCF-NL-LDLPRY-KTKER
LKYKLL-QAIQQ-TQGF
>Entomophthora_muscae_GENC01026301.1 .
LKVRRDNI-VNDALIGL-----------ELVAMSNPKDLKK-QLVVEFVG-------EQG
IDEGGVSKEF---FQLI----VEEIFNPDF--------GMFTHQEDTN------------
----------------TMWFNSTP------------------------------------
-----FENEAQFTLIGIIIGLAIYNNIILAVNFPM--VVYRKLMG---------------
--------GRGSFYDLQD----------------------WNPTLYRS--------LKSM
LEYQEP---DME-EVFM-
--QTFKISYKNV-------------------------FDE-VVEHE-LKQX--------
GSDLA---
------------------------------VGQENKQ-EFVDL-YSDFLLNKN-IERQFK
-AFKKG---FEMVTDE--SPLKLLFRPE-EIELLVCG-----------------------
----SRKFD-----------FVEL----EKSTEYEGGYTKD-------------------
------------------------TQII-KDFWSIVH--------------------DLP
EESKRKLLEF-------TTGSDRVP------------VGG-----LS-RLKLLI--TRHG
PDS-------------------------------DRLPTSHTCF-NV-LLLPEY-NSKEK
LEERLL-KAINY-SKGF
>Batrachochytrium_dendrobatidis_XM_006680517.1 .
IEVRRDHV-IRDALFQL--------------EGKSTHDLKK-QLRISFTG-------EEG
IDEGGVQKEF---FQLV----VRDMFSSSY--------GMFKYNDESR------------
----------------MCWLANMS------------------------------------

-DLRDSETLEEYNLMGRIIGLAIYNGVVLDIHFPL--ALYKKLLD---------------
--------ISPDLDDLAE---------------------LDPDLCRG--------LKQL
LEFEG---DIE-EVYG-
--RTFVAEIETS--------------------------VGE-RQTLE-LHEG--------
GANRP---
------------------------------VTAENRQ-EFVDL-LVDFLMNKS-ISSAFQ
-AFRDG---FDLVLEG--SALQ-LFRPE-ELQELICG-----------------------
----SPLLD-----------FYAL----EKTTQYDG-FDKD-------------------
------------------------SFVI-KSFWKVVH--------------------EFT
EEQKKLLLFF-------ATGSDRVP------------VGG-----LS-KLQFII--AKNG
PDS-------------------------------DRVPTSHTCY-NV-LLLCEY-ASIEK
LKDRLL-TALANSNCGF
>Entomophthora_muscae_GEMZ01017570.1 .
LEIRRDYI-IRDALFQL--------------SSKSPQDLKK-QLRVQFIG-------EEG
VDEGGVQKEF---FQLL----VREMFDPKY--------GMFTTQEDQR------------
----------------LYWFNPNP------------------------------------
--LDDDIALEEYRLIGRLIGLAIYNSVILDLHFPG--ALYKKLMG---------------
--------NAVGLDDLEV---------------------VDPSLGRG--------LRCL
LXFQD---DVE-AAYG-
--WTFQIEYEV--------------------------VGE-RFKYD-LKPN--------
GANIP---
------------------------------LKNDNRE-EFVEL-YIDFILNKA-IERQFK
-AFREG---FDHVCAG--SAIQ-LFRPE-EVEQLVCG-----------------------
----SSDLD-----------FEAL----EKVTQYDGGFTAA-------------------
------------------------TPVI-MFFWEVVH--------------------SFT
EAQKKMLLFF-------ATGSDRVP------------IGG-----LS-KLQFVI--AKNG
PDS-------------------------------DRLPTSHTCF-NV-LLLCEY-GSLEK
LRERLL-TAIHN-AEGF
>Mucor_irregularis_GFBC01027739.1 .
LEIRRDHI-ISDTLLQL--------------EEKSIHDLKK-QLRVQFLG-------EEG
VDEGGVQKEF---FQLI----VREIFDPKY--------GMFTFNEESR------------
----------------LCWFTPNQ------------------------------------
--VLDDINIREYKLVGLLLGLAVYNSVILDLHFPL--ALYKKLMD---------------
--------VPIGLSDLRQ---------------------LDPSLGNG--------LERL
LTFDG---DTV-SEYD-
--RFFQVDIQS--------------------------FGH-VFTCD-LKQN--------
GAKTQ---
------------------------------LTNENKS-EFVDL-YTHFVLSKS-VEKQFE
-AFREG---FQLVCQD--SAIK-IFRPE-EVEQLICG-----------------------
----SSDLD-----------FNAL----ETSTVYDGGWTKD-------------------
------------------------SDII-KYFWEIVH--------------------SFS
YDEKKKLLFF-------ATGSDRAP------------IGG-----LS-KLQFVI--AKNG
GDS-------------------------------DRLPTSHTCY-NV-LLLCEY-SSKEK
LKERLL-TSISN-AEGF
>Rhizopus_oryzae_GDUK01017940.1 .
LEIRRDHI-ISDTLLQL--------------EEKSIHDLKK-QLRVQFLG-------EEG
VDEGGVQKEF---FQLI----VREIFDPKY--------GLFVYNEESR------------
----------------LCWFSSNP------------------------------------
--VLDETNTREYKLVGLLLGLAVYNSVILDLHFPL--ALYKKLMN---------------
--------VDVDLSDLKQ---------------------LDLSLGKG--------LEML
LQYDG---DIE-AEYD-
--RFFQVNMES--------------------------FGH-VFTYD-LKPM--------
GSEIQ---
------------------------------LTNENRS-EFVNL-YTKFILDTS-VQKQFE
-AFKEG---FLLVCQD--SAIK-MFRPE-EVEQLICG-----------------------
----CSDLD-----------FEAL----EKSTVYDGGWTKD-------------------
------------------------SDII-KYFWEIVH--------------------SFS
YEDKKKLLFF-------ATGSDRAP------------IGG-----LS-KLQFVI--AKNG

GDS-------------------------------DRLPTSHTCY-NV-LLLCEY-SSKEK
LRERLL-TSISN-AEGF
>Debaryomyces_fabryi_XM_015609234.1 .
VRVRREYI-VQDSLRCI---------------KINPTNLKK-SLRVQFID-------EPG
VDAGGIKKEW---FLLL----TRALFSPQA--------GIFVNIEDSN------------
----------------LLWFNIVP------------------------------------
-----IENYEMYYLFGAILGLAIYNSTILNLKFPT--VLYKLLLG---------------
--------KPVDLSDYQK----------------------LYPVSASN--------LLKL
REYNDE---ELR-AME--
--LTFEASYTDL--------------------------LGK-VHTKQ-LIPK--------
GRHTV---
------------------------------VTSQNRE-TYIEK-YAKFFMSDG-IANQVN
-FFVKG---FSTVIGG--NALS-LFLPQ-EIELLLCG-----------------------
--SDEEKID-----------VAIL----RSITKYTG-WKDNDHAMT---------------
------------------------SSAV-KWFWEYMD--------------------SLS
YKQHKKVLAF-------VTGSDRVP------------ATG-----IQ-NLNFRI--SRLA
NGKDS-----------------------------NRLPVAHTCF-NE-LALYEY-SSKEK
MISKLT-MAVNE-SSGF
>Debaryomyces_hansenii_XM_460872.1 .
VRVRREYI-VQDSLRCI---------------KMNPTNLKK-SLRVQFID-------EPG
VDAGGIKKEW---FLLL----TRELFSPQA--------GIFVNIEDSN------------
----------------LLWFNVVP------------------------------------
-----IENYEMYYLFGAILGLAIYNSTILNLKFPT--ALYKLLLG---------------
--------KPVDLSDYQK----------------------LYPVSASN--------LLKL
REYSNE---ELM-TME--
--LTFEVSYTDF--------------------------LGK-VHTKQ-LIPK--------
GTNTI---
------------------------------VTVENRE-KYIEK-YAKFFMYDG-ITNQVN
-FFVKG---FSNVIGG--NALS-LFLPQ-EIELLLCG-----------------------
--SDEEKID-----------VAIL----RSITKYSG-WKDNDHATN---------------
------------------------SSPV-KWFWEYMD--------------------NLS
YKQHKKVLAF-------VTGSDRVP------------ATG-----IQ-NLNFRI--SRLA
NGNDS-----------------------------NRLPVAHTCF-NE-LALYEY-SSREK
MLSKLT-TAVNE-SSGF
>Hyphopichia_burtonii_XM_020219851.1 .
VQVRRNYI-VQDSLRAI---------------QMNNSNLKK-SLRVKFLG-------EPG
IDAGGLKKEW---FLLL----TKELFSFEA--------GMLINVEDSN------------
----------------YYWFNISK------------------------------------
-----NDNYDLYYLFGSILGLAIYNSTILELKFSM--ILYKLLLG---------------
--------LPIGLSDYQE----------------------LYPISCDN--------LFKL
RDYDDD---TLG-LLD--
--LTFEVSFNDS--------------------------RGK-VITKE-LIPG--------
GSKVK---
------------------------------VSSDNRE-YYIDK-YSRFFMVDG-FGNKID
-SFING---FNNVIGG--NALS-LFSAE-EIQLLLCG-----------------------
--SEESKLD-----------IDIL----KSITKYNG-WLSREVAIE---------------
------------------------SSII-KWFWDFLE--------------------KLP
YQKQKKFLLF-------VTGSDRLP------------ATG-----IQ-NLNFKI--TLLN
NGKKS-----------------------------HRLPVAHTCF-NE-LALYNY-ETQDV
MVQKIS-QAVEG-SSGF
>Pichia_sorbitophila_FO082048.1 .
VKVRREFI-VQDSLRCI---------------KLNTPNLKK-SLRVQFVN-------ELG
IDAGGLKKEW---FLLL----TRSLFSPEA--------GMFVNVEDSN------------
----------------YLWFSISP------------------------------------
-----AENNEMYFLFGAILGLAIYNSTILNLKFPT--ALYKLLLN---------------
--------IPVGLDDYRQ----------------------LYPLTAKN--------LVTL
KNYDSD---VLE-DLD--
--LNFEISFKDV--------------------------FGK-IHSKE-LIPN--------

GRHIR---
------------------------------VTKENAD-QYIRC-YSKFFMHDG-VASQIS
-FFIKG---FSTVIGG--NALS-LFLPE-EIELLLCG-----------------------
--SDEETLD-----------INTL----KSITKYAG-WKDQEQASN--------------
------------------------SIVI-RWFWEYLE-------------------ALS
FKEQRKFLLF-------VTGSDRVP------------ATG-----LQ-NLNFKI--TRLS
NGRDS-----------------------------HRLPVAHTCF-NE-LALYDY-SSKSK
FIQKLN-TAVNE-SSGF
>Metschnikowia_bicuspidata_XM_018858799.1 .
VRVRRDHI-VQDSLMCI---------------ELNQNNLKK-SLRVQFIN-------EPG
VDAGGLKKEW---FLLL----TRALFSSRT--------GMLHNVEQSN------------
----------------LLWFNVIP------------------------------------
-----MRDLEIYYLFGAVLGLAIYNSTILDLNFPL--GLYKILLD---------------
--------LPVGLADYKD----------------------LYPEAARN--------LFKL
REYTAD---ELD-AVN--
--LVFDVTFLDV--------------------------YGK-YHHRN-LIEN--------
GSNVA---
------------------------------VTVHNRE-MYIDK-YARFFLVEG-IEAQLR
-ALKNG---FSSVVDG--NAFS-LFLPD-EIQLLLCG-----------------------
--SEQKSFD-----------VDIL----KSVTKYSG-WGRKEDAEN--------------
------------------------SLTV-KWFWEYMS-------------------GLT
FQQQKQLLLF-------VTGSDRVP------------ATG-----IQ-NLSLRI--SRLS
NGRDS-----------------------------DRLPTAHTCF-NE-LAIYEY-SSREK
MIDKLS-KAIYM-LAGF
>Candida_auris_XM_018315906.1 .
VRVRRDYI-VQDSLRCI---------------QLNPNNLKK-SLRVQFIN-------EPG
VDAGGLKKEW---FLLL----TRALFSPHA--------GMLSYVEDSN------------
----------------FLWFNVVP------------------------------------
-----VDNFEMYYLFGAILGLAIYNLTILDLKFPI--TMYKILLG---------------
--------LPIGLADYQE----------------------IFPMSARN--------LFRL
RDYSAE---EIE-SLD--
--LTFEVTFSDP--------------------------FGT-RYTKD-LIPG--------
GSNLV---
------------------------------VDGDNRE-LYIDK-YARFFLWDG-MSKQLA
-AFKGG---FSNVVDG--NAFS-LFLPE-EIQLLLCG-----------------------
--SEESKFD-----------VDVL----QSVTNYSG-WASKEEATE--------------
------------------------SSTV-KWFWEYVS-------------------GLT
YKQQKKLLLF-------ITGSDRVP------------ATG-----IQ-NLTLKI--SRLK
TAGGDS----------------------------DRLPVAHTCF-NE-LALYDY-ISKKK
LAEKLD-KAVNM-SAGF
>Clavispora_lusitaniae_XM_002614672.1 .
VRIRREYI-VQDLLRCI---------------QLNPGNLKK-SLKVQFVN-------EPG
VDAGGLKKEW---FLLL----TRALFSPLA--------GMLGNVDDSN------------
----------------FLWFNVIP------------------------------------
-----VENAEMYYLFGAVLGLAIYNLTILDLKFPV--ALYKLLLG---------------
--------MPLGLADYKD----------------------IFPVAAEN--------LFKL
RDFSPE---DLE-MLE--
--LTFEVTFHDA--------------------------FGR-LHHRE-LVPG--------
GREIA---
------------------------------VNSENRE-MYIDK-YARFFLTDG-MKRQLQ
-LFTAG---FRSVVDG--NSFS-LFLPE-EIQFLLCG-----------------------
--SEETRFD-----------IDVL----KSITHYTG-WPSKEEALA--------------
------------------------SPIV-QWFWQYVT-------------------DLT
FDQQKRLLLF-------ITGSDRVP------------ATG-----AQ-NLTLKI--SRLR
SGEDS-----------------------------DRLPVAHTCF-NE-LSIYQY-SSREK
MVDKLT-KAVNM-LAGF
>Candida_intermedia_LT635765.1 .
VRVRRDYI-VQDSLRCI---------------QLNPANLKK-SLKVQFIN-------EPG

VDAGGLKKEW---FLLL----TKALFSPKT--------GMLYNVKDSN------------
----------------LLWFNVIP------------------------------------
-----IDNFEMYYLFGAVLGLAIYNSTILELKFPT--AMYKILLG---------------
--------LPVGLADYQE----------------------IFPEAAEN--------LFRL
RNYSEE---ELE-SIG--
--LNFEVSFMDV--------------------------FGR-AHQRE-LIPG--------
GTDVD---
------------------------------VTTNNRE-KYIDK-YARFFLMDG-MFRQLH
-AFKNG---FSSVVDG--NAFS-LFLPE-EMQLLLCG-----------------------
--SEESRFD-----------VDVL----QSVTSYTG-WSSKEEALK--------------
------------------------SPTV-KWFWEYVS--------------------GLT
FKQQKKVLMF-------ITGSDRVP------------ATG-----IQ-NLSLKI--SRLN
NGIDS-----------------------------DRLPVAHTCF-NE-LSIYEY-SSREK
MIDKLT-KAVNM-LAGF
>Meyerozyma_guilliermondii_XM_001487443.1 .
VRVRREYI-VQDSLHCI---------------KTNTANLKK-SLRVQFVN-------EPG
VDAGGLKKDW---FLNL----TKVIFDPSA--------GMLTNVDESN------------
----------------LLWFNLVP------------------------------------
-----VDNHEIYYLFGSILGLAIYNSTILDLRFPS--AMYKLLLG---------------
--------HPVGFGDYQQ----------------------LFPESAIN--------LSKL
LDYPPN---VIE-SLG--
--LTFEVNFKDL--------------------------FGR-LRQKE-LVAG--------
GSRRY---
------------------------------VSDENKH-EYVER-YCQFFMYDG-IANQVQ
-LFVKG---FSTVVSG--NALS-LFLPE-EIELLLCG-----------------------
--NDEGKLN-----------VEIL----RSITKYVG-FNGEPNQ----------------
------------------------SQLV-TWFWEYAN--------------------GLT
YSQQKKLLRF-------VTGSDRVP------------ATG-----LQ-NMAFKI--S-MA
GSRDT-----------------------------ERLPIAHTCF-NE-LALYNY-KSKEK
MVRKLN-IAINE-SAGF
>Candida_tenuis_AEIM01000013.1 .
VRVRRSHF-VQDSLNCI---------------LANQQNLKK-SLKVQFVG-------EAG
IDAGGLRKEW---FLLL----TKAMLSPET--------GMLVNVEESN------------
----------------YHWFNLVP------------------------------------
-----INNVDNYYLFGAVLGLAVYNSTILELNFPI--AFYKMLLK---------------
--------IPLGFSDFEQ----------------------LHPDLSRN--------LFKI
KSLSDE---ELN-MLD--
--LSFEISIFDL--------------------------FHN-VVNRE-LVPD--------
GKNIQ---
------------------------------VTSKNRD-LYIEK-YAKFRLTDG-VAEQTD
-SLLKG---FCSVTSG--NGLS-LFSPE-EIQLLLCG-----------------------
--NEEGKLD-----------LEIL----RSVTKYLG-WKDSEEAQN--------------
------------------------SQLI-NWLWEYLN--------------------ELS
YKEQKKFLSF-------VTGSDRIP------------ATG-----IQ-NLNLTI--K--K
AGSES-----------------------------ERLPTAHTCF-NE-LEIHRY-ATKEK
LYEKLS-MAIQG-SSGF
>Candida_tanzawaensis_XM_020207813.1 .
VKVRREYV-VQDSLNCI---------------KSNPTNLKK-SLRVQFLN-------EPG
IDAGGLRKEW---FSLL----TKGIFSPQT--------GMLYNVEDSN------------
----------------YLWFNVKP------------------------------------
-----IENFEMYHLFGAILGLAIYNSTILDLKFPL--AIYKLLLG---------------
--------QLVGLADYQQ----------------------LFPVSYKN--------LMAL
KKFDEA---NLS-SLD--
--LTFEVTYSDA--------------------------FGR-PHTAE-LIAG--------
GKKIQ---
------------------------------VNVSNLG-LYIQK-YCDFFIKDG-IKPQLD
-AFTTG---FARVIGG--NGLS-LFLAE-EIHLLICG-----------------------
--NEESDID-----------IDIL----KSVTQYAG-WSGGKEEATN-------------

```
------------------------SQVI-QWFWEYFQ--------------------SLN
ANQRKKFLVF-------VTGSDRVP------------ATG-----IQ-NLTFKI--SLMG
KDT-------------------------------NRLPVAHTCF-NE-LALYNY-RSKAI
LVDKVS-RAVNE-SAGF
>Scheffersomyces_stipitis_XM_001387054.1 .
VKVRRENI-VQDSISAI---------------KNNSNNLKK-SLRVQFVN-------EPG
VDVGGLKKEW---FLLL----TRALFNPQA--------GMVYNIEDSN------------
----------------YLWFNLVP------------------------------------
-----IENFEMYYLLGAVLGLAIYNSTILDLHFPM--ALYKILLD---------------
--------KPVGLDDYKQ----------------------LFPVSYGN--------LMKL
KKYSTE---------------------------------------------ELL-ALD--
--LTFEVSYQDL--------------------------FGK-TYSAE-LIKD--------
GRKIF-------------------------------------------------------
------------------------------VTAETRK-SYIEK-YTQFFLQEG-IKKQIT
-AFSSG---FKNVIGG--NGLS-LFLPE-EIQLLLCG-----------------------
--SEEGGID-----------VDVL----KSVTKYVG-WKTPDDGAD--------------
------------------------STVV-QWFWEYMC--------------------EIN
TQERKRLLMF-------VTGSDRVP------------ATG-----IQ-NLSFKI--SSQG
KDS-------------------------------NRLPVAHTCF-NE-LGLYNY-SSKEK
LVDKLV-TAVNE-SAGF
>Candida_orthopsilosis_HE681721.1 .
IKVRRDRI-VQDSMKAI---------------KLNLTNLKK-SLKVQFVN-------EPG
VDAGGLRKEW---FILL----TKEIFHPQA--------GLFHNVDDSN------------
----------------LLWFNMFP------------------------------------
-----LEDPEMYFLFGAVLGLAIYNSTILDLQFPV--ALYKILLG---------------
--------KSLDKEDYKQ----------------------LYPESYKC--------LTQL
RAMGDR---------------------------------------------QLK-DLE--
--LTFEVTVKDT--------------------------FGK-IYTRE-LITN--------
GSKVP-------------------------------------------------------
------------------------------VEKDNVD-DYVNK-YMKYFMREG-IKQQLD
-SFVEG---FNSVIAG--NALS-LFLEE-EIQLLLCG-----------------------
--SDDHRID-----------IDVL----QSITKYIG-WPSATDAVN--------------
------------------------SNII-KWFWEYLV--------------------TLS
NSQRKKLLVF-------VTGSDRVP------------ATG-----IQ-NLPFKV--SLLG
HGLDS-----------------------------ERLPIAHTCF-NE-LAIYNY-NSKAK
MIEKLN-KAINE-SSGF
>Candida_parapsilosis_HE605205.1 .
IKIRRDHI-VQDSMKAI---------------KLNLTNLKK-SLKVQFIN-------EPG
VDAGGLRKEW---FILL----TKEIFHPQA--------GLFHNVEDSN------------
----------------LLWFNIFP------------------------------------
-----LEDREMYFLFGAVLGLAIYNSTILDLQFPI--ALYKILLG---------------
--------KSLDKENYKQ----------------------LYPESYKN--------LYQL
RAMDDN---------------------------------------------QLK-HLE--
--LTFEVTIKDT--------------------------FGE-PHTRE-LVPN--------
GSKTN-------------------------------------------------------
------------------------------VSKANVD-DYINK-YMTYFMREG-IKQQLD
-AFIEG---FNTVIAG--NALS-LFLEE-EIQLLLCG-----------------------
--SEDHNID-----------FDVL----RSITKYIG-WPSATDAAN--------------
------------------------SCII-KWFWEHLA--------------------TMN
NSQRKKLLVF-------VTGSDRVP------------ATG-----IQ-NLPFKV--TLLG
HGLDS-----------------------------ERLPIAHTCF-NE-LTIYNY-NSKAK
MIDKLN-KAVNE-SSGF
>Spathaspora_passalidarum_XM_007377074.1 .
VKVRRERI-VQDSLECI---------------KLNSNNLKK-SLKVHFIN-------EPG
VDAGGLRKEW---FLLL----TRAIFHPQT--------GMLHNVEDSN------------
----------------YLWFNIVP------------------------------------
-----VENFEMYYLFGAVLGLAIYNSTILDLQFPL--ALYKILMG---------------
--------KLLDHNDYKT----------------------IFPVSYKN--------LMSL
```

KTMTAE---ELA-ELE--
--LTFEVSYSDA--------------------------FGT-NLTGD-LIPT--------
GSQIS---
------------------------------VTADNLD-EYIEK-YIGFFIKDG-IKRQVR
-SFHEG---FIHVIGG--NALS-LFLPE-EIELLLCG-----------------------
--SDDSGID-----------VDLL----KSVTKYSG-WKPSEDAQN--------------
------------------------SNII-KWFWEYMN--------------------DMS
NKERRKLLGF-------VTGSDRVP------------ATG-----IQ-NLVFKI--TLLK
AYDS------------------------------NRLPVAHTCF-NE-LAIYEY-SSKAK
LVGKLS-KAIFE-SAGF
>Candida_tropicalis_XM_002547964.1 .
IRVRRDHI-VQDSLQHI---------------KMNPDNLKK-SLRVQFMN-------EPG
VDAGGLKKEW---FLLL----TKEIFHPQSG-------GMFHNVEDSN------------
----------------LLWFNVIP------------------------------------
-----AENPDMYYLFGAILGLAIYNSTILDLQFPI--ALYKLLLG---------------
--------RPLDQDDYKQ----------------------LYPVSYKN--------LLNL
KTLSTS---DLL-LLD--
--LTFEVSYSDI--------------------------FDK-TYTVE-LIPD--------
GANTK---
------------------------------VKMHNLD-EYIEK-YTSFFMKDG-IRRQVD
-AFKTG---FNNVIGG--NALS-LFLPE-EIQLLLCG-----------------------
--SEDHGID-----------VDVL----KSVTKYIG-WRSPEDAAN--------------
------------------------SKII-TWFWDYMT--------------------KIS
NKEKKKLLIF-------VTGSDRVP------------ATG-----IQ-NLPFKI--SLLN
NGQDS-----------------------------TRLPIAHTCF-NE-LALYNY-TTKEK
FVEKLN-KAINE-SAGF
>Candida_dubliniensis_XM_002419306.1 .
IRVRRDHI-VQDSLQYI---------------KTNSDNLKK-SLRVQFVN-------EPG
VDAGGLKKEW---FLLL----TKEIFHPQS--------GMFHNVDDSN------------
----------------FLWFNIIP------------------------------------
-----VENPDMYYLFGAILGLAIYNSTILDLQFPI--ALYKILLK---------------
--------RGLDREDYRK----------------------LYPVSFKN--------LINL
KTMTNK---EIL-DLD--
--LTFEVSYSDM--------------------------FGK-HHTTE-LIAN--------
GANTQ---
------------------------------VTKLNLE-EYIDK-YTGFFMKDG-IEKQVD
-AFITG---FNNVIGG--NALS-LFLPE-EIQLLLCG-----------------------
--SDDHRID-----------VDVL----KSVTKYIG-WRSSEDAVD--------------
------------------------SKII-TWFWDNMN--------------------KMS
NKERKKLLIF-------ITGSDRVP------------ATG-----IQ-NLPFKI--SLLN
NGHDS-----------------------------HRLPIAHTCF-NE-LALYNY-STKEK
FVEKLN-KAVNE-SAGF
>Naumovozyma_dairenensis_JUTY01004063.1 .
IRVRRDHI-VQDSLQYI---------------KTNSDNLKK-SLRVQFAN-------EPG
VDAGGLKKEW---FLLL----TKEIFHPQS--------GMFHNVDDSN------------
----------------FLWFNIIP------------------------------------
-----VENPDMYYLFGAILGLAIYNSTILDLQFPI--ALYKILLK---------------
--------RGLDRDDYRK----------------------LYPVSYKN--------LINL
KSMSNK---EIL-DLD--
--LTFEVSYSDM--------------------------FGK-NHTTE-LVTN--------
GANIQ---
------------------------------VTKSNLE-EYIDK-YTGFFMKDG-IAKQVD
-AFITG---FNNVIGG--NALS-LFLPE-EIQLLLCG-----------------------
--NDDHRID-----------VDVL----KSVTKYIG-WRSSEDAVD--------------
------------------------SKII-TWFWDHMN--------------------KMS
NKERKKLLIF-------ITGSDRVP------------ATG-----IQ-NLPFKI--SLLN
NGHDS-----------------------------HRLPIAHTCF-NE-LALYNY-STKEK
FIEKLN-KAVNE-SAG-

>Candida_albicans_XM_718015.1 .
IRVRRDHI-VQDSLQYI---------------KTNSDNLKK-SLRVQFAN-------EPG
VDAGGLKKEW---FLLL----TKEIFHPQS--------GMFHNVDDSN------------
----------------FLWFNIIP------------------------------------
-----VENPDMYYLFGAILGLAIYNSTILDLQFPI--ALYKILLK---------------
--------RGLDRDDYRK----------------------LYPVSYKN--------LINL
KSMSNK---EIL-DLD--
--LTFEVSYSDM--------------------------FGK-NHTTE-LVTN--------
GANIQ---
------------------------------VTKSNLE-EYIDK-YTGFFMKDG-IAKQVD
-AFITG---FNNVIGG--NALS-LFLPE-EIQLLLCG-----------------------
--NDDHRID-----------VDVL----KSVTKYIG-WRSSEDAVD--------------
------------------------SKII-TWFWDHMN--------------------KMS
NKERKKLLIF-------ITGSDRVP------------ATG-----IQ-NLPFKI--SLLN
NGHDS------------------------------HRLPIAHTCF-NE-LALYNY-STKEK
FIEKLN-KAVNE-SAGF
>Lodderomyces_elongisporus_XM_001526046.1 .
ISVRRDHV-VQDSLRCL---------------QNNLGSFKK-SLRVLFLN-------EPG
IDAGGVRKEW---FMLL----TKEIFNPLS--------GMFSNIEDSN------------
----------------LLWFALGP------------------------------------
-----TEREDMYQLFGSILGLALYNSTVLELNFPQ--ALYKVLAG---------------
--------KSLNQLDYKT----------------------LHPTIYRS--------LSSL
RKVDAN---ELN-TLG--
--LTFEVTYKDV--------------------------LGT-TQTKE-LIEG--------
GADVL---
------------------------------VDESNLE-QYIDL-YTLFFLRDG-VSRQLD
-AFIDG---FKNVIGG--NALS-LFDEE-EIELLLCG-----------------------
--HTDHGID-----------VEIL----ESVTKYTG-WPSAQEAKN--------------
------------------------STVI-KWFWEIMQ--------------------SMD
QNHRKLLMSF-------VTGSDRVP------------ATG-----IQ-NLPFRI--HLLN
DNQDS------------------------------CRLPLAHTCF-NE-LAIYNY-SSKEK
LSQKLY-RAMEE-SSGF
>Wickerhamomyces_anomalus_XM_019181002.1 .
IDVRRTHI-SNDSLRCI---------------KEQAGDLQK-ALKVHFVG-------EPG
VDVGGLKKEW---FSLL----TAEIFSQEN--------GMFEYNDESH------------
----------------LCWFAAMP------------------------------------
----LERNDELYYMVGVVLGLAMYNSTILDLRFPL--ALYKKLLG---------------
--------KKVDLDDYSE----------------------LFPQTGLG--------LSKL
LATKE--DVS-DYG--
--LYFETTYKDL--------------------------LGN-PITKE-LVPN--------
GSKIP---
------------------------------VNLLNRH-EYVGK-WVNFYMNES-IKAQFD
-SFHTG---FHSVIGG--NALS-LFAPK-EIEMIICG-----------------------
DNLNDSKLD-----------VESF----RSITKYNG-WASPTDANE--------------
------------------------SNIV-SWFWEWFG--------------------RLS
NSQHKKFLQF-------VTGSDRIP------------ATG-----IH-TMQFKI--TRMP
YYGPHNA----------------------------IRFPVAHTCF-NE-LCLYEY-RSKDE
LYHKLN-WAINE-SRGF
>Wickerhamomyces_ciferrii_XM_011278463.1 .
IKVRRNHI-SNDSLRCI---------------KEQKDDLKK-SLKVEFIG-------EPG
IDAGGLKKEW---FGLL----TKELFQQEN--------GMFYYNEESK------------
----------------LCWFAVLP------------------------------------
----LEKNDELYFMLGAVLGLAIYNSTILGLHFPL--ALYKKILG---------------
--------KKVGIDDYLE----------------------LFPDTARG--------LLKL
YEINND---ELE-NLD--
--LFFETTYKNY--------------------------FDE-PITKE-LIPN--------
GSKIK---
------------------------------VNKSNLD-NYINE-WVNFYLNDS-IESQFK

-NFQNG---FHNVIGG--NSLS-LFSPK-EIELLICG-----------------------
-NNDTCKID-----------INEF----KSITKYQG-WSTRDLATN--------------
------------------------STLI-KWFWEWFE--------------------NLN
YQQQKKFLQF-------ITGSDRIP------------ATG-----IH-TMNIKI--TKMP
NYGPSIMNKSS-----------------------SRLPVAHTCF-NE-LCIYGY-QTREE
MWNKLD-WAINE-SKGF
>Cyberlindnera_fabianii_LK052887.1 .
--VRRTHI-TNDSLRCI---------------KNHQGDLKK-VLKVEFAG-------EPG
IDAGGLKKEW---FQLL----TRELFSQNA--------GMFFYNDESH------------
----------------LCWFSPAP------------------------------------
----LEGNDELYYLVGVILGLAIYNSTILDLKFPV--ALYKKLMG---------------
--------KKVTFEDYNQ----------------------LFPSTGKG--------LSTL
LVSKD--SIE-DMG--
--IYFETTFINL--------------------------LGE-PVVKE-LIPG--------
GSDVL---
------------------------------VTNDNRR-EYIER-WVDYYMNKS-VERQFK
-SFYNG---FHNIIGG--NALS-LFSPR-EIELVICG-----------------------
--NNESKID-----------TESF----KAITKYSG-WGKVGPEVMT-------------
------------------------IEIV-QWFWEWFG--------------------KLN
YDRQAKFLVF-------VTGSDRIP------------ATG-----VS-TMTFKM--TKLT
DYSGSG----------------------------GRLPCAHTCF-NE-LCLYDY-SSKRQ
FWDKMD-MAITE-SEGY
>Cyberlindnera_jadinii_XM_020215058.1 .
FHIRRSHI-TDDSLHCI---------------KQHQNELKK-ALKIEFVG-------EPG
VDAGGLKKEW---FQLL----IRDLFKQDN--------GMFLFNSESR------------
----------------LCWFAPEP------------------------------------
----SEKNDELFFLVGVILGLAIYNSTILDLKFPL--ALYKKLLG---------------
--------KSIHLDDYTE----------------------LCPSTGEG--------LRKL
LHCDHD---EVE-EMG--
--IYFETSFQSG--------------------------PGE-VITRE-LCPN--------
GSKRL---
------------------------------VTHESRR-EYVNR-WVDFYMNRS-IEEQFN
-SFNRG---FQMIIGG--NAIS-LFAPR-EIEMIICG-----------------------
--NGDTRID-----------VSSL----KSITKYNG-WSSSEEASN--------------
------------------------SQLI-KWFWEWVE--------------------TLN
NAKQKQFLCF-------VTGSGRIP------------ATG-----IT-TMSFKI--TRLK
SYGTS-----------------------------TRLPMAHTCF-NE-LCLFEY-RSREE
MFRKLN-ISISE-SEGF
>Saccharomycopsis_fibuligera_CP015982.1 .
VRVRREYI-THDSLRCI---------------KDSAKDLKK-SLKVEFIG-------EPG
IDAGGLKKEW---FLIL----TKKLFDPNH--------GLFYNVKESN------------
----------------FLWFTLNP------------------------------------
---NTTENLELYYLVGVVLGLAIYNSTILDLKFPP--ALYKLLLN---------------
--------KPVNFDDYSQ----------------------IYPETAKN--------LKKM
ADYTKS---DFA-EVFAD
--TNFEITYKNQ--------------------------FDE-VHTVE-LVPN--------
GSAMP---
------------------------------VTSINKN-KFLKK-YADFYLKIG-IKDKFT
-SFKSG---FENVING--NAFS-LFGAE-EIEMILCG-----------------------
SDEGNRKFD-----------LESL----RSVTRYNG-FGDNTNIGE--------------
------------------------VSVV-KWFWGFFE--------------------SLT
IKQQRRLMLF-------ITGTDRIP------------ATG-----IV-SMNFKI--TRLG
GDS-------------------------------NRLPVAHTCF-NE-LCIYEY-ESEAK
FIDKLT-TAMNE-SEGF
>Ascoidea_rubescens_XM_020189731.1 .
IRVRRNFI-TTDSLFSI---------------KKHKNDLKK-SLRVEFIG-------ESG
LDAGGLKKDW---FLLL----TKNLFNPEN--------AMFFFSDESN------------
----------------LTWFTLKP------------------------------------

----IDNNYELYHLVGVVLGLAIYNSTILDLNFPS--ALYKKLLN---------------
--------KPVNINDYAI----------------------LYPQTAHG--------LNAL
LNYNND--DFE-DIFS-
--LSFVVTYKDL--------------------------FGN-ILTAD-LIPN--------
GSNIN---
------------------------------VTNKNKH-LYVHK-YVDFFLNKA-IHLQFN
-AFKSG---FDNVING--NAIS-LFSWD-EIQLLLCG-----------------------
SDEQHGKLD-----------VVSL----RRISKYNG-WDSVQQAQN--------------
------------------------SNVV-NWFWHYFE--------------------NLS
YLHQKKLLLF-------VTGSDRLP------------AIG-----IT-SLNFKI--TKLG
DDS-------------------------------NNLPIAHTCF-NE-ICLYEY-SSEEK
FLQKLE-FAIFE-SEGF
>Babjeviella_inositovora_XM_019128121.1 .
VRVRRTHI-VPDSLRCI---------------QDNRANFKK-GLRIEFVG-------EPG
IDVGGLRKEW---FLLL----SRDLFNADN--------GMFTMSQESN------------
----------------LLWFSLAP------------------------------------
-----LENDEMYYLFGVVLGLAIYNSTILDLSVFP-FALFRALLG---------------
--------KPLDLEDYSE----------------------LYPETANG--------LRHL
LSYDG---NVE-DLS--
--LTFEATYRDV--------------------------FGS-VQTRE-LVPK--------
GGDIS---
------------------------------VTSENRV-EYVDR-YVDFFLMQG-LLEQFR
-PFKKG---FDNVTGG--NALS-LFTAH-EIQLLLCG-----------------------
--SSEGSLD-----------VKIL----RSVSKYAG-WGSVERAEE--------------
------------------------ANIV-VWFWDYFE--------------------EMP
YREQKLLLRF-------VTGSDRVP------------ATG-----VH-TMNFKV--VKLS
GHH-------------------------------ERLPIAHTCF-NE-LCLYEY-RSKEV
LQQKLS-VAINE-SSGF
>Komagataella_phaffii_CP014716.1 .
VRVRRGLI-ANDSLKCI---------------KNHTNDLQK-SLKVEFVD-------EPG
VDAGGLKKEW---FVLL----TRELFHPNK--------GLFSYDETSK------------
----------------LAWFTISN------------------------------------
-----IDHEELYYLVGVVLGLAIYNSTILDLRLPF--VLFKKLLN---------------
--------KKPSLEDFCE----------------------LYPENGGS--------LRKL
LKLQDD--EIW-DNME-
--IYFDVTYSDL--------------------------LGA-IKTEE-LVPG--------
GSSIK---
------------------------------VNNSNKN-EYVSR-YLDFYLNKV-ISNSFN
-SFYKG---FYSVIGG--NALS-LFSPH-EIQLIVLG-----------------------
DDNDGKKVD-----------TEIL----KSVTNYNG-WDSREAAVT--------------
------------------------SKQV-AWFWEFFD--------------------SIS
YKQQKKLLLF-------ITGSDRIP------------ATG-----IQ-NLPFKI--TRLK
GNK-------------------------------ERFPIAHTCF-NE-LCIYEY-PDKNT
MWRKLE-YGMNE-SEGF
>Komagataella_pastoris_CP014585.1 .
VRVRRGLI-ANDSLKCI---------------KNHTNDLQK-SLKVEFVD-------EPG
VDAGGLKKEW---FVLL----TRELFHPNK--------GLFSYDETSK------------
----------------LAWFTISN------------------------------------
-----IDHEELYYLVGVVLGLAIYNSTILDLRLPF--VLFKKLLN---------------
--------KKPSLEDFCE----------------------LYPENGGS--------LRKL
LKLQDD--EIW-DNME-
--IYFEVTYSDL--------------------------LGT-IKTDE-LVPG--------
GSSIK---
------------------------------VNNSNKN-EYISR-YLDFYLNKI-ISNSFN
-SFYKG---FYSVIGG--NALS-LFSPH-EIQLIVLG-----------------------
DDNDGKKVD-----------TEIL----KSVTNYNG-WDSREAAIT--------------
------------------------SKQV-SWFWEFFD--------------------SIS
YKQQKKLLLF-------ITGSDRIP------------ATG-----IQ-NLPFKI--TRLK

GNK-------------------------------NRFPIAHTCF-NE-LCLYEY-PDKNT
MWRKLE-YGMNE-SEGF
>Pachysolen_tannophilus_CAHV01000221.1 .
IRVRREHI-SNDSLRCI---------------QQHPNDLRK-SLRVEFIN-------EPG
IDAGGLKKEW---FLLL----SKELFNPDK--------GLFTYNETSC------------
----------------LGWFSISP------------------------------------
-----IENDEMYYLVGVVLGLAIYNSTILDLQFPR--AFYKKLLG---------------
-------NTNVGLDDFIE----------------------LYPEAGKN--------LKKI
LHYHKND--DLK-SLE--
--LYFEVTFRDI--------------------------FGL-IHTRE-LIPN--------
GSKIQ---
------------------------------VTFENKI-EYINK-YLLFYLDEI-IKTQLD
-AFKRG---FDQVIGG--NALS-LFTAE-EIELILIG---------------------DD
SKFGDKFLN-----------VDIL----KVVSKYQG-GLTEN------------------
------------------------SKLV-LWFWEYVE--------------------TLN
YKQQKKLLLF-------ITGSDRLP------------ATG-----IQ-SLNFKI--TLLN
GSK-------------------------------SRFPVAHTCF-NE-LCIYNY-DLKQD
FYNKLD-FAVNE-SEGF
>Dekkera_bruxellensis_AHIQ01000073.1 .
IRVRRSFI-TEDSLRSI---------------KEHRHDFKK-SLKIEFVN-------EPG
VDGGGLKKEW---FLLL----TKELFDPSK--------KLFSYYNPSN------------
----------------LCYFSISN------------------------------------
-----VNNEELYYLVGAVLGLAIYNSTILDLKLPR--ALYKKLLG---------------
--------HKVTLDDFTQ----------------------LDQNAGKG--------LKKL
LLLE---NVE-ELS--
--IYFEVSFKDV--------------------------EGQ-LITHE-LISN--------
GSNVL---
------------------------------VNDTNKY-EYVER-YLNFFLSDI-CKSRFD
-SFQRG---FYNVVGG--NALS-LFTPX-EIEQILIG-----------------------
-DSNEGAID-----------TMMM----RSITRYKG-FESN-------------------
------------------------DKVI-SWFWHYFD--------------------SCV
PEVQKKILFF-------VTGTYRLP------------ATG-----LP-SLXFKI--SRIX
AERGC-----------------------------SRLPTSHTCF-NE-LCLSDY-ESEXE
FRNKME-IAINN-CEGF
>Ambrosiozyma_kashinagacola_IAAG01000935.1 .
IRVRRSHI-TSDSLRSI---------------KAHQSSFQK-LLRIEFVD-------EPG
IDAGGLKKEW---FLLL----SKDLFSADK--------GLFAVNDESG------------
----------------LTWFAISN------------------------------------
-----IDNTELYYLVGVVLGLAIYNSTILDLKLPN--AMYKMLMG---------------
--------KRVTLNDYIQ----------------------LNPDTGHG--------LKRL
LKMK---DIE-ELD--
--LTYEVAYKDM--------------------------FGD-VQTAE-LIPN--------
GATTK---
------------------------------VTNDNKY-DYVDQ-YYLFFMRKL-IEKQFS
-AFSKG---FQNVVGG--NALS-LFTPE-EIELILIG-----------------------
DDYNNQPLD-----------LDVL----RSVTKYSN-CSQE-------------------
------------------------DQSV-QWFWDYFG--------------------KLS
HSRQKKLLLF-------VTGSDRVP------------ATG-----LP-SLNFKI--TKLQ
STT-------------------------------ERLPIAHTCF-NE-MCLYDY-ASQEM
FDHKMD-LAIEC-SEGF
>Ogataea_methanolica_IAAJ01004582.1 .
IRVRRTSI-TMDSLRAI---------------KSHQSDFKK-LLRIEFVD-------EPG
IDAGGLKKEW---FLLL----TKDLFHPEK--------GLFSYNEESQ------------
----------------LGYFAVSP------------------------------------
-----LDNSELYYLVGVVLGLAIYNSTILDLRLPK--ALFKKLMH---------------
--------KKPTLNDFIE----------------------LNPETGHG--------LKRL
AKMR---GAE-SLD--
--LYFEVTYKDI--------------------------FGD-LKTVQ-LIKN--------

GSDLK--
------------------------------VTDKNKH-AYIAA-YYSYFLDSL-VEHPFK
-SFSNG---FYNVIGG--NALS-LFTPE-EIELILLG-----------------------
DDNNHQKMD-----------INIL----RSVTEYNG-FKPT-------------------
------------------------DILI-QWFWEFFE--------------------DLS
IKDQKKLLLF-------VTGSDRLP------------ATG-----LP-SLHMKI--TRIE
HRDI------------------------------TRLPIAHTCF-NE-LCLFNY-ESQEV
FVDKLR-MAIEC-SEGF
>Ogataea_parapolymorpha_AEOI01000011.1 .
VRVRRNFI-TPDSLRCI---------------KSHQTDFKK-LLRVEFVD-------EPG
IDAGGLKKEW---FLLL----TKDLFNADK--------GLFSYDETSG------------
----------------LCWFALSN------------------------------------
-----LDNDELYYLVGVVLGLAIYNSTILDLRLPK--ALYKKLMG---------------
--------QKVTLNDFIQ----------------------LNPDVGHN--------FKRL
LKLE---DVT-DLE--
--LFFTVSYADI--------------------------FGV-MHHED-LIRE--------
GSSIR---
------------------------------VTNENKY-EYVER-YCSFFLNDK-VKAPFK
-SFLNG---FQHVIGG--NALS-LFTPE-EIELILIG-----------------------
EDYRNQKID-----------IEIL----KSVTKYNG-FSET-------------------
------------------------DTVV-NWFWDYFE--------------------RKS
AADQRKILLF-------ITGSDRLP------------ATG-----LP-SLTFKI--TKLV
DTNP------------------------------NRLPIAHTCF-NE-LCLYVY-PSKEI
FVSKLD-KAIWY-SEGF
>Ogataea_polymorpha_XM_018355154.1 .
VRVRRNFI-TPDSLRCI---------------KSHQTDFKK-LLRVEFVD-------EPG
IDAGGLKKEW---FLLL----TKDLFNADK--------GLFSYDETSG------------
----------------LCWFSISN------------------------------------
-----LDNDELYYLVGVVLGLAIYNSTILDLRLPK--ALYKKLMG---------------
--------QKVTLNDFIQ----------------------LNPDVGHN--------FKRL
LKLE---DVS-DLE--
--LFFTVSYADI--------------------------FGV-MHHED-LVRG--------
GSSIR---
------------------------------VTNENKY-EYVER-YCSFFLNDK-VKAPFK
-SFLNG---FQHVIGG--NALS-LFTPE-EIELILIG-----------------------
EDYRNQKID-----------IEIL----KSVTKYSG-FFET-------------------
------------------------DTVV-NWFWDYFE--------------------RKS
AADQRRILLF-------ITGSDRLP------------ATG-----LP-SLTFKI--TKLV
DSNP------------------------------NRLPIAHTCF-NE-LCLYVY-PSKEI
FESKLY-KAIWY-SEGF
>Kazachstania_naganishii_HE978322.1_a.
IKVRRNEI-MNDSLRAL---------------RQHQGDFLK-SLRVEFVN-------EPG
IDAGGLRKEW---FMLL----TREMLSKQN--------GLFRYVEESR------------
----------------YCWFEFQP----------------------------------DK
LSKQSLASQSFYFLFGVVVGLAIYNGVILDLKFPR--ALYRKMCS---------------
--------EKLTFADYFE----------------------LFPQTGKN--------LKLM
LNYSAE---DFT-DVFG-
--LTFETTYKTRNRKTK---------------------KLT-TITEE-LCEN--------
GSSLD---
------------------------------VTLKNKS-RYVDL-WIDYYMNSS-VDKQFD
-YFLEG---FHQVFGGC-NSIG-LFNSE-ELERLLCG-----------------------
-DIQENKYD-----------FDML----RSVTKYVNGYADD-------------------
------------------------SPVV-QWFWDILF--------------------QWD
PKMQSKFLQF-------VTGSDRIP------------ATG-----IT-TLPFKI--SRLK
GPTD------------------------------SMLPIAHTCF-NE-VSLGNF-TNRDV
LGQKLF-LAVTE-SQGF
>Kluyveromyces_marxianus_CP009303.1 .
VKVRRTHV-TQDSLLSI---------------QRHPHDLKK-SLRVEFAN-------EPG

IDAGGLKKEW---XLLL----TRELFHPNH--------GLFQYVEESR------------
----------------LSWFAYGT----------------------------------SG
LKLHGESNTQLYYLFGVVLGLAIYNSTILDVHFAK--ALYKKLCH---------------
--------EKLEFKDYEE----------------------LYPETARN--------LQKM
LDYEEP---DFE-DVFG-
--LTFETTYYDIL-------------------------SAK-SITKP-LCSG--------
GESIP---
------------------------------VTLQNKR-DFVDK-WVKFYMEDE-VAASFS
-SFQSG---FSRVIGDG-LAFP-MFRSC-EVERLICG-----------------------
--SIEQDMD-----------FTQL----RAVTKYHGGYNDK-------------------
------------------------TPII-EWLWELLP--------------------QLE
HEKQQXFLHF-------VTGSDRVP------------VTG-----LA-TLPFKI--TRTV
TGAD------------------------------TQLPTAHTCF-NE-LCLYEY-GSRDD
LKNRLL-IALEL-YEGY
>Kluyveromyces_lactis_CP021243.1 .
IKVRRSHV-TQDSLTSI---------------QRHPHDLKK-SLRVEFSN-------EPG
IDAGGLKKEW---FLLL----TRELFHPNH--------GLFQYVEESR------------
----------------LSWFAYGN----------------------------------TG
LKLHGENNNELYYLFGVVLGLAIYNSTILDLHFAR--AMYKKLCN---------------
--------ERITFEDYEE----------------------LYPETARN--------LKKM
LSYSEL---DFS-DIFG-
--LSFETTYYDVA-------------------------SGI-NVTKP-LCDG--------
GESIP---
------------------------------VTSENKT-EFVER-WVNFYMNEG-VSSSFS
-AFQSG---FTRVIGDG-LAFP-MFKSC-EVERLICG-----------------------
--SIEQDMD-----------FEQL----RAVTKYQGGFHNQ-------------------
------------------------TPVV-EWLWEILP--------------------QLS
HEQQRQFLHF-------VTGSDRVP------------VTG-----LA-TLPFKV--TRTT
SGSH------------------------------DQLPTAHTCF-NE-LCLYEY-ESRDT
LLLRLV-TALEM-YEGY
>Kluyveromyces_wickerhamii_AEAV01000326.1 .
IRVRRSHV-TQDSLAWI---------------QRHPNDLKK-SLRIEFAN-------EPG
IDAGGLKKEW---FLLL----TRELFHPDH--------GLFQSVQESR------------
----------------LSWFTFGT----------------------------------AG
LRIHGESTDELYYLFGVVLGLAIYNSTILDLHFAR--ALYKKLCN---------------
--------EPVTFEDYQE----------------------LYPDTAKS--------LLKM
LDYQEP---DFD-DLFA-
--LTFETTYTDVT-------------------------TGA-RVTRP-LCPD--------
GNRVP---
------------------------------LTLQNKH-HFVAA-WVDFYLNTE-VAPSFR
-AFKNG---FDRVIGEG-LAFP-MFKSP-EVERLICG-----------------------
--SKDQQID-----------FAQL----RAVTKYLGGYCDS-------------------
------------------------SDVI-QWLWHILS-------------------SSLS
THHQRQFLHF-------VTGSDRVP------------VTG-----LA-TLPFKI--SRTC
SGPP------------------------------NQLPTSHTCF-NE-LCLYEY-PSRET
LLHQLT-TALDM-YEGY
>Kluyveromyces_aestuarii_AEAS01000241.1 .
--VRRDHV-TQDSIACI---------------QNHPQDLKK-SLRVEFVN-------EPG
IDAGGLKKEW---FLLL----TRELFHPGH--------GLFQNIPESR------------
----------------LCWFTLGS----------------------------------SG
LELHGESTYELYYLFGVVVGLAMYNGTILDLRFGR--VLYKKLCG---------------
--------EKVGFEDYQE----------------------LYPETARN--------LLKL
YDYNDP---DFT-EVFD-
--LYFETTYSDVA-------------------------TDK-KITKP-LCPE--------
GSQLP---
------------------------------VTLGNRT-SFIEH-WIDFYLNKE-VETTFN
-AFKNG---FKRVIGDS-LTFL-MFTSE-EVERVICG-----------------------
--SVQQEMD-----------FSQL----RTVTKYHGGFCDS-------------------

------------------------STVV-QWLWQLLP--------------------SLS
QKRQQKFLHF-------VTGSDRIP------------VTG-----LT-NLPFKI--TRIT
MGSS------------------------------SQLPTSHTCF-NE-LCLYEY-TSREQ
LSEKL------------
>Eremothecium_cymbalariae_XM_003644374.1 .
VRVRRNFV-TQDSLRSI----------------QSQQKDLKK-SLRIEFVN-------EPG
IDAGGLRKEW---FLLL----TRDLFNPNN--------GLFVYIPESR------------
----------------LSWFSISS-------------------------------SLDSE
LLQGQNNSSELYYLFGVVLGLAIYNSTILDLKFPR--ALYKKLCS---------------
--------EKLSLDDFME----------------------LYPETGEN--------LLKM
LEYNES--DFE-ETFG-
--LNFETSFPDCLD------------------------ETK-VYYHE-LCPD--------
GGNRA---
-----------------------------VTQENKD-EYFKL-WMDFYLNRS-IEKSFE
-SFRSG---FFHVIEA--NSFK-LFGSE-EIEQLVCG-----------------------
--SHEQGLD-----------VDML----RSVTKYQGGFDDN-------------------
------------------------SPVV-QWFWEIIQ--------------------EFD
YEKQRKLLQF-------VTGSDRVP------------ATG-----VT-TIPFRI--SRIR
SGA-------------------------------DRLPLSHTCF-NE-ICLHDY-IDKHT
LRSKLL-VAIDE-SEGY
>Ashbya_gossypii_NM_210147.1 .
IRVRREFV-TTDSLRSI----------------QNQQKDLKK-SLRIEFVN-------EPG
IDAGGLRKEW---FLLL----TRDLFNPNN--------GLFVYYVPESR------------
----------------LSWFSIME-------------------------------SIEHE
LLQGEGSSSELYYLFGVVLGLAIYNSTILDLKFPR--AFYKKICG---------------
--------EVLSVNDFLQ----------------------LYPETGTN--------MLKM
LEYDGE--DFE-DIFA-
--LTFETCFPDRFD------------------------ESK-IHYRQ-LCPD--------
GSTQA---
-----------------------------VTRENKH-EYFRL-WMDFYLNRS-IAPGFE
-SFRNG---FFHVIEG--NSFR-LFGSE-ELEQLVCG-----------------------
--SNEQSLD-----------VSML----RSVTRYQGGFDDN-------------------
------------------------SPVV-QWFWEILS--------------------EME
YPQQRKLLHF-------VTGSDRVP------------ATG-----VT-TIPFRI--SRIR
SGA-------------------------------DRLPLSHTCF-NE-ICLHEY-KDKET
LRNKLI-IALEE-SQGY
>Eremothecium_sinecaudum_XM_018131820.1 .
IRIRRNFV-TQDSLLGI----------------QAQQKDLKK-SLRIEFVN-------EPG
IDAGGLRKEW---FLLL----TRDLFNPNN--------GLFVYYVAQSR------------
----------------LSWFSITS-------------------------------SMNPE
LLQGQNNSLELYYLFGLVLGLAIYNSTILDLKFPR--ALYKKLCG---------------
--------ENLSVNDFLE----------------------LYPETGGN--------LLKM
LEYDGD--DFE-DLFC-
--LTFETSFSDCLN------------------------ENV-VHYQE-LCEN--------
GRNRP---
-----------------------------VTQHNKH-EFFKL-WMDFYLNKS-IEASFE
-SFKCG---FFHVIEG--NTFQ-LFGSE-EVEQLVCG-----------------------
--SNEQNLD-----------VDML----RSVTKYQGGFEDT-------------------
------------------------TPVV-NWFWEILQ--------------------EFD
YSKQRKLLQF-------VTGSDRVP------------ATG-----VT-TIPFRI--SKLK
SGA-------------------------------DRLPISHTCF-NE-ICLYEY-ETKEM
LREKIL-IAIEE-SEGY
>Lachancea_kluyveri_AACE03000004.1 .
IRVRRDHV-AQDSLRCI----------------QNHQRDLKK-SLRVEFIN-------EPG
VDAGGLRKEW---FLLL----TRDLFNPSN--------GLFVYYVEESR------------
----------------FFWFGITR-------------------------------ATDGE
LLQGHKNNSELYYLCGVVLGLAIYNSTILDLNFPR--SLYKKLCG---------------
--------EKLTMNDFLE----------------------LYPITGQN--------MLKM

CEYDGE---DFE-DIFT-
--LNHETSFQDC--------------------------WGK-VHRRE-LCSG--------
GSNKA---
------------------------------VNNQNKY-EYTKL-WMDFYMSKS-IAASFE
-SFHNG---FKHVIES--DSFK-LFDSE-EVEQLVCG-----------------------
--SPDKAVD-----------VRML----QSVAHYSSGWSD--------------------
------------------------STIV-KWFWEIFE--------------------NYS
YREQRKLLQF-------VTGSDRVP------------ATG-----IS-TMQFKV--TRLG
NDS-------------------------------IKLPLAHTCF-NE-LCLYEY-SSMEK
LHNKLS-IAINE-SEGY
>Lachancea_thermotolerans_XM_002556434.1 .
MRVRRDYV-SQDSLRCI---------------ETHHADLKK-SLRVEFVN-------EPG
IDAGGLRKEW---FLLL----TRELFNPSS--------GLFIVVEESR------------
----------------FSWFNMAY----------------------------------KD
VDFGPENAEKLYYLFGIVLGLAIYNGTILDLCFPM--ALYKKLCG---------------
--------EPLNERDFLE----------------------LYPTTGKN--------LIKM
MEYDGD---DFE-DVFC-
--LNFEITYSNTW-------------------------ATR-IHRQE-LCDG--------
GAKRH---
------------------------------VTRENRS-EYVRL-WMDFHMNRA-IKSSFA
-VFLNG---FRRVVES--DAFK-LFTSE-ELEQLLCG-----------------------
--SHDRDID-----------VNVL----KSVAKYGASITAE-------------------
------------------------SRIV-RWFWDTFQ--------------------DFN
FEQKCKLLEF-------VTASDRIP------------ATG-----IS-TIPFKI--SKLG
GDS-------------------------------EKLPLVHTCF-NE-ICLYDY-NSQPK
LRHKLI-LAMNE-SEGY
>Lachancea_waltii_AADM01000336.1 .
VKVRRDSI-TQDSLRCI---------------ETNLSDLKK-SLKIEFVD-------EPG
IDAGGLRKEW---FLLL----TRELFNPDN--------GLFTVIEESR------------
----------------FSWFNIAY----------------------------------TD
LDAGKENAHKLYFLLGVVLGLAIYNGTILDLSFPM--ALYKKMKG---------------
--------EPLKRADFLQ----------------------LYPISGNN--------LLKL
LEYDND---DFE-EIFS-
--LTFEITYPGAW-------------------------DTW-LHKKE-LCSG--------
GSTRH---
------------------------------VTRENRE-EYINL-WMDFHMTQA-IGESFV
-NFSNG---FQRVVDN--HAFE-LFTPE-EVELLLCG-----------------------
--SHEKEID-----------VSVL----KSVTKYSIGLNRE-------------------
------------------------SRIV-QWLWEIIY--------------------MFS
HAERCKLLEF-------VTGSDRIP------------ATG-----IS-TVPFKI--TRLG
PDS-------------------------------ENLPLAHTCF-NE-LCIYEY-KSMEK
LRVKLI-LAMNE-SEGY
>Vanderwaltozyma_polyspora_XM_001643865.1 .
IKVRRSHI-THDSLNYI---------------KNHPQDLLK-SLRVEFVD-------EPG
VDAGGLRKEW---FILL----TRKLFDPMN--------GLFIVNEESR------------
----------------LAWFSIQP------------------------------------
-HDFDEQNDELFYLFGVILGLAIFNSTILDLRFPK--AFYKKLIH---------------
--------EPLSFVDYAE----------------------LYPENAKN--------LLKL
CAYNED---DFC-EVFD-
--LTFETTFESSELSIENNGDRSGEP------------KRN-KITVE-LCEG--------
GKNIQ---
------------------------------VTNENKN-QFIKL-WFNFYMNKS-IERQYN
-QFEKG---FHEVFSQC-DCTK-LFNSE-ELERLACG-----------------------
-DNEQDSID-----------IMML----RSVTKYIGGYNDQ-------------------
------------------------SQVI-SWFWEILQ--------------------GWD
HKLQKKMLQF-------VTGSDRIP------------ATG-----IS-TLPFKI--ARVG
PNLHHVENGLLE----------------------EPLPTAHTCF-NE-ICLWDY-KSKEE
LERRLI-YSISE-SEGF

>Tetrapisispora_phaffii_XM_003684460.1 .
IKVRRTHI-TSDSLKSI---------------KNHPKDFLK-SLRVEFID-------EPG
IDAGGLRKEW---FLLL----SKSLFSPMN--------GLFVYNKQSY------------
----------------LSWFNINP------------------------------------
-GSVDDKNEEMYYLFGLVLGLAIFNSTILDIQFPK--ALYKKLCH---------------
--------EQLKFGDYME---------------------LYPETGKS--------LIKL
LNYNEP--DFE-EAFS-
--LNFETTYDNPMFESVPDKITDS--------------QNR-KVTVE-LCEN--------
GSLTP---
------------------------------VTNENKD-KFIQK-WFDFYMNKS-IEKQFT
-KFKTG---FYEVVSQC-ESSK-LFNSE-ELEKLICG----------------------
-ERESESYD-----------FSML----RSVCTYTGGYTNN-------------------
------------------------DTII-NWFWEILE--------------------SWD
PELQKKLLQF-------VTGSDRIP------------STG-----IS-TLTFKI--SKLT
INPYTTYNKTTI----------------------EPLPLAHTCF-NE-LCLWEF-KNKEV
LEKKLK-IAITE-SEGY
>Tetrapisispora_blattae_XM_004179448.1 .
ISIRRNHV-TKDSLQAI---------------RNHKKDLLK-SLKVEFID-------EPG
VDAGGLKKEW---FYLL----TGSLFNPIN--------GLFVYVEESR------------
----------------LEWFTIHE-------------------PGHHK-PTENKKYLFKD
PPALSEKISELYYLFGVIVGLAIFNSNILDLKFPK--AFYKKLSG---------------
--------ESLNFDDFTE---------------------LYPETARN--------LVKM
LEYEGN--DFE-DVFG-
--LTFETTYLDISPGNAPNSNDKKGTSIMHSK------KKI-YRTVE-LCEN--------
GQSIK---
------------------------------INNSNKH-KYVSL-WMDFYLNTS-IQEPFE
-RFQAG---FKHVFAQC-MSCK-LFNSE-ELERLICG----------------------
-SSQKDSYD-----------FAML----RSVTRYNGGFSDT-------------------
------------------------SQVV-QWFWEILQ--------------------SWP
SVLQKKFLIF-------VSGSDRVP------------ATG-----MS-TLPFKI--TRIY
SKSKP-----------------------------QSLPLAHTCF-NE-LCLWDY-ESKTI
LEQKLK-YAVLE-AEGF
>Zygosaccharomyces_bailii_HG316459.1 .
VKVRRRHI-TSDSLRCI---------------RDHQKDLMK-SLRVEFVN-------EPG
VDAGGLRKEW---FLLL----TRSVFSPLN--------GLFNYITESR------------
----------------LSWFAISP--------------------------------INDA
PHEGSPNNGQLYYLFGTVVGLAIFNSTILDLQFPR--AFYKKLCA---------------
--------EPVNFNDYLQ---------------------LYPETGQN--------LIKM
LNYEED--DFT-DVFG-
--LTFEITYRDFNKELLGI-------------------LPN-VTTME-LCPN--------
GKHKL---
------------------------------VTQENKH-EFVQL-WQDFFMNKS-IESQFK
-QFVSG---FRQVFALC-ESIK-LFDHE-ELERLVCG----------------------
-NQEKNCYE-----------FQML----RSVTIYVGGFTDR-------------------
------------------------SQVV-SWFWEIVQ--------------------SWN
FQLQRKLLQF-------ATGSDRVP------------PGG-----MS-TLPFKI--SRLG
CKDS------------------------------NKLPLAHTCF-NE-VCLWEY-SSKQK
LEHKLW-WAVTQ-SEGY
>Zygosaccharomyces_parabailii_CP019504.1_a.
VKVRRRHI-TSDSLRCI---------------RDHQKDLMK-SLRVEFVN-------EPG
VDAGGLRKEW---FLLL----TRSVFSPLN--------GLFNYITESR------------
----------------LSWFAISP--------------------------------INDA
PHEGSPNNGQLYYLFGTVVGLAIFNSTILDLQFPR--AFYKKLCG---------------
--------EPVNFNDYLQ---------------------LYPETGQN--------LIKM
LNYEED--DFT-DVFG-
--LTFEITYRDFNKELLGI-------------------LPN-VTTME-LCPN--------
GKHKL---
------------------------------VTQENKH-EFVQL-WQDFFMNKS-IESQFK

-QFVSG---FRQVFALC-ESIK-LFDHE-ELERLVCG----------------------
-NQEKNCYE-----------FQML----RSVTIYVGGFTDR-------------------
------------------------SQVV-SWFWEIVQ--------------------SWN
FQLQRKLLQF-------ATGSDRVP------------PGG-----MS-TLPFKI--SRLG
CKDS------------------------------NKLPLAHTCF-NE-VCLWEY-SSKQK
LEHKLW-WAVTQ-SEGY
>Zygosaccharomyces_rouxii_XM_002499032.1 .
IRVRRSNI-TNDSLRSI---------------ENHQRDLMK-SLRVEFVN-------EPG
VDAGGLRKEW---FLLL----TRSLFNPLN--------GLFSYVEESR------------
----------------LSWFAIIP---------------------------------IRSG
SQDGCPDHSQLYYLFGIVIGLAIFNSTILDLEFPR--AFYKKLCG---------------
--------ELLNFDDYMQ----------------------LYPETGQN--------LIKM
LDYNKE---DFT-DVFA-
--LTFEATYEDRSKELLGH-------------------KPC-IVSVE-LCRN--------
GRYRK---
------------------------------VTQENKY-EFVQL-WQDFFMNKS-VEAQFK
-QFASG---FRQVFVLC-ESIR-LFNHE-ELARLICG----------------------
-NREKNCFE-----------FQML----RSVTRYVGGFNDK-------------------
------------------------SRVV-VWFWEILQG--------------------WD
FRLQRKLLQF-------ATGSDRVP------------PGG-----MS-TLPFKI--SRLG
SKDS------------------------------DKLPLAHTCF-NE-VCLWEY-SSKEK
LEQKLW-WAVTQ-SEGY
>Kazachstania_africana_XM_003957816.1 .
IKVRRSHI-AQDSLRCI---------------QSRQADLLK-SLRVEFVN-------EEG
IDAGGLKKEW---FLLL----TKTLFSPIH--------GLFQYISESR------------
----------------FCWFSIFP---------------------------------ISE
EPNGLLTNEKLYYLFGVVLGLAIFNGIILDLQFPS--AFYKKICN---------------
--------EPLNFSDYYQ----------------------IYPETAQN--------LKKM
LDYQDD---NFC-EVFG-
--LTFETTVESMASATQRNDSLS---------------NPG-FVTVS-LSRN--------
GSSKY---
------------------------------VTQANKH-NFVDL-WVDYYMNKS-IKKQFG
-QFMTG---FKQVFASC-SSIQ-LFNSE-ELERLLCG----------------------
-DKEQNKYD-----------FTLL----RSVTKYAGGFQDD-------------------
------------------------STVV-IWFWEVID--------------------QWT
KVLQRKVLQF-------VTGSDRIP------------ATG-----IS-ALPFKI--TKCS
SRQN------------------------------EELPVAHTCF-NE-ICLWDY-CSKEV
LERKLL-LAINE-SQEF
>Saccharomyces_exiguus_AL409700.1 .
-----------------------------------QGDLLK-SLRIEFIN-------EPG
IDAGGLRKEW---FILL----TKSLFSPTT--------GLFDHIKDSR------------
----------------LCWFPIVA---------------------------------SSK
SKEKVQSQEEYYYLLGVVIALAMFNGNILDLSFPK--ALYKKMCN---------------
--------ETLTFADYTE----------------------LYPETASN--------LLKM
LEYSKD---DFD-DVFC-
--LTFETTYHNKLTANNKGSKXSK--------------GSN-TVTVE-LCKN--------
GSNIP---
------------------------------VTQSNKS-DYVKL-WIDFYLTKS-VAASFE
-RFMDG---FSRVFSNC-KSIG-LFNSE-ELERLLCG----------------------
-DDEHTKYD-----------FQML----RSVTKYQGGFTNE-------------------
------------------------TSVV-NWFWEILE--------------------DWD
YKMQGKVLQF-------ITSSNRVP------------ATG-----IS-TLTFKI--SRLG
ARDN------------------------------NDLPIAHTCF-NE-LCIWEY-SSKEK
LENKLM-LAVTE-SEGF
>Candida_glabrata_XM_447326.1 .
VRVRRSNI-SSDSLQSI---------------KEHQGDLFK-SLKVEFVG-------EPG
IDAGGLRKEW---FMLL----TKTLFDPRT--------SLFVYVPESR------------
----------------LIWFSNSA---------------------------------QNE

ASIPDKETMELYYLFGVVMALAIFNSTILNLHFPK--ALYKKICG---------------
--------EPLKFDDYAE----------------------IYPETAQN--------LQKL
LEYDGD---DFS-EVFA-
--LNFETTFINDMWEINPKN------------------IKK-YNTVE-LCAN--------
GKNMS---
------------------------------VTSSNKE-EYVKL-WIDFYLNKN-ISTTFE
-KFLLG---FKRVFSVS-KSIK-LFNFE-ELQRLVCG-----------------------
-DQDTNKYD-----------FGML----KSVTKYIGGMNEN-------------------
------------------------MPVA-KWFWEIAQ--------------------EWD
ITKQKKLMRF-------VTGSDGVP------------ATG-----MS-TLPFKL--SLLG
SSDS------------------------------DKLPIAHTCF-NE-LCLWQY-RSKEK
LESKLK-WAVTE-SEGF
>Naumovozyma_castellii_XM_003673728.1 .
IRVRRERI-TNDSLKCI---------------RQHQGDLLK-SLRVEFIN-------EPG
IDAGGLKKEW---FLLL----TKSLFNPMN--------GLFKYLEDSR------------
----------------LSWFAIQP---------------------------------ICE
EESDIESQKELYYLFGVVLALALFNSTILDLKFPK--ALYKKLCK---------------
--------EPLNFEDYRE----------------------LYPETAKN--------LEKM
LSYPGK---DFE-DLFC-
--LSFTTTYQDATLGIFGSQS-----------------QTD-KVTVE-LCKG--------
GKGIN---
------------------------------VTQENKQ-RFVDL-WVDFYLTKS-ISNSFD
-HFQTG---FNRVCGKC-YSIS-LFDSD-ELEKLVCG-----------------------
-DEGKENYD-----------FKIL----RSITKYGGGFNDQ-------------------
------------------------SKIV-EWFWKIVE--------------------SWS
NQLQRKLLLF-------VTGSDRIP------------ATG-----IS-TLPFKV--TRLG
TKDK------------------------------EDLPLSHTCF-NE-LCLWEY-TTEEK
LKNKLL-CAITE-SEGF
>Naumovozyma_dairenensis_XM_003671230.1 .
---LSIEFID-------EPG
IDAGGLRKEW---FLLL----TKSLFNPMN--------GLFVSVEESN------------
----------------LSWIAIHD---------------------------------IQT
LNKSSTFKNELFFLFGVVVALAIFNSTILDLQFPR--TFYKKLCN---------------
--------EPLTFDDYRE----------------------IYPVTAQN--------LMKM
LQYEND---DFE-EVFG-
--LTFETTYKDPLKYALDNEKSK---------------GDG-MVSVE-LCKN--------
GSKMK---
------------------------------VTQANKQ-KFINL-WVNFYLNES-IINQIS
-QFRSG---FDRVFART-KSIS-LLNSE-ELERLLCG-----------------------
-DETQQSYD-----------FKML----RSVTKYSGGFSDD-------------------
------------------------SRVV-AWFWEIVE--------------------GWN
YKLQRKLLSF-------ITGSDRIP------------ATG-----IS-TLNFKI--SRLG
SKDK------------------------------NNLPLSHTCF-NE-LCLWEY-SSRGK
LEKKLL-YAITE-SEGF
>Naumovozyma_dairenensis_XM_003980093.1 .
IRVRRNRI-TNDSLQCI---------------QQHQGDLLK-SLRIEFID-------EPG
IDAGGLRKEW---FLLL----TKSLFNPMN--------GLFVSVEESN------------
----------------LSWIAIHD---------------------------------IQT
LNKSSTFKNELFFLFGVVVALAIFNSTILDLQFPR--TFYKKLCN---------------
--------EPLTFDDYRE----------------------IYPVTAQN--------LMKM
LQYEND---DFE-EVFG-
--LTFETTYKDPLKYALDNEKSK---------------GDG-MVSVE-LCKN--------
GSKMK---
------------------------------VTQANKQ-KFINL-WVNFYLNES-IINQIS
-QFRSG---FDRVFART-KSIS-LLNSE-ELERLLCG-----------------------
-DETQQSYD-----------FKML----RSVTKYSGGFSDD-------------------
------------------------SRVV-AWFWEIVE--------------------GWN
YKLQRKLLSF-------ITGSDRIP------------ATG-----IS-TLNFKI--SRLG

SKDK------------------------------NNLPLSHTCF-NE-LCLWEY-SSRGK
LEKKLL-YAITE-SEGF
>Saccharomyces_pastorianus_ABPO01000056.1 .
IKIRREVI-SHDSLRCI---------------KAHQGDLLK-SLRVEFVN-------EPG
IDAGGLRKEW---FFLL----TKSLFNPMN--------GLFVYIKESS------------
----------------RSWFAIDP-------------------------------PNFD
KSKKSNSQLELYYLFGVVIALAIFNSTILDLQFPK--AFYKKLCL---------------
--------EPLSFEDYSE---------------------LFPETSRN--------LIKM
LNYTED---DFE-EVFS-
--LTFETTYKNNNWIISDNKS-----------------TRE-YVTVE-LCEN--------
GKNIP---
------------------------------ITQKNKH-DFVTK-WVEFYLEES-IKPQFN
-RFISG---FKRVFAEC-NSIK-LFNFE-ELERLVCG----------------------
-DAEQTKFD-----------FKSL----RSVTKYVGGFSDD-------------------
------------------------SKIV-RWFWEIIE--------------------DWD
YPLQKKLLQF-------VTASDRIP------------ATG-----IS-TIPFKI--SQLG
THDS------------------------------NDLPLAHTCF-NE-VCLWGY-SSKKK
MEQKLL-WAVNE-SEGY
>Saccharomyces_eubayanus_XM_018366134.1 .
IKIRREVI-SHDSLRCI---------------KAHQGDLLK-SLRVEFVN-------EPG
IDAGGLRKEW---FFLL----TKSLFNPMN--------GLFVYIKESS------------
----------------RSWFAIDP-------------------------------PNFD
KSKKSNSQLELYYLFGVVIALAIFNSTILDLQFPK--AFYKKLCL---------------
--------EPLSFEDYSE---------------------LFPETSRN--------LIKM
LNYTED---DFE-EVFS-
--LTFETTYKNNNWIISDNKS-----------------TRE-YVTVE-LCEN--------
GKNIP---
------------------------------ITQKNKH-DFVTK-WVEFYLEES-IKPQFN
-RFISG---FKRVFAEC-NSIK-LFNFE-ELERLVCG----------------------
-DAEQTKFD-----------FKSL----RSVTKYVGGFSDD-------------------
------------------------SKIV-RWFWEIIE--------------------DWD
YPLQKKLLQF-------VTASDRIP------------ATG-----IS-TIPFKI--SQLG
THDS------------------------------NDLPLAHTCF-NE-VCLWGY-SSKKK
MEQKLL-WAVNE-SEGY
>Saccharomyces_bayanus_AACG02000013.1 .
IKIRREVI-SHDSLRCI---------------KEHQGDLLK-SLRVEFVN-------EPG
IDAGGLRKEW---FFLL----TKSLFNPMN--------GLFVYIKESS------------
----------------RSWFAIDP-------------------------------PNFD
KSKKTNPQLELYYLFGVVMALAIFNSTILDLQFPK--AFYKKLCL---------------
--------EPLSFEDYSE---------------------LFPETSKN--------LIKM
LNYTED---DFE-EVFS-
--LTFETTYKNNNWIISDNKS-----------------TRE-YVTVE-LCEN--------
GKSIP---
------------------------------ITQKNKH-DFVTK-WVEFYLEES-IKPQFN
-KFISG---FKRVFAEC-NSIK-LFNFE-ELERLVCG----------------------
-DAEQTKFD-----------FKSL----RSVTKYVGGFSDD-------------------
------------------------SKIV-RWFWEIIE--------------------DWD
YPLQKKLLQF-------VTASDRIP------------ATG-----IS-TIPFKI--SQLG
THDS------------------------------NDLPLAHTCF-NE-VCLWGY-SSKKK
LEQKLL-WAVNE-SEGY
>Saccharomyces_kudriavzevii_AACI02000462.1 .
IRVRREVI-SHDSLRCI---------------KEHQGDLLK-SLRVEFVN-------EPG
IDAGGLRKEW---FFLL----TKSLFNPMN--------GLFVYIKESS------------
----------------CSWFAINP-------------------------------PNFD
KSKKNNCQLELYYLFGVVMALAIFNSTILDLQFPK--AFYKKLCS---------------
--------EPLSFEDYSE---------------------LFPETSGN--------LIKM
LNYTKN---DFE-DVFS-
--LTFETTYRNNNWILDDNKS-----------------SRE-YVTVE-LCEN--------

GKNVS---
------------------------------ITQNNKH-EFVTK-WVEFYLEKS-IEPQFN
-KFILG---FKRVFAEC-SSIK-LFNFE-ELERLVCG-----------------------
-DEEQTKFD-----------FKSL----RSVTKYVGGFSDN-------------------
------------------------SKVV-HWFWEIIE--------------------NWD
YPLQKKLLQF-------ITASDRIP------------ATG-----IS-TIPFKI--SQLG
SHDS------------------------------NDLPLAHTCF-NE-VCLWGY-SSKKK
LEQKLL-WAINE-SEGY
>Saccharomyces_paradoxus_AABY01000022.1 .
IKVRRDVI-SHDSLRCI---------------KEHQGDLLK-SLRIEFVN-------EPG
IDAGGLRKEW---FFLL----TKSLFNPMN--------GLFIYIKESS------------
----------------RSWFAIDP--------------------------------PNFD
KSKKKNSQLELYYLFGVVMALAIFNSTILDLQFPK--ALYKKLCS---------------
--------ESLSFEDYSE----------------------LFPETSRN--------LIKM
LNYTKD---DFE-DVFS-
--LTFETTYRNNNWILNDSKS-----------------SKE-YVTVE-LCEN--------
GKNLP---
------------------------------ITQSNKH-DFVTK-WVEFYLEKS-IEPQYN
-KFVSG---FKRVFAEC-NSIK-LFNSE-ELERLVCG-----------------------
-DEEQTKFD-----------FKSL----RSVTKYVGGFSDD-------------------
------------------------SKVV-RWFWEIIE--------------------SWD
YTLQKKLLQF-------ITASDRIP------------ATG-----IS-TIPFKI--SLLG
SHDS------------------------------DDLPLAHTCF-NE-ICLWSY-SSKKK
LELKLL-WAINE-SEGY
>Saccharomyces_cerevisiae_NM_001181694.3 .
IKVRRDVI-SHDSLRCI---------------KEHQGDLLK-SLRIEFVN-------EPG
IDAGGLRKEW---FFLL----TKSLFNPMN--------GLFIYIKESS------------
----------------RSWFAIDP--------------------------------PNFD
KSKGKNSQLELYYLFGVVMGLAIFNSTILDLQFPK--ALYKKLCS---------------
--------EPLSFEDYSE----------------------LFPETSRN--------LIKM
LNYTED---NFE-DVFS-
--LTFETTYRNNNWILNDSKS-----------------SKE-YVTVE-LCEN--------
GRNVP---
------------------------------ITQSNKH-EFVMK-WVEFYLEKS-IEPQYN
-KFVSG---FKRVFAEC-NSIK-LFNSE-ELERLVCG-----------------------
-DEEQTKFD-----------FKSL----RSVTKYVGGFSDD-------------------
------------------------SRAV-CWFWEIIE--------------------SWD
YPLQKKLLQF-------VTASDRIP------------ATG-----IS-TIPFKI--SLLG
SHDS------------------------------DDLPLAHTCF-NE-ICLWNY-SSKKK
LELKLL-WAINE-SEGY
>Saccharomyces_pastorianus_BPO01002116.1 .
IKVRRDVI-SHDSLRCI---------------KEHQGDLLK-SLRIEFVN-------EPG
IDAGGLRKEW---FFLL----TKSLFNPMN--------GLFIYIKESS------------
----------------RSWFAIDP--------------------------------PNFD
KSKGKNSQLELYYLFGVVMGLAIFNSTILDLQFPK--ALYKKLCS---------------
--------EPLSFEDYSE----------------------LFPETSRN--------LIKM
LNYTED---NFE-DVFS-
--LTFETTYRNNNWILNDSKS-----------------SKE-YVTVE-LCEN--------
GRNVP---
------------------------------ITQSNKH-EFVMK-WVEFYLEKS-IEPQYN
-KFVSG---FKRVFAEC-NSIK-LFNSE-ELERLVCG-----------------------
-DEEQTKFD-----------FKSL----RSVTKYVGGFSDD-------------------
------------------------SRAV-CWFWEIIE--------------------SWD
YPLQKKLLQF-------VTASDRIP------------ATG-----IS-TIPFKI--SLLG
SHDS------------------------------DDLPLAHTCF-NE-ICLWNY-SSKKT
CSSKLH-AVV-------
>Sugiyamaella_lignohabitans_CP014501.1 .
IRVNRNSL-LQDSFEAI---------------DKNDEHLKK-GLKVEFIG-------EPG

```
IDAGGLKKEW---FLLL----FKELFDPSN--------ALFIEDEESH------------
----------------YCWFDASS------------------------------------
-----EHALKYYKLTGVAIGLALYNSTILDVNLPS--VLFKKLLS---------------
--------SPYSIQDFTK----------------------LYPSYGAN--------LQKL
LNYEGD---------------------------------------------DIE-EVFS-
--LDFSICSQDPAD------------------------RSK-IKETV-LIPN--------
GKTIL-------------------------------------------------------
------------------------------VNKSNRQ-DYVKR-VMAHYLDIS-VKRQFE
-AFKQG---FYKVAGG--NALS-LFRPE-EIELLIRG-----------------------
---SLEAID-----------VDAL----RSVTRYQH-WGPKGYDAEQ-------------
------------------------DIVV-KWFWKFFK--------------------NLD
PLNQRKLLIF-------VTGSDRIP------------ATG-----IV-NMPFRI--SKIG
EDS-------------------------------DRFPISHTCF-NQ-LCLYKY-SSREK
LTAKIM-TAINE-SEGF
>Yarrowia_lipolytica_XM_502861.1 .
IKVKRESL-LADSLSEI---------------ERNNGNYKK-SLRVEFVG-------EPG
IDVGGIRKEW---FLLL----KTALFNPAR--------NLFVEDEESH------------
----------------YCWFNPGC------------------------------------
--KAQDGDLREYRLAGVITGLALYNSSMLDLPLPP--VVFKKLLG---------------
--------CPVGLEDFTV----------------------VNPTVGRS--------LSQL
LKFTES---------------------------------------------EVE-SLN--
--LSYNVIIDS---------------------------EGV-KKSCD-LISN--------
GSHVS-------------------------------------------------------
------------------------------VTTRNRR-DYVSR-LVKFFLETS-ISTTFA
-EYKKG---FHSVVGG--NALS-LFRPE-ELEALVKG-----------------------
---SSEPLD-----------IGTL----KTVTRYQN-FTTEDPES---------------
------------------------ELVV-RWFWKWAE--------------------NLE
EEKQKKLLRF-------ITGSDRIP------------ATG-----IS-NMNFKI--TFSG
PDC-------------------------------DRFPVSHTCF-NE-LCVYGY-SSRQK
FIDKIV-MAMNE-SEGF
>Pichia_kudriavzevii_XM_020689061.1 .
LSIRRNDI-IHDSLMQI---------------RSHPTEVRK-LLKVEFVN-------EPG
IDAGGLKKEW---FSLL----TKELFDPLN--------GLISYSNENS------------
----------------VAYLTTHH------------------------------------
--NPRKIAYESYHLLGIVLGMAIYNSIILDIHFPP--VIYKKLLG---------------
--------FESSFDDLKE----------------------IEPLLWKN--------LKSL
INLK-----------------------------------------------DAG-TLG--
--LSFEVTVEDV--------------------------TGQ-LRNYE-LIKG--------
GSNIK-------------------------------------------------------
------------------------------VTNDNRL-EYILK-YSKFLLDGI-IDKQFG
-QFKRG---FDHVMGT--PSFT-LFTPN-EIQKLVAG----------------------D
ETFENDKYD-----------VDIL----ESICKLKN-CKKS-------------------
------------------------DNVV-VWFWEYFR--------------------DLS
VKKQRKLMRF-------VAGTDRIP------------ATG-----LQ-SMQLKI--SKLY
DTGRFS----------------------------NRLPVSHTCF-NE-LCLWNY-ESKEV
LRDKLD-MAISE-SVGF
>Schizosacch_pombe_NM_001022455.1 .
IRVRRDRL-LEDSLRQI---------------NDRNKDFRK-ALKVEFLG-------EEG
IDAGGLKREW---LLLL----TRKVFSPEF--------GLFVNCEESSN-----------
----------------YLWFNYSH------------------------------------
----RSKEIDYYHMSGILMGIAIHNSINLDVQMPR--AFYKKLLQ---------------
--------LPLSFNDLDD----------------------FQPSLYRG--------LKEL
LLFEG----------------------------------------------DVK-NTYG-
--LNFTINLKA---------------------------VEG-FRTVE-LKEG--------
GSELS-------------------------------------------------------
------------------------------VDNENRK-EYVLR-YVDYLLNTT-VKKQFS
-AFFDG---FMKVCGG--NAIS-LFQDN-EISKLIRG-----------------------
---SEEVID-----------WELL----KNVCVYDX------------------------
```

------------------------RKII-LWFWDLIS--------------------HYS
LKMQKLFLIF-------VTGSDRIP------------ATG-----AH-NFQLRI--SVLG
PDS-------------------------------DQLPISHTCF-NH-LCIWEY-SSREK
LKKKLD-TALLE-TNGF
>Schizosacch_octosporus_XM_013164275.1 .
LRVRRNHL-LADSLRQV---------------SKPGIDLKK-SLKVEFVN-------EEG
VDAGGLKKEW---LLLL----SREIFDPSF--------GLFESYPDEQMD----------
----------------YVWFNTNN------------------------------------
----NSVNKEYYHLVGVLMGMAVYNSVNLDVRLPP--VCYKKLLG---------------
--------LPLTLNDMAD----------------------FRPSLTNG--------LREL
LEFEG--DVE-ETYG-
--LNFTINVPT---------------------------GTG-YETVE-LIKD--------
GSHIS---
------------------------------LNNKNRK-EYVVA-YSDYLLNTS-VKSPFS
-TFQEG---FLKVCGG--NALS-LFQEQ-EIEKLVRG-----------------------
---NEEHIN-----------WDLL----RATCTYDM-YNRESICRQSGRVHERRSH----
---------------MLNNHSVQKISAI-AWLWDVIF--------------------SLT
EDKQRKLLVF-------MTGSDRIP------------ATG-----IQ-SIQLRV--SILG
PDT-------------------------------EKLPIAHTCF-NQ-LCIWEY-STREK
LERKLN-RAIYE-TKGF
>Schizosacch_cryophilus_XM_013165721.1 .
LRVRRTHL-LADSLRQV---------------SKPGIDLKK-SLKVEFVN-------EEG
VDAGGLKKEW---LLLL----SREIFDPSF--------GLFESYPDEQLD----------
----------------YVWFSSRR------------------------------------
----KTTNKEYYHLVGVLMGMAVYNSVNLDVKLPT--ACYKKLLG---------------
--------LPLTLNDVAE----------------------FRPSLTHG--------LRML
LEFDG--DVE-EMYG-
--LNFTVNVPT---------------------------ETG-YETVE-LIKD--------
GAQIS---
------------------------------LNNENKK-EYVVA-YSDYLLNKS-IQSQFS
-TFQEG---FLKVCGG--NALS-LFQEQ-EMEKLVRG-----------------------
---NEELIN-----------WDLL----RATCTYDM-YNRETVCRQNSRAHERRTH----
---------------MLNDHSVKKIPVI-AWLWETIF--------------------SLP
EEKQRRLLVF-------MTGSDRIP------------ATG-----IQ-SIQLRV--SILG
PNN-------------------------------EKLPIAHTCF-NQ-LCIWEY-SSKEK
LKQKLN-RAIYE-TKGF
>Mitosporidium_daphniae_XM_013383421.1 .
VRIPRSPL-REMLHASI-----------IQLLNASPPQLRG-RIKVEFQG-------EPG
IDAGGLLREW---LQLL----IHALMDPTL--------GLFQADICGFELNAP-------
----------------CVWFHSGE------------------------------------
--NCSQGVFELVHLLGTILGLSLNNGIVLDHRLPF--ALFKKLLSPDTNP-------PAC
VSAHGESMSSITLDDLWE----------------------IAPTLARG--------LGQL
LDYHGEA--EVE-DVFQ-
--LQFTAGFSK-------------------------PGPEK-REIID-LTNR--------
GSEIA---
------------------------------VTGENRE-IFISL-YLEHIFHHR-PGNHWT
-KFRDS---FYSTLSG--STIA-LLSPQ-ELKQLLSG-----------------------
---DPAEIS-----------IDLL----RSVALYEG-YLSA-------------------
------------------------EHPVSLWFWDILT--------------------SYS
AEMRRKFLIF-------VTGVGRLP------------AALX--------WVIRL--WGRD
ASWKNLSGG-------------------------EYRPLGRTCF-NQ-LCLHKY-PDMET
LRRLLT-NAIVE-SEGF
>Puccinia_graminis_XM_003324209.1 .
LSIRRSHL-VEDSLRQI---------------ASSQSELKK-LLKITFVD-------EEG
VDGGGLKKEW---FLLL----IRQLVAPEY--------GMFLHDQDQH------------
----------------QIWFNPAS------------------------------------
------QELEEFKLIGTVLGLAIYNRATLDFGLPL--IGYRKLLG---------------
-------FSVNRLSDLAT----------------------LKPEVAKS--------LRWL

LEYDGD---DFE-EICS-
--RNFVGDYDA---------------------------YGT-VVEVP-LIPN--------
GENIP---
------------------------------VTKSNRA-EFVKL-YCDYILNKS-IEKQFQ
-AFSEG---FNSIAAG--NGLS-LFQPE-EIELLVIG-----------------------
-STYDSKLA-----------IEDL----KAITHYEG-FQPT-------------------
------------------------DLTI-QYFWIVVE--------------------NFG
FEDQKKLLRF-------ITGTDRIP------------ATG-----IS-GLNLKI--TRSV
XS--------------------------------ERLPESHTCF-NQ-LILSDF-SSVQS
LDQKLR-LAINE-SQGF
>Puccinia_graminis_XM_003890082.1 .
LSIRRSHL-VEDSLRQI---------------ASSQSELKK-LLKITFVD-------EEG
VDGGGLKKEW---FLLL----IRQLVAPEY--------GMFLHDQDQH------------
----------------QIWFNPAS------------------------------------
------QELEEFKLIGTVLGLAIYNRATLDFGLPL--IGYRKLLG---------------
-------FSVNRLSDLAT----------------------LKPEVAKS--------LRWL
LEYDGD---DFE-EICS-
--RNFVGDYDA---------------------------YGT-VVEVP-LIPN--------
GENIP---
------------------------------VTKSNRA-EFVKL-YCDYILNKS-IEKQFQ
-AFSEG---FNSIAAG--NGLS-LFQPE-EIELLVIG-----------------------
-STYDSKLA-----------IEDL----KAITHYEG-FQPT-------------------
------------------------DLTI-QYFWIVVE--------------------NFG
FEDQKKLLRF-------ITGTDRIP------------ATG-----IS-GLNLKI--TRSV
RSSLDSNPTNNNRRRRHLSNQLRSMAFN------ERLPESHTCF-NQ-LILSDF-SSVQS
LDQKLR-LAINE-SQGF
>Puccinia_triticina_ADAS01003916.1 .
LSIRRSHL-VEDSLRQI---------------ASSQSELKK-LLKITFVD-------EEG
VDGGXLKKEW---FLLL----IRQLVAPEY--------GMFLHDPDQH------------
----------------QIWFNPAS------------------------------------
------QEFEEFRLIGTVLGLAIYNRVTLDFGLPL--VGYRKLLG---------------
--------LPVNRSDLAA----------------------LKPEVAKN--------LGWL
LEYDGD---DFE-EICC-
--RNFVGDYDA---------------------------YGA-IVEVP-LIPN--------
GENIP---
------------------------------VTKSNRA-EFVKL-YCDYILNKS-IEKQFK
-AFSDG---FNSIAAG--NGLS-LFQPE-EIELLVIG-----------------------
-STYNSKLD-----------IEDL----KAITLYDG-FSPT-------------------
------------------------DLTV-QYFWIVID--------------------SFS
FEDQKKLLRF-------ITGTDRIP------------ATG-----IS-GLNLKI--TRSX
----------------------------------ERLPESHTCF-NQ-LILSDF-SSVQS
LDEKLR-LAINE-SEGF
>Puccinia_striiformis_GAIR01009636.1 .
--SFVD-------EEG
VDGGGLKKEW---FLLL----IRELVAPEY--------GMFLHDSELN------------
----------------QIWFNPAS------------------------------------
------TELEEFRLIGIVIGLAIYNRATLDFGLPL--VGYRKLLG---------------
-------SPVTELTDLAS----------------------LKPEVANS--------LKWI
LEYDGD---DLE-EICS-
--RNFVGDYDA---------------------------YGT-VREVP-LLPN--------
GENLP---
------------------------------VTKSNRT-EFVKL-YCDYVLNKS-ITQQFQ
-AFSDG---FHSIAAG--NGLS-LFQPE-EIELLVIG-----------------------
-SSYDSKLS-----------VEDL----KAITTYDG-FLST-------------------
------------------------DSTI-QYFWSVVN--------------------SFD
FEDQKKLLRF-------ITGTDRIP------------ATG-----IS-RLTLKI--SKSX
----------------------------------ERLPESHTCF-NQ-LILSEF-NSVHS
LHEKLS-LAINE-SLGF

>Phakopsora_pachyrhizi_GACM01001996.1 .
LRVRRSHL-VEDSLRQI---------------EAGRSELKM-LLKIGFVN-------EEG
VDGGGLKREW---FLLL----IRRLVAPEY--------GMFIHDEETN------------
----------------QIWFNPAS------------------------------------
------SEFEEFKLIGTVIGLAIYNCVTLDINLPL--VCYKKLLK---------------
-------SPTSGLEDVQT----------------------FRPSVARG--------LKWI
LDYEGN---DLE-EVCG-
--RNMVGDYES---------------------------YGT-IVEVP-LIEG--------
GADIP---
------------------------------VSNKNRK-EYVKL-YSDFIVNKS-VEKQFT
-AFAQG---FNTVAAG--NALS-LFQPQ-EIELLVRG-----------------------
---SEEALD-----------FNQL----QLTTVYDG-YSRN-------------------
------------------------DETI-VEFWNYFK--------------------DLD
VPGQRKALGF-------ITGSDRIP------------AVG-----TS-GLDFKI--SKLG
DGTD------------------------------GRLPVSHTCF-NQ-LMLPDY-KRRSL
IAKKMN-IAIGD-SEGF
>Melampsora_larici_populina_XM_007404837.1 .
LNIRRNHL-VEDSLRQI---------------ERSRGELKK-LLKICFVG-------EDG
LDGGGLKKEW---FLLL----IRQLVGPEY--------GMFIHDPDSN------------
----------------QLWFNPAS------------------------------------
------QELGEFRLIGTVIGLAIYNRATLDISLPL--VCYKKLLG---------------
-------QNQVSLADLEI----------------------MRPLTARG--------LRQL
LEWDHPE--MVE-EIFC-
--RSMVGEYEDF--------------------------DGT-VIEVP-LVPN--------
GSNIP---
------------------------------VTGSNRE-EFVEL-YVDFLLNVS-VQHQFM
-AFKEG---FDAVAAG--NALS-LFQPE-EIELVVSG-----------------------
---SRERLD-----------VDEL----SSRTEYEN-YQSK-------------------
------------------------DLTI-LSFWSYMK--------------------SLN
RSEEKKILSF-------ITGTDRIP--------------------TC-GLKFKI--SREF
DL--------------------------------NRLPSSHTCF-NQ-LVLPDL-LTVQK
VSEKLW-IAMNE-SEGF
>Verruconis_gallopava_XM_016362775.1 .
LTVRRDYI-IEDSFKVI-----------RQAVGSMSKVTSK-KLRVHFEG-------EEA
VDAGGPQKEW---FLIL----TEKLFDPEL--------GLFYLSDSG-------------
----------------YFWFNSAC------------------------------------
-----KQPNEYFELVGAVFGLAIYNQTSLFAPFPP--FLFKKLAAAAPRQSN--------
VGILWRHLWRPTLQDYAQ----------------------LSPDFARV--------LKQG
LE---DPS-VMEG-
--LAFEIPVWQ---------------------------NGQ-SVYHA-LEPD--------
GSNRI---
------------------------------INAGNID-EFVEK-AVSYVLQDS-INNQFR
-AFARG---FHSICGG--NALS-LFRGE-ELELLVRG-----------------------
----SEDFD-----------LETL----RAAAVYDN-WKRLGTDEDLQTEDEVVQT----
------------------------YPLV-NMFWNYFQ--------------------AAD
IKERRRILRF-------ITGTDRLP------------PAG-----IV-NLVIKI--SRQQ
MKKGE-----------------------------YRYPSARTCF-NQ-ILLPDC-FEKQS
IFDKVFKNALDMAGTGF
>Saitoella_complicata_XM_019170827.1 .
LRIRRANL-MEDSMRQL--------------RAVEGGELKK-GLRIQFEG-------EDG
VDAGGLRKEW---FLIL----VREIFDPKY--------GLFVEEESSG------------
----------------VVWFDPAS------------------------------------
---TSKEALEKYYLAGVVVGLAIYNSTILDVRLPR--ALFTKLMG---------------
--------GRLGLEDMEG----------------------WKPEFVKG--------LRQL
LEFEG--DVE-SVLG-
--LDFTVHDI----------------------------YG---SLRE-LVPN--------
GSRLS---
------------------------------VTNANRK-EYVDA-YVAYLLNTS-VNAQYS

-TFRDG---FWRVCGG--NAMG-LFNAE-EVEGLVCG-----------------------
LGMGEEGVD-----------VGAI----RAVAVYEG-YRRGPAGPSPTVINPEK------
------------------------DPLI-TWFWAFFS--------------------SLP
GADQRRLLRF-------VTGSDRVP------------ATG-----AA-NLAFKI--SVMP
EVNGGE----------------------------DRFPIAHTCF-NQ-IVLPRY-KRREK
MEGMLR-RAMWE-----
>Spizellomyces_punctatus_XM_016754113.1 .
LRIRRSAL-IEDSLNQL---------------QSRHFDLKK-KLRIEFVN-------EDG
VDAGGLTKEW---FLLL----VRDLFDPQY--------GMFTFDDDSH------------
----------------LCWFNPAS------------------------------------
-----FENTEEFRLVGTIIGLAIHNSNILDVHFPP--ACYKKLLG---------------
--------HVCGLEDLKK----------------------LRPALGRG--------LEQL
LSYDGD---DVE-TVFC-
--RDFVAEYEA---------------------------FGE-VKQVP-LVPN--------
GDRIP---
------------------------------VTKDNKQ-EFVDR-YVNWVLNDS-IETQFT
-AFKAG---FGYVCGG--NALS-LFRPA-EIELMVCG-----------------------
----GTELD-----------IHGL----EGVTEYEG-FTSS-------------------
------------------------DRTV-RNFWDIVN--------------------AYP
TEMKRKLLLF-------VTGTDRIP------------ATG-----IQ-NMAFKV--SCLG
EDS-------------------------------ENLPISHTCF-NQ-ICLYRY-SNREK
LEEKLS-KAVVW-SSGF
>Homoloaphlyctis_polyrhiza_AFSM01000760.1_R .
ITVRRSHL-ISDSLNQI---------------QAKTADLKK-KLRIEFAD-------EEG
VDAGGLTKEW---FLLL----VRQLFDPQY--------GMFTFDEDSK------------
----------------LCWFNQAG------------------------------------
-----FENAVQFQLVGTVVGLSLYNTTIIDVQLPL--ACFKKLLN---------------
--------VPVX------------------------------PALARG--------LQLM
LDYTED---DIE-QVFC-
--RTFVAEFER---------------------------FGE-VIQVP-LVPN--------
GENIL---
------------------------------VNHSNKK-QFVDL-LVDWILNKS-VAEQFA
-AFKDG---FNHVCGG--NALS-LFRPE-EIELMVRG-----------------------
----SSHLD-----FEGLQLTADL----DGCVSCVG-WLVV-------------------
------------------------YMTL-KWFWQIIT--------------------AFS
EAMKRKFLLF-------VTGTDRVP------------PTG-----LE-ALRIKL--SCMG
DDS-------------------------------EKLPVAHTCF-NQ-ICLYRY-ASKQK
LETKLV-QAIEW-SSGF
>Allomyces_macrogynus_ACDU01000860.1 .
LTIRRSHL-VQDSMDQL---------------SDPDLDLKK-RLRVEFAG-------EEG
IDAGGLTKEW---LMLL----VRDLFDPSY--------GLWLVPNEGNP-----------
----------------TCWFHPAC------------------------------------
----PADMHQEYYLVGVVVGLAVYHSTILDVPLAS--ACYKKLLG---------------
--------NSVGLEDLAS----------------------LDVALAHG--------LQQL
LDYPGD---DVE-TVFC-
--RDFVAEYGGY--------------------------GGE-RVRVP-LIPD--------
GENTP---
------------------------------VTSSNKH-AYVAA-YVDFILTKS-VTAAFD
-NFRRG---FLRVLQG--NALS-LFRAE-EIELLVRG-----------------------
----TADID-----------LGPL----QATAEYDG-FRSN-------------------
------------------------DTTV-RYLWRILH--------------------AFD
PDMKRQFLRF-------LTGTDRIP------------ATG-----VG-NVHLKI--TCVD
PNPDS-----------------------------DRLPTAHTCF-NQ-LCLAAY-TSKSR
LERKLR-MAILE-SQGF
>Rhodosporidium_toruloides_GEEN01008143.1 .
LRVRRDRL-VDDSLRQI---------------SLHRTDLKK-PLRITWEG-------EEG
IDAGGLRKEW---FLLL----CRQLFDPQF--------GMFLHDPDSN------------
----------------LCWFNPAA------------------------------------

-----IGMEDDFWMVGVIVGLAVYNEATLDVPLPL--ATYKKLAS---------------
--------ESLTLRDLAQ----------------------VQPSLARG--------LQQL
LDYDKG---DVE-GTFM-
--RSYVGTYEA---------------------------WGE-LVEVE-LVEG--------
GAEIA---
------------------------------VTEENRQ-DYVQR-FVDFILSTS-VSSQWD
-AFAEG---FIEVCAG--NALS-LFKAQ-ELELVVRG-----------------------
---SPEPLD-----------VGAL----KGVTVYEG-FSPD-------------------
------------------------EPTI-QYFWSVFA--------------------SLQ
PDRQRRLLAF-------CTASDRVP------------ATG-----VS-GLQLRL--QCLG
DDN-------------------------------DRLPQSHTCF-NT-LSMWRY-GTREK
VERMLV-RVMED-SEGF
>Rhodotorula_graminis_XM_018415422.1 .
LRVRREHL-VADSLRQI---------------SLNRFNLKK-PLRVKWEG-------EEG
IDAGGLRKEW---FLLL----CRQLFDPQF--------GMFVPDPDSN------------
----------------LCYLNPGA------------------------------------
-----LGMEDDFWLVGVVVGLAVYNSATLDLPLPL--AIYKKLSF---------------
--------EPLGLADLAQ----------------------VQPALARG--------LQQL
LDYDGADG---TVE-DVFC-
--RAFVGAYEA---------------------------WGE-TVEEE-LVEG--------
GREVA---
------------------------------VTEENRK-DYVRL-LVDFLLSKS-VSPQFD
-AFAEG---FHEVCAG--SALS-LFKAQ-ELELVVRG-----------------------
---STEALD-----------VDAL----RGVTVYEG-FAPD-------------------
------------------------EPTI-DAFWSTFH--------------------AFS
PEQQRRLLAF-------ITASDRLP------------ATG-----TA-GLTLKL--QCSG
DDT-------------------------------ARLPSASTCF-NT-LILPRY-RTRVV
VESMLV-RAVED-SEGF
>Rhizopus_oryzae_GDUK01014586.1 .
LRVSRDNL-IEDSLRQL---------------AQNKLDLKK-SLRIEFIG-------EEG
VDAGGLRKEW---FLLL----VRSLFDPQY--------SMFTYDEDSN------------
----------------LCWFNPAS------------------------------------
-----FENEDQFFLVGVVLGLAIYNSTILDIHLPT--ACYKKLLN---------------
--------MPVGLSDLGS----------------------FRPALKRG--------FDQL
LEFDG--DVE-NIFC-
--RSFVAEIDK---------------------------FGQ-RICIP-LIPN--------
GEHIM---
------------------------------VTKENRQ-QFVSL-YADFVLNTS-VERQFG
-AFKRG---FYHVCGG--NALS-IFQPE-EIELLVRG-----------------------
---SDGPLE-----------IDDL----KSQTEYIG-FDEN-------------------
------------------------EETI--XFWSIIK--------------------AME
PKMQRKLLMF-------VTGSDRIP------------ATG-----AT-QMHLKI--TCGN
NGDS------------------------------DRLPSAHTCF-NQ-LVLYKY-HTKEK
LKRMLY-TAILE-SQGF
>Mucor_irregularis_GFBC01022114.1 .
LRVSRENL-IEDSLRQL---------------AQNELDLKK-SLRIEFIG-------EDG
VDAGGLRKEW---FLLL----VRSLFDPQY--------GMFTFDEDSN------------
----------------LCWFNPAS------------------------------------
-----FENEDQFFLVGVVLGLAIYNSTILDVHLPT--ACYRKLLN---------------
--------LPIGLSDLKS----------------------FRPALTKG--------FEQL
LEFEG--DVE-NVFC-
--RSFVAEIEA---------------------------FGA-RQCVP-LVPK--------
GDQLM---
------------------------------VTQENKQ-EFVAL-YADFILNKS-VERQFG
-AFKQG---FYHVCGG--NALS-LFRPE-EIELLVRG-----------------------
---SDEPLE-----------IDDL----KGQTEYIG-FDEN-------------------
------------------------EETI-VNFWSIMK--------------------AME
PQMQRKLLMF-------VTGSDRIP------------ATG-----AT-QMNLKI--TCGN

NGDS------------------------------DRLPSAHTCF-NQ-LILYKY-HTKQK
LEKMLI-TAIQE-SQGF
>Phycomyces_blakesleeanus_XM_018428259.1 .
LRVRREHL-IEDSLRQL---------------AQNELDLKK-SLRIEFIG-------EEG
VDAGGLRKEW---FLLL----VRSLFDPQY--------GMFTYDEDSN------------
----------------LCWFNPAS------------------------------------
-----FENKDQYFLVGVVLGLAIYNSTILDIHLPT--ACYKKLFH---------------
--------QPVDLTDLGV---------------------FRPSLTHG--------FQQL
LEFEG--DVE-NVFC-
--RSFVAEFEN---------------------------FGQ-RKCVS-LLPD--------
GAQKM---
------------------------------VTNENRQ-EFVDL-YVNYVLNSS-VERQFD
-AFQRG---FYHVCGG--NALS-LFRPE-EIELLVRG-----------------------
---SDEPLE-----------IDEL----RRQTEYNG-FEED-------------------
------------------------ERTI-VDFWSIMK--------------------EME
PEKQRRLLMF-------FTGSDRIP------------ATG-----AS-NMHLKI--TWGG
NVL------------------------------DRLPSAHTCF-NQ-LVLYKY-ENKKK
LKRMLE-MAMME-SQGF
>Gigaspora_margarita_GBYF01078344.1 .
LKVRRDYL-IEDSLSQI---------------SENEMELKK-SLRIEFVG-------EVG
VDGGGLRKEW---FLLL----VRHLFDPKF--------GMFTWDEDSR------------
----------------LCWFNPAS------------------------------------
-----FEPSDQFYLVGVVIGLAIYNSTILDVHFPL--ACYKKLLN---------------
--------VPVGLEDLKE---------------------FRPAFARG--------LEQL
LSFEG--DVE-STFC-
--RDFVGEYEV---------------------------FDE-ITRIP-LKPN--------
GQNIP---
------------------------------VTNDNRK-EYVER-YVNFILNES-VTKQFE
-PFRRG---FNHVCSG--NALS-LFQPE-EIELLVRG-----------------------
---SAEPLE-----------IEQL----RSVTIYEG-FRED-------------------
------------------------EVTI-INFWAIFK--------------------AMN
PIMQRKLLTF-------VTGTDRIP------------GTG-----CA-NLSFKI--SYYG
EDC------------------------------ERFPIAHTCF-NQ-IFLYRY-RTKRK
LEEKLL-RAITE-SEGF
>Pneumocystis_jirovecii_XM_018375896.1 .
LRVRRECI-IEDSLRQI---------------SNNEMDLKK-SLRIEFVG-------EDG
VDIGGLRKEW---FLLL----CREVFDPLY--------GMFVWNEETN------------
----------------YCWFNPAS------------------------------------
-----FESSDQYFLVGVVLGLAIYNSTILDIHFPL--ACYKKLLD---------------
--------IPCGLDDLKV---------------------FRPSLVKG--------FQHL
LTFEG--NVE-DTFC-
--RDFVGEYEA---------------------------FGN-VYRLP-LCKN--------
GEKIA---
------------------------------VTNLNRE-EYVKR-YTSFILNTS-ISKQFE
-PFKRG---FYHVCGG--NALS-LFRPE-EIELLIRG-----------------------
---SPESLD-----------VDQL----RSVTVYDS-LNSLEINPEE-------------
------------------------ESVI-EWFWSIFK--------------------EMK
PILQRKLLAF-------VTGSDRIP------------ATG-----AA-NLSFKI--SILG
NDC------------------------------DRYPIAHTCF-NQ-LCLYRY-KTHQK
LYKFLI-TAISD-SEGF
>Chaetomium_thermophilum_XM_006690725.1 .
LRVRRECL-VEDSLSAV---GEAIGASAAAGGGVGILDVKK-ELRIEFMG-------EEG
VDAGGLRKEW---FLLL----CRELFDPGN--------GMFLYDPDSH------------
----------------YCYFNPFS------------------------------------
-----LEPSQQYFLVGVVLGLAIYNSTILDISLPP--FAFRKLLA---------------
--SPSSNPHSSTLDDLAE---------------------YRPTLARG--------LRQL
LDYPNPD--EVE-DVFA-
--LSFAITVDR---------------------------YGV-ADTVE-LCPG--------

GSRRP---
------------------------------VTGANRR-EFVDL-YVRYLLDTS-VAKQFE
-PFKRG---FWSIVGX--NVLS-LFQPD-EVELLIRG--------------SNGSSGNSL
NGDSLPPLD-----------VAAL----KTAATYDG-WERYRPKSPSGRPPFNPAED---
------------------------EPTI-RWFWETFE--------------------SAS
PVAQRKLLAF-------VTGSDRVP------------AAG-----AG-SLRIAL--HCLG
DDC-------------------------------GRFPTARTCF-NV-LGLWRC-GTKER
LEEVLW-RAVWE-SEGF
>Ascosphaera_apis_AARE01002810.1_R .
LKVRRQCL-VEDSLTSV-----------SEVVATGQEDIKK-GLRIEFIG-------EEG
VDAGGLRKEW---FLLL----TRDIFNPDH--------GLFLYDEDSQ------------
----------------YAYFNPYC------------------------------------
-----LESSEQFYLVGVLLGLAIYNSTILDIALPP--FLFRKLLASTPRSIH------SP
GMAAPNQSWKPTLEDLAE----------------------YRPSVAKG--------LQRL
LDYEG--DAR-DLC--
--LDFVIESER---------------------------YGE-RIVTP-LCTG--------
GENRP---
------------------------------VTNSNKR-EYVNL-YVRHLLEES-VARQFN
-PFRNG---FFTVCAG--NALH-LFRPE-EIELLVRG-----------------------
---SDEPLD-----------IHSL----RSVAAYAG-FGSNPTS----------------
------------------------DRIV-RWFWDFFS--------------------RSS
PKDQRKILSF-------ITGSDRIP------------ATG-----AA-NLIMKI--SLMG
NDC-------------------------------ERYPVAHTCY-NQ-IGLYRY-PTRQI
FEEKLW-RAVVD-SQGF
>Endocarpon_pusillum_XM_007805859.1 .
LKVRRDCL-VEDSLRRI-----------SEVVGSGQGDIKK-GLRIDFIG-------EEG
VDAGGLRKEW---FLLL----IREVFDEAH--------GLFVYDEDSR------------
----------------FCYFNPHC------------------------------------
-----FESSEQYFLVGVVTGLAIYNSTILDIAFPP--FVFKKLLASTPVTTN-------V
TPSTPRLAHGSSLEDLAE----------------------LHPRLARG--------LSQL
LEYTG--DVQ-QTFC-
--RDFVVEEDR---------------------------YGE-SVQVP-LLPG--------
GEKVP---
------------------------------VTNSNCR-EYVEL-YIHYLLDIS-VSRQYE
-PFKRG---FFTVCGG--NALS-LFRPE-EIELLIRG-----------------------
---SDEPLD-----------IHSL----RAVAVYDG-WPQGRTPDQ--------------
------------------------EPQA-QWFWEAFE--------------------SAN
PANQRKLLAF-------ITGSDRIP------------AMG-----AT-NLVIKI--QLLE
TTKRSGIIDT------------------------ERFPTARTCF-NT-LVLHPY-RSKAQ
LEKKLW-MAVSE-SEGF
>Epichloe_typhina_AFSE01000078.1 .
FNIRRDCL-VEDSLAAV-----------SGVIGSGSEDIKK-GLRITFKG-------EEG
IDGGGLRKEW---FLLL----VREVFNPDH--------XMFLYDEDSQ------------
----------------YCYFNPST------------------------------------
-----FETSDQFFLVGVVMGLAIYNSTILDVALPP--FAFRKLLAAAPAHGL-------G
MSSRPRPWMQYTLDDLAE----------------------YRPQVAHG--------LRQL
LDFEG--DVE-STFC-
--LDFVIDTEK---------------------------YGT-TVQVP-LCPG--------
GERKP---
------------------------------VTNGNRR-EYVEL-YVRYLLDTA-VTRQFE
-PFKRG---FYTVCGG--NAFS-LFRPE-EIELLIRG-----------------------
---SDEALD-----------IAAL----RAVAEYDN-WGHPHPDDN--------------
------------------------IDVV-VWFWETFQ--------------------DAT
SKNQRKLLSF-------ITGSDRIP------------ATG-----AA-MLPIKI--SCLG
EDS-------------------------------GRYPIARTCF-NM-ISLWKY-GSKQR
LEGMLW-RAVFE-SEGF
>Epichloe_brachyelytri_AFRB01000025.1_R .
--IRRDCL-VEDSLAAV-----------SGVIGSGSEDIKK-GLRITFKG-------EEG

IDGGGLRKEW---FLLL----VREVFNPDH--------GMFLYDEDSQ------------
----------------YCYFNPST------------------------------------
-----FETSDQFFLVGVVMGLAIYNSTILDVALPP--FAFRKLLAAAPAHGL-------G
MSSRPRPWMQYTLDDLAE----------------------YRPQVAHG--------LRQL
LDFEG--DAE-STFC-
--LDFVIDTEK---------------------------YGT-TVQVP-LCPG--------
GERKP---
------------------------------VTNGNRR-EYVEL-YVRYLLDTA-VTRQFE
-PFKRG---FYTVCGG--NAFS-LFRPE-EIELLIRG-----------------------
---SDEALD-----------IAAL----RAVAEYDN-WGHPHPDDN--------------
------------------------IDVV-VWFWETFQ--------------------DAT
SKNQRKLLSF-------ITGSDRIP------------ATG-----AA-MLPIKI--SCLG
EDS-------------------------------GRYPIARTCF-NM-ISLWEY-RSKQR
LEGMLW-RAVFE-SEGF
>Epichloe_glyceriae_AFRG01000296.1_R .
--IRRDCL-VEDSLAAV-----------SEVIGSGSEDIKK-GLRITFRG-------EEG
IDGGGLRKEW---FLLL----VREVFNPDH--------GMFLYDEDSQ------------
----------------YCYFNPST------------------------------------
-----FETSDQFFLVGVVMGMAIYNSTILDVALPP--FAFRKLLAAAPAHGL-------G
MSSRPRPWMQYTLDDLAE----------------------YRPQVAHG--------LRQL
LDFEG--DVE-STFC-
--LDFVIDTEK---------------------------YGT-TVQVP-LCPG--------
GERKP---
------------------------------VTNGNRR-EYVEF-YVRYLLDTA-VTRQFE
-PFKRG---FYTVCGG--NAFS-LFRPE-EIELLIRG-----------------------
---SDEALD-----------IAAL----RAVAEYDN-WGHSHPDDN--------------
------------------------IDVV-VWFWETFQ--------------------DAT
SKNQRKLLSF-------ITGSDRIP------------ATG-----AA-MLPIKI--SCLG
EDS-------------------------------GRYPIARTCF-NM-ISLWEY-GSKQR
LEGMLW-RAVFE-SEGF
>Epichloe_festucae_AFRX01000288.1_R .
--IRRDCL-VEDSLAAV-----------SGAIGSGSEDIKK-GLRITFKG-------EEG
IDGGGLRKEW---FLLL----VREVFNPDH--------GMFLYDEDSQ------------
----------------YCYFNPST------------------------------------
-----FETSDQFFLVGVVMGLAIYNSTILDVALPP--FAFRKLLAAAPAHGL-------G
MSSRPRPWMQYTLDDLAE----------------------YRPQVAHG--------LHQL
LDFEG--DVE-STFC-
--LDFVIDTEK---------------------------YGT-TVQVP-LCPG--------
GERKP---
------------------------------VTNGNRR-EYVEL-YVRYLLDTA-VTRQFE
-PFKRG---FYTVCGG--NAFS-LFRPE-EIELLIRG-----------------------
---SDEALD-----------IAAL----RAVAEYDN-WGHSHPDDN--------------
------------------------IDVV-VWFWETFQ--------------------DAT
SKNQRKLLSF-------ITGSDRIP------------ATG-----AA-MLPIKV--SCLG
EDS-------------------------------GRYPIARTCF-NM-ISLWKY-GSKQR
LEGMLW-RAVFE-SEGF
>Epichloe_amarillans_AFRF01000120.1 .
--IRRDCL-VEDSLAAV-----------SGAIGSGSEDIKK-GLRITFKG-------EEG
IDGGGLRKEW---FLLL----VREVFNPDH--------XMFLYDEDSQ------------
----------------YCYFNPST------------------------------------
-----FETSDQFFLVGVVMGLAIYNSTILDVALPP--FAFRKLLAAAPAHGL-------G
MSSRPRPWMQYTLDDLAE----------------------YRPQVAHG--------LRQL
LDFEG--DVE-STFC-
--LDFVIDTDK---------------------------YGT-TVQVP-LCPG--------
GERKP---
------------------------------VTNGNRR-EYVEL-YVRYLLDTA-VTRQFE
-PFKRG---FYTVCGG--NAFS-LFRPE-EIELLIRG-----------------------
---SDEALD-----------IAAL----RAVAEYDN-WGHSHPDDN--------------

------------------------IDVV-VWFWETFQ--------------------DAT
SKNQRKLLSF-------ITGSDRIP------------ATG-----AA-MLPIKV--SCLG
EDS-------------------------------GRYPIARTCF-NM-ISLWKY-GSKQR
LEGMLW-RAVFE-SEGF
>Neotyphodium_gansuense_AFRE01000099.1_R .
--IRRDCL-VEDSLAAV-----------SEVIGSGSEDIKK-GLRITFKG-------EEG
IDGGGLRKEW---FLLL----VREVFNPDH--------GMFLYDEDSQ------------
----------------YCYFNPNT------------------------------------
-----FETSDQFFLVGVVMGLAIYNSTILDVALPP--FAFRKLLAAAPAHGL-------G
ISSRPRPWMHYTLDDLAE----------------------YRPQVAHG--------LRQL
LEFEG--DVE-STFC-
--LEFVIDTEK---------------------------YGT-TVQVP-LCPG--------
GERKP---
------------------------------VTNGNRR-EYVEF-YVRYLLDTA-VTRQFE
-PFKRG---FYTVCGG--NAFS-LFRPE-EIELLIRG-----------------------
---SDEALD-----------IAAL----RAVAEYDN-WGHSDPDGN--------------
------------------------IDVV-VWFWETFQ--------------------DAT
SKNQRKLLSF-------ITGSDRIP------------ATG-----AA-MLPIKI--SCLG
EDS-------------------------------GRYPIARTCF-NM-ISLWEY-GSKQR
LEGMLW-RAVFE-SEGF
>Periglandula_ipomoeae_AFRD01000398.1 .
LHVRRDCL-VEDSLAAV-----------SEVIGSGSEDIKK-GLRITFKG-------EEG
IDGGGLRKEW---FLLL----VREVFNPDH--------XLFLYDEDSQ------------
----------------YCYFNPTT------------------------------------
-----FETSNQFFLVGVVMGLAIYNSTILDVALPP--FAFRKLLAAAPAHGL-------G
ISSRPRPWMQYTLNDLAE----------------------YRPRVAHG--------LRQL
LDFDG--DVE-NTFC-
--LDFVIDTER---------------------------YGT-TVQVP-LCPG--------
GERKP---
------------------------------VTNSNRR-EYVDL-YVRYLLDTA-VTRQFE
-PFKRG---FYTVCGG--NAFS-LFRPE-EIELLIRG-----------------------
---SDETLD-----------IASL----RAVTEYDN-WGHARPDGN--------------
------------------------IDVV-AWFWETFE--------------------EAT
AKNQRKLLSF-------ITGSDRIP------------ATG-----AA-MLPIKI--SCLG
EDT-------------------------------GRYPIARTCF-NM-ISLWEY-GSKQR
LEGMLW-RAVFE-SEGF
>Aciculosporium_take_AFQZ01000561.1_R .
LSVRRDCL-VEDSLAAV-----------SEVIGSGSEDIKK-ALRITFKG-------EEG
IDGGGLRKEW---FLLL----IREVFNPDH--------GMFLYDEDSQ------------
----------------YCYFNPST------------------------------------
-----FETSDQFFLVGVVMGLAIYNSTILDVALPP--FAFRKLLAAAPAHGL-------G
ISSRPRPWMQYTLDDLAE----------------------YRPRVAHG--------LRQL
LDFEG--DVE-NTFC-
--LDFVIDTER---------------------------FGT-TVSIP-LCPG--------
GERKP---
------------------------------VTNSNRR-EYVDL-YIRYLLDTA-VTRQFE
-PFKRG---FYTVCGG--NAFS-LFRPE-EIELLIRG-----------------------
---SDEALD-----------IAAL----RAVAEYDN-WGHSHPDDN--------------
-----------------------VDVV-VWFWETFQ--------------------EAT
PKDQRKLLSF-------ITGSDRIP------------ATG-----AA-MLPIKI--SCLG
QDS-------------------------------GRYPTARTCF-NT-ISLWEY-GSKQK
LEKMLW-RAVFE-SEGF
>Claviceps_fusiformis_AFRA01000046.1 .
LSVRRDCL-VEDSLAAV-----------SEVIGSGTEDIKK-ALRITFKG-------EEG
IDGGGLRKEW---FLLL----VREVFNPDH--------XLFLYDEDSQ------------
----------------YCYFNPST------------------------------------
-----FETSDQFFLVGVVMGLAIYNSTILDVALPP--FAFRKLLAAAPAHGL-------G
MSSRPRPWMQYTLDDLAE----------------------YRPRVAQG--------LRQL

LEFEG--DVE-NTFC-
--LDFVIDTDK---------------------------FGT-TVQVP-LCPG--------
GERKA---
------------------------------VTNSNRR-EYVEL-YTRYLLDTA-VTRQFE
-PFKRG---FYTVCGG--NAFS-LFRPE-EIELLIRG-----------------------
---SDEALD-----------IAAL----RAVAEYDN-WGHPHPDDN--------------
------------------------VDVV-VWFWETFQ--------------------EAT
PKDQRKLLSF-------ITGSDRIP------------ATG-----AA-MLPIKI--SCLG
ADS-------------------------------GRYPIARTCF-NM-LSLWEY-GSKKR
LETMLW-RAVFE-SEGF
>Pochonia_chlamydosporia_XM_018280726.1 .
LTVRRDCL-VEDSLTAV-----------SEVIGSGSEDIKK-GLRITFSG-------EEG
IDGGGLRKEW---FLLL----IREVFNPDH--------GMFLYDEDSQ------------
----------------YCYFNPAT------------------------------------
-----FETSDQFFLVGVVMGLAIYNSTILDVALPP--FAFRKLLAAAPAHGL-------G
MSSRPRPSMQYTLDDLAE----------------------YRPRVARG--------LRQL
LDFEG--DVE-NTFC-
--LDFVIDTEK---------------------------YGT-KVQVP-LCPG--------
GERKA---
------------------------------VTNSNRR-EYVDL-YVRYVLDTA-VTRQFE
-PFKRG---FYTVCGG--NAFS-LFRPE-EIELLIRG-----------------------
---SDEALD-----------IASL----RAVAEYDN-WGNRQPEGS--------------
------------------------SPVV-SWFWETFQ--------------------EAA
PANQRKLLSF-------ITGSDRIP------------ATG-----AA-MLPIKI--SCLG
EDV-------------------------------GRFPIARTCF-NM-ISLWQY-GSKQK
LEAMLW-RAVFE-SEGF
>Metarhizium_acridum_ADNI01000878.1_R .
LDVRRECL-VEDSLAAV-----------SEVIGSGSEDVKK-GLRITFRG-------EEG
IDAGGLRKEW---FLLL----IREVFNPDH--------GMFLYDEDSQ------------
----------------YCYFNPAT------------------------------------
-----FETSDQFFLVGVVMGLAIYNSTILDVALPP--FAFRKLLAAAPAHGL-------G
MSSRPRPSMQYTLEDLAE----------------------YRPRVARG--------LRQL
LDFEG--DVE-SAFS-
--LDFVIDTEK---------------------------YGT-TVQVP-LCHG--------
GERKA---
------------------------------VTNSNRR-EYVDL-YIRYVLDTA-VTRQFE
-PFKRG---FYTVCGG--NAFS-LFRPE-EIELLIRG-----------------------
---SDEALD-----------IASL----RAVAEYDN-WESRQPDGS--------------
------------------------SPVV-GWFWETFQ--------------------EAT
PGNQRKLLSF-------ITGSDRIP------------ATG-----AA-MLPIKI--SCLG
EDV-------------------------------GRYPIARTCF-NM-LSLWRY-GSKQK
LEGMLW-RAVFE-SEGF
>Metarhizium_majus_XM_014719252.1 .
LDVRRECL-VEDSLAAV-----------SEVIGSGSEDVKK-GLRITFRG-------EEG
IDGGGLRKEW---FLLL----IREVFNPDH--------GMFLYDEDSQ------------
----------------YCYFNPAT------------------------------------
-----FETSDQFFLVGVVMGLAIYNSTILDVALPP--FAFRKLLAAAPAHGL-------G
MSSRPRPSMQYTLEDLAE----------------------YRPRVARG--------LRQL
LDFEG--DVE-SVFS-
--LDFVIDTEK---------------------------YGT-TVQVP-LCHG--------
GERKA---
------------------------------VTNSNRR-EYVDL-YIRYVLDTA-VTRQFE
-PFKRG---FYTVCGG--NAFS-LFRPE-EIELLIRG-----------------------
---SDEALD-----------IASL----RAVAEYDN-WESRQPDGS--------------
------------------------SPVV-GWFWETFQ--------------------EAT
PGNQRKLLLF-------ITGSDRIP------------ATG-----AA-MLPIKI--SCLG
EDV-------------------------------GRYPIARTCF-NM-ISLWQY-GSKQK
LEGMLW-RAVFE-SEGF

>Metarhizium_anisopliae_ADNJ01000572.1 .
LDVRRECL-VEDSLAAV-----------SEVIGSGSEDVKK-GLRITFRG-------EEG
IDGGGLRKEW---FLLL----IREVFNPDH--------XMFLYDEDSQ------------
-----------------YCYFNPAT-------------------------------------
-----FETSDQFFLVGVVMGLAIYNSTILDVALPP--FAFRKLLAAAPAHGL-------G
MSSRPRPSMQYTLEDLAE----------------------YRPRVARG--------LRQL
LDFEG---DVE-SAFS-
--LDFVIDTEK----------------------------YGT-TVQVP-LCHG--------
GERKA---
------------------------------VTNSNRR-EYVDL-YIRYVLDTA-VTRQFE
-PFKRG---FYTVCGG--NAFS-LFRPE-EIELLIRG----------------------
---SDEALD-----------IASL----RAVAEYDN-WESRQPDGS--------------
------------------------SPVV-GWFWETFQ--------------------EAT
PGNQRKLLLF-------ITGSDRIP------------ATG-----AA-MLPIKI--SCLG
EDV-------------------------------GRYPIARTCF-NM-ISLWQY-GSKQK
LEGMLW-RAVFE-SEGF
>Metarhizium_robertsii_XM_007825659.2 .
LDVRRECL-VEDSLAAV-----------SEVIGSGSEDVKK-GLRITFRG-------EEG
IDGGGLRKEW---FLLL----IREVFNPDH--------GMFLYDEDSQ------------
-----------------YCYFNPAT-------------------------------------
-----FETSDQFFLVGVVMGLAIYNSTILDVALPP--FAFRKLLAAAPAHGL-------G
MSSRPRPSMQYTLEDLAE----------------------YRPRVARG--------LRQL
LDFEG---DVE-SAFS-
--LDFVIDTEK----------------------------YGT-TVQVP-LCHG--------
GERKA---
------------------------------VTNSNRR-EYVDL-YIRYVLDTA-VTRQFE
-PFKRG---FYTVCGG--NAFS-LFRPE-EIELLIRG----------------------
---SDEALD-----------IASL----RAVAEYDN-WESRQPDGS--------------
------------------------SPVV-GWFWETFQ--------------------EAT
PGNQRKLLLF-------ITGSDRIP------------ATG-----AA-MLPIKI--SCLG
EDV-------------------------------GRYPIARTCF-NM-ISLWQY-GSKQK
LEGMLW-RAVFE-SEGF
>Claviceps_paspali_AFRC01000329.1_R .
LNVRRDCL-VEDSLAAV-----------SEVIGSGTEDIKK-ALRITFKG-------EEG
IDGGGLRKEW---FLLL----VREVFNPDS--------GLFLYDEDSH------------
-----------------YCYFNPNT-------------------------------------
-----FETSDQFFLVGVVMGLAIYNSTILDVALPP--FAFRKLLAAAPAHGL-------G
MSSRPRPWMQYTLDDLAE----------------------YRPRVARG--------LRQL
LDFEG---DVE-STFC-
--LDFAIDTDK----------------------------FGT-TVQIP-LCPG--------
GERKV---
------------------------------VTNSNRR-EYVEL-YIKYLLDTA-VSRQFE
-PFKRG---FYTVCGG--NAFS-LFRPE-EIELLIRG----------------------
---SDEVLD-----------IAAL----RAVAQYDN-WEHSQPDGN--------------
------------------------IDVV-AWFWETFQ--------------------EAT
PKDQRKLLSF-------ITGSDRIP------------ATG-----AA-MLHIKM--SCLG
EDS-------------------------------GRYPIARTCF-NM-LSLWKY-ASKQK
LEKMLW-TAVFE-SEGF
>Purpureocillium_lilacinum_XM_018324046.1 .
LDVRRDCL-VDDSLQAV-----------SEVIGSGSEDIKK-ALRITFAG-------EEG
IDGGGLRKEW---FLLL----VRDVFNPDH--------GMFLYDEDSQ------------
-----------------YCYFNPSS-------------------------------------
-----FETSDQFFLVGVVMGLAIYNSTILDVALPP--FTFRKLLAAAPAHGQ-------G
ASAHPKPSMKYTLEDLAE----------------------YKPRLAKG--------LRQL
LDYDG---DVE-ETFA-
--LDFVLDMDR----------------------------YGT-AVQVP-LCHG--------
GERKP---
------------------------------VTNSNRR-EYVDL-YVRHMLDAA-VSRQFE

-PFKRG---FYTVCGG--NAFS-LFRPE-EIELIVRG-----------------------
---SDEALD-----------ISSL----RAVAEYDN-WGTRQPDGK--------------
------------------------EPVV-SWFWQSFQ--------------------DAT
PADQRKLLLF-------ITGSDRIP------------AMG-----AA-MLPIKI--SCLG
DDC-------------------------------GRYPIARTCF-NM-LSLWRY-QSKAR
LEAMLW-RAVRE-SEGF
>Nectria_haematococca_XM_003051960.1 .
LNVRRDCL-VDDSLKAV-----------SEVIGSGSEDIKK-GLRITFKG-------EEG
VDAGGLRKEW---FLLL----VREVFNPDH--------GMFIYDEDSQ------------
----------------HCYFNPNS------------------------------------
-----FETSDQFFLVGVVMGLAIYNSTILDLALPP--FAFRKLIASAPTQGT-------G
ASSHPRPPMRYTLEDLAE----------------------YRPGLARG--------LRQL
LEYEG--NVE-ETFA-
--LDFVIETEK---------------------------YGT-TVEVP-LCPG--------
GERIP---
------------------------------VTNNNRR-EYVDL-YVRYIIDVS-VTRQFE
-PFKRG---FYTVCGG--NALS-LFRPE-EIELLVRG-----------------------
---SDEALD-----------IDSL----RGVAEYDN-WGSKKPDGS--------------
------------------------EPVI-DWFWETFK--------------------AAS
PEDQRKLLLF-------ITGSDRIP------------AMG-----AA-VLPIKI--SCLG
EDE-------------------------------GRYPIARTCF-NM-LSLSRY-ESKER
LEKLLW-TAVRE-SEGF
>Fusarium_virguliforme_GBJV01005365.1 .
LNVRRDCL-VDDSLKAV-----------SEVIGSGSEDIKK-GLRITFKG-------EEG
VDAGGLRKEW---FLLL----VREVFNPDH--------GMFIYDEDSQ------------
----------------HCYFNPNS------------------------------------
-----FETSDQFFLVGVVMGLAIYNSTILDLALPP--FAFRKLIASAPTQGT-------G
ASSHPRPPMRYTLEDLAE----------------------YRPGLARG--------LRQL
LEYEG--NVE-ETFA-
--LDFVIETEK---------------------------YGT-TVEVP-LCPG--------
GERIP---
------------------------------VTNNNRR-EYVDL-YVRYMIDVS-VTRQFE
-PFKRG---FYTVCGG--NALS-LFRPE-EIELLVRG-----------------------
---SDEALD-----------IDSL----RGVAEYDN-WGTKKPDGS--------------
------------------------EPVI-DWFWETFQ--------------------AAT
PEDQRKLLLF-------ITGSDRIP------------AMG-----AA-VLPIKI--SCLG
EDE-------------------------------GRFPIARTCF-NM-LSLSRY-GSKER
LERMLW-TAVRE-SEGF
>Fusarium_pseudograminearum_XM_009254105.1 .
LNVRRDCL-VDDSLKAV-----------SEVIGSGSEDIKK-GLRITFNG-------EEG
VDAGGLRKEW---FLLL----VREVFNPDH--------GLFLYDEDSQ------------
----------------YCYFNPNA------------------------------------
-----FETSDQFFLIGVVMGLAIYNSTILDVALPP--FAFRKLIASAPTHGT-------G
ASAHAKPPMRYTLEDLAE----------------------YRPRLARG--------LRQL
LEYEG--NVE-ETFC-
--LDFVVDMDK---------------------------YGT-QVQVP-LCRG--------
GERIP---
------------------------------VTNSNRR-EYVDL-YVRHVIDVS-VTRQFE
-PFKRG---FYTVCGG--NALS-LFRPE-EIELLVRG-----------------------
---SDEELD-----------INSL----RGVAEYDN-WGNKKPDGS--------------
------------------------EPVI-DWFWETFQ--------------------EAT
SQDQRKLLSF-------ITGSDRIP------------AMG-----AA-VLPIKI--SCLG
EDE-------------------------------GRYPIARTCF-NM-LSLSRY-ASKER
LEKMLW-TAVRE-SEGF
>Fusarium_graminearum_XM_011326620.1 .
LNVRRDCL-VDDSLKAV-----------SEVIGSGSEDIKK-GLRITFNG-------EEG
VDAGGLRKEW---FLLL----VREVFNPDH--------GLFLYDEDSQ------------
----------------YCYFNPNA------------------------------------

-----FETSDQFFLIGVVMGLAIYNSTILDVALPP--FAFRKLIASAPTHGT-------G
ASAHPKPPMRYTLEDLAE----------------------YRPRLARG--------LRQL
LEYEG--NVE-ETFC-
--LDFVVDMDK---------------------------YGT-QVQVP-LCRG--------
GERIP---
------------------------------VTNSNRR-EYVDL-YVRHVIDVS-VTRQFE
-PFKRG---FYTVCGG--NALS-LFRPE-EIELLVRG-----------------------
---SDEELD-----------INSL----RGVAEYDN-WGNKKSDGS--------------
------------------------EPVI-DWFWETFQ--------------------EAT
SQDQRKLLSF-------ITGSDRIP------------AMG-----AA-VLPIKI--SCLG
EDE-------------------------------GRYPIARTCF-NM-LSLSRY-ASKER
LEKMLW-TAVRE-SEGF
>Fusarium_culmorum_LT598661.1_a.
LNVRRDCL-VDDSLKAV-----------SEVIGSGSEDIKK-GLRITFNG-------EEG
VDAGGLRKEW---FLLL----VREVFNPDH--------XLFLYDEDSQ------------
----------------YCYFNPNA------------------------------------
-----FETSDQFFLIGVVMGLAIYNSTILDVALPP--FAFRKLIASAPTHGT-------G
ASAHPKPPMRYTLEDLAE----------------------YRPRLARG--------LRQL
LEYEG--NVE-ETFC-
--LDFVVDMDK---------------------------YGT-QVQVP-LCRG--------
GERIP---
------------------------------VTNSNRR-EYVDL-YVRHVIDVS-VTRQFE
-PFKRG---FYTVCGG--NALS-LFRPE-EIELLVRG-----------------------
---SDEELD-----------INSL----RGVAEYDN-WGNKKPDGS--------------
------------------------EPVI-EWFWETFQ--------------------EAT
SQDQRKLLSF-------ITGSDRIP------------AMG-----AA-VLPIKI--SCLG
EDE-------------------------------GRYPIARTCF-NM-LSLSRY-ASKER
LEKMLW-TAVRE-SEGF
>Fusarium_fujikuroi_HF679026.1 .
LNVRRDCL-VDDSLKAV-----------SEVIGSGSEDIKK-GLRITFSG-------EEG
VDAGGLRKEW---FLLL----AREVFNPXX------------------------------

-----XETSDQFFLVGVVMGLAIYNSTILDVALPP--FAFRKLIASAPTHGT-------G
ASAHPKPPMRYNLEDLAE----------------------YRPRLARG--------LRQL
LEYEG--NVE-ETFC-
--LDFVIDVDK---------------------------YGT-QVQVP-LCPG--------
GERIP---
------------------------------VTNSNRR-EYVDL-YVRHIIDVS-VTRQFE
-PFKRG---FYTVCGG--NALS-LFRPE-EIELLVRG-----------------------
---SDEALD-----------INSL----RGVAEYDN-WGIKRPDGS--------------
------------------------EPVI-DWFWETFQ--------------------AAT
SQDQRKLLLF-------ITGSDRIP------------AMG-----AA-VLPIKI--SCLG
EDE-------------------------------GRFPIARTCF-NM-LSLSRY-KSKER
LEKLLW-TAVHE-SEGF
>Fusarium_oxysporum_XM_018386738.1 .
LTVRRDCL-VDDSLKAV-----------SEVIGSGSEDIKK-GLRITFSG-------EEG
VDAGGLRKEW---FLLL----AREVFNPDH--------GLFLYDEDSQ------------
----------------YCYFNPNA------------------------------------
-----FETSDQFFLVGVVMGLAIYNSTILDVALPP--FAFRKLIASAPTHGT-------G
ASAHPRPPMRYTLEDLAE----------------------YRPRLARG--------LRQL
LEYEG--NVE-DTFC-
--LDFVIDMDK---------------------------YGT-QVQVP-LCPG--------
GERIP---
------------------------------VTNSNRR-EYVDL-YVRYIIDVS-VTRQFE
-PFKRG---FYTVCGG--NALS-LFRPE-EIELLVRG-----------------------
---SDEALD-----------INSL----RGVAEYDN-WGTKKPDGS--------------
------------------------EPVI-DWFWETFQ--------------------AAT
SQDQRKLLLF-------ITGSDRIP------------AMG-----AA-VLPIKI--SCLG

```
EDE-------------------------------GRFPIARTCF-NV-LSLSRY-KSKER
LEKLLW-TAVHE-SEGF
>Fusarium_verticillioides_XM_018892904.1 .
LNVRRDCL-VDDSLKAV-----------SEVIGSGSEDIKK-GLRITFSG-------EEG
VDAGGLRKEW---FLLL----AREVFNPDH--------GLFLYDEDSQ------------
----------------FCYFNPNA------------------------------------
-----FETSDQFFLVGVVMGLAIYNSTILDVALPP--FAFRKLIASAPTHGT-------G
ASAHPKPPMRYTLEDLAE----------------------YRPRLARG--------LRQL
LEYEG----------------------------------------------NVE-ETFC-
--LDFVIDMDK---------------------------YGT-QVQVP-LCPG--------
GERIP-------------------------------------------------------
------------------------------VTNSNRR-EYVDL-YVRHIIDVS-VTRQFE
-PFKRG---FYTVCGG--NALS-LFRPE-EIELLVRG-----------------------
---SDEELD-----------INSL----RGVAEYDN-WGTKKPDGS--------------
------------------------EPVI-DWFWETFQ--------------------AAS
SQDQRKLLLF-------ITGSDRIP------------AMG-----AA-VLPIKI--SCLG
EDE-------------------------------GRFPIARTCF-NM-LSLSRY-NSKER
LEKLLW-TAVHE-SEGF
>Fusarium_verticillioides_XM_018892905.1 .
LNVRRDCL-VDDSLKAV-----------SEVIGSGSEDIKK-GLRITFSG-------EEG
VDAGGLRKEW---FLLL----AREVFNPXX------------------------------
------------------------------------------------------------
-----XETSDQFFLVGVVMGLAIYNSTILDVALPP--FAFRKLIASAPTHGT-------G
ASAHPKPPMRYTLEDLAE----------------------YRPRLARG--------LRQL
LEYEG----------------------------------------------NVE-ETFC-
--LDFVIDMDK---------------------------YGT-QVQVP-LCPG--------
GERIP-------------------------------------------------------
------------------------------VTNSNRR-EYVDL-YVRHIIDVS-VTRQFE
-PFKRG---FYTVCGG--NALS-LFRPE-EIELLVRG-----------------------
---SDEELD-----------INSL----RGVAEYDN-WGTKKPDGS--------------
------------------------EPVI-DWFWETFQ--------------------AAS
SQDQRKLLLF-------ITGSDRIP------------AMG-----AA-VLPIKI--SCLG
EDE-------------------------------GRFPIARTCF-NM-LSLSRY-NSKER
LEKLLW-TAVHE-SEGF
>Trichoderma_reesei_XM_006961613.1 .
LDVRRDCL-VDDSLTAV-----------SSVIGSGGEDLKK-GLRISFQG-------EEG
IDAGGLRKEW---FLLL----IREVFNPDY--------GMFIYDDDSQ------------
----------------YCYFNPNS------------------------------------
-----FEPSDQFFLVGVVMGLAIYNSTILDVALPP--FAFRKLLASAPSPPG-------T
MAAQHRSPMRYTLDDLAE----------------------YRPRLANG--------LKQL
LAFDG----------------------------------------------DVE-ETFC-
--LDFVIETDR---------------------------YGS-KVQMP-LCPG--------
GESKP-------------------------------------------------------
------------------------------VTNENRR-EYVDL-YVRYVLDTA-VKRQFE
-PFKRG---FYTVCGG--NAFS-LFRPE-EIELLIRG-----------------------
---SDESLD-----------IASL----RAVAEYDN-WESKQPDET--------------
------------------------EPVV-GWFWETFQ--------------------QAT
PRDQRKLLTF-------VTGSDRIP------------AMG-----AA-SLTIKL--SCLG
EDC-------------------------------GRFPIARTCF-NM-LSLWRY-TSKER
LESMLW-RAVHE-SEGF
>Trichoderma_virens_XM_014103183.1 .
LDVRRDCL-VDDSLTAV-----------SSVIGSGGEDLKK-GLRISFQG-------EEG
IDAGGLRKEW---FLLL----IREVFNPDY--------GMFIYDDDSQ------------
----------------YCYFNPNS------------------------------------
-----FEPSDQFFLVGVVMGLAIYNSTILDVALPP--FAFRKLLASAPSPPG--------
SIITHRSPMRYTLDDLAE----------------------YLPRLANG--------LRQL
LAFDG----------------------------------------------DVE-ETFC-
--LDFVIETDK---------------------------YGS-KVQVP-LCPG--------
```

GESKP---
------------------------------VTNENRR-EYVDL-YVRHVLDVA-VKRQFE
-PFKRG---FYTVCGG--NAFS-LFRPE-EIELLIRG-----------------------
---SDEPLD-----------IASL----RAVAEYDN-WENKQPDET--------------
------------------------EPVV-GWFWETFQ--------------------QAT
PSDQRKLLTF-------VTGSDRIP------------AMG-----AA-SLTIKL--SCLG
DDC-------------------------------GRYPIARTCF-NM-LSLWRY-TSKQR
LESMLW-RAVHE-SEGF
>Trichoderma_atroviride_XM_014091713.1 .
LDIRRDCL-VDDSLTAV-----------SSVIGSGGEDLKK-GLRISFQG-------EEG
IDAGGLRKEW---FLLL----IREVFNPDY--------GMFIYDDDSQ------------
----------------YCYFNPNS------------------------------------
-----FEPSDQFFLVGVVMGLAIYNSTILDVALPP--LAFRKLLASAPSLAG-------T
MPQQHRPVIRYTLDDLSE----------------------YRPRLANG--------LRQL
LAFDG--DVE-ETFC-
--LDFVIETDK---------------------------YGS-KVQVP-LCPG--------
GENKP---
------------------------------VTNENRR-EYVDL-YVRYVLETA-VKRQFE
-PFKRG---FYTVCGG--NAFS-LFQPE-EIELLIRG-----------------------
---SDEPLD-----------IASL----RAVAEYDN-WESKTPDET--------------
------------------------EPVV-SWFWETFQ--------------------QAT
PSDQRKMLTF-------ITGSDRIP------------AMG-----AA-SLTIKL--SCLG
DDC-------------------------------GR------------------------

>Trichoderma_gamsii_XM_018807822.1 .
LDIRRNCL-VDDSLTAV-----------SSVIGSGGEDLKK-GLRISFQG-------EEG
IDAGGLRKEW---FLLL----VREVFNPDY--------GMFIYDDDSQ------------
----------------YCYFNPNS------------------------------------
-----YEPSDQFFLVGVVMGLAIYNSTILDVALPP--FAFRKLLASAPSLAG-------T
MPQQHRPVMRYTLDDLSE----------------------YRPRLANG--------LRQL
LAFDG--DVE-ETFC-
--LDFVIETDK---------------------------YGS-KAQVP-LCPG--------
AENKP---
------------------------------VTNENRR-EYVDL-YVRYVLDTA-VKRQFE
-PFKRG---FYTVCGG--NAFS-LFRPE-EIELLIRG-----------------------
---SDESLD-----------IASL----RAVAEYDN-WESKTPDET--------------
------------------------EPVV-GWLWETFQ--------------------QAT
PSDQRKLLTF-------ITGSDRIP------------AMG-----AA-SLTIKL--SCLG
DDC-------------------------------GRYPIARTCF-NM-LSLWRY-ESKEK
LEYMLW-RAVHE-SEGF
>Verticillium_albo_atrum_XM_003007375.1 .
LDVRRDCL-VEDSLAAV-----------SEVIGSGGEDIKK-RLRIEFRG-------EEG
YDAGGLCKEW---FFSS----SVRVFNPEH--------GLFTYDEESQ------------
----------------LCYFNPNS------------------------------------
-----FETSDQFFLVGVVIGLAIYNSTILDVALPP--FAYRKLLAAAPISPV-------P
SSAHPRPIMTYNLEDLAE----------------------WRPRLAAG--------LKQL
LDYDG--DVE-ETFG-
--LDFVVPVDK---------------------------YGT-VLQVP-LCPG--------
GEWKP---
------------------------------VTNANRR-EFVDC-YVRYLLDHA-VTRQFE
-PFKRG---FYTVCGG--TALS-LFRPE-EIELLVRG-----------------------
---SATALD-----------IDSL----RAAAEYDN-WGSKNPDGT--------------
------------------------EPVI-GWFWDTFK--------------------SAK
PSEQRKLLSF-------ITGSDRIP------------AMG-----AA-LLPIKI--SCLG
EDE-------------------------------ERYPIARTCF-NM-LSLRRY-GSQER
LEHMLW-TAVHE-SEGF
>Verticillium_dahliae_XM_009657972.1 .
LDVRRDCL-VEDSLAAV-----------SEVIGSGGEDIKK-RLRIEFRG-------EEG

YDAGGLRKEW---FLLL----VREVFNPEH--------GLFTYDEESQ------------
----------------LCYFNPNS------------------------------------
-----FETSDQFFLVGVVIGLAIYNSTILDVALPP--FAYRKLLAAAPISSI-------P
SSAHPRPVMTYNLEDLAE----------------------WRPRLAAG--------LKQL
LDYDG--DVE-ETFG-
--LDFVVPVEK---------------------------YGT-VLQVP-LCPG--------
GEWKS---
------------------------------VTNANRR-EFVDC-YVRYLLDHA-VTRQFE
-PFKRG---FYTVCGG--NALS-LFRPE-EIELLVRG-----------------------
---SDTALD-----------IDSL----RAAAEYDN-WGTKNPDGT--------------
------------------------EPVI-GWFWDTFK--------------------SAK
PSEQRKLLSF-------ITGSDRIP------------AMG-----AA-LLPIKI--SCLG
EDE-------------------------------ERYPIARTCF-NM-LSLRRY-GSQER
LEHMLW-TAVHE-SEGF
>Colletotrichum_gloeosporioides_XM_007273143.1 .
LDVRRECL-VDDSLKAV-----------SEVIGSGSEDIKK-GLRIVFKG-------EEG
LDAGGLKKEW---FLLL----VREVFNPDH--------GMFIYDEDSQ------------
----------------FCYFNPNS------------------------------------
-----FETSDQYFLVGVVMGLAIYNSTILDVALPP--FAFRKLLASAPSSST-------G
PSAHPRPSMNYTLEDLAE----------------------YRPRLAAG--------LRQL
LEFDG--DVE-ETFQ-
--LDFVVDVEK---------------------------YGI-ITQVP-LCPG--------
GERKA---
------------------------------VTNANKW-EYVNL-YVRYLLETA-VTRQFE
-PFKRG---FYTVCGG--NALS-LFRPE-EIELLVRG-----------------------
---SDTALD-----------VDAL----RGVAEYDN-WGTKTPDGV--------------
------------------------EPVI-GWFWETFK--------------------EAT
PDEQRKLLSF-------ITGTDRVP------------AMG-----AA-LLPIKI--TCLG
EDE-------------------------------NRYPIARTCF-NM-LSLRRY-GSRER
LEQMLW-TAVHE-SEGF
>Colletotrichum_fioriniae_XM_007589639.1 .
LDVRRECL-VDDSLKAV-----------SEVIGSGSEDIKK-GLRIVFKG-------EEG
LDAGGLKKEW---FLLL----VREVFNPEH--------GMFIYDEDSH------------
----------------FCYFNPNA------------------------------------
-----FETSDQFFLVGVVMGLAIYNSTILDVALPP--FAFRKLLASAPSSSS-------G
PSAHPRPTMNYTLEDLAE----------------------YRPRLASG--------LKQL
LEYDG--DVE-ATFQ-
--LDFVVDIEK---------------------------YGT-VMQVP-LCPG--------
GERKP---
------------------------------VTNANRA-EYVTL-YVRYLLETA-VARQFE
-PFKRG---FYTVCGG--NALS-LFRPE-EIELLVRG-----------------------
---SDTALD-----------VDAL----RGVAEYDN-WGSKNPDGV--------------
------------------------EPVI-GWFWDAFK--------------------NAT
PDEQRKLLSF-------ITGTDRVP------------AMG-----AA-LLPIKI--TCLG
DDE-------------------------------NRYPIARTCF-NM-LALRRY-GSRER
LEHMLW-TAVHE-SEGF
>Colletotrichum_higginsianum_XM_018305094.1 .
LDVRRECL-VDDSLKAV-----------SEVIGSGSEDIKK-GLRIVFKG-------EEG
LDAGGLKKEW---FLLL----VREVFNPEH--------XMFIYDEDSQ------------
----------------FCYFNPNS------------------------------------
-----FETSDQYFLVGVVLGLAIYNSTILDVALPP--FAFRKLLASAPSASA-------G
SSAHPRPTMNYTLEDLAE----------------------YRPRLAAG--------LRQL
LEYDG--DVE-ETFQ-
--LDFVVDVEK---------------------------YGI-VMQVP-LCPG--------
GEAKP---
------------------------------VTNANRG-EYVSL-YVRYLLETA-VTRQFE
-PFKRG---FYTVCGG--NALS-LFRPE-EIELLVRG-----------------------
---SDTALD-----------VDAL----RGVAEYDN-WGTRNPDGV--------------

------------------------EPVV-GWFWDTFK--------------------EAS
ADDQRKLLSF-------ITGTDRVP------------AMG-----AA-LLSIKI--TCLG
EDE-------------------------------NRYPIARTCF-NM-LALRRY-ESRER
LEHMLW-TAVYE-SEGF
>Colletotrichum_graminicola_XM_008095886.1 .
LDVRRECL-VDDSLKAV-----------SEVIGSGSEDIKK-GLRIVFKG-------EEG
LDAGGLKKEW---FLLL----VREVFNPEHV-------GMFIYDEDSQ------------
----------------FCYFNPNS------------------------------------
-----FETSDQFFLVGVVLGLAIYNSTILDVALPP--FAFRKLLASAPTAWA-------G
PSAHPRPTMNYTLEDLAE----------------------YRPRLAAG--------LRQL
LEYDG--NVE-TTFQ-
--LDFVVDVEK---------------------------YGT-VMQVP-LCPG--------
GETKP---
------------------------------VTNANRG-EYVGL-HVRYLLETA-VARQFE
-PFKRG---FYTVCGG--NALS-LFRPE-EIELLVRG-----------------------
---SDTALD-----------VDAL----RGVAEYDN-WGTRNPDGV--------------
------------------------EPVV-GWFWDTFK--------------------DAS
PDKQRKLLSF-------ITGTDRVP------------AIG-----AA-LLPIKI--TCLG
DDE-------------------------------DRYPIARTCF-NM-LALRRY-ETRER
LERMLW-TAVHE-SEGF
>Corollospora_maritima_GDFX01001137.1 .
LHVRRECL-VEDSLKTV-----------SEVVGSGSEDIQK-RLRIVFAG-------EEG
VDHGGLRKEW---FLLL----VREVFSPDH--------GMFIYDEDSQ------------
----------------YCYFNPHS------------------------------------
-----FETSDQFFLAGVVLGLAIYNSTILDVPLPP--FAFRKLVASGPPPSP-------G
PATHSRSTMAYSLDDLAE----------------------YRPALARG--------LRQL
LDYDG--DVQ-EAFS-
--LDFVIAVDR---------------------------YGT-TLQVP-LCPE--------
GERKP---
------------------------------VNNSNRR-EYVEL-YVRYLLDTA-VSRQFE
-PFKRG---FYTICSG--NALS-LFRPE-EIELLIRG-----------------------
---SDEALD-----------VGSL----KAGAEYDN-WTTESPDGP--------------
------------------------EPAI-GWFWAAFE--------------------KAS
PSHQRKLLLF-------ITGSDRIP------------ATG-----AS-SLHIKI--SCLG
DDV-------------------------------GRYPIARTCF-NT-LGLWRY-GTRER
LESMLW-TAVHG-SEGF
>Blumeria_graminis_ABSB02000807.1_R .
LSVRRDCL-IEDSLKAI-----------SEVVGTGGEEIKK-SLRITFKG-------EEG
IDAGGLRKEW---FLLL----VRDVFNPEH--------GMFTYDEDSR------------
----------------MCYFNPHS------------------------------------
-----FETSDQFFLVGVILGLAIYNSTILDVALPQ--FAFRKLLAASPPPAP-------G
AATHTKPSMTYSLDDLAQ----------------------YRPALANG--------LRQL
LEYDG--DVE-STFL-
--RDFVAEFDR---------------------------YGQ-VVRVP-LCPD--------
GENRT---
------------------------------VTNANRR-EFVEL-YVRYLLDTA-VTRQFE
-PFKRG---FFTVCGG--NALS-LFRPE-EIELLIRG-----------------------
---SDEPLD-----------ITSL----RAVCECEN-WGTHINPGD--------------
------------------------EPVL-DWFWETFE--------------------SSK
PNDQRKLLSF-------VTGSDRIP------------AMG-----AT-NLIIKL--SCAG
DDE-------------------------------NRFPVARTCF-NQ-LSLPRY-SSKQR
LESMLW-RAVVE-SEGF
>Erysiphe_pisi_CACN01004189.1_R .
LTVRRDCL-VEDSLKAV-----------SEVVGSGGEEIKK-GLRIKFKG-------EEG
IDAGGLRKEW---FLLL----VRDVFNPEH--------GMFTYDEDSR------------
----------------YCYFNPNS------------------------------------
-----FETTDQFFLVGVVLGLAIHNSTILDVALPP--FAFRKLLAAAPPPSP-------G
GTSHAKPLMTYSLADLAQ----------------------YRPALANG--------LRQL

```
LEYEG----------------------------------------------DVE-STFC-
--RDFLADVDR---------------------------YGQ-ITQVP-LCPN--------
GEKRT-------------------------------------------------------
------------------------------VTNANRR-EFVDL-YVRYLLDTA-VSRQFE
-PFKRG---FFTVCGG--NALS-LFRPE-EIELLIRG-----------------------
---SDENLD-----------IASL----RAVCIYDN-WSQENPNED--------------
------------------------EPVV-QWFWETFQ--------------------AAK
SSDQRKLLSF-------ITGSDRIP------------AMG-----AT-NLVIKL--SCLG
GDG-------------------------------MRFPIARTCF-NQ-LSLWQY-STREK
LEHMLW-RAVYE-SEGF
>Glarea_lozoyensis_XM_008082440.1 .
LKIRRECL-VEDSLKGV-----------SEVVGSGSEEIKK-GLRIEFKG-------EEG
IDAGGLRKEW---FLLL----VRDVFNPEH--------GMFTYDEDSQ------------
----------------FCYFNPNS------------------------------------
-----FETTDQFFLVGVVLGLAIYNSTILDVALPP--FAFRKLLAAGPPTPL-------G
ATSHAKPIMAYSLEDLAE----------------------YRPALAAG--------LRQL
LDFEG----------------------------------------------DVE-ATFC-
--RDFVADVEK---------------------------YGQ-TIQVP-LCPG--------
GENRS-------------------------------------------------------
------------------------------VTNSNRR-EFVDL-YVRYLLDGS-VVRQFE
-PFKRG---FFTVCGG--NALS-LFRPE-EIELLIRG-----------------------
---SDEPLD-----------IASL----RAVAVYEH-WGVPVKEAAN-------------
------------------------QSTV-VWFWDSFA--------------------GAS
PKDQRKLLSF-------ITGSDRIP------------AMG-----AT-NLIIKV--SCLG
NDC-------------------------------SRFPQARTCF-NM-VSLWRY-SSKEK
LERMLW-RAVNE-SEGF
>Pseudogymnoascus_destructans_XM_012890571.1 .
LKVRRDCL-VEDSLKGV-----------SEVVGASGEEIKK-GLRIEFKG-------EEG
IDAGGLRKEW---FLLL----VRDVLNPEH--------GMFVYDEESH------------
----------------VCYFNPSS------------------------------------
-----FETSDQFFLVGVVLGLAIYNSTILDVALPP--FAFRKLLAAGPSSLP-------G
SPSHAKPTMVYTLDDLAE----------------------YRPSLAHG--------LRQL
LEFDG----------------------------------------------DVE-ETFC-
--RDFVADVEK---------------------------YGK-IHQVP-LCPD--------
GEKRA-------------------------------------------------------
------------------------------VNNSNRR-EFVNL-YVKYLLDTS-VVRQFE
-PFKRG---FYTVCSG--NALS-LFRPE-EIELLVRG-----------------------
---SDEPLD-----------ISSL----EAVSVCEN-WGVPNPAEM--------------
------------------------EPVI-QWFWQSFK--------------------NAD
PKDQRKLLSF-------ITGSDRIP------------AMG-----AA-NLVIKL--SCLG
DDS-------------------------------LRFPIARTCF-NM-LLLWRY-SSRTK
FERLLW-RAVNE-SEGF
>Pseudogymnoascus_verrucosus_XM_018275735.1 .
LKVRRDCL-VEDSLKGV-----------SEVVGGGGEEIKK-GLRIEFKG-------EEG
IDAGGLRKEW---FLLL----VRDVLNPEH--------GMFVYDEESH------------
----------------VCYFNPSS------------------------------------
-----FETSDQFFLVGVVLGLAIYNSTILDVALPS--FAFRKLLAAGPSSLP-------G
SPSHAKPTMVYTLDDLAE----------------------YRPSLAHG--------LRQL
LEFDG----------------------------------------------DVE-ETFC-
--RDFVADVEK---------------------------YGK-IHQVP-LCPD--------
GEKRA-------------------------------------------------------
------------------------------VNNSNRR-EFVNL-YVKYLLDTS-VVRQFE
-PFKRG---FYTVCGG--NALS-LFRPE-EIELLIRG-----------------------
---SDEPLD-----------ISSL----EAVSVCEN-WGVPNPAET--------------
------------------------ELVI-QWFWQSFK--------------------NAD
PKDQRKLLSF-------ITGSDRIP------------AMG-----AA-NLVIKL--SCLG
DDS-------------------------------LRFPIARTCF-NM-LSLWRY-SSRTK
FERLLW-RAVNE-SEGF
```

```
>Phialocephala_scopiformis_XM_018211827.1 .
LKIRRECL-VEDSLRSV-----------SEVIGSGGEEIKK-GLRIEFKG-------EEG
IDAGGLRKEW---FLLL----VRDVFNPEH--------GMFTYDEDSG------------
----------------FCYFNPNS------------------------------------
-----FETTDQYFLVGIVLGLAIYNSTILDVALPP--FAFRKLLAAGPPAAP-------G
ATSHAKPSMTYSLDDLAE----------------------FRPALANG--------LRKL
LEFDG----------------------------------------------DVE-NTFC-
--RDFVADIER---------------------------YGQ-TTQFP-LCPD--------
GEKRA-------------------------------------------------------
------------------------------VTNANRR-EFVDL-YVRYLLDTA-VTRQFE
-PFKRG---FFTVCGG--NALS-LFRPE-EIELLIRG-----------------------
---SDEPLD-----------IASL----RAVSVCEN-WGVPNPAET--------------
------------------------EPVI-RWFWQSFQ--------------------AAK
PGDQRKLLTF-------ITGSDRIP------------AMG-----AT-NLVIKL--SCLG
DDG-------------------------------PRFPVARTCF-NM-LSLWRY-GSKQK
LESMLW-RAVYE-SEGF
>Marssonina_brunnea_XM_007296882.1 .
LKIRRECL-VEDSLKGV-----------GAVVGTGGEEIKK-GLRIEFRG-------EEG
IDAGGLRKEW---FLLL----VRDVFNPEH--------GMFAYDDDSG------------
----------------FCYFNPNS------------------------------------
-----FETTDQFFLVGVVLGLAIYNSTILDVALPP--FAFRKLLAAAPPTAA-------G
ASSHTKLSMTYSLEDLAE----------------------YRPALAQG--------LRQL
LEFEG----------------------------------------------DVE-ATFC-
--RDFVADIDR---------------------------YGQ-ITQVA-LCPD--------
GDKKA-------------------------------------------------------
------------------------------VTNANRR-EFVDL-YVRYLLDTA-VARQFE
-PFKRG---FFTVCGG--NALS-LFRPE-EIELLVRG-----------------------
---SDDPLD-----------IASL----RAVSVCEN-WGVPNAKET--------------
------------------------EPVV-QWFWKSFE--------------------AAT
PEDQRKLLSF-------ITGSDRIP------------AMG-----AT-NLVIKL--SCLG
DDC-------------------------------SRFPVARTCF-NM-LSLWRY-SSVEK
LERMLW-RAVNE-SEGF
>Sclerotinia_homoeocarpa_JW834334.1 .
LKIRRDCL-VEDSLKGV-----------SEVVGTGGEEIKK-GLRIEFKG-------EEG
IDAGGLRKEW---FLLL----VRDVFNPDH--------GMFSYDEDSH------------
----------------FCYFNPNS------------------------------------
-----FETTDQFFLVGVVLGLAIYNSTILDVALPP--FAFRKLLAAAPAAAP-------G
ATSHVKPTMTYSLEDLAE----------------------YRPSLAHG--------LRKL
LEFDG----------------------------------------------DVE-STFC-
--RDFVADVDR---------------------------YGQ-QVQVP-LCPD--------
GDQKA-------------------------------------------------------
------------------------------VTNSNRR-EFVDL-YVRYLLDHA-VARQFE
-PFKRG---FFTVCGG--NALS-LFRPE-EIELLIRG-----------------------
---SDEPLD-----------IASL----RAVAVCEN-WEIPNAADR--------------
------------------------EPVI-QWFWDTFQ--------------------KAD
PKDQRKLLSF-------ITGSSCIP------------AMG-----AT-SLVIKL--SCLG
DDS-------------------------------PRFPVARTCF-NM-LSLWRY-ATREK
LEDRLW-RAVHE-SEGF
>Botryotinia_fuckeliana_XM_001545523.1 .
LRVRRECL-VEDSLKGV-----------SEVVGTGGEEIKK-SLKIEFKG-------EEG
IDAGGLRKEW---FLLL----VREVFNPDH--------GMFSYDEDSH------------
----------------FCYFNPNS------------------------------------
-----FETTDQYFLVGAVLGLAIYNSTILDVALPP--FAFRKLLAAAPAAAP-------G
ATSHAKPSMTYSLEDLAE----------------------FRPALAHG--------LRQL
LEFDG----------------------------------------------DVE-ETFC-
--RDFVADVDR---------------------------YGQ-TVQIP-LCPD--------
GDKKP-------------------------------------------------------
------------------------------VTNSNRR-EFVDL-YVRYLLDNA-VARQFE
```

-PFKRG---FFTVCGG--NALS-LFRPE-EIELLIRG-----------------------
---SDEPLD-----------ISSL----RAVSVCEN-WGAKDAAER--------------
------------------------EPVI-QWFWESFQ--------------------KAD
PKDQRKLLSF-------ITGSSCIP------------AMG-----AA-SLIIKL--NCLG
DDS-------------------------------ERYPVARTCF-NA-LSLWRY-ATREK
LEGRLW-RAVHE-SEGF
>Sclerotinia_sclerotiorum_XM_001588915.1 .
LKVRRECL-VEDSLKGV-----------SEVVGTGGEEIKK-GLRIEFKG-------EEG
IDAGGLRKEW---FLLL----VRDVFNPDH--------GMFSYDEDSH------------
----------------FCYFNPNS------------------------------------
-----FETTDQYFLVGVVLGLAIYNSTILDVALPP--FAFRKLLAAAPAAAP-------G
ATSHAKPSMTYSLEDLAE----------------------FRPSLAHG--------LRQL
LEFDG--DVE-TTFC-
--RDFVADVDR---------------------------YGQ-TIQVP-LCPD--------
GDKKP---
------------------------------VTNSNRR-EFVDL-YVRYLLDNA-VARQFE
-PFKRG---FFTVCGG--NALS-LFRPE-EIELLIRG-----------------------
---SDEPLD-----------ITSL----RAVSVCEN-WGAPNAAER--------------
------------------------EPVI-QWFWESFQ--------------------KAD
PKDQRKLLSF-------ITGSSCIP------------AMG-----AT-SLVIKL--SCLG
DDS-------------------------------ERFPVARTCF-NM-LSLWRY-ASREK
LEGRLW-RAVHE-SEGF
>Pestalotiopsis_fici_XM_007837919.1 .
MSVRRECL-VEDSLAKV-----------SEVVGSGSEDIKK-GLRIEFRG-------EEG
VDAGGLRKEW---FQLL----VKDVFNPDH--------GLFVFDEDSQ------------
----------------YCYFNPHT------------------------------------
-----FETTDQYFLVGVVLGLAIYNSAILDVAFPP--FAFRKLLSAAPASAS-------S
GSIIQRSTMTYTLDDLAE----------------------FRPRLASG--------LRQL
LEFDG--DVE-NTFC-
--LDFAVEVDK---------------------------YGS-RSRVP-LCLG--------
GDTKP---
------------------------------VTNANRR-EYVDL-YVKYLLDES-VKRQFE
-PFKRG---FFTVCAG--NALS-LFRPE-EIELLIRG-----------------------
---SDEPLD-----------IASL----RAVCKYSD-WDNSKDGKEPTQ-----------
------------------------EPVI-DWFWESFA--------------------AAT
PQDQRRLLSF-------ITGSDRIP------------AMG-----AA-TLIIKI--SCLG
EDT-------------------------------ERYPTARTCF-NM-LNLFKY-NSKEK
LETLLW-RAVHE-SEGF
>Daldinia_eschscholzii_AIID01008878.1 .
LNIRRECL-VEDSLKQV-----------SEVVGSGGEEIKK-GLRIEFRG-------EEG
IDGGGLRKEW---FLLL----VREVFNPDH--------GLFVYDEDSN------------
----------------FCYFNPNT------------------------------------
-----FETSDQYFLVGVVLGLAIYNSTILDVAFPP--FAFRKLLASAPPPTP-------G
TPAHSRPTMSYTLDDLAE----------------------FRPVLARG--------LRQL
LDYEG--DVQ-STFC-
--LDFVIEMEK---------------------------YGT-RMRVP-LCPG--------
GESKM---
------------------------------VTNANRR-EYVDL-YVRYLLDTS-VSRQFE
-PFKRG---FFTVCAG--NALT-LFKPE-EIELLIRG-----------------------
---SDEPLD-----------IAAL----QGAAAYTN-WPSNRSAEQ--------------
------------------------HPTV-QWFWDTFK--------------------RAS
IADQRRLLSF-------ITGSDRIP------------AMG-----AA-SLVIKI--NCLG
VDE-------------------------------GRFPSARTCF-NI-LSLYCY-TSRQR
LEESLW-RAVNE-SEGF
>Eutypa_lata_XM_007796270.1 .
-------------LKQV-----------SEVVGSGSEDIKK-ALRIEFQG-------EEG
VDAGGLRKEW---FLLL----VREVFNPDH--------GLFAYDEDSQ------------
----------------FCYFNSNS------------------------------------

-----FETSDQFFLVGVVLGLAIYNSTILDVALPP--FAFRKLLASAPVPGA-------G
AAAHPRPTMSYTLDDLAE----------------------YRPVLARG--------LRQL
LEYEG--DVQ-ATFC-
--LDFVVEVEK---------------------------YGA-RLRAP-LLPG--------
GESRM---
------------------------------VTNANRR-EYVDL-YVRYLLDTS-VSRQFE
-PFKRG---FFTVCGG--NALS-LFRPE-EIELLVRG-----------------------
---SDEPLD-----------ILSL----KASTEYTN-WRPSGAIASKNSNANANATSTGG
AGSGGVGAAMSNLEPEQGLEDEEQEPTL-RWFWTTFE--------------------RAT
PQDQRKLLSF-------ITGSDRIP------------ATG-----AA-SLVIRI--HCLG
EDE-------------------------------GRFPTARTCF-NQ-LSLYRC-RTRER
LEASLW-RAVRE-SEGF
>Arthrinium_malaysianum_GEGW01005435.1 .
LSVRRDCL-VEDSLTQV-----------SEVVGSGSEDIKK-GLRIEFRG-------EEG
IDAGGLRKEW---FLLL----VREVFNPDH--------GLFVFDEDSQ------------
----------------YCYFNPNT------------------------------------
-----FETSDQYFLVGVLLGLAIYNSTILDIALPP--FAFRKLLAAAPQSSA------SS
TAIHQRSIMTYTLDELAE----------------------YRPRLARG--------LRQL
LEYEG--DVQ-STFC-
--LDFVIDVDK---------------------------YGD-RVRVP-LCPG--------
GDTKM---
------------------------------VTNSNRR-EYVDL-YVRYLLDTS-VSRQFE
-PFKRG---FFTVCAG--NALS-LFRPE-EIELLVRG-----------------------
---SDEPLD-----------ITSL----RAVATYSS-WNGSDKPAPEPEN----------
------------------------EPAV-EWFWETFG--------------------AAT
PQDQRRLLSF-------ITGSDRIP------------ATG-----AA-SLVVKI--TCLG
PDI-------------------------------GRYPTARTCF-NV-LNLYRY-QSRER
LETSLW-RAVNE-SEGF
>Magnaporthe_oryzae_XM_003717771.1 .
LNVRRDCL-VEDSLKAV-----------SEVIGSGGEDIKK-GLRITFRG-------EEG
VDAGGLRKEW---FLLL----VREVFNPDH--------GLFVYDDDSH------------
----------------YCYFNPNS------------------------------------
-----FETSDQYFLVGVVFGLAIYNSTILDVAFPP--FLFRKLLAASPQPSG-------A
SPAHSRPPMTYTLEDLAE----------------------FHPMLAKG--------LRQL
LEFEG--DVE-ETFC-
--LDFVVDVPK---------------------------YGA-IERVP-LCQG--------
GERRA---
------------------------------VTNANRR-EYVDL-HVRYILDTA-VARQFD
-PFKRG---FFTVCGG--NALS-LFRPE-EIELLVRG-----------------------
---SEETLD-----------VVSL----KSAAEYDG-WMRNNKKRPEESSPSGGDGTEV-
---------------------AEEVPTL-RWFWDSFQ--------------------RAS
PEDQRKLLIF-------ITGSDRIP------------AMG-----AA-SLSIKI--LCLG
DDC-------------------------------DRYPTARTCF-NT-LALWRY-ESKEK
LERMLW-GAVFE-SEGF
>Gaeumannomyces_graminis_XM_009231703.1 .
LTVRRDCL-VEDSLRAV-----------SEVIGSGGEDIKK-GIRITFKG-------EEG
VDAGGLRKEW---FLLL----VREVFNPDH--------GLFIYDDDSH------------
----------------FCYFNPYS------------------------------------
-----FETSDQYFLVGVVLGLAIYNSTILDIALPP--FAFRKLLAAAPMPAK-------S
ASTHPRTIMTYTLDDLAE----------------------FRPRLARG--------LRQL
LEYDG--DVE-STFC-
--LDFVVDVDK---------------------------YGS-IERVP-LTPG--------
GENKA---
------------------------------VTNSNRR-EYVDL-YVRHLLDTA-VFRQFD
-PFKRG---FFTVCGG--NALS-LFRPE-EIELLVRG-----------------------
---SAESLD-----------ISSL----RTAAEYDG-WTTDAEDT---------------
------------------------EPTV-RWFWELLE--------------------EAN
PAEQRHLLVF-------VTGSDRIP------------ATG-----AA-SLVIRL--LCLG

DDC-------------------------------ERYPTARTCF-NT-LALWRY-RSKKK
LARKLW-DAVHE-SEGF
>Podospora_anserina_CU638743.1 .
LNIRRDCL-VEDSLKAV-----------SEVIGGGGEDIKK-GLKIVFKG-------EEG
VDAGGLRKEW---FLLL----VREVFGRDH--------GMFLYDEDSG------------
----------------YCYFNPNS------------------------------------
-----LEPSEQFFLVGVVLGLAIYNSTILDVALPP--FAFRKLLMAAPPPPL-----AGQ
VAHQQRQTMNYTLEDLAE----------------------FRPRLARG--------LRQL
LEYSDD---DLE-EVFC-
--LDFVVDVEK---------------------------YGV-VERVP-LCPG--------
GERRP---
------------------------------VTNTNKR-EYVEL-YVRYLLDGS-VTRQFE
-PFKRG---FFTVCGG--NALS-LFRPE-EIELLVRG-----------------------
---SDEALD-----------IDSL----RAVAQYEG-WGKEVADPGED------------
------------------------EPVV-RWFWSSFE--------------------RAS
PKDQRKLLSF-------ITGSDRIP------------ATG-----AA-SLVVKI--SCLG
DDI-------------------------------GRYPTARTCF-NA-LGLWKY-GTRER
LEGMLW-GAVNG-SEGF
>Myceliophthora_thermophila_XM_003666797.1 .
LNIRRECL-VDDSLKAV-----------SEVIGSGGEDIKK-GLRINFKG-------EEG
VDAGGLRKEW---FLLL----VREVFNPDH--------GMFLYDEDSN------------
----------------YCYFNPNS------------------------------------
-----LESSEQFFLVGVVFGLAIYNSTILDVALPP--FAFRKLLAAAPPPSV--------
PTAQPRQPMTYTLDDLAE----------------------YRPRLAHG--------LRQL
LEFEG--DVE-STFG-
--LDFTIDTTR---------------------------YGA-VERVL-LCPG--------
GDRRP---
------------------------------VTNANRR-EYVDA-YVRHVLDTS-VARQFE
-PFKRG---FYTVCSX--SALS-LFRPE-EIELLVRG---------------------SA
GASDQTPLD-----------VPSL----RAVAQYDG-WKTATPDSYGDGDGDGDGDNDD-
----------------------TKEPTV-RWFWEAFE--------------------SAA
PRDQRRLLAF-------ITGSDRIP------------ALG-----AA-SLGIRI--SCLG
DEC-------------------------------GRFPTARTCF-NS-VGLWRS-TDRER
FVSTLW-RAVWE-SEGF
>Thielavia_terrestris_CP003014.1 .
LNIRRECL-VEDSLKAV-----------SEVIGSGGEEIKK-GLKINFQG-------EEG
VDAGGLRKEW---FLLL----VREVFNPDH--------GMFLYDEDSH------------
----------------YCYFNPNS------------------------------------
-----LEPSEQFFLVGVVLGLAIYNSTILDIALPP--FAFRKLLLAAPAPST--------
PSPQPRQQMTYTLDDLAE----------------------YRPRLAHG--------LRQL
LAFEG--DVE-STFA-
--LDFAIDTQR---------------------------YGV-VERVA-LCPG--------
GENRP---
------------------------------VTNANRR-EYVDA-YVRHALDTS-VARQFE
-PFKRG---FFTVCGG--GALS-LFRPE-EIDLLVRG----------------------S
SGYADQPLD-----------VDAL----RAGAQYDG-WDCNXPAED--------------
------------------------EPTV-RWFWDALA--------------------RAP
PPAQRRLLAF-------VTGSDRVP------------AMG-----AA-SLRVRL--VCLG
DDC-------------------------------GRFPTARTCF-NS-LGLWRC-KDRER
FEAMLW-RAVEE-SEG-
>Sordaria_macrospora_XM_003344922.1 .
LNVRRDCL-VDDSLKAV-----------SEVIGSGSEDIKK-GLKINFKG-------EEG
IDAGGLRKEW---FLLL----VREVFNPDH--------GMFVYDEDSQ------------
----------------YCYFNPAS------------------------------------
-----LEPSEQYFLVGVVFGLAIYNSTILDVALPP--FAFRKLLSAAPPPTL--------
ATSQPRQPMTYSLDDLAE----------------------YRPRLASG--------LRQL
LEYDG--DVE-STFC-
--LDFVVDIER---------------------------YGS-TERVA-LCPN--------

GERRP---
------------------------------VTNANRR-EYVDL-YVRYLLDTA-VTRQFE
-PFKRG---FYTVCGG--NALS-LFRPE-EIELLVRG-----------------------
---SDEALD-----------ISAL----KSAATYDN-WSTKNPVETG-------------
------------------------EPMV-RWFWELFE--------------------EAS
PADQRKLLLF-------ITGSDRIP------------AGG-----AA-ALSIRI--SCLG
DDC-------------------------------GRYPTARTCF-NS-LALWRY-ADKET
LRKVLW-TAVLE-SEGF
>Neurospora_tetrasperma_XM_009853944.1 .
LNVRRDCL-VDDSLKAV-----------SEVIGSGGEDIKK-GLKINFKG-------EEG
IDAGGLRKEW---FLLL----VREVFNPDH--------GMFVYDEDSQ------------
----------------YCYFNPAS------------------------------------
-----LEPSEQYFLVGVVFGLAIYNSTILDVALPP--FAFRKLLMAAPPATL--------
ATSQPRQPMTYSLDDLAE----------------------YRPRLASG--------LRQL
LEYDG--DVE-STFC-
--LDFVVDIER---------------------------YGS-TERVS-LCPN--------
GERRP---
------------------------------VTNANRR-EYVDL-YVRYLLDTA-VTRQFE
-PFKRG---FYTVCGG--NALS-LFRPE-EIELLVRG-----------------------
---SDESLD-----------ISAL----KSAATYDN-WSTKNPVET--------------
------------------------EPTV-RWFWELFE--------------------EAS
PADQRKLLLF-------ITGSDRIP------------AGG-----AA-ALSIRI--ACLG
EDC-------------------------------GRYPTARTCF-NS-LALWKY-GSRER
LKEVLW-MAVLE-SEGF
>Neurospora_crassa_XM_011395283.1 .
LDVRRDCL-VDDSLKAV-----------SEVIGSGGEDIKK-GLKINFKG-------EEG
IDAGGLRKEW---FLLL----VREVFNPDH--------GMFVYDEDSQ------------
----------------YCYFNPAS------------------------------------
-----LEPSEQYFLVGVVFGLAIYNSTILDVALPP--FAFRKLLMAAPPPTL--------
ATSQPRQPMTYSLDDLAE----------------------YRPRLASG--------LRQL
LEYDG--DVE-STFC-
--LDFVVDIER---------------------------YGS-TERVS-LCPN--------
GERRP---
------------------------------VTNANRR-EYVDL-YVRYLLDTA-VTRQFE
-PFKRG---FYTVCGG--NALS-LFRPE-EIELLVRG-----------------------
---SDESLD-----------ISAL----KSAATYDN-WSTKNPVET--------------
------------------------EPTV-GWFWELFE--------------------EAS
PADQRKLLLF-------ITGSDRIP------------AGG-----AA-ALSIRI--ACLG
EDC-------------------------------GRYPTARTCF-NS-LALWKY-GSRER
LKEVLW-TAVLE-SEGF
>Sporothrix_schenckii_XM_016727191.1 .
LTVRRDCL-VEDSLKGV-----------SAAIGSGTDDIKK-GLRIIFSG-------EEG
IDAGGLRKEW---FLLLRFFTLKILDPSLTNL------GMFVYDEDSH------------
----------------YCYFNPAS------------------------------------
-----FETSDQFFLVGVVLGLAIYNSTILDVALPP--FAFRKLLFAAPTSTA------GG
QTTSPRPHMAYTLADLAE----------------------FRPLLAKG--------LQQL
LDYNG--DVE-STFG-
--LEFTVSIER---------------------------FGQ-VDAIP-LCPG--------
GESRA---
------------------------------VNKSNRE-EYVRL-YTRYLLDTA-VSRQFE
-PFKRG---FFTVCGC--NALG-LFQPD-EIDLLVRG-----------------------
---SAEPLD-----------VAEL----RLAASYEN-WPSTNPADT--------------
------------------------EPTV-KWFWDMFE--------------------EAS
SEDQRKLLIF-------ITGSDRIP------------VTG-----PA-SLSIRI--LCLG
DNT-------------------------------GRFPTARTCF-NV-LTLYRY-TSRET
MQAMLW-GAVYG-SEGF
>Grosmannia_clavigera_XM_014320344.1 .
LRVRRDCL-VDDSLTAV-----------SEAVGSGGEDLKK-GLRIVFRG-------EEG

IDAGGLRKEW---FLLL----VREVFNPDH--------GMFSYNDDSR------------
----------------YCYFNPNS------------------------------------
-----FETSDQFFLVGVVLGLAIYNSTILDIALPP--FAFRKLLLAGPEAPA------GM
ITGMTRPPISYTLADLAE----------------------YRPGLAKG--------LQQL
LDYEG--DVE-SAFS-
--LDFTIPAEK---------------------------YGV-MEQVP-LCVD--------
GEKRP---
------------------------------VTGSNRR-EYVHL-YVRYLLDTG-VSQQFE
-PFKRG---FFTVCDS--NALL-LFRPE-EIELLVRG-----------------------
---SDERLD-----------ITSL----RMAASYDN-WGAHSSPES--------------
------------------------EPTI-RWFWQAFE--------------------EAS
PQDQRKLLTF-------ITGSDRIP------------ATG-----PA-SLSIRI--LCLG
NDT-------------------------------GRYPTARTCF-NT-LTLYRY-RSKEA
LQKRLW-EAVNE-SEGF
>Scedosporium_apiospermum_XM_016786594.1 .
LDVRRDCL-VEDSLKGV-----------SEIIGSGSEDIKK-SLRIAFAG-------EEG
IDHGGLRKEW---FLLL----IREVFNLDN--------GMFVYDEDSG------------
----------------YCYFNPNS------------------------------------
-----FETSDQFFLVGVVMGLAIYNSTILDMPLPP--FTFRKLLATGPAPAP-------G
SAAHPRPLLSYTLDDLAE----------------------LRPRLANG--------LRQL
LDYTG--DVE-ETFG-
--LDFAIDTDR---------------------------YGT-TVQVP-LVPG--------
GADRP---
------------------------------VTNENRR-EYVDF-G---------------
------------------NALS-LFRPE-EIELLVRG-----------------------
---SDDALD-----------IDAL----RGVAEYEN-WDTEKPDET--------------
------------------------EPVV-QWFWETFA--------------------KAP
PEDQRKLLSF-------ITGSDRIP------------AMG-----TS-SLKIKI--SCLG
DDC-------------------------------DRFPIARTCF-NM-IVLHRY-ASREK
LERLVW-TAVRE-SEGF
>Beauveria_bassiana_XM_008600591.1 .
LTVRRECL-VDDSLMAV-----------SEVIGSGTDDIKK-GLRIKFRG-------EEG
IDGGGLRKEW---FQLV----IRDVFNPDC--------GMFLYDEDSQ------------
----------------FCYFNPNS------------------------------------
-----LESTDQYFLVGVVLGLAIYNSTILDVPLPP--FAFKKLLAAAPGHGM-------S
SLAHAPPHLKYTLDDLAE----------------------YRPRLARG--------LQQL
LEYDG--DVE-STFA-
--LDFMLENDR---------------------------YGS-SSTVL-LCEG--------
GERRP---
------------------------------VTNANKR-EYVDL-YIRYVLDVS-VRRQFE
-PFRRG---FYNVCGG--NAFS-LFRPE-EIELLIRG-----------------------
---SDEPLD-----------VDSL----RAVAEYQN-WDKKQPDGV--------------
------------------------EPVV-GWFWETFK--------------------QAE
PGDQRRLLSF-------ITGSDRIP------------ATG-----AA-SLRIKL--SCLG
DDC-------------------------------LRFPVARTCF-NQ-ISLWRY-RTREK
LEYVLW-RAVKE-SEGF
>Cordyceps_militaris_XM_006669686.1 .
LTVRRECL-VDDSLMAV-----------SEVIGSGTDDIKK-GLRIKFRG-------EEG
IDGGGLRKEW---FQLV----IRDVFNPDY--------GMFLYDEDSK------------
----------------FCYFNPNS------------------------------------
-----LESTDQYFLVGVVLGLAIYNSTILDVPLPP--FAFKKLLAAAPGHGM-------T
SLAHAPPHLRYTLDDLAE----------------------YRPSLARG--------LQQL
LEYDG--DVE-STFA-
--LDFIIENDR---------------------------FGS-LTTVP-LCDG--------
GERRP---
------------------------------VTNANRR-EYVDL-YIRYVLDAS-VRRQFD
-PFRRG---FYNVCGG--NAFS-LFRPE-EIELLIRG-----------------------
---SDEPLD-----------VDSL----RAVAEYQN-WDSKQPDGA--------------

------------------------EPVA-G------------------------------
PGDQRRLLSF-------ITGSDRIP------------ATG-----AT-SLRIKL--SCLG
DDC-------------------------------LRFPVARTCF-NL-LSLWRY-RTREK
LEYVLW-RAVKE-SEGF
>Arthrobotrys_oligospora_XM_011124985.1 .
LRVRRDCL-VEDSLKRI-----------SESVGGAGGDIKK-GLKIEFEG-------EDG
IDIGGLRKEW---FLLL----IRDIFDPRH--------GMFVYDEDSR------------
----------------YCYFNPHT------------------------------------
-----FEATEQYFLVGVLLGMAIYNSTILDVALPP--VVFKKLLSPSSTVST--PSKFST
PSPTARLPFTSTLEDLAL----------------------FRPALANG--------LRQL
LEFEG--DVE-STFC-
--RDFVVEVDR---------------------------YGQ-NMQVP-LVPG--------
GENKP---
------------------------------VTNENKR-EFVDL-YAQYLLDTS-VARQFE
-PFKRG---FWTVCGG--NALT-LFQPD-EIELLVRG-----------------------
---SDEPLD-----------VTAL----RAVATYEN-WSTSSPHE---------------
------------------------VNLV-RWFWETFE--------------------SAT
PTMQRQLLGF-------ITGSDRIP------------AMG-----IA-NMVLKL--VYGG
PASVHGRKKGV-----------------------ERYPIARTCF-NS-ITLWGW-GDKET
LERVLW-RAVRE-SEGF
>Dactylellina_haptotyla_XM_011117033.1 .
LRVRRDCL-VDDSLKKI-----------SESVGGGTGDIKK-GLKIEFEG-------EDG
IDIGGLRKEW---FLLL----IRDIFDPRH--------GMFVYDEDSR------------
----------------YCYFNPNT------------------------------------
-----FEATEQYFLVGVLLGMAIHNSTILDVALPP--VVFKKLLNPAASIST--PNKFST
PSPTARLPFTSTLDDLAF----------------------FRPALARG--------LRQL
LEFDG--DVE-STFC-
--RDFVVEVER---------------------------YGQ-MMQVP-LVPG--------
GENRP---
------------------------------VTNENRK-EFVDL-YAQYLLDTS-VARQFE
-PFKRG---FWTVCGG--NALS-LFQPD-EIELLVRG-----------------------
---SDEPLD-----------ITAL----RAVATYEN-WSSSSPSE---------------
------------------------INVV-RWFWETFE--------------------AAA
PEMQRMLLSF-------ITGSDRIP------------AMG-----IA-NMVLKV--VYGG
LPSVHGSKKA------------------------ERYPIARTCF-NS-ITLWGW-GSKEI
LERVLW-RAVRE-SEGF
>Tuber_melanosporum_XM_002836093.1 .
LKVRRECL-VEDSLKGI-----------SEGVGS-LDDIKK-GLRIEFIG-------EDG
VDAGGLKKEW---FLLV----ARDVFDPSY--------GMFVYDDDSQ------------
----------------YCYFNPNS------------------------------------
-----LESSEEFFLVGVLFGLAIYNSTILDVALPP--YIFKKLLHFTVPHSL--------
AVSSIRPPLHHTLEDLAV----------------------FRPSLAHG--------LRQL
LEFEG--DVE-STFC-
--RDFVAETER---------------------------FGQ-VIRVP-LCPN--------
GENRP---
------------------------------VTSSNRR-EFVDL-YLNYLLNSS-VAKQFE
-PFKRG---FYTVCGG--NALA-LFRPE-EIELLIRG-----------------------
---SDEALD-----------VSAL----KAVAIYDG-WGGGNPAEN--------------
------------------------DPIV-KWFWNFFE--------------------KIT
PKEQRMLLSF-------ITGSDRIP------------AMG-----AT-NLIIKV--VCLG
QDS-------------------------------NRFPVARTCF-NQ-ICLWRY-KRREK
LEALLW-RAVTE-SEGF
>Zymoseptoria_brevis_GCVP01012254.1 .
LRVRRECM-VDDSLRQI-----------SAAVGAGQEELKK-GLRVQFSG-------EEG
VDAGGPRKEW---FLML----VRDIFDPNH--------GMFVYDEDSQ------------
----------------TCYFNANS------------------------------------
-----FETSDQYYLVGALLGLAIYNSTILDISLPS--FAFRKLLAAAPTSSN--PSSNIT
SLTGTKNQMTYTLSDLAE----------------------FRPSLASG--------LQQL

LDFDG--DVE-ATYC-
--RDFVAPIDR---------------------------YGS-LTYVP-LISN--------
GENIP---
------------------------------VTNSNRH-DFVDA-YVRYLLDTA-VARQFE
-PFKRG---FFTVCAG--NALS-LFRAE-EIELLIRG-----------------------
---SDESLD-----------VDSL----RAVAVYEN-WRLPTPPHSLVVR-PAEH-----
------------------------VDVV-RWFWEVFR--------------------SAT
PERQRMLLTF-------ITGTDRIP------------AVG-----AT-SLVLRI--MAGG
DGWGGGGREER-----------------------ERFPVARTCF-NM-LVLWRY-ESKEV
LKGKLW-RAVEE-SEGF
>Zymoseptoria_tritici_LT853692.1 .
LRVRRECM-VDDSLRQI-----------SAAVGAGQEELKK-GLRVQFSG-------EEG
VDAGGPRKEW---FLML----VRDIFDPNH--------GMFVYDEDSQ------------
----------------TCYFNANS------------------------------------
-----FETSDQYYLVGALLGLAIYNSTILDISLPS--FAFRKLLAAAPTSSN--PSSNIT
SLTGTKNQMTYTLSDLAE----------------------FRPSLASG--------LQQL
LDFDG--DVE-ATYC-
--RDFVAPIDR---------------------------YGS-LTYVP-LISN--------
GENIP---
------------------------------VTNSNRH-DFVDA-YVRYLLDTA-VARQFE
-PFKRG---FFTVCAG--NALS-LFRAE-EIELLIRG-----------------------
---SDESLD-----------VDSL----RAVAVYEN-WRLPTPPHSLVVR-PAEH-----
------------------------VDVI-RWFWEVFR--------------------SAT
PERQRMLLTF-------ITGTDRIP------------AVG-----AT-SLVLRI--MAGG
DGWGGGGREER-----------------------ERFPVARTCF-NM-LVLWRY-ESKEV
LKGKLW-RAVEE-SEGF
>Zymoseptoria_pseudotritici_GCJY01000829.1 .
LRVRRECM-VDDSLRQI-----------SAAVGAGQEELKK-GLRVQFSG-------EEG
VDAGGPRKEW---FLML----VRDIFDPNH--------GMFVYDEDSQ------------
----------------TCYFNANS------------------------------------
-----FETSDQYYLVGALLGLAIYNSTILDISLPS--FAFRKLLAAAPSSSN-NPSSNIT
SLTGTKNQMTYTLSDLAE----------------------FRPSLASG--------LQQL
LDFDG--DVE-ATYC-
--RDFVAPIDR---------------------------YGS-LTYVP-LISN--------
GENIP---
------------------------------VTNSNRH-DFVDA-YVRYLLDTA-VARQFE
-PFKRG---FFTVCAG--NALS-LFRAE-EIELLIRG-----------------------
---SDESLD-----------VDSL----RAVAVYEN-WRLPTPPHSLVVR-PAEH-----
------------------------VDVI-RWFWEVFR--------------------SAT
PERQRMLLTF-------ITGTDRIP------------AVG-----AT-SLVLRI--MAGG
DGWGGGGREER-----------------------ERFPVARTCF-NM-LVLWRY-ESKEV
LKGKLW-RAVEE-SEGF
>Mycosphaerella_graminicola_AFIR01000719.1 .
LRVRRECM-VDDSLRQI-----------SAAVGAGQEELKK-GLRVQFSG-------EEG
VDAGGPRKEW---FLML----VRDIFDPNH--------GMFVYDEDSQ------------
----------------TCYFNANS------------------------------------
-----FETSDQYYLVGALLGLAIYNSTILDISLPS--FAFRKLLAAAPSSSN-NPSSNIT
SLTGTKNQMTYTLSDLAE----------------------FRPSLASG--------LQQL
LDFDG--DVE-ATYC-
--RDFVAPIDR---------------------------YGS-LTYVP-LISN--------
GENIP---
------------------------------VTNSNRH-DFVDA-YVRYLLDTA-VARQFE
-PFKRG---FFTVCAG--NALS-LFRAE-EIELLIRG-----------------------
---SDESLD-----------VDSL----RAVAVYEN-WRLPTPPHSLVVR-PAEH-----
------------------------VDVI-RWFWEVFR--------------------SAT
PERQRMLLTF-------ITGTDRIP------------AVG-----AT-SLVLRI--MAGG
DGWGGGGREER-----------------------ERFPVARTCF-NM-LVLWRY-ESKEV
LKGKLW-RAVEE-SEGF

```
>Zymoseptoria_ardabiliae_GCJX01000519.1 .
LRVRRECM-VDDSLRQI-----------SAAVGAGQEELKK-GLRVQFTG-------EEG
VDAGGPRKEW---FLML----VRDIFDPNH--------GMFVYDEDSQ------------
----------------TCYFNANS------------------------------------
-----FETSDQYYLVGALLGLAIYNSTILDISLPS--FAFRKLLAAAPTSSN--PSSNIT
SLTGTKNQMTYTLSDLAE----------------------FRPSLASG--------LQQL
LDFDG----------------------------------------------DVE-ATYC-
--RDFVAPIDR---------------------------YGS-LTYVP-LIPN--------
GENIP-------------------------------------------------------
------------------------------VTNSNRH-DFVDA-YVRYLLDTA-VARQFE
-PFKRG---FFTVCAG--NALS-LFRAE-EIELLIRG-----------------------
---SDESLD-----------VDSL----RAVAVYEN-WRLPNPPHALVVR-PAEH-----
------------------------IDVI-RWFWDVFR--------------------DAT
PERQRMLLTF-------ITGTDRIP------------AVG-----AT-SLVLRI--MAGG
DGWGGGGREER-----------------------ERFPVARTCF-NM-LVLWRY-DSREV
LEGKLW-RAVEE-SEGF
>Sphaerulina_musiva_XM_016904773.1 .
LRVRRDCI-VEDSLRQI-----------SEAVGSGQEDLKK-GMRVHFAG-------EEG
VDAGGPRKEW---FLML----VRDIFDPNH--------GMFVYDDDSN------------
----------------TCYFNPSS------------------------------------
-----FETSDQYHLVGVLLGLAIYNSTILDVALPP--FAFRKLLAAAPASITGTSNHNVS
SLTGTKGQMTYTLQDLAE----------------------FRPSLAAG--------LQQL
LDYDG----------------------------------------------DVQ-ETYC-
--RDFVASIER---------------------------YGT-IQSVP-LVPN--------
GENIA-------------------------------------------------------
------------------------------VTNTNRR-EFVDA-YVRYMLDTA-VARQFE
-PFKRG---FFTVCAG--NALS-LFRAE-EIELLIRG-----------------------
---SDEALD-----------VDSL----RAVAQYEN-WRHFQPPHPLILN-PAEE-----
------------------------APVI-GWFWELFR--------------------EAS
PDKQRKLLTF-------ITGSDRIP------------AVG-----AT-SLILRI--QAGG
DGWGGGGRDER-----------------------ERFPVARTCF-NM-LVLWRY-DYREQ
LEEKLW-RAVDE-SEGF
>Mycosphaerella_eumusae_GDIK01008829.1 .
LRIRRECM-VDDSLRQI-----------SEAVGSGQEELKK-GLRVHFTD-------EEG
VDAGGPRKEW---FLML----VRDIFDPNH--------GMFVYDDESN------------
----------------TCYFNPNS------------------------------------
-----FETSDQYFLVGALLGLAIYNSTILDVALPA--FAFRKLLAAAPSSVTSAANTNVS
SLTGTKGQMTYTLNDLAE----------------------FRPSLAAG--------LQKL
LDFDG----------------------------------------------DVE-ATYC-
--RDFVASVER---------------------------YGV-HTDVP-LKPN--------
GANIP-------------------------------------------------------
------------------------------VTNANRH-EFVDA-YVRYMLDTA-VTRQFE
-PFKRG---FFQVCQG--NALS-LFRAE-EIELLIRG-----------------------
---SDESLD-----------VDSL----RAVAQYEN-WRHFQPPHQAIQK-PAES-----
------------------------VPVI-AWFWELFA--------------------EAS
PEKQRKLLTF-------ITGTDRIP------------AVG-----AT-SLILRI--QAGG
DGWGGGGLDER-----------------------SRFPVARTCF-NM-LVLWRY-DYREQ
LEEKLW-RAVDE-SEGF
>Pseudocerc_fijiensis_XM_007928208.1 .
LRVRRECM-VDDSLRQI-----------SEAVGSGQEELKK-GLRVHFTD-------EEG
VDAGGPRKEW---FLML----VRDIFDPNH--------GMFVYDDESN------------
----------------TCYFNPNS------------------------------------
-----FETSDQYFLVGALLGLAIYNSTILDVALPP--FAFRKLLAAAPSSVTSAANTNVS
SLTGTKGQMTYTLNDLAE----------------------FRPSLAAG--------LQKL
LDFDG----------------------------------------------DVE-ATYC-
--RDFVASVER---------------------------YGV-TTDVP-LKAN--------
GTNIP-------------------------------------------------------
------------------------------VTNANRH-EFVDA-YVRYMLDTA-VVRQFE
```

-PFKRG---FFQVCQG--NALS-LFRAE-EIELLIRG-----------------------
---SDESLD-----------VDSL----RAVAQYEN-WRHFQPPHQAIQK-PAES-----
------------------------VPVI-TWFWELFA--------------------EAS
PEKQRKLLTF-------ITGTDRIP------------AVG-----AT-SLILRI--QAGG
DGWGGGGLDER----------------------SRFPVARTCF-NM-LVLWRY-DYREQ
LEEKLW-RAVYE-SEGF
>Pseudocerc_musae_GDIN01007046.1 .
LRVRRECM-VDDSLRQI-----------SEAVGAGQEELKK-GLRVHFTD-------EEG
VDAGGPRKEW---FLML----VRDIFDPNH--------GMFVYDDESN------------
----------------TCYFNPNS------------------------------------
-----FETSDQYFLVGALLGLAIYNSTILDVALPP--FAFRKLLAAAPSSVTSAANTNVS
SLTGTKGQMTYTLNDLAE----------------------FRPSLAAG--------LQKL
LDFDG---DVE-ATYC-
--RDFVASVER---------------------------YGV-TTDVP-LKAN--------
GANIP---
------------------------------VTNANRH-EFVDA-YVRYMLDTA-VTRQFE
-PFKRG---FFQVCQG--NALS-LFRAE-EIELLIRG-----------------------
---SDESLD-----------VDSL----RAVAQYEN-WRHFQPPHQAIQK-PAES-----
------------------------VPVI-AWFWELFA--------------------EAS
PEKQRKLLTF-------ITGTDRIP------------AVG-----AT-SLILRI--QAGG
DGWGGGGRDER----------------------SRFPVARTCF-NM-LVLWRY-DYREQ
LEEKLW-RAVDE-SEGF
>Baudoinia_panamericana_XM_007673556.1 .
LRIRRDCM-VDDSLRQI-----------SEAVGAGHEELKK-GLRVHFEG-------EEG
VDAGGPRKEW---FLML----VRDIFDPNH--------GMFVYDDESH------------
----------------TCYFNPNS------------------------------------
-----FETSDQYYLVGALLGLAIYNSTILDVALPP--FAFRKLLSAAPSSAT---TGTVA
SITGTKGQMTYTVSDLAE----------------------FRPSLAAG--------LQQL
LDFDG---DVE-ATYC-
--RDFVAPVER---------------------------YGT-LVNIP-LIPN--------
GEGTP---
------------------------------VTNANRQ-EFVDA-YIRYLLDTS-VARQFE
-PFKRG---FFTVCAG--NALS-LFRAE-EIELLVRG-----------------------
---SDEQLD-----------IDAL----RAVAVYEN-WKDPHHPNHTPMAHPEDH-----
------------------------EQVI-RYFWNAFA--------------------DAP
PTKQRKLLTF-------ITGTDRIP------------AVG-----AT-SLVLRI--VAGG
DGWGGGGKAEQ----------------------QRFPIARTCF-NM-LVLWHY-KDRAV
LEQKLW-RAVEE-SEGF
>Aureobasidium_melanogenum_GEEO01015597.1 .
LRVRRECM-VDDSLKQI-----------SSAVGSGPEELKK-GLKVHFTD-------EEG
VDAGGLRKEW---FLTV----VRDIFDPNH--------GMFLYDDDSG------------
----------------FCYFNPSS------------------------------------
-----FETSDQYYLVGALLGLAIYNSTILDVALPP--FAFRKLLASAPPSSQ-----NPA
LPSTSRTQVSYTLEDLAE----------------------WRPALAKG--------LRDL
LEFDG---DVE-TTYC-
--RDFVASIER---------------------------YGA-VTDIP-LKNN--------
GEAIP---
------------------------------VTNENRR-EYVDL-YVRYLLDQS-VSRQFE
-PFKRG---FFTVCAG--NALS-LFRAE-EIELMVRG-----------------------
---SDEALD-----------VDSL----KAVAVYEN-WREVSPPHKPLSN-PADQ-----
------------------------VPVI-SWFWDCFR--------------------SAT
PEAQRKLLGF-------ITGSDRIP------------AVG-----AT-NLVLRI--VCGG
DGTAGQGQNAGKDK-------------------ERFPIARTCF-NM-LVLWGY-ESKEK
LEEKLW-RAVSE-SEGF
>Aureobasidium_subglaciale_XM_013493020.1 .
LRVRRECM-VDDSLKQI-----------SGAVGSGPEELKK-GLKVHFTD-------EEG
VDAGGLRKEW---FLTV----VRDIFDPNH--------GMFLYDDDSG------------
----------------FCYFNPSS------------------------------------

-----FETSDQYYLVGALLGLAIYNSTILDVALPP--FAFRKLLASAPPSTQ-----NPT
FPSSGRNQLTYTLEDLAE----------------------WRPVLAKG--------LRDL
LEFDG--DVE-ATYC-
--RDFVASVER---------------------------YGA-VTDTP-LKTG--------
GEGIP---
------------------------------VTNENRR-EYVDA-YVRYLLDTS-VARQFE
-PFKRG---FFTVCAG--NALS-LFRAE-EIELMVRG-----------------------
---SDEALD-----------VDSL----KAVAVYEN-WREASPPHKPLPN-PADQ-----
------------------------VPVI-SWFWDCFR--------------------SAT
PESQRKLLGF-------VTGSDRIP------------AVG-----AT-NLVLRI--VCGG
DGTAGQGQDGATDK--------------------ERFPIARTCF-NM-LVLWGY-ESKEK
LEEKLW-RAVSE-SEGF
>Aureobasidium_namibiae_XM_013570446.1 .
LRVRRECM-VDDSLKQI-----------SGAVGSGPEELKK-GLKVHFTD-------EEG
VDAGGLRKEW---FLTV----VRDIFDPNH--------GMFLYDDDSG------------
----------------FCYFNPSS------------------------------------
-----FETSDQYYLVGALLGLAIYNSTILDVALPP--FAFRKLLASAPPPAQ-----NSA
LPSSGRTQVTYTLEDLAE----------------------YRPVLAKG--------LRDL
LDFDG--DVE-ATYC-
--RDFVASTER---------------------------YGV-VTDVP-LIPG--------
GEAIP---
------------------------------VTNTNCR-EYVDA-YVRYLLDTS-VTRQFE
-PFKRG---FFTVCAG--NALS-LFRAE-EIELMVRG-----------------------
---SDEALD-----------VNSL----KAVAVYEN-WREVAPPHKPLPN-PADQ-----
------------------------VPVI-SWFWDCFR--------------------GAT
PEAQRKLLGF-------ITGSDRIP------------AVG-----AT-NLVLRI--VCGG
DGTAGQGQNAGTDQ--------------------ERFPIARTCF-NM-LVLWGY-ESKAK
LEEKLW-RAVSE-SEGF
>Coniosporium_apollinis_XM_007784739.1 .
LRVRRECM-VDDSLRRI-----------SEVVGGGQEEIKK-GLRVQFVG-------EEG
VDAGGLRKEW---FLLL----VRDIFDPNH--------GMFVYDEDSH------------
----------------FCYFNPNS------------------------------------
-----FETSDQYFLVGALLGLAIYNSTILDVALPP--FAFKKLLASAPNST--------T
AVPSVRQPLHYTLEDLAE----------------------FRPALARG--------LRAL
LDHDG--DVE-MDFC-
--RDFVADVER---------------------------YGA-VTQVP-LCPN--------
GAKRP---
------------------------------VTNENRQ-EFVDL-YVRYLLDTS-VSRQFE
-PFKRG---FFTVCGG--NALS-LFRPE-EIELLVRG-----------------------
---SDEPLD-----------VASI----KAVAVYEN-WRD--ASNHKIPH-PEET-----
------------------------EPVI-RWFWEFFG--------------------AAD
AKDQRKILSF-------ITGSDRIP------------AVG-----AT-NLVIKI--TCLG
EDC-------------------------------ERFPVARTCF-DM-VQLYRY-KSRRK
LVEKLW-RAVCE-SEGF
>Neofusicoccum_parvum_XM_007589006.1 .
LRVRRECL-VEDSLRRI-----------SEVVGGGSEDIKK-GLRVQFVG-------EEG
IDAGGLRKEW---FLML----VRELFDPNH--------GMFVYDEDSQ------------
----------------FCYFNPNT------------------------------------
-----FEQSDQYFLVGAVLGLALYNSTILDVALPP--FAFKKLLASAPTKN--------G
VAPASRNRIDYTLDDLAE----------------------FRPRLAKG--------LRQL
LEFEG--DVE-ATFC-
--RDFVAEIER---------------------------YGV-VQEVP-LCKN--------
GANRP---
------------------------------VTNENRK-EFVDL-YVRYFLDTA-VARQFD
-PFRRG---FFTVCGG--NALS-LFRSE-EIELMVRG-----------------------
---SDEPLD-----------VASV----RAVAVYEG-WSDPAHGSRKVDR-PGEQ-----
------------------------ILVL-RWFWEFFE--------------------SAS
PADQRKILSF-------ITGSDRIP------------AVG-----AT-NLVIKI--GCLG

DDC-------------------------------DRFPVARTCF-NM-LQLYKY-KSRQK
LIEKLW-RAVVE-SEGF
>Diplodia_corticola_XM_020270195.1 .
LRVRRECL-VEDSLRRI-----------SEVVGGGGEEIKK-GLRVQFVG-------EEG
IDAGGLRKEW---FLML----VRELFDPNH--------GMFVYDEDSQ------------
----------------FCYFNPNT------------------------------------
-----FEQSDQFFLVGAVLGLALYNSTILDVALPP--FAFKKLLASAPTKN--------G
VAPASRNRIDYTLDDLAE----------------------FRPRLAKG--------LRQL
LEFDG--DVE-ATFC-
--RDFVAEVER---------------------------YGV-VHQVP-LCKN--------
GENRA---
------------------------------VTNANRK-EFVDL-YVRYFLDTA-VARQFD
-PFRRG---FFTVCGG--NALS-LFRSE-EIELMVRG-----------------------
---SDEPLD-----------VASL----RAVAVYEG-WSDPAHGGRKVDR-PGEH-----
------------------------ILVL-RWFWEFFE--------------------SAS
PTDQRKILSF-------ITGSDRIP------------ALG-----AT-NLVIKI--GCLG
DDV-------------------------------DRFPVARTCF-NM-LQLYKY-NSRQK
LVDRLW-RAVVE-SEGF
>Aspergillus_nidulans_XM_652956.1 .
LRVRRECL-VEDSLQRV-----------SEVLGSSPEEMKK-GLRIGFVG-------EEG
VDAGGLRKEW---FLLL----VREVFDPHH--------GLFIYDEDSR------------
----------------YCYFNPYC------------------------------------
-----LESSEQFFLVGVVLGLAIYNSIILDIALPP--FAFKKLLAGAPQTTG-------P
QPLTTRPTYKCSLEDLAE----------------------YRPVLARG--------LRAL
LEFEG--DVA-ETFC-
--HTFVADVDR---------------------------YGE-VVSVP-LCPG--------
GEKKP---
------------------------------VTNANRR-EFVDL-FVHYHLDTA-VARQFE
-PFRRG---FFSVCGG--NALS-LFRPE-EIELLVRG-----------------------
---SDEQLD-----------VKSL----RAVATYLN-WGTPKPES---------------
------------------------VPVV-RWFWEYFE--------------------RAN
PEAQRKILSF-------VTSSDRIP------------AMG-----AT-SLNIQL--ICLG
DDS-------------------------------PRFPTAHTCF-NR-LGLYRY-KTREK
FERLLS-EAVLN-SEGF
>Aspergillus_flavus_XM_002374397.1 .
LRVRRDCL-VDDSLRSV-----------SEVVGSSQEEIKK-GLRIEFVG-------EEG
VDAGGLRKEW---FLLL----VREIFDPHH--------GLFIYDEDSQ------------
----------------FCYFNPYC------------------------------------
-----FESSEQFFLVGVLLGLAIYNSTILDINLPP--FAFKKLLAAAPQTTG-------P
QPATTRSTYKCNLDDLAE----------------------YRPPLAKG--------LRAL
LDFEG--DVA-ETFC-
--YDFVAQMDR---------------------------YGE-VVAVP-LCTG--------
GDKRP---
------------------------------VTNANRR-EFVDL-YVHYLLDTA-VTRQFE
-PFKRG---FFTVCGG--NALS-LFRPE-EIELLVRG-----------------------
---SDEPLD-----------VASL----RAVATYDN-WSDPRPEM---------------
------------------------VPVV-QWFWDFFE--------------------HTQ
PQAQRKILSF-------ITGSDRIP------------AMG-----AT-SLIIRV--ACLG
DDS-------------------------------SRFPTARTCF-NM-LGLYRY-TTREQ
LEQRLW-GAVLN-SEGF
>Aspergillus_sojae_BACA01000971.1_R .
LRVRRDCL-VDDSLRSV-----------SEVVGSSQEEIKK-GLRIEFVG-------EEG
VDAGGLRKEW---FLLL----VREIFDPHH--------GLFIYDEDSQ------------
----------------FCYFNPYC------------------------------------
-----FESSEQFFLVGVLLGLAIYNSTILDINLPP--FAFKKLLAAAPQMTG-------P
QPATTRSTYKCNLDDLAE----------------------YRPALAKG--------LRAL
LDFEG--DVA-ETFC-
--YDFVAQMDR---------------------------YGE-VVAVP-LCTG--------

GDKRP---
------------------------------VTNANRR-EFVDL-YVHYLLDTA-VTRQFE
-PFKRG---FFTVCGG--NALS-LFRPE-EIELLVRG-----------------------
---SDEPLD-----------VASL----RAVATYDN-WSDPRPEM---------------
------------------------VPVV-QWFWDFFE--------------------HTQ
PQAQRKILSF-------ITGSDRIP------------AMG-----AT-SLIIRV--ACLG
DDS-------------------------------SRFPTARTCF-NM-LGLYRY-TTREQ
LEQRLW-GAVLN-SEGF
>Aspergillus_nomius_XM_015549606.1 .
LRVRRDCL-VDDSLRSV-----------SEVVGSSQEEIKK-GLRIEFIG-------EEG
VDAGGLRKEW---FLLL----VREIFDPHH--------GLFIYDEDSQ------------
----------------FCYFNPYC------------------------------------
-----FESSEQFFLVGVLLGLAIYNSTILDINLPP--FAFKKLLAAAPQTTG-------P
QPATTRSTYKCSLDDLAE----------------------YRPALAKG--------LRAL
LDFEG--DVA-ETFC-
--YDFVAQMDR---------------------------YGE-VVAVP-LCTG--------
GDKRP---
------------------------------VTNANRR-EFVDL-YVHYLLDTA-VTRQFE
-PFKRG---FFTVCGG--NALS-LFRPE-EIELLVRG-----------------------
---SDEPLD-----------VASL----RAVATYDN-WSDPRPEM---------------
------------------------VPVV-QWFWNFFE--------------------HTQ
PQAQRKILSF-------ITGSDRIP------------AMG-----AT-SLIIRV--ACLG
DDS-------------------------------SRFPTARTCF-NM-LGLYRY-TTREQ
LERRLW-GAVLN-SEGF
>Aspergillus_terreus_XM_001212943.1 .
LKVRRDCL-VEDSLRGV-----------SEVVGSSQEEIKK-GLRIEFVG-------EEG
IDAGGLRKEW---FLLL----VREVFDPEH--------GLFIYDEDSQ------------
----------------FCYFNPYC------------------------------------
-----FESSEQFFLVGVLLGLAIYNSTILDIALPP--FAFKKLLAAAPPTTG-------P
QPSTTRSPFKCSLDDLAE----------------------YRPALAKG--------LRAL
LEFEG--DVA-ETFC-
--YDFVAHVDR---------------------------YGE-VLPVP-LCPG--------
GEKRP---
------------------------------VTNANRR-EFVDL-YVHYLLDTA-VTRQFE
-PFKRG---FFTVCGG--NALS-LFRPE-EIELLVRG-----------------------
---SDEALD-----------VASL----RAVATYDN-WGVARPET---------------
------------------------VPVV-RWFWDFFQ--------------------HTR
PQAQRKILSF-------ITGSDRIP------------AMG-----AT-SLTIRV--ACLG
EDAS------------------------------SRFPTARTCF-NM-LGLYRY-ETREQ
LERKLW-AAVAN-SEGF
>Aspergillus_clavatus_XM_001269695.1 .
LKVRRDCL-VDDSLRSV-----------SEVVGSSEEEIKK-GLRIEFVG-------EEG
VDAGGLRKEW---FLLL----VREIFDPHH--------GLFLYDEDSQ------------
----------------FCYFNPFC------------------------------------
-----FESSEQFFLVGVLLGLAIYNSTILDIALPP--FAFKKLLAAAPPTSG-------P
QPTTSRSNFKCTLDDLAE----------------------YRPALAKG--------LRGL
LEFDG--DVM-DTFC-
--YDFVAHVDR---------------------------YGE-VVAVP-LCPG--------
GEKRP---
------------------------------VTNANRR-EFVDL-YVHYMLDTA-VARQFE
-PFKRG---FFTVCGG--NALS-LFRPE-EIELLVRG-----------------------
---SDEALD-----------VASL----RAVATYDN-WSHPRPEN---------------
------------------------VPVV-RWFWDFFE--------------------QAD
PQAQRKILSF-------ITGSDRIP------------AMG-----AT-SLTIRV--ACLG
DDTS------------------------------SRYPIARTCF-NT-LGLYRY-ATRQT
LERMLW-DAVGN-SEGF
>Aspergillus_fumigatus_XM_745120.1 .
LKVRRDCL-VDDSLRSV-----------SEVVGSNQEEIKK-GLRIEFVG-------EEG

VDAGGLRKEW---FLLL----VREIFDPHH--------GLFLYDEDSR------------
----------------FCYFNPYC-------------------------------------
-----FESSEQFFLVGVLLGLAIYNSTILDIALPP--FAFKKLLAAAPQTSG-------P
QPSSARSNYRCTLDDLAE----------------------YRPALAKG--------LRAL
LEFDG--DVA-DTFC-
--YDFVAHVDR---------------------------YGE-AVAVP-LCPG--------
GETRP---
-----------------------------VTNANRR-EFVDL-YVHYMLDTA-VTRQFE
-PFKRG---FFTVCGG--NALS-LFRPE-EIELLVRG-----------------------
---SDEALD-----------VASL----RAVATYDN-WSHPRPEN---------------
------------------------IPVV-RWFWEFFE--------------------NTD
PQAQRKILSF-------ITGSDRIP------------AMG-----AT-SLTIRL--ACLG
DDAS------------------------------SRYPIARTCF-NT-LGLYRY-PTREK
LERMLW-EAVGN-SEGF
>Neosartorya_fischeri_XM_001265199.1 .
LKVRRDCL-VDDSLRSV-----------SEVVGSNQEEIKK-GLRIEFVG-------EEG
VDAGGLRKEW---FLLL----VREIFDPHH--------GLFLYDEDSQ------------
----------------FCYFNPYC-------------------------------------
-----FESSEQFFLVGVLLGLAIYNSTILDIALPP--FAFKKLLAAAPQTSG-------P
QPSSGRSNYRCTLDDLAE----------------------YRPALAKG--------LRAL
LEFDG--DVA-DTFC-
--YDFVAHVDR---------------------------YGE-AVAVP-LCPG--------
GETRP---
-----------------------------VTNANRR-EFVDL-YVHYMLDTA-VTRQFE
-PFKRG---FFTVCGG--NALS-LFRPE-EIELLVRG-----------------------
---SDEALD-----------VASL----RAVATYDN-WSHPRPEN---------------
------------------------IPVV-RWFWEFFE--------------------NTD
PQAQRKILSF-------ITGSDRIP------------AMG-----AT-SLTIRL--ACLG
DDAS------------------------------SRYPIARTCF-NT-LGLYRY-PTRER
LERMLW-EAVGN-SEGF
>Aspergillus_aculeatus_XM_020196080.1 .
LKVRRDCL-VEDSLRRV-----------SEVVGSSHEEIKK-GLRIEFVG-------EEG
VDAGGLRKEW---FLLL----VREVFDPHH--------GLFIYDEDSR------------
----------------YCYFNPYC-------------------------------------
-----FESSEQFFLVGVLLGLAIYNSTILDVALPP--FAFKKLLAAA------------P
QWSTSRSVYKASLDDLAE----------------------FRPQLAKG--------LRAL
LDFEG--DVA-ETFC-
--YDFVAQVDR---------------------------YGE-VVPVP-LCAG--------
GEDRP---
-----------------------------VTNANRR-EFVDL-YVQHLLDTA-VTRQFE
-PFKRG---FFTVCGG--NALS-LFRPE-EIELLVRG-----------------------
---SDEPLD-----------VASL----RAVATYEN-WSVPRPET---------------
------------------------VPVV-RWFWEIFG--------------------QAA
PQSQRKILSF-------ITGSDRIP------------AMG-----AT-SLSIRL--GCLG
DDTS------------------------------SRFPTARTCF-NQ-LGLYRY-ETREK
LERVLW-EAVLN-SEGF
>Aspergillus_kawachii_BACL01000208.1 .
LKVRRDCL-VEDSLRGV-----------SEVVGSSQEEIKK-GLRIEFLG-------EEG
VDAGGLRKEW---FLLL----VREVFDPHH--------GLFIYDDDSR------------
----------------YCYFNPYC-------------------------------------
-----FESSEQFFLVGVLLGLAIYNSTILDIALPP--FAFKKLLAAA------------P
LSSITRPTYKCGLDDLAE----------------------LRPALAKG--------LRAL
LDYEG--DVA-ETFC-
--YDFVAQVDR---------------------------YGE-TVSVP-LCAG--------
GENRP---
-----------------------------VTNANRR-EFVDL-YVHFLLDTA-VTRQFE
-PFKRG---FFTVCGG--NALS-LFRPE-EIELLVRG-----------------------
---SDEPLD-----------VASL----RAVATYDN-WSNARPET---------------

------------------------EPVV-RWFWDFFE--------------------QTQ
PQAQRKILSF-------VTGSDRIP------------AMG-----AT-SLSIRL--VCLG
DET-------------------------------SRFPTARTCF-NQ-LGLYKY-ETREK
LERMLW-DAVLN-SEGF
>Aspergillus_niger_XM_001388740.2 .
LKVRRDCL-VEDSLRGV-----------SEVVGSSQEEIKK-GLRIEFLG-------EEG
VDAGGLRKEW---FLLL----VREVFDPHH--------GLFIYDDDSR------------
----------------YCYFNPYC------------------------------------
-----FESSEQFFLVGVLLGLAIYNSTILDIALPP--FAFKKLLAAA------------P
LSSITRPMYKCSLDDLAE----------------------LRPALAKG--------LRAL
LDYEG--DVA-ETFC-
--YDFVAQVDR---------------------------YGE-TVSVP-LCAG--------
GENRP---
------------------------------VTNANRR-EFVDL-YVHFLLDTA-VTRQFE
-PFKRG---FFTVCGG--NALS-LFRPE-EIELLVRG-----------------------
---SDEPLD-----------VASL----RAVATYDN-WSNARPET---------------
------------------------EPVV-RWFWDFFE--------------------QTQ
PQAQRKILSF-------VTGSDRIP------------AMG-----AT-SLSIRL--VCLG
DET-------------------------------SRFPTARTCF-NQ-LGLYRY-ETREK
LERMLW-DAVLN-GEGF
>Penicillium_janthinellum_KF280651.1 .
LKVRRECL-VEDSMRAV-----------GESVGAGSEEIKK-GLRIEFVG-------EEG
VDAGGLRKEW---FLLL----VREVFDPX----------LFIYDDDSQ------------
----------------YCYFNPYC------------------------------------
-----FESSEQFYLVGVLLGLAIYNSTILDVDLPP--FAFRKLLAAAPQNTG------SH
PPNPHTSRFKCTLDDLAE----------------------FRPALAKG--------LRGL
LEFEG--DVA-ETFC-
--YDFVAQVDR---------------------------YGE-VVTVP-LCTG--------
GEKRA---
------------------------------VTNSNRR-EFVDL-YVRYLLDTA-VARQFE
-PFKRG---FFTVCGC--NSLS-LFRSE-EIELLVRG-----------------------
---SDEPLD-----------VTSL----RAVATYDN-WSNQRPEA---------------
------------------------SAVV-QWFWDFFE--------------------RAQ
PQAQRKILSF-------VTGSDRIP------------AMG-----AT-SLSIRL--ACLG
DDC-------------------------------SRYPIARTCF-NT-LGLYRY-GSREK
LEKLLW-DAVVN-SEGF
>Penicillium_raistrickii_GFHR01015184.1 .
---LRIEFLG-------EEG
IDAGGLRKEW---FLLL----VREVFDPHH--------GLFIYDDDSQ------------
----------------YCYFNPYC------------------------------------
-----FESSEQFFLVGVLLGLAIYNSTILDVDLPP--FAFKKLLALAPHSSG------PQ
TSISSRSGFKCTLEDLAE----------------------YRPALAKG--------LRGL
LEFEG--DVA-ETFC-
--YDFVAQVDR---------------------------YGE-IVNVP-LCAN--------
GENRP---
------------------------------VTNANRR-EFVDL-YVSQLLDTA-VARQFE
-PFKRG---FFTVCGG--NALS-LFRPE-EIELLVRG-----------------------
---SDEPLD-----------VNSL----RAVATYDN-WSHPRPDS---------------
------------------------VPVV-RWFWEFFE--------------------KSL
PQAQRKILSF-------ITGSDRIP------------AMG-----AT-SLVIRL--ACLG
DET-------------------------------SRYPIARTCF-NT-LGLYRY-TSREK
FQQLLW-DAVVN-SEGF
>Penicillium_chrysogenum_XM_002561187.1 .
LKVRRECL-IEDSLKGV-----------SEVVGTGQEEIKK-GLRIEFSG-------EEG
IDAGGLRKEW---FLML----VREVFDPLH--------GLFIYDDDSQ------------
----------------YCYFNPYC------------------------------------
-----FESSEQFFLVGVLLGLAIYNSTILDVDLPP--FAFKKLLSSAPYSNG------PQ
AATSLRSTFKCTLEDLAE----------------------YRPTLAKG--------LRGL

```
LEFEG----------------------------------------------NVA-ETFC-
--YDFVAQVDR---------------------------YGE-IISVP-LCPN--------
GENRP-------------------------------------------------------
------------------------------VTNSNKR-EFVDL-YVQYLLDTA-VARQFE
-PFKRG---FFTVCGG--NALS-LFRPE-EIEMLVRG-----------------------
---SDEPLD-----------VSSL----RAVATYDN-WSHQRPES---------------
------------------------LPVV-RWFWDFFE--------------------ESQ
PQAQRKILSF-------ITGSDRIP------------AMG-----AT-SLVIRL--ACLG
DDC-------------------------------PRYPIARTCF-NT-LGLYRY-PTREK
FQRLLW-DAVVN-SEGF
>Penicillium_expansum_XM_016744429.1 .
LKVRRECL-IEDSLKGV-----------SEVVGTGQEEIKK-GLRIEFSG-------EEG
IDAGGLRKEW---FLLL----VREVFDPLH--------GLFIYDDDSQ------------
----------------YCYFNPYC------------------------------------
-----FESSEQFFLVGVLLGLAIYNSTILDVDLPP--FAFKKLLSAAPHANG------PQ
TATSMRSTFKCTLEDLAE----------------------YRPALAKG--------LRGL
LEFEG----------------------------------------------NVA-ETFC-
--YDFVAQVDR---------------------------YGE-IVSVP-LCSN--------
GENRP-------------------------------------------------------
------------------------------VTNSNKR-EFVDL-YVQYLLDTA-VARQFE
-PFKRG---FFTVCGG--NALS-LFRPE-EIELLIRG-----------------------
---SDEALD-----------VSSL----RAVATYDN-WSHPRPES---------------
------------------------LPVV-RWFWDFFE--------------------KSQ
PQDQRKILSF-------ITGSDRIP------------AMG-----AT-SLIIRL--ACLG
DDC-------------------------------PRYPIARTCF-NT-LGLYRY-PTREK
FQRLLW-DAVLN-SEGF
>Penicillium_digitatum_XM_014681755.1 .
LKVRRECL-IEDSLKGV-----------SEVVGTGQEEIKK-GLRIEFSG-------EEG
IDAGGLRKEW---FLLL----VREVFDPLH--------GLFVYDDDSQ------------
----------------YCYFNPYC------------------------------------
-----FESSEQFFLVGVLLGLAIYNSTILDVDLPP--FAFKKLLSAAPHANG------PQ
TATSMRSTFKCTLEDLAE----------------------YRPALAKG--------LRGL
LEFEG----------------------------------------------NVA-ETFC-
--YDFVAQVDR---------------------------YGE-IISVP-LCSN--------
GENRP-------------------------------------------------------
------------------------------VTNSNRR-EFVDL-YVHYLLDTA-VARQFE
-PFKRG---FFTVCGG--NALS-LFRPE-EIEMLIRG-----------------------
---SDEALD-----------VNSL----KAVATYDN-WSHPRPES---------------
------------------------VPVV-RWFWDFFE--------------------KSQ
PQDQRKILSF-------ITGSDRIP------------AMG-----AT-SLVIRL--ACLG
DDC-------------------------------PRYPIARTCF-NT-LGLYRY-PTREK
FQRLLW-DAVVN-SEGF
>Rasamsonia_emersonii_XM_013472791.1 .
LRVRRDCL-VEDSLRGV-----------SEVVGSGPEEVKK-GLRIEFIG-------EEG
VDAGGLRKEW---FLLL----VREVFDPNH--------XLFIYDDDSH------------
----------------FCYFNPFC------------------------------------
-----FESSEQFFLVGVLLGLAIYNSTILDVALPP--FAFKKLLTSAPAPSV------SS
SMSTSRNTYTCTLDDLAE----------------------YRPALAKG--------LRAL
LEFEG----------------------------------------------DVQ-ETFC-
--YDFVAQIDR---------------------------YGQ-RIEVP-LCPG--------
GEKRP-------------------------------------------------------
------------------------------VTNANRH-EFVNL-YVQYLLDTA-VQKQFE
-PFKRG---FFTVCGG--NALS-LFRPE-EIELLVRG-----------------------
---SDEPLD-----------VASL----RAVATYEN-WPSADPES---------------
------------------------EPAV-RWFWEFFA--------------------ESS
AQDQRKILSF-------ITGSDRIP------------AMG-----AT-SLSIRI--SCLG
EDS-------------------------------PRYPIARTCF-NT-LGLFRY-STRKK
LESKLW-GAVVN-SEGF
```

```
>Talaromyces_atroroseus_XM_020267671.1 .
LKVRRDCL-IEDSLRGV-----------SEVVGAGSEDIKK-SLRIEFIG-------EEG
VDAGGLRKEW---FLLL----VREVFDPNH--------GLFVYDEDSQ------------
----------------YCYFNPFC------------------------------------
-----FESSEQFFLVGVLLGLAIYNSTILDIALPP--FAFKKLLAAAPATSI--------
PAAAQKPPHVSSLDDLSE----------------------YRPALAKG--------LRAL
LDFDG----------------------------------------------HVQ-ETFC-
--YDFVAQVDR---------------------------YGQ-NVKVP-LCPN--------
GEKKP-------------------------------------------------------
------------------------------VTNTNRH-EFVSL-YVQYLLDTA-VQRQFE
-PFKRG---FYTVCGG--NALA-LFRPE-EIELMVRG-----------------------
---SDEPLD-----------VPTL----RAVATYEN-WPAPKAADLQVHATTQPSSSSTS
--------------KEPPTTDPPSEPVI-TWFWDFFT--------------------RSS
PSNQRKLLSF-------VTGSDRIP------------ATG-----AA-SLSIRL--SCLG
EDC-------------------------------ERYPIAHTCF-NK-LGLFRY-ASKQK
LERKLW-DAICN-SEGF
>Talaromyces_stipitatus_XM_002483969.1 .
LKVRRDCL-VEDSLRGV-----------SEVVGAGSEDIKK-SLRIEFIG-------EEG
VDAGGLRKEW---FLLL----VREVFDPNH--------GLFVYDDDSQ------------
----------------FCYFNPYC------------------------------------
-----FESSEQFFLVGVLLGLAIYNSTILDVALPP--FAFKKLLAAAPSTNM--------
PASAQRQPHTSSLDDLAE----------------------YRPALAKG--------LRAL
LEFDG----------------------------------------------DVQ-ETFC-
--YDFVAEVDK---------------------------YGQ-HISVP-LCLN--------
GENKP-------------------------------------------------------
------------------------------VTNDNRH-EFVNL-YVQYLLDTA-VQRQFE
-PFKRG---FYTVCGG--NALS-LFRPE-EIELMVRG-----------------------
---SDEPLD-----------VPTL----RAVATYEN-WPKPLPPLPPSGTDHTYNSDNNN
------------------TETEPAEPTI-SWFWDFLA--------------------RST
PTNQRKLLSF-------VTGSDRIP------------ATG-----AA-SLSIRI--SCLG
EDS-------------------------------SRYPIAHTCF-NK-LGLFRY-GSRQK
LERMLW-DAICN-SEGF
>Penicillium_marneffei_XM_002150212.1 .
LRVRRDCL-VEDSLRGV-----------SEVVGAGSEDIKK-SLRIEFIG-------EEG
VDAGGLRKEW---FLLL----VREVFDPNH--------GLFTYDDDSQ------------
----------------FCYFNPYC------------------------------------
-----FESSEQFFLVGVLLGLAIYNSTILDIALPP--FAFKKLLAAAPSTSI--------
PAAAQRQPHTSSLDDLAE----------------------YRPALAKG--------LRAL
LEFDG----------------------------------------------DVQ-ETFC-
--YDFVAQVDR---------------------------YGQ-VVNVP-LCPN--------
GENKP-------------------------------------------------------
------------------------------VTNTNRH-EFVSL-YVQYILDIS-VQRQFE
-PFKRG---FYTVCGG--NALA-LFRPE-EIELMVRG-----------------------
---SDEPLD-----------VPTL----RAVATYEN-WPTSQPKPNDKNNKDANT-----
------------------EAEPTSEPTI-CWFWDFLA--------------------RST
PTNQRKLLSF-------VTGSDRIP------------ATG-----AA-SLSIRI--SCLG
EDS-------------------------------SRYPIAHTCF-NK-LGLFRY-ASRQK
LERMLW-DAICN-SEGF
>Xylona_heveae_XM_018331309.1 .
LKVRRDCL-VEDSLRGV-----------SEVVGASQEEIKK-GLRIEFRN-------EEG
VDAGGLRKEW---FLLL----VREVFDPNH--------GLFMYDEDSH------------
----------------YCYFNPNC------------------------------------
-----FETSDQFFLVGVVLGLAIYNSTILDVALPP--FAFKKLLASAPSTST-------P
AMSVPRVKMTYSLEDLAE----------------------YRPALASG--------LRQL
LEFEG----------------------------------------------DVQ-ATFC-
--RDFVAEIDR---------------------------YGQ-IVQVP-LCDG--------
GESRP-------------------------------------------------------
------------------------------VTNENRQ-EFVQL-YVQYLLDVA-VARQFE
```

-PFKRG---FFTVCGG--NALS-LFRPE-EIELLVRG-----------------------
---SDEPLD-----------VASL----RAVAIYEN-WGEGSTPAIS-------------
------------------------DPVL-VWFWDLFS--------------------HAN
PKDQRKLLSF-------VTGSDRIP------------AMG-----AT-NLVIKL--VCLG
DDC-------------------------------DRFPIARTCF-NS-LCLFRY-ATREK
LESKLW-RAVME-SEGF
>Byssoonygena_ceratinophila_GDRB01005084.1 .
LKVRRECL-VEDSLRGV-----------SEVVGAGEEDIKK-GLRIEFVG-------EEG
VDAGGLRKEW---FLLL----VREVFDPLN--------GLFLYDDDSR------------
----------------YCYFNPYC------------------------------------
-----FESSEQFFLVGLVLGLAIYNSTILDIALPP--FAFRKLLASARPSTI-------P
TLATPYQPFRCTLDDLAE----------------------YRPALARG--------LRKL
LDYDG---DVQ-ETFC-
--QDFVVQVER---------------------------YGD-TVEVP-LCPG--------
GEKRP---
------------------------------VTNSNRR-EFVDL-YVRFMLDDA-VARQFE
-PFKRG---FFTVCGG--NALH-LFRPE-EIELLVRG-----------------------
---SDEALD-----------IPSL----RAVAVYEH-WPVANPDR---------------
------------------------EPVV-NWFWEFFA--------------------RIS
PQDQRKILSF-------ITGSDRIP------------AMG-----AT-NLVIRL--LFLG
QDS-------------------------------ERFPIARTCF-NM-LSLYRY-KTRQK
FESKLW-RAVVE-SEGF
>Chrysosporium_queenslandicum_GDRC01009885.1 .
LKVRRECL-VEDSLRGV-----------GEVVGGGQEDIKK-GLRIEFVG-------EEG
VDAGGLRKEW---FLLL----VREVFDPLN--------GLFLYDDDSQ------------
----------------YCYFNPYC------------------------------------
-----FESSEQFFLVGLVLGLAIYNSTILDIALPP--FAFRKLLASARPSSI-------P
TLSTPYQPFRCTLDDLAE----------------------YRPALARG--------LHQL
LEYDG---DVE-ETFC-
--QDFVVQVER---------------------------YGE-TVEIP-LCVG--------
GEKRP---
------------------------------VTNSNRR-EFVDL-YVKYMLDEA-VARQFE
-PFKRG---FFTVCGG--NALH-LFRPE-EIELLVRG-----------------------
---SDEALD-----------IPSL----RAVAVYEH-WPVPNPER---------------
------------------------EPVV-NWFWEFFG--------------------RVS
PQDQRKILSF-------ITGSDRIP------------ATG-----AT-NLVIRL--LCLG
QDS-------------------------------ERFPIARTCF-NM-LSLYRY-KTRQK
LEAKLW-RAVVE-SEGF
>Amauroascus_mutatus_GDQZ01005014.1 .
LKVRRDCL-VEDSLRGV-----------SEVVGSGQEEIKK-GLRIEFVD-------EEG
VDAGGLRKEW---FLLL----VREVFDPLH--------GLFLYDDDSR------------
----------------YCYFNPYC------------------------------------
-----FESSEQFFLVGVVLGLAIYNSTILDIALPP--FAFRKLLASARSNAV-------P
ALSTPHQPFRCTLDDLAE----------------------YRPVLARG--------LQQL
LEYDG---DVQ-ETFC-
--QDFVVQIER---------------------------YGQ-AVEVL-LCPG--------
GDKRP---
------------------------------VTNSNRR-EFVDL-YVKYLLDDA-VSRQFE
-PFKRG---FFTVCGG--NALH-LFRPE-EIELLVRG-----------------------
---SDEALD-----------IPSL----RAVALYEH-WPVANPER---------------
------------------------EPVI-NWFWEFFA--------------------RVS
PQDQRKILSF-------ITGSDRIP------------AMG-----AT-NLVIRL--LYLG
QDS-------------------------------ERFPIARTCF-NM-LSLYRY-KSRQK
LELKLW-RAVVE-SEGF
>Amauroascus_niger_GDRA01002944.1 .
LKVRRECL-AEDSLRGV-----------SEVVGSGEEEIKK-GLRIEFVG-------EEG
VDAGGLRKEW---FLLL----VREVFDPLH--------GLFLYDEDSQ------------
----------------YCYFNPYC------------------------------------

```
-----FESSEQFFLVGVVLGLAIYNSTILDIALPP--FAFRKLLASARSSGV-------P
SLSTPHQPFRCTLDDLAE----------------------YHPALARG--------LRQL
LEYDG----------------------------------------------DVQ-ETFY-
--RDFVVQIER---------------------------YGE-TLEVP-LCAG--------
GEKRP-------------------------------------------------------
------------------------------VTNSNRR-EFVDL-YVKYMLDDA-VSRQFE
-PFKRG---FFTVCGG--NALH-LFRPE-EIELLVRG-----------------------
---SDEALD-----------IPSL----RAVAVYEN-WPVANPER---------------
------------------------EPVV-NWVWEFFA--------------------RVS
PQDQRKILSF-------ITGSDRIP------------AMG-----AT-NLVIRL--VYLG
QDS-------------------------------ERFPIARTCF-NM-LSLYRY-KTRQK
LESKLW-RAVVE-SEGF
>Uncinocarpus_reesii_XM_002583591.1 .
LKVRRECL-VEDSLRGV-----------SEVVGSGQEEIKK-GLRIEFVG-------EEG
VDAGGLRKEW---FLLL----VREVFDPLN--------XLFLYDEDSG------------
----------------YCYFNPYC------------------------------------
-----FESSEQFFLVGLVFGLAIYNSTILDVALPP--FAFRKLLASARPNNI-------P
TLSTPCQPFRCTLDDLAE----------------------YRPTLARG--------LRQL
LEFDG----------------------------------------------DVE-TTFC-
--QDFVINVER---------------------------YGE-TMEVA-LCPG--------
GEKRP-------------------------------------------------------
------------------------------VTNSNRR-EFVDL-YVKYMLDGA-VSRQFE
-PFKRG---FFTVCGG--NALH-LFRPE-EIELLVRG-----------------------
---SDEALD-----------IPSL----RAVAVYEH-WPAANPER---------------
------------------------EPVV-NWFWDFFA--------------------RVS
PRDQRKILSF-------ITGSDRIP------------AMG-----AT-NLVIRL--LCLG
QDS-------------------------------ERFPIARTCF-NM-LSLYRY-QTREK
FESKLW-RAVVE-SEGF
>Coccidioides_immitis_XM_012358455.1 .
LKVRRECL-VEDSLRGV-----------SEVVGSGQEEIKK-GLRIEFVG-------EEG
VDAGGLRKEW---FLLL----VREVFDPLN--------GSFLYDNDSR------------
----------------YCYFNPYC------------------------------------
-----FESSEQFFLVGVVLGLAIYNSTILDVAFPP--FAFRKLLASARPNNV-------P
TLSTPYQPFRCTLDDLAE----------------------YRPALAKG--------LRQL
LEYDG----------------------------------------------DVE-ETFC-
--QDFVIQVER---------------------------YGE-TIEVP-LCPG--------
GEKRP-------------------------------------------------------
------------------------------VTNSNRW-EFVDL-YVKYMLDDA-VSRQFE
-PFKRG---FFTVCGG--NALH-LFRPE-EIELLVRG-----------------------
---SDEALD-----------IPSL----RAVAVYEH-WPTANPDR---------------
------------------------DPVV-NWFWEFFT--------------------RVN
PQDQRKILSF-------ITGSDRIP------------AMG-----AT-NLVICL--LYLG
QDS-------------------------------ERFPIARTCF-NM-LSLYRY-KTRQK
LESKLW-RAVVE-SEGF
>Coccidioides_posadasii_XM_003071351.1 .
LKVRRECL-VEDSLRGV-----------SEVVGSGQEEIKK-GLRIEFVG-------EEG
VDAGGLRKEW---FLSL----VREVFDPLN--------GSFLYDNDSR------------
----------------YCYFNPYC------------------------------------
-----FESSEQFFLVGVVLGLAIYNSTILDVAFPP--FAFRKLLASARPNNV-------P
TLSTPYQPFRCTLDDLAE----------------------YRPALAKG--------LRQL
LEYDG----------------------------------------------DVE-ETFC-
--QDFVIQVER---------------------------YGE-TIEVP-LCPG--------
GEKRP-------------------------------------------------------
------------------------------VTNSNRW-EFVDL-YVKYMLDDA-VSRQFE
-PFKRG---FFTVCGG--NALH-LFRPE-EIELLVRG-----------------------
---SDEALD-----------IPSL----RAVAVYEH-WPTANPDR---------------
------------------------DPVV-NWFWEFFT--------------------RVN
PQDQRKILSF-------ITGSDRIP------------AMG-----AT-NLVICL--LYLG
```

QDS-----------------------------------ERFPIARTCF-NM-LSLYRY-KTRQK
LESKLW-RAVVE-SEGF
>Ajellomyces_dermatitidis_XM_002623822.1 .
LKVRRECL-AEDSLRSV-----------SEVVGTGEGEIKK-GLRIEFLG-------EEG
VDAGGLRKEW---FLLL----VREIFDPLH--------GLFVYDEQSQ------------
----------------YCYFNPYC------------------------------------
-----FESSEQFFLVGVLLGLAIYNSTILDIALPP--FAFRKLLACAPPNNV-------P
TLSTPQQTFKCTLEDLAE----------------------YQPTLAKG--------LRDL
LTYDG--DVQ-ETFC-
--LDFVVQIER---------------------------YGE-KLVVP-LRPG--------
GERKP---
------------------------------VTNSNRR-EYVDL-YVKYLLDTA-VARQFE
-PFKRG---FFTVCGG--NAMH-LFRPE-EIELLVRG-----------------------
---SDEALN-----------IPSL----QAVAVYEH-WRTDHPEK---------------
------------------------EPVV-TWFWDFFS--------------------RAS
PRDQRKVLSF-------ITGSDRIP------------AMG-----AT-NLSIRL--VCLG
QDS-----------------------------------ERFPTARTCF-NM-IALYRY-KSREK
LEEKLW-RAVVD-SEGF
>Ajellomyces_capsulatus_XM_001541458.1 .
LKVRRECL-AEDSLRSV-----------SEVVGTGQGEIKK-GLRIEFLG-------EEG
VDAGGLRKEW---FLLL----VREIFDPLH--------GLFVYDEQSQ------------
----------------YCYFNPYC------------------------------------
-----FESSEQFFLVGVLLGLAIYNSTILDIALPP--FAFRKLLACAPPNNV-------P
ALSTPQQSFKCTLDDLAE----------------------YQPALAKG--------LRDL
LNYEG--DVQ-ETFC-
--LDFVVQIER---------------------------YGE-RLVIP-LRPG--------
GETRP---
------------------------------VTNSNRR-EYVDL-YVKYLLDTA-VSRQFE
-PFKRG---FFTICEG--NALH-LFRPE-EIELLVRG-----------------------
---SDEALD-----------IPSL----QAVAVYEH-WHTDHPEK---------------
------------------------EPVV-TWFWDFFS--------------------RVS
PRDQRKILSF-------ITGSDRIP------------AMG-----AT-NLSIRL--VCLG
QDS-----------------------------------DRFPTARTCF-NL-ISLYRY-KSREK
LEQKLW-RAVVD-SEGF
>Paracoccidioides_brasiliensis_XM_010760910.1 .
LKVRRECL-AEDSLRSV-----------SEVVGTGQEEIKK-GLRIEFLG-------EEG
VDAGGLRKEW---FLLL----VREIFDPLH--------GLFVYDEQSQ------------
----------------YCYFNPFC------------------------------------
-----FESSEQFFLVGVLLGLAIYNSTILDIALPP--FAFRKLLSCAPSNNV-------P
ALSTPQQVFKCTLQDLAE----------------------YQPALARG--------LRNL
LEYDG--DVQ-EAFC-
--LDFVIQVDR---------------------------YGK-KLELP-LRPG--------
GEKKP---
------------------------------VTNFNRR-EYVDL-YVKYLLDTA-VARQFE
-PFKRG---FFTVCGG--NALH-LFRPE-EIELLVRG-----------------------
---SDEPLD-----------IPSL----QAVAVYEH-WQCEKPEQ---------------
------------------------EPVV-IWFWEFFA--------------------RVS
PADQRKILSF-------ITGSDRIP------------AMG-----AT-NLMIRL--VCLG
QDS-----------------------------------DRFPTARTCF-NM-VALYRY-TTREK
LEEKLW-RAVVD-SEGF
>Paracoccidioides_lutzii_XM_002797063.1 .
LKVRRECL-AEDSLRSV-----------SEVVGTGQEEIKK-GLRIEFLG-------EEG
VDAGGLRKEW---FLLL----VREIFDPLH--------GLFVYDEQSQ------------
----------------YCYFNPFC------------------------------------
-----FESSEQFFLVGVLLGLAIYNSTILDIALPP--FAFRKLLSCAPSNNV-------P
ALSTPQQVFKCTLQDLAE----------------------YQPALARG--------LRNL
LEYDG--DVQ-EAFC-
--LDFVIQVDR---------------------------YGK-KLVLP-LRPG--------

GEKKP---
------------------------------VTNSNRR-EYVDL-YVKYLLDTA-VARQFE
-PFKRG---FFTVCGG--NALH-LFRPE-EIELLVRG-----------------------
---SDEPLD-----------IPSL----QAVAVYEH-WQCEKPEQ---------------
------------------------EPVV-IWFWEFFA--------------------RVS
PADQRKILSF-------ITGSDRIP------------AMG-----AT-NLMIRL--VCLG
QDS-------------------------------DRFPTARTCF-NM-VALYRY-MTREK
LEEKLW-RAVVD-SEGF
>Arthroderma_otae_XM_002849460.1 .
LKVRRDCL-VEDSLQSV-----------SAVIGSGEEDIKK-GLRIEFAG-------EEG
IDAGGLRKEW---FLLL----VREVFDPLH--------GLFLYDEDSQ------------
----------------YCYFNPYC------------------------------------
-----FESSEQFFLVGVVLGLAIYNSTILDVALPP--FAFRKLLAYSPSNIA-------P
TLSSPPQPFKPNLDDLAE----------------------LRPALAKG--------LRQL
LEYDG--DVT-ETFC-
--QDFVIQVER---------------------------YGE-MIQIP-LCPG--------
GEKKQ---
------------------------------VTNDNRR-EFVDL-YVRYIIDGA-VSRQFE
-PFRRG---FFTVCGG--NALH-LFKPE-EIELLIRG-----------------------
---SEEPLD-----------IQSL----RGVAMYEH-WPTSSPDR---------------
------------------------EPVV-NWFWEFFE--------------------SIS
AKDQRKILGF-------ITGSDRLP------------AMG-----AV-NLVIRL--MCLG
PDS-------------------------------ERFPTARTCF-NA-LGLYRY-KTRRK
LEEKLW-RAVAE-SEGF
>Nannizzia_gypsea_XM_003170153.1 .
LKVRRDCL-AEDSLQSV-----------SAVIGSAEEDIKK-GLRIEFAG-------EEG
IDAGGLRKEW---FLLL----VREVFDPLY--------GLFLYDEDSQ------------
----------------YCYFNPYC------------------------------------
-----FESSEQFFLVGVVLGLAIYNSTILDIALPP--FAFRKLLAYSPSNVA-------P
TLSSPPQPFKPTLDDLAE----------------------FRPALAKG--------LRLL
LEYDG--DVA-ETFC-
--QDFVVQVER---------------------------YGE-VVQVP-LCAG--------
GENQA---
------------------------------VTNENRR-EFVDL-YVRYIIDGA-VSRQFE
-PFRRG---FFTVCGG--NALH-LFKPQ-EIELLIRG-----------------------
---SEEPLD-----------IPSL----RGVAMYEH-WPASSPDR---------------
------------------------EPVV-NWFWEFFE--------------------RIP
AKDQRKILSF-------ITGSDRLP------------AMG-----AV-NLVIRL--MCLG
PDS-------------------------------ERFPTARTCF-NA-LGLYRY-KTRQK
FEEKLW-RAVAE-SEGF
>Trichophyton_rubrum_XM_003233883.1 .
LNVRRDCL-AEDSLQSV-----------SSVIGSAEEDIKK-GLRIEFAG-------EEG
IDAGGLRKEW---FLLL----VREVFDPLH--------GLFLYDEDSQ------------
----------------YCYFNPYC------------------------------------
-----FESSEQFFLVGVVLGLAIYNSTILDIALPP--FAFRKLLAYSPSNVA-------P
TLSSPPQPFKPTLDDLAE----------------------FRPALAKG--------LRQL
LEYDG--DVA-ETFC-
--QDFVVQVER---------------------------YGE-MIQVP-LCAG--------
GENQP---
------------------------------VTNENRR-DFVDL-YVRYIIDGA-VCRQFE
-PFRRG---FFTVCGG--NALH-LFKPE-EIELLIRG-----------------------
---SEEPLD-----------IPSL----RGVAMYEH-WPASSPDR---------------
------------------------EPVV-TWFWDFFE--------------------RIP
AKDQRKILSF-------ITGSDRLP------------AMG-----AV-NLVIRL--MCLG
PDS-------------------------------ERFPTARTCF-NA-LGLYRY-KTRQK
FEEKLW-RAVVD-SEGF
>Arthroderma_benhamiae_ABSU01000004.1_R .
LKVRRDCL-AEDSLQSV-----------SSVIGSAEEDIKK-GLRIEFAG-------EEG

IDAGGLRKEW---FLLL----VREVFDPLA--------GLFLYDEDSQ------------
----------------YCYFNPYC------------------------------------
-----FESSEQFFLVGVVLGLAIYNSTILDIALPP--FAFRKLLAYSPSNVT-------P
TLSSPPQPFKPTLDDLAE----------------------FRPALAKG--------LRQL
LEYDG--DVA-ETFC-
--QDFVVQVER---------------------------YGE-TIQVP-LCAG--------
GENQP---
------------------------------VTNENRR-EFVDL-YVRYIIDGA-VCRQFE
-PFRRG---FFTVCGG--NALH-LFKPE-EIELLIRG-----------------------
---SEEPLD-----------IPSL----RGVAMYEH-WPASSPDR---------------
------------------------EPVV-NWFWDFFE--------------------RTP
AKDQRKILSF-------ITGSDRLP------------AMG-----AV-NLVIRL--MCLG
PDS-------------------------------ERFPTARTCF-NA-LGLYRY-KTRQK
FEEKLW-RAVVD-SEGF
>Trichophyton_verrucosum_XM_003025169.1 .
LKVRRDCL-AEDSLQSV-----------SSVIGSAEEDIKK-GLRIEFAG-------EEG
IDAGGLRKEW---FLLL----VREVFDPLH--------XLFLYDEDSQ------------
----------------YCYFNPYC------------------------------------
-----FESSEQFFLVGVVLGLAIYNSTILDIALPP--FAFRKLLAYSPSNVT-------P
TLSSPPQPFKPTLDDLAE----------------------FRPALAKG--------LRQL
LEYDG--DVA-GTFC-
--QDFVVQVER---------------------------YGE-TIQVP-LCAG--------
GENQP---
------------------------------VTNENRR-EFVDL-YVRYIIDGA-VCRQFE
-PFRRG---FFTVCGG--NALH-LFKPE-EIELLIRG-----------------------
---SEEPLD-----------IPSL----RGVAMYEH-WPASSPDR---------------
------------------------EPVV-NWFWDFFE--------------------RIP
AKDQRKILSF-------ITGSDRLP------------AMG-----AV-NLVIRL--MCLG
PDS-------------------------------ERFPTARTCF-NA-LGLYRY-KTRQK
FEEKLW-RAVVD-SEGF
>Cyphellophora_europaea_XM_008717003.1 .
LRVRRDCL-VEDSLRNV-----------SEVVGSGGGDIKK-GLRIDFVG-------EEG
IDSGGLRKEW---FLLL----VREIFDPTH--------GLFVYDEDSQ------------
----------------YCYFNPYC------------------------------------
-----FESSEQFFLVGVLLGLAIYNSTILDIAFPP--FVFKKMLASAPSAGD-------K
LTSTPKISHGYTLEDLAE----------------------FRPALAKG--------LRQL
LDFEG--DVQ-ETFC-
--RDFVIEMDR---------------------------YGE-KVEVP-LCPG--------
GSTRA---
------------------------------VTNSNRK-EFVDS-YIRYMLDTA-VARQYE
-PFKRG---FFTVCGG--NALS-LFRPE-EIELLVRG-----------------------
---SDEPLD-----------IGSL----RAVAVYEG-WSKSKDAPQPDR-----------
------------------------QPQV-IWFWDFFA--------------------RVS
PTDQRKILSF-------ITGSDRIP------------ALG-----TT-NLIIKI--SLLR
EKEDPMDGKGWKEP--------------------ERFPIARTCF-NS-IGLYQY-GSRKK
LEEKLW-TAVTG-SEGF
>Phialophora_attae_XM_018147197.1 .
LKVRRECL-VEDSLRGV-----------SEVVGSGGSDIKK-GLKIDFVG-------EEG
IDAGGLRKEW---FLLL----VREIFDPNH--------GLFVWDEDSQ------------
----------------HCYFNPHC------------------------------------
-----FESSEQFFLVGVLFGLAIYNSTILDVAFPP--FVFKKLLASAPSTGD-------K
LTSTPKVSHGYTLEDLAQ----------------------FRPALAQG--------LRQL
LDFEG--NVE-ETFC-
--RDFVVETDR---------------------------YGV-KAEMP-LCTG--------
GEKRA---
------------------------------VTNSNRR-EFVDL-YIHYLLDTA-VARQYE
-PFKRG---FFTVCGG--NALS-LFRPE-EIELLVRG-----------------------
---SDEPLD-----------IASL----RAVAIYEN-WPTGRQHPPPEH-----------

------------------------QPQV-MWFWEFFA--------------------RVT
PADQRKILSF-------ITGSDRIP------------ALG-----TT-HLVIKV--QRLL
EEVPQHLPDSKLVLG-------------------ERFPTARTCF-NS-IGLYQY-RTRQK
LEEKLW-MAVVG-SEGF
>Exophiala_oligosperma_XM_016409904.1 .
LKVRRECL-VEDSLRNV-----------SEVVGSGGSDIKK-GLRIDFQG-------EEG
VDAGGLRKEW---FLLL----VREIFDPNH--------GLFVYDDDSH------------
----------------FCYFNPYC------------------------------------
-----FESSEQFFLVGVLLGLAIYNSTILDIAFPP--FVFKKMLASAPMTGD-------K
LTSTTRVGHSYTLEDLAE----------------------FRPALARG--------LRQL
LEFEG--DVE-ETFY-
--RDFVVEMER---------------------------YGE-VIRVP-LCPG--------
GEKRP---
------------------------------VTNSNRR-EFVDL-YIHYLLDTS-VTRQYE
-PFKRG---FFTVCGG--NALS-LFRPE-EIELLIRG-----------------------
---SDEPLD-----------IASL----RAVATYEG-WSKNEGPPDK-------------
------------------------QPQV-IWFWDFFG--------------------RAT
PGDQRKILTF-------ITGSDRIP------------AMG-----AT-NLIIRI--QLLR
LKDEVDGLGRGPPRKSI-----------------ERFPIARTCF-NT-LSLYRY-SSRQK
LEQKLW-TAVTE-SEGF
>Exophiala_spinifera_XM_016386071.1 .
LKVRRECL-VEDSLRNV-----------SEVVGSGGSEIKK-GLRIDFQG-------EEG
VDAGGLRKEW---FLLL----VREIFDPNH--------GLFVYDDDSH------------
----------------FCYFNPYC------------------------------------
-----FESSEQFFLVGVLLGLAIYNSTILDIAFPP--FVFKKMLASAPLTGD-------K
ISSTPRVGHSYTLEDLAE----------------------FRPALARG--------LRQL
LEFDG--DVE-ETFC-
--RDFVVEMER---------------------------YGE-IIQVP-LCPG--------
GEKRP---
------------------------------VTNANRR-DFVDM-YIHYLLDTS-VTRQYE
-PFKRG---FFTVCGG--NALS-LFRPE-EIELLIRG-----------------------
---SDEPLD-----------IASL----RAVATYEG-WPKNEGPPDK-------------
------------------------QPQV-IWFWDFFG--------------------RAS
PGDQRKILTF-------ITGSDRIP------------AMG-----AT-NLIIRI--QLLR
TKDEVDGLGRAPRTSV------------------ERFPIARTCF-NT-LSLYRY-SSRQK
LEQKLW-TAVTE-SEGF
>Exophiala_mesophila_XM_016365137.1 .
LKVRRDCL-VEDSLRNV-----------SEAVGSGGNDLKK-GLRIDFLG-------EEG
IDAGGLRKEW---FLLL----VREIFDPYH--------GLFVYDDDSQ------------
----------------YCYFNPYC------------------------------------
-----FESSEQFFLVGVLLGLAIYNSTILDVAFPP--FVFKKMLASAPSSGD-------K
LMSTPKVSHGYTLEDLAE----------------------FRPALARG--------LRQL
LEYEG--DVQ-ETFC-
--RDFVAEMDR---------------------------YGE-ILQIP-LFPG--------
GEKRA---
------------------------------VTNSNRK-EFVDL-YVHYLLDTA-VARQYE
-PFKRG---FFTVCGG--NALS-LFRPE-EIELLVRG-----------------------
---SDEHLD-----------VASL----RAVATYEG-WPKEDGPPDQ-------------
------------------------QPQV-IWFWDFFS--------------------RVS
PLDQRKILGF-------ITGSDRIP------------AMG-----AT-NLVIRV--QLLR
ESQEVDVYGRPLWQPV------------------ERFPIARTCF-NS-ISLYKY-GSREK
LEQKLW-TAVTE-SEGF
>Exophiala_aquamarina_XM_013402362.1 .
LRVRRDCL-VEDSLRSV-----------SEAVGSGGNDLKK-GLRIDFQG-------EEG
IDAGGLRKEW---FLLL----VREIFDPNH--------GLFVYDDDSQ------------
----------------YCYFNPFC------------------------------------
-----FESSEQFFLVGVLLGLAIYNSTILDIAFPP--FVFRKMLASAPLIGD-------K
LTSTPRIGHGYSLDDLAE----------------------YRPALARG--------FRQL

```
LEFEG----------------------------------------------DVQ-ETFC-
--RDFVAEMDR---------------------------YGE-VVQVP-LFPG--------
GEKRA-------------------------------------------------------
------------------------------VTNSNRK-EFVDL-YVHYLLDTA-VTRQYE
-PFKRG---FFTVCGG--NALS-LFRPE-EIELLVRG-----------------------
---SDESLD-----------VASL----RAVATYEG-WPKQEGPAEQ-------------
------------------------QPQV-VWFWDFVC--------------------KVS
PSDQRRILSF-------ITGSDRIP------------AMG-----AT-NLVIRI--QLIR
GKDEFDAQGRPTNLPI------------------EKFPIARTCF-NT-ISLFKY-ESRQK
LEYKLW-MAVTN-SEGF
>Rhinocladiella_mackenziei_XM_013417230.1 .
LKVRRDCL-VEDSLRGV-----------SEVVGSGGNDIKK-GLRIDFQG-------EEG
IDAGGLRKEW---FLLL----VREIFDPYH--------GLFVYDEDSR------------
----------------YCYFNPYC------------------------------------
-----FESSEQFFLVGVLLGLAIYNSTILDVAFPP--FVFKKMLASAPSTGD-------K
LMSTPKVGHGCTLEDLAE----------------------FRPALARG--------FRQL
LEFEG----------------------------------------------DVE-ETFC-
--RDFVAEMDR---------------------------YGE-IVRVP-LCPG--------
GEKRA-------------------------------------------------------
------------------------------VTNSNRR-EFVDL-YVHYLLETA-VARQYE
-PFKRG---FFTVCGG--NALS-LFRPE-EIELLVRG-----------------------
---SDEPLD-----------ITSL----RAVATYEG-WPKNEGPPDQ-------------
------------------------QPQV-VWFWEFFA--------------------RVS
PADQRKILGF-------ITGSDRIP------------AMG-----AT-NLIIRI--QLIR
SKDETDPLGRPTNKPI------------------ERFPIARTCF-NT-LSLYRY-SSREK
LEHKLW-MAVTG-SEGF
>Exophiala_dermatitidis_XM_009155562.1 .
LKVRRDCL-VEDSLRSV-----------SEVVGSGGSDIKK-GLRIDFQG-------EEG
IDAGGIRKEW---FLLL----VREIFDPNH--------GLFVYDDDSG------------
----------------YCYFNPYC------------------------------------
-----FESSEQFFLVGVLLGLAIYNSTILDVAFPP--FVFKKLLASAPSTGD-------K
LTSTPKVGHGFTLEDLAE----------------------FRPALARG--------FKQL
LEFEG----------------------------------------------DVE-ETFC-
--RDFVAEMDR---------------------------YGE-IVQVP-LCPG--------
GEKRA-------------------------------------------------------
------------------------------VTNANRR-EFVDL-YIHYLLETS-VARQYE
-PFKRG---FFTVCGG--NALS-LFRPE-EIELLVRG-----------------------
---SDEPLD-----------IASL----RAVATYDG-WPKNEGPPEK-------------
------------------------QSQV-VWFWDFFA--------------------RVS
PEDQRKILSF-------ITGSDRIP------------AMG-----AT-NLNIRI--QLIR
SKDEFDGLGRPTNKPV------------------ERFPIARTCF-NT-LSLYRY-SSREK
LEHKLW-MAVTG-SEGF
>Capronia_epimyces_XM_007740526.1 .
LKVRRDCL-VEDSLRSV-----------SEVVGSGGNDIKK-GLRIDFQG-------EEG
IDAGGIRKEW---FLLL----VREIFDPHH--------GLFVYDDDSQ------------
----------------YCYFNPFC------------------------------------
-----FESSEQFFLVGVLLGLAIYNSTILDVAFPP--FVFKKLLASAPLTGD-------K
LTSTPKVSHGFTLEDLAE----------------------FRPALARG--------FRQL
LEFEG----------------------------------------------DVE-ETFC-
--RDFVAELDR---------------------------YGE-IVQVP-LCPG--------
GEKRA-------------------------------------------------------
------------------------------VTNANRR-EFVDL-YIRYLLDTA-VARQYE
-PFKRG---FFTVCGG--NALS-LFRPE-EIELLVRG-----------------------
---SDEPLD-----------IASL----RAVATYEG-WPKNDGPPEQ-------------
------------------------QPQV-VWFWDFFA--------------------RVS
AEDQRKILGF-------ITGSDRIP------------AMG-----AT-NLIIRI--QLLR
SKDEFDAYGRPMSKPIEI----------------ERFPIARTCF-NT-LSLYRY-SSREK
LEHKLW-MAVTG-SEGF
```

>Capronia_coronata_XM_007727579.1 .
LKVRRDCL-VEDSLRSV-----------SEVVGSGGSDIKK-GLRIDFQG-------EEG
IDAGGIRKEW---FLLL----VREIFDPNH--------GLFVYDDDSQ------------
----------------YCYFNPYC------------------------------------
-----FESSEQFFLVGVLLGLAIYNSTILDVAFPP--FVFKKLLASAPATGD-------K
LTSTPKVSHACSLDDLAE----------------------FRPALARG--------FRQL
LDFEG--DVE-ETFC-
--RDFVAEMDR---------------------------YGE-IVQVP-LCPG--------
GEKRA---
------------------------------VTNANRR-DFVDL-YTHYLLETA-VARQYE
-PFKRG---FFTVCGG--NALS-LFRPE-EIELLVRG-----------------------
---SDEPLD-----------IASL----RAVATYEG-WPKTEGPPEQ-------------
------------------------QPQV-VWFWDFFA--------------------RAS
AEDQRKILGF-------ITGSDRIP------------AMG-----AT-NLIIRI--QLLR
SKDEMDGFGRPINKPI------------------ERFPIARTCF-NT-LSLYRY-SSREK
LEHKLW-MAVTG-SEGF
>Exophiala_xenobiotica_XM_013463240.1 .
LKVRRDCL-VEDSLRNV-----------SEVVGSGGSDIKK-GLRIDFQG-------EEG
VDAGGLRKEW---FLLL----VREIFDPNH--------GLFVYDDDSQ------------
----------------YCYFNPFC------------------------------------
-----FESSEQFFLVGVLLGLAIYNSTILDVAFPP--FVFKKMLASAPSTGD-------K
LTSTPRVGHGFTLDDLAE----------------------FRPALARG--------FRQL
LEFEG--DVE-ETFC-
--RDFVAEMDR---------------------------YGE-IIQVP-LCPG--------
GEKRP---
------------------------------VTNSNRR-EFVDL-YIHYLLDTA-VTRQYE
-PFKRG---FFTVCGG--NALS-LFRPE-EIELLVRG-----------------------
---SDEPLD-----------IASL----RAVSTYEG-WPKNEGPPEK-------------
------------------------QPQV-VWFWDFFS--------------------RAA
PADQRKILTF-------ITGSDRIP------------AMG-----AT-NLVIRI--QLLR
NKDDFDGLGRPVNKQV------------------ERFPIARTCF-NT-LSLYRY-SNRQK
LEQKLW-TAVTE-SEGF
>Cladophialophora_carrionii_XM_008732842.1 .
LKVRRDCL-VEDSLRGV-----------SEVVGSGGSEIKK-GLRIDFQG-------EEG
VDAGGLRKEW---FLLL----TREIFDPYH--------GLFVYDDDSQ------------
----------------YCYFNPFC------------------------------------
-----FESSEQFFLVGVLLGLAIYNSTILDVAFPP--FVFKKMLASAPSTGD-------K
LTSTPKVAHGYALEDLAE----------------------FRPVLAKG--------LRQL
LEFEG--DVE-ATFC-
--RDFVAEMDR---------------------------YGE-IVQVP-LCPG--------
GEKRA---
------------------------------VTNSNRR-EFVDL-YVHYLLDTA-VARQYE
-PFKRG---FFTVCGG--NALS-LFRPE-EIELLVRG-----------------------
---SDEPLD-----------IASL----RAVATYEG-WPKAEGPPEQ-------------
------------------------QPQV-IWFWEFFA--------------------RVS
PTDQRKILSF-------ITASDRIP------------AMG-----AT-NLVIRI--QLIR
EKEEFDLFGAPANKPS------------------ERFPIARTCF-NT-LSLYRY-ASRQK
LEEKLW-TAVQC-SEGF
>Cladophialophora_yegresii_XM_007763736.1 .
LKVRRDCL-VEDSLRGV-----------SEVVGSGGNEIKK-GLRIDFQG-------EEG
VDAGGLRKEW---FLLL----TREIFDPYH--------GLFVYDDDSQ------------
----------------YCYFNPFC------------------------------------
-----FESSEQFFLVGVLLGLAIYNSTILDVAFPP--FVFKKMLASAPLAGD-------K
LTSTPKVAHGYALEDLAE----------------------FRPALAKG--------LRQL
LEFEG--DVE-TIFC-
--RDFVAEMDR---------------------------YGE-IIQVP-LCPG--------
GEKRA---
------------------------------VTNSNRR-EFVDL-YVHYLLDTA-VARQYE

-PFKRG---FFTVCGG--NALS-LFRPE-EIELLVRG-----------------------
---SDEPLD-----------IASL----RAVATYEG-WPKAEGAPEQ-------------
------------------------QPQV-MWFWEFFA--------------------RVS
PADQRKILSF-------ITASDRIP------------AMG-----AT-NLVIRI--QLIR
EKEEFDMLGTATNKPS------------------ERFPIARTCF-NT-LSLYRY-ASRLK
LEEKLW-TAVQC-SEGF
>Cladophialophora_psammophila_XM_007748090.1 .
LRVRRECL-VEDSLRSV-----------SEVVGSGGSDIKK-GLRIDFQG-------EEG
VDAGGLRKEW---FLLL----VREIFDPHH--------GLFVYDDDSR------------
----------------YCYFNPFC------------------------------------
-----FESSEQFFLVGVLVGLAIYNSTILDVAFPP--FVFKKMLASAPSTGD-------K
LSSIPKVGHGFTLEDLAE----------------------FRPALARG--------FRQL
LEFEG--DVE-ETFC-
--RDFVAEMDR---------------------------YGD-VIQVP-LCPG--------
GERRA---
------------------------------VTNSNRR-EFVDL-YIHYLLDTA-VARQYE
-PFKRG---FFTVCGG--NALS-LFRPE-EIELLVRG-----------------------
---SDEPLD-----------IASL----RAVATYEG-WPKQEGPPEQ-------------
------------------------QPQV-VWFWDFFA--------------------RVS
PVDQRKILSF-------ITASDRIP------------AMG-----AT-NLVIRI--QLIR
AKEEIDEFGRPTNKPV------------------ERFPIARTCF-NT-LSLYRY-VSREK
LEEKLW-TAVQG-SEGF
>Cladophialophora_bantiana_XM_016763807.1 .
LKVRRECL-VEDSLRSV-----------SEVVGSGGSDIKK-GLRIDFQG-------EEG
VDAGGLRKEW---FLLL----VREIFDPHH--------GLFVYDDDSR------------
----------------YCYFNPFC------------------------------------
-----FESSEQFFLVGVLVGLAIYNSTILDVAFPP--FVFKKMLASAPSTGD-------K
LSSIPKVGHGFTLEDLAE----------------------FRPALARG--------LRQL
LEFEG--DVE-ETFC-
--RDFVAEMDR---------------------------YGD-VVQVP-LCPG--------
GEKRA---
------------------------------VTNSNRR-EFVDL-YIHYLLDTA-VARQYE
-PFKRG---FFTVCGG--NALS-LFRPE-EIELLVRG-----------------------
---SDEPLD-----------IGSL----RAVATYEG-WPKQEGPPEQ-------------
------------------------QPQV-VWFWDFFA--------------------RVS
PVDQRKILSF-------ITASDRIP------------AMG-----AT-NLVIRI--QLIH
AKEEMDEFGRPTNKPV------------------ERFPIARTCF-NT-LSLYRY-VSREK
LEEKLW-TAVQG-SEGF
>Fonsecaea_pedrosoi_XM_013422568.1 .
LKVRRECL-VEDSLRSV-----------SEVVGSGGSDIKK-GLRIDFQG-------EEG
VDAGGLRKEW---FLLL----VREIFDPHH--------GLFVYDDDSQ------------
----------------YCYFNPFC------------------------------------
-----FESSEQFFLVGVLVGLAIYNSTILDVAFPP--FVFKKMLASAPATGD-------K
LSSIPKVGHGFTLEDLAE----------------------FRPALARG--------FRQL
LEFEG--DVE-ETFC-
--RDFVVEMDR---------------------------YGD-IIQIP-LCPG--------
GEKRA---
------------------------------VNNSNRR-EFVDL-YIHYLLDTA-VARQYE
-PFKRG---FFTVCGG--NALS-LFRPE-EIELLVRG-----------------------
---SDEPLD-----------IASL----RAVATYEG-WPKQEGPPEN-------------
------------------------QPQV-VWFWEFFA--------------------RVS
PGDQRKILSF-------ITASDRIP------------AMG-----AT-NLVIRI--QLIR
AKEEMDEFGRPTNKPV------------------ERFPIARTCF-NT-LSLYRY-ASREK
LEEKLW-MAVMG-SEGF
>Cladophialophora_immunda_XM_016399905.1 .
LKVRRECL-VEDSLRSV-----------SEVVGSGGSDIKK-GLRIDFQG-------EEG
VDAGGLRKEW---FLLL----VREIFDPHH--------GLFVYDDDSL------------
----------------YCYFNPFC------------------------------------

-----FESSEQFFLVGVLVGLAIYNSTILDVAFPP--FVFKKMLASAPSTSD-------K
LSSIPKVGHGFTLEDLAE----------------------FRPALARG--------FRQL
LEFEG--DVE-ETFC-
--RDFVAEMDR---------------------------YGD-VVQVP-LCPG--------
GEKRA---
------------------------------VTNSNRR-EFVDL-YIHYLLDTA-VARQYE
-PFKRG---FFTVCGG--NALS-LFRPE-EIELLVRG-----------------------
---SDEPLD-----------IASL----RAVATYEG-WPKQEGPPEQ-------------
------------------------QPQV-VWFWDFFA--------------------RVS
PVDQRKILSF-------ITASDRIP------------AMG-----AT-NLVIRI--QLIR
AKEEMDDFGRPTNKPV------------------ERFPIARTCF-NT-LSLYRY-ASREK
LEEKLW-MAVQG-SEGF
>Fonsecaea_erecta_XM_018841764.1 .
LKVRRECL-VEDSLRGV-----------SEVVGSGGSDIKK-GLRIDFQG-------EEG
VDAGGLRKEW---FLLL----VREIFDPHH--------GLFVYDDDSH------------
----------------YCYFNPFC------------------------------------
-----FESSEQFFLVGVLVGLAIYNSTILDVAFPP--FVFKKMLASAPSTSD-------K
LSSVPKVGHGFTLEDLAE----------------------FRPALARG--------FRQL
LEFEG--DVE-ETFC-
--RDFVAEMDR---------------------------YGD-IIQVP-LCPG--------
GEKRA---
------------------------------VTNSNRR-EFVDL-YIHYLLDTA-VARQYE
-PFKRG---FFTVCGG--NALS-LFRPE-EIELLVRG-----------------------
---SDEPLD-----------IASL----RAVATYEG-WPKQEGPPEQ-------------
------------------------QPQV-IWFWEFFA--------------------RVS
PGDQRKILSF-------ITASDRIP------------AMG-----AT-NLIIRI--QLIR
AKEEMDALGRSTNKPV------------------ERFPIARTCF-NT-LSLYRY-ASREK
LEEKLW-MAVQG-SEGF
>Fonsecaea_multimorphosa_XM_016778461.1 .
LKVRRDCL-VEDSLRSV-----------SEVVGSGGSDIKK-GLRIDFQG-------EEG
VDAGGLRKEW---FLLL----VREIFDPHH--------GLFVYDDDSH------------
----------------YCYFNPFC------------------------------------
-----FESSEQFFLVGVLVGLAIYNSTILDVAFPP--FVFKKMLASAPSTSD-------K
LSSVPKVGHGFTLEDLAE----------------------FRPALARG--------FRQL
LEFEG--DVE-ETFC-
--RDFVAEMDR---------------------------YGD-VIQVP-LCPG--------
GEKRA---
------------------------------VTNSNRR-EFVDL-YIHYLLDTA-VARQYE
-PFKRG---FFTVCGG--NALS-LFRPE-EIELLVRG-----------------------
---SDEPLD-----------IASL----RAVATYEG-WPKQEGPPEQ-------------
------------------------QPQV-VWFWEFFA--------------------RVS
PMDQRKILSF-------ITASDRIP------------AMG-----AT-NLVIRI--QLIR
AKEEMDELGRPTNDPV------------------ERFPIARTCF-NT-LSLYRY-ASREK
LEEKLW-MAVQG-SEGF
>Hanseniaspora_valbyensis_LXPE01000042.1 .
IQVDRNNF-FNDLMYHL-----------------NSSSGS---SFAVEYKG-------EQG
RGYG-LKKDF---LSEA----SKMFFYDL---------NLFSVSGNGD------------
----------------GLVPNQEF--------------------------LCHSILENLG
LTETLFTNEQLVKNAGKILAFLLLEGFTIPYKFSY--WFWRKFLL---------------
-----------TLFDLYH----------------------FNPKKTHI-------YKNEF
IDLQYHDY---ELYFNLE--
--GLFKLNEEELKSLDL---------------------YME-YEGIE-LVPN--------
GNEIP---
------------------------------VTYENLH-FYIKK-ITDFKLNNKGLDKFME
-YFVKG---FKLVVQX-------------DISLIVNG-----------------------
DSLEKFPID---------DFISNL---------KLQGYNED-------------------
------------------------DKTI-KDLIYILKN-------------------DFT
NDQLIKFFHF-------ITSLRAPP------------FNG-----FS-SLNPLI--CITK

ITDPDFIDSK------------------------HHLPSSSTCF-NL-LRLPDY-KNKDI
LFKKLT-IAIED-KGG-
>Hamiltosporidium_tvaerminnensis_ACSZ01009 .
LQVNRENI-LDNFLNLVSSF-------------DLKKTLKENIWYVTFGT-------ELC
AGIG-PTLEF---FTLF----GKSMNRSRFF-------EMFKSTETS-------------
------------------YMNKFA------------------------------------
--ECPEYIEKVYFYTGIIMAKCIIYNTTLGLEFID--SFYLYLTK---------------
--------DCFTFEDLKI----------------------CDSELHKS--------LSFF
ATSG---KSS-FHG--
--YNFTWATNN---------------------------NGI-YEEFE-LVEN--------
GCNIS---
------------------------------LTSDNLE-EYLQK-ITEFKLFKR-MEKYLD
-KMKEG---FCFILGD--SFSN-MFNYR-DLSTMLQG-----------------------
----EPDFQ-----------VEDF----KQCTSYVGDYNSN-------------------
------------------------HPVI-IWFWNYMS--------------------SLE
AIKKKKLFLF-------LTTFEKIP------------FGG-----FK-SQRFLEY-KFTI
MPIENS----------------------------KSLPIIHTCS-NL-LELPKY-ESEEI
LRSKFE-YCL-------
>Rhizophagus_irregularis_JAQX01011177.1 .
--VQKENL-ANWTREKF----------------HRYADAKT-TFGITFAG-------EMG
IGAG-VTRDF---FTQM----S-QYFQIVGS-------NMWTDLETSPN-----------
-------PEDYVTNEYGLYPTLFD--------------------------------EKYA
ISDEGTRVLKDFQILGMLTAKSLADKQIMEVPLNK--MFLKRFIF---------------
---GEHPSVELSNYDFTK----------------------VDDQSRKDMK------IKKL
LGLINQINPTITKIAN-----------------------------------HLR-DIQ--
--NNFSDCLESFYI------------------------PAG-RSWHQ-NNLV--------
SIDLPE--
-----------------------------TETQNIFY-LYLNK-IKEVVFETG-IHEQIS
-KFREG---FNHVFPLD-HLKN-FYTVE-EIHKVVFH-----------------------
--NKKEDWS-----------ADVI------RRAFFVNNESI-------------------
------------------------RIEV-DKISQILS--------------------KFN
IDEQRKMLRF-------FTGSTRLP------------IGG-----WT-KLKPEL--AVIE
DL--------------------------------GAYPIGRTCF-NK-LSIPRN-NSLQE
LEEKL------------
>Gigaspora_margarita_GBYF01016133.1 .
--ISMDNL-FGWVRQYF----------------EKYAVTGS-RLSVQFEG-------VGG
IGDG-VAREF---FSKL----SLEFSQICR--------KMWVHDVKYKKP----------
-----YKPSDPIDNDYGLYPALMND-------QIDEKPDGESKEGVESVDESNEVPKKET
LKSEELSVLENFKLLGQYVAKVIYDDRMIDILLHPEHLLRKLPRG---------------
--TDLNEQDCLKVADLIDS---------------------VYPSFGSS--------IRKL
LNNNDLDEKDC--IPY-DIMT-
--GRTTSVVGPD--------------------------GKK-REQPY-NIDN--------
GNDSDE--
-----------------------------WVTRKNIG-RYVAA-CRDWFFDRG-IALQSK
-AFKEG---FNRVFPI--EVMR-EYTAT-EFALYFLH-----------------------
-QGGDGDWS-----------IETL---------RQYVQSYD-------------------
----------------QNTT----NEEV-EKVIQIMA--------------------EYD
TDNRKKFLRF-------ATGSSKLP------------IGG-----FE-ALNLKV---FRD
SGLDE-----------------------------RALPKARTCF-NR-LIIPPN-NSKEE
LVRKLE-FAF-------
>Entomophthora_muscae_GENB01020500.1 .
VKVHRDEN-LLEWAMQV---------------MKVHCNRKS-VLEVGFTG-------EEG
TGLG-PTLEF---FALV----ASELQRSDL--------CMWLCDDETTIT----------
---------QTSDVNEEVSPISIK------------------------------------
---------ESSKPIGYYVNRXLPQDSEVCEKASK-HFWFLGVF----------------
--------IAKVLQDMRL----------------------VDLPLSDS--------FLQL
LCHNKMLSSSTTHKRSTT---------------------------------ASE-DLMA-
--------------------------------------XG--YEYAE-LIPN--------

GGDID---
------------------------------VTINNLE-EYCES-LINFSLQDG-IAKQLD
-AFHRG---FCEVFPL--QKLA-AFSPS-EARMMICG-----------------------
--EQHPQWT-----------REDL---------INYTEPKLG------------------
----------------FSKD----SPGF-LRFVNVLM--------------------SLT
GPERKAFLQF-------TTGCSSLP------------PGG-----LA-NLHPRL--TVVR
KVDAGD----------------------------GSYPSVNTCV-HY-LKLPDY-QTEEI
MKERLL-TATKE--KGF
>Anaeromyces_robustus_MCFG01000340.1 .
VRVHRDKI-IESLSIVM----------------NMYCSSQA-LLEIEFYD-------EVG
TGLG-PTLEF---YNLV----CKEICKKSL--------NIWRDNGFKNNS----------
-------DEIYISPKNGLFPKPLA------------------------------------
SNASESKCCKYFNVLGSFVGKALLDSRIINISFNP--VFLYLVVNYPL-LSKHLIGRNSK
HPKLLRKWKDIGIFIIDM----------------------IDQDLAKS--------LRHL
KKYIQIKDQYIQ----------------------EGKSEDDIKSIEVDG-AKID-DLC--
--LDFTL-------------------------------PG--YPEIE-LIPD--------
GENVS---
------------------------------VNINNLE-EYIIK-IIDYTVCCG-ISKQIK
-AFRDG---FNKVFLL--TDLQ-IFEVK-ELLQMVSS-----------------------
---NEEDWS-----------KETL---------MKTVKADHG------------------
----------------YSMK----SQSV-INLIEVMS--------------------EMN
KDERKEFLEF-------ITGTSRLP------------LGG-----FK-NLNPPF--TVVC
KTTDSDENPD------------------------DFLPSVMTCA-NY-LKLPDY-KTKDI
LKERLT-TAIQE-GRG-
>Piromyces_finnis_MCFH01000003.1 .
VRVHRDKI-IESLSIVM----------------NMYCSSQA-LLEIEFYD-------EVG
TGLG-PTLEF---YNLV----CKEICKKSL--------NIWRDNGFESNS----------
------GEECYLSPKTGLFPKPLG---------------------------------VNA
PEAKVKKCCKYFSMLGSFVGKALLDSRIINIAFNP--VFLYLVVNYPL-LNKYLIGRNHK
HPKLLKKWRDVGIFIIDM----------------------VDPDLAKS--------LRYL
EKFIQIKDQYIE----------------------EGKSEEEIKSIEVDG-AKVD-DLC--
--FDFVL-------------------------------PG--YPEIE-LIPD--------
GENVS---
------------------------------VTINNLE-DYIVK-IIDFTLCSG-ISKQVK
-AFREG---FNKVFLI--TDLQ-IFEVK-EILQMISN-----------------------
---SEEDWT-----------KETL---------MKTIKADHG------------------
----------------YSMK----SQSV-INLIEVMS--------------------EMN
KDEKKEFLEF-------ITGTSRLP------------LGG-----FK-NLNPPF--TVVC
KTTSSDENPD------------------------DFLPSVMTCA-NY-LKLPDY-KTKEI
LKERLT-TAIKE-GRG-
>Pecoramyces_ruminatium_ASRE01012983.1 .
VRVHRDKI-IESLSIVM----------------NMFCSSQA-LLEIEFNG-------EVG
TGLG-PTLEF---YNLV----CKEICKKSL--------NIWRDNGFENKD----------
-------GNTYLCPKDGLFPKPLA------------------------------------
TNAPESKCCKYFNILGSFVGKALLDSRIINISFNP--VFLYLVVNYPL-LSKYLIGRNSK
HPKLLRKWKDVGLFIINM----------------------IDQDLAKS--------LRHL
MKYIIIKDKYIE----------------------EDKTEDEIKLIEVDG-AKID-DLC--
--LDFTL-------------------------------PG--YPEIE-LIPD--------
GEKIS---
------------------------------VNINNLE-EYIIK-VIDFTICCG-ISKQVK
-AFREG---FNKVFLI--TDLQ-IFDIK-ELLQMVSS-----------------------
---NEEDWS-----------KESKAL-------MKTIKADHG------------------
----------------YSMK----SQTV-INLIEVMS--------------------EMN
KNEKKEFLEF-------ITGTSRLP------------LGG-----FK-NLNPPF--TVVC
KTTSSDENPD------------------------DFLPSVMTCA-NY-LKLPDY-KTKDI
LKERLT-TAIKE-GRG-
>Neocallimastix_californiae_MCOG01000061.1 .
VRVHRDKI-IESLSIVM----------------NMFCSSQA-LLEIEFNG-------EVG

TGLG-PTLEF---YNLV----CKEICKKSL--------NIWRDNGYEGKD----------
-------ENIYLSPKSGLFPKPLA------------------------------------
INASESKCCKYFNMFGSFVAKALLDSRIINISFNP--VFLYLVVNYPL-LSKYLIGRNSS
HTKLLRKWKDIGLFIINM----------------------IDQDLAKS--------LKHL
LKYIQIKDQYIK----------------------EERSEVEIKSIEVDG-AKIE-DLC--
--LDFTL-------------------------------PG--YSDIE-LIPN--------
GENVT---
------------------------------VNINNLE-EYIIK-IIDFTLCSG-ISKQVK
-AFREG---FNKVFLV--TDLQ-IFNIK-ELLQMISS-----------------------
---NEEDWT-----------KESL---------MKTIKADHG------------------
----------------YSMK----SQTV-INLIEVMS--------------------EMD
KEEKKEFLEF-------ITGTSRLP------------LGG-----FK-NLNPPF--TVVC
KTTSSDENPD------------------------EFLPSVMTCA-NY-LKLPDY-KTKER
LKERLT-TAIKE-GRG-
>Anaeromyces_robustus_MCFG01000015.1 .
VRVNRNHI-LESMVKIM----------------PLYGVKNS-LLEIEFYN-------EVG
TGLG-PTLEF---YSLV----SNEVCKSKY--------KLWRDTSNNIGG----------
-------SMNSILEKEXLFPAPIN--------------------------------PDNL
DTNAGKKLLKIFNCLGIFVAKAMLDFRTIDLPLNT--YFVKLIKD----NVNNLDFSELY
CLSSNKNYLEMGLKIIEQ----------------------VDPPLMNS--------LNQI
MKYSNLKKEIYSNTSL-----------------SPDELHTMVQNIRIDD-SSIQ-DLC--
--LDFTL-------------------------------PG--YPDIE-LIEN--------
GSEVE---
------------------------------VTNWNVE-EYLKE-IINFTIGKG-VSKQIE
-AFRKG---FNSVFPI--SNLS-IFDND-ELVLLFGG-----------------------
--SENEDWS-------------XL---------INCIHADHG------------------
----------------YTMD----SPQI-IYLVEVMS--------------------EMT
DSEKRDFIQF-------VTGCPKLP------------LGG-----FK-NLKPPF--TVVC
KTTEASHKPD------------------------EYLPSVMTCA-NY-LKIPQY-SSKEV
LKEKLE-IAYKE-GRG-
>Catenaria_anguillulae_MCFL01000020.1 .
VRIDRPHI-LSSAYRTF----------------DLFGAEYS-MLEIEYRG-------EVG
TGLG-PTLEF---YSLT----VQEIANRK---------DLWQTAAVP-------------
----------------FLLPRGAS--------------VTDAEVDQVRAESEKPQVDKKM
VPKDKLTHAHILFLLGQVMAKALMDDRTVDVPLHP--AFLKLVFS---------------
-----QQPSAADMDGLRA----------------------VDPVVAKS--------MQAV
LDMS---DPS-ALH--
--LHFEY-------------------------------DG-----KP-LIPG--------
GEDVP---
-----------------------------VNTAEDAR-RYVAL-VADRVAGVEGLGAAAR
-CVRLG---FERVLPV--APLASLFSAD-ELLTLFSG----------------------S
ATEDNQYWT-----------HQAL-------LSPGGMQADHG------------------
----------------YSLD----SPAV-RWLVDWMV--------------------ALP
ASDRRTFLTF-------LTGAPRLP------------LGG-----WK-ALRPKF--TVVH
RSAPNPD---------------------------TYLPSVMTCA-NY-LKMPSY-SSREL
LDQRMR-MAMAE-GQG-
>Allomyces_macrogynus_ACDU01002927.1 .
VRISRDRI-LGAAVKVM----------------ELFAPEYS-MLEIEYQN-------EVG
TGLG-PTLEF---YALV----VAELAKCA---------DLWQAGAAP-------------
----------------YLMPRAGV--VTKADVEAVQTAVADAAKTTSPLEHRTAVLEAIG
KCGARRPAAQYLRMLGQLMAKALLDDRLVDLPLHP--QFLEMVLS---------------
SASSEAEATDATLDDVRA----------------------IDPTLAKS--------LAAM
LHMADAETDPNSPTL------------------------------------AID-ALT--
--VQFEF-------------------------------LG-----TA-LVPG--------
GSDRA---
-----------------------------VRTPTDVR-EYVRL-VCNHVAGHATHAPAAT
-LVRDG---FASVLPV--TSVSALFGTD-EIVALVSS---------------------SR
AGDNDEHWT-----------PAAI---------TAGMRADHG------------------

----------------YHAS----SRVV-QDLVLEMS--------------------AMD
AARRRAFVQW-------LTGAPRLP------------LGG-----WT-ALKPPF--TVVE
RTVPVGEVSD------------------------AYLPSVMTCA-NY-LKLPSY-SSREV
LSVRLK-VAVEE-GQG-
>Smittium_culicis_LSSM01003176.1 .
ILVNRSQS-FDSAFLTL----------------NKFANVDN-VLEFQFVD-------EVG
TGLG-PTIEM---YSIV----CNEFLRKDL--------GIWYSNNNLNSP----------
-------NSDYILPPNGLFPLPFN----------------------------------SE
DENEISKYHDMFSFLGLFVAKSIVDERPIDLPIHP--SFFLLLLN---------------
SIDSIKFPMISPIKIISH----------------------IDPTLANS--------LNIL
QSCITKKSEIYSKKNL----------------TQDEKNDEVLKINLDDG-SSID-DLG--
--LDFTL-------------------------------PG--NPAYL-LRPN--------
GADIT---
------------------------------VNILNLN-LYLEL-VYDAFASTG-TKLSTS
-AFKHG---FTKLLPI--ETLA-YHSIN-ELSVIVGFNGSSNEDDTQSSPGDNAGNDNRY
KSIDDDAWS-----------SESL---------STNIVADHG------------------
----------------YSQD----SPVF-KMFISWLS--------------------NLS
RTDRRKFLNF-------VTGSPRKAQ-----------ISG-----FG-ALNPPL--TVVL
RHCQGSLSPD------------------------DYLPTVMTCA-NY-IKLPNY-SSIEA
LNKNWT-QAIKE-G---
>Smittium_mucronatum_LSSL01002305.1 .
ILINRDKA-FESARLTM----------------LKFAGVDN-ILEFQFVD-------EVG
TGLG-PTLEL---YSSV----CAGFLRKDL--------NIWYTNGDKNFN----------
-----DSDSVFISN--GLFPLPIN-----------------------------------SQ
DYKITERYEEMFSFIGLFVAKSIIDERPIDLPIHP--FFFLLLLG---------------
SINDKKLSSIDPIEIVSS----------------------IDPVLAKS--------LVVL
RTFNSKSSDNSSFTDS-----------------QDVDEDSVDSSTRI-G--FVE-DLG--
--LDFTF-------------------------------PG--NPDYL-LRPN--------
GHEIS---
------------------------------VNKLNLN-LYLEL-IYDAFVGRG-IHLAIS
-GFKAG---FSKLLPV--ETLC-YYSID-ELSIMFGF---------------NSDAKGAN
QSAEDDAWS-----------AQSL---------SLNIVADHG------------------
----------------YSSD----SPAF-KMFVDWLS--------------------GLD
RMDRRKFLNF-------VTGAPRLPX-----------IAG-----FG-ALSPPL--TVVL
RHSSHPLSPD------------------------DYLPTVMTCA-NY-IKLPNY-SSIEV
LDKRWR-QAIDE-GS--
>Capniomyces_stellatus_LUVW01000009.1 .
IMISRKSM-FESGKIAM----------------DKFSNLNV-VLDIEYQD-------EVG
TGLG-PTLEF---YSSV----CIDFTRKKH--------ALWVNIDSVSVSDS---VSDSD
SDSDSADNNAFVDFGNGLFPRPIS----------------------------KRQMSNFQ
YLCHVNKYKELFEYLGKFVAKAIVDDRPIDLPLHL--HFFNLLLHQEP--PQLSSNPHVL
ASQGDEGRDIEEMLDVAKS---------------------IDPQFAAS--------VERL
YAMVTTKLDLYHQLSS----------------AKCAETNLVVEKELGELKQLVD-SLA--
--LDFTL-------------------------------PG--HSGYE-LVEN--------
GSNIP---
------------------------------VTVENIQ-RYVPL-VLAEYVREG-ASVAVE
-SFKVG---FESVAKSV-DVLR-MYTLS-ELVHVFGS----------------------S
GLRECGIWE-----------RASL---------ARYVRADHG------------------
----------------YTME----SLPV-IYFLEWVE--------------------SLD
LQGKRKFLRF-------VTGSSTLPX-----------YLG-----FG-ALNPPL--TIVL
RHXLQPSSAD------------------------DYLPSVMTCA-NY-IKLPNY-SSFDV
LKMRWE-QALDE-GQA-
>Entomophthora_muscae_GENC01016730.1 .
IQVPRESI-LEAAKRIA----------------RLYSKDRS-VLEIQFRD-------EVG
SGQG-PTLEF---FSCV----FQEFCKKAH--------GLWRESQASDS-----------
---------EFVNIHHGLFPACLP------------------------------------
SHASHIHKSLLFKTLGWIAAKSLIDARVTDLPLSE--AFLELLMN---------------
KTCDIPQTNLNTLKLVRS----------------------FDPTFADS--------LFNI

```
NEMASNDAD------------------------------------------SIA-SLG--
--LTFVF-------------------------------PG---TTNE-LMEN--------
GTNII-------------------------------------------------------
------------------------------VSKKNVD-KFIKD-TLELLTNSG-IRYALS
-SFEEG---FNDVFPL--QKLG-IFTPR-ELTTIFGS-----------------------
---GEEDWS-----------AEVI---------HSAIKAEHG------------------
----------------YTSE----SLTV-IRLVTIIS--------------------SFE
IEQRRSFLQF-------ITGSRKLP------------IGG-----FR-ALNPNL--TIVR
KICDATCVPD------------------------NYLPSVNTCF-NY-LKLPDY-SSEEV
MKNNLL-KAMEE-GKMS
>Rozella_allomycis_ATJD01001143.1 .
-ELNRNTI-LKDFKLAM----------------QKYAKEKT-VLEFTYQS-------EIG
TGTG-PTLEF---YSLI----SKELQKNYL--------NIWRSEDSSS------------
---------EFVSTKNGLFPSFSL------------------------------------
------SNLDLFKSIGMLCAKAISDSRILDLPLNP--IFLNLVFK---------------
------RQIVNDISTLHT----------------------IDPILASS--------LINL
KKCS-----------------------------------------------DIK-SLD--
--LYFVL-------------------------------PG---SNIE-LIPN--------
GSSKR-------------------------------------------------------
------------------------------VKNSNIN-NYIKL-IIEKTIYSV--QPMVD
-EFSIG---FNSVIPI--ERMK-IFTAN-QILSLLNG-----------------------
---DDQPWS-----------IECV--------LTSSIKADHG------------------
----------------FTIE----SPCL-QNLICLLS--------------------SLN
NEERSLFLRF-------TTGSSNLP------------VGG-----LK-ALSPPL--TVVK
KIVPDPD---------------------------VYLPSAMTCT-NY-LKLPDY-SSYEV
LKEKLL-VAINE-G---
>Conidiobolus_incongruus_JNEM01010040.1 .
-QITREDI-LANAQSIL----------------HSLASKDA-ILEIQFTG-------EVG
TGRG-PTSEF---FSLS----FQELQMIQY--------DLWRVDSSLNNSS---------
---------EYVYNQKGLFPRPLQ---------------------------------FNP
DNPSHIKKYQHMRLLGGLVAKALADNRIIDLPLNH--HFIRKVFG------------TFN
TSQVPLTTLEQKLKLLST----------------------IDPMTYQS--------LNGL
YELSNSPTAS-----------------------------------------EID-NLG--
--LSFEI-------------------------------PG--YNDIQ-LDTS--------
HSDVS-------------------------------------------------------
--------------------------------SKNIK-EYFDL-VLDWTLTIG-IQWAID
-AFTEG---FNIYSDV--KYMK-IFTDD-EISNLLGS-----------------------
--GDQEDWS-----------LATL---------IESIQTNHG------------------
----------------YTMA----SPII-TQFLETLS--------------------NFD
QAQKRLFLQF-------VTGSPRLP------------IGG-----FK-ALNPIF--TIVR
KDPEFPLTSD------------------------DYLPSVMTCR-NY-FKLPQY-SSREV
LKMRLL-QAIEE-G---
>Conidiobolus_coronatus_JXYT01004414.1 .
-QITRDDI-LANAQSIL----------------HSSNYNDA-ILEIQFTG-------EVG
TGRG-PTAEF---FSLS----FQELQMIKY--------DLWRVDSSLDSNS---------
---------EYVYNQKGVFPRPLQ---------------------------------FNS
NNPNHSKRYQQMKLLGGLVAKALADNRIIDLPLNQ--HFIRSVFN------------TFN
SSQVSFPSLDQNLKLLSI----------------------IDPITYQS--------LKHL
VDLSNSHSAS-----------------------------------------QID-DMG--
--LSFEI-------------------------------PG--YNDIE-LDSA--------
QSD---------------------------------------------------------
------------------------------VTSTNVK-RYLEL-VLDWTLNTG-IQWALD
-AFTEG---FNIYSDI--EYMK-IFTDD-EITNLLGS-----------------------
---DQEDWS-----------LNTL---------IESIQTNHG------------------
----------------YTMA----SPII-TQFLETIS--------------------NFD
QTQKRLFLQF-------VTGSPRLP------------IGG-----FK-ALNPTF--TIVR
KDPESPNKSD------------------------DYLPSVMTCR-NY-FKLPQY-SSPEI
LKTRLL-QAIEE-G---
```

>Candida_apicola_LBNK01000007.1 .
-RLSRNRL-LEGAVRML----------------EKSSTSSA-VVEVMFFD-------EAG
VGRG-PTQEF---FSLA----CKELSSPNT--------KMWKETPF--------------
----------------GLFPAPHA------------------------------------
--FPSDEVLRYFSALGMLISRALLDRRTLDLTLHP--AFFEAVVT---------------
-------KEEPSVDRLKR----------------------LDPELAKT--------LETI
LATPPA---EIA-DMG--
--LDFTM-------------------------------PG---SGED-LLPS--------
GSEIP---
------------------------------VTKENYV-DYVKH-VCEYSLGSG-ISSQIE
-AFVKG---FDSTMSI--TNLE-CFMYD-ELAELCGS-----------------------
--SKDQDWS-----------LDAL---------RAAIVPSHG------------------
----------------YHSD----SKPF-TDFLEVLG--------------------NLD
YTDRRLFLMF-------ATGSPNLP------------VGG-----FK-ALTPPF--TVVI
KYPEDESLGYD-----------------------VYLPSVMTCT-NY-FKLPAY-SNKEI
MRLRLR-TAFSE-GAG-
>Starmerella_bombicola_BBSW01000012.1 .
-RLSRDNI-LEGAIRLL----------------ELSSKNPA-VVEVMFYA-------EEG
TGKG-PTQEF---FALV----CKALSSPSL--------KMWKFTQS--------------
----------------GLFPESNL------------------------------------
--NPDPAILRNFKAMGMLVARALLDKRILDFHLNP--AFFEAVLS---------------
-------ETGPSVDKLRR----------------------VDPGLAAS--------MDKL
LDAPPH---ELV-SIG--
--LDFTL-------------------------------PG---TDIE-LLPS--------
GADIE---
------------------------------VTAENVG-DYVHL-ICDYTVGSG-IQSAID
-AFRLG---FSEMIPI--SALK-SFMLD-ELTTLCGA-----------------------
--DSPEDWS-----------ISTL---------RDSFTADHG------------------
----------------YHIS----SEPV-CRFLNILS--------------------SFD
STERRRFLIF-------ATGSPHLP------------VGG-----FK-ALSPPF--TVVV
KHPDNEELDAD-----------------------QYLPSVMTCA-NY-FKLPAY-SSEKV
MRRQLT-KAISE-GSG-
>Naumovozyma_dairenensis_JUTY01004661.1 .
VRISRKMM-LQSAVKVL----------------GMYGSTPG-ILEIEYFD-------EEG
SGLG-PTLEF---YSTV----SKEFSKKKL--------RLWRDEEPRQAS----------
DVDVDVDDESYVVNKYGLFPKPMD--------------------------------KTQL
SSENGRKVLYFFSSLGKFIARALLDSRIIDFNFNP--VFLLLIQLLNK---TNGINSTSR
TNTLKLSKKMATISNLRL----------------------VDPTLADS--------LQHL
NKYIELF-----------------------------ESNSDIHNVTVDG-ARVE-DLA--
--LFFEL-------------------------------PG--NPDYE-LIPN--------
GSDTL---
------------------------------VTADNLE-LYINK-VIEATLFSG-ILTQTK
-AFMDG---FSKVFPI--NSLI-IFSSR-ELVELFGN-----------------------
---AEEDWS-----------MDTL---------TSSIVANHG------------------
----------------YTKE----SEAI-KSLIDILM--------------------NFS
IEEKREFLQF-------LTGAPKLP------------IGG-----FK-ALRPEL--TVVR
KHAEDGLKDD------------------------DYLPSVMTCA-NY-LKLPNY-SSKEM
MKEKLI-QAMKE-GAGA
>Candida_africana_GEVV02006702.1 .
VRISRKMM-LQSAVKVL----------------GMYGSTPG-ILEIEYFD-------EEG
SGLG-PTLEF---YSTV----SKEFSKKKL--------RLWRDEEPRQAS----------
DVDVDV-DESYVVNKYGLFPKPMD--------------------------------KTQL
SSENGRKVLYFFSSLGKFIARALLDSRIIDFNFNP--VFLLLIQLLNK---TNGINSTSR
TNTLKLSKKMATISNLRL----------------------VDPTLADS--------LQHL
NKYIELF-----------------------------ESNSDIHNVTVDG-ARVE-DLA--
--LFFEL-------------------------------PG--NPDYE-LIPN--------
GSDTL---
------------------------------VTADNLE-LYINK-VIEATLFSG-ILTQTK

-AFMDG---FSKVFPI--NSLI-IFSSR-ELVELFGN-----------------------
---AEEDWS-----------MDTL---------TSSIVANHG------------------
----------------YTKE----SEAI-KSLIDILM--------------------NFS
IEEKREFLQF-------LTGAPKLP------------IGG-----FK-ALRPEL--TVVR
KHAEDGLKDD------------------------DYLPSVMTCA-NY-LKLPNY-SSKEM
MKEKLI-QAMKE-GAGA
>Candida_albicans_XM_712835.1 .
VRISRKMM-LQSAVKVL----------------GMYGSTPG-ILEIEYFD-------EEG
SGLG-PTLEF---YSTV----SKEFSKKKL--------RLWRDEEPRQAS----------
DVDVDV-DESYVVNKYGLFPKPMD--------------------------------KTQL
SSENGRKVLYFFSSLGKFIARALLDSRIIDFNFNP--VFLLLIQLLNK---TNGINSTSR
TNTSKLSKKMATISNLRL----------------------VDPTLADS--------LQHL
NKYIELF-----------------------------ESNSDIHNVTVDG-ARVE-DLA--
--LFFEL-------------------------------PG--NPDYE-LIPN--------
GSDTL---
------------------------------VTADNLE-LYINK-VIEATLFSG-ILTQTK
-AFMDG---FSKVFPI--NSLI-IFSSR-ELVELFGN-----------------------
---AEEDWS-----------MDTL---------TSSIVANHG------------------
----------------YTKE----SEAI-KSLIDILM--------------------NFS
IEEKREFLQF-------LTGAPKLP------------IGG-----FK-ALRPEL--TVVR
KHAEDGLKDD------------------------DYLPSVMTCA-NY-LKLPNY-SSKEM
MKEKLI-QAMKE-GAGA
>Candida_dubliniensis_XM_002421667.1 .
VRISRKMM-LQSAVKVL----------------GMYGSTPG-ILEIEYFD-------EEG
SGLG-PTLEF---YSTV----SKEFSKKKL--------RLWRDDEPRQTV----------
SID----DESYVVNKCGLFPRPMD--------------------------------KAQL
SSENGRKVLYFYSSLGKFIARALLDSRIIDFNFNP--VFLLLIQLLNK---TSGISYTSK
TSTSKLSKKMATISNLRL----------------------VDPTLADS--------LEHL
NKYIQLF-----------------------------EFNSDIHNVTVDG-ASVE-DLA--
--LFFEL-------------------------------PG--NPDYE-LIPN--------
GSDTL---
------------------------------VTADNLE-LYINK-VIEATLFSG-ILTQTK
-AFMDG---FSKVFPI--NSLI-IFSSR-ELVGLFGN-----------------------
---AEEDWT-----------MDTL---------TSSIAANHG------------------
----------------YTKD----SEAI-KSLIDILM--------------------NFN
IEEKREFLQF-------LTGAPKLP------------IGG-----FK-ALRPEL--TVVR
KHAEDGLKDD------------------------DYLPSVMTCA-NY-LKLPNY-SSKEV
MKEKLI-QAMKE-GAGA
>Candida_maltosa_AOGT01000880.1 .
VRISRKMM-LQSAVKVL----------------GMYGSTPG-ILEIEYFD-------EVG
SGLG-PTLEF---YSTV----SKEFSKKKL--------KLWRDEDPGMIN----------
-------EDSYVINKQGLFPVPMD--------------------------------KAQI
SSENGRKVLYFFSSLGKFIARALLDSRIIDFNFNT--VFLLLIQLLNK---NNG--ATSK
SIHQKLLKKVATLQNLRL----------------------VDPGLADS--------LEHL
NKYLTLF-----------------------------KDAEDINKVTVDG-ATVH-DLA--
--LFFEV-------------------------------PG--NADYE-LIPN--------
GSETQ---
------------------------------VTPDNLE-LYINK-VIEASLFSG-IINQTK
-AFMDG---FSKVFPI--NSLI-IFSPP-ELVELFGN-----------------------
---SEEDWS-----------HDTL---------AAGIIANHG------------------
----------------YTKD----SPAI-KSLINILI--------------------NFS
KDEKRDFLQF-------LTGASKLP------------IGG-----FK-ALRPEL--TVVR
KHAEDGLNDD------------------------DYLPSVMTCA-NY-LKLPNY-SSENV
MKERLL-KAIRD-GAGA
>Candida_sojae_LMTL01000128.1 .
VRISRKMM-LQSAVKVL----------------GMYGSTPS-ILEIEYFD-------EVG
TGLG-PTLEF---YSTV----SKEFSKKKL--------RLWRDEEPGATD----------
-------DDAYVINKQGLFPMPMD--------------------------------KSQV

SSENGRKVLYFFASLGKFIARALLDSRIIDFNFNA--VFLLLIQLLNK---GNGSGNSKN
HHHQKFLKKMANLNKLRL----------------------VDSALADS--------LEHL
NKYVVQF-----------------------------KSSDDIHQVRVDG-ATVQ-DLA--
--LFFGL-------------------------------PG--NPDYQ-LIPN--------
GGDIQ---
------------------------------VTADNLE-LYISK-VIEATLYNG-IINQTK
-AFMEG---FSKVFPI--NALI-IFSPP-ELVELFGN-----------------------
---AEEDWS-----------YDSL---------TSGIIANHG------------------
----------------YTKD----SPAI-KSLINILV--------------------NFD
KDEKREFLQF-------LTGAPKLP------------IGG-----FK-ALRPEL--TVVR
KHAEDGLKDD------------------------DYLPSVMTCA-NY-LKLPNY-SSEKL
MKEKLL-KAIRD-GAGA
>Candida_tropicalis_XM_002546136.1 .
VRISRKMM-LQSAVKVL----------------GMYGSTPS-ILEIEYFD-------EVG
TGLG-PTLEF---YSTV----SKEFSKKKL--------KLWRDEEPGAAD----------
-------DNAYVVNKQGLFPMPMD--------------------------------KSQI
ASENGRKVLYFFASLGKFIARALLDSRIIDFNFNL--VFLLLIQMLNK---NYG--GSSK
SSQQKLLKSMANLNKLRV----------------------VDPGLADS--------LEHL
YKYVKQF-----------------------------KNCEDIHQVTVDG-ATVQ-DLA--
--LFFEL-------------------------------PG--NPDYE-LIPN--------
GGNTP---
------------------------------VTAENLE-LYLNK-VLEATLYSG-IINQTK
-AFMEG---FSKVFPI--NSLI-IFSPP-ELVELFGN-----------------------
---AEEDWS-----------YDSL---------TSGIIANHG------------------
----------------YTKE----SPAI-KSLINILV--------------------NFD
KDEKREFLQF-------LTGAPKLP------------IGG-----FK-ALRPEL--TVVR
KHAEDGLKDD------------------------DYLPSVMTCA-NY-LKLPNY-SSEKV
MKDKLL-KAIRD-GAGA
>Spathaspora_arborariae_AYLH01000100.1 .
VRISRQMM-LQSAVKVL----------------GLYGSTPG-ILEIEYFD-------EVG
SGLG-PTLEF---YSTV----LKEFCKGKL--------RLWRDEGDG-------------
---------EFVEYKLGLFPVALD--------------------------------KNQL
NSENGKKVVYFFSILGKFIARALLDSRIIDFNFNP--IFLKLIQLLNV---------MTN
KLPLKTIKKFTTLNSLKL----------------------VDRNLGDS--------IEHL
QKYINQF---------------------------PNVKPEHRDDIIING-STIK-DLC--
--LHFEL-------------------------------PG--NPEYE-LIPN--------
GSDIP---
------------------------------ITSSNLE-QYINK-VIEATLYTG-IIHQTK
-AFMDG---FSKVFPI--TSLI-IFSPQ-ELVELFGN-----------------------
---AEEDWS-----------FEAI---------SSSINANHG------------------
----------------YTKQ----LTSI-THLTNILV--------------------NFS
LEEKRKFLQF-------ITGAPKLP------------IGG-----FK-ELTPNL--TVVR
KHAEDGLTDD------------------------DYLPSVMTCA-NY-LKLPNY-SSEKI
MKEKLL-QAIKE-GADA
>Spathaspora_girioi_LQMS01002368.1 .
VRISREMM-LASAVKVL----------------NLYGSIPG-ILEIEYFD-------EVG
SGLG-PTLEF---YSTV----SKEFARKKL--------KLWRDDESESDG----------
--------EGFVENKLGFFPVPLD--------------------------------KHQL
GSENGKKILYFFSILGKFIARALLDSRIIDFQFNP--VFLKLIQLLNQ---------VTT
KLSSKLIKKFTSLTNLKL----------------------VDRELADS--------IERL
QKYTTNT--DP-TVIK-ELC--
--LHFVL-------------------------------PG--NPDYE-LTPN--------
GHDIP---
------------------------------ITRENLQ-SYLNK-IIETTLYTG-IIHQTK
-AFMDG---FSTVFPI--NSLI-IFQPY-ELAELFGN-----------------------
---SPEDWS-----------PAII---------SSSINANHG------------------
----------------YTKD----SPTI-KSLINILI--------------------NLS
PLEKRQFLQF-------LTGAPKLP------------IGG-----FK-CLNPNL--TVVR

KHAEDGLSDD------------------------DYLPSVMTCA-NY-LKLPNY-SSEAV
MKKKLI-QAINE-GAEA
>Spathaspora_passalidarum_XM_007374033.1 .
VRIARQMM-LQSAVKVL----------------GLYGSTPG-ILEIEYFD-------EVG
SGLG-PTLEF---YSTV----SKEFGKRKL--------KMWRDHGDPSDS----------
--------EGYVDNKYGLFPVPMD--------------------------------KLGI
ASENGKKMVYFFSILGKFIARALLDSRIIDFNFNP--VFLKLVQLLNL-------LGNAQ
KLNAKSIKKFTTMANLRI----------------------VDPALADS--------VEHL
VKYIKQF---------------------------SDVKPEHRDGITVDG-CTIK-DLS--
--IYFEL-------------------------------PG--NPEYE-LIPN--------
GGETQ---
------------------------------VTADNLE-LYVSK-ILEATLYTG-IIHQIK
-AFMEG---FSKVFPI--NSLV-IFSPQ-ELVELFGN-----------------------
---AEEDWS-----------FDAL---------TSSINANHG------------------
----------------YSKE----SQSI-KSLTNILV--------------------NFN
HDEQRAFLQF-------LTGAPKLP------------IGG-----FK-ALRPPL--TVVR
KHAEDDLKDD------------------------DYLPSVMTCA-NY-LKLPNY-SSEDV
MREKLL-QAIRE-GAGA
>Spathaspora_xylofermentans_NDXA01000025.1 .
VRISRKVM-LQSALKVL----------------GMYGSTPG-ILEIEYFD-------EVG
SGLG-PTLEF---YSTV----SKEFSKKKL--------KLWRDQDADTLD----------
-------GESYVVHKQGLFPMPMD--------------------------------KNAV
ASENGKKVLYLFSSLGKFIARSLLDSRIIDFNFNP--VFLKLVQLLNQ---------FAN
KSSTKLVRKMATVSNLRI----------------------VDTEWADS--------IEHL
YKYVNQF---------------------------NNVPVEQRDSIKVEG-ATIK-DLE--
--IYFEL-------------------------------PG--APEYE-LIPN--------
GNELQ---
------------------------------VTALNLE-QYINK-VLEAVLYTG-IIHQTK
-ALMDG---FSKVFPI--NSLI-IFSPQ-ELVELFGN-----------------------
---AEEDWS-----------YDTL---------SSAMIANHG------------------
----------------YTKE----SPSI-KSLIEILY--------------------EFN
IDERRAFLQF-------LTGAPKLP------------IGG-----FK-ALTPEL--TVVR
KHAEDGLKDD------------------------DYLPSVMTCA-NY-LKLPNY-SSKEV
MRERLI-QAIRE-GADA
>Spathaspora_gorwiae_LQMZ01002412.1 .
VRISRKMM-LQSAVKVL----------------GMYGSTPG-ILEIEYFD-------EVG
SGLG-PTLEF---YSTV----SKEFSKRKL--------RLWRDDDDESQD----------
-DDAKDKEEAYIVNKQGLFPIAMD--------------------------------KSMI
SSENGKKVLYFFSCLGKFIARALLDSRIIDFNFNP--VFLKLVQLLNQ---------YHN
KSSIKILKKIVNMGNLRL----------------------VDSKLTDS--------LEHL
YKYVEIF---------------------------KNIPVEQHDSIMIDG-ATIK-DLA--
--IYFEL-------------------------------PG--NPQYE-LIPN--------
GNEIQ---
------------------------------ITSKNLE-HYINK-IMESILYTG-IIQQTK
-SFMDG---FSKVFPI--NSLI-IFTPQ-ELVELFGN-----------------------
---SQEDWS-----------YDAL---------ANSIIANHG------------------
----------------YTKE----SPTI-KTLIDILI--------------------SFN
HDEKRAFLQF-------LTGAPKLP------------IGG-----FK-SLRPEL--TVVR
KHAEDGLKDD------------------------DYLPSVMTCA-NY-LKLPNY-STKEL
MKEKLL-QAISE-GAGA
>Spathaspora_hagerdaliae_LQHL01001739.1 .
VRISRKMM-LQSAVKVL----------------GMYGSTPG-ILEIEYFD-------EVG
SGLG-PTLEF---YSTV----SKEFCKRKL--------RLWRDEEGENRD----------
NHEEVVEEEAYIVNKQGLFPIAMD--------------------------------KSMI
SSENGKKVLYFFSCLGKFIARALLDSRIIDFNFNP--VFLKLVQLLNQ---------YHN
KSSIKILKKIVNMGNLRL----------------------VDAKLTDS--------LEHL
YKYIELF---------------------------KSVPVEEHDSIIVDG-ATIK-DLA--
--IYFEL-------------------------------PG--NPQYE-LIPQ--------

GNEIQ---
------------------------------ITSKNLE-QYINK-IMESILYTG-IIQQTK
-SFMDG---FSKVFPI--NSLI-IFSPQ-ELVELFGN-----------------------
---SQEDWS-----------YDAL---------ANSIIANHG------------------
----------------YTKE----STTI-KTLIDILI--------------------SFN
HDEKRAFLQF-------LTGAPKLP------------IGG-----FK-SLRPEL--TVVR
KHAEDGLKDD------------------------DYLPSVMTCA-NY-LKLPNY-STKEL
MKEKLL-QAIRE-GAGA
>Candida_metapsilosisCBZN020000090.1 .
VRISRKMM-LQSALKVL----------------GMYGSTPG-ILEIEYFD-------EVG
SGLG-PTLEF---YSTV----SKEFSKRKL--------KLWRDNNKYNGD----------
-------TEGYVDNKQGLFPSPMD--------------------------------KQQL
NNENGKKILYFYSSLGKFIARALLDSRIIDFNFNP--VFLKLVQFLNE---KHSSSQSQQ
LNQKLMMKKIVSLQNLKM----------------------VDPELAKS--------LLHL
QKYVNAF---------------------------DTVAPEQRNLIEIDG-VTIR-DLG--
--IYFEL-------------------------------PG--YPKYE-LIPN--------
GSETL---
------------------------------VEAENLE-LYISK-VFENTLFTG-VIHQTK
-AFMDG---FSKVFPI--TSLV-IFSPQ-ELVELFGN-----------------------
---CEEDWS-----------YDTL---------SSAIIANHG------------------
----------------YSKD----SPEI-KDLIDILI--------------------GLN
LEEKRQFLQF-------LTGSPKLP------------IGG-----FK-ALRPEL--TVVR
KRAEDGLKDD------------------------DYLPSVMTCA-NY-LKIPKY-SSKLV
MKEKLM-QAVKE-GAGA
>Candida_orthopsilosis_XM_021355901.1 .
VRISRKMM-LQSALKVL----------------GMYGSTPG-ILEIEYFD-------EVG
SGLG-PTLEF---YSTV----SKEFSKRKL--------KLWRDDNRYDGD----------
-------AEGYVENKHGLFPSPMD--------------------------------KQQV
NNENGKKILYFFSSLGKFIARALLDSRIIDFNFNP--IFLKLVQFLNQ----KNTSSQSQ
PLNQKLMKKIVSLQNLKL----------------------VDPELAKS--------LQHL
QKYAKVF---------------------------ENVPPEQRNFVEIDG-AKLG-DLG--
--IYFEL-------------------------------PG--YPKYE-LIPN--------
GSDTL---
------------------------------VQADNLD-LYITK-VLENTLFTG-VIHQTK
-AFMDG---FSKVFPI--SSLM-IFSPQ-ELVELFGN-----------------------
---CEEDWS-----------FDAL---------SSAIVANHG------------------
----------------YSKD----SPEI-KELINILI--------------------DFD
LEEKRQFLQF-------LTGSPKLP------------IGG-----FK-ALRPEL--TVVR
KHAEDGLKDD------------------------DYLPSVMTCA-NY-LKIPKY-SSKEV
MREKLL-QAVKE-GAGA
>Candida_parapsilosis_HE605205.1_a.
VRISRKMM-LQSALKVL----------------GMYGSTPG-ILEIEYFD-------EVG
SGLG-PTLEF---YSTV----SKEFSKRKL--------KLWRDEDRYDGD----------
-------AEGYVENKHGLFPSPMD--------------------------------KQQV
NNENGKKILYFFSSLGKFIARALLDSRIIDFNFNP--VFLKLVQFFNQ----KNSSSQLQ
PMNQKLMKKIVSLQNLRM----------------------VDPELAKS--------LLHL
QKYARGF---------------------------ETVAPEQRDFVEIDG-AKLG-DLG--
--IYFEL-------------------------------PG--YPKYE-LIPN--------
GSDTL---
------------------------------VQAGNLD-LYITK-VLEHTLFTG-VVHQAK
-AFMDG---FSKVFPI--TSLV-IFSPQ-ELVELFGN-----------------------
---CEEDWS-----------YDAL---------SSAIVANHG------------------
----------------YSKD----SPEI-KDLIQILI--------------------DFN
LEEKRQFLQF-------LTGSPKLP------------IGG-----FK-ALRPEL--TVVR
KHAEDGLKDD------------------------DYLPSVMTCA-NY-LKIPKY-SSRDV
MKEKLL-QAVKE-GAG-
>Priceomyces_haplophilus_BCIF01000006.1 .
IRLSRKLI-LQSALKVL----------------SMYGSTPG-VLEVEYFD-------EVG

SGLG-PTLEF---YATV----SKEFSRKRL--------RLWRDSDFSSGD----------
-------EEGFVVCNSGLFPAPLD--------------------------------KTQI
TSENGRKVLYFFNSLGKFIARALLDSRIVDFHFNP--SFLKMVQSLNL--------NGHH
KSSARDIKSMVSLSCLRL----------------------IDAELANS--------IEHL
LKYVKQF---------------------------PEVEEDKRKLVEVDG-VTLH-ELS--
--LTFVL-------------------------------PG--YPSYE-LIPN--------
GEETL---
------------------------------VDDRNLE-QYITE-VMEATLYTG-IIHQAK
-AFSEG---FSKVFPF--NSLA-IFSPD-ELVRIFGR-----------------------
---TEEDWS-----------MKTL---------NAVIKANHG------------------
----------------YSKD----SEAI-KRLINILS--------------------SFN
EEDKSSFLQF-------LTGATKLP------------IGG-----FK-ALRPEL--TVVR
KHPEKGFKDD------------------------DYLPSVMTCA-NY-LKLPNY-SSEAL
MKERIL-QAVRE-GAGA
>Sugiyamaella_xylanicola_MQSX01000052.1 .
VRLSRKLI-LQSAIKVI----------------SMYGSTPG-ILEIEYFE-------EVG
SGLG-PTLEF---YSMV----SKEFSKKKL--------KLWRSEHSIDEN----------
-------EDDYVDSSTGLFPIPMD--------------------------------KHNL
SSENGKKILQLFASLGKFIARAMIDSRIIDFNFNL--MFIMMIQLLNS-----------N
KRSLKDVKKIANISCLRI----------------------VDPHLASS--------VEHL
LKYLKEF---------------------------PNFNENERNKITVDG-ATLE-QLS--
--LTFTL-------------------------------PG--YPEYE-LIPH--------
GDEVM---
------------------------------VDASNVE-LYINK-VLDATLYSG-IINQTN
-AFMEG---FSKVFPV--ESLT-IFSPE-EVLNLWGS-----------------------
---AEEDWS-----------IDTL---------TSCIHANHG------------------
----------------YTKE----SDIV-KGLVNVLA--------------------SFG
NSERRLFLQF-------LTGAPRLP------------IGG-----FK-ALKPEF--TVVR
KQPESGMKDD------------------------DYLPSVMTCA-NY-LKLPNY-SSEDV
MRRRLL-QAMND-GAGA
>Candida_homilentoma_BCGB01000002.1 .
VRLSRKLI-LQSAVKVL----------------QLYGSTPG-VLEIEYFD-------EVG
SGLG-PTLEF---YSAV----SHEFAKKKL--------KLWRDEGLDG------------
---------EYVHYRPGLFPMPID--------------------------------KLDL
MCENARKVLYFFATLGKFVARALLDLRIIDFNFNP--VFLRLVQFFNE----------HQ
KPSNKDLKKVVNLTTLRA----------------------VDPLLADG--------MAHL
QKYVEAF---------------------------KTVPSEERHLVQIDG-CTLE-DLS--
--LTFTL-------------------------------PG--YDYYE-LIAD--------
GSDVA---
------------------------------ITPDNLE-LYVEK-VLDATLYSG-IVNQTK
-AFMEG---FLKVFPI--VSLV-IFSPQ-ELVEMFGS-----------------------
---AEEDWS-----------EATL---------TSAVRANHG------------------
----------------YTKE----SEAI-GRLIRILIS--------------------FN
EIEKRAFLQF-------LTGAPKLP------------VGG-----FK-SLRPEL--TVVR
KLAEDGLVDD------------------------DYLPSVMTCA-NY-LKLPNY-SSEEV
MRKKLL-QAVYE-GAGA
>Hyphopichia_burtonii_XM_020221781.1 .
VRLSRKSI-LQSAVKVL----------------QLYGSTPG-VLEIEYFD-------EVG
SGLG-PTLEF---YATV----SREFCKKKL--------KLWRDNSDDNQE----------
------DLESYVQSNTGLFPYPLD--------------------------------KNQV
FSENGRKMIYFFATLGKFIARALLDSRIIDFNFNP--VFLKLVQFFNQ----------GA
KPTNRDLKKLTSLNSLRL----------------------VDSTLANS--------LEHL
IKYIEAY---------------------------KDVPIDQRSAVVIDD-CTLD-DLS--
--LTFTL-------------------------------PG--YPDYE-LIPN--------
GDEIF---
------------------------------INHENVE-NYVNK-VLEATLYSG-IVHQTK
-AFMEG---FSKVFPI--NSLI-IFSPQ-ELVELLGS-----------------------
---AEEDWS-----------LSTI---------VNSVHANHG------------------

----------------YTKE----SEAI-KRLINILVN--------------------FN
DIEKRSFLQF-------LTGSPKLP------------VGG-----FK-SLRPEF--TVVR
KLAEPGSSDD------------------------DYLPSVMTCA-NY-LKLPNY-SSEEI
MKAKLV-QAVNE-GAGA
>Debaryomyces_hansenii_XM_460177.1 .
VRLSRNLI-LQSAVKVL----------------GLYGSSPG-ILEIEYFD-------EVG
SGLG-PTLEF---YATV----SKEFSKKKL--------RLWRDYDLANNN----------
-------EDDYIFSKTGLFPAPLD--------------------------------KSQA
SSENGRKVLYFFSNLGKFIARALLDSRIIDFNFNP--VFLKFVQFFNQ--------NGIQ
KAARRDVKKLANMATLRL----------------------VDPELADS--------MEHL
LKYAEQF---------------------------STVEEHDRDNIKIDD-CTIN-DLS--
--LSFIV-------------------------------PG--YPKYE-LIPN--------
GDDTP---
------------------------------VTSSNLE-TYINK-VLEATLYSG-IVHQTK
-AFMDG---FSKVFPI--TSLV-IFSPE-ELVELFGS-----------------------
---AEEDWS-----------IETL---------PSAVNANHG------------------
----------------YTKE----SESI-TRLINILV--------------------GFN
DIEKRAFLQF-------LTGAPKLP------------IGG-----FK-ALRPVF--TVVR
KQADSGLKDD------------------------DYLPSVMTCA-NY-LKLPNY-SSESI
MREKLL-QAVNE-GAGA
>Debaryomyces_fabryi_XM_015609934.1 .
VRLSRNLM-LQSAVKVL----------------GLYGSSPG-ILEIEYFD-------EVG
SGLG-PTLEF---YATV----SKEFSKKKL--------KLWRDYGLADSD----------
-------EEDYIFSKTGLFPAPLD--------------------------------KSQV
SSENGRKVLYFFSNLGKFIARALLDSRIIDFNFNP--VFLKFVQFFNK--------NGIQ
KAARRNIKKLANMATLRL----------------------VDPELADS--------MLHL
LKYVERF---------------------------STVEEHERDNIKVDD-CTIN-DLA--
--LSFVV-------------------------------PG--YPQYE-LIPN--------
GEDTP---
------------------------------VTSSNLE-TYINK-VLEATLYSG-IVHQTK
-AFMDG---FSKVFPI--NSLI-IFSPE-ELVGLFGS-----------------------
---AEEDWS-----------IETL---------PAAINANHG------------------
----------------YNKE----SEAI-TRLINILV--------------------GFN
DIEKRAFLQF-------LTGAPKLP------------IGG-----FK-ALRPAF--TVVR
KQAENGLKDD------------------------DYLPSVMTCA-NY-LKLPNY-SSESV
MREKLL-QAVNE-GAGA
>Wickerhamia_fluorescens_BCGE01000004.1 .
VRLSRKLI-LQSAIKVL----------------GMYGSTPG-ILEIEYFD-------EVG
SGLG-PTLEF---YASV----SKEFTKKKL--------KLWRDDDTFEND----------
-------GEAYITNKKGLYPTPLD--------------------------------KNQL
QSENGRKVLFFFSTLGKFIARALLDSRIIDFNFNP--VFLKLVQQLNQ--------LESQ
KQVPRDFKKIANLPSLRN----------------------VDPILADS--------MEHL
LKYVKQF---------------------------PQIAESEWDKIKVKG-STIR-DMS--
--LYFEL-------------------------------PG--HPEYE-LIPG--------
GSEIQ---
------------------------------ICASNLE-AYINR-VLEVSLYSG-VVHQTK
-AFMEG---FSKVFPI--SSLI-VFSPQ-ELVELFGN-----------------------
---AEEDWS-----------YDTL---------KSSISANHG------------------
----------------YTKE----SDII-KSLVRCLT--------------------KFS
HSQKRLFLQF-------LTGSPKLP------------IGG-----FK-SLRPNF--TVVR
KYAELNLTDD------------------------DYLPSVMTCV-SY-LKLPKY-SSENI
MQEKLI-QAMNE-GAGA
>Candida_tanzawaensis_XM_020211719.1 .
VRLSRKMI-LQSAVKVL----------------GLYGSTPG-ILEIEYFD-------EVG
SGLG-PTLEF---YSTV----SKEFVRKKL--------KLWRDDDLGNMD----------
-------EEAFIWNEHGLFPAPLD--------------------------------KHQV
NSENGRKVLYFFSSLGKFIARALLDSRIIDFNFST--VFLKLVQYFNY----------NE
KPGNRDLKKLANIPSLKL----------------------VDPKLASS--------IEHL

LKYLKQY---------------------------PRFGAVERDSIEIEG-ATLK-DLS--
--IFFEL-------------------------------PG--YPEYE-LIPN--------
GSEIQ---
------------------------------VDAANLE-NYINK-VLEATLYTG-IVQQTK
-AFMDG---FSKVFPI--SSLV-LFSPQ-ELVELFGN-----------------------
---AEEDWS-----------LDTL---------TSAINANHG------------------
----------------FTKD----SDAI-KSLINILV--------------------SFT
NEEKRLFLQF-------LTGAPKLP------------IGG-----FK-ALRPEL--TVVR
KRAEDNLQDD------------------------DYLPSVMTCA-NY-LKLPNY-SSEDI
MREKLI-QAIHE-GAGA
>Scheffersomyces_stipitis_XM_001385835.1 .
VRISRKFI-LQSAIKVL----------------GLYGSTPR-ILEIEYFD-------EVG
SGLG-PTLEF---YATV----SKEFSKKKL--------KLWRDNDPLEVD----------
-------DEAYVSNALGLFPSPLD--------------------------------KHQI
STENGRKVLSFFSSLGKFVARALLDSRIVDFNFNP--VFLKLVQYFNL--------HGVQ
RINHRNLKKMTNISSLRM----------------------VDPALADS--------VEHL
LKYVKQF---------------------------PQVGEIERDSIVIDG-STIK-DLS--
--LYFEL-------------------------------PG--YPEFE-LIAG--------
GSEIQ---
------------------------------VTATNLE-LYINR-ILESTLFTG-IVHQAK
-SFMDG---FSNVFPV--NTLI-IFSSE-ELAGLFGG-----------------------
---AEEDWS-----------IDTI---------TSAVYANHG------------------
----------------FTKE----SDAI-KSLITILV--------------------QFN
EEERRAFLQF-------LTGAPKLP------------IGG-----FK-ALRPEL--TVVR
KHAEEGLKDD------------------------DYLPSVMTCA-NY-LKLPNY-SSEDL
MRKRLL-QAINE-GAGA
>Scheffersomyces_lignosus_BCGS01000005.1 .
VRISRKMI-LQSAVKVL----------------GLYGSTPG-TLEIEYFD-------EVG
SGLG-PTLEF---YATV----SKEFSKKKL--------KLWRDNDSSNTE----------
-------EEAYVSNALGFFPSPLD--------------------------------KNQI
ANENGRKVLYFYSSLGKFIARALLDSRIVDFNFNT--VFLKLVQYFNH--------NGPQ
KTSHRNLKKMANIASLRL----------------------VDPELADS--------LEHL
YKYVKQF---------------------------PQVEESQRDTIVVDG-ATIK-DLS--
--LYFEL-------------------------------PG--YPDFE-LFAN--------
GSEIQ---
------------------------------VDASNLE-LYITR-ILESTLYTG-IVHQTK
-AFMDG---FSKVFPV--NSLI-IFSPE-ELAGLFGG-----------------------
---AEEDWS-----------VDTL---------TSAISANHG------------------
----------------YTKE----SDAI-KSLIKILV--------------------EFT
DTERRTFLQF-------LTGAPKLP------------IGG-----FK-ALRPEL--TVVR
KHAEGNLKDD------------------------DYLPSVMTCA-NY-LKLPEY-SSEQL
MRQRLL-QAINE-GAGA
>Scheffersomyces_shehatae_BDMP01000005.1 .
VRISRKMI-LQSAVKVL----------------GLYGSTPG-TLEIEYFD-------EVG
SGLG-PTLEF---YATV----SKEFSKKKL--------KLWRDNDSSNTE----------
-------EEAYVSNALGFFPSPLD--------------------------------KHQI
ANENGRKVLYFYSSLGKFIARALLDSRIVDFNFNT--VFLKLVQYFNH--------NGAQ
KTSHRNLKKMANIASLRL----------------------VDPELADS--------LEHL
YKYVKQF---------------------------PQVEESQRDSIVVDG-ATIK-DLS--
--LYFEL-------------------------------PG--YPDFE-LFAN--------
GSEIQ---
------------------------------VDASNLE-LYITR-ILESTLYTG-IVHQTK
-AFMDG---FSKVFPV--NSLI-IFSPE-ELAGLFGG-----------------------
---AEEDWS-----------VDTL---------TSAISANHG------------------
----------------YTKE----SDAI-KSLIKILV--------------------EFT
DTERRAFLQF-------LTGAPKLP------------IGG-----FK-ALRPEL--TVVR
KHAEGDLKDD------------------------DYLPSVMTCA-NY-LKLPEY-SSEQL
MRQRLL-QAINE-GAGA

>Lodderomyces_elongisporus_XM_001527597.1 .
VRISRNMM-LQSAIKVL-----------------ELYGSTPG-ILEIEYFD-------EAG
SGLG-PTLEF---YATV----SKEFIRKKL--------KLWRDQSLSNFG----------
SKKGLELNDPYIINSNGLFPAPMD---------------------------------KHQI
LSDNGKKILYFCSSLGKFVARALLDSRIIDFNFNP--IFLKLIQFLNR-------KTLQA
HTTPKQSRKFATLSNLHL----------------------VDPELARS--------LEHL
QKYIKVY----------------------------ERTPELQKRDLRIDD-ATIE-DLS--
--LYFVL--------------------------------PG--YPNYE-LIPN--------
GREVQ---
------------------------------VDHTNLE-AYIHK-VVDATLFTG-VISQIK
-AFMEG---FSKVFPI--NSLI-IFSYK-ELVELFGN-----------------------
---AEEDWS-----------FDAL---------SSAINANHG------------------
----------------YNKE----SEGI-KNLISVLV--------------------EFD
KEEKRQFLQF-------LTGSPRLP------------IGG-----FK-SLRPEL--TVVR
KMAEDGLKDD------------------------DYLPSVMTCA-NY-LKLPNY-SSKDV
MKRKLL-QAISE-GAGA
>Pichia_sorbitophila_FO082047.1 .
VRISRKLM-LQSAVKVL-----------------GMYGSSPG-IFEIEYFD-------EVG
SGLG-PTLEF---YAVV----SREFSRKKL--------KLWRDESDSESS----------
-------SDNYVVSFNGLFPSPLH---------------------------------KTQL
NTENGRKVLFFFQVLGKFVARALLDSRILDLRLNP--LFLRIVQVLSS--------EDSE
KISKEKRKGLCNLSTLKM----------------------VDPKLARS--------IEHL
LKYVDAY----------------------------LKLLPEERESHEVDG-CTLE-DLS--
--LYFVL--------------------------------PG--YPKYE-LIQA--------
GENVQ---
------------------------------VDSKNIE-VYINK-VLDATLFAG-VASQVQ
-AFAEG---FSTVFPI--SSLC-IFSPE-ELAGLFGS-----------------------
---SEEDWS-----------YETL---------LSLMHANHG------------------
----------------YSKD----SDSF-KRLLNILG--------------------SFN
DVERRCFLQF-------LTGSPRLP------------IGG-----FK-ALRPEF--TVVK
KLAENGLKDD------------------------DYLPSVMTCA-NY-LKLPNY-SSQEV
MKVKLL-QAVKE-GADA
>Millerozyma_acaciae_BCKO01000005.1 .
VRISRKLI-LQSAVKVL-----------------SLYGSSPG-ILEIEYFD-------EEG
SGLG-PTLEF---YATV----SKEFAKRNL--------KLWRDYCDSFTA----------
-----EDQDSFVFSPSGLFPAPLD---------------------------------KNQL
SSENGRKVLYFFHTLGKFVARALLDSRILDLNFNL--QFLKIIQSLNQ--------SDLQ
GSEKENTSVLVSMACLKL----------------------VDPELAKS--------LEHL
LKFVEEY----------------------------QRQPEGNREGTTVNG-ATIE-DLS--
--IFFTL--------------------------------PG--YPLYE-LIPN--------
GDNIQ---
------------------------------VCAFNIE-LYINK-VLDATLYSG-VIKQIK
-AFVDG---FSKVFPI--TSLA-IFSSE-ELSGLFGS-----------------------
---NEEDWS-----------FKTL---------YSAIQTNHG------------------
----------------YNKE----SDTF-KRLLNILV--------------------SFN
TTEKRTFLQF-------LTGSPRLP------------IGG-----FK-SLRPEF--TVVR
KVAENGLKDD------------------------DYLPSVMTCA-NY-LKLPNY-SSEAV
MRDKLL-QAIKE-GANA
>Candida_aaseri_LKAN01000038.1 .
IRIPRNLI-LQSAINVM-----------------NLYGASAG-VLEIEYLH-------EVG
TGLG-PTLEF---YSNV----SKEFSKRRL--------KLWRDDEPDGPL----------
--------EGFINHKEGLFPQPMD---------------------------------ESTI
LSENGKKVLFLFSTLGTFIARAMIDSRLIDFKFNP--LFLKIIQIFNE--------HSLN
KQGFKDLKKINNLNSLRI----------------------VDKQLADS--------MEHL
MKYVELM----------------------------KNSPEQERPYIEVDD-LTLE-DLC--
--LTFVL--------------------------------PG--Y-DYE-LIEN--------
GKNIP---
------------------------------ITYKNIE-LYISK-VIEATLYNG-IIPQTK

-ALMEG---FSKVFPI--TSLT-LFSSQ-ELASLFGN-----------------------
---NVEDWS-----------YATL---------ESAIHANHG------------------
----------------FSKE----SPAI-NHLIEILI--------------------DFN
ERERRMFLQF-------LTGAPRLP------------IGG-----FK-SLNPEL--TVVR
KYPEDQLKDD------------------------DYLPSVMTCA-NY-LKLPNY-SSKEM
MKQKLI-KAIEE-GADA
>Yamadazyma_sp._MVNT01000007.1 .
VRISRKLI-LQSALKVL----------------NLYGTSPG-VLEIEYFD-------EVG
SGLG-PTLEF---YSSV----SKEFSKRKL--------KLWRDDDPEGDE----------
--------DDFVNYKTGLFPSPMD--------------------------------VEEI
QTENGKKVLYLFSMLGKFIARTLLDSRIIDFRFNT--LFLKIVQIFNE--------HSLN
KEGFRDLKKVTNLNSLRI----------------------VDPSLADS--------LEHL
MKYVELY---------------------------KDLPEGERDGALVDG-CTLD-DLA--
--LSFTL-------------------------------PG--YPDYK-LVEN--------
GETLS---
------------------------------VTPENIE-TYISK-VIEATLYNG-IIPQTK
-AFMSG---FSKVFPI--SSLI-VFSSE-ELVDLFGN-----------------------
---ASEDWS-----------PETL---------HSSINANHG------------------
----------------YSKD----SDAV-KTLIDILV--------------------HFN
DIERRSFLQF-------LTGAPKLP------------VGG-----FK-ALNPEF--TVVR
KYPESGFKDD------------------------DYLPSVMTCA-NY-LKLPNY-STEAK
MKQKIL-QAISE-GAGA
>Candida_tenuis_XM_006684961.1 .
VRISRKLI-LQSALKVL----------------NLYGTSPG-VLEIEYFD-------EVG
SGLG-PTLEF---YSSV----SKEFSKRKL--------RLWRDDDPDGDE----------
--------EDFVDYKTGLFPSPID--------------------------------MNEI
QTENGKKKLYFFRMLGKFIARTLLDSRIIDFRFNP--LFLKLVQIFNE--------HSMN
KEGFSDLKKINNLNSLRI----------------------VDPSLADS--------LEHL
MKYVELY---------------------------KNVPEHERDLILVDN-CTLE-DLS--
--LSFSL-------------------------------PG--YPSYQ-LIAD--------
GDNIA---
------------------------------ITSKNIE-SYISK-VIEATLCNG-IIPQTK
-AFMNG---FSKVFPI--SSLI-VFSPN-ELVNLFGN-----------------------
---AVEDWS-----------LETL---------HSSINANHG------------------
----------------YSKD----SDAV-RTLIDILV--------------------HFN
DIERRSFLQF-------LTGAPKLP------------VGG-----FK-ALKPEF--TVVR
KYPEGGSKDD------------------------DYLPSVMTCA-NY-LKLPNY-STEEI
MKRKIL-QAISE-GAGA
>Candida_carpophila_BCGK01000006.1 .
VRISRKLI-LQSAIKVL----------------NMYGATPG-ILEIEYFG-------EVG
SGMG-PTLEF---YASV----SNEFARKKL--------KMWRDDSPYDLE----------
--------NEYIVNSEGLFPAPMS--------------------------------SQQL
NSANGRKVLYLFGMLGKFVARALLDSRIVDFHFNP--FFWQIIHG---------------
--NIERKAMKYSEAHLKM----------------------VDARLASS--------VDHL
KHYLPSK-------------------------------GDSQEEIVYDG-CTIS-DLS--
--LTFSL-------------------------------PG--YPDYN-LIPN--------
GAHIA---
------------------------------VDQSNLE-EYISK-LIEATLHEG-VQHQLE
-AFMEG---FSSVFPI--DALT-VFQPC-EIVELLGS-----------------------
---ADEDWS-----------LQTL---------RDATKANHG------------------
----------------YTKD----SKAI-EQLLNIMS--------------------SFN
HGERRAFLQF-------ITGSPKLP------------IGG-----FK-TLKPEF--TVVR
KMPEDNYTSD------------------------DYLPSVMTCA-NY-LKLPEY-SSEAL
MRRRLL-QAVSE-GAGE
>Meyerozyma_guilliermondii_XM_001483068.1 .
VRISRKLI-LHSAIKVL----------------NMYGATPG-ILEIEYFG-------EVG
SGMG-PTLEF---YASV----SNEFSRKKL--------KMWRDDSPSDAE----------
--------NEYITNSEGLFPAPMS--------------------------------SEQL

```
NNANGRKVLYLFGMLGKFVARALLDSRIVDFNFNP--FFWQMIQA---------------
--KIERKAMKYSEAHLKM----------------------VDARLASS--------VDHL
KHYLRSK-------------------------------GDSQEEIAFDG-CTIS-DLS--
--LTFSL-------------------------------PG--YPDYD-LIPN--------
GANIA-------------------------------------------------------
------------------------------VDQSNLE-EYISR-LIEATLHEG-VQHQLE
-AFMEG---FSSVFPI--DALT-VFQPY-EIVELLGS-----------------------
---ADEDWS-----------LQTL---------RGATKANHG------------------
----------------YTKD----SNAI-EQLLNIMS--------------------TFS
SEERRAFLQF-------ITGSPKLP------------IGG-----FK-ALKPEF--TVVR
KMPEDNFSSD------------------------DYLPSVMTCA-NY-LKLPEY-SSEAL
MKRRLL-QAVSE-GAGE
>Meyerozyma_caribbica_BADS01000002.1 .
VRISRKLI-LHSAIKVL----------------NMYGATPG-ILEIEYFG-------EVG
SGMG-PTLEF---YASV----SNEFSRKKL--------KMWRDDSPSDAE----------
--------NEYITNSEGLFPAPMS--------------------------------SEQL
NNANGRKVLYLFGMLGKFVARALLDSRIVDFNFNP--FFWQMIQA---------------
--KIERKAMKYSEAHLKM----------------------VDARLASS--------VDHL
KHYLRSK-------------------------------GDSQEEIAFDG-CTIS-DLS--
--LTFSL-------------------------------PG--YPDYD-LIPN--------
GANIA-------------------------------------------------------
------------------------------VDQSNLE-EYISR-LIEATLHEG-VQHQLE
-AFMEG---FSSVFPI--DALT-VFQPY-EIVELLGS-----------------------
---ADEDWS-----------LQTL---------RGATKANHG------------------
----------------YTKD----SNAI-VQLLNIMS--------------------TFS
SEERRAFLQF-------ITGSPKLP------------IGG-----FK-ALKPEF--TVVR
KMPEDNFSSD------------------------DYLPSVMTCA-NY-LKLPEY-SSEAL
MKRRLL-QAVSE-GAGE
>Clavispora_lusitaniae_XM_002614588.1 .
ARISREQF-FPSAMKVL----------------LVYGTSPE-VLEIEYFN-------EVG
SGLG-PTLEF---YSTT----SREFCRKEL--------HMWRDDPFISDN----------
----DTNGNPYVFNSSGLFPAPLD--------------------------------PRKI
GTENGRKILFLFSQLGKFVARALLDSRIVDFEFNP--LFLCMVRNS--------EMFEGD
LNDSERKYPDKLSSALEL----------------------VDPKLAKS--------LEQL
KSLLSRV-----------------------------------ELKGNHY-DQIE-ELS--
--LCFVL-------------------------------PG--YSNYE-LIPN--------
GANIS-------------------------------------------------------
------------------------------VTSENID-QYIEK-VIDASVFSG-VFEQMK
-SFVKG---FSEVFPI--SALS-IFTTD-ELNEVFGS-----------------------
---SHEDWS-----------RDAL---------SEAIKPNHG------------------
----------------YNHD----SIAI-ERLINVLV--------------------NFS
GKERRKFLQF-------LTGSPKLP------------IGG-----FK-AMRPEF--TVVR
KYAEDNFISD------------------------DYLPSVMTCA-NY-LKLPDY-SLEEI
MHQKLI-DAITE-GANA
>Candida_intermedia_BCGD01000001.1 .
VRISRKHI-LQSALKVL----------------QLYGSSPG-VLEIEYFD-------EVG
SGLG-PTLEF---FSSV----SMEFSKKSL--------YMWIDENPGDFD----------
--------DEFVFSPRGLFPRPMD--------------------------------RQMV
NSENGKKVLFLFSALGTFLARSLIDSRIVDFSFNR--IFLRLMQD---------IDFNPL
SAYSNNPDLSHYLVILGE----------------------VDPQLASS--------LDYL
SHFLSS------------------------------------ETAAIDG-VSIE-ELS--
--LVFVL-------------------------------PG--YQKFE-LVPG--------
GADIA-------------------------------------------------------
------------------------------VTSGNLR-RYIEL-IVEATVYSG-VINQVK
-AFMQG---FSKVFPI--SSLT-IFSPN-ELGDIFGS-----------------------
---SEEDWS-----------IDTL---------VDAMRANHG------------------
----------------YSSD----SKAI-HYLVVVLN--------------------GFS
ETNRRKFLQF-------LTGSPKLP------------IGG-----FK-AIRPEF--TVVK
```

KRPEDGLTSD------------------------DYLPSVMTCA-NY-LKLPDY-SLENV
MREKLL-RAITE-GAGA
>Metschnikowia_fructicola_ANFW02000036.1 .
VRLARDKI-FKSAIKIL----------------QQFGLQPP-ILEMEYFD-------EVG
SGLG-PTLEF---YASV----SKAFCKTEL--------GMWRHNSIDE------------
---------EYADSSQGLFPAPIN---------------------------------AQK
LDPRNKNILFYFRMLGVFVARALLDSRIMDFNFNP--LFISLIQD----------PRSLD
DILGEKAEITTQLELLRG----------------------VDADLARS--------LKHL
TKYLDVYC-----------------------------GNSQDKLVEIDG-MQLS-DLS--
--LYFNV-------------------------------PG--HEKLN-LVQD--------
GGNIE---
------------------------------VTSENLE-VYVRK-STEFLLVSG-VAKQVT
-AFREG---FSSVFPI--ESLN-IFTPQ-ELREIFGS-----------------------
---GEEDWS-----------RETI---------SDSIKANHG------------------
----------------YTQD----SKAI-VMLVNVLQ--------------------DLN
LSERREFLQF-------LTGSPRLP------------IGG-----FK-AMRPEF--TVVR
KHPDAGLSSD------------------------DYLPSVMTCA-NY-LKLPDY-ALETL
LRSKLL-HAIRE-GAGA
>Metschnikowia_australis_MVNQ01000021.1 .
VRLSRDKI-FKGAIKVL----------------QQYGLLPT-ILEIEYYD-------EVG
SGLG-PTLEF---YASV----SKAFCKKES--------HMWRQSENDN------------
---------VYAASTQGLFPSPIN---------------------------------NAG
NDESERNVLLLFRMLGVFVSRALLDSRIIDFNFNT--LFISLIQD----------PHFLD
DVFTNKKDVSELIEVLRD----------------------VDLEYARG--------MQYL
TKYLCAFK-----------------------------GTPNDNLVEFDG-TKLS-DLS--
--LYFTV-------------------------------PG--YADID-LVED--------
GSNIA---
------------------------------VTPQNLE-SYIRK-VTKHLLLGG-IGKQVQ
-SFREG---FSSVFPI--ESLS-IFTAK-ELKEIFGS-----------------------
---GEEDWS-----------RETI---------SKSIKANHG------------------
----------------FSQD----SKAI-CMLVNVLQ--------------------DLD
LSARREFLQF-------LTGSPRLP------------IGG-----FK-AMRPEF--TVVR
KYPDAGLSGD------------------------VYLPSVMTCA-NY-LKLPDY-TLEQL
LKTKLL-HAIRE-GAGA
>Metschnikowia_bicuspidata_XM_018856960.1 .
VRLSRDTI-FKGAIKVL----------------QQYGLLPT-ILEIEYYD-------EVG
SGLG-PTLEF---YASV----SKAFCKKEL--------LMWRHSEIDG------------
---------VYAASTQGLFPSPIS---------------------------------VMG
NDEKERKVLFLFNMLGVFVARALLDSRIIDFNFNT--LFISLIQD----------PHFLD
DIFMNKKDVSEMIEVLRD----------------------VDLDYARG--------MQYL
TKFLYDFR-----------------------------GTPDDDRVEFDG-TELS-DLS--
--LYFTV-------------------------------PG--YADIE-LVEN--------
GSNIA---
------------------------------VTPQNLE-SYIRK-VTEHLLLGG-IGKQVQ
-SFREG---FTSVFPI--ESLS-IFTAK-ELKEIFGS-----------------------
---GEEDWS-----------RQTI---------STSIKANHG------------------
----------------FSQD----SRAI-NMLVNVLQ--------------------ELD
LSERRDFLQF-------LTGSPRLP------------IGG-----FK-AMRPEF--TVVR
KHPDAGLSGD------------------------DYLPSVMTCA-NY-LKLPDY-SLEHL
LKTKLL-HAIRE-GAGA
>Candida_auris_XM_018314231.1 .
VRISRSIL-FQSALKVL----------------EKFGSSPG-ILEIEYYN-------EVG
SGLG-PTLEF---YSMV----SREFQRSNL--------HLWRNLDSYESC----------
-----DSMATYVNSKMGLFPQVMH--------------------------------QLPS
KRKYNSKILHLFFCLGKFLARSLFDSRIVDIELHP--LTFTILRL-------------LE
KGLMHEWNEHLTLETLSS----------------------VDESLARS--------LQSL
LDIASCHQ------------------------------------ASQEK-DTIQ-SLE--
--LFFTL-------------------------------PG--MPEYE-LILN--------

GANTR---
------------------------------VTHENIT-EYVDA-ILAAILKDG-VIRQIE
-KLAEG---FSTVFPM--KSLN-VFSPE-ELCKVFGS-----------------------
---GEEDWA-----------EATI---------LGAIQANHG------------------
----------------YTKS----SASV-IRLAKILS--------------------SFD
QEHRRKFLQF-------LTGSPRLP------------IGG-----FK-SLRPEF--TVVK
KNPDEDLGSD------------------------NYLPSVMTCA-LY-LKLPDY-SLIDI
MRSKLI-RAITE-GADS
>Wickerhamomyces_anomalus_XM_019183795.1 .
VRVPRNQL-LQSSFKVL----------------DKLGSHPS-ILEVEFTN-------EVG
TGLG-PTLEF---YALV----SKLYCQKSL--------GIWRQHDEIDGD----------
---------DYVNNKNGLFPSPIV------------------------------------
--SDGKERLSSFKYLGKFVARALLDNRIVDFYFNK--VFFQIAQS---------------
--IINKEPIIKDLEVLKL----------------------VDEGLYNS--------LIYL
QKNK--DLVE-GLE--
--INFTL-------------------------------PG--FPSIE-LIEN--------
GSDIN---
------------------------------VDSNNVD-DYISK-VVDFTIGLG-VEKQVE
-SFIKG---FSEVFPY--SSML-IFSPN-ELVELLGN-----------------------
---ANEDWS-----------YETL---------ISAIHADHG------------------
----------------YTVD----SPSV-QNLITLMT--------------------KFS
KQERRLFLQF-------LTGSPRLP------------IGG-----FK-NLKPVF--TVVL
KHSEGDLKPD------------------------NYLPSVMTCA-NY-LKLPNY-SSSDI
LEERLM-KAVNE-GAGA
>Wickerhamomyces_ciferrii_XM_011275821.1 .
LRVDRSQL-FQSLLKIL----------------DKYASRPS-ILEIEFVG-------EAG
TGLG-PTLEF---YSNV----SKEFLRKQY--------GLWRDENDHN------------
---------DYISNANGLFPVPIR----------------------------------EK
SINKIKDHSKYFRNLGKFISRALIDNRIVDFHFNK--VFFEIASS---------------
IVNKQEIPKYILYKKLKI----------------------VDPQMFKS--------LDYM
MKNK--HQLS-NLT--
--INFTL-------------------------------PG--YNKIE-LIPN--------
GTNIY---
------------------------------VDETNFD-KYIDK-IVEFTLGGN-VKKQIE
-SFIIG---FSEIFPY--SSLT-IFSSK-ELVEMMGN-----------------------
---ANEDWS-----------YETI---------IGSIHADHG------------------
----------------YTID----SPSV-QRLIEFMT--------------------RLN
KTERRLFLQF-------LTGSPRLP------------IGG-----FK-MMRPVF--TVVL
KHGESGLKSD------------------------DFLPSVMTCA-NY-LKLPDY-SSQGI
LEERLL-KAIHE-GGGA
>Cyberlindnera_fabianii_LK052922.1 .
VRVSRQHL-LKSSIKVL----------------EKLGAHPS-ILEVEFVD-------EAG
TGLG-PTLEF---YSNV----CYAFVERKY--------GLWRTDGEQSG-----------
---------KHVYCVKGLFPAPIG-----------------------------------P
SDPNSSNKLSLFKNLGKFIARALIDGRILDFNFNP--VFFEACAR---------------
--EIDESFGEVGLQTLKH----------------------IDNHLYTS--------LLYI
EQNP--ALVA-DLG--
--LTFVL-------------------------------PG--Y-DIE-LIEN--------
GSDIC---
------------------------------VDSTNCQ-QYVDL-VVDFTLGKG-IKEQVE
-NFVVG---FSEVFPY--YSML-VFTPS-ELVELFGN-----------------------
---SNEDWS-----------YETL---------LSSLHADHG------------------
----------------YNLD----SPAV-QNLIRIMS--------------------SFD
PTERRAFLKF-------MTGSPRLP------------IGG-----FK-NLKPEF--TVVR
KHEEDGLKPD------------------------DYLPSVMTCA-NY-LKLPDY-SSLEV
MKTRLS-KAISD-GADV
>Cyberlindnera_jadinii_XM_020217128.1 .
VRVSRMHL-LKSAFKVL----------------DKLASHPS-ILEVEFND-------EVG

TGLG-PTLEF---YSNV----CHCFAEKSL--------KLWRDNESSED-----------
---------GYVYSQLGLFPAPIS-----------------------------------P
NDPNFDDRMMNFKYLGMFIARALLDGRILDFSFNP--VFFELCAL---------------
--EIEQKYDAFPLEKLKD----------------------IDLQLYKS--------LKYL
KEKQGDD---DFVA-ELG--
--INFTV-------------------------------PG--Y-DIE-LVEGG-------
AERIV---
------------------------------SSAGDVQ-EYIEL-VIDWTLNKG-IQDQLE
-SFILG---FSEVFPY--YSML-VFSPL-EIVKLTGE-----------------------
---SSEDWS-----------YQTL---------ASCIHADHG------------------
----------------YNID----SKSV-QNLIKLMT--------------------SFT
HDERRSFLKF-------LTGSPRLP------------IGG-----FK-NLKPVF--TVVL
KRAEDGLKPD------------------------DYLPSVMTCA-NY-LKLPDY-SSLDM
LTSKVK-TAIYE-GAED
>Sporopachydermia_quercuum_BCGN01000004.1 .
ARVSRENI-LGCAMKIL----------------LLYGSSPN-VLEIEYFD-------EEG
SGLG-PTLEF---YSKV----SKELCRKQL--------KLWRENNSDDLX----------
----------FVYSKNGLFPAPLD--------------------------------KAQL
SSNNGQKLLKLFSVLGKFVARSLLDSRLIDLPFNE--VFFHLAHR----MEERDGSDLVF
DDDDEDADILDQLELVSR----------------------VDIKLGRS--------LRML
YRYVEADKLKKQKSNGIPVEH---------------DENDISGKLAIDG-VEVE-DLA--
--LDFTL-------------------------------PG--YPEIE-LKEN--------
GEMIN---
------------------------------VTLANIE-EFIRL-VIDLTIGSG-VQAQLK
-AFREG---FSEVFPY--SAML-VFFPE-EMVKLFGN-----------------------
---ADEDWS-----------LPTL---------ANSLKADHG------------------
----------------YMMS----SPAI-TDLLSIMS--------------------EFS
IEERRLFLQF-------VSGSPRLP------------VGG-----FK-ALKPTF--TIVC
KQCEAPLKSN------------------------DYLPSVMTCA-NY-LKLPSY-SSREV
MKAKLI-QAMNE-GSDS
>Nadsonia_fulvescens_LXPB01000087.1 .
IQVSRDHI-LVSAVKLL----------------EKFGSTNN-FIEVAFKD-------EVG
TGLG-PTLEF---YSNV----SKEFSKKNL--------GMWREGDNAES-----------
---------EHVYDKAGLFPAPMD--------------------------------DVLE
EKSIGKSVLKWFKVLGRFVARAMLDSRIIDVSFNP--MFFRLIGK---------------
------GSISSSIGTVML----------------------VDSSLGKS--------LLVL
QNYVKEKELRELNK-------------------------EPVEGITICG-STVD-DLS--
--LDFTL-------------------------------AG--YPSIN-LKDN--------
GSEIP---
------------------------------VTIHNIE-EYIAL-TVDMTVGSG-VRRQLD
-SFASG---FSDVFPC--SSLK-SFTSE-ELVSMFGK-----------------------
---SQENWS-----------RDIL---------QDSIKADHG------------------
----------------YTME----STSI-VNLMDIMS--------------------SFE
QHEQRSFLQF-------ITGSPNLP------------IGG-----FK-SLSPALT-VVRK
SSDDAILGSD------------------------DYLPSVMTCA-NY-LKLPNY-STKDI
MRSRII-QAIHE-G---
>Babjeviella_inositovora_XM_019129554.1 .
VRIKREQM-FPSALKVL----------------ALYGAHPS-VLEIEFSD-------EVG
TGLG-PTLEF---YAEV----SKEFTRREL--------KLWRCEDFDVS-----------
----------EGYVGPGLFPAPLT-----------------------------------G
LEAEISVKLKQFAMLGKFIAKSLLDGRIVDFRFNP--AFFRLTRT---------------
----------TDLDAVKE----------------------VDPQLWRS--------LLQL
SG--ANLE-DLS--
--LSFTL-------------------------------PG--YFDYP-LREN--------
GSAID---
------------------------------VGPANID-CYIAA-VVDATVGVG-VEKQLR
-AFVKG---FSEVIPY--ESMH-MFTPE-EKVNLFGG-----------------------
-SGELEDWS-----------FEAI---------RSAVKAAHG------------------

----------------YTSD----HPTV-INFVLVLS--------------------ELD
PALRRNFVQF-------LTGSPMLP------------LGG-----FK-ALKPEF--TIVL
KHNEDGLGPD------------------------DYLPSVMTCA-NY-LKLPNY-SSVGV
MRERML-VAINE-GLGA
>Saccharomycopsis_fibuligera_CP012818.1 .
VRISRKHI-LQSGIKLL----------------DMYGNHPS-VLEIEYFN-------EEG
SGLG-PTLEF---YSTI----SREFCRKSL--------KLWRKDD--NEE----------
--------NPYVHYPTGLFPIPMN--------------------------------DTIA
NGSDGKRVLSLFKVLGKLIARALLDSRLVDFAFNK--FFFELASI----------IDATG
TIKYNKLPRTTMIGKVAN----------------------VDDGLAKS--------LLHL
HKYLEIRDAQKLL-------------------------SIEAERIQVDG-ATLE-DLA--
--LTFTL-------------------------------PG--YPEIE-LVEN--------
GDTVS---
------------------------------VTMVNLA-EYIDD-VVNFTIGSG-VVRQIN
-AFIDG---FSQVFPY--NAIT-IFTPG-ELISMFGN-----------------------
---GEEDWS-----------FETL---------YSSIHANHG------------------
----------------YTVD----SESV-IRLLEIMT--------------------SFN
KDERRRFLQF-------MTGSPKLP------------IGG-----FK-ALRPEF--TVVR
KPSEDGFTAD------------------------DYLPSVMTCA-NY-LKLPEY-SSKDK
MRARLL-QSINE-GLG-
>Saccharomycopsis_malanga_BCGJ01000013.1 .
IRISRSHV-LQSGIKLL----------------DLYGAHPS-VLEIEYFG-------EEG
SGLG-PTLEF---YSSM----SREFCKKSL--------GLWRYDNHDDAA----------
--------NEYVHYSSGLFPAPMD------------------------------------
-DADSNKVLKLFTMLGKMTARALLDSRIVDFAFNK--LFFEIANL------------LEG
GNTSSEHNRLFMLAKVST----------------------VDKHLASS--------LSHL
QKYVNN--------------------------------------NNVDG-TTLE-DLS--
--ITFTL-------------------------------PG--YPQIP-LVQG--------
GDDIL---
------------------------------VDVHNVE-EYIDK-VIDMTVGNG-VHTQIQ
-SFING---FSLVFPY--TSMT-IFTPS-ELVSLFGN-----------------------
---GEEDWS-----------LETL---------RAAVHADHG------------------
----------------YSPS----SPTV-VSLLEIMA--------------------QFN
KLERRSFLQF-------LTGSPRLP------------IGG-----FA-SLRPEF--TVVR
KPSEKGFVPD------------------------DYLPSVMTCA-NY-LKLPEY-SSKEK
LRERLA-QSISE-GLGA
>Nakazawaea_peltata_BCGQ01000004.1 .
IRISRNKL-FECALKVL----------------RLYASIPS-LIEIEYFD-------EVG
TGLG-PTLEF---YANT----SRAFAMKKR--------QMWRDSDHKQQM----------
-ENDGDDTGEYVTQ--PLFPRPLG---------------------------------AAS
ETAASERTLDYFHLLGSFIARALLDSRIIDFRFNP--LFLQLANE----RVLEGNGGSDA
EAPGSVDNLDLQLQRLLR----------------------VDSSLYKS--------LLHL
LKYLEQYENCST--------------------------ELEKGQVTVDG-LTLE-DLS--
--LTFEL-------------------------------PG--YPDYD-LYGKWKN-----
GGDVSSEAVD--
------------------------------ITPSNLL-PYISC-VLDLTVGSG-ISRQID
-LFTTG---FSKVFPY--SSMT-VFSAD-ELTMLFGN-----------------------
---QEEDWS-----------ISVL---------LAAIKADHG------------------
----------------YNLE----SASV-RNLIEIMS--------------------EFS
KHQRRMFLQF-------LTGSPRLP------------IGG-----FK-NLRPKLT-VVKK
ESDELSLGAD------------------------DYLPSVMTCA-NY-LKLPNY-SSKEV
MRGRVL-QLITM-GSDS
>Komagataella_pastoris_CP014584.1 .
VRLSRKKL-LQSSIKVL----------------ETYGDVPS-ILEIEYFG-------EEG
TGLG-PTLEF---YANV----SKEFSKNKL--------GMWRTSDKEA------------
---------IYVNSKTGLFPRPIS-----------------------------------L
DVNEAHKPLHLFKVLGIFVARSLLDSRIIDFEFNP--FFFHTAKN-------------MV
LGIQEKLNRRVLLDRLDE----------------------IDPVLSKS--------LRYL

```
LKFVEQYPSVP---------------------------LDLRDSITVDD-SQLS-NLS--
--LNYVM-------------------------------PG--YPEIE-LIED--------
GVDTE-------------------------------------------------------
------------------------------ITHENLE-DYIDE-VLDYTLQYG-IEQQVR
-SFIDG---FSAVFPY--SSLL-IFSAS-ELVKLFGN-----------------------
---SSEDWS-----------YETV---------MASIHADHG------------------
----------------YSIE----SAAV-QNLIQLMS--------------------DFT
KDEQRMFLQF-------LTGSPRLP------------IGG-----FK-ELKPQL--TVVR
KTTEDGLLPD------------------------NYLPSVMTCA-NY-LKLPNY-TNKEI
MRQRIM-QAIRE-GAG-
>Komagataella_phaffii_XM_002489975.1 .
VRLSRKKL-LQSSIKVL-----------------ETYGDVPS-ILEIEYFG-------EEG
TGLG-PTLEF---YANV----SKEFTKNKL--------GMWRTSDKEA------------
---------IYVSSKTGLFPRPIA-----------------------------------L
DVNEIHKPLHLFKVLGIFVARSLLDSRIIDFEFNP--FFFHTAKN-------------MV
LGVQEKLNRRVLLDRLDE----------------------IDPVLSKS--------LRYL
LKFVEQYPNVP---------------------------PDLRDSITVED-SPLS-NLS--
--LNYVL-------------------------------PG--YPDIE-LTED--------
GADTE-------------------------------------------------------
------------------------------ITHENLE-DYIDE-VLDYTLQYG-IEQQVR
-SFIDG---FSTVFPY--SSLL-IFSAS-ELVKLFGN-----------------------
---SSEDWS-----------YDTV---------MASVHADHG------------------
----------------YSIE----SAAV-QNLIQLMS--------------------EFT
KDEQRMFLQF-------LTGSPRLP------------IGG-----FK-ELKPQL--TVVR
KTTEDGLLPD------------------------NYLPSVMTCA-NY-LKLPNY-TNKEI
MRQRIM-QAIKE-GAGA
>Candida_succiphila_BCGL01000001.1 .
FRLSRKHL-FQGAFKVL-----------------EKYGAAPG-IIEFEYFE-------EVG
TGLG-PTLEF---YSEI----SKEFVKTKH--------HLWRYGDSNNGD----------
--------PTYVHSRTGIFPAPLP-----------------------------------K
NDPRRKEVLHLFEVLGIFIGRSLLDSRLIDFSFNP--LFFELAHS----------IANEV
DENEEIVDRNTLLDRLSI----------------------VDPALAKS--------TRFL
TRYLNEYNSIPDDA------------------------ADDLASVEIEG-CSLA-DLS--
--LTFSL-------------------------------PG--YDNIE-LVRH--------
GSEKE-------------------------------------------------------
------------------------------VTPGNLD-FYVDK-VLDLSVGSG-VVDQIK
-AFIKG---FSKVFPY--SSLI-VLSPQ-ELVNLMGN-----------------------
---ANEDWS-----------SETL---------HSAVKADHG------------------
----------------YSSA----STSV-IRLIKIMS--------------------EFD
TQDRRKFLQF-------LTGSPKLP------------MGG-----FK-ALSPEL--TVVL
KRSEDGLKSD------------------------DYLPSVMTCA-NY-LKLPDY-SSEEV
MKRRII-QAVNE-GAGA
>Kuraishia_capsulata_CBUD020000055.1 .
VRISRKHL-LQSAFKVL-----------------DMYGSLPS-VLEIEYFD-------EVG
TGLG-PTLEF---YANV----SKAFAKKKL--------KIWRTDGQSAGD----------
---------DYIDFRTGLFPSPLS------------------------------------
--DFNEKSLELFRFLGKFIARSLLDSRIVDFNFNP--LFFTLANE------------IDS
LDVPISASRSVRLDRVAM----------------------VDSQLASS--------LKQL
DEYSKLFKSVA---------------------------SQSRSTATLNG-VTLQ-DLA--
--LTYVL-------------------------------PG--YPNVN-LIEN--------
GDSVD-------------------------------------------------------
------------------------------VTPENLD-DYIDK-IIDLTIGSG-VRRQIE
-AFVEG---FSKVFPY--SAMM-IFSSQ-ELVRLFGN-----------------------
---GEEDWS-----------LETL---------FSSINADHG------------------
----------------YTID----SVSV-RCLLEIMS--------------------QFS
KEERRMFLQF-------MTGSPRLP------------VGG-----FK-ALHPSF--TVVL
KNNEDGFKAD------------------------DFLPSVMTCA-NY-LKLPNY-SSKEM
MNQRIL-QAMHE-GSGA
```

>Alloascoidea_hylecoeti_BCKZ01000029.1 .
VRISRRQL-IQSAMKVM----------------DLYGAEPN-VLEIEYFD-------EAG
TGLG-PTQEF---YANV----SKEFCQKKL--------KLWRDGEGENDN----------
---INDNKSRYVFSKFGLFPAPMD--------------------------------ENMS
KSENGKKIIGFFNILGKFIARSLLDXRIVDFNFSX--LAHRAILG---------------
------NEIPIGLESIKL----------------------VDIGLYKN--------LKFL
MEYVDEKRRLERNGID----------------------INEDSDVLINN-TPLG-QLS--
--IPYTI-------------------------------PG--YPDIE-LGSK--------
HENED---
------------------------------LNINNVE-EYIEF-IIDITVGSG-VSRQIK
-AFYEG---FSLVFPY--SAMI-SFYPQ-ELVNFFGQ-----------------------
---GEEDWS-----------YETL---------SSSIKADHG------------------
----------------YTSE----SQTI-HNLLEIMS--------------------KLS
LKERRKFLQF-------LSGSPKLP------------IGG-----FK-ALKPQF--TVVR
KHGEGKLKAD------------------------DYLPSVMTCA-NY-LKLPDY-SSIEL
MKKRLL-QAMHE-GLGA
>Pachysolen_tannophilus_LZCH01000212.1 .
IRISRKNL-LQCAIKVL----------------DLYATIPS-LIEIEYVD-------EVG
TGLG-PTLEF---YANV----SKEFSRKKV--------RLWREDGDQSNE----------
----------FVNYKTGLFPVPMD--------------------------------DKFS
ESKNGAKILQLFNILGRFIARALLDSRIIDFAFNP--LFFSIAKN----------FIESD
TKKHGYHSKLALLEEVEL----------------------VDPDLARS--------LRHL
TKYVEQYDKVE-----------------------------KKEAVTVDG-CTLE-DLS--
--LTFSL-------------------------------PG--YPYVE-LISN--------
GADVP---
------------------------------VNSSNLE-EFIDK-VIDLTIGSG-VRCQIH
-AFVDG---FSKVFPY--SSMS-IFSPE-ELVTLFGN-----------------------
---GAEDWS-----------YETL---------LSSIHADHG------------------
----------------YNIE----SKSV-ASLLEIMS--------------------EFD
KEERRKFLQF-------LTGSPRLP------------IGG-----FK-NLRPNL--TVVC
KRSEDGLKPD------------------------DYLPSVMTCA-NY-LKLPDY-SSKMV
MKKRIV-HAFNE-GSGA
>Candida_boidinii_MSSA01000082.1 .
LRVSRRRI-LQGAMKIL----------------ETYGSIPG-LLEIEYFD-------EVG
TGLG-PTLEF---YSLV----SKEFNRSVL--------CMWRDDDNNSGX---------N
ELENASIDEKYVNSPLGLFPRPIT-----------------------------------K
DSKNYKKNLYMFSTLGKFIARALLDSRIIDFVFNP--LFFEICNL---------------
--XFKNFSRFAKIDYVKL----------------------VDPVLAKS--------LLLL
NKYLIEYEEIDKLAIKNGFD--------------SSKVASLKNDVKIDD-CSLE-QLS--
--LVFTL-------------------------------PG--YLDVN-LCEGN-------
GENID---
------------------------------ITFNNLA-KYIDS-VVDITIGNG-VLPQAQ
S-FIEG---FSKVFPY--SSMI-IFNPK-ELVTLLGS-----------------------
---GEEDWS-----------FETL---------ISIIRADHG------------------
----------------YTTE----SPTV-RNLLELMS--------------------QFN
SEERRKFLQF-------LTGSPRLP------------VGG-----FK-SLNPEL--TVVL
KHSEDGLKSD------------------------DYLPSVMTCA-NY-LKLPDY-SSKEV
LKRRLK-QAVNE-GAGS
>Brettanomyces_naardenensis_MDSA01000003.1 .
LRLSRNKL-LQGAAKVM----------------DGYASVPG-LLEIEYFD-------EAG
TGLG-PTLEF---YANV----SKEFCKKSL--------QMWRGSDENSED----------
----------YVNYTSGLFPRPLN-----------------------------------P
SVPHYHRILHWFSVLGKFVARSLLDSRIIDFAFNP--LFFEISRN---------------
---GEQADTRTMLDRLRL----------------------VDPALARS--------LDHL
SLYL--------------------------------------NDAHSEG-ATLA-DLS--
--LNFVL-------------------------------PG--YPDVI-LIPH--------
GESIP---
------------------------------IGPDNLR-QYISA-VVEYTVGKG-IRSQIE

-SFAEG---FSTVFPY--SSMI-IFSPD-ELVRILGN----------------------
---DDEDWS-----------LETL---------LDVVHADHG------------------
----------------YTLE----SPTV-QSLLEIMS--------------------SLD
KDSRRKFLQF-------LTGSPKLP------------IGG-----FK-ALHPEF--TVVL
KQAEDNLKPD------------------------DYLPSVMTCA-NY-LKLPEY-SSKEV
LKRRLM-KAMNE-GANA
>Brettanomyces_custersianus_MDVK01000012.1 .
LRLSRKKL-FQGAVKVM----------------DGYASIPG-LIEIEYFD-------EAG
TGLG-PTLEF---YANV----SKEFCRKRL--------QMWRDSDGEPMD----------
----------FVRCTEGLFPRPIN-----------------------------------R
TSRYYHQTVHYFSVLGKFVARAMLDSRLIDFSFNP--VLFKLSRR---------------
--NSAPTDEAELLDLVRK----------------------IDVELYRS--------LREL
QAMND--DEIE-GLE--
--LTYVL-------------------------------PG--YEDVE-LVKN--------
GKHTP---
------------------------------ITGENIR-DYIYS-IASYTAGQG-VWVEIA
-AFKQG---FSAVFPY--SSMT-IFSPD-ELVKILGN----------------------
---GEEDWS-----------LAEL---------EKVVHADHG------------------
----------------YTLQ----SATV-QNLLDVMS--------------------KFD
KDQRRKFLQF-------LTGSPKLP------------IGG-----FK-SLHPEF--TVVC
KQAEGGLKPD------------------------DYLPSVMTCA-NY-LKLPDY-SSEQI
LKRRLI-QAMNE-GADA
>Brettanomyces_anomalus_MDSB01000004.1 .
FRLSRKKL-FQGAIKVL----------------DGYASAPG-LLEIEYFD-------EAG
TGMG-PTLEF---YANV----SKEFCKKEL--------HIWRDLEGSDGL----------
----------YVNNLTGLFPRPID-----------------------------------S
SNPMYQKCLHYFSALGKFIARALLDNRIVDFAFNP--LFFELSKF---------------
---LNLPNEARLLDILKG----------------------VDPELFNS--------LTFL
ISYLN--------------------------------------KKDKKE-TSLP-DLS--
--LSYVL-------------------------------PG--YNDVK-LIRD--------
GEKIQ---
------------------------------VTEDNLE-NYIRA-VVQATVGNG-VWKQIQ
-AFKEG---FSTVFPY--SSMS-IFSSN-ELTKLLGS----------------------
---ADEDWS-----------IETL---------LGVVKADHG------------------
----------------YTVE----SASF-QNLLEVLS--------------------SFD
KSDRRKFLQF-------LTGSPKLP------------IGG-----FK-ALHPEF--TVVR
KPAEGNLKPD------------------------NYLPSVMTCA-NY-LKLPDY-SSKNI
LRQRLT-KAMNE-GANS
>Brettanomyces_bruxellensis_MDGX01000080.1 .
FRLSRKKL-FQGAIKVL----------------DGYASAPG-LLEIEYFD-------EAG
TGMG-PTLEF---YANV----SKEFCKKEL--------YLWRDTDGSNSL----------
----------YVNYRTGLFPRPMD-----------------------------------R
KARIYQKSLHHFSILGKFIARALLDNRIVDFAFNP--LFFELSKF---------------
---PELPGETKLLDILKG----------------------VDPELFKS--------LSFL
ISYLD--------------------------------------DNVKKE-ANLT-DLS--
--LSYVL-------------------------------PG--YNDIQ-LVEN--------
GANVQ---
------------------------------VTEVNLE-AYVND-VVQATVGKG-VWEQIQ
-AFKKG---FSTVFPY--SSMA-IFSSD-ELTKLLGS----------------------
---ADEDWS-----------LETL---------LGVVKADHG------------------
----------------YTVE----SPSF-QNLLEVLS--------------------SFD
KNDRRKFLQF-------LTGSPKLP------------IGG-----FK-ALHPEF--TVVR
KPAEGKLKPD------------------------NYLPSVMTCA-NY-LKLPEY-SSKDV
LRQRLI-KAMNE-GANT
>Ambrosiozyma_kashinagacola_IAAG01004394.1 .
LRISRKRL-LQSAIKVL----------------DSYGTSPG-LLEIEYFD-------EAG
TGLG-PTLEF---YANV----SKEFSKSRL--------HMWRDNRKNFSTND-VYDNPPE
SENTTDDANLYVENESGLFPRPLT-----------------------------------P

MIPHYEKILHVYSVLGKFVARSLLDSRIIDFAFNP--MFFQLALE-------------LS
LNSPRIERRSALVKRLRS----------------------VDSTLAQS--------LVHL
IKYLEKYNGLTE---------------------------HERESIEVDG-CKLA-DLS--
--LNFVL-------------------------------PG--YDDIK-IKTG--------
GEDIE---
------------------------------VTHENLE-EYIDT-VIDFTIGSG-VKAQIE
-AFVDG---FSKVLPY--LPMT-IFSST-EMVRLLGN------------------------
---EDEDWS-----------FETL---------FAAIHADHG------------------
----------------YTAE----SQSV-QRLLEIMT--------------------EYD
KDERRLFLQF-------LTGSPRLP------------VGG-----FK-ALKPDL--TVVL
KHAEDNLKSD------------------------DYLPSVMTCA-NY-LKLPDY-SSKEV
MKSRLG-KAMRE-GANA
>Ambrosiozyma_monospora_BCIP01000003.1 .
LRISRKKL-LQSAIKVM----------------DTYGTSPG-LLEIEYFD-------EAG
TGLG-PTLEF---YANV----SKEFSKSRV--------HMWRDNKKFNYA---TSKFESD
DDSDTDSAQPYVDNQYGLFPRPLT-----------------------------------P
LTPYYEKIIHGFSVLGKFVARSLLDSRIIDFNFNP--LFFQLSSE-------------LQ
NGIPLSTRRSALIERLKL----------------------VDPALANS--------LIHL
SKYESKYPGLTP---------------------------EERERVEVDG-CKLS-DLS--
--LNFVL-------------------------------PG--YPEVR-IKTN--------
GENVD---
------------------------------ITHENLS-DYIDT-VIDFTIGTG-IKAQID
-SFLEG---FSKVFPF--LSMT-VFSSS-EIVRLLGN------------------------
---EEEDWS-----------YETL---------LNAVHADHG------------------
----------------YSSE----SPSI-QNLLTIMS--------------------EFD
KKERRMFLQF-------LTGSPKLP------------IGG-----FK-SLQPDL--TVVL
KRPEDDLQSD------------------------DYLPSVMTCA-NY-LKLPSY-SSIXV
MREKLR-KAMNE-GANA
>Candida_arabinofermentans_LWUO01000192.1 .
LRLSRKHL-LKGAIKVL----------------DGYGTIPG-LLEIEYFD-------EAG
TGLG-PTLEF---YANV----SKEFSKTRL--------SMWRTSKVEKEX-----SIGSE
DTLEEDIGDAFVNNPTGLFPRPLT-----------------------------------E
STRHYQKIIHLFSVLGKFVARSLLDSRIIDFTFNP--LLFQLGRD-------------LS
VSAKVFESRESQIETLRL----------------------VDPFLANS--------LAHL
TQYLTKYEGLSD---------------------------ADKAAVQIDG-CSLE-DLA--
--LNFVL-------------------------------PG--YQDVQ-LKED--------
GEKID---
------------------------------VTHENLE-EYIDK-VIDMSIGSG-VRGSIE
-AFVSG---FSVVFPF--SSMS-IFYPD-EMVRLLGN------------------------
---GEEDWS-----------YDTL---------ISVLHADHG------------------
----------------YSID----SPSI-QRLLQILT--------------------DFT
TEERRKFLQF-------ITGSPKLP------------IGG-----FK-SLHPDL--TVVL
KHTENGLKPD------------------------DYLPSVMTCA-NY-LKLPDY-SSIEV
MKARLL-KAMTE-GANS
>Ogataea_methanolica_IAAJ01001981.1 .
LRLSRKHL-LKGAIKVM----------------DGYGTIPG-LLEIEYFN-------EVG
TGLG-PTLEF---YANV----SKEFCKSRL--------MMWRNTKNEEDX-------TND
SDSDQHSDDPYISSPTGLFPRPLA-----------------------------------E
SVRHYSKIIHLFNVLGKFVARSLLDSRIIDFAFNP--LFFQIAMD--------------I
DCNNKIKNRAASIEKLRL----------------------IDPTLAKS--------LSHL
NKYLVSYEGLTSP---------------------------EEMSIVTVD-NCLLEDLG--
--LNFVL-------------------------------PG--YETIE-LKPN--------
GSEIE---
------------------------------ITHSNLE-EYIDN-VLDKMIGSG-VQPQIK
-AFVDG---FSAVFPY--SSMM-IFSPN-ELVKLLGN------------------------
---GEEDWS-----------FETL---------LTVVHADHG------------------
----------------YSTE----SPSI-QRLLEIMA--------------------EFS
KEERRKFLQF-------LTGSPKLP------------IGG-----FK-SLHPDL--TVVL

```
KHSEDGLKPD------------------------DYLPSVMTCA-NY-LKLPDY-SSKEV
MRLKIE-KAMNE-GANS
>Ogataea_parapolymorpha_XM_014076945.1 .
LRLSRKKL-LQGAIKVM----------------DCYGTIPG-VLEIEYFD-------EAG
TGLG-PTLEF---YANV----SKEFAKAKL--------KMWRTNGRTYSD----------
SASSSDSDSEYVKFETGLFPRPSA------------------------------------
--KPSSKVLHLFSVLGKFVARSLLDSRIIDFEFNP--LFFEIAKE-------------LD
TKSWKELSRKKSIERLSR----------------------VDADLAKS--------LGHL
TRYLDAYAEVEE---------------------------SERPEILIEG-CKLS-DLS--
--LNFTL-------------------------------PG--YEDVE-LCLN--------
GEEVE-------------------------------------------------------
------------------------------VDHTNLE-RYVDK-VIDLTIKSG-VRSQIH
-SFVQG---FSAVFPY--SSMT-IFSSA-ELVKLLGN-----------------------
---GEEDWS-----------YETL---------AGVIHADHG------------------
----------------YSMD----SPSV-QRLMEIMS--------------------EFT
ADERRKFLQF-------LTGSPKLP------------IGG-----FK-SLSPDF--TVVL
KHPEDGLKPD------------------------NYLPSVMTCA-NY-LKLPDY-SSKSV
MKQRLQ-TAMTE-GANS
>Ogataea_polymorpha_XM_018353674.1 .
LRLSRKKL-LQGAIKVM----------------DCYGTIPG-VLEIEYFD-------EAG
TGLG-PTLEF---YANV----SKEFAKAKL--------NMWRTNGRTYFD----------
STSSSDSESEYVKFETGLFPRPNA------------------------------------
--KPSSKVLHLFSVLGKFVARSLLDSRIIDFEFNP--LFFEIAKE-------------LD
TKSRKNLSRKKSIERLSR----------------------VDADLAKS--------LSHL
TRYLDAYAEVEE---------------------------SERSEILIDG-CKLS-DLS--
--LNFTL-------------------------------PG--YEDVE-LCLN--------
GEEVE-------------------------------------------------------
------------------------------VDHTNLE-RYVDS-VIDLTIKSG-VRSQIQ
-SFVQG---FSAVFPY--SSMT-IFSSA-ELVKLLGN-----------------------
---GEEDWS-----------YETL---------ASVIHADHG------------------
----------------YSID----SPSV-QRLMEIMS--------------------EFN
ADERRKFLQF-------LTGSPKLP------------IGG-----FK-SLSPDF--TVVL
KHPEDGLKPD------------------------NYLPSVMTCA-NY-LKLPDY-SSKSV
MKQRLQ-TAMTE-GANS
>Candida_sorboxylosa_BCGC01000001.1 .
LRIARDKM-FASAVKVM----------------ETYATNPG-LIEIEFFD-------EIG
SGMG-PTLEF---YSSV----SKEFRKLHL--------FMWRSDXYGDKN----------
----SVTKDVYVSDSNGLFPRPLN-----------------------------------K
ANPHYQSALLYFNVLGKFVARSLLDSRLVDFHFNP--LFFQLAMN---------------
---HTQLXRDESLLLMKK----------------------VDKHLASS--------MNHL
IKYFDDLH-------------------------------------------DVS-NLM--
--LTFVV-------------------------------PG--YEDIE-LVPN--------
GRDMS-------------------------------------------------------
------------------------------VTNXNFE-DYVDK-IISFTLFDG-IKAQLX
-SFRDG---FSMIFPF--ECMK-IFSNE-EITKLLGL---------------------ES
TDEIEQNWS-----------VNNL---------QDVIHADHG------------------
----------------YTKS----STQV-QWLIEILN--------------------GFD
LEKRRKFLQF-------LTGSPRLP------------VGG-----FK-GLSPPF--TVVL
KHPEHDTDDID-----------------------GYLPSVMTCA-NY-LKLPKY-SNIET
MAAKIS-KAIES-DYG-
>Pichia_kudriavzevii_BBOI01000487.1 .
LRISRERI-FSSAVKVM----------------ESYATNPG-LLEIEFFD-------EVG
SGLG-PTLEF---YSEV----SKSFSRVKL--------HMWRSDQYCKQQ---------N
TDVSKDKHEVHIHNAHGLFPRPLH-----------------------------------K
MDPHLNNTLLYFKVLGKFLARSFIDSRLIDFHFNP--LFFQLAMK-------------YA
CFEPMRVSLEESIELIKL----------------------IDESLSSS--------LRHL
NLYLTEFRHTP---------------------------EEERADFKIMG-ATVS-DLM--
--LTFVL-------------------------------PG--YEEIK-LIPG--------
```

GDDVS---
------------------------------VTAENLD-LYISA-IVEYTLYDG-VSQQIK
-SFVDG---FSEVFPF--SSLK-LFSPE-ELTRLSGN-----------------------
---AVENWS-----------VETL---------LAVVRSDHG------------------
----------------YTNH----SQQI-EWLIDIMS--------------------KFE
KEERRKFLKF-------ITGSPRLP------------FNG-----FK-GLSPPF--TVVL
KHTEDNLRPD------------------------DYLPSVMTCA-NY-LKLPRY-SSREV
MLAKIK-QAMNE-GTNA
>Candida_ethanolica_ANNA01000639.1 .
LRISRDKI-FSSAIKVM----------------ENYATNPG-LIEIEYFD-------EVG
SGLG-PTLEF---YSNV----SREFSKLKL--------WMWRSDKYSTIK----------
--SVNDVSDVYIHDVNGIFPRPMS-----------------------------------K
LDSHLTNTLLYFKVLGKFLARSFLDSRLVDFHFNP--LFFEIAMN-------------YA
ISNDFKYDIDESIKKIRK----------------------IDETLGSS--------LQHL
QLYLKEFHKLP---------------------------QNEWNHVKISG-AKVE-DLM--
--LTFVL-------------------------------PG--YEEIK-LIPN--------
GEEVE---
------------------------------VNSSNLE-DYLNK-IIDFTIYNG-IKSQIE
-SFVNG---FSEIFPF--TSMI-LFSSN-ELTRLSGN-----------------------
---ETENWS-----------VETL---------ISMIHADHG------------------
----------------YNSK----SDQI-QWLIEIMS--------------------KFD
NENRRKFLKF-------ITGSPRLP------------FDG-----FK-GLSPPF--TVVL
KHCENDLKPD------------------------DYLPSVMTCA-NY-LKLPSY-SSLEI
MHAKIV-QAMNE-GNN-
>Pichia_membranifaciens_XM_019162530.1 .
LRISRDRI-FSSAVKVM----------------ENYATNPG-LIEIEYFG-------EVG
SGLG-PTLEF---YSNV----SKEFGRLKL--------RMWRSDQYSKLQ----------
-----YNNELYVHDDHGLFPRPLH-----------------------------------K
LDPHLNNTLLYFKVLGKFLARSFLDSRIVDFHFNP--LFFEIAMK-------------YS
ATKSFVHDISDSIKKIKS----------------------VDQSLSSS--------LQHL
KLYLDEFARIP---------------------------EADWEEVQISG-CKVT-DLM--
--LTFVL-------------------------------PG--YEEIK-LIPD--------
GDEIG---
------------------------------VNSSNLG-MYINK-IIDFTIYGG-IENQIK
-SFVSG---FSEIFPF--TSMI-LFSAE-ELTRLSGN-----------------------
---DVENWN-----------VETL---------ITMIHADHG------------------
----------------YNNK----SSQV-EWLIQIMS--------------------NFD
KDERRKFLKF-------ITGSPRLP------------FEG-----FK-GLSPPF--TVVL
KHCEDKLQPD------------------------DYLPSVMTCA-NY-LKLPCY-TSKEV
MYTKIV-QAMNE-GNDS
>Hanseniaspora_osmophila_LPNM01000006.1 .
IVIDRKDM-FDQALKYS----------------SSISDPAN-VLSVKYTN-------EKG
EGSG-PTREF---YSIL----SKMFTRKSL--------NMWRGSLDSNPQ----------
---------DHNVKHXELFPQPLV-----------------------------------F
TERENNAVASLFKFLGTFLARSLLDLEMVDFRFNE--VFFQLLDT----YVENGCENKNL
LKLMETLSIPDGIDLITK----------------------IDKGIGKS--------FRFI
YEQGLKN----------------------------------TSDNTGTC-KDLL-ELN--
--IPYNL-------------------------------PG--Y-DSF-AMPS--------
SSASTEEY--
------------------------------VSNANYK-EYLRT-CINMMLCEG-ILPCLQ
-NFYEG---FNTVLPY--EALL-FLTPK-ETSLYFGK-----------------------
---IDEDWS-----------FETI---------SKSLNADHG------------------
----------------YTME----SNEV-VNLINIMT--------------------SLS
KNERRLFLQF-------ATSSPRLP------------LGG-----FQ-KLRPKL--LIAL
KPPADNCSPD------------------------DTLPSVMTCV-NY-FKLPQY-SSIDV
MRKRIL-QAITE-GSG-
>Hanseniaspora_vineae_JFAV02000305.1 .
IVIDRKNM-FQQALTYS----------------SSISDPTN-ILSVKYTN-------EKG

```
EGSG-PTREF---YSII----SKMFTKKSL--------GMWRGSFDSSVL----------
---------EKAVKQXELFPQPLV-----------------------------------S
TEKNNDDVATLFKFLGTFLARSLLDLEMVDFRFNE--VFFQILNS----YVKNSCENKRQ
VDLMETLSIPDGIDLITK----------------------LDKGIGNS--------FRYI
YEQGLRN----------------------------------TVNETGES-KDLL-ESN--
--IPYNL-------------------------------PG--Y-DSF-VMSS--------
SATSADEY----------------------------------------------------
------------------------------VSNANYE-NYLKT-CINVMLFEG-ILPCLQ
-NFLDG---FNRVLPY--KALL-FLTPK-EASLYFGK-----------------------
---TDEDWS-----------FETI---------SKSLNADHG------------------
----------------YTME----SNEV-VNLINIMT--------------------NLS
KDERRLFLQF-------ATSSPRLP------------LGG-----FQ-KLRPKL--LIAL
KPPADNCSPD------------------------NTLPSVMTCV-NY-FKLPQY-SDIDI
MRKRIL-QAITE-GSG-
>Kluyveromyces_lactis_XM_452136.1 .
LRVSRDSL-FLSGIKIL----------------KKYGSSPN-VLEIEYKD-------EEG
TGLG-PTLEF---YSLM----SKEFARKSL--------KMWKTNSDTSDD----------
---------EEEYVTGALFPGPLL----------------------------------GT
TEENNEKTLKLFENLGTLVARSMLDNRILDFRFNA--VFFELMHK------------CCK
NDRLDLEDFETCIHLVGQ----------------------FDVQLGKS--------LAFL
YDNRHDD--------------------------------------------SIK-NLD--
--LYFML-------------------------------PG--Y-DIE-LLEG--------
GSKIL-------------------------------------------------------
------------------------------VESFNVE-EYLIR-VFDQFLGKG-VDMQLA
-AFRNG---FSKSFPY--SSLL-ILTPT-ELSDLFGA-----------------------
---VAEDWS-----------VQTL---------YSSIHADHG------------------
----------------YSMD----SPII-TDLIDILS--------------------NFS
TQQKRLFLQF-------ITGSPKLP------------LGG-----FK-NLKPKF--TVVL
KHPDGNISAD------------------------HCLPSVMTCA-NY-LKLPKY-SDKLV
LKDRIA-QAMTE-GSGA
>Kluyveromyces_dobzhanskii_CCBQ010000045.1 .
LRVSRDSL-FLSGIKIL----------------EKYGSSTN-VLEIEYKD-------EEG
TGLG-PTLEF---YSLM----SKEFADKSL--------NMWRTNGDTDDI----------
---------VNNYITGTLFPGPLL----------------------------------GS
TDEENEKIIKLFENLGTLVARSMLDNRILDFRFNP--LFFELMHK------------QCR
DEQIDLEDLDTCLDLVGK----------------------LDVQLGRS--------LSFL
YNNRHND--------------------------------------------SLK-DLN--
--LFFML-------------------------------PG--Y-NIE-LLEG--------
GSRIM-------------------------------------------------------
------------------------------VEPFNVQ-EYLIR-VFDQLLGKG-IERQLT
-AFRDG---FSKSFSY--SSLL-ILNPA-ELSDLFGA-----------------------
---VAEDWS-----------VQTL---------YSFIHADHG------------------
----------------YSMD----SPII-TYLIDILS--------------------NFS
TEEKRLFLQF-------ITGSPKLP------------LGG-----FK-NLKPKF--TVVL
KHPDGNISPN------------------------HCLPSVMTCA-NY-LKLPKY-SDKSV
LRDRLV-HAMTE-GSGA
>Kluyveromyces_marxianus_CP009305.1 .
LRVSRETL-FLSGIKIL----------------EKYGSSPN-VLEIEYKD-------EEG
TGLG-PTLEF---YSLM----SKEFAKKSL--------NMWRIDSTAAYDL---------
---------EEKYVTNTLFPGPLV----------------------------------GA
DEEENGKVLKLFENLGTFVARSMQDNRILDFRFNE--MFFELMHK------------QCR
GEQLDLQDFETCINMIQL----------------------VDPQLGKS--------LRFL
YENRKDP--------------------------------------------SIE-SLD--
--LYFML-------------------------------PG--Y-NVE-LLEG--------
GSKIL-------------------------------------------------------
------------------------------VSSENVE-EYLIR-IFDQILAKG-IQRQLN
-AFVTG---FSKSFPY--SSLL-ILTPE-ELSGLFGA-----------------------
---VEEDWS-----------AETL---------FSCIHADHG------------------
```

----------------YSMD----SPTI-TNLIHTLA--------------------TFS
PQERRLFLQF-------ITGSPKLP------------LGG-----FK-NLKPKF--TVVL
KHPDDNTSPD------------------------ECLPSVMTCA-NY-LKLPKY-SDKTM
LRNRLV-QAMTE-GSGA
>Kluyveromyces_wickerhamii_AEAV01000035.1 .
LRVSRDSL-FLSGMKIL----------------EKYGSSPN-VLEIEYKS-------EEG
TGLG-PTLEF---FALM----SKEFARKSL--------HIWTLKDDIADGP---------
---------GEDFVSGLLFPRPLL------------------------------------
-VSEEDRILRLFENLGTLVARSMLDNRILDFRFNE--VFFELLHR------------RCK
GEPFNFHEYEYCIDLIKK----------------------IDLQLGKS--------LTYL
LDRKGDE--RIR-ELD--
--LYFTL-------------------------------PG--Y-DIE-LVED--------
GSNVL---
------------------------------VEAHNLE-EYLIR-IFEQLLGQG-INKQLD
-AFMKG---FSKSFPY--SSLL-ILMPH-ELSDLFGG-----------------------
---VEEDWS-----------VQTL---------YECLNADHG------------------
----------------YSMD----STSI-TDLIDILS--------------------QFS
KQERRIFLQF-------ITGSPKLP------------LGG-----FK-NLKPKF--TVVL
KHPEGESSPD------------------------HCLPSVMTCA-NY-LKLPKY-SSRAI
LQQRIV-QAMTE-GSGA
>Kluyveromyces_aestuarii_AEAS01000105.1 .
LRVTRDSL-FLSGIKIL----------------EKYGSSPD-ILEIEYKD-------EEG
TGLG-PTLEF---YTLM----SGEFVKKSL--------DMWRVETASSGEK---------
---------SSEYVSNSLFPRPMT------------------------------------
-SSSNEKLLKLFENLGTFVARSMLDNRILDFRFSP--IFFELLHK------------RSK
GERLDFENLDYPIELVHS----------------------IDPQLGKS--------LLYL
YEHRMDK--SIE-ELH--
--LNFTL-------------------------------PG--D-EVE-LLTG--------
GAEIF---
------------------------------VTSQNVE-EYLIR-TMEYLLDKG-INRQLD
-AFIRG---FSKSFPY--SSLL-VLSPE-ELADLCGG-----------------------
---IEEDWS-----------VQTL---------YSCISADHG------------------
----------------YSMD----SPAI-HDLISILS--------------------EFS
KQERRIFLQF-------ITGSPKLP------------IGG-----FK-NLKPKF--TVVL
KHPEEGMQAD------------------------QYLPSVMTCA-NY-LKLPKY-SHKSI
MHQRLL-HAMTE-GAGA
>Lachancea_meyersii_FJUM01000026.1 .
LRISRDDI-FLSALKIL----------------RKYGSSPN-ILEIEYSN-------EVG
TGLG-PTMEF---YAIV----SKEFAKKSL--------RLWRTDNFSSHQ----------
---------KDELVEGLLFPAPLG------------------------------------
ALEDQERVLALFQQLGVFVARSMLDNRILDFRFSQ--AFFELAHM------------YAA
DQPMLLEDKELALSILGM----------------------VDPQLEKS--------LNFL
QKHKNDD--VLE-SLS--
--LTFHL-------------------------------PG--Y-NVN-LVAN--------
GLETA---
------------------------------VTKNNFD-QYLDS-ILDQIVGAG-VEKQIR
-SFMQG---FSHAFPY--RSLM-ILTPE-ELTQFFGG-----------------------
---VEEDWS-----------TETL---------IANIEADHG------------------
----------------YNHD----SDII-QDLISLMS--------------------SFS
DQERRQFLQF-------LTGSPKLP------------VGG-----FK-TLSPKF--TVVR
KHTEENMTAD------------------------EFLPSVMTCA-NY-LKLPLY-SNKAI
LKSRVI-QAMTE-GSGA
>Lachancea_lanzarotensis_CDLU01000020.1 .
LKISRDNM-FLSALKIF----------------SKYGSSPN-ILEIEYLE-------EVG
TGLG-PTMEF---YAII----SREFAKKSL--------GLWRSDQYPLNK----------
---------QDELVEGLLFPAPLR------------------------------------
ASKDQKGTLELFKQLGAFVARSMLDNRILDFRFNV--AFFELAHL------------YAD
GENLSLDQIGPTFSLMHK----------------------VDPQLTKS--------LQFL

LEHKDND--ILE-SLS--
--LTFRL-------------------------------PG--Y-DLD-LIPN--------
GTNFA---
------------------------------VTKDNLT-EYIDL-VIDQMLGAG-VEKQIR
-SFTDG---FSQAFPY--RSLL-ILTPE-ELTELFGR-----------------------
---VEEDWS-----------PETL---------LAHVEADHG------------------
----------------YTQD----SDII-HDLISLMT--------------------SFS
AQERRFFLQF-------LTGSPKLP------------LGG-----FK-SLSPKL--TVVR
KHTEENMTAD------------------------EYLPSVMTCA-NY-LKLPMY-SDRDI
LRSRII-QAMTE-GSGA
>Lachancea_nothofagi_FJUQ01000007.1 .
LRISREAL-FLSSLKIL----------------SKYGSSPN-ILEIEYLD-------EVG
TGLG-PTMEF---YAMT----SKEFTRASL--------ELWRVDDYGSCK----------
---------KSEFVEGALFPSPLL------------------------------------
AAGDHEKTLELFKKLGVFVARSMLDNRILDFRFSR--AFFELAHL------------YAA
EERFTFEDKESALQFLSI----------------------VDPQLAKS--------LRFL
HVHKDDT--VLE-SLS--
--LTFYL-------------------------------PG--Y-DVE-LIEN--------
GMEIA---
------------------------------VTKTNFD-MYFSC-VMDQVLGSG-VEKQIR
-SFMEG---FSQAFPY--RSLL-ILTPD-ELIELFGR-----------------------
---VEEDWS-----------AETL---------YANVEADHG------------------
----------------YTQD----SSTV-RDLISLMS--------------------FFS
DNERRLFLQF-------LTGSPKLP------------VGG-----FK-SLNPKL--TVVR
KHTEEDMGPD------------------------EYLPSVMTCA-NY-LKLPKY-SSKEV
LRSRIY-QAMNE-GSGA
>Lachancea_dasiensis_FJUP01000011.1 .
LRVSRDSI-FLSALKIL----------------SKYGSSPS-ILEIEFMH-------EVG
TGLG-PTMEF---YAIV----SREFGRRVL--------GLWRCDNYSAH-----------
---------EDNFVEGLLFPAPLA------------------------------------
ASKDHDRTLELYKQLGLFVARSMLDNRILDFRFNR--AFFELAHL------------YAE
EGNLDFGDTEFMLHLLDM----------------------VDPQLSNS--------LRFL
LEHKYDD--LIE-TLS--
--LTFQL-------------------------------PG--Y-NVE-LIPN--------
GASIA---
------------------------------VTKENFG-TYFRC-VLNETIGPG-VENQIR
-SFMSG---FSQTFPY--RSLL-ILSPV-ELTELFGR-----------------------
---TEEDWT-----------VSTL---------VANVEADHG------------------
----------------YTND----SETI-RDLISLMV--------------------SFS
YKERRLFLQF-------LTGSPKLP------------VGG-----FK-SLNPKL--TIVR
KHTEGDLGPD------------------------HYLPSVMTCA-NY-LKLPKY-SNKNI
MECRIK-QAMKE-GSGA
>Lachancea_thermotolerans_XM_002552937.1 .
LKVSRGAV-FLSALKIL----------------DKYGSTPN-ILEVEFLN-------EVG
TGLG-PTLEF---FAIV----SKEFAYKSL--------RMWRSENFNSCK----------
---------EGELVEEHLFPAPLL------------------------------------
ASSNQEKTLELFHKLGTFVSRSMLDDRILDFRFSK--AFFELAHM------------YAR
NEKIRFDDFDLTIDYMSM----------------------VDPHLAKS--------MKYL
FTLKNDH--DFE-SLS--
--LTFYL-------------------------------SG--Y-NIE-LIEN--------
GKDVA---
------------------------------VTIENFP-DYFER-LSDQVLGTG-VKMQIQ
-SFIEG---FSRTFPY--TSLL-ALTPD-ELTDLFGS-----------------------
---VEEDWT-----------HETL---------YAYVDADHG------------------
----------------YSHD----SNII-HDLISIMT--------------------SFS
KAERRLFLEF-------LTGSPKLP------------IGG-----FK-SLNPRM--TVVR
KHTENDLEPD------------------------HYLPSVMTCA-NY-LKLPKY-SNKDV
LKSRVL-QAMKE-GLGA

>Lachancea_quebecensis_CZLH01000060.1 .
LKVSRGAI-FLSALKIL----------------DKYGSSPN-ILEVEFLN-------EVG
TGLG-PTLEF---FAMV----SKEFAHKSL--------KMWRTENFKSWK----------
---------GSELVEEHLFPAPLL------------------------------------
ASSHQDKTLELFQKLGTFVSRSMLDDRILDFRFSK--AFFELAHM------------YAQ
SEKIRFDDFDMTIKYMSM----------------------VDPHLAKS--------MKYL
STLEDDH---DFD-SLS--
--LTFYL-------------------------------SG--Y-DIE-LIEN--------
GKDLA---
------------------------------VTVENFH-DYFLR-VSDQVLGAG-VEKQLH
-SFIEG---FSRTFPY--TSLL-ALTPE-ELTDLFGS-----------------------
---VEEDWT-----------PETL---------YAYVDADHG------------------
----------------YSHD----SNII-HDLISIMT--------------------SFS
KAERRLFLEF-------LTGSPKLP------------IGG-----FK-SLNPRM--TVVR
KHTENDLEPD------------------------HYLPSVMTCA-NY-LKLPKY-SNKDV
LKSRVQ-QAMNE-GLG-
>Lachancea_waltii_AADM01000093.1 .
LRVSRDDL-LLSAIKIL----------------TKYGSSPN-ILEIEYQN-------EVG
TGLG-PTLEF---YAIV----SKEFARKSL--------GLWRCDNYAFHD----------
---------KTGFVEGLLYPAPLR------------------------------------
AANDEKRILELFSQLGAFVSRSMLDDRLLDFRFNR--AFFELSHM------------RCR
GETLSFADLEFALQYLDM----------------------VDPQLTKS--------LRYV
LGLKNDQ---DIE-SLA--
--LTFYL-------------------------------PG--Y-DVE-LVEN--------
GKNTP---
------------------------------VTASNLR-EYLEK-VLDQVLGTG-VEKQIS
-SFIQG---FSQAFPY--SSLL-ILTPD-ELTKLFGR-----------------------
---IEEDWT-----------TGTL---------LSYIDADHG------------------
----------------YNQD----SEII-HNLVTVMS--------------------SFT
DSERRLFLQF-------LTGSPKLP------------IGG-----FK-SLSPKL--TVVR
KHTEDNLEPD------------------------QYLPSVMTCA-NY-LKLPKY-SSLEV
LRSRLI-QAMTE-GGG-
>2_Lachancea_mirantina_FJUN01000018.1 .
LRISRENA-FLSALKIL----------------AKYGSTPN-MLEVEYQN-------EVG
SGLG-PTLEF---YATI----SKEFAKKSH--------LMWRRNDYGLSG----------
---------SDNFETSLLFPAPLA------------------------------------
NNADSPKILELFEHLGNFVSRSMLDNRILDFRFSE--AFFQLAHT------------SAR
GDKVDLTNRAVCLDLLHL----------------------VDPQIEKS--------IAYL
LDQKNAS---EVE-NLA--
--LTFYL-------------------------------SG--Y-DIE-LVTG--------
GKNIV---
------------------------------VTSENLE-VYLEK-LLDQLLGSG-VQKQIQ
-SFIDG---FSRAFPY--ASLL-IFSPA-ELTNLFGM-----------------------
---IEEDWT-----------TETL---------YSYINADHG------------------
----------------YTMD----SVSI-HDFVSILS--------------------DFI
PEERRLFLQF-------LTGSPKLP------------IGG-----FE-SLNPKF--TVVM
KHADEGMQPD------------------------HYLPSVMTCA-NY-LKLPKY-SSKSV
MRSRII-QAMKE-G---
>Lachancea_kluyveri_AACE03000003.1 .
LRISRDNM-FLSALKII----------------NKYGSTPN-ILEIEYQN-------EVG
TGLG-PTLEF---YASV----SKDFAKKSL--------RMWRCNDYTTNK----------
---------TEGFVEELLFPAPMS------------------------------------
STEDESRILELFGYLGTFVARSMLDNRILDFRFSK--VFFQLMHL------------HAK
GQRLDLNQPEQLIDIVSM----------------------IDAQIGKS--------LQYL
YDNQNSS---NLE-SLA--
--LTFTV-------------------------------PG--Y-DIE-LVAN--------
GGDIV---
------------------------------VNSANVG-TYIEK-VIDQILQSG-IEKQIK

-SFIQG---FSKAFPY--TSLS-ILTPE-ELSDLYGR-----------------------
---VEEDWS-----------EKTL---------YSFINADHG------------------
----------------YNMD----SATI-HDLVSVLS--------------------SFN
RQEQRLFLQF-------LTGSPKLP------------IGG-----FK-SLKPRL--TVVL
KHTEDNLTPD------------------------EYLPSVMTCA-NY-LKLPKY-SCREI
LRSRIV-QAMNE-GSG-
>Lachancea_fermentati_FJUO01000031.1 .
LRISRKTI-FLSALKII----------------NKYGSTPN-ILEIEYQD-------EVG
TGLG-PTLEF---YAVV----SKEFARKSL--------GMWRHENCDSHD----------
---------QKEFVDTSLFPAPIL------------------------------------
ASKDEDRIVELFKHLGNFVSRSMLDNRILDFRFNR--AFFEITHS------------VCR
GGTLDLDDPKLLLGLLSL----------------------VDNQIAKS--------LGYL
LDQQEQE--DFQ-TMC--
--LTFTL-------------------------------PG--Y-DIE-LVEN--------
GREVI---
------------------------------VDSSNVQ-EYIKK-VIDQMLVTG-IERQVK
-SFIEG---FSRAFPY--SSLL-ILTPE-ELTRLFGQ-----------------------
---VEEDWS-----------TETL---------YSCMNADHG------------------
----------------YTMD----SPSV-NDLILVMT--------------------TFS
LEERRLFLQF-------LTGSPKLP------------IGG-----FK-SLNPRL--TVVL
KHPEEGQKPD------------------------EYLPSVMTCA-NY-LKLPKY-SNKEI
LRSRIS-QAMNE-GSG-
>Lachancea_cidri_FJUT01000004.1 .
LRISRKNI-FLSALKII----------------SKYASTPN-ILEIEYQH-------EVG
TGLG-PTLEF---YAVV----SKEFARKSL--------GMWRYDNYGSCE----------
---------EEDFVETLLFPAPLS------------------------------------
ASHDEARIVELFKHLGNFVSRSMLDNRILDFRFNK--AFFEVAHA------------HCR
GEKLNLDDPKSLLNLLVM----------------------LDSQIAKS--------LSYL
LAHKEED--NLE-SLA--
--LTFTL-------------------------------PG--Y-DIE-LVEN--------
GREIL---
------------------------------VDSENVD-SYIMK-TIDQMLGSG-VEKQIE
-SFIQG---FSNGFPY--SSLL-ILAPE-ELAKLFGQ-----------------------
---IEEDWS-----------TETL---------YSCVNADHG------------------
----------------YNMD----SSSV-HDLISVMS--------------------TFT
PEERRLFLQF-------LTGSPKLP------------IGG-----FK-SLNPRL--TVVL
KHPEEGQQAD------------------------EYLPSVMTCA-NY-LKLPKY-SSKEV
LKSRIV-QAMNE-GSG-
>Ashbya_gossypii_NM_208312.2 .
LRISRENM-FLSALKIL----------------SKYGSSPS-VLEIEYQD-------EVG
IGMG-PTLEF---YASV----SKEFARKSL--------GMWHGSSVPGDD----------
------------FLDGLLFPAPLS------------------------------------
PKRNGAKIYELFTHLGTFVARSMLDNRILDFRFNR--VFFDLMHT------------AAR
REELDFAHPAKLLESLRL----------------------IDPQLAKS--------LAFV
REPRNWP--DLE-SMA--
--LSFTV-------------------------------PG--Y-DFD-LVEG--------
GANTP---
------------------------------VTAANAE-LYVAR-VLDAFLGSG-VRQQLR
-AFMEG---FSRAFPY--SSLL-VLTPD-ELSELYGR-----------------------
---VEEDWS-----------VETL---------YSYITADHG------------------
----------------YSMD----SPTL-HDLIAVMA--------------------AFD
QHHRRLFLQF-------LTGSPKLP------------VGG-----FK-NLKPHL--TVVL
KHPEGDLSPD------------------------QYLPSVMTCA-NY-LKLPKY-SSREV
LRARIV-HAIHE-GSGA
>Saccharomycetaceae_sp._CP006021.1 .
LRISRENM-FLSALKIL----------------SKYGSSPS-VLEIEYQD-------EVG
IGMG-PTLEF---YASV----SKEFSRKSL--------NMWHGSHVSGGD----------
------------FIDDLLFPAPLS------------------------------------

PQRDEAKLYELFTHLGTFVARSMLDNRILDFRFNK--VFFELMHS------------AAR
HEELDFAHPTKLLELLRL----------------------IDPQLAKS--------LAYI
REPRNWP--DLE-SLA--
--LSFTV-------------------------------PG--Y-DID-LIEG--------
GANTP---
------------------------------VTPANAE-LYVTS-VLDAFLGSG-VRQQLR
-AFMEG---FSRAFPY--SSLL-VLTPE-ELSELYGR------------------------
---VEEDWS-----------LETL---------YSYITADHG------------------
----------------YSMD----SPTL-HDLIAVMA--------------------AFD
KHHRRLFLQF-------LTGSPKLP------------VGG-----FK-NLKPHL--TVVL
KHPEGGLSPD------------------------QYLPSVMTCA-NY-LKLPKY-SSREV
LRARIV-HAIHE-GSGA
>Eremothecium_cymbalariae_XM_003644934.1 .
LRILRENM-FLSALKIL----------------SKYGSSPN-VLEIEYQD-------EVG
TGMG-PTLEF---YASV----SKEFARKSL---------QMWHCPDNDTDD----------
----------DGFVKGLLFPSPLS------------------------------------
QDKNEVKILELFSHLGTFIARSMLDNRILDFRFNR--VFFELMHC------------RVR
GEQLDFTHPEILFELLEI----------------------IDPQLESS--------LKYI
WNNRDSA--SLE-ALS--
--LYFVL-------------------------------PG--Y-KLQ-LIEN--------
GANIP---
------------------------------VTSENVA-LYISE-VFDAFLGSG-IDRQLT
-SFIEG---FSRAFPY--SSLL-VLTPN-ELCDLFGR------------------------
---VEEDWS-----------IETL---------YSCITADHG------------------
----------------YSMD----SPTL-HNLIDVMF--------------------AFE
KHERRLFLQF-------LTGSPKLP------------IGG-----FK-NLKPHL--TVVL
KHPEGDLTPD------------------------QYLPSVMTCA-NY-LKLPKY-TTKDV
LRSRIV-QAMNE-GSGA
>Eremothecium_coryli_AZAH01000012.1 .
LRISRENI-FLSALKIL----------------SKYGPTPN-ILEIEYQD-------EVG
TGMG-PTLEF---YAIV----SKEFARKSL---------HLWHCDPRGEAE----------
------------YVDGLLFPAPLS------------------------------------
PERDTDKVLELFSHLGTFIARSMLDNRILDFRFNR--IFFELMHA------------KVR
NEKLHALPLESMLSLLKE----------------------VDPRLESS--------LRYV
SDHRDPT--SLQ-SFS--
--LFFTA-------------------------------PG--Y-NIE-LIEN--------
GVNIP---
------------------------------VTPANVD-LYLSK-VCDAFLGTG-IERQLD
-SFIEG---FSKAFPY--SSLL-IFSPS-EICDLYGR------------------------
---VEEDWS-----------PETL---------LSCIIADHG------------------
----------------YSMD----SPTL-CDLIHVMA--------------------SFD
NHQRRLFLQF-------LTGSPKLP------------IGG-----FK-NLKPRL--TVVL
KHPEGDLTAD------------------------QYLPSVMTCA-NY-LKLPKY-SSRDI
LRSRII-QAMQE-GSG-
>Eremothecium_sinecaudum_XM_018130421.1 .
LRIARETI-FLSALKIL----------------TKYGSSPN-ILEIEYQD-------EVG
TGMG-PTLEF---YSSV----SKEFARKSL---------HMWHIGSDNEGE----------
------------YISGLLYPGPIS------------------------------------
PSQDRAKVLELFTHLGTFVARSMLDNRIVDFRFNR--VFFELMHS------------KIK
NEPLDFNSTEKMFKLLGA----------------------IDPQLESS--------LRYI
HTHRDSE--TLE-ALS--
--LNFTV-------------------------------PG--Y-DID-LIEG--------
GSSTA---
------------------------------ITTSNVD-LYSSK-LFDAFLGSG-VESQLS
-NFIDG---FSKAFPY--SSLL-VFAPS-ELSDLFGS------------------------
---VEEDWS-----------TETL---------FSFIMADHG------------------
----------------YTMD----SPTL-HDLIVVMS--------------------SFS
LEERRNFLQF-------LTGSPKLP------------MGG-----FK-NLKPHL--TVVL

KQPEGDLTSD------------------------QYLPSVMTCA-NY-LKLPKY-SNREI
LRARII-QAINE-GSGA
>Tetrapisispora_phaffii_XM_003684941.1 .
VKLSRDNL-FASGLKTI----------------DKYGSNRN-TLEIEYID-------EEG
TGLG-PTLEF---YASM----SLEFAKPLL--------DIWLCDEEVGNT----------
---------DTKFIEQELFPKPLS------------------------------------
TCSDANKTIELFRILGKFIAKSMLDGRIVDFRFKE--VFFQLLHE---------RIVADD
TKNSSRKQILYSLDILSL----------------------VDTNLSQS--------LKYL
YNNMSDND---IIK-GLD--
--LNFVL-------------------------------PG--KQIVE-IIDG--------
GNNIL---
------------------------------VTSDNVE-KYITG-VIDQMLYTG-IEKQLD
-AFIKG---FSECFPY--KNLL-ILTPK-ELVELFGK-----------------------
---EEEDWS-----------ESTL---------YSSIKAEHG------------------
----------------YTMD----SQTV-HDLINILK--------------------YFN
INQRRLFSQF-------LTGSPKLP------------IGG-----FK-CLKPKL--TVVL
KTPEDNLTPD------------------------QVLPSVMTCA-NY-LKLPKY-SSKEI
LKSRID-QAMRE-GSGA
>Vanderwaltozyma_polyspora_XM_001644641.1 .
LRITRSDL-FASGLKIL----------------ENYGSNPN-IIEIEYQD-------EAG
TGLG-PTLEF---YATM----SRDFAKKSL--------DIWSFGETEDE-----------
---------NEIYIQRSLFPRPLE------------------------------------
TCTDQTKTLELFTYLGEFVAKAILDGRILDFRFNR--LMFELIEE------------SYN
ETSIRNSDVLTQLKRLSV----------------------VDYQLAKS--------LQYI
FDQKNNNK---AIS-DLE--
--LTFVL-------------------------------PG--Y-GLE-LIEH--------
GKNVP---
------------------------------ITSANVE-EYISR-VLSMMIEDG-VKKQLN
-CFKEG---FSRVFPY--RNML-ILTPN-ELVTMFGE-----------------------
---AEEDWS-----------AETL---------YSCITAEHG------------------
----------------YTMD----SQTI-HDLVHIIS--------------------NFD
NQDRRLFLQF-------ITGSPKLP------------IGG-----FK-SLKPKL--TVVL
KHAEDGSSPD------------------------EYLPSVMTCA-NY-LKLPKY-SDKLI
MSSRIS-QAIKE-GSGA
>Candida_nivariensis_CAPV01000093.1 .
LRISRKDL-FPTGLKVL----------------EKFGGDSA-IIEIEYID-------EVG
TGLG-PTLEF---YATM----SKEFARKSL--------KMWHSTNSTDDS----------
------DDEFSAYISNLLYPAPMM-----------------------------------Y
HDSEQAKILDLFKQLGVLVARSLYDNRILDFRFNS--VFFTLLHA-------HARESHKD
VLSIEPAHVYNQLALLEK----------------------IDSQLSSS--------LKYI
YDNRENRN---LIE-QLS--
--VSYTL-------------------------------PG--Y-NSD-LIED--------
GSNKF---
-----------------------------LDDVTSVE-KYISL-VLDMFLGSG-IANQLE
-KFIEG---FSRVFPY--KNLL-LFLPE-ELCDLFGR-----------------------
---VHEDWS-----------PQTL---------YSYLIADHG------------------
----------------YTMD----SPPV-KELISIMT--------------------GFN
EQEKRVFLQF-------LTGSPKLP------------LGG-----FK-ALKPKF--TVVL
KHAEDGLQPD------------------------DYLPSVMTCA-NY-LKLPKY-SSKEV
MRKKII-QAMEE-GAG-
>Nakaseomyces_delphensis_CAPT01000034.1 .
LRISRKDL-FPTGIKIL----------------EKFGGDSA-IMEVEYID-------EVG
TGLG-PTLEF---YAIM----SKEFAQKSL--------KMWHLTSSTDDS----------
------NEEFSAYIPNLLYPAPMI-----------------------------------Y
KGTEREKILDLFKQLGVLVARSLYDNRILDFRFNS--VFFLLLHG-------HSRKDAND
EFSTEPAHVYNQLLLLEK----------------------VDIQLATS--------LKYI
YDNRENRS---LIE-QLS--
--VSYTL-------------------------------PG--Y-NSE-LIDD--------

```
GANKF-----------------------------------------------------------
-----------------------------IDDRTSVE-KYISL-VLDKFIGSG-VDAQLD
-SFIEG---FSRVFPY--KNLL-LFLPG-ELCDLFGR-----------------------
---VQEDWS-----------PQTL---------YSYLIADHG------------------
----------------YTMD----SPPV-KDLIAIMT--------------------EFN
DYEKRMFLQF-------LTGSPKLP------------LGG-----FK-ALKPKF--TVVL
KHAEDGLGPD------------------------DYLPSVMTCA-NY-LKLPKY-SSKAV
MKRKIF-QAMQE-GAG-
>Candida_bracarensis_CAPU01000122.1 .
LRISRKDL-FPTGLKVL----------------DKFGGDSA-VMEYAYIE-------EVG
TGLG-PTLEF---YATM----SKEFAKKSL--------KMWHSTSVAEKV----------
-TNTNEIDEIDNYVTTLLYPAPMV-----------------------------------Y
QDSEKAKILDLFKQLGVLVARSLYDNRILDFRFNS--IFFTLLHN-------LVRKVYND
ALSTEPVHVYNLLSMLEE----------------------IDNQLAKS--------LKYI
YDNRENKS--------------------------------------------MIE-QLS--
--VSYTL--------------------------------PG--Y-NLE-LIDG--------
GKNKF-----------------------------------------------------------
-----------------------------LDDATSVE-KYISL-VLDMFLGSG-VTEQLE
-KFIEG---FSRVFPY--RNLL-LFQPG-ELCDLFGR-----------------------
---VQEDWS-----------PQTL---------YSYLIADHG------------------
----------------YTMD----SSPV-KDLISIMT--------------------EFD
EQEKRVFLQF-------LTGSPKLP------------LGG-----FK-ALKPKF--TVVL
KHAEDGLHPD------------------------DYLPSVMTCA-NY-LKLPKY-SSKEI
MVKKIM-QAMQE-GAG-
>Candida_glabrata_XM_449789.1 .
LRIPRKDM-FSTGVKVL----------------KRFGGDST-VMEVEYID-------EVG
TGLG-PTLEF---YAII----SKEFARKSL--------NMWHCLDDNMKH--------VA
DGTPNDNTMSEVYVLNPLFPAPMI-----------------------------------P
EEKKRDEVLDLFNQLGVFVARSLYDNRILDFRFNP--VFFMLCHA-------ITKNEGLS
TLTLDSDQVYQQLSILAN----------------------IDSQLAAS--------LRYI
YNNRTNTP--------------------------------------------LIK-SLS--
--LTFTL--------------------------------PG--Y-NFE-LVEH--------
GNDHA-----------------------------------------------------------
-----------------------------VDDLTSVE-KYISL-VLDMFLGTG-IAPQLN
-SFIDG---FSRLFPY--KNML-LFSPN-EIADLFGR-----------------------
---VEEDWS-----------PETL---------FTYLVADHG------------------
----------------YTMD----SPTI-RDLISIIS--------------------TFD
NQEKRMFLQF-------LTGSPKLP------------LGG-----FK-SLKPKL--TVVL
KHAEDGLQPD------------------------DYLPSVMTCA-NY-LKLPKY-SSREV
MRQQIL-TAMTE-GADS
>Saccharomyces_cerevisiae_NM_001179576.3 .
LRISRKTI-FATGLKIL----------------SKYGSSPD-VLEIEYQE-------EAG
TGLG-PTLEF---YSVV----SKYFARKSL--------NMWRCNSYSYRS----------
---EMDVDTTDDYITTLLFPEPLN------------------------------------
PFSNNEKVIELFGYLGTFVARSLLDNRILDFRFSK--VFFELLHR-----------MSTP
NVTTVPSDVETCLLMIEL----------------------VDPLLAKS--------LKYI
VANKDDNM--------------------------------------------TLE-SLS--
--LTFTV--------------------------------PG--NDDIE-LIPG--------
GCNKS-----------------------------------------------------------
------------------------------LNSSNVE-EYIHG-VIDQILGKG-IEKQLK
-AFIEG---FSKVFSY--ERML-ILFPD-ELVDIFGR-----------------------
---VEEDWS-----------MATL---------YTNLNAEHG------------------
----------------YTMD----SSII-HDFISIIS--------------------AFG
KHERRLFLQF-------LTGSPKLP------------IGG-----FK-SLNPKF--TVVL
KHAEDGLTAD------------------------EYLPSVMTCA-NY-LKLPKY-TSKDI
MRSRLC-QAIEE-GAGA
>Saccharomyces_pastorianus_ABPO01001141.1 .
LRISRKTI-FATGLKIL----------------SKYGSSPD-VLEIEYQE-------EAG
```

TGLG-PTLEF---YSVV----SKYFARKSL--------NMWRCNSYSYRS----------
---EMDVDTTDDYITTLLFPEPLN------------------------------------
PFSNNEKVIELFGYLGTFVARSLLDNRILDFRFSK--VFFELLHR-----------MSTP
NVTTVPSDVETCLLMIEL----------------------VDPLLAKS--------LKYI
VANKDENM---TLE-SLS--
--LTFTV-------------------------------PG--NDDIE-LIPG--------
GCNKS---
------------------------------LNSSNVE-EYIHG-VIDQILGKG-IEKQLK
-AFIEG---FSKVFSY--ERML-ILFPD-ELVDIFGR-----------------------
---VEEDWS-----------MATL---------YTNLNAEHG------------------
----------------YTMD----SSII-HDFISIIS--------------------AFG
KHERRLFLQF-------LTGSPKLP------------IGG-----FK-SLNPKF--TVVL
KHAEDGLTAD------------------------EYLPSVMTCA-NY-LKLPKY-TSKDI
MRSRLC-QAIEE-GAGA
>Saccharomyces_kudriavzevii_AJIG01002459.1 .
LRISRKTI-FATGLKIL----------------SKYGCSXD-VLEIEYQE-------EAG
TGLG-PTLEF---YSVV----SKYFARRSL--------NMWRCNSYNYRS----------
---EMDIDTTDDYITTLLFPKPLN------------------------------------
LSADNEKIIELFGYLGTFVSRSLLDNRILDFRFSK--VFFELLHR-----------MCTP
DVTTVPGDVGSCLLMIDL----------------------VDPLLAKS--------LKYL
VANKNNNT---ALE-ELS--
--LTFTV-------------------------------PG--NDDIE-LVPD--------
GNNKS---
------------------------------LNSSNIE-EYIHA-VIDQILGKG-IEKQLV
-AFSEG---FSKVYSY--ERML-ILFPE-ELVDIFGR-----------------------
---VEEDWS-----------TGTL---------YTNLNAEHG------------------
----------------YTMD----SSII-HDFISIIS--------------------AFN
KHERRLFXXF-------LTGSPKLP------------IGG-----FK-SLKPRF--TVVL
KHAEDGLTAD------------------------EYLPSVMTCA-NY-LKLPKY-TSKDI
MRSRLC-QAIEE-GAGA
>Saccharomyces_eubayanus_XM_018366434.1 .
LRISRKTV-FATGLKIL----------------SKYGGSPD-VLEIEYQE-------EAG
TGLG-PTLEF---YSLV----SKYFARKSL--------NMWRCNSYSYRS----------
---EMNMDTTDDYITTLLFPEPLD------------------------------------
PTANNEKILELFGYLGTFVARSLLDNRILDFRFSR--VFFELLHR-----------MCTP
NLTTVPSDVESCLSMIEL----------------------VDPLLAKS--------LKYI
VANKNNNN---TLE-ELF--
--LTFTV-------------------------------PG--NDDIE-LIPD--------
GSNKF---
------------------------------LNSSNVE-EYICA-IIDQILGKG-IERQLK
-AFSEG---FSKVFLY--ERML-ILFPE-ELVDMFGR-----------------------
---VEEDWS-----------VGTL---------YTNLNAEHG------------------
----------------YTMD----SSII-HDFISIIS--------------------TFN
KQERRLFLQF-------LTGSPKLP------------IGG-----FK-SLNPKF--TVVL
KHAEDGLTAN------------------------EYLPSVMTCA-NY-LKLPKY-SNKDI
MRSRLC-QAIEE-GAGA
>Saccharomyces_bayanus_AACA01000313.1 .
LRISRKTI-FATGLKIL----------------SKYGGSPD-VLEIEYQE-------EAG
TGLG-PTLEF---YSLV----SKYFARKSL--------NMWRSNSYSYRS----------
---EMSMDTTEDYITTLLFPEPLN------------------------------------
LTADNEKILELFGYLGTFVARSLLDNRILDFRFSR--VFFELLHR-----------MCTP
NVTTVPSDVESCLLMIEL----------------------VDPLLAKS--------LKYI
FANKNNNN---ALE-ELF--
--LTFTV-------------------------------PG--NDDIE-LVPG--------
GSSKF---
------------------------------LNSSNVE-EYIHA-VIDQILGKG-IERQLK
-AFSEG---FSKVFLY--ERML-ILFPE-ELVDMFGR-----------------------
---VQEDWS-----------VGTL---------YTNLNAEHG------------------

----------------YTMD----SSII-HDFISIIS--------------------TFN
KKERRLFLQF-------LTGSPKLP------------IGG-----FK-SLNPKF--TVVL
KHAEDGLTSD------------------------EYLPSVMTCA-NY-LKLPKY-SNKDI
MRSRLC-QAIEE-GAGA
>Nakaseomyces_bacillisporus_CAPX01000095.1 .
LRVSRSGL-FATGLKVL----------------DKYGASPH-ILEIEYHN-------EAG
TGLG-PTLEF---YSTI----SKDFANKSL--------GMWRCDESNSST----------
------------YVDTLLFPSYTS-----------------------------------E
PDESFPKKIDLFKLLGTFIGRSMLDNRIVDFRFNV--LFFEIMHN----------IVRDK
MWSVEGCGIPEAFRLLSL----------------------LDEDVAKN--------LFYV
WENRNDDS---LLN-SLT--
--LSFKD-------------------------------ID--------------------
-GDMP---
------------------------------VNSKNVK-RYIWN-QINEKIGTG-ILPFAR
-AFIDG---FTKVFPY--SDLL-ILSPN-ELVDMLGR-----------------------
---VEEDWS-----------IETL---------YNCMNADHG------------------
----------------YTMD----SKII-DDLISLLS--------------------NFS
WTEKRKFLQF-------ITGSPKLP------------IGG-----FK-GLKPKF--TVVL
KHAENGLTPD------------------------QYLPSVMTCA-NY-LKLPKY-SSKEI
MYARLT-QAMDE-GSGA
>Candida_castellii_CAPW01000064.1 .
LRIDRDNL-FAAGVKIL----------------EKYGSSPH-TLEIEYQN-------EEG
TGLG-PTLEF---YSKM----SKSFSSNEL--------DLWRNDTTKDSG----------
------------YVENLLFPKPAR-----------------------------MIYNTRI
QQDTLDKKMQLFNTLGTLLARSILENRILDFRFNA--TFFEMMHNIVR-CNKATSDNNYY
QNLKEAYGLEESFRLLTQ----------------------IDVQVARS--------LRYI
WDNREQDV---LLS-DLS--
--LTYIS-------------------------------DR--T-PPD-GEP---------

------------------------------LNKANAI-HYVWQ-QLDAALGSG-IMPLLV
-SFIEG---FNRVFPY--TNVL-VLEPK-ELVELFGR-----------------------
---AEEDWS-----------TETL---------FGAITADHG------------------
----------------YTMD----SNTV-HDLIEVMS--------------------NFT
LEQRREFLQF-------LTGAPKLP------------IGG-----FK-ELKPQF--TVVL
KHAEENMAAD------------------------QYLPSVMTCA-NY-LKVPKY-SSSEI
LRSRLI-QAMQE-GSDA
>Kazachstania_africana_XM_003957072.1 .
LKISRDTM-FLTGLKIL----------------EKYGSSPS-VLEIEYQE-------EVG
TGLG-PTLEF---YAMI----SKAFAKKNL--------NMWLQSGYAPQS----------
------DSEQSTYVENLLYPNPID-----------------------------------L
SKAESSKILDLFEYLGTFAARSMIDNRILDFRFNR--LFFHVAHH----------LARHG
NLTSLFNNIDDGINLVKE----------------------LDSQVANS--------LKYV
HDNKDNSD---VID-QLY--
--LNFVL-------------------------------PG--T-DIE-LIEN--------
GRSVH---
------------------------------VQAFNAD-EYIRQ-ILSQLMGKG-IKSQVE
-SFMRG---FSKVFPY--KNLL-ILSPS-ELIDLFGS-----------------------
---VKEDWS-----------PELL---------YTSMSADHG------------------
----------------YTMS----SPAI-ADLISIMS--------------------NFS
RQEKRLFLQF-------LTGSPKLP------------LGG-----FK-ALKPKF--TVVL
KHAEGDLKPD------------------------QYLPSVMTCA-NY-LKLPKY-SSKEI
MHKRIK-QAMEE-GAGA
>Kazachstania_saulgeensis_FXLY01000002.1 .
LRIPRDKI-FLTGLKIL----------------DKYGSNPS-ILEMEYQD-------EVG
TGLG-PTLEF---YATM----SKDFSRQSL--------GMWRVDNYNSKI----------
--DESDGDTNQEYVTQLLFPMSID-----------------------------------P
LVEKRGKIKELFFYLGEFIARSMLDNRILDFNFNT--LFFSLTHE----------LVRNQ
KLPDYMANVEQSIDMISY----------------------IDKQIGNS--------LKYM

YENKANAG---KIE-ELY--
--LTFIV-------------------------------PG--T-TIP-LIDN--------
GDETS---
------------------------------VTSENIL-EYINL-LISGLIGDG-IKQQLL
-AFIDG---FSKVFPY--SNLL-ILSPE-ELIDIFGR-----------------------
---VEEDWS-----------KETL---------YSCIVSDHG------------------
----------------YTMD----STII-HDLISIMS--------------------SFN
PKDKRLFTQF-------LTGSPRLP------------FGG-----FK-ALKPKF--TVVL
KHAEDGLSPD------------------------QYLPSVMTCA-NY-LKLPKY-SNQEI
MRTRIQ-QAIEE-GAGA
>Kazachstania_naganishii_HE978314.1 .
LRIPRKNM-LLTALKVL----------------DKYGSVPS-VLEFEYQD-------EVG
TGLG-PTLEF---YATV----SREFAKKNL--------NLWRTDTYEESR----------
-------AEENEYITKPLFPSALT-----------------------------------G
DSEGRARIIELFGTWARLSPGPLWITESLTSVSNR--LFFILADR-----------LSRP
SMNNQCEATEDNLELIKL----------------------VDPQIAES--------LRYV
YNNIDNDT---ELS-EMY--
--LSFVL-------------------------------PG--S-DIE-LVEN--------
GRAVL---
------------------------------VNSGSAY-HYITK-VISYMIGDG-VKDQIR
-AFIDG---FSKVFPY--SNVS-ILTPE-ELVDWFGG-----------------------
---VEEDWS-----------PQVL---------YGCIEANHG------------------
----------------YTMD----SDTI-HQFISIMT--------------------ELN
ARERRLFLQF-------LTGSPKLP------------LGG-----FK-VLKPRL--TVVL
KHAEDGLTPD------------------------QYLPSVMTCA-NY-VKLPKY-SSKEV
MRDRIK-QAIEE-GAGA
>Naumovozyma_dairenensis_XM_003672597.1 .
LRISRNTI-FLTGLKIL----------------SKYGSSPS-ILEIEYQD-------EIG
TGLG-PTLEF---YATL----SKEFAKKPL--------KMWRTDDYASKV----------
----EEQGDDTDYISELLFPAPID------------------------------------
LKADNAKLLELFEFLGTFIGRSMLDSRILDFRFNK--LFFEFTHK----------RYRKM
DLVDTCNDFESLCTKLSL----------------------VDAQLSRS--------LHYL
YENKGDDD---KIS-SLT--
--LTFVL-------------------------------PG--T-DIE-LIEN--------
GANIL---
------------------------------VNSINVE-DFIAK-VLSYVLDTG-IVQQLD
-AFIKG---FSKVFPY--VGLL-SLMPD-ELVELFGR-----------------------
---VEEDWS-----------PKTL---------YSCISADHG------------------
----------------YTID----SPTI-HELIAIMA--------------------DFD
GQERRLFTQF-------LTGSPKLP------------IGG-----FR-GLKPKF--TVVL
KHAEYDLTPD------------------------QYLPSVMTCA-NY-LKLPKY-SDQQI
MRSRLK-QAMEE-GSGA
>Naumovozyma_castellii_XM_003673402.1 .
LRVSRKNM-FLTGLKII----------------GKYGSSPS-VLEIEYID-------EVG
SGLG-PTLEF---YASI----SKEFAKNSL--------HLWRSEDFDKKA----------
--NATDGESSSDYVKDLLFPAPLD------------------------------------
SKCDNAKTLELFESLGTFIARSMLDSRILDFRFNK--LFFELLHK----------RFTKV
ALLDNLGDLKGMLQMLSR----------------------VDEQLAKS--------LEYL
YDHKDDSV---SLE-ALS--
--LTFVL-------------------------------PG--Y-NIE-LIPD--------
GTNTF---
------------------------------VNATNVE-DFIQK-VLESTLGRG-IEKQLD
-AFIDG---FSKVFPY--VALL-ILRPE-ELTELFGR-----------------------
---IDEDWS-----------TETL---------YNCMNADHG------------------
----------------YTMD----SQTI-HDLISIVT--------------------QFT
VQERRLFCQF-------LTGSPKLP------------IDG-----FK-GLKPKF--TVVL
KHAEDGLTPD------------------------QYLPSVMTCA-NY-LKLPKY-SSRDV
MRARIL-QAIEE-GAGA

>Tetrapisispora_blattae_XM_004182163.1 .
LRLSRKTL-FLSALKIL----------------NKYGSSTS-ILEMEYQN-------EVG
TGTG-PTLEF---YANV----SREFAKKSL--------NMWRDQNSGKED----------
-------EFESPYIIDSLFPKPLN-----------------------------------P
KNMNVERVIELFKYLGIFIARSMIDNRILDFRFNP--AFFEICQK-----------YSKN
YQDPNDSDNKTRFQLVEL----------------------IDNQVFKT--------LKYL
ETNSENDL---LLK-ELT--
--LTFVF-------------------------------PG--S-DLE-LIPN--------
GNEVY---
------------------------------VDSTNVK-QYIDL-LLGQMIGSG-ISKQVE
-SFIEG---FSEVFQF--SSLQ-MLTSY-ELTNIFGC-----------------------
---FEEDWN-----------EKTL---------FTYINAEHG------------------
----------------YTLD----SPTI-HDLIKVMC--------------------NLD
ETQRRKFVQF-------LTGAPKLP------------IGG-----FK-SLNPRL--TVVL
KHAEDGLKPD------------------------QCLPSVMTCR-NY-LKLPKY-STFDV
MRSRIL-QAIDE-GSGA
>Torulaspora_delbrueckii_XM_003678768.1 .
LRVSRETL-FLSGLKIL----------------DKYGSTAS-VLEIEYQG-------EVG
TGLG-PTLEF---YACI----SREFTRRNL--------NMWRCENFKSSA----------
------DVEVPDYVETSLFPTPLD------------------------------------
PSKDNKRVIELFRYLGIFVARSMLDNRILDFYFSR--VFFQLTHR----------RYRTK
GKDILVEDIEGGLELLAL----------------------VDEHLASS--------LCYL
YENREKAS---ELE-QMT--
--LTFTL-------------------------------PG--L-ELD-LIEN--------
GKDIN---
------------------------------VTSKNVE-EYIKL-VLNYTLDVG-IRQQLD
-SFIEG---FSVVFPY--SSLL-ILTPE-ELVEMHGR-----------------------
---VEEDWS-----------SSTL---------YASINADHG------------------
----------------YSMG----SNSI-HELVSIMS--------------------SFE
RQDRRLFLQF-------LTGSPKLP------------IGG-----FK-SLKPRL--TVVL
KHPEDGLGPD------------------------AYLPSVMTCA-NY-FKLPKY-SSGEM
MRSRIV-QAMNE-GSQA
>Candida_versatilis_LAVI01000092.1 .
LRLSRGTM-FLSGLKIL----------------NKYGASPS-VLEIEYQD-------EVG
TGLG-PTLEF---YASM----SREFSKKSL--------GMWRYEAFGSTS----------
-------TQESEYVNALLFPAPLT------------------------------------
SSQDESKVLELFHHLGTFVARSMLDNRILDLNFNK--VFFELTHR------------ISD
KASIKAKDTQDMLHLLSL----------------------VDPQLERS--------LKTL
YNEP---DLE-SLT--
--LTFTL-------------------------------PG--Y-DVE-LVEN--------
GKSIL---
------------------------------VNSTNLS-DYVDR-VLDYVLGAG-IHHQLN
-SFIEG---FSKVFPY--TSLL-LLTPD-EIVHMCGR-----------------------
---INEDWS-----------SETL---------YGSLVADHG------------------
----------------YTMD----SSTI-HDLICVMS--------------------TFD
DQERRLFLQF-------LTGSPKLP------------IGG-----FK-ALSPRL--TVVL
KHPEEGMNPD------------------------AYLPSVMTCA-NY-FKLPKY-TSQQT
MRSRIT-QAMQE-GSGA
>Zygosaccharomyces_rouxii_XM_002494959.1 .
LRISRSTM-FLSGLKIL----------------NKYGSSPS-VLEIEYQD-------EVG
TGLG-PTLEF---YASM----SREFSKKSL--------GMWRYDTLGSTT----------
-------TQESDYVNALLFPAPLT------------------------------------
SSQDEPKVLELFHHLGTFVARSMLDNRILDLNFNK--VFFELTHR------------ISD
RASVKARDTRDMLHLLSL----------------------VDPQLERS--------LKTL
YNET---DLE-SLT--
--LTFTL-------------------------------PG--Y-DVE-LVEN--------
GKSIL---
------------------------------VNSANLS-DYVER-VLDYVLGAG-IERQLN

-SFIEG---FSKVFPY--TSLL-LLTPE-EIVHMCGR----------------------
---IQEDWS-----------SETL---------YGSLVADHG-----------------
----------------YTMD----SSTI-HDLIYVMS--------------------TFN
DQERRLFLQF-------LTGSPKLP------------IGG-----FK-ALRPRL--TVVL
KHPDEGMNPD-----------------------AYLPSVMTCA-NY-FKLPKY-TSQQT
MRSRIT-QAMQE-GSGA
>Zygosaccharomyces_parabailii_CP019493.1 .
LRISRNTM-FLSGLKIL----------------NKYGSSPS-VLEIEYQD-------EVG
TGLG-PTLEF---YATM----SQGFSKKSL--------GMWRYDAYGSGQ----------
----LDAEGNSPYVNGLLFPAPLT------------------------------------
STQNEVKILELFHYLGTFVARSMLDTRILDLNINK--IFFELAHR----------ICNRH
GVPNKEMDEQDMLRLLSF----------------------VDPQLARS--------LRAL
YTEA--DLE-SLT--
--LNFTL------------------------------PG--Y-DIE-LVHE--------
GKSLL---
------------------------------VSSNNLN-QYVKR-VLDYTLGTG-IEKQLT
-SFIDG---FSTVFPY--SSLL-LLTPE-ELVHMCGR----------------------
---TMEDWS-----------SETL---------YASLVADHG-----------------
----------------YSMD----SSTI-HDLIYVMS--------------------TFN
DREKRLFLQF-------LTGSPKLP------------IGG-----FK-ALNPKL--TVVL
KHPEDGLQAD-----------------------EYLPSVMTCA-NY-FKLPKY-SSQTV
MRSRIL-QAMQE-GSG-
>Zygosaccharomyces_bailii_HG316458.1 .
LRISRNTM-FLSGLKIL----------------NKYGSSPS-VLEIEYQD-------EVG
TGLG-PTLEF---YATM----SQGFSKKSL--------GMWRYDAYGSGQ----------
----LDAEGNSPYVNGLLFPASLT------------------------------------
STQNEVKILELFHYLGTFVARSMLDTRILDLNINK--IFFELAHR----------ICNRH
GVPNKEMDEQDMLRLLSF----------------------VDPQLARS--------LRAL
YTEA--DLE-SLT--
--LNFTL------------------------------PG--Y-DIE-LVHE--------
GKSLL---
------------------------------VSSNNLN-QYVKR-VLDYTLGTG-IEKQLT
-SFIDG---FSTVFPY--SSLL-LLTPE-ELVHMCGR----------------------
---TMEDWS-----------SETL---------YASLVADHG-----------------
----------------YSMD----SSTI-HDLIYVMS--------------------TFN
DREKRLFLQF-------LTGSPKLP------------IGG-----FK-ALNPKL--TVVL
KHPEDGLQAD-----------------------EYLPSVMTCA-NY-FKLPKY-SSQTV
MRSRIL-QAMQE-GSGA
>Wickerhamiella_domercqiae_BCGM01000001.1 .
-RIARERL-LQGTLKLL----------------EMSKSLPS-QVEIEFFE-------EAG
TGLG-PTQEF---FALA----SHEFASQK---------NMWRDQQDQ-------------
----------------GLFPRAET------------------------------------
--GKTEERMEFFIGLGRLVARAILDRRLLDLRLSS--GFFTLVLD--------------
-----VXPTASALDLLEK----------------------VDRGLAFS--------LQHI
LETK--DPE-SLA--
--LDFTL------------------------------PG---TSTE-LVSG--------
GMEMD---
------------------------------VTNENKY-EYAQR-VAEMTLYSPSVIDAVR
-CFSKG---FGESVPV--SALH-AFLPE-ELATMFGG----------------------
-AGLNEDWS-----------ASTL---------KGAIRADHG-----------------
----------------YTME----SRPV-QELIEIMA--------------------QLT
PHQRRAFLAF-------MTGSPNLP------------FGG-----FR-SLQPPF--TVVL
KHPETEGGDRD----------------------LYLPSVMTCA-NY-LKLPEY-SSPEI
MHERLL-MAIQE-GAG-
>3_Candida_versatilis_BCJV01000001.1 .
-RIGRDGL-LQGGLKLL----------------EMSSASPA-IVDIEFYG-------EVG
TGLG-PTQEF---YADI----SREFALQK---------DMWRDGD---------------
----------------GLFPRAYA-----------------------------------Q

```
DDPRLENVCVKFKGLGRLIARALLDQRLLDFRFNL--GFFSVLLG---------------
-------LPSANILDL------------------------VDPMLLKF--------LNEL
PNQT-----------------------------------------------NIA-SLG--
--LDFTL-------------------------------PG---TNLE-MLPA--------
GADQD-------------------------------------------------------
------------------------------LTPNRVF-EYIYL-VKDWTLERG-IRHQLD
-AFVSG---FSASVSI--DALH-AFSPH-ELVMLFGG-----------------------
-GGNDADWS-----------IQTL---------QAVIKADHG------------------
----------------YTVD----SEPV-RTLVRVMS--------------------ALC
REERRQFLQF-------MTGSPNLP------------LGG-----FK-ALQPQF--TVVF
KHPENDAQPD------------------------LTLPSVMTCA-NY-LKLPAY-SSEDV
LRRQLR-TAISE-GSG-
>Candida_infanticola_LWLF01000022.1 .
VNVPRQDL-FANFVEVL----------------HMIGNSPA-ILEVEFTD-------EAG
TGLG-PTQEF---YAKV----SQEFASVSL--------DLWLGEDVNG------------
----------FLHHNQGLFPKPIA------------------------------------
---DFTRKVFEFRNLGRFISRAFMDQRILDFRFNP--AFFLAVLK---------------
----SQGFLQSTTLTLKD----------------------VDSDLYDN--------LQKL
DKE------------------------------------------------TVE-SLE--
--LTFVL-------------------------------PG--DEDVE-ICAD--------
GKQKL-------------------------------------------------------
------------------------------VTADNLD-EYKQR-VESFMLEDG-VKFQID
-QFVAG---FSENLPI--ASLQ-ALLPV-EICRMCGQ-----------------------
---SDEDWS-----------VETL---------SCSIHVDHG------------------
----------------YSSD----SPAV-QNLILVMS--------------------ALT
LEERRAFLQF-------TTGSPNLP------------IGG-----FK-ALQPPF--TVVL
KHPEDEKIDRD-----------------------QYLPSVMTCA-NY-LKLPNY-SSRAI
MARQIR-VAVEE-GS--
>Homoloaphlyctis_polyrhiza_AFSM01003790.1 .
------------------------------------------TLEIEFED-------EVG
TGLG-PTLEF---FSAA----SKGLRLCQX--------QIWRSDNGRGQL----------
--------SGVLDPANGLYPCPIS---------------------------------SNL
SRAETSRVADLFRCLGAFAGKALMDSRLLDLHFSP--LFLDQVMX---------------
--------------ALKM----------------------VDPGLANS--------LASL
FQLADAHNTDLNALNN-----------------------------------EIA-DLC--
--LETTL-------------------------------PG--YPSIQ-LQPA--------
TESIQT------------------------------------------------------
------------------------------ITTQQQLRDYLWN-VMDMTIAQG-VQLQIK
-SFRQG---FSAIYADRFEKFR-CMSGA-EIATLIGG-----------------------
--EGEEDWS-----------ISX------------SIKADHG------------------
----------------YHTD----SRTI-QDLIQMMS--------------------AFT
IDQRRQFLQF-------VTGSPKLP------------IGG-----FK-ALSPPL--TVVR
KSASMSVXTD------------------------MYLPSVMTCV-NY-LKVPEY-SSAVV
MKRQFE-IAMRE-G---
>Spizellomyces_punctatus_XM_016754372.1 .
VRIARQRI-LDSMIKVM----------------ELYGSTQA-LLEVEFFD-------EVG
TGLG-PTLEF---YANV----CRDLRKKDX--------KIWRDDDSLDSQAV---KDDNR
STKAGLVPDDYLNPALGFFPAPLT--------------------------------PAEV
DTEKGRKILMLYKALGTFVAKALLDSRIVDIPFSA--MFLEMVVG-----EEEEEESAAE
AALGTAGRKGAEFHLLRH----------------------VDPSLYKS--------LLDL
KKYVHIKRSLEADPTL-----------------SPAERAARISNITVKG-ARLE-DLF--
--LDFTL-------------------------------PG--YPSAE-LIPN--------
GKDIA-------------------------------------------------------
------------------------------LTLDNLE-HYIDR-VVEMTVGEG-VQRQVE
-AFRRG---FDRVFPA--ADLR-SFTVQ-ELAVLVGG-----------------------
--AEEEDWA-----------YDVL---------IDSIKADHG------------------
----------------YNSD----SRTI-KHLATFMS--------------------TLN
PIQRREFLQF-------VTGSPKLP------------LGG-----FK-ALNPSL--TVVR
```

KNVEAGKKPD------------------------DYLPSVMTCV-NY-LKVPDY-SELEV
MKMRFE-VAVRE-GQGC
>Linderina_pennispora_MCFD01000002.1 .
VRISRHRM-LESALKVM----------------ELYGTAKT-ILEVEYFD-------EVG
TGLG-PTLEF---YATV----SRTLRDKSL--------ELWX------------------

--SPTERAIQLFKFMGHFVAKGLIDGRNLDLPLHE--EFWAAVQR---------------
-XDDTKTGLSWSWAQLEH----------------------VDPAQTRS--------LQYL
QQFVDQRTEIYQREDL-----------------TADQKMTEAEAIRDPKTDASVDDLS--
--LDFTL-------------------------------PG--YPDIE-LREG--------
GSSIP---
------------------------------VTINNLP-AYIDQ-VAQWTLGKG-IQRQVH
-AFCDG---FDKIFPV--RDLS-IFAPG-ELSRLVGP-----------------------
-SSDSEDWG-----------MATL---------LGAVKADHG------------------
----------------FSLA----SPAV-KMFLDFME--------------------SLD
STGRRRFLRF-------VTGSPQLP------------FGG-----FR-ALHPPL--TLVH
KPHEAPLTPD------------------------DYLPSVMTCA-NY-IKLPNY-STPEI
LAERWS-RAVTE-G---
>Rhodotorula_toruloides_XM_016417872.1 .
VRISRDRI-LESAYKVF----------------ELYGSSRA-SLEVEFFN-------EVG
SGLG-PTLEF---YALV----SKEFARKAL--------GLWRAGDHSDTS----------
---------DYVHAKMGLFPVPLA----------------------------------DV
STDAGKKALKIFHVLGQFVAKALMDSRIIDVNFSR--AFMRLVLE---------------
------HDLPLNLASIKASLIA------------------VDPDLGKS--------LAHL
QEYVAEKEAVEADESR-----------------TEEQRVADLEAIQVRG-ATVS-DLT--
--LDFTL-------------------------------PG---FDLE-LKDG--------
GKDIA---
------------------------------VDIHNVE-EYIEL-VLDWTLRRG-VQAQIN
-EFKKG---FSTVFPV--RDLQ-TFTPA-ELVMITAA-----------------------
---VDEDWS-----------LETL---------TNATKADHG------------------
----------------FTMD----SRPV-REFLAHMA--------------------GLT
VEERREFLSF-------MTGSPRLP------------IGG-----FA-ALDPPL--TIVR
KDGGD-----------------------------AVLPSVMSCV-NY-VKLPDY-SSGEV
LKERIL-LAVRE-GAGG
>Rhodotorula_graminis_XM_018417113.1 .
VRISRDRI-LESAYKVF----------------ELYGSSRA-SLEVEFFD-------EVG
SGLG-PTLEF---YALV----SKEFARKTL--------GLWREGDHADKS----------
---------DYVHTRTGLFPMPLA----------------------------------DS
TTDAGKKVLRVFRILGQFVAKAMMDTRIIDVNFSR--TLMRLVLE---------------
------QDVPLSIASVTA----------------------VDPELGSS--------LSHL
QEYVAAKDEIEADEAA-----------------SEDDRRSKLDAVQVRG-ATVA-DLV--
--LDFTL-------------------------------PG---RNIE-LKEG--------
GAEIP---
------------------------------VTLDNLA-EYIDL-VIEWTLKRG-IEAQVA
-EFQRG---FSTVFPV--RDLQ-TFTPA-ELVMMTAA-----------------------
---VEEDWT-----------VETL---------TNSAKADHG------------------
----------------FTMD----SRPV-RDFLSIMA--------------------DFS
AEERREFLSF-------ITGSPRLP------------IGG-----FG-ALSPPL--TIVR
KDGGD-----------------------------AALPSVMTCV-NY-VKLPNY-SSRAV
VRERIL-MAVRE-GADG
>Microbot_violaceum_AEIJ01000314.1 .
VRIARDRV-LESTHKVF----------------ELYGSSRA-ALEVEFFD-------EVG
SGLG-PTLEF---YALA----SKEFARKNL--------RLWRDNDGHDDA----------
-------SSTYVHSPTGLFPRPIK----------------------------------NT
TSDSNAKVLKHFRVLGQFVAKAMMDSRIIDVHFSR--SFMKLVX----------------
-----------SLGFLQS----------------------IDRGLAKS--------LSIL
QDFVDVRRAILDDDTK-----------------SEVEQQSAIEAINVHG-SSIA-DLA--
--LDFTL-------------------------------PG---YDIA-LKPE--------

GDSIS---
------------------------------VTADNVE-EYISL-VLDWTLRRG-VASQVS
-EFQTG---FSAVFPV--RDMQ-SFTPS-ELVMMTSA-----------------------
---LQEDWS-----------IEGL---------TSATKADHG------------------
----------------FTMD----SRPV-RDLLSIMS--------------------ELS
LEDRREFLSF-------ITGSXRLP------------IGG-----FA-ALSPTL--TIVR
KDGGD-----------------------------PSLPSVMTCV-NY-FKLPDY-TSRQI
VKERIM-TAIRE-GGG-
>Microbot_lychnidis_dioicae_CZCF01000038. 1..
VRIARDRV-LESTHKVF----------------ELYGSSRA-ALEVEFFD-------EVG
SGLG-PTLEF---YALA----SKEFARKNL--------RLWRDNDGHDDA----------
-------SSTYVHSPTGLFPRPIK----------------------------------DT
ISDSNAKVLKHFRVLGQFVAKAMMDSRIIDVHFSR--SFMKLVLD---------------
------QELPLAIAXLQS----------------------IDRGLAKS--------LSIL
QDFVDARRAILDDDTK-----------------SEVEQQSAIEAITVHG-SSIV-DLA--
--LDFTL-------------------------------PG---YDIA-LKPE--------
GDSIS---
------------------------------VTADNVE-EYISL-VLEWTLRRG-VASQVS
-EFQTG---FSAVFPV--RDMQ-SFTPS-ELVMMTSA-----------------------
---LQEDWS-----------IEGL---------TSATKADHG------------------
----------------FTMD----SRPV-RDLLSVMS--------------------ELS
LEDRRVFLSF-------ITGSXRLP------------IGG-----FA-ALSPTL--TIVR
KDGGD-----------------------------PSLPSVMTCV-NY-FKLPDY-TSRQI
VKERIM-TAIRE-GGG-
>Cystobasidiopsis_lactophilus_BCIO01000002 1..

---------------LV----SKEFSRKDL-------KLWRSSDDASNP-----------
--------SIFINNACGLFPAPIM--------------------------------DENV
ESEGGRKVTSMFRVLGQFIAKGLMDSRIIDIPFGK--TFMKLVLD---------------
------QEIPLTTSSVKT----------------------VDPVLGSS--------LDHV
MAYVTAKQEILADASS-----------------SDQAKSQAVQGIKIKG-ASVD-DLS--
--LDFTL-------------------------------PG--YPHIL-LKPD--------
GDSVS---
------------------------------VTIDNVE-EYVQL-VIDYTLKKG-XHRQVQ
-AFREG---FSEVFPV--KDLA-CFTPE-ELVLLFGN-----------------------
---PSEDWT-----------FESL---------HESIRADHG------------------
----------------FSMD----SRIV-TELLHLIS--------------------TYE
KPQQRSFLSF-------ATGSPRLP------------VGG-----FR-ALHPPL--TVVR
KLPESGHQPD------------------------EYLPSVMTCQ-NX-IKLPEY-SSSDV
LAARFD-VAVHE-GQG-
>Cystobasidium_pallidum_BCIL01000001.1 .
VRISRQKL-FESAFKVV----------------ELYGSSKS-LLEVEYFD-------EVG
TGLG-PTLEF---YSLV----SKEYRRKEL--------GIWREGDSPADS----------
---------EFVSNSRGLYPAPID--------------------------------TIEM
SSEASEKYDMLFKTLGTLVAKAQMDSRIIDFPFNK--VFMKLVLG---------------
------QAVPLTTVSVRA----------------------VDPTLAQS--------LEHL
QKYLAAAEEIRSHATL-----------------ADAQKAEQINSLVIDD-ARVE-DLA--
--LDFTL-------------------------------PG---YDLE-LKEG--------
GKDMH---
------------------------------VTMNNLS-EYLDL-VLDWTLLKG-TKTAVA
-KFKEG---FNLVFPV--GHLH-TFTPD-ELVKLFGD-----------------------
---AEEDWS-----------FTTI---------SDHIKADHG------------------
----------------YTLG----SASI-TNFISFMS--------------------ELD
PVHRRKALSF-------MTGASRLP------------IGG-----FG-GLNPPL--TVVR
KVPEDGASPD------------------------SVLPSVMTCV-SF-VKL---------

>Erythrobasidium_yunnanense_BCJC01000005.1 .
VRISRQRL-FESAFKVF----------------ELYGSSKA-LLEVEYFD-------EVG

TGLG-PTLEF---YSLV----SKDFRRSXL--------GLWREGDSPAGS----------
---------EYVSHSRGLFPAPVE---------------------------------VNKL
DEAAWAKLSSHFKVLGTVVAKGLMDSRILDLPFSK--MFMKLMLN---------------
------QSIPLTIASVRF----------------------VDASLAQS--------LEHI
QKYVDEKEAILAVDGI------------------SDEDKQAAIGGIEIDG-AGIQ-DLS--
--LDFTL--------------------------------PG---YDVE-LKSG--------
GKEID---
------------------------------LTMDNVE-EYIKL-VLDWTLIKG-VKPMVQ
-KFREG---FSEVFPV--RDLH-SFRAE-ELVLLFGN-----------------------
---EEEDWS-----------ETTI---------KDSIKADHG------------------
----------------YSPT----SNAI-NNLVAILT--------------------TFD
TDMRRKFLSF-------STGAARLP------------LGG-----FG-ALNPPL--TVVR
KAPEDSLSAD------------------------DILPSASTCA-NF-FKLPDY-STRDI
MRERLE-LAVRE-GAG-
>Erythrobasidium_hasegawianum_BCII01000005 1..
VRISRQRL-FESAFKVF-----------------ELYGSSKA-LLEVEYFD-------EVG
TGLG-PTLEF---YSLV----SKDFRRESL--------GLWREGDSPPSS----------
---------EYVSHSRGLFPAPVE---------------------------------VNKL
DDAAWAKLSSHFKVLGTVVAKGLMDSRILDLPFSK--MFMKLMLN---------------
------QSIPLTIASVRF----------------------VDLSLAQS--------LEHI
QKYADEKEAILAAEGI------------------SDEDKQAAIGGIEIDG-AGIQ-DLS--
--LDFTL--------------------------------PG---YDVE-LKSG--------
GKEID---
------------------------------LTMDNVE-EYIKL-VLDWTLVKG-VKPMVQ
-KFREG---FSAVRXV--RDLH-SFRAE-ELVLLFGN-----------------------
---EEEDWS-----------ETTI---------KDSIKADHG------------------
----------------YSPT----STAI-SNLVAILT--------------------TFD
TDMRRKFLSF-------STGAARLP------------LGG-----FG-ALNPPL--TVVR
KAPEDSLSAD------------------------DILPSASTCA-NF-FKLPDY-STRDI
MRQRLE-LAVRE-GAG-
>Trichosporon_laibachii_BCKV01000005.1 .
VRISRAQL-LESCAKVL-----------------QVYGTSNG-ILEVEYFD-------EIG
TGLG-PTLEF---YSLA----SKEFARRSL--------KLWRDEDDTKEG----------
---------AYVFHPHGLFPSPST---------------------------------PNEP
SIETAGSRLAYFKTLGLFVGRALLDSRIIDLNLNK--VFLKLLLG---------------
------KPVKKNIATLKL----------------------LDVPLARS--------LERL
QTYLQARKEIEALVSL------------------PPSSRRAKLAALTING-AKLQ-DLS--
--LDFTF--------------------------------PG---YDIE-LQP---------
GGRMI---
-----------------------------DVDDSNLE-EYLSL-VLDKTLGSG-VQPMVK
-AFQEG---FSMIFPI--QDLQ-IFSPE-ELGLLCGN-----------------------
---VDEDWS-----------RETL---------EQSIKADHG------------------
----------------YNAD----SRAV-KNLIEVMT--------------------SYN
VDERRQFLQF-------ITGAPKLP------------IGG-----FRNGFNPPF--TVVR
KPHEPPIKAD------------------------QMLPSVMTCA-QY-LKLPDY-STIDA
LRAQLN-RAIVD-GSG-
>Trichosporon_gracile_BCJO01000001.1 .
VRISRAQL-LESCAKVL-----------------EVYGTSNG-ILEVEYFD-------EIG
TGLG-PTLEF---YSLA----SKEFARTSL--------KLWRDEDDTKEG----------
---------PYVYHPHGLFPAPVL---------------------------------PDEP
SIETAGSRLAYFKTLGLFVGRALLDSRIIDLNLNK--VFLKLLLG---------------
------KPVKKSIGTLKL----------------------IDVPLARS--------LERL
QTYLQARKEIEALVSL------------------PPSSRRAKLAALTING-AKLE-DLS--
--LDFTL--------------------------------PG---YDIE-LKT---------
GGRMT---
-----------------------------DVDDSNLE-EYLDL-VLDKTLGSG-VQLMVK
-AFQEG---FSKIFPI--QDLQ-IFSPE-ELGLLCGN-----------------------
---VDEDWS-----------RESK---------FQSIKADHG------------------

----------------YNAD----SRAV-KNLIEVMS--------------------SYK
PEERRQFLQF-------ITGAPKLP------------IGG-----FRSGFNPPF--TVVR
KPHEPPIKAD------------------------QMLPSVMTCA-QY-LKLPDY-STVDV
LRAQLN-RAIVD-GSG-
>Cutaneotrichosp_cutaneum_LTAL01000158.1 .
VRISRAQL-LESCAKVL----------------EVYGTSNG-ILEVEYFD-------EIG
TGLG-PTLEF---YSLA----SKEFARRSL--------KLWRDEDETKEG----------
---------AYVYHPHGLFPAPAL--------------------------------PNEP
SIDTTGSRLAYFKTLGLFVGRALLDSRIIDLSLNK--IFLKLLLG---------------
------KPVKKSISTLKL----------------------VDATLARS--------LERL
QSYLQSRKEIEALVGL-----------------PASSRRAKLAALTING-AKLA-DLS--
--LDLTL------------------------------PG---YDIE-LRP---------
GGRLV---
-----------------------------DVDDSNLE-EYLEL-VLDKTLGSG-VQPMVK
-AFQEG---FSMIFPI--QHLE-IFSPE-ELGLLCGN-----------------------
---VDEDWS-----------RESL---------EQSIKADHG------------------
----------------YNAD----SRAI-KNLIEVMS--------------------SYT
IEERRQFLQF-------ITGAPKLP------------IGG-----FRNGLNPPF--TVVR
KPHEPPIKAD------------------------QMLPSVMTCA-QY-LKLPDY-SSIDV
LRAQLN-RAIVD-GSG-
>Trichosporon_veenhuisii_BCKJ01000003.1 .
VRISRAQL-LESCAKVL----------------EVYGTSNG-ILEVEYFD-------EIG
TGLG-PTLEF---YSLA----SKEFARRSL--------KMWRDEDETKEG----------
---------AYVYHPHGLFPSPAL--------------------------------PNEP
SIDTPGSRLAYFKTLGLFVGRALLDSRIIDLSFNK--VFLNLLLG---------------
------KPVKKTITTLKL----------------------VDATLARS--------LERL
QSYLQSRKEIEQLVSL-----------------PASSRRAKLAALTING-AKLA-DLS--
--LDLTL------------------------------PG---YDIE-LKP---------
GGRMI---
-----------------------------DVDDSNLE-EYLEL-VLDKTLGSG-VQPMVK
-AFQEG---FSMIFPI--QHLE-IFSPE-ELGLLCGN-----------------------
---VDEDWS-----------QSAL---------EQSIKADHG------------------
----------------YNAD----SRAI-KNLIEVMS--------------------SYT
IEERRQFLQF-------ITGAPKLP------------IGG-----FRNGLNPPF--TVVR
KPHEPPIKAD------------------------QMLPSVMTCA-QY-LKLPDY-SSIDV
LRAQLN-RAIVD-GSG-
>NWGS3B_2_Trichosporon_brassicae_BCJI01000001.1 .
VRISRAQL-LESCAKVL----------------EVYGTSNG-VLEVEYFD-------EIG
TGLG-PTLEF---YSLA----SKEFARRSL--------KLWRDEDDTKQG----------
---------NYVFHPGGLFPAPAL--------------------------------PNEP
SIETAGSRLAFFKTLGLFVGRALLDSRIIDLHFNK--VYLKLLLG---------------
------RPVKKSIATLKQ----------------------VDVPLARS--------LERL
QSYLEARNEIEALVSL-----------------PASSRRAKLAALTIGG-AKLQ-DLS--
--LDFTL------------------------------PG---YDIE-LKA---------
GGRMI---
-----------------------------DVDDSNLE-EYLGL-VLDKTLGSG-VQPMIK
-AFQEG---FTMIFPI--QDLQ-IFSPE-ELGLLCGN-----------------------
---VEEDWS-----------RESL---------EQSIKADHG------------------
----------------YNAD----SRGI-KNLIEVMA--------------------SYT
VQERREFLQF-------ITGAPKLP------------IGG-----FRNGLNPPL--TVVR
KPHEPPIKAD------------------------QMLPSVSTCA-QY-LKLPDY-SSIDV
LRKQLN-RAIVD-GCG-
>Trichosporon_domesticum_BCFW01000002.1 .
VRISRAQL-LESCAKVL----------------EVYGTSNG-ILEVEYFD-------EIG
TGLG-PTLEF---YSLA----SQAFARRSL--------KLWRDEDESKEG----------
---------SYVFHPHGLFPAPSV--------------------------------ANEP
GTDVTGSRLAYFKTLGLFVGRALLDSRIIDINFNK--VFLKLLLG---------------
------RPVKKSIATLKL----------------------IDPSMARS--------LERF

```
QSYLEARREIESLVSL-----------------PASSRRAKLAALTING-AKLQ-ELS--
--IDFTL-------------------------------PG---YDIE-LKE---------
GGRLI-------------------------------------------------------
-----------------------------DVDDSNLE-EYIEL-VLDKTLGSG-VQPMVK
-AFQEG---FSMIFPV--HDLQ-IFSPE-ELGLLCGN-----------------------
--NVEEDWS-----------RESECQK------YSSIKADHG------------------
----------------YNAD----SRSV-KNLIEVMS--------------------SYN
AQERRDFLQF-------ITGAPKLP------------IGG-----FRNGLNPPF--TVVR
KPHEPPIKAD------------------------QMLPSVSTCA-QY-LKLPDY-SSVDV
LRTQLN-RAMLD-GSG-
>Trichosporon_montevideense_BCFV01000010.1 .
VRISRAQL-LESCAKVL----------------EVYGTSNG-ILEVEYFD-------EIG
TGLG-PTLEF---YSLA----SQAFARRSL--------KLWRDEDESKEG----------
---------SYVFHPHGLFPAPSV--------------------------------ANEP
GTDVTGSRLAYFKTLGLFVGRALLDSRIIDINFNK--VFLKLLLG---------------
------RPVKKSIATLKL----------------------IDPSMARS--------LERF
QSYLEARREIESLVSL-----------------PASSRRAKLAALTING-AKLQ-ELS--
--IDFTL-------------------------------PG---YDIE-LKE---------
GGRLI-------------------------------------------------------
-----------------------------DVDDSNLE-EYIEL-VLDKTLGSG-VQPMVK
-AFQEG---FSMIFPV--QDLR-IFSPE-ELGLLCGN-----------------------
--NVEEDWS-----------RESL---------EQSIKADHG------------------
----------------YNAD----SRSV-KNLIEVMS--------------------SYN
AQERRDFLQF-------ITGAPKLP------------IGG-----FRNGLNPPF--TVVR
KPHEPPIKAD------------------------QMLPSVSTCA-QY-LKLPDY-SSVDV
LRTQLN-RAMLD-GSG-
>Trichosporon_porosum_BCJG01000003.1 .
VRISRAQL-LESCAKVL----------------ELYGTSNG-ILEVEYFD-------EIG
TGLG-PTLEF---YALA----SKEFARRSL--------KLWRDEDESKEG----------
---------PYAFHPHGLFPAPAL--------------------------------VNEP
SKDTPGSRLSYFKTLGLFVGRALLDSRIIDVNMNT--VFIKRLLG---------------
------KPVKKNIATLKL----------------------VDMTLARS--------LERL
QSYLESRKEIEAMVSL-----------------PPSSRRAKLAALTIGG-AKLQ-DLS--
--LDFTL-------------------------------PG---YDIE-LKL---------
GGRLF-------------------------------------------------------
-----------------------------DVDDSNLE-EYLEL-VLDKTLGSG-ITQQVK
-AFQEG---FSMIFPV--QDLQ-IFSPE-ELGLLCGN-----------------------
---TDEDWS-----------KESE---------YSSIKADHG------------------
----------------YNAD----SRTV-KNLIEIMS--------------------SYD
LEERRQFLQF-------ITGAPKLP------------IGG-----FRNGLTPPF--TVVR
KPHEPPIKAD------------------------QMLPSVMTCA-LY-LKLPDY-STIET
LRSQLN-RAIVD-GSG-
>Trichosporon_gamsii_BCJN01000003.1 .
VRISRAQL-LESCAKVL----------------ELYGTSTG-ILEVEYFD-------EIG
TGLG-PTLEF---YSLA----SKEFARRSL--------KLWRDEDESKEG----------
---------AYVFHPHGLFPAPAL--------------------------------PEEP
SVDTPGSRLSYFKTLGLFVGRALLDSRIIDVTLNK--VFLKRLLG---------------
------KPVKKNIATLKC----------------------VDMTLARS--------LERL
QSYLESRKEIEAMVSL-----------------PPSSRRAKLATLAIGG-AKLQ-DLS--
--LDFTV-------------------------------PG---YDIE-LKP---------
NGRLT-------------------------------------------------------
-----------------------------DVDDSNLE-EYLEL-VLDKTLGSG-IMQQVK
-AFQEG---FSMIFPI--QDLQ-IFSPE-ELGLLCGN-----------------------
---SDEDWS-----------KETL---------EQSIKADHG------------------
----------------YNAD----SRTV-KNLIEIMS--------------------NYT
VEERRAFLQF-------ITGAPKLP------------IGG-----FRNGLNPPF--TVVR
KPHEPPIKAD------------------------QMLPSVMTCA-LY-LKLPDY-STIDA
LRAQLS-RAIQD-GSG-
```

>Vanrija_humicola_BCJF01000005.1 .
VRISRAQL-LESCAKVL----------------ELYGTSNG-ILEVEYFD-------EIG
TGLG-PTLEF---YSLA----SKEFARRSL--------KMWRDEDETREG----------
---------NYVYHPSGLFPAPAA--------------------------------ANEP
SKETAGSRLSYFKTLGLFVGRALLDSRIIDVNLNR--VLIKLLLS---------------
------KPVKKNIATLKL----------------------VDLSLARS--------LERL
QSYLQSRQEIEALASL-----------------PPSSRRNKLATLTIGG-AKLH-DLS--
--LDFTL-------------------------------PG---YDIE-LKP---------
GGRLI---
-----------------------------EVDDSNLE-EYLEL-VLDKTLGSG-VAQQVK
-AFQEG---FSMIFSI--QDLQ-IFSPE-ELGLLFGN-----------------------
---TEEDWL--------T--SSAL---------EQTIRADHG------------------
----------------YNAD----SRAV-RTLIEVMS--------------------SYS
KEERRAFLQX-------ITGAPKLP------------IGG-----FR-GLNPAF--TVVR
KPHEPPFRAD------------------------DYLPSVMTCA-QY-LKLPDY-SSKEV
LSAQLG-RAMHD-GGG-
>Trichosporon_inkin_JXYM01000003.1 .
VRISRAQL-LESCVKVL----------------ELYGTSNG-ILEVEYFD-------EIG
TGLG-PTLEF---YALA----SKEFARRSL--------HMWRDDDASKEG----------
---------PYVFAPKGLFPAPTA----------------------------------EK
VRNAPSSKHSYFKTLGLFVGRALLDSRIIDVNLNK--VFLKLLLG---------------
------KPVRKNIATLKE----------------------VDETLARS--------LERL
QTYLQTRKEIESLTGL-----------------PASSRRNKLSALTIGG-AKLH-DLS--
--LDFTL-------------------------------PG---YDIE-LKP---------
GGRMI---
-----------------------------DVDDSNLE-EYLEL-VFDKTLGSG-IAEQVK
-EFQTG---FSMIFPI--RDLG-IFSPD-ELGLLCGN-----------------------
---TDEDWS-----------KETL---------GQAIKADHG------------------
----------------YNAD----SRSI-RNLIDILS--------------------AFD
SAERREFLQF-------MTGSPKLP------------IGG-----WR-SLNPPF--TVVR
KPHEPPNKAD------------------------AMLPSVMTCA-QY-LKLPDY-SSKEV
LASQLW-RAIRD-GSG-
>Trichosporon_faecale_JXYK01000002.1 .
VRISRAQL-LESCVKVL----------------ELYGTSNG-ILEVEYFD-------EIG
TGLG-PTLEF---YSLA----SKEFSRRSL--------HMWRDENTSKEG----------
---------PYVFAPNGLFPAPTS----------------------------------EK
IKTAPSSKHSYFKTLGLFIGRALLDSRIIDVNLNK--VFLKLLLG---------------
------KPVRKNIATLKQ----------------------VDETLARS--------LERL
QTYLQARQEIESLTAL-----------------PASSRRNKLSALTIGG-AKLQ-DLS--
--LDFTL-------------------------------PG---YDIE-LKP---------
GGRMI---
-----------------------------DVDDSNLE-EYLEL-VLDKTLRSG-IAEQVQ
-EFRAG---FSMIFPI--NDLG-IFSPE-ELALLCGN-----------------------
---TDEDWS-----------KETL---------EQAIKADHG------------------
----------------YNAD----SRSI-RNLIEILS--------------------AFD
SSERREFLQF-------MTGSPKLP------------IGG-----WR-SLNPPF--TVVR
KPHEPPIKAD------------------------SMLPSVMTCA-QY-LKLPDY-SSKEV
LATQLW-RAIRD-GSG-
>Trichosporon_coremiiforme_JXYL01000027.1 .
VRISRAQL-LESCVKVL----------------ELYGTSNG-ILEVEYFD-------EIG
TGLG-PTLEF---YSLA----SKEFSRRSL--------HMWRDEDTSKEG----------
---------PYVFAPNGLFPGPTS----------------------------------EK
IKNAPSSKHSYFKTLGLFIGRALLDSRIIDVNLNK--VFLKLLLG---------------
------KPVRKNIVTLKQ----------------------VDETLARS--------LERL
QTYLQARQEIESFTAL-----------------PASSRRNKLSALTIGG-AKLQ-DLS--
--LDFTL-------------------------------PG---YDIE-LKP---------
GGRMV---
-----------------------------DVDDSNLE-EYLEL-VLDKTLGSG-IAEQVK

-EFQVG---FSMIFPI--NDLG-IFSPE-ELALLCGN-----------------------
---TDEDWS-----------KETL---------EQTIKADHG------------------
----------------YNAD----SRSI-RNLIEILS--------------------AFD
SSERREFLQF-------MTGSPKLP------------IGG-----WR-SLNPPF--TVVR
KPHEPPIKAD------------------------SMLPSVMTCA-QY-LKLPDY-SSKEV
LATQLW-RAIRD-GSG-
>Trichosporon_asahii_XM_014324352.1 .
VRISRAQL-LESCVKVL----------------ELYGTSNG-ILEVEYFD-------EIG
TGLG-PTLEF---YSLA----SKEFSRRSL--------HMWRDEDTSKEG----------
---------PYVFAPNGLFPAPTS----------------------------------EK
IKNAPSSKHSYFKTLGLFIGRALLDSRIIDVNLNK--VFLKLLLG---------------
------KPVRKNIATLKQ----------------------VDETLARS--------LERL
QTYLQARQEIECLTAL-----------------PASSRRNKLSALTIGG-AKLQ-DLS--
--LDFTL-------------------------------PG---YDIE-LKP---------
GGRMI---
-----------------------------DVDDSNLE-EYLEL-VLDKTLGSG-IAEQVK
-ELQVG---FSMIFPI--NDLG-IFSPE-ELALLCGN-----------------------
---TDEDWS-----------KETL---------EQTIKADHG------------------
----------------YNAD----SRSI-RNLIEILS--------------------AFD
SSERREFLQF-------MTGSPKLP------------IGG-----WR-SLNPPF--TVVR
KPHEPPIKAD------------------------SMLPSVMTCA-QY-LKLPDY-SSKEV
LATQLW-RAIRD-GSGS
>Trichosporon_guehoae_BCJX01000003.1 .
VRISRAQL-LESCVRVL----------------ELYGTTNG-ILEVEYFD-------EIG
TGLG-PTLEF---YSLA----SKEFARRSL--------NLWRDDDESKEG----------
---------QYVFHPNGLFPAPAV--------------------------------PNEP
PKDTAGSRLAYFRTLGLFVGRALLDSRIIDLNMNK--VFLKLLLG---------------
------MPVKKSIATLKL----------------------VDHQLARS--------LERL
QTYLQTRNEIEALVSL-----------------PASTRRNKLAALTIGG-AKLH-DLS--
--LDFTL-------------------------------PG---YDIE-LKPG--------
GRLID---
------------------------------VDDSNLE-EYLEL-VLDKTLGSG-VAQQVK
-EFRDG---FSMIFPI--QDLQ-IFSPE-ELGLLCGN-----------------------
---AEEDWT-----------RESL--------GILYVSPSHG------------------

-----------------LTGAPRLP------------TGG-----WR-ALNPPF--TVVR
KPTEAGGIYS--------------------------LPSAMTCA-SF-LKLSDY-PTKEL
LQEQLH-RAARD-A---
>Cryptococcus_curvatus_BCJH01000005.1 .
VRISRAQL-LESCVKVL----------------ELYGTSNG-ILEVEYFD-------EIG
TGLG-PTLEF---YAMA----SREFARRQL--------RLWRDEDESHEG----------
---------QYVWHPHGLFPAPIQ-----------------------------------P
GEASDSPRLSFFKTLGVFVGRALLDSRIIDLNFNK--VFLKRVLG---------------
------QRAKPTIATLRL----------------------VDEPLARS--------LERL
QTYLHTRQEIEALTSL-----------------PASSRRSKLAALTIGG-AKLQ-DLA--
--LDFTL-------------------------------PG---YSIE-LKA---------
GGRAI---
-----------------------------DVDDSNLE-EYIEL-VLDKTIGSG-IEQQVK
-QFRDG---FSSIFSV--TDLE-IFSPE-ELGLLCGN-----------------------
---ADEDWS-----------KAAL---------SQAIKADHG------------------
----------------YNSD----SRVV-RDLIEVMS--------------------SYT
KEERRLFLQF-------MTGAPKLP------------LGG-----FK-GLNPQF--TVVR
KPHEPPFKAD------------------------DYLPSXMTCA-SY-LKLPDY-SSKTV
LGTQLM-RCMSD-GQG-
>Trichosporon_cutaneum_BCKU01000045.1 .
VRISRSQL-LESCVKVL----------------ELYGKADG-ILEVEYFD-------EIG
TGLG-PTLEF---YAMA----SKEFARRSL--------KLWRDEDESQEG----------
---------VHVWHPHGLFPAPFK----------------------------------TD

EPTKDSQRLSYFKTLGVFVGRALLDSRIIDLHFNR--VFLKMVLS---------------
------RPVKKSIATLKL----------------------VDAPLARS--------LERL
QIYLQARHEIEELAAL-----------------PPSTRRSKLAALTIGG-AKLH-DLS--
--LDFTL-------------------------------PG---YDIE-LKP---------
GGRSI---
-----------------------------DVDDTNLA-EYIDL-VLELALGSG-VIRQVQ
-AFQAG---FSSIFSV--SDLQ-IFSPD-ELALLFGN-----------------------
---AEEDWS-----------QESE---------YQAIKADHG------------------
----------------YNSD----SRVV-RDLIDVMT--------------------SYS
KEERRAFLQF-------MTGAPKLP------------LGG-----FK-GLNPQF--TVVR
KPHEAPFKAD------------------------DYLPSVMTCA-TY-LKLPNY-SSRAV
LASQLM-RSMKD-GQG-
>Cutaneotrichosp_cutaneum_LRUG01000040.1 .
VRISRSQL-LESCVKVL----------------ELYGTSNG-ILEVEYFD-------EIG
TGLG-PTLEF---YAIA----SKEFARRSL--------KLWRDEDESMDG----------
---------AYVWHPHGLFPAPLK----------------------------------PD
EPSKDNTRLSYFKTLGLFVGRALLDSRIIDLNFNH--VFLKMVLN---------------
------RPVKKSIATLKQ----------------------IDAPLARS--------LERL
QAYLQARKEIEELTAL-----------------PPSTRRSKLATLTIGG-AKLH-DLS--
--LDFTL-------------------------------PG---YDIE-LKP---------
GGRSI---
-----------------------------DVDDSNLA-EYIDL-VLDMTVGSG-IARQVK
-EFQDG---FSMIFSV--SDLQ-IFSPD-ELGLLCGN-----------------------
---ADEDWS-----------RESL---------EQAIKADHG------------------
----------------YNSD----SRVV-RDLIDIMN--------------------SYS
KEERRAFLQF-------MTGAPKLP------------LGG-----FK-GLSPQF--TVVR
KPHEAPFKAD------------------------DYLPSVMTCA-TY-LKLPNY-SSRAV
LAAQLM-RSMKD-GQG-
>Cutaneotrichosp_curvatus_MATS01000113.1 .
VRISRAQL-LESCVKVL----------------ELYGKADG-ILEVEYFD-------EIG
TGLG-PTLEF---YALA----SKEFARRSL--------KLWRDEDESHEG----------
---------SHVWHPHGLFPAPLK----------------------------------KD
EAGRDSQRLSYFKTLGVFVGRAMLDSRIIDLNFNR--VFLKMVLN---------------
------KPVKKSIATLKL----------------------VDAPLARS--------LERL
QSYQQARQEIEGLAAL-----------------PPSTRRSKLAALTIGG-AKLH-DLS--
--LDFTL-------------------------------PG---YDIE-LKSG--------
GRSID---
------------------------------VDDSNLG-EYIEL-VLDLTLGSG-VHHQLQ
-AFQEG---FSSIFPV--VDLQ-IFSPD-ELALLCGN-----------------------
---AEEDWS-----------QESE---------YQAIKADHG------------------
----------------YNSD----SRVV-RDLIDVMT--------------------SYS
KEERRAFLQF-------MTGAPKLP------------LGG-----FK-GLNPQF--TVVR
KPHEAPFKAD------------------------DYLPSGTTCA-TY-LKLPNY-SSRAV
LSSQLM-RSMKD-GQG-
>Trichosporon_oleaginosus_XM_018425038.1 .
VRISRAQL-LESCVKVL----------------ELYGKADG-ILEVEYFD-------EIG
TGLG-PTLEF---YALA----SKEFARRSL--------KLWRDEDESHEG----------
---------SHVWHPHGLFPAPLK----------------------------------KD
EAGRDSQRLSYFKTLGVFVGRAMLDSRIIDLNFNR--VFLKMVLN---------------
------KPVKKSIATLKL----------------------VDAPLARS--------LERL
QSYQQARQEIEGLAAL-----------------PPSTRRSKLAALTIGG-AKLH-DLS--
--LDFTL-------------------------------PG---YDIE-LKSG--------
GRSID---
------------------------------VDDSNLG-EYIEL-VLDLTLGSG-VHHQLQ
-AFQEG---FSSIFPV--VDLQ-IFSPD-ELALLCGN-----------------------
---AEEDWS-----------QETL---------EQAIKADHG------------------
----------------YNSD----SRVV-RDLIDVMT--------------------SYS
KEERRAFLQF-------MTGAPKLP------------LGG-----FK-GLNPQF--TVVR

KPHEAPFKAD------------------------DYLPSVMTCA-TY-LKLPNY-SSRAV
LSSQLM-RSMKD-GQGS
>Cryptococcus_neoformans_XM_770762.1 .
VRISRTQL-LESCAKVM----------------EMYATFPG-TLEVEYFD-------EIG
TGLG-PTLEF---YALA----SKEFARRNL--------HVWRDEDPSIAG----------
---------NYVHHPHGLFPSPLP----------------------------------RA
SAELVASRLKWFKTLGQFIGRSMLDSRIIDISFNK--VFLKLLLD---------------
------KPIKKSLSTLRA----------------------VDPSLARS--------LERL
QGYYCVRKEIEALPI------------------PASSRRTKLAALTVGD-AKLA-DLA--
--LDFTL-------------------------------PG---YDIE-LKPG--------
GAHIE---
------------------------------VDDSNLG-EYLEK-VLDWTLGSG-VAEQVK
-AFQDG---FSSIFPI--KSIK-IFSLD-ELYLLFGN-----------------------
---ADEDWS-----------RETL---------EVAIKADHG------------------
----------------YNQD----SRAV-QNLIEVMS--------------------SYN
KEQRRQFLQF-------MTGAPKLP------------IGG-----FR-GLTPPF--TVVR
KPHEPPYKAD------------------------DYLPSVMTCA-LY-LKMPDY-SSKEV
LAAQFE-RAMRD-GRGS
>Cryptococcus_neoformans_XM_570576.1 .
VRISRTQL-LESCAKVM----------------EMYATFPG-TLEVEYFD-------EIG
TGLG-PTLEF---YALA----SKEFARRNL--------HVWRDEDPSIAG----------
---------NYVHHPHGLFPSPLP----------------------------------RA
SAELVASRLKWFKTLGQFIGRSMLDSRIIDISFNK--VFLKLLLD---------------
------KPIKKNLSTLRA----------------------VDPSLARS--------LERL
QGYYCVRKEIEALPI------------------PASSRRTKLAALTVGD-AKLA-DLA--
--LDFTL-------------------------------PG---YDIE-LKPG--------
GAHIE---
------------------------------VDDSNLG-EYLEK-VLDWTLGSG-VAEQVK
-AFQDG---FSSIFPI--KSIK-IFSLD-ELYLLFGN-----------------------
---ADEDWS-----------RETL---------EVAIKADHG------------------
----------------YNQD----SRAV-QNLIEVMS--------------------SYN
KEQRRQFLQF-------MTGAPKLP------------IGG-----FR-GLTPPF--TVVR
KPHEPPYKAD------------------------DYLPSVMTCA-LY-LKMPDY-SSKEV
LAAQFE-RAMRD-GRGS
>Cryptococcus_neoformans_XM_012194029.1 .
VRISRTQL-LESCAKVM----------------EMYATFPG-TLEVEYFD-------EIG
TGLG-PTLEF---YALA----SKEFARRNL--------QVWRDEDVSIAG----------
---------NYVHHPHGLFPSPLP----------------------------------RA
SAELMASRLKWFKTLGQFIGRSMLDSRIIDISFNK--VFLKLLLD---------------
------KPIKKSLSTLKA----------------------VDPSLARS--------LERL
QGYYNVRKEIEALPI------------------PASSRRTKLAALTVGD-AKLA-DLA--
--LDFTL-------------------------------PG---YDIE-LKPG--------
GAHIE---
------------------------------VDDSNLG-EYLEK-VLDWTLGSG-VAEQVK
-AFQDG---FSSIFPI--KSIK-IFSLD-ELYLLFGN-----------------------
---ADEDWS-----------RETL---------EVAIKADHG------------------
----------------YNQD----SRAV-QNLIEVMS--------------------SYN
KEQRRQFLQF-------MTGAPKLP------------IGG-----FR-GLTPPF--TVVR
KPHEPPYKAD------------------------DYLPSVMTCA-LY-LKMPDY-SSKEV
LAAQFE-RAMRD-GRGS
>Cryptococcus_gattii_XM_003193674.1 .
VRISRTQL-LESCAKVM----------------EMYATFPG-TLEVEYFD-------EIG
TGLG-PTLEF---YALA----SKEFARRNL--------HVWRDEDPSIAG----------
---------NYVHHPHGLFPSPLP----------------------------------RA
SAELMASRLKWFKTLGQFIGRSMLDSRIIDISLNK--VFLKLLLD---------------
------KPVKKNLSTLRG----------------------VDPSLARS--------LERL
QGYYCARKEIEALPI------------------PASSRRTKLAALTVGD-AKLA-DLA--
--LDFTL-------------------------------PG---YDIE-LKPG--------

```
GAHIE-------------------------------------------------------
------------------------------VDDSNLG-EYLEK-VLDWTLGSG-VVEQVK
-AFQDG---FSSIFPI--KSIK-IFSLD-ELYLLFGN-----------------------
---ADEDWS-----------RETL---------EAAIKADHG------------------
----------------YNQD----SRAV-QNLIEVMS--------------------SYN
KEQRRQFLQF-------MTGAPKLP------------IGG-----FK-GLTPPF--TVVR
KPHEPPYKAD------------------------DYLPSVMTCA-LY-LKMPDY-SSKEV
LAAQFE-RAMRD-GRGS
>Cryptococcus_amylolentus_XM_019138889.1 .
VRISRTQL-LESCAKVM----------------EMYATFPG-TLEVEYFD-------EIG
TGLG-PTLEF---YALA----STEFARRNL--------NIWRDEDSSIPG----------
---------TYVHHPHGLFPSPLA-----------------------------------G
FGDAVAPKLRWFKTLGLFIGRSMLDSRIIDIRLSK--IFFKLLLG---------------
------RPVKKNITTLKA----------------------VDPQLARS--------LERL
QAYSSARREIEALPL------------------PASSRRAKIASLTVGG-AKLG-DLA--
--LDFTL-------------------------------PG---YNIE-LKS---------
GGAHI-------------------------------------------------------
-----------------------------EVDDGNLD-EYLEL-VLDWTLGSG-VAQQVK
-AFQEG---FSGIFPI--KSLK-IFSLE-ELQLVFGN-----------------------
---ADEDWS-----------RETL---------EQSIKADHG------------------
----------------YNSE----SRAV-QNLIEVMS--------------------SYT
KEQRRHFLQF-------MTGAPKLP------------IGG-----FR-GFTPPF--TVVR
KPHEPPYKAD------------------------DYLPSVMTCA-LY-LKMPDY-SNKEV
LATQFD-RAMRD-GKGS
>Tsuchiyaea_wingfieldii_XM_019176735.1 .
VRISRTQL-LESCAKVM----------------EMYATFPG-TLEVEYFD-------EIG
TGLG-PTLEF---YALA----STEFARRNL--------NIWRDEDSSLPG----------
---------TYVHHPHGLFPSPLA-----------------------------------G
FGDAVAPKLRWFKTLGLFIGRSMLDSRIIDIGLSK--IFFKLLLG---------------
------RPVKKNITTLKA----------------------VDPQLARS--------LERL
QAYSSARREIEALPL------------------PASSRRAKIASLTVGG-AKLG-DLA--
--LDFTL-------------------------------PG---YNIE-LKS---------
GGAHI-------------------------------------------------------
-----------------------------EVDDGSLD-EYLEL-VLDWTLGSG-VAQQVK
-AFQEG---FSGIFPI--KSLK-IFSLE-ELQLVFGN-----------------------
---ADEDWS-----------RETL---------EQSIKADHG------------------
----------------YNSE----SRAV-QNLIEVMT--------------------SYT
KEQRRHFLQF-------MTGAPKLP------------IGG-----FR-GFTPPF--TVVR
KPHEPPYKAD------------------------DYLPSVMTCA-LY-LKMPDY-STKEV
LATQFD-RAMRD-GKGS
>Bulleromyces_albus_BCIX01000001.1 .
VRISRPQL-LESCAKVM----------------ELYGTSSG-MLEIEYFD-------EIG
TGLG-PTLEF---YSIA----SREFARRSL--------SLWRDEDDTKAG----------
---------NYVHFPKGLFPAPIS----------------------------------GS
DAAITXXXLSWFKIIGLFVGRALLDSRIIDVNLNK--VFLRAVLG---------------
------QPIKKNVATLRL----------------------VDPALARS--------LEGL
QSYLQTRKDIESLQL------------------PAAAKRNKLASLTIHG-VKLV-DLS--
--LDFTL-------------------------------PG--YPNVQ-LKPD--------
GALVD-------------------------------------------------------
------------------------------VDDSNLE-EYLDR-VLEMTLGEG-IDKQVK
-AFKDG---FSMIFSV--DDMK-IFSPD-ELGLLFGN-----------------------
---ADEDWS-----------KESL---------EQAIKADHG------------------
----------------YNLD----SVAV-QNLIQVMS--------------------GYD
KEQRRQFLQX-------ITGAPKLP------------IGG-----FR-GLSPPF--TVVK
KPHEAPFRAD------------------------DYLPSVMTCA-QY-LKMPDY-SNKEV
LAAQIQ-RAMLD-GGG-
>Papiliotrema_laurentii_JDSR01000898.1 .
VRISRSQL-LESCAKVL----------------ELYGTSSG-ILEVEYFD-------EIG
```

TGLG-PTLEF---YSIA----SKEFARKSL--------GLWRDEDESKPS----------
---------EYVFHPRGLYPAPVR---------------------STE-SAEVSAASLEK
NVLIIRAQLGWYKTLGLFTGRALLDSRIIDVNLNK--VFLKLILG---------------
------RPVKRTISNLKL----------------------VDMGLARS--------LERL
QNYVQTRKEIEALKL-------------------PAGARRNKLMQLTVSG-AKLA-DLS--
--LDFTL--------------------------------PG---YDIE-LKP---------
GGKHI---
-----------------------------DVDDHNIE-EYLEL-VLDMTLGGG-IARQVK
-AFQEG---FSMIFSI--DDMR-IFSPE-ELGLLFGN-----------------------
---TEEDWS-----------KESL---------EHTIKADHG------------------
----------------YNLD----SRSV-QNLVEVMS--------------------SYD
KEQRRQFLQX-------ITGAPKLP------------IGG-----FR-GLNPPF--TVVR
KPHEPPFRAD------------------------DYLPSVMTCA-QY-LKLPDY-SSKEV
LAAQIQ-RAVLD-GGG-
>Cryptococcus_flavescens_CAUG01000238.1 .
VRISRGQL-LESCAKVL-----------------ELYGTSNG-ILEVEYFD-------EIG
TGLG-PTLEF---YSIA----SKEFARRSL--------KLWRDEDESKTG----------
---------DYVFHPRGLYPAPX-------------------------------------
-VADARSNMSWYKTLGLFIGRALLDSRIIDVHLNK--VFLRLVLG---------------
------RPVKKTIANLKL----------------------VDAPLARS--------LERL
QAYLYARKEIEALQL-------------------PPGARRNKLMQLTVSG-ANLA-DLS--
--LDFTL--------------------------------PG---YNIE-LKP---------
GGAHV---
-----------------------------DVDDHNLE-EYLEL-VLDWTLGAG-IARQVK
-HFQEG---FSQIFSV--EDLK-IFSPE-ELALLFGN-----------------------
---TEEDWS-----------RDTL---------EHIIKADHG------------------
----------------YNLD----SRTV-QNLIQVMT--------------------EYT
KDERRQFLQL-------ITGAPKLP------------IGG-----FR-GLNPPF--TVVR
KPHEAPFRAD------------------------DYLPSVMTCA-QY-LKLPDY-STKEA
LAAQLQ-RAILD-GGG-
>Tremella_fuciformis_LBGW01003092.1 .
VRISRAQL-LESCVKVM-----------------ELYGTSGG-ILEVEYFD-------EIG
TGLG-PTLEF---YSIA----SKEFARRTL--------KIWRDEDESKSG----------
---------TYVFHPRGLFPAPVX-------------------------------VCSAV
NHDCRRSRMSWYRILGLFVGRALLDSRIIDVNLNT--LFLRLILG---------------
------IPVKKTIGNLKL----------------------VDPALARS--------LERL
QNYAIARKEIEALQL-------------------PASTRRNKLAALTVGD-AKLA-DLS--
--LDFTL--------------------------------PG---YSID-LKP---------
RGAFT---
-----------------------------DVDDSNLE-EYIEK-VLDLTLGSG-IMAQVR
-AFREG---FSMIFPI--TDLG-IFSSD-ELGVLFGN-----------------------
---AEEDWS-----------KDVL---------EQAIKADHG------------------
----------------YTSE----SRAV-QNLIEVMS--------------------GYT
QEQRRHFLQX-------ITGAPKLP------------IGG-----FR-GLQPPF--TVVR
KPHEAPFKAD------------------------DYLPSVMTCA-QY-LKMPDY-STKEI
LAAQLQ-RAMQD-GGG-
>Tremella_mesenterica_XM_007000466.1 .
VRISRAQL-LESCVKVM-----------------ELYGTASG-ILEVEYFD-------EIG
TGLG-PTLEF---YSIA----SREFARRAL--------QIWRDEDEAKPG----------
---------PYVFHPRGLFPAPVG--------------------------------PGEP
ASSVAGSRLSWFKTLGLFVGRALLDSRIIDVNLNK--LFLKLILG---------------
------KTVKKSISNLKL----------------------VDPALARS--------LERL
QSYLIARKEIEALAL-------------------PASSRRNKLSALTVGG-AKLT-DLS--
--LDFTL--------------------------------PG---YAID-LRP---------
RGAFV---
-----------------------------DVDDSNLE-EYIDK-VLDLTLGSG-IHDQVK
-AFQEG---FSMIFPI--QDMA-IFSPE-ELGVLFGN-----------------------
---ADEDWS-----------RETL---------EQAIKADHG------------------

----------------YNLD----SRAV-QNLLEVMS--------------------GYD
KDERRHFLQF-------ITGAPKLP------------IGG-----FR-GLQPPF--TVVR
KPHEAPFRAD------------------------DYLPSVMTCA-QY-LKMPDY-TTKEI
LAAQLK-RAMQD-GGGS
>Kwoniella_mangroviensis_XM_019146588.1 .
VRISRSQL-LESCSKVL----------------EIYGTSTG-ILEIEYFD-------EIG
TGLG-PTLEF---YSLA----SKEFARRAL--------SIWRDEDETKEG----------
---------NYVFHPKGLFPSPLN-----------------------------------E
GSSGSESRLSWFKTLGLFAGRALLDTRIIDVNLNR--VFLKLILG---------------
------QPVKKTIATLKA----------------------VDTGLARS--------LERL
QAYSYARKEIEALKL------------------PASTRRTKLAALTVGG-AKLA-DLS--
--LDFTL------------------------------PG---YNIE-LKP---------
GGSHV---
-----------------------------DVDDSNLE-EYLER-VLEMTLGSG-VEKQVK
-AFQEG---FSMVFSI--RDMR-IFSPE-ELGLLFGN----------------------
---AEEDWS-----------RETL---------EQSLKADHG------------------
----------------YNLD----SRAV-QNLLEVMV--------------------GYD
KEQRRQFLQF-------ITGAPKLP------------IGG-----FK-GLTPPF--TVVR
KPHEPPFKAD------------------------DYLPSVMTCA-QY-LKMPDY-STKEI
LAAQIE-RAMKD-GGGS
>Kwoniella_bestiolae_XM_019192519.1 .
VRISRSQL-LESCAKVL----------------EIYGTSTG-ILEIEYFD-------EIG
TGLG-PTLEF---YSLA----SKEFARRAL--------GIWRDEDETKDG----------
---------NYVFHPKGLFPAPLN-----------------------------------E
SVNGSESKLSWYKTLGLFAGRALLDSRIIDVNLNR--VFLKLILG---------------
------QPVKKTIATLKA----------------------IDTALARS--------LERL
QTYLFARKEIEALKL------------------PASTRRTKLAALTVGG-AKLA-DLS--
--LDFTL------------------------------PG---YSIE-LKP---------
GGSHI---
-----------------------------DVDDSNLE-EYLEK-VLEMTLGTG-VEKQVK
-AFQEG---FSLVFSV--REMR-IFSPE-ELGLLFGN----------------------
---AEEDWS-----------RETL---------EQSLKADHG------------------
----------------YNLD----SRAV-QNLLEVMV--------------------GYD
KEQRRQFLQF-------ITGAPKLP------------IGG-----FK-GLTPPF--TVVR
KPHEPPFKAD------------------------DYLPSVMTCA-QY-LKMPDY-STKEV
LAAQIE-RAMKD-GGGS
>Kwoniella_dejecticola_XM_018408555.1 .
VRISRSQL-LESCAKVL----------------EIYGTSQG-ILEIEYFD-------EIG
TGLG-PTLEF---YSLA----SKEFARKSL--------GIWRDEDESKES----------
---------EYVFHPKGLYPAPLD-----------------------------------E
KQSGIEAKLSWFKTLGLFAGRALLDTRIIDVNLNK--VFLKAILG---------------
------QPIKKNIATLKA----------------------VDTHLARS--------LERL
QNYLFARKEIEALKL------------------PASTRRTKLAALTVGG-AKLA-DLS--
--LDFTL------------------------------PG---YNIE-LVP---------
GGSHV---
-----------------------------DVDDSNLE-DYLTQ-VLEMTLGTG-VERQVK
-AFQEG---FSMVFSI--RDMR-IFSPD-ELGLLFGN----------------------
---ADEDWS-----------RETL---------EQSLKADHG------------------
----------------YNLD----SRAV-QNLLEVMV--------------------GYD
KEQRRQFLQF-------ITGAPKLP------------IGG-----FK-GLTPQF--TVVR
KPHEPPFKAD------------------------DYLPSVMTCA-QY-LKMPDY-STKEI
LAAQIE-RAMKD-GGGS
>Kwoniella_pini_XM_019154120.1 .
VRISRSQL-LESCAKVL----------------EIYGTSQG-ILEIEYFD-------EIG
TGLG-PTLEF---YSLA----SKEFARKNL--------GIWRDEDESKES----------
---------DYVFHPKGLYPAPLD-----------------------------------E
KQSGFESKLSWFKTLGLFAGRALLDTRIIDVNLNR--VFLKAILG---------------
------QPIKKNIPTLKA----------------------IDAHLARS--------LERL

QTYLFARKEIEALKL------------------PASSRRTKLAALTVGG-ARLA-DLS--
--LDFTL-------------------------------PG---YSIE-LVP---------
GGSLI---
-----------------------------DVDDSNLE-DYLDK-VLEMTLGSG-VERQVK
-AFQEG---FSSVFSV--RDMR-IFSPD-ELGLLFGN-----------------------
---ADEDWS-----------RETL---------EQSLKADHG------------------
----------------YNLD----SRAV-QNLLEVMV--------------------GYD
REQRRQFLQF-------ITGAPKLP------------IGG-----FK-GLTPQF--TVVR
KPHEPPFKAD------------------------DYLPSVMTCA-QY-LKMPDY-STKEI
LAAQIE-RAMKD-GGGS
>Phanerochaete_carnosa_XM_007400881.1 .
VRISRKHI-LESAVKVF----------------ELYGSSSS-VLEVEYFE-------EVG
TGLG-PTLEF---YSLV----SKEFARRDL--------KIWRDADDTKPG----------
---------AYVHHPAGLFPAPIP--------------------------------PEDI
ISDGGLKRTHIFRVIGQFVAKAMLDSRIIDLSLNK--VFLKLILG---------------
------DEIPLTIENLKR----------------------VDLELAAS--------LVKL
RGMTVHTGQS------------------------------DKIRRKLGM-VNVE-DLA--
--LDFTI-------------------------------PG---YDIE-LRPG--------
GRVLA---
------------------------------VTSENVE-EYIAE-VIDAIIGKG-AMLQAK
-AFREG---FSKVFPI--ADLQ-AFTGD-ELVMLFGN-----------------------
---SDEDWS-----------IETL---------NEAIKADHG------------------
----------------FNVE----SRAI-HDLLDIMS--------------------EYD
LPMRRSYLQF-------ITGSPKLP------------IGG-----FR-GLNPPL--TVVR
KPHESPLTAD------------------------DYLPSVMTCV-NY-LKLPEY-SSKAV
MKEKLT-IAMRE-GVGS
>Sparassis_latifolia_GELB01006616.1 .
VRISRQHI-LESAVKVF----------------ELYGSSSS-ILEVEYFE-------EVG
TGLG-PTLEF---YSLV----SKEFARRDL--------KIWRDADSSLPG----------
---------PYVHHPLGLFPAPIS--------------------------------VASI
ANDGGQKRTHIFRVIGQFVAKAMLDSRIIDLSMNK--IFLKLILG---------------
------EDVPLTVDSLRR----------------------VDPDLAAS--------LAQV
QSFAATKSQNDK---------------------------LRRKLAMVEE-VNVE-DLA--
--LDFTI-------------------------------PG---YDIE-LMPG--------
GRNVS---
------------------------------VTAENVD-QYIQD-VIDAIIGKG-AQLQAK
-AFREG---FSKVFPI--TDLQ-AFTAD-ELVMLFGN-----------------------
---GDEDWS-----------LETL---------SEAVKADHG------------------
----------------FNVE----SRAI-RDLLEVMS--------------------GYD
APVRRSFLQF-------ITGSPKLP------------IGG-----FR-GLNPPL--TVVR
KPHEAPLTAD------------------------DYLPSVMTCV-NY-LKLPEY-SSKAV
MKEKLG-IAMKE-GVGS
>Trametes_versicolor_XM_008040555.1 .
VRISRKHI-LESAVKVF----------------ELYGSSSS-ILEVEYFE-------EVG
TGLG-PTLEF---YSLV----SKEFARKDL--------KVWRDADTSMPS----------
---------PYVFHPLGLFPAPIS--------------------------------PDDI
AKDGVQKRTHLIRVIGQFVAKAMLDSRIIDLSFNK--IFLKLVLG---------------
------EEVPLNLESLKR----------------------VDAELAAS--------LSKV
QSLAAAGKSQNEKLRRK----------------------LAALEDMDTG-DEVE-NLG--
--LDFTV-------------------------------PG---YDIE-LRPG--------
GRDIS---
------------------------------VTADNVD-EYTQE-VIDAIVGKG-AQAQAQ
-AFREG---FSKVFPI--ADLQ-AFTAD-ELAMLFGN-----------------------
---ADEDWS-----------AETL---------GESLKADHG------------------
----------------FNVE----SRAI-RDLIEIMS--------------------EYD
ASARRSYLQF-------ITGSPKLP------------IGG-----FK-GLNPPL--TVVR
KPHEAPLTAD------------------------DYLPSVMTCV-NY-LKLPEY-STKAV
MREKLS-VAMRE-GVGS

>Coriolopsis_gallica_GBYM01000198.1 .
VRISRKHI-LESAVKVF----------------ELYGSSSS-ILEVEYFE-------EVG
TGLG-PTLEF---YSLV----SKEFARRDL--------KIWRDADPTLTG----------
---------PYVHHPLGLFPAPIS--------------------------------PDDI
AKDGVQKRTHIIRVIGQFVAKAMLDSRIIDLSFNK--IFLKLALG---------------
------EQVPLTVDTLER----------------------VDPELAGS--------LYKI
QQMAAGNSQAEKLRR------------------------KLAALEDVPQ-VNIE-DLG--
--LDFTV-------------------------------PG---YDIE-LKAG--------
GRDIT---
------------------------------VNADNVD-EYVRE-VIDAIIGKG-AQAQAQ
-AFREG---FSKVFPI--ADLQ-AFTAD-ELAMLFGN-----------------------
---ADEDWS-----------VETL---------SEALKADHG------------------
----------------FHVE----SRAI-RDLIEIMS--------------------EYD
GPTRRSYLQF-------ITGSPKLP------------IGG-----FK-GLNPPL--TVVR
KPHEAPLTAD------------------------DYLPSVMTCV-NY-LKLPEY-STKAV
MREKLL-VAMRE-GVGS
>Dichomitus_squalens_XM_007368125.1 .
VRISRKHI-LESAVKVF----------------ELYGSSSS-ILEVEYFE-------EVG
TGLG-PTLEF---YSLV----SKEFARRDL--------KIWRDADSSIPG----------
---------PHVHHPLGLFPAPIS--------------------------------PDDI
AKDGVQKRTHIIRVIGQFVAKAMLDSRIIDLSFNK--IFLKLVLG---------------
------EEVPLTIDNLKR----------------------VDPELAIS--------LSKI
QGLAAANSQNEKLRQ------------------------KLAAIEGLEE-LNVD-DLG--
--LDFTV-------------------------------PG---YDIE-LRPG--------
GRDIA---
------------------------------VTADNVD-EYIRE-VINAIIGKG-AQAQAQ
-AFREG---FSKVFPI--SDLQ-AFTAD-ELAMLFGN-----------------------
---ADEDWS-----------VETL---------SEALKADHG------------------
----------------FNVE----SRAI-RDLVEIMA--------------------GYD
APTRRSYLQF-------ITGSPKLP------------IGG-----FK-GLNPPL--TVVR
KPHEAPLTAD------------------------DYLPSVMTCV-NY-LKLPEY-SSKKV
MSEKLL-IAMRE-GVGS
>Gloeophyllum_trabeum_XM_007867103.1 .
VRISRKHI-LESAVKVF----------------ELYGSSSS-ILEVEYFE-------EVG
TGLG-PTLEF---YSLV----SKEFARRDL--------KIWRDEDPTNPG----------
---------VYVHHPSGLFPAPIS--------------------------------SDDI
ANDGGQKRTHIFRVIGQFVAKAMLDSRIIDLSFNK--IFVKLVLG---------------
------EHVPLTVESLKH----------------------VDVELAQS--------LIKL
RSLASSA--------------------------------GSKQKLVQES-VSVE-DLA--
--LDFTV-------------------------------PG---YDIE-LRPG--------
GREIP---
------------------------------VTSDNVE-DYIEA-VIDAIIGKG-VQRQAK
-ALRDG---FSKVFPI--SDLQ-AFSAD-ELVMLFGN-----------------------
---SDEDWS-----------IETL---------SEALKADHG------------------
----------------FNVE----SRAI-RNLLEIMS--------------------EFD
APSRREYLQF-------ITGSPKLP------------IGG-----FR-GLNPPL--TVVR
KPHEAPLTAD------------------------DYLPSVMTCV-NY-LKLPDY-TTKAI
MREKLL-IAMRE-GVGS
>Stereum_hirsutum_XM_007304413.1 .
VRISRKHI-LESAMKVF----------------ELYGSSSS-ILEVEYFE-------EVG
TGLG-PTLEF---YSLV----SKEFARKDL--------KIWRDEDPSRSG----------
---------THVFHPKGLFPAPIS--------------------------------PEDI
ANDGGQKRTHIIRVIGQFVAKAMLDSRIIDMSFNK--VFLRIVLG---------------
------EEIPLTIESLKL----------------------VDLDLANS--------LEKL
RGFANTAKDGRR---------------------------DKLQELVQAE-VDIE-DLA--
--LDFTV-------------------------------PG---YDIE-LRSD--------
GKNTP---
------------------------------VTKDNVE-EYIQE-VIDAIIGHG-AKLQAK

-AFKDG---FSKVFPI--TDLQ-AFTVD-ELIMLFGN-----------------------
---SDEDWS-----------SETL---------SEALKADHG------------------
----------------FNVE----SRAI-RSLIEIMS--------------------DYD
ASARREYLQF-------ITGSPKLP------------IGG-----FR-GLNPPL--TVVR
KPHEAPLSAD------------------------DYLPSVMTCV-NY-LKLPEY-STKEV
MREKLR-VAMKE-GVGS
>Heterobasidion_irregulare_XM_009550991.1 .
VRISRKHI-LESAMKVF----------------ELYGSSSS-ILEVEYFE-------EVG
TGLG-PTLEF---YSLV----SKEFARRDL--------KIWRDEDSMKTG----------
---------VYVFHPCGLFPAPIS--------------------------------PQDI
ANDGGQKRTHIFRVIGQFVAKALLDSRIIDMSFNK--VFVRLALG---------------
------EEVPLTLDTLKL----------------------VDPDLATS--------LEKL
HSLAGATVNSSRDKVYLTPATSLT---------TEPDRENKLHKVVDDR-VKIE-DLA--
--LDFTI-------------------------------PG---YDIE-LRPE--------
GKNMT---
------------------------------VTASNVE-EYIHE-VIDAIVGHG-AKLQTK
-AFREG---FSKVFPI--ADLQ-AFSID-ELVMLFGN-----------------------
---AEEDWS-----------RETL---------SESIKADHG------------------
----------------FTVE----SRAI-RDLIDIMS--------------------DYD
LPTRREYLQF-------LSGSPKLP------------IGG-----FR-GLNPPL--TVVR
KPHEAPLSAD------------------------DYLPSVMTCV-NY-LKLPEY-SSKES
MREKLQ-LAMRE-GVEC
>Peniophora_sp._LOAU01000036.1 .
VRISRKHI-LESAMKVF----------------ELYGSSSS-ILEVEYFE-------EVG
TGLG-PTLEF---YALV----SREFARRDL--------KVWRDEDSNKPG----------
---------VHVWHPRGLFPAPIT--------------------------------PEEI
AADGGAKKTHIVRVIGQFVAKALLDSRIIDVSFNK--VFLRLALG---------------
------QPVPLTIEYLKM----------------------VDVELAQS--------LEKL
RAMAVQEPAAR----------------------------DKLTGRIEES-VKIE-DLA--
--LDFTL-------------------------------PG---YDIE-LRPG--------
GREMA---
------------------------------VTAENVE-EYITD-VIGAIIGEG-ARLQAR
-AFSEG---FSKVFPI--DDLH-AFTID-ELAMLFGN-----------------------
---AEEDWS-----------AESTF--------LSLIKADHG------------------
----------------FNAD----SRTI-RNLIEIMS--------------------AYD
APTRRHFLQF-------ITGSPKLP------------IGG-----FR-GLNPAL--TVVR
KPSEAPLTAD------------------------DYLPSVMTCV-NY-LKLPDY-SGREV
MAERMR-VAMIE-GVG-
>Irpex_lacteus_MQVO01005826.1 .
VRISRKHL-LESAVKVF----------------ELYGSSSS-ILEVEYFE-------EVG
TGLG-PTLEF---YSLV----SKEFARRDL--------KIWRDADTTYPG----------
---------PYAFHPLGLFPSPIA--------------------------------PEDL
ARDGGQKRTHIFRVIGQFVAKAMLDSRIIDLHVNK--IFLKLILG---------------
------ENVPLTIDNLKR----------------------VDAELAES--------LLKL
RNLVSNNTQ------------------------------SEKMRRKLGM-VSIE-DLA--
--LDFTI-------------------------------PG---YDIE-LRTG--------
GRDLA---
------------------------------VTSENVE-EYIAE-VLDAIIGKG-AALQAK
-AFREG---FSKVFPI--ADLK-AFTAD-ELVMLFGN-----------------------
---SDEDWS-----------IETL---------SEVVKADHG------------------
----------------FNVE----SRAI-HDLLEIMS--------------------EYD
APTRRNFLQF-------VTGSPKLP------------IGG-----FR-GLNPSF--TVVR
KAHETPLTAD------------------------DYLPSVMTCV-NY-LKLPDY-STKEV
MRERLL-TAMKE-GVG-
>Punctularia_strigosozonata_XM_007382801.1 .
VRISRKHI-LESAMKVF----------------ELYGSSSS-VLEVEYFE-------EVG
TGLG-PTLEF---YSLV----SREFARRTL--------KLWRDADVNSPG----------
---------LYVQHPTGLFPAPIS--------------------------------PDDA

GNDGTQKRTHIFRVIGQFVAKAMLDSRIIDMSYNK--IFLKLVLG---------------
------EQVPLTLETLKL----------------------VDADLASS--------LSKL
RRMAKAKEEQE----------------------------PAENVDKAES-VNVE-DLA--
--LDFTI-------------------------------PG---YDIE-LKPG--------
GRDIA---
------------------------------VTADNVE-EYIES-VIDAIIGSG-AQPQVQ
-AFREG---FSKVFPI--TDLQ-AFSAD-ELAMLFGN-----------------------
---TDEDWS-----------VETL---------SEALKADHG------------------
----------------FNVE----SRAI-RNLVEIMA--------------------AYD
APTRRGYLQF-------ITGSPKLP------------IGG-----FR-GLNPPL--TVVR
KPHEPPLTAD------------------------DYLPSVMTCV-NY-LKLPDY-SSKEV
MREKLH-TAMKE-GVGS
>Boletopsis_grisea_GEZR01001261.1 .
VRISRKHI-FESAVKVF----------------ELYGSSSS-ILEVEYFE-------EVG
TGLG-PTLEF---YSLV----SKEFARRDL--------KIWRDADPTKPG----------
---------VYVHHPAGLFPAPIS--------------------------------SDEI
ATEHGQKRIHIFKVMGQFIAKAMLDSRIIDVSLNK--VFLKFVLG---------------
------ETVPLTLESLKL----------------------VDADLASS--------LEKL
QRMAQAKC-------------------------------VDKVSSKVGL-VDVA-DLA--
--LDFTI-------------------------------PG---YDIE-LKPG--------
GRDIA---
------------------------------VTSDNAS-GYIEA-VIDAIIGKG-AQPQAK
-ALREG---FSKVFPI--SDLR-TFTVD-ELVMLFGN-----------------------
---SDEDWS-----------VETL---------NEVLKADHG------------------
----------------FTGD----SRAI-HHLIEIMS--------------------EYD
VSSRRSCLQF-------ITGSPKLP------------IGG-----FR-GLNPPL--TVVR
KPHEPPLTAD------------------------DYLPSVMTCV-NY-LKLPDY-SSKET
MRQKVK-VAMQE-GVGS
>Sphaerobolus_stellatus_JOMA01000216.1 .
VRISRKHI-LESAVKVF----------------ELYGSSSS-ILEVEYFE-------EVG
TGLG-PTLEF---YSLV----SKEFARRDL--------KIWRDADTTAPG----------
---------PYVHHPQGLFPAPLS-----------------------------------X
TCFFFRKRTHLFQIIGQFVAKALLDSRIVDMSFNK--IFIKLVVG---------------
------EEVPQTIETLAL----------------------VDPELASS--------LSKL
X---IE-DLA--
--LDFTL-------------------------------PG---YDME-LXPD--------
GHNIA---
------------------------------VTADNVE-EYISL-VIDAIIGKG-AEAQTK
-AFKEG---FSKVFHI--DDLK-IFSAE-ELVMMFGN-----------------------
---GEEDWS-----------XTAL---------SEALKADHG------------------
----------------FNVE----SRAI-RNLVELMA--------------------SFD
LNVRRQYLQF-------ITGSPKLP------------IGG-----FR-GLNPPL--TVVR
KPHEAPLSAD------------------------DYLPSVMTCV-NY-LKLPEY-SSKVV
MGEKLS-IAMRE-GVG-
>Serpula_lacrymans_XM_007320701.1 .
VRISRKHI-LESAVKVF----------------ELYGSSSS-VLEVEYFE-------EVG
TGLG-PTLEF---YSLV----SKEFARRDL--------KIWRDADPTYPG----------
---------NYVHHPAGLYPAPIS--------------------------------PDDI
ANDGGQKRTHIFSVIGQFLAKAMLDSRIIDISFNK--IFVKLVLG---------------
------EEVPVTIATLKL----------------------VDSELANS--------LHKL
QDLASLSG-------------------------------PTAKGMALDT-VHIE-DLA--
--LDFTI-------------------------------PG---YDIE-LRPD--------
GRNMQ---
------------------------------VTSDNAH-EYIHE-VLEAIVGKG-VQLQAK
-AFREG---FSKVFPI--SDLQ-TFSPD-ELVMLFGN-----------------------
---SDEDWS-----------IETL---------SESIKADHG------------------
----------------FNVE----SRAI-RDLIELMS--------------------EFD
ATTRRDYLQF-------ITGSPKLP------------IGG-----FR-GLNPPF--TVVR

KPHEAPLTAD------------------------HYLPSVMTCV-NY-LKLPEY-SSKKV
MREKVY-TAMKE-GVGS
>Coniophora_puteana_XM_007772246.1 .
VRISRKHI-LESAVKVF----------------ELYGSSSS-ILEVEYFD-------EVG
TGLG-PTLEF---YSLV----SKEFARRDL--------KIWRDADATRSG----------
---------TYVHHPTGLYPAPIS--------------------------------PDDV
SNDGSQKRTHILRVVGQFVAKAMLDSRIIDMSLNK--VFIKQVLG---------------
------EDVPVTIDTLRL----------------------VDKDLANS--------LAKL
QGYASCKGQAEKLEQ------------------------IRNKVALMDI-VNID-DLA--
--LDFTI-------------------------------PG---YDIE-LRPG--------
GRDIP---
------------------------------VTSDNAE-QYIQE-VLNAIIGSG-ARAQAK
-AFREG---FSKVFSI--SDLH-SFSPD-ELVMLFGN-----------------------
---TDEDWS-----------LETL---------TESIKADHG------------------
----------------FHMD----SRAI-HDLMDIMS--------------------EYD
VTSRRDFLQF-------ITGSPKLP------------IGG-----FR-GLNPPF--TVVR
KPHEAPLTAD------------------------DYLPSVMTCV-NY-LKLPEY-SSKKV
MKEKLR-VAMKE-GVGS
>Pisolithus_tinctorius_JMDO01000648.1 .
VRISRKHI-LESAVKVF----------------ELYGSSSS-ILEVEYFE-------EVG
TGLG-PTLEF---YSLV----SKEFARRDL--------KIWRDADPTSPG----------
---------VHVHHPAGLFPAPIS------------------------------------
PVDIARKRTHILRVIGQYVAKAMLDSRIIDLSFNK--IFIKLVLG---------------
------DEVPFTVDSLRVGDSFQLCILTT--------PRLVDNDLANS--------LAKL
QGYAALKGQNEKVSVATPGARSCFLSGLQ----------LFRKVALMDI-VNVE-DLA--
--LDFTL-------------------------------PG---YDIE-LXPG--------
GRDIP---
------------------------------VTSENVE-QYIHD-VLDAIIGSG-VQPQAK
-AFREG---FSKVFPV--SDLQ-AFSAD-ELVMLFGN-----------------------
---SDEDWX-----------XAAL---------SEAIKADHG------------------
----------------FHGE----SRSI-RELIELMT--------------------EFD
PPTRRDYLQF-------ITGSPKLP------------IGG-----FR-GLNPPL--TVVR
KPAEPPNTPD------------------------AYLPSVMTCV-NY-LKLPEY-SSKTI
MRERLL-TAIKE-GVG-
>Scleroderma_citrinum_JMDU01002705.1 .
VRIARKHI-LESAVKVF----------------ELYGSSSS-ILEVEYFE-------EVG
TGLG-PTLEF---YSLV----SKEFARRDL--------KIWRDADPSSPG----------
---------IHVYHPAGLYPAPIS----------------PDDIANDG-GQVNNSSVLIG
KSVFVRKRTHILRVIGQYIAKAMLDSRIIDLSFNK--VFIKLVLG---------------
------EEVPLTIDSLRVRDYSGILYLLT---------NAPSWLITTS--------PTLL
LSFRAMHPSKARMRRCGSTLCMINSDVWTNV--------LFRKIALIDI-VNVE-DLA--
--LDFTL-------------------------------PG---YDIE-LXPG--------
GRDIP---
------------------------------VTSKNVE-QYVDE-VLDAIIGSG-VQPQAK
-AFREG---FSKVFPI--TDLQ-AFSAD-ELVILFGN-----------------------
---SDEDWT-----------SESL---------SEALKADHG------------------
----------------FNGE----SRSI-RELIELMT--------------------EFD
SCTRRNYLQF-------ITGSPKLP------------IGG-----FR-GLNPPL--TVVR
KPAESPLTPD------------------------DYLPSVMTCV-NY-LKLPEY-SSKKV
MKERLR-TAMKE-GVG-
>Suillus_luteus_JMSM01000071.1 .
VRISRKHI-LESAVKVF----------------ELYGSSSS-ILEVEYFD-------EVG
TGLG-PTLEF---YSLV----SKEFARRDL--------KIWRDADSAAPG----------
---------IYVHHPTGLYPAPIS------------------------------------
PTSNGRKRTHILRVVGQYVAKAMLDSRIIDLSVNK--VFIKLVLG---------------
------EEVPLTIESLKVRNIVRDKTLYS----------HDLSSLWIT--------NLX-
---------------------------------------LFRKVALKDI-VNIE-DLA--
--LDFTL-------------------------------PG---YDIE-LXPG--------

GGNIL---
------------------------------VTSENAE-EYIHE-VLDAIIGAG-VQPQAR
-AFREG---FSKVFPI--SDLQ-AFSAD-ELVMLFGN-----------------------
---SDEDWG-----------VESM---------NEAIKADHG------------------
----------------FHSE----SRAI-RELVGIMS--------------------DFD
AFARRDFLQF-------ITGSPKLP------------IGG-----FR-GLNPPL--TVVR
KPHEPPLMAD------------------------DYLPSVMTCV-NY-LKLPEY-SSKVV
MREKLR-IAMKE-GVG-
>Hydnomerulius_pinastri_JMSK01000840.1 .
VRISRKHI-LESAVKVF----------------ELYGSSSS-ILEVEYFE-------EVG
TGLG-PTLEF---YSLV----SKEFARRDL--------KLWRDADAGSPG----------
---------IYVHHPAGLYPAPIS------------------------------------
----XRKRTHILRVIGQYVAKAMLDSRIIDLSFNK--VFIKLVLG---------------
------EEIPLTIESLRVCQHVLCGHITR---PLLSWSTTISPTLWLS--------FKAM
RRKDRAKRFVFFASSLCILTPHKQ---------------LFRKVALMDI-VNVE-DLA--
--LDFTL-------------------------------PG---YDIE-LXPG--------
GRDTP---
------------------------------VTSENAD-QYIHE-VLDAIIGTG-AQRQAK
-AFREG---FSKVFPI--TDLQ-AFSAD-ELVMLFGN-----------------------
---SDEDWS-----------VESL---------NEAIKADHG------------------
----------------FNGE----SRSI-RDLVELMS--------------------EYD
AFARRDYLQF-------ITGSPKLP------------IGG-----FR-GLNPPL--TVVR
KPHEAPLTAD------------------------DYLPSVMTCV-NY-LKLPEY-SSKKV
MKEKLG-TAMKE-GVG-
>Fibroporia_radiculosa_XM_012330090.1 .
VRISRQHI-LESAVKVF----------------ELYGSSSS-ILEVEYFE-------EVG
TGLG-PTLEF---YSLV----SKEFARKDL--------KIWRDADPLLSS----------
---------PFVHHPLGLFPAPIS----------------------------------SS
NVTNELGQTHIFRVIGQFVAKALLDSRIIDLTLNK--VFLKLILG---------------
------DEVPLTIDNLRR----------------------VDPDLAAS--------LGQV
QSFASINQNEK----------------------------IRRKLGMIEN-VSIE-DLA--
--LDFTL-------------------------------PG---YDIE-LRPG--------
GRDMP---
------------------------------VTSQNID-QYIHE-VLDAILGKG-AQAQAK
-AFREG---FSKVFPI--SDLQ-AFTTD-ELAMLFGN-----------------------
---ADEDWS-----------LETL---------SEALKADHG------------------
----------------FNVE----SRAI-RNLLEIMA--------------------EFD
TQTRRSYLQF-------ITGSPRLP------------IGG-----FR-GLNPAL--TVVR
KPHEAPLTAD------------------------DYLPSVMTCV-NY-LKLPDY-SSKSV
MREKLQ-TAMHE-GVGS
>Clavaria_fumosa_CVRD01110896.1 .
VRISRKHI-LESAVKVF----------------ELYGSSSS-VLEVEYFE-------EVG
TGLG-PTLEF---YSLV----SKEFARRDL--------KIWRDADLIVPG----------
---------IYVQHPDGLYPAPIS------------------------------------
AEDKSRKKTHVFRVIGQFVAKAMLDSRIVDLHFNK--IFLKLVLG---------------
------DEVPLTLSSLKVTRTP------------------IHGESWLSN-------ILYA
SSSTRNSRIHFLSSRHSQVRTVTRFAYSTLLHFSHEAS-LARKVAIAEG-ITVE-DLA--
--LDFTI-------------------------------PG---YDID-LXPG--------
GKNIP---
------------------------------VTSDNID-EYINE-VLDAIIGGG-AQQQAN
-AFRDG---FSKVFPI--NDLQ-AFSPD-ELVMLFGN-----------------------
---SEEDWS-----------TECL---------SEALKADHG------------------
----------------FNVD----SRAI-RDLVEILS--------------------EYD
VSSRRACLQF-------ITGSPKLP------------IGG-----FR-GLNPPL--TVVR
KPHEAPLTAD------------------------DYLPSVMTCV-NY-LKLPEY-SSKAV
MREKLR-TAMRE-GVG-
>Hypsizygus_marmoreus_GBCL01003428.1 .
VRISRKHI-LESAVKVF----------------ELYGSSSS-VLEVEYFE-------EVG

TGLG-PTLEF---YSLV----SKEFARKDL--------KLWRDSDATSSS----------
---------AYVGHPSGLYPAPLS--------------------------------PEDV
ASDGGQKRTHLLRVIGQFVAKAMLDSRIIDLHFNK--IFLKLVLG---------------
------EEVPLTITSLKL----------------------VDVELANS--------LEKV
QSIVAAKNQVQTDK-------------------------LQRKIAMVES-VSIE-DLA--
--LDFTI-------------------------------PG---YDIE-LKPG--------
GRDMA---
------------------------------VTSENVD-EYVSE-VLDAILGKG-TQIQAK
-AFRDG---FSKVFPI--QDLQ-AFSAD-ELVMLFGN-----------------------
---SDEDWS-----------VETL---------AESLKADHG------------------
----------------FNVE----SRAI-HDLVEIMS--------------------SYD
APTRRAFLQF-------ITGSPKLP------------IGG-----FR-GLNPLL--TVVR
KPHEAPLAAD------------------------DYLPSVMTCV-NY-LKLPEY-SSKAV
MREKLR-IAIQE-GVGS
>Termitomyces_sp._BCLB01000014.1 .
VRISRKHI-LESAVKVF----------------ELYGSSSS-ILEVEYFE-------EVG
TGLG-PTLEF---YSLV----SKEFARKDL--------KIWRDTDALGST----------
---------PYVGHPSGLYAAPLS-----------PEDVASDGGLEIA-DCFRATNMTDH
LNPEISKRTHLLRVIGQFVAKAMLDSRIIDLTFNK--VFLKLVLG---------------
------EEVPLTIASLKVCAYSFNSTISSQ--FNIPSFQLVDIDLANS--------LEKL
RSIASSKLQPQNNR-------------------------VCNISKSLNF-PCFE--IA--
--ITKSC-------------------------------KG---ISLN-XVPG--------
GKEIS---
------------------------------VTSDNVD-EYINA-VLDAILGKG-TMIQAK
-AFRDG---FSKVFPV--TDLR-AFSAD-ELVMLFGN-----------------------
---SEEDWS-----------XTAL---------SEALKADHG------------------
----------------FNVE----SRAI-HDLIEIIS--------------------AYD
NPTRRAFLQF-------ITGSPKLP------------IGG-----FR-GLNPSL--TVVR
KLHEPPLTAD------------------------DYLPSVMTCV-NY-LKLPEY-TSKKV
MKEKLR-IAMQE-GVG-
>Schizophyllum_commune_XM_003033304.1 .
VRISRKHL-LESAVKVF----------------ELYGSSSS-VLEVEYFE-------EVG
TGLG-PTLEF---YALV----SKEFARKDL--------KLWRDADAASKS----------
---------LYVDHPAGLYPAPIG--------------------------------TEDL
DNDGGHKRTHLLRVIGQFVAKAMLDSRIIDLSFNK--VFMKLVLG---------------
------EEVPLTIATLKL----------------------VDQGLADS--------LVKI
QNIVSGKQPQDKVCTDKSYQ-------------------LAKKVAMVEG-VTIE-DLA--
--LDFTL-------------------------------PG---YDIE-LRPG--------
GRNVA---
------------------------------VTAANVD-EYIHD-ILDAILGKG-AQEQAA
-AFREG---FSKVFPI--SDLR-AFTAD-ELVLLFGN-----------------------
---GDEDWS-----------IETL---------TEALKADHG------------------
----------------FNSE----SRSI-RNLVEIMA--------------------NYD
PPTRRMYLQF-------ITGSPKLP------------IGG-----FR-GLNPPL--TVVR
KPHEAPLKAD------------------------DYLPSVMTCV-NY-LKLPEY-STKDV
MREKLR-VAMLE-GVGS
>Agrocybe_aegerita_JW842373.1 .
VRISRKHI-LESAVKVF----------------ELYGSSSS-ILEVEYFE-------EVG
TGLG-PTLEF---YSLV----SKEFARKDL--------KIWRDADASGPG----------
---------VYVDHPKGLYPAPIS--------------------------------TEDI
INDGGQKRTHILRVIGQFAAKAMLDSRIIDMTFNK--VFLKLVLG---------------
------EELPLTISTLRL----------------------VDVELANS--------LAKV
QNMATQHNAVPKDK-------------------------LSRKVAKIEQ-VSFE-DLS--
--LDFTI-------------------------------PG---YDIE-LRPG--------
GRDIL---
------------------------------VTVENVD-EYVHE-VLDAILGKG-AALQAK
-AFREG---FSKVFPI--TDLR-AFSSD-ELIMLFGN-----------------------
---AEEDWS-----------IETL---------SEALKADHG------------------

----------------FNVE----SRAI-RDLIEVMA--------------------DYD
ASTRRAYLQF-------ITGSPKLP------------IGG-----FR-GLNPSL--TVVR
KPHEAPLTAD------------------------DYLPSVMTCV-NY-LKLPEY-SSKQV
MKEKLR-IAMQE-GVGS
>Moniliophthora_roreri_XM_007844679.1 .
VRISRKHI-LESAVKVF----------------ELYGSSSS-VLEVEYFE-------EVG
TGLG-PTLEF---YSLV----SKEFARKDL--------KIWRDADHTNAG----------
---------TYVNHPSGLYPAPLT--------------------------------AEDI
ASDGGQKKTHIFRVIGQFVAKAMLDSRIIDLSLNK--IFLKLILG---------------
------EEVPLTIVSLKV----------------------IDVELANS--------LAKV
LNIATGNTQSPNDK-------------------------VAKKIALLES-VSVE-DLA--
--LDFTL-------------------------------PG---YDID-LRPG--------
GKDIA---
------------------------------VTSQNAH-EYVQE-VLDVILGKG-AQLQAK
-AFREG---FSKVFPI--SDLQ-PFSVD-ELVMLFGN-----------------------
---ADEDWS-----------IETL---------SEALKADHG------------------
----------------FHPE----SRAI-RDLMEIMS--------------------EYD
PQTRREYLQF-------ITGSPKLP------------IGG-----FR-GLNPPL--TVVR
KPYEAPLTAD------------------------DYLPSVMTCV-NY-LKLPEY-SSKAV
MKEKIR-VAIKE-GIGS
>Leucocoprinus_comp38870_c0_seq1_GEHF0100419 .
VRISRKHM-LESAIKVF----------------ELYGSSSS-ILEVEYFE-------EVG
TGLG-PTLEF---YSLV----SKEFARRDL--------KVWRDADTAGSG----------
---------TYVSHPTGLYPAPVQ--------------------------------ADEP
NLDGGHKQIHILRIIGQFIAKAMLDSRIIDLSFNK--VFLKLILD---------------
------EEVPLTLASLRL----------------------VDAELANS--------LEKL
RNIAAEQNRAQKDK-------------------------LLSKIAKIES-ANVE-DLS--
--LDFTL-------------------------------PG---YDIE-LHPG--------
GRSIP---
------------------------------VTANNVE-EYIQE-VLDAILGKG-IQTQVQ
-AFKEG---FSKVFPI--NDLK-TFTAE-ELVMLFGN-----------------------
---SEEDWG-----------IETL---------SEAIKADHG------------------
----------------FNVE----SRAI-RDLIEIMS--------------------DYD
TSTRRAYLQF-------ITGSPKLP------------IGG-----FR-GLNPLF--TVVR
KPHEAPLKAD------------------------DYLPSVMTCV-NY-LKLPDY-SSKDV
MREKLG-VAMQE-GVGS
>Agaricus_bisporus_XM_006461736.1 .
VRISRKHI-LESAVKVF----------------ELYGSSSS-ILEVEYFE-------EVG
TGLG-PTLEF---YSLV----SKEFARKDL--------KIWRDSDTTEAG----------
---------VYVNHPMGLYPAPLH--------------------------------SDDC
AGEGGQKRTHIIRVIGQFVAKAMLDSRIIDLSFNK--VFLKRVLH---------------
------EDVPLSITSLRL----------------------VDAELANS--------LEKL
RNIAAEQSLAQKDK-------------------------LLSKIVKIES-VSVE-ELS--
--LDFTL-------------------------------PG---YDIE-LRPG--------
GKNTA---
------------------------------VTSSNVE-DYISD-ILDAVLGKG-VEVQVQ
-AFREG---FSKVFPV--SDLK-TFTAD-ELVMLFGN-----------------------
---SDEDWS-----------TETL---------SEALKADHG------------------
----------------FNVE----SRAI-RDLIEVMS--------------------DYD
RPTRRACLQF-------ITGSPKLP------------IGG-----FR-GLNPPL--TVVR
KPHEVPLKAD------------------------DYLPSVMTCV-NY-LKLPDY-SSKAV
MKDKLG-IAMQE-GVGS
>Agaricus_bisporus_XM_007327131.1 .
VRISRKHI-LESAVKVF----------------ELYGSSSS-ILEVEYFE-------EVG
TGLG-PTLEF---YSLV----SKEFARKDL--------KIWRDSDTTEAG----------
---------VYVNHPMGLYPAPLH--------------------------------SDDC
AGEGGQKRTHIIRVIGQFVAKAMLDSRIIDLSFNK--VFLKRVLH---------------
------EDVPLSIASLRL----------------------VDAELANS--------LEKL

```
RNIAAEQSLAQKDK-------------------------LLSKIVKIES-VSVE-ELS--
--LDFTL--------------------------------PG---YDIE-LRPG--------
GKNTA-------------------------------------------------------
------------------------------VTSSNVE-DYISD-ILDAVLGKG-VEVQVQ
-AFREG---FSKVFPV--SDLK-TFTAD-ELVMLFGN-----------------------
---SDEDWS-----------TETL---------SEALKADHG------------------
----------------FNVE----SRAI-RDLIEVMS--------------------DYD
RPTRRACLQF-------ITGSPKLP------------IGG-----FR-GLNPPL--TVVR
KPHEAPLKAD------------------------DYLPSVMTCV-NY-LKLPDY-SSKAV
MKDKLG-IAMQE-GVGS
>Leucocoprinus_gongylophorus_HAAN01020641.1 .
VRISRKHI-LESAVKVF----------------ELYGSSSS-ILEVEYFE-------EVG
TGLG-PTLEF---YSLV----SKEFARKDL--------KIWRDADVVGAG----------
---------VYVSHPFGLYPAPVQ--------------------------------PDE
PTKGGHKGTHILRIIGQFIAKAMLDSRTIDLSLNK--VFLKLVLD---------------
------EEVPLTIASLRL---------------------VDTELTNS--------LEKL
KTMAAAEQSRAHKDK------------------------LSSKIAKMES-ANIE-DLS--
--LDFTL--------------------------------PG---YDIE-LCPG--------
GKNIV-------------------------------------------------------
------------------------------VTANNVD-EYIKE-IINAILGDG-VREQVK
-AFKEG---FSKVFPV--IDLK-TFTAD-ELVMLFGN-----------------------
---SEEDWS-----------TETI---------SEALKADHG------------------
----------------FNVE----SRAI-RDLIEIMA--------------------DYG
SSTRRAYLQF-------ITGSPKLP------------IGG-----FR-GLNPPF--TVVR
KPHEAPLKAD------------------------DYLPSVMTCV-NY-LKLPDY-SSKTV
MREKLR-IAMEE-GVGS
>Coprinopsis_cinerea_XM_002910849.1 .
VRISRKHI-LESAVKVF----------------ELYGSSSS-VLEVEYFE-------EVG
TGLG-PTLEF---YSLV----SKEFARKDL--------KIWRDNDASGPG----------
---------SYVSHPHGLYPAPIS-------------------------------REDI
ISDGGQKRTHILRVIGQFVAKAMLDSRIIDLSFSK--VFLKIVLG---------------
------EEVPITIASLKL---------------------VDPSLAKS--------LSQL
QAIASEPTEPPTDP-------------------------LQRKIAEIEK-VNIE-DLA--
--LDFTI--------------------------------PG---YDIE-LRPG--------
GRDIP-------------------------------------------------------
------------------------------VTSANVY-DYIDE-VLDAILGKG-IQVQAK
-AFREG---FSKVFPI--DDLR-AFTAD-ELVMLFGN-----------------------
---TEEDWC-----------IETL---------SESIKADHG------------------
----------------FNVD----SRSI-RYLLEIMA--------------------EFD
VPTRRAYLQF-------ITGSPKLP------------IGG-----FK-GLNPPL--TVVR
KPHEAPLTAD------------------------DYLPSVMTCV-NY-LKLPEY-SSKEV
MREKLC-IAIQE-GVGS
>Volvariella_volvacea_AMXZ01000273.1 .
VRISRKHI-LESAVKVF----------------ELYGSSSS-VLEVEYFE-------EVG
TGLG-PTLEF---YSMV----SKEFARKDL--------KIWRDMDASGSG----------
---------PFVSHPYGLYPAPLS------------PEDIASDGGQVL-HYCLL---YLP
HFANHRKRTHILRVVGQFVAKAMLDSRIIDLSFNK--TFLKLVLG---------------
------EEIPVSVASLKVDRPLQPFETSY-----LTDPCLVDPDLANS--------LTKL
QGMAVRKTQVPNDKVSEFVLHVCLQEPL-----------LSRKIAQLEN-DDIE-NLS--
--LDFTL--------------------------------PG---YDIE-LXPG--------
GRDIA-------------------------------------------------------
------------------------------VTSENVN-EYIDA-VLDAIIGSG-VQVQVK
-AFKEG---FSKVFPV--SDLQ-AFTAD-ELVMLFGN-----------------------
---SDEDWS-----------LESL---------SEAIKADHG------------------
----------------FNVE----SRAI-HDLMDIMS--------------------EFD
VTTRRNFLQF-------ITGSPKLP------------IGG-----FR-GLNPPL--TVVR
KPHEHPLTAD------------------------DYLPSVMTCV-NY-LKLPEY-SSKSV
MLEKLR-LAIKE-GVG-
```

```
>Amanita_phalloides_MEHY01000026.1 .
VRISRKHI-LESAVKVF----------------ELYGSSSS-ILEVEYFE-------EVG
TGLG-PTLEF---YSLV----SKEFARKDL--------KIWRDVDPHGTS----------
---------PYVHHPQGLFPSPIS---------------------------------PED
VANXXXKRTHVLRVIGQFVAKALLDSRIIDLNFNK--IFLKHILG---------------
------EEVPLTIASLKVAIAGYMAEL-------LMLTQLVDIDLANS--------LAKL
QSMAAGQNSNDKVCSVAWFFPSVDFYKNH---------QLARKIALVEA-GRIE-DLA--
--LDFTI-------------------------------PG---HDIE-LXPG--------
GRDIV-------------------------------------------------------
------------------------------VTSENVR-EYIDH-ILDAILGKG-IQLQAK
-AFKEG---FSKVFPV--PDLQ-AFSAD-ELAMLFGN-----------------------
---SDEDWN------------XAL---------TEAVKADHG------------------
----------------FNVD----SKTI-RDLIEVMS--------------------EYD
IPMRRAFLQF-------ITGSPKLP------------IGG-----FR-GLNPPL--TVVR
KPHEAPLTAD------------------------DYLPSVMTCV-NY-LKLPEY-SSRHV
MKEKLQ-MAIQE-GIG-
>Amanita_polypyramis_JNHY01003435.1 .
VRISRKHM-LESAVKVF----------------ELYGSSSS-ILEVEYFE-------EVG
TGLG-PTLEF---YSLV----SKEFARKDL--------KIWRDADHHGTG----------
---------SYVHHPQGLFPAPIS-----------------------------------P
EDVANXKRTHILRVIGQFVAKAMLDSRIIDLNFNK--IFLKQVLG---------------
------GEVPLTIASLKVALLN------------------LDIGLTAN--------INAA
CRHRPCQLSGKITEYGCRAKIESEHCQSFYNSLTDQDGKLARKIALVEA-GKIE-DLA--
--LDFTI-------------------------------PG---YDIE-LXPG--------
GRDIA-------------------------------------------------------
------------------------------VSSENVN-EYING-VLDAILGKG-TQIQAK
-AFKEG---FSKVFPV--SDLQ-AFSAD-ELAMLFGN-----------------------
---SDEDWN-----------IDSL---------VEAIKADHG------------------
----------------FNVD----SKAI-RDLIEIMS--------------------EYD
IPMRRAFLQF-------ITGSPKLP------------IGG-----FR-GLNPPL--TVVR
KPHEAPFTAD------------------------DYLPSVMTCV-NY-LKLPEY-SSSGV
MKEKLQ-TAVQE-GVG-
>Amanita_jacksonii_AYNK01004035.1 .
VRISRKHI-LESAVKVF----------------ELYGSSSS-ILEVEYFE-------EVG
TGLG-PTLEF---YSLV----SKEFARKDL--------KIWRDADPHGSG----------
---------PYVHHPQGLYPAPIS------------------------------------
PEDVARKRTHVLRVIGQFVAKAMLDSRIIDLNFNK--IFLKHVLG---------------
------EEVSLTIAALKVTLSQRR----------SMFSANVNLVGHRA--------SFSR
KITKYGCGAELEGERREARETDLTDERH----------KLARKIALVEE-GSIE-DLA--
--LDFTI-------------------------------PG---YDIE-LXPG--------
GRDIA-------------------------------------------------------
------------------------------VTSDNVE-EYINE-VLDAILGKG-VQIQAK
-TFKEG---FSKVFPV--SDLQ-AFSAD-ELAMLFGN-----------------------
---SDEDWS-----------IECV---------STSKAKLFG------------------
----------------X-------------------------------------------
---------------------------------------G-----FR-GLNPPL--TVVR
KPHEAPLSAD------------------------DYLPSVMTCV-NY-LKLPEY-SARSV
MKEKLQ-TAIQE-GVG-
>Cylindrobasidium_torrendii_JYFH01000078.1 .
VRISRKHI-LESAVKVF----------------ELYGSSSS-VLEVEYFE-------EVG
TGLG-PTLEF---YSLV----SREFARKDL--------KLWRDADASATTM---------
---------QFVHHPTGLYPAPLS------------PEAIAGDGGQYV-PLNVQ---RNP
SDDMYSKRTQLFKVTGQFVAKAMLDSRIIDLSFNK--VFMKLVLG---------------
------EEVPVTMATLKL----------------------VDVDLANS--------LTKL
QEMADKQNQVDPSDT------------------------LAVKTAMVEG-INVE-DLA--
--LDFTV-------------------------------PG---YDI-----X--------
GRDIP-------------------------------------------------------
------------------------------VTADNVN-EYIAA-VLDVIVGQG-VALPVK
```

```
-AFREG---FSKVFPI--MDLQ-AFSAD-ELVMLFGN-----------------------
---SDEDWS-----------SETL---------SEAMKADHG------------------
----------------FNPE----SRSI-RHLLEIFS--------------------EFD
VTERRNCLQF-------VTGSPKLP------------IGG-----FR-GLNPPL--TVVR
KPHEAPLTAD------------------------DYLPSVMTCV-NY-LKLPEY-SSKEV
MKAKLL-TAIRE-GTG-
>Armillaria_fuscipes_LWUH01002426.1 .
VRISRKHI-LESAVKVF----------------ELYGSSSS-VLEVEYFE-------EVG
TGLG-PTLEF---YSLV----SKEFARKDL--------KLWRDVDLPGSG----------
---------VYVNHPVGLYPAPIS---------------------------------PEDI
ASDGG-QYVHVFRVIGQFVAKAMLDSRIIDLSFNK--VFLKLVLG---------------
------EEVPLTVATLKVKYIYYTPRSR------LTVPSLVDADLGNS--------LTKV
LNIVNTQKNQVHSDKVLNPLIYVISLTRF----------LSRKIALVES-VDIE-DLA--
--LDYTI-------------------------------PG---YDIE-LXPG--------
GRDIT-------------------------------------------------------
------------------------------VTADNVH-EYVEL-VLDAIIGKG-AALQAK
-AFREG---FSKVFPI--SDLQ-AFSAD-ELVMLFGN-----------------------
---SDEDWS-----------IESM---------LSRVKADHG------------------
----------------FNPE----SRAI-RDLVEIFA--------------------DYD
TPTRRDCLQF-------ITGSPKLP------------IGG-----FR-GLNPPL--TVVR
KPHEAPLTAD------------------------DYLPSVMTCV-NY-LKLPDY-SSKEV
MREKLR-IAIKE-GVG-
>Armillaria_ostoyae_GAHM01014859.1 .
VRISRKHI-LESAVKVF----------------ELYGSSSS-VLEVEYFE-------EVG
TGLG-PTLEF---YSLV----SKEFARKDL--------KLWRDADLPGSG----------
---------VYVNHPVGLYPAPIS---------------------------------PEDI
ASDGGQKRTHVFRVIGQFVAKAMLDSRIIDLSFNK--VFLKLVLG---------------
------EEVPITVATLKL----------------------VDADLGNS--------LTKV
LNIVNTQKNQVHPDK------------------------LSRKIAMVES-VDIE-DLA--
--LDFTI-------------------------------PG---YDIE-LRPG--------
GRDIA-------------------------------------------------------
------------------------------VTAENVH-EYVEE-VLDAIIGKG-AALQAK
-AFREG---FSKVFPI--SDLQ-AFSAD-ELVMLFGN-----------------------
---SDEDWS-----------IETL---------SEALKADHG------------------
----------------FNPE----SRAI-RDLVEIFA--------------------DYD
APTRRDCLQF-------ITGSPKLP------------IGG-----FR-GLNPPL--TVVR
KPHEAPLTAD------------------------DYLPSVMTCV-NY-LKLPDY-SSKDV
MREKLR-VAIKE-GVGS
>Pleurotus_ostreatus_MAYC01000084.1 .
VRISRKHI-LESAVKVF----------------ELYGSSSS-ILEVEYFE-------EAG
TGLG-PTLEF---YSLV----SREFARKDL--------KIWRDSDTSGTG----------
---------MYVQHPTGLYPAPLS------------AEDIASDGGQCV-FSPPHWHFSDQ
LLRWCSKRTHILRVVGQFVAKAMLDSRIIDLHFNK--VFLKLVLG---------------
------EEVPLTIQTLKVVIAIRKSPRAY----IYIVPLLVDSGLASS--------LAKL
QNMVGKRQGSTDKVGATPQVLDTQFTWL-----------LSRKLALLDS-VNIE-DLA--
--LDFTV-------------------------------PG---YDIE-LXPG--------
GREKA-------------------------------------------------------
------------------------------VTSDNVE-EYIQA-VLETIIGKG-AQVQVK
-AFREG---FSKVFPI--TDLQ-TFSAD-ELVMLFGN-----------------------
---SDEDWS-----------IESL---------QEALKADHG------------------
----------------FNSE----SRSI-HDLISVMS--------------------EFD
APTRRDYLQF-------ITGSPKLPIG----------IQG-----FR-GLNPPL--TVVR
KPHEAPFTAD------------------------DYLPSVMTCV-NY-LKLPEY-SSKAV
LREKVR-IAMKE-GVG-
>Rigidoporus_microporus_GDMN01021679.1 .
VRISREHI-LQSAFKVF----------------ELYGSSSS-ILEVEYFD-------EVG
TGLG-PTLEF---YSLV----SKEFARRDL--------KIWRDVDTTYPG----------
---------VYVHHPHGLFPAPIA---------------------------------KSE
```

GGKNNEKRIYVFRTIGQFVAKAMLDSRIIDMSFNK--IFLKLILG---------------
------EEVPLTIASLKH----------------------VDPGLANS--------LSKM
QACVPSDNEDP----------------------------DSESEYPDQL-MNIE-DLM--
--LDFTL-------------------------------PG---YDIE-LKPN--------
GRNIP---
------------------------------VTRANVK-EYIHE-VIDAIIGEG-AMQQAK
-AFREG---FSKVFPV--TDLL-AFSAD-ELVILFGS-----------------------
---AEEDWS-----------METL---------GEALKADHG------------------
----------------FNVE----STAI-RDLVTIMS--------------------EYD
AQSRRNFLQF-------ITGSPKLP------------IGG-----FR-GLNPPL--TVVR
KPHEPPLTAD------------------------DYLPSVMTCV-NY-LKLPQY-STKEV
MREKLD-VAMKE-GVGS
>Fomitiporia_mediterranea_XM_007261146.1 .
VRISRKHI-LESAFKVF----------------ELYGSSSS-ILEVEYFD-------EVG
TGLG-PTLEF---YSLV----SREFARRDL--------KIWRDADATYPG----------
---------IYVHHPHGLFPAPVS---------------------------------ADL
VGDGGEKRTYVFKIIGQFVAKAMLDSRIIDMSFNR--IFLKMILG---------------
------EEIPLTINTLRL----------------------VDPPLANS--------LFKL
E-ALAHN--------------------------------AGSDDAKMQY-GSIE-DLM--
--LDFTL-------------------------------PG---YDIE-LRPG--------
GKEMA---
------------------------------VTSDNVK-EYIQG-VIDATIGSG-AELQAK
-AFREG---FSKVFPV--TDLQ-AFTAD-ELGVLFGS-----------------------
---AVEDWS-----------VDTL---------MESMKADHG------------------
----------------FNME----SPAI-RDLVSIMS--------------------EYD
EISRRRFLQF-------ITGSPKLP------------IGG-----FR-GLNPPF--TVVR
KPHEAPYTAD------------------------DYLPSVMTCV-NY-LKLPQY-SSRDV
MREKLE-TAIME-GVG-
>Rhizoctonia_solani_CDGK01004317.1 .
VRISRHHI-LESAVKVL----------------ELYGSSSS-VLEVEYFE-------EVG
TGLG-PTLEF---YSLV----SKEFARREL--------KMWRDADSSVEG----------
---------PYVQRPLGLFPSP--------------------------------------
-----XRLAKLWSILGQFVGKALLDSRIIDMSFNA--LFLKYILN---------------
------EEVPLTIASLKV--
---------------------------------------SSRWTLDLFA-QCLP-YAL--
--VDPTL-------------------------------AN---SLSK-LX-G--------
GRDVS---
------------------------------VTNSNVE-EYVRE-VIDVVIGRG-VQNQVQ
-AFRTG---FSKVFAV--TDLQ-SFSSE-ELDLLFGN-----------------------
---ADEX---------------AL---------TDALKADHG------------------
----------------FNVD----SGAI-RDLISIMN--------------------SYD
DPTRRAFLQF-------ITGSPKLP------------IGG-----FR-GLSPQL--TVVR
KPHEPPLKAD------------------------DYLPSVMTCV-NY-LKLPEY-STKNV
MDARLK-TAMME-GGG-
>Auricularia_delicata_AFVO01002089.1_R .
VRIYRKHV-LESAYRVF----------------ELYGSSSS-VLEVEYFE-------EVG
TGLG-PTLEF---YAMV----SRELARRSL--------KIWRDADSTVPG----------
---------TYVHHPLGLFPAPIK--------------------------------ADQV
ETPHCRRRLQQFHLVGQFIAKALLDSRIIDMSFNK--VFMKYVX----------------
-----------------L----------------------VDPALAKS--------LAKV
EAFAKAKQVVDADQALASEAKT-----------------AATADLRING-VSLG-DLA--
--LDFTV-------------------------------PG---YX------D--------
GRNTT---
------------------------------VTIDNVE-QYLDL-VLDMLLGSG-IAAAVQ
-AFRDG---FSKVFPV--TDMQ-IFSAD-ELVTIFGN-----------------------
---ADEDWS-----------VESL---------NEAIKADHG------------------
----------------FNVD----SRAI-RDLISVMA--------------------EYD
APTRREFLQF-------ITGSPKLP------------IIG-----YR-GLNPPF--TVVR

KPHESPLTPD------------------------DYLPSVMTCV-NY-LKLPEY-SSKAV
MAAKLR-TAMKE-GVG-
>Auricularia_subglabra_XM_007352069.1 .
VRIYRKHV-LESAYRVF----------------ELYGSSSS-VLEVEYFE-------EVG
TGLG-PTLEF---YAMV----SRELARRSL--------KIWRDADSTVPG----------
---------TYVHHPLGLFPAPIK--------------------------------ADQV
ETEGGKRRLQQFHLVGQFIAKALLDSRIIDMSFNK--VFMKYVLD---------------
------EEVPLTIGTLEL----------------------VDPALAKS--------LAKV
EAFAKAKQVVDADQALASEAKT-----------------AATADLRING-VSLG-DLA--
--LDFTV-------------------------------PG---YDID-LIED--------
GRNTT---
------------------------------VTIDNVE-QYLDL-VLDMLLGSG-IAAAVQ
-AFRDG---FSKVFPV--TDMQ-IFSAD-ELVTIFGN-----------------------
---ADEDWS-----------VETL---------NEAIKADHG------------------
-----------------FNVD----SRAI-RDLISVMA-------------------EYD
APTRREFLQF-------ITGSPKLP------------IGG-----YR-GLNPPF--TVVR
KPHESPLTPD------------------------DYLPSVMTCV-NY-LKLPEY-SSKAV
MAAKLR-TAMKE-GVGS
>Xanthophyllomyces_dendrorhous_LN483166.1 .
VRISRSHL-LESAIKVF----------------ELYGKPSS-ILEVEYFE-------EVG
TGLG-PTLEF---YALA----SKEFGRRDL--------KMWRDADADLPG----------
---------DFVHRPQGLFPRPYA--------------------------------ADEA
SKTX-----QLFRVLGQFVAKALLDSRIIDMSFNT--IFLKYVLG---------------
------QEVVPSIEILKA----------------------VDRRLGMS--------LNKV
QEFANAKAMLVDE--------------------------DLSEIKLADG-SRLS-DFC--
--LDFTL-------------------------------PG---YDIE-LKEG--------
GGNIA---
------------------------------VDMDNVE-EYIEK-VLDLTLVSG-IKTQVN
-AFREG---FSAIFPV--RDLQ-AFSAD-EVX-LFGS-----------------------
---AEEDWS-----------VESL---------TEVMKADHG------------------
-----------------FNIE----SRTI-SNLTEVMS-------------------QYD
APTRREFLQF-------VTGAPKLP------------IGG-----FR-GLNPPL--TVVR
KGHEPPLKAD------------------------DYLPSVMTCV-QY-LKLPDY-STKEI
LQEKLF-TAMRE-GGG-
>Wallemia_ichthyophaga_XM_009269672.1 .
VRISREYV-LESAIKVF----------------ELYGGSSS-ILEIEYFE-------EVG
TGLG-PTLEF---YSLV----SKEFARKDL--------KLWRGSSDSSES----------
---------PYVYSQTGLFPMPMS--------------------------------QKEI
ESDRGEERIKLFRILGQFLAKGLLDSRIIDISFNP--LLMNLVMG---------------
------LNIPKSVAGVKL----------------------VDPLVGKS--------IEEV
QKIVGST----------------------------------ELSKGQIA-DKID-NLA--
--LDFTL-------------------------------PG--QPEYQ-LVEK--------
GENVD---
------------------------------VTLDNVG-DFIDK-VIDATLVSG-VTKQVD
-AFVEG---FSKILSV--EDMK-IFTPE-ELTSLFGN-----------------------
---ADEDWT-----------TETL---------SEVIKADHG------------------
-----------------FNND----SPAL-RRLIEALS-------------------EFD
KVARRDFLQF-------LTGSPKLP------------IGG-----FR-GLHPQL--TVVR
KSPEAGYKAD------------------------DSLPSVMTCA-NY-LKLPDY-SDKDV
LKQKLT-IAMSE-GKG-
>Wallemia_sebi_XM_006955606.1 .
VRISREYV-LESAIKVF----------------ELYGGSSS-ILEIEYFE-------EVG
TGLG-PTLEF---YSLV----SKEFARKSL--------RMWRGSQDSSES----------
---------PYVFSPTGLFPEPMS--------------------------------DKES
ESDKGKERIKLFRILGQFLAKGLLDSRIIDISFNP--LFMKLVLG---------------
------LSIPKSVAGVKL----------------------VDPFVGKS--------MMEV
QKIVDSK-----------------------------------LSTSEIT-EKVS-ALA--
--LDFTL-------------------------------PG--KPEYE-LVEN--------

```
GTNVD-------------------------------------------------------
------------------------------VTIDNVG-EYLDK-IIEATLGSG-VSKQVE
-AFVEG---FSKILRI--EDMK-IFTAE-ELTSLFGN-----------------------
---ADEDWT-----------NETL---------SEIIKADHG------------------
----------------FNND----SPAL-RRLVEVMS--------------------EFD
NVSRRDFLQF-------LTGSPKLP------------IGG-----FR-GLHPQL--TVVR
KSPEAGYKAD------------------------DSLPSVMTCA-NY-LKLPDY-SNKDI
LKQKLT-IAMSE-GKG-
>Wallemia_mellicola_AFQX01000001.1 .
VRISREYV-LESAIKVF----------------ELYGGSSS-ILEIEYFE-------EVG
TGLG-PTLEF---YSLV----SKEFARKSL--------RMWRGSQDSSES----------
---------PYVFSPTGLFPEPMS--------------------------------DKES
ESDKGKERIKLFRILGQFLAKGLLDSRIIDISFNP--LFMKLVLG---------------
------LSIPKSVAGVKL----------------------VDPFVGKS--------MMEV
QKIVDSK-----------------------------------LSTSEIT-EKVS-ALA--
--LDFTL-------------------------------PG--KPEYE-LVEN--------
GTNVD-------------------------------------------------------
------------------------------VTIDNVG-EYLDK-IIEATLGSG-VSKQVE
-AFVEG---FSKILRI--EDMK-IFTAE-ELTSLFGN-----------------------
---ADEDWT-----------NETL---------SEIIKADHG------------------
----------------FNND----SPAL-RRLVEVMS--------------------EFD
NVSRRDFLQF-------LTGSPKLP------------IGG-----FR-GLHPQL--TVVR
KSPEAGYKAD------------------------DSLPSVMTCA-NY-LKLPDY-SNKDI
LKQKLT-IAMSE-GKG-
>Mixia_osmundae_XM_014709622.1 .
VRISRARL-LESAVKVF----------------ELYGGSRA-MLEVEYFE-------EVG
TGLG-PTLEF---FSLV----SKEFARSDL--------KLWREGDVGSIS----------
---------AYVYNLSGLFPAPLS--------------------------------SQEA
ETEAGRKRLAMFTVLGQFVAKGLMDSRILDLSFSR--SFSRLVQG---------------
------ERLPETISSVKS----------------------IDRALGNS--------LTDL
QAYADAKTAVKARELG------------------QEAEREAMYDIRVRD-ARVE-DLA--
--LDFTL-------------------------------PG---YGYD-LKEN--------
GSDCA-------------------------------------------------------
------------------------------VDIENVE-EYIRL-VIQATVSTG-IAAQVC
-AFRDG---FSTVFPI--QDMN-CFTPD-ELVHICGS-----------------------
---AEEDWS-----------MKTL---------TDGIRADHG------------------
----------------FNMD----SSTI-RNFLTILT--------------------AYD
ASERRDFLQF-------MTGSSKLP------------IGG-----FR-ALTPAL--TVVR
KPAEAPLTSD------------------------QYLPSVMTCQ-NY-LKLPAY-SSLEI
MSERLH-TAIKE-GSGA
>Cronartium_ribicola_AWVX01046301.1 .
VRISRQKI-LESLMKVF----------------EIYSNCRA-MLEVEYFD-------EVG
TGLG-PTLEF---FSLA----SRAFSERQY--------GMWRDHETENNS----------
--------SLYVYTKAGLFPSPMD--------------------------------EKMS
KTEKGIEKLKKFKVLGQFMAKALMDSRIIDLSLSK--TFAKSILD---------------
------YEIPLNIDSIKL----------------------IDKSLAAS--------LEHL
QKYLIEKDKVL-----------------------IEDPNADIESIRVDG-ATVE-DLA--
--LEFVL-------------------------------PG---YEVE-MG----------
GKEEM-------------------------------------------------------
------------------------------VRMGNLD-EFIKL-VIDWTLSKG-VERQIQ
-AFKNG---FSTVFPI--RDLR-SFTPE-EIVNIFGN-----------------------
--AEQEDWS------------XAL---------TATMRADHG------------------
----------------YNME----SRAI-RDLIEIMS--------------------SYD
VTSRRLFFLY--------SGAPKLP------------IGG-----FR-GLHPPL--TVVR
KAAEPGHTAD------------------------EYLPSVMTCV-NX-LKLPEY-SSSAI
SKEKLN-VAMRE-GA--
>Cronartium_quercuum_AXDN01014665.1 .
VRISRQKI-LESLMKVF----------------EIYSNCRA-MLEVEYFD-------EVG
```

```
TGLG-PTLEF---FSLA----SRAFSERQY--------GMWRDHETENNS----------
--------SLYVYTKAGLFPSPMD---------------------------------EKMS
KTEKGIEKLKKFKVLGQFMAKALMDSRIIDLSLSK--TFAKSILD---------------
------YEIPLNIDSIKL----------------------IDKSLAAS--------LEHL
QKYLIEKDKVL------------------------IEDPNADIESIRVDG-ATVE-DLA--
--LEFVL--------------------------------PG---YEVE-MG----------
GKEEM-------------------------------------------------------
------------------------------VRMGNLD-EFIKL-VIDWTLSKG-VERQIQ
-AFKNG---FSTVFPI--RDLR-SFTPE-EIVNIFGN-----------------------
--AEQEDWSA----------ESAL---------TAAMRADHG------------------
----------------YNME----SRAI-RDLIEIMS--------------------SYD
VTSRREFLQF-------XSGAPKLP------------IGG-----FR-GLHPPL--TVVR
KAAEPGHTAD------------------------EYLPSVMTCV-NX-LKLPEY-SSSAI
SKEKLN-VAMRE-GA--
>Melampsora_medusae_MWRK01010727.1 .
VRISRQKI-LESLIKVF-----------------ELYSSCRA-MLEVEYFD-------EVG
TGLG-PTLEF---FSLA----SKAFAERQY--------KMWRDHEGSSDS----------
---------VHVFSRTGLFPAPMD--------------------------------QEMC
ESESGVERLKKFKVLGQFMAKALMDSRIVDISLSR--SFAKLVLD---------------
------YEVPQTIESVKL----------------------VDSALAGS--------LEHL
MQYVVEKRRLEAELS--------------------GSELEEAIKVIRIDG-ATVE-DLA--
--LEFVL--------------------------------PG--YDEIQ-MKPD--------
GSDKT-------------------------------------------------------
------------------------------VTIGNIE-EFIDL-VIDWTLSKG-VAKQLE
-VFKSG---FSAVFPI--RDLK-SFTPD-EIVNIFGN-----------------------
--AEMEDWS-----------LPAL---------TSSMRADHG------------------
----------------YNME----SRAI-RDLIHIMS--------------------TYD
VTSRREFLQF-------ITGAPKLP------------IGG-----FR-GLHPPL--TVVR
KAAESGHTPD------------------------EYLPSVMTCV-QY-LKLPEY-SSADV
ASQKLD-IAMRE-GA--
>Melampsora_abietis-canadensis_MWRH0102148 .
VRISRQKI-LESLIKVF-----------------ELYSSCRA-MLEVEYFD-------EVG
TGLGXPTLEF---FSLA----SKAFAERQY--------KMWRDHEGSSDS----------
---------VHVFSRTGLFPAPMD--------------------------------QEMC
ESDSGVERLKKFKVLGQFMAKALMDSRIVDISLSR--SFAKLVLD---------------
------YEVPQTIESVKL----------------------VDSALAGS--------LEHL
MQYVVEKRRLEAELS--------------------GSELEEAIKVIRIDG-ATVE-DLA--
--LEFVL--------------------------------PG--YDEIQ-MKPD--------
GSDKT-------------------------------------------------------
------------------------------VTIGNIE-EFIDL-VIDWTLSKG-VAKQLE
-VFKSG---FSAVFPI--RDLK-SFTPD-EIVNIFGN-----------------------
--AEMEDWS-----------LPAL---------TSSMRADHG------------------
----------------YNME----SRAI-RDLIHIMS--------------------TYD
VTSRREFLQF-------ITGAPKLP------------IGG-----FR-GLHPPL--TVVR
KAAESGHTPD------------------------EYLPSVMTCV-QY-LKLPEY-SSADV
ASQKLD-IAMRE-GA--
>Melampsora_larici_populina_XM_007410495.1 .
VRISRQKI-LESLIKVF-----------------ELYSSCRA-MLEVEYFD-------EVG
TGLG-PTLEF---FSLA----SKAFAERQY--------KMWRDHEGSSDS----------
---------VHVFSKTGLFPAPMD--------------------------------QEMC
ESESGLERLKKFKVLGQFMAKALMDSRIVDISLSR--SFAKLVLD---------------
------YEVPQTIESVKL----------------------VDSALAGS--------LEHL
MQYVVEKRRLEAELS--------------------GSELEEAIKVIRIDG-ATVE-DLA--
--LEFVL--------------------------------PG--YDEIE-MKPD--------
GSDKT-------------------------------------------------------
------------------------------VTIGNIE-EFIDL-VIDWTLSKG-VAKQLE
-VFKSG---FSTVFPI--RDLK-SFTPD-EIVNIFGN-----------------------
--AEMEDWS-----------AETL---------TSSMRADHG------------------
```

----------------YNME----SRAI-RDLIQIMS--------------------TYD
VTSRREFLQF-------ITGAPKLP------------IGG-----FR-GLHPPL--TVVR
KAAESGHVPD------------------------EYLPSVMTCV-NY-LKLPEY-SSTDV
ATQKLD-IAMRE-GADS
>Uromyces_appendiculatus_GACI01007196.1 .
VRISRQKI-LESLIKVF----------------ELYSSCRA-MLEVEYFD-------EVG
TGLG-PTLEF---FSLA----SKAFAEKQH--------QMWRDHESDSQA----------
---------LHVFSRTGLFPAPMD--------------------------------DRMA
QAEKGIERLRKFKVLGQFMAKALMDSRIVDISLSR--SFARLVLD---------------
------YSLPHTIASVRL----------------------VDKALATS--------LEHL
NKYIIAKRAIEADKSLL-----------------EDQAHAAIQQIRIDD-ATVN-DLA--
--LEFTL-------------------------------PG---YDIE-IKPG--------
TSGTL---
------------------------------VTIENIE-EFIEL-VIYWTLSKG-VARQIE
-MFKSG---FSMVFPI--RDLK-SFTPE-EIVNIFGN-----------------------
--VESEDWS-----------PEAL---------TSAMRADHG------------------
----------------YNMD----SPAI-RGLITIMS--------------------SYD
VPSRRQFLQF-------ITGAPKLP------------IGG-----FR-GLHPAL--TVVR
KAAEPGHSPD------------------------EYLPSVMTCV-NY-LKLPEY-SSQEV
AAEKLS-LAVKE-GADS
>Uromyces_viciae-fabae_JNCO01003240.1 .
VRISRQKI-LESLIKVF----------------ELYSSCRA-MLEVEYFD-------EVG
TGLG-PTLEF---FSLA----SKAFAEKQY--------QMWRDHESDSQA----------
---------LHVFSRTGLFPAPMD--------------------------------DHTA
QTEKGVERLRKFKVLGQFMAKALMDSRIVDISLSR--SFARLVLD---------------
------YSLPLTIDSVRL----------------------VDKALANS--------LDHL
NKYVIAKRAIEANQSLS-----------------EDEARAAIQKIRIDD-ATAD-DLA--
--LEFSL-------------------------------PG---YDIE-IKPG--------
SSATL---
------------------------------VSIDNIE-EFIEL-VIYWTLSKG-VSRQIE
-MFQAG---FSMVFPI--RDLK-SFTPD-EIVNIFGN-----------------------
--AESEDWS-----------PTAL---------TSAMRADHG------------------
----------------YNMD----SSAI-RGLISIMS--------------------SYD
VPSRRQFLQF-------IXGAPKLP------------IGG-----FR-GLHPAL--TVVR
KAAEPGHSPD------------------------EYLPSVMTCV-NX-LKLPEY-SSQSV
AAEKLS-LAIRE-GA--
>Puccinia_psidii_LKHF01006257.1 .
VRISRQKI-LESLIKVF----------------ELYSSCRA-MLEVEYFD-------EVG
TGLG-PTLEF---FSLA----SKAFAERQH--------QMWRDHENDSQS----------
---------IHIFSRTGLFPAPNQ----------------------------------TA
QTEKGIERLRKFKVLGQFMAKALMDSRIVDISLSR--SFARLVLD---------------
------YDLPLTIASVGL----------------------VDKALAAS--------LSHL
NKYVIIKRTILADQTLT-----------------QSAQSSTIQEIRVDD-ATVE-DLA--
--LEFAL-------------------------------PG---YDVE-LKSS--------
NNENL---
------------------------------VTIDNIE-EFIEL-VISWTLSKG-VARQIE
-MFKSG---FSMVFPI--RDLK-SFTPD-EIVNIFGN-----------------------
--AESEDWS------------XAL---------TSAMRADHG------------------
----------------YNME----SAAI-RGLIQIMS--------------------SYD
VPSRRQFLQF-------IXGAPKLP------------IGG-----FR-GLHPPL--TVVR
KAPEPGHTAD------------------------EYLPSVMTCV-N---------------
----
>Puccinia_sorghi_LAVV01007183.1 .
VRISRQKI-LESLIKVF----------------ELYSSCRA-MLEVEYFD-------EVG
TGLG-PTLEF---FSLA----SKAFAERQY--------HMWRDHESDSQA----------
---------IHVFSHTGLFPSPMD--------------------------------DRTA
ESDKGIERLRKFKVLGQFMAKALMDSRIVDISLSR--SFARLVLD---------------
------YPLPLTIASVGL----------------------VDKALAAS--------LEHL

```
NKYVIAKHAIEADASLS-----------------GSEARVAIQQIRIDD-ATVD-DLA--
--LEFIL-------------------------------PG---YDVE-MKPD--------
SSETL-------------------------------------------------------
------------------------------VTIDNIE-EFIQL-VIYWTLSKG-VTRQIE
-MFKAG---FSMVFPI--KDLK-SFTPD-EIVNIFGN-----------------------
--AESEDWS------------XAL---------TSAMRADHG------------------
----------------YNMD----SPAI-RGLINILS--------------------SYD
VPSRRQFLQF-------ITGAPKLP------------IGG-----FR-GLHPAL--TVVR
KAAEPGHSPD------------------------EYLPSVMTCV-NX-LKLPEY-SSQEV
AAVKLS-IAMKE-GA--
>Puccinia_triticina_AZRP01001592.1 .
VRISRQKI-LESLIKVF----------------ELYSSCRA-MLEVEYFD-------EVG
TGLG-PTLEF---FSLA----SKAFAERQY--------QMWRDHESDSQA----------
---------LHVFSRTGLFPSPMD--------------------------------DRTA
ESDRGIERLRKFKVLGQFMAKALMDSRIVDISLSR--SFARLVLD---------------
------YPLPLTIASVGL----------------------VDKSLAAS--------LEHL
NKYVIAKHAIEADASRS-----------------ETEAREAIQQIRIDD-ATVE-DLA--
--LEFIL-------------------------------PG---YDVE-MKPD--------
SSETL-------------------------------------------------------
------------------------------VTIDNIE-EFIEL-VIYWTLSKG-VTRQIE
-MFKAG---FSMVFPI--RDLK-SFTPD-EIVNIFGN-----------------------
--AESEDWSI----------EGTL---------YPSMRADHG------------------
----------------YNMD----SAAI-RGLINIMS--------------------SYD
VPSRRQFLQF-------ISGAPKLP------------IGG-----FR-GLHPAL--TVVR
KAAEPGHSPD------------------------EYLPSVMTCV-NX-LKLPEY-SSQDV
AAEKLT-IAIKE-GA--
>Puccinia_striiformis_GAIR01012268.1 .
---------------------------------------RA-MLEVEYFD-------EVG
TGLG-PTLEF---FSLA----SKAFAEKQY--------HMWRDHESDSQS----------
---------LHVFSRTGLFPSPMD--------------------------------DRTA
ETEKGIERLRKFKVLGQFMAKALMDSRIVDISLSR--SFARLVLD---------------
------YPLPLTIASVGL----------------------VDKSLAAS--------LEHL
NKYIVAKHAIEADKSLS-----------------ESEASAAIQQIRIDD-ATVD-DLA--
--LEFIL-------------------------------PG---YDVE-MKPD--------
SSETL-------------------------------------------------------
------------------------------VTIDNIE-EFIEL-VIYWTLSKG-VNRQIE
-MFKAG---FSMVFPI--RDLK-SFTPD-EIVNIFGN-----------------------
--AESEDWS-----------PETL---------TSAMRADHG------------------
----------------YNMD----SAAI-RGLINIMS--------------------TYD
VPSRRQFLQF-------ITGAPKLP------------IGG-----FR-GLHPAL--TVVR
KAAEPGHSPD------------------------EYLPSVMTCV-NY-LKLPEY-STQGV
AADKLT-TAIQE-GADS
>Puccinia_horiana_LWDY01000015.1 .
VRISRQKI-LESLIKVF----------------ELYSSCRA-MLEVEYFD-------EVG
TGLR-PTLEF---FSLA----SKAFAERQY--------QMWRDHESDSQA----------
---------LHVFSHTGLFPSPMD--------------------------------DRTA
ESEKGIERLRKFKVLGQFMAKALMDSRIVDISLSR--SFARLVLD---------------
------YPLPLTIASVGL----------------------VDTALAVS--------LEHL
NKYVVAKHAIEADTSLS-----------------ESEARTAIQSIRIDD-ATVD-DLA--
--LEFIL-------------------------------PG---YDVE-MKPN--------
SSETL-------------------------------------------------------
------------------------------VTINNIE-EFIQL-VIYWTLSKG-VTRQIE
-MFRTG---FSMVFPI--RDLK-SFTPE-EIVNIFGN-----------------------
--AESEDWS-----------PLAL---------TSAMRADHG------------------
----------------YNMD----SLAI-RGLINIMS--------------------SYD
VPSRRQFLQF-------IXGAPKLP------------IGG-----FR-GLHPAL--TVVR
KAAEPGHSPD------------------------EYLPSVMTCV-NV-------------
-----------------
```

>Puccinia_graminis_XM_003326706.1 .
VRISRQKI-LESLIKVF----------------ELYSSCRA-MLEVEYFD-------EVG
TGLG-PTLEF---FSLA----SKAFAERQY--------QMWRDHESDSQA----------
---------LHVFSRTGLFPSPMD--------------------------------DRTA
ESEKGIERLRKFKVLGQFMAKALMDSRIVDISLSR--SFARLVLD---------------
------YPLPLTIASVGL----------------------VDKSLAAS--------LEHL
NKYVIAKHAIEADSSLS-----------------ESETHAAIQQIRIDD-ATVD-DLA--
--LEFIL-------------------------------PG---YDVE-MKPD--------
SSATL---
------------------------------VTIDNIE-EFIEL-VIYWTLSKG-VTRQIE
-MFKAG---FSMVFPI--RDLK-SFTPD-EIVNIFGN-----------------------
--AESEDWS-----------PEAL---------TSAMRADHG------------------
----------------YNMD----SAAI-RGLINIMS--------------------TYD
VPSRRQFLQF-------ITGAPKLP------------IGG-----FR-GLHPAL--TVVR
KAAEPGHSPD------------------------EYLPSVMTCV-NY-LKLPEY-SSQDV
AAEKLT-IAIKE-GADS
>Puccinia_graminis_XM_003889834.1 .
VRISRQKI-LESLIKVF----------------ELYSSCRA-MLEVEYFD-------EVG
TGLG-PTLEF---FSLA----SKAFAERQY--------QMWRDHESDSQA----------
---------LHVFSRTGLFPSPMD--------------------------------DRTA
ESEKGIERLRKFKVLGQFMAKALMDSRIVDISLSR--SFARLVLD---------------
------YPLPLTIASVGL----------------------VDKSLAAS--------LEHL
NKYVIAKHAIEADSSLS-----------------ESETHAAIQQIRIDD-ATVD-DLA--
--LEFIL-------------------------------PG---YDVE-MKPD--------
SSATL---
------------------------------VTIDNIE-EFIEL-VIYWTLSKG-VTRQIE
-MFKAG---FSMVFPI--RDLK-SFTPD-EIVNIFGN-----------------------
--AESEDWS-----------PEAL---------TSAMRADHG------------------
----------------YNMD----SAAI-RGLINIMS--------------------TYD
VPSRRQFLQF-------ITGAPKLP------------IGG-----FR-GLHPAL--TVVR
KAAEPGHSPD------------------------EYLPSVMTCV-NY-LKLPEY-SSQDV
AAEKLT-IAIKE-GADS
>Malassezia_caprae_LFFV01000193.1 .
VRIARSTL-LASAVKVL----------------DLYANAQT-VLEVEYFD-------EVG
SGLG-PTLEF---YALV----SRELQRTEL--------GLWRHDSSVQS-----------
---------EYVHAP-ALFPAPVN------------------------------------
-GDATERVCTLFRTLGQLIAKALLDGRIVDVPFHP--LFWRAVLR---------------
------RRVPRTLSTLRQ----------------------VDASLAQS--------LEAM
QRMNAD---EFE-ALD--
--LDGTL-------------------------------AG----TDL-VLPGW-------
RSDEK---
------------------------------VSQRRVH-EYVQA-VVDMCLADG-IAPQLD
-AFRGG---FDAVLPL--VALD-TFQSK-ELVTLFGQ-----------------------
---SQEDWD-----------LATL---------QRTIVADHG------------------
----------------LTSE----SSHF-QDLLGILA--------------------AFS
LEERRTFLQW-------LTGAPRLP------------VGG-----FA-ALQPPL--TVVR
RQPEPPLGPD------------------------DYLPSVMTCV-NY-LKLPCY-SDRET
MKARLH-TAMYE-GLTS
>Malassezia_dermatis_LFFX01000006.1 .
VRIARSTL-LASAVKVL----------------DLYANAQT-VLEVEYFD-------EVG
SGLG-PTLEF---YALV----SRELQRTEL--------GLWRHDSSVQS-----------
---------EYVQAP-ALFPAPAS------------------------------------
-SDAVERVRTLFRTLGQLVAKALLDGRIVDVPFHP--LFWRAVLR---------------
------RRVPRTLSTLRQ----------------------VDAGLAQS--------LEAM
QRMSAD---EIE-ALN--
--LDGTL-------------------------------PG----MDL-VLPGW-------
RPDET---
------------------------------VTQSRVH-EYVQA-VVDMCLADG-IAQQLD

-AFRGG---FDAVLPL--VVLD-VFQSK-ELVTLFGQ-----------------------
---SQEDWD-----------LATL---------QRTVVADHG------------------
-----------------FTSE----SSHF-QDLLGILA--------------------VFS
LEERRTFLQW-------LTGAPRLP------------VGG-----FA-ALQPPF--TVVR
RQPEPPLEPN------------------------DYLPSVMTCV-NY-LKLPCY-SDRET
MKARLH-TAMYE-GLTS
>Malassezia_sympodialis_XM_018884846.1 .
VRIARSTL-LASAVKVL-----------------NLYANAQT-VLEVEYFD-------EVG
SGLG-PTLEF---YALV----SHELQRTEL--------GLWRHDSAAQS-----------
---------EYVQAP-ALFPAPAS------------------------------------
-SDATERVRTLFRALGQLIAKALLDGRIVDVPFHP--LFWRAVLR---------------
------RRVPRTLSTLRQ----------------------VDASLAQS--------LEAM
ERMSAD---EVE-ALH--
--LEGTL--------------------------------PG----TDL-VLPGW-------
RADEP---
------------------------------VTQSRVH-EYVQA-IVDMCLADG-IAQQLD
-AFRDG---FDAVLPL--EVLD-VFQSK-ELATLFGQ-----------------------
---SQEDWD-----------LATL---------QRTVVADHG------------------
-----------------FTSE----SAHF-QDLLGILA--------------------AFS
LEERRTFLQW-------LTGAPRLP------------VGG-----FA-ALQPPL--TVVR
RQHEPPLQPN------------------------DYLPSVMTCV-NY-LKLPCY-SDRET
MKARLH-TAMYE-GLTS
>Malassezia_equina_LFFY01000082.1 .
VRIARSTL-LASAVKVL-----------------DLYANAQT-VLEVEYFD-------EVG
SGLG-PTLEF---YALV----SRELQRTEL--------GLWRHDSSVQS-----------
---------EYVQAP-ALFPAPVS------------------------------------
-SEGTERVRTLFRALGQLIAKALLDGRIVDVPFHP--LLWRAVLR---------------
------RRVPRTLATLRQ----------------------VDASLAQS--------LEAM
ERMSAN---EIE-ALD--
--LDGTL--------------------------------PG----TDL-ALPGW-------
RADES---
------------------------------VTHGRVH-EYVQA-VVDMCLADG-IATQID
-AFREG---FDNVLPL--VALD-VFASK-ELVTLFGQ-----------------------
---SQEEWD-----------LATL---------QRTIVADHG------------------
-----------------FSSE----STHV-QDLLAILA--------------------DFS
LQERRTFLQW-------LTGAPRLP------------VGG-----FA-ALQPPL--TVVR
RQHEPPLVPN------------------------DYLPSVMTCV-NY-LKLPCY-SDRET
MRARLH-TAMYE-G---
>Malassezia_nana_LFGD01000002.1 .
VRIARATL-LASAIKVL-----------------DLYANAQT-VLEVEYFD-------EVG
SGLG-PTLEF---YALV----SRELQRTEL--------GLWRHDSSVQS-----------
---------EYVQSV-GLFPAPVG------------------------------------
-DDDRKRVLTHFRALGQLIAKALLDGRIVDVPFHV--LFWRAVLR---------------
------RRVPRTLSTLRQ----------------------VDPSMAQS--------LEAL
ERMSAD---DIA-ALE--
--LDGTL--------------------------------PG----TSL-VLPGW-------
RREDT---
------------------------------VTQERVH-DYIQA-IIDMCLAEG-IAPQLD
-AFREG---FDGVLPL--VALD-VFQSK-ELVTLFGQ-----------------------
---SQEDWD-----------LATL---------QRTVVPDHG------------------
-----------------FTSE----SSHF-QDLLAILA--------------------SFS
LDERRTLLQW-------LTGSPRLP------------VGG-----FA-ALQPPL--TVVR
RQPEPPLQPN------------------------DYLPSVMTCV-NY-LKLPCY-SDRET
MKARLR-TAMYE-GLTS
>Malassezia_pachydermatis_XM_018136504.1 .
VRIARDNL-LPSAVKVL-----------------DLYSRGSS-ILEIEYFD-------EVG
SGMG-PTLEF---YALV----SKALQRVEV--------GMWRHDSHTES-----------
---------EYVSSPHGLFPMVTE------------------------------------

----DKDVVRLFRTLGQLVAKSLVDARIIDVPFHP--LFWRAVLG---------------
------RRVPCTLATLGS----------------------VDPALETS--------LSSL
LRMPTD---EVA-ALS--
--LEPCF-------------------------------PG----TDC-VLPGW-------
DQNTP---
------------------------------IDGANIK-TYVDA-VITMALQSG-IEKQLD
-AFRDG---IAYVLPL--SALD-VFQSK-ELVVLFGQ-----------------------
---SQEDWD-----------EATL---------LRTVVPDHG------------------
----------------FTSD----STHF-KDLLAILA--------------------SFS
LEERRTFLQW-------LTGSPRLP------------VGG-----FT-ALQPPL--TVVR
RLPDAPLQPD------------------------DYLPSVMTCV-NY-LKLPCY-SSREK
MKERLW-TAMHE-GLTS
>Malassezia_restricta_AAXK01001990.1 .
VRIARNQL-LESAIRVL----------------ELYAHTPS-LLEVEYFD-------EPG
SGLG-PTLEF---YTLV----SQAFQAMP---------DLWRGEPSM-------------
----------------PLFPKPSG------------------------------------
-------DASLFRVLGQFVAKSIVDLRMIDVPFHP--LFWRAVLG---------------
------RRVPHTLETLEA----------------------LDAVQARS--------LRAL
QTMPAD---ELE-RLE--
--LDGTL-------------------------------PG---TERE-LVQG--------
----P---
------------------------------LTSANIN-AYIQA-VVDVSLHDG-IAPQLD
-AFRAG---FESVLSL--DALR-VFASH-ELSALFGQ-----------------------
---ATEDWS-----------EATL---------LRTIVPDHG------------------
----------------FSSD----SEPF-RALLSILV--------------------EFD
AFERRTFLQW-------LTGAPRLP------------IGG-----FA-ALQPPF--TVVR
RQPEPPLQPD------------------------DYLPSVMTCV-NY-LKLPCY-SSRDV
MRQKLC-LAMHE-G---
>Malassezia_japonica_LFDB01000090.1 .
VRIARDSL-LPSAVKVL----------------ELYSAGAY-LLEVEYFD-------EVG
SGLG-PTLEF---YALV----SKEFARADL--------GLWRADETSEVGG---------
--------VTYVHPKQGLFPVRIG------------------------------------
-----DKGAKLFTTLGQFVAKSLLDARIIDVPLNP--VFVRKVLG---------------
------QRVEPTLATLRQ----------------------VDAALARS--------LEAL
QTMPAA---ELE-ALA--
--MDYAV-------------------------------PG---SGES-LGDG--------
V---
------------------------------VTAANVD-AYVRD-VASAVLD---IGGAVD
-AFRTG---FAGVLSL--DTLR-VFAAE-ELVLLLGH-----------------------
---ADEDWS-----------EAAL---------RRALVPDHG------------------
----------------FSGE----SSSF-LDLVAILA--------------------AYT
PEERRAFLQW-------LTGSPRLP------------IGG-----FA-GLHPPL--TVVR
RDHEAPLQPD------------------------DYLPSVMTCV-NY-LKLPCY-SSRDV
MQRRLQ-TAVRE-GLTS
>NWGS3B_2_Malassezia_yamatoensis_LFCX01000042.1 .
VRIARNKL-LPSAIKVL----------------EQYGSGRY-ALEVEYFD-------EVG
SGSG-PTLEF---YALV----SQAFARADL--------GIWRGDQRDERD----------
--------STYLDTRHGLYPTSDT------------------------------------
-SISSSTYTSLFMTLGRFIAKALLDDRLIDVPLHP--VFFRQVLR---------------
------QPIEQKIDTIQD----------------------IDPTFARS--------LMAL
KDMPAS---DLN-DLQ--
--LPYAM-------------------------------PG---TDTQ-LSQE--------
AAL---
------------------------------VSDSNVH-QYVKD-VVHKVLD---IEPALN
-AFREG---FTQVMPL--EHLR-IFSAE-ELVQLVAH-----------------------
---HEEDWS-----------ESAL---------RRAILPDHG------------------
----------------FTGE----SREI-LDLIAIMT--------------------SFD
IQERRMFLQW-------LTGAKRLP------------MGG-----FQ-ALQPPL--TVVR

RDHEPPLTPD------------------------DYLPSVMTCV-NY-LKLPRY-TTREV
MRQRLY-TAVHE-GLTS
>Malassezia_slooffiae_LFGK01000536.1 .
VRISRESI-LPSAIKVL----------------ELYGKGPS-ILEVEYFE-------EVG
SGLG-PTLEF---YALV----SREFVRADL--------DLWRVEKTSRVGD---------
--------AEYVHNTHGVFPAPLP------------------------------------
PGKKAARSLQLFYSLGQFVAKSLLDTRIIDIHFSP--LFLSAVLG---------------
------VEPPATLAALSF----------------------VDPSLARS--------LALM
PTMPAE---QLE-TMA--
--LDFTL-------------------------------PG--AAHVE-LVEG--------
GAQRS---
------------------------------VTAENVG-EYVER-TLHCVLHES-VREQVE
-SFRSG---FGSILPL--DALR-VFRAD-ELAMLFGQ-----------------------
---SEEDWS-----------EATL---------RRSLVPDHG------------------
----------------FNSE----SRQF-QDLIAILA--------------------GFS
VEERRTFLQW-------LTGSPRLP------------IGG-----FA-GLHPPL--TVVK
RQHEEPLKPD------------------------DYLPSVMTCA-NY-LKVPFY-SSREM
LHERLV-KAMNE-GLTS
>Malassezia_cuniculi_LFFW01000010.1 .
VRIARENI-LQSAVKVM----------------ELYGSSKS-ILEVEYFD-------EVG
SGLG-PTLEF---YSLV----SREFAREDL--------KMWRASSTSTDVNG--------
--------TVYVHASHGFFPAALP-----------------------------------Q
SESMRKNILSMFTVLGTFVAKSLLDSRIIDVPFSP--YFLLAVLG---------------
------EHIPLSLEALAN----------------------IDPEVARS--------LGSI
AQLSAQ---DVE-SLA--
--LDFTL-------------------------------PG--APHIE-LCDS--------
GSERS---
------------------------------VTAENRD-EYISL-VLEYSMRTT-VEAQVN
-AFRRG---FSRVVSL--RALN-VLRPD-ELASMFGQ-----------------------
---CAEDWT-----------EDTL---------RRAIVPDHG------------------
----------------FSAS----SREI-SDLISIMS--------------------EFG
EKDRRTFLQW-------LTGSPRLP------------VGG-----FT-ALQPPL--TVVR
RQHEEPLKPD------------------------DYLPSVMTCV-NY-LKLPCY-SSRDT
MRTRLE-TAMRE-GLTA
>Tilletiaria_anomala_XM_013390685.1 .
VRISRRRL-LDSAVKIF----------------DLYASNSS-ILEVEYFD-------EVG
TGLG-PTLEF---YSLV----SQDFALKEN--------NIWLDEQTNPQ-----------
--------SLHVSAAHGLYPIPIS--------------------------------SSAS
TTLEGSKRLGLFRTLGTFVAKALLDSRIVQIDFSR--LFISAILG---------------
------RKVAYNLDTLAQ----------------------VDGGLASS--------LKAI
QSVAPE---DVA-TLH--
--LDFTL-------------------------------PG--DSNYE-LVPG--------
GSDKK---
------------------------------VSGNNVN-DYISA-VIDAYLDSG-IQPVVE
-SFRAG---FEKVFML--SSLK-PFTAD-ELVLLFGN-----------------------
---SQEDWS-----------AETL---------LSNIKPDHG------------------
----------------FTPE----SEPY-RNLLSIMQ--------------------DFT
DQERRTFLQW-------LTGSPKLP------------IGG-----FK-ALHPQL--TVVK
RPHEAPLKPD------------------------DYLPSVMTCV-NY-LKLPSY-SGKEV
MRQRLN-TAMLE-GGTA
>Tilletia_indica_MAPX01000244.1 .
VRISRSRL-FESAMKVF----------------ELYGHNTS-VLEVEYFE-------EVG
TGLG-PTLEF---YALV----SREFARRDL--------GIWRDDGHGTSG----------
--------PKFVTPTGGLFPLPQS--------------------------------ASDL
ETEEGKHRVAIFKTLGQFVAKALLDSRIIDCNFSP--VFMKAVLN---------------
------TKIPVTTASLRA----------------------VDPMLARS--------MDQL
LEMDAE---TLD-AMS--
--VDFTL-------------------------------PG--HPGVE-LVED--------

```
GKQKT-------------------------------------------------------
------------------------------VTVNNIQ-EYVDG-VITQTMRDG-IQPLVR
-AFRKG---FNLIMPL--QALS-SFTAD-ELVMLFGN-----------------------
---MDEDWS-----------ETTL---------LASIKPDHG------------------
----------------FNAE----SASF-RDLVAIMA--------------------DFD
TTKRREFLQW-------LTGSPKLP------------IGG-----FA-GLHPQL--TIVK
RPHEAPLAPD------------------------DYLPSVMTCV-NY-LKMPGY-SSRDK
MRRRLE-TAMSE-GSG-
>Tilletia_walkeri_LWDG01000124.1 .
VRISRSRL-FESAMKVF----------------ELYGHNTS-VLEVEYFE-------EVG
TGLG-PTLEF---YALV----SREFARRDL--------GIWRDDGHGTSG----------
--------PKFVTPTGGLFPLPQS--------------------------------ASDL
ETEEGKHRVAIFKTLGQFVAKALLDSRIIDCNFSP--VFMKAVLN---------------
------TKIPVTTASLRA---------------------IDPMLARS--------MDQL
LEMDAE---------------------------------------------TLD-AMS--
--LDFTL-------------------------------PG--HPGVE-LVED--------
GKQKT-------------------------------------------------------
------------------------------VTVNNVQ-EYVDG-VIIQTMRDG-IQPLVR
-AFRKG---FNLIMPL--QALS-SFTAD-ELVMLFGN-----------------------
---MDEDWS-----------ETTL---------LASIKPDHG------------------
----------------FNAE----SASF-RDLVAIMA--------------------DFD
TTKRREFLQW-------LTGSPKLP------------IGG-----FA-GLHPQL--TIVK
RPHEAPLAPD------------------------DYLPSVMTCV-NY-LKMPGY-SSRDK
MRHRLE-TAMSE-GSG-
>Tilletia_controversa_LWDE01000224.1 .
VRISRSRL-FESAMKVF----------------ELYGHNTS-VLEVEYFE-------EVG
TGLG-PTLEF---YALV----SREFARRDL--------GIWRDDGHGASG----------
--------PKFVTPTGGLFPAPQS--------------------------------ASDM
ETEEGKHRLAVFKTLGQFVAKALLDSRIIDCNFSP--VFMKAVLN---------------
------ANIPVTIASLRT---------------------VDPALARS--------MDQL
LEMDAE---------------------------------------------MLA-SMS--
--LDFTL-------------------------------PG--HPGVE-LVED--------
GKQKA-------------------------------------------------------
------------------------------VDLENVQ-EYVDA-IISQTMWDG-IQPQVR
-AFRRG---FNLIMPL--QALS-SFTPD-ELVMLFGN-----------------------
---MDEDWS-----------ETTL---------LASIKPDHG------------------
----------------FNSE----SASF-RDLVAIMA--------------------NFD
VTKRREFLQW-------LTGSPKLP------------IGG-----FA-GLHPQL--TIVK
RPHEAPLAPD------------------------DYLPSVMTCV-NY-LKMPGY-SSREK
MRGRLE-TAMSE-GSG-
>Tilletia_horrida_LAXH01000020.1 .
VRISRSRL-FESAMKVF----------------ELYGHNSS-VLEVEYFE-------EVG
TGLG-PTLEF---YSLV----SREFARRDL--------EIWRDDGHGEAG----------
--------PKFVSPAGGLFPSPES--------------------------------APSF
ETEEGKHKLAIFKTLGQFVAKALLDSRIIDCNFSP--VFMKAVLN---------------
------VKIPTTLSSLRL---------------------VDSALARS--------MDQV
LEMDTD---------------------------------------------TLA-SMA--
--LDFTL-------------------------------PG--HAHIE-LGSD--------
GKNKT-------------------------------------------------------
------------------------------VTTENVQ-EYVNA-VIDQTVNRG-IQLQVR
-AFRRG---FNTIMPL--RALS-SFTAD-ELVMLFGN-----------------------
---TEEDWS-----------EATL---------LASIKPDHG------------------
----------------FNSE----SASF-RDLVAIMA--------------------NFD
SRKRREFLQW-------LTGSPKLP------------IGG-----FA-GLHPQL--TIVK
RPHEAPLAPD------------------------DYLPSVMTCV-NY-LKMPGY-SSRDK
MRRRLE-TAMSE-GSG-
>Salmacisia_buchloeana_MOEQ01000226.1 .
VRISRSRL-FESAMKVF----------------ELYGHSTS-VLEVEYFE-------EVG
```

```
TGLG-PTLEF---YALV----SREFARRDL--------GLWRDDGSAGGS----------
--------GNYVNTSKGLFPAPMS--------------------------------AAQL
ETDEGKQRLAMFRTLGQFVAKALLDSRIIDCNFST--LFMKAVLS---------------
------ASVPATLGSVRA----------------------VDPGLARS--------MEQV
LAMDAN---------------------------------------------TLS-GMA--
--LDFTL-------------------------------PG--HPSIE-LVEG--------
GKSKT-------------------------------------------------------
------------------------------VDKDNVH-EYVDA-IVRQTVRDG-IQPAVR
-AFRRG---FNSILPL--QALS-SFTPE-ELVMLFGN-----------------------
---MDEDWS-----------EATL---------LAAIKPDHG------------------
----------------FNSE----SAAF-RDLVSIMS--------------------SFD
VRKRREFLQW-------LTGSPKLP------------IGG-----FA-GLNPQL--TMVK
RPHEAPLMPD------------------------DYLPSVMTCV-NY-LKMPGY-SSREK
MRLRLE-TAMAE-GSG-
>Meira_nashicola_BCJU01000002.1 .
VRISRSNI-LASAFKVF----------------ELYGTNSS-LLEVEYFD-------EVG
TGLG-PTLEF---YAMV----SREFAKKST--------HMWRSDEGRSGE----------
--------SEYVYSTNGLFPAPCG----------------------------QDDVNADS
SGPVGKSRAHAFKIMGQFVAKALFDSRIVDLNLST--MFMRLVLN---------------
------ECVPNTLDMLRQ----------------------VDATLAQS--------LEKI
KLMEKD---------------------------------------------ELE-SLS--
--LDFTL-------------------------------PG--YVDYE-LHKD--------
GKNEQ-------------------------------------------------------
------------------------------VDSTNVA-LYVEE-VITHTLNNG-VKPLVR
-AFREG---FNLIFPI--NAMS-TFTPE-EVVMLFGN-----------------------
---QEEDWS-----------ESTL---------LSSVKPDHG------------------
----------------YNVD----SQTF-RDVISVMS--------------------TFT
AVERRDFLQW-------LTGSPKLP------------IGG-----FA-GLHPHL--TIVK
RTPDTNQMAD------------------------QTLPSVMTCV-NF-CKMPPF-STRSI
MKERLS-LAMRE-GQA-
>Ceraceosorus_bombacis_CCYA01000221.1 .
VRIQRSNI-FPSAMKVL----------------ELYGTTSA-LLEVEYFE-------EVG
TGLG-PTLEF---YALV----SREFAKKSV--------LMWRDDGGGPED----------
--------SEYVSSPLGLFPTVLS-----------------AKDAKAACSSPNASTTASS
PDKSDAVRLQQFTVLGQFVAKALLDSRIIDIHFSP--VFMRLVLN---------------
------QRLPETLGMLAA----------------------VDPSLARS--------MDKL
RGLDEE---------------------------------------------TLA-GMG--
--LDFTL-------------------------------PG--HSEYE-LKAG--------
GKEIA-------------------------------------------------------
------------------------------VERHNLE-EYIQE-VISHTLVHG-VKPFVR
-AFRRG---FTLIFPI--SAMA-SFTAD-ELVMLFGN-----------------------
---TEEDWG-----------ESTL---------LSAIKPDHG------------------
----------------FNSE----SAAF-RDIISIMA--------------------SFG
LNDRRRFLQW-------LTGSPKLP------------IGG-----LA-GLHPQL--TIVK
RPHEAPLTPD------------------------DYLPSVMTCV-NY-LKLPSY-STKEV
MRNRLE-TAMTE-GGTS
>Tilletiopsis_pallescens_BCHO01000001.1 .
VRVPRARL-LDSAFKVF----------------ELYGAGSA-VLEIEYHE-------EVG
TGLG-PTLEF---YSLV----SKEFARKKL--------GAWRDSNESDST----------
--------GEFVVARQGLFPAPLA--------------------------------QSDV
ETETGKSRLQIFRTLGQFVAKALIDSRIVDLNFSP--VFLKSVLN---------------
------QHIPTNLGTLSA----------------------VDSTMAHS--------LESL
RLMDAE---------------------------------------------TLV-ELA--
--LDFTL-------------------------------PG--YPEME-LHEG--------
GKDES-------------------------------------------------------
------------------------------VTKDTLE-QYISE-VASMALQEG-IAPMVR
-SFRQG---FNTIFPL--RAMT-SFTAD-ELVMLFGN-----------------------
---SEEDWS-----------EDTL---------QRSVKPDHG------------------
```

```
----------------LTSE----SSTF-KNMISIMA--------------------NFD
AKDRRSFLQW-------LTGAPKLP------------IGG-----FG-GLHPQL--TVVK
RPHEAPLGPD------------------------DYLPSVMTCV-NY-LKMPLY-SSKET
MKKRLD-IAMRE-GSTS
>Pseudozyma_flocculosa_XM_007881284.1 .
VRISRSSL-LPSAFKVL----------------ELYGSTSS-VLEVEYFE-------EVG
TGLG-PTLEF---YSLV----SKEFARKDL--------RLWRDDDATGTH----------
--------TEFVRSAKGLFPAPFD--------------------------------AAQL
ESAESRKRIHSFKILGQFVAKALLDSRIIDCNFSP--VFLRAVLN---------------
------QPMAASLNTMSA----------------------VDPALARS--------LRSM
QGMSAE---------------------------------------------DVA-GLA--
--LDFTV-------------------------------PG--HPDME-LHAG--------
GRDES-------------------------------------------------------
------------------------------VDASNLD-RYVAE-VLEASLGSG-IKPAVR
-AFRQG---FNLIFPI--AAMS-SFTAE-ELAMLFGN-----------------------
---TDEDWS-----------EATL---------MSSIKPDHG------------------
----------------LNAE----SASF-KDVVAIMA--------------------SFS
VSERRNFLQW-------LTGSPKLP------------IGG-----FA-GLHPQL--TVVK
RPHEPPLTAD------------------------DYLPSVMTCV-NY-LKMPLY-SSRET
MRRRLE-VAMKE-GSTS
>Moesziomyces_aphidis_AWNI01000038.1 .
VRISRANL-LASAFKVF----------------ELYGSNSS-VLEVEYFE-------EVG
TGLG-PTLEF---YSLV----SKEFARRDL--------ELWRDSRSGGSAEDG-------
--------SAYVFSPQGLFPAPLITAQGGEAKA------------------ATASASAEG
GSNSASKRLQAFRILGQFVAKALLDSRIIDCNFSA--VFMRAVLN---------------
------QHVAPTLATLSA----------------------VDATLARS--------LASL
REMKPD---------------------------------------------EVE-AVG--
--LDFTL-------------------------------PG--YEAIE-LHAG--------
GRDES-------------------------------------------------------
------------------------------VTGANVE-QYVSE-VLDMTLQRG-IRPAVR
-AFRQG---FNLIFPI--SAMS-SFTAD-ELVMLFGN-----------------------
---TDEDWS-----------ESTL---------VASIKPDHG------------------
----------------LNAD----SASF-RDIVAIMA--------------------SFG
ADERREFLQW-------LTGSPKLP------------IGG-----FA-GLHPQL--TIVK
RPHEAPLTPD------------------------DYLPSVMTCV-NY-LKMPSY-SSRDK
MRERLH-TAMKE-GSTS
>Pseudozyma_antarctica_XM_014799908.1 .
VRISRANL-LASAFKVF----------------ELYGSNSS-VLEVEYFE-------EVG
TGLG-PTLEF---YSLV----SKEFARRDL--------ELWRDSRAGGTAEDG-------
--------SAYVFSPQGLFPAPLITAQGDEGKT-----------TTTATAATTGSANGEG
GSSSVSKRLQAFRILGQFVAKALLDSRIIDCNFSA--VFMRAALN---------------
------QHVAPTLATLSA----------------------VDATLARS--------LASL
REMKPD---------------------------------------------EVE-AVG--
--LDFTL-------------------------------PG--YEAIE-LHPG--------
GRDES-------------------------------------------------------
------------------------------VTGANVE-QYVSE-VLDMTLQRG-IRPAVR
-AFRQG---FNLIFPI--SAMS-SFTAD-ELVMLFGN-----------------------
---TDEDWS-----------ESTL---------VASIKPDHG------------------
----------------LNAD----SASF-RDIVAIMA--------------------SFG
ADERREFLQW-------LTGSPKLP------------IGG-----FA-GLHPQL--TIVK
RPHEAPLTPD------------------------DYLPSVMTCV-NY-LKMPSY-SSRDK
MRQRLH-TAMKE-GSTS
>Kalmanozyma_brasiliensis_XM_016434715.1 .
VRISRGNL-LASAFKVF----------------ELYGSNSS-VLEVEYFE-------EVG
TGLG-PTLEF---YSLV----SKEFARRDL--------KLWRDSRAGGSSEDG-------
--------SQYVFSPLGLFPAPLLVAGSELEQG-----------------ATASTSESGE
ASGSIQKRLQAFRILGQFVAKALLDSRIIDCNFSP--VFMRAALN---------------
------QHVAPTLATLSA----------------------VDPTLARS--------LSSM
```

RQMPAE---EIE-SLG--
--LDFTL-------------------------------PG--YESIE-LHPG--------
GREET---
------------------------------VTGANVE-QYVSE-VLDLTLQKG-VRAAVR
-AFRQG---FSLIFPI--SAMT-SFTAD-ELVMLFGN-----------------------
---TEEDWS-----------EATL---------LASLKPDHG------------------
----------------LNSD----SPTF-RDIITIMA--------------------SFD
VSQRREFLQW-------LTGSPKLP------------IGG-----FS-GLHPQL--TIVK
RPHEAPLTPD------------------------DYLPSVMTCV-NY-LKMPNY-SSRDK
MRERLQ-TAMKE-GSTS
>Sporisor_iseilematis_ciliati_MJEU01000018 1..
VRISRGNL-LASAFKVF----------------ELYGSNSS-VLEVEYFE-------EVG
TGLG-PTLEF---YSLV----SKEFARRDL--------KLWRDSRAGGSDEDG-------
--------SQYVFSPLGLFPAPLLVSDSTDGDA-------------GKSASPSAGSTSSE
AAGSVQKRLQAFRILGQFVAKALLDSRIIDCNFSP--VFMDAVLN---------------
------QHVAPTLATLAC----------------------VDATLARS--------LASM
RKMSAD---EVE-SLG--
--LDFTL-------------------------------PG--YEGIE-LHAG--------
GRDET---
------------------------------VTGANVE-QYISE-VLDMTLHKG-IRAAVR
-AFRQG---FNLIFPI--SAMS-SFTAD-ELVMLFGN-----------------------
---TQEDWS-----------EKTL---------VASVKPDHG------------------
----------------LNAD----SPTF-RDIVAIMA--------------------SFD
VSQRREFLQW-------LTGSPKLP------------IGG-----FS-GLHPQL--TIVK
RPHEAPLTPD------------------------DYLPSVMTCV-NY-LKMPNY-SSRDK
MRERLQ-TAMKE-GSTS
>Sporisor_reilianum_FQ311434.1 .
VRISRGNL-LASAFKVF----------------ELYGSNSS-VLEVEYFE-------EVG
TGLG-PTLEF---YSLV----SKEFARRDL--------KLWRDSRAGGSDEDG-------
--------SQYVFSPSGLFPAPLLTEDDQSKAA------------------GSSGAEGSE
AAGSVQKRLQAFRILGQFVAKALLDSRIIDCNFSA--VFMGAVLN---------------
------QHVAPTLATLAC----------------------VDAPLARS--------LASM
RKMAAD---EIE-SLG--
--LDFTL-------------------------------PG--YESIE-LHAG--------
GRDET---
------------------------------VTGANVE-RYICE-VVDMTLHKG-IRAAVR
-AFRQG---FNLIFPI--SAMS-SFTAD-ELVMLFGN-----------------------
---TEEDWS-----------ESTL---------VASVKPDHG------------------
----------------LNAD----SPTF-RDIVAIMA--------------------AFD
VSQRREFLQW-------LTGSPKLP------------IGG-----FS-GLHPQL--TIVK
RPHEAPLTPD------------------------DYLPSVMTCV-NY-LKMPNY-SSRDK
MRERLQ-TAMKE-GS--
>Sporisor_scitamineum_LK056689.1 .
VRISRGNL-LASAFKVF----------------ELYGSNSS-VLEVEYFE-------EVG
TGLG-PTLEF---YSLV----SKEFARRDL--------KLWRDSRAGGSEEDG-------
--------SQYVFSPLGLFPAPLLVTESSTDGN--------------QGKTGSTSDGSSE
TAGSVQKRLQAFRILGQFVAKALLDSRIIDCNFSP--VFMGAVLN---------------
------QHVAPTLATLSC----------------------VDATLARS--------LASM
RKMPAD---EIE-SLG--
--LDFTL-------------------------------PG--YESIE-LHPG--------
GRDET---
------------------------------VTGDNVE-RYISE-VLDMTLHKG-IRAAVR
-AFRQG---FNLIFPI--SAMS-SFTAD-ELVMLFGN-----------------------
---TEEDWS-----------EKTL---------VASVKPDHG------------------
----------------LNAD----SPTF-RDIVAIMA--------------------AFD
VSQRREFLQW-------LTGSPKLP------------IGG-----FS-GLHPQL--TIVK
RPHEAPLTPD------------------------DYLPSVMTCV-NY-LKMPNY-SSRDK
MRERLQ-TAMKE-GS--

>Ustilago_maydis_XM_011392545.1 .
VRISRGNL-LASAFKVF----------------ELYGSNSS-VLEVEYFE-------EVG
TGLG-PTLEF---YSLV----SKEFARRDL--------KLWRDSRAGGSDADG-------
--------SAYVFSPLGLFPAPLLADDAKDDDS--------------------------D
DAGSVQKRLQAFRILGQFVAKALLDSRIIDCNFSP--VFLRAVLN---------------
------QHVAPTLATLSR----------------------VDATLARS--------LSAM
RKMSAG---EIE-SLG--
--LDFTL-------------------------------PG--YESIE-LHAC--------
GRDEN---
------------------------------VTSANVE-RYISE-VLDMTLHKG-IRAAVR
-AFRQG---FNLIFPI--SAMS-SFTAD-ELVMLFGN-----------------------
---TEEDWS-----------ETTL---------VASVKPDHG------------------
----------------LNAD----SPTF-RDIVAIMA--------------------SFD
VDQRREFLQW-------LTGSPKLP------------IGG-----FS-GLHPQL--TIVK
RPHEAPLTPD------------------------DYLPSVMTCV-NY-LKMPNY-SSRDK
MRERLQ-TAMKE-GSTS
>Pseudozyma_hubeiensis_XM_012333099.1 .
VRISRGNL-LASAFKVF----------------ELYGSNSS-VLEVEYFE-------EVG
TGLG-PTLEF---YSLV----SKEFARRDL--------KLWRDGRAGGSDEDG-------
--------SAYVYSPLGLFPAPLLIDSAKEDQS----------------KDSSPSGASDA
ADGSVQKRLQAFRILGQFVAKALLDSRIIDCNFSP--VFMRAVLN---------------
------QHVAPTLATLSC----------------------VDATLARS--------LSAM
RKMPAD---EIE-SLG--
--LDFTL-------------------------------PG--HETIE-LHAG--------
GRDES---
------------------------------VTGVNVE-RYISE-VLDWTLRKG-IRAAVR
-AFRRG---FNLIFPI--SAMS-SFTAD-ELVMLFGN-----------------------
---TEEDWT-----------ETTL---------VASVKPDHG------------------
----------------LNAD----SPTF-RDIVAIMA--------------------AFD
VNERREFLQW-------LTGSPKLP------------IGG-----FS-GLHPQL--TIVK
RPHEAPLTPD------------------------DYLPSVMTCV-NY-LKMPNY-STRDK
MRERLH-TAMKE-GATS
>Ustilago_vetiveriae_MAIM01000010.1 .
VRISRGNL-LASAFKVF----------------ELYGSNSS-VLEVEYFE-------EVG
TGLG-PTLEF---YSLV----SKEFARRDL--------KLWRDSRAGGSEEDG-------
--------SAYVFSPLGLFPAPLFTENVKDEPG---------------RAASPSPAGASD
RASPVQKRLQAFRILGQFVAKALLDSRIIDCNFSP--VFMRAVLN---------------
------LHVAPTLATLAC----------------------VDPTLARS--------LSAM
RKMAAD---ELD-SLG--
--LDFTL-------------------------------PG--HDTIE-LHPG--------
GRDEN---
------------------------------VTETNVE-RYISE-VLDMTLQKG-IRAAVR
-AFRQG---FNLIFPI--SAMS-SFTAD-ELVMLFGN-----------------------
---TEEDWS-----------ETTL---------VASVKPDHG------------------
----------------LNAD----SPTF-RDIVSIMA--------------------AFD
VGQRREFLQW-------LTGSPKLP------------IGG-----FS-ALHPQL--TIVK
RPHEAPLTPD------------------------DYLPSVMTCV-NY-LKMPSY-SSRDK
MRERLQ-TAMKE-GSTS
>Ustilago_esculenta_JTLW01000034.1 .
VRISRGNL-LASAFKVF----------------ELYGSNSS-VLEVEYFE-------EVG
TGLG-PTLEF---YSLV----SKEFARRDL--------NLWRDSRASGSDEDG-------
--------SPYVFSPLGLFPAPLISTSEEKTGS----------------------SDGGD
GSGSVQKRLQTFRILGQFVAKALLDSRIVDCNFSP--ILMRAVLN---------------
------QHVAPTLATLSA----------------------VDATLARS--------LASM
RKMQPD---EIE-SLG--
--LDFTL-------------------------------PG--YESVE-LHPG--------
GREEN---
------------------------------VTGVNVE-QYISE-VLEMTLQKG-IRPAVR

-AFRQG---FNLIFPI--TAMS-SFTAD-ELVMLFGN-----------------------
---TEEDWS-----------ETTL---------LASVKPDHG------------------
----------------LNAD----SPTF-RDIVAIMA--------------------SFD
VSQRREFLQW-------LTGSPKLP------------IGG-----FS-GLHPQL--TIVK
RPHEAPLTPD------------------------DYLPSVMTCV-NY-LKMPSY-SSRDK
MRERLQ-TAMKE-GSTS
>Ustilago_trichophora_LVYE01000065.1 .
VRISRSNL-LASAFKVF----------------ELYGSNSS-VLEVEYFE-------EVG
TGLG-PTLEF---YSLV----SKEFARRDL--------KLWRDNRAGGSDEDG-------
--------SQYVFSPSGLFPAPLITTGADAEES------------------KTASTEGGD
GPGSVQKRLQAFRILGQFVAKALLDSRIIDCNFSP--VFMRSVLN---------------
------QHVAPTLATLSA----------------------VDATLARS--------LASM
QKMQPD---EIE-SLG--
--LDFTL-------------------------------PG--YESIE-LHAG--------
GRDEN---
------------------------------VTGANME-RYISE-VLDMTLQKG-IRPAVR
-AFRQG---FNLIFPI--AAMS-SFTAE-ELVMLFGN-----------------------
---SEEDWS-----------EKTL---------LASVKPDHG------------------
----------------LNAE----SPTF-RDIVAIMA--------------------SFD
VSQRREFLQW-------LTGSPKLP------------IGG-----FS-GLHPQL--TIVK
RPHEAPLTPD------------------------DYLPSVMTCV-NY-LKMPNY-SSRNK
MRERLQ-TAMKE-GSTS
>Pseudozyma_tsukubaensis_MAIP01000102.1 .
VRISRGNL-LASAFKVF----------------ELYGSNSS-VLEVEYFE-------EVG
TGLG-PTLEF---YSLV----SKEFARREL--------KLWRDSRAEGSDEDG-------
--------SQYVFSPLGLFPAPLISADAEEAKA--------------NPPASSVSPGVGD
GASSAQKRLQAFRILGQFVAKALLDSRIIDCNFSP--VFMRAVLN---------------
------QHVAPTLATLSA----------------------VDATLARS--------LASM
RTMQPD---EIE-SLG--
--LDFTL-------------------------------PG--YDSIE-LHAG--------
GKDEN---
------------------------------VTGDNVE-QYISE-VLDMTLHKG-IRPAVR
-AFRQG---FNLIFPI--AAMS-SFTAD-ELVMLFGN-----------------------
---TEEDWS-----------ETTL---------LASVKPDHG------------------
----------------LNAD----SPTF-RDIVAIMA--------------------SFD
ATQRREFLQW-------LTGSPKLP------------IGG-----FS-GLHPQL--TIVK
RPHEAPLTPD------------------------DYLPSVMTCV-NY-LKMPNY-SSRDK
MRERLH-TAMKE-GSTS
>Melanopsichium_pennsylvanicum_HG529635.1 .
VRISRGNL-LASAFKVF----------------ELYGSNSS-VLEVEYFE-------EVG
TGLG-PTLEF---YSLV----SKEFARRDL--------KLWRDSRAGGSDEDG-------
--------SQYVFSPLGLFPAPLITIGTDDQEA------------------KAADSEISD
ASGSAQKRLQAFRVLGQFVAKALLDSRIIDCNFSP--IFMRAVLN---------------
------QHVAPTLATLSA----------------------VDAMLARS--------LASM
RQMKPD---EVE-SLG--
--LDFTL-------------------------------PG--YESIE-LHAG--------
GREEN---
------------------------------VTGDNVE-RYISE-VLDMTLGKG-IRAAVR
-AFRQG---FNIILPM--AAMS-SFTVD-ELVMLFGN-----------------------
---TEEDWS-----------ETTL---------LASVKPDHG------------------
----------------MNAD----SPAF-RDMIGIMA--------------------SFN
VGERREFLQW-------LTGSPKLP------------IGG-----FS-GLHPQL--TIVK
RPHEAPLTPD------------------------DYLPSVMTCV-NY-LKMPNY-SSRDK
MRQRLQ-TAMKE-GS--
>Ustilago_cynodontis_LZZZ01000257.1 .
VRISRANL-LASAFKVF----------------DLYGSNSS-VLEVEYFE-------EVG
TGLG-PTLEF---YSLV----SKEFARRDL--------KLWRDSRAGGSNEDG-------
--------SQYVFSPLGLFPAPLMQDNTGADSE-----------KGKSAGTAISVDDISS

GAGSTQKRLQAFRILGQFVAKALLDSRIIDCNFSP--VFMRAVLN---------------
------QHVAPTLATLAA----------------------VDATLARS--------LASM
RKMQPD---EIE-SLG--
--LDFTL-------------------------------PG--YESIE-LHAG--------
GRDEN---
------------------------------VTGSNVE-QYISE-VLDMTLQRG-IRPAVR
-AFRQG---FNLIFPI--SAMS-SFTAD-ELVMLFGN-----------------------
---TEEDWS-----------ESTL---------LGSVKPDHG------------------
----------------LNAD----SPTF-RDIVAIMA--------------------SFD
VSQRREFLQW-------LTGSPKLP------------IGG-----FS-GLHPQL--TIVK
RPHEAPLTPD------------------------DYLPSVMTCV-NY-LKMPNY-SSRDK
MRERLQ-TAMKE-GSTS
>Ustilago_xerochloae_MAIN01000175.1 .
VRISRANL-LASAFKVF----------------DLYGSNSS-VLEVEYFE-------EVG
TGLG-PTLEF---YSLV----SKEFARRDL--------KLWRDSRAGGSNEDG-------
--------SQYVFSPFGLFPAPLTQDNTSADSE------------GKSAATAISVDDISS
GAGSTQKRLQAFRILGQFVAKALLDSRIIDCNFSP--VFMRAVLN---------------
------QHVAPTLATLAA----------------------VDPTLARS--------LASM
RKMHPD---EIE-SLG--
--LDFTL-------------------------------PG--YETIE-LHAG--------
GRDEN---
------------------------------VTGANVE-QYISE-VLDMTLQRG-IRPAVR
-AFRQG---FNLIFPI--SAMS-SFTAD-ELVMLFGN-----------------------
---TEEDWS-----------ESTL---------LGSVKPDHG------------------
----------------LNAD----SPTF-RDIVAIMA--------------------AFD
VSQRREFLQW-------LTGSPKLP------------IGG-----FS-GLHPQL--TIVK
RPHEAPLTPD------------------------DYLPSVMTCV-NY-LKMPNY-SSRDK
MRERLH-TAMKE-GSTS
>Ustilago_hordei_CAGI01000136.1 .
VRISRANL-LASAFKVF----------------DLYGSNSS-VLEVEYFE-------EVG
TGLG-PTLEF---YSLV----SKEFARRDL--------KLWRDSRAGGSNEDG-------
--------SQYVFSPLGLFPAPLTQDTSAVSTE------------GKSAETAITVDDISS
GSGSTPKRLHAFRILGQFVAKALLDSRIIDCDFSP--VFMRAVLN---------------
------QHVAPTLATLAA----------------------VDATLARS--------LESM
RKMPSD---EIE-SLG--
--LDFTL-------------------------------PG--YESIE-LHAG--------
GRDEN---
------------------------------VTGANVE-QYIFE-VLDMTLQRG-IRPAVR
-AFRQG---FNLIFPI--SAMS-SFTAD-ELVMLFGN-----------------------
---TEEDWS-----------ELTL---------LASVKPDHG------------------
----------------LNAD----SPTF-RDIVAIMA--------------------SFD
VSQRREFLQW-------LTGSPKLP------------IGG-----FS-GLHPQL--TIVK
RPHEAPLTPD------------------------DYLPSVMTCV-NY-LKMPNY-SSRDK
MRERLH-TAMKE-GSTS
>Ustilago_bromivora_LT558127.1 .
VRISRANL-LASAFKVF----------------DLYGSNSS-VLEVEYFE-------EVG
TGLG-PTLEF---YSLV----SKEFARRDL--------KLWRDSRAGGSNEDG-------
--------SQYVFSPLGLFPAPLTQDTSAVSAE------------GKSAETAITVDDISS
GSGSTPKRLHAFRILGQFVAKALLDSRIIDCDFSP--VFMRAVLN---------------
------QHVAPTLATLAA----------------------VDATLARS--------LESM
RKMPSD---EIE-SLG--
--LDFTL-------------------------------PG--YESIE-LHAG--------
GREEN---
------------------------------VTGANVE-QYISE-VLDMTLQRG-IRPAVR
-AFRQG---FNLIFPI--SAMS-SFTAD-ELVMLFGN-----------------------
---TEEDWS-----------ELTL---------LASVKPDHG------------------
----------------LNAD----SPTF-RDIVATMA--------------------SFD
VSQRREFLQW-------LTGSPKLP------------IGG-----FS-GLHPQL--TIVK

RPHEAPLTPD------------------------DYLPSVMTCV-NY-LKMPNY-SSRDK
MRERLH-TAMKE-GS--
>Schizosacch_cryophilus_XM_013169457.1 .
IRIPRKNL-FNYALQVL----------------SSYGSSDK-VLEIEYED-------EVG
SGLG-PTLEF---YTTV----SKEFTNTSL--------DIWRNEQAGS------------
---------QYVFHASGLFPSPIS---------------------------------TAE
ENEENKRKLSLFSALGKFVARSIYDSRIISLQFNP--LLFTRTIP---------------
----------LTISSVAK----------------------VDKGLANS--------LRYL
QSLNSEVG------------------------------------NPGFD-IDIE-SLG--
--LDFTL-------------------------------PG--YSSIE-LIPN--------
GSNIS---
------------------------------VNKINLE-DYIKC-IIDFTVGKG-IRRQLE
-SFQEG---FSSVFAY--SSLQ-VLTEY-ELSSLFGT-----------------------
---IDEDWS-----------YETL---------IKSVVADHG------------------
----------------YTID----SPPI-QRLLQLMS--------------------SMN
FREQRDFLQF-------ITGSRKLP------------IGG-----FA-GMNPPF--TIVR
RLNEPPYRPD------------------------DYLPTVMTCV-NY-LKLPEY-SSVEV
LANRLS-KAILE-GQGS
>Schizosacch_octosporus_XM_013163264.1 .
IRIPRKDM-FNYALQVL----------------SSYGSSDK-VLEIEYED-------EVG
SGLG-PTLEF---YTTV----SREFTNTAL--------DIWRNEQGDS------------
---------QYVFHASGLFPSPIS---------------------------------MAE
GSDESKRKLSLFSALGKFVARSIYDSRIVSLQCNP--LFFARAIP---------------
----------LTISSVAK----------------------VDKGLANS--------LRYL
ESLISGED------------------------------------GHIND-IDIE-SLG--
--LDFTL-------------------------------PG--YSSIE-LIPN--------
GSTVS---
------------------------------VNKSNLK-DYIEH-IIDFTVGKG-VRRQIE
-CFQEG---FSSVFAY--SSLK-VLTEY-ELSSLFGT-----------------------
---IDEDWS-----------YETL---------LKFVVADHG------------------
----------------YTIE----SAPI-QRLLQLMS--------------------SMN
FREQRDFLQF-------ITGSRKLP------------IGG-----FA-GMNPPF--TIVR
RLNEPPYKPD------------------------DYLPTVMTCV-NY-LKLPEY-SSVDV
LASRLS-KAILE-GQGS
>Schizosacch_pombe_NM_001020061.2 .
IRISRKKI-FNYALHLL----------------ATYAASEN-ILEIEYED-------EVG
SGLG-PTLEF---YTSV----SKEFTLNSL--------DIWRNDQPNS------------
---------KFVYQASGLFPSPIP---------------------------------LLG
SSPENERKISLFFALGQFVARSIYDSRIISIQFNP--LFFARNIP---------------
----------LTISSVAK----------------------VDKGLANS--------LRYL
EKLIPGKNPT----------------------------------NAETD-IKLE-DLH--
--LDFTL-------------------------------PG--FPSIE-LIPD--------
GASTP---
------------------------------VTTFNVS-DYLNY-VIDYTVGKG-VQQQLE
-AFQNG---FSSVFPY--TSLQ-VLTEH-ELVTLFGT-----------------------
---VDEDWS-----------YATL---------MKSIVADHG------------------
----------------YTME----SPTI-QRLLTLMS--------------------QMN
FQEQRDFLQF-------ITGSRKLP------------IGG-----FA-GLNPPL--TVVR
RLNEPPYVPD------------------------DYLPSVMTCV-NY-LKLPEY-SSSEV
LGSRLS-KAILE-GQGS
>Schizosacch_japonicus_XM_002173100.2 .
LRISRNQI-FEYAKQVL----------------FSFGSSKN-IIEIEYED-------EVG
SGLG-PTQEF---YTTV----SREFTRKHH--------HMWRDDNQTS------------
---------NYVHSATGLFPAPMS-----------------------------------H
AIGANEKICSLFKMLGQFIARSLYDSRLISVQLNP--LIFEETIP---------------
----------LTLSSVSK----------------------VDKELGKS--------LRFL
ERLLARQQAGF--------------------------------TAVPTE-TKIE-DLC--
--LDFTL-------------------------------PG--YPSVE-LIPN--------

GRNVQ---
------------------------------VSHKNAS-KYISS-IIDFTVGKG-VQLQIQ
-NFREG---FSTVFNY--ESLR-IITSH-ELSMLFGK-----------------------
---VEEDWS-----------FETI---------AKSIVADHG------------------
----------------YNME----NTTI-HNLIEVMS--------------------NFS
FSEQRDFLQF-------ITGSRKLP------------IGG-----FS-SLHPAL--TVVR
KLNEPPYVPD------------------------DYLPSVMTCV-NY-LKLPEY-SSKEI
LKQKLM-LAMKE-GQGS
>Pneumocystis_jirovecii_XM_018374063.1 .
IRISRDHI-MESTIKIM----------------DLYGTSSF-LLEIEYFD-------EVG
TGLA-PTLEF---YSIA----SHEFTRKSL--------GLWRNVDDLSGN----------
---------DFVFSPNGLFPMPLN--------------------------------CDFK
DSDIEKKKISLFKVMGKFIARSILDSRIIDISINP--MFFRIASD---------------
-----VNDVRLSIESISE----------------------IDENLAKS--------LRFL
LQFEIAKKKILNKDICENEKE------------------QLLKDIKVQG-MSID-EFS--
--LNFTL-------------------------------PG--FPDVH-LITN--------
GDEKI---
------------------------------VTIYTIG-EYIDL-IIDFTIGKG-VREQIN
-AFRDG---FSSVLPY--SSLA-LFTPE-ELSMLFGQ-----------------------
---GKEDWS-----------IETL---------IDSIKADHG------------------
----------------YNMD----SRSI-QNFLDILA--------------------NMN
DMERRQFLQF-------ITGSPKLP------------IGG-----FK-SLNPPL--TVVC
KTHEPPLTPN------------------------DYLPSVMACV-NY-LKLPDY-TTKKI
MKSKLF-LAIKE-GQGS
>Pneumocystis_murina_XM_007876380.1 .
IRISRDHI-LESTIKIM----------------DLYGSSPF-LLEIEYFD-------EVG
TGLG-PTLEF---YSTA----SHEFMKKNH--------GLWRNVDDLSED----------
---------EFVFSPNGLFPAPLG--------------------------------TSST
DLDDKWKRCSLFKVLGKLIARSMLDSRIVDILINP--MFFRIASG---------------
-----TNEVKLSIESISE----------------------IDKDLANS--------LYFL
MQFDIAKKKILNKNICEKEQE------------------LRLNDIRIQD-VSVE-DLS--
--LNFTL-------------------------------PG--FPNIY-LVSN--------
GHEKS---
------------------------------VTIHNIG-EYIDL-IIDFTIGKG-VKEQIN
-AFRDG---FSSVLPY--SSLA-LFTPE-ELSILFGQ-----------------------
---GKEDWS-----------IETL---------LDSIKADHG------------------
----------------YNMD----SRSI-RNFLDILT--------------------NMN
DMERRQFLQF-------ITGSPKLP------------IGG-----FK-NLTPPL--TVVC
KSHEPPLTPN------------------------DYLPSVMVCV-NY-LKLPDY-TTKKI
MKTKLF-LAMKE-GQGS
>Pneumocystis_carinii_XM_018371190.1 .
IRISRDHI-LESTIKIM----------------DLYGSSSF-LLEIEYFD-------EVG
TGLG-PTLEF---YSTV----SHEFMRKNL--------GLWRNVDDFSEN----------
---------EFVSSPNGLFPAPLS--------------------------------TDST
YLDEKRKKCNLFKVLGKFIARSMLDSRIVDILINP--MFFRIAFG---------------
-----MNEVKLSIESIAE----------------------IDRDLANS--------LYFL
MQFDTAKKKILNKNICDKEME------------------LRLKDIRVHD-VSVE-DLS--
--LNFTL-------------------------------PG--FPDIH-LANN--------
GYEKL---
------------------------------VTIHNID-EYIDL-IIDFTIGKG-VKEQIN
-AFRDG---FSSVLPY--SSLS-LFTPE-ELSILFGQ-----------------------
---DKEDWS-----------IETL---------LDSIKADHG------------------
----------------YNID----SRSI-RNFLDILT--------------------NMN
DMERRQFLQF-------ITGSPKLP------------IGG-----FK-NLNPPL--TVVC
KSHEPPLTPN------------------------DYLPSVMVCA-NY-LKLPDY-TTKKI
MKTKLF-LAIKE-GQGS
>Neolecta_irregularis_LXFE01000149.1 .
VRMQRDRM-LDSALKIF----------------RTYGSPAA-ILEVEYYD-------EVG

TGLG-PTLEF---YATV----SKEFSQKSL--------SLWRGSNSSAGG----------
---------SYVHTSEGLFPNPIN---------------------------------VLTA
KPETLX-----FKGLGQFIARSMLDSRIIDIHFNP--LFLRLAQT---------------
-----PASVIKSVGLIRH----------------------VDKDLFRA--------LCLL
DTYIAEKKRIKMLNIDAVSKG------------------SLLDQVQIDD-ISLE-DLC--
--LHFTL--------------------------------PG--FSDVE-LXPN--------
GAETA---
------------------------------VTIDNVE-SFVHA-VIDFTVGSG-IQRQID
-AFREG---FSSIFSF--DSLR-TLFVD-ELIMLFGQ-----------------------
---ADEDWS-----------IESL---------TTSIKADHG------------------
----------------FHSE----SVTI-KRLIEVMS--------------------EFD
TSQRRQLLQF-------LTGSPRLP------------IGG-----FQ-SLSPPL--TVVC
KPNEPPLTSD------------------------DYLPSVMTCV-NY-LKLPDY-SSKDV
LRKRIL-SAAVE-G---
>Lobosporangium_transversale_MCFF01000010. 1..
VRISRQKA-LESAVRVM-----------------DLYGASQA-MLEVEFFD-------EVG
TGLG-PTLEF---YSLV----SKEFCKKSL--------KLWRDADSESLT----------
---------EYVSAPQGLFPRPMAYHRTDSDDEKYV-----SSTMYHDLVEVDRHLNLFS
FFFICRKILKLFRSLGQFIAKAMLDSRIIDVPLSP--LFVNQLLG---------------
------HNLKTHLQLVSL----------------------IDPVLARS--------LESL
HAFVTEKKRIYGMKLPSKERE------------------VALKNIKLHG-TKLE-DMS--
--LDFTL--------------------------------AG--YPHIE-LXTG--------
GASVP---
------------------------------VTIYNVE-EYIRL-TVDMTVGRG-VEAQLS
-AFRAG---FNSVFSI--QDLA-GFTSE-ELVNLFGL-----------------------
---GEEDWS-----------YLAL---------VDTVKADHG------------------
----------------FRSE----SRAF-KNLLTIMS--------------------EFN
NQERRQFLQF-------ITGSPKLP------------IGG-----FK-NLHPPF--TVVC
KPFEAPLKAD------------------------DYLPSVMTCA-NY-LKMPDY-SCKEV
TLAKFK-MAYEE-GQG-
>Mortierella_alpina_LDAW01000698.1 .
VRISREKA-LESAVKVM-----------------ELYGASQG-MLEVEYFD-------EVG
TGLG-PTLEF---YSVV----SKEFCKKAV--------KLWRDADSEALS----------
---------DYVLAPHGLYPRPMV------------------------------------
-CSEARKILKLFRSLGQFIAKAMLDSRIIDVPLSA--LFVSQLLG---------------
------RSRKPHLQLVAS----------------------IDPVLAQS--------LRSL
QSFVVEKKRVYGMNLPSKERE------------------MALKNIELDG-SRLE-DMS--
--LDFTL--------------------------------PG--YSRID-LXSG--------
GANIP---
------------------------------VTIYNVE-EYIDL-AVDMTVGRG-IQAQTA
-AFQEG---FNRVFSI--QDLA-GFRAE-ELVNLFGS-----------------------
---GEEDWS-----------ESAL---------MDSIKADHG------------------
----------------FRSD----SPAF-INLLQVMS--------------------EFS
VEERRQFLQF-------ITGSPKLP------------IGG-----FK-NLHPPF--TVVC
KHFPQRAD--------------------------DYLPSVMTCA-NY-LKMPEY-SCKEV
TLAKFR-MAYEE-GQG-
>Mortierella_elongata_LYLZ01000147.1 .
VRISRQKA-LESAVRVM-----------------DLYGSHQA-MLEVEYFE-------EVG
TGLG-PTLEF---YSVV----SKEFCKKFL--------KLWRDGDSEGAT----------
---------KYVTAPHGLYPRPMATVPSHADNHRYVFDA--VFMVTEFLSAYITCRIDRL
NLQFHRKILKLFKSLGQFIAKAMLDSRIIDVPLSA--LFVSQLLG---------------
------RNLKPRLQHVAS----------------------IDPVLARS--------LQSL
QAYVAEKKRIYGLNLPSKERE------------------SALKNIELQG-AKLD-DMG--
--LDFTL--------------------------------VG--YPEIE-LXAG--------
GTNIP---
------------------------------VTIYNVE-EYIQL-TVDMTVGRG-VEAQAA
-AFRDG---FNCVFSI--QDLA-GFRSE-ELVRLFGS-----------------------
---GDEDWS------------XAL---------VDTVKADHG------------------

----------------FRSE----SRAF-KNLLRAMS--------------------ELD
KEERRQFLQF-------ITGSPKLP------------IGG-----FK-NLHPAF--TVVC
KPFEAPLKAD------------------------DYLPSVMTCA-NY-LKMPDY-STKEV
MMAKFK-TAYEE-GQG-
>Basidiobolus_meristosporus_JNEO01002071.1 .
VRISRSRM-LESAVKVM----------------DLYGSQQS-ILEVEYFE-------EAG
TGLG-PTLEF---YATV----SKEFCKKSL--------KLWRDDDDSDPSR---------
---------KYVTSQLGLFPRPVS--------------------------------ESYL
ESENGIKILNLFKAMGKFVAKALIDSRIIDLPFNP--VFIKTLLN---------------
-----PAEDIKNLRSVMH----------------------IDSVLGNS--------LKVI
KHFIREKQRIMQNPDLNESERQ-----------------EAVNNLRHNG-VSLD-DLC--
--LDFTL------------------------------PG--YPEIE-FVEN--------
GANVA---
------------------------------VTIENVE-EYLDG-VLEHTVGSG-VTTKVA
-AFRDG---FNTVFPL--KHLN-AFSPE-ELVMLFGQ-----------------------
---AEEDWS-----------PEVL---------LDAIKADHG------------------
----------------YTLE----SRPI-QYLIDIMS--------------------SFT
AEQRREFLQF-------VTGSPKLP------------IGG-----FK-NLLPAF--TVVC
KPNELPLTPD------------------------DYLPSVMTCV-NY-LKMPNY-STEEI
MRKKLE-KAMKE-GQG-
>Basidiobolus_heterosporus_JNET01034159.1 .
VRISRSRM-LESAVKVM----------------DLYGSQQS-ILEVEYFE-------EAG
TGLG-PTLEF---YATV----SKEFCKKSL--------KLWRDDEDSNSSR---------
---------KYVTSPLGLFPRPVS--------------------------------ESYL
KSENGIKILNLFKAMGKFVAKALIDTRIIDLPFNP--IFIKTLLS---------------
-----PEENITNLRSVMH----------------------IDSVLGNS--------LKTI
KHFVREKRQILQNPALNETERQ-----------------EALDHLRHQG-VSLE-DLC--
--LDFTL------------------------------PG--YPEIE-LIEN--------
GANVA---
------------------------------VTVQNVD-EYLDG-VLEYTVGSG-IAKKVA
-AFRDG---FNTVFPL--KHLN-AFSPE-ELVMLFGQ-----------------------
---AEEDWS-----------SEVL---------LDAIKADHG------------------
----------------YTLE----SRPI-KYLIDIMS--------------------NFT
SEERREFLQF-------VTGSPKLP------------IGG-----FK-NLLPAF--TVVC
KPNELPLTPD------------------------DYLPSVMTCV-NY-LKMPNY-STKEI
MRKKLE-KAMKE-GQG-
>Umbelopsis_isabellina_JNEQ01000027.1 .
VRISRSRM-LESAVKVL----------------ELYGKSQS-VLEVEYFD-------EEG
TGLG-PTLEF---YAII----SKQFCKKSL--------HMWRDDDSNEES----------
---------AYVSTKLGLFPRPMS--------------------------------EAMA
KTEKGRKIINLFNVLGQFVSKAMLDSRNIDIPFNP--AFLSKVLG---------------
------QSTMTDITAVME----------------------VDPTLGNS--------LLDL
QSFVTQKHAIYANQQAPYD--------------------EALANIEVRG-AKIS-DFC--
--LDFTV------------------------------PG--DSDME-LKPN--------
GSQIP---
------------------------------VTIHNVD-EYLEL-VVDAVTGSS-IARQVE
-AFRNG---FDGVFPV--NDLQ-LFLPE-ELVSFFGK-----------------------
---TEEDWT-----------YETL---------TDAVKADHG------------------
----------------YNME----SKAI-KYLLEIMT--------------------DFS
EDERREFLQF-------ITGSPKLP------------IGG-----WK-NLSPMF--TIVR
KPHEPPLTSD------------------------DYLPSVMTCV-NY-LKLPDY-SSKEA
MRKRLL-TSVTE-GKG-
>NWGS3C_2_Absidia_repens_MCGE01000004.1 .
VRMMRNQI-LESAIKML----------------DLFASSTS-SLEIEFVD-------EEG
TGLG-PTLEF---YALT----SKEFCKKSL--------GLWRDDDDTYNSLQQHQDTQAN
DDDSNVV--RYVSSPHGLFPKPLI----------------------------NNDGTSIV
PDRDIQRILTLFKTLGRFIAKAMLDFRIVDIPFSV--SFFDLVFS---------------
-----KNIPAIDLLGVSF----------------------KNGQLIFS-------ELFNI

LYSL---ILQ--
--VDPTI-------------------------------AK----SIN-LLX---------
GDKIP---
------------------------------VTIHNLS-NYVEL-LLDAIVGSG-VSKQID
-AFRSG---FNDSLQI--DDLK-IITSE-ELVSLFGS-----------------------
---SNEDWS-------------XL---------TDTIKADHG------------------
----------------FSME----STTL-KNLLVILS--------------------EFD
TKGRRDFLQF-------TTGSPRLP------------IGG-----WK-SLRPAF--TVVR
KIAEAPLSAD------------------------DYLPSVMTCA-NY-LKMPDY-SDKNM
MLKRLQ-TAMDE-G---
>Absidia_caerulea_GFAW01003102.1 .
VLVRRDQI-LEYAVKIM----------------DVFGDSQP-VLEIEYAD-------EEG
SGLG-PTLEF---YALA----SKEFCKKST--------NMWRQGDNMAVEGGDSGDNG--
---------LYVVNQQGLFPAPLP---------------------------------KRT
DAKARKKTIQLFKSLGQFVAKSMLDFRIIDIPFNA--AFFELLLF---------------
-------GDANPVEFIKQ----------------------VDPVLGKS--------VSHL
LEYVDKKRAIELQTNLTPKERG-----------------VRIENIEVNG-AKIQ-DLC--
--LDFTL-------------------------------PG--QANVD-LKRG--------
GSQLS---
------------------------------VTMDTVE-QYIHL-LADMIAGSG-VKDQVS
-AFREG---FNGLLCV--EDLK-VLTSQ-ELVTLFGS-----------------------
---TTEDWS-----------YSTL---------FNTIKADHG------------------
----------------FTME----SAAI-KNLLEILS--------------------EMD
TQDQRGFLQF-------TTGSPRLP------------IGG-----WK-AMRPTF--TVVR
KNCDAPLTAD------------------------DYLPSVMTCA-NY-LKMPDY-SNKKI
MHKQLM-KSIQE-GT--
>Lichtheimia_corymbifera_CBTN010000003.1 .
VRIMRSQM-LESAIKIL----------------DLFGSSQS-VLEIEYNG-------EEG
TGLG-PTLEF---YAAT----SREFCKKSL--------NMWRDDDDDDNNTGG-------
---------EYIVAKHGLFPRPLS------------------------------------
-KSGSTKIINLFKTLGQFMAKAMLDFRIIDLPFSA--AFFKVAIG---------------
-------HEQPSMSLVAV--------------------------SLDT--------MCFL
X---IE-DLC--
--LDFTL-------------------------------PG--ETDLE-LKAG--------
GADIP---
------------------------------VTINNVE-EYIDL-LQDKLAGSG-IAKQMD
-AFRQG---FDQLFAL--DDLK-ILTYN-ELVSLYGV-----------------------
---SSEDWS-----------YAXL----------SRLTTDSQ------------------
----------------WKVSRS--KTCLPSCLKMMMN---------------DASFCNSP
LEALDCQLEVN------NTDSFTWSTYWS-------AIIG-----WK-AMRPVF--TVVC
KTAEAPLSPD------------------------DYLPSVMTCA-NY-LKMPDY-SSKEK
MRTRLL-TSMRE-G---
>Phycomyces_blakesleeanus_XM_018443934.1 .
VRIIRSQM-LESAIKIL----------------DLFGSSQS-VLEVEYTG-------EEG
TGLG-PSLEF---YAST----SKEFCKKSI--------NLWRDGGNDPSS----------
---------LYVDASRGLFPKTLP---------------------------------KNA
NGKSAKKILNLFKTLGQFVAKAMLDFRIIDIPFSV--AFFKLALD---------------
-------ESVDQDKLIKE----------------------IDPTLSKS--------LDTL
QSYIKQKNALYKDTTKDMMQQI-----------------KEAQNITVDN-ARLE-DLC--
--LEFTL-------------------------------PG--DPTIE-LKPG--------
GSEIP---
------------------------------VTINNVE-EYVNL-LKDSLVGSG-IAQQLD
-VFRKG---FNGLFAI--DDLK-ILSHQ-ELVSLFGQ-----------------------
---SSEDWT-----------YGTL---------ADTIKADHG------------------
----------------FTME----SPAF-KNLLEILS--------------------EMD
DDSRRDFLQF-------TTGSPRLP------------IGG-----WK-AIRPVF--TVVR
KVPEAPLHAD------------------------DYLPSVMTCA-NY-LKMPDY-TNKIV
MEQRIF-KSMKE-GKNS

```
>Rhizopus_delemar_JNEC01012710.1 .
VRIVRSQM-LESAIKIF----------------DLFGSSPG-VLEIEYVG-------EEG
TGLG-PTLEF---YAST----SKEFSKHSI--------NMWRGSIKNETG----------
----------YVDDPLGLFPKPLA----------------------------------KS
NSKSVKKTVQLFRTLGQFVAKAMLDFRIIDIRFNS--AFFKIALE---------------
-----DAKPSHELLVVSK----------------------IEKDVSQR--------CLKV
FN------------------------------------------LQVEG-VQIQ-DLC--
--LDFTL-------------------------------PGDATYELK-VXPD--------
GAEIP-------------------------------------------------------
------------------------------VTIQNVE-QYVEA-IIDAVTGSG-VSQQIE
-AFKEG---FNDLFAI--EDLK-LLTYS-ELVSLFGT-----------------------
---SEEDWS-----------LSTL---------ADTIKADHG------------------
----------------FTIE----SKSV-QYLLEILS--------------------EMD
NSQRREFLQF-------TTGSPRLP------------IGG-----WK-ALRPVF--TVVR
KVPESSLSPD------------------------DYLPSVMTCA-NY-LKMPEY-SSKEI
MRQKLE-ISMKE-G---
>Rhizopus_oryzae_JNDZ01010326.1 .
VRIVRSQM-LESAIKIF----------------DLFGSSPG-VLEIEYVG-------EEG
TGLG-PTLEF---YAST----SKEFSKHSI--------NMWRGSIKNETG----------
----------YVDDPLGLFPKPLA----------------------------------KS
NSKSVKKTVQLFRTLGQFVAKAMLDFRIIDIRFNS--AFFKIALE---------------
-----DAKPSHELLMVSK------------------IENGGELEVLXS--------MSQK
MQ--------------------------------------EIESLQVEG-VQIQ-DLC--
--LDFTL-------------------------------PGDATYELK-VXPD--------
GAEIP-------------------------------------------------------
------------------------------VTIQNVE-QYVEA-IIDAVTGSG-VSQQIE
-AFKEG---FNDLFAI--EDLK-LLTYS-ELVSLFGT-----------------------
---SEEDWS-----------LSTL---------ADTIKADHG------------------
----------------FTIE----SKSV-QYLLEILS--------------------EMD
KSQRREFLQF-------TTGSPRLP------------IGG-----WK-ALRPVF--TVVR
KVPESSLSPD------------------------DYLPSVMTCA-NY-LKMPEY-SSKEI
MRQKLE-ISMKE-G---
>Mucor_irregularis_GFBC01014357.1 .
VRIMRNQM-LESAIKIL----------------DLFGSSPT-VLEIEYMG-------EEG
TGMG-PTLEF---YAST----SKEFSKHSL--------NMWRGSNKSKKG----------
----------YVDAPYGLFPKPLA----------------------------------KS
SSRSTKKIVHLFKTLGQFIAKGLLDFRIIDIPFSP--AFFKVALD---------------
-----HVQPSPELFME------------------------IDPVLAKS--------VNML
QAYIDQKREINADSKLSAAEKS-----------------AAIKEIEVDG-AKIE-DLC--
--LDFTL-------------------------------PG--DETYE-LKTG--------
GSEIP-------------------------------------------------------
------------------------------VTINNVE-EYIEL-LQDTIAGSG-ISQQID
-AFREG---FNCLFAI--DDLK-LLTYS-ELVSLFGK-----------------------
---ADEDWS-----------YATL---------ADAIRADHG------------------
----------------FAMD----SEPV-KYLLEILS--------------------SMD
PTEKRQFIQF-------TTGSPRLP------------IGG-----WK-ALRPVF--TVVR
KMPEEPLSAD------------------------DYLPSVMTCA-NY-FKMPAY-SSKEV
TRTRLL-TAMKE-G---
>Mucor_circinelloides_LGTF01001125.1 .
VRIMRTQM-LESAVKIL----------------DLFGSSPT-VLEIEYMG-------EEG
TGMG-PTLEF---YAAT----SKEFSKHSL--------NMWRGSNKAEKG----------
----------HVEAPFGLFPKTCV----------------------------------KP
GSRSAKKINHLFKTLGQFIAKGLLDFRIIDIPFSP--AFFKVALD---------------
-----HVDPSPELLMVSSDS---------ANMMQCTKYLEIDPQLAKS--------IRML
TSY------------------------------------------VKDG-AKLE-DLC--
--LDFTL-------------------------------PG--DSNYE-LXTG--------
GSEIA-------------------------------------------------------
------------------------------VTVNNVE-EYIDL-VQDAIAGSG-IAQQIN
```

```
-AFREG---FNGLFAI--DDLK-LLTHS-ELVSLFGK-----------------------
---ASEDWS-----------YTTL---------ANAIRADHG------------------
-----------------FTME----SDPV-RFLLEILS--------------------NFT
DVEKRQFLQF-------TTGSPRLP------------IGG-----WK-ALRPVF--TVVC
KIPEAPLTPD------------------------DYLPSVMTCA-NY-FKMPAY-STKEI
MRERLL-TSMKE-G---
>Tortispora_caseinolytica_LSKT01000031.1 .
VRVSRNHM-LQNAVKVM----------------ESYGKGPH-VLEIEYFG-------EVG
IGLG-PTLEF---YSLI----SSEFTLKKL--------RMWRDENPSSES----------
---------RHVTAPNGLFPVPMS---------------------------------LKMT
ETESGKFLLRMFEVLGIFVARALLDSRIIDIPFNP--NFFRSVVE---------------
-----ESCSAPSMLQERQLT--------------------LDALAQIA--------PEHA
NALKRVIEL------------------------------AETEDEKTKQ-ESIA-NLY--
--LDFTL------------------------------PG--FPEYE-LIEG--------
GSDIA-------------------------------------------------------
-----------------------------VSVDNVD-LYIKR-VIEVALREG-IQRQIS
-AFCDG---FSKVFPV--QALE-SFTAE-ELVMICSN-----------------------
---REIDFS-----------MPTL---------LGEVKAEHG------------------
-----------------YTSE----SVKF-RWLLETLS--------------------QMD
TNEKRSFLQF-------MTGSPNLP------------IGG-----LS-VLTPPM--TVVC
KEPDQGYSAD------------------------DYLPSVMTCA-NY-LKLPPY-SSIEV
LKKQLK-KAMSD-GLG-
>Galactomyces_candidum_CCBN010000012.1 .
VRVSRNHL-LQSAIKVL-----------------DLYGASPY-VLEVEFFD-------DVG
TGSG-PTLEF---YASV----SKQFTRKKL--------SIWRNEDTDPKN----------
---------QFVFNKQGLYPLPYS---------------------------------PEFF
DTKAGAKVLGFFKSLGIFIARAMLDSRIIDINFNP--VFFRNSLS---------------
-----SSVGGNSIGTVSL----------------------VDKQLGQS--------LSML
QKFVELK--------------------------------KQGVVNPEVDG-AHIE-DLA--
--LDFTY------------------------------PG--QSNLL-LKEN--------
GANIN-------------------------------------------------------
-----------------------------VTLDNIE-SYIDS-VVDMTIGFG-VETQIN
-AFREG---FSQVFPY--AALH-AFNPE-ELVVMCGQ-----------------------
---GENDWS-----------YELL---------CSAAKADHG------------------
-----------------YSLD----SRIV-RELFEVMS--------------------TFT
KAEQKQFLQF-------VTGSPNLP------------IGG-----FK-ALSPPF--TIVC
RESEMPLTPD------------------------DYLPTVMTCV-NY-FKLPNY-SSKEV
LKERLK-TAISE-GSG-
>Saprochaete_clavata_CBXB010000038.1 .
VRIPREHM-FYSGIKLM----------------ELYGTSPY-ALEVEFFD-------DVG
TGLG-PTLEF---YATF----SKDFTQKSL--------NMWRNDDGDSQS----------
---------KYVFSKNGLFPLPHD---------------------------------GKFL
STKQGKHLLSLFRSLGIFVARSLIDSRIIDLNFNP--LFFRIAQF---------------
------GTSHQNIGMLSL----------------------IDEHLEKS--------MKFL
QRFVDARDK------------------------------QGSLGEIRVDN-VSID-DLS--
--LDFTL------------------------------PG--RSDIE-LIPG--------
GSSIS-------------------------------------------------------
-----------------------------VNIDNVD-EYIKL-VLDKTLGSG-IAKQIA
S-FKEG---VSTLIPY--SALH-SFTPE-ELVVLCGQ-----------------------
---EEADWT-----------YETL---------CNSIKADHG------------------
-----------------YTLD----SPVV-RNLLEVMS--------------------EFT
KEERKAFLQF-------STGSRNLP------------IGG-----FP-SLRPPF--TVVC
RDSDPPYTPN------------------------DYLPTVMTCV-NY-FKLPNY-SSKEI
LRERLL-TAMHE-GSG-
>Sugiyamaella_lignohabitans_XM_018879843.1 .
VRVSRNHI-LHSAIKVM-----------------NLCGSSPN-ILEVEFFD-------EVG
TGLG-PTLEF---YSSV----SKEFSLKKN--------KMWREGDSHKDS----------
---------DYVFGNQGLFPAPLS---------------------------------KHQL
```

```
ENTNSRKVLQLFKTLGTFIARALLDSRIIDINLNP--IFFALSRS---------------
-----NSGLKPSISMVSA----------------------VDKQLGNS--------LLMI
ERAARKQRQEKDTV------------------------STEQEAEESIQ-VLIE-DLA--
--LDFTL-------------------------------PG--YPDIN-LIED--------
GSHIP-------------------------------------------------------
------------------------------VTIDNVN-EYLDL-VVDMTIGSG-ISKQIE
Q-FQTG---FSEVFPY--SALN-AFSPQ-ELAVLCGQ-----------------------
---GEEDWS-----------YETL---------YESIKADHG------------------
----------------YTKG----SKII-RELLEVMT--------------------SFN
PQERRAFLQF-------MTGSPNLP------------IGG-----FR-ALNPVF--TVVW
KQNEEPFSRD------------------------DYLPSVMTCA-NY-LKLPNY-SSKEV
LEERLK-TAMYE-GSGA
>3_Blastobotrys_adeninivorans_CBZY010000004. 1..
VRVSRKHI-MHSAVKVM----------------DLCGSSPN-ILEVEFFD-------EVG
TGLG-PTLEF---YSSV----SREFSLKKF--------KLWREDESSANS----------
---------QYAFGSLGLFPAPMN--------------------------------EARA
QSANGKKVLQLFRTLGIFVARSMLDSRIIDLKINP--VFFRVAGS---------------
-----NVSVTSSLGTVAA----------------------VDRQLAQS--------LSLV
QKYAAG----------------------------------------EGE-TTVD-DLA--
--LDFVL-------------------------------PG--YDDIE-LIPN--------
GSKTP-------------------------------------------------------
------------------------------VTSDNVE-LYIER-VVDMTLKSG-VERQIE
-AFKEG---FSTVFPY--SALH-AFTPD-ELAILCGQ-----------------------
---GEEDWS-----------YETL---------YDAVKADHG------------------
----------------YTKD----SRVV-KELLEVMS--------------------QMN
TKQRRAFLQF-------MTGSPNLP------------IGG-----FK-ALHPEF--TVVC
KHDDDSISPD------------------------NHLPSVMTCA-NY-LKLPNY-SSRQV
LKEKLF-TAVYE-GAGA
>Yarrowia_deformans_BCIW01000014.1 .
VRISRKHM-FQSAIKVM----------------ELYGSSPS-VLEVEYFD-------EVG
TGLG-PTLEF---YATV----SHQFAHKSL--------EMWRDDTPTSGSGGSE------
---------AFAFSQQGLFPIPLI------------------------------------
TCSNPEKVLNRFKVLGTFVARSMIDSRLIDIHFST--TFFRLACL---------------
-----QLSFSPTSETLLA----------------------IDEQLGKS--------LRQL
QKFSPG---------------------------------------------EIE-HLG--
--LDFTL-------------------------------PG--FPQAE-LKPN--------
GSQIP-------------------------------------------------------
------------------------------VTGDNVQ-EYVDL-IVDQTVGSG-VSQQIE
-AFNTG---FSEVFPF--SAVC-AFSPA-ELVLMCGP-----------------------
---SVEDWS-----------LDTL---------SETVHADHG------------------
----------------FDQR----SKTF-MNLLEVMS--------------------EFD
ESQRRQFLSF-------VTGSPKLP------------IGG-----FK-ALKPGL--TVVR
KASENGLGPD------------------------DYLPSVMTCV-NY-LKCPDF-STKEL
LKSRLL-QAISE-GGGA
>Yarrowia_keelungensis_BCJD01000001.1 .
VRISRKHM-FQSAIKVM----------------ELYGSSPS-VLEVEYFD-------EVG
TGLG-PTLEF---YATV----SHQFAHKSL--------EMWRDDTPTSGSGGSE------
---------AFAFSQQGLFPIPLI------------------------------------
TCSNPEKVLNRFKVLGTFVARSMIDSRLIDIHFST--TFFRLACL---------------
-----QLSFSPTSETLVA----------------------IDEQLGKS--------LRQL
QKMSPD---------------------------------------------EVE-HLG--
--LDFTL-------------------------------PG--FAQAE-LKPN--------
GSQIS-------------------------------------------------------
------------------------------VTGDNVQ-EYIDL-IVDQTVGSG-VSQQIE
-AFKAG---FSEVFPF--SAVC-AFSPA-ELVLMCGP-----------------------
---SVEDWS-----------LDTL---------SEAVHADHG------------------
----------------FDQR----SKTF-MNLLEVMS--------------------EFD
ESQRRQFLSF-------VTGSPKLP------------IGG-----FK-ALKPGL--TVVR
```

KASENGLGPD------------------------DYLPSVMTCV-NY-LKCPDF-STKEL
LKSRLL-QAISE-GGGA
>Yarrowia_lipolytica_XM_503583.1 .
VRISRKHM-FQSAIKVM----------------ELYGSSPS-VLEVEYFD-------EVG
TGLG-PTLEF---YATV----SHQFAHKSL--------EMWRDDTPTSGAGGSE------
---------AFAFSQQGLFPIPLI------------------------------------
TCSNNEKVLHRFKVLGTFVARSMIDSRLIDIHFST--TFFRLACL---------------
-----QLSFSPTSETLLA---------------------IDEQLGKS--------LRQL
QRMSPG---EIE-HLG--
--LDFTL-------------------------------PG--FPQAE-LKPN--------
GSQIP---
------------------------------VNGDNVQ-EYVDL-IVDQTVGSG-VSQQIE
-AFKTG---FSEVFPF--SAVC-AFSPA-ELVLMCGP-----------------------
---SVEDWS-----------LDTL---------SEAVHADHG------------------
-----------------FDQR----SKTF-MNLLEVMS--------------------EFD
ESQRRQFLSF-------VTGSPKLP------------IGG-----FK-ALKPGL--TVVR
KASENGLGPD------------------------DYLPSVMTCV-NY-LKCPDF-STKEL
LRSRLL-QAISE-GGGA
>Taphrina_wiesneri_BAVU01000106.1 .
VRISRPRM-LESAMKVM----------------ELYGSSPS-ILEVEYFD-------EVG
TGLG-PTLEF---YATV----SKEISKKKL--------NLWRDFGSVSPS----------
---------DYVFSQNGLFPRPLP------------------------------AFENEQ
NSETTKRSLLLYKTIGTFVARSMIDSRLIDIPFNP--TFFRLLNN---------------
-----ANSLLLTIGAIYG---------------------IDKDLARS--------LSLV
NKFALAYKDLMSELGQTHSEF-----------------DSHATEIVFDG-VRVE-DLG--
--LDFTY-------------------------------PG--DPELE-LIAD--------
GAQTD---
------------------------------VTIFNVS-AYLEK-VVDFTIGSG-ILRQLQ
-AFEEG---FSQVFPY--TALR-AFTAQ-ELTMFFGR-----------------------
---SEEDWS-----------TATL---------LDSIKADHG------------------
-----------------YTME----SSTI-RNLVLVLS--------------------ELD
ETERREFLQF-------MTGSPKLP------------IGG-----FK-SLTPQF--TVVC
KPHEAPMTAD------------------------DYLPSVMTCV-NY-LKMPDY-SNIEV
LRSKLF-TAMRE-GGG-
>Taphrina_deformans_CAHR02000067.1 .
VRISRPRM-LESAMKVM----------------ELYGSSPS-ILEVEYFD-------EVG
TGLG-PTLEF---YSTV----SREISRKKL--------SLWRDYSSSSQT----------
---------EYVFSQNGLFPKPLP------------------------------AFENEQ
NLETTKKSLLLFKTIGTFVARSMIDSRLIDIPFNP--TFFRFLNS---------------
-----ASSLPPTIGAIYG---------------------IDKDLAKS--------LSLV
NKFALAYQDLVAKLGHAHTDL-----------------SALATNIVLDG-VRVE-DLG--
--LDFTY-------------------------------PG--SPELE-LIDN--------
GAQTD---
------------------------------VTIFNVS-TYLET-VVDFTIGSG-VLRQLQ
-AFEEG---FTQVFPY--TALR-AFTAQ-ELTMFFGR-----------------------
---SKEDWS-----------PAIL---------MDSIKADHG------------------
-----------------YTMD----SSTI-RNLVLVLS--------------------ELN
ETDRREFLQF-------MTGSPKLP------------IGG-----FK-SLTPQF--TVVC
KPHEAPMSAD------------------------DYLPSVMTCV-NY-LKMPDY-SSIEV
LRSKLF-TAMRE-GGG-
>Taphrina_flavorubra_BAVW01000086.1 .
VRISRPRM-LESAMKVM----------------ELYGSSPS-VLEVEYFD-------EVG
TGLG-PTLEF---YSVV----STEISRKKF--------NLWRDYHSSSSQ--S-------
---------EYVFSPSGLFPAPLP------------------------------NFEDNE
TSETTRKTLLLFKTIGTFVARSMIDSRLIDIPLNP--TFFRLLNN---------------
-----SNSLPPTIGAIYG---------------------IDKDLAKS--------LSLV
NKFATAYQDLVDKLGESSDEL-----------------NSCATGIKVDG-VRVE-DLG--
--LDFTY-------------------------------PG--SPEIE-LMVN--------

GAQTD---
------------------------------VTIFNVA-KYLEK-VVDLTIGSG-VLRQLQ
-AFEEG---FSQVFPY--TALR-AFTAQ-ELTMLFGR-----------------------
---SEEDWT-----------TKTL---------LESIKADHG------------------
----------------YTMD----SLTI-RNLVLVLS--------------------EMT
EVDRREFLQF-------MTGSPKLP------------IGG-----FK-SLTPQF--TVVC
KPHEAPMTAD------------------------DYLPSVMTCV-NY-LKMPDY-SGIEV
LKARLF-TAMRE-GGG-
>Taphrina_populina_BAVX01000011.1 .
VRISRERM-LESACKVM----------------LLYGSSPA-ILEVEYYD-------EVG
TGLG-PTLEF---YSTI----SREFSKSKL--------RMWRNDHAEI------------
---------EFVFSPNGLFPQPLA-------------------------VSIRGEERIAK
YVKYVQAIVHLFRILGTFVARAMIDSRLIDLAFNP--AFFRICSD---------------
-----SSQSPPTIGALWT----------------------VDKSLARS--------LTLL
QQFSDACKALQDDRNLSAEDL-----------------AEGIAAIRIDD-VRVE-DLG--
--LDFTY-------------------------------PG--KPELE-LMPN--------
GAEMD---
------------------------------VTIHNVS-EYLTR-VLDLTLGSG-ISRQVA
-AFKEG---FSMVFPF--TALR-AFTPS-ELTMFFGR-----------------------
---ANEDWS-----------FETL---------SESIKADHG------------------
----------------FTKD----SKTV-QNMILVLS--------------------SLD
DVDRRDFLQF-------ITGSPKLP------------IGG-----FK-NLTPQF--TVVR
KPYEAPLTAD------------------------DYLPSVMTCV-NY-LKLPDY-SNIDV
LRTKLF-MAVKE-GGG-
>Protomyces_lactucaedebilis_MCFI01000011.1 .
VRISRPRL-LESAIKVM----------------ELYGASPS-VLEVEYFD-------EVG
TGLG-PTLEF---YSSV----SLELSKNRL--------HLWRADDEEA------------
---------EYAFSTAGLFPAPLP----------------------------------SC
NEEKTKVTLALFKMLGTFVARSMLDSRMIDLALSP--MLFKVDFT---------------
----------PTWEDVAQ----------------------VDPALGKS--------LALL
AQFADAYQAVLDADMTDS-QR-----------------GKAVRAITFEN-CTVE-DLG--
--LDFAY-------------------------------PG--NPEVE-LMPG--------
GSQVE---
------------------------------VTIHNVA-DYVTK-VTDATLGSG-VARQIA
-AFRKG---FSAVFPY--SALR-AFTPL-ELSMLFGS-----------------------
---GEEDWS-----------AETL---------ADNIKADHG------------------
----------------YTMD----SKTI-QNLISVLS--------------------QLD
KIDRRAFLQF-------ITGSPKLP------------IGG-----FK-ALTPQF--TIVC
RPHEAPMTAD------------------------DYLPSVMTCV-NY-LKMPDY-SSREV
LQTKLV-LAMRE-GSG-
>Saitoella_complicata_XM_019166789.1 .
VRISRAHL-FESVKKVM----------------ELYGSSPS-VLEVEYFD-------EVG
TGLG-PTLEF---YTSG----SREFCKKSL--------GLWRENDSDPQS----------
---------EYAFGTNGLFPVPMS--------------------------------ESEL
SSSKGKNILAHFKILGTFVARSMLDSRMIDVSFNP--AFFRVSDE---------------
-----QGAGMVNIAAIQE----------------------VDKNLAQS--------MRLV
NKFVHVRNKVMHDQSLNAGEK-----------------RQALAAITVDQ-VSID-DLA--
--LDFTL-------------------------------PG--YPMFE-LIEN--------
GASVA---
------------------------------VDINDVG-EYVER-VVDCTVGSG-VRQQVK
-AFREG---FSAVFPY--EALK-SFSAE-ELVMLFGQ-----------------------
---TEEDWS-----------VETL---------SDSIKADHG------------------
----------------FNLD----SRTI-IDLLSVLS--------------------SFT
LSERRDFLQF-------ITGSPKLP------------IGG-----FK-ALTPPF--TIVC
KPHDPPLTAD------------------------DYLPSVMTCV-NY-LKLPDY-SNADV
LREKLM-VAMKE-GQGS
>Lipomyces_starkeyi_LSGR01000054.1 .
VRIARSQM-LQSAIKVM----------------ELYGSSPS-ILEVEYFD-------EVG

TGLG-PTLEF---YATV----SREFAKKKL--------RLWRENESDGEH----------
---------EYAFGSHGLYPLPMS--------------------------------ESKA
AGESGKKVLHLFKILGQFVARALLDSRLLDIKFNP--MFFRARQD---------------
-------SVAPSLGSVKT----------------------VDAHLARS--------LKLL
QKFSAAKNKILADGTLSDEEK-----------------DVRVENIEIDG-VKVD-DLA--
--LDFTL-------------------------------PG--QPEFE-FIEG--------
SANAP---
------------------------------VTIHNVD-QYVKK-VIDATLGSG-VEKQLN
-AFREG---FSSVFSF--AAMS-AFTPE-ELVMLCGQ-----------------------
---IDEDWS-----------LESX---------FDSVKADHG------------------
----------------YTMS----SKTV-QNLLEVMT--------------------QYD
VNQRRAFLQF-------ITGSPNLP------------IGG-----FK-SLTPTF--TIVC
KPSEPPYSPD------------------------DYLPSVMTCV-NY-LKIPDY-SSKEV
LRARVT-KAIEE-GSG-
>Pyronema_omphalodes_CATG01001464.1 .
VRISRDRL-LDSAFKVM----------------DIYGASTS-TLEIEYFD-------EVG
TGLG-PTLEF---YANV----SKAFAQKHL--------KLWRGSDARGPN----------
---------QYINEANGLFPAPMD--------------------------------EKSA
ESESGKQILKNFKVIGKFVARSMIDSRIIDIWFNP--TFFRTGTN---------------
-----DAPVPPSLGAVNS----------------------VDSDLGSR--------LKTL
RKFAAAKKEIENNPALTTFDK-----------------DQALRELRING-ERVE-DWC--
--LDFVC-------------------------------PG--YDEIE-LIPN--------
GADTS---
------------------------------VTIFNVD-EYIER-VIDMTVGSG-VKRQVE
-AFRAG---FTEVFPY--NALR-SFTPS-ELVMLFGM-----------------------
---SEEDWS-----------LENI---------TDSIKADHG------------------
----------------FNMD----SPSI-RNLLHVMS--------------------EYT
STERRQFLQF-------VTGSPKLP------------IGG-----FK-ALHPML--TVVC
KPAEPPYSSD------------------------DYLPSVMTCV-NY-LKMPDY-STIDI
LREKLA-YSMTE-GAG-
>Epichloe_typhina_AMDI01000260.1 .
VRISRLKI-LESALKVM----------------DLYGASQS-ILEVEYFE-------EVG
TGLG-PTLEF---YSTV----SKEFSKKKL--------KLWREMDSVGPD----------
---------EFVSGQTGLFPRPLN--------------------------------QEEL
SAANGERILHLFKMLGKFVARSMIDSRIIDIHLNP--IFFRIGDA---------------
----PSTGIKPSLGAVKL----------------------VDPGLARS--------LKTI
KKFALAKKEIDEDPNRTAAQK-----------------VTDTESIVIDN-VRLE-DLC--
--LDFTL-------------------------------PG--YPHIE-LEDN--------
GSHKR---
------------------------------VTIENVD-TYLEN-VIDMTLGSG-VRQQVD
-AFRTG---FSQVFPY--SALS-AFTPD-ELVSLFGR-----------------------
---VDEDWS-----------LESL---------TDSIKADHG------------------
----------------FNMD----SRSV-KNLLQTMS--------------------DFD
VPHRRDFLQF-------TTGSPKLP------------IGGG----FR-SLTPMF--TVVC
KPSEHPYTSD------------------------DYLPSVMTCV-NY-LKLPDY-TSIET
MKKQLF-KAMKE-GQG-
>Epichloe_elymi_AMDJ01000060.1 .
VRISRLKI-LESALKVM----------------DLYGASQS-ILEVEYFE-------EVG
TGLG-PTLEF---YSTV----SKEFSKKKL--------KLWREMDSVGPD----------
---------EFVSGQTGLFPRPLN--------------------------------QEEL
SAANGERILHLFKMLGKFVARSMIDSRIIDIHLNP--IFFRIGDA---------------
----PSTGIKPSLGAVKL----------------------VDPGLARS--------LKTI
KKFALAKKEIDEDPNRTAAQK-----------------VTDTESIVIDN-VRLE-DLC--
--LDFTL-------------------------------PG--YPHIE-LEDN--------
GSHKR---
------------------------------VTIENVD-TYLEN-VIDMTLGSG-VRQQVD
-AFRTG---FSQVFPY--SALS-AFTPD-ELVSLFGR-----------------------
---VDEDWS-----------LESX---------TDSIKADHG------------------

```
----------------FNMD----SRSV-KNLLQTMS--------------------DFD
VPHRRDFLQF-------TTGSPKLP------------IGG-----FR-SLTPMF--TVVC
KPSEHPYTSD------------------------DYLPSVMTCV-NY-LKLPDY-TSIET
MKKQLF-KAMKE-GQG-
>Epichloe_festucae_NDBD01003194.1 .
VRISRLKI-LESALKVM----------------DLYGASQS-ILEVEYFE-------EVG
TGLG-PTLEF---YSTV----SKEFSKKKL--------KLWRELDSVGPD----------
---------EFVSGQTGLFPRPLN--------------------------------QEEL
SAANGERILHLFKMLGKFVARSMIDSRIIDIHLNP--IFFRIGDA---------------
----PSTGIKPSLGAVKL----------------------VDPGLARS--------LKTI
KKFALAKKEIDEDPNRTAAQK-----------------VTDTESIVIDN-VRLE-DLC--
--LDFTL-------------------------------PG--YPHIE-LEDN--------
GSHKR-------------------------------------------------------
------------------------------VTIENVD-TYLEN-VIDMTLGSG-VRQQVD
-AFRTG---FSQVFPY--SALS-AFTPD-ELVSLFGR-----------------------
---VDEDWS-----------LESL---------TDSIKADHG------------------
----------------FNMD----SRSV-KNLLQTMS--------------------DFD
VPHRRDFLQF-------TTGSPKLP------------IGG-----FR-SLTPMF--TVVC
KPSEHPYTSD------------------------DYLPSVMTCV-NY-LKLPDY-TSIET
MKKQLF-KAMKE-GQG-
>Epichloe_glyceriae_AFRG01000113.1 .
VRISRLKI-LESALKVM----------------DLYGASQS-ILEVEYFE-------EVG
TGLG-PTLEF---YSTV----SKEFSKKKL--------KLWREMDSVGPD----------
---------EFVSGQTGLFPRPLN--------------------------------QEEL
SAANGERILHLFKMLGKFVARSMIDSRIIDIHLNP--IFFRIGDA---------------
----PSTGIKPSLGAVKL----------------------VDPGLARS--------LKTI
KKFALAKKEIDEDPNRTAAQK-----------------VTDTESIVIDN-VRLE-DLC--
--LDFTL-------------------------------PG--YPHIE-LEDN--------
GSHKR-------------------------------------------------------
------------------------------VTIENVD-TYLEN-VIDMTLGSG-VRQQVD
-AFRTG---FSQVFPY--SALS-AFTPD-ELVGLFGK-----------------------
---VDEDWS-----------LESX---------TDSIKADHG------------------
----------------FNMD----SRSV-KNLLQTMS--------------------DFD
VPHRRDFLQF-------TTGSPKLP------------IGG-----FR-SLTPMF--TVVC
KPSEHPYTSD------------------------DYLPSVMTCV-NY-LKLPDY-TSIET
MKKQLF-KAMKE-GQG-
>Epichloe_brachyelytri_AFRB01000294.1 .
VRISRLKI-LESALKVM----------------DLYGASQS-ILEVEYFE-------EVG
TGLG-PTLEF---YSTV----SKEFSKKKL--------KLWREMDSVRPD----------
---------EFVSGQTGLFPRPLN--------------------------------QEEL
SAANGERILHLFKMLGKFVARSMIDSRIIDIHLNP--IFFRIGDA---------------
----PSTGIKPSLGAVKL----------------------VDPGLARS--------LKTI
KKFALAKKEIDEDPNRTAAQK-----------------VTDTESIVIDN-VRLE-DLC--
--LDFTL-------------------------------PG--YPHIE-LEDN--------
GSHKR-------------------------------------------------------
------------------------------VTIENVD-TYLEN-VIDMTLGSG-VRQQVD
-AFRTG---FSQVFPY--SALS-AFTPD-ELVSLFGR-----------------------
---VDEDWS-----------LESX---------TDSIKADHG------------------
----------------FNMD----SRSV-KNLLQTMS--------------------DFD
IPHRRDFLQF-------TTGSPKLP------------IGG-----FR-SLTPMF--TVVC
KPSEHPYTSD------------------------DYLPSVMTCV-NY-LKLPDY-TSIET
MKKQLF-KAMKE-GQG-
>Epichloe_bromicola_LBNI01000067.1 .
VRISRLKI-LESALKVM----------------DLYGASQS-ILEVEYFE-------EVG
TGLG-PTLEF---YSTV----SKEFSKKKL--------KLWREMDSVGPD----------
---------EFVSGQTGLFPRPLN--------------------------------QEEL
SAANGERILHLFKMLGKFVARSMIDSRIIDIHLNP--IFFRIGDA---------------
----PSTGIKPSLGAVKL----------------------VDPGLARS--------LKTI
```

KKFALAKKEIDEDPNRTAAQK-----------------VTDTESIVIDN-VRLE-DLC--
--LDFTL-------------------------------PG--YPHIE-LEDN--------
GSHKR---
------------------------------VTIENVD-TYLEN-VIDMTLGSG-VRQQVD
-AFRTG---FSQVFPY--SALS-AFTPD-ELVSLFGR-----------------------
---VDEDWS------------XAL---------TDSIKADHG------------------
----------------FNMD----SRSV-KNLLQTMS--------------------DFD
LPHRRDFLQF-------TTGSPKLP------------IGG-----FR-SLTPMF--TVVC
KPSEHPYTSD------------------------DYLPSVMTCV-NY-LKLPDY-TSIET
MKKQLF-KAMKE-GQG-
>Epichloe_uncinata_LELE01000466.1 .
VRISRLKI-LESALKVM----------------DLYGASQS-ILEVEYFE-------EVG
TGLG-PTLEF---YSTI----SKEFSKKKL--------KLWREMDSVGPD----------
---------EFVSGQTGLFPRPLN--------------------------------QEEL
SAANGERILHLFKMLGKFVARSMIDSRIIDIHLNP--IFFRIGDA---------------
----PSTGIKPSLGAVKL----------------------VDPGLARS--------LKTI
KKFALAKKEIDEDPNRTAAQK-----------------VTDTESIVIDN-VRLE-DLC--
--LDFTL-------------------------------PG--YPHIE-LEDN--------
GSHKR---
------------------------------VTIENVD-TYLEN-VIDMTLGSG-VRQQVD
-AFRTG---FSQVFPY--SALS-AFTPD-ELVSLFGR-----------------------
---VDEDWS-------------XL---------TDSIKADHG------------------
----------------FNMD----TRSV-KNLLQTMS--------------------DFD
LPHRRDFLQF-------TTGSPKLP------------IGG-----FR-SLTPMF--TVVC
KPSEHPYTSD------------------------DYLPSVMTCV-NY-LKLPDY-TSIET
MKKQLF-KAMKE-GQG-
>Epichloe_sylvatica_LCTT01000989.1 .
VRISRLKI-LESALKVM----------------ELYGASQS-ILEVEYFE-------EVG
TGLG-PTLEF---YSTV----SKEFSKKKL--------KLWREMDSVGPD----------
---------EFVSGQTGLFPRPLN--------------------------------QEEL
SAANGERILHLFKMLGKFVARSMIDSRIIDIHLNP--IFFRIGDA---------------
----PSTGIKPSLGAVKL----------------------VDPGLARS--------LKTI
KKFALAKKEIDEDPNRTAAQK-----------------VTDTESIVIDN-VRLE-DLC--
--LDFTL-------------------------------PG--YPHIE-LEDN--------
GSHKR---
------------------------------VTIENVD-TYLEN-VIDMTLGSG-VRQQVD
-AFRTG---FSQVFPY--SALS-AFTPD-ELVSLFGR-----------------------
---VDEDWS-----------LESX---------SSTIKADHG------------------
----------------FNMD----SRSV-KNLLQTMS--------------------DFD
VPHRRDFLQF-------TTGSPKLP------------IGG-----FR-SLTPMF--TVVC
KPSEHPYTSD------------------------DYLPSVMTCV-NY-LKLPDY-TSIET
MKKQLF-KAMKE-GQG-
>Periglandula_ipomoeae_AFRD01000044.1 .
VRISRMKI-LESALKVM----------------DLYGASQS-ILEVEYFE-------EVG
TGLG-PTLEF---YSTV----SKEFSKKKL--------KLWREMDSVGQD----------
---------EYVSGQTGLFPRPLN--------------------------------QEEL
STANGERILHLFKMLGKFVARSMIDSRIIDIHFNP--IFFCIGDA---------------
----PSTGIKPSLGAVKI----------------------VDPGLARS--------LKTI
KKFALAKKEIDEDPNRSAAQK-----------------VTDTESIVIDN-VRLE-DLC--
--LDFTL-------------------------------PG--YPHIE-LQDN--------
GSHKR---
------------------------------VTIENVD-TYLEK-VIDMTLGSG-VRQQVD
-AFRSG---FSQVFPY--SALS-AFTPD-ELVSLFGK-----------------------
---VDEDWS-----------LESX----------PSIKADHG------------------
----------------FNMD----SRSV-KNLLQTMS--------------------EFD
VAHRRDFLQF-------TTGSPKLP------------IGG-----FR-SLTPMF--TVVC
KPSEHPYTSD------------------------DYLPSVMTCV-NY-LKLPDY-TSIDT
MKKQLF-KAMKE-GQG-

>Atkinsonella_texensis_LBND01000703.1 .
VRISRLKI-LESALKVM----------------DLYGASQS-ILEVEYFE-------EVG
TGLG-PTLEF---YSTV----SKEFSKKKL--------KLWREMDSVSQD----------
---------EYVNGRTGLFPRPLN--------------------------------QEEL
SAANGERILHLFRMLGKFVARSMIDSRIIDIHFNP--IFFRIGDA---------------
----PSTGIKPSLGAVKI----------------------VDPGLARS--------LKTI
KKFVLAKKEIDEDPNRSAAQK-----------------VTDTESIVIDN-VRLE-DLC--
--LDFTL-------------------------------PG--YPHIE-LEDN--------
GSHKR---
------------------------------VTIENVE-AYLEK-VVDMTLGSG-VRQQMD
-AFRTG---FSQVFPY--SALS-AFTPD-ELVSLFGK-----------------------
---VDEDWS-----------LESL---------TDSIKADHG------------------
----------------FNMD----SRSV-KNLLHTMS--------------------EFD
MAHRRDFLQF-------TTGSPKLP------------IGG-----FR-SLTPMF--TVVC
KPSEHPYTSD------------------------DYLPSVMTCV-NY-LKLPDY-TSIET
MKRQLF-KAMKE-GQG-
>Balansia_obtecta_JFZS01000040.1 .
VRISRLKI-LESALKVM----------------DLYGASQS-ILEVEYFE-------EVG
TGLG-PTLEF---YSTV----SKEFSKKKL--------KLWREMDSVSQD----------
---------EYVSGRTGLFPRPLN--------------------------------QEEL
SAANGERILHLFRMLGKFVARSMIDSRIIDIHFNP--IFFRIGDA---------------
----PSSGIKPSLGAVKI----------------------VDPGLAQS--------LKTI
KKFVLAKKEIDEDPNRSAAQK-----------------VTDTESIVIDN-VRLE-DLC--
--LDFTL-------------------------------PG--YPHIE-LEDN--------
GSHKR---
------------------------------VTIENVE-AYLEK-VIDMTLGSG-VRQQVD
-AFRTG---FSQVFPY--SALS-AFTPD-ELVSLFGK-----------------------
---VDEDWS-----------LESL---------TDSIKADHG------------------
----------------FNMD----SRSV-KNLLQTMS--------------------EFD
IAHRRDFLQF-------TTGSPKLP------------IGG-----FR-SLTPMF--TVVC
KPSEHPYTSD------------------------DYLPSVMTCV-NY-LKLPDY-TSIET
MKRQLF-KAMKE-GQG-
>Aciculosporium_take_AFQZ01000215.1 .
VRISRQKI-LESALKVM----------------DLYGASQS-ILEVEYFE-------EVG
TGLG-PTLEF---YSTV----SKEFSKKKL--------KLWREMDSVGQA----------
---------EFVNGQFGLFPRPLN--------------------------------QEEL
SAANGERILHLFRMLGKFVARSMIDSRIIDIHFNA--IFFRIEDG---------------
----PSTGIKPSLGAVKI----------------------VDPGLARS--------LKTI
KKFALAKKEIDEDPNRTAAQK-----------------VKDAESIVIDN-VRLE-DLC--
--LDFTL-------------------------------PG--YPHIE-LEDN--------
GSHKR---
------------------------------VNIENVD-MYLEK-VIDMTLGSG-VRQQVD
-AFRGG---FSQVFPY--SALS-AFTPN-ELVSLFGR-----------------------
---VEEDWS------------XAL---------TDSIKADHG------------------
----------------FNMD----SRSV-KNLLQTMS--------------------EFD
ASHRRDFLQF-------TTGSPKLP------------IGG-----FR-SLTPMF--TVVC
KPSEHPYTSD------------------------DYLPSVMTCV-NY-LKLPDY-TSIET
MKKQLL-KAMKE-GQG-
>Claviceps_paspali_AFRC01000359.1 .
VRISRQKI-LESALKVM----------------DLYGASQS-ILEVEYFE-------EVG
TGLG-PTLEF---YSTV----SKEFSKKKL--------RLWREMDSVGHA----------
---------EFVSGQFGLFPRPLN--------------------------------HDEL
SAANGERILHLFRMLGKFVARSMIDSRIIDIHFNP--IFFRIGDA---------------
----PSTGIKPSLGAVKI----------------------VDPGLARS--------LKTI
KKFALAKKEIDEDPNRTAARK-----------------VADTEAIVIDN-VRLE-DLC--
--LDFTL-------------------------------PG--YPHIE-LEDN--------
GSHKR---
------------------------------VNIENVE-TYLEK-VIDMTLGSG-VRQQVE

-AFRAG---FSQVFPY--SALS-AFTPD-ELVSLFGR-----------------------
---VDEDWS-----------LESL---------TDSIKADHG------------------
----------------FNMD----SRSV-KNLLQTMS--------------------EFD
ITHRRDFLQF-------TTGSPKLP------------IGG-----FR-SLTPMF--TVVC
KPSEHPYTSD------------------------DYLPSVMTCV-NY-LKLPDY-TSIEI
MRKQLF-KAMKE-GQG-
>Claviceps_purpurea_CAGA01000021.1 .
VRISRQKI-LESALKVM----------------DLYGASQS-ILEVEYFE-------EVG
TGLG-PTLEF---YSTV----SKEFSKKKL--------KLWREMDSVGQA----------
---------EFVSGQFGLFPRPLN---------------------------------QEEL
SAANGERILHLFKMLGKFVARSMIDSRIIDIHFNP--IFFRIGDA---------------
----PSTGIKPSLGAVKV----------------------VDPGLARS--------LKAI
KKFALAKKEIDEDPNRSAAQK-----------------VADAESIVIDN-VRLE-DLC--
--LDFTL-------------------------------PG--YPHIE-LEDN--------
GSQKR---
------------------------------VNIENVD-RYLEK-VIDMTLGTG-VRQQVE
-AFRTG---FSQVFPY--SALS-AFTPD-ELVSLFGR-----------------------
---VDEDWS-----------LEAL---------TDSIKADHG------------------
----------------FNMD----SRSV-KNLLQTMS--------------------QFS
LPHRREFLQF-------TTGSPKLP------------IGG-----FR-SLTPMF--TVVC
KPSEHPYTSD------------------------DYLPSVMTCV-NY-LKLPDY-TSIET
MKTQLF-KAMKE-GQG-
>Claviceps_fusiformis_AFRA01000286.1 .
VRISRQKI-LESALKVM----------------DLYGAAQS-ILEVEYFE-------EVG
TGLG-PTLEF---YSTV----SKEFSKKKL--------KLWREMDSVGHA----------
---------EFVSGQFGLFPRPLN---------------------------------QEEL
SAPNGERILHLFKMLGKFVARSMIDSRIIDIHFNP--IFFRIGDA---------------
----PSTGIKPSLGAVKI----------------------VDPGLARS--------LKTI
KKFALAKKEIDEDPNRTAARK-----------------VLDTESIVIDN-VRLE-DLC--
--LDFTL-------------------------------PG--YPHIE-LVEN--------
GSHKR---
------------------------------VNIENVD-LYLEK-VIDMTLGSG-VRKQVD
-AFRTG---FSQVFPY--SALS-AFTPD-ELVSLFGR-----------------------
---VDEDWS-----------XTAL---------TDSIKADHG------------------
----------------FNMD----SRSV-KNLLQTMS--------------------QFS
ISNRRDFLQF-------TTGSPKLP------------IGG-----FR-SLTPMF--TVVC
KPSEHPYTSD------------------------DYLPSVMTCV-NY-LKLPDY-TSIET
MKKQLF-KAMKE-GQG-
>Ustilaginoidea_virens_JHTR01000024.1 .
VRISRLKI-LESALKVM----------------DLYGASQS-IIEVEYFE-------EVG
TGLG-PTLEF---YSTV----SKEFSRKKL--------KLWREMDSAGSN----------
---------EFVNGQNGLFPRPLS---------------------------------QEEL
STPNGERILHLFKMLGKFVARSMIDSRIIDIHLNP--IFFRIGDA---------------
----PTTGIKPSLGAVKV----------------------VDPGLARS--------LKTI
KKFALAKREIDEDPNRTAAQK-----------------VLDTESIVIDN-VRLD-DLC--
--LDFTL-------------------------------PG--YPNIE-LEDN--------
GSHKR---
------------------------------VTIENVD-AYLEK-VIDTTLGSG-VRHQVD
-AFRAG---FSQVFPY--SALS-AFTPD-ELVRLFGK-----------------------
---VEEDWS-----------TLAL---------TDSIKADHG------------------
----------------FNMD----SRTV-KNLLQTMS--------------------EFD
TQHRRDFLQF-------TTGSPKLP------------IGG-----FR-SLTPMF--TVVC
KPSEHPYTSD------------------------DYLPSVMTCV-NY-LKLPDY-TTTET
MKKQLF-KAMKE-GQG-
>Aschersonia_aleyrodis_AZGY01000007.1 .
VRISRTKI-LESALKVM----------------DLYGASQS-ILEVEYFE-------EVG
TGLG-PTLEF---YSTV----SKEFSKKKL--------KLWREMDSVGSE----------
---------EFVSGQNGLFPRPLN---------------------------------QEEL

SAPNGERILHLFKMLGKFVARSMIDSRIIDIHFNP--IIFRIGDA---------------
----PTTGIKPSLGAVKI----------------------VDPGLARS--------LKTI
KKFALAKKEIDEDPSRTPAQK-----------------VADTEAIIIDN-VRLD-DLC--
--LDFTL--------------------------------PG--YPNIE-LEDN--------
GSQKR---
------------------------------VTIENVD-TYLDK-VIDMTLGSG-VRQQVD
-AFRTG---FSQVFPY--SALS-AFTPD-ELVSLFGK-----------------------
---VDEDWS-----------LESL---------TDSIKADHG------------------
----------------FNMD----SRSV-KNLLHTMS--------------------DFD
AQQRRDFLQF-------TTGSPKLP------------IGG-----FR-SLTPMF--TVVC
KPSEHPFTSD------------------------DYLPSVMTCV-NY-LKLPDY-SSIAT
MKNQLF-KAMKE-GQG-
>Pochonia_chlamydosporia_XM_018281494.1 .
VRISRLKI-LESALKVM----------------DLYGAAQS-ILEVEYFE-------EVG
TGLG-PTLEF---YSTV----SKEFSKKKL--------KLWREMDSVGTD----------
---------EFVTSQNGLFPRPLN---------------------------------PEEL
STPNGERILHMFKMLGKFVARSMIDSRIIDIHFNP--IFFRIGDA---------------
----PMTGIRPSLGAVKI----------------------VDPGLARS--------LKTI
KKFALAKKEIDEDPNRTAAQK-----------------VADTESIVIDS-VRLD-DLC--
--LDFTL--------------------------------PG--YPNIE-LEDH--------
GSHKR---
------------------------------VTIENVD-SYLEK-VIDMTLGSG-VRQQVD
-AFRTG---FSQVFPY--SALS-AFTPD-ELVSLFGK-----------------------
---VEEDWS-----------LETL---------TDSIKADHG------------------
----------------FNMD----SRSV-KNLLQTMS--------------------EFD
VQQRRDFLQF-------TTGSPKLP------------IGG-----FR-SLTPMF--TVVC
KPSEHPYTSD------------------------DYLPSVMTCV-NY-LKLPDY-TTIET
MKKQLY-KAMKE-GQGA
>Metarhizium_rileyi_AZHC01000001.1 .
VRISRLKI-LESALKVM----------------DLYGASQS-ILEVEYFE-------EVG
TGLG-PTLEF---YSTV----SKEFSKKKL--------KLWREMDSVGPD----------
---------EFVTGQNGLFPRPLN---------------------------------QEEL
SAPNGERILHLFKMLGKFVARSMIDSRIIDLHFNP--IFFRIGDA---------------
----PTTGIRPSLGAVKV----------------------VDPGLARS--------LKTI
KRFALAKKEIDEDPNRTAAQK-----------------VADTESIVIDK-VRLD-DLC--
--LDFTL--------------------------------PG--YPNIE-LEDH--------
GSHKR---
------------------------------VTIENVD-AYLEK-VVDMTLGSG-VRQQID
-AFRSG---FSQVFPY--SALS-AFTPD-ELVSLFGK-----------------------
---VEEDWS-----------LESL---------TDSIKADHG------------------
----------------FNMD----SRSV-KNLLQSMS--------------------EFD
VQQRRDFLQF-------TTGSPKLP------------IGG-----FR-SLTPMF--TVVC
KPSEHPYTSD------------------------DYLPSVMTCV-NY-LKLPDY-TTIGT
MKNQLY-KAMRE-GQG-
>Metarhizium_album_AZHE01000005.1 .
VRISRLKI-LESALKVM----------------DLYGASQS-ILEVEYFE-------EVG
TGLG-PTLEF---YSTV----SKEFSKKKL--------KLWREMDSVGSE----------
---------EFVTGQTGLFPRPLN---------------------------------QEEL
SAPNGERILHLFKMLGKFVARSMIDSRIIDIHFNP--IFFRIGDA---------------
----PSTGIKPSLGAVKI----------------------VDPGLARS--------LKTI
KKFALAKKEIDEDPNRTPAQK-----------------VADTESIVIDN-VRLD-DLC--
--LDFTL--------------------------------PG--YPNIE-LEDH--------
GSHKR---
------------------------------VTIENVD-IYLEK-VIDVTLGSG-VRQQVD
-AFRSG---FSQVFPY--SALS-AFTPD-ELVSLFGK-----------------------
---VEEDWS-----------LESL---------TDSIKADHG------------------
----------------FNMD----SRSV-KNLLQSMS--------------------EFD
AQQRRDFLQF-------TTGSPKLP------------IGG-----FR-SLTPMF--TVVC

KPSEEPYTSD------------------------DYLPSVMTCV-NY-LKLPDY-TTIET
MKKQLY-KAMKE-GQG-
>Metarhizium_acridum_XM_007811540.1 .
VRISRLKI-LESALKVM----------------DLYGASQS-ILEVEYFE-------EVG
TGLG-PTLEF---YSTV----SKEFSKKKL--------KLWREMDSVGSD----------
---------EFVTGQTGLFPRPLN--------------------------------QEEL
SAPNGERILHLFKMLGKFVARSMIDSRIIDIHFNP--IFFRIGDA---------------
----PSTGIKPSLGAVKI----------------------VDPGLARS--------LKTI
KKFALAKKEIDEDPNRTAAQK-----------------VADTESIVIDN-VRLD-DLC--
--LDFTL-------------------------------PG--YPNIE-LEDH--------
GSHKR---
------------------------------VTIENVD-IYLEK-VIDMTLGSG-VRQQVD
-AFRSG---FSQVFPY--SALS-AFTPD-ELVSLFGK-----------------------
---VEEDWS-----------LETL---------TDSIKADHG------------------
----------------FNMD----SRSV-KNLLQSMS--------------------EFD
VQQRRDFLQF-------TTGSPKLP------------IGG-----FR-SLTPMF--TVVC
KPSEDPYTSD------------------------DYLPSVMTCV-NY-LKLPDY-TTIET
MKKQLY-KAMKE-GQGA
>Metarhizium_robertsii_ADNJ02000001.1 .
VRISRLKI-LESALKVM----------------DLYGASQS-ILEVEYFE-------EVG
TGLG-PTLEF---YSTV----SKEFSKKKL--------KLWREMDSVGSD----------
---------EFVTGQTGLFPRPLN--------------------------------QEEL
SAPNGERILHLFKMLGKFVARSMIDSRIIDIHFNP--IFFRIGDA---------------
----PSTGIKPSLGAVKI----------------------VDPGLARS--------LKTI
KKFALAKKEIDEDPNRTAAQK-----------------VADTESIVIDN-VRLD-DLC--
--LDFTL-------------------------------PG--YPNIE-LEDH--------
GSHKR---
------------------------------VTIENVD-IYLEK-VIDMTLGSG-VRQQVD
-AFRSG---FSQVFPY--SALS-AFTPD-ELVSLFGK-----------------------
---VEEDWS-----------LESM---------LSSIKADHG------------------
----------------FNMD----SRSV-KNLLQSMS--------------------EFD
VQQRRDFLQF-------TTGSPKLP------------IGG-----FR-SLTPMF--TVVC
KPSEDPYTSD------------------------DYLPSVMTCV-NY-LKLPDY-TTIQT
MKKQLY-KAMKE-GQG-
>Metarhizium_brunneum_XM_014691533.1 .
VRISRLKI-LESALKVM----------------DLYGASQS-ILEVEYFE-------EVG
TGLG-PTLEF---YSTV----SKEFSKKKL--------KLWREMDSVGSD----------
---------EFVTGQSGLFPRPLN--------------------------------QEEL
SAPNGERILHLFRMLGKFVARSMIDSRIIDIHFNP--IFFRIGDA---------------
----PSTGIKPSLGAVKI----------------------VDPGLARS--------LKTI
KKFALAKKEIDEDPNRTAAQK-----------------VADTESIVIDN-VRLD-DLC--
--LDFTL-------------------------------PG--YPNID-LEDH--------
GSHKR---
------------------------------VTIENVD-IYLEK-VIDMTLGSG-VRQQVD
-AFRSG---FSQVFPY--SALS-AFTPD-ELVSLFGK-----------------------
---VEEDWS-----------LETL---------TDSIKADHG------------------
----------------FNMD----SRSV-KNLLQSMS--------------------EFD
VQQRRDFLQF-------TTGSPKLP------------IGG-----FR-SLTPMF--TVVC
KPSEDPYTSD------------------------DYLPSVMTCV-NY-LKLPDY-TTIQT
MKKQLY-KAMKE-GQGA
>Metarhizium_majus_XM_014727329.1 .
VRISRLKI-LESALKVM----------------DLYGASQS-ILEVEYFE-------EVG
TGLG-PTLEF---YSTV----SKEFSKKKL--------KLWREMDSVGSD----------
---------EFVTGQTGLFPRPLN--------------------------------QEEL
SAPNGERILHLFKMLGKFVARSMIDSRIIDIHFNP--IFFRIGDA---------------
----PSTGIKPSLGAVKI----------------------VDPGLARS--------LKTI
KKFALAKKEIDEDPNRTAAQK-----------------VADTESIVIDN-VRLD-DLC--
--LDFTL-------------------------------PG--YPNIE-LEDH--------

GSHKR---
------------------------------VTIENVD-IYLEK-VIDMTLGSG-VRQQVD
-AFRSG---FSQVFPY--SALS-AFTPD-ELVSLFGK-----------------------
---VEEDWS-----------LETL---------TDSIKADHG------------------
----------------FNMD----SRSV-KNLLQSMS--------------------EFD
VQQRRDFLQF-------TTGSPKLP------------IGG-----FR-SLTPMF--TVVC
KPSEDPYTSD------------------------DYLPSVMTCV-NY-LKLPDY-TTIQT
MKKQLY-KAMKE-GQGA
>Metarhizium_anisopliae_AZNF01000004.1 .
VRISRLKI-LESALKVM----------------DLYGASQS-ILEVEYFE-------EVG
TGLG-PTLEF---YSTV----SKEFSKKKL--------KLWREMDSVGSD----------
---------EFVTGQTGLFPRPLN--------------------------------QEEL
SAPNGERILHLFKMLGKFVARSMIDSRIIDIHFNP--IFFRIGDA---------------
----PSTGIKPSLGAVKI----------------------VDPGLARS--------LKTI
KKFALAKKEIDEDPNRTAAQK-----------------VADTESIVIDN-VRLD-DLC--
--LDFTL-------------------------------PG--YPNIE-LEDH--------
GSHKR---
------------------------------VTIENVD-IYLEK-VIDMTLGSG-VRQQVD
-AFRSG---FSQVFPY--SALS-AFTPD-ELVSLFGK-----------------------
---VEEDWS-----------LSAL---------TDSIKADHG------------------
----------------FNMD----SRSV-KNLLQSMS--------------------EFD
VQQRRDFLQF-------TTGSPKLP------------IGG-----FR-SLTPMF--TVVC
KPSEDPYTSD------------------------DYLPSVMTCV-NY-LKLPDY-TTIQT
MKKQLY-KAMKE-GQG-
>Metarhizium_guizhouense_AZNH01000001.1 .
VRISRLKI-LESALKVM----------------DLYGASQS-ILEVEYFE-------EVG
TGLG-PTLEF---YSTV----SKEFSKKKL--------KLWREMDSVGSD----------
---------EFVTGQTGLFPRPLN--------------------------------QEEL
SAPNGERILHLFKMLGKFVARSMIDSRIIDIHFNP--IFFRIGDA---------------
----PSTGIKPSLGAVKI----------------------VDPGLARS--------LKTI
KKFALAKKEIDEDPNRTAAQK-----------------VADTESIVIDN-VRLD-DLC--
--LDFTL-------------------------------PG--YPNIE-LEDH--------
GSHKR---
------------------------------VTIENVD-IYLEK-VIDMTLGSG-VRQQVD
-AFRSG---FSQVFPY--SALS-AFTPD-ELVSLFGK-----------------------
---VEEDWS-----------LSAL---------TDSIKADHG------------------
----------------FNMD----SRSV-KNLLQSMS--------------------EFD
VQQRRDFLQF-------TTGSPKLP------------IGG-----FR-SLTPMF--TVVC
KPSEDPYTSD------------------------DYLPSVMTCV-NY-LKLPDY-TTIQT
MKKQLY-KAMKE-GQG-
>Drechmeria_coniospora_LAYC01000001.1 .
VRISRLKM-LESALKVM----------------DLYGASQS-VLEVEYFE-------EVG
TGLG-PTLEF---YATV----SKEFSKKKL--------KLWREVDSLGND----------
---------EFVSGQSGLFPRPLS--------------------------------QEAL
ATANGERILQMFKMLGKFVARSMIDSRIIDIHFNP--IFFRIGDT---------------
----PSTGVKPSLGAVKM----------------------VDPGLARS--------LKVI
KKFALAKKEIDEDANRTAVQK-----------------VADTADLVVDN-VRLE-DLC--
--LDFTL-------------------------------PG--YPNIE-LEVN--------
GSHKR---
------------------------------LTMENVD-LYLER-VIDLTLGSG-VRQQVD
-AFRAG---FSTVFPY--SALS-AFTPD-ELVTLFGR-----------------------
---VDEDWS-----------XTAL---------TDSIKADHG------------------
----------------YNMD----SQSV-KNLLQTMA--------------------EFD
VQQRREFLQF-------TTGSPKLP------------IGGG----FR-SLTPMF--TVVC
KPSEHPYMSD------------------------DYLPSVMTCV-NY-LKLPDY-STVSS
MKKQLF-TAMKE-GQG-
>Purpureocillium_lilacinum_XM_018321216.1 .
VRISRLKI-LESALKVM----------------DLYGASQS-ILEVEYFE-------EVG

TGLG-PTLEF---YSTV----SKEFSKKKL--------KLWREVDSIGSD----------
---------EFVSGPVGLFPRPLS---------------------------------QEEL
SAPNGERILHLFKMLGKFVARSMIDSRIVDIHFNP--IFFRIGDG---------------
----PSTGVKPSLGAVKL----------------------VDPGLARS--------LKVI
KKFALAKKEIDEDAKRSPAQK------------------VADTTDLVVDN-VRLE-DLC--
--LDFTL---------------------------------PG--YPNIE-LEDN--------
GSHKR---
------------------------------VTMENVE-TYLDK-VIDMTLGTG-VRQQVD
-AFRSG---FSQVFPY--SALS-AFTPD-ELVSLFGK-----------------------
---VDEDWS-----------LETL---------TDSIKADHG------------------
----------------YNMD----SRSV-KNLLQTMA--------------------SFD
TPQRRDFLQF-------TTGSPKLP------------IGG-----FR-SLTPMF--TVVC
KPSEHPYTSD------------------------DYLPSVMTCV-NY-LKLPDY-TTVEI
MRKQLS-TAMKE-GQGA
>Tolypocladium_ophioglossoides_LFRF0100002 .
VRISRLKI-LESALKVM-----------------DLYGASQS-ILEVEYFE-------EVG
TGLG-PTLEF---YSTV----SKEFSKKKL--------KLWREVDSVGSD----------
---------EFVFGQTGLFPRPLS---------------------------------QEEL
SAPNGERILHLFRMLGKFVARSMIDSRIIDIHLNP--IFFRIGDG---------------
----PSTGVKPSLGAVKI----------------------VDPGLARS--------LKAI
KKFSLAKKEIDEDPNRTPAQK------------------VADTTDLVVDG-VKLE-DLC--
--LDFTL---------------------------------PG--FPNIE-LEVN--------
GSHNR---
------------------------------VTIENVD-SYLDK-VIDMTLGSG-VRQQVD
-AFRTG---FSKVFPY--SALS-AFTPD-ELVSLFGK-----------------------
---VDEDWS-----------LESL---------TDSIKADHG------------------
----------------YNMD----SRSV-KNLLQTMA--------------------EFD
TQQRRDFLQF-------TTGSPKLP------------IGG-----FR-SLTPMF--TVVC
KPSEHPYTSD------------------------DYLPSVMTCV-NY-LKLPDY-TTIDS
MRKQLF-TAIKE-GQG-
>Tolypocladium_sp._JPIJ02000026.1 .
VRISRLKI-LESALKVM-----------------DLYGASQS-ILEVEYFE-------EVG
TGLG-PTLEF---YSTV----SREFSKKKL--------KLWREVDSVGSD----------
---------EFVFGQTGLFPRPLS---------------------------------PEEL
SAPNGERILHLFRMLGKFVARSMIDSRIIDIHLNP--IFFRIGDG---------------
----PSTGVKPSLGAVKI----------------------VDPGLARS--------LKAI
KKFSLAKKEIDEDPNRSPAQK------------------VADTTDLAVDG-VKLE-DLC--
--LDFTL---------------------------------PG--FPNIE-LEVN--------
GSHKR---
------------------------------VTIENVD-SYLDG-VIDMTLGSG-VRQQVD
-AFRTG---FSKVFPY--SALS-AFTPD-ELVSLFGK-----------------------
---VDEDWS-----------LESL---------TDSIKADHG------------------
----------------YNMD----SRSV-KNLLQTMA--------------------EFD
TQQRRDFLQF-------TTGSPKLP------------IGG-----FR-SLTPMF--TVVC
KPSEHPYTSD------------------------DYLPSVMTCV-NY-LKLPDY-TSIDS
MRKRLF-TAIKE-GQG-
>2_Albophoma_yamanashiensis_BCKH01000007.1 .
VRISRLKI-LESALKVM-----------------DLYGASQS-ILEVEYFE-------EVG
TGLG-PTLEF---YSTV----SKEFSKRKL--------RLWREVDSAGPD----------
---------EFVFGQTGLFPRPLS---------------------------------QEEL
STPNGERILHLFRMLGKFVARSMIDSRIIDIHLNP--IFFRIGDG---------------
----PSTGVKPSLGAIKM----------------------VDPVLARS--------LKAI
KKFSLAKKEIDEDPNRSPAQK------------------VADTTDIAVDG-VKLE-DLC--
--LDFTL---------------------------------PG--FPNIE-LEVN--------
GSHKR---
------------------------------VTIENVD-SYLDA-VIDMTLGSG-VRQQVD
-AFRAG---FSKVFPY--SALS-AFTPD-ELVSLFGK-----------------------
---VDEDWS-----------LTAL---------TDSIKADHG------------------

----------------YNMD----SRSV-KNLLQTMA--------------------EFD
AQQRRDFLQF-------TTGSPKLP------------IGG-----FR-SLTPMF--TVVC
KPSEHPYTSD------------------------DYLPSVMTCV-NY-LKLPDY-TSIET
MKKRLY-TAIKE-GQG-
>Nilaparvata_lugens_JRMI01001108.1 .
VRISRNKI-LESALKVM----------------DLYGASQS-ILEVEYFE-------EVG
TGLG-PTLEF---YSTV----SKEFSKKKL--------KLWREMDCVGHE----------
---------EFISGQTGLFPRPLS--------------------------------QEEV
TTPNGERILHLFKMLGKFVARSMIDSRIIDIHFNP--IFFRIGDG---------------
----PSTGVKPSLGAVRI----------------------VDPGLARS--------LKVI
KKFSLAKKEIDEDPKRTPAQK-----------------VADTTGLVVDQ-VRLE-DLC--
--LDFTL-------------------------------PG--YPHIE-LEAN--------
GSHKR---
------------------------------VTIENVD-AYLDK-VIDTTLGAG-VRQQVE
-AFRCG---FSTVFPY--SALS-AFTPD-ELVSLFGK-----------------------
---VEEDWS-----------LESL---------TDSIKADHG------------------
----------------YNMD----SRSV-KHLLQTMS--------------------EFS
AQQRRDFLQF-------TTGSPKLP------------IGG-----FR-SLTPMF--TVVC
KPSEAPYTSD------------------------DYLPSVMTCV-NY-LKLPDY-TTVHM
MKKQLF-MAMKE-GQG-
>Cerataphis_brasiliensis_AOFP01003619.1 .
VRISRNKI-LESALKVM----------------DLYGASQS-ILEVEYFE-------EVG
TGLG-PTLEF---YSTV----SKEFSKKKL--------KLWREMDCMGHE----------
---------EFISGQTGLFPRPLS--------------------------------QEEV
TTPNGERILHLFKMLGKFVARSMIDSRIIDLHFNP--IFFRIGDS---------------
----PSTGVKPSLGAVRM----------------------VDPGLARS--------LKAI
KKFSLAKKEIDEDPKRTPAQK-----------------VADTTGLVVDQ-VRLE-DLC--
--LDFTL-------------------------------PG--YPHIE-LEAN--------
GSYKR---
------------------------------VTIENVD-AYLDK-VIDTTLESG-VRQQVD
-AFRCG---FSTVFPY--SALS-AFTPD-ELVSLFGQ-----------------------
---VEEDWS-----------LESL---------TDSIKADHG------------------
----------------YNMD----SRSV-KHLLQTMS--------------------EFD
AQQRRDFLQF-------TTGSPKLP------------IGG-----FR-SLTPMF--TVVC
KPSEAPYTSD------------------------DYLPSVMTCV-NY-LKLPDY-TTVHM
MKKQLF-MAMKE-GQG-
>Ophiocord_sinensis_GAGW01007013.1 .
VRISRQKI-LESALKVM----------------DLYGASQS-ILEVEYFE-------EVG
TGLG-PTLEF---YSTV----SQEFSKKKL--------KLWREVDSMGTD----------
---------EFVFGQTGLFPRPLG--------------------------------QEEV
GSPNGERILHLFKMLGKFVARSMIDSRIIDIHLNP--TFFRIGDG---------------
----PSTGVKPSLGAVKM----------------------VDPGLARS--------LKVI
KKFSLAKKEIDEDPSRSPAQK-----------------VADTTDLVVDN-VRLE-DLC--
--LDFTL-------------------------------PG--YPSIE-LGVN--------
GSHKR---
------------------------------VTIENVD-SYLDD-VIDMTLGSG-VRHQVD
-AFRAG---FSQVFPY--SALS-AFTPD-ELVSLFGK-----------------------
---VNEDWS-----------LETL---------TDSIKADHG------------------
----------------YNMD----SRSV-RNLLQSMS--------------------DFD
ALQRREFLQF-------TTGSPKLP------------IGG-----FR-SLTPMF--TVVC
KPSEAPYMSD------------------------DYLPSVMTCV-NY-LKLPDY-TTAGI
MKKQLF-TAMKE-GQG-
>Hirsutella_thompsonii_APKB01000076.1 .
VRISRLKI-LESALKVM----------------DLYGASQS-ILEVEYFE-------EVG
TGLG-PTLEF---YSTV----SMEFSKKKL--------KLWREVDTTGSN----------
---------EFIYGQAGLFPRPLS--------------------------------PEEV
TSPNGERILHLFKMLGKFVARSMIDSRIIDLHFNP--IFFRIGDG---------------
----PSTGVKPSLGAVKM----------------------VDPGLARS--------LKVI

```
KKFSLAKKEIDEDPKRSAAQK-----------------VADTVNLVVDN-VRLE-DLC--
--LDFTL-------------------------------PG--FPSIE-LEVN--------
GSHKR-------------------------------------------------------
------------------------------VTIENVD-SYLEK-VIDMTLGSG-VRHQVD
-AFRTG---FSQVFPY--SALS-AFTPD-ELVSLFGK-----------------------
---ADEDWS------------XAL---------TDSIKADHG------------------
----------------YNMD----SRSV-RNLLQTMS--------------------EFS
AQQRREFLQF-------TTGSPKLP------------IGG-----FR-SLTPMF--TVVC
KPSESPYTSD------------------------DYLPSVMTCV-NY-LKLPDY-TTVDI
MKKQLF-TAMKE-GQG-
>Hirsutella_minnesotensis_JPUM01000159.1 .
VRISRLKI-LESALKVM----------------DLYGASQS-ILEVEYFE-------EVG
TGLG-PTLEF---YSTV----SKEFSKKKL--------KLWREVDSTGSD----------
---------EFVYGQAGLFPRPLS--------------------------------PEEV
SSPNGERILHLFKMLGKFVARSMIDSRIIDLHFNP--IFFRIGDG---------------
----PSTGVRPSLGAVKM----------------------VDPGLARS--------LKVI
KKFSLAKKEIDEDPKRSPAQK-----------------VADTVDLVVDN-VRLE-DLC--
--LDFTL-------------------------------PG--FPSIE-LEVN--------
GSHKR-------------------------------------------------------
------------------------------VTIENVD-SYLEK-VIDMTLGSG-VRHQVD
-AFRAG---FSQVFPY--SALS-AFTPD-ELVSLFGK-----------------------
---VDEDWS-----------LESX---------LSRPTTATT------------------
----------------WTAE----VSET-FCRPVNSPLSSDASSCNSLRAALN-----FR
LEVKSPLLSR-------QLRGSPSR------------KLG-----FR-SLTPMF--TVVC
KPSESPYTSD------------------------DYLPSVMTCV-NY-LKLPDY-TTVEI
MKKQLF-TAMKE-GQG-
>Ophiocord_unilateralis_LAZP01000026.1 .
VRISRLKI-LESALKVM----------------DLYGASQS-ILEVEYFE-------EVG
TGLG-PTLEF---YSTV----SREFSKKKL--------KLWREVDSSMSA----------
---------EFVSGQAGLFPRPLS--------------------------------PEEL
SSPNGERILHLFKMLGKFVARSMIDSRIIDIHFNP--IFFRIGDT---------------
----PSTGVKPSLGAVKL----------------------VDPGLARS--------LKAI
KNFSLAKKEIDEDPGRSPAQK-----------------VADTTNLTVDN-VRLE-DMC--
--LDFTL-------------------------------PG--YPNIE-LEVN--------
GSHNR-------------------------------------------------------
------------------------------VTMENVD-LYLDR-VIDMTLGFG-VRRQVD
-AFRTG---FSQVFPY--SALS-AFTPD-ELVSLFGR-----------------------
---VDEDWS-----------XTAL---------TDSIKADHG------------------
----------------YNMD----SRSV-KHLLQTMS--------------------EFD
AQQRREFLQF-------TTGSPKLP------------IAG-----FR-SLTPMF--TVVC
KPSEAPYTSD------------------------DYLPSVMTCV-NY-LKLPDY-TTVGT
MKKQLF-TAMKE-GQG-
>Ophiocord_polyrhachis_furcata_LKCN0100001 .
VRISRLKI-LESALKVM----------------DLYGASQS-ILEVEYFE-------EVG
TGLG-PTLEF---YSTV----SREFSKKRL--------KLWREVDSSGSA----------
---------EFVSGQSGLFPRPLS--------------------------------PEEL
TSPNGERILHLFRMLGKFVARSMIDSRIIDIHFNP--IFFRIGDT---------------
----PSTGVKPSLGAVKM----------------------VDPGLARS--------LKAI
KNFSLAKREIDEDPGRSPAQK-----------------VADTTNLTIDN-VKLE-DLC--
--LDFTL-------------------------------PG--YPNIE-LEVN--------
GSHTR-------------------------------------------------------
------------------------------VTMENVD-LYLDR-VIDMTLGSG-VRRQVD
-AFRTG---FSQVFPY--SALS-AFTPD-ELVSLFGR-----------------------
---VEEDWT-----------LESX---------ANSVDSPDR------------------
----------------FYQG----RPRL-QHGPECQAS------------------VADD
ERVRRSAAAIPSVHDG-PAADWRVPSQSCHGDFADWLLAG-----FR-SLTPMF--TVVC
KPSEAPFTSD------------------------DYLPSVMTCV-NY-LKLPDY-TTVGA
MRKQLF-TAMKE-GQG-
```

>Escovopsis_weberi_LGSR01000002.1 .
VRISRNKI-LESALKVM----------------DLYGASQS-ILEVEYFE-------EVG
TGLG-PTLEF---YSTV----SNEFSKKKL--------KLWREVDSNGPD----------
---------EFVTSQNGLFPRPLR--------------------------------LEEV
STPNGDRILHLFKVLGKFVARSMIDSRIIDIHFNP--IFFRIGDA---------------
-----STSVKPSLGAVKI----------------------VDPGLAQS--------LKLI
KKFALAKKEIDEDPARSPAQK-----------------VADTERIVVDN-VRLE-DLC--
--LDFTL-------------------------------PG--YPDIE-LEDN--------
GSHKR---
------------------------------VTLENVD-AYLEK-VIDMTLGSG-VRSQID
-AFRTG---FSQVFPY--SALR-AFTPA-ELVALFGK-----------------------
---IEEDWS------------XAL---------MDSIKADHG------------------
----------------YNMD----SRSV-KNLLQTMS--------------------DLS
PKHRRDFLQF-------TTGSPKLP------------IGG-----FK-SLTPMF--TVVC
KPSEPPYTSD------------------------DYLPSVMTCV-NY-LKLPDY-TTLET
LKKQLF-TAIKE-GQG-
>Trichoderma_asperellum_JNNP01001195.1 .
VRISRLKI-LESALKVM----------------ELYGASQS-ILEVEYFE-------EVG
TGLG-PTLEF---YSTV----SKEFSKKKL--------KLWREVDSAGSD----------
---------EFVMGQTGLFPRPLS--------------------------------PEEA
TTPNGERILHLFKALGKFVARSMIDSRIIDIHFNP--IFFRIGDT---------------
----SLTGVKPSLGAVKI----------------------VDPGLARS--------LKTI
KKFVLAKKEIDENPNLTPAQK-----------------VADTEAIMIDN-ARLE-DFC--
--LDFTL-------------------------------PG--YPEIE-LEEH--------
GSLKR---
------------------------------VTIENVD-TYLEK-VIDMTLGAG-VKRQID
-AFRTG---FSQVFPY--TALR-AFTPD-ELVSLFGQ-----------------------
---VEEDWS-----------LESL---------MDSIKADHG------------------
----------------YNMD----SRSV-KNLLQAMS--------------------ELD
LKQRRDFLQF-------TTGSPKLP------------IGGKFFT-FR-CLTPMF--TVVC
KPSEPPYTSD------------------------AYLPSVMTCV-NY-LKLPDY-TTMDI
LKKQLY-TAIRE-GQG-
>Trichoderma_hamatum_ANCB02000345.1 .
VRISRLKI-LESALKVM----------------ELYGASQS-ILEVEYFE-------EVG
TGLG-PTLEF---YSTV----SKEFSKKKL--------KLWREVDSAGSD----------
---------EFVMGQTGLFPRPLS--------------------------------PEEA
TTPNGERILHLFKALGKFVARSMIDSRIIDIHFNP--IFFRIGDT---------------
----SLTGVKPSLGAVKI----------------------VDPGLARS--------LKTI
KKFVLAKKEIDENPNLTPAQK-----------------VADTEAIMIDN-AKLE-DFC--
--LDFTL-------------------------------PG--YPEIE-LEEH--------
GSLKR---
------------------------------VTIENVD-TYLEK-VIDMTLGTG-VKRQID
-AFRTG---FSQVFPY--TALR-AFTPD-ELVSLFGQ-----------------------
---VEEDWS-----------LESL---------MDSIKADHG------------------
----------------YNMD----SRSV-KNLLQAMS--------------------ELD
LKQRRDFLQF-------TTGSPKLP------------IGG-----FK-SLTPMF--TVVC
KPSEPPYTSD------------------------AYLPSVMTCV-NY-LKLPDY-TTMDI
LKKQLY-TAIRE-GQG-
>Trichoderma_gamsii_XM_018807352.1 .
VRISRLKI-LESALKVM----------------ELYGASQS-ILEVEYFE-------EVG
TGLG-PTLEF---YSTV----SKEFSKKKL--------KLWREVDSAGSD----------
---------EFVMGQTGLFPRPLS--------------------------------PEEA
TTPNGERILHLFKALGKFVARSMIDSRIIDLHFNP--IFFRIGDA---------------
----SLTGVKPSLGAVKI----------------------VDPGLARS--------LKTI
KKFVLAKKEIDENPNLTPAQK-----------------VADTEAIMIDN-VRLE-DFC--
--LDFTL-------------------------------PG--YPEIE-LDEH--------
GSSKR---
------------------------------VTIENVD-TYLEK-VIDMTLGTG-VKRQID

-AFRTG---FSQVFPY--TALR-AFTPD-ELVSLFGQ-----------------------
---VEEDWS-----------LETL---------MDSIKADHG------------------
----------------FNMD----SRSV-KNLLQAMS--------------------ELD
LKQRRDFLQF-------TTGSPKLP------------IGG-----FK-SLTPMF--TVVC
KPSEPPYTSD------------------------AYLPSVMTCV-NY-LKLPDY-TTMDI
LKKQLY-TAIRE-GQGA
>Trichoderma_atroviride_BCFX01000002.1 .
VRISRLKI-LESALKVM----------------ELYGASQS-ILEVEYFE-------EVG
TGLG-PTLEF---YSTV----SKEFCKKKL--------KLWREVDSAGSD----------
---------EFVMGQTGLFPRPLS---------------------------------PEEA
TTPNGERILHLFKALGKFVARSMIDSRIVDIHFNP--IFFRIGDP---------------
----SLTGVKPSLGAVKL----------------------VDPGLARS--------LKTI
KKFVLAKKEIDENPNLTPAQK-----------------VADTEAIMIDN-ARLE-DFC--
--LDFTL-------------------------------PG--YPEIE-LEEH--------
GSSKR---
------------------------------VTMENVD-TYLEK-VIDMTLGTG-VKRQID
-AFRTG---FSQVFPY--TALR-AFTPD-ELVSLFGQ-----------------------
---VEEDWS------------XAL---------MDSIKADHG------------------
----------------FNMD----SRSV-KNLLQAMS--------------------ELD
LKQRRDFLQF-------TTGSPKLP------------IGG-----FK-SLTPMF--TVVC
KPSEPPYTSD------------------------AYLPSVMTCV-NY-LKLPDY-TTMDI
LKKQLY-TAIRE-GQG-
>Trichoderma_pleuroti_MDJU01000067.1 .
VRISRLKI-LESALKVM----------------ELYGASQS-ILEVEYFE-------EVG
TGLG-PTLEF---YSTV----SKEFSKKKL--------KLWREVDSAGSD----------
---------EFVTGQTGLFPRPLS---------------------------------SEEA
TTPNGERILHMFKALGKFVARSMIDSRIIDIHLNP--IFFRIGDA---------------
----SLTGVKPSLGAVKI----------------------VDPGLARS--------LITI
KKFVLAKKEIDEDPARTPAQK-----------------VADTEGIMIDS-MRLD-DLC--
--LDFTL-------------------------------PG--YPDIE-LQEH--------
GSLKR---
------------------------------VTIENVD-LYLER-VIDMTLGSG-VKRQID
-AFRTG---FSQVFPY--TALR-AFTPD-ELVSLFGR-----------------------
---VEEDWS-------FV--DVAL---------MDSIKADHG------------------
----------------YNMD----SRSV-KNLLQTMS--------------------ELD
LKQRRDFLQF-------TTGSPKLP------------IGG-----FK-SLTPMF--TVVC
KPSEPPYTSD------------------------AYLPSVMTCV-NY-LKLPDY-TTMDI
LKKQAV-HCNQG-RAG-
>Trichoderma_virens_XM_014100202.1 .
VRISRLKI-LESALKVM----------------ELYGASQS-ILEVEYFE-------EVG
TGLG-PTLEF---YSTV----SKEFSKKKL--------KLWREVDSAGSD----------
---------EFVTGQTGLFPRPLS---------------------------------PEET
TTPNGERILHMFKALGKFVARSMIDSRIIDIHLNP--IFFRIGDA---------------
----SLTGVKPSLGAVKI----------------------VDPVLARS--------LITI
KKFVLAKKEIDEDPARTPAQK-----------------VADTEGIMIDN-MRLE-DLC--
--LDFTL-------------------------------PG--YPDIE-LQEH--------
GSLKR---
------------------------------VTIENVD-LYLER-VIDMTLGSG-VKRQID
-AFRTG---FSQVFPY--TALR-AFTPD-ELVSLFGR-----------------------
---VEEDWS-----------LETL---------MDSIKADHG------------------
----------------YNMD----SRSV-KNLLQTMS--------------------ELD
LKQRRDFLQF-------TTGSPKLP------------IGG-----FK-SLTPMF--TVVC
KPSEPPYTSD------------------------AYLPSVMTCV-NY-LKLPDY-TTMDI
LKKQLY-TAIRE-GQGA
>Trichoderma_harzianum_JOKZ01000208.1 .
VRISRLKI-LESALKVM----------------ELYGASQS-ILEVEYFE-------EVG
TGLG-PTLEF---YSTV----SKEFSKKKL--------KLWREVDSAGSD----------
---------EFVTGQTGLFPRPLS---------------------------------AEEA

```
TTPNGERILHMFKALGKFVARSMIDSRIIDIHLNP--IFFRIGDA---------------
----SLTGVKPSLGAVKI----------------------VDPGLARS--------LITI
KKFVLAKKEIDEDPARTPAQK-----------------VADTEGIMIDN-MRLE-DLC--
--LDFTL--------------------------------PG--YPDIE-LQEH--------
GSLKR-------------------------------------------------------
------------------------------VTIENVD-LYLEK-VIDMTLGSG-VKRQID
-AFRTG---FSQVFPY--TALR-AFTPD-ELVSLFGR-----------------------
---VEEDWS-----------LESL---------MDSIKADHG------------------
----------------YNMD----SRSV-KNLLQTMS--------------------ELD
PKQRRDFLQF-------TTGSPKLP------------IGG-----FK-SLTPMF--TVVC
KPSEPPYTSD------------------------AYLPSVMTCV-NY-LKLPDY-TTMDI
LKKQLY-TAIRE-GQGA
>Trichoderma_parareesei_LFMI01000600.1 .
VRISRLKI-LESALKVM----------------DLYGASQS-ILEVEYFE-------EVG
TGLG-PTLEF---YSTV----SKEFSKKKL--------KLWREVDSAGSD----------
---------EFVTGQTGLFPRPLS---------------------------------PEET
TTPNGERILHLFKALGKFVARSMIDSRIIDIHFNP--IFFRIGDA---------------
----SLTGVKPSLGAVKV----------------------VDPVLARS--------LITI
KKFVLAKKEIDEDPARTPAQK-----------------VADTERIMIDN-MRLE-DLC--
--LDFTL--------------------------------PG--YPEIE-LVEH--------
GSLKR-------------------------------------------------------
------------------------------VTMENVD-VYLEK-VIDMTLGSG-VKRQID
-AFRAG---FSQVFPY--TALR-AFTPD-ELVSLFGR-----------------------
---VEEDWS-----------LESE---------YSSIKADHG------------------
----------------YNMD----SRSV-KNLLQTMS--------------------ELD
AKQRRDFLQF-------TTGSPKLP------------IGG-----FK-SLTPMF--TVVC
KPSEPPYTSD------------------------AYLPSVMTCV-NY-LKLPDY-STMEV
LKKQLY-TAIRE-GQGA
>Trichoderma_koningii_BCGH01000005.1 .
VRISRLKI-LESALKVM----------------DLYGASQS-ILEVEYFE-------EVG
TGLG-PTLEF---YSTV----SKEFSKKKL--------KLWREVDSAGSD----------
---------EFVTGQTGLFPRPLS---------------------------------PEET
TTPNGERILHLFKALGKFVARSMIDSRIIDIHFNP--IFFRIGDA---------------
----SLTGVKPSLGAVKI----------------------VDPVLARS--------LITI
KKFVLAKKEIDEDPARTPAQK-----------------VADTERIMIDN-MRLE-DLC--
--LDFTL--------------------------------PG--YPEIE-LLEH--------
GSHKR-------------------------------------------------------
------------------------------VTIDNVD-VYLEK-VIDMTLGSG-VKRQID
-AFRAG---FSQVFPY--TALR-AFTPD-ELVSLFGR-----------------------
---VEEDWS-----------LESL---------MDSIKADHG------------------
----------------YNMD----SRSV-KNLLQTMS--------------------ELD
AKQRRDFLQF-------TTGSPKLP------------IGG-----FK-SLTPMF--TVVC
KPSEPPYTSD------------------------AYLPSVMTCV-NY-LKLPDY-STMEV
LKRQLY-TAIRE-GQGA
>Trichoderma_reesei_XM_006962445.1 .
VRISRLKI-LESALKVM----------------DLYGASQS-ILEVEYFE-------EVG
TGLG-PTLEF---YSTV----SKEFSKKKL--------KLWREVDSAGSD----------
---------EFVTGQTGLFPRPLS---------------------------------PEET
TTPNGERILHLFKALGKFVARSMIDSRIIDIHFNP--IFFRIGDA---------------
----SLTGVKPSLGAVKV----------------------VDPGLARS--------LITI
KKFVLAKKEIDEDPARTPAQK-----------------VADTERIMIDN-MRLE-DLC--
--LDFTL--------------------------------PG--YPEIE-LVEH--------
GSLKR-------------------------------------------------------
------------------------------VTMENVD-VYLEK-VIDMTLGSG-VKRQID
-AFRAG---FSQVFPY--TALR-AFTPD-ELVSLFGR-----------------------
---VEEDWS-----------LETL---------MDSIKADHG------------------
----------------YNMD----SRSV-KNLLQTMS--------------------ELD
AKQRRDFLQF-------TTGSPKLP------------IGG--------NLTPMF--TVVC
```

```
KPSEPPYTSD------------------------AYLPSVMTCV-NY-LKLPDY-STMEV
LKRQLY-TAIRE-GQGA
>Stachybotrys_chartarum_ASEQ01002789.1 .
VRISRLKI-LESALKVM----------------DLYGASQS-MLEVEYFE-------EVG
TGLG-PTLEF---YSTV----SKEFSKKKL--------MLWRDMESTGAV----------
---------DFVSGQAGLFPRPLS--------------------------------DEEA
SSPNGERILHLFKILGKFVARSMIDSRIIDIHFNP--FFFRIGDA---------------
----PATSVKPSLGAVKL----------------------VDAGLARS--------LKAI
KKFALVKKEIDEDPLRTPAQK-----------------VADTANASIDN-VKLE-DLC--
--LDFTL-------------------------------PG--YPSIE-LEDN--------
GSQKR-------------------------------------------------------
------------------------------VTIDNVD-SYLDK-VLDMTLGSG-VRRQVD
-AFRAG---FSQVFPY--SALN-AFTPD-ELVSLFGR-----------------------
---VDEDWS-----------LESL---------MDSIKADHG------------------
----------------YNMD----SKTV-KNLLQTMS--------------------EFT
AAQRRDFLQF-------TTGSPKLP------------IGG-----FK-SLTPMF--TVVC
KPSEHPYTSD------------------------DYLPSVMTCV-NY-LKLPNY-STIEI
LKKQLS-VAVKE-GQGA
>Stachybotrys_chlorohalonata_APWP01002673. 1..
VRISRLKI-LESALKVM----------------DLYGASQS-MLEVEYFE-------EVG
TGLG-PTLEF---YSTV----SKEFSKKKL--------MLWRDMESTGAV----------
---------DFVSGQAGLFPRPLS--------------------------------DEEA
SSPNGERILHLFKILGKFVARSMIDSRIIDIHFNP--FFFRIGDA---------------
----PATSVKPSLGAVKL----------------------VDAGLARS--------LKVI
KKFALVKKEIDEDPLRTPAQK-----------------VADTANASVDN-VKLE-DLC--
--LDFTL-------------------------------PG--YPSIE-LEDN--------
GSQKR-------------------------------------------------------
------------------------------VTIDNVD-SYLDK-VLDMTLGSG-VRRQVD
-AFRAG---FSQVFPY--SALN-AFTPD-ELVSLFGR-----------------------
---VDEDWS-----------LESL---------MDSIKADHG------------------
----------------YNMD----SKTV-KNLLQTMS--------------------EFT
APQRRDFLQF-------TTGSPKLP------------IGG-----FK-SLTPMF--TVVC
KPSEHPYTSD------------------------DYLPSVMTCV-NY-LKLPNY-STIEI
LKKQLS-VAVKE-GQGA
>Stachybotrys_echinata_BCHF01000003.1 .
VRISRLKI-LESALKVM----------------DLYGASQS-MLEVEYFE-------EVG
TGLG-PTLEF---YSTV----SKEFSKKKL--------MLWRDMDSAGSV----------
---------DFVSGQAGLFPRPLS--------------------------------DEEA
SSPNGERILHLFKMLGKFVARSMIDSRIIDIHFNP--IFFRIGDA---------------
----TSIGVKPSLGAVKL----------------------VDPGLARS--------LKAI
KKFALVKKEIDEDPRRTPAQK-----------------VADTANITIDN-VKLE-DLC--
--LDFTL-------------------------------PG--YPGIE-LEDN--------
GSHKR-------------------------------------------------------
------------------------------VTIDNVD-SYLDK-VLDMTLGSG-VRRQVD
-AFRAG---FSQVFPY--SALS-AFTPD-ELVGLFGR-----------------------
---VDEDWS-----------LESL---------MDSIKADHG------------------
----------------YNMD----SKTV-KNLLQTMS--------------------EFT
ATQRRDFLQF-------TTGSPKLP------------IGG-----FK-SLTPMF--TVVC
KPSEHPYTSD------------------------DYLPSVMTCV-NY-LKLPDY-STIET
MRKQLS-VAIKE-GQGA
>Ilyonectria_destructans_MPHF01000080.1 .
VRISRQKI-LESALKVM----------------ELYGASQS-ILEVEYFE-------EVG
TGLG-PTLEF---YSTV----SKEFFKKKL--------KLWREMDSNGPD----------
---------EFVSGGTGLFPRPLS--------------------------------EEEA
GTPNGERILHLFKMLGKFVARSMIDSRIIDIHFNP--IFFRIGDG---------------
----APTGVKPSLGAVKI----------------------VDPGLARS--------LKAI
KKFALAKKAIDEDPGRTAAQK-----------------VTDTENITIDD-VKLD-DLC--
--LDFTL-------------------------------PG--YPSIE-LEDN--------
```

GSQKR---
------------------------------VSIDNVE-SYLEK-VIDMTLGSG-VRLQVD
-AFQTG---FSQVFPY--SALS-AFTPD-ELVTLFGR-----------------------
---VDEDWS------------LESL--------LDSLKADHG------------------
----------------YNMD----SKSV-KNLLHIMS--------------------QYD
PSQRRDFLQF-------TTGSPKLP------------IGG-----FK-SLTPMF--TVVC
KPSEHPYASD------------------------DYLPSVMTCV-NY-LKLPDY-TSMET
MKKQLS-MAVKE-GQGA
>Dactylonectria_macrodidyma_JYGD01000481.1 .
VRISRQKI-LESALKVM----------------ELYGASQS-ILEVEYFE-------EVG
TGLG-PTLEF---YSTV----SKEFFKKKL--------KLWREMDSNGPD----------
---------EFVSGGTGLFPRPLG--------------------------------EEEA
GTPNGERILHLFKMLGKFVARSMIDSRIIDIHFNP--IFFRIGDA---------------
----ASTGVKPSLGAVKI----------------------VDPGLARS--------LKAI
KKFALAKKEIDEDPERTPAQK-----------------VTDTEDITIDG-VKLD-DLC--
--LDFTL-------------------------------PG--YPNIE-LEDN--------
GSQKR---
------------------------------VSIDNVE-SYLEK-VIDMTLGSG-VRLQVD
-AFQTG---FSQVFPY--SALS-AFTPD-ELVTLFGR-----------------------
---VEEDWS-----------LSAL---------LDSLKADHG------------------
----------------YNMD----SKSV-KNLLQIMS--------------------QFT
PSQRRDFLQF-------TTGSPKLP------------IGG-----FK-SLTPMF--TVVC
KPSEHPYVSD------------------------DYLPSVMTCV-NY-LKLPDY-TDMET
MKKQLS-TAVKE-GQGA
>Neonectria_ditissima_LDPL01000002.1 .
VRISRQKI-LESALKVM----------------ELYGASQS-ILEVEYFE-------EVG
TGLG-PTLEF---YSTV----SKEFFKKKL--------KLWREMDSNGPE----------
---------EFVSGGTGLFPRPLS--------------------------------DEEA
GTPNGERILHLFKMLGKFVARSMIDSRIIDIHLNP--IFFRIGDG---------------
----SPTGVKPSLGAVKI----------------------VDPGLARS--------LKAI
KKFALAKKAIDEDPGRTPAQK-----------------VADTEDIVIDG-VKLE-DLC--
--LDFTL-------------------------------PG--YPNIE-LEDN--------
GSQKR---
------------------------------VGIDNVE-SYLEK-VIDMTLGSG-VRRQVD
-AFQAG---FSQVFPY--SALS-AFTPD-ELVTLFGR-----------------------
---VDEDWS-----------LSAL---------LDSLKADHG------------------
----------------YNMD----SKSV-KNLLQIMS--------------------QFD
PSQRRDFLQF-------TTGSPKLP------------IGG-----FK-SLTPMF--TVVC
KPSEHPYASD------------------------DYLPSVMTCV-NY-LKLPDY-TSMEK
MKKQLS-TAVKE-GQGA
>Calonectria_pseudoreteaudii_MOCD01001086. 1..
VRISRQKI-LESALKVM----------------ELYGASQS-ILEVEYFE-------EVG
TGLG-PTLEF---YSTV----SKEFFKKKL--------KLWREMDSNGPE----------
---------EFVSGGAGLFPRPLS--------------------------------DEEA
LTPNGERILHLFKMLGKFVARSMIDSRIIDIHFNP--IFFRIGDG---------------
----SATGVKPSLGAVKI----------------------VDPGLARS--------LKTI
KKFALAKKAIDEDPERTPAQK-----------------VADTEEIVIDE-VKLD-DLC--
--LDFTL-------------------------------PG--YPSIE-LEDN--------
GAQKR---
------------------------------VTIDNVD-SYLEK-VIDMTLGSG-VRRQVD
-AFQVG---FSQVFPY--SALS-AFTPD-ELVTLFGR-----------------------
---VDEDWS------------XAL---------LDSIKADHG------------------
----------------YNMD----SKSV-KNLLQIMS--------------------EFD
PAQRRDFLQF-------TTGSPKLP------------IGG-----FK-SLTPMF--TVVC
KPSEHPYTSD------------------------DYLPSVMTCV-NY-LKLPDY-TNVET
MRKQLS-TAVKE-GQGA
>Fusarium_tucumaniae_MAED01001024.1 .
VRISRQKI-LESALKVM----------------ELYGASQS-ILEVEYFE-------EVG

TGLG-PTLEF---YSTV----SKEFSKRKL--------KLWREMDSNGSD----------
---------DFVSGATGLFPRPQS--------------------------------DEEA
GTPNGERILHLFKMLGKFVARSMIDSRIIDLHFNP--IFFRIGDA---------------
----TSCGVKPSLGAVKI----------------------VDPGLARS--------LKAI
KRFALAKKEIDEDPARTAAQK-----------------VADTENIVIDG-VKID-DLC--
--LDFTL-------------------------------PG--YPSIE-LESN--------
GSQKR---
------------------------------VTIDNVD-TYLEK-VIDMTLGSG-VRRQVD
-AFRTG---FAQVFPY--SALS-AFTPD-ELVTLFGR-----------------------
---VDEDWS-----------LESM---------FDSIKADHG------------------
----------------YNMD----SKTV-KNLLHTMS--------------------SFD
AAERRDFLQF-------TTGSPKLP------------IGG-----FK-SLTPMF--TVVC
KPSEHPYTSD------------------------DYLPSVMTCV-NY-LKLPDY-SDIDI
MRKQLF-TAVRE-GQGA
>Fusarium_phaseoli_MAEB01001185.1 .
VRISRQKI-LESALKVM----------------ELYGASQS-ILEVEYFE-------EVG
TGLG-PTLEF---YSTV----SKEFSKRKL--------KLWREMDSNGSD----------
---------DFVSGATGLFPRPQS--------------------------------GEEA
GTPNGERILHLFKMLGKFVARSMIDSRIIDLHFNP--IFFRIGDA---------------
----TSCGVKPSLGAVKI----------------------VDPGLARS--------LKAI
KRFALAKKEIDEDPARTAAQK-----------------VADTENIVIDG-VKID-DLC--
--LDFTL-------------------------------PG--YPSIE-LESN--------
GSQKR---
------------------------------VTIDNVD-TYLEK-VIDMTLGSG-VRRQVD
-AFRTG---FAQVFPY--SALS-AFTPD-ELVTLFGR-----------------------
---VDEDWS-----------LESM---------FDSIKADHG------------------
----------------YNMD----SKTV-KNLLHTMS--------------------SFD
AAERRDFLQF-------TTGSPKLP------------IGG-----FK-SLTPMF--TVVC
KPSEHPYTSD------------------------DYLPSVMTCV-NY-LKLPDY-SDIDI
MRKQLF-TAVKE-GQGA
>Fusarium_virguliforme_MADX01007039.1 .

-------LEF---YSTV----SKEFSKRKL--------KLWREMDSNGSD----------
---------DFVSGATGLFPRPQS--------------------------------DEEA
GTPNGERILHLFKMLGKFVARSMIDSRIIDLHFNP--IFFRIGDA---------------
----TSCGVKPSLGAVKI----------------------VDPGLARS--------LKAI
KRFALAKKEIDEDPARTAAQK-----------------VADTENIVIDG-VKID-DLC--
--LDFTL-------------------------------PG--YPSIE-LESN--------
GSQKR---
------------------------------VTIDNVD-TYLEK-VIDMTLGSG-VRRQVD
-AFRTG---FAQVFPY--SALS-AFTPD-ELVTLFGR-----------------------
---VDEDWS-----------LESM---------FDSIKADHG------------------
----------------YNMD----SKTV-KNLLHTMS--------------------SFD
AAERRDFLQF-------TTGSPKLP------------IGG-----FK-SLTPMF--TVVC
KPSEHPYTSD------------------------DYLPSVMTCV-NY-LKLPDY-SDIDI
MRKQLF-TAVKE-GQGA
>Nectria_haematococca_XM_003046776.1 .
VRISRQKI-LESALKVM----------------ELYGASQS-ILEVEYFE-------EVG
TGLG-PTLEF---YSTV----SKEFSKRKL--------KLWREMDSNGSD----------
---------EFVSGATGLFPRPQS--------------------------------DEEA
GTPNGERILHLFKMLGKFVARSMIDSRIIDLHFNP--IFFRIGDA---------------
----TLSGVKPSLGAVKI----------------------VDPGLARS--------LKAI
KRFALAKKEIDEDPARTAAQK-----------------VADTENIVIDG-VKID-DLC--
--LDFTL-------------------------------PG--YPSIE-LESN--------
GSQKR---
------------------------------VTIDNVD-SYLEK-VIDMTLGSG-VRRQVD
-AFRTG---FSQVFPY--SALS-AFTPD-ELVTLFGR-----------------------
---VDEDWS-----------LETL---------LDSIKADHG------------------

----------------YNMD----SKSV-KNLLHTMS--------------------DFD
AAQRRDFLQF-------TTGSPKLP------------IGG-----FK-SLTPMF--TVVC
KPSEHPYTSD------------------------DYLPSVMTCV-NY-LKLPDY-STIDI
MKKQLF-TAVKE-GQGA
>Fusarium_graminearum_XM_011330230.1 .
VRISRQKI-LESALKVM----------------ELYGASQS-ILEVEYFE-------EVG
TGLG-PTLEF---YSTV----SKEFSKKKL--------KLWREVDSNDSD----------
---------EFVSGATGLFPRPQS--------------------------------DEEA
GTPNGERILHLFKMLGKFVARSMIDSRIIDLHFNP--IFFRIGDA---------------
-----ITGVKPSLGAVKI----------------------VDPGLARS--------LKAI
KQFSLAKKEIDEDPSRTAAQK-----------------VADTENITIEG-VKLD-DLC--
--LDFTL-------------------------------PG--YPNIQ-LEDN--------
GSQKR---
------------------------------VTIDNVD-TYLEK-VIDMTLGSG-VRRQVD
-AFRAG---FSQVFPY--SALS-AFTPD-ELVTLFGR-----------------------
---VDEDWS-----------LESL---------LDSIKADHG------------------
----------------YNMD----SKTV-KNLLHTMS--------------------QFN
ASERRDFLQF-------TTGSPKLP------------IGG-----FK-SLTPMF--TVVC
KPSEEPYTSD------------------------DYLPSVMTCV-NY-LKLPDY-STVEA
MKKQLS-TAVKE-GQGA
>Fusarium_meridionale_LHUB01000185.1 .
VRISRQKI-LESALKVM----------------ELYGASQS-ILEVEYFE-------EVG
TGLG-PTLEF---YSTV----SKEFSKKKL--------KLWREVDSNDSD----------
---------EFVSGATGLFPRPQS--------------------------------DEEA
GTPNGERILHLFKMLGKFVARSMIDSRIIDLHFNP--IFFRIGDA---------------
-----ITGVKPSLGAVKI----------------------VDPGLARS--------LKAI
KQFSLAKKEIDEDPSRTAAQK-----------------VADTENITIEG-VKLD-DLC--
--LDFTL-------------------------------PG--YPNIQ-LEDN--------
GSQKR---
------------------------------VTIDNVD-TYLEK-VIDMTLGSG-VRRQVD
-AFRAG---FSQVFPY--SALS-AFTPD-ELVTLFGR-----------------------
---VDEDWS-----------LESL---------LDSIKADHG------------------
----------------YNMD----SKTV-KNLLHTMS--------------------QFN
ASERRDFLQF-------TTGSPKLP------------IGG-----FK-SLTPMF--TVVC
KPSEEPYTSD------------------------DYLPSVMTCV-NY-LKLPDY-STVEA
MKKQLS-TAVKE-GQGA
>Fusarium_asiaticum_LHTY01000097.1 .
VRISRQKI-LESALKVM----------------ELYGASQS-ILEVEYFE-------EVG
TGLG-PTLEF---YSTV----SKEFSKKKL--------KLWREVDSNESD----------
---------EFVSGATGLFPRPQS--------------------------------DEEA
GTPNGERILHLFKMLGKFVARSMIDSRIIDLHFNP--IFFRIGDA---------------
-----ITGVKPSLGAVKI----------------------VDPGLARS--------LKAI
KQFSLAKKEIDEDPSRTAAQK-----------------VADTENITIEG-VKLD-DLC--
--LDFTL-------------------------------PG--YPNIQ-LEDN--------
GSQKR---
------------------------------VTIDNVD-TYLEK-VIDMTLGSG-VRRQVD
-AFRAG---FSQVFPY--SALS-AFTPD-ELVTLFGR-----------------------
---VDEDWS-----------LESL---------LDSIKADHG------------------
----------------YNMD----SKTV-KNLLHTMS--------------------QFN
ASERRDFLQF-------TTGSPKLP------------IGG-----FK-SLTPMF--TVVC
KPSEEPYTSD------------------------DYLPSVMTCV-NY-LKLPDY-STVEA
MKKQLS-TAVKE-GQGA
>Fusarium_pseudograminearum_XM_009262748.1 .
VRISRQKI-LESALKVM----------------ELYGASQS-ILEVEYFE-------EVG
TGLG-PTLEF---YSTV----SKEFSKKKL--------KLWREVDSNDSD----------
---------EFVSGATGLFPRPQS--------------------------------DEEA
GTPNGERILHLFKMLGKFVARSMIDSRIIDLHFNP--IFFRIGDA---------------
-----ITGVKPSLGAVKI----------------------VDPGLARS--------LKAI

KQFSLAKKEIDEDPSRTAAQK-----------------VADTENITIEG-VKLD-DLC--
--LDFTL-------------------------------PG--YPNIQ-LEDN--------
GSQKR---
------------------------------VTIDNVD-TYLEK-VIDMTLGSG-VRRQVD
-AFRAG---FSQVFPY--SALS-AFTPD-ELVTLFGR-----------------------
---VDEDWS-----------LETL---------LDSIKADHG------------------
----------------YNMD----SKTV-KNLLHTMS--------------------QFN
PSERRDFLQF-------TTGSPKLP------------IGG-----FK-SLTPMF--TVVC
KPSEEPYTSD------------------------DYLPSVMTCV-NY-LKLPDY-SNVEA
MKKQLS-TAVKE-GQGA
>Fusarium_sambucinum_LSRD01000023.1 .
VRISRQKI-LESALKVM----------------ELYGASQS-ILEVEYFE-------EVG
TGLG-PTLEF---YSTV----SKEFSKKKL--------KLWREVDSNDSD----------
---------EFVSGATGLFPRPQS--------------------------------DEEA
GTPNGERILHLFRMLGKFVARSMIDSRIIDLHFNP--IFFRIGDA---------------
-----ITGVKPSLGAVKI----------------------VDPGLARS--------LKAI
KQFALAKKEIDEDPNRTPAQK-----------------VADTENITIDG-VELD-DLC--
--LDFTL-------------------------------PG--YPNIQ-LEDN--------
GSQKR---
------------------------------VTIDNVD-TYLEK-VIDMTLGSG-VRRQVD
-AFRAG---FSQVFPY--SALS-AFTPD-ELVTLFGR-----------------------
---VDEDWS-----------LESL---------LDSIKADHG------------------
----------------YNMD----SKTV-KNLLHTMS--------------------EFN
ASERRDFLQF-------TTGSPKLP------------IGG-----FK-SLTPMF--TVVC
KPSEEPYTSD------------------------DYLPSVMTCV-NY-LKLPDY-STIEA
MRKQLS-TAVKE-GQGA
>Fusarium_poae_LYXU01000004.1 .
VRISRQKI-LESALKVM----------------ELYGASQS-ILEVEYFE-------EVG
TGLG-PTLEF---YSTV----SKEFSKKKL--------KLWREVDSNDSD----------
---------EFVSGATGLFPRPQS--------------------------------DEEA
GTPNGERILHLFRMLGKFVARSMIDSRIIDLHFNP--IFFRIGDA---------------
-----ITGVKPSLGAVKI----------------------VDPGLARS--------LKAI
KQFALAKKEIDEDPNRTPAQK-----------------VADTENITIDG-VKLE-DLC--
--LDFTL-------------------------------PG--YPNIQ-LEDN--------
GSQKR---
------------------------------VTIDNVD-TYLEK-VVDMTLGSG-VRRQVD
-AFRAG---FSQVFPY--SALS-AFTPD-ELVTLFGR-----------------------
---VDEDWS-----------LESL---------LDSIKADHG------------------
----------------YNMD----SKTV-KNLLHTMS--------------------QFN
ASERRDFLQF-------TTGSPKLP------------IGG-----FK-SLTPMF--TVVC
KPSEEPYTSD------------------------DYLPSVMTCV-NY-LKLPDY-STIEA
MKKQLS-TAVKE-GQGA
>Fusarium_equiseti_CBMI010000893.1 .
VRISRQKI-LESALKVM----------------ELYGAAQS-ILEVEYFE-------EVG
TGLG-PTLEF---YSTV----SREFSKKKL--------KLWREVDTNDSD----------
---------EFVSGATGLFPRPQS--------------------------------DEEA
GTPNGERILHLFKMLGKFVARSMIDSRIIDLHFNP--IFFRIGDA---------------
-----VSGVKPSLGAVRM----------------------VDPGLARS--------LKAI
KQFALAKKEIDEDPSRTPAQK-----------------VADTENITIDG-VKLD-DLC--
--LDFTL-------------------------------PG--YPNIQ-LEDN--------
GSQKR---
------------------------------VTIDNVD-AYLEK-VIDMTLGSG-VRRQVD
-AFRAG---FSQVFPY--SALS-AFTPD-ELVTLFGR-----------------------
---VDEDWS-----------LESL---------LDSIKADHG------------------
----------------YNMD----SKTV-KNLLHTMS--------------------GFN
ASERRNFLQF-------ITGSPKLP------------IGG-----FK-SLTPMF--TVVC
KPSEDPYTSD------------------------DYLPSVMTCV-NY-LKLPDY-STIEI
MKKQLF-TAVNE-GQGA

>Fusarium_oxysporum_XM_018399339.1 .
VRISRQKI-LESALKVM----------------ELYGASQS-ILEVEYFE-------EVG
TGLG-PTLEF---YSTV----SKEFSKRKL--------KLWREVDSSGSD----------
---------EFVSGATGLFPRPQS--------------------------------DEEA
GTPNGERILHLFKMLGKFVARSMIDSRIIDLHFNP--IFFRIGDA---------------
----VSSGVKPSLGAVKI----------------------VDPGLARS--------LKAI
KQFALAKKEIDEDPNRTPAQK-----------------VADTENITIDG-VKLD-DLC--
--LDFTL-------------------------------PG--YPNIQ-LEDN--------
GSQKR---
------------------------------VTIDNVD-SYLEK-VIDMTLGSG-VRRQVD
-AFRAG---FSQVFPY--SALS-AFTPD-ELVTLFGR-----------------------
---VDEDWS-----------LETL---------LDSIKADHG------------------
----------------YNMD----SKTV-KNLLHTMS--------------------EFD
ASQRRDFLQF-------TTGSPKLP------------IGG-----FK-SLTPMF--TVVC
KPSEHPYISD------------------------DYLPSVMTCV-NY-LKLPDY-STIEI
MRKQLF-TAVKE-GQGA
>Fusarium_verticillioides_XM_018904474.1 .
VRISRQKI-LESALKVM----------------ELYGASQS-ILEVEYFE-------EVG
TGLG-PTLEF---YSTV----SKEFSKRKL--------KLWREVDSNGSD----------
---------EFVSGATGLFPRPQS--------------------------------DEEA
GTPNGERILHLFKMLGKFVARSMIDSRIIDLHFNP--IFFRIGDA---------------
----VSSGVKPSLGAVKI----------------------VDPVLARS--------LKAI
KQFALAKKEIDEDPNRTPAQK-----------------VADTQSITIDG-VKLD-DLC--
--LDFTL-------------------------------PG--YPNIQ-LEDN--------
GSQKR---
------------------------------VTIDNVD-SYLEK-VIDMTLGSG-VRRQVD
-AFRAG---FSQVFPY--SALS-AFTPD-ELVTLFGR-----------------------
---VDEDWS-----------LETL---------LDSIKADHG------------------
----------------YNMD----SKTV-KNLLHTMS--------------------EFD
ASQRRDFLQF-------TTGSPKLP------------IGG-----FK-SLTPMF--TVVC
KPSEHPYTSD------------------------DYLPSVMTCV-NY-LKLPDY-STIEI
MRKQLF-TAVKE-GQGA
>Fusarium_proliferatum_FCQG01000021.1 .
VRISRQKI-LESALKVM----------------ELYGASQS-ILEVEYFE-------EVG
TGLG-PTLEF---YSTV----SKEFSKRKL--------KLWREVDSNGSD----------
---------EFVSGATGLFPRPQS--------------------------------DEEA
GTPNGERILHLFKMLGKFVARSMIDSRIIDLHFNP--IFFRIGDA---------------
----ASTGVKPSLGAVKI----------------------VDPVLARS--------LKAI
KQFALAKKEIDEDPNRTPAQK-----------------VADTESITIDG-VKLD-DLC--
--LDFTL-------------------------------PG--YPNIQ-LEDN--------
GSQKR---
------------------------------VTIDNVD-SYLEK-VIDMTLGSG-VRRQVD
-AFRAG---FSQVFPY--SALS-AFTPD-ELVTLFGR-----------------------
---VDEDWS-----------LESL---------LDSIKADHG------------------
----------------YNMD----SKTV-KNLLHTMS--------------------EFD
ASQRRDFLQF-------TTGSPKLP------------IGG-----FK-SLTPMF--TVVC
KPSEHPYTSD------------------------DYLPSVMTCV-NY-LKLPDY-STIEI
MRKQLF-TAVKE-GQGA
>Fusarium_fujikuroi_HF679024.1_a.
VRISRQKI-LESALKVM----------------ELYGASQS-ILEVEYFE-------EVG
TGLG-PTLEF---YSTV----SKEFSKRKL--------KLWREVDSNGSD----------
---------EFVSGATGLFPRPQS--------------------------------DEEA
GTPNGERILHLFKMLGKFVARSMIDSRIIDLHFNP--IFFRIGDA---------------
----VSTGVKPSLGAVKI----------------------VDPVLARS--------LKAI
KQFALAKKEIDEDPNRTPAQK-----------------VADTESITIDG-VKLD-DLC--
--LDFTL-------------------------------PG--YPNIQ-LEDN--------
GSQKR---
------------------------------VTIDNVD-SYLEK-VIDMTLGSG-VRRQVD

-AFRAG---FSQVFPY--SALS-AFTPD-ELVTLFGR-----------------------
---VDEDWS-----------LESL---------LDSIKADHG------------------
----------------YNMD----SKTV-KNLLHTMS--------------------EFD
ASQRRDFLQF-------TTGSPKLP------------IGG-----FK-SLTPMF--TVVC
KPSEHPYTSD------------------------DYLPSVMTCV-NY-LKLPDY-STIEI
MRKQLF-TAVKE-GQG-
>Fusarium_temperatum_LJGR01000002.1 .
VRISRQKI-LESALKVM----------------ELYGASQS-ILEVEYFE-------EVG
TGLG-PTLEF---YSTV----SKEFSKRKL--------KLWREVDSNSSD----------
---------EFVSGATGLFPRPQS--------------------------------DEEA
GTPNGERILHLFKMLGKFVARSMIDSRIIDLHFNP--IFFRIGDA---------------
----VSSGVKPSLGAVKI----------------------VDPVLARS--------LKAI
KQFALAKKEIDEDPNRTPAQK-----------------VADTESITIDG-VKLD-DLC--
--LDFTL-------------------------------PG--YPNIQ-LEDN--------
GSQKR---
------------------------------VTIDNVD-SYLEK-VIDMTLGSG-VRRQVD
-AFRAG---FSQVFPY--SALS-AFTPD-ELVTLFGR-----------------------
---VDEDWS-----------LESL---------LDSIKADHG------------------
----------------YNMD----SKTV-KNLLHTMS--------------------EFD
ASQRRDFLQF-------TTGSPKLP------------IGGG----FK-SLTPMF--TVVC
KPSEHPYTSD------------------------DYLPSVMTCV-NY-LKLPDY-STIET
MRKQLF-TAVKE-GQGA
>Fusarium_nygamai_LBNR01000005.1 .
VRISRQKI-LESALKVM----------------ELYGASQS-ILEVEYFE-------EVG
TGLG-PTLEF---YSTV----SKEFSKRKL--------KLWREVDSNGSD----------
---------EFVSGATGLFPRPQS--------------------------------DEEA
GTPNGERILHLFKMLGKFVARSMIDSRIIDLHFNP--IFFRIGDA---------------
----VSSGVKPSLGAVKI----------------------VDPVLARS--------LKAI
KQFALAKKEIDEDPNRTPAQK-----------------VADTESITIDG-VKLD-DLC--
--LDFTL-------------------------------PG--YPNIQ-LEDN--------
GSQKR---
------------------------------VTIDNVD-SYLEK-VIDMTLGSG-VRRQVD
-AFRAG---FSQVFPY--SALS-AFTPD-ELVTLFGR-----------------------
---VDEDWS-----------LESL---------LDSIKADHG------------------
----------------YNMD----SKTV-KNLLHTMS--------------------EFD
ASQRRDFLQF-------TTGSPKLP------------IGGG----FK-SLTPMF--TVVC
KPSEHPYTSD------------------------DYLPSVMTCV-NY-LKLPDY-STIET
MRKQLF-TAVKE-GQGA
>Fusarium_circinatum_JRVE01000093.1 .
VRISRQKI-LESALKVM----------------ELYGASQS-ILEVEYFE-------EVG
TGLG-PTLEF---YSTV----SKEFSKRKL--------KLWREVDSNGSD----------
---------EFVSGATGLFPRPQS--------------------------------DEEA
GTPNGERILHLFKMLGKFVARSMIDSRIIDLHFNP--IFFRIGDA---------------
----VSSGVKPSLGAVKI----------------------VDPVLARS--------LKAI
KQFALAKKEIDEDPNRTPAQK-----------------VADTESITIDG-VKLD-DLC--
--LDFTL-------------------------------PG--YPNIQ-LEDN--------
GSQKR---
------------------------------VTIDNVD-SYLEK-VIDMTLGSG-VRRQVD
-AFRAG---FSQVFPY--SALS-AFTPD-ELVTLFGR-----------------------
---VDEDWS-----------LESL---------LDSIKADHG------------------
----------------YNMD----SKTV-KNLLHTMS--------------------EFD
ASQRRDFLQF-------TTGSPKLP------------IGGG----FK-SLTPMF--TVVC
KPSEHPYTSD------------------------DYLPSVMTCV-NY-LKLPDY-STIEA
MRKQLF-TAVKE-GQGA
>Fusarium_mangiferae_FCQH01000006.1 .
VRISRQKI-LESALKVM----------------ELYGASQS-ILEVEYFE-------EVG
TGLG-PTLEF---YSTV----SKEFSKRKL--------KLWREVDSNGSD----------
---------EFVSGATGLFPRPQS--------------------------------DEEA

GTPNGERILHLFKMLGKFVARSMIDSRIIDLHFNP--IFFRIGDA---------------
----VSSGVKPSLGAVKI----------------------VDPVLARS--------LKAI
KQFALAKKEIDEDPNRTPAQK-----------------VADTESITIDG-VKLD-DLC--
--LDFTL-------------------------------PG--YPNIQ-LEDN--------
GSQKR---
------------------------------VTIDNVD-SYLEK-VIDMTLGSG-VRRQVD
-AFRAG---FSQVFPY--SALS-AFTPD-ELVTLFGR-----------------------
---VDEDWS-----------LESL---------LDSIKADHG------------------
----------------YNMD----SKTV-KNLLHTMS--------------------EFD
ASQRRDFLQF-------TTGSPKLP------------IGG-----FK-SLTPMF--TVVC
KPSEHPYTSD------------------------DYLPSVMTCV-NY-LKLPDY-STIQI
MRKQLF-TAVKE-GQGA
>Torrubiella_hemipterigena_CDHN01000006.1 .
VRISRTKI-LESALKVM----------------DLYGASQS-ILEVEYFE-------EVG
TGLG-PTLEF---YSTV----SQEFAKKKL--------RLWRDMDSIGSE----------
---------EFVNSQVGLFPRPLG--------------------------------VEEL
STPNGERILHLFKILGKFVARSMIDSRIIDIHLNP--IFFRIADQ---------------
----TGRGVKPSLGAVKL----------------------VDPGLARS--------LKMI
RKFAQAKKEVDEDPTRTPAQK-----------------VADTEAICVDG-IKLE-DLC--
--LDFTL-------------------------------PG--YPGVE-LEEN--------
GSQKR---
------------------------------VTMDNVE-AYLDG-VVEFTLGTG-VSQQID
-AFRTG---FSQVFPY--TALS-AFTPD-ELVSLFGR-----------------------
---VEEDWS-----------LETL---------TDSIKADHG------------------
----------------YNMD----SRSV-KNLLQAMS--------------------QFD
DVQRREFLQF-------ITGSPKLP------------IGG-----FR-SLTPMF--TVVC
KPSEHPYTSD------------------------DYLPSVMTCV-NY-LKLPDY-SSLDV
MIQRLS-TAMKE-GQGA
>Lecanicillium_fungicola_FWCC01000041.1 .
VRISRQKI-LESALKVM----------------ELYGASQS-ILEVEYFE-------EVG
TGLG-PTLEF---YSTV----SNEFAKKKL--------RLWRDVDGNGSD----------
---------EFISGASGLFPRPLS--------------------------------AEEV
STPNGERILHLFKMLGKFVARSMIDSRIIDIHFNP--TFFRITDV---------------
----ASAGVKPSLGAVKV----------------------VDPGLARS--------LKTI
MKYSTAKKEIDEDPSRTPAQK-----------------VADTESISIDG-VRLD-DLC--
--LDFTL-------------------------------PG--YPNIE-LETN--------
GSQKR---
------------------------------VSIENVD-SYLDK-VIDMTLGSG-VRSQVD
-AFRAG---FSQVFPY--TALS-AFTPA-ELVALFGK-----------------------
---VNEDWT-----------ATAL---------TDSIKADHG------------------
----------------YNMD----SHSV-KNLLQVMS--------------------EFN
LSERREFLQF-------ITGSPKLP------------IGG-----FR-SLTPMF--TVVC
KPSEHPYTSD------------------------DYLPSVMTCV-NY-LKLPDY-TTHET
MRKRIL-TAMKE-GQGA
>Cordyceps_confragosa_LUKN01001594.1 .
VRISRLKI-LESALKVM----------------ELYGASQS-ILEVEYFE-------EVG
TGLG-PTLEF---YSTV----SKEFAKKKL--------RLWRDIDNTGSD----------
---------EFVSGPSGLFPRPLS--------------------------------AEEL
STPNGERVLHLFKILGKFIARSMIDSRIIDIHFNP--TFFRIGDA---------------
----AAVGVKPSLGAVKV----------------------VDPGLARS--------LKTI
KKYSVAKKKIDEHPSRTPAQK-----------------VADTENISIDG-VRLD-DLC--
--LDFTL-------------------------------PG--YPGIE-LEPN--------
GSQKR---
------------------------------VTIENVD-AYLDK-VIDMTLGSG-VRVQVE
-AFKIG---FSQVFPY--DALS-AFTPV-ELVALFGK-----------------------
---VNEDWS-----------LESX---------GSSIKADHG------------------
----------------YNMD----SRSV-KNLLQVMS--------------------EFD
LSERREFLQF-------ITGSPKLP------------IGG-----FR-SLTPMF--TVVC

```
KPSEHPYTSD------------------------DYLPSVMTCV-NY-LKLPDY-TDHDT
MRKRLL-TAMKE-GQGA
>Cordyceps_cicadae_AEIW01001170.1 .
VRISRLKI-LESALKVM----------------ELYGASQS-ILEVEYFE-------EVG
TGLG-PTLEF---YSSV----SKEFAKKKL--------RLWRDVDNTGSD----------
---------EFVSGPSGLFPRPLS--------------------------------AEEL
GTPNGERILHLFKMLGKFIARSMIDSRIIDIHFNP--TFFRISDA---------------
----AAAGVKPSLGAVKV----------------------VDPGLARS--------LKAI
KKYSIAKKEIDEDPTRTPAQK-----------------VADTEDICIEG-VRLD-DLC--
--LDFTL-------------------------------PG--YPGIE-LEPN--------
GPQKR-------------------------------------------------------
------------------------------VTIDNVD-VYLDR-VIDMTLGSG-VRAQVD
-AFRIG---FSQVFPY--TALS-AFTPA-ELVTLFGK-----------------------
---VNEDWS-----------LESL---------TDSIKADHG------------------
----------------FNMD----SRSV-KNLLQVMS--------------------EFD
LSERREFLQF-------ITGSPKLP------------IGG-----FR-SLTPMF--TVVC
KPSEHPYTSD------------------------DYLPSVMTCV-NY-LKLPDY-TDHDT
MRKRLL-TAMKE-GQGA
>Isaria_fumosorosea_XM_018851238.1 .
VRISRLKI-LESALKVM----------------ELYGASQS-ILEVEYFE-------EVG
TGLG-PTLEF---YSSV----SKEFAKKKL--------RLWRDVDNTGSD----------
---------EFVSGPSGLFPRPLS--------------------------------TEEL
GTPNGERILHLFKMLGKFIARSMIDSRIIDIHFNP--TFFRISDA---------------
----AAAGVKPSLGAVKV----------------------VDPGLARS--------LKAI
KKYSIAKKEIDEDPTRTPAQK-----------------VADTENICIDG-VRLD-DLC--
--LDFTL-------------------------------PG--YPGIE-LEPN--------
GPQNR-------------------------------------------------------
------------------------------VTIDNVD-VYLDR-VIDMTLGSG-VRAQVD
-AFRTG---FSQVFPY--TALS-AFTPA-ELVTLFGK-----------------------
---VNEDWS-----------LETL---------TDSIKADHG------------------
----------------FNMD----SRSV-KNLLQVMS--------------------EFD
LSERREFLQF-------ITGSPKLP------------IGG-----FR-SLTPMF--TVVC
KPSEHPYTSD------------------------DYLPSVMTCV-NY-LKLPDY-TDHDT
MRKRLL-TAMKE-GQGA
>Paecilomyces_hepiali_LNDK02000121.1 .
VRISRLKI-LESALKVM----------------ELYGASQS-ILEVEYFE-------EVG
TGLG-PTLEF---YSTV----SKEFAKKKL--------RLWRDIDNTGSD----------
---------EFVSGPSGLFPRPLS--------------------------------SEEL
GTPNGERVLHLFKILGKFIARSMIDSRIIDIHFNS--TFFRISDA---------------
----AAAGVKPSLGAVKV----------------------VDPGLARS--------LKAI
MKYSIAKKEIDEDPNRTPARK-----------------VADTENISIEG-VRLD-DLC--
--LDFTL-------------------------------PG--YPGIE-LETN--------
GSQKR-------------------------------------------------------
------------------------------VTIDNVD-AYLDK-VIDMTLGSG-VRAQVD
-AFRTG---FSQVFPY--TALS-AFTPA-ELVALFGK-----------------------
---VNEDWS--------L--ESTL---------NSSIKADHG------------------
----------------YNMD----SRSV-KNLLQVMS--------------------EFD
LNERREFLQF-------ITGSPKLP------------IGG-----FR-SLTPMF--TVVC
KPSEHPYTSD------------------------DYLPSVMTCV-NY-LKLPDY-TDHAT
MGKRLL-TAMKE-GQGA
>Isaria_farinosa_JMNC01000648.1 .
VRISRLKI-LESALKVM----------------ELYGASQS-ILEVEYFE-------EVG
TGLG-PTLEF---YSTV----SKEFAKKKL--------RLWRDIDNTGSN----------
---------EFVSGPSGLFPRPLS--------------------------------AEEL
NTPNGERVLHLFRILGKFIARSMIDSRIIDIHFNP--TFFRISEA---------------
----AAAGVKPSLGAVKV----------------------VDPGLARS--------LKAI
KRYSIAKKEIDEDPSRTPAQK-----------------VADTENISIDG-VRLD-DLC--
--LDFTL-------------------------------PG--YPGIE-LEAN--------
```

GSQKR---
------------------------------VTIDNVD-TYLDK-VIDMTLGSG-VRAQVD
-AFRTG---FSQVFPY--TALS-AFTPA-ELVALFGK-----------------------
---VNEDWS-----------YSAL---------TDSIKADHG------------------
----------------YNMD----SRSV-KNLLQVMS--------------------EFD
LSERREFLQF-------ITGSPKLP------------IGG-----FR-SLTPMF--TVVC
KPSEHPYTSN------------------------DYLPSVMTCV-NY-LKLPDY-TDHDT
MRKRLL-TAMKE-GQGA
>Cordyceps_militaris_XM_006665520.1 .
VRISRLKI-LESALKVM----------------ELYGASQS-ILEVEYFE-------EVG
TGLG-PTLEF---YSTV----SKEFAKKKL--------RLWRDVDNTGSD----------
---------EFVSGPSGLFPRPLS--------------------------------AEEL
STPNGERILHLFKIIGKFIARSMIDSRIIDIHFNP--TFFRISDV---------------
----VAPGVKPSLGAVKV----------------------VDPGLARS--------LKAI
KKYSIAKKDIDEDPNRTPAQK-----------------VADTENISIDG-VRLD-DLC--
--LDFTL-------------------------------PG--YPGIE-LEAN--------
GSQKR---
------------------------------VTIDNVD-AYLDK-VIDMTLGSG-VRAQVD
-AFRTG---FSQVFPY--TALS-AFTPA-ELVALFGK-----------------------
---VNEDWS-----------LETL---------TDSIKADHG------------------
----------------FNMD----SRSV-KDLLQVMS--------------------EFD
LSERREFLQF-------ITGSPKLP------------IGG-----FR-SLTPMF--TVVC
KPSEHPYTSD------------------------DYLPSVMTCV-NY-LKLPDY-TDHDT
MRRRLL-TAMKE-GQGA
>Cordyceps_brongniartii_AZHA01000002.1 .
VRISRLKI-LESALKVM----------------ELYGASQS-ILEVEYFE-------EVG
TGLG-PTLEF---YSNV----SKEFAKKKL--------RLWRDIDSPGSD----------
---------EFVSGPSGLFPRPLS--------------------------------AEEL
GTPNGERILHLFKMLGKFIARSMIDSRIIDIHFNP--IFFRISDA---------------
----AAAGVKPSLGAVKV----------------------VDPGLARS--------LKAI
KKYSIAKKAIDEDPNRTPAQK-----------------VAHTENICIDG-VRLD-DLC--
--LDFTL-------------------------------PG--YPGIE-LETN--------
GSQKR---
------------------------------VTIDNVD-AYLDK-VIDMTLGSG-VRAQVD
-AFGAG---FSQVFPY--TALS-AFTPA-ELVALFGK-----------------------
---VNEDWS-----------LESL---------TDSIKADHG------------------
----------------FNMD----SRSV-KNLLQVMS--------------------EFA
LSERREFLQF-------ITGSPKLP------------IGG-----FR-SLTPMF--TVVC
KPSEHPYTSD------------------------DYLPSVMTCV-NY-LKLPDY-TDHDT
MRRRLL-TAMKE-GQGA
>Beauveria_bassiana_XM_008598655.1 .
VRISRLKI-LESALKVM----------------ELYGASQS-ILEVEYFE-------EVG
TGLG-PTLEF---YSTV----SKEFAKKKL--------RLWRDIDSPGSD----------
---------EFVSGPSGLFPRPLS--------------------------------AEEL
GTPNGERILHLFKMLGKFIARSMIDSRIIDIHFNP--IFFRISDA---------------
----AAAGVKPSLGAVKV----------------------VDPGLARS--------LKAI
KKYSIAKKAIDEDPNRTPAQK-----------------VADTENICIDG-VRLD-DLC--
--LDFTL-------------------------------PG--YPGIE-LETN--------
GSQKR---
------------------------------VTIDNVD-AYLDK-VIDMTLGSG-VRAQVD
-AFGAG---FSQVFPY--SALS-AFTPA-ELVALFGK-----------------------
---VNEDWS-----------LETL---------TDSIKADHG------------------
----------------FNMD----SRSV-KNLLQVMS--------------------EFA
LSERREFLQF-------ITGSPKLP------------IGG-----FR-SLTPMF--TVVC
KPSEHPYTSD------------------------DYLPSVMTCV-NY-LKLPDY-TDHDT
MRRRLL-TAMKE-GQGA
>Beauveria_rudraprayagi_JMNB01004186.1 .
VRISRLKI-LESALKVM----------------ELYGASQS-ILEVEYFE-------EVG

TGLG-PTLEF---YSTV----SKEFAKKKL--------RLWRDIDSSGSD----------
---------EFVSGPSGLFPRPLS--------------------------------AEEL
GTPNGERILHLFKMLGKFIARSMIDSRIIDIHFNP--IFFRISDA---------------
----AAAGVKPSLGAVKV----------------------VDPGLARS--------LKAI
KKYSIAKKAIDEDPNRTPAQK-----------------VADTENICIDG-VRLD-DLC--
--LDFTL-------------------------------PG--YPGIE-LETN--------
GSQKR---
------------------------------VTIDNVD-AYLDK-VIDMTLGSG-VRAQVD
-AFGAG---FSQVFPY--SALS-AFTPA-ELVALFGK-----------------------
---VNEDWS-----------LESL---------TDSIKADHG------------------
----------------FNMD----SRSV-KNLLQVMS--------------------EFA
LSERREFLQF-------ITGSPKLP------------IGG-----FR-SLTPMF--TVVC
KPSEHPYTSD------------------------DYLPSVMTCV-NY-LKLPDY-TDHDT
MRRRLL-TAMKE-GQGA
>Verticillium_albo_atrum_XM_003001605.1 .
VRISRSKI-LESALKVM----------------ELYGASQS-ILEVEYFE-------EVG
TGLG-PTLEF---YSTV----SKEFSKKKL--------KLWREMDSHD-D----------
---------EYVSGVSGLFPRPLS--------------------------------ADEA
ASANGERICQLFKTLGKFVARSMIDSRIIDLNFNP--TFFRITDG---------------
---ASISGIKPSLGAVKV----------------------VDPGLARS--------LKTI
KKFSIAKKEIDEDPTRTPAQK-----------------VADTEAIVIDD-VRLE-DLC--
--LDFTL-------------------------------PG--YPEIE-LVAN--------
GSHVR---
------------------------------VTMDNVD-SYLDR-VIDMTLGSG-VRRQVD
-AFRAG---FSQVFPY--SALS-AFTPD-ELVSLFGR-----------------------
---VDEDWS-----------LETL---------MDSIKADHG------------------
----------------YNMD----SKSV-RNLLQAMS--------------------ELT
PNERRDFLQF-------TTGSPKLP------------IGG-----FK-SLTPMF--TVVC
KPSEAPFTSD------------------------DYLPSVMTCV-NY-LKLPDY-TDLDI
MRKQLT-MAIRE-GQG-
>Verticillium_dahliae_XM_009652377.1 .
VRISRSKI-LESALKVM----------------ELYGASQS-ILEVEYFE-------EVG
TGLG-PTLEF---YSTV----SKEFSKKKL--------KLWREMDSHD-D----------
---------EYVSGVSGLFPRPLS--------------------------------ADEA
ASANGERICQLFKTLGKFVARSMIDSRIIDLNFNP--TFFRITDG---------------
---ASISGIKPSLGAVKV----------------------VDPGLARS--------LKTI
KKFSLAKKEIDEDPTRTPAQK-----------------VADTEAIVIDD-VRLE-DLC--
--LDFTL-------------------------------PG--YPEIE-LVAN--------
GSHVR---
------------------------------VTMDNVD-SYLDR-VIDMTLGSG-VRRQVD
-AFRAG---FSQVFPY--SALS-AFTPD-ELVSLFGR-----------------------
---VDEDWS-----------LETL---------MDSIKADHG------------------
----------------YNMD----SKSV-RNLLQAMS--------------------ELT
PNERRDFLQF-------TTGSPKLP------------IGG-----FK-SLTPMF--TVVC
KPSEAPFTSD------------------------DYLPSVMTCV-NY-LKLPDY-TSLDI
MRKQLT-MAIRE-GQGA
>Verticillium_longisporum_CVQH01001114.1 .
VRISRSKI-LESALKVM----------------ELYGASQS-ILEVEYFE-------EVG
TGLG-PTLEF---YSTV----SKEFSKKKL--------KLWREMDSHD-D----------
---------EYVSGVSGLFPRPLS--------------------------------ADEA
ASANGERICQLFKTLGKFVARSMIDSRIIDLNFNP--TFFRITDG---------------
---ASISGIKPSLGAVKV----------------------VDPGLARS--------LKTI
KKFSIAKKEIDEDPTRTPAQK-----------------VADTEAIVIDD-VRLE-DLC--
--LDFTL-------------------------------PG--YPEIE-LVAN--------
GSHVR---
------------------------------VTMDNVD-SYLDR-VIDMTLGSG-VRRQVD
-AFRAG---FSQVFPY--SALS-AFTPD-ELVSLMPRM----------------------
---VEDNFS-----------AAAL---------MDSIKADHG------------------

----------------YNMD----SKSV-RNLLQAMS--------------------ELT
PNERRDFLQF-------TTGSPKLP------------IGG-----FK-SLTPMF--TVVC
KPSEAPFTSD------------------------DYLPSVMTCV-NY-LKLPDY-TSLDI
MRKQLT-MAIRE-GQGA
>Verticillium_alfalfae_ABPE01003279.1 .
VRISRSKI-LESALKVM----------------ELYGASQS-ILEVEYFE-------EVG
TGLG-PTLEF---YSTV----SKEFSKKKL--------KLWREMDSHD-D----------
---------EYVSGVSGLFPRPLS--------------------------------ADEA
ASANGERICQLFKTLGKFVARSMIDSRIIDLNFNP--TFFRITDG---------------
---ASISGIKPSLGAVKV----------------------VDPGLARS--------LKTI
KKFSIAKKEIDEDPTRTPAQK-----------------VADTEAIVIDD-VRLE-DLC--
--LDFTL-------------------------------PG--YPEIE-LVAN--------
GSHVR---
------------------------------VTMDNVD-SYLDR-VIDMTLGSG-VRRQVD
-AFRAG---FSQVFPY--SALS-AFTPD-ELVSLFGR-----------------------
---VDEDWS-----------LESX---------HGYNMTARR------------------
----------------LQHD----SKSV-RNLLQAMS--------------------ELT
PNERRDFLQF-------TTGSPKLP------------IGG-----FK-SLTPMF--TVVC
KPSEAPFTSD------------------------DYLPSVMTCV-NY-LKLPDY-TDLDI
MRKQLT-MAIRE-GQGA
>Reticulascus_tulasneorum_LSAY01000010.1 .
VRISRSKI-LESALKVM----------------ELYGASQS-ILEVEYFE-------EVG
TGLG-PTLEF---YSTV----SKEFSKKKL--------KLWREMDSNDSD----------
---------EFVSGPSGLFPRPLS--------------------------------EEEI
TTPNGERILQMFKMLGKFVARSMIDSRIIDLNFNP--TFFRIGEA---------------
---SAEPSVKPSFGAVRI----------------------VDPGLARS--------MKAI
KKFALAKKEIDEDHTRAAAQK-----------------VADTEDIVIDG-VKIE-DLC--
--LDFTL-------------------------------PG--YPEIE-LVSN--------
GSQVR---
------------------------------VTIDNVD-SYLER-VTDMTLGSG-VRRQVD
-AFRAG---FSQVFPY--SALS-AFTPD-ELVTLFGR-----------------------
---VDEDWS--------------L---------MDSIKADHG------------------
----------------FNMD----SKTV-KNLLQTMS--------------------ELT
ATERREFLQF-------TTGSPKLP------------IGG-----FR-SLTPMF--TVVC
KPSEPPFMSD------------------------DYLPSVMTCV-NY-LKLPDY-TSIDT
MRKQLF-TAIKE-GQGA
>Colletotrichum_orbiculare_AMCV01005424.1 .
VRISRSKI-LESALKVM----------------ELYGASQS-ILEVEYFE-------EVG
TGLG-PTLEF---YSTV----SKEFAKKKL--------KLWREMDSNDSE----------
---------EYISGASGLFPRPLS--------------------------------DEDA
TSPNGERILQLFKTLGKFVARSMIDSRIIDLNFNP--IFFRIGDG---------------
---SSASGVKPSLGAVKV----------------------VDPGLARS--------LKTI
KKFAFAKKEIDEDPSRTPAQK-----------------VTDTESITIDG-VKLD-DLC--
--LDFTL-------------------------------PG--YPEIE-LSPG--------
GAQTR---
------------------------------VTTDNVD-AYLER-VIDITLGSG-VRRQVD
-AFRTG---FSQVFPY--SALS-AFTPD-ELVTLFGR-----------------------
---VEEDWS------------XAL---------MDSIKADHG------------------
----------------YNMD----SKSV-KNLLQTMS--------------------ELS
PAARRDFLQF-------TTGSPKLP------------IAG-----FK-SLTPMF--TVVC
KPSEAPYTSD------------------------DYLPSVMTCV-NY-LKLPDY-TNIDV
MRKQLS-TAIKE-GQGA
>Colletotrichum_gloeosporioides_XM_007275846. 1..
VRISRSKI-LESALKVM----------------ELYGASQS-ILEVEYFE-------EVG
TGLG-PTLEF---YSTV----SKEFAKKKL--------KLWREMDSNDSE----------
---------EYISGASGLFPRPLS--------------------------------DEDS
TSPNGERILQLFKTLGKFVARSMIDSRIIDLNFNP--TFFRIGDS---------------
---SAAPGVKPSLGAVKV----------------------VDPGLARS--------LKTI

```
KKFALAKKEIDEDPSRTPAQK-----------------VADTEDITIDG-VKLD-DLC--
--LDFTL-------------------------------PG--YPEIE-LMHN--------
GAQIR-------------------------------------------------------
------------------------------VTIDNVD-SYLER-VIDMTLGTG-VRRQVD
-AFRTG---FSQVFPY--SALS-AFTPD-ELVTLFGR-----------------------
---VEEDWS-----------LETL---------MDSIKADHG------------------
----------------YNMD----SKSV-KNLLQTMS--------------------ELT
PAERRDFLQF-------TTGSPKLP------------IGG-----FK-SLTPMF--TVVC
KPSEAPYTSD------------------------DYLPSVMTCV-NY-LKLPDY-TSINV
MRKQLS-TAIKE-GQGA
>Colletotrichum_incanum_LFIW01001556.1 .
VRISRSKI-LESALKVM----------------ELYGASQS-ILEVEYFE-------EVG
TGLG-PTLEF---YSTV----SKEFSKKKL--------KLWREMDSNDSE----------
---------EYISGASGLFPRPLS--------------------------------DDEA
GAPNGERILQLFKTLGKFVARSMIDSRIIDLNFNP--TFFRIGDG---------------
---SSAPGVKPSLGAVKV----------------------VDPGLARS--------LKTI
KKFAVAKKEIDEDPSRTPAQK-----------------VADTEDIAIDG-VKLD-DLC--
--LDFTL-------------------------------PG--YPEIE-LIPN--------
GGQIR-------------------------------------------------------
------------------------------VTIDNVD-SYLER-VVDMTLGTG-VRRQVD
-AFRTG---FSQVFPY--SALS-AFTPD-ELVTLFGR-----------------------
---VEEDWS-----------LESL---------MDSIKADHG------------------
----------------YNMD----SKSV-KNLLQTMS--------------------ELS
PSERRDFLQF-------TTGSPKLP------------IGG-----FK-SLTPMF--TVVC
KPSEHPYTSD------------------------DYLPSVMTCV-NY-LKLPDY-TSIGV
MRKQLS-TAIKE-GQGA
>Colletotrichum_tofieldiae_LFIV01000002.1 .
VRISRSKI-LESALKVM----------------ELYGASQS-ILEVEYFE-------EVG
TGLG-PTLEF---YSTV----SKEFSKKKL--------KLWREMDSNDSE----------
---------EYISGASGLFPRPLS--------------------------------DDEA
GAPNGERILQLFKTLGKFVARSMIDSRIIDLNFNP--TFFRIGDG---------------
---SSAPGVKPSLGAVKV----------------------VDPGLARS--------LKTI
KKFAVAKKEIDEDPSRTPAQK-----------------VADTEDIAIDG-VKLD-DLC--
--LDFTL-------------------------------PG--YPEIE-LIPN--------
GSQAR-------------------------------------------------------
------------------------------VTIDNVD-SYLER-VVDMTLGTG-VRRQVD
-AFRTG---FSQVFPY--SALS-AFTPD-ELVTLFGR-----------------------
---VEEDWS-----------LESL---------MDSIKADHG------------------
----------------YNMD----SKSV-KNLLQTMS--------------------ELS
PSERRDFLQF-------TTGSPKLP------------IGG-----FK-SLTPMF--TVVC
KPSEHPYTSD------------------------DYLPSVMTCV-NY-LKLPDY-TSIGV
MRKQLS-TAIKE-GQGA
>Colletotrichum_higginsianum_XM_018303045.1 .
VRISRSKI-LESALKVM----------------ELYGASQS-ILEVEYFE-------EVG
TGLG-PTLEF---YSTV----SKEFAKKKL--------KLWREMDSNDSE----------
---------EYISGASGLFPRPLS--------------------------------DDEA
GAPNGERILQLFKTLGKFVARSMIDSRIIDLNFNP--TFFRIGDD---------------
---SSAPGVKPSLGAVKV----------------------VDPGLARS--------LKTI
KKFAVAKKEIDEDPSRTPAQK-----------------VANTEDIVIDG-VKLD-DLC--
--LDFTL-------------------------------PG--YPEIE-LIPD--------
GGQVR-------------------------------------------------------
------------------------------VTIDNVD-SYLER-VIDMTLGTG-VRRQVD
-AFRTG---FSQVFPY--SALS-AFTPD-ELVTLFGR-----------------------
---VDEDWS-----------LESL---------MDSIKADHG------------------
----------------YNMD----SKSV-KNLLQTMS--------------------ELS
PSERRDFLQF-------TTGSPKLP------------IGG-----FK-SLTPMF--TVVC
KPSEHPYMSD------------------------DYLPSVMTCV-NY-LKLPDY-TGIEV
MRKQLS-TAIKE-GQGA
```

>Colletotrichum_graminicola_XM_008096094.1 .
VRISRSKI-LESALKVM----------------ELYGASQS-ILEVEYFE-------EVG
TGLG-PTLEF---YSTV----SKEFSKKKL--------KLWREMDSNDSE----------
---------EYISGASGLFPRPLS--------------------------------DDEA
SAPNGERILQLFRTLGKFVARSMIDSRIIDLNFNP--TFFRIGDG---------------
---SSAPGVKPSLGAVKV----------------------VDPGLARS--------LKTI
KKFAVAKKEIDEDPSRTPAQK-----------------VADTEDIAIDG-MKLD-DLC--
--LDFTL-------------------------------PG--YPEIE-LIPN--------
GGQIR---
------------------------------VTIDNVD-SYLER-VVDMTLGTG-VRRQVD
-AFRTG---FSQVFPY--SALS-AFTPD-ELVTLFGR-----------------------
---VEEDWS-----------LETL---------MDSIKADHG------------------
----------------YNMD----SKSV-KNLLQTMS--------------------ELS
PPERRDFLQF-------TTGSPKLP------------IGG-----FK-SLTPMF--TVVC
KPSEHPYMSD------------------------DYLPSVMTCV-NY-LKLPDY-TSIEV
MRKQLS-TAIKE-GQG-
>Colletotrichum_orchidophilum_MJBS01000051 1..
VRISRSKI-LESALKVM----------------ELYGASQS-ILEVEYFE-------EVG
TGLG-PTLEF---YSTV----SREFSKKKL--------KLWREMDSNDSE----------
---------EYISGASGLFPRPLS--------------------------------DDEA
TAPNGERILQLFKTLGKFVARSMIDSRIIDLNFNP--TFFRIGDG---------------
---SSAPGVKPSLGAVKV----------------------VDPGLARS--------LKTI
KKFAVAKKEIDEDPSRTPAQK-----------------VADTEDIGIDG-VKLD-DLC--
--LDFTL-------------------------------PG--FPEIE-LMPN--------
GGQVR---
------------------------------VTIDNVD-SYLER-VIDMTLGSG-VRRQVD
-AFRLG---FSQVFPY--SALS-AFTPD-ELVTLFGR-----------------------
---VDEDWS-----------LESL---------MDSIKADHG------------------
----------------YNMD----SKTV-KNLLQTMS--------------------ELS
APERRDFLQF-------TTGSPKLP------------IGG-----FK-SLTPMF--TVVC
KPSEHPYTSD------------------------DYLPSVMTCV-NY-LKLPDY-TDIES
MRKQLS-TAIKE-GQGA
>Colletotrichum_fioriniae_XM_007595785.1 .
VRISRSKI-LESALKVM----------------ELYGASQS-ILEVEYFE-------EVG
TGLG-PTLEF---YSTV----SREFSKKKL--------KLWREMDSNDSE----------
---------EYISGASGLFPRPLS--------------------------------DDEA
VAPNGERILQLFKTLGKFVARSMIDSRIIDLNFNP--TFFRIGDG---------------
---SSAPGVKPSLGAVKV----------------------VDPGLARS--------LKTI
KKFAVAKKEIDEDPSRTPAQK-----------------VADTEDIVIDG-VKLD-DLC--
--LDFTL-------------------------------PG--FPEIE-LMPN--------
GGQIR---
------------------------------VTIDNVD-AYLER-VIDMTLGTG-VRRQVD
-AFRTG---FSQVFPY--SALS-AFTPD-ELVTLFGR-----------------------
---VDEDWS-----------LETL---------MDSIKADHG------------------
----------------YNMD----SKTV-KNLLQTMS--------------------ELS
APERRDFLQF-------TTGSPKLP------------IGG-----FK-SLTPMF--TVVC
KPSEHPYTSD------------------------DYLPSVMTCV-NY-LKLPDY-TNIEA
MRKQLS-TAIKE-GQG-
>Colletotrichum_salicis_JFFI01001510.1 .
VRISRSKI-LESALKVM----------------ELYGASQS-ILEVEYFE-------EVG
TGLG-PTLEF---YSTV----SREFSKKKL--------KLWREMDSNDSE----------
---------EYISGASGLFPRPLS--------------------------------DDEA
IAPNGERILQLFKTLGKFVARSMIDSRIIDLNFNP--TFFRIGDG---------------
---SSAPGVKPSLGAVKV----------------------VDPGLARS--------LKTI
KKFAVAKKEIDEDPSRTPAQK-----------------VSDTEDIVIDG-VKLD-DLC--
--LDFTL-------------------------------PG--FPEIE-LMPN--------
GGQVR---
------------------------------VTIDNVD-SYLES-VIEMTLGTG-VRRQVD

-AFRTG---FSQVFPY--STLS-AFTPD-ELVTLFGR-----------------------
---VDEDWS-----------LESL---------MDSIKADHG------------------
----------------YNMD----SKTV-KNLLQTMS--------------------ELS
APERRDFLQF-------TTGSPKLP------------IGG-----FK-SLTPMF--TVVC
KPSEHPYTSD------------------------DYLPSVMTCV-NY-LKLPDY-TNIEA
MRKQLS-TAIKE-GQGA
>Colletotrichum_acutatum_LVCK01000416.1 .
VRISRSKI-LESALKVM----------------ELYGASQS-ILEVEYFE-------EVG
TGLG-PTLEF---YSTV----SREFSKKKL--------KLWREMDSNDSE----------
---------EYISGASGLFPRPLS--------------------------------DDEA
IAPNGERILQLFKTLGKFVARSMIDSRIIDLNFNP--TFFRIGDG---------------
---SSAPGVKPSLGAVKV----------------------VDPGLARS--------LKTI
KKFAVAKKEIDEDPSRTPAQK-----------------VADTEDIVIDG-VKLD-DLC--
--LDFTL-------------------------------PG--FPEIE-LMPN--------
GGQIR---
------------------------------VTIDNVD-SYLER-VIDMTLGTG-VRRQVD
-AFRTG---FSQVFPY--SALS-AFTPD-ELVTLFGR-----------------------
---VDEDWS-----------LESL---------MDSIKADHG------------------
----------------YNMD----SKTV-KNLLQTMS--------------------ELS
APERRDFLQF-------TTGSPKLP------------IGG-----FK-SLTPMF--TVVC
KPSEHPYTSD------------------------DYLPSVMTCV-NY-LKLPDY-TNIEA
MRKQLS-TAIKE-GQGA
>Colletotrichum_nymphaeae_JEMN01001641.1 .
VRISRSKI-LESALKVM----------------ELYGASQS-ILEVEYFE-------EVG
TGLG-PTLEF---YSTV----SREFSKKKL--------KLWREMDSNDSE----------
---------EYISGASGLFPRPLS--------------------------------DDEA
IAPNGERILQLFKTLGKFVARSMIDSRIIDLNFNP--TFFRIGDG---------------
---SSAPGVKPSLGAVKV----------------------VDPGLARS--------LKTI
KKFAVAKKEIDEDPSRTPAQK-----------------VADTEDIVIDG-VKLD-DLC--
--LDFTL-------------------------------PG--FPEIE-LTPN--------
GGQIR---
------------------------------VTIDNVD-SYLER-VIDMTLGTG-VRRQVD
-AFRTG---FSQVFPY--SALS-AFTPD-ELVTLFGR-----------------------
---VDEDWS-----------LESL---------MDSIKADHG------------------
----------------YNMD----SKTV-KNLLQTMS--------------------ELS
APERRDFLQF-------TTGSPKLP------------IGG-----FK-SLTPMF--TVVC
KPSEHPYTSD------------------------DYLPSVMTCV-NY-LKLPDY-TNIEA
MRKQLS-TAIKE-GQGA
>Colletotrichum_simmondsii_JFBX01000926.1 .
VRISRSKI-LESALKVM----------------ELYGASQS-ILEVEYFE-------EVG
TGLG-PTLEF---YSTV----SREFSKKKL--------KLWREMDSNDSE----------
---------EYISGASGLFPRPLS--------------------------------DDEA
IAPNGERILQLFKTLGKFVARSMIDSRIIDLNFNP--TFFRIGDG---------------
---SSAPGVKPSLGAVKV----------------------VDPGLARS--------LKTI
KKFAVAKKEIDEDPSRTPAQK-----------------VADTEDIVIDG-VKLD-DLC--
--LDFTL-------------------------------PG--FPEIE-LTPN--------
GGQIR---
------------------------------VTIDNVD-SYLER-VIDMTLGTG-VRRQVD
-AFRTG---FSQVFPY--SALS-AFTPD-ELVTLFGR-----------------------
---VDEDWS-----------LESL---------MDSIKADHG------------------
----------------YNMD----SKTV-KNLLQTMS--------------------ELS
APERRDFLQF-------TTGSPKLP------------IGG-----FK-SLTPMF--TVVC
KPSEHPYTSD------------------------DYLPSVMTCV-NY-LKLPDY-TNIEA
MRKQLS-TAIKE-GQGA
>Microdochium_bolleyi_LSSP01000005.1 .
VRISRSKI-LESALKVM----------------ELYGASQS-ILEVEYFE-------EVG
TGLG-PTLEF---YSTV----SREFSKKKL--------KLFRDTDTHDSE----------
---------EFAGGPNGLFPRPMS--------------------------------EES-

-----AVITHLFKMLGKFVARSMIDSRIIDINFNP--IFFRIGDE---------------
-----ASSVKPTLGAVRI----------------------VDPGLANS--------LKHV
KKFALAKKAIDEDPNRTAAQK-----------------VADTENIVVDN-VSLE-DLA--
--LDFTL--------------------------------PG--YPEIE-LIPG--------
GSSEA---
------------------------------VTIYNVD-TYLDK-VIDMTLGDG-VKTQIN
-AFRTG---FSQVFPY--SALS-AFTPD-ELVSLFGR-----------------------
---VEEDWS-----------XAAL---------MDSIKADHG------------------
----------------YNMD----SKTV-RHLLQTMS--------------------ELT
PAERRDFLQF-------TTGSPKLP------------IGG-----FR-SLTPMF--TVVC
KPSEPPYTSD------------------------DYLPSVMTCV-NY-LKLPDY-SSIEI
MKKQLF-TATRE-GQGA
>Arthrinium_malaysianum_GEGN01024218.1 .
VRISRSKI-LQSALKVM----------------ELYGASQS-ILEVEYFE-------EVG
TGLG-PTLEF---YSTV----SKEFSKRKL--------KLWREADSSESD----------
---------EFISGPHGLFPRPLA---------------------------------DED
SSANTERILQLFKMLGKFVARSMIDSRIIDLNFNP--IFFRIGDE---------------
-----GAGVKPSLGAVKI----------------------VDPGLANS--------LKLI
KKFALAKKAIDEDPARTAAQK-----------------VADTEQIVIDH-SRLE-DLA--
--LDFTL--------------------------------PG--HPEIE-LKPG--------
GAHIP---
------------------------------VTIYNVD-SYLEK-VIDMTLGSG-VKRQVD
-AFRAG---FAQVFPY--SALR-AFTPD-ELVTLFGR-----------------------
---VEEDWS-----------LETL---------MDSIKADHG------------------
----------------FNMD----SRSV-KNLLQTMS--------------------ELT
APQRRDFLQF-------TTGSPKLP------------IGG-----FK-SLTPMF--TVVC
KPSETPYTSD------------------------DYLPSVMTCV-NY-LKLPDY-TTIER
MRRQLF-TAIRE-GQG-
>Termitomyces_clypeatus_GAFV01010203.1 .
VRISRSKI-LQSALKVM----------------ELYGASQS-ILEVEYFE-------EVG
TGLG-PTLEF---YSTV----SKEFSKRKL--------KLWREADSSESD----------
---------EFISGPHGLFPRPLA---------------------------------DED
SSANTERILQLFKMLGKFVARSMIDSRIIDLNFNP--IFFRIGDE---------------
-----GAGVKPSLGAVKI----------------------VDPGLANS--------LKLI
KKFALAKKAIDEDPARTAAQK-----------------VADTEQIVIDH-SRLE-DLA--
--LDFTL--------------------------------PG--HPEIE-LKPG--------
GAHIP---
------------------------------VTIYNVD-SYLEK-VIDMTLGSG-VKRQVD
-AFRAG---FAQVFPY--SALR-AFTPD-ELVTLFGR-----------------------
---VEEDWS-----------LETL---------MDSIKADHG------------------
----------------FNMD----SRSV-KNLLQTMS--------------------ELT
APQRRDFLQF-------TTGSPKLP------------IGG-----FK-SLTPMF--TVVC
KPSETPYTSD------------------------DYLPSVMTCV-NY-LKLPDY-TTIER
MRRQLF-TAI-------
>Pseudomassariella_vexata_MCFJ01000010.1 .
VRISRTKI-LESAVKVM----------------ELYGASQS-ILEVEYFE-------EVG
TGLG-PTLEF---YSTV----SKEFSKKKL--------KLWREMDSNDSE----------
---------VFISGPHGLFPRPMA--------------------------------KDDV
NS---ERILQLFKMLGKFVARSMIDSRIIDLNFNP--IFFRIGDG---------------
-----TTGVKPSLGAVKI----------------------VDAGLANS--------LKHI
KKFASAKKEIDEDPNRDAAQK-----------------VADTEQIVIDN-IKIE-DLA--
--LDFTL--------------------------------PG--FPDIE-LIPN--------
GSHSP---
------------------------------VTIYNVD-MYLDK-VVDMTLGSG-VKRQVD
-AFRAG---FSQVFPY--SALS-AFTPD-ELVALFGR-----------------------
---VDEDWS-----------XXAL---------MDSIKADHG------------------
----------------FNMD----SKSV-KNLLQTMS--------------------ELT
AAQRRDFLQF-------TTGSPKLP------------IGG-----FK-SLTPMF--TVVC

KPSEPPYTSD------------------------DYLPSVMTCV-NY-LKLPDY-TTIDS
MRRQLF-TAIKE-GQGA
>Pestalotiopsis_fici_XM_007838867.1 .
VRISRAKI-LESALKVM----------------ELYGESRS-ILEVEYFE-------EVG
TGLG-PTLEF---YSTV----SKEFSKKKI--------KLWREMDSNDSE----------
---------EFISGPHGLFPRPLA--------------------------------TDNA
AN--IDRVLFLFKMLGKFVARSMIDSRIIDLNFNP--IFFRIGDA---------------
-----GHGVKPSLGSVKI----------------------VDRGLANS--------LKLI
KKFVSAKQEIDQDPSRTAAQK-----------------VADTEQIVVDG-TTID-DLA--
--LDFTL-------------------------------PG--YPDIE-LAPQ--------
GTHTP---
------------------------------VTIYNVE-SYLDK-VIDMTLGSG-VKRQID
-AFQTG---FSQVFPY--SALS-AFTPD-ELVSLYGR-----------------------
---VEEDWS-----------LETL---------MDSIKADHG------------------
----------------FNMD----SRSV-KNLLQTMS--------------------EMT
PAQRRDFLQF-------TTGSPKLP------------IGG-----FK-SLTPMF--TVVC
KPSEPPYTPD------------------------DYLPSVMTCV-NY-LKLPDY-TTIEK
MKKQLF-TATRE-GQGA
>Rosellinia_necatrix_BBSO02000384.1 .
VRISRSKI-LESALKVM----------------ELYGSSQS-ILEVEYFE-------EVG
TGLG-PTLEF---YSTV----SKEFSKRKL--------KLWREEDSHDSD----------
---------EFVSAPNGLFPRPLN--------------------------------QDDP
SS---EIILQLFKMLGKFVARSMIDSRIIDIHLNP--TFFRIGEA---------------
------TGVKPSLGAVKI----------------------VDPGLANS--------LKHI
KKFALAKKTIVEDPKRTPAQK-----------------VADTEKIVIED-TMLE-DLA--
--LDFTL-------------------------------PG--YPDIE-LIPN--------
GNHIQ---
------------------------------VTIDNVE-SYLDK-VIDMTLGRG-VRRQVD
-AFRTG---FSQVFPY--SALS-AFTPD-ELVTLFGR-----------------------
---ADEDWT-----------LESL---------TDSIKADHG------------------
----------------FNMD----SKSV-KNLLQAMS--------------------ELT
PTQRRDFLQF-------TTGSPKLP------------IGG-----FK-SLTPMF--TVVC
KPSEPPYTSD------------------------DYLPSVMTCV-NY-LKLPDY-TSIDT
LKRQLF-MAIKE-GQGA
>Kretzschmaria_deusta_MLHU01000491.1 .
VRISRSKI-LESALKVM----------------ELYGSSQS-ILEVEYFE-------EVG
TGLG-PTLEF---YSTV----SREFSKRKL--------KLWREEDSHDSD----------
---------EFVSAPNGLFPRPLN--------------------------------QDDP
SS---EIILQLFKMLGKFVARSMIDSRIIDIHLNP--TFFRIGEG---------------
------TGVKPSLGAVKI----------------------VDPGLANS--------LKHI
KKFVSAKKAIVEDPNRTPAQK-----------------VADTEHIVVDG-TMLD-DLA--
--LDFTL-------------------------------PG--FPDIE-LIPR--------
GSHIQ---
------------------------------VTIDNVE-SYLDK-VIDMTLGRG-VKRQVD
-AFRAG---FSQVFPY--SALS-AFTPD-ELVTLFGR-----------------------
---AEEDWS-----------LESL---------TDSIKADHG------------------
----------------FNME----SKSV-KNLLQTMS--------------------ELT
LPQRRDFLQF-------TTGSPKLP------------IGG-----FK-SLTPMF--TVVC
KPSEPPYTSD------------------------DYLPSVMTCV-NY-LKLPNY-TSIDT
LKRQLF-TAIKE-GQGA
>Didymobotryum_rigidum_BCKI01000004.1 .
VRISRAKI-LESALKVM----------------ELYGSSQS-ILEVEYFD-------EVG
TGLG-PTLEF---YSTV----SKEFSKKKL--------KLWREMDSNDSE----------
---------EFVSGRNGLFPRPLS--------------------------------DEDP
TA---GIIMQLFKMLGKFVARSMIDSRIIDLNFNP--TFFRIGEG---------------
-----SKGVKPSLGAVKI----------------------VDPGLANS--------LKHI
KKFVLAKKAIAEDPRRTPAQK-----------------VADIEKIVVDD-MKLE-DLA--
--LDFTL-------------------------------PG--YPDIE-LVAG--------

GSHKQ---
------------------------------VTIENVE-SYLDK-VIDLTLGRG-VIRQVE
-AFRTG---FSQVFPY--TALS-AFTPD-ELVSLFGR-----------------------
---FEEDWS-----------LESL---------MDSIKADHG------------------
----------------FNMD----SKSV-KNLLQTMS--------------------ELT
SAQRRDFLQF-------TTGSPKLP------------IGG-----FK-SLTPMF--TVVC
KPSEPPYTSD------------------------DYLPSVMTCV-NY-LKLPDY-TSIDS
MRRQLF-TAIKE-GQGA
>Endocalyx_cinctus_BCKC01000006.1 .
VRISRSKI-LESALKVM----------------ELYGSSQS-ILEVEYFE-------EVG
TGLG-PTLEF---YSTV----SKEFSKKKL--------KLWREMDSNDSE----------
---------EFISGPNGLFPKPIS--------------------------------DDDP
TS---NIILQLFKMLGKFVARSMIDSRIIDLNFNP--IFFRIGEG---------------
-----SSAVKPSLGAVKI----------------------VDPGLANS--------LKHI
KKFALAKKAIDEDPNRTAAQK-----------------VADTERIVVDN-STLD-DLA--
--LDFTL-------------------------------PG--YPDIE-LIPG--------
GSHTP---
------------------------------VTIYNVE-SYLDK-VIDMTLGRG-VRRQVE
-AFRMG---FSQVFPY--SALS-AFTPD-ELVTLFGR-----------------------
---VEEDWS-----------LESKF--------YDSIKADHG------------------
----------------FNMD----SRTV-KNLLQTMS--------------------ELT
PSQRRDFLQF-------TTGSPKLP------------IGG-----FK-SLTPMF--TVVC
KPSEPPYTSD------------------------DYLPSVMTCV-NY-LKLPDY-TSIDS
MKRQLF-TAIKE-GQGA
>Xylaria_sp._JWIU01000023.1 .
VRISRSKI-LESALKVM----------------ELYGSSQS-ILEVEYFE-------EVG
TGLG-PTLEF---YSTV----SKEFCKKKL--------KLWREMDSNDSE----------
---------EFISGPSGLFPRPMS--------------------------------DDDP
TV---PVILQLFKMLGKFVARSMIDSRIIDLNFNP--IFFRIGEG---------------
-----TSGVKPSLGAVKV----------------------VDPGLANS--------LKHI
KNFALAKKAIDQDPNRTPAQK-----------------VADTQRIIVEN-TKLE-DLA--
--LDFTL-------------------------------PG--YPDIE-LVPG--------
GSQMP---
------------------------------VSIYNVE-SYLEK-VIDMTLSRG-VKRQVE
-AFRAG---FSQVFPY--SALS-AFTPD-ELVTLFGR-----------------------
---VEEDWS-----------LESL---------MDSIKADHG------------------
----------------FNMD----SRSV-RNLLQTMS--------------------ELT
PAQRRDFLQF-------TTGSPKLP------------IVG-----FK-SLTPMF--TVVC
KPSEPPYTAD------------------------DYLPSVMTCV-NY-LKLPDY-TSIKS
MRRQLF-VAIKE-GQGA
>Hypoxylon_sp._MDGY01000007.1 .
VRISRSKI-LESALKVM----------------ELYGSSQS-ILEVEYFE-------EVG
TGLG-PTLEF---YSTV----SKEFSKKKL--------RLWRDMDSNDST----------
---------EFISGPTGLFPRPIS--------------------------------EDDS
SA---NLTIQLFKMLGKFVARSMIDSRIIDLNFNP--IFFRIGEG---------------
-----SSGVKPSLGAVKS----------------------VDSGLANS--------LKHI
KKFASAKKAIDEDPHRTPAQK-----------------VADTERIVVDN-GTLE-DLA--
--LDFTL-------------------------------PG--YPEIE-LIAN--------
GSQIP---
------------------------------VSIYNVE-SYLEH-VIDLTLGRG-VKRQVE
-AFRTG---FSQVFPY--SALS-AFTPD-ELVALFGR-----------------------
---VDEDWS-----------TPAL---------MDSIKADHG------------------
----------------FNMD----SRTV-RNLLQTMS--------------------ELT
PAQRRDFLQF-------TTGSPKLP------------ISG-----FK-SLTPMF--TVVC
KPSEPPYTSD------------------------DYLPSVMTCV-NY-LKLPDY-STIDS
MRRQLF-TAIRE-GQGA
>Daldinia_eschscholzii_AKGB01000797.1 .
VRISRSKI-LESALKVM----------------ELYGSSQS-ILEVEYFE-------EVG

```
TGLG-PTLEF---YSTV----SKEFSKKKL--------RLWRDMDSNDSD----------
---------EFISGPTGLFPRPLS---------------------------------DDDA
SA---SVILQLFKMLGKFVARSMIDSRIIDLNFNP--IFFRIGEG---------------
-----SSSVKPSLGAVKI----------------------VDPGLANS--------LKHI
KKFAMAKKAIDEDPNRTPAQK-----------------VADTEKIVVDN-GTLE-DLA--
--LDFTL-------------------------------PG--YPEIE-LIPG--------
GSHIP-------------------------------------------------------
-----------------------------VTIYNVE-SYLDK-VIDMTLGRG-VKRQVE
-AFRTG---FSQVFPY--SALS-AFTPD-ELVALFGR----------------------
---VEEDWS-----------LESL---------MDSIKADHG------------------
----------------FNMD----SKSV-RNLLQTMS--------------------ELS
PAQRRDFLQF-------TTGSPKLP------------IGG-----FK-SLTPMF--TVVC
KPSEPPYTSD-----------------------DYLPSVMTCV-NY-LKLPDY-SSIDS
MKRQLF-TAIRE-GQGA
>Chaetomium_globosum_XM_001227186.1 .
VRISRAKI-LESAVKVM----------------ELYGASQS-ILEVEYFD-------EVG
TGLG-PTLEF---YSTV----SKEFCKKKL--------KLWRDNDPNGDD----------
---------EFVFGPNGLFPRPLS---------------------------------ETFA
ASEEGEKILQLYKMLGKFVARSMIDSRIIDVNFNP--IFFRIGAE---------------
-----LTAVRPSLGAIKS----------------------VDPMVARS--------LMIV
KKFALAKKAIDEDPNRSAAQK-----------------VTDTENIVVDK-IRID-DLY--
--LDFTL-------------------------------PG--YPEIE-LIPE--------
GAQTQ-------------------------------------------------------
-----------------------------VTIDNVD-LYLEK-VIDMTLGSG-VRRQVD
-AFQAG---FSQVFPY--SALS-AFTPD-ELCTLFGR----------------------
---VDEDWS-----------LETL---------MDSVKADHG------------------
----------------YNMD----SKTV-RNLLQAMS--------------------EFT
AAQRRDFLQF-------TTGSPKLP------------IGG-----FK-KLTPMF--TVVC
KPSEAPYTSD-----------------------DYLPSVMTCV-NY-LKLPDY-SDIGI
LRKQLF-TAVKE-GQGA
>Thielavia_terrestris_XM_003650276.1 .
VRISRAKI-LESAVKVM----------------ELYGASQS-ILEVEYFD-------EVG
TGLG-PTLEF---YSTV----SKEFCKKKL--------KLWRDNDPNGDD----------
---------EYVSGPNGLFPRPLS---------------------------------DEYA
ASEEGEKILQLFKMLGKFVARSMIDSRIVDINFNP--IFFRIGDE---------------
-----STAVRPSLGAVKS----------------------VDPVIARS--------LMTV
KKFALAKKAIDEDPNRTPAQK-----------------VIDTENIVVDK-IRIE-DLY--
--LDFTL-------------------------------PG--YPEIE-LIPN--------
GSQIQ-------------------------------------------------------
-----------------------------VTIDNVD-LYLER-VIDMTLGSG-VRRQVD
-AFQAG---FSQVFPY--SALS-AFTPD-ELCTLFGR----------------------
---VEEDWS-----------LETL---------MDSIKADHG------------------
----------------YNMD----SKTV-RNLLQAMS--------------------EFT
PAQRRDFLQF-------TTGSPKLP------------IGG-----FK-KLTPMF--TVVC
KPSEAPYTSD-----------------------DYLPSVMTCV-NY-LKLPDY-SNIDI
LKKRLF-TAIKE-GQGA
>Myceliophthora_thermophila_XM_003663951.1 .
VRISRSKI-LESAVKVM----------------ELYGASQS-ILEVEYFD-------EVG
TGLG-PTLEF---YSTV----SKEFCKKKL--------KLWRDNDPNGHD----------
---------EYVSATNGLFPRPLS---------------------------------DEFA
ASEEGEKILQLFKILGKFVARSMIDSRIIDINFNP--IFFRIGDE---------------
-----SAAVRPSLGAIKS----------------------VDPVVAKS--------LMVV
KKFVSAKKAIEEDPNRSAAQK-----------------VIDIENIVIDK-IRID-DLY--
--LDFTL-------------------------------PG--YPEIE-LIPN--------
GAQTQ-------------------------------------------------------
-----------------------------VTIDNVD-LYLEK-VIDMTLGSG-VRRQID
-AFRAG---FSQVFPY--SALS-AFTPD-ELCSLFGR----------------------
---VDEDWS-----------LETL---------MDSVKADHG------------------
```

----------------YNMD----SKTV-RNLLQAMS--------------------EFT
PAQRRDFLQF-------TTGSPKLP------------IGG-----FK-KLTPMF--TVVC
KPSEAPYTSD------------------------DYLPSVMTCV-NY-LKLPDY-SSIDV
LREKLF-TAIKE-GQG-
>Chaetomium_thermophilum_XM_006694686.1 .
VRISRNKI-MESAVKVM----------------ELYGASQS-ILEVEYFD-------EVG
TGLG-PTLEF---YSTV----SKEFCKKKL--------KMWRDNDPNGDD----------
---------EYVSGSNGLFPRPIS--------------------------------DDFA
ASEEGQKVLQLFKTLGKFVARSMIDSRIIDINFNP--IFFRIGDE---------------
-----STAVRPSLGAIKQ----------------------VDPVVARS--------LMLI
KKFALAKKEIDEDPTRSPAQK-----------------VVDMENIVIDK-IKID-DLY--
--LDFTL------------------------------PG--YPEIE-LIPN--------
GSQTR---
------------------------------LTIDNVE-EYLER-VIDMTLGSG-VRRQVD
-AFRQG---FSQVFPY--SALS-AFTPD-ELCSLFGR----------------------
---VEEDWS-----------LETL---------MDSVKADHG------------------
----------------YNMD----SKTV-KNLLQVMS--------------------EMT
PQQRRDFLQF-------TTGSPKLP------------IGG-----FK-KLTPMF--TVVC
KASEPPYTSD------------------------DYLPSVMTCV-NY-LKLPDY-SSIDI
LRQRLF-TAIKE-GQGA
>Madurella_mycetomatis_LCTW02000001.1 .
VRIARAKI-LESAVKVM----------------ELYGASQS-ILEVEYFD-------EVG
TGLG-PTLEF---YSTV----SKEFCKKKL--------RLWRDNDPNGDG----------
---------EFVSGPNGLFPRPLS--------------------------------EEFA
ASEEGEKILQLFKMLGKFVARSMIDSRIIDLNFNP--IFFRIGNE---------------
-----SAAVRPSLGAVKS----------------------VDPVVARS--------LMTI
KKFALAKKAIDEDPNRNAAQK-----------------VLDTANIVIDK-IQID-DLY--
--LDFTL------------------------------PG--YPEIE-LVPN--------
GSHTR---
------------------------------LTIDNVD-LYLEK-VIDMTLGTG-VRRQVD
-AFRAG---FSQVFPY--SALS-AFTPD-ELCTLFGR----------------------
---VDEDWS-----------XSAL---------MDSVKADHG------------------
----------------YNMD----SKSV-RNLLQTMS--------------------ELT
PSQRRDFLQF-------TTGSPKLP------------IGG-----FK-SLTPMF--TVVC
KPSEAPYTSD------------------------DYLPSVMTCV-NY-LKLPDY-SSVEI
LKQRLF-TAIKE-GQGA
>Podospora_anserina_XM_001906184.1 .
VRIARPKI-LESAMKVM----------------ELYGASQS-ILEVEYFE-------EVG
TGLG-PTLEF---YSTV----SKEFCKKKL--------KLWRDHDPSDNG----------
---------EFVSGPNGLFPRPVS--------------------------------EDFL
ATEEGEKTLQLFKILGKFVARSMIDSRIIDINFNP--IFFRIGNE---------------
-----HNAVRPSLGAIKS----------------------VDPMVARS--------LMVI
KKFAMAKKAIEEDPNRDAAQK-----------------VHDLENIAFDK-IRLD-DLY--
--LDFTL------------------------------PG--YPDID-LIPN--------
GSQTR---
------------------------------LTISNVD-LYLER-VIDMTLGSG-VRRQVD
-AFRTG---FSQVFPY--SALS-AFTPD-ELCSLFGR----------------------
---VEEDWS-----------LETL---------MDSIKADHG------------------
----------------YNMD----SKSV-RNLLQTMS--------------------QLT
PAQRRDFLQF-------TTGSPKLP------------IGG-----FK-SLTPMF--TVVC
KPSEAPYTSD------------------------DYLPSVMTCV-NY-LKLPDY-TTIDV
LKKRLF-TAIKE-GQGA
>Sordaria_macrospora_XM_003349751.1 .
VRISRSKI-LESAVKVM----------------ELYGASQS-ILEVEYFE-------EVG
TGLG-PTLEF---YSTV----SKEFSKRKL--------KLWRDNELNGDD----------
---------EFVSGPTGLFPRPLS--------------------------------DEFA
TSEEGEKIMQLFKVLGKFVARSMIDSRIIDINFNA--LFFRVGDA---------------
------TATRPTLATIKS----------------------VDPVIARS--------LMTI

KKFSLAKKEIDEDPNRSAVQK-----------------VTDTENIMIDN-FKID-DLY--
--LDFTL-------------------------------PG--YPEVE-LIPN--------
GSQTR---
------------------------------LTIENVD-IYLEK-VIDMTLGSG-VRRQIE
-AFRAG---FSQVFPY--SALS-SFTPD-ELCSLFGR-----------------------
---VDEDWS-----------LETL---------NDSIKADHG------------------
----------------YNMD----SKSV-RNLLQIMS--------------------ELT
LAERRDFLQF-------TTGSPKLP------------IGG-----FK-SLNPMF--TVVC
KPSEAPYTSD------------------------DYLPSVMTCV-NY-LKLPNY-SDIHV
LKKQLF-TAMKE-GQG-
>Neurospora_pannonica_CAPQ020019039.1 .
VRISRSKI-LESAVKVM----------------ELYGASQS-ILEVEYFE-------EVG
TGLG-PTLEF---YSTV----SKEFSKRKL--------KLWRDNELNGDD----------
---------EFASGPTGLFPRPLS--------------------------------DEFA
SSEEGEKILQLFKMLGKFVARSMIDSRIIDINFNS--LFFRVGDS---------------
------TATRPTLATIKS----------------------VDPVVARS--------LMTI
KKFSLAKKEIDEDPNRSAVQK-----------------VTDTENITIDN-FKID-DLY--
--LDFTL-------------------------------PG--YPEVE-LIPN--------
GSQTR---
------------------------------LTIENVD-LYLEK-VIDMTLGSG-VRRQIE
-AFRSG---FSQVFPY--SALS-SFTPD-ELCTLFGR-----------------------
---VDEDWS-----------LESL---------NDSIKADHG------------------
----------------YNMD----SKSV-RNLLQTMS--------------------ELT
LAERRDFLQF-------TTGSPKLP------------ITG-----FK-SLNPMF--TVVC
KPSEVPYTSD------------------------DYLPSVMTCV-NY-LKLPDY-SDIHV
LKKQLF-TAMKE-GQGA
>Neurospora_terricola_CAPR020035383.1 .
VRISRSKI-LESAVKVM----------------ELYGASQS-ILEVEYFE-------EVG
TGLG-PTLEF---YSTV----SKEFSKRKL--------KLWRDNELNGDD----------
---------EFVSGPTGLFPRPLS--------------------------------DEFA
SSEEGEKILQLFKVLGKFVARSMIDSRIIDINFNS--LFFRVGDS---------------
------TATRPTLATIKS----------------------VDPVVARS--------LMTI
KKFSLAKKEIDEDPNRSAVQK-----------------VRDTENITIDN-FKID-DLY--
--LDFTL-------------------------------PG--YPEVE-LIPN--------
GSQTR---
------------------------------LTIENVD-LYLEK-VIDMTLGSG-VRRQIE
-AFRSG---FSQVFPY--SALS-SFTPD-ELCTLFGR-----------------------
---VDEDWS-----------LESL---------NDSIKADHG------------------
----------------YNMD----SKSV-RNLLQTMS--------------------ELT
LAERRDFLQF-------TTGSPKLP------------IGG-----FK-TLNPMF--TVVC
KPSEAPYTSD------------------------DYLPSVMTCV-NY-LKLPDY-SDIHV
LKKQLF-TAMKE-GQGA
>Neurospora_sublineolata_CAPP020009646.1 .
VRISRSKI-LESAVKVM----------------ELYGASQS-ILEVEYFE-------EVG
TGLG-PTLEF---YSTV----SKEFSKRKL--------KLWRDNELNGDD----------
---------DFVSGPTGLFPRPLS--------------------------------DEFA
SSEEGEKILQLFKVLGKFVARSMIDSRIIDINFNP--LFFRVGDS---------------
------TATRPTLATIKL----------------------VDPVVARS--------LMTI
KKFWLAKKEIDEDPNRSAVQK-----------------VTDTENITVDN-FKID-DLY--
--LDFTL-------------------------------PG--YPEVE-LVPN--------
GSQTR---
------------------------------LTIENVD-LYLEK-VIDTTLGSG-VRRQIE
-AFRSG---FSQVFPY--SALS-SFTPD-ELCTLFGR-----------------------
---VDEDWS-----------LESL---------NDSIKADHG------------------
----------------YNMD----SKSV-RNLLQIMS--------------------ELT
LAERREFLQF-------TTGSPKLP------------ITG-----FK-SLNPMF--TVVC
KPSEAPYTSD------------------------DYLPSVMTCV-NY-LKLPDY-SDMHV
LKKQLF-TAMKE-GQGA

>Neurospora_tetrasperma_AFCY01000802.1 .
VRISRSKI-LESAVKVM----------------ELYGASQS-ILEVEYFE-------EVG
TGLG-PTLEF---YSTV----SKEFSKRKL--------KLWRDNELNGDD----------
---------DFVSGPTGLFPRPLS---------------------------------DEFA
SSEEGEKILQLFKVLGKFVARSMIDSRIIDINFNP--LFFRVGDS---------------
------TATRPTLATIKS----------------------VDPVVARS--------LMTI
KKFSLAKKEIDEDPNRSAVQK-----------------VADTENITIDN-FKID-DLY--
--LDFTL--------------------------------PG--YPEVE-LVSN--------
GSQTR---
------------------------------LTIENVD-LYLEK-VIDMTLGSG-VRRQIE
-AFRSG---FSQVFPY--SALS-SFTPD-ELCTLFGR----------------------
---VDEDWS-----------LESL---------NDSIKADHG-----------------
----------------YNMD----SKSV-RNLLQIMS--------------------ELT
LAERRDFLQF-------TTGSPKLP------------IGG-----FK-SLNPMF--TVVC
KPSEAPYTSD------------------------DYLPSVMTCV-NY-LKLPDY-SDIGV
LKKQLF-TAMKE-GQGA
>Neurospora_crassa_XM_011396715.1 .
VRISRSKI-LESAVKVM----------------ELYGASQS-ILEVEYFE-------EVG
TGLG-PTLEF---YSTV----SKEFSKRKL--------KLWRDNELNGDD----------
---------DFVSGPTGLFPRPLS---------------------------------DEFA
SSEEGEKILQLFKVLGKFVARSMIDSRIIDINFNP--LFFRVGDS---------------
------TATRPTLATIKS----------------------VDPVVARS--------LMTI
KKFSLAKKEIDEDPNRSAVQK-----------------VADTENITIDN-FKID-DLY--
--LDFTL--------------------------------PG--YPEVE-LVSN--------
GSQTR---
------------------------------LTIENVD-LYLEK-VIDMTLGSG-VRRQIE
-AFRSG---FSQVFPY--SALS-SFTPD-ELCTLFGR----------------------
---VDEDWS-----------LETL---------NDSIKADHG-----------------
----------------YNMD----SKSV-RNLLQIMS--------------------ELT
LAERRDFLQF-------TTGSPKLP------------IGG-----FK-SLNPMF--TVVC
KPSEAPYTSD------------------------DYLPSVMTCV-NY-LKLPDY-SDIGV
LKKQLF-TAMKE-GQGA
>Magnaporthe_grisea_LOEM01000306.1 .
VRISRSKI-LESAVKVM----------------ELYGANQS-ILEVEYFE-------EVG
TGLG-PTLEF---YSTV----SKEFSKKKL--------KLWRDADPHDSD----------
---------EFVSSPNGLFPRPIS---------------------------------EEDG
SGSNSDRVLGLFKILGKFVARSMIDSRIIDINLNP--IFFRIGDE---------------
-----KSGVKPSLGAVKM----------------------VDPALARS--------LMLI
KKFSLAKKEIDEDPCRTPAQK-----------------VADTERITLNN-ISIE-DLS--
--LDFTL--------------------------------PG--QPHIE-LIPN--------
GAQIN---
------------------------------LTMDNVD-LYLDK-VIDMTLGSG-VRRQVD
-AFRQG---FSQVFPY--AALS-AFTPD-ELVSLFGR----------------------
---IDEDWS--------L--ESTL---------MDSIKADHG-----------------
----------------YNMD----SLSV-RNLLQFMS--------------------ELS
PAERRDFLQF-------TTGSPKLP------------VTG-----FK-SLNPMF--TVVC
KPSEEPYTSD------------------------DYLPSVMTCV-NY-LKLPNY-TDINV
LRKRMT-TAMKE-GQGA
>Magnaporthe_oryzae_XM_003720001.1 .
VRISRSKI-LESAVKVM----------------ELYGANQS-ILEVEYFE-------EVG
TGLG-PTLEF---YSTV----SKEFSKKKL--------KLWRDADPHDSD----------
---------EFVSSPNGLFPRPIS---------------------------------EEDG
SGSNSDRVLGLFKILGKFVARSMIDSRIIDINLNP--IFFRIGDE---------------
-----KSGVKPSLGAVKM----------------------VDPALARS--------LMLI
KKFSLAKKEIDEDPCRTPAQK-----------------VADTERITLNN-ISIE-DLS--
--LDFTL--------------------------------PG--QPHIE-LIPN--------
GAQIN---
------------------------------LTMDNVD-LYLDK-VIDMTLGSG-VRRQVD

-AFRQG---FSQVFPY--AALS-AFTPD-ELVSLFGR-----------------------
---IDEDWS-----------LETL---------MDSIKADHG------------------
----------------YNMD----SLSV-KNLLQFMS--------------------ELS
PAERRDFLQF-------TTGSPKLP------------IGG-----FK-SLNPMF--TVVC
KPSEEPYTSD------------------------DYLPSVMTCV-NY-LKLPNY-TDINV
LRKRMS-TAMKE-GQGA
>Gaeumannomyces_tritici_ADBI01000624.1 .
VRISRSKI-LESAVKVM----------------ELYGANQS-ILEVEYFE-------EVG
TGLG-PTLEF---YSTV----SKEFAKKKL--------KLWRDTDPHDSD----------
---------DFVASPNGLFPRPIS---------------------------------EDEG
SGGNSERILGLFKMLGKFVARSMIDSRIIDINLNP--IFFRIGDE---------------
-----KSGVRPSLGAVKA----------------------VDPVLAQS--------LMLI
KKFSLAKKEIDEDPRRTPALK-----------------VADTESIMVNH-TRIE-DLS--
--LDFTM--------------------------------PG--QPHIE-LVPN--------
GSHVN---
------------------------------LTIENVD-LYLEK-VLDMTLGSG-VRRQID
-AFRKG---FSQVFPY--SALS-AFTPD-ELVSLFGR-----------------------
---VEEDWS-----------XXAL---------MDSIKADHG------------------
----------------YNMD----SRNV-KNLLQFMS--------------------ELT
PAERRDFLQF-------TTGSPKLP------------IGG-----FK-SLNPIF--TVVC
KPSEEPYTSN------------------------DYLPSVMTCV-NY-LKLPDY-TSIDV
LKRRLM-TAMKE-GQGA
>Gaeumannomyces_graminis_XM_009225229.1 .
VRISRSKI-LESAVKVM----------------ELYGANQS-ILEVEYFE-------EVG
TGLG-PTLEF---YSTV----SKEFAKKKL--------KLWRDTDPHDSD----------
---------DFVASPNGLFPRPIS---------------------------------EDEG
SGGNSERILGLFKMLGKFVARSMIDSRIIDINLNP--IFFRIGDE---------------
-----KSGVRPSLGAVKA----------------------VDPVLAQS--------LMLI
KKFSLAKKEIDEDPRRTPALK-----------------VADTESIMVNH-TRIE-DLS--
--LDFTM--------------------------------PG--QPHIE-LVPN--------
GSHVN---
------------------------------LTIENVD-LYLEK-VLDMTLGSG-VRRQID
-AFRKG---FSQVFPY--SALS-AFTPD-ELVSLFGR-----------------------
---VEEDWS-----------LETL---------MDSIKADHG------------------
----------------YNMD----SRNV-KNLLQFMS--------------------ELT
PAERRDFLQF-------TTGSPKLP------------IGG-----FK-SLNPIF--TVVC
KPSEEPYTSN------------------------DYLPSVMTCV-NY-LKLPDY-TSIDV
LKRRLM-TAMKE-GQGA
>Coniochaeta_ligniaria_MNPN01000106.1 .
VRISRSKI-LESAVKVM----------------ELYGASQS-ILEVEYFE-------EVG
TGLG-PTLEF---YSTV----SKEFSKKKL--------KLWRDSDPGEGE----------
---------EFVNGPNGLFPRPWS---------------------------------NDFL
ISEDGEKILQLFKMLGKFVARSMIDSRIIDINFNP--IFFRIGDE---------------
-----TSGVRPSLGAVKA----------------------VDPMLAKA--------LNLI
KKFAIAKKEIDEDPNRTPAQK-----------------VGDTEKVVLNN-TKIN-DLC--
--LDFTL--------------------------------PG--FPEIE-LVPN--------
GAQMS---
------------------------------LSMDNVD-LYLEK-VIEMTLGTG-VRRQVD
-AFRTG---FSQVFPY--SALS-AFTPD-ELCTLFGG-----------------------
---TDEDWS-----------IESL---------MDSIKADHG------------------
----------------YNMD----SKSV-KNLLQTMS--------------------EMT
PTQRRDFLQF-------TTGSPKLP------------IGG-----FK-SLTPMF--TVVC
RPSEAPYSSD------------------------DYLPTVMTCV-NY-LKLPDY-TDLTV
MRKQLF-IAIKE-GQGA
>Togninia_minima_XM_007919854.1 .
VRISRSKI-LESALKVM----------------ELYGASQS-ILEVEYFE-------EVG
TGLG-PTLEF---YSTV----SKEFSKKKL--------RLWRDTDHHESD----------
---------EFVSGPSGLFPRPMS---------------------------------EDDA

LSPNGERIMQLFKMLGKFVARSMIDSRIIDINFNP--IFFRIGDE---------------
-----SSGIRPSLGAMKI----------------------VDPVLARS--------LLLV
KKFALAKKEITEDPDRNAAQK-----------------VADTENIVIDNGIRIE-DLA--
--LDFTL-------------------------------PG--YPEIE-LIPN--------
GSQIM---
------------------------------LMLDNVD-LYLEK-VIDMTLGTG-VRRQVD
-AFRAG---FSQVFPY--SALS-AFTPD-ELVTLFGR-----------------------
---VDEDWS-----------LETL---------MDSVKADHG------------------
----------------YNMD----SKSV-RNLLQTMC--------------------ELS
AAQRRDFLQF-------TTGSPKLP------------IGG-----FR-SLTPMF--TVVC
KPSEPPYTSD------------------------DYLPSVMTCV-NY-LKLPDY-TDIDI
MKKRLF-TAIKE-GQGA
>Phaeoacremonium_minimum_AORD01001041.1 .
VRISRSKI-LESALKVM----------------ELYGASQS-ILEVEYFE-------EVG
TGLG-PTLEF---YSTV----SKEFSKKKL--------RLWRDTDHHESD----------
---------EFVSGPSGLFPRPMS--------------------------------EDDA
LSPNGERIMQLFKMLGKFVARSMIDSRIIDINFNP--IFFRIGDE---------------
-----SSGIRPSLGAMKI----------------------VDPVLARS--------LLLV
KKFALAKKEITEDPDRNAAQK-----------------VADTENIVIDNGIRIE-DLA--
--LDFTL-------------------------------PG--YPEIE-LIPN--------
GSQIM---
------------------------------LMLDNVD-LYLEK-VIDMTLGTG-VRRQVD
-AFRAG---FSQVFPY--SALS-AFTPD-ELVTLFGR-----------------------
---VDEDWS-----------XSAL---------MDSVKADHG------------------
----------------YNMD----SKSV-RNLLQTMC--------------------ELS
AAQRRDFLQF-------TTGSPKLP------------IGG-----FR-SLTPMF--TVVC
KPSEPPYTSD------------------------DYLPSVMTCV-NY-LKLPDY-TDIDI
MKKRLF-TAIKE-GQGA
>Chrysoporthe_deuterocubensis_LJDD01000008 1..
VRISRSKI-LESALKVM----------------DLYGSSQS-ILEVEYFE-------EVG
TGLG-PTLEF---YSTV----SKEFATKKL--------KMWRDADGSHSDPD--------
---------EYVSAPNGLFPRPYG--------------------------------EDGF
SSTSGERVLQLFRTLGKFVARSMIDSRIIDINLNP--IFFRIGDE---------------
-----SSGIRPSLGAMKL----------------------VDPVLARS--------LVLV
KKFALAKKEIAEDPFRNATQK-----------------VNDIQNITIDNGVRIE-DLS--
--LDFTL-------------------------------PG--YADIE-LVPN--------
GSQVM---
------------------------------LTIDNVD-QYLDR-VIDMTLGSG-VRRQVD
-AFRTG---FSQVFPY--QALS-AFTPE-ELCTLFGR-----------------------
---IDEDWS-----------LASL---------MDSVKADHG------------------
----------------FNMD----SKSV-RNLLQTMS--------------------EFT
PPQRRDFLQF-------TTGSPKLP------------IGG-----FK-SLTPMF--TVVC
KPSEGPYSSD------------------------DYLPSVMTCV-NY-LKLPDY-TDINT
LKKQLY-TAMRE-GQGA
>Chrysoporthe_cubensis_LJCY01000118.1 .
VRISRSKI-LESALKVM----------------DLYGSSQS-ILEVEYFE-------EVG
TGLG-PTLEF---YSTV----SKEFATKKL--------KMWRDADGSHSDPD--------
---------EYVSAPNGLFPRPYG--------------------------------EDGF
SSTSGERVLQLFRTLGKFVARSMIDSRIIDINLNP--IFFRIGDE---------------
-----SSGIRPSLGAMKL----------------------VDPVLARS--------LVLV
KKFALAKKEIAEDPFRNATQK-----------------VNDIQNITIDNGVRIE-DLS--
--LDFTL-------------------------------PG--YADIE-LVPN--------
GSQVM---
------------------------------LTIDNVD-QYLDR-VIDMTLGSG-VRRQVD
-AFRTG---FSQVFPY--QALS-AFTPE-ELCTLFGR-----------------------
---IDEDWS-----------LASL---------MDSVKADHG------------------
----------------FNMD----SKSV-RNLLQTMS--------------------EFT
PPQRRDFLQF-------TTGSPKLP------------IGG-----FK-SLTPMF--TVVC

```
KPSEGPYSSD-----------------------DYLPSVMTCV-NY-LKLPDY-TDINT
LKKQLY-TAMRE-GQGA
>Chrysoporthe_austroafricana_JYIP01003195. 1..
VRISRSKI-LESALKVM----------------DLYGSSQS-ILEVEYFE-------EVG
TGLG-PTLEF---YSTV----SKEFATKKL--------KMWRDADGSHSDPD--------
---------EYVSAPNGLFPRPYG--------------------------------EDGF
SSTSGERVLQLFRTLGKFVARSMIDSRIIDINLNP--IFFRIGDE---------------
-----SSGIRPSLGAMKL----------------------VDPVLARS--------LVLV
KKFALAKKEIAVDPFRNATQK-----------------VNDIQNITIDNGVRIE-DLS--
--LDFTL-------------------------------PG--YADIE-LVPN--------
GSQVM-------------------------------------------------------
------------------------------LTIDNVD-QYLDR-VIDMTLGSG-VRRQVD
-AFRTG---FSQVFPY--QALS-AFTPE-ELCTLFGR-----------------------
---IDEDWS-----------LASL---------MDSVKADHG------------------
-----------------FNMD----SKSV-RNLLQTMS--------------------EFT
PPQRRDFLQF-------TTGSPKLP------------IGG-----FK-SLTPMF--TVVC
KPSEGPYSSD-----------------------DYLPSVMTCV-NY-LKLPDY-TDINT
LKKQLY-TAMRE-GQGA
>Valsa_mali_JUIY01000465.1 .
VRISRAKI-LESALKVM----------------DLYGSSQS-ILEVEYFE-------EVG
TGLG-PTLEF---YSTV----SKEFAMKKL--------RLWRDADQAESD----------
---------EYVSGPNGLFPRPLS--------------------------------DDDA
SGSYGARILQLFRTLGKFVARSMIDARIIDINFNP--IFFRIGDE---------------
-----TSGIKPSLGAMKL----------------------VDPVLARS--------LVMV
KKFALAKKAIAEDPNRDATQK-----------------VNDIQNITVDNGVHID-DLS--
--LDFTL-------------------------------PG--YPEIE-LVPR--------
GSHIT-------------------------------------------------------
------------------------------LTIDNVD-QYLDR-VIDMTLGSG-VRRQVD
-AFRAG---FSQVFPY--ESLR-AFTPD-ELCSLFGR-----------------------
---TNEDWT-----------LASL---------MDSIKADHG------------------
-----------------FNMD----SKSV-RNLLQTMS--------------------ELT
PGQRRDFLQF-------TTGSPKLP------------IGG-----FK-SLTPMF--TVVC
KPAEAPLGSD-----------------------DYLPSVMTCA-NY-LKLPDY-SDVKI
LQKRLF-TAIKE-GQGA
>Diaporthe_helianthi_MAVT01004708.1 .
VRISRAKI-LESALKVM----------------DLYGSSQS-ILEVEYFE-------EVG
TGLG-PTLEF---YSTV----SKEFAMKKL--------RLWRDADQNESD----------
---------EYVSGPTGLFPRPLS--------------------------------EDDV
STPNGERILQLFRTLGKFTARSMIDSRIIDINFNP--IFFRIGDE---------------
-----TSGIKPSLGAMKL----------------------VDPVLARS--------LVLV
KKFALAKKTIAEDPNKDATQK-----------------VNDIQNITVDNNVRVE-DLS--
--LDFTL-------------------------------PG--YPEID-LVPN--------
GSQIM-------------------------------------------------------
------------------------------LTIDNVD-NYLER-VIDMTLGSG-VRRQVD
-AFRTG---FSQVFPY--SALR-AFTPD-ELCTLFGR-----------------------
---TDEDWS-----------TTAL---------MDSIKADHG------------------
-----------------FNMD----SKSV-RNLLQTMS--------------------EMT
PVQRREFLQF-------TTGSPKLP------------IGG-----FK-SLTPMF--TVVC
KPAEAPYTSD-----------------------DYLPSVMTCA-NY-LKLPDY-TDVKV
LQKRLF-TATKE-GQGA
>Diaporthe_ampelina_LCUC01000012.1 .
VRISRAKI-LESALKVM----------------DLYGSSQS-ILEVEYFE-------EVG
TGLG-PTLEF---YSTV----SKEFAMKKL--------RLWRDADQNESD----------
---------EYVSGPTGLFPRPLG--------------------------------EDDV
STPNGERILQLFRTLGKFTARSMIDSRIIDINFNP--IFFRIGDE---------------
-----TSGIKPSLGAMKL----------------------VDPVLARS--------LVLV
KKFALAKKTIVEDPNRDATQK-----------------VNDIQNITVDNGVRVE-DLS--
--LDFTL-------------------------------PG--YPEID-LVSN--------
```

GSQIM---
------------------------------LTIDNVD-NYLDR-VIDMTLGSG-VRRQVD
-AFRTG---FSQVFPY--NALR-AFTPD-ELCTLFGR-----------------------
---TDEDWT-----------LASL---------MDSIKADHG------------------
----------------FNMD----SKSV-RNLLQTMS--------------------EMT
PVQRREFLQF-------TTGSPKLP------------IGG-----FK-SLTPMF--TVVC
KPAEAPYTSD------------------------DYLPSVMTCA-NY-LKLPDY-TDVKI
LQKRLF-TATKE-GQGA
>Diaporthe_longicolla_AYRD02012300.1 .
VRISRAKI-LESALKVM----------------DLYGSSQS-ILEVEYFE-------EVG
TGLG-PTLEF---YSTV----SKEFAMKKL--------RLWRDADQNESD----------
---------EYVSGPTGLFPRPLS--------------------------------EDDV
STPNGERILQLFRTLGKFTARSMIDSRIIDINFNP--IFFRIGDE---------------
-----TSGIKPSLGAMKL----------------------VDPVLARS--------LLLV
KKFALVKKTIAEDPNRDATQK-----------------VNDIQNISVDNGVRVE-DLS--
--LDFTL-------------------------------PG--YPEID-LVPN--------
GSQIM---
------------------------------LTIDNVD-NYLER-VIDMTLGSG-VRRQVD
-AFRTG---FSQVFPY--SALR-AFTPD-ELCTLFGR-----------------------
---TEEDWS-----------LASL---------MDSIKADHG------------------
----------------FNMD----SKSV-RNLLQTMS--------------------EMT
PVQRREFLQF-------TTGSPKLP------------IGG-----FK-SLTPMF--TVVC
KPAEAPYTSD------------------------DYLPSVMTCA-NY-LKLPDY-TDVKV
LQKRLF-TATKE-GQGA
>Knoxdaviesia_capensis_LNGK01000010.1 .
VRIGRPKI-MESALKVM----------------DLYGACQS-ILEVEYFE-------EVG
TGLG-PTLEF---YSTI----SKEFAKKKL--------RLWREADSNDVD----------
---------EFVSGSLGLFPRPMS--------------------------------EEET
TTPNGVRILHLFTVLGKFVARSMLDSRIIDINFNP--IFFRIGDS---------------
---GAVSGIKPSLGAIKA----------------------VDPALARS--------LKAI
RKFALAKKEIDEDPVRSAAQK-----------------VADTEAIVIDD-SRIE-DLC--
--LDFTL-------------------------------PG--Y-DIE-LVPD--------
GSQIK---
------------------------------VTIENVD-QYLEC-VTDMTLGAG-VRHQVQ
-AFRNG---FSAVFPY--SALS-AFTPD-ELVSLFGR-----------------------
---VDEDWS-----------LESK---------FSSIKADHG------------------
----------------FNMD----SRSV-KNLLQVMS--------------------ELT
LAERRDFLQF-------TTGSPKLP------------IGG-----FK-TLNPMF--TVVC
KPSEHPYTSD------------------------DYLPSVMTCV-NY-LKLPDY-TSLEV
MRKQLF-LAMRE-GQGA
>2_Knoxdaviesia_proteae_LNGL01000042.1 .
VRIGRPKI-MESALKVM----------------DLYGACQS-ILEVEYFE-------EVG
TGLG-PTLEF---YSTI----SKEFAKKKL--------RLWREADSNDVD----------
---------EFVSGSLGLFPRPMS--------------------------------EEET
TTPNGVRILHLFTVLGKFVARSMLDSRIIDINFNP--IFFRIGDS---------------
---GAVSGIKPSLGAIKA----------------------VDPTLARS--------LKAI
RKFALAKKEIDEDPVRSAAQK-----------------VADTEAIVIDD-SRIE-DLC--
--LDFTL-------------------------------PG--Y-DIE-LVPD--------
GSQIK---
------------------------------VTIENVD-QYLEC-VTDMTLGAG-VRRQVQ
-AFRNG---FSAVFPY--SALS-AFTPD-ELVSLFGR-----------------------
---VDEDWS------------XAL---------MDSIKADHG------------------
----------------FNMD----SRSV-KNLLQVMS--------------------ELT
LAERRDFLQF-------TTGSPKLP------------IGGKI---YK-TLNPMF--TVVC
KPSEHPYTSD------------------------DYLPSVMTCV-NY-LKLPDY-TSLEV
MRKQLF-LAMRE-GQGA
>Basipetospora_chlamydospora_BCHP01000005. 1..
VRIGRPKI-FESAFKVM----------------ELYGACQS-VLEVEYFG-------EVG

TGLG-PTLEF---YSSV----SREFAKKKF--------KLWREVDSYDSD----------
---------EFISGATGLFPRPMS--------------------------------EEEA
SSPNGERVLQLFTTLGKFVARSMLDSRIIDLNFNP--IFFRIGDD---------------
---SSPVGIKPSLGAVKV----------------------VDPGMASS--------LKTI
KKFAQAKKEIDEDPSRTPAEK-----------------VMLTEHITIDG-TKID-DLC--
--LDFTL-------------------------------PG--YPDIE-LVPN--------
GSQVR---
-----------------------------VTISNVE-EYLDH-VIDMTLGGG-VRRQVD
-AFRAG---FSHVFPY--SALS-AFTPD-ELVSLFGN-----------------------
---VDEDWS-----------LTAL---------MDSIKADHG------------------
----------------YNMD----SRSV-KNLLQTMS--------------------ELT
LSQRREFLRF-------TTGSPKLP------------IGG-----FK-ALNPMF--TVVC
KPSEHPYTSD------------------------DYLPSVMTCV-NY-LKLPDY-TSIEN
MRKQLY-TAMKE-GQGA
>Corollospora_maritima_GDFX01001174.1 .
VRIGRPKI-LESALKVM-----------------ELYGASQS-ILEVEYFE-------EVG
TGLG-PTLEF---YSTI----SKEFSKKKL--------KLWREVDAADSD----------
---------EFVNGSTGLFPRPMS--------------------------------DDEA
SSANGERVLHLFSSLGKFVARSMLDSRIIDINFNP--IFFRIGDD---------------
---SATSGVKPSLGAVKS----------------------VDPILARS--------LKAI
KRFALAKKEIDEDPGRTPARK-----------------VADTETIVIDG-SRID-DLC--
--LDFTL-------------------------------PG--YPDID-LVPN--------
GSQVR---
-----------------------------VTIANVE-EYLQK-VIDMTLGAG-VRRQID
-AFRAG---FTAVFPY--SALS-AFTPD-ELVSLFGR-----------------------
---VEEDWS-----------LETL---------MDSIKADHG------------------
----------------YNMD----SKSV-RNLLQTMS--------------------ELD
LNQRREFLQF-------TTGSPKLP------------IGG-----FK-ALTPMF--TVVC
KPSEHPYTSD------------------------DYLPSVMTCV-NY-LKLPDY-TSIDI
MRKQLF-TAMKE-GQG-
>Scedosporium_aurantiacum_JUDQ01000249.1 .
VRIGRAKI-LESALKVM-----------------ELYGASQS-VLEVEYFE-------EVG
TGLG-PTLEF---YSTI----SKEFSKKKL--------KLWREVDSNESD----------
---------EFVNGLTGLFPRPMS--------------------------------DEEA
SSPNGERVLHLFTSLGKFVARSMLDSRIIDINFNP--IFFRIGDE---------------
---SSPVGIRPSLGAVKV----------------------VDPGLGRS--------LKAI
KRFSIAKKEIDEDPSRTPTQK-----------------VMHTEDIVIDG-SKID-DLC--
--LDFTL-------------------------------PG--FPDIE-LVPS--------
GSQIR---
-----------------------------VTISNVD-EYLDR-VIDMTLGSG-VRRQID
-AFRAG---FSHVFPY--SALS-AFTPD-ELVSLFGK-----------------------
---VEEDWS-----------SAAL---------MDSLKADHG------------------
----------------YNMD----SRSV-KNLLQTMS--------------------ELT
LSQRREFLQF-------TTGSPKLP------------IGG-----FK-SLTPMF--TVVC
KPSEHPYTSD------------------------DYLPSVMTCV-NY-LKLPDY-TSIDV
MKKQLF-TAMKE-GQGA
>Scedosporium_apiospermum_XM_016789899.1 .
VRIGRAKI-LESALKVM-----------------ELYGASQS-VLEVEYFE-------EVG
TGLG-PTLEF---YSTI----SKEFSKKKL--------KLWREVDSNESD----------
---------EFVNGLTGLFPRPMS--------------------------------DEEA
SSPNGERVLHLFTSLGKFVARSMLDSRIIDINFNP--IFFRIGDE---------------
---SSPVGIRPSLGAVKV----------------------VDPGLGRS--------LKAI
KRFSIAKKEIDEDPSRTPTQK-----------------VMHTEDIVIDG-SKID-DLC--
--LDFTL-------------------------------PG--FPDIE-LVPN--------
GSQMR---
-----------------------------VTISNVD-EYLDR-VIDMTLGSG-VRRQID
-AFRAG---FSHVFPY--SALS-AFTPD-ELVSLFGK-----------------------
---VEEDWS-----------LETL---------MDSLKADHG------------------

----------------YNMD----SRSV-KNLLQTMS--------------------ELT
LSQRREFLQF-------TTGSPKLP------------IGG-----FK-SLTPMF--TVVC
KPSEHPYTSD------------------------DYLPSVMTCV-NY-LKLPDY-TTIDV
MKKQLF-TAMKE-GQGA
>Aureobasidium_pullulans_MSJF01000011.1 .
VRIGRAKI-LESALKVM----------------ELYGASQS-VLEVEYFE-------EVG
TGLG-PTLEF---YSTI----SKEFSKKKL--------KLWREVDSNEAD----------
---------EFVNGLTGLFPRPMS---------------------------------DEEA
SSPNGERVLHLFTSLGKFVARSMLDSRIIDINFNP--IFFRIGDE---------------
---SSTVGIRPSLGAVKV----------------------VDPGLGRS--------LKAI
KRFSIAKKEIDEDPSRTPTQK-----------------VMHTEDIVIDG-SKID-DLC--
--LDFTL--------------------------------PG--FPDIE-LVPN--------
GSQIR---
------------------------------VAISNVD-EYLDR-VVDMTLGSG-VRRQID
-AFRAG---FSHVFPY--SALS-AFTPD-ELVSLFGK-----------------------
---VEEDWS-----------XTAL---------MDSLKADHG------------------
----------------YNMD----SRSV-KNLLQTMS--------------------ELT
LSQRREFLQF-------TTGSPKLP------------IGG-----FK-SLTPMF--TVVC
KPSEHPYTSD------------------------DYLPSVMTCV-NY-LKLPDY-TTIDV
MKKQLF-TAMKE-GQGA
>Sarocladium_oryzae_LOPT01001467.1 .
VRISRTKI-LESALKVM----------------DLYGASQS-ILEVEYFE-------EVG
TGLG-PTLEF---YSTV----SREFSKKKL--------KLWREMDSSSSD----------
---------EFVSSQSGLFPRPLS---------------------------------DEEA
ITPNGERILHLFKMLGKFVARSMLDSRIIDLHFSP--IFFRIGET---------------
----ATSGIKPSLGAVKV----------------------VDPGLARS--------LKTI
KKFAMAKKEIDEDPNRTAAQK-----------------VTDTEGIIIDN-VRLD-DLC--
--LDFTL--------------------------------PG--YPNIE-LLPN--------
GSQER---
------------------------------VTIENVD-VYLQK-VIDMTLGGG-VRRQVD
-AFRAG---FSQVFPY--SALS-AFTPN-ELVNLFGR-----------------------
---VEEDWS-----------IESM---------YSSIKADHG------------------
----------------YNMD----SKTV-RNLLQTMS--------------------SFD
AAQRREFLQF-------ITGSPKLP------------MTG-----FK-SLTPMF--TVVC
KPSEHPYTSD------------------------DYLPSVMTCV-NY-LKLPNY-TTAEV
MKKQLF-TAMKE-GQGA
>Gliomastix_tumulicola_BCHX01000004.1 .
VRISRSKI-LESALKVM----------------DLYGTSQS-MLEVEYFE-------EVG
TGLG-PTLEF---YSIV----SLEFSKKKL--------KLWRDMDSSADS----------
---------EYVTSQSGLFPRPLS---------------------------------AQEV
STPNGERILHLFRMLGKFIARSMIDSRIIDLHFNP--TFFRTGES---------------
----AASGIKPSLGAVKT----------------------VDPGLARS--------LMTI
KKFEMAKKEINEDPNRTAAQK-----------------VADVDNIVIDS-MKLE-DLC--
--LDFTL--------------------------------PG--YPEIE-LEEG--------
GAQKR---
------------------------------VTIDNVE-SYLEK-VIDATLGSG-VRRQVD
-AFRSG---FSSVFPY--SALS-AFTPE-ELVNLFGR-----------------------
---VEEDWS------------XAL---------IDSMKADHG------------------
----------------FNMD----SASV-QNLLHLMS--------------------EFN
ATQRRDFLQFS-------TGSPKLP------------IGG-----FR-NLTPMF--TVVC
KPSEDPYTSD------------------------DYLPSVMTCV-NY-LKLPAY-SSREV
MKRQLS-KAIKE-GQGA
>Geosmithia_morbida_FNXU01000010.1 .
VRISRSKI-LESALKVM----------------DLYGASQS-ILEVEYFE-------EVG
TGLG-PTLEF---YSTV----SREFAKKKV--------RLWRDMDSSSTS----------
---------EYVSGPMGLFPRPMS---------------------------------EKES
STPNGERILHLFKMLGKFVARSMIDSRIIDIHFNP--IFFRLAGE-------AATTATAT
ASPPSSTNLRPSLASVKM----------------------VDPGLASS--------LMTI

QKFVLAKKEIANDANRTPAQK-----------------VQDMENITIDGGVTID-DLC--
--LDFTL-------------------------------PG--YPEIE-LEDG--------
GSHKR---
------------------------------VTIDNVG-SYLDK-VVDATLGSG-VRRQVD
-AFRAG---FSTVFPY--SALS-SFTPD-ELVSLFGK-----------------------
---ADEDWS-----------XKAL---------LDSIKADHG------------------
----------------YNMD----SKTV-RNLLQLMS--------------------QFD
STQRRDFLQF-------TTGSPKLP------------IGG-----FK-SLTPMF--TVVC
KPSEEPYTSD------------------------DYLPSVMTCV-NY-LKLPNY-SDMDS
MTKQMS-VAMKE-GQGA
>Acremonium_chrysogenum_JPKY01000023.1 .
VRISRSKI-LESALKVM----------------DLYGASQS-ILEVEYFE-------EVG
TGLG-PTLEF---YSTV----SMEFSKKKL--------RLWRDMDASTSS----------
---------EFASGHAGLFPRPLS--------------------------------DQEA
STPNGQRILHLFKMLGKFVARSMLDSRIVDIHFNP--IFFRIGET---------------
----NSSGIKPSLASVKM----------------------VDPGLARS--------LMTI
KNFALAKRAIDEDPNRTPAQK-----------------VADLENIVIDN-VKLD-DLC--
--LDFTL-------------------------------PG--YPEIE-LEEG--------
GSHKR---
------------------------------VTIDNVA-SYLEK-VVDVTLGSG-VRRQVD
-AFRAG---FSTVFPY--SALS-AFTPE-ELVNLFGK-----------------------
---ADEDWS-----------LESL---------VDSIKADHG------------------
----------------FNMD----SPSV-RNLLHLMS--------------------NFS
SSERRDFLQF-------TTGSPKLP------------IGG-----FK-SLTPMF--TVVC
KPSEEPYTSD------------------------DYLPSVMTCV-NY-LKLPDY-SSIET
MKKQLS-TAMKE-GQGA
>Clonostachys_rosea_JYFM02000461.1 .
VRISRTKI-LESALKVM----------------DLYGASQS-ILEVEYFE-------EVG
TGLG-PTLEF---YSTV----SLEFSKKKL--------KLWRDIDSDVSS----------
---------EFATVQTGLFPRPIS--------------------------------DQEA
STPNGERILHLFKMLGKFVARSMIDSRIVDIHLNP--IFFRIGEA---------------
----TSAGVKPSLGAVKM----------------------VDPGLAQS--------LMTI
KDFAIAKKQIDEDPNKTPAQK-----------------VDELANISIDN-VRLE-DLC--
--LDFTL-------------------------------PG--YPEIE-LEDG--------
GAHTR---
------------------------------VTIDNVE-SYLAK-VIDVTLGSG-VRRQVD
-AFRSG---FSSVFPY--SALS-AFTPD-ELVNLFGR-----------------------
---TDEDWS-----------LESL---------LDSIKADHG------------------
----------------YNME----SPSV-RNLLQLMS--------------------GFD
ATERRDFLQF-------TTGSPKLP------------IGG-----FK-SLTPMF--TVVC
KPSEEPYTSD------------------------DYLPSVMTCV-NY-LKLPNY-SGVEV
MNRQLS-KAMKE-GQGA
>Ophiostoma_novo_ulmi_AMZD01000139.1 .
VRISRSKI-LESAVKVM----------------ELYGASQS-LLEVEYFG-------EVG
TGLG-PTLEF---YSTV----SREFSKKKL--------KLWRDSDLGDS-----------
---------EFVSSASGLFPRPLS--------------------------------DEGA
ASANGQRIMSLFRALGMFVARSMLDTRIIDINFNP--IFFRIGDE---------------
-----SSGVRPSLGAVKA----------------------VDPALARS--------LLMI
KQFTAARKAILDDPTRTDSEK-----------------VQAIAAITVND-MSID-DLS--
--LDFTL-------------------------------PG--YPEIE-LVPG--------
GSDIS---
------------------------------VSIGNVS-TYIDR-VISMTLGGG-VRRQVD
-AFRKG---FSEVFPY--PALS-AFTPS-ELVSLFGR-----------------------
---IEEDWS-----------LESL---------VDSIKADHG------------------
----------------FTMD----SRSV-KNLLQAMS--------------------EMT
LSERRGFLQF-------TTGSPKLP------------IGG-----FR-SLSPMF--TVVC
KPSEAPYTSD------------------------DYLPSVMTCV-NY-LKLPDY-TSIEV
LKKRLS-TAVQE-GQGA

>Sporothrix_brasiliensis_AWTV01000009.1 .
VRISRSKI-LESAVKVM----------------ELYGTSQS-ILEVEYFE-------EVG
TGLG-PTLEF---YSTV----SREFSKKKL--------RLWRDNDLGDS-----------
---------EFVSGPNGLFPRPIS--------------------------------AKDA
EGTNGQRLMSLFKALGRFVARSMLDSRIIDINFNP--IFFRIGNG---------------
-----SQGWRPSLGAVKV----------------------VDPTLARS--------LLLI
KKFANAKKVIEEGVDLSATEK-----------------AAGIQAITIDD-TRID-DLS--
--LDFTL-------------------------------PG--YPDIE-LIPG--------
GCHIS---
------------------------------VTMSNVD-TYVDK-VVAMTLEGG-VRLQVD
-AFRRG---FSEVFPY--SALS-AFTPD-ELVSLFGH-----------------------
---VDEDWS-----------KIAL---------SDSIKADHG------------------
----------------FNMD----SRNV-KNLLQVMS--------------------EMS
LIERRDFLQF-------ATGSPRLP------------IGG-----FR-SLTPMF--TVVC
KPSEAPYSSD------------------------DYLPSVMTCV-NY-LKLPDY-TSIDV
LRKQLF-TAIKE-GQGA
>Sporothrix_schenckii_XM_016727908.1 .
VRISRSKI-LESAVKVM----------------ELYGTSQS-ILEVEYFE-------EVG
TGLG-PTLEF---YSTV----SREFSKKKL--------RLWRDNDLGDS-----------
---------EFVSGPNGLFPRPIS--------------------------------AEDA
EGTNGERLMSLFKALGRFVARSMLDSRIIDINFNP--IFFRIGNE---------------
-----SQGMRPSLGAVKV----------------------VDPTLARS--------LLLI
KKFANAKKVIEEGVDLSATEK-----------------AAGIQAITIDD-TSID-DLS--
--LDFTL-------------------------------PG--YPDIE-LIPG--------
GCHIS---
------------------------------VTMSNVD-TYVDK-VVAMTLEGG-VRLQVD
-AFRKG---FSEVFPY--SALS-AFTPD-ELVSLFGH-----------------------
---VDEDWS-----------LETL---------SDSIKADHG------------------
----------------FNMD----SRNV-KNLLQVMS--------------------EMS
LVERREFLQF-------ATGSPRLP------------IGG-----FR-SLTPMF--TVVC
KPSEAPYSSD------------------------DYLPSVMTCV-NY-LKLPDY-TSIDV
LRKQLF-TAIKE-GQGA
>Sporothrix_globosa_LVYW01000001.1 .
VRISRSKI-LESAVKVM----------------ELYGTSQS-ILEVEYFE-------EVG
TGLG-PTLEF---YSTV----SREFSKKKL--------RLWRDSDLGES-----------
---------EFVSGPNGLFPRPIS--------------------------------AKDA
EGTNGERLMSLFKALGRFVARSMLDSRIIDINFNP--IFFRIGNG---------------
-----SQGVRPSLGAVKV----------------------VDPTLARS--------LLLI
KKFANAKKAIEEDTDRSAPEK-----------------AAGIQAITIND-TLID-DLS--
--LDFTL-------------------------------PG--YPDIE-LTPG--------
GCHIS---
------------------------------VTMSNVD-TYVDQ-VVAMTLEGG-VRLQVD
-AFRRG---FSEVFPY--SALS-AFTPD-ELVSLFGH-----------------------
---VDEDWS--------L--ESKL---------ISSIKADHG------------------
----------------FNMD----SRSV-KNLLQVMS--------------------EMS
PIERRDFLQF-------ATGSPRLP------------IGG-----FR-SLTPMF--TVVC
KPSEAPYSSD------------------------DYLPSVMTCV-NY-LKLPDY-TSIEV
LRKQLF-TAIKE-GQG-
>Sporothrix_pallida_JNEX02000234.1 .
VRISRSKI-LESAVKVM----------------ELYGASQS-ILEVEYFE-------EVG
TGLG-PTLEF---YSTV----SREFSKKKL--------KLWRDTDLGDS-----------
---------EFISGPNGLFPRPIS--------------------------------DEDA
AGTNGERLLSLFSALGKFVARSMLDSRIIDINFNP--IFFRIGEG---------------
-----SSAVRPSLGAVKV----------------------VDPALARS--------LLLI
KKFASAKRAIEEDTARPAADK-----------------AADIQALTVNN-THIN-DLS--
--LDFTL-------------------------------PG--YPDIE-LIPE--------
GRHIG---
------------------------------VTMDNVD-TYVDK-VVAMTLEGG-VRRQMD

-AFRKG---FSQVFPY--SALS-AFTPD-ELVSLFGR-----------------------
---VEEDWS-----------LESL---------MDSIKADHG------------------
----------------FNMD----SRSV-KNLLQVMS--------------------EMT
LVERRDFLQF-------ATGSPRLP------------IGG-----FR-SLTPMF--TVVC
KPSEAPYTSD------------------------DYLPSVMTCV-NY-LKLPDY-TSIDA
LRKQLS-TAIKE-GQGA
>Graphilbum_fragrans_LLKO01000004.1 .
VRISRSKI-LESAIKVM----------------ELYGASQS-ILEVEYFE-------EVG
TGLG-PTLEF---YSTV----SREFAKRKL--------KLWRDNDMNDS-----------
---------EFVSSPSGLFPRPLS--------------------------------DEDA
GAINGERILSLFRALGKFVARSMLDSRIIDIHFNP--IFFRIGDD---------------
-----KSGIRPSLGAVKV----------------------VDPALARS--------LLLI
KKFAIAKKEIDEDPQQSAAEK-----------------AIKARNIRINN-SCVE-DLS--
--LDFTL-------------------------------PG--HPEIE-LIPD--------
GAQES---
------------------------------VTIDNVD-LYIER-VVAMTLGSG-VRRQVD
-AFRKG---FSQVFPY--SALS-SFTPD-ELVSLFGR-----------------------
---IDEDWS-----------LESL---------TDSVKADHG------------------
----------------FNMD----SRSV-KNLLQVMS--------------------ELT
PEERRDFLQF-------ATGSPKLP------------IGG-----FR-SLTPMF--TVVC
KPSEAPYTSD------------------------DYLPSVMTCV-NY-LKLPDY-SDMDV
LRKQLF-TAIKE-GQGA
>Ceratocystiopsis_minuta_LZPB01000185.1 .
VRISRSKI-LESAVKVM----------------ELYGASQS-ILEVEYFE-------EVG
TGLG-PTLEF---YSTV----SREFSKKKL--------KLWRENDSNDS-----------
---------EFVSNANGLFPRPLS--------------------------------EIDA
SGTNGERIMTLFQALGKFVARSMLDSRIIDLNFSP--IFFRIGDE---------------
-----AYGIRPSLGAVKA----------------------VDPPLARS--------LLLI
KKFAVAKKEVEEQSHLTAAEK-----------------ATAIRDITVNG-THIE-DLS--
--LDFTL-------------------------------PG--YPENE-LVPG--------
GAQMS---
------------------------------VTIDNVD-KYLDK-VVAMTLGSG-VHRQVD
-AFRKG---FSQVFPY--SALS-AFTPD-ELVSLFGR-----------------------
---VEEDWS-----------XVAL---------LDSIKADHG------------------
----------------FNMD----SPSV-KNLLQVMS--------------------EMT
PSERRDFLQF-------TTGSPKLP------------IGG-----FR-NLTPMF--TVVC
KPSEAPYSSD------------------------DYLPSVMTCV-NY-LKLPNY-TSIGV
LRKQLF-TAIRE-GQGA
>Sporothrix_insectorum_AZHD01000009.1 .
VRISRSKI-LESAVKVM----------------ELYGASQS-ILEVEYFE-------EVG
TGLG-PTLEF---YSTV----SREFAKRKL--------RLWRDNDLDDS-----------
---------EFVSNPNGLFPRPLS--------------------------------ETDA
AGTNGERIMSLFRALGKFVARSMLDSRIIDINFNP--IFFRIGDE---------------
-----SSGVRPSLGAVKV----------------------VDPTLARS--------LLLI
KEFVSVKKDIEADRHLTSAEK-----------------AAAVQHITVNN-THIE-DLS--
--LDFTM-------------------------------PG--YPEIE-LVSG--------
GAQIS---
------------------------------VTIDNVE-DYIEK-VVAKTLGSG-VRRQVD
-AFRKG---FSQVFPY--SALS-AFTPD-ELVSLFGR-----------------------
---IEEDWS------------XAL---------MDSIKADHG------------------
----------------FNMD----SRSV-KNLLQVMS--------------------ELA
PAERRDFLQF-------TTGSPKLP------------IGG-----FR-SLTPMF--TVVC
KPSEAPYTSD------------------------DYLPSVMTCV-NY-LKLPDY-TSIEV
LRKQMF-TAIRE-GQGA
>2_Raffaelea_quercivora_BCFZ01000003.1 .
VRISRTKI-LESAVKVM----------------ELYGASQS-MLEVEYFE-------EVG
TGLG-PTLEF---YSTV----SREFAKKKL--------KLWRDSDMSES-----------
---------EFVNTPNGLFPRPIG--------------------------------DEDA

TGTNGERILSLFRALGKFVARSMLDSRIIDINFNP--IFFRIGDE---------------
-----TSGVRPSLGAIKV----------------------VDPTLARS--------LQLI
KKFALAKKEIEEDPRRSASQK-----------------VADMQAITVNN-THID-DLS--
--LDFTL--------------------------------PG--YPEIE-LVDN--------
GAQVN---
------------------------------VTIENVD-VYIDR-VVAMTLGSG-VYRQVD
-AFRKG---FSQVFPY--STLC-AFTPD-ELASLFGR-----------------------
---VDEDWS-----------XTAL---------LDSVKADHG------------------
----------------FNMD----SRSV-KNLLQVMS--------------------ELT
LSERRDFLQF-------TTGSPKLP------------IGG-----FR-SLTPMF--TVVC
KPSEAPYVSD------------------------DYLPSVMTCV-NY-LKLPDY-TSIDV
LRKRLF-TAIKE-GQGA
>Leptographium_procerum_JRUC01000554.1 .
VRISRSKI-LESAVKVM----------------ELYGASQS-MLEVEYFE-------EVG
TGLG-PTLEF---YSTV----SREFSKTKL--------KLWRDNDFSDS-----------
---------EYVTGPNGLFPRPLS--------------------------------NDDA
VGPNGERILSLFRALGKFVARSMLDSRIIDINFNP--IFFQIGDE---------------
-----STGVRPSLGAIKV----------------------VDPALARS--------LQLI
KKFATAKKEVEEDPYRTASQK-----------------VADIQAITVNG-TNID-DLS--
--LDFTL--------------------------------PG--YPEIE-LVTG--------
GAQLN---
------------------------------VTIENVD-VYIDR-VVALTLGSG-VRHQVD
-AFRKG---FSEVFPY--STLS-AFTPD-ELSSLFGR-----------------------
---VEEDWS-----------LESL---------VDSVKADHG------------------
----------------FNMD----SSSV-KNLLQVMS--------------------ELS
LAERRDFLQF-------TTGSPKLP------------IGG-----FR-SLSPMF--TVVC
KPSEPPFVSD------------------------DYLPSVMTCV-NY-LKLPDY-TTVDV
LRRRLF-TAIKE-GQGA
>Leptographium_lundbergii_LDEF01000040.1 .
VRISRSKI-LESAVKVM----------------ELYGASQS-MLEVEYFE-------EVG
TGLG-PTLEF---YSTV----SREFSKKRL--------KLWRDNDFGDS-----------
---------EFVTSPNGLFPRPLS--------------------------------IEDA
TGPNDERILSLFRALGKFVARSMLDSRIIDINFNP--IFFRIGDE---------------
-----STGVRPSLGAIKV----------------------VDPTLARS--------LQLI
KKFAIAKREVEEDPYRSVSQK-----------------MVDIQAITVNG-TNID-DLS--
--LDFTL--------------------------------PG--YPEIE-LVAG--------
GAQMN---
------------------------------VTIENVD-VYIER-VVALTLGSG-VRRQVD
-AFRNG---FSQVFPY--STLS-AFTPD-ELSSLFGR-----------------------
---VEEDWS-----------LESL---------VDSVKADHG------------------
----------------FNMD----SSSV-KNLLQVMS--------------------ELT
LAERRDFLQF-------TTGSPKLP------------IGG-----FR-SLTPMF--TVVC
KPSEPPFVSD------------------------DYLPSVMTCV-NY-LKLPDY-TTVDV
LRKQLF-TAIKE-GQGA
>Grosmannia_clavigera_XM_014319907.1 .
VRISRSKI-LESAVKVM----------------ELYGASQS-MLEVEYFE-------EVG
TGLG-PTLEF---YSTV----SREFSKKKL--------KLWRDNDFGDS-----------
---------EFVTSPNGLFPRPLS--------------------------------NEGG
TGPNDERILSLFRALGKFVARSMLDSRIIDINFNP--IFFRIGDE---------------
-----STGVRPSLGAIKV----------------------VDPTLARS--------LQLI
KKFAIAKREVEEDPHRSVSQK-----------------VVDIQAITVDG-TNID-DLS--
--LDFTL--------------------------------PG--YPEIE-LVTG--------
GAHMN---
------------------------------VTIENVD-VYIER-VVALTLGSG-VRRQVD
-AFRNG---FSQVFPY--STLS-AFTPD-ELSSLFGR-----------------------
---IEEDWS-----------LETL---------VDSVKADHG------------------
----------------FNMD----SSSV-KNLLQVMS--------------------ELT
LAERRDFLQF-------TTGSPKLP------------IGG-----FR-SLTPMF--TVVC

KPSEPPFVSD------------------------DYLPSVMTCV-NY-LKLPDY-TTVDV
LRKRLF-TAIKE-GQGA
>Huntiella_savannae_LCZG01000066.1 .
VRIARSKM-FESALKVM----------------NLYGAGQS-ILEIEYFD-------EVG
TGLG-PTLEF---YSTI----SREFALKKL--------RLWRDDDNDDN-----------
---------EYVNGRTGLFPKPMS--------------------------------EEEA
NGQNGSRILNMFTMLGKFVARSMLDSRLIDINFNP--LFFRIGEG---------------
-------DVRPSLGAIKS----------------------VDAALATS--------LKLI
KKFVVAKREIEEDYSRNAADK-----------------AVAIENVRIED-CSIE-DLC--
--LDFTV-------------------------------PG--HSNIE-MIPK--------
GADTS---
------------------------------VTMANVD-KYLDL-VIDFTLGSG-VSRQID
-AFRAG---FTTVFPY--TALN-AFTPD-ELVLLFGR-----------------------
---SEEDWS-----------LETL---------LDSIKADHG------------------
----------------FNMD----SRSI-QNLLHLMS--------------------SYT
IEERRDFLQF-------TTGSPKLP------------IGG-----FK-ALKPMF--TVVR
RPHEAPYTAD------------------------DYLPSVMTCA-NY-LKLPDY-SNIDS
MRGRLG-VAIKE-GQGA
>Huntiella_bhutanensis_MJMS01000155.1 .
VRIARSKM-FESALKVM----------------NLYGAGQS-ILEIEYFD-------EVG
TGLG-PTLEF---YSTI----SREFALKKL--------RLWRDDDNDDN-----------
---------EYVNGRTGLFPKPMS--------------------------------EEEA
NGQNGSRILNMFTMLGKFVARSMLDSRLIDINFNP--LFFRIGEG---------------
-------DVRPSLGAVKS----------------------VDLALATS--------LKLI
KKFVVAKREIDEDYSRNAADK-----------------AVAIENVRVED-CSIE-DLC--
--LDFTV-------------------------------PG--HSNIE-MIPK--------
GADTS---
------------------------------VTMANVD-KYLDL-VIDFTLGSG-VSRQID
-AFRAG---FTTVFPY--TALN-AFTPD-ELVLLFGR-----------------------
---SEEDWS-----------LETL---------LDSIKADHG------------------
----------------FNMD----SRSI-QNLLHLMS--------------------SYT
TEERRDFLQF-------TTGSPKLP------------IGG-----FK-ALKPMF--TVVR
RPHEAPYTAD------------------------DYLPSVMTCA-NY-LKLPDY-SNIDS
MRGRLG-VAIKE-GQGA
>Huntiella_omanensis_JSUI01006514.1 .
VRIARSKM-FESALKVM----------------NLYGAGQS-ILEIEYFD-------EVG
TGLG-PTLEF---YSTI----SREFALKKL--------RLWRDDDNDDN-----------
---------EYVNGRTGLFPKPMS--------------------------------EEEA
NGQNGSRILNMFTMLGKFVARSMLDSRLIDINFNP--LFFRIGEG---------------
-------DVRPSLGAVKS----------------------VDAALATS--------LKLI
KKFVVAKREIEEDYSRSAADK-----------------AVAIENVRIEG-CSIE-DLC--
--LDFTV-------------------------------PG--HSHIE-MIPK--------
GADTS---
------------------------------VTMANVD-KYLDL-VIDFTLGSG-VSRQID
-AFRAG---FTTVFPY--TALN-AFTPD-ELVLLFGR-----------------------
---SEEDWS-----------LETL---------LDSIKADHG------------------
----------------FNMD----SRSI-QNLLHLMS--------------------SYT
IEERRDFLQF-------TTGSPKLP------------IGG-----FK-ALKPMF--TVVR
RPHEAPYTAD------------------------DYLPSVMTCA-NY-LKLPDY-SNIES
MRGRLG-VAIKE-GQGA
>Huntiella_moniliformis_JMSH01000118.1 .
VRIARSKM-FESALKVM----------------NLYGAGQS-ILEIEYFD-------EVG
TGLG-PTLEF---YSTI----SREFALKKL--------RLWRDDDNDDN-----------
---------EYVSGRTGLFPKPMS--------------------------------QEEA
NGQNGSRILNMFTMLGKFVARSMLDSRLIDINFNP--LFFRIGEG---------------
-------DVRPSLGAIKS----------------------VDSTLASS--------LKLI
KKFVVAKREIDEDSTRSAADK-----------------AVAIENVRVDD-CSID-DLC--
--LDFTV-------------------------------PG--HSNIE-MIPK--------

GADTS---
------------------------------VTMTNVD-KYLDL-VIDFTLGSG-VSRQID
-AFRAG---FTTVFPY--TALN-AFTPD-ELVLLFGR-----------------------
---SEEDWS-----------LETL---------LDSIKADHG-------------------
----------------FNMD----SRSI-QNLLQLMS--------------------SYT
TEERRDFLQF-------TTGSPKLP------------IGG-----FK-ALKPMF--TVVR
RPHEAPYTAD------------------------DYLPSVMTCA-NY-LKLPDY-SNIDS
MRGRLG-VAVKE-GQGA
>Thielaviopsis_musarum_LKBB01000109.1 .
VRIGRSKM-FESALKVM----------------NLYGSGQS-ILEVEYFE-------EVG
TGLG-PTLEF---YATI----SREFAKKKL--------KLWRDLDTPDDE----------
---------HYVMGATGLFPRPMT--------------------------------KAQL
SSSNGSRILHLFTMLGKFVARSMLDSRLIDIHMNP--IFFRIGDG---------------
----SMSGVRPSLGAVKS----------------------VDPTLARS--------LKLI
KKFALAKKEIDDEPELTDAEK-----------------TSLYEDVVVEG-CSLD-DLC--
--LDFTL-------------------------------PG--YPNID-LSLK--------
GSQNR---
------------------------------VTVANVD-KYLDL-VIDMTLGSG-VRRQID
-AFRAG---FTTVFPY--TALN-AFTPD-ELVSLFGR-----------------------
---VDEDWS------------XAL---------MDSIKADHG------------------
----------------FNMD----SRSI-KNLLQVMS--------------------TLS
DTERRDFLQF-------TTGSPKLP------------IGG-----FR-SLKPMF--TVVC
RPSEAPNTPD------------------------DYLPSVMTCV-NY-LKLPDY-SNLDT
MRKRLM-TAIRE-GQGA
>Thielaviopsis_punctulata_LAEV01000930.1 .
VRIGRSKM-FESALKVM----------------NLYGSGQS-ILEVEYFE-------EVG
TGLG-PTLEF---YATI----SREFAKKKL--------KLWRDIDTSEDE----------
---------LYVTGPTGLFPRPMT--------------------------------KAQL
SSSNGTRILHLFTMLGKFVARSMLDSRLIDIHLNP--IFFRIGDG---------------
----SMSGIRPSLGAIKS----------------------VDPALARS--------LKMI
KKFVVAKKEIDDDSSLTPEEK-----------------TAMYDSVMVDG-CSID-DLC--
--LDFTL-------------------------------PG--YPNID-LLHR--------
GAQKR---
------------------------------VTMANVD-KYLDL-VIDMTLGSG-VRRQID
-AFRAG---FTTVFPY--TALN-AFTPD-ELVSLFGR-----------------------
---VDEDWS-----------LSAL---------MDSIKADHG-------------------
----------------FNMD----SRSV-KNLLQVMS--------------------TLS
DTERRDFLQF-------TTGSPKLP------------IGG-----FR-SLKPMF--TVVC
RPSEAPNTPD------------------------DYLPSVMTCV-NY-LKLPDY-SDLET
MRKRLM-TAIKE-GQGA
>Ceratocystis_fagacearum_MKGJ01000036.1 .
VRIGRAKM-FESALKVM----------------NLYGAGQS-ILEIEYFE-------EVG
TGLG-PTLEF---YATI----SREFAKKKL--------KLWRENDSADD-----------
---------TYVSGPAGLFPKPIS--------------------------------EEEA
SGSNCERILHMFAMLGKFVARSMLDSRLIDINFNP--IFFRIGES---------------
-------GIRPSLGAVKS----------------------VDPALARS--------LKLI
KKFVLAKKEIDEDPTRTPTQK-----------------VSDTENATVDD-CTIE-DLC--
--LDFTL-------------------------------PG--FSDIE-LLPN--------
GSEIQ---
------------------------------VTMSNVE-QYLDR-VIDMTLGSG-VRRQID
-AFRSG---FTTVFPY--SALN-AFTPD-ELVELFGR-----------------------
---VDEDWS-----------LESL---------MDSIKADHG-------------------
----------------FNMD----SRSV-KNLLQLMS--------------------SFS
PEERRDFLQF-------TTGSPKLP------------IGG-----FK-ALKPMF--TVVC
RPSEAPHTPD------------------------DYLPSVMTCA-NY-LKLPDY-SDATM
MRNRLM-VAIKE-GQGA
>Endoconidiophora_polonica_LXKZ01000184.1 .
VRIGRSKI-FESALKVM----------------NLYGAGQS-ILEVEYFE-------EVG

TGLG-PTLEF---YATI----SREFAKKKL--------KLWRETDSLDD-----------
---------EYVSGPCGLFPKPIS--------------------------------DEES
GTPNGERILHMFAMLGKFVARSMLDSRIIDVNFNP--IFFRIGEG---------------
---SMSSGVRPSLGAVKS----------------------VDPGLSRS--------LKLI
KKFVVAKKDIDEDPSRLPAQK-----------------VVDTENISIDD-CTID-DLC--
--LDFTL-------------------------------PG--FPDIE-LIPN--------
GSQTR---
------------------------------VTMSNVE-QYLDR-VVDMTLGSG-VRRQID
-SFRAG---FTTVFPY--SALN-AFTPD-ELVSLFGR-----------------------
---VDEDWS-----------XSAL---------MDSIKADHG------------------
----------------FNMD----SRSV-KNLLQLMS--------------------SLD
ADQRRDFLQF-------TTGSPKLP------------IGG-----FK-ALKPMF--TVVC
RPSEAPHTPD------------------------DYLPSVMTCV-NY-LKLPDY-SDMNM
MRKQLM-TAIKE-GQGA
>Endoconidiophora_laricicola_LXGT01000011. 1..
VRIGRSKI-FESALKVM-----------------NLYGAGQS-ILEVEYFE-------EVG
TGLG-PTLEF---YATI----SREFAKKKL--------KLWRETDSLDD-----------
---------EYVSGPCGLFPKPIS--------------------------------DEES
GTPNGERILHMFAMLGKFVARSMLDSRIIDVNFNP--IFFRIGEG---------------
---SMSSGVRPSLGAVKS----------------------VDPGLSRS--------LKLI
KKFVVAKKDIDEDPSRLPAQK-----------------VVDTENISIDD-CTID-DLC--
--LDFTL-------------------------------PG--FPDID-LIPN--------
GSQTR---
------------------------------VTMSNVE-QYLDR-VVDMTLGSG-VRRQID
-SFRAG---FTTVFPY--SALN-AFTPD-ELVSLFGR-----------------------
---VDEDWS------------XAL---------MDSIKADHG------------------
----------------FNMD----SRSV-KNLLQLMS--------------------SLD
ADQRRDFLQF-------TTGSPKLP------------IGG-----FK-ALKPMF--TVVC
RPSEAPHTPD------------------------DYLPSVMTCV-NY-LKLPDY-SDMNM
MRKQLM-TAIKE-GQGA
>Davidsoniella_virescens_LJZU01000179.1 .
VRIGRSKI-FESALKVM-----------------NLYGAGQS-ILEVEYFE-------EVG
TGLG-PTLEF---YATI----SREFAKKKL--------KLWRETDSLDE-----------
---------EYVSGPCGLFPRPIS--------------------------------DEEA
ATPNGERILHMFAMLGKFVARSMLDSRIIDVNFNP--IFFRIGEG---------------
---SMSSGVRPSLGAVKN----------------------ADPGLARS--------LKLI
KKFVQAKKDIDEDPSRLPAQK-----------------VVDTENITIDN-CTIN-DLY--
--LDFTL-------------------------------PG--FPDIE-LIPN--------
GSQTR---
------------------------------VTMSNVE-QYLDR-VVDMTLGCG-VRRQID
-SFRAG---FTTVFPY--SALN-AFTPD-ELVSLFGR-----------------------
---VDEDWS-----------LESL---------HPSIKADHG------------------
----------------FNMD----SRSV-KNLLQLMS--------------------GLD
ANERRDFLQF-------TTGSPKLP------------IGG-----FK-ALKPMF--TVVC
RPSEAPHTPD------------------------DYLPSVMTCV-NY-LKLPDY-SNMKT
MRKQLM-TAIKE-GQGA
>Chalaropsis_thielavioides_BCGU01000009.1 .
VRIGRAKI-FESALKVM-----------------NLYGAGQS-ILEVEYFE-------EVG
TGLG-PTLEF---YATI----SREFAKKKL--------KLWREADSADD-----------
---------EFISGPTGLFPRPMS--------------------------------DAEA
ETPNGRRILHMFAMLGKFVARSMLDSRLIDINFNP--IFFRIGEG---------------
---AMTAGVRPSLGAIKS----------------------VDPDLARS--------LKLI
KKFALAKKEVEEDATRSPAQK-----------------VSALESITVNG-CHID-DLC--
--LDFTL-------------------------------PG--FPGVE-LIPN--------
GDQTR---
------------------------------VTIDNVE-NYLDC-MIHMTLGSG-VRRQID
-SFRAG---FTTVFPY--SALN-AFTPD-ELVSLFGR-----------------------
---VDEDWS-----------LESL---------MDSIKADHG------------------

----------------FNMD----SRSI-RNLLQLMS--------------------DLN
ASERRDFLQF-------TTGSPKLP------------IGG-----FK-ALKPMF--TVVC
RPSEAPNTPD------------------------DYLPSVMTCV-NY-LKLPDY-SNMDI
MRKQLM-TAIKE-GQGA
>Ceratocystis_albifundus_JSSU01001277.1 .
VRIGRAKI-FESALKVM----------------NLYGAGQS-ILEVEYFE-------EVG
TGLG-PTLEF---YATI----SREFAKKKL--------KLWRETDSHDD-----------
---------EYVNGSTGLFPRPMS--------------------------------DAEA
DTPNGRRILHMFAMLGKFVARSMLDSRLIDINFNP--IFFRIGDG---------------
---PMAAGVRPSLGAVKS----------------------VDPDLARS--------LKLI
KKFALAKKEIEEDATRSAAQK-----------------VDDLEKITING-CHID-DLC--
--LDFTL-------------------------------PG--FPDIE-LIPN--------
GDQTR---
------------------------------VTIDNVE-SYLDS-MIHMTLGSG-VRRQID
-SFRAG---FTTVFPY--SALN-AFTPY-ELVSLFGR-----------------------
---VDEDWS-----------LESL---------MDSIKADHG------------------
----------------FNMD----SRSI-RNLLQLMS--------------------NLN
ASERRDFLQF-------TTGSPKLP------------IGGHLSG-FK-ALKPMF--TVVC
RPSEAPNTPD------------------------DYLPSVMTCV-NY-LKLPDY-SDMEI
MRKQLM-TAIKE-GQGA
>Ceratocystis_harringtonii_MKGM01000017.1 .
VRIGRAKI-FESALKVM----------------NLYGAGQS-ILEVEYFE-------EVG
TGLG-PTLEF---YATI----SREFAKKKL--------KLWRETDSSDD-----------
---------EYVNGSTGLFPRPMS--------------------------------DAEA
DTPNGRRILHMFSMLGKFVARSMLDSRLIDINFNP--IFFRIGDG---------------
---PMTAGVRPSLGAVKS----------------------VDPDLARS--------LKLI
KKFALAKKEIEEDATRSAAQK-----------------VDDLEKITING-CHID-DLC--
--LDFTL-------------------------------PG--FPDVE-LIPN--------
GDQTR---
------------------------------VTIDNVE-NYLDS-MIHMTLGSG-VRRQID
-SFRAG---FTTVFPY--SALN-AFTPD-ELVSLFGR-----------------------
---VDEDWS-----------LESL---------MDSIKADHG------------------
----------------FNMD----SRSI-RNLLQLMS--------------------NLN
ISERRDFLQF-------TTGSPKLP------------IGG-----FK-ALKPMF--TVVC
RPSEAPNTPD------------------------DYLPSVMTCV-NY-LKLPDY-SDMET
MHKQLM-TAIKE-GQGA
>Ceratocystis_fimbriata_APWK02000061.1 .
VRIGRAKI-FESALKVM----------------NLYGAGQS-ILEVEYFE-------EVG
TGLG-PTLEF---YATI----SREFAKKKL--------KLWRETDSPDD-----------
---------EYVNGSTGLFPRPMS--------------------------------DAEA
DTPNGRRILHMFAMLGKFVARSMLDSRLIDINFNP--KFFRIGDG---------------
---PMTAGVRPSLGAVKS----------------------VDPDLARS--------LKLI
KKFALAKKEIEEDATRSAAQK-----------------VDDLEKITING-CHID-DLC--
--LDFTL-------------------------------PG--FPDVE-LIPN--------
GDQTR---
------------------------------VTIDNVE-NYLDS-MIHMTLGSG-VRRQID
-SFRAG---FTTVFPY--SALN-AFTPD-ELVSLFGR-----------------------
---VDEDWS-----------LESL---------MDSIKADHG------------------
----------------FNMD----SRSI-RNLLQLMS--------------------NLN
VSERRDFLQF-------TTGSPKLP------------IGG-----FK-ALKPMF--TVVC
RPSEAPNTPD------------------------DYLPSVMTCV-NY-LKLPDY-SDMET
MRKQLM-TAIKE-GQGA
>Ceratocystis_platani_LBBL01000106.1 .
VRIGRAKI-FESALKVM----------------NLYGAGQS-ILEVEYFE-------EVG
TGLG-PTLEF---YATI----SREFAKKKL--------KLWRETDSPDD-----------
---------EYVNGSTGLFPRPMS--------------------------------DAEA
DTPNGRRILHMFAMLGKFVARSMLDSRLIDINFNP--IFFRIGDG---------------
---PMTAGVRPSLGAVNS----------------------VDPDLARS--------LKLI

KKFALAKKEIEEDATRSAAQK-----------------VDDLEKITING-CHID-DLC--
--LDFTL-------------------------------PG--FPDVE-LIPN--------
GDQTR---
------------------------------VTIDNVE-NYLDS-MIHMTLGSG-VRRQID
-SFRAG---FTTVFPY--SALN-AFTPD-ELVSLFGR-----------------------
---VDEDWS-----------LESL---------MDSIKADHG------------------
----------------FNMD----SRSI-RNLLQLMS--------------------NLN
VSERRDFLQF-------TTGSPKLP------------IGG-----FK-ALKPMF--TVVC
RPSEAPNTPD------------------------DYLPSVMTCV-NY-LKLPDY-SDMET
MRKQLM-TAIKE-GQGA
>Ceratocystis_eucalypticola_LJOA01000187.1 .
VRIGRAKI-FESALKVM----------------NLYGAGQS-ILEVEYFE-------EVG
TGLG-PTLEF---YATI----SREFAKKKL--------KLWRETDSPDD-----------
---------EYVNGSTGLFPRPMS--------------------------------DAEA
DTPNGRRILHMFAMLGKFVARSMLDSRLIDINFNP--IFFRIGDG---------------
---PMTAGVRPSLGAVKS----------------------VDPDLARS--------LKLI
KKFALAKKEIEEDATRSAAQK-----------------VDDLEKITING-CHID-DLC--
--LDFTL-------------------------------PG--FPDVE-LIPN--------
GDQTR---
------------------------------VTIDNVE-NYLDS-MIHMTLGSG-VRRQID
-SFRAG---FTTVFPY--SALN-AFTPD-ELVSLFGR-----------------------
---VDEDWS-----------LESL---------MDSIKADHG------------------
----------------FNMD----SRSI-RNLLQLMS--------------------NLN
VSERRDFLQF-------TTGSPKLP------------IGG-----FK-ALKPMF--TVVC
RPSEAPNTPD------------------------DYLPSVMTCV-NY-LKLPDY-SDMET
MRKQLM-TAIKE-GQGA
>Arthroderma_benhamiae_XM_003017024.1 .
VRISRTRI-LESAMKVL----------------ELYGSSPS-VLEIEYFE-------EVG
TGLG-PTLEF---YSTV----SKEFAKRKL--------KLWRDSDASGDG----------
---------EYVDNKLGLFPAPMS--------------------------------QEQV
TQEAGKKQLQYFKALGKFVARSMLDSRIIDIGFNP--LFFSVGRG---------------
----AYTKKPPSIGSVKR----------------------VDAELANS--------LKFL
KKFADKAAAIKADASLSAAEA-----------------ACAMEQCEIDD-TKLA-DLG--
--LDFTL-------------------------------PG--YPHIK-LIPN--------
GQNTP---
------------------------------ITMSNVG-LYIER-VIDMTLGTG-IKAQLD
-AFAAG---FSQVFLY--SSLK-TFTPD-ELVMLFGQ-----------------------
---VDEDWS-----------IEKL---------MDSIKADHG------------------
----------------FNMD----SRSV-RNLLATLS--------------------EFN
LQQRRDFLQF-------VTGSPKLP------------IGG-----FK-GLTPMF--TVVC
RPSEPPYTPD------------------------DYLPSVMTCV-NY-LKLPDY-SSAEV
LLNKLS-IAMRE-GQGA
>Trichophyton_rubrum_XM_003235823.1 .
VRISRTRI-LESAMKVL----------------ELYGSSPS-VLEIEYFE-------EVG
TGLG-PTLEF---YSTV----SKEFAKRKL--------KLWRDSDASGDG----------
---------EYVDNKLGLFPAPMS--------------------------------QEQV
TQEAGKKQLQYFKALGKFVARSMLDSRIIDIGFNP--LFFSVGRG---------------
----AYTKKPPSIGSVKR----------------------VDAELANS--------LRFL
KKFADQAVAIKADASLSAAEA-----------------ACAMEQCEIDD-TKLA-DLG--
--LDFTL-------------------------------PG--YPHIK-LIPN--------
GQDTP---
------------------------------ITMSNVG-LYIDR-VIDMTLGTG-IKAQLD
-AFAAG---FSQVFLY--SSLK-TFTPD-ELVMLFGQ-----------------------
---VDEDWS-----------IETL---------MDSIKADHG------------------
----------------FNMD----SRSV-RNLLATLS--------------------EFN
LQQRRDFLQF-------VTGSPKLP------------IGG-----FK-GLTPMF--TVVC
RPSEPPYTPD------------------------DYLPSVMTCV-NY-LKLPDY-SSAEV
LLNKLS-IAMRE-GQGA

```
>Trichophyton_verrucosum_XM_003021485.1 .
VRISRTRI-LESAMKVL----------------ELYGSSPS-VLEIEYFE-------EVG
TGLG-PTLEF---YSTV----SKEFAKRKL--------KLWRDSDSSGDG----------
---------EYVDNKLGLFPAPMS--------------------------------QEQV
TQEAGKKQLQYFKALGKFVARSMLDSRIIDIGFNP--LFFSVGRG---------------
----AYTKKPPSIGSVKR----------------------VDAELANS--------LKFL
KKFADKAAAIKTDSSLSAAEA-----------------ACAMEQCEVDD-TKLA-DLG--
--LDFTL-------------------------------PG--YPHIK-LIPN--------
GQNTP-------------------------------------------------------
------------------------------ITMSNVE-LYIDR-VIDMTLGTG-IKAQLD
-AFAAG---FSQVFLY--SSLK-TFTPD-ELVMLFGQ-----------------------
---VDEDWS-----------IEKL---------MDSIKADHG------------------
----------------FNMD----SRSV-RNLLATLS--------------------EFN
LQQRRDFLQF-------VTGSPKLP------------IGG-----FK-GLTPMF--TVVC
RPSEPPYTPD------------------------DYLPSVMTCV-NY-LKLPDY-SSAEV
LLNKLS-IAMRE-GQGA
>Trichophyton_interdigitale_FUFL01001129.1 .
VRISRTRI-LESAMKVL----------------ELYGSSPS-VLEIEYFE-------EVG
TGLG-PTLEF---YSTV----SKEFAKRKL--------KLWRDSESSGDG----------
---------EYVDNKLGLFPAPMS--------------------------------QEQV
LQEAGKKQLQYFKALGKFVARSMLDSRIIDIGFNP--LFFSVGRG---------------
----AYAKKPPSIGSVKR----------------------VDAELANS--------LKFL
KKFADKAAAIKVDTSLSATEA-----------------ACAMEQCEVDD-TKLA-DLG--
--LDFTL-------------------------------PG--YPHIK-LIPN--------
GQNTP-------------------------------------------------------
------------------------------ITMSNVE-LYIDR-VIDMTLGTG-IKAQLD
-AFAAG---FSQVFLY--SSLK-TFTPD-ELVMLFGQ-----------------------
---VDEDWS-----------IESL---------MDSIKADHG------------------
----------------FNMD----SRSV-RNLLATLS--------------------EFN
LQQRRDFLQF-------VTGSPKLP------------IGG-----FK-GLTPMF--TVVC
RPSEPPYTPD------------------------DYLPSVMTCV-NY-LKLPDY-SSAEV
LLNKLS-IAMRE-GQGA
>Trichophyton_equinum_ABWI01001052.1 .
VRISRTRI-LESAMKVL----------------ELYGSSPS-VLEIEYFE-------EVG
TGLG-PTLEF---YSTV----SKEFAKRKL--------KLWRDSESSGDG----------
---------EYVDNKLGLFPAPMS--------------------------------QEQV
LQEAGKKQLQYFKALGKFVARSMLDSRIIDIGFNP--LFFSVGRG---------------
----AYAKKPPSIGSVKR----------------------VDAELANS--------LKFL
KKFADKAAAIKADTSLSATEA-----------------ACAMEQCEVDD-TKLA-DLG--
--LDFTL-------------------------------PG--YPHIK-LIPN--------
GQNTP-------------------------------------------------------
------------------------------ITMSNVE-LYIDR-VIDMTLGTG-IKAQLD
-AFAAG---FSQVFLY--SSLK-TFTPD-ELVMLFGQ-----------------------
---VDEDWS-----------IESL---------MDSIKADHG------------------
----------------FNMD----SRSV-RNLLATLS--------------------EFN
LQQRRDFLQF-------VTGSPKLP------------IGG-----FK-GLTPMF--TVVC
RPSEPPYTPD------------------------DYLPSVMTCV-NY-LKLPDY-SSAEV
LLNKLS-IAMRE-GQGA
>Trichophyton_tonsurans_ACPI01000776.1 .
VRISRTRI-LESAMKVL----------------ELYGSSPS-VLEIEYFE-------EVG
TGLG-PTLEF---YSTV----SKEFAKRKL--------KLWRDSESSGDG----------
---------EYVDNKLGLFPAPMS--------------------------------QEQV
LQEAGKKQLQYFKALGKFVARSMLDSRIIDIGFNP--LFFSVGRG---------------
----AYAKKPPSIGSVKR----------------------VDAELANS--------LKFL
KKFADKAAAIKADTSLSATEA-----------------ACAMEQCEVDD-TKLA-DLG--
--LDFTL-------------------------------PG--YPHIK-LIPN--------
GQNTP-------------------------------------------------------
------------------------------ITMSNVE-LYIDR-VIDMTLGTG-IKAQLD
```

-AFAAG---FSQVFLY--SSLK-TFTPD-ELVMLFGQ----------------------
---VDEDWS-----------IESL---------MDSIKADHG------------------
----------------FNMD----SRSV-RNLLATLS--------------------EFN
LQQRRDFLQF-------VTGSPKLP------------IGG-----FK-GLTPMF--TVVC
RPSEPPYTPD------------------------DYLPSVMTCV-NY-LKLPDY-SSAEV
LLNKLS-IAMRE-GQGA
>Nannizzia_gypsea_XM_003169628.1 .
VRISRTRI-LESAMKVL----------------ELYGSSPS-ILEIEYFE-------EVG
TGLG-PTLEF---YSTV----SKEFSKRKL--------KLWRDSDASNDG----------
---------EYVHNKLGLFPAPMS---------------------------------QDQV
TQEAGKKQLQYFKALGKFVARSMLDSRIIDIGFNP--LFFSVGRG---------------
----AYTTKAPSIGSVKR----------------------VDAELANS--------LKFL
KKFADKAAAIEVDTSLSTAEA-----------------ASAMEKFEVDD-TKLA-DLG--
--LDFTL-------------------------------PG--YPHIK-LIPN--------
GQNIP---
-----------------------------ITMSNVD-LYINK-IIDMTLGSG-IKPQLD
-AFAAG---FSQVFLY--SSLK-TFTPD-ELVMLFGQ----------------------
---VDEDWS-----------IETL---------MDSIKADHG------------------
----------------FNMD----SRSV-RNLLATLS--------------------EFN
LQQRRDFLQF-------VTGSPKLP------------IGG-----FK-GLTPMF--TVVC
RPSEPPYTPD------------------------DYLPSVMTCV-NY-LKLPDY-SSAEV
LLNKLS-IAMRE-GQGA
>Arthroderma_otae_XM_002847246.1 .
VRISRTRI-LDSAMKVL----------------ELYGSSPS-VLEIEYFE-------EVG
TGLG-PTLEF---YSTV----SKEFSKRKL--------KLWRDSDTNDDG----------
---------EYVNNKLGLFPAPMS---------------------------------HEQT
NQEAGKKQLQYFKALGKFVARSMLDSRIIDIGFNP--LFFSAGRG---------------
----VYAKQPPSIGSVKR----------------------VDPELAHS--------LMFL
KRFADKAAAIKADPSLSADEI-----------------TEAMEQCEVDD-TKLA-DLG--
--LYFTL-------------------------------PG--YPNIK-LMPD--------
GHNTL---
-----------------------------ITMSNVE-LYIKK-VIDMTLVSG-VKPQLD
-AFAAG---FSQVFLY--SSLK-TFTPE-ELVMLFGQ----------------------
---VDEDWS-----------IETL---------MDSIKADHG------------------
----------------FNMD----SRSV-RNLLATLS--------------------EFT
LQQRREFLQF-------VTGSPKLP------------IGG-----FK-GLTPMF--TVVC
RPSEPPYTPD------------------------DYLPSVMTCV-NY-LKLPDY-SSAEV
LLDKLS-IAMRE-GQGA
>Onygena_corvina_JWPT01000027.1 .
VRISRTRI-LDSAMKVL----------------ELYGSSPS-VLEIEYFE-------EVG
TGLG-PTLEF---YSTV----SREFSKKRL--------KLWRDSNVDGDG----------
---------EYVDNNLGLFPTPMS---------------------------------YEQS
TQEAGKKQLQLFRALGKFVARSMLDSRIIDIGFNP--TFFSAGRG---------------
----DCAMNTASISSVKR----------------------VDPALAQS--------LNLL
KRYADKAAEIKADKSLSASEI-----------------IDMLEQCEINN-IKLT-DLD--
--LDFIL-------------------------------PG--YPNVS-LMLD--------
GHKTP---
-----------------------------VTMRNVE-LYVTK-VIDMTVGSG-IKPQLD
-AFASG---FSQVFPY--SSLK-SFTPD-ELVMLFGQ----------------------
---VDEDWS-----------ELAL---------TDSIKADHG------------------
----------------FNMD----SRSV-RNLLTTMS--------------------EFS
LRQRRDFLQF-------VTGSPKRP------------PTG-----FK-GLTPMF--TVVC
RPSEPPYTPD------------------------DYLPSVMTCV-NY-LKLPDY-SSAQV
LLDKLG-MAMRE-GQGA
>Zymoseptoria_brevis_GCVP01008288.1 .
VRISRSRI-LESAIKVM----------------ELYGSSSS-VLEVEYFE-------EVG
TGLG-PTLEF---YATV----SKEFSKKKI--------KLWRENESSDGS----------
---------TYAFGKRGLFPAPMS---------------------------------EAMS

```
RDENGTRVLHLFKMLGKFIARSMLDSRIIDVSFNP--TFFRIGDG---------------
-----SATVTPSLGAVAT----------------------VDEGLAKS--------LKML
RKYVSAKQRIEEDDRLTAVQQ-----------------AAKIEQVRVND-ARLE-DLG--
--LDFTL-------------------------------PG--YPEIE-LQPH--------
GSTTN-------------------------------------------------------
------------------------------VTMDNVG-VYVKK-VIEFTLGTG-VQRQID
-AFRAG---FSQVFPY--SALK-AFTPE-ELVMLFGR-----------------------
---VEEDWS-----------LETL---------TDAIKADHG------------------
----------------YNLD----SLSV-RNLLQMMS--------------------ELP
APAKRDFLQF-------VTGSPKLP------------IGG-----FK-SLTPLF--TVVC
KPSEPPYTSD------------------------DYLPSVNTCA-HY-LKMPNY-SSVDV
LRARFK-IAMEE-GQG-
>Zymoseptoria_pseudotritici_JYJD01001238.1 .
VRISRSRI-LESAIKVM----------------ELYGSSSS-VLEVEYFE-------EVG
TGLG-PTLEF---YATV----SKEFSKKKI--------KLWRENESADGS----------
---------TYAFGKRGLFPAPMS--------------------------------EAMS
RDENGTRVLHLFKMLGKFIARSMLDSRIIDVSFNP--TFFRIGDG---------------
-----SATVTPSLGAVAT----------------------VDEGLAKS--------LKML
RKYASAKQRIEEDDRLTAVQQ-----------------AAKIEQVRVND-ARLE-DLG--
--LDFTL-------------------------------PG--YPEIE-LQPH--------
GSTTN-------------------------------------------------------
------------------------------VTMDNVG-VYVKK-VIEFTLGTG-VQRQID
-AFRAG---FSQVFPY--SALK-AFTPE-ELVMLFGR-----------------------
---VEEDWS-----------LETL---------TDAIKADHG------------------
----------------YNLD----SLSV-RNLLQMMS--------------------ELP
APAKRDFLQF-------VTGSPKLP------------ITG-----FK-SLTPLF--TVVC
KPSEPPYTSD------------------------DYLPSVNTCA-HY-LKMPNY-SSVDV
LRARFK-IAMEE-GQGA
>Zymoseptoria_ardabiliae_AFIU01000490.1 .
VRISRSRI-LESAIKVM----------------ELYGSSSS-VLEVEYFE-------EVG
TGLG-PTLEF---YATV----SKEFSKKKI--------KLWRENESSDGS----------
---------TYAFGKRGLFPAPMS--------------------------------EAMS
RDENGTRVLHLFKMLGKFIARSMLDSRIIDVSFNP--TFFRIGDG---------------
-----SATVTPSLGAVAT----------------------VDEGLAKS--------LKML
RQYASAKQRIEEDDRLTAVQQ-----------------AAKIEQVRVND-ARLE-DLG--
--LDFTL-------------------------------PG--YPEIE-LQPH--------
GSTTN-------------------------------------------------------
------------------------------VTMDNVG-DYVKK-VIEFTLGTG-VQRQID
-AFRAG---FSQVFPY--SALK-AFTPE-ELVMLFGR-----------------------
---VEEDWS-----------LETL---------TDAIKADHG------------------
----------------YNLD----SLSV-RNLLQMMS--------------------ELP
APAKRDFLQF-------VTGSPKLP------------IGG-----FK-SLTPLF--TVVC
KPSEPPYTSD------------------------DYLPSVNTCA-HY-LKMPNY-SSVDV
LRARFK-IAMEE-GQGA
>Zymoseptoria_tritici_GCJU01006400.1 .
VRISRSRI-LESAIKVM----------------ELYGSSSS-VLEVEYFE-------EVG
TGLG-PTLEF---YATV----SKEFSKKKI--------KLWRENESSDGS----------
---------TYAFGKRGLFPAPMS--------------------------------EAMS
RDENGTRVLHLFKMLGKFIARSMLDSRIIDVSFNP--TFFRIGDG---------------
-----SATVTPSLGAVAT----------------------VDEGLAKS--------LKML
RKYASAKQRIEEDDRLTAVQQ-----------------AAKIEQVRVND-ARLE-DLG--
--LDFTL-------------------------------PG--YPEIE-LQPH--------
GSTTN-------------------------------------------------------
------------------------------VTMDNVG-VYVKK-VIEFTLGTG-VQRQID
-AFRAG---FSQVFPY--SALK-AFTPE-ELVMLFGR-----------------------
---VEEDWS-----------LETL---------TDAIKADHG------------------
----------------YNLD----SLSV-RNLLQMMS--------------------ELP
APAKRDFLQF-------VTGSPKLP------------IGG-----FK-SLTPLF--TVVC
```

KPSEPPYTSD------------------------DYLPSVNTCA-HY-LKMPNY-SSVEV
LRARFK-IAMEE-GQG-
>Mycosphaerella_graminicola_XM_003856419.1 .
VRISRSRI-LESAIKVM----------------ELYGSSSS-VLEVEYFE-------EVG
TGLG-PTLEF---YATV----SKEFSKKKI--------KLWRENESSDGS----------
---------TYAFGKRGLFPAPMS--------------------------------EAMS
RDENGTRVLHLFKMLGKFIARSMLDSRIIDVSFNP--TFFRIGDG---------------
-----SATVTPSLGAVAT----------------------VDEGLAKS--------LKML
RKYASAKQRIEEDDRLTAVQQ-----------------AAKIEQVRVND-ARLE-DLG--
--LDFTL-------------------------------PG--YPEIE-LQPH--------
GSTTN---
------------------------------VTMDNVG-VYVKK-VIEFTLGTG-VQRQID
-AFRAG---FSQVFPY--SALK-AFTPE-ELVMLFGR-----------------------
---VEEDWS-----------LETL---------TDAIKADHG------------------
----------------YNLD----SLSV-RNLLQMMS--------------------ELP
APAKRDFLQF-------VTGSPKLP------------IGG-----FK-SLTPLF--TVVC
KPSEPPYTSD------------------------DYLPSVNTCA-HY-LKMPNY-SSVEV
LRARFK-IAMEE-GQGA
>Zymoseptoria_passerinii_AFIY01001257.1 .
VRISRSRI-LESAIKVM----------------ELYGSSSS-VLEVEYFE-------EVG
TGLG-PTLEF---YATV----SKEFSKKKI--------KLWRENESSDGS----------
---------VYAFGKRGLFPAPMS--------------------------------EAMS
RDENGMRVLHLFKMLGKFIARSMLDSRIIDVSFNP--TFFRIGDG---------------
-----SATVTPSLGAVAT----------------------VDEGLAKS--------LKML
RQYASAKQRIEEDTRLTSAQQ-----------------ATKIEQVRVND-ARLE-DLG--
--LDFTL-------------------------------PG--YPEIE-LQPH--------
GSTTN---
------------------------------VTMDNVG-VYVKK-VIDFTLGIG-VQRQID
-AFRAG---FSQVFPY--SALK-AFTPE-ELVMLFGR-----------------------
---VEEDWS-----------LETL---------TDAIKADHG------------------
----------------YNLD----SMSV-RNLLQMMS--------------------ELP
APAKRDFLQF-------VTGSPKLP------------IGG-----FK-SLTPLF--TVVC
KPSEPPYTSD------------------------DYLPSVNTCA-HY-LKMPNY-SSVAV
LRARFK-IAMEE-GQGA
>Phaeocryptopus_gaeumannii_MWSP01000216.1 .
VRISRSRI-LESAIKVM----------------ELYGSSSS-VLEVEYFE-------EVG
TGLG-PTLEF---YATV----SKEFSKKKT--------KLWRENESYEG-----------
---------EFAFGKRGLFPAPMS--------------------------------EETS
RNENGQRVLHLFRMLGKFVARSMLDSRIIDVSLNP--TFFRIGDS---------------
------ATVTPSLGAVAS----------------------VDEDLAKS--------LKIL
RKYANAKQRIEEDGDLSSTQK-----------------AEKIERIRVND-AKID-DLA--
--LDFTL-------------------------------PG--YPHIE-LVDH--------
GSSTN---
------------------------------VTIENVS-QYIKS-VIDFTLGVG-VQKQVD
-AFRAG---FSLVFPY--SALK-AFTPD-ELVMLFGR-----------------------
---VEEDWS-----------LETL---------MDSIKADHG------------------
----------------FNLD----SKSV-RNLLQVMS--------------------EFS
PQAKRDFLQF-------VTGSPKLP------------IGG-----FK-ALTPMF--TVVC
KPSEPPYTSD------------------------DYLPSVMTCQ-HY-FKLPDY-SSADV
LRQRLG-TATKE-GQGA
>Lecanosticta_acicola_AWYC02001244.1 .
IRISRSRI-LESAIKVM----------------ELYGSSSS-VLEVEYFE-------EVG
TGLG-PTLEF---YSTV----SKEFSKKKT--------KLWRENESADRD----------
---------EYAFGKRGLFPAPMS--------------------------------EEMS
RDENGQRVLHLFKMLGKFVARSMLDSRIIDVSFNP--TFFRIGDG---------------
-----STTVTPSLGAVAT----------------------VDHDLANS--------LKIL
RKYANAKQRIDEDTKLSPAQK-----------------AQKAEHIRIND-LKID-DLA--
--LDFTL-------------------------------PG--YPHIE-LQPH--------

GASID---
------------------------------VTIDNVG-AYVKK-VIEFTLGAG-VQKQHE
-AFSAG---FSQVFPY--SALK-AFTPD-ELVMLFGR-----------------------
---VEEDWR-----------LETL---------MDSIKADHG------------------
----------------YNLD----SKSV-RNLLQTMS--------------------ELS
PQARRDFLQF-------VTGSPKLP------------IAG-----FK-SLTPMF--TVVC
KPAEPPYASD------------------------DYLPSVMTCV-NY-LKMPDY-SSLDV
LKQKLN-VAIKE-GQGA
>Mycosphaerella_laricina_AWYE02000435.1 .
VRISRSRI-LESAIKVM----------------ELYGQSSS-VLEVEYFE-------EVG
TGLG-PTLEF---YSTV----SKEFSKKKT--------KLWRENESFDDA----------
---------EFAFGKRGLFPAPMS--------------------------------EETS
KNENGQRVLHLFKMLGKFVARSMLDSRIIDVSFNP--TFFRVGDS---------------
------ATVPPSLGAVST----------------------VDQDLAKS--------LKML
KKYASAKQRINDDIRLSGVQK-----------------QQKVQQIRIND-AKID-ELG--
--LDFTL-------------------------------PG--YPDIE-LVPR--------
GATID---
------------------------------VTMDNVD-SYLKK-VIDFTLGAG-VERQVE
-AFRAG---FSQVFPY--SALK-AFTPD-ELVMLFGR-----------------------
---VDEDWS-----------LETL---------MDSIKADHG------------------
----------------YNLE----SKSV-RNLLQFMS--------------------ELA
PTSRRDFLQF-------ITGSPKLP------------IGG-----FK-ALTPMF--TVVC
KPAEPPHTSD------------------------DYLPSVMTCV-NY-LKMPDY-TDINV
LREKMN-VAIKE-GQGA
>Passalora_fulva_AMRR01002339.1 .
VRISRGRI-LESALKVM----------------ELYGSSSS-MLEVEYFE-------EVG
TGLG-PTLEF---YSTV----SKEFSKKKT--------KLWRENESFEND----------
---------EFAFGKRGLFPAPMN--------------------------------EETS
RNENGQRVLHLFKMLGKFVARSMLDSRIIDISFNP--TFFRIGDS---------------
------TAVAPSLGAVAT----------------------VDEDLAKS--------LKML
KKYATTKQRIDEDIQLSAAQK-----------------LQKTQQIRVND-AKVE-DLG--
--LGFTL-------------------------------PG--YPDIE-LLPH--------
GSTID---
------------------------------VTIDNVD-LYIKK-VIDFTLGAG-IEKQVE
-AFRAG---FSQVFPY--SATK-AFTPD-ELVMLFGR-----------------------
---VDEDWS-----------LETL---------MDSIKADHG------------------
----------------FNLD----SKSV-RNLLQFMS--------------------ELP
APTRRDFLQF-------ITGSPKLP------------LTG-----FK-ALTPMF--TVVC
KPSEPPYTSD------------------------DYLPSVMTCV-NY-LKMPDY-SSLDI
LKEKLN-VAIKE-GQGA
>Mycosphaerella_arachidis_LIHB01000057.1 .
VRISRSRI-LESALKVM----------------ELYGSSSS-MLEVEYFE-------EVG
TGLG-PTLEF---YSTV----SKEFSKKKT--------KLWRENESFEND----------
---------EFAFGKRGLFPAPMS--------------------------------EETS
HNENGQRVLHLFKMLGKFVARSMLDSRIIDVSFNP--TFFRVGDS---------------
------TAVAPSLGAVAT----------------------VDEDLAKS--------LKML
KKYAIAKQRVDEDIRLSAVQK-----------------LQKIQQIRIND-AKVE-ELG--
--LEFTL-------------------------------PG--YPDIE-LLPN--------
GSTID---
------------------------------VTIDNVD-LYVKK-VIDFTLGAG-VEKQVE
-AFRAG---FSQVFPY--SATK-AFTPD-ELVMLFGR-----------------------
---VDEDWS-----------LETL---------MDSIKADHG------------------
----------------FNLD----SKSV-RNLLQFMS--------------------ELP
EPNRRDFLQF-------ITGSPKLP------------IAG-----FK-ALTPMF--TVVC
KPSEPPYTSD------------------------DYLPSVMTCV-NY-LKMPDY-SSLDV
LREKLN-VAIKE-GQGA
>Dothistroma_pini_MWSO01000104.1 .
VRISRSRI-LESALKVM----------------ELYGSSSS-MLEVEYFE-------EVG

TGLG-PTLEF---YSTV----SKEFSKKKT--------KLWRENESFEDD----------
---------QFAFGKRGLFPAPMS---------------------------------EETS
RNENGQRVLHLFKMLGKFVARSMLDSRNIDVSFNP--TFFRVGDS---------------
------SAVAPSLGAVAT----------------------VDQDLAKS--------LKML
KKYATAKQRIDEDTRLSPVQK-----------------LQKIQQIRIND-AKVE-ELG--
--LEFTL--------------------------------PG--YPDIE-LIPN--------
GSTTD---
------------------------------VNIENVD-VYVKK-VIEFTLGAG-VQKQVD
-AFRAG---FSQVFPY--SATK-AFTPD-ELVMLFGR-----------------------
---VEEDWS-----------LETL---------MDSIKADHG------------------
----------------FNLD----SKSV-RNLLQFMF--------------------ELP
APTRREFLQF-------ITGSPKLP------------IGG-----FK-ALTPMF--TVVC
KPADPPYTSD------------------------DYLPSVMTCV-NY-LKMPDY-SSLDV
LKEKLD-VAIKE-GQGA
>Dothistroma_septosporum_AIEN01000020.1 .
VRISRSRI-LESALKVM-----------------ELYGSSSS-MLEVEYFD-------EVG
TGLG-PTLEF---YSTV----SKEFCKKKT--------KLWRENESFEND----------
---------EFAFGKRGLFPAPMS---------------------------------EETS
RNENGQRVLHLFKMLGKFVARSMLDSRIIDVSFNP--TFFRIGDS---------------
------SAVAPSLGAVAT----------------------VDQDLAKS--------LKML
KKYATAKQRIDEDPRLSAVQK-----------------FQKMQQICIND-AKVE-ELG--
--LEFTL--------------------------------PG--YPDID-LIPN--------
GATID---
------------------------------VDIGNVD-IYVKK-VIEFTLGAG-VQKQVD
-AFRAG---FSQVFPY--SATK-AFTPD-ELVMLFGR-----------------------
---VEEDWS-----------LETL---------MDSIKADHG------------------
----------------FNLD----SKSV-RNLLQFMS--------------------ELP
APTRRDFLQF-------ITGSPKLP------------IGG-----FK-ALTPMF--TVVC
KPAEPPYTSD------------------------DYLPSVMTCV-NY-LKMPDY-SSLDV
LKEKLD-VAIQE-GQGA
>Cercospora_sojina_AHPQ01000119.1 .
VRISRSRI-LESAIKVM-----------------ELYGSSSS-ILEVEYFE-------EVG
TGLG-PTLEF---YSTV----SKEFSKKKT--------KLWRENESADSD----------
---------EFAFGKRGLFPAPMN---------------------------------EEMA
RNENGQRVLHLFKMLGKFVARSMLDSRIIDVSFNP--TFFRIGEG---------------
-----STTVTPSLGAVAA----------------------VDADLAKS--------LKVL
RKYASAKQRIDEDADLTPSEK-----------------SDKIEQIRIND-AKIE-DLA--
--LDFTL--------------------------------PG--HPNIE-LLPN--------
GATIN---
------------------------------VTIDNVA-QYIKK-VIDFTLGTG-VQRQID
-AFRAG---FTQVFPY--SAVR-AFTPD-ELVMLFGR-----------------------
---VEEDWS-----------LETL---------MDSIKADHG------------------
----------------YNLD----SKSV-RNLLQVMS--------------------EFP
PQSKRDFLQF-------VTGSPKLP------------IGG-----FK-SLTPMF--TVVC
KPAEPPHGSD------------------------DFLPSVMTCV-NY-LKMPDY-STLEV
LQKRLD-TAIKE-GQGA
>Cercospora_canescens_ANSM01004771.1 .
VRISRSRI-LESAIKVM-----------------ELYGSSSS-VLEVEYFE-------EVG
TGLG-PTLEF---YSTV----SKEFSKKKT--------KLWRENESADGD----------
---------DFAFGKRGLFPAPMN---------------------------------EEMA
RNENGQRVLHLFKMLGKFVARSMLDSRIIDVSFNP--TFFRIGEG---------------
-----SLTVTPSLGAVAA----------------------VDADLAKS--------LKIL
RKYASAKQRIDEDTNLTPSQK-----------------SEKIEEIRISD-AKLE-DLA--
--LDFTL--------------------------------PG--HPNIE-LLPN--------
GATIN---
------------------------------VTIDNVG-QYIKK-VIDFTLGTG-VQRQID
-AFRAG---FTQVFPY--SALR-AFTPD-ELVMLFGR-----------------------
---VEEDWS-----------LETL---------MDSIKADHG------------------

----------------YNLD----SKSV-RNLLQVMS--------------------GFS
PQSKRDFLQF-------VTGSPKLP------------IGG-----FK-SLTPMF--TVVC
KPAEPPYGSD------------------------DFLPSVMTCV-NY-LKMPDY-STLEV
LQQRLD-TAIKE-GQGA
>Sphaerulina_populicola_AIDU01000703.1 .
VRISRSRI-LESAIKVM----------------ELYGSSSS-VLEVEYFE-------EVG
TGLG-PTLEF---YSSV----SKEFSKKKT--------KLWRENESADGD----------
---------EFAFGKRGLFPAPMS--------------------------------EETA
RNENGQRVLHLFKMLGKFVARSMLDSRIIDVSFNP--TFFRIGDA---------------
-----STSVTPSLGAVAA----------------------VDADLAKS--------LKML
RQFVTAKQRIDDNPNLTPAQK-----------------AQKAEQIRVND-ANID-DLA--
--LDFTL------------------------------PG--HASIE-LLPN--------
GATIN--
-----------------------------VTIENIA-EYIKK-VIDFTLGAG-VQRQVD
-AFRAG---FSQVFPY--SALK-AFTPD-ELVMLFGR----------------------
---VDEDWS-----------LETL---------MDSIKADHG------------------
----------------FNLD----SKSV-RNLLQVMS--------------------EFP
PQSKRDFLQF-------VTGSPKLP------------IGG-----FK-SLTPMF--TVVC
KPAEPPHGSD------------------------DFLPSVMTCV-NY-LKMPDY-STLEV
LQKRLG-TAIRE-GQGA
>Sphaerulina_musiva_XM_016910196.1 .
VRISRSRI-LESAIKVM----------------ELYGSSSS-VLEVEYFE-------EVG
TGLG-PTLEF---YSTV----SKEFSKKKT--------KLWRENESADGD----------
---------EFAFGKRGLFPAPMS--------------------------------EETA
RNENGQRVLHLFKMLGKFVARSMLDSRIIDVSFNP--TFFRIGDA---------------
-----STTVTPSLGAVAA----------------------VDADLAKS--------LKML
RQFVTAKQRIDDNPNLTPAQK-----------------AQKAEQIRVND-AKID-DLA--
--LDFTL------------------------------PG--HASIE-LLPN--------
GATIN--
-----------------------------VTIENIA-EYIKK-VIDFTLGAG-VQRQVD
-AFRAG---FSQVFPY--SALK-AFTPD-ELVMLFGR----------------------
---VDEDWS-----------LETL---------MDSIKADHG------------------
----------------FNLD----SKSV-RNLLQVMS--------------------EFP
PQSKRDFLQF-------VTGSPKLP------------IGG-----FK-SLTPMF--TVVC
KPAEPPHGSD------------------------DFLPSVMTCV-NY-LKMPDY-STLEV
LQKRLG-TAIRE-GQGA
>Baudoinia_panamericana_XM_007674762.1 .
VRISRTRI-LESAIKVM----------------ELYGGSQS-ILEVEYFE-------EVG
TGLG-PTLEF---YSTV----SKEFSKKKT--------KLWRENDSGDHG----------
---------DYAFGRRGLFPAPMD--------------------------------ETLA
NNENGKKVLHLFTMLGKFIARSMIDSRIIDVSFNP--TFFRVGDG---------------
-----TATVAPSLGAVAA----------------------VDEDLAKA--------LKML
KKYASAKQRIEGDDGLSASQK-----------------RQKIDEIRIND-AKIE-DLG--
--LDFTL------------------------------PG--YPHID-LIER--------
GSSTN--
-----------------------------VTVDNVG-QYINA-VVDCTLGSG-VQRQVD
-AFRAG---FSQVFPY--SALK-AFTPD-ELVMLFGR----------------------
---VEEDWS-----------LETL---------MDSIKADHG------------------
----------------YNLD----SKSV-RNLLQVMS--------------------ELS
PQSKRDFLQF-------VSGSPKLP------------IGG-----FK-ALTPMF--TVVC
KPSEPPLTSD------------------------DYLPSVMTCV-NY-LKMPDY-TSMEV
LKERLG-VAIRE-GQGA
>Acidomyces_richmondensis_JPDO01000171.1 .
VRISRSRI-LESAVKVM----------------ELYGGSQS-ILEVEYFE-------EVG
TGLG-PTLEF---YATV----SKEFSKKKT--------KLWRENESADSG----------
---------EYAFGNRGLFPAPMD--------------------------------EEMA
KNENGQRILHLFKMLGTFVARSMLDSRIIDISFNP--TFFRIGDG---------------
-----SATVTPSLGAVAA----------------------VDQDLAKS--------LKML

KKYVSAKQRIEENDSLSELQK-----------------QGKIQDIRIND-ARVD-DLG--
--LDFTL-------------------------------PG--YPHME-LIPN--------
GSNTN---
------------------------------VSIDNVD-LYIQK-VIDMTLGSG-VQRQVD
-AFRAG---FSEVFPY--SALK-AFTPD-ELVMLFGR-----------------------
---IEEDWS-----------LETL---------MDSIKADHG------------------
----------------YNLD----SKSV-RNLLQVMS--------------------ELS
PQAKRDFLQF-------VTGSPKLP------------IGG-----FK-ALTPMF--TVVC
KPSEPPLVSD------------------------DYLPSVMTCV-NY-LKMPDY-SSLEV
LRGKLA-VAIKE-GQGA
>Ramularia_collo-cygni_CZLF01000095.1 .
VRISRTRI-LESAIKVM----------------ELYGSSSS-VLEVEYFE-------EVG
TGLG-PTLEF---YSTV----SKEFCKQST--------KLWRDSDSQGDD----------
---------EYAFGKQGLFPRPMS--------------------------------ETMA
SNENGQRVLHLFKMLGKFVARSMLDARIIDVSFNQ--TFFRIGDS---------------
-----ATTVTPSLGAVAT----------------------VDEDLAKS--------LKML
RKYENAKKRIEEDPRLSATQK-----------------AMKMEQIRVND-AKIE-DLA--
--INFTL-------------------------------PG--YPEIE-LVDN--------
GVNLD---
------------------------------VNLDNVG-LYIKK-VIDFTLGAG-VQRQVD
-AFRAG---FSLVFPY--TAMK-SFTPK-ELVGLFGR-----------------------
---VDEDWS-----------LATL---------MDSIKADHG------------------
----------------YNLD----SKSV-RNLLQVMS--------------------EYA
PEQKRAFLQF-------VTGSPKLP------------VSG-----FK-ALTPMF--TVVC
KPSEPPYSSD------------------------DYLPSVMTSV-QY-LKLPDY-SSVEI
LKAKLN-VAMSD-GQGA
>Rachicladosp_antarcticum_NAJO01000003.1 .
VRISRSRI-LESAIKVM----------------EMYGSSPS-VLEVEYFE-------EVG
TGLG-PTLEF---YSTV----SKDFSKKKT--------KLWRENESTGTD----------
---------DYAFGKRGLFPAPMD--------------------------------EAMS
KSENGQRILQLFKVLGKFVARSMLDSRIIDVSLNP--TFFRVGDG---------------
-------GLSLNLAAVAT----------------------VDQDLAKS--------LKML
KKYVTARERIEEDSRLNLSQK-----------------ISKVQGIRVND-ARIE-DLG--
--LDFTL-------------------------------PG--YPHIE-LAEH--------
GASTD---
------------------------------VTIHNLS-TYIRK-VIEVTLLTG-VQRQIS
-AFRAG---FSQVFPY--TALK-AFTPD-ELVMLFGR-----------------------
---VKEDWS-----------LETL---------MDSIKADHG------------------
----------------FNLD----SKNV-RNLLQVMS--------------------EMP
EEERRGFLQF-------VTGSPKLP------------IGG-----FK-SLTPMF--TVVC
KPAEPPYTSD------------------------EYLPSVMTCV-NY-LKMPDY-SSLKV
LKDKLG-VACRE-GQG-
>Cladosporium_sphaerospermum_MSJI02000485. 1..
VRISRNRI-LESAIKVM----------------EMYGSSPS-MLEVEYFE-------EVG
TGLG-PTLEF---YSTV----SKEYSRKKT--------KLWRENESSGSG----------
---------EYAFGRRGLFPAPMN--------------------------------EETS
KTENGQRVLHLFKILGKFIARSMLDSRIIDVSLNP--TFFRVGDG---------------
-------GLPINLGAVAT----------------------VDQDLARS--------LKIL
KKYVSAKQRIDEDTRLSDVQK-----------------AAKIDQIRIND-AKIE-DLG--
--LDFTL-------------------------------PG--YSNIE-LTPS--------
GADVP---
------------------------------VTIENVN-SYIKK-VVEVTLSSG-VQRQVD
-AFRSG---FSQVFPY--SALK-AFTPD-ELVMLFGR-----------------------
---VEEDWS-----------LETL---------MDSIKADHG------------------
----------------YNLD----SKSV-RNLLQIMS--------------------ELP
EEGRRGFLQF-------ITGSPKLP------------IGG-----FK-ALTPMF--TVVC
KPAEAPYTSD------------------------EFLPSVMTCV-NY-LKMPDY-SDIDI
MRARLD-VAVKE-GQG-

>Preussia_sp._LJJI01000327.1 .
VRISRTRI-LESAIKVM----------------EMYGSSPS-MLEVEYFE-------EVG
TGLG-PTLEF---YSTV----SKEYSRKKT--------KLWRENESAGGG----------
---------EYAFGRRGLFPAPMS--------------------------------EEIS
KTENGQRVLHLFKILGKFIARSMLDSRIIDVSLNP--TFFRVGDG---------------
-------GLPLNISAVAT----------------------VDQDLARS--------LKVL
KKYVAAKQRIDDDERLSNVQK-----------------AAKIDQIRIND-AKIE-DLG--
--LDFTL-------------------------------PG--YPNIE-LTAG--------
GANVD---
------------------------------VTIENVS-SYIRK-VVEVTLSSG-VQRQVD
-AFRAG---FSQVFPY--TALK-AFTPD-ELVMLFGR-----------------------
---VEEDWS-----------LETL---------MDSIKADHG------------------
----------------YNLD----SKSV-RNLLQVMS--------------------ELS
EDGRRGFLQF-------ITGSPKLP------------IGG-----FK-ALTPMF--TVVC
KPAEAPYTSD------------------------QFLPSVMTCV-NY-LKMPDY-SDIDI
LRARLD-TAVKE-GQG-
>Leptoxyphium_fumago_LSHF01000090.1 .
VRISRSRI-LESAIKVM----------------ELYGSSAS-ILEVEYFE-------EVG
TGLG-PTLEF---YSTV----SKEFSKKKT--------KMWRENESFAFSSN--------
---------EFAFARNGLFPAPMN--------------------------------EEMA
GNENGQRILHLFKILGKLVARSMLDSRIIDIPFNP--TFFRSGSG---------------
-----WGTVTPSLGAVVT----------------------VDQDLAKQ--------LRWV
KKYANAKDRIETDESLSAAEK-----------------SEKLNNIRIND-AKIE-ELG--
--LDFTL-------------------------------PG--YSHIE-LIPN--------
GSNVD---
------------------------------VTLDNVE-TFIDK-VIDFTLRSG-VQRQIE
-AFAEG---FSKVFPY--SALR-AFTPN-ELVMLFGR-----------------------
---VEEDWS-----------LSTL---------MDSIKADHG------------------
----------------YNLD----SKSV-RNLLQLMS--------------------ELT
PEERRDFLQF-------VTGSPKLP------------IGG-----FK-SLTPMF--TVVC
RPSEAPLTPD------------------------DYLPSVMTCV-NY-LKMPDY-SSIDV
MREKLN-LAFKE-GQGA
>Pseudocerc_musae_GDIN01012977.1 .
---LEVEYFD-------EVG
TGLG-PTLEF---YSTV----SKEFSKRKT--------KLWRENESFDNS----------
---------EYAFGKRGLFPAPMS--------------------------------EEMA
RKENGQKVLNMFKMLGKFVARSMLDSRIIDVSFNP--TFFRVGES---------------
-----TTTVTPSLGAVAA----------------------VDEGLAHS--------LKVL
MKYANAKQRIDEDVTLSPDQK-----------------SFKAEQIRIND-ALIE-DLG--
--LDFTL-------------------------------PG--HPHIE-LLPH--------
GSTIN---
------------------------------VTIDNVG-LYIKK-VIDFTLQSG-VQRQVE
-AFHTG---FSLVFPY--SALR-AFTPD-ELCMLFGH-----------------------
---GEEDWS-----------LETL---------MDSIKADHG------------------
----------------FNLD----SRSV-RNLLQAMS--------------------EFS
SQDRRDFLQF-------VTGSPKLP------------IGG-----FK-SLTPMF--TVVR
KSADAPYTAD------------------------DYLPSVMTCV-NY-LKMPDY-SSLEV
LKSRFN-TASKE-GQG-
>Mycosphaerella_eumusae_GDIK01009756.1 .
---LEVEYFD-------EVG
TGLG-PTLEF---YSTV----SKEFSKKKT--------KLWRENESFDNS----------
---------EYAFGKRGLFPAPMS--------------------------------EEMA
RKENGQKVLNMFKMLGKFVARSMLDSRIIDVSFNP--TFFRVGES---------------
----TTTTVTPSLGAVAA----------------------VDEGLAHS--------LKVL
KKYANAKQRIDEDATLSPDQK-----------------SSKAEQIRIND-ALIE-DLG--
--LDFTL-------------------------------PG--HPHIE-LLPH--------
GSTIN---
------------------------------VTIDNVG-QYIKK-VIDFTLQSG-VQRQVE

-AFQTG---FSLVFPY--SALR-AFTPD-ELCMLFGH----------------------
---GEEDWS-----------LETL---------MDSIKADHG-----------------
----------------FNLD----SRSV-RNLLQAMS--------------------EFS
PQDRRDFLQF-------VTGSPKLP------------IGG-----FK-SLTPMF--TVVR
KSADAPYTSD------------------------DYLPSVMTCV-NY-LKMPDY-SSLEV
LKSRFN-TASKE-GQG-
>Pseudocerc_fijiensis_XM_007921992.1 .
VRISRSRM-LESAMKVM----------------ELYGSSSS-MLEVEYFD-------EVG
TGLG-PTLEF---YSTV----SKEFSKKKT--------KLWRENESLDNS----------
---------EYAFGKRGLFPAPMS---------------------------------EEMA
RKENGQKVLNMFKMLGKFVARSMLDSRIIDVSFNP--TFFRVGAS---------------
-----TTTVTPSLGAVAA----------------------VDEGLAHS--------LKVL
RKYANAKQRIDEDATLSPDQK-----------------ASKAEQIRIND-AHIE-DLG--
--LDFTL-------------------------------PG--HPDIE-LLPY--------
GSTIN---
------------------------------VTIDNVG-QYIKK-VIDFTLQSG-VQRQVE
-AFRTG---FSLVFPY--SALR-AFTPD-ELCMLFGH----------------------
---GEEDWS-----------LETL---------MDSIKADHG-----------------
----------------FNLD----SRSV-RNLLQAMS--------------------EFS
SQDRRDFLQF-------VTGSPKLP------------IGG-----FK-SLTPMF--TVVR
KSADAPYTSD------------------------DYLPSVMTCV-NY-LKMPDY-SSLEV
LKSRFN-TASKE-GQGA
>Pseudocerc_musae_GDIN01012980.1 .
VRISRSRM-LESAMKVM----------------ELYGSSSS-MLEVEYFD-------EVG
TGLG-PTLEF---YSTV----SKEFSKRKT--------KLWRENESFDNS----------
---------EYAFGKRGLFPAPMS---------------------------------EEMA
RKENGQKVLNMFKMLGKFVARSMLDSRIIDVSFNP--TFFRVGES---------------
-----TTTVTPSLGAVAA----------------------VDEGLAHS--------LKVL
MKYANAKQRIDEDVTLSPDQK-----------------SFKAEQIRIND-ALIE-DLG--
--LDFTL-------------------------------PG--HPHIE-LLPH--------
GSTIN---
------------------------------VTIDNVG-LYIKK-VIDFTLQSG-VQRQVE
-AFHTG---FSLVFPY--SALR-AFTPD-ELCMLFGH----------------------
---GEEDWS-----------LETL---------MDSIKADHG-----------------
----------------FNLD----SRSV-RNLLQAMS--------------------EFS
SQDRRDFLQF-------VTGSPKLP------------IGG-----FK-SLTPMF--TVVR
KSADAPYTAD------------------------DYLPSVMTCV-NY-LKMPDY-SSLEV
LKSRFN-TASKE-GQG-
>Pseudocerc_pini_densiflorae_AWYD02003823. 1..
VRISRSRM-LESAMKVM----------------ELYGSSSS-MLEVEYFD-------EVG
TGLG-PTLEF---YSTV----SKEFSKKKT--------KLWRENESFDNS----------
---------EYAFGKRGLFPAPMS---------------------------------EEMA
RKENGQKVLNMFKMLGKFVARSMLDSRIIDVSFNP--TFFRVGES---------------
-----TTTVTPSLGAVAA----------------------VDEGLAHS--------LKVL
KKYANAKQRIDEDASLSPDQK-----------------SSKAEQIRIND-ALIE-DLG--
--LDFTL-------------------------------PG--HPHIE-LLPH--------
GSTIN---
------------------------------VTIDNVG-QYIKK-VIDFTLQSG-VQRQVE
-AFQTG---FSLVFPY--SALR-AFTPD-ELCMLFGH----------------------
---GEEDWS-----------LETL---------MDSIKADHG-----------------
----------------FNLD----SRSV-RNLLQAMS--------------------EFS
SQDRRDFLQF-------VTGSPKLP------------IGG-----FK-SLTPMF--TVVR
KSADAPYTSD------------------------DYLPSVMTCV-NY-LKMPDY-SSLEV
LKSRFN-TASKE-GQGA
>Mycosphaerella_eumusae_GDIK01009758.1 .
VRISRSRM-LESAMKVM----------------ELYGSSSS-MLEVEYFD-------EVG
TGLG-PTLEF---YSTV----SKEFSKKKT--------KLWRENESFDNS----------
---------EYAFGKRGLFPAPMS---------------------------------EEMA

RKENGQKVLNMFKMLGKFVARSMLDSRIIDVSFNP--TFFRVGES---------------
----TTTTVTPSLGAVAA----------------------VDEGLAHS--------LKVL
KKYANAKQRIDEDATLSPDQK-----------------SSKAEQIRIND-ALIE-DLG--
--LDFTL-------------------------------PG--HPHIE-LLPH--------
GSTIN---
------------------------------VTIDNVG-QYIKK-VIDFTLQSG-VQRQVE
-AFQTG---FSLVFPY--SALR-AFTPD-ELCMLFGH-----------------------
---GEEDWS-----------LETL---------MDSIKADHG------------------
----------------FNLD----SRSV-RNLLQAMS--------------------EFS
PQDRRDFLQF-------VTGSPKLP------------IGG-----FK-SLTPMF--TVVR
KSADAPYTSD------------------------DYLPSVMTCV-NY-LKMPDY-SSLEV
LKSRFN-TASKE-GQG-
>Aureobasidium_pullulans_LVWM01000019.1 .
VRISRSRI-LESAIKVM----------------ELYGSSPS-ILEVEYFE-------EVG
TGLG-PTLEF---YSTV----SREFSKKKI--------KLWRENESIAGD----------
---------DYAFGMRGLFPAPMS--------------------------------DEQS
NKENGKRVLHLFKMLGKFVARSMLDSRIIDVSFNP--TFFRIGAG---------------
-----NSAVKPSLGAVAS----------------------VDRDLANS--------LRPL
VRCARAKQAIQEDGNLTAEQK-----------------SQAIKNIRIEK-ASIE-DLG--
--LDFTL-------------------------------PG--YPGVE-LQPN--------
GSEID---
------------------------------VTIENVE-LYVEK-VIDFTLGCG-VQKQVD
-AFSAG---FSQVFPY--SALK-AFTPD-ELVMLFGR-----------------------
---ADEDWS-----------LETL---------MDSIKADHG------------------
----------------FNLD----SASV-KNLLQTMS--------------------EFT
LPERRDFLQF-------VTGSPKLP------------IGG-----FK-SLTPMF--TVVC
KPNELPLIPD------------------------DYLPSVMTCV-NY-LKMPNY-STQEV
LRAKLS-VAIKE-GQG-
>Aureobasidium_subglaciale_XM_013487103.1 .
VRISRSRI-LESAVKVM----------------ELYGSSPS-ILEVEYFE-------EVG
TGLG-PTLEF---YSTV----SKEFSKKKI--------KLWRENESIAGD----------
---------DYAFGLGGLFPAPMS--------------------------------DEQA
NDEYGKKVLHLFKMLGKFVARSMLDSRIIDVSFSP--TFFRIGAG---------------
-----NSAVKPSLGAVAS----------------------VDKDLANS--------LKPL
VRCARAKQEIQEDPSLTPEQK-----------------VQAISNIRIKD-ASIE-DLG--
--LDFTL-------------------------------PG--YPAIE-LQPK--------
GAEID---
------------------------------VTIDNVE-LYVEK-VIDFTLGRG-VQKQVD
-AFSAG---FSQVFPY--SALK-AFTPD-ELVMLFGR-----------------------
---ADEDWS-----------LETL---------MDSIKADHG------------------
----------------FNLD----SASV-KNLLQTMS--------------------ELT
MSERRDFLQF-------VTGSPKLP------------IGG-----FK-SLTPMF--TVVC
KPNEPPLMPD------------------------DYLPSVMTCV-NY-LKMPNY-SSQEM
LKAKLS-LAIKE-GQGA
>Aureobasidium_melanogenum_MWII01000005.1 .
VRISRSRI-LESAIKVM----------------ELYGSSPS-ILEVEYFE-------EVG
TGLG-PTLEF---YSTV----SKEFSKKKI--------KLWRENESIAGD----------
---------DYAFGMRGLFPAPMS--------------------------------EAQS
NDEHGKKVLHLFKMLGKFVARSMLDSRIIDVSFNP--TFFRISAG---------------
-----NSTIKPSLGAVAS----------------------VDKDLANS--------LKPL
VRCARAKQDIQEDETMTAEQK-----------------TQAIKGIKIKD-ASIE-DLG--
--LDFTL-------------------------------PG--YPTIE-LQPN--------
GSEID---
------------------------------VTIYNVE-MYVEK-VIDFTLGQG-VQKQVD
-AFCAG---FSQVFPY--SALK-AFTPD-ELVMLFGR-----------------------
---AEEDWS-----------LETL---------MDSIKADHG------------------
----------------FNLD----SASV-KNLLQTMS--------------------EFK
MPERRDFLQF-------VTGSPKLP------------IGGG----FK-SLTPMF--TVVC

KPNEPPLMPD------------------------DYLPSVMTCV-NY-LKMPNY-SSQEV
LRAKLS-VAIKE-GQG-
>Aureobasidium_namibiae_XM_013573060.1 .
VRISRSRI-LESAIKVM----------------ELYGSSPS-ILEVEYFE-------EVG
TGLG-PTLEF---YSTV----SREFSKKKI--------KLWRENESIAGD----------
---------DYAFGMRGLFPAPMS--------------------------------EEQS
KDDHGKKVLHLFKMLGKFVARSMLDSRIIDVSFNP--TFFRIGAG---------------
-----NSTIKPSLGAVAS----------------------VDKDLANS--------LKPL
VRCARAKQEIQDDAGLSSEQK-----------------TQAIEDIRIKD-ASIE-DLG--
--LDFTL-------------------------------PG--YPTIE-LQPN--------
GAEID---
------------------------------VTIDNVE-QYVEK-VIDFTLGQG-VQRQVD
-AFCAG---FSQVFPY--SALK-AFTPD-ELVMLFGR-----------------------
---AEEDWS-----------LETL---------MDSIKADHG------------------
----------------FNLD----SASV-KNLLQTMS--------------------EFT
LPERRDFLQF-------VTGSPKLP------------IGAG----FK-SLTPMF--TVVC
KPNEPPLMPD------------------------DYLPSVMTCV-NY-LKMPNY-TSQEM
LRAKLS-VAIKE-GQGA
>Peltaster_fructicola_LJAO01000003.1 .
VRISRHRI-LESAIKVM----------------EMYGSSPS-VLEVEYFE-------EVG
TGLG-PTLEF---YSTV----SKEFSKKKT--------KLWRENESQTG-----------
---------DFAFGRKGLFPAPMT--------------------------------EKSS
KTETGQRVLHLFKMLGKFVARSMLDSRIIDVSFNP--AFFRFGAD---------------
-------NAILSIAAVAS----------------------VDQDLARS--------LKAL
QSFSDAKQQIEHSGISKAAQ------------------TQKVANIRVND-AKLD-DLA--
--LDFTL-------------------------------PG--YEKLE-LIEN--------
GSNIA---
------------------------------VTMDNLD-DYIEK-VVDLTLGSG-IRRQVK
-AFAAG---FNQVFPF--DALQ-AFTPD-ELVMLFGR-----------------------
---TSEDWT-----------LETL---------MDSIKADHG------------------
----------------YNLD----SKSV-RNLLHVMS--------------------ELT
EEEKRAFLQF-------VTGSPKLP------------ITG-----FK-SLTPMF--TVVC
KPAEAPLTSD------------------------DYLPSVMTCV-NY-LKMPDY-STIEV
LKAKLH-TAVHE-GQG-
>Cladophialophora_carrionii_XM_008733729.1 .
VRISRHRI-LESAMKVM----------------DMYGASPS-VLEVEYFE-------EVG
TGLG-PTLEF---YSSV----SKEFSKKKL--------KMWRENDSADKD----------
---------QFAFGKNGLFPIPTS--------------------------------PEES
GKEAGKKLALLFKTLGKFVARSMLDSRIIDITFSP--TFFRIGGI---------------
------TNAVPSIGLLRT----------------------IDQDLANS--------LSQL
QQIVKVKAKIELDSSMSEEEK-----------------AQAIQNLNIHG-AKVE-DLA--
--LDFTL-------------------------------PG--YPEIE-LIPD--------
GSNVT---
------------------------------VNVDNVQ-QYIDR-VLDLSLGSG-VRPQIE
-AFQTG---FSQVFSY--SSLR-AFTPD-ELVMLFGR-----------------------
---TQEDWS-----------IETL---------MDSVKADHG------------------
----------------FNMD----SKSV-KNLLQTMS--------------------ELS
LSQRRDFLQF-------VTGSPKLP------------IGG-----FK-SLTPMF--TVVC
KPSEPPYTSD------------------------DYLPSVMTCV-NY-LKLPDY-TSQDV
LKERLF-VAIRE-GQGA
>Phialophora_americana_JYCC01000058.1 .
VRISRHRI-LESAMKVM----------------DMYGASPS-VLEVEYFE-------EVG
TGLG-PTLEF---YSSV----SKEFSKKKL--------KMWRENDSAEKD----------
---------QFAFGKNGLFPIPMS--------------------------------PEES
GKEAGKKLALLFKTLGKFVARSMLDSRIIDITFSP--TFFKIGGI---------------
------TNAVPSIGLLRT----------------------IDQDLANS--------LSQL
QQIVKAKEKIELDSSMSAEEK-----------------AQAIENLTIHG-AKVE-DLA--
--LDFTL-------------------------------PG--YPEIE-LIPD--------

GSSVT--
------------------------------VNLDNVQ-QYIDR-VLDLSLGSG-VRPQIE
-AFQTG---FSQVFSY--SSLR-AFTPD-ELVMLFGR-----------------------
---TQEDWS-----------IETL---------MDSVKADHG------------------
----------------FNMD----SKSV-KNLLQTMS--------------------ELS
PSQRRDFLQF-------VTGSPKLP------------IGG-----FK-SLTPMF--TVVC
KPSEPPYTSD------------------------DYLPSVMTCV-NY-LKLPDY-TSQHV
LKERLF-VAIRE-GQGA
>Phialophora_verrucosa_MSED01000007.1 .
VRISRHRI-LESAMKVM----------------DMYGASPS-VLEVEYFE-------EVG
TGLG-PTLEF---YSSV----SKEFSKKKL--------KMWRENDSADKD----------
---------QFAFGKNGLFPIPTS--------------------------------PEES
GKEAGKKLALLFKTLGKFVARSMLDSRIIDITFSP--TFFKIGGI---------------
------TNAVPSIGLLRT---------------------IDQDLANS--------LSQL
QQIVKAKAKIEADSSTSKEEK-----------------AQAIENLNIHG-AKVE-DLA--
--LDFTL-------------------------------PG--YPEIE-LIPN--------
GSNVT--
------------------------------VNLDNVQ-QYIDR-VLDLSLGSG-VRPQIE
-AFQTG---FSQVFSY--SSLR-AFTPD-ELVMLFGR-----------------------
---TQEDWS-----------IETL---------MDSVKADHG------------------
----------------FNMD----SKSV-KNLLQTMS--------------------ELT
PSQRRDFLQF-------VTGSPKLP------------IGG-----FK-SLTPMF--TVVC
KPSEPPYTSD------------------------DYLPSVMTCV-NY-LKLPDY-TSQHV
LKERLF-VAIRE-GQGA
>Cladophialophora_yegresii_XM_007763456.1 .
VRISRHRI-LESAMKVM----------------DMYGASPS-VLEVEYFE-------EVG
TGLG-PTLEF---YSSV----SKEFSKKKL--------KMWRENDSADKD----------
---------QFAVGKNGLFPIPTS--------------------------------PEEG
GKEAGKKLSLLFKTLGKFVARSMLDSRIIDITFSP--TFFKIGGI---------------
------TNAVPSIGLLRT---------------------IDQDLANS--------LSQL
QHIVKAKAKIELDSSMSEEEK-----------------AEAMQNLNIHG-AKIE-DLA--
--LDFTL-------------------------------PG--YPDIE-LISE--------
GSNVT--
------------------------------VNLDNVQ-QYIDR-VLDLSLGSG-VRPQIE
-AFQTG---FSQVFSY--SSLR-AFTPD-ELVMLFGR-----------------------
---TQEDWS-----------IETL---------MDSVKADHG------------------
----------------FNMD----SKSV-KNLLQTMS--------------------ELS
LSQRRDFLQF-------VTGSPKLP------------IGG-----FK-SLTPMF--TVVC
KPSEPPYTSD------------------------DYLPSVMTCV-NY-LKLPDY-TTQDV
LKKRLF-VAIRE-GQGA
>Exophiala_calicioides_BCHZ01000001.1 .
VRISRHRI-LESAMKVM----------------DMYGASPS-VLEVEYFE-------EVG
TGLG-PTLEF---YSSV----SKEFSRKKL--------KMWRENDSADTD----------
---------QFAFGKNGLFPTPMS--------------------------------EADT
RTEGGKKLTLLFKTLGKFVARSMLDSRIIDISFSP--TFFKIAGI---------------
------TSVVPSIGLLRT---------------------VDHDLSNS--------LSQL
QQFVKAKTKIEMDSSQTKVEK-----------------AQALDNLTIHG-AKVE-DLM--
--LDFTL-------------------------------PG--YPGIE-LIEN--------
GANVF--
------------------------------VTKDNVQ-KYIER-VLDLSLGSG-VRPQIE
-AFQAG---FSQVFAF--PTLK-AFTPD-ELVMLFGR-----------------------
---VDEDWS-----------IETL---------MDSVKADHG------------------
----------------FNMD----SRSV-KNLLQTMS--------------------EFT
PSQRRDFLQF-------VTGSPKLP------------IGG-----FK-SLTPMF--TVVC
KPSEPPYSTD------------------------DYLPSVMTCV-NY-LKLPDY-TSPSV
LKERLF-VAIRE-GQGA
>Rhinocladiella_mackenziei_XM_013415685.1 .
VRISRHRI-LESAMKVM----------------GMYGASPS-VLEVEYFE-------EVG

TGLG-PTLEF---YSTV----SKEFSKKKL--------KMWRENDSADND----------
---------EFAFGKNGLFPTPMS--------------------------------EAET
HTEGGKKLLLLFKVLGKFVARSMLDSRIIDISFSP--TFFKIGGI---------------
------STAVPSIGLLRT----------------------IDQDLANS--------LSQL
QQFVKAKTKVELDPSMTKTAK-----------------AQAIDNLTIRG-ARIE-DLM--
--LDFTL-------------------------------PG--YPGIE-LIEN--------
GSNVA---
-----------------------------VTADNVE-TYIER-VLDMSLGSG-VRPQIE
-AFQAG---FSQVFSY--SSLK-AFTPD-ELVMLFGR-----------------------
---VDEDWS-----------IETL---------MDSVKADHG------------------
----------------FNMD----SKSV-KNLLQTMS--------------------ELT
ASERRDFLQF-------VTGSPKLP------------IGG-----FK-SLTPMF--TVVC
KPSEPPYASD------------------------DYLPSVMTCV-NY-LKLPDY-TSQHI
LKERLF-VAIRE-GQGA
>Exophiala_dermatitidis_XM_009162170.1 .
VRISRHRI-LESAMKVM----------------DMYGASPS-VLEVEYFE-------EVG
TGLG-PTLEF---YSTV----SREFSKKKL--------KMWRENDSSDHD----------
---------QFAFGKNGLFPAPMS--------------------------------EVDS
RGDAGKKLLLLFKVLGKFVARSMLDSRIIDISFSP--TFFKVGGG---------------
-----ISTVVPSIGLLRT----------------------IDRDLANS--------LSQL
QQFVKAKTKIELDSSLTQAEK-----------------AEAIVNIMIHD-AKVE-DLM--
--LDFTL-------------------------------PG--YPGIE-LIEN--------
GSNVT---
-----------------------------VTIDNVQ-KYIDR-VLDLSLGSG-VRPQIE
-AFQAG---FSEVFSY--SSLK-AFTPD-ELVMLFGR-----------------------
---VDEDWS-----------METL---------MDSVKADHG------------------
----------------YNMD----SKSV-KNLLQTMS--------------------ELT
PAQRRDFLQF-------VTGSPKLP------------IGG-----FK-SLTPMF--TVVC
KPSEPPYTSD------------------------DYLPSVMTCV-NY-LKLPDY-SSQEV
LKERLF-VAIRE-GQGA
>Capronia_coronata_XM_007724864.1 .
VRISRHRI-LESAMKVM----------------DMYGASPS-VLEVEYFE-------EVG
TGLG-PTLEF---YSSV----SKEFSKKKL--------KMWRENDSGHQD----------
---------QFAFGKNGLFPAPMS--------------------------------EADT
HGEPGKKLLLLFKTLGKFVARSMLDSRIIDISFSP--TFFKIGGI---------------
------SNTMPSIGLLRT----------------------VDRDLANS--------LAQL
QQFVKAKTNIELDSSLTKAER-----------------ANAIDNITIHD-ARVE-DLM--
--LDFTL-------------------------------PG--YPGIE-LIEN--------
GSNVA---
-----------------------------VTIDNVQ-NYIDR-VLDLSLGSG-VRPQIE
-AFQTG---FSQVFSY--ASLR-AFTPD-ELVMLFGR-----------------------
---VDEDWS-----------METL---------MDSVKADHG------------------
----------------YNMD----SKTV-KNLLQTMS--------------------ELT
PAQRRDFLQF-------VTGSPKLP------------IGG-----FK-SLTPMF--TVVC
KPSEPPYASD------------------------DYLPSVMTCV-NY-LKLPDY-SSQEV
LKARLF-VAIRE-GQGA
>Capronia_epimyces_XM_007731698.1 .
VRISRHRI-LESAMKVM----------------DMYGASPS-VLEVEYFE-------EVG
TGLG-PTLEF---YSSV----SKEFSKKKL--------KMWRENDSAHQD----------
---------QFAFGSNGLFPAPMS--------------------------------DADI
PGEAGKKLLLLFKILGKFVARSMLDSRIIDISFSP--TFFKVGGV---------------
------STTVPSIPLLRT----------------------VDRDLANS--------LAQL
QRFVRAKTKIELDSSLTKIEK-----------------AQAIDNITIHD-ARVE-DLM--
--LDFTL-------------------------------PG--YPGIE-LIEN--------
GSNVG---
-----------------------------VTIDNVQ-KYIDR-VLDLSLGSG-VRSQIE
-AFQAG---FSQVFSY--SSLK-AFTPD-ELVMLFGR-----------------------
---VEEDWS-----------IETL---------MDSVKADHG------------------

----------------YNMD----SKSV-KNLLQTMS--------------------ELN
PAQRRDFLQF-------VTGSPKLP------------IGG-----FK-SLTPMF--TVVC
KPSEPPYTSD------------------------DYLPSVMTCV-NY-LKLPDY-SSQAV
LKERMF-VAIRE-GQGA
>Exophiala_alcalophila_BCHY01000001.1 .
VRISRHRI-LESAMKVM----------------DMYGASPS-VLEVEYFE-------EVG
TGLG-PTLEF---YSTV----SKEFSKKKL--------KMWRENDSAADD----------
---------PFAFGKNGLFPTPMS--------------------------------EADT
HSEGGKKLLLLFKTLGKFVARSMLDSRIIDISFSP--TFFKIAGM---------------
------STTTPSIGLLRT----------------------IDHDLANS--------LSQL
QQFVKAKTQIERDESMTKDEK-----------------ARAVENVSIHD-AKIE-DLM--
--LDFTL-------------------------------PG--YPEIE-LVEN--------
GSNVA---
------------------------------VTLENVQ-RYIER-VLDLSLGSG-VRSQTE
-AFQAG---FSQVFSY--SSLK-AFTPD-ELVMLFGR-----------------------
---VEEDWT-----------IETL---------MDSVKADHG------------------
----------------FNMD----SKSV-KNLLQTMS--------------------EMT
KSQRRDFLQF-------VTGSPKLP------------IGG-----FK-SLTPMF--TVVC
KPSEPPYASD------------------------DYLPSVMTCV-NY-LKLPDY-TSQAT
LKQRLF-VAIRE-GQGA
>Exophiala_sideris_JYBR01000046.1 .
VRISRHRI-LESAMKVM----------------DMYGASPS-VLEVEYFE-------EVG
TGLG-PTLEF---YSTV----SKEFSKKKL--------KMWRENDSATGE----------
---------QFAFGKNGLFPAPIS--------------------------------QGNA
NTEAGKKLLLLFKTLGKFVARSMLDSRIIDIAFNP--TFFKVGAI---------------
------SSMVPTIGLLRT----------------------IDQDLANS--------LSQL
HQFVNAKTKIELDSSMTKLEK-----------------QQAIEDLTIRG-AKVE-DLM--
--LDFTL-------------------------------PG--YPDIE-LMEN--------
GSDIS---
------------------------------VTMGNVQ-HYIEQ-VLDASLGRG-VRTQIE
-SFQAG---FSQVFSY--ATLK-AFTPD-ELVMLFGR-----------------------
---VEEDWS-----------IETL---------MDSVKADHG------------------
----------------FNMD----SKSV-RNLLQTMS--------------------EMS
PALRRDFLMF-------VTGSPRLP------------MGG-----FK-SLTPMF--TVVC
KPSEPPYTSD------------------------DYLPSVMTCV-NY-LKLPDY-TSQAV
LKEKLF-VAIRE-GQGA
>Exophiala_xenobiotica_XM_013464573.1 .
VRISRHRI-LESAMKVM----------------DMYGGSPS-VLEVEYFE-------EVG
TGLG-PTLEF---YSTV----SKEFCKKKL--------KMWRENDSADTD----------
---------QFAFGKTGLFPAPMS--------------------------------EADV
HTDAGKKLLLLFKTLGKFVARSMLDSRIIDISFSP--TFFKVGGI---------------
------SSVVPSIGLLRT----------------------IDQDLANS--------LSQL
QQFVKAKAKIELDSSLTKVEK-----------------AEAIEDLTILG-AKIE-DLM--
--LDFTL-------------------------------PG--YPGIE-LVEN--------
GANVN---
------------------------------VDMENVQ-KYIDQ-VLDVSLGRG-VRSQVE
-AFQVG---FSQVFSY--TTLK-AFTPD-ELVMLFGR-----------------------
---VEEDWS-----------IETL---------MDSVKADHG------------------
----------------FNMD----SKSV-KNLLQTMS--------------------ELK
PSQRRDFLQF-------VTGSPKLP------------IGG-----FK-SLTPMF--TVVC
KPSEPPYTSD------------------------DYLPSVMTCV-NY-LKLPDY-SSQAV
LKEKLF-VAIRE-GQGA
>Exophiala_spinifera_XM_016381791.1 .
VRISRHRI-LESAMKVM----------------DMYGASPS-VLEVEYFE-------EVG
TGLG-PTLEF---YSTV----SKEFSKKKL--------KMWRENDSAETD----------
---------QFAFGRMGLFPAPMS--------------------------------QADA
HTDAGKKLLLLFKTLGKFVARSMLDSRIIDISFSP--TFFRVGGI---------------
------SNVPPSIGLLRT----------------------VDQDLANS--------LSQL

QHYVKAKTKIELDSSLTELEK-----------------AEAIQNLTIRG-AKVE-DLM--
--LDFTL-------------------------------PG--YPSIE-LCEN--------
GSDIN---
------------------------------VTMENVQ-DYIDR-VLDLSLGRG-VRSQIE
-AFQTG---FSQVFSF--ATLQ-AFTPD-ELVMLFGR-----------------------
---VEEDWS-----------METL---------TDSVKADHG------------------
----------------FNMD----SKSV-KNLLQTMS--------------------ELN
PAQRRDFLQF-------VTGSPKLP------------IGG-----FK-SLTPMF--TVVC
KPSEAPYTSD------------------------DYLPSVMTCV-NY-LKLPDY-SSQAV
LKERLF-VAIRE-GQGA
>Exophiala_oligosperma_XM_016408011.1 .
VRISRHRI-LESAMKVM----------------DMYGASPS-VLEVEYFE-------EVG
TGLG-PTLEF---YSTV----SKEFSKKKL--------KMWRENDSAATD----------
---------QFAFGKNGLFPAPMS--------------------------------EADT
HTDAGKKLLLLFKTLGKFVARSMLDSRIIDISFSP--TFFRVGGI---------------
------SNVAPSIGLLRT----------------------VDQDLANS--------LAQL
QQYVKAKTKIELDASLTELEK-----------------ANNIENLTIRG-AKVE-DMM--
--LDFTL-------------------------------PG--YPSID-LREK--------
GSDIS---
------------------------------VTTENVQ-DYIDQ-VLDMSLGRG-VRSQIE
-AFQTG---FSQVFSF--TTLK-AFTPD-ELVMLFGR-----------------------
---VEEDWS-----------METL---------TDSVKADHG------------------
----------------FNMD----SKSV-KNLLQTMS--------------------ELN
PAQRRDFLQF-------VTGSPKLP------------IGG-----FK-SLTPMF--TVVC
KPSEAPYTSD------------------------DYLPSVMTCV-NY-LKLPDY-SSQAV
LKKRLF-VAIRE-GQGA
>Fonsecaea_erecta_XM_018841616.1 .
VRLSRHRI-LESAMKVM----------------DMYGAGPG-VIEVEYFE-------EVG
TGLG-PTLEF---YSSV----SKEFCQKKL--------KMWRENDSGESD----------
---------EFAFGKNGLFPAPMS--------------------------------PEES
RNEAGKKLLQLFSTLGKFVARSMLDSRIIDISFSP--TFFKVGGA---------------
------GSATPSIGLLRT----------------------IDQDLANS--------LSQL
QQFLKAKARIELDSSMTEDEK-----------------VRALEDLTIRG-AKVE-DLM--
--LDFTL-------------------------------PG--YPNIE-LIPD--------
GANVS---
------------------------------VTMENVQ-NYVER-VLDLSLGSG-IRAQIG
-AFQSG---FSQVFSY--ASLR-AFTPD-ELVMLFGR-----------------------
---TEEDWS-----------IETL---------MDSVKADHG------------------
----------------YNMD----SKSV-KNLLQTMS--------------------ELT
APQRRDFLQF-------VTGSPKLP------------IGG-----FK-SLTPMF--TVVC
KPSEPPYSSD------------------------DYLPSVMTCV-NY-LKLPDY-TSQKV
LRERLF-VAIRE-GQGA
>Cladophialophora_bantiana_XM_016762555.1 .
VRISRHRI-LESGMKVM----------------DMYGASPS-VLEVEYFE-------EVG
TGLG-PTLEF---YSTV----SKEFCKKKL--------KMWRENDSADND----------
---------PFAFGKNGLFPTPMS--------------------------------AEES
RSEAGKKLLLLFKTLGKFVARSMLDSRIIDIAFSP--TFFKMGGV---------------
------SNAAPSIGLLRT----------------------IDQDLANS--------LSQL
QQFLKAKTKIELDSCMTEIEK-----------------AQAIEQLTIHG-AKVE-DLM--
--LDFTL-------------------------------PG--YPGIE-LIPN--------
GSNVT---
------------------------------VTMENIQ-QYIER-VLDLSLGSG-VRAQIE
-AFQTG---FSQVFSY--SSLR-AFTPD-ELVMLFGR-----------------------
---TEEDWS-----------IETL---------MDSVKADHG------------------
----------------YNMD----SKSV-KNLLQTMS--------------------ELT
PAQRRDFLQF-------VTGSPKLP------------IGG-----FK-SLTPMF--TVVC
KPSEPPYTSD------------------------DYLPSVMTCV-NY-LKLPDY-TSQKV
LKERLF-VAIRE-GQGA

>Cladophialophora_psammophila_XM_007746520.1 .
VRISRHRI-LESGMKVM----------------DMYGASPS-VLEVEYFE-------EVG
TGLG-PTLEF---YSTV----SKEFCKKKL--------KMWRENDSADND----------
---------QFAFGKNGLFPTPMS--------------------------------AEES
QSEAGKKLLLLFKTLGKFVARSMLDSRIIDIAFSP--TFFKMGGV---------------
------SNAAPSVGLLRT----------------------IDQDLANS--------LSQL
QQFLKAKTKIELDSCMTEIEK-----------------ARAIEQLTIHG-AKVE-DLM--
--LDFTL-------------------------------PG--YPGIE-LIPN--------
GSNVT---
------------------------------VTMENIQ-QYIER-VLDLSLGSG-VRAQIE
-AFQSG---FSQVFSY--SSLR-AFTPD-ELVMLFGR-----------------------
---TEEDWS-----------IETL---------MDSVKADHG------------------
----------------YNMD----SRSV-KSLLQTMS--------------------ELT
PAQRRDFLQF-------VTGSPKLP------------IGG-----FK-SLTPMF--TVVC
KPSEPPYTSD------------------------DYLPSVMTCV-NY-LKLPDY-TSQKV
LKERLF-VAIRE-GQGA
>Cladophialophora_immunda_XM_016396424.1 .
VRISRHRI-LESAMKVM----------------DMYGASPS-VLEVEYFE-------EVG
TGLG-PTLEF---YSTV----SKEFCKKKL--------KMWRENDSGEND----------
---------QFAFGKNGLFPTPMS--------------------------------PEES
RGEAGKKLLLLFKTLGKFVARSMLDSRIIDIAFSP--TFFKMGGA---------------
------SNAAPSIGLLRT----------------------IDQDLANS--------LSQL
QQFLKAKARIEVDSSMTEDEK-----------------AQTIEDLTIRG-AKVE-DLM--
--LDFTL-------------------------------PG--YPSIE-LVPN--------
GSNVS---
------------------------------VTMENIQ-HYVER-VLDLSLGSG-VRAQIE
-AFQTG---FSQVFSY--SSLS-AFTPD-ELVMLFGR-----------------------
---TEEDWS-----------IETL---------MDSVKADHG------------------
----------------YNMD----SKSV-KNLLQTMS--------------------ELT
PAQRRDFLQF-------VTGSPKLP------------IGG-----FK-SLTPMF--TVVC
KPSEPPYTSD------------------------DYLPSVMTCV-NY-LKLPDY-TSQKV
LKERLF-VAIRE-GQGA
>Fonsecaea_multimorphosa_XM_016776856.1 .
VRISRHRI-LESAMKVM----------------DMYGASPS-VLEVEYFE-------EVG
TGLG-PTLEF---YSTV----SKEFCKKKL--------KMWRENDSGEND----------
---------QFAFGKNGLFPIPMG--------------------------------PDES
QSEPGKKLLLLFKTLGKFVARSMLDSRIIDIAFSP--TFFKMGGA---------------
------SNAPPSIGLLRT----------------------IDQDLANS--------LSQL
QQFLKAKARIELDSSMTEDEK-----------------RHAIEDLSIRG-AKVE-DLM--
--LDFTL-------------------------------PG--YPNIE-LIPD--------
GANVS---
------------------------------VTMENVQ-RYVER-VLDLSLGSG-VRAQIE
-AFQTG---FSQVFSY--SSLR-AFTPD-ELVMLFGR-----------------------
---TKEDWS-----------IETL---------MDSVKADHG------------------
----------------YNMD----SKSV-KNLLQTMS--------------------ELT
APQRRDFLQF-------VTGSPKLP------------IGG-----FK-SLTPMF--TVVC
KPSEPPYTSD------------------------DYLPSVMTCV-NY-LKLPDY-TSQKV
LRERLF-VAIRE-GQGA
>Fonsecaea_nubica_LVCJ01000030.1 .
VRISRHRI-LESAMKVM----------------DMYGASPS-VLEVEYFE-------EVG
TGLG-PTLEF---YSTV----SKEFCKKKL--------KMWRENDSGENE----------
---------QFAFGKNGLFPIPMS--------------------------------SEES
RSEAGKKLLLLFKTLGKFVARSMLDSRIIDIAFSP--TFFKMGGA---------------
------SNVAPSIGLLRT----------------------VDQDLANS--------LSQL
QQFLKAKAGIELDSSLTEDEK-----------------TQAIGDLTIRG-AKVE-DLM--
--LDFTL-------------------------------PG--YPSIE-LIPD--------
GSNVS---
------------------------------VTMKNVQ-QYVER-VLDLSLGSG-VRAQIE

-AFQTG---FSQVFSY--SSLR-AFTPD-ELVMLFGR-----------------------
---TDEDWS-----------IETL---------MDSVKADHG------------------
----------------YNMD----SKSV-KNLLQTMS--------------------ELT
LPQRRDFLQF-------VTGSPKLP------------IGG-----FK-SLTPMF--TVVC
KPSEPPYTSD------------------------DYLPSVMTCV-NY-LKLPDY-TSQAV
LKERLF-VAIRE-GQG-
>Fonsecaea_monophora_LVKK01000060.1 .
VRISRHRI-LESAMKVM----------------DMYGASPS-VLEVEYFE-------EVG
TGLG-PTLEF---YSTV----SKEFCKKKL--------KMWRENDSGENE----------
---------QFAFGKNGLFPIPMS--------------------------------SEES
RSEAGKKLLLLFKTLGKFVARSMLDSRIIDIAFSP--TFFKMGGA---------------
------SNVAPSIGLLRT----------------------VDQDLANS--------LSQL
QHFLKAKAGIELDSSLTEDEK-----------------TQAIGDLTIRG-AKVE-DLM--
--LDFTL-------------------------------PG--YPSIE-LIPD--------
GSNVS---
------------------------------VTMKNVQ-QYVER-VLDLSLGSG-VRAQIE
-AFQTG---FSQVFSY--SSLR-AFTPD-ELVMLFGR-----------------------
---TDEDWS-----------IETL---------MDSVKADHG------------------
----------------YNMD----SKSV-KNLLQTMS--------------------ELT
LPQRRDFLQF-------VTGSPKLP------------IGG-----FK-SLTPMF--TVVC
KPSEPPYTSD------------------------DYLPSVMTCV-NY-LKLPDY-TSQAV
LKERLF-VAIRE-GQG-
>Fonsecaea_pedrosoi_XM_013425741.1 .
VRISRHRI-LESAMKVM----------------DMYGASPS-VLEVEYFE-------EVG
TGLG-PTLEF---YSTV----SKEFCKKKL--------KMWRENDSGENE----------
---------QFAFGKNGLFPIPMS--------------------------------SEES
RSEAGKKLLLLFKTLGKFVARSMLDSRIIDIAFSP--TFFKMGGA---------------
------SNVAPSIGLLRT----------------------VDQDLANS--------LSQL
QHFLKAKAGIELDSSLTEDEK-----------------TQAIGDLTIRG-AKVE-DLM--
--LDFTL-------------------------------PG--YPSIE-LIPD--------
GSNVS---
------------------------------VTMKNVQ-QYVER-VLDLSLGSG-VRAQIE
-AFQTG---FSQVFSY--SSLR-AFTPD-ELVMLFGR-----------------------
---TDEDWS-----------IETL---------MDSVKADHG------------------
----------------YNMD----SKSV-KNLLQTMS--------------------ELT
LPQRRDFLQF-------VTGSPKLP------------IGG-----FK-SLTPMF--TVVC
KPSEPPYTSD------------------------DYLPSVMTCV-NY-LKLPDY-TSQAV
LKERLF-VAIRE-GQGA
>Exophiala_aquamarina_XM_013410984.1 .
VRISRHRI-LESAMKVM----------------DMYGASPS-VLEVEYFE-------EVG
TGLG-PTLEF---YSNV----SKEFCKKKL--------KMWRENDSGDND----------
---------QFAFGKNGLFPAPLS--------------------------------TAES
RSEAGKKLMLLFKTLGKFVARSMLDSRIIDVSFSP--TFFKVGEN---------------
-------SAAPSLGLLRT----------------------IDHDLANS--------LSQL
QQFVKAKIKIETDSTMTHSAK-----------------AQAINDLTIRG-ARIE-DLM--
--LDFTL-------------------------------PG--YPDIE-LAEN--------
GQEIP---
------------------------------VTMDNVQ-DYVDK-VLDLSLGSG-VRRQVE
-AFQTG---FSQVFAY--PTLK-AFTPD-ELVMLFGR-----------------------
---VNEDWS-----------IETL---------MDSVKADHG------------------
----------------FNMD----SKSV-KNLLQTMS--------------------ELT
PAQRRDFLQF-------VTGSPKLP------------IGG-----FK-SLTPMF--TVVC
KPSEPPYSSD------------------------DYLPSVMTCV-NY-LKLPDY-STQAK
LKERLF-VAIRE-GQGA
>Exophiala_mesophila_XM_016371495.1 .
VRISRTRI-LESAMKVM----------------DMYGASPS-VLEVEYFE-------EVG
TGLG-PTLEF---YSSV----SKEFSKKKL--------KMWRENDSADKD----------
---------QFAFGKNGLFPAPMS--------------------------------EAEA

```
HSEAGKKLLLLFKGLGKFVARSMLDSRIIDVSFNA--TFFKVGSF---------------
-------SIKPTIGLLRS----------------------VDQDLANS--------LSQL
QQYIKAKAKIQSDASLTKAEK-----------------IEGIANLTIQG-ARVE-DLM--
--LDFTL-------------------------------PG--YPHIE-LLED--------
GAETV-------------------------------------------------------
------------------------------VTGDNVQ-DYVDK-VLDLSLNTG-VRRQIE
-AFQSG---FSQVFAF--STLT-AFTPN-ELVMLFGR-----------------------
---NDEDWS-----------IETL---------TDSVKADHG------------------
----------------YNMD----SKSV-KNLLTCMS--------------------ELT
QAQRRDFLQF-------VTGSPKLP------------IGG-----FK-SLTPMF--TVVC
KPSEPPYSSD------------------------DYLPSVMTCV-NY-LKLPDY-TSEEV
LKERLF-VAIRE-GQGS
>Phialophora_attae_LFJN01000013.1 .
VRISRGKI-LESAMKVL----------------EMYGASPS-ILEVEYFN-------EVG
TGLG-PTLEF---YSTV----SKEFSKRKL--------KMWRENEITEG-----------
---------DFAFGKNGLFPAPMT--------------------------------ESMT
RGNNGKKLLQLFSLMGKFVARSMLDARIIDISFNQ--TFFKLAAN---------------
------ASVPLTIGLLRT----------------------IDQDLANS--------ISQL
QEVADASAAILA-SKTTAAEK-----------------MKAYDALEIKG-AKLE-DLA--
--LDFTL-------------------------------PG--HSNIE-LITD--------
GANVA-------------------------------------------------------
------------------------------VTAYNVQ-KYIDC-VLDMSLGQG-VRKQIE
-SFQAG---FSEVFSY--SSLQ-AFTPE-ELVMLFGR-----------------------
---VEEDWS-----------IETL---------MDSVKADHG------------------
----------------FNMD----SKSV-RNLLQTMS--------------------ELT
PEGRRDFLQF-------VTGSPKLP------------IGG-----FK-SLTPMF--TVVC
KPSEPPYASD------------------------DYLPSVMTCV-NY-LKLPDY-STQEV
LKERLT-TATRE-GQGA
>Cyphellophora_europaea_XM_008720495.1 .
VRISRSKI-LESAMKVM----------------EMYGASPS-VLEVEYFQ-------EVG
TGLG-PTLEF---YSTV----SKEFSKKKL--------KMWRENDSANTE----------
---------DFAFGRNGLFPAPMS--------------------------------EAAA
SADSGKKLLLFFKMLGKFVARSMIDSRIIDIAFSP--TFFKVPAK---------------
------STMSLSIGLLRT----------------------IDQDLANS--------MAQL
QQFANARANIEA-AKLPAAQK-----------------EQALSELSIKG-ARIE-DLM--
--LDFTL-------------------------------PG--YPSIE-LVED--------
GADVS-------------------------------------------------------
------------------------------VTMDNVQ-DYIDR-VLNMSLGQG-VRRQIE
-AFQAG---FSQVFSF--SSLQ-AFTPN-ELVMLFGR-----------------------
---VEEDWS-----------IETL---------MDSVKADHG------------------
----------------FNMD----SKSV-KNLLQTMS--------------------ELT
QEQRRDFLQF-------VTGSPKLP------------IGG-----FK-SLTPMF--TVVC
KPSEPPYSSD------------------------DYLPSVMTCV-NY-LKLPDY-SDQEV
LKKRLG-VAIKE-GQGA
>Herpotrichiellaceae_sp._AMYF01000020.1 .
VRIARTKM-LESAMKVM----------------DHFGPSPS-ILEVEYFD-------EVG
TGLG-PTLEF---YSTV----SKEFSKKQL--------KLWRENDTDAKD----------
---------QYAFGKNGLFPAPMS--------------------------------EEDK
KTEKGKKILECFKVLGKFVARSMLDSRIIDVNFSP--TFFKIGDD---------------
--------MKTSVALLRT----------------------VDQDLANS--------VSQL
QAYANRRANIEADTTMNASAK-----------------ATALSNLTIRG-AHLE-DLG--
--LDFTL-------------------------------PG--YPSIS-MIAN--------
GAEVD-------------------------------------------------------
------------------------------VTNENIS-LYIEK-VLDFTLGSG-VQSQIQ
-AFRNG---FSQVFPY--NSLK-AFTPN-ELVMLFGR-----------------------
---VEEDWS-----------IETL---------MDSIKADHG------------------
----------------FNMD----SKSV-KNLLQVMS--------------------ELT
AAQRRDFLQF-------ITGSPRLP------------IGG-----FK-NLTPMF--TVVC
```

KPSEPPYTSD------------------------DYLPSVMTCA-NY-LKLPDY-SDVSV
LKDKLE-TAIRE-GQGA
>Arthrobotrys_oligospora_XM_011119066.1 .
VRISRNRI-FDSAVKVM----------------ELYGASPS-VLEVEYFE-------EVG
TGLG-PTLEF---YSSV----SREFARKTQ--------NMWREGDSFADTDK--------
---------EYTFGAQGLFPAPMD--------------------------------AKYA
ETEAGKRILQRFKILGKFVARAMLDSRLIDISFNAN--FFRGGDD---------------
-----AEAVAPSYGAVKT----------------------VDKGLAQS--------LNLL
RKFAVAKSVVDEMKGLPAAER-----------------NIYLSEIRVEG-VTVD-DLA--
--LDFTL-------------------------------PG--YPHIE-LVKG--------
GADKQ---
------------------------------VTINNVG-DYVDK-VIDLTLGSG-VRRQVD
-AFRSG---FSQVFPY--AALK-AFTPD-ELVMMFGR-----------------------
---VDEDWS-----------LETL---------MDSIKADHG------------------
----------------FNMD----SKSV-RNLLQVMS--------------------SFT
LQQRREFLQF-------VTGSPKLP------------IGG-----FK-NLTPMF--TVVC
KPSESPLVSD------------------------DYLPSVMTCV-NY-LKLPDY-TCIEV
LRQRLH-TAVHE-GSGA
>Dactylellina_haptotyla_XM_011111723.1 .
VRISRNRI-FDSAVKVM----------------ELYGASPS-VLEVEYFE-------EVG
TGLG-PTLEF---YSSV----SREFARKNQ--------NMWRDSDAFSEK----------
---------EYTFGAQGLFPAPMD--------------------------------AKFA
ETEGGKKVLQRFKILGKFVARAMLDSRLIDISFNP--NFFRGGED---------------
-----AEAVAPSFGAVKT----------------------VDKTLAQS--------LSLL
RKFAVAKSVIDEMKGLPSSEK-----------------NKMLSEVRMDG-VLVD-DLA--
--LDFTL-------------------------------PG--YPHIE-LIKD--------
GANKA---
------------------------------VTINNVG-DYVEK-VIDLTLGSG-VQRQVD
-AFRAG---FSQVFPY--AALK-AFTPY-ELVMMFGR-----------------------
---VNEDWS-----------LETL---------MDSIKADHG------------------
----------------FNMD----SKSV-RNLLQVMS--------------------EYT
PQERREFLQF-------VTGSPKLP------------IGG-----FK-NLTPMF--TVVC
KPSEAPLVSD------------------------DYLPSVMTCV-NY-LKLPDY-TSAHV
LKQRLH-TAVHE-GSGA
>Ramalina_peruviana_MSTJ01000079.1 .
VRISRTRI-LESAYKVL----------------ELYAGSPS-ILEIEYFH-------EVG
TGLG-PTLEF---YSSV----SKEFSKKKT--------KLWRLDDTDEQD----------
---------EYAFSKYGLFPAPMT--------------------------------AEMA
ESEAGRKILTNFRTLGKFIARSMLDSRIIDVSLNP--TFFRVGDQ---------------
-----PSTVPLSLGAVKT----------------------VDEQLAKS--------LKLL
KQYANAKTAIESNRGLSATQR-----------------SREIKKITIDG-AAVE-DLG--
--LDFTL-------------------------------PG--YPQIE-LVED--------
GANKA---
------------------------------VTIENAG-EYVDK-VIDLTLGSG-VQRQVD
-QFREG---FSEIFAY--SALR-AFTPN-ELVMLFGR-----------------------
---VEEDWS------------XAL---------CDSIKADHG------------------
----------------YNMD----SKSV-RNLLQVMS--------------------ELT
IPERRDFLQF-------VTGSPKLP------------IGG-----FK-SLNPMF--TVVC
KPAEAPYSSD------------------------DFLISVMTCA-NY-VKLPDY-SDINI
LRKQLH-IATSE-GQGA
>Penicillium_polonicum_MDYM01000015.1 .
VRISRSRI-LDSALKVM----------------ELYGSSPS-ILEVEYFE-------EVG
TGLG-PTLEF---YSTV----SREFSKKKL--------KIWRDTDGSSTA----------
---------EYAFGKRGLFPAPMS--------------------------------DEQA
AQDMGKKQLNIFKVLGKFVARSMLDSRIIDISFNP--AFFRIADT---------------
-----LSSVAPSLGTVKL----------------------VDHDLAKS--------LIML
KEFVNAKNAIEANKSLSPASQ-----------------SEAVQNITVHG-ASVD-DLG--
--LDFTL-------------------------------PG--YPAIE-LIPG--------

GADVP---
------------------------------LTIENVD-TYIER-VIDMTLGSG-VRRQVD
-AFRAG---FSQVFPF--SSLR-AFTPS-ELVMLFGQ-----------------------
---AEEDWS-----------IETL---------MDSIKADHG------------------
----------------FNMD----SRSV-RNLLQTMS--------------------ELD
KQQRRDFLQF-------VTGSPKLP------------IGG-----FK-SLTPIF--TVVC
RPSEHPYTPD------------------------DYLPSVMTCV-NY-LKLPDY-SDLDV
LKKRLS-VAIKE-GQGA
>Penicillium_freii_LLXE01000153.1 .
VRISRSRI-LDSALKVM----------------ELYGSSPS-ILEVEYFE-------EVG
TGLG-PTLEF---YSTV----SREFSKKKL--------KIWRDTDGSSTA----------
---------EYAFGKRGLFPAPMS--------------------------------DEQA
AQDMGKKQLNIFKVLGKFVARSMLDSRIIDISFNP--AFFRIADT---------------
-----LSSVAPSLGTVKL----------------------VDHDLAKS--------LIML
KEFVNAKNAIEANKSLSPASQ-----------------SEAVQNITVHG-ASVD-DLG--
--LDFTL-------------------------------PG--YPAIE-LIPG--------
GADVP---
------------------------------LTIENVD-TYIER-VIDMTLGSG-VRRQVD
-AFRAG---FSQVFPF--SSLR-AFTPS-ELVMLFGQ-----------------------
---AEEDWS-----------IETL---------MDSIKADHG------------------
----------------FNMD----SRSV-RNLLQTMS--------------------ELD
NQQRRDFLQF-------VTGSPKLP------------IGG-----FK-SLTPIF--TVVC
RPSEHPYTPD------------------------DYLPSVMTCV-NY-LKLPDY-SDLDV
LKKRLS-VAIKE-GQGA
>Penicillium_solitum_JYNM02000002.1 .
VRISRSRI-LDSALKVM----------------ELYGSSPS-ILEVEYFE-------EVG
TGLG-PTLEF---YSTV----SREFSKKKL--------KIWRDTDGGSTA----------
---------EYAFGKRGLFPAPMS--------------------------------DEQA
AQDMGKKQLNIFKVLGKFVARSMLDSRIIDISFNP--AFFRIADT---------------
-----LSSVAPSLGTVKL----------------------VDHDLAKS--------LIML
KEFVNAKNAIEANKSLSPASQ-----------------SEAVQNITVHG-ASVD-DLG--
--LDFTL-------------------------------PG--YPAIE-LIPG--------
GADVP---
------------------------------LTIENVD-TYIER-VIDMTLGSG-VRRQVD
-AFRAG---FSQVFPF--SSLR-AFTPS-ELVMLFGQ-----------------------
---AEEDWS-----------IETL---------MDSIKADHG------------------
----------------FNMD----SRSV-RNLLQTMS--------------------EMD
KQQRRDFLQF-------VTGSPKLP------------IGG-----FK-SLTPIF--TVVC
RPSEHPYTPD------------------------DYLPSVMTCV-NY-LKLPDY-SDLDV
LKKRLS-VAIKE-GQGA
>Penicillium_camemberti_CBVV010000298.1 .
VRISRSRI-LDSALKVM----------------ELYGSSPS-ILEVEYFE-------EVG
TGLG-PTLEF---YSTV----SREFSKKKL--------KIWRDTDGSSTT----------
---------EYAFGKRGLFPAPMS--------------------------------DEQA
AQDMGKKQLNIFKVLGKFVARSMLDSRIIDISFNP--AFFRIADT---------------
-----LSSVAPSLGTVKL----------------------VDHDLAKS--------LIML
KEFVDAKNAIEANKSLSSASQ-----------------SEALQNITVHG-ASVD-DLG--
--LDFTL-------------------------------PG--YPAIE-LIPG--------
GADVP---
------------------------------LTIENVD-TYIER-VIDMTLGSG-VRHQVD
-AFRAG---FSQVFPF--SSLR-AFTPS-ELVMLFGQ-----------------------
---AEEDWS-----------IETL---------MDSIKADHG------------------
----------------FNMD----SRSV-RNLLQTMS--------------------ELD
NQQRRDFLQF-------VTGSPKLP------------IGG-----FK-SLTPIF--TVVC
RPSEHPYTPD------------------------DYLPSVMTCV-NY-LKLPDY-SDLDV
LKKRLS-VAIKE-GQGA
>Penicillium_fuscoglaucum_CBXP010000325.1 .
VRISRSRI-LDSALKVM----------------ELYGSSPS-ILEVEYFE-------EVG

TGLG-PTLEF---YSTV----SREFSKKKL--------KIWRDTDGSSTT----------
---------EYAFGKRGLFPAPMS--------------------------------DEQA
AQDMGKKQLNIFKVLGKFVARSMLDSRIIDISFNP--AFFRIADT---------------
-----LSSVAPSLGTVKL---------------------VDHDLAKS--------LIML
KEFVDAKNAIEANKSLSSASQ-----------------SEALQNITVHG-ASVD-DLG--
--LDFTL-------------------------------PG--YPAIE-LIPG--------
GADVP---
------------------------------LTIENVD-TYIER-VIDMTLGSG-VRHQVD
-AFRAG---FSQVFPF--SSLR-AFTPS-ELVMLFGQ-----------------------
---AEEDWS-----------IETL---------MDSIKADHG------------------
----------------FNMD----SRSV-RNLLQTMS--------------------ELD
NQQRRDFLQF-------VTGSPKLP------------IGG-----FK-SLTPIF--TVVC
RPSEHPYTPD------------------------DYLPSVMTCV-NY-LKLPDY-SDLDV
LKKRLS-VAIKE-GQGA
>Penicillium_solitum_MJCB01000701.1 .
VRISRSRI-LDSALKVM----------------ELYGSSPS-ILEVEYFE-------EVG
TGLG-PTLEF---YSTV----SREFSKKKL--------KIWRDTDGSSTA----------
---------EYAFGKRGLFPAPMS--------------------------------DEQA
DQDIGKKQLNIFKVLGKFVARSMLDSRIIDISFNP--AFFRIADT---------------
-----LSSVAPSLGTVKL---------------------VDHDLAKS--------LIML
KEFVNAKNAIEANKSLSPASQ-----------------SEAVQNITVHG-ARVD-DLG--
--LDFTL-------------------------------PG--YPSIE-LIPG--------
GADVP---
------------------------------LTIENVD-TYIER-VIDMTLGNG-VRRQVD
-AFRTG---FSQVFPF--SSLR-AFTPS-ELVMLFGQ-----------------------
---AEEDWS-----------IETL---------MDSIKADHG------------------
----------------FNMD----SRSV-RNLLQTMS--------------------EMD
NQQRRDFLQF-------VTGSPKLP------------IGG-----FK-SLTPIF--TVVC
RPSEHPYTPD------------------------DYLPSVMTCV-NY-LKLPDY-SDLDV
LKKRLS-VAIKE-GQGA
>Penicillium_nordicum_LHQQ01000030.1 .
VRISRSRI-LDSALKVM----------------ELYGSSPS-ILEVEYFE-------EVG
TGLG-PTLEF---YSTV----SREFSKKKL--------KIWRDTDGSSTG----------
---------EYAFGKRGLFPAPMS--------------------------------DAQA
AQDMGKKQLNIFKVLGKFVARSMLDSRIIDISFNP--AFFRIADT---------------
-----LSSVAPSLGTVKL---------------------VDHDLAKS--------LVML
KEFVNAKNAVEADKSLSPASQ-----------------SEAVQNITVHG-ASVD-DLG--
--LDFTL-------------------------------PG--YPAIE-LIPG--------
GTNVP---
------------------------------LTIENVD-IYIER-VIDVTLGSG-VRRQVD
-AFRAG---FSQVFPF--SSLR-AFTPS-ELVMLYGQ-----------------------
---AEEDWS-----------IETL---------MDSIKADHG------------------
----------------FNMD----SRSV-RNLLQTMS--------------------ELD
NQQRRDFLQF-------VTGSPKLP------------IGG-----FK-SLTPIF--TVVC
RPSEHPYTPD------------------------DYLPSVMTCV-NY-LKLPDY-SDLDV
LKKRLS-VAIKE-GQGA
>Penicillium_verrucosum_LAKW01002444.1 .
VRISRSRI-LDSALKVM----------------ELYGSSPS-ILEVEYFE-------EVG
TGLG-PTLEF---YSTV----SREFSKKKL--------KIWRDTDGSSTG----------
---------EYAFGKRGLFPAPMS--------------------------------DAQA
AQDMGKKQLNIFKVLGKFVARSMLDSRIIDISFNP--AFFRIADT---------------
-----LSSVAPSLGTVKL---------------------VDHDLAKS--------LVML
KEFVNAKNAVEADKSLSPASQ-----------------SEAVQNITVHG-ASVD-DLG--
--LDFTL-------------------------------PG--YPAIE-LIPG--------
GTNVP---
------------------------------LTIENVD-IYIER-VIDVTLGSG-VRRQVD
-AFRAG---FSQVFPF--SSLR-AFTPS-ELVMLFGQ-----------------------
---AEEDWS-----------IETL---------MDSIKADHG------------------

```
----------------FNMD----SRSV-RNLLQTMS--------------------ELD
NQQRRDFLQF-------VTGSPKLP------------IGG-----FK-SLTPIF--TVVC
RPSEHPYTPD------------------------DYLPSVMTCV-NY-LKLPDY-SDLDV
LKKRLS-VAIKE-GQGA
>Penicillium_italicum_JMDK01000046.1 .
VRISRSRI-LDSALKVM----------------ELYGSSPS-ILEVEYFE-------EVG
TGLG-PTLEF---YSTV----SREFSKKKL--------KIWRDTDGSAHA----------
---------EYAFGKRGLFPAPMS--------------------------------DEQA
AQDFGKKQLNIFKVLGKFVARSMLDSRIIDISFNP--AFFRIADT---------------
-----LSSVAPSLGTVKL----------------------VDHDLANS--------LIML
KEFVNAKNAIEADKSLSPAHK-----------------SEAVQNITVHG-ANVD-DLG--
--LDFTL------------------------------PG--YPAIE-LIPG--------
GADVQ-------------------------------------------------------
------------------------------LTIENVD-TYIER-VIDMTLGSG-VQRQVD
-AFRAG---FSQVFPF--SSLR-AFTPS-ELVMLFGQ-----------------------
---AEEDWS-----------IETL---------MDSIKADHG------------------
----------------FNMD----SRSV-RNLLQTMS--------------------ELD
NQQRRDFLQF-------VTGSPKLP------------IGG-----FK-SLTPIF--TVVC
RPSEHPYTPD------------------------DYLPSVMTCV-NY-LKLPDY-SDLDV
LRKRLS-VAIKE-GQGA
>Penicillium_expansum_XM_016746509.1 .
VRISRSRI-LDSALKVM----------------ELYGSSPS-ILEVEYFE-------EVG
TGLG-PTLEF---YSTV----SREFSKKKL--------KIWRDTDESSNA----------
---------EYAFGKRGLFPAPMS--------------------------------DEQA
AQDIGKKQLNIFKVLGKFVARSMLDSRIIDISFNP--AFFRIADT---------------
-----LSSVAPSLGTVKL----------------------VDHDLAKS--------LLML
KEFVNAKNAIEADRSLSPALK-----------------SEAVQNITVHG-ANVD-DLG--
--LDFTL------------------------------PG--YPAIA-LIPG--------
GADVQ-------------------------------------------------------
------------------------------LTIENVD-TYIER-VIDMTLGSG-VQRQVD
-AFRAG---FSQVFPF--SSLC-AFTPS-ELVMLFGQ-----------------------
---AEEDWS-----------IETL---------MDSIKADHG------------------
----------------FNMD----SRSV-RNLLQTMS--------------------ELD
NQQRRDFLQF-------VTGSPKLP------------IGG-----FK-SLTPIF--TVVC
RPSEHPYTPD------------------------DYLPSVMTCV-NY-LKLPDY-SDLDV
LKKRLS-VAIKE-GQGA
>Penicillium_digitatum_XM_014679946.1 .
VRISRSRI-LDSALKVM----------------ELYGSSPS-ILEVEYFE-------EVG
TGLG-PTLEF---YSTV----SREFSKKKL--------KIWRDTDGSSTT----------
---------EYAFGTRGLFPAPMS--------------------------------DEQA
AQDVGKKQLNIFKVLGKFVARSMLDSRIIDISFNP--AFFRIADT---------------
-----LSSVAPSLGTVKL----------------------VDHDLANS--------LIML
KEFVNAKIVIEADMSLSPARK-----------------SEAVQNITVHG-ARVD-DLG--
--LDFTL------------------------------PG--YPAIE-LISG--------
GADVQ-------------------------------------------------------
------------------------------LTIENVD-TYVER-VIDMTLGSG-VRRQVD
-AFRAG---FSQVFPF--SSLR-AFTPS-ELVMLFGQ-----------------------
---AEEDWS-----------IETL---------MDSIKADHG------------------
----------------FNMD----SRSV-RNLLQTMS--------------------ELD
NQQRRDFLQF-------VTGSPKLP------------IGG-----FK-SLTPIF--TVVC
RPSEPPYTPD------------------------DYLPSVMTCV-NY-LKLPDY-SDLDV
LKKRLS-VAIKE-GQGA
>Penicillium_paneum_CBXN010000147.1 .
VRISRSRI-LDSALKVM----------------ELYGSSPS-ILEVEYFE-------EVG
TGLG-PTLEF---YSTV----SREFSKKKL--------KIWRDTDGNSAS----------
---------EYAFGKSGLFPAPMS--------------------------------DEQA
AQDIGKRQLNIFKVLGKFVARSMLDSRIIDISFNP--AFFRIADT---------------
-----LSSVAPSLGTVKL----------------------VDHDLAKS--------LIML
```

KEFVNAKNAIEADKSLSPALK-----------------SEAVRNITVHG-ANVD-DLG--
--LDFTL-------------------------------PG--YPAIE-LIPG--------
GAHVQ---
------------------------------LTIENID-TYIER-VIDMTLGSG-VRRQVD
-AFRAG---FSQVFPF--SSLC-AFTPS-ELVMLFGQ-----------------------
---ADEDWS-----------IETL---------MDSIKADHG------------------
----------------FNMD----SRSV-RNLLQTMS--------------------ELD
SQQRRDFLQF-------VTGSPKLP------------IGG-----FK-SLTPIF--TVVC
RPSEHPYTPD------------------------DYLPSVMTCV-NY-LKLPDY-SDLDV
LKKRLS-VAIKE-GQGA
>Penicillium_carneum_CBXS010001756.1 .
VRISRSRI-LDSALKVM----------------ELYGSSPS-ILEVEYFE-------EVG
TGLG-PTLEF---YSTV----SREFSKKKL--------KIWRDTDGSSXT----------
---------EYAFGKXGLFPAPMS--------------------------------DEQA
XQDIGKRQLNIFKVLGKFVARSMLDSRIIDISFNP--AFFRIADT---------------
-----LSSVAPSLGTVKL----------------------VDHDLAKS--------LIML
KEFVNAKNVIEANXSLSPAXK-----------------XEAVQNITVHG-AXVD-DLG--
--LDFTL-------------------------------PG--YPXIE-LXXD--------
GANVP---
------------------------------LTIENID-TYIER-VIDMTLGSG-VQRQVD
-AFRAG---FSQVFPF--SSLC-AFTPS-ELVMLFGQ-----------------------
---ADEDWS-----------IETL---------MDSIKADHG------------------
----------------FNMD----SRSV-RNLLQTMS--------------------ELD
NQQRRDFLQF-------VTGSPKLP------------IGG-----FK-SLTPIF--TVVC
RPSEHPYTPD------------------------DYLPSVMTCV-NY-LKLPDY-SDLDV
LKKRLS-VAIKE-GQGA
>Penicillium_roqueforti_MSQC01000680.1 .
VRISRSRI-LDSALKVM----------------ELYGSSPS-ILEVEYFE-------EVG
TGLG-PTLEF---YSTV----SREFSKKKL--------KIWRDTDGSSTT----------
---------EYAFGKSGLFPAPMS--------------------------------DEQA
SQDIGKRQLNIFKVLGKFVARSMLDSRIIDISFNP--AFFRIADT---------------
-----LSSVAPSLGTVKL----------------------VDHDLAKS--------LVML
KEFVNAKNVIEANQSLSPALK-----------------SEAVQNITVHG-ASVD-DLG--
--LDFTL-------------------------------PG--YPAIE-LISD--------
GANVP---
------------------------------LTIENID-TYIER-VIDMTLGSG-VRRQVD
-AFRAG---FSQVFPF--SSLC-AFTPS-ELVMLFGQ-----------------------
---ADEDWS-----------IETL---------MDSIKADHG------------------
----------------FNMD----SRSV-RNLLQTMS--------------------ELD
NQQRRDFLQF-------VTGSPKLP------------IGG-----FK-SLTPIF--TVVC
RPSEHPYTPD------------------------DYLPSVMTCV-NY-LKLPDY-SDLDV
LKKRLS-VAIKE-GQGA
>Penicillium_nalgiovense_CBXQ010000081.1 .
VRISRSRI-LDSALKVM----------------ELYGSSPS-ILEVEYFE-------EVG
TGLG-PTLEF---YSTV----SREFSRKKL--------KIWRDTDGSSGT----------
---------EYAFGKRGLFPAPIS--------------------------------DEQA
AQEIGKKQLNIFKVLGKFVARSMLDSRIIDISFNP--AFFRIADT---------------
-----LSSVAPSLGTVKL----------------------VDQDLAKS--------LIML
KEFVNAKNAVETNKSLSPAHK-----------------IEAVQNITVHG-AHVD-DLG--
--LDFTL-------------------------------PG--YPAIE-LIPG--------
GADVP---
------------------------------LTIENVD-IYIER-VIDMTLGSG-VRRQVD
-AFRAG---FSQVFPF--SSLR-AFTPS-ELVMLFGQ-----------------------
---AEEDWS-----------IETL---------MDSIKADHG------------------
----------------FNMD----SRSV-RNLLQTMS--------------------EMD
HQQRRDFLQF-------VTGSPKLP------------IGG-----FK-SLTPIF--TVVC
RPSEHPYTPD------------------------DYLPSVMTCV-NY-LKLPDY-SDLDV
LKKRLS-VAIKE-GQGA

>Penicillium_chrysogenum_XM_002557649.1 .
VRISRSRI-LDSALKVM----------------ELYGSSPS-ILEVEYFE-------EVG
TGLG-PTLEF---YSTV----SREFSKKKL--------KIWRDTDGSSGT----------
---------EYAFGKRGLFPAPMS--------------------------------DGQA
GQEIGKKQLNIFKVLGKFVARSMLDSRIIDISFNP--AFFRIADT---------------
-----LSSVAPSLGTVKL----------------------VDQDLAKS--------LFML
KEFVDAKNAVEANRSLSPAHK-----------------IEAVQNITVHG-AHVD-DLG--
--LDFTL-------------------------------PG--YPAIE-LIPG--------
GTDVP---
------------------------------LTIENVD-IYIER-VIDMTLDSG-VRRQVD
-AFRAG---FSQVFPF--SSLR-AFTPS-ELVMLFGQ-----------------------
---ADEDWS-----------IETL---------MDSIKADHG------------------
----------------FNMD----SRSV-RNLLQTMS--------------------ELD
HQQRRDFLQF-------VTGSPKLP------------IGG-----FK-SLTPIF--TVVC
RPSEHPYTPD------------------------DYLPSVMTCV-NY-LKLPDY-SDLDV
LKKRLS-VAIKE-GQGA
>Penicillium_flavigenum_MLQL01000041.1 .
VRISRSRI-LDSALKVM----------------ELYGSSPS-ILEVEYFE-------EVG
TGLG-PTLEF---YSTV----SREFSKKKL--------KIWRDTDGSSGT----------
---------EYAFGQRGLFPAPMS--------------------------------DEQA
AQEIGKKQLNIFKVLGKFVARSMLDSRIIDISFNP--AFFRIADT---------------
-----LSSVAPSLGTVKL----------------------VDQDLAKS--------LFML
KEFVNAKNAVEANMSLSPAHK-----------------IEAVQNITVHG-AHVD-DLG--
--LDFTL-------------------------------PG--YPAIE-LIPG--------
GTDVP---
------------------------------LTIENVD-IYIER-VIDMTLGSG-VRRQVD
-AFRAG---FSQVFPF--SSLR-AFTPS-ELVMLFGQ-----------------------
---AEEDWS-----------IETL---------MDSIKADHG------------------
----------------FNMD----SRSV-RNLLQTMS--------------------ELD
HQQRRDFLQF-------VTGSPKLP------------IGG-----FK-SLTPIF--TVVC
RPSEHPYTPD------------------------DYLPSVMTCV-NY-LKLPDY-SDLDV
LKKRLS-VAIKE-GQGA
>Penicillium_vulpinum_MDYP01000011.1 .
VRISRSRI-LDSALKVM----------------ELYGSSPS-ILEVEYFE-------EVG
TGLG-PTLEF---YSTV----SREFSKKKL--------KIWRDTDGTAAA----------
---------EYAFGKRGLFPAPMS--------------------------------DEQA
TQDIGKKQLNIFKVLGKFVARSMLDSRIIDISFNP--AFFRIADT---------------
-----LSSVAPSLGTVKL----------------------VDQDLGKS--------LLML
QEFVNAKNTIEADKLLSPADK-----------------SEAVQNITVQG-AKVD-DLG--
--LDFTL-------------------------------PG--YPAIE-LIPG--------
GTDVS---
------------------------------LTIENVD-TYIER-VIDMTLGSG-VRRQVD
-AFRAG---FSQVFPF--SSLR-AFTPS-ELVMLFGQ-----------------------
---AKEDWT-----------IETL---------MDSIKADHG------------------
----------------FNMD----SRSV-RNLLQTMS--------------------EMD
GQERRDFLQF-------VTGSPKLP------------IGG-----FK-SLTPIF--TVVC
RPSEHPYTPD------------------------DYLPSVMTCV-NY-LKLPDY-SDLDV
LKKRLS-VAIKE-GQGA
>Penicillium_griseofulvum_LHQR01000065.1 .
VRISRSRI-LDSAIKVM----------------ELYGSSPS-ILEVEYFE-------EVG
TGLG-PTLEF---YSTV----SREFSKKKL--------KIWRDTDGSSAV----------
---------EYAFGKRGLFPAPMS--------------------------------DEQA
AQDIGKKQLNIFKVLGKFVARSMLDSRIIDISFNP--AFFRIADT---------------
-----LSSVAPSLGTVKL----------------------VDHDLGKS--------LLML
KEFVNAKNAIEADKSLSPARK-----------------LEAVQNIMVQG-AKVD-DLG--
--LDFTL-------------------------------PG--YPTIE-LIPG--------
GAEVQ---
------------------------------LTIENVD-TYIER-VIDMTLGSG-VRRQVD

-AFRAG---FSQVFPF--SSLR-AFTPS-ELVMLFGQ-----------------------
---AKEDWS-----------IETL---------MDSIKADHG------------------
----------------FNMD----SRSV-RNLLQTMS--------------------EMD
SQQRRDFLQF-------VTGSPKLP------------IGG-----FK-SLTPIF--TVVC
RPSEHPYTPD------------------------DYLPSVMTCV-NY-LKLPDY-SDLDV
LKKRLS-VAIKE-GQGA
>Penicillium_coprophilum_MDDG01000001.1 .
VRISRSRI-LDSAIKVM----------------ELYGSSPS-ILEVEYFE-------EVG
TGLG-PTLEF---YSTV----SREFSKKKL--------KIWRDTDGSSAA----------
---------EYAFGKRGLFPAPMS---------------------------------DEQA
DQDIGKKQLNIFKVLGKFVARSMLDSRIIDISFNP--AFFRIADT---------------
-----LSSVAPSLGTVKL----------------------VDHDLGKS--------LLML
KEFADAKNAIEADKSLSPARK-----------------SEAVQNITVQG-AKVD-DLG--
--LDFTL-------------------------------PG--YPAIE-LIPG--------
GSEVQ---
------------------------------LTIENVD-TYIER-VIDMTLGSG-VRRQVD
-AFRAG---FSQVFPY--SSLR-AFTPS-ELVMLFGQ-----------------------
---AKEDWS-----------IETL---------MDSIKADHG------------------
----------------FNMD----SRSV-RNLLQTMS--------------------ELD
NQQRRDFLQF-------VTGSPKLP------------IGG-----FK-SLTPIF--TVVC
RPSEHPYTPD------------------------DYLPSVMTCV-NY-LKLPDY-SDLDV
LKKRLS-VAIKE-GQGA
>Penicillium_antarcticum_MDYN01000030.1 .
VRISRARI-LDSAMKVM----------------ELYGSSPS-ILEVEYFE-------EVG
TGLG-PTLEF---YSTV----SKEFSKKKL--------KIWRETEGTSSGA---------
---------EYAFGKRGLFPAPMS---------------------------------DQQA
AQDSGKKQLNIFKVLGKFVARSMLDSRIIDISFNP--AFFRIADT---------------
-----LSSVAPSLGTVKL----------------------VDQDLAKS--------LIML
QQFVNAKDAIEGDDSLSPGHK-----------------TEAVQEITVHG-VKVD-DLS--
--LDFTL-------------------------------PG--YPAIE-LIPG--------
GSDVP---
------------------------------LTIENVA-IYIER-VIDMTLGSG-VRRQVD
-AFRAG---FSQVFPF--SSLR-AFTPN-ELVMLFGQ-----------------------
---AKEDWT-----------IETL---------MDTIKADHG------------------
----------------FNMD----SRSV-RNLLQTMS--------------------ELD
NQQRRDFLQF-------VTGSPKLP------------IGG-----FK-SLTPIF--TVVC
RPSEHPYTPD------------------------DYLPSVMTCV-NY-LKLPDY-SDLDV
LKKRLS-VAIKE-GQGA
>Penicillium_capsulatum_JPLR01000027.1 .
VRISRARI-LDSAMKVM----------------ELYGSSPS-ILEVEYFE-------EVG
TGLG-PTLEF---YSTV----SKEFSKKKL--------KIWRETEGASSGA---------
---------EYAFGKRGLFPAPMS---------------------------------DQQA
AQDSGKKQLNIFKVLGKFVARSMLDSRIIDISFNP--AFFRIADT---------------
-----LSSVAPSLGTVKL----------------------VDEELAKS--------LIML
QQFVHAKDAIESDNTLSPGHK-----------------TEAVQEITVHG-VKVD-DLS--
--LDFTL-------------------------------PG--YPAIE-LIPG--------
GSDVP---
------------------------------LTIENVD-TYIER-VIDMTLGSG-VRRQVD
-AFRAG---FSQVFPY--SSLR-AFTPS-ELVMLFGQ-----------------------
---AKEDWT-----------IETL---------MDTIKADHG------------------
----------------FNMD----SRSI-RNLLQTMS--------------------ELD
NQQRRDFLQF-------VTGSPKLP------------IGG-----FK-SLTPIF--TVVC
RPSEHPYTPD------------------------DYLPSVMTCV-NY-LKLPDY-SDLDV
LKKRLS-VAIKE-GQGA
>Penicillium_arizonense_LXJU01000022.1 .
VRISRARI-LDSAMKVM----------------ELYGSSPS-ILEVEYFE-------EVG
TGLG-PTLEF---YSTV----SKEFSKKKL--------KIWRETEGASSGA---------
---------EYAFGKRGLFPAPMS---------------------------------DQQA

AQDSGKKQLNIFKVLGKFVARSMLDSRIIDISFNP--AFFRIADT---------------
-----LSSVAPSLGTVKL----------------------VDEDLAKS--------LIML
QQFVHAKDAIESDNTLSPGHK-----------------TEAVQEITVHG-VKVD-DLS--
--LDFTL-------------------------------PG--YPAIE-LIPG--------
GSDVP---
------------------------------LTIENVD-TYIER-VIDMTLGSG-VRRQVD
-AFRTG---FSQVFPY--SSLR-AFTPS-ELVMLFGQ-----------------------
---AKEDWT-----------IETL---------MDTIKADHG------------------
----------------FNMD----SRSI-RNLLQTMS--------------------ELD
NQQRRDFLQF-------VTGSPKLP------------IGG-----FS-CLTPIF--TVVC
RPSEHPYTPD------------------------DYLPSVMTCV-NY-LKLPDY-SDLDV
LKKRLS-VAIKE-GQGA
>Penicillium_decumbens_MDYL01000013.1 .
VRISRARI-LDSAMKVM----------------ELYGSSPS-VLEVEYFE-------EVG
TGLG-PTLEF---YSTV----SKEFSKKKI--------KIWRETDGTSSTA---------
---------EYAFGKRGLFPAPMS--------------------------------DEQA
SQESGKKQLNLFKVLGKFVARSMLDSRIIDISFNP--AFFRIADT---------------
-----LSSVAPSLGTVKA----------------------VDEDLAKS--------LVML
KQFFKAKRSIEEDKSISTHEK-----------------SRAVQNIIVDG-VRVE-DLS--
--LDFTL-------------------------------PG--YPAIE-LIPG--------
GSDVS---
------------------------------VTIDNVE-QYVDR-VIDMTLGSG-VRRQVD
-AFRAG---FSQVFPY--SALR-AFTPN-ELVMLFGQ-----------------------
---AEEDWS-----------IETL---------TDSIKADHG------------------
----------------FNMD----SRSV-RNLLQTMS--------------------ELD
KQQRRDFLQF-------VTGSPKLP------------ILG-----FK-NLTPIF--TVVC
RPSEPPYTAD------------------------DYLPSVMTCV-NY-LKLPDY-SSLEF
LKERLS-VAIKE-GQGA
>Penicillium_sclerotiorum_MJCA01000018.1 .
VRISRSRI-LDSAIKVM----------------ELYGSSPS-ILEVEYFE-------EVG
TGLG-PTLEF---YSTA----SKEFSKKKL--------KIWRDTDGGTSSA---------
---------DYAFGKRGLFPAPMS--------------------------------EVQA
VSEAGKKQLNTFKVLGKFVARSMLDSRIIDISFNP--AFFRIADT---------------
-----LSSVAPSVGTVKA----------------------VDEDLAKS--------LIML
KRFVKAKSSIKEDDTLSSYEK-----------------RKAIQNIMVDG-VRVE-DLS--
--LDFTL-------------------------------PG--YPSIE-LIPG--------
GADVD---
------------------------------LTIKNVD-QYVDR-VIDMTLGSG-VRRQVD
-AFQAG---FSQVFPY--SALR-AFTPS-ELVMLFGQ-----------------------
---ADEDWS-----------IETL---------MDSIKADHG------------------
----------------FNMD----SKSV-RSLLQTMS--------------------EMD
TQQRRDFLQF-------VTGSPKLP------------IGG-----FK-SLTPIF--TVVC
RPSEPPYTPD------------------------DCLPSVMTCV-NY-LKLPDY-STPQV
LKDRLS-VAIKE-GQGA
>Mycosphaerella_populi_MWPW01000056.1 .
VRISRARI-LDSAMKVM----------------ELYGSSPS-VLEVEYFE-------EVG
TGLG-PTLEF---YSTV----SKEFSKKKL--------KIWRETDDLSGA----------
---------EYAFGKRGLFPAPMS--------------------------------EEQA
SSEAGKKQLNIFKVLGKFVARSMLDSRIIDISFNP--AFFRIADT---------------
-----LSSVAPSLGTVKA----------------------VDDDLAKS--------LVML
KEFVKAKNSIEEDRSVSSHDK-----------------PKAIENIMIDG-VRVE-DLS--
--LDFTL-------------------------------PG--YPSIE-LIPG--------
GSDIS---
------------------------------LTINNVD-QYVDR-VIDMTLSSG-VRHQVE
-AFRTG---FSQVFPF--SALR-AFTPS-ELVMLFGQ-----------------------
---AEEDWS-----------IETL---------MDSIKADHG------------------
----------------FNMD----SKSV-RNLLQTMS--------------------ELD
NQQRRDFLQF-------VTGSPKLP------------IGG-----FK-SLTPIF--TVVC

RPSEPPYTPD------------------------DYLPSVMTCV-NY-LKLPDY-SNLDV
LKERLS-VAFKE-GQGA
>Penicillium_paxilli_AOTG01000136.1 .
LRISRARI-LDSAMKVM----------------ELYGSSPS-VLEVEYFE-------EVG
TGLG-PTLEF---YSTV----SKEFSKTKL--------KIWRETDGHSATP---------
---------EYAFGKRGLFPSPMS--------------------------------EEQA
SQESGKKQLNIFKILGKFVARSMLDSRIIDISFNP--AFFRIADT---------------
-----LSSVAPSLGTVKL---------------------VDEDLAKS--------LIML
KQFVKAKKAINKNNSLSEQQK-----------------TDAIQEIEVDG-VRVD-DLS--
--LDFTL-------------------------------PG--YPSIE-LVPS--------
GADRL---
------------------------------LNIDNVD-EYIER-VIDLTLGSG-VRRQVD
-AFRAG---FSQVFPF--SSLR-AFTPN-ELVMLFGQ-----------------------
---AEEDWS-----------IETL---------MDSIKADHG------------------
----------------FNMD----SKSV-RNLLQTMS--------------------ELD
KQQRRDFLQF-------VTGSPKLP------------IGG-----FK-SLTPIF--TVVC
RPSEPPYTPD------------------------DYLPSVMTCV-NY-LKLPDY-SSLEV
LGERLS-VAIKE-GQGA
>Penicillium_citrinum_LKUP01000373.1 .
VRISRARI-LDSAMKVM----------------ELYGSSPS-VLEVEYFE-------EVG
TGLG-PTLEF---YSTV----SKEFSKKKL--------KIWRETDGHSGTP---------
---------EYAFGKRGLFPCPMS--------------------------------DEQA
SQDSGKKQLNIFKILGKFVARSMLDSRIIDISFNP--AFFRIADT---------------
-----LSSVAPSLGTVKA---------------------VDEDLAKS--------LIML
KQFVKAKKNIKKDQNLSEQQK-----------------ATAIQQIEVDG-VRVD-DLS--
--LDFTL-------------------------------PG--YPSIE-LVPN--------
GADVS---
------------------------------LNIDNVD-HYIER-VIDMTLGSG-VRRQVD
-AFRTG---FSQVFPF--SSLR-AFTPN-ELVMLFGQ-----------------------
---ADEDWS-----------IETL---------MDSIKADHG------------------
----------------FNMD----SKSV-RNLLQTMS--------------------ELD
KQQRRDFLQF-------VTGSPKLP------------IGAG----FK-SLTPIF--TVVC
RPSEPPYTPD------------------------DYLPSVMTCV-NY-LKLPDY-SSLNV
LGERLS-VAIKE-GQGA
>Penicillium_steckii_MLKD01000027.1 .
VRISRARI-LDSAMKVM----------------ELYGSSPS-VLEVEYFE-------EVG
TGLG-PTLEF---YSTV----SKEFSKKKL--------KIWRETDGHSATP---------
---------EYAFGKRGLFPCPMS--------------------------------DEQA
SQDSGKKQLNIFKVLGKFVARSMLDSRIIDISFNP--AFFRIADT---------------
-----LSSVAPSLGTVKA---------------------VDEDLAKS--------LIML
KQFVKAKKNIQKDQDLSEQQK-----------------ISAIQQIEVDG-VRVD-DLS--
--LDFTL-------------------------------PG--YPSIE-LVPN--------
GADVS---
------------------------------LNINNVD-HYIER-VIDMTLGSG-VRRQVD
-AFRTG---FSQVFPF--SSLR-AFTPN-ELVMLFGQ-----------------------
---ADEDWS-----------IETL---------MDSIKADHG------------------
----------------FNMD----SKSV-RNLLQTMS--------------------ELD
KQQRRDFLQF-------VTGSPKLP------------IGG-----FK-SLTPIF--TVVC
RPSEPPYTPD------------------------DYLPSVMTCV-NY-LKLPDY-SSLNV
LGERLS-VAIKE-GQGA
>Penicillium_expansum_JARG01000001.1 .
VRISRSRI-LDSAMKVM----------------ELYGSSPS-VLEVEYFE-------EVG
TGLG-PTLEF---YSTV----SKEFSRKKL--------KIWRETETSPTA----------
---------DFAFGKRGLFPAPMS--------------------------------EEQA
SQEYGKKQLGIFKVLGKFVARSMLDSRIIDISFNP--AFFRIADT---------------
-----LSSVAPSLGTVKC---------------------VDEDLAKS--------LLML
KQFVTAKEVICNDKTMSASQK-----------------AQAMNNIQIDG-VRVE-DLS--
--LDFTL-------------------------------PG--YPSIE-LVPD--------

GSNVA---
------------------------------LTIENVD-QYVEN-VIDMTLGRG-VQRQVD
-AFRAG---FSQVFPY--SALR-AFTPN-ELVMLFGQ-----------------------
---AEEDWS-----------IETL---------MDSIKADHG------------------
----------------FNMD----SRSV-RNLLQTMC--------------------DLD
SQERRDFLQF-------VTGSPKLP------------IGG-----FK-SLTPIF--TVVC
RPSEPPYTPD------------------------DYLPSVMTCV-NY-LKLPDY-STLEV
LKQRLS-VAIKE-GQGA
>Penicillium_janthinellum_GBSQ01005963.1 .
VRISRSRI-LESAMKVM----------------ELYGSSPS-VLEVEYFE-------EVG
TGLG-PTLEF---YSTV----SKEFSKKKL--------KIWREHDHSSAA----------
---------EFAFGKRGLFPAPMS--------------------------------EEQA
SQEPGKKQLNLFKILGKFVARSMLDSRIIDISFNP--AFFRIADT---------------
-----LSSVAPSLGTVKA----------------------VDEDLARS--------LIML
KQFVKAKDAIKHDKTLTSHQM-----------------AQAMENIQVDG-VRVE-DLG--
--LDFTL-------------------------------PG--YPAIE-LIHD--------
GSNVP---
------------------------------LTIENVD-QYVDS-VIDMTLGSG-VRRQVD
-AFRAG---FSQVFPF--SALR-AFTPN-ELVMLFGQ-----------------------
---AEEDWS-----------IETL---------MDSIKADHG------------------
----------------FNMD----SKSV-RNLLHTMC--------------------ELD
SQQRRDFLQF-------VTGSPKLP------------IGG-----FK-SLTPIF--TVVC
RPSEPPYTPD------------------------DYLPSVMTCV-NY-LKLPDY-SSLDV
LKERLS-VAIKE-GQG-
>Penicillium_brasilianum_LJBN01000224.1 .
VRIARSRI-LDSAMKVM----------------ELYGSSPS-VLEVEYFE-------EVG
TGLG-PTLEF---YSTV----SKEFSKKKL--------KIWRESDASPAV----------
---------EYAFGKGGLFPAPMS--------------------------------EEQA
AQDTGKKQLKLFKILGKFVARSMLDSRIIDISFNP--AFFRIADT---------------
-----LSSVAPSLGTVKA----------------------VDEDLAKS--------LIML
KQFVKAKDAIQNDKTLSSHQR-----------------ASAVENIRVDG-VRVE-DLS--
--LDFTL-------------------------------PG--YPSIE-LTED--------
GSNVP---
------------------------------LTIENVD-QYVES-VIDMTLGSG-VQRQVD
-AFRAG---FSQVFPF--SALR-AFTPN-ELVMLFGR-----------------------
---AEEDWS-----------IETL---------MDSIKADHG------------------
----------------FNMD----SKSV-RNLLHTMC--------------------ELD
PQQRRDFLQF-------VTGSPKLP------------IGG-----FK-SLTPIF--TVVC
RPSEPPYTAD------------------------DYLPSVMTCV-NY-LKLPDY-SSLDV
LKERLS-VAIKE-GQGA
>Penicillium_subrubescens_MNBE01000157.1 .
VRISRSRI-LDSAMKVM----------------ELYGSSPS-VLEVEYFE-------EVG
TGLG-PTLEF---YSTV----SKEFSRKKL--------KIWRESDATSGV----------
---------EYAFGKRGLFPAPMS--------------------------------EEQA
TQESGKKQLNLFKILGKFVARSMLDSRIIDISFNP--AFFRIADT---------------
-----LSSVAPSLGTVKA----------------------VDEDLAKS--------LIML
KQFVNAKDAIKSDKSLSSHQK-----------------ASAIENIQVDG-VRVE-DLS--
--LDFTL-------------------------------PG--YPGIE-LTAD--------
GANVP---
------------------------------LTIDNVD-QYVDG-IIEMTLGSG-VRRQVD
-AFRAG---FSQVFPF--SALR-AFTPT-ELVMLFGR-----------------------
---AEEDWS-----------IETL---------MDSIKADHG------------------
----------------FNMD----SKSV-RNLLHTMC--------------------DLD
LQQRRDFLQF-------VTGSPKLP------------LTG-----FK-SLTPIF--TVVC
RPSEPPYTPD------------------------DYLPSVMTCV-NY-LKLPDY-SSLDV
LKERLS-VAIKE-GQGA
>Talaromyces_purpureogenus_LIAB01000692.1 .
VRISRSRI-LDSAMKVM----------------ELYGSSPS-VLEVEYFE-------EVG

```
TGLG-PTLEF---YSTV----SKEFSRKKL--------KIWRETDATSGV----------
---------EYAFGKRGLFPAPMS--------------------------------EEQA
MQESGKKQLNLFKILGKFVARSMLDSRIIDISFNP--AFFRIADT---------------
-----LSSVAPSLGTVKA----------------------VDEDLAKS--------LIML
KQFVNAKDAIKTDKSLSSHQK------------------ASAIENIQVDG-VRVE-DLS--
--LDFTL--------------------------------PG--YPGIE-LTAD--------
GSNVP-------------------------------------------------------
------------------------------LTIDNVD-QYVDG-VIDMTLGSG-VRRQVD
-AFRSG---FSQVFPF--SALR-AFTPN-ELVMLFGR-----------------------
---AEEDWS-----------IETL---------MDSIKADHG------------------
----------------FNMD----SKSV-RNLLHTMC--------------------DLD
PQQRRDFLQF-------VTGSPKLP------------IGG-----FK-SLTPIF--TVVC
RPSEPPYTPD------------------------DYLPSVMTCV-NY-LKLPDY-SSLDV
LKERLS-VAIKE-GQGA
>Penicilliopsis_zonata_MRBM01000129.1 .
VRISRTRI-LESAMKVM-----------------ELYGSSPS-VLEVEYFE-------EVG
TGLG-PTLEF---YSTV----SKEFSKRKL--------RIWRDGDSSAGD----------
---------EYAFGKRGLFPAPMS--------------------------------DDQA
TQESGKKQLQLFKMLGKFVARSMLDSRIIDVSFNP--TFFRIAGN---------------
-----PSMVQPSLGTVKT----------------------VDKDLANS--------LLLL
KRFAQAKKSVDDNKSIPPSQK------------------ALVLAEIAIDD-VQVE-DLG--
--LDFTL--------------------------------PG--YPAIE-LIKD--------
GSNIP-------------------------------------------------------
------------------------------VTIENVG-FYVER-VVDMTLGSG-VQRQVD
-AFRQG---FSQVFPY--SALR-AFTPN-ELVMLFGR-----------------------
---TEEDWA-----------XSAL---------MESIKADHG------------------
----------------FNMD----SKSV-RNLLQTMS--------------------ELS
TQQRRDFLQF-------VTGSPKLP------------IGG-----FK-SLTPIF--TVVC
RPSEPPYTPD------------------------DYLPSVMTCV-NY-LKLPDY-SSLDV
LRERLS-VAIKE-GQGA
>Aspergillus_chevalieri_BCIE01000012.1 .
VRISRSRI-LESAMKVM-----------------ELYGSSPS-ILEIEYFE-------EVG
TGLG-PTLEF---YSTI----SKEFSKKKL--------KMWRENDCNHDD----------
---------EYAFGKCGLFPAPMS--------------------------------DAQA
SQESGKKQLQLFKILGKFVARSMLDSRIIDVFFNS--AFFRIADS---------------
-----SSSVAPSLGTVKA----------------------VDHDLANS--------LMLL
KRFASAKKAVEDNKDLSASDK------------------AQALQQVEVDG-VKVD-DLG--
--LDFTL--------------------------------PG--YPSID-LIED--------
GSNAL-------------------------------------------------------
------------------------------VTIENVD-LYVDR-VVDMTLGSG-VRRQVE
-AFRSG---FSQVFAY--SSLK-TFTPS-ELVILFGQ-----------------------
---SEEDWS-----------IETL---------MDSIKADHG------------------
----------------FNMD----SRSV-RNLLQTMS--------------------EFN
LQQRRDFLQF-------VTGSPKLP------------IGG-----FK-SLTPIF--TVVC
RPSEPPYTPD------------------------DYLPSVMTCV-NY-LKLPDY-SSLDV
LRERLS-VAIKE-GQGA
>Aspergillus_cristatus_MAQV01000010.1 .
VRISRSRI-LESAMKVM-----------------ELYGSSPS-ILEIEYFE-------EVG
TGLG-PTLEF---YSTI----SKEFSKKKL--------KMWRENDCNHGD----------
---------EYAFGKRGLFPAPMS--------------------------------DAQA
SQESGKKQLQLFKILGKFVARSMLDSRIIDVFFNP--AFFRIADS---------------
-----SSSVAPSLGTVKA----------------------VDHDLANS--------LMLL
KRFASAKKAVED-KDLSASDK------------------PQALQQVEVDG-VKVD-DLG--
--LDFTL--------------------------------PG--YPSID-LIED--------
GSNTS-------------------------------------------------------
------------------------------VTIENVD-LYVDR-VVDMTLGSG-VRHQVE
-AFRSG---FSQVFAY--SSLK-TFTPS-ELVLLFGQ-----------------------
---SEEDWS-----------IETL---------MDSIKADHG------------------
```

```
----------------FNMD----SRSV-RNLLQTMS--------------------EFD
LQQRRDFLQF-------VTGSPKLP------------IGG-----FK-SLTPIF--TVVC
RPSEPPYTPD------------------------DYLPSVMTCV-NY-LKLPDY-SSLDV
LRERLS-VAIKE-GQGA
>Aspergillus_ruber_AWRT01000129.1 .
VRISRSRI-LESAMKVM----------------ELYGSSPS-ILEIEYFE-------EVG
TGLG-PTLEF---YSTI----SKEFSKKKL--------KIWRENDCNHGD----------
---------GYAFGKRGLFPAPMS--------------------------------DAQA
ARESGKKQLQLFKILGKFVARSMLDSRIIDVFFNP--AFFRIADS---------------
-----SSSVTPSLGTVKA----------------------VDHDLANS--------LMLL
KRFAVAKKAVED-KGLSASDK-----------------AQALQQVEIDG-VKVD-DLG--
--LDFTL-------------------------------PG--YPSID-LIKD--------
GSNTS-------------------------------------------------------
------------------------------VTIDNVD-VYVDR-VVDMTLGSG-VRLQVE
-AFRAG---FSQVFAY--SSLK-TFTPS-ELVILFGQ-----------------------
---SEEDWS-----------IETL---------MDSIKADHG------------------
----------------FNMD----SRSV-RNLLQTMS--------------------EFD
LQQRRDFLQF-------VTGSPKLP------------IGG-----FK-SLTPIF--TVVC
RPSEHPYTPD------------------------DYLPSVMTCV-NY-LKLPDY-SSLDV
LRERLS-VAIKE-GQGA
>Aspergillus_glaucus_LSTL01000123.1 .
VRISRSRI-LESAMKVM----------------ELYGSSSS-ILEIEYFE-------EVG
TGLG-PTLEF---YSTI----SKEFSKKKL--------KMWRENDCNHGD----------
---------EYAFGKRGLFPAPMS--------------------------------DAQS
SQESGKKQLQLFKILGKFVARSMLDSRIIDVFFNP--AFFRIADS---------------
-----SSSVTPSLGTVKA----------------------VDHDLANS--------LMLL
KRFASAKKAVED-KGLSASDK-----------------AQALQQVEVDS-VKVD-DLG--
--LDFTL-------------------------------PG--YPSID-LIKD--------
GSNTS-------------------------------------------------------
------------------------------VTIENVD-LYVDR-VVDMTLGSG-VCRQVE
-AFRAG---FSQVFAY--SSLK-TFTPS-ELVILFGQ-----------------------
---SKEDWS-----------IETL---------MDSIKADHG------------------
----------------FNMD----SRSV-RNLLQTMS--------------------EFD
MQQRRDFLQF-------VTGSPKLP------------IGG-----FK-SLTPIF--TVVC
RPSEHPYTPD------------------------DYLPSVMTCV-NY-LKLPDY-SSLDV
LRERLS-VAIKE-GQGA
>Aspergillus_flavus_LOAN01000318.1 .
VRISRSRI-LESAMKVM----------------ELYGSSPS-VLEVEYFE-------EVG
TGLG-PTLEF---YSTV----SKEFSKKKL--------KIWRENDCNDAE----------
---------EFAFGKRGLFPAPMS--------------------------------EQYA
ASESGKKQLHLFKVLGKFVARSMLDSRIIDVSFNP--AFFRIADS---------------
-----SFSVAPSLGTVKA----------------------VDQDLANS--------LLLL
KRFANAKAEVENKALSEAQTR------------------QALLNVEVDG-VKVE-DLS--
--LDFTL-------------------------------PG--YPSIE-LIKD--------
GSNVP-------------------------------------------------------
------------------------------VTIENVD-LYVER-VVDMTLSSG-VQRQVE
-AFREG---FSQVFPY--SALR-TFTPA-ELVMLFGR-----------------------
---AEEDWS-----------IESK---------YTSIKADHG------------------
----------------FNMD----SRSV-RNLLQTMS--------------------ELD
AQQRRDFLQF-------VTGSPKLP------------IGG-----FK-SLTPIF--TVVC
RPSEPPYTPD------------------------DYLPSVMTCV-NY-LKLPDY-SSLDV
LRTRLS-VAIQE-GQGA
>Aspergillus_oryzae_XM_001819021.2 .
VRISRSRI-LESAMKVM----------------ELYGSSPS-VLEVEYFE-------EVG
TGLG-PTLEF---YSTV----SKEFSKKKL--------KIWRENDCNDAE----------
---------EFAFGKRGLFPAPMS--------------------------------EQYA
ASESGKKQLHLFKVLGKFVARSMLDSRIIDVSFNP--AFFRIADS---------------
-----SFSVAPSLGTVKA----------------------VDQDLANS--------LLLL
```

KRFANAKAEVENKALSEAQTR------------------QALLNVEVDG-VKVE-DLS--
--LDFTL-------------------------------PG--YPSIE-LIKD--------
GSNVP---
------------------------------VTIENVD-LYVER-VVDMTLSSG-VQRQVE
-AFREG---FSQVFPY--SALR-TFTPA-ELVMLFGR-----------------------
---AEEDWS-----------IETL---------MDSIKADHG------------------
----------------FNMD----SRSV-RNLLQTMS--------------------ELD
AQQRRDFLQF-------VTGSPKLP------------IGG-----FK-SLTPIF--TVVC
RPSEPPYTPD------------------------DYLPSVMTCV-NY-LKLPDY-SSLDV
LRTRLS-VAIQE-GQGA
>Aspergillus_nomius_CBWY010000531.1 .
VRISRSRI-LESAMKVM----------------ELYGSSPS-VLEVEYFE-------EVG
TGLG-PTLEF---YSTV----SKEFSKKKL--------KIWRENDCNDAE----------
---------EFAFGKRGLFPAPMS--------------------------------EQYA
SSEPGKKQLHLFKVLGKFVARSMLDSRIIDVSFNP--AFFRIADS---------------
-----SFSVAPSLGTVKA----------------------VDQDLANS--------LLLL
KRFANAKAAVENKGLSEPQKR------------------EALLNVEVDG-VRVE-DLS--
--LDFTL-------------------------------PG--YPSIE-LIKD--------
GSDVP---
------------------------------VTIENVD-LYVER-VVDMTLSSG-VQRQVE
-AFREG---FSQVFPY--SALR-TFTPA-ELVMLFGR-----------------------
---AEEDWS------------XAL---------MDSIKADHG------------------
----------------FNMD----SRSV-RNLLQTMS--------------------ELD
AQQRRDFLQF-------VTGSPKLP------------IGG-----RK-SLTPIF--TVVC
RPSEPPYTPD------------------------DYLPSVMTCV-NY-LKLPDY-SSLDV
LRTRLS-VAIQE-GQGA
>Aspergillus_bombycis_LYCR01000045.1 .
VRISRSRI-LESAMKVM----------------ELYGSSPS-VLEVEYFE-------EVG
TGLG-PTLEF---YSTV----SKEFSKKKL--------KIWRENDCNDVE----------
---------EFAFGKRGLFPAPMS--------------------------------EQYA
SSESGKKQLHLFKVLGKFVARSMLDSRIIDISFNP--AFFRIADS---------------
-----SFSVAPSLGTVKA----------------------VDQHLANS--------LLLL
KRFANAKVAVENKGLSEHQKR------------------HALLNVEVDG-VKVE-DLS--
--LDFTL-------------------------------PG--YPAIE-LIKD--------
GSNVP---
------------------------------VTIENVD-LYVER-VVDMTLSSG-VQRQVE
-AFREG---FSQVFPY--SALR-TFTPA-ELVMLFGR-----------------------
---AEEDWS-----------IESK---------YTSIKADHG------------------
----------------FNMD----SRSV-RNLLQTMS--------------------QLD
AQQRRDFLQF-------VTGSPSIA------------STG-----FK-SLTPIF--TVVC
RPSEPPYTPD------------------------DYLPSVMTCV-NY-LKLPDY-SSLDV
LRTRLS-VAIQE-GQGA
>Aspergillus_nomius_XM_015556650.1 .
--KVEYFE-------EVG
TGLG-PTLEF---YSTV----SKEFSKKKL--------KIWRENDCNDAE----------
---------EFAFGKRGLFPAPMS--------------------------------EQYA
SSEPGKKQLHLFKVLGKFVARSMLDSRIIDVSFNP--AFFRIADS---------------
-----SFSVAPSLGTVKA----------------------VDQDLANS--------LLLL
KRFANAKAAVEN-KGLSEPQK-----------------REALLNVEVDG-VKVE-DLS--
--LDFTL-------------------------------PG--YPSIE-LIED--------
GSDVP---
------------------------------VTIENVD-LYVER-VVDMTLSSG-VQRQVE
-AFREG---FSQVFPY--SALR-TFTPA-ELVMLFGR-----------------------
---AEEDWS-----------IETL---------MDSIKADHG------------------
----------------FNMD----SRSV-RNLLQTMS--------------------ELD
AQQRRDFLQF-------VTGSPKLP------------IGG-----FK-SLTPIF--TVVC
RPSEPPYTPD------------------------DYLPSVMTCV-NY-LKLPDY-SSLDV
LRTRLS-VAIQE-GQGA

```
>Aspergillus_sclerotiorum_CBWZ010000014.1 .
VRISRSRI-LESAMKVM----------------ELYGSSPS-ILEVEYFE-------EVG
TGLG-PTLEF---YSTV----SKEVSKKKL--------KIWRENDCNNGE----------
---------EYAFGKRGLFPSPMS---------------------------------DDQA
ASESGKKQLQLFKVLGKFVARSMLDSRIIDISFNP--AFFRIADL---------------
-----SSSVAPSLGTVKA----------------------VDQDLANS--------LLLL
KRFANAKKAVLGNKFHSDAQK-----------------KHALEQVEVDD-VKVE-DLS--
--LDFTL-------------------------------PG--YPAID-LIPN--------
GSNIP-------------------------------------------------------
------------------------------VTMENVD-LYVDR-VVDMTLGSG-VQRQVD
-AFRTG---FSQVFPY--AALR-TFTPS-ELVMLFGR-----------------------
---AEEDWT-----------XSAL---------MDSIKADHG------------------
----------------FNMD----SRSV-RNLLQTMS--------------------ELG
VQQRRDFLQF-------VTGSPKLP------------IGG-----FK-SLTPIF--TVVC
RPSEPPYTPD------------------------DYLPSVMTCV-NY-LKLPDY-SSLDV
LRERLT-VAIKE-GQGA
>2_Aspergillus_westerdijkiae_LKBE01000025.1 .
VRISRSRI-LESAMKVM----------------ELYGSSPS-ILEVEYFE-------EVG
TGLG-PTLEF---YSTV----SKEFSKKKL--------KIWRENDCNNSE----------
---------EYAFGQRGLFPSPMS---------------------------------EEQA
ASESGKKQLQLFKVLGKFVARSMLDSRIIDISFNP--AFFRIADL---------------
-----TSSVAPSLGTVKA----------------------VDHDLANS--------LLLL
KRFANSKKAIIDNKAYSGAQK-----------------KHALQQVELDG-VQVD-DLG--
--LDFTL-------------------------------PG--YPTID-LISN--------
GSNIP-------------------------------------------------------
------------------------------VTIENVD-LYVDR-VVDMTLGSG-VQRQVD
-AFRTG---FSQVFPY--AALR-TFTPS-ELVMLFGR-----------------------
---AEEDWT-----------IESL---------MDSIKADHG------------------
----------------FNMD----SRSV-RNLLQTMS--------------------ELS
AQQRRDFLQF-------VTGSPKLP------------IGGKG---FK-SLTPIF--TVVC
RPSEPPYTPD------------------------DYLPSVMTCV-NY-LKLPDY-SSLDV
LRDRLT-VAIKE-GQGA
>Aspergillus_calidoustus_CDMC01000020.1 .
VRISRSRI-LESAMKVM----------------ELYGSSPS-VLEVEYFE-------EVG
TGLG-PTLEF---YSTV----SKEFSKKKL--------KMWRENDCAGAG----------
---------EFAFGNSGLFPAPMS---------------------------------EAQL
STESGKKQLSLFKTLGKFVARSMLDSRIIDISFNP--AFFRIADT---------------
-----SSTVAPSLGTVKA----------------------VDHDLAKS--------LLML
KRFANAKKAIEQ-RGLSKAQK-----------------TQALTEVEVDG-VRVE-DLS--
--LDFTL-------------------------------PG--YPAIE-LIKN--------
GSDTA-------------------------------------------------------
------------------------------VTMDNID-LYVDR-VVDMTLGSG-VQAQVE
-AFRTG---FSQVFPY--SALQ-TFTPS-ELVMLFGR-----------------------
---AEEDWT-----------IESL---------MDSIKADHG------------------
----------------FNMD----SRSV-RNLLQTMS--------------------ELD
TQQRRDFLQF-------VTGSPKLP------------IGG-----FK-SLTPIF--TVVC
RPSEPPYTPD------------------------DYLPSVMTCV-NY-LKLPDY-STLDV
LRERLA-IAIKE-GQGA
>Aspergillus_ustus_JOMC01000101.1 .
VRISRSRI-LESAMKVM----------------ELYGSSPS-VLEVEYFE-------EVG
TGLG-PTLEF---YSTV----SKEFSKRKL--------KMWRENECAGPG----------
---------EFAFGNRGLFPAPMS---------------------------------EAQV
SSESGKKQLSLFKTLGKFVARSMLDSRIIDISFNP--AFFRIADT---------------
-----STTVAPSLGTVKA----------------------VDQDLAKS--------LLLL
KRFANAKKAIEV-KGLSKAQK-----------------TQALSQVEVDD-VRVE-DLS--
--LDFTL-------------------------------PG--YPTIE-LIQN--------
GSDVP-------------------------------------------------------
------------------------------VTINNVD-LYVDR-VVDMTLGSG-VQAQVE
```

-AFRAG---FSQVFPY--SALQ-TFTPS-ELVMLFGR-----------------------
---AEEDWT------------XAL---------MDSIKADHG------------------
----------------FNMD----SRSV-RNLLQTMS--------------------ELD
TQQRRDFLQF-------VTGSPKLP------------IGGITG--FK-SLTPIF--TVVC
RPSEPPYTPD------------------------DYLPSVMTCV-NY-LKLPDY-STLEV
LRERLA-VAIKE-GQGA
>Aspergillus_rambellii_JZBS01002735.1 .
VRISRSRI-LESAMKVM----------------ELYGSSPS-VLEVEYFE-------EVG
TGLG-PTLEF---YSTV----SKEFSKKKL--------KLWRENDCNNTE----------
---------EYAFGNRGLFPAPMS--------------------------------EEQL
SSESGKKQLHLFKTLGKFVARSMLDSRIIDISFNP--AFFRIADT---------------
-----SSVVAPSLGTVKA----------------------VDQDLANS--------LLLL
KRFATAKKAIEEKKFLSGYEK-----------------AQALTAVEVDG-VTVE-DLS--
--LDFTL-------------------------------PG--YPAIE-LIKN--------
GSNIS---
------------------------------VTMDNVD-LYVDR-VVDMTLGDG-VQAQVE
-AFRAG---FSQVFSY--SALQ-TFTPN-ELVMLFGR-----------------------
---AEEDWT-----------IESL---------MDSIKADHG------------------
----------------FNMD----SRSV-RNLLQTMS--------------------ELN
TQQRRDFLQF-------VTGSPKLP------------IGG-----FK-SLTPIF--TVVC
RPSEPPYTPD------------------------DYLPSVMTCV-NY-LKLPDY-STLDV
LRGRLD-VAIKE-GQGA
>Aspergillus_ochraceoroseus_JYKN01001773.1 .
VRISRSRI-LESAMKVM----------------ELYGSSPS-VLEVEYFE-------EVG
TGLG-PTLEF---YSTV----SKEFSKKKL--------KLWRENDCNNTE----------
---------EYAFGNRGLFPAPMS--------------------------------EEQL
SSESGKKQLHLFKTLGKFVARSMLDSRIIDISFNP--AFFRIADT---------------
-----SSVVAPSLGTVKA----------------------VDQDLANS--------LLLL
KRFATAKKAIEEKKFLSGYEK-----------------AQALTAVEVDG-VTVE-DLS--
--LDFTL-------------------------------PG--YPAIE-LVKN--------
GSNIP---
------------------------------VTMDNVD-LYVDR-VVDMTLGDG-VQAQVE
-AFRAG---FSQVFPY--SALQ-TFTPN-ELVMLFGR-----------------------
---AEEDWT-----------IESL---------MDSIKADHG------------------
----------------FNMD----SRSV-RNLLQTMS--------------------ELN
TQQRRDFLQF-------VTGSPKLP------------IGG-----FK-SLTPIF--TVVC
RPSEPPYTPD------------------------DYLPSVMTCV-NY-LKLPDY-STLDV
LRGRLD-VAIKE-GQGA
>Aspergillus_nidulans_XM_654258.1 .
VRISRSRI-LESAMKVM----------------ELYGSSPS-VLEVEYFE-------EVG
TGLG-PTLEF---YSTV----SKEFSKKKL--------KMWRENDCGDSE----------
---------EYAFGTRGLFPAPLS--------------------------------EEQL
ASEFGKKQLQLFKTLGKFVARSMLDSRIIDISFNP--AFFRIADT---------------
-----SSPVAPSLGTVKA----------------------VDQDLAKS--------LLLL
KRFANAKKALEA-KNLPKAKK-----------------TQALMAIEVDG-VHVE-DLS--
--LDFTL-------------------------------PG--YPAIE-LIKN--------
GSNIP---
------------------------------VTIENVD-VYVDR-VVDMTLGSG-VQAQVE
-AFRTG---FSQVFPH--SALQ-TFTPN-ELAMLFGR-----------------------
---AEEDWS-----------IETL---------MDSIKADHG------------------
----------------FNMD----SRSV-RNLLQTMS--------------------ELD
TQQRRDFLQF-------VTGSPKLP------------IGG-----FK-SLTPIF--TVVC
RPSEPPYLPD------------------------DYLPSVMTCV-NY-LKLPDY-SSLDV
LRERLS-VAIKE-GQGA
>Aspergillus_awamori_BCGT01000004.1 .
VRISRSRI-LDSAMKVM----------------ELYGSSPS-VLEVEYFE-------EVG
TGLG-PTLEF---YSTV----SKEFSKKKL--------KLWRENESVHGD----------
---------EYAFGKRGLFPAPMS--------------------------------EEQA

ASESGKKQLQLFKCLGKFVARSMLDSRIIDISFNP--AFFRIAAS---------------
-----SSSVAPSLGTVKA----------------------VDHDLAKS--------LLLL
KSFADAKKAVDDNRTLSKAQK-----------------AQALQQIEVGG-VKVE-DLS--
--LDFTL-------------------------------PG--YPAIE-LIKN--------
GSNIP---
------------------------------VTNENVD-LYVER-VIDMTLGSG-VQRQVE
-AFRAG---FSQVFPY--SALR-TFTPQ-ELVMLFGR-----------------------
---AEEDWT-----------IESL---------MDSIKADHG------------------
----------------FNMD----SRSV-RNLLQTMS--------------------ELN
PQQRRDFLQF-------VTGSPKLP------------IGG-----FK-SLTPIF--TVVC
RPSEPPYTPD------------------------DYLPSVMTCV-NY-LKLPDY-SSLDV
LRERLS-VAIKE-GQGA
>Aspergillus_luchuensis_MRBP01000021.1 .
VRISRSRI-LDSAMKVM----------------ELYGSSPS-VLEVEYFE-------EVG
TGLG-PTLEF---YSTV----SKEFSKKKL--------KLWRENESVHGD----------
---------EYAFGKRGLFPAPMS--------------------------------EEQA
ASESGKKQLQLFKCLGKFVARSMLDSRIIDISFNP--AFFRIAAS---------------
-----SSSVAPSLGTVKA----------------------VDHDLAKS--------LLLL
KSFADAKKAVDDNRTLSKAQK-----------------AQALQQIEVGG-VKVE-DLS--
--LDFTL-------------------------------PG--YPAIE-LIKN--------
GSNIP---
------------------------------VTNENVD-LYVER-VIDMTLGSG-VQRQVE
-AFRAG---FSQVFPY--SALR-TFTPQ-ELVMLFGR-----------------------
---AEEDWT-----------IESL---------MDSIKADHG------------------
----------------FNMD----SRSV-RNLLQTMS--------------------ELN
PQQRRDFLQF-------VTGSPKLP------------IGG-----FK-SLTPIF--TVVC
RPSEPPYTPD------------------------DYLPSVMTCV-NY-LKLPDY-SSLDV
LRERLS-VAIKE-GQGA
>Aspergillus_tubingensis_LJXU01000034.1 .
VRISRSRI-LDSAMKVM----------------ELYGSSPS-VLEVEYFE-------EVG
TGLG-PTLEF---YSTV----SKEFSKKKL--------KLWRENESVHGD----------
---------EYAFGKRGLFPAPMS--------------------------------EEQA
ASESGKKQLQLFKCLGKFVARSMLDSRIIDISFNP--AFFRIAAS---------------
-----SSSVAPSLGTVKA----------------------VDHDLAKS--------LLLL
KSFADAKKAVDDNRILSKAQK-----------------AQALQQIEVGG-VKVE-DLS--
--LDFTL-------------------------------PG--YPAIE-LIKN--------
GSNIP---
------------------------------VTNENVD-LYVER-VIDMTLGSG-VQRQVE
-AFRAG---FSQVFPY--SALR-TFTPQ-ELVMLFGR-----------------------
---AEEDWT-----------IESL---------MDSIKADHG------------------
----------------FNMD----SRSV-RNLLQTMS--------------------ELN
PQQRRDFLQF-------VTGSPKLP------------IGG-----FK-SLTPIF--TVVC
RPSEPPYTPD------------------------DYLPSVMTCV-NY-LKLPDY-SSLDV
LRERLS-VAIKE-GQGA
>Aspergillus_niger_XM_001394604.2 .
VRISRSRI-LDSAMKVM----------------ELYGSSPS-VLEVEYFE-------EVG
TGLG-PTLEF---YSTV----SKEFSKKKL--------KLWRENESVHGD----------
---------EYAFGKRGLFPAPMS--------------------------------EEQA
TSESGKKQLQLFKVLGKFVARSMLDSRIIDISFNP--AFFRIAAS---------------
-----SSSVAPSLGTVKA----------------------VDHDLAKS--------LLLL
KSFANAKKAVDDNRTLSKAQK-----------------TQALQQIEVGG-VKVE-DLS--
--LDFTL-------------------------------PG--YPAIE-LVKN--------
GSNIP---
------------------------------VTNENVD-LYVDR-VIDMTLGSG-VQRQVE
-AFRAG---FSQVFPY--SALR-TFTPQ-ELVMLFGR-----------------------
---AEEDWT-----------IETL---------MDSIKADHG------------------
----------------FNMD----SRSV-RNLLQTMS--------------------ELS
PQQRRDFLQF-------VTGSPKLP------------IGG-----FK-SLTPIF--TVVC

RPSEPPYTPD------------------------DYLPSVMTCV-NY-LKLPDY-SSLDV
LRERLS-VAIKE-GQGA
>Aspergillus_brasiliensis_LJXV01000010.1 .
VRISRSRI-LDSAMKVM----------------ELYGSSPS-VLEVEYFE-------EVG
TGLG-PTLEF---YSTV----SKEFSKKKL--------KLWRENESVHGD----------
---------EYAFGKRGLFPAPMS--------------------------------EEQA
TSESGKKQLQLFKVLGKFVARSMLDSRIIDISFNP--AFFRIASS---------------
-----SSSVAPSLGTVKA----------------------VDHDLARS--------LLLL
KSFAIAKKAVDDDRTLSKAQK-----------------TQALQQIEVGG-VKVE-DLS--
--LDFTL-------------------------------PG--YPAIE-LIKN--------
GSNIP---
------------------------------VTIENVD-LYVDR-VIDLTLGSG-VQRQVE
-AFRAG---FSQVFPY--AALR-TFTPQ-ELVMLFGR-----------------------
---AEEDWT-----------IESL---------MDSIKADHG------------------
----------------FNMD----SRSV-RNLLQTMS--------------------ELS
PQQRRDFLQF-------VTGSPKLP------------IGG-----FK-SLTPIF--TVVC
RPSEPPYTPD------------------------DYLPSVMTCV-NY-LKLPDY-SSLDV
LRERLS-VAIKE-GQGA
>Aspergillus_carbonarius_AHIG01000080.1 .
VRISRSRI-LDSAMKVM----------------ELYGSSPS-VLEVEYFE-------EVG
TGLG-PTLEF---YSTV----SKEFSKKKL--------KIWRENDCVNGD----------
---------EYAFGKRGLFPAPIS--------------------------------EEQA
ASESGKKHLQLFKVLGKFVARSMLDSRIIDISFNP--AFFRIANS---------------
-----SSSVVPSLGTVKA----------------------VDQDLANS--------LLLL
KAFANAKKAVDDNRTLSKTQK-----------------SQALQQVEVGG-VTVE-DLS--
--LDFTL-------------------------------PG--YPAIE-LIKN--------
GSNMP---
------------------------------VTIENVD-LYVDR-VVDMTLGSG-VQRQVE
-AFRAG---FSQVFPY--SALR-TFTPN-ELVMLFGR-----------------------
---AEEDWT-----------IESL---------MDSIKADHG------------------
----------------FNMD----SRSV-RNLLQTMS--------------------ELS
APQRRDFLQF-------VTGSPKLP------------IAG-----FK-SLTPIF--TVVC
RPSEPPYTPD------------------------DYLPSVMTCV-NY-LKLPDY-SSLDV
LRERLS-VAIKE-GQGA
>Aspergillus_wentii_LJSE01000088.1 .
VRISRTRI-LESAMKVM----------------ELYGSSPS-ILEVEYFE-------EVG
TGLG-PTLEF---YSTV----SKEFSKKKL--------KIWRENDCMNDS----------
---------EYAFGKRGLFPSPMS--------------------------------EEQA
SQESGKKQLQLFKILGKFVARSMLDSRIIDVSFNP--AFFRIADS---------------
-----SSFVAPSLGTVKA----------------------VDQDLANS--------LLLL
KRFANAKIAIDQNKALSAAQK-----------------AQALLDVEIDG-VKVE-DLS--
--LDFTL-------------------------------PG--YPSIE-LVND--------
GSNVS---
------------------------------VTIENVD-LYVDR-VVDMTLGSG-VQRQVE
-AFRTG---FSQVFPY--SALR-AFTPS-ELVMLFGR-----------------------
---VEEDWS------------XAL---------MDSIKADHG------------------
----------------FNMD----SRSV-RNLLQTMS--------------------ELS
TQQRRDFLQF-------VTGSPKLP------------IGG-----FK-SLIPIF--TVVC
RPSEPPYTPD------------------------DYLPSVMTCV-NY-LKLPDY-SSLDV
LRERLS-VAIKE-GQGA
>Aspergillus_aculeatus_XM_020195675.1 .
VRISRSRI-LESAIKVM----------------ELYGSSPS-VLEVEYFE-------EVG
TGLG-PTLEF---YSTV----SKEFSKKKL--------KIWRENDCANGE----------
---------EYAFGKRGLFPAPMS--------------------------------EAQG
ASEAGKKQLHLFKILGKFVARSMLDSRIIDISFNP--AFFRIADS---------------
-----SSFVAPSLGTVKA----------------------VDQDLANS--------LMLL
KRYANVKKAIEEDKGMSVTQK-----------------AQALQAVEING-VKVE-DLS--
--LDFTL-------------------------------PG--YPAIE-LVKN--------

GSNIP---
------------------------------VVIENVD-LYVDR-VVDMTLGSG-VQRQVE
-AFRAG---FSQVFPY--SALR-TFTPN-ELVMLFGR-----------------------
---AEEDWT-----------IETL---------MDSIKADHG------------------
----------------FNMD----SRSV-RNLLQTMS--------------------ELN
AQQRRDFLQF-------VTGSPKLP------------IGG-----FK-SLTPIF--TVVC
RPSEPPYTPD------------------------DYLPSVMTCV-NY-LKLPDY-SSLDT
LRERLS-VAIKE-GQGA
>Aspergillus_clavatus_XM_001268083.1 .
VRISRSRI-LESAMKVM----------------ELYGSSPS-VLEVEYFE-------EVG
TGLG-PTLEF---YSTV----SKEFSKKKL--------KIWRENDCNNDE----------
---------EFAFGRRGLFPAPMS--------------------------------EEQA
ASESGKRQLSLFKTLGKFVARSMLDSRIIDISFNP--AFFRIADS---------------
-----GSSVAPSLGTVKA----------------------VDRDLANS--------LLLL
KRFASVKKTIEGDKSLSKAQK-----------------SQALQNVEVDG-VKVE-DLS--
--LDFTL-------------------------------PG--YPSIE-LIDN--------
GSNIP---
------------------------------VTTDNVD-QYVDR-VVDMTLGRG-VQHQVD
-AFRAG---FSQVFPY--SALR-TFTPS-ELVMLFGR-----------------------
---AEEDWT-----------IETL---------MDSIKADHG------------------
----------------FNMD----SKSV-RNLLQTMS--------------------ELD
TQQRRDFLQF-------VTGSPKLP------------IGG-----FK-SLTPIF--TVVC
RPSEPPYTPD------------------------DYLPSVMTCV-NY-LKLPDY-SCLDV
LRERLS-VAIKE-GQGA
>Aspergillus_fumigatus_XM_745684.2 .
VRISRSRI-LESAMKVM----------------ELYGSSPS-VLEVEYFE-------EVG
TGLG-PTLEF---YSTV----SKEFSKKKL--------KIWRENDCHNDE----------
---------EFAFGKRGLFPAPMS--------------------------------EEQA
NSESGKKQLSLFKTLGKFVARSMLDSRIIDISFNP--AFFRIADS---------------
-----SSSVAPSLGTVKA----------------------VDQDLANS--------LMLL
KRFANAKRAIDRDRTLSAAAK-----------------SQALQNVEIDG-VRVE-DLS--
--LDFTL-------------------------------PG--YPSIE-LIDN--------
GSNVP---
------------------------------VTIENVD-TYVDR-VVDMTLGSG-VQRQVE
-AFRTG---FSQVFPY--SALR-TFTPN-ELVMLFGR-----------------------
---AEEDWT-----------IETL---------MDSIKADHG------------------
----------------FNMD----SKSV-RNLLQTMS--------------------ELD
TQQRRDFLQF-------VTGSPKLP------------IGG-----FK-SLTPTF--TVVC
RPSEPPYTPD------------------------DYLPSVMTCV-NY-LKLPDY-SSLEV
LRERLS-VAIRE-GQGA
>Aspergillus_lentulus_BCLY01000001.1 .
VRISRSRI-LESAMKVM----------------ELYGSSPS-VLEVEYFE-------EVG
TGLG-PTLEF---YSTV----SKEFSKKKL--------KIWRENDCHNDE----------
---------EFAFGKRGLFPAPMS--------------------------------EEQA
ASESGKKQLSLFKTLGKFVARSMLDSRIIDISFNP--AFFRIADS---------------
-----SSSVAPSLGTVKA----------------------VDQDLANS--------LLLL
KRFANAKRAIDRDRTLSAAAK-----------------SQALQNVEIDG-VRVE-DLS--
--LDFTL-------------------------------PG--YPSIE-LIDD--------
GSNVP---
------------------------------VTIENVD-KYVDR-VVDMTLGSG-VQRQVE
-AFRTG---FSQVFPY--SALR-TFTPN-ELVMLFGR-----------------------
---AEEDWT-----------IESKL--------PSSIKADHG------------------
----------------FNMD----SKSV-RNLLQTMS--------------------ELD
TQQRRDFLQF-------VTGSPKLP------------IGG-----FK-SLTPIF--TVVC
RPSEPPYTPD------------------------DYLPSVMTCV-NY-LKLPDY-SSLEV
LRERLS-VAIRE-GQGA
>Neosartorya_fischeri_XM_001258003.1 .
VRISRSRI-LESAMKVM----------------ELYGSSPS-VLEVEYFE-------EVG

TGLG-PTLEF---YSTV----SKEFSKKKL--------KIWRENDCHNDE----------
---------EFAFGKRGLFPAPMS--------------------------------EEQA
ASESGKKQLSLFKTLGKFVARSMLDSRIIDISFNP--AFFRIADS---------------
-----SSSVAPSLGTVKV----------------------VDQDLANS--------LLLL
KRFANAKRAIDRDGTLSAAAK-----------------SQALQNVEIDG-VRVE-DLS--
--LDFTL------------------------------PG--YPSIE-LMDN--------
GSNVP---
------------------------------VTIENVD-KYVDR-VVDMTLGSG-VQRQVE
-AFRTG---FSQVFPY--SALR-TFTPN-ELVMLFGR-----------------------
---AEEDWT-----------IETL---------MDSIKADHG------------------
----------------FNMD----SKSV-RNLLQTMS--------------------ELD
TQQRRDFLQF-------VTGSPKLP------------IGG-----FK-SLTPIF--TVVC
RPSEPPYTPD------------------------DYLPSVMTCV-NY-LKLPDY-SSLEV
LRERLS-VAIRE-GQGA
>Talaromyces_leycettanus_JSYV01000764.1 .
VRISRTRI-LDSAMKVM----------------ELYGSSPS-VLEVEYFE-------EVG
TGLG-PTLEF---YSTV----SKEFCKKKL--------KIWRENDSNADD----------
---------EYAFGKRGLFPAPMS--------------------------------DEQA
ASESGKKQLHLFKILGKFVARSMLDSRIIDISFNP--AFFRIADS---------------
-----SSTVTPSLGTVKA----------------------VDHDLANS--------LLLL
KRFANAKQAIEDNKHLSAVEK-----------------SQAVRNIEFEG-VKVE-DLG--
--LDFTL------------------------------PG--YPAIE-LIKN--------
GSNIP---
------------------------------VTIENVA-LYVDR-VVDLTLGSG-VRRQVD
-AFRTG---FSQVFPY--TALR-AFTPN-ELVMLFGR-----------------------
---AEEDWS-----------AIAL---------MDSIKADHG------------------
----------------FNMD----SKSV-RNLLQTMS--------------------ELN
PQQRRDFLQF-------VTGSPKLP------------IAG-----FK-SLTPIF--TVVC
RPSEPPYTPD------------------------DYLPSVMTCV-NY-LKLPDY-SSLEV
LKERLS-VAIKE-GQGA
>Thermoascus_crustaceus_BCIC01000002.1 .
VRISRSRI-LESAMKVM----------------ELYGSSPS-ILEVEYFE-------EVG
TGLG-PTLEF---YSTV----SKEFSKKKL--------KIWRENESNEQD----------
---------EYAFGKYGLFPAPMS--------------------------------DDQA
VTETGKKQLHLFKVLGKFVARSMLDSRIIDISFNP--TFFRIGDS---------------
-----SSTVTPSLGTVKA----------------------VDHDLANS--------LLLL
KRFANAKTAIEQDNSLSATQK-----------------SQALQNVEVDG-VKVE-DLG--
--LDFTL------------------------------PG--YPAIE-LIKD--------
GSNIP---
------------------------------VTIENVD-LYVDR-VVDLTLGSG-VRRQVD
-AFRAG---FSQVFPY--SSLR-AFTPN-ELVMLFGR-----------------------
---VEEDWS------------XAL---------MDSIKADHG------------------
----------------FNMD----SKSV-RNLLQTMS--------------------EFT
PQQRRDFLQF-------VTGSPKLP------------IGG-----FK-SLTPMF--TVVC
RPSEPPYTSD------------------------DYLPSVMTCV-NY-LKLPDY-SSVEV
LRERLL-VAIQE-GQGA
>Xeromyces_bisporus_CCCX01000073.1 .
VRISRARI-LESAMKVM----------------ELYGSSPS-ILEVEYFE-------EVG
TGLG-PTLEF---YSTV----SREFSKKKL--------KIWRENDYGHNN----------
---------EYAFGQHGLFPAPMS--------------------------------DSQA
TQESGKKQLQLFKVLGKFVARSMLDSRIIDISFNP--TFFRITDT---------------
-----SSAVTPSLGTVKT----------------------VDQGLANS--------LLLL
QRFTDAKKAIEHDKALSTAMK-----------------SRALRNIEVDG-VKVE-DLS--
--LDFTL------------------------------PG--YPAIE-LIQN--------
GANIP---
------------------------------VTIENVD-LYVDR-VVDMTLGSG-VRRQVD
-AFRKG---FSQVFPY--KALR-AFTPN-ELVMLFGR-----------------------
---AEEDWT-----------IESL---------MDSIKADHG------------------

----------------FNMD----SRSV-RNLLQTMS--------------------ELN
VQQRRDFLQF-------ITGSPKLP------------IGG-----FK-NLTPIF--TVVC
RPSDPPYTPD------------------------DYLPSVMTCV-NY-LKLPDY-SSIDV
LRKRLS-VAMKE-GQGA
>Rasamsonia_emersonii_XM_013472257.1 .
VRISRSRI-LESAMKVM----------------ELYGSSPS-ILEVEYFE-------EVG
TGLG-PTLEF---YSTV----SKEFSKKKL--------KIWRENESNEDD----------
---------EYAFGKHGLFPAPMS--------------------------------DEQA
ASEAGKKQLQLFRTLGKFVARSMLDSRIIDINFNP--TFFRVGTT---------------
-------SVPPTLGTVRT----------------------VDQNLANS--------LLLL
KRFANAKKQISEDVSLSADEK-----------------VRAIQNIQVDG-VSVE-DLG--
--LDFTL-------------------------------PG--YPSIE-LIPN--------
GSNVA---
------------------------------VTIDNVD-VYVDR-VIDMTLGSG-VRRQIE
-AFRAG---FSQVFPY--SALR-TFTPN-ELVMLFGR-----------------------
---VEEDWS-----------IETL---------MDSIKADHG------------------
----------------FNMD----SKSV-RNLLQIMS--------------------ELN
HQQRRDFLQF-------VTGSPKLP------------IGG-----FK-SLTPMF--TVVC
RPSEPPYKSD------------------------DYLPSVMTCV-NY-LKLPDY-SSLEV
L----------------
>Byssochlamys_spectabilis_BAUL01000350.1 .
VRISRSRI-LESAMKVM----------------ELYGSSPS-VLEVEYFE-------EVG
TGLG-PTLEF---YSTV----SKEFSKKKL--------KIWRENESNDRD----------
---------EYAFGKLGLFPAPMS--------------------------------DEQA
NSEAGKKQLQLFKTLGKFVARSMLDSRIIDISFNP--TFFRIGDS---------------
-----ASTVTPSLGTVKA----------------------VDQDLANS--------LLLV
KRFANAKKVIDEDKTLSPAQK-----------------VQYLRQIEVDG-VKVE-DLA--
--LDFTL-------------------------------PG--YPAIE-LIKN--------
GANIP---
------------------------------VMIDNVD-LYLDR-VIDMTLGGG-VRRQVD
-AFRSG---FSQVFAY--SSLR-AFTPN-ELVMLFGR-----------------------
---IEEDWS-----------IESL---------MDSIKADHG------------------
----------------FNMD----SRSV-RNLLQTMS--------------------ELN
PQERRDFLQF-------VTGSPKLP------------IGG-----FK-SLTPMF--TVVC
RPSEPPYTSD------------------------DYLPSVMTCV-NY-LKLPDY-SSLDI
LRQRLS-VAIKE-GQGA
>Talaromyces_islandicus_CVMT01000008.1 .
VRISRSRI-LESAMKVM----------------ELYGSSPS-ILEVEYFE-------EVG
TGLG-PTLEF---YSTV----SKEFSKKKL--------KLWRGNESDSRN----------
---------DYVFGRHGLFPAPMS--------------------------------DRQA
ESESGKRQLQLFKALGKFVARSMLDSRIIDISFNP--TFFRVVSS---------------
-------SFTPSLGSVKT----------------------VDRDLANS--------LLLV
KRFVNAKKRVSEDLSLSTNQK-----------------SQAVQDIEVDG-VRVD-DLG--
--LDFTL-------------------------------PG--YPSIE-LISN--------
GSNIA---
------------------------------VTIENVD-TYLER-VIDLTLGSG-VRPQVE
-AFRSG---FSQVFPY--SALR-AFTPN-ELVMLFGR-----------------------
---VDEDWS-----------IESL---------MDSIKADHG------------------
----------------FNMD----SKSV-RNLLQTMS--------------------ELD
AQQRRDFLQF-------VTGSPKLP------------IGG-----FK-SLTPMF--TVVC
RPSEPPYTSD------------------------DYLPSVMTCV-NY-LKLPDY-SSLEV
LRERLI-VAIQE-GQGA
>Talaromyces_wortmannii_MJVA01000858.1 .
VRISRSRI-LESAMKVM----------------ELYGSSPS-ILEVEYFE-------EVG
TGLG-PTLEF---YSTV----SKEFSKKKL--------KLWRSNESDSRS----------
---------DYVFGKHGLFPAPMS--------------------------------DRQA
ESESGKRQLQLFKALGKFVARSMLDSRIIDISFNP--TFFRVVSP---------------
-------SFTPSLGSVKT----------------------VDRDLANS--------LLLV

KRFVNAKKRISEDVSLSLDQK-----------------SQAVEDIEIDG-VRVD-DLG--
--LDFTL-------------------------------PG--YPSIE-LISN--------
GSNIA---
------------------------------VTIENVD-MYLER-VIDLTLGSG-VRAQVE
-AFRSG---FSQVFPY--SALR-AFTPN-ELVMLFGR-----------------------
---VDEDWS-----------IESL---------MDSIKADHG------------------
-----------------FNMD----SKSV-RNLLQTMS--------------------ELD
SQQRRDFLQF-------VTGSPKLP------------IGG-----FK-SLTPMF--TVVC
RPSEPPYTSD------------------------DYLPSVMTCV-NY-LKLPDY-SSLEV
LRERLI-VAIQE-GQGA
>Talaromyces_piceae_JNNX01000003.1 .
VRISRSRI-LESAMKVM----------------ELYGSSPS-ILEVEYFE-------EVG
TGLG-PTLEF---YSTV----SKEFSKKRL--------KLWRVNETDDHS----------
---------DYVFGKHGLFPAPMS--------------------------------DQQA
ESEQGKRQLQLFKTLGKFVARSMLDSRIIDINFNS--TFFRVGHP---------------
-------TFTPSLGSVKT----------------------VDRDLANS--------LLLV
KRFVNAKKHISEDASLSPAQK-----------------HQAIQNIEIDG-VRVD-DLG--
--LDFTL-------------------------------PG--YPYIE-LVPN--------
GSNIS---
------------------------------LAIDNVD-LYLER-VIDFTLGSG-VRHQVE
-AFRSG---FSQVFPY--SALR-AFTPN-ELVMLFGR-----------------------
---VEEDWS-----------IESD---------QSRPWIQHG------------------

-------------------------------------G-----FK-SLTPMF--TVVC
RPSEPPYTSD------------------------DYLPSVMTCV-NY-LKLPDY-SSLQV
LRERLI-VAIQE-GQGA
>Talaromyces_atroroseus_XM_020265708.1 .
VRISRSRI-LESAMKVM----------------ELYGSSAS-ILEVEYFE-------EVG
TGLG-PTLEF---YSTV----SREFSKKKL--------KIWRENESSGTS----------
---------EYAFGKHGLFPSPMS--------------------------------EQQA
ESEMGKKQLHLFKSLGKFVARSMLDSRIIDINFNP--TFFRTGSS---------------
-------GFTPSLGSAKT----------------------VDQDLANS--------LLLV
KRFASAKKQIVDNISLSADQK-----------------LKAIQEIEIDG-VQVD-DLG--
--LDFTL-------------------------------PG--YPAIE-LVAN--------
GSNIN---
------------------------------LTIDNVD-LYLDR-VIDLTLGSG-VQRQIE
-AFRSG---FSQVFPY--SALR-AFTPN-ELVMLFGQ-----------------------
---IEEDWS-----------METL---------MDSIKADHG------------------
-----------------FNMD----SKGV-RNLLQTMS--------------------GLD
AQQRRDFLQF-------VTGSPKLP------------IGG-----FK-SLTPMF--TVVC
RPSEPPYTPD------------------------DYLPSVMTCV-NY-LKLPDY-SSPEA
LRERLL-VAIRE-GQGA
>Talaromyces_stipitatus_XM_002478911.1 .
VRISRSRI-LDSAMKVM----------------ELYGSSAS-ILEVEYFE-------EVG
TGLG-PTLEF---YSTV----SKEFSKKKL--------KIWRENESADKN----------
---------EYAFGKHGLFPAPMS--------------------------------VQQA
ESDPGKKQLHLFKSLGKFVARSMLDSRIIDINFNP--TFFRVGTN---------------
-------GFVPSLGAVKT----------------------VDESLANS--------LQLV
KRFAKVKSEITQDASLTAGQK-----------------IMAIEELEIDG-VRVE-DLG--
--LDFTL-------------------------------PG--YPTIE-LIEN--------
GSNIN---
------------------------------VDLDNVE-LYLDR-VIDFTLGKG-VERQIE
-AFRAG---FSQVFPY--SALR-AFTPN-ELVMLFGR-----------------------
---IEEDWS-----------METL---------MDSIKADHG------------------
-----------------FNMD----SKSV-RNLLQTMS--------------------ELD
PQQRRDFLQF-------VTGSPKLP------------IGG-----FK-SLTPMF--TVVC
RPSEPPYTPD------------------------DYLPSVMTCV-NY-LKLPDY-SSLEV
LRQRLL-IATKE-GQG-

```
>Penicillium_marneffei_XM_002146614.1 .
VRISRSRI-LDSAMKVM----------------ELYGSSAS-ILEVEYFE-------EVG
TGLG-PTLEF---YSTV----SKEFSKKKL--------KIWRENESVDKS----------
---------EYAFGKYGLFPAPMS---------------------------------DQQA
ESDLGKKQLLLFKSLGKFVARSMLDSRIIDINFNP--TFFRVGTP---------------
-------GFVPSLGAVKT----------------------VDRDLANS--------LQLV
KRFVDAKFEIEEDDDLTWDQK-----------------MMALEELEIDG-VRVE-DLG--
--LDFTL-------------------------------PG--YPAIE-LIKD--------
GSNTS-------------------------------------------------------
------------------------------VQTSNID-MYLDR-VIDFTLGEG-VERQIE
-AFRSG---FSQVFSY--LALR-SFTPN-ELVMLFGR----------------------
---IEEDWS-----------METL---------MDSIKADHG------------------
----------------FNMD----SKSV-RNLLQTMS--------------------ELD
PQQRRDFLQF-------VTGSPKLP------------IGG-----FK-SLTPMF--TVVC
RPSEPPYMPD------------------------DYLPSVMTCV-NY-LKLPDY-SSLEV
LRERLL-VATKE-GQGA
>Talaromyces_verruculosus_LHCL01000007.1 .
VRISRSRI-LDSAMKVM----------------ELYGSSAS-ILEVEYFE-------EVG
TGLG-PTLEF---YSTV----SKEFSKKKL--------KIWRENESVDKN----------
---------EYAFGKHGLFPAPMN---------------------------------EQQA
ESELGKKQLLLFKSLGKFVARSMLDSRIIDINFNP--TFFRVGTP---------------
-------GFVPSLGAVKT----------------------VDEDLANS--------LQLV
KRFADAKFEIEEDCHLTFDQK-----------------MMALEELEIDG-VRVE-DLG--
--LDFTL-------------------------------PG--YPAIE-LMKD--------
GSNIS-------------------------------------------------------
------------------------------VQTSNVD-MYLER-VIDFTLGEG-VERQIE
-AFRSG---FSQVFAY--AALR-SFTPN-ELVMLFGR----------------------
---IEEDWS-----------MESL---------MDSIKADHG------------------
----------------FNMD----SKSV-RNLLQTMS--------------------ELD
SQQRRDFLQF-------VTGSPKLP------------IGG-----FK-SLTPMF--TVVC
RPSEPPYTPD------------------------DYLPSVMTCV-NY-LKLPDY-SSLEV
LRERLL-VATKE-GQG-
>Talaromyces_pinophilus_LSFK01000010.1 .
VRISRSRI-LDSAMKVM----------------ELYGSSAS-ILEVEYFE-------EVG
TGLG-PTLEF---YSTV----SKEFSKKKL--------KIWRENESADKN----------
---------EYAFGKHGLFPAPMS---------------------------------EQQA
ESELGKKQLLLFKSLGKFVARSMLDSRIIDINFNP--TFFRVGTP---------------
-------GFVPSLGAVKT----------------------VDEDLANS--------LQLV
KRFADAKFEIEEDDHLTFDQK-----------------MMALEELEIDG-VRVE-DLG--
--LDFTL-------------------------------PG--YPAIE-LMKD--------
GSNIS-------------------------------------------------------
------------------------------VQTSNID-MYLER-VIDFTLGEG-VERQIE
-AFRSG---FSQVFAY--AALR-SFTPN-ELVMLFGR----------------------
---IEEDWS-----------MESL---------MDSIKADHG------------------
----------------FNMD----SKSV-RNLLQTMS--------------------ELD
PQQRRDFLQF-------VTGSPKLP------------IGG-----FK-SLTPMF--TVVC
RPSEPPYTPD------------------------DYLPSVMTCV-NY-LKLPDY-SSLEV
LRERLL-VATKE-GQGA
>Amauroascus_mutatus_LJPJ01000482.1 .
VRISRTRI-LDSAMKVM----------------ELYGSSSS-VLEVEYFE-------EVG
TGLG-PTLEF---YSTV----SKELCKKKL--------KLWREQDSNES-----------
---------DYIYSKLGLFPAPLG---------------------------------PEQL
IQDSGKKILSYFKGLGKFVARSMLDSRIIDIAFNP--IFFQIGNN---------------
-----FSTFKPSIGAVKA----------------------VDLDLAKS--------LMIV
KQFADAKYAIDCNQSLTAAEK-----------------EHALENCEVDG-IQIG-DLG--
--LDFTL-------------------------------PG--YPNID-LLPD--------
GSNTP-------------------------------------------------------
------------------------------VTMDNVH-LYVDN-VIDMTLGAG-VRPQID
```

-VFRSG---FSEVFAY--SALK-AFTPN-ELVMLFGQ-----------------------
---VEEDWS-----------IESL---------MDSIKADHG------------------
----------------FNMD----SRSV-RNLLETMS--------------------KFT
LQQRRDFLQF-------VTGSPKLP------------IGG-----FK-SLTPMF--TVVC
RPSEPPYTSD------------------------DYLPSVMTCV-NY-LKLPDY-SSPEI
LRKQLD-VAMHE-GQGA
>Amauroascus_niger_LJPK01000498.1 .
VRISRTRI-LDSAMKVM----------------ELYGSSPS-VLEVEYFE-------EVG
TGLG-PTLEF---YSTV----SKEFCKKKL--------KLWREQDSGDT-----------
---------EFVYSKPGLFPASLG--------------------------------PEQL
TQDSGKKTLNYFKGLGKFVARSMLDSRIIDVAFNP--TFFQIGNN---------------
-----FSTFKPSIGAVKA----------------------VDPELAKS--------IMIV
KQFADAKHAIESDPSLTVEGK-----------------AHALKNCEVEG-VHIG-DLG--
--LDFTL-------------------------------PG--YPNIH-LLPD--------
GANIP---
------------------------------VTMENVH-LYVDN-VIDMTLGAG-VRAQID
-AFRSG---FSQVFPY--SALK-AFTPN-ELVMLFGQ-----------------------
---VEEDWC-----------IESL---------MDSIKADHG------------------
----------------FNMD----SRSV-RNLLETMS--------------------KFT
LQQRRDFLQF-------VTGSPKLP------------IGG-----FK-SLTPMF--TVVC
RPSDPPYTSD------------------------DYLPSVMTCV-NY-LKLPDY-SSAEI
LRKQLD-VAMHE-GQGA
>Coccidioides_posadasii_XM_003065801.1 .
VRISRTRI-LDSAMKVM----------------ELYGSSPS-VLEVEYFE-------EVG
TGLG-PTLEF---YSTV----SKEFCKKKL--------KLWREQDSADG-----------
---------EYVYSKLGLFPAPLG--------------------------------PEQL
AQDSGKKILNYFKGLGKFVARSMLDSRIIDIAFNP--TFFQIGNN---------------
-----FSTFKPSVGAIKA----------------------VDPDLAKS--------LLMV
KHFADAKGAIESSLSHTSEEK-----------------EYALKTCEVDG-ARIG-DLG--
--LDFTL-------------------------------PG--YPNIH-LLPD--------
GANTP---
------------------------------VTIENVH-VYVEK-VIDVTLGTG-VRPQID
-AFRSG---FSQVFAY--SALK-SFTPN-ELVMLFGH-----------------------
---VEEDWS-----------IETL---------MDSIKADHG------------------
----------------FNMD----SRSV-RNLLETMS--------------------NFT
LQQRRDFLQF-------VTGSPKLP------------IGG-----FK-SLTPMF--TVVC
RPSDPPYTSD------------------------DYLPSVMTCV-NY-LKLPDY-SSAEV
LRKQLD-VAIHE-GQGA
>Coccidioides_immitis_XM_001247572.1 .
VRISRTRI-LDSAMKVM----------------ELYGSSPS-VLEVEYFE-------EVG
TGLG-PTLEF---YSTV----SKEFCKKKL--------KLWREQDSADG-----------
---------EYVYSKLGLFPAPLG--------------------------------PEQL
AQDSGKKILNYFKGLGKFVARSMLDSRIIDIAFNP--TFFQIGNN---------------
-----FSTFKPSVGAIKA----------------------VDPDLAKS--------LLMV
KQFADAKGAIESSLSHASEEK-----------------EHALKTCEVDG-ARIG-DLG--
--LDFTL-------------------------------PG--YPNIH-LLPD--------
GANTP---
------------------------------VTIENVH-VYVEK-VIDVTLGTG-VRPQID
-AFRSG---FSQVFAY--SALK-SFTPN-ELVMLFGH-----------------------
---VEEDWS-----------IETL---------MDSIKADHG------------------
----------------FNMD----SRSV-RNLLETMS--------------------NFT
LQQRRDFLQF-------VTGSPKLP------------IGG-----FK-SLTPMF--TVVC
RPSDPPYTSD------------------------DYLPSVMTCV-NY-LKLPDY-SSAEV
LRKQLD-VAMHE-GQGA
>Byssoonygena_ceratinophila_GDRB01005418.1 .
VRISRTRI-LDSALKVM----------------ELYGSSSS-ILEVEYFE-------EVG
TGLG-PTLEF---YSTV----SKEFCKKKL--------KIWREQESGDG-----------
---------EYVYSKLGLFPAPLS--------------------------------PEQS

TQDSGKKVLAYFKGLGKFVARSMLDSRIIDIAFNP--TFFQIGNN---------------
-----FSTFSPSIGAIKA----------------------VDPDLAKS--------LLTV
KQFADAKLTVQSSPSLTTEEK-----------------ERTLKNFEVDG-VLIG-DLG--
--LDFTL-------------------------------PG--FPHIE-LLPD--------
GSNVP---
------------------------------VTMDNVQ-LYVDN-VIDMTLGTG-IRAQID
-AFRSG---FSQVFLY--SALK-AFTPS-ELVMLFGH-----------------------
---VEEDWS-----------IETL---------MDSVKADHG------------------
----------------FNMD----SRSV-RNLLDTMS--------------------KFT
LQQRRDFLQF-------VTGSPKLP------------IGG-----FK-SLTPMF--TVVC
RPSEPPYTSD------------------------DYLPSVMTCV-NY-LKLPDY-SSTEI
LRKQLN-VAMHE-GQG-
>Chrysosporium_queenslandicum_GDRC01005858.1 .
VRISRTRI-LDSALKVM----------------ELYGSSPS-VLEVEYFE-------EVG
TGLG-PTLEF---YSTV----SKEFCKKKL--------KLWREQDSNNG-----------
---------EYVYNKLGLFPAPLG--------------------------------PEQL
TQDAGKKILNFFKGLGKFVARSMLDSRIIDIAFNP--TFFQIGNN---------------
-----FSTFNPSIGAIKA----------------------VDPDLAKS--------LLIV
KQFADAKVAVESDASLTAEEQ-----------------EVALENYEVDG-VQIE-DLG--
--LDFTL-------------------------------PG--YPTIH-LLPD--------
GPNIP---
------------------------------VTMQNVQ-LYVDS-VIDMTLGIG-VRSQID
-AFRSG---FSQVFPY--SALK-AFTPN-ELVMLFGH-----------------------
---VEEDWS-----------IETL---------MDSIKADHG------------------
----------------FNMD----SRSV-RNLLETMS--------------------KFT
LQQRRDFLQF-------VTGSPKLP------------IGG-----FK-SLTPMF--TVVC
RPSEPPYTSD------------------------DYLPSVMTCV-NY-LKLPDY-TSAEI
LQKQLD-VAMHE-GQG-
>Uncinocarpus_reesii_XM_002541708.1 .
VRISRTRI-LDSALKVM----------------ELYGSSPS-VLEVEYFE-------EVG
TGLG-PTLEF---YSTV----SKELCKKKL--------RLWREHDSSDG-----------
---------EYVYSKLGLFPAPLS--------------------------------PEQA
VQDSGKKVVNYFKGLGKFVARSMLDSRIIDIAFNP--TFFRIANN---------------
-----FSTFTPSIGAIKA----------------------VDPDLAKS--------LLVV
KQFANAKIAIDNDASLTPEEK-----------------EKTLRECEVGG-AHLR-DLG--
--LDFTL-------------------------------PG--YPHIQ-LLPD--------
GADIS---
------------------------------VTLENVQ-LYVDK-VIDMTLGIG-VRSQID
-AFRSG---FSQVFSY--SALK-AFTPN-ELVMLFGQ-----------------------
---VEEDWS-----------IETL---------MDSIKADHG------------------
----------------FNMD----SRSV-RNLLETMS--------------------KFT
LQQRRDFLQF-------VTGSPKLP------------IGG-----FK-SLTPMF--TVVC
RPSEPPYTSD------------------------DYLPSVMTCV-NY-LKLPDY-SSADI
LRKQLD-VAMHE-GQGA
>Paracoccidioides_lutzii_XM_015846430.1 .
VRISRSRI-LDSAMKVM----------------ELYGSSPS-VLEVEYFE-------EVG
TGLG-PTLEF---YSTV----SKEFSKKKL--------KLWREHDSNQLD----------
---------EYVFSRLGLFPAPMS--------------------------------REQI
SSEVGKKHLQYFRALGKFVARSMLDSRIIDIAFNP--TFFRLGMN---------------
-SASPSTTITPTIATIKA----------------------VDPDLAKS--------LMVV
KHFATAQTAINSEDSLSDEEK-----------------AVALEEFTIDG-LRVE-DLG--
--LDFTL-------------------------------PG--YPNIQ-LIPH--------
GSSIA---
------------------------------VTMDNVG-TYVDK-VIDMTLGSG-VQAQIE
-AFRIG---FSQVFPY--SSLQ-SFTPD-ELVMLFGQ-----------------------
---VEEDWS-----------IETL---------MDSMKADHG------------------
----------------FNMD----SRSV-RNLLQVMS--------------------EFT
KQERRDFLQF-------VTGSPKLP------------IGG-----FK-SLTPMF--TVVC

RPSDPPYLPD------------------------DYLPSVMTCV-NY-LKLPDY-SNIDI
LRKRLN-VAIHE-GQGA
>Paracoccidioides_brasiliensis_XM_010765698.1 .
VRISRSRI-LDSAMKVM----------------ELYGSSPS-VLEVEYFE-------EVG
TGLG-PTLEF---YSTV----SKEFSKKKL--------KLWREHDSNQLD----------
---------EYVFSRLGLFPAPMS--------------------------------REQI
SSEVGKKQLQYFRALGKFVARSMLDSRIIDIAFNP--TFFRLGMN---------------
-SASPSTTITPTIATIKA----------------------VDPDLAKS--------LMVV
KHFATAQTAINSEDALSDEEK-----------------AVALEEFTIDG-LRVE-DLG--
--LDFTL-------------------------------PG--YPNIQ-LIPH--------
GSSIA---
------------------------------VTMDNVG-TYVDK-VIDMTLGSG-VQAQIE
-AFRIG---FSQVFPY--SSLQ-SFTPD-ELVMLFGQ-----------------------
---VEEDWS-----------IETL---------MDSMKADHG------------------
----------------FNMD----SRSV-RNLLQVMS--------------------EFT
KQERRDFLQF-------VTGSPKLP------------IGG-----FK-SLTPMF--TVVC
RPSDPPYLPD------------------------DYLPSVMTCV-NY-LKLPDY-SNIDI
LRKRLN-VAIHE-GQGA
>Ajellomyces_capsulatus_XM_001538091.1 .
VRISRSRI-LDSAMKVM----------------ELYGSSPS-VLEVEYFD-------EVG
TGLG-PTLEF---YSTV----SREFSRKKL--------KLWREHNSHDKS----------
---------DFVFNKLGLFPAPMS--------------------------------REQV
SSEAGKKQLQYFKGLGKFVARSMLDSRIIDIAFNP--IFFRIGGV---------------
----TPSAVIPTVTTIKA----------------------VDPELANS--------LMLL
KRFTAAQAAINADDSLSADGK-----------------ARALDELTVDG-VRVE-DLS--
--LDFTL-------------------------------PG--YPSIQ-MIPH--------
GSSIA---
------------------------------VTMDNVG-SYISK-VIDMTLGSG-VQAQID
-AFRAG---FSQVFPY--SSLQ-SFTPD-ELVMLFGQ-----------------------
---VEEDWS-----------IETL---------MDSMKADHG------------------
----------------FNMD----SKSV-RNLLHVMS--------------------EFT
KQERRDFLQF-------VTGSPKLP------------IGG-----FK-SLTPMF--TVVC
RPSDPPYMPD------------------------DYLPSVMTCV-NY-LKLPDY-STIDI
LRQRLN-VAIHE-GQGA
>Emmonsia_parva_LDEV01001474.1 .
VRISRSRI-LDSAMKVM----------------ELYGSSPS-VLEVEYFE-------EVG
TGLG-PTLEF---YSTV----SKEFSKKKL--------KLWREHDSHDKN----------
---------DYVFSKLGLFPAPMS--------------------------------REQA
SSETGKKQLQYFKGLGKFVARSMLDSRIIDIAFNP--IFFRIGGA---------------
----TSSTVTPTIATIKA----------------------VDPELANS--------LTLV
KQFATAQASINADDALNAEEK-----------------ARALDEFTVDG-VRVE-DLG--
--LDFTL-------------------------------PG--YPSIQ-MIPH--------
GSSIA---
------------------------------VTMDNVG-SYVNK-VIDMTLGSG-VQAQLD
-AFRAG---FSQVFPY--SSLQ-SFTPD-ELVMLFGQ-----------------------
---VEEDWS-----------IESL---------MDSMKADHG------------------
----------------FNMD----SKSV-RNLLQVMS--------------------EFT
KQERRDFLQF-------VTGSPKLP------------IGG-----FK-SLTPMF--TVVC
RPSDPPYLPD------------------------DYLPSVMTCV-NY-LKLPDY-TNIDI
LRERLN-VAIHE-GQGA
>Blastomyces_percursus_LGTZ01000623.1 .
VRISRSRI-LDSAMKVM----------------ELYGSSPS-VLEVEYFE-------EVG
TGLG-PTLEF---YSTV----SKEFSKKKL--------KLWREHDSHDKN----------
---------DYVFSKLGLFPAPMS--------------------------------REQA
SSESGKKQLQYFKGLGKFVARSMLDSRIIDIAFNP--IFFRIGGA---------------
----TSSTVTPTIATVKA----------------------VDPELASS--------LTLV
KQFATAQASINADDSLSTEEK-----------------ARALDGITVDG-VRVE-DLG--
--LDFTL-------------------------------PG--YPSIQ-MIPH--------

GSSIA---
------------------------------VTMDNVG-SYVNK-VIDMTLGSG-VQAQLD
-AFRAG---FSQVFPY--SSLQ-SFTPD-ELVMLFGQ-----------------------
---VEEDWS-----------XAAL---------MDSMKADHG------------------
----------------FNMD----SKSV-RNLLQVMS--------------------EFT
KQERRDFLQF-------VTGSPKLP------------IGG-----FR-SLTPMF--TVVC
RPSDPPYLPD------------------------DYLPSVMTCV-NY-LKLPDY-TNIDI
LRERLN-VAMHE-GQGA
>Ajellomyces_dermatitidis_XM_002627337.1 .
VRISRSRI-LDSAMKVM----------------ELYGSSPS-VLEVEYFE-------EVG
TGLG-PTLEF---YSTV----SKEFSKKKL--------KLWREHDSNDKN----------
---------DYVFHKLGLFPAPMS--------------------------------REQA
SSEAGKKQLQYFKGLGKFVARSMLDSRIIDIAFNP--IFFRIGGA---------------
----TSSTVTPTIATVKA----------------------VDPELANS--------LTLV
KRFVTAQASINADDSLSAEEK-----------------ARALDELTVDG-VRVE-DLG--
--LDFTL-------------------------------PG--HPSIQ-MIPH--------
GSSIA---
------------------------------VTMDNVG-SYVSK-VIDMTLGSG-VQAQLD
-AFRAG---FSQVFPY--SSLQ-SFTPD-ELVMLFGQ-----------------------
---VEEDWS-----------IETL---------MDSMKADHG------------------
----------------FNMD----SKSV-RNLLQVMS--------------------EFT
KQERRDFLQF-------VTGSPKLP------------IGG-----FK-SLTPMF--TVVC
RPSDPPYLPD------------------------DYLPSVMTCV-NY-LKLPDY-TNIDI
LRERLN-VAMHE-GQGA
>Emmonsia_crescens_LCZI01000988.1 .
VRISRSRI-LDSAMKVM----------------ELYGSSPS-VLEVEYFE-------EVG
TGLG-PTLEF---YSTV----SREFSKKKL--------KLWREHDSHDQD----------
---------DYVFSKLGLFPAPMS--------------------------------REQV
SSESGKKQLQYFKGLGKFVARSMLDSRIIDIAFNP--IFFRIGGA---------------
----TPSTVTPTVATIKA----------------------VDPELANS--------LMLV
KRFATAQAAINADDSLSAEEK-----------------AHALDDLTIDG-VRVE-DLG--
--LDFTL-------------------------------PG--YPSIQ-MIPH--------
GSSIA---
------------------------------VTVDNVG-SYVDK-VIDMTLGSG-VQAQID
-AFRAG---FSQVFPY--SSLQ-SFTPD-ELVMLFGQ-----------------------
---IEEDWS------------XAL---------MDSMKADHG------------------
----------------FNMD----SRSV-RNLLQVMS--------------------EFT
KQERRDFLQF-------VTGSPKLP------------IGG-----FK-SLTPMF--TVVC
RPSDPPYLPD------------------------DYLPSVMTCV-NY-LKLPDY-SNINI
LRQRLN-VAIHE-GQGA
>Emergomyces_pasteuriana_LGRN01000013.1 .
VRISRSRI-LDSAMKVM----------------ELYGSSPS-VLEVEYFE-------EVG
TGLG-PTLEF---YSTV----SREFSKKKL--------KLWREHDSQDQH----------
---------DYVFSKLGLFPAPMS--------------------------------REQV
SSEAGKKQLQYFKGLGKFVARSMLDSRIIDIAFNP--IFFRIGGA---------------
----TPSTITPTIATVKA----------------------IDPELGNS--------LMLM
KRFATAQATINADDTLNAEEK-----------------TRALDELTVDG-VRVE-DLG--
--LDFTL-------------------------------PG--YPSIQ-MIPH--------
GSSIA---
------------------------------VTVDNVG-SYVDK-VIDMTLGSG-VQAQID
-AFRAG---FSQVFPY--SSLQ-SFTPD-ELVMLFGQ-----------------------
---VEEDWS-----------IESL---------MDSMKADHG------------------
----------------FNMD----SRSV-RNLLQVMS--------------------EFT
KQERRDFLQF-------VTGSPKLP------------IGG-----FK-SLTPMF--TVVC
RPSDPPYLPD------------------------DYLPSVMTCV-NY-LKLPDY-SNIDI
LRKRLN-VAIHE-GQGA
>Emergomyces_orientalis_MOWL01000067.1 .
VRISRSRI-LDSAMKVM----------------ELYGSSPS-VLEVEYFE-------EVG

```
TGLG-PTLEF---YSTV----SREFSKKKL--------KLWREHDSHDQG----------
---------DYVFSKLGLFPTPMS---------------------------------REQV
SSEAGKKQLQYFKGLGKFVARSMLDSRIIDIAFNP--IFFRIGGA---------------
----TPSTITPTVATIKA----------------------VDPELANS--------LMLV
KRFATAQATINADDSVNTEEK------------------ARKLDELTIDG-VRVE-DLG--
--LDFTL--------------------------------PG--YPSIQ-MIPH--------
GSSIA-------------------------------------------------------
------------------------------VTVDNVG-SYVDK-VIDMTLGSG-VQAQID
-AFRAG---FSQVFPY--SSLQ-SFTPD-ELVMLFGQ-----------------------
---VEEDWS-----------IESM---------LSSMKADHG------------------
----------------FNMD----SRSV-RNLLQVMS--------------------EFT
KQERRDFLQF-------VTGSPKLP------------IAG-----FK-SLTPMF--TVVC
RPSDPPYLPD------------------------DYLPSVMTCV-NY-LKLPDY-SNIDI
LRKRLN-VAIHE-GQGA
>Bipolaris_zeicola_XM_007720086.1 .
VRISRSRI-LESAMKVM-----------------QLYGHSAS-VLEVEYFE-------EVG
TGLG-PTLEF---YSTV----SREFSKKKL--------KLWRENESNDSD----------
---------EYAFGKRGLYPAPMS---------------------------------EEEA
NSENGEKRLELFKVLGKFVARSMLDSRIIDISFNP--TFFRISDG---------------
----SSTAVVPSLGAIKT----------------------VDEGLASS--------LLLL
KQFADAKKKVE-DSDLSPDEK------------------AVALQEIVIHD-CTVE-DLA--
--LDFTL--------------------------------PG--YDSIE-LIEN--------
GANTA-------------------------------------------------------
------------------------------VTIENVD-LYVDK-VIDFTLGSG-VERQAN
-AFREG---FTEVFPY--SALK-AFTPD-ELVMLFGR-----------------------
---TDEDWS-----------LETL---------VDSIKADHG------------------
----------------YNLD----SKSV-RNLLSTMS--------------------QFN
AQERRDFLQF-------ITGSPKLP------------IGG-----FK-ALTPMF--TVVC
KPSEPPFTSD------------------------DYLPSVMTCV-NY-LKMPDY-SSVEI
LREKLS-VAIRE-GQGA
>Bipolaris_victoriae_XM_014704843.1 .
VRISRSRI-LESAMKVM-----------------QLYGHSAS-VLEVEYFE-------EVG
TGLG-PTLEF---YSTV----SREFSKKKL--------KLWRENESNDSD----------
---------EYAFGKRGLYPAPMS---------------------------------EEEA
NSENGEKRLELFKVLGKFVARSMLDSRIIDISFNP--TFFRISDG---------------
----SSTAVVPSLGAIKT----------------------VDEGLASS--------LLLL
KQFADAKKKVE-DSDLSPEEK------------------AVALQEIVIHD-CTVE-DLA--
--LDFTL--------------------------------PG--YDSIE-LIEN--------
GANTA-------------------------------------------------------
------------------------------VTIENVD-LYVDK-VIDFTLGSG-VERQAN
-AFREG---FTEVFPY--SALK-AFTPD-ELVMLFGR-----------------------
---TDEDWS-----------LETL---------VDSIKADHG------------------
----------------YNLD----SKSV-RNLLSTMS--------------------QFN
AQERRDFLQF-------ITGSPKLP------------IGG-----FK-ALTPMF--TVVC
KPSEPPFTSD------------------------DYLPSVMTCV-NY-LKMPDY-SSVEI
LREKLS-VAIRE-GQGA
>Bipolaris_oryzae_XM_007691874.1 .
VRISRSRI-LESAMKVM-----------------QLYGHSAS-VLEVEYFE-------EVG
TGLG-PTLEF---YSTV----SREFSKKKL--------KLWRENESNDSD----------
---------EYAFGKRGLYPAPMS---------------------------------EEEA
NSENGEKRLELFKVLGKFVARSMLDSRIIDISFNP--TFFRISDG---------------
----SSTAVVPSLGAIKT----------------------VDEGLASS--------LLLL
KQFADAKKKVE-DSDLSPEEK------------------VVALQEIVIHD-CTVE-DLA--
--LDFTL--------------------------------PG--YDSIE-LIEN--------
GANTA-------------------------------------------------------
------------------------------VTIENVD-SYVDK-VIDFTLGSG-VERQAN
-AFREG---FTEVFPY--SALK-AFTPD-ELVMLFGR-----------------------
---TDEDWS-----------LETL---------VDSIKADHG------------------
```

-----------------YNLD----SKSV-RNLLSTMS--------------------QFN
AQERRDFLQF-------ITGSPKLP------------IGG-----FK-ALTPMF--TVVC
KPSEPPFTSD------------------------DYLPSVMTCV-NY-LKMPDY-SSVEI
LREKLS-VAIRE-GQGA
>Bipolaris_maydis_XM_014223865.1 .
VRISRSRI-LESAMKVM----------------QLYGHSAS-VLEVEYFE-------EVG
TGLG-PTLEF---YSTV----SREFSKKKL--------KLWRENESNDSD----------
---------EYAFGKRGLYPAPMS--------------------------------EEEA
NSENGEKRLELFKVLGKFVARSMLDSRIIDISFNP--TFFRINDG---------------
----SSTAVVPSLGAIKT----------------------VDEGLASS--------LLLL
KQFADAKKKVE-DSDLSPEEK-----------------AVALQEIAIHD-CTVE-DLA--
--LDFTL-------------------------------PG--YDSIE-LIDN--------
GANTA---
------------------------------VTIENVD-LYVDK-VIDFTLGSG-VERQAN
-AFREG---FTEVFPY--SALK-AFTPD-ELVMLFGR-----------------------
---TDEDWS-----------LETL---------VDSIKADHG------------------
-----------------YNLD----SKSV-RNLLFTMS--------------------QFN
AQERRDFLQF-------ITGSPKLP------------IGG-----FK-ALTPMF--TVVC
KPSEPPFTSD------------------------DYLPSVMTCV-NY-LKMPDY-SSVEI
LREKLS-VAIRE-GQGA
>Cochliobolus_sativus_XM_007701908.1 .
VRISRSRI-LESAMKVM----------------QLYGHSAS-VLEVEYFE-------EVG
TGLG-PTLEF---YSTV----SREFSKKKL--------KLWRENESNDSD----------
---------EYAFGKRGLYPAPMS--------------------------------EEEA
NSENGEKRLELFKVLGKFVARSMLDSRIIDISFNP--TFFRINDG---------------
----SSTAVVPSLGAIKT----------------------VDEGLASS--------LLLL
KQFADAKKKVD-DSDLSPEEK-----------------AVALQEIVIHD-CTVE-DLA--
--LDFTL-------------------------------PG--YHSIE-LIEN--------
GANTT---
------------------------------VTIENVD-LYVDK-VIDFTLGSG-VERQAN
-AFREG---FTEVFPY--SALK-AFTPD-ELVMLFGR-----------------------
---TDEDWS-----------LETL---------VDSIKADHG------------------
-----------------YNLD----SKSV-RNLLSTMS--------------------QFN
AQERRDFLQF-------ITGSPKLP------------IGG-----FK-ALTPMF--TVVC
KPSEPPFTSD------------------------DYLPSVMTCV-NY-LKMPDY-SSVEI
LREKLS-VAIRE-GQGA
>Setosphaeria_turcica_XM_008025968.1 .
VRISRSRI-LESAMKVM----------------QLYGHSAS-VLEVEYFE-------EVG
TGLG-PTLEF---YSTV----SREFSKKKL--------KLWRENESNDGD----------
---------EYAFGKRGLYPAPMS--------------------------------EEEA
NSENGEKRLELFKVLGKFVARSMLDSRIIDISFNP--TFFRISDG---------------
----SSAAVVPSLGAIKT----------------------VDEGLAKS--------LVLL
KQFADAKKNVE-DSDLSPEEK-----------------AAALEKIVIHD-CTVD-DLA--
--LDFTL-------------------------------PG--YDSIE-LIEN--------
GANTP---
------------------------------VTISNVD-RYVDK-VIDFTLGSG-VERQAN
-AFREG---FTEVFPY--SALK-AFTPD-ELVMLFGR-----------------------
---TDEDWT-----------LETL---------MDSIKADHG------------------
-----------------YNLD----SKSV-RNLLATMS--------------------EFN
AQERRDFLQF-------ITGSPKLP------------IGG-----FK-ALTPMF--TVVC
KPSEPPFTSD------------------------DYLPSVMTCV-NY-LKMPDY-SSVEV
LREKLS-VAIRE-GQGA
>Curvularia_lunata_JFHG01000587.1 .
VRISRSRI-LESAMKVM----------------QLYGHSAS-VLEVEYFE-------EVG
TGLG-PTLEF---YSTV----SREFSKKKL--------KLWRENESNDND----------
---------EYAFGKRGLYPAPMS--------------------------------EEEA
NSENGEKRLELFKVLGKFVARSMLDSRIIDISFNP--TFFRISDG---------------
----SSTAVMPSLGAIKT----------------------VDEGLAKS--------LLLL

KQFADAKKKVE-DSDMSPEEK-----------------AVALQEIVISD-CTVE-DLA--
--LDFTL-------------------------------PG--YDSIE-LIEN--------
GANTP---
------------------------------VTIDNVD-TYVDK-VIDFTLGSG-VERQAN
-AFREG---FTEVFPY--SALK-AFTPD-ELVMLFGR-----------------------
---TDEDWS-----------LEAL---------MDSIKADHG------------------
----------------YNLD----SKSV-RNLLATMS--------------------EFN
VQERRDFLQF-------ITGSPKLP------------IGG-----FK-ALTPMF--TVVC
KPSEPPYTSD------------------------DYLPSVMTCV-NY-LKMPDY-SSIEV
LREKLS-VAIRE-GQGA
>Curvularia_papendorfii_JXCC01000251.1 .
VRISRSRI-LESAMKVM----------------QLYGHSAS-VLEVEYFE-------EVG
TGLG-PTLEF---YSTV----SREFSKKKL--------KLWRENESNDND----------
---------EYAFGKRGLYPAPMS--------------------------------EEEA
NSENGEKRLELFKVLGKFVARSMLDSRIIDISFNP--TFFRISDG---------------
----SSTAVMPSLGAIKT----------------------VDEGLAKS--------LLLL
KQFADTKKKVE-DSDMSPEEK-----------------AVALQEIVISD-CTVE-DLA--
--LDFTL-------------------------------PG--YDSIE-LIEN--------
GANTP---
------------------------------VTIDNVD-TYVDK-VIDFTLGSG-VERQAN
-AFREG---FTEVFPY--SALK-AFTPD-ELVMLFGR-----------------------
---TDEDWS-----------LEAL---------MDSIKADHG------------------
----------------YNLD----SKSV-RNLLATMS--------------------EFN
VQERRDFLQF-------ITGSPKLP------------IGG-----FK-ALTPMF--TVVC
KPSEPPYTSD------------------------DYLPSVMTCV-NY-LKMPDY-SSIEV
LREKLS-VAIRE-GQGA
>Stemphylium_lycopersici_LGLR01000185.1 .
VRISRSRI-LESAMKVM----------------QLYGHSAS-VLEVEYFE-------EVG
TGLG-PTLEF---YSTV----SREFSKKKL--------KLWRENESNDDD----------
---------EYAFGKRGLYPAPMS--------------------------------TEEA
NSENGEKRLELFKVLGKFVARSMLDSRIIDVSFNP--TFFRIGDG---------------
----SSAAVVPSLGAIKT----------------------VDEGLAKS--------LVLL
KQFADAKNKVE-NSEMGPEEK-----------------AAALRGIVIQD-CTVD-DLA--
--LDFTL-------------------------------PG--YGSIE-LIEN--------
GADTP---
------------------------------VTIENVD-TYVDK-VIDFTLGSG-VQRQAN
-AFRDG---FTEVFPY--SALK-AFTPD-ELVMLFGR-----------------------
---TDEDWT-----------LESL---------MDSIKADHG------------------
----------------YNLD----SKSV-RNLLATMS--------------------EFD
AQERRDFLQF-------ITGSPKLP------------IGG-----FK-ALTPMF--TVVC
KPSEPPFTSD------------------------DYLPSVMTCV-NY-LKMPDY-SSVEI
LREKLS-VAIRE-GQGA
>Pyrenophora_teres_XM_003299277.1 .
VRISRSRI-LESAMKVM----------------QLYGHSPS-VLEVEYFE-------EVG
TGLG-PTLEF---YSTV----SREFSKKKL--------KLWRENESNDTD----------
---------EFAFGKRGLYPAPMS--------------------------------AEEA
STENGEKRLELFKVLGKFVARSMLDSRIIDVSFNP--TFFRIGDG---------------
----SSAAVVPSLGAIKA----------------------VDEGLAKS--------LLLL
KQFADAKKKID-VSNMSEAKK-----------------TAALQDIVIQD-CRVE-DLA--
--LDFTL-------------------------------PG--YGSIE-LIPK--------
GADTP---
------------------------------VSIDNVD-LYVDK-VIDFTLGSG-VQRQAN
-AFREG---FTEVFPY--SALK-AFTPD-ELVMLFGR-----------------------
---TDEDWS-----------LETL---------MDSIKADHG------------------
----------------YNLD----SKSV-RNLLATMS--------------------EFD
AQERRDFLQF-------ITGSPKLP------------IGG-----FK-ALTPMF--TVVC
KPSEPPYTSD------------------------DYLPSVMTCV-NY-LKMPDY-SSITV
LREKLS-VAIRE-GQGA

>Pyrenophora_tritici_repentis_XM_001936785.1 .
VRISRSRI-LESAMKVM----------------QLYGHSPS-VLEVEYFE-------EVG
TGLG-PTLEF---YSTV----SREFSKKKL--------KLWRENESNDTD----------
---------EFAFGKRGLYPAPMS--------------------------------AEEA
STENGEKRLELFKVLGKFVARSMLDSRIIDVSFNP--TFFRIGDG---------------
----SSAAVVPSLGAIKA----------------------VDEGLAKS--------LLLL
KQFADAKKKIE-ISNISEANK-----------------TAALQEIVIQD-CRVE-DLA--
--LDFTL-------------------------------PG--YGSIE-LIPK--------
GADTP---
------------------------------VSIDNVD-LYVDK-VIDFTLGSG-VQRQAN
-AFRDG---FTEVFPY--SALK-AFTPD-ELVMLFGR-----------------------
---TDEDWS-----------LETL---------MDSIKADHG------------------
----------------YNLD----SKSV-RNLLATMS--------------------EFD
AQERRDFLQF-------ITGSPKLP------------IGG-----FK-ALTPMF--TVVC
KPSEPPYTSD------------------------DYLPSVMTCV-NY-LKMPDY-SSITV
LREKLS-VAIRE-GQGA
>Pyrenophora_seminiperda_ATLS01000185.1 .
VRISRSRI-LESAMKVM----------------QLYGHSPS-VLEVEYFE-------EVG
TGLG-PTLEF---YSTV----SREFSKKKL--------KLWRENESNDSD----------
---------EYAFGKRGLYPAPMS--------------------------------AEEA
ATENGEKRLELFKVLGKFVARSMLDSRIIDVSFNP--TFFRISDG---------------
----GSAAVAPSLGAIKA----------------------VDEDLAKS--------LLLL
KQFADAKKKIE-ISNMSEAKK-----------------TAALQNIVIQD-CHVE-DLA--
--LDFTL-------------------------------PG--YDSIE-LIPK--------
GTDTL---
------------------------------VSIENVD-LYVEK-VIDFTLGSG-VQRQAN
-AFREG---FTEVFPY--SALK-AFTPD-ELVMLFGR-----------------------
---TDEDWS--------L--ESAL---------MDSIKADHG------------------
----------------YNLD----SKSV-RNLLATMS--------------------EFD
AQERRDFLQF-------ITGSPKLP------------IGG-----FK-ALTPMF--TVVC
KPSEPPYTSD------------------------DYLPSVMTCV-NY-LKMPDY-SSITV
LREKLS-VAIRE-GQGA
>Alternaria_brassicicola_ACIW01002902.1 .
VRISRSRI-LESAMKVM----------------QLYGHSAS-VLEVEYFE-------EVG
TGLG-PTLEF---YSTV----SREFSKKKL--------KLWRENESNDSD----------
---------EYAFGKRGLYPAPMS--------------------------------AAEA
NTENGEKRLELFKILGKFVARSMLDSRIIDVSFNP--TFFRISDG---------------
----TSAAVAPSLGAIKA----------------------VDEDLAKS--------LLLL
KQFADAKKKVD-NDNISATAK-----------------SAALQDIVIHD-CRVE-DLA--
--LDFTL-------------------------------PG--YGSIE-LIPN--------
GADTP---
------------------------------VTIENVD-MYVDK-VIDFTLGSG-VQRQAD
-AFRAG---FTEVFPY--SALK-AFTPD-ELVMLFGR-----------------------
---TDEDWS-----------LESL---------MDSIKADHG------------------
----------------YNLD----SKSV-RNLLATMS--------------------EFD
AQERRDFLQF-------ITGSPKLP------------LHG-----FK-ALTPMF--TVVC
KPSEPPFTSD------------------------DYLPSVMTCV-NY-LKMPDY-STVEI
LREKLS-VAIRE-GQGA
>Alternaria_consortialis_BCGG01000002.1 .
VRISRSRI-LESAMKVM----------------QLYGHSAS-VLEVEYFE-------EVG
TGLG-PTLEF---YSTV----SREFSKKKL--------KLWRENESNDSD----------
---------EYAFGKRGLYPAPMS--------------------------------AEEA
STENGEKRLELFKILGKFVARSMLDSRIIDVSFNP--TFFRISDG---------------
----TSAAVAPSLGAIKA----------------------VDEDLAKS--------LLLL
KQFADAKKKVD-NDSMSATAK-----------------AAALQDIVIQD-CRVE-DLA--
--LDFTL-------------------------------PG--YGSIE-LIAN--------
GADTP---
------------------------------VTIENVD-TYVEK-VIDFTLGSG-VQRQAD

-AFRAG---FTEVFPY--SALK-AFTPD-ELVMLFGR-----------------------
---TDEDWS-----------LESL---------MDSIKADHG-----------------
----------------YNLD----SKSV-RNLLATMS--------------------EFD
VQERRDFLQF-------ITGSPKLP------------IGG-----FK-ALTPMF--TVVC
KPSEPPFTSD------------------------DYLPSVMTCV-NY-LKMPDY-STVEI
LREKLS-VAIRE-GQGA
>Alternaria_arborescens_AIIC01000035.1 .
VRISRSRI-LESAMKVM----------------QLYGHSAS-VLEVEYFE-------EVG
TGLG-PTLEF---YSTV----SREFSKKKL--------KLWRENESNDSD----------
---------EYAFGKRGLYPAPMS---------------------------------AEEA
STENGEKRLELFKILGKFVARSMLDSRIIDVSFNP--TFFRISDG---------------
----TSAAVAPSLGAIKA----------------------VDEDLAKS--------LLLL
KQFADAKKKVD-NDNMSATAK-----------------SAALQDIVIQD-CRVE-DLA--
--LDFTL------------------------------PG--YDSIE-LIPN--------
GADTP---
------------------------------VTIENVD-TYVEK-VIDFTLGSG-VQRQAD
-AFRAG---FTEVFPY--SALK-AFTPD-ELVMLFGR-----------------------
---TDEDWS-----------LESL---------MDSIKADHG-----------------
----------------YNLD----SKSV-RNLLATMS--------------------EFD
AQERRDFLQF-------ITGSPKLP------------IGG-----FK-ALTPMF--TVVC
KPSEPPFTSD------------------------DYLPSVMTCV-NY-LKMPDY-STVEI
LREKLS-VAIRE-GQGA
>Alternaria_alternata_XM_018528584.1 .
VRISRSRI-LESAMKVM----------------QLYGHSAS-VLEVEYFE-------EVG
TGLG-PTLEF---YSTV----SREFSKKKL--------KLWRENESNDSD----------
---------EYAFGKRGLYPAPMS---------------------------------AEEA
STENGEKRLELFKILGKFVARSMLDSRIIDVSFNP--TFFRISDG---------------
----TSAAVAPSLGAIKA----------------------VDEDLAKS--------LLLL
KQFADAKKKVD-NDNMSATAK-----------------SAALQDIVIQD-CRVE-DLA--
--LDFTL------------------------------PG--YDSIE-LIPN--------
GADTP---
------------------------------VTIENVD-TYVEK-VIDFTLGSG-VQRQAD
-AFRAG---FTEVFPY--SALK-AFTPD-ELVMLFGR-----------------------
---TDEDWS-----------LETL---------MDSIKADHG-----------------
----------------YNLD----SKSV-RNLLATMS--------------------EFD
AQERRDFLQF-------ITGSPKLP------------IGG-----FK-ALTPMF--TVVC
KPSEPPFTSD------------------------DYLPSVMTCV-NY-LKMPDY-STVEI
LREKLS-VAIRE-GQGA
>Leptosphaeria_maculans_XM_003839790.1 .
VRISRARI-LESAMKVM----------------QLYGHSAS-VLEVEYFE-------EVG
TGLG-PTLEF---YSTV----SKEFSKKKL--------KLWRENESSSTD----------
---------EYAFGKRGLYPAPMS---------------------------------AEEA
NGENGEKRLELFKVLGKFVARSMLDSRIIDVSFNP--TFFRIGDG---------------
----SNTAVLPSLGAIKT----------------------VDNDLANS--------LVLL
KQFADAKNSVMEDNTMSAAQK-----------------DKALQSIVIQD-CRVE-DLA--
--LDFTL------------------------------PG--YSSIE-LIPN--------
GSDTA---
------------------------------VTIHNVD-MYVDK-VIDFTLGSG-VQRQAD
-AFRAG---FTEVFPY--SALK-AFTPD-ELVMLFGR-----------------------
---TDEDWS-----------LETL---------MDSIKADHG-----------------
----------------YNLD----SKSV-RNLLQTMS--------------------EFD
AQERRDFLQF-------ITGSPKLP------------IGG-----FK-ALTPMF--TVVC
KPSEPPYTSD------------------------DYLPSVMTCV-NY-LKMPDY-STVEI
LRQKLS-VAIRE-GQGA
>Pyrenochaeta_lycopersici_GAJI01023387.1 .
VRISRSRI-LESAMKVM----------------QLYGHSTS-VLEVEYFE-------EVG
TGLG-PTLEF---YSTV----SKEFSKKKL--------KLWRENDSNEND----------
---------EYAFGKGGLYPAPMS---------------------------------AEEA

```
NTENGEKKLELFKVLGKFVARSMLDSRIIDVSFNP--TFFRIGDG---------------
----SSTAVTPSLGAVLT----------------------VDQDLGKS--------LVLL
KQFADAKKQIDANTEMSAAEK-----------------ADAINNIVIQD-CRVE-DLA--
--LDFTL-------------------------------PG--YASIE-LIEQ--------
GADTP-------------------------------------------------------
------------------------------VTIENVG-LYVDK-VLDFTLGSG-VQRQAN
-AFHAG---FTEVFPY--SALK-AFTPD-ELVMLFGR-----------------------
---SDEDWS-----------LETL---------MDSIKADHG------------------
----------------YNLD----SKSV-RNLLQTMS--------------------EFD
MQQRRDFLQF-------ITGSPKLP------------IGG-----FK-SLTPMF--TVVC
KPSEPPYTSD------------------------DYLPSVMTCV-NY-LKMPDY-SDVDV
LREKLS-IAIKE-GQG-
>Pleosporales_sp._AJMS01005549.1 .
VRIARSRI-LESAMKVM----------------QLYGHSTS-MLEVEYFE-------EVG
TGLG-PTLEF---YATV----SREFSKKKL--------KLWRENESADDS----------
---------EYAFGKRGLFPAPMS--------------------------------AQEG
ASVNGERRAELFKTLGKFVARSMMDSRIIDVSFNP--TFFRLGES---------------
-------DATVTI---KS----------------------VDEGLAKS--------LKFL
KQFADAKEQVLADGTLSDSAK-----------------QEALQNIVIQD-SKVE-DLA--
--LDFTL-------------------------------PG--YSSIE-LMEN--------
GSDTA-------------------------------------------------------
------------------------------VTIDNVD-TYIDK-VTDLTLGSG-VQRQVD
-AFRAG---FSEVFPY--SALK-AFTPD-ELVMLFGR-----------------------
---TDEDWS-----------LESL---------MDSIKADHG------------------
----------------YNLD----SKSV-RNLLQTMS--------------------EFT
DAQRRDFLQF-------ITGSPKLP------------IGG-----FK-NLTPMF--TVVC
KPSEPPYTSD------------------------DYLPSVMTCV-NY-LKMPDY-STVDI
LREKLS-VAIKE-GQGA
>Paraphoma_sp._BCLK01000265.1 .
VRISRSRI-LESAMKVM----------------QLYGHSAS-VLEVEYFE-------EVG
TGLG-PTLEF---YSTV----SREFSKKKL--------KLWRENESTDDG----------
---------DYAFGKRGLFPAPMG--------------------------------AEEA
ASENGERRLELFKVLGKFVARSMMDSRIIDVSFNP--TFFRVGDS---------------
-----NTTVEPSLGAIKS----------------------VDQDLAKS--------LSLV
KQFADAKKLVDEDSSLTAADK-----------------IAALHNITVQD-CRVE-DLA--
--LDFTL-------------------------------PG--YSAIE-LIAN--------
GGETS-------------------------------------------------------
------------------------------VTIDNVD-TYVEK-VLDFTLGSG-VQRQVD
-AFRAG---FSEVFPY--SALK-AFTPD-ELVMLFGR-----------------------
---TDEDWS-----------LESTL--------MDSIKADHG------------------
----------------YNLD----SKSV-RNLLQTMS--------------------EFT
PQQRRDFLQF-------ITGSPKLP------------IGG-----FK-NLTPMF--TVVC
KPSEPPYTSD------------------------DYLPSVMTCV-NY-LKMPDY-STVDI
LRDKLS-VAIRE-GQGA
>Hymenoscyphus_laetus_LLCA01000059.1 .
VRISRSRI-LESAMKVM----------------QLYGSSTS-LLEVEYFE-------EVG
TGLG-PTLEF---YSTV----SREFSKKKL--------KLWRENESSE-G----------
---------EYAFGKRGLFPAPMS--------------------------------AAEA
ASVNGERRLELFKTLGKFVARSMMDSRIIDVSFNP--TFFRIGDS---------------
-----DTTVLPSLGAIKS----------------------VDEGLAKS--------LKLL
KQFADAKYLVDTNDSLTSVQK-----------------TQALKDIFVQD-CRVE-DLA--
--LDFTL-------------------------------PG--YDTIE-LIDN--------
GANTA-------------------------------------------------------
------------------------------VTINNVD-KYFEH-VLDFTLGSG-VQRQVD
-AFRVG---FSEVFPY--SALK-AFTPD-ELVMLFGR-----------------------
---TDEDWT------------XAL---------MDSIKADHG------------------
----------------YNLD----SKSV-RNLLQTMS--------------------EFK
DQQRRDFLQF-------ITGSPKLP------------IGG-----FK-NLTPMF--TVVC
```

KPSEPPYSSD------------------------DYLPSVMTCV-NY-LKMPDY-STVDI
LREKLS-VAIRE-GQGA
>Phaeosphaeria_nodorum_XM_001802599.1 .
VRISRSRI-LESAMKVM----------------QLYGSSTS-LLEVEYFE-------EVG
TGLG-PTLEF---YSTV----SREFSKKKL--------KLWRENESSE-G----------
---------EYAFGKRGLFPAPMS--------------------------------AAEA
ASINGERRLELFKTLGKFVARSMMDSRIIDVSFNP--TFFRIGDS---------------
-----DTTVLPSLGAIKS----------------------VDEGLAKS--------LNLL
KQFADAKYLVDTNDSLTSAEK-----------------TQALKDILVQD-CRVE-DLA--
--LDFTL-------------------------------PG--YDTIE-LIDN--------
GANTA---
------------------------------VTINNVD-KYFEH-VLDFTLGSG-VQRQVD
-AFRVG---FSEVFPY--SALK-AFTPD-ELVMLFGR-----------------------
---TDEDWT-----------LETL---------MDSIKADHG------------------
----------------YNLD----SKSV-RNLLQTMS--------------------EFK
DQQRRDFLQF-------ITGSPKLP------------IGG-----FK-NLTPMF--TVVC
KPSEPPYSSD------------------------DYLPSVMTCV-NY-LKMPDY-STVDI
LREKLS-VAIRE-GQGA
>Helminthosporium_solani_AWWW01001204.1 .
VRISRSRI-LESAMKVM----------------HLYGHSPS-ILEVEYFD-------EVG
TGLG-PTLEF---YSTV----SKEFSKKKL--------KLWRENESNPSD----------
---------EYAFGKRGLFPAPMS--------------------------------AEQA
ENENGKKVLELYRVLGKFAARSMLDSRIIDVSFNP--TFFRVGEG---------------
-----SAAVVPSLGAVKS----------------------VDHDLAKS--------LKLL
KRFADARKSIDENAELSPAQK-----------------TAARKSVVIQD-CRVE-DLG--
--LDFTL-------------------------------PG--YA-ID-LMEG--------
GSDTP---
------------------------------VTIENVD-KYADA-VVDLTLGSG-VRRQVD
-SFRAG---FSEVFPY--SALR-AFTPA-ELVMLFGR-----------------------
---VEEDWS-----------LESAL--------MDSIKADHG------------------
----------------YNLD----SKSV-RNLLQAMS--------------------EFS
ATQRRDFLQF-------ITGSPKLP------------ITG-----FK-ALTPMF--TVVC
KPSEPPFTSD------------------------DYLPSVMTCV-NY-LKMPDY-STMEI
LKEKLS-VAIQE-GQGA
>Paraphaeosphaeria_sporulosa_XM_018183348.1 .
VRISRARI-LESAMKVM----------------HLYGSSPS-VLEVEYFE-------EVG
TGLG-PTLEF---YSSV----SKEFSKKKL--------KLWRENESSGDD----------
---------EYAFGKRGLFPAPMS--------------------------------TAQA
GTENGTKILELFKTLGKFVARSMLDSRIIDVSFNP--TFFRVGDG---------------
-----TVAVAPSLGVIKS----------------------VDYDLAKS--------LKLL
KQFADAKKQIEGDDSLTAAEK-----------------ASALKDVVFQD-CHVE-DLG--
--LDFTL-------------------------------PG--YP-IE-LVED--------
GADKQ---
------------------------------VTIENVE-MYVEK-VLDLTLGSG-VQRQVE
-AFRAG---FSEVFPY--SALR-AFTPD-ELVMLFGR-----------------------
---VDEDWS-----------LETL---------MDSIKADHG------------------
----------------YNLD----SKSV-RNLLQTMS--------------------EFS
ATERRDFLQF-------ITGSPKLP------------IGG-----FK-SLTPMF--TVVC
KPSEPPYTSD------------------------DYLPSVMTCV-NY-LKMPDY-SSMQI
LREKLR-VAIQE-GQGA
>Corynespora_cassiicola_JAQF01000344.1 .
VRISRSRI-LESAMKVM----------------HLYGHSPS-VLEVEYFE-------EVG
TGLG-PTLEF---YSTV----SKEFSKKKL--------KLWRENESNEDD----------
---------EFAFGKRGLFPAPMS--------------------------------ADQA
DTENGKKILELFRVLGKFVARSMLDSRIIDVSFNP--TFFRIGEG---------------
-----SA-VTPSLGSVKS----------------------VDYDLAKS--------LKLL
KQFADAKKQIDEDGNLTPAQK-----------------VQAIQEIVIHD-SHVE-DLG--
--LDFTL-------------------------------PG--YP-IE-LMEN--------

GADTA---
------------------------------VTIDNVD-VYVNK-VLDSTLGSG-VQRQVD
-AFRAG---FSEVFPY--SALR-AFTPD-ELVMLFGR-----------------------
---TDEDWT-----------LEAL---------MDSIKADHG------------------
----------------FNLD----SKSV-RNLLQTMS--------------------EFS
GPERRDFLQF-------ITGSPKLP------------IGG-----FK-SLTPMF--TVVC
KPSEPPFTPD------------------------DYLPSVMTCV-NY-LKMPDY-SSIDV
LREKLG-VAIQE-GQGA
>Clohesyomyces_aquaticus_MCFA01000335.1 .
VRISRTRI-LESAVKVM----------------ELYGSSPS-VLEVEYFE-------EVG
TGLG-PTLEF---YSTV----SKEFSKKKL--------KLWRENESGDGD----------
---------EYAFGKRGLFPAPMS--------------------------------AEQA
DTENGKKVLHLFKMLGKFVARSMLDSRIIDVSFNP--TFFRIGDG---------------
-----AATVTPSLGSVKS----------------------VDNDLARA--------LKLL
KRFAKSKKQIDEDGSLTAAQK-----------------VQAIQDIVIDD-SRVE-DLG--
--LDFTL-------------------------------PG--YPSID-LVEK--------
GADKS---
------------------------------VTIDDVD-TYVEK-VLDYTLGTG-VQRQVD
-AFRAG---FSQVFPY--SALK-AFTPD-ELVMLFGR-----------------------
---VDEDWS-----------LESL---------MDSIKADHG------------------
----------------YNLD----SKSV-RNLLHAMS--------------------EFD
PTQRRDFLQF-------ITGSPKLP------------IGGNC---FS-CLTPMF--TVVC
KPSEPPFTSD------------------------DYLPSVMTCV-NY-LKMPDY-STMDI
LRNKLK-TAIRE-GQGA
>Beverwykella_pulmonaria_BCHH01000017.1 .
VRISRTRI-LESAVKVM----------------ELYGSSPS-VLEVEYFE-------EVG
TGLG-PTLEF---YSTV----SKEFSKKKL--------KLWRENESPNDD----------
---------EYAFGKRGLFPAPMS--------------------------------AEQA
ETENGKKVLHLFKMLGKFVARSMLDSRIIDVSFNP--TFFRIGDG---------------
-----SATVAPSLGSIRS----------------------VDHDLAKS--------LLLL
KQFADARKQIDEDCNLDAAQK-----------------VQAIQNIVIQG-SHVE-DLG--
--IDFTL-------------------------------PG--YP-ID-LVEK--------
GSEKP---
------------------------------VTIDNVE-AYVDK-VLDFTLGSG-VQRQVD
-AFRAG---FSQVFPY--TALK-AFTPD-ELVMLFGR-----------------------
---VDEDWS-----------LESL---------MDSIKADHG------------------
----------------YNLD----SKSV-RNLLQTMS--------------------ELS
ATQRRDFLQF-------ITGSPKLP------------IGG-----FK-SLTPMF--TVVC
KPSEPPYTSD------------------------DYLPSVMTCV-NY-LKMPDY-SSMDI
LREKLN-VAIQE-GQGA
>Rhytidhysteron_rufulum_AJFL01001386.1 .
VRISRTRI-LESALKVM----------------DLYGSSPS-VLEVEYFE-------EVG
TGLG-PTLEF---YSTV----SKEFSKKKL--------KLWRENESSERD----------
---------EYAFGKRGLFPAPMS--------------------------------EKQA
SQENGMKVLQLFTALGKFVARSMLDSRIIDVSFNP--TFFRIGDG---------------
-----SAAVTPSLGSVKS----------------------VDADLAKA--------LKLL
KKFANAKKKIDEDGTLSPAQK-----------------VKAIQDITIDS-AHVE-DLG--
--LDFTL-------------------------------PG--YPDIE-LIEN--------
GAETP---
------------------------------VTIDNVS-LYVDK-VLSFTLGSG-VQKQVD
-AFRAG---FSQVFPY--SALK-AFTPD-ELVMLFGR-----------------------
---VEEDWS-----------LEAL---------MDSIKADHG------------------
----------------YNLD----SKSV-RNLLQTMS--------------------ELS
PTERRDFLQF-------VTGSPKLP------------IGG-----FK-SLTPMF--TVVC
KPSEPPYTSD------------------------DYLPSVMTCV-NY-LKMPDY-SSMDI
LREKLN-VAIKE-GQGA
>Hysterium_pulicare_AJFK01000147.1 .
VRISRTRI-LESAVKVM----------------ELYGSSPS-VLEVEYFE-------EVG

TGLG-PTLEF---YSTV----SKEFSKKKL--------KLWRENESSDSD----------
---------EYAFGKRGLFPAPMS---------------------------------EEQA
KGENGKKVLHLFEMLGKFVARSMLDSRIIDVSFNP--TFFRIGDG---------------
-----SAAVTPSLGSVKS----------------------VDSDLARA--------LKLL
KKFANAKKMVDENGNLDPAQK-----------------VQAIQTIEIDS-AHVE-DLG--
--LDFTL--------------------------------PG--YPSIE-LVEH--------
GSETS---
------------------------------VTIDNVA-TYVDK-VLDYTLGAG-VQRQVD
-AFRAG---FSKVFPY--SALK-AFTPD-ELVMLFGR-----------------------
---VDEDWS-----------LESL---------MDSIKADHG------------------
----------------YNLD----SKSV-RNLLQTMS--------------------ELS
PTQRRDFLQF-------VTGSPKLP------------IGG-----FK-SLTPMF--TVVC
KPSEPPYTSD------------------------DYLPSVMTCV-NY-LKMPDY-SSMGV
LREKLN-VAIQE-GQGA
>Glonium_stellatum_LKAO01001677.1 .
VRISRMRI-LESAIKVM-----------------ELYGSSPS-VLEVEYFE-------EVG
TGLG-PTLEF---YSTV----SKEFSKKKL--------KLWRENESNDND----------
---------EYAFGKRGLFPAPMS---------------------------------EEQS
NSENGKRVLHLFKMLGKFVARSMLDSRIIDVSFNP--TFFRIGDG---------------
-----TAAVTPSLGAVKS----------------------VDSDLAKA--------LKLL
KQFANAKKKIDENGNLNPAQK-----------------VQAIQDIQIDG-AHVE-DLG--
--LDFTL--------------------------------PG--YPAIE-LIER--------
GADSS---
------------------------------VTIDNVS-LYIDK-VLDYTLGSG-VQRQVD
-AFRAG---FSQVFPY--SALK-AFTPD-ELVMLFGR-----------------------
---VEEDWS------------XAL---------MDSIKADHG------------------
----------------YNLD----SKSV-RNLLQTMS--------------------ELS
LPQRRDFLQF-------VTGSPKLP------------IGG-----FK-SLTPMF--TVVC
KPSEPPYTSD------------------------DYLPSVMTCV-NY-LKMPDY-SSMEI
LREKLN-VAIQE-GQGA
>Lepidopterella_palustris_LKAR01000880.1 .
VRISRSRI-LESAIKVM-----------------ELYGSSPS-VLEVEYFE-------EVG
TGLG-PTLEF---YSTV----SKEFSKKKL--------KLWRENESNDND----------
---------EYAFGKRGLFPAPMS---------------------------------EEQA
NSENGKRILQLFKMLGKFVARSMLDSRIIDVSFNP--TFFRIGDG---------------
-----TAAVTPSLGSVKS----------------------VDSNLAQA--------LKLL
KQFANAKKRIDENGKLSAAQK-----------------VQAIQNIEIDG-AHVE-DLG--
--LDFTL--------------------------------PG--YPSIE-LVDN--------
GADTP---
------------------------------LTIDNVG-VYVDK-VLDYTLGLG-VQKQVD
-AFRAG---FSQVFPY--TALK-AFTPD-ELVMLFGR-----------------------
---IEEDWS------------XAL---------MDSIKADHG------------------
----------------YNLD----SKSV-RNLLQTMS--------------------EFS
PTQRRDFLQF-------VTGSPKLP------------IGG-----FK-SLTPMF--TVVC
KPSEPPHTSD------------------------DYLPSVMTCV-NY-LKMPDY-STVEI
LREKLG-VAIQE-GQGA
>Epicoccum_nigrum_NCTX01000293.1 .
VRISRSRI-LESAMKVM-----------------QLYGHSPS-VLEVEYFE-------EVG
TGLG-PTLEF---YSTV----SREFSKKKL--------KIWRENESNSSD----------
---------EYAFGKRGLFPAPLS---------------------------------AQQA
TSEDGEKKLEMFKVLGKFVARSMLDSRIVDVSFNP--TLFRLSNS---------------
----SSTLITPSLATIKT----------------------VDEDLAKS--------LRLL
KKFADAKQKIDQDQNKTEAQR-----------------LDASRNIRFHD-VAVE-DLS--
--LDFTL--------------------------------PG--YPAIE-LIDN--------
GAHTA---
------------------------------VTIDNVG-LYVEK-VLDLTLGSG-VKRQIE
-AFSAG---FSEVFPY--TALR-AFTPD-ELVMLFGR-----------------------
---SDEDWS-----------LESK---------YTSIKADHG------------------

----------------YNLD----SKSV-RNLLTTMS--------------------QYT
PEERRDFLQF-------ITGSPKLP------------IGG-----FK-SLTPMF--TVVC
KPAESPYTSD------------------------DYLPSVMTCV-NY-LKMPDY-STLDV
LREKLN-VAVQE-GQGA
>Epicoccum_sorghinum_MIEO01000322.1 .
VRISRSRI-LESALKVM----------------QLYGHSPS-VLEVEYFE-------EVG
TGLG-PTLEF---YSTV----SRAFSQKKL--------KLWRENESSSTG----------
---------EYAFGKRGLFPAPIS--------------------------------AQQA
GSEDGEKKLEMFKVLGKFVARSMLDSRIIDMSFNP--TLFRLSDS---------------
----SSTVITPSLATIKT----------------------VDEDLAKS--------LQLL
KKFADAKQQIDQDRDRTEAQR-----------------VEASRNIRFHD-CTVE-DLA--
--LDFTL------------------------------PG--YPAIE-LIDN--------
GAHTS---
-----------------------------VTIENVG-LYIEK-VLDLTLGSG-VKRQIE
-AFSAG---FSEVFPY--SALR-AFTPD-ELVMLFGR-----------------------
---NDEDWS-----------LESL---------MDSIKADHG------------------
----------------YNLD----SKSV-RNLLTTMS--------------------QYT
PEERRDFLQF-------ITGSPKLP------------IGG-----FK-SLTPMF--TVVC
KPAEAPYTSD------------------------DYLPSVMTCV-NY-LKMPDY-STLDV
LREKLN-VAVKE-GQGA
>Ascochyta_rabiei_JYNV01000254.1 .
VRISRSRI-LESAMKVM----------------QLYGHSPS-VLEVEYFE-------EVG
TGLG-PTLEF---YSTV----SREFSKKKL--------KIWRENDSNGTD----------
---------EYAFGKRGLFPAPMS--------------------------------AQQA
ASEDGEKKLEMFKVLGKFVARSMLDSRIIDVSFNP--TLFRLSDS---------------
----SSTVITPSLATIRT----------------------VDEDLAKS--------LMLL
KKFADAKKQIDVDQSKTDAQK-----------------LDAAQSIRFQD-CAVD-DLA--
--LDFTL------------------------------PG--YPAIE-LIED--------
GAQTT---
-----------------------------VTIENVG-LYVEK-VLDFTLGSG-VKRQID
-AFSAG---FTEVFPY--SALR-AFTPD-ELVMLFGR-----------------------
---NDEDWS-----------KSAL---------MDSIKADHG------------------
----------------FNLD----SKSV-RNLLQTMS--------------------QYT
PEERREFLQF-------ITGSPKLP------------IGG-----FK-SLTPMF--TVVC
KPAEPPYTSD------------------------DYLPSVMTCV-NY-LKMPDY-STLDM
LREKLS-VAVKE-GQGA
>Stagonosporopsis_tanaceti_JUDZ01009347.1 .
VRISRSRI-LESAMKVM----------------QLYGHSPS-VLEVEYFE-------EVG
TGLG-PTLEF---YSTV----SREFSKKKL--------KIWRENDSNGTD----------
---------EYAFGKRGLFPAPMS--------------------------------AQQA
ASDDGEKKLEMFKVLGKFVARSMLDSRIIDVSFNP--TLFRLSDS---------------
----SSTAITPSLATIRT----------------------VDEDLAKS--------LRLL
KRFADAKRQIDLDQDKTEAQR-----------------AEAAQEIRFQD-CTVE-DLA--
--LDFTL------------------------------PG--YPSIE-LIEN--------
GAHTA---
-----------------------------VTIHNVG-LYVDN-VLDLTLGSG-VKRQID
-AFGAG---FSEVFPY--SALR-AFTPD-ELVMLFGR-----------------------
---NDEDWS------------XAL---------MDSIKADHG------------------
----------------YNLD----SKSV-RNLLTTMS--------------------QYT
PEERRVFLQF-------ITGSPKLP------------IGG-----FK-SLTPMF--TVVC
KPAEPPYNSD------------------------DYLPSVMTCV-NY-LKMPDY-STLDV
LREKLN-VAVNE-GQGA
>Phoma_herbarum_BCGR01000025.1 .
VRISRSRI-LESAMKVM----------------QLYGHSPS-VLEVEYFE-------EVG
TGLG-PTLEF---YSTV----SREFSKKKL--------KIWRENDSNGQD----------
---------EFAFGKRGLFPAPMS--------------------------------AQQA
TSEDGEKKLEMFKVLGKFVARSMLDSRIIDVSFNP--TLFRLSDS---------------
----SSTVITPSLATIKT----------------------VDEDLAKS--------LRLL

KKFADAKKQIDHDQNMTSAQR-----------------SEAAQGIRFQD-CAVE-DLA--
--LDFTL-------------------------------PG--YPAIE-LIEN--------
GSHTA---
------------------------------VTIDNVG-LYVEK-VLDLTLGSG-VKRQID
-AFSAG---FSEVFPY--SALR-AFTPD-ELVMLFGR-----------------------
---NDEDWS-----------LESKL--------MDSIKADHG------------------
----------------YNLD----SKSV-RNLLTTMS--------------------QYT
PEERRDFLQF-------ITGSPKLP------------IGG-----FK-NLTPMF--TVVC
KPADPPYTSD------------------------DYLPSVMTCV-NY-LKMPDY-STLDV
LREKLN-VAVKE-GQGA
>Endocarpon_pusillum_XM_007804357.1 .
VRISRTRI-LESALKVM----------------EMYGGSPS-VLEVEYFE-------EVG
TGLG-PTLEF---YSTV----SKEFSKKKL--------KMWRENESNSHD----------
---------EYAFGQLGLFPVPMS--------------------------------QKQS
ESEAGKKLLNLFKMLGNFVARSMLDSRIIDVSFNP--TFFRLANG---------------
------HIKAPSIALVKT----------------------VDHDLANS--------LVQV
KKFVIAKGQVEEDLHMSAAQK-----------------AHALQDVCVQG-ARVD-DLM--
--LDFTL-------------------------------PG--YPSIE-LIPN--------
GSSTP---
------------------------------VTIDNVD-LYIER-VLDMTVGAG-VAKQIE
-AFKSG---FSQVFSY--SSLR-AFTPD-ELIMLFGR-----------------------
---VEEDWS-----------LETL---------MDSIKADHG------------------
----------------FNMD----SKSV-KNLLQTMS--------------------ELT
PTQRRDFLQF-------VTGSPKLP------------IGG-----FK-SLTPMF--TVVC
KPSEPPYSSD------------------------DYLPSVMTCV-NY-LKLPDY-TDLNV
LRNRLN-VAIQE-GQGA
>Phaeomoniella_chlamydospora_LCWF01000144. 1..
VRISRNRI-LESAVKVM----------------ELYGASPS-VLEVEYFE-------EVG
TGLG-PTLEF---YSAV----SKEFSKKKL--------KLWRENDSSDRD----------
---------EYAFGKLGLFPAPMS--------------------------------DDQA
TQESGKRSLHLFKMLGKFVARSMLDSRIIDVSFNP--IFFRLANE---------------
------KSLKASLTLVKS----------------------IDHDLANS--------LSQV
NQFAKAKASIEHDVHLSAIEK-----------------VQALHAITVHG-AHIE-DLM--
--LDFTL-------------------------------PG--YPAIE-LIPN--------
GSNIV---
------------------------------VTIDNVD-KYVER-VLDLTLGSG-VQKQIK
-AFQEG---FSLVFSY--DSLR-AFTPS-ELVMLFGR-----------------------
---VEEDWS-----------ETAL---------TDSIKADHG------------------
----------------FNMD----SKSV-KNLLQAMS--------------------ELD
RAQRRDFLQF-------VTGSPKLP------------IGG-----FK-SLTPMF--TVVC
KPSEAPYTSD------------------------DYLPSVMTCV-NY-LKLPDY-TNLEI
LRQRLY-TAIRE-GQGA
>Xylona_heveae_XM_018332920.1 .
VRISRTRI-LESAIKVM----------------ELYGASPS-ILEVEYFE-------EVG
TGLG-PTLEF---YSTV----SKEFSKKKL--------KLWRENESASRD----------
---------EYAFGKKGLFPAPMS--------------------------------EKQA
DSENGRRVLHLFKMLGKFVARSMLDSRIIDVSFNP--TFFRVGDG---------------
-----PVTVVPSLGAVKT----------------------VDDDLAKS--------LKLF
KQFMTEKRKIDEDQTFDPGQK-----------------ARAVANVRVHG-ARVE-DLE--
--LDFTL-------------------------------PG--YPSIE-LIPN--------
GSNVP---
------------------------------VTIENVG-QYVER-VIDLTLGSG-VQKQVK
-AFTTG---FSQVFPY--SALS-AFTPG-ELVMLFGR-----------------------
---VEEDWS-----------LETL---------TDSIKADHG------------------
----------------YNMD----SKSV-RNLLQAMS--------------------EMS
IPERRDFLQF-------VTGSPKLP------------IGG-----FK-SLTPMF--TVVC
KPSEPPYTSD------------------------DYLPSVMTCV-NY-LKLPDY-TTLDT
LRQKLN-VAVKE-GQGA

>Diplodia_scrobiculata_LAEG01002239.1 .
VRISRTKI-LESAIKVM----------------ELYGSSPS-VLEVEYFE-------EVG
TGLG-PTLEF---YSTV----SKEFSKKKL--------KLWRENESPEKD----------
---------EFAFGKRGLFPAPMS--------------------------------AEQA
ETEDGKKVLHLFKILGKFVSRSMLDSRIIDVSFNP--TFFRIGDN---------------
------AAVRPSLGAVKT----------------------VDADLARA--------LVLL
KKFAKEKKKIDEDPNMTPAQK-----------------VSAIEAIEVDG-AHVE-DLG--
--LDFTL-------------------------------PG--YPNIE-LVED--------
GANTS---
------------------------------VTIENVA-LYVEK-VLDITLGSG-VQKQVD
-AFRTG---FSQVFPY--SALR-AFTSD-ELVMLFGR-----------------------
---TDEDWT-----------LETL---------MDSIKADHG------------------
----------------YNLD----SKSV-RNLLQVMS--------------------ELT
AAQRRDFLQF-------VTGSPKLP------------IGG-----FK-SLTPMF--TVVC
KPSEPPYTSD------------------------DYLPSVMTCV-NY-LKMPDY-SSLDI
LKEKLF-VAIRE-GQGA
>Diplodia_sapinea_JHUM01000518.1 .
VRISRTKI-LESAIKVM----------------ELYGSSPS-VLEVEYFE-------EVG
TGLG-PTLEF---YSTV----SKEFSKKKL--------KLWRENESPEKD----------
---------EFAFGKRGLFPAPMS--------------------------------AEQA
ETEDGKKVLHLFKILGKFVSRSMLDSRIIDVSFNP--TFFRIGDN---------------
------AAVRPSLGAVKT----------------------VDADLARA--------LVLL
KKFAKEKKKIDEDPNMTPAQK-----------------VSAIEAIEVDG-AHVE-DLG--
--LDFTL-------------------------------PG--YPNIE-LVED--------
GANTS---
------------------------------VTIENVA-LYVEK-VLDITLGSG-VQKQVD
-AFRTG---FSQVFPY--SALR-AFTSD-ELVMLFGR-----------------------
---TDEDWT-----------LETL---------MDSIKADHG------------------
----------------YNLD----SKSV-RNLLQVMS--------------------ELT
AAQRRDFLQF-------VTGSPKLP------------IGG-----FK-SLTPMF--TVVC
KPSEPPYTSD------------------------DYLPSVMTCV-NY-LKMPDY-SSLDI
LKEKLF-VAIRE-GQGA
>Diplodia_seriata_MSZU01000080.1 .
VRISRTKI-LESAIKVM----------------ELYGSSPS-VLEVEYFE-------EVG
TGLG-PTLEF---YSTV----SKEFSKKKL--------KLWRENESPEKD----------
---------EFAFGKRGLFPAPMS--------------------------------AEQA
ETEDGKKVLHLFKILGKFVSRSMLDSRIIDVSFNP--TFFRIGDN---------------
------AAVRPSLGAVKT----------------------VDADLARA--------LVLL
KKFAKEKKKIDEDPNMTPAQK-----------------VSAIEAIEVDG-AHVE-DLG--
--LDFTL-------------------------------PG--YPNIE-LVED--------
GANTS---
------------------------------VTIENVA-LYVEK-VLDITLGSG-VQKQVD
-AFRTG---FSQVFPY--SALR-AFTSD-ELVMLFGR-----------------------
---TDEDWT-----------LETL---------MDSIKADHG------------------
----------------YNLD----SKSV-RNLLQVMS--------------------ELT
AAQRRDFLQF-------VTGSPKLP------------IGG-----FK-SLTPMF--TVVC
KPSEPPYTSD------------------------DYLPSVMTCV-NY-LKMPDY-SSLDV
LKEKLF-VAIRE-GQGA
>Diplodia_corticola_XM_020269456.1 .
VRISRTKI-LESAIKVM----------------ELYGSSPS-VLEVEYFE-------EVG
TGLG-PTLEF---YSTV----SKEFSKKKL--------KLWRENESPEKD----------
---------EFAFGKRGLFPAPIS--------------------------------AEQA
ETEDGKKVLHLFKILGKFVARSMLDSRIIDVSFNP--TFFRIGDN---------------
------TAVKPSLGAVKT----------------------VDADLARA--------LVLL
KKFAKEKKRIDEDPNMTPALK-----------------VSAIEAIEVDG-AHVE-DLG--
--LDFTL-------------------------------PG--YPNIE-LVQD--------
GANTS---
------------------------------VTIENVA-LYVEK-VLDITLGSG-VQKQVD

-AFRTG---FSQVFPY--SALR-AFTSD-ELVMLFGR-----------------------
---TDEDWT-----------LETL---------MDSIKADHG------------------
----------------YNLD----SKSV-RNLLQVMS--------------------ELT
ASQRRDFLQF-------VTGSPKLP------------IGG-----FK-SLTPMF--TVVC
KPSEPPYTSD------------------------DYLPSVMTCV-NY-LKMPDY-SSLDT
LKEKLF-VAIKE-GQGA
>Lasiodiplodia_theobromae_MDYX01000014.1 .
VRISRNKI-LESAVKVM----------------ELYGSSPS-VLEVEYFE-------EVG
TGLG-PTLEF---YSTV----SKEFSKKKL--------KLWRENESNEKD----------
---------EFAFGKRGLFPAPMS---------------------------------AEQA
ETEDGKKVLHLFKILGKFVARSMLDSRIIDVSFNP--TFFRIGDN---------------
------TAVKPSLGAVKT----------------------VDSDLARA--------LVLL
KKFAKEKKKIDEDPNMPPAEK-----------------VSAIEAIEVDG-AHVE-DLG--
--LDFTL-------------------------------PG--YPNIE-LVED--------
GANTS---
------------------------------VTIENVG-SYVDK-VLDITLGSG-VQKQVD
-AFRTG---FSQVFPY--SALR-AFTSD-ELVMLFGR-----------------------
---TEEDWT-----------LETL---------MDSIKADHG------------------
----------------YNLD----SKSV-RNLLQVMS--------------------ELD
ASQRRDFLQF-------VTGSPKLP------------IGG-----FK-ALTPMF--TVVC
KPSEPPYTSD------------------------DYLPSVMTCV-NY-LKMPDY-SSLDI
LKEKLF-VAIKE-GQGA
>Botryosphaeria_dothidea_MDSR01000108.1 .
VRISRTKI-LESAVKVM----------------ELYGSSPS-VLEVEYFE-------EVG
TGLG-PTLEF---YSTV----SKEFSKKKL--------KLWRENESNEKD----------
---------EFAFGKRGLFPAPMS---------------------------------TEQG
ETEDGKKVLHLFKILGKFVARSMLDSRIIDVSFNP--TFFRIGDN---------------
------AAVPPSLGAVKT----------------------VDSDLARA--------LLLL
KKFAKEKKAVDEDPNLTPAQK-----------------VSAIEAIEIDG-AHVE-DLG--
--LDFTL-------------------------------PG--YPGIE-LVPD--------
GANTS---
------------------------------VTIENVA-QYVEE-VLDMTLGGG-VQKQVD
-AFRAG---FSQVFPY--SALR-AFTSD-ELVMLFGR-----------------------
---TDEDWS-----------LETL---------MDSIKADHG------------------
----------------YNLD----SKSV-RNLLQVMS--------------------ELT
QSQRRDFLQF-------VTGSPKLP------------IGG-----FK-ALTPMF--TVVC
KPSEPPYTSD------------------------DYLPSVMTCV-NY-LKMPDY-SSLEV
LKEKLF-VAIKE-GQGA
>Macrophomina_phaseolina_AHHD01000424.1 .
VRISRNKI-LESAIKVM----------------ELYGSSPS-VLEVEYFE-------EVG
TGLG-PTLEF---YSTV----SKEFSKKKL--------KLWRENESNEND----------
---------EFAFGKRGLFPAPMS---------------------------------AEQA
ETEDGKKVLHLFRILGKFVARSMLDSRIIDVSFNP--TFFRVGDN---------------
------AAVPPSLGAVKT----------------------VDSDLAKA--------LVLL
KKFAKEKKTVDENPNLTPAEK-----------------VSAIEAIEIDG-AHVE-DLG--
--LDFTL-------------------------------PG--YPSIE-LIPE--------
GANTS---
------------------------------VTIDNVA-TYVQS-VLDMTLGSG-VQKQVD
-AFRAG---FSQVFPY--SALR-AFTSD-ELVMLFGR-----------------------
---TEEDWS-----------LETL---------MDSIKADHG------------------
----------------YNLD----SKSV-RNLLQVMS--------------------ELT
PSQRRDFLQF-------VTGSPKLP------------IGG-----FK-ALTPMF--TVVC
KPSEPPYTSD------------------------DYLPSVMTCV-NY-LKMPDY-SSLDV
LKEKLF-VAIKE-GQGA
>Neofusicoccum_parvum_XM_007588688.1 .
VRISRNKI-LESAIKVM----------------ELYGSSPS-VLEVEYFE-------EVG
TGLG-PTLEF---YSTV----SKEFSKKKL--------KLWRENESNEKD----------
---------DFAFGKRGLFPAPLG---------------------------------AEQA

DTEDGKKVLHLFKILGKFVARSMLDSRIIDVSFNP--TFFRVGDS---------------
------TAVPPSLGAVKT----------------------VDADLAKA--------LVLL
KKFSKEKKKIDENRNLTAAQK------------------VPAIEAITVDG-AHVE-DLG--
--LDFTL--------------------------------PG--YPAIE-LIPD--------
GVNTA---
------------------------------VTIDNVA-QYVEK-VLDMTLGSG-VQKQAE
-AFRTG---FTQVFPY--SALR-AFTSD-ELVMLFGR-----------------------
---TEEDWS-----------LETL---------MDSIKADHG------------------
----------------YNLD----SKSV-RNLLQVMS--------------------ELT
ASQRRDFLQF-------VTGSPKLP------------IGG-----FK-ALTPMF--TVVC
KPSEPPYTSD------------------------DYLPSVMTCV-NY-LKMPDY-STLDI
LKEKLF-VAIKE-GQGA
>Phyllosticta_citricarpa_LOEN01003997.1 .
VRISRNKI-LESAVKVM----------------ELYGSSPS-VLEVEYFE-------EVG
TGLG-PTLEF---YSTV----SKEFSKKKL--------KLWRENDSNEKD----------
---------EFAFGKGGLFPAPLS---------------------------------AESA
ESEDGKKVIHLFKILGKFVARSMLDSRIIDVSFNP--TFFRIGDK---------------
------AAVAPSLGAVKS----------------------VDADLAKA--------LLML
KKFANAKKRINEDPHMTPVQK------------------AHAVEEIQVDG-AHVE-DLG--
--LDFTL--------------------------------PG--YPSVE-LIDN--------
GADTS---
------------------------------VTIDNVG-QYVER-VIDMTLGGG-VQRQVE
-SFRAG---FSQVFPY--SALR-AFTSD-ELVMLFGR-----------------------
---NEEDWS-----------LETL---------MDSVKADHG------------------
----------------YTLD----SKSV-RNLLQVMS--------------------EFT
PSQRRDFLQF-------VTGSPKLP------------IGG-----FK-ALTPMF--TVVC
KPSEPPFTSD------------------------DYLPSVMTCV-NY-LKMPDY-SSLDM
LREKLF-VAIKE-GQGA
>Phyllosticta_capitalensis_LOEO01001092.1 .
VRISRNKI-LESAVKVM----------------ELYGSSPS-VLEVEYFE-------EVG
TGLG-PTLEF---YSTV----SKEFSKKKL--------KLWRENDSNDKD----------
---------EFAFGKGGLFPAPMS---------------------------------PESA
ETEDGKKVIVLFKILGKFVARSMLDSRIIDVSFNP--TFFRIGDK---------------
------AAVAPSLGAVRS----------------------VDSDLAKA--------LLML
KKFANAKKKIDEDPHMSPVQK------------------AHAVEEIQVDG-ARVE-DLG--
--LDFTL--------------------------------PG--YPSVE-LVEN--------
GADTS---
------------------------------VTIDNVA-QYVER-VIDMTLGSG-VQKQVE
-SFRAG---FSQVFPY--SALR-AFTSD-ELVMLFGR-----------------------
---NEEDWS-----------LETL---------MDSVKADHG------------------
----------------YTLD----SKSV-RNLLQVLS--------------------EFT
PSQRREFLQF-------VTGSPKLP------------IGG-----FK-ALTPMF--TVVC
KPSEPPYTSD------------------------DYLPSVMTCV-NY-LKMPDY-SSLEM
LREKLI-VAIKE-GQGA
>Cryomyces_antarcticus_AYQD01000717.1 .
VRLSRSRI-LESAIKVM----------------ELYGSSAS-VLEVEYFE-------EVG
TGLG-PTLEF---YSTV----SKEFSKKKT--------KLWRENESYDND----------
---------EYAFGKRGLFPAPMS---------------------------------EDQA
NDENGQRVLHLYKMLGKFVARSMLDSRIIDISFNP--TFFRIGDG---------------
-----SATVTPSLGAVGS----------------------VDHDLANS--------LKLL
KQFASAKRAIQEDGTLTAAQK------------------VQAASEIEIAG-AHVE-DLG--
--LDFTL--------------------------------PG--YPHIE-LQPN--------
GADIH---
------------------------------VTIDNVG-LYVQQ-VIEFTLGRG-VQKQVD
-AFRAG---FSQVFPY--SALK-AFTPD-ELVMLFGR-----------------------
---VDEDWS-----------LESL---------MDSIKADHG------------------
----------------YNLD----SKSV-RNLLQTMS--------------------ELD
APARRDFLQF-------VTGSPKLP------------IGG-----FK-SLTPMF--TVVC

KPSEAPFTSD------------------------DYLPSVMTCV-NY-LKMPDY-SSLEV
LRKRLH-VAINE-GQGA
>Coniosporium_apollinis_XM_007785784.1 .
VRISRNRI-LESAIKVM----------------ELYGSSPS-VLEVEYFE-------EVG
TGLG-PTLEF---YSTV----SKEFSKKRI--------KLWRENESDDKS----------
---------EFAFGKQGLFPAPMS--------------------------------EEQA
NSENGKRILQLFKVLGKFIARSMLDSRIVDVSFNP--TFFRVGDI---------------
------TAVAPSLGAVKS----------------------VDDGLAKS--------LKML
KQFATAKKKIDEDGRLSAAQK-----------------VQAAQNIKVHG-THVE-DLG--
--LDFTL-------------------------------PG--YPTIE-LVPN--------
GSNMA---
------------------------------VTIDNVA-LYLEK-VLDFTLGSG-VQRQVD
-AFRAG---FSAVFPY--SALK-AFTPD-ELVMLFGR-----------------------
---VEEDWS-----------LETL---------MDSIKADHG------------------
----------------YNLD----SKSV-RNLLQTMS--------------------ELS
PTRRRDFLQF-------VTGSPKLP------------IGG-----FK-SLTPMF--TVVC
KPSEPPLTSD------------------------DYLPSVMTCV-NY-LKMPDY-SSIEV
LGRKLQ-VAMSE-GQGA
>Cladonia_metacorallifera_AXCT02000171.1 .
VRISRARM-LESALKVM----------------ELYGGSAS-VLEVEYFD-------EVG
TGLG-PTLEF---YSTV----SKEFSKKKT--------KLWRENDANEND----------
---------EFAFGKSGMFPAPLT--------------------------------AEQA
ETEGGKRILHQFKMLGKFIARSMLDSRIIDVSLNP--TFFRIGDQ---------------
-----PSTVPLSLGAVKT----------------------VDSQLAIS--------LKLL
KQFANSKKDIER-KHLSVAQK-----------------AQAARKVMING-ACIE-DLG--
--LDFTL-------------------------------PG--YPSIE-LISN--------
GGNTP---
------------------------------VTLDNVG-TYVEK-VIDMTLGSG-VQRQVD
-HFRAG---FSEVFPY--PALK-AFTPS-ELVMLFGR-----------------------
---VEEDWS-----------IESL---------LDSIKADHG------------------
----------------FNMD----SKSV-RNLLQTMS--------------------ELS
PPQRRDFLQF-------VTGSPKLP------------IGG-----FK-SLNPMF--TVVC
KPSEPPYTSD------------------------DFLISVMTCA-NY-VKLPDY-SNIEI
LRKRLL-TAIQE-GQGA
>Umbilicaria_pustulata_JYIL01001411.1 .
VRISRTRI-LESAVKVM----------------ELYGASPS-VLEVEYFE-------EVG
TGLG-PTLEF---YSTV----SKEFSKKKI--------KLWRENESNEDD----------
---------EYAFGKLGLFPAPMS--------------------------------AEQA
DHENGKKILHLFKMLGKFVARSMLDSRIIDVSFNP--TFFRIGDG---------------
-----PTTVPPSLGAVNA----------------------VDSDLAKS--------LKLV
KQFASAKKEIDENGNLTAAQK-----------------VNAAYKIEVHG-VHVE-DLG--
--LDFTL-------------------------------PG--YSSIE-LVHN--------
GSNVP---
------------------------------VTIDNVG-LYVEK-VIDFTLGGG-VRRQVD
-AFRSG---FSQVFPY--SALR-AFTPN-ELVMLFGR-----------------------
---VDEDWA-----------IESL---------MDSIKADHG------------------
----------------FNMD----SKSV-RNLLQTMS--------------------ELI
PPQRRDFLQF-------VTGSPKLP------------IGG-----FK-SLTPMF--TVVC
KPSEPPYSSD------------------------DYLPSVMTCV-NY-LKLPDY-SNLDV
MRARLG-TAIRE-GQGA
>Umbilicaria_muehlenbergii_JFDN01000248.1 .
VRISRTRI-LESAVKVM----------------ELYGASPS-VLEVEYFE-------EVG
TGLG-PTLEF---YSTV----SKEFSKKKI--------KLWRENESNEDD----------
---------EYAFGKLGLFPAPMS--------------------------------AEQA
DHENGKKVLHLFKMLGKFVARSMLDSRIIDVSFNP--TFFRIGDG---------------
-----PATVPPSLGAVNA----------------------VDSDLAKS--------LKLV
KQFANAKKEIDENGKLTAAQK-----------------VNAAYKIEIHG-VHVE-DLG--
--LDFTL-------------------------------PG--YPSIE-LVHN--------

GSNVP---
------------------------------VTIDNVG-LYVEK-VVDFTLGGG-VRRQVD
-AFRSG---FSQVFPY--SALR-AFTPN-ELVMLFGR-----------------------
---VDEDWT-----------IESL---------MDSIKADHG------------------
----------------FNMD----SKSV-RNLLQTMS--------------------ELS
PPQRRDFLQF-------VTGSPKLP------------IGG-----FK-SLTPMF--TVVC
KPSEPPYTSD------------------------DYLPSVMTCV-NY-LKLPDY-SNLDV
MRARLG-TAIRE-GQGA
>Ochroconis_constricta_AZYM01000063.1 .
IRISRQKM-LESAMKVM----------------DLYGSSPS-ILEVEYFE-------EVG
TGLG-PTLEF---YSTV----SKEFAKKKL--------KLWRENESGENS----------
---------EYAFGKRGLFPAPMS--------------------------------EDMS
EGENGKRVLNMFTSMGKFVARSMLDSRIIDLNFNP--TFFRIGDT---------------
-----AGAVSPSLGAMQT----------------------VDSDLANS--------LKLL
KKFANRKKEIDEDGSLTAAQK-----------------VDAIQAIEISG-AHVE-DMA--
--LDFTL-------------------------------PG--YPSIE-LLPS--------
GSSTA---
------------------------------VTIDNVA-TYIDL-VLDFTLGTG-VQRQVD
-AFRAG---FSQVFPY--AALH-AFTPD-ELVMLFGR-----------------------
---VEEDWS-----------METL---------MDSVKADHG------------------
----------------FNLD----SRSV-RNLLGVMA--------------------EFG
AEERRDFLQF-------VTGSPKLP------------IGG-----FK-ALTPMF--TVVC
KPSEAPYTSD------------------------DYLPSVMTCV-NY-LKLPDY-SSMEV
MRRKLS-TAIRE-GQGA
>Verruconis_gallopava_XM_016358037.1 .
IRISRQKM-LESAMKVM----------------DLYGSSPS-ILEVEYFE-------EVG
TGLG-PTLEF---YSTV----SKEFAKKKL--------KLWRENESQEDS----------
---------EYAFGKRGLFPAPMS--------------------------------EEMA
ETENGKRIIHLFTSLGKFVARSMLDSRIIDLNFNP--TFFRIGDT---------------
-----PEAVSPSLGAINS----------------------VDSDLANS--------LKLL
KKFASAKKAIDENGGLTPARK-----------------VEAIQNIEISG-VHVE-DMA--
--LDFTL-------------------------------PG--YSSIE-LVPD--------
GSNIP---
------------------------------VTIDNVG-QYIDA-VLDFTLGSG-VRRQVD
-AFRAG---FSQVFPY--AALY-AFTPD-ELVMLFGR-----------------------
---TDEDWS-----------LETL---------MDSIKADHG------------------
----------------YNLD----SKSV-RNLLQIMS--------------------EFN
LTERRDFLQF-------VTGSPKLP------------IGG-----FK-ALTPMF--TVVC
KPSEAPFTSD------------------------DYLPSVMTCV-NY-LKLPDY-SSLDV
MRAKLA-IAIKE-GQGA
>Venturia_carpophila_MECS01000028.1 .
VRISRQKM-LESAMKVM----------------DIYGSSPS-ILEVEYFE-------EVG
TGLG-PTLEF---YSTV----SKEFAKKKL--------KLWRENESSDAS----------
---------EYAFGSRGLFPAPMS--------------------------------EEFA
NSDNGKRIVNMFTSLGKFVARSMLDSRIIDLNFNS--TFFRIGDT---------------
-----KGAVAPSLGAVRS----------------------VDNYLANS--------LKLL
KKFATAKKDIDENGSMTPAEK-----------------VQAIQDIQIDD-TSVE-DMG--
--LNFTL-------------------------------PG--YE-IE-LVPD--------
GGNVP---
------------------------------VTIDNVA-SYVEK-VLDFTLGAG-VRPQVE
-AFRAG---FSQVFPY--TALQ-AFTPD-ELVMLFGR-----------------------
---VDEDWT------------XAL---------MDSIKADHG------------------
----------------FNLD----SRSI-RNLLQVMS--------------------ELS
DQDRRDFLQF-------VSGSPKXX-------------XG-----FK-ALTPMF--TVVC
KPSEPPYTSD------------------------DYLPSVMTCV-NY-LKLPDY-SNVDV
MRAKIG-TAIRE-GQGA
>Venturia_pyrina_JEMP01000020.1 .
VRISRQKM-LESAMKVM----------------DIYGSSPS-ILEVEYFE-------EVG

TGLG-PTLEF---YSTV----SKEFAKKKL--------KLWRENESPDAS----------
---------EYAFGSRGLFPAPMS--------------------------------EEFA
KSDNGKRIVNMFTSLGKFVARSMLDSRIIDLNFNS--TFFRIGDT---------------
-----KGAVAPSLGAVRS----------------------VDNYLANS--------LKLL
KKFATAKKEIDENTSMSPAEK-----------------VQAIQDIKIDD-SNLE-DMG--
--LNFTL-------------------------------PG--YP-IE-LVAD--------
GTNVP---
------------------------------VTIDNVA-SYVEK-VLDFTLGVG-VRPQVD
-AFRAG---FSQVFPY--TALQ-AFTPD-ELVMLFGR-----------------------
---VEEDWT-----------MAAL---------MDSIKADHG------------------
----------------FNLD----SRSI-RNLLQVMS--------------------ELS
DQERRDFLQF-------VSGSPKLP------------IGG-----FK-ALTPMF--TVVC
KPSEPPYTSD------------------------DYLPSVMTCV-NY-LKLPDY-SDVAV
MRAKIG-TAIRE-GQGA
>Venturia_inaequalis_JRQC01000052.1 .
VRISRLKM-LESAMKVM----------------DIYGSSPS-ILEVEYFE-------EVG
TGLG-PTLEF---YSTV----SKEFAKKKL--------KLWRENESPDAS----------
---------EYAFGSRGLFPAPMS--------------------------------EEFA
KSDNGKRIINMFTSLGKFVARSMLDSRIIDLNFNS--TFFRIGDT---------------
-----KGAVAPSLGAVRS----------------------VDNYLANS--------LKLL
KKFATAKKEIDENGSMTPAEK-----------------VQAIQNIMIDD-TNVE-DMG--
--LNFTL-------------------------------PG--YP-IE-LVAD--------
GTNVP---
------------------------------VTIDNVA-SYVEK-VLDFTLGVG-VRPQVD
-AFRAG---FSQVFPY--TALQ-AFTPD-ELVMLFGR-----------------------
---VEEDWT------------XAL---------MDSIKADHG------------------
----------------FNLD----SRSI-RNLLQVMS--------------------ELS
DQERRDFLQF-------VSGSPKLP------------IGG-----FK-ALTPMF--TVVC
KPSEPPYTSD------------------------DYLPSVMTCV-NY-LKLPDY-SDVEV
MRAKIG-TAIRE-GQGA
>Tuber_melanosporum_XM_002839053.1 .
VRISRLRI-LESAIKVM----------------DLYGASPS-VLEVEYFE-------EVG
TGLG-PTLEF---YSSV----SRAFARKKI--------KLWRENESSPES----------
---------EFAFGQQGLFPAPMS--------------------------------DPMA
ESENGCKVLHLFTMLGKFVARSMLDSRIIDISFNP--TFFRTGEN---------------
----VDEAVAPSLGAVKT----------------------VDKDLASS--------LKLL
RKFALAKEEIDNNRVLTQGGK-----------------AKKAAQIMFDG-VHVE-ELG--
--LDFTL-------------------------------PG--YPHIE-LIPG--------
GSDVM---
------------------------------VTIDNVK-GYVDK-VIDLTLGSG-VRRQAE
-AFRSG---FSQVFPY--SALR-AFTPD-ELVMLFGR-----------------------
---NEEDWS-----------LETL---------MDSIKADHG------------------
----------------FNMD----SKSV-RNLLTAMS--------------------EFS
AQERRDFLQF-------VTGSPKLP------------IGG-----FK-SLTPLF--TVVC
KPSEPPYTSD------------------------DYLPSVMTCV-NY-LKLPDY-STFEI
LKKRLS-IAIQE-GQGA
>Symbiotaphrina_buchneri_BCIG01000028.1 .
VRISRSRI-LESAVKVM----------------ELYGASPS-ILEVEYFE-------EVG
TGLG-PTLEF---YSTV----SREFAKKKL--------RLWRENESSDSS----------
---------EFAFGKGGLFPAPIS--------------------------------PEQV
GSENGKKILHLFKMLGKFVARSMLDSRIIDVPFNP--TFFRIGDS---------------
------AAVPPSLGAVKV----------------------VDDDLARS--------LKLL
KQFAVAKRRIDEDDRLSAAEK-----------------VKRTNEIVIQG-VTVE-DLG--
--LDFTL-------------------------------PG--YPEID-LVPG--------
GSDTS---
------------------------------VTIDNVD-SYLER-VIDLTLGGG-VQKQVE
-AFRNG---FSQVFPY--SALR-AFTPD-ELVMLFGR-----------------------
---MEEDWS-----------LESL---------MDSIKADHG------------------

```
----------------FNMD----SRSV-KNLLQTMS--------------------ELS
VSERRDFLQF-------VTGSPKLP------------IGG-----FK-SLTPMF--TVVC
KPSEPPYTSD------------------------DYLPSVMTCV-NY-LKLPDY-STLEV
MKARLK-VAIRE-GQGA
>Blumeria_graminis_AOLT01005729.1 .
VRISRSKI-LESALKVM----------------ELYGASQS-ILEVEYFE-------EVG
TGLG-PTLEF---YSTV----SKEFSKKKL--------QLWRESDSNNTD----------
---------EFAFGVNGLFPAPMS--------------------------------EEQS
SNENGKRILHLFKMLGKFVARSMIDSRIIDVSFNP--TFFRIGDA---------------
-----FKTVSPSLGAVKA----------------------VDPQLSSS--------LKLI
KRFVMAKRAIDMNPVLTAIEK-----------------VDQSERLKIDG-ISID-DLS--
--LDFTL-------------------------------PG--YSSIE-LLPG--------
GSKVA-------------------------------------------------------
------------------------------VTIDKVE-QYLDK-VIDMTLGSG-VQRQID
-AFRAG---FTQVFPY--TALS-AFTPD-ELVMLFGR-----------------------
---AEEDWS-----------LESL---------MDSIKADHG------------------
----------------FNMD----SKSV-RNLLQTMS--------------------ELD
LPTRREFLQF-------TTGSPKLP------------IGG-----FK-SLTPMF--TVVR
KPSEAPYTSD------------------------DYLPSVMTCV-NY-LKLPDY-TNLEI
MRERMT-TAIRE-GQGA
>Rhynchosporium_agropyri_FJUX01000013.1 .
VRIARSKI-LESALKVM----------------ELYGASQS-ILEVEYFE-------EVG
TGLG-PTLEF---YSTV----SKEFSKKIL--------KLWRETESNDAD----------
---------EFAFGAHGLFPAPMS--------------------------------EEQA
SKENGKRILHLFKMLGKFVARSMIDSRIIDVSFNP--TFFRIGEQ---------------
-----STAVSPSLGAVKT----------------------VDPQLAKS--------LKLI
KKYALAKKAIDENPNLTPAQK-----------------VLHAEELTIDG-MRVD-DLG--
--LDFTL-------------------------------PG--YPTIE-MVPS--------
RRNET-------------------------------------------------------
------------------------------VTIDTVQ-LYLDK-VIDFTLGSG-VQRQVD
-AFRAG---FTQVFPY--SSLS-AFTPD-ELVMLFGR-----------------------
---IEEDWT-----------LESH---------QGRSRLQHG------------------
-------------------Q----EREL-ATDYERVD--------VATPRFPSVYHWSKA
PYWRAFPHIF-------LPCSSLIL------------IAG-----FK-SLTPMF--TVVC
KPSEPPYSSD------------------------DYLPSVMTCV-NY-LKLPDY-TDLEV
MRRRMG-TAIKE-GQGA
>Rhynchosporium_secalis_FJVC01000226.1 .
VRIARSKI-LESALKVM----------------ELYGASQS-ILEVEYFE-------EVG
TGLG-PTLEF---YSTV----SKEFSKKIL--------KLWRETESNDAD----------
---------EFAFGAHGLFPAPMS--------------------------------EEQA
SKENGKRILHLFKMLGKFVARSMIDSRIIDVSFNP--TFFRIGEQ---------------
-----STAVSPSLGAVKT----------------------VDPQLAKS--------LKLI
KKYALAKKAIDENPNLTPAQK-----------------VLHAEELTIDG-MRVD-DLG--
--LDFTL-------------------------------PG--YPTIE-MVPS--------
RRNET-------------------------------------------------------
------------------------------VTIDTVQ-LYLDK-VIDFTLGSG-VQRQVD
-AFRAG---FTQVFPY--SSLS-AFTPD-ELVMLFGR-----------------------
---IEEDWT-----------LESH---------QGRSRLQHG------------------
-------------------Q----EREL-ATDYERVD--------VATPRFPSVYHWSKA
PYRRAFPHIF-------LPCSSLIL------------IAG-----FK-SLTPMF--TVVC
KPSEPPYSSD------------------------DYLPSVMTCV-NY-LKLPDY-TDLEV
MRRRMG-TAIKE-GQGA
>Rhynchosporium_commune_FJUW01000012.1 .
VRIARSKI-LESALKVM----------------ELYGASQS-ILEVEYFE-------EVG
TGLG-PTLEF---YSTV----SKEFSKKIL--------KLWRETESNDAD----------
---------EFAFGAHGLFPAPMS--------------------------------EEQA
SKENGKRILHLFKMLGKFVARSMIDSRIIDVSFNP--TFFRIGEQ---------------
-----STAVSPSLGAVKT----------------------VDPQLAKS--------LKLI
```

```
KKYALAKKAIDENPNLTPAQK-----------------VLHAEELTIDG-MKID-DLG--
--LDFTL-------------------------------PG--YPTIE-MVPS--------
RRNET-------------------------------------------------------
------------------------------VTIDTVQ-LYLDK-VIDFTLGSG-VQRQVD
-AFRAG---FTQVFPY--SSLS-AFTPD-ELVMLFGR-----------------------
---IEEDWT-----------LESH---------QGRSRLQHG------------------
-------------------Q----EREL-ATDYERVD--------VATPRFPSVYHWSKA
PYWRAFPHIF-------LPCSSLIS------------IAG-----FK-SLTPMF--TVVC
KPSEPPYSSD------------------------DYLPSVMTCV-NY-LKLPDY-TDLEV
MRRRMG-TAIKE-GQGA
>Cadophora_malorum_FKJQ01000051.1 .
VRISRSKI-LESALKVM----------------ELYGASQS-ILEVEYFE-------EVG
TGLG-PTLEF---YSTV----SKEFSKKKL--------KLWRETEANDTD----------
---------EYAFGVRGLFPAPMS--------------------------------EEQA
SNENGKRILHLFKMLGKFVARSMIDSRIIDVSFNP--TFFRIGDE---------------
-----STTVSPSLGAVKT----------------------VDPQLAKS--------LKLI
KKYAVAKKAIDENPNLTPAQK-----------------VANAQELEIDG-MKID-DLG--
--LDFTL-------------------------------PG--Y-PIE-LIPN--------
GGRTT-------------------------------------------------------
------------------------------VDIDNVQ-LYLDK-VIDFTLGTG-VQRQVN
-AFRAG---FTQVFPY--SALS-AFTPD-ELVMLFGR-----------------------
---IEEDWS-----------LESKSFFPQ----LSSHINVLLSWIPSRLIM---------
----------------ASTW----TARA-RTYFKPVN-----RYQSVEISCSLPLVVPSF
QLAVSISHIAI------VRVKLLIS------------MLG-----FK-SLTPMF--TVVC
KPSEPPYSSD------------------------DYLPSVMTCV-NY-LKLPDY-TDLEV
MRRRMG-TAIKE-GQGA
>Hymenoscyphus_scutula_LKTO01000077.1 .
VRISRSKI-LESAVKVM----------------ELYGASQS-ILEVEYFE-------EVG
TGLG-PTLEF---YSTV----SKEFSKKKL--------KLWRETDANEAD----------
---------EYAFGARGLFPAPMS--------------------------------EDQA
SDENGKKILHLFKMLGKFVARSMIDSRIIDVSFNP--TFFRIGDG---------------
-----SNPVTPSLGAVKT----------------------VDAQLAKS--------LKLI
KKFAISKKAIDENPNLTAAQK-----------------VAEAETLRVDD-VSID-DLG--
--LDFTL-------------------------------PG--YSSIE-LLPN--------
GSTIS-------------------------------------------------------
------------------------------VTVDNVE-LYLEK-VIDYTLGTG-VQRQVD
-AFRAG---FTQVFPY--SALS-AFTPD-ELVMLFGR-----------------------
---IEEDWS-----------LESXNQSPW----LQHGQERQESSPNHEAFT---------
----------------LGTS---GLPSV---YHWPQA-----------------------
SYWRVKPFRF-------LQFYSL--------------AVG-----FK-SLTPMF--TVVC
KPSEPPYASD------------------------DYLPSVMTCV-NY-LKLPDY-SDLEV
MRRRMN-TAIKE-GQGA
>Hymenoscyphus_fraxineus_LLCC01000474.1 .
VRISRSKI-LESAVKVM----------------ELYGASQS-ILEVEYFE-------EVG
TGLG-PTLEF---YSTV----SKEFSKKKL--------KLWRETDANDAD----------
---------EYAFGARGLFPAPMS-------------------------------EEQAA
SEDIGKPILRLFKMLGKFVARSMIDSRIIDVSFNP--TFFRIGDG---------------
-----SNPVTPSLGAVKT----------------------VDAQLAKS--------LKLI
KKFAVAKKAIDENPNLTAAQK-----------------VAEAEALRIDD-VSID-DLG--
--LDFTL-------------------------------PG--YSSIE-LLPN--------
GSTLA-------------------------------------------------------
------------------------------VTIDNVE-LYLEK-VIDFTLGSG-VQRQVD
-AFRAG---FTQVFPY--SALS-AFTPD-ELVMLFGR-----------------------
---IEEDWS-----------LESIQSRPI----MDSIWIARASEISS-------------
----------------RPAS----CHPRNVGTSFNLP--------------------LVA
PSSLLEVSQF-------LRISLKIYSR----------HIG-----FK-SLTPMF--TVVC
KPSEPPYASD------------------------DYLPSVMTCV-NY-LKLPDY-SNLEV
MKRRMG-TAIKE-GQGA
```

>Erysiphe_pisiCACN01002001.1 .
VRISRSKI-LESAIKVM----------------ELYGASQS-ILEVEYFE-------EVG
TGLG-PTLEF---YSTV----SKEFSKKKL--------KLWRETDCNDSD----------
---------EFAFGARGLFPAPMS--------------------------------EEQA
TTENGKRILHLFKMLGKFVARSMIDSRIIDVSFNP--TFFRIGDA---------------
-----SKAVSPSLGAVKT----------------------VDPQLATS--------LKLI
KKFVTGKKAIDENPNLNPAQK-----------------VSQAEELKIDG-VSID-DLG--
--LDFTL-------------------------------PG--YSAIE-LLPN--------
GSQIS---
------------------------------VSIDKVE-EYLNK-VIDMTLGTG-VQRQIN
-AFRTG---FTQVFPY--SALS-AFTPD-ELVMLFGR-----------------------
---VEEDWS-----------LESIPLKLT----MDLTWIARARICY--------------
----------------KLVS----SITLHVANFYNLP--------------------LAA
LSCLLEVCYS------NLMYFLGMHQW----------SLG-----FK-SLTPMF--TVVR
KPSEPPYTSD------------------------DYLPSVMTCV-NY-LKLPDY-TNLEV
MRERMT-TAIRE-GQGA
>Erysiphe_necator_JNVN01001413.1 .
VRISRSKI-LESAIKVM----------------ELYGASQS-ILEVEYFE-------EVG
TGLG-PTLEF---YSTV----SKEFSKKKL--------KLWRETDCNNTD----------
---------EFAFGARGLFPAPMS--------------------------------EEQA
TTENGKRILHLFKMLGKFVARSMIDSRIIDVSFNP--TFFRIGDA---------------
-----SKAVSPSLGAVKT----------------------VDPQLAKS--------LKLI
KKFVTGKKAIDENPNLTPAQK-----------------VREAEQLRVDG-ARID-DLG--
--LDFTL-------------------------------PG--YSSIE-LLSN--------
GSQVS---
------------------------------VSIDRVE-EYLNK-VIDMTLGSG-VQRQIN
-AFRTG---FTQVFPY--SALS-AFTPD-ELVMLFGR-----------------------
---VEEDWS------------XAL---------MDSIKADHG------------------
----------------FNMD----SKSI-KNLLQTMS--------------------ELD
LTARREFLQF-------TTGSPKLP------------IGG-----FK-SLTPMF--TVVR
KPSEPPYTSD------------------------DYLPSVMTCV-NY-LKLPDY-TNLEV
MRERMT-TAIRE-GQGA
>Geotrichum_candidum_JMRO01000019.1 .
VRISRSKI-LESALKVM----------------ELYGASQS-ILEVEYFE-------EVG
TGLG-PTLEF---YSTV----SKEFCKKKL--------KLWRETDSNEND----------
---------EYAFGTRGLFPAPMS--------------------------------DEQA
SNENGKRILHLFKMLGKFVARSMIDSRIIDVSFNP--TFFRIGDQ---------------
-----TKPVNPSLGAVKT----------------------VDPQLAKS--------LKLI
KRFAIGKKDIDEDPHLSPAEK-----------------VARAEVLEVDG-VRID-DLG--
--LDFTL-------------------------------PG--YSSIE-LLPN--------
GSQIP---
------------------------------VTIDNVE-LYLEK-VIDMTLGKG-VQRQVE
-AFRTG---FTQVFPY--SALS-AFTPD-ELVMLFGR-----------------------
---VEEDWS-----------LESAL--------MDSIKADHG------------------
----------------FNMD----SKSV-KNLLQTMS--------------------ELS
MQERREFLQF-------TTGSPKLP------------IGG-----FK-ALTPMF--TVVC
KPSEPPYVSD------------------------DYLPSVMTCV-NY-LKLPDY-TSLEV
MRRRMF-TAIKE-GQGA
>Cairneyella_variabilis_AYLM01000311.1 .
VRISRSKI-LESALKVM----------------ELYGASQS-QLEVEYFD-------EVG
TGLG-PTLEF---YSTV----SKEFSKKKL--------KLWRESDGNDMD----------
---------EFAFGSRGLFPAPMS--------------------------------EEQA
SNENGKRILHLFKMLGKFAARSMIDERIVDVSFNP--TFFRINDD---------------
-----SKTVVPSLGAVNA----------------------VDPQLATS--------LKLI
KKFATEKKAIDENPNLTPAQK-----------------VAKAESLEIDG-SRID-DLG--
--LDFTL-------------------------------PG--YSSIE-LLPN--------
GSHIS---
------------------------------VTIENVD-LYLEK-VIDMTLGSG-VQRQVD

```
-AFRAG---FTQVFPY--SALS-AFTPD-ELVMMFGR-----------------------
---SEEDWS-----------LESL---------MDSIKADHG------------------
----------------FNMD----SKSV-KNLLQTMS--------------------ELS
LPERRDFLQF-------TTGSPKLP------------IGGG----FK-KLTPMF--TVVC
KPSEPPYTSD------------------------DYLPSVMTCV-NY-LKLPDY-TSLGV
MRQKLS-MAIKE-GQGA
>Calycina_herbarum_LLEY01000103.1 .
VRISRSKI-LESAVKVM----------------ELYGASQS-ILEVEYFE-------EVG
TGLG-PTLEF---YSTV----SKEFSKKKL--------KLWRETDANEAD----------
---------EYAFGARGLFPAPMS---------------------------------EDQA
SDENGKKILHLFKMLGKFVARSMIDSRIIDVSFNP--TFFRIGDG---------------
-----SNPVTPSLGAVKT----------------------VDAQLAKS--------LKLI
KRFAVSKKAIDENPNLTAAQK-----------------VSEAEALRVDD-VLID-DLG--
--LDFTL-------------------------------PG--YSSIE-LLPN--------
GSTIS-------------------------------------------------------
------------------------------VTIDNVE-LYLEK-VIDYTLGSG-VQRQVD
-AFRAG---FTQVFPY--SALS-AFTPD-ELVMLFGR-----------------------
---IEEDWS-----------LESKL--------YDSIKADHG------------------
----------------FNMD----SKSV-KNLLQTMS--------------------ELS
LSARRDFLQF-------TTGSPKLP------------IGG-----FK-SLTPMF--TVVC
KPSEPPYASD------------------------DYLPSVMTCV-NY-LKLPDY-SDLEV
MRRRMN-TAIKE-GQGA
>Hymenoscyphus_infarciens_LLCB01000108.1 .
VRISRSKI-LESAVKVM----------------ELYGASQS-ILEVEYFE-------EVG
TGLG-PTLEF---YSTV----SKEFSKKKL--------KLWRETDANEAD----------
---------EYAFGARGLFPAPMS---------------------------------EDQA
SDENGKKILHLFKMLGKFVARSMIDSRIIDVSFNP--TFFRIGDG---------------
-----SNPVTPSLGAVKT----------------------VDAQLAKS--------LKLI
KKFAVSKKAIDENPNLTAAQK-----------------VAEAEALRVDD-VSID-DLG--
--LDFTL-------------------------------PG--YSSIE-LLPN--------
GSTIS-------------------------------------------------------
------------------------------VTIDNVE-LYLEK-VINYTLGSG-VQRQVD
-AFRAG---FTQVFPY--SALS-AFTPD-ELVMLFGR-----------------------
---IEEDWX-------------AL---------MDSIKADHG------------------
----------------FNMD----SKSV-KNLLQTMS--------------------ELS
LSERRDFLQF-------TTGSPKLP------------IGG-----FK-SLTPMF--TVVC
KPSEPPYASD------------------------DYLPSVMTCV-NY-LKLPDY-SDLEV
MRRRMN-TAIKE-GQGA
>Hymenoscyphus_fructigenus_LKUV01000020.1 .
VRISRSKI-LESAVKVM----------------ELYGASQS-ILEVEYFE-------EVG
TGLG-PTLEF---YSTV----SKEFSKKKL--------KLWRETDANEAD----------
---------EYAFGARGLFPAPMS---------------------------------EDQA
SDENGKKILHLFKMLGKFVARSMIDSRIIDVSFNP--TFFRIGDG---------------
-----SNPVTPSLGAVKT----------------------VDAQLAKS--------LKLI
KKFAVSKKAIDENPNFTAAQK-----------------VAEAEALRVDD-VSID-DLG--
--LDFTL-------------------------------PG--YSSIE-LLPN--------
GSTIS-------------------------------------------------------
------------------------------VTIDNVE-LYLEK-VIDYTLGGG-VQRQVD
-AFRAG---FTQVFPY--SALS-AFTPD-ELVMLFGR-----------------------
---IEEDWS-----------LESKL--------YSSIKADHG------------------
----------------FNMD----SKSV-KNLLQTMS--------------------ELS
LSERRDFLQF-------TTGSPKLP------------IGG-----FK-SLTPMF--TVVC
KPSEPPYASD------------------------DYLPSVMTCV-NY-LKLPDY-SDLEV
MRRRMN-TAIKE-GQGA
>Hymenoscyphus_salicellus_LLCD01001710.1 .
VRISRSKI-LESAVKVM----------------ELYGASQS-ILEVEYFE-------EVG
TGLG-PTLEF---YSTV----SKEFSKKKL--------KLWRETDANEAD----------
---------EYAFGARGLFPSPMS---------------------------------EDQA
```

```
SDENGRKILHLFKMLGKFVARSMIDSRIIDVSFNP--TFFRIGDG---------------
-----SNPVTPSLGAVKT----------------------VDAQLAKS--------LKLI
KKFAVSKKAIDENPNLTAAQK-----------------VAEAEALRVDD-VSID-DLG--
--LDFTL-------------------------------PG--YSSIE-LLPN--------
GSTIS-------------------------------------------------------
------------------------------VTIDNVE-LYLEK-VIDCTLGTG-VQRQVD
-AFRAG---FTQVFPY--SALS-AFTPD-ELVMLFGR-----------------------
---IEEDWS-----------LESKL--------YSSIKADHG------------------
----------------FNMD----SKSV-KNLLQTMS--------------------ELS
LSERRDFLQF-------TTGSPKLP------------IGG-----FK-SLTPMF--TVVC
KPSEPPYASD------------------------DYLPSVMTCV-NY-LKLPDY-SDLEV
MRRRMN-TAIKE-GQGA
>Hymenoscyphus_repandus_LLCE01000077.1 .
VRISRSKI-LESALKVM----------------ELYGASQS-ILEVEYFE-------EVG
TGLG-PTLEF---YSTV----SKEFSKKKL--------KLWRETDANDAD----------
---------DYAFGMSGLFPAPMS--------------------------------EDQA
SNENGKRILHLFKMLGKFTARSMIDSRIIDVSFNS--TFFRIGDG---------------
-----SNPVTPSLGAVKS----------------------VDAQLAKS--------LKLI
KKFAVAKKTIDEDPNLTAAQK-----------------VATAEALQVDK-VSID-DLG--
--LDFTL-------------------------------PG--YSSIE-LLPN--------
GSTIA-------------------------------------------------------
------------------------------VTIDNVE-LYLEK-VIDMTLGSG-VQRQID
-AFRIG---FTQVFPY--SALS-AFTPD-ELVMLFGR-----------------------
---IEEDWT-----------LESL---------MDSIKADHG------------------
----------------FNMD----SKSV-KNLLQTMS--------------------ELS
LPERRDFLQF-------TTGSPKLP------------IGG-----FK-SLTPMF--TVVC
KPSEAPYASD------------------------DYLPSVMTCV-NY-LKLPDY-SDLDV
MRRRMN-TAIKE-GQGA
>Glarea_lozoyensis_XM_008087657.1 .
VRISRSKI-LESALKVM----------------ELYGASQS-ILEVEYFE-------EVG
TGLG-PTLEF---YSTV----SKEFSKKKL--------KLWRETDTNEND----------
---------EYAFGLSGLFPAPMS--------------------------------EDQA
LNENGKRILHLFKMLGKFTARSMIDSRIIDVSFNP--TFFRIGDG---------------
-----SNPVNPSLGAVKT----------------------VDAQLAKS--------LKLI
KKFAVAKKAIDENPKLSPAQK-----------------VDEAERLQIDK-VSID-DLG--
--LDFTL-------------------------------PG--YSTIE-LLPN--------
GANIA-------------------------------------------------------
------------------------------VTIENVE-LYLDK-VIDMTLGSG-VQRQVD
-AFRAG---FTQVFPF--SALS-AFTPD-ELVMLFGR-----------------------
---IEEDWS-----------LETL---------MDSIKADHG------------------
----------------FNMD----SKSV-KNLLQTMS--------------------ELS
LPERRDFLQF-------TTGSPKLP------------IGG-----FK-SLTPMF--TVVC
KPSEPPYTSD------------------------DYLPSVMTCV-NY-LKLPDY-TDLDV
MRRRMN-TAIKE-GQGA
>Amorphotheca_resinae_JZSE01000418.1 .
VRISRSKI-LESALKVM----------------ELYGASQS-ILEVEYFE-------EVG
TGLG-PTLEF---YSTV----SKEFSKKKL--------KLWRETDVNDND----------
---------EYAFGVRGLFPAPMS--------------------------------EEHA
SNENGKRILHLFKMLGKFVARSMIDSRIIDVSFNP--TFFRIGDG---------------
-----SKTVTPSLGAVKT----------------------VDPGLASS--------LKLI
KKFVVAKKAIDEDPNLSAAEK-----------------VAKAEALTIEN-CRVD-DLG--
--LDFTL-------------------------------PG--YPTIE-LLPN--------
GSQIA-------------------------------------------------------
------------------------------VTIDNVE-LYLEK-VIDMTLGSG-VQRQVD
-AFRAG---FTQVFPY--SALS-AFTPD-ELVMLFGR-----------------------
---VEEDWSX----------HAAL---------MDSIKADHG------------------
----------------FNMD----SKSV-KNLLQTMS--------------------ELT
LAERRDFLQF-------TTGSPKLP------------IGG-----FK-SLTPMF--TVVC
```

KPSEAPYTSD------------------------DYLPSVMTCV-NY-LKLPDY-SSLSV
MRRRMN-TAIKE-GQGA
>Oidiodendron_maius_JMDP01000076.1 .
VRISRSKI-LESALKVM----------------ELYGASQS-ILEVEYFE-------EVG
TGLG-PTLEF---YSTV----SKEFSKKKL--------KLWRETDSHDSD----------
---------EYAFGVRGLFPAPMN--------------------------------EEQA
TNENGKRILHLFKMLGKFVARSMIDSRIIDVSFNP--TFFRIGDK---------------
-----SKAVTPSLGAVKT----------------------VDPGLATS--------LKLI
KKFVIAKKSIDQNPGLTAVEK-----------------VAQAEALTVEN-CRID-DLG--
--LDFTL-------------------------------PG--YSAIE-LITD--------
GSQIS---
------------------------------VTIDNVE-LYLDK-VIDMTLGSG-VQRQVD
-AFRAG---FTQVFPY--SALS-AFTPD-ELVMLFGR-----------------------
---IEEDWS-----------LEAAL--------MDSIKADHG------------------
----------------FNMD----SKSV-KNLLQTMS--------------------ELT
LSERREFLQF-------TTGSPKLP------------IGG-----FR-TLTPMF--TVVC
KPSEAPYTSD------------------------DYLPSVMTCV-NY-LKLPDY-SSLDV
MRRRLT-TAIKE-GQGA
>Ascocoryne_sarcoides_AIAA01000184.1 .
VRISRSKI-LESALKVM----------------ELYGASQS-ILEVEYFE-------EVG
TGLG-PTLEF---YSTV----SKEFSKKKL--------KLWRETDANDAD----------
---------EYAFGVCGLFPSPMS--------------------------------EDQS
TNENGKRILHLFKMLGKFVARSMIDSRIIDVSFNP--TFFRLGDG---------------
-----SMTVTPSLGAVKT----------------------VDPQLATS--------LKLI
KKFVIAKKAVDENPNLTPAQK-----------------VAEAEALQVDG-VHID-DLG--
--LDFTL-------------------------------PG--YSSIE-LIAG--------
GAQTA---
------------------------------VTIENVD-RYLER-VIDMTLGSG-VQRQID
-AFRTG---FTQVFPY--TALS-AFTPD-ELVMLYGR-----------------------
---IEEDWS-----------LESL---------MDSIKADHG------------------
----------------FNMD----SKSV-KNLLQTMS--------------------ELT
LPERRDFLQF-------TTGSPKLP------------IGG-----FK-SLTPMF--TVVC
KPSEPPYSSD------------------------DYLPSVMTCV-NY-LKLPDY-TSLDV
MRRRMS-TAIKE-GQGA
>Phialocephala_subalpina_FJOG01000002.1 .
VRISRSKI-LESALKVM----------------ELYGASQS-ILEVEYFE-------EVG
TGLG-PTLEF---YSTV----SKEFSKKKL--------KLWRETDANDAD----------
---------EYAFGIRGLFPAPMS--------------------------------EEQA
SNENGKRILHLFKMLGKFVARSMIDSRIIDVSFNP--TFFRIGDG---------------
-----SNPVTPSLGAVKT----------------------VDPWLAKS--------LKLI
KKFAVGKKAIDENPNLTPAQK-----------------VAHAEALEIDN-VHID-DLG--
--LDFTL-------------------------------PG--YSSID-LLPN--------
GSQMS---
------------------------------VSIDNVE-LYLER-VIDMTLGSG-VQRQVD
-AFRAG---FTQVFPY--SALS-AFTPD-ELVMLFGR-----------------------
---IEEDWS-----------LESEL--------MDSIKADHG------------------
----------------FNMD----SRSV-KNLLQTMS--------------------ELT
LPERRDFLQF-------TTGSPKLP------------IGG-----FK-SLTPMF--TVVC
KPSEPPYTSD------------------------DYLPSVMTCV-NY-LKLPDY-TSLDV
MRRRMN-IAIKE-GQGA
>Phialocephala_scopiformis_XM_018213352.1 .
VRISRSKT-LESALKVM----------------ELYGASQS-ILEVEYFE-------EVG
TGLG-PTLEF---YSTV----SKEFSKKKL--------KLWRETDANDAD----------
---------EYAFGFRGLFPAPMS--------------------------------EEQA
SNENGKRILHLFKMLGKFVARSMIDSRIIDVSFNP--TFFRIGDG---------------
-----SKTVTPSLGAVKT----------------------VDPQLAKS--------LKLI
KKFATGKKSIDENPSLTPAQK-----------------VAHAEALEING-VHID-DLG--
--LDFTL-------------------------------PG--YSSIE-LLAN--------

GSQIA---
------------------------------VTIDNVE-LYLER-VIDYTLGVG-VQKQVD
-AFRAG---FTQVFPY--SALS-AFTPD-ELVMLFGR-----------------------
---IEEDWS-----------LETL---------MDSIKADHG------------------
----------------FNMD----SKSV-KNLLQTMS--------------------ELS
LPERRDFLQF-------TTGSPKLP------------IGG-----FK-SLTPMF--TVVC
KPSEPPYTSD------------------------DYLPSVMTCV-NY-LKLPDY-TSLDV
MRRRMS-TAIKE-GQGA
>Pseudogymnoascus_verrucosus_XM_018271463.1 .
VRISRSKI-LESALKVM----------------ELYGASQS-MLEVEYFD-------EVG
TGLG-PTLEF---YSTV----SKEFSKKKL--------KLWRETEGNDSD----------
---------EYAFGLRGLFPAPMS--------------------------------EEQA
QHENGKKILHLFKMLGKFVARSMIDSRIIDVSFNP--TFFRIGDE---------------
-----STTVTPSLGAVKT----------------------VDAQLAAS--------LKLI
KKFVHAKKAVDENGSLTAAEK-----------------VAAAENILVAG-VRID-ELG--
--LDFTM-------------------------------PG--YSTIE-LIHN--------
GSHTA---
------------------------------VTIDNVD-LYLEK-VIDMTLGSG-VQRQVD
-AFRTG---FTQVFPY--SALS-AFTPN-ELVMLFGR-----------------------
---VDEDWS-----------LETL---------MDSIKADHG------------------
----------------FNMD----SKSV-KNLLQTMS--------------------ELS
DTQRRDFLQF-------TTGSPKLP------------IGG-----FK-NLTPLF--TVVC
KPSEPPYTSD------------------------DYLPSVMTCV-NY-LKLPDY-TDLEV
MKRRMD-TAIKE-GQGA
>Pseudogymnoascus_destructans_XM_012884782.1 .
VRISRSKI-LESALKVM----------------ELYGASQS-MLEVEYFD-------EVG
TGLG-PTLEF---YSTV----SKEFSKKKL--------KLWRETEGNDSD----------
---------EYAFGLRGLFPAPMS--------------------------------EEQA
QHENGKKILHLFKMLGKFVARSMIDSRIIDVSFNP--TFFRIGDE---------------
-----STTVTPSLGAVKT----------------------VDAQLAAS--------LKLI
KKFVLAKKAVDENGSFTAAEK-----------------VAAAENILVAG-VRID-ELG--
--LDFTM-------------------------------PG--YSSIE-LIHN--------
GSHTA---
------------------------------VTIDNVD-LYLEK-VIDMTLGSG-VQRQVD
-AFRTG---FTQVFPY--SALS-AFTPN-ELVMLFGR-----------------------
---VDEDWS-----------LETL---------MDSIKADHG------------------
----------------FNMD----SKSV-KNLLQTMS--------------------ELS
ATQRRDFLQF-------TTGSPKLP------------IGG-----FK-NLTPLF--TVVC
KPSEPPYTSD------------------------DYLPSVMTCV-NY-LKLPDY-TDLEV
MKRRMD-TAIKE-GQGA
>Marssonina_brunnea_XM_007295221.1 .
VRISRSKI-LESALKVM----------------ELYGASQS-ILEVEYFD-------EVG
TGLG-PTLEF---YSTV----SKEFSKKKL--------KLWREMETSDSD----------
---------EYAFGTRGLFPAPMS--------------------------------EEQA
SNENGKRILHIFKMLGKFVARSMIDSRIIDVSFNP--TFFRIGDE---------------
-----SKTVPPSLGAVKT----------------------VDPQLAKS--------LKLI
KKYAVAKKAIDENPNLTPAQK-----------------VANAEALTIDG-MRID-DLG--
--LDFTL-------------------------------PG--Y-AID-LLPH--------
GSSIS---
------------------------------VTIDNVQ-LYLDR-VIDMTLGSG-VARQVD
-AFRTG---FTQVFPY--TALS-AFTPD-ELVMLFGR-----------------------
---IEEDWT-----------LETL---------MDSIKADHG------------------
----------------FNMD----SKSV-RNLLQTMS--------------------ELT
LPERRDFLQF-------TTGSPKLP------------IGG-----FK-SLTPMF--TVVC
KPSEPPYTSD------------------------DYLPSVMTCV-NY-LKLPDY-TDLEV
MRRRMT-TAIKE-GQG-
>Rutstroemia_echinophila_JWJA01006435.1 .
VRISRSKI-LESALKVM----------------ELYGASQS-ILEVEYFE-------EVG

TGLG-PTLEF---YSTV----SKEFSKKKL--------KLWRETDANETD----------
---------EYAFGARGLFPAPMS--------------------------------EDQA
SNENGKRIIHLFKMLGKFVARSMIDSRIIDVSFNP--TFFRIGDE---------------
-----SKPVTPSLGAVKT----------------------VDAQLAKS--------LKMI
KKFAVAKKAIAEDGTLTAAQK-----------------VAATEALEIDG-AHIE-DLS--
--LDFTL-------------------------------PG--Y-SID-LLPN--------
GSQIS---
------------------------------VTIDNVD-LYLEK-VVDMTLGSG-VQRQVD
-AFRTG---FTQVFPY--SALS-AFTPD-ELVMLFGR-----------------------
---IEEDWS-----------LESL---------MDSIKADHG------------------
----------------FNMD----SKSV-KNLLQTMS--------------------ELS
LSERRDFLQF-------TTGSPKLP------------IGG-----FK-SLTPIF--TVVC
KPSEPPYVSD------------------------DYLPSVMTCV-NY-LKLPDY-TDLEV
MRRRMS-TAIKE-GQGA
>Rutstroemia_sydowiana_JWJB01011171.1 .
VRISRSKI-LESALKVM----------------ELYGASQS-ILEVEYFE-------EVG
TGLG-PTLEF---YSTV----SKEFSKKKL--------KLWRETDANEAD----------
---------EYAFGARGLFPAPMS--------------------------------EEQA
SNENGKRILHLFKMLGKFVARSMIDSRIIDVSFNP--TFFRIGDE---------------
-----SKPVTPSLGAVKT----------------------VDSQLAQS--------LKLI
KKFAVAKKAIAEDGTLTAAHK-----------------VTATEALEIDG-AHIE-DLS--
--LDFTL-------------------------------PG--Y-SID-LLPN--------
GSQVS---
------------------------------VTIDNVD-LYLEK-VIDMTLGSG-VQRQVD
-AFRTG---FTQVFPY--SALS-AFTPD-ELVMLFGR-----------------------
---IEEDWS-----------LESL---------MDSIKADHG------------------
----------------FNMD----SKSV-KNLLQTMS--------------------ELS
LSERRDFLQF-------TTGSPKLP------------ITG-----FK-SLTPIF--TVVC
KPSEPPYASD------------------------DYLPSVMTCV-NY-LKLPDY-TDLEV
MRRRMN-TAIKE-GQGA
>Sclerotinia_homoeocarpa_LNKV01000010.1 .
VRISRSKI-LESALKVM----------------ELYGASQS-ILEVEYFE-------EVG
TGLG-PTLEF---YSTV----SKEFSKKKL--------KLWRETDANDAD----------
---------EYAFGTRGLFPAPMS--------------------------------EEQA
SNENGKRILHLFKMLGKFVARSMIDSRIIDVSFNP--TFFRIGDE---------------
-----SKPVTPSLGAVKT----------------------VDEQLAKS--------LKMI
KKFAVAKKAIVENGALTPAQK-----------------VAAIEALEIDG-ARIE-DLS--
--LDFTL-------------------------------PG--Y-PID-LLPN--------
GSHIA---
------------------------------VTIDNVD-LYLEK-VIDMTLGSG-VQRQVD
-AFRAG---FTQVFPY--SALS-AFTPD-ELVMLFGR-----------------------
---IEEDWS-----------LESX---------YRSIKADHG------------------
----------------FNMD----SKSV-KNLLQTMS--------------------ELS
LSERRDFLQF-------TTGSPKLP------------IGG-----FK-SLTPMF--TVVC
KPSEPPYTSD------------------------DYLPSVMTCV-NY-LKLPDY-SNLDV
MRRRMN-TAIKE-GQGA
>Botryotinia_fuckeliana_XM_001557027.1 .
VRISRSKI-LESALKVM----------------ELYGASQS-ILEVEYFE-------EVG
TGLG-PTLEF---YSSV----SKEFSKKKL--------KLWREND-NDTD----------
---------EYAFGVRGLFPAPMS--------------------------------EEQS
LNENGKRILHLFKMLGKFVSRSMIDSRIIDVSFNP--TFFRIGDE---------------
-----LTPVTPSLGTVKT----------------------VDPQLAKS--------LKMI
KKFSIAKKAIAEDSTLTATQK-----------------VLATEALKIDG-AKIE-DLS--
--LDFTL-------------------------------PG--Y-AID-LLPN--------
GSSIS---
------------------------------VTIDNVD-LYLEK-VIDMTLGSG-VQKQID
-AFREG---FTQVFPY--SALR-AFTPD-ELVMLFGR-----------------------
---VEEDWS-----------LETL---------TDSIKADHG------------------

----------------FHMD----SKSV-KNLLQTMS--------------------ELS
LPDRRDFLQF-------TTGSPKLP------------IGG-----FK-SLTPMF--TVVC
KPSEPPYSSD------------------------DYLPSVMTCV-NY-LKLPDY-TDLDV
MRRRMS-TAIRE-GQGA
>Botrytis_paeoniae_LBGX01000691.1 .
VRISRSKI-LESALKVM----------------ELYGASQS-ILEVEYFE-------EVG
TGLG-PTLEF---YSSV----SKEFSKKKL--------KLWREND-NDTD----------
---------EYAFGVRGLFPAPMS--------------------------------EEQS
LNENGKRILHLFKMLGKFVSRSMIDSRIIDVSFNP--TFFRIGDE---------------
-----SNPVTPSLGTVKT----------------------VDPQLAKS--------LKMI
KKFSVAKKAIAEDSSLTATQK-----------------VLATEALEIDG-AKIE-DLS--
--LDFTL-------------------------------PG--Y-AID-LLPD--------
GSSIS---
------------------------------VTIDNVD-LYLEK-VIDMTLGSG-VQKQID
-AFREG---FTQVFPY--SALR-AFTPD-ELVMLFGR-----------------------
---VEEDWS-----------LESM---------FSSLKADHG------------------
----------------FHMD----SKSV-KNLLQTMS--------------------ELS
LPDRRDFLQF-------TTGSPKLP------------IGG-----FK-SLTPMF--TVVC
KPSEPPYSSD------------------------DYLPSVMTCV-NY-LKLPDY-TDLDV
MRRRMS-TAIRE-GQGA
>Monilinia_fructicola_NGKE01000019.1 .
VRISRSKI-LESALKVM----------------ELYGASQS-ILEVEYFE-------EVG
TGLG-PTLEF---YSTV----SKEFSKKKL--------KLWRETDANDTD----------
---------EYAFGARGLFPAPMS--------------------------------EEQS
LNENGKRILHLFKMLGKFVSRSMIDSRIIDVSFNP--TFFRIGDA---------------
-----SNPVTPSLGAVKT----------------------VDPQLAKS--------LKMI
KKFSIAKKAIAEDSTLTAAQK-----------------VLATEALEIDG-AKIE-DLS--
--LDFTL-------------------------------PG--Y-AID-LLPN--------
GSNIP---
------------------------------VTIENVD-LYLEK-VIDMTLGSG-VQRQVD
-AFRAG---FTQVFPY--SALK-AFTPD-ELVMLFGR-----------------------
---VEEDWS-----------LESL---------MDSIKADHG------------------
----------------FHMD----SKSV-KNLLQTMS--------------------ELS
LPERRDFLQF-------TTGSPKLP------------IGG-----FK-SLTPMF--TVVC
KPSEPPYSSD------------------------DYLPSVMTCV-NY-LKLPDY-TDLDV
MRRRMN-TAIKE-GQGA
>Monilinia_aucupariae_NGKF01000026.1 .
VRISRSKI-LESALKVM----------------ELYGASQS-ILEVEYFE-------EVG
TGLG-PTLEF---YSTV----SKEFSKKKL--------KLWRETDANDAD----------
---------EYAFGARGLFPAPMS--------------------------------EEQS
LNENGKRILHLFKMLGKFVSRSMIDSRIIDVSFNP--TFFRIGDA---------------
-----SNPVTPSLGAVKT----------------------VDPQLAKS--------LKLI
KRFSVAKKAIAEDSTLTATQK-----------------VLATEALEIDG-AKIE-DLS--
--LDFTL-------------------------------PG--Y-PID-LLPG--------
GSNIP---
------------------------------VTIENVD-LYLEK-VIDMTLGSG-VQRQVD
-AFRAG---FTQVFPY--SALR-AFTPD-ELVMLFGR-----------------------
---IEEDWS-----------XTAL---------MDSIKADHG------------------
----------------FHMD----SKSV-KNLLQTMS--------------------ELS
LPERRDFLQF-------TTGSPKLP------------IGG-----FK-SLTPMF--TVVC
KPSEPPYSSD------------------------DYLPSVMTCV-NY-LKLPDY-TDLDV
MRRRMN-TAIKE-GQGA
>Sclerotinia_sclerotiorum_XM_001595958.1 .
VRISRSKI-LESALKVM----------------ELYGASQS-ILEVEYFE-------EVG
TGLG-PTLEF---YSTV----SKEFSKKKL--------KLWREND-NDAD----------
---------EYAFGARGLFPAPMS--------------------------------EEQS
LNENGKRILHLFKMLGKFVSRSMIDSRIIDVSFNP--TFFRIGDE---------------
-----SNPVTPSLGAVKT----------------------VDPQLAKS--------LKMI

KKFSVAKKAIAEDSTLTATQK-----------------VLATEALEIDG-AKIE-DLS--
--LDFTL-------------------------------PG--Y-DID-LLPN--------
GSSIP---
------------------------------VTIDNVD-LYLEK-VIDMTLGSG-VQRQVD
-AFRAG---FTQVFPY--SALR-AFTPD-ELVMLFGR-----------------------
---IEEDWS-----------LETL---------TDSIKADHG------------------
----------------FHMD----SKSV-KNLLQTMS--------------------ELT
LPERRDFLQF-------TTGSPKLP------------IGG-----FK-SLTPMF--TVVC
KPSEPPYSSD------------------------DYLPSVMTCV-NY-LKLPDY-TDLDV
MRRRMN-TAIRE-GQGA
>Sclerotium_cepivorum_NGKD01000085.1 .
VRISRSKI-LESALKVM----------------ELYGASQS-ILEVEYFE-------EVG
TGLG-PTLEF---YSTV----SKEFSKKKL--------KLWREND-NDTD----------
---------EYAFGARGLFPAPMS--------------------------------EEQS
LNENGKRILHLFKMLGKFVSRSMIDSRIIDVSFNP--TFFRIGDE---------------
-----SNPVTPSLGAVKT----------------------VDPQLAKS--------LKMI
KKFSVAKKAIAEDSTLTATQK-----------------VLATEALEIDG-AKIE-DLS--
--LDFTL-------------------------------PG--Y-DID-LLPN--------
GSSIP---
------------------------------VTIDNVD-LYLEK-VIDMTLGSG-VQRQVD
-AFRAG---FTQVFPY--SALR-AFTPD-ELVMLFGR-----------------------
---IEEDWS-----------LESL---------TDSIKADHG------------------
----------------FHMD----SKSV-KNLLQTMS--------------------ELT
LPERRDFLQF-------TTGSPKLP------------IGG-----FK-SLTPMF--TVVC
KPSEPPYSSD------------------------DYLPSVMTCV-NY-LKLPDY-TDLDV
MRRRMN-TAIRE-GQGA
>Ciborinia_camelliae_LGKQ01000844.1 .
VRISRSKI-LESALKVM----------------ELYGASQS-ILEVEYFE-------EVG
TGLG-PTLEF---YSTV----SKEFSKKKL--------KLWRETDANDTD----------
---------EYAFGARGLFPAPMS--------------------------------EEQS
LNENGKRILHLFKMLGKFVSRSMIDSRIIDVSFNP--TFFRIGDE---------------
-----SNPVTPSLGAVKT----------------------VDPQLAKS--------LKMI
KKFSVAKKAIAEDSSLTATQK-----------------VLATEALEIDG-AKIE-DLS--
--LDFTL-------------------------------PG--Y-AID-LLPN--------
GSHVS---
------------------------------VTMDNVD-LYLEK-VIDMTLGSG-VQRQVD
-AFRAG---FTQVFPY--SALR-AFTPD-ELVMLFGR-----------------------
---IEEDWS------------TAL---------TDSIKADHG------------------
----------------FHMD----SKSV-KNLLQTMS--------------------ELS
LPERRDFLQF-------TTGSPKLP------------IGG-----FK-SLTPMF--TVVC
KPSEPPYSSD------------------------DYLPSVMTCV-NY-LKLPDY-TDLDV
MRRRMN-TAIKE-GQGA
>Sclerotinia_borealis_AYSA01000125.1 .
VRISRSKI-LESALKVM----------------ELYGASQS-ILEVEYFE-------EVG
TGLG-PTLEF---YSTV----SKEFSKKKL--------KLWRETDANDID----------
---------EYAFGARGLFPAPMS--------------------------------EEQS
LNENGKRILHLFKMLGKFVSRSMIDSRIIDVSFNP--TFFRIGDE---------------
-----STPVTPSLGAVKT----------------------VDPQLAKS--------LKMI
KKFSVAKKAIAEDSTLTATQK-----------------VLATEALEIDG-AKIE-DLS--
--LDFTL-------------------------------PG--Y-AID-LLPN--------
GSHIS---
------------------------------VTVENVD-LYLEK-VIDMTLGSG-VQRQVD
-AFRAG---FTQVFPY--SALR-TFTPD-ELVMLFGR-----------------------
---IEEDWS-----------LESL---------TDSIKADHG------------------
----------------FHMD----SKSV-KNLLQTMS--------------------ELG
LPDRRDFLQF-------TTGSPKLP------------IGG-----FK-SLTPMF--TVVC
KPSEPPYSSD------------------------DYLPSVMTCV-NY-LKLPDY-TDLDV
MRRRMN-TAIKE-GQGA

>Myriosclerotinia_scirpicola_NGKG01000013. 1..
VRISRSKI-LESALKVM----------------ELYGASQS-ILEVEYFE-------EVG
TGLG-PTLEF---YSTV----SKEFSKKKL--------KLWRETDTNDND----------
---------EYAFGARGLFPAPMS--------------------------------EEQS
LNENGKRILHLFKMLGKFVSRSMIDSRIIDVSFNP--TFFRIGDE---------------
-----SNPVTPSLGAVKT----------------------VDPQLAKS--------LKMI
KKFSVAKKAIAEDSTLTATQK-----------------VLATEALEIDG-AKIE-DLS--
--LDFTL-------------------------------PG--Y-SID-LLPN--------
GSQIP---
------------------------------VTIDNVD-LYLEK-VIDMTLGSG-VQRQVD
-AFRAG---FTQVFPY--SALR-AFTPD-ELVMLFGR-----------------------
---IEEDWS--------L--ESTF---------HDSIKADHG------------------
----------------FHMD----SKSV-KNLLQTMS--------------------ELS
LPERRDFLQF-------TTGSPKLP------------IGGRIFPGFK-SLTPMF--TVVC
KPSEPPYSSD------------------------DYLPSVMTCV-NY-LKLPDY-TDLDV
MRRRMN-TAIKE-GQGA
>Myriosclerotinia_duriaeana_NGKI01000005.1 .
VRISRSKI-LESALKVM----------------ELYGASQS-ILEVEYFE-------EVG
TGLG-PTLEF---YSTV----SKEFSKKKL--------KLWRETDTNDND----------
---------EYAFGARGLFPAPMS--------------------------------EEQS
LNENGKRILHLFKMLGKFVSRSMIDSRIIDVSFNP--TFFRIGDE---------------
-----SNPVTPSLGAVKT----------------------VDPQLAKS--------LKMI
KKFSVAKKAIAEDSTLTATQK-----------------VLATEALEIDG-AKIE-DLS--
--LDFTL-------------------------------PG--Y-SID-LLPN--------
GSQIP---
------------------------------VTIDNVD-LYLEK-VIDMTLGSG-VQRQVD
-AFRAG---FTQVFPY--SALR-AFTPD-ELVMLFGR-----------------------
---IEEDWS-----------LESM---------FDSIKADHG------------------
----------------FHMD----SKSV-KNLLQTMS--------------------ELS
LPERRDFLQF-------TTGSPKLP------------IGGG----FK-SLTPMF--TVVC
KPSEPPYSSD------------------------DYLPSVMTCV-NY-LKLPDY-TDLDV
MRRRMN-TAIKE-GQGA
>Myriosclerotinia_curreyana_NGKJ01000060.1 .
VRISRSKI-LESALKVM----------------ELYGASQS-ILEVEYFE-------EVG
TGLG-PTLEF---YSTV----SKEFSKKKL--------KLWRETDTNDTD----------
---------EYAFGARGLFPAPMS--------------------------------EEQS
LNENGKRILHLFKMLGKFVSRSMIDSRIIDVSFNP--TFFRIGDE---------------
-----SNPVTPSLGAVKT----------------------VDPQLAKS--------LKMI
KKFSVAKKAIAEDSTLTATQK-----------------VLATEALEIDG-AKIE-DLS--
--LDFTL-------------------------------PG--Y-SID-LLPN--------
GSQIP---
------------------------------VTTDNVD-LYLEK-VVDMTLGSG-VQRQVD
-AFRAG---FTQVFPY--SALR-AFTPD-ELVMLFGR-----------------------
---IEEDWS-----------LESM---------FDSIKADHG------------------
----------------FHMD----SKSV-KNLLQTMS--------------------ELS
LPERRDFLQF-------TTGSPKFP------------IGWRIFLGFK-SLTPMF--TVVC
KPSEPPYSSD------------------------DYLPSVMTCV-NY-LKLPDY-TDLDV
MRRRMN-TAIKE-GQGA

Supplementary File 2

>Aspergillus_niger_XM_001392187.2 .
VKVRRNNIFEDSYAEI-----------------------MRQSASDLKK-RLMIKFDG--
--------EDGLDYGGLSREFFFLL-SHEMF-NPFY--------------------CLFE
YSAHDNY------------------------TLQINPHS---------------------
-------------------------------GVNPEHLNYFKFIGRVVGLAIFH----RR
FLDSFFIGAFYKMMLR-----------------------------------KKVSLQDME
G---VDEDLHRNLAWTLEN------------------------------DIE-GII-ELT
--FSVDDEK-----FGE------------RTTIDLKPG-GRDIP----------VTNENK
G-EYVELV--TEWKIVKR-VEEQFNAFMS--GFNELI---PADLVN--------------
--------VFDERELELLIGG---------IADID-VDDW--------------------

---------------KKHTDYRG-YQEQ--------------------------DEVIQN
FWKIVR-TWDAEQKSRLLQFTTGTSRIPVNGFKDLQGSDG-PRRFTIEKSG---------
----------------DPIALPKSHTCFNRLDLPPYKTHDVLEHKLSIAVEE-TLGF
>Penicillium_chrysogenum_XM_002560600.1 .
VKVRRNNIFEDSYAEI-----------------------MRQSASDLKK-RLMIKFDG--
--------EDGLDYGGLSREFFFLL-SHEMF-NPFY--------------------CLFE
YSAHDNY------------------------TLQINPHS---------------------
-------------------------------GVNPEHLNYFKFIGRVVGLAIFH----RR
FLDSFFIGAFYKMMLR-----------------------------------KKVSLQDME
G---VDEDLHRNLAWTLDN------------------------------DID-GIV-ELT
--FSVDDEK-----FGE------------RRTIDLIPG-GRDIP----------VTNENK
P-QYIELV--TEWKIMKR-VEEQFDAFMS--GFNELI---PPDLVN--------------
--------VFDERELELLIGG---------IADID-VEDW--------------------

---------------KKHTDYRG-YQEQ--------------------------DEVIQN
FWKIVR-TWDAEQKSRLLQFTTGTSRIPVNGFKDLQGSDG-PRRFTIEKSG---------
----------------DPAALPKSHTCFNRLDLPPYKTHDALEHKMSIAVEE-TLGF
>Coccidioides_immitis_XM_001243223.2 .
VKIRRSAIFEDSYAEI-----------------------MRQSPSDLKK-RLMIKFDG--
--------EDGLDYGGLSREFFFLL-SHEMF-NPFY--------------------CLFE
YSAHDNY------------------------TLQINPHS---------------------
-------------------------------GVNPEHLNYFRFIGRVVGLAIFH----RR
FLDSFFIGAFYKMMLR-----------------------------------KKVTLQDME
G---VDEDFHRNLTWTLEN------------------------------DIE-GVF-ELT
--FAVDDEQ-----FGE------------HKTIDLIPN-GRDIA----------VTNENK
R-QYVELV--TEWKIQKR-VEEQFNAFIT--GFNELI---PADLVN--------------
--------VFDERELELLIGG---------IADID-VDDW--------------------

---------------KKHTDYRG-YQEQ--------------------------DEVIQN
FWKIIR-TWDAEQKSRLLQFATGTSRIPVNGFKDLQGSDG-PRRFTIEKSG---------
----------------DINALPKSHTCFNRLDLPPYKTYEALQNKLSIAVEE-TVGF
>Mycosphaerella_graminicola_XM_003848197.1 .
VKVRRTHIFEDSYAEI-----------------------MRQSPNDLKK-RLMIKFDG--
--------EDGLDYGGLSREFFFLL-SHEMF-NPFY--------------------CLFE
YSAHDNY------------------------TLQINPHS---------------------
-------------------------------GINPEHLGYFKFIGRVVGLAIFH----RR
FLDAFFIGAFYKMILR-----------------------------------KKVNLLDME
G---VDAEFHRTLSWAMEN------------------------------DIT-DVI-YST
--FSVEDER-----FGE------------KVTVDLKPG-GRDIE----------VDNDNK
K-EYVELI--TEWRIQKR-VEEQFNAFVA--GFHELI---PADLVN--------------
--------VFDERELELLIGG---------IADID-VDDW--------------------

---------------KKHTDYRG-YTES--------------------------DLVVQN
FWKCIR-GWDAEQKSRLLQFATGTSRIPVNGFKDLQGSDG-PRRFTIEKSG---------

```
----------------EETQLPKSHTCFNRLDLPPYKSYEALNTKLTWAVEE-TVGF
>Pyrenophora_tritici_repentis_AAXI01000200 .
VKVRRTHIFEDSYHEI-----------------------MRQSAADLKK-RLMIKFDG--
--------EDGLDYGGLSREFFFLL-SHEMF-NPFY--------------------CLFE
YSAHDNY------------------------TLQINPHS---------------------
-------------------------------GINPEHLNYFKFIGRVVGLAIFH----RR
FLDAFFIGAFYKMILR-----------------------------------KKVALQDME
G---VDADFHRNLEWMLXN------------------------------DIT-DAL-ELT
--FATDDER-----FGE------------TVSIELKPG-GDEIE----------VTNENK
H-EYVEXI--TEWRIQKR-VEEQFQAFIT--GFHELI---PADLVN--------------
--------VFDERELELLIGG---------IADID-VEDW--------------------
------------------------------------------------------------
---------------KKHTDYRG-YTEN--------------------------DEVIQN
FWKCIR-SWDAEQKSRLLQFATGTSRIPVNGFKDLQGSDG-PRRFTIEKAG---------
----------------EPNQLPKSHTXFNRLDLPPYKTFEALNQKLTIAVEE-TVGF
>Sclerotinia_sclerotiorum_XM_001595569.1 .
VKIRRSHIFEDSYAEI-----------------------MRQSATDLKK-RLMIKFDG--
--------EDGLDYGGLSREFFFLL-SHEMF-NPFY--------------------CLFE
YSAHDNY------------------------TLQINPHS---------------------
-------------------------------GINPEHLNYFKFIGRVVGLAIFH----RR
FLDAFFIGALYKMMLS-----------------------------------KAVSLQDME
G---VDADFHRSLQWMLDN------------------------------PIE-GVL-DQT
--FSTEDER-----FGV------------TNVEDLKPG-GRDIE----------VTDENK
K-EYVDLM--VKWRIQKR-IDEQFQAFIN--GFHELI---PAELVN--------------
--------VFDERELELLIGG---------IAEID-VDDW--------------------
------------------------------------------------------------
---------------KKHTDYRG-YTES--------------------------DEVIKF
FWQTIR-SWDGEQKSRLLQFATGTSRIPVNGFKDLQGSDG-PRRFTIEKQG---------
----------------EPNNLPKSHTCFNRLDLPPYKTLEQLQTKLTMAVEE-TMGF
>Fusarium_verticillioides_XM_018890483.1 .
IKVRRSHIFEDSFAEI-----------------------TRQSATDLKK-RLMIKFDG--
--------EDGLDYGGLSREFFFLL-SHEMF-NPFY--------------------CLFE
YSAHDNY------------------------TLQINPHS---------------------
-------------------------------GINPEHLNYFKFIGRVVGLAIFH----RR
FLDAFFIGALYKMMLG-----------------------------------KAVALADME
G---VDADFHRSLQWMLDN------------------------------DISGGIL-EQT
--FSTEDER-----FGV------------MTTEDLIPD-GRNID----------VTNENK
K-EYVDLM--VKWRIEKR-IAEQFQAFKE--GFQELI---PQDLIN--------------
--------VFDERELELLIGG---------IAEID-VDDW--------------------
------------------------------------------------------------
---------------KKHTDYRG-YTES--------------------------DEVVQN
FWATVR-SWDGEQKSRLLQFTTGTSRIPVNGFKDLQGSDG-PRRFTIEKAG---------
----------------EITNLPKAHTCFNRLDLPPYKSLEMLQQKLTIAVEE-TMGF
>Chaet_globosum_XM_001226583.1_AAFU01001138.1 .
IKVRRSHIFEDSFAEI-----------------------SRQSATDLKK-RLMIKFDG--
--------EDGLDYGGLSREFFFLL-SHEMF-NPFY--------------------CLFE
YSAHDNY------------------------TLQINPHS---------------------
-------------------------------GINPEHLNYFKFIGRVVGLAIFH----RR
FLDAFFIGALYKMVLG-----------------------------------KXVVLADMX
G---VDADFHRSLQWMLDN------------------------------DISGGIL-EQT
--FSTEDER-----FGV------------ITVEDLIPN-GRNID----------VTNDNK
K-EYVDLM--VKWRIQKR-IAEQFEAFKE--GFQDLI---PQDLIN--------------
--------VFDERELELLIGG---------IAEID-VDDW--------------------
------------------------------------------------------------
---------------KKHTDYRG-YTES--------------------------DEVIQF
FWQTVR-SWDGEQKSRLLQFTTGTSRIPVNGFKDLQGSDG-PRRFTIEKAG---------
----------------ELGNLPKAHTCFNRLDLPPYKTLEMLQGKLTMAVEE-TMGF
>T_melanosporum_XM_002842107.1_CABJ01000520..1 .
```

```
VKVRRNHIFEDSYAEI-----------------------MRQTPNDLKK-RLMVKFDG--
--------EDGLDYGGLSREFFFLL-SHEMF-NPFY--------------------CLFE
YSAHDNY------------------------TLQINPHS---------------------
-------------------------------GINPEHLNYFKFIGRVVGLAIFH----RR
FLDAFFIGAFYKMILK-----------------------------------KKVVLADME
G---VDADFHRNLTWMLEN------------------------------DIT-DIL-DLT
--FSTEDSR-----FGE------------TVTIDLKPN-GQNIE----------VTNDNK
R-EYVDLV--TGWRIEKR-VQEQFKAFVD--GFHDLI---PADLIN--------------
--------VFDERELELLIGG---------IADID-VEDW--------------------
------------------------------------------------------------
---------------KKHTDYRG-YTES--------------------------DDVIAN
FWKXVR-SWDAEQKSRLLQFTTGTSRIPVNGFKDLQGSDG-PRRFTIEKAG---------
----------------DIGQLPKSHTCFNRLDLPPYKSFDVLNQKLSLAVEE-TMGF
>Yarrowia_lipolytica_HG934063.1 .
IKVRRDHIFEDSYQEI-----------------------MRQTPQDLQK-RLMIKFDG--
--------EEGLDYGGVSREFFFLL-SHEMF-NPFY--------------------CLFE
YSAHDNY------------------------TLQINPHS---------------------
-------------------------------GINPEHLNYFKFIGRCVGLAIFH----RR
FLDAFFIGAFYKMILK-----------------------------------KKVMLEDME
G---VDADYHRNLEWALDN------------------------------DIT-DVL-DLT
--FSVEDDQ-----FGE------------IVTIDLKPD-GRNIE----------VTNDNK
I-EYVELV--TEWRISKR-VEEQFQAFVS--GFYELI---PQELVN--------------
--------VFDERELELLIGG---------IADID-VDDW--------------------
------------------------------------------------------------
---------------KKHTDYRG-YSES--------------------------DEVIKW
FWQCIR-SWDSEQKSRLLQFTTGTSRIPVNGFKDLQGSDG-PRRFTIEKAG---------
----------------EAQHLPKSHTCFNRVDLPPYKNYEDLVKKLSMAVEE-TVGF
>Candida_albicans_XM_709377.2 .
IKVRRDHIFEDSYQEI-----------------------MRQTPEDLKK-RLMIKFDG--
--------EEGLDYGGVSREFFFLL-SHDMF-NPFY--------------------CLFE
YSSHDNY------------------------TLQINPNS---------------------
-------------------------------GINPEHLNYFKFIGRVVGLGVFH----RR
FLDAFFVGALYKMMLH-----------------------------------KKVVLQDME
G---VDAEFYRSLKWILDN------------------------------DIT-GIL-DLT
--FSAEEES-----FGE------------IVEVDLKPG-GRDIE----------VTEENK
H-EYVELI--TEWRISKR-VEEQFKAFID--GFNELI---PQELVN--------------
--------VFDERELELLIGG---------LAEID-CEDW--------------------
------------------------------------------------------------
---------------KKHTDYRG-YQEN--------------------------DQVIQW
FWKCIN-EWDSEQKARLLQFTTGTSRIPVNGFKDLQGSDG-PRRFTIEKAG---------
----------------EANQLPKSHTCFNRVDLPPYTDYESLKQKLTLAVEE-TVGF
>Saccharomyces_cerevisiae_NM_001179019.2 .
IKVRRKNIFEDAYQEI-----------------------MRQTPEDLKK-RLMIKFDG--
--------EEGLDYGGVSREFFFLL-SHEMF-NPFY--------------------CLFE
YSAYDNY------------------------TIQINPNS---------------------
-------------------------------GINPEHLNYFKFIGRVVGLGVFH----RR
FLDAFFVGALYKMMLR-----------------------------------KKVVLQDME
G---VDAEVYNSLNWMLEN------------------------------SID-GVL-DLT
--FSADDER-----FGE------------VVTVDLKPD-GRNIE----------VTDGNK
K-EYVELY--TQWRIVDR-VQEQFKAFMD--GFNELI---PEDLVT--------------
--------VFDERELELLIGG---------IAEID-IEDW--------------------
------------------------------------------------------------
---------------KKHTDYRG-YQES--------------------------DEVIQW
FWKCVS-EWDNEQRARLLQFTTGTSRIPVNGFKDLQGSDG-PRRFTIEKAG---------
----------------EVQQLPKSHTCFNRVDLPQYVDYDSMKQKLTLAVEE-TIGF
>Kluyveromyces_lactis_XM_454562.1 .
IRVRRKNIFEDSYQEI-----------------------MRQTPEDLKK-RLMIKFDG--
--------EEGLDYGGVSREFFFLL-SHEMF-NPFY--------------------CLFE
```

YSAHDNY------------------------TIQINPNS---------------------
-------------------------------GINPEHLNYFKFIGRVVGLGVFH----RR
FLDAFFVGALYKMMLR-----------------------------------KKVVLQDME
G---VDSEVYNSLKWILEN------------------------------SID-GIL-DLT
--FNVDDER-----FGE------------LVVVDLKPN-GREIE----------VTDENK
K-EYVELY--TQWRIADR-VQEQFKAFMD--GFNELV---PEDLVN--------------
--------VFDERELELLIGG---------IAEID-VEDW--------------------

---------------KKHTDYRG-YQES--------------------------DEVIQW
FWKCIT-EWDNEQRARLLQFTTGTSRIPVNGFKDLQGSDG-PRRFTIEKAG---------
----------------EVQQLPKSHTCFNRVDLPPYTDYESFKQKLTLAVEE-TIGF
>Schizosacch_pombe_NM_001019819.2 .
IKVRRNHIFEDSYAEI-----------------------MRQSATDLKK-RLMIKFDG--
--------EDGLDYGGLSREYFFLL-SHEMF-NPFY--------------------CLFE
YSSVDNY------------------------TLQINPHS---------------------
-------------------------------GINPEHLNYFKFIGRVIGLAIFH----RR
FVDAFFVVSFYKMILQ-----------------------------------KKVTLQDME
S---MDAEYYRSLVWILDN------------------------------DIT-GVL-DLT
--FSVEDNC-----FGE------------VVTIDLKPN-GRNIE----------VTEENK
R-EYVDLV--TVWRIQKR-IEEQFNAFHE--GFSELI---PQELIN--------------
--------VFDERELELLIGG---------ISEID-MEDW--------------------

---------------KKHTDYRS-YSEN--------------------------DQIIKW
FWELMD-EWSNEKKSRLLQFTTGTSRIPVNGFKDLQGSDG-PRKFTIEKAG---------
----------------EPNKLPKAHTCFNRLDLPPYTSKKDLDHKLSIAVEE-TIGF
>Schizosacch_pombe_NM_001021694.2 .
VKVRRDHIFEDSYAEI-----------------------MRYSAHDLKK-RLMIRFDG--
--------EDGLDYGGLSREFFFLL-SHKMF-DPIY--------------------CLFE
YSAVDNY------------------------TLQINPHS---------------------
-------------------------------SINPEHLNYFRFIGRVIGLAIFH----RR
FLDAFFVVSLYKKLLR-----------------------------------KKVSLADME
S---IDAEFYRSLKWVLEN------------------------------DIT-GIL-DLT
--FSVEEDH-----FGE------------VRTVELITN-GENIE----------VTEENK
K-KYVDLV--TEWRVSKR-VEQQFNAFYS--GFVELV---SPDLVN--------------
--------VFDERELELLIGG---------ISDVD-VEDW--------------------

---------------KSHTEYRT-YIAT--------------------------DPVIKW
FWEIIA-GWKNEDRSKLLQFATGTSRIPVNGFRDLQGSDG-PRKFTIEKAG---------
----------------TPDQLPVAHTCFNRLDLPDYPSKDTLHEKLSLAVEN-TVGF
>Pneumocystis_carinii_XM_018371258.1 .
IKIRRNHIFEDSYAEI-----------------------MRQSPNDLKK-RFMVKFDG--
--------EDGLDYGGLSREFFFLL-SHEMF-NPFY--------------------CLFE
YSSVDNY------------------------TLQINPHS---------------------
-------------------------------GINPEHLNYFKFIGRVLGLAIFH----RR
FLDAFFIVSFYKMILK-----------------------------------KKVTLADME
S---VDAEFFRSLTWILEN------------------------------DIT-NVL-ELT
--FSTEDDR-----FGE------------VMTIDLKPN-GRNIE----------VTNDNK
K-EYVELV--AYWRVFKR-VEEQFNAFQS--GFNELI---PHELIS--------------
--------VFDERELELLIGG---------ITEMD-MDDW--------------------

---------------KKHTDYRG-YTES--------------------------DEIIQW
FWKCVR-SWDSERKSRLLQFITGTSRVPVNGFKDLQGSDG-PRRFTIERAG---------
----------------EITQLPKSHTCFNRVDLPQYPTYEMLVQKLTLAVEE-TVGF
>Puccinia_graminis_XM_003889329.1 .
VKIRRSHIFEDSYAEI-----------------------MRQQPNDLKK-RLMIKFDG--
--------EDGLDYGGVSREFFFLL-SHEMF-NPFY--------------------CLFE
YSAVDNY------------------------TLQINPHS---------------------
-------------------------------GVNPEHLNYFKFIGRVLALAIFH----RR

```
FLDAYFITSFYKMILK-----------------------------------KKIALADME
S---VDAEIFRSLTWMLEN------------------------------DIT-DVI-ENS
--FSVEDEK-----FGE------------VVTIDLREN-GRNIP----------VTEANK
K-DYIELI--TQWRIEKR-VADQFKAFLS--GFHELI---PQELIN--------------
--------VFDERELELLIGG---------MSDID-VDDW--------------------
------------------------------------------------------------
---------------IKHTDYRG-YQPD-------------------------DQVIKW
FWQAVR-AWPAEKKSRLLQFTTGTSRIPVNGFKDLQGSDG-PRRFTIEKAG---------
----------------EITQLPKSHTCFNRLDLPPYPNFDQLEQKISFAIEE-TLGF
>Cryptococcus_neoformans_XM_012198185.1 .
MKVSRDNIFEGSYTEI-----------------------MRQTPNDLKK-RLMIKFEG--
--------EDGLDYGGLSREFFFLL-SHEMF-NPFY--------------------CLFE
YSAHDNY------------------------TLQINPNS---------------------
-------------------------------GVNPEHLNYFKFIGRVVGLGIFH----RR
FLDAYFIVSFYKMILG-----------------------------------KKIALQDLE
S---VDAGLFRGLTWMLEN------------------------------DIT-GVI-EDT
--FSITEEH-----FGE------------VVTVDLMPG-GRDVE----------VTEDNK
K-DYVDLV--TEYRISKR-VSEQFQAFMS--GFNELI---PQELIN--------------
--------VFDERELELLIGG---------MSEID-VDDW--------------------
------------------------------------------------------------
---------------QKHTDYRG-YNPS-------------------------DEVVEW
FWKIVK-NWPAEKKSRLLQFTTGTSRIPVNGFKDLQGSDG-PRRFTIEKAG---------
----------------EVTQLPKSHTCFNRIDLPAYKSYEALEQKLTIAVEE-TVGF
>Ustilago_maydis_XM_011388165.1 .
IKVRRTHIFEDSYAEI-----------------------MRQQPNDLKK-RLMIKFDG--
--------EDGLDYGGLSREFFFLL-SHEMF-NPFY--------------------CLFE
YSAHDNY------------------------TLQINPHS---------------------
-------------------------------GINPEHLNYFKFIGRVLGLAIFH----RR
FLDAYFIVSFYKMILK-----------------------------------KKITLSDLE
S---VDADYHRSLQWMLDN------------------------------SIE-GIV-EET
--FTAVEDK-----FGE------------MVTVELKKG-GEEVE----------VTDENK
K-EYVDLM--TEWRISKR-VEEQFKAFIS--GFTELI---PQDLIN--------------
--------VFDERELELLIGG---------MSEID-VDDW--------------------
------------------------------------------------------------
---------------KKFTDYRG-FTEQ-------------------------DQVVQW
FWQCVR-AWPTEKKSRLLQFATGTSRIPVNGFKDLQGSDG-PRRFTIEKSG---------
----------------DVNQLPKSHTCFNRIDLPPYPSFETLESKLALAIEE-GMGF
>Laccaria_bicolor_XM_001876255.1 .
VKVRRSRVLEDSYASV-----------------------MGFTGEDLKR-RLMVNFDG--
--------EDGLDYGGVSREWFFLL-SHEIF-NPSY--------------------GLFE
YSTHDNY------------------------TLQINPAS---------------------
-------------------------------GINPDHLSYFKFIGRCLGLAIFH----RR
FLDAYFVPSFYKMILG-----------------------------------KHMALADLE
S---VDSDLHRSLVWMLEN------------------------------DIT-DVL-DET
--FTTAEER-----FGE------------LVTIELKPG-GEEVP----------VTEENK
K-EYVDSV--VAYRISKR-VKEQFDAFME--GLLELV---PRDLIN--------------
--------VFDERELELLIGG---------MSEID-MDDW--------------------
------------------------------------------------------------
---------------TKFTDYRG-YEKT-------------------------DQVIEW
FWQCIR-SWPAERKSRLLQFTTGTSRVPVNGFKDLQGSDG-PRRFTIEKSG---------
----------------DPMGLPRSHTCFNRLDLPPYQDYESLETKLLFAIEE-TEGF
>Laccaria_bicolor_XM_001878532.1 .
IKIRRNHIFEDSYAEI-----------------------MRQTPNDLKK-RLMIKFDG--
--------EDGLDYGGLSREFFFLL-SHEMF-NPFY--------------------CLFE
YSAHDNY------------------------TLQINPAS---------------------
-------------------------------GVNPEHLNYFKFIGRCLGLGIFH----RR
FLDAYFIVSFYKMILK-----------------------------------KKVTLSDLE
S---VDAELHRGMTWMLEN------------------------------DIT-DII-DET
```

--FTTTEER-----FGE------------MVTIDLKPG-GADVP----------VTEENK
K-EYVDHV--VDYRISKR-VNEQFEAFMS--GFSELI---PQELIT--------------
--------VFDERELELLIGG---------MSEID-VDDW--------------------

---------------TKFTDYRG-YEMN--------------------------DEVIQW
FWKCVR-SWPPERKSRLLQFATGTSRIPVNGFKDLQGSDG-PRRFTIEKSG---------
----------------DPSQLPKSHTCFNRIDLPPYKDYASLEHKLTLAVEE-TVGF
>Entomophthora_muscae_GEND01005691.1 .
IKVRRDHIFEDAFVEV-----------------------MKHSPSDLKK-RLMIKFEG--
--------EDGLDYGGLSREFFYLL-SHEMF-NPVY--------------------CLFQ
YSSHNNY------------------------TLQINPYS---------------------
-------------------------------AINPEHLNYFKFIGRVLGLAVFH----RR
FLDAFFVVSIYKMILK-----------------------------------KKTTLADME
S---IDVEYWRSLEWMLNN------------------------------DIE-DIL-ELD
--FSVEEDR-----FGE------------KVQIDLITD-GRNIP----------VTNENK
R-EYVELV--TQWKINNR-IREQFTAFFE--GFHQLI---PDDLIV--------------
--------VFDERELELLIGG---------IAEID-VEDW--------------------

---------------KKHTDYRG-YTEQ--------------------------DEVIQW
FWKCVR-TFDNEKKSRLLQFTTGTSRIPVNGFKDLQGSDG-PRRFTIEKAG---------
----------------EIAQLPKSHTCFNRIDLPPYKSYDVLVQKLTFAVEE-TLGF
>Mortierella_alpina_ADAG01000979.1 .
MKVRRSHIFEDAYHEI-----------------------MRQSPTDLKK-RLMIKFEG--
--------EDGLDYGGLSXEFFFLL-SHEMF-NPFY--------------------CLFE
YSAHDNY------------------------TLQINPHS---------------------
-------------------------------SINSEHLNYFKFIGRVVGLAIFH----RR
LLDAFFIVSFYKMILK-----------------------------------KKVTLADLE
S---VDADVYRNLNWLLDD------------------------------DTAAETL-DTT
--FSTNDER-----FGE------------IVTIDLKEN-GRDIE----------VTEENK
K-EYVELM--TEWRITRR-VEEQFKAFAE--GFHQLI---PQELVT--------------
--------VFDERELELLMGG---------ISEID-CDDW--------------------

---------------KKHTDYRG-YTEQ--------------------------DEVVQW
FWKCIR-SWDSEKKARLLQFTTGTSRIPVNGFKDLQGSDG-PRRFTIEKAG---------
----------------EIGQLPKSHTXFNRIDLPPYKSYDVLVNKLTMAVEE-TVGF
>Rhizopus_oryzae_GDUK01018643.1 .
IKVRRDHIFEDAYAEI-----------------------MRQVPADLKK-RLMIKFDG--
--------EDGLDYGGLSREFFFLL-SHEMF-NPFY--------------------CLFE
YSAHDNY------------------------TLQINPHS---------------------
-------------------------------SINPEHLNYFRFIGRVVGLAIFH----RR
FLDAFFIVSFYKMILN-----------------------------------KKVLVADME
S---VDADFYRSLKWIIDN------------------------------DIT-DVL-DLT
--FSVDDDK-----FGE------------VVTVDLKED-GRNIE----------VTEENK
K-EYVDLV--TEWRISKR-VDEQFKAFKE--GFNQLI---PQDLIN--------------
--------VFDERELELLIGG---------IAEID-VDDW--------------------

---------------KKHTDYRG-YTEQ--------------------------DDVIQW
FWKCIR-SWDSEKKSRLLQFTTGTSRIPVNGFKDLQGSDG-PRRFTIEKAG---------
----------------EITQLPKAHTCFNRIDMPPYKSYEALVAKLTMAVEE-TVGF
>Rhizopus_oryzae_GDUK01018646.1 .
IKVRRDHIFEDAYAEI-----------------------MRQVPADLKK-RLMIKFDG--
--------EDGLDYGGLSREFFFLL-SHEMF-NPFY--------------------CLFE
YSAHDNY------------------------TLQINPHS---------------------
-------------------------------GINPEHLNYFRFIGRVVGLSIFH----RR
FLDAFFIVSFYKMVLN-----------------------------------KKILVADME
S---VDAEFHRSLMWILDN------------------------------DIT-DIL-DLT
--FSTDDDR-----FGE------------VVTVDLIPN-GQNIE----------VTEENK
K-EYVNLI--TEWRIHRR-VEEQFKAFKE--GFNQLI---PQELIN--------------

```
--------VFDERELELLIGG---------IAEID-VDDW--------------------
------------------------------------------------------------
---------------KKHTDYRG-YTEQ--------------------------DDVIQW
FWKCIR-SWDSEKKARLLQFTTGTSRIPVNGFKDLQGSDG-PRRFTIEKSG---------
----------------EITQLPKAHTCFNRIDMPPYKSYETLVAKLTMAVEE-TVGF
>Phycomyces_blakesleeanus_XM_018443014.1 .
IKIRRETIFEDAYAEV----------------------MRQSPSDLKK-RLMIKFDS--
--------EDGLDYGGLSREFFFLL-SHEMF-NPFY--------------------CLFE
YSAHDNY------------------------TLQINPHS---------------------
-------------------------------GINPEHLNYFRFIGRVVGLSIFH----RR
FLDSFFIVSFYKMILS-------------------------------------KRVAVADME
S---VDAEYHRSLMWMLNN------------------------------DIT-DVL-DLT
--FSTEDDR-----FGE------------TVTVDLKPD-GQNIP----------VTEENK
K-EYVNLV--TEWRISRR-VEEQFKAFKE--GFNQLI---PHDLVN--------------
--------VFDERELELLIGG---------ISEID-VEDW--------------------
------------------------------------------------------------
---------------KKHTDYRG-YTEQ--------------------------DDVIQW
FWKCIK-TWDSEKKSRLLQFTTGTSRIPVNGFKDLQGSDG-PRRFTIEKSG---------
----------------EVTQLPKAHTCFNRIDMPPYKSYEALVAKLTMAVEE-TVGF
>Phycomyces_blakesleeanus_XM_018439955.1 .
IKVRRDHIFEDAYAEV----------------------MRQSPADLKK-RLMIKFEG--
--------EDGLDYGGLSREFFFLL-SHEMF-NPFY--------------------CLFE
YSAHDNY------------------------TLQINPHS---------------------
-------------------------------GINPEHLNYFRFIGRVVGLSIFH----RR
FLDAFFIVSFYKMILN-------------------------------------KRVAVVDME
S---VDAEFHRSLKWILDN------------------------------DIT-DVL-DLT
--FSTDDDR-----FGE------------LVTVDLKPD-GQNIE----------VTEENK
K-EYVDLI--TEWRISKR-VEEQFKAFKD--GFNQLI---PQDLIN--------------
--------VFDERELELLIGG---------IAEID-VDDW--------------------
------------------------------------------------------------
---------------KKHTDYRG-YTEQ--------------------------DDVIQW
FWKCVS-SWDSEKKSRLLQFTTGTSRIPVNGFKDLQGSDG-PRRFTIEKAG---------
----------------EVTMLPKAHTCFNRIDMPPYKTYESLVAKLTLAVEE-TMGF
>Spizellomyces_punctatus_XM_016750836.1 .
VTVRRSNIFEDAYSEI----------------------MRYPAPELQK-RLMIKFHG--
--------EDGLDYGGLSREFFFLL-SHEMF-NPFY--------------------CLFE
YSAHDNY------------------------TLQINPNS---------------------
-------------------------------GVNPEHLNYFKFIGRVVGLAIFH----QR
FLDAFFITAFYKMILK-------------------------------------KKIVMKDME
S---VDAEHWRSLQWMLDN------------------------------DIT-GVL-DLT
--FSAEEEV-----FGV------------VRTVDLKPD-GQNIP----------VTEENK
Q-EYVELI--VEWRVCKR-VEEQFKAFQQ--GFHELV---PPDLIT--------------
--------VFDERELELLIGG---------IADID-VDDW--------------------
------------------------------------------------------------
---------------KKHTDYRG-YQET--------------------------DEVVQW
FWKCVR-SWDSEKKARLLQFVTGTSRIPVNGFKDLQGSDG-PRRFTIEKAG---------
----------------EGEQLPKSHTCFNRLDLPPYRGMDILEKKLTMAIEE-TIGF
>Allomyces_macrogynus_ACDU01000955.1 .
IKVSRNAIFEDSYNEI----------------------MRLPVAELKK-KLVIIFDK--
--------EDGLDYGGLSREFFFLL-SHEMF-NPFY--------------------CLFE
YSAHDNY------------------------TLQINPNS---------------------
-------------------------------SINAEHLNYFRFIXRIVGLAIFH----RR
FLDAFFVVSFYKTILK-------------------------------------KKITLADME
S---VDADFHRSLEWMLNN------------------------------DIT-DIL-DLT
--MSTEDNR-----FGE------------VVTIDLVPG-GRDIP----------VTEENK
R-EYVEKI--TEWRIVKR-VEEQSNAFRQ--GLFDFV---PEDLIT--------------
--------VFDERELELLIGG---------LAEID-VDDW--------------------
------------------------------------------------------------
```

---------------VKHTDYRG-YTES--------------------------DEVVLW
FWKCIR-SWENEKRARFLQFATGTSRIPVNGFKDLQGSDG-PRRFTIEKAAG--------
----------------DDQALPKAHTCFNRIDLPPYKSYDVLVQKLSLAVEE-TMGF
>Allomyces_macrogynus_ACDU01000360.1 .
IHVSRRSLLEDAYNVI-----------------------TRLPTSELKK-RLMITFDR--
--------EEGLDVGGISREFFLLL-SQEMF-NPNY--------------------GLFE
YAELDKL------------------------TLKINPSS---------------------
-------------------------------AINTEHLAYFRFIGRMVGLAIFH----GM
LLDALFVRSFYKTILG-----------------------------------LKITLSDME
S---VDAKYYQSLKWMLEN------------------------------DIT-DVF-YET
--MSTEDHR-----FGE------------VVTIDLVPG-GRDIP----------VTEKNK
K-EYVDKI--TEWRIVKS-VEEQLNEFQL--GLSELV---PASVIT--------------
--------VFGERDLELLING---------LAEFD-VDDW--------------------

---------------VQHTDYIG-YTAS--------------------------DPVVVW
FWQCVR-SWDNEKRARILQFATGTSRIPSSGFKYLQGANG-PCKFTIKRAGS--------
----------------DDQALPLAHTCFNHIDLPPYKSYDVLVQKLLMAVDE-TVGF
>Allomyces_macrogynus_ACDU01000930.1 .
FSVSRKLLFETSIAAL-----------------------MRLPVHELKK-RPSITMDG--
--------EEGVDVGGVSREFFLLL-SQEMF-NPDY--------------------GLFE
YAESDKR------------------------TLKINPKS---------------------
-------------------------------AVNPEHLTYFRFIGRVVGLAIFH----GF
FLDALFVGSFYKTILK-----------------------------------QKITVSDME
S---FDAAHYRSLKWMLEN------------------------------DIT-DVL-DET
--MSTEDHR-----FGE------------VVTINLVPG-GRDIA----------VTEKNK
K-EYVDKI--TEWHIVKS-VEKQLNAFRQ--GLFELV---PANVIS--------------
--------VFDERELELLING---------LAEFD-VDDW--------------------

---------------VTHSVYLG-YTAS--------------------------EPVIIW
FWQCVR-SWDNEKRARLLQFATGTSRIPSSGFRYLQGTDG-PCKFTIERARG--------
----------------DVQALPKAHTCFNRIDLPPYQSYAMLERKLLMAVEE-TMGF
>Rozella_allomycis_ATJD01000996.1 .
IVVRRDHIFEDAYHEI-----------------------SRRNPSDLKK-KIAFKFQG--
--------EEGLDYGGVSREFFFLL-SHEMF-NPFY--------------------CLFE
YSAHDNY------------------------TLQINANS---------------------
-------------------------------SVNPEHLNYFTFIGRVLGMAIFH----QR
FLDAFFVVSFYKMILK-----------------------------------MKCTPQDLE
S---IDAEFYRNLNWMLEN------------------------------DIT-DVL-DLT
--FTVEDER-----FGE------------RVQIDLKPN-GSNIP----------VTEENK
R-EYVQLV--AEWKIEKR-VATQFAAFKK--GFNDFI---PDDLIC--------------
--------IFDEKELELLIGG---------IAEVD-VDDW--------------------

---------------IKNTEYKG-YKGD--------------------------EQVIQW
FWRMIR-EFDQEKKARMLQFVTGTSRIPVNGFKDLQGSDG-PRKFTIEKVG---------
----------------DVTSLPKSHTCFNRLDLPPYLDYDVLVSKVTLAIEE-TMGF
>Anaeromyces_robustus_MCFG01000098.1 .
IEVNRKKLFEDAYRII-----------------------MSYTPDELKQ-KIQIKFIG--
--------EEGLDYGGLTREFFYLL-SHEMF-NPDY--------------------SLFQ
YSHENSY------------------------TLKINPNS---------------------
-------------------------------GINPEHLYYFKFIGRVMGMSIFH----NQ
YLDISFTVPLYKGLLN-----------------------------------KKPNFNDLE
S---DDPQLYKNFKWILEN------------------------------EITEDYG--LT
--FTKDEED----CFGV------------KRTIDLKPD-GANIP----------VTEENK
Q-EYIELM--IEHSINTG-VEEQLNVLRI--GLYEVI---PSDLIS--------------
--------IFDERELELLISG---------VNEID-VDDW--------------------

---------------QNNTDYRS-YKKD--------------------------DKTVVY
FWKCVK-EFDNEMRSRLLQFVTGTSRIPVTGFKDLQGSDG-PRHFTIEKVG---------

```
----------------NPEDLPKSHTXFNRIDLPPYTSYEQLKQKLILAIEE-GITF
>Pecoramyces_ruminatium_ASRE01005267.1 .
IEVNRNKLFEDAYRII-----------------------MSYTPTELKQ-KIQIKFIG--
--------EEGLDYGGLKREFFFLL-SHEMF-NPDY--------------------SLFQ
YSHENSY------------------------TLKINPQS---------------------
-------------------------------GINPEHLFYFKFIGRVVGMAIFH----DQ
FLDISFTVPLYKGLLN-----------------------------------KKPNFNDLE
S---DDPTLYKNFKWLLEN------------------------------EVTEDMG--FC
--FAKDEED----CFGV------------KQTIELKPD-GANIP----------VTDENK
Q-EYVKLM--IEHSINAG-VEEQLKVFRN--GLYEII---PSDLIS--------------
--------IFDERELELLISG---------VSEIN-VDDW--------------------
------------------------------------------------------------
---------------ENNTDYRT-YKKD--------------------------DKTVIY
FWKVVR-EFENEMRARLLQFVTGTSRIPVTGFKDLQGSDG-PRHFTIEKAG---------
----------------SPDDLPKSHTXFNRIDLPPYNSYEQLKQKLTLAIEE-GITF
>Piromyces_finnis_MCFH01000002.1 .
IEVDRKRLFEDAFRII-----------------------MSCTPNELKQ-KLQIKFIG--
--------EEGLDYGGLAREFFFLL-SHEMF-NPNY--------------------SLFQ
YSHETSY------------------------TLKINPKS---------------------
-------------------------------GINPEHLDYFKFIGRVVGMAIFH----NH
YLDISFTVPLYKGLLG-----------------------------------KKPGFKDLE
S---DDPQLYKSFKWILES------------------------------EITEDYG--LT
--FTKDEED----CFGV------------KQTIELKPN-GENIP----------VTEENK
Q-EYIXLM--INYSINNG-VEEQLKYFRN--GLYEVI---PDDLLK--------------
--------IFDERELXLLIGG---------ISNID-CDDW--------------------
------------------------------------------------------------
---------------QNNTDYRS-YKKD--------------------------DKTVIY
FWKVVR-EFEPEMRARLLQFVTGTSRIPVTGFRDLQGSDG-PRHFTIEKVG---------
----------------NQEDLPKSHTXFNRIDLPPYNSYELLKQKLTLAIEE-GITF
>Pecoramyces_ruminatium_ASRE01001545.1 .
-KIRRSNLFDDAFNCV----------------------MYEYKPVDLKK-RIKIKFTD--
--------EPGIDAGGLRRDFFLNL-SHELF-NPMY--------------------SLFE
YLYEGSY------------------------NLKINPNS---------------------
-------------------------------GVNENHLQYFKFTGRVIALAILH----QE
HLDVSFTVPFYKSILD-----------------------------------KPLVFEDMA
N---IDPDVYRNLKWLLDN------------------------------DGADALC--YN
--FTRDTED----CFGD------------KKTIKLKEG-GSHID----------LTDENK
Q-EYVXTV--KSYDEDDK-TEEQLKAIKE--GINEIV---PLSKIKE-------------
--------FFDERDLXLLISG---------IKKID-VDDW--------------------
------------------------------------------------------------
---------------QANTEYKD-YTKN--------------------------DKTIVN
FWRVVR-EYDDEMKSRLLQFATGTTCVPVTGFKDLRGANK-LQKFTLQKID---------
----------------NTNLLPAAHTXFNRIDLPAYETYEQLKEKLTFAIEEGMQMG
>Pecoramyces_ruminatium_ASRE01009082.1 .
ITINRNELFSDAYKAI-----------------------MTISPDELKN-LLKIKYVG--
--------EEGIDAGGLLRDFFYNL-AKEIG-NPDY--------------------LLFQ
YSSDNSY------------------------DLNINKHS---------------------
------------------------------SRIHSNYLGYYKFVGRMLGLTILH----KQ
TLPISFSILFYKKLLN-----------------------------------KPVTFSDLK
Y---IDSELYKNLNWLKEN------------------------------DGSENLF--LT
--FELEETD----CFGQ------------HNVVELKPN-GRNIN----------VNDSNK
N-EYIDLI--VQKKLKSN-DEEQMIAIRE--GFYELI---PNDVSE--------------
--------ILNEIDLNYLISG---------TNEID-INDW--------------------
------------------------------------------------------------
---------------KNNTIYIG-YNEN--------------------------DTTVVN
FWKCIE-DFSDEKRKRLLLFVTGNTKLPVTGFKDLQGRNGKIGHFTIRKLG---------
----------------KINDLPKSHTCCNYLSIPPYTSYTQLKQKLLFSITEGTDSF
>Pecoramyces_ruminatium_ASRE01008212.1 .
```

```
MQISRKNLFMDTFYFI-----------------------MDKTPEELKK-RLEIQYRG--
--------EIGIDAGGLLRDLFYHF-SKEIG-NPSY--------------------TLFQ
YTHDDSY------------------------ELEINPNS---------------------
-------------------------------GIEPDHLKYFRFIGRMIGLAIFH----KQ
FLSITFNILFYKRLLN-----------------------------------KEPEFSDLE
Y---IDAEMFKNLKXFRNN------------------------------DGAEKLS--LT
--FEINVVD----CFGK------------TKNIELKPN-GSNIN----------VTDENK
N-EYMELL--VRSKLDYLNDKEQFDALKQ--GFYEII---PQTINT--------------
--------ILNEIDLKVLISG---------INEID-VDDW--------------------
------------------------------------------------------------
---------------EKNTVYDK-YKKD--------------------------DPTIKY
FWECVR-EFSNENRTKLLLFATGNSQVPVTGFKDLQGSGR-IQHFKIKKNG---------
----------------TEDDLPKSHTSFNCIELPPYTSYTTLKQKLLYAISEGVGTF
>Pecoramyces_ruminatium_ASRE01009846.1 .
IKVNRNNLFNDAYNNI-----------------------MSRPPKDFKN-ILRIEYKE--
--------EEGLDAGGLLRDFFYHI-SKEIG-NPNY--------------------SLFQ
YPNNNSY------------------------ELEINPNS---------------------
------------------------------SIADPEHLKYFRFIGRIIGLAIFN----KQ
YLPLSFTLLFYKKLLN-----------------------------------KPLEISDLE
Y---VDSQLFKSLQQIRXY------------------------------KGVEDLN--LT
--FSMDIED----CFSN------------RKTIELIPN-GINIS----------VTDSNK
N-KYIXLI--AKNKLNDTNDKEQMEALKQ--GFYEII---PNNINL--------------
--------LLNEVDXKFLISG---------INEID-IDDW--------------------
------------------------------------------------------------
---------------ENNTDYDG-YKKD--------------------------DITIIN
FWKVLVRNFSDENRKKLLLFATGNSQVPITGFKDLQGNNE-IQHFNIKKIG---------
----------------KEDDLPISHTSFNRIDLPPYTSFTIMKQKLLLAISEGMGGM
>Anaeromyces_robustus_MCFG01000521.1 .
ITINRNNLFYNAYEIF-----------------------MNHSPEDLKK-VLRIRYEG--
--------EEGLDAGGLLRDFFYQL-SKEIG-NPNY--------------------SLFQ
YSNDNLY------------------------ELSINPAS---------------------
------------------------------YTVYPNHLNYFKFIGRILGLSIFH----KQ
YLSVNFTILFYKKLLD-----------------------------------IPLEISDLE
F---IDPEIYKNINWLKEN------------------------------EETENLC--LT
--FSIDTED----CFGT------------HKKVELKPN-GASID----------VNDSNK
N-EYIRLL--VKYKLNNLNDKEQFEAIKE--GFYEII---PKNIKS--------------
--------IINEFDLKVLISG---------INEID-VNDW--------------------
------------------------------------------------------------
---------------ENNTDYEG-YTKN--------------------------DITIVN
FWKCVR-DFKPEKQMKLLLFATGNSQVPVTGFKDLQGSGK-IQHFKLKRVG---------
----------------TPNDLPISHTXFNRIDLPPYTTYTQLKQKLLLAISEGMGGF
>Pecoramyces_ruminatium_ASRE01015036.1 .
ISLRRLDLFVEAYYNI-----------------------ENKSPEELKK-RLKINYEG--
--------ETGIDAGGLLRDFFYNL-SKEIG-NPNY--------------------SLFQ
YSHEDSY------------------------ELEINPYS---------------------
------------------------------GIFEPDHLEYFKFVGRIMGLAVFH----DK
YLPLPFSIIFYKKFLN-----------------------------------KPLEFTDLE
L---IDPEFYKNILWLRNN------------------------------EGAENLN--LT
--FEIEEVD----CFNN------------RETIELKPN-GANID----------VDDFNK
Y-EYIRXL--AKYKLNYTKDKEQFQZIRD--GFYEII---PENIIS--------------
--------SFDEFDXQFLISG---------INEID-IDDW--------------------
------------------------------------------------------------
---------------ENNTVYEN-YNKC--------------------------DTTIIN
FWKVVR-EFSQENRTRLLLFTTGTTQVPVTGFKDLQGNGK-IEHFKIKRIE---------
----------------SNKNLPISHTXFNRIDLPPYTTYTQQKEKLLLAISNGIGSF
>Pecoramyces_ruminatium_ASRE01002525.1 .
ISLKRLNLFGEAYNNI-----------------------GNKSLEELKK-RLKIKYKG--
--------EIGIDAGGLNRDFFYNL-SKEIG-NPNY--------------------SLFQ
```

```
YSHEDSY------------------------ELEINPKS---------------------
------------------------------GIFEPDHLKYFKFVGRIMGLAVFH----DK
YLPLPFSIIFYKKFLN-----------------------------------KPLEFIDLE
L---VDPEFYKNILWLKDN------------------------------EGAENLN--L-
--FEIEEVD----CFNN------------RETIELKPN-GSNID----------VNDNNK
Y-EYIRLL--VNHKLNYLNAKEQFQAIRE--GFYEII---PEDIIS--------------
--------SFDEFDLKVLISG---------INEID-IDDW--------------------
------------------------------------------------------------
---------------EENTVYEN-YNKY--------------------------DITIIN
FWKVVR-EFSEENRTRLLLFTTGTTQVPVTGFKDLQGNGK-TEQFKIKRIE---------
----------------SNKNLPISHTXFNRIDLPSYTSYTQLKEKLLIAIHEGIGSF
>Schizosacch_pombe_NM_001019355.2 .
LKVSRATTFEDAYDII-----------------------SKLSVSDMKK-KLLIRFRN--
--------EDGLDYGGVSREFFYIL-SHAIF-NPGY--------------------SLFE
YATDDNY------------------------GLQISPLS---------------------
-------------------------------SVNPDFRSYFRFVGRVMGLAIYH----RR
YLDVQFVLPFYKRILQ-----------------------------------KPLCLEDVK
D---VDEVYYESLKWIKNN------------------------------DVDESLC--LN
--FSVEENR-----FGE------------SVTVDLIPN-GRNIA----------VNNQNK
M-NYLKAL--TEHKLVTS-TEEQFNALKG--GLNELI---PDSVLQ--------------
--------IFNENELDTLLNG---------KRDID-VQDW--------------------
------------------------------------------------------------
---------------KRFTDYRS-YTET--------------------------DDIVIW
FWELLS-EWSPEKKAKLLQFATGTSRLPLSGFKDMHGSDG-PRKFTIEKVG---------
----------------HISQLPKAHTCFNRLDIPPYNSKEELEQKLTIAIQE-TAGF
>Mit_daphniae_XM_013382979.1_JMKJ01000144.1 .
VPIRRDNLLEDSFNVI-----------------------SNLTLSSLKK-KPQIIFEG--
--------EEGLDYGGISREFFFLL-AKEIF-NPYY--------------------SLFE
YSSQDNY------------------------TLQISPGS---------------------
-------------------------------YINPEHMAYFYFVGRILGLAAFH----GF
LIDAYFVPAFYKRLLD---------------------------------RAACTMLSDLE
A---IDSELYSSLIWMLNN------------------------------PIH-DQI-FET
--MSVEEVR-----FGGGD----------PIIVDLIPN-GSSIS----------VTDDNK
R-LYIEKL--VEWKTYKR-ISEQMHQINR--GFYEIV---PRELIS--------------
--------IFDARELEVVISG---------VTQID-ISDW--------------------
------------------------------------------------------------
---------------KENTIYKN-CTES--------------------------DPLIIW
FWKVVH-SYPAEKKIRLLQFVTGSSKLPLNGFRDLQGSDG-PRKFTIEKTLS--------
----------------PPSHLPVSHTCFNRLDLPSYNSMEMLKKRLDKSLEN-ILGF
>Fonticula_alba_XM_009496587.1 .
IRVRRSRLLDDSYRAV-----------------------IRADPQHLRR-PLNVTFAG--
--------ESALDYGGVQREWFAAV-SKLAF-SPDY--------------------GLFE
YAKSGQY------------------------LLDVSRVS---------------------
-------------------------------GVNPSHLEYFRFVGRLIGMAILH----DK
YLDAFFVTSFYKRLLG-----------------------------------HSVALSDLE
S---IDAEHYRSFNWMLSN------------------------------PIE-GVI-YEN
--FSAAYEY-----LGQ--------------VRRVELD----IE----------VTDDNK
H-EYVEKY--VAWRCAHG-TEAQMTAILH--GLHEVI---PRSMLQ--------------
--------AFEPSELELLIGG---------RDDID-LDDW--------------------
------------------------------------------------------------
---------------FRNTIYKK-YTPVD-------------------------PQIV-W
FWEVIR-SWDPSKRRKLLQFVTGSERVPATGFGDLVGSSG-IRRFCIEKWDASH------
----------------SGDSLPQSHTCFNRIDIPPYTSKAQLLEKLEVAIEH-ATSF
>Encephalitozoon_cuniculi_NM_001042086.1 .
VYVDRSDVLRSSYFQV-----------------------MAKSPEEFRTRRLEIKLTG--
--------EEGLDYGGLTREWLVLL-AKDLL-DPNF--------------------ALFE
FATEDKT------------------------TVVPCKNS---------------------
-------------------------------YVNPEHLSYFKFVGRIIAKAIMD----GN
```

FINLHLSKFIYQYILG-----------------------------------KSCDLQDLE
S---ADPEFHKSLVWIRDN------------------------------PVD-KSL-GIT
--FSFDDVS-----FGV------------HRTVELVEG-GAHVF----------VDDSNK
A-EYVKLA--TQYRLFNG-IELQLSALKS--GLFEIL---GSKALE--------------
--------MFDESELELLICG---------IPDID-VDDW--------------------

---------------KNNTLYYG-YAEN--------------------------SKTVIW
FWRAVK-SLDSVSRAKLLQFVTGTSTLPFEGFSHLQGNNE-VQKFSIHKVSD--------
----------------RIDSLPTAHTCFNQLVLPEYSSYENLLKYLTLAINECSTGF
>Ordospora_colligata_XM_014708285.1 .
VYVDRDDVLRSSYFQV-----------------------MAKSPEDFRTKRFEIKLAG--
--------EEGLDYGGITREWLLLL-AKDLL-DPNF--------------------ALFE
FSTEDKT------------------------VAVPCKNS---------------------
-------------------------------HVNPEHLSYFKFVGRIMAKAIME----GY
FLNLQLPKFVYKHILG-----------------------------------KTCGLDDLK
S---VDNEFYKSLIWIRDH------------------------------SVD-ESL-GLM
--FSFTEVS-----FGV------------NITTDLIDN-GRDVF----------VSESNK
K-EYVRVA--AWHRLFNG-IEAQLSALKA--GIFEIL---GEDALD--------------
--------MFDENELELLVCG---------IPEIN-VDDW--------------------

---------------KSNTLYYG-YTES--------------------------SKTIIW
FWKAVK-CLDSVQKAKLLQFATGTSTLPFEGFSHLQGNNT-IQKFSIHKMPD--------
----------------RMDSLPTAHTCFNQLVLPSYSSYETMLKCLTTAINECSTGF
>Nosema_ceranae_ACOL01000841.1 .
ITVRRSSILNDSFFQV-----------------------MRKSSKELKAKRIQIKFSG--
--------EQGIDFGGLTKEWLELV-MQEAL-KPDQ--------------------GLFV
YSSDKRN------------------------SLHPFKNS---------------------
-------------------------------KIDPDHLSFFRFIGRMLAKIIIE----GF
NISIHFDKSVYKYLLG-----------------------------------LKCTLQDLE
E---IDPQFYNSLMWIKNN------------------------------KIE-NIL-NLT
--FSVENNN-----FGF------------NEVIDLIPE-GRQVL----------VTDDNK
N-DYIDLV--VENRLIKS-VEKQLNAMRE--GLFEMI---DEDNIC--------------
--------IFNEKELELLICG---------IPDIN-IDDW--------------------

---------------KNNTEYIG-YTSH--------------------------SRNISW
FWKAVE-GFTPEEKAKLLQFCTGSSRVPFEGFKNLQSTNG-YQKFSINKI----------
----------------STNRLPSAHTCFNQLDLPEYTSYEELRKNILYAINECQGGF
>Fonticula_alba_XM_009497911.1 .
LTVDRDTVLEDSFTAM-----------------------QFLKPERVRLAPIQITFRG--
--------EPAVDAGGVTREWFNIL-TRQMF-DPNY--------------------ALFE
NVSGKKV------------------------TLQPNRLS---------------------
------------------------------HLASANHLAYFRFVGRIIGRAIFD----AR
VLDVHFTQSLYKHMLG-----------------------------------RPISHQDMA
A---VDPSFHQSLVWILEN------------------------------SID-DVL-DLT
--FSHDTDD-----LGQ------------TVAVDLVPG-GSAIP----------VTDANK
H-DYVRLV--AELRLSKA-IESQIGAFLQ--GLHEII---PRHFLS--------------
--------IFSEQELESLIAG---------MPDID-VDDW--------------------

---------------RNNTLYTN-YSPA--------------------------SPQVQW
FWRAVR-SFSNEERAQLLQFVTGSSRIPLEGFSHLEGSNG-RQRFSISKDFR--------
----------------SNNRLPAAHTCFNQLDLPEYDSYEQLRRNLLTAISECTTGF
>Mitosporidium_daphniae_XM_013383512.1 .
LRIRRDRLFEDSFREI-----------------------MTLPATALAERPLSIAFDG--
--------EEGVDAGGLTREWFSLL-AQQML-NAGY--------------------ALFL
PVGGGQGGSGA--------------------VFYPNRLS---------------------
-------------------------------AVNPEHLDYFEFVGRIIGKAVVE----SQ
LLACHFACAFYKILLD-----------------------------------IPLELSDLE
A---VDASYYRSLAWMLSQA-----------------------------DPE-QLE-ELS

L-FMVTELDD----FGR------------PRMVDLIPN-GSTIP----------VTA-DR
IEEYVSLT--VDVRLRGA-LEAQFDAIRR--GLFGVL---SQEILA--------------
--------IFTEKELELFISG---------LALIDDIDDW--------------------

---------------KRNTEYGTGYTAT--------------------------SPQVVW
FWRAVR-SMSYEERSQLLQFVTGSMRVPLGGFSRLLGSNGALQRFSIHRDTG--------
----------------GAHRLPQAHTCFNQLDIPEYESYEQLRRALLVAIREGTTGF
>Nematocida_parisii_XM_013204697.1 .
LMVQRGAVFEDTFHQL-----------------------MRLNGEQVRNAKFNIKFAG--
--------EEGVDAGGLTREWYSEL-SKEMF-NANY--------------------ALFT
PIGS---------------------------SYQPNHIS---------------------
-------------------------------HINPEHLVYFKFIGRIIGKAVYD----EM
TVDCHFTRAFYKRVLS-----------------------------------IPVDLTDVE
A---LDPEFHRSLVWILEN------------------------------DIE-NVL-EMT
--FSLEQDR-----FGI------------TEVIDLKEN-GRNIA----------VTNENK
R-EYVELV--CRFKLVRV-IERQLSAFAE--GFFEIL---DVDMLR--------------
--------MFNEKELELLISG---------LPEID-VDDW--------------------

---------------RNNTIYFG-YTSD--------------------------SQVIRW
YWRAVR-NFSMEERAKLLQFATGTSKLPLEGFAGLRCQNG-NQKFQIHKASG--------
----------------GSSRLPTAHTCFNQLDLPEYDSYEQLVKALLFSLEECTSGF
>Aspergillus_niger_XM_001401916.2 .
LSVRRDQVFLDSFKSL-----------------------YFKTADELKYGKLNVRFHG--
--------EEGVDAGGVTREWFQVL-ARGMF-NPNY--------------------ALFI
PVASDRT------------------------TFHPNRLS---------------------
-------------------------------GVNSEHLMFFKFIGRIIGKALYE----GR
VLDCHFSRAVYKCILG-----------------------------------RSVSIKDME
T---LDLDYYKSLLWMLEN------------------------------DIT-DII-TET
--FAVETDD-----FGE------------KQVIDLIEN-GRNIP----------VTEENK
E-EYIQRV--VDYRLVGS-VKEQLDNFLK--GFHEII---PSDLIS--------------
--------IFNEQELELLISG---------LPEIE-VDDW--------------------

---------------KVNTEYHN-YSAS--------------------------SPQIQW
FWRAVR-SFDKEERAKLLQFVTGTSKVPLNGFKELEGMNG-VSRFNIHRDYG--------
----------------NKDRLPSSHTCFNQLDLPEYESYETLRQRLYTAMTAGSEYF
>Penicillium_chrysogenum_XM_002564676.1 .
LSVRRSEVFLDSFKSL-----------------------YFKSADELKYGKLNVRFHG--
--------EEGVDAGGVTREWFQVL-ARGMF-NPNY--------------------ALFI
PVAADRT------------------------TFHPNRLS---------------------
-------------------------------GVNSEHLMFFKFIGRIIGKALYE----GR
VLDCHFSRAVYKNILG-----------------------------------RSVSIKDME
T---LDLDYYKSLLWMLEN------------------------------DIT-DII-TET
--FAIETDD-----FGE------------KQVIDLKPG-GRDIP----------VTQENK
E-EYVQRV--VEYRLVES-VREQLDNFLK--GFHEII---PPELIS--------------
--------IFNEQELELLISG---------LPEID-VDEW--------------------

---------------KNNTEYHN-YSAS--------------------------SSQIQW
FWRAVR-SFDKEERAKLLQFVTGTSKVPLNGFKELEGMNG-VSKFNIHRDYG--------
----------------HKDRLPSSHTCFNQLDLPEYESYEDLRQRLYTAVTTGSEYF
>Coccidioides_immitis_XM_001247708.2 .
LSVRRDQVFLDSFKSL-----------------------YFKTADEMKYGKLSVRFHG--
--------EEGVDAGGVTREWFQVL-ARGMF-NPNY--------------------ALFI
PVASDRT------------------------TFHPNRLS---------------------
-------------------------------GVNQEHLMFFKFIGRIIGKAIYE----GR
VLDCHFSRAVYKRILG-----------------------------------KSVSIKDME
T---LDLDYYKSLLWMLEN------------------------------DIT-DIL-TEN
--FSVEVEA-----FGE------------KQVIDLVEN-GRNIP----------VTQENK
E-EYVQLV--VEHRLVGS-VKEQLDNFLK--GFHDII---PADLIS--------------

```
--------IFNEQELELLISG---------LPEID-VDDW--------------------
------------------------------------------------------------
---------------KNNTDYHN-YSAS--------------------------SPQIQW
FWRAVR-SFDKEERAKLLQFVTGTSKVPLNGFRELEGMNG-FSKFNIHRDYG--------
----------------NKDRLPSSHTCFNQLDLPEYESYETLRQRLYTAMTAGSEYF
>Pyrenophora_tritici_repentis_XM_001935731.1 .
LSVRRDQVFLDSFKSL-----------------------YFKSADEMKYGKLSIRFHG--
--------EEGVDAGGVTREWFQSI-SRQMF-NADY--------------------ALFV
PVASDRT------------------------TFHPNRLS---------------------
-------------------------------SINPEHLMFFKFIGRIIGKALYE----GR
VLDCHFSRAVYKQIMG-----------------------------------KQVSLKDME
T---LDLEYYKSLEWMIHN------------------------------EIT-DII-TET
--FSVEVEA-----FGE------------MQTVDLIEN-GRNIP----------VTEDNK
H-EYVRLI--TEHRLTGA-VHEQLENFLK--GFHDIV---PAELVS--------------
--------IFSEQELELLISG---------LPDIN-VDDW--------------------
------------------------------------------------------------
---------------KNNTEYHN-YTAA--------------------------SPQIQW
FWRAVR-TFEKEEQAKLLQFVTGTSKVPLNGFKELEGMNG-FSKFNIHRDYG--------
----------------SKDRLPSSHTCFNQLDLPEYETYEDLRKALYTAMTAGGEYF
>Tuber_melanosporum_XM_002835388.1 .
LNVRRDQVFLDSYKSM-----------------------YYKNGDEIKYAKLSIRFHG--
--------EEGVDAGGVTREWFQVM-ARQMF-NPDY--------------------ALFI
PVASDRT------------------------TFHPSRMS---------------------
-------------------------------GVNPEHLSFFKFIGRIIGKALYE----GR
VLDCHFSRAVYKRILG-----------------------------------KSVSLKDME
T---LDLDYYKSLVWMLEN------------------------------DIT-DII-TET
--FSVETDD-----FGD------------KKIIDLVPD-GRNVP----------VTDDNK
H-EYVRLL--VEYRLLTS-VQEQMENFLV--GFHDIV---PAELIS--------------
--------IFNEQELELLISG---------LPEID-VDDW--------------------
------------------------------------------------------------
---------------RNNTEYHN-YSAS--------------------------SPQIQW
FWRAVR-SFDKEERAKLLQFVTGTSKVPLNGFKELEGMNG-FSKFNIHRDYG--------
----------------SKDRLPSSHTCFNQIDLPEYESYESLRQNILTAITQGAEYF
>Mycosphaerella_graminicola_XM_003857457.1 .
LSIRRDQVFLDSFKSL-----------------------YYKSGNEIKYGKLNIRFIG--
--------EEGVDAGGVSREWFAAM-ARQMF-NPDY--------------------ALFN
PVASDRT------------------------TFHPNTLS---------------------
-------------------------------EVNPEHLMFFKFIGRIIGKALYE----NR
VLDCHFSRAVYRRILG-----------------------------------KSVSLKDME
T---LDLDYYKSLVWILEN------------------------------DIT-DVT-FET
--FSVDVDK-----FGV------------TETVDLISN-GRNIP----------VTEENK
H-EYVRHV--VDYRLVTS-VKNQLDNFLQ--GFHEII---PAELVS--------------
--------IFNEQELELLISG---------LPDID-VDDW--------------------
------------------------------------------------------------
---------------KNNTDYTN-YQPT--------------------------SPQIQW
FWRAVR-SFDKEEKAKLLQFVTGTSKVPLNGFKELEGMNG-FSKFNIHRDYS--------
----------------NKERLPSSHTCFNQLDLPEYESYEALRHQLYTAITAGSEYF
>Sclerotinia_sclerotiorum_XM_001588216.1 .
LSVRREQVFHDSFKSL-----------------------YFQTPDQMKYGKLSIRFHG--
--------EEGVDAGGVTREWFQVL-SRQMF-DPGY--------------------ALFI
PVSSDRT------------------------TFHPNQLS---------------------
-------------------------------SINEEHLMFFKFIGRIIGKALYE----GR
VLDCHFSRAVYKRILG-----------------------------------KAVSVKDME
S---LDPDYYKSLIWMLEN------------------------------DIT-DII-TET
--FSVDNDK-----FGV------------VETIDFIEN-GRNVA----------VTEENK
H-EYVRLM--VEWKLTGS-VKAQLDEFLK--GFHDII---PAELVS--------------
--------IFNEQELELLISG---------LPEID-VDDW--------------------
------------------------------------------------------------
```

---------------KSNTEYHN-YSAS--------------------------SPQIQW
FWRAVR-SYDKEERAKLLQFVTGTSKVPLNGFKELEGMNG-FSRFNIHRDYG--------
----------------NKERLPSSHTCFNQLDLPEYESYETLRAQVLTAITAGSEYF
>Fusarium_verticillioides_XM_018887114.1 .
LSVRREQVFHDSFKSL-----------------------YFKSGDEMKFGKLNIRFHG--
--------EEGVDAGGVTREWFQVL-SRQMF-DPNY--------------------VLFT
PVSSDRT------------------------TFHPNKLS---------------------
-------------------------------GINDEHLMFFKFIGRIIGKALYE----GR
VLDCYFSRAVYKRILG-----------------------------------KSVSVKDME
S---FDPDYYKSLCWMLDN------------------------------DIT-DII-TET
--FSVENDE-----FGA------------TTVVDLIPN-GREIA----------VTEENK
H-DYVRLV--VEHKLLSS-VKEQMAHFLQ--GFHDII---PAELIS--------------
--------IFNEQELELLISG---------LPDID-IDDW--------------------

---------------KSNTEYHN-YTPS--------------------------SQQIQW
FWRALR-SFDKEERAKLLQFVTGTSKVPLNGFKELEGMNG-VNRFNIHRDYG--------
----------------NKDRLPSSHTCFNQLDLPEYESYDHLRSQIMKAITAGSEYF
>Chaet_globosum_XM_001228090.1_AAFU01000362.1 .
LSVRRDQVFHDSFKSL-----------------------YFKSGPEMKFGKLNIRFHG--
--------EEGVDAGGVTREWFQVL-ARQMF-DPNY--------------------ALFI
PVSSDRT------------------------TFHPNKLS---------------------
-------------------------------GINDEHLMFFKFIGRIIGKALYE----GR
LLDCYFSRAVYKRILG-----------------------------------KPVSVKDME
S---FDPDYYKSLVWMLEN------------------------------DIT-DII-VET
--FSVEDDE-----FGV------------TKVVDLIEN-GRNIP----------VTEDNK
H-EYVRLI--VEHKLLSS-VKEQMENFLK--GFHDII---PEDLIA--------------
--------IFTEQELELLISG---------LPDID-VDDW--------------------

---------------KSNTEYHN-YTAA--------------------------SQQIQW
FWRAVR-SFDKEERAKLLQFVTGTSKVPLNGFKELEGMNG-VNRFNIHRDYG--------
----------------NKERLPSSHTCFNQLDLPEYESYDILRSQLLKAITA-GNDY
>Yarrowia_lipolytica_XM_500551.2 .
LNVRRDQVFLDSYKSM-----------------------YFKSAAEIRSGKLNIHFSG--
--------EEGVDAGGVTREWYQVL-ARQMF-NPDY--------------------ALFT
PVASDTT------------------------TFHPNRTS---------------------
-------------------------------WVNPEHLSFFKFIGRIIGKAIFD----QR
LLDCHFSRAVYKKILG-----------------------------------RGVSLKDME
T---LDIEYHKSLVWMLEN------------------------------DIT-DII-TET
--MSIETED-----YGE------------KKTIDLMPD-GRNIA----------VDESNK
A-EFVQRV--VEYRLITS-VEEQLEHFLQ--GFHDII---PKELVS--------------
--------IFNEQELELLICG---------LPEID-VDDW--------------------

---------------RNNTVYTN-YSAS--------------------------SPQIQW
FWRSIR-SFDDEERAKLLQFVTGTSKVPLDGFKELEGMNG-PTKFNIHRAYG--------
----------------NNERLPSSHTCFNQLDLPEYDSYETLRGSLLLAITEGREGF
>Schizosacch_pombe_NM_001020331.1 .
ITVRRDHVFLDSYRAL-----------------------HFKDADEVKFSKLNIHFRD--
--------EEGVDAGGVTREWLQVL-ARQMF-NPDY--------------------ALFL
PVTGDAT------------------------TFHPNRDS---------------------
-------------------------------SVNPDHLSFFKFTGRIIGKALYD----GR
LLDCHFSRAVYKHMLH-----------------------------------RSVSVKDIE
S---LDPDYYKSLVWMLNN------------------------------DIT-DII-TEE
--FAVEKDV-----FGE------------KTVVDLIPN-GRNIP----------VTELNK
Q-NYVNRM--VDYKLRES-VKDQLKSLLD--GFSDII---PSHLIQ--------------
--------IFNEQELELLISG---------LPEID-IDDW--------------------

---------------KNNTEYHG-YNVS--------------------------SPQVQW
FWRAVR-SFDEEERAKLLQFATGTSKVPLNGFKELEGMSG-FQRFNIHKSYG--------

----------------SLNRLPQSHTCFNQLDLPEYDTYEQLRSMLLTAINEGSEGF
>Pneumocystis_carinii_XM_018370458.1 .
LNVRREMIFLDSYLAL-----------------------YFKSGDEMKYSKLNIRFHG--
--------EEGVDAGGLTREWYQAL-ARQMF-NPDY--------------------ALFI
PVAADRT------------------------TFHPNRRS---------------------
-------------------------------DVNQDHLSFFKFIGRIIGKALYD----NR
LLDSHFSRAVYKKILG-----------------------------------KPVSLKDIE
T---LDLEYYKSLVWMLEN------------------------------DIT-DVI-TET
--FSVETEN-----YGA------------TETVDLVPG-GRTIL----------VTEENK
H-EYVKAV--IEYRLIDS-VKDQLDNFLV--GFYDII---PPDLIQ--------------
--------IFNEQELELLISG---------LPDID-VDDW--------------------

---------------RHNTEYFN-YTAS--------------------------SPQIQW
FWRAVR-SFDDEQRAKLLQFATGTSKVPLNGFKELEGMQG-IQKFSIHRDPT--------
----------------SSDRLPQSHTCYNQIDLPEYGSYEALRSALLTAINEGSEGF
>Candida_albicans_XM_710225.1 .
VSVRRDQVFLDSYRSL-----------------------FFKPKDEFRNSKLEINFKG--
--------EQGIDAGGVTREWYQVL-SRQMF-NPDY--------------------ALFT
PVVSDET------------------------TFHPNRTS---------------------
-------------------------------YINPEHLSFFKFIGRIIGKAIYD----NC
FLDCHFSRAVYKRILG-----------------------------------KPQSLKDME
T---LDLEYFKSLMWMLEN------------------------------DIT-DVI-TED
--FSVETDD-----YGE------------HKIIDLIPN-GRNIP----------VTEENK
N-EYVKKV--VEYRLQTS-VEEQMENFLI--GFHEII---PKDLVA--------------
--------IFDEKELELLISG---------LPDID-VSDW--------------------

---------------QNHTSYNN-YSPL--------------------------SLQIQW
FWRAVK-SFDNEERARLLQFATGTSKVPLNGFKELSGASG-TCKFSIHRDYG--------
----------------STDRLPSSHTCFNQIDLPAYDCYETLRGSLLMAITEGHEGF
>Kluyveromyces_lactis_XM_451611.1 .
ITVLRDQVFLDSYRAL-----------------------FFKSNDEIKNCKLDITFKG--
--------EAGVDEGGVTREWYQVL-SRQMF-NPDY--------------------ALFI
PVGTDNT------------------------KFRPNRTS---------------------
-------------------------------GINPEHLSFFKFVGMIIGKAISD----NC
FLDCHFSREVYKNILG-----------------------------------KPVSLKDME
S---LDLEYYKSLNWMLEN------------------------------DIT-YVI-DET
--FSVDTDD-----YGE------------HKTIDLIPN-GRNIP----------VTEENK
K-EYVQKI--VEYKLQES-VKDHMQNLLQ--GFYAVI---DKDLIS--------------
--------IFDEQELELLISG---------LPDID-VDDW--------------------

---------------KNNTTYVN-YTPT--------------------------CKQINY
FWRAVR-SFDKEERAKLLQFVTGTSKLPLNGFKDLSGING-DSKFSIHRDYG--------
----------------STERLPSSHTCFNQLDLPAYDSYEQLRGSLLLAINEGHEGF
>Saccharomyces_cerevisiae_CP004732.2 .
ITVRREQVFLDSYRAL-----------------------FFKTNDEIKNSKLEITFKG--
--------ESGVDAGGVTREWYQVL-SRQMF-NPDY--------------------ALFL
PVPSDKT------------------------TFHPNRTS---------------------
-------------------------------GINPEHLSFFKFIGMIIGKAIRD----QC
FLDCHFSREVYKNILG-----------------------------------RPVSLKDME
S---LDPDYYKSLVWILEN------------------------------DIT-DII-EET
--FSVETDD-----YGE------------HKVINLIEG-GKDIV----------VTEANK
Q-DYVKKV--VEYKLQTS-VKEQMDNFLV--GFYALI---SKDLIT--------------
--------IFDEQELELLISG---------LPDID-VDDW--------------------

---------------KNNTTYVN-YTAT--------------------------CKEVSY
FWRAVR-SFDAEERAKLLQFVTGTSKVPLNGFKELSGVNG-VCKFSIHRDFG--------
----------------SSERLPSSHTCFNQLNLPPYESYETLRGSLLLAINEGHEGF
>Laccaria_bicolor_XM_001877693.1 .

```
LNVRRARVFEDSFQYL-----------------------QRKTGDQIKHGKLSVRFYD--
--------EEGVDAGGVTREWFQIL-ARQMF-DPNN--------------------ALFQ
PCAADKL------------------------TYQPNKNS---------------------
-------------------------------WVNPEHLSFFKFVGRVIGKAIYD----GR
LLDAYFARSLYRQLLG-----------------------------------KPVDYKDVE
W---VDPEYYKSLCWILEN------------------------------DPT--VL-DLN
--FSVEADA-----FGV------------NQIIPLKEG-GESIS----------VTQENK
R-EFVQHS--AQYRLYSS-IKDQIESLST--GFYEII---PKDLIT--------------
--------IFNEQELELLISG---------TPDID-VDEW--------------------
------------------------------------------------------------
---------------RAATEYNG-YTSS--------------------------DPNIVW
WWRALK-SFNRDERAKVLSFATGTSRVPLSGFVDLQGVQG-VQRFSIHRAYG--------
----------------ESDRLPQAHTCFNQIDLPQYSSYEMLRQQLLMAINEGGEGF
>Ustilago_maydis_XM_011391602.1 .
LSVRRNSVFEDSFRYF-----------------------SRKTGPEVKHGKLNVRFTN--
--------EEGIDAGGVTREWFQVL-ARAMF-NPDY--------------------ALFQ
PCAADRT------------------------TYQPNRMS---------------------
-------------------------------YVNPDHLSFFKFVGRIIGKAIYD----GR
LLDAYFTRSFYKHILG-----------------------------------KPVDYRDLE
S---IDPEYFKSLEWMLSN------------------------------DIT-DIL-DLT
--FSVDDEE-----FGE------------TKVVDLKPN-GTSIS----------VTEANK
Q-EYVRLV--TEQRLTKS-IKSQIDAFLG--GFNEII---PSDLIR--------------
--------IFSEQELELLISG---------LPDID-VDAW--------------------
------------------------------------------------------------
---------------KNNTELHG-YSSG--------------------------DAVVQW
WWRAVR-SFDQTEKAKLLQFITGTSKVPLEGFAHLQGVQG-TQRFNIHKAYG--------
-----------------ADRLPAAHTCFNQLDLPQYESYEKLRSSLLLAMNEGGEGF
>Puccinia_graminis_XM_003319784.2 .
LNVRRPHVFEDSFHSL-----------------------ARRTGDELKYGKLSVRFYD--
--------EEGVDAGGVTREWLTIL-VKQML-DPNY--------------------ALFT
GSAADSK------------------------TYQPNRAS---------------------
-------------------------------AVNPDHLGFFTFCGRVIGKALYD----GR
VVDAYFTLAFYKHLLG-----------------------------------IPVGLSDLE
S---VDPDHHRSLKWMLDN------------------------------DID-GIF-ELT
--FSVEADD-----FGS------------TRIVDLKPG-GQEIP----------VTNENK
A-EYVQLL--VQNRLTVS-IREQIDAFKK--GFDEII---PRDLVR--------------
--------IFSATELQLLLNG---------LPDIN-VEDW--------------------
------------------------------------------------------------
---------------RANTELHQ-FQQS--------------------------DSTVTW
FWRAVR-SFGQEERAKLLQFATGSSRVPLEGFGALQGAQG-ATKFSLVNAH---------
----------------TKNVLPSAHTCFNQIDLPSYDSYEELRRMFLIAINEGSEGF
>Cryptococcus_neoformans_XM_012196494.1 .
LNIRRQYVFEDSFLAL-----------------------QRWNGEELKYGKLSVKFRH--
--------EDGVDVGGVTREWYSVL-AQQIF-DPNF--------------------ALFE
PCAADQQ------------------------TYQPNKTS---------------------
-------------------------------WINDVHLSYFKFVGRVIGKAVYD----GR
LLDAYFNRAFYKQILG-----------------------------------RTVDMRDLE
S---IDPEYHKSLQWMLDN------------------------------DIT-GVI-DQE
--FTIEDDQ-----FGE------------KKIVELKEN-GANIP----------VTEENK
E-EYVRLV--VSYRLDNS-IRDQIKSFLE--GFYDII---PQELIQ--------------
--------IFEPDQLELLISG---------ITTVD-VDEL--------------------
------------------------------------------------------------
---------------KNATQLNG-WKAT--------------------------DPEVAW
FWRALR-SFSQEERSRFLMFVTSSSRVPLGGFSQLQGSSG-TQPLQLQKLHG--------
----------------KEGGLPQASTCFNLLLLPTYASYEQLRERLQFAITE-TGGF
>Rhizopus_oryzae_GDUK01004271.1 .
LSIRRDYIFEDTYQQL-----------------------QDRTGNEIRYGKLKVHFQD--
--------EEGVDEGGVSREWFSAL-ARQMF-DPNY--------------------ALFI
```

TSAADKL------------------------TYLPNRAS---------------------
-------------------------------GVNPDHLSYFKFVGRVIGKAIHD----GR
LLDAYFTRSFYKLILG-----------------------------------RSIDYKDLE
A---IDPTYYKSLVWMLEN------------------------------DIT-NVI-DLT
--FSVETDD-----FGT------------TKTIDLKPD-GRNIP----------VTEENK
H-EYVYLI--AQQRLVLA-IKPQVDAFLE--GFHEII---PSSLIS--------------
--------IFNEQELELLISG---------LPDID-IDDW--------------------

---------------KANTVYQG-YNFQ--------------------------SPQIQW
FWRAVR-SFDEEERAKLLQFATGTSKVPLGGFSALQGSNG-LQKFQIHKEFS--------
----------------DINRLPSAHTCFNQIDLPQYQNYEDLRRNLFKAISECSTGF
>Allomyces_macrogynus_ACDU01002042.1 .
LNLRRQNVFEQSYQQM-----------------------QHLSGEELKNGKLNVRFYG--
--------EEGVDAGGLTREWFSVL-ARQIF-NPDY--------------------ALFK
TSAVDKA------------------------TYQPNRAS---------------------
-------------------------------WVNPEHLHYFKFVGRFIGKAIYD----QR
LLDCYFTRSFYKHLLG-----------------------------------KAVDIRDME
A---VDPSYYKSLEWILEN------------------------------EIN-DIM-DLT
--FSVETDD-----FGK------------TKVIDLKPD-GRNIA----------VTDENK
H-EYVRLV--VEQRLTLA-IRDQIAAFTE--GFFDMV---PRDLVS--------------
--------IFNEQELELLISG---------MPEID-VDDW--------------------

---------------RNNTEYHH-FTAS--------------------------APVIQW
FWRAVR-SFDQEHRAKLVQYVTGTSKVPMEGFRALQGSTG-VQKFQIVRDPG--------
----------------GTHRLPSAHTCFNQLDLPEYESYEKLRTMLLKAVDEASEGF
>Rozella_allomycis_ATJD01000704.1 .
ITVHRQYVFEESFHQV-----------------------MSRSSEELKYGKLNVRFYN--
--------EDGVDAGGVTRDWFSTL-SIQMF-NPDY--------------------ALFK
TSAVDKI------------------------TYQPNRSS---------------------
-------------------------------WVNPEHLLYFKFCGRIIGKAIYD----GK
LLDCFFTRSFYKQILG-----------------------------------LQIDYKDME
A---IDPEYFKSLVWILEN------------------------------NIT-DIL-DLT
--FSIESDD-----FGK------------RRIVDLITN-GRNIP----------VTEQNK
V-DYVKLV--SEHRLLNA-IKSQVDAFKQ--GFYEII---PQNLIS--------------
--------IFNEQELELLISG---------LPDID-IDDW--------------------

---------------RNNTEYQG-XISS--------------------------SPQIQW
FWRAVR-SFTQEERAKLIQFVTGTSKVPLEGFSKLQGSSG-VQRFQIHKAYGNDA-----
----------------STVRLPSAHTXFNQLDLPPYESYEQTRELLLLAINEC-AGF
>Phycomyces_blakesleeanus_XM_018428041.1 .
LNVRRQYVFEDSYHQL-----------------------QGRTGEEIKHGKLNVRFYD--
--------EEGVDAGGVTREWFSVL-ARQMF-DPNY--------------------ALFI
TSAADKL------------------------TYQPNRAS---------------------
-------------------------------WVNPDHLSFFKFVGRLIGKAIYD----GR
LLDAYFTRSFYKHILG-----------------------------------RQVDYRDVE
A---IDPSYYKSLVWMLEN------------------------------NIT-DVV-DLT
--FSIDTDD-----FGT------------AKTIDLKPN-GRDIP----------VTEQNK
H-EYVYLV--TEQKLTTA-IKDQINAFLQ--GFHDII---PASLIQ--------------
--------IFNEQELELLISG---------LPDID-IDDW--------------------

---------------RNNTDYET-YNVS--------------------------SIQIQW
FWRAVR-SFDQEERAKLLQFATGTSKVPLKGFAHLQGSSG-LQKFQIHKDFG--------
----------------GENRLPSAHTCFNQIDLPMYTSYESLRANLFKAINECSTGF
>Rhizopus_oryzae_GDUK01028786.1 .
LNVRRQYVFEDSYHQL-----------------------QGRTGDEIKYGKLSVLFYD--
--------EEGLDAGGVTREWFSVL-ARQMF-DPNY--------------------ALFI
TSAADKL------------------------TYQPNRAS---------------------
-------------------------------AVNPDHLSFFKFVGRVIGKAIYD----GR

```
LLDAYFTRSFYKHILG-----------------------------------RPVDYRDVE
A---IDPEYYKSLVWMLEN------------------------------DIT-DII-DLT
--FSIETDY-----FGT------------KETVDLKPD-GRNIP----------VTEANK
H-EYVTLV--TEQKLTTA-IKDQINAFVQ--GFHDII---PAHLIQ--------------
--------IFNEQELELLISG---------LPDID-IDDW--------------------
------------------------------------------------------------
---------------KNNTEYEG-YSAS--------------------------SPPIQW
FWRAVR-SFDQEERAKLLQFATGTSKVPLEGFAHLQGSSG-IQKFQIHKDFG--------
----------------GEKRLPSAHTCFNQIDLPQYDSYESLRANLFKAINECSTGF
>Mortierella_alpina_ADAG01001083.1 .
MNVRREMVFVDSFSQL-----------------------QSRSGEEIKYSKLNVHFHG--
--------EEGVDAGGVTREWFQVL-ARQMF-NPDY--------------------ALFK
TSAADKL------------------------TYQPNRAS---------------------
-------------------------------WVNSDHLLFFKFIGRVIGKAIYD----GR
LLDAYFTRSFYKHILG-----------------------------------RPVDYRDVE
A---VDPEYYKSLVWMLEN------------------------------DIT-DIV-EET
--FSVETDD-----FGN------------TKVVDLKPN-GRNIP----------VTEQNK
H-EYVKYI--TEQKLTLA-IKDQIHAFLQ--GFHEVI---PAHLIS--------------
--------IFNEQELELLISG---------LPDID-VDEW--------------------
------------------------------------------------------------
---------------KNCTEYQN-YSSS--------------------------SPQIQN
FWRAVR-SFDQTERAKLLQFVTGTSKVPLGGFSQLQGISG-VQKFQIHKDFS--------
----------------STKRLPSAHTXFNQLDLPEYETYEELRQQLLTAISECSTGF
>Entomophthora_muscae_GEMZ01017396.1 .
LNVRRQYVFEDSFRQF-----------------------QGRSGQDIKYGKLNVKFHD--
--------EEGVDAGGVTREWFSVL-SRQMF-NPNY--------------------ALFK
SSANDKV------------------------TYQPNRTS---------------------
-------------------------------WVNSDHLLYFKFVGRIIGKAIYD----DH
LFDAYFTRSFYKHILG-----------------------------------KPVEIRDLE
A---IDPEFYKSMVWMLEN------------------------------DIT-DVV-YAN
--FCVETDD-----FGR------------KLVIELKPG-GQDIT----------VTQENK
F-EYVQLV--TEQKLYGA-IKDQIEHFLR--GFHEVI---PKELIS--------------
--------IFNEQELELLISG---------LPDID-IDDW--------------------
------------------------------------------------------------
---------------KNNTDYEG-YTTS--------------------------SPQIQW
FWRAVR-SFDQSERAKLLQFVTGTSKVPLEGFAHLQGSTG-VQKFQIHRDFA--------
----------------SVKRLPSAHTCFNQLDIPMYESYDDLRRQLLLAIEECNTGF
>Spizellomyces_punctatus_XM_016753891.1 .
INVRRQYVFEDSYHQL-----------------------QGRSGDEIKFSKLNVRFYD--
--------EEGVDAGGVTREWFSVL-ARQMF-NPDY--------------------ALFR
PSAVDKV------------------------TYQPNRLS---------------------
-------------------------------YINPDHLLYFKFVGRIIGKAIYD----GR
LLDAYFTRSFYKSMIE-----------------------------------VPVDYKDME
A---VDPEYHKSLEWILQN------------------------------DNV-EVL-DLT
--FSTEIDE-----FGK------------KQTIDLKPN-GRDIQ----------VTEENK
H-EYVKLV--VEQRLMTA-IRAQIDAFLS--GFHDII---PKDLVK--------------
--------IFNEQELELLISG---------MPDID-IDDW--------------------
------------------------------------------------------------
---------------KNNTEYQN-YTSS--------------------------SPQVQW
FWRAVR-SFSQEERAKLVQFATGTSKVPLEGFAQLQGANG-VQKFQIHKDFS--------
----------------SADRLPSAHTCFNQLDLPQYESYEQLRSNLLLAISEGGTGF
>Pecoramyces_ruminatium_ASRE01028691.1 .
IAVRRQYVFEDSFHKL-----------------------QGKTGNEIKYSKLNVRFVE--
--------EEGVDVGGVTREWFSAL-ARQMF-NPDY--------------------ALFK
PSAQDRV------------------------TYQPNRNS---------------------
-------------------------------WINPDHLSFFKFVGRIIGKAIYD----GR
ALDCYFTRSFYKHILN-----------------------------------IAVDYKDIE
A---IDPEYFKSLEWILHN------------------------------DIT-DVL-DLT
```

--FSLEIDE-----FGK------------KSIIDLKPD-GRNIP----------VTEENK
V-EYVKLV--TEQRLTVA-IKKQIKAFLD--GFHDII---PHSLIS--------------
--------IFNEQELELLISG---------LPEID-IDDW--------------------

---------------KNNTVYEN-YSSS--------------------------SPQVQW
FWRAVR-SFTQEERAKLIQFTTGTSKVPLEGFSNLQGVNG-IQKFQIHKDFG--------
----------------SIERLPSAHTXFNQLDIPAYESYEHLRKALLLAINECSVGF
>Anaeromyces_robustus_MCFG01000004.1 .
IAVRRQYVFEDSFHKL-----------------------QGKTGNEIKYGKLNVRFME--
--------EEGVDVGGVTREWFSAL-ARQMF-NPDY--------------------ALFK
PSAVDRV------------------------TYQPNRNS---------------------
-------------------------------WINPDHLSFFKFVGRIIGKAIYD----GR
LLDCYFTRSFYKHILN-----------------------------------IDVDYKDIE
A---IDPEYFKSLEWILHN------------------------------DIT-DVL-DLT
--FSLEIDE-----FGK------------KSIIDLKPD-GRNIP----------VTEENK
V-EYVKLV--TEQRLTVA-IKKQIEAFLN--GFHDII---PHSLIS--------------
--------IFNEQELELLISG---------LPEID-IDDW--------------------

---------------KNNTVYEN-YSSS--------------------------SPQVQW
FWRAVR-SFTQEERAKLIQFTTGTSKVPLEGFSNLQGVNG-IQKFQIHKDFG--------
----------------SIERLPSAHTXFNQLDIPAYESYEHLRKALLLAINECSVGF
>Piromyces_finnis_MCFH01000037.1 .
IAVRRQYVFEDSFHKL-----------------------QGKTGNEIKYSKLNVRFAE--
--------EEGVDVGGITREWFSAL-ARQMF-NPDY--------------------ALFK
PSAVDRV------------------------TYQPNRNS---------------------
-------------------------------WINPDHLSFFKFVGRIIGKAIYD----GR
CLDCYFTRSFYKHILN-----------------------------------IDVDYKDIE
A---IDPEYFKSLEWILHN------------------------------DIT-DVL-DLT
--FSLEIDE-----FGK------------KSIIDLKPD-GRNIP----------VTEDNK
V-EYVKLV--TEQRLTVA-IKKQIEAFLD--GFHDII---PHSLIS--------------
--------IFNEQELELLISG---------LPDID-IDDW--------------------

---------------KNNTVYEN-YSSS--------------------------SPQVQW
FWRAVR-SFTQEERAKLIQFTTGTSKVPLEGFSSLQGVNG-IQKFQIHKDFG--------
----------------SIERLPSAHTWFNQLDIPAYESYEHLRKALLLAINECSVGF
>Rhizopus_oryzae_GDUK01021469.1 .
ITVKRGQVLLDGYQQL-----------------------SSLPTSAWKG-KIRVNFINEL
GME-----EAGIDRGGPFKEFITTL-ISEAF-KPNY--------------------GLFE
ATSQN--------------------------SFYPSPSS---------------------
------------------------------AVHGKNHIQLFEFIGKAIGKAVCE----GI
LLDVQFASFLLAKLLG-----------------------------------RNVFLEELK
E---LDEDVWKNLIYVKNYEG----------------------------DVE-VL--GLT
--FEVDEDV-----FGK------------IESHELKYR-GKHVP----------VVNSNR
I-EYVYLM--ADYKLNQR-AKNQTNAFIQ--GFKTVV---SESWIR--------------
--------LFSPPELQRVLTG--------EDKDFD-VLDM--------------------

---------------RKHTVYEDGYFDE--------------------------HPVIRS
FWQIVE-QFTFEEKKTLLKFVTGCSKPPLGGFSYLQPPFT-IRMVSTELDGPASIRMIKS
VLKMNT----------KSGRLPTSSTCFNLLKLPAYTRKAQLKEKLSYSINS-NTGF
>P_blakesleeanus_XM_018428618.1_AMYC01000237.1 .
ITVRRNFVLEDGLRGL-----------------------SNLSPTAWKG-TIRVSFVNEL
GIE-----EAGIDQGGPFKDFISLL-VAEVF-KPSC--------------------ELFA
ATPKTN-------------------------LFYPAASS---------------------
------------------------------HIIGASHVAYFELIGKIIGKAVYE----GI
LIDAQFASFLLSKLLG-----------------------------------RNVFLEELR
E---LDEDIWRNLTFVKHQD------------------------------NIE-DLG--LT
--FATDEQI-----NGK------------VVTHELKFL-GSQTA----------VTDSNK
V-EYVYLM--ADYKLNQQ-AKEQTKAFIN--GFRSVI---FDGWIK--------------

--------VFSPPELQRVISG--------EDTDFD-VHDL--------------------

---------------RRHTDYQNGYFDQ--------------------------HPVIKL
MWQIVD-GLTSQEKRAFLKFVTGCPKPPLGGFDYLQPPFT-IRMVSPDKDQQSMEGLGIV
KSFFKINGLQN-----KGGRLPTSSTCFNLLKLPAYTKKSLLREKILY---------
>Mortierella_alpina_LDAW01000451.1 .
VKVRRGYVLEDGYQNL-----------------------GQLSASGWKN-TIRVKFVNEV
GAD-----EAGIDQGGPFKEFMESF-LEAGF-SPNL--------------------NLFT
TTTASMN------------------------MLYPSPTS---------------------
-----------------------------HYTHPGTGLELFRLFGRMLGKAMYE----GL
LVEVKFANFFLCKILG-----------------------------------RTVFLDEMR
S---FDEQVFKNLMFLKKYEG----------------------------DVE-DL--GLT
--FSLDEEV-----FGT------------HRTVELLTG-GKDIE----------VTKDNR
I-NYIFQV--SDYKLNKQ-IQDQSRAFIE--GFRSII---PQPWIS--------------
--------IFSPQELHRVMAG--------EDVDFD-VQDL--------------------

---------------RAHTDYQNGYFDQ--------------------------HPVIRN
LWSVLE-DFSSEEKRAFLKFVTSCSKPPLGGFKHLHPPFS-IRLVMNPTTTDQNHGGSST
Q---------------AXARLPTSSTCFNLLKLPPFSSKPVLKDKLRYAIMS-NTGF
>Spizellomyces_punctatus_XM_016754283.1 .
VVVHRNAVLEDGYRQL-----------------------ARVPPQQLKQ-AIRVKFVNEL
GLE-----EAGIDQSGIFKEFLEEM-CKRAF-STNL--------------------NLFH
TTPDG--------------------------YVTPSPTS---------------------
-------------------------------FIHEEHLQLLEFVGKIFGKALYE----GI
AIDIPFANFIYAKLLG-----------------------------------RYNYLDELP
S---LDPQLYKNLTFLKRYEG----------------------------DSE-DL--GLT
--FTIDQDI-----FGN------------VVSKEIKPG-GAAIS----------VTNENK
F-EYVHLM--ADYRLNRE-CRDQFKALIR--GFRSII---SEKWLG--------------
--------FFSPTELQKLMCG--------ENVEFD-VKDL--------------------

---------------RAHVRYEGGYFDQ--------------------------HKTIRS
LWQVVS-DFGPKDKAAFLKFVTSCSKPPVGGFQYLNPPFT-IRYVAESDEGGTQTENPVV
AGARLIGSAFGITLGKDMNRLPTASTCFNMLKLPAYKKKSTLKEKLLYAINS-GAGF
>Rozella_allomycis_ATJD01000205.1 .
INIQRGNEFESGFQQM------------------------NSLGHKWKQ-KIRISFLDEF
QQV-----EEGVDGGGLYKEFLNNL-LKIAL-SPNY--------------------GLFK
VNHLG--------------------------QVYPNNNS---------------------
---------------------FSSHGIKLKFITIDDYLTLFSFLGRVIGKALCD----RI
LIDVPFVRFFIAKWIG--------------------------KHISCNKFYIKCLVDDLA
L---LDQDLYKNLLFLRDYKG----------------------------NVE-DL--NLT
--FSIADDGKL---YLRLIKDLNF-----NSSKNLIPN-GSEIS----------VTNDNR
L-NYIYLL--ANEKLNNQ-IRHQTLAFLD--GLSNLV---SLEWLK--------------
--------IFTQSELQLLLSG--------TTTPID-LNDW--------------------

---------------KKHCVYQGDYSQLGHE-----------------------DPTIKL
FWEIIS-EMSEVEKRKVLKFVTSCPRPPLLGFSQLEPKFG-IQSTG--------------
----------------TDDRLPSSSTCFNLLKLPRYTSKSVLKGKLLYAINS-EAGF
>Aspergillus_niger_XM_001388703.2 .
ANIRRESIFKDAFDEF------------------------YELGDGLKE-PIQITFIDKF
NTT-----EAGIDGGGVTKEFLTSV-TSEAF-KSTSDL------------------NLFE
ENDQH--------------------------LLYPNPAA---------------------
----------VEQRRELLRQLGFVENTAEWNENVRDLLRRYEFLGRIIGKCLYE----GI
LVDVNFAPFFLLKWAL-----------------------TGGTGSAQRETAYRANLNDLK
D---LDQGLYQGLLQLKNYPG----------------------------DVE-DF--SLN
--FTVTDTIPLP--DGGNR----------TTTRDLKSH-GSDIP----------VTNQNR
L-VYISYI--ARYRLQVQ-PALQTNAFLQ--GLGQII---QPSWLS--------------
--------MFNQTELQTLVSG--------DSGDID-VADL--------------------

---------------RRNTLYGGVYTIGDDKEE---------------------HPTVKL
FWEVME-KMSNEERQKVLRFVTSTPRAPLLGFSHLNPRFS-IRDSSE-------------
----------------DQERLPSTSTCVNLLKLPRYTNANILREKLLYAVNS-GAGF
>Coccidioides_immitis_XM_001245550.2 .
ANIRRENLFEDAFEQF------------------------YPLGEGLKE-PIQITFIDKF
DTV-----EAGIDGGGVTKEFLTSI-INDAF-DPSGVL------------------SLFS
ENDQH--------------------------LLYPNPTA---------------------
----------VEQRKALLRQAGVPERSAEWNQQIRELLKRYEFLGRVIGKCLYE----GI
LVDVNFAGFFLLKWAL-----------------------TGGSSLASKESAYRANLNDIR
D---LDESLYQGLLQLKNYPG----------------------------NVE-DF--SLN
--FTVTDTVTVPG-TGPDDPEKAQ-----TITRELKPN-GSNIA----------VTNQNR
L-VYISYI--ARHRLQAQ-PYLQTNAFLQ--GVGQII---QPSWLS--------------
--------MFNQGELQRLVGG--------DSGEID-VADL--------------------

---------------RRNTVYSGVYTLGDDMEE---------------------HMTVKL
FWQVME-SMPNTDRQKVLKFVTSTPRAPLLGFSHLNPRFS-IRDSSS-------------
----------------DEERLPSTSTCANLLKLPRYTSARTLHEKLMYAINS-GAGF
>Penicillium_chrysogenum_XM_002561175.1 .
ADIRRESVFEDAFSQY------------------------YGLGDGLKE-PIQISFIDQF
GAM-----EAGIDGGGVTKEFLTSV-TAEAF-KTDDYE------------------SMFA
ENDHH--------------------------LLYPSPVA---------------------
----------VDQLKKVLSEAGLTSSSPEWQNDVRGLLRRYEFLGRIIGKCLYE----GI
LVDVNFAPFFLLKWAL-----------------------TGGSRSAVKESSYRANLNDLK
D---LDEGLYQGLLQLKNYPG----------------------------DVE-DF--GLD
--FTINNVIRMP--GGQNR----------TVTAELKPS-GSQTP----------VTSKNR
L-VYISYV--ARYRLQLQ-PALQTNAFLQ--GLGQII---QPAWLS--------------
--------MFNQSELQTLVSG--------DKADID-VEDL--------------------

---------------RRNTLYGGVYVIGDDNLE---------------------HPTIAL
FWQVMH-EMTNEERQKVIRFVTSTPRAPLLGFSHLRPHFS-IRDSSD-------------
----------------DQERLPSTSTCVNLLKLPRYSDADTLRSKLLYAVSS-GAGF
>Pyrenophora_triticirepentis_XM_001931824.1 .
ARIRRENEFDDAFEQF------------------------YELGQGLKE-PIQITFMDQF
GAA-----EAGIDGGGVTKEFLTSV-TNRAF-MPTDYI------------------DMFV
ENDQH--------------------------LLYPNPAA---------------------
----------LEEHKEALRQAGLRENSPEYRAQVTELLQRYEFLGRIIGKCLYE----GI
LVDVNFAPFFLRKWAL-----------------------TGGAGSAPNESGYRPTLNDLR
D---LDEELYQGLHKLKTYPG----------------------------DVE-DF--SLN
--FTVTDTVVVDHATSPKKTK--------AITKELKPD-GSNTP----------VTNQNR
L-VYISYM--ARHRLQNQ-PYAQTTAFLR--GLSTMI---QPSWLS--------------
--------MFNQSELQTLISG--------TRTSID-VEDL--------------------

---------------RRNTIYGGTYVIGDDGQE---------------------HPTIQI
LWKVMK-EMSDDERRAVLKFVTSTPRAPLLGFGTLNPRFS-IRDAGS-------------
----------------DQERLPSTSTCVNLLKLPMYRDEKTLKEKLLYSVFS-GAGF
>Chaetomium_globosum_XM_001226851.1 .
AQIQRGRMFDDALESF------------------------WGLQDGLKE-PIQITFVDEF
GMP-----EAGIDGGGVTKEFLTSV-TTEAF-TPNE--------------------GLFI
ANSKN--------------------------SYYPNPCS---------------------
----------IDQSKNALQEAQIPENSEEWAETITSRLRQYEFLGRVIGKCLYE----GI
LIDVSFAGFFLLKWAT----------------------------SAGASDTYRANINDLR
E---LDEELYQGMLRLKNYPG----------------------------NVG-DL--SLD
--FTITDQISL---PGEPFR---------TTTRDLVPN-GENVV----------VTNENR
L-LYISYV--ARHRLIIQ-PYAQTRAFLR--GLGMII---DPSWLS--------------
--------MFNQNELQRLVGG--------DSSEID-VEDL--------------------

---------------RKHTAYSGVYEIGDDGQE---------------------HPTVKL
FWEVMY-QLEDRERRDVLKYVTSTPRAPLLGFSQLSPPFS-IRDGGR-------------

```
----------------DQERLPSASTCVNLLKLPRYDDAETLKRKLLYAVTS-GAGF
>Fusarium_verticillioides_XM_018889203.1 .
AQISRESLFDDAYKQF------------------------YEIGDDLKG-PIQITFVDQF
GAA-----EAGIDGGGVTKEFLISV-ATEAF-SAEGGR------------------GMFT
SNEKG--------------------------LLYPDPTA---------------------
----------LDVIREELRHAGMTEADSTFRDMISDLLRRYEFLGRIIGKCMYE----GI
LVDLVFAGFFLLKWTS---------------------------TGPNDENTYKGSVNDLR
D---MDEDLYRGMLRLKNHPG----------------------------DVS-EM--GVD
--FTIEDQVSD---PKDSVK---------TVTRKLIAN-GDQIH----------VTNDNR
L-LYISYV--ARHRLVVQ-PAPQTSAFLR--GLRSII---RPSWLS--------------
--------MFNQSELQRLVGG--------DSSEID-VEDL--------------------
------------------------------------------------------------
---------------RKHTIYGGLYQIGDDGEE---------------------HDTIKL
FWKVMH-SFTDDQRRAVLKYVSSTPRAPLLGFSQLKPLFS-IRDGGT-------------
----------------DEERLPSTSTCVNLLKLPRYTTEATLREKLLYAIQS-GAGF
>Sclerotinia_sclerotiorum_XM_001598229.1 .
ARIRRESVFEDAFEAF------------------------YPLSEGLKE-PIQITFIDSF
GTA-----EAGIDGGGVTKEFLTSA-TNEAF-RGPLGYGSRT--------------SLFV
TNDQN--------------------------LLYPNPTA---------------------
----------TEQDYEFLRSNGITETNPDWKAHSAQLRNRMQFLGRIVGKCLYE----GI
LVDINFAGFFLLKWSA---------------------------AFSGSEATSRGNLNDLR
D---LDEGLYQGLLKLKNYPG----------------------------NVE-DF--SLD
--FTITDTIPSHI-PGGRPK---------IITRDLMPN-GSNIP----------VTNENR
L-LYIAYV--VRHRLHIQ-PYELTQAFLR--GLGQII---NPSWLS--------------
--------MFNQVELQTLISG--------ETSEIN-IDDL--------------------
------------------------------------------------------------
---------------RRNTRYGGVYEIGDDGLE---------------------HPTVMM
FWQVMK-EFEDEDRRKVLKYVTSTPRAPLLGFSSLVPRFS-IRDGSL-------------
----------------DEKRLPSASTCVNLLKLPRYQSKEKLKEKLLYAINA-GAGF
>Tuber_melanosporum_XM_002841937.1 .
ATIRRNRVFEDAYEQF------------------------WPLGEGLKE-PIQITFIDRF
GVE-----EAGIDGGGVTKEFLTGV-CSDAF-TPSK--------------------NLFL
ENEQH--------------------------LLYPNPTS---------------------
----------IEELKEELIKTRGPYQEVIGDEV-KPLSKRYEFLGRVLGKCLYE----GI
LVDVAFAPFFLLKWSQ---------------------------QASDSRASMAIGVNDLR
D---LDEGLYRGLVKLKNYTG----------------------------DVESDF--GLN
--FTISSRIPR---HDK------------TITVELKPG-GETIP----------VTNANK
L-EYVHLV--SRYRLSVQ-AHIQTSAFLK--GLSSII---NPSWLS--------------
--------MFNQSELQTLVGG-------DINTPID-VEDL--------------------
------------------------------------------------------------
---------------RRNTIYGGVYQLGEDGIE---------------------HESIRL
FWEVMR-SLGDEERRKVLKFVTSVARAPLLGFGVLRPRFS-IRDAGE-------------
----------------DQGRLCSASTCVNLLKLPRYRDPRILREKLLYSVNS-NAGF
>Mycosphaerella_graminicola_AFIV01000278.1 .
AKIKRTQEFQDAYEQF------------------------YELGADLKE-PIQITFVDQW
DMP-----EAGIDGGGVTKEFLTSI-ISQAF-DPDANNME----------------QFFV
ENDQH--------------------------LLHPNPTA---------------------
----------FETLKYRLISAGHRPDSDEVRTQIRELYRQYEFLGRIIGKCLYE----GI
LVDVSFAGFFLKKWAL-----------------------TGGTGSAPSESHYRANINDLR
E---LDESLYRGLLQVKYAS-----------------------------DAS-DL--GMT
--FSVNDIVTSP--PPSKPH---------VLEVDLIPN-GANTP----------VTNENR
L-LYINAL--SRYRLQTQ-STAQSRAFLR--GLGDMI---QPSWLS--------------
--------MFNQSELQTLIGG--------ASAGID-VQDL--------------------
------------------------------------------------------------
---------------RRNTLYGGTYVIGTDGLE---------------------HPSIQH
FWRVME-TLPDEDRRKVLKFVTSTPRGPLLGFGHLNPRFS-IRDSGR-------------
----------------DENRFPTTSTCVNLLKLPMYGSEERLRRMLLAAVNS-GAGF
>Yarrowia_lipolytica_XM_500283.1 .
```

APVIRGREFESAYEKL------------------------YSYGSELKK-PLSIRFFNDF
G-P-----EAGIDGGGLTKEFLTSV-TDDAF-NTSR--------------------GLFA
ATDNH--------------------------LLYPNPTT---------------------
-----------------------------------HDTKQLAFLGNLVGKGLYE----NI
LIEHGFASFFLQKWTQ---------------------------------GSMRSSIDDLY
S---LDPNLYESLASLKQIYAT---------------------------QGDADL--GLT
--FSIDYADS----EGN------------VRTRDLIRN-GSEVP----------VTKANY
L-RYIYEV--ANFKLNTS-IRTQTDAFLG--GLYQLI---DPSWVS--------------
--------MFNADELQMLISG--------GHANVD-VWDL--------------------

---------------KTHTNYGG-YLDT--------------------------DQTIKD
FWTVFE-SFEEEDKRLLLKFVTSVSKAPLQGFKALNPSFA-IRNAGR-------------
----------------QVDRFPTASTCVNLLKLPDYQDIDQLRKKLLYAIRS-HAGF
>Candida_albicans_XM_704929.2 .
AKINRDSILEDAYNAY------------------------HRQGANFKN-RLQVEFFNQY
G-K-----EAGIDGGGITKEFLTCV-VKEGF-NPDNAF------------------ELFK
ETGDN--------------------------QLYPNDKI---------------------
---------------------FEILYVGMDREFQQIKLDYIRFLGMIVGKCLYE----NV
LIDVSFAPFFLNKWCN---------------------------------DGMKNSINDLS
Y---LDNELFKNLMKLTKMTN----------------------------DELKQL--ELT
--FSINLKI-----DNK------------SYNLDLLPN-GANVE----------VDLSNI
L-NYIHQL--ANYKLNQS-LKIQTKYFLE--GLYSMI---SKSWLS--------------
--------MFDCFELQMLISG--------GKDDIN-IDDW--------------------

---------------KNNVEYGG-YLDD--------------------------DPAVIM
FWEIVE-EMTPQERCKLIKFVTSVSRAPLLGFGSLAPKFG-IRNSGN-------------
----------------DSVRLPTASTCVNLLKLPNYRDKKTMREKLLYAINT-EAGF
>Saccharomyces_cerevisiae_NM_001181006.1 .
AIISRDNVLEDAFNAF------------------------NSIGERFKA-SLDVTFINEF
G-E-----EAGIDGGGITKEFLTTV-SDEGFKDPKH--------------------ELFR
TNDRY--------------------------ELYPSVVY---------------------
---------------------------------DATKLKYIWFLGKVVGKCLYE----HV
LIDVSFADFFLKKLLN-------------------------------YSNGFLSSFSDLG
S---YDSVLYNNLIKLLNMTT----------------------------DEIKSL--DLT
--FEIDEPE-----SS-------------AKVVDLIPN-GSKTY----------VTKDNV
L-LYVTKV--TDYKLNKR-CFKPVSAFHG--GLSVII---APHWME--------------
--------MFNSIELQMLISG--------ERDNID-LDDL--------------------

---------------KSNTEYGG-YKEE--------------------------DQTIVD
FWEVLN-EFKFEEKLNFLKFVTSVPQAPLQGFKALDPKFG-IRNAGT-------------
----------------EKYRLPTASTCVNLLKLPDYRNKTILREKLLYAINS-GARF
>Kluyveromyces_lactis_XM_454612.1 .
STIRREHALEDAYNAY------------------------GAVGEMFKE-KLGIQFVNEF
G-P-----EAGIDGGGITKEFLQTL-VQEGFINQPF--------------------KLFD
TTSFN--------------------------KLYPSKNV---------------------
---------------------------------SPQNLKYISFMGKILGKCLYE----NI
LVDVELADFFLKKILN-------------------------------VENNMNVPFNDLY
S---LDPEYYRNLMKLLEMSE----------------------------NELAYM--DLY
--FEVPDAP-----NG--------------KRIPLIKD-GLSTK----------VTQKNV
F-EYITRI--SHYKLNLQ-LYTVTSRFIT--GLSYMI---PAHWLR--------------
--------MFTSYELKTLISG--------SEKDFD-LEDL--------------------

---------------KKNTSYGG-FSES--------------------------SVTIQH
FWQVLS-SFTPEERRKFLKFVFSVPTAPLKGFHSLNPLFG-IRNAGD-------------
----------------ETDRLPTASTCINLLKLPDYQNYHTLREKLLTAINS-DSRF
>Allomyces_macrogynus_ACDU01006624.1 .
IVVRRDHVVEDGFAKI------------------------HPLGSRLKG-RLAISFLSEL
GME-----EAGIDGGGVFKEFFTML-ANEAF-DPKM--------------------GLFD

```
RNPEQ--------------------------ELYPSVHG---------------------
----------------------------------RTKLPMYEFLGRVIGKVLYD----GI
LVEATFAPFFINKWLG-----------------------------------MRNFVDDMP
S---MDPELYKHLMFLKNYGS----------------------------DVA-DL--ALD
--FTITYDE-----QGQ------------THTVDLIPG-GRDVP----------VTKDNR
I-KYMYLV--ANYKLNVL-IHRQTMAFMD--GLKSLV---QPEWLR--------------
--------MFSQAELSTIISG--------ARADLD-VADM--------------------
------------------------------------------------------------
---------------RSHTQYTGGYADS--------------------------HPVILD
FWAVVE-RMGPEDRRRLLRFITSCTRAPLLGFKELQPALC-IRHAGD-------------
----------------DQERLPTSSTCVNLLKLPAYRSRRVMEAKLLYSIRS-GAGF
>Entomophthora_muscae_GEND01016789.1 .
VNIRRDNVFEDGFAQL------------------------NPLGSGLKG-RVSITMVSSL
GMP-----EAGIDGGGVFKEFLNAL-IKQAF-APET--------------------DLFL
TTPAE--------------------------LLYPSPHR---------------------
------------------------------ANREHPRLRQYEFLGRMVGKALFE----GI
LIDASFARFFLSKWLG-----------------------------------RRSFLDDLP
S---LDDELYAGLMSLLRYDG----------------------------DPE-DM--ALN
--FTLVDREP----SG-------------PVNVELVRG-GEKLP----------VTAANK
F-EYXRLV--ADYRLNIQ-LQRQSRAFLG--GLIDIL---PPRWLQ--------------
--------MFDPAELQMVISG--------GRQPID-VQEL--------------------
------------------------------------------------------------
---------------QTHITYSGVYHAE--------------------------HPTIVA
FWSVVH-GLETDTLALLIKFITSCSRPPLQGFKALSPPMC-IRDSGR-------------
----------------DDGRLPTASTCVNLLKLPIYSSPEALKNKLLYAIHS-ESGF
>Puccinia_graminis_XM_003322600.1 .
ASIRRQFVSEDGFTHL------------------------NALGPRLKE-TIEIKFIDPY
GLE-----EAGIDGGGVFKEFLTSL-TKEVF-DVNK--------------------GLWL
VNKNQ--------------------------EIYPNPHS---------------------
------------------------------YSRQPLSLEWYKFLGRVLGKALYE----GI
LIDIDFADFFLNKWLG-----------------------------------KQSYLDDLA
S---LDPELYQGLIFLKHYKG----------------------------DVEADL--SLN
--FTVTNNE-----FDA------------SETIELIPE-GSKTS----------VTAQNR
I-NYIYLM--SNYKLNIQ-LESQCAAFFK--GLNDII---ELKWLR--------------
--------MFNQLELKVLVGG-------LDGQDLD-IDDM--------------------
------------------------------------------------------------
---------------QQWTVYGG-WDES--------------------------HPTIRI
FWKVLR-EFDNLTKRKLLRFVTSCARPPLLGFKELRPSFA-IRSSGV-------------
----------------DRSRLPSASTCVNLLKLPEYQTEAELREKVLYAINA-GAGF
>Laccaria_bicolor_XM_001885008.1 .
VQVRRGMVAQDGFDRL--------------------------GEVDLKA-PIEITFIDQF
GQE-----EAGIDGGGVFKEFFTSL-CKEVF-DTDR--------------------GLWL
ENKKN--------------------------ELYPNPHA---------------------
------------------------------YATEAHSLNWYRFIGRILGKAMYE----GI
LVDVAFAGFFLAKWLG-----------------------------------KQSFLDDLV
S---LDPDLYNGLIFLKHYTG----------------------------NTD-DL--SLN
--FTIAVDE-----IGV------------TKIIDLIPN-GSNIA----------VTKENR
L-KYIYLV--SHYRLTKQ-IRRQSDAFFE--GLSEMI---DPKWLR--------------
--------MFNQQEVQILIGG--------VNSLID-LDDL--------------------
------------------------------------------------------------
---------------RRHTNYGGLYDDK--------------------------HENIVD
FWNVVN-TFDQDQRRALLRFVTSCSRPPLLGFKELVPNFS-IRDAGA-------------
----------------DPQRLPTASTCINLLKLPRYPNARILKSKLLQAISS-GAGF
>Ustilago_maydis_XM_011388361.1 .
VKIRRDHVAQDGFVSL------------------------FPLGAELKK-PLEIIFVDQF
GQP-----EAGIDGGGLFKEFLTSL-VREAF-DTNR--------------------GLWK
ATDAQ--------------------------ELYPNPHT---------------------
------------------------------YATSSDQLEWYEFLGRVIGKALYE----GI
```

LVDAKFAGFFLSKMLG-----------------------------------KQSYLDDLG
SIDSLDKELYKGLISLKNYQG----------------------------NVE-DL--SLN
--FTVTDEE-----FGV------------SMTRELVPG-GANIP----------VTNLNR
M-EYIFRI--SHYRLSTQ-IQHQCTAFFN--GLADIV---NPRWLR--------------
--------NFNREELSILISG--------TEDPVD-IDDL--------------------

---------------RKHTVLGG-YHEA--------------------------DLTVQH
FWKVLE-GFDQRMRKAFLKFVTSSPNPPLLGFSQLNPLFA-IRKAGD-------------
----------------DTSRLPTASTCVNMLKLPDYADEQTCEDKLRYAIQS-EAGF
>Cryptococcus_neoformans_XM_012196594.1 .
AKIRRDHVAQDGFDEL------------------------SNLGPALKG-RVDITFVDQY
GIT-----EAGIDGGGLYKEFLTIL-SKEVF-DSNR--------------------GLWL
VTDQN--------------------------ELYPNPHS---------------------
------------------------------YASESHNLSWYRFIGQVLGKAIYD----GI
LVDVTFAAFFLAKWLG-----------------------------------RQSYLDDLA
S---LDKDLYKGLIILKNDP-----------------------------KPE-DM--ALT
--FSTTIEE-----FGV------------QRQIDLVPG-GSDIP----------VTAENR
H-EYIQLV--CKYKLDKQ-IAAQSKAFFI--GLSDLL---DAKWLR--------------
--------MFDQQELQQLIGG--------EEKPID-LKDL--------------------

---------------KAHCNFDG-FPN---------------------------DVTPAL
FWKVVQ-EFTEEQKRALLRFVTSCSRPPLLGFSQLNPQFG-VRFNGG-------------
----------------DMDRLPSASACFNLLKLPGYTTEATLRAKLLQAINS-GAGF
>Pneumocystis_carinii_XM_018371944.1 .
VTIRRNNIFNDGFESL------------------------YTIGKDIKK-PINIVFIDQY
GLP-----EVGIDGGGITKEFLTCI-CKQAL-DVNF--------------------GLFH
ETSEH--------------------------MLYPNPHS---------------------
------------------------------YACESSQLQCFEFLGKLIGKCIYE----SV
LLDVTFAPFFLTKILG-----------------------------------KKSYLDDLV
I---LDFELYKGLMFLKRYTG----------------------------DVQNDF--SLN
--FTIIEQE-----FGK------------TTTIELIPG-GSDIS----------VTNTNR
L-QYIYTM--ADYRLNKV-ISKQSYAFAK--GLFDII---DIKWLS--------------
--------MFSSQELQKIIGG--------SSLPID-IDDL--------------------

---------------RNNSVYGG-FHDN--------------------------DPTIEL
FWSVLY-EFSVFERQTFLKFVTSVSRPPLLGFKDLKPLFC-IRDGGD-------------
----------------DTNRLPTASTCINLLILPRYNDKATMKR-------------
>Schizosacch_pombe_NM_001018810.2 .
AVIRRNRIFDDGFDAF------------------------YNFGKLLKG-PIRITFVDEH
GVV-----EEGIDGGGLTKEFLTSI-CKTVF-DINY--------------------GLFS
ETKAH--------------------------LLYPNTHA---------------------
------------------------------YAQDVERLRCYEFLGMLIGKCIYE----GI
QIDAAFASFFVAKWLG-----------------------------------HPSYFDDLT
S---LDPNLYEGLVFLKNYDG----------------------------DVENDM--ALN
--FTVVHEE-----FGV------------RNVIDLIPN-GSNIS----------VTNENR
L-QYIHLV--SNYYLNAR-LSRQCRAFTN--GFTQII---DPHWLA--------------
--------MFHESEIQILVGG--------DPVPID-IDDL--------------------

---------------RRHTVYAGGYEPN--------------------------SPTIVL
FWEVLR-EFEEEDKRSFVKFVTSVARPPILGFKALMPSFC-IRVNGE-------------
----------------DETRLPTASTCVNLLKLPMYSTKQTLRDKLLTAVRS-GVGF
>Phycomyces_blakesleeanus_XM_018427911.1 .
VTIHRNNVLEDGFTQL------------------------YPLGANLKK-RIAISFVDEF
GLL-----EAGIDGGGVFKEFLTCL-GHEAF-DTNY--------------------GLFL
ATPDQ--------------------------LLYPNPSA---------------------
---------------------------LISNESLAEKLVFYEFLGLIIGKALYE----GI
LLDVAFAEFFLKRCLG-----------------------------------KVNYLDDLP
S---LDPELYKGLIEVKNFQG----------------------------NVE-DL--CLD

```
--FSLAETA-----DGK------------SKVIELIPG-GSDIA----------VTEKNR
I-RYVHLV--ANYRLNVQ-IAKQCKAFFR--GLSTIV---DIKWLR--------------
--------MFNERELQILLGG--------ASVPID-IEDL--------------------
------------------------------------------------------------
---------------RRHTVYAG-YREN--------------------------DPTVKD
LWKALE-SFDNTQRMKFVKFVTSCSRPPLLGFKELRPQLC-IREAGS-------------
----------------DSGRLPTSSTCVNLLKLPKFASYNILRQKLLYAINA-DAGF
>Rhizopus_oryzae_GDUK01016278.1 .
ATIRRNHIFEDGFNAF------------------------HKLESALKD-KVAISFVDEF
GLE-----EAGIDGGGVFKEFLTGL-SHDAF-NVNY--------------------GLFV
ATPEQ--------------------------LLYPNPNS---------------------
------------------------------FATEPLQLEYFRFLGLIIGKAVYE----GI
LLDIPFASFFLKKCLG-----------------------------------KVNYLDDLS
S---LDPELYRGLLTLKNYDG----------------------------NVE-DL--SLD
--FTITHDE-----LGK------------SKTVELIPN-GSQIA----------VTNQNR
I-QYIYLV--ANYRLNIQ-IAKQCRAFFK--GLSTIV---DIKWLR--------------
--------MFNEQELQVLLGG--------ASIPID-LDDL--------------------
------------------------------------------------------------
---------------RANTVLAG-YMEH--------------------------DATVQN
FWKVLE-SFDNTLRMKFVKFVTSCSRPPLLGFKELVPKFC-IRNAGV-------------
----------------DDERLPTSSTCVNLLKLPNFSSFERLKE-------------
>Anaeromyces_robustus_MCFG01000106.1 .
ATIRRDRIFEDGYDQL------------------------NSLXSNLRQ-KVQISFINEQ
GLP-----EAGIDGGGVFKEFLTXI-THQAF-DMNY--------------------GLFL
STSNQ--------------------------CLYPNPHS---------------------
---------------------YARQGIYIYLFKIDERLRYYEFLGRILGKALYE----GI
LVDVFFASFFLSKWVX-----------------------------------INILVDDLP
S---LDEELYKGLLYIKNYKG----------------------------DIR-DL--SLT
--FSIDDERK----LNI------------SXSIDLIAN-GSSIP----------VTDENK
I-QYIYHM--ANYKLNTQ-IYPQCKAFFR--GLSDLI---EPRWLQ--------------
--------MFSQQELQTLISG-------SNSASVD-IIDL--------------------
------------------------------------------------------------
---------------KMNTEYSGVYDEN--------------------------HPTIKL
FWQVVN-DLSEEQKRKLIKFVTSCPRPPLLGFKTLQPKFA-IRDSGN-------------
----------------DQERLPTSSTCINLLKLPVYRTYDVMKSKLIYSISS-DSGF
>Spizellomyces_punctatus_XM_016755140.1 .
VTIRRQYVFKDGYTHL------------------------NALGSRLKN-RVAITFISEQ
GLV-----EAGIDGGGVFKEFLTTL-CRQAF-DLNY--------------------GLFQ
ATTDQ--------------------------LLYPSPHS---------------------
------------------------------YATQETQLKHLEFLGRILGKALYE----GI
LVDVGFANFFLAKWLG-----------------------------------RTSYLDDLP
S---LDPDLYQGLLFLKNYQG----------------------------DVR-DL--GLT
--FSVDDTE-----FGA------------QKTIELIPN-GSTIP----------VTNTNR
I-KYIYLM--AHWKLNTR-IERQCKAFFG--GLVDLI---DPSWVR--------------
--------MFNQQELQILLSG--------TPTPIS-LTSL--------------------
------------------------------------------------------------
---------------REHTTYAGGYTST--------------------------HPTILL
FWTVLE-QFDEEYRRGLLRFVTSCAREPLLGFGELRPGFC-IRFAGD-------------
----------------EEDRLPTASTCVNLLKLPAYKSLEVMRA-------------
>Mortierella_alpina_ADAG01001035.1 .
AQIHRGSAFEDGYEQL------------------------NQLGKKLKG-RIAISFIDQY
GIP-----EAGIDGGGVFKEFLTSL-VLQAF-DTNY--------------------GLFL
STSDQ--------------------------LLYPNPHR---------------------
------------------------------FAQEMTQLKHYEFLGRILGKALYE----GI
LIDAAFAGFFLGKCLG-----------------------------------QVNYLDDLP
S---LDPDLYKGLMFLKNYEG----------------------------NVE-DL--SLY
--FTVDDEG-----KGX------------TITRELIPN-GGNTL----------VTRANR
I-RYIYLT--AHYRLNTQ-IDRQCRAFFR--GLSDLI---DPKWLW--------------
```

--------MFNQQELQVMLGG--------AQTAIS-LADL--------------------

---------------EKNVVYSN-FSRT--------------------------HPTIEH
FWSVVQ-SMKEEDRRLLIKFITSCARPPLLGFAELNPRLC-IRNAGQ-------------
----------------EEDRLPTSSTCMNLLKLPAFTSRERLKEKLMYAIHS-QAGF
>Chaetomium_globosum_XM_001227186.1 .
VRISRAKILESAVKVM-------------------------ELYGASQS-ILEVEYFD--
--------EVGTGLG-PTLEFYSTV-SKEFC-KKKL--------------------KLWR
DNDPNGDD----------------EFVFGPNGLFPRPLS---------------------
-------------------------ETFAASEEGEKILQLYKMLGKFVARSMID----SR
IIDVNFNPIFFRIGAE--------------------------------LTAVRPSLGAIK
S---VDPMVARSLMIVKKFALAKKAIDEDPNRSAAQKVTDTENIVVDKIRID-DL--YLD
--FTL---------PG-------------YPEIELIPE-GAQTQ----------VTIDNV
D-LYLEKV--IDMTLGSG-VRRQVDAFQA--GFSQVF---PYSALS--------------
--------AFTPDELCTLFGR--------VDEDWS-LETL--------------------

--------------MDSVKADHG-YNMD--------------------------SKTVRN
LLQAMS-EFTAAQRRDFLQFTTGSPKLPIGGFKKLTPMFT-VVCKPSEAPYT--------
----------------SDDYLPSVMTCVNYLKLPDYSDIGILRKQLFTAVKE-GQGA
>Fusarium_verticillioides_XM_018904474.1 .
VRISRQKILESALKVM-------------------------ELYGASQS-ILEVEYFE--
--------EVGTGLG-PTLEFYSTV-SKEFS-KRKL--------------------KLWR
EVDSNGSD----------------EFVSGATGLFPRPQS---------------------
-------------------------DEEAGTPNGERILHLFKMLGKFVARSMID----SR
IIDLHFNPIFFRIGDA--------------------------------VSSGVKPSLGAVK
I---VDPVLARSLKAIKQFALAKKEIDEDPNRTPAQKVADTQSITIDGVKLD-DL--CLD
--FTL---------PG-------------YPNIQLEDN-GSQKR----------VTIDNV
D-SYLEKV--IDMTLGSG-VRRQVDAFRA--GFSQVF---PYSALS--------------
--------AFTPDELVTLFGR--------VDEDWS-LETL--------------------

--------------LDSIKADHG-YNMD--------------------------SKTVKN
LLHTMS-EFDASQRRDFLQFTTGSPKLPIGGFKSLTPMFT-VVCKPSEHPYT--------
----------------SDDYLPSVMTCVNYLKLPDYSTIEIMRKQLFTAVKE-GQGA
>Sclerotinia_sclerotiorum_XM_001595958.1 .
VRISRSKILESALKVM-------------------------ELYGASQS-ILEVEYFE--
--------EVGTGLG-PTLEFYSTV-SKEFS-KKKL--------------------KLWR
END-NDAD----------------EYAFGARGLFPAPMS---------------------
-------------------------EEQSLNENGKRILHLFKMLGKFVSRSMID----SR
IIDVSFNPTFFRIGDE--------------------------------SNPVTPSLGAVK
T---VDPQLAKSLKMIKKFSVAKKAIAEDSTLTATQKVLATEALEIDGAKIE-DL--SLD
--FTL---------PG-------------Y-DIDLLPN-GSSIP----------VTIDNV
D-LYLEKV--IDMTLGSG-VQRQVDAFRA--GFTQVF---PYSALR--------------
--------AFTPDELVMLFGR--------IEEDWS-LETL--------------------

--------------TDSIKADHG-FHMD--------------------------SKSVKN
LLQTMS-ELTLPERRDFLQFTTGSPKLPIGGFKSLTPMFT-VVCKPSEPPYS--------
----------------SDDYLPSVMTCVNYLKLPDYTDLDVMRRRMNTAIRE-GQGA
>Pyrenophora_tritici_repentis_XM_001936785.1 .
VRISRSRILESAMKVM-------------------------QLYGHSPS-VLEVEYFE--
--------EVGTGLG-PTLEFYSTV-SREFS-KKKL--------------------KLWR
ENESNDTD----------------EFAFGKRGLYPAPMS---------------------
-------------------------AEEASTENGEKRLELFKVLGKFVARSMLD----SR
IIDVSFNPTFFRIGDG-------------------------------SSAAVVPSLGAIK
A---VDEGLAKSLLLLKQFADAKKKIE-ISNISEANKTAALQEIVIQDCRVE-DL--ALD
--FTL---------PG-------------YGSIELIPK-GADTP----------VSIDNV
D-LYVDKV--IDFTLGSG-VQRQANAFRD--GFTEVF---PYSALK--------------
--------AFTPDELVMLFGR--------TDEDWS-LETL--------------------

--------------MDSIKADHG-YNLD--------------------------SKSVRN
LLATMS-EFDAQERRDFLQFITGSPKLPIGGFKALTPMFT-VVCKPSEPPYT--------
----------------SDDYLPSVMTCVNYLKMPDYSSITVLREKLSVAIRE-GQGA
>Mycosphaerella_graminicola_XM_003856419.1 .
VRISRSRILESAIKVM-------------------------ELYGSSSS-VLEVEYFE--
--------EVGTGLG-PTLEFYATV-SKEFS-KKKI--------------------KLWR
ENESSDGS----------------TYAFGKRGLFPAPMS---------------------
-------------------------EAMSRDENGTRVLHLFKMLGKFIARSMLD----SR
IIDVSFNPTFFRIGDG--------------------------------SATVTPSLGAVA
T---VDEGLAKSLKMLRKYASAKQRIEEDDRLTAVQQAAKIEQVRVNDARLE-DL--GLD
--FTL---------PG-------------YPEIELQPH-GSTTN----------VTMDNV
G-VYVKKV--IEFTLGTG-VQRQIDAFRA--GFSQVF---PYSALK--------------
--------AFTPEELVMLFGR--------VEEDWS-LETL--------------------

--------------TDAIKADHG-YNLD--------------------------SLSVRN
LLQMMS-ELPAPAKRDFLQFVTGSPKLPIGGFKSLTPLFT-VVCKPSEPPYT--------
----------------SDDYLPSVNTCAHYLKMPNYSSVEVLRARFKIAMEE-GQGA
>Tuber_melanosporum_XM_002839053.1 .
VRISRLRILESAIKVM-------------------------DLYGASPS-VLEVEYFE--
--------EVGTGLG-PTLEFYSSV-SRAFA-RKKI--------------------KLWR
ENESSPES----------------EFAFGQQGLFPAPMS---------------------
-------------------------DPMAESENGCKVLHLFTMLGKFVARSMLD----SR
IIDISFNPTFFRTGEN-------------------------------VDEAVAPSLGAVK
T---VDKDLASSLKLLRKFALAKEEIDNNRVLTQGGKAKKAAQIMFDGVHVE-EL--GLD
--FTL---------PG-------------YPHIELIPG-GSDVM----------VTIDNV
K-GYVDKV--IDLTLGSG-VRRQAEAFRS--GFSQVF---PYSALR--------------
--------AFTPDELVMLFGR--------NEEDWS-LETL--------------------

--------------MDSIKADHG-FNMD--------------------------SKSVRN
LLTAMS-EFSAQERRDFLQFVTGSPKLPIGGFKSLTPLFT-VVCKPSEPPYT--------
----------------SDDYLPSVMTCVNYLKLPDYSTFEILKKRLSIAIQE-GQGA
>Coccidioides_immitis_XM_001247572.1 .
VRISRTRILDSAMKVM-------------------------ELYGSSPS-VLEVEYFE--
--------EVGTGLG-PTLEFYSTV-SKEFC-KKKL--------------------KLWR
EQDSADG-----------------EYVYSKLGLFPAPLG---------------------
-------------------------PEQLAQDSGKKILNYFKGLGKFVARSMLD----SR
IIDIAFNPTFFQIGNN--------------------------------FSTFKPSVGAIK
A---VDPDLAKSLLMVKQFADAKGAIESSLSHASEEKEHALKTCEVDGARIG-DL--GLD
--FTL---------PG-------------YPNIHLLPD-GANTP----------VTIENV
H-VYVEKV--IDVTLGTG-VRPQIDAFRS--GFSQVF---AYSALK--------------
--------SFTPNELVMLFGH--------VEEDWS-IETL--------------------

--------------MDSIKADHG-FNMD--------------------------SRSVRN
LLETMS-NFTLQQRRDFLQFVTGSPKLPIGGFKSLTPMFT-VVCRPSDPPYT--------
----------------SDDYLPSVMTCVNYLKLPDYSSAEVLRKQLDVAMHE-GQGA
>Aspergillus_niger_XM_001394604.2 .
VRISRSRILDSAMKVM-------------------------ELYGSSPS-VLEVEYFE--
--------EVGTGLG-PTLEFYSTV-SKEFS-KKKL--------------------KLWR
ENESVHGD----------------EYAFGKRGLFPAPMS---------------------
-------------------------EEQATSESGKKQLQLFKVLGKFVARSMLD----SR
IIDISFNPAFFRIAAS--------------------------------SSSVAPSLGTVK
A---VDHDLAKSLLLLKSFANAKKAVDDNRTLSKAQKTQALQQIEVGGVKVE-DL--SLD
--FTL---------PG-------------YPAIELVKN-GSNIP----------VTNENV
D-LYVDRV--IDMTLGSG-VQRQVEAFRA--GFSQVF---PYSALR--------------
--------TFTPQELVMLFGR--------AEEDWT-IETL--------------------

--------------MDSIKADHG-FNMD--------------------------SRSVRN
LLQTMS-ELSPQQRRDFLQFVTGSPKLPIGGFKSLTPIFT-VVCRPSEPPYT--------

```
----------------PDDYLPSVMTCVNYLKLPDYSSLDVLRERLSVAIKE-GQGA
>Penicillium_chrysogenum_XM_002557649.1 .
VRISRSRILDSALKVM-------------------------ELYGSSPS-ILEVEYFE--
--------EVGTGLG-PTLEFYSTV-SREFS-KKKL--------------------KIWR
DTDGSSGT----------------EYAFGKRGLFPAPMS---------------------
-------------------------DGQAGQEIGKKQLNIFKVLGKFVARSMLD----SR
IIDISFNPAFFRIADT--------------------------------LSSVAPSLGTVK
L---VDQDLAKSLFMLKEFVDAKNAVEANRSLSPAHKIEAVQNITVHGAHVD-DL--GLD
--FTL---------PG-------------YPAIELIPG-GTDVP----------LTIENV
D-IYIERV--IDMTLDSG-VRRQVDAFRA--GFSQVF---PFSSLR--------------
--------AFTPSELVMLFGQ--------ADEDWS-IETL--------------------
------------------------------------------------------------
--------------MDSIKADHG-FNMD--------------------------SRSVRN
LLQTMS-ELDHQQRRDFLQFVTGSPKLPIGGFKSLTPIFT-VVCRPSEHPYT--------
----------------PDDYLPSVMTCVNYLKLPDYSDLDVLKKRLSVAIKE-GQGA
>Yarrowia_lipolytica_XM_503583.1 .
VRISRKHMFQSAIKVM-------------------------ELYGSSPS-VLEVEYFD--
--------EVGTGLG-PTLEFYATV-SHQFA-HKSL--------------------EMWR
DDTPTSGAGGSE------------AFAFSQQGLFPIPLI---------------------
-----------------------------TCSNNEKVLHRFKVLGTFVARSMID----SR
LIDIHFSTTFFRLACL--------------------------------QLSFSPTSETLL
A---IDEQLGKSLRQLQRMSPG---------------------------EIE-HL--GLD
--FTL---------PG-------------FPQAELKPN-GSQIP----------VNGDNV
Q-EYVDLI--VDQTVGSG-VSQQIEAFKT--GFSEVF---PFSAVC--------------
--------AFSPAELVLMCGP--------SVEDWS-LDTL--------------------
------------------------------------------------------------
--------------SEAVHADHG-FDQR--------------------------SKTFMN
LLEVMS-EFDESQRRQFLSFVTGSPKLPIGGFKALKPGLT-VVRKASENGLG--------
----------------PDDYLPSVMTCVNYLKCPDFSTKELLRSRLLQAISE-GGGA
>Ustilago_maydis_XM_011392545.1 .
VRISRGNLLASAFKVF-------------------------ELYGSNSS-VLEVEYFE--
--------EVGTGLG-PTLEFYSLV-SKEFA-RRDL--------------------KLWR
DSRAGGSDADGS------------AYVFSPLGLFPAPLLADDAKD---------------
-------------------------DDSDDAGSVQKRLQAFRILGQFVAKALLD----SR
IIDCNFSPVFLRAVLN---------------------------------QHVAPTLATLS
R---VDATLARSLSAMRKMSAG---------------------------EIE-SL--GLD
--FTL---------PG-------------YESIELHAC-GRDEN----------VTSANV
E-RYISEV--LDMTLHKG-IRAAVRAFRQ--GFNLIF---PISAMS--------------
--------SFTADELVMLFGN--------TEEDWS-ETTL--------------------
------------------------------------------------------------
--------------VASVKPDHG-LNAD--------------------------SPTFRD
IVAIMA-SFDVDQRREFLQWLTGSPKLPIGGFSGLHPQLT-IVKRPHEAPLT--------
----------------PDDYLPSVMTCVNYLKMPNYSSRDKMRERLQTAMKE-GSTS
>Mortierella_alpina_LDAW01000698.1 .
VRISREKALESAVKVM-------------------------ELYGASQG-MLEVEYFD--
--------EVGTGLG-PTLEFYSVV-SKEFC-KKAV--------------------KLWR
DADSEALS----------------DYVLAPHGLYPRPMV---------------------
------------------------------CSEARKILKLFRSLGQFIAKAMLD----SR
IIDVPLSALFVSQLLG---------------------------------RSRKPHLQLVA
S---IDPVLAQSLRSLQSFVVEKKRVYGMNLPS-KEREMALKNIELDGSRLE-DM--SLD
--FTL---------PG-------------YSRIDLXSG-GANIP----------VTIYNV
E-EYIDLA--VDMTVGRG-IQAQTAAFQE--GFNRVF---SIQDLA--------------
--------GFRAEELVNLFGS--------GEEDWS-ESAL--------------------
------------------------------------------------------------
--------------MDSIKADHG-FRSD--------------------------SPAFIN
LLQVMS-EFSVEERRQFLQFITGSPKLPIGGFKNLHPPFT-VVCKHF--PQR--------
----------------ADDYLPSVMTCANYLKMPEYSCKEVTLAKFRMAYEE-GQGS
>Phycomyces_blakesleeanus_XM_018443934.1 .
```

VRIIRSQMLESAIKIL-------------------------DLFGSSQS-VLEVEYTG--
--------EEGTGLG-PSLEFYAST-SKEFC-KKSI--------------------NLWR
DGGNDPSS----------------LYVDASRGLFPKTLP---------------------
--------------------------KNANGKSAKKILNLFKTLGQFVAKAMLD----FR
IIDIPFSVAFFKLALD----------------------------------ESVDQDKLIK
E---IDPTLSKSLDTLQSYIKQKNALYKDTTKDMMQQIKEAQNITVDNARLE-DL--CLE
--FTL---------PG-------------DPTIELKPG-GSEIP----------VTINNV
E-EYVNLL--KDSLVGSG-IAQQLDVFRK--GFNGLF---AIDDLK--------------
--------ILSHQELVSLFGQ--------SSEDWT-YGTL--------------------

--------------ADTIKADHG-FTME--------------------------SPAFKN
LLEILS-EMDDDSRRDFLQFTTGSPRLPIGGWKAIRPVFT-VVRKVPEAPLH--------
----------------ADDYLPSVMTCANYLKMPDYTNKIVMEQRIFKSMKE-GKNS
>Rhizopus_oryzae_GDUK01012222.1 .
VRIVRSQMLESAIKIF-------------------------DLFGSSPG-VLEIEYVG--
--------EEGTGLG-PTLEFYAST-SKEFS-KHSI--------------------NMWR
GSIKNETG-----------------YVDDPLGLFPKPLA---------------------
---------------------------KSNSKSVKKTVQLFRTLGQFVAKAMLD----FR
IIDIRFNSAFFKIALE----------------------------------DAKPSHELLV
E---IDPVLVKSLNLLQLFIKQKNIIYADKTKSMSQKMQEIESLQVEGVQIQ-DL--CLD
--FTL---------PG-------------DATYELKPD-GAEIP----------VTIQNV
E-QYVEAI--IDAVTGSG-VSQQIEAFKE--GFNDLF---AIEDLK--------------
--------LLTYSELVSLFGT--------SEEDWS-LSTL--------------------

--------------ADTIKADHG-FTIE--------------------------SKSVQY
LLEILS-EMDNSQRREFLQFTTGSPRLPIGGWKALRPVFT-VVRKVPESSLS--------
----------------PDDYLPSVMTCANYLKMPEYSSKEIMRQKLEISMKE-GQNS
>Spizellomyces_punctatus_XM_016754372.1 .
VRIARQRILDSMIKVM-------------------------ELYGSTQA-LLEVEFFD--
--------EVGTGLG-PTLEFYANV-CRDLR-KKDX--------------------KIWR
DDDSLDSQAVKDDNRSTKAGLVPDDYLNPALGFFPAPLT---------------------
-------------------------PAEVDTEKGRKILMLYKALGTFVAKALLD----SR
IVDIPFSAMFLEMVVG-----------------EEEEEESAAEAALGTAGRKGAEFHLLR
H---VDPSLYKSLLDLKKYVHIKRSLEADPTLSPAERAARISNITVKGARLE-DL--FLD
--FTL---------PG-------------YPSAELIPN-GKDIA----------LTLDNL
E-HYIDRV--VEMTVGEG-VQRQVEAFRR--GFDRVF---PAADLR--------------
--------SFTVQELAVLVGG-------AEEEDWA-YDVL--------------------

--------------IDSIKADHG-YNSD--------------------------SRTIKH
LATFMS-TLNPIQRREFLQFVTGSPKLPLGGFKALNPSLT-VVRKNVEAGKK--------
----------------PDDYLPSVMTCVNYLKVPDYSELEVMKMRFEVAVRE-GQGC
>Candida_albicans_XM_712835.1 .
VRISRKMMLQSAVKVL-------------------------GMYGSTPG-ILEIEYFD--
--------EEGSGLG-PTLEFYSTV-SKEFS-KKKL--------------------RLWR
DEEPRQASDVDVDVDE--------SYVVNKYGLFPKPMD---------------------
-------------------------KTQLSSENGRKVLYFFSSLGKFIARALLD----SR
IIDFNFNPVFLLLIQL---------------LNKTNGINSTSRTNTSKLSKKMATISNLR
L---VDPTLADSLQHLNKYIELF------------ESNSDIHNVTVDGARVE-DL--ALF
--FEL---------PG-------------NPDYELIPN-GSDTL----------VTADNL
E-LYINKV--IEATLFSG-ILTQTKAFMD--GFSKVF---PINSLI--------------
--------IFSSRELVELFGN--------AEEDWS-MDTL--------------------

--------------TSSIVANHG-YTKE--------------------------SEAIKS
LIDILM-NFSIEEKREFLQFLTGAPKLPIGGFKALRPELT-VVRKHAEDGLK--------
----------------DDDYLPSVMTCANYLKLPNYSSKEMMKEKLIQAMKE-GAGA
>Cryptococcus_neoformans_XM_012194029.1 .
VRISRTQLLESCAKVM-------------------------EMYATFPG-TLEVEYFD--
--------EIGTGLG-PTLEFYALA-SKEFA-RRNL--------------------QVWR

DEDVSIAG----------------NYVHHPHGLFPSPLP---------------------
---------------------------RASAELMASRLKWFKTLGQFIGRSMLD----SR
IIDISFNKVFLKLLLD---------------------------------KPIKKSLSTLK
A---VDPSLARSLERLQGYYNVRKEIEALPIPA-SSRRTKLAALTVGDAKLA-DL--ALD
--FTL---------PG-------------Y-DIELKPG-GAHIE----------VDDSNL
G-EYLEKV--LDWTLGSG-VAEQVKAFQD--GFSSIF---PIKSIK--------------
--------IFSLDELYLLFGN--------ADEDWS-RETL--------------------

--------------EVAIKADHG-YNQD--------------------------SRAVQN
LIEVMS-SYNKEQRRQFLQFMTGAPKLPIGGFRGLTPPFT-VVRKPHEPPYK--------
----------------ADDYLPSVMTCALYLKMPDYSSKEVLAAQFERAMRD-GRGS
>Puccinia_graminis_XM_003889834.1 .
VRISRQKILESLIKVF-------------------------ELYSSCRA-MLEVEYFD--
--------EVGTGLG-PTLEFFSLA-SKAFA-ERQY--------------------QMWR
DHESDSQA----------------LHVFSRTGLFPSPMD---------------------
-------------------------DRTAESEKGIERLRKFKVLGQFMAKALMD----SR
IVDISLSRSFARLVLD---------------------------------YPLPLTIASVG
L---VDKSLAASLEHLNKYVIAKHAIEADSSLSESETHAAIQQIRIDDATVD-DL--ALE
--FIL---------PG-------------Y-DVEMKPD-SSATL----------VTIDNI
E-EFIELV--IYWTLSKG-VTRQIEMFKA--GFSMVF---PIRDLK--------------
--------SFTPDEIVNIFGN-------AESEDWS-PEAL--------------------

--------------TSAMRADHG-YNMD--------------------------SAAIRG
LINIMS-TYDVPSRRQFLQFITGAPKLPIGGFRGLHPALT-VVRKAAEPGHS--------
----------------PDEYLPSVMTCVNYLKLPEYSSQDVAAEKLTIAIKE-GADS
>Pneumocystis_carinii_XM_018371190.1 .
IRISRDHILESTIKIM-------------------------DLYGSSSF-LLEIEYFD--
--------EVGTGLG-PTLEFYSTV-SHEFM-RKNL--------------------GLWR
NVDDFSEN----------------EFVSSPNGLFPAPLS---------------------
-------------------------TDSTYLDEKRKKCNLFKVLGKFIARSMLD----SR
IVDILINPMFFRIAFG--------------------------------MNEVKLSIESIA
E---IDRDLANSLYFLMQFDTAKKKILNKNICD-KEMELRLKDIRVHDVSVE-DL--SLN
--FTL---------PG-------------FPDIHLANN-GYEKL----------VTIHNI
D-EYIDLI--IDFTIGKG-VKEQINAFRD--GFSSVL---PYSSLS--------------
--------LFTPEELSILFGQ--------DKEDWS-IETL--------------------

--------------LDSIKADHG-YNID--------------------------SRSIRN
FLDILT-NMNDMERRQFLQFITGSPKLPIGGFKNLNPPLT-VVCKSHEPPLT--------
----------------PNDYLPSVMVCANYLKLPDYTTKKIMKTKLFLAIKE-GQGS
>Schizosacch_pombe_NM_001020061.2 .
IRISRKKIFNYALHLL-------------------------ATYAASEN-ILEIEYED--
--------EVGSGLG-PTLEFYTSV-SKEFT-LNSL--------------------DIWR
NDQPNS------------------KFVYQASGLFPSPIP---------------------
--------------------------LLGSSPENERKISLFFALGQFVARSIYD----SR
IISIQFNPLFFARNIP-------------------------------------LTISSVA
K---VDKGLANSLRYLEKLIPGKNPTNAE-----------------TDIKLE-DL--HLD
--FTL---------PG-------------FPSIELIPD-GASTP----------VTTFNV
S-DYLNYV--IDYTVGKG-VQQQLEAFQN--GFSSVF---PYTSLQ--------------
--------VLTEHELVTLFGT--------VDEDWS-YATL--------------------

--------------MKSIVADHG-YTME--------------------------SPTIQR
LLTLMS-QMNFQEQRDFLQFITGSRKLPIGGFAGLNPPLT-VVRRLNEPPYV--------
----------------PDDYLPSVMTCVNYLKLPEYSSSEVLGSRLSKAILE-GQGS
>Kluyveromyces_lactis_XM_452136.1 .
LRVSRDSLFLSGIKIL-------------------------KKYGSSPN-VLEIEYKD--
--------EEGTGLG-PTLEFYSLM-SKEFA-RKSL--------------------KMWK
TNSDTSDD----------------EEEYVTGALFPGPLL---------------------
---------------------------GTTEENNEKTLKLFENLGTLVARSMLD----NR

```
ILDFRFNAVFFELMHK------------------------CCKNDRLDLEDFETCIHLVG
Q---FDVQLGKSLAFLYDNR--------------------------HDDSIK-NL--DLY
--FML---------PG-------------Y-DIELLEG-GSKIL----------VESFNV
E-EYLIRV--FDQFLGKG-VDMQLAAFRN--GFSKSF---PYSSLL--------------
--------ILTPTELSDLFGA--------VAEDWS-VQTL--------------------
------------------------------------------------------------
--------------YSSIHADHG-YSMD--------------------------SPIITD
LIDILS-NFSTQQKRLFLQFITGSPKLPLGGFKNLKPKFT-VVLKHPDGNIS--------
----------------ADHCLPSVMTCANYLKLPKYSDKLVLKDRIAQAMTE-GSGA
>Saccharomyces_cerevisiae_NM_001179576.3 .
LRISRKTIFATGLKIL-------------------------SKYGSSPD-VLEIEYQE--
--------EAGTGLG-PTLEFYSVV-SKYFA-RKSL--------------------NMWR
CNSYSYRSEMDVDT----------TDDYITTLLFPEPLN---------------------
-----------------------------PFSNNEKVIELFGYLGTFVARSLLD----NR
ILDFRFSKVFFELLHR-----------------------MSTPNVTTVPSDVETCLLMIE
L---VDPLLAKSLKYIVANKD-------------------------DNMTLE-SL--SLT
--FTV---------PG-------------NDDIELIPG-GCNKS----------LNSSNV
E-EYIHGV--IDQILGKG-IEKQLKAFIE--GFSKVF---SYERML--------------
--------ILFPDELVDIFGR--------VEEDWS-MATL--------------------
------------------------------------------------------------
--------------YTNLNAEHG-YTMD--------------------------SSIIHD
FISIIS-AFGKHERRLFLQFLTGSPKLPIGGFKSLNPKFT-VVLKHAEDGLT--------
----------------ADEYLPSVMTCANYLKLPKYTSKDIMRSRLCQAIEE-GAGA
>Piromyces_finnis_MCFH01000003.1 .
VRVHRDKIIESLSIVM-------------------------NMYCSSQA-LLEIEFYD--
--------EVGTGLG-PTLEFYNLV-CKEIC-KKSL--------------------NIWR
DNGFESNSGEE-------------CYLSPKTGLFPKPLG---------------------
--------------------------VNAPEAKVKKCCKYFSMLGSFVGKALLD----SR
IINIAFNPVFLYLVVN-------------YPLLNKYLIGRNHKHPKLLKKWRDVGIFIID
M---VDPDLAKSLRYLEKFIQIKDQYIEE-----GKSEEEIKSIEVDGAKVD-DL--CFD
--FVL---------PG-------------YPEIELIPD-GENVS----------VTINNL
E-DYIVKI--IDFTLCSG-ISKQVKAFRE--GFNKVF---LITDLQ--------------
--------IFEVKEILQMISN--------SEEDWT-KETL--------------------
------------------------------------------------------------
--------------MKTIKADHG-YSMK--------------------------SQSVIN
LIEVMS-EMNKDEKKEFLEFITGTSRLPLGGFKNLNPPFT-VVCKTTSSDEN--------
----------------PDDFLPSVMTCANYLKLPDYKTKEILKERLTTAIKE-GRG-
>Anaeromyces_robustus_MCFG01000340.1 .
VRVHRDKIIESLSIVM-------------------------NMYCSSQA-LLEIEFYD--
--------EVGTGLG-PTLEFYNLV-CKEIC-KKSL--------------------NIWR
DNGFKNNSDE--------------IYISPKNGLFPKPLA---------------------
-----------------------------SNASESKCCKYFNVLGSFVGKALLD----SR
IINISFNPVFLYLVVN-------------YPLLSKHLIGRNSKHPKLLRKWKDIGIFIID
M---IDQDLAKSLRHLKKYIQIKDQYIQE-----GKSEDDIKSIEVDGAKID-DL--CLD
--FTL---------PG-------------YPEIELIPD-GENVS----------VNINNL
E-EYIIKI--IDYTVCCG-ISKQIKAFRD--GFNKVF---LLTDLQ--------------
--------IFEVKELLQMVSS--------NEEDWS-KETL--------------------
------------------------------------------------------------
--------------MKTVKADHG-YSMK--------------------------SQSVIN
LIEVMS-EMNKDERKEFLEFITGTSRLPLGGFKNLNPPFT-VVCKTTDSDEN--------
----------------PDDFLPSVMTCANYLKLPDYKTKDILKERLTTAIQE-GRG-
>Pecoramyces_ruminatium_ASRE01012983.1 .
VRVHRDKIIESLSIVM-------------------------NMFCSSQA-LLEIEFNG--
--------EVGTGLG-PTLEFYNLV-CKEIC-KKSL--------------------NIWR
DNGFENKDGN--------------TYLCPKDGLFPKPLA---------------------
-----------------------------TNAPESKCCKYFNILGSFVGKALLD----SR
IINISFNPVFLYLVVN-------------YPLLSKYLIGRNSKHPKLLRKWKDVGLFIIN
M---IDQDLAKSLRHLMKYIIIKDKYIEE-----DKTEDEIKLIEVDGAKID-DL--CLD
```

--FTL---------PG-------------YPEIELIPD-GEKIS----------VNINNL
E-EYIIKV--IDFTICCG-ISKQVKAFRE--GFNKVF---LITDLQ--------------
--------IFDIKELLQMVSS--------NEEDWS-KESKAL------------------

--------------MKTIKADHG-YSMK--------------------------SQTVIN
LIEVMS-EMNKNEKKEFLEFITGTSRLPLGGFKNLNPPFT-VVCKTTSSDEN--------
----------------PDDFLPSVMTCANYLKLPDYKTKDILKERLTTAIKE-GRG-
>Anaeromyces_robustus_MCFG01000015.1 .
VRVNRNHILESMVKIM-------------------------PLYGVKNS-LLEIEFYN--
--------EVGTGLG-PTLEFYSLV-SNEVC-KSKY--------------------KLWR
DTSNNIGGSM--------------NSILEKEXLFPAPIN---------------------
-------------------------PDNLDTNAGKKLLKIFNCLGIFVAKAMLD----FR
TIDLPLNTYFVKLIKD----------------NVNNLDFSELYCLSSNKNYLEMGLKIIE
Q---VDPPLMNSLNQIMKYSNLKKEIYSNTSLSPDELHTMVQNIRIDDSSIQ-DL--CLD
--FTL---------PG-------------YPDIELIEN-GSEVE----------VTNWNV
E-EYLKEI--INFTIGKG-VSKQIEAFRK--GFNSVF---PISNLS--------------
--------IFDNDELVLLFGG-------SENEDWS---XL--------------------

--------------INCIHADHG-YTMD--------------------------SPQIIY
LVEVMS-EMTDSEKRDFIQFVTGCPKLPLGGFKNLKPPFT-VVCKTTEASHK--------
----------------PDEYLPSVMTCANYLKIPQYSSKEVLKEKLEIAYKE-GRG-
>Allomyces_macrogynus_ACDU01003217.1 .
VRISRDRILGAAVKVM-------------------------ELFAPEYS-MLEIEYQN--
--------EVGTGLG-PTLEFYALV-VAELA-KCG---------------------EMWQ
AGAA--------------------PYLMPHAGVVTKADVEAVQMAVADAAKTA-------
---------------SLLEHRTAVLEAIGKCGARRPAAQYLRMLGQLMAKALLD----DR
LVDVPLHPRFLGMVFS----------------------------STDETEPLDATVDDVR
V---IDPTLAKSLGAMLHMADAELDA------------------TAPTLAID-AL--TVQ
--FEY---------LG----------------TALVPG-GSDRAV---------RTPTDV
R-EYVQLV--CNHVAGHAAHSAAAELVRD--GFASVL---PVTSVSA-------------
--------LFGTDEIAALVSS---SRAGDNDEHWT-PAAI--------------------

--------------TAGMRADHG-YHAS--------------------------SRVVQD
LVLEMS-TMDAPRRRAFVQWLTGAPRLPLGGWSALKPPFT-VVERTVPLGEV--------
----------------SDAYLPSVMTCANYLKLPGYSSREVLSVRLKVAVEE-GQGS
>Allomyces_macrogynus_ACDU01002927.1 .
VRISRDRILGAAVKVM-------------------------ELFAPEYS-MLEIEYQN--
--------EVGTGLG-PTLEFYALV-VAELA-KCA---------------------DLWQ
AGAA--------------------PYLMPRAGVVTKADVEAVQTAVADAAKTT-------
---------------SPLEHRTAVLEAIGKCGARRPAAQYLRMLGQLMAKALLD----DR
LVDLPLHPQFLEMVLS---------------------------SASSEAEATDATLDDVR
A---IDPTLAKSLAAMLHMADAETDP------------------NSPTLAID-AL--TVQ
--FEF---------LG----------------TALVPG-GSDRAV---------RTPTDV
R-EYVRLV--CNHVAGHATHAPAATLVRD--GFASVL---PVTSVSA-------------
--------LFGTDEIVALVSS---SRAGDNDEHWT-PAAI--------------------

--------------TAGMRADHG-YHAS--------------------------SRVVQD
LVLEMS-AMDAARRRAFVQWLTGAPRLPLGGWTALKPPFT-VVERTVPVGEV--------
----------------SDAYLPSVMTCANYLKLPSYSSREVLSVRLKVAVEE-GQGS
>Entomophthora_muscae_GENC01016730.1 .
IQVPRESILEAAKRIA-------------------------RLYSKDRS-VLEIQFRD--
--------EVGSGQG-PTLEFFSCV-FQEFC-KKAH--------------------GLWR
ESQASDS-----------------EFVNIHHGLFPACLP---------------------
-----------------------------SHASHIHKSLLFKTLGWIAAKSLID----AR
VTDLPLSEAFLELLMN---------------------------KTCDIPQTNLNTLKLVR
S---FDPTFADSLFNINEMASNDAD------------------------SIA-SL--GLT
--FVF---------PG--------------TTNELMEN-GTNII----------VSKKNV
D-KFIKDT--LELLTNSG-IRYALSSFEE--GFNDVF---PLQKLG--------------

--------IFTPRELTTIFGS--------GEEDWS-AEVI--------------------

--------------HSAIKAEHG-YTSE--------------------------SLTVIR
LVTIIS-SFEIEQRRSFLQFITGSRKLPIGGFRALNPNLT-IVRKICDATCV--------
----------------PDNYLPSVNTCFNYLKLPDYSSEEVMKNNLLKAMEE-GKMS
>Rozella_allomycis_ATJD01001143.1 .
-ELNRNTILKDFKLAM-------------------------QKYAKEKT-VLEFTYQS--
--------EIGTGTG-PTLEFYSLI-SKELQ-KNYL--------------------NIWR
SEDSSS------------------EFVSTKNGLFPSFSL---------------------
-----------------------------------SNLDLFKSIGMLCAKAISD----SR
ILDLPLNPIFLNLVFK------------------------------------RQIVNDIS
TLHTIDPILASSLINLKKCS-----------------------------DIK-SL--DLY
--FVL---------PG--------------SNIELIPN-GSSKR----------VKNSNI
N-NYIKLI--IEKTIYS--VQPMVDEFSI--GFNSVI---PIERMK--------------
--------IFTANQILSLLNG--------DDQPWS-IECVL-------------------

--------------TSSIKADHG-FTIE--------------------------SPCLQN
LICLLS-SLNNEERSLFLRFTTGSSNLPVGGLKALSPPLT-VVKKIVPD-----------
----------------PDVYLPSAMTCTNYLKLPDYSSYEVLKEKLLVAINE-GQLS
>Fonticula_alba_XM_009498309.1 .
ELVTRNPALESQYTHLFPYTDTGSRFAVHRRGLRASSQLITKELSSSGR-APHIRFDQ--
--------EVGTGSG-PTMEFFHKA-SREFA-RRGL--------------------GLWL
GDDQFVEHVPRSPDAMA-------PAEAPNRAPYFAMQWLFAPSAGLGPDSDLTDVPLTI
GAADAAGPFVSVPDAGLYPRPTLSQNDARAFGQEHLVHARFFALGQLVGCSLLN----AT
ILELPLSAAFWRAVVFGLDADVPMAAGPTGAATSPGKPWADAVSGVMGATPRAAVCMDLL
EE--VDPRLHMSMQRLFRFVCDKRRILADTGLSRAEQDRQIQALTLDGQPLE-AL--ALD
--FTL---------PG-------------APHIELLPR-GSDIP----------VTALNV
D-RYLALV--IQNLTKHWSIAARVAAFRAGLGTFLVV-EELIPLVAGGLMRSVDD-----
--------VPGQAPMDLLLGG--------ATESWT-LEYL--------------------

--------------AEHIRPSYG-FTAD--------------------------SPAFKA
LLRVLS-ELDDTQRAAFLAFTTGASRLPIGGLASIQPPLS-VVNKTSSHVASDSSAAGPT
----------------DDTSLPSSMTCSHTLKIPNYSSEAILRERLLTAIYE-GTGS
>Fonticula_alba_XM_009498131.1 .
VDPRRPVRSTMHTVNA-------------------------LSSEDLRF-RFLVGYEDSS
RPAGSSSRPQTVDLGGVTRGYLSDV-ARDLL-GLVPHLTAAEVWQSDPLADSSLAQRLYS
TVSRRLQETVAPGG----------EPGSATSSPGPSPGLAGALAPDIAEEIAEFERLHSE
FRSHLWTAYGLGFNSGTATDIFPS-VTAVGEGFGPASARLFSFIGRLVGVCLLHPG--KF
LFPLPLSLSLFKLLLD------------------------------------QPLNAWDLK
HH--VDRSLFNSLEYLTDVDEA--------------------------MLS-SL--VLS
--FSNTLPPFPSP-AGPRGPVGPGLGATSFTTFNLAPD-GSNID----------VNNDNL
F-AFFTFH--ISGRLGEF-SLPAIEAFVK--GVAMVV---PLSLLR--------------
--------CFSARELQTLTCG---------LHTVD-PEQLLALLDIQQPLNCPFAATGCP
PAPKDATMEHSPPDSPDGPDSPEGDLDCLEAGPLGRQVALPVGLRPAALPGIITRNAFGR
GPAGGLGLGSGGMAARRLSVAVP-AVCHCQR-----------------------RLAAWF
RWCLSPPQMGPEDLGRFLAFATGTPRMPVGGRLATNHSGR-LTVRPIL------------
----------------SAGYLPTAHTCSGDLDIPVFPSREAMLQGLLRSM-E-TTGF
>Ustilago_maydis_XM_011387749.1 .
LRIDRHAAWRQSRTLL---------------------LQLLESGAHVRG--LRVEFAG--
--------EQAADGGGPAREWFATVASNCSF-RD----------------------VLGP
HGW----------------------------FIATCPVQ---------------------
---------------------------------EKESQDAAAFLGMLLALAALHTT--KL
ALPFSLPSVAFK-IAN-----------------------------APKLEQVQLTPVDLE
F---ADPALAKSLQSVLDWTLTDQGREQDADK-----------------LFD-STF-SLS
--WSTNIWTAQ---DG-------------VKTVDLIPG-GRHRA----------VKVRER
R-EFVSKL--ISRVLIGS-VSEQVMAFRD--GWQSIM---ASHWLSGAPAAGVKSASDVL
SRGGAALSLFSPEEINQAIVSVASSSTADLATALD-VADI--------------------

```
---------------KVSTELIL-ATSADRDRG---------------------ARVAEW
FWTTWS-TLAPHRQRRLLAFITGAQDVPASGARGIGLRIH-LVNPSVDDATDGDA-----
----------------RSWPLPWSSTCTSTLFLPVYPAKQVLEDKLNVAIEH-YQGF
>Encephalitozoon_cuniculi_NM_001041082.1 .
ITVSRETVYRDSI-EI----------------------FKKINVLDARK-QLRITFRN--
--------EEGVDSGGIRKEYFQLL-SQEIK-EDE---------------------RLFE
HTEN---------------------------RIWIRPHE---------------------
-----------------------------------GDGEGYEAIGRIIAIALYN----NV
VLNIPFPSLFFKKLLD-----------------------------------RRPTLDDLR
E---ISSGIATSLRNLRRLSRD---------------------------EVD-SL--DLR
--FVVEHSV-----DGI------------PRSYPLVKN-GEDIK----------LTSENM
R-LFIEKY--VEFHTDAL-IKPQFESIKR--GFYSII---DKDKLA--------------
--------YLDPKELEKIMMG---------SNTFD-IKAI--------------------
------------------------------------------------------------
---------------RSTTTYSG-FRED--------------------------SPIIVY
FWEIFE-AFNRKKRKKLLQFITGNDRIPVSGPASLKLVIM-RNGC---------------
----------------DTDRLPSSQTCFNTLLLPEYSSKDKLEGKLETALEL-TAGF
>Ordospora_colligata_XM_014708456.1 .
VTVDRETVYRDSI-EI----------------------FKHLNPQDARK-QLRTTFRN--
--------EEGVDSGGIRKEYFQLL-SHEIR-EDN---------------------KLFE
HVEN---------------------------KIWIKAGA---------------------
-----------------------------------KDMCEYEAVGRIIAIALYN----NI
VLSIPFPRLFFKKLLE-----------------------------------KKPTIEDLE
E---ISPDVSMSLKKLRMLSKE---------------------------EID-FL--DLR
--FVSEYYE-----EGN------------VLIHALVEN-GENIR----------LTEDNI
G-LFIEKY--SEFYTDVL-VRPQFEALKK--GFYSVM---EKCKLV--------------
--------YLNPKELEKIMMG---------SNGFD-IKEI--------------------
------------------------------------------------------------
---------------RSATTYSG-FTEE--------------------------SPVIVH
FWEIFE-AFDKKKRKKLLQFITGNDRIPVSGSVSLKLVIM-RNGC---------------
----------------DTDRLPSSQTCFNTLLLPEYSSKDKLENKLETALEL-TAGF
>Nosema_ceranae_XM_002995198.1 .
ITVNRENIYSESLKIL-----------------------LKIRFEDMHK-QLRITFKN--
--------EEGVDSGGIRKEYFQLL-SHEIK-HDN---------------------SLFI
IAEN---------------------------IIWLKNDC---------------------
-----------------------------------LDFDKYYCIGKILGIALYN----NV
VLNIPFPSFFFKKLLN-----------------------------------KKTTFNDLK
E---IDHSLYLSLSKLKKLSAK---------------------------EIE-NL--ELT
--FTVIYTTT----TGE------------VKSYNLDKR-NREIK----------VTKANL
H-SFINKY--SDFILNKL-IKKQFNAIKE--GFYFVI---NKNILT--------------
--------PVNSKELEKIILG---------SNNLN-VDEI--------------------
------------------------------------------------------------
---------------KSTTSYSG-YKND--------------------------STIIKY
FWEIFE-SYSKKMKKKLIQFITGHDRIPIAGAGSLKLVIM-KNGC---------------
----------------DTERLPSSQTCFNTLLLPEYSSKEKLEGKLRTALEM-TAGF
>Hamiltosporidium_tvaerminnensis_ACSZ01001332. .
ITVGRSTIYRDTMNIL-----------------------KNLDDSEMKK-QLKITFDN--
--------EEGVDSGGIRKEYFQLL-SEEIT-NDH---------------------GLFK
IKNN---------------------------YLWFKITD---------------------
--------------------------------RASDNLREYETIGKLIGIALYN----DV
VLNIPFPSVFFKKLLS-----------------------------------KRTNTSDLQ
Q---ICPDIFQSLYNLKKCQKE---------------------------ELK-EL--EQT
--FEIAYST-----NDI------------EITHFLVEN-GNKVM----------VDCENV
N-EFIEKY--ADFILNES-ISAEFESLKK--GFFSIV---KHSTVS--------------
--------YLHSKELEKIIVG---------TTFID-VEAI--------------------
------------------------------------------------------------
---------------HKHALNTG-FDEN--------------------------SSTLQN
FWEIFT-EFDSENRKKLLQFITGNDRIPVSGPESLKLVIM-KNGC---------------
```

```
----------------DTDRLPSSQTCFNTLLLPEYSSKEKLKSKLLSAIHM-TKGF
>Nematocida_parisii_AEOO01000020.1 .
LEVGRESVVEDSLRLL-----------------------ESLEEGTAWK-QLKIKFVG--
--------EDGIDSGGIKKEFFQIL-SQKTL-GEW---------------------DIFR
ESNG---------------------------CLWFNHFS---------------------
------------------------------DEELEKRKTQYKILGSILGLAAYN----GA
VLCFYFPQVFYKRLLG-----------------------------------YSGTFEDLK
T---VEPTIYQTLTQIKEMSPE---------------------------EVS-SL--SLE
--YTL--------------------------------N-GKVHE----------VSHHNI
E-EFTQVY--CKELLETR-LEPAFALIKE--GLWRIC---GDTFIK--------------
--------SLLPCELSILIGG---------MECIN-MEEL--------------------
------------------------------------------------------------
---------------ERYTIYNG-YRRD--------------------------SELIQS
FWEIFK-QYDITMQKKFLRFVTGTDRAPSGGLSRMALVFM-RNGG---------------
----------------DTDRLPSSQTCFNTFLIPEYSNKKKLKEKLDLAISN-TEGF
>Rozella_allomycis_ATJD01000494.1 .
MNVRREFILQDTFAIL-----------------------SNARIEDLMK-PFSVKFLN--
--------EDAVDHGGVTKEYFYLI-TRELL-KDSN--------------------GIFV
WLEESK-------------------------YYWFNSNS---------------------
-----------------------------------NDYEKFKFAGIIFGLAVFN----SC
IVEGHFPLAFFKTVLG-----------------------------------VTCSTEDYS
K---LFPTIHKNLNELLLLAENCETDKD---------------------PIK-DL--DLT
--FSITRKK-----DSS------------IQNVDLVPG-GSFLP----------VDKFNV
K-KYVKDY--LRFIAEKE-ISEQIDHFQE--GVGSVL---CPSVISVISNFKS-------
--------IFRPLELKLIMMG-------ESCNSLEDLEFF--------------------
------------------------------------------------------------
---------------KSDTIYKD-YTPV--------------------------DNTVVY
FWEILD-SFNLDDRKLFFKFLSGSDRIPFKGLSSIPFTIQ-RVN----------------
----------------DTSRLPVSHTCFNLLDLPDYNDKHILTQKLLFSIHN-TEGF
>Aspergillus_niger_XM_001388740.2 .
LKVRRDCLVEDSLRGV--------------------SEVVGSSQEEIKK-GLRIEFLG--
--------EEGVDAGGLRKEWFLLL-VREVF-DPHH--------------------GLFI
YDDDSR-------------------------YCYFNPYC---------------------
----------------------------------FESSEQFFLVGVLLGLAIYN----ST
ILDIALPPFAFKKLLA------------------------AAPLSSITRPMYKCSLDDLA
E---LRPALAKGLRALLDYEG----------------------------DVA-ETF-CYD
--FVAQVDR-----YGE------------TVSVPLCAG-GENRP----------VTNANR
R-EFVDLY--VHFLLDTA-VTRQFEPFKR--GFFTVC---GGNALS--------------
--------LFRPEEIELLVRG--------SDEPLD-VASL--------------------
------------------------------------------------------------
---------------RAVATYDN-WSNARPET----------------------EPVVRW
FWDFFE-QTQPQAQRKILSFVTGSDRIPAMGATSLSIRLV-CLGD---------------
----------------ETSRFPTARTCFNQLGLYRYETREKLERMLWDAVLN-GEGF
>Penicillium_chrysogenum_XM_002561187.1 .
LKVRRECLIEDSLKGV--------------------SEVVGTGQEEIKK-GLRIEFSG--
--------EEGIDAGGLRKEWFLML-VREVF-DPLH--------------------GLFI
YDDDSQ-------------------------YCYFNPYC---------------------
----------------------------------FESSEQFFLVGVLLGLAIYN----ST
ILDVDLPPFAFKKLLS------------------SAPYSNGPQAATSLRSTFKCTLEDLA
E---YRPTLAKGLRGLLEFEG----------------------------NVA-ETF-CYD
--FVAQVDR-----YGE------------IISVPLCPN-GENRP----------VTNSNK
R-EFVDLY--VQYLLDTA-VARQFEPFKR--GFFTVC---GGNALS--------------
--------LFRPEEIEMLVRG--------SDEPLD-VSSL--------------------
------------------------------------------------------------
---------------RAVATYDN-WSHQRPES----------------------LPVVRW
FWDFFE-ESQPQAQRKILSFITGSDRIPAMGATSLVIRLA-CLGD---------------
----------------DCPRYPIARTCFNTLGLYRYPTREKFQRLLWDAVVN-SEGF
>Coccidioides_immitis_XM_012358455.1 .
```

LKVRRECLVEDSLRGV--------------------SEVVGSGQEEIKK-GLRIEFVG--
--------EEGVDAGGLRKEWFLLL-VREVF-DPLN--------------------GSFL
YDNDSR-------------------------YCYFNPYC---------------------
----------------------------------FESSEQFFLVGVVLGLAIYN----ST
ILDVAFPPFAFRKLLA-------------------SARPNNVPTLSTPYQPFRCTLDDLA
E---YRPALAKGLRQLLEYDG----------------------------DVE-ETF-CQD
--FVIQVER-----YGE------------TIEVPLCPG-GEKRP----------VTNSNR
W-EFVDLY--VKYMLDDA-VSRQFEPFKR--GFFTVC---GGNALH--------------
--------LFRPEEIELLVRG--------SDEALD-IPSL--------------------

---------------RAVAVYEH-WPTANPDR----------------------DPVVNW
FWEFFT-RVNPQDQRKILSFITGSDRIPAMGATNLVICLL-YLGQ---------------
----------------DSERFPIARTCFNMLSLYRYKTRQKLESKLWRAVVE-SEGF
>Sclerotinia_sclerotiorum_XM_001588915.1 .
LKVRRECLVEDSLKGV--------------------SEVVGTGGEEIKK-GLRIEFKG--
--------EEGIDAGGLRKEWFLLL-VRDVF-NPDH--------------------GMFS
YDEDSH-------------------------FCYFNPNS---------------------
----------------------------------FETTDQYFLVGVVLGLAIYN----ST
ILDVALPPFAFRKLLA-------------------AAPAAAPGATSHAKPSMTYSLEDLA
E---FRPSLAHGLRQLLEFDG----------------------------DVE-TTF-CRD
--FVADVDR-----YGQ------------TIQVPLCPD-GDKKP----------VTNSNR
R-EFVDLY--VRYLLDNA-VARQFEPFKR--GFFTVC---GGNALS--------------
--------LFRPEEIELLIRG--------SDEPLD-ITSL--------------------

---------------RAVSVCEN-WGAPNAAER---------------------EPVIQW
FWESFQ-KADPKDQRKLLSFITGSSCIPAMGATSLVIKLS-CLGD---------------
----------------DSERFPVARTCFNMLSLWRYASREKLEGRLWRAVHE-SEGF
>Fusarium_verticillioides_XM_018892904.1 .
LNVRRDCLVDDSLKAV--------------------SEVIGSGSEDIKK-GLRITFSG--
--------EEGVDAGGLRKEWFLLL-AREVF-NPDH--------------------GLFL
YDEDSQ-------------------------FCYFNPNA---------------------
----------------------------------FETSDQFFLVGVVMGLAIYN----ST
ILDVALPPFAFRKLIA-------------------SAPTHGTGASAHPKPPMRYTLEDLA
E---YRPRLARGLRQLLEYEG----------------------------NVE-ETF-CLD
--FVIDMDK-----YGT------------QVQVPLCPG-GERIP----------VTNSNR
R-EYVDLY--VRHIIDVS-VTRQFEPFKR--GFYTVC---GGNALS--------------
--------LFRPEEIELLVRG--------SDEELD-INSL--------------------

---------------RGVAEYDN-WGTKKPDGS---------------------EPVIDW
FWETFQ-AASSQDQRKLLLFITGSDRIPAMGAAVLPIKIS-CLGE---------------
----------------DEGRFPIARTCFNMLSLSRYNSKERLEKLLWTAVHE-SEGF
>Tuber_melanosporum_XM_002836093.1 .
LKVRRECLVEDSLKGI--------------------SEGVGS-LDDIKK-GLRIEFIG--
--------EDGVDAGGLKKEWFLLV-ARDVF-DPSY--------------------GMFV
YDDDSQ-------------------------YCYFNPNS---------------------
----------------------------------LESSEEFFLVGVLFGLAIYN----ST
ILDVALPPYIFKKLLH-------------------FTVPHSLAVSS-IRPPLHHTLEDLA
V---FRPSLAHGLRQLLEFEG----------------------------DVE-STF-CRD
--FVAETER-----FGQ------------VIRVPLCPN-GENRP----------VTSSNR
R-EFVDLY--LNYLLNSS-VAKQFEPFKR--GFYTVC---GGNALA--------------
--------LFRPEEIELLIRG--------SDEALD-VSAL--------------------

---------------KAVAIYDG-WGGGNPAEN---------------------DPIVKW
FWNFFE-KITPKEQRMLLSFITGSDRIPAMGATNLIIKVV-CLGQ---------------
----------------DSNRFPVARTCFNQICLWRYKRREKLEALLWRAVTE-SEGF
>Mycosphaerella_graminicola_AFIR01000719.1 .
LRVRRECMVDDSLRQI--------------------SAAVGAGQEELKK-GLRVQFSG--
--------EEGVDAGGPRKEWFLML-VRDIF-DPNH--------------------GMFV

YDEDSQ-------------------------TCYFNANS---------------------
----------------------------------FETSDQYYLVGALLGLAIYN----ST
ILDISLPSFAFRKLLA-------------AAPSSSNNPSSNITSLTGTKNQMTYTLSDLA
E---FRPSLASGLQQLLDFDG----------------------------DVE-ATY-CRD
--FVAPIDR-----YGS------------LTYVPLISN-GENIP----------VTNSNR
H-DFVDAY--VRYLLDTA-VARQFEPFKR--GFFTVC---AGNALS--------------
--------LFRAEEIELLIRG--------SDESLD-VDSL--------------------

---------------RAVAVYEN-WRLPTPPHSLVVRPAEH-------------VDVIRW
FWEVFR-SATPERQRMLLTFITGTDRIPAVGATSLVLRIM-AGGDGWGGGGRE-------
----------------ERERFPVARTCFNMLVLWRYESKEVLKGKLWRAVEE-SEGF
>Mortierella_alpina_LDAW01000728.1 .
LRVNRINLIEESLTQL------------------------SRNEMDLKK-SLRIEFVG--
--------EDGVDAGGLRKEWFLLL-VRQLF-DPQY--------------------GMFI
YDDQSS-------------------------YCWFNPAS---------------------
----------------------------------FESLDQFYLVGVVIGLAIYN----ST
ILDLPLPLAVYKKLLN-----------------------------------TPVALEDLA
T---FRPDLAKGFDQLLNYEDD---------------------------DVE-DVF-CLN
--FVGVYEA-----YGE------------SREVPLIPD-GENIP----------VTNQNR
R-QYVERY--ANFIMNSS-ISDQFESFRR--GFYLVC---GGHALS--------------
--------LFRPEEIEFLVRG--------SAEPLD-IDQL--------------------

---------------RSVTIYEG-FNDD--------------------------HDVIRN
FWSIFK-EFEDKNQRRLLQFITASDRYPATGIANLSFKIT-CMGSH--------------
----------------DSNRYPTTHTCFNQLCLYNYKGREKLKNMLLRAMNE-SEGF
>Phycomyces_blakesleeanus_XM_018428259.1 .
LRVRREHLIEDSLRQL------------------------AQNELDLKK-SLRIEFIG--
--------EEGVDAGGLRKEWFLLL-VRSLF-DPQY--------------------GMFT
YDEDSN-------------------------LCWFNPAS---------------------
----------------------------------FENKDQYFLVGVVLGLAIYN----ST
ILDIHLPTACYKKLFH-----------------------------------QPVDLTDLG
V---FRPSLTHGFQQLLEFEG----------------------------DVE-NVF-CRS
--FVAEFEN-----FGQ------------RKCVSLLPD-GAQKM----------VTNENR
Q-EFVDLY--VNYVLNSS-VERQFDAFQR--GFYHVC---GGNALS--------------
--------LFRPEEIELLVRG--------SDEPLE-IDEL--------------------

---------------RRQTEYNG-FEED--------------------------ERTIVD
FWSIMK-EMEPEKQRRLLMFFTGSDRIPATGASNMHLKIT-WGGN---------------
----------------VLDRLPSAHTCFNQLVLYKYENKKKLKRMLEMAMME-SQGF
>Rhizopus_oryzae_GDUK01014586.1 .
LRVSRDNLIEDSLRQL------------------------AQNKLDLKK-SLRIEFIG--
--------EEGVDAGGLRKEWFLLL-VRSLF-DPQY--------------------SMFT
YDEDSN-------------------------LCWFNPAS---------------------
----------------------------------FENEDQFFLVGVVLGLAIYN----ST
ILDIHLPTACYKKLLN-----------------------------------MPVGLSDLG
S---FRPALKRGFDQLLEFDG----------------------------DVE-NIF-CRS
--FVAEIDK-----FGQ------------RICIPLIPN-GEHIM----------VTKENR
Q-QFVSLY--ADFVLNTS-VERQFGAFKR--GFYHVC---GGNALS--------------
--------IFQPEEIELLVRG--------SDGPLE-IDDL--------------------

---------------KSQTEYIG-FDEN--------------------------EETI-X
FWSIIK-AMEPKMQRKLLMFVTGSDRIPATGATQMHLKIT-CGNNG--------------
----------------DSDRLPSAHTCFNQLVLYKYHTKEKLKRMLYTAILE-SQGF
>Spizellomyces_punctatus_XM_016754113.1 .
LRIRRSALIEDSLNQL------------------------QSRHFDLKK-KLRIEFVN--
--------EDGVDAGGLTKEWFLLL-VRDLF-DPQY--------------------GMFT
FDDDSH-------------------------LCWFNPAS---------------------
----------------------------------FENTEEFRLVGTIIGLAIHN----SN

```
ILDVHFPPACYKKLLG-----------------------------------HVCGLEDLK
K---LRPALGRGLEQLLSYDGD---------------------------DVE-TVF-CRD
--FVAEYEA-----FGE------------VKQVPLVPN-GDRIP----------VTKDNK
Q-EFVDRY--VNWVLNDS-IETQFTAFKA--GFGYVC---GGNALS--------------
--------LFRPAEIELMVCG---------GTELD-IHGL--------------------
------------------------------------------------------------
---------------EGVTEYEG-FTSS--------------------------DRTVRN
FWDIVN-AYPTEMKRKLLLFVTGTDRIPATGIQNMAFKVS-CLGE---------------
----------------DSENLPISHTCFNQICLYRYSNREKLEEKLSKAVVW-SSGF
>Allomyces_macrogynus_ACDU01000860.1 .
LTIRRSHLVQDSMDQL------------------------SDPDLDLKK-RLRVEFAG--
--------EEGIDAGGLTKEWLMLL-VRDLF-DPSY--------------------GLWL
VPNEGNP------------------------TCWFHPAC---------------------
---------------------------------PADMHQEYYLVGVVVGLAVYH----ST
ILDVPLASACYKKLLG-----------------------------------NSVGLEDLA
S---LDVALAHGLQQLLDYPGD---------------------------DVE-TVF-CRD
--FVAEYGG-----YGGE-----------RVRVPLIPD-GENTP----------VTSSNK
H-AYVAAY--VDFILTKS-VTAAFDNFRR--GFLRVL---QGNALS--------------
--------LFRAEEIELLVRG---------TADID-LGPL--------------------
------------------------------------------------------------
---------------QATAEYDG-FRSN--------------------------DTTVRY
LWRILH-AFDPDMKRQFLRFLTGTDRIPATGVGNVHLKIT-CVDPNP-------------
----------------DSDRLPTAHTCFNQLCLAAYTSKSRLERKLRMAILE-SQGF
>Yarrowia_lipolytica_XM_502861.1 .
IKVKRESLLADSLSEI------------------------ERNNGNYKK-SLRVEFVG--
--------EPGIDVGGIRKEWFLLL-KTALF-NPAR--------------------NLFV
EDEESH-------------------------YCWFNPGC---------------------
-------------------------------KAQDGDLREYRLAGVITGLALYN----SS
MLDLPLPPVVFKKLLG-----------------------------------CPVGLEDFT
V---VNPTVGRSLSQLLKFTES---------------------------EVE-SL--NLS
--YNVIIDS-----EGV------------KKSCDLISN-GSHVS----------VTTRNR
R-DYVSRL--VKFFLETS-ISTTFAEYKK--GFHSVV---GGNALS--------------
--------LFRPEELEALVKG--------SSEPLD-IGTL--------------------
------------------------------------------------------------
---------------KTVTRYQN-FTTEDPES----------------------ELVVRW
FWKWAE-NLEEEKQKKLLRFITGSDRIPATGISNMNFKIT-FSGP---------------
----------------DCDRFPVSHTCFNELCVYGYSSRQKFIDKIVMAMNE-SEGF
>Puccinia_graminis_XM_003890082.1 .
LSIRRSHLVEDSLRQI------------------------ASSQSELKK-LLKITFVD--
--------EEGVDGGGLKKEWFLLL-IRQLV-APEY--------------------GMFL
HDQDQH-------------------------QIWFNPAS---------------------
-----------------------------------QELEEFKLIGTVLGLAIYN----RA
TLDFGLPLIGYRKLLG----------------------------------FSVNRLSDLA
T---LKPEVAKSLRWLLEYDGD---------------------------DFE-EIC-SRN
--FVGDYDA-----YGT------------VVEVPLIPN-GENIP----------VTKSNR
A-EFVKLY--CDYILNKS-IEKQFQAFSE--GFNSIA---AGNGLS--------------
--------LFQPEEIELLVIG------STYDSKLA-IEDL--------------------
------------------------------------------------------------
---------------KAITHYEG-FQPT--------------------------DLTIQY
FWIVVE-NFGFEDQKKLLRFITGTDRIPATGISGLNLKIT-RSVRSSLDSNPTNNNRRRR
HLSNQLRSMA------FNERLPESHTCFNQLILSDFSSVQSLDQKLRLAINE-SQGF
>Schizosacch_pombe_NM_001022455.1 .
IRVRRDRLLEDSLRQI------------------------NDRNKDFRK-ALKVEFLG--
--------EEGIDAGGLKREWLLLL-TRKVF-SPEF--------------------GLFV
NCEESSN------------------------YLWFNYSH---------------------
---------------------------------RSKEIDYYHMSGILMGIAIHN----SI
NLDVQMPRAFYKKLLQ-----------------------------------LPLSFNDLD
D---FQPSLYRGLKELLLFEG----------------------------DVK-NTY-GLN
```

```
--FTINLKAV----EG-------------FRTVELKEG-GSELS----------VDNENR
K-EYVLRY--VDYLLNTT-VKKQFSAFFD--GFMKVC---GGNAIS--------------
--------LFQDNEISKLIRG--------SEEVID-WELL--------------------
------------------------------------------------------------
---------------KNVCVYDF-YDQNAISNSISESEPSMTASKYLCHSFVSKRKIILW
FWDLIS-HYSLKMQKLFLIFVTGSDRIPATGAHNFQLRIS-VLGP---------------
----------------DSDQLPISHTCFNHLCIWEYSSREKLKKKLDTALLE-TNGF
>Candida_albicans_XM_718015.1 .
IRVRRDHIVQDSLQYI------------------------KTNSDNLKK-SLRVQFAN--
--------EPGVDAGGLKKEWFLLL-TKEIF-HPQS--------------------GMFH
NVDDSN-------------------------FLWFNIIP---------------------
----------------------------------VENPDMYYLFGAILGLAIYN----ST
ILDLQFPIALYKILLK-----------------------------------RGLDRDDYR
K---LYPVSYKNLINLKSMSNK---------------------------EIL-DL--DLT
--FEVSYSDM----FGK------------NHTTELVTN-GANIQ----------VTKSNL
E-EYIDKY--TGFFMKDG-IAKQVDAFIT--GFNNVI---GGNALS--------------
--------LFLPEEIQLLLCG-------NDDHRID-VDVL--------------------
------------------------------------------------------------
---------------KSVTKYIG-WRSSEDAVD---------------------SKIITW
FWDHMN-KMSNKERKKLLIFITGSDRVPATGIQNLPFKIS-LLNNGH-------------
----------------DSHRLPIAHTCFNELALYNYSTKEKFIEKLNKAVNE-SAGF
>Saccharomyces_cerevisiae_NM_001181694.3 .
IKVRRDVISHDSLRCI------------------------KEHQGDLLK-SLRIEFVN--
--------EPGIDAGGLRKEWFFLL-TKSLF-NPMN--------------------GLFI
YIKESS-------------------------RSWFAIDP---------------------
-------------------------PNFDKSKGKNSQLELYYLFGVVMGLAIFN----ST
ILDLQFPKALYKKLCS-----------------------------------EPLSFEDYS
E---LFPETSRNLIKMLNYTED---------------------------NFE-DVF-SLT
--FETTYRNN----NWILNDSKSSKE---YYVTVELCEN-GRNVP---------ITQSNK
H-EFVMKW--VEFYLEKS-IEPQYNKFVS--GFKRVFA--ECNSIK--------------
--------LFNSEELERLVCG------DEEQTKFD-FKSL--------------------
------------------------------------------------------------
---------------RSVTKYVGGFSDD--------------------------SRAVCW
FWEIIE-SWDYPLQKKLLQFVTASDRIPATGISTIPFKIS-LLGSH--------------
----------------DSDDLPLAHTCFNEICLWNYSSKKKLELKLLWAINE-SEGY
>Kluyveromyces_lactis_CP021243.1 .
IKVRRSHVTQDSLTSI------------------------QRHPHDLKK-SLRVEFSN--
--------EPGIDAGGLKKEWFLLL-TRELF-HPNH--------------------GLFQ
YVEESR-------------------------LSWFAYGN---------------------
---------------------------TGLKLHGENNNELYYLFGVVLGLAIYN----ST
ILDLHFARAMYKKLCN-----------------------------------ERITFEDYE
E---LYPETARNLKKMLSYSEL---------------------------DFS-DIF-GLS
--FETTYYDVA---SGI------------NVTKPLCDG-GESIP----------VTSENK
T-EFVERW--VNFYMNEG-VSSSFSAFQS--GFTRVIG--DGLAFP--------------
--------MFKSCEVERLICG-------SIEQDMD-FEQL--------------------
------------------------------------------------------------
---------------RAVTKYQGGFHNQ--------------------------TPVVEW
LWEILP-QLSHEQQRQFLHFVTGSDRVPVTGLATLPFKVT-RTTSG--------------
----------------SHDQLPTAHTCFNELCLYEYESRDTLLLRLVTALEM-YEGY
>Mitosporidium_daphniae_XM_013383421.1 .
VRIPRSPLREMLHASI--------------------IQLLNASPPQLRG-RIKVEFQG--
--------EPGIDAGGLLREWLQLL-IHALM-DPTL--------------------GLFQ
ADICGFELNAP--------------------CVWFHSGE---------------------
-------------------------------NCSQGVFELVHLLGTILGLSLNN----GI
VLDHRLPFALFKKLLS-------------------PDTNPPACVSAHGESMSSITLDDLW
E---IAPTLARGLGQLLDYHGEA--------------------------EVE-DVF-QLQ
--FTAGFSK-----PGPEK----------REIIDLTNR-GSEIA----------VTGENR
E-IFISLY--LEHIFHHR-PGNHWTKFRD--SFYSTL---SGSTIA--------------
```

--------LLSPQELKQLLSG--------DPAEIS-IDLL--------------------

---------------RSVALYEG-YLSAE-------------------------HPVSLW
FWDILT-SYSAEMRRKFLIFVTGVGRLPAAXVIRLWGRDA-SWKNLS-------------
----------------GGEYRPLGRTCFNQLCLHKYPDMETLRRLLTNAIVE-SEGF
>Penicillium_chrysogenum_XM_002557878.1 .
LVIRRDDILSDALSQL-----------------------WRREKRELMR-PLKVQMGMDE
G-------EEGLDHGGVQQEFFRLL-MGQAF-DPSY--------------------GMFT
VDTRHR-------------------------VSWFQPCS---------------------
----------------------------------LEPLYKFELIGLLMSIAIYN----GL
TLPVNLPTAFYRKMLG----------------------------------LKVKHLDHIR
D---GWPELSQGLDTLLAWKDG---------------------------DVG-DIF-TRT
--YEFSFEA-----FGS------------IETIDMXPA-EEAAL----------VTNKNR
H-QFVKDY--IFWLTDKS-IRPQFEAFQR--GFNTCL---DRSALS--------------
--------IFSPEALKTVVEG---------IQSID-VEEL--------------------

---------------ENHTRYEGGFGPD--------------------------HRVIRD
FWDIIR-EYPNEKRAQLLEFVTASDRVPVNGISSIMFVIQ-KNGV---------------
----------------GDLRLPTSLTCFGRLLLPEYSSKTTLAQKLDKALEN-AQGF
>Aspergillus_niger_XM_001394283.2 .
LMVRRDNILRDALNQL-----------------------WRRERRELMR-PLKVQMGMDE
G-------EEGLDHGGVQQEFFRVL-MAEAL-DPSF--------------------GMFT
TDARTR-------------------------VSWFQPCS---------------------
----------------------------------WEPLYKFELLGLLMSLAVYN----GL
TLPVNFPTAFYRKLLG----------------------------------LKVKHLEHIQ
D---GWPELTKGLGDLLTWEDG---------------------------DVG-DIF-MRT
--YEFSFEA-----FGV------------VETVDMXPA-DEASL----------VTNSNR
A-QFVKDY--IFWLTDKS-VRPQFEAFAQ--GFYTCL---DRTALS--------------
--------IFTPEALKTVVEG---------IQTID-IREL--------------------

---------------ERHARYEGGFDPS--------------------------HRVIRD
FWSVVR-RYPAEKKAQLLEFVTASDRVPVNGIASIMFVIQ-KNGV---------------
----------------GDARLPTSLTCFGRLLLPEYSSRSVLEEKLSKALEN-ARGF
>Coccidioides_immitis_XM_001247392.1 .
LIVRRDNVLTDTLNQL-----------------------WRREKQELMR-PLRVQMGMDE
G-------EEGADQGGVQQELFRVV-MAEIL-DPAY--------------------GMFT
LSDRDH-------------------------TSWFQPCS---------------------
----------------------------------FEPLYKFELAGLLMSLAIYN----GI
TLPVNFPVAFYMKLLD----------------------------------FEVKKLDDIR
S---GWEDLARGLSELLSWSDG---------------------------DVG-DIF-LRT
--YEFSFDA-----FGK------------VVTVDMXPE-HEAPL----------VTNANR
E-QFVNDY--IFWLTDKS-IRPQYEAFAR--GFYTCL---DRTALS--------------
--------IFTPQALKSVVEG---------IQEID-MDEL--------------------

---------------EKHTRYEG-YNPE--------------------------DRVIRD
FWDVVK-TYPQEKRSRLLEFVTASDRVPVKGVSSLLFIIM-KNGV---------------
----------------GDERLPTSGTCFGRLLLPEYSSRQALEENFDRALEY-CKGF
>Sclerotinia_sclerotiorum_XM_001585574.1 .
IVVSRENLLSDAFNVL-----------------------WRREERELMR-PLKVSLGGEI
G-------EEGVDMGGVQQEFFRIA-MTEAL-NPDY--------------------GVFT
IDGTTK-------------------------MTWFQPGS---------------------
----------------------------------PEPLWKFELIGTIMSLAVYN----GM
TLPITFPKAFYRKLQG----------------------------------ESITELHHIS
D---GWPELAKGLTDLLEWDENKG-------------------------AVE-DIF-CRT
--YEFSQFQ-----FGK------------PVSREMXPGEVEAPL----------VTNENR
N-NYVSDY--INWLTNIS-IQPQFEAFKK--GFFACL---DPRSIS--------------
--------IFDSDTLQSLVEG---------VQEID-IVEM--------------------

```
---------------QRGTQYIG-YEAG--------------------------DRVIQD
FWSVVK-EYDLEKKKKLLEFTTASDRVPMGGMRNFQLRIM-KNGA---------------
----------------TDEQLPSSYTCFQNLLLPNYSSRQILKERFDIALEH-SKGF
>Chaetomium_globosum_XM_001227135.1 .
LDIRRGTVLQDALDQL-----------------------WRREERELLK-PLKVHLGEAT
G-------EEGFDSGGVQQEFFRLA-IGEAL-NPDH--------------------GAFT
VDERTR-------------------------MTWFLPGS---------------------
----------------------------------MEDEWKFELIGLLVSLAVYN----GL
TLPVTFPKAFYRKLLG----------------------------------EPVTELHHIA
D---GWPDLASGLTSLLEWDEKDG-------------------------AVE-DVF-ART
--YEFSVSA-----FGQ------------HITREMXPAEDEAPL----------VTSANR
N-AYVSDY--IRYLTDVS-VRPQYEAFAR--GFHACL---HPKSLS--------------
--------LLTPSLLQSVVEG---------VQEID-IAEL--------------------
------------------------------------------------------------
---------------KRHARYVG-WDAS--------------------------HRTVKD
FWSVVK-RYDDAMKRKLLEFVTASDRVPVGGVKNLMFIIQ-RNGEEED------------
----------------PAGRLPTSYTCYGTLLLPEYRDKEMLRQRLGMALEN-AQGF
>Fusarium_verticillioides_XM_018894887.1 .
LEIGRSNVARDAFDQL-----------------------WRREKRELLR-PLKVHLGENS
G-------EEGFDSGGVQQEFFRLA-IAECL-DPQY--------------------GAFT
IDERTR-------------------------MAWFTPGS---------------------
----------------------------------LTEDWKYELVGLLMSLALYN----GL
TLPITFPKALYRKLLG----------------------------------KPVEELHHIA
D---GWPDLASGLTTLLEWDEKDG-------------------------LVE-DIF-ART
--YEFSVES-----LGT------------IVTRDMXPD-DAPLV-----------TNENR
D-DYIIDY--IRHLTDVS-IRRQYLAFEQ--GFNSCL---DRKSLS--------------
--------LLSPSTLQSLVEG---------VQEID-IVEL--------------------
------------------------------------------------------------
---------------KRYARYVG-WDAS--------------------------HRTIKD
FWSIVK-RYDERMKQRLLEFVTSSDRVPVGGMKNLQFVIQ-KNGEEDG------------
----------------AGGHLPTAYTCYGTLLLPEYRDKEVLRERLGMALEN-AQGF
>Tuber_melanosporum_XM_002837154.1 .
VEVRRETVLQDALNQV-----------------------FGREIRELKR-PLKVRFA-NE
G-------EEGVDHGGVQQDFFIVA-IREAL-RSDY--------------------GLFT
TDEQTR-------------------------MNWFSVTP---------------------
----------------------------------IEPIHKYELLGLLVGLAVYN----GV
TLPITFPKILYKKLLG----------------------------------GKAEGLEDIE
D---GWCQLAKGFKQLLEWNDG---------------------------DVG-DVF-LRT
--YDFSYDF-----FGQ------------VKYVNMXPK-EEAPL----------VTNENR
E-AFVRDY--ISWLTDRS-IRRQYQAFEK--GFFAVI---DRKSLS--------------
--------LFTPSNFQSLTEG---------IQDID-ISEL--------------------
------------------------------------------------------------
---------------EKAARYEDGYSPT--------------------------HRVIKD
FWAIVR-GFSAERRRQLLEFVTASGRVPVNGISSIMFVIQ-RNGP---------------
----------------DSDRVPTSLTCFGRLLLPEYSRRAKLRDKLKLALEN-GKGF
>Pyrenophora_tritici_repentis_XM_001931912.1 .
LDVSRENPLKDTLDQL-----------------------WGQDKRMLLK-PLKVRMGREE
G-------EVGQDHGGVTYEFFRVV-LGEAF-QPEN--------------------GMFT
IDPETR-------------------------MMWFQPYS---------------------
----------------------------------LEPCWKFEMLGILFSLAVYN----GI
TLPVTFPLAFYNYLWT-------------------------NGYTVSNPMTLTERISFIS
D---GWPTLAKGFEQLLTWPDP---------------------------NVE-DIF-SLD
--SVFPYQV-----YGQ------------RYAHNLXPN-LDAAP----------VTNENR
E-EYIDNY--ITTLTHAS-ISPQLSAFNK--GFRTCI---SPPSLS--------------
--------LFTPTSLRNLIEG---------NTHIS-LTDL--------------------
------------------------------------------------------------
---------------KRCVRYEDGYTPT--------------------------HSTIRM
FWDIVE-RYDQDDARRLLEFVTASDRVPVTGYESITFAIH-KIGG---------------
```

```
----------------APRSLPSSSTCFGKLYLPEYESREGMEGKLGLAIRN-SRGF
>Mycosphaerella_graminicola_AFIU01000081.1 .
VSVSRNNILQDAFDQL-----------------------WQRRKSELRR-PLRVRLGEVD
EL------EVGHDLGGVQIEFFNLV-CREVF-SEQX--------------------ELFT
TDSTTG-------------------------CSYFRPGS---------------------
-----------------------------------LQPLYMFELIGLLMALAIYN----GV
TLPVNLPWVFYYFLAH--------------------------NGEGRGSPRDHITLEKIV
D---CWPTTARSFRSILDN------------------------------DV--DGL-ELE
--YSFPLEA-----NGL------------RLAAHLXTT-RNPKT----------VNSANK
D-NYVQDH--IKWLTCYS-VLPQLTAFLK--GFHSAG-LFEPLTLQ--------------
--------LFGIEALKAYVEG---------SDTLD-INDL--------------------
------------------------------------------------------------
---------------RAATRYDG-YDAK--------------------------SRYITS
FWDIVT-SWSQEKQKQLLKFVTAAERIPITGASQLTFVIK-RGHPE--------------
----------------SVNKLPTSSTCFGTLMLPKYASVETLKEKLSKAVEYGAVGF
>Allomyces_macrogynus_ACDU01000604.1 .
LSVRRDHVVRDTLVQL-----------------------ASVPAAELRK-QLKVQFVD--
--------EVAVDAGGVQKEWLHLV-MRKVV-APEY--------------------GMFV
PVDNNR-------------------------GVWFRPWV---------------------
-----------------------------GTPAPKEVLEEYELIGKLVGLAVFH----SI
LINVPFPLALYKKLAN-----------------------------------ERVTLADLT
E---IDPDLGKGLADLAAYAND---------------------------DLE-DVY-CRN
--FTVDVPMQ----PGL------------TVTVPLVPD-GANVA----------LTRANR
M-EYIAQA--VEFYLHRA-VEVPFRAFRR--GFEMVT---RAVRVX--------------
--------LFTASEMQALIAG---------DETLD-FHAL--------------------
------------------------------------------------------------
---------------EQRTTYDG-FTLD--------------------------SPVVQX
FWSIVH-DLDLVQHRQLLQFVTGSDRAPVGGLAHLPFVLV-RAGT---------------
----------------DSVRLPSAHVCFNALLLPEYAERDMLRHKLLAAIAN-GEGF
>Rhizopus_oryzae_GDUK01017940.1 .
LEIRRDHIISDTLLQL-----------------------EEKSIHDLKK-QLRVQFLG--
--------EEGVDEGGVQKEFFQLI-VREIF-DPKY--------------------GLFV
YNEESR-------------------------LCWFSSNP---------------------
-------------------------------VLDETNTREYKLVGLLLGLAVYN----SV
ILDLHFPLALYKKLMN-----------------------------------VDVDLSDLK
Q---LDLSLGKGLEMLLQYDG----------------------------DIE-AEY-DRF
--FQVNMES-----FGH------------VFTYDLKPM-GSEIQ----------LTNENR
S-EFVNLY--TKFILDTS-VQKQFEAFKE--GFLLVC---QDSAIK--------------
--------MFRPEEVEQLICG---------CSDLD-FEAL--------------------
------------------------------------------------------------
---------------EKSTVYDGGWTKD--------------------------SDIIKY
FWEIVH-SFSYEDKKKLLFFATGSDRAPIGGLSKLQFVIA-KNGG---------------
----------------DSDRLPTSHTCYNVLLLCEYSSKEKLRERLLTSISN-AEGF
>Mortierella_alpina_LDAW01000107.1 .
LTIRRDFIIEDALVQL-----------------------QNKSHEDLKK-QLQIKFVN--
--------EEGIDEGGVQKEFFQLA-MHELT-DPKY--------------------GMFT
TNEESR-------------------------LCWFAQSP---------------------
-------------------------------LADELALDEYNMVGRLIGIAIYN----GV
ILDIHFPLALYKKLAL-----------------------AAEPQGDPRKLDEQWDLDDLM
E---LDPTLAKGLRQLESFEG----------------------------DVL-EAY-DRT
--FQIDYES-----MGQ------------TFQYDLVPN-GASVP----------LTNSNR
F-EFVKAY--LKFYFTTS-VAKQFNAFSE--GFHLVT---LGSAIQ--------------
--------LFRPEEVEQLICG---------SPILD-FNAL--------------------
------------------------------------------------------------
---------------EQITQYEGGFHAK--------------------------SRIIRW
FWEIVH-AYEEKEKKRLLFFATGSDRVPIGGLGQLSFTIS-KNGP---------------
----------------DSMRLPTSHTCYNTLMLCAYSSKEKLQERLTTAIGN-SEGF
>Entomophthora_muscae_GEMZ01017570.1 .
```

LEIRRDYIIRDALFQL-----------------------SSKSPQDLKK-QLRVQFIG--
--------EEGVDEGGVQKEFFQLL-VREMF-DPKY--------------------GMFT
TQEDQR-------------------------LYWFNPNP--------------------
-------------------------------LDDDIALEEYRLIGRLIGLAIYN----SV
ILDLHFPGALYKKLMG-----------------------------------NAVGLDDLE
V---VDPSLGRGLRCLLXFQD----------------------------DVE-AAY-GWT
--FQIEYEV-----VGE------------RFKYDLKPN-GANIP----------LKNDNR
E-EFVELY--IDFILNKA-IERQFKAFRE--GFDHVC---AGSAIQ--------------
--------LFRPEEVEQLVCG---------SSDLD-FEAL--------------------

---------------EKVTQYDGGFTAA--------------------------TPVIMF
FWEVVH-SFTEAQKKMLLFFATGSDRVPIGGLSKLQFVIA-KNGP---------------
----------------DSDRLPTSHTCFNVLLLCEYGSLEKLRERLLTAIHN-AEGF
>Rozella_allomycis_ATJD01001080.1 .
LEIRRDHIIRDALFQL-----------------------DLKSPQDLKK-QLRVQFVG--
--------EEGVDEGGVQKEFFQLV-FREMF-DQKY--------------------GMFR
LYEDSN-------------------------LCWFMNNP--------------------
-------------------------------EDDEVALDEYRLVGRLIGLAIYN----NV
ILDVHFPLALYKKLLN-----------------------------------RPISFQDLL
Q---LDPSMAQGFNQLLNFKE----------------------------SVQ-EFY-DRT
--FEIEYEI-----FGN------------RKCVELKPG-GSKIS----------LTNENR
K-EFVDLY--IDFVFNKS-VFKQFEAFRG--GFESVC---KGTAIT--------------
--------VFRPEELEQLVCG---------SSDLD-FEAL--------------------

---------------EANTAYDGGYSPD--------------------------TTVXRW
FWEVVH-AFSEKEKKLLLFFTTGTDRVPIGGLSKLNFVIA-RNGP---------------
----------------DCDRLPTSHTCFNVLLLNEYSSKEKLRERLLTAIQN-AEGF
>Phycomyces_blakesleeanus_XM_018428460.1 .
LEIKRISFVEDAWDQV------------------------SRKWTDIKK-PLKVRFVEGG
--------EEGMDQGGVQKEFFGVL-FEKLL-SAEL--------------------GLFE
MDPESR-------------------------LYWIRPYL--------------------
---------------------------------DTDTVRHYEMTGVMIGLAIYN----GI
MINLPFPDIFWKVLVA----------------------PTEQEVDAQADNHSLFTLSDLN
T---DWPSLASGLEQLLQWPD----------------------------EVQ-DVF-DRN
--YEISIQV-----FGQG-----------IITVPLIPG-GESIP----------VTNNNR
E-AFVQDY--CTYFMYRA-QRDAILALRR--GVRSVI---GSRALD--------------
--------LFTAAEIQVVACG---LRQGPGSQDLC-MEDL--------------------

---------------ESVTDYDDGYHAD--------------------------HPTIRQ
FWSVVHNSLSPDQKRELLLFVTASDRVPIGGLKELSFIIQ-RNGP---------------
----------------DSDRLPTALTCFSRLLLPEYSSEEKLEERLVTAIEN-AKGF
>Rhizopus_oryzae_GDUK01009237.1 .
LEIRREHFVEDTFQQV------------------------SRKWSDLRK-PLKVKFIEGG
--------EEGMDQGGVQKEFFGVL-FEKLV-SSEL--------------------GLFS
QDESTR-------------------------LCWIRPVS--------------------
----------------------------------NLDRRTYEMVGVMMGLAIYN----GV
MMNLQFPKVLWKSLVM----------------------PSEAMLEAVAERHHLFTLDDLE
E---GWPALGQGLKQLLDWQDG---------------------------DVE-DVF-CRD
--YEISLEV-----FGQG-----------VVTQPLMPS--PVVP----------VTNANR
E-AYVRDY--CTYFMYTA-QKEQILALRR--GLWSVI---GSRALH--------------
--------LCTADELEMVACG---QRQGPDAIELN-MADL--------------------

---------------ESVAEYDE-YTSD--------------------------HPTIRQ
FWSVIHHDLTAEQKRQLLLFVTASDRVPVGGLKELSFYIQ-RNGP---------------
----------------DSDRLPTALTCFSRLLLPEYSSRRKLRDRLITAIEN-TKGF
>Mortierella_alpina_LDAW01000968.1 .
LELTRAHLVEEAFEQI------------------------TKKHADLKK-PLKVAFVDVG
--------EEGMDQGGVTKEFFQIM-VEKVF-DSQF--------------------GLFK

ELEEQR-------------------------SWWFEGTM---------------------
---------------------DGSSHVEMSEEDARVRLVEYELVGVLVGLALYN----GV
ILGVRFPSVVYRKLLN-----------------------------------WQVGLDTFI
E---SFXALGHGLEQMLTWTDG---------------------------DVY-DVF-MRE
--FEISYEH-----FGQ------------VTTIPLVPG-GQDLP----------VTNANR
E-EYVQAY--IEHYVHTH-IRQEFEAFQR--GFEKIC---SGPALK--------------
--------LLRPEELELLLCG---------NSDLD-MHDL--------------------

---------------EASCMYDDGYSTS--------------------------HTLIKE
FWQIVHEEMTAEQHKQLLVFVTGSDRVPIRGLKDLMFVIQ-RNGP---------------
----------------DSDRLPTALTCFSRLLLPEYADKDKLKERLVTAIEN-SHGF
>Allomyces_macrogynus_ACDU01003958.1 .
LAVRRTHLVADTWAQL------------------------AAKPGEWKK-PLKVKFAN--
--------EDGMDQGGVQKEFFQVL-LAQLM-DPLY--------------------GLFT
YDESTR-------------------------YSWLNAAS---------------------
----------------------------------LEPVRQFELVGIVLGLALYN----GV
IVDVRFPRLLYRRLLG-----------------------------------DAPTLDDVK
C---TWPDLGRGLQQLLDWDDG---------------------------DVE-DVF-MRS
--FDVSVAA-----YGA------------VRTVELKPG-GSNLP----------VTNANR
H-EYVALY--VDWVCRKS-VEPQFRALRR--GFLKVC---GGYALG--------------
--------LCRPEEVEQLLCG--------QEVDLD-MSAL--------------------

---------------EKGCGYDDGYHAA--------------------------HPTIRD
FWSVVH-AMDLPHKKMLLEFVTASDRVPLKGLGSLTFVVQ-RNGP---------------
----------------DCDRLPTALTCFGRLLLPEYAGKEKMKRFLVTAIEN-AKGF
>Spizellomyces_punctatus_XM_016749649.1 .
LEIRRQHLVQDALDQI------------------------RKKGADLKK-PLKVRFIGGG
--------EEGMDQGGVQKEFFQVI-VSMLL-DPAY--------------------GMFL
YDQETR-------------------------YCWINGAS---------------------
----------------------------------LESEKEFELVGTIVGLALYN----GV
ILDVNFPKVLYKKLLD-----------------------------------EAPTLEDVK
D---AWPTLGRGLQQLLEWSDG---------------------------DVG-DVF-LRT
--FEISYDI-----YGQ------------VKHFPLVEG-GEDIL----------VTNDNR
K-EYVDLY--IHHYVVES-VRRQFSAFRR--GFHKVC---GGNALK--------------
--------MCRASELELMICG-------TSTTDLD-FTEL--------------------

---------------EQGAQYDDGYGPD--------------------------HEVIQW
FWEIVHSDMELEQKKKLLNFVTASDRVPLKGLGGLTFVIQ-RNGP---------------
----------------DTDRLPTALTCFGRLLLPEYSDKDKLRDRLVTAIEN-AKGF
>Piromyces_finnis_MCFH01000020.1 .
LEVRRAHLVEDVLNQI------------------------SKKEKDLKK-PLKVKFVGGG
--------EEGMDQGGVQKEFFQII-TAQLL-DQQY--------------------GMFT
YDTETR-------------------------YSWINGAS---------------------
----------------------------------LESEKHFELVGIVIGLALYN----GV
ILAVNFPRLMYKRLLD-----------------------------------EEPTLEDIK
L---AFPALGKGLEQMLNWTDG---------------------------DVG-DIF-MRS
--FQISYEV-----YGQ------------VKTYNLVEN-GENIL----------VTNENR
E-EFVKLY--IHHLVIDS-VSRQFRAFRR--GFYKVC---GGYALK--------------
--------MCRAEELELLISG---------SVELD-YEEL--------------------

---------------EKATEYDDGYSKD--------------------------HIVIKX
FWEIVH-AMTYDQKKKLLMFVTASDRVPLKGLGNLTFVIQ-RNGP---------------
----------------DTDRLPTALTCFGRLLLPEYSTKEKLKNRLLTAIEN-AKGF
>Anaeromyces_robustus_MCFG01000023.1 .
LEVRRAHLVEDVLNQI------------------------SKKEKDLKK-PLKVKFVGGG
--------EEGMDQGGVQKEFFQII-TAQLL-DQQY--------------------GMFT
YDTETR-------------------------YSWINGAS---------------------
----------------------------------LESEKHFELVGIVIGLALYN----GV

```
ILAVNFPRLMYKRLLD-----------------------------------EEPTLEDIK
L---AFPALGKGLEQMLNWTDG---------------------------DVG-DIF-MRS
--FQISYEV-----YGQ------------VKTYNLVEN-GENIL----------VTNENR
E-EFVKLY--IHHLVIDS-INRQFTAFRR--GFYKVC---GGYALK--------------
--------MCRAEELELLISG---------SVELD-YEEL--------------------
------------------------------------------------------------
---------------EKATEYDDGYSKD--------------------------HIVIKX
FWEIVH-AMTYDQKKKLLMFVTASDRVPLKGLGNLTFVIQ-RNGP---------------
----------------DTDRLPTALTCFGRLLLPEYSTKEKLKNRLLTAIEN-AKGF
>Pecoramyces_ruminatium_GFSU01009987.1 .
LEVRRAHLVEDALNKI------------------------SKKEKDLKK-PLKVKFVGGG
--------EEGMDQGGVQKEFFQII-TAQLL-DQQY--------------------GMFT
YDTETR-------------------------YSWINGAS---------------------
----------------------------------LESEKHFELVGIVIGLALYN----GV
ILAVNFPRLMYKRLLD-----------------------------------EEPTLEDIK
L---AFPALGKGLEQMLNWTDG---------------------------DVG-DIF-MRS
--FQISYEV-----YGQ------------VKTYNLVEN-GENIL----------VTNENR
E-EFVKLY--IHHLVIDS-ISRQFTAFRR--GFYKVC---GGYALK--------------
--------MCRAEELELLISG---------SVELD-YEEL--------------------
------------------------------------------------------------
---------------EKATEYDDGYSKD--------------------------HIVIKN
FWEIVH-AMTYDQKKKLLMFVTASDRVPLKGLGNLTFVIQ-RNGP---------------
----------------DTDRLPTALTCFGRLLLPEYSTKEKLKNRLLTAIEN-AKGF
>Hamiltosporidium_tvaerminnensis_ACSZ01000311.1 .
LQIDRNNIIESTIDAF----------------------KNFSNEIFSQK-NWFVKYSN--
--------EEGSGI-GLLYEFFNIF-GKCID-ESDYF-------------------APFK
SLEFI--------------------------IICVSSPE---------------------
--------------------------------EQNKIEQLYYFTGVIMAKSIFM----NS
VMSFEFINSFYLYLLQ-----------------------------------EKFNIEDLK
D---IDFEYYKNLISLR--------------------------------NVE-NHDPDLT
--FDISVK------IGQK-----------IISRNLIEN-GSEIK----------VTKDNL
E-DYIQKM--AEFKMFKG-MEVYLDKLKE--GFQSLF---YHCFYN--------------
--------MFTHSDLKIVIEG---------EKRIN-INEW--------------------
------------------------------------------------------------
---------------KAATRYRGKYYTD--------------------------HQLIVW
FWIFLG-KSDEITKRKLLFFTTGLSYLPIGGFKSSKFIDH-PFTITSDD-----------
----------------RKDLFPRSQTCSNLLILPLYDQEDVLLDKIEHAIEVINYGF
>Hamiltosporidium_tvaerminnensis_ACSZ01008657.1 .
LDIDRKNVIKSTIDCF----------------------VKPNKGDFLQN-YWKVKFKD--
--------EEGRGI-GLLYDFFGSY-GKALI-DEDCF-------------------EPFR
TLYST--------------------------TIIVSFET---------------------
--------------------------------EYKEILEKYYYTGAIMAKSIIY----NS
PMGIGFNDYVYLYLIE-----------------------------------KTLGLEDLK
I---VDPTFYKNLISYRTHE-----------------------------DL--DSQ-DLD
--FTATASV-----KGT------------NKAFDLVEN-GSSMQ----------VTKNNL
E-SYIQKM--TEFKIYKN-MKPYLDKLEE--GFKLVF---ENDFKE--------------
--------LFTPLDFRNILEG---------NEVVD-VDEW--------------------
------------------------------------------------------------
---------------KSYTKYIDGFDVN--------------------------HQVIKW
FWNFVE-KTDTVTRKKLIYFITGLERLPIGGFGSDKFREN-CFKIASVE-----------
----------------GTNLLPTSQTCINLLILPLYEDENTFIEKLTFAIEYRGYGF
>Hamiltosp_tvaerminnensis_ACSZ01009899.1 .
LQVNRENILDNFLNLV----------------------SSFDLKKTLKENIWYVTFGT--
--------ELCAGI-GPTLEFFTLF-GKSMN-RSRFF-------------------EMFK
STE----------------------------TSYMNKFA---------------------
-------------------------------ECPEYIEKVYFYTGIIMAKCIIY----NT
TLGLEFIDSFYLYLTK-----------------------------------DCFTFEDLK
I---CDSELHKSLSFFATSG-----------------------------KSS-FHG--YN
```

```
--FTWATNN-----NGI------------YEEFELVEN-GCNIS----------LTSDNL
E-EYLQKI--TEFKLFKR-MEKYLDKMKE--GFCFIL---GDSFSN--------------
--------MFNYRDLSTMLQG---------EPDFQ-VEDF--------------------
------------------------------------------------------------
---------------KQCTSYVGDYNSN--------------------------HPVIIW
FWNYMS-SLEAIKKKKLFLFLTTFEKIPFGGFKSQRFLEY-KFTIMPIE-----------
----------------NSKSLPIIHTCSNLLELPKYESEEILRSKFEYCLDS-IT--
>Fonticula_alba_XM_009498620.1 .
IVVHRDHILDSSVIAV-----------------------NKLRREDIER-KLVVKFHG--
--------EDGLDFGGVARGSAPLR-NKLL--DKDY--------------------AYFR
DLESESDHL----------------------GHAIDLTH---------------------
---------------------------IRSLDDDDDSRQFYSFFGRVLGLCLRN----RI
TIDVCFPLHIYKHLLG-----------------------------------AEVTPEDLK
S---FDPGLYNSYQLMRNPE-----------------------------VLE-HVE--YN
--FVQTVKDD----LTNE-----------VYDVDLIEN-GSEIL----------VTLENV
D-LFIQRA--SEYLLTER-YGQKLQYLRD--GFNFVT---PINIVKS-------------
--------FFGPHELESIIAG----------NDIS-IEDW--------------------
------------------------------------------------------------
---------------QANTVYIDC-NAN--------------------------HPSVRL
FWAYMS-QGDFALRSQVLKFVTGLTRPPFGGFARLFGYGR-LEPFKIQLVNATP------
-----------------PAIPFPSASTCHNRLHLPAFQDMETLAAKFEVSLQN-TSFQ
>Nematocida_parisii_AEOO01000022.1 .
LEVSRDSILYSFYKLY----------------------ANPYYKKDLYK-RITVKFVG--
--------EMGEDHGALRKEFFELA-GNELVQDRRFILSG----------------GLFD
LATVEEM------------------------TEKIDPAM---------------------
------------------------------EALSISDTEFYAFVGFFIGHVIFQ----QV
QISVRFSRTLYMALLK-----------------------------------KKGTSNDI-
----ANETLKTSLDWIKNNSV----------------------------D----------
------------------------------QMEFVLKS-GKK------------VTDANK
E-EFI-----EEFIYEET-YGKRVGYPAMAQGFSKAV---SDDIYN--------------
---------FQPHQLERLLSG---------VDHIS-IKHL--------------------
------------------------------------------------------------
---------------KTLAIYRECTFQT---------------------------EEVMN
FWHILE-NSNEEFRRNVLRFITGSSSLQSIPGSHSECIII-TQMN---------------
-----------------IQGFLPTANTCFRRIVLYKYKSYSELKNKLERAVKE-NGGF
>Mitosporidium_daphniae_JMKJ01000083.1 .
IKVCRNNIFQSSFKAF-----------------KGVLRTEGNTTPLLHASNFKVSFLN--
--------EEGIDLDGFSREWASLM-AIQLQ-VSEL--------------------SIFN
SYKE---------------------------GFYFRRDG---------------------
-----------------------------------KNKEYAKFCGAFLGLAISK----EL
TLDCRFSGLFYHILTS-------------------------------DPKNIQLSLEDMK
L---IDTTLYTGFSNSDSNLLN---------------------------ELE-DFYKDGD
DKKKLDFFKT----FKRH-----------LIASSDEKI-GTHLPREENGEISRSIVLTND
--EFNALKDELTHEIFYSALEDTIKPFLD--GLNMFI---PPEKLQ--------------
--------QFRIEDLRKKVEG--------EFSEIN-DKAF--------------------
------------------------------------------------------------
------------EKWKAITVWTDCTNSGIGIKKKTWKKKL--------------KTQKDW
FWEIVY-EFSEKEKRDLVQFWTGSGNIPENLEVIHGGTV---------------------
------------------NCLPSSHTCVFTLELPYYKNEVKVSVDGIETIKG-AKEI
>Mitosporidium_daphniae_XM_013383569.1 .
IKVRRDDIFRSSFTAF-----------------KRILSIPKEHSALLHIHSFEVEFLN--
--------EEGVDAGGLSREWATLM-AAQLQ-VSEL--------------------SIFN
SYKE---------------------------GFYFRRDG---------------------
-----------------------------------KNKEYAKFCGAFLGLAISK----EL
TLDCRFSDLFYRILTS-------------------------------DPKNIPLLFKDME
L---IDTDLHRGFSNSDSNLLN---------------------------DLE-NFYKDGD
DKKKLDFFKA----CKRY-----------LIVSPDEKI-GAHLPRDKDGETFTSIVLTDD
--DLNSLRDELTHEIYYSALKDTIERFLD--GVHIFI---PPEELQ--------------
```

```
--------LFSIEGLRKKVEG--------EFSEIN-DKAF--------------------
------------------------------------------------------------
------------EKWKAITVWKYLTNGKTGIEEETWKNGL--------------KIQKDW
FWEIIQ-KFSEKEKRDLLQFWTGSRNIPKTLEVNHGGTV---------------------
------------------NYFPSSSTCLLSLNLPYYKKE-KVAVDGIETIKG-AKEI
>Nosema_ceranae_JPQZ01000457.1_ACOL01000628.1 .
VTVDRKNILKSSFNSL-----------------------KNVDLNCLRANNVYISFKD--
--------EIGQDVGGLSRDWLNSV-LKSLL-DNGA--------------------GLFV
QASSYPG------------------------NIIPKCLA---------------------
-------------------------------QDNYSNLIYYKFLGRLLGLLIVK----KH
NYNIQFEKLIYKKILN-----------------------------------IPCTISDYC
A---ADSEFFNNLVKLKSC------------------------------EEA-LFE----
--------------FTA------------SSGKELLKG-GQNIK----------VVKDNV
N-LYIVLT--LLDKYEAS-INTAVEYIKE--GFYDII---PQKFLS--------------
--------NWNADELELLLCG---------KELID-ISYW--------------------
------------------------------------------------------------
---------------KRHTQYEG-YLPN--------------------------DKVIVW
FWEYVE-TLSDEGRSRLLNFVTACTRISADRSNSDSGRQV-NEDRIMTISRV--------
----------------NNESTPTASTCIKILFLPCYKTEKKLNFNV-----------
>Mitosporidium_daphniae_JMKJ01000543.1 .
LIIRNGFALQDGLRHF----------------------VLSREGYFSSS-PWFIIFTDEN
GTP-----EPGADGGGLYRQFLSES-LSSVL-DPKMKFFEEIHSK-----------ENYL
SSALIF-------------------------PSFEEPCS---------------------
----------------------DFLLSLFNDPFLCDVDTIYRYMGHLLGRILARLPLSGL
HINMRFPLSFWCQLLS-------------------------------LPSLPECKINEMR
E---IDSFYLLQLLSLDHT------------------------------LKSKSIS----
-----------------------------PDELAIFNDEGL-------------LVDENN
FPCYLFSS--IQKKVSRP----QIAKIAL--GFFSLV---PPEIFS--------------
--------IFNATEISCIIDG--------RYTXID-LNDW--------------------
------------------------------------------------------------
---------------RENTVYVLPYTSQ--------------------------DPTIVL
FWNLLS-KWEGKDLSSLLRFCTGYGSGPYLGFKEFNPLFC-IGYGGA-------------
----------------SPERLPTASTCVHMLTLPEYASEDVIEIRLRTAIGC-Q---
>Nosema_ceranae_ACOL01000093.1 .
LEVRRGHILDSTVIYI------------------------LQHVDKLKTKKLHASFID--
--------EIGQDYGALMREFIYET-SLEILNDPRLEVC-----------------NDFV
DVKPNDFC-----------------------NQNLLSET---------------------
-----------VKEIANRHEQICEFTDDFKGKNRLTDEAFYIYLGVFIALLLIC----NE
NIDYTFSLAFYENLLL-----------------------------------RRYTMRLIQ
-----DVQYQKSLNWMCQN------------------------------E----------
------------------------------VTEDILP--G--------------VTSTNK
S-DLIYSI--FYDKLFLQ-KKVPYDFISK--GFYSVI---PEDFRD--------------
--------MFESDELSMLISN---------NKYLE-VSHL--------------------
------------------------------------------------------------
---------------KEFVLYNL-CSES--------------------------TEEIIW
LWEILK-SKDQIFLRKFLKFITGSGSIPMLINNSS-FKIV-IEQNN--------------
----------------LKDSLLRASACLNKLVLSKYSNKQTLEEILDFSILN-TEGF
>Hamiltosporidium_tvaerminnensis_ACSZ01004659.1 .
IIARRGFLIQSTAGQI----------------------IGAQVADLMKD--IHIYFID--
--------EPGEDYGALLREYFYET-SFEIAQDYRMQEVE----------------NIFD
VLPLNTSNSVD--------------------NSYIPPTN---------------------
---IEAVKKTYFNYESAEPENPFFENIENKGKNCLDDKSFFKFVGIFLALAIEH----QK
NIGVNFSLAFYENILQ-----------------------------------RNFTISHIQ
-----DIQFQSSMVWLLEN------------------------------DY--DLS---S
--EDVDVNK---------------------NSESISP--GNNR-----------QIQNNR
K-DYVTSL--ISKCFYKS-KISSYESIRN--GFYYVC---VPEFSE--------------
--------IFNCYDLLYILHG---------NETVT-PALI--------------------
------------------------------------------------------------
```

```
---------------KQNLVFTN-CTNS--------------------------TKEIIY
LFKILD-KKNEKYLRKFLSFVTGTGTVLFNAIKFGKFTIY-IEQEN--------------
----------------KKISLIRASSCINKLYIGKYDSLEQFEGIIDFCLYN-TEGF
```
